# Global, regional, and national life expectancy, all-cause mortality, and cause-specific mortality for 249 causes of death, 1980–2015: a systematic analysis for the Global Burden of Disease Study 2015

**DOI:** 10.1016/S0140-6736(16)31012-1

**Published:** 2016-10-08

**Authors:** GBD 2015 Mortality and Causes of Death Collaborators, Haidong Wang, Mohsen Naghavi, Christine Allen, Ryan M Barber, Zulfiqar A Bhutta, Austin Carter, Daniel C Casey, Fiona J Charlson, Alan Zian Chen, Matthew M Coates, Megan Coggeshall, Lalit Dandona, Daniel J Dicker, Holly E Erskine, Alize J Ferrari, Christina Fitzmaurice, Kyle Foreman, Mohammad H Forouzanfar, Maya S Fraser, Nancy Fullman, Peter W Gething, Ellen M Goldberg, Nicholas Graetz, Juanita A Haagsma, Simon I Hay, Chantal Huynh, Catherine O Johnson, Nicholas J Kassebaum, Yohannes Kinfu, Xie Rachel Kulikoff, Michael Kutz, Hmwe H Kyu, Heidi J Larson, Janni Leung, Xiaofeng Liang, Stephen S Lim, Margaret Lind, Rafael Lozano, Neal Marquez, George A Mensah, Joe Mikesell, Ali H Mokdad, Meghan D Mooney, Grant Nguyen, Elaine Nsoesie, David M Pigott, Christine Pinho, Gregory A Roth, Joshua A Salomon, Logan Sandar, Naris Silpakit, Amber Sligar, Reed J D Sorensen, Jeffrey Stanaway, Caitlyn Steiner, Stephanie Teeple, Bernadette A Thomas, Christopher Troeger, Amelia VanderZanden, Stein Emil Vollset, Valentine Wanga, Harvey A Whiteford, Timothy Wolock, Leo Zoeckler, Kalkidan Hassen Abate, Cristiana Abbafati, Kaja M Abbas, Foad Abd-Allah, Semaw Ferede Abera, Daisy M X Abreu, Laith J Abu-Raddad, Gebre Yitayih Abyu, Tom Achoki, Ademola Lukman Adelekan, Zanfina Ademi, Arsène Kouablan Adou, José C Adsuar, Kossivi Agbelenko Afanvi, Ashkan Afshin, Emilie Elisabet Agardh, Arnav Agarwal, Anurag Agrawal, Aliasghar Ahmad Kiadaliri, Oluremi N Ajala, Ali Shafqat Akanda, Rufus Olusola Akinyemi, Tomi F Akinyemiju, Nadia Akseer, Faris Hasan Al Lami, Samer Alabed, Ziyad Al-Aly, Khurshid Alam, Noore K M Alam, Deena Alasfoor, Saleh Fahed Aldhahri, Robert William Aldridge, Miguel Angel Alegretti, Alicia V Aleman, Zewdie Aderaw Alemu, Lily T Alexander, Samia Alhabib, Raghib Ali, Ala'a Alkerwi, François Alla, Peter Allebeck, Rajaa Al-Raddadi, Ubai Alsharif, Khalid A Altirkawi, Elena Alvarez Martin, Nelson Alvis-Guzman, Azmeraw T Amare, Adeladza Kofi Amegah, Emmanuel A Ameh, Heresh Amini, Walid Ammar, Stephen Marc Amrock, Hjalte H Andersen, Benjamin O Anderson, Gregory M Anderson, Carl Abelardo T Antonio, Atsede Fantahun Aregay, Johan Ärnlöv, Valentina S Arsic Arsenijevic, Al Artaman, Hamid Asayesh, Rana Jawad Asghar, Suleman Atique, Euripide Frinel G Arthur Avokpaho, Ashish Awasthi, Peter Azzopardi, Umar Bacha, Alaa Badawi, Maria C Bahit, Kalpana Balakrishnan, Amitava Banerjee, Aleksandra Barac, Suzanne L Barker-Collo, Till Bärnighausen, Lars Barregard, Lope H Barrero, Arindam Basu, Sanjay Basu, Yibeltal Tebekaw Bayou, Shahrzad Bazargan-Hejazi, Justin Beardsley, Neeraj Bedi, Ettore Beghi, Haileeyesus Adamu Belay, Brent Bell, Michelle L Bell, Aminu K Bello, Derrick A Bennett, Isabela M Bensenor, Adugnaw Berhane, Eduardo Bernabé, Balem Demtsu Betsu, Addisu Shunu Beyene, Neeraj Bhala, Ashish Bhalla, Sibhatu Biadgilign, Boris Bikbov, Aref A Bin Abdulhak, Brian J Biroscak, Stan Biryukov, Espen Bjertness, Jed D Blore, Christopher D Blosser, Megan A Bohensky, Rohan Borschmann, Dipan Bose, Rupert R A Bourne, Michael Brainin, Carol E G Brayne, Alexandra Brazinova, Nicholas J K Breitborde, Hermann Brenner, Jerry D Brewer, Alexandria Brown, Jonathan Brown, Traolach S Brugha, Geoffrey Colin Buckle, Zahid A Butt, Bianca Calabria, Ismael Ricardo Campos-Nonato, Julio Cesar Campuzano, Jonathan R Carapetis, Rosario Cárdenas, David O Carpenter, Juan Jesus Carrero, Carlos A Castañeda-Orjuela, Jacqueline Castillo Rivas, Ferrán Catalá-López, Fiorella Cavalleri, Kelly Cercy, Jorge Cerda, Wanqing Chen, Adrienne Chew, Peggy Pei-Chia Chiang, Mirriam Chibalabala, Chioma Ezinne Chibueze, Odgerel Chimed-Ochir, Vesper Hichilombwe Chisumpa, Jee-Young Jasmine Choi, Rajiv Chowdhury, Hanne Christensen, Devasahayam Jesudas Christopher, Liliana G Ciobanu, Massimo Cirillo, Aaron J Cohen, Valentina Colistro, Mercedes Colomar, Samantha M Colquhoun, Cyrus Cooper, Leslie Trumbull Cooper, Monica Cortinovis, Benjamin C Cowie, John A Crump, James Damsere-Derry, Hadi Danawi, Rakhi Dandona, Farah Daoud, Sarah C Darby, Paul I Dargan, José das Neves, Gail Davey, Adrian C Davis, Dragos V Davitoiu, E Filipa de Castro, Pieter de Jager, Diego De Leo, Louisa Degenhardt, Robert P Dellavalle, Kebede Deribe, Amare Deribew, Samath D Dharmaratne, Preet K Dhillon, Cesar Diaz-Torné, Eric L Ding, Kadine Priscila Bender dos Santos, Edem Dossou, Tim R Driscoll, Leilei Duan, Manisha Dubey, Bruce Bartholow Duncan, Richard G Ellenbogen, Christian Lycke Ellingsen, Iqbal Elyazar, Aman Yesuf Endries, Sergey Petrovich Ermakov, Babak Eshrati, Alireza Esteghamati, Kara Estep, Imad D A Faghmous, Saman Fahimi, Emerito Jose Aquino Faraon, Talha A Farid, Carla Sofia e Sa Farinha, André Faro, Maryam S Farvid, Farshad Farzadfar, Valery L Feigin, Seyed-Mohammad Fereshtehnejad, Jefferson G Fernandes, Joao C Fernandes, Florian Fischer, Joseph R A Fitchett, Abraham Flaxman, Nataliya Foigt, F Gerry R Fowkes, Elisabeth Barboza Franca, Richard C Franklin, Joseph Friedman, Joseph Frostad, Thomas Fürst, Neal D Futran, Seana L Gall, Ketevan Gambashidze, Amiran Gamkrelidze, Parthasarathi Ganguly, Fortuné Gbètoho Gankpé, Teshome Gebre, Tsegaye Tsewelde Gebrehiwot, Amanuel Tesfay Gebremedhin, Alemseged Aregay Gebru, Johanna M Geleijnse, Bradford D Gessner, Aloke Gopal Ghoshal, Katherine B Gibney, Richard F Gillum, Stuart Gilmour, Ababi Zergaw Giref, Maurice Giroud, Melkamu Dedefo Gishu, Giorgia Giussani, Elizabeth Glaser, William W Godwin, Hector Gomez-Dantes, Philimon Gona, Amador Goodridge, Sameer Vali Gopalani, Richard A Gosselin, Carolyn C Gotay, Atsushi Goto, Hebe N Gouda, Felix Greaves, Harish Chander Gugnani, Rahul Gupta, Rajeev Gupta, Vipin Gupta, Reyna A Gutiérrez, Nima Hafezi-Nejad, Demewoz Haile, Alemayehu Desalegne Hailu, Gessessew Bugssa Hailu, Yara A Halasa, Randah Ribhi Hamadeh, Samer Hamidi, Jamie Hancock, Alexis J Handal, Graeme J Hankey, Yuantao Hao, Hilda L Harb, Sivadasanpillai Harikrishnan, Josep Maria Haro, Rasmus Havmoeller, Susan R Heckbert, Ileana Beatriz Heredia-Pi, Pouria Heydarpour, Henk B M Hilderink, Hans W Hoek, Robert S Hogg, Masako Horino, Nobuyuki Horita, H Dean Hosgood, Peter J Hotez, Damian G Hoy, Mohamed Hsairi, Aung Soe Htet, Maung Maung Than Htike, Guoqing Hu, Cheng Huang, Hsiang Huang, Laetitia Huiart, Abdullatif Husseini, Inge Huybrechts, Grace Huynh, Kim Moesgaard Iburg, Kaire Innos, Manami Inoue, Veena J Iyer, Troy A Jacobs, Kathryn H Jacobsen, Nader Jahanmehr, Mihajlo B Jakovljevic, Peter James, Mehdi Javanbakht, Sudha P Jayaraman, Achala Upendra Jayatilleke, Panniyammakal Jeemon, Paul N Jensen, Vivekanand Jha, Guohong Jiang, Ying Jiang, Tariku Jibat, Aida Jimenez-Corona, Jost B Jonas, Tushar Kant Joshi, Zubair Kabir, Ritul Kamal, Haidong Kan, Surya Kant, André Karch, Corine Kakizi Karema, Chante Karimkhani, Dimitris Karletsos, Ganesan Karthikeyan, Amir Kasaeian, Marzieh Katibeh, Anil Kaul, Norito Kawakami, Jeanne Françoise Kayibanda, Peter Njenga Keiyoro, Laura Kemmer, Andrew Haddon Kemp, Andre Pascal Kengne, Andre Keren, Maia Kereselidze, Chandrasekharan Nair Kesavachandran, Yousef Saleh Khader, Ibrahim A Khalil, Abdur Rahman Khan, Ejaz Ahmad Khan, Young-Ho Khang, Sahil Khera, Tawfik Ahmed Muthafer Khoja, Christian Kieling, Daniel Kim, Yun Jin Kim, Brett M Kissela, Niranjan Kissoon, Luke D Knibbs, Ann Kristin Knudsen, Yoshihiro Kokubo, Dhaval Kolte, Jacek A Kopec, Soewarta Kosen, Parvaiz A Koul, Ai Koyanagi, Norun Hjertager Krog, Barthelemy Kuate Defo, Burcu Kucuk Bicer, Andreas A Kudom, Ernst J Kuipers, Veena S Kulkarni, G Anil Kumar, Gene F Kwan, Aparna Lal, Dharmesh Kumar Lal, Ratilal Lalloo, Tea Lallukka, Hilton Lam, Jennifer O Lam, Sinead M Langan, Van C Lansingh, Anders Larsson, Dennis Odai Laryea, Asma Abdul Latif, Alicia Elena Beatriz Lawrynowicz, James Leigh, Miriam Levi, Yongmei Li, M Patrice Lindsay, Steven E Lipshultz, Patrick Y Liu, Shiwei Liu, Yang Liu, Loon-Tzian Lo, Giancarlo Logroscino, Paulo A Lotufo, Robyn M Lucas, Raimundas Lunevicius, Ronan A Lyons, Stefan Ma, Vasco Manuel Pedro Machado, Mark T Mackay, Jennifer H MacLachlan, Hassan Magdy Abd El Razek, Mohammed Magdy, Abd El Razek, Marek Majdan, Azeem Majeed, Reza Malekzadeh, Wondimu Ayele Ayele Manamo, John Mandisarisa, Srikanth Mangalam, Chabila C Mapoma, Wagner Marcenes, David Joel Margolis, Gerard Robert Martin, Jose Martinez-Raga, Melvin Barrientos Marzan, Felix Masiye, Amanda J Mason-Jones, João Massano, Richard Matzopoulos, Bongani M Mayosi, Stephen Theodore McGarvey, John J McGrath, Martin McKee, Brian J McMahon, Peter A Meaney, Alem Mehari, Man Mohan Mehndiratta, Fabiola Mejia-Rodriguez, Alemayehu B Mekonnen, Yohannes Adama Melaku, Peter Memiah, Ziad A Memish, Walter Mendoza, Atte Meretoja, Tuomo J Meretoja, Francis Apolinary Mhimbira, Renata Micha, Anoushka Millear, Ted R Miller, Mojde Mirarefin, Awoke Misganaw, Charles N Mock, Karzan Abdulmuhsin Mohammad, Alireza Mohammadi, Shafiu Mohammed, Viswanathan Mohan, Glen Liddell D Mola, Lorenzo Monasta, Julio Cesar Montañez Hernandez, Pablo Montero, Marcella Montico, Thomas J Montine, Maziar Moradi-Lakeh, Lidia Morawska, Katherine Morgan, Rintaro Mori, Dariush Mozaffarian, Ulrich O Mueller, Gudlavalleti Venkata Satyanarayana Murthy, Srinivas Murthy, Kamarul Imran Musa, Jean B Nachega, Gabriele Nagel, Kovin S Naidoo, Nitish Naik, Luigi Naldi, Vinay Nangia, Denis Nash, Chakib Nejjari, Subas Neupane, Charles R Newton, John N Newton, Marie Ng, Frida Namnyak Ngalesoni, Jean de Dieu Ngirabega, Quyen Le Nguyen, Muhammad Imran Nisar, Patrick Martial Nkamedjie Pete, Marika Nomura, Ole F Norheim, Paul E Norman, Bo Norrving, Luke Nyakarahuka, Felix Akpojene Ogbo, Takayoshi Ohkubo, Foluke Adetola Ojelabi, Pedro R Olivares, Bolajoko Olubukunola Olusanya, Jacob Olusegun Olusanya, John Nelson Opio, Eyal Oren, Alberto Ortiz, Majdi Osman, Erika Ota, Raziye Ozdemir, Mahesh PA, Amanda Pain, Jeyaraj D Pandian, Puspa Raj Pant, Christina Papachristou, Eun-Kee Park, Jae-Hyun Park, Charles D Parry, Mahboubeh Parsaeian, Angel J Paternina Caicedo, Scott B Patten, George C Patton, Vinod K Paul, Neil Pearce, João Mário Pedro, Ljiljana Pejin Stokic, David M Pereira, Norberto Perico, Konrad Pesudovs, Max Petzold, Michael Robert Phillips, Frédéric B Piel, Julian David Pillay, Dietrich Plass, James A Platts-Mills, Suzanne Polinder, C Arden Pope, Svetlana Popova, Richie G Poulton, Farshad Pourmalek, Dorairaj Prabhakaran, Mostafa Qorbani, Justice Quame-Amaglo, D Alex Quistberg, Anwar Rafay, Kazem Rahimi, Vafa Rahimi-Movaghar, Mahfuzar Rahman, Mohammad Hifz Ur Rahman, Sajjad Ur Rahman, Rajesh Kumar Rai, Zhale Rajavi, Sasa Rajsic, Murugesan Raju, Ivo Rakovac, Saleem M Rana, Chhabi L Ranabhat, Thara Rangaswamy, Puja Rao, Sowmya R Rao, Amany H Refaat, Jürgen Rehm, Marissa B Reitsma, Giuseppe Remuzzi, Serge Resnikoff, Antonio L Ribeiro, Stefano Ricci, Maria Jesus Rios Blancas, Bayard Roberts, Anna Roca, David Rojas-Rueda, Luca Ronfani, Gholamreza Roshandel, Dietrich Rothenbacher, Ambuj Roy, Nawal K Roy, George Mugambage Ruhago, Rajesh Sagar, Sukanta Saha, Ramesh Sahathevan, Muhammad Muhammad Saleh, Juan R Sanabria, Maria Dolores Sanchez-Niño, Lidia Sanchez-Riera, Itamar S Santos, Rodrigo Sarmiento-Suarez, Benn Sartorius, Maheswar Satpathy, Miloje Savic, Monika Sawhney, Michael P Schaub, Maria Inês Schmidt, Ione J C Schneider, Ben Schöttker, Aletta E Schutte, David C Schwebel, Soraya Seedat, Sadaf G Sepanlou, Edson E Servan-Mori, Katya A Shackelford, Gavin Shaddick, Amira Shaheen, Saeid Shahraz, Masood Ali Shaikh, Marina Shakh-Nazarova, Rajesh Sharma, Jun She, Sara Sheikhbahaei, Jiabin Shen, Ziyan Shen, Donald S Shepard, Kevin N Sheth, Balakrishna P Shetty, Peilin Shi, Kenji Shibuya, Min-Jeong Shin, Rahman Shiri, Ivy Shiue, Mark G Shrime, Inga Dora Sigfusdottir, Donald H Silberberg, Diego Augusto Santos Silva, Dayane Gabriele Alves Silveira, Jonathan I Silverberg, Edgar P Simard, Abhishek Singh, Gitanjali M Singh, Jasvinder A Singh, Om Prakash Singh, Prashant Kumar Singh, Virendra Singh, Samir Soneji, Kjetil Søreide, Joan B Soriano, Luciano A Sposato, Chandrashekhar T Sreeramareddy, Vasiliki Stathopoulou, Dan J Stein, Murray B Stein, Saverio Stranges, Konstantinos Stroumpoulis, Bruno F Sunguya, Patrick Sur, Soumya Swaminathan, Bryan L Sykes, Cassandra E I Szoeke, Rafael Tabarés-Seisdedos, Karen M Tabb, Ken Takahashi, Jukka S Takala, Roberto Tchio Talongwa, Nikhil Tandon, Mohammad Tavakkoli, Bineyam Taye, Hugh R Taylor, Braden J Te Ao, Bemnet Amare Tedla, Worku Mekonnen Tefera, Margreet Ten Have, Abdullah Sulieman Terkawi, Fisaha Haile Tesfay, Gizachew Assefa Tessema, Alan J Thomson, Andrew L Thorne-Lyman, Amanda G Thrift, George D Thurston, Taavi Tillmann, David L Tirschwell, Marcello Tonelli, Roman Topor-Madry, Fotis Topouzis, Jeffrey Allen Towbin, Jefferson Traebert, Bach Xuan Tran, Thomas Truelsen, Ulises Trujillo, Abera Kenay Tura, Emin Murat Tuzcu, Uche S Uchendu, Kingsley N Ukwaja, Eduardo A Undurraga, Olalekan A Uthman, Rita Van Dingenen, Aaron van Donkelaar, Tommi Vasankari, Ana Maria Nogales Vasconcelos, Narayanaswamy Venketasubramanian, Ramesh Vidavalur, Lakshmi Vijayakumar, Salvador Villalpando, Francesco S Violante, Vasiliy Victorovich Vlassov, Joseph A Wagner, Gregory R Wagner, Mitchell T Wallin, Linhong Wang, David A Watkins, Scott Weichenthal, Elisabete Weiderpass, Robert G Weintraub, Andrea Werdecker, Ronny Westerman, Richard A White, Tissa Wijeratne, James D Wilkinson, Hywel C Williams, Charles Shey Wiysonge, Solomon Meseret Woldeyohannes, Charles D A Wolfe, Sungho Won, John Q Wong, Anthony D Woolf, Denis Xavier, Qingyang Xiao, Gelin Xu, Bereket Yakob, Ayalnesh Zemene Yalew, Lijing L Yan, Yuichiro Yano, Mehdi Yaseri, Pengpeng Ye, Henock Gebremedhin Yebyo, Paul Yip, Biruck Desalegn Yirsaw, Naohiro Yonemoto, Gerald Yonga, Mustafa Z Younis, Shicheng Yu, Zoubida Zaidi, Maysaa El Sayed Zaki, Faiez Zannad, Diego E Zavala, Hajo Zeeb, Berihun M Zeleke, Hao Zhang, Sanjay Zodpey, David Zonies, Liesl Joanna Zuhlke, Theo Vos, Alan D Lopez, Christopher J L Murray

## Abstract

**Background:**

Improving survival and extending the longevity of life for all populations requires timely, robust evidence on local mortality levels and trends. The Global Burden of Disease 2015 Study (GBD 2015) provides a comprehensive assessment of all-cause and cause-specific mortality for 249 causes in 195 countries and territories from 1980 to 2015. These results informed an in-depth investigation of observed and expected mortality patterns based on sociodemographic measures.

**Methods:**

We estimated all-cause mortality by age, sex, geography, and year using an improved analytical approach originally developed for GBD 2013 and GBD 2010. Improvements included refinements to the estimation of child and adult mortality and corresponding uncertainty, parameter selection for under-5 mortality synthesis by spatiotemporal Gaussian process regression, and sibling history data processing. We also expanded the database of vital registration, survey, and census data to 14 294 geography–year datapoints. For GBD 2015, eight causes, including Ebola virus disease, were added to the previous GBD cause list for mortality. We used six modelling approaches to assess cause-specific mortality, with the Cause of Death Ensemble Model (CODEm) generating estimates for most causes. We used a series of novel analyses to systematically quantify the drivers of trends in mortality across geographies. First, we assessed observed and expected levels and trends of cause-specific mortality as they relate to the Socio-demographic Index (SDI), a summary indicator derived from measures of income per capita, educational attainment, and fertility. Second, we examined factors affecting total mortality patterns through a series of counterfactual scenarios, testing the magnitude by which population growth, population age structures, and epidemiological changes contributed to shifts in mortality. Finally, we attributed changes in life expectancy to changes in cause of death. We documented each step of the GBD 2015 estimation processes, as well as data sources, in accordance with Guidelines for Accurate and Transparent Health Estimates Reporting (GATHER).

**Findings:**

Globally, life expectancy from birth increased from 61·7 years (95% uncertainty interval 61·4–61·9) in 1980 to 71·8 years (71·5–72·2) in 2015. Several countries in sub-Saharan Africa had very large gains in life expectancy from 2005 to 2015, rebounding from an era of exceedingly high loss of life due to HIV/AIDS. At the same time, many geographies saw life expectancy stagnate or decline, particularly for men and in countries with rising mortality from war or interpersonal violence. From 2005 to 2015, male life expectancy in Syria dropped by 11·3 years (3·7–17·4), to 62·6 years (56·5–70·2). Total deaths increased by 4·1% (2·6–5·6) from 2005 to 2015, rising to 55·8 million (54·9 million to 56·6 million) in 2015, but age-standardised death rates fell by 17·0% (15·8–18·1) during this time, underscoring changes in population growth and shifts in global age structures. The result was similar for non-communicable diseases (NCDs), with total deaths from these causes increasing by 14·1% (12·6–16·0) to 39·8 million (39·2 million to 40·5 million) in 2015, whereas age-standardised rates decreased by 13·1% (11·9–14·3). Globally, this mortality pattern emerged for several NCDs, including several types of cancer, ischaemic heart disease, cirrhosis, and Alzheimer's disease and other dementias. By contrast, both total deaths and age-standardised death rates due to communicable, maternal, neonatal, and nutritional conditions significantly declined from 2005 to 2015, gains largely attributable to decreases in mortality rates due to HIV/AIDS (42·1%, 39·1–44·6), malaria (43·1%, 34·7–51·8), neonatal preterm birth complications (29·8%, 24·8–34·9), and maternal disorders (29·1%, 19·3–37·1). Progress was slower for several causes, such as lower respiratory infections and nutritional deficiencies, whereas deaths increased for others, including dengue and drug use disorders. Age-standardised death rates due to injuries significantly declined from 2005 to 2015, yet interpersonal violence and war claimed increasingly more lives in some regions, particularly in the Middle East. In 2015, rotaviral enteritis (rotavirus) was the leading cause of under-5 deaths due to diarrhoea (146 000 deaths, 118 000–183 000) and pneumococcal pneumonia was the leading cause of under-5 deaths due to lower respiratory infections (393 000 deaths, 228 000–532 000), although pathogen-specific mortality varied by region. Globally, the effects of population growth, ageing, and changes in age-standardised death rates substantially differed by cause. Our analyses on the expected associations between cause-specific mortality and SDI show the regular shifts in cause of death composition and population age structure with rising SDI. Country patterns of premature mortality (measured as years of life lost [YLLs]) and how they differ from the level expected on the basis of SDI alone revealed distinct but highly heterogeneous patterns by region and country or territory. Ischaemic heart disease, stroke, and diabetes were among the leading causes of YLLs in most regions, but in many cases, intraregional results sharply diverged for ratios of observed and expected YLLs based on SDI. Communicable, maternal, neonatal, and nutritional diseases caused the most YLLs throughout sub-Saharan Africa, with observed YLLs far exceeding expected YLLs for countries in which malaria or HIV/AIDS remained the leading causes of early death.

**Interpretation:**

At the global scale, age-specific mortality has steadily improved over the past 35 years; this pattern of general progress continued in the past decade. Progress has been faster in most countries than expected on the basis of development measured by the SDI. Against this background of progress, some countries have seen falls in life expectancy, and age-standardised death rates for some causes are increasing. Despite progress in reducing age-standardised death rates, population growth and ageing mean that the number of deaths from most non-communicable causes are increasing in most countries, putting increased demands on health systems.

**Funding:**

Bill & Melinda Gates Foundation.

## Introduction

Comparable information about deaths and mortality rates broken down by age, sex, cause, year, and geography provides a starting point for informed health policy debate. However, generating meaningful comparisons of mortality involves addressing many data and estimation challenges, which include reconciling marked discrepancies in cause of death classifications over time and across populations; adjusting for vital registration system data with coverage and quality issues; appropriately synthesising mortality data from cause-specific sources, such as cancer registries, and alternative cause of death identification tools, such as verbal autopsies; and developing robust analytical strategies to estimate cause-specific mortality amid sparse data.^1^, ^2^, ^3^, ^4^, ^5^, ^6^ The annual Global Burden of Disease (GBD) analysis provides a standardised approach to addressing these problems, thereby enhancing the capacity to make meaningful comparisons across age, sex, cause, time, and place.

**Research in context**

**Evidence before this study**

In 2012, the Global Burden of Disease 2010 study was published, providing results from the first complete revision of the Global Burden of Disease (GBD) since the first assessment in 1993. The study reported on mortality and causes of death between 1990 and 2010 in 187 countries. In response to demand for up-to-date information on the health of populations to inform health policy debates, annual updates of the GBD study are now prepared, with the first of these, the GBD 2013 study, published in 2015. For the first time, collaborative teams undertook subnational assessments for China, Mexico, and the UK as part of this study.

**Added value of this study**

The GBD 2015 assessment of mortality and causes of death provides new and more robust evidence on the health of populations worldwide through the inclusion of subnational data from an expanded group of countries, including Brazil, India, Japan, Kenya, Saudi Arabia, South Africa, Sweden, and the USA, in addition to updates for China, Mexico, and the UK. This study complies with the Guidelines for Accurate and Transparent Health Estimates Reporting (GATHER) recommendations. Estimation of mortality levels, patterns, and distribution for several new causes, including Ebola virus disease, further disaggregations of carcinoma and leukaemia, motor neuron disease, and mortality attributable to environmental heat and cold exposure have been added for the GBD 2015 study. Furthermore, this analysis extends the concept of sociodemographic status first reported in GBD 2013, with important changes to computational methods, resulting in a new Socio-demographic Index (SDI) for a more robust positioning of countries and territories on the development continuum.

**Implications of all the available evidence**

This study provides the most comprehensive assessment to date of patterns and levels of mortality worldwide, expanding on previous analyses by further investigating the main determinants of epidemiological patterns and trends across geographies and over time. The GBD 2015 study entails a complete reanalysis of trends for each cause of death from 1990 to 2015; the time series published here supersedes the results of the GBD 2013 study. The expansion of geographic units, from 296 in GBD 2013 to 519 for GBD 2015, is envisaged to continue so as to sustain comparability over time and across all geographies. The comparison of estimates of observed mortality levels with patterns expected based on the SDI provides an in-depth understanding of national health challenges and priority areas for intervention.

Previous iterations of the GBD study showed substantial reductions in under-5 mortality, largely driven by decreasing rates of death from diarrhoeal diseases, lower respiratory infections, malaria, and, in several countries, neonatal conditions and malnutrition.^7^, ^8^, ^9^, ^10^, ^11^ Non-communicable diseases (NCDs) and injuries claimed increasingly more lives throughout the world, although age-standardised death rates fell for many causes and countries.^7^ Examination of epidemiological convergence among high-income, middle-income, and low-income countries showed the importance of evaluating both absolute and relative changes in mortality, as solely focusing on absolutes can mask rising relative inequality among certain age groups and causes. The GBD 2015 study expands on these analyses by further evaluating the drivers of epidemiological patterns across countries and over time. Such mortality trends are generally shaped by a combination of factors, including changes in income per capita, educational attainment, fertility, shifts in clinical care and health system responsiveness, emergent health threats such as disease outbreaks or increasing rates of obesity, and geography-specific health contexts. An in-depth understanding of national health gains and priority areas for intervention can be provided by comparing estimates of expected mortality patterns. These results are of particular importance amid debates on financing and policy options for the newly adopted Sustainable Development Goals, which include both ambitious targets for maternal and child health and a much broader health agenda also encompassing NCDs and injuries.

The GBD 2010 study presented results for 187 countries, encompassing all those with a population greater than 50 000 in the year 2000.^12^ In the GBD 2013 study, collaborative teams produced subnational assessments for the UK, Mexico, and China, expanding the number of geographies included in the GBD analysis to 296.^7^, ^13^, ^14^, ^15^ The value of such subnational assessments to local decision makers^16^ has driven further geographical disaggregation for GBD 2015 including in Brazil, India, Japan, Kenya, Saudi Arabia, South Africa, Sweden, and the USA, in addition to updates for China, Mexico, and the UK. The expansion of the geographical units in the GBD studies will continue in a way that will sustain the comparability over time for the period 1990 to present and across all geographic entities.

As with all revisions of the GBD, the GBD 2015 study provides an update for the entire time series from 1990 to 2015 based on newly identified data sources released or collected since GBD 2013. In response to published commentaries and unpublished seminars and communications about GBD methods, various methodological refinements have been implemented.^17^, ^18^ Additionally, in the GBD 2015 cycle, a major effort towards data and code transparency has been made. As with each GBD cycle, the full time series published here supersedes previous GBD studies. This detailed assessment of causes of death allows the exploration of key questions including what are the leading causes of deaths in each geography, which causes are increasing or decreasing, what is the expected pattern of change in causes of death with the epidemiological transition and how does this expected pattern over time diverge across geographies.

## Methods

### Overview

**Figure 1:**
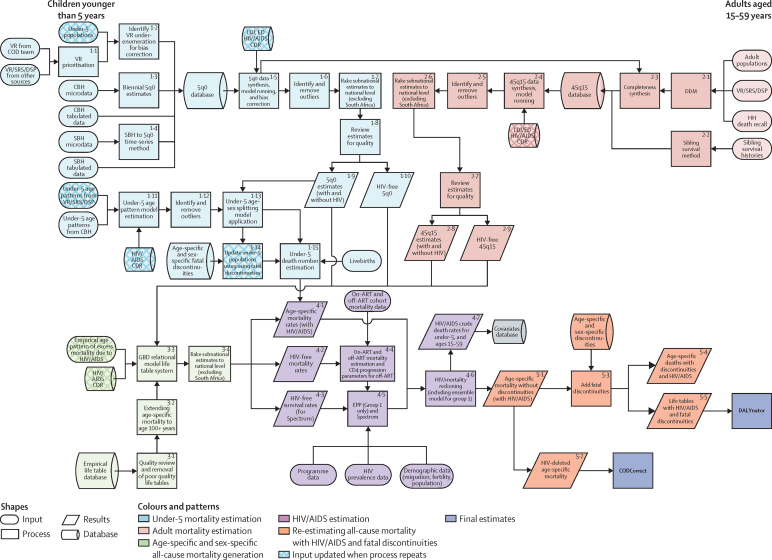
Estimation of all-cause mortality by age and sex and HIV/AIDS incidence, prevalence, and mortality for GBD 2015 Data and analyses are indicated by shape and the flow chart is colour coded by major estimation component. The process depicted is performed twice to bring in updated under-5 population estimates and crude death rates due to HIV/AIDS. The inputs that are updated in the second run of the process are shown by patterned boxes in this flow chart. Because of the very large and changing effects of HIV/AIDS on all-cause mortality in several countries with large HIV epidemics and limited data on all-cause mortality, the estimation of HIV/AIDS and all-cause mortality are closely linked and are presented jointly here. GBD=Global Burden of Disease. 5q0=probability of death from birth to age 5 years. 45q15=probability of death from age 15 to 60 years. ART=antiretroviral therapy. CBH=complete birth histories. CDR=crude death rate. COD=causes of death. DSP=disease surveillance points. ED=educational attainment in years per capita above age 15 years and mother's educational attainment in years per capita for children younger than 5 years. EPP=Estimation and Projection Package. HIV CDR=crude death rate due to HIV/AIDS. LDI=lagged distributed income per capita. SBH=summary birth history. SRS=Sample Registration System. VR=vital registration.

**Figure 2:**
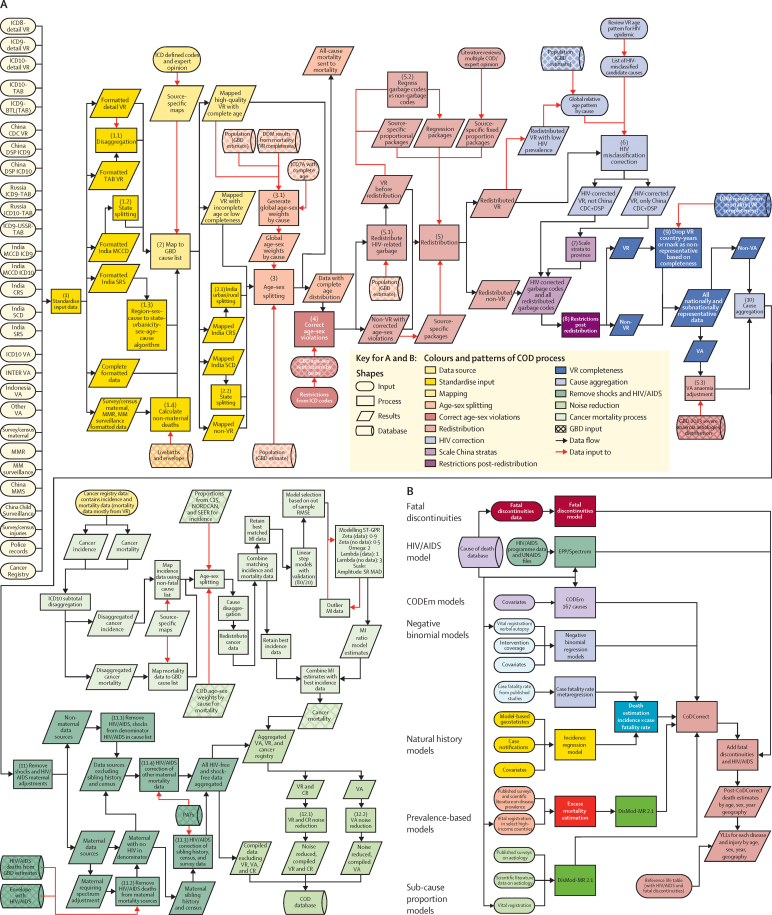
Development of the GBD 2015 cause of death database Figure shows (A) different strategies used to model different causes and to (B) combine them into a consistent set of cause-specific deaths for each location, age, sex, and year. Data and analytical processes are indicated by shape and the flow chart is colour coded by major estimation component. GBD=Global Burden of Disease. BTL=basic tabulation list. CDC=Center for Disease Control and Prevention. COD=cause of death. CODEm=Cause Of Death Ensemble model. CR=cancer registry. CRS=civil registration system. DSP=disease surveillance points. ICD=International Classification of Diseases. MI=mortality/incidence ratio. MCCD=medical certification of causes of death. MM=maternal mortality. MMR=maternal mortality ratio. MMS=maternal mortality surveillance. PAF=population-attributable fraction. SCD=survey of causes of death. SEER=Surveillance, Epidemiology, and End Results Program. SRS=Sample Registration System. SR MAD=super-region median average deviation. ST-GPR=spatiotemporal Gaussian process regression. VA=verbal autopsy. VR=vital registration. YLL=years of life lost.

GBD employs various analytical tools and a diverse set of data sources to generate comparable estimates of deaths and mortality rates broken down by age, sex, cause, year, and geography. Multiple publications show more detail on the various aspects of the methods.^7^, ^8^, ^12^, ^19^ Part 1 of the methods appendix (pp 4–51) is a structured and succinct explanation of each step. Figure 1 shows all of the inputs, analytical processes, and outputs from the analysis of all-cause mortality and HIV/AIDS mortality, included because of its important effects on all-cause mortality in countries with large HIV epidemics, and figure 2 does the same for cause-specific mortality. Each input or process is numbered for reference, with part 2 of the methods appendix (pp 52–70) providing explanation for each step. The GBD analytical approach to estimation is guided by standardised solutions to some general analytical problems: inconsistent case definitions or coding over time or across geographies; missing data; conflicting data for the same year and geography; and population groups (eg, the poor, minorities, and vulnerable groups) who are often missed in administrative data sources. In this Article, we provide only a very high-level summary. This analysis adheres to the new Guidelines for Accurate and Transparent Health Estimates Reporting (GATHER) proposed by the World Health Organization (WHO) and others, which includes recommendations on documentation of data sources, estimation methods, and statistical analysis.^20^ Table 1 shows the precise ways in which we have adhered to each element of the GATHER agreement.

### Geographic units

We have organised geographies into a set of hierarchical categories: seven super-regions; 21 regions nested within the seven super-regions; and 195 countries and territories nested within the 21 regions (table 2). Details on the classification of each geographical unit into each level of this hierarchy are provided in the methods appendix (pp 670–83). Compared with GBD 2013, we have added seven territories—American Samoa, Bermuda, Greenland, Guam, the Northern Mariana Islands, Puerto Rico, and the Virgin Islands—because of the availability of high-quality vital registration data. These territories were not previously included in the national totals of the USA, UK, or Denmark, and were included only in GBD 2013 regional totals. We have further disaggregated data for selected countries or territories into subnational units: 26 states and one district for Brazil, 34 provinces and municipalities for China, 31 states and union territory groupings for India that include 62 rural and urban units, 47 prefectures for Japan, 47 counties for Kenya, 32 states and districts for Mexico, 13 regions for Saudi Arabia, nine provinces for South Africa, two regions for Sweden, 13 regions for the UK (Northern Ireland, Scotland, Wales, England, and nine subregions of England), and 51 states and districts for the USA. At the first subnational unit level, we have 256 geographic units. Subnational level 1 geographies in the GBD 2015 analysis include countries that have been subdivided into the first subnational level, such as states or provinces. The subnational level 2 category applies only to India and England. In this Article we present national, territory, and previously published subnational units in the UK.^13^

### GBD cause list

The GBD cause list is the crucial organising framework for the analysis of causes of death and premature mortality, as well as disease incidence and prevalence and years lived with disability.^21^ The GBD cause list has evolved during the 25 years of the GBD study to become a list of causes that have public health and medical care importance either because they are major causes of lost health or because of policy relevance.^7^, ^21^, ^22^, ^23^, ^24^ Because different levels of cause aggregation are appropriate for different purposes and users, the GBD cause list is organised hierarchically (table 2). At each level of the cause hierarchy, the set of causes is mutually exclusive and collectively exhaustive.^21^ At the first level of the cause list, there are three broad causes: communicable, maternal, neonatal, and nutritional diseases; NCDs; and injuries. At the second level of the hierarchy, these three causes are broken down into 21 cause groups such as neoplasms (cancers) or cardiovascular diseases. Levels 3 and 4 of the cause list provide more disaggregated causes. Based on policy interest and by approval of the GBD Scientific Council, we have added eight causes to the GBD cause list: Ebola virus disease, motor neuron disease, environmental heat and cold exposure, squamous-cell carcinoma, acute lymphoid leukaemia, chronic lymphoid leukaemia, acute myeloid leukaemia, and chronic myeloid leukaemia. Bulimia nervosa has also been added as a cause of death. In total, there are now three causes at Level 1, 21 at Level 2, 166 at Level 3, and 261 at Level 4. Some causes, such as acne, medication overuse headache, and cutaneous leishmaniasis, are not considered causes of death according to the rules of the International Classification of Diseases (ICD), so the number of causes included in this analysis of causes of death is three at Level 1, 21 at Level 2, 144 at Level 3, and 200 at Level 4. The full GBD cause list, including those for which we estimate deaths, is available in the methods appendix (pp 684–90).

### Time periods

Because of the greater availability of data on all-cause mortality than cause-specific mortality, the all-cause mortality analysis for GBD 2015 covered 1970 to 2015. The cause of death analysis of GBD 2015 covered 1980 to 2015. A complete set of age-specific, sex-specific, cause-specific, and geography-specific death numbers and rates were generated. We present results covering different periods. However, for the main global and national results, we have focused on trends in the past decade, from 2005 to 2015, and detailed findings in 2015. Data visualisation tools are available online and provide results for each year from 1990 to 2015.

### All-cause mortality and HIV/AIDS mortality

Because of the very large and changing effects of HIV/AIDS on all-cause mortality in several countries with large HIV epidemics and scarce data on all-cause mortality, especially in eastern and southern Africa,^11^ the estimation of HIV/AIDS mortality and all-cause mortality are closely linked and presented jointly in figure 1. We divided the estimation effort into five distinct components: estimation of under-5 mortality rate (5q0); estimation of the adult mortality rate (45q15); age-specific mortality estimation; HIV/AIDS mortality estimation; and addition of the effects of events such as wars, pandemics, and disasters, which can cause abrupt discontinuities in death numbers (fatal discontinuities). Because of the interdependencies in the estimation of HIV/AIDS incidence, prevalence, and mortality and all-cause mortality, the estimation steps shown in figure 1 were repeated, with the HIV/AIDS crude death rates produced in step 4.7 used as covariates in steps 1.5, 1.11, 2.4, and 3.3 in the flow diagram.

### Under-5 mortality estimation

**Figure 3:**
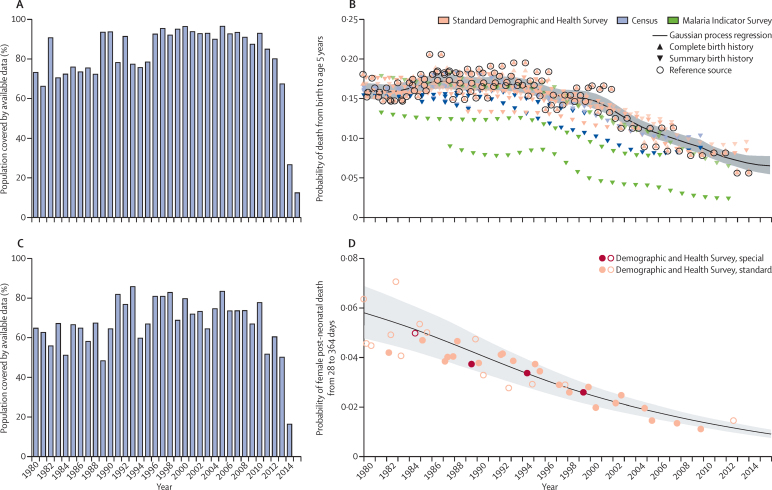
Examples of under-5 mortality data availability and estimation (A) Percentage of global under-5 population covered by under-5 mortality data for each year, 1980–2015. The percentage of under-5 population covered was calculated by dividing the population of children aged 0–4 years in locations covered by available under-5 mortality data by the total global under-5 population. Because of lags in reporting of both vital registration data and the release of household survey or census data, the availability of data was much lower for 2014 and 2015 than for previous years. (B) Country-specific example of data and under-5 mortality estimates in Zambia, 1980–2015. The black line shows Gaussian process regression fit with 95% uncertainty interval shown in grey. Black circles denote reference data. Triangles denote complete birth history data. Inverted triangles denote summary birth history data. Transparent symbols are the data post-adjustment for non-sampling error. Hollow shapes represent data identified as outliers. (C) Percentage of global under-5 population covered by under-5 age-specific and sex-specific data for each year, 1980–2015. The percentage of under-5 population covered was calculated by dividing the population of children aged 0–4 years in locations covered by available under-5 age-specific and sex-specific data by the total global under-5 population. Because of lags in reporting both vital registration data and the release of household survey or census data, the availability of data was much lower for 2014 than for previous years, and no data existed for 2015. (D) Country-specific example of probability of female post-neonatal mortality in Bangladesh, 1980–2015. Standard Demographic and Health Surveys generally include large population samples and standard sets of questions. Special Demographic and Health Surveys can survey smaller, more targeted populations, such as women who have given birth. The black line shows probability of death, with 95% uncertainty interval shown in grey. Solid circles represent data sources. Hollow circles represent outliers. The post-neonatal period is 28–364 days.

Seven types of primary data contributed to the estimation process (oval shapes in figure 1). The most important set of inputs were the data for estimating the overall level of under-5 mortality (5q0) that were obtained from vital registration systems, surveys, and censuses. Figure 3A provides information about the proportion of the 519 geographies included in the analysis for which data were available in each year from 1980 to 2015. Because of lags in reporting of both vital registration data and the release of household survey or census data, the availability of data was much lower for 2014 and 2015 than for previous years. Different data types, such as summary or complete birth histories, were processed to yield estimates for each year of the under-5 death rate; country-specific and year-specific details of the measurements are provided in the methods appendix (pp 4–19). Figure 3B shows the nature of the data and estimation process for under-5 mortality using the example of Zambia, as well as the uncorrected and bias-adjusted datapoints for each source. We used spatiotemporal Gaussian process regression to synthesise the sources and simultaneously correct for biases in specific source types.^8^ Bias corrections were made by comparison to reference sources, which for Zambia were the Demographic and Health Surveys. Further details of this estimation process are provided in the methods appendix (pp 4–19).

Because there are many sources for measuring under-5 mortality, such as summary birth histories from censuses and surveys, that do not provide sex and specific age group detail, we first estimated under-5 mortality and then split it into mortality for four age groups: early neonatal (0–6 days), late neonatal (7–28 days), post-neonatal (29–364 days), and ages 1–4 years. Splitting into these age groups was based on a statistical model using the analysis of available data that provide breakdowns by age and sex. Figure 3C shows the availability by country–year of data used to build the model to estimate mortality for specific age–sex groups younger than age 5 years. We modelled the ratio of male-to-female probability of death from birth to age 5 years as a function of both sexes' combined under-5 mortality rate and country and regional random effects. We further disaggregated sex-specific probability of death between birth and age 5 by modelling the ratio between age-and-sex-specific probability of deaths in the early neonatal, late neonatal, post-neonatal, and 1–4 year age groups and sex-specific probability of death between birth and age 5 years. This model allowed for the association between these age-and-sex-specific probabilities and the under-5 death rate to be non-linear, and included other covariates consisting of the death rate due to HIV/AIDS in children younger than 5 years, average years of schooling among females of reproductive age, and country and regional random effects. More details, including the equations are provided in the methods appendix (p 18). Figure 3D shows an example of the empirical fit for the post-neonatal period for Bangladesh. This model was applied to all countries to generate the under-5 estimates for each geography–year.

With the estimated mortality by detailed age group, we generated both deaths and population estimates for the respective age groups for each location, sex, and year.

### Adult mortality estimation

**Figure 4:**
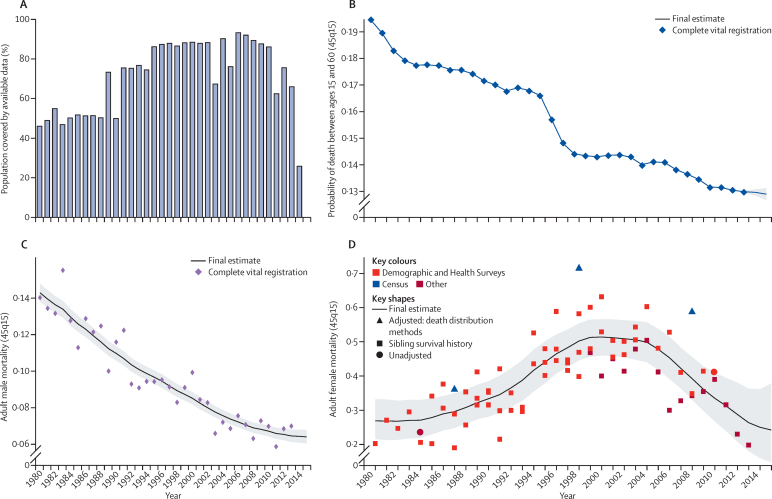
Examples of adult mortality data availability and estimation (A) Percentage of global adult population covered by adult mortality data from vital registration systems, sibling survival surveys, sample registration systems, or censuses, 1980–2015. The percentage of available data was calculated by dividing the population of adults aged 15–59 years in locations covered by available adult mortality data by the total global population aged 15–49 years. Because of lags in reporting both vital registration data and the release of household survey or census data, the availability of data was much lower for 2014 than for previous years, and no data existed for 2015. Country-specific examples of (B) vital registration data and adult male mortality (45q15) estimation for a country with complete vital registration and large population (USA), 1980–2015; (C) vital registration data and adult male mortality (45q15) estimation for a country with complete vital registration and small population (Iceland), 1980–2015; and (D) sibling survival data and adult female mortality (45q15) estimation (Malawi), 1980–2015. Black line shows final estimates of adult mortality among males or females in each country, with 95% uncertainty interval shown in grey. Squares show sibling survival histories. 45q15=probability of death from age 15 years to 60 years.

Measurements of adult mortality (45q15) were mainly derived from vital registration data and household surveys that ask about the birth and death of siblings.^25^ In a smaller set of cases, information was obtained from censuses or surveys about household deaths in a defined interval before the interview. Figure 4A shows the number of geographies for which data in each year were available for adult mortality estimation. Vital registration data were assessed for completeness with death distribution methods optimised for performance.^26^, ^27^ We generated a best estimate of the completeness of vital registration in each geography over time by combining estimated completeness of registration for under-5 deaths with the results for different intercensal periods of the application of three death distribution methods. These sources were combined by use of spatiotemporal Gaussian process regression—details are provided in the methods appendix (p 21). Data from sibling histories were corrected for known biases, including selection bias, zero reporter bias, and recall bias.^7^, ^25^ Our sibling history method can also deal with data sparsity in many sibling survival modules (ie, sibling history questions and variables from surveys). The predictive validity of the sibling history analytical methods has been assessed with simulated data and shown to be unbiased.^25^ Additionally, we compared estimates of adult mortality rates from sibling survival data with completeness-adjusted vital registration data in countries from which both sources are available and found no systematic biases from sibling survival method (methods appendix p 292).^7^, ^25^, ^26^ We synthesised vital registration data corrected for completeness and adjusted sibling history data into a best time series estimate of adult 45q15 using spatiotemporal Gaussian process regression. Examples of the application of these steps in three types of settings are shown in figure 4.

The spatiotemporal Gaussian process regression method used to fit the model to the available data included lag distributed income per capita, educational attainment, and the estimated HIV/AIDS death rate as covariates. Because the estimation of the HIV/AIDS death rate used the estimate of HIV-free mortality rate by age and sex as an input, the entire estimation loop was repeated once, which dealt with this interconnection. Step 2.9 in figure 1 deals with situations in which an inconsistency exists between the spatiotemporal Gaussian process regression-estimated adult mortality rate and the separately estimated crude death rate due to HIV/AIDS. When the HIV/AIDS death rate as estimated from the natural history model is too high compared with demographic sources, there is a risk that HIV-free death rates are depressed to implausibly low levels. In step 2.9, we scaled the HIV/AIDS crude death rate by imposing a maximum proportion of deaths that can be attributed to HIV/AIDS, as shown in our version of UNAIDS' Spectrum model, which estimates HIV/AIDS prevalence and deaths by age and sex. Our adult mortality estimation is for ages 15–60 years (45q15), but other adult age groups that can be calculated for other purposes include 35q15 (ages 15–50 years, corresponding to the reproductive age period), and 20q50 (ages 20–70 years).

### Age-specific mortality

In demographic estimation, measures of child mortality, adult mortality, or both are used alongside a model life table system to predict age-specific mortality.^27^, ^28^, ^29^, ^30^ The UN mostly still uses the Coale-Demeny model life tables, which were based on 192 empirical tables gathered before 1963, and in a few cases they use the 33-year-old UN Model Life Table for Developing Countries.^31^, ^32^ Murray and colleagues^33^ developed the Modified Logit Model Life Table system that is used by WHO to estimate age-specific mortality, which captures a much wider range of age patterns of mortality through the year 2000. The GBD approach uses three inputs to generate age-specific mortality: 5q0, 45q15, and a relevant empirical reference pattern of mortality by age.^7^ The reference in the GBD system was selected on the basis of empirical age patterns that are closest to the population in space and time.^7^ The reference was developed with a database of 16 507 age patterns of mortality from settings that meet explicit inclusion criteria as described in the methods appendix (pp 34–42). Table 3 shows a summary of the availability of empirical age–sex patterns of mortality in the GBD database.

To account for the effect of HIV/AIDS on the age pattern of mortality, the GBD model life table system for locations affected by HIV/AIDS and without high-quality vital registration data used a two-step process whereby we first estimated an HIV-free age pattern of mortality assuming that deaths due to HIV/AIDS were removed. This was accomplished by use of the HIV-free and without-fatal-discontinuity 5q0 and 45q15 estimates, crude death rates due to HIV/AIDS in age groups 0–4 and 15–59 years, and the methods detailed in the methods appendix (p 44), which reconcile the potential disconnect between HIV/AIDS mortality implied in the spatiotemporal Gaussian process regression estimates of all-cause mortality and those estimated by the Estimation and Project Package (EPP)-Spectrum. We then added the excess mortality due to HIV/AIDS to specific age groups to match the with-HIV/AIDS 5q0 and 45q15 by using the estimated age pattern of excess mortality due to HIV/AIDS for generalised and concentrated epidemics. These age patterns of excess mortality were based on ICD-10-coded vital registration data from various countries, including high-income countries with good-quality vital registration data and other middle-income nations that are affected by HIV/AIDS such as South Africa, Thailand, and Trinidad and Tobago. A list of country–years for which we obtained the empirical age pattern of HIV/AIDS excess mortality rate is shown in the methods appendix (p 313).

**Figure 5:**
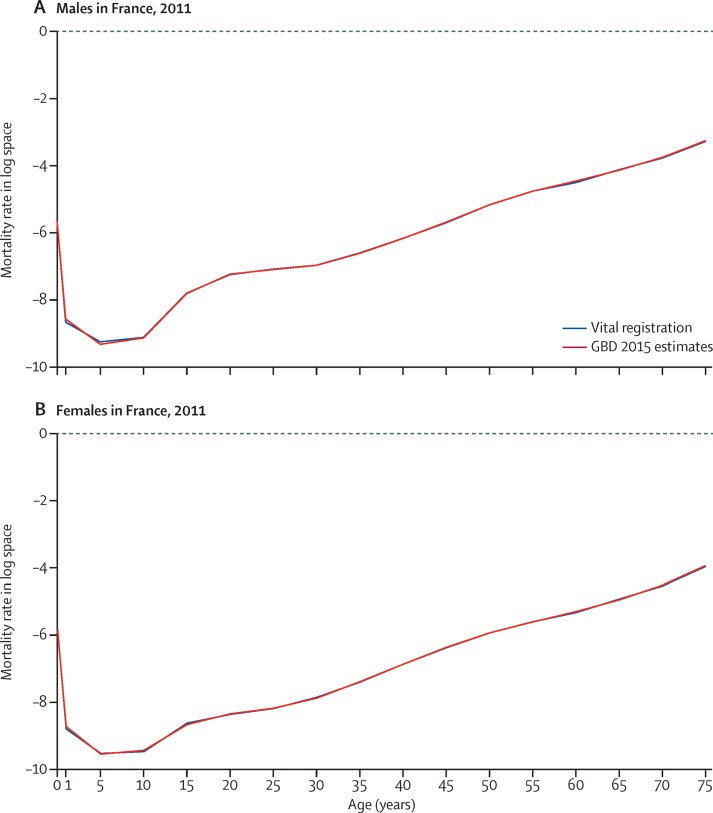
Age-specific mortality estimation with GBD life table method versus observed data excluded from the model Country-specific examples for (A) males and (B) females in France, 2011. The red line shows the GBD 2015 life table system estimates of age-specific mortality rates from birth through age 75 years in log space, compared with observed age-specific mortality (blue line). Year 2011 selected for illustration purposes. GBD=Global Burden of Disease.

Figure 5 shows examples of the life table system estimates of age-specific mortality compared with observed patterns for males and females in France in 2011. There was a very close fit between the estimated age-specific mortality and the observed mortality.

### HIV/AIDS estimation

Because HIV/AIDS estimation is so closely connected to all-cause mortality estimation, we discuss HIV/AIDS estimation separately here rather than in the later section about estimating other causes of death. We divided geographies into two broad groups: countries with larger epidemics and incomplete or non-existent vital registration systems and the remaining geographies. For the first group of geographies for which we had necessary information about the transmission of HIV/AIDS among adults and children and other programme information, we fitted a modified version of EPP-Spectrum^11^, ^34^ to the data on prevalence collated by UNAIDS from antenatal clinic serosurveillance and household surveys. EPP-Spectrum is a natural history model of the HIV/AIDS epidemic that has two distinct components. In the EPP component, data on the prevalence of HIV are used to back-estimate incidence of HIV. In the Spectrum component, the estimated incidence and a set of assumptions are used to estimate prevalence and deaths by age and sex. These assumptions are informed by published or unpublished cohort studies on the initial CD4 distribution of new HIV infections, rates of decline in CD4 counts, death rates on and off antiretroviral therapy (ART) differentiated by age, sex, and CD4 count, and prevention of mother-to-child transmission (PMTCT) coverage data, as well as other demographic assumptions, such as the HIV-free death rate. We have modified EPP-Spectrum to enhance the internal consistency between EPP and Spectrum and to more accurately reflect published cohort data on CD4 progression and death rates on and off ART.

**Figure 6:**
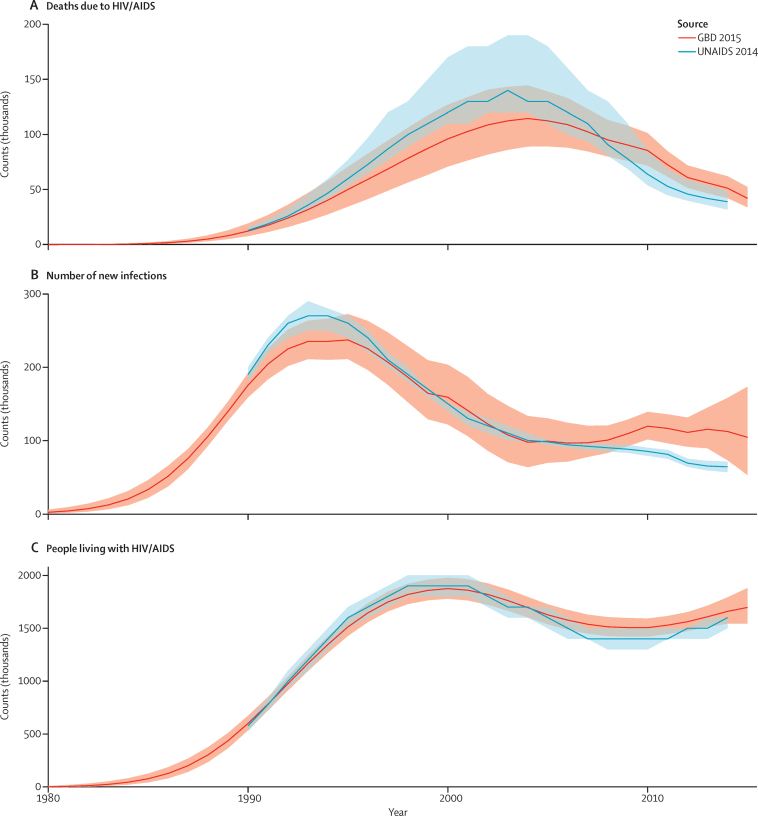
Comparisons of GBD 2015 estimates and UNAIDS 2014 estimates for Zimbabwe Country-specific example comparing estimates of deaths due to HIV/AIDS (A), new HIV infections (B), and people living with HIV/AIDS (C) in Zimbabwe from GBD 2015 and UNAIDS 2014. Curves show the estimation process of a particular country and highlight the differences in results from the GBD and UNAIDS analysis of the same prevalence data. Numbers are reported in thousands. Uncertainty intervals are shown in red and blue shading. GBD=Global Burden of Disease. UNAIDS=The Joint United Nations Programme on HIV and AIDS.

At this point in the estimation process for the first group of geographies, we generated two estimates of HIV/AIDS. One is informed by available data on all-cause mortality and the statistically related association between all-cause mortality and the HIV/AIDS crude death rate; the other is the EPP-Spectrum natural history model. In some locations, these estimates can be quite different. Given the inherent uncertainties in both methods, for GBD 2015 we have adopted an ensemble model which is the average of HIV/AIDS deaths for each age–sex–year from the two approaches. Figure 6 shows the results of this process using Zimbabwe as an example for incidence, prevalence, and deaths from HIV/AIDS. For comparison, we provide prevalence data from surveys and the UNAIDS estimates from their 2014 round of estimation.^35^, ^36^

For the second group of geographies, we estimated mortality due to HIV/AIDS on the basis of vital registration data if available. Estimates of incidence and prevalence for HIV were based on calibrating Spectrum to match the observed numbers of HIV/AIDS deaths after accounting for under-registration of the vital registration system. This calibration method is based on tracking incidence cohorts through Spectrum and adjusting incidence to fit the observed deaths for that cohort in each year in a specific age group (methods appendix p 44). Using the methods discussed in the age-specific mortality section, with-HIV/AIDS all-cause mortality was estimated for these countries. Depending on the subgroup categorisation within the second group of geographies (methods appendix pp 44–45), we generated the HIV/AIDS-specific mortality either by applying spatiotemporal Gaussian process regression to HIV/AIDS cause-specific data from the vital registration system if the quality of vital registration was deemed high (methods appendix pp 21–26) or by using cohort incidence bias-adjusted mortality estimates from Spectrum.

### Fatal discontinuities

The fifth stage of estimation (figure 1) re-estimates all-cause mortality by incorporating the effects of HIV/AIDS and fatal discontinuities. To incorporate fatal discontinuities from natural disasters (eg, the 2011 Japan earthquake and tsunami), wars, pandemics, wildfires (eg, the Australian bushfires in 2009), or major transportation accidents (eg, the Al Ayyat train accident in Egypt in 2002), we used death counts reported in a wide range of international databases such as the International Disaster Database, the Uppsala Conflict Data Program, the International Institute for Strategic Studies Armed Conflict Databases, the Robert S Strauss Center, and various internet sources for more recent events such as the ongoing Syrian and Yemeni conflicts (databases are listed in the methods appendix [p 270], and additional sources are downloadable from the online source tool).^37^, ^38^, ^39^, ^40^ When multiple sources for the same fatal discontinuity event exist, we prioritised data from vital registration systems if it had the highest estimate, and gave least priority to data from internet searches. We constructed uncertainty on the basis of high and low estimates when available. We generated regional and cause-specific uncertainty intervals in relative terms and applied them to fatal discontinuities when only the mean estimate was provided by a specific source. The fatal discontinuity section of the methods appendix (pp 270–73) provides more detail on how we assigned fatal discontinuity deaths to different GBD causes as appropriate and how we applied a cause-specific age–sex splitting model to arrive at age-and-sex-specific deaths due to specific fatal discontinuity events.

Given that all-cause mortality analysis requires estimates of crude death rates from HIV/AIDS as initial inputs and that the ensemble model changes the HIV/AIDS-specific and with-HIV/AIDS age-specific mortality rates, the all-cause mortality and HIV/AIDS estimation processes were performed twice. Crude death rates due to HIV/AIDS and under-5 population estimates were generated in the first run of the processes and propagated the second run of the processes to make the HIV/AIDS-specific and all-cause mortality processes more consistent.

### Causes of death estimation

The GBD cause list relies on categorical attribution of deaths to a single underlying cause in accordance with the principles outlined in the ICD. The core principle of the ICD is to assign each death to only the underlying cause of death; ie, the cause that initiated the series of events leading to death. We used the ICD principle of underlying cause of death for the primary tabulations in this Article. Data from vital registration sources, verbal autopsy studies, and other sources all adhere to the same principle that one death can only have one cause. The categorical attribution of causes of death differs from a counterfactual approach, which answers the question “in the absence of the disease of interest how many deaths would not have occurred?”, similar to how we estimate burden due to risk factors in GBD. The categorical attribution of causes of death also differs from excess mortality in people with a disease followed up over time in a cohort study or through linkage of a disease registry to vital registration data. The excess mortality in such studies might contain deaths that are assigned as the underlying cause, those that are causally related to the disease, and those that are due to confounding, such as by a common underlying risk that predisposes to the disease but where there are also additional pathways to death. These counterfactual and excess mortality relationships are important and need to be quantified by considering the underlying risk, such as elevated fasting plasma glucose.

Figure 2 shows the steps in the estimation of causes of death, which are divided into seven categories: cause of death database development (figure 2A), Cause of Death Ensemble modelling (CODEm), negative binomial models for rare causes, natural history models, subcause proportion models, prevalence-based models, and CodCorrect (figure 2B). For each component, we discuss the steps, with more extensive detail provided in the methods appendix (pp 52–283). Details about the modelling of HIV/AIDS and fatal discontinuities are also described in detail in the methods appendix (pp 255–64, 270–73).

### Cause of death database development

**Figure 7:**
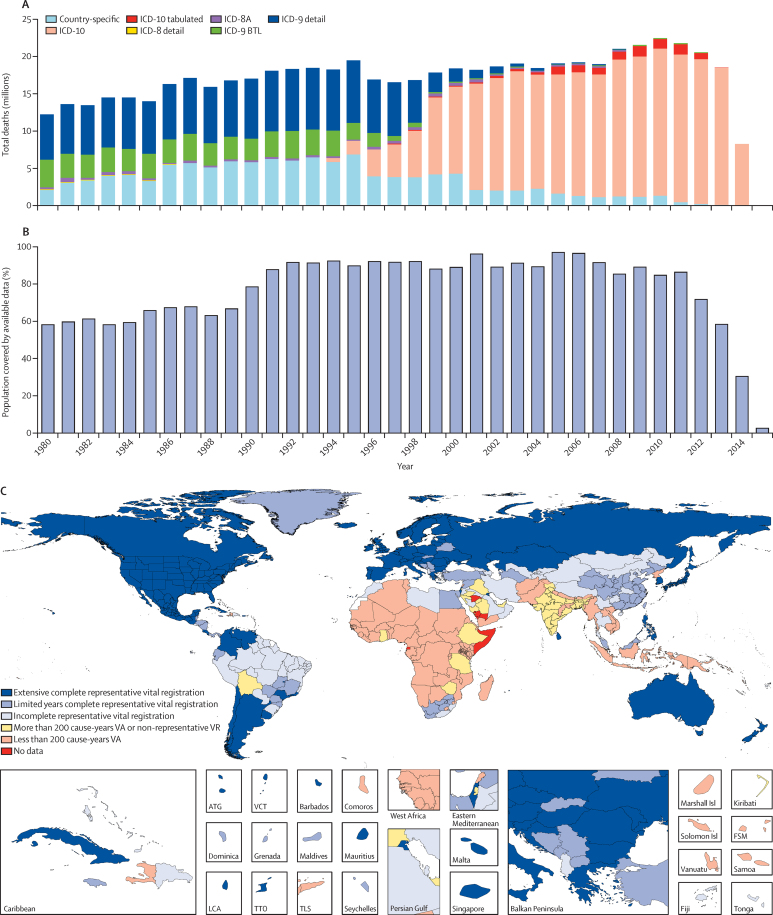
Availability and quality of cause of death data in the GBD 2015 database (A) Total deaths with a WHO-standard death certificate available in the GBD 2015 cause of death database classified by the variant of the International Classification of Diseases used for reporting. Cause of death data have been reported in national variants of ICD-8, ICD-9, and ICD-10 during the interval 1980–2015. Because of lags in reporting of both vital registration data and the release of household survey or census data, the availability of data was much lower for 2014 than for previous years and no data existed for 2015. (B) Percentage of global population covered by cause-specific data in the cause of death database for GBD 2015, 1980–2015; the percentage of available data was calculated by dividing the population of locations covered by available cause-specific data by the total global population. This figure is computed using vital registration, verbal autopsy, maternal, cancer, and injury sources. (C) Overall classification of each GBD subnational level 1 geography by availability and quality of cause of death data for the period 1980 to 2015. Countries have been assigned on the basis of the available time series of data into one of six categories. The figure uses GBD subnational level 1 geographies because subnational level 2 cannot be easily seen on a map. Extensive complete representative vital registration was defined as 25 total years or more of vital registration data with an estimated 95% completeness or above. All geographies that do not meet the threshold for extensive complete representative vital registration are classified as one of the following: limited years of complete representative vital registration, defined as 5 years or more of vital registration data with an estimated 95% completeness or above; incomplete representative vital registration, defined as at least 1 year of vital registration data with an estimated 70% completeness or above; more than 200 cause-years VA or non-representative VR, defined as more than 200 cause-years of verbal autopsy or at least 1 year of vital registration with an estimated 50% completeness or above; less than 200 cause-years of VA; or no data. Cause-years are defined as the number of years for each cause for which data are available. GBD=Global Burden of Disease. ICD=International Classification of Diseases. BTL=basic tabulation list. VA=verbal autopsy. VR=vital registration. ATG=Antigua and Barbuda. VCT=Saint Vincent and the Grenadines. LCA=Saint Lucia. TTO=Trinidad and Tobago. TLS=Timor-Leste. FSM=Federated States of Micronesia.

Figure 2A shows the detailed steps from data inputs and processing to the finalisation of the cause of death database. The methods appendix (pp 52–70) includes details on each step. Cause of death data collected through vital registration systems are available from governments and coded to different variants of the ICD including various national ICD variants. Multiple sources were used in addition to vital registration data, including verbal autopsy data, cancer registries, maternal mortality surveillance, census and survey data on maternal death, census and survey data on selected injuries, and police records for some injuries. Figure 2A shows how each type of data were processed to deal with the challenges of different coding schemes, different age group reporting, variation in certification, misclassification of HIV/AIDS deaths, misclassification of maternal HIV/AIDS deaths, and incorporation of population-based cancer registry data. The first and second steps in the cause of death database development were standardisation of multiple data formats to a single GBD standard, then the mapping of each ICD or verbal autopsy variant to the GBD cause map. Figure 7A shows the number of deaths captured for each year in the GBD causes of death database by coding version. In step 3, we split a small subset of data reported in non-GBD-standard age formats into GBD age categories using the global relative age pattern of mortality for each cause as estimated from the pooled data that provide full age detail. In step 4, based on expert judgment, some causes were not allowed for certain age–sex groups, for example, male uterine cancer.

In step 5, deaths assigned to causes that cannot be underlying causes of death (ie, garbage coded) were reassigned to their likely underlying cause of death.^4^, ^7^ These redistribution algorithms are based on three approaches. For some garbage codes, such as senility or old age, deaths were proportionately reassigned to all causes that are not garbage codes for a country–age–sex–year. For HIV/AIDS in many countries, deaths from HIV/AIDS have been misclassified as opportunistic infections, tuberculosis, cancer, digestive diseases, and immune deficiencies. In step 6, using methods developed by Birnbaum and colleagues,^41^ these deaths were identified and reclassified as HIV/AIDS in select countries with evidence of misclassification. In step 7, data from the China Center for Disease Control and Prevention (CDC) vital registration system were re-weighted to take into account potential selection bias caused by a larger fraction of deaths being captured in hospital than out of hospital in some locations.^14^ Step 8 ensured that the process of redistributing garbage codes or identifying misclassified HIV/AIDS deaths would not assign deaths to causes in an age–sex–country–year that violated age–sex or other restrictions.

Step 9 excluded vital registration sources that were less than 50% complete in a given geography from the database, because of the potential for selection bias in highly incomplete sources. Sources estimated to be 50–70% complete were identified as non-representative, which was information that we used in the building of the cause of death statistical model to increase the estimated data variance for these datapoints. All included sources were corrected to be 100% complete by multiplying the cause fraction in a source for a country–age–sex–year by the estimate of all-cause mortality for that country–age–sex–year. Step 10 aggregated causes of death from most to least detailed levels of the GBD hierarchy, ensuring deaths for a given cause were representative of all branches of the hierarchy that fall beneath it. In step 11, deaths due to HIV/AIDS and various types of fatal discontinuities were removed before cause fractions were computed. Because of the very large effects of fatal discontinuities, such as wars and natural disasters in some cases, and the impact of HIV/AIDS in countries with large epidemics, we converted cause fractions to be cause fractions excluding HIV/AIDS and fatal discontinuities in the denominator. Deaths from HIV/AIDS and the fatal discontinuities were added back during the final stages of the modelling process. Because many sources on maternal mortality identify deaths during pregnancy and the post-partum period and not maternal deaths, the separation of HIV/AIDS deaths during pregnancy and HIV/AIDS deaths aggravated by pregnancy was more complicated (methods appendix p 66).

Figure 7B provides information about the fraction of the 519 geographies in the analysis for which cause of death data were available in each year from 1980 to 2015 for any cause, including maternal death and injuries. Data availability by geography–year by cause is shown in the methods appendix (pp 318–401). To facilitate understanding of the range of quality and availability of data for each geography, we classified geographies into six categories: extensive complete representative vital registration (vital registration data that are 95% complete and cover more than 25 years); moderate data (vital registration data that are 95% complete but cover fewer than 25 years); incomplete representative vital registration (all other geographies with some representative vital registration data); extensive verbal autopsy and other sources (covering more than 20% of cause-years); limited verbal autopsy or other data (all others with some data available); and no data for any cause (methods appendix pp 691–710). Figure 7C shows this designation for each class of country.

### CODEm

**Figure 8:**
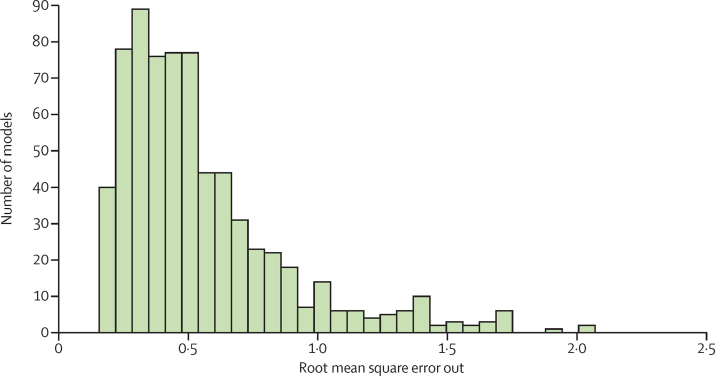
Distribution of out-of-sample model performance for CODEm models used for GBD 2015 Model performance was assessed by use of the root mean square error of the ensemble model predictions of the log of the age-specific death rates for a cause assessed with 15% of the data held out from the statistical model building. The figure shows the distribution of root mean square error across the set all models for all causes. Model performance varies substantially across causes. GBD=Global Burden of Disease. CODEm=cause of death ensemble modelling.

Figure 2B shows the analytical flow chart for modelling different causes of death and combining them into internally consistent estimates of cause-specific mortality that sum to all-cause mortality with uncertainty levels. 167 individual causes of death were modelled using CODEm. Developed for GBD 2010,^5^ CODEm tests a large number of model specifications, comparing different functional forms and permutations of relevant covariates for each cause of death. Models that met requirements for direction and significance of the regression coefficients were then evaluated for out-of-sample predictive validity through multiple iterations of cross-validation testing. We then combined these models into an ensemble, weighting them such that top performing models (in terms of out-of-sample prediction error on levels and trends) contributed the most to the final prediction. Out-of-sample predictive validity testing was also used to select the psi parameter that determines the number of models and their weight in the final ensemble (figure 8).

For each cause of death, we ran independent CODEm models by sex and for countries with extensive complete vital registration representation and all other countries. We included all datapoints for the other categories of geographies, whereas for countries with extensive complete vital registration representation, we included only datapoints from those countries, so that heterogeneous data from other countries did not inflate the uncertainty interval.

### Negative binomial models

For ten causes of death, the number of events are so low, including many zero counts in countries with high income per capita or high educational attainment, that CODEm out-of-sample predictive validity testing was unstable. For these rare causes of death, which included other intestinal infectious diseases, upper respiratory infections, diphtheria, varicella and herpes zoster, malaria, schistosomiasis, cysticercosis, cystic echinococcosis, ascariasis, and iodine deficiency, we used negative binomial regression to develop simple models to predict deaths. More details are available in the methods appendix (pp 185–200; negative binomial models).

### Natural history models

For some causes, deaths are rarely recorded in either vital registration data or verbal autopsy data. Partly, this is because of the geographical location of the deaths or because of the potential for systematic bias in vital registration data or verbal autopsy data. For 14 causes, we have developed natural history models in which incidence and case-fatality rates are modelled separately and combined to yield estimates of cause-specific mortality. We developed natural history models for typhoid fever, paratyphoid fever, whooping cough, measles, visceral leishmaniasis, African trypanosomiasis, yellow fever, syphilis (congenital), and acute hepatitis A, B, C, and E. Additionally, for malaria in sub-Saharan Africa, we have used a natural history model based on the incidence estimated by the Malaria Atlas Project and age–sex-specific case-fatality rates estimated from available data. Further details on the development of these natural history models are available in the methods appendix (pp 201–26; natural history models).

### Subcause proportion models

For meningitis, maternal disorders, liver cancer, cirrhosis, and chronic kidney disease, we estimated detailed causes for each of these cause groupings by modelling the proportion of the cause grouping (parent cause) due to each of the component causes. We used this approach because the available data on the specific causes can come from sources other than vital registration, such as end-stage renal disease registries, or from too few places to model the death rates directly. For these causes, the parent cause was first estimated with CODEm and the fraction of the parent due to each component cause for each age–sex–geography–year was generally estimated with DisMod-MR 2.1, a Bayesian meta-regression method developed for the GBD studies.^42^, ^43^ Details for each cluster of causes analysed in this way are shown in the methods appendix (pp 233–52; subcause proportion models).

### Prevalence-based models

For Alzheimer's disease and other dementias and atrial fibrillation and flutter, there is evidence of marked changes over time in the propensity of individuals who completed death certificates to list these causes as underlying causes of death.^44^, ^45^ These changes created increases in the reported death rates. Conversely, prevalence surveys do not show a matching increase in age-specific disease prevalence. Garbage code redistribution algorithms used in the development of the cause of death database have so far not accurately captured this shift over time in the certification of underlying causes of death. For these two causes, we based our estimates on prevalence surveys and estimates of excess mortality based on deaths certified in countries with the greatest proportion of deaths allocated to the correct underlying cause of death in recent years. In both cases, more detail is available in the methods appendix (pp 227–32; prevalence-based models). We developed models for prevalence and excess mortality using DisMod-MR 2.1.

### CodCorrect

Depending on the specific data availability and details of individual causes, we adopted different modelling strategies for each cause. We generated a set of underlying cause of death estimates, with uncertainty intervals, that equalled all-cause mortality, with uncertainty intervals, for each age–sex–year–geography and cause and all-cause mortality at the individual draw level.^24^ In CodCorrect, for each draw from the posterior distribution of each cause, the sum of cause-specific estimates is rescaled to equal the draw from the all-cause distribution (methods appendix p 285).

### Pathogen counterfactual analysis

We used a counterfactual analysis approach to estimate aetiology-specific population attributable fraction for mortality due to lower respiratory infections and diarrhoeal diseases. This approach involved analysing changes in mortality on the basis of the estimated prevalence of each pathogen and relative risk of developing disease given pathogen exposure.

The prevalence of each pathogen in diarrhoeal cases was extracted from a systematic literature review and modelled with DisMod-MR 2.1. The odds ratios of an episode of diarrhoea given exposure to the pathogen were estimated from a reanalysis of the Global Enteric Multicentre Study (GEMS) that used the TaqMan Array Card (TAC), which is based on a quantitative polymerase chain reaction diagnostic (qPCR).^46^, ^47^ We attributed mortality to all pathogens, even if the odds ratio was not significant in all age groups. We corrected the estimated prevalence for each pathogen on the basis of conventional laboratory techniques, such as bacterial culture or enzyme-linked immunosorbent assay (ELISA), to be consistent with the new qPCR method. Cholera mortality was estimated by modelling the under-reporting to the WHO cholera case notification system and applying this correction factor to estimate the number of cholera cases and deaths (methods appendix p 281). The incidence and mortality of *Clostridium difficile* was modelled with natural history and incidence data in DisMod-MR 2.1.

We estimated attributable mortality due to respiratory syncytial virus and influenza with a similar approach to that for diarrhoea. We used a counterfactual approach whereby the prevalence in patients with lower respiratory infection was extracted from a systematic literature review and modelled with DisMod-MR 2.1. The odds ratios of lower respiratory infections given pathogen presence were obtained from a meta-analysis by Shi and colleagues.^48^ We adjusted the population attributable fraction for lower respiratory infection mortality due to respiratory syncytial virus and influenza for the relative case-fatality rate of viral to bacterial pneumonia episodes by age. *Haemophilus influenzae* type b and pneumococcal pneumonia (*Streptococcus pneumonia*) were estimated with a vaccine probe approach whereby the attributable fraction was calculated as the ratio of vaccine efficacy against non-specific pneumonia to vaccine efficacy against pathogen-specific and serotype-specific pneumonia. Studies that report vaccine efficacy against vaccine-type invasive pneumococcal disease were adjusted for the relative efficacy against vaccine-type clinical pneumococcal pneumonia using a uniform distribution of uncertainty around this ratio.^49^, ^50^

### Socio-demographic Index and epidemiological transition analysis

In this Article, we built on GBD 2013^51^ concepts by improving the interpretability of sociodemographic status and characterising and describing this relationship in more detail for years of life lost due to premature mortality (YLLs), as well as highlighting changes in age-standardised death rates, population age structure, and YLL rates. We have made two important changes to the GBD 2013 computation. First, we have used only lag-dependent income per capita, average educational attainment in the population over age 15 years, and the total fertility rate. We excluded the mean age of the population because it is directly affected by death rates. Second, we have applied the methods used to compute the Human Development Index to generate an interpretable scale, resulting in the Socio-demographic Index (SDI).^52^ The Human Development Index method weights each component equally and rescales each component on a zero-to-one scale with zero being the lowest value observed in the time period 1980 to 2015 and 1 being the highest value observed. The final composite SDI value is the geometric mean of each of the components. The SDI ranges from 0·060 in Mozambique in 1987 to 0·978 in Washington, DC, USA, in 2015. The correlation of the SDI with the sociodemographic status principal component analysis used in GBD 2013 was 0·982. The very high correlation is because the principal component analysis yields weights that are nearly equal across components. The advantage of the index is that 1 can be interpreted as the level of SDI at which a geography has the highest observed log income per capita and educational attainment and lowest fertility rate. We tested whether alternative lags of the components of SDI would provide a better predictor of outcomes such as life expectancy and age-specific probabilities of death. Using lag distributed income per capita, educational attainment, and the total fertility rate in the current year was the most predictive of these mortality outcomes (methods appendix p 286).

**Figure 9:**
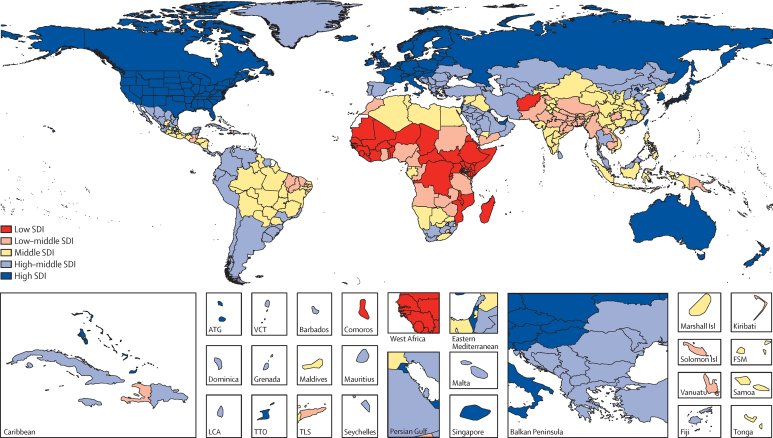
SDI quintiles by GBD subnational level 1 geography, 2015 SDI is calculated for each geography as a function of lag-dependent income per capita, average educational attainment in the population older than age 15 years, and the total fertility rate. SDI units are interpretable; a zero represents the lowest level of income per capita and educational attainment and highest total fertility rate observed during 1980–2015, whereas a one represents the highest income per capita and educational attainment and lowest total fertility rate observed in the same period. Cutoffs on the SDI scale for the quintiles have been selected on the basis of examination of the entire distribution of geographies 1980–2015. GBD=Global Burden of Disease. SDI=Socio-demographic Index. ATG=Antigua and Barbuda. VCT=Saint Vincent and the Grenadines. LCA=Saint Lucia. TTO=Trinidad and Tobago. TLS=Timor-Leste. FSM=Federated States of Micronesia.

To report on aggregate results, we divided geographies into SDI quintiles in 2015. Quintile cutoffs were based on the entire distribution of geography–years from 1980 to 2015, excluding populations smaller than 1 million. Figure 9 shows a map of the SDI level in 2015 categorised into five groups including subnational geographies. Because SDI includes educational attainment and the total fertility rate, some countries which have very high income, such as Saudi Arabia, are classified in the second quintile of SDI because of lower educational attainment and higher fertility rates.^53^

To capture the average relationships for each age–sex–cause group, we used spline regression of death rates on SDI (methods appendix pp 285–86). To ensure a coherent set of estimated death rates for Levels 1, 2, and 3 in the GBD cause hierarchy for each level of SDI, the Level 2 death rates were rescaled such that for each age–sex–cause bin, the sum of Level 2 death rates equalled the Level 1 death rate. This procedure was repeated for Level 3 and Level 2 causes. These rates were used as the expected death rates by age–sex–cause and SDI. Various summary measures have been computed on the basis of the age–sex–cause-specific predictions based on SDI, including age-standardised death rates, age-standardised YLL rates, and life expectancy at birth.

To further characterise how patterns of crude death rates and death numbers change with SDI, we have computed the average population age structure associated with each SDI level. These population age structures have then been used to estimate how crude death rates and death numbers by cause are expected to change with rising SDI.

### Decomposition of changes in global deaths

To analyse the drivers of change in the numbers of deaths by cause or geography, we decomposed change from 2005 to 2015 into three explanatory components: change due to growth of the total population; change in the population structure by age or sex; and change in age-specific, sex-specific, and cause-specific rates. We refer to all changes in age-specific, sex-specific, and cause-specific death rates not explained by demographic change (population growth and ageing) as the epidemiological change. The observed change in the total number of deaths equals the net change of these three components.

Decomposition analyses for 1980 to 2015 and 2000 to 2015 are shown in the results appendix (pp 6–7). The decomposition analysis uses methods developed in demographic research by Das Gupta.^54^ As an example, we describe our approach to decomposition for the 2005 to 2015 period. We used counterfactual scenarios to calculate two different sets of numbers for death. In the first scenario, for population growth, the number of deaths in 2015 was the number expected if the total population increased from 2005 as observed, but the age–sex-specific population structure and rates of death were the same in 2015 as in 2005. In the second scenario, for population growth and ageing, the number of deaths in 2015 was the number expected according to the 2015 age–sex-specific population structure, but with the age–sex-specific rates of death held constant to 2005. The difference between the number of deaths observed in 2005 and those estimated for 2015 with the population growth scenario is the change in the number of deaths exclusively from population growth. The difference between the scenario for population growth alone and the scenario for population growth and ageing is the change in the number of deaths exclusively attributable to population ageing.

### Attribution of changes in life expectancy to changes in causes of death

When considering the estimated levels and changes in all-cause and cause-specific mortality rates for each geographical area covered by GBD 2015, it is important to understand the relative contribution of changes in mortality due to each cause to the overall changes in life expectancy at birth during the same period. To examine the changes in life expectancy at birth between 2005 and 2015, we have applied the state-of-the-art life expectancy cause-specific decomposition method developed by Beltran-Sanchez, Preston, and Canudas-Romo.^55^

### YLL computation

We computed YLLs using the standard GBD methods whereby each death is multiplied by the normative standard life expectancy at each age. The normative standard life expectancy at birth is 86·59 years, which is based on the lowest observed death rates for each 5-year age group in populations larger than 5 million. For GBD 2015, we computed age-standardised mortality rates and YLL rates from the updated world population age standard developed for GBD 2013.^7^ Details of the GBD world population age standard are available in the methods appendix (pp 286–287 and 314).

### Uncertainty analysis

To account for uncertainties that arise from sample sizes of data, adjustments to sources of all-cause mortality, model specifications in spatiotemporal Gaussian process regression and model life table systems, and cause-specific model specifications and estimation, we have estimated uncertainty intervals in key steps of the all-cause mortality and cause-specific mortality estimation processes. We have produced 1000 draws of all mortality metrics, including under-5 mortality rate, adult mortality rate, age-specific mortality rate and envelope, and cause-specific mortality rates and death numbers for each location by sex for all years covered by each analytical step from the posterior distribution in the estimation process. This allowed the quantification and propagation of uncertainty into the final quantities of interest. Because of computational time limitations, we have not propagated uncertainty in covariates used in cause of death models, nor have we been able to propagate uncertainty in garbage code redistribution algorithms into the final results. Our tests on the estimation of under-5 mortality rates show that the incorporation of uncertainty for included first stage model covariates such as crude death rate due to HIV/AIDS does not have a significant impact on the final estimates (data not shown).

### Role of the funding source

The funder of the study had no role in study design, data collection, data analysis, data interpretation, or writing of the report. All authors had full access to the data in the study and had final responsibility for the decision to submit for publication.

## Results

### Global life expectancy and mortality

Global life expectancy at birth increased by 10·2 years, rising from 61·7 years (95% uncertainty interval [UI] 61·4–61·9) in 1980 to 71·8 years (71·5–72·2) in 2015 (table 4), equating to an average gain of 0·29 years per year. By 2015, male life expectancy had risen by 9·4 years, increasing from 59·6 years (59·3–60·0) in 1980 to 69·0 years (68·6–69·4), whereas female life expectancy improved by 11·1 years, climbing from 63·7 years (63·3–64·1) to 74·8 years (74·4–75·2). On average, an additional 0·27 and 0·32 years of life were gained per year for males and females, respectively, since 1980. Global gains in life expectancy were generally gradual but steady, although catastrophic events, including the Rwandan genocide and North Korean famines, and escalating mortality due to HIV/AIDS, had worldwide effects on longevity. Slower gains were achieved for life expectancy at 50 years, or the average number of additional years of life 50 year olds can anticipate at a given point in time. On average, 50-year-old females saw an increase of 4·5 additional years of life since 1980, and 50-year-old males experienced an increase of 3·5 years. Annual estimates of life expectancy, by sex and geography are shown in the results appendix (pp 36–47).

Global mortality trends showed a 16·4% (95% UI 14·3–18·5) increase in total deaths between 1990 and 2015, whereas age-standardised rates of mortality fell by 28·5% (27·3–29·8) during this time. The trend was similar from 2005 to 2015, with total deaths increasing by 4·1% (2·6–5·6) and age-standardised death rates decreasing by 17·0% (15·8–18·1). In 2015, 55·8 million deaths (55·0 million to 56·6 million) occurred worldwide, an increase of 7·9 million deaths since 1990 and 2·2 million deaths since 2005 (table 4). From 1990 to 2015, total deaths rose by 21·9% (19·1–24·8) for males and 10·3% (7·6–13·1) for females, whereas age-standardised death rates fell by 26·2% (24·5–27·9) for males and 32·1% (30·4–33·8) for females. In 2015, 30·9 million (30·3 million to 31·5 million) males and 24·9 million (24·5 million to 25·5 million) females died, representing an increase of 5·6 million male deaths and 2·3 million female deaths since 1990. Differences in total deaths by sex widened over time, with increasingly more males dying than females; this gap grew from 2·7 million in 1990 to 4·1 million in 2005 and 6·0 million in 2015.

Age-standardised rates of YLLs per 100 population, a measure of premature mortality, fell 34·1% (95% UI 32·5–35·6) for males and 42·1% (40·6–43·5) for females between 1990 and 2015. Notably, the pace of decline in YLL rates was faster from 2005 to 2015 (19·9%, 95% UI 18·3–21·5 for males and 26·3%, 24·6–27·9 for females) than from 1990 to 2005 (17·7%, 16·2–19·2 for males and 21·4%, 20·0–22·7 for females).

### Evolution of global and super-region life expectancy, probabilities of death, and SDI

**Figure 10:**
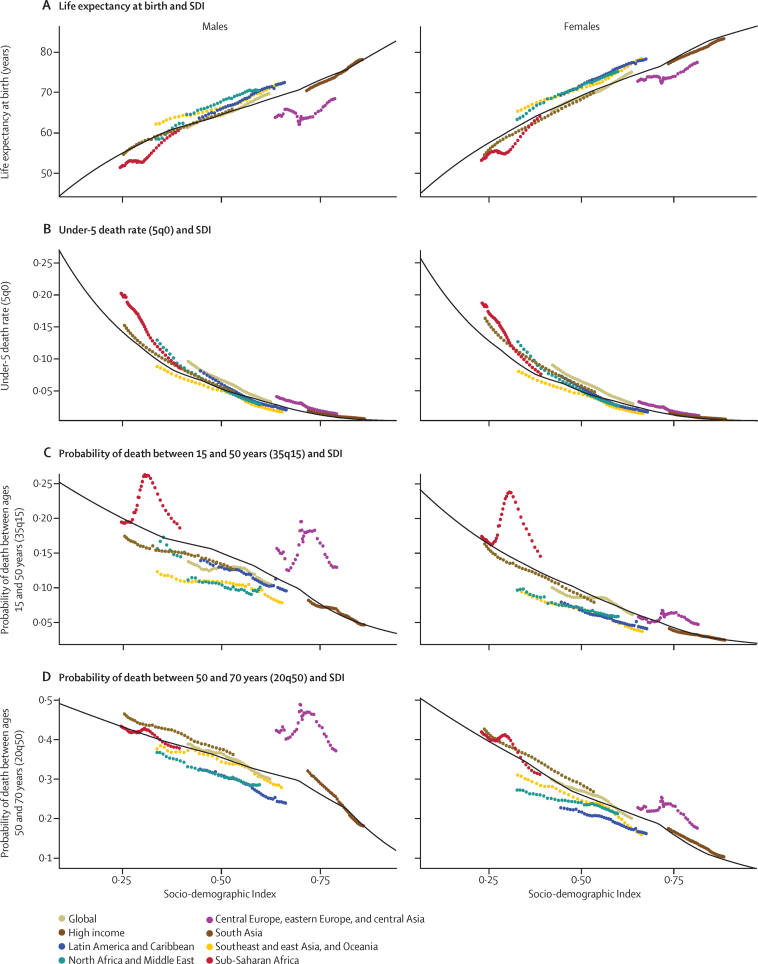
Co-evolution of life expectancy and probabilities of death with SDI globally and for GBD super-regions, 1980 to 2015 (A) Life expectancy at birth and SDI; (B) under-5 death rate (5q0) and SDI; (C) probability of death between 15 and 50 years of age (35q15) and SDI; and (D) probability of death between 50 and 70 years of age (20q50) and SDI. Coloured lines show global and super-region values. Each point in a line represents 1 year, starting at 1980 and ending at 2015. In all super-regions, SDI has increased year on year so progress in SDI is associated with later years for a given super-region. Black lines show trajectories expected for each geography on the basis of SDI alone. GBD=Global Burden of Disease. SDI=Socio-demographic Index. 5q0=probability of death from birth to age 5 years. 35q15=probability of death from age 15 years to 50 years. 20q50=probability of death from age 50 years to 70 years.

The differences between observed life expectancy and mortality rates and those expected on the basis of SDI show the complex interactions between gains in SDI and improved health over time. Figure 10 summarises the trends in observed and expected life expectancy or mortality at the global level and for each GBD super-region from 1980 to 2015. Some regions have higher than expected levels, whereas others have lower levels than expected.

By 2015, global life expectancy had increased faster than expected based on changes in SDI for both sexes, equating to an increase of an additional 3·06 years for males and 2·78 years for females (figure 10A); however, before 2005, gains in life expectancy were lower than expected, particularly for females. Observed life expectancy consistently exceeded expected levels over time in southeast and east Asia and Oceania; Latin America and the Caribbean; and north Africa and the Middle East. Furthermore, for the latter two super-regions, gains for male life expectancy improved following the 1980s and 1990s, when observed levels of longevity were closer to expected life expectancy based on SDI. By contrast, observed life expectancy was generally lower than expected based on SDI in high-income countries and central Europe, eastern Europe, and central Asia. For high-income countries, however, observed male life expectancy converged with expected levels around 2005, whereas the gap between observed and expected life expectancy based on SDI widened for females in this super-region. In south Asia, where average SDI more than doubled between 1980 and 2015, observed male life expectancy consistently met or slightly exceeded expected levels, whereas female life expectancy gradually moved closer to expected levels based on SDI. Amid its escalating HIV/AIDS epidemic, sub-Saharan Africa recorded widening gaps between observed and expected life expectancies for both sexes between 1988 and 1999. From 2001 to 2015, during which the region's average SDI rose by 31%, observed life expectancy quickly increased, particularly among females, nearing expected levels.

Overall, global and regional trends for observed under-5 mortality steadily moved closer to expected levels, based on rising SDI, and in some super-regions, such as Latin America, the Caribbean, and north Africa and the Middle East, observed rates of under-5 mortality became lower than expected (figure 10B). Substantial progress occurred in sub-Saharan Africa, with the gap between observed and expected 5q0 decreasing from 0·055 in 1980 to 0·006 in 2015. Observed under-5 mortality was consistently lower than expected, given rising SDI, in southeast Asia, east Asia, and Oceania, whereas the opposite was seen for central and eastern Europe and central Asia, with observed under-5 mortality exceeding expected levels from 1980 to 2015. With the exception of south Asia, super-region under-5 mortality trends did not substantially differ by sex. Observed levels of male under-5 mortality in south Asia gradually neared expected rates of under-5 mortality over time, whereas female under-5 mortality remained above expected levels between 1980 and 2000; by 2015, however, this gap had narrowed considerably.

Regional trends for observed and expected 35q15, which represents the probability of dying between the ages of 15 and 50 years, were much more variable than life expectancy at birth or 5q0 (figure 10C). The 35q15 age band corresponds to the reproductive period; for the analysis of changes in mortality with SDI, we include results for these age groups because of their very strong association with mortality from HIV/AIDS during the reproductive age period. Except for three super-regions (high income; sub-Saharan Africa; and central Europe, eastern Europe, and central Asia), observed levels of 35q15 remained lower than would be expected based on SDI between 1980 and 2015. However, relative trends, in terms of proximity to expected levels of 35q15 over time and by sex, shifted considerably. Although observed rates of female 35q15 were lower than expected from 1980 to 2015 in three super-regions (Latin America and the Caribbean; north Africa and the Middle East; and southeast and east Asia and Oceania), each super-region registered improvements in 35q15 over time and moved closer to expected levels by 2015. In sub-Saharan Africa, observed 35q15 for both sexes increased to well above expected levels between 1988 and 2000, a trend largely attributable to HIV/AIDS. By 2004, however, gains in SDI quickened in sub-Saharan Africa, and observed 35q15 began to fall closer to expected levels at a similar pace. Central and eastern Europe and central Asia experienced the most divergent patterns for 35q15 by sex. For males in this super-region, observed 35q15 remained far above expected levels of mortality based on SDI from 1980 to 2015, but observed 35q15 climbed between 1986 and 1994. This rapid rise in observed male mortality between the ages of 15 and 50 years, relative to SDI, occurred in tandem with the collapse of the Soviet Union and the widespread economic hardships that followed. The gap between observed and expected male 35q15 began to gradually narrow during the late 1990s, corresponding with rises in SDI; nonetheless, gains stalled by 1999. For females in central and eastern Europe and central Asia, observed 35q15 closely followed expected levels from 1980 to 1990, after which observed mortality jumped and remained higher than expected 35q15, based on SDI, through to 2015.

Results were similarly heterogeneous for observed and expected trends for 20q50, or the probability of dying between the ages 50 and 70 years, particularly by sex and rising SDI (figure 10D). First, based on gains in SDI alone, expected levels of 20q50 differed substantially by sex. For males, expected reductions for 20q50 were quite gradual relative to improvements in SDI, until the 80th percentile, after which expected 20q50 steeply fell. For females, expected 20q50 followed a fairly linear trend with rising SDI. Two regions—Latin America and the Caribbean and north Africa and the Middle East—experienced observed levels of 20q50 that were lower than expected from 1980 to 2015 for both sexes; however, for females in north Africa and the Middle East, observed 20q50 shifted closer to expected levels of mortality after 1999. After largely following expected rates of 20q50 from 1989 to 1998, southeast and east Asia and Oceania saw observed male 20q50 drop below expected levels. Observed female 20q50 generally remained lower than expected for this super-region, although observed levels approached expected rates from 2000 to 2003 before declining again. Although south Asia recorded large gains in SDI over time, from an average of the 25th percentile in 1980 to the 54th in 2015, observed rates of 20q50 remained higher than expected for both sexes over time. Aside from a jump in observed 20q50 rates between 1995 and 2007, in sub-Saharan Africa, observed 20q50 for both sexes mainly followed the expected rates given rising SDI. Similar to the results for 35q15, observed levels of male and female 20q50 in central and eastern Europe and central Asia followed a dissonant pattern over time. For males, although observed 20q50 surpassed expected rates between 1980 and 2015, observed 20q50 escalated from 1986 to 1994, rapidly increasing the gap between observed and expected rates of mortality for several years. The difference between observed and expected 20q50 widened for females in central and eastern Europe and central Asia during this time, albeit with a much smaller magnitude of change. Notably, observed levels of male 20q50 consistently exceeded expected rates for high-income countries between 1980 and 1997 before converging. Conversely, observed 20q50 for females in high-income countries remained higher than expected from 1980 to 2015.

### Global causes of death

Table 5 shows the global estimates of total deaths and age-standardised death rates by cause for 2005 and 2015, as well as the percentage change in mortality from 2005 to 2015. Annual mortality estimates from 1990 to 2015 and more detailed age–sex results can be viewed online.

Broadly, communicable, maternal, neonatal, and nutritional diseases, known as Group 1 causes for GBD, accounted for 20·2% (95% UI 19·7–20·7) of global deaths in 2015 (11·3 million, 95% UI 10·9 million to 11·6 million), NCDs caused 71·3% (70·9–72·0) of deaths (39·8 million, 39·2 million to 40·5 million), and injuries resulted in 8·5% (7·9–8·5) of deaths (4·7 million, 4·4 million to 4·9 million). Between 2005 and 2015, Group 1 causes saw significant reductions for both total deaths (decrease of 19·7% [17·8–21·6]) and age-standardised rates (decrease of 29·6% [27·9–31·3]). For NCDs, total deaths rose by 14·3% (12·6–16·0), an increase of 5·0 million deaths (4·4 million to 5·6 million) since 2005, but age-standardised rates decreased from 719·1 deaths (711·9–727·3) per 100 000 in 2005 to 624·7 deaths (615·8–634·5) per 100 000 in 2015 (decrease of 13·1%, 11·9–14·3). Injuries caused about 4·7 million deaths in both 2005 and 2015, but the age-standardised rates due to injuries significantly declined during this time, decreasing by 15·8% (12·4–18·7) from 78·6 deaths (73·5–80·8) per 100 000 in 2005 to 66·2 deaths (61·5–68·7) per 100 000 in 2015.

### Communicable, maternal, neonatal, and nutritional diseases

Marked reductions in total deaths and age-standardised death rates were achieved for many of the world's most important communicable diseases. Total HIV/AIDS deaths fell 33·4% (95% UI 30·0–36·2), from 1·8 million (95% UI 1·7 million to 1·9 million) in 2005 to 1·2 million (1·1 million to 1·3 million) in 2015, and age-standardised death rates dropped even more rapidly (reduction of 42·1%, 39·1–44·6). Globally, HIV/AIDS mortality peaked in 2005, underscoring the continued expansion of ART and PMTCT. Malaria deaths decreased by 37·4% (27·8–47·0), falling to 730 500 (555 800–904 000) in 2015. Age-standardised death rates due to malaria fell slightly more rapidly (43·1%, 34·7–51·8) during this time; nonetheless, this rate of decline only partly represents the sustained gains against malaria, given that mortality peaked in 2003, claiming 1·2 million lives (1·0 million to 1·4 million) that year. Age-standardised death rates due to diarrhoeal diseases fell 32·2% (27·7–36·5) from 2005 to 2015, although total deaths fell more slowly (20·8%, 15·4–26·1) to 1·3 million deaths (1·2 million to 1·4 million). Other communicable diseases that had significant reductions in mortality included tetanus (decreased by 47·5% [95% UI 39·0–54·6], to 56 700 deaths [48 200–80 000]), measles (decreased by 75·0% [58·8–84·5], to 73 400 deaths [26 100–161 400]), and African trypanosomiasis (decreased by 75·3% [67·9–81·4], to 3510 deaths [1790–5660]).

Amid these gains, less pronounced progress occurred for several communicable diseases, and fatalities climbed rapidly for others, such as Ebola virus disease. Tuberculosis, which killed fewer people than HIV/AIDS in 2005 (1·3 million, 95% UI 1·2 million to 1·7 million), essentially matched HIV/AIDS's toll by 2015, causing 1·1 million deaths (0·91 million to 1·4 million). Deaths due to tuberculosis decreased by 17·4% (11·3–24·4) between 2005 and 2015; however, age-standardised tuberculosis death rates dropped by 33·8% (28·7–39·6). Total mortality due to lower respiratory infections remained fairly constant from 2005 to 2015 (between 2·8 million and 2·7 million deaths), although age-standardised death rates fell by 19·5% (16·9–22·3); a similar trend was observed for meningitis. Deaths due to hepatitis and age-standardised death rates decreased (deaths fell by 14·0% [10·0–17·9], to 106 000 [101 000–111 000], and death rates fell by 28·0% [24·7–31·1]), which was mainly driven by significant reductions in deaths due to acute hepatitis A (decrease of 34·0% [24·2–43·5], to 11 000 [7000–16 000]) since 2005. Mortality due to other types of hepatitis improved less rapidly. Dengue deaths increased by 48·7% (15·1–90·9), resulting in 18 400 deaths (11 800–22 700) in 2015, and Chagas disease, which largely affects populations in Latin America, claimed 8000 lives (7500–8600) that year. Deaths due to leishmaniasis increased, albeit not significantly, between 2005 and 2015, causing 24 200 deaths (17 100–32 500) in 2015. The peak of the west African Ebola virus disease outbreak occurred in 2014, causing 12 800 deaths (10 300–15 300) that year. In 2015, 5500 people (4400–6600) died from Ebola virus disease, mainly in Guinea, Liberia, and Sierra Leone.

Among the leading causes of global maternal mortality, most showed significant reductions in both total deaths and age-standardised death rates between 2005 and 2015. Deaths due to maternal haemorrhage decreased by 16·6% (95% UI 3·2–28·8), claiming 16 600 (3300–29 800) fewer lives in 2015, and deaths due to abortion, miscarriage, and ectopic pregnancies dropped by 23·1% (11·1–33·9), to 32 000 (25 000–40 000); age-standardised death rates fell by 25·0% (12·9–35·9) for maternal haemorrhage and by 30·7% (19·8–40·4) for abortion, miscarriage, and ectopic pregnancies. For neonatal disorders, total deaths fell by 18·5% (16·4–20·4) and age-standardised death rates fell by 22·8% (−24·6 to −20·9) from 2005 to 2015, to 2·2 million (2·1 million to 2·2 million). Preterm birth complications caused 282 200 (215 000–353 500) fewer deaths in 2015 than in 2005 (reduction of 25·9%, 20·6–31·3) and age-standardised rates dropped by 29·8% (24·8–34·9). Total deaths and age-standardised death rates due to neonatal encephalopathy also decreased significantly during this time, albeit at a more moderate pace. Overall, these trends probably reflect a combination of decreasing fertility rates, improved maternal care, and safer delivery practices in many settings.

Notably less progress occurred for nutritional deficiencies, which caused 405 700 deaths (95% UI 331 700–495 600) in 2015. In 2015, iron-deficiency anaemia led to 54 200 deaths (35 100–72 900) and protein-energy malnutrition caused 323 200 deaths (264 900–400 800); in combination, nutritional deficiencies accounted for 3·6% (2·7–4·1) of lives lost to Group 1 disorders. Age-standardised death rates significantly decreased for nutritional deficiencies (decreased by 24·3%, 14·3–32·9).

### Non-communicable diseases

In 2015, the leading causes of NCD deaths were cardiovascular disease (17·9 million, 95% UI 17·6 million to 18·3 million), cancers (8·8 million, 8·6 million to 8·9 million), and chronic respiratory diseases (3·8 million, 3·7 million to 3·9 million). The global death toll due to cancers increased by 17·0% (95% UI 14·8–19·3) between 2005 and 2015, although age-standardised rates of death fell by 10·0% (8·3–11·6). Tracheal, bronchus, and lung cancer (total deaths 1·7 million, 1·7 million to 1·8 million) were the leading causes of cancer deaths, and also had the highest age-standardised death rate (26·6 deaths [25·9–27·4] per 100 000) among cancers in 2015. For several cancers, total deaths increased by 20% or more between 2005 and 2015, including tracheal, bronchus, and lung cancer (20·1% [16·7–24·0], to 1·7 million deaths [1·7 million to 1·8 million); colon and rectum cancer (23·2% [20·6–26·0], to 832 000 deaths [811 700–854 500]); malignant skin melanoma (27·2% [20·0–32·6], to 59 800 deaths [47 600–72 700]); pancreatic cancer (30·8% [28·3–33·6], to 411 600 deaths [403 600–420 700]); and prostate cancer (31·9% [28·2–35·4], to 365 900 deaths [303 500–459 600]). Breast and ovarian cancers, which largely, if not exclusively, affect females, caused significantly more deaths in 2015 than in 2005 (breast cancer increased by 21·3% [14·9–27·2], to 534 000 deaths [502 000–553 000]; ovarian cancer increased by 20·4% [16·5–24·4], to 161 000 deaths [157 000–167 000]); however, age-standardised death rates for both cancers significantly declined during this time (breast cancer decreased by 6·8% [2·5–11·5] and ovarian cancer decreased by 7·9% [4·9–10·8]). The largest reductions in death rates from 2005 to 2015 were recorded for oesophageal cancer, which fell by 26·8% (22·9–30·3) and Hodgkin's lymphoma, which fell by 23·9% (20·1–27·7). At the same time, significant increases occurred in age-standardised death rates due to non-melanoma skin cancer (increased by 7·6%, 3·4–11·1) and mesothelioma (increased by 7·8%, 3·6–11·6).

Global cardiovascular disease deaths rose by 12·5% (95% UI 10·6–14·4) between 2005 and 2015, whereas age-standardised rates of death due to cardiovascular disease fell 15·6% (14·2–16·9). These reductions were largely driven by declining mortality rates due to cerebrovascular disease (ie, stroke; decreased by 21·0%, 19·2–22·8) since 2005. Globally, deaths due to ischaemic heart disease increased by 16·6% (14·6–18·6) from 2005 to 2015 to 8·9 million deaths (8·8 million to 9·1 million), whereas age-standardised mortality rates for ischaemic heart disease decreased at a more moderate pace (fell by 12·8%, 11·4–14·2). Ischaemic heart disease and stroke accounted for 15·2 million deaths (15·0 million to 15·6 million) in 2015, equating to 85·1% (84·7–85·5) of all deaths due to cardiovascular disease that year. Among respiratory conditions, age-standardised death rates fell by 22·9% (20·0–25·4) for chronic obstructive pulmonary disease (COPD) and by 31·3% (19·4–38·9) for asthma; total deaths due to these causes did not significantly differ from 2005 to 2015. By contrast, for interstitial lung disease and pulmonary sarcoidosis, significant increases occurred in total deaths, which rose by 51·5% (37·9–60·5) to 121 800 deaths (94 100–135 200), and age-standardised rates, which rose by 14·1% (4·1–20·9) from 2005 to 2015.

Mortality patterns were similar for other leading NCD causes of death. Age-standardised mortality rates decreased for all subtypes of cirrhosis, yet total deaths increased to 1·3 million in 2015 (95% UI 1·2 million to 1·4 million). Total mortality also increased from 2005 to 2015 for diabetes, which rose by 32·1% (95% UI 27·7–36·3), to 1·5 million deaths (1·5 million to 1·6 million), and chronic kidney disease, which rose by 31·7% (27·7–35·6), to 1·2 million deaths (1·1 million to 1·3 million); by contrast, changes in age-standardised death rates due to diabetes and chronic kidney disease were not statistically significant. Chronic kidney disease due to diabetes mellitus caused significantly more deaths in 2015 than in 2005 (an increase of 39·5% [35·4–43·5], to 418 000 deaths [389 000–441 000]), and age-standardised death rates also rose 6·4% (3·3–9·3). Global deaths due to Alzheimer's disease and other dementias increased by 38·2% (36·2–40·1), to 1·9 million deaths (1·6 million to 2·2 million), which was largely driven by population ageing, given that age-standardised mortality decreased by 2·7% (1·7–3·7). Notably, both total deaths and age-standardised death rates due to alcohol use disorders significantly dropped from 2005 to 2015, falling by 12·6% (7·0–16·7), to 138 000 deaths (131 000–144 000), and 29·2% (24·7–32·4), respectively. However, drug use disorders claimed increasingly more lives, resulting in a rise of 31·8% (20·4–39·4; rising to 170 000 deaths, 152 000–179 000) since 2005. Deaths due to opioid use disorders accounted for 71·9% (69·5–73·3) of these drug-related deaths in 2015, increasing by 29·6% (18·2–37·2) to a total of 122 100 deaths (109 500–129 700) that year.

### Injuries

In 2015, transport injuries caused 1·5 million deaths (95% UI 1·4 million to 1·5 million), unintentional injuries resulted in 1·8 million (1·6 million to 1·9 million), and intentional injuries, including self-harm and interpersonal violence, led to 1·2 million (1·1 million to 1·3 million). Although total deaths did not significantly change between 2005 and 2015, age-standardised death rates fell by 16·2% (11·8–20·8) for transport injuries, 17·8% (13·4–20·4) for unintentional injuries, and 16·3% (12·6–20·1) for intentional injuries. Age-standardised death rates for road injuries and most types of road injuries, including pedestrian, cyclist, and motor vehicle injuries, significantly decreased from 2005 to 2015. Notably, deaths due to falls increased by 20·9% (14·6–27·2) between 2005 and 2015 (to 527 000 deaths, 468 000–555 000), which probably reflects global shifts in ageing rather than a rise in injury risk given that age-standardised death rates fell by 5·5% (0·8–10·1). For drowning, there were significant reductions in both total deaths (decreased by 19·7% [14·2–23·6], to 324 000 deaths [286 000–347 000]) and age-standardised death rates (decreased by 28·5%, 23·8–31·8). Total deaths due to self-harm and interpersonal violence remained relatively unchanged since 2005, but age-standardised deaths fell by 16·3% (11·2–21·5) and 16·4% (13·1–19·1), respectively. Assault by firearms, which accounted for 42·4% (40·4–43·9) of all interpersonal violence deaths, claimed 173 100 lives (149 300–183 200) in 2015, and in contrast with all other causes of interpersonal violence, total deaths significantly increased since 2005 (rose by 6·3%, 2·4–11·0).

**Figure 11:**
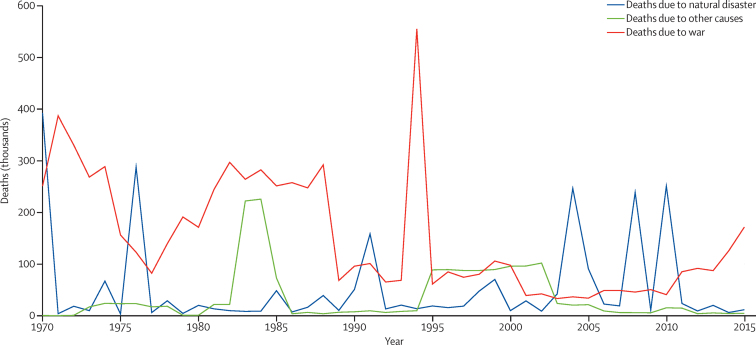
Global deaths due to fatal discontinuities by cause group for each year, 1980–2015 Numbers shown are total deaths. Fatal discontinuities are events that lead to abrupt changes in deaths in a geography. The causes for these fatal discontinuities include wars, natural disasters, industrial accidents, large transport accidents, epidemics, famines, or other injuries.

Mortality trends due to natural disasters and war were highly irregular (figure 11). By decade, the numbers of war deaths were higher in the 1970s and 1980s, then fell in the 1990s and in the first decade of the 21st century. Conversely, between 2010 and 2015, mortality due to war (collective violence and legal intervention) increased, rising to 171 300 (88 100–251 100) in 2015. More than 40·6% (34·8–45·1) of these deaths occurred in Syria and Yemen (70 000 deaths, 33 000–107 000). These numbers of war fatalities remain much lower than those recorded in 1993 and 1994, when more than 626 000 lives were lost to the Rwandan genocide, the Iraq civil war, ongoing armed conflict in Bosnia and Herzegovina, and other occurrences of collective violence; nonetheless, the rising number of casualties in the Middle East represents the largest increase in war deaths since 1995. Because of deaths in Afghanistan, Iraq, Syria, and Yemen, war deaths have increased during 2014–15. Between 2004 and 2010, natural disasters claimed thousands of lives, including 226 000 from the Indonesian earthquake and tsunami in 2004; 74 700 from earthquakes in India and Pakistan, as well as 1870 from Hurricane Katrina in the USA in 2005; 87 900 from an earthquake in China and 138 000 from a cyclone in Myanmar in 2008; and 223 000 from the earthquake in Haiti in 2010. In 2015, natural disasters caused 11 800 deaths (7160–16 400), mainly due to the Nepal earthquake and floods in India.

**Figure 12:**
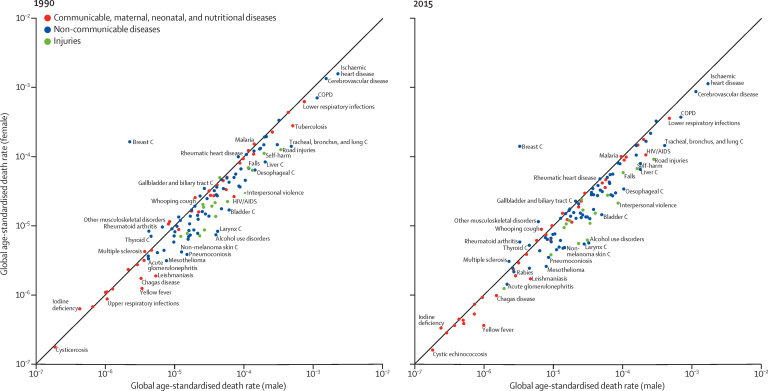
Global age-standardised death rates for males versus females, by GBD cause Level 3, 1990 and 2015 The y-axis and x-axis are shown on a log scale to enable comparisons between males and females spanning a wide range of values. Black lines show where death rates are identical for males and females. Causes that only affect one sex, including maternal disorders, chlamydia, cervical, uterine, ovarian, prostate, testicular cancers, and gynaecological diseases are not shown. GBD=Global Burden of Disease. COPD=chronic obstructive pulmonary disease. C=cancer.

In general, age-standardised death rates for males and females by cause are highly correlated at the global level (figure 12). For most causes, male age-standardised death rates are higher than for females. Death rates are notably higher in males for many cancers including tracheal, bronchus, and lung, liver, oesophageal, bladder, and larynx cancer, and mesothelioma. Male age-standardised death rates are also higher for many injuries including road injuries, self-harm, falls, and interpersonal violence. For a small number of causes, female age-standardised rates are higher than for males, including breast cancer, rheumatic heart disease, gallbladder and biliary cancer, whooping cough, rheumatoid arthritis, thyroid cancer, and multiple sclerosis, and other musculoskeletal disorders.

### Decomposition analysis of changes in global mortality

**Figure 13:**
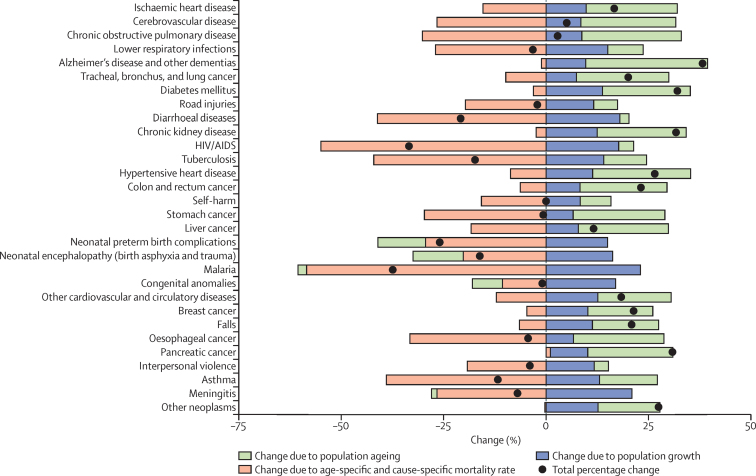
Global decomposition of changes in leading 30 causes of death, 2005 to 2015 Changes due to population growth, population ageing, and changes in age-specific mortality rates are shown. Causes are reported in descending order by total number of deaths for all ages and both sexes combined in 2015. The black circle shows the overall median percentage change in global deaths from 2005 to 2015. Causes with increases in overall death rates have a circle to the right of the zero, whereas a circle to the left of the zero denotes causes with decreases in overall death rates. The contributions of population growth, ageing, and change in age-specific death rates sum to the total change in numbers of deaths.

Drivers of global changes in mortality—population growth, ageing, and changes to age-standardised rates of cause-specific mortality—varied substantially by cause from 2005 to 2015 (figure 13). Among the leading 30 causes of death worldwide, changes in total death tolls ranged from a reduction of 37·4% (95% UI 27·8–47·0) for malaria to an increase of 38·2% (36·2–40·1) for Alzheimer's disease and other dementias. Population growth accounted for increases in numbers of deaths across all 30 causes, but its contribution ranged from less than 9% for several types of cancer, including lung cancer and liver cancer, to 22·9% for malaria. In fact, for malaria, as well as a subset of other Group 1 causes (ie, preterm birth complications, neonatal encephalopathy, and meningitis), population growth was the only factor that hindered further reductions in mortality. Population ageing led to increasing mortality for most causes, but its relative contribution ranged from less than 4% for interpersonal violence and diarrhoeal diseases to 29·6% for Alzheimer's disease and other dementias. Shifts in population age structures also accounted for more than 23% of increased mortality due to cardiovascular disease (ischaemic heart disease and stroke), 24·2% for COPD, and at least 20% for several types of cancer (eg, pancreatic cancer and oesophageal cancer). Conversely, for some causes, namely neonatal conditions and causes that largely affect children, such as malaria, population ageing contributed to decreasing levels of mortality. Except for pancreatic cancer, changes in age-specific and cause-specific rates of death drove reductions in deaths due to the 28 other leading causes. Declines attributable to changes in age-specific and cause-specific mortality rates markedly differed, with several causes experiencing reductions of more than 40% (eg, malaria [58·2%], HIV/AIDS [54·8%], tuberculosis [41·9%], and diarrhoeal diseases [41·0%]) and others showing much smaller decreases (eg, chronic kidney disease [2·4%] and diabetes [3·1%]). Notably, patterns were less distinct for most injuries because population growth and shifts in age-specific and cause-specific mortality rates had relatively similar contributions to changes in deaths due to road injuries, self-harm, and interpersonal violence. Falls were the exception, with a combination of population growth and ageing mainly driving its rising death toll.

### Global YLLs

**Figure 14:**
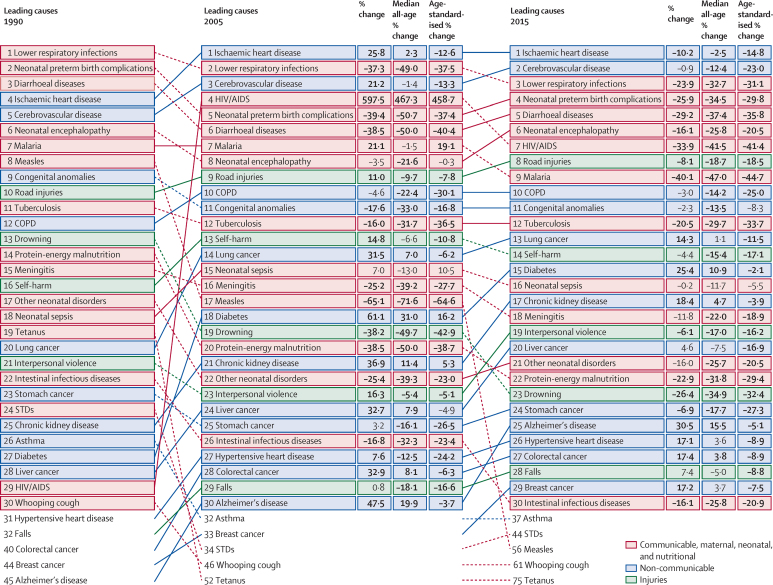
Leading 30 Level 3 causes of global YLLs for both sexes combined for 1990, 2005, and 2015, with percent change in number of YLLs, and all-age and age-standardised rates Causes are connected by lines between time periods. For the time periods 1990 to 2005 and 2005 to 2015, three measures of change are shown: percent change in the number of YLLs, percent change in the all-age YLL rate, and percent change in the age-standardised YLL rate. Statistically significant changes are shown in bold. YLLs=years of life lost. COPD=chronic obstructive pulmonary disease. STDs=sexually transmitted diseases excluding HIV. An interactive version of this figure is available online at http://vizhub.healthdata.org/gbd-compare.

From 2005 to 2015, changes in the relative ranks of YLLs, emphasised the increasing complexity of global mortality patterns (figure 14). The top three causes of YLLs—ischaemic heart disease, stroke (which includes both ischaemic and haemorrhagic stroke), and lower respiratory infections—all saw reductions in age-standardised rates between 2005 and 2015, but changed minimally in rankings. In 2005, the causes ranked fourth to eighth were all communicable diseases (HIV/AIDS [fourth], diarrhoeal diseases [sixth], and malaria [seventh]) or neonatal disorders (preterm birth complications [fifth] and neonatal encephalopathy [eighth]). By 2015, both total and age-standardised rates of YLLs had significantly decreased for all of these causes, but their relative rankings did not substantially change; the exceptions were HIV/AIDS, which fell to seventh, and malaria, which dropped to ninth. Road injuries, COPD, and congenital anomalies, ranked as ninth, tenth, and 11th leading causes of YLLs in 2005, remained largely the same in terms of ranks in 2015, with only road injuries moving up by one spot to rank eighth. YLLs due to road injuries fell significantly between 2005 and 2015, both in terms of total YLLs, which decreased by 8·1% (95% UI 3·3–13·3), and age-standardised rates, which decreased by 18·5% (14·3–23·1). Among the top ten leading causes, premature mortality due to malaria decreased the most, with total YLLs falling by 40·1% (29·4–50·2) and age-standardised rates dropping 44·7% (34·9–54·1).

More pronounced shifts in YLL ranks and percentage changes between 2005 and 2015 occurred beyond the leading 11 causes, particularly for several NCDs. Total YLLs due to diabetes rose 25·4% (95% UI 20·4–30·0) and diabetes advanced from being ranked 18th to 15th. Similar increases occurred for chronic kidney disease (18·4% [13·8–23·1] for total YLLs and rising from 21st to 17th) and Alzheimer's disease and other dementias (30·5% [28·6–32·4] for total YLLs and increasing from 30th to 25th). Several types of cancer, including colon and rectum cancer, breast cancer, pancreatic cancer, and brain cancer, showed significant increases in total YLLs and relative ranks by 2015; however, none of these cancers had significant increases in age-standardised rates of YLLs. Among NCDs, only asthma (ranked 32nd in 2005 and 37th in 2015) and rheumatic heart disease (ranked 41st in 2005 and 43rd in 2015) had significant reductions for both total YLLs and age-standardised rates. By contrast, larger declines for total YLLs and age-standardised rates occurred for several leading Group 1 causes, including tuberculosis (20·5% [14·9–26·0] and 33·7% [29·1–38·3], respectively); protein-energy malnutrition (22·9% [4·8–38·1] and 29·4% [13·0–43·0], respectively); and most notably, measles (75·0% [58·9–84·4] and 76·7% [61·8–85·5], respectively). In general, changes in total YLLs and age-standardised rates due to injuries suggested reduced levels of premature mortality; the main exception was early deaths due to war, which climbed from the 92nd leading cause of YLLs in 2005 to 38th in 2015, and increased more than 350% for total YLLs and age-standardised rates of early death. Additional comparisons for changes in YLLs across different years between 1990 and 2015 can be explored online.

### Causes of child death

Globally, under-5 deaths decreased by 27·2% (95% UI 25·2–29·0) between 2005 and 2015, reaching 5·8 million deaths (95% UI 5·6 million to 6·0 million). Group 1 causes, which include communicable, maternal, neonatal, and nutritional conditions, led to 80·8% (79·5–82·3; 4·7 million deaths, 4·6 million to 4·8 million) of under-5 deaths, NCDs caused 13·8% (12·6–14·9; 804 000 deaths, 733 000–868 000), and injuries accounted for 5·4% (4·6–6·0; 313 000 deaths, 265 000–348 000) of deaths. Of the selected causes shown in table 6, neonatal disorders, which can affect children beyond the neonatal period (ie, infants younger than 1 month), caused 37·2% (36·0–38·3) of under-5 deaths, equating to 2·2 million deaths (2·1 million to 2·2 million) in 2015; these causes included preterm birth complications, neonatal encephalopathy, neonatal sepsis, and other neonatal disorders. 62 Group 1 causes of under-5 deaths were lower respiratory infections (12·1% [11·2–13·1], 703 000 deaths [651 000–763 000]); diarrhoeal diseases (8·5% [7·7–9·5], 499 000 deaths [447 000–558 000]); malaria (8·1% [5·7–10·7], 474 000 deaths [333 000–624 000]); nutritional deficiencies (3·3% [2·6–4·3], 193 000 deaths [147 000–248 000]); and meningitis (3·0% [2·3–3·9], 173 000 deaths [137 000–229 000]). Congenital anomalies, which include congenital heart anomalies, led to 8·5% (7·7–9·5) of under-5 deaths in 2015 (497 000 deaths, 444 000–555 000), and other NCDs, such as sudden infant death syndrome, accounted for 9·0% (8·1–9·9) of under-5 deaths that year (522 000 deaths, 470 000–580 000).

Neonatal deaths, which accounted for 45·0% (44·9–45·2) of total under-5 deaths in 2015, decreased by 20·3% (95% UI 18·7–21·8), falling from 3·3 million (3·2 million to 3·3 million) in 2005 to 2·6 million (2·6 million to 2·7 million) in 2015. Neonatal causes, mainly preterm complications and neonatal encephalopathy, accounted for 77·5% (76·0–79·5) of deaths among neonates (766 000 deaths, 700 000–854 000), followed by neonatal sepsis, congenital anomalies, and lower respiratory infections. Tetanus had the largest reduction in neonatal deaths between 2005 and 2015, falling 57·7% (50·0–64·2) to 19 900 deaths (17 000–23 400), followed by malaria, which fell by 55·9% (41·1–67·8) to 13 900 deaths (8 930–19 800). Smaller improvements were achieved in the reduction of neonatal deaths from sepsis and congenital anomalies.

Among children aged 1–59 months, total deaths fell by 32·1% (95% UI 29·7–34·2) from 2005 to 2015, with 1·5 million (1·2 million to 1·8 million) fewer children in this age group dying in 2015. Lower respiratory infections, diarrhoeal diseases, and malaria caused 57·0% (49·7–64·6) of deaths among children aged 1–59 months, leading to a total of 1·8 million deaths (1·6 million to 2·1 million). Other leading causes of death for this age group included congenital anomalies (254 000 deaths, 224 000–297 000), nutritional deficiencies (193 000 deaths, 147 000–248 000), and meningitis (147 000 deaths, 117 000–196 000). Deaths due to measles fell by 75·1% (59·6–84·5), the largest reduction among this age group, to 62 600 deaths (22 400–136 000), followed by tetanus, which fell by 55·2% (39·3–66·4) to 5560 deaths (4140–7760), and HIV/AIDS, which fell by 51·9% (49·6–54·2), to 88 900 deaths (84 300–93 700). Nearly all of these leading causes of death showed some form of reduction from 2005 to 2015; neonatal sepsis and congenital anomalies were exceptions, both of which had, albeit not significant, increases in deaths since 2005.

### Lower respiratory infections and diarrhoea by pathogens

In 2015, we estimated that 1·3 million deaths (95% UI 1·2 million to 1·4 million) were caused by diarrhoeal diseases, including 499 000 (447 000–558 000) in children younger than 5 years, representing 8·6% (7·7–9·5) of all deaths in this age group. Reductions in under-5 deaths due to diarrhoeal diseases exceeded the rate of change for all other age groups. Rotaviral enteritis (rotavirus) was the leading cause of diarrhoeal death in children younger than 5 years globally (29·3% [24·6–35·9%], 146 500 deaths [118 000–183 500]) in 2015 followed by cryptosporidiosis (*Cryptosporidium*; 12·1% [2·8–26·9], 60 400 deaths [13 700–134 500]) and shigellosis (*Shigella*; 11·0% [5·5–18·7], 54 900 deaths [27 000–94 700]). Rotavirus was also the leading cause of mortality due to diarrhoea in all ages (15·2% [12·9–18·1], 199 200 deaths [165 500–241 200]), followed by *Shigella* (12·5% [6·4–21·2], 164 300 deaths [85 000–278 700]) and other *Salmonella* infections (6·9% [2·7–13·9], 90 300 deaths [34 100–183 100]; table 7). Adenovirus was an important cause of mortality due to diarrhoea in children younger than 5 years, accounting for nearly 10% of such deaths in this age group (9·2 [3·3–19·7], 46 000 deaths [16 200–97 700]). Mortality due to *Clostridium difficile* was the lowest among all diarrhoea causes, but was a major cause of diarrhoea mortality in high-income countries. Moreover, it was the only cause for which the attributable fraction increased from 2005 to 2015 (increased by 36·3%, 11·3–65·6). During this same time period, the only attributable fraction to significantly decrease was for rotavirus (decreased by 14·1%, 6·3–20·5; table 7).

We estimated that 2·7 million (95% UI 2·5 million to 2·9 million) deaths occurred in 2015 due to lower respiratory infections, of which 704 000 (651 000–763 000) occurred among children younger than 5 years, representing 12·1% of deaths in this age group. From 2005 to 2015, the number of deaths due to lower respiratory infections decreased by 3·25% (−0·45 to 6·94) globally in all age groups, but decreased by 36·9% (31·6–42·0) in children younger than 5 years. Pneumococcal pneumonia and *H influenzae* type b together accounted for nearly 65% of deaths due to lower respiratory infections in children younger than 5 years (table 7). The attributable fraction of deaths due to lower respiratory infection caused by pneumococcal pneumonia was highest in children younger than 5 years (55·8%, 32·5–75·0) and all ages (55·4%, 31·5–79·1). The percentage of under-5 deaths due to *H influenzae* type b decreased by 60·7% (56·8–65·7), from 13·4% (−0·8 to 24·7) in 2005 to 8·3% (−0·5 to 15·9) in 2015, with 58 700 deaths (−3130 to 115 000) recorded in 2015. Respiratory syncytial virus (5·2% [2·9–8·6], 36 400 deaths [20 400–61 500]) and influenza (1·4% [0·8–2·4], 10 200 deaths [5700–16 800]) together accounted for less than 10% of deaths due to lower respiratory infections in children younger than 5 years, and the remaining 29% of such deaths in this age group remain unattributed.

### Expected changes in disease profile with higher SDI

**Figure 15:**
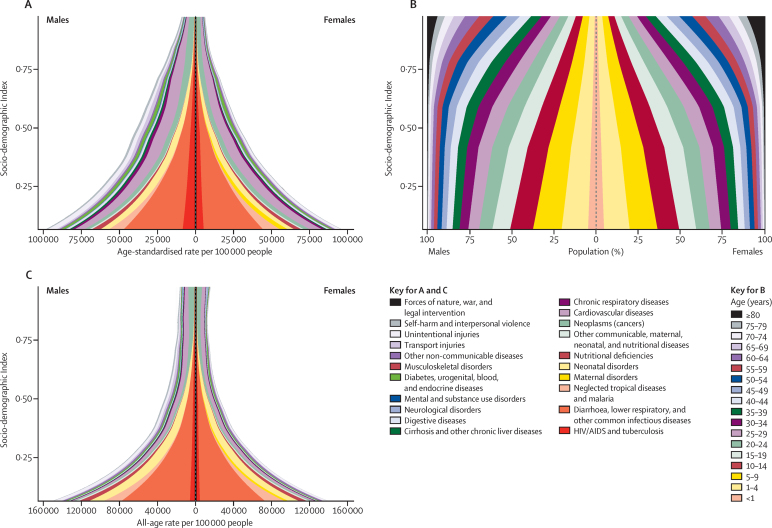
Expected relationship between age-standardised YLL rates per 100 000 people for the 21 GBD Level 2 causes and SDI (A), the expected relationship between population and SDI (B), and the expected relationship between all-age YLL rates per 100 000 people for the 21 GBD Level 2 causes and SDI (C), by sex The stacked curves in A and C represent the average relationship between SDI and each cause of YLLs observed across all geographies over the time period 1980 to 2015. In each figure, the y-axis spans from the lowest SDI up to the highest SDI. To the left of the midline are male rates, and the female rates are to the right; higher rates are further from the midline. GBD=Global Burden of Disease. SDI=Socio-demographic Index. YLL=years of life lost.

Figure 15 shows the changes in patterns of premature mortality as they relate to age-standardised rates of YLLs, population age structure, and total YLLs per 100 000 population. With increasing SDI, age-standardised YLL rates narrowed (figure 15A), from a total of 98 742 YLLs per 100 000 for males and 96 381 YLLs per 100 000 for females at low SDI, to a low of 9172 YLLs per 100 000 and 6239 YLLs per 100 000, respectively, at high SDI. The cause composition of YLLs substantially shifted as well. At lower SDI, premature mortality was largely due to communicable diseases that disproportionately affect children, such as diarrhoeal disease and lower respiratory infections, measles, and meningitis, but with increasing SDI, YLLs due to these causes markedly decreased. Age-standardised YLL rates due to HIV/AIDS and tuberculosis, neglected tropical diseases and malaria, neonatal disorders, and maternal disorders also rapidly decreased as SDI increased. Nonetheless, the gains achieved for neglected tropical diseases and malaria with rising SDI were somewhat attenuated because the rates of premature mortality due to dengue and Chagas disease increased. For a subset of NCDs, several causes had reductions in age-standardised YLL rates amid improving SDI, including chronic respiratory diseases; digestive diseases; diabetes, urogenital, blood, and endocrine diseases; and unintentional injuries. However, for other causes, the relationship between increasing SDI and premature mortality was less obvious. Age-standardised YLL rates due to cardiovascular disease gradually increased for both sexes until SDI reached 0·40, after which rates declined slowly until an SDI of 0·7, and more rapidly thereafter. For cancers, age-standardised rates of YLLs rose steadily between SDI levels of 0·60 and 0·72, and then largely plateaued. Notably, changes in age-standardised YLL rates and SDI affected the relative ratio of early death by sex for a subset of causes. Cancers, for example, began to exact a larger toll for males than females at SDI levels of about 0·40; this was mainly associated with rising mortality due to lung cancer. A similar trend occurred for cardiovascular disease, with the age-standardised YLL rates of males exceeding those of females beyond an SDI of 0·27. Injuries generally caused higher rates of age-standardised YLLs among males than females across all levels of SDI, but the largest imbalance occurred between SDI levels of 0·35 and 0·72.

Increases in SDI had sizeable implications for population age structure (figure 15B), and in combination with age-standardised rates of cause-specific YLLs, these factors shape the magnitude—and types—of early deaths worldwide (figure 15C). At the lowest levels of SDI (where SDI equals 1), 49·8% of the population was younger than 15 years and 0·23% was older than 80 years, whereas 11·3% were younger than 15 years and 9·3% were older than 80 years at the highest levels of SDI (where SDI equals 100). Differences by sex with increasing SDI were minimal, except for in the oldest age groups starting from SDI of 0·40. At highest SDI, the 80 years and older age group consisted of far more females (10·9%) than males (6·2%). Below an SDI of 0·50, Group 1 causes, especially infectious diseases such as diarrhoeal diseases and lower respiratory infections, accounted for most premature mortality (as much as 72·5%, or roughly 141 513 YLLs per 100 000). Between SDI levels of 0·32 and 0·58, premature mortality from communicable causes, nutritional deficiencies, and maternal disorders sharply decreased, whereas YLLs per 100 000 due to NCDs and injuries remained relatively unchanged or slowly increased; notably, as SDI increased, early death due to neonatal disorders decreased at a much slower pace than did other Group 1 causes. At SDI of 0·50 and above, NCDs and injuries accounted for a larger portion of total YLLs per 100 000 than did communicable, maternal, neonatal, and nutritional causes. With further improvements in SDI, YLLs per 100 000 generally plateaued—or even increased for some causes—as population age structures began to shift faster than the falls in age-standardised rates of death. The stark contrast between the absolute and relative causes of YLLs per 100 000 for SDI levels lower than 0·40 and higher than 0·60 accentuates the complex disease profile of the remaining levels of SDI: relatively high levels of premature mortality due to a broad mixture of causes, ranging from neonatal disorders to cardiovascular disease.

### Attribution of change in life expectancy to changes in major causes of death

**Figure 16:**
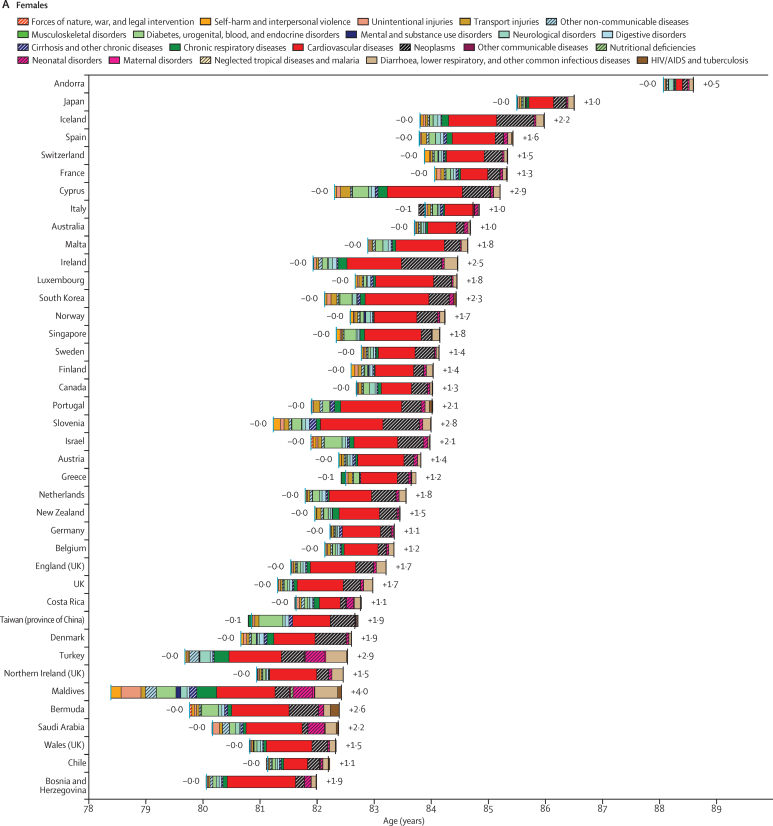

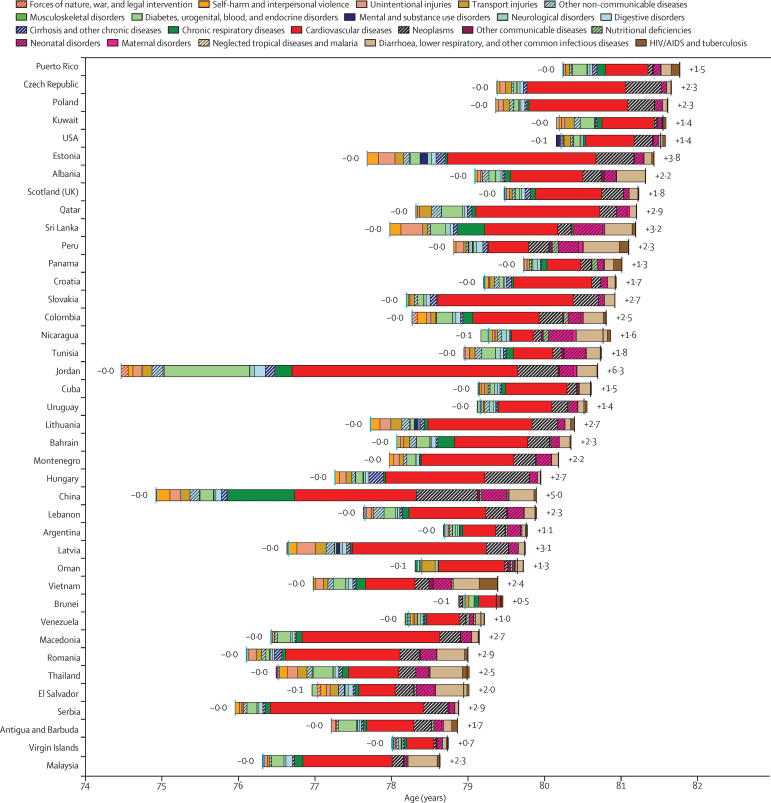

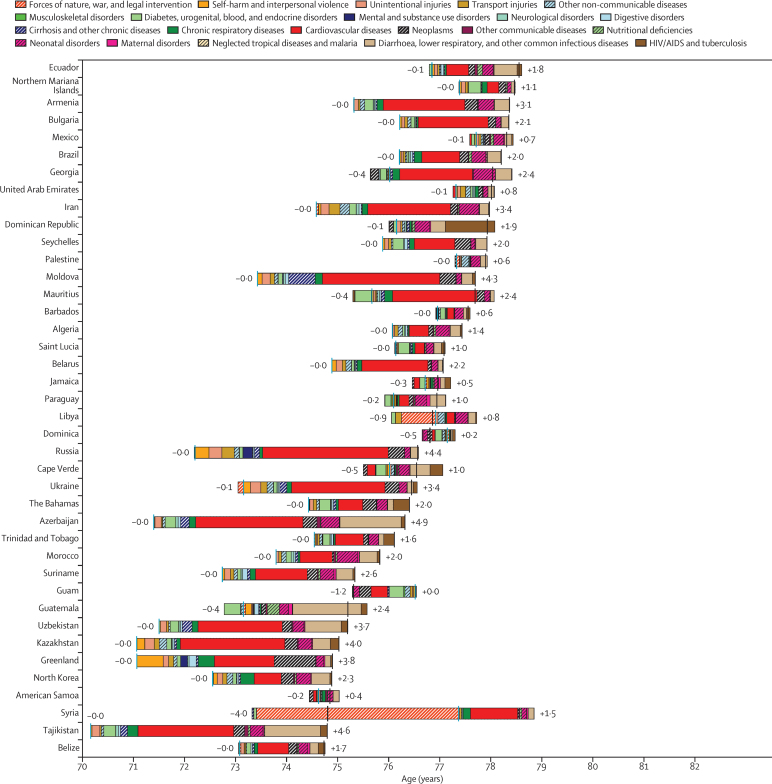

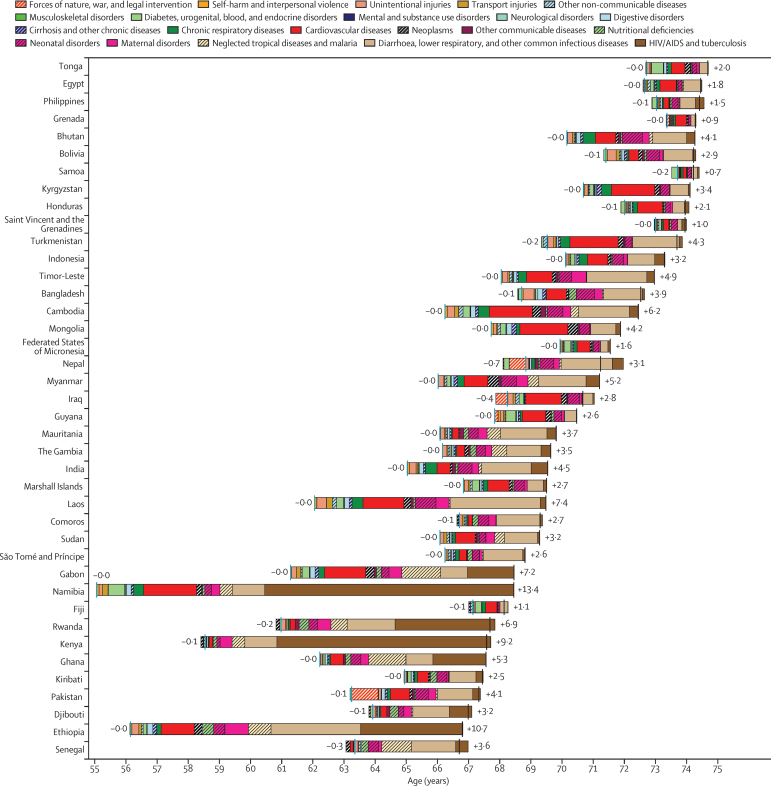

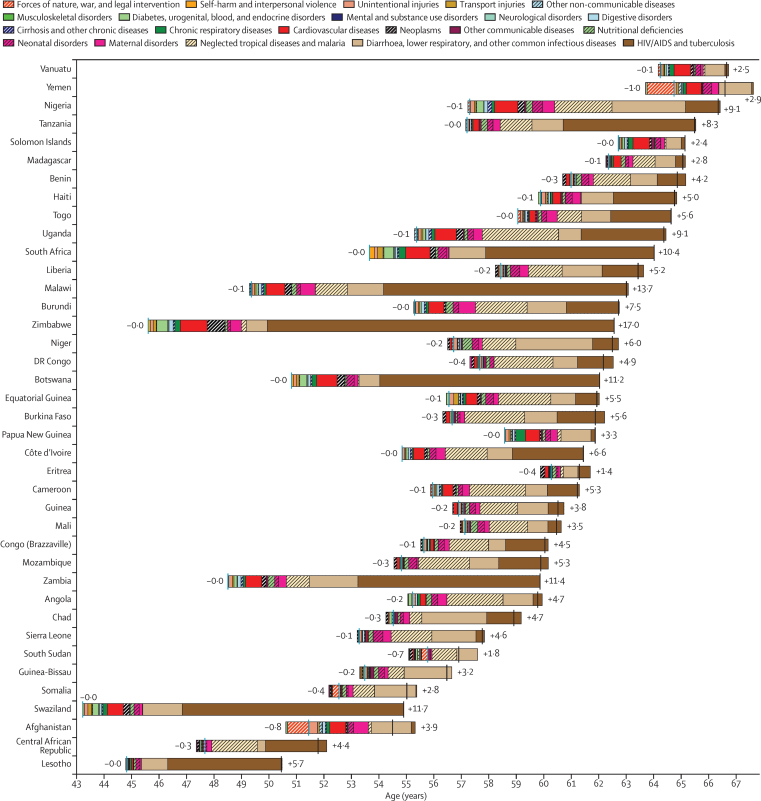

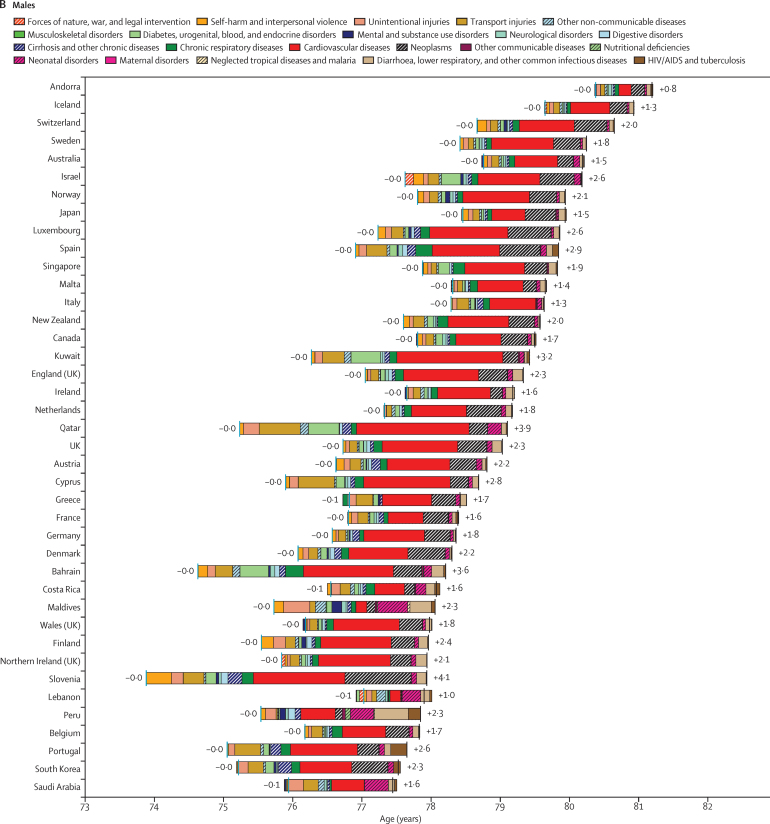

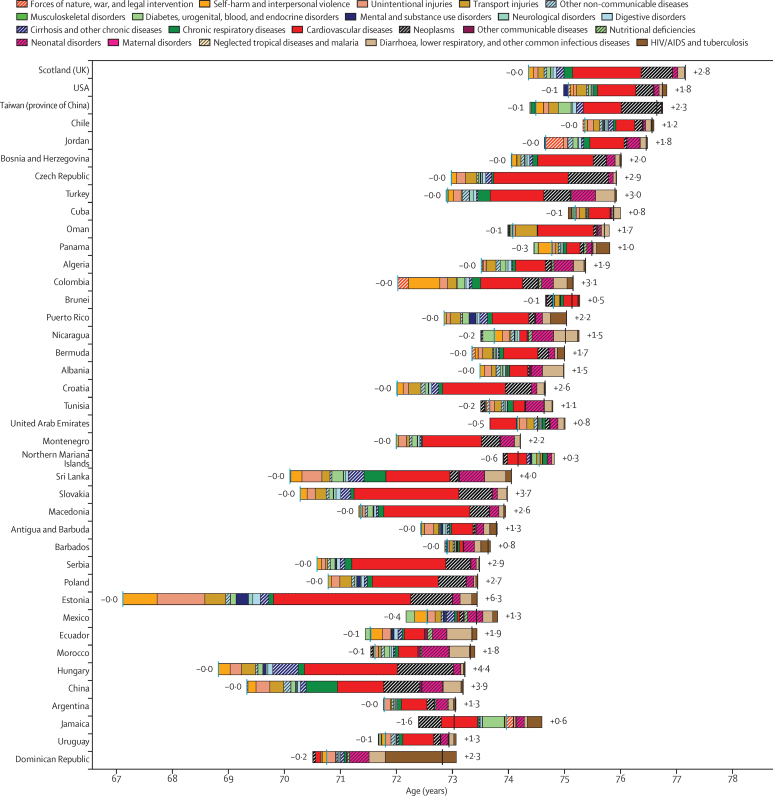

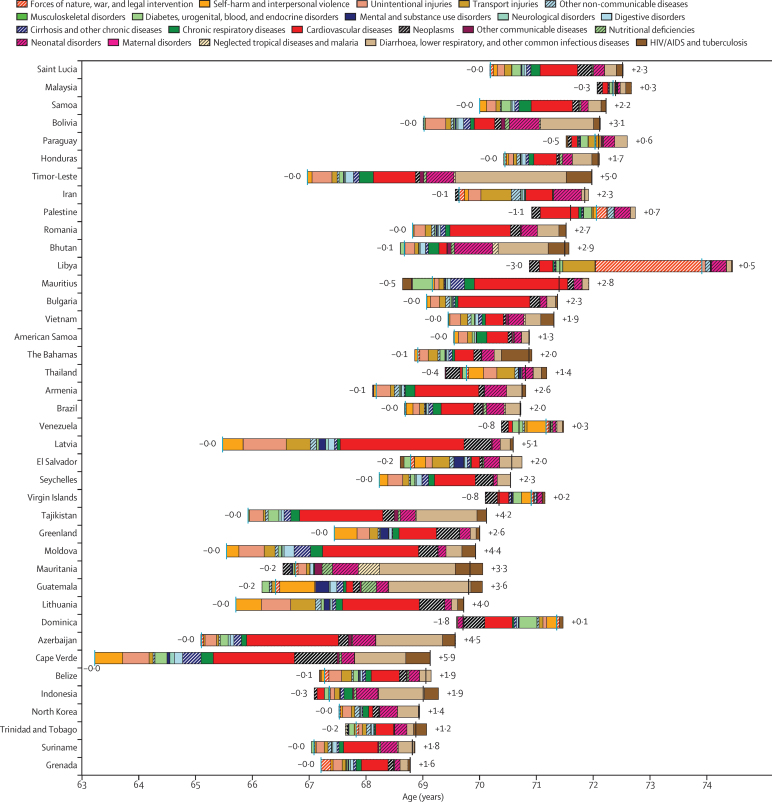

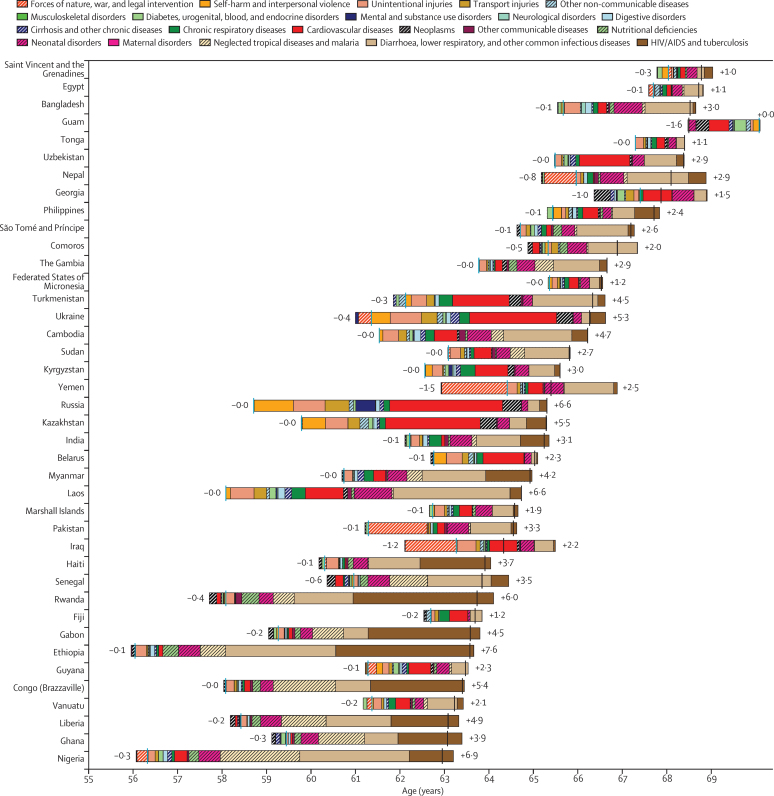

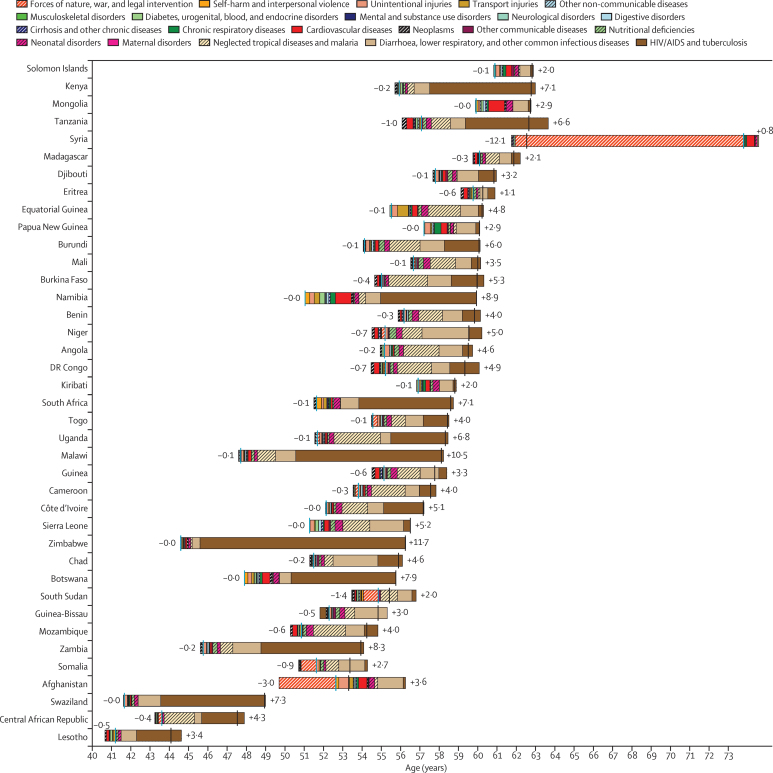
Attribution of changes in life expectancy at birth to changes in major groups of causes of death, 2005 to 2015 Changes are shown for countries and territories (and subnational units in the UK) for females (A) and males (B). Locations are ordered by decreasing life expectancy at birth in 2015. Blue lines show life expectancy at birth in 2005 and black lines show life expectancy at birth in 2015. Causes to the left of the 2005 life expectancy values reflect causes that contributed to reductions in life expectancy from 2005 to 2015. Causes to the right of the 2005 life expectancy values contributed to increases in life expectancy from 2005 to 2015.

Figure 16 shows changes in life expectancy from 2005 to 2015, as attributable to changes in Level 2 causes of death, for each country, territory, and subnational geography. In 2015, Andorra had the highest life expectancy at birth for males (81·2 years, 95% UI 80·8–81·6) and females (88·4 years, 88·1–88·7), whereas Lesotho experienced the lowest life expectancy at birth for both sexes (44·1 years, 38·6–51·8 for males and 50·4 years, 43·6–58·5 for females). Overall, several countries in sub-Saharan Africa had the largest gains in longevity since 2005. By 2015, Zimbabwe had the fastest progress for both sexes, with life expectancy increasing by 11·7 years (5·5–18·3) to 56·3 years (51·1–62·6) for males and 17·0 years (10·1–23·3) to 62·5 years (56·3–68·3) for females. Furthermore, female life expectancy increased by more than 10 years for eight countries in sub-Saharan Africa (South Africa, Ethiopia, Botswana, Zambia, Swaziland, Namibia, Zimbabwe, and Malawi); this progress was largely attributable to marked reductions in mortality from HIV/AIDS. Death rates due to HIV/AIDs peaked from 2003 to 2005 for many of these countries, after which sizeable gains in prolonged life occurred through to 2015. For some countries, including Laos, decreases in death rates from various communicable diseases, such as diarrhoeal diseases and lower respiratory infections, were related to improved life expectancy, whereas several countries in central and eastern Europe recorded gains in longevity that were mainly associated with relative reductions in cardiovascular disease deaths.

Nonetheless, seven countries and territories had higher life expectancies for both sexes combined (nine for males only, four for females only) in 2005 than in 2015 and many others had minimal progress due to rising numbers of war-related causalities. By 2015, average life expectancy for both sexes fell by 1·3 years (−0·3 to 2·8) in Libya, 1·1 years (−0·2 to 2·4) in Dominica, and 7·3 years (1·8–12·1) in Syria; however, these reductions were far more pronounced among males in these countries, with male life expectancy reduced by 11·3 years (3·7–17·4) in Syria, 2·5 years (0·2–4·9) in Libya, and 1·6 years (−0·3 to 3·6) in Dominica. For Syria and Libya, rising mortality due to war was the main driver of such losses in longevity, whereas NCDs, including cancers and cardiovascular disease, led to reduced male life expectancy in Dominica. Six other geographies also had decreases in male life expectancy since 2005, with losses of 0·9 years (−0·6 to 2·5) in Jamaica, 1·6 years (−1·2 to 4·2) in Guam, 0·5 years (−2·4 to 3·2) in Palestine, 0·4 years (–1·3 to 2·0) in the Northern Mariana Islands, 0·6 years (−0·7 to 2·0) in the Virgin Islands, and 0·5 years (−1·2 to 2·1) in Venezuela. Increased mortality from cancers, cardiovascular disease, diabetes, and chronic kidney disease was associated with reduced life expectancy among males in Jamaica and Guam, whereas increased death rates due to interpersonal violence largely contributed to reduced longevity for males in Venezuela. For several countries and territories, overall life expectancy increased from 2005 to 2015, but heightened mortality due to natural disasters, interpersonal violence, and war offset gains achieved against other causes of death since 2005. In Yemen, for example, male and female life expectancy rose by 1·0 years and 1·9 years, respectively, yet rising war-related deaths resulted in reductions in life expectancy of 1·5 years for males and 1·0 years for females, attenuating further improvement in life expectancy. Similar results emerged for other countries in which war has claimed increasingly more lives, including Afghanistan, Iraq, Somalia, and South Sudan. The 2015 earthquake in Nepal largely contributed to the 0·7 years lost for females and 0·8 years for males; nonetheless, overall life expectancy improved by 2·1 years (−0·4 to 4·6) and 2·4 years (0·0–4·6) for males and females in Nepal, respectively—gains mainly attributable to reductions in mortality from diarrhoeal diseases and lower respiratory infections.

Inequalities in life expectancy by sex generally increased over time. In 2005, the difference between male and female life expectancy was 5·0 years (65·7 years, 95% UI 65·5–65·9 for males and 70·7 years, 70·5–71·0 for females), which widened to 5·8 years in 2015 (69·0 years, 68·3–69·4 for males and 74·8 years, 74·4–75·2 for females). For several countries, including Russia, Estonia, and Latvia, differences between male and female life expectancy narrowed more rapidly; these gains could be attributed to reduced mortality due to cardiovascular diseases, cancer, and injuries. Yet, inequalities in life expectancy grew in many countries and territories, often driven by uneven progress in health by sex and increasing male deaths due to a subset of NCDs. For example, Georgia recorded a widening gap between male and female life expectancy, rising from 8·6 years (7·7–9·6) in 2005 to 10·2 years (8·9–11·4) in 2015. In other places (eg, Syria), rising mortality from interpersonal violence or war disproportionately affected males.

### Leading causes of YLLs and deviations from expected levels based on SDI

**Figure 17:**
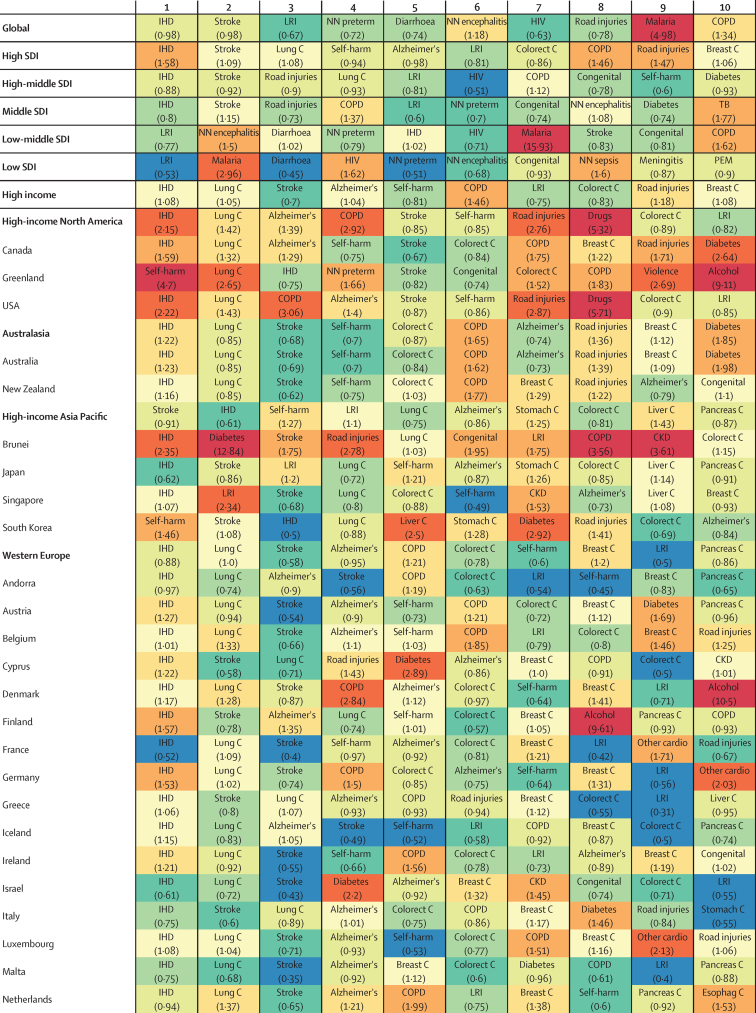

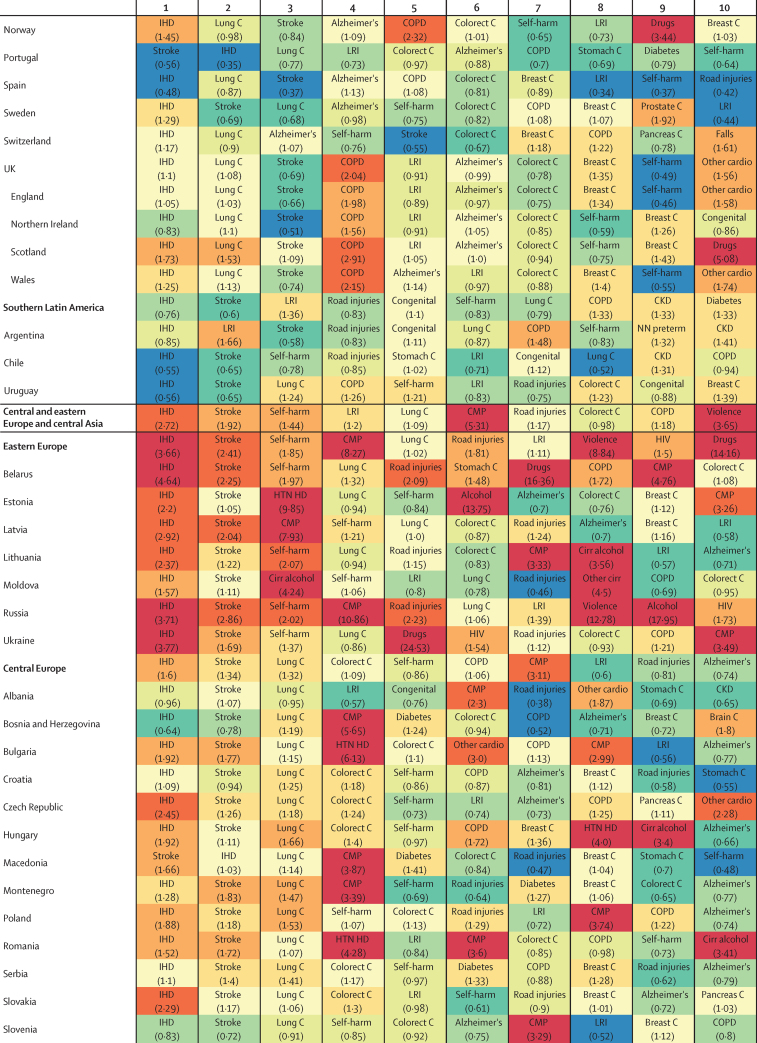

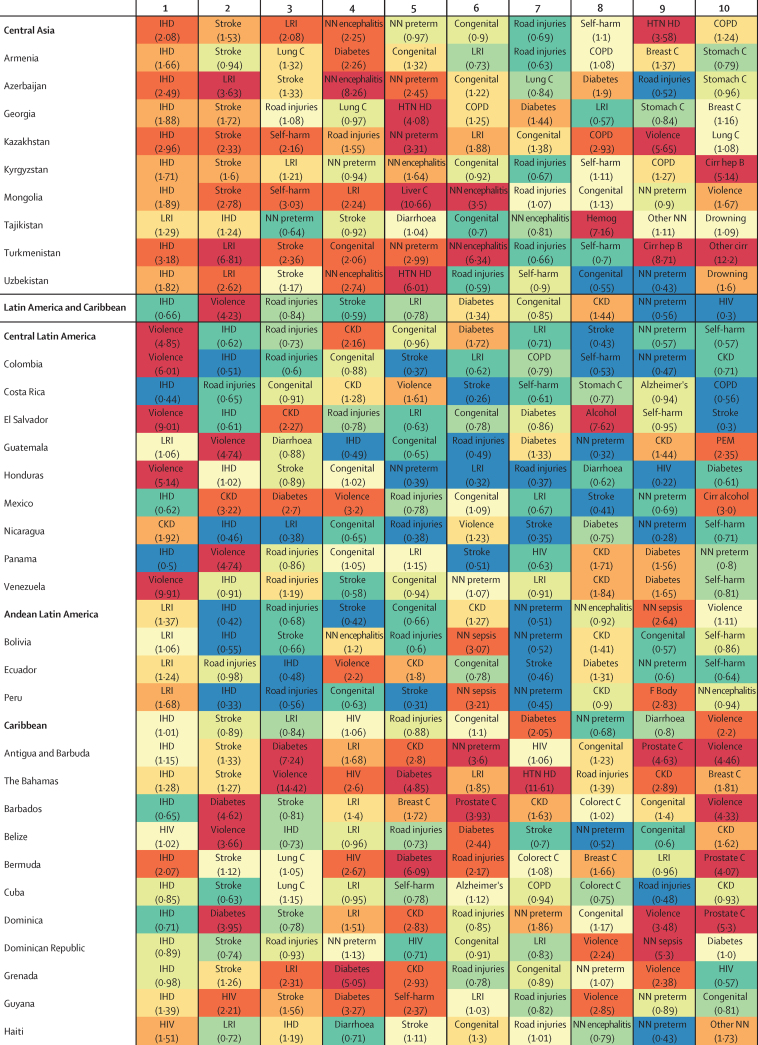

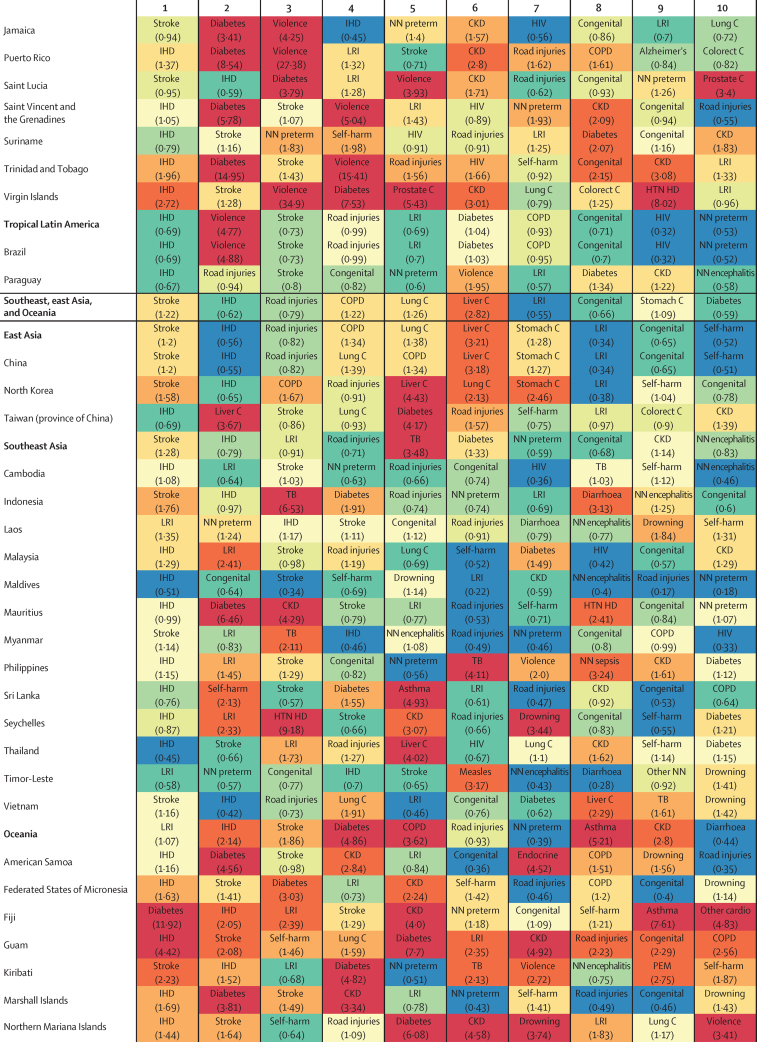

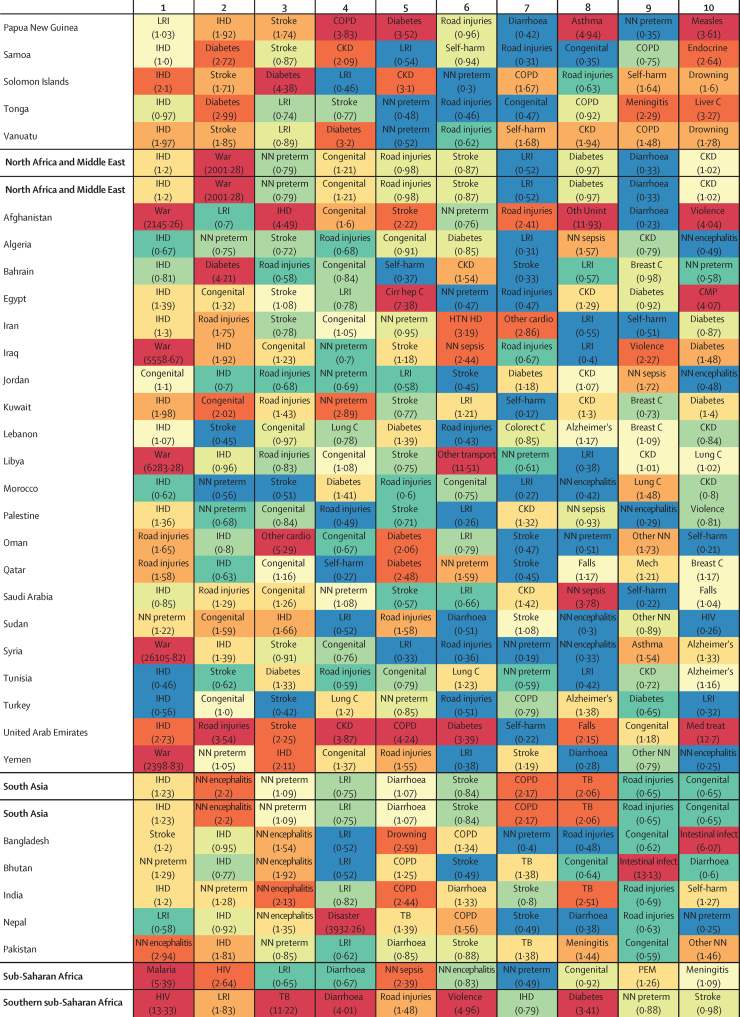

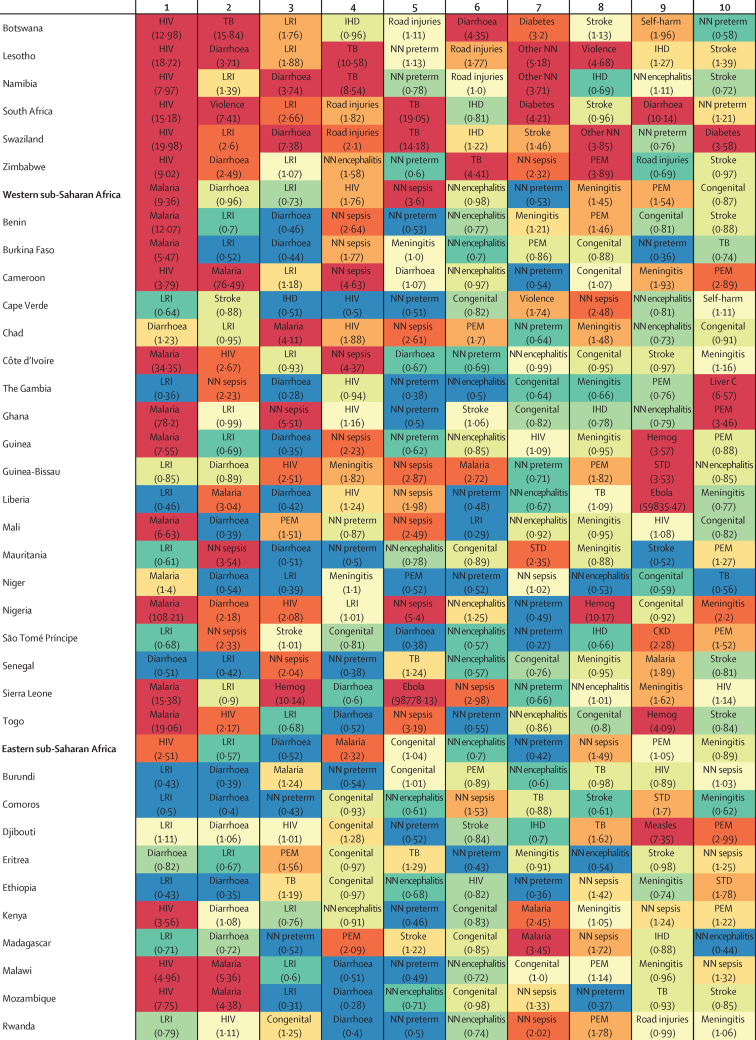

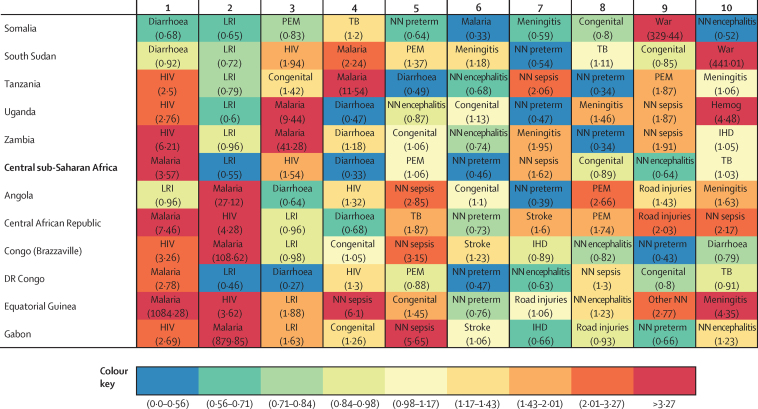
Leading ten causes of YLLs with the ratio of observed YLLs to YLLs expected on the basis of SDI in 2015, by location The ratio of observed YLLs to YLLs expected based on SDI is provided in parentheses for each cause, and cells are colour coded by ratio ranges (calculated to place a roughly equal number of cells into each bin). Shades of blue represent much lower observed YLLs than expected levels based on SDI, whereas red shows that observed YLLs exceed expected levels. SDI=Socio-demographic Index. YLL=years of life lost. IHD=ischaemic heart disease. LRI=lower respiratory infection. NN enceph=neonatal encephalitis. COPD=chronic obstructive pulmonary disease. Congenital=congenital disorders. C=cancer. Alzheimer=Alzheimer's disease and other dementias. HTN HD=hypertensive heart disease. Cirr hepB=cirrhosis due to hepatitis B. NN preterm=neonatal preterm birth complications. CKD=chronic kidney disease. TB=tuberculosis. Intestinal infect=intestinal infectious disease. NN sepsis=neonatal sepsis. Endocrine=endocrine, metabolic, blood, and immune disorders. Other cardio=other cardiovascular diseases. CMP=cardiomyopathies. Haemog=haemoglobinopathies and haemolytic anaemias. Cirr alcohol=cirrhosis due to alcohol use. Violence=interpersonal violence. Alcohol=alcohol use disorders. Other cirr=cirrhosis due to other causes. Cirr hepC=cirrhosis due to hepatitis C. Drugs=drug use disorders. F Body=pulmonary aspiration and foreign body in airway. PEM=protein-energy malnutrition. Mech=exposure to mechanical forces. Other transport=other transport injuries. Med treat=adverse effects of medical treatment. Leish=leishmaniasis. Other NN=other neonatal disorders. Iron=iron-deficiency anaemia. Whooping=whooping cough.

Distinct, yet notably varied, patterns emerged across and within GBD regions when we compared observed YLLs due to leading causes with the levels of premature mortality expected on the basis of SDI. Figure 17 shows the ratios of observed and expected YLLs for the ten leading causes by geography in 2015, colour coded by the magnitude of differences between observed and expected YLLs.

Globally, ischaemic heart disease and stroke were the leading two causes of premature mortality in 2015; 119 countries and territories also had ischaemic heart disease or stroke as the leading cause of YLLs that year. Three geographical regions featured countries that largely diverged from this trend: Latin America and the Caribbean, where interpersonal violence or lower respiratory infections frequently accounted for the most YLLs; north Africa and the Middle East, where war was the primary cause of early death in several countries; and sub-Saharan Africa, where HIV/AIDS or malaria was the leading cause of YLLs in 28 countries. Furthermore, lung cancer consistently ranked among the top three causes of YLLs in high-income countries; road injuries were a major cause of early death in Latin American countries; and neonatal disorders were frequently among leading five causes of YLLs in south Asia and sub-Saharan Africa.

Of the ten leading causes of premature mortality globally, lower respiratory infections resulted in the most countries (122) recording observed YLLs lower than those expected on the basis of SDI. This finding was particularly prevalent in east Asia, where China and North Korea had YLL ratios less than 0·40. Other leading causes for which observed YLLs were much lower than expected included stroke in Andean Latin America and neonatal preterm birth complications in Oceania. By contrast, HIV/AIDS led to the highest discrepancies for observed and expected YLLs, particularly affecting southern sub-Saharan Africa. Diarrhoeal diseases, particularly in southern sub-Saharan Africa, and neonatal encephalopathy due to birth asphyxia and trauma, particularly in southern Asia, also resulted in large differences for observed and expected YLLs.

### Regional, country, territory, and selected subnational results

For many high-income countries, observed YLLs due to stroke—a cause consistently among the leading three causes of early death—fell below the levels expected on the basis of SDI in 2015. Spain, France, Malta, and Israel had particularly low ratios for observed versus expected YLLs from stroke, all falling below 0·45; 26 countries, including Portugal, Argentina, and Uruguay had ratios lower than 0·80. A subset of countries, including Japan, South Korea, and Chile, also had lower observed YLLs from ischaemic heart disease than expected on the basis of SDI. Early death due to drug use disorders exceeded expected levels in the USA (5·71), Scotland (5·08), and Norway (3·44), and a similar pattern was found for YLLs due to alcohol use disorders in Denmark (10·50) and Finland (9·61). Within the GBD high-income super-region, Brunei, Greenland, and the USA had some of the largest deviations between observed and expected YLLs across causes. Within the UK, observed YLLs from self-harm and stroke were often substantially lower than expected for most regions, whereas observed levels of premature mortality due to COPD were higher than expected.

Throughout Latin America and the Caribbean, observed YLLs due to interpersonal violence far exceeded those expected on the basis of SDI, with 19 countries and territories recording ratios higher than 3·00. Furthermore, interpersonal violence ranked as the first or second leading cause of early death for seven of 11 countries in central and Tropical Latin America. For these two geographical regions, discrepancies between observed and expected YLLs from interpersonal violence were highest in Venezuela (9·91) and Brazil (4·88), respectively. Observed YLLs were also higher than expected for diabetes, especially in the Caribbean; chronic kidney disease, particularly in Mexico (3·22); and prostate cancer for several Caribbean islands and territories. Although ischaemic heart disease was a leading cause of early death for many Latin American countries, several had ratios of observed and expected YLLs lower than 0·60, including Peru (0·33), Panama (0·50), and Colombia (0·51). Similar results were found for stroke (eg, a ratio of 0·26 for Costa Rica); road injuries (eg, Honduras [0·37] and Cuba [0·48]); and for preterm birth complications (Haiti [0·43] and Guatemala [0·32]).

In 2015, leading causes of early death, as well as their observed and expected levels, were markedly heterogeneous in southeast and east Asia and Oceania. For many countries and territories, lower respiratory infections ranked among the ten leading causes of premature mortality, but observed YLLs fell below the levels expected on the basis of SDI (eg, 0·22 in the Maldives, 0·34 in China, and 0·46 in the Solomon Islands). For others, such as Malaysia and Laos, observed YLLs due to lower respiratory infections were higher than expected. Within the region, 18 countries and territories had ischaemic heart disease as their leading cause of YLLs in 2015, but their ratios of observed versus expected YLLs ranged from less than 0·50 in Thailand to more than 4·00 in Guam. Particularly in Oceania, observed levels of YLLs due to diabetes and chronic kidney disease consistently exceeded expected YLLs, and premature mortality due to liver cancer was higher than expected in Taiwan (province of China), Thailand, China, North Korea, Vietnam, and Tonga. Observed YLLs due to communicable diseases were at least twice as high as expected for some countries, including measles in Papua New Guinea (3·61) and tuberculosis in Indonesia (6·53) and the Philippines (4·11). At the same time, several countries and territories recorded cause-specific YLLs that were substantially lower than expected, such as preterm birth complications in Papua New Guinea (0·35); road injuries in Sri Lanka (0·47) and Samoa (0·31); and self-harm in Malaysia (0·52) and China (0·51).

Patterns of early death in south Asia reflect the region's diversity of countries and their relative stages of development. Although lower respiratory infections, diarrhoeal diseases, and congenital anomalies remained among the leading causes of premature mortality throughout south Asia, observed levels of YLLs were generally lower than those expected on the basis of SDI. However, for most countries in south Asia, observed YLLs due to neonatal encephalopathy were more than twice as high as expected (eg, 2·94 in Pakistan and 2·13 in India). Notably, observed YLLs from intestinal infections, such as typhoid fever, were above expected levels in Bangladesh (6·07). Observed levels of YLLs exceeded expected levels for a subset of NCDs, including ischaemic heart disease in Pakistan (1·81), and COPD in India (2·44). In 2015, an earthquake claimed more than 8700 lives in Nepal, and since the occurrence of earthquakes has minimal linkages to SDI, the ratio for observed and expected YLLs was extremely high for natural disasters. At the same time, Nepal and Bangladesh had substantially fewer than expected YLLs due to preterm birth complications (0·25 and 0·40, respectively). Observed levels of early death from stroke were also lower than expected in Bhutan (0·49) and Nepal (0·49).

In central Europe, eastern Europe, and central Asia, except for a subset of causes and countries, observed YLLs generally met or exceeded the levels expected on the basis of SDI. YLL ratios due to ischaemic heart disease and hypertensive heart disease were more than 2·00 for 17 countries, and observed premature mortality due to cardiomyopathy and myocarditis was at least three times higher than expected levels in Russia (10·86), Latvia (7·93), and Bosnia and Herzegovina (5·65). Alcohol and drug use disorders were among the ten leading causes of early death throughout the region, and observed levels often exceeded expected YLLs (eg, 24·53 for drug use disorders in the Ukraine and 17·95 for alcohol use disorders in Russia). Early death from cirrhosis due to alcohol use was more common than expected in several countries, including Moldova (4·24) and Hungary (3·40), whereas levels of YLLs due to interpersonal violence far exceeded expected levels in Russia (12·78) and Kazakhstan (5·65). Group 1 causes often led to higher levels of observed YLLs than expected in central Asia, such as neonatal encephalopathy in Azerbaijan (8·26) and lower respiratory infections in Turkmenistan (6·81). Notably, several countries had much lower than expected observed YLLs due to road injuries, including Albania (0·38) and Macedonia (0·47).

In north Africa and the Middle East, large discrepancies occurred between observed YLLs and those expected on the basis of SDI, underscoring the region's rapid development and inequalities in wealth. Furthermore, because of the region's escalating rates of war-related mortality, which is not strictly related to SDI, ratios of observed versus expected YLLs from war were extremely high. The United Arab Emirates (UAE) and Afghanistan had the most causes for which observed levels of YLLs exceeded expected YLLs; these causes ranged from ischaemic heart disease to interpersonal violence for Afghanistan, and included chronic kidney disease, COPD, diabetes, and road injuries for the UAE. Many countries in the region recorded substantially lower YLLs than expected for several causes: 13 had ratios less than 0·60 for lower respiratory infections, including Iraq (0·40) and Palestine (0·25); eight countries had ratios less than 0·60 for stroke, including Lebanon (0·45) and Turkey (0·42); and six had ratios less than 0·60 for preterm birth complications, including Egypt (0·47) and Syria (0·19).

For every country in sub-Saharan Africa, the leading cause of early death was one of four communicable diseases—HIV/AIDS, malaria, lower respiratory infection, or diarrhoeal diseases—yet patterns in observed and expected levels of YLLs strikingly differed within the continent. In southern sub-Saharan Africa, HIV/AIDS was the leading cause of premature mortality and resulted in exceedingly more YLLs than expected given SDI; Namibia, with a YLL ratio for HIV/AIDS of 7·97, was the lowest among these countries, whereas Swaziland had the highest ratio (19·98). Early death due to tuberculosis also surpassed expected levels, especially in South Africa (19·05), as did observed YLLs due to violence (eg, 7·41 in South Africa). Observed YLLs due to preterm birth complications were somewhat lower than expected in southern sub-Saharan Africa (eg, 0·58 in Botswana), but such levels were largely surpassed by countries in western and eastern sub-Saharan Africa. In 2015, 13 countries in western sub-Saharan Africa and 14 countries in eastern sub-Saharan Africa had YLL ratios lower than 0·60 for preterm birth complications. In western sub-Saharan Africa, observed premature mortality due to malaria was at least twice as high as expected in 13 of 19 countries in the region, with the highest YLL ratios in Nigeria (108·21) and Ghana (78·2). Neonatal sepsis caused more YLLs than expected based on SDI in several western sub-Saharan African countries, including Ghana (5·51). Despite the fact that the Ebola virus disease epidemic claimed more lives in 2014 than in 2015, Ebola virus disease remained among the leading cause of YLLs in Sierra Leone and Liberia. Although still among the leading causes of early death in western sub-Saharan Africa, YLLs due to lower respiratory infections and diarrhoeal diseases were much lower than expected for most countries, with YLL ratios as low as 0·29 in Mali and 0·28 in The Gambia, respectively. Similar trends emerged for eastern sub-Saharan Africa, although the toll of HIV/AIDS was generally higher; observed levels of premature mortality due to HIV/AIDS were at least five times higher than expected in Zambia (6·21) and Mozambique (7·75). Within eastern sub-Saharan Africa, eight of 15 countries had YLL ratios less than 0·60 for YLLs due to diarrhoeal diseases (eg, Rwanda [0·40] and Uganda [0·47]), emphasising the region's rising SDI. Furthermore, every country in eastern sub-Saharan Africa had observed YLLs due to preterm birth complications that were below expected levels, including Ethiopia (0·36) and Kenya (0·46). Nonetheless, YLL ratios for malaria were quite high for several countries, particularly Zambia (41·28) and Tanzania (11·54). Among countries in central sub-Saharan Africa, malaria resulted in many more YLLs than expected based on SDI, with Angola, Congo (Brazzaville), Equatorial Guinea, and Gabon reporting ratios that exceeded 20·00. Neonatal sepsis consistently caused more early deaths than expected within the region. Observed YLLs due to preterm birth complications fell below expected levels in Angola (0·39), Congo (Brazzaville; 0·43), and the Democratic Republic of the Congo (0·47). Notably, the Democratic Republic of the Congo, which had the world's sixth-highest death toll due to diarrhoeal diseases in 2015, had far fewer YLLs due to diarrhoea than expected (0·27).

## Discussion

### Main findings

Over the past 35 years, global life expectancy, age-standardised death rates, and age-standardised YLL rates have all substantially improved year on year, with the single exception of 1994 (high mortality from the Rwandan genocide, the Iraq civil war, and ongoing armed conflict in Bosnia and Herzegovina that year was enough to change the trend in global life expectancy). During 2005–15, life expectancy increased in 188 of 195 countries and territories and those increases were faster than expected on the basis of SDI improvements in 120 countries and territories. Such gains were mainly driven by marked reductions in mortality due to HIV/AIDS, malaria, infectious diseases such as lower respiratory infections and diarrhoea, neonatal disorders, cardiovascular disease, and cancers. Running counter to the general improvement, age-standardised death rates for 13 causes increased significantly in the past decade. In addition to these causes with increases, for some leading causes of death such as diabetes and chronic kidney disease, age-standardised death rates have stagnated globally and increased in some countries. Since 2011, global deaths from war have risen massively due to conflicts in Syria, Yemen, and Libya. Furthermore, gains in life expectancy were reversed for a subset of countries, with Syrian men bearing the largest toll (a 12·1 year reduction in life expectancy from 2010 to 2015).

Our analysis of the average relationships between age, sex, and cause-specific mortality and SDI suggest that the epidemiological transition does not affect all causes uniformly. Not all causes of death or YLLs improve as SDI increases. In fact, some causes such as cardiovascular disease and a subset of cancer types, initially tend to increase with rising SDI and then decline. This inverted U pattern might be related to changes in risk factors that initially increase disease incidence and then, with rising SDI, risk factor management and disease treatment might act to reduce age-specific mortality. The change in death numbers and population rates with the epidemiological transition was driven by both expected changes in age-specific rates and the highly regular shifts in population age structure that occur with rising SDI. Previous attempts to characterise the shifts in disease pattern with development^56^, ^57^, ^58^ have not been as comprehensive and have not been based on a comprehensive database such as that provided by GBD. Across causes and within geographical regions, we found huge country-level heterogeneities when observed levels of premature mortality were compared with those expected on the basis of SDI. The average patterns of age-specific mortality associated with a given level of SDI are a useful starting point for benchmarking a country's mortality pattern, but many other factors, including public health programmes, access to medical care, and inequalities could account for deviations from the pattern expected on the basis of SDI alone.

Nearly 40 years ago, Samuel Preston observed a shifting relationship between life expectancy and income per capita;^58^ in a series of subsequent analyses, the crucial role of technological change has been supported by this changing relationship.^58^, ^59^, ^60^, ^61^ With global life expectancy or other mortality metrics improving faster than expected on the basis of SDI, this general trend was tempered by stagnant or increasing mortality rates due to a subset of causes such as diabetes, cardiovascular disease, and some types of cancer. The overall shift in life expectancy masked larger gains for children and minimal progress for older adults compared with that expected on the basis of SDI. In fact, figures 10C and 10D show that global progress has been much less impressive for the probability of death from ages 15–50 years than for children and that progress in reducing the probability of death from ages 50–70 years only seems to be noticeable at high levels of SDI. In the younger adult age groups, temporal patterns over the past 25 years were profoundly shaped by the unfolding of the HIV epidemic in eastern and southern sub-Saharan Africa and roll-out of ART and PMTCT and the rise and subsequent fall in adult mortality in eastern Europe and central Asia. This is an important difference from previous notions of the inevitability of technological shifts driving up levels of health faster than expected on the basis of income and education alone.^58^, ^59^, ^60^, ^61^, ^62^ This finding comes at a time when two very different views of the future of health can be envisioned: rising threats such as climate change, food insecurity, water shortages, pandemics, human security, continued increases in obesity, or antimicrobial resistance that could undermine past health gains; and the realisation of the huge potential of new medical and public health breakthroughs driven by genomics, nanotechnology, and other technical developments.^63^, ^64^, ^65^, ^66^, ^67^, ^68^ Future health scenario construction will crucially depend on how the balance of these forces is played out.

The analysis of trends in numbers of global deaths by cause broken down into changes due to population growth, population ageing, and changes in age-standardised death rates highlights the critical importance of ageing in changing the mixture of causes of death and disease that health systems will have to manage. Likewise, the marked difference in patterns between age-standardised rates to crude rates of YLLs shows how ageing amplifies the speed of the shifts from communicable, maternal, neonatal, and nutritional diseases to NCDs. The combination of demographic and epidemiological change can also lead to extremely rapid changes in disease profiles in some countries. China is a clear example of having an accelerated change in disease profiles: from 1990 to 2015, the percentage of YLLs due to NCDs increased from 50·0% (48·5–53·0) to 77·3% (76·5–78·1). The potential rapidity of these changes presents challenges to many ministries of health in terms of human resources for health planning and policy formulation. Understanding where this potential is about to unfold will be aided by systematic demographic and epidemiological forecasts grounded in the patterns recorded in GBD.

### Communicable diseases

Although our methods for the assessment of HIV/AIDS mortality changed substantially from GBD 2013, our general findings at the global level have remained similar across iterations of GBD.^11^ With the global epidemic of HIV/AIDS mortality peaking in 2005, at 1·8 million deaths (95% UI 1·7 million to 1·9 million), deaths due to HIV/AIDS had decreased by 2015, dropping to 1·2 million (1·1 million to 1·3 million). Yet despite major progress, especially amid the scale-up of ART and PMTCT in sub-Saharan Africa, HIV/AIDS remains the leading cause of YLLs in 16 countries and among top five causes of mortality in 38 countries. Large numbers of individuals continue to die from HIV/AIDS each year in eastern and southern sub-Saharan Africa, where many countries still experience large-scale epidemics, even with the rapid scale-up of ART. We estimate that 17·8% (13·6–20·6) of these HIV/AIDS deaths in 2015 were due to tuberculosis in HIV-positive people, but little data exist for the immediate causes of other HIV/AIDS deaths. Continued high death rates in sub-Saharan Africa highlight the critical importance of improved quality of care and early initiation of therapy, irrespective of disease progression.^69^ In the face of calls for the end of HIV/AIDS by 2030, more rapid progress is urgently needed.^70^, ^71^, ^72^, ^73^ Stagnating development assistance for health for HIV/AIDS programmes amplifies the challenge of reducing HIV/AIDS mortality.^74^, ^75^

Our results suggest a notable decrease in malaria mortality in sub-Saharan Africa since 2003, consistent with documented reductions in incidence and prevalence, as well as observed increases in effective treatment and preventive interventions.^76^ We found that malaria mortality peaked in 2003, which is slightly earlier than reported in previous GBD estimates. However, the timing of peak malaria mortality varied across countries (eg, 2005 in Angola and 2000 in Uganda), which might reflect a combination of factors. Estimates presented here for sub-Saharan Africa are based on an estimation method that maps from all untreated cases to mortality and will be refined in further iterations of GBD (eg, the introduction of an intermediate step of severe malaria).

The population attributable fraction of diarrhoeal aetiologies increased for most aetiologies compared with GBD 2013. This is mainly because of two factors. First, we used the new TAC diagnostic for the detection of pathogens in GEMS compared with the conventional laboratory diagnostic methods used in GBD 2013. The TAC method is more sensitive and specific than the conventional methods, such as bacterial culture or ELISA, which tends to increase the odds ratios used in the attributable fraction estimation by correcting for the pathogen misclassification.^77^, ^78^ Second, we used a correction factor to adjust for false negatives and false positives of the prevalence of pathogens in patients with diarrhoea and to make it comparable with the odds ratios from the TAC diagnostic method. We corrected our modelled prevalence estimates for the imperfect sensitivity and specificity of the laboratory diagnostic results compared with TAC because most studies reported diarrhoea based on previous diagnostics. Therefore, the correction for the prevalence of the pathogens widened our uncertainty of the final estimates. Although qPCR is a well established diagnostic for diarrhoeal pathogens, the application of TAC remains a novel approach and further testing of the appropriate cutoffs for continuous measures of pathogen presence is needed.

The attributable fractions for *Aeromonas,* amoebiasis (*Entamoeba histolytica*), and *H influenzae* type b were not significant in children younger than 5 years at the global level. Indeed, this is biologically implausible given that these pathogens do not have a protective effect against mortality. The odds ratios of diarrhoea given the presence of *Aeromonas* and amoebiasis were not significant in children aged 0–1 years and 1–2 years but were significant in the 2–5-year age group, highlighting that these pathogens might not be significant contributors across all age groups. The attributable fraction for *H influenzae* type b is based on a meta-analysis of randomised controlled trials of vaccine efficacy where the CI of the pooled estimate was not statistically significant. This is a potential limitation and future analyses could use alternative meta-analytical methods, such as log-log meta-analysis or imposition of a non-negative population attributable fraction prior, to prevent negative population attributable fraction estimates.

Our estimation of the fraction of deaths due to lower respiratory infection attributable to pneumococcal pneumonia relies on data from vaccine efficacy studies that show a decrease in all pneumonia and among invasive (bacteraemic) pneumococcal disease in children and adults. However, at least one study that used a urine antigen test in elderly adults found that the relative vaccine efficacy of pneumococcal conjugate vaccine against invasive pneumococcal disease was 66% greater than against pneumococcal pneumonia.^49^ For GBD 2015, we corrected the estimated pneumococcal conjugate vaccine efficacy against invasive pneumococcal disease by this ratio in both child and adult age groups. This is a change in our GBD 2015 methods, although no studies have used this diagnostic test to detect non-invasive pneumococcal pneumonia in children. To reflect the uncertainty of this adjustment, we used a uniform distribution around the point estimate of the ratio. This adjustment contributed to an increase in our estimates of pneumococcal pneumonia mortality because the vaccine efficacy against pneumococcal pneumonia could be lower than has previously been estimated.^49^ Given the much larger attributable fraction after the adjustment was made and wide uncertainty of the final estimates, further studies to confirm and precisely quantify the difference in vaccine efficacy between invasive and non-invasive disease are needed. Data availability limitations, particularly for vaccine efficacy data across age groups, hindered our ability to conduct pathogen attribution analyses for *H influenzae* type b among populations aged 5 years and older; a similar lack of data made estimating population attributable fractions and pathogen-specific mortality for neonates analytically infeasible.

While mortality rates from most infectious diseases fell, dengue emerged as a notable counterexample. The combination of new data and an improved dengue trend covariate (methods appendix p 82) enabled our model to better capture these trends. Compared with GBD 2013 estimates, our GBD 2015 dengue mortality estimates are higher for the most recent decade, yielding a pronounced upward trend that is more consistent with case reports, GBD 2013 incidence estimates, and expert consensus.^79^ The increasing geographic range of dengue and, in some areas (eg, Latin America) increasing transmission intensity, contribute to growing concerns about other viruses that are transmitted by *Aedes* mosquitoes, including the chikungunya and Zika viruses.^80^ Progress in dengue vaccine development, including the licensure of the CYD-TDV vaccine in 2015,^81^ is promising, but the eventual impact of such vaccines remains unclear.

In GBD we follow the ICD principles of underlying cause of death. According to this principle, a single underlying cause is assigned to each death. In some cases, somewhat arbitrary rules are used to deal with interactions between pathogens or disease processes—eg, all deaths from opportunistic infections, such as fungal infections, in HIV-positive patients are assigned to HIV/AIDS as the underlying cause. Underlying cause assignment is distinct from the notion of excess mortality, whereby a condition or disease can be associated with increased risk of death from other pathophysiological processes. In some cases, the underlying cause of death rules embedded in the ICD could lead to lower death estimates for some neglected tropical diseases. For example, anaemia is a sequela of various different conditions, some of which are parasitic. Hookworm infections can cause iron-deficiency anaemia,^82^ which is then recorded as the underlying cause of death. Similarly, infections by *Clonorchis* spp have been associated with increased risk of cholangiocarcinoma^83^ and are classified as a Group 1 carcinogen by the International Agency for Research on Cancer (IARC);^84^ however, the cause of death will be listed as cholangiocarcinoma and will be captured within the total cancer estimates. Alternative post-hoc analyses might be useful for some of these neglected tropical diseases to characterise the excess mortality that could be related to the disease.

Pandemics were incorporated into GBD as fatal discontinuities in the same way as armed conflicts and natural disasters.^7^, ^85^ In late 2013 an epidemic of Ebola virus disease started in west Africa. Slow national and international responses allowed the virus to spread throughout 2014 and to become a Public Health Emergency of International Concern,^86^ which was eventually brought under control in 2015 after a sustained multilateral effort. These estimates of mortality are of the direct deaths attributable to Ebola virus disease and do not account for the full effects of mechanisms such as the breakdown of health systems or critical infrastructure and their subsequent potential health repercussions.^87^, ^88^ Previous estimates of mortality^86^ combined with these destabilising effects on wider health-care provisioning within the three most affected countries have already resulted in appropriate introspection among the international community on the ability of institutions to cope with pandemic threats.^89^ Correctly establishing the relative public health importance of pandemic preparedness investments is non-trivial, because even if there are no deaths in a year from a potential pandemic cause, such as pandemic influenza, substantial excess mortality risk remains.^90^ The need for better surveillance and preparedness infrastructures to help to mitigate the risk posed by potential pandemic infectious diseases has been strongly advocated^91^ and has been underscored again in 2016 with the rapid spread of Zika virus across the Americas and the declaration of another Public Health Emergency of International Concern.^92^

In GBD 2015, we have not estimated the burden of drug-resistant tuberculosis as a cause distinct from overall tuberculosis. Nor have we estimated deaths related to drug-resistant bacteria or malaria. The challenge that antimicrobial resistance poses both to current and future health-care systems is becoming increasingly documented and quantified,^93^ with an estimated 3·3% of new tuberculosis cases and 20% of previously treated cases having multidrug resistance.^94^ Incorporating the proportion of cases caused by pathogens with drug resistance therefore represents a significant, but increasingly important, objective for future estimates.^95^ Within the GBD framework, drug resistance might be best assessed by examining antimicrobial resistance as a risk factor rather than estimation of subtypes of each pathogen.

Researchers have long foreseen health effects caused by changes in the environment, with a particular focus on climate change.^96^ To date, our results do not show these trends being realised. Instead they show a substantial global trend of improvement in SDI and notable improvements in many of the infectious disease causes that would be most sensitive to climate change such as malaria,^76^ in a process known as the environmentalist's paradox.^97^ This lack of any association so far could be related to time lags between global environmental change and health outcomes and is widely hypothesised to be at the expense of degradation of ecosystem services.^98^ It might also simply be much harder to detect changes against the backdrop of rapid improvements that are expected with SDI. The value expected from SDI might therefore help to identify which countries or subnational areas are improving at rates that are slower than expected.

### Non-communicable diseases

From 2005 to 2015 age-standardised death rates due to most types of cancer decreased, a reflection of risk factor reduction, as well as improvements in some settings of health systems equipped for early diagnosis and effective treatment of cancers. With respect to YLLs due to cancer, lung cancer remains the leading cause globally and for most countries, with YLLs increasing by almost 15% over the past decade. Given that most cases of lung cancer can be prevented,^99^ this observation stresses the importance of tobacco and air pollution control. For other types of cancer, the geographic pattern is more diverse and reflects the vast differences in risk factors, as well as health system capacity. Whereas colorectal, breast, and pancreatic cancer are the leading causes of cancer YLLs for most high-income countries, stomach, liver, and oesophageal cancer dominate in countries with low SDI. It is encouraging that global YLLs due to stomach cancer have decreased over the past decade, with the substantial decrease in age-specific death rates counterbalancing population growth and ageing. Oesophageal cancer is the other example for which YLLs decreased from 2005 to 2015, with the decrease in age-specific death rates offsetting the increase due to an ageing and growing population. Translating the observed changes in liver cancer mortality into public health planning and policy requires knowledge of the underlying aetiologies, as risk factors differ substantially between locations. This has become an even higher priority now that effective treatment for hepatitis C is available.^100^ The increase in numbers of liver cancer deaths due to hepatitis B from 2005 to 2015 was not significant, which contrasted with the significant increases in deaths, often 20–30%, observed for many other types of cancer: this relative progress might be at least partly attributable to hepatitis B vaccination. A similar development for liver cancer due to hepatitis C can be achieved in the coming decades if hepatitis C treatment, which currently has high costs per patient,^100^ becomes accessible in high prevalence populations.

Data newly available to GBD 2015 improved our understanding of global patterns of cardiovascular disease. High proportions of death due to cardiovascular disease have been observed in Oceania. Previously, very few data sources were available from Kiribati and Fiji,^101^ but with additional data from Tonga, Guam, American Samoa, and the Northern Mariana Islands, it seems that 27–30% of deaths in this region were due to cardiovascular disease causes. Newly available data from India show that, similar to surveillance data previously available from Bangladesh, cardiovascular disease accounts for a large and increasing proportion of deaths. Updated data from China now show a clear trend toward decreasing risk of cardiovascular disease death in all age groups since 2010, when comprehensive vital registration became available. In GBD 2015 we examined age-standardised YLL rates by level of SDI. YLL rates for ischaemic heart disease were lowest in countries with the lowest SDI when country populations were examined by SDI, rising steadily at higher levels of SDI, and only reduced for populations in the quarter of countries with the highest SDI. This pattern was more pronounced among males than females and differs from that seen for stroke, where YLL rates decrease gradually at higher levels of SDI and then fall steeply for the highest SDI populations. The result is that for overall cardiovascular disease, YLL rates were lowest in both the lowest and highest sociodemographic groups with an increase for those in the middle of the sociodemographic rankings. One hypothesis is that medical care in the highest SDI populations might have increased life expectancy to the point where cardiovascular disease is most prevalent, while people in the lowest sociodemographic group are dying from other conditions before reaching an age where they would develop ischaemic heart disease and stroke.^102^ In this scenario, people living in countries categorised in the middle range of the sociodemographic rankings are surviving long enough to develop ischaemic heart disease but might not have access to optimal medical or surgical treatment.

Deaths due to cirrhosis increased globally from 2005 to 2015, whereas age-standardised cirrhosis mortality rates fell during the same period. Underlying the global picture, though, are notably distinct regional trends, with substantial reductions in cirrhosis mortality in east Asia and central Europe, for example, and increases in cirrhosis mortality in central Asia and north Africa and the Middle East. These trends largely reflect changes in the major risk factors for the disease, with some cirrhosis risk factors, such as chronic hepatitis B infection, decreasing in the face of widespread vaccination, and other risk factors, such as alcohol consumption and chronic hepatitis C, increasing in some parts of the world.^103^ The decades-long lag between hepatitis B infection and cirrhosis death, and the increasing use of the hepatitis B vaccine, suggest that the potential effect of vaccination on cirrhosis mortality is only beginning to become apparent, and hepatitis B-attributable cirrhosis deaths should continue to fall. Notably, with nearly a quarter of cirrhosis deaths being due to chronic hepatitis C infection, improvements in blood screening and new short-course oral treatments for hepatitis C have the potential to reduce cirrhosis mortality in the future.^103^, ^104^

Globally, total deaths from Alzheimer's disease and other dementias increased by almost 40% from 2005 to 2015. This increase is due to population increases and ageing accompanied by a small but significant decrease in age-standardised death rates from Alzheimer's disease and other dementias, possibly reflecting the reduced burden of cardiovascular disease and the contribution of vascular brain injury to the dementia syndrome.^105^ A small reduction in mortality rates is consistent with reports of a fall in the prevalence of dementia from two cross-sectional surveys a decade apart in the UK and a decline in incidence of dementia reported from the Framingham Cohort Study in the USA.^106^, ^107^ There is also evidence that increased education and healthier lifestyles can reduce disease incidence and delay disease onset,^105^, ^107^, ^108^ partly reflecting the reduced burden of cardiovascular disease and the contribution of vascular brain injury to the dementia syndrome. Although the reductions in rates are welcome news, the substantial increase in the number of deaths from Alzheimer's disease and other dementias and the associated increase in prevalence present challenges for health systems and social support systems that need to address the needs of these patients and their families.

We estimate small numbers of death due to mental disorders as the underlying cause. We include schizophrenia as a cause of death because high-quality vital registration data every year show a stable, small number of deaths from this cause. Many more deaths occur in people with schizophrenia in excess of what would be expected on the basis of general population mortality rates. These deaths are certified and coded to other diseases and injuries as the underlying cause, such as self-harm, unintentional injuries, infectious diseases, substance use, cardiovascular disease, and cancers, for which excess mortality in people with schizophrenia has been reported.^109^, ^110^, ^111^ Most deaths from self-harm can be attributed to underlying mental and substance disorders such as depression, anxiety disorders, schizophrenia, bipolar disorder, and alcohol and drug use disorders, as has been quantified based on GBD 2010 estimates.^112^ In future iterations of GBD, it might be useful to more systematically and regularly quantify the excess mortality associated with several mental disorders.

The estimate of 1·5 million deaths (95% UI 1·5 million to 1·6 million) due to diabetes, plus 418 000 deaths (389 000–441 000) from chronic kidney disease due to diabetes mellitus, still underestimates the full impact of diabetes on all-cause mortality because of the increased risk of ischaemic heart disease, stroke, and tuberculosis associated with diabetes.^113^, ^114^, ^115^ In the GBD framework, computation of deaths attributable to elevated fasting plasma glucose more comprehensively captures the effects of diabetes and pre-diabetes on mortality—see the GBD comparative risk assessment for 2015.^116^ Given the strong association between diabetes and obesity and the global rise of obesity, the finding that age-standardised death rates for diabetes, including chronic kidney disease due to diabetes mellitus, are not increasing suggests that other protective factors such as treatment might have an effect.^117^, ^118^ Spatial patterns show marked variation in death rates, even in places with relatively similar prevalence such as Mexico and the USA.^119^ These variations in death rates could be related to variation in cause of death certification, with some medical communities more likely than others to list diabetes as the underlying cause of death, or they might be due to treatment effects. Given the importance of diabetes as a cause of death already, and the likely global rise in prevalence of diabetes, more research is needed to understand the determinants of variation in diabetes death rates.^119^

In this analysis we have estimated that there were more deaths due to chronic kidney disease than in previous analyses because of improved estimates within countries with large populations such as China, India, and Russia. We have also improved our chronic kidney disease subtype estimation strategy by implementing consistent data source inclusion, as well as estimation strategy across the four subtypes. A further improvement is that we have narrowed the definition of deaths due to “chronic kidney disease other” so that deaths formerly included in this category are now attributed to original disease cause, such as polycystic kidney disease. Thus chronic kidney disease subtype mortality results will differ notably from those of previous analyses. Our results indicate that, globally, deaths from chronic kidney disease increased among both males and females, but age-standardised death rates remained relatively unchanged in the past decade. Likely contributors include an increase in the burden of chronic kidney disease risk factors such as diabetes mellitus and hypertension. In 2015 Latin America had the highest chronic kidney disease death rates in the world. Within Mexico, the country with the highest chronic kidney disease death rate, more than half of patients with incident end-stage renal disease have an underlying diagnosis of diabetes mellitus.^120^ Unique aetiological contributors, such as those suspected in chronic kidney disease due to other causes, have been shown to cause chronic kidney disease deaths mostly in younger adults in El Salvador and Nicaragua, as well as Sri Lanka.^121^ Efforts to delay deaths due to end-stage renal disease currently depend on renal replacement therapy in the form of either maintenance dialysis or renal transplantation. These costly interventions require appropriate medical infrastructure, as well as government subsidisation, to be accessible to the general population. Therefore, these interventions are currently accessible mainly to populations in high-income countries.^122^ If deaths due to chronic kidney disease continue to increase globally, further research into possible ways to prevent chronic kidney disease, such as with population-level or individual-level approaches, is warranted. Given that many chronic kidney disease risk factors overlap with cardiovascular risk factors,^123^, ^124^, ^125^ closer collaboration between these two specialties could foster preventive strategies in the future.

### Injuries and fatal discontinuities

Experience and evidence from studies on intervention suggest that there is a potential to reduce road injury deaths through a range of interventions. Drunk driving bans, seat belt laws, road engineering including traffic calming, safety devices in vehicles, speed limits, mandatory helmet usage, bans on mobile phone use while driving, and separation of vulnerable road users from vehicles have all been shown to be effective.^126^, ^127^, ^128^, ^129^, ^130^ Progress in reducing road injury can be rapid. From 2005 to 2015, western European countries such as Spain (43·8%, 95% UI 39·9–47·3), Portugal (39·6%, 35·4–43·6), and Switzerland (18·8%, 11·8–25·4), had significant reductions in total deaths. Such rapid decreases indicate that not only are there specific interventions that can work,^131^, ^132^ but also that population-level reductions are possible in a short period. A reverse trend is apparent in low-income and middle-income countries, partly because the growth in motorisation and traffic density is outpacing the reductions associated with infrastructural development and levels of law enforcement. This trend is particularly the case for major fast-growing BRICS economies (Brazil, Russia, India, China, and South Africa).^133^, ^134^, ^135^

Global death rates due to interpersonal violence have decreased since 2005, but regional trends were much more diverse. Reductions were largest in Asian and European countries, but rates of deaths due to interpersonal violence in Latin America and southern sub-Saharan Africa remained quite high. Interpersonal violence can often be mitigated or reduced by addressing underlying drivers or risks, such as the accessibility of weapons and use of alcohol and psychoactive drugs.^136^, ^137^, ^138^, ^139^ In 2015, self-harm was the second-leading cause of death from injury. Nearly half of all self-harm deaths occur in India and China, but the trends in these countries have reversed, decreasing significantly in China but rising in India from 1990 to 2015. Over the past two decades China and India have both experienced rapid economic growth and urbanisation, and therefore the opposing trends might be explained by other factors. Evidence from previous research has shown that a combination of preventive approaches addressing multiple factors, such as increased public awareness, programmes based on behavioural change and coping strategies, physician education, and decreased access to means of self-harm is needed.^140^, ^141^, ^142^, ^143^

Age-standardised death rates from drowning fell by nearly 30% globally during the past decade, with greatest reductions occurring in China, southeast Asia, central Asia, and eastern and central Europe. The highest rates of death due to drowning in 2015 were in island nations of Oceania and in the Indian Ocean, southeast Asia, Afghanistan, Bangladesh, and sub-Saharan African countries. Globally, drowning is a leading cause of death in children younger than 5 years. There is potential to reduce drowning deaths by preventive measures such as installing barriers, controlling access to water, education, swimming lessons and safe boating practices, and shipping and ferry regulations.^144^, ^145^, ^146^ However, change in access to open water with increasing urbanisation might be a more important factor driving down drowning deaths than specific prevention measures.

For GBD 2015 we systematically collected data on major transport accidents, natural and man-made disasters, wildfires, pandemics, and wars. Although the accuracy of the death numbers due to some of these causes vary by country (ie, they are usually more accurate in stable or higher income countries), they tend to be more reliable than are the estimates for wars. Indeed, it has been challenging to accurately document the number of casualties from wars and deaths resulting from malnutrition, infections, or disruption in health services during wars.^147^, ^148^, ^149^, ^150^ The challenge is due to scarcity of vital statistics during wars, the increase in refugee populations who are displaced internally or externally, and the fact that surveys can only capture mortality rates among those who have remained in their households during the time of interviews. Unfortunately, these estimates can also be challenging to capture with a future census because refugees might have settled in other countries. For example, it is estimated that more than 3 million Syrians have settled in neighbouring countries or elsewhere.^151^, ^152^ Nonetheless, we believe that providing such estimates, even with a wide UI, will draw attention to the devastating effects of war on health and hopefully lead to better methods to estimate morbidity and mortality from wars.

### Measurement challenges and opportunities

The assessment of all-cause mortality in GBD 2015 includes important innovations that have substantially increased uncertainty for adult mortality in some countries. We believe this is a much more accurate reflection of our knowledge of age-specific mortality in countries without vital registration or sample registration systems. The most important changes included processing of sibling history data using single years rather than pooling data for 5-year intervals, and the propagation of uncertainty in HIV/AIDS crude death rates used in the first stage of the spatiotemporal Gaussian process regression model into the estimation of 45q15. In addition to these changes, we have greatly improved our parameter selection process for both 5q0 and 45q15 Gaussian process regression to reflect data density and quality of data partly represented by the data variance. Combined, these changes led to an increased uncertainty interval for 45q15. In many countries in Africa, the width of the uncertainty interval for 45q15 more than doubled. The increase in the uncertainty interval for 45q15 directly leads to higher uncertainty intervals for our age-specific mortality rate estimates. This partly helped with the removal of an arbitrary matching algorithm from GBD 2013, which picked the pairs of draws of all-cause mortality and HIV-specific mortality estimates that were consistent. With the widened uncertainty interval in all-cause mortality estimates, we are able to apply the ensemble model to combine draw-level HIV/AIDS mortality estimates from the demographic estimation process and the epidemiological model of EPP-Spectrum for all 1000 draws.

Cause of death data for low SDI countries in sub-Saharan Africa were limited to a small number of mostly local verbal autopsy data often with small sample size. Members of the INDEPTH network of demographic surveillance systems have been collecting verbal autopsy data in some sites for many years.^153^ In the development of the GBD cause of death database, we have been able to make only limited use of INDEPTH verbal autopsy data collected in multiple sites. Some INDEPTH members like Matlab routinely release physician-certified verbal autopsy data in full detail whereas others have published periodic results in journal articles. In 2015, 22 INDEPTH sites published INTERVA model predictions of individual causes of death for the period 1992 to 2012.^153^ No primary data from the verbal autopsy interviews have been released to date. Unfortunately, INTERVA model predictions are highly inaccurate. The only validation study comparing INTERVA to an objectively defined cause of death standard at the individual level found it accurately assigned the cause of death in 23·8% of adult deaths, 30·3% of child deaths, and 19·4% of neonatal deaths; at the population level, estimated cause-specific mortality fractions were not better than random guessing.^154^, ^155^ This potentially important resource for global health estimation is being underused because of the limitations of current versions of the INTERVA tool for cause ascertainment. Given that injury death assignment might (although there are no objective validation data on this issue) be more plausibly established from INTERVA, we have chosen to use the INTERVA results from INDEPTH for eight types of injuries: transport injuries; falls; drowning; fire, heat, and hot substances; poisonings; venomous animal contact; self-harm; and interpersonal violence. Hopefully, a large number of deaths from INDEPTH verbal autopsies will be assessed using physician-certified verbal autopsy or more robust computer algorithms in the future.

In GBD 2013, for select causes, we developed separate CODEm models for countries with long series of complete vital registration data in order that UI estimation in these settings was not affected by UI in settings with either less complete or lower quality data. For GBD 2015, we standardised this approach. We defined countries with extensive complete vital registration representation as those with vital registration equal to or more than 95% complete for more than 25 years. We also modified the ranges of psi used in testing different ensemble models to be higher, effectively allowing CODEm to evaluate out-of-sample ensembles made up of fewer models. In countries with nearly complete high-quality time series, smaller numbers of models in the ensemble allow the models to follow the data more closely with narrower UIs for many but not all causes.

It is extremely difficult to properly inform national and global policy responses to reduce mortality when available cause of death information is very sparse, outdated, or unreliable. Fortunately, there is now evidence of increased investment and technical support being offered to countries to improve their vital registration systems. Important new partnerships have been formed between major bilateral donors and philanthropic organisations,^156^ building on earlier efforts of the Health Metrics Network and the Australian development assistance program, AusAID.^157^, ^158^ Regional UN-led partnerships, particularly in Africa and the Asia-Pacific region, are also helping governments to understand the essential role of good vital registration systems in development, and advocating for change. The interventions now being offered in many countries include targeted and strategic training of doctors to correctly fill out death certificates that identify the underlying cause of death; improving practices and exploiting information technology advances to more effectively register deaths, consolidate and validate data, and transfer information more efficiently to policy-relevant destinations; and, perhaps most importantly, facilitating the widespread adoption of automated verbal autopsy methods to cost-effectively provide information on causes of death in populations for which these data are unavailable.^2^ Although the initial focus of these efforts is on improving data systems, a substantial impact on data quality and availability can be reasonably expected within the next 3–5 years.^159^ This will not only improve the evidence base for guiding local policy decisions, but will also lead to reduced uncertainty in global comparative mortality assessments, such as the GBD study.

WHO led the process of developing new guidelines for reporting on global health estimation.^20^ For GBD 2015, we invested substantial resources to ensure that the GBD 2015 studies, including this Article, are compliant with the GATHER recommendations. Figure 1, Figure 2 provide detailed workflow diagrams. In appendices and the Global Health Data Exchange, we also provide detailed documentation of all sources used in the analysis. For each step in the workflow diagrams, computer code is either on GitHub, such as CODEm 2015 and DisMod-MR 2.1,^160^, ^161^ or available on the IHME website for download. Enhanced transparency and documentation highlight the multiple interconnections between analyses, for example, the strong connections between HIV/AIDS incidence, prevalence, and mortality estimation and all-cause mortality estimation in countries with large epidemics. Across this assessment, there are hundreds of separate analytical steps; for each we documented input data, code, and information on model development and performance.

### Limitations

Here we highlight some broad cross-cutting limitations to the GBD mortality and cause of death analysis. The analysis of all-cause mortality in countries without vital registration systems is critically dependent on the validity of sibling history data for measuring adult mortality. Although we show, with appropriate corrections for survivor bias (methods appendix pp 21–24), that sibling history data are unbiased when compared with vital registration data, these comparisons are not available for sub-Saharan Africa, where sibling history data are of key importance. Sibling histories in some African countries might underestimate mortality due to the practice of adoption in some countries, but empirical studies in these settings have not confirmed any consistent underestimation of adult mortality.^162^

All-cause mortality in settings with vital registration is corrected for under-registration using the three available demographic methods for the detection of under-registration: generalised growth balance, synthetic extinct generations, and a hybrid approach of these two methods. However, these methods, as shown in simulation studies,^26^ are unbiased but imprecise. To further stabilise our estimates of vital registration completeness over time, we synthesise raw estimates of completeness from death distribution methods and implied child death registration completeness by comparing vital registration to GBD under-5 mortality estimates into one coherent time series. Despite these efforts, estimates of completeness can change between GBD revisions as new census data become available or new surveys are released. We propagate uncertainty into the all-cause mortality in the analysis of completeness, but these UIs might be underestimates in some settings because of the scarcity of available data.

Because of the close connection between the estimation of all-cause mortality and HIV/AIDS incidence, prevalence, and mortality in countries with large epidemics, all limitations pertaining to HIV modelling also apply to our estimates of all-cause mortality. For GBD 2015, we used an ensemble model for HIV in which the demographic model and the natural history model of HIV/AIDS death are combined. Despite these attempts to triangulate on the magnitude of the HIV/AIDS epidemic, we assume that the CD4 progression rate, off-ART death rates, and on-ART death rates for age–sex–CD4 categories are the same throughout sub-Saharan Africa. Because of other factors, such as co-infections with tropical diseases or nutritional status, CD4 progression rates can vary across populations. It is also likely that the quality of and access to ART programmes varies across communities, and thus on-ART death rates might well vary. Because of the strong assumptions made in the natural history models (assumptions also made by UNAIDS^163^) about the consistency of these parameter values across countries, we might be underestimating the true variation in HIV/AIDS death rates and all-cause mortality.

Our cause of death analysis depends substantially on the validity of medical certification of causes of death and physician-certified verbal autopsies. Although efforts to redistribute garbage codes to likely underlying causes of death help to enhance comparability, our findings are affected by systematic bias in medical certification of causes of death. We see in the rapid increase in certain causes of death on death certificates—such as Alzheimer's and other dementias, or atrial fibrillation—evidence of diagnostic trends that are generally incompatible with time series data even after garbage code redistribution. For these reasons, we used other methods to estimate these particular causes of death. However, these patterns suggest that garbage coding practices not only vary by country but also across time.

Our results depend critically on the validity of our approach to garbage code redistribution. As we further utilised statistical methods to establish redistribution algorithms over the development of the GBD 2015 results, we saw sizeable changes in major causes of death. For example, changes in how left-sided heart failure and right-sided heart failure are analysed substantially changed the number of deaths reassigned to pneumoconiosis, haemoglobinopathies, or COPD. We believe these changes reflect improvements in our methods, but they also show how some causes of death, even though they result in lower absolute levels of mortality, can be profoundly affected by the redistribution of large garbage codes such as heart failure or sepsis. The sensitivity of our findings to how major garbage codes, such as heart failure, sepsis, or ICD-X59 (exposure to unspecified factor), are redistributed emphasises the importance of more systematic research on garbage code practices and improvements in primary data collection to avoid deaths being certified to garbage codes.

We used CODEm to model all causes for which sufficient numbers of deaths are observed in vital registration, sample registration, or verbal autopsy datasets. For these 167 causes, we conducted rigorous out-of-sample validation exercises and documented prediction error and UI coverage. However, for the remaining causes, particularly those modelled with natural history models, the design of validation tests was severely limited by data sparseness. In the absence of better data collection, particularly for causes of death that mostly occur in data-sparse geographies, our options for more robust model validation will remain limited. The analysis of causes of death in India also has important ramifications for global estimates. The three major sources of cause of death information for India are the Medical Certification of Causes of Death (MCCD), largely collected in urban areas; the Survey of Causes of Death (Rural; SCD[R]); and verbal autopsy data collected for deaths recorded in the Sample Registration System (SRS) from 2001 to 2013. All three systems are or were maintained by the Registrar-General of India. Unfortunately, whereas the MCCD and SCD(R) data have been released in considerable age, sex, and cause detail for states in India, the SRS data have been released only for large aggregates and not at the state level, although various academic works using data for 2002–04 have been published for specific causes with varying levels of detail. The rich resource of the SRS verbal autopsy data could be much more informative if the standard WHO table of ICD code, age, and sex were publicly released.

Available data from some countries in sub-Saharan Africa pose substantial challenges for cause of death analysis. Only extremely scarce verbal autopsy data are currently available for large populations. Given substantial gradients in mortality within Nigeria, for example, there is substantial risk of over-interpreting the limited verbal autopsy data. Civil registration data are collected in Nigeria, but the completeness is low and the level of garbage coding is very high. New data for countries in the sub-Saharan African region would substantially narrow a major source of uncertainty in sub-Saharan African causes of death.

It is possible that some of the heterogeneity reported in observed and expected causes of early death in different geographies has been artificially created by variations in data quality and the use of different methods. The GBD group makes extensive efforts to try to reduce the effects of variable data quality, and we have used standardised methods for each cause that are the same for all countries.

Lastly, although our estimates of uncertainty intervals reflect multiple sources of uncertainty, they do not include every possible source of uncertainty. For all-cause mortality, we include uncertainty due to the following: sampling error in the underlying data; non-sampling error associated with particular child or adult mortality data types; HIV/AIDS crude death rate; and reference age pattern of mortality used in the GBD model life table. We do not, however, include uncertainty in the measurement of income per capita or educational attainment, and we do not include uncertainty from the choice of our model specification for the first stage of the spatiotemporal Gaussian process regression analysis. For causes of death, we capture sampling uncertainty, model specification uncertainty through the creation of CODEm ensembles, and non-sampling error from subnational or non-representative datasets. We do not, however, capture uncertainty due to garbage code redistribution, uncertainty in the covariates used in the models, or uncertainty due to cause of death assignment in verbal autopsy data. With each iteration of the GBD we have sought to include more comprehensively different sources of uncertainty, and we expect this evolution to continue in the future.

### Comparison of different global health estimates

The GBD 2015 estimates for under-5 deaths due to diarrhoea and lower respiratory infection differ from estimates by other agencies, such as the WHO Department of Evidence, Information, and Research and the Maternal and Child Epidemiology Estimation (MCEE) group (results appendix pp 3985–89).^164^ In 2015, we estimated the number of under-5 deaths as 5·8 million (95% UI 5·7 million to 6·0 million), which was lower than the MCEE's estimate of 5 945 000 (95% CI 5 707 000–6 395 000) for that year.^165^ Our estimates of under-5 deaths due to diarrhoea for 2015 were also lower, at 499 000 (447 000–558 000), than those of the MCEE group (526 000), although we attributed more diarrhoeal deaths among neonates. For 2015, our results for under-5 lower respiratory infection deaths were notably lower than the estimates produced by MCEE, especially for children aged 1 to 59 months (ie, 557 000 [488 000–633 000]) from GBD 2015 and 760 000 from MCEE. Based on the latest estimates for aetiologies of diarrhoea and lower respiratory infection produced by the Child Health Epidemiology Research Group (CHERG), which is now known as MCEE,^166^ we found that our estimates for rotavirus are similar for the year 2010. However, in GBD 2015, the estimates for *Cryptosporidium* were five times higher than those of CHERG, and the estimates for *Shigella* were 2·5 times higher. These differences might arise from variations in estimation approaches, as well as the use of various sources and types of data. For example, CHERG generated estimates of pathogen-specific diarrhoea based on the proportion of hospitalised cases of diarrhoea that tested positive for each pathogen,^167^ whereas the GBD study uses a counterfactual approach that includes odds ratios of disease given exposure and corrects pathogen prevalence estimates using the results from a PCR analysis of samples from GEMS.^78^ Estimates of deaths due to rotavirus in GBD 2015 and CHERG were similar, which is not surprising given the good diagnostic validity of ELISA for rotavirus. However, GBD 2015 estimates for bacterial pathogens were generally higher, which reflects the relatively low sensitivity and specificity of conventional diagnostic methods for these pathogens.^77^ For pneumonia, estimates for bacterial aetiologies from GBD 2015 were similar to those from CHERG in terms of total lower respiratory infection deaths and generating similarly large wide uncertainty intervals. However, *H influenzae* type b estimates were lower than the CHERG results for GBD 2015, and our estimates for under-5 deaths due to pneumococcal pneumonia deaths were higher than the CHERG estimates. These differences probably stem from our efforts to correct for vaccine efficacy against bacteraemic pneumococcal disease to instead represent the efficacy against pneumococcal pneumonia. This correction greatly increases the attributable fraction, and uncertainty, for pneumococcal pneumonia.

IARC last produced cancer estimates by country, age, sex, and cancer site for 2012 (GLOBOCAN).^168^ GBD and GLOBOCAN definitions are compatible for 25 cancer types. For these cancer sites, the total estimated number of deaths from GLOBOCAN is 7 498 760 in 2012. By comparison, the GBD 2015 estimate was 7 823 429 (7 374 053–8 241 385) for 2012. Worldwide, the largest differences between estimates were for larynx cancer, other pharynx cancers, thyroid cancer, nasopharynx cancer, and myeloma, with differences of 20–85%. GLOBOCAN has been a source for descriptive global cancer epidemiology for many years. However, GBD analyses have some advantages over the past GLOBOCAN estimates. One advantage is that the annual GBD updates allow for the incorporation of new data rapidly. We expect the availability of cancer registry data in low-income and middle-income countries to increase due to the Global Initiative for Cancer Registry Development, which is led by IARC. These data, in addition to increasing the availability of cause of death data, will lead to improvements in cancer estimates, especially for regions with sparse data, and will allow policy makers to adjust health-care strategies in accordance with the latest evidence. The GBD study also provides the unique opportunity for direct comparison of the cancer burden with that of other diseases and therefore for health system investments guided by objective, comprehensive estimates rather than incomplete, unreliable data and advocacy. Furthermore, the GBD 2015 study is compliant with the GATHER guidelines, including reporting uncertainty intervals for each cancer estimate.

A comparison of GBD 2013, GBD 2015, and the two most recent UNAIDS assessments of global HIV/AIDS mortality over time is shown in the results appendix p 4). Although, as noted, GBD methods have changed substantially, our results at the global level are fairly consistent with previous iterations of GBD analyses. UNAIDS, in their latest revision, noticeably changed their assessment of peak global HIV/AIDS mortality from 2·24 million deaths in 2005 to 2·0 million deaths,^169^ rendering increased consistency between the latest UNAIDS statistics and GBD 2015. Although it is encouraging that thoughtful analyses of the epidemic and its mortality consequences are converging, additional analysis is needed to discern the differences between the estimates from GBD and UNAIDS, and the changes that occurred in the estimation series by GBD or UNAIDS. It is important to understand differences and changes in models and underlying assumptions such as on-ART and off-ART mortality and their effects on the metrics of HIV/AIDS incidence, prevalence, and mortality provided by GBD and UNAIDS. GBD 2015 mostly used epidemiological and programmatic data provided by UNAIDS in its 2015 iteration. The significant difference between GBD and UNAIDS might mark the important difference in assumptions for on-ART and off-ART mortality, CD4 progression ratio, and background mortality rates. In the GBD studies, we have also found that results are highly sensitive to the assumptions of initial CD4 count used in both the analysis of pre-ART cohort data and the initial population distribution of CD4 count for new infections.^11^

The other major effort to estimate age-specific all-cause mortality and life expectancy other than the annual GBD is the biennial United Nations Population Division assessment published in the World Population Prospects.^170^ Although the UN calculates estimates for each age–sex–country–year, the UN Population Division publishes data for most metrics only in 5-year intervals. The UN has yet to publish since the introduction of the new WHO GATHER guidelines and, in most cases, the statistical method used to generate estimates of age-specific mortality is unclear for any given country and hard to reproduce with no codes made available. A comparison of GBD 2015 life expectancy estimates and the UN Population Division estimates for the midpoint of 2010–15 is available in the results appendix (p 5). For the 189 countries for which both series provide estimates, GBD 2015 tends to have higher estimates of life expectancy at birth for both males (111 countries) and females (112 countries). The differences are even more prominent for the time period 1980–85, for which GBD life expectancy is higher for 139 countries and territories for males and 134 for females. Although the correlation between the two sets of estimates is 0·94 for males and 0·97 for females for the period 1980 to 2015, there are systematic differences in all four GBD geographical regions in Africa, north Africa and the Middle east, south Asia, and Oceania, for which the correlation coefficient is less than 0·9. In southern sub-Saharan Africa, the UN Population Division estimates are 0·1 year (95% UI −3·9 to 3·6) lower for males and 1·7 years (−6·3 to 3·3 years) lower for females on average in these countries around the peak of the HIV/AIDS epidemic in 2002. This difference exists mainly because their estimates are based on estimated under-5 death rates and model life tables, based on patterns of mortality in the Coale-Demeny model life table system and the United Nations Model Life Tables for Developing Countries, that are in turn based on observations of age patterns of mortality before 1980.^31^, ^32^, ^170^ Unlike UN estimates, GBD estimates of adult mortality rates take into account corrected sibling history data collected in household surveys, which provide direct measurements of adult mortality. The empirical information about adult mortality, used as an entry parameter to GBD's model life table system, certainly helps to improve the accuracy of our mortality age pattern assessment, even in countries affected by the HIV/AIDS epidemic.

Another important difference emerged between the GBD and UN Population Division assessments: mortality and population numbers among youths. For the period 2010–14, the UN Population Division estimates that 6·3 million deaths occurred globally among people aged 5–14 years, whereas GBD estimates this number at 4·6 million.^170^ The difference is mostly driven by the differences between the Coale-Demeny life tables used by the UN compared with our life tables, which use much more recent empirical patterns of mortality, and different assessments of under-5 mortality rate that come from differences in both treatment of data and data synthesis methods. Hill and colleagues^171^ reported that, based on complete birth histories in the Demographic and Health Surveys (DHS), which are mostly based in low-income and middle-income countries, mortality among youths might also be much higher than was estimated in GBD 2010. As a data source, long-term recall by mothers of their 5–14-year-old children's deaths, using complete birth histories, has not been validated. We examined the estimates of mortality in India in the 5–14 years age groups from the SRS, which directly measures age-specific mortality in a sample of 7597 villages in rural areas and census enumeration blocks in urban areas in India. We compared these estimates with those from the GBD 2015 and found that estimates from the two series are highly concordant, with a correlation coefficient of 0·95 for the 5–14 years age group and a mean relative difference of −2·2% for the probability of death between the ages of 10 and 14 years in the period 1980 to 2012. Our estimated age pattern of mortality for India is mainly informed by data from the SRS, with exceptions in children younger than 5 years, for whom our under-5 mortality rate data synthesis takes into account under-5 mortality rate estimates from other sources, including the India DHS. As shown in the methods appendix (pp 293–96), for the 5–9 years age group, GBD 2015 estimates of probability of death are mostly consistent with those extracted from the DHS. For the 10–14 years age group, GBD estimates are consistent with both DHS and SRS.

In 2014, WHO published cause-specific mortality estimates at global, regional, and country levels for the years 2000 and 2012.^172^ WHO used GBD 2010 mortality results for all but 12 cause groups, for which WHO and UN agencies have historically produced estimates.^172^, ^173^, ^174^ These 12 causes are tuberculosis, HIV/AIDS and other sexually transmitted infections, malaria, whooping cough, measles, schistosomiasis, maternal disorders, cancers, alcohol and drug use disorders, epilepsy, conflict and natural disasters, and road traffic accidents.^172^, ^173^ WHO has also generated estimates of YLLs, but comparisons of WHO estimates with those of GBD are limited because WHO chose to use the highest projected life expectancy for 2050—91·9 years—rather than the GBD 2010 normative standard life expectancy of 86·0 years for 2010.^172^ GBD 2010 did not include mortality estimates for the year 2012, so WHO estimates for that year were interpolated.

### Conclusion

A key goal, if not the fundamental goal, of a health system is to prolong life, especially healthy life, into old age. To do so, decision makers in health need comprehensive and disaggregated evidence on comparative mortality levels in populations, particularly for causes of death that are largely preventable through political action, either through improving health services or strengthening prevention programmes. Traditionally, this evidence has been limited to the findings of relatively conventional and straightforward mortality analyses. As our results show, more novel approaches can provide much more detailed and systematic descriptions of how survival status and cause of death patterns vary according to measures such as SDI, which are becoming increasingly relevant for policy as overall mortality levels fall. Overall population health is likely to improve more rapidly in places where the relationships between determinants of health and cause-specific mortality patterns are understood—especially areas where addressing of these gradients is a key priority for health and development policy. Our analyses provide important new evidence on where such gradients in survival among populations are greatest, and for which causes of death. Thus, although we found continued reductions in major communicable diseases such as HIV/AIDS and malaria in response to concerted global action, and further, albeit more modest reductions in the risk of death from NCDs and injuries, the comprehensive analysis of the effect of SDI on health, across and among countries, is likely to be much more relevant for accelerating global health progress.

Correspondence to: Prof Christopher J L Murray, 2301 5th Avenue, Suite 600, Seattle, WA 98121, USA cjlm@uw.edu

**This online publication has been corrected. The corrected version first appeared at thelancet.com on January 5, 2017**

## Figures and Tables

**Table 1:** GATHER checklist with description of compliance and location of information in the GBD 2015 mortality and causes of death study

	**GATHER checklist item**	**Description of compliance**	**Reference**
**Objectives and funding**			
1	Define the indicators, populations, and time periods for which estimates were made	Narrative provided in paper and methods appendix describing indicators, definitions, and populations	Main text (Methods—Geographic units, GBD cause list, Time periods) and methods appendix (pp 4–70)
2	List the funding sources for the work	Funding sources listed in paper	Summary (Funding)
**Data inputs**			
For all data inputs from multiple sources that are synthesised as part of the study			
3	Describe how the data were identified and how the data were accessed	Narrative description of data seeking methods provided	Main text (Methods) and methods appendix (pp 4–283)
4	Specify the inclusion and exclusion criteria; identify all ad-hoc exclusions	Narrative about inclusion and exclusion criteria by data type provided	Main text (Methods) and methods appendix (pp 4–283)
5	Provide information on all included data sources and their main characteristics; for each data source used, report reference information or contact name or institution, population represented, data collection method, years of data collection, sex and age range, diagnostic criteria or measurement method, and sample size, as relevant	An interactive, online data source tool that provides metadata for data sources by component, geography, cause, risk, or impairment has been developed	Online data citation tools
6	Identify and describe any categories of input data that have potentially important biases (eg, based on characteristics listed in item 5)	Summary of known biases by cause included in methods appendix	Methods appendix (pp 4–283)
For data inputs that contribute to the analysis but were not synthesised as part of the study			
7	Describe and give sources for any other data inputs	Included in online data source tool	Online data citation tools
For all data inputs			
8	Provide all data inputs in a file format from which data can be efficiently extracted (eg, a spreadsheet as opposed to a PDF), including all relevant metadata listed in item 5; for any data inputs that cannot be shared due to ethical or legal reasons, such as third-party ownership, provide a contact name or the name of the institution that retains the right to the data	Downloads of input data available through online tools, including data visualisation tools and data query tools; input data not available in tools will be made available upon request	Online data visualisation tools, data query tools, and the Global Health Data Exchange
**Data analysis**			
9	Provide a conceptual overview of the data analysis method; a diagram may be helpful	Flow diagrams of the overall methodological processes, as well as cause-specific modelling processes, have been provided	Main text (Methods, Figure 1, Figure 2) and methods appendix (pp 4–287)
10	Provide a detailed description of all steps of the analysis, including mathematical formulae; this description should cover, as relevant, data cleaning, data pre-processing, data adjustments and weighting of data sources, and mathematical or statistical models	Flow diagrams and corresponding methodological write-ups for each cause, as well as the demographics and causes of death databases and modelling processes, have been provided	Main text (Methods, Figure 1, Figure 2) and methods appendix (pp 4–287)
11	Describe how candidate models were evaluated and how the final models were selected	Provided in the methodological write-ups	Methods appendix (pp 71–283)
12	Provide the results of an evaluation of model performance, if done, as well as the results of any relevant sensitivity analysis	Provided in the methodological write-ups	Methods appendix (pp 71–283)
13	Describe methods for calculating uncertainty of the estimates; state which sources of uncertainty were, and were not, accounted for in the uncertainty analysis	Provided in the methodological write-ups	Methods appendix (pp 71–283)
14	State how analytic or statistical source code used to generate estimates can be accessed	Access statement provided	Code is provided in an online repository
**Results and discussion**			
15	Provide published estimates in a file format from which data can be efficiently extracted	GBD 2015 results are available through online data visualisation tools, the Global Health Data Exchange, and the online data query tool	Main text, methods appendix, and online data tools (data visualisation tools, data query tools, and the Global Health Data Exchange)
16	Report a quantitative measure of the uncertainty of the estimates (eg, uncertainty intervals)	Uncertainty intervals are provided with all results	Main text, methods appendix, and online data tools (data visualisation tools, data query tools, and the Global Health Data Exchange)
17	Interpret results in light of existing evidence; if updating a previous set of estimates, describe the reasons for changes in estimates	Discussion of methodological changes between GBD rounds provided in the narrative of the Article and methods appendix	Main text (Methods and Discussion) and methods appendix (pp 4–287)
18	Discuss limitations of the estimates; include a discussion of any modelling assumptions or data limitations that affect interpretation of the estimates	Discussion of limitations provided in the narrative of the main paper, as well as in the methodological write-ups in the methods appendix	Main text (Limitations) and methods appendix (pp 4–283)

GBD 2015=Global Burden of Disease 2015 Study. GATHER=Guidelines for Accurate and Transparent Health Estimates Reporting.

**Table 2:** Number of geographies and causes at each hierarchical level for GBD 2015

		**Number of geographies**
**Geographical levels**		
Super-region		7
Regions		21
Nations and territories		195
Subnational level 1		480
Subnational level 2		519
**Cause levels**		
Level 1		
	Total causes	3
	YLD causes	3
	YLL causes	3
Level 2		
	Total	21
	YLD	21
	YLL	21
Level 3		
	Total causes	166
	YLD causes	161
	YLL causes	144
Level 4		
	Total causes	261
	YLD causes	256
	YLL causes	200

Nations and territories includes countries, territories, and non-sovereign states. Subnational level 1 includes countries that, in the GBD analysis, have been subdivided into the first subnational level such as states or provinces. Subnational level 2 applies only to India and England. In India, states have been divided into urban and rural units. England has been divided into nine regions. For each level, the number of geographies includes the geographies at that level plus the number of most-detailed geographies at each higher level such that at each level of the hierarchy, all geographies create a collectively exhaustive and mutually exclusive set covering the world. Likewise, the GBD cause list is mutually exclusive and collectively exhaustive. The three Level 1 GBD causes consist of communicable, maternal, neonatal, and nutritional disorders; non-communicable diseases; and injuries. Level 2 causes consist of 21 cause groups, such as neoplasms and cardiovascular diseases. Levels 3 and 4 consist of disaggregated causes, such as liver cancer and cerebrovascular disease (Level 3), and liver cancer due to hepatitis C and ischaemic stroke (Level 4). GBD=Global Burden of Disease. YLD=years lived with disability. YLL=years of life lost.

**Table 3:** Distribution of empirical life tables by GBD super-region and decade, 1950–2015

	**1950–59**	**1960–69**	**1970–79**	**1980–89**	**1990–2000**	**2000–14**
Central Europe, eastern Europe, and central Asia	61	145	240	386	477	555
High income	434	498	611	2481	2399	3056
Latin America and Caribbean	56	170	280	879	948	1416
North Africa and Middle East	2	5	16	27	32	61
South Asia					45	145
Southeast and east Asia and Oceania	3	30	76	171	148	310
Sub-Saharan Africa				2	60	282
Total	556	848	1223	3946	4109	5825

Numbers show available empirical life tables in the GBD 2015 database. All life tables included in the database meet quality inclusion criteria whereby the observed age-specific mortality rate in an empirical life table conforms to the age pattern of mortality as described by the Gompertz–Makeham law of mortality and that observed in countries with high-quality vital and civil registration systems. GBD=Global Burden of Disease.

**Table 4:** Global life expectancy at birth and at age 50, age-standardised death rates, age-standardised YLL rate, and total deaths, by sex, 1980–2015

	**Life expectancy at birth (years)**		**Life expectancy at age 50 (years)**		**Age-standardised death rate (per 100 000)**		**Age-standardised YLL rate (per 100)**		**Total deaths (millions)**		
	Male	Female	Male	Female	Male	Female	Male	Female	Male	Female	Both sexes
1980	59·6 (59·3–60·0)	63·7 (63·3–64·1)	23·1 (22·9–23·2)	26·4 (26·2–26·7)	1536·1 (1513·0–1558·5)	1194·6 (1172·4–1217·9)	49·8 (49·0–50·5)	40·8 (40·1–41·6)	23·5 (23·2–23·9)	21·6 (21·2–22·0)	45·2 (44·6–45·7)
1981	59·9 (59·6–60·2)	64·1 (63·7–64·4)	23·1 (22·9–23·3)	26·5 (26·3–26·8)	1525·0 (1502·7–1547·1)	1177·5 (1155·9–1199·6)	49·1 (48·4–49·8)	40·0 (39·3–40·7)	23·7 (23·4–24·1)	21·7 (21·3–22·1)	45·5 (44·9–46·0)
1982	60·3 (59·9–60·6)	64·5 (64·1–64·9)	23·2 (23·0–23·4)	26·7 (26·5–26·9)	1499·7 (1478·4–1521·2)	1155·8 (1134·3–1177·4)	48·1 (47·4–48·7)	39·1 (38·4–39·7)	23·8 (23·5–24·1)	21·7 (21·3–22·1)	45·5 (45·0–46·1)
1983	60·5 (60·1–60·9)	64·7 (64·3–65·2)	23·3 (23·1–23·5)	26·7 (26·5–26·9)	1486·1 (1463·7–1507·7)	1146·6 (1125·7–1167·7)	47·4 (46·7–48·2)	38·5 (37·8–39·2)	24·0 (23·6–24·4)	22·0 (21·6–22·4)	46·0 (45·5–46·6)
1984	60·8 (60·4–61·2)	65·1 (64·6–65·5)	23·4 (23·2–23·5)	26·8 (26·6–27·0)	1472·0 (1451·3–1493·7)	1132·5 (1112·4–1153·3)	46·7 (46·0–47·5)	37·7 (37·0–38·4)	24·2 (23·9–24·6)	22·1 (21·8–22·5)	46·4 (45·8–47·0)
1985	61·2 (60·9–61·6)	65·5 (65·1–65·9)	23·4 (23·3–23·6)	26·9 (26·7–27·1)	1453·9 (1434·0–1474·9)	1116·4 (1097·3–1135·7)	45·6 (45·0–46·3)	36·8 (36·2–37·4)	24·3 (24·0–24·7)	22·2 (21·9–22·6)	46·6 (46·0–47·1)
1986	61·7 (61·3–62·0)	66·0 (65·6–66·4)	23·6 (23·4–23·8)	27·1 (26·9–27·3)	1428·4 (1409·1–1450·0)	1091·5 (1073·4–1111·2)	44·5 (43·9–45·2)	35·7 (35·2–36·3)	24·3 (24·0–24·7)	22·1 (21·8–22·5)	46·5 (45·9–47·0)
1987	62·0 (61·6–62·3)	66·3 (65·9–66·7)	23·7 (23·5–23·9)	27·2 (27·0–27·4)	1411·7 (1392·5–1432·2)	1077·6 (1059·9–1097·0)	43·8 (43·2–44·4)	35·0 (34·5–35·6)	24·5 (24·1–24·8)	22·3 (21·9–22·6)	46·7 (46·2–47·3)
1988	62·1 (61·7–62·4)	66·5 (66·2–66·9)	23·7 (23·5–23·8)	27·2 (27·0–27·4)	1409·9 (1389·3–1430·2)	1068·2 (1051·0–1087·0)	43·5 (42·8–44·1)	34·5 (34·0–35·0)	24·9 (24·5–25·2)	22·5 (22·1–22·9)	47·3 (46·8–47·9)
1989	62·4 (62·0–62·7)	66·9 (66·5–67·2)	23·6 (23·5–23·8)	27·3 (27·1–27·5)	1401·9 (1380·9–1422·3)	1055·5 (1039·0–1073·2)	42·8 (42·2–43·4)	33·8 (33·3–34·3)	25·1 (24·7–25·4)	22·6 (22·3–23·0)	47·7 (47·2–48·2)
1990	62·5 (62·2–62·8)	67·1 (66·7–67·4)	23·7 (23·5–23·9)	27·4 (27·2–27·6)	1381·1 (1359·0–1401·6)	1035·1 (1019·1–1052·0)	42·4 (41·8–43·0)	33·2 (32·8–33·7)	25·3 (24·9–25·7)	22·6 (22·3–23·0)	47·9 (47·4–48·5)
1991	62·6 (62·3–62·9)	67·3 (66·9–67·6)	23·8 (23·6–24·0)	27·5 (27·3–27·7)	1371·9 (1349·3–1393·1)	1025·1 (1010·2–1041·2)	42·1 (41·4–42·7)	32·8 (32·4–33·3)	25·6 (25·2–26·0)	22·7 (22·4–23·1)	48·3 (47·8–48·8)
1992	62·8 (62·5–63·1)	67·5 (67·2–67·8)	23·8 (23·7–24·0)	27·6 (27·4–27·7)	1363·9 (1342·2–1384·4)	1016·1 (1001·2–1032·0)	41·6 (41·0–42·2)	32·3 (31·9–32·8)	25·8 (25·4–26·2)	22·9 (22·5–23·2)	48·7 (48·2–49·2)
1993	62·8 (62·5–63·1)	67·6 (67·3–67·9)	23·8 (23·6–24·0)	27·5 (27·3–27·7)	1367·1 (1347·1–1386·3)	1016·0 (1001·6–1031·0)	41·5 (40·9–42·1)	32·0 (31·6–32·5)	26·3 (25·9–26·6)	23·2 (22·8–23·5)	49·4 (48·9–49·9)
1994	62·6 (62·2–63·0)	67·7 (67·4–68·0)	23·8 (23·6–24·0)	27·6 (27·4–27·7)	1375·7 (1353·5–1399·0)	1014·1 (999·2–1029·5)	42·0 (41·2–43·0)	31·9 (31·5–32·4)	27·0 (26·5–27·5)	23·5 (23·2–23·8)	50·5 (49·9–51·2)
1995	63·1 (62·8–63·4)	68·0 (67·7–68·3)	23·9 (23·8–24·1)	27·7 (27·5–27·8)	1351·0 (1333·4–1368·4)	1000·7 (987·4–1014·9)	40·9 (40·4–41·4)	31·3 (30·9–31·7)	26·8 (26·5–27·2)	23·5 (23·2–23·9)	50·4 (49·9–50·9)
1996	63·4 (63·1–63·6)	68·3 (68·0–68·5)	24·1 (24·0–24·3)	27·9 (27·7–28·0)	1330·2 (1314·1–1347·0)	986·6 (974·0–999·9)	40·2 (39·7–40·7)	30·8 (30·5–31·2)	26·9 (26·6–27·2)	23·6 (23·3–23·9)	50·4 (50·0–50·9)
1997	63·7 (63·4–63·9)	68·5 (68·3–68·8)	24·3 (24·2–24·4)	28·0 (27·9–28·1)	1312·0 (1296·7–1327·7)	975·1 (962·9–986·9)	39·6 (39·1–40·0)	30·4 (30·0–30·7)	27·0 (26·6–27·3)	23·6 (23·3–23·9)	50·6 (50·1–51·1)
1998	63·9 (63·6–64·1)	68·8 (68·5–69·0)	24·4 (24·3–24·5)	28·1 (28·0–28·2)	1301·7 (1286·8–1316·9)	966·6 (954·7–978·8)	39·1 (38·7–39·5)	29·9 (29·6–30·3)	27·2 (26·8–27·5)	23·8 (23·5–24·1)	50·9 (50·5–51·4)
1999	64·0 (63·7–64·2)	68·9 (68·7–69·1)	24·4 (24·3–24·6)	28·1 (28·0–28·3)	1297·1 (1282·1–1312·1)	963·9 (952·1–976·1)	38·8 (38·4–39·2)	29·6 (29·3–30·0)	27·6 (27·2–27·9)	24·1 (23·8–24·4)	51·6 (51·2–52·1)
2000	64·2 (64·0–64·4)	69·1 (68·9–69·4)	24·5 (24·4–24·6)	28·2 (28·1–28·3)	1284·9 (1270·1–1299·5)	954·8 (943·4–966·3)	38·3 (37·9–38·7)	29·2 (28·8–29·6)	27·9 (27·5–28·2)	24·3 (24·0–24·6)	52·1 (51·7–52·6)
2001	64·4 (64·2–64·7)	69·4 (69·1–69·6)	24·6 (24·5–24·7)	28·3 (28·2–28·4)	1272·5 (1258·1–1286·7)	944·1 (932·9–955·5)	37·7 (37·3–38·1)	28·7 (28·4–29·1)	28·1 (27·8–28·4)	24·5 (24·2–24·8)	52·6 (52·1–53·1)
2002	64·6 (64·4–64·9)	69·6 (69·4–69·8)	24·6 (24·5–24·8)	28·4 (28·2–28·5)	1265·8 (1251·2–1280·3)	933·4 (923·0–944·4)	37·2 (36·8–37·7)	28·2 (27·9–28·6)	28·5 (28·2–28·9)	24·7 (24·4–25·0)	53·2 (52·7–53·7)
2003	65·0 (64·8–65·2)	70·0 (69·7–70·2)	24·8 (24·7–24·9)	28·5 (28·4–28·7)	1240·0 (1225·5–1254·6)	915·4 (905·3–925·9)	36·4 (36·0–36·8)	27·5 (27·2–27·9)	28·6 (28·2–28·9)	24·7 (24·4–25·0)	53·3 (52·9–53·8)
2004	65·3 (65·1–65·5)	70·3 (70·1–70·5)	25·0 (24·8–25·1)	28·7 (28·6–28·9)	1216·5 (1202·2–1231·6)	895·9 (885·5–906·9)	35·8 (35·3–36·2)	26·9 (26·6–27·3)	28·7 (28·4–29·1)	24·7 (24·4–25·0)	53·5 (53·0–54·0)
2005	65·7 (65·5–65·9)	70·7 (70·5–71·0)	25·1 (25·0–25·3)	28·9 (28·8–29·0)	1195·0 (1180·9–1209·1)	878·1 (868·3–888·2)	34·9 (34·5–35·3)	26·1 (25·8–26·4)	28·9 (28·5–29·2)	24·8 (24·5–25·1)	53·6 (53·1–54·1)
2006	66·2 (65·9–66·4)	71·2 (71·0–71·5)	25·4 (25·3–25·5)	29·2 (29·1–29·3)	1163·8 (1150·2–1176·9)	852·4 (842·9–862·3)	33·8 (33·4–34·2)	25·2 (24·9–25·5)	28·7 (28·4–29·1)	24·6 (24·3–24·8)	53·3 (52·8–53·7)
2007	66·6 (66·3–66·8)	71·7 (71·5–72·0)	25·6 (25·4–25·7)	29·4 (29·3–29·5)	1141·3 (1127·7–1154·8)	830·4 (820·5–840·6)	33·0 (32·6–33·4)	24·4 (24·1–24·7)	28·8 (28·4–29·2)	24·5 (24·2–24·8)	53·3 (52·8–53·7)
2008	66·8 (66·6–67·1)	72·1 (71·9–72·4)	25·7 (25·6–25·8)	29·6 (29·5–29·7)	1127·2 (1112·9–1140·8)	814·0 (803·9–823·4)	32·5 (32·0–32·9)	23·7 (23·4–24·1)	29·1 (28·7–29·5)	24·5 (24·2–24·8)	53·6 (53·1–54·1)
2009	67·3 (67·0–67·6)	72·6 (72·4–72·9)	25·9 (25·7–26·0)	29·9 (29·7–30·0)	1103·4 (1090·0–1116·6)	791·7 (782·3–801·5)	31·5 (31·1–31·9)	22·9 (22·6–23·2)	29·1 (28·7–29·5)	24·4 (24·1–24·7)	53·5 (52·9–54·0)
2010	67·5 (67·2–67·8)	72·9 (72·6–73·2)	26·0 (25·8–26·1)	30·0 (29·9–30·2)	1091·6 (1077·2–1105·6)	777·8 (767·4–788·0)	31·0 (30·6–31·5)	22·4 (22·0–22·7)	29·5 (29·1–29·9)	24·5 (24·2–24·8)	54·0 (53·4–54·6)
2011	68·0 (67·7–68·3)	73·4 (73·1–73·8)	26·2 (26·0–26·3)	30·3 (30·1–30·4)	1068·1 (1053·8–1083·1)	756·1 (745·4–767·2)	30·1 (29·7–30·5)	21·5 (21·2–21·9)	29·5 (29·0–29·9)	24·4 (24·0–24·7)	53·8 (53·3–54·4)
2012	68·3 (68·0–68·6)	73·9 (73·5–74·2)	26·3 (26·1–26·5)	30·5 (30·3–30·6)	1051·9 (1037·1–1067·9)	739·9 (728·9–751·9)	29·4 (29·0–29·9)	20·8 (20·5–21·2)	29·7 (29·2–30·2)	24·4 (24·0–24·8)	54·1 (53·5–54·7)
2013	68·6 (68·2–68·9)	74·2 (73·9–74·6)	26·4 (26·2–26·6)	30·6 (30·4–30·8)	1037·9 (1021·4–1055·4)	725·8 (714·1–738·9)	28·8 (28·3–29·3)	20·2 (19·9–20·6)	30·0 (29·5–30·5)	24·5 (24·1–25·0)	54·5 (53·8–55·1)
2014	68·8 (68·4–69·1)	74·5 (74·1–74·9)	26·5 (26·3–26·6)	30·7 (30·5–30·9)	1029·7 (1012·5–1048·3)	715·8 (703·8–729·6)	28·4 (27·9–28·9)	19·8 (19·4–20·2)	30·4 (29·9–31·0)	24·8 (24·3–25·2)	55·2 (54·4–55·9)
2015	69·0 (68·6–69·4)	74·8 (74·4–75·2)	26·6 (26·4–26·8)	30·9 (30·7–31·1)	1018·6 (1000·4–1037·1)	703·4 (691·0–717·8)	27·9 (27·4–28·5)	19·3 (18·9–19·7)	30·9 (30·3–31·5)	24·9 (24·5–25·5)	55·8 (55·0–56·6)

Data in parentheses are 95% uncertainty intervals. Age-standardised rates are standardised using the GBD world population standard. YLLs=years of life lost. GBD=Global Burden of Disease.

**Table 5:** Global deaths in 2005 and 2015 for all ages and both sexes combined and age-standardised death rates, with percentage change between 2005 and 2015 for 249 causes

				**All age deaths (thousands)**			**Age-standardised mortality rate (per 100 000)**		
				2005	2015	Percentage change, 2005–15	2005	2015	Percentage change, 2005–15
**All causes**				**53 618·5(53 139·8 to 54 075·8)**	**55 792·9 (54 984·1 to 56 640·3)**	**4·1 (2·6 to 5·6)**	**1024·0 (1015·1 to 1032·6)**	**850·1 (838·3 to 862·4)**	**−17·0 (−18·1 to −15·8)**
**Communicable, maternal, neonatal, and nutritional diseases (Group 1 causes)**				**14 023·9 (13 734·8 to 14 335·3)**	**11 263·6 (10 922·7 to 11 594·5)**	**−19·7 (−21·6 to −17·8)**	**226·2 (221·3 to 231·6)**	**159·3 (154·4 to 163·9)**	**−29·6 (−31·3 to −27·9)**
	HIV/AIDS and tuberculosis			3139·5 (2938·6 to 3469·8)	2305·2 (2092·7 to 2578·0)	−26·6 (−30·1 to −23·0)	51·6 (48·0 to 57·4)	31·9 (28·8 to 35·9)	−38·2 (−41·2 to −35·2)
		Tuberculosis		1347·6 (1152·9 to 1658·7)	1112·6 (909·8 to 1392·8)	−17·4 (−24·4 to −11·3)	24·2 (20·8 to 29·9)	16·0 (13·1 to 20·1)	−33·8 (−39·6 to −28·7)
		HIV/AIDS		1791·9 (1703·8 to 1886·6)	1192·6 (1130·8 to 1270·3)	−33·4 (−36·2 to −30·0)	27·4 (26·0 to 28·8)	15·8 (15·0 to 16·9)	−42·1 (−44·6 to −39·1)
			HIV/AIDS—tuberculosis	351·8 (281·7 to 400·4)	211·7 (161·9 to 245·0)	−39·8 (−44·3 to −34·4)	5·4 (4·4 to 6·2)	2·8 (2·2 to 3·3)	−48·2 (−52·1 to −43·5)
			HIV/AIDS resulting in other diseases	1440·1 (1349·6 to 1546·7)	980·8 (914·7 to 1063·6)	−31·9 (−35·6 to −27·7)	21·9 (20·5 to 23·6)	13·0 (12·1 to 14·1)	−40·6 (−43·8 to −36·9)
**Diarrhoea, lower respiratory, and other common infectious diseases**				**5773·1 (5548·3 to 6004·8)**	**4959·8 (4711·6 to 5179·4)**	**−14·1 (−17·1 to −11·0)**	**99·3 (95·5 to 103·1)**	**73·2 (69·5 to 76·4)**	**−26·3 (−28·7 to −23·8)**
	Diarrhoeal diseases			1657·2 (1565·0 to 1756·1)	1312·1 (1233·6 to 1391·3)	−20·8 (−26·1 to −15·4)	28·1 (26·7 to 29·6)	19·1 (18·0 to 20·2)	−32·2 (−36·5 to −27·7)
	Intestinal infectious diseases			208·6 (118·0 to 344·1)	178·5 (100·9 to 293·7)	−14·4 (−20·7 to −8·7)	3·0 (1·7 to 5·0)	2·4 (1·4 to 4·0)	−20·3 (−26·1 to −15·0)
		Typhoid fever		172·9 (94·6 to 293·2)	148·8 (81·9 to 249·7)	−14·0 (−20·6 to −8·1)	2·5 (1·4 to 4·3)	2·0 (1·1 to 3·4)	−19·8 (−25·7 to −14·0)
		Paratyphoid fever		33·9 (15·6 to 65·1)	29·2 (13·7 to 56·3)	−14·1 (−21·8 to −6·2)	0·5 (0·2 to 0·9)	0·4 (0·2 to 0·8)	−20·3 (−27·4 to −13·2)
		Other intestinal infectious diseases		1·8 (0·6 to 3·3)	0·6 (0·3 to 1·2)	−64·0 (−75·5 to −43·9)	0·0 (0·0 to 0·0)	0·0 (0·0 to 0·0)	−67·7 (−77·7 to −51·2)
	Lower respiratory infections			2828·5 (2628·6 to 2965·8)	2736·7 (2500·3 to 2860·8)	−3·2 (−6·9 to 0·4)	51·7 (47·9 to 54·1)	41·6 (38·0 to 43·5)	−19·5 (−22·3 to −16·9)
	Upper respiratory infections			3·8 (3·3 to 4·2)	3·1 (2·8 to 3·5)	−18·0 (−28·5 to −5·2)	0·1 (0·1 to 0·1)	0·0 (0·0 to 0·1)	−32·2 (−40·4 to −22·0)
	Otitis media			3·9 (3·5 to 4·3)	3·2 (2·9 to 3·7)	−17·7 (−27·3 to −4·5)	0·1 (0·1 to 0·1)	0·0 (0·0 to 0·1)	−27·8 (−38·5 to −14·5)
	Meningitis			407·7 (351·1 to 457·1)	379·2 (322·7 to 444·7)	−7·0 (−15·4 to 5·2)	6·3 (5·5 to 7·1)	5·2 (4·5 to 6·1)	−17·2 (−24·3 to −6·7)
		Pneumococcal meningitis		112·1 (93·3 to 135·2)	112·9 (93·4 to 141·8)	0·7 (−8·7 to 13·8)	1·7 (1·5 to 2·1)	1·6 (1·3 to 1·9)	−10·8 (−18·4 to 0·7)
		*Haemophilus influenzae* type b meningitis		110·6 (88·3 to 135·8)	71·5 (56·7 to 91·8)	−35·4 (−43·6 to −24·7)	1·7 (1·3 to 2·0)	1·0 (0·8 to 1·3)	−41·1 (−48·3 to −31·4)
		Meningococcal meningitis		74·3 (58·7 to 91·8)	73·3 (58·0 to 93·2)	−1·3 (−13·1 to 15·4)	1·1 (0·9 to 1·4)	1·0 (0·8 to 1·3)	−11·6 (−21·8 to 3·0)
		Other meningitis		110·6 (94·9 to 128·6)	121·5 (101·8 to 144·2)	9·8 (1·1 to 22·0)	1·8 (1·5 to 2·1)	1·7 (1·4 to 2·0)	−4·6 (−11·8 to 5·9)
	Encephalitis			147·0 (135·3 to 163·0)	149·5 (137·6 to 167·0)	1·7 (−4·7 to 8·1)	2·4 (2·2 to 2·7)	2·1 (1·9 to 2·4)	−11·7 (−17·1 to −6·4)
	Diphtheria			5·6 (3·0 to 11·0)	2·1 (1·1 to 4·7)	−61·3 (−85·0 to −2·0)	0·1 (0·0 to 0·2)	0·0 (0·0 to 0·1)	−64·2 (−86·2 to −9·2)
	Whooping cough			99·6 (36·8 to 226·3)	58·7 (20·3 to 126·6)	−41·0 (−77·9 to 65·1)	1·4 (0·5 to 3·3)	0·8 (0·3 to 1·7)	−45·1 (−79·4 to 53·8)
	Tetanus			108·0 (90·9 to 151·1)	56·7 (48·2 to 80·0)	−47·5 (−54·6 to −39·0)	1·7 (1·4 to 2·4)	0·8 (0·7 to 1·1)	−53·2 (−59·7 to −45·9)
	Measles			293·7 (110·6 to 611·4)	73·4 (26·1 to 161·4)	−75·0 (−84·5 to −58·8)	4·2 (1·6 to 8·8)	1·0 (0·4 to 2·2)	−76·7 (−85·5 to −61·6)
	Varicella and herpes zoster			9·6 (8·5 to 11·0)	6·4 (5·4 to 7·8)	−33·5 (−44·4 to −19·2)	0·2 (0·2 to 0·2)	0·1 (0·1 to 0·1)	−45·8 (−54·7 to −34·7)
**Neglected tropical diseases and malaria**				**1298·5 (1082·7 to 1509·1)**	**843·1 (669·9 to 1019·7)**	**−35·1 (−43·6 to −26·7)**	**19·5 (16·3 to 22·6)**	**11·5 (9·1 to 13·9)**	**−41·3 (−48·9 to −33·8)**
	Malaria			1167·0 (952·1 to 1378·1)	730·5 (555·8 to 904·0)	−37·4 (−47·0 to −27·8)	17·4 (14·2 to 20·6)	9·9 (7·5 to 12·3)	−43·1 (−51·8 to −34·7)
	Chagas disease			7·5 (7·2 to 7·8)	8·0 (7·5 to 8·6)	7·7 (0·1 to 15·9)	0·1 (0·1 to 0·2)	0·1 (0·1 to 0·1)	−16·5 (−22·4 to −10·3)
	Leishmaniasis			23·1 (14·8 to 33·2)	24·2 (17·1 to 32·5)	4·9 (−8·5 to 21·5)	0·3 (0·2 to 0·5)	0·3 (0·2 to 0·4)	−7·2 (−18·7 to 7·1)
		Visceral leishmaniasis		23·1 (14·8 to 33·2)	24·2 (17·1 to 32·5)	4·9 (−8·5 to 21·5)	0·3 (0·2 to 0·5)	0·3 (0·2 to 0·4)	−7·2 (−18·7 to 7·1)
	African trypanosomiasis			14·2 (7·6 to 23·1)	3·5 (1·8 to 5·7)	−75·3 (−81·4 to −67·9)	0·2 (0·1 to 0·4)	0·0 (0·0 to 0·1)	−78·4 (−83·7 to −72·0)
	Schistosomiasis			7·8 (7·0 to 8·9)	4·4 (3·8 to 4·9)	−44·2 (−52·7 to −35·6)	0·1 (0·1 to 0·2)	0·1 (0·1 to 0·1)	−55·8 (−62·7 to −48·8)
	Cysticercosis			0·6 (0·5 to 0·7)	0·4 (0·3 to 0·5)	−37·0 (−43·5 to −28·0)	0·0 (0·0 to 0·0)	0·0 (0·0 to 0·0)	−47·7 (−53·1 to −40·2)
	Cystic echinococcosis			1·8 (1·7 to 1·9)	1·2 (1·1 to 1·3)	−32·6 (−35·7 to −29·4)	0·0 (0·0 to 0·0)	0·0 (0·0 to 0·0)	−44·6 (−47·0 to −42·1)
	Dengue			12·3 (8·6 to 15·1)	18·4 (11·8 to 22·7)	48·7 (15·1 to 90·9)	0·2 (0·1 to 0·2)	0·3 (0·2 to 0·3)	34·0 (4·4 to 70·9)
	Yellow fever			6·3 (1·3 to 16·9)	5·1 (1·1 to 14·2)	−18·8 (−33·5 to −0·8)	0·1 (0·0 to 0·2)	0·1 (0·0 to 0·2)	−25·7 (−39·2 to −9·4)
	Rabies			32·1 (28·0 to 36·1)	17·4 (14·8 to 20·6)	−45·8 (−52·2 to −39·0)	0·5 (0·4 to 0·6)	0·2 (0·2 to 0·3)	−52·9 (−58·4 to −47·3)
	Intestinal nematode infections			3·8 (3·3 to 4·3)	2·7 (2·4 to 3·1)	−28·5 (−36·9 to −19·7)	0·1 (0·1 to 0·1)	0·0 (0·0 to 0·0)	−34·7 (−42·3 to −26·7)
		Ascariasis		3·8 (3·3 to 4·3)	2·7 (2·4 to 3·1)	−28·5 (−36·9 to −19·7)	0·1 (0·1 to 0·1)	0·0 (0·0 to 0·0)	−34·7 (−42·3 to −26·7)
	Ebola virus disease			0·0 (0·0 to 0·0)	5·5 (4·4 to 6·6)	32 659·1 (32 659·1 to 32 659·1)	0·0 (0·0 to 0·0)	0·1 (0·1 to 0·1)	28 636·1 (28 636·1 to 28 636·1)
	Other neglected tropical diseases			21·9 (14·0 to 27·7)	21·8 (12·7 to 27·6)	−0·6 (−14·5 to 15·9)	0·4 (0·2 to 0·4)	0·3 (0·2 to 0·4)	−13·0 (−24·9 to 1·1)
**Maternal disorders**				**350·8 (327·9 to 376·6)**	**275·3 (243·8 to 315·5)**	**−21·5 (−30·3 to −10·7)**	**5·1 (4·7 to 5·5)**	**3·6 (3·2 to 4·1)**	**−29·1 (−37·1 to −19·3)**
	Maternal haemorrhage			99·7 (87·6 to 113·0)	83·1 (67·0 to 101·5)	−16·6 (−28·8 to −3·2)	1·4 (1·3 to 1·6)	1·1 (0·9 to 1·3)	−25·0 (−35·9 to −12·9)
	Maternal sepsis and other maternal infections			24·7 (20·4 to 29·9)	17·9 (13·4 to 23·9)	−27·7 (−41·7 to −10·8)	0·4 (0·3 to 0·4)	0·2 (0·2 to 0·3)	−35·0 (−47·6 to −19·8)
	Maternal hypertensive disorders			63·7 (54·9 to 74·3)	46·9 (37·1 to 59·6)	−26·4 (−36·7 to −13·1)	0·9 (0·8 to 1·1)	0·6 (0·5 to 0·8)	−32·9 (−42·5 to −20·9)
	Maternal obstructed labour and uterine rupture			26·9 (22·1 to 32·0)	23·1 (17·2 to 30·0)	−13·9 (−27·4 to 1·5)	0·4 (0·3 to 0·5)	0·3 (0·2 to 0·4)	−22·5 (−34·5 to −8·5)
	Maternal abortion, miscarriage, and ectopic pregnancy			41·2 (34·7 to 49·6)	31·7 (24·6 to 39·7)	−23·1 (−33·9 to −11·1)	0·6 (0·5 to 0·7)	0·4 (0·3 to 0·5)	−30·7 (−40·4 to −19·8)
	Indirect maternal deaths			38·2 (31·6 to 45·4)	30·8 (23·1 to 40·5)	−19·4 (−32·4 to −1·9)	0·6 (0·5 to 0·7)	0·4 (0·3 to 0·5)	−27·1 (−38·8 to −11·1)
	Late maternal deaths			7·9 (5·3 to 11·5)	6·7 (4·3 to 10·0)	−15·1 (−26·2 to −1·1)	0·1 (0·1 to 0·2)	0·1 (0·1 to 0·1)	−23·3 (−33·3 to −10·6)
	Maternal deaths aggravated by HIV/AIDS			2·8 (1·8 to 3·8)	2·3 (1·4 to 3·3)	−15·8 (−33·1 to 8·0)	0·0 (0·0 to 0·1)	0·0 (0·0 to 0·0)	−24·8 (−40·4 to −3·7)
	Other maternal disorders			45·7 (39·0 to 53·9)	32·7 (26·3 to 40·5)	−28·4 (−37·1 to −18·3)	0·7 (0·6 to 0·8)	0·4 (0·3 to 0·5)	−35·3 (−43·3 to −26·2)
**Neonatal disorders**				**2653·5 (2583·4 to 2728·1)**	**2163·2 (2095·1 to 2232·5)**	**−18·5 (−20·4 to −16·4)**	**37·3 (36·4 to 38·4)**	**28·8 (27·9 to 29·7)**	**−22·8 (−24·6 to −20·9)**
	Neonatal preterm birth complications			1088·0 (1010·9 to 1217·7)	805·8 (736·2 to 898·6)	−25·9 (−31·3 to −20·6)	15·3 (14·2 to 17·1)	10·7 (9·8 to 12·0)	−29·8 (−34·9 to −24·8)
	Neonatal encephalopathy (birth asphyxia and trauma)			882·8 (800·7 to 974·3)	740·4 (667·6 to 829·2)	−16·1 (−23·8 to −8·0)	12·4 (11·3 to 13·7)	9·9 (8·9 to 11·0)	−20·5 (−27·8 to −12·8)
	Neonatal sepsis and other neonatal infections			352·3 (252·0 to 465·7)	351·7 (249·2 to 459·1)	−0·2 (−16·2 to 20·3)	5·0 (3·5 to 6·6)	4·7 (3·3 to 6·1)	−5·5 (−20·6 to 13·9)
	Haemolytic disease and other neonatal jaundice			68·3 (42·9 to 106·1)	45·1 (30·1 to 67·3)	−34·0 (−47·9 to −17·1)	1·0 (0·6 to 1·5)	0·6 (0·4 to 0·9)	−37·6 (−50·6 to −21·6)
	Other neonatal disorders			262·0 (190·3 to 343·0)	220·2 (167·6 to 276·8)	−16·0 (−34·1 to 5·6)	3·7 (2·7 to 4·8)	2·9 (2·2 to 3·7)	−20·5 (−37·7 to −0·1)
Nutritional deficiencies				460·8 (380·3 to 541·6)	405·7 (331·7 to 495·6)	−11·9 (−22·9 to 0·6)	7·8 (6·5 to 9·0)	5·9 (4·8 to 7·2)	−24·3 (−32·9 to −14·3)
	Protein-energy malnutrition			379·7 (314·3 to 457·1)	323·2 (264·9 to 400·8)	−14·9 (−27·3 to −0·2)	6·4 (5·3 to 7·6)	4·7 (3·9 to 5·8)	−26·3 (−35·8 to −14·5)
	Iodine deficiency			2·1 (1·6 to 2·9)	2·0 (1·5 to 2·7)	−3·1 (−31·4 to 40·7)	0·0 (0·0 to 0·0)	0·0 (0·0 to 0·0)	−18·4 (−41·6 to 15·7)
	Iron-deficiency anaemia			48·4 (31·9 to 63·1)	54·2 (35·1 to 72·9)	12·1 (−2·1 to 28·0)	0·8 (0·5 to 1·1)	0·8 (0·5 to 1·0)	−5·1 (−16·8 to 7·8)
	Other nutritional deficiencies			30·7 (21·7 to 43·7)	26·3 (20·7 to 33·6)	−14·1 (−33·1 to 1·1)	0·6 (0·4 to 0·8)	0·4 (0·3 to 0·5)	−30·0 (−45·1 to −18·7)
**Other communicable, maternal, neonatal, and nutritional diseases**				**347·7 (291·2 to 419·8)**	**311·3 (257·9 to 372·7)**	**−10·5 (−15·6 to −4·6)**	**5·6 (4·7 to 6·6)**	**4·4 (3·6 to 5·2)**	**−21·5 (−26·0 to −16·6)**
	Sexually transmitted diseases excluding HIV			135·5 (81·2 to 207·2)	108·0 (64·6 to 165·7)	−20·3 (−28·3 to −12·4)	2·0 (1·2 to 3·0)	1·5 (0·9 to 2·2)	−26·1 (−33·7 to −19·0)
		Syphilis		134·1 (79·9 to 205·7)	106·8 (63·4 to 164·6)	−20·3 (−28·5 to −12·4)	1·9 (1·2 to 3·0)	1·4 (0·9 to 2·2)	−26·1 (−33·8 to −18·8)
		Chlamydial infection		0·2 (0·1 to 0·2)	0·2 (0·1 to 0·2)	−5·3 (−17·0 to 10·6)	0·0 (0·0 to 0·0)	0·0 (0·0 to 0·0)	−23·5 (−32·9 to −11·3)
		Gonococcal infection		0·8 (0·7 to 0·9)	0·7 (0·5 to 0·8)	−15·7 (−26·7 to −4·7)	0·0 (0·0 to 0·0)	0·0 (0·0 to 0·0)	−31·3 (−40·1 to −22·6)
		Other sexually transmitted diseases		0·4 (0·3 to 0·4)	0·3 (0·2 to 0·4)	−16·8 (−27·6 to −6·1)	0·0 (0·0 to 0·0)	0·0 (0·0 to 0·0)	−31·7 (−40·3 to −23·0)
	Hepatitis			123·0 (118·6 to 127·6)	105·8 (100·7 to 110·8)	−14·0 (−17·9 to −10·0)	2·1 (2·0 to 2·2)	1·5 (1·4 to 1·6)	−28·0 (−31·1 to −24·7)
		Acute hepatitis A		16·9 (10·9 to 23·1)	11·2 (6·9 to 15·9)	−34·0 (−43·5 to −24·2)	0·2 (0·2 to 0·3)	0·2 (0·1 to 0·2)	−39·4 (−47·9 to −30·4)
		Hepatitis B		71·4 (62·0 to 80·1)	65·4 (56·4 to 73·7)	−8·4 (−14·1 to −2·2)	1·3 (1·1 to 1·4)	0·9 (0·8 to 1·1)	−26·4 (−30·9 to −21·6)
		Hepatitis C		2·8 (0·6 to 6·4)	2·5 (0·5 to 5·9)	−9·8 (−24·4 to 8·6)	0·1 (0·0 to 0·1)	0·0 (0·0 to 0·1)	−29·6 (−41·0 to −15·1)
		Acute hepatitis E		31·9 (23·0 to 42·3)	26·7 (18·5 to 36·6)	−16·4 (−26·1 to −6·6)	0·5 (0·4 to 0·7)	0·4 (0·3 to 0·5)	−26·3 (−34·3 to −17·9)
	Other infectious diseases			89·1 (61·3 to 102·3)	97·5 (61·3 to 112·9)	9·4 (−2·3 to 23·2)	1·5 (1·1 to 1·7)	1·4 (0·9 to 1·6)	−6·4 (−16·8 to 5·2)
**Non-communicable diseases**				**34 835·6 (34 441·3 to 35 277·1)**	**39 804·2 (39 210·8 to 40 452·2)**	**14·3 (12·6 to 16·0)**	**719·1 (711·9 to 727·3)**	**624·7 (615·8 to 634·5)**	**−13·1 (−14·3 to −11·9)**
	Neoplasms			7492·8 (7378·4 to 7616·5)	8764·6 (8591·1 to 8945·6)	17·0 (14·8 to 19·3)	149·2 (147·1 to 151·7)	134·3 (131·6 to 137·0)	−10·0 (−11·6 to −8·3)
		Lip and oral cavity cancer		110·2 (107·6 to 112·9)	146·0 (141·6 to 150·6)	32·5 (28·5 to 37·0)	2·2 (2·1 to 2·2)	2·2 (2·1 to 2·3)	1·4 (−1·6 to 4·8)
		Nasopharynx cancer		55·8 (45·9 to 58·8)	63·0 (51·1 to 67·0)	12·8 (5·1 to 19·4)	1·0 (0·8 to 1·1)	0·9 (0·7 to 1·0)	−10·9 (−16·8 to −5·7)
		Other pharynx cancer		51·9 (50·5 to 53·2)	64·4 (61·6 to 67·1)	24·1 (18·4 to 29·7)	1·0 (1·0 to 1·0)	1·0 (0·9 to 1·0)	−5·0 (−9·3 to −0·6)
		Oesophageal cancer		459·3 (445·1 to 474·2)	439·0 (422·6 to 456·9)	−4·4 (−9·0 to 0·7)	9·2 (8·9 to 9·5)	6·7 (6·5 to 7·0)	−26·8 (−30·3 to −22·9)
		Stomach cancer		824·5 (806·8 to 843·0)	818·9 (795·5 to 843·7)	−0·7 (−3·7 to 2·6)	16·7 (16·3 to 17·0)	12·7 (12·4 to 13·1)	−23·8 (−26·0 to −21·4)
		Colon and rectum cancer		675·5 (664·9 to 688·2)	832·0 (811·7 to 854·5)	23·2 (20·6 to 26·0)	14·0 (13·8 to 14·2)	13·0 (12·7 to 13·4)	−6·7 (−8·7 to −4·6)
		Liver cancer		726·7 (636·0 to 762·2)	810·5 (749·7 to 862·8)	11·5 (5·9 to 20·4)	13·9 (12·3 to 14·6)	12·1 (11·2 to 12·9)	−13·1 (−17·4 to −6·7)
			Liver cancer due to hepatitis B	263·1 (224·4 to 282·5)	265·3 (241·0 to 290·5)	0·8 (−5·6 to 12·5)	4·8 (4·1 to 5·1)	3·8 (3·5 to 4·2)	−20·2 (−25·2 to −11·5)
			Liver cancer due to hepatitis C	137·8 (126·1 to 146·3)	167·1 (153·9 to 177·9)	21·3 (17·3 to 26·2)	2·8 (2·6 to 3·0)	2·6 (2·4 to 2·8)	−7·4 (−10·4 to −3·8)
			Liver cancer due to alcohol use	194·5 (168·8 to 208·3)	245·2 (225·1 to 266·6)	26·1 (18·5 to 37·1)	3·8 (3·3 to 4·1)	3·7 (3·4 to 4·0)	−3·1 (−8·6 to 4·8)
			Liver cancer due to other causes	131·3 (114·1 to 141·6)	132·9 (119·8 to 144·4)	1·2 (−4·2 to 9·7)	2·5 (2·2 to 2·7)	2·0 (1·8 to 2·2)	−21·1 (−25·2 to −15·0)
		Gallbladder and biliary tract cancer		124·5 (120·8 to 127·8)	140·5 (131·4 to 147·2)	12·9 (7·3 to 18·2)	2·6 (2·5 to 2·7)	2·2 (2·1 to 2·3)	−14·7 (−19·0 to −10·6)
		Pancreatic cancer		314·6 (310·1 to 319·0)	411·6 (403·6 to 420·7)	30·8 (28·3 to 33·6)	6·5 (6·4 to 6·6)	6·4 (6·3 to 6·6)	−0·9 (−2·8 to 1·2)
		Larynx cancer		93·1 (90·9 to 95·7)	105·9 (102·7 to 109·5)	13·8 (10·5 to 17·5)	1·8 (1·8 to 1·9)	1·6 (1·6 to 1·7)	−12·8 (−15·3 to −10·0)
		Tracheal, bronchus, and lung cancer		1434·5 (1406·5 to 1463·5)	1722·5 (1673·7 to 1772·7)	20·1 (16·7 to 24·0)	29·0 (28·5 to 29·6)	26·6 (25·9 to 27·4)	−8·1 (−10·7 to −5·2)
		Malignant skin melanoma		47·0 (38·7 to 58·6)	59·8 (47·6 to 72·7)	27·2 (20·0 to 32·6)	0·9 (0·8 to 1·1)	0·9 (0·7 to 1·1)	−1·8 (−7·1 to 2·4)
		Non-melanoma skin cancer		36·3 (35·4 to 37·1)	51·9 (49·9 to 53·8)	42·9 (37·4 to 47·8)	0·8 (0·7 to 0·8)	0·8 (0·8 to 0·9)	7·6 (3·4 to 11·1)
			Squamous-cell carcinoma	36·3 (35·4 to 37·1)	51·9 (49·9 to 53·8)	42·9 (37·4 to 47·8)	0·8 (0·7 to 0·8)	0·8 (0·8 to 0·9)	7·6 (3·4 to 11·1)
		Breast cancer		439·8 (418·8 to 461·9)	533·6 (502·2 to 553·1)	21·3 (14·9 to 27·2)	8·5 (8·1 to 8·9)	7·9 (7·5 to 8·2)	−6·8 (−11·5 to −2·5)
		Cervical cancer		225·4 (213·9 to 237·8)	238·6 (225·3 to 252·4)	5·8 (−0·5 to 13·8)	4·2 (4·0 to 4·4)	3·5 (3·3 to 3·7)	−17·7 (−22·5 to −11·5)
		Uterine cancer		81·8 (78·5 to 85·4)	89·9 (86·1 to 94·3)	10·0 (3·8 to 17·4)	1·6 (1·6 to 1·7)	1·4 (1·3 to 1·4)	−16·1 (−20·8 to −10·7)
		Ovarian cancer		133·8 (130·8 to 138·6)	161·1 (156·5 to 166·5)	20·4 (16·5 to 24·4)	2·6 (2·6 to 2·7)	2·4 (2·3 to 2·5)	−7·9 (−10·8 to −4·9)
		Prostate cancer		277·4 (230·8 to 348·9)	365·9 (303·5 to 459·6)	31·9 (28·2 to 35·4)	6·1 (5·1 to 7·7)	6·0 (5·0 to 7·6)	−1·7 (−4·5 to 0·8)
		Testicular cancer		8·6 (8·3 to 9·1)	9·4 (8·8 to 9·9)	8·4 (1·3 to 14·4)	0·1 (0·1 to 0·1)	0·1 (0·1 to 0·1)	−8·9 (−14·7 to −3·9)
		Kidney cancer		104·5 (102·4 to 106·9)	136·9 (133·0 to 141·3)	31·0 (27·0 to 34·6)	2·1 (2·1 to 2·1)	2·1 (2·0 to 2·2)	0·4 (−2·6 to 3·2)
		Bladder cancer		150·6 (147·9 to 153·2)	188·0 (182·8 to 192·7)	24·8 (21·4 to 28·3)	3·2 (3·2 to 3·3)	3·0 (2·9 to 3·1)	−6·4 (−9·0 to −3·8)
		Brain and nervous system cancer		190·4 (173·3 to 201·2)	228·8 (209·5 to 244·7)	20·1 (12·7 to 27·2)	3·5 (3·2 to 3·6)	3·3 (3·0 to 3·6)	−3·6 (−9·3 to 1·8)
		Thyroid cancer		25·5 (24·4 to 27·6)	31·9 (28·9 to 33·2)	24·8 (14·8 to 31·0)	0·5 (0·5 to 0·6)	0·5 (0·5 to 0·5)	−4·8 (−12·2 to 0·0)
		Mesothelioma		23·2 (22·7 to 23·8)	32·4 (31·2 to 33·4)	39·6 (34·4 to 44·3)	0·5 (0·5 to 0·5)	0·5 (0·5 to 0·5)	7·8 (3·6 to 11·6)
		Hodgkin lymphoma		25·4 (22·7 to 29·7)	23·9 (21·8 to 29·0)	−6·0 (−10·6 to −1·3)	0·5 (0·4 to 0·5)	0·3 (0·3 to 0·4)	−23·9 (−27·7 to −20·1)
		Non-Hodgkin's lymphoma		179·3 (160·6 to 191·6)	231·4 (195·7 to 243·8)	29·0 (18·1 to 35·2)	3·5 (3·1 to 3·7)	3·5 (3·0 to 3·7)	0·0 (−8·0 to 4·4)
		Multiple myeloma		77·5 (75·6 to 79·7)	101·1 (97·7 to 104·1)	30·5 (26·3 to 34·5)	1·6 (1·6 to 1·6)	1·6 (1·5 to 1·6)	−1·3 (−4·4 to 1·7)
		Leukaemia		303·5 (297·3 to 311·8)	353·5 (344·6 to 363·1)	16·5 (13·5 to 19·6)	5·6 (5·5 to 5·7)	5·3 (5·1 to 5·4)	−5·7 (−7·9 to −3·3)
		Acute lymphoid leukaemia		97·5 (90·4 to 108·0)	110·5 (101·2 to 118·4)	13·3 (7·1 to 19·0)	1·6 (1·5 to 1·8)	1·6 (1·4 to 1·7)	−3·0 (−8·1 to 1·7)
		Chronic lymphoid leukaemia		51·5 (49·3 to 53·9)	60·7 (57·9 to 64·6)	17·9 (12·5 to 23·4)	1·1 (1·0 to 1·1)	1·0 (0·9 to 1·0)	−10·1 (−14·0 to −6·1)
		Acute myeloid leukaemia		119·0 (110·5 to 126·7)	147·1 (137·3 to 157·0)	23·5 (19·3 to 27·8)	2·2 (2·1 to 2·3)	2·2 (2·1 to 2·3)	−0·4 (−3·5 to 2·7)
		Chronic myeloid leukaemia		35·4 (33·4 to 38·2)	35·2 (33·4 to 37·7)	−0·7 (−4·8 to 3·8)	0·7 (0·6 to 0·7)	0·5 (0·5 to 0·6)	−22·0 (−25·3 to −18·5)
		Other neoplasms		292·1 (270·9 to 302·6)	372·2 (335·9 to 392·1)	27·4 (21·4 to 32·6)	5·5 (5·1 to 5·7)	5·5 (5·0 to 5·8)	1·2 (−3·6 to 5·3)
	Cardiovascular diseases			15 933·7 (15 732·1 to 16 161·6)	17 921·0 (17 590·5 to 18 276·8)	12·5 (10·6 to 14·4)	338·1 (333·8 to 342·9)	285·5 (280·2 to 291·2)	−15·6 (−16·9 to −14·2)
		Rheumatic heart disease		333·2 (313·1 to 349·5)	319·4 (297·3 to 337·3)	−4·1 (−8·2 to −0·1)	6·4 (6·0 to 6·7)	4·8 (4·5 to 5·1)	−24·7 (−27·9 to −21·6)
		Ischaemic heart disease		7648·4 (7551·5 to 7774·3)	8917·0 (8751·6 to 9108·8)	16·6 (14·6 to 18·6)	163·1 (160·8 to 165·6)	142·1 (139·5 to 145·2)	−12·8 (−14·2 to −11·4)
		Cerebrovascular disease		6020·9 (5920·2 to 6127·1)	6326·1 (6175·2 to 6492·9)	5·1 (2·7 to 7·5)	127·9 (125·8 to 130·1)	101·0 (98·6 to 103·6)	−21·0 (−22·8 to −19·2)
			Ischaemic stroke	2760·8 (2682·7 to 2837·5)	2978·0 (2880·8 to 3068·8)	7·9 (5·2 to 10·6)	61·3 (59·5 to 62·9)	48·9 (47·3 to 50·4)	−20·2 (−22·1 to −18·1)
			Haemorrhagic stroke	3260·1 (3169·2 to 3359·8)	3348·2 (3240·9 to 3500·1)	2·7 (−0·6 to 6·4)	66·7 (64·9 to 68·7)	52·1 (50·4 to 54·5)	−21·9 (−24·3 to −19·0)
		Hypertensive heart disease		760·5 (711·9 to 823·7)	962·4 (873·6 to 1024·5)	26·5 (17·5 to 32·3)	16·2 (15·2 to 17·5)	15·4 (13·9 to 16·4)	−4·9 (−11·9 to −0·6)
		Cardiomyopathy and myocarditis		328·8 (315·8 to 338·8)	353·7 (339·5 to 370·6)	7·6 (3·8 to 11·4)	6·4 (6·2 to 6·6)	5·4 (5·2 to 5·7)	−16·1 (−18·9 to −13·2)
		Atrial fibrillation and flutter		142·8 (117·3 to 172·0)	195·3 (159·5 to 236·2)	36·8 (34·0 to 39·6)	3·4 (2·8 to 4·1)	3·3 (2·7 to 4·0)	−3·4 (−4·9 to −1·9)
		Aortic aneurysm		134·8 (131·7 to 138·3)	168·2 (163·6 to 172·8)	24·8 (19·5 to 28·5)	2·9 (2·8 to 3·0)	2·7 (2·6 to 2·8)	−6·6 (−10·4 to −3·8)
		Peripheral vascular disease		38·1 (36·7 to 39·6)	52·5 (49·7 to 55·7)	37·7 (30·1 to 46·5)	0·9 (0·8 to 0·9)	0·9 (0·8 to 0·9)	−0·3 (−5·9 to 6·3)
		Endocarditis		68·8 (60·4 to 74·7)	84·9 (74·7 to 93·0)	23·4 (18·5 to 28·2)	1·4 (1·2 to 1·5)	1·3 (1·2 to 1·4)	−4·6 (−8·1 to −1·1)
		Other cardiovascular and circulatory diseases		457·4 (446·7 to 470·6)	541·4 (521·7 to 561·2)	18·4 (14·0 to 22·5)	9·6 (9·3 to 9·8)	8·6 (8·3 to 8·9)	−10·3 (−13·5 to −7·4)
	Chronic respiratory diseases			3709·1 (3631·6 to 3796·0)	3795·5 (3683·9 to 3910·4)	2·3 (−0·8 to 5·4)	79·0 (77·3 to 80·9)	61·0 (59·3 to 62·8)	−22·8 (−25·1 to −20·4)
		Chronic obstructive pulmonary disease		3100·5 (2997·7 to 3194·9)	3188·3 (3083·8 to 3292·5)	2·8 (−0·6 to 6·9)	67·0 (64·8 to 69·0)	51·7 (50·0 to 53·4)	−22·9 (−25·4 to −20·0)
		Pneumoconiosis		31·9 (28·4 to 36·3)	36·1 (31·5 to 40·7)	13·2 (6·4 to 22·4)	0·7 (0·6 to 0·8)	0·6 (0·5 to 0·7)	−14·4 (−19·5 to −7·7)
			Silicosis	10·2 (9·4 to 11·4)	10·4 (9·4 to 11·7)	2·0 (−9·0 to 15·4)	0·2 (0·2 to 0·2)	0·2 (0·1 to 0·2)	−21·8 (−30·2 to −11·8)
			Asbestosis	2·8 (2·0 to 3·2)	3·6 (2·5 to 4·2)	28·4 (17·6 to 39·5)	0·1 (0·0 to 0·1)	0·1 (0·0 to 0·1)	−2·1 (−10·2 to 6·2)
			Coal workers pneumoconiosis	2·7 (2·4 to 3·0)	2·5 (2·2 to 2·9)	−7·3 (−21·5 to 6·4)	0·1 (0·1 to 0·1)	0·0 (0·0 to 0·0)	−29·9 (−40·4 to −19·6)
			Other pneumoconiosis	16·1 (13·3 to 19·7)	19·5 (15·8 to 23·2)	21·2 (11·0 to 33·5)	0·3 (0·3 to 0·4)	0·3 (0·3 to 0·4)	−9·4 (−16·9 to −0·2)
		Asthma		449·9 (362·1 to 518·0)	397·1 (363·0 to 438·7)	−11·7 (−21·2 to 3·4)	8·8 (7·1 to 10·2)	6·1 (5·6 to 6·7)	−31·3 (−38·9 to −19·4)
		Interstitial lung disease and pulmonary sarcoidosis		80·4 (61·2 to 92·8)	121·8 (94·1 to 135·2)	51·5 (37·9 to 60·5)	1·7 (1·3 to 2·0)	2·0 (1·5 to 2·2)	14·1 (4·1 to 20·9)
		Other chronic respiratory diseases		46·4 (33·9 to 55·6)	52·1 (38·0 to 61·0)	12·2 (1·4 to 23·0)	0·8 (0·6 to 1·0)	0·8 (0·6 to 0·9)	−5·9 (−13·8 to 2·5)
	Cirrhosis and other chronic liver diseases			1171·7 (1131·9 to 1236·7)	1292·1 (1239·9 to 1371·7)	10·3 (7·0 to 13·7)	21·6 (20·9 to 22·8)	18·8 (18·0 to 19·9)	−13·1 (−15·6 to −10·5)
		Cirrhosis and other chronic liver diseases due to hepatitis B		341·4 (316·2 to 374·1)	371·1 (341·6 to 410·0)	8·7 (4·7 to 12·7)	6·3 (5·9 to 6·9)	5·4 (5·0 to 6·0)	−14·6 (−17·5 to −11·6)
		Cirrhosis and other chronic liver diseases due to hepatitis C		287·4 (268·1 to 307·8)	325·6 (301·8 to 349·9)	13·3 (10·4 to 16·5)	5·4 (5·0 to 5·8)	4·8 (4·4 to 5·1)	−11·7 (−13·9 to −9·3)
		Cirrhosis and other chronic liver diseases due to alcohol use		310·1 (289·4 to 333·4)	347·9 (322·7 to 374·6)	12·2 (8·4 to 16·7)	5·6 (5·2 to 6·0)	5·0 (4·6 to 5·3)	−11·6 (−14·5 to −8·2)
		Cirrhosis and other chronic liver diseases due to other causes		232·8 (217·6 to 254·6)	247·5 (230·3 to 272·2)	6·3 (3·4 to 9·8)	4·3 (4·0 to 4·7)	3·6 (3·4 to 4·0)	−14·8 (−17·1 to −12·2)
	Digestive diseases			1113·2 (1081·1 to 1186·7)	1203·0 (1150·5 to 1270·0)	8·1 (3·7 to 12·6)	22·0 (21·4 to 23·4)	18·5 (17·7 to 19·5)	−16·1 (−19·3 to −12·7)
		Peptic ulcer disease		294·3 (278·0 to 322·0)	267·5 (249·4 to 290·0)	−9·1 (−15·3 to 0·0)	5·8 (5·5 to 6·3)	4·1 (3·8 to 4·4)	−29·3 (−34·1 to −22·1)
		Gastritis and duodenitis		49·9 (40·2 to 60·9)	49·7 (41·8 to 60·1)	−0·5 (−10·9 to 12·4)	1·0 (0·8 to 1·2)	0·8 (0·6 to 0·9)	−23·0 (−31·0 to −12·9)
		Appendicitis		49·9 (42·9 to 56·4)	50·1 (41·2 to 56·0)	0·5 (−9·0 to 10·7)	0·9 (0·8 to 1·0)	0·7 (0·6 to 0·8)	−17·0 (−24·5 to −9·0)
		Paralytic ileus and intestinal obstruction		233·4 (210·7 to 272·0)	264·3 (242·4 to 307·4)	13·2 (7·7 to 19·7)	4·6 (4·1 to 5·3)	4·0 (3·7 to 4·7)	−11·4 (−15·5 to −6·6)
		Inguinal, femoral, and abdominal hernia		56·6 (40·0 to 63·8)	59·8 (41·1 to 69·2)	5·8 (−3·1 to 16·8)	1·1 (0·8 to 1·3)	0·9 (0·6 to 1·1)	−18·1 (−25·2 to −9·3)
		Inflammatory bowel disease		42·6 (38·3 to 47·3)	47·4 (43·5 to 51·7)	11·2 (2·9 to 18·8)	0·9 (0·8 to 0·9)	0·7 (0·7 to 0·8)	−14·6 (−20·4 to −9·1)
		Vascular intestinal disorders		84·3 (79·0 to 91·2)	105·8 (99·0 to 114·3)	25·6 (20·4 to 30·5)	1·8 (1·7 to 2·0)	1·7 (1·6 to 1·8)	−6·1 (−10·0 to −2·7)
		Gallbladder and biliary diseases		96·4 (91·1 to 103·1)	111·7 (105·3 to 120·0)	15·9 (10·2 to 21·3)	2·0 (1·9 to 2·1)	1·8 (1·7 to 1·9)	−11·7 (−15·8 to −7·7)
		Pancreatitis		109·6 (103·0 to 117·2)	132·7 (122·9 to 144·0)	21·1 (14·3 to 28·1)	2·0 (1·9 to 2·2)	1·9 (1·8 to 2·1)	−3·9 (−9·2 to 1·5)
		Other digestive diseases		96·3 (86·5 to 116·2)	114·0 (101·9 to 131·1)	18·3 (8·1 to 28·9)	2·0 (1·8 to 2·4)	1·8 (1·6 to 2·0)	−9·5 (−16·6 to −2·1)
	Neurological disorders			1671·0 (1445·9 to 1889·8)	2258·9 (1937·8 to 2574·4)	35·2 (33·2 to 37·2)	38·7 (33·1 to 44·1)	37·6 (32·2 to 43·0)	−2·7 (−3·7 to −1·6)
		Alzheimer's disease and other dementias		1380·8 (1152·7 to 1599·4)	1908·2 (1586·7 to 2229·1)	38·2 (36·2 to 40·1)	33·2 (27·6 to 38·7)	32·3 (26·9 to 37·8)	−2·7 (−3·7 to −1·7)
		Parkinson's disease		82·4 (80·0 to 85·6)	117·4 (113·9 to 121·3)	42·4 (37·3 to 46·9)	1·9 (1·8 to 2·0)	2·0 (1·9 to 2·0)	4·2 (0·5 to 7·6)
		Epilepsy		119·0 (111·9 to 127·6)	124·9 (119·3 to 131·0)	5·0 (−2·1 to 12·3)	1·9 (1·8 to 2·0)	1·7 (1·6 to 1·8)	−9·2 (−15·1 to −3·3)
		Multiple sclerosis		16·5 (14·9 to 18·1)	18·9 (17·3 to 20·0)	14·8 (7·2 to 20·7)	0·3 (0·3 to 0·3)	0·3 (0·2 to 0·3)	−9·7 (−15·3 to −5·1)
		Motor neuron disease		27·6 (26·8 to 28·8)	35·2 (33·9 to 36·7)	27·6 (20·6 to 31·1)	0·5 (0·5 to 0·6)	0·5 (0·5 to 0·6)	−1·2 (−6·5 to 1·6)
		Other neurological disorders		44·8 (43·6 to 47·8)	54·3 (53·0 to 57·5)	21·3 (17·5 to 24·5)	0·8 (0·8 to 0·8)	0·8 (0·8 to 0·8)	−1·0 (−4·1 to 1·7)
	Mental and substance use disorders			305·9 (293·5 to 314·9)	324·9 (308·6 to 337·4)	6·2 (1·9 to 10·4)	5·1 (4·9 to 5·3)	4·5 (4·3 to 4·7)	−12·6 (−16·0 to −9·2)
		Schizophrenia		19·1 (17·9 to 20·0)	16·9 (15·9 to 18·0)	−11·4 (−18·8 to −2·9)	0·3 (0·3 to 0·4)	0·2 (0·2 to 0·3)	−29·2 (−34·9 to −22·9)
		Alcohol use disorders		157·4 (147·4 to 163·3)	137·5 (131·5 to 144·0)	−12·6 (−16·7 to −7·0)	2·7 (2·5 to 2·8)	1·9 (1·8 to 2·0)	−29·2 (−32·4 to −24·7)
		Drug use disorders		128·8 (124·0 to 133·6)	169·9 (152·1 to 179·2)	31·8 (20·4 to 39·4)	2·1 (2·0 to 2·2)	2·3 (2·1 to 2·5)	11·5 (2·0 to 17·7)
			Opioid use disorders	94·2 (90·5 to 99·7)	122·1 (109·5 to 129·7)	29·6 (18·2 to 37·2)	1·5 (1·5 to 1·6)	1·7 (1·5 to 1·8)	10·0 (0·5 to 16·5)
			Cocaine use disorders	7·4 (5·0 to 7·9)	11·1 (8·5 to 12·2)	49·7 (33·6 to 75·4)	0·1 (0·1 to 0·1)	0·1 (0·1 to 0·2)	26·4 (12·9 to 48·0)
			Amphetamine use disorders	7·3 (4·3 to 8·2)	12·2 (8·4 to 14·2)	67·5 (25·6 to 118·9)	0·1 (0·1 to 0·1)	0·2 (0·1 to 0·2)	42·3 (7·2 to 85·6)
			Other drug use disorders	19·9 (18·6 to 22·8)	24·5 (22·7 to 27·3)	23·0 (12·7 to 32·1)	0·3 (0·3 to 0·4)	0·3 (0·3 to 0·4)	2·6 (−5·6 to 9·5)
		Eating disorders		0·6 (0·4 to 0·8)	0·7 (0·5 to 0·9)	7·7 (0·8 to 17·3)	0·0 (0·0 to 0·0)	0·0 (0·0 to 0·0)	−4·4 (−10·2 to 3·5)
			Anorexia nervosa	0·6 (0·4 to 0·7)	0·6 (0·4 to 0·8)	5·6 (−1·2 to 14·1)	0·0 (0·0 to 0·0)	0·0 (0·0 to 0·0)	−6·0 (−11·8 to 1·1)
			Bulimia nervosa	0·0 (0·0 to 0·1)	0·1 (0·0 to 0·1)	33·2 (19·1 to 58·1)	0·0 (0·0 to 0·0)	0·0 (0·0 to 0·0)	14·6 (3·3 to 34·2)
	Diabetes, urogenital, blood, and endocrine diseases			2635·3 (2534·5 to 2716·0)	3409·3 (3287·5 to 3516·5)	29·4 (26·1 to 32·7)	52·9 (51·0 to 54·3)	52·9 (51·0 to 54·5)	−0·1 (−2·3 to 2·3)
		Diabetes mellitus		1150·2 (1120·9 to 1176·8)	1519·0 (1470·3 to 1576·0)	32·1 (27·7 to 36·3)	23·6 (23·0 to 24·2)	23·7 (23·0 to 24·6)	0·4 (−2·7 to 3·6)
		Acute glomerulonephritis		12·7 (9·9 to 14·4)	11·8 (7·8 to 13·3)	−6·9 (−23·8 to 2·0)	0·2 (0·2 to 0·3)	0·2 (0·1 to 0·2)	−25·3 (−38·9 to −18·2)
		Chronic kidney disease		937·7 (866·2 to 970·8)	1234·9 (1131·7 to 1282·4)	31·7 (27·7 to 35·6)	19·0 (17·7 to 19·7)	19·2 (17·7 to 20·0)	1·2 (−1·9 to 4·0)
			Chronic kidney disease due to diabetes mellitus	299·4 (278·7 to 314·2)	417·8 (388·7 to 441·4)	39·5 (35·4 to 43·5)	6·1 (5·7 to 6·4)	6·5 (6·1 to 6·9)	6·4 (3·3 to 9·3)
			Chronic kidney disease due to hypertension	408·5 (377·1 to 427·6)	549·5 (501·6 to 575·6)	34·5 (30·0 to 38·7)	8·4 (7·8 to 8·8)	8·7 (7·9 to 9·1)	2·4 (−0·9 to 5·5)
			Chronic kidney disease due to glomerulonephritis	205·6 (184·9 to 217·9)	237·7 (212·6 to 255·9)	15·6 (10·9 to 20·2)	4·0 (3·6 to 4·2)	3·6 (3·3 to 3·9)	−9·0 (−12·6 to −5·4)
			Chronic kidney disease due to other causes	24·2 (20·2 to 28·6)	30·0 (25·0 to 35·2)	23·9 (17·8 to 30·2)	0·5 (0·4 to 0·6)	0·5 (0·4 to 0·5)	−2·2 (−6·9 to 2·5)
		Urinary diseases and male infertility		201·1 (188·2 to 215·7)	261·7 (243·2 to 277·6)	30·1 (23·7 to 36·6)	4·2 (3·9 to 4·5)	4·1 (3·8 to 4·4)	−1·3 (−6·1 to 3·6)
			Interstitial nephritis and urinary tract infections	149·8 (139·3 to 160·9)	196·4 (181·5 to 211·0)	31·1 (25·3 to 37·3)	3·2 (2·9 to 3·4)	3·1 (2·9 to 3·4)	−1·1 (−5·5 to 3·7)
			Urolithiasis	14·9 (12·5 to 18·1)	16·1 (14·0 to 20·3)	7·8 (0·0 to 21·2)	0·3 (0·3 to 0·4)	0·2 (0·2 to 0·3)	−16·5 (−22·6 to −5·8)
			Other urinary diseases	36·4 (30·8 to 42·7)	49·2 (41·2 to 55·4)	35·1 (20·4 to 49·9)	0·7 (0·6 to 0·9)	0·8 (0·6 to 0·9)	4·2 (−7·1 to 15·3)
		Gynaecological diseases		7·6 (6·1 to 8·6)	7·9 (5·9 to 9·2)	2·9 (−10·1 to 24·5)	0·1 (0·1 to 0·2)	0·1 (0·1 to 0·1)	−17·4 (−28·0 to −1·0)
			Uterine fibroids	2·1 (1·2 to 2·7)	2·3 (1·2 to 3·0)	9·5 (−12·5 to 34·7)	0·0 (0·0 to 0·0)	0·0 (0·0 to 0·0)	−13·3 (−30·5 to 5·8)
			Polycystic ovarian syndrome	0·7 (0·2 to 1·3)	0·6 (0·2 to 1·0)	−17·9 (−37·0 to 10·1)	0·0 (0·0 to 0·0)	0·0 (0·0 to 0·0)	−30·9 (−47·0 to −7·9)
			Endometriosis	0·0 (0·0 to 0·1)	0·1 (0·0 to 0·1)	24·2 (−0·6 to 59·4)	0·0 (0·0 to 0·0)	0·0 (0·0 to 0·0)	5·7 (−15·2 to 35·4)
			Genital prolapse	1·1 (0·6 to 1·8)	0·9 (0·6 to 1·5)	−19·5 (−36·0 to 12·9)	0·0 (0·0 to 0·0)	0·0 (0·0 to 0·0)	−39·0 (−51·1 to −15·9)
			Other gynaecological diseases	3·6 (2·7 to 4·5)	4·0 (3·0 to 4·8)	9·9 (−5·8 to 34·4)	0·1 (0·0 to 0·1)	0·1 (0·0 to 0·1)	−10·0 (−23·1 to 9·9)
		Haemoglobinopathies and haemolytic anaemias		215·5 (173·5 to 274·0)	226·9 (177·2 to 306·2)	5·3 (−9·4 to 24·1)	3·6 (2·9 to 4·5)	3·2 (2·5 to 4·3)	−9·8 (−21·2 to 5·1)
			Thalassaemias	19·7 (16·5 to 23·3)	16·8 (13·9 to 20·2)	−14·5 (−27·7 to 2·9)	0·3 (0·3 to 0·4)	0·2 (0·2 to 0·3)	−23·6 (−34·1 to −10·3)
			Sickle cell disorders	108·3 (78·5 to 159·8)	114·8 (78·3 to 183·2)	6·0 (−20·1 to 40·4)	1·6 (1·2 to 2·3)	1·6 (1·1 to 2·5)	−2·7 (−26·0 to 28·3)
			Glucose-6-phosphate dehydrogenase deficiency	27·5 (23·5 to 32·1)	33·0 (28·0 to 38·9)	19·9 (11·6 to 29·2)	0·4 (0·4 to 0·5)	0·5 (0·4 to 0·5)	1·2 (−5·9 to 8·7)
			Other haemoglobinopathies and haemolytic anaemias	60·0 (53·2 to 66·5)	62·3 (54·7 to 71·1)	3·9 (−3·4 to 10·2)	1·2 (1·1 to 1·3)	1·0 (0·9 to 1·1)	−19·6 (−25·6 to −15·0)
		Endocrine, metabolic, blood, and immune disorders		110·5 (108·1 to 113·5)	147·3 (142·5 to 151·7)	33·2 (28·0 to 37·2)	2·1 (2·1 to 2·2)	2·2 (2·2 to 2·3)	5·6 (1·7 to 8·7)
	Musculoskeletal disorders			76·2 (69·2 to 80·5)	90·1 (82·5 to 94·6)	18·2 (10·8 to 24·3)	1·5 (1·4 to 1·6)	1·4 (1·3 to 1·5)	−8·3 (−13·5 to −3·8)
		Rheumatoid arthritis		26·5 (23·2 to 29·3)	30·0 (27·0 to 34·6)	13·2 (5·0 to 23·5)	0·6 (0·5 to 0·6)	0·5 (0·4 to 0·5)	−14·0 (−20·0 to −6·4)
		Other musculoskeletal disorders		49·7 (45·5 to 53·1)	60·1 (53·1 to 63·5)	20·9 (11·5 to 27·0)	1·0 (0·9 to 1·0)	0·9 (0·8 to 1·0)	−5·0 (−11·5 to −0·5)
	Other non-communicable diseases			726·6 (626·8 to 869·3)	744·6 (667·8 to 811·6)	2·5 (−10·6 to 11·7)	10·9 (9·4 to 13·0)	10·2 (9·2 to 11·1)	−6·5 (−17·7 to 1·5)
		Congenital anomalies		634·2 (535·9 to 773·4)	627·8 (567·3 to 694·4)	−1·0 (−15·4 to 9·0)	9·2 (7·8 to 11·2)	8·4 (7·6 to 9·3)	−8·0 (−21·1 to 1·1)
			Neural tube defects	76·5 (56·7 to 107·0)	64·6 (47·9 to 83·4)	−15·5 (−34·9 to 4·4)	1·1 (0·8 to 1·5)	0·9 (0·6 to 1·1)	−20·5 (−38·8 to −1·7)
			Congenital heart anomalies	319·0 (267·4 to 378·1)	303·3 (268·8 to 335·4)	−4·9 (−18·9 to 6·5)	4·6 (3·9 to 5·4)	4·1 (3·6 to 4·5)	−11·4 (−24·4 to −0·9)
			Cleft lip and cleft palate	3·3 (2·6 to 3·8)	1·3 (1·1 to 1·7)	−59·0 (−66·4 to −50·1)	0·0 (0·0 to 0·1)	0·0 (0·0 to 0·0)	−61·3 (−68·3 to −52·9)
			Down's syndrome	26·6 (17·9 to 42·7)	26·5 (19·1 to 36·9)	−0·2 (−25·0 to 30·6)	0·4 (0·3 to 0·6)	0·4 (0·3 to 0·5)	−10·1 (−31·7 to 16·9)
			Other chromosomal abnormalities	20·6 (9·8 to 49·3)	22·7 (12·9 to 40·5)	10·3 (−21·8 to 41·0)	0·3 (0·1 to 0·7)	0·3 (0·2 to 0·5)	3·3 (−26·6 to 31·3)
			Other congenital anomalies	188·3 (158·7 to 231·9)	209·4 (188·9 to 241·3)	11·2 (−3·7 to 24·5)	2·8 (2·3 to 3·4)	2·8 (2·6 to 3·3)	2·7 (−10·6 to 14·6)
		Skin and subcutaneous diseases		71·6 (48·1 to 90·4)	97·6 (66·6 to 128·8)	36·2 (29·5 to 46·4)	1·5 (1·0 to 1·8)	1·5 (1·0 to 2·0)	3·7 (−1·6 to 12·5)
			Cellulitis	12·6 (7·8 to 17·4)	16·9 (10·4 to 23·0)	34·2 (22·2 to 48·2)	0·3 (0·2 to 0·3)	0·3 (0·2 to 0·3)	3·5 (−5·5 to 14·1)
			Pyoderma	30·9 (21·3 to 43·5)	44·1 (31·1 to 62·8)	42·7 (33·0 to 53·3)	0·6 (0·4 to 0·8)	0·7 (0·5 to 1·0)	12·5 (4·7 to 21·4)
			Decubitus ulcer	25·1 (14·5 to 30·7)	32·4 (19·3 to 40·0)	29·3 (20·4 to 42·4)	0·6 (0·3 to 0·7)	0·5 (0·3 to 0·7)	−5·7 (−12·6 to 4·4)
			Other skin and subcutaneous diseases	3·1 (2·2 to 4·2)	4·2 (3·0 to 5·8)	35·4 (26·9 to 46·0)	0·1 (0·0 to 0·1)	0·1 (0·0 to 0·1)	5·7 (−1·2 to 14·2)
			Sudden infant death syndrome	20·8 (16·9 to 33·7)	19·2 (15·9 to 27·5)	−7·9 (−23·0 to 8·9)	0·3 (0·2 to 0·5)	0·3 (0·2 to 0·4)	−13·5 (−27·7 to 2·3)
**Injuries**				**4759·0 (4451·4 to 4893·1)**	**4725·1 (4398·5 to 4905·2)**	**−0·7 (−4·3 to 3·5)**	**78·6 (73·5 to 80·8)**	**66·2 (61·5 to 68·7)**	**−15·8 (−18·7 to −12·4)**
	Transport injuries			1494·1 (1444·8 to 1550·6)	1466·6 (1394·8 to 1536·5)	−1·8 (−7·4 to 3·3)	24·2 (23·4 to 25·0)	20·2 (19·3 to 21·2)	−16·2 (−20·8 to −11·8)
		Road injuries		1392·5 (1341·0 to 1446·0)	1361·7 (1294·0 to 1428·1)	−2·2 (−7·8 to 2·6)	22·5 (21·7 to 23·3)	18·8 (17·9 to 19·7)	−16·4 (−21·1 to −12·3)
			Pedestrian road injuries	581·4 (546·3 to 631·3)	560·6 (525·8 to 617·1)	−3·6 (−10·7 to 2·8)	9·6 (9·1 to 10·5)	7·8 (7·4 to 8·6)	−18·6 (−24·6 to −13·2)
			Cyclist road injuries	63·4 (58·7 to 68·9)	58·7 (54·4 to 64·4)	−7·5 (−15·2 to 1·2)	1·0 (1·0 to 1·1)	0·8 (0·8 to 0·9)	−21·9 (−28·5 to −14·6)
			Motorcyclist road injuries	245·7 (215·2 to 263·8)	257·1 (230·6 to 290·9)	4·6 (−4·7 to 16·5)	3·8 (3·4 to 4·1)	3·5 (3·1 to 3·9)	−8·9 (−17·0 to 1·6)
			Motor vehicle road injuries	483·1 (445·9 to 536·6)	464·2 (417·8 to 508·3)	−3·9 (−9·3 to 2·1)	7·7 (7·1 to 8·5)	6·4 (5·7 to 7·0)	−17·2 (−21·8 to −12·1)
			Other road injuries	18·9 (13·1 to 22·5)	21·1 (14·0 to 24·8)	11·3 (−5·4 to 34·9)	0·3 (0·2 to 0·4)	0·3 (0·2 to 0·3)	−4·5 (−18·7 to 14·7)
		Other transport injuries		101·5 (93·2 to 114·0)	104·9 (90·5 to 127·2)	3·3 (−6·4 to 14·7)	1·7 (1·5 to 1·9)	1·4 (1·2 to 1·8)	−12·6 (−20·7 to −3·2)
	Unintentional injuries			1887·3 (1701·0 to 1969·6)	1838·7 (1634·6 to 1939·1)	−2·6 (−5·7 to 3·2)	32·3 (29·1 to 33·6)	26·5 (23·6 to 28·0)	−17·8 (−20·4 to −13·4)
		Falls		436·1 (402·3 to 451·2)	527·2 (467·8 to 554·5)	20·9 (14·6 to 27·2)	8·6 (7·9 to 8·9)	8·1 (7·2 to 8·5)	−5·5 (−10·1 to −0·8)
		Drowning		403·1 (349·2 to 424·7)	323·8 (285·8 to 347·5)	−19·7 (−23·6 to −14·2)	6·3 (5·4 to 6·6)	4·5 (4·0 to 4·8)	−28·5 (−31·8 to −23·8)
		Fire, heat, and hot substances		195·2 (161·4 to 209·0)	176·0 (145·1 to 189·6)	−9·9 (−14·8 to −2·5)	3·3 (2·7 to 3·5)	2·5 (2·1 to 2·7)	−23·5 (−27·7 to −17·7)
		Poisonings		100·8 (71·4 to 117·4)	86·4 (58·6 to 101·0)	−14·3 (−24·1 to −1·1)	1·6 (1·2 to 1·9)	1·2 (0·8 to 1·4)	−26·7 (−34·3 to −16·2)
		Exposure to mechanical forces		202·6 (175·4 to 214·0)	200·6 (157·6 to 216·7)	−1·0 (−11·7 to 7·0)	3·3 (2·8 to 3·5)	2·8 (2·2 to 3·0)	−15·1 (−24·1 to −8·9)
			Unintentional firearm injuries	32·7 (23·8 to 35·6)	32·0 (23·3 to 35·1)	−2·1 (−7·1 to 3·3)	0·5 (0·4 to 0·6)	0·5 (0·3 to 0·5)	−17·0 (−20·9 to −12·7)
			Unintentional suffocation	34·9 (27·1 to 39·4)	35·6 (25·6 to 40·3)	2·0 (−11·6 to 15·8)	0·6 (0·4 to 0·6)	0·5 (0·4 to 0·6)	−9·2 (−21·2 to 2·5)
			Other exposure to mechanical forces	135·0 (113·9 to 144·3)	133·0 (101·1 to 145·1)	−1·5 (−13·6 to 8·5)	2·2 (1·9 to 2·3)	1·9 (1·4 to 2·0)	−16·2 (−26·2 to −8·3)
		Adverse effects of medical treatment		97·3 (74·1 to 107·8)	99·8 (77·3 to 109·0)	2·5 (−2·6 to 9·3)	1·7 (1·4 to 1·9)	1·5 (1·1 to 1·6)	−15·6 (−19·2 to −11·4)
		Animal contact		104·4 (67·1 to 114·9)	94·0 (55·8 to 132·3)	−9·9 (−19·7 to 18·4)	1·7 (1·1 to 1·9)	1·3 (0·8 to 1·8)	−22·5 (−31·0 to 1·6)
			Venomous animal contact	88·2 (54·1 to 98·6)	79·6 (44·2 to 115·9)	−9·8 (−21·0 to 20·5)	1·4 (0·9 to 1·6)	1·1 (0·6 to 1·6)	−22·3 (−32·1 to 3·7)
			Non-venomous animal contact	16·2 (13·1 to 18·7)	14·4 (11·4 to 16·4)	−10·8 (−19·7 to 10·9)	0·3 (0·2 to 0·3)	0·2 (0·2 to 0·2)	−23·5 (−30·6 to −5·8)
		Foreign body		145·7 (118·7 to 175·9)	151·6 (132·6 to 169·8)	4·1 (−4·7 to 13·6)	2·5 (2·1 to 3·0)	2·2 (1·9 to 2·4)	−12·3 (−18·4 to −6·1)
			Pulmonary aspiration and foreign body in airway	116·6 (93·0 to 148·1)	124·0 (105·5 to 143·2)	6·3 (−4·9 to 15·7)	2·0 (1·7 to 2·5)	1·8 (1·5 to 2·1)	−10·8 (−18·5 to −4·6)
			Foreign body in other body part	29·0 (18·1 to 43·2)	27·6 (20·3 to 34·4)	−5·0 (−22·7 to 11·2)	0·5 (0·3 to 0·7)	0·4 (0·3 to 0·5)	−18·8 (−34·3 to −6·2)
		Environmental heat and cold exposure		53·4 (39·9 to 57·2)	45·2 (33·5 to 49·5)	−15·4 (−21·1 to −9·1)	0·9 (0·7 to 1·0)	0·7 (0·5 to 0·7)	−31·3 (−35·8 to −26·6)
		Other unintentional injuries		148·8 (141·4 to 157·7)	134·2 (124·8 to 147·8)	−9·8 (−16·7 to −1·6)	2·4 (2·3 to 2·5)	1·9 (1·7 to 2·0)	−22·3 (−28·2 to −15·4)
	Self-harm and interpersonal violence			1253·0 (1125·6 to 1288·8)	1236·7 (1130·1 to 1287·9)	−1·3 (−5·7 to 3·0)	20·3 (18·2 to 20·9)	17·0 (15·5 to 17·7)	−16·3 (−20·1 to −12·6)
		Self-harm		827·6 (725·4 to 855·5)	828·1 (745·8 to 868·7)	0·1 (−6·2 to 6·1)	13·7 (12·0 to 14·2)	11·5 (10·3 to 12·1)	−16·3 (−21·5 to −11·2)
		Interpersonal violence		425·3 (388·7 to 439·6)	408·6 (370·5 to 431·5)	−3·9 (−7·0 to −0·1)	6·5 (6·0 to 6·8)	5·5 (5·0 to 5·8)	−16·4 (−19·1 to −13·1)
			Assault by firearm	162·9 (142·8 to 168·8)	173·1 (149·3 to 183·2)	6·3 (2·4 to 11·0)	2·4 (2·1 to 2·5)	2·3 (2·0 to 2·4)	−6·0 (−9·4 to −1·8)
			Assault by sharp object	104·4 (97·5 to 110·7)	89·5 (83·3 to 97·3)	−14·3 (−18·8 to −9·1)	1·6 (1·5 to 1·7)	1·2 (1·1 to 1·3)	−25·6 (−29·4 to −21·2)
			Assault by other means	158·0 (143·1 to 167·5)	145·9 (129·6 to 159·7)	−7·6 (−12·6 to −1·5)	2·5 (2·3 to 2·6)	2·0 (1·8 to 2·2)	−20·6 (−24·8 to −15·4)
	Forces of nature, war, and legal intervention			124·7 (82·5 to 166·5)	183·1 (100·0 to 263·8)	46·8 (−14·2 to 120·5)	1·9 (1·3 to 2·6)	2·4 (1·3 to 3·5)	27·4 (−25·5 to 90·7)
		Exposure to forces of nature		90·8 (53·0 to 128·0)	11·8 (7·2 to 16·4)	−87·0 (−87·9 to −86·1)	1·4 (0·8 to 2·0)	0·2 (0·1 to 0·2)	−88·5 (−89·3 to −87·6)
		Collective violence and legal intervention		33·8 (25·5 to 43·0)	171·3 (88·1 to 251·1)	406·0 (236·0 to 524·5)	0·5 (0·4 to 0·7)	2·3 (1·2 to 3·3)	347·5 (192·4 to 455·8)

Data in parentheses are 95% uncertainty intervals.

**Table 6:** Selected causes of global child deaths for both sexes combined in 2005 and 2015, with percentage change between 2005 and 2015

		**Neonates age <1 month**		**Children age 1–59 months**		**Under-5 totals**	
		2015 (thousands)	Percentage change, 2005–15	2015 (thousands)	Percentage change, 2005–15	2015 (thousands)	Percentage change, 2005–15
All causes		2621·5 (2562·0–2680·8)	−20·3 (−21·8 to −18·7)	3199·4 (3093·9–3309·8)	−32·1 (−34·2 to −29·7)	5820·9 (5673·0–5965·2)	−27·2 (−29·0 to −25·2)
Communicable, maternal, neonatal, and nutritional diseases		2331·6 (2272·8–2394·0)	−21·6 (−23·2 to −19·9)	2371·7 (2267·7–2473·5)	−37·1 (−39·9 to −34·4)	4703·4 (4569·9–4845·5)	−30·3 (−32·4 to −28·1)
	HIV/AIDS	..	..	88·9 (84·3–93·7)	−51·9 (−54·2 to −49·6)	88·9 (84·3–93·7)	−51·9 (−54·2 to −49·6)
	Diarrhoeal diseases	44·0 (38·6–50·6)	−38·5 (−46·3 to −29·1)	454·9 (404·4–510·2)	−33·9 (−42·4 to −23·4)	498·9 (447·5–557·6)	−34·3 (−42·3 to −24·9)
	Intestinal infectious diseases	..	..	42·2 (22·5–73·2)	−20·0 (−29·8 to −8·9)	42·2 (22·5–73·2)	−20·0 (−29·8 to −8·9)
	Lower respiratory infections	152·9 (140·4–166·6)	−35·9 (−40·8 to −30·7)	551·0 (502·2–600·5)	−37·1 (−43·0 to −30·9)	703·9 (651·4–763·0)	−36·9 (−42·0 to −31·6)
	Meningitis	25·8 (18·3–35·9)	−15·6 (−31·9 to 9·3)	147·3 (117·1–196·0)	−17·9 (−32·0 to 4·9)	173·1 (137·1–228·9)	−17·6 (−31·0 to 4·0)
	Whooping cough	..	..	54·5 (18·8–117·0)	−41·0 (−77·8 to 63·5)	54·5 (18·8–117·0)	−41·0 (−77·8 to 63·5)
	Tetanus	19·9 (17·0–23·5)	−57·7 (−64·2 to −50·0)	5·6 (4·1–7·8)	−55·2 (−66·4 to −39·3)	25·5 (21·8–30·9)	−57·2 (−63·8 to −49·1)
	Measles	..	..	62·6 (22·4–135·8)	−75·1 (−84·5 to −59·6)	62·6 (22·4–135·8)	−75·1 (−84·5 to −59·6)
	Malaria	13·9 (8·9–19·8)	−55·9 (−67·8 to −41·1)	460·2 (324·1–604·9)	−42·3 (−54·1 to −29·0)	474·1 (333·3–623·7)	−42·8 (−54·6 to −29·4)
	Neonatal preterm birth complications	765·9 (700·0–854·3)	−25·9 (−31·5 to −20·5)	39·9 (32·7–48·3)	−25·9 (−39·3 to −8·4)	805·8 (736·2–898·6)	−25·9 (−31·3 to −20·6)
	Neonatal encephalopathy (birth asphyxia and trauma)	707·8 (638·4–789·7)	−16·3 (−23·8 to −8·0)	32·6 (24·8–43·0)	−11·9 (−34·0 to 16·4)	740·4 (667·6–829·2)	−16·1 (−23·8 to −8·0)
	Neonatal sepsis and other neonatal infections	336·3 (237·4–441·5)	−0·5 (−16·9 to 20·7)	15·4 (10·0–20·6)	7·8 (−18·0 to 40·6)	351·7 (249·2–459·1)	−0·2 (−16·2 to 20·3)
	Other neonatal disorders	180·0 (133·9–229·4)	−16·4 (−35·1 to 6·2)	40·3 (29·2–52·3)	−14·1 (−39·2 to 19·9)	220·2 (167·6–276·8)	−16·0 (−34·1 to 5·6)
	Nutritional deficiencies	..	..	192·8 (147·2–248·1)	−24·3 (−40·4 to −4·1)	192·8 (147·2–248·1)	−24·3 (−40·4 to −4·1)
	Syphilis	31·5 (17·5–49·2)	−28·4 (−34·5 to −21·5)	59·0 (32·9–95·7)	−16·5 (−28·7 to −5·4)	90·5 (50·6–144·5)	−21·1 (−30·4 to −12·5)
	Other communicable diseases	53·6 (39·3–75·5)	−30·9 (−44·1 to −16·4)	124·7 (108·2–141·5)	−21·7 (−30·2 to −10·3)	178·3 (151·7–211·3)	−24·7 (−32·3 to −16·2)
Non-communicable diseases		267·4 (234·3–290·8)	−7·1 (−18·4 to 0·8)	537·2 (488·1–592·6)	−8·0 (−18·0 to 2·9)	804·5 (733·8–868·9)	−7·7 (−17·3 to 1·0)
	Congenital anomalies	242·6 (213·6–263·6)	−6·0 (−18·4 to 2·5)	254·0 (223·6–297·4)	−0·4 (−17·2 to 13·7)	496·6 (444·4–554·6)	−3·2 (−17·8 to 7·6)
	Sudden infant death syndrome	1·9 (1·6–2·6)	−9·7 (−25·5 to 4·9)	17·3 (14·2–24·9)	−7·7 (−23·1 to 9·7)	19·2 (15·9–27·5)	−7·9 (−23·0 to 8·9)
	Other non-communicable diseases	22·9 (17·3–37·3)	−17·3 (−29·3 to 3·8)	265·9 (232·7–315·0)	−14·3 (−25·5 to 1·6)	288·8 (251·6–348·9)	−14·6 (−25·3 to −0·1)
Injuries		22·5 (17·0–25·9)	−13·9 (−23·1 to −2·7)	290·5 (247·2–323·4)	−17·5 (−25·9 to −5·0)	313·0 (265·2–348·4)	−17·3 (−25·3 to −5·2)
	Road injuries	2·4 (1·7–3·4)	−27·8 (−49·9 to 2·4)	47·1 (40·9–54·2)	−16·0 (−30·0 to 4·1)	49·5 (43·0–56·8)	−16·7 (−30·1 to 2·5)
	Drowning	1·4 (0·9–1·9)	−20·3 (−44·4 to 16·9)	54·7 (43·4–64·3)	−37·1 (−47·0 to −23·9)	56·1 (44·4–65·7)	−36·8 (−46·6 to −23·8)
	Other injuries	18·7 (14·0–21·7)	−11·2 (−20·8 to 1·6)	188·7 (155·8–216·0)	−9·8 (−20·2 to 6·2)	207·4 (170·2–236·6)	−9·9 (−19·9 to 5·3)

Data in parenthesis are 95% uncertainty intervals. The selected causes are the major causes of death within each Level 1 group that accounted for deaths in children younger than 5 years. Neonates were defined as children younger than 1 month. Childhood was defined as ages 1–59 months.

**Table 7:** Global counterfactual deaths and population attributable fractions for diarrhoea and lower respiratory infection pathogens for 2005 and 2015, with percentage change between 2005 and 2015

	**Children younger than 5 years**					**All ages**				
	2005		2015		Percentage change, 2005–15	2005		2015		Percentage change, 2005–15
	Deaths (thousands)	Population attributable fractions (%)	Deaths(thousands)	Population attributable fractions (%)		Deaths(thousands)	Population attributable fractions (%)	Deaths (thousands)	Population attributable fractions (%)	
**Diarrhoea**										
Cholera	46·8 (32·2 to 64·5)	6·2 (4·3 to 8·4)	28·8 (20·6 to 39·7)	5·8 (4·1 to 7·9)	−38·4 (−49·9 to −24·3)	98·7 (70·7 to 130·3)	6·0 (4·3 to 7·7)	68·4 (50·4 to 87·1)	5·2 (3·8 to 6·6)	−30·7 (−38·9 to −21·1)
Other *Salmonella* infections	60·1 (18·6 to 131·3)	7·9 (2·5 to 17·6)	38·5 (12·2 to 84·2)	7·7 (2·5 to 16·6)	−35·9 (−76·9 to 76·3)	116·8 (44·3 to 241·9)	7·0 (2·7 to 14·5)	90·3 (34·1 to 183·1)	6·9 (2·7 to 13·9)	−22·7 (−71·8 to 116·1)
Shigellosis	83·0 (42·8 to 147·3)	10·9 (5·7 to 18·9)	54·9 (27·0 to 94·7)	11·0 (5·5 to 18·7)	−33·8 (−68·3 to 40·9)	195·4 (101·5 to 328·5)	11·8 (6·2 to 19·6)	164·3 (85·0 to 278·7)	12·5 (6·4 to 21·2)	−15·9 (−59·2 to 77·4)
Enteropathogenic *Escherichia coli* infection	15·2 (0·7 to 41·7)	2·0 (0·1 to 5·4)	11·3 (0·7 to 32·0)	2·3 (0·1 to 6·2)	−26·0 (−85·5 to 283·4)	16·0 (0·6 to 44·6)	1·0 (0·0 to 2·6)	12·0 (0·6 to 34·1)	0·9 (0·0 to 2·6)	−25·1 (−83·8 to 249·8)
Enterotoxigenic *Escherichia coli* infection	38·2 (15·7 to 72·5)	5·0 (2·1 to 9·3)	23·6 (9·6 to 44·3)	4·7 (2·0 to 8·9)	−38·1 (−74·0 to 42·7)	91·8 (40·3 to 167·0)	5·5 (2·5 to 10·1)	74·1 (29·9 to 137·9)	5·6 (2·3 to 10·4)	−19·3 (−66·5 to 91·2)
*Campylobacter* enteritis	46·1 (10·5 to 94·1)	6·1 (1·4 to 12·4)	30·9 (8·3 to 62·5)	6·2 (1·7 to 12·5)	−32·9 (−68·2 to 48·8)	52·7 (11·1 to 111·0)	3·2 (0·7 to 6·8)	37·5 (6·3 to 81·6)	2·9 (0·5 to 6·2)	−28·9 (−69·8 to 67·0)
Amoebiasis	26·1 (−37·8 to 173·9)	3·4 (−4·9 to 22·3)	15·5 (−32·4 to 102·4)	3·1 (−6·3 to 20·7)	−40·8 (−467·1 to 881·9	79·7 (4·9 to 316·8)	4·8 (0·3 to 18·8)	67·9 (5·6 to 236·7)	5·2 (0·4 to 18·1)	−14·8 (−90·9 to 837·6)
Cryptosporidiosis	78·7 (21·2 to 179·0)	10·3 (2·9 to 22·8)	60·4 (13·7 to 134·5)	12·1 (2·8 to 26·9)	−23·2 (−80·6 to 188·8)	83·0 (14·5 to 201·5)	5·0 (0·9 to 11·8)	64·8 (11·1 to 154·2)	4·9 (0·8 to 11·6)	−21·9 (−81·6 to 208·8)
Rotaviral enteritis	259·7 (211·2 to 323·5)	34·2 (29·3 to 41·5)	146·5 (118·0 to 183·5)	29·3 (24·6 to 35·9)	−43·6 (−52·1 to −33·0)	336·1 (281·3 to 403·7)	20·3 (17·4 to 24·0)	199·2 (165·5 to 241·2)	15·2 (12·9 to 18·1)	−40·7 (−48·0 to −32·3)
Aeromonas	11·8 (−72·2 to 90·6)	1·5 (−9·5 to 11·8)	7·3 (−48·3 to 59·1)	1·4 (−9·7 to 12·0)	−37·9 (−96·0 to 287·7)	67·8 (−10·4 to 189·9)	4·1 (−0·6 to 11·4)	56·8 (−4·0 to 151·3)	4·3 (−0·3 to 11·6)	−16·2 (−112·7 to 475·3)
*Clostridium difficile*	0·9 (0·8 to 1·1)	0·1 (0·1 to 0·1)	0·8 (0·7 to 0·9)	0·2 (0·1 to 0·2)	−10·5 (−24·0 to 2·3	6·7 (5·9 to 7·7)	0·4 (0·3 to 0·5)	9·4 (7·9 to 11·5)	0·7 (0·6 to 0·9)	40·1 (29·6 to 49·9)
Norovirus	20·7 (5·0 to 46·3)	2·7 (0·7 to 6·3)	14·8 (4·2 to 33·7)	3·0 (0·8 to 6·7)	−28·5 (−71·9 to 86·3)	36·3 (5·6 to 82·5)	2·2 (0·3 to 5·0)	29·7 (4·8 to 67·6)	2·3 (0·4 to 5·2)	−18·3 (−70·8 to 131·3)
Adenovirus	68·5 (24·8 to 141·9)	9·0 (3·3 to 18·6)	46·0 (16·2 to 97·7)	9·2 (3·3 to 19·7)	−32·8 (−77·2 to 90·1)	95·2 (35·4 to 191·4)	5·7 (2·1 to 11·6)	70·2 (25·4 to 145·4)	5·4 (2·0 to 10·9)	−26·2 (−74·5 to 110·8)
**Lower respiratory infections**										
Influenza	16·3 (9·6 to 26·0)	1·5 (0·8 to 2·4)	10·2 (5·7 to 16·8)	1·4 (0·8 to 2·4)	−37·8 (−44·2 to −31·7)	81·3 (56·3 to 116·8)	2·9 (1·9 to 4·2)	83·1 (55·7 to 122·1)	3·0 (2·0 to 4·4)	2·3 (−4·4 to 8·7)
Pneumococcal pneumonia	642·0 (386·2 to 848·6)	57·6 (35·5 to 74·1)	393·0 (228·4 to 532·3)	55·8 (32·5 to 75·0)	−38·8 (−45·7 to −32·1)	1692·3 (1061·1 to 2245·6)	59·8 (37·7 to 79·2)	1517·4 (857·9 to 2183·8)	55·4 (31·5 to 79·1)	−10·3 (−22·4 to −0·8)
*Haemophilus influenzae* type b	149·5 (−8·9 to 277·9)	13·4 (−0·8 to 24·7)	58·7 (−3·1 to 114·5)	8·3 (−0·5 to 15·9)	−60·7 (−65·7 to −56·8)	149·5 (−8·9 to 277·9)	5·3 (−0·3 to 9·9)	58·7 (−3·1 to 114·5)	2·1 (−0·1 to 4·2)	−60·7 (−65·7 to −56·8)
Respiratory syncytial virus	58·4 (33·2 to 97·6)	5·2 (3·0 to 8·7)	36·4 (20·4 to 61·5)	5·2 (2·9 to 8·6)	−37·8 (−44·4 to −30·6)	95·8 (61·5 to 142·6)	3·4 (2·2 to 5·1)	82·0 (53·9 to 117·6)	3·0 (2·0 to 4·3)	−14·3 (−23·1 to −5·2)

Data in parentheses are 95% uncertainty intervals. Numbers for each cause represent the reduction in deaths that is estimated to occur if a pathogen were eliminated. Numbers should not be summed across pathogens because of interactions between pathogens.

## Supplementary Material

Supplementary appendix

Supplementary video New results from the Global Burden of Disease Study 2015 examine causes of death and categorise regions according to the Socio-demographic Index, or ‘SDI’.
