# Department of Error

**DOI:** 10.1016/S0140-6736(16)32605-8

**Published:** 2017-01-07

*GBD 2015 Mortality and Causes of Death Collaborators. Global, regional, and national life expectancy, all-cause mortality, and cause-specific mortality for 249 causes of death, 1980–2015: a systematic analysis for the Global Burden of Disease Study 2015.* Lancet *2016: **388:** 1459–1544—*In this Article, Tea Lallukka, Anoushka Millear, and Amanda Pain should have been listed as authors. The affiliation details for Monica Cortinovis and Giorgia Giussani have been updated. An affiliation and funding statement have been added for Simon I Hay. These corrections have been made to the online version as of Jan 5, 2017.

