# Supplementary material for: Global, regional, and national life expectancy, all-cause mortality, and cause-specific mortality for 249 causes of death, 1980–2015: a systematic analysis for the Global Burden of Disease Study 2015
Source: Lancet. 2016 Oct 8;388(10053):1459–544. doi: 10.1016/S0140-6736(16)31012-1 (PMC5388903; doi:10.1016/S0140-6736(16)31012-1)
Supplement: Supplementary appendix [file mmc1.pdf]

THE LANCET

Supplementary appendix

This appendix formed part of the original submission and has been peer reviewed. We post it as supplied by the authors.

Supplement to: GBD 2015 Mortality and Causes of Death Collaborators. Global, regional, and national life expectancy, all-cause mortality, and cause-specific mortality for 249 causes of death, 1980–2015: a systematic analysis for the Global Burden of Disease Study 2015. *Lancet* 2016; **388:** 1459–544.

Appendix to Global, regional, and national life expectancy, all-cause and cause-specific mortality for 249 causes of death, 1980–2015: a systematic analysis for the Global Burden of Disease Study 2015

This appendix provides methodological detail, supplemental figures and more detailed results for all-cause mortality, causes of death, and years of life lost. The appendix is organized into broad sections following the structure of the main paper.

This study complies with the Guidelines for Accurate and Transparent Health Estimates Reporting (GATHER) recommendations, and this appendix is more comprehensive and encyclopedic than previous Global Burden of Disease appendices. It includes detailed tables, figures, cause modeling write-ups, and information on data in an effort to maximize transparency in our estimation processes and provide a comprehensive description of analytical steps. Components of this document are the same as described in the appendix to our GBD 2013 mortality and causes of death paper; much more of this appendix is new text. We intend this to be a living document, to be updated with each annual iteration of the Global Burden of Disease.

Contents

Part 1. GBD 2015 all-cause mortality and HIV estimation process……………………………….4
Part 2. Cause of death database……………………………………………………………………………….52
Part 3. Causes of death modeling methods……………………………………………………………….71
Part 4. Central computation………………………………………………………………………………….284
Part 5. Data sources and abbreviations…………………………………………………………………288
Part 6. Figures and tables……………………………………………………………………………………..291

Figures and Tables

Appendix Figure 1: Number of all-cause mortality data sources by country, 1950–2015 291

Appendix Figure 2: Sibling history correction for zero-survivorship, by sex 292

Appendix Figure 3: Comparison of GBD 2015 adolescent mortality in India to sample registration system, Demographic Health Surveillance System, and United Nations Population Division, for age groups 5-10, 5-15, 10-15, and 15-20, 1990–2015 293

Appendix Table 1: GBD 2015 geographies with HIV/AIDS modeling strategy group 297

Appendix Table 2: Number of years with all-cause mortality data points by geography and decade, 1950-2015 309

Appendix Table 3: Total number of country-years used in HIV/AIDS excess mortality age pattern estimation .... .313

Appendix Table 4: GBD world population age standard.. 314

Appendix Table 5: Total number of site years by cause and source type for 2015, excluding studies on lower respiratory infection and diarrhea etiologies 315

Appendix Table 6: Causes of death source list by geography, source type, and study site, with years, age range, and sample size, excluding sources on lower respiratory infection and diarrhea etiologies 318

Appendix Table 7: List of International Classification of Diseases (ICD) codes mapped to the Global Burden of Disease cause list for causes of death.. 402

Appendix Table 8: Restrictions on age and sex by cause for GBD 2015 407

Appendix Table 9: HIV/AIDS-related garbage code redistribution packages 410

Appendix Table 10: CODEm covariates used, level of covariate, and expected direction of covariate by cause, sex, and age 411

Appendix Table 11: CODEm predictive validity results by cause, location type, sex, and age 575

Appendix Table 12: Comparison of GBD 2013 and GBD 2015 covariates and level of covariates used in cause of death modeling.. 585

Appendix Table 13: Modeling strategy for individual cause of death models in GBD 2015 632

Appendix Table 14: CoDCorrect cause hierarchy with levels 636

Appendix Table 15: Percent change after CoDCorrect by cause for all ages, both sexes, 2015 641

Appendix Table 16: Socio-Demographic Index R-squared values with lags up to 10 years 649

Appendix Table 17: Socio-Demographic Index coefficients with and without year covariate 650

Appendix Table 18: Socio-Demographic Index groupings by geography, based on 2015 values................ 651

Appendix Table 19: Socio-Demographic Index values for all estimated GBD 2015 locations, 1980–2015 663

Appendix Table 20: GBD 2015 geography hierarchy with levels 670

Appendix Table 21: Causes included in the 2015 Global Burden of Disease Study 684
Appendix Table 22: Classification of each geography into six data availability categories 691
Appendix Table 23: Under-5 and adult mortality data sources used in GBD 2015 711

Part I. GBD 2015 All-Cause Mortality and HIV Estimation Process

Background

The goal of the all-cause mortality estimation process for the GBD is to produce the most accurate time series estimates for 1970 to 2015 for all-cause deaths and death rates broken down into the GBD age groups and by sex for all 591 geographies in the GBD 2015. This task is necessarily complicated by the diversity of data sources available for all-cause mortality for different age groups, the known biases in some sources, and the powerful effects of the HIV epidemic in countries with large epidemics on the age pattern of mortality. In this appendix, we divide the analysis of all-cause mortality into five distinct but interconnected areas: child mortality, adult mortality between ages 15 and 60, estimation of a complete set of age-specific death rates, estimation of HIV mortality, and final estimates of age-specific mortality including HIV and fatal discontinuities. While HIV is a cause of death, we present some information on the epidemiological modeling of HIV in this section because of the close inter-dependency between HIV mortality estimation and the all-cause mortality estimation process.

If all countries had complete vital registration systems recording the event of death and periodic censuses, the task of estimating all-cause mortality rates would be much easier. However, in many countries with low or moderate levels of socio-demographic status, information systems on mortality are incomplete and multiple sources must be used to infer the patterns of mortality. The analytical building blocks of the process, as shown in Figure 1 in the main text are repeated through two full iterations. The reason for the iterations is the interdependence between the HIV epidemiological models on estimates of HIV-free all-cause mortality and the use of estimated HIV crude death rates as an input to the 5q0, 45q15, and age-specific mortality models. In the following sections, we provide more detail on each analytical module identifying primary input data and analytical processes.

Section 1. Child mortality

Data sources

Vital registration from Causes of Death team

Approximately 62% of deaths data from vital registration (VR) systems used as input for our all-cause mortality modeling were provided by the GBD causes of death (CoD) research team and were aggregated into total age-sex-specific all-cause mortality for each location-year. This aggregation occurred after the data were adjusted and mapped to the GBD cause list.

Data intended for use in causes of death modeling are assessed for quality with respect to consistency of cause fractions, diagnostic accuracy and missing data, whereas for all-cause mortality modeling it is more important that data are fully representative of the given estimation area and are consistent with other all-cause mortality data sources. Thus, there are cases in which VR data prepared for cause-specific modeling cannot be used in all-cause modeling or must be adjusted based on degree of completeness before being used.

In our vetting of CoD VR data, we dropped points where there was a larger than 1% difference from corresponding points in the WHO database. There were instances where VR data used in cause-specific mortality analysis had been collapsed to Basic Tabulation List (BTL) format rather than in full cause classification list format (e.g., ICD-9). In some of these cases, we elected to use WHO data instead.

Vital registration, sample registration systems, and Disease Surveillance Points from other sources

We endeavored to include all available data from vital registration systems as inputs in our all-cause mortality estimation process. To achieve this, we utilized a number of multi-country vital registration sources, including the WHO Mortality Database, the Human Mortality Database, United Nations Demographic Yearbooks and OECD databases. These multi-country sources are regularly updated in our systems when new data are added. Beyond multi-country sources, for all ongoing national VR systems (for example, the USA National Vital Statistics System), where possible, we cataloged all data sources from each system.

Some countries that do not have a well-performing VR system implement sample registration systems that are incomplete by design. We made use of these data, paying close attention to the proper weighting of sampled data and consistency with other representative sources. We have systematically extracted data from the Sample Registration System Statistical Report series published by the Registrar General of India. For the Disease Surveillance Points system of China, we obtained both national and provincial level DSP data through a data usage agreement with the Chinese Center for Disease Control and Prevention. Census data are systematically extracted from Demographic Yearbook series, Integrated Public Use Microdata Series (IPUMS), and statistical reports from the national statistical bureaus.

Under-5 populations and live births

For most GBD locations, live births come from the World Population Prospects 2015 (WPP 2015). For subnational locations, we often use interpolated census birth numbers scaled to the national estimates. For locations not estimated in the WPP 2015, we use interpolated census birth counts.

Complete birth history microdata

Complete birth histories, the preferred method for data collection on child mortality in the absence of vital registration, rely on administering surveys to mothers. The questionnaires ask about all living and deceased children, including date of birth, survival status, and date of death. These modules are included in many routine survey series, including the World Fertility Surveys (WFS), Demographic and Health Surveys (DHS), Multiple Indicator Cluster Surveys (MICS), and many national survey programs. When available, we download and use microdata that has individual-level survey responses as opposed to using tabulated results.

Complete birth history tabulated data

In some instances, tabulated records from reports become available before survey microdata, and we incorporate these data points into our database of 5q0 data as well. However, as microdata become available, we update with point estimates from our processed microdata rather than the tabulated report estimates.

Summary birth history microdata

Summary birth history questionnaires are a shorter alternative to complete birth histories. Instead of asking in detail about each child, summary birth histories simply ask mothers how many children they have given birth to and how many of the children have died. The questionnaires are shorter and can be more easily attached to other surveys. Often, Censuses and MICS surveys contain summary birth histories. For GBD, we have complied all available SBH data with micro-level data that enables us to apply the updated Summary birth history method that leads to more accurate and timely assessment of U5MR.[1]

Summary birth history tabulated data

In cases where we don't have access to the micro-level data on summary birth history modules from surveys and censuses, we utilize the reported estimates of U5MR from survey or census reports and outlier the first two data points based on mothers in ages 15-19 and 20-24. In cases where tabulated proportions of child died by mother's age group are available, we apply the Maternal Age Cohort method as updated by Rajaratnam and colleagues.[1]

Under-5 age-sex patterns from VR/SRS/DSP

Vital registration systems are the primary source of data for the under-5 age pattern of mortality in developed countries. Often, these data group under-5 deaths into several age groups- Early Neonatal (0-6 days), Late Neonatal (7-27 days), Post Neonatal (28-364 days), and 1 to 4 years. Some country-years of data have other age groupings with less specificity, with the Early and Late Neonatal age groups combined, or all of the under-1 age groups combined. Sample Registration Systems (SRS) also provide data for the age-sex pattern of under-5 mortality in several countries (notably India and Bangladesh), as well as the Disease Surveillance Points (DSP) system in China.

Under-5 age-sex patterns from complete birth history

In many countries without vital registration systems, complete birth history surveys can be used to obtain age-sex patterns of mortality in under-5 age groups. These sources are described above in the "Complete birth history microdata" section. For all complete birth history microdata sources, we apply direct estimation methods to obtain probabilities of death for each of the under-5 age groups. Within each survey, if each observation is a child recalled by a mother, observations are grouped into 5-year groups in time to provide a data point of probability of death for each of the under-5 age-sex groups. Recall is cut off 15 years before the survey, limiting data points estimated from the survey to the 15 years prior. All of these estimates are then in the database of estimates for the age-sex pattern of under-5 mortality.

1.1 VR prioritization

Our continual evaluation of VR data sources has led us to develop a general hierarchy of preferred VR sources. When considering which of multiple sources to use for a given location-year, we first prefer to use WHO data from GBD cause-specific mortality estimation, then unadjusted WHO data, then Human Mortality Database (HMD) data, then UN Demographic Yearbook data. There were exceptions to this hierarchy where we had reason to believe that there were quality issues with a certain source. For instance, where available we preferred to use HMD VR over WHO data for Germany, Taiwan, and Spain deaths. Single-country VR sources were evaluated based on consistency with other data sources and also VR system documentation.

1.2 Identify VR under-enumeration for bias correction

The approach to estimating the completeness of vital registration systems for deaths under age 5 is the same as that of the previous two GBD studies.

In many countries with vital registration (VR) systems to record deaths, complete and/or summary birth histories are also conducted. By comparing the under-5 death rates from these sources to the levels from VR or sample registration systems, we can assess the completeness of under-5 death registration. Completeness can evolve over time as seen with the likely declines in completeness in Central Asia in the 1990s or the increases in completeness in other settings.[2,3] We estimate VR completeness where VR data are available using a model that allows for completeness to vary over time. This assessment is undertaken in two steps: we first assess whether VR is biased, and then we assess time-varying completeness.

In the first step, a country-level regression of $\log_{10}({}_5q_0)$ on year with a binary indicator variable for ${}_5q_0$ estimates derived from VR systems is used to determine whether or not a VR system is biased (see equation). If the coefficient for the VR indicator variable is statistically significant at the .05 α-level, we deem the VR system to be biased.

$$log_{10}({}_5q_0)_t = \alpha + \beta_1 * t + \beta_2 * I_{VR} + \xi_t$$

Where: t is time (a continuous variable);

I_{VR} is an indicator for ${}_5q_0$ estimates derived from VR systems; and

ξ_t is in error term.

Second, for all countries with biased VR systems, we estimate the bias, allowing for completeness to evolve over time. We first apply Loess regression to all non-VR ${}_5q_0$ estimates in a given country; we then calculate the difference between the Loess predicted $\log_{10}({}_5q_0)$ and the observed $\log_{10}({}_5q_0)$ estimate from VR in a given year. Since we believe that completeness changes relatively slowly over time, the bias in any given year is defined as the mean difference between the predicted $\log_{10}({}_5q_0)$ and the observed $\log_{10}({}_5q_0)$ from VR systems over the adjacent five-year period. This allows for flexibility in the bias correction over time while still maintaining the premise that the completeness of VR systems does not change abruptly. Loess predictions can be unreliable out of sample, so for country-years outside of the range of non-VR data used to generate the predicted ${}_5q_0$, we use the mean bias from the nearest five

years of bias estimates from VR points that are within the timespan of the non-VR data. We then correct the VR estimates of ${}_5q_0$ using the bias correction as shown in the equation:

$$ {}_5q_0^{corr.} = {}_5q_0^{obs.} * 10^{\widehat{bias}} $$

Where: ${}_5q_0^{corr.}$ is the corrected estimate of ${}_5q_0$;
${}_5q_0^{obs.}$ is the observed estimate of ${}_5q_0$; and
$\widehat{bias}$ is the bias estimate described above.

Once the biased estimates have been adjusted, we also approximate the variance of the bias estimate. This variance is approximated using the median absolute deviation (MAD) comparing the biased VR estimates to the Loess-based estimate of $\log_{10}({}_5q_0)$. As with the bias estimation above, the MAD is estimated over a five-year time period. This MAD times 1.4826 is an approximation of standard deviation used to add variance to the biased VR data when included in our final Gaussian Process Regression (GPR) model described in detail later.

In addition to countries where there are both VR estimates and survey estimates of under-5 mortality, there are countries for which only VR data are available, and the VR systems are considered biased. This is a problem particularly in English-speaking Caribbean countries, so for these countries we have adjusted ${}_5q_0$ estimates from VR using the regional average VR bias in a given year for those countries with both VR and survey ${}_5q_0$ estimates. The countries for which VR systems have been adjusted using this method include Antigua and Barbuda, Bahamas, Barbados, Bermuda, Dominica, Grenada, Saint Lucia, and Saint Vincent and the Grenadines. While there is no direct evidence on the level of VR bias in these countries, assuming they are complete when similar countries in the region have under-registration seems unwarranted.

1.3 Biennial 5q0 estimates

Complete birth history 5q0 computation

Microdata (individual-level survey data) from complete birth histories yield direct calculation of death numbers and probabilities of death in the under-5 age group. Observations are grouped into two-year intervals such that biennial estimates of 5q0 are obtained from these survey data. In GBD 2015, we have unpooled surveys for our analysis, whereas surveys were pooled by series in GBD 2013. Instead of grouping observations from all DHS complete birth history questionnaires from a country into one full set of observations and all MICS observations from multiple survey years into another full set of observations, we analyzed each survey separately (e.g., DHS 2012, DHS 1996, MICS 2002). This allowed for greater ability to address known data quality issues in specific surveys. To compensate for the decreased sample size and to generate greater stability in the unpooled data points, we created 2-year estimates of under-5 mortality, pooling observations over 2-year periods instead of single years.

Tabular complete birth history processing

In some instances, microdata from surveys were not available. If survey reports could be obtained but the microdata were not available for us to do our own calculations to obtain 5q0, we used report data point estimates. These estimates were added directly to the under-5 mortality database.

1.4 Summary birth history time series method

Summary birth history method from microdata

Rajaratnam and colleagues have developed an updated summary birth history method that is able to provide more accurate and timely estimates of U5MR from micro data on SBH from surveys and censuses.[1]

Summary birth history analysis from tabular data

When only tabular data is available for the numbers of children ever born and number of children that have died by mother's age, we apply the Maternal Age Cohort model from the method developed by Rajaratnam and colleagues.[1]

1.5 5q0 data synthesis, model running, and bias correction

1.5.A Data synthesis using ST-GPR and bias correction

We apply the child mortality estimation methodology as reported by Wang et al.[4] Based on the under-five mortality data synthesis model for the Global Burden of Disease Study 2010 and 2013, we have incorporated data bias adjustment into the modeling process. Specifically, we have included a fixed effect for source type across all locations to detect systematic differences in the level of child mortality, controlling for covariates for one source type versus another. The groups of sources to make this adjustment are listed in the table below. In addition, we include a random effect for each country-source. By choosing a reference source country-by-country or using the mean of a set of sources, we can adjust on a country-by-country basis for the problem of compositional bias created by substantial source-specific non-sampling error. Once the systematic difference in sources is removed, we are able to avoid estimating false trends due to partial overlap of sources with different levels of non-sampling variance. We then apply the combination of non-linear mixed effects model, spatial-temporal regression and Gaussian process regression to synthesize raw child mortality data after data bias adjustment to obtain consistent time series estimates of mortality with 95% uncertainty intervals for every country.

Table: Source types used in child mortality bias correction

Data Source Type
Complete Birth History-Demographic and Health Survey
Complete Birth History-AIDS Indicator Survey and Malaria Indicators Survey
Complete Birth History-World Fertility Survey
Complete Birth History-Multiple Indicator Cluster Survey
Complete Birth History-Census
Complete Birth History-Other survey Series
Summary Birth History-Demographic and Health Survey
Summary Birth History-Multiple Indicator Cluster Survey
Summary Birth History-Other survey series
Summary Birth History-AIDS Indicator Survey and Malaria Indicators Survey
Summary Birth History-Census
Summary Birth History-World Fertility Survey
Vital Registration/Sample Registration/Surveillance- complete
Vital Registration/Sample Registration/Surveillance- incomplete
Household Death Recall-Other survey series
Household Death Recall-Census
Household Death Recall – incomplete Vital Registration/Sample Registration/Surveillance

1.5.B Mixed effect non-linear model and the bias adjustment for raw U5MR sources

In this stage, we used a nonlinear mixed effects regression to estimate data bias and provide first stage predictions.

The nonlinear mixed effects regression model is

$$ {}_5m_{0cys} = \exp[(\beta_1 + \gamma_{1c}) * \log\left(LDI_{cy}\right) + (\beta_2 + \gamma_{2c}) * education_{cy} + \gamma_c + \gamma_{cs} + \alpha_t] + \beta_3 * HIV_{cy} + \varepsilon_{cys} $$

where *c* is country, *y* is year, *s* is source, and *t* is source type; each source was categorized into one of 17 source types across all countries, as listed in the table above.

Additionally,

${}_5m_0$ is under five mortality rate

LDI is lagged distributed income per capita

education is mean years of education for women of reproductive age (15-49 years)

HIV is death rate due to HIV in age groups 0-4

γ is a random effect

α is a fixed effect on source type across countries

β_i is a fixed covariate coefficient

ε is the residual

For each country, we rely on expert opinion to choose a source, or combination of sources, which are believed to be the least biased. If a country has vital registration which we deem to be complete (described in detail in an earlier section), this is the reference source. If a country does not have complete vital registration, but has DHS estimates from complete birth histories, these were chosen as the reference source. If a country has neither of these types of data or DHS surveys are deemed unreliable, we assigned the surveys conducted after 1980, in combination, as the reference (incomplete vital registration data is not included). Additionally, in many countries we chose other surveys as the reference. For accurate estimation, it is important to have local knowledge on specific data sources' accuracy. All-cause mortality experts draw from their familiarity with data quality to help us to choose the reference category.

Each data source has an associated random effect as well as a source type fixed effect. The values of these random and fixed effects for the reference sources are deemed to be the true deviation from unbiased mortality level. In countries with multiple high-quality sources, the mean of the random and fixed effects from these sources is taken as this true deviation. We adjusted all other sources by including these reference values for the random and fixed effects values instead of those estimated for each individual source, as shown below.

$$adjusted_5m_{0,cys} = \exp[(\beta_1 + \gamma_{1c}) * \log\left(LDI_{cy}\right) + (\beta_2 + \gamma_{2c}) * education_{cy} + \gamma_c + \gamma_{ref,c} + \alpha_{ref,c}] + (\beta_3 + \gamma_{3c}) * HIV_{cy} + \varepsilon_{cys}$$

The exception to this correction is incomplete vital registration data, which was adjusted upwards using a five year rolling mean of the difference between incomplete vital regression and a Loess of the already-adjusted survey data, described above in section 1.2.

1.5.C Spatio-temporal smoothing

The spatio-temporal stage smooths the residuals between the predicted time series of $_5q_0$ and the adjusted raw data over time and across countries in the same GBD region. The predicted time series for this smoother was obtained from the equation below; no random effects or survey type fixed effects are included.

$$predicted_5m_{0,cy} = \exp[\beta_1 * \log\left(LDI_{cy}\right) + \beta_2 * education_{cy} + \alpha_{intercept}] + \beta_3 * HIV_{cy}$$

We first found the residuals between the predicted time series, above, and the adjusted points. We then applied a combination of smoothing functions to these residuals. For each country year, we weighted all the data points in this region based on their proximity to this country-year in space and time. We gave 99% of the weight to in-country residuals, and 1% of the weight to out-of-country residuals. Additionally,

we used a modified tricubic window, as specified below, to give more weight to points closer in time, and less weight to points further in time.

$$w_t = \left(1 - \left(\frac{|r_t - r_{est}|}{1 + argmax_t|r_t - r_{est}|}\right)^{\lambda}\right)^3$$

The r_t and r_{est} terms are, respectively, the year of interest and the year of the residual being weighted. The $argmax_t|r_t - r_{est}|$ term is the maximum distance between the year of interest and a residual within the region. The λ parameter in this weighting function dictates how quickly the weights fall off as the distance in time increases: a larger λ implies that the assigned weights will diminish slowly with time, while a smaller λ allows the weights to diminish more rapidly with time.

For most countries, we chose λ = 0.8. We then created one estimate of the smoothed residuals using a linear fit to this weighted data; this is similar to a Loess fit. Additionally, we created a second estimate of the smoothed residuals by calculating the weighted average of this data.

We then combined these two estimates for a final estimate of the smoothed residuals. In data-dense countries, more weight was given to the local linear fit; in data sparse countries, more weight was given to the weighted average. The equation for this is as follows.

$$final\ smoothed\ residual = k * linear\ estimate + (1-k) * weighted\ average$$

where $k = \frac{number\ of\ in\ country\ data\ points}{number\ of\ in\ country\ data\ points + number\ of\ country\ years\ with\ no\ data}$

Finally, the smoothed residuals were added back to the predictions from above; this smoothed approximation to the adjusted data was used as the prior for the Gaussian process regression, described below.

Third stage: Gaussian process regression (GPR)

The output of the space-time local smoother was used as a prior for the Gaussian process regression, which produced a final time series of point estimates, as well as confidence bounds. Parameters for the GPR were chosen through cross-validation described in section 1.5.E.

The model for the Gaussian process regression is shown below, where μ_t is the true $\log_{10}({}_5q_0)$ at time t, f(t) is the baseline mortality risk, and S_t captures excess mortality due to war and disasters. S_t is estimated independently of f(t). M and C describe the Gaussian process, giving the mean and covariance, respectively.

$$\mu_t = f(t) + S_t$$

$$f(t) \sim GP(M, C)$$

We specified a prior distribution for f(t) from the spatio-temporal regression, and a likelihood function which describes the data generation process; the specified prior distributions and likelihood function are described below. We then used Markov Chain Monte Carlo (MCMC)[5] to approximate the posterior

distribution of f(t) which also incorporates information from the observed empirical estimates of adult mortality. An MCMC chain of length 5000 was produced; the first 3000 samples were discarded and the remaining 2000 were thinned by a factor of 2 for a total of 1000 simulations retained. The reported best estimates and confidence intervals were generated from the mean and the 2.5th and 97.5th percentiles of the 1000 samples, respectively.

The prior distribution of f(t) can be described in terms of the mean prior—the prior for M—and the covariance prior—the prior for C. We utilized the second stage predictions as the mean prior and used a Matern covariance function to describe the covariance prior. The parameters of the Matern covariance function were selected through cross-validation and are region-specific.

For cross-validation, data were divided as follows: for each region, a number X between 10 and 20 was sampled and the most recent X years of data in that region were assigned to the testing set. Then a number X between 5 and 10 was sampled, a country from within the region was sampled, and a year where there is data in that country was sampled. All data within X years of the selected year in the selected country were assigned to the testing set. This was repeated as many times as there are countries in the region; because iterations of this procedure were independent, the data selected for the testing set may overlap. Any data that were not selected for the testing set were included in the training set.

For each testing and training division, the second stage model is fit on the training data. Then, the third stage model is also fit on the training data using each combination of scale and squared amplitude values tested for a total of 25 sets of predictions. The testing data are matched to the predictions in the corresponding country and year for each of the 25 sets of predictions. For each match we calculated the absolute relative error of the prediction compared to the empirical estimate in the testing set. We also classified each empirical estimate in the testing set as being covered or not covered by each corresponding prediction. The determination of coverage was made by calculating total variance—the sum of the variance of the empirical estimate (described below) and the variance of the GPR estimate—and then calculating a 95% confidence interval around the prediction based on this total variance and assuming a normal distribution. If the empirical estimate was within this confidence interval, it was classified as covered, and otherwise not.

Once this procedure has been carried out for all 100 testing and training divisions of the data we calculated the mean absolute relative error and the mean coverage for each combination of GPR parameters across all 100 sets of predictions. The ideal set of parameters would produce estimates with low mean absolute relative error and mean coverage close to 0.95. We used the function described in the equation below to calculate a loss metric which incorporates both the coverage and the absolute relative error into a single measure to assess performance. Parameter combinations with lower values of this loss metric are considered preferable.

$$\text{Loss} = \begin{cases} \text{if coverage} \leq 0.95\text{:} \;\; (0.95 - \text{coverage})/5 + (\text{absolute relative error}) \\ \text{if coverage} > 0.95\text{:} \;\; (\text{coverage} - 0.95)/1 + \left(\text{absolute relative error}\right) \end{cases}$$

The optimal parameters may differ from country to country. To allow for this, we calculated the loss function described in the equation above separately for each of the 22 GBD geographic regions.

1.5.D Likelihood

The likelihood describes the probability of observing the data given a particular set of parameters. As shown in the equation below, we used a normal model for describing the probability of observing a particular value of $\log({}_5q_0)$ where the mean is given by f(t) and the variance by V_t, the data variance.

$$\log_{10}({}_5q_{0_t}) \sim \text{Normal}(f(t), V_t)$$

Data variance was calculated for each empirical observation of ${}_5q_0$ and incorporated both sampling and non-sampling variation. The method for calculating the data variance depended on the type of data:

1. For estimates derived from complete vital registration data we assumed that there was no non-sampling variance and included only sampling variance as computed from a binomial model. We set N equal to the national population aged 0 to 5 years and p equal to the mortality rate, ${}_5m_0$. We calculated the variance of ${}_5m_0$ from p(1-p)/N and then transformed this to the variance of $\log_{10}({}_5q_0)$ using the delta method.[6]
2. For estimates derived from incomplete vital registration data, we wanted to include not only sampling variance but also the non-sampling variance that arises from uncertainty in the completeness estimate. For these data, the total data variance was given by the sum of the sampling variance (calculated as for complete vital registration data) and the variance of the completeness estimate;
3. For estimates derived from complete birth histories we generate 1000 simulations of ${}_5q_0$, convert these estimates into $\log_{10}$ space and calculate the sampling variance from these 1,000 simulations;
4. For estimates derived from summary birth histories we use the standard error from the mean residuals;
5. For estimates not covered under the above 4 calculations the missing data variance is determined as the maximum standard error from non-VR points in the country, if the data variance is still missing it is calculated as the maximum standard error from non-VR data in the GBD region.
6. Finally, for each source type, we calculate the within-source-type variance of the source-specific random effect. This additional non-sampling variance is then converted to $\log_{10}$ space and added to the variance as calculated above for all data points not classified as complete vital registration.

1.5.E Hyper-parameter selection for under-5 mortality rate ST-GPR

In GBD 2015, we expanded the scope of our parameter selection to include variables used in space-time smoothing in addition to scale and amplitude used in the Gaussian process regression. We have applied rigorous out-of-sample predictive validity testing to select space-time and GPR parameters, and the process is carried out in the following steps:

1. For space-time smoothing, we tested ζ, space weight, values of 0.7, 0.8, 0.9, and 0.99 and λ, time weight, values of 0.1 to 0.9 in increments of 0.1. We test five values of the scale—10, 15, 20, 25, 30 --- and five values of the squared amplitude—1, 1.5, 2, 2.5, and 3 times the mean squared error of the residuals from the second-stage prediction model. Because we tested combinations of both space-time and GPR parameters, this led to a total of 900 combinations tested in each process.

2. We divided the data into testing and training sets 100 times. Data were divided as follows: for each region, a number X between 10 and 20 was sampled and the most recent X years of data in that region were assigned to the testing set. Then a number N between 5 and 10 was sampled, a country from within the region was sampled, and a year where there is data in that country was sampled. All data within N years of the selected year in the selected country were assigned to the testing set. This was repeated as many times as there are countries in the region; because iterations of this procedure were independent, the data selected for the testing set may overlap. Any data that were not selected for the testing set were included in the training set.

3. The space-time smoothing and Gaussian process regression are fit on the training set using each set of parameters and estimates for every location are generated for the entire time period;

4. Within a given iteration we calculate the absolute relative error of the final GPR estimates compared to each empirical estimate in the testing set. We also classify each empirical estimate in the testing set as covered or not covered and calculate the percent of the data covered by the 95% uncertainty interval of the GPR estimates while considering the uncertainty of the data themselves. The determination of coverage is made by calculating total variance—the sum of the data variance and the variance of the GPR estimate—and then calculating a 95% uncertainty interval around the GPR estimate based on this total variance and assuming a normal distribution. If the empirical estimate is within this uncertainty interval, it is classified as covered, and otherwise not. For each combination of parameters, we calculate the mean absolute relative error and the mean coverage across all iterations from all countries within a particular group. The loss function described below is then calculated for each parameter combination, and the parameter combination with the lowest loss is selected for each group;

if coverage <=.95, loss = absolute relative error + ((1-coverage)-0.05) / 5
if coverage > 0.95, loss = absolute relative error + (0.05 - (1-coverage))/1

5. For U5MR, parameter selection occurs at the location level, i.e. different parameters for each location. While there are data sparse locations, all locations have some data on U5MR for the time period we provide estimates for.

In some cases, we restricted the universe of possible parameters. Most of these restrictions occurred for the λ parameter. For many locations with complete VR, we knew that higher lambda values would result in a larger confidence interval than would be appropriate. We also included a lower limit of λ for some locations with either incomplete VR or no VR, so that confidence intervals would reflect the uncertainty of these data. For example, in Western Europe, High-Income North America, High-Income-Asia-Pacific, and Australasia, we set the condition that λ must be less than 0.5 and the condition that ζ be .99. We also made some other manual exceptions to λ, ζ, and scale where results did not pass the common sense test.

We set the differentiability to 0.8 in countries with only complete VR data, excepting those in the Caribbean, Oceania, and the country of Mauritius, and to 2.0 in other all other locations. We used a

lower differentiability in countries with complete VR data because in these countries we want the final estimates to follow the data closely even if the trend described is not smooth. In contrast, in countries where the data are less reliable we don't want the final estimates to be overly influenced by individual data points.

1.6 Identify and remove outliers

There are several important quality-control steps in reviewing child mortality data and estimates. First, data points from years in which fatal discontinuities occurred are outliered, unless they are VR data points with sufficient information that the fatal discontinuities can simply be subtracted out of the VR data. The intent is to capture the underlying mortality risk rather than large stochastic variations. These fatal discontinuities are then added on in a later step (see section 5). Secondly, we outlier data sources with quality concerns such as the Afghanistan DHS from 2010. Our extensive collaborator network allows for review of sources, and collaborators can raise concerns over known issues with data sources about which they have expert knowledge.

1.7 Rake subnational estimates to national level (excluding South Africa)

The estimation process for 5q0 does not enforce consistency between subnational estimates and national estimates. To ensure consistency throughout the GBD hierarchy, we rescaled the subnational estimates to the national level by population-weighting to get an implied national estimate from the subnational estimates, creating a scalar of the national-level estimate from GPR to the aggregated subnational estimates, and then multiplying all of the subnational estimates by this scalar to obtain the scaled estimates. In most cases, we considered national-level estimates to be more reliable, so we chose this strategy of subnational scaling. In locations with high-quality vital registration data, this scaling has a minimal effect, but the effect can be greater in locations with more subnational units and variable-quality data. In South Africa, it was essential that the state-specific mortality patterns be consistent with HIV models is essential, since such a large part of the trend is driven by deaths due to HIV/AIDS. In this case, instead of scaling provincial-level estimates to national-level GPR estimates, we aggregated province-level GPR estimates to generate the national-level estimates.

1.8 Review estimates for quality

Estimates of U5MR from the ST-GPR process are reviewed with comparison to UNICEF estimates from their 2015 revision and GBD 2013 results. Any change and difference will be traced to either changes in available data or changes induced by the improved parameter selection process. Revision is made after the review process and through expert consultation with country experts and GBD mortality collaborator network.

1.9 Under-5 mortality rates with HIV

The U5MR ST-GPR process generates U5MR for all GBD 2015 locations that is inclusive of the impact of all causes of death excluding fatal discontinuities, which are added in a separate step (see section 5).

1.10 HIV-free 5q0

As a result of the Non-linear mixed effects model, we are able to generate HIV free 5q0 counterfactuals where the crude death rate due to HIV in age group 0-4 is set to zero. This is a crucial input to the GBD model life table system as described in section 3.

1.11 Under-5 age pattern model estimation

The process used to break down under-5 mortality into age- and sex- specific groups has been previously described.[7] The current process is largely similar but has been modified to improve the accuracy of predictions for countries affected by HIV/AIDS. As pointed out by Bradshaw et al., neonatal mortality tends to be overestimated if the all-cause child mortality rate is used as the only predictor.[8] We use a two-stage modeling process to generate sex-specific estimates of early neonatal (days 0 to 6), late neonatal (days 7 to 27), post-neonatal (the remainder of the first year), and childhood (ages 1 to 4) mortality. First, the ratio of male to female under-5 probability of death is estimated, then age- and sex-specific mortality estimates are generated using this ratio. To fit models to obtain estimates, data from vital registration, sample vital registration, and complete birth histories are converted to mortality risks for specific age groups. Sources have differing levels of age specificity and at least include infant (composed of early neonatal, late neonatal, and post-neonatal) and child mortality, but can include all 4 smaller age groups. The two models – first the sex model, then the age-specific and sex specific model – are fit on the data.

The sex model predicts the ratio of male probability of death under age 5 ($_5q_0$) to female $_5q_0$ for each country *i* in region *j* in year *t*. The data are ordered by observed $_5q_0$, and categorized into 20 evenly sized bins. Then, the model is fit to the data as described in the equation below.

$$\left(\frac{\text{Male } _5q_0}{\text{Female } _5q_0}\right)_{\text{jit}} = \beta + \gamma_{_5q_0 \text{ bin}} + \gamma_\text{j} + \gamma_\text{i} + \varepsilon_{\text{jit}}$$

The ratio is predicted by nested location and region random effects γ_i and γ_j, a random effect on the $_5q_0$ bin, and an intercept term, β. A Loess regression is then used to smooth the estimated $\gamma_{_5q_0 \text{ bin}}$ on $_5q_0$, creating a continuous $\gamma'_{_5q_0 \text{ bin}}$. Then, the equation below is used to predict the ratio of male to female $_5q_0$:

$$\left(\frac{\text{Male } _5q_0}{\text{Female } _5q_0}\right)_{\text{jit}} = \hat{\beta} + \gamma'_{_5q_0 \text{ bin}}\left({_5q_0}_{\text{jit}}\right) + \hat{\gamma}_\text{j} + \hat{\gamma}_\text{i}$$

The male and female $_5q_0$ values are found using the system of equations that includes the model above and equation below, where r_{birth} is the sex-ratio at birth.

$$_5q_0 = \left(\frac{1}{1+\text{r}_{\text{birth}}}\right) * (\text{female}_5q_0) + \left(\frac{\text{r}_{\text{birth}}}{1+\text{r}_{\text{birth}}}\right) * (\text{male}_5q_0)$$

Age-specific models are then fit for each age group on sex-specific data. A separate model is fit for each age group yielding five models for each sex: early neonatal, late neonatal, postneonatal, infant, and child. The log of the probability that an under-5 death occurs in a given age group is modeled instead of the mortality risk, simplifying the scaling process and restricting risks to be between 0 and 1. Because evidence suggests HIV has differential effects on different under-5 age groups,[8,9] the crude death rates from HIV/AIDS in the under-5 age group were included in the model. We used crude death rate due to

HIV from the GBD 2015 model (see section 3). The inclusion of this covariate improves both the fit and prediction of the model in countries with HIV. In addition, in this version of GBD, we added two new covariates to improve model fit. First, we included the maternal education covariate that is also used in the 5q0 first-stage model. Second, we used the completeness of the source-specific 5q0 estimate for the data-point used in the regression. This completeness was calculated by taking the source-specific 5q0 point estimate and dividing by the final 5q0 estimate from GPR. The functional form of the model is below.

$$\log(\Pr(\text{death at age y}|\text{u5 death})_{\text{jit}}) = \beta_1 + \beta_2 * HIV_{it} + \beta_3 * Mat.Ed._{it} + \beta_4 * Completeness_{sit} + \gamma_{5q_0\text{ bin}} + \gamma_{\text{j}} + \gamma_{\text{i}} + \varepsilon_{\text{jit}}$$

Similar to the sex model, the sex-specific age prediction uses $_5q_0$ bins and smooths the random effect on the bin using $_5q_0$. The prediction equation for age *y* in country in region *j* at time *t* is seen below, with nested random effects on country ($\hat{\gamma}_{\text{i}}$) and region ($\hat{\gamma}_{\text{j}}$), an intercept term ($\hat{\beta}_1$), a smoothed random effect on $_5q_0$ bin ($\hat{\gamma}'_{5q_0\text{ bin}}({}_5q_{0_{\text{jit}}})$), a coefficient on the under-5 crude death rate from HIV ($\hat{\beta}_2$), a coefficient on maternal education ($\hat{\beta}_3$), and a coefficient on completeness ($\hat{\beta}_4$):

$$\log(\Pr(\text{death at age y}|\text{u5 death})_{\text{jit}}) = \hat{\beta}_1 + \hat{\beta}_2 * HIV_{it} + \hat{\beta}_3 * Mat.Ed._{it} + \hat{\beta}_4 * 1 + \hat{\gamma}'_{5q_0\text{ bin}}({}_5q_{0_{\text{jit}}}) + \hat{\gamma}_{\text{j}} + \hat{\gamma}_{\text{i}} \quad 13$$

Note that for prediction, the completeness coefficient gets multiplied by 1 instead of a source-specific completeness, as we seek to predict based on a hypothetically complete source.

Once each of these predictions is made by age group, they are rescaled such that the probabilities of death in the Early Neonatal, Late Neonatal, Post Neonatal, and 1-4 year age groups aggregate to the 5q0 estimates from the under-5 model.

1.12 Identify and remove outliers

There are several criteria for removing outliers for the under-5 age-sex pattern model. First, sources may be marked as outliers if they contain low population numbers or very few deaths. If data come from vital registration and the under-5 population of the country is less than 20,000 person-years, then the data are outliered. If the total number of deaths in a VR system among both sexes under-5 is less than 200, the data are also outliered. VR data that are considered incomplete are marked as outliers. To be considered incomplete, the 9-year rolling average of the VR data 5q0 value is compared to the 9-year rolling average of the 5q0 estimates. Then, for a given data-year, the value of 5q0 in the raw data is compared to our final 5q0 estimate. A value of 90% would be considered incomplete and outliered, unless the ratio of the 9-year rolling average above is above 90% complete. Any data that are chosen as outliers as part of the 5q0 analysis are also marked as outliers in the age-sex pattern analysis. If the female-to-male ratio of 5q0 in the raw data is less than .5 or greater than 2, the data are outliered because of an implausible sex ratio. If a country has both VR and CBH data, they are typically both used, unless the two conflict, in which case the VR data are used. CBH data points more than 15 years before the survey are outliered. Lastly, some data points are manually outliered. For example, the definition of live birth changed in some Eastern European countries in the 1990s, leading to inconsistencies. For these examples, age group data in ages that would include childbirth deaths (early neonatal, neonatal, and ages 1-4) are outliered if the definition of live birth contains a minimum weight, as it did in some of these locations.

1.13 Under-5 age-sex splitting model application

The prediction method from the age-specific model is described above in 1.11. First, the results of the sex model are applied, yielding sex-specific 5q0 estimates. Once age-sex-specific predictions of the log conditional probability of death are made, these are exponentiated and rescaled so that they some to 1. First, the under-1 and 1-4 conditional probabilities are scaled to add to 1. Then, the early neonatal, late neonatal, and post neonatal conditional probabilities are scaled to the under-1 conditional probability. Then, the probabilities of death can be calculated so that they properly aggregate to the final 5q0 prediction. For example, to calculate the probability of death in the early neonatal age group, the rescaled conditional probability of early neonatal death given under-5 death is multiplied by the probability of under-5 death. Then, to obtain the probability of death in the late neonatal age group, the rescaled conditional probability of death in the late neonatal age group given under-5 death is multiplied by the probability of under-5 death and then divided by the probability of survival to the beginning of the age group, and so on. Equations below represent this process, where enn represents early neonatal and lnn represents late neonatal.

$$q_{enn} = \Pr(death\ in\ enn \mid u5\ death) * 5q0$$

$$q_{lnn} = \Pr(death\ in\ lnn \mid u5\ death) * 5q0/(1 - q_{enn})$$

The rest of the older age groups are also calculated in this manner, yielding probabilities of death in each of the under-5 age-sex groups.

1.14 Update under-5 populations using fatal discontinuities

To obtain denominators for vital registration death numbers and to estimate death numbers for age groups under-5, we need to obtain age-specific populations for the under-5 age groups. Taking final probability of death estimates including impacts of fatal discontinuities from the first run of the all-cause mortality process as the mortality risks, we take our input birth numbers and create person-year estimates of population as described in section 1.15. These person-year estimates are then the input as populations for the final run of the estimation process.

1.15 Under-5 death number estimation

Assigning under-5 deaths to GBD age-sex groups

To estimate the number of under-5 deaths, we run an estimation process that ages birth cohorts through our estimated probabilities of death. This process separates our yearly birth numbers for each location into week-sized cohorts and ages each of these cohorts through our mortality estimates in week-long steps to estimate the number of person-years and deaths in each of the early neonatal, late neonatal, post neonatal, and 1-4 years age groups.

Section 2. Adult mortality

Data sources

Adult population estimates

To calculate adult mortality rate using household death recall, age specific populations in age group 15 to 59 from the corresponding survey or census sources are used. This is also true in calculating adult mortality rate using reported deaths from Sample Registration System from India and Disease Surveillance Point system from China. For data from vital registration systems, we currently have two major sources for population in the corresponding age groups:

1. Population estimates from the World Population Prospect 2015 Revision by the United Nations Population Division. This provides majority of the population estimates used in GBD2015.
2. For the 37 countries covered by the Human Mortality Database, we use population exposure from this source instead of WPP2015.

For subnational locations, interpolation and extrapolation based on rate of change are used together with age specific population from censuses. Raking is applied to ensure consistency between subnational and national populations.

VR/SRS/DSP

See section 1 descriptions of vital registration sources for information on how VR, SRS, and DSP data is identified and prioritized.

Household recall of deaths

Household recall is ascertained from large survey series such as the Malawi 2010 DHS. A survey series must include a module asking about the number of deaths of household members within a given recall time period, along with a list of household members who have not died over this period of time. In addition, these survey series must be considered nationally representative, include survey weights (if applicable), and include the sex and age (either current or at death) of all household members.

Sibling survival histories

Data for sibling survival histories is primarily taken from large survey series in which respondents are asked about the status of their siblings, alive and dead. Some examples of sibling survival history sources include the Laos 2011-12 Multiple Indicator Cluster Survey and many DHS sources. To generate estimates of sibling survival, each questionnaire must contain a module with a full accounting of all siblings (children born to the same mother) of all respondents, along with data on the year of death (if applicable), sex, and age at death or year of birth. Additionally, the surveys must not have significant missingness in terms of responses to the sibling survival history module, as was the case in certain surveys such as the Mexico Health and Aging Study 2012.

2.1 Completeness Assessment: Death Distribution Methods and completeness estimates synthesis

Vital registration systems may not capture all adult deaths. It is important to assess the quality, or in other words, the completeness, of available VR data. Demographers have long been applying a suite of death distribution methods (DDMs) including generalized growth balance (GGB), synthetic extinct generation (SEG), and a combined approach (GGBSEG) to assess completeness.[10–14] These methods compare the age distribution of the population recorded in two censuses with the age distribution of deaths recorded between these two censuses and attempt to estimate completeness of VR.
Recent modifications of these DDM methods provide estimates of completeness that, based on careful simulation studies, are more accurate and robust than traditional methods; nevertheless, these methods generate completeness estimates with substantial uncertainty intervals.[15]

For the GBD study, the process for estimating completeness of death registration for adults is based on estimates of adult completeness from three death distribution methods updated by Murray and colleagues in 2010 as well as information about child completeness.[15] These two sources of information are combined to generate a series of estimates of source- and country-specific adult death registration completeness from 1970 to 2015. The underlying assumption of this process is that completeness of systems will change gradually, and consequently, assessments of completeness for a given year should be informed by DDM estimates for prior and future years in that country. Further, completeness is likely to be similar among countries within a region, and we can inform estimates of completeness with levels of completeness estimated for countries in the same region or super-region by borrowing strength over space as well as time. To do this, we use a two-stage model, whereby we first predict adult completeness based on child completeness and then use a spatial-temporal regression model to incorporate information about adult completeness from the application of DDM methods.

Child completeness is calculated as the ratio of observed child mortality to estimated child mortality for a given source, country, and year. For a particular country-source, estimates of child completeness are only available for years where that data source is present, but a complete time series of child completeness estimates is produced based on a smoothing process. For country sources with no more than three years of data, a constant level of child completeness at the level of the mean of those years that are available is assumed. For country sources with more than three years of data, Loess regression is used to fill in the time series. In order to be conservative in our out-of-sample estimates of child completeness, instead of using the Loess predictions to forecast and backcast we simply hold child completeness constant before the first observation and after the last observation. Additionally, when there is a gap of more than five years we linearly interpolate between the observations on either side of this gap instead of using the Loess predictions to fill in these years.

2.2 Sibling survival method

In countries where sources including vital registration and household death recall are scarce, sibling histories provide important information on the levels and trends of adult mortality rates. The sibling survival technique employed by the GBD study is largely based on the work by Obermeyer et al. (2010) [16] with a few improvements to their methods: (1) use of appropriate survival weights that account for the study design; (2) implementation of a correction to account for the mortality experience of families not represented because none of the siblings were alive and eligible to respond to the survey; and (3)

refinements for adjusting for recall bias. We validated these methodological developments in a range of simulation environments, and we have also developed ways of adjusting for recall bias and handling sparse data in survey designs where the age range of the respondents is narrower than the age range desired for estimation.[16]

Selection bias refers to the underrepresentation of high mortality sibships in the sample population—sibships with higher rates of mortality are less likely to be represented in the survey because fewer of them are likely to have survived to be selected into the sample. A method to correct for this underrepresentation, proposed by Gakidou and King[17], incorporates a sibship-level weight, $Wj = Bj / Sj$, where Bj is the original sibship size and Sj is the number of siblings in sibship j who survive to the time of the survey. When each observation in the dataset being analyzed is at the sibship level, this Gakidou-King (GK) weight can be used to compute a weighted average of the proportions of siblings deceased as reported by each respondent. In the absence of any sibships where all siblings have died, this correction algebraically corrects for the underrepresentation of high-mortality sibships in the survey sample.

When the dataset is expanded to the sibling level (i.e., one observation for each sibling as opposed to sibship), the number of observations listed in the dataset for each sibship corresponds to the original sibship size, Bj, and so the numerator of Wj is already accounted for. The resulting sibling-level weight is therefore $Wi = 1/Sj$ for sibling i in sibship j.[18,19]

Since the analysis reported here is carried out at the sibling level, we use Wi rather than Wj . This improves on previous applications of the method where the sibship-level weight was inappropriately applied to data that had been expanded to the sibling level.

Further, the number of surviving siblings in the family must also be tailored to the eligibility criteria for respondents of the given survey.[18] In applying Gakidou and King's elucidation of the survivorship correction, Sj/Bj represents the probability that a sibling in sibship j survives and is eligible to be selected in the survey. For Demographic and Health Surveys (DHS), respondents must be women between the ages of 15 and 49 and so the Sj in this case would be the number of surviving women in a sibship j who are between the ages of 15 and 49 at the time of the survey. In this analysis, the value of Sj has been chosen to be consistent with the eligibility criteria of each survey.

The sampled population excludes sibships in which there are not any eligible siblings to respond to the surveys; thus, we cannot report on the mortality experiences of these siblings. The zero-survivor correction estimates the number of sibling deaths that are missing from the sample by age and sibship size, and then adds these siblings to the observed sample before calculating age-specific mortality rates. This correction is applied to sibships with one or two females. The correction uses the relationship between the true number of sibships with one (or two) females and the cumulative probability of dying before the time of the survey to estimate the number of missing sibling deaths. For one-sibling sibships,

$$K^1_{obs} = K^1_{true} * (1 - \ {}_a q^1_0)$$
$$K^1_{miss} = K^1_{true} * \ {}_a q^1_0$$

Where: K_{obs}^1 is the number of sibships with one sister that are observed in the sampled population;
K_{true}^1 is the true number of sibships with one sister in the population;
K_{miss}^1 is the number of sibships with one sister that are not represented in the sampled population due to zero-survivor bias;
${}_aq_0^1$ is the cumulative probability of death for five-year age-group a; and
$(1 - {}_aq_0^1)$ is the probability that the sister has survived to the time of the survey.

From these two equations, it follows that the number of sibships with only one sister that are not represented in the population due to zero-survivor bias is equal to:

$$K_{miss}^1 = \frac{K_{obs}^1}{1 - {}_aq_0^1} * {}_aq_0^1$$

We multiply this estimate of the number of missing sibships by the number of females in the sibship (which in this case is one) to get an estimate of the number of females in each age group that are missing from the sample because they have died. We then expand this number so that we have one observation per missing sibling, assign birth and death dates to these missing siblings based on the distribution in the observed siblings, and append them to our existing dataset. This process is also carried out for families with two sisters:

$$K_{obs}^2 = K_{true}^2 * (1 - {}_aq_0^1 * {}_aq_0^2)$$
$$K_{miss}^2 = K_{true}^2 * {}_aq_0^1 * {}_aq_0^2$$
$$\therefore K_{miss}^2 = \frac{K_{obs}^2}{1 - {}_aq_0^1 * {}_aq_0^2} * {}_aq_0^1 * {}_aq_0^2$$

Where: K_{obs}^2 is the number of sibships with two sisters that are observed in the sampled population;
K_{true}^2 is the true number of sibships with two sisters in the population;
K_{miss}^2 is the number of sibships with two sisters that are not represented in the sampled population due to zero-survivor bias;
${}_aq_0^1$ is the cumulative probability of death for the first sister in five-year age-group a;
${}_aq_0^2$ is the cumulative probability of death for the second sister in five-year age-group a.

If there is only one sister within the 15 to 49 age range, the equations are different than above because the second sister does not contribute to the probability of the sibship being observed in the sample:

$$K_{obs}^2 = K_{true}^2 * (1 - {}_aq_0^1)$$
$$K_{miss}^2 = K_{true}^2 * {}_aq_0^1 * {}_aq_0^2 + K_{true}^2 * {}_aq_0^1 * (1 - {}_aq_0^2)$$
$$\therefore K_{miss}^2 = \frac{K_{obs}^2}{1 - {}_aq_0^1} * {}_aq_0^1 * {}_aq_0^2 * \frac{K_{obs}^2}{1 - {}_aq_0^1} * {}_aq_0^1 * (1 - {}_aq_0^2)$$

Both this analysis and Obermeyer et al take into account time prior to the survey in the logistic regression to model mortality.[16] This current analysis, however, provides an updated method for recall

bias adjustment. After ${}_{45}q_{15}$ has been estimated for each of the four surveys, the estimates are combined and paired up for all periods where they overlap. This overlap occurred when there were at least two surveys carried out in the same country within 15 years of each other. In GBD2013, we estimated adult mortality from sibling histories for three five-year periods prior to the survey date. This is changed in GBD2015 where single year 45q15 estimates from sibling survival were generated using the same methodology to account for the changing level and trends of 45q15 within the 15 year period covered by sibling survival module. This generates pairs of estimates in years where there are overlapping surveys. For each of these pairs, we calculate the difference in the years of recall as the interval between when the two surveys were conducted and we also calculate the magnitude of the difference between the two estimates of ${}_{45}q_{15}$. We then estimate the linear regression model shown in the below equation to quantify the relationship between years of recall and level of mortality separately for each sex of sibling:

$$\Delta({}_{45}q_{15})_{i,j} = \beta * \Delta(\text{survey date})_{i,j} + \xi$$

Where: $\Delta({}_{45}q_{15})_{i,j}$ is the difference in ${}_{45}q_{15}$; and
$\Delta(\text{survey date})_{i,j}$ is the difference in survey date for survey pair *j* in country *i*.

Upper and lower uncertainty intervals were also derived. The coefficient on recall period represents the effect of recall bias and was used to adjust the ${}_{45}q_{15}$ estimates to account for that bias.

2.3 Completeness data synthesis

Once we have obtained a full series of under-5 completeness estimates for each country source we fit the model described in the equation:

$$\log_{10}\left(c_{i,s,t}^{\text{adult}}\right) = \alpha + \beta_1 * \log_{10}\left(c_{i,s,t}^{\text{child}}\right) + \gamma_1^{SR} + \gamma_2^{SR} * \log_{10}\left(c_{i,s,t}^{\text{child}}\right) + \gamma_1^{R} + \gamma_2^{R} * \log_{10}\left(c_{i,s,t}^{\text{child}}\right) + \eta_{i,s} + \xi_{i,s,t}$$

Where: $c_{i,s,t}^{\text{adult}}$ is completeness of adult deaths registration in country *i*, source *s*, at time *t*;
$c_{i,s,t}^{\text{child}}$ is completeness of child deaths registration in country *i*, source *s*, at time *t*;
γ terms are random effects at the region (R) and super-region (SR) level;
$\eta_{i,s}$ is a random effect at the country and source level; and
$\xi_{i,s,t}$ is an error term.

This model relates adult completeness to under-5 completeness and includes super-region and region-level random effects to allow for differences in both the average level of adult completeness and the relationship between child and adult completeness at these levels. The country-source random effect captures the fundamental difference in level of completeness between different data sources.

A $\log_{10}$ transformation is employed to make over- and under-completeness symmetric (e.g., 50% complete and 200% complete are symmetric around 0 when $\log_{10}$ transformed) and to simplify calculation of the variance of completeness estimates in $\log_{10}$ space, which is needed for the adult mortality estimation process. Also, to avoid allowing outlying DDM-derived estimates of adult completeness from unduly influencing the predictions from the model in the equation above, for any

given set of three DDM estimates (GGB, SEG, GGBSEG) calculated from a single pair of censuses, the estimate that is furthest from 1 (i.e., complete) is excluded.

For each country-source $\log_{10}(c_{i,s,t}^{adult})$ is predicted from coefficients estimated in the model above and child completeness. Not every country can be used to fit this model as DDM cannot be applied in some cases due to lack of appropriate census data. However, because the coefficients used for prediction are at the region and super-region level, predictions from this model can be generated for all countries where estimates of child completeness are available. We do not believe that the same relationship between adult and child completeness exists for registration-based sources as for recall-based sources, so the above model is applied only to registration-based sources (primarily VR data but also sample registration systems). For sources that only include household death recall, we set an arbitrary value of 1 for the first stage values instead of making predictions from completeness from child age groups for the aforementioned reason. However, it should be noted that this set value by no means reflects the true completeness of adult age groups in the household death recall sources and it will change once we apply the spatial-temporal regression in the second-stage estimation.

In the second stage, we calculate the residuals from the first stage and apply spatial-temporal smoothing to these residuals. The predicted residuals are then added back onto the first-stage predictions, generating the second-stage predictions. Spatial-temporal smoothing is carried out in the same way as in the adult mortality estimation process with three modifications: the λ and ζ parameters are set to 2.0 and 0.95; only the fixed effect local regression variant is used; and the residuals are not held constant out of sample. The registration-based sources and the non-registration-based sources are handled separately in this step.

The variance of the completeness estimates must also be calculated, as this information is utilized in the adult mortality estimation process. To do this, we approximate the variance based on the median absolute deviation (MAD) compared to the second-stage estimates. We calculate variances at the regional level and do so separately for registration-based sources and other sources. Then, for each country-source-year, we generate 10,000 simulations from a normal distribution with mean equal to the second-stage prediction for that year and variance calculated as just described. For non-registration-based sources we believe that both under- and over-reporting are possible (over-reporting may occur due to telescoping of events outside of the recall period into the recall period), and so for these sources we now exponentiate the 10,000 simulations and find the mean, which serves as the final prediction for completeness. For registration-based sources we believe that only underreporting is possible, so for these sources we first truncate any simulations above 1 to 1 and then exponentiate the 10,000 simulations to find the truncated mean, which will serve as the final prediction for completeness. In both cases, before exponentiating the simulations, the variance of the simulations is calculated, and used as the variance of the completeness estimates in the adult mortality estimation process.

The final completeness estimates are used to adjust, where appropriate, the corresponding country-source-years before these data are used in the adult mortality estimation process. For countries in which we believe males and females have differential completeness, we carry out the above process separately by sex. For a small number of data points, completeness cannot be estimated using the procedure described above due to a lack of appropriate census data, and the original growth balance

method is the only viable option.[12] In previous papers, we have included a selected number of data points derived from household recall of deaths to which the Brass growth balance method had been applied. Our simulation studies suggest this method is extremely imprecise, so we have excluded these points from the analysis. This accounts for why the number of household recall of deaths data points has decreased in this analysis compared to Lozano et al.[20]

2.4 45q15 data synthesis using non-linear mixed effects model and ST-GPR

2.4.A. Overview of adult ($_{45}q_{15}$) mortality estimation

For each country, we generated a time series of $_{45}q_{15}$ estimates. We modeled the underlying mortality risk separately from excess mortality due to fatal discontinuities. To model the underlying mortality risk we relied on a three-stage process that incorporates all data in our database for each country after excluding data identified as outliers or that refer to years identified to contain mortality shocks from conflict or natural disaster. In the first stage, we applied a nonlinear mixed effects model which used covariates to explain variation in $_{45}m_{15}$. In the second stage, we exploited spatial and temporal correlation in the residuals from the first stage regression by performing a smoothing process on these residuals. The smoothed residuals were then added back into the first stage regression predictions to produce an updated time-series of $_{45}q_{15}$ for each country. In the third stage, we applied Gaussian process regression (GPR) which synthesizes information from the second stage predictions and the observed data. After applying this procedure to generate estimates of the underlying mortality risk, we modeled abrupt changes in mortality by estimating the excess risk of mortality in years identified as containing a conflict or natural disaster. This estimated excess mortality risk was then added to the underlying mortality risk to produce our final time series of $_{45}q_{15}$ estimates. All analyses were carried out in Stata 13.1, R 3.0.2, and the PyMC package, version 2.0, in Python 2.5.4. A more complete description of the various stages of our modeling strategy is given below.

2.4.B. First stage nonlinear mixed effects regression

The first stage nonlinear regression models country/year/sex-specific adult mortality rate using key covariates: lag distributed GDP per capita, mean years of education in age group 15 to 59, and crude death rate due to HIV/AIDS in age group 15 to 59. We use the nonlinear mixed effects model specified in the equation below. This model was fit separately for males and females.

$$_{45}\mathrm{m}_{15}^{\mathrm{observed}} = \exp(\beta_1 + \beta_2 \cdot \mathrm{Edu} + \beta_3 \cdot \ln(\mathrm{LDI}) + \gamma_{\mathrm{country}}) + \beta_4 \cdot \mathrm{HIV} + \mathcal{E}$$

Edu is the mean years of education for the age group 15 to 59; LDI is lagged distributed income; $\gamma_{\mathrm{country}}$ is a country-level random effect; and HIV is the crude mortality rate from HIV for ages 15 to 59. We initialized the model with starting values for each of the β coefficients equal to 0. We tested our model with different starting values, including values from a hierarchical linear mixed effects model, and found that this model was not sensitive to starting values.

The final stage one predictions were based on predictions from the model, the above equation excluding the country random effect. We excluded the country random effect to facilitate modeling spatial trends in mortality in the second stage. The model predictions were then converted from $_{45}m_{15}$ to $_{45}q_{15}$ to be put into the second stage model described next.

2.4.C. Second stage spatial-temporal smoothing of residuals

The first stage regression model reflects the explanatory power of the set of covariates but, as might be expected, fails to explain all of the variation in ${}_{45}q_{15}$. The residuals from the first stage regression are correlated in both time and space, indicating that ${}_{45}q_{15}$ is correlated in time and space in ways that are not fully captured by the covariates included in this regression. We exploited this remaining pattern of variation by applying a local regression to the residuals from the first stage regression, effectively smoothing across time and space.

The local regressions were fitted separately for each of the 21 GBD regions which have been constructed so that countries within each region share similar epidemiological profiles.[[21]] We applied two variations of local regression to the first stage residuals, both of which utilized the same weighting scheme to incorporate temporal and spatial relatedness. In both variants, a set of weighted linear regressions, one for each country-year of interest, were undertaken. When carrying out the regression for a given country-year of interest, all residuals in the dataset were weighted with respect to this country and year. We first weighted residuals with respect to time using a weighting function similar to that utilized in Loess regression:

$$w_t = \left(1 - \left(\frac{|r_t - r_{est}|}{1 + \text{argmax}_t|r_t - r_{est}|}\right)^{\lambda}\right)^3$$

The r_t and r_{est} terms are, respectively, the year of interest and the year of the residual being weighted. The $\text{argmax}_t|r_t - r_{est}|$ term is the maximum distance between the year of interest and a residual within the region. The λ parameter in this weighting function dictates how quickly the weights fall off as the distance in time increases: a larger λ implies that the assigned weights will diminish slowly with time, while a smaller λ allows the weights to diminish more rapidly with time.

We then weighted residuals with respect to space by modifying the time weights described below. Weights for residuals within the country of interest were multiplied by a factor of:

$$\frac{\zeta \sum_{i \notin c_{est}} w_i}{(1-\zeta) \sum_{i \in c_{est}} w_i}$$

where c_{est} is the country of interest and w_i are the time weights described above. As a result of this modification, 100·ζ % of the total weight was placed on residuals within the same country, and the remaining 100·(1-ζ) % of the weight was placed on residuals from other countries in the region. For countries with no residuals (i.e. countries with no data) the above factor is 0 and there was no re-weighting: consequently all of the weight remains, by necessity, in other countries in the region. We set λ to 0.8 and ζ to 0.8 based on an examination of country plots under various different settings of both parameters through iterative testing.

The first local regression variant, which we call linear local regression, is described below and is a weighted linear regression of the residuals on year and an indicator of the residual from the country currently being estimated (if this indicator cannot be estimated, i.e. because there are no residuals in a given country, it is dropped):

$$r_{est} = \beta_0 + \beta_1 t + \beta_2 c_{est} + \mathcal{E}$$

The second variant, which we call fixed effects local regression, used a weighted linear regression with no covariates. This is equivalent to a simple weighted average of the residuals.

Linear local regression incorporates information from covariates, in this case year, but extrapolation is heavily based on this covariate and in settings with sparse data this can result in implausible out-of-sample predictions. In contrast, fixed effects local regression does not incorporate an explicit time-trend and does not suffer from this problem in extrapolation, but also is less adequate at fitting the data in countries with many observations. We therefore chose to combine the estimates from both variants. As described in the below equation, we calculated the data density, d_c, for each country we estimated, c_{est}, and then calculated a weighted average of the predictions from the linear local regression and fixed effects local regression where d_c% of the weight is assigned to the linear local regression and the rest to the fixed effects local regression. In this way, the final estimates for countries with more vital registration data are more heavily informed by the linear local regression and the final estimates for countries with less (or no) vital registration data are predominately informed by the fixed effects local regression, as is appropriate given the strengths and weaknesses of these two variants.

$$\mathrm{d_c} = 100 * \left(\frac{\#\ \text{VR points in } \mathrm{c_{est}}}{\text{Maximum}\ \#\ \text{VR points in any country in the region}}\right)$$

Residuals were logit-transformed before undergoing this smoothing; once the final estimates of the smoothed residuals were obtained for every country-year, these estimates were added back into the logit transform of the first stage regression predictions. This sum was then reverse-logit transformed; by carrying out the first two stages in logit-space we restricted the predictions to between 0 and 1. These predictions are called the second-stage predictions.

2.4.D. Model

The third stage of our prediction method is a Gaussian process regression (GPR) based on the model given in below where μ_t is the true $\log_{10}({}_{45}q_{15})$ at time t, $f(t)$ is the baseline mortality risk, and S_t captures excess mortality due to war and disasters. S_t is estimated independently of $f(t)$ as described in a later section. M and C describe the Gaussian process, giving the mean and covariance, respectively.

$$\mu_t = f(t) + S_t$$

$$f(t) \sim GP(M, C)$$

For the Dominican Republic, Peru, and Madagascar, a slightly different model, described in the equation below, is used. For these countries, measurements from sibling histories and from vital registration are at different levels and the direction of the bias in each source is unknown. We therefore used a model which includes a bias term for each source (β_S).

$$\mu_t = f(t) + \beta_s + S_{t,s}$$

$$\beta_s \sim \text{Normal}(0, 0.01^2)$$

$$f(t) \sim GP(M, C)$$

Gaussian process regression is a method of Bayesian inference. We specified a prior distribution for $f(t)$, and a likelihood function which describes the data generation process; the specified prior distributions

and likelihood function are described in subsequent sections. We then used Markov Chain Monte Carlo (MCMC) to approximate the posterior distribution of *f(t)* which also incorporates information from the observed empirical estimates of adult mortality. An MCMC chain of length 5,000 was produced; the first 3,000 samples were discarded and the remaining 2,000 were thinned by a factor of 2 for a total of 1,000 simulations retained. The reported best estimates and confidence intervals were generated from the mean and the 2.5th and 97.5th percentiles of the 1,000 samples, respectively.

2.4.E. Priors

The prior distribution of *f(t)* can be described in terms of the mean prior—the prior for *M*—and the covariance prior—the prior for *C*. We utilized the second stage predictions as the mean prior and used a Matérn covariance function to describe the covariance prior. This covariance function incorporates three parameters: the amplitude, which controls the amount by which realizations of the Gaussian process distribution can deviate from the mean function, the scale, which controls the distance over which the function is correlated, and the degree of differentiability, which influences the smoothness of the samples from the Gaussian process.

We set the differentiability to 0.8 in countries with only complete VR data and to 2.0 in other countries. We used a lower differentiability in countries with complete VR data because in these countries we want the final estimates to follow the data closely even if the trend described is not smooth. In contrast, in countries where the data are less reliable we don't want the final estimates to be overly influenced by individual data points.

We selected prior values for the amplitude and scale based on model performance as assessed by out-of-sample predictive validity. We tested five values of the scale—10, 15, 20, 25, 30—and five values of the squared amplitude—1, 2, 3, 4, and 5 times the mean squared error of the residuals from the second stage prediction model—for a total of 25 combinations of parameters. The out-of-sample predictive validity was assessed for each parameter combination by repeatedly dividing the data available into training and testing sets, fitting the model on the training sets and comparing the predictions to the corresponding data in the testing set.

We divided the data into testing and training sets 100 times. Data were divided as follows: for each region, a number X between 10 and 20 was sampled and the most recent X years of data in that region were assigned to the testing set. Then a number X between 5 and 10 was sampled, a country from within the region was sampled, and a year where there were data in that country was sampled. All data within X years of the selected year in the selected country were assigned to the testing set. This was repeated as many times as there are countries in the region; because iterations of this procedure were independent, the data selected for the testing set may overlap. Any data that were not selected for the testing set were included in the training set.

For each testing and training division, the second stage model was fit on the training data. Then the third stage model was also fit on the training data using each combination of scale and squared amplitude values tested for a total of 25 sets of predictions. The testing data were matched to the predictions in the corresponding country and year for each of the 25 sets of predictions. For each match

we calculated the absolute relative error of the prediction compared to the empirical estimate in the testing set. We also classified each empirical estimate in the testing set as being covered or not covered by each corresponding prediction. The determination of coverage was made by calculating total variance—the sum of the variance of the empirical estimate (described below) and the variance of the GPR estimate—and then calculating a 95% confidence interval around the prediction based on this total variance and assuming a normal distribution. If the empirical estimate was within this confidence interval, it was classified as covered, and otherwise not.

Once this procedure was carried out for all 100 testing and training divisions of the data, we calculated the mean absolute relative error and the mean coverage for each combination of GPR parameters across all 100 sets of predictions. The ideal set of parameters would produce estimates with low mean absolute relative error and mean coverage close to 0.95. We used the function described below to calculate a loss metric which incorporates both the coverage and the absolute relative error into a single measure to assess performance. Parameter combinations with lower values of this loss metric were considered preferable.

$$\text{Loss} = \begin{cases} \text{if coverage} \leq 0.95\text{:} \;\; (0.95 - \text{coverage})/5 + (\text{absolute relative error}) \\ \text{if coverage} > 0.95\text{:} \;\; (\text{coverage} - 0.95)/1 + \left(\text{absolute relative error}\right) \end{cases}$$

The optimal parameters may differ from country to country. To allow for this, we calculated the loss function described above separately for seven distinct groups of countries. These groups of countries are defined by the type of data which are available for estimating mortality: countries with complete VR only, countries with VR only (some or all of which is incomplete), countries with VR and other data, countries without VR data but with sibling histories, and the remaining countries. The first two groups listed are further subdivided into 'small' and 'large' population countries based on a population threshold of 50,000 people between ages 15 and 49 years. For countries with no data, the highest of all selected scales and squared amplitudes from among all other countries was used.

2.4.F. Likelihood

The likelihood describes the probability of observing the data given a particular set of parameters. As shown in the below equation, we used a normal model for describing the probability of observing a particular value of log $({}_{45}q_{15})$ where the mean is given by *f(t)* and the variance by V_t, the data variance.

$$\log_{10}({}_{45}q_{15_t}) \sim \text{Normal}(f(t), V_t)$$

Data variance was calculated for each empirical observation of ${}_{45}q_{15}$ and incorporated both sampling and non-sampling variation. The method for calculating the data variance depended on the type of data: for estimates derived from complete vital registration data we assumed that there was no non-sampling variance and included only sampling variance as computed from a binomial model. We set *N* equal to the national population aged 15 to 59 years and *p* equal to the mortality rate, ${}_{45}m_{15}$. We calculated the variance of ${}_{45}m_{15}$ from *p(1-p)/N* and then transformed this to the variance of $\log_{10}({}_{45}q_{15})$ using the delta method. For estimates derived from incomplete vital registration data, we wanted to include not only sampling variance but also the non-sampling variance that arises from uncertainty in the completeness estimate. For these data, the total data variance was given by the sum of the sampling variance

(calculated as for complete vital registration data) and the variance of the completeness estimate (calculated as described in section 2.3).

For estimates derived from sample registration systems or surveys where completeness has been assessed in the normal ways, the above procedures for complete and incomplete vital registration were followed, but the national population size was replaced with the appropriate population size for the given system or survey. For estimates derived from sources other than sibling histories where completeness could not be assessed or was assessed by the growth balance method, the highest data variance generated by the above three procedures within the same region was assigned. Finally, for estimates derived from sibling history data, the mean absolute deviation (MAD) estimator of the variance was calculated with reference to the first stage predictions, as described in the equation:

$$\theta^2_{r,s} = \left(1.4826 \cdot \mathrm{MAD}_{r,s}\right)^2 = 1.4826 \cdot \mathrm{median}\left(\left|\log_{10}\left({}_{45}q_{15}^{\mathrm{observed}}\right) - \log_{10}\left({}_{45}q_{15}^{\mathrm{predicted}}\right)\right|\right)^2$$

2.4.G. Hyper-parameter selection for adult mortality rate ST-GPR

Similar to the new parameter selection process for U5MR, we have also expanded the scope of our parameter selection for adult mortality rate (45q15) to include variables used in space-time smoothing. The out-of-sample predictive validity testing used to select space-time and GPR parameters for adult mortality rate follows.

For space-time smoothing, we tested ζ values of 0.7, 0.8, 0.9, and 0.99 and λ values of 0.1 to 0.9 in increments of 0.1. We test five values of the scale—5, 7.5, 10, 12.5, 15, 17.5, 20 -- and five values of the squared amplitude—1, 1.5, 2, 2.5, and 3 times the mean squared error of the residuals from the second-stage prediction model. Because we tested combinations of both space-time and GPR parameters, this led to 1,260 combinations of ST-GPR parameters being tested in the process.

We divided the data into testing and training sets 100 times. Data were divided as follows: for each region, a number X between 10 and 20 was sampled and the most recent X years of data in that region were assigned to the testing set. Then a number N between 5 and 10 was sampled, a country from within the region was sampled, and a year where there is data in that country was sampled. All data within N years of the selected year in the selected country were assigned to the testing set. This was repeated as many times as there are countries in the region; because iterations of this procedure were independent, the data selected for the testing set may overlap. Any data that were not selected for the testing set were included in the training set.

We then ran Space-time smoothing and Gaussian process regression with the training data set using each set of parameters being tested to generate estimates for each location and every year in both the training and testing data set.

Within a given iteration we calculate the absolute relative error of the GPR estimate compared to each empirical estimate in the testing set. We also classify each empirical estimate in the testing set as covered or not covered. The determination of coverage is made by calculating total variance—the sum of the data variance and the variance of the GPR estimate—and then calculating a 95% uncertainty interval around the GPR estimate based on this total variance and assuming a normal distribution. If the empirical estimate is within this uncertainty interval, it is classified as covered, and otherwise not. For each combination of parameters, we calculate the mean absolute relative error and the mean coverage

across all iterations from all countries within a particular group. The loss function described below is then calculated for each parameter combination, and the parameter combination with the lowest loss is selected for each group;

$$\text{loss} = (0.95 - \text{coverage}) / 5 + (\text{absolute relative error})$$

Countries with at least 20 years of VR data were assigned their own parameters. Other locations were grouped based on how much and what type of data they had available. A set of parameters was then applied to all locations in that group. A summary of these groups is in the following table:

Group	Conditions
Sparse data, with complete VR only	Only complete VR, more than 10 years of VR, with VR after 1980
Spare data, VR only	At least some incomplete VR, more than 10 years VR, with VR after 1980
Sparse data, VR +	At least some incomplete VR, with either fewer than 10 years of VR or all VR before 1980
Sparse data, other	No VR or sibling data
Sibling data, small	No VR, less than 21 years of sibling data
Sibling data, large	No VR, more than 21 years of sibling data
No data	No data

For countries with no data, the highest of all selected scales and squared amplitudes from among all other countries was used.

In some cases, we restricted the universe of possible parameters. Most of these restrictions occurred for the λ parameter. For many locations with complete VR, we knew that higher lambda values would result in a larger confidence interval than would be appropriate. We also included a lower limit of λ for some locations with either incomplete VR or no VR, so that confidence intervals would reflect the uncertainty of these data. For example, in locations where we only had sibling history data, we restricted lambda to being greater than 0.5. We also made some other manual exceptions to λ, ζ, and scale where results did not pass the common sense test.

We set the differentiability to 0.8 in countries with only complete VR data, excepting those in the Caribbean, Oceania, and the country of Mauritius, and to 2.0 in other all other locations. We used a lower differentiability in countries with complete VR data because in these countries we want the final estimates to follow the data closely even if the trend described is not smooth. In contrast, in countries where the data are less reliable we don't want the final estimates to be overly influenced by individual data points.

2.4.H. Accounting for the uncertainty in HIV covariate in the first stage model for high HIV/AIDS burden locations

To account for the uncertainty in estimated crude death rate due to HIV, a key covariate for the first stage model described in section 2.4.B, we generated draw level crude death rate due to HIV for Group 1 locations (detailed in section 4.6 and Appendix Table 1) and repeated the first stage model and ST-GPR processes described in sections 2.4B-2.4F 250 times using 250 unique draw level crude death rate due to HIV derived from our EPP-Spectrum process. We repeated this process 250 times to balance between computation intensity and obtained increases in the estimated uncertainty interval for 45q15. For each one of the 250 ST-GPR runs, 4 draws were selected out of 1,000 to form the final 1,000 draws of 45q15 for each location over time.

2.5 Identify and remove outliers

To arrive at sensible level and trends in adult mortality rate, certain outliers were excluded from our ST-GPR regression process. In general, we used the following process to outlier influential raw data points that otherwise lead to erroneous without fatal discontinuity 45q15 estimates:

1. Raw input 45q15 data points from years affected war, natural disasters, and other fatal discontinuities as defined in GBD 2015 are excluded from the analysis described in section 2.4.
2. Examination of survey/registration data quality leads certain raw data to be outliered. Such examples include the Afghanistan 2010 Mortality Survey that was not national representative, and some of the vital registration data from Serbia that didn't include deaths from Kosovo.
3. Visual inspection of raw input data on 45q15 and estimated time series estimates of 45q15 from ST-GPR by GBD researchers and country experts through the GBD collaborator network. Data points are outliered when it is unexplainably different from other adjacent points from the similar source indicating a data reporting issue and compilation error in the direct sources where such data was obtained. Some subnational level single year 45q15 estimates from sibling survival methods are excluded for unreasonably high or low estimates due to small sample sizes.

2.6 Rake subnational estimates to national level (excluding South Africa)

First, we randomize the order of the 1,000 subnational-level draws and the national draws separately to avoid any correlation between subnational and national draws that may have been introduced in prior processes.

GBD 2015 provides estimates of 45q15 for 561 locations at both subnational and national level. While it is absolutely essential to use input data from the subnational level in informing the level and trend of 45q15 for the corresponding location, it is often the case that we tend to have more robust data at the national level, more data sources and data sources that cover longer time periods. To have consistent estimates between aggregated subnational level estimates and the separately estimated national level estimates, we rake the subnational 45q15 to match our national level 45q15 estimate by using the following formulas:

$$_{45}q_{15}^{s'} = 1 - e^{-45 \cdot {}_{45}M_{15}^{S} \cdot r}$$

and

$$r = \frac{\ln(1-{}_{45}q_{15}^{N})/-45}{\sum_{s=1}^{n}\ln(1-{}_{45}q_{15}^{s})\cdot\frac{p_s}{P_N}/(-45)}$$

In the above equations, s refers to subnational locations within a country N, p is population in age group 15 to 59, and 45q15 are estimates of adult mortality rate from the ST-GPR process, and ${}_{45}q_{15}^{s'}$ is the post-raking 45q15 for subnational locations.

2.7 Review estimates for quality

The preliminary estimates of adult mortality rates are reviewed by both the researchers who work on the demographic estimation process and the GBD all-cause mortality collaborator network. Concerns regarding quality of certain survey and data points are raised and reviewed which lead to revision of the database where applicable.

2.8 45q15 estimates with HIV

The results of the adult mortality rate ST-GPR process are estimates of probability of death from all causes of death except fatal discontinuities for all locations covered in GBD 2015 for 1970 to 2015.

2.9 HIV-free 45q15

As a result of the non-linear mixed effects model used in the first stage before ST-GPR, we also generate HIV-free counterfactual 45q15 by removing the impact of HIV, as specifically measured by crude death rate due to HIV/AIDS estimated in the EPP-Spectrum process (described in the HIV/AIDS model in Part 3), from all-cause 45q15 without fatal discontinuity.

Section 3. Model life table system

Overview

For many purposes, estimates of age-specific mortality rates are useful. In settings without complete VR systems, data are often available for some summary measures of mortality such as under-5 mortality rate (henceforth ${}_5q_0$) from complete or summary birth histories and mortality from ages 15 to 60 from sibling histories (${}_{45}q_{15}$, and commonly described as adult mortality rate). Model life tables, which are structured relationships between levels of age-specific mortality at different ages, can be used to generate an estimate of age-specific mortality for detailed age-groups from summary measures of mortality on children and adults (${}_5q_0$ and ${}_{45}q_{15}$, respectively). Model life tables have many other applications as well, such as simplifying the task of mortality projection or updating life tables from the most recent empirically observed data. This is true even for countries with good VR systems due to the fact that compilation and publication of VR data may take years.

An ideal model life table system has several desirable attributes. First, a model life table system should be parsimonious and require only a few entry parameters to generate a full life table with age specific mortality rates. Second, it should adequately capture the range of age patterns of mortality observed in

real populations and yield high predictive validity, not just measured by summary indices such as life expectancy at birth, but more importantly by age-specific mortality rates. Third, it should provide satisfactory estimates of age-specific mortality for countries with high levels of mortality, especially those with substantial HIV/AIDS epidemics. Finally, a model life table should generate age-specific mortality with a plausible time trend, and the partial derivative of age-specific mortality should be positive with respect to entry parameters such as ${}_5q_0$ and ${}_{45}q_{15}$.

A simple and somewhat flexible model life table system was proposed by Brass.[18] He observed that in general the logit of the *lx* (proportion of a hypothetical birth cohort still alive at age x) column in a life table could be represented as a linear transformation of the logit of the *lx* column of a reference standard life table. Murray et al noted that as a population moved further away from the levels of mortality in the reference standard, the assumption of linearity in logit *lx* space was violated.[19] A series of age-specific modification factors were proposed that allowed for the characteristic bending of the logit *lx* function compared to the standard. Murray et al also assessed the ability of this modified logit life table system to predict age-specific mortality rates. The modified logit life table system with its built-in optional mechanism of predicting adult mortality (${}_{45}q_{15}$) from child mortality (${}_5q_0$) has been extensively used by the World Health Organization since the early 2000s.

This system, however, suffers from two major limitations. First, when adult mortality is very high relative to child mortality, such as in the presence of an HIV/AIDS epidemic, the age patterns of mortality generated do not fit well to the observed data. Second, when the modified logit life table system is applied to time series of ${}_5q_0$ and ${}_{45}q_{15}$, paradoxical trends can be generated where adult and child mortality are declining, but predicted age-specific death rates in some age groups are increasing. We have extended the modified logit life table system to deal with these two major limitations. Wang et al provide a more complete discussion of the development of this approach.[2,4] We summarize these developments in brief here. There are four distinct steps.

3.1 Building empirical model life table database: data sources and quality review

Relational model life table systems critically depend on the empirical database used to generate model life table standards and test the predictive validity of model life tables. For both all-cause mortality and cause-specific mortality analysis in GBD, we have amassed a comprehensive database on human mortality from censuses, Vital Registration systems, sample vital registration systems such as SRS from India and Disease Surveillance Point system from China, and the Human Mortality Database. These aforementioned data sources provide a total of 38,460 empirical life tables. After removing duplicated empirical life tables between Human Mortality Database and vital registration sources, we have closely examined every single empirical life table by applying the following quality control process to further remove life tables with irregular/impossible age pattern of mortality:

a. Empirical life tables with Irregular age pattern of mortality such as probability of death in age group 1-4 higher than first year of year are dropped;
b. Empirical life tables where age pattern of mortality doesn't conform to Gompertz law of mortality between age 40 and 80 are dropped.

Murray et al. used a database of 3,566 life tables covering 63 countries.[22] We have expanded this

database to include 16,507 empirical life tables. Two major data sources provide about 97.0% of these life tables. The Human Mortality Database (HMD)[23] has over 8,400 life tables from 37 countries or areas dating back to 1,751. A total of 4,147 life tables from this database dating between 1950 and 2014 are included in our database. We excluded life tables of poor quality and those from years affected by wars and pandemic. The other major source of empirical life tables is the collection in which VR data have been evaluated using death distribution methods, as revised by Murray and colleagues (2010) and Wang et al (2014).[24,25]

It is our view that accuracy of the age pattern of mortality in the raw data is to a certain degree correlated with the completeness of the data. We did make certain exceptions for the purpose of including age pattern of mortality from regions that otherwise won't be represented in our database with the strict completeness threshold. In all cases, rigorous assessment of the quality of these data is conducted. In total, we included 11,867 life tables from various VR sources including subnational VR from Mexico, Brazil, and United States, Disease Surveillance Point system, and sample registration system that are adjusted by GBD's completeness assessment process as detailed in section 2.1.

In building the empirical life table database for the model life table system used by GBD, we examined additional data sources that we decided to exclude from our database. These included VR sources deemed incomplete and life tables with implausible patterns or high levels of stochastic fluctuation among age groups.

In addition, all life tables in our database with crude death rate due to HIV/AIDS in adult age groups over 0.1% are excluded in the process described in section 3.3.

3.2 Extending age-specific mortality to age 100+

To extrapolate age-specific mortality beyond age 85, the Gompertz law of mortality and other functional model age pattern of mortality methods are generally used.[26,27] Here, we have developed a new model with better predictive validity than existing methods. Age-group dummies and probability of dying from age 80 to 84 in logarithmic scales are used to estimate the difference in age-specific probability of dying in logit scale between two consecutive age-groups, as described in the following equation:

$$\mathrm{logit}\left({}_5q_x^{\mathrm{j,t,g}}\right) - \mathrm{logit}\left({}_5q_{x+5}^{\mathrm{j,t,g}}\right) = \alpha^{\mathrm{g}} + \beta_{\mathrm{x}}^{\mathrm{g}} \cdot \mathrm{age} + \gamma^{\mathrm{g}} \cdot \mathrm{logit}\left({}_5q_{80}^{\mathrm{j,t,g}}\right) + \eta_{\mathrm{j}}^{\mathrm{g}} + \xi_{\mathrm{x}}^{\mathrm{j,t,g}}$$

Here, *j* refers to country, *g* refers to sex, and *t* refers to time. Parameters are estimated using data from selected countries in Human Mortality Database with high quality VR data in the oldest old age groups above age 80.[23] The parameters estimated from the above model are then used to generate age-specific probability of death from age 85 to 109.

3.3 GBD relational model life table system with a flexible standard selection mechanism

The relational model life table system is based on the logic that in order to capture the very high levels of younger adult mortality seen in populations with high HIV prevalence, we needed to develop a model life table for a counterfactual population without HIV and then add on the effects of HIV by age and sex.

This system is captured in three components. We first estimated counterfactual levels of $_5q_0$ and $_{45}q_{15}$ in the absence of HIV. The empirical approach taken for this step is explained below. Then a full set of age-specific death rates were generated using the model life table system from the counterfactual levels of child and adult mortality. Finally, the increase in mortality, specific to each age group, associated with HIV was estimated.

3.3.A. Model for populations free of HIV/AIDS

In terms of estimating a set of age-specific death rates from $_5q_0$ and $_{45}q_{15}$ using a relational model life table, we have undertaken several innovations. A key change from previous relational model life table systems is the shift to modeling q_x in logit space rather than the l_x in the same space. Modeling q_x enables us to more precisely capture the different impacts of changes from the two entry parameters ($_5q_0$ and $_{45}q_{15}$) on different age-groups. The following equation provides the life table for populations not affected by HIV/AIDS.

$$\mathrm{logit}({}_nq_x^c) = \mathrm{logit}({}_nq_x^s) + \beta_x^1 \cdot \left(\mathrm{logit}({}_5q_0^c) - \mathrm{logit}({}_5q_0^s)\right) + \beta_x^2 \cdot \left(\mathrm{logit}({}_{45}q_{15}^c) - \mathrm{logit}({}_{45}q_{15}^s)\right) + \xi_x$$

Where: $\mathrm{logit}({}_{45}q_{15}^s)$ is the logit transformation of the $_{45}q_{15}$ in the standard life table (development of the standard life table is presented below);

$\mathrm{logit}({}_{45}q_{15}^c)$ is the logit transformation of the $_{45}q_{15}$ value for a country without HIV or the counterfactual level of $_{45}q_{15}$ in the absence of HIV in a country affected by HIV/AIDS;

$\mathrm{logit}({}_5q_0^s)$ is the logit transformation of the $_5q_0$ in the standard population;

$\mathrm{logit}({}_5q_0^c)$ is the logit transformation of $_5q_0$ for a country without HIV or the counterfactual level of $_5q_0$ in the absence of HIV in a country affected by HIV/AIDS;

$\mathrm{logit}({}_nq_x^s)$ is the logit of the probability of death in the standard population from age x to x + n;

$\mathrm{logit}({}_nq_x^c)$ is the logit transformation of the probability of death from age x to x + n in a country without HIV or the counterfactual level of $_nq_x$ in the absence of HIV in a country affected by HIV/AIDS;

β_x^1 and β_x^2 are coefficients that vary by age x and which measure the impact of differences in child and adult mortality rates between a target life table and the standard life table on the estimated age pattern of mortality. In other words, both coefficients determine how much the estimated age pattern of mortality deviates from the standard by age and from linearity.

This equation proposes that the logit transformed age specific probability of dying in a target life table (*c*) can be represented as a function of the corresponding logit transformed age specific probability of dying in a standard life table (*s*) and the differences in probability of dying from age 0 to 5 in logit scale and the difference in probability of dying from age 15 to 60 in logit scale between a pairs of life tables: c and s. Life table *c* is the estimated HIV-free life table either for a country affected by the epidemic or not. The model is based on an empirical observation where the differences in age specific probabilities of dying in logit scale between two life tables are highly correlated with differences in $_5q_0$ or $_{45}q_{15}$ in logit scale when HIV/AIDS epidemic is not present.

Coefficients β_x^1 and β_x^2 are estimated using the following equation:

$$logit\ {}_nq_x^c - logit\ {}_nq_x^s = \beta_x^1 * (logit\ {}_5q_0^c - logit\ {}_5q_0^s) + \beta_x^2 * (logit_{45}q_{15}^c - logit_{45}q_{15}^s) + \xi_x$$

In estimating the parameters, we use country-time specific and region (i.e. global burden of disease region) specific standard for each life table not affected by HIV/AIDS in our database (other aggregated standard life tables by different geographical or epidemiological clustering criteria are also possible). Country-time specific standard life tables are used whenever an empirical life table from the same country within a 15-year time frame is available in our database. Region specific standard life tables are generated by collapsing all zero-HIV life tables in our database from the same global burden of disease region by sex. We then pair up all zero-HIV life tables in our database with the generated region specific life tables.

The estimated $\hat{\beta}_x^1$ and $\hat{\beta}_x^2$ are shown in the table below. We limit the effects of ${}_5q_0$ and ${}_{45}q_{15}$ to certain age groups to avoid implausible outputs when ${}_5q_0$ and ${}_{45}q_{15}$ from a population change in opposite directions. With the results in the table below**,** we can generate full life tables for populations not affected by HIV/AIDS. The values of ${}_5q_0$ and ${}_{45}q_{15}$ serve as points of entry (or entry parameters) for this model life table system.

Table: Model life table coefficients

	**Diff. in ${}_5q_0$ (logit scale)**		**Diff. in ${}_{45}q_{15}$ (logit scale)**	
**Age**	**Male**	**Female**	**Male**	**Female**
0	0.984	0.977	--	--
1-4	1.023	1.057	--	--
5-9	0.814	0.763	--	--
10-14	0.464	0.360	0.389	0.514
15-19	0.156	0.148	0.777	0.783
20-24	0.053	0.148	0.807	0.853
25-29	0.039	0.132	0.766	0.917
30-34	0.023	0.109	0.791	0.912
35-39	--	--	0.880	1.080
40-44	--	--	0.912	0.986
45-49	--	--	0.930	0.922
50-54	--	--	0.903	0.871
55-59	--	--	0.857	0.855
60-64	--	--	0.792	0.841
65-69	--	--	0.780	0.857
70-74	--	--	0.785	0.898
75-79	--	--	0.777	0.898
80-84	--	--	0.675	0.807

Our procedure of standard life table computation takes into account empirical relationships between differences in age pattern of mortality, geography, and time. To generate a standard, we first calculated the Mahalanobis distance between the target life table and all zero-HIV empirical life tables of the same sex in our database based on ${}_5q_0$ and ${}_{45}q_{15}$ (in logit scale). The Mahalanobis distance between two sets of ${}_5q_0$ and ${}_{45}q_{15}$ are defined as:

$$D_M^i(Q^i) = \sqrt{(Q^i - O)^{\tau} S^{-1} (Q^i - O)}$$

Where *O* is a multivariate vector representing entry parameters $_5q_0$ and $_{45}q_{15}$ in logit scale. $Q^i = (logit(\,_5q_0^i), logit(_{45}q_{15}^i))$ is a multivariate vector that corresponds to an empirical life table *i* in our life table database. We chose Mahalanobis distance over Euclidean distance due to the fact that $_5q_0$ and $_{45}q_{15}$ are highly correlated in logit space (the correlation coefficients are 0.58 and 0.87 for males and females, respectively), and Mahalanobis distance takes the covariance matrix of $_5q_0$ and $_{45}q_{15}$ in logit scale into consideration when calculating the distance between any pair of life tables. We then keep the potentially most similar life tables as measured by the Mahalanohis distance. The number of life tables retained is based on the numbers of empirical life tables from the same country included in our database. For example, for developed countries with empirical life tables for every year, only the 10 most similar life tables and all life tables from the same country are kept for additional analysis.

In the second step, instead of obtaining a simple arithmetic mean of all selected life tables, we applied empirical weights to each selected life table and computed the weighted average. We examined all possible pairs of life tables of the same sex in our empirical database by country, GBD region, and GBD super-region. For each pair of life tables, we computed the sum of difference in $_nq_x$ in logit space. We then obtained the mean of such values by sex, time difference in years of observation, and whether the pair of life tables are from the same country, same GBD region, or same GBD super-region. The reciprocal of the squared means were used as empirical weights. The figure below shows the empirical weights by lag in time and geographic region for males. This process gives more weight to life tables closer in terms of time and geographic location.

Figure: Empirical weights by lag in time and geographic region for males.

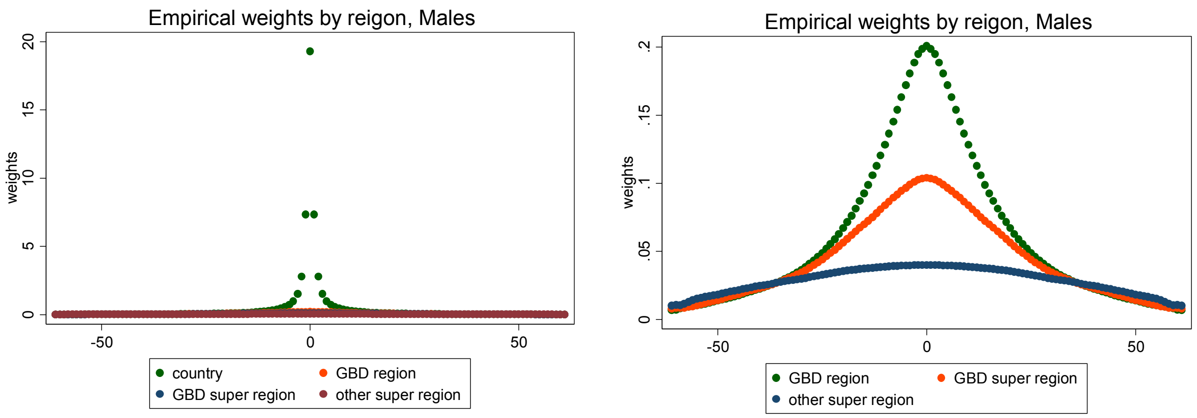

The counterfactual life table, *c*, or the final life table, *p*, when HIV/AIDS is non-existent in the population, is estimated using a standard life table, *s*, which is generated using the aforementioned procedure and coefficients $\hat{\beta}_x^{1,g}$ and $\hat{\beta}_x^{2,g}$ as shown in the model life table coefficients table.

3.3.B. Introducing AIDS

Currently, perhaps the most challenging issue for all existing model life table systems is providing plausible age specific mortality estimates given an HIV/AIDS epidemic. Both widely used model life table systems, the Coale-Demeny model life tables and the Modified Logit Life Table system, are largely based on empirical life tables from the pre-HIV era. In addition, they do not provide an integrated solution to the problem of incorporating HIV/AIDS.

In GBD 2013 and GBD 2015, we apply a two-step process where we first estimate HIV/AIDS counterfactual age pattern of mortality first using the method described in step 3.3A with HIV counterfactual 5q0 and 45q15 as entry parameters, and then in the second step add excess mortality due to HIV/AIDS in summary age group of under-5 and age 15 to 59 to HIV free age specific mortality estimated from step 1. HIV counterfactual 5q0 and 45q15 are estimated using the first stages models employed in the Gaussian Process Regressions for 5q0 and 45q15 discussed in sections 1 and 2.

For GBD 2013 and GBD 2015, we apply an innovative way of adding excess mortality due to HIV to specific age groups by the type of the HIV/AIDS epidemic: concentrated or generalized. In this step, we take all available vital registration data with ICD10 coded causes of death. Then by sex, and type of epidemic, we run Seemingly Unrelated Regression (SUR) on the relative mortality risk due to HIV using constant only as shown in the equation below. The relative risks here are defined as the ratio HIV specific mortality in a specific age group and age group 40-44.

$$R_i = \alpha_i + \xi_i$$

Where *i* indicates different age groups 0, 1-4, 4-9, 10-14, etc.

$$R_i = {}_nM_x^{HIV} \Big/ {}_5M_{40}^{HIV}$$

We then repeatedly re-sample from the variance and covariance matrix of the estimated constants and residuals to get the mean and 95% uncertainty intervals of estimated relative risks by sex, age and types of epidemic. The figure below shows the relative risks for generalized epidemic.

Figure: Relative risks for generalized epidemic

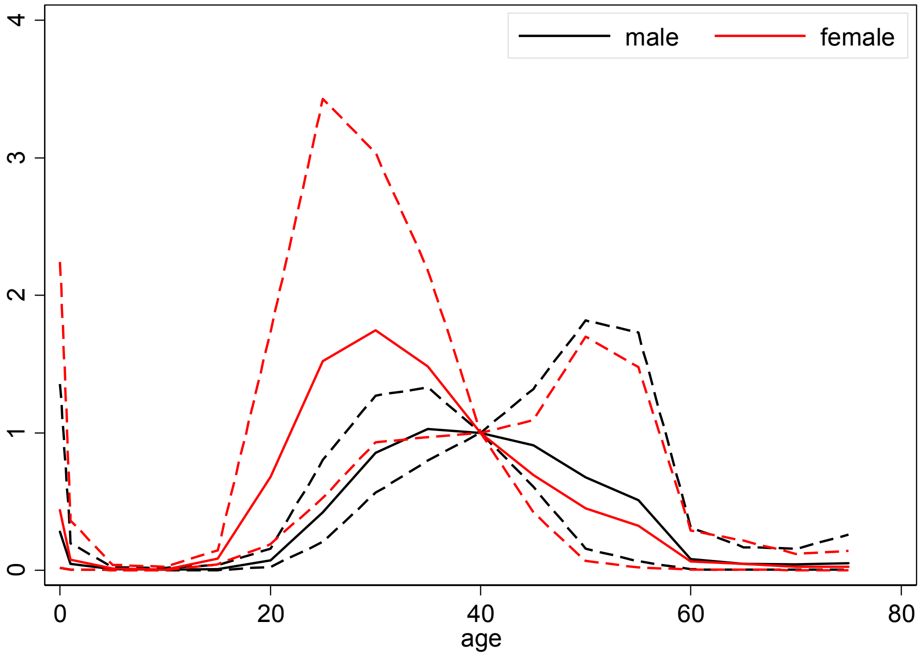

3.3.E. Uncertainty in the estimated life tables

One important attribute of this study is the inclusion of uncertainty in every single step of the GBD mortality estimation process. We have integrated uncertainty from the entry parameters (${}_5q_0$ and ${}_{45}q_{15}$) and the coefficients of each model discussed in this section based on the method illustrated by King et al.[28] Essentially, the model life table process described above is repeated 1,000 times based on randomly paired entry parameters (thus different standard life tables) and model coefficients. As a result of this process, in countries where uncertainties in estimated entry parameters are high, we can have wide uncertainty intervals in the estimated deaths, which is the final product of the GBD mortality process.

3.4 Rake subnational life tables to national level (excluding South Africa)

Since data is scarcer for subnational units than it is at the national level in most countries, estimates of subnational mortality require adjustment to be consistent with national estimates. To generate consistent time series estimates of all-cause mortality including mortality rates and death numbers, we raked subnational-level estimates to separately-estimated national level estimates. For the mortality envelope, we applied a national scalar such that the sum of the subnational location mortality envelopes matched the mortality envelope separately estimated for the national level.

The only exception to this subnational-to-national raking process is South Africa, where we aggregate subnational level estimates as our national level estimates. Based on our observation in previous iterations of GBD, the high level of heterogeneity in the HIV/AIDS epidemic at the subnational level in South Africa introduced a challenge to accurate estimation of both all-cause and HIV cause-specific mortality in South Africa at the national level. Since our EPP-Spectrum model (described in the HIV/AIDS modelling process in Part 3) generated estimates at the province level for South Africa, we produced province-level all-cause mortality estimates using all available subnational sources of child and adult mortality rates and aggregated province-level results as our final national-level estimates for South Africa. The number of data sources were found to be comparable at the national and provincial levels.

Section 4. HIV/AIDS estimation

Input data

The section on HIV/AIDS modeling in Part 3 of this appendix contains details on the input data used for estimating HIV/AIDS.

4.1 Age-specific mortality (with and without HIV)

The with-HIV and without-HIV age-specific mortality rates that are inputs to the HIV/AIDS estimation are part of the model life table process described in section 3.

4.2 HIV-free mortality rate

The age-specific mean HIV-free mortality rates produced by the model life table process are used as inputs to the on/off ART survival process described in the HIV modeling section in Part 3 of this appendix.

4.3 HIV-free survival rates (for Spectrum)

The age-specific HIV-free survival probabilities from the model life table process were used to create inputs for Spectrum. However, because the model life table process produces 5-year age group estimates and Spectrum models in single-year age bins, we interpolated 5-year age group survival into single-year age group survival. Life tables generally include a variable called lx, which represents a synthetic cohort, aged through the mortality observed in the table. At age 0, 100,000 people may be alive, and at each age group, the probability of survival is applied to the number who survive the previous age group. In order to interpolate single-year survival, we fit a spline to the 5-year lx values to interpolate single-year lx values. Then, we back-calculated the single-year probabilities of survival. This method was considered better than assuming constant probability of survival for each year in a 5-year age group and generated smoother single-year estimates. These single-year probabilities of HIV-free survival were then used as inputs to Spectrum.

4.4 On- and off-ART mortality estimation process and CD4 progression parameters for off-ART

Detailed descriptions of the data and process related to on- and off-ART mortality estimation and CD4 progression parameters can be found in the HIV/AIDS estimation write-up in Part 3 of this appendix.

4.5 EPP and Spectrum

For the GBD analysis of HIV, we used variants of two tools developed by UNAIDS: a) the estimates and projections package (EPP) which is meant to generate a time series estimate of HIV incidence consistent with observed prevalence data in ages 15-49 and b) the Spectrum natural history model that uses an estimated time series of HIV incidence, demographic inputs including HIV-free mortality and population, assumptions about CD4 progression rates, and assumptions about on and off ART HIV death rates by age, sex, and CD4 rate to predict incidence, prevalence, and death by age, sex, and year. We modified both tools for use in the GBD. Additional detailed information about EPP and Spectrum can be found in the HIV/AIDS estimation write-up in Part 3 of this appendix.

4.6 HIV/Mortality Reckoning

The Reckoning process is intended as a method of reconciling separate estimates of HIV mortality (and its resulting effect on estimates of HIV-free and all-cause mortality) due to two separate estimation processes within the GBD all-cause and HIV estimation framework: those from the model life table system as a way to capture the impact of HIV on age pattern of all-cause mortality, and the those from the natural history model of EPP-Spectrum as used by GBD and UNAIDS. In addition, we also utilize space-time GPR smoothed VR data on HIV specific mortality for countries with good quality VR instead of using mortality estimates from Spectrum based on back-calculated incidence using case report data.

As part of the HIV/AIDS estimation process, all GBD 2015 locations were assigned to a modeling strategy group, depending on the level of HIV within the country and the availability and quality of HIV and VR data. Groups were used to determine which sources to use for HIV-specific mortality data and how to calculate final estimates of HIV and all-cause mortality.

Group 1 includes much of Sub-Saharan Africa and other locations where HIV prevalence survey data is available to generate incidence curves using EPP. In Group 1 locations, demographic assessments depend substantially on the sibling history data analysis which have large uncertainty intervals and for which there may be local variation in biases in sibling history responses. All Group 1 locations except for India are classified as Group 1A, while India and its states are classified as Group 1B, where we maintained more influence from the SRS system used in all-cause mortality estimation.

Table: Group 1 Locations

Angola	DR Congo	Guinea Bissau	Niger	Swaziland
Benin	Djibouti	Haiti	Nigeria	Tanzania
Botswana	Dominican Republic	India (2B)	Papua New Guinea	Togo
Burkina Faso	Equatorial Guinea	Kenya	Rep of the Congo	Uganda
Burundi	Eritrea	Lesotho	Rwanda	Zambia
Cameroon	Ethiopia	Liberia	Senegal	Zimbabwe
Cape Verde	Gabon	Malawi	Sierra Leone	
Central African Rep	Gambia	Mali	Somalia	
Chad	Ghana	Mozambique	South Africa	
Cote D'Ivoire	Guinea	Namibia	South Sudan	

Group 2A locations have at least 25 years of complete vital registration since 1980, as assessed by GBD's VR completeness synthesis method described in section 2.3. Since these locations have high-quality vital registration data, all-cause mortality estimates are largely driven by national data and the vital registration system is a robust source of data for HIV deaths.

Table: Group 2A Locations

Antigua and Barbuda	Croatia	Ireland	Mexico	Slovenia
Argentina	Cuba	Israel	Moldova	Spain
Australia	Czech Republic	Italy	Netherlands	South Korea
Austria	Denmark	Japan	Philippines	Sweden
Barbados	Estonia	Kazakhstan	Poland	Switzerland
Belgium	Finland	Kuwait	Portugal	Trinidad and Tobago
Bermuda	France	Latvia	Puerto Rico	Ukraine
Bulgaria	Germany	Lithuania	Romania	United Kingdom
Canada	Greece	Luxembourg	Russia	United States of America
Chile	Guatemala	Macao	Saint Lucia	Uruguay
Colombia	Hong Kong	Malta	St. Vincent and the Grenadines	Venezuela
Costa Rica	Hungary	Mauritius	Singapore	

Remaining locations with any VR make up Group 2B, and locations without any VR data are Group 2C. Exceptions were made for Madagascar, which was reclassified from Group 2B to 2C due to data quality concerns, and Cambodia, which was treated as a group 2B instead of 2C because of its available HIV prevalence data from the 2005 DHS survey (see the HIV/AIDS modeling write-up in Part 3 for more details). All subnational locations were classified according to the national-level group.

Table: Group 2B Locations

Albania	Cambodia	Honduras	Nicaragua	Sri Lanka
Algeria	China	Iran	Northern Mariana Isl	Suriname
American Samoa	Cyprus	Iraq	Oman	Syria
Armenia	Dominica	Jamaica	Palestine	Taiwan
Azerbaijan	Ecuador	Jordan	Panama	Tajikistan
Bahamas	Egypt	Kiribati	Paraguay	Thailand
Bahrain	El Salvador	Kyrgyzstan	Peru	Tonga
Belarus	Fiji	Malaysia	Qatar	Tunisia
Belize	FYR Macedonia	Maldives	Sao Tome and Principe	Turkey
Bolivia	Georgia	Mongolia	Saudi Arabia	Turkmenistan
Bosnia and Herzegovina	Greenland	Montenegro	Serbia	U.S. Virgin Isl
Brazil	Guam	Morocco	Seychelles	Uzbekistan
Brunei	Guyana	Myanmar	Slovakia	

Table: Group 2C Locations

Afghanistan	Indonesia	Mauritania	Timor-Leste
Andorra	Laos	Nepal	United Arab Emirates
Bangladesh	Lebanon	North Korea	Vanuatu
Bhutan	Libya	Pakistan	Vietnam
Comoros	Madagascar	Samoa	Yemen
Federated States of Micronesia	Marshall Islands	Solomon Islands	

For HIV mortality data, Group 2A locations used mortality output from the ST-GPR process due to the high quality of their vital registration systems. Group 1A and 1B locations used Spectrum output, while Group 2B and 2C locations used output from cohort incidence bias adjusted (CIBA) deaths due to HIV/AIDS from the Spectrum model (for more information, see the HIV/AIDS modeling write-up in Part 3 of this appendix).

Outputs were modeled to include an under-1 age group without the early-, late-, and post-neonatal groups. To attribute under-1 deaths from Spectrum to these neonatal groups, we make the assumption that all HIV deaths that occur in the first year of a child's life occur in the post-neonatal stage (after 28 days), since the literature on HIV in these age groups is still unclear but seems to indicate higher mortality in the post-neonatal stage, and there is no clear evidence to guide alternative methods of age-splitting under-1 deaths due to HIV.[29,30]

Envelope Calculation

In general, the all-cause and HIV-deleted envelopes were generated by synthesizing the results from the with-HIV and HIV-free life tables, along with selected HIV mortality from ST-GPR, Spectrum output, or

cohort incidence bias adjusted Spectrum output (CIBA-Spectrum). We used with-HIV and HIV-free lifetables in order to ascertain the implied HIV from the model life table system.

For age groups under age 5, we used the under-5 results from the age-sex process described in section 1. In Group 1A and 1B locations, we generated a scalar based on Spectrum results of HIV-specific and non-HIV deaths to generate HIV-deleted envelope deaths based on the all-cause results from the envelope. In other locations, we directly subtracted mortality from ST-GPR or Spectrum, after capping HIV at 90% of the all-cause envelope, to generate the HIV-deleted envelope. As mentioned previously, we made the assumption that all under-1 HIV-specific deaths occur in the post-neonatal stage.

In all groups except Group 1A, for all ages above 5 and under 15, we subtracted mortality from Spectrum or ST-GPR directly from the all-cause with-HIV envelope, after capping HIV at 90% of the all-cause envelope. In Group 1A, we took the HIV-free mortality and add mortality from Spectrum directly to generate the all-cause with-HIV envelope.

For ages above 15 and below 80, we applied separate approaches for Group 1A and 1B locations compared to Group 2A, 2B, and 2C locations. Group 1A and 1B locations used HIV mortality determined by an ensemble model where we averaged the implied HIV mortality from the model life table process and the HIV mortality output by Spectrum, which are intrinsically linked by the draw-level HIV-free mortality age pattern. Group 2A, 2B, and 2C locations used HIV mortality directly from ST-GPR and CIBA-Spectrum. For all locations except Group 1A, we subtracted the calculated HIV mortality from the all-cause envelope to generate HIV-deleted envelope deaths. In Group 1A, we added the HIV mortality to HIV-free mortality from the MLT process to calculate all-cause mortality, thus effectively allowing all-cause mortality from the demographic estimation process to be changed based on our ensemble HIV estimates. This reflects our view about the inherent uncertainty in all-cause mortality estimates largely based on sibling survival data in Group 1A locations and the various assumptions on mortality and program data required in both EPP and Spectrum.

For the age group 80 and above, we first approximated the over-80 mortality rate by using our lifetable output and divided lx by Tx from the age 80-84 values in the country-specific lifetables. We then calculated a scalar from the approximated all-cause mortality rate from the lifetable to the all-cause mortality rate by taking the envelope deaths/populations. We applied this scalar to both the with-HIV and HIV-free lifetables to rescale these numbers and the implied HIV death rate to the envelope space. Finally, HIV-deleted and all-cause mortality ere determined by the same approaches as used for other ages above 15.

Lifetable Calculation

Generally, lifetables were calculated using a consistent approach to those used in calculating the envelope. There are, however, a few differences in calculation, particularly related to sex and location aggregation.

First, for the granular age groups above 80, which are contained in the life tables but not the all-cause envelope, we used the ratio of all-cause deaths to HIV-deleted deaths in the 80+ group in the envelope process, and applied this to the lifetable results. For Group 1A locations, we took the HIV-free lifetable results and multiplied by the ratio to obtain all-cause lifetable results. In all other locations, we took the

all-cause lifetable and divided by the ratio to obtain the HIV-free lifetable results, consistent with their handling in the envelope process.

For aggregate locations, we generated aggregated mx and ax values by weighting mx by population and ax by deaths (here, calculated by mx * population). From this, we population- and death-weighted regional mx and ax values, which we used to generate region-level lifetables.

In the under-5 age groups, we applied a similar approach to calculating the envelope. However, when aggregating the all-cause lifetable for countries with subnational locations, we substituted the national-level under-5 results instead of using the aggregated mx and ax values from the subnational units. We did this in all aggregate countries except South Africa, where we aggregated the national results from subnational results, consistent with our approach in 5q0, 45q15, and the model life tables. We substituted in the under-5 national aggregates because we want to preserve the estimated 5q0 results from our 5q0 data synthesis and age/sex splitting modeling processes, which are not necessarily equivalent to those generated by the aggregated mx and ax values from the model life table process. For HIV-deleted lifetables, we aggregated subnational units using the regular weighting scheme.

To generate lifetables for both sexes combined, we used the populations available through the highest age group possible to weight the male and female mx and ax values, using the population of the highest age group for all further age groups when we did not have population data throughout the ages contained in the lifetable. For instance, if a country only had ages through age 80+ for a given year, we would use the 80+ populations to weight mx and ax for ages 80-85, 85-90, and onwards. For aggregating to regional aggregates, given the heterogeneity of population data across countries, we chose to use the 80+ populations to weight mx and ax.

4.7 HIV crude death rates for under-5 and ages 15-59

As a result of the envelope and lifetable calculation steps, the Reckoning produces final HIV death rates, based on the results of the Envelope Calculation portion of the Reckoning process.

Section 5. Age-specific mortality estimation for all GBD age-groups: with and without HIV

5.1 Age-specific mortality without discontinuities (with HIV/AIDS)

Age-specific mortality rates without fatal discontinuities were generated using the model life table system (section 3), age-sex model (section 1.13), and HIV reckoning process (section 4.6) depending on the location groups detailed in section 4.6 and in Appendix Table 1.

For mortality rates in age groups younger than age 5, age specific mortality was taken from our age-sex model that split U5MR into mortality for age groups enn, lnn, pnn and ages 1-4. For Group 1B, 2A, 2B, and 2C locations, age-specific mortality rates in ages 5 and older were generated using the GBD model life table system described in section 3. For Group 1A locations, age specific mortality with HIV was produced as the sum of HIV free mortality rates generated in the first step of the model life table system described in section 3 and the results of the ensemble model for HIV-specific morality described in section 4.6.

5.2 HIV-deleted age-specific mortality

HIV-deleted age-specific mortality was used in cause of death analyses (CodCorrect) in GBD 2015 to avoid the spill-over effect of HIV mortality into other causes, particularly for locations affected by high HIV burden. We used the difference between the age-specific mortality with HIV, without fatal discontinuities as described in section 5.1 and the HIV-specific mortality as a result of the HIV reckoning process described in section 4.6.

5.3 Add fatal discontinuities

In our mortality estimation process, we excluded data from years with fatal discontinuities to ensure that these sudden idiosyncratic increases in mortality would not affect long-term trends in mortality for a given country. Part 3 of this appendix describes the process by which we identify fatal discontinuities, the data sources we used, the method by which we split the deaths according to age and sex, and how we made draws to create uncertainty for these estimates. This section details how we added the deaths due to fatal discontinuities to the all-cause mortality envelope and life tables.

To incorporate deaths due to fatal discontinuities into the mortality envelope, 1,000 draws of deaths due to fatal discontinuities were added pairwise to 1,000 draws of the with-HIV mortality envelope for each location, sex, and age group. Ninety-five percent uncertainty intervals were calculated as in other processes, taking the 97.5% and 2.5% quantiles of the summed draws.

To incorporate fatal discontinuity deaths into the life table, we created a ratio of deaths with fatal discontinuities to deaths without fatal discontinuities and applied the ratio as a scalar to the without-fatal-discontinuity mortality rate (${}_nm_x$) produced in the HIV Reckoning step, discussed in section 4.6. For under 1 age groups, we have scalars for early neonatal (enn), late neonatal (lnn) and post neonatal (pnn) ages. In order to calculate the probability of death for the under 1 age group (${}_1q_0$), we pulled results from the under-5 age and sex pattern of mortality process (section 1.13) , calculated with-fatal discontinuity mx for enn, lnn, and pnn, back-calculated qx for enn, lnn, and pnn, and then aggregated to ${}_1q_0$ using the equations in section 1.13. In addition to the granular under-1 age groups, we also used the ${}_4q_1$ generated by the age-sex model. Because we only had mortality and fatal discontinuity death numbers for the aggregate age group 80+, we applied the scalars for this group to the granular age groups of the life table up to 100 in order to calculate the life table with fatal discontinuities for older age groups.

5.4 Age-specific deaths with discontinuities and HIV/AIDS

From the process described in section 5.3, we produced location-, sex-, year-, and age- specific death numbers including fatal discontinuities.

5.5 Life tables with HIV/AIDS and fatal discontinuities

From the process described in section 5.4, we produced location-, sex-, and year-specific child mortality (5q0), adult mortality (45q15), and life expectancy at birth and age 50. Additionally, we produced pred-ex, an input to the DALYnator, described in Part 4 of this appendix. Pred-ex compares the age of death of those who die in each age group to the life expectancy observed in the standard life table in order to calculate YLLs.

References

1 Rajaratnam JK, Tran LN, Lopez AD, Murray CJL. Measuring under-five mortality: validation of new low-cost methods. *PLoS Med* 2010; **7**: e1000253.

2 Wang H, Dwyer-Lindgren L, Lofgren KT, *et al.* Age-specific and sex-specific mortality in 187 countries, 1970-2010: a systematic analysis for the Global Burden of Disease Study 2010. *Lancet* 2012; **380**: 2071–94.

3 Hill K. Estimating census and death registration completeness. *Asian Pac Popul Forum* 1987; **1**: 8–13, 23–4.

4 Wang H, Liddell CA, Coates MM, *et al.* Global, regional, and national levels of neonatal, infant, and under-5 mortality during 1990-2013: a systematic analysis for the Global Burden of Disease Study 2013. *Lancet* 2014; **384**: 957–79.

5 Gelman A, Carlin JB, Stern HS, Rubin DB. Bayesian Data Analysis, Second Edition. CRC Press, 2003.

6 Klein LR. A Textbook of Econometrics. Evanst: Row, 1974.

7 Lozano R, Wang H, Foreman KJ, *et al.* Progress towards Millennium Development Goals 4 and 5 on maternal and child mortality: an updated systematic analysis. *The Lancet* 2011; **378**: 1139–65.

8 Kerber KJ, Lawn JE, Johnson LF, *et al.* South African child deaths 1990-2011: have HIV services reversed the trend enough to meet Millennium Development Goal 4?. [Miscellaneous Article]. *AIDS October 23, 2013* 2013; **27**: 2637–48.

9 Marston M, Becquet R, Zaba B, *et al.* Net survival of perinatally and postnatally HIV-infected children: a pooled analysis of individual data from sub-Saharan Africa. *Int J Epidemiol* 2011; **40**: 385–96.

10 Hill K. Estimating census and death registration completeness. *Asian Pac Popul Forum* 1987; **1**: 8–13, 23–4.

11 Bennett NG, Horiuchi S. Estimating the completeness of death registration in a closed population. *Population Index* 1981; **47**: 207–21.

12 Brass W, Coale AJ. Methods of Analysis and Estimation. In: Brass W, ed. The Demography of Tropical Africa. Princeton: Princeton University Press, 1968.

13 Preston S, Coale AJ, Trussell J, Weinstein M. Estimating the completeness of reporting of adult deaths in populations that are approximately stable. *Popul Index* 1980; **46**: 179–202.

14 Preston S, Hill K. Estimating the completeness of death registration. *Population Studies* 1980; **34**: 349–66.

15 Murray CJL, Rajaratnam JK, Marcus J, Laakso T, Lopez AD. What can we conclude from death registration? Improved methods for evaluating completeness. *PLoS Med* 2010; **7**: e1000262.

16 Obermeyer Z, Rajaratnam JK, Park CH, *et al.* Measuring Adult Mortality Using Sibling Survival: A New Analytical Method and New Results for 44 Countries, 1974–2006. *PLOS Med* 2010; **7**: e1000260.

17 Gakidou E, King G. Death by survey: estimating adult mortality without selection bias from sibling survival data. *Demography* 2006; **43**: 569–85.

18 Masquelier B. Adult Mortality From Sibling Survival Data: A Reappraisal of Selection Biases. *Demography* 2012; **50**: 207–28.

19 Rogers RG, Crimmins EM, editors. Adult Mortality in Africa. In: International Handbook of Adult Mortality, 2011 edition. Dordrecht Netherlands ; New York: Springer, 2011: 151–70.

20 Lozano R, Wang H, Foreman KJ, *et al.* Progress towards Millennium Development Goals 4 and 5 on maternal and child mortality: an updated systematic analysis. *The Lancet* 2011; **378**: 1139–65.

21 Schnuelle P, Lorenz D, Trede M, Van Der Woude FJ. Impact of renal cadaveric transplantation on survival in end-stage renal failure: evidence for reduced mortality risk compared with hemodialysis during long-term follow-up. *J Am Soc Nephrol* 1998; **9**: 2135–41.

22 Murray CJL, Ferguson BD, Lopez AD, Guillot M, Salomon JA, Ahmad O. Modified logit life table system: Principles, empirical validation, and application. *Population Studies* 2003; **57**: 165–82.

23 Human Mortality Database. Berkeley, USA: University of California; Rostock, Germany: Max Planck Institute for Demographic Research. http://www.mortality.org/ (accessed April 11, 2011).

24 Murray CJL, Rajaratnam JK, Marcus J, Laakso T, Lopez AD. What can we conclude from death registration? Improved methods for evaluating completeness. *PLoS Med* 2010; **7**: e1000262.

25 Wang H, Liddell CA, Coates MM, *et al.* Global, regional, and national levels of neonatal, infant, and under-5 mortality during 1990–2013: a systematic analysis for the Global Burden of Disease Study 2013. *Lancet* 2014; **384**: 957–79.

26 Gompertz B. On one uniform law of mortality from birth to extreme old age, and on the law of sickness. *Journal of the Institute of Actuaries and Assurance Magazine* 1871; **16**: 329–44.

27 Thatcher AR. The long-term pattern of adult mortality and the highest attained age. *Journal of the Royal Statistical Society: Series A (Statistics in Society)* 1999; **162**: 5–43.

28 King G, Tomz M, Wittenberg J. Making the most of statistical analyses: improving interpretation and presentation. *American Journal of Political Science* 2000; **44**: 347–61.

29 Brocklehurst P, French R. The association between maternal HIV infection and perinatal outcome: a systematic review of the literature and meta-analysis. *BJOG: An International Journal of Obstetrics and Gynaecology* 1998; **105**: 836–48.

30 Kim H-Y, Kasonde P, Mwiya M, *et al.* Pregnancy loss and role of infant HIV status on perinatal mortality among HIV-infected women. *BMC Pediatrics* 2012; **12**: 138.

Part 2: Cause of death database

Background

Figures 2A and 2B show the high-level view of data inputs, analytical steps, and outputs of the cause of death analysis frame. The complexity of the overall process can be usefully divided into three broad phases: data inputs on the event of death going into the cause of death database that are analyzed using CODEm; data inputs on precursors to death that are modeled through a variety of strategies; and the integration of these streams of analysis into a single set of cause of death estimates by age, sex, year, and geography with uncertainty through the CodCorrect algorithm. The process for cancer and for HIV/AIDS is somewhat different, and described in more detail in Part 3.

Cause of death data identification

0.1 Overview of data types

The cause of death database contains six types of data sources: vital registration, verbal autopsy, cancer registry, police records, sibling history, surveillance, and survey/census. The highest-quality data will have detailed characteristics of each demographic group and detailed causes of death across the time series. Countries with complete vital registration systems are considered to be high-quality. For counties with incomplete vital registration systems, vital statistics for causes of death can be supplemented with other data types to provide cause-specific estimates.

0.2 ICD detail

A majority of the cause of death data is vital registration data obtained from either the WHO Mortality Database, country-specific mortality databases operated by official offices, provided by trusted country collaborators. It is considered to be the highest-quality data, as it is the most comprehensive. Each cause is coded directly to the most detailed cause of death when possible, whereas cause codes in ICD tabulated data are coded to aggregated cause groups. The cause of death database contains 8,044 location-years of detailed data from 1980 to 2014, which includes underlying causes of deaths coded with 3-5 digit se codes, by country, year, sex, and age groups. Detail causes are coded to one of the following ICD detail coding systems: ICD8, ICD9, or ICD10. Each coding system has a similar cause hierarchy and cause list that has continually developed over time. ICD10 is the current standard and most exhaustive cause list. Within the cause lists 5-digit codes are truncated to 4-digit codes to condense the cause lists. Updates to ICD detail occur biannually as WHO releases new versions or as country collaborators provide additional data. Updates to data from WHO increasingly include ICD10 cause of death data, as it is the most current classification of cause of death, while updates to ICD8 and ICD9 detailed lists are less common. In the case of overlapping data, preference is given to data from pre-determined country collaborators, which is updated annually.

0.3 ICD tabulations lists

The ICD tabulation lists include the ICD8 List A (ICD8A), ICD9 Basic Tabulation List (BTL), ICD10 Mortality Tabulation, Russia Tabulation list, and India Medical Certification of Cause of Death (MCCD). These data sources make up 2,664 location-years from 1980 to 2014 in the cause of death database. All are condensed versions of the ICD9 and ICD10 detail lists, with some differences in the format of cause lists depending on the data source. ICD8A, ICD9 BTL, and ICD10 Mortality Tabulation cause of death are assigned to subtotal groups, referred to as chapters, and cause groups respective to ICD detail groups. Additionally ICD9 BTL includes ICD9 detail codes for some cancers and a custom tabulation scheme for

the former USSR countries. The Russia Tabulation lists and India MCCD cause lists each have custom nomenclatures based on ICD detail cause codes.

Two of the drawbacks in data using tabulation lists are discrepancies in the accuracy of death counts and lack of detail to due to aggregated cause groups. There are instances where the sum of deaths in chapter subtotals are not equal to the sum of cause groups within the chapter. To account for any missing or duplicate deaths reported within the cause groupings, death counts are systematically adjusted, by calculating the differences between subtotals and sub-causes within the cause groups. Any differences are assigned to a remainder cause group. To account for the lack of cause code detail, select cause groups are disaggregated (Step 1.1) to create a complete cause list. Updates to ICD Tabulation lists obtained from WHO occur less frequently compared to ICD detailed lists, as more countries are reporting deaths in ICD detail. In instances of overlapping data, preference is given first to data from country collaborators' data from WHO, then preference to ICD detail data from WHO, before choosing to use ICD tabulation lists.

China DSP/ China CDC

The two primary sources of data for China are surveillance data from the China Disease Surveillance Points (DSP) system and vital registration collected by the Chinese Center for Disease Control and Prevention (CDC). In the China DSP data, deaths were reported across 145 disease surveillance points used from 1991 to 2003, and 161 points used from 2004 to 2007. While China DSP with ICD10 code is considered surveillance data, it provides national coverage and cause detail. Thus it receives similar processing and treatments to the China CDC vital registration from 2008 to 2012. From 2008 to 2012, all of the deaths and cause of death information from the Disease Surveillance Points system and other system points throughout China were collected and reported via the Mortality Registration and Reporting System, an online reporting system of the Chinese CDC. The deaths in these data are reported at the strata level, a metric that is specific to China. Counties are stratified by urban and rural classification, but definitions of urbanity vary across counties. In Step 7.1 we use a method developed to scale up deaths from strata level to the province level.

India CRS

The India Civil Registration System (CRS) is a continuous systematic recording of deaths and births. We consider these data to be similar to vital registration as the coverage is national. Usable cause-specific death data are in the form of a custom cause list and exist between 1983 and 1995, with 1983-1984 and 1986-1987 including deaths data specific to rural or urban locations. Due to the lack of complete India subnational locations we state-split the data after age-sex splitting to fill in data gaps (Step 1.2). Though this system is continuous we have not found any cause-specific data past 1995. The more recent years of the report only include data for all-cause mortality.

India MCCD

The India Medical Certification of Cause of Death (MCCD) is the largest data source we have for India, with nationally representative data in a majority of the urban states and also union territories beginning in 1980. Deaths reported in this data source have been medically certified and are considered vital registration. The causes of death are reported in a tabulation list with a unique numbering scheme that conform to ICD9 and ICD10 detail codes, which must be disaggregated. Similar to the CRS, MCCD is state-split to fill in data gaps (Step 1.2 State Splitting); however, in these data state splitting occurs prior to age-sex splitting.

0.4 Verbal autopsy

VA coded to ICD 10 and VA coded to other lists

In countries without vital registration systems, verbal autopsy studies are a viable data source to inform cause of death. Data are obtained by trained interviewers who use a standardized questionnaire to ask relatives about the signs, symptoms, and demographic characteristics of recently deceased family members. Based on the answers to the questionnaires a cause of death is assigned.

Verbal autopsy data are highly heterogeneous: studies use different instruments, different cause lists from single causes to full ICD cause lists, different methods for assigning cause of death based on a completed verbal autopsy, different recall periods, and different age groups, quite apart from cultural differences in the interpretation of specific questions. The validity of cause of death must be considered when mapping to a GBD cause. Verbal autopsies are likely accurate in assigning cause of death to road injury or homicide, but less accurate for causes requiring medical certification, such as cardiovascular causes. Studies can also occur once in a particular country or as part of an extended network, such as INDEPTH. INDEPTH is a continuous surveillance source with several Demographic Surveillance Systems sites that collect data which is coded to ICD detail causes.

INTERVA modeled VA

In previous years, INTERVA modeled VA was excluded from our analysis. Verbal autopsies used in our analysis are non-INTERVA, as they use questionnaires and modules consistent with WHO standards. The Population Health Metrics Research Consortium (PHMRC) published a study that shows results of INTERVA-modeled VA are not compelling enough to be credible, thus we have decided to exclude data for all causes due to low validations with the exception of injuries in Sub-Saharan Africa.[1] We lack data in sub-Saharan Africa and use INTERVA to fill in gaps and stabilize injuries patterns.

India SCD and SRS

Deaths reported in verbal autopsy studies in rural Indian states can be accounted for in the Survey of Causes of Death (SCD) from 1980 to 1994 and in the Sample Registration System (SRS) post-1998 in urban and rural states. Data in the SCD were collected through a verbal autopsy survey from a sample of villages. To expand our estimates to more states and causes we used methods of state splitting post-mapping to GBD causes (Step 1.2). Like the SCD, SRS also records deaths in a sample of villages but also includes urban blocks in its sample. This survey is collected by the Registrar-General of India, and since 1999 SRS has merged with SCD to provide ongoing coverage on both urban and rural areas.[2] The maternal data reported in SRS are given in MMR, which we convert to deaths by state and urbanity by using our estimates for the SRS population and neonatal mortality rates.

0.5 Other data types

Maternal mortality data

In locations with low-quality vital registration or no vital registration, maternal mortality metrics can be found in surveillance, surveys, census, and sibling history data sources. The best data have death counts due to maternal causes and the total number of deaths for women within the reproductive ages of 10 to 54 (previously 15 to 49) by year. If a data source is missing these components, it is necessary to create a complete cause list using live births and all-cause mortality deaths (Step 1.4). Though death counts is the preferred metric, maternal mortality is often measured using maternal mortality ratio (MMR), which is

easily converted to deaths using live births. An additional adjustment that must be applied to the China Maternal and Child Surveillance data is scaling data from the strata to the province level (Step 7).

Surveys and censuses reporting fraction of deaths due to selected injuries

Surveys and censuses are often used in countries with less developed vital registration systems, or in countries with adequate vital registration these data source are supplementary. Much like the verbal autopsies, the validity of cause of death is a concern due to lack of medical certification at the time of death. For these data sources we keep only causes related to maternal mortality and injuries. The remaining causes are accounted for as a remainder of total deaths in the sample size.

Police records

In most countries, police and crime reports are an important source of information for some types of injury deaths, notably road injuries and interpersonal violence. Our police data comes from reports on road traffic and crime trends. The police reports used in this analysis were obtained from published studies, national agencies, and institutional surveys such as the UN Crime Trends survey and United Nations Office on Drugs and Crime Global study on Homicides. We can assess whether police reports were likely to be complete and cover the entire country if police trends are close to trends seen in vital registration. Data are excluded in instances where police data for road traffic injuries are significantly lower than our vital registration. The threshold for exclusion is less than 80% of the cause fraction of the road traffic injuries in vital registration. Police data that meet our inclusion criteria and provide complete coverage are uploaded to the database for injuries causes.

0.6 Population-based cancer registries

Cancer registries with incidence

Data on cancer incidence was sought from individual population based cancer registries as well as from databases that include multiple registries, for example “Cancer Incidence in Five Continents” (CI5) (NID 133224), NORDCAN (NID: 113386), or EUREG (114368). Cancer registries were identified through the membership list of the International Association of Cancer Registries (IACR), through the GBD collaborator network, or through the GHDx. Registries were excluded if they were not representative of the coverage population, if they did not contain incidence data tabulated by cancer site, if the data were limited to years prior to 1980, if the source did not provide details on the population covered, or if the list of cancer sites included was not comprehensive.

Cancer registries with incidence and high quality mortality

In addition to incidence, some high-quality cancer registries also report cancer mortality data. These data were also extracted and used as inputs into the mortality-to-incidence model.

Step 1. Standardize input data

The input data to the Cause of death (CoD) database are received in various formats and must be standardized to run through central CoD machinery to then upload to the database. Raw data inputs come from data sources such as mortality databases, literature reviews, or reports. Usable data sources must have a clear sample size of the number of deaths in the population and exhaustive cause lists. The complexity of the cleaning process varies drastically across data sources. For vital registration micro-data with the location, age, sex, year, and ICD-coded cause of every death, very little effort is necessary to standardize it into a consistent structure. Other sources may require weeks of careful review to

accurately extract scans of hardcover cause of death reports into spreadsheets that can be transformed and standardized.

At this point, data are assigned source identifiers so that they can be linked to the Global Health Data Exchange (GHDx) and cited appropriately. Any aggregate age and sex categories are flagged for age-sex splitting. The methods of cause-of-death assignment and data collection are reviewed to determine which source type to assign; for example, we distinguish sibling history data from surveys with a verbal autopsy module. Only data at the most detailed level of the Global Burden of Disease location hierarchy are used. Documentation from the source is reviewed to determine if the population is representative of the location or only a subset of the population in that location. Data sources representing a subset of the population are flagged as non-representative; this flag is used by CODEm to increase the variance associated with such data points.

Finally, diagnostics are reviewed at this stage to avoid sending cleaning errors downstream. We review cause-specific deaths for each demographic group to ensure the data are reasonable. For example, it is unlikely that male breast cancer deaths are higher than female breast cancer or deaths from neonatal causes occur in age groups over one year. All deaths totals are compared with the sum of cause-specific deaths to ensure the observed deaths are accounted for and sample size is complete.

1.1 Disaggregation

Causes of death in tabulated vital registration data are condensed into aggregated groups, some of which can be mapped directly to GBD causes while other aggregated cause groups are not informative and cannot be mapped to GBD causes. To correct for this, aggregated causes were mapped and split onto multiple ICD9 and ICD10 detail causes, or targets, based on the ICD groupings within the aggregated causes. Both ICD9 and ICD10 detail codes serve as targets because they are the highest quality vital registration data and enabled the calculation of proportions used to split the aggregated cause data into detailed causes. The proportions of deaths from nearby countries within the super region were used to fill in data gaps as they were likely to have similar cause of death trends.

We determined the targets based on detail causes missing from the tabulated cause list. For example, in ICD9 BTL, the tabulated cause list includes a viral diseases group. In the hierarchy of causes, this group consists of measles, yellow fever, encephalitis, hepatitis, rabies, other infectious diseases, garbage code, and remainder of viral diseases. We did not consider this list to be an exhaustive list of viral diseases based on the range of ICD detail codes given in the ICD9 BTL documentation. To make the cause list exhaustive and inclusive of other viral diseases, we split the remainder of viral diseases group into: other meningitis, other infectious diseases, herpes, dengue, other neglected tropical diseases, and garbage code. After a list of targets was determined, the aggregated deaths were disaggregated to the target causes using ICD9 and ICD10 detail proportions generated at the super region level for the corresponding sex and age groups across all years in the time series. For example, in ICD9 detail data, 54.8% of deaths in males in Latin American and Caribbean within the target group for BTL Viral Diseases were designated “other meningitis”, so 54.8% of deaths in the tabulated group, “remainder of viral diseases”, were assigned to “other meningitis” for any country within that particular super region. For any cause and demographic group where we lacked ICD detail, global proportions were used.

1.2 State splitting

Two important sources for cause of death estimation in India are the Medical Certification of Causes of Death (MCCD) report, which reports medically certified deaths from health facilities in mostly urban areas [3], and the Survey of Causes of Death (SCD), which collects information via verbal autopsy on about one-half of 1% of all rural deaths in India, based on populations living in about 1,300 primary health care centers spread throughout the country.[4] For both of these reports, data missingness impedes estimation of trends at the state level. We used a first-order, log-linear model of the four-way contingency table of deaths by sex, age, state, and year to estimate the missing state-years. We fit the model to all available data for MCCD and SCD separately for each cause, including state-specific all-age measurements and age-specific national measurements. From this, we produced estimates for each combination of sex, age, state, and year. We then used these estimates wherever the raw data did not include sex-, age-, state-specific death counts.

For MCCD, the model was fit separately for ICD10- and ICD9-based reports using the tabulated cause list present in the data. In the SCD report, the model was fit for each GBD cause in the data. As data from the SCD reports were relatively sparse, the pooling of like causes together led to an improved model fit.

1.3 Region-sex-cause to state-urbanicity-age-sex-cause algorithm

We also made use of the Special Survey of Deaths in 2001–2003 and 2004–2005, a representative, national and subnational verbal autopsy study under the Sample Registration System in India. Data for the top 10 causes of death were available by region and sex, but we require data by urban and rural state in India. To achieve this, we first split the regions into states in proportion to the number of GBD-estimated deaths in each state. We then determined the urbanicity of the resulting state-cause-deaths by applying the SRS distributions of urban and rural deaths by cause in India. We then used a relative-rate splitting algorithm that accounts for the population structure of urban and rural areas in the state. Finally, we applied the SRS all-India age distribution to the all-age urban-rural-state-data with a similar approach, accounting for the age-specific population structure of urban and rural areas in each state.

1.4 Calculate non-maternal deaths

In cases when maternal mortality metrics do not include both deaths due to maternal causes and deaths due to non-maternal causes for women of reproductive ages, live births and all-cause mortality estimates can be used to calculate deaths. Many studies report maternal deaths as the maternal mortality ratio (MMR). MMR is the number of maternal deaths per 100,000 live births and can be used to calculate deaths when it has been derived from primary data and not estimated. Maternal deaths were calculated using MMR and live births, if live births were missing we substituted live birth estimates and used the following equation:

$$\text{Maternal deaths} = (\text{MMR}/100{,}000) * \text{Live births}$$

If a study was non-representative we extracted sample size and live births from that study. After maternal deaths were calculated, we used the difference from all-cause mortality estimates to determine non-maternal deaths.

A more accurate and data inclusive method of calculating maternal and non-maternal deaths incorporates coverage and splits deaths for a range of years into individual years. If there were live births in the study we adjusted the coverage.

$$\text{Coverage} = \text{live births} / \text{GBD estimated live births}$$

After coverage was calculated, totals deaths were scaled to be more representative. This gives a more accurate death count since the envelope assumes representative coverage. Using all-cause mortality as an all-cause total, non-maternal deaths were subsequently calculated.

$$\text{Maternal envelope with coverage} = \text{maternal envelope} * \text{coverage}$$

An additional adjustment can be applied to maternal data spanning over a range of consecutive years, which allows for more data inclusion. The years within specified year ranges are separated into individual years and total deaths within the year range were split between each individual year using the fixed proportions of maternal deaths from vital registration in that particular country. We only used vital registration to inform the proportions because it was both high quality and representative.

Step 2. Map to GBD cause list

In GBD 2015 we developed 411 maps to translate causes found in the input data to the GBD 2015 cause list. This included 40 maps for vital registration data, 279 for verbal autopsy data sources, and 92 for other data types. The largest and most universal maps used were those for ICD9 and ICD10 detail vital registration data. The input data causes varied from 3-4 digit ICD codes to custom cause lists with cause names such as cholera or hepatitis. Our mapping process made it possible to compare these various data sources across demographic groups.

Appendix Table 12. shows the ICD10-detail and ICD9-detail codes included in the mapping of each GBD cause.

2.1 India urban/rural splitting

Another source of data for urban and rural state estimation of cause of death trends in India is the Civil Registration System (CRS), from which we retrieved usable data over the period 1983-1995. From 1983-1987, data were available for the urban and rural populations of each state in the system. However, after 1988, only state-level data were available.

We can only use data at the most detailed location level that we estimated for cause of death models. As a result, in order to use of all years of the CRS data split state-level mortality into urban and rural state-level mortality. To do this, we assumed the same relative rate of cause-specific urban and rural mortality in 1988-1995 as was present in the data in 1983-1987. We applied the same algorithm that is used for age-sex splitting, modified for this purpose:

$$D_{s,u,y,a,x,c} = R_{s,u,c} N_{s,u,y,a,x} \frac{D_{s,,y,a,x,c}}{\sum_{u=urban}^{u=rural} (R_{s,u,c} N_{s,u,y,a,x})}$$

Where:

$D_{s,u,y,a,x,c}$ = Number of observed deaths in state s, urbanicity u (either rural or urban), year y, age a, sex x, and cause c

$R_{s,u,c}$ = Death rate in state s, urbanicity u, cause c from 1983-1987; data were not suitable for age or sex specific rates.

$N_{s,u,y,a,x}$ = GBD-estimated population in state s, urbanicity u, year y, age a, sex x

$D_{s,,y,a,x,c}$ = Number of observed deaths in state s, year y, age a, sex x, and cause c (the deaths that are being split)

The result was a full time series of CRS data for both rural and urban populations of each state in the CRS system.

2.2 State splitting

This step is described in step 1.2, above.

Step 3. Age-sex splitting

3.1 Generate global age-sex weights by cause

Different sources, particularly verbal autopsy studies, report deaths for a wide range of age-groups with varying intervals. For the analysis of causes of death, we mapped these different age intervals to the GBD standard set of age-groups. The approach to undertake this mapping was the same as in the prior two GBD studies, GBD 2013 and GBD 2010.

In the process of assembling a consolidated demographic database, perhaps the most impairing source of inconsistency is the aggregation of age groups. It is conventional to report such data in broad age groupings such as "0-4, 5-14, 15-49," or to report data with both sexes together. The issue of comparability between age-sex groups arose when assembling the GBD cause of death database. The compiled database included 22 distinct tabulation formats for infants and 141 distinct tabulation formats for non-infants. We developed a tool, which we call age-sex splitting, that takes aggregated age groupings, and likewise the "both sexes combined" grouping, and divides them into what their constituent age groups would likely have been using respective cause-specific and country-specific age distributions. The analytical framework for the GBD includes three infant age categories: Early neonatal (0-6 days), late neonatal (7-27 days) and post neonatal (28 days to one year), and 17 non-infant age categories starting with age one to four years, then proceeding in five-year age groups until the terminal age group of 80+. We treat unknown ages and sexes in the same manner we treated the "all ages combined" age category and "both sexes combined" sex group. Through this process, we were able to directly compare all data sources on even terms.

The approach to age splitting is based on the following formula. The key assumption underlying this formula is that the relative risk of death by age group compared to a reference age group is invariant across populations. While this assumption is likely violated in specific cases, there is a strong biologically based pattern of the relative risk of death for a cause by age that is observed for most causes. The basic formula is as follows:

$$D_a = R_a N_a \left(\frac{D_a^{a+x}}{\sum_a^{a+x}(R_a N_a)}\right)$$

Where:

D_a= the number of deaths from a cause in age group *a*

R_a= the relative risk of death in age group *a* compared to a reference group

N_a= the country-year-sex-specific population in age group *a*

D_a^{a+x}= the number of deaths in the age group *a* to *a+x*

With the assumption of invariant relative risks of death by age with respect to a reference age group, this equation can be used, along with population distribution by age, to split an aggregate number of deaths for the age groups *a* to *a+x* into specific deaths for each age group within the aggregate interval.

In some cases, deaths are reported for an aggregate age group for both sexes combined. The task in this case is more complicated, but the same principle can be applied. In this case we assumed that the relative risks of death by and sex are constant.

$$D_{as} = R_{as}N_{as}\left(\frac{D_{as}^{a+x,s}}{\sum_{a}^{a+x}(R_{as}N_{as})}\right)$$

Where:

D_{as}= the number of deaths from a cause in age group *a,* sex *s*

R_{as}= the relative risk of death in age group *a* compared to a reference group for sex *s*

N_{as}= the country-year-sex-specific population in age group *a* for sex *s*

$D_{a}^{a+x,s}$= the number of deaths in the age group *a* to *a+x* for sex *s*

This equation can be used to split data aggregated over age and sex. The assumption, however, of invariant relative risks across age and sex is a stronger assumption. Fortunately, data pooled across sexes are less common in the published or unpublished cause of death data.

The relative risk of death in a particular age group for a given sex is derived from the global distribution of cause-specific mortality rates found in available vital registration data. Location-years from the following code systems are used, provided they report the requisite age- and sex-detail: ICD7, ICD8, ICD9 BTL, ICD10 tabulated, ICD9, and ICD10. Upon compiling these data, we mapped them to GBD causes, and aggregated up to cause level 3. This is the level at which a particular cause is split – that is, any daughter cause of a level 3 parent is split using the age distribution of that parent (so, chronic kidney disease due to diabetes would be split using the age pattern of chronic kidney disease).

We next adjusted separately for estimated adult and child vital registration completeness. Location-year-age-sex-specific deaths and population were then aggregated across all location-years, in order to produce cause-specific mortality rates by age and sex. These were used to determine the risk of death at any age relative to any reference age group.

Step 4. Correct age-sex violations

Occasionally, data sources will include deaths by a cause for which there is medical consensus that death is impossible for the sex and age. For example, there may be some number of deaths due to cervical cancer in males, or deaths due to maternal causes in ages under 10. We have constructed a conservative list of age-sex restrictions. When deaths violate these restrictions, we redistribute them proportionally onto all causes.

4.1 GBD age-sex restrictions by cause

All restrictions are included in Appendix Table 13. Age-sex restrictions by cause.

Step 5. Redistribution

A crucial aspect of enhancing the comparability of data for cause of death is to deal with uninformative, so-called garbage codes. Garbage codes are codes to which deaths were assigned that cannot or should not be considered as the underlying cause of death, for example: heart failure, ill-defined cancer site, senility, ill-defined external causes of injuries, and septicaemia. The methods for redistributing these garbage-coded deaths were outlined in detail in Naghavi et al,[5] and the underlying algorithm for redistributing deaths assigned to these codes has not changed since GBD 2013.

5.1 Redistribute HIV-related garbage

The list of garbage codes known to be used to code deaths caused by HIV/AIDS can be found in Appendix Table 14. HIV-related garbage code redistribution packages.

Due to the disparate nature of HIV/AIDS mortality across space and time, dynamic redistribution of HIV/AIDS-related garbage codes was needed. To inform this redistribution, we generated target proportions for each garbage group by age band (Under 1 month, 1-59 months, 5-19 years, 20-49 years, 50-59 years, 60-69 years, 70-79 years, and 80+ years), 5-year time interval, and sex. The garbage groups will either target HIV or a remainder target. The allotment of deaths to either of these is based on the regional increase in the mortality rate of all codes in the group relative to the rates seen in 1980-1984 – an increase greater than 5% is assumed to be HIV/AIDS-related, and the proportion of those deaths exceeding 5% are redistributed to HIV/AIDS. Any increase ≤ 5% is then assigned to the remainder target.

5.2 Regress garbage codes versus non-garbage

As in GBD 2013, the statistical analysis used to determine proportions for garbage code redistribution for ill-defined cancer sites, ill-defined external causes of injury, unspecified stroke, heart failure, hypertension, and atherosclerosis was based on the approach outlined by Ahern et al.[6] For each redistribution package, we defined the “universe” of data as all deaths coded to either the package’s garbage codes or the package’s redistribution targets for each country, year, age, and sex. We then ran a regression based on the following equation, separately for each target group and sex:

$$TG_{crt} = \alpha + \beta_1 Gar_{crt} + \beta_2 Age_{crt} Gar_{crt} + \theta_r Gar_{crt} + \gamma_r + \varepsilon_{ct}$$

TG_{crt} = percentage of deaths within the given garbage code’s universe which were coded to a given target group, by country

Gar_{crt} = percentage of deaths within the given garbage code’s universe which were coded to a given set of garbage codes

α = constant

β_1 = slope coefficient describing the association between Gar_{crt} and G_{crt}

β_2 = slope coefficient describing the association between the interaction $Age_{crt}Gar_{crt}$ and G_{crt}

γ_r = region specific random intercept (or super region if the random effect on region is not significant)

θ_r = region specific random slope (or super region if the random effect on region is not significant)

ε_{ct} = standard error, normally distributed and calculated by bootstrapping

This regression was adjusted from GBD2013 to include fixed effects on the interaction of garbage and age to ensure smooth age patterns. We made this decision after investigating diagnostic visualizations that showed unlikely gaps between proportions assigned to different age groups.

Once proportions were produced for each country, sex, age, and target group, certain adjustments were made to conform our packages to the best medical evidence available. In some cases, we implemented restrictions on the proportions that the regressions could yield. For example, we did not allow any redistribution onto Chagas disease outside of Latin America and the Caribbean, or suicide under the age of 15. In other cases, we capped the proportion for some targets to the level that would be produced from proportional redistribution; for example, hemoglobinopathies and hemolytic anemias were restricted to the level of proportional redistribution in the redistribution of left heart failure. Occasionally, further adjustments were made on a case-by-case basis per country, age, sex, and target group to suppress the impact of outliers based on existing epidemiological evidence and expert judgment.

5.3 VA anemia adjustment

To compensate for the over-representative cause fractions from anemia found in verbal autopsy studies, we redistributed these deaths based on the causal attribution of severe anemia from the GBD 2013 study. The proportions were country-year-age-sex specific.

Step 6. HIV/AIDS misclassification correction

In many location-years, certain causes of death known to be comorbid with HIV/AIDS (e.g., tuberculosis, other infectious diseases) are seen to have age-patterns that diverge from those observed in location-years without widespread HIV/AIDS epidemics, and are in fact more reflective of HIV/AIDS mortality trends. In order to identify these instances, a global relative age pattern is generated using all VR deaths in countries with observed HIV prevalence less than 1% using the following:

$$RR_{asc} = {}^{R_{asc}}/_{\bar{x}(R_{65sc}, R_{70sc}, R_{75sc})}$$

Where $RR_{asc}$is the relative death rate for age group *a*, sex *s*, and cause *c*; $R_{asc}$is the rate for that age group; and $\bar{x}(R_{65sc}, R_{70sc}, R_{75sc})$ is the mean of the rates in ages 65-69, 60-74, and 75-79 for that sex and cause. This is preferable to comparing mortality rates because we are able to isolate divergence in age pattern while accounting for varying levels of overall mortality by fixing death rates to age groups that are unlikely to be confounded by the presence of HIV. Expected deaths for an identified cause were then determined to be:

$$ED_{lyasc} = \bar{x}\left(R_{ly65sc}, R_{ly70sc}, R_{ly75sc}\right) * p_{lasc} * RR_{asc}$$

Where ED_{lasc} are deaths for location *l*, year *y*, age group *a*, sex *s*, and cause *c*; $\bar{x}(R_{l65sc}, R_{l70sc}, R_{l75sc})$ is the mean of the rates for ages 65-69, 60-74, and 75-79 for that location-year-sex-cause; p_{lasc} is the

population for that location-year-age-sex-cause; and RR_{asc} is the global standard relative rate determined in the previous step for that age-sex-cause. The expected deaths remain attributed to that particular cause, while the difference between observed and expected are reallocated to HIV/AIDS.

Step 7. Scale strata to province

Over time, a higher proportion of deaths have been registered in China through the expansion of the Disease Surveillance Point (DSP) system and provincial and county efforts to increase cause of death registration. With the expansion of coverage, it is possible that province aggregates do not accurately represent the population distribution between urban and rural areas in each year. For this reason, we stratified the data preparation by urban and rural status for each county within each province. Stratification was based on the median level of urbanization across counties within each province as recorded in the 2010 China census. In the provinces of Tibet and Hainan, all counties were placed into one strata based on largely homogeneous urbanization levels within each province. This yielded a total of 62 analytical province-strata. Macao and Hong Kong were not included in this stratification system as the VR systems there are independent from that on the mainland; no weighting scheme needs to be carried out in these complete VR systems with quality data on causes of death.

Within each province-strata, a larger proportion of deaths in-hospital might be reported than that of deaths outside of hospital because of the internet hospital reporting system. To avoid bias, we reweighted in-hospital and out-of-hospital deaths based on the age-sex-province-specific fraction of deaths in and out of hospital in the DSP system. DSP data have been used to establish these percentages because, in these communities, there is a concerted effort to identify all out-of-hospital deaths. Province-strata death rates are combined to produce overall province death rates by weighting each strata by population in each age-sex-year group. Province death rates are rescaled so that all-cause mortality equals the estimated death rate in each age-sex-year estimated in the life-table analysis. The Bayesian noise reduction algorithm was used to deal with zero counts and small number issues for rare causes.[7]

Step 8. Restrictions post-redistribution

Some causes of death can only be reliably assigned through an autopsy by a trained physician. For example, it is unlikely that a verbal autopsy would reliably distinguish between ischemic and hemorrhagic stroke.

In this step, it is ensured that the detail of the cause list at this point in the data prep process is reasonable given the detail of the original data source and the methods by which the cause of death was assigned. Two primary corrections are applied. First, any cause which is purely an artifact of the redistribution machinery targeting too detailed a cause is aggregated up to the parent cause. Second, a “bridge map” is applied over a certain set of sources to ensure that these sources do not contain causes which could not reliably be determined by the methodology. These two corrections are applied to ICD9-BTL, ICD10-tabulated, USSR tabulated ICD9, India MCCD reports, China-DSP-tabulated-ICD9, India SCD reports, and all verbal autopsy sources.

Step 9. Drop VR country years or mark as non-representative based on completeness

Lozano and colleagues[8] describe the negative impact that low-completeness vital registration (VR) data can have on cause of death modeling for the GBD 2010. In particular, in settings where a data source does not capture all deaths in a population, the cause composition of deaths captured might be different from those that are not. However, a completeness sensitivity test found that low-completeness VR data had little impact on the cause-specific mortality trends at the global level.

For GBD 2015, we investigated the impact of these data at the country and subnational levels using the more thorough diagnostic visualizations available to us. It was determined that these data produced unlikely trends in the models affected. Despite the minimal impact on global trends, better models were produced by eliminating or marking as non-representative data with extremely low completeness. VR completeness was estimated using the Distribution of Death Methods (DDM) described in Part 1 of the appendix.

For this round, vital registration location-years with completeness below 50% were dropped, while location-years with completeness between 50-69% were marked as non-representative.

The following country-years were dropped from the database:

Location Years Below 50% Completeness

Location	Years Below 50% Completeness
'Asir	1999 - 2012
Bahah	1999 - 2012
Eastern Province	1999 - 2003
Ha'il	1999 - 2012
Haiti	1981, 1999, 2002 - 2004
Iran	1980 - 1985, 1987
Jawf	1999 - 2012
Jizan	1999 - 2012
Makkah	1999 - 2005
Maranhão	1985
Najran	1999 - 2012
Northern Borders	1999 - 2012
Papua New Guinea	1980
Qassim	1999 - 2003
Riyadh	1999 - 2012
Tabuk	1999 - 2002
Turkey	1983, 1984, 1987 - 1995

Step 10. Cause aggregation

The cause list is organized in a top down hierarchical format containing 4 levels. The first group, or level 1, sums all causes. Following all cause-mortality are level 2 causes, which include 3 broad groupings of causes of deaths: communicable, maternal, neonatal, and nutritional diseases; non-communicable diseases; and injuries. Within those level 2 groupings are finer levels used for modeling. Level 3, or parent causes, are aggregated, meaning the mortality estimate for a parent cause in the hierarchy represents the sum of the causes under that rubric. Sub-causes within level 3 causes – level 4 – are more

detailed. For example, the parent cause "intestinal infectious diseases" contains the 3 sub-causes: typhoid fever, paratyphoid fever, and other intestinal infectious diseases. Included in the parent cause estimate are deaths mapped directly to the parent and any level 4 sub-causes. In data where there was not enough information to assign a level 4 cause, we aggregated to the level 3 parent cause. Exceptions to aggregating the level 4 sub-causes to the parent are instances when certain sub-causes are not present. The United Nations Crime Trends police data only identifies homicides, aggregating homicides to injuries would not accurately represent all injuries.

Step 11. Remove shocks and HIV/AIDS maternal adjustments

For GBD 2015, CODEm models use an HIV/AIDS- and shock-free envelope, as described in Part 1 of the appendix. In order to be comparable, cause fractions must also be HIV/AIDS- and shock-free. Cause fractions were uploaded to the Causes of Death database as the number of deaths due to the cause over an adjusted sample in which the number of deaths due to HIV/AIDS, collective violence and legal intervention, and exposure to forces of nature were removed.

11.1 Remove HIV/AIDS, shocks from denominator where HIV/AIDS in cause list

The first step to generate HIV- and shock-free cause fractions was to remove any deaths from the sample which were directly coded to HIV/AIDS, collective violence and legal intervention, or exposure to forces of nature. The resulting equation for a cause fraction uploaded to the database is simple:

$$CF_{l,t,a,x,c} = \frac{D_{l,t,a,x,c}}{D_{l,t,a,x} - D_{l,t,a,x,hiv} - D_{l,t,a,x,war} - D_{l,t,a,x,disaster}}$$

In this equation, $CF_{l,t,a,x,c}$ is the cause fraction for a location (l), year (t), age (a), sex (x), and cause (c), $D_{l,t,a,x,c}$ is the number of deaths observed in the sample for the same, $D_{l,t,a,x}$ is the total number of deaths observed in the sample in the location, year, age and sex, and $D_{l,t,a,x,hiv}$, $D_{l,t,a,x,hiv}$, and $D_{l,t,a,x,disaster}$ are the number of deaths observed in the sample for HIV/AIDS, collective violence and legal intervention, and exposure to forces of nature, respectively.

Cause fractions for HIV/AIDS and shock causes were also uploaded to the database for use in separate estimation processes described in Part 1 of the appendix. In this case, cause fractions followed the standard equation, with variables following the same explanation as above:

$$CF_{l,t,a,x,c} = \frac{D_{l,t,a,x,c}}{D_{l,t,a,x}}$$

11.2 Remove HIV/AIDS deaths from maternal mortality sources

HIV-free cause fractions were also uploaded for sources on mortality due to maternal causes. In these cases, the sample of all deaths observed in the study is likely to contain some amount of deaths due to HIV/AIDS and shocks, but the sample only includes cause information on maternal deaths. To account for the presence of HIV/AIDS and shocks in the entire sample, we assumed the same proportion of total deaths due to HIV/AIDS by location, age, sex, and year as provided from the estimation of HIV/AIDS and all-cause mortality described in Part 1 of the appendix.

Maternal mortality studies were only corrected for HIV/AIDS if the sample of total deaths was provided in the data source. Where sources only provided the Maternal Mortality Rate (MMR), we applied the

rate to the HIV- and shock-free envelope produced by the analysis described in Part 1 of the appendix and thus did not need to adjust cause fractions at this point in the process.

Where a correction was applied, we applied the following equation:

$$CF_{l,t,a,x,mat} = D_{l,t,a,x,mat} * \frac{E[D_{l,t,a,x,hiv\_shock\_free}]}{E[D_{l,t,a,x}]}$$

In this equation, X is the resulting cause fraction due to maternal causes for the location (l), year (t), age (a), and sex (x); $D_{l,t,a,x,mat}$ is the number of observed deaths in the sample due to maternal causes, $E[D_{l,t,a,x}]$ is the GBD estimate of all-cause mortality in the location, year, age, and sex, and $E[D_{l,t,a,x,hiv\_shock\_free}]$ is the GBD estimate of HIV- and shock- free mortality in the location, year, age, and sex.

11.3 HIV/AIDS correction of sibling history, census, and survey data

As described in our analysis from GBD 2013, many studies have failed to find increased mortality in HIV-positive pregnant mothers, but those who have advanced HIV are known to have increased baseline mortality. Prior to GBD 2013, we did not distinguish between deaths in HIV+ women that were caused by pregnancy and those for which the pregnancy was incidental to their death. In order to more explicitly quantify the contribution of pregnancy to death in HIV+ women, and therefore more accurately estimate the maternal death count, we completed two additional analyses for GBD 2013. First, we determined the population attributable fraction (PAF) of HIV/AIDS to pregnancy-related death. Second, we determined the proportion of pregnancy-related deaths in HIV-positive persons that are aggravated by pregnancy and are therefore by definition maternal deaths.

$$PAF = \frac{p(RR-1)}{1+p(RR-1)}$$

Where PAF is the population attributable fraction, p denotes the prevalence of HIV in pregnancy, and RR is relative risk of mortality in HIV+ vs HIV- pregnant females.

To recap our analysis for GBD 2013, we used the paper published by Calvert and Ronsmans to identify sources[9] that could inform Step 1 of our HIV-correction analysis. We independently reviewed each of the component studies in Calvert and Ronsmans' review and extracted data directly, not from the systematic review paper. We only identified one additional study that was not used in Calvert and Ronsmans' analysis. We have, however, not used all the studies included in that review. Specific details are as follows: 1) Figueroa-Damian, et.al was excluded for not including any postpartum deaths at all. 2) In the case of Ryder, et al. and Zvandasara, et al. we excluded those deaths > 12 months after delivery. 3) We excluded the results from Chilongozi, et al. from the site that did not include any HIV-negative patients. 4) Leroy, et al. was not in the bibliography. We could not locate it for review so it was excluded. 5) Kourtis et al. was extracted with adjustment of the denominator based on the average number of hospitalizations per delivery in each group. 6) Ticconi, et al. was excluded for being both non-representative and including subgroup data from mothers with malaria infection. A total of 21 sources were included in our analysis of the increased mortality risk of HIV+ versus HIV- women in pregnancy.[9] We performed DerSimonian-Laird random effects meta-analysis to derive a pooled estimate of RR of death during pregnancy given HIV positivity.[10] The pooled effect size was 6·40 (95% UI 3·98 – 10·29) which was then used to calculate an HIV PAF for each country, age group and year. In order to

determine the proportion of those HIV-related deaths that were attributable to maternal causes, we performed a second systematic literature review. This time we sought evidence for the excess mortality risk of pregnancy in those women who are already HIV-positive. Most studies have failed to find such an effect, but most also did not stratify their study population by stage of HIV or ART status. Only two studies did this stratification, with a pooled effect size of 1·13 (95% UI 0·73 − 1·77).

An updated literature review to inform the relative risk of mortality in pregnancy in HIV-positive versus HIV-negative women had 14 hits, but no usable sources. We completed this search on May 7, 2015, using the following two search strings:

> (""HIV""[Mesh] OR ""Acquired Immunodeficiency Syndrome""[Mesh]) AND (""Pregnancy""[Mesh] OR ""Postpartum Period""[Mesh]) AND ""Mortality""[Mesh]
>
> “HIV”(MeSH) AND (“pregnant”(Title/Abstract) OR “pregnancy”(Title/Abstract) OR “postpartum”(Title/Abstract) OR ""post partum""(Title/Abstract)) AND (“mortality”(Title/Abstract) OR “death”(Title/Abstract))"

Prevalence of HIV in pregnant women was calculated using UNAIDS’ Spectrum model. Spectrum is a compartmental HIV progression model used to generate age-specific incidence, prevalence, and death rates from pre-calculated incidence curves and assumptions about intervention scale-up and local variation in epidemiology. For each location, we used UNAIDS’ age-specific ratios of fertility in women living with HIV to fertility in women not living with HIV. In most locations, this ratio is assumed to be greater than one in women aged 15-24 and less than one and decreasing as age increases beyond 24. Since Spectrum assumes fertile ages of 15-49, we used the ratio of HIV prevalence in pregnant women to HIV prevalence in the general population at either end of that range to extend estimates to age bands 10-14 and 50-54.

Unlike GBD 2013, when we applied the PAF correction to the envelope of maternal deaths predicted by CODEm, we instead applied country-year-age-group-specific PAF to maternal mortality input data prior to modeling in CODEm. This ensured that both the numerator and denominator of all CF data were internally consistent in their exclusion of background HIV/AIDS mortality. The cause fractions for maternal deaths in sibling history, survey, and census data were therefore adjusted as follows:

$$CF_{l,t,a,x,mat_{adj}} = CF_{l,t,a,x,mat} * (1 - prop_{hiv_{l,t,a,x}})$$

$$prop_{hiv_{l,t,a,x}} = PAF_{l,t,a,x,hivpos} * (1 - \pi_{mat})$$

$$CF_{l,t,a,x,mat_{hiv}} = CF_{l,t,a,x,mat} * prop_{maternalhiv_{l,t,a,x}}$$

$$prop_{maternalhiv_{l,t,a,x}} = PAF_{l,t,a,x,hivpos} * \pi_{mat}$$

Where:

> $\pi_{mat} = .13/1.13$ = The proportion of HIV/AIDS deaths during pregnancy that were exacerbated by the pregnancy.
>
> $PAF_{l,t,a,x,hivpos}$ = The population-attributable fraction (PAF) that describes the percentage of all maternal deaths that were HIV-related for the location (l), year (t), age (a), and sex (x=Female)).

$CF_{l,t,a,x,mat}$ = The proportion of deaths due to all maternal causes before HIV/AIDS correction for the location, year, age, and sex.

$prop_{hiv_{l,t,a,x}}$ = The proportion of deaths in pregnancy for the location, year, age, and sex that are estimated to be incidental deaths due to HIV/AIDS, and therefore not a maternal cause of death.

$prop_{maternalhiv_{l,t,a,x}}$ =The proportion of deaths in pregnancy for the location, year, age, and sex that are estimated to be HIV-positive and maternal deaths which are aggravated by HIV/AIDS.

$CF_{l,t,a,x,mat_{adj}}$ = The proportion of deaths due to maternal causes after the adjustment for the location, year, age, and sex.

$CF_{l,t,a,x,mat_{hiv}}$ = The proportion of deaths due to maternal deaths aggravated by HIV/AIDS after the adjustment for the location, year, age, and sex.

11.4 HIV/AIDS correction of other maternal mortality data

Although there are a specific subset of codes in ICD-10 that correspond to HIV/AIDS deaths aggravated by pregnancy, these codes are sparsely used and unreliable. We therefore adapted the method above to also correct VR and VA sources for the systematic exclusion of HIV-related maternal deaths. This correction was calculated in the same manner, using the same input data as above, with the only difference that HIV correction of VR and VA sources resulted in a net increase in maternal CF. Maternal deaths aggravated by HIV/AIDS are calculated as the following:

$$CF_{l,t,a,x,mat_{hivvr}} = CF_{l,t,a,x,matvr} * prop_{maternalhiv_{l,t,a,x}}$$

$$prop_{maternalhiv_{l,t,a,x}} = \frac{PAF_{l,t,a,x,hivpos} * \pi_{mat}}{1 - PAF_{l,t,a,x,hivpos} * \pi_{mat}}$$

Where all symbols are the same as described above.

Step 12. Noise reduction

To deal with problems of zero counts in vital registration, verbal autopsy, cancer registries, or sibling histories for a given age group in a given year, we use a Bayesian noise reduction algorithm. For this algorithm, we assume a normal prior and a normal data likelihood. We estimate the normal prior for a given country series of data by estimating a negative binomial for the fraction of deaths in each age group due to each respective cause with dummy variables for age and year. With two notable exceptions (detailed below), these regressions are country-specific, so borrowing strength over age is only within a data type in a country. The variance of the prior, τ^2, is estimated from the negative binomial regression, taking into account the variance-covariance matrix of the regression coefficients. For the data variance, we use the Wilson approximation which provides an estimate of σ^2 even in cases with a zero count of cause-specific deaths. The posterior estimate for each data point is:

$$Mean = \left(\frac{\tau^2}{\tau^2 + \sigma^2} X + \frac{\sigma^2}{\tau^2 + \sigma^2} \mu\right)$$

$$Variance = \left(\frac{\tau^2 \sigma^2}{\tau^2 + \sigma^2}\right)$$

Where X is the mean of the data and μ is the mean of the prior. This approach to noise reduction avoids the problem that zero counts in an *ln* rates model or a logit cause fraction model will be dropped from the regression and lead to upward bias in the estimates. This is particularly important in two settings: high-income countries with small numbers of cause-specific deaths, and in the analysis of sibling history data where for any given age group in any given year the number of deaths reported in the survey that are pregnancy-related or the number of deaths from all causes in that age group may be small.

Regarding the exceptions to the regression, the first is that country-years with populations under 1 million are pooled with the region data in order to prevent overdispersion and provide a stronger signal. Additionally, verbal autopsy data diverge from the above description in two ways. First, all data for a given super-region are pooled together and a study dummy variable is added, allowing for different studies and surveillance sites to borrow strength from one another within a super-region. Second, unless the data are part of a time series (e.g., Matlab HDSS), there is no year component to the regression.

13. COD database and outlier identification

Death rates for different causes of death generally have a stable age pattern. In large populations, these patterns will not change very rapidly over time. We can assume a relatively stable pattern in death rates for all causes except for some epidemic diseases and specific types of injuries. Rare causes in large populations and prevalent causes in small populations usually have stochastic patterns. To correct for these stochastic patterns we implement a noise reduction process, explained in Step 12.

In vital registration data, we infrequently find one or more data points for specific geography/age/sex/years that lie very far from the stable pattern of death rates. In these situations, the model will usually ignore the data point(s). If the model fails to ignore these data, dramatic jumps or drops can occur in the death rates. When there is no logical explanation for variation in the death rates to this degree, we outlier the data point(s). The selection of data points to outlier occurs after data have been prepped for modeling, as well as during preliminary reviews of the models.

In non-vital-registration sources, data collection methods and data quality can vary widely from source to source. Where data points in each age-sex-geography-year are very sparse, extreme data points can have a bad effect on regional estimation. In these situations we investigate the study's methods and outlier lower-quality data points.

Identifying outliers in the cause of death data occurs prior to finalization of models for each cause. We do not automate the selection of outliers, but investigate the source of the offending data as well as

reviewing other data sources for the same cause, geography, and year. Ultimately, outliers are identified based on the judgement of the modeler and senior faculty and are reversible to allow for decisions to be revisited in the future.

References

1 Lozano R, Freeman MK, James SL, *et al.* Performance of InterVA for assigning causes of death to verbal autopsies: multisite validation study using clinical diagnostic gold standards. *Popul Health Metr* 2011; **9**: 50.

2 Office of the Registrar General and Census Commissioner. India Special Survey of Deaths. India, 2004.

3 Office of the Registrar General and Census Commissioner. India Medical Certification of Cause of Death Reports 1990-2010. India, 2014.

4 Office of the Registrar General and Census Commissioner. India Medical Certification of Cause of Death Reports 1980-1986. India.

5 Naghavi M, Makela S, Foreman K, O'Brien J, Pourmalek F, Lozano R. Algorithms for enhancing public health utility of national causes-of-death data. *Popul Health Metr* 2010; **8**: 9.

6 Ahern RM, Lozano R, Naghavi M, Foreman K, Gakidou E, Murray CJ. Improving the public health utility of global cardiovascular mortality data: the rise of ischemic heart disease. *Popul Health Metr* 2011; **9**: 8.

7 GBD 2013 Mortality and Causes of Death Collaborators. Global, regional, and national age-sex specific all-cause and cause-specific mortality for 240 causes of death, 1990-2013: a systematic analysis for the Global Burden of Disease Study 2013. *Lancet* 2015; **385**: 117–71.

8 Lozano R, Naghavi M, Foreman K, *et al.* Global and regional mortality from 235 causes of death for 20 age groups in 1990 and 2010: a systematic analysis for the Global Burden of Disease Study 2010. *Lancet* 2012; **380**: 2095–128.

9 Calvert C, Ronsmans C. The contribution of HIV to pregnancy-related mortality: a systematic review and meta-analysis. *AIDS* 2013; **27**: 1631–9.

10 DerSimonian R, Laird N. Meta-analysis in clinical trials. *Control Clin Trials* 1986; **7**: 177–88.

Part 3: Causes of death modeling methods

1. CODEm

1.1 Overview of method

CODEm is a framework for modeling most cause-specific death rates in the GBD using five core principles: 1) Identify and use all the available data in the modeling process. Though data may vary in quality it all contains some signal of the true epidemiological process. 2) Develop a diverse set of plausible models to use for estimation. That is, build a number of models capturing well-documented associations to make estimates. 3) Assess the predictive validity of each plausible individual model and of an ensemble of models created from the pool of plausible models. 4) Choose the models and ensemble model with the best performance in the out-of-sample predictive validity tests.

For some causes, separate models were run for different age ranges when there was reason to believe that the relation between covariates and death rates might be different in different age ranges, for example, in children compared with adults. Separate models are developed for countries with extensive, complete, and representative VR for every cause such that uncertainty can better reflect the more complete vital registration in these locations. A complete listing of countries with extensive, complete, and representative VR can be found in Appendix Table 37.

1.2 Model pool development

As many factors covary with a particular cause of death, a large range of plausible statistical models are developed for each cause. For the CODEm framework, four families of statistical models are developed using covariates (see 2x2 table in Foreman et al).[i] These are mixed effects linear models of the natural log of the death rate, mixed effects linear models of the logit of the cause fraction, spatiotemporal Gaussian process regression (ST-GPR) models of the log of the death rate, and ST-GPR of the logit of the cause fraction. All plausible relationships between covariates and relevant cause are identified, and all possible permutations of selected covariates are tested in linear models where the logit cause fraction or log death rate is the response variable. Because we test all permutations of covariates, multicollinearity between covariates may produce implausible signs on coefficients or unstable coefficients. All models where the sign on the coefficient is in the direction expected based on the literature and where the coefficient is statistically significant at $p < 0.05$ are retained. We run covariate selection for both cause fractions and death rates and then create both mixed effects only and ST models for each set of covariates. For a detailed explanation of the covariate selection algorithm see Foreman et al 2012.[1]

1.3 Testing model pool on 15% sample

The performance of all component models and ensembles is evaluated using out-of-sample predictive validity tests. Thirty percent of the data are excluded from the initial model fits, and half of that (15% of total) is used to evaluate and rank component models and then build ensembles. Data are held out from the analysis using the pattern of missingness for each cause in the cause of death database. Out-of-sample predictive validity testing is repeated until stable model results have been obtained. The out-of-sample performance tests include the root mean squared error of the log of the cause-specific death rate, the direction of the trend in the prediction compared to the data, and the validity of the 95% UI. For every model, we show the in-sample root mean squared error of the log

death rates (RMSE) and the out-of-sample performance in the 15% of data not used in the model building process.

1.4 Ensemble development

After component models are ranked on their out-of-sample predictive validity they are weighted based on their ranking and each component model contributes a portion to the final estimate. How much each submodel contributes is a function of its relative ranking as well as the value of psi chosen, which dictates that distribution of rankings (see Foreman et al 2012 for the details of psi distribution).[1]

1.5 Testing ensembles

Using the second half of the holdout data (15% of total), the differently weighted ensembles and different values of psi are tested using the same predictive validity metrics as the component models. For every model, we show the in-sample root mean squared error of the log death rates (RMSE) and the out-of-sample performance in the 15% of data not used in the model building process. The ensemble with the best average trend and RMSE is chosen as the final ensemble weighting scheme.

1.6 Final estimation

After a model weighting scheme has been chosen, each model contributes a number of draws proportional to its weight such that 1,000 draws are created. The mean of the draws is used as the final estimate for the CODEm process and 95% UI are created from the 0.025 and 0.975 quantiles of the draws. The final assessment of ensemble model performance is the validity of the UIs; ideally, the 95% UI for a model would capture 95% of the data out-of-sample. Higher coverage suggests that UIs are too large and lower than 95% suggest UIs are too narrow.

1.7 Causes for which CODEm is used – criteria for those where CODEm is not used

CODEm is used to model 167 causes, which are described in detail below. CODEm is unsuitable for use in modeling some causes, including those with very low death counts, when cause-specific death record availability is inadequate, or when there are marked biases or variability for cause of death certification over time which could not be fully accounted for with the current garbage code redistribution algorithms. Criteria for causes where CODEm is not used are discussed in further detail in Section 3 below.

1.8 Covariates used for each CODEm model plus CODEm model hyper parameters

For details on covariates used for CODEm models, see Appendix Table 15. CODEm covariates used, level of covariate, and expected direction of covariate by cause, sex, age, and location.

1.9 Fit statistics for CODEm models

For details on fit statistics for CODEm models, see Appendix Table 16. CODEm predictive validity results by cause, sex, age, and location.

CODEm was used for 167 causes of death. Descriptions for each of these causes are below.

[i] Foreman KJ, Lozano R, Lopez AD, Murray CJ. Modeling causes of death: an integrated approach using CODEm. Population Health Metrics 2012; 10: 1.

Otitis Media

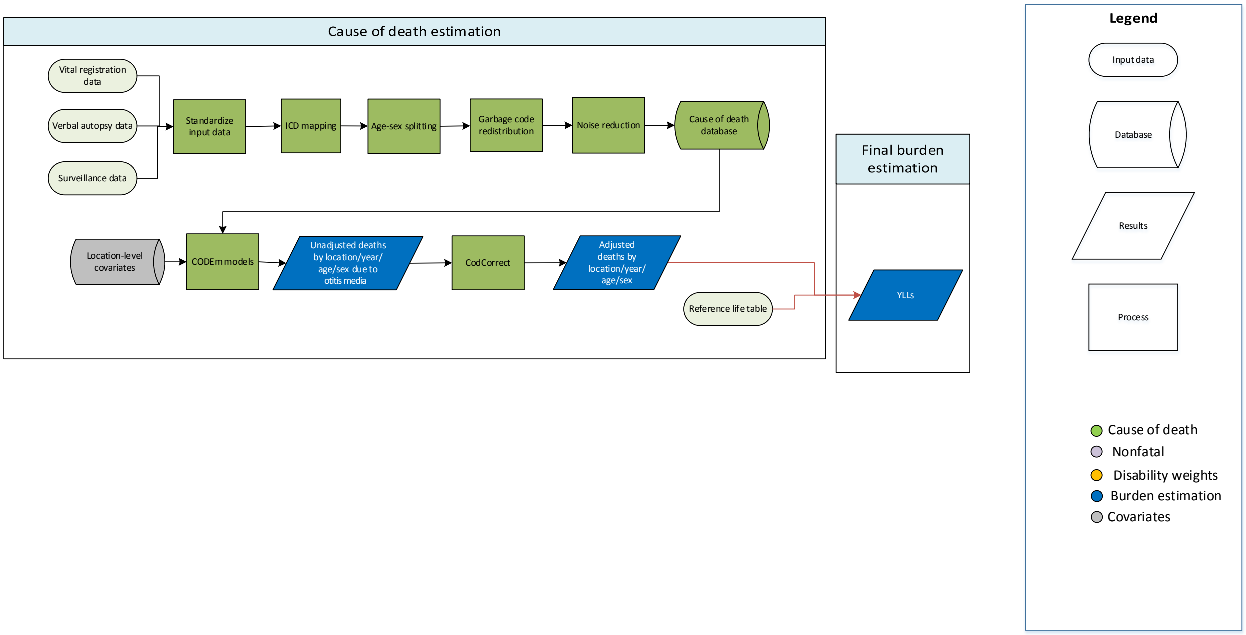

Input data

Vital registration, verbal autopsy and surveillance data were used. We outliered data that were largely conflicting with the majority of data from other studies conducted either in the same or different countries (with similar sociodemographic characteristics) in the same region.

Modeling strategy

A general CODEm modeling strategy was used. There were no substantive changes from GBD 2013 in terms of modeling strategy.

Encephalitis

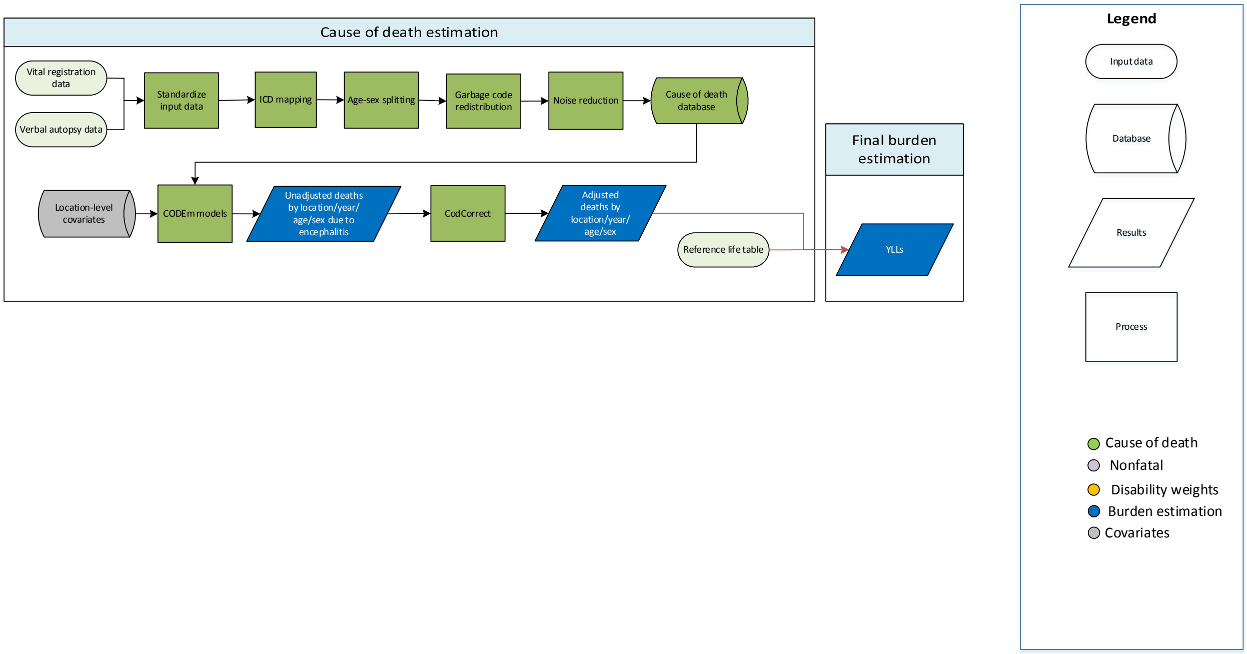

Input data

Vital registration and verbal autopsy data were used to model this cause. We outliered data in instances where garbage code redistribution and noise reduction, in combination with small sample sizes, resulted in unreasonable cause fractions when compared to regional, super-regional, and global rates, and data that violated well-established time or age trends. Outliering methods were consistent across both vital registration and verbal autopsy data.

Modeling strategy

We modeled deaths due to encephalitis with a standard CODEm model using the cause of death database and location-level covariates as inputs. We hybridized separate global and data-rich models to acquire unadjusted results, which were adjusted using CodCorrect to reach final years of life lost (YLLs) due to encephalitis.

In GBD 2013, the encephalitis model was modeled using two age categories – under 5 and 5 years and above – because the mortality trends differed substantially between children and adults and a significant number of data sources only had data for under-5-year-olds. With the addition of new data sources for GBD 2015, this modeling process was deemed unnecessary and the encephalitis model covered the entire age range. Another significant change was the addition of the Japanese encephalitis covariate, which is a binary covariate indicating if the location is known to be endemic for Japanese encephalitis. The covariate was modeled according to data from the Centers for Disease Control and Prevention.[1]

Reference

1 Centers for Disease Control (CDC). CDC health information for international travel 2016: the yellow book. New York City, United States: Oxford University Press, USA, 2016.

Tetanus

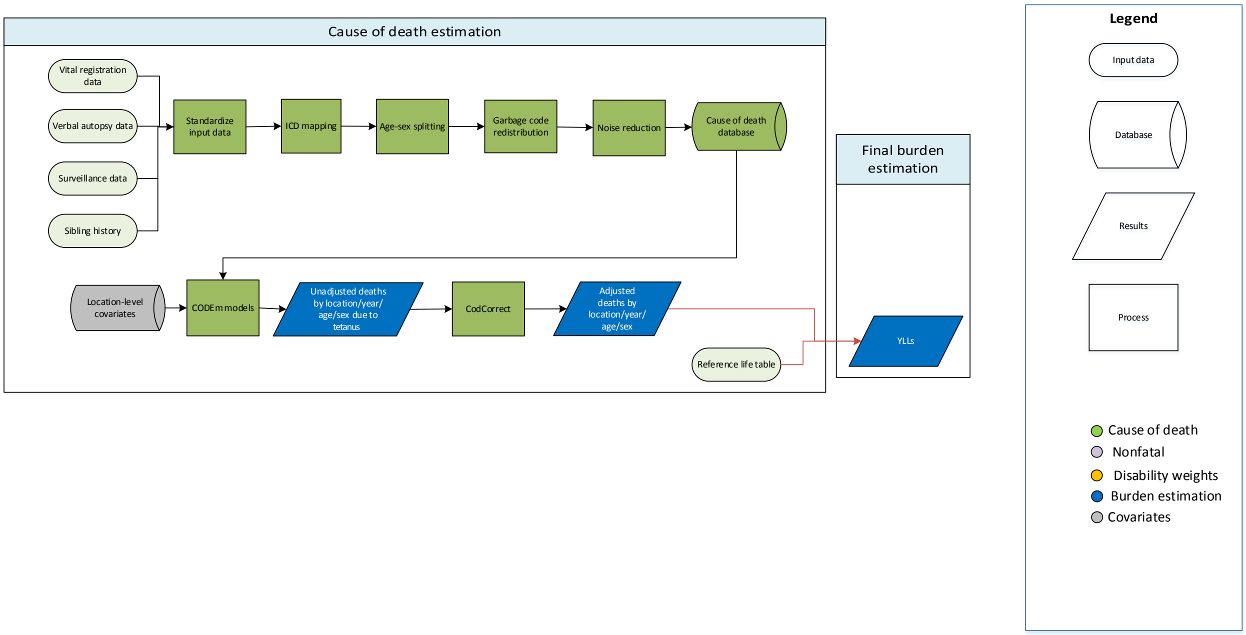

Input data

Vital registration, verbal autopsy, sibling history, and surveillance data were used. Data were outliered if they largely conflicted with the majority of data from other studies conducted either in the same or different countries with similar sociodemographic characteristics in the same region.

Modeling strategy

A general CODEm modeling strategy was used. We ran separate CODEm models for under 1 year and 1-80 years. There were no substantive changes from GBD 2013 in terms of modeling strategy.

Malaria

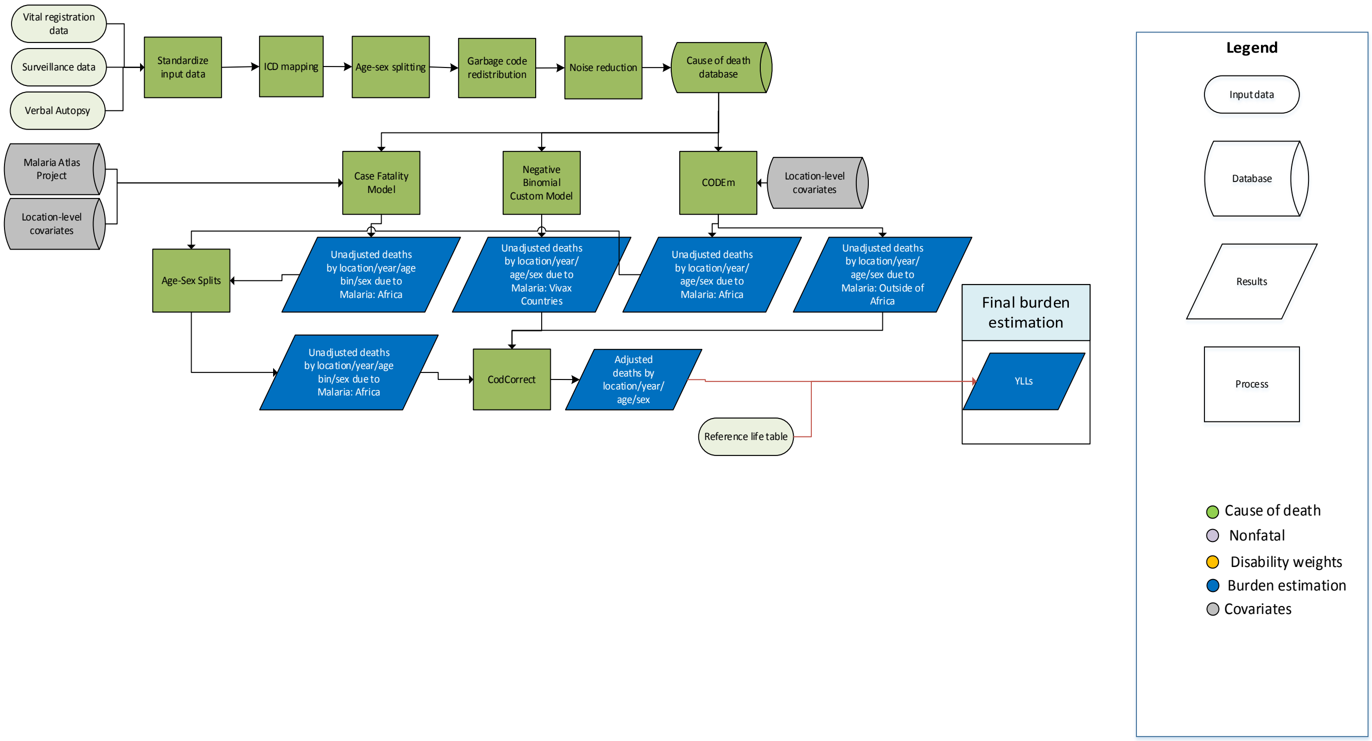

Input data

For the Outside of Africa and *P. vivax* models, data include vital registration, verbal autopsy, and surveillance data from the cause of death (COD) database. Unlike other causes of death, we did not redistribute deaths to malaria. For the Africa models, we only used COD data (mostly verbal autopsy) where we have been able to successfully geo-reference (e.g., find latitude and longitude) the site. Systematic literature reviews for malaria were not conducted.

Our outlier criteria excluded data points that (1) were implausibly high or low relative to global or regional patterns, (2) substantially conflicted with established age or temporal patterns, or (3) significantly conflicted with other data sources conducted from the same locations or locations with similar characteristics (i.e., local Socio-Demographic Index).

Unlike other causes of death we directly used epidemiologic measures in our estimation process – whereas causes modeled with a traditional CODEm approach generally use epidemiologic measures as a covariate, if at all. We received spatio-temporal cubes of incidence in three broad age-bins (0-5, 5-14 and 15+) from 1980 to 2015 as well as time series (at the country level) estimates of any antimalarial use from the Malaria Atlas Project (MAP) at the University of Oxford. Separately, we updated our measure of drug efficacy from GBD 2013.

Modeling strategy

As described above, the malaria modeling strategy was carried out in three parts.

For countries where the main/exclusive strain of malaria was *P. vivax*, deaths were estimated using a simple negative binomial model where the outcome is study deaths and the exposure of sample sizes. We used fixed effects on year and indicator effects for age and sex on the other side of the equation.

In locations outside of Africa, we continued to use a traditional CODEm approach. It must be noted that "outside of Africa" also includes some countries on the continent of Africa including Algeria, Egypt, Morocco, Comoros, Mauritius, Cape Verde, Sao Tome, Principe, and South Africa. Thus, our split was both a function of location (e.g., in/out Africa) as well as epidemiologic profile (significantly fewer cases and deaths). For GBD 2015, we strictly matched MAP's designation of malarial Africa.

To estimate the fatal burden of disease due to malaria in Africa, we first used a traditional CODEm approach to generate age-patterns for later use. Relative to GBD 2013, the covariate list was significantly updated and now reflects the central role of MAP data. Specifically, we used MAP estimates of *P. falciparum* prevalence in 2- to 10-year-olds, its conversion to malaria incidence in three broad age bands, untreated incidence given antimalarial coverage and efficacy, drug efficacy, lag distributed income, health systems access, and the interaction between incidence and drug efficacy as covariates for these models.[1] Where appropriate, we have extracted site-specific values of these covariates which is used by CODEm to improve the accuracy at each stage.

Separately, for mortality data that were geo-referenced, we aggregated the data to match the age groups of the MAP incidence cube and then extracted the age-bin-specific incidence values. Using national-level estimates of drug efficacy and antimalarial use, we calculated untreated incidence (our denominator) where:

Untreated incidence = incidence * (1-antimalarial coverage * (1-drug efficacy))

Death rate was derived from the COD data and was combined with incidence in the following form as the outcome variable: logit(death rate/untreated incidence rate). We modeled logit case fatality rate (cfr) in a mixed effects regression with fixed effects on log mortality rate and sex with random effects on the site of the study. The regression was weighted using the sample size (e.g., number of deaths) in the study to recognize the relative information contributions of each data point. We ran this model separately by age bin and collated the results at the end. All regressions were run in STATA 13 (specifically using the mixed function).

Predictions of logit case fatality were made at the national level using 1,000 realizations of each model (to propagate uncertainty) while omitting the location based random effects. It was not possible to propagate covariate uncertainty. These draws were back-transformed and multiplied by national-level estimates of untreated cases to get deaths by sex and age-bin. The age-bins were subsequently split into GBD age groups by incorporating the observed cause-specific mortality pattern previously generated by the CODEm models and the underlying population pyramid.

The results from the *P. vivax*, Outside of Africa, and Africa models were collated and incorporated in the CodCorrect algorithm.

References

1 Bhatt S, Weiss DJ, Cameron E, *et al.* The effect of malaria control on Plasmodium falciparum in Africa between 2000 and 2015. *Nature* 2015; **526**: 207–11.

Chagas disease

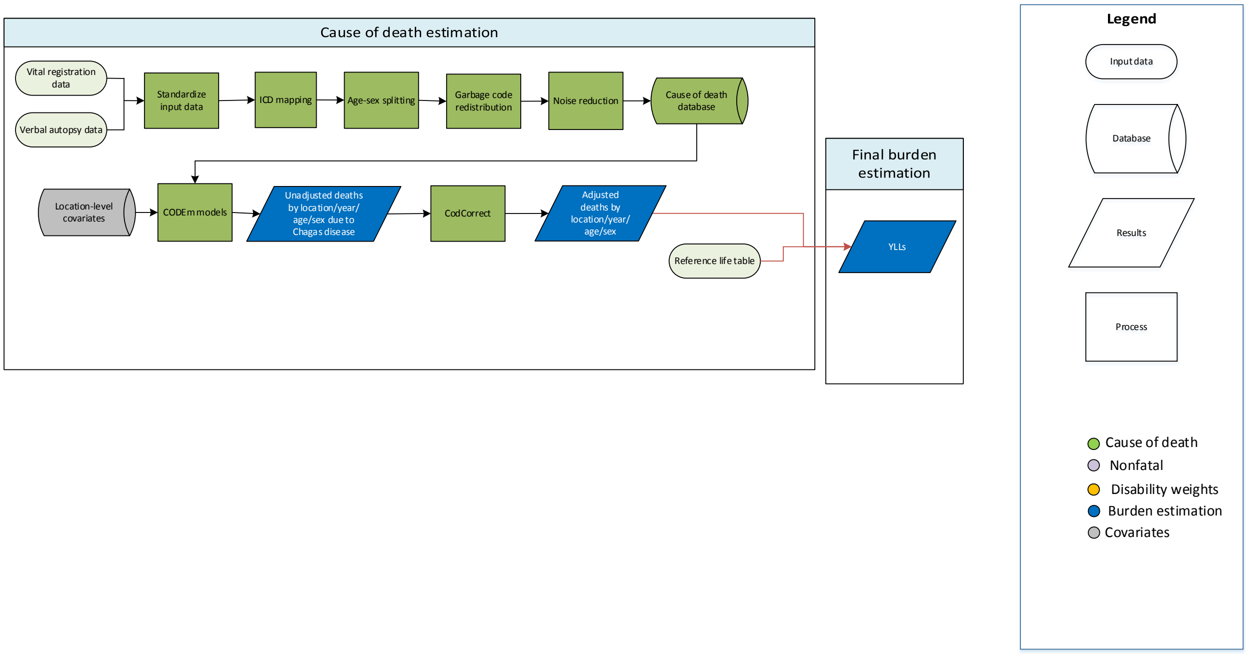

Input data

We modeled Chagas mortality using all available data in the cause of death database. No data were outliered for this cause.

Modeling strategy

We modeled Chagas mortality using a two-model hybrid approach: 1) a CODEm model of all Chagas-endemic countries of Latin America using all data in the CoD database; and 2) estimates of mortality from imported cases in non-endemic, data-rich countries. Where Chagas deaths were reported in non-endemic data-rich countries, we produced non-zero estimates by drawing from a beta distribution defined based on number of reported deaths and the underlying sample size. Estimates of Chagas mortality in endemic countries were drawn from the CODEm model.

We have made no substantive changes in the modeling strategy from GBD 2013 for Chagas endemic countries. We have added mortality from imported cases for GBD 2015.

Dengue

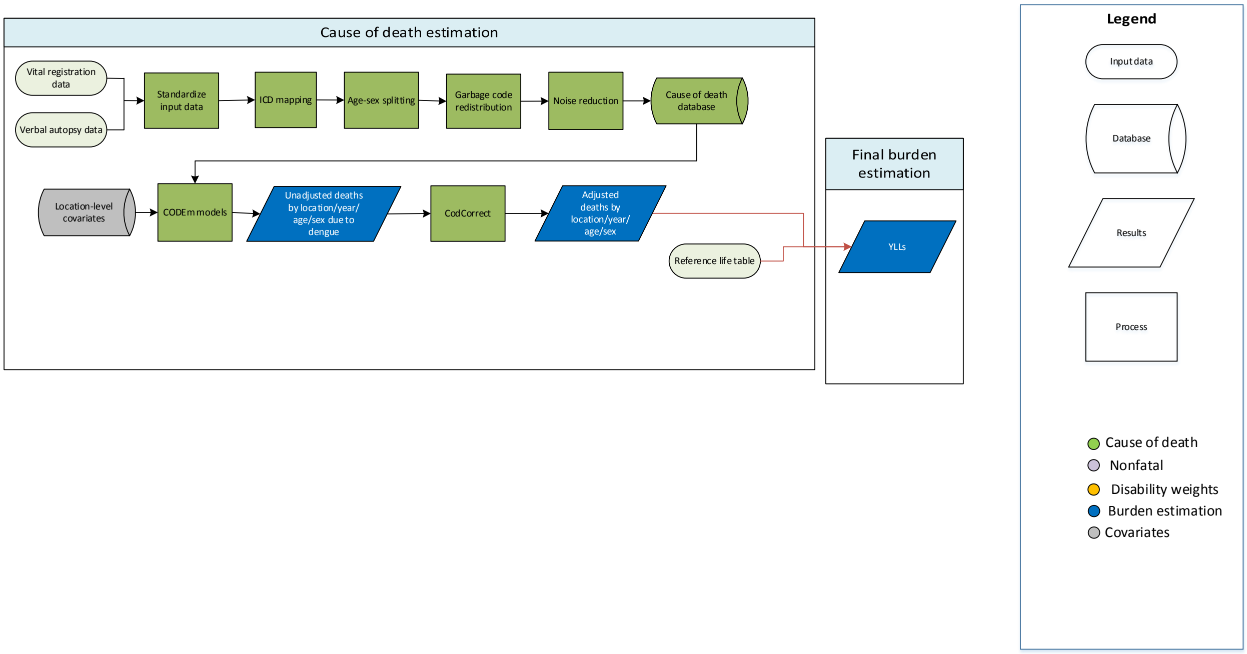

Input data

We modeled dengue mortality using all available data in the cause of death database. Data points were outliered if they reported an improbably low number of dengue deaths (e.g., zero dengue deaths in a hyper-endemic country) or an improbably high number of dengue deaths.

Modeling strategy

We modeled dengue mortality using three-model hybrid approach: 1) a global CODEm model of all locations, using all data in the CoD database; 2) a CODEm model restricted to data-rich countries; and 3) estimates of mortality from imported cases in non-endemic, data-rich countries. Where dengue deaths were reported in non-endemic data-rich countries, we produced non-zero estimates by drawing from a beta distribution based on number of reported deaths and the underlying sample size. Estimates of dengue mortality in endemic data-rich countries were drawn from the data rich CODEm model. Finally, estimates in other endemic countries were drawn from the global CODEm model.

The three-model hybrid approach is new for GBD 2015, and GBD 2013 used a single global CODEm model for all locations. We have also revised a covariate that we use to inform trends in dengue mortality. For GBD 2013 we used a covariate that represented anomalies in dengue case reports (i.e., standard deviations above or below the mean reported incidence for a given location). This covariate was largely intended to inform the model about epidemic years and not to inform trends. This covariate was therefore agnostic about countries and years for which no case reports were available. Based on collaborator feedback, and to fully leverage information about trends, we have updated this covariate. It is now based on a mixed-effects model of reported incidence and yields informed estimates for countries and years with no reports. The result is dramatically improved trend estimates in the current model.

Rabies

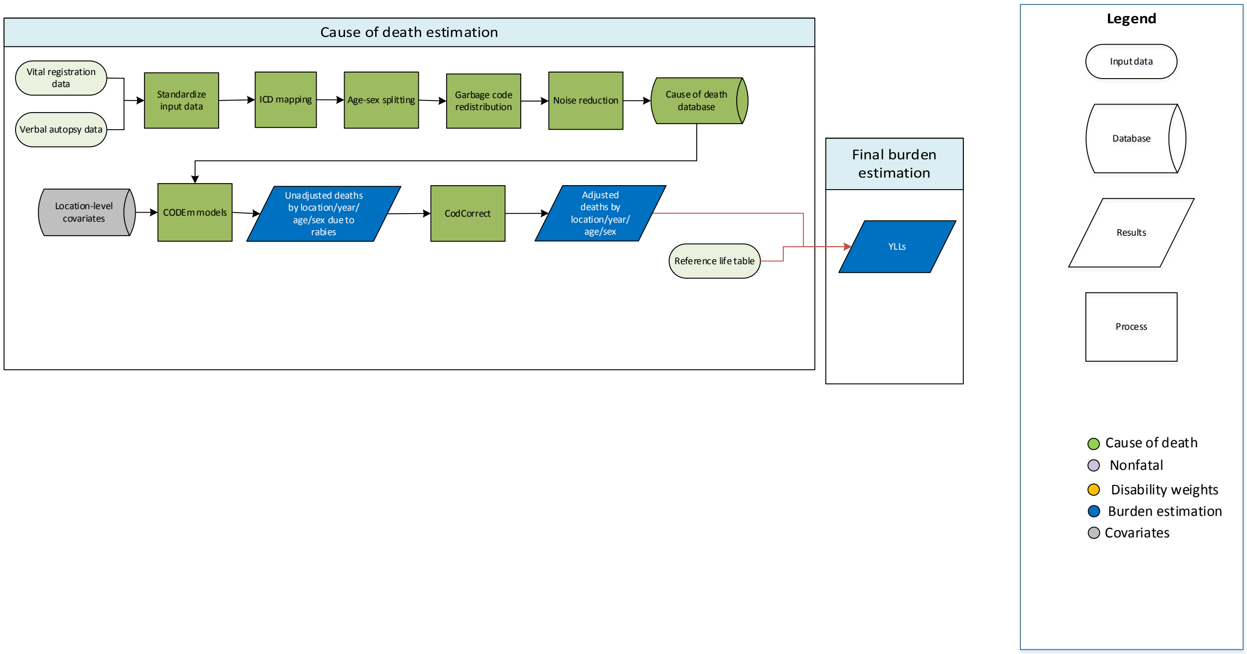

Input data

We modeled rabies mortality using all available data in the cause of death database. Data points were outliered if they reported an improbable number of rabies deaths (e.g., zero rabies deaths in a hyper-endemic country) or if their inclusion in the model yielded distorted trends. In some cases multiple data sources for the same location differed dramatically both in their quality and reported rabies mortality (e.g., a verbal autopsy and vital registration source). In these cases the lower-quality data source was outliered.

Modeling strategy

We modeled rabies mortality using a two-model hybrid approach: 1) a global CODEm model of all locations, using all data in the CoD database; and 2) a CODEm model restricted to data-rich countries.

We have made two substantive changes in the modeling strategy from GBD 2013. First, we have changed from a single global model to the hybrid global/data rich model approach. Second, we conducted an exploratory analysis to determine the most predictive covariates for rabies and have updated the covariates used in the CODEm model accordingly.

Other neglected tropical diseases (NTDs)

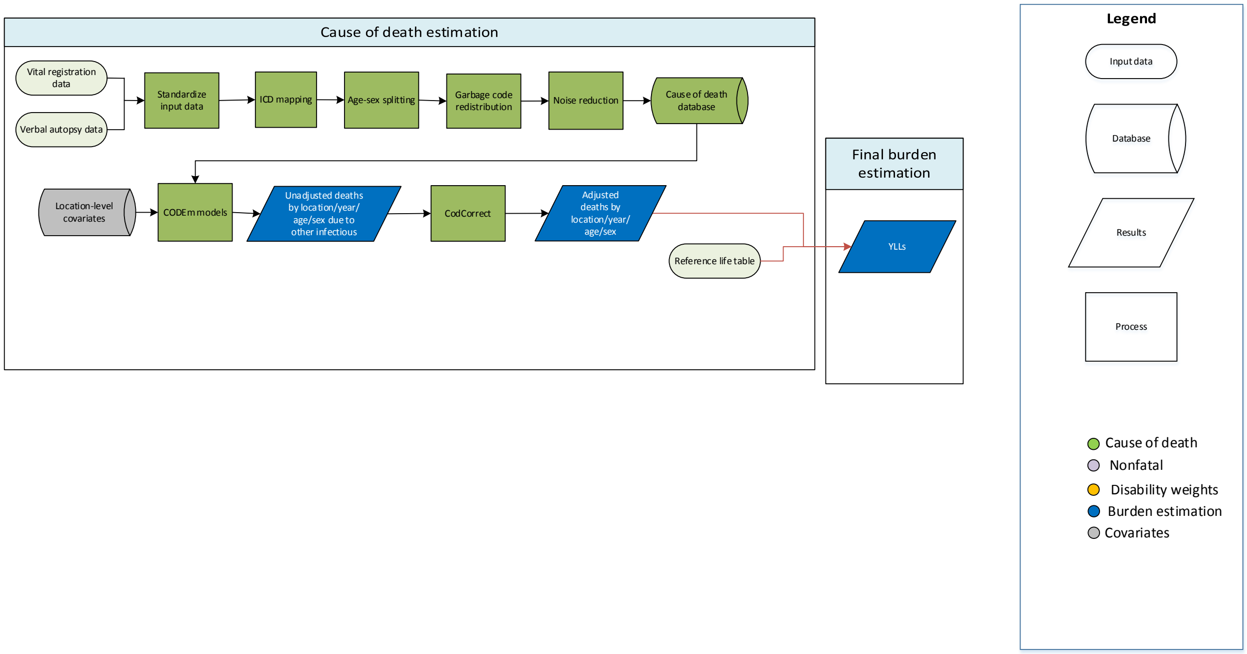

Input data

We modeled other infectious disease mortality using all available data in the cause of death database. Data points were outliered if they reported an improbable number of deaths or if their inclusion in the model yielded distorted trends.

Modeling strategy

We modeled other infectious disease mortality using a two-model hybrid approach: 1) a global CODEm model of all locations, using all data in the CoD database; and 2) a CODEm model restricted to data-rich countries.

Since GBD 2013 we have switched from a single global model to the hybrid global/data-rich model approach. We have otherwise made no substantive changes in the modeling strategy for other infectious disease from GBD 2013.

Neonatal conditions

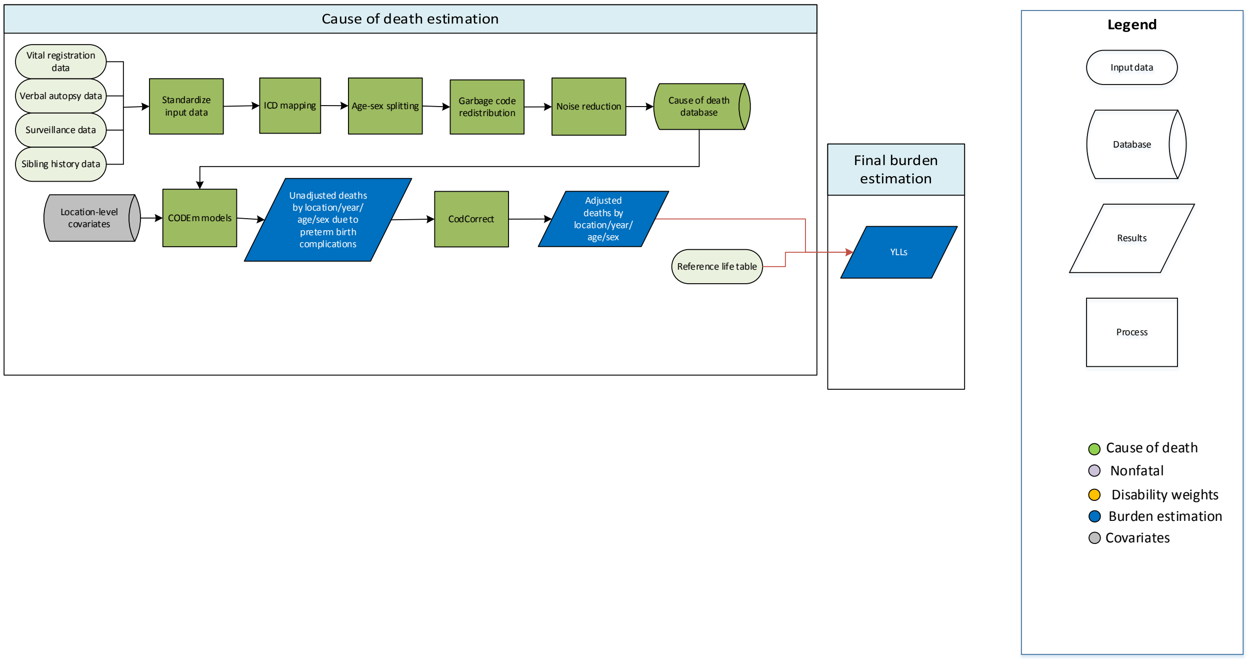

Input data

For preterm birth complications and neonatal encephalopathy, vital registration, verbal autopsy, surveillance, and sibling history data were used to estimate number of deaths from each condition. For sepsis and other neonatal infections, vital registration, surveillance, and sibling history data were used. And for neonatal hemolytic disease and other neonatal conditions, vital registration and surveillance data were used. For all neonatal causes of death, vital registration was by far the most common data type. We only modeled deaths among males and females under age 5. Data points were selected as outliers if they were implausibly high, low, or significantly conflicted with established age or temporal patterns.

Modeling strategy

For GBD 2013, an ensemble modeling approach was used via CODEm. The same was done for GBD 2015. In GBD 2013, we included skilled birth attendance as a covariate to account for the direct association with neonatal mortality. However, skilled birth attendance is already incorporated in the health system access covariate, which is a composite estimated using a principal component analysis of antenatal clinics, DTP3 immunization, measles immunization, in-facility delivery, and skilled birth attendance. Furthermore, skilled birth attendance and in-facility delivery are high correlated, so only one of these should be included in the model. This year, we opted to include in-facility delivery as a covariate and remove skilled birth attendance, which improved our model fits slightly.

Varying levels of data quality may have affected our results. Validation studies suggest that verbal autopsy methods tend to be less accurate for neonatal cause of death ascertainment.[1–4] This implies that in regions such as sub-Saharan Africa or South Asia, where the data primarily come from verbal

autopsy studies, the distribution of sub-causes within all neonatal conditions may be less accurate. Furthermore, validation studies suggest that verbal autopsy methods tend to be particularly poor at ascertaining deaths from neonatal sepsis. With this in mind, for GBD 2015 all verbal autopsy data were excluded for neonatal sepsis.

References

1 Anker M, Black RE, Coldham C, *et al.* A Standard Verbal Autopsy Method for Investigating Causes of Death in Infants and Children. Geneva, Switzerland: World Health Organization Department of Communicable Disease Surveillance and Response; The Johns Hopkins School of Hygiene and Public Health; The London School of Hygiene and Tropical Medicine, 1999.

2 Kalter HD, Gray RH, Black RE, Gultiano SA. Validation of postmortem interviews to ascertain selected causes of death in children. *Int J Epidemiol* 1990; **19**: 380–6.

3 Quigley MA, Armstrong Schellenberg JR, Snow RW. Algorithms for verbal autopsies: a validation study in Kenyan children. *Bull World Health Organ* 1996; **74**: 147–54.

4 Snow RW, Armstrong JR, Forster D, *et al.* Childhood deaths in Africa: uses and limitations of verbal autopsies. *The Lancet* 1992; **340**: 351–5.

Nutritional deficiencies

Nutritional deficiencies

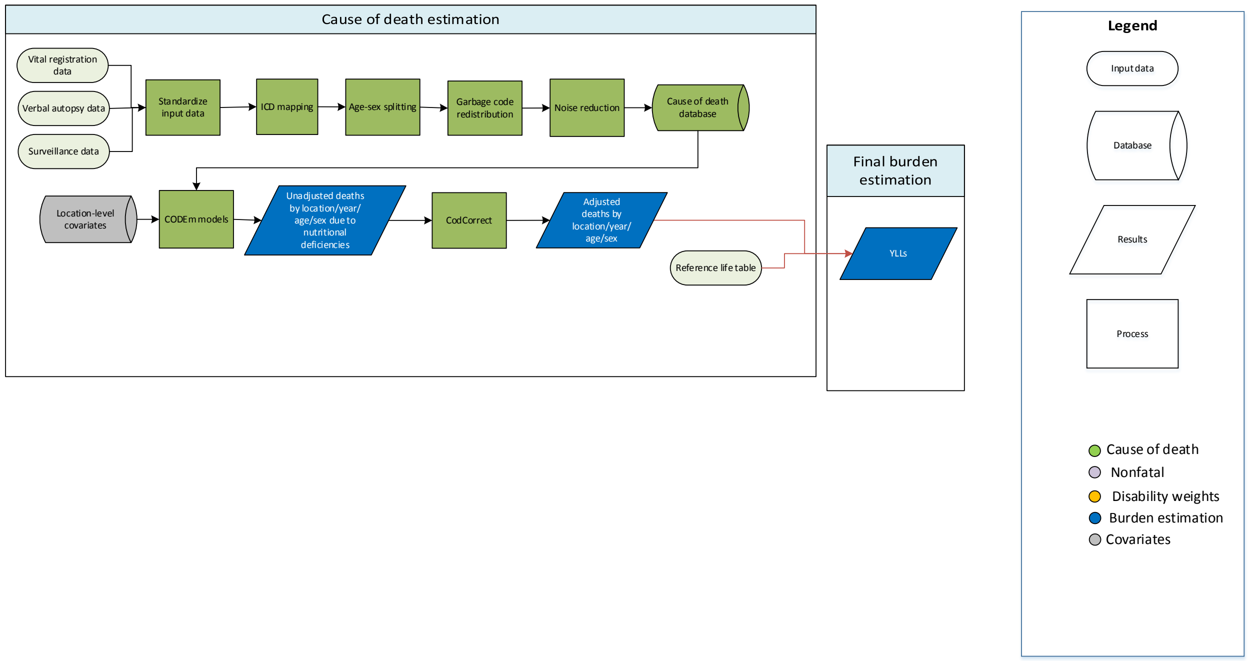

Input data

Vital registration, verbal autopsy and surveillance data were used. We outliered data that were largely conflicting with the majority of data from other studies conducted either in the same or different countries (with similar sociodemographic characteristics) in the same region.

Modeling strategy

We used a general CODEm strategy to model nutritional deficiencies. There were no substantive changes from GBD 2013 in terms of modeling strategy.

Protein energy malnutrition

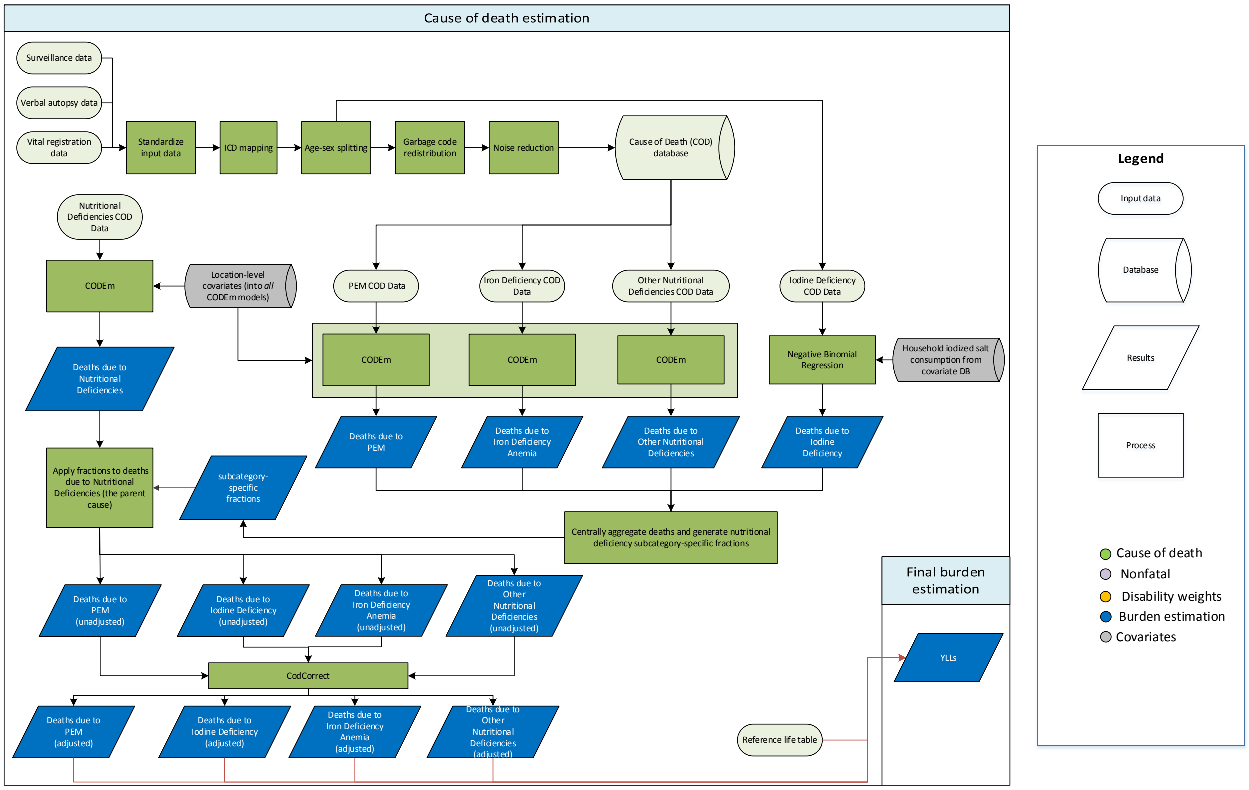

Input data

Vital registration, verbal autopsy, and surveillance data were used. We outliered data that were largely conflicting with the majority of data from other studies conducted either in the same or different countries (with similar sociodemographic characteristics) in the same region

Modeling strategy

We estimated mortality due to protein-energy malnutrition (PEM) in two steps. CODEm was first used to generate mortality estimates for total nutritional deficiencies. The sub-categories of nutritional deficiencies, namely, PEM, iodine deficiency, iron-deficiency anemia, and other nutritional deficiencies, were modeled separately. CODEm was used to model all sub-categories except for iodine deficiency, which we modeled using a negative binomial regression model given the small number of deaths attributable to it. Estimated deaths from four nutritional sub-categories were then aggregated centrally at the 1,000 draw level to generate cause fractions for each sub-category. These cause fractions were then applied centrally to the total nutritional deficiencies CODEm estimates to generate mortality estimates for PEM, iodine deficiency, iron-deficiency anemia, and other nutritional deficiencies, respectively.

Iron deficiency anemia

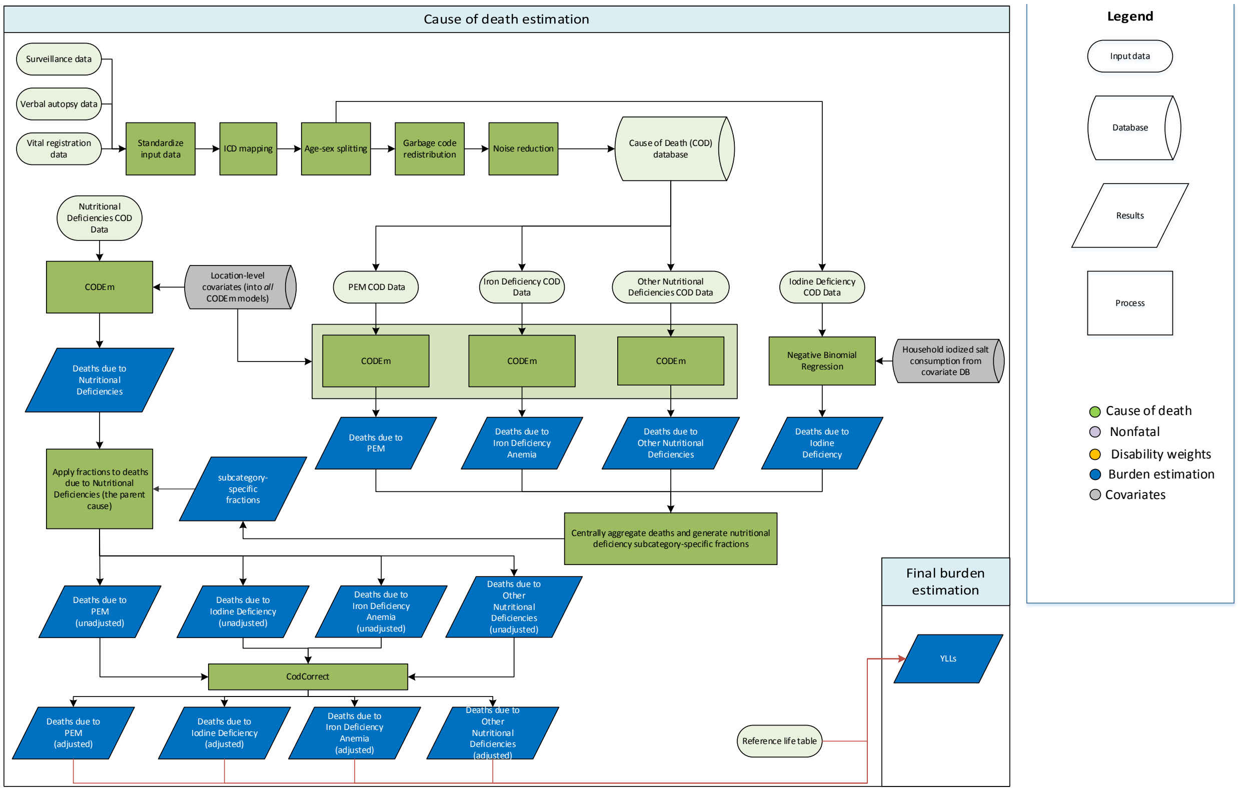

Input data

Vital registration, verbal autopsy, and surveillance data were used. We outliered data that were largely conflicting with the majority of data from other studies conducted either in the same or different countries (with similar sociodemographic characteristics) in the same region.

Modeling strategy

We estimated mortality due to iron-deficiency anemia in two steps. CODEm was first used to generate mortality estimates for total nutritional deficiencies. The sub-categories of nutritional deficiencies, namely protein-energy malnutrition, iodine deficiency, iron-deficiency anemia, and other nutritional deficiencies, were modeled separately. CODEm was used to model all sub-categories except for iodine deficiency, which we modeled using a negative binomial regression model given the small number of deaths attributable to it. Estimated deaths from four nutritional sub-categories were then aggregated centrally at the 1,000 draw level to generate cause fractions for each sub-category. These cause fractions were then applied centrally to the total nutritional deficiencies CODEm estimates to generate mortality estimates for protein energy malnutrition, iodine deficiency, iron-deficiency anemia, and other nutritional deficiencies respectively.

Other nutritional deficiencies

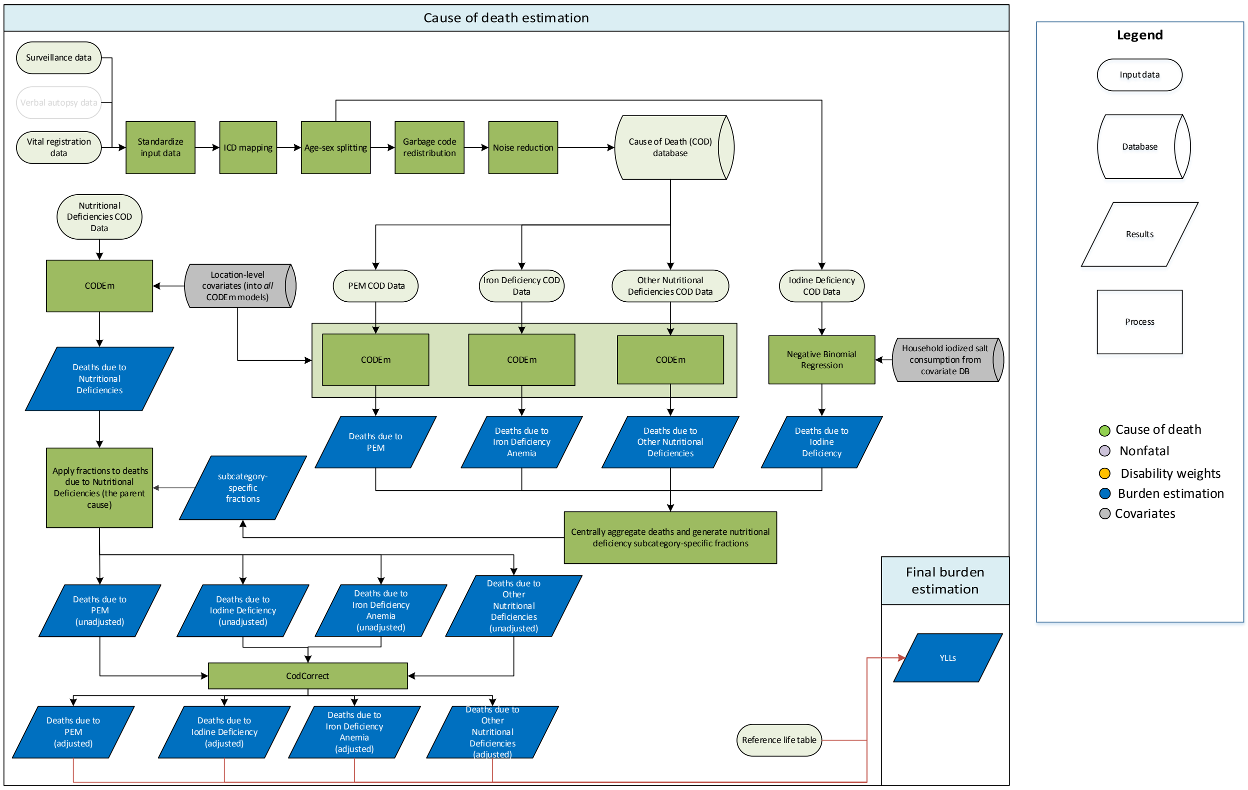

Input data

Vital registration and surveillance data were used. We outliered data that were largely conflicting with the majority of data from other studies conducted either in the same or different countries (with similar sociodemographic characteristics) in the same region.

Modeling strategy

We estimated mortality due to other nutritional deficiencies in two steps. CODEm was first used to generate mortality estimates for total nutritional deficiencies. The sub-categories of nutritional deficiencies, namely protein-energy malnutrition (PEM), iodine deficiency, iron-deficiency anemia, and other nutritional deficiencies, were modeled separately. CODEm was used to model all sub-categories except for iodine deficiency, which we modeled using a negative binomial regression model given the small number of deaths attributable to it. Estimated deaths from four nutritional sub-categories were then aggregated centrally at the 1,000 draw level to generate cause fractions for each sub-category. These cause fractions were then applied centrally to the total nutritional deficiencies CODEm estimates to generate mortality estimates for PEM, iodine deficiency, iron-deficiency anemia and other nutritional deficiencies, respectively.

Hepatitis

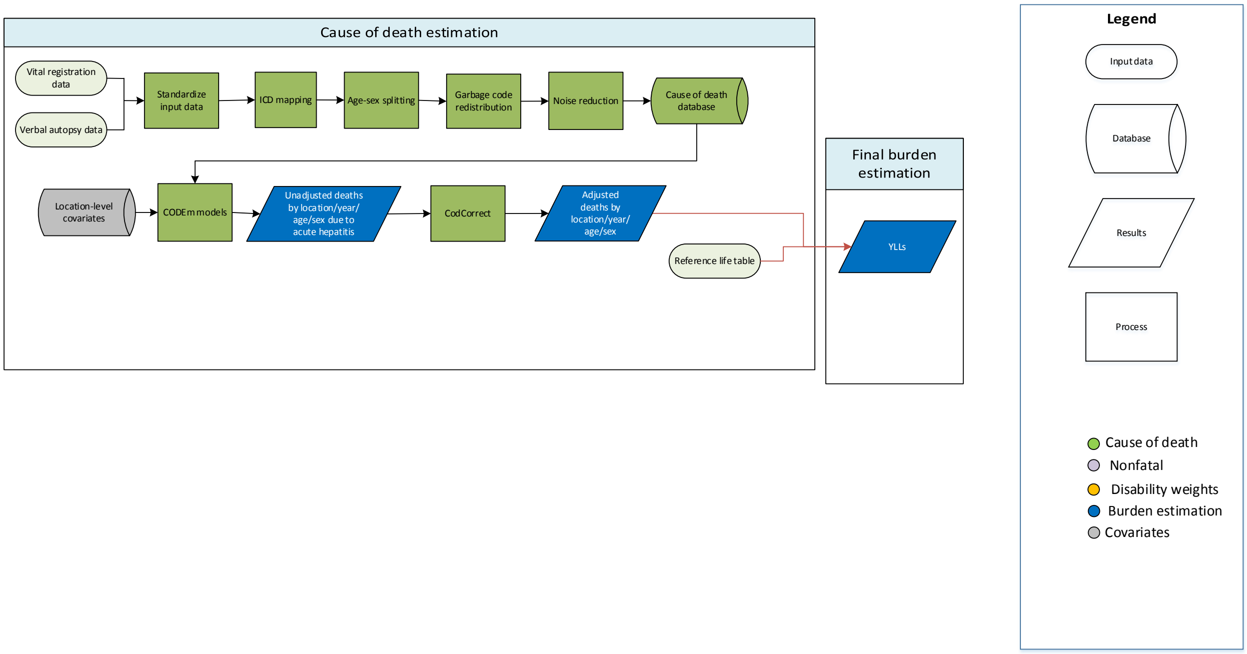

Input data

We modeled hepatitis mortality using all available data in the cause of death database. Data points were outliered if they reported an improbable number of hepatitis deaths or if their inclusion in the model yielded distorted trends. In some cases multiple data sources for the same location differed dramatically both in their quality and reported rabies mortality (e.g., a verbal autopsy and vital registration source). In these cases the lower-quality data source was outliered.

Modeling strategy

We modeled hepatitis mortality using a two-model hybrid approach: 1) a global CODEm model of all locations, using all data in the CoD database; and 2) a CODEm model restricted to data-rich countries. Since GBD 2013 we have switched from a single global model to the hybrid global/data-rich model approach. We have otherwise made no substantive changes in the modeling strategy for hepatitis from GBD 2013.

Other Infectious Diseases

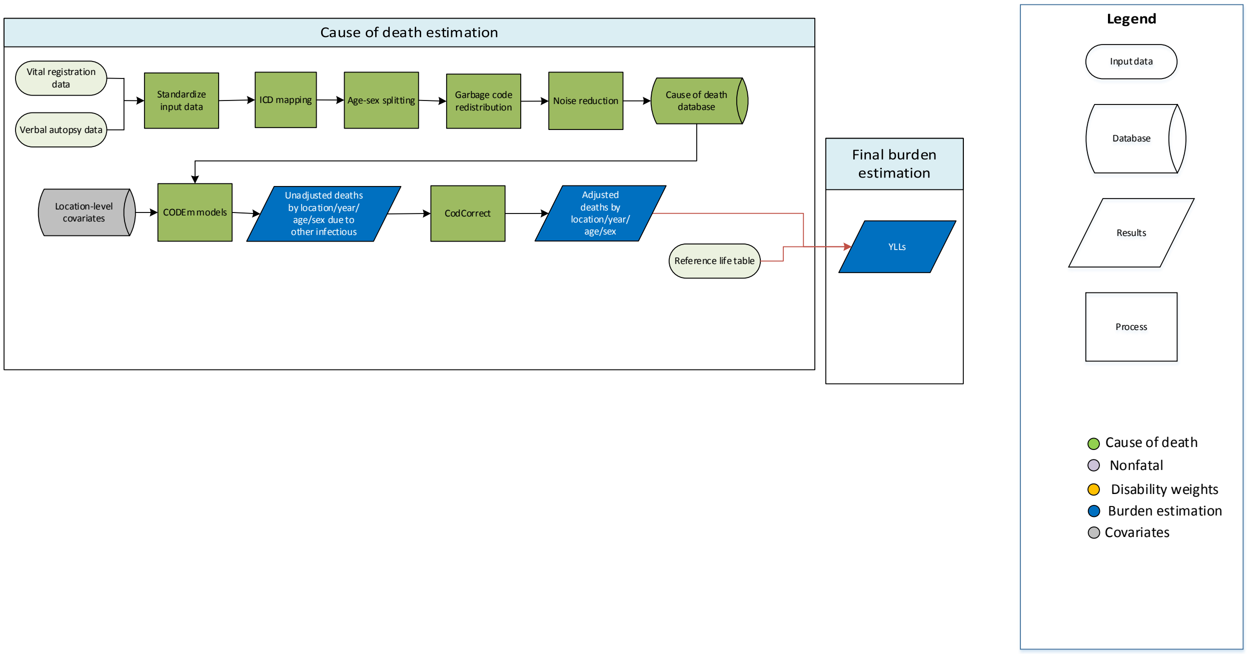

Input data

We modeled other infectious disease mortality using all available data in the cause of death database. Data points were outliered if they reported an improbable number of deaths or if their inclusion in the model yielded distorted trends.

Modeling strategy

We modeled other infectious disease mortality using a two-model hybrid approach: 1) a global CODEm model of all locations, using all data in the CoD database; and 2) a CODEm model restricted to data-rich countries.

Since GBD 2013 we have switched from a single global model to the hybrid global/data-rich model approach. We have otherwise made no substantive changes in the modeling strategy for other infectious disease from GBD 2013.

Cancers

Input data and methodological summary for all cancers except for liver cancer and non-melanoma skin cancer

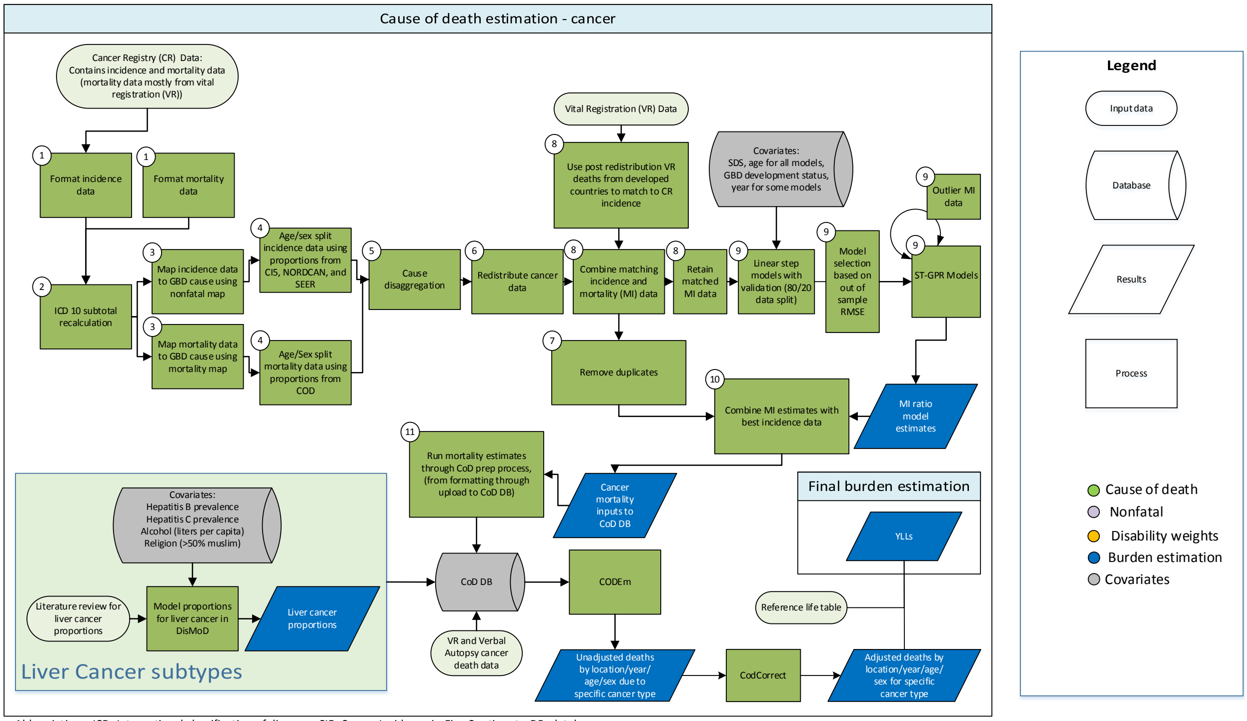

Abbreviations: ICD: International classification of diseases; CI5: Cancer Incidence in Five Continents, DB: database

Data

The Cause of Death (COD) database contains multiple sources of cancer mortality data. These sources include vital registration, verbal autopsy, and cancer registry data. The cancer registry mortality estimates that are uploaded into the COD database stem from cancer registry incidence data that have been transformed to mortality estimates through the use of mortality-to-incidence (MI) ratios.

Data seeking processes

Cancer mortality data in the cause of death database other than cancer registry data

Sources for cancer mortality data other than cancer registry data are described in the COD database description (Part 2).

Cancer registry data

All cancer registry data used for GBD 2010 were also included for GBD 2013, and the majority of these data were also used for GBD 2015 unless superseded by newer data (see step 7 in flow chart and below). Most new data were added based on availability and collaborator recommendation. Some new data were acquired and approved for GBD 2013 but were received after the deadline for adding new data to GBD 2013. More than half (56%) of the final incidence data and 35% of the final MI model input data came from the Cancer Incidence in Five Continents series (CI5).[1–10]

Cancer registry data were most often downloaded from a publicly available webpage or provided by collaborators. Most cancer registries only report cancer incidence. However, if a cancer registry also reported cancer mortality, mortality data were also extracted from the source to be used in the mortality-to-incidence estimation.

Inclusion and exclusion criteria

Only population-based cancer registries were included, and only those that included all cancers (no specialty registries), data for all age groups, and data for both sexes. Pathology-based cancer registries were included if they had a defined population. Hospital-based cancer registries were not included.

Cancer registry data were excluded from either the final incidence data input or the MI model input if a more detailed source (e.g., providing more detailed age or diagnostic groups) was available for the same population. Preference was given to registries with national coverage over those with only local coverage, except those from countries where the GBD study provides subnational estimates; thus some data were excluded because newly acquired national registry data could replace a regionally representative predecessor.

Data were excluded from the final incidence data input if the coverage population was unknown.

Bias of categories of input data

Cancer registry data can be biased in multiple ways. A high proportion of ill-defined cancer cases in the registry data requires redistribution of these cases to other cancers, which introduces a potential for bias. Changes between coding systems can lead to artificial differences in disease estimates; however, we adjust for this bias by mapping the different coding systems to the GBD causes. Underreporting of cancers that require advanced diagnostic techniques (e.g., leukemia and brain, pancreatic, and liver cancer) can be an issue in cancer registries from low-income countries. On the other hand, misclassification of metastatic sites as primary cancer can lead to overestimation of cancer sites that are common sites for metastases like brain or liver. Since many cancer registries are located in urban areas, the representativeness of the registry for the general population can also be problematic. The accuracy of mortality data reported in cancer registries usually depends on the quality of the vital registration system. If the vital registration system is incomplete or of poor quality, the mortality-to-incidence ratio can be biased to lower ratios.

Methods

Overall methodological process

See cancer flowchart.

Steps of analysis and data transformation processes

Cancer registry data went through multiple processing steps before integration with the COD database. First, the original data were transformed into standardized files, which included standardization of format, categorization, and registry names (#1 in flowchart).

Second, some cancer registries report individual codes as well as aggregated totals [e.g., C18, C19, and C20 are reported individually but the aggregated group of C18-C20 (colorectal cancer) is also reported in the registry data]. The data processing step "subtotal recalculation" (#2 in flowchart) verifies these totals and subtracts the values of any individual codes from the aggregates.

In the third step (#3 in the flowchart), cancer registry incidence data and cancer registry mortality data are mapped to GBD causes. A different map is used for incidence and for mortality data because of the assumption that there are no deaths for certain cancers. One example is basal cell carcinoma of the skin. In the cancer registry incidence data, basal cell carcinoma is mapped to non-melanoma skin cancer (basal cell carcinoma). However, if basal cell skin cancer is recorded in the cancer registry mortality data, the deaths are instead mapped to non-melanoma skin cancer (squamous cell carcinoma) under the assumption that they were indeed misclassified squamous cell skin cancers. Other examples are benign or in situ neoplasms. Benign or in situ neoplasms found in the cancer registry incidence dataset were simply dropped from that dataset. The same neoplasms reported in a cancer registry mortality dataset were mapped to the respective invasive cancer (e.g., melanoma in situ in the cancer registry incidence dataset was dropped from the dataset; melanoma in situ in the cancer registry mortality dataset was mapped to melanoma).

In the fourth data processing step (#4 in the flowchart) cancer registry data were standardized to the GBD age groups. Age-specific incidence rates were generated using CI5, SEER, and NORDCAN data, while age-specific mortality rates were generated from the CoD data through a method described in Part 2. Age-specific weights were then generated by applying the age-specific rates to a given registry population that required age-splitting to produce the expected number of cases/deaths for that registry by age. The expected number of cases/deaths for each sex, age, and cancer were then normalized to 1, creating final, age-specific proportions. These proportions were then applied to the total number of cases/deaths by sex and cancer to get the age-specific number of cases/deaths.

In the rare case that the cancer registry only contained data for both sexes combined, the now-age-specific cases/deaths were split and re-assigned to separate sexes using the same weights that are used for the age-splitting process. Starting from the expected number of deaths, proportions were generated by sex for each age (e.g., if for ages 15 to 19 years old there are six expected deaths for males and four expected deaths for females, then 60% of the combined-sex deaths for ages 15-19 years would be assigned to males and the remaining 40% would be assigned to females).

In the fifth step (#5 in the flowchart) data for cause entries that are aggregates of GBD causes were redistributed. Examples of these aggregated causes include some registries reporting ICD10 codes C00-C14 together as, "lip, oral cavity, and pharyngeal cancer." These groups were broken down into sub-causes that could be mapped to single GBD causes. In this example, those include lip and oral cavity cancer (C00-C08), nasopharyngeal cancer (C11), cancer of other parts of the pharynx (C09-C10, C12-C13), and "Malignant neoplasm of other and ill-defined sites in the lip, oral cavity, and pharynx" (C14). To redistribute the data, weights were created using the same "rate-applied-to-population" method employed in age-sex splitting (see step four above). For the undefined code (C14 in the example) an "average all cancer" weight was used, which was generated by adding all cases from SEER/NORDCAN/CI5 and dividing the total by the combined population. Then, proportions were

generated by sub-cause for each aggregate cause as in the sex-splitting example above (see step four). The total number of cases from the aggregated group (C00-C14) was then recalculated for each subgroup and the undefined code (C14). C14 was then redistributed as a "garbage code" in step six. Distinct proportions were used for C44 (non-melanoma skin cancer) and C46 (Kaposi's sarcoma). Population data were not used to redistribute data for these ICD codes. Non-melanoma skin cancer processing is described under section "Input data and methodological summary for non-melanoma skin cancer (squamous-cell carcinoma)." C46 entries were redistributed as "other cancer," HIV, and C80 (other and unknown cancers) using proportions described in Part 2.

In the sixth step (#6 in the flowchart) unspecified codes ("garbage codes") were redistributed. Redistribution of cancer registry incidence and mortality data mirrored the process of the redistribution used in the cause of death database (Part 2).

In the seventh step (#7 in the flowchart) duplicate or redundant sources were removed from the processed cancer registry dataset. Duplicate sources were present if, for example, the cancer registry was part of the CI5 dataset but we also had data from the registry directly. Redundancies occurred and were removed as described in "Inclusion and Exclusion Criteria," where more detailed data were available, or when national registry data could replace regionally representative data. From here, two parallel selection processes were run to generate input data for the MI models and to generate incidence for final mortality estimation. Higher priority was given to registry data from the most standardized source when creating the final incidence input (generally CI5 data), whereas preference was given to registry data from sources with matching mortality and incidence for the MI model input (in order to reduce confounding due to oppositional input biases when matching the two data types).

In the eighth step (#8 in the flowchart) the processed incidence and mortality data from cancer registries were matched by cancer, age, sex, year, and location to generate MI ratios. Because some cancer registries do not report mortality data – even though high-quality vital registration system data are available to the registry's coverage area – processed vital registration mortality data from the CoD database were matched to the registry's incidence data for some countries. This was the case for certain registries in the following countries: Australia, Austria, Belgium, Bulgaria, Denmark, Estonia, Finland, Hungary, Iceland, Ireland, New Zealand, Norway, South Korea, and Switzerland.

The ninth step involved creating and selecting the MI models. All models were run separately by cancer, and the best model was selected from the following list (see Table below).

1. $\text{logit}\left(MI\ ratio_{c,a,s,t}\right) = \alpha + \beta_1 SDS_{c,t} + \sum_a^A \beta_2 I_a + \beta_3 I_s + \theta_c + \epsilon_{c,a,s,t}$
2. $\text{logit}\left(MI\ ratio_{c,a,s,t}\right) = \alpha + \beta_1 SDS_{c,t} + \sum_a^A \beta_2 I_a + \beta_3 I_s + \beta_4 t + \theta_c + \epsilon_{c,a,s,t}$
3. $\text{logit}\left(MI\ ratio_{c,a,s,t}\right) = \alpha + \beta_1 SDS_{c,t} + \sum_a^A \beta_2 I_a + \beta_3 I_s + \beta_4 DS + \theta_c + \epsilon_{c,a,s,t}$
4. $\text{logit}\left(MI\ ratio_{c,a,s,t}\right) = \alpha + \beta_1 SDS_{c,t} + \sum_a^A \beta_2 I_a + \beta_3 I_s + \beta_4 DS + \beta_5 t + \theta_c + \epsilon_{c,a,s,t}$
5. $\text{logit}\left(MI\ ratio_{c,a,s,t}\right) = \alpha + \beta_1 SDS_{c,t} + \sum_a^A \beta_2 I_a + \beta_3 I_s + \theta_c + \lambda_{SR}(SDS_{c,t}) + \beta_4 t + \epsilon_{c,a,s,t}$
6. $\text{logit}\left(MI\ ratio_{c,a,s,t}\right) = \alpha + \beta_1 SDS_{c,t} + \sum_a^A \beta_2 I_a + \beta_3 I_s + \theta_c + \lambda_{SR}(SDS_{c,t}) + \epsilon_{c,a,s,t}$
7. $\text{logit}\left(MI\ ratio_{c,a,s,t}\right) = \alpha + \beta_1 SDS_{c,t} + \sum_a^A \beta_2 I_a + \beta_3 I_s + \theta_c + \lambda_{SR}(SDS_{c,t}) + \beta_4 DS + \epsilon_{c,a,s,t}$
8. $\text{logit}\left(MI\ ratio_{c,a,s,t}\right) = \alpha + \beta_1 SDS_{c,t} + \sum_a^A \beta_2 I_a + \beta_3 I_s + \theta_c + \lambda_{SR}(SDS_{c,t}) +$
   $\beta_4 t + \beta_5 DS + \epsilon_{c,a,s,t}$

c: country; a: age group; t: time (years); s: sex
I: indicator variable
DS: binary variable for development status
θ_c: random effect by country (intercept)
$\lambda_{SR}(SDSc, t)$: random effect modifier between SDI and super-region (slope)
$\epsilon_{c,a,s,t}$: error term

Table: MI models

All models were tested at multiple stages before creating the final model output. Models were initiated with an SDI covariate (Socio-Demographic Index) and first tested using the complete input dataset (Part 4). If after that initial test the SDI covariate's coefficient was negative (as expected), the next step was to outlier any data point for which the residual from the prediction was greater than three times the MAD from the mean residual. Next, data were marked as outliers due to a random effect criterion: if the country-level random effect for a lower-income country was lower than the random effect for the USA, all data points for that country were marked as outliers. This process was run iteratively until all lower-income countries had country-level random effects greater than that of the USA. All data points marked outliers were dropped from the final dataset, and that dataset was used to create the final model predictions.

If the SDI coefficient was found to be positive (unexpected) after the initial SDI test, it was assumed to indicate an excess of unrealistic data in the input dataset. To remove these unrealistic data, SDI was temporarily removed from the model formula. The model proceeded as above without SDI until all unrealistic data points were removed and the SDI coefficient was found to be negative. Unrealistic data were marked as outliers using the same residual MAD and random effect methodology described above. Once SDI was established as negative (expected) the model proceeded as usual.

To select the best model formula, the initial model results were tested by comparing mean MI predictions and the mean root-mean-squared error (RMSE) values of 10 random samples of 80%/20% splits from the input dataset. Mean MI predictions were compared between developing and developed countries. Models were eliminated if the mean MI for developing countries was lower than the mean MI ratio for developed countries. For RMSE testing, the dataset was split into an 80% dataset for model development and a 20% dataset for model testing. The process was repeated 10 times. The best model for each cancer was selected based on the lowest mean out-of-sample RMSE from those models remaining after checking the mean MI. The table below contains the final models selected for each cancer.

Cancer	Final model number (see numbering above)
Ovarian cancer	1
Uterine cancer	1
Gallbladder cancer	1
Kidney cancer	1

Larynx cancer	1
Acute lymphoid leukemia	1
Chronic myeloid leukemia	1
Lip and oral cavity cancer	1
Pancreatic cancer	1
Hodgkin lymphoma	2
Acute myeloid leukemia	2
Chronic lymphoid leukemia	2
Malignant skin melanoma	2
Bladder cancer	3
Brain and nervous system cancer	3
Esophageal cancer	3
Tracheal, bronchus, and lung cancer	3
Mesothelioma	3
Multiple myeloma	3
Other cancer	3
Prostate cancer	4
Testicular cancer	4
Breast cancer	4
Colorectal cancer	4
Leukemia	4
Liver cancer	4
Non-Hodgkin lymphoma	4
Non-melanoma skin cancer (squamous cell carcinoma)	4
Stomach cancer	4
Nasopharynx cancer	6
Cervical cancer	7
Other pharynx cancer	8
Thyroid cancer	8

Table: Final model selections

Once the best models were selected, data points were manually outliered based on the results of the first run of the model algorithm. Data points were outliered if they clearly influenced the model in an unrealistic way. For example, a data point was marked as an outlier if it created a single-year, single-age-group spike in model predictions. This was mainly the case in countries with a small number of cases or deaths, or in age groups with small numbers of cases or deaths. Manual outliers were removed from the input dataset prior to initiating the second run of the model algorithm.

After best models were selected, all final outliers were dropped from the data input, and final linear predictions were created, the final linear predictions and residuals were used as input for space-time smoothing. Space-time smoothing is a spatiotemporal regression to smooth residuals over space, time, and age.[11–13] The weighted residuals were added to the linear model predictions and used as priors for

the third stage, a Gaussian process regression (GPR) implementing a Matern covariance function.[13–18] GPR is a nonparametric technique for interpolating non-linear trends that has been used extensively in the estimation of time series data. Final MI ratio predictions with 95% uncertainty intervals were obtained by back-transforming 1,000 draws from the posterior distribution.

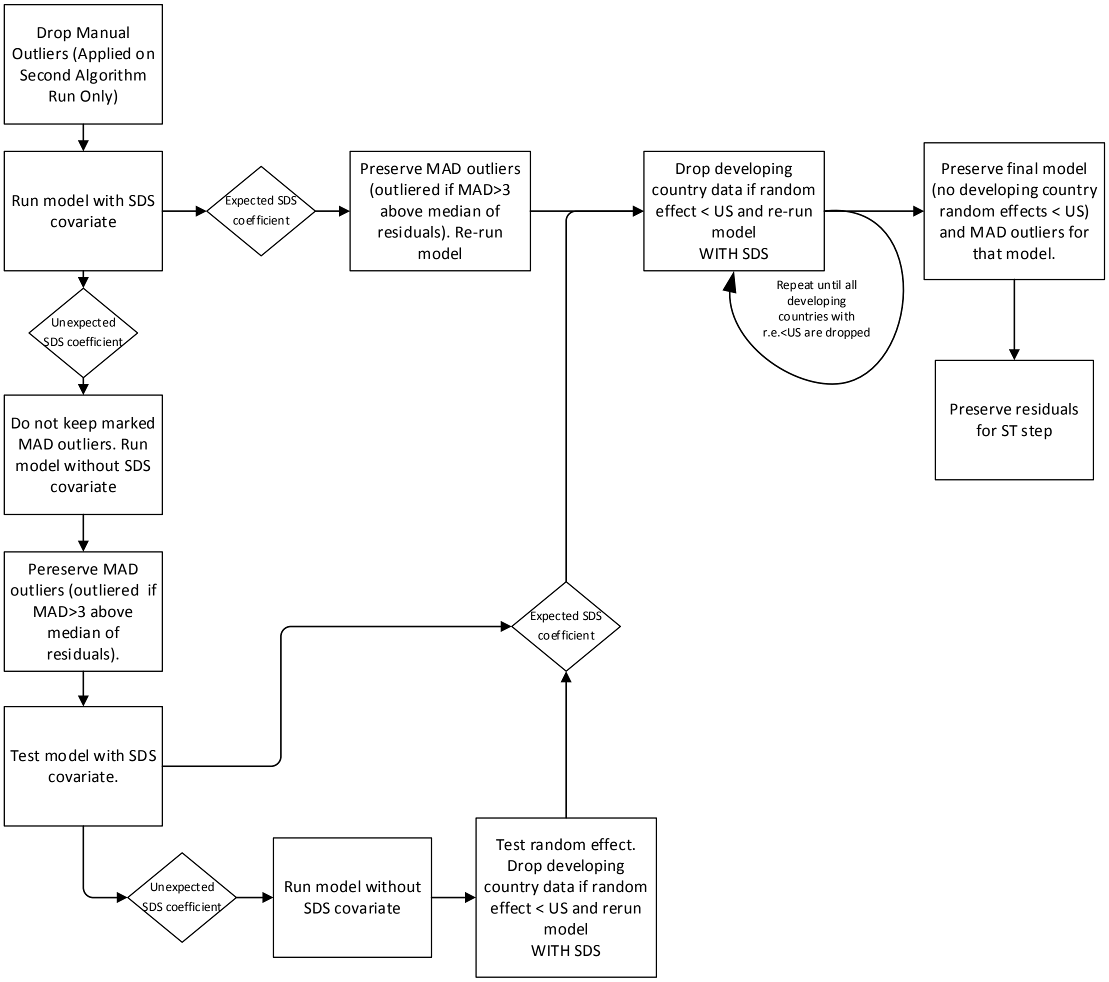

Figure: MI model estimation algorithm

Step 9 has undergone a revision compared to GBD 2010 and GBD 2013. In GBD 2010 and GBD 2013 only one model was used to predict all MI ratios, whereas for GBD 2015 we generated multiple models and chose a best model based on out-of-sample validation. Another major difference is that LDI (lagged distributed income) was used as a covariate in previous versions and was replaced by SDI for GBD 2015.

Final MI ratios were matched with the cancer registry incidence dataset in the ninth step (#10 in the flowchart) to generate mortality estimates (Incidence * Mortality/Incidence = Mortality). The final mortality estimates were then uploaded into the COD database (#11 in the flowchart).

After transforming cancer registry incidence data to mortality estimates, the modeling strategy followed the general CODEm process as described in Part 3.

Results

Interpretation of results

Cancer mortality estimates for GBD 2015 can differ from the GBD 2013 results for multiple reasons. First, compared to GBD 2013 more cancer mortality data were added to the cause of death database. Second, we added sources for cancer registry data, which were transformed into mortality estimates by using the MI ratio. Third, mapping of cancer ICD codes to the GBD cancer causes was updated slightly based on collaborator comments. One example is that mapping for the ICD10 code D46 (myelodysplastic syndrome) was changed from “other cancer” to “undefined cancer” for later redistribution to non-Hodgkin lymphoma and leukemia. The one major mapping change was the addition of subtypes for leukemia and non-melanoma skin cancer. Fourth, the method to redistribute undefined causes of death or undefined cancers changed compared to GBD 2013. Models for redistribution are now performed regionally rather than by super-region. Fifth, we updated and refined the mortality-to-incidence ratio estimation compared to GBD 2013. Whereas for GBD 2010 and GBD 2013 a single model was used to estimate the MI ratios for each location, by cancer, sex, and age, we developed multiple plausible models for GBD 2015 and chose the best model based on out-of-sample validation. Sixth, we reviewed the covariate inputs for the CODEm models and changed covariates when updated or improved covariates were available. Seventh, many covariates used in CODEm models were updated for GBD 2015 (Part 4).

The other group producing country-level cancer mortality estimates is the International Agency for Research on Cancer (IARC) with their GLOBOCAN database. Significantly different methods between the GBD study and GLOBOCAN can lead to differences in results. Whereas estimates in GLOBOCAN are based on the assumption that there are “In theory, […] as many methods as countries,”[19] the cancer estimation process for the GBD study follows a coherent, well-documented method for all cancers, which allows cross-validation of models as well as determination of uncertainty. Another major difference is the ability in the GBD study to adjust single cause estimates to the all-cause mortality, which is being determined independently. This also allows us to adjust individual causes of death to the all-cause mortality envelope which permits us to correct for the underdiagnosis of cancer in countries with inadequate diagnostic resources. Redistribution of a fraction of undefined causes of death to certain cancers is another methodical advantage the GBD study has over GLOBOCAN, and estimates for cancer mortality can therefore differ substantially in countries with a large proportion of undefined causes of deaths in their vital registration data or a large proportion of undefined cancer cases in their cancer registry data.

Limitations

There are certain limitations to consider when interpreting the GBD mortality cancer estimates. First, even though every effort is made to include the most recently available data for each country, data-seeking resources are not limitless and new data cannot always be accessed as soon as they are made available. It is therefore possible that the GBD study does not include all available data sources for cancer incidence or cancer mortality. Second, different redistribution methods can potentially change the cancer estimates substantially if the data sources used for the estimated location contain a large number of undefined causes; however, neglecting to account for these undefined deaths would likely

introduce an even greater bias in the disease estimates. Third, using mortality-to-incidence ratios to transform cancer registry incidence data to mortality estimates requires accurate MI ratios. For GBD 2015 the methodology to estimate MI ratios was improved with development of multiple different models and implementation of model cross-validation, but the method is still sensitive to underdiagnosis of cancer cases or underascertainment of cancer deaths. However, given that the majority of data used for the cancer mortality estimation come from vital registration data and not cancer registry data this is not a major limitation.

Non-melanoma skin cancer (squamous-cell carcinoma)

Data

Data seeking processes

The input data were identified and processed using the same methods as all other cancers described above.

Inclusion and exclusion criteria

Inclusion and exclusion criteria followed the same methods as described for other cancers (see above).

Bias of categories of input data

The potential biases of the input data are the same as for other cancers (see above).

Methods

Overall methodological process

The GBD produces estimates for non-melanoma skin cancer via two subgroups: non-melanoma skin cancer (basal cell carcinoma) and non-melanoma skin cancer (squamous cell carcinoma). While some cancer registries report non-melanoma skin cancer at the four- or five- digit level required to distinguish between the subtypes (e.g. “C44.01” vs. “C44.02”, “173.01” vs. “173.02), most registries report these cancers at the three-digit level as “C44” or “173” (“Other and unspecified malignant neoplasm of skin”). Because of this, those incident cases that were reported at this three-digit level were split to “basal cell carcinoma” and “squamous cell carcinoma” based on proportions reported by Karagas et al during the cause disaggregation step (step #5 in the flowchart).[20] Since mortality estimates are produced for squamous cell carcinoma under the assumption that basal cell carcinoma causes almost no deaths, all mortalities reported as “C44” or “173” were simply mapped to the “squamous cell carcinoma” GBD cause. Apart from this additional step for some incident cases, the remainder of the cancer registry processing was the same as for other cancers as described above.

Steps of analysis and data transformation processes

Non-melanoma skin cancer (squamous cell carcinoma) mortality estimation followed the same steps as the other cancers (see flowchart and description above) except for step #5 in the flowchart as described above.

Model selection

The modeling strategy for non-melanoma skin cancer (squamous cell carcinoma) followed the general CODEm process.

Model performance and sensitivity

The modeling performance and sensitivity for non-melanoma skin cancer (squamous cell carcinoma) mirrored that of the general CODEm process.

Uncertainty intervals

Uncertainty was determined using standard CODEm methodology.

Results

Interpretation of results

Non-melanoma skin cancer mortality estimates are not available from other sources. GLOBOCAN, for example, does not report deaths due to non-melanoma skin cancer. Even though the data availability for non-melanoma skin cancer is poor, the fact that it is the most common incident cancer with rates expected to rise makes it a necessity to include the disease in the GBD framework.

[4–13]Limitations

Cancer registry data for non-melanoma skin cancer incidence have to be interpreted with caution due to a substantial amount of underreporting or rules that only the first non-melanoma skin cancer has to be registered. Many cancer registries therefore do not include non-melanoma skin cancers at all. For vital registration data we make the assumption that there are no deaths due to non-melanoma skin cancer (basal cell carcinoma), therefore all deaths attributed to basal cell carcinoma were included instead as squamous cell carcinoma. Based on collaborator recommendations we will test this assumption formally for GBD 2016.

References

1 Cancer Incidence in Five Continents.Vol I. Geneva, Switzerland: Union Internationale Contre le Cancer, 1966.

2 Cancer Incidence in Five Continents.Vol II. Geneva, Switzerland: Union Internationale Contre le Cancer, 1970.

3 Cancer Incidence in Five Continents.Vol III. Lyon, France: IARC, 1976.

4 Cancer Incidence in Five Continents.Vol IV. Lyon, France: IARC, 1982.

5 Cancer Incidence in Five Continents.Vol V. Lyon, France: IARC, 1987.

6 Cancer Incidence in Five Continents.Vol VI. Lyon, France: IARC, 1992.

7 Cancer Incidence in Five Continents.Vol VII. Lyon, France: IARC, 1997.

8 Cancer Incidence in Five Continents.Vol VIII. Lyon, France: IARC, 2002.

9 Cancer Incidence in Five Continents.Vol IX. Lyon, France: IARC, 2007.

10 Cancer Incidence in Five Continents.Vol X. Lyon, France: IARC, 2013.

11 Wang H, Liddell CA, Coates MM, *et al.* Global, regional, and national levels of neonatal, infant, and under-5 mortality during 1990-2013: a systematic analysis for the Global Burden of Disease Study 2013. *Lancet* 2014; **384**: 957–79.

12 Forouzanfar MH, Foreman KJ, Delossantos AM, *et al.* Breast and cervical cancer in 187 countries between 1980 and 2010: a systematic analysis. *Lancet* 2011; **378**: 1461–84.

13 Foreman KJ, Lozano R, Lopez AD, Murray CJ. Modeling causes of death: an integrated approach using CODEm. *Popul Health Metr* 2012; **10**: 1.

14 Murray CJL, Rosenfeld LC, Lim SS, *et al.* Global malaria mortality between 1980 and 2010: a systematic analysis. *Lancet* 2012; **379**: 413–31.

15 GBD 2013 Mortality and Causes of Death Collaborators. Global, regional, and national age-sex specific all-cause and cause-specific mortality for 240 causes of death, 1990-2013: a systematic analysis for the Global Burden of Disease Study 2013. *Lancet* 2015; **385**: 117–71.

16 Murray CJL, Ortblad KF, Guinovart C, *et al.* Global, regional, and national incidence and mortality for HIV, tuberculosis, and malaria during 1990-2013: a systematic analysis for the Global Burden of Disease Study 2013. *Lancet* 2014; **384**: 1005–70.

17 Rajaratnam JK, Marcus JR, Flaxman AD, *et al.* Neonatal, postneonatal, childhood, and under-5 mortality for 187 countries, 1970-2010: a systematic analysis of progress towards Millennium Development Goal 4. *Lancet* 2010; **375**: 1988–2008.

18 Rasmussen CE, Williams CKI. Gaussian processes for machine learning. Cambridge, Mass.: MIT Press, 2006.

19 International Agency for Research on Cancer, World Health Organization. GLOBOCAN estimated cancer incidence, mortality, and prevalence worldwide in 2012. Lyon, France: IARC, 2014 http://globocan.iarc.fr/Default.aspx (accessed April 19, 2016).

20 Karagas MR, Greenberg ER, Spencer SK, Stukel TA, Mott LA. Increase in incidence rates of basal cell and squamous cell skin cancer in New Hampshire, USA. New Hampshire Skin Cancer Study Group. *Int J Cancer* 1999; **81**: 555–9.

Cardiovascular Diseases

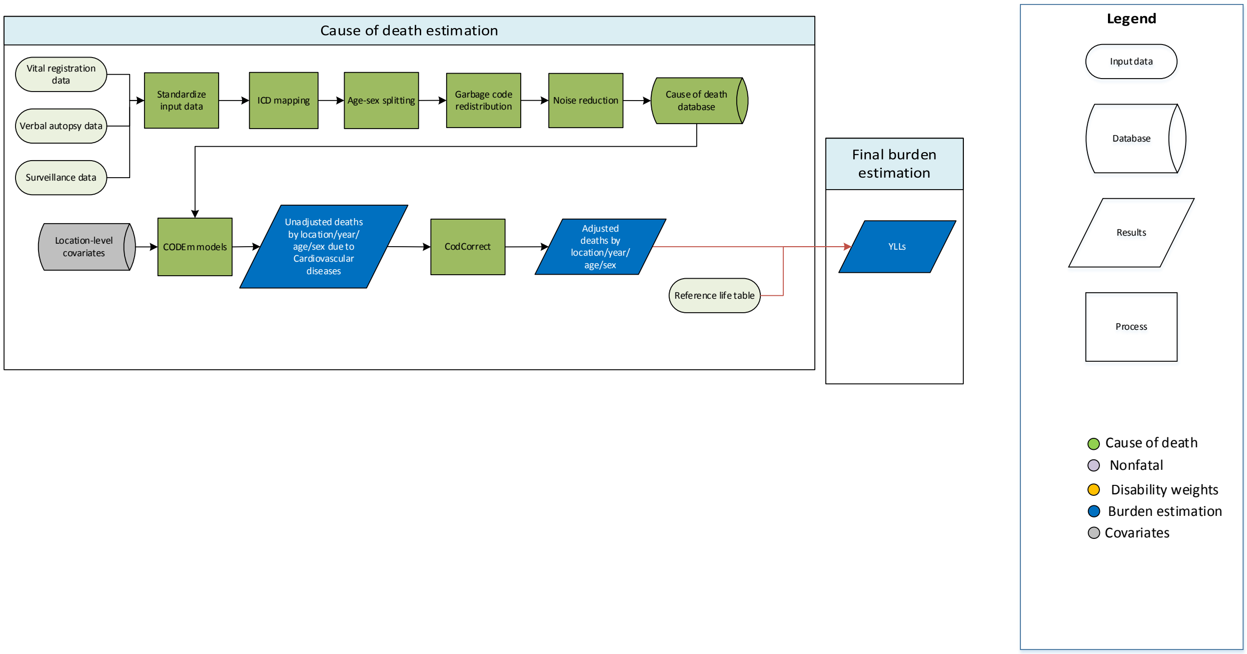

Input data

Vital registration, verbal autopsy, and surveillance data were used to model this cause. We outliered non-representative subnational verbal autopsies in a number of Indian states. We also outliered verbal autopsy data sources that were implausibly low in all age groups and ICD8 and ICD9 BTL data points that were inconsistent with the rest of the data and created implausible time trends.

Modeling strategy

We used a standard CODEm approach to model deaths from cardiovascular diseases. We have included two new variables, Socio-Demographic Index and the SEV scalar for rheumatic heart disease, as possible covariates for selection in the ensemble modeling process. Otherwise, there have been no substantive changes from the approach used in GBD 2013.

Rheumatic Heart Disease

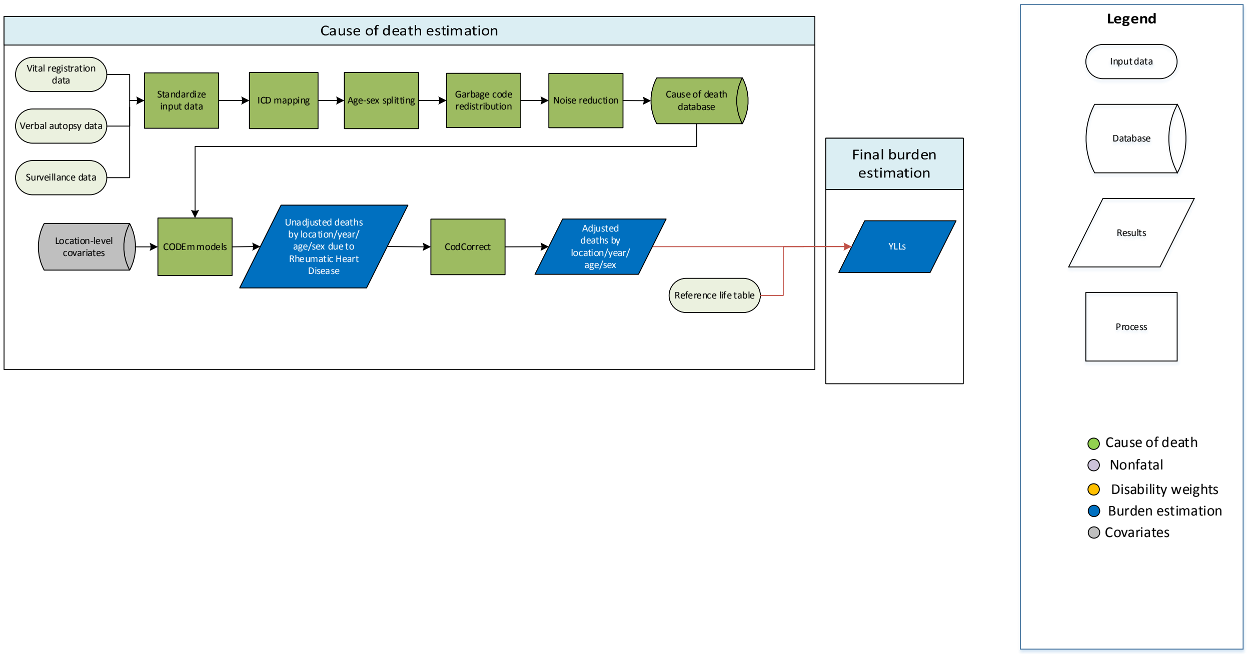

Input data

Vital registration, verbal autopsy, and surveillance data were used to model rheumatic heart disease. We outliered ICD8 and ICD9 BTL data points which were inconsistent with the rest of the data and created implausible time trends. We also outliered data points which were too high after the redistribution process in a number of age groups.

Modeling strategy

We used a standard CODEm approach to model deaths from rheumatic heart disease. We have included two new variables, Socio-Demographic Index and the SEV scalar for rheumatic heart disease, as possible covariates for selection in the ensemble modeling process. Otherwise, there have been no substantive changes from the approach used in GBD 2013.

Ischemic Heart Disease

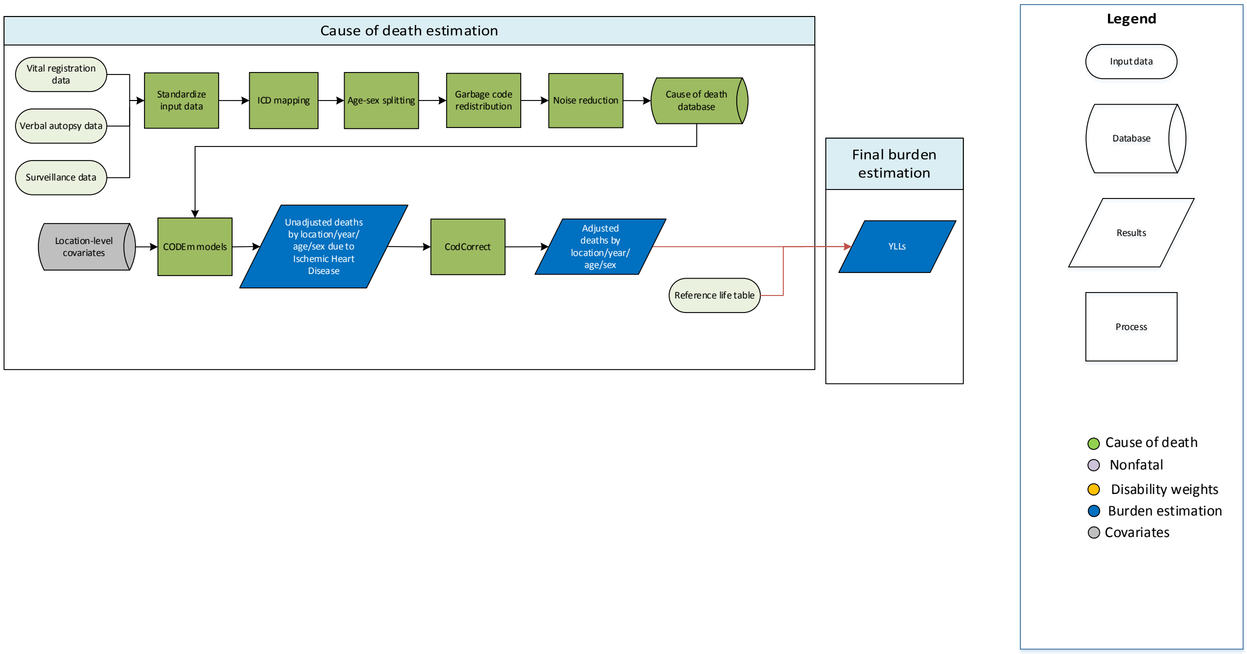

Input data

Vital registration, verbal autopsy, and surveillance data were used to model ischemic heart disease. We outliered verbal autopsy data in countries and subnational locations where high-quality vital registration data were also available. We also outliered non-representative subnational verbal autopsy data points, ICD8 and ICD9 BTL data points which were inconsistent with the rest of the data and created implausible time trends, and data in a number of Indian states identified by experts as poor-quality.

Modeling strategy

We used a standard CODEm approach to model deaths from ischemic heart disease. We have included two new variables, Socio-Demographic Index and the SEV scalar for ischemic heart disease, as possible covariates for selection in the ensemble modeling process. Otherwise, there have been no substantive changes from the approach used in GBD 2013.

Cerebrovascular Disease

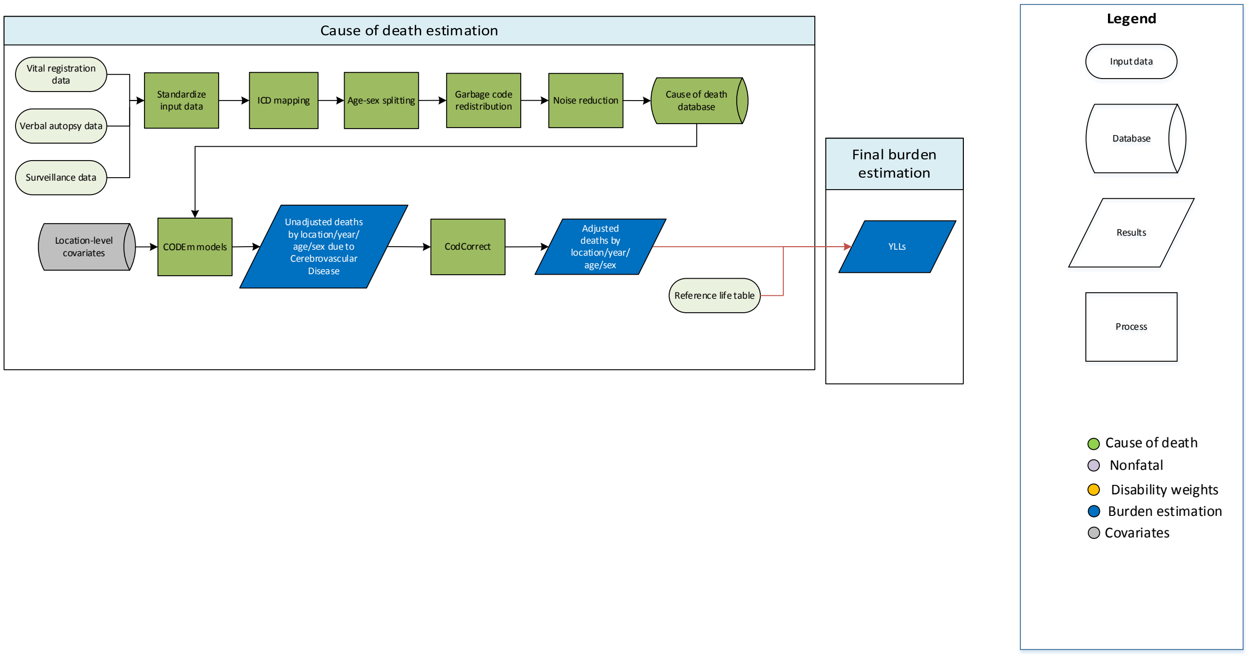

Input data

Verbal autopsy and vital registration data were used to model this cause. We outliered non-representative subnational verbal autopsy data points. We reassigned deaths from verbal autopsy reports for cerebrovascular disease to the parent cardiovascular disease for both sexes for those under 20 years of age. We also outliered ICD8, ICD9 BTL, and ICD10 Tabulated data points which were inconsistent with the rest of the data and created implausible time trends. Data points from sources which were implausibly low in all age groups and data points that were causing the regional estimates to be improbably high were outliered.

Modeling strategy

We used a standard CODEm approach to model deaths from cerebrovascular disease. We have included two new variables, Socio-Demographic Index and the SEV scalar for cerebrovascular disease, as possible covariates for selection in the ensemble modeling process. Otherwise, there have been no substantive changes from the approach used in GBD 2013.

Ischemic Stroke

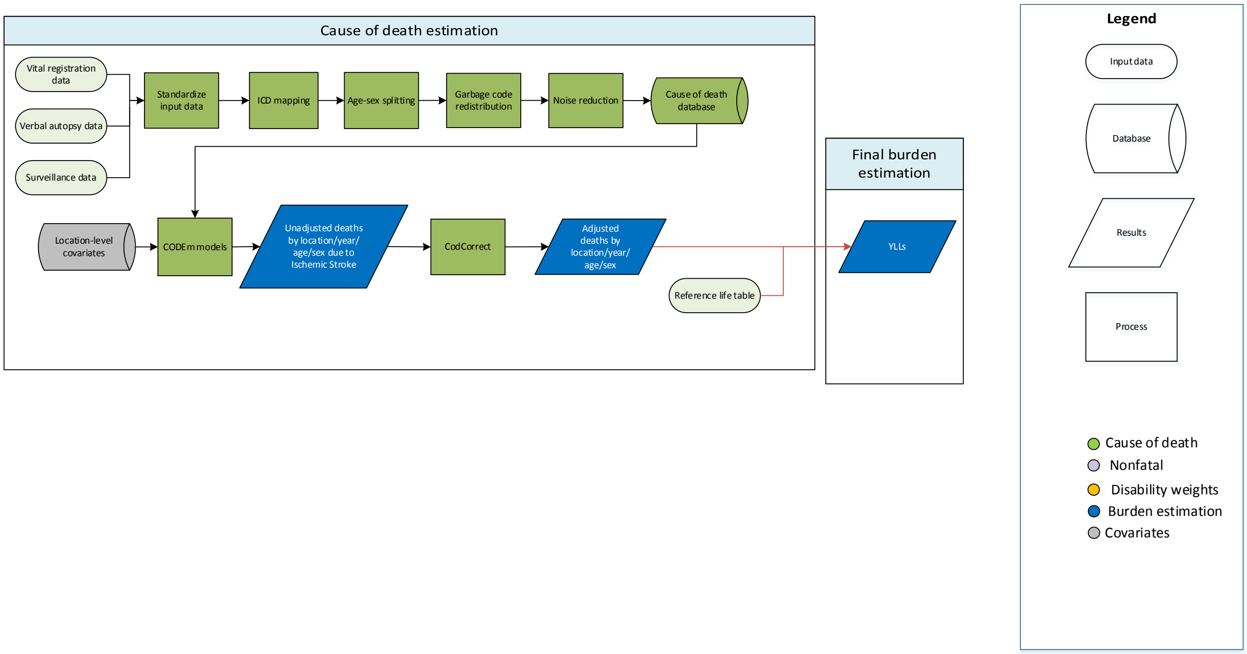

Input data

Vital registration, verbal autopsy, and surveillance data were used to model ischemic stroke. We reassigned deaths from verbal autopsy reports for ischemic stroke to the parent cardiovascular disease for both sexes for those under 20 years of age. We outliered ICD8 data points which were inconsistent with the rest of the data and created implausible time trends.

Modeling strategy

We used a standard CODEm approach to model deaths from ischemic stroke. In locations with limited data on ischemic stroke, the subtype-specific deaths were estimated by squeezing both ischemic and hemorrhagic stroke to the overall cerebrovascular envelope. We have included two new variables, Socio-Demographic Index and the SEV scalar for ischemic stroke, as possible covariates for selection in the ensemble modeling process. Otherwise, there have been no substantive changes from the approach used in GBD 2013.

Hemorrhagic Stroke

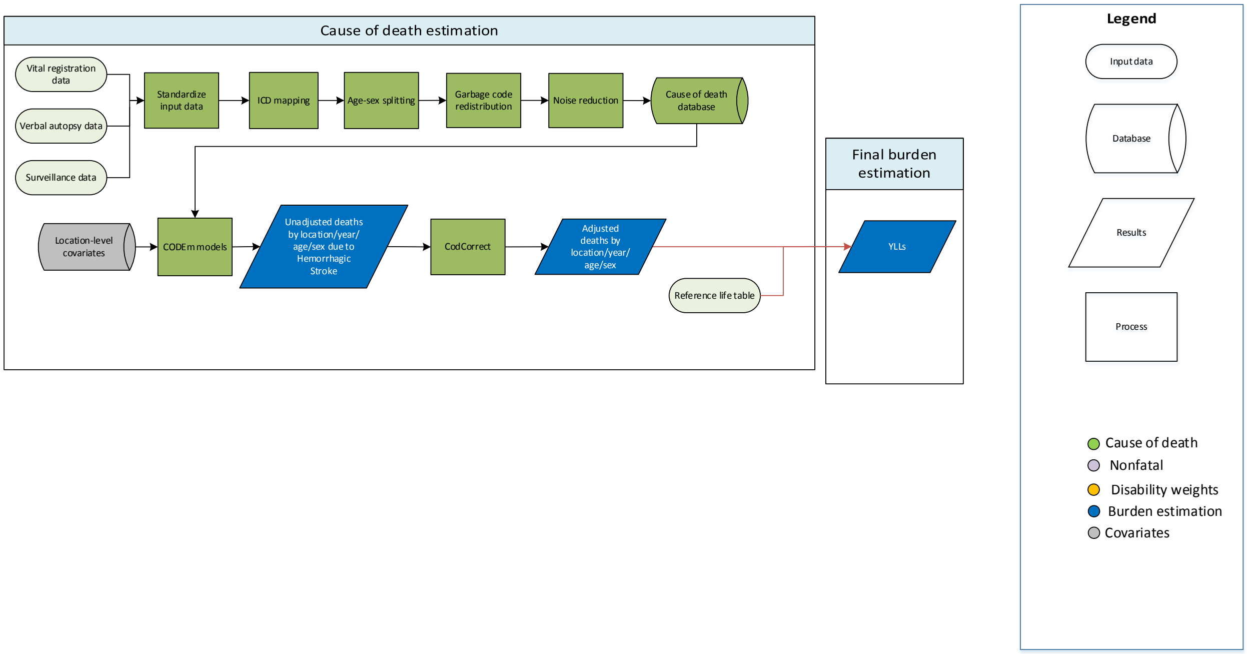

Input data

Vital registration, verbal autopsy, and surveillance data were used to model hemorrhagic stroke. We reassigned deaths from verbal autopsy reports for hemorrhagic stroke to the parent cardiovascular disease for both sexes for those under 20 years of age. We outliered ICD8 data points which were inconsistent with the rest of the data and created implausible time trends.

Modeling strategy

We used a standard CODEm approach to model deaths from hemorrhagic stroke. In locations with limited data on hemorrhagic stroke, the subtype-specific deaths were estimated by squeezing both ischemic and hemorrhagic stroke to the overall cerebrovascular envelope. We have included two new variables, Socio-Demographic Index and the SEV scalar for hemorrhagic stroke, as possible covariates for selection in the ensemble modeling process. Otherwise, there have been no substantive changes from the approach used in GBD 2013.

Hypertensive Heart Disease

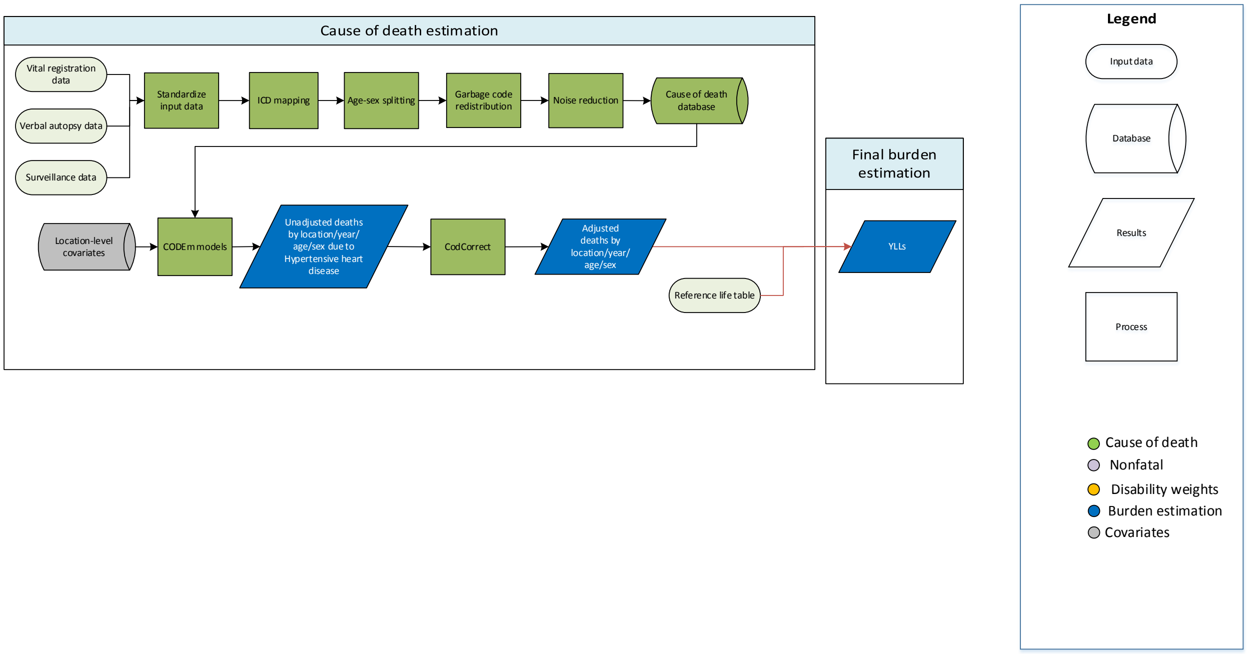

Input data

Vital registration, verbal autopsy, and surveillance data were used to model hypertensive heart disease. We outliered ICD9 BTL data points, which were inconsistent with the rest of the data and created implausible time trends.

Modeling strategy

We used a standard CODEm approach to model deaths from hypertensive heart disease. We have included two new variables, Socio-Demographic Index and the SEV scalar for hypertensive heart disease, as possible covariates for selection in the ensemble modeling process. Otherwise, there have been no substantive changes from the approach used in GBD 2013.

Cardiomyopathy and Myocarditis

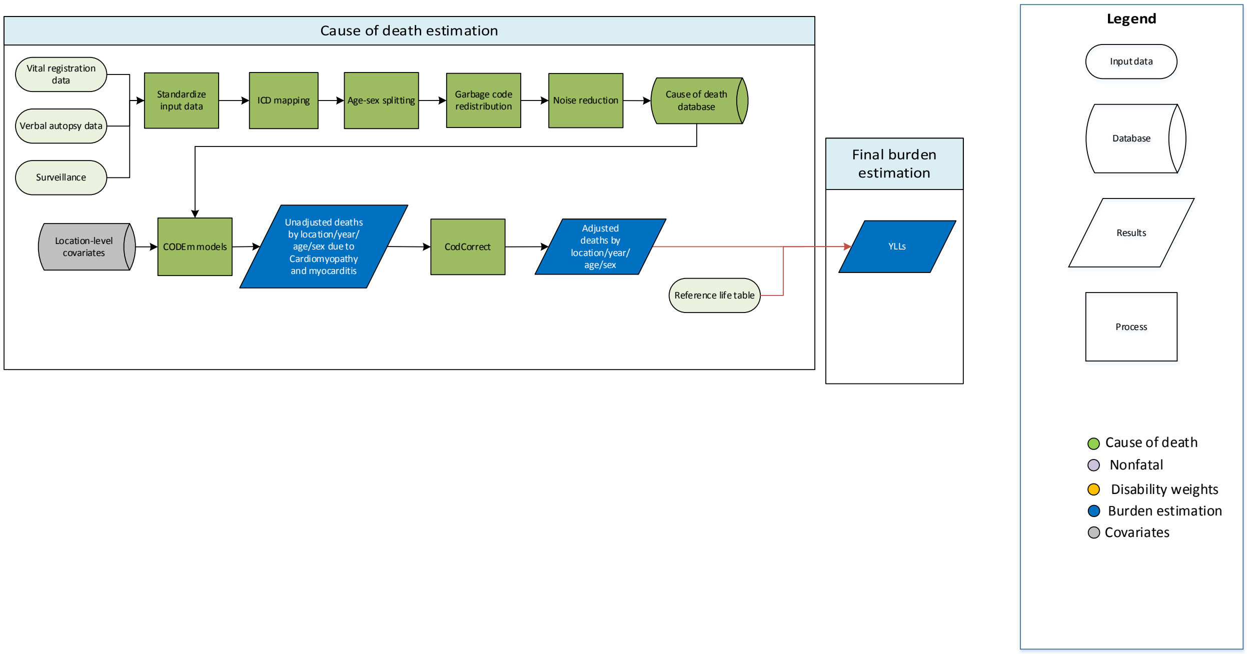

Input data

Vital registration, verbal autopsy, and surveillance data were used to model cardiomyopathy and myocarditis. We outliered data points in Central Asia and Central and Eastern Europe due to implausibly high values which we attributed to variation in local coding practices. We also outliered ICD8 data points in countries where they were discontinuous with other data in the time series.

Modeling strategy

We used a standard CODEm approach to model deaths from cardiomyopathy and myocarditis. We have included two new variables, Socio-Demographic Index and the SEV scalar for cardiomyopathy and myocarditis, as possible covariates for selection in the ensemble modeling process. Otherwise, there have been no substantive changes from the approach used in GBD 2013. Finally, local differences in coding practices may explain some of the geographic variation that we see for deaths due to cardiomyopathy; we plan to explore this issue further in future iterations of GBD.

Aortic Aneurysm

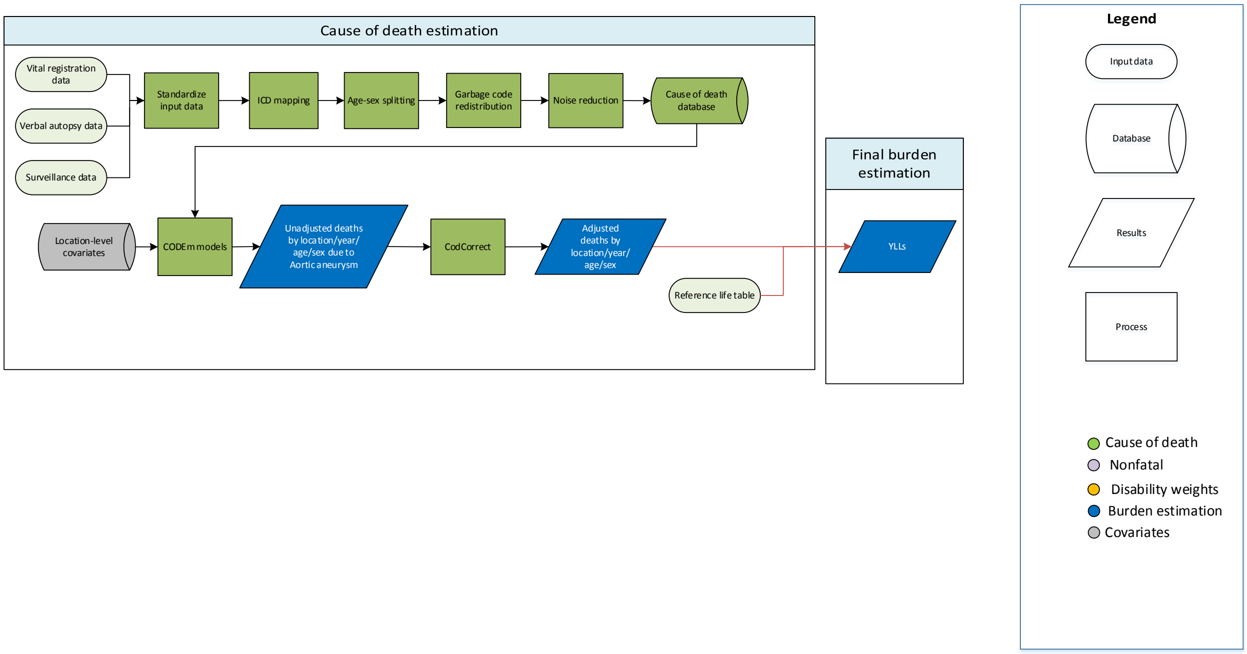

Input data

Vital registration, verbal autopsy, and surveillance data were used to model this cause. We outliered data in Oman as they were improbably high in comparison with the rest of the region. We also outliered ICD8 data that were discontinuous with the rest of the time series and created implausible time trends.

Modeling strategy

We used a standard CODEm approach to model deaths from aortic aneurysm. We have included two new variables, Socio-Demographic Index and the SEV scalar for aortic aneurysm, as possible covariates for selection in the ensemble modeling process. Otherwise, there have been no substantive changes from the approach used in GBD 2013.

Peripheral Vascular Disease

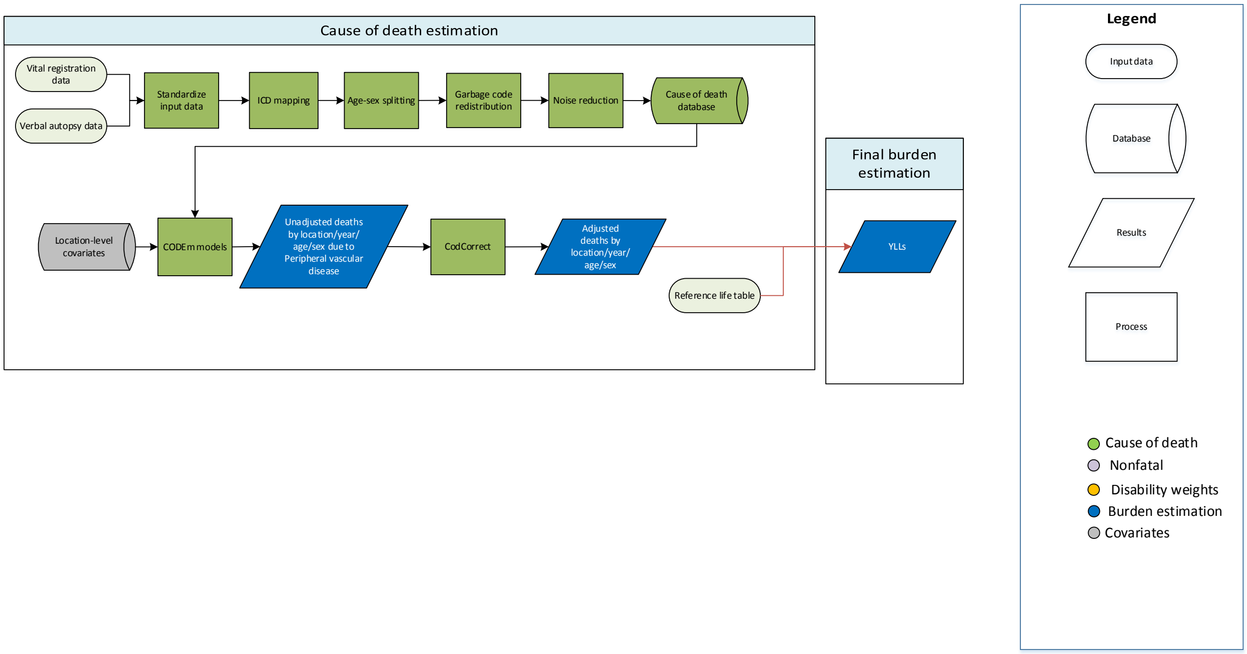

Input data

Vital registration and verbal autopsy data were used to model peripheral vascular disease. We outliered all data points with <1 death in Egypt per expert review.

Modeling strategy

We used a standard CODEm approach to model deaths from peripheral vascular disease. We have included two new variables, Socio-Demographic Index and the SEV scalar for peripheral vascular disease, as possible covariates for selection in the ensemble modeling process. Otherwise, there have been no substantive changes from the approach used in GBD 2013.

Endocarditis

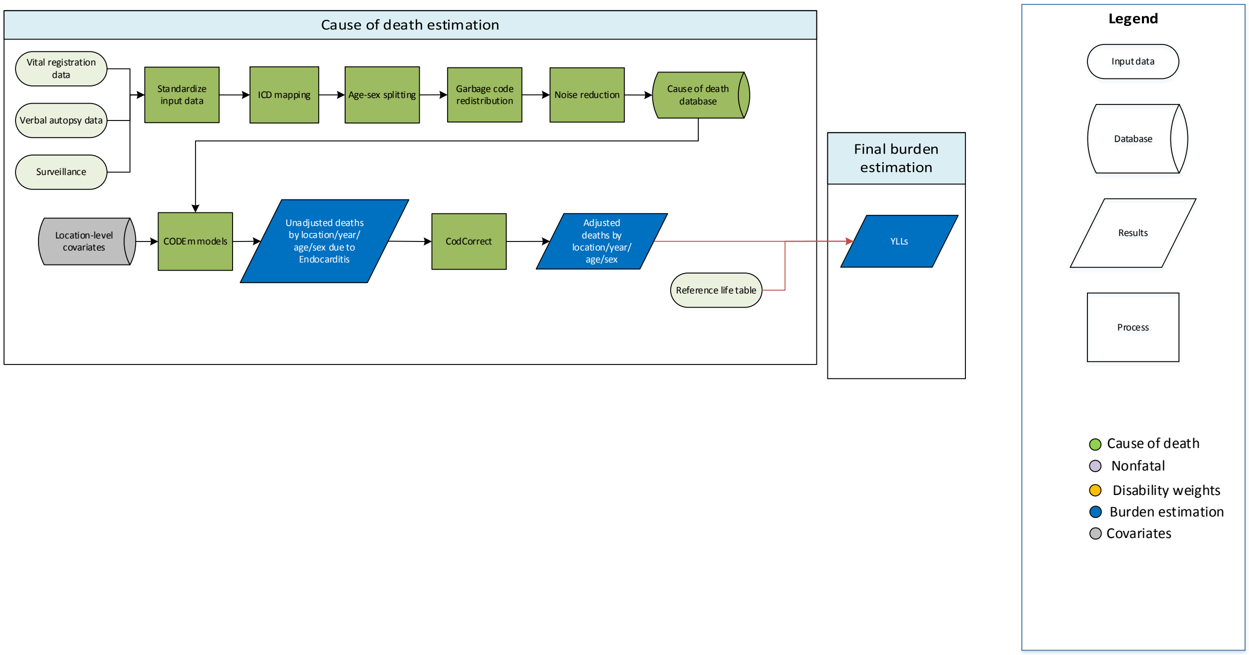

Input data

Vital registration, verbal autopsy, and surveillance data were used to model endocarditis. We outliered vital registration data in Mozambique as these were non-representative for sub-Saharan Africa and were causing regional estimates to be implausibly low. We also outliered ICD8 data that were discontinuous from the rest of the data series and created an implausible time trend.

Modeling strategy

We used a standard CODEm approach to model deaths from endocarditis. We have included two new variables, Socio-Demographic Index and the SEV scalar for endocarditis, as possible covariates for selection in the ensemble modeling process. Otherwise, there have been no substantive changes from the approach used in GBD 2013.

Other Cardiovascular and Circulatory Diseases

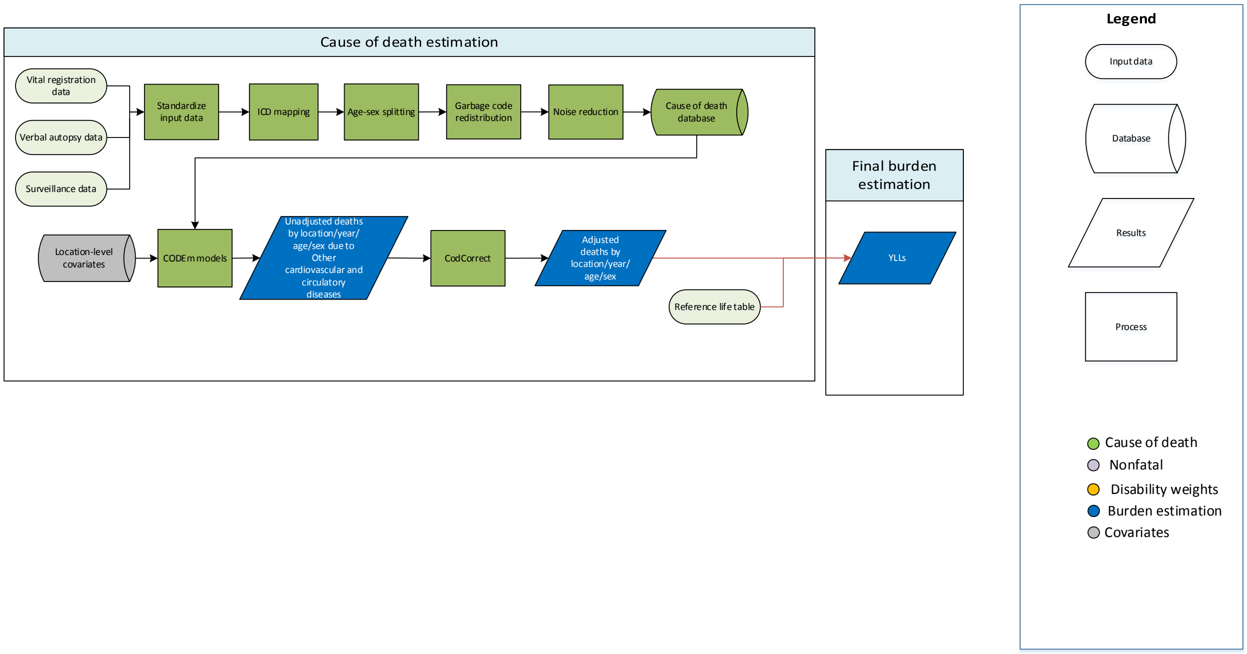

Input data

Vital registration, verbal autopsy, and surveillance data were used to model other cardiovascular and circulatory diseases. We outliered ICD8 and ICD9 BTL data points that were inconsistent with the rest of the data and created implausible time trends. We also outliered ICD8 data points which were not nationally representative.

Modeling strategy

We used a standard CODEm approach to model deaths from other cardiovascular and circulatory diseases. We have included two new variables, Socio-Demographic Index and the SEV scalar for other cardiovascular and circulatory diseases, as possible covariates for selection in the ensemble modeling process. Otherwise, there have been no substantive changes from the approach used in GBD 2013.

Chronic Respiratory Diseases

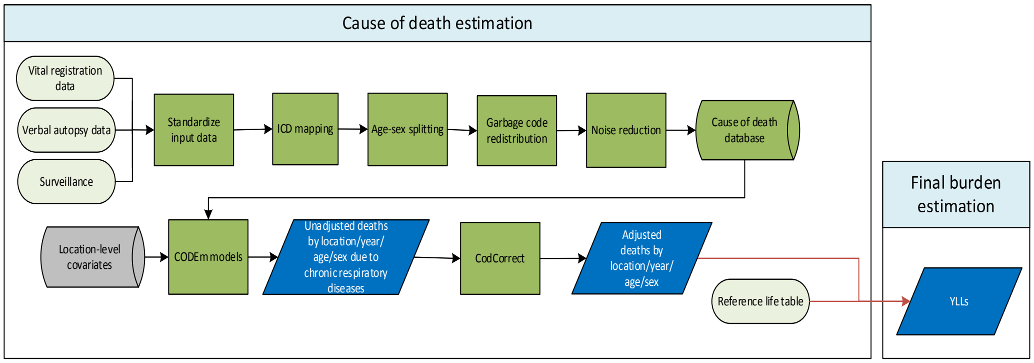

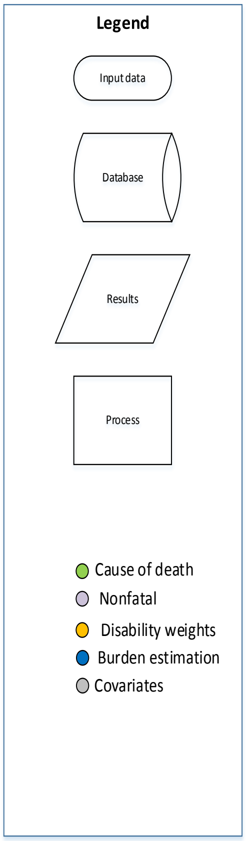

Input data

Sources used to estimate chronic respiratory disease mortality included vital registration, verbal autopsy, and surveillance data from the cause of death (COD) database. Our outlier criteria excluded data points that (1) were implausibly high or low, (2) substantially conflicted with established age or temporal patterns, or (3) significantly conflicted with other data sources conducted from the same locations or locations with similar characteristics (i.e., Socio-Demographic Index).

For GBD 2015, there were two significant changes in the data preparation process that affect Chronic Respiratory Diseases and its children causes. First, the algorithm package that redistributes heart-failure-related garbage codes has been updated to take into account the “side” of the heart failure – with right heart failure denoting an underlying respiratory disease. Second, verbal autopsy data are no longer used to inform children causes as they are thought to be unreliable below this cause level. Practically, this has

a larger influence on the uncorrected children models than the parent Chronic Respiratory Diseases model discussed here.

Modeling strategy

The standard CODEm modeling approach was applied to estimate deaths due to chronic respiratory diseases. Chronic respiratory diseases served as the parent cause to chronic obstructive pulmonary disease, pneumoconiosis (including silicosis, asbestosis, coal worker's pneumoconiosis, other pneumoconiosis), asthma, interstitial lung disease and pulmonary sarcoidosis, and other chronic respiratory diseases. Functionally, this means the death estimates for Chronic Respiratory Diseases serve as an envelope into which the children causes are squeezed by the CodCorrect algorithm. This approach allows us to use a broader range of data – specifically verbal autopsy data – which cannot be accurately mapped to a cause further down in the hierarchy.

Separate models were conducted for male and female mortality, and the age range for both models was 0 to 80+ years. The same covariates from GBD 2013 were used, with the addition of the Socio-Demographic Index (SDI) covariate. Although all covariates in this model received updates for GBD 2015, cumulative cigarettes, smoking prevalence, and health systems access received the larger overhauls. The updates to the smoking-based covariates were particularly helpful in developing these models. Beyond changes in the underlying covariates, there were no substantial deviations from the GBD 2013 approach.

Chronic Obstructive Pulmonary Disease

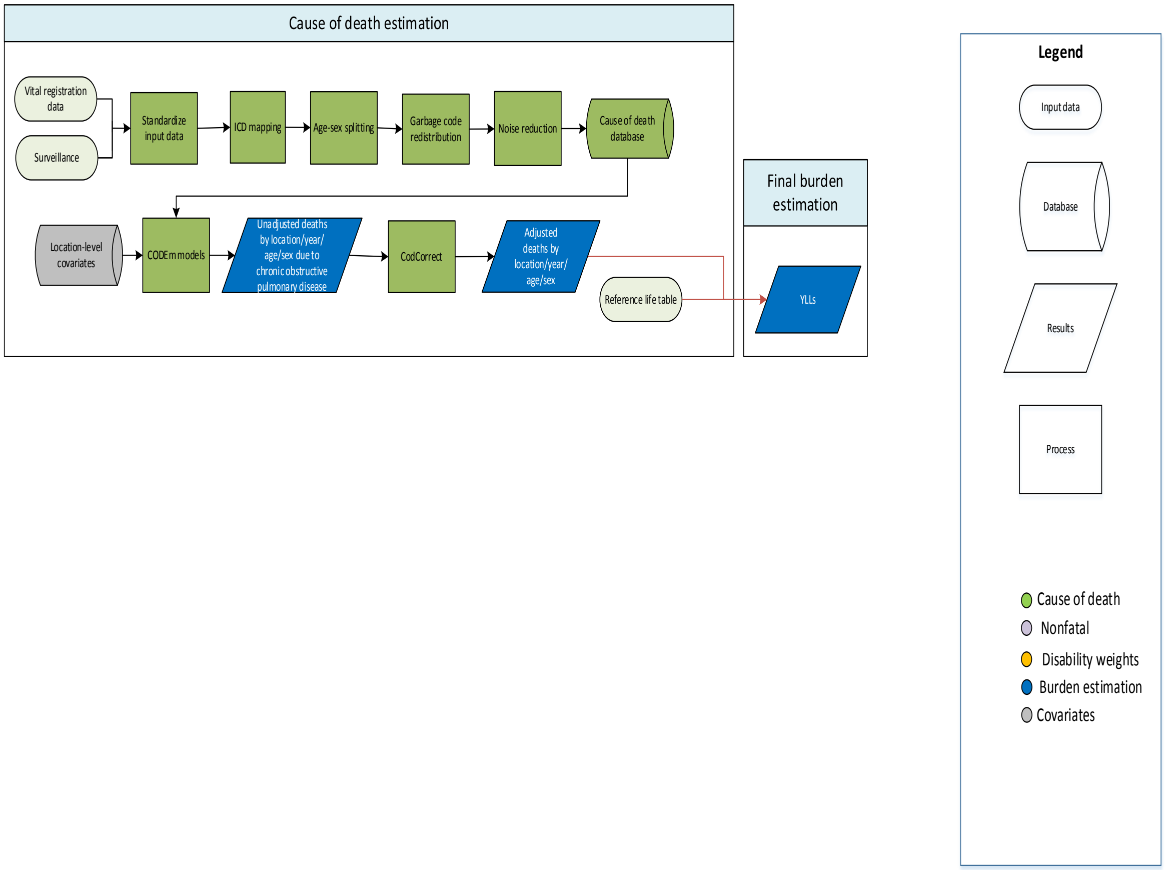

Input data

Data used to estimate chronic obstructive pulmonary disease (COPD) mortality included vital registration and surveillance data from the cause of death (COD) database. Our outlier criteria excluded data points that (1) were implausibly high or low, (2) substantially conflicted with established age or temporal patterns, or (3) significantly conflicted with other data sources conducted from the same locations or locations with similar characteristics (i.e., Socio-Demographic Index). The main consequences of this protocol are the mapping of state-level data from India MCCD ICD10 to the chronic respiratory parent due to implausibly high values and the outliering of some Thailand vital registration data from the late 90s that implied an unreasonable peak of COPD during the covered time frame.

Notable differences in the data processing strategy relative to GBD 2013 include 1) Verbal autopsy data have been excluded from this model and mapped to the Chronic Respiratory Disease parent cause as we no longer believe that verbal autopsy accurately captures deaths due to specific respiratory diseases, and 2) The heart failure redistribution package has been updated to account for the "side" of the heart. As a result, the amount of heart failure being attributed to chronic respiratory diseases is now largely

based on proportions of left and right heart failure (with right heart failure signifying an underlying respiratory condition). In general, this has reduced the level of COPD deaths – all else being equal.

Modeling strategy

The standard CODEm modeling approach was applied to estimate deaths due to COPD. Separate models were conducted for male and female mortality, and the age range for both models was 28 days to 80+ years. The mortality estimates from the COPD models were ultimately fit into the chronic respiratory diseases envelope.

While the core covariates have remained unchanged, covariates relating to population density and proportion of population living between 500 meters and 1,500 meters of elevation have been removed because they increased model run time without substantially contributing to the model results. Conversely, 10-year cumulative cigarette consumption has been added to the model to better capture any smoking-related lag effects on COPD, along with the Socio-Demographic Index (SDI) covariate.

Pneumoconiosis diseases:

Pneumoconiosis, silicosis, asbestosis, coal worker's pneumoconiosis, and other pneumoconiosis

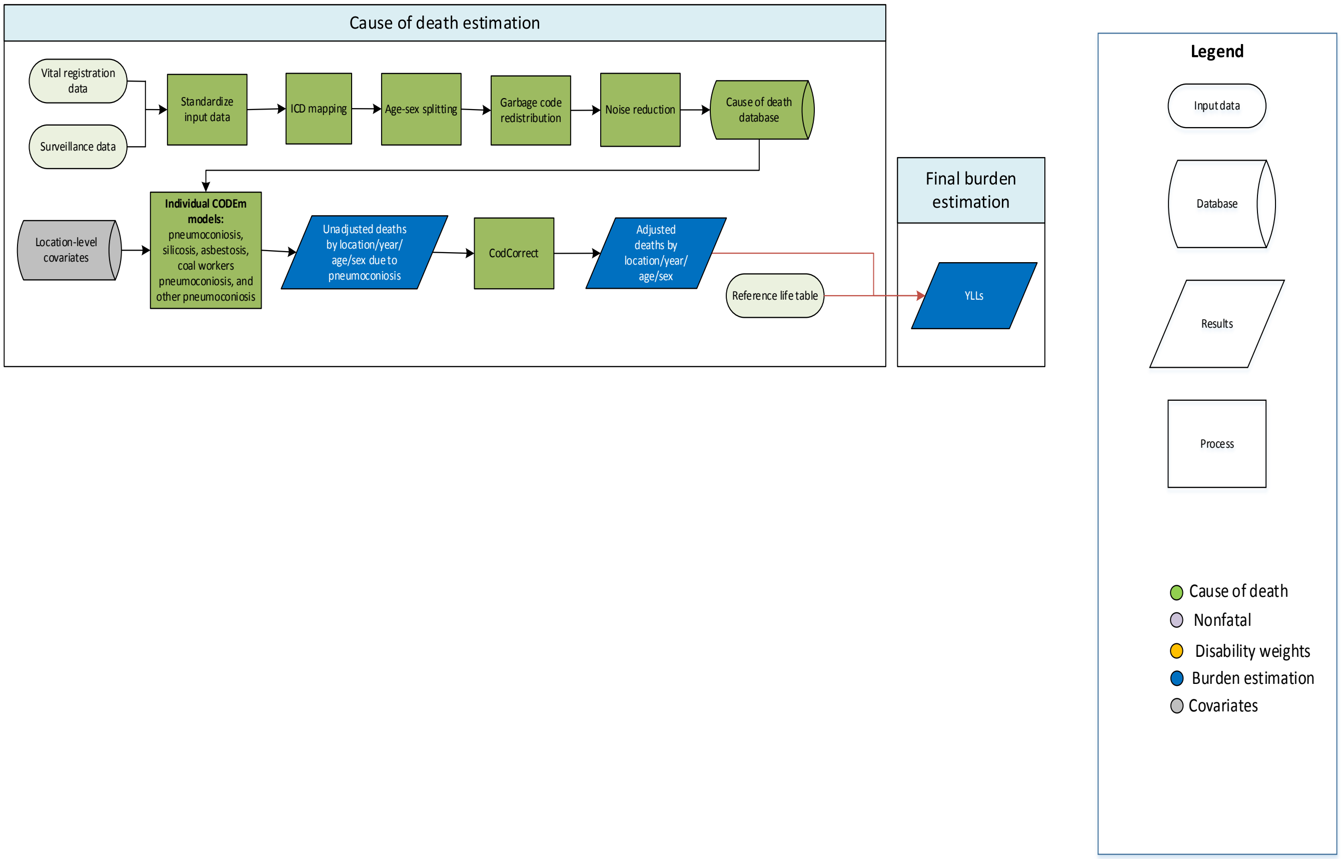

Input data

Data used to estimate pneumoconiosis diseases mortality included vital registration and surveillance data from the cause of death (COD) database. Our outlier criteria excluded data points that (1) were implausibly high or low, (2) substantially conflicted with established age or temporal patterns, or (3) significantly conflicted with other data sources conducted from the same locations or locations with similar characteristics (i.e., Socio-Demographic Index).

The major change in data processing relative to GBD 2013 is the removal of pneumoconiosis and its children causes from possible targets for heart failure redistribution. All else being equal, this results in a slight drop in uncorrected estimates relative to GBD 2013. The decision to alter "targeting" was made to improve the stability of the model and avoid artificial inflation of death estimates from the garbage code redistribution process.

Modeling strategy

The standard CODEm modeling approach was applied to estimate deaths due to pneumoconiosis diseases. Separate models were conducted for male and female mortality, and the age range for both models was 1–80+ years. The mortality estimates from pneumoconiosis disease models were ultimately fit into the chronic respiratory envelope, which is the parent cause for pneumoconiosis disease. The pneumoconiosis model serves as an envelope for silicosis, asbestosis, coal worker's pneumoconiosis, and other pneumoconiosis. This two-stage parent-child relationship is applied by the CodCorrect algorithm.

The covariates used in GBD 2013 have been updated and carried over to GBD 2015, with the addition of the Socio-Demographic Index (SDI) covariate.

Asthma

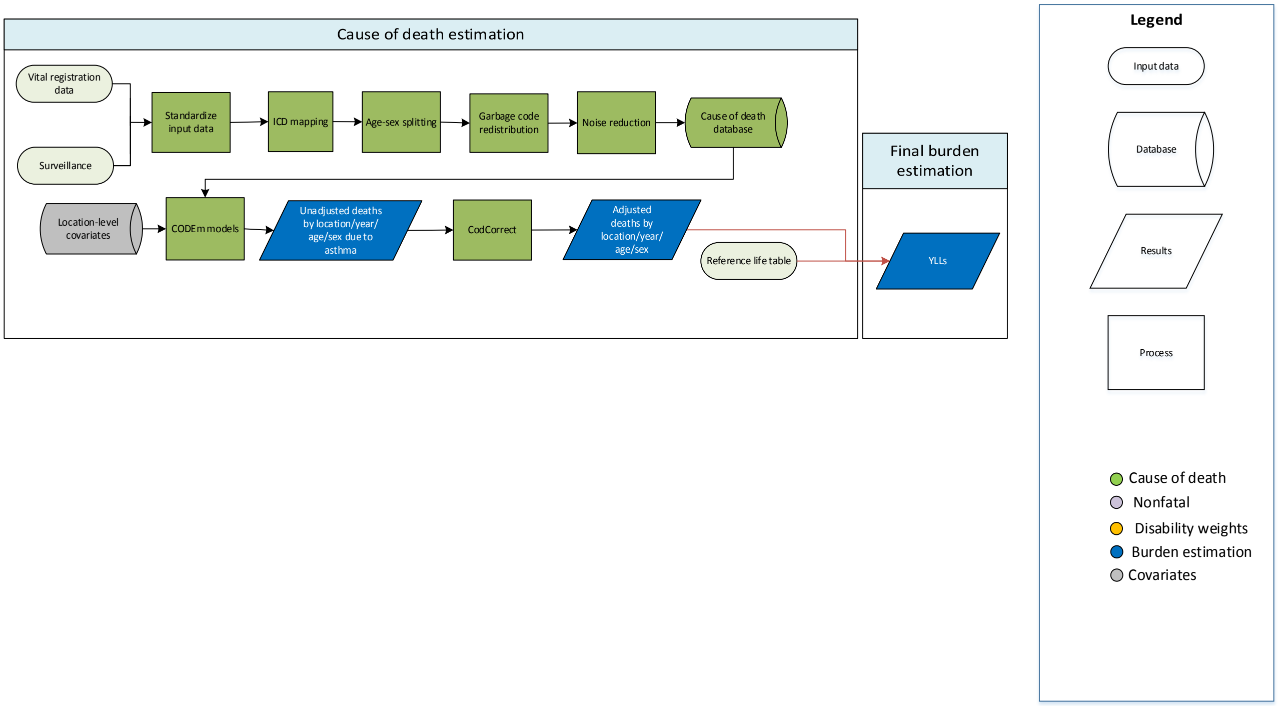

Input data

Data used to estimate asthma mortality included vital registration and surveillance data from the cause of death (COD) database. Verbal autopsy data were not included and were instead mapped to the parent model (Chronic Respiratory Diseases). Our outlier criteria excluded data points that (1) were implausibly high or low relative to global or regional patterns, (2) substantially conflicted with established age or temporal patterns, or (3) significantly conflicted with other data sources conducted from the same locations or locations with similar characteristics (i.e., Socio-Demographic Index).

Modeling strategy

The standard CODEm modeling approach was applied to estimate deaths due to asthma. Separate models were conducted for male and female mortality, and the age range for both models was 1–80+ years. The mortality estimates from the asthma models were ultimately fit into the chronic respiratory diseases envelope.

Notable differences between the GBD 2013 strategy and this iteration are across the board updates in smoking-based covariates, the removal of elevation and population density covariates due to lack of informative contributions, and the inclusion of the Socio-Demographic Index (SDI) covariate and the SEV-scalar (disease-specific values that reflect the combined effect of all GBD risks) for asthma.

Interstitial lung disease and pulmonary sarcoidosis

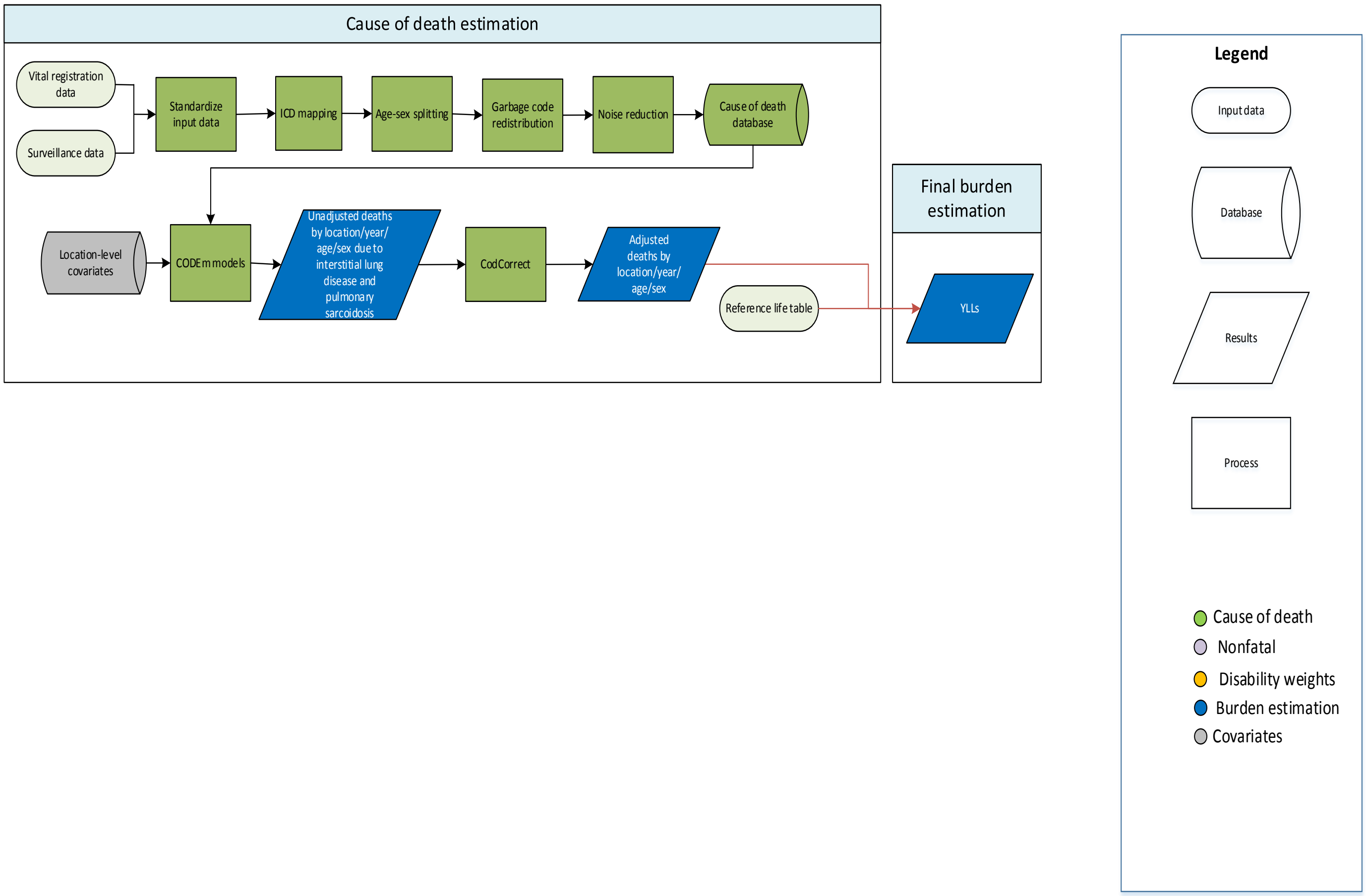

Input data

Data used to estimate interstitial lung disease and pulmonary sarcoidosis mortality included vital registration and surveillance data from the cause of death (COD) database. Our outlier criteria excluded data points that (1) were implausibly high or low, (2) substantially conflicted with established age or temporal patterns, or (3) significantly conflicted with other data sources conducted from the same locations or locations with similar characteristics (i.e., Socio-Demographic Index).

The main difference in data relative to GBD 2013 is the improved the heart failure redistribution package. This method has been updated to account for the “side” of the heart that failed when redistributing garbage coded deaths due to heart failure. As a result, the amount of heart failure being attributed to chronic respiratory diseases is now largely based on proportions of left and right heart

failure (with right heart failure signifying an underlying respiratory condition). In general, this has reduced the level of COPD deaths – all else being equal.

Modeling strategy

The standard CODEm modeling approach was applied to estimate deaths due to interstitial lung disease and pulmonary sarcoidosis. Separate models were conducted for male and female mortality, and the age range for both models was 1–80+ years. The mortality estimates from the interstitial lung disease and pulmonary sarcoidosis models were ultimately fit into the chronic respiratory envelope. We applied the same set of covariates from GBD 2013, adding the in the associated SEV scalar and Socio-Demographic Index (SDI) covariates. Otherwise, there were no substantial changes from GBD 2013.

Other chronic respiratory diseases

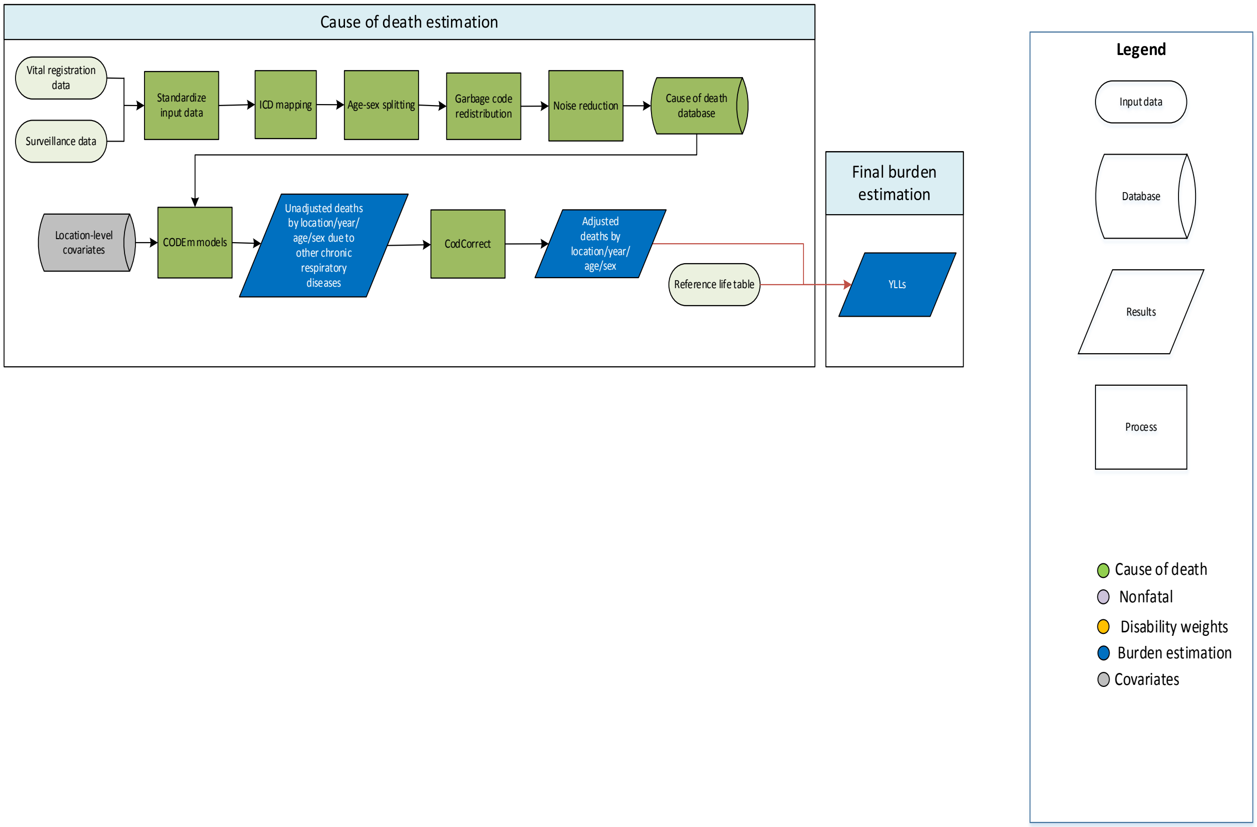

Input data

Data used to estimate other chronic respiratory diseases included vital registration and surveillance data from the cause of death (COD) database. Our outlier criteria excluded data points that (1) were implausibly high or low, (2) substantially conflicted with established age or temporal patterns, or (3) significantly conflicted with other data sources conducted from the same locations or locations with similar characteristics (i.e., Socio-Demographic Index).

Modeling strategy

The standard CODEm modeling approach was applied to estimate deaths due to other chronic respiratory diseases. Separate models were conducted for male and female mortality, and the age range for both models was 0 days to 80+ years. Like other respiratory causes, the mortality estimates from other chronic respiratory diseases were ultimately fit into the chronic respiratory envelope.

Besides general updates to the 2013 covariate set (specifically, the health systems and smoking-related ones), the modeling strategy remained unchanged from GBD 2013. For GBD 2015, we included two new covariates: the Socio-Demographic Index (SDI) covariate, and a standardized exposure variable (SEV) scalar (disease-specific values that reflect the combined effect of all GBD risks) for other respiratory

diseases. However, as SEVs are essentially covariate/risk aggregate measures, no substantial changes were expected.

Digestive Diseases

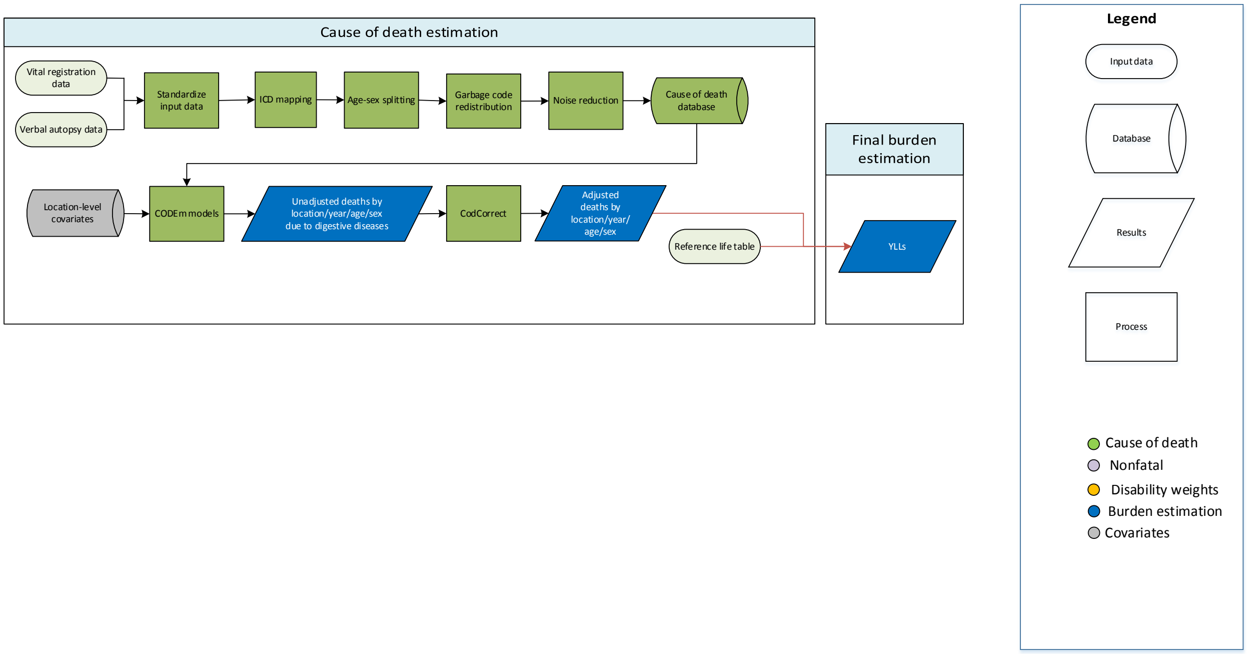

Input data

Vital registration and verbal autopsy data were used to model this cause. We outliered data in instances where garbage code redistribution and noise reduction, in combination with small sample sizes, resulted in unreasonable cause fractions; and data that violated well-established time or age trends. Outliering methods were consistent across both vital registration and verbal autopsy data.

Modeling strategy

We modeled deaths due to all digestive diseases with a standard CODEm model using the cause of death database and location-level covariates as inputs. The model followed standard parameters, with the exception that the start age of the model was 1 year old instead of 0. We hybridized separate global and data-rich models to acquire unadjusted results, which we finalized and adjusted using CodCorrect to reach final YLLs due to digestive diseases. There were no significant changes in the modeling process between GBD 2013 and GBD 2015.

Peptic ulcer disease

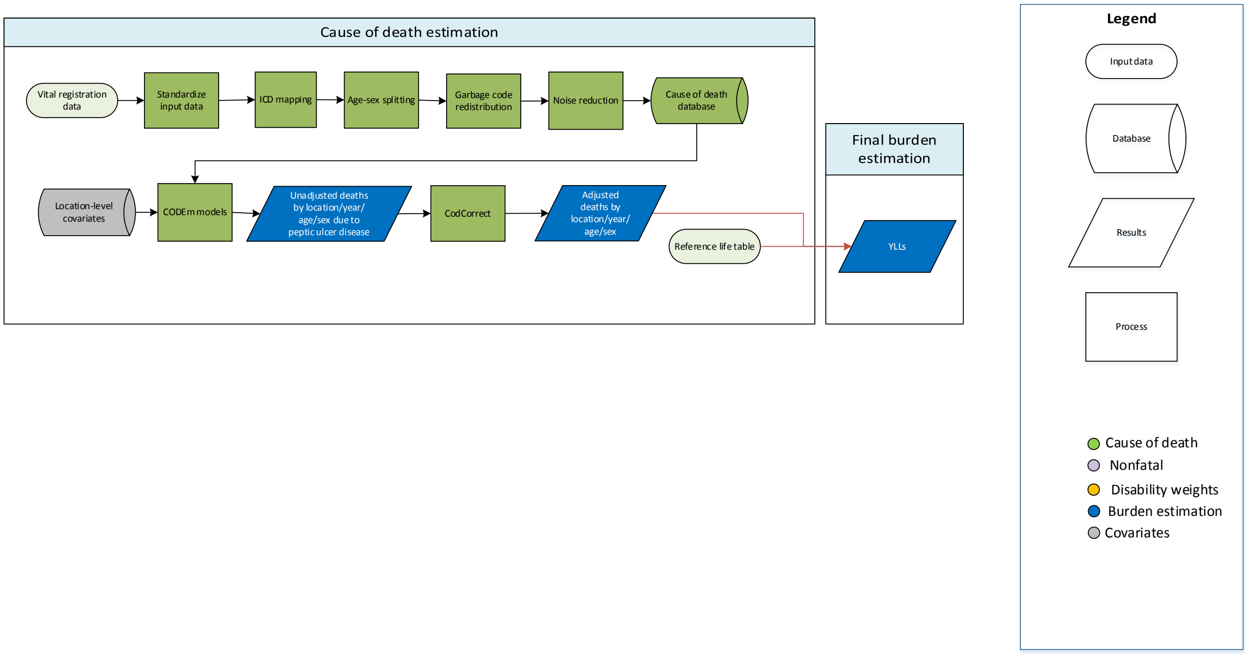

Input data

Vital registration data were used to model this cause. We outliered data in instances where garbage code redistribution and noise reduction, in combination with small sample sizes, resulted in unreasonable cause fractions, and data that violated well-established time or age trends.

Modeling strategy

We modeled deaths due to peptic ulcer disease with a standard CODEm model using the cause of death database and location-level covariates as inputs. The model followed standard parameters, with the exception that the start age of the model was 1 year old instead of 0. We hybridized separate global and data-rich models to acquire unadjusted results, which we finalized and adjusted using CodCorrect to reach final YLLs due to peptic ulcer disease. There were no significant changes in the modeling process between GBD 2013 and GBD 2015.

Gastritis and duodenitis

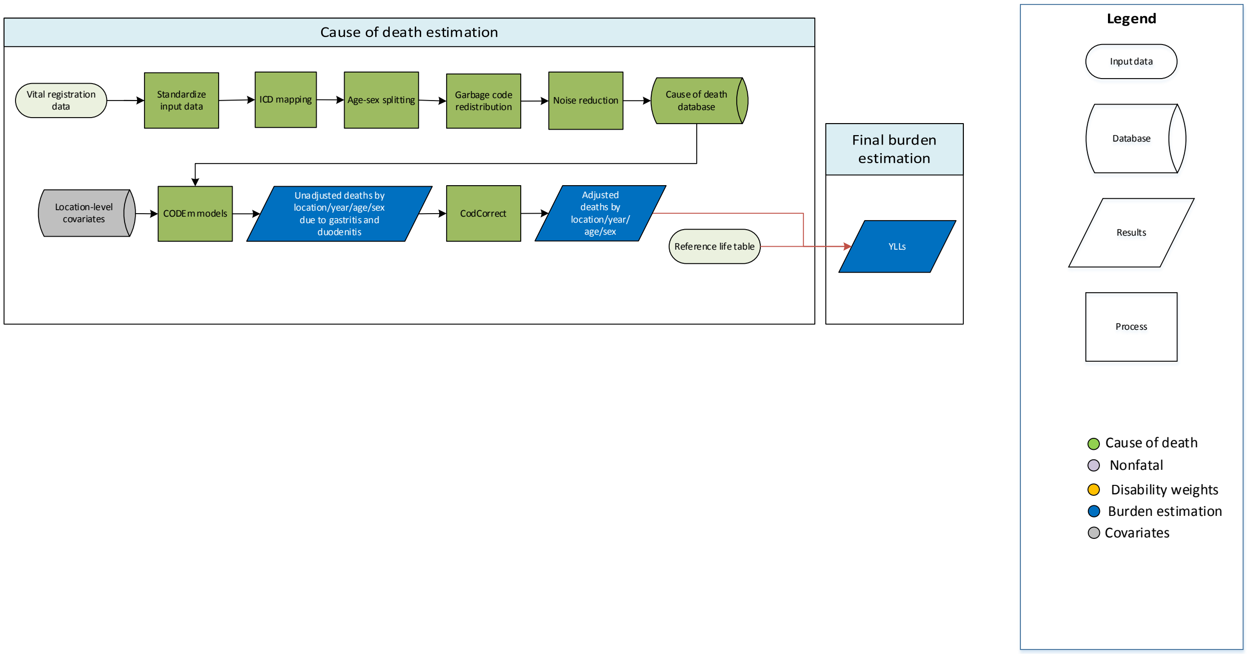

Input data

Vital registration data were used to model this cause. We outliered data in instances where garbage code redistribution and noise reduction, in combination with small sample sizes, resulted in unreasonable cause fractions, and data that violated well-established time or age trends.

Modeling strategy

We modeled deaths due to gastritis and duodenitis with a standard CODEm model using the cause of death database and location-level covariates as inputs. The model followed standard parameters, with the exception that the start age of the model was 1 year old instead of 0 and the linear floor rate was lowered to 0.001 in order to better capture low data. We hybridized separate global and data-rich models to acquire unadjusted results, which we finalized and adjusted using CodCorrect to reach final YLLs due to gastritis and duodenitis. There were no significant changes in the modeling process between GBD 2013 and GBD 2015.

Appendicitis

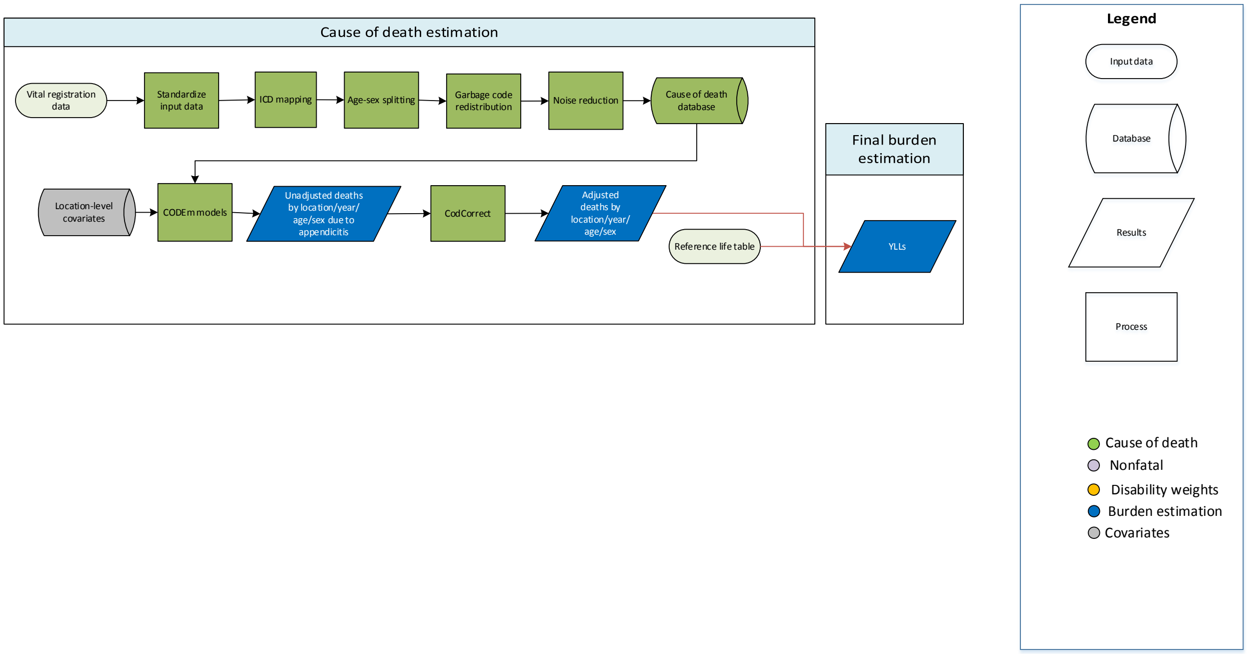

Input data

Vital registration were used to model this cause. We outliered data in instances where garbage code redistribution and noise reduction, in combination with small sample sizes, resulted in unreasonable cause fractions; and data that violated well-established time or age trends.

Modeling strategy

We modeled deaths due to appendicitis with a standard CODEm model using the cause of death database and location-level covariates as inputs. The model followed standard parameters, with the exception that the start age of the model was 1 year old instead of 0 and the linear floor rate was lowered to 0.0001 in order to better capture low data. We hybridized separate global and data-rich models to acquire unadjusted results, which we finalized and adjusted using CodCorrect to reach final YLLs due to appendicitis. There were no significant changes in the modeling process between GBD 2013 and GBD 2015.

Paralytic Ileus and Intestinal Obstruction

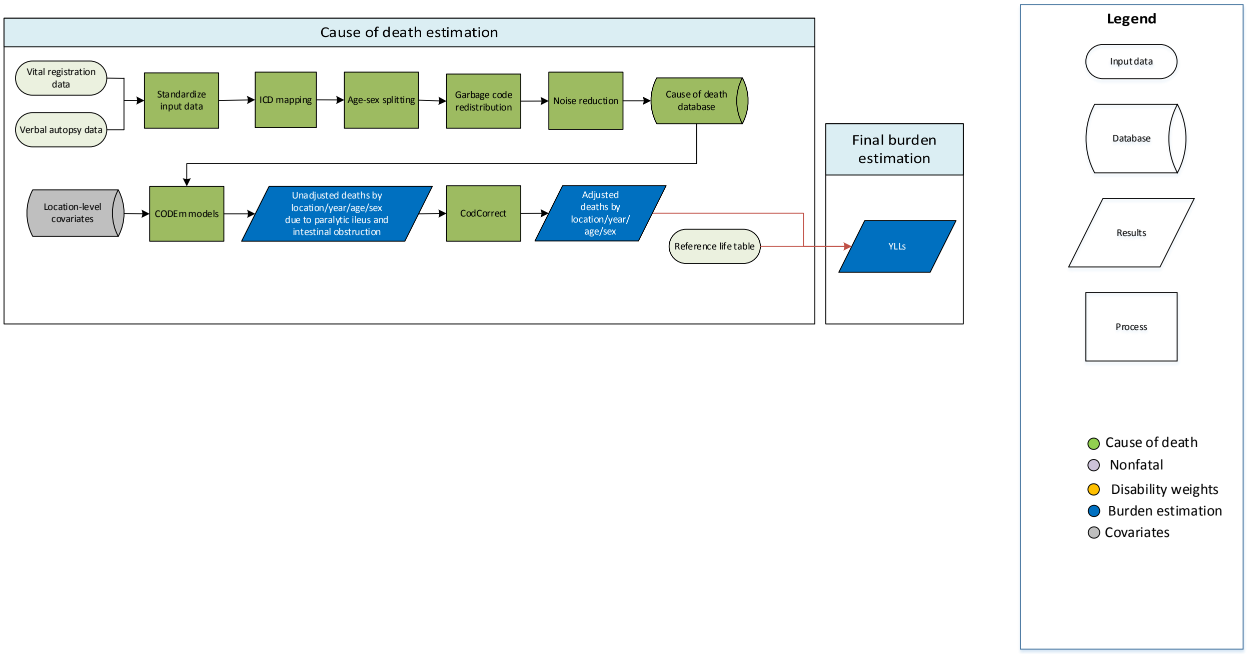

Input data

Vital registration and verbal autopsy data were used to model this cause. We outliered all VA data in children under the age of 1 because it is not possible to accurately diagnose paralytic ileus or intestinal obstruction in this age group; and data that violated well-established time or age trends; and data in instances where garbage code redistribution and noise reduction, in combination with small sample sizes, resulted in unreasonable cause fractions. Outliering methods were consistent across both vital registration and verbal autopsy data.

Modeling strategy

We modeled deaths due to paralytic ileus and intestinal obstruction with a standard CODEm model using the cause of death database and location-level covariates as inputs. The model followed standard parameters, with the exception that the linear floor rate was lowered to 0.0001 in order to better capture low data. We hybridized separate global and data-rich models to acquire unadjusted results, which we finalized and adjusted using CodCorrect to reach final YLLs due to paralytic ileus and intestinal obstruction. There were no significant changes in the modeling process between GBD 2013 and GBD 2015.

Inguinal, Femoral, and Abdominal Hernias

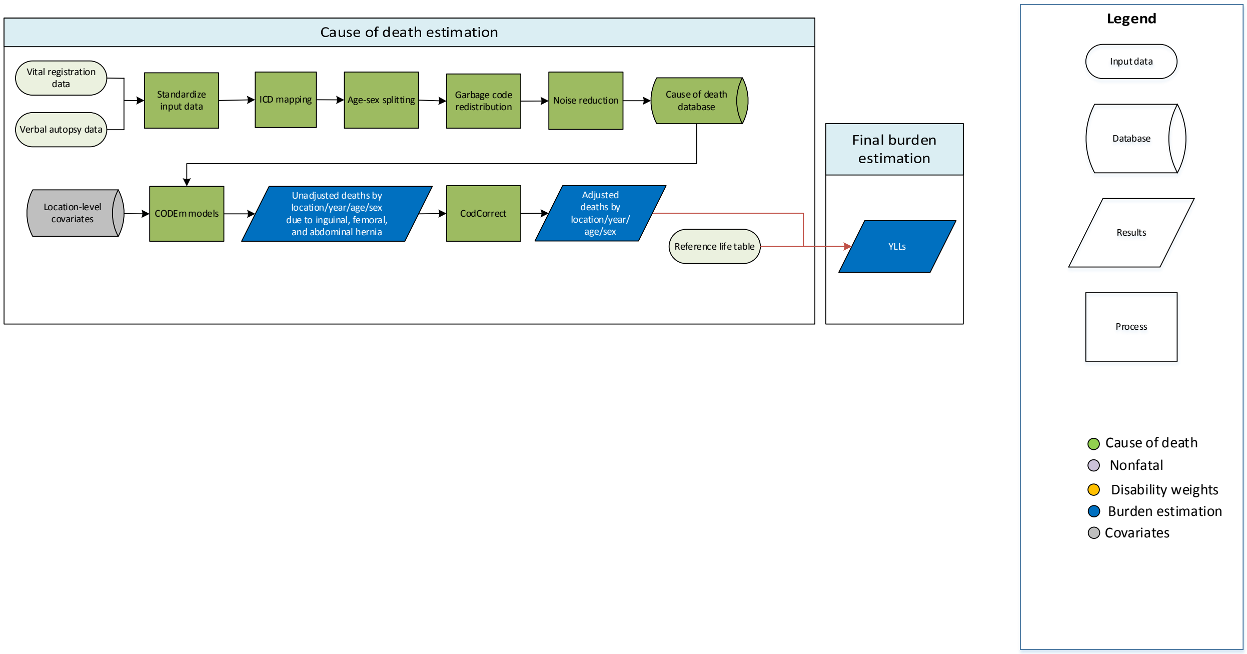

Input data

Vital registration and verbal autopsy data were used to model this cause. We outliered data in instances where garbage code redistribution and noise reduction, in combination with small sample sizes, resulted in unreasonable cause fractions; and data that violated well-established time or age trends. Outliering methods were consistent across both vital registration and verbal autopsy data.

Modeling strategy

We modeled deaths due to inguinal, femoral, and abdominal hernias with a standard CODEm model using the cause of death database and location-level covariates as inputs. The model followed standard parameters, with the exception that the start age of the model was 1 year old instead of 0 and the linear floor rate was lowered to 0.0001 in order to better capture low data. We hybridized separate global and data-rich models to acquire unadjusted results, which we finalized and adjusted using CodCorrect to reach final YLLs due to inguinal, femoral, and abdominal hernias. There were no significant changes in the modeling process between GBD 2013 and GBD 2015.

Inflammatory Bowel Disease

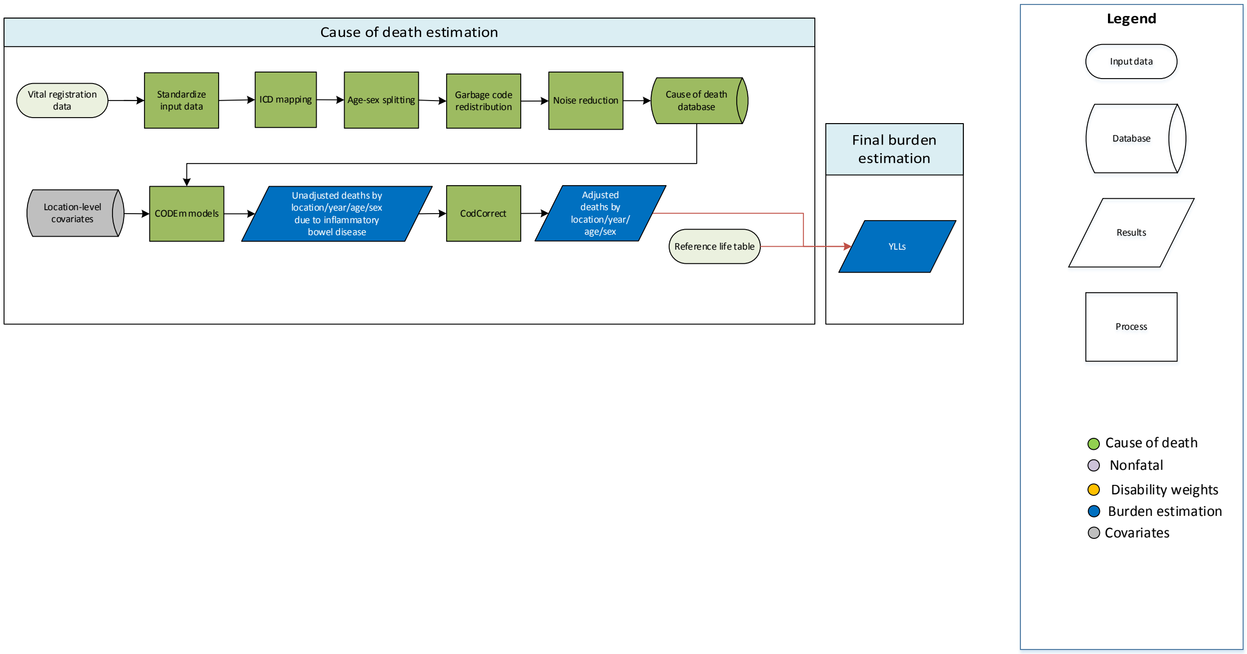

Input data

Vital registration data were used to model this cause. We outliered data in instances where garbage code redistribution and noise reduction, in combination with small sample sizes, resulted in unreasonable cause fractions, and data that violated well-established time or age trends.

Modeling strategy

We modeled deaths due to inflammatory bowel disease with a standard CODEm model using the cause of death database and location-level covariates as inputs. The model followed standard parameters, with the exception that the start age of the model was 1 year old instead of 0 and the linear floor rate was lowered to 0.0001 in order to better capture low data. We hybridized separate global and data-rich models to acquire unadjusted results, which we finalized and adjusted using CodCorrect to reach final YLLs due to inflammatory bowel disease. There were no significant changes in the modeling process between GBD 2013 and GBD 2015.

Vascular Intestinal Disorders

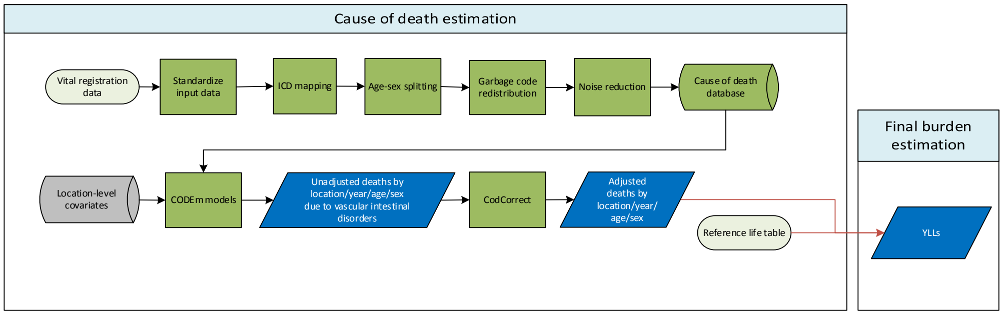

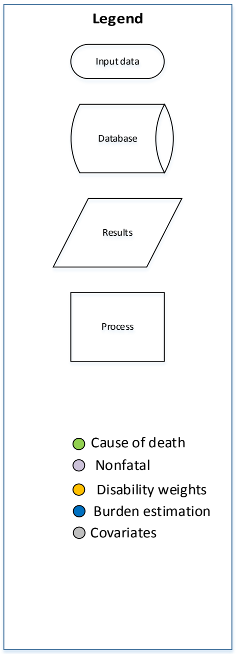

Input data

Vital registration data were used to model this cause. We outliered data in instances where garbage code redistribution and noise reduction, in combination with small sample sizes, resulted in unreasonable cause fractions; and data that violated well-established time or age trends.

Modeling strategy

We modeled deaths due to vascular intestinal disorders with a standard CODEm model using the cause of death database and location-level covariates as inputs. The model followed standard parameters, with the exception that the start age of the model was 1 year old instead of 0 and the linear floor rate was lowered to 0.0001 in order to better capture low data. We hybridized separate global and data-rich models to acquire unadjusted results, which we finalized and adjusted using CodCorrect to reach final YLLs due to vascular intestinal disorders. There were no significant changes in the modeling process between GBD 2013 and GBD 2015.

Gallbladder and biliary diseases

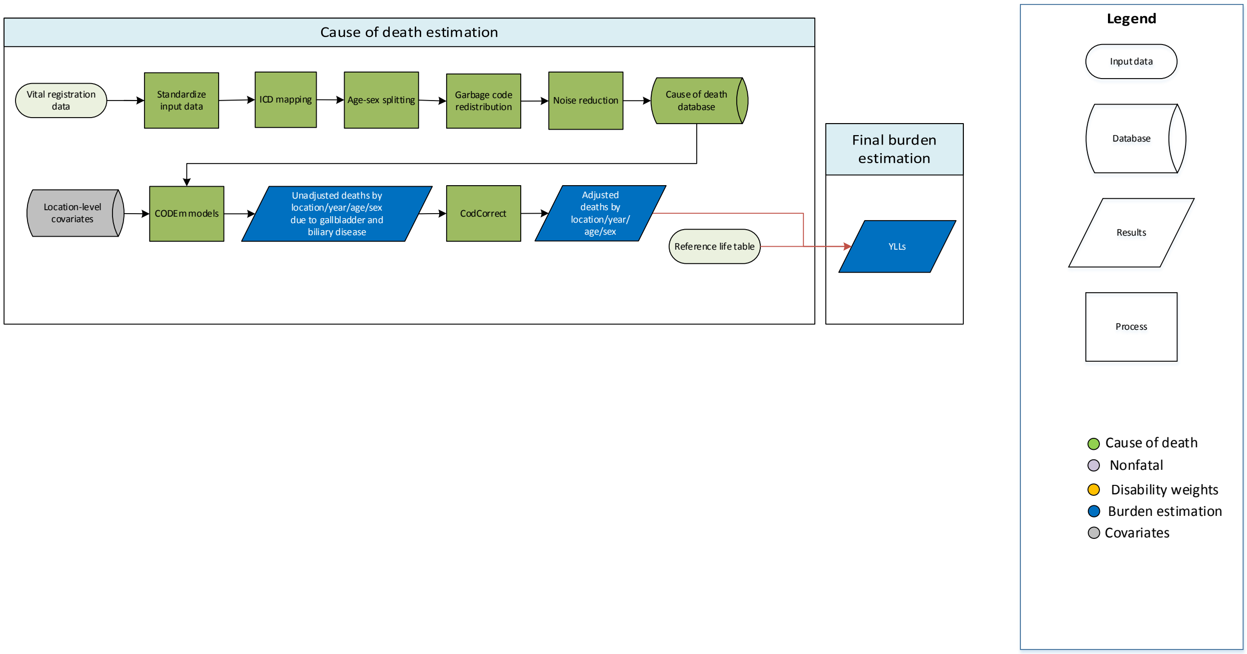

Input data

Vital registration data were used to model this cause. We outliered data in instances where garbage code redistribution and noise reduction, in combination with small sample sizes, resulted in unreasonable cause fractions; and data that violated well-established time or age trends.

Modeling strategy

We modeled deaths due to gallbladder and biliary diseases with a standard CODEm model using the cause of death database and location-level covariates as inputs. The model followed standard parameters, with the exception that the start age of the model was 1 year old instead of 0 and the linear floor rate was lowered to 0.0001 in order to better capture low data. We hybridized separate global and data rich models to acquire unadjusted results, which we finalized and adjusted using CodCorrect to reach final YLLs due to gallbladder and biliary diseases. There were no significant changes in the modeling process between GBD 2013 and GBD 2015.

Pancreatitis

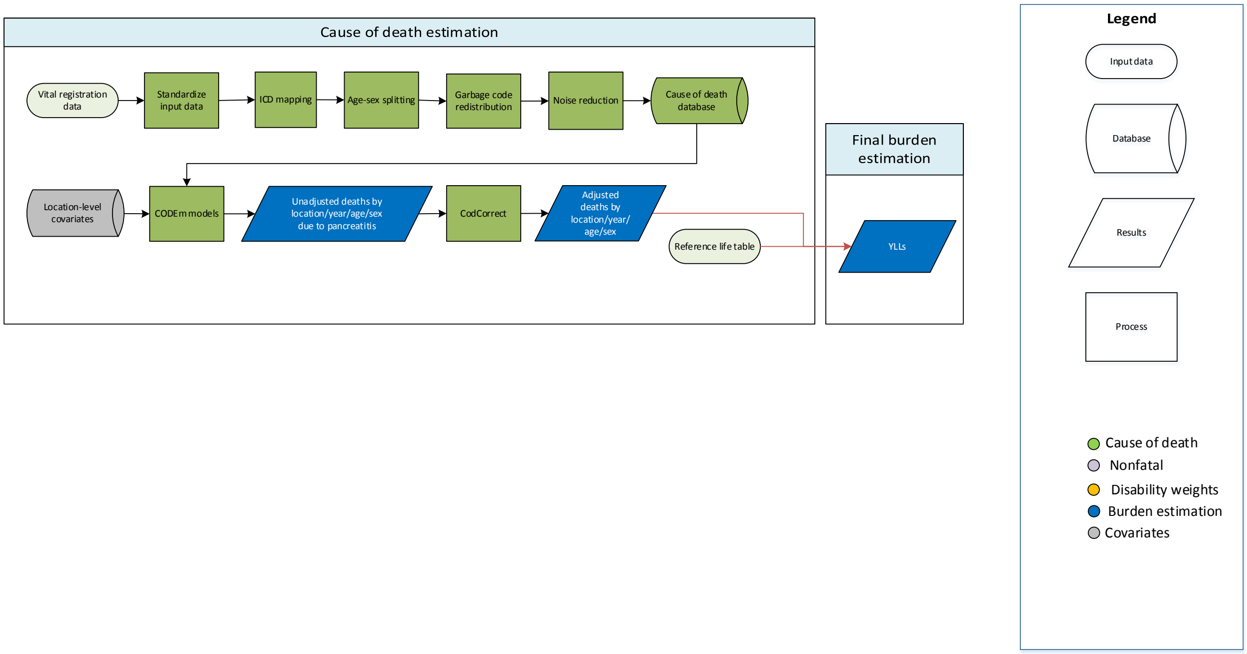

Input data

Vital registration data were used to model this cause. We outliered data in instances where garbage code redistribution and noise reduction, in combination with small sample sizes, resulted in unreasonable cause fractions, and data that violated well-established time or age trends.

Modeling strategy

We modeled deaths due to pancreatitis with a standard CODEm model using the cause of death database and location-level covariates as inputs. The model followed standard parameters, with the exception that the start age of the model was 1 year old instead of 0 and the linear floor rate was lowered to 0.0001 in order to better capture low data. We hybridized separate global and data-rich models to acquire unadjusted results, which we finalized and adjusted using CodCorrect to reach final YLLs due to pancreatitis. There were no significant changes in the modeling process between GBD 2013 and GBD 2015.

Other digestive diseases

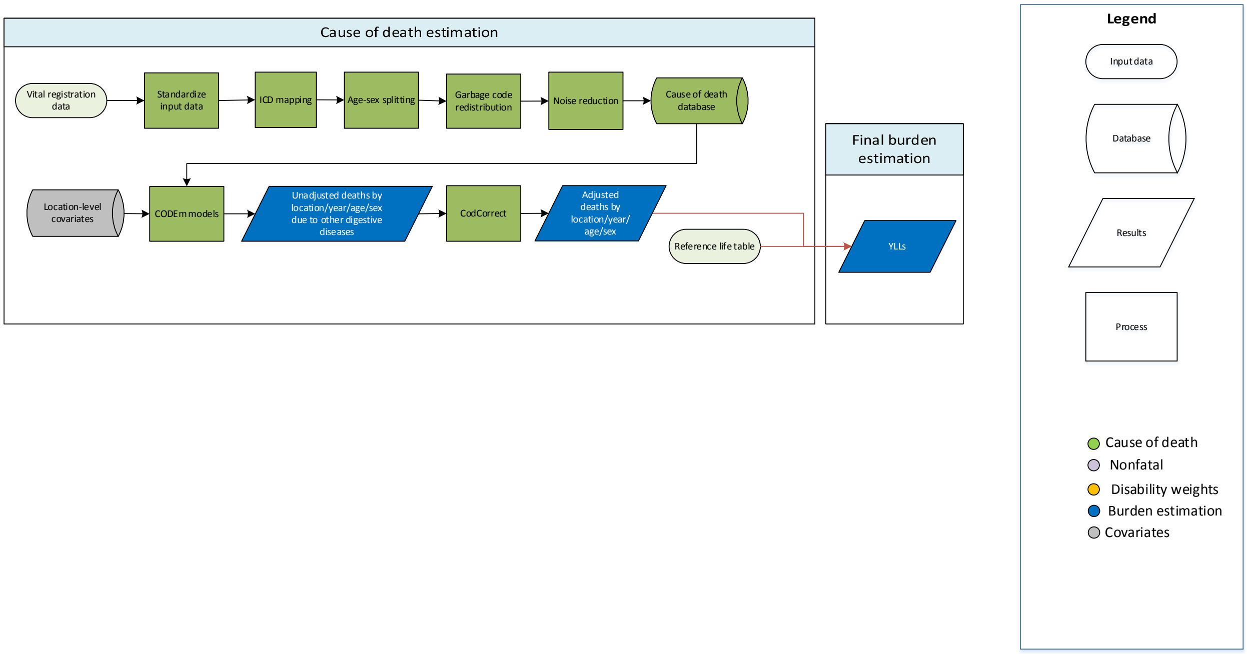

Input data

Vital registration data were used to model this cause. We outliered data in instances where garbage code redistribution and noise reduction, in combination with small sample sizes, resulted in unreasonable cause fractions; and data that violated well-established time or age trends.

Modeling strategy

We modeled deaths due to other digestive diseases with a standard CODEm model using the cause of death database and location-level covariates as inputs. The model followed standard parameters, with the exception that the start age of the model was 1 year old instead of 0. We hybridized separate global and data-rich models to acquire unadjusted results, which we finalized and adjusted using CodCorrect to reach final YLLs due to other digestive diseases. There were no significant changes in the modeling process between GBD 2013 and GBD 2015.

Parkinson Disease

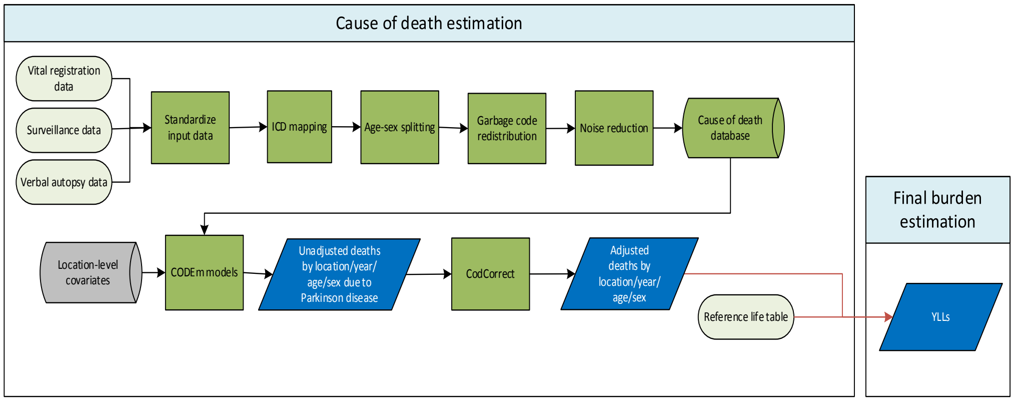

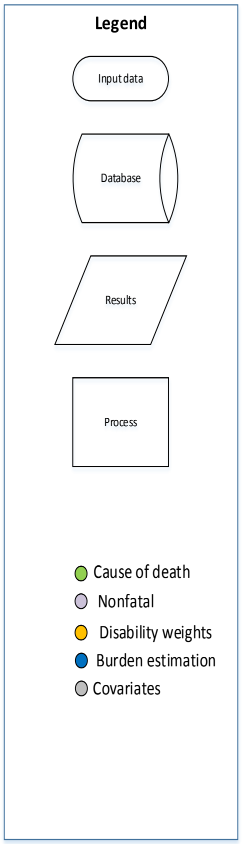

Input data

Data used to estimate Parkinson disease included vital registration, surveillance, and verbal autopsy data from the cause of death (COD) database. Our outlier criteria excluded data points that (1) were implausibly high or low, (2) substantially conflicted with established age or temporal patterns, or (3) significantly conflicted with other data sources conducted from the same locations or locations with similar characteristics (i.e., Socio-Demographic Index).

Regarding data preparation, a change from GBD 2013 is that Parkinson disease no longer receives garbage-coded deaths during the redistribution process. This change slightly reduces the number of uncorrected deaths but otherwise preserves the observed age and temporal patterns.

Modeling strategy

The standard CODEm modeling approach was applied to estimate deaths due to Parkinson disease. Separate models were conducted for male and female mortality, and the age range for both models was

20–80+ years. There were no substantial changes from GBD 2013. The covariates used in GBD 2013 have been retained for this iteration, with the addition of the Socio-Demographic Index (SDI) covariate.

Epilepsy

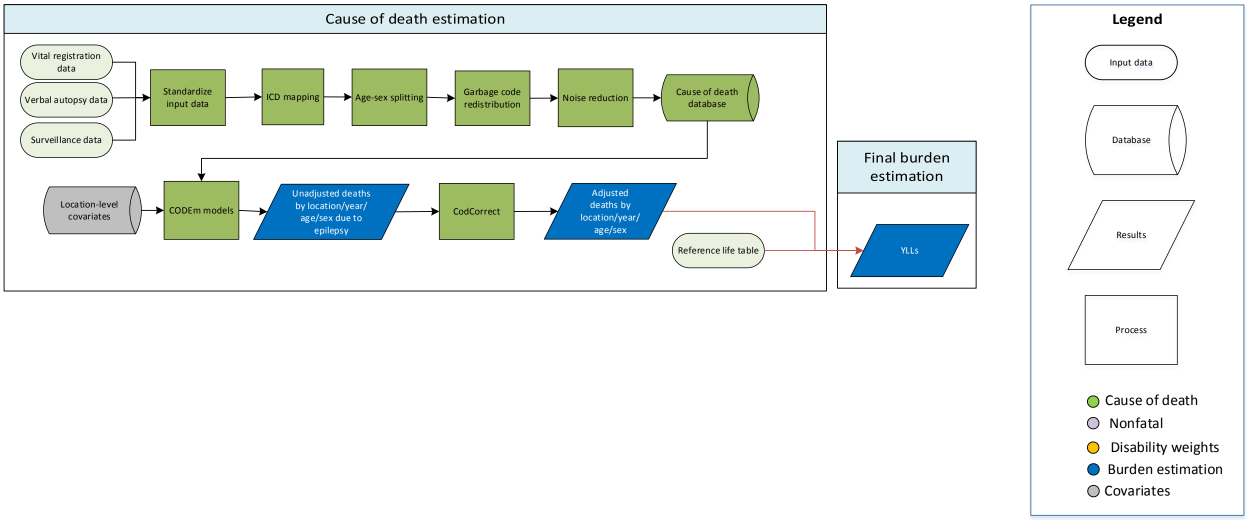

Input data

Data used to estimate epilepsy mortality included vital registration (VR), verbal autopsy, and China disease surveillance point data from the cause of death (COD) database. Our outlier criteria excluded data points that were implausibly high or low relative to global or regional patterns, substantially conflicted with established age or temporal patterns, or significantly conflicted with other data sources based from the same locations or locations with similar characteristics (i.e., Socio-Demographic Index).

Based on these criteria, we excluded ICD-9 BTL data for Sri Lanka, Fiji, and Kiribati as the estimates varied from year to year between zero and high values. We also outliered all VR data for Eastern Cape for ages 15-74 as this was a single province in South Africa for which the HIV correction (i.e., removing excess deaths due to a cause during the HIV/AIDS epidemic compared to pre-epidemic years) was inadequate and caused this province to be an extremely high outlier globally.

Modeling strategy

The standard CODEm modeling approach was applied to estimate deaths due to epilepsy. We applied the same covariates used in GBD 2013 but added the Socio-Demographic Index (SDI) variable created for this GBD cycle and the standardized exposure variable scalar (SEV-Scalar) for epilepsy. This covariate reflected the level of epilepsy burden attributed to alcohol (the only risk estimated for epilepsy). Otherwise, there were no changes from the GBD 2013 modeling strategy.

Multiple Sclerosis

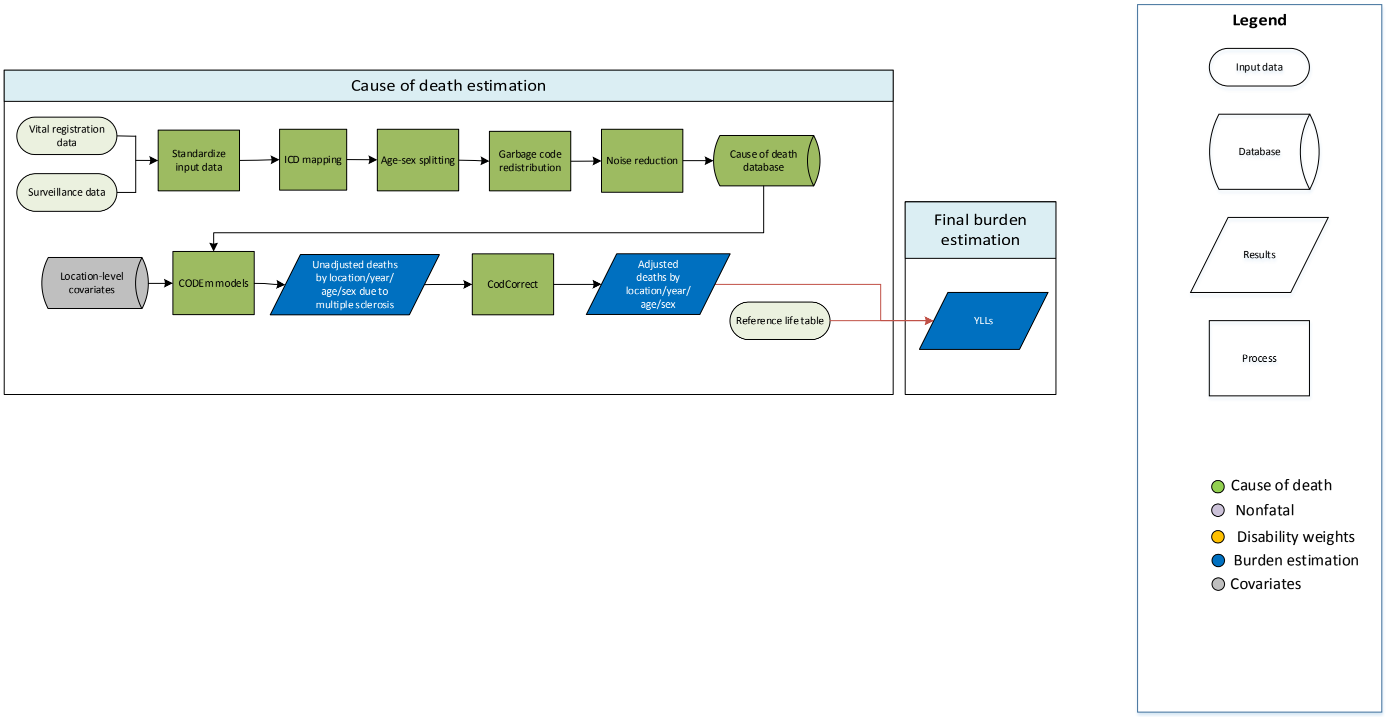

Input data

Data used to estimate multiple sclerosis included vital registration and surveillance data from the cause of death (COD) database. Our outlier criteria excluded data points that (1) were implausibly high or low, (2) substantially conflicted with established age or temporal patterns, or (3) significantly conflicted with other data sources conducted from the same locations or locations with similar characteristics (i.e., Socio-Demographic Index).

Modeling strategy

The standard CODEm modeling approach was used to estimate deaths due to multiple sclerosis. Separate models were conducted for male and female mortality, and the age range for both models was 20–80+ years. There were no substantial changes from GBD 2013. The covariates used in GBD 2013 have been retained for this iteration, with the addition of the Socio-Demographic Index (SDI) covariate.

Motor Neuron Disease

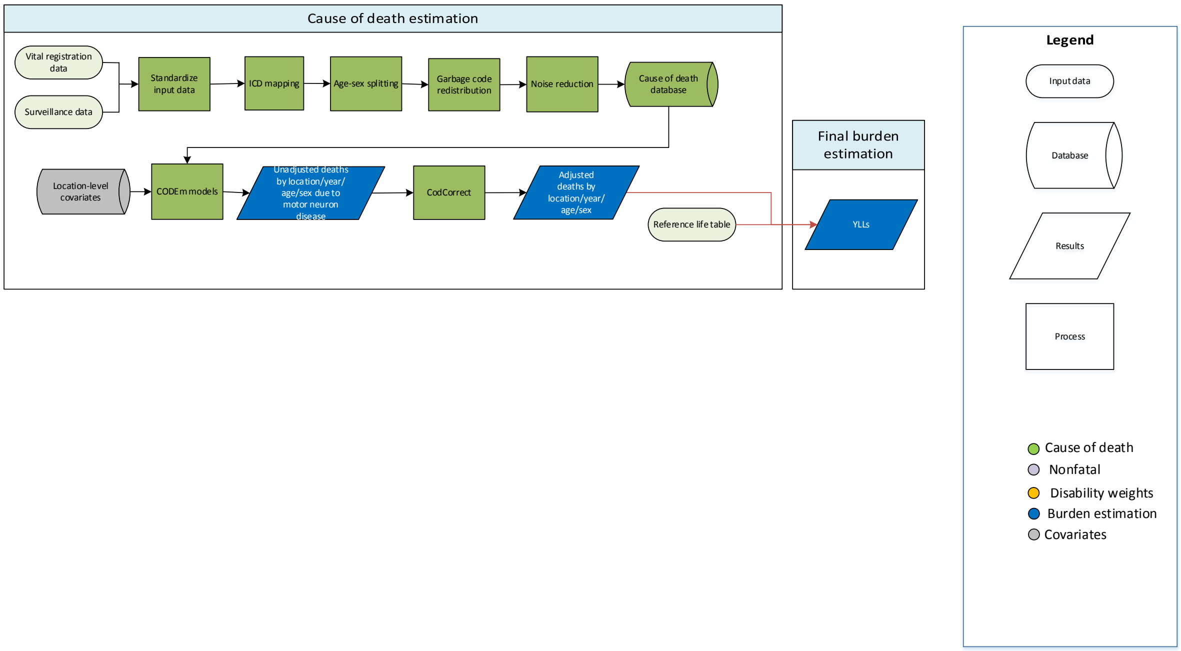

Input data

Data used to estimate motor neuron disease mortality included vital registration and surveillance data from the cause of death (COD) database. Our outlier criteria excluded data points that (1) were implausibly high or low, (2) substantially conflicted with established age or temporal patterns, or (3) significantly conflicted with other data sources conducted from the same locations or locations with similar characteristics (i.e., Socio-Demographic Index).

Modeling strategy

Previously, deaths due to motor neuron disease (MND) were classified and modeled under Other Neurological Disorders. For GBD 2015, we elevated MND deaths to their own cause. We used the standard CODEm modeling approach to generate estimates of deaths due to MND for ages 0 days–80+ years. The covariate structure of the MND model is very similar to other neurological causes and takes into account geographic factors (e.g., latitude), infrastructure (water and sanitation, health systems access), and socioeconomic variables (e.g. lag distributed income).

Other Neurological Disorders

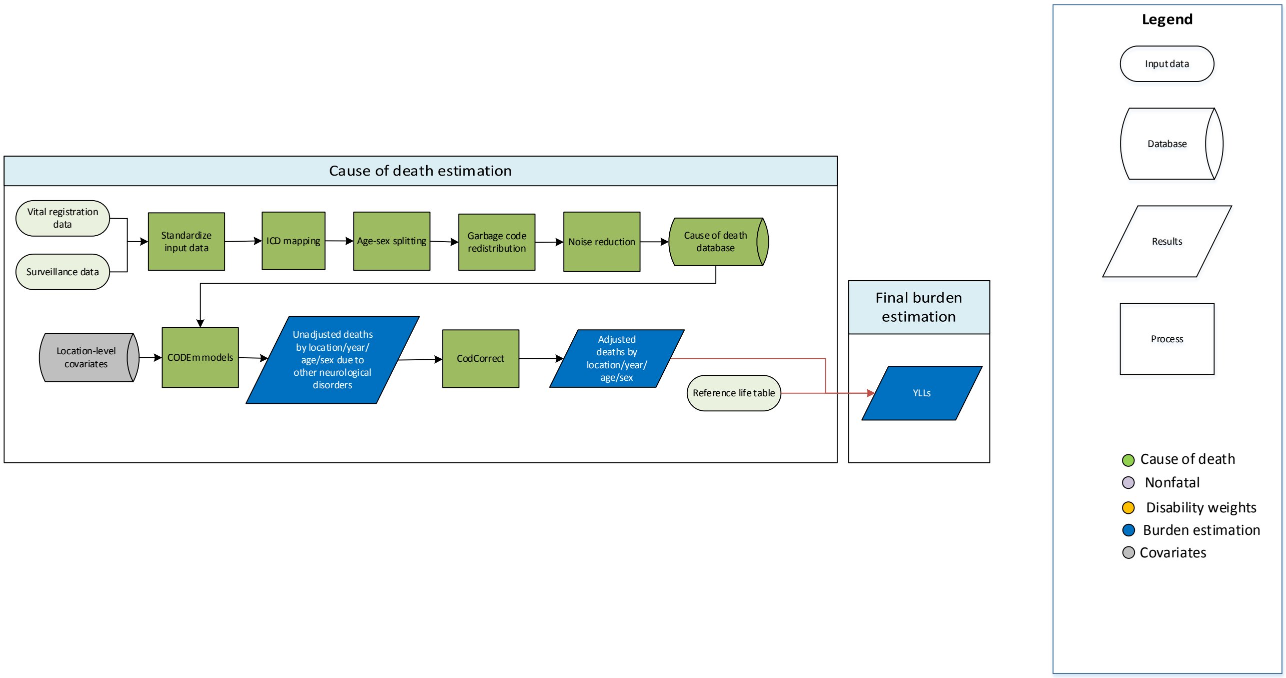

Input data

Data used to estimate other neurological disorders included vital registration and surveillance data from the cause of death (COD) database. Our outlier criteria excluded data points that (1) were implausibly high or low, (2) substantially conflicted with established age or temporal patterns, or (3) significantly conflicted with other data sources conducted from the same locations or locations with similar characteristics (i.e., Socio-Demographic Index).

Relative to GBD 2013, the main data related change to other neurological disorders is the removal of deaths due to motor neuron disease (MND) – a function of MND becoming a separate cause. This resulted in a reduction of our mortality estimates for other neurological disorders.

Modeling strategy

The standard CODEm modeling approach was applied to estimate deaths due to other neurological disorders. Male and female CODEm models were run for deaths occurring between ages 28 days to 80+ years.

Although the covariate list remains essentially unchanged from GBD 2013, a number of covariates have received substantial updates (all received some updates). Metabolic values (e.g., BMI), smoking-based covariates, health systems access, and meat-based dietary energy consumption received the greatest overhaul of the covariates informing this model. The Socio-Demographic Index (SDI) covariate was added to the GBD 2015 covariate set.

Schizophrenia

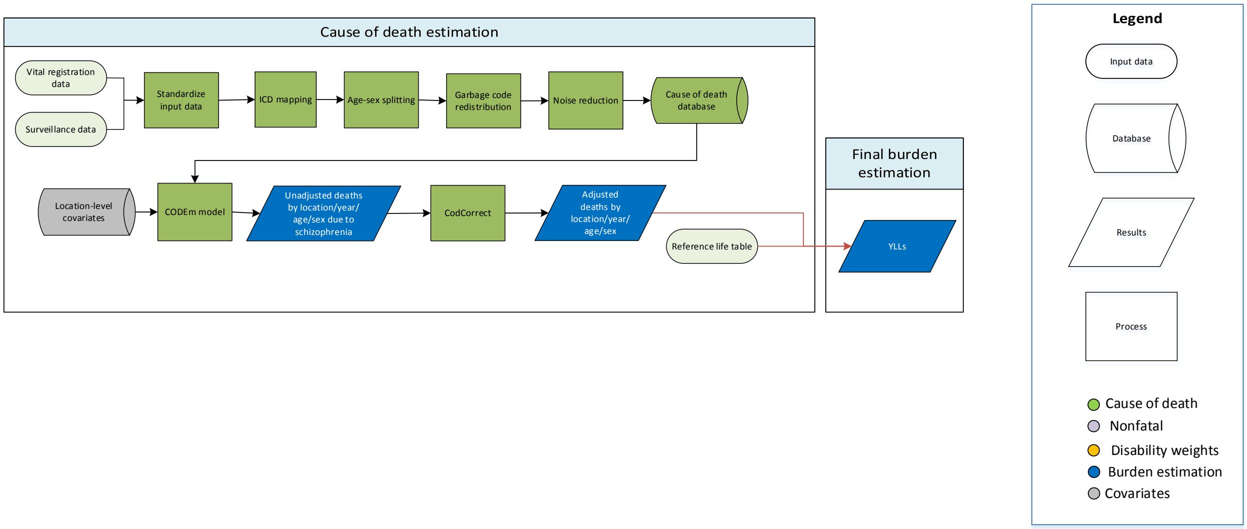

Input data

Data used to estimate deaths due to schizophrenia included centrally prepped vital registration and surveillance sources from the cause of death (COD) database. Per our outlier criteria, data from countries with sparse yet heterogeneous data were excluded as the data exaggerated fluctuations in deaths and gave implausible regional patterns. Excluded data were typically from lower-income countries.

Modeling strategy

Cause of death modeling for schizophrenia followed the general CODEm strategy. Age was restricted to deaths occurring between 25 and 80+ years. There were no substantial changes from GBD 2013; the same set of covariates were used with the addition of the Socio-Demographic Index (SDI) variable.

Limitations in modelling schizophrenia stem from cause attribution issues; for instance, varying practices in coding deaths can lead to misattribution (i.e., miscoding schizophrenia deaths to injuries). This trend can explain some of the geographical variations observed for schizophrenia mortality rates, which we will explore further in upcoming iterations of GBD.

Alcohol use disorders

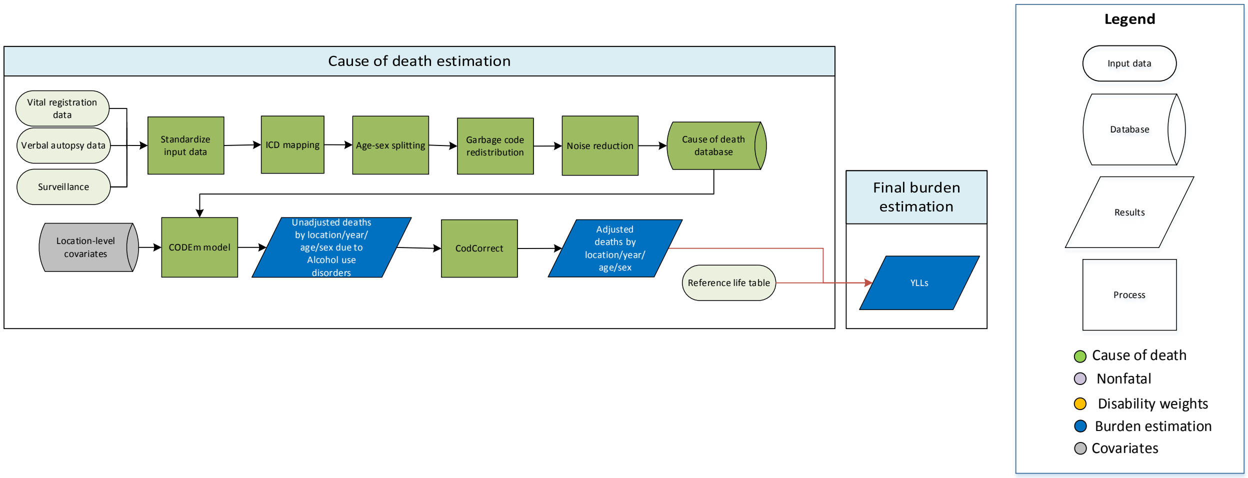

Input data

Data used to estimate deaths due to alcohol use disorders included centrally prepped vital registration, Chinese disease surveillance point (DSP) and verbal autopsy sources from the cause of death (COD) database. Per our outlier criteria, data from countries with sparse yet heterogeneous data were excluded as the data exaggerated fluctuations in deaths and gave implausible regional patterns. Excluded data were typically from lower-income countries.

Modeling strategy

Cause of death modeling for alcohol use disorders followed the general CODEm strategy. Age was restricted to deaths occurring between 15 and 80+ years. There were no substantial changes from GBD 2013; however, a review of model covariates was undertaken based on empirical evidence and expert feedback, which resulted in a new set of model covariates for GBD 2015. Additions included a prevalence of binge alcohol drinking covariate and the Socio-Demographic Index (SDI) variable. Based on expert consultation, we excluded the mean body mass index (BMI), mean cholesterol per capita, mean systolic blood pressure (mmHg), and red meat (kcal per capita) covariates from GBD 2015 as they provided no observable benefit to the model.

Limitations in modeling alcohol use stem from cause attribution issues; for instance, varying practices in coding deaths related to substance use disorders can lead to misattribution (i.e., miscoding alcohol-related deaths to cardiovascular diseases). This poses challenge to our redistribution strategy; however, we will continue to refine our approach in future iterations of GBD.

Drug use disorders

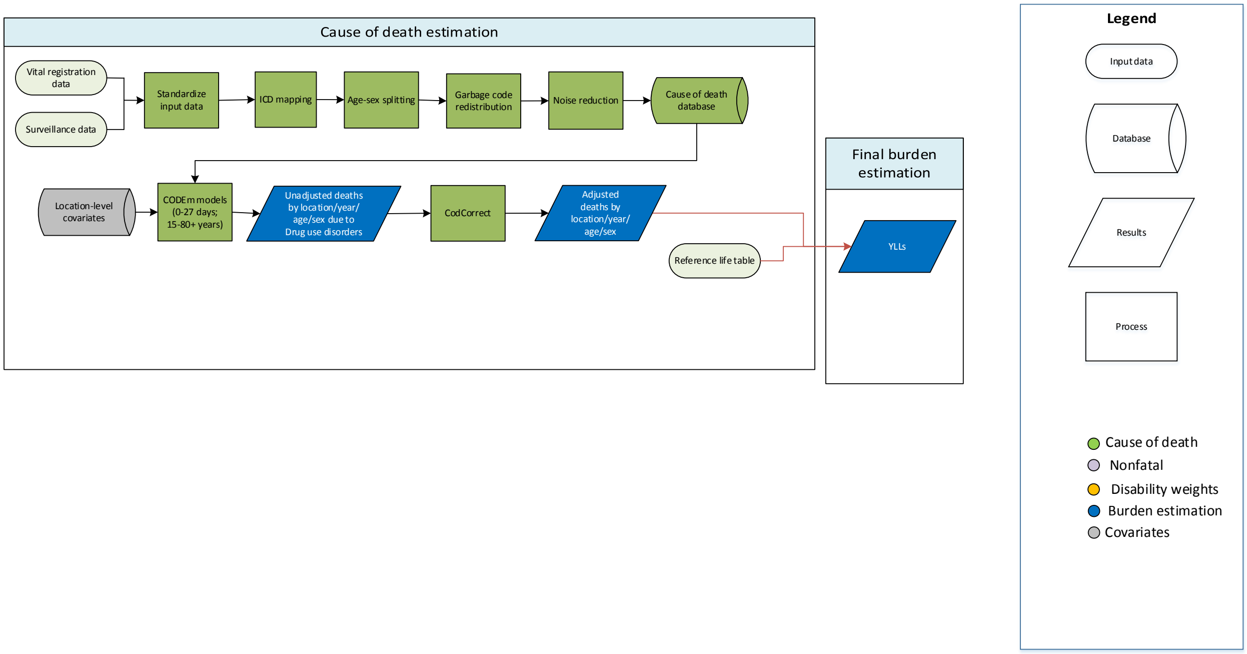

Input data

Data used to estimate deaths due to drug use disorders included centrally prepped vital registration and surveillance sources from the cause of death (COD) database. Per our outlier criteria, data from countries with sparse yet heterogeneous data were excluded as the data exaggerated fluctuations in deaths and gave implausible regional patterns. Excluded data were typically from lower-income countries.

Modelling strategy

Drug use disorders were modelled using the general CODEm approach and included the child models opioid use disorders, cocaine use disorders, amphetamine use disorders, and other drug use disorders. Drug use disorders were modelled separately in CODEm for deaths occurring between age 0 days and 27 days, and deaths occurring between age 15 and 80+ years.

In GBD 2013, the parent drug use disorders CODEm model was split proportionately into its constituent parts (opioid use disorders, cocaine use disorders, amphetamine use disorders, and other drug use disorders) through a custom process that used region/year/age/sex-specific vital registration data. In GBD 2015, sufficient data permitted a revised strategy to model individual drug use disorders through CODEm within the parent drug use envelope.

In GBD 2015, we sought to refine our redistribution strategy to more accurately represent unspecified poisoning deaths. Upon close review of ICD-coded deaths for unspecified poisoning, we identified a specific age-pattern, in which the death rate spiked in ages 15 to 50 years. Noting this trend, we conducted further review of data and found that these deaths were likely due to drug use disorders. We then redistributed unspecified poisoning deaths to drug use disorders in accordance with the appropriate age and sex pattern. Based on high-quality ICD-9 and ICD-10 data, we then reassigned these deaths to the drug use disorder sub-causes.

Opioid use disorders

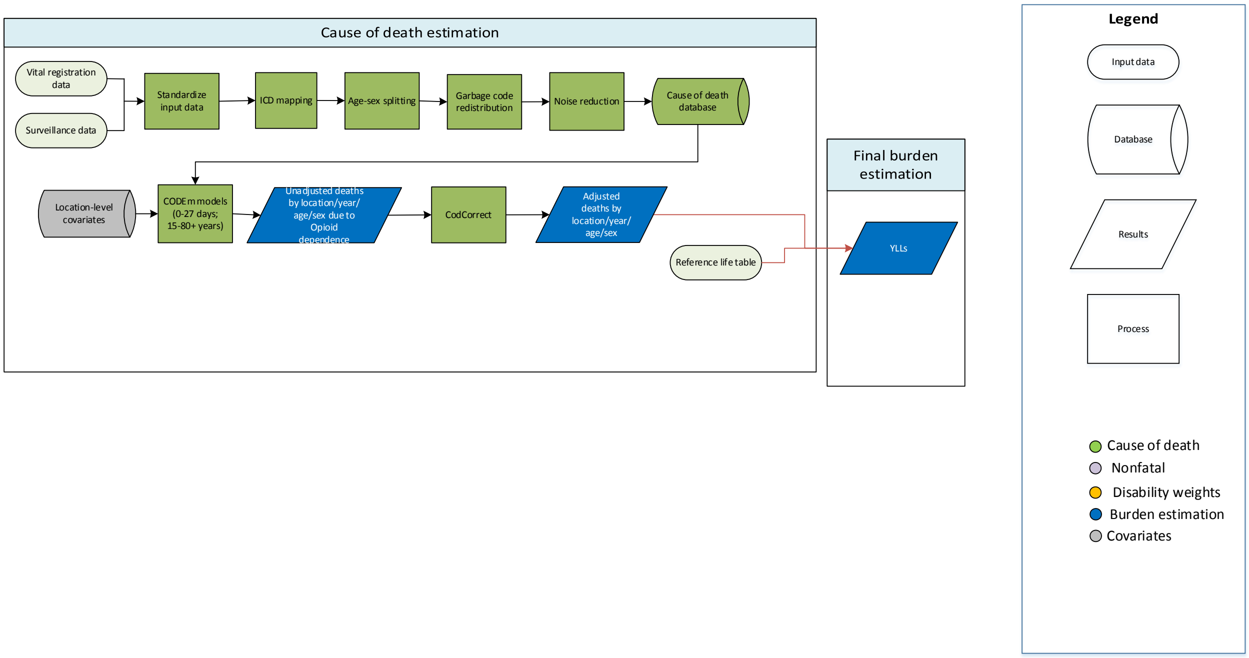

Input data

Data used to estimate deaths due to opioid use disorders included centrally prepped vital registration and China Disease Surveillance Points (DSP) sources from the cause of death (COD) database. Per our outlier criteria, data from countries with sparse yet heterogeneous data were excluded as the data exaggerated fluctuations in deaths and gave implausible regional patterns. Excluded data were typically from lower-income countries.

Modelling strategy

Opioid use disorders were modelled using the general CODEm approach and came under the parent drug use disorders model. Opioid use disorders were modelled separately in CODEm for deaths occurring between ages 0 days and 27 days, and deaths occurring between ages 15 and 80+ years.

In GBD 2013, the parent drug use disorders CODEm model was split proportionately into its constituent parts (opioid use disorders, cocaine use disorders, amphetamine use disorders, and other drug use disorders) through a custom process that used region/year/age/sex-specific vital registration data. In GBD 2015, sufficient data permitted a revised strategy to model individual drug use disorders through CODEm within the parent drug use envelope.

In GBD 2015, we sought to refine our redistribution strategy to more accurately represent unspecified poisoning deaths. Upon close review of ICD-coded deaths for unspecified poisoning, we identified a specific age-pattern, in which the death rate spiked in ages 15 to 50 years. Noting this trend, we conducted further review of data and found that these deaths were likely due to drug use disorders. We then redistributed unspecified poisoning deaths to drug use disorders in accordance with the appropriate age and sex pattern. Based on high-quality ICD-9 and ICD-10 data, we then reassigned these deaths to the drug use disorder sub-causes.

Through literature review, we also found that opioid-related dependency was a large contributor to deaths previously attributed to the residual "other drug use" category.[1–9] Noting this evidence, we redistributed a large portion of the "other drug use" deaths to the "opioid use disorder" category.

References

1 Australian Bureau of Statistics. Drug Induced Deaths, Australia, 1991-2001. 2003; published online July 15. http://www.abs.gov.au/ausstats/abs@.nsf/productsbytopic/E32F06E91C80389DCA256D640001E0FF?OpenDocument#6.%20TYPES%20OF%20DRUGS; (accessed April 20, 2016).

2 Darke S. The life of the heroin user: typical beginnings, trajectories and outcomes. Cambridge, UK: Cambridge University Press, 2010.

3 Darke S, Duflou J, Torok M. The comparative toxicology and major organ pathology of fatal methadone and heroin toxicity cases. *Drug Alcohol Depend* 2010; **106**: 1–6.

4 Darke S, Kaye S, Duflou J. Comparative cardiac pathology among deaths due to cocaine toxicity, opioid toxicity and non-drug-related causes. *Addict Abingdon Engl* 2006; **101**: 1771–7.

5 Kaye S, McKetin R, Duflou J, Darke S. Methamphetamine and cardiovascular pathology: a review of the evidence. *Addict Abingdon Engl* 2007; **102**: 1204–11.

6 Stenhouse G, Stephen D, Grieve JHK. Blood free morphine levels vary with concomitant alcohol and benzodiazepine use. *J Clin Forensic Med* 2004; **11**: 285–8.

7 Karch SB. Karch's Pathology of Drug Abuse, Third Edition. Boca Raton, Florida: CRC Press, 2002.

8 Karch SB. Karch's Pathology of Drug Abuse, Fourth Edition. Boca Raton, Florida: CRC Press, 2009.

9 Coffin PO, Galea S, Ahern J, Leon AC, Vlahov D, Tardiff K. Opiates, cocaine and alcohol combinations in accidental drug overdose deaths in New York City, 1990-98. *Addict Abingdon Engl* 2003; **98**: 739–47.

Cocaine use disorders

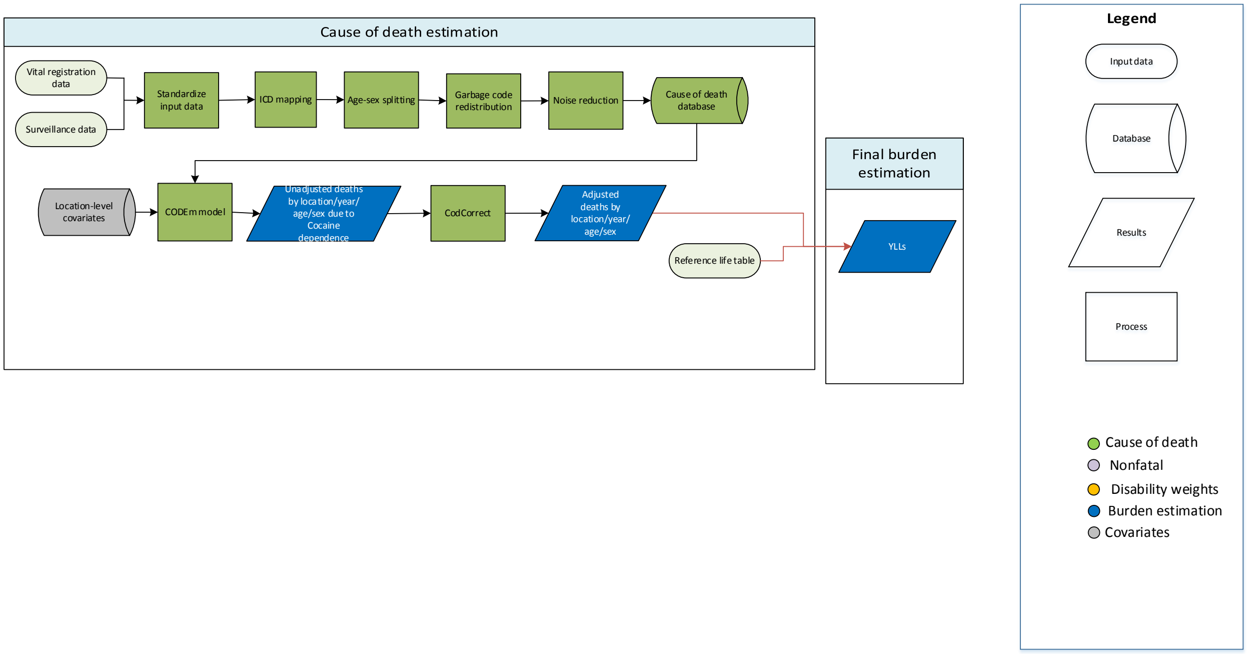

Input data

Data used to estimate deaths due to cocaine use disorders included centrally prepped vital registration and surveillance sources from the cause of death (COD) database. Per our outlier criteria, data from countries with sparse yet heterogeneous data were excluded as the data exaggerated fluctuations in deaths and gave implausible regional patterns. Excluded data were typically from lower-income countries.

Modeling strategy

Cocaine use disorders were modelled using the general CODEm approach and came under the drug use disorders parent model. Age was restricted to deaths occurring between 15 and 80+ years.

In GBD 2013, the parent drug use disorders CODEm model was split proportionately into its constituent parts (opioid use disorders, cocaine use disorders, amphetamine use disorders, and other drug use disorders) through a custom process that used region/year/age/sex-specific vital registration data. In GBD 2015, sufficient data permitted a revised strategy to model individual drug use disorders through CODEm within the parent drug use envelope.

In GBD 2015, we sought to refine our redistribution strategy to more accurately represent unspecified poisoning deaths. Upon close review of ICD-coded deaths for unspecified poisoning, we identified a specific age-pattern, in which the death rate spiked in ages 15 to 50 years. Noting this trend, we conducted further review of data and found that these deaths were likely due to drug use disorders. We

then redistributed unspecified poisoning deaths to drug use disorders in accordance with the appropriate age and sex pattern. Based on high-quality ICD-9 and ICD-10 data, we then reassigned these deaths to the drug use disorder sub-causes.

Amphetamine use disorders

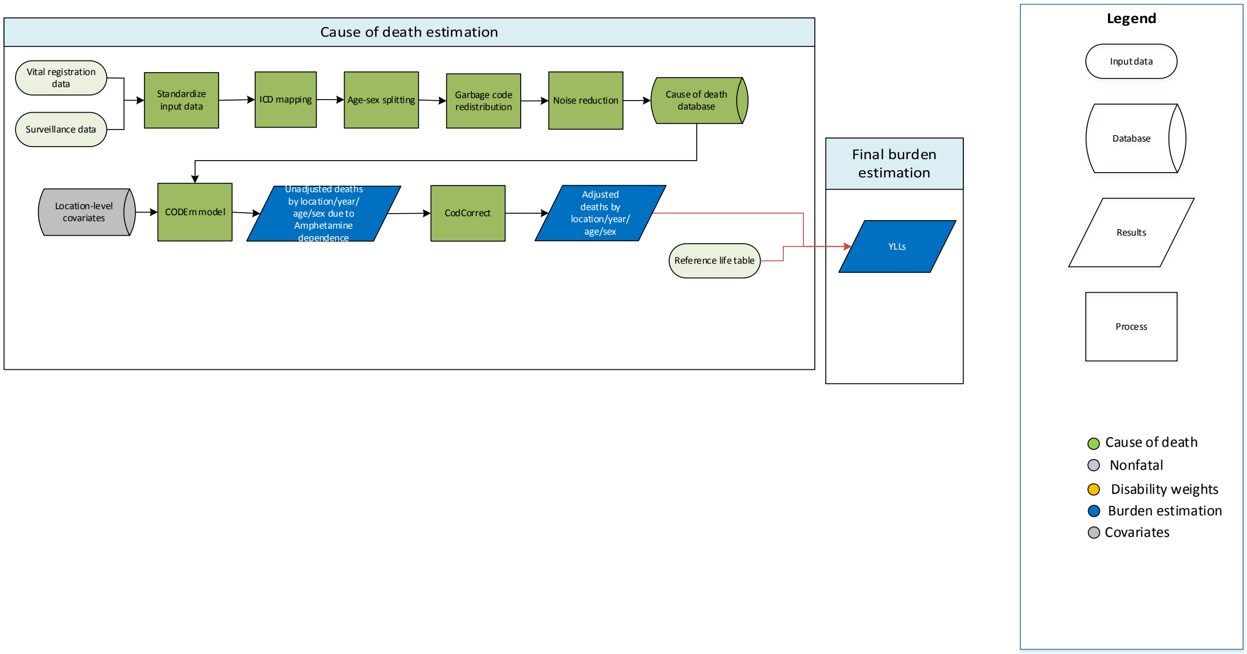

Input data

Data used to estimate deaths due to amphetamine use disorders included centrally prepped vital registration and surveillance sources from the cause of death (COD) database. Per our outlier criteria, data from countries with sparse yet heterogeneous data were excluded as the data exaggerated fluctuations in deaths and gave implausible regional patterns. Excluded data were typically from lower-income countries.

Modeling strategy

Amphetamine use disorders were modeled using the general CODEm approach and came under the drug use disorders parent model. Age was restricted to deaths occurring between 15 and 80+ years.

In GBD 2013, the parent drug use disorders CODEm model was split proportionately into its constituent parts (opioid use disorders, cocaine use disorders, amphetamine use disorders, and other drug use disorders) through a custom process that used region/year/age/sex-specific vital registration data. In GBD 2015, sufficient data permitted a revised strategy to model individual drug use disorders through CODEm within the parent drug use envelope.

In GBD 2015, we sought to refine our redistribution strategy to more accurately represent unspecified poisoning deaths. Upon close review of ICD-coded deaths for unspecified poisoning, we identified a specific age-pattern, in which the death rate spiked in ages 15 to 50 years. Noting this trend, we conducted further review of data and found that these deaths were likely due to drug use disorders. We then redistributed unspecified poisoning deaths to drug use disorders in accordance with the

appropriate age and sex pattern. Based on high-quality ICD-9 and ICD-10 data, we then reassigned these deaths to the drug use disorder sub-causes.

Other drug use disorders

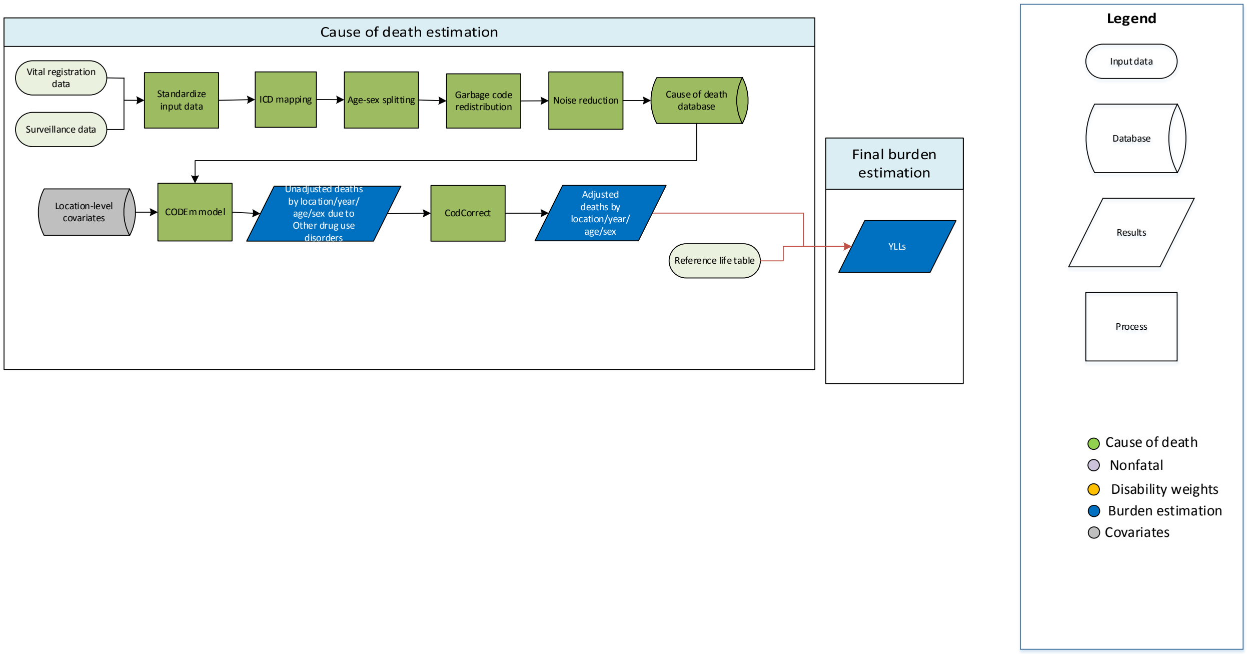

Input data

Data used to estimate deaths due to other drug use disorders included centrally prepped vital registration and surveillance sources from the cause of death (COD) database. Per our outlier criteria, data from countries with sparse yet heterogeneous data were excluded as the data exaggerated fluctuations in deaths and gave implausible regional patterns. Excluded data were typically from lower-income countries.

Modelling strategy

Other drug use disorders were modelled using the general CODEm approach and came under the drug use disorders parent model. Age was restricted to deaths occurring between 15 and 80+ years.

In GBD 2013, the parent drug use disorders CODEm model was split proportionately into its constituent parts (opioid use disorders, cocaine use disorders, amphetamine use disorders, and other drug use disorders) through a custom process that used region/year/age/sex-specific vital registration data. In GBD 2015, sufficient data permitted a revised strategy to model individual drug use disorders through CODEm within the parent drug use envelope.

In GBD 2015, we sought to refine our redistribution strategy to more accurately represent unspecified poisoning deaths. Upon close review of ICD-coded deaths for unspecified poisoning, we identified a

specific age-pattern, in which the death rate spiked in ages 15 to 50 years. Noting this trend, we conducted further review of data and found that these deaths were likely due to drug use disorders. We then redistributed unspecified poisoning deaths to drug use disorders in accordance with the appropriate age and sex pattern. Based on high-quality ICD-9 and ICD-10 data, we then reassigned these deaths to the drug use disorder sub-causes.

Anorexia nervosa

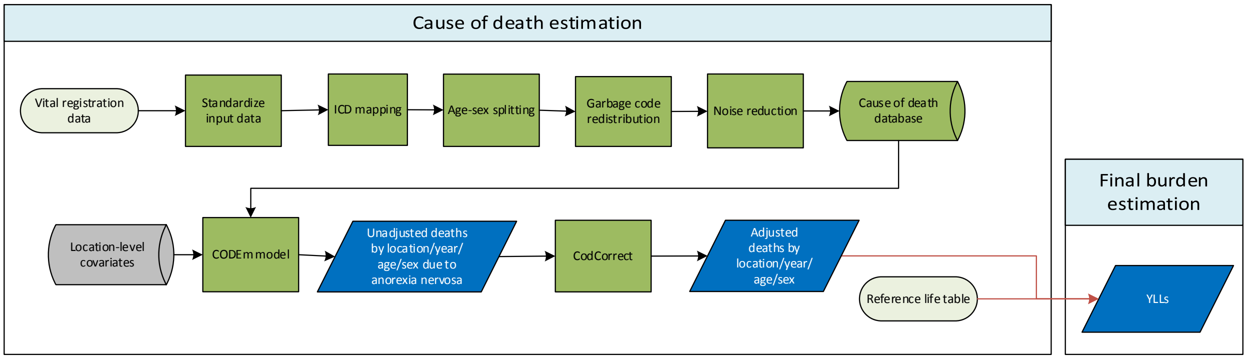

Input data

Data used to estimate anorexia nervosa mortality included centrally prepped vital registration data from the cause of death (COD) database. Per our outlier criteria, data points from sub-Saharan Africa were excluded as these did not result in a plausible model fit. Additionally, we excluded data from countries with small populations and sparse yet heterogeneous data as these sources exaggerated fluctuations in deaths and gave implausible regional patterns. Data were also excluded from countries including but not limited to Caribbean countries, Oceanic countries, some Central Latin American countries, and other developing countries (particularly those with small populations).

Modeling strategy

Anorexia nervosa was modeled using the standard CODEm approach and came under the eating disorders parent model. Age was restricted to deaths occurring between 5 and 49 years based on expert advice and patterns of prevalence seen in the non-fatal model. Only one location-level covariate, Socio-Demographic Index, was included in the anorexia nervosa model. A range of other covariates were tested including lagged distributed income, education, maternal education, sanitation, body mass index, and malnutrition. However, these covariates did not improve the model fit and were omitted from the final version.

In GBD 2013, anorexia nervosa deaths were extrapolated from the eating disorders model, which was modeled through a negative binomial approach. In GBD 2015, we changed this strategy and modeled anorexia nervosa deaths through a standard CODEm approach under the overarching eating disorders model as there was no benefit observed from applying the custom approach. As such, it was decided that all eating disorders (eating disorders, anorexia nervosa, and bulimia nervosa) would be modeled using CODEm.

The rate of deaths due to anorexia nervosa is relatively low although we suspect that in many countries, particularly lower-income countries, the coding of deaths to eating disorders is inconsistent. For example, a sharp increase in deaths was seen in the raw data from age 50 onward, which we believe is due to the incorrect assignments of deaths to anorexia nervosa, e.g., deaths from starvation due to dementia being incorrectly assigned to anorexia nervosa. This causes issues when trying to discern

plausible patterns in age and geography. We will explore this further in future iterations of GBD in order to determine how to optimally reflect deaths due to anorexia nervosa.

Bulimia nervosa

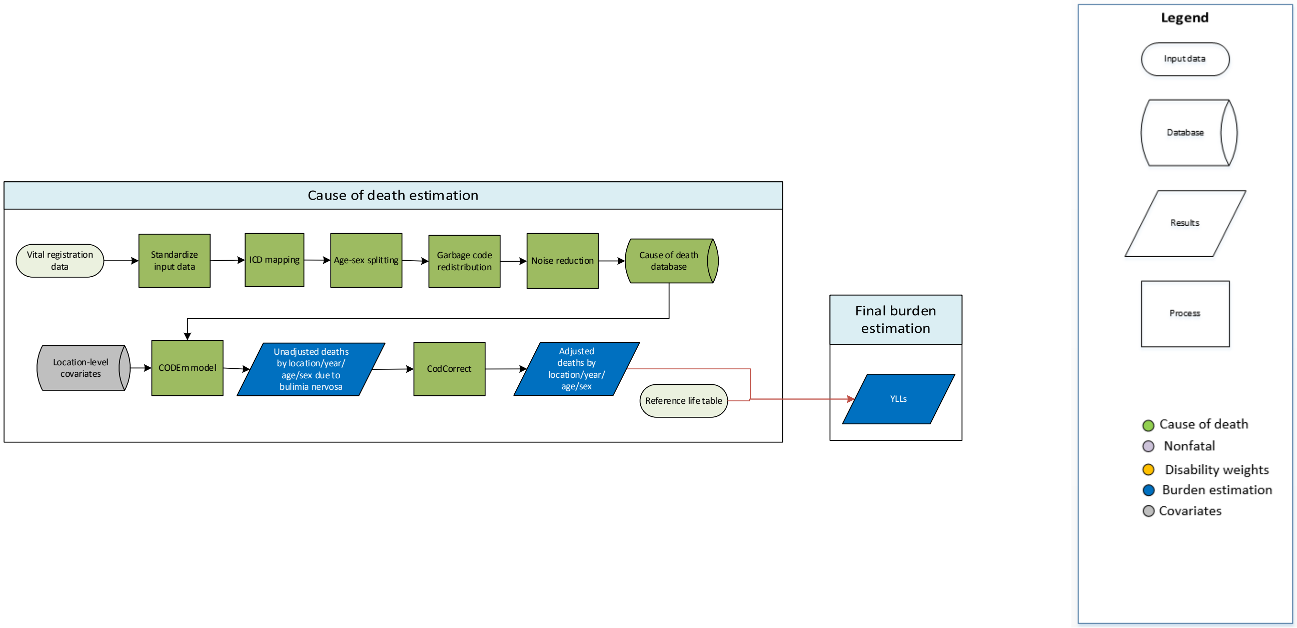

Input data

Data used to estimate bulimia nervosa mortality included centrally prepped vital registration data from the cause of death (COD) database. Per our outlier criteria, data points from sub-Saharan Africa, Greenland, Malta, and Brunei were excluded as these did not result in a plausible model fit. Additionally, we excluded data from countries with small populations and sparse yet heterogeneous data, as these sources exaggerated fluctuations in deaths and gave implausible regional patterns.

Modeling strategy

Bulimia nervosa was modeled using the standard CODEm approach and comes under the eating disorders parent model. Age was restricted to deaths occurring between 5 and 49 years based on expert advice and patterns of prevalence seen in the non-fatal model. Only one location-level covariate, Socio-Demographic Index, was included in the bulimia nervosa model. A range of other covariates were tested including lagged distributed income, education, maternal education, sanitation, body mass index, and

malnutrition. However, these covariates did not improve the model fit and were omitted from the final version.

In GBD 2013, bulimia nervosa was not modeled as a distinct cause of death. Any deaths due to bulimia nervosa were attributed to the eating disorders model. We changed this approach in GBD 2015, recognizing bulimia nervosa as an individual cause of death and therefore modeled it as a standard CODEm model under the overarching eating disorders model. This decision was based on observing deaths due to bulimia nervosa in high-quality vital registration data, such as data from the US. These data also include eating disorders not otherwise specified.

The rate of deaths due to bulimia nervosa is relatively low although we suspect that in many countries, particularly lower-income countries, the coding of deaths to eating disorders is inconsistent. Furthermore, the inclusion of deaths due to eating disorders not otherwise specified may add more noise to the model. This causes issues when trying to discern plausible patterns in age and geography. We plan to explore this further in future iterations of GBD in order to determine how to better reflect deaths due to bulimia nervosa.

Diabetes Mellitus

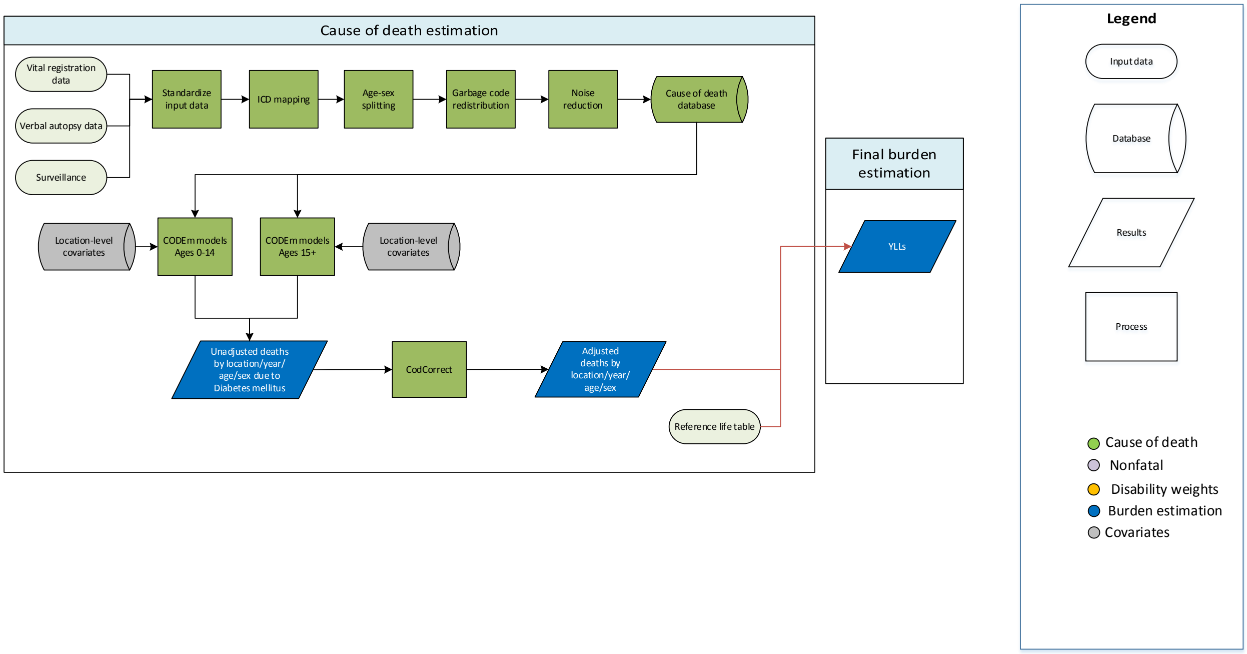

Input data

Verbal Autopsy Data: We outliered VA data points in urban Indian states where high-quality vital registration data were also available. We also outliered data points where the VA data were implausible in all age groups as we determined that these data sources were unreliable.

Vital Registration Data: We outliered all data in four urban Indian states where the source of the data was unreliable according to expert opinion. We also outliered ICD9BTL data points which were inconsistent with the rest of the data series and created unlikely time trends.

Modeling strategy

We used a slight variation on the standard CODEm approach to model deaths from diabetes mellitus. Since deaths in younger age groups are almost exclusively due to Type 1 diabetes while deaths in older ages are primarily due to Type 2, we used two models to estimate overall diabetes deaths. The first is for deaths in 0-14 year olds; the second is for deaths in 15-80+ year olds. In previous iterations of GBD, we used a similar approach, but the two models had age ranges of 0-24 and 25-80+. This change was made due to the increasing prevalence of Type 2 diabetes at younger ages, and thus the increasing likelihood that diabetes-related deaths in the 15-24 year age groups are due to Type 2 diabetes.

We have included two new variables, Socio-Demographic Index and the SEV scalar for diabetes mellitus, as possible covariates for selection in the ensemble modeling process for the model in older ages.

Acute Glomerulonephritis

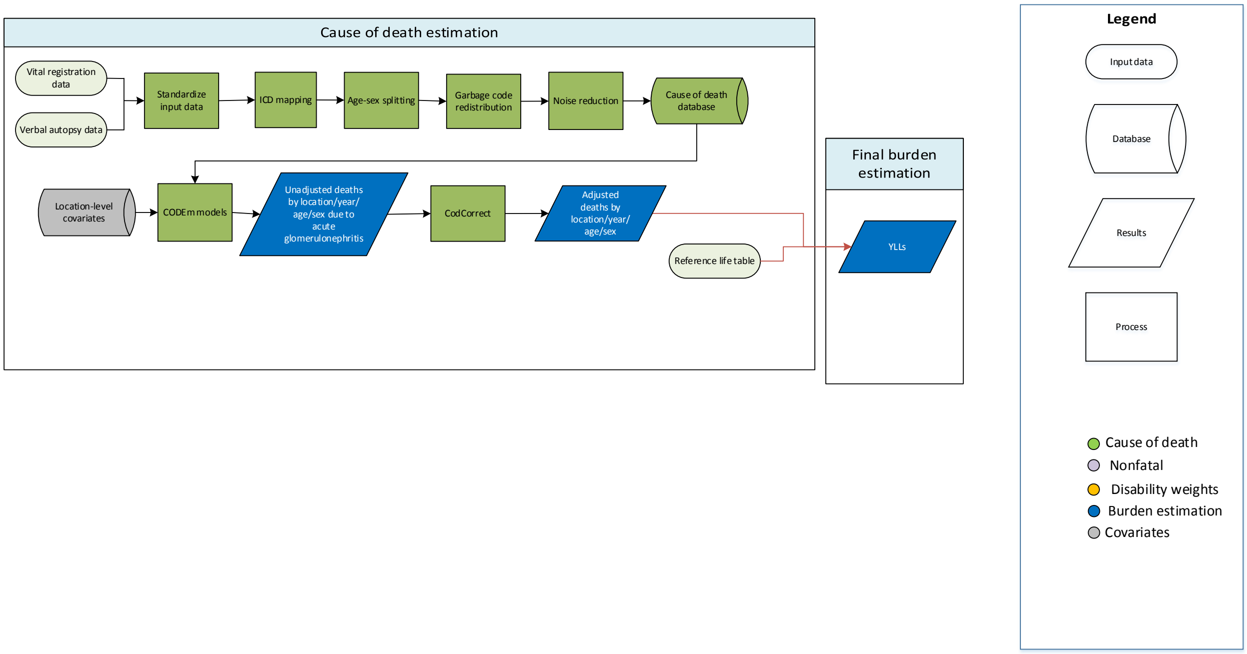

Input data

Data informing the acute glomerulonephritis estimates include vital registration. Mortality secondary to acute glomerulonephritis is uncommon, thus no literature review was conducted for this cause to identify registry, survey, or literature data. Outliers were identified by systematic examination of data points for all location-years. Data points were determined to be outliers if the data point was extremely low or high out of proportion for the region/year.

Modeling strategy

The estimation strategy used for fatal acute glomerulonephritis is largely similar to methods used in GBD 2013. Vital registration data were standardized, and mapped according to the GBD causes of death ICD mapping method. These data were then age-sex split, and appropriate redistribution of data was performed using garbage coding. After applying noise reduction, these data were then uploaded to the COD database. Age-restrictions for death estimations secondary to acute glomerulonephritis include 28 days for lower bounds, 80+ for upper bound. Iterations of models were assessed at the location/year/age-group/sex level to determine whether data points merited exclusion via outliering. The estimates are limited by a paucity of data for regions such as Eastern and Central sub-Saharan Africa.

Urinary diseases and male infertility

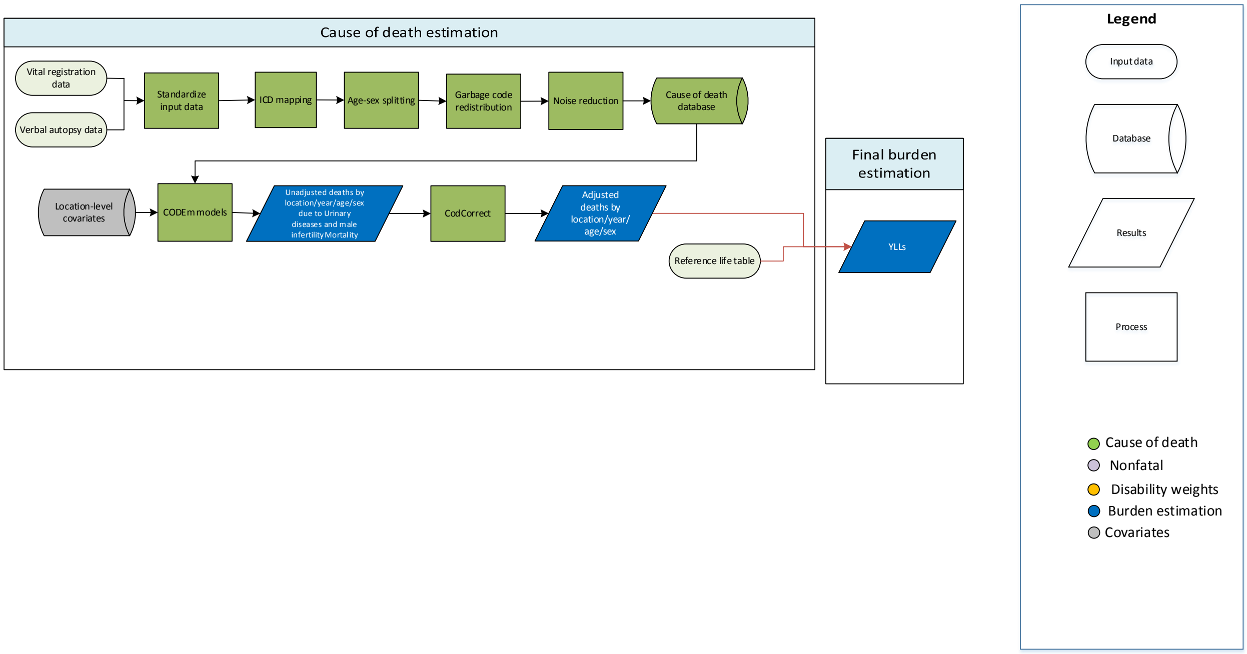

Input data

Data informing the urinary diseases and male infertility estimates include vital registration and verbal autopsy data. Mortality secondary to urinary diseases and male infertility is uncommon, thus no literature review was conducted for this cause to identify registry, survey, or literature data. Outliers were identified by systematic examination of data points for all location-years. Data points were determined to be outliers if the data point was extremely low or high out of proportion for the region/year. No crosswalks were used for this data analysis.

Modeling strategy

The estimation strategy used for fatal urinary diseases and male infertility is largely similar to methods used in GBD 2013. Vital registration data were standardized, and mapped according to the GBD COD ICD mapping method. These data were then age-sex split, and appropriate redistribution of data was performed using garbage coding. After applying noise reduction, these data were then uploaded to the COD database. Country-level covariates included in the model are the following: “education,” “health system access 2,” “sanitation,” and “SDI.” Age-restrictions for death estimations secondary to urinary diseases and male infertility include 0 days for lower bounds, 80+ for upper bound. Iterations of models were assessed at the location/year/age-group/sex level to determine whether data points merited exclusion via outliering. The estimates are limited by a paucity of data for regions such as Eastern and Central sub-Saharan Africa. The results of this disease differ by gender as no “male infertility” estimates were performed among women.

Interstitial Nephritis and Urinary Tract Infections

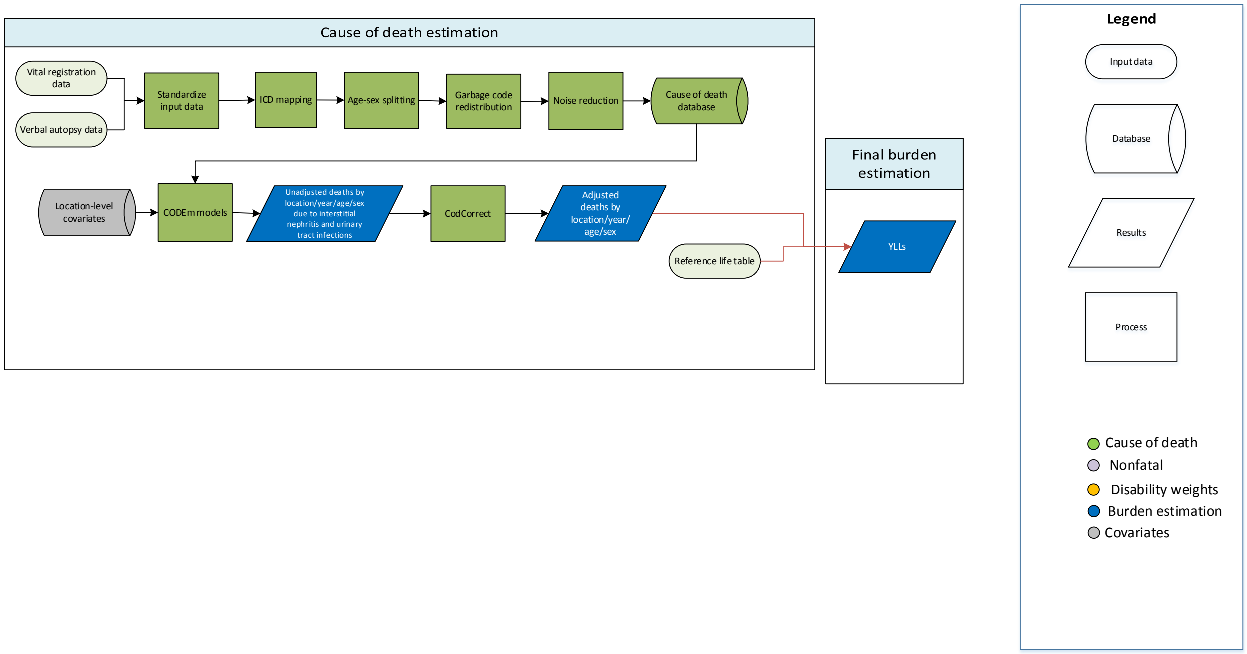

Input data

Data informing the interstitial nephritis estimates include vital registration and verbal autopsy data. Mortality secondary to interstitial nephritis is uncommon, thus no literature review was conducted for this cause to identify registry, survey, or literature data. Outliers were identified by systematic examination of data points for all location-years. Data points were determined to be outliers if the data point was extremely low or high out of proportion for the region/year. No crosswalks were used for this data analysis.

Modeling strategy

The estimation strategy used for fatal interstitial nephritis is largely similar to methods used in GBD 2013. Vital registration data were standardized, and mapped according to the GBD COD ICD mapping method. These data were then age-sex split, and appropriate redistribution of data was performed using garbage coding. After applying noise reduction, these data were then uploaded to the COD database. Country-level covariates included in the model are the following: “education,” “health system access 2,” “sanitation,” and “SDI.” Age-restrictions for death estimations secondary to interstitial nephritis include 0 days for lower bounds, 80+ for upper bound. Iterations of models were assessed at the location/year/age-group/sex level to determine whether data points merited exclusion via outliering. The estimates are limited by a paucity of data for regions such as Eastern and Central sub-Saharan Africa.

Urolithiasis

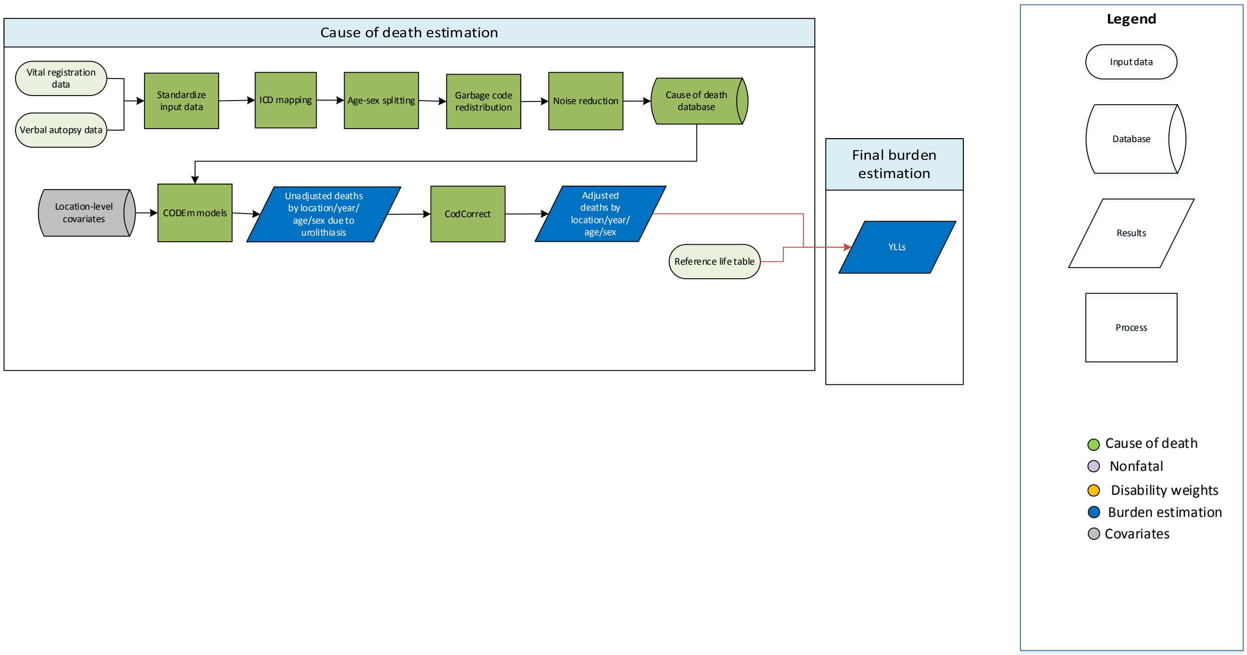

Input data

Data that informed the urolithiasis estimates include vital registration and verbal autopsy data. Mortality secondary to urolithiasis is uncommon, thus no literature review was conducted for this cause to identify registry, survey, or literature data. Outliers were identified by systematic examination of data points for all location-years. Data points were determined to be outliers if the data point was extremely low or high out of proportion for the region/year.

Modeling strategy

The estimation strategy used for fatal urolithiasis is largely similar to methods used in GBD 2013. Vital registration data were standardized, and mapped according to the GBD COD ICD mapping method. These data were then age-sex split, and appropriate redistribution of data was performed using garbage coding. After applying noise reduction, these data were then uploaded to the COD database. Age-restrictions for death estimations secondary to urolithiasis include 5 years for lower bounds, 80+ for upper bound. Iterations of models were assessed at the location/year/age-group/sex level to determine whether data points merited exclusion via outliering. The estimates are limited by a paucity of data for regions such as Western sub-Saharan Africa.

Other urinary diseases

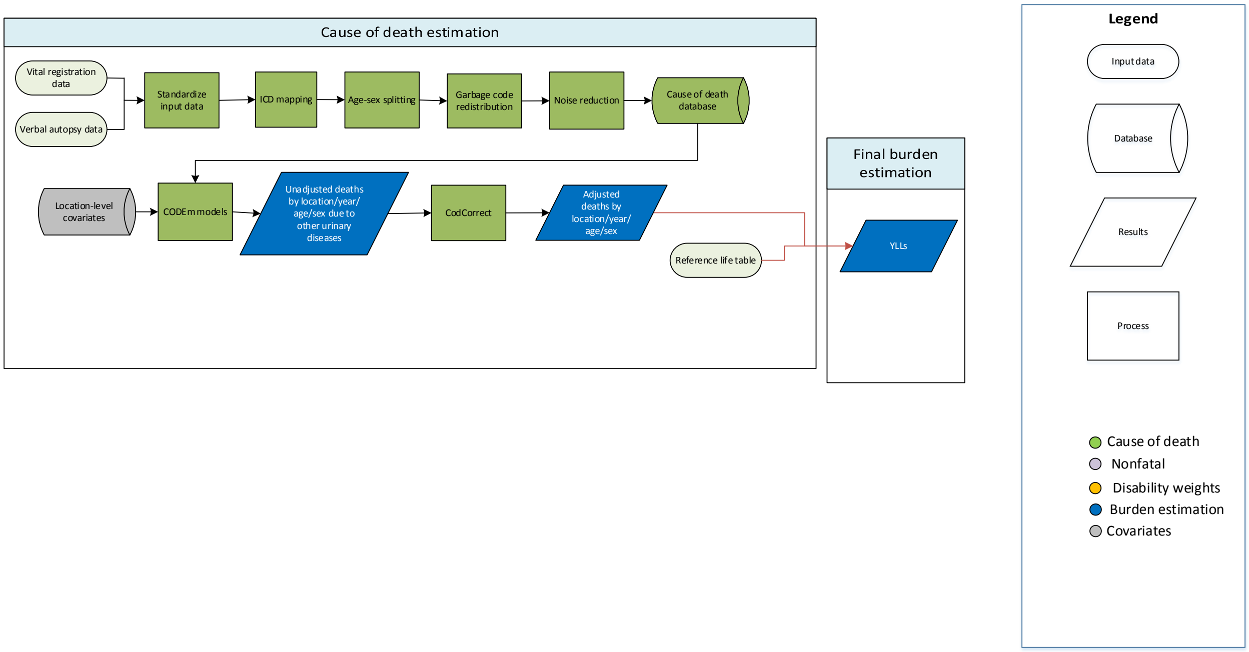

Input data

Data informing other urinary diseases include vital registration and verbal autopsy data. Mortality secondary to other urinary diseases is uncommon, thus no literature review was conducted for this cause to identify registry, survey, or literature data. Outliers were identified by systematic examination of data points for all location-years. Data points were determined to be outliers if the data point was extremely low or high out of proportion for the region/year.

Modeling strategy

The estimation strategy used for other urinary diseases is largely similar to methods used in GBD 2013. Vital registration data were standardized, and mapped according to the GBD COD ICD mapping method. These data were then age-sex split, and appropriate redistribution of data was performed using garbage coding. After applying noise reduction, these data were then uploaded to the COD database. Age-restrictions for death estimations secondary to urinary diseases and male infertility include 0 days for lower bounds, 80+ for upper bound. Iterations of models were assessed at the location/year/age-group/sex level to determine whether data points merited exclusion via outliering. The estimates are limited by a paucity of data for regions such as Eastern and Central sub-Saharan Africa.

Gynecological conditions

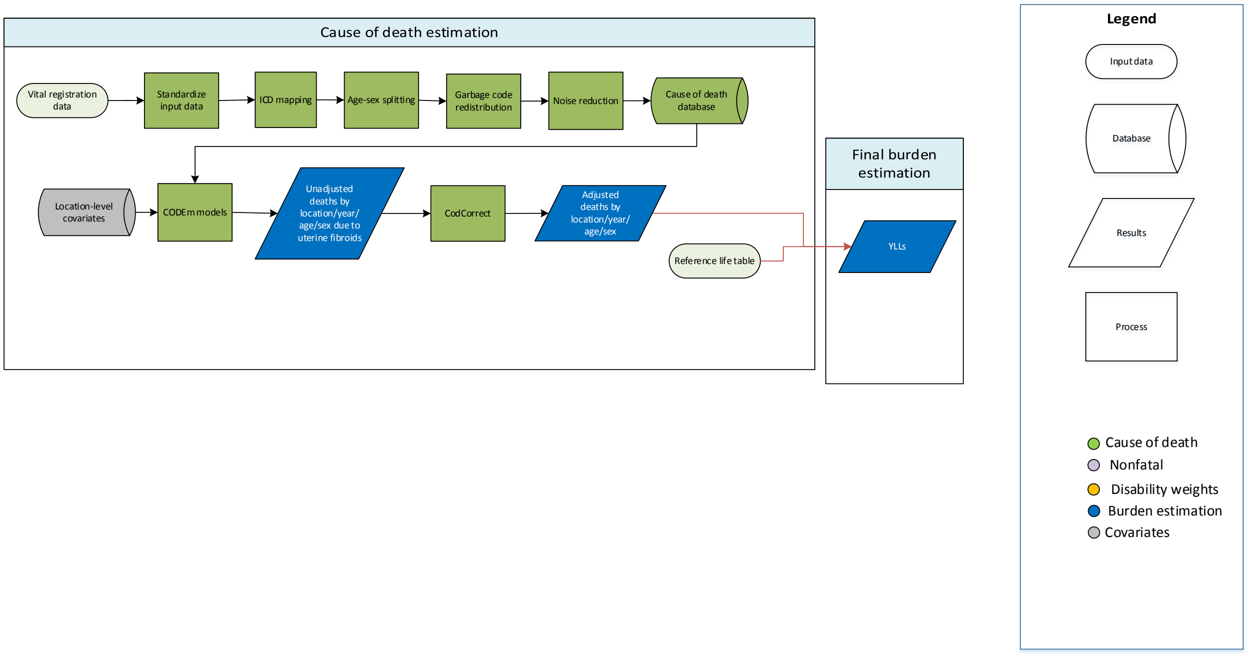

Input data

Vital registration data were used to estimate deaths for each of the five fatal gynecological conditions. We only modeled deaths among women. Data points were selected as outliers if they were implausibly high, low, or significantly conflicted with established age or temporal patterns.

Modeling strategy

For GBD 2013, after we estimated mortality due to all gynecological diseases using CODEm, we used a proportional allocation method to attribute deaths to uterine fibroids, endometriosis, genital prolapse, polycystic ovarian syndrome, and other gynecological diseases. We assumed no deaths from premenstrual syndrome and primary infertility. When possible, we used the region-age-specific proportion of mortality derived from the raw data for each sub-cause of death and applied these proportions to the overall gynecological death estimates, then to super-region-age-specific estimates, and last to global estimates. For GBD 2015, we again assume no deaths from premenstrual syndrome and primary infertility. However, CODEm was used to model each of the remaining subcategories individually. This was possible due to new data that were added this cycle, and this modeling strategy improved model fit over the old approach.

This year, we have reassigned deaths due to leiomyomas and other benign uterine tumors to our uterine fibroids cause. This has contributed to an overall increase in the estimated number of deaths from uterine fibroids and subsequently contributed to an increase in deaths from all gynecological conditions.

Endocrine, metabolic, blood, and immune disorders

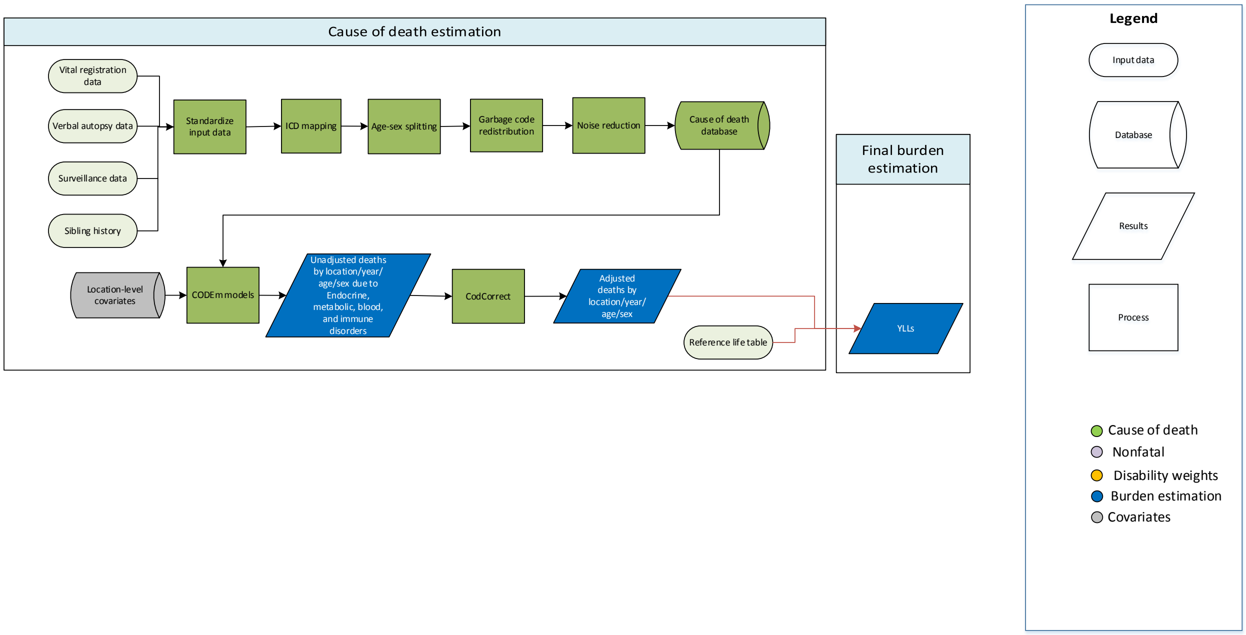

Input data

Vital registration, verbal autopsy, sibling history, and surveillance data were used. Data were outliered if they largely conflicted with the majority of data from other studies conducted either in the same or different countries (with similar sociodemographic characteristics) in the same region.

Modeling strategy

A general CODEm modeling strategy was used. There were no substantive changes from GBD 2013 in terms of modeling strategy.

Musculoskeletal disorders

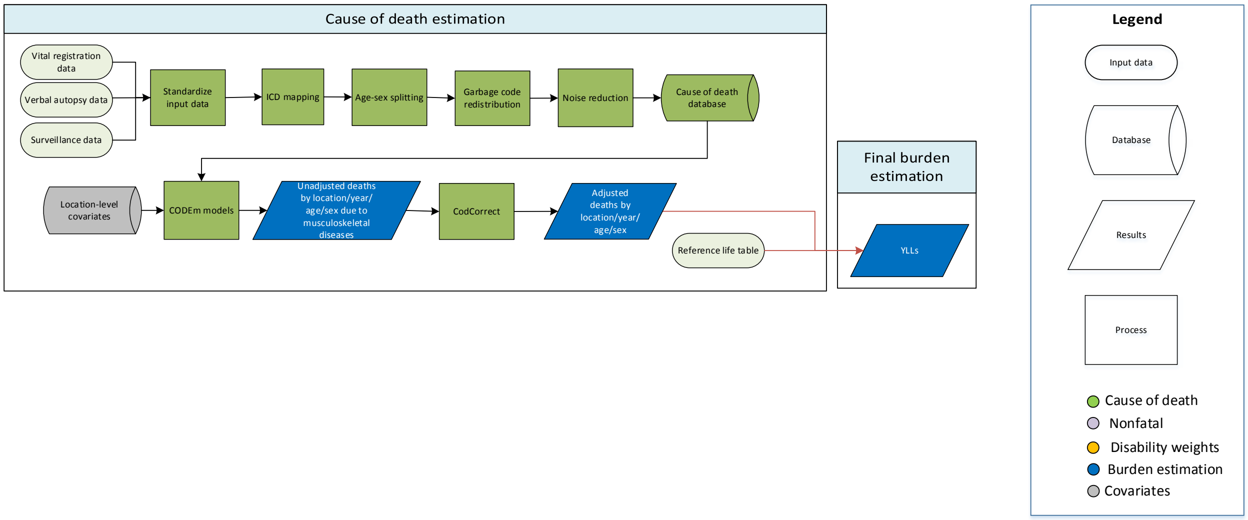

Input data

Data used to estimate mortality from musculoskeletal disorders (MSK) included vital registration, verbal autopsy (VA), and China disease surveillance point data from the cause of death (COD) database. Our outlier criteria excluded data points that were implausibly high or low relative to global or regional patterns, substantially conflicted with established age or temporal patterns, or significantly conflicted with other data sources based from the same locations or locations with similar characteristics (i.e., Socio-Demographic Index).

Based on these criteria, in GBD 2015 we excluded VA data from India and Bangladesh since they did not sufficiently capture MSK deaths and overall, estimates for the subcontinent were disproportionately high. In GBD 2013, a large amount of vital registration data for the oldest age groups in Mexican states were outliered. For GBD 2015, we retained all these data, which led to notably high estimates for some states, particularly in Oaxaca for males.

Modeling strategy

The standard CODEm modeling approach was applied to estimate deaths due to musculoskeletal disorders. We applied the same covariates used in GBD 2013, excluding the four covariates for proportions of population at 0-15, 15-30, 30-45, and 45 and over latitudes, which had no observable benefit to the model. We also added the Socio-Demographic Index (SDI) variable created for GBD 2015. Otherwise, there were no changes from the GBD 2013 modeling strategy.

The variation in mortality levels may be influenced by variations in coding practices with regard to the osteoporosis codes. For GBD 2016 we plan to examine the contribution of osteoporosis to all musculoskeletal mortality more closely.

Rheumatoid arthritis

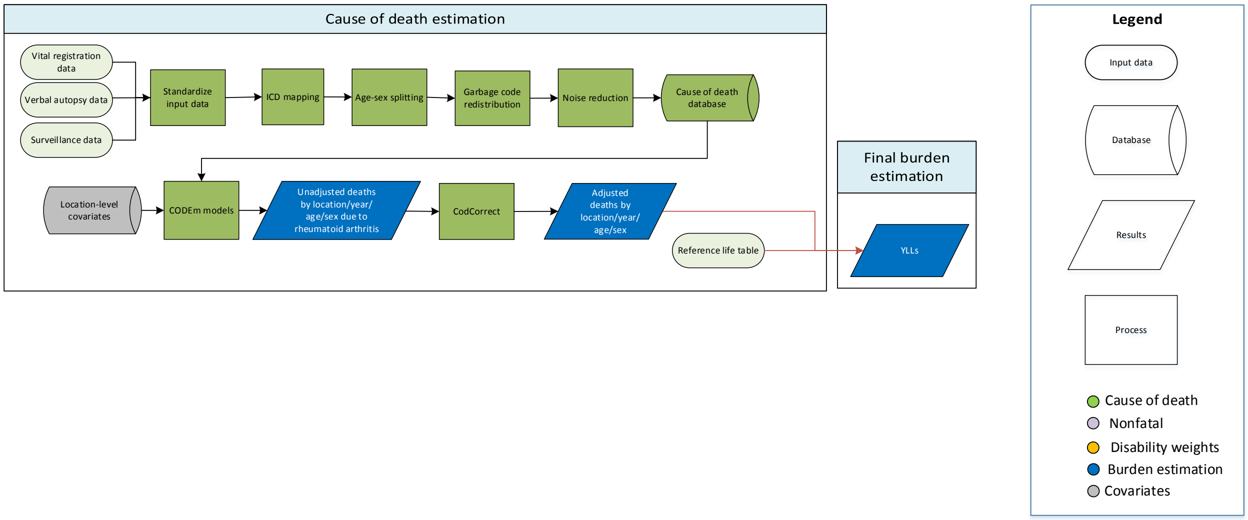

Input data

Data used to estimate rheumatoid arthritis mortality included vital registration, verbal autopsy, and China disease surveillance point data from the cause of death (COD) database. Our outlier criteria excluded data points that were implausibly high or low relative to global or regional patterns, substantially conflicted with established age or temporal patterns, or significantly conflicted with other data sources based from the same locations or locations with similar characteristics (i.e., Socio-Demographic Index).

In GBD 2015, we re-included many vital registration data points for the oldest age groups in Mexican states that were dropped in GBD 2013. We also outliered all data points for men in Tibet, as these led to disproportionately high estimates.

Modeling strategy

The standard CODEm modeling approach was applied to estimate deaths due to rheumatoid arthritis. We applied the same covariates used in GBD 2013 but excluded the four covariates for proportions of population at 0-15, 15-30, 30-45, and 45 and over latitudes, which had no observable benefit to the model. We added the Socio-Demographic Index (SDI) variable created for GBD 2015. Otherwise, there were no changes from the GBD 2013 modeling strategy.

Other musculoskeletal disorders

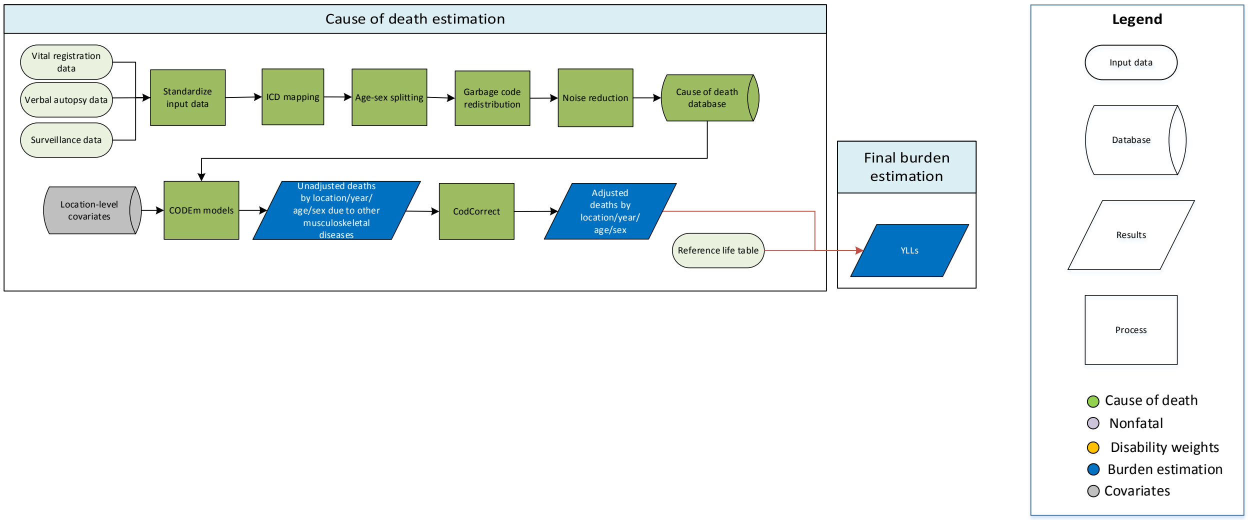

Input data

Data used to estimate mortality of other musculoskeletal disorders (MSK) included vital registration, verbal autopsy (VA), and China disease surveillance point data from the cause of death (COD) database. Our outlier criteria excluded data points that were implausibly high or low relative to global or regional patterns, substantially conflicted with established age or temporal patterns, or significantly conflicted with other data sources based from the same locations or locations with similar characteristics (i.e., Socio-Demographic Index). Based on these criteria, we excluded VA studies from Eastern and Western sub-Saharan Africa as VA was not sufficient in capturing other MSK deaths and estimates for the regions were disproportionately high.

Modeling strategy

The standard CODEm modeling approach was applied to estimate deaths due to other musculoskeletal disorders. We applied the same covariates used in GBD 2013, excluding the four covariates for proportions of population at 0-15, 15-30, 30-45, and 45 and over latitudes, which had no observable benefit to the model. We added the Socio-Demographic Index (SDI) variable created for GBD 2015. Otherwise, there were no changes from the GBD 2013 modeling strategy.

Congenital anomalies

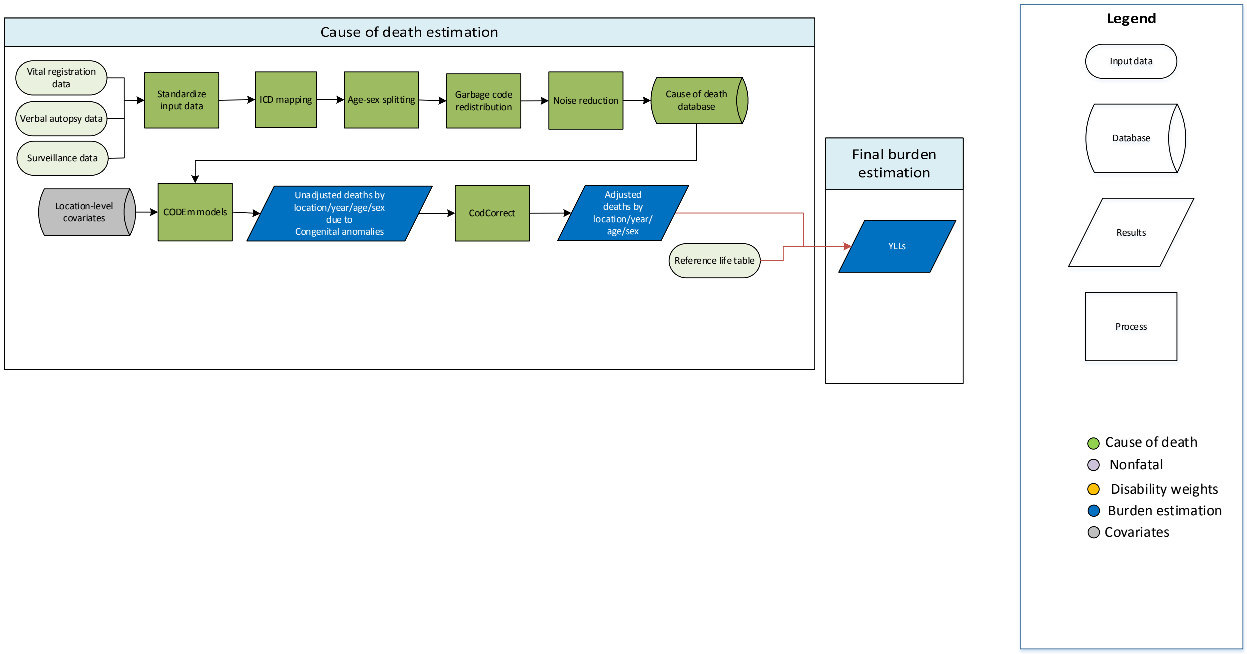

Input data

Input data for estimating mortality due to congenital anomalies was centrally extracted, processed, and stored in causes of death (COD) database. Vital registration (VR) was the dominant data type, followed by verbal autopsy (VA) and surveillance. Those COD data sources that specified the sub-cause of birth defect were included in estimation of both the parent congenital anomalies model as well as in sub-type-specific models. Data exclusions were limited. We outliered all VA data in those over 5 years old as the age patterns were unreliable and led to poor model performance in the under-5 age groups. We also excluded some data sources from the parent model where only a subset of sub-causes were specified (e.g., congenital heart disease, neural tube defects, and other congenital anomalies) and the sum of the sub-causes clearly represented systematic underreporting of one of the sub-causes. Systematic underreporting was suspected when sex- and age-specific rates were more than an order of magnitude lower than neighboring or comparable locations. These data sources were still included for those sub-cause specific models where reporting was reliable.

Modeling strategy

All congenital anomalies were estimated using cause-of-death ensemble modeling (CODEm). This was unchanged from GBD 2013. Specific causes included neural tube defects, congenital heart disease, Down syndrome, other chromosomal disorders, and other congenital anomalies. We assumed no mortality from either Klinefelter syndrome or Turner syndrome.

Skin and subcutaneous diseases

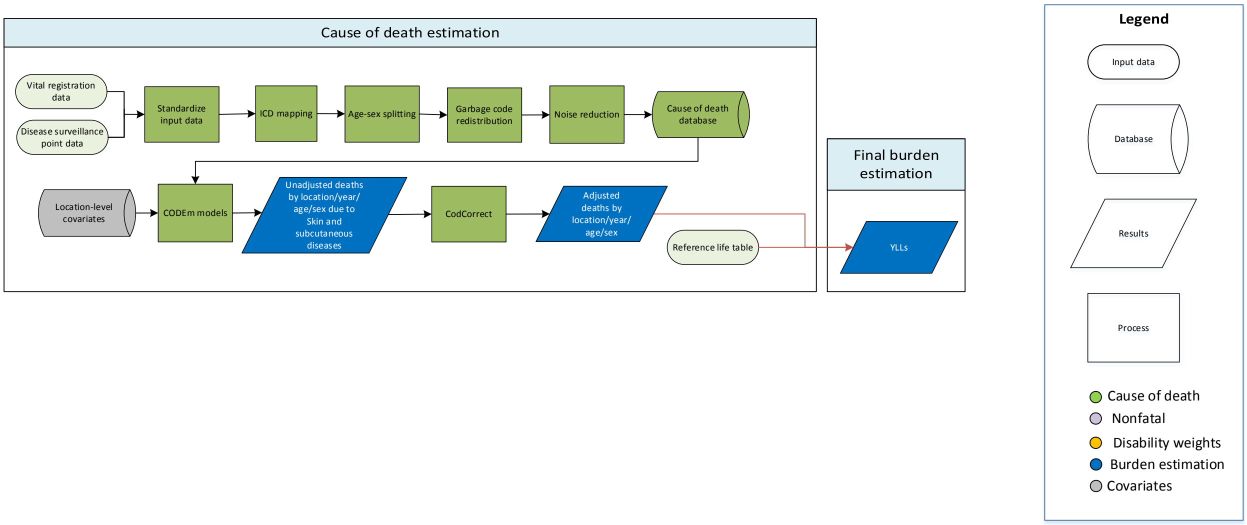

Input data

Data used to estimate mortality of skin and subcutaneous diseases consisted of vital registration and China disease surveillance point (DSP) sources from the cause of death (COD) database. Outlier criteria excluded data points that were implausibly high or low relative to global or regional patterns and data from countries with small populations.

Modeling strategy

The standard CODEm modeling approach was used to estimate deaths due to skin and subcutaneous diseases. CODEm parameters were centrally defined. We evaluated over 30 models at different stages of the modeling process as updates were made to the data and the modeling coefficients. COD models were evaluated by comparing age-standardized death rates per 100,000 people to GBD 2013 best model for 1990, and 2013 individually for males and females. We also compared age-standardized annualized rate of change for death rate per 100,000 people to GBD 2013.

We applied the same covariates used in GBD 2013, excluding the four covariates for proportions of population at latitudes 0-15, 15-30, 30-45, and 45 and over. We also added the standardized exposure variable scalar (SEV-Scalar) for unsafe sanitation and Socio-Demographic Index (SDI) variable created for GBD 2015. Otherwise, there were no changes from the GBD 2013 modeling strategy.

Cellulitis

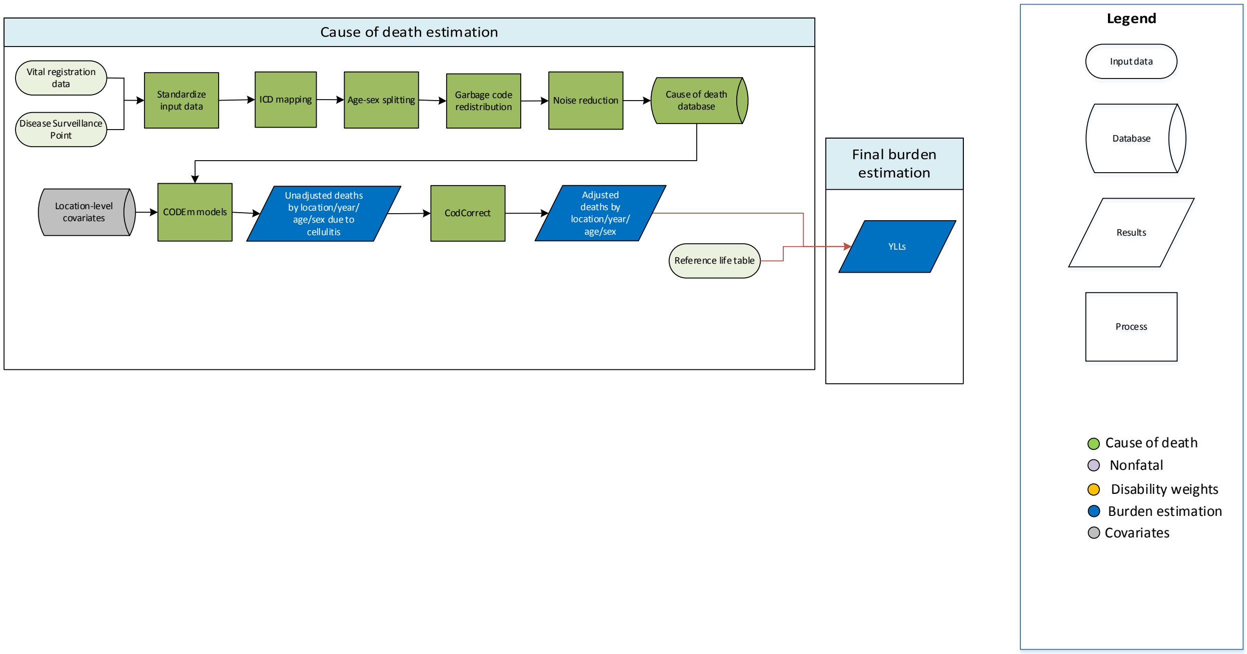

Input data

Data used to estimate cellulitis mortality consisted of vital registration and Chinese disease surveillance point (DSP) data from the cause of death (COD) database. Outlier criteria excluded data points that were implausibly high or low relative to global or regional patterns and data from countries with small populations.

Modeling strategy

The standard CODEm modeling approach was used to estimate deaths due to cellulitis. CODEm parameters were centrally defined. We evaluated over 30 models at different stages of the modeling process as updates were made to the data and the modeling coefficients. COD models were evaluated by comparing age-standardized death rates per 100,000 people to GBD 2013 best model for 1990, and 2013 individually for males and females. We also compared age-standardized annualized rate of change for death rate per 100,000 people to GBD 2013.

We applied the same covariates used in GBD 2013, excluding the four covariates for proportions of population at latitudes 0-15, 15-30, 30-45, and 45 and over. We also added the standardized exposure variable scalar (SEV-Scalar) for unsafe sanitation and Socio-Demographic Index (SDI) variable created for GBD 2015. Otherwise, there were no changes from the GBD 2013 modeling strategy.

Pyoderma

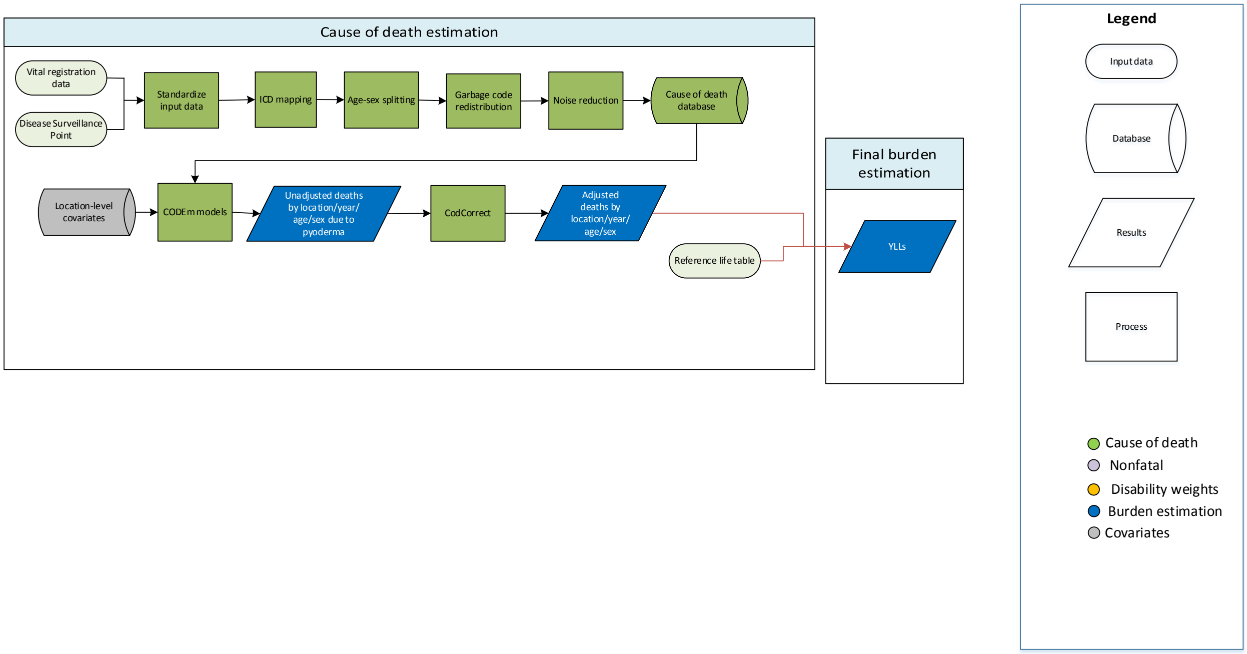

Input data

Data used to estimate pyoderma mortality included centrally prepped vital registration and China disease surveillance point sources from the cause of death (COD) database. Outlier criteria excluded data points that were implausibly high or low relative to global or regional patterns and data from countries with small populations.

Modeling strategy

The standard CODEm modeling approach was used to estimate deaths due to pyoderma. CODEm parameters were centrally defined. We evaluated over 30 models at different stages of the modeling process as updates were made to the data and the modeling coefficients. COD models were evaluated by comparing age-standardized death rates per 100,000 people to GBD 2013 best model for 1990, and 2013 individually for males and females. We also compared age-standardized annualized rate of change for death rate per 100,000 people to GBD 2013.

We applied the same covariates used in GBD 2013, excluding the four covariates for proportions of population at latitudes 0-15, 15-30, 30-45, and 45 and over. We also added the standardized exposure variable scalar (SEV-Scalar) for unsafe sanitation and Socio-Demographic Index (SDI) variable created for GBD 2015. Otherwise, there were no changes from the GBD 2013 modeling strategy.

Decubitus ulcer

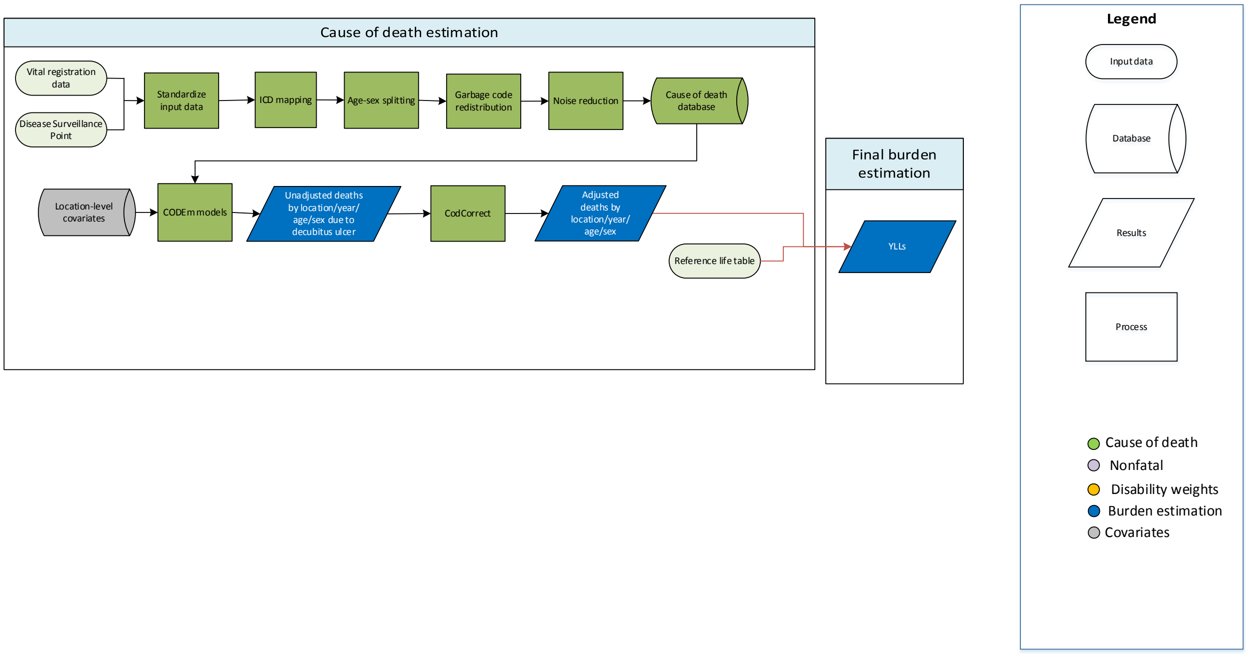

Input data

Data used to estimate mortality of decubitus ulcer consisted of vital registration and China disease surveillance point (DSP) sources from the cause of death (COD) database. Outlier criteria excluded data points that were implausibly high or low relative to global or regional patterns and data from countries with small populations.

Modeling strategy

The standard CODEm modeling approach was used to estimate deaths due to decubitus ulcer. CODEm parameters were centrally defined. We evaluated more than 30 models at different stages of the modeling process as updates were made to the data and the modeling coefficients. COD models were evaluated by comparing age-standardized death rates per 100,000 people to GBD 2013 best model for 1990, and 2013, individually for males and females. We also compared age-standardized annualized rate of change for death rate per 100,000 people to GBD 2013.

We applied the same covariates used in GBD 2013, excluding the four covariates for proportions of population at latitudes 0-15, 15-30, 30-45, and 45 and over. We also added the standardized exposure variable scalar (SEV-Scalar) for unsafe sanitation and Socio-Demographic Index (SDI) variable created for GBD 2015. Otherwise, there were no changes from the GBD 2013 modeling strategy.

Other skin and subcutaneous diseases

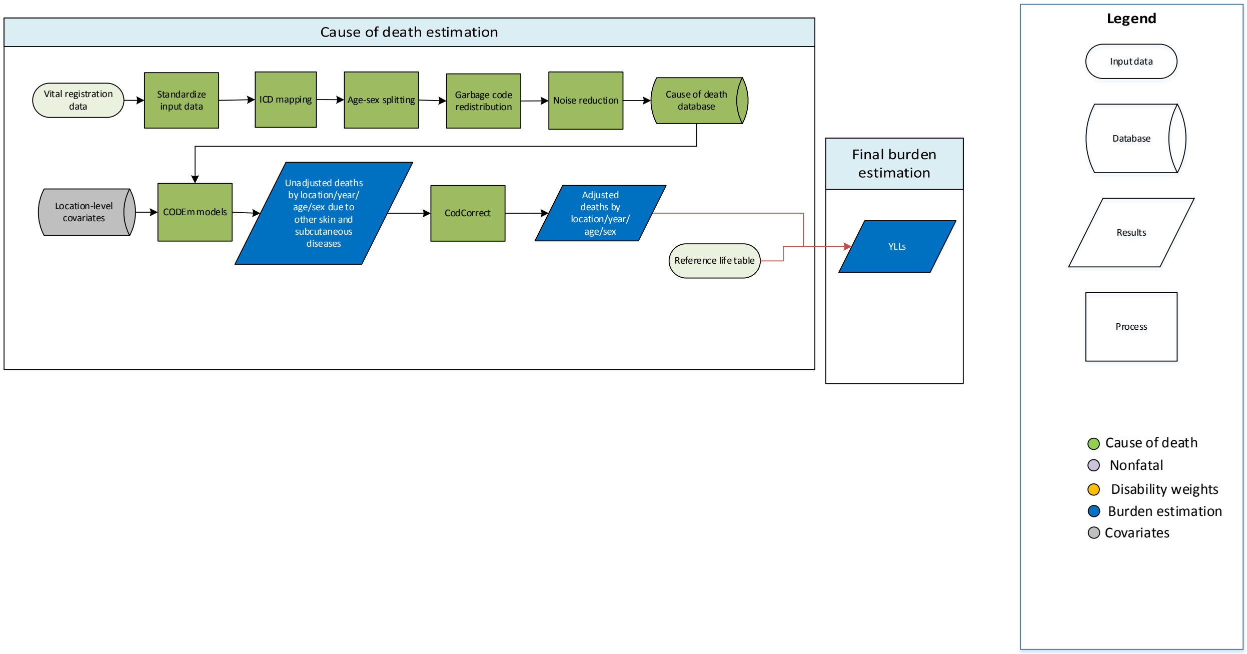

Input data

Data used to estimate mortality of other skin and subcutaneous diseases consisted of vital registration sources from the cause of death (COD) database. Outlier criteria excluded data points that were implausibly high or low relative to global or regional patterns and data from countries with small populations.

Modeling strategy

The standard CODEm modeling approach was used to estimate deaths due to other skin and subcutaneous diseases. CODEm parameters were centrally defined. We evaluated more than 30 models at different stages of the modeling process as updates were made to the data and the modeling coefficients. COD models were evaluated by comparing age-standardized death rates per 100,000 people to GBD 2013 best model for 1990, and 2013 individually for males and females. We also compared age-standardized annualized rate of change for death rate per 100,000 people to GBD 2013.

We applied the same covariates used in GBD 2013, excluding the four covariates for proportions of population at latitudes 0-15, 15-30, 30-45, and 45 and over. We also added the standardized exposure variable scalar (SEV-Scalar) for unsafe sanitation and Socio-Demographic Index (SDI) variable created for GBD 2015. Otherwise, there were no changes from the GBD 2013 modeling strategy.

Sudden Infant Death Syndrome (SIDS)

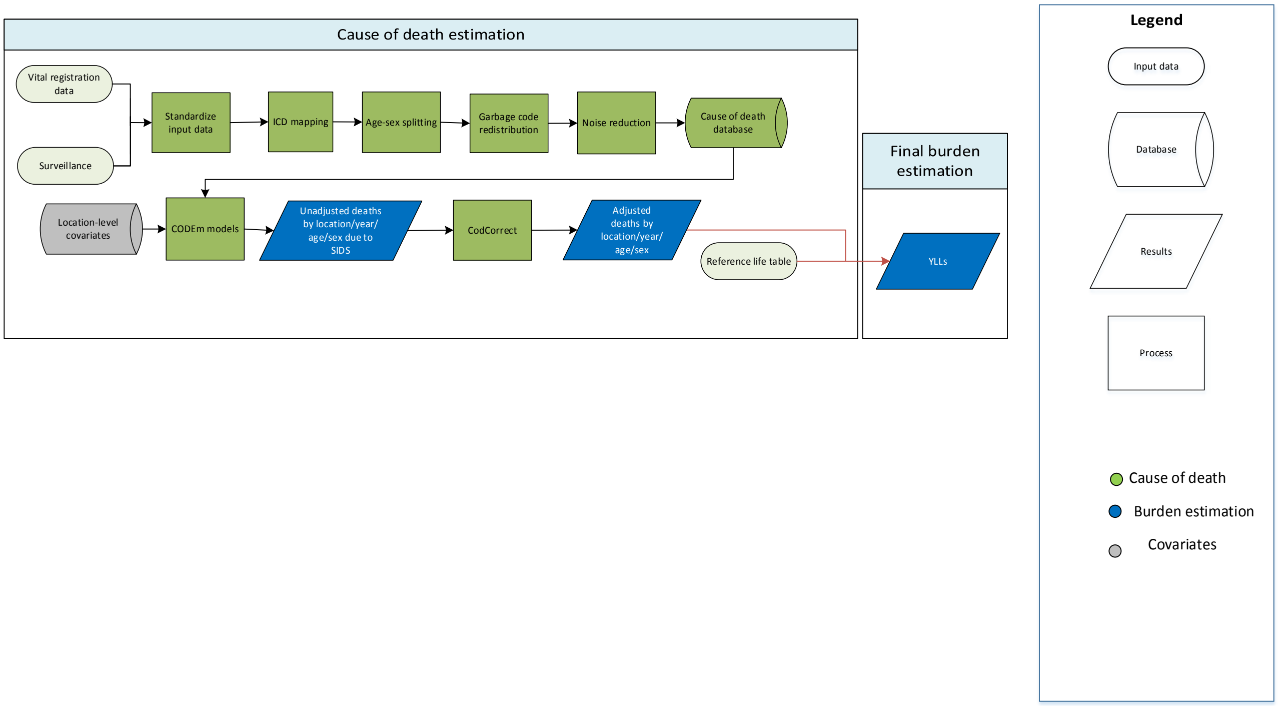

Input data

Vital registration data were used to estimate deaths due to sudden infant death syndrome (SIDS). Data points were selected as outliers if they met the following criteria: (1) implausibly high values relative to country time trends or global or regional patterns, based on the assumption that there are not "outbreaks" of SIDS, or (2) substantially conflicting with established age or temporal patterns.

Modeling strategy

The standard CODEm modeling approach was applied to estimate deaths due to SIDS. We ran CODEm models for ages 7-27 and 28-364 days because we believe that deaths assigned to SIDS in other age groups are mis-assigned and therefore treated as garbage codes. Surveillance data and verbal autopsy data were not used as inputs to this model because these sources do not use data collection methods that can accurately diagnose deaths due to SIDS.

Notable differences between the GBD 2013 strategy and this iteration include updates across the board to smoking-related covariates, total fertility rate, and Socio-Demographic Index covariates. The addition of American Samoa to the Oceania region is also of note, as well as the shift to including more ICD detail codes in the input data for some countries that previously reported only aggregated codes.

Injuries

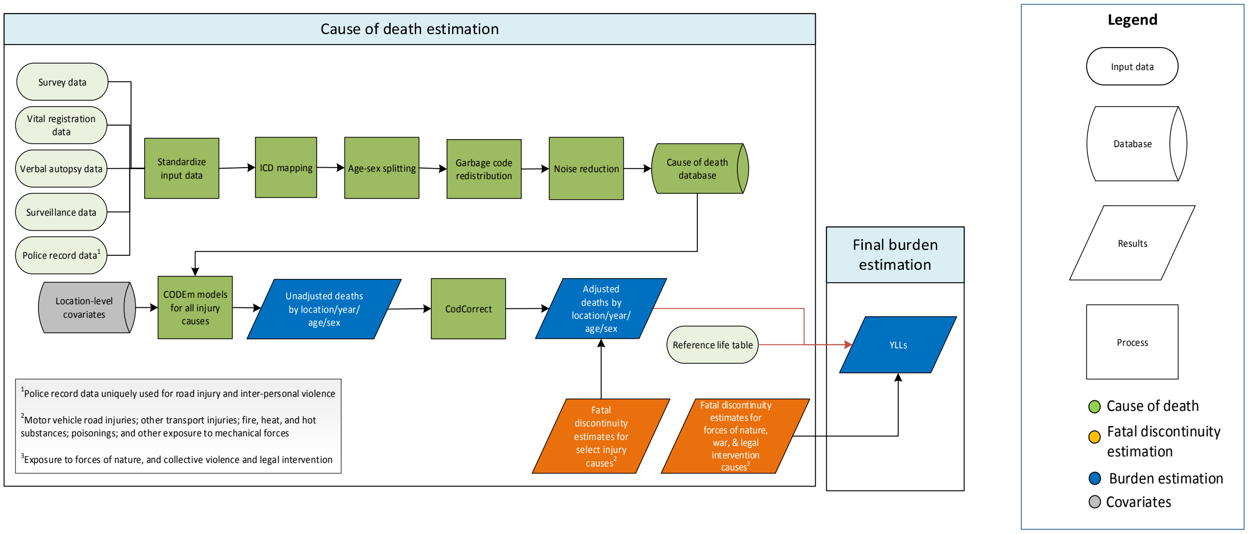

Input data

In GBD 2015, we estimated injury mortality from vital registration, verbal autopsy, mortality surveillance, censuses, surveys, and police record data. Police and crime reports were data sources uniquely used for the estimation of deaths from road traffic injury and interpersonal violence. The police data were collected from published studies, national agencies, and institutional surveys such as the United Nations Crime Trends Survey and the WHO Global Status Report on Road Safety Survey. For countries with vital registration data we did not use police records, except if the recorded number of road injury and interpersonal violence deaths from police records exceeded that in the vital registration.

Infrequently, data points were marked as outliers. Outlier criteria excluded data points that (1) were implausibly high or low relative to global or regional patterns, (2) substantially conflicted with established age or temporal patterns, or (3) significantly conflicted with other data sources conducted from the same locations or locations with similar characteristics (i.e., Socio-Demographic Index).

Modeling strategy

Overview

In GBD 2015, the standard CODEm modeling approach was applied to estimate deaths due to all causes of injury, excluding “Exposure to forces of nature” and “Collective violence and legal intervention,” which fall under the aggregate cause “Forces of nature, war, and legal intervention.” Similar to GBD 2013, both of these causes were modeled solely outside of the CODEm process as mortality shock regressions (now referred to as fatal discontinuities estimation); this process is detailed further in the fatal discontinuities estimation in the appendix. New to GBD 2015, fatal discontinuity was estimated for five injury causes also modeled in CODEm. These causes included “Motor vehicle road injuries,” “Other transport injuries,” “Fire, heat, and hot substances,” “Poisonings,” and “Other exposure to mechanical

forces." Final fatal discontinuity estimations for these causes were merged with CODEm results post-CodCorrect to produce final cause of death results.

Refer to the Table at the end of this section for a complete list of the cause-of-injury categories, modeling strategies, and covariate changes from GBD 2013.

GBD injury codes and categories

The International Classification of Diseases (ICD) was used to classify injuries because it is the standard diagnostic tool for epidemiology. In GBD, injury incidence and death are defined as ICD-9 codes E000-E999 and ICD-10 chapters V to Y. There is one exception: deaths and cases of alcohol poisoning and drug overdoses are classified under drug and alcohol use disorders. In GBD 2013, injury causes were organized into 26 mutually exclusive and collectively exhaustive external cause-of-injury categories. For GBD 2015, "Environmental exposure to heat and cold" was added to the list of cause-of-injury categories. These are deaths defined by ICD-10 codes W88-W94, W99, X30-32, and X39. In GBD 2013, these deaths were included in the cause-of-injury categories "Other unintentional injuries" and "Exposure to forces of nature, disaster." For GBD 2015, this change resulted in a decrease of deaths attributed to "Other unintentional injuries."

Another cause-of-injury category affected by a redistribution strategy change was "Unintentional poisoning." Similar to GBD 2013, we redistributed deaths coded as unspecified poisoning among the homicide, self-harm, unintentional poisoning, and drug use disorder causes. However, in GBD 2015, we sought to refine our redistribution strategy to more accurately represent unspecified poisoning deaths. Upon close review of ICD-coded deaths for unspecified poisoning, we identified a specific age-pattern, in which the death rate spiked in ages 15–50 years. Noting this trend, we conducted further review of data and found that these deaths were likely due to drug use disorders. We then redistributed many unspecified poisoning deaths from unintentional poisoning to drug use disorders, and based on high-quality ICD-9 and ICD-10 data, re-assigned these deaths to the drug use disorder sub-causes. Therefore, our estimates for unintentional poisoning decreased notably from GBD 2013.

Preparation of data

The preparation of cause of death data includes age splitting, age-sex splitting, smoothing, and outlier detection. These steps are described in detail by Naghavi et al and Lozano et al.[1,2] The concept of "garbage codes" and redistribution of these codes was proposed in the GBD 1990.[3] Garbage codes are causes of death that should not be identified as specific underlying causes of death but have been entered as the underlying cause of death on death certificates. A classic example of these types of codes in injuries chapters are "Exposure to unspecified factor" (X59 in ICD-10 and E887 in ICD-9) and all undetermined intent codes (Y10-Y34 in ICD-10 and E980-E988 in ICD-9). Other examples of garbage codes in injuries are the coding of an injury death to intermediate codes like septicemia or peritonitis or as an ill-defined and unknown cause of mortality (R99). Approximately 2% of total deaths in countries with vital registration data are assigned to these three injury garbage code categories.

Splitting into sublevel causes

In countries with non-detail ICD code data, cause-of-injury categories were proportionally split into sublevel cause-of-injury categories. The sublevel cause-of-injury causes were created in the CodCorrect process. One of the countries with non-detail ICD code data is South Africa, and in GBD 2013 the

proportions of sublevel cause-of-injury were based on vital registration data. For GBD 2015 the proportions were based on the paper by Matzopoulos et al. 2015.[4]

Limitations and model assumptions

We added police data for road injuries and interpersonal violence to help predict level and age patterns in countries with sparse or absent cause of death data even though we know from countries with near-complete vital registration data that police records tend to underestimate the true level of deaths. However, we applied police data estimates in instances where reported deaths were higher than vital registration numbers.

For the cause-of-injury category "Unintentional suffocation" we suspect that varying practices in coding deaths to sudden infant death syndrome (which end up in "Unintentional suffocation") can explain some of the differences we see and we plan to explore that further in the next iteration of GBD.

Table – Injury Cause List

ID	Cause	Modeling Strategy	Covariate changes from GBD 2013
1	Transport injuries	CODEm	+Socio-Demographic Index (SDI)
1.1	Road injuries	CODEm	+Standardized exposure variable (SEV) scalar for road injuries, SDS
1.1.a	Pedestrian road injuries	CODEm	+SEV scalar for pedestrian road injuries, SDS
1.1.b	Cyclist road injuries	CODEm	+SEV scalar for cyclist road injuries, SDS
1.1.c	Motorcyclist road injuries	CODEm	+SEV scalar for motorcyclist injuries, SDS
1.1.d	Motor vehicle road injuries	CODEm and fatal discontinuity estimation	+SEV scalar for motor vehicle road injuries, SDS
1.1.e	Other road injuries	CODEm	+SEV scalar for other road injuries, SDS
1.2	Other transport injuries	CODEm and fatal discontinuity estimation	+SEV scalar for other transport injuries, SDS
2	Unintentional injuries	CODEm	+Cumulative cigarettes (5 years), Diabetes fasting plasma glucose (mmol/L), Education (years per capita), Health system access, Mal-nutrition, Indoor air pollution, Population density (500–1000 ppl/sqkm, prop), Smoking prevalence<br><br>-Population over 65 and Population under 30
2.1	Falls	CODEm	+SEV scalar for falls, SDS
2.2	Drowning	CODEm	+SEV scalar for drowning, SDS
2.3	Fire, heat, and hot substances	CODEm and fatal discontinuity estimation	+SEV scalar for fire, heat, and hot substances, SDS
2.4	Poisonings	CODEm and fatal discontinuity estimation	+SEV scalar for poisonings, SDS
2.5	Exposure to mechanical forces	CODEm	+SDI
2.5.a	Unintentional firearm injuries	CODEm	+SEV scalar for unintentional firearm injuries, SDS

2.5.b	Unintentional suffocation	CODEm	+SEV scalar for unintentional suffocation, SDS
2.5.c	Other exposure to mechanical forces	CODEm and fatal discontinuity estimation	+SEV scalar for other exposure to mechanical forces, SDS
2.6	Adverse effects of medical treatment	CODEm	SDI
2.7	Animal contact	CODEm	+SEV scalar for animal contact, SDS
2.7.a	Venomous animal contact	CODEm	+SEV scalar for venomous animal contact, SDS
2.7.b	Non-venomous animal contact	CODEm	+SEV scalar for non-venomous animal contact, SDS
2.8	Foreign body	CODEm	+SDI
2.8.a	Pulmonary aspiration and foreign body in airway	CODEm	+SEV scalar for pulmonary aspiration and foreign body in airway, SDS
2.8.b	Foreign body in other body part	CODEm	+SEV scalar for foreign body in other body part, SDS
2.9	Environmental exposure to heat and cold	CODEm	N/A
2.10	Other unintentional injuries	CODEm	+SEV scalar for other unintentional injuries, SDS
3	Self-harm and interpersonal violence	CODEm	+Elevation over 1500 m and under 100m, population density under 150 ppl/sqkm, vehicles 2 wheels per capita, vehicles 4 wheels per capita<br><br>-Abortion legality, opium cultivation
3.1	Self-harm	CODEm	+SEV scalar for self-harm, SDI, major depressive disorder prevalence
3.2	Interpersonal violence	CODEm	+SEV scalar for interpersonal violence, SDS
3.2.a	Assault by firearm	CODEm	+SEV scalar for assault by firearm, SDS
3.2.b	Assault by sharp object	CODEm	+SEV scalar for assault by sharp object, SDS
3.2.c	Assault by other means	CODEm	+SEV scalar for assault by other means, SDS
4	Forces of nature, war, and legal intervention		
4.1	Exposure to forces of nature	Fatal discontinuity estimation for disaster (appended post-CodCorrect)	N/A
4.2	Collective violence and legal intervention	Fatal discontinuity estimation for war (appended post-CodCorrect)	N/A

References

1 Lozano R, Naghavi M, Foreman K, *et al.* Global and regional mortality from 235 causes of death for 20 age groups in 1990 and 2010: a systematic analysis for the Global Burden of Disease Study 2010. *The Lancet* 2012; **380**: 2095–128.

2 Global, regional, and national age–sex specific all-cause and cause-specific mortality for 240 causes of death, 1990–2013: a systematic analysis for the Global Burden of Disease Study 2013. *The Lancet* 2015; **385**: 117–71.

3 Murray CJL, Lopez AD, Harvard School of Public Health, World Health Organization, World Bank. The global burden of disease: a comprehensive assessment of mortality and disability from diseases, injuries, and risk factors in 1990 and projected to 2020. Cambridge, MA: Published by the Harvard School of Public Health on behalf of the World Health Organization and the World Bank : Distributed by Harvard University Press, 1996.

4 Matzopoulos R, Prinsloo M, Wyk VP, Gwebushe N, Mathews S, et al. Injury-related mortality in South Africa: a retrospective descriptive study of postmortem investigations. *Bull World Health Organ* 2015; **93**: 303–13.

2. Causes modeled outside of CODEm

2.1 Overview

A number of causes required alternative modeling strategies to those used for CODEm, as they were not compatible with CODEm estimation infrastructure and processes. Such unsuitability included having very low death counts; inadequate availability of cause-specific death records; and marked biases or variability for cause of death certification over time which could not be fully accounted for with current garbage code redistribution algorithms. The inclusion of these causes in CODEm often renders its out-of-sample predictive validity testing, a key advantage of using CODEm for cause of death estimation, unstable, or CODEm simply fails to generate plausible mortality rates in the absence of enough VR or VA data. Due to increased data availability and redistribution algorithm refinements, we were able to incorporate several new causes, which were modeled separately for GBD 2013, into CODEm for this iteration of the GBD study; with each annual update of GBD, we aim to add more causes within the CODEm estimation space. For GBD 2015, we used alternative modeling approaches for these causes, including negative binomial models, natural history models, sub-cause proportion models, and prevalence-based models.

2.2 Negative binomial models

For 10 rare causes of death, there were too few observed deaths in the cause of death database to produce stable estimates. For these causes, we ran negative binomial regression models with either a constant or constant multiplied by the mean assumption for the dispersion parameter, using reverse step-wise model building. We selected between the two model dispersion assumptions on the basis of best fit to the data, using the same method as GBD 2013. For GBD 2015 we also tested zero-inflated Poisson models for these rare causes of death, but rejected them after finding that they did not substantially affect the mean predictions but produced unrealistically large UIs. Descriptions of the modeling process for each of these causes are provided below.

Other Intestinal Infectious Diseases

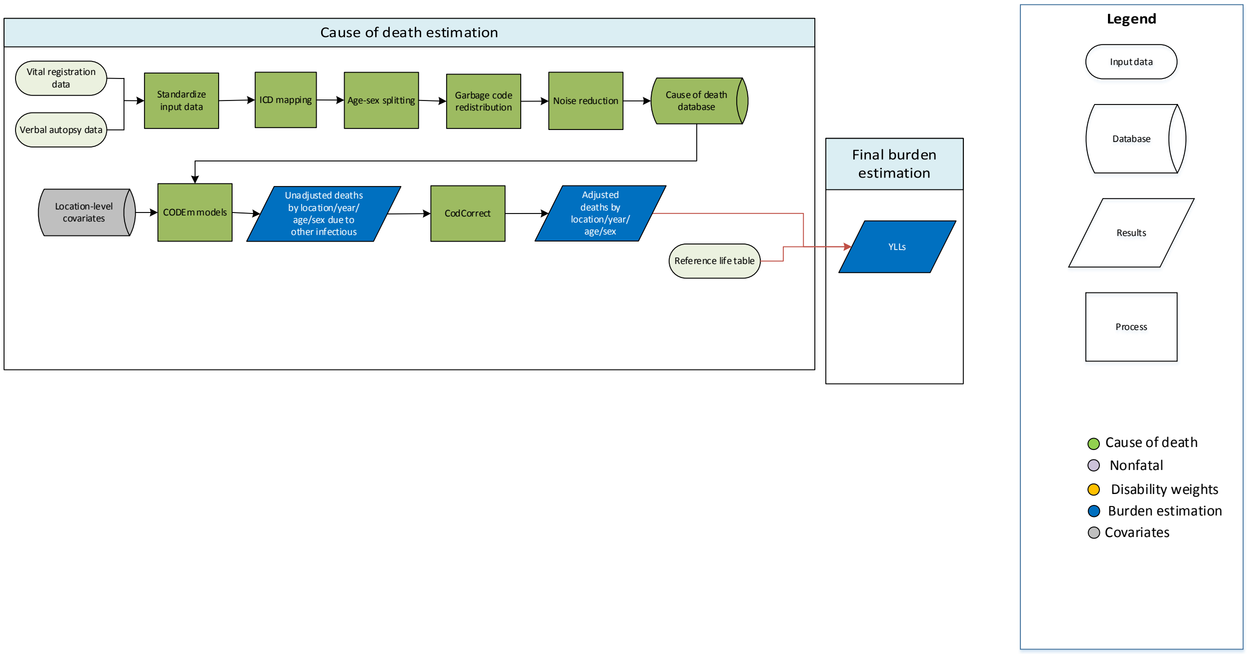

Input data

We modeled other intestinal infectious disease mortality using all available data in the cause of death database. Data points were outliered if they reported an improbable number of deaths or if their inclusion in the model yielded distorted trends. In some cases multiple data sources for the same location differed dramatically both in their quality and reported rabies mortality (e.g., a verbal autopsy and vital registration source). In these cases the lower-quality data source was outliered.

Modeling strategy

We modeled other intestinal infectious disease mortality using a two-model hybrid approach: 1) a global CODEm model of all locations, using all data in the CoD database; and 2) a CODEm model restricted to data-rich countries.

Since GBD 2013 we have switched from a single global model to the hybrid global/data-rich model approach. We have otherwise made no substantive changes in the modeling strategy for other intestinal infectious diseases from GBD 2013.

Upper respiratory infections

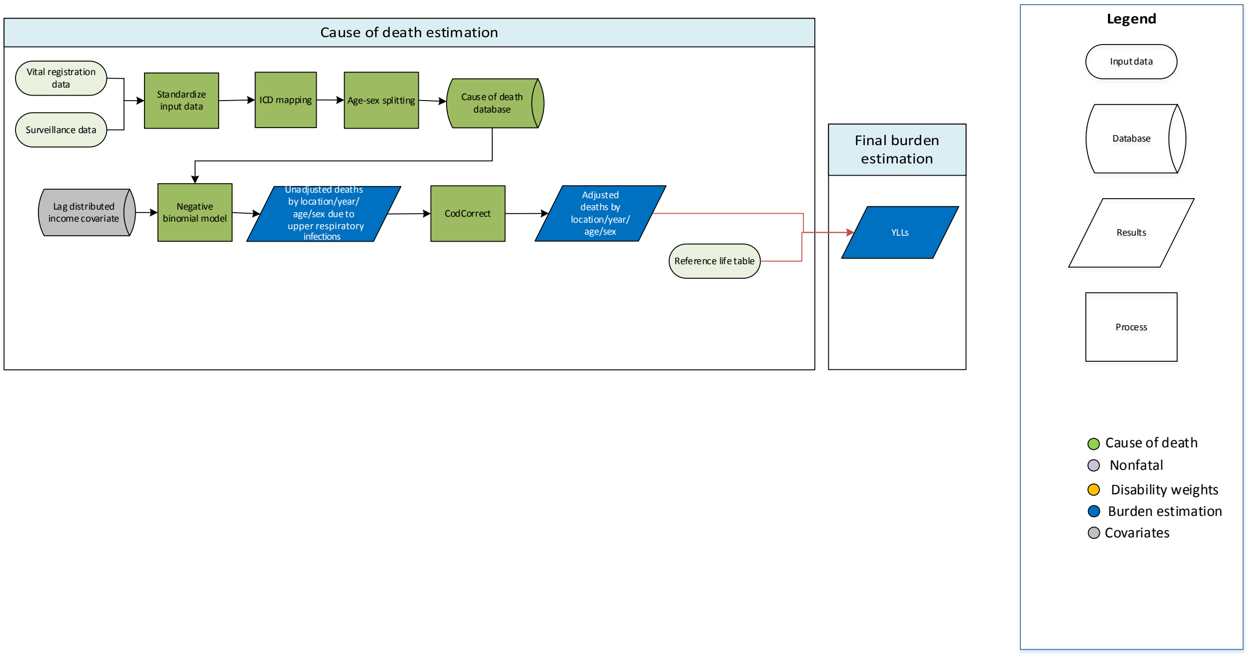

Input data

Vital registration and surveillance data from the cause of death database were used. Data with very high cause fractions (those greater than the 97th percentile values) were excluded in the regression.

Modeling strategy

Due to a small number of deaths, mortality from upper respiratory infections was modeled using a negative binomial regression, which is more appropriate than a Poisson count model as it accounts for greater variance (over-dispersion) in the data. By utilizing the exposure option in Stata, we model cause fractions with a negative binomial model. We tested both rate- and cause fraction-based models but selected a cause fraction model due to better model performance. Using the input data mentioned above, we modeled mortality from upper respiratory infections using the lag distributed income covariate and age dummy variables and the exposure set to the total number of deaths in the study. Uncertainty was estimated by taking 1,000 iterations of the predictions based on the variance covariance matrix and a random sample from a gamma distribution. The fit of the model was evaluated using diagnostic plots of predicted versus observed values.

Diphtheria

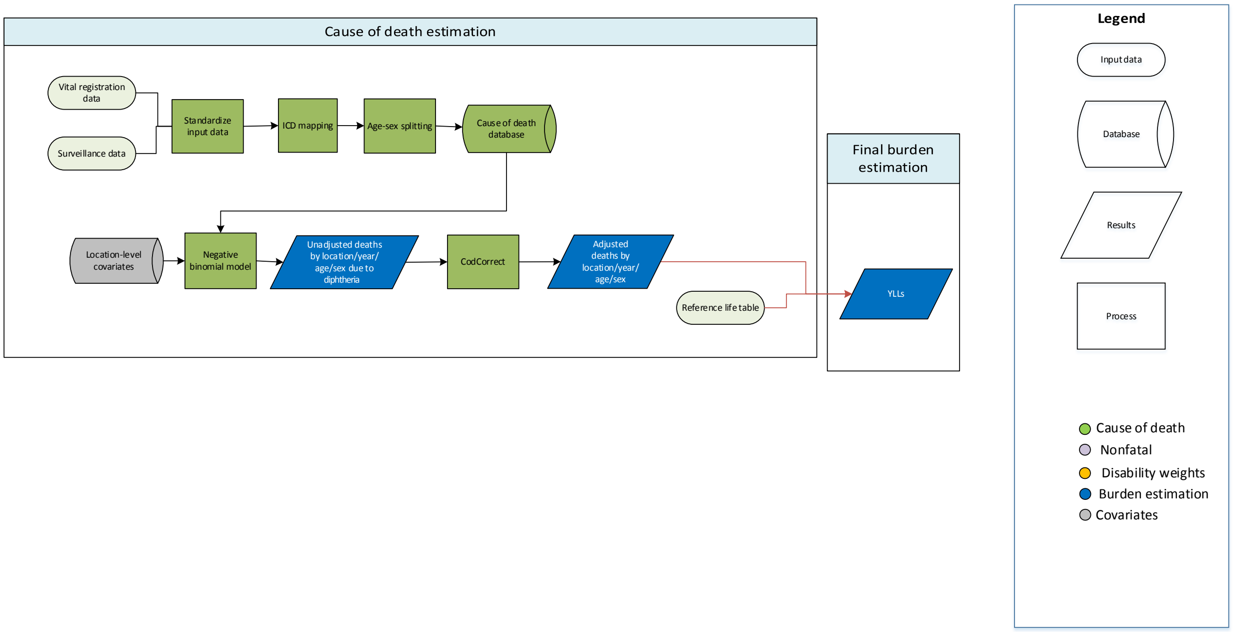

Input data

Vital registration and surveillance data from the cause of death database were used. Data with very high cause fractions (those greater than the 99th percentile values) were excluded in the regression.

Modeling strategy

Due to the small number of deaths, diphtheria mortality was modeled using a negative binomial regression, which is more appropriate than a Poisson count model as it accounts for greater variance (over-dispersion) in the data. By utilizing the exposure option in Stata, we model cause fractions with a negative binomial model. We tested both rate- and cause fraction-based models but selected a cause fraction model due to better model performance. Using the input data mentioned above, we modeled mortality due to diphtheria with the DPT3 coverage covariate and age dummy variables and the exposure set to the total number of deaths in the study. Uncertainty was estimated by taking 1,000 iterations of the predictions based on the variance covariance matrix and a random sample from a gamma distribution. The fit of the model was evaluated using diagnostic plots of predicted versus observed values.

Varicella

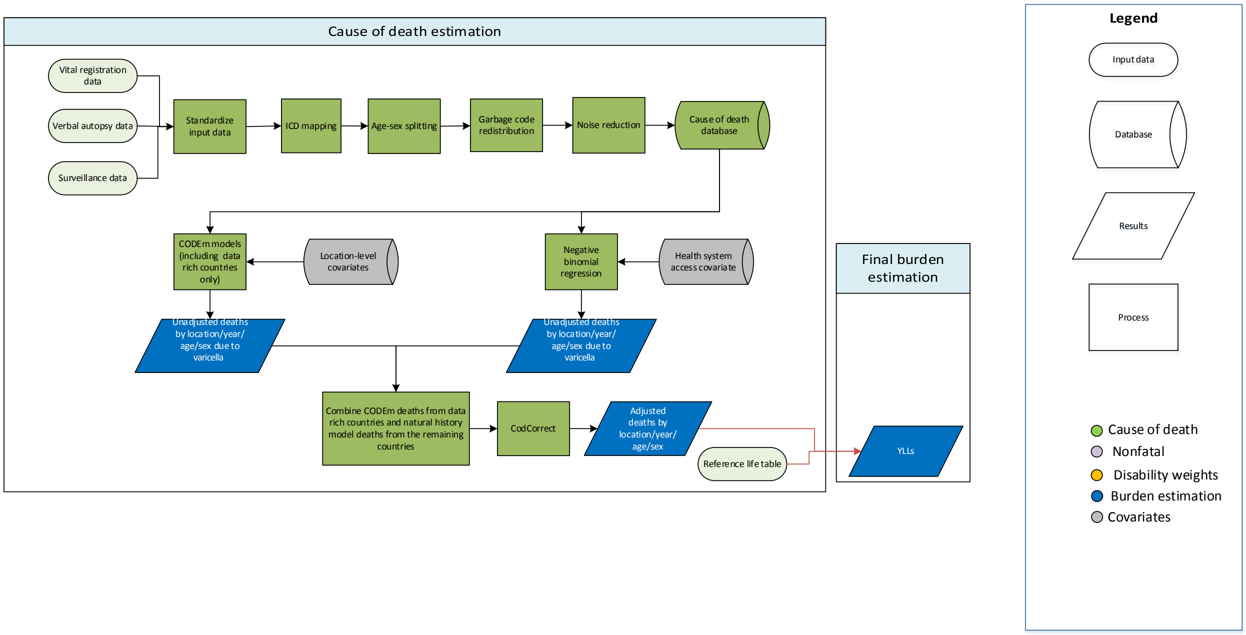

Input data

Vital registration, verbal autopsy and surveillance data from the cause of death database were used. Data with very high cause fractions (those greater than the 99th percentile values) were excluded in the negative binomial regression.

Modeling strategy

Mortality was modeled two ways. For data-rich countries (i.e., countries with vital registration more than 95% complete for more than 25 years), we used CODEm. For the remaining countries, since CODEm did not predict well in data-sparse areas, we used a negative binomial regression to model varicella mortality. By utilizing the exposure option in Stata, we modeled cause fractions with a negative binomial model. We tested both rate- and cause fraction-based models but ultimately selected a rate model due to better model performance. Using the input data mentioned above, we modeled mortality due to varicella using the health system access covariate and age dummy variables with the exposure set to the location-year-age-sex-specific population. Uncertainty was estimated by taking 1,000 iterations of the predictions based on the variance covariance matrix and a random sample from a gamma distribution. The fit of the model was evaluated using diagnostic plots of predicted versus observed values.

In GBD 2013, we used a negative binomial regression to model varicella mortality for all countries. For GBD 2015, we switched to using CODEm for data-rich countries because it fit the data better in those countries.

Schistosomiasis

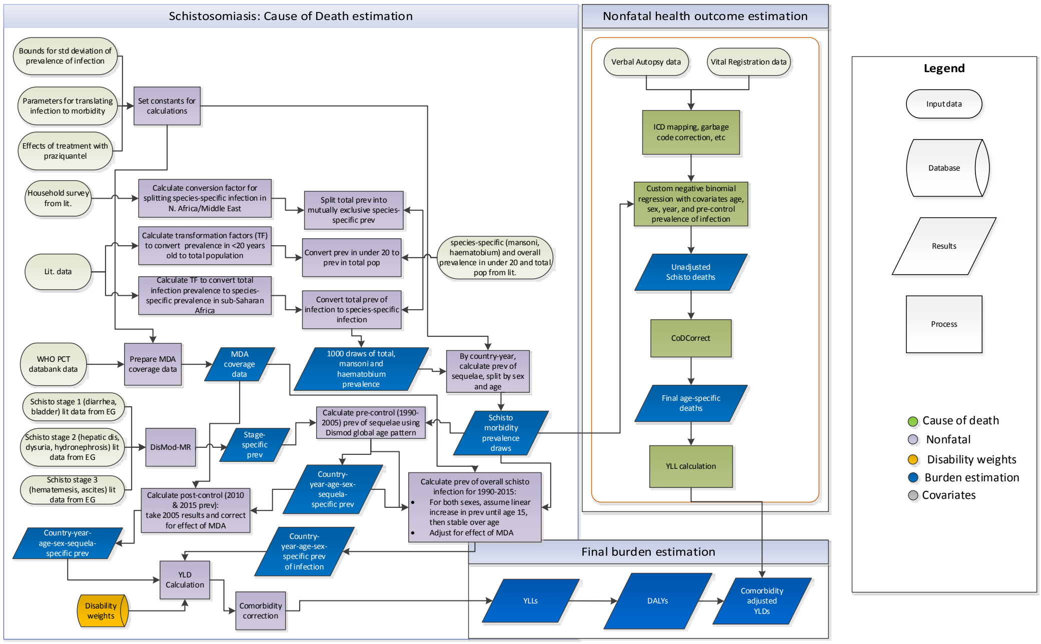

Input data

To estimate mortality due to schistosomiasis, data on deaths and prevalence of infection were used. The prevalence data were prepared in GBD 2010, and further information on this is available in the nonfatal write-up for this cause. Country-year-age-sex-specific verbal autopsy and vital registration data were used in the mortality model. We did not conduct a systematic literature review for schistosomiasis for GBD 2015.

Modeling strategy

To estimate deaths due to schistosomiasis, a negative binomial regression model of country-year-age-sex-specific deaths on natural log-transformed prevalence of total schistosomiasis infection was used. The negative binomial regression was selected due to its suitability for modeling count data. In addition, there are relatively low number of deaths attributable to schistosomiasis. Time trends in the data for Egypt and China were very non-linear. Therefore, in the model, time trends for Egypt and China were allowed to differ from the global time trend by adding fractional polynomials for these two countries. A multivariate normal distribution using the mean and variance-covariance matrix from the model was used to generate 1,000 draws of deaths due to schistosomiasis.

Models were evaluated by assessing the AIC and plotting the predicted deaths against time, age, and sex. In addition, the Cause of Death Visualization tool was used to evaluate time trends across locations, age, and sex. A map of the global distribution of schistosomiasis across age-groups was also used to assess the changes in death rates over time. The final model was selected based on how well the estimated numbers fit the input data and how plausible the predicted distribution of disease was over time and with age.

In addition to the additional data from CoD, we added new information on the main covariate, infection prevalence, used in the model. Details of this are included in the nonfatal write-up for this cause; briefly, data from the Brazil Ministry of Health were used to split the national Brazil prevalence into 19 out of the 27 Brazil subnational locations for which we estimate burden of disease. In addition, for locations for which we added subnational estimation, the national prevalence was applied to subnational prevalence due to lack of information to split the prevalence accordingly. These included Kenya, Saudi Arabia, and South Africa. Finally, in GBD 2013, the negative binomial model applied a different time trend for Brazil and the Philippines. This time trend was not applied in GBD 2015 due to poor model performance with its inclusion.

Cysticercosis

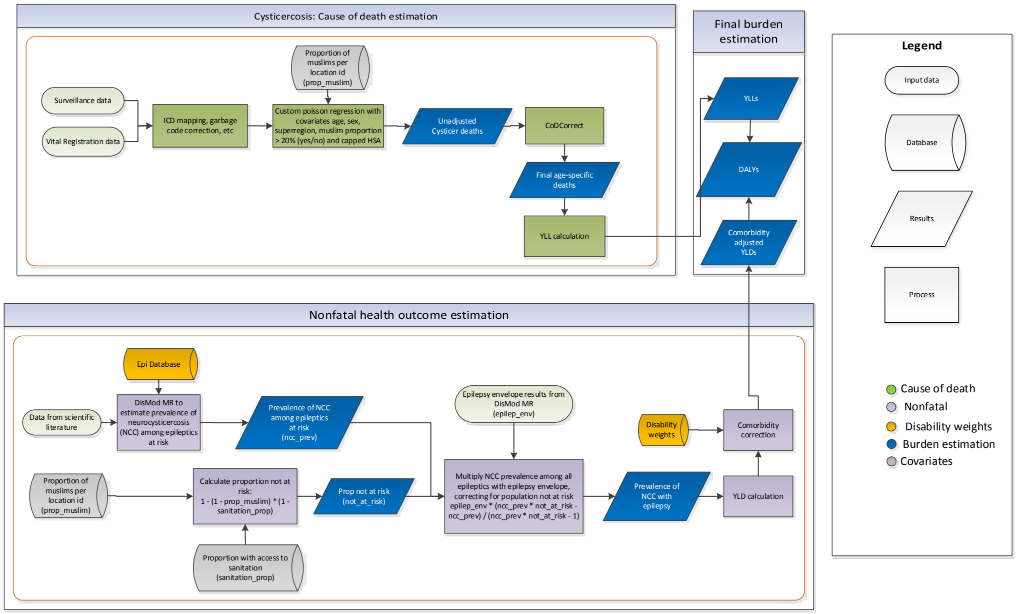

Input data

The model for mortality due to cysticercosis relied on vital registration and surveillance data (from endemic countries). In addition, we used data from the Pew Research Center on percentage of population that is Muslim by country. The primary covariates adjusted for in the model were pigs per capita and proportion of Muslim (binary, 20% vs <=20%). Other covariates adjusted for in the model were age, sex, GBD super-regions, and capped health system access (HSA).

Modeling strategy

Globally, deaths due to cysticercosis are relatively low. Therefore, a Poisson model was used to model cysticercosis deaths due to its suitability for count data. One thousand draws of deaths estimates were generated using a multivariate normal distribution using the mean and variance-covariance matrix from the Poisson regression. There were no data on cysticercosis deaths from South Asia. Therefore, in the predictions of deaths we applied the super-region coefficient for (Southeast Asia, East Asia, and Oceania) to South Asia.

We applied the same modeling strategy as used in GBD 2013, with the addition of estimates for new subnational locations. Since the Pew Research Center only has data on proportion of Muslims by country, we applied the national proportions to subnational locations. We understand that this does not account for sometimes large expected difference in proportions of Muslims within a country, but were limited by data availability.

Cystic Echinococcosis (CE)

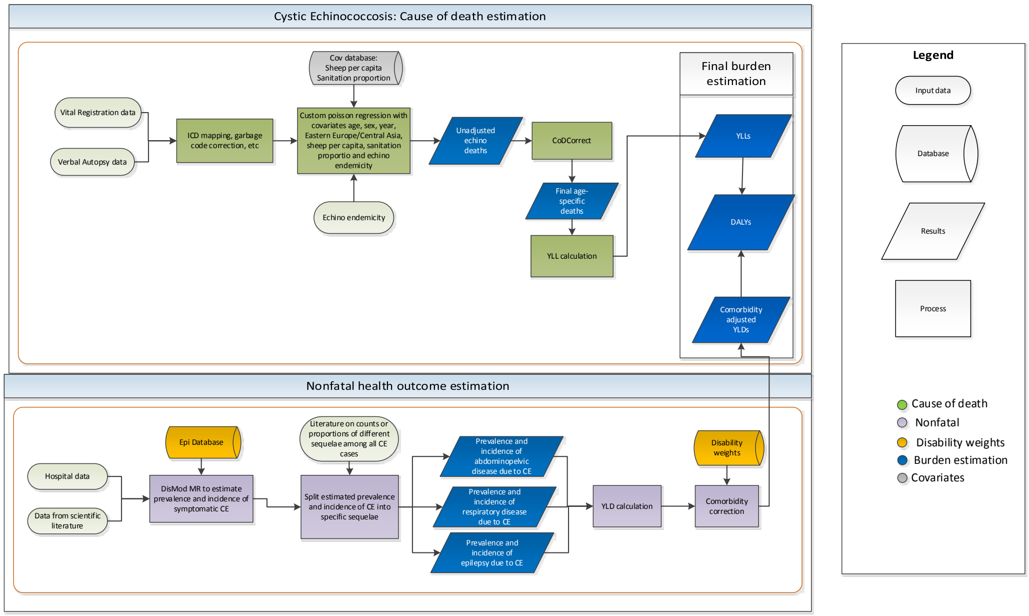

Input data

There are limited data sources on deaths due to cystic echinococcosis (CE) and we did not use alveolar due to *E. multilocularis* or cystic echinococcosis due to *E. granulosus*. To model mortality due to CE, we used vital registration data. In addition, we incorporated data on echinococcosis endemicity provided by one of our echinococcosis collaborators. Sheep per capita was used as a covariate because sheep are the main intermediate host for the *Echinococcosus granulosus* tapeworm, which leads to CE in humans when its eggs are ingested. Other covariates include proportion of population with access to sanitation, echinococcosis endemicity, and a region indicator for Eastern Europe and Central Asia. The echinococcosis endemicity covariate had four categories: 0=no cases/no data, 1=sporadic/mostly imported, 2=endemic/limited data, 3=highly endemic.

Modeling strategy

The cause of death ensemble model (CODEm) was not employed for modeling deaths from CE due to the paucity of data on deaths from CE. We therefore used a Poisson regression of deaths on age, sex, year, and geography.

Using a multivariate normal distribution with mean and variance-covariance matrix from the Poisson regression, we generated 1,000 draws of estimates for the countries endemic for CE. The final model was selected based on how well the estimated numbers fit the input data and how plausible the predicted distribution of disease was over time and with age.

Ascariasis

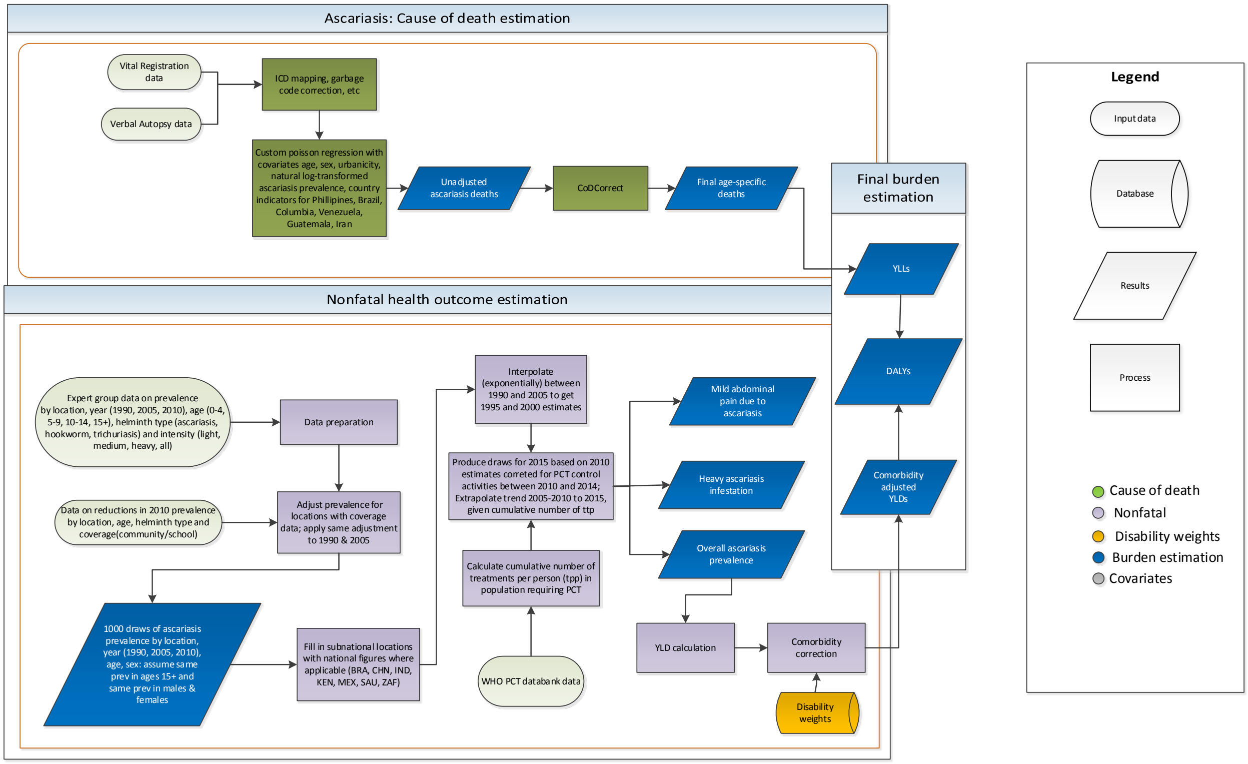

Input data

To estimate mortality due to ascariasis, data on deaths and prevalence of infection were used. Country-year-age-sex-specific verbal autopsy and vital registration data were used in the mortality model. We did not conduct a systematic literature for ascariasis. The model was adjusted for age, sex, urbanicity (proportion of land with population density > 1000 / km^2), and country indicators for Philippines, Brazil, Columbia, Venezuela, Guatemala, and Iran. Other epidemiologically relevant covariates (sanitation and capped health system access) were also added in the model.

Modeling strategy

To estimate deaths due to ascariasis, a negative binomial regression model of country-year-age-sex-specific deaths on natural log-transformed age-standardized prevalence of ascariasis was used. A multivariate normal distribution using the mean and variance-covariance matrix from the model was used to generate 1,000 draws of deaths due to ascariasis.

Iodine deficiency

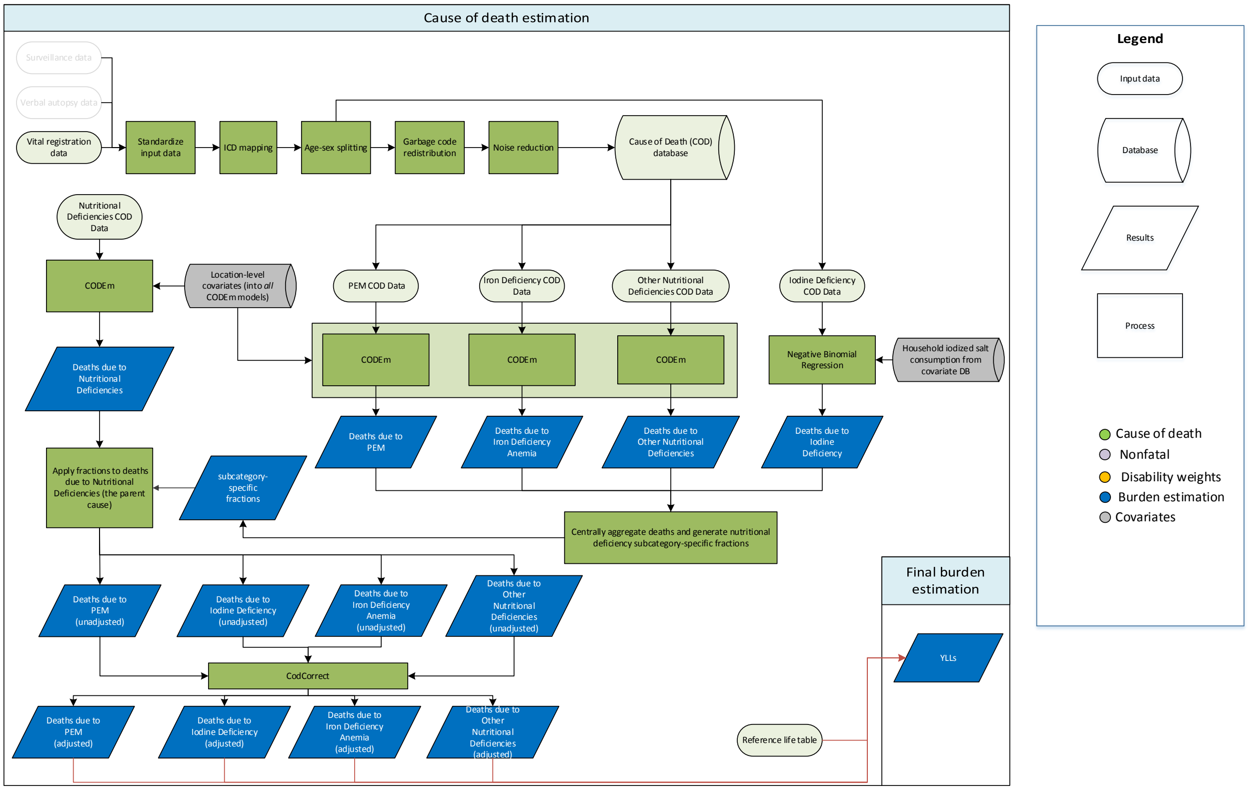

Input data

Vital registration data were used.

Modeling strategy

We estimated mortality due to iodine deficiency in two steps. CODEm was first used to generate mortality estimates for total nutritional deficiencies. The sub-categories of nutritional deficiencies, namely protein-energy malnutrition, iodine deficiency, iron-deficiency anemia, and other nutritional deficiencies, were modeled separately. CODEm was used to model all sub-categories except for iodine deficiency, which we modeled using a negative binomial regression model given the small number of deaths attributable to it. A negative binomial model is more appropriate than a Poisson count model as it accounts for greater variance (over-dispersion) in the data. By utilizing the exposure option in Stata, we model cause fractions with a negative binomial model. We tested both rate- and cause fraction-based models but selected a cause fraction model due to better model performance. We used vital registration data with proportion of household iodized salt consumption as a country-level covariate and dummy variables on age and sex to model mortality from iodine deficiency. Uncertainty was estimated by taking 1,000 iterations of the predictions based on the variance covariance matrix and a random sample from a gamma distribution. Estimates from the four nutritional sub-categories were then aggregated centrally at the 1,000 draw level to generate cause fractions for each sub-category. These

cause fractions were then applied centrally to the total nutritional deficiencies CODEm estimates to generate final sub-category mortality estimates.

2.3 DisMod-MR 2.1

Until GBD 2010, non-fatal estimates were based on a single data source on prevalence, incidence, remission, or a mortality risk selected by the researcher as most relevant to a particular geography and time. For GBD 2010, we set a more ambitious goal: to evaluate all available information on a disease that passes a minimum quality standard. That required a different analytical tool that would be able to pool disparate information presented in varying age groupings and from data sources using different methods. The DisMod-MR 1.0 tool used in GBD 2010 evaluated and pooled all available data, adjusted data for systematic bias associated with methods that varied from the reference and produced estimates by world regions with uncertainty intervals. For GBD 2013, the improved DisMod-MR 2.0 had increased computational speed allowing computations that were consistent between all disease parameters at the country rather than region level. The hundred-fold increase in speed of DisMod-MR 2.0 was partly due to a more efficient re-write of the code in C++ but also by changing to a model specification using log rates rather than a negative binomial model used in DisMod-MR 1.0. In cross-validation tests, the log rates specification worked as well or better than the negative binomial specification.[i] For GBD 2015, the computational engine (DisMod-MR 2.1) remained substantively unchanged but we re-wrote the 'wrapper' code that organized the flow of data and settings at each level of the analytical cascade. The sequence of estimation occurred at five levels: global, super-region, region, country, and, where applicable, subnational geographical units (see flow diagram of DisMod-MR 2.1 cascade, below). The super-region priors were generated at the global level with mixed-effects, non-linear regression using all available data; the super-region fit, in turn, informed the region fit, and so on down the cascade. The wrapper gave analysts the choice to branch the cascade in terms of time and sex at different levels depending on data density. The default used in most models was to branch by sex after the global fit but to retain all years of data until the lowest level in the cascade. For GBD 2015, we generated fits for the years 1990, 1995, 2000, 2005, 2010, and 2015.

In updating the 'wrapper,' we consolidated the code base into a single language, Python, to make the code more transparent and efficient and to better deal with subnational estimation. The computational engine is limited to three levels of random effects; we differentiated estimates at the super-region, region, and country level. In GBD 2013, the subnational units of China, Mexico, and the UK were treated as 'countries' such that a random effect was estimated for every geography with contributing data. However, the lack of a hierarchy between country and subnational units meant that the fit to country data contributed as much to the estimation of a subnational unit as the fits for all other countries in the region. We found inconsistency between the country fit and the aggregation of subnational estimates when the country's epidemiology varied from the average of the region. Adding an additional level of random effects required a prohibitively comprehensive rewrite of the underlying DisMod-MR engine. Instead, we added a fifth layer to the cascade, with subnational estimation informed by the country fit and country covariates, plus an adjustment based on the average of the residuals between the subnational unit's available data and its prior. This mimicked the impact of a random effect on estimates between subnationals.

For GBD 2015 we improved how country covariates differentiate non-fatal estimates for diseases with sparse data. The coefficients for country covariates were re-estimated at each level of the cascade. For a

given geography, country coefficients were calculated using both data and prior information available for that geography. In the absence of data, the coefficient of its parent geography was used, in order to utilize the predictive power of our covariates in data sparse situations.

Figure - GBD 2015 DisMod-MR 2.1 analytical cascade

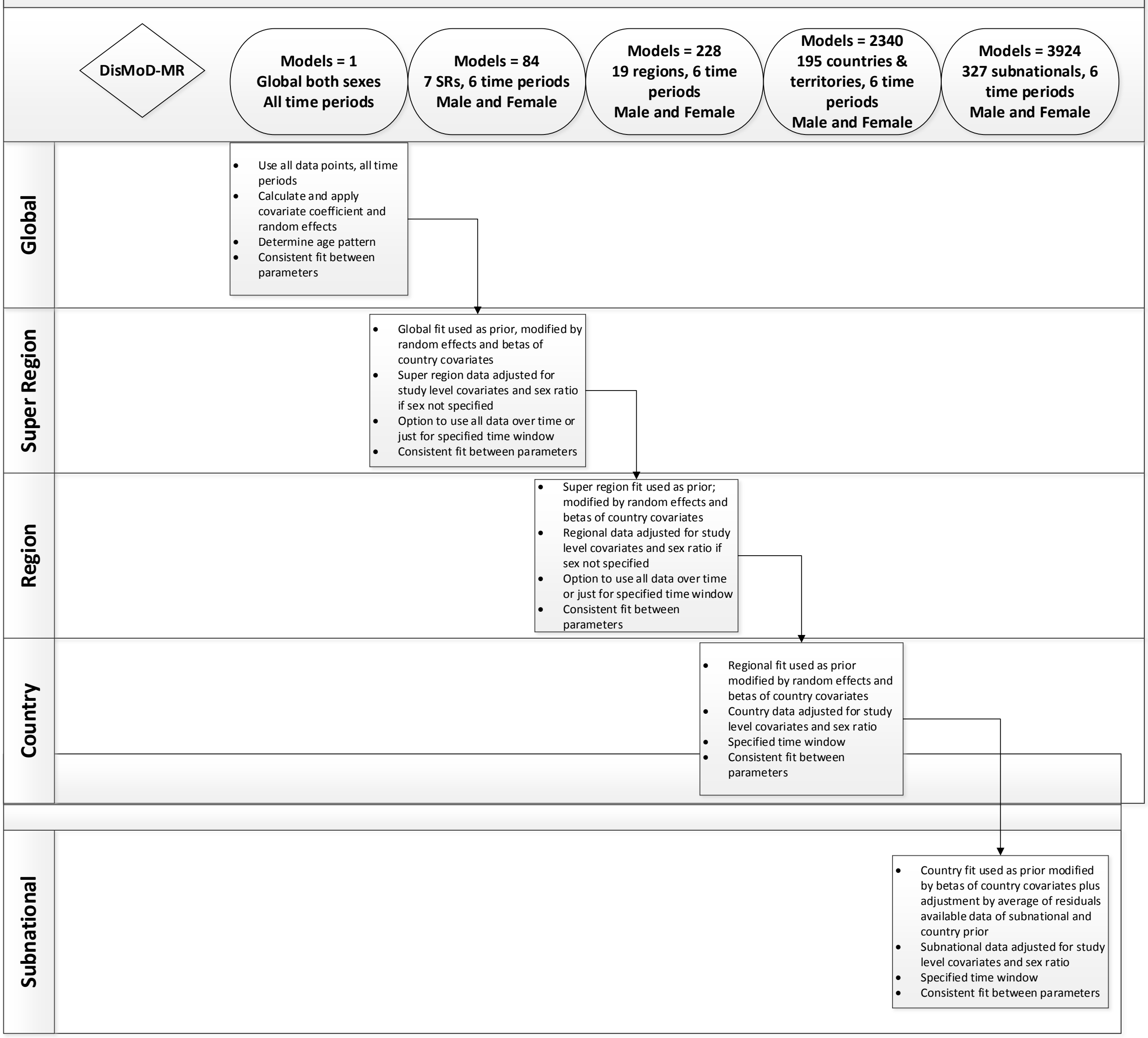

DisMod-MR 2.1 likelihood estimation

Analysts have the choice of using a Gaussian, log-Guassian, Laplace, or log-Laplace likelihood function in DisMod-MR 2.1. The default log-Gaussian equation for the data likelihood is:

$$-log[p(y_j|\Phi)] = \log(\sqrt{2\pi}) + \log(\delta_j + s_j) + \frac{1}{2}\left(\frac{\log(a_j + \eta_j) - \log(m_j + \eta_j)}{\delta_j + s_j}\right)^2$$

where, yj is a 'measurement value' (i.e. data point); Φ denotes all model random variables; ηj is the offset value, eta, for a particular 'integrand' (prevalence, incidence, remission, excess mortality rate, with-condition mortality rate, cause-specific mortality rate, relative risk, or standardised mortality ratio), and aj is the adjusted measurement for data point j, defined by:

$$a_j = e^{(-u_j - c_j)} y_j$$

where uj is the total 'area effect' (i.e. the sum of the random effects at three levels of the cascade: super-region, region, and country) and cj is the total covariate effect (i.e. the mean combined fixed effects for sex, study level, and country level covariates), defined by:

$$c_j = \sum_{k=0}^{K[I(j)]-1} \beta_{I(j),k} \hat{X}_{k,j}$$

with standard deviation

$$s_j = \sum_{l=0}^{L[I(j)]-1} \zeta_{I(j),l} \hat{Z}_{k,j}$$

where k denotes the mean value of each data point in relation to a covariate (also called x-covariate); I(j) denotes a data point for a particular integrand, j; βI(j),k is the multiplier of the kth x-covariate for the ith integrand; $\hat{X}_{k,j}$ is the covariate value corresponding to the data point j for covariate k; l denotes the standard deviation of each data point in relation to a covariate (also called z-covariate); ζI(j),k is the multiplier of the lth z-covariate for the ith integrand; and δj is the standard deviation for adjusted measurement j, defined by:

$$\delta_j = log\left[y_j + e^{(-u_j - c_j)}\eta_j + c_j\right] - log\left[y_j + e^{(-u_j - c_j)}\eta_j\right]$$

Where mj denotes the model for the jth measurement, not counting effects or measurement noise and defined by:

$$m_j = \frac{1}{B(j) - A(j)} \int_{A(j)}^{B(j)} I_j(\text{a})\ \text{da}$$

where A(j) is the lower bound of the age range for a data point; B(j) is the upper bound of the age range for a data point; and I(j) denotes the function of age corresponding to the integrand for data point j.

The source code for DisMod-MR 2.1 as well as the wrapper code are available at http://ihmeuw.org/dismod-ode.

[i] A Flaxman, C Murray, T Vos (Eds.) Integrated meta-regression framework for descriptive epidemiology. University of Washington Press, Seattle, WA; 2014

2.4 Natural history models

For some causes where cause of death data may be systematically biased due to either misclassification or because the disease exists in focal communities without vital registration or verbal autopsy studies, we have developed natural history models. In natural history models incidence and case-fatality rates are modeled separately, and then combined to produce estimates of cause-specific mortality. The 14 causes where natural history models have been used include: typhoid fever, paratyphoid fever, whooping cough, measles, visceral Leishmaniasis, African trypanosomiasis, yellow fever, congenital syphilis, and acute hepatitis A, B, C, and E. Descriptions of the modeling process for each of these causes follow.

Typhoid fever

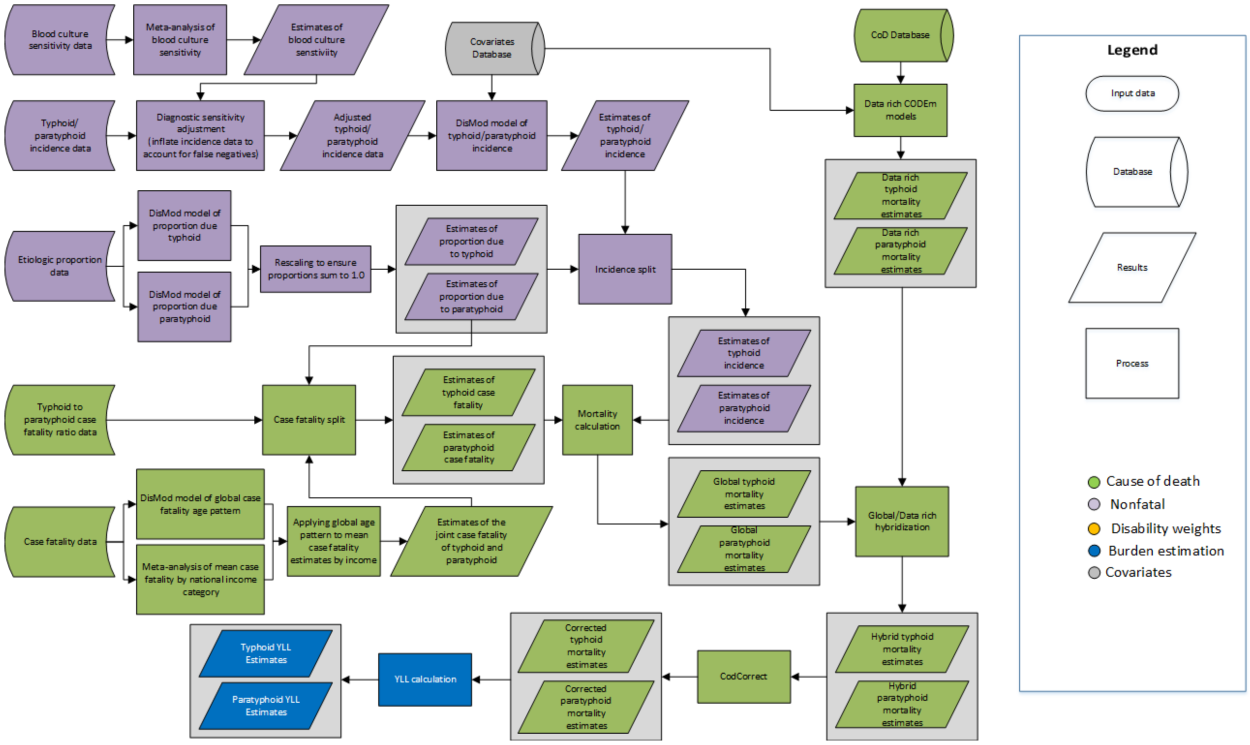

Input data

Our incidence dataset included a combination of data from prospective cohort studies and national surveillance systems. Similarly, data on proportions due to typhoid and paratyphoid included a combination of prospective cohort studies and national surveillance systems. Case fatality data were from national surveillance systems and hospital databases. Our data-rich model uses vital registration data in our CoD database.

Modeling strategy

We model typhoid deaths using a hybrid approach in which we model deaths in data-rich countries using CODEm with VR data, and model deaths in other countries using a natural history approach. For the natural history model we first model total incidence of typhoid and paratyphoid combined. Second, we model the proportion of this total due to typhoid and the proportion due to paratyphoid. Third, we estimate case fatality by age and national income category for typhoid and paratyphoid combined. Fourth, we use data on the relative fatality of typhoid and paratyphoid to split the joint case fatality estimates into typhoid- and paratyphoid-specific case fatality estimates. Finally, we estimate cause-specific mortality rates as the product of incidence and case fatality.

Total incidence was modeled using DisMod-MR, using the proportion of the population with access to clean water, and the proportion of the population living in the Indian Ocean monsoon belt as covariates. We performed a crosswalk using a study-level covariate indicating sources that were based on passive versus active surveillance, with active surveillance as the reference. This adjusts for incomplete case capture by passive surveillance. Incidence data were inflated to account for poor diagnostic sensitivity, based on a meta-analysis of the sensitivity of blood culture, the most common diagnostic used for typhoid. Similarly, we used two DisMod models to estimate etiologic proportions: one for the proportion of total incidence due to typhoid, and one for the proportion due to paratyphoid.

Case fatality data were too limited to allow for a complete DisMod model, or to allow for varying estimates by time and space. We had sufficient data, however, to estimate case fatality by age and by three categories of national income. We used DisMod to extract a global age-pattern in case fatality, and meta-regression to estimate the mean case fatality by income category. Finally, we estimated the relative risk of death from typhoid relative to paratyphoid based on data from Chinese surveillance and used that relative risk to estimate case fatality separately for typhoid and paratyphoid, by age and income.

Finally, we estimated typhoid mortality as the product of total incidence, the proportion of the total due to typhoid, and case fatality for typhoid. We propagated uncertainty through every step of the modeling process by pulling 1,000 draws from the distribution of each model component (e.g., incidence, proportion due to typhoid, overall case fatality, case fatality age pattern, relative fatalness of typhoid versus paratyphoid), and performing all calculations at the draw level.

Our model for GBD 2013 used the natural history approach for all countries. We have implemented the hybrid approach for GBD 2015 and now estimate typhoid deaths in data-rich countries based on a CODEm model of VR data.

Paratyphoid fever

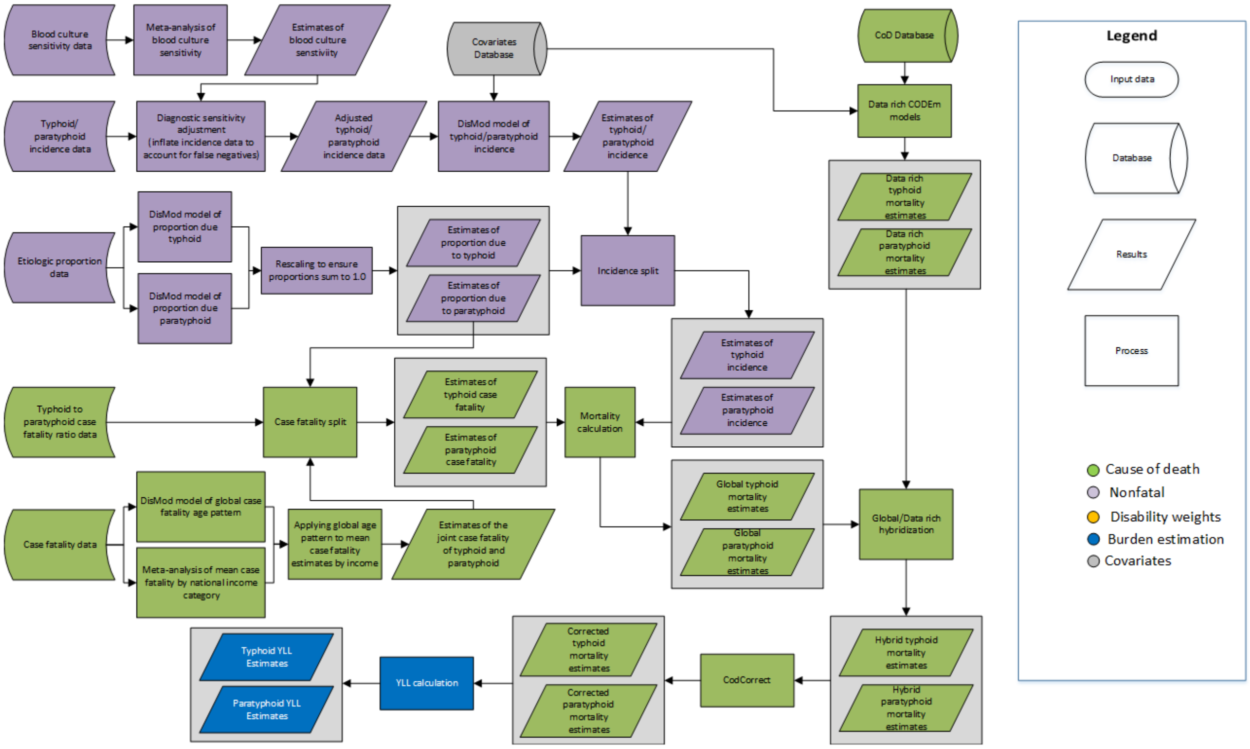

Input data

Our incidence dataset included a combination of data from prospective cohort studies and national surveillance systems. Similarly, data on proportions due to typhoid and paratyphoid included a combination of prospective cohort studies and national surveillance systems. Case fatality data were from national surveillance systems and hospital databases. Our data-rich model uses vital registration data in our CoD database.

Modeling strategy

We model paratyphoid deaths using a hybrid approach in which we model deaths in data-rich countries using CODEm with VR data, and model deaths in other countries using a natural history approach. For the natural history model we first model total incidence of typhoid and paratyphoid combined. Second, we model the proportion of this total due to typhoid and the proportion due to paratyphoid. Third, we estimate case fatality by age and national income category for typhoid and paratyphoid combined. Fourth, we use data on the relative fatality of typhoid and paratyphoid to split the joint case fatality estimates into typhoid- and paratyphoid-specific case fatality estimates. Finally, we estimate cause-specific mortality rates as the product of incidence and case fatality.

Total incidence was modeled using DisMod-MR, using the proportion of the population with access to clean water and the proportion of the population living in the Indian Ocean monsoon belt as covariates. We performed a crosswalk using a study-level covariate indicating sources that were based on passive versus active surveillance, with active surveillance as the reference. This adjusts for incomplete case capture by passive surveillance. Incidence data were inflated to account for poor diagnostic sensitivity, based on a meta-analysis of the sensitivity of blood culture, the most common diagnostic used for paratyphoid. Similarly, we used two DisMod models to estimate etiologic proportions: one for the proportion of total incidence due to typhoid, and one for the proportion due to paratyphoid.

Case fatality data were too limited to allow for a complete DisMod model, or to allow for varying estimates by time and space. We had sufficient data, however, to estimate case fatality by age and by three categories of national income. We used DisMod to extract a global age-pattern in case fatality, and meta-regression to estimate the mean case fatality by income category. Finally, we estimated the relative risk of death from typhoid relative to paratyphoid based on data from Chinese surveillance and used that relative risk to estimate case fatality separately for typhoid and paratyphoid, by age and income.

Finally, we estimated paratyphoid mortality as the product of total incidence, the proportion of the total due to paratyphoid, and case fatality for paratyphoid. We propagated uncertainty through every step of the modeling process by pulling 1,000 draws from the distribution of each model component (e.g., incidence, proportion due to paratyphoid, overall case fatality, case fatality age pattern, relative fatalness of typhoid versus paratyphoid), and performing all calculations at the draw level.

Our model for GBD 2013 used the natural history approach for all countries. We have implemented the hybrid approach for GBD 2015 and now estimate paratyphoid deaths in data-rich countries based on a CODEm model of VR data.

Whooping cough

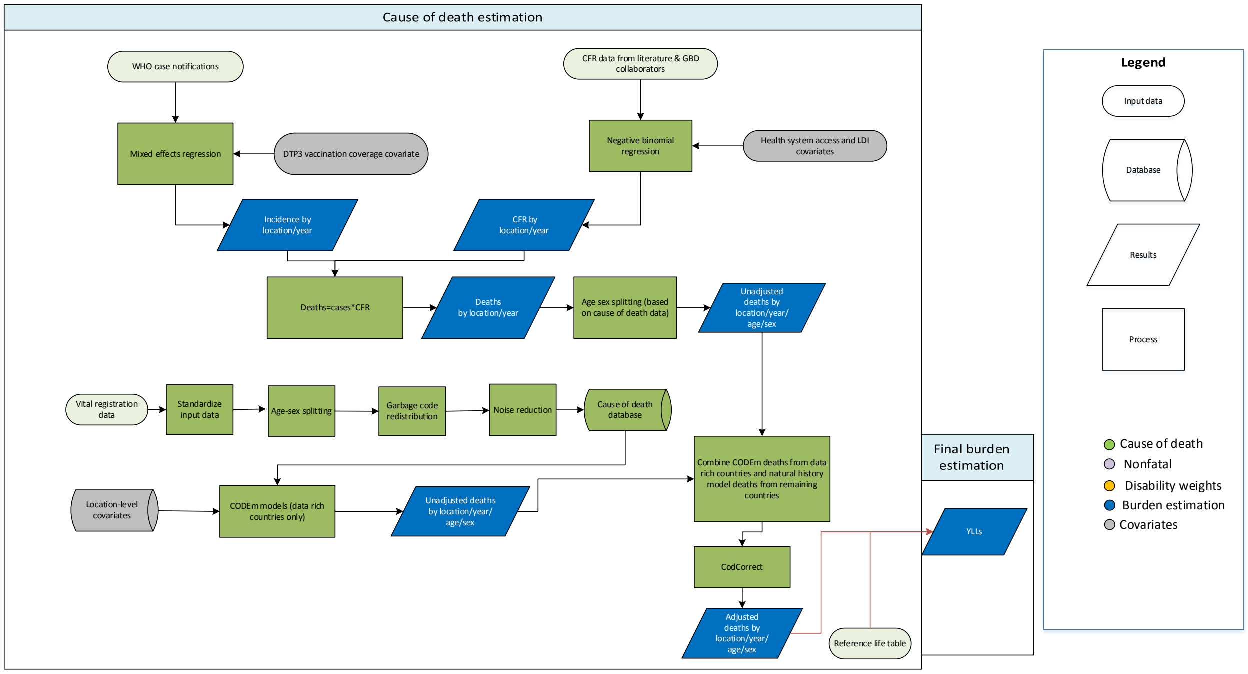

Input data

Vital registration data from the cause of death database were used for data-rich countries. For the natural history model, we used WHO case notifications data, historical case notifications for the UK back to 1940, and case fatality data identified by collaborators and through systematic literature reviews for GBD 2010 and GBD 2013. The PubMed search query for GBD 2013 was: (whooping cough [Title/Abstract]) OR (pertussis [Title/Abstract]) AND (case fatality [Title/Abstract]) AND ("2009"[Date - Publication]: "2013"[Date - Publication]). The literature review was not updated for GBD 2015.

Modeling strategy

Mortality was modeled two ways. For data-rich countries (i.e., countries with vital registration more than 95% complete for more than 25 years), we used CODEm with DPT3 vaccination coverage, lagged distributed income, and education as country-level covariates. We estimated for the age range post-neonatal to 59 years. For the remaining countries, we used a natural history-based model since CODEm did not predict well for those countries. First, we modeled incidence using case notifications from the WHO (1985-2014) with the DTP3 vaccination coverage covariate. Historical data of UK cases and UK DTP3 coverage rates (both back to 1940) were also used in the incidence model. More precisely, log-transformed reported incidence rates were regressed on the log of the fraction unvaccinated with a random effect on country. The random effect by country allowed for registration completeness to vary by country. The results of this model were then used to predict incidence as a function of vaccine coverage. We used a value of the random effect that matched the highest random effect in a high

income region, Switzerland (which has a pertussis monitoring system which should capture cases well). Uncertainty was estimated by taking 1,000 iterations of the predictions based on the variance covariance matrix. Second, we modeled CFR using a negative binomial model with the health system access and LDI covariates. Uncertainty was estimated by taking 1,000 iterations of the predictions based on the variance covariance matrix and a random sample from a gamma distribution. Finally, whooping cough deaths were calculated at the 1,000 draw level as

$$deaths = incidence * CFR$$

We first estimated overall number of deaths, which were then assigned an age-sex distribution based on the age-sex-specific patterns found in the cause of death data.

Measles

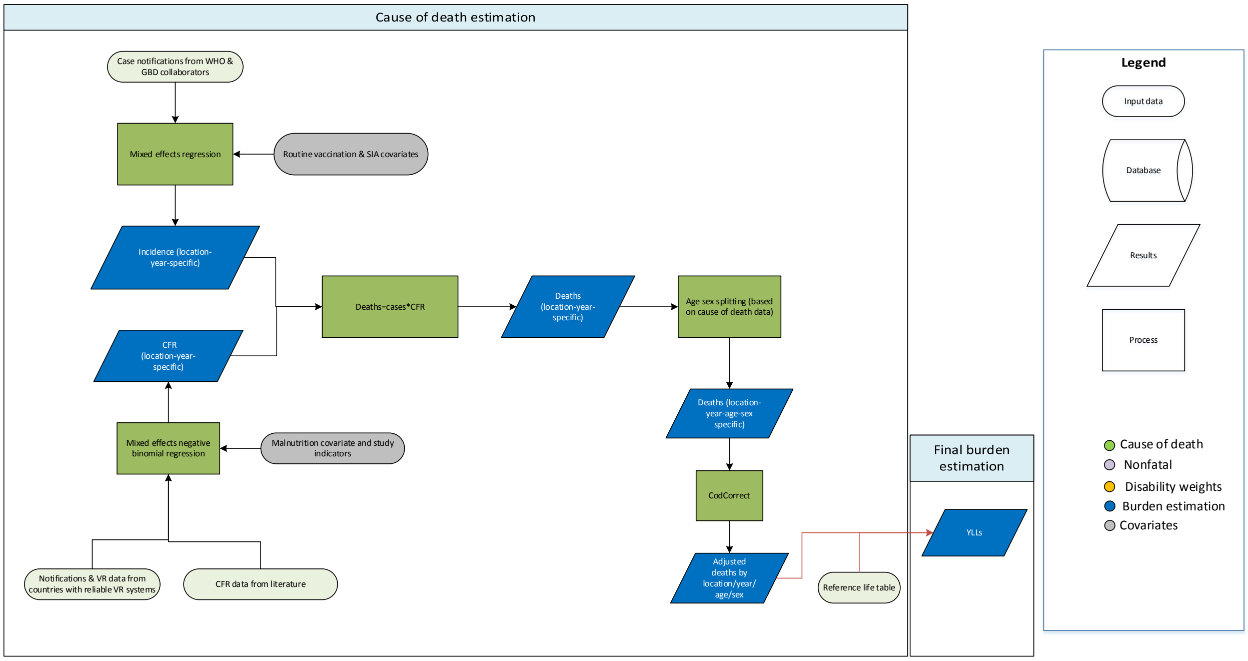

Input data

The input data for the measles incidence model were measles case notifications from the World Health Organization (WHO) and the GBD collaborators. We included notifications data from 1995 to 2014 in the mixed-effects regression. We dropped data prior to 1995 because of sparse and noisy data in earlier years. Case fatality rates (CFRs) were derived from the case notifications and vital registration (VR) data in countries in the following three super-regions: high-income; Central Europe/Eastern Europe/Central Asia; and Latin America and Caribbean. These CFRs were used in combination with CFR data identified by collaborators and through systematic literature reviews for GBD 2010 and GBD 2013. The PubMed search query for GBD 2013 was: (measles[Title/Abstract]) AND (case fatality[Title/Abstract]) AND ("2009"[Date - Publication] : "2013"[Date - Publication]). The literature review was not updated for GBD 2015. Studies were included if they reported CFR, number of deaths, and number of cases. Studies were excluded if they included non-representative minority samples only.

Modeling strategy

Measles mortality was modeled using a natural-history-based model. First, incidence was modeled using routine vaccination rates and supplementary immunization activities (SIAs) data. More precisely, log-transformed incidence rates were regressed on the log of the proportion unvaccinated, SIA variables lagged by one, two, three, four, and five years, with super-region, region, and country random effects. The results of this mixed effects regression model were then used to predict location-year-specific incidence as a function of vaccine coverage and SIAs. Uncertainty was estimated by taking 1,000 iterations of the predictions based on the variance covariance matrix. For locations in the following three super-regions, namely, high-income, Central Europe/Eastern Europe/Central Asia and Latin America and Caribbean, we directly used reported measles cases as incidence cases. Second, CFR was

modeled using a mixed effects negative binomial regression with the malnutrition covariate and study indicators (hospital-based or not; outbreak or not; and rural or urban/mixed), with country random effects. Uncertainty was estimated by taking 1,000 iterations of the predictions based on the variance covariance matrix and uncertainty in country random effects. The fit of the model was evaluated using diagnostic plots of predicted versus observed values. Finally, estimated incidence was multiplied by CFR (both at the 1,000 draw level) to get overall number of deaths, which were then assigned an age-sex distribution based on the age-sex-specific patterns found in the COD data.

Visceral leishmaniasis

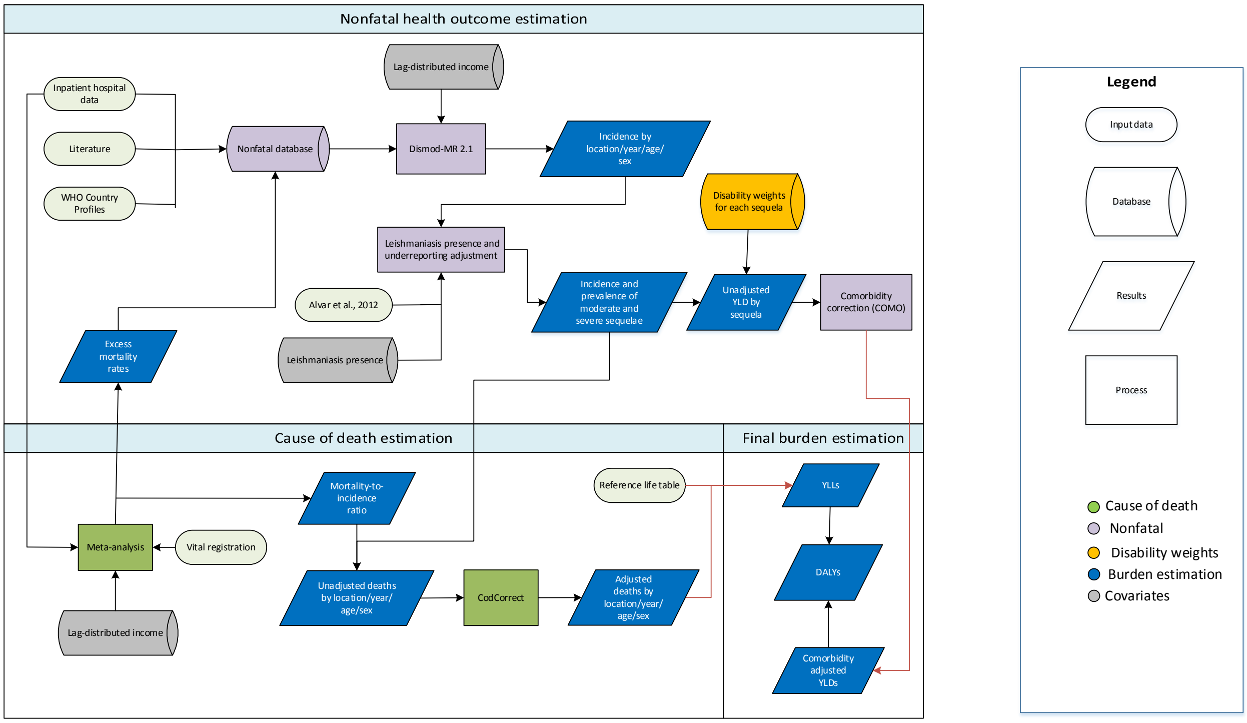

Input data

No mortality data from GBD 2015 were used in the estimation of visceral leishmaniasis. Details on the collection of incidence and prevalence data are detailed in the GBD 2015 nonfatal health study.

Modeling strategy

In GBD 2013, we estimated country-year-specific MI ratios by running a linear regression of the logit of the MI ratio on the log of income per capita using vital registration and inpatient hospital data from Brazil and Spain, two countries in which we had both reliable mortality and incidence data at the national level. Those ratios were used here. This ratio was used in two ways; first, in assuming a duration of 3 months, we were able to derive excess mortality for use in DisMod. Second, the product of the M:I ratio and cases then estimated by DisMod were used as death estimates for CoDCorrect.

Human African Trypanosomiasis (HAT)

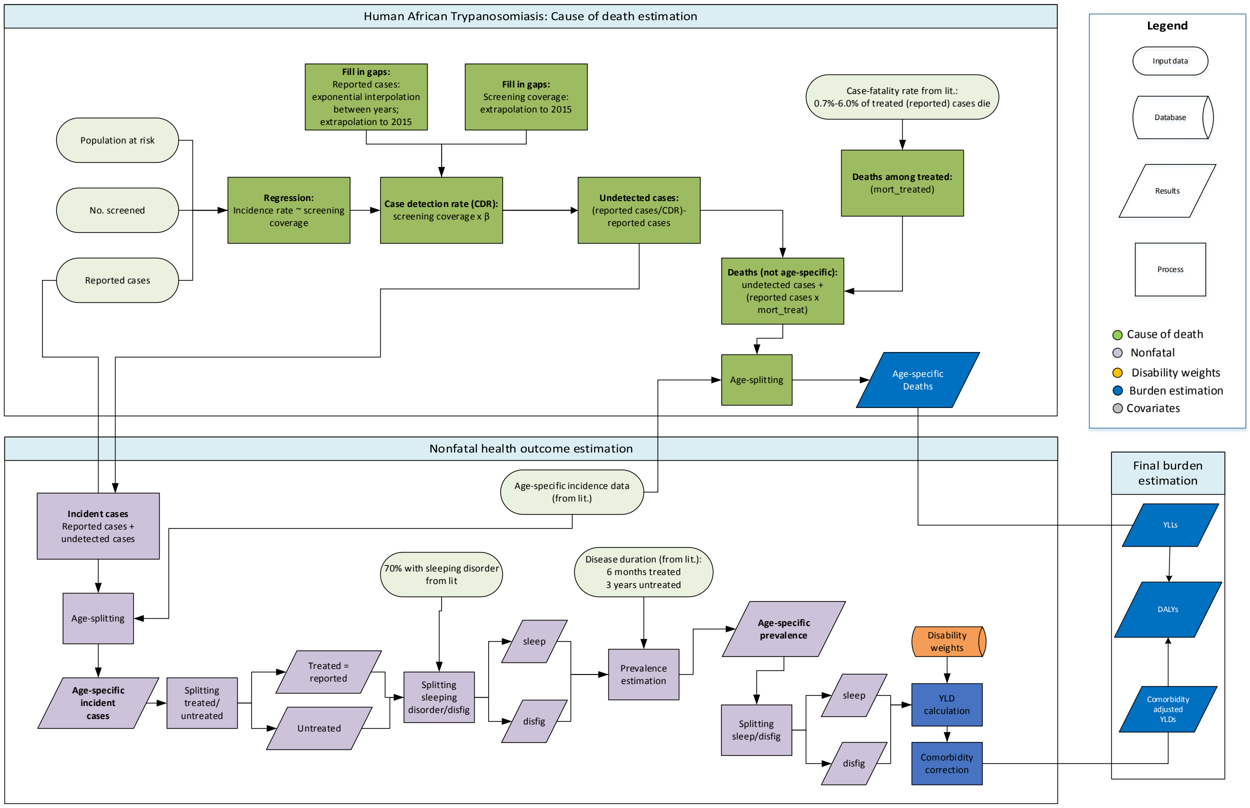

Input data

A literature search was done for GBD 2013 and for GBD 2015. The GBD 2015 search was conducted between 1/1/2013 and 8/10/2015, and the number of initial hits was 87. Of these, five sources were extracted for data.

Additional input data used to estimate mortality due to HAT included a) population at risk estimates from GBD 2010 ArcGIS analysis using geocoded case notifications for 2000 to 2009[1] and population Count Grid estimates from Gridded Population of the World 3,[2,3] b) population screened from 1997 to 2004,[4] c) historical data from GBD 2010 on total number of HAT cases reported[1,4,5] and d) cases reported annually to the WHO[6] – for Kenya, a study on cases reported subnationally[7] was used to split the national cases into five counties (HomaBay, Migori, Busia, Bungoma, Kakamega). In addition, age-specific incidence data from active screening undertaken in the Democratic Republic of Congo[8] and Uganda[9] were used to inform age pattern for deaths.

Modeling strategy

The cause of death model for HAT involved seven main steps. First, a multi-level mixed-effects linear regression of natural log-transformed incidence rate (ratio of HAT cases reported to population at risk) on natural log-transformed screening coverage (ratio of number screened for HAT to population at risk), with country random effects, was performed. Gaps were then filled using exponential interpolation between years and extrapolation from 2014 to 2015 for reported cases; for screening coverage only

extrapolation from 2014 to 2015 was done. One thousand draws of mortality among treated cases were then generated, assuming that 0.7% - 6.0% of all treated (reported) cases die.[10–12] This was followed by the generation of 1,000 draws of case detection rate (CDR), given the expected screening coverage from the regression. Undetected deaths were then estimated as the difference between the ratio of reported cases to CDR and reported cases (reported cases/CDR – reported cases). Estimates of death were obtained by adding the reported cases (scaled by mortality among treated) to the undetected cases. Without information on sex-specific incidence or deaths, equal death rates between both sexes was assumed. Finally, an age-pattern was applied to the mortality estimates using the incidence studies from DRC and Uganda.[8,9]

A few changes from GBD 2013 were made to the estimation of deaths due to HAT. To begin with, new data on reported cases from WHO[6] (years 2013 and 2014 for 23 locations) were included. In addition, based on available historical data post-1980, the following countries (years) were included in the estimation: Botswana (1983), Ethiopia (1980-1983), Guinea-Bissau (1980-1983, 1985-1987), Rwanda (1980, 1982-1988), and Sierra Leone (1981-1982). Five subnational locations (out of 49) for Kenya were also added in the estimation for GBD 2015. Finally, the age-split proportion was corrected such that a 0.32/0.68 proportion was used for adults/children. In GBD 2013, this proportion was 0.25/0.75 for adults/children.

References:

1 Simarro PP, Cecchi G, Paone M, *et al.* The Atlas of human African trypanosomiasis: a contribution to global mapping of neglected tropical diseases. *Int J Health Geogr* 2010; **9**: 57.

2 Gridded Population of the World (GPW), Version 3: Population Count Grid. Palisades, NY: Socioeconomic Data and Applications Center (SEDAC); Center for International Earth Science Information Network (CIESIN) at Columbia University; Centro Internacional de Agricultura Tropical (CIAT), 2005.

3 Gridded Population of the World (GPW), Version 3: Population Count Grid, Future Estimates. Palisades, NY: Socioeconomic Data and Applications Center (SEDAC); Center for International Earth Science Information Network (CIESIN) at Columbia University; United Nations Food and Agriculture Programme (FAO); Centro Internacional de Agricultura Tropical (CIAT), 2005.

4 Weekly Epidemiological Record (WER). World Health Organization, 2006.

5 World Health Organization, Department of Communicable Disease Surveillance and Response (CDS). WHO Report on Global Surveillance of Epidemic-prone Infectious Diseases. World Health Organization, 2000.

6 World Health Organization. Human African Trypanosomiasis. Global Health Observatory data repository. http://apps.who.int/gho/data/node.main.A1635?lang=en (accessed April 21, 2016).

7 Rutto JJ, Karuga JW. Temporal and spatial epidemiology of sleeping sickness and use of geographical information system (GIS) in Kenya. *J Vector Borne Dis* 2009; **46**: 18–25.

8 Lutumba P, Makieya E, Shaw A, Meheus F, Boelaert M. Human African trypanosomiasis in a rural community, Democratic Republic of Congo. *Emerging Infect Dis* 2007; **13**: 248–54.

9 Fèvre EM, Odiit M, Coleman PG, Woolhouse MEJ, Welburn SC. Estimating the burden of rhodesiense sleeping sickness during an outbreak in Serere, eastern Uganda. *BMC Public Health* 2008; **8**: 96.

10 Balasegaram M, Harris S, Checchi F, Ghorashian S, Hamel C, Karunakara U. Melarsoprol versus eflornithine for treating late-stage Gambian trypanosomiasis in the Republic of the Congo. *Bull World Health Organ* 2006; **84**: 783–91.

11 Odiit M, Kansiime F, Enyaru JC. Duration of symptoms and case fatality of sleeping sickness caused by Trypanosoma brucei rhodesiense in Tororo, Uganda. *East Afr Med J* 1997; **74**: 792–5.

12 Priotto G, Kasparian S, Mutombo W, *et al.* Nifurtimox-eflornithine combination therapy for second-stage African Trypanosoma brucei gambiense trypanosomiasis: a multicentre, randomised, phase III, non-inferiority trial. *Lancet* 2009; **374**: 56–64.

Yellow fever

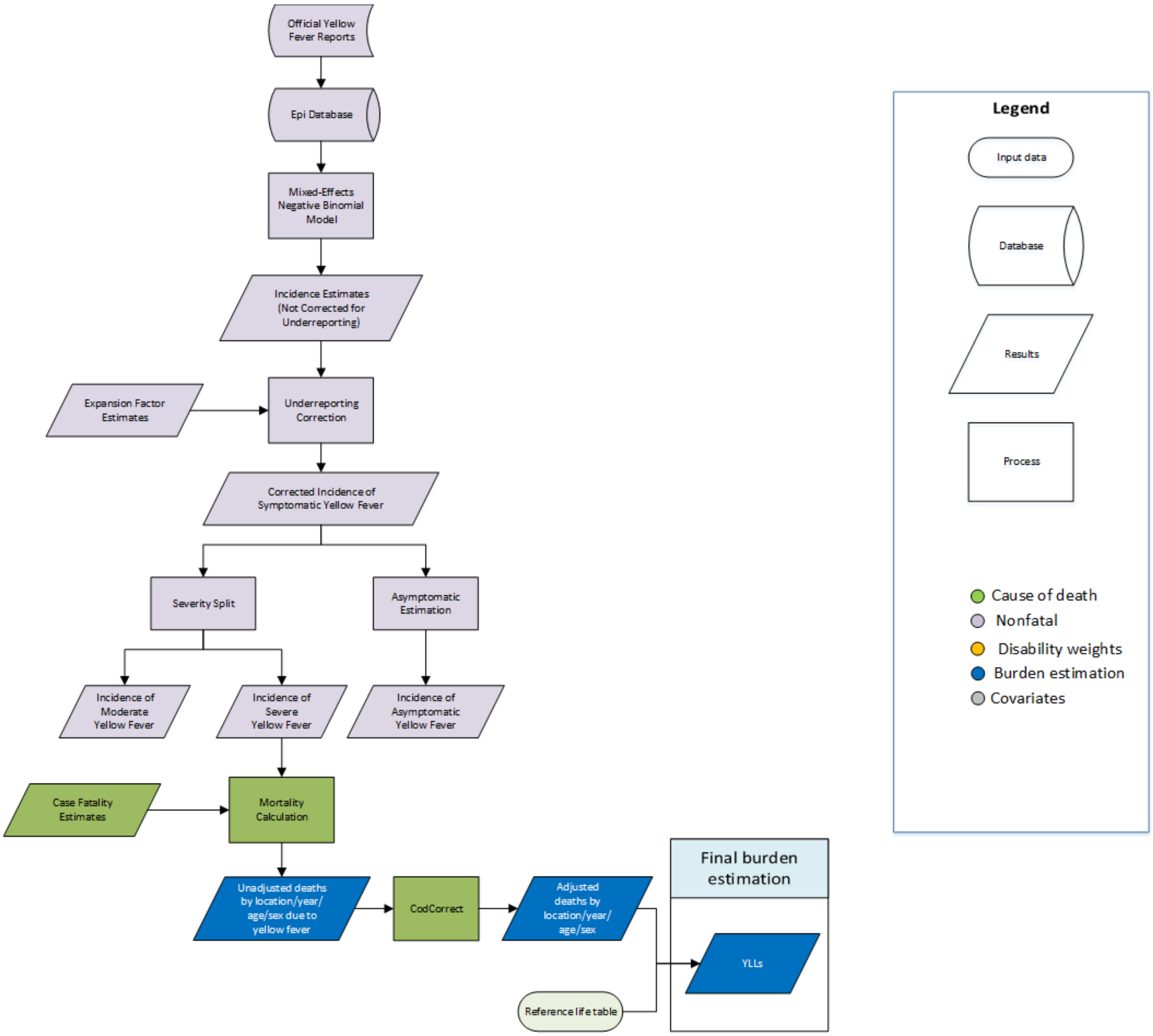

Input data

Case data come from official case reports filed with the World Health Organization. Data on case fatality come from published studies of yellow fever fatality. Data on deaths in non-endemic countries is restricted to only vital registration data.

Modeling strategy

We model yellow fever deaths using a hybrid approach. For countries in which yellow fever is endemic, we use a natural history approach in which we estimate deaths as the product of cases and case fatality. For non-endemic countries, we allow for deaths among imported cases where we have vital registration data indicating yellow fever deaths. That is, we assume no yellow-fever deaths in non-endemic

countries; however, where yellow fever deaths are reported in vital registration data, we accept those as true imported yellow fever deaths.

We model reported cases using a mixed-effects negative binomial model, with fixed effects for year and random effects for super-region, region, and country. We assume that yellow fever cases are underreported and that this underreporting mirrors that of dengue (a disease for which we have better data on underreporting). With that, we estimate symptomatic cases as the product of our base case estimates and dengue expansion factors (i.e., the factor by which you must multiply reported cases to derive true cases). Based on published estimates, we assume that 27% of symptomatic cases will be severe.[1]

We performed a meta-analyses of case fatality using data from published studies of yellow fever fatality. Studies tend to report deaths among those with severe infection (e.g., hospitalized cases), rather than among all cases. We assume that no deaths occur with asymptomatic infection or among those with only moderate symptoms. With that, we estimate deaths as the product of severe cases and case fatality.

We have improved our method for correcting for underreporting of yellow fever. In estimating yellow fever deaths for GBD 2013, our model assumed that all severe cases were reported and that reported cases reflected only severe cases. With that, we adjusted our base estimates upward to account for non-severe cases, and based our mortality estimates off these adjusted numbers. Based on feedback from collaborators, we believe that this adjustment was inadequate to fully account for underreporting. Accordingly, we have adopted the expansion factor based method described above for GBD 2015.

Moreover, we have adopted the hybrid approach for GBD 2015. Whereas we previously allowed no yellow fever deaths in non-endemic countries, we now accept deaths reported in vital registration data as true imported deaths.

Reference

1 Johansson MA, Vasconcelos PFC, Staples JE. The whole iceberg: estimating the incidence of yellow fever virus infection from the number of severe cases. *Trans R Soc Trop Med Hyg* 2014; 108: 482–7.

Sexually transmitted infections excluding HIV

This write-up covers causes including congenital syphilis, which was run as a natural history model.

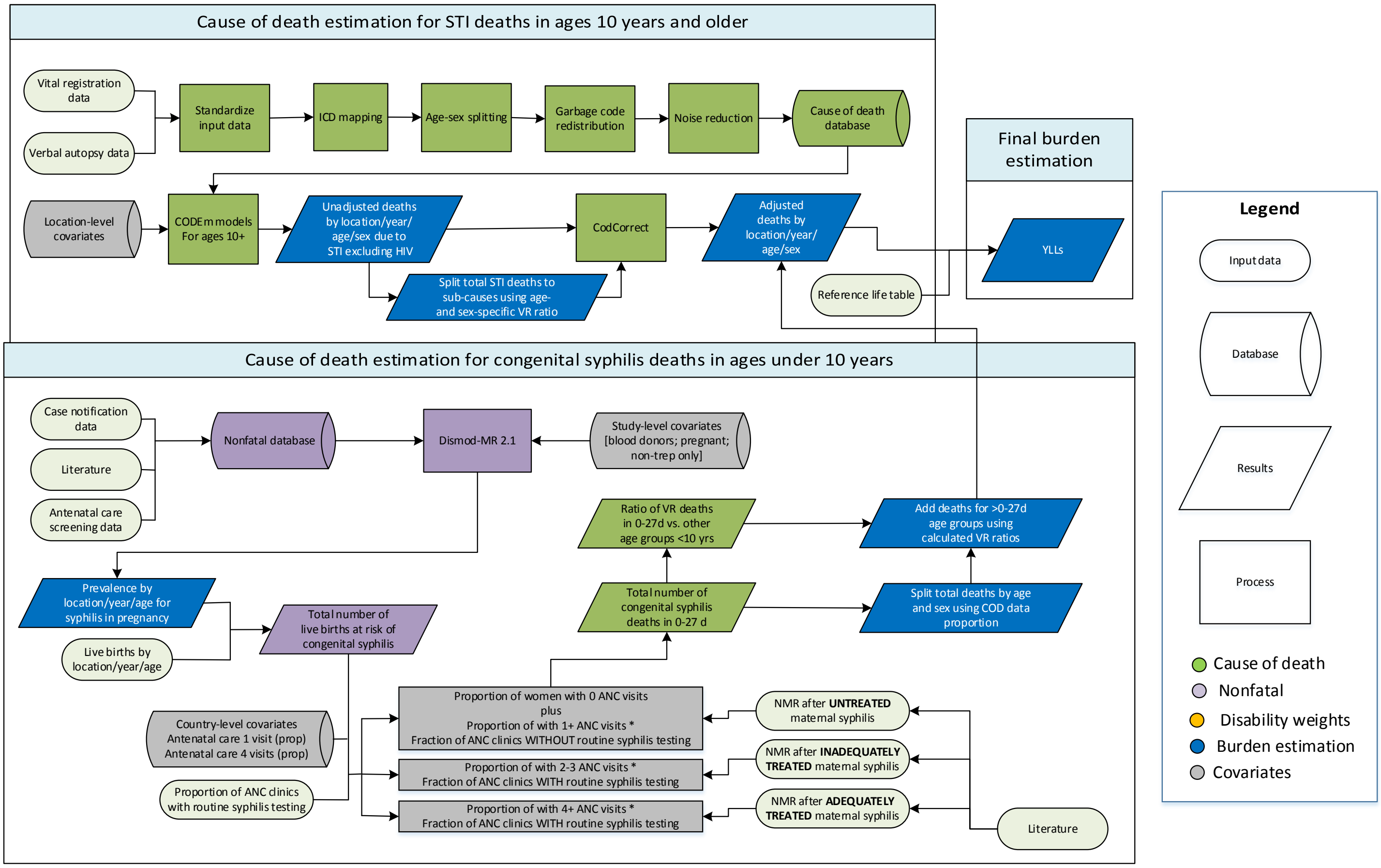

Input data

CODEm models for males and females 10 years and older were informed from centrally prepped data stored in the cause of death (COD) database. All data from all geographies were reviewed. Outliers were identified as those data where age patterns or temporal patterns were inconsistent with neighboring age groups or locations or where sparse data were predicting implausible overall temporal or age patterns for a given location.

Four different types of data were used for the NHM model. First, we used literature, survey, and report data described below to estimate early syphilis in pregnancy. Second, we used GBD 2015 estimates of antenatal care (ANC) coverage data from our covariates database and live births estimates from our demographics analysis. Third, we used published data from the Global Health Observatory on proportion of ANC clinics that test for syphilis and the proportion of women testing positive who receive treatment. Fourth, we used the results of a systematic literature review completed for GBD 2010 to inform excess mortality of neonates born with syphilis.

We completed data-rich (DR) and global CODEm models for ages 10 years and over for males and females separately. Nine covariates were used in each CODEm model, including 1) syphilis prevalence in pregnancy from DisMod-MR 2.1 analysis described below; 2) coverage of one antenatal care (ANC) visit, 3) coverage of four ANC visits; 4) age-specific fertility rate; 5) total fertility rate; 6) health system access, a principal components analysis of ANC, in-facility delivery, skilled birth attendance, and vaccine coverage; 7) national income per capita (LDI); 8) years of education per capita; and 9) abortion legality, a categorical variable that ranges from 1 (always illegal) to 7 (always legal on demand).

The overall CODEm model for STI was split into the sub-causes using vital registration (VR) data from the COD database. Trichomoniasis and HSV-2 are assumed not to cause mortality. Chlamydia is further assumed not to cause death in males. Cause-specific mortality rate VR data for each age-group, sex, and year were summed and scaled to match the total STI cause-specific mortality rate predicted by CODEm. This VR pattern was applied globally to all locations.

Our NHM for congenital syphilis began with estimation of early syphilis prevalence in all age-groups, both sexes, all GBD locations, and in each year from 1990 to 2015. Systematic literature review was combined with review of all publicly available UN GASS country progress reports, antenatal clinic surveillance data from country-specific and UNAIDS reports, targeted searches of national ministry of health (MoH) websites, and other published data provided by GBD collaborators. A Pubmed search was completed on June 1, 2015, using the following search string:

> ("syphilis"[MeSH] OR "Treponema pallidum"[MeSH]) NOT "Yaws"[MeSH] AND ("prevalence"[MeSH] OR "incidence"[MeSH]) AND "1990"[PDAT] : "2015"[PDAT] AND "humans"[MeSH]

A total of 1,265 titles and abstracts were found, of which 178 had suitable data for inclusion in our dataset. In addition to standard demographic identifiers, all data were extracted to include number of cases, prevalence or incidence rate, and sample size. Laboratory testing modality for each study was specified as including treponemal test, non-treponemal test, both treponemal and non-treponemal, or direct spirochete detection (e.g., darkfield microscopy). Usage of treponemal and non-treponemal testing was the reference category. Our final dataset included data from 113 distinct geographies. Data from blood donor and pregnant populations were included, but all other high-risk groups were excluded.

Modeling strategy

We modeled early syphilis in DisMod-MR 2.1, restricting incidence before the age of 10 and after the age of 65 and assuming excess mortality from early syphilis is very low ($\leq$ 1 death per 10,000 person-years). Study-level covariates identified data in non-reference categories (pregnant, blood donors) and using non-reference testing modalities (e.g., treponemal test only, non-treponemal test only) and were crosswalked to the reference category within the DisMod-MR 2.1 cascade, predicting specific conversion values for each country. The latter specification of country-level crosswalks was chosen because the bias between non-reference populations and testing modalities was assumed to differ by geography and as a function of, for example, endemicity of acute infectious agents like malaria.

Age-specific prevalence results were paired with age-specific live birth results to generate total number of births at risk of congenital syphilis. To estimate the actual number of congenital syphilis births, we combined information on ANC coverage from GBD 2015 covariates analyses with ANC syphilis testing

and treatment data from GHO. Adequate treatment was assumed to confer no risk of congenital syphilis mortality. Those with four ANC visits or 1-3 ANC visits with testing and treatment were assumed to have received adequate treatment. Inadequate treatment occurred in those women with 1-3 ANC visits without either testing or treatment or those women with one ANC visit but with testing and treatment. Those women with only one or fewer ANC visits and no syphilis testing or treatment were assumed to be untreated. Untreated and inadequate treatment proportions were combined with potential congenital syphilis births to estimate total neonatal syphilis births. Each categorical risk category was combined with corresponding neonatal excess mortality rates derived in GBD 2010. This total number of neonatal syphilis deaths was then split between 0-6 days and 7-27 days age groups using sex and age-specific VR data from the COD database. Congenital syphilis deaths beyond the neonatal period were likewise estimated using sex- and age-specific VR data. This VR pattern was applied globally to all locations.

The primary limitation of our estimation of STI deaths in those over 10 years old is data availability, especially from countries where VR systems are not available. Even in countries with VR, there may be some variation in practices for coding deaths to STI as the underlying cause, especially given the potentially variable presentation of many of the conditions in this category. Such variation is more likely to lead to underestimation of STI deaths than overestimation. Sub-cause estimation is similarly limited by data availability in those locations without VR data, and our estimates are thus based on the overall pattern of deaths in generally higher-income geographies. The primary implication of this limitation is that it decreases the resolution with which we can decompose the relationship between mortality from HIV and other STI. Our NHM for congenital syphilis was a significant improvement over GBD 2013 but still is limited by data availability issues, especially on the coverage and effectiveness of ANC interventions to prevent congenital syphilis. We do not have information on the proportion of women that tested positive who may have received treatment elsewhere, or information on the coverage of treatment for neonates, infants, and children born with congenital syphilis. Both limitations could potentially have led to lower estimates of congenital syphilis deaths. On the other hand, our DisMod-MR 2.1 analysis suggested that pregnant women may in fact have higher syphilis prevalence than the general population, which would have led to higher estimates. We have also not quantified the number of stillbirths associated with congenital syphilis. We will continue to work to address these shortcomings more thoroughly in future GBD analyses.

Acute hepatitis A

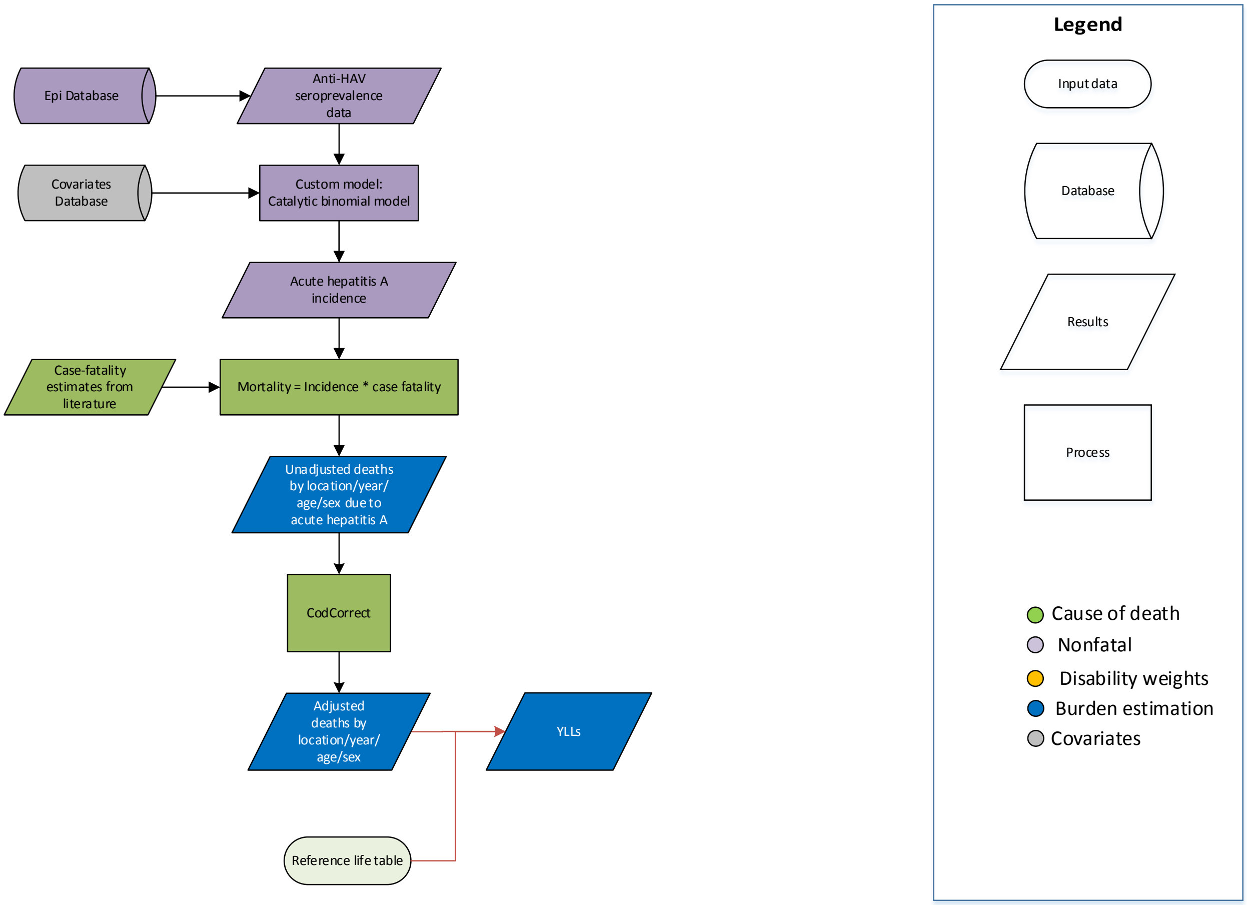

Input data

We use anti-HAV seroprevalence data from population-based studies and surveys for the incidence model.

Modeling strategy

Virus-specific CoD data for acute hepatitis are inconsistently reported and of questionable accuracy. We therefore use a two-part modeling strategy for acute hepatitis A, B, C, and E. First, we develop a parent acute hepatitis mortality model using CODEm and all acute hepatitis mortality data within the CoD database. Second, we develop four separate natural history models to estimate deaths from acute hepatitis A, B, C, and E, respectively. Finally, we rescale the virus-specific death estimates from the four natural history models to fit within the envelope defined by the parent acute hepatitis CoD model.

Given its reasonably stable force of infection among susceptible people across age groups, we used a catalytic binomial model to estimate incidence of acute hepatitis A based on anti-HAV seroprevalence. The catalytic binomial model is a binomial generalized linear model with a complementary log-log link, and an offset term for log-age. Since anti-HAV is a lifetime marker of past infection, and a given individual can only be infected once, seroprevalence at age *t* is equal to the cumulative incidence (CI) over *t* years. Assuming constant force of infection, we can estimate the incidence rate (IR) as,

$$CI = 1 - e^{-IR \cdot t}$$

We can rearrange this equation to solve for the log-IR:

$$\ln(IR) = \frac{\ln(-\ln(1 - CI))}{\ln(t)}$$

Thus, by using the complimentary log-log link for CI (i.e., ln(-ln(1-CI)) with an offset for log-age, we are able to model the incidence rate of infection from seroprevalence data. To inform the model in the absence of data we use a predictive covariate derived from principal components analysis of lag-distributed income (LDI) and the proportion of the population with access to improved water. We use a mixed effects model with fixed effects on the aforementioned PCA-derived covariate, and nested hierarchical random effects on super-region, region, country, and subnational geographies.

Our overall approach has not changed from that used in GBD 2013. However, whereas we previously used a fixed-effects only catalytic binominal model for GBD 2013, we have incorporated random effects into the model for GBD 2015, improving the spatial structure of the model and allowing the model to better follow data.

Acute hepatitis B

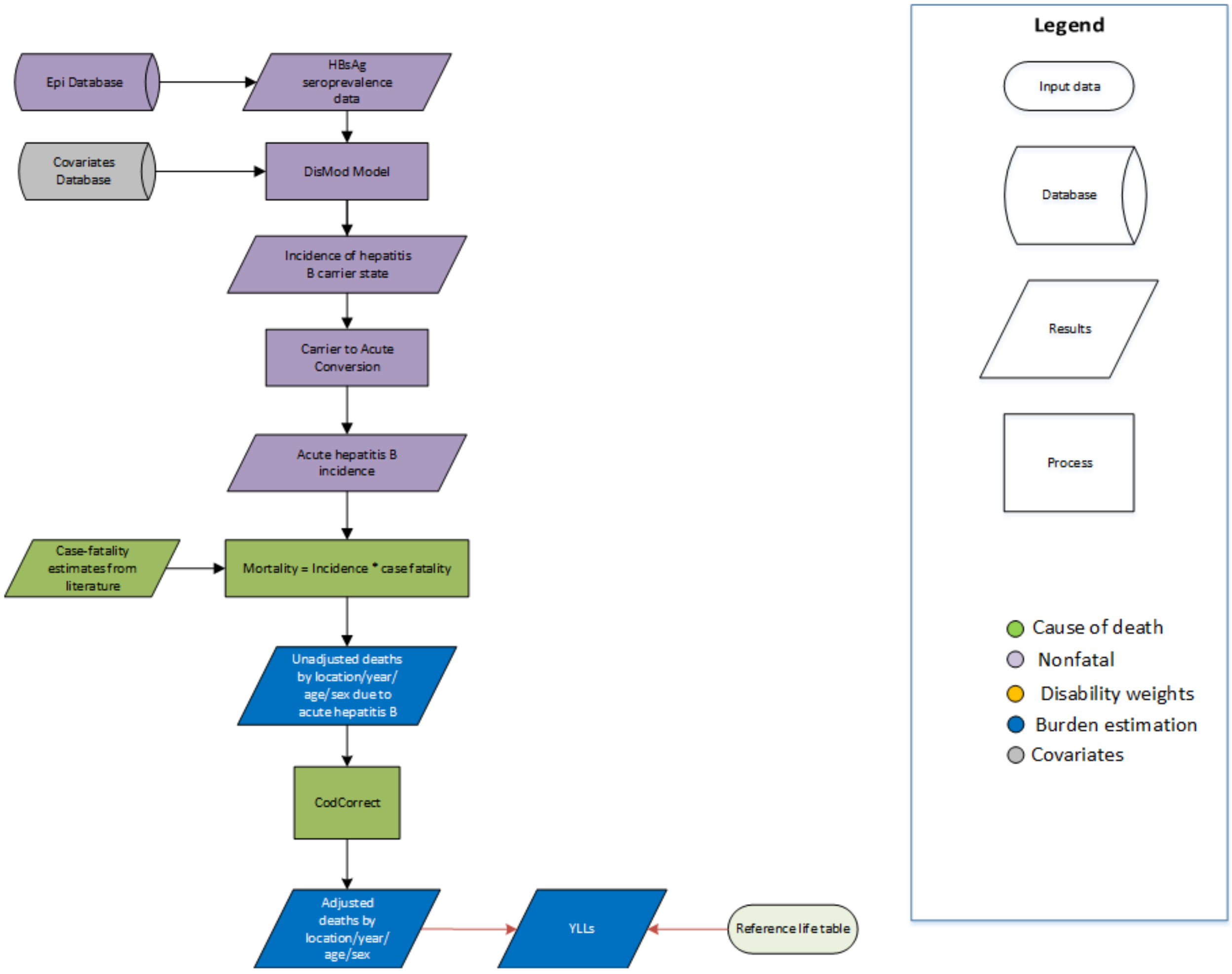

Input data

We use hepatitis B surface antigen (HBsAg) seroprevalence data from population-based studies and surveys for the incidence model.

Modeling strategy

Virus-specific CoD data for acute hepatitis are inconsistently reported and of questionable accuracy. We therefore use a two-part modelling strategy for acute hepatitis A, B, C, and E. First, we develop a parent acute hepatitis mortality model using CODEm and all acute hepatitis mortality data within the CoD database. Second, we develop four separate natural history models to estimate deaths from acute hepatitis A, B, C, and E, respectively. Finally, we rescale the virus-specific death estimates from the four natural history models to fit within the envelope defined by the parent acute hepatitis CoD model.

We model the incidence of chronic HBsAg carriage using a full DisMod model of HBsAg seroprevalence. We then convert incidence of chronic carriage to total incidence of hepatitis B infection by dividing age-specific estimates of the incidence of chronic carriage by age-specific estimates of the probability of infection resulting in carriage. We then multiply these incidence estimates by published estimates of case fatality to estimate acute hepatitis B deaths.

We have made no substantive changes in the modeling strategy from GBD 2013.

Acute hepatitis C

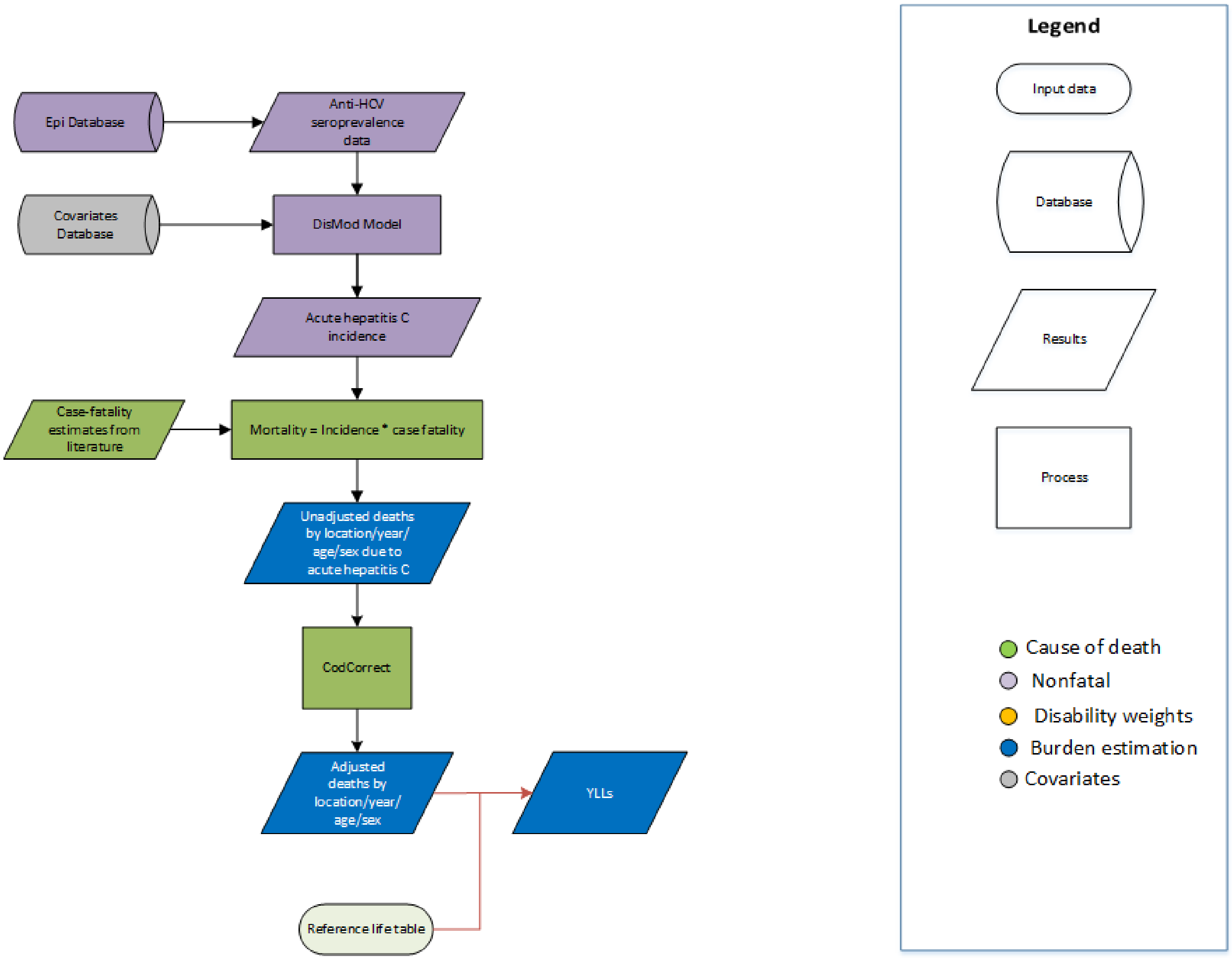

Input data

We use anti-HCV seroprevalence data from population-based studies and surveys for the incidence model.

Modeling strategy

Virus-specific CoD data for acute hepatitis are inconsistently reported and of questionable accuracy. We therefore use a two-part modeling strategy for acute hepatitis A, B, C, and E. First, we develop a parent acute hepatitis mortality model using CODEm and all acute hepatitis mortality data within the CoD database. Second, we develop four separate natural history models to estimate deaths from acute hepatitis A, B, C, and E, respectively. Finally, we rescale the virus-specific death estimates from the four natural history models to fit within the envelope defined by the parent acute hepatitis CoD model.

We model the incidence of hepatitis C using a full DisMod model of anti-HCV seroprevalence and estimate acute hepatitis C deaths as the product of these incidence estimates and published estimates of acute hepatitis C case fatality.

We have made no substantive changes in the modeling strategy from GBD 2013.

Acute hepatitis E

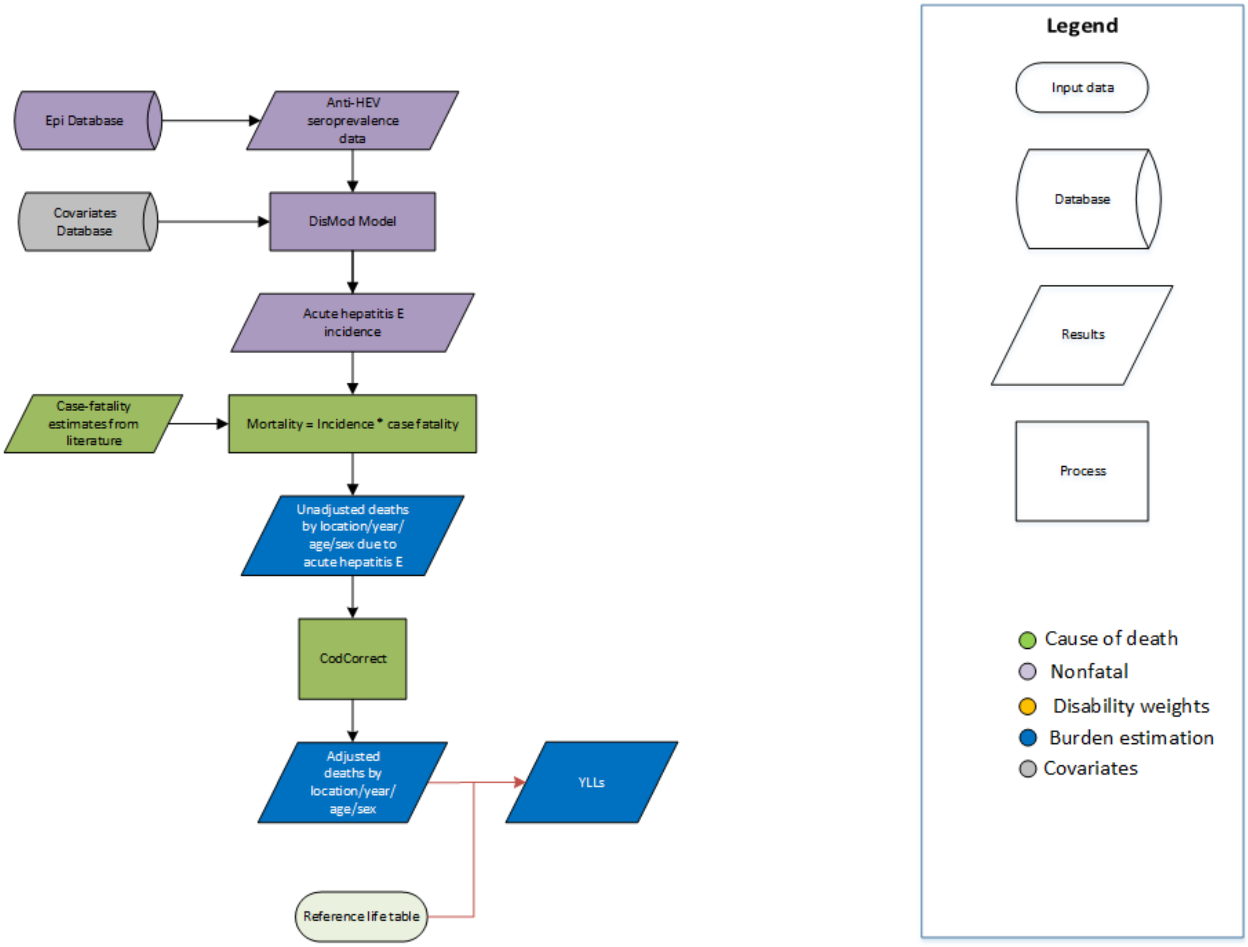

Input data

We use anti-HEV seroprevalence data from population-based studies and surveys for the incidence model.

Modeling strategy

Virus-specific CoD data for acute hepatitis are inconsistently reported and of questionable accuracy. We therefore use a two-part modeling strategy for acute hepatitis A, B, C, and E. First, we develop a parent acute hepatitis mortality model using CODEm and all acute hepatitis mortality data within the CoD database. Second, we develop four separate natural history models to estimate deaths from acute hepatitis A, B, C, and E, respectively. Finally, we rescale the virus-specific death estimates from the four natural history models to fit within the envelope defined by the parent acute hepatitis CoD model.

We model the incidence of hepatitis E using a full DisMod model of anti-HEV seroprevalence and estimate acute hepatitis E deaths as the product of these incidence estimates and published estimates

of acute hepatitis E case fatality, accounting for the proportion of infections that occur in pregnant women and the higher case fatality of hepatitis E among pregnant women. Since HEV genotypes tend to be less pathogenic and less virulent, we restrict hepatitis E deaths to countries in regions where genotypes 1 and 2 predominate (i.e., Central Asia, Central sub-Saharan Africa, East Asia, Eastern sub-Saharan Africa, North Africa and Middle East, Oceania, South Asia, Southeast Asia, Southern sub-Saharan Africa, and Western sub-Saharan Africa).[1]

We have made no substantive changes in the modeling strategy from GBD 2013.

Reference

1 Rein DB, Stevens GA, Theaker J, Wittenborn JS, Wiersma ST. The global burden of hepatitis E virus genotypes 1 and 2 in 2005. *Hepatology* 2012; **55**: 988–97.

2.5 Prevalence-based models

The modeling strategies for Alzheimer's and other dementias and atrial fibrillation are distinct from those used for other causes modeled as natural history models. These models use prevalence estimates and excess mortality rates (EMR) generated through DisMoD MR2.1, rather than incidence and case-fatality rates.

Alzheimer Disease and Other Dementias

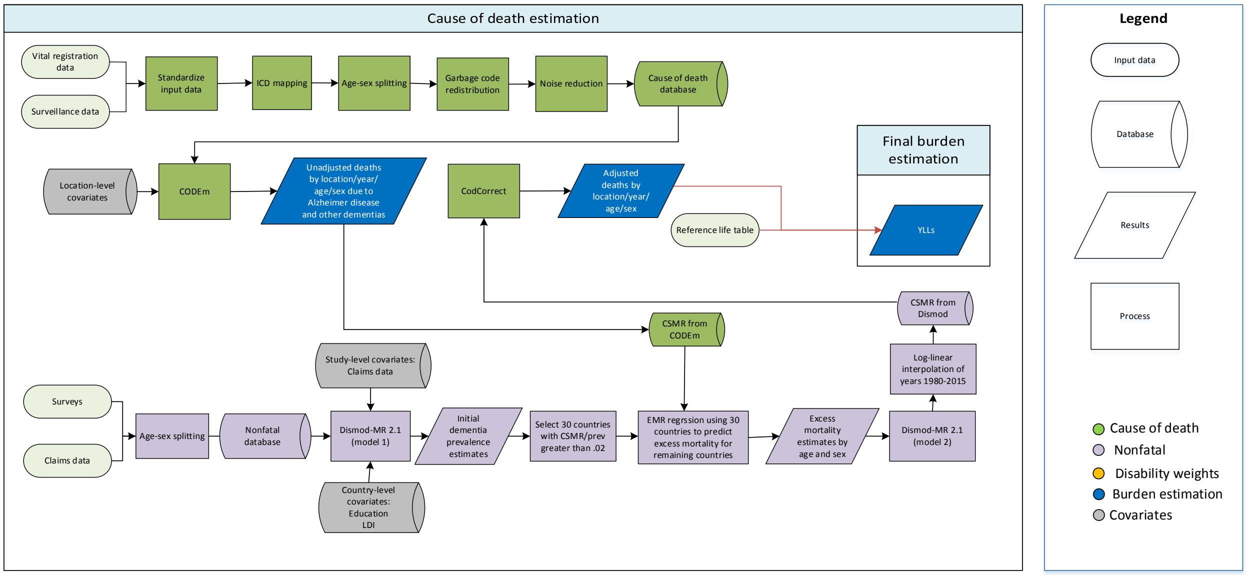

Input Data

In GBD 2015, data used to estimate deaths due to Alzheimer disease and other dementias (dementias hereafter) included mortality data from vital registration systems and prevalence data from surveys and claims sources.

An updated systematic review was conducted from January 2013 to October 2015, and search terms[1] were set to capture studies for all dementia, including its sub-types. The search yielded 1,399 initial hits and 27 were marked for extraction. Inclusion criteria comprised studies that reported prevalence, incidence, remission rate, excess mortality rate, relative risk of mortality, standardized mortality ratio, or with-condition mortality rate. Studies with no clearly defined sample were excluded.

Modeling Strategy

Overview

Dementia mortality rates have increased more than five-fold since 1980 in high-quality vital registration systems such as in the US and Scandinavia. We have not seen an equivalent increase in prevalence and incidence data sources. If at all, there has been a modest decline in incidence and prevalence of dementia in studies in the UK and the US that used comparable survey methods. Also, the greater than 20-fold variation in mortality rates of dementia between countries is much greater the four-fold difference in prevalence and incidence between countries. As it is unlikely that case fatality from dementia has dramatically increased over the time period and that it would differ by a very large margin between countries, the hypothesis is that certifying and coding practices have changed over time and at a different pace between countries. To avoid spurious large trends over time in the fatal component of the burden of dementia, we decided for GBD 2013 to make dementia mortality rates consistent with the rates observed in 2015 relative to prevalence of countries that are most likely to certify or code

[1] ((dementia[Title/Abstract]) AND ((incidence[Title/Abstract]) or (prevalence[Title/Abstract])) AND ('2013'[Date - Publication] : '2015'[Date - Publication]))

dementia as an underlying cause of death. This approach was applied for GBD 2015, described further below.

Modeling steps

First, we ran a CODEm model for dementia and extracted the mortality rates by age, sex, and geography for 2015.

Second, we ran a DisMod-MR 2.1 model with all data on incidence, prevalence, and mortality risk (RR, SMR, or with-condition mortality rates) and a setting of zero remission and extracted 2015 prevalence by age, sex, and geography. The most substantial new source used for GBD 2015 was medical claims data, which provided data by age and sex for years 2000, 2010, and 2012 across all US states. To account for potential systematic differences between claims and survey data, we crosswalked for each year of claims data.

Third, we selected 30 countries with a cause-specific mortality rate to prevalence ratio greater than 0.02 (excluding small island nations and those without vital registration).

Fourth, we used a mixed effects regression with dummies on age group and sex to predict excess mortality (i.e., the ratio of cause-specific mortality rate and prevalence) by age and sex, the results of which are found in the Table below.

Table: Fixed effect coefficients of EMR regression. Outcome: ln(EMR)

Independent variables	Coef	Std. error	P value	95% Confidence Interval	
Female	-0.235	0.020	0.000	-0.274	-0.196
Age 60-64	0.707	0.034	0.000	0.640	0.774
Age 65-69	0.817	0.034	0.000	0.750	0.884
Age 70-74	1.120	0.034	0.000	1.053	1.188
Age 75- 80	1.471	0.034	0.000	1.404	1.539
Age 80+	2.198	0.034	0.000	2.131	2.266
Constant	-4.978	0.074	0.000	-5.122	-4.834
Random effect parameters					
Variance(constant)	0.143	0.038		0.085	0.240
Variance(residual)	0.034	0.003		0.029	0.040

We also fit a variation of the main EMR regression including the natural log of lagged distributed income (lnldi) as an additional covariate. The coefficient estimate and the corresponding confidence interval were then used to set a prior on the relationship between lnldi and EMR in DisMod-MR 2.1, where lnldi was a country-level covariate. This helped to capture location-specific variation in EMR for locations not included in the regression.

Fifth, these estimates were added to a second DisMod-MR 2.1 model as pertaining to the full 1990–2015 estimation period. For the 30 countries included in the regression, we retained their age- and sex-specific ratios and entered those also as pertaining to the full 1990–2015 estimation period.

Sixth, we took the predictions of cause-specific mortality by age, sex, geography, and year that DisMod-MR 2.1 calculated as being consistent with the data on incidence, prevalence, and the priors on excess mortality from step five. As mentioned, log lag distributed income per capita was used as a country-level covariate on EMR. The prior bounds of this latter selection were calculated using an iteration of the main EMR regression with log LDI as an additional covariate. We excluded data for standardized mortality ratio, with-condition mortality rate, relative risk as we wanted to estimate cause-specific mortality rates that were consistent with the level of excess mortality from the 30 chosen countries in 2015.

Seventh, because DisMod-MR 2.1 only produces estimates in five-year intervals from 1990 to 2015, we expanded the time series by log-linear interpolation. The trend from 1990–1995 was used to back-cast values for the 1980–1990 time period.

Atrial Fibrillation and Flutter

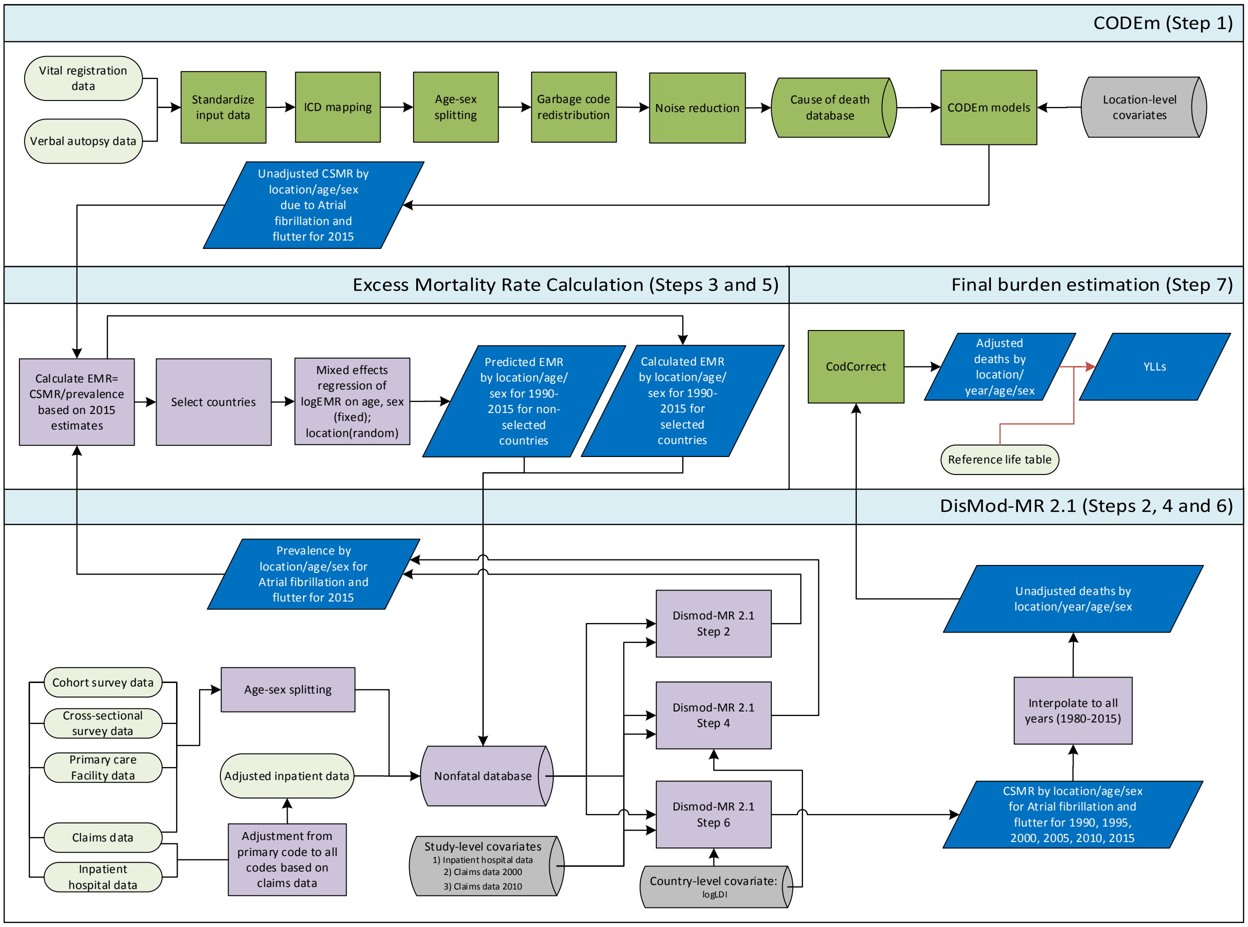

Input data
Vital Registration Data: We outliered ICD8 and ICD9 data points that were discontinuous from other data in the time series and created an unlikely time trend. We also outliered data points that were implausibly low in multiple age groups.

Modeling strategy
In order to address changes in coding practices for atrial fibrillation, we used an integrated approach that combined DisMod-MR and CODEm models to estimate deaths from atrial fibrillation and flutter. This approach allowed us to adjust estimates to more accurately reflect the number of deaths for which atrial fibrillation was the true underlying cause of death.

The modeling steps are illustrated in the above flowchart. In Step 1, we estimated deaths for atrial fibrillation using a standard CODEm approach. In Step 2, we estimated prevalence rates in DisMod-MR using data from published reports of cross-sectional and cohort surveys and primary care facility data. We also used claims data covering inpatient and outpatient visits for the United States along with inpatient hospital data from 22 countries. As the inpatient hospital data only included information from the primary code for each visit, prevalence rates for these data were adjusted based on the age- and

sex-specific proportions of atrial fibrillation in the primary codes vs. secondary codes in the US claims data. In Step 3, we calculated the excess mortality rate (EMR) for 2015 (defined as the cause-specific mortality rate (CSMR) estimated from CODEm divided by the prevalence rate from DisMod-MR). We then selected 27 countries based on four conditions: 1) availability of VR data; 2) prevalence rate ≥ 0.005; 3) CSMR ≥ 0.00002; and, 4) EMR ≥ 0.001. Using information from these countries as input data, we ran a linear mixed-effects regression of logEMR on sex, age, and location. Sex and age were treated as fixed effects for the regression, while location was considered a random effect. We then predicted age- and sex-specific EMR using the results of this regression for all non-selected countries. Countries included in the regression were assigned their directly calculated values. These EMR data points were assigned to the time period 1990–2015 and uploaded into the nonfatal database in order to be used in modeling. In Step 4, we reran DisMod-MR including the EMR estimated in Step 3 and using log-transformed lagged distributed income (LDI) as a country-level covariate. Based on information from other regressions, we set the bounds at -1.5 to -0.25.

In Steps 5 and 6, we repeated the process in Steps 3 and 4. In this iteration, we selected 31 countries that were included in the mixed-effects regression. The criteria were: 1) availability of VR data; 2) prevalence rate ≥ 0.004; 3) CSMR ≥ 0.00002; and, 4) EMR ≥ 0.002. The CSMR from the DisMod-MR model in Step 6 was used as the finalized output. As DisMod-MR only generates estimates for six years (1990, 1995, 2000, 2005, 2010, 2015), we interpolated the missing years to generate death estimates for all years (1980–2015). These results were then uploaded into the Cause of Death database. Finally, in Step 7, the unadjusted death estimates were run through the CodCorrect process to generate adjusted deaths and YLLs were generated by the DALYnator using a standard reference life table.

2.6 Sub-cause proportion models

For several causes for which death is rare, we first modelled the parent cause in the GBD hierarchy with CODEm and then allocated deaths to specific causes using proportions of the parent cause for each age-sex-geography-year for each sub-cause. For these causes, we identified no significant predictors in negative binomial regressions. This approach was taken because the available data on these specific causes may come from sources other than VR, such as end-stage renal disease registries, or come from too few places to model the death rates directly. Details for each cluster of causes analyzed in this way are below.

Meningitis

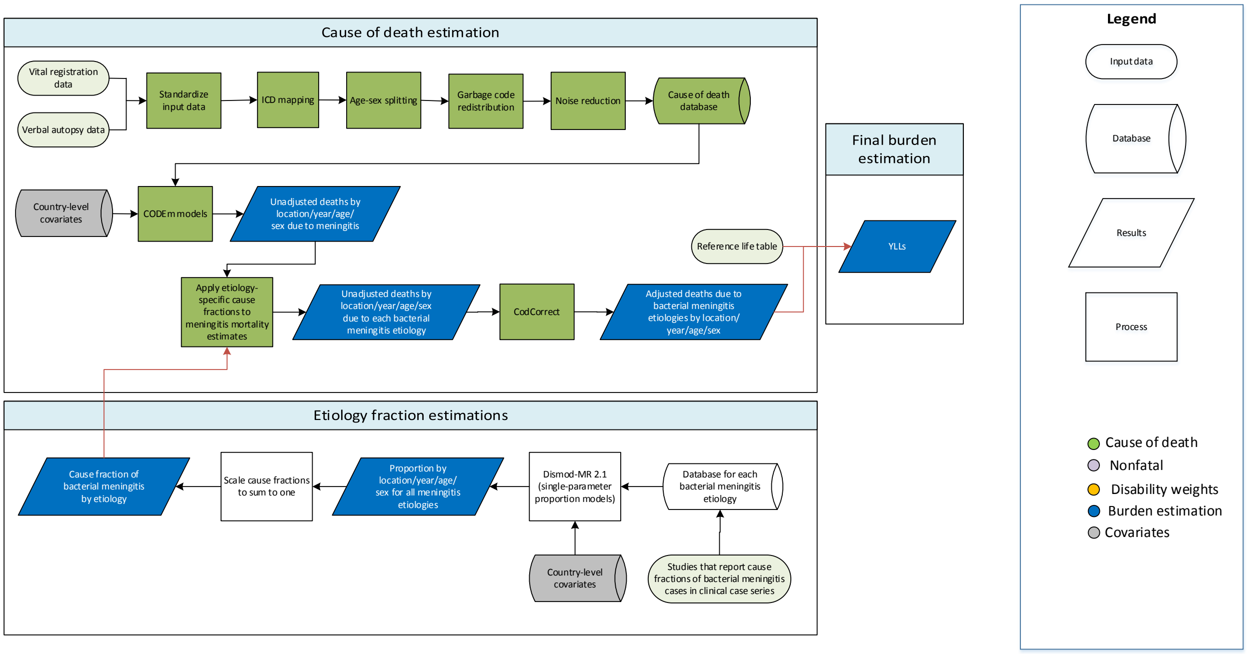

Input data

Input data for the all-meningitis model came from the cause of death database which includes vital registration and verbal autopsy data. We outliered data in instances where garbage code redistribution and noise reduction, in combination with small sample sizes, resulted in unreasonable cause fractions when compared to regional, super-regional, and global rates, and data that violated well-established time or age trends. Outliering methods were consistent across both vital registration and verbal autopsy data.

Input data that informed the etiology splits came from a systematic review. In the GBD 2010 study, we conducted a systematic review of literature to capture studies of incidence for all four etiologies of bacterial meningitis (meningococcal, pneumococcal, H influenza type B, and other bacterial meningitis). It was assumed that viral meningitis does not lead to mortality, therefore only deaths due to bacterial meningitis were considered. The inclusion criteria of the systematic review stipulated that: (1) the publication year must be between 1980 and 2010; (2) “caseness” was based on diagnoses by antigen test, blood test, cerebrospinal fluid test, polymerase chain reaction test, or latex agglutination test; (3) sufficient information must be provided on study method and sample characteristics to assess the quality of the study; and (4) study samples must be representative of the general population. No limitation was set on the language of publication. For GBD 2013, the search strategy was replicated to capture epidemiological studies published between 2010 and 2013. This was repeated for GBD 2015 for studies published between 2013 and 2015, but only excess mortality was extracted. There were no bias corrections such as crosswalks or study-level covariates for the input data used for the etiology splits.

Modeling strategy

We modeled deaths due to all bacterial meningitis with two CODEm models, separately for each sex and two age categories – under 5 and 5 years and above – because the mortality trends differ substantially between children and adults, and there are a significant number of data sources that only have data for under-5-year-olds. The two models used the same covariates and otherwise standard CODEm parameters. The final sex-specific models for deaths due to all bacterial meningitis were a hybridized model of separate global and data rich models.

To obtain estimates for each of the four etiologies of bacterial meningitis – meningococcal, pneumococcal, H influenza type B, and other bacterial – we ran four single-parameter proportion models using DisMod-MR 2.1. The meningococcal meningitis proportion model used two country-level covariates to inform the model – proportion of the population living within the meningitis belt, and proportion of the population covered by the meningococcal meningitis type A vaccine (an initiative called Menafrivac). The pneumococcal meningitis model was informed by PCV3 coverage, and the H influenza type B meningitis model was informed by HIB3 coverage. The other bacterial meningitis proportion model did not use any country-level covariates. Since DisMod-MR 2.1 estimates in 5-year intervals, the etiological proportions for years between the intervals were interpolated at the draw level. Additionally, DisMod-MR 2.1 only produces estimates beginning in 1990, while cause of death estimates begin in 1980. Values at the draw level from 1990 were used for the years 1980–1989. The four proportion models were forced to sum to 1 at the draw level for each location, year, sex, and age combination. We applied these proportions to the all bacterial meningitis cause of death models to produce estimates for each of the four etiologies assuming that the etiological proportions derived from incidence studies apply equally to deaths.

Maternal disorders

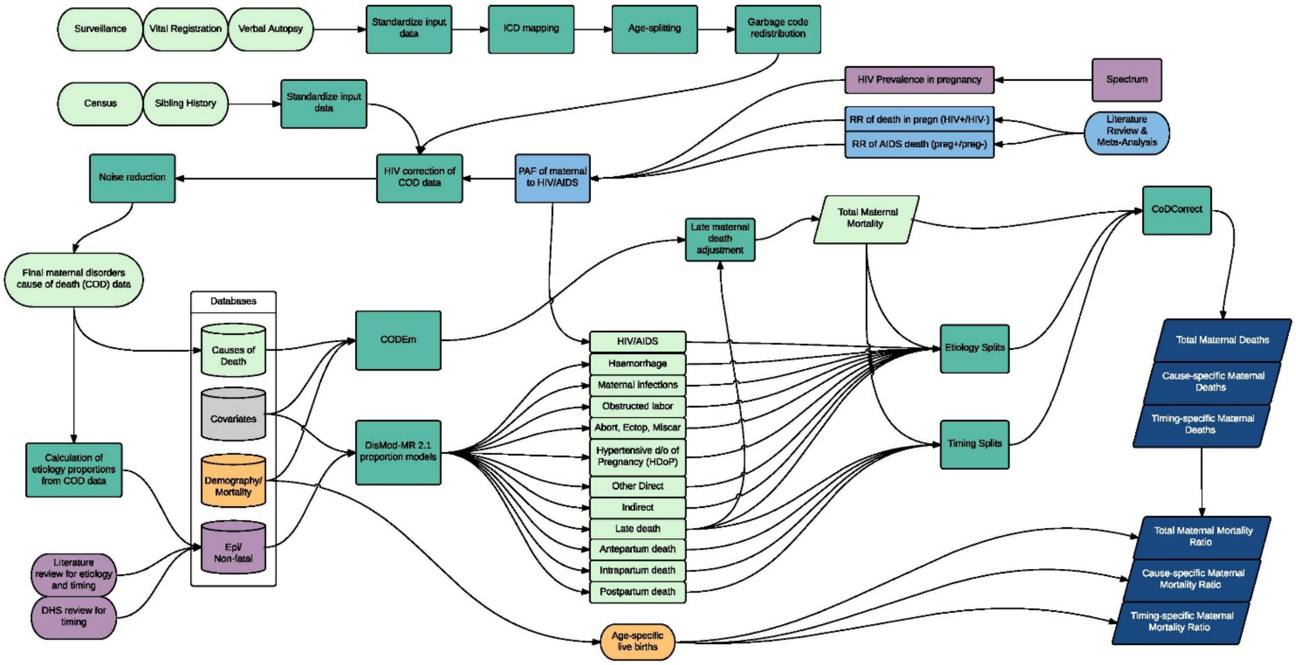

Input data

CODEm models were informed by centrally prepped data stored in the cause of death (COD) database. All data were corrected for incidental HIV deaths. Spectrum outputs of HIV prevalence in pregnancy were combined with relative risk of mortality during pregnancy (HIV+ vs. HIV-negative) to calculate PAFs. A proportion of these deaths are incidental and a proportion are maternal. PAFs were applied to all sibling history and census data to remove incidental HIV deaths. An updated literature review to inform the relative risk of mortality in pregnancy in HIV-positive versus HIV-negative women produced 14 leads but no usable sources. We completed this search on May 7, 2015, using the following two search strings:

> (""HIV""[Mesh] OR ""Acquired Immunodeficiency Syndrome""[Mesh]) AND (""Pregnancy""[Mesh] OR ""Postpartum Period""[Mesh]) AND ""Mortality""[Mesh]

> “HIV”(MeSH) AND (“pregnant”(Title/Abstract) OR “pregnancy”(Title/Abstract) OR “postpartum”(Title/Abstract) OR ""post partum""(Title/Abstract)) AND (“mortality”(Title/Abstract) OR “death”(Title/Abstract))"

All data from all geographies were reviewed in CODEm models. Outliers were identified as those data where age patterns or temporal patterns were inconsistent with neighboring age groups or locations or where sparse data were predicting implausible overall temporal or age patterns for a given location.

DisMod-MR 2.1 etiology proportion models were informed by two sources of data. First, we completed a systematic literature review on May 7, 2015, using two different search strings:

To inform etiology and timing models: (""maternal mortality""[Title/Abstract] OR ""maternal death""[Title/Abstract] OR ""MM""[Title/Abstract] OR ""confidential enquiry""[Title/Abstract] OR ((obstetric[Title/Abstract] OR pregnancy[Title/Abstract]) AND (etiology[Title/Abstract] OR “cause pattern” [Title/Abstract]) AND (death[Title/Abstract] OR mortality[Title/Abstract])) AND (""2013""[PDAT] : ""2015""[PDAT])) AND ""humans""[MeSH Terms] NOT (fetal[Title/Abstract] OR newborns[Title/Abstract] OR newborn[Title/Abstract] OR neonatal[Title/Abstract] OR “case report” [Title/Abstract] OR “case study” [Title/Abstract] OR pathogenesis[Title/Abstract] OR thromboprophylaxis[Title/Abstract])

To identify all sources on country-specific maternal mortality: (“maternal mortality” [Title/Abstract] OR “maternal death*”[Title/Abstract] OR “MMR” [Title/Abstract]) AND (“Afghanistan”[Title/Abstract] OR “Albania”[Title/Abstract] OR “Algeria”[Title/Abstract] OR “Andorra”[Title/Abstract] OR “Angola”[Title/Abstract] OR “Antigua and Barbuda”[Title/Abstract] OR “Argentina”[Title/Abstract] OR “Armenia”[Title/Abstract] OR “Azerbaijan”[Title/Abstract] OR “Bahrain”[Title/Abstract] OR “Bangladesh”[Title/Abstract] OR “Barbados”[Title/Abstract] OR “Belarus”[Title/Abstract] OR “Belize”[Title/Abstract] OR “Benin”[Title/Abstract] OR “Bhutan”[Title/Abstract] OR “Bolivia”[Title/Abstract] OR “Bosnia and Herzegovina”[Title/Abstract] OR “Botswana”[Title/Abstract] OR “Brazil”[Title/Abstract] OR “Brunei”[Title/Abstract] OR “Bulgaria”[Title/Abstract] OR “Burkina Faso”[Title/Abstract] OR “Burundi”[Title/Abstract] OR “Cambodia”[Title/Abstract] OR “Cameroon”[Title/Abstract] OR “Cape Verde”[Title/Abstract] OR “Central African Republic”[Title/Abstract] OR “Chad”[Title/Abstract] OR “China”[Title/Abstract] OR “Colombia”[Title/Abstract] OR “Comoros”[Title/Abstract] OR “Congo”[Title/Abstract] OR “Costa Rica”[Title/Abstract] OR “Croatia”[Title/Abstract] OR “Cuba”[Title/Abstract] OR “Cyprus”[Title/Abstract] OR “Côte d’Ivoire”[Title/Abstract] OR “Democratic Republic of the Congo”[Title/Abstract] OR “Djibouti”[Title/Abstract] OR “Dominica”[Title/Abstract] OR “Dominican Republic”[Title/Abstract] OR “Ecuador”[Title/Abstract] OR “Egypt”[Title/Abstract] OR “El Salvador”[Title/Abstract] OR “Equatorial Guinea”[Title/Abstract] OR “Eritrea”[Title/Abstract] OR “Ethiopia”[Title/Abstract] OR “Federated States of Micronesia”[Title/Abstract] OR “Fiji”[Title/Abstract] OR “Gabon”[Title/Abstract] OR “Georgia”[Title/Abstract] OR “Ghana”[Title/Abstract] OR “Grenada”[Title/Abstract] OR “Guatemala”[Title/Abstract] OR “Guinea”[Title/Abstract] OR “Guinea-Bissau”[Title/Abstract] OR “Guyana”[Title/Abstract] OR “Haiti”[Title/Abstract] OR “Honduras”[Title/Abstract] OR “India”[Title/Abstract] OR “Indonesia”[Title/Abstract] OR “Iran”[Title/Abstract] OR “Iraq”[Title/Abstract] OR “Jamaica”[Title/Abstract] OR “Jordan”[Title/Abstract] OR “Kazakhstan”[Title/Abstract] OR “Kenya”[Title/Abstract] OR “Kiribati”[Title/Abstract] OR “Kuwait”[Title/Abstract] OR “Kyrgyzstan”[Title/Abstract] OR “Laos”[Title/Abstract] OR “Latvia”[Title/Abstract] OR “Lebanon”[Title/Abstract] OR “Lesotho”[Title/Abstract] OR “Liberia”[Title/Abstract] OR “Libya”[Title/Abstract] OR “Lithuania”[Title/Abstract] OR “Macedonia”[Title/Abstract] OR “Madagascar”[Title/Abstract] OR “Malawi”[Title/Abstract] OR “Malaysia”[Title/Abstract] OR “Maldives”[Title/Abstract] OR “Mali”[Title/Abstract] OR “Malta”[Title/Abstract] OR “Marshall Islands”[Title/Abstract] OR “Mauritania”[Title/Abstract] OR “Mauritius”[Title/Abstract] OR “Moldova”[Title/Abstract] OR “Mongolia”[Title/Abstract] OR “Montenegro”[Title/Abstract] OR “Morocco”[Title/Abstract] OR “Mozambique”[Title/Abstract] OR “Myanmar”[Title/Abstract] OR “Namibia”[Title/Abstract] OR “Nepal”[Title/Abstract] OR “Nicaragua”[Title/Abstract] OR “Niger”[Title/Abstract] OR “Nigeria”[Title/Abstract] OR “North Korea”[Title/Abstract] OR “Oman”[Title/Abstract] OR “Pakistan”[Title/Abstract] OR “Palestine”[Title/Abstract] OR “Panama”[Title/Abstract] OR “Papua New Guinea”[Title/Abstract] OR “Paraguay”[Title/Abstract] OR “Peru”[Title/Abstract] OR “Philippines”[Title/Abstract] OR “Qatar”[Title/Abstract] OR “Romania”[Title/Abstract] OR “Russia”[Title/Abstract] OR “Rwanda”[Title/Abstract] OR “Saint Lucia”[Title/Abstract] OR “Saint Vincent and the Grenadines”[Title/Abstract] OR “Samoa”[Title/Abstract] OR “Saudi Arabia”[Title/Abstract] OR “Senegal”[Title/Abstract] OR “Serbia”[Title/Abstract] OR “Seychelles”[Title/Abstract] OR “Sierra Leone”[Title/Abstract] OR “Singapore”[Title/Abstract] OR “Solomon Islands”[Title/Abstract] OR “Somalia”[Title/Abstract] OR “South Africa”[Title/Abstract] OR “Sri Lanka”[Title/Abstract] OR

“Sudan”[Title/Abstract] OR “Suriname”[Title/Abstract] OR “Swaziland”[Title/Abstract] OR “Syria”[Title/Abstract] OR “São Tomé and Príncipe”[Title/Abstract] OR “Taiwan”[Title/Abstract] OR “Tajikistan”[Title/Abstract] OR “Tanzania”[Title/Abstract] OR “Thailand”[Title/Abstract] OR “The Bahamas”[Title/Abstract] OR “The Gambia”[Title/Abstract] OR “Timor-Leste”[Title/Abstract] OR “Togo”[Title/Abstract] OR “Tonga”[Title/Abstract] OR “Trinidad and Tobago”[Title/Abstract] OR “Tunisia”[Title/Abstract] OR “Turkmenistan”[Title/Abstract] OR “Uganda”[Title/Abstract] OR “Ukraine”[Title/Abstract] OR “United Arab Emirates”[Title/Abstract] OR “Uruguay”[Title/Abstract] OR “Uzbekistan”[Title/Abstract] OR “Vanuatu”[Title/Abstract] OR “Venezuela”[Title/Abstract] OR “Vietnam”[Title/Abstract] OR “Yemen”[Title/Abstract] OR “Zambia”[Title/Abstract] OR “Zimbabwe”[Title/Abstract]) AND (""2013""[PDAT] : ""2015""[PDAT])) AND ""humans""[MeSH] NOT (“demographic and health survey*”[Title/Abstract] OR DHS[Title/Abstract] OR “reproductive health survey*” [Title/Abstract] OR RHS[Title/Abstract])

A total of 893 sources were reviewed for their title and abstract. Of those selected for full text review, 29 had usable data for etiology-specific maternal mortality models. All data were prepped as “proportion” of total maternal deaths due to that cause. Because many sources do not include the entire cause list, a series of study covariates were used to facilitate crosswalking back to the reference definition. The reference definition ***includes*** “other” direct obstetric complications, indirect maternal deaths, and late maternal death. The second source of data was from the COD database. All etiology-specific COD data were processed to be “proportion” data by calculating the cause-specific deaths divided by the total maternal deaths for the matching data source, year, age, and location. Owing to the large volume of total COD data and small sample sizes in many locations, COD data were collapsed around each of the five-year periods for which DisMod-MR 2.1 makes distinct estimates (1990, 1995, 2000, 2005, 2010, and 2015). Late maternal death data were only included for the subset of locations where they were reliably coded in raw VR. All data were uploaded to the nonfatal database.

Modeling strategy

Overall maternal mortality was estimated with CODEm. Etiology-specific estimates were derived by multiplying the proportion outputs from DisMod-MR 2.1 by the total maternal deaths for that age-group, location, and year, which were scaled in relation to each other to equal one. HIV-related maternal deaths were estimated for all locations using the PAF approach described above for mortality data processing. Incidental HIV deaths during pregnancy were by definition excluded.

DisMod-MR 2.1 proportion models for each sub-cause of maternal mortality were all single-parameter models. Study covariates included those listed above that were used to crosswalk back to a reference definition where all specific sub-causes of maternal mortality were included in every study. Country covariates were specific for each model and included abortion legality (for abortion, ectopic pregnancy, and miscarriage), log-transformed lag-distributed income (for sepsis and late maternal death), and logit-transformed in-facility delivery proportion (for hemorrhage, hypertensive disorders of pregnancy, and obstructed labor). The time window was set at +/- 2 years for all models except late, which was +/- 5 years. The narrower window ensured that any given year of VR data only informed a single estimate.

Sexually transmitted infections excluding HIV

This write-up covers includes all sexually transmitted diseases excluding HIV, which was run as a sub-cause proportion model.

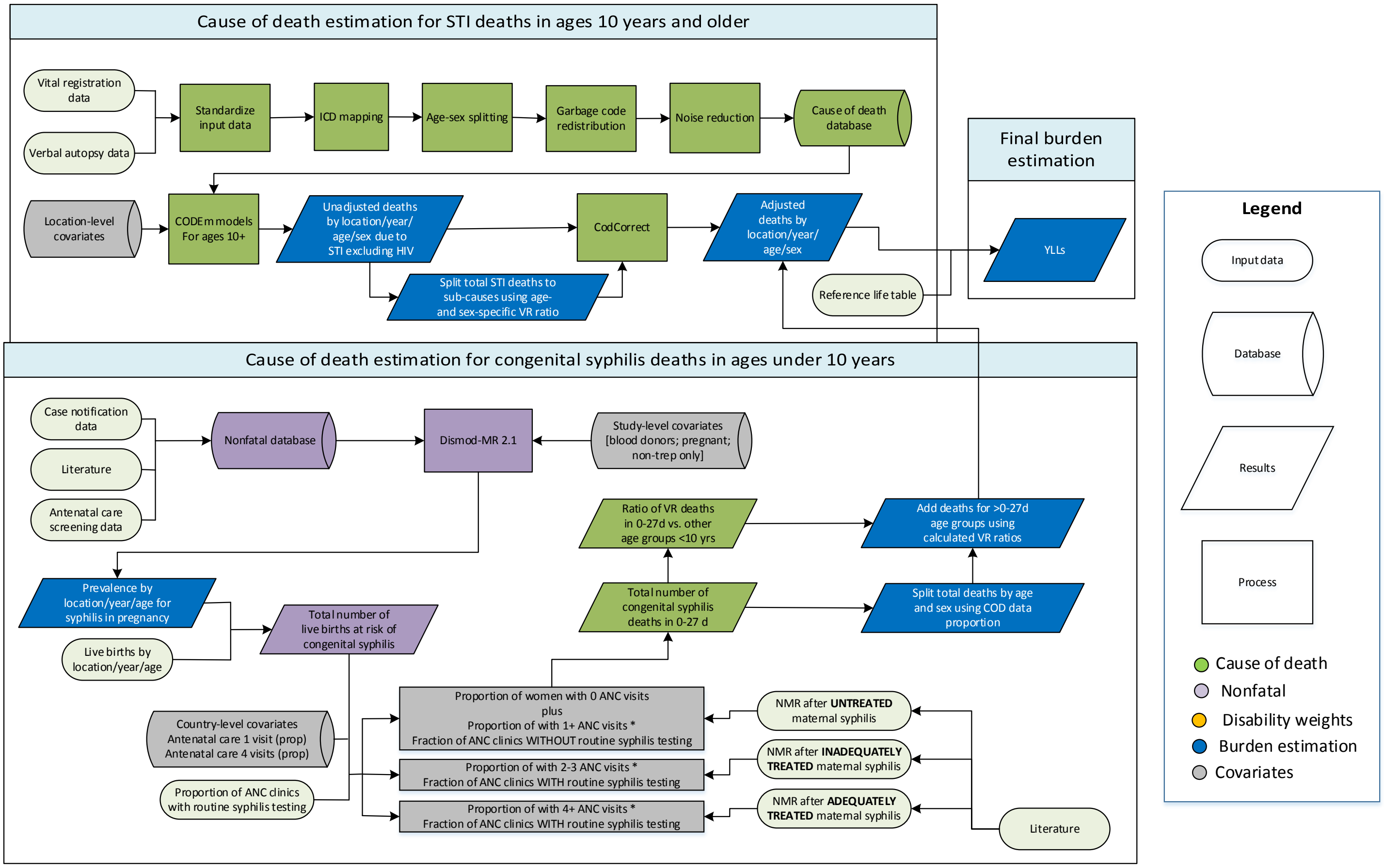

Input data

CODEm models for males and females 10 years and older were informed from centrally prepped data stored in the cause of death (COD) database. All data from all geographies were reviewed. Outliers were identified as those data where age patterns or temporal patterns were inconsistent with neighboring age groups or locations or where sparse data were predicting implausible overall temporal or age patterns for a given location.

Four different types of data were used for the NHM model. First, we used literature, survey, and report data described below to estimate early syphilis in pregnancy. Second, we used GBD 2015 estimates of antenatal care (ANC) coverage data from our covariates database and live births estimates from our demographics analysis. Third, we used published data from the Global Health Observatory on proportion of ANC clinics that test for syphilis and the proportion of women testing positive who receive treatment. Fourth, we used the results of a systematic literature review completed for GBD 2010 to inform excess mortality of neonates born with syphilis.

We completed data-rich (DR) and global CODEm models for ages 10 years and over for males and females separately. Nine covariates were used in each CODEm model, including 1) syphilis prevalence in pregnancy from DisMod-MR 2.1 analysis described below; 2) coverage of one antenatal care (ANC) visit, 3) coverage of four ANC visits; 4) age-specific fertility rate; 5) total fertility rate; 6) health system access, a principal components analysis of ANC, in-facility delivery, skilled birth attendance, and vaccine coverage; 7) national income per capita (LDI); 8) years of education per capita; and 9) abortion legality, a categorical variable that ranges from 1 (always illegal) to 7 (always legal on demand).

The overall CODEm model for STI was split into the sub-causes using vital registration (VR) data from the COD database. Trichomoniasis and HSV-2 are assumed not to cause mortality. Chlamydia is further assumed not to cause death in males. Cause-specific mortality rate VR data for each age-group, sex, and year were summed and scaled to match the total STI cause-specific mortality rate predicted by CODEm. This VR pattern was applied globally to all locations.

Our NHM for congenital syphilis began with estimation of early syphilis prevalence in all age-groups, both sexes, all GBD locations, and in each year from 1990 to 2015. Systematic literature review was combined with review of all publicly available UN GASS country progress reports, antenatal clinic surveillance data from country-specific and UNAIDS reports, targeted searches of national ministry of health (MoH) websites, and other published data provided by GBD collaborators. A Pubmed search was completed on June 1, 2015, using the following search string:

> ("syphilis"[MeSH] OR "Treponema pallidum"[MeSH]) NOT "Yaws"[MeSH] AND ("prevalence"[MeSH] OR "incidence"[MeSH]) AND "1990"[PDAT] : "2015"[PDAT] AND "humans"[MeSH]

A total of 1,265 titles and abstracts were found, of which 178 had suitable data for inclusion in our dataset. In addition to standard demographic identifiers, all data were extracted to include number of cases, prevalence or incidence rate, and sample size. Laboratory testing modality for each study was specified as including treponemal test, non-treponemal test, both treponemal and non-treponemal, or direct spirochete detection (e.g., darkfield microscopy). Usage of treponemal and non-treponemal testing was the reference category. Our final dataset included data from 113 distinct geographies. Data from blood donor and pregnant populations were included, but all other high-risk groups were excluded.

Modeling strategy

We modeled early syphilis in DisMod-MR 2.1, restricting incidence before the age of 10 and after the age of 65 and assuming excess mortality from early syphilis is very low ($\leq$ 1 death per 10,000 person-years). Study-level covariates identified data in non-reference categories (pregnant, blood donors) and using non-reference testing modalities (e.g., treponemal test only, non-treponemal test only) and were crosswalked to the reference category within the DisMod-MR 2.1 cascade, predicting specific conversion values for each country. The latter specification of country-level crosswalks was chosen because the bias between non-reference populations and testing modalities was assumed to differ by geography and as a function of, for example, endemicity of acute infectious agents like malaria.

Age-specific prevalence results were paired with age-specific live birth results to generate total number of births at risk of congenital syphilis. To estimate the actual number of congenital syphilis births, we combined information on ANC coverage from GBD 2015 covariates analyses with ANC syphilis testing

and treatment data from GHO. Adequate treatment was assumed to confer no risk of congenital syphilis mortality. Those with four ANC visits or 1-3 ANC visits with testing and treatment were assumed to have received adequate treatment. Inadequate treatment occurred in those women with 1-3 ANC visits without either testing or treatment or those women with one ANC visit but with testing and treatment. Those women with only one or fewer ANC visits and no syphilis testing or treatment were assumed to be untreated. Untreated and inadequate treatment proportions were combined with potential congenital syphilis births to estimate total neonatal syphilis births. Each categorical risk category was combined with corresponding neonatal excess mortality rates derived in GBD 2010. This total number of neonatal syphilis deaths was then split between 0-6 days and 7-27 days age groups using sex and age-specific VR data from the COD database. Congenital syphilis deaths beyond the neonatal period were likewise estimated using sex- and age-specific VR data. This VR pattern was applied globally to all locations.

The primary limitation of our estimation of STI deaths in those over 10 years old is data availability, especially from countries where VR systems are not available. Even in countries with VR, there may be some variation in practices for coding deaths to STI as the underlying cause, especially given the potentially variable presentation of many of the conditions in this category. Such variation is more likely to lead to underestimation of STI deaths than overestimation. Sub-cause estimation is similarly limited by data availability in those locations without VR data, and our estimates are thus based on the overall pattern of deaths in generally higher-income geographies. The primary implication of this limitation is that it decreases the resolution with which we can decompose the relationship between mortality from HIV and other STI. Our NHM for congenital syphilis was a significant improvement over GBD 2013 but still is limited by data availability issues, especially on the coverage and effectiveness of ANC interventions to prevent congenital syphilis. We do not have information on the proportion of women that tested positive who may have received treatment elsewhere, or information on the coverage of treatment for neonates, infants, and children born with congenital syphilis. Both limitations could potentially have led to lower estimates of congenital syphilis deaths. On the other hand, our DisMod-MR 2.1 analysis suggested that pregnant women may in fact have higher syphilis prevalence than the general population, which would have led to higher estimates. We have also not quantified the number of stillbirths associated with congenital syphilis. We will continue to work to address these shortcomings more thoroughly in future GBD analyses.

Liver cancer

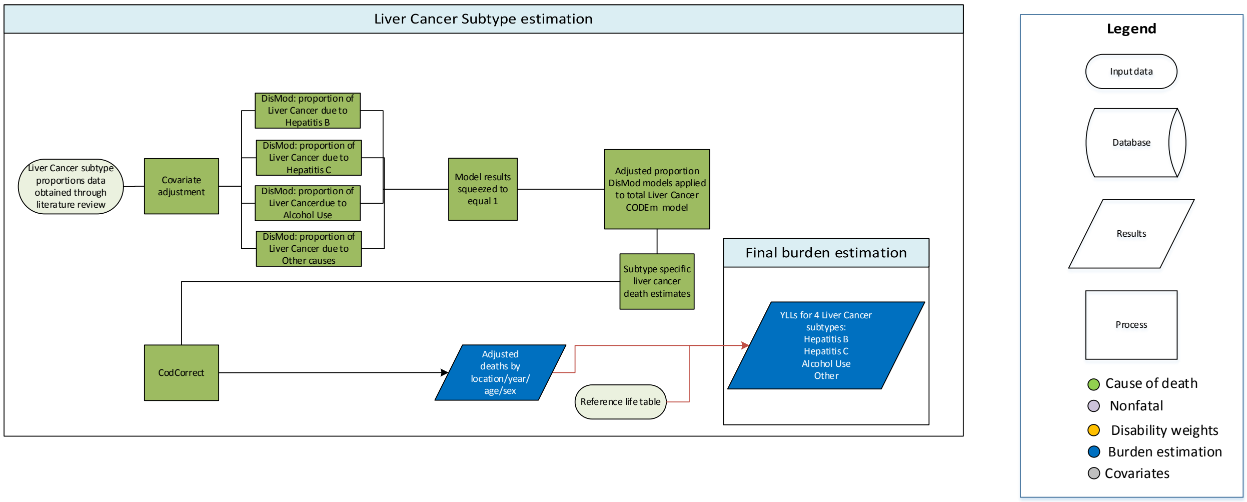

Input data

Data seeking processes

The input data for the parent cause (liver cancer) were identified and processed using the same methods as all other cancers described above. To estimate the number of liver cancers for each of these sub-causes, DisMod-MR 2.1 was used to model the proportion of liver cancers due to the four subgroups. All publications used in GBD 2013 were included, and sources were supplemented with a systematic review of the published literature on the etiology of liver cancer. The literature search was performed in Pubmed on 8/13/15 with the following search string: (("Carcinoma, Hepatocellular"[Mesh] AND "etiology"[Subheading] AND ("epidemiology"[Subheading] OR "epidemiology"[All Fields] OR "epidemiology"[MeSH Terms])) AND ("Hepatitis B"[Mesh] OR "Hepatitis C"[Mesh] OR ("ethanol"[MeSH Terms] OR "ethanol"[All Fields] OR "alcohol"[All Fields] OR "alcohols"[MeSH Terms] OR "alcohols"[All Fields]) OR autoimmune[All Fields])). The duration was restricted to articles published between 2013 and 2015. 385 articles were found, of which eight studies were added for liver cancer due to alcohol use (49 studies included from GBD 2013), 11 studies were added for liver cancer due to hepatitis B and C (99 studies included for GBD 2013), and 7 studies were added for liver cancer due to other causes (25 studies included for GBD 2013).

Inclusion and exclusion criteria

Articles were included if it was possible to identify at least one of the causes (alcohol, hepatitis B, hepatitis C, or “other causes”) as the only etiology.

Bias of categories of input data

The potential biases of the input data are the same as for other cancers (see above).

Modeling strategy

Overall methodological process

The modeling strategy for the parent cause "liver cancer" followed the general CODEm process. To estimate the fraction of liver cancer due to each etiology for each age-sex-geography-year DisMod-MR 2.1 was used.

Steps of analysis and data transformation processes

If the etiology was reported to be due to multiple causes, the cases due to multiple causes were split based on the proportion of cases in the individual etiologies reported in the publication. If cases were reported to be due to cryptogenic causes, these cases were removed from the denominator. A study covariate was used for publications that only assessed liver cancer in a cirrhotic population. The reference or "gold standard" that was used for crosswalking was the compilation of all studies that assessed the etiology of liver cancer in a general population. Smoothness (xi) was set at 0.1 to 0.3, heterogeneity (zeta) was set at 0.5 to 1. Time window for fit was 10 years, minimum coefficient of variation for global, super-region, region, and country was 0.4, 0.2, 0.1, and 0.1.

For liver cancer due to hepatitis C and hepatitis B, a prior value of 0 was set between age 0 and 0.01. For liver cancer due to alcohol a prior value of 0 was set for ages 0 to 5 years and a prior maximum value of 0.8 for ages older than 5.

For liver cancer due to hepatitis C, hepatitis C prevalence was used as a covariate with a predefined minimum of 0 and maximum of 10. Alcohol and hepatitis B prevalence were used as covariates with a pre-specified covariate of -1 to 0. A positive prior was set on the slope from age 0 to age 60.

For liver cancer due to hepatitis B, hepatitis B prevalence was used as a covariate with a predefined minimum of 0 and maximum of 10. Alcohol and hepatitis C prevalence were used as covariates with a pre-specified covariate of -1 to 0.

For liver cancer due to alcohol, alcohol (liters per capita) was used as a covariate with a predefined minimum of 0 and maximum of 10. Hepatitis B prevalence and hepatitis C prevalence were used as covariates with a pre-specified covariate of -1 to 0. A negative prior was set on the slope from age 0 to age 70.

For liver cancer due to other causes, hepatitis C prevalence, alcohol, and hepatitis B prevalence were used as covariates with a pre-specified covariate of -1 to 0.

To ensure coherent results between the cirrhosis and the liver cancer etiologies, the results from the liver cancer etiology models were transformed into covariates that were then used in the cirrhosis etiology models. The results from the cirrhosis etiology models were then used in the liver cancer proportion models.

The DisMod proportions for the underlying liver cancer etiologies were then squeezed to 100% and these final proportions were applied to the parent cause, "liver cancer," to derive the estimates for the liver cancer etiologies.

Uncertainty intervals

Uncertainty was determined using standard DisMod methodology.

Results

Interpretation of results

Results for the liver cancer subtype mortality can differ from GBD 2013 for multiple reasons. First, additional sources were added based on an updated literature review. Second, to ensure consistency between cirrhosis and liver cancer etiologies the results from each model for cirrhosis and for liver cancer subtypes were converted into covariates and used in the respective other model. Third, DisMod methods were updated for GBD 2015 (Section 3).

Limitations

The etiological proportion models for the liver cancer sub-causes depend on the availability of data sources that inform DisMod about the local patterns of liver cancer etiologies as well as age patterns. Unfortunately, not many data sources provide data on the etiology of liver cancer by sex or by different age groups. Age patterns were therefore determined based on the assumption that there are no cases of liver cancer due to hepatitis B, hepatitis C, or alcohol in young age groups.

Cirrhosis

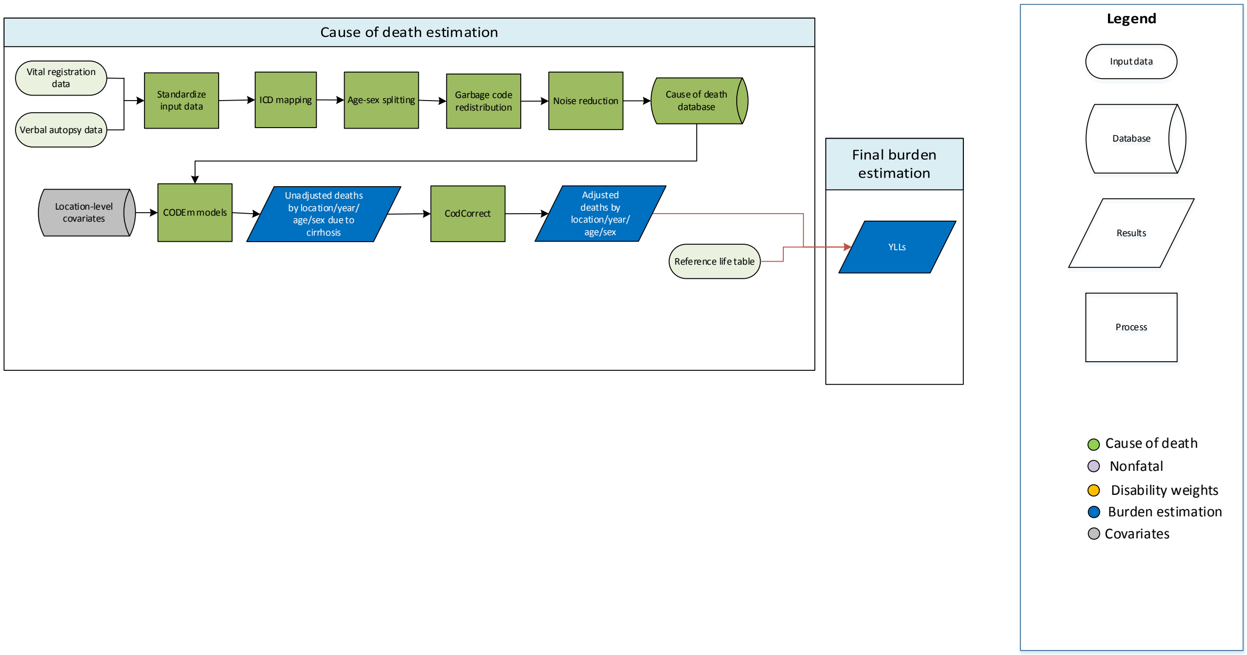

Input data

We modeled cirrhosis mortality using all available data in the cause of death database. Data points were outliered if they reported an improbable number of deaths or if their inclusion in the model yielded distorted trends.

Modeling strategy

We modeled cirrhosis mortality using a two-model hybrid approach: 1) a global CODEm model of all locations, using all data in the CoD database; and 2) a CODEm model restricted to data-rich countries.

Since GBD 2013 we have switched from a single global model to the hybrid global/data-rich model approach. We have otherwise made no substantive changes in the modeling strategy for cirrhosis from GBD 2013.

Cirrhosis by etiology

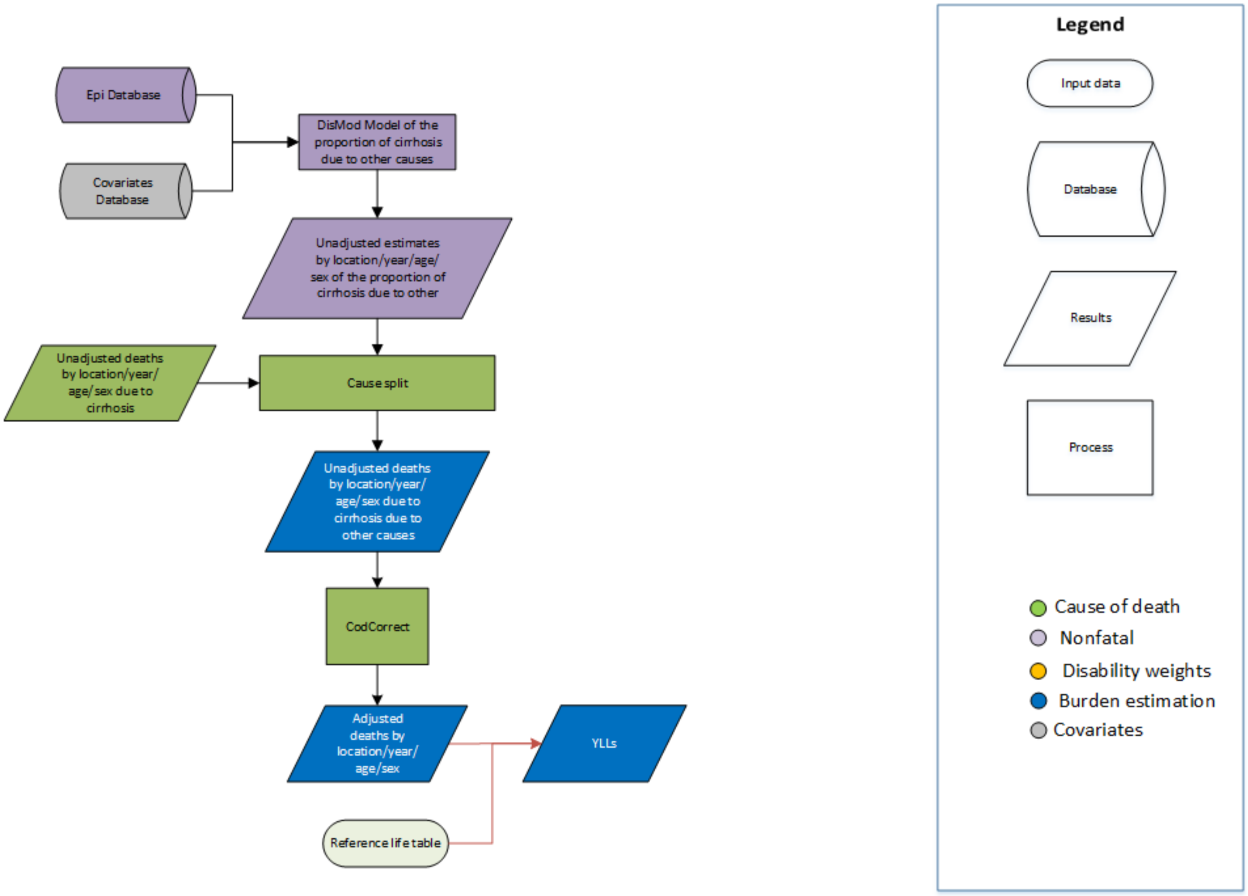

Input data

We conducted a literature review for studies reporting etiologies of cirrhosis patients.

Modeling strategy

We first modeled all cirrhosis mortality using all available data in the cause of death database and a hybrid CODEm model. To estimate mortality from cirrhosis due to alcohol, cirrhosis due to hepatitis B, cirrhosis due to hepatitis C, and cirrhosis due to other causes, we developed etiological proportion models using DisMod and used the results of these models to split the parent cirrhosis mortality estimates.

Given the similar etiologies for liver cancer and cirrhosis we integrated the etiology models for these two causes. We have more data for liver cancer etiologies than we do for cirrhosis. Therefore, we first developed four single-parameter DisMod models, each to estimate the proportion of liver cancer due to a given cause (i.e., alcohol, hepatitis B, hepatitis C, and other). These models included as covariates alcohol consumption (liters per capita), hepatitis B surface antigen (HBsAg) seroprevalence, and hepatitis C (anti-HCV IgG) seroprevalence. Moreover, the model for the proportion due to alcohol

included a binary covariate indicating countries with a predominantly Muslim population (thought to be associated with very low alcohol consumption). Estimates from these liver cancer models were then used as covariates (along with alcohol, HBsAg, and anti-HCV) in the four corresponding cirrhosis etiology models. Estimates from these cirrhosis models were then similarly used as covariates in the corresponding liver cancer models. Proportions from the four etiology models were then rescaled to sum to one at the draw level, and used to split the parent cirrhosis mortality estimates.

To better integrate our liver cancer and cirrhosis models, we implemented the aforementioned covariate cycle approach as an improvement for GBD 2015.

Chronic Kidney Disease

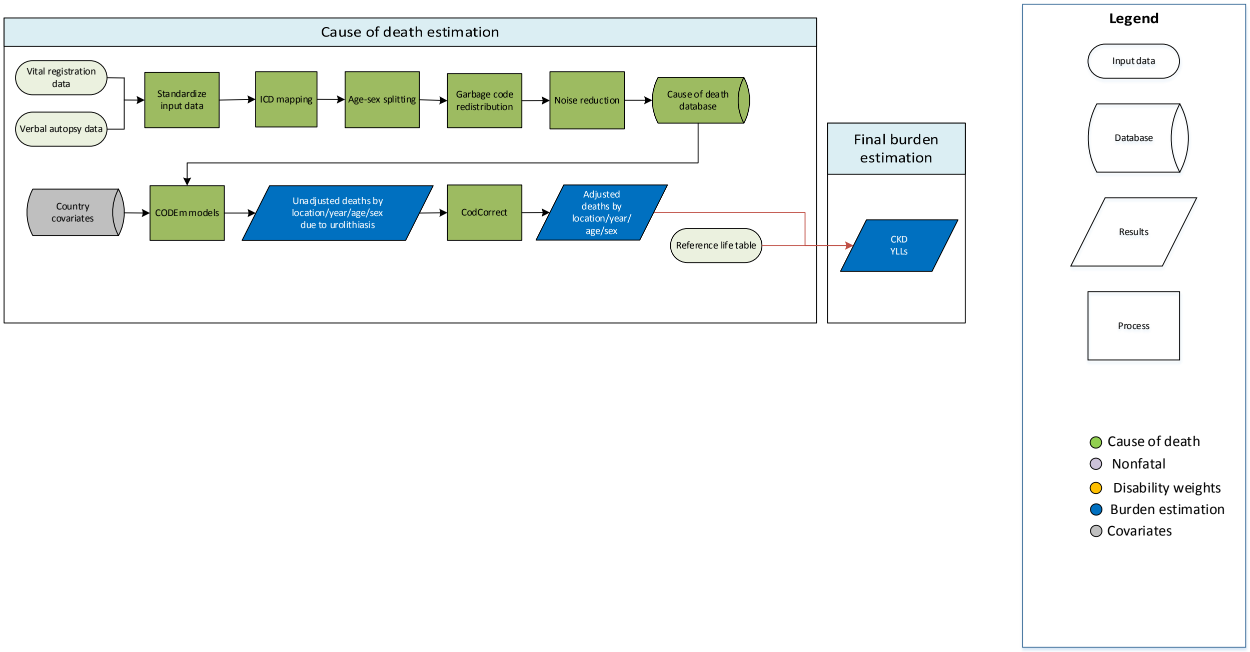

Input data

Mortality estimates for chronic kidney disease (CKD) were informed using vital registration and verbal autopsy data from the COD database. These data were uploaded into the COD estimation tool, CODEm, to estimate CKD deaths by age-group and gender from 1990 to 2015. Data points were selected for outliering if they deviated significantly from other age- and gender-grouped data points within the region.

Modeling strategy

The modeling strategy for GBD 2015 CKD estimation largely follows the methods used for GBD 2013. The major changes include increased data sources, specifically for China and India. As China's and India's populations are very large, this has resulted in notably higher global estimates of CKD mortality when compared to results from the GBD 2013 analysis.

Vital registration data were standardized, and mapped according to the GBD COD ICD mapping method. These data were then age-sex split, and appropriate redistribution of data was performed using garbage coding. After applying noise reduction, these data were then uploaded to the COD database.

Country covariates included in the models are the following: "animal fat," "diabetes," "education," "health system access," "LDI," "mean BMI," "mean cholesterol," "mean systolic blood pressure," "red meat," "total kcal," "whole grains," and "SDI." Iterations of models were assessed at the location/year/age-group/sex level to determine whether data points merited exclusion via outliering.

Chronic Kidney Disease subtypes

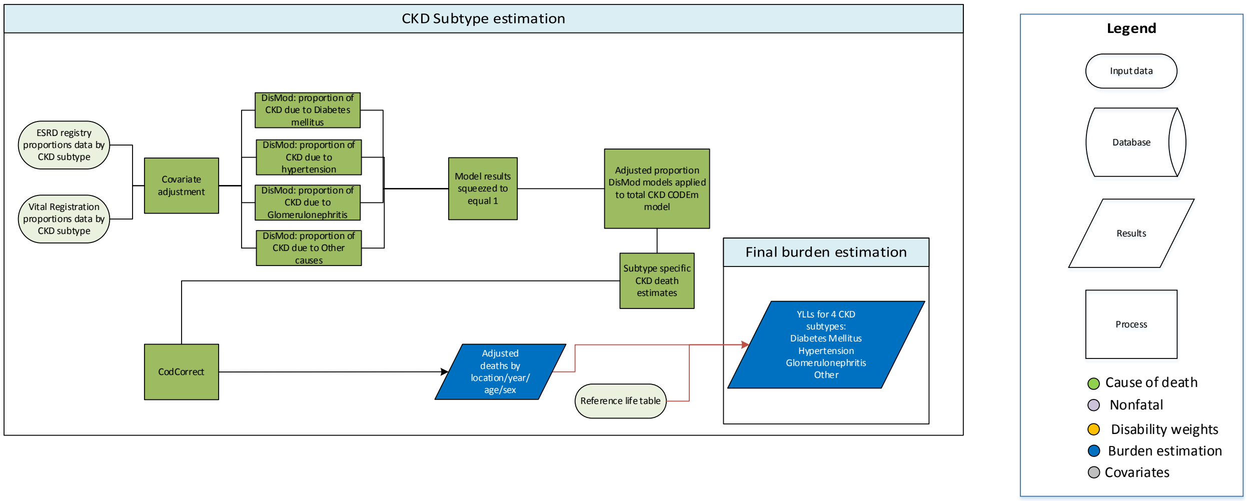

Input data

The estimation strategy for CKD subtypes of 1) diabetes mellitus 2) hypertension 3) glomerulonephritis and 4) “other” has changed significantly from the GBD 2013 analysis to achieve consistency of method among the four subtypes as well as full use of all available data. Details of this improved method are detailed below.

Data sources which informed these estimates include vital registration (VR) data from the COD database and end-stage renal disease (ESRD) registry subtype data from ESRD registries. ESRD deaths as listed in the COD database were determined used ICD coding. Raw proportions of deaths by CKD subtype were extracted from the COD database. Vital registration data by CKD subtype were prepared following standard central COD database standardization, ICD mapping, age-sex splitting, and redistribution. Prevalent or incident proportions of CKD subtypes were extracted from renal registries. These data were reviewed for appropriateness for inclusion into the CKD subtype mortality analysis. Registry inclusion was determined based on whether 1) the registry data offered complete capture of dialysis activity within the country or subnational region, 2) the baseline extraction criteria were available, such as ages described, gender data 3) all subtypes were available for extraction 4) subtypes were provided in enough etiology detail to allow for recalculation of our definition of “other.” GBD 2015 “other” category excludes cystic disease deaths, urolithiasis deaths, deaths from genetic/hereditary causes, nephritis, and deaths from kidney cancer. As all categories were not explicitly listed in all registries, baseline criteria for inclusion based on ability to re-calculate “other” category included ability to exclude “cystic diseases,” “urinary/urolithiasis,” and “nephritis.”

Modeling strategy

ESRD and vital registration proportion data were uploaded to the nonfatal database, then the VR proportions were adjusted to the registry proportions by using a study-level covariate. For each subtype, the proportions from the VR data were combined to the proportions from registry data. The VR data were adjusted to the ESRD proportions by use of a covariate where registry data equals 0, and VR data

equals 1. We adjusted the VR data to the registry data as we determined the registry data to be more accurate, as most national registries have 100% capture of ESRD activity within their country. This method relies on the assumption that death rates among CKD subtypes do not significantly differ in a clinically meaningful way from incidence/prevalence of ESRD by subtype.

Once these data types were uploaded and assigned a covariate value, they were then uploaded to the disease modeling tool DisMod, which estimates proportions of each subtype by location/year/age/sex. These models are then centrally squeezed so that estimates across the subtypes equal 1. These adjusted proportions are then applied to the parent CKD CODEm model to result in death estimates by CKD subtype that are internally consistent to the parent CKD death estimates.

Hemoglobinopathies and hemolytic anemias

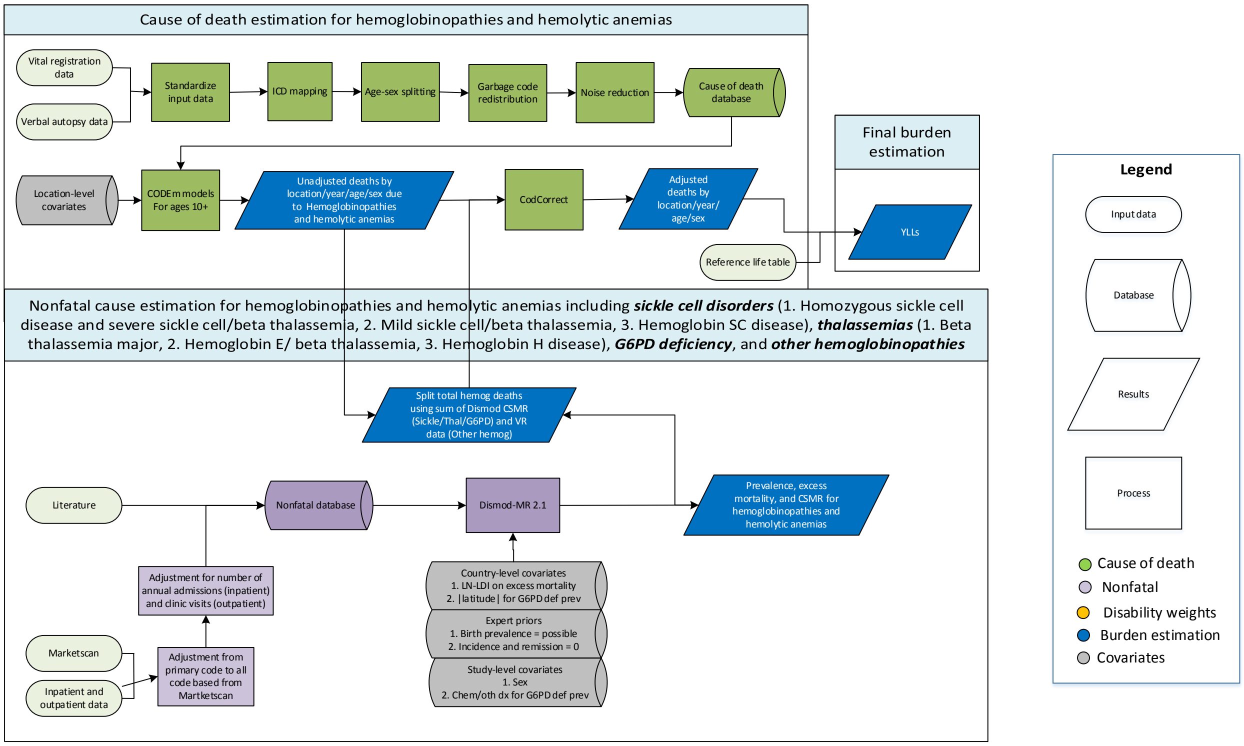

Input data

CODEm models were informed by centrally prepped data stored in the cause of death (COD) database. All data from all geographies were reviewed. Outliers were identified as those data where age patterns or temporal patterns were inconsistent with neighboring age groups or locations or where sparse data were predicting implausible overall temporal or age patterns for a given location.

Each datum for sickle cell disease was used for one of three mutually exclusive conditions: 1) homozygous sickle cell disease and severe sickle cell/beta thalassemia, 2) Mild sickle cell/beta thalassemia, or 3) Hemoglobin SC disease. We similarly extracted data for thalassemias using three mutually exclusive disease states: 1) Beta thalassemia major, 2) Hemoglobin E/beta thalassemia, and 3) Hemoglobin H disease. G6PD deficiency was estimated as a single model.

Three sources of data were used for DisMod-MR 2.1 models. First, we re-extracted all literature sources in GBD 2013, so elected not to perform an additional review of literature published in the last two years, and included data on prevalence, excess-mortality rate, or with-condition mortality rate. Age-specific survival probabilities from cohort studies were converted to corresponding with-condition mortality rates. We also included additional sources recommended by GBD 2015 collaborators or identified during the process of re-extraction. G6PD deficiency is an X-linked recessive genetic disease, and genetic homozygosity served as the reference definition for our DisMod-MR 2.1 models in 2015. This is a change from GBD 2013 when we quantified G6PD deficiency on reagent tests as the reference category. Second, we extracted ICD-9-coded Marketscan data from the United States, correcting for multiple outpatient and inpatient visits as determined from patient linkage analysis. Third, we used ICD-9 and ICD-10 inpatient and outpatient hospital data from all those locations where it was available. We calculated the

ratio of prevalence that would be derived from only using the primary discharge ICD code versus that derived from using any of the discharge diagnosis codes. We applied this as a correction factor for those sources where only a single ICD code is given for each discharge. Of note, there were no hospital data available for Hemoglobin E/beta-thalassemia, Hemoglobin H disease, or G6PD deficiency. All prevalence data from Marketscan, hospital sources, and literature were uploaded to the nonfatal database. With the addition of hospital and Marketscan sources, our dataset was significantly larger than that used in GBD 2013.

Modeling strategy

We completed seven separate DisMod-MR 2.1 models as listed above. Each used log-transformed lag-distributed income as a country-level covariate on excess mortality, which had the effect of predicting higher excess mortality in those locations with lower national income. The only study covariate used for most models was for gender. Genetic G6PD deficiency is far more common in males, but for all others the male to female ratio is nearly equivalent. Our G6PD deficiency model included additional study covariates to crosswalk from non-genetic diagnostic tests (e.g., chemical reagent testing) back to the reference definition. Incidence and remission were both set to be zero.

We completed data-rich (DR) and global CODEm models for males and females separately. The sum of CSMR from all seven DisMod-MR 2.1 models was used as a predictive covariate for CODEm model development. CODEm results were then split between sickle cell disorders, thalassemias, G6PD deficiency, and other hemoglobinopathies and hemolytic anemias using summed and scaled CSMR outputs from the same models. Other hemoglobinopathies and hemolytic anemias did not have a separate DisMod-MR 2.1 model, but was instead informed by location-specific VR data.

The primary limitation of our estimation is data availability. We elected a hybrid approach of CODEm and DisMod-MR 2.1 to improve the quality of estimates in data-poor locations, but in most of these locations data are still relatively sparse for nonfatal models, which leads to relatively large uncertainty. Further adding to the uncertainty is the fact that hemoglobinopathies dramatically increase the risk of mortality due to infectious agents such as malaria, lower respiratory infections, and diarrhea, as well as increasing the risk of maternal mortality. In locations with poor diagnostic capabilities and high infectious burden, it is thus very plausible that mortality due to hemoglobinopathies may be even higher. Secondly, our specification of seven distinct entities for DisMod-MR 2.1 models does not align perfectly with the cause categories in the central COD prep, which limits the extent to which CSMR data from the COD database can inform nonfatal models. We will continue to work to expand our dataset and consolidate the GBD analysis of hemoglobinopathies going forward.

2.7 HIV/AIDS and tuberculosis

Tuberculosis

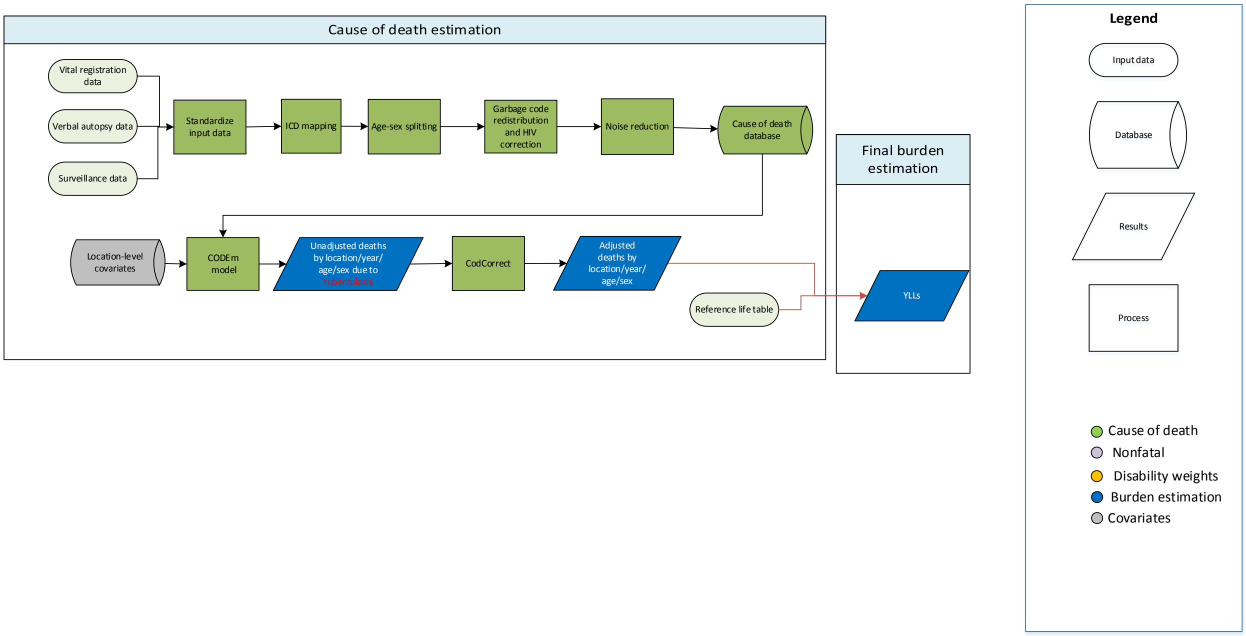

Input data

Input data for modeling tuberculosis mortality among HIV-negative individuals include vital registration, verbal autopsy, and surveillance data. Vital registration data were adjusted for garbage coding (including ill-defined codes, and the use of intermediate causes) following GBD algorithms and misclassified HIV deaths (i.e., HIV deaths being assigned to other underlying causes of death such as tuberculosis or diarrhea because of stigma or misdiagnosis). This correction was done based on examining changes in the age pattern of diseases over time.

Verbal autopsy data in countries with age-standardized HIV prevalence greater than 5% were removed because of a high probability of misclassification, as verbal autopsy studies have poor validity in distinguishing HIV deaths from HIV-TB deaths. We also outliered data that were largely conflicting with the majority of data from other studies conducted either in the same or different countries (with similar sociodemographic characteristics) in the same region.

Modeling strategy

A general CODEm modeling strategy was used.

HIV/AIDS

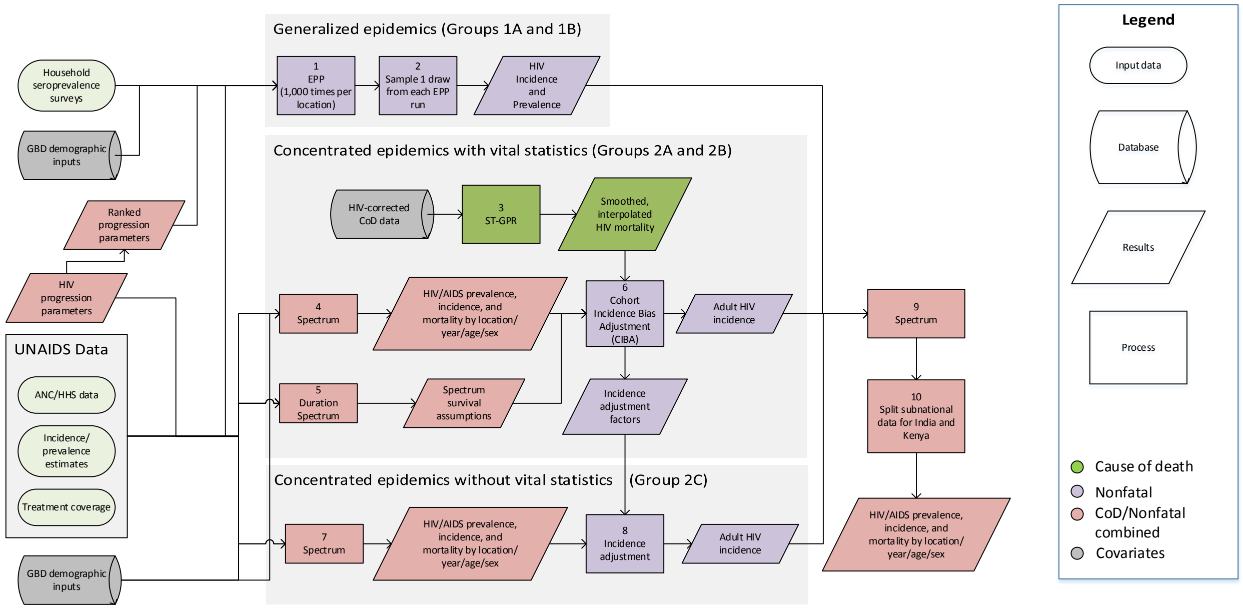

Input data

Household seroprevalence surveys

Geographically representative HIV seroprevalence survey results were used as inputs to the model for countries with generalized HIV epidemics where available.

GBD demographic inputs

Location-specific population, fertility, and HIV-free survival rates from GBD 2015 (see Part 1 for details on the generation of these data) and migration data from UNAIDS were used as inputs in modeling all locations.

UNAIDS data

Antenatal care, incidence, prevalence, and treatment coverage data from UNAIDS were used in modeling for all locations.

On-ART literature data

Data were identified by using search terms "HIV," "mortality," and "antiretroviral therapy" in PubMed searches across the literature. To be included, studies must include only HIV-positive people who receive antiretroviral therapy (ART) but who were ART-naïve prior to the study. In addition, studies must report either a duration-specific mortality proportion or a hazard ratio across age or sex, and must not include children.

For duration-specific survival data, studies must report uncertainty on mortality estimates or provide stratum-specific sample sizes and must include duration-specific data to allow for calculation of 0-6, 7-12, or 13-24 month conditional mortality. In addition, studies must either report separate mortality and

loss-to-follow-up (LTFU) curves, be corrected for LTFU using vital registration data, or be conducted in a high-income setting. Finally, studies must report the percent of participants who are male, the median age of participants, and either data with specific data on the number of CD4 T lymphocytes (CD4 counts) or the median CD4 count used for the data.

Hazard ratio data for ages or sexes can only be used if the hazard ratios are controlled for other variables of interest (age, sex, and CD4 category).

Changes for GBD 2015

In GBD 2013, we identified 102 papers for extraction. For GBD 2015, we included 13 additional studies informing the duration-specific mortality estimation process and 26 studies informing the age and sex hazard ratio estimation process (some studies were used and counted in both). We also added one study to our LTFU analysis. In addition, we updated our data from the Antiretroviral Therapy Cohort Collaboration (ART-CC) with country-specific data pre- and post-2001 for enhanced use in estimating time trends for high-income countries. We excluded nine hazard ratio and four duration-specific mortality studies used in GBD 2013 which reported results on populations already present in other extracted studies. The inclusion of new ART-CC data necessitated the exclusion of four additional studies used in GBD 2013.

We also included on-ART cohort mortality data from 10 high-income nations with collaboration from ART-CC. These countries include Austria, Denmark, France, Germany, Italy, the Netherlands, Spain, Switzerland, the United Kingdom, and the United States. We excluded the US data because they were not fully representative of the complete with-HIV on-ART population at the time.

Off-ART literature data

In GBD 2013, to characterize uncertainty in the progression and death rates, we systematically reviewed the literature on mortality without ART. We searched terms related to pre-ART or ART-naive survival since seroconversion.[1] After screening, we identified 13 cohort studies that included the cohorts used by UNAIDS from which we extracted survival at each one-year point after infection. Screening for additional, recently published studies for GBD 2015 identified no new cohort studies for inclusion in this analysis.

Burden estimation

The files compiled by UNAIDS for their HIV/AIDS estimation process were our main source of data for producing estimates of HIV burden. These files are typically country-specific and contain both demographic data (population, fertility, migration, and HIV-free survival rates) and HIV-specific information. In all cases except migration, we substituted in our own, internally consistent demographic estimates. The HIV-specific information includes what is needed to run both the Spectrum and Estimation and Project Package (EPP) models. Spectrum requires data on AIDS mortality among people living with HIV with and without ART, CD4 progression among people living with HIV not on ART, ART coverage among adults and children, coverage of breastfeeding among women living with HIV, prevention of mother-to-child transmission coverage, and CD4 thresholds for treatment eligibility. EPP uses many of the same assumptions as Spectrum but fits a simpler model to HIV prevalence data from surveillance sites and large household surveys. We extracted all of these data from UNAIDS' proprietary formats.

Between GBD 2013 and 2015, we received new files for all locations in which UNAIDS produces estimates except Indian subnational locations and locations identified as high-income. For many of the missing countries, we had UNAIDS files from the previous estimation process, which we used again. After combining these two sources, we were left with a set of 41 locations for which we had never received a UNAIDS file, many of them smaller countries with less severe HIV epidemics. In those places, we generated regional averages of all needed inputs. This enabled us to run Spectrum for every GBD location.

In several cases, we have modified the structure or data in the UNAIDS files. In South Africa, which has been estimated at the province level in GBD 2015, we split the national-level UNAIDS file into nine provincial datasets. We used GBD 2015 demographic inputs for the provinces. These provinces are already fit as separate subpopulations in EPP, so we extracted the prevalence data for the individual provinces and assumed national rates for all other Spectrum inputs. The only location where GBD 2015 demographic inputs are not used is Moldova, where UNAIDS data are provided at the subnational level but GBD 2015 demographic inputs are only generated at the national level. Child ART coverage rates were implausibly high in the South Africa Spectrum data in 2015, so we extrapolated using the rate of increase over the past five years. Additionally, we identified that the ratio of fertility in HIV-positive women to HIV-negative women was negative in Indonesia. We used linear extrapolation to replace this value.

We used all available sources of vital registration data from the GBD Causes of Death database after garbage code redistribution and HIV/AIDS mis-coding correction, excluding countries with seroprevalence surveys and antenatal clinic data (Group 1 countries as described in Part 1)[2,3]. There are two different cause of death data sources for HIV/AIDS in China: the Disease Surveillance Point (DSP) system and the Notifiable Infectious Disease Reporting (NIDR) system. Both systems are administered by the Chinese Center for Disease Control and Prevention, but the reported number of deaths due to HIV is significantly lower in DSP. Therefore, we have used the provincial-level ratio of deaths due to HIV/AIDS from NIDR to those from DSP, choosing the larger ratio between years 2013 and 2014, and scaled the reported deaths in the DSP system, which is in turn used in the Space-Time Gaussian Process Regression (ST-GPR) process.

Modeling strategy

In GBD 2015, our general modeling strategy for estimating HIV incidence, prevalence, and mortality is similar in many ways to the strategy used in GBD 2013. In GBD 2015, we continue to use the Spectrum program rewritten in Python for GBD 2013 to facilitate faster and more flexible execution necessary for our more intensive computational needs. We made several changes to Spectrum's assumptions comparing to the Spectrum software used by UNAIDS. A key change in GBD 2015 is the application of EPP using an open-source computer program in R written by Jeffrey Eaton.[4] We ran EPP for all group 1 countries in order to produce incidence curves that were consistent with the demographic and epidemiological assumptions used in GBD 2015. This differed from GBD 2013, where we used the incidence curves provided by UNAIDS.

On-ART

First, we corrected reported probabilities of death for loss to follow-up using an update of the approach developed by Verguet and colleagues.[5] Verguet and colleagues used tracing and follow-up studies to empirically estimate the relationship between death in LTFU and the rate of LTFU.

After extracting the survival data into duration-specific conditional mortality, we used DisMod-MR 2.0 to synthesize the data into estimates of conditional probability of death over initial CD4 count.[1] We modeled the data separately by duration and added a fixed effect on whether the study was conducted prior to 2002. Each analysis was conducted separately for high-income countries, GBD low-income countries outside of sub-Saharan Africa, and sub-Saharan Africa.

To create estimates of age-specific hazard ratios, we synthesized hazard ratio data in five broad age groups: 15-25, 25-35, 35-45, 45-55, 55-100, and modeled the data using DisMod-MR 2.0.

To create estimates of sex-specific hazard ratios, we use the *metan* function in Stata to create estimates of relative risks separately by region, using female age groups as the reference group.

The age and sex hazard ratios were applied to the CD4-specific mortality rates, accounting for the distribution of ages and sexes in the mortality data. We then subtracted HIV-free mortality from the model life table process to calculate HIV-specific mortality, and used 1,000 draws from the posterior distribution for each age, sex, and CD4 category for conditional probabilities of death for 0-6 months, 7-12 months, and 13-24 months after initiation of ART as inputs into Spectrum.

Changes for GBD 2015

In GBD 2015, our primary methodological change was the analysis of on-ART mortality using a fixed effect on studies before/after 2002, only in the high-income region, to estimate conditional probability of death in DisMod-MR 2.0. By doing so, we incorporated changes over time in the quality of on-ART care which may improve on-ART mortality. This change was also complemented by the inclusion of time-split data from ART-CC, which allowed us to incorporate this time trend across the large cohort. We then used the estimated post-2002 on-ART mortality through the rest of the on-ART estimation process.

Off-ART

Following UNAIDS assumptions, no-ART mortality is modeled as shown in the figure below.[1]

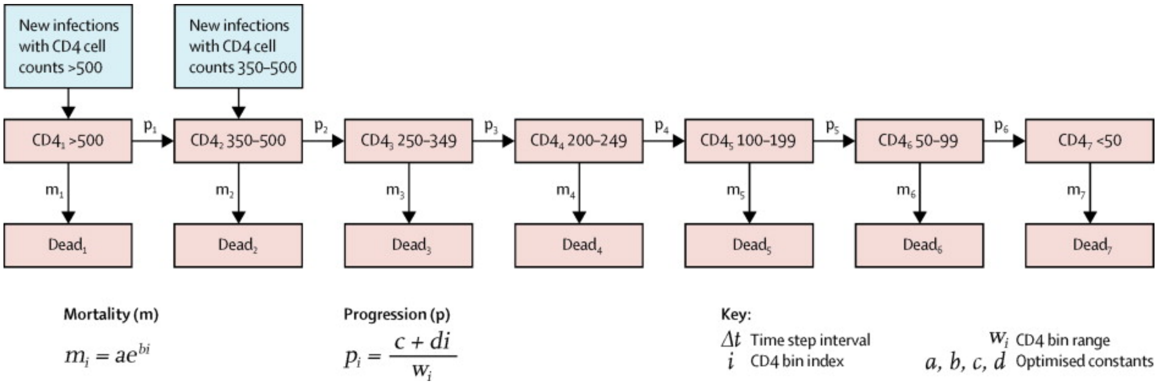

The death and progression rates between CD4 categories vary by age according to four age-groups, 15–24 years, 25–34 years, 35–44 years, and 45 years or older. We modeled the logit of the conditional probability of death between years in these studies using the following formula:

$$logit\ (m_{ijk}) = \beta_0 + \sum_{i=1}^{4} \beta_{1i}\, a_i + \sum_{j=1}^{12} \beta_{2j} t_j + u_k + \varepsilon_{ijk}$$

In the formula, m is conditional probability of death from year t_j to t_{j+1}, a_i is an indicator variable for age group at seroconversion (15–24 years, 25–34 years, 35–44 years, and 45 years or older), t_j is an indicator variable of year since seroconversion, and u_κ is a study-level random effect.

By sampling the variance-covariance matrix of the regression coefficients and the study-level random effect, we generated 1,000 survival curves for each age group that capture the systematic variation in survival across the available studies. For each of the 1,000 survival curves, we use a framework modeled after the UNAIDS optimization framework in which we find a set of progression and death rates that minimizes the sum of the squared errors for the fit to the survival curve.[6,7]

Burden estimation overview

UNAIDS uses two key analytical components in their epidemiological estimation. EPP is used to estimate incidence trajectories that are consistent with prevalence surveys and other prevalence measurements such as antenatal clinic serosurveillance. Spectrum is a compartmental HIV progression model used to generate age-specific incidence, prevalence, and death rates from the EPP incidence curves and assumptions about intervention scale-up and local variation in epidemiology.

For GBD 2013, we created an exact replica of Spectrum in Python. This enabled us to run thousands of iterations of the model at once on our computing cluster and allowed for more flexible input data structures. Additionally, in order to generate estimates with more realistic ranges of uncertainty than those in UNAIDS 2012, we adjusted all input data by uniformly sampled factors between 0.9 and 1.1. These changes, along with our new estimation of with- and without-ART mortality and CD4 progression parameters, persist into GBD 2015.

We have made several substantial improvements elsewhere in the process for GBD 2015. Of particular note, we have integrated EPP into the modeling process when feasible, enabling more robust and internally consistent incorporation of parameter uncertainty in generalized epidemics, and we have vastly improved the accuracy of the incidence adjustment used to fit Spectrum to high-quality vital registration data. Details of the impacts are included in the descriptions of the appropriate country strategies.

Due to the substantial differences in the quality and types of data available across different countries, we used three different methodologies to produce year-, age-, and sex-specific estimates of HIV incidence, prevalence, and mortality.

Countries with seroprevalence surveys and antenatal clinic data (Groups 1A and 1B)

We identified 43 countries – as well as 48 subnational locations from India, Kenya, Mozambique, and South Africa – with at least one geographically representative HIV seroprevalence survey. In order to ensure that our estimates of incidence and prevalence in these places were consistent with our estimates of HIV progression, we used a version of EPP written in R and C++ by Jeffrey Eaton to create new fits to the prevalence data in the UNAIDS files. By substituting in our own assumptions about HIV progression, we were able to ensure that the implied relationship between incidence and mortality/prevalence in EPP is similar to that in Spectrum.

In these locations, most of which experience generalized HIV epidemics, we expect estimates of HIV burden to exhibit substantial uncertainty. To reflect this, we induced a perfect correlation between the previously independent draws of HIV mortality with and without ART and CD4 progression. We paired the draws of the three parameter sets internally and with each other in the following way: we sorted

without-ART mortality and CD4 progression internally by age (not CD4), meaning the highest draw of HIV mortality without ART for age a_i and CD4 category c_l will be paired with the highest draw of HIV mortality without ART for age a_k and CD4 category c_l. In the same way, we sorted with-ART mortality internally by age, sex, CD4 count at treatment initiation, and duration on treatment. After this sorting process, the lowest indexed draw of each parameter has the highest values and vice versa. This means that we will use the most extreme possible parameter sets in EPP and Spectrum and should see a commensurate expansion in the range of the uncertainty.

To ensure that this expanded uncertainty is replicated in EPP, we fit the model once for every set of paired draws of the progression parameters for every location. This means that the first iteration of EPP for Uganda sees the highest draws of all three sets of progression parameters. Such a procedure is necessary because EPP currently has no mechanism for incorporating uncertainty in any inputs except prevalence data. This process (Process 1 in the HIV/AIDS Estimation Flowchart), produced 1,000 sets of EPP output for each of the locations that make up the 47 countries in the group. Every set of EPP outputs contains 500 consistent draws of HIV incidence and prevalence in adults aged 15-49. In many cases, the algorithm used to fit EPP, incremental mixture importance sampling, failed, resulting in fewer than 1,000 sets of EPP results.

For every location in the group, we sampled one of the 500 incidence/prevalence draws from each of the sets of EPP results (Process 2 in the HIV/AIDS Estimation Flowchart). By sampling one draw from each set, we ensured that the distribution of progression parameters dictating the relationship between incidence and prevalence was exactly the same as the distribution of the sorted parameters generated in the previous step. In locations where not all 1,000 iterations of EPP fit successfully, we sampled one draw from every iteration that did succeed and then resampled with replacement from that set of draws. To maintain the link between the input progression draws and the resulting incidence and prevalence draws from EPP, we replaced any parameter draw associated with a failed run of EPP with the parameter draw that that failed draw was replaced with. At the end of this process, for every location in the set of 47 countries, we were left with 1,000 linked draws of adult incidence and prevalence and the exact progression parameters that generated those draws.

We then ran these results, along with the previously described demographic and HIV-specific inputs, through Spectrum to produce location-, year-, age-, and sex-specific estimates of HIV incidence, prevalence, and mortality (Process 9 in the HIV/AIDS Estimation Flowchart).

Countries with vital registration data (Group 2A and 2B)

Vital registration is one of the highest-quality sources of data on HIV burden in many countries, so generating estimates that are consistent with these data, with necessary adjustment to account for any potential underreporting, is critical. We identified 116 countries – as well as 208 subnational locations from Brazil, China, Japan, Mexico, Saudi Arabia, Sweden, United Kingdom, and United States – with vital registration data or sample registration systems such as DSP in China.

We imputed missing years of data to generate a complete time series for HIV from the estimated start year of the epidemic using ST-GPR. We analyzed mortality trends using ST-GPR starting in 1981, the year that HIV was first identified in the United States.[8] For ST-GPR, we adjusted the lambda (time weight) and GPR scale according to the completeness of vital registration data, based on whether a country had 10 or more years of complete VR data as analyzed by the Death Distribution Methods (DDM) model described in Part 1. We produced separate splines by country/age group, up to the peak year of death

rate. We then ran a linear regression with random effects on region, age, and sex. Following this, we ran space-time residual smoothing, in which time, age, and space weights are used to inform smoothing of the residuals between data points and the linear regression estimate. From this process, we generated space-time estimates with the applied weights, along with the median absolute deviation (MAD) of the space-time estimates from the data. The MAD was calculated at various levels of the geographic hierarchy (e.g., subnational and national), and was added into the data variance term. The data variance and space-time estimates were then analyzed using Gaussian Process Regression to return a final estimate of mortality along with uncertainty.

Although Spectrum produces HIV mortality estimates that are within the realm of possibility in most countries using the incidence curves provided in the UNAIDS 2012/2015 country files, it is a deterministic model that has not yet been integrated into an optimizable framework. Therefore, in order to "fit" it to vital registration data, we need to adjust input incidence. For GBD 2013, we used a process that assumed several different durations between HIV infection and HIV death and adjusted incidence based on death some number of years in the future. Although that method worked relatively well and substantially reduced the disconnect between Spectrum and the VR data, it required very rigid and unrealistic assumptions about these survival durations. For GBD 2015, we have improved the performance of this method, allowing Spectrum to fit to the VR data more closely.

To improve the fit of this process, we restructured Spectrum to add compartments that identify groups of people living with HIV by year of infection (Process 5 in the HIV/AIDS Estimation Flowchart). With this version of Spectrum we can output, among many other metrics, HIV deaths by year, age, sex, and infection cohort. This enables us to adjust incidence to fit to death much more precisely and without making any rigid assumptions about the time from HIV infection to HIV death.

We have incorporated these improvements into a cohort incidence bias adjustment (CIBA) process. First, we ran Spectrum normally to produce 1,000 draws of incidence, prevalence and mortality (Process 4 in the HIV/AIDS Estimation Flowchart). Then, by year, age, and sex, we took the ratio of VR deaths to Spectrum deaths to quantify the amount of bias in Spectrum. Using the mean duration data from the new version of Spectrum, for every year-, age-, and sex-specific infection cohort, we calculated the share of all HIV deaths observed over the course of the projection period in that cohort that would occur in each year after the year of infection. For example, projecting from 1970 through 2015, we identified the cohort of men infected in 1992 at the age of 16, calculated the total number of HIV deaths in that cohort in all subsequent years through the end of 2015, and divided the annual number of deaths by that total. This showed us the distribution of deaths among that cohort over the projection period. In the most extreme case (infections in 2014), we could only produce one point of that distribution (2015), so that single value is exactly 1.0; 100% of the deaths observed in that cohort occurred in 2015.

We then used these distributions of death to weigh the ratio of VR deaths to Spectrum deaths, meaning that ratios in the years where we expect the largest share of deaths were weighed most heavily. We then multiplied the initial size of that cohort from the normal run of Spectrum by the sum of the combined ratios to get a new estimate of new cases in that year/age/sex combination.

We can write this method mathematically in the following way:

$$r_t = \frac{VR_t}{D_t}$$

$$\rho_t^{t-i} = \frac{d_t^{t-i}}{\sum_{k=t-i+1}^{n} d_k^{t-i}}$$

$$\alpha^{t-i} = \sum_{k=t-i+1}^{n} r_k * \rho_k^{t-i}$$

$$n_{\text{adjusted}}^{t-i} = \alpha^{t-i} * n^{t-i}$$

VR_t is the number of HIV/AIDS deaths in year t from ST-GPR, and D_t is the number of HIV/AIDS deaths from the first run of Spectrum. In the second equation, d_t^{t-i} is the number of HIV/AIDS deaths among members of infection cohort $t-i$ in year t, with $i \geq 1$, from the new, duration-tracking version of Spectrum, and n is final year of the projection. Therefore, ρ_t^{t-i} is the share of observed deaths in cohort $t-i$ that we expect to occur in year t. It follows, that α^{t-i} is the weighted adjustment ratio described above, which we multiply by the estimated initial size of infection cohort $t-i$ as calculated in the first stage Spectrum run to get the adjusted nubmer of new cases, $n_{\text{adjusted}}^{t-i}$. This process is run separately for every sex and single-age pair.

CIBA (Process 6 in the HIV/AIDS Estimation Flowchart) allows ratios in each year after a given infection year to influence the final adjustment to incidence. The size of that influence is determined by the relative importance of that year in the cohort-year's distribution of deaths over time. The result is a new set of 1,000 draws of incidence and a set of 1,000 ratios of post-adjustment incidence to pre-adjustment incidence. We perform this adjustment using mean durations from the new version of Spectrum in order to try to shift the mean of the regular distribution of deaths.

Finally, to produce location-, year-, age-, and sex-specific estimates of HIV incidence, prevalence and mortality, we ran the new estimates of incidence and all previously input data through Spectrum (Process 9 in the HIV/AIDS Estimation Flowchart).

Countries without survey data and vital registration data (Group 2C)

The remaining 24 countries – as well as nine subnational locations from China and Saudi Arabia – had neither geographically representative seroprevalence surveys nor reliable vital registration systems. To produce estimates of HIV burden in these countries, we assumed that Spectrum is similarly biased as in other Group 2 countries. This involved running Spectrum (Process 7 in the HIV/AIDS Estimation Flowchart), adjusting incidence using 1,000 adjustment ratios randomly sampled from the entire set of CIBA results (Process 8), and rerunning Spectrum using the new draws of adjusted incidence (Process 9). As above, the estimates of incidence, prevalence, and mortality were incorporated into the rest of the machinery via the reckoning process.

Originally, Cambodia, which does have a prevalence survey, was included in this group because we have not yet coded the machinery necessary to reproduce the Asian Epidemic Model used by UNAIDS to model prevalence and incidence in Southeast Asian countries.[9] The 2005 DHS survey in Cambodia made clear that we were underestimating the burden due to HIV there by not using survey data during the modeling process. In order to more accurately represent the epidemic, we used the mortality profile from Thailand and scaled it by 80%, the ratio of estimated prevalence rate in Thailand in 2005 and the prevalence rate from the DHS survey in Cambodia. We then treated the scaled death series as VR data and added Cambodia to the 2B group that is run through CIBA.

Subnational splitting for India and Kenya

Spectrum results for India and Kenya subnational locations are modeled at higher levels of geography than our GBD locations. For example, Spectrum results for India are produced at the state level, while GBD 2015 estimates were produced at the state urban-rural level. Similarly, Spectrum is modeled at the province level, while we compute Kenyan subnational estimates for the 47 counties. To split the Spectrum results into more granular results for processing, we assign each GBD subnational unit to a Spectrum modeling unit. From this, we generate age/sex/year-specific proportions for population, HIV-specific death, and HIV-free mortality.

After this subnational splitting, results were incorporated into the all-cause mortality estimation machinery via the reckoning process described in Part 1.

HIV/AIDS resulting in other diseases

There are two Level 4 causes under the HIV/AIDS Level 3 cause in the GBD 2015 cause hierarchy. The modeling process for HIV/AIDS-tuberculosis is detailed in Part 3. We computed deaths for HIV resulting in other diseases by subtracting HIV/AIDS-tuberculosis deaths from all HIV deaths at the 1,000 draw level.

References

1 Murray CJL, Ortblad KF, Guinovart C, *et al.* Global, regional, and national incidence and mortality for HIV, tuberculosis, and malaria during 1990–2013: a systematic analysis for the Global Burden of Disease Study 2013. *The Lancet* 2014; **384**: 1005–70.

2 Global, regional, and national age–sex specific all-cause and cause-specific mortality for 240 causes of death, 1990–2013: a systematic analysis for the Global Burden of Disease Study 2013. *The Lancet* 2015; **385**: 117–71.

3 Birnbaum JK, Murray CJ, Lozano R. Exposing misclassified HIV/AIDS deaths in South Africa. *Bull World Health Organ* 2011; **89**: 278–85.

4 jeffeaton/epp. GitHub. https://github.com/jeffeaton/epp (accessed April 21, 2016).

5 Verguet S, Lim SS, Murray CJL, Gakidou E, Salomon JA. Incorporating Loss to Follow-up in Estimates of Survival Among HIV-Infected Individuals in Sub-Saharan Africa Enrolled in Antiretroviral Therapy Programs. *J Infect Dis* 2013; **207**: 72–9.

6 Ghys PD, Zaba B, Prins M. Survival and mortality of people infected with HIV in low and middle income countries: results from the extended ALPHA network. *AIDS* 2007; **21 Suppl 6**: S1–4.

7 Hallett TB, Zaba B, Todd J, *et al.* Estimating incidence from prevalence in generalised HIV epidemics: methods and validation. *PLoS Med* 2008; **5**: e80.

8 CDC. Pneumocystis Pneumonia --- Los Angeles. MMWR Weekly. 1981; published online June 5. http://www.cdc.gov/mmwr/preview/mmwrhtml/june_5.htm (accessed April 21, 2016).

9 Brown T, Peerapatanapokin W. The Asian Epidemic Model: a process model for exploring HIV policy and programme alternatives in Asia. *Sex Transm Infect* 2004; **80 Suppl 1**: i19–24.

HIV/AIDS-Tuberculosis

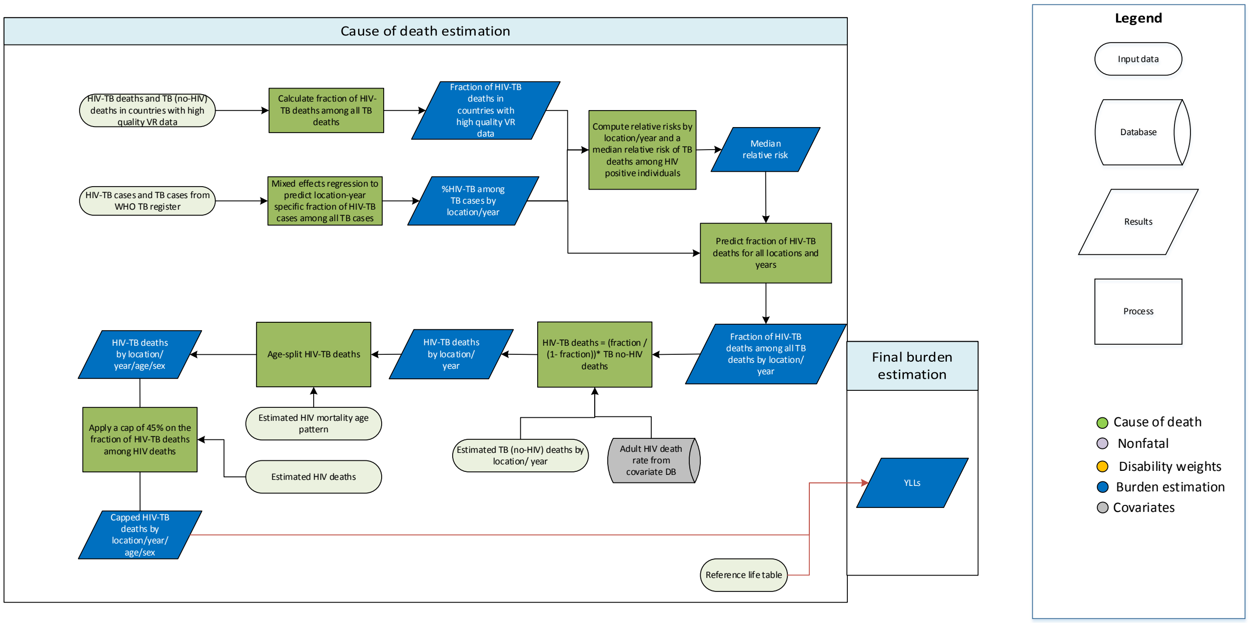

Input data

Input data include: (i) 144 country-years of high-quality vital registration data where cause of death data for directly coded HIV/AIDS-tuberculosis (HIV-TB) and tuberculosis (TB) were available, and (ii) the number of TB cases (new and re-treatment) recorded as HIV-positive and the number of TB cases (new and re-treatment) with an HIV test result recorded in the TB register from the World Health Organization (WHO). We excluded data from countries with 10 HIV-TB deaths or less. We also excluded implausible data (e.g., proportion of HIV-TB cases among all TB cases greater than 1), and data that were largely conflicting with the majority of data for other years from the same country.

Modeling strategy

To determine TB deaths in HIV-positive individuals, we first computed the fraction of HIV-TB deaths among all TB deaths using 144 country-years of high-quality vital registration data. We also calculated the proportion of HIV-TB cases among all TB cases (number of TB cases recorded as HIV-positive/number of TB cases with an HIV test result recorded in the WHO TB register), which we used as input data to predict the proportions of HIV-TB cases among all TB cases for all locations and years using an adult HIV death rate covariate and country random effects. We estimated the fraction of HIV-TB deaths among all TB deaths, defined by

$$D = \frac{P(RR)}{P(RR) + (1 - P)}$$

where *P* is the proportion of HIV-TB cases among all TB cases and *RR* is the relative risk of TB deaths in HIV positive individuals, defined by:

$$RR = \frac{DP - D}{DP - P}$$

where *D* is the fraction of HIV-TB deaths among all TB deaths. We took the median relative risk (RR) from each calculation to get the global RR. We then applied the global RR and the predicted proportions of HIV-TB cases among all TB cases to get the fractions of HIV-TB deaths among all TB deaths for all locations and years. Location-year-specific HIV-TB deaths were then calculated using the following equation:

$$death_{HIVTB} = \frac{D}{1-D} death_{TB}$$

where $death_{TB}$ is *TB deaths among HIV negative individuals* were location-year specific deaths from the CODEm TB no-HIV model. Finally, we applied the age-sex pattern of the HIV mortality estimates to these HIV-TB deaths to generate location-year-age-sex-specific HIV-TB deaths. As the HIV-TB deaths were estimated based on the fraction of HIV-TB deaths among all TB deaths, the total number of HIV-TB deaths could exceed the total number of HIV cases in some locations. To avoid this, we applied a cap of 45% on the fraction of HIV-TB deaths among HIV deaths, based on a review by Cox et al., 2010,[1] and a systematic review and meta-analysis by Ford et al., 2016.[2]

There were changes from GBD 2013 in terms of modeling strategy. In GBD 2013, we estimated the relationship between the logit-transformed fraction of HIV-TB deaths among HIV deaths and the log-transformed TB death rate in a mixed effects regression. The regression coefficients were then used to predict the fraction of HIV-TB deaths in all countries. This approach did not use all available data and also underestimated HIV-TB deaths. For GBD 2015, we made use of all available data and improved our modeling strategy as described above.

References

1 Cox JA, Lukande RL, Lucas S, Nelson AM, Van Marck E, Colebunders R. Autopsy causes of death in HIV-positive individuals in sub-Saharan Africa and correlation with clinical diagnoses. *AIDS Rev* 2010; **12**: 183–94.

2 Ford N, Matteelli A, Shubber Z, *et al.* TB as a cause of hospitalization and in-hospital mortality among people living with HIV worldwide: a systematic review and meta-analysis. *J Int AIDS Soc* 2016; **19**: 20714.

2.8 Shock models

Ebola

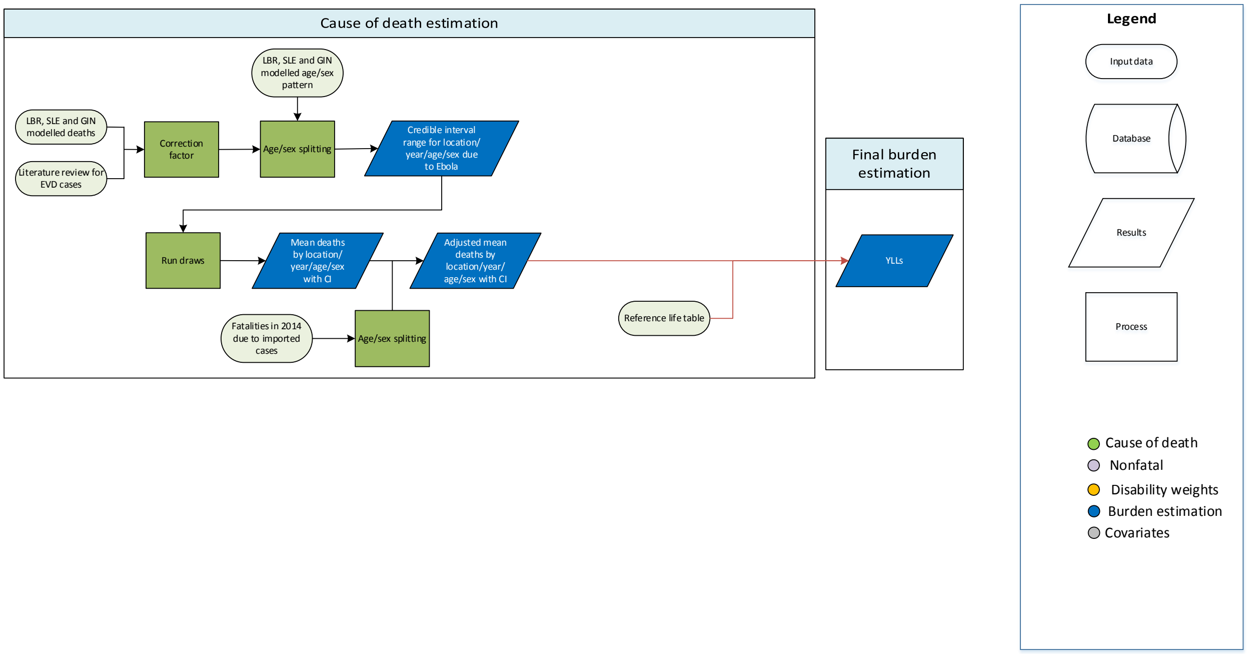

Input data

The input data for deaths due to Ebola virus disease (EVD) came in two forms: (i) modeled estimates for the West African outbreak from 2013 to 2015 provided by the World Health Organization (WHO) focused specifically on the three worst affected countries (Liberia, Guinea, and Sierra Leone) and (ii) literature searches for reported deaths due to EVD not captured by the West African dataset. This is further explained below:

i. WHO estimates for Liberia, Guinea, and Sierra Leone, 2014 to 2015
    1. Researchers from Imperial College London (UK), as part of the WHO Ebola response team, provided modeled estimates for the number of fatalities that result from a given number of reported cases (provided by line lists from the WHO). This method was used in a variety of papers to generate baseline estimates of case fatality rates and other key epidemiological measures while correcting for the lag period between initially reporting a case and the final outcome of that case (whether it be death or survival). The full data cleaning and methodology are reported elsewhere.[1,2] Bespoke estimates were provided for GBD for Liberia, Sierra Leone, and Guinea and were stratified by age, sex, and year. Death data from Guinea ranged from February 18, 2014, until September 27, 2015, with data from Liberia ranging from March 20,

2014 to May 4, 2015, and data from Sierra Leone ranging from May 21 until September 28, 2015.

ii. Literature searches for reported deaths due to EVD outside of Liberia, Guinea, and Sierra Leone

1. In order to capture the small number of fatalities that occurred in countries outside of the core three mentioned above, WHO Situation Reports were consulted. Fatalities were reported in the USA (specifically Texas), Mali, and Nigeria.[3] All deaths occurred in 2014. Additional age and sex information could only be obtained for the death that occurred in the US.

Using a previous review of historical outbreaks,[4,5] original articles describing the progression of historical outbreaks were consulted. This initial review was also updated to include the 2014 outbreak that occurred in the Democratic Republic of the Congo in 2014.[6] This resulted in datasets describing each outbreak with variable degrees of detail; some fully describing the age and sex breakdown of all deaths [e.g.,[7]] and others simply providing the final total. Only confirmed or probable deaths were included; suspected EVD deaths were omitted. Outbreaks that spanned multiple years, in the absence of sufficient data providing an accurate breakdown, were apportioned between the years by evenly assigning a uniform number of deaths to each month of the outbreak's duration.

Modeling strategy

Data on deaths resulting from imported cases from 2014 were used as specific count data as it was assumed to be an accurate representation of the cases and outbreaks in these countries, all of which were on high alert for importation of cases.[8,9]

The other input data were processed prior to inclusion in GBD to account for any potential underreporting of deaths. The United Nations Mission for Ebola Emergency Response surveys suggested that such underreporting of deaths ranged from 1.43 – 2x the reported number.[10] In order to capture this potential variation, all input data were multiplied by the lower and upper limit of this estimated correction factor; these numbers then provided the lower and upper bounds from which draw values were taken. For outbreaks where no data were supplied for age and/or sex, the pattern observed in the West African outbreak (for which there was the most comprehensive data) was used to apportion these total values.

One thousand draws were taken from a normal distribution fitted between these lower and upper bound values, which generated mean estimates stratified by age, sex, location, and year along with credible intervals for these numbers. These estimates were then adjusted by including the count data for imported cases from 2014.

Data on Ebola outbreaks prior to 2014 are sparse, and as a result many values derived from the West African outbreak were assumed to be valid for historic outbreaks as well. This may mask significant differences that exist between these outbreaks, some of which were caused by different species of *Ebolavirus*. In order to minimize this problem we chose to implement a data-driven approach – for those outbreaks where sufficiently detailed historical data could be obtained, this was used in preference to any assumed age/sex breakdown.

Similarly, we only have one estimate for potential underreporting, taken from December 2014 in Sierra Leone. It could well be that this rate varies considerably both over time within an outbreak as well as

between different outbreaks. As more studies are undertaken in the years following the peak of the outbreak, it is likely that this correction factor will become better parameterized in future GBD iterations.

References

1 West African Ebola Epidemic after One Year — Slowing but Not Yet under Control. *N Engl J Med* 2015; **372**: 584–7.

2 Ebola Virus Disease in West Africa — The First 9 Months of the Epidemic and Forward Projections. *N Engl J Med* 2014; **371**: 1481–95.

3 Ebola Situation Reports. World Health Organization.

4 Pigott DM, Golding N, Mylne A, *et al.* Mapping the zoonotic niche of Ebola virus disease in Africa. *eLife* 2014; **3**: e04395.

5 Mylne A, Brady OJ, Huang Z, *et al.* A comprehensive database of the geographic spread of past human Ebola outbreaks. *Sci Data* 2014; **1**: 140042.

6 Maganga GD, Kapetshi J, Berthet N, *et al.* Ebola virus disease in the Democratic Republic of Congo. *N Engl J Med* 2014; **371**: 2083–91.

7 Rosello A, Mossoko M, Flasche S, *et al.* Ebola virus disease in the Democratic Republic of the Congo, 1976-2014. *eLife* 2015; **4**. DOI:10.7554/eLife.09015.

8 Fasina FO, Shittu A, Lazarus D, *et al.* Transmission dynamics and control of Ebola virus disease outbreak in Nigeria, July to September 2014. *Euro Surveill Bull Eur Sur Mal Transm Eur Commun Dis Bull* 2014; **19**: 20920.

9 Althaus CL, Low N, Musa EO, Shuaib F, Gsteiger S. Ebola virus disease outbreak in Nigeria: Transmission dynamics and rapid control. *Epidemics* 2015; **11**: 80–4.

10 Sierra Leone: Ebola emergency Weekly Situation Report No. 7. UN for Ebola Emergency Response, National Emergency Response Centre, 2014.

Fatal Discontinuities

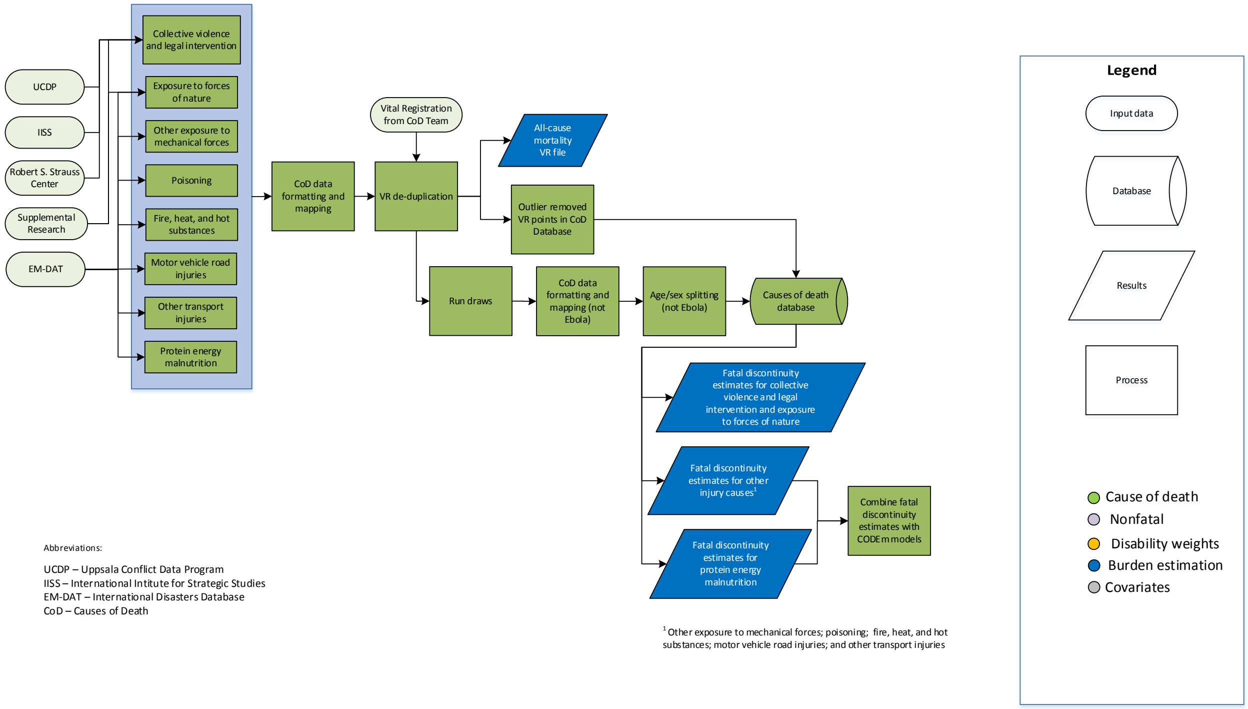

Input data

Collective Violence and Legal Intervention

Data for collective violence and legal intervention comes from the Uppsala Conflict Data Program (UCDP), International Institute for Strategic Studies, and Robert S. Strauss Center for International Security and Law. The table below provides details about the various datasets we utilized from these sources, the dates they were last accessed, and the years for which we used the data provided.

Data source name	Date accessed	Years of data accessed	Type of data included
**Uppsala Conflict Data Program**[1]			
Battles	9/23/15	1989-2015	Armed conflict: incompatibility that concerns government and/or territory over which the use of armed force between the military forces of two parties, of which at least one is the government of a state, has resulted in at least 25 battle-related deaths each year
Non-state	11/4/15	1989-2015	The use of armed force between two organized armed groups, neither of which is the government of a state, which results in at least 25 battle-related deaths each year
One-sided	11/3/15	1989-2015	The use of armed force by the government of a state or by a formally organized group against civilians which results in at least 25 deaths in a year
Africa Georeferenced Event Dataset	11/4/15	1989-2008	UCDP battles; non-state; one-sided for African countries
PRIO Battles Deaths Dataset	9/23/15	1970-1989	Armed conflict (civil wars, etc.)
**International Institute for Strategic Studies**			
Armed Conflict Dataset	9/25/15	1997-Present	Insurgency, Inter-state, Intra-state
**Robert S. Strauss Center For International Security And Law**			
Armed Conflict Location and Event Dataset (ACLED)	9/15/15	1997-2015	Actions of opposition groups, governments, and militias across Africa, specifying the exact location and date of battle events, transfers of military control, headquarter establishment, civilian violence, and rioting
Social Conflict Analysis Database (SCAD)	9/15/15	1990-2015	Protests, riots, strikes, inter-communal conflict, government violence against civilians, and other forms of social conflict (covers Africa and Latin America)

Supplemental online research was conducted for recent conflicts where the databases above were not up-to-date. Where there was large variance in death estimates in recent years, we averaged estimates from all sources at the country-year level.

For country-years where multiple sources provided estimates, we prioritized sources in the following order: (1) country vital registration (VR) data, if death estimates were highest of all sources; (2) UCDP; (3) IISS; (4) country VR if death estimates were not the highest of all sources; (5) Strauss Center; (6) online supplemental research; and (7) combined average country data where applicable.

Exposure to Forces of Nature and Other Injury Causes

Data for disaster events which caused greater than 50 deaths due to exposure to forces of nature; poisonings; fire, heat, and hot substances; motor vehicle road injuries; other transport injuries; and other exposure to mechanical forces came from the Centre for Research on the Epidemiology of

Disasters' International Disaster Database (EM-DAT). Data from EM-DAT were last accessed January 3, 2016. Supplemental online research was conducted for events where EM-DAT was not up-to-date.

For country-years where multiple sources provided estimates, we prioritized sources in the following order: (1) country VR data, if death estimates were highest of all sources; (2) EM-DAT; (3) country VR if death estimates were not the highest of all sources; (4) online supplemental research. Exceptions were made where it was clear that vital registration systems had been compromised by the event being measured. In those cases, such as in the United States following Hurricane Katrina in 2005, supplemental research was prioritized over VR data.

In locations where we produced estimates at the subnational level for GBD 2015, deaths due to all fatal discontinuity causes were assigned to the relevant subnational location(s) when that information could be obtained either through the data sources mentioned above or through additional online research. If no subnational location could be found, the deaths were split proportionally by population across all subnational locations.

A systematic literature review was not used to identify input data for fatal discontinuities, though some literature sources were identified through online supplemental research.

Modeling strategy

All input data for fatal discontinuity causes were run through the Causes of Death data formatting and mapping process detailed in Part 2.

VR de-duplication

For country-years where deaths due to fatal discontinuity causes were recorded in both VR and other utilized data sources, the higher of the two estimates were taken in the case of deaths due to war and collective violence and exposure to forces of nature.

For the other injury causes that also have a CODEm model, a process was established to avoid duplication of fatal discontinuity deaths in the two models. First, location-years with greater than 50 deaths due to the cause were identified. If these location-cause-years had vital registration death estimates that were greater than 40% higher than the immediately surrounding years, the identified years were reviewed. Those that represented a true diversion from the trend of VR and could be linked to a specific fatal discontinuity event were marked as outliers in the VR data and the difference between the outlier year and the average of the surrounding years was included in the relevant cause in the fatal discontinuities database. The deaths from the identified events were subtracted from the all-cause VR estimates used in the all-cause mortality estimation process.

Uncertainty analysis for input and draw-level input to age-sex splitting

Uncertainty intervals for deaths due to collective violence and legal intervention were generated using UCDP high and low death estimates. In cases where low and high estimates are not provided by the original source, regional average relative uncertainty interval by type of fatal discontinuity is applied to the mean input that is available.

We assumed a normal distribution using the mean deaths and standard deviation based on high and low estimates. The standard deviation was capped at the mean divided by 1.96 in order to ensure that 95%

of the 1,000 draws generated were greater than 0. Any negative draws were dropped from final calculations of means and uncertainty intervals.

Age-sex splitting

All compiled data were run through the causes of death age-sex splitting process detailed in Part 2.

Changes from GBD 2013

Only collective violence and legal intervention and exposure to forces of nature were modeled as fatal discontinuities (previously called mortality shocks) in GBD 2013. GBD 2015 also includes fatal discontinuity models for protein energy malnutrition (previously included as "famine" in exposure to forces of nature) and additional injury models (motor vehicle road injuries; other transport injuries; exposure to fire, heat, and hot substances; poisoning; and other exposure to mechanical forces). These new causes (unlike collective violence and legal intervention and exposure to forces of nature) are also modeled in CODEm and thus have two models (a shock model and a CODEm model) that are combined to produce the final estimates of deaths for these causes.

The VR de-duplication process and the use of the causes of death age-sex splitting process for fatal discontinuity causes were added in GBD 2015.

Reference

1 UCDP/PRIO Armed Conflict Dataset Codebook. Uppsala Conflict Data Program (UCDP); Centre for the Study of Civil Wars, International Peace Research Institute, Oslo (PRIO), 2013.

2.9 Pathogen counterfactuals

Lower Respiratory Infections

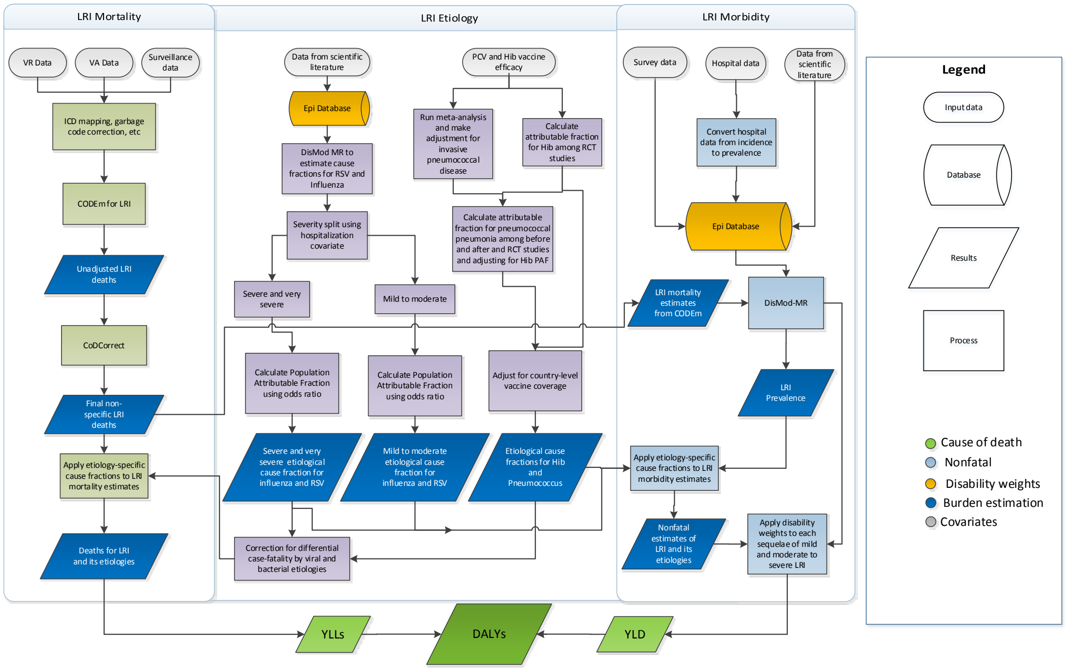

Input data

Lower respiratory infection (LRI) mortality was estimated in CODEm. We estimated LRI mortality separately for males and females and for children under 5 years and older than 5 years. We used all available data from vital registration systems and verbal autopsy. We checked for and excluded outliers from our data by country or region. We also excluded ICD9-coded mortality data in Sri Lanka (1982, 1987–1992), ICD9-coded neonatal mortality data in Guatemala (1980, 1981, 1984, 2000–2004), and Civil Registration System data in many Indian states (1986–1995).

We conducted a systematic review of scientific literature for the proportion of LRI that tested positive for influenza and respiratory syncytial virus (RSV). We had inclusion criteria to select studies that were published between January 1990 and June 2015, studies that had a sample size of at least 100, studies that were at least one year in duration, and studies describing lower respiratory infections, pneumonia, or bronchiolitis as the case definition. During our literature review from January 2012 through June 2015, we identified 1,164 studies, of which 137 met our inclusion criteria and were included. We excluded studies that described pandemic H1N1 influenza solely and studies that used influenza-like illness as the case definition. We assigned an age range based on the prevalence-weighted mean age of LRI in the appropriate year/sex/location if the ages of the study participants were not reported. Overall, we included 471 data points for influenza and 362 data points for RSV.

We also conducted a systematic literature review of studies on the Hib vaccine and PCV effectiveness studies against X-ray-confirmed pneumonia and against pneumococcal and Hib disease until January 2016. For PCV studies, we also extracted, if available, the distribution of pneumococcal pneumonia serotypes and the serotypes included in the PCV used in the study. We did not include any new studies for Hib, and three new studies were included for pneumococcal pneumonia since GBD 2013. We excluded observational and case-control studies due to implausibly high vaccine efficacy estimates. Hib trial data were exclusively from children <5 years so we did not include the effect of Hib on ages over 5 years of age. PCV trial data are also frequently limited to younger age populations. To understand the contribution of pneumococcal pneumonia in older populations, we also included PCV efficacy studies that used before-after approaches to understand the impact of introduction of PCV at the population level.

For influenza and RSV, we included several study-level covariates in these models. For these covariates, we extracted data from studies included in the literature review if the study used diagnostic PCR, if the sample was from hospitalized cases, and if the study investigated RSV or influenza exclusively from other etiologies.

We used country-level covariates to inform our CODEm models. We included the following covariates in our LRI models: diphtheria-tetanus-pertussis vaccine coverage, years of education per capita, health system access, income per capita, prevalence of children malnutrition (<2 standard deviations below global mean of weight for age), prevalence of exposure to indoor air pollution (solid fuel use), outdoor air pollution level of $PM_{2.5}$, smoking prevalence, pneumococcal conjugate vaccine (PCV) coverage, *Haemophilus influenzae* type B (Hib) vaccine coverage, access to improved water, access to improved sanitation, and Socio-Demographic Index.

Modeling strategy

We evaluated our LRI cause of death models using in and out of sample predictive performance. We estimated LRI etiologies separately from overall LRI mortality using two distinct counterfactual modeling strategies to estimate population attributable fractions (PAFs), described in detail below. We did not attribute etiologies to neonatal pneumonia deaths due to a dearth of reliable data in this age group. We calculated uncertainty of our PAF estimates from 1,000 draws of each parameter using normal distributions in log space.

We modeled the proportion data using the meta-regression tool DisMod-MR to estimate the proportion of LRI cases that are positive for influenza and RSV, separately, by location/year/age/sex.

We calculated the population attributable fraction (PAF) from the proportion of severe LRI cases positive for influenza and RSV. We assumed that hospitalized LRI cases are a proxy of severe cases. We used the following formula to estimate PAF:[1]

$$PAF = Proportion * (1-1/OR)$$

Where *Proportion* is the proportion of LRI cases that test positive for influenza or RSV and *OR* is the odds ratio of LRI given the presence of the pathogen. We used an odds ratio of 5.1 (3.19 – 8.14) for influenza and 9.79 (4.98 – 19.27) for RSV from a recently published meta-analysis.[2] These odds ratios are marginally different from those used in GBD 2013.

As the case-fatality of viral causes of pneumonia is lower than for bacterial causes, we adjusted for differential case-fatality by determining the etiological fractions for mortality attributable to RSV and influenza (below table). We measured the etiologic fractions by applying a relative case-fatality adjustment based on in-hospital case-fatality, which we coded to specific pneumonia etiologies. Hospital admissions data of this type were limited to data from the USA, Austria, Brazil, and Mexico. We generated the pooled estimate of the case-fatality differential between bacterial (pneumococcus, Hib) and viral etiologies (RSV, influenza) using DisMod-MR.

For *Streptococcus pneumoniae* (pneumococcal pneumonia) and *Haemophilus influenzae* type B (Hib), we calculated the population attributable fraction using a vaccine probe design.[3,4] The ratio of vaccine effectiveness against nonspecific pneumonia to pathogen-specific disease represents the fraction of pneumonia cases attributable to each pathogen.

To estimate the PAF for Hib and pneumococcal pneumonia, we calculated the ratio of vaccine effectiveness against nonspecific pneumonia to pathogen-specific pneumonia (Equations 1 and 3). We were able to get a study-level estimate of PAF from a meta-analysis of these ratios. We then adjusted this estimate by vaccine coverage and expected vaccine performance to estimate country- and year-specific PAF values (Equations 2 and 4). For pneumococcal pneumonia, we adjusted the PAF even further using the final Hib PAF estimate and by vaccine serotype coverage. Finally, we used an age distribution of PAF modeled in DisMod to determine the PAF by age.

1) $PAF_{Base} = 1 - \frac{VE}{VE_i}$

2) $Hib\ PAF = PAF_{Base} * \frac{(1-Cov_{Hib}*VE_{Optimal})}{(1-PAF_{Base}*Cov_{Hib}*VE_{Optimal})}$

Where VE is the vaccine efficacy against nonspecific pneumonia, VE_i is the vaccine efficacy against invasive Hib disease, Cov_{Hib} is the country-level coverage of Hib vaccine and $VE_{Optimal}$ is the assumed optimal vaccine effectiveness (0.8, assumed). Optimal vaccine effectiveness is an adjustment for imperfect vaccine performance. We only used randomized-controlled trials (RCTs) in the Hib PAF estimation.

3) $PAF_{Base} = 1 - \frac{VE_{Pneumonia} * (1 - PAF_{Hib} * VE_{Hib\ Optimal})}{VE_{Pneumococcus} * Cov_{Serotype}}$

4) $PAF_{Final} = \frac{PAF_{Base} * \left(1 - Cov_{PCV} * VE_{PCV\ Optimal}\right)}{\left(1 - PAF_{Hib} * Cov_{Hib} * VE_{Hib\ Optimal}\right) * \left(1 - \frac{PAF_{Base} Cov_{PCV} * VE_{PCV\ Optimal}}{\left(1 - PAF_{Hib} * Cov_{Hib} * VE_{Hib\ Optimal}\right)}\right)}$

Where $VE_{Pneumonia}$ is the vaccine efficacy against nonspecific pneumonia, PAF_{Hib} is the final PAF for Hib, $VE_{Hib\ Optimal}$ is the optimal Hib effectiveness (0.8, assumed), $VE_{Pneumococcus}$ is the vaccine efficacy against serotype-specific pneumococcal pneumonia, $Cov_{Serotype}$ is the serotype-specific coverage, Cov_{PCV} is the vaccine coverage for region specific serotypes, $VE_{PCV\ Optimal}$ is the optimal vaccine effectiveness (0.8, assumed), and Cov_{Hib} is the Hib coverage by country.

For Hib, we assumed that the vaccine efficacy against invasive Hib disease is the same against Hib pneumonia. For pneumococcal pneumonia, a recent study in adults found that the vaccine efficacy against invasive pneumococcal disease may be significantly higher than against pneumococcal pneumonia.[5] We used this ratio to adjust estimates of vaccine efficacy against invasive pneumococcal disease from other studies. However, recognizing that the study is unique in that it uses a urine antigen test among adults, we added uncertainty around our adjustment using a wide uniform distribution (0.3-1.0). This is an important change from our etiology estimation in GBD 2013.

There are several important differences from the approach to LRI etiology estimation in GBD 2015 compared to GBD 2013. There are new data used in the CODEm models and in the LRI proportion DisMod models. Most significantly, we introduced the adjustment of pneumococcal conjugate vaccine efficacy. Most studies report the vaccine efficacy against invasive, serotype-specific pneumococcal disease; however, a recent study found that this may be an overestimate of the vaccine efficacy against serotype-specific pneumococcal pneumonia.[5] Using a large range of uncertainty, we corrected vaccine efficacy estimates against invasive disease to our serotype-specific pneumonia case definition. This has increased the estimates of pneumococcal pneumonia mortality in a meaningful way.

Table: The median values for the ratio of viral to bacterial pneumonia case fatality ratio by age is shown. These estimates are modeled using hospital-based, ICD-coded admissions and mortality for etiology-specified pneumonia. Values in parentheses represent 95% Uncertainty Interval.

Age group	Ratio
Early Neonatal	0.34 (0.15-0.53)
Late Neonatal	0.34 (0.15-0.53)
Post Neonatal	0.34 (0.15-0.53)
1 to 4	0.28 (0.14-0.42)
5 to 9	0.31 (0.1-0.52)
10 to 14	0.33 (0.17-0.49)
15 to 19	0.37 (0.15-0.59)
20 to 24	0.46 (-0.08-1)
25 to 29	0.44 (0.07-0.82)
30 to 34	0.46 (0.13-0.78)
35 to 39	0.5 (0.11-0.89)
40 to 44	0.61 (-0.23-1.45)
45 to 49	0.5 (0.1-0.89)
50 to 54	0.44 (0.18-0.71)
55 to 59	0.42 (0.13-0.71)
60 to 64	0.42 (0-0.85)
65 to 69	0.39 (0.13-0.65)
70 to 74	0.38 (0.17-0.58)
75 to 79	0.37 (0.16-0.58)
80 plus	0.35 (0.2-0.5)

References

1 Miettinen OS. Proportion of disease caused or prevented by a given exposure, trait or intervention. *Am J Epidemiol* 1974; **99**: 325–32.

2 Shi T, McLean K, Campbell H, Nair H. Aetiological role of common respiratory viruses in acute lower respiratory infections in children under five years: A systematic review and meta-analysis. *J Glob Health* 2015; **5**: 010408.

3 Feikin DR, Scott JAG, Gessner BD. Use of vaccines as probes to define disease burden. *Lancet Lond Engl* 2014; **383**: 1762–70.

4 O'Brien KL, Wolfson LJ, Watt JP, *et al.* Burden of disease caused by Streptococcus pneumoniae in children younger than 5 years: global estimates. *Lancet Lond Engl* 2009; **374**: 893–902.

5 Bonten MJM, Huijts SM, Bolkenbaas M, *et al.* Polysaccharide conjugate vaccine against pneumococcal pneumonia in adults. *N Engl J Med* 2015; **372**: 1114–25.

Diarrheal diseases

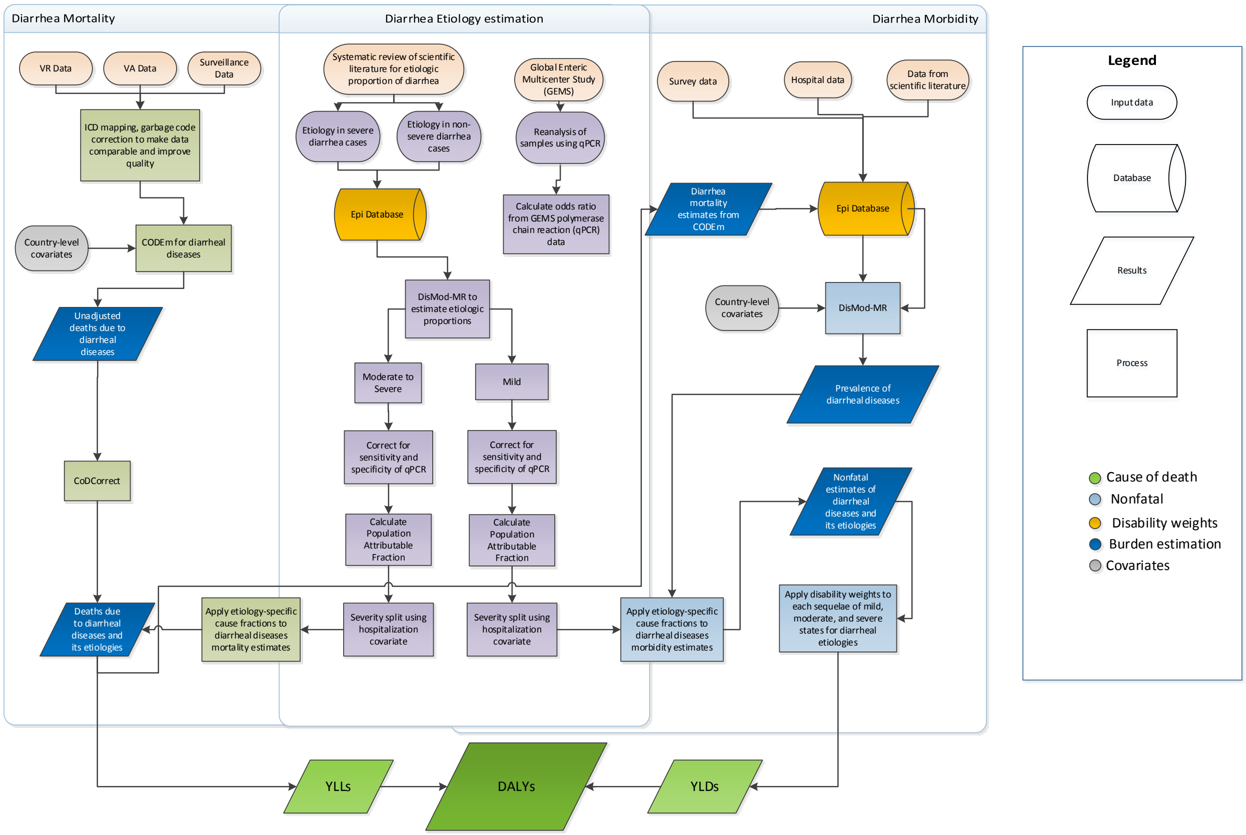

Input data

Diarrheal disease mortality was estimated in CODEm. We estimated diarrhea mortality separately for males and females and for children under 5 years and older than 5 years. We used all available data from vital registration systems and verbal autopsy. We checked for and excluded outliers from our data by country or region. We also excluded early neonatal mortality data in the Philippines (1994–1998) and India Civil Registration System data for several states (1986–1995).

We conducted a systematic literature review for the proportion of diarrhea cases that tested positive for the pathogens listed above. We updated our review of literature to include studies published between January 2012 and May 2015. We used inclusion criteria of diarrhea as the case definition, studies with a sample size of at least 100, and studies with at least one year of follow-up. We excluded studies that reported on diarrheal outbreaks exclusively and those that used acute gastroenteritis with or without diarrhea. We identified 2,847 studies, of which 152 met our criteria of inclusion and were included. We extracted data points for location, sex, year, and age. We assigned an age range based on the prevalence-weighted mean age of diarrhea in the appropriate year/sex/location if the age of the study participants was not reported.

Our literature review extracted the proportion of any enteropathogenic *Escherichia coli* (EPEC) without differentiating between typical (tEPEC) and atypical (aEPEC). In order to be consistent with the odds ratios that we obtained, we adjusted our proportion estimates of any EPEC to typical EPEC only. This adjustment was informed by a subset of our literature review that reported both atypical and typical EPEC. We estimated a ratio by super-region of tEPEC to any EPEC and adjusted our proportion estimates accordingly. We found that the majority of EPEC diarrhea cases were positive for atypical EPEC, consistent with other published work.[1]

We used the Global Enteric Multicenter Study (GEMS), a seven-site, case-control study of moderate-to-severe diarrhea in children under 5 years,[2] to calculate odds ratios for the diarrheal pathogens. We analyzed raw data for a systematic reanalysis, representative of the distribution of cases and controls by age and site, of roughly half of the 22,000 original GEMS samples that were tested for the presence of pathogen using quantitative polymerase chain reaction (qPCR).[3]

We used country-level covariates to inform our CODEm models. We included covariates for years of education per capita, income per capita, prevalence of malnutrition (weight for age z score <2 standard deviations), population density above 1,000 people per square kilometer, population density below 150 people per square kilometer, access to improved sanitation, access to improved water, Socio-Demographic Index, and rotavirus vaccine coverage.

We modeled *Vibrio cholerae* independently from the other etiologies because of its epidemic tendency. We conducted a systematic review of literature for studies published between January 1980 and June 2015 that reported the proportion of diarrhea cases that tested positive for cholera or the case fatality of cholera. We excluded studies specifically about outbreaks and with less than one year of follow-up.

We also modeled *Clostridium difficile* independently from the etiologies because it was not included as a pathogen in GEMS. We conducted a systematic literature review for the prevalence and incidence of *C. difficile* between January 1990 and May 2015. We used inpatient and outpatient hospital visits coded for *C. difficile* as our incidence data. However, nearly all of the hospital data came from Western countries.

Modeling strategy

We evaluated our diarrheal disease cause of death models using in and out of sample predictive performance. We estimated diarrheal disease etiologies separately from overall diarrhea mortality using a counterfactual strategy for enteric adenovirus, *Aeromonas*, *Entamoeba histolytica* (amoebiasis), *Campylobacter enteritis*, cryptosporidiosis, typical enteropathogenic *Escherichia coli* (t-EPEC), enterotoxigenic *Escherichia coli* (ETEC), norovirus, non-typhoidal salmonella infections, rotavirus, and *Shigella*. *Vibrio cholerae* and *Clostridium difficile* were modeled separately.

We dichotomized the continuous qPCR test result using the value of the cycle threshold (Ct) that accurately discriminated between cases and controls. We used the lower Ct value that

represented the smallest false positive samples (positive in non-diarrhea samples) when we had multiple Ct values for the cutpoint. The Ct values range from 0 to 35 cycles representing the relative concentration of the target gene in the stool sample. A low value indicates a higher concentration of the pathogen while a value of 35 indicates the absence of the target in the sample.

The case definition for each pathogen is a Ct value that is below the established cutoff point. We used a mixed effects conditional logistic regression model to calculate the odds ratio by age for each of our pathogens.

We modeled the proportion data using the meta-regression tool DisMod-MR to estimate the proportion of positive diarrhea cases for each separate etiology by location/year/age/sex and to adjust for the covariates.

The population attributable fraction (PAF) was calculated from the proportion of severe diarrhea cases that are positive for each etiology. This is a counterfactual approach, meaning that the PAF represents the relative reduction in diarrhea mortality if there was no exposure to a given etiology. As diarrhea can be caused by multiple pathogens and the pathogens may co-infect, PAFs can overlap and add up to more than 100%. We calculated the PAF from the proportion of severe diarrhea cases that are positive for each etiology. We assumed that hospitalized diarrhea cases are a proxy of severe and fatal cases. We defined severe cases as those presenting with severe dehydration, sunken eyes, convulsion, shock, and coma.[4] We used the following formula to estimate PAF:[5]

$$PAF = Proportion * (1 - \frac{1}{OR})$$

Where *Proportion* is the proportion of diarrhea cases positive for an etiology and *OR* is the odds ratio of diarrhea given the presence of the pathogen. We used the estimated sensitivity and specificity of the laboratory diagnostic technique used in the GEMS study compared to the qPCR case definition to adjust our proportion before we computed the PAF:[6]

$$Proportion_{True} = \frac{(Proportion_{Observed} * Specificity - 1)}{(Sensitivity + Specificity - 1)}$$

We used this correction for the "proportion" to account for the fact that the proportions we used are based on a new test that is not consistent with the case definition from GEMS (qPCR versus GEMS conventional laboratory testing for pathogens).

For cholera specifically, we used the literature review to estimate expected number of cholera cases for each country-year using the incidence of diarrhea, estimated using DisMod-MR, and the proportion of diarrhea cases that are positive for cholera. We assigned cholera PAF using odds ratios from the qPCR results. We compared this expected number of cholera cases to the number reported to the World Health Organization.[7] We modeled the underreporting fraction to correct the cholera case notification data for all countries. We used the age-specific

proportion of positive cholera samples in DisMod and our incidence estimates to predict the number of cholera cases for each sex/year/location. Finally, we modeled the case fatality ratio of cholera using DisMod-MR and to estimate the number of cholera deaths.

For *C. difficile*, we modeled incidence and mortality in DisMod-MR for each age, sex, year, location. DisMod-MR is a Bayesian meta-regression tool that uses spatio-temporal information as priors to estimate prevalence, incidence, remission, and mortality for *C. difficile* infection. DisMod-MR uses a compartmental model to relate prevalence, incidence, remission, and mortality. We set remission in our model to 1 month.

There are several key updates to the approach to diarrhea etiology estimation in GBD 2015 compared to GBD 2013. There are new data used in the CODEm models and in the diarrhea proportion DisMod models. We also introduced qPCR diagnostic results from a retest of a systematic sample by age and site from the original GEMS stool samples. The qPCR diagnostic is more sensitive in detecting some pathogens, particularly bacterial ones, than the conventional laboratory test diagnostics used in the primary GEMS analysis and in GBD 2013. These qPCR results impact the odds ratios of diarrhea given pathogen detection and also the exposure which is the proportion of diarrhea cases positive for each pathogen. We adjusted the proportion estimates from the literature review by the sensitivity and specificity of the laboratory diagnostics to the new qPCR case definition to be consistent with the odds ratios. In general, this increased the attributable fractions for each pathogen.

References

1 Ochoa TJ, Barletta F, Contreras C, Mercado E. New insights into the epidemiology of enteropathogenic Escherichia coli infection. *Trans R Soc Trop Med Hyg* 2008; **102**: 852–6.

2 Kotloff KL, Nataro JP, Blackwelder WC, *et al.* Burden and aetiology of diarrhoeal disease in infants and young children in developing countries (the Global Enteric Multicenter Study, GEMS): a prospective, case-control study. *Lancet Lond Engl* 2013; **382**: 209–22.

3 Liu J, Gratz J, Amour C, *et al.* A laboratory-developed TaqMan Array Card for simultaneous detection of 19 enteropathogens. *J Clin Microbiol* 2013; **51**: 472–80.

4 World Health Organization, Department of Child and Adolescent Health and Development. Handbook IMCI integrated management of childhood illness. Geneva: World Health Organization, Deparment of Child and Adolescent Health and Development, 2005.

5 Miettinen OS. Proportion of disease caused or prevented by a given exposure, trait or intervention. *Am J Epidemiol* 1974; **99**: 325–32.

6 Reiczigel J, Földi J, Ozsvári L. Exact confidence limits for prevalence of a disease with an imperfect diagnostic test. *Epidemiol Infect* 2010; **138**: 1674–8.

7 World Health Organization. Global Health Observatory data repository: Cholera. http://apps.who.int/gho/data/node.main.174?lang=en (accessed April 20, 2016).

Part 4: Central Computation

1. CodCorrect

1.1 Objective of CodCorrect

As mentioned in the main text, the Causes of Death models are cause-specific. As such, there is no guarantee that the sum of these models will equal the results of the all-cause mortality estimates or that model results of child causes add up to the parent model results. The CoDCorrect process is used to make the Causes of Death and all-cause mortality estimates internally consistent using a very simple algorithm.

1.2 Algorithm and levels

The core algorithm remains the same as it did in GBD 2013. The equation can be written as follows:

$$CD_{lyasjd} = D_{lyasjd}\left(\frac{PD_{lyasjd}}{\sum_{j=1}^{j=k} D_{lyasjd}}\right)$$

Where CD_{lyasjd} is the corrected number of deaths for a location *l*, year *y*, age *a*, sex *s*, cause *j*, and draw *d*. PD_{lyasjd} is the parent cause deaths for a location *l*, year *y*, age *a*, sex *s*, cause *j*, and draw *d*. D_{lyasjd} is the uncorrected number of deaths estimated from a cause-specific model for a location *l*, year *y*, age *a*, sex *s*, cause *j*, and draw *d*.

The CoDCorrect process starts by rescaling the Level 1 causes to match the all-cause mortality estimates (which is used for PD_{lyasjd} in the above equation). Level 2 causes are then rescaled to their corrected parent causes. This continues until all levels of the hierarchy have been rescaled. Causes and their levels within the CoDCorrect hierarchy can be found in Appendix Table 19.

Unlike in GBD 2013, HIV is not included in the CoDCorrect process for GBD 2015. To account for this change, Level 1 CoDCorrect causes are rescaled to HIV-deleted mortality estimates which are produced as part of the mortality and HIV estimation process. Results from the GBD version of Spectrum are added to the post-CoDCorrect death estimates, along with fatal discontinuities and imported cases, to generate the full set of death estimates.

1.3 Diagnostic results of CodCorrect by cause and location

For more detail on diagnostic results of CodCorrect by cause and location see Appendix Table 20.

2. Years of Life Lost (YLLs) calculation

Years of life lost due to premature mortality (YLLs) were computed for 596 geographies and 36 years. The YLL is a metric that is computed by multiplying the number of estimated deaths by the standard life expectancy at age of death. The metric therefore highlights premature deaths by applying a larger weight to deaths that occur at younger age groups. We propagated uncertainty from CoDCorrected deaths for all demographics. The core equation can be written as follows:

$$YLL = \sum_{c=1,a=0,s=1}^{\infty} d_{cas} e_a$$

3. Socio-Demographic Index (SDI) analysis

3.1 Development of revised SDI indicator

The Socio-Demographic Index (SDI) was calculated using the Human Development Index (HDI) methodology, wherein an index value was determined for each covariate input (log lag dependent income per capita (LDI), average educational attainment in the population over age 15, and total fertility rate (TFR)):

$$I_{Cly} = {(C_{ly} - \min(C))}/{(\max(C) - \min(C))}$$

Where I_{cly} – the index for covariate *C*, location *l*, and year *y* – is equal to the difference between the value of that covariate in that location-year and the minimum observed value of the covariate in any location over the time interval divided by the observed range. An additional innovation for GBD 2015 was to incorporate subnational locations where estimated (resulting in 519 unique administrative units) for the entire estimation period of 1980 to 2015. The Socio-Demographic Index is then the geometric mean of these three indices.

For LDI and TFR, we noted diminishing gains in life expectancy at birth and 5q0 at the higher and lower terminals, respectively. Due to the significance of these values in indexing, we aimed to identify the point at which increasing income or reducing fertility no longer resulted in improved child mortality or life expectancy. We tested various restrictions, and found that capping LDI at 60,000 and setting a TFR floor at 1 resulted in improved correlations with resultant health indicators.

We further aimed to validate the use of SDI by regressing it in a variety of forms against life expectancy at birth, 5q0, 35q15, and 20q50. We found that SDI generally is as capable of predicting these demographic indicators as the previous SDS, and also as the inputs. We also found that in incorporating year, we did not substantially reduce the coefficients for SDI (Appendix Table 24). Additionally, in testing lags of 2-10 years, we found the version with no lag to be the most predictive (Appendix Table 23).

3.2 Age-sex-specific relationships between vigintile and death rates

In order to evaluate the relationship between SDI and mortality, we fit a simple least-squares regression using a smoothing spline on SDI for every cause in levels 1, 2, and 3 of the GBD cause hierarchy:

$$\ln(m_{l,y,a,s,c}) = \sum_{i=0}^{d+k} \beta_i B_{i,d}(SDI) + \gamma_U + \gamma_E + \gamma_C + \gamma_O + \varepsilon_{l,y,a,s,c}$$

where:

- $\ln(m_{l,y,a,s,c})$ is the log mortality rate in location *l* and year *y*, and for age *a*, sex *s*, and cause *c*
- $\sum_{i=0}^{d+k} \beta_i B_{i,d}(SDI)$ is resultant parametric curve, of degree *d* and interior knots *k*, of a linear combination of basis splines $B_{i,d}(SDI)$
- γ_U is a dummy variable for the United States
- γ_E is a dummy variable for the GBD region Eastern Europe
- γ_C is a dummy variable for the GBD region Central Asia
- γ_O is a dummy variable for the GBD region Oceania
- $\varepsilon_{l,y,a,s,c}$ is the error term for location *l* and year *y*, and for age *a*, sex *s*, and cause *c*

We ran regressions separately by age, sex, and cause, using all location-years. Dummy variables were included for locations that were identified in modeling to skew fit due to significant deviation from levels of mortality observed elsewhere at similar levels of SDI. Because of the inclusion of 51 states and the District of Columbia, the United States collectively had an undue influence on the shape of the relationship, so a separate dummy variable was included for the U.S. Because of the mortality crisis in Eastern Europe and Central Asia after the collapse of the Soviet Union, we included a dummy variable to adjust for the mean difference in these regions.

We used the same modeling set up but used the logit of the share of population in each age group as the dependent variable to estimate a smoothed relationship between population age-structure and SDI. Predictions for each age group at each level of SDI were rescaled to sum to 100%.

3.3 Computation of summary measures ASDR, life expectancy, etc.

Due to the more reliable estimation at more aggregate levels of cause specificity, we imposed a top-down hierarchical scaling scheme, in which the level 1 causes were scaled to the predictions for all-cause mortality, then level 2 causes were scaled to their scaled level 1 parents, and once more for level 3 to level 2. Having a complete set of age-specific mortality rates, we were then able to produce a full set of age-standardized death rates for every SDI level. We evaluated this relationship at each .01 value of SDI. Using the predicted age-specific death rates and predicted population structure at each single unit level of SDI, we computed the all-age death rates expected at each level. Age-specific all-cause mortality rates were used to construct an abridged life table for each single unit value of SDI from .01 to 0.99. Using the abridged life table, we computed life expectancy and probability of death for ages 0 to 5 (5q0), 15 to 50 (35q15), and 50 to 70 (20q50). Additionally, this provided a mean age at death for each GBD age group, allowing for implementation of the GBD life standard to calculate expected years of life lost (YLLs) by cause, age, sex, and SDI level.

4. Calculation of the population age standard

The World Health Organization (WHO) last released a global age standard in 2001. The world has experienced substantial change in global demography since then, inciting the need for a new standard population for computing age-standardized rates. We calculated a new global age standard for GBD

2013. This same standard was used for GBD 2015. The paragraph below explains how we created this new age standard.

The old WHO standard used population data from the United Nations Population Division's World Population Prospects (WPP) 1998 revision. It was calculated using the non-weighted average across all countries of each age-specific population percentage in each 5-year age group up to the terminal group of 100+, using estimates from the years 2000-2025. We mirrored this technique with three key adaptations: (1) we used population data from the WPP 2012 revision for years 2010-2035; (2) we used the interpolated population in age group 0-1 provided in the WPP 2012 revision to separate ages 0-1 and 1-4; and (3) we applied the non-weighted mean proportion across countries of populations in the early neonatal (0-6 days), late neonatal (7-27 days), and post neonatal (28-364 days) age groups from the GBD 2013 study in years 2010-2013 to the new age 0-1 standard to get standard populations in these age groups. This new standard reflects an up-to-date age structure that facilitates more relevant comparisons between countries when computing age-standardized rates.

5. SEV scalar covariates

The "SEV scalar" covariates used in various models are created from the Summary Exposure Variables (SEV). For a given cause, these reflect the average exposure to all of the risk factors related to that cause. SEVs are calculated using the following formula: 1 / (1-PAF), where PAF is the population attributable fraction. They are log-transformed. In cause of death models, the SEV covariate allows a modeler to reflect the exposure of all GBD risks for a cause in a single summary variable rather than using exposure to each of the individual risks, separately.

Part 5. Data sources and abbreviations

Data input sources

GBD 2015 incorporated a large number and wide variety of input sources to estimate mortality, causes of death and illness, and risk factors for 195 countries and territories from 1990-2015. These input sources are accessible through an interactive citation tool available in IHME's GHDx.

Users can retrieve citations for a specific GBD component, cause or risk, and geography by choosing from the available selection boxes. They can then view and access GHDx records for input sources and export a CSV file that includes the GHDx metadata, citations, and information about where the data were used in GBD. Additional metadata for each input source are available through the citation tool, as required by the GATHER statement.

The citation tool is accessible through the GHDx at http://ghdx.healthdata.org/global-burden-disease-study-2015.

List of abbreviations

5q0: probability of death from birth to age 5 years

20q50: probability of death from age 50 years to 70 years

35q15: probability of death from age 15 years to 50 years

45q15: probability of death from age 15 years to 60 years

ART: antiretroviral therapy

BTL: basic tabulation list

CBH: complete birth histories

CDR: crude death rates

CHERG: Child Health Epidemiology Research Group

CKD: chronic kidney disease

CKD-DM: chronic kidney disease deaths attributable to diabetes

COD: causes of death

CODEm: cause of death ensemble modeling

COPD: chronic obstructive pulmonary disease

CR: cancer registry

CRS: Civil Registration System

CVD: cardiovascular disease

DALY: disability-adjusted life year

DSP: Disease Surveillance Points

ELISA: enzyme-linked immunosorbent assay

EPEC: enteropathogenic *E. coli*

EPP: Estimation and Projection Package

ETEC: enterotoxigenic *E. coli*

GATHER: Guidelines for Accurate and Transparent Health Estimates Reporting

GBD: Global Burden of Disease

GEMS: Global Enteric Multicenter Study

Hib: *Haemophilus influenzae* type B

HIV CDR: Crude death rate due to HIV/AIDS

IARC: International Agency for Research on Cancer

ICD: International Classification of Disease

IHD: ischemic heart disease

LDI: lag distributed income per capita

LRI: lower respiratory infection

MAP: Malaria Atlas Project

MCCD: Medical Certification of Causes of Death

MCEE: Maternal and Child Epidemiology Estimation group

MI: mortality/incidence ratio

MM: maternal mortality

MMR: maternal mortality ratio

MMS: Maternal Mortality Surveillance

NCD: non-communicable disease

PAF: population-attributable fraction

PMTCT: prevention of mother-to-child transmission

RMSE: root mean square error

RSV: respiratory syncytial virus

SBH: summary birth history

SCD(R): Survey of Causes of Death (Rural)

SDI: Socio-Demographic Index

SEER: Surveillance, Epidemiology, and End Results Program

SRS: Sample Registration System

ST-GPR: spatiotemporal Gaussian process regression

TAC: TaqMan Array Card

TB: tuberculosis

UI: uncertainty interval

UN: United Nations

VA: verbal autopsy

VR: vital registration

WHO: World Health Organization

YLD: years lived with disability

YLL: years of life lost

Appendix Figure 1. Number of all−cause mortality data sources by country, 1950−2015

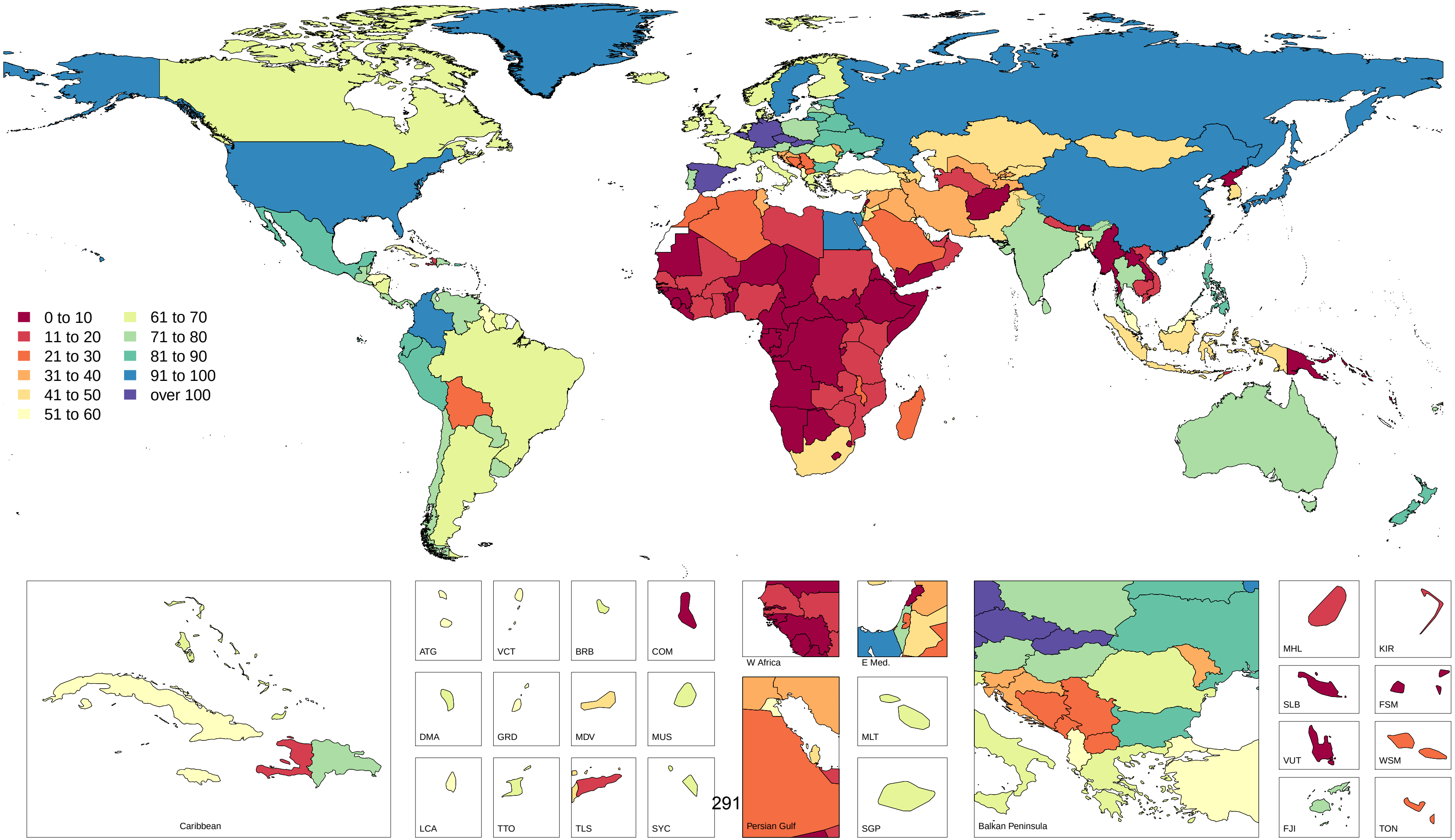

Appendix Figure 2: Sibling history correction for zero-survivorship, by sex

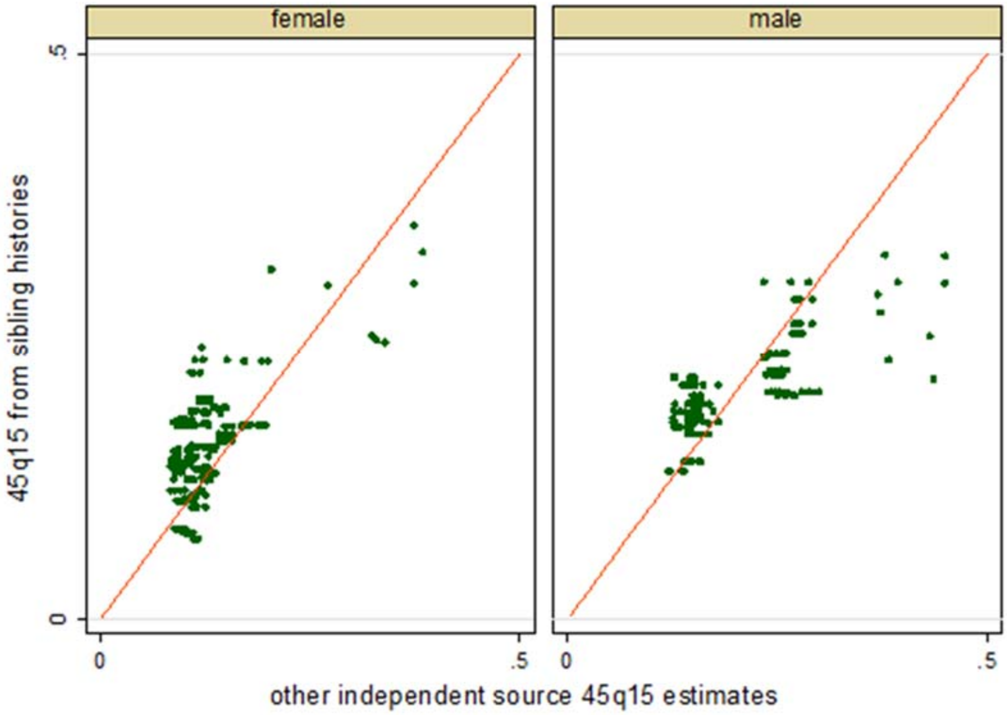

Appendix Figure 3: Comparison of GBD 2015 adolescent mortality in India to sample registration system, Demographic and Health Surveys (DHS), and UN Population Division's World Population Prospects (WPP) 2015

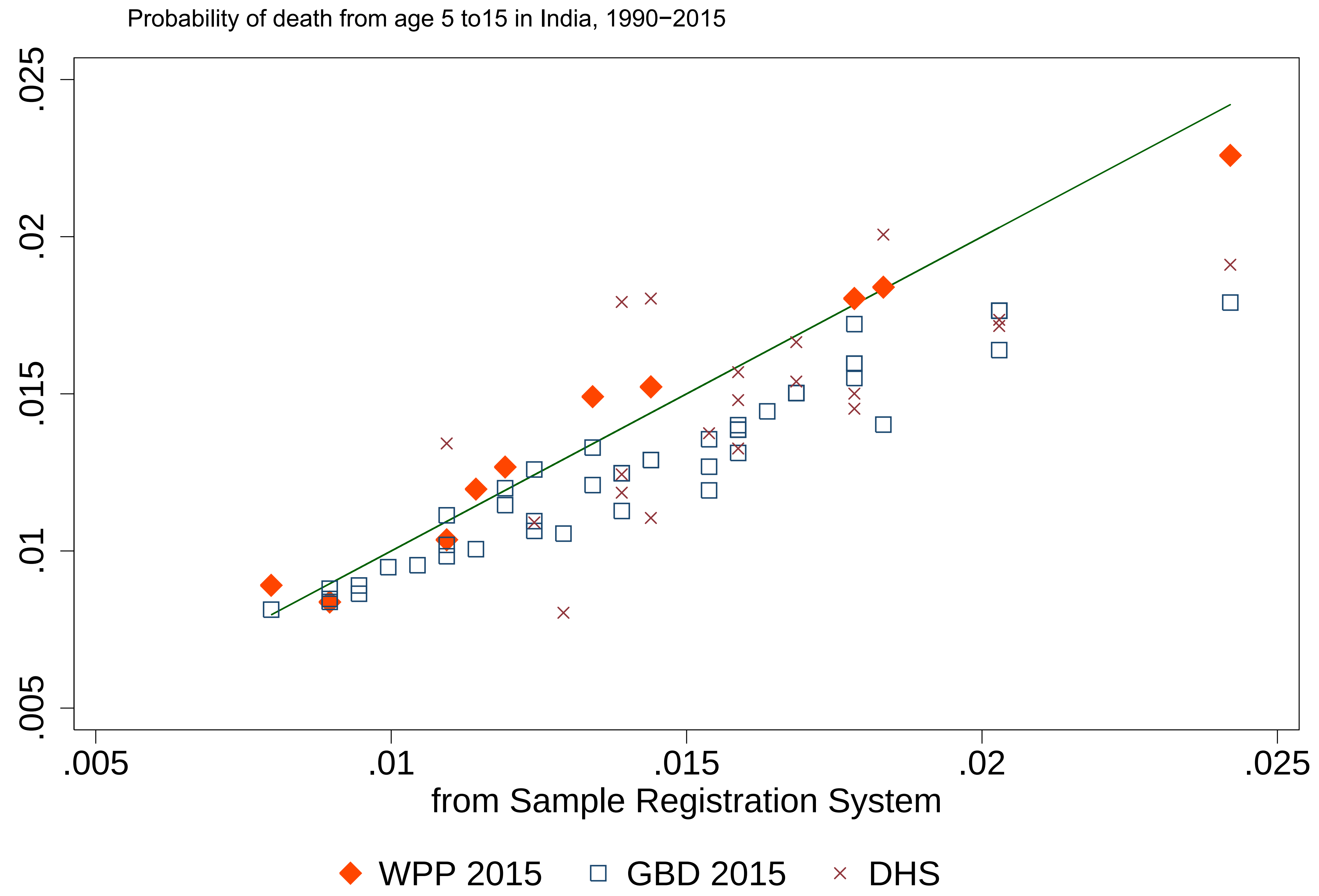

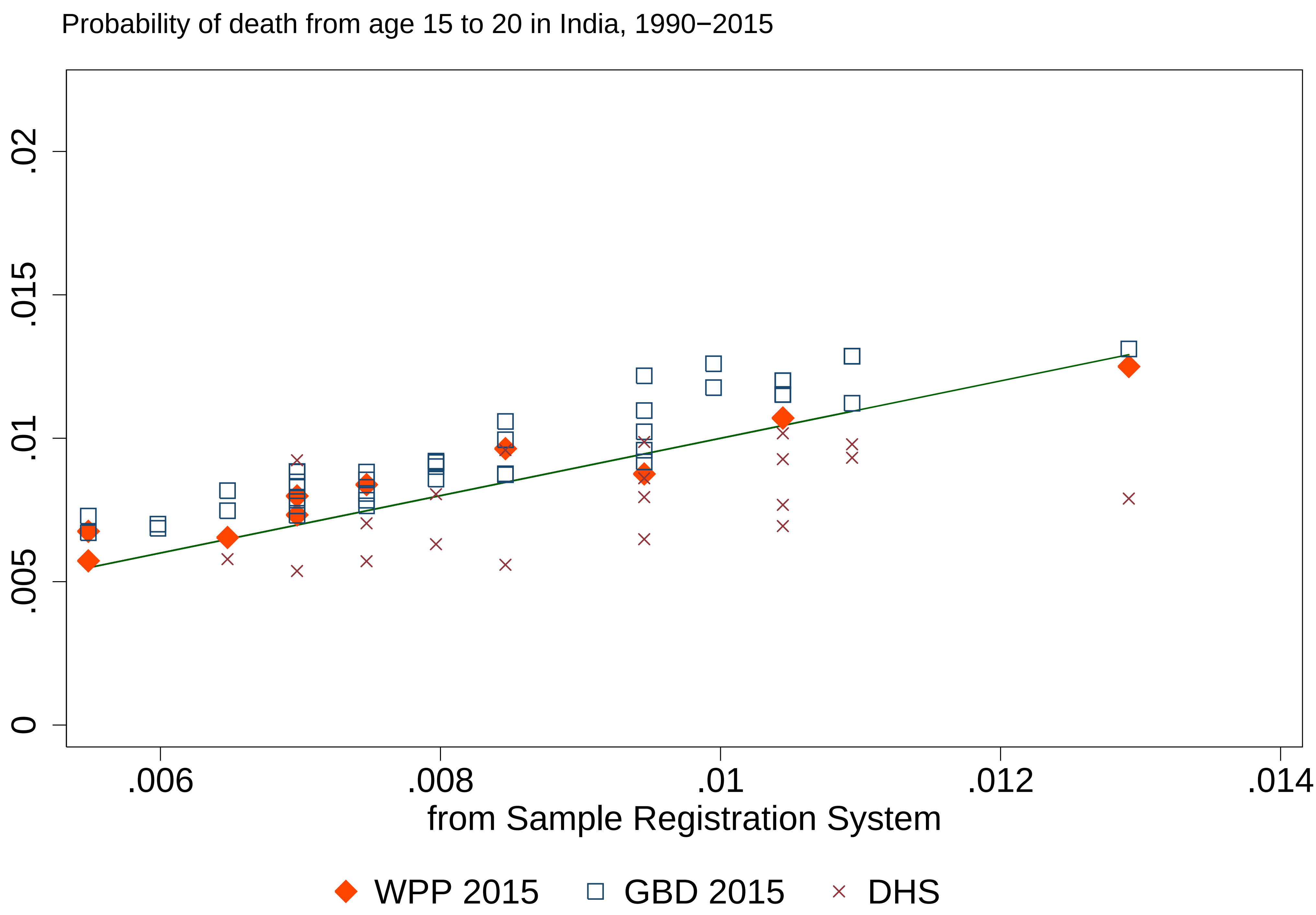

Probability of death from age 15 to 20 in India, 1990−2015
0
.005
.01
.015
.02
.006
.008
.01
.012
.014
from Sample Registration System
WPP 2015
GBD 2015
DHS

Probability of death from age 10 to 15 in India, 1990−2015

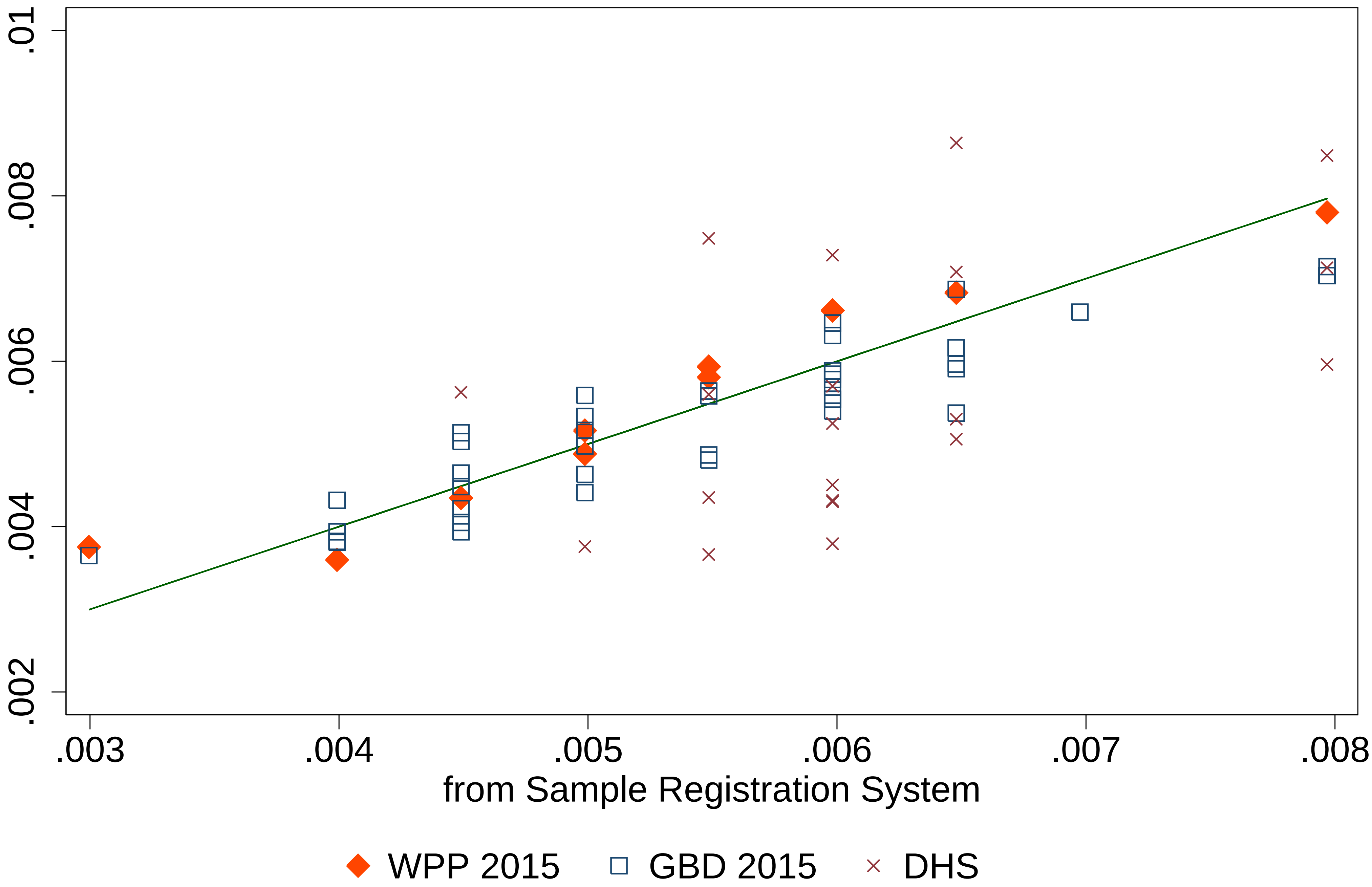

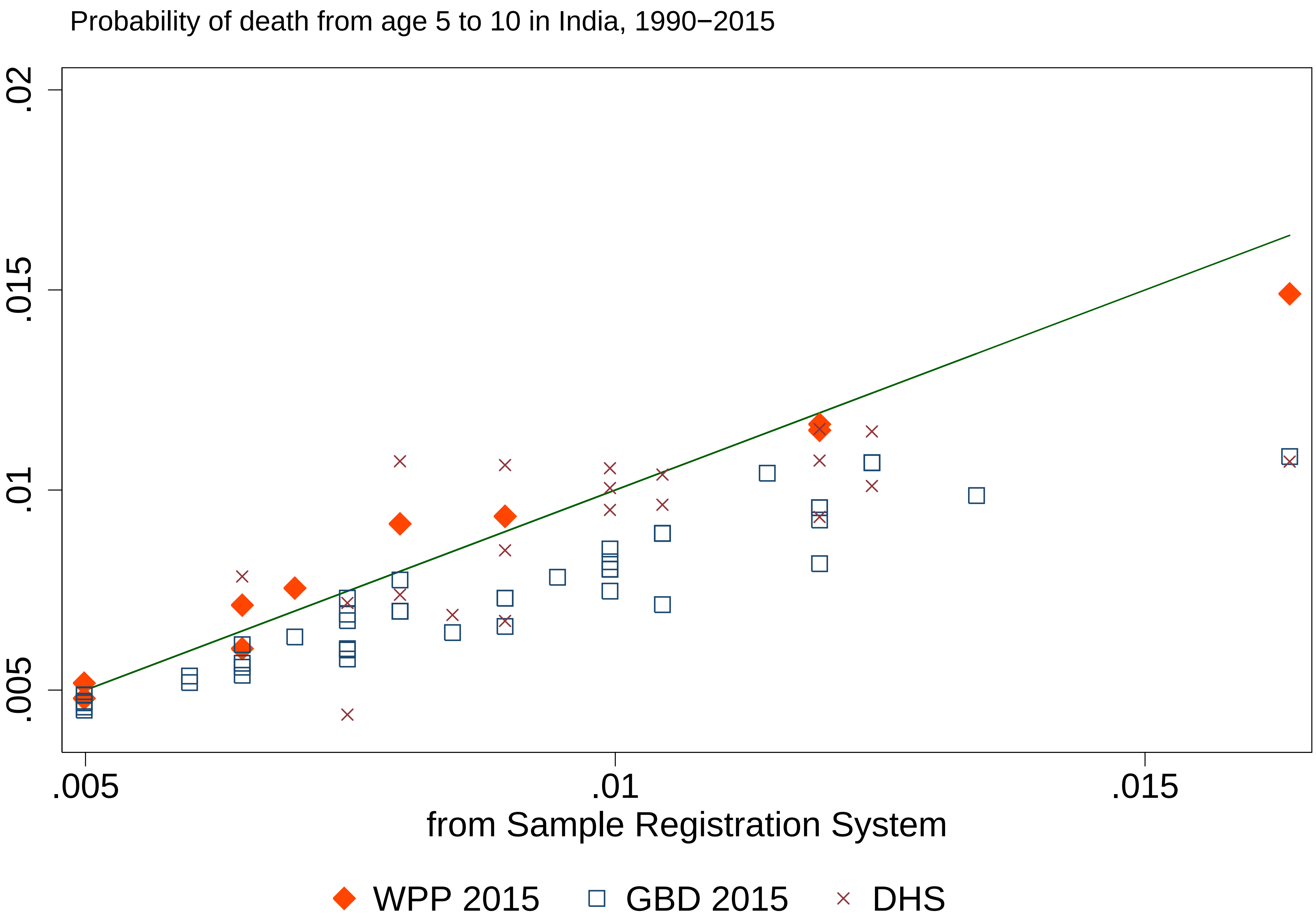

Probability of death from age 5 to 10 in India, 1990−2015
.005
.01
.015
.02
.005
.01
.015
from Sample Registration System
WPP 2015
GBD 2015
DHS

Appendix Table 1: GBD 2015 geographies with HIV/AIDS modeling strategy group	
**Location**	**Group**
Afghanistan	2C
Albania	2B
Algeria	2B
American Samoa	2B
Andorra	2C
Angola	1A
Antigua and Barbuda	2A
Argentina	2A
Armenia	2B
Australia	2A
Austria	2A
Azerbaijan	2B
Bahrain	2B
Bangladesh	2C
Barbados	2A
Belarus	2B
Belgium	2A
Belize	2B
Benin	1A
Bermuda	2A
Bhutan	2C
Bolivia	2B
Bosnia and Herzegovina	2B
Botswana	1A
**Brazil**	*
Acre	2B
Alagoas	2B
Amapa	2B
Amazonas	2B
Bahia	2B
Ceara	2B
Distrito Federal	2B
Espirito Santo	2B
Goias	2B
Maranhao	2B
Mato Grosso	2B
Mato Grosso do Sul	2B
Minas Gerais	2B
Para	2B
Paraiba	2B
Parana	2B
Pernambuco	2B
Piaui	2B
Rio Grande do Norte	2B
Rio Grande do Sul	2B
Rio de Janeiro	2B
Rondonia	2B

Appendix Table 1: GBD 2015 geographies with HIV/AIDS modeling strategy group	
**Location**	**Group**
Roraima	2B
Santa Catarina	2B
Sao Paulo	2B
Sergipe	2B
Tocantins	2B
Brunei	2B
Bulgaria	2A
Burkina Faso	1A
Burundi	1A
Cambodia	2B
Cameroon	1A
Canada	2A
Cape Verde	1A
Central African Republic	1A
Chad	1A
Chile	2A
**China**	*
Anhui	2B
Beijing	2B
Chongqing	2B
Fujian	2B
Gansu	2B
Guangdong	2B
Guangxi	2B
Guizhou	2B
Hainan	2B
Hebei	2B
Heilongjiang	2B
Henan	2B
Hong Kong Special Administrative Region of China	2B
Hubei	2B
Hunan	2B
Inner Mongolia	2B
Jiangsu	2B
Jiangxi	2B
Jilin	2B
Liaoning	2B
Macao Special Administrative Region of China	2C
Ningxia	2B
Qinghai	2B
Shaanxi	2B
Shandong	2B
Shanghai	2B
Shanxi	2B
Sichuan	2B
Tianjin	2B
Tibet	2B

Appendix Table 1: GBD 2015 geographies with HIV/AIDS modeling strategy group

Location	Group
Xinjiang	2B
Yunnan	2B
Zhejiang	2B
Colombia	2A
Comoros	2C
Congo	1A
Costa Rica	2A
Cote d'Ivoire	1A
Croatia	2A
Cuba	2A
Cyprus	2B
Czech Republic	2A
Democratic Republic of the Congo	1A
Denmark	2A
Djibouti	1A
Dominica	2B
Dominican Republic	1A
Ecuador	2B
Egypt	2B
El Salvador	2B
Equatorial Guinea	1A
Eritrea	1A
Estonia	2A
Ethiopia	1A
Federated States of Micronesia	2C
Fiji	2B
Finland	2A
France	2A
Gabon	1A
Georgia	2B
Germany	2A
Ghana	1A
Greece	2A
Greenland	2B
Grenada	2B
Guam	2B
Guatemala	2A
Guinea	1A
Guinea-Bissau	1A
Guyana	2B
Haiti	1A
Honduras	2B
Hungary	2A
Iceland	2A
**India**	*
Andhra Pradesh, Rural	1B
Andhra Pradesh, Urban	1B

Appendix Table 1: GBD 2015 geographies with HIV/AIDS modeling strategy group	
**Location**	**Group**
Arunachal Pradesh, Rural	1B
Arunachal Pradesh, Urban	1B
Assam, Rural	1B
Assam, Urban	1B
Bihar, Rural	1B
Bihar, Urban	1B
Chhattisgarh, Rural	1B
Chhattisgarh, Urban	1B
Delhi, Rural	1B
Delhi, Urban	1B
Goa, Rural	1B
Goa, Urban	1B
Gujarat, Rural	1B
Gujarat, Urban	1B
Haryana, Rural	1B
Haryana, Urban	1B
Himachal Pradesh, Rural	1B
Himachal Pradesh, Urban	1B
Jammu and Kashmir, Rural	1B
Jammu and Kashmir, Urban	1B
Jharkhand, Rural	1B
Jharkhand, Urban	1B
Karnataka, Rural	1B
Karnataka, Urban	1B
Kerala, Rural	1B
Kerala, Urban	1B
Madhya Pradesh, Rural	1B
Madhya Pradesh, Urban	1B
Maharashtra, Rural	1B
Maharashtra, Urban	1B
Manipur, Rural	1B
Manipur, Urban	1B
Meghalaya, Rural	1B
Meghalaya, Urban	1B
Mizoram, Rural	1B
Mizoram, Urban	1B
Nagaland, Rural	1B
Nagaland, Urban	1B
Orissa, Rural	1B
Orissa, Urban	1B
Punjab, Rural	1B
Punjab, Urban	1B
Rajasthan, Rural	1B
Rajasthan, Urban	1B
Sikkim, Rural	1B
Sikkim, Urban	1B
Tamil Nadu, Rural	1B

Appendix Table 1: GBD 2015 geographies with HIV/AIDS modeling strategy group

Location	Group
Tamil Nadu, Urban	1B
Telangana, Rural	1B
Telangana, Urban	1B
The Six Minor Territories, Rural	1B
The Six Minor Territories, Urban	1B
Tripura, Rural	1B
Tripura, Urban	1B
Uttar Pradesh, Rural	1B
Uttar Pradesh, Urban	1B
Uttarakhand, Rural	1B
Uttarakhand, Urban	1B
West Bengal, Rural	1B
West Bengal, Urban	1B
Indonesia	2C
Iran	2B
Iraq	2B
Ireland	2A
Israel	2A
Italy	2A
Jamaica	2B
**Japan**	*
Aichi	2A
Akita	2A
Aomori	2A
Chiba	2A
Ehime	2A
Fukui	2A
Fukuoka	2A
Fukushima	2A
Gifu	2A
Gunma	2A
Hiroshima	2A
Hokkaido	2A
Hyogo	2A
Ibaraki	2A
Ishikawa	2A
Iwate	2A
Kagawa	2A
Kagoshima	2A
Kanagawa	2A
Kochi	2A
Kumamoto	2A
Kyoto	2A
Mie	2A
Miyagi	2A
Miyazaki	2A
Nagano	2A

Appendix Table 1: GBD 2015 geographies with HIV/AIDS modeling strategy group	
**Location**	**Group**
Nagasaki	2A
Nara	2A
Niigata	2A
Oita	2A
Okayama	2A
Okinawa	2A
Osaka	2A
Saga	2A
Saitama	2A
Shiga	2A
Shimane	2A
Shizuoka	2A
Tochigi	2A
Tokushima	2A
Tokyo	2A
Tottori	2A
Toyama	2A
Wakayama	2A
Yamagata	2A
Yamaguchi	2A
Yamanashi	2A
Jordan	2B
Kazakhstan	2A
**Kenya**	*
Baringo	1A
Bomet	1A
Bungoma	1A
Busia	1A
Elgeyo-Marakwet	1A
Embu	1A
Garissa	1A
HomaBay	1A
Isiolo	1A
Kajiado	1A
Kakamega	1A
Kericho	1A
Kiambu	1A
Kilifi	1A
Kirinyaga	1A
Kisii	1A
Kisumu	1A
Kitui	1A
Kwale	1A
Laikipia	1A
Lamu	1A
Machakos	1A
Makueni	1A

Appendix Table 1: GBD 2015 geographies with HIV/AIDS modeling strategy group

Location	Group
Mandera	1A
Marsabit	1A
Meru	1A
Migori	1A
Mombasa	1A
Murang'a	1A
Nairobi	1A
Nakuru	1A
Nandi	1A
Narok	1A
Nyamira	1A
Nyandarua	1A
Nyeri	1A
Samburu	1A
Siaya	1A
TaitaTaveta	1A
TanaRiver	1A
TharakaNithi	1A
TransNzoia	1A
Turkana	1A
UasinGishu	1A
Vihiga	1A
Wajir	1A
WestPokot	1A
Kiribati	2B
Kuwait	2A
Kyrgyzstan	2B
Laos	2C
Latvia	2A
Lebanon	2C
Lesotho	1A
Liberia	1A
Libya	2C
Lithuania	2A
Luxembourg	2A
Macedonia	2B
Madagascar	2C
Malawi	1A
Malaysia	2B
Maldives	2B
Mali	1A
Malta	2A
Marshall Islands	2C
Mauritania	2C
Mauritius	2A
**Mexico**	*
Aguascalientes	2A

Appendix Table 1: GBD 2015 geographies with HIV/AIDS modeling strategy group	
**Location**	**Group**
Baja California	2A
Baja California Sur	2A
Campeche	2A
Chiapas	2A
Chihuahua	2A
Coahuila	2A
Colima	2A
Distrito Federal	2A
Durango	2A
Guanajuato	2A
Guerrero	2A
Hidalgo	2A
Jalisco	2A
Mexico	2A
Michoacan de Ocampo	2A
Morelos	2A
Nayarit	2A
Nuevo Leon	2A
Oaxaca	2A
Puebla	2A
Queretaro	2A
Quintana Roo	2A
San Luis Potosi	2A
Sinaloa	2A
Sonora	2A
Tabasco	2A
Tamaulipas	2A
Tlaxcala	2A
Veracruz de Ignacio de la Llave	2A
Yucatan	2A
Zacatecas	2A
Moldova	2A
Mongolia	2B
Montenegro	2B
Morocco	2B
Mozambique	1A
Myanmar	2C
Namibia	1A
Nepal	2C
Netherlands	2A
New Zealand	2A
Nicaragua	2B
Niger	1A
Nigeria	1A
North Korea	2C
Northern Mariana Islands	2B
Norway	2A

Appendix Table 1: GBD 2015 geographies with HIV/AIDS modeling strategy group	
**Location**	**Group**
Oman	2B
Pakistan	2C
Palestine	2B
Panama	2B
Papua New Guinea	1A
Paraguay	2B
Peru	2B
Philippines	2A
Poland	2A
Portugal	2A
Puerto Rico	2A
Qatar	2B
Romania	2A
Russia	2A
Rwanda	1A
Saint Lucia	2A
Saint Vincent and the Grenadines	2A
Samoa	2C
Sao Tome and Principe	2B
**Saudi Arabia**	*
'Asir	2C
Bahah	2C
Eastern Province	2B
Ha'il	2C
Jawf	2C
Jizan	2C
Madinah	2B
Makkah	2B
Najran	2C
Northern Borders	2C
Qassim	2B
Riyadh	2C
Tabuk	2B
Senegal	1A
Serbia	2B
Seychelles	2B
Sierra Leone	1A
Singapore	2A
Slovakia	2B
Slovenia	2A
Solomon Islands	2C
Somalia	1A
**South Africa**	*
Eastern Cape	1A
Free State	1A
Gauteng	1A
KwaZulu-Natal	1A

Appendix Table 1: GBD 2015 geographies with HIV/AIDS modeling strategy group	
**Location**	**Group**
Limpopo	1A
Mpumalanga	1A
North-West	1A
Northern Cape	1A
Western Cape	1A
South Korea	2A
South Sudan	1A
Spain	2A
Sri Lanka	2A
Sudan	1A
Suriname	2B
Swaziland	1A
**Sweden**	*
Stockholm	2A
Sweden except Stockholm	2A
Switzerland	2A
Syria	2B
Taiwan	2B
Tajikistan	2B
Tanzania	1A
Thailand	2B
The Bahamas	2B
The Gambia	1A
Timor-Leste	2C
Togo	1A
Tonga	2B
Trinidad and Tobago	2A
Tunisia	2B
Turkey	2B
Turkmenistan	2B
Uganda	1A
Ukraine	2A
United Arab Emirates	2C
**United Kingdom**	*
East Midlands	2A
East of England	2A
Greater London	2A
North East England	2A
North West England	2A
Northern Ireland	2A
Scotland	2A
South East England	2A
South West England	2A
Wales	2A
West Midlands	2A
Yorkshire and the Humber	2A
**United States**	*

Appendix Table 1: GBD 2015 geographies with HIV/AIDS modeling strategy group

Location	Group
Alabama	2A
Alaska	2A
Arizona	2A
Arkansas	2A
California	2A
Colorado	2A
Connecticut	2A
Delaware	2A
District of Columbia	2A
Florida	2A
Georgia	2A
Hawaii	2A
Idaho	2A
Illinois	2A
Indiana	2A
Iowa	2A
Kansas	2A
Kentucky	2A
Louisiana	2A
Maine	2A
Maryland	2A
Massachusetts	2A
Michigan	2A
Minnesota	2A
Mississippi	2A
Missouri	2A
Montana	2A
Nebraska	2A
Nevada	2A
New Hampshire	2A
New Jersey	2A
New Mexico	2A
New York	2A
North Carolina	2A
North Dakota	2A
Ohio	2A
Oklahoma	2A
Oregon	2A
Pennsylvania	2A
Rhode Island	2A
South Carolina	2A
South Dakota	2A
Tennessee	2A
Texas	2A
Utah	2A
Vermont	2A
Virginia	2A

Appendix Table 1: GBD 2015 geographies with HIV/AIDS modeling strategy group	
**Location**	**Group**
Washington	2A
West Virginia	2A
Wisconsin	2A
Wyoming	2A
Uruguay	2A
Uzbekistan	2B
Vanuatu	2C
Venezuela	2A
Vietnam	2C
Virgin Islands, U.S.	2B
Yemen	2C
Zambia	1A
Zimbabwe	1A

*estimated at the sub-national level and aggregated to produce national estimates

Appendix Table 2: Number of years with data points by country and decade							
	1950s	1960s	1970s	1980s	1990s	2000s	2010s
**High-income North America**							
Canada	10	10	10	10	10	10	5
Greenland	16	18	20	16	11	10	4
United States	10	10	10	20	20	20	6
**Australasia**							
Australia	10	10	10	10	10	14	6
New Zealand	10	10	10	12	20	20	6
**High-income Asia Pacific**							
Brunei	10	7	9	8	8	15	6
Japan	10	10	11	20	20	20	7
Singapore	10	10	10	10	10	10	8
South Korea	0	0	6	12	12	10	6
**Western Europe**							
Andorra	5	0	0	0	4	14	2
Austria	15	10	10	10	10	10	8
Belgium	20	21	21	20	20	20	6
Cyprus	0	1	7	10	13	10	4
Denmark	11	10	10	10	10	13	5
Finland	12	10	10	10	10	10	6
France	11	10	10	10	10	10	4
Germany	8	20	20	20	20	20	6
Greece	9	10	10	10	10	10	5
Iceland	11	10	10	10	10	10	6
Ireland	10	10	10	10	10	10	8
Israel	10	10	10	10	14	20	4
Italy	11	10	10	10	10	12	5
Luxembourg	10	12	10	10	10	10	8
Malta	10	10	10	10	10	10	8
Netherlands	10	10	10	10	10	10	6
Norway	11	10	10	10	10	10	6
Portugal	15	10	11	11	10	9	6
Spain	20	20	20	20	20	20	6
Sweden	11	10	10	17	20	20	8
Switzerland	11	11	10	10	10	12	8
United Kingdom	10	10	10	10	10	10	4
**Southern Latin America**							
Argentina	10	10	6	11	11	10	7
Chile	10	10	11	12	11	11	8
Uruguay	10	10	11	11	11	14	6
**Eastern Europe**							
Belarus	2	20	20	14	11	14	7
Estonia	2	20	20	14	10	10	4
Latvia	2	20	20	11	10	10	4
Lithuania	2	20	20	13	10	10	7
Moldova	0	0	0	8	11	11	6
Russia	2	20	20	11	11	20	8
Ukraine	2	20	20	14	11	14	6
**Central Europe**							
Albania	10	10	0	3	12	17	2
Bosnia and Herzegovina	0	0	0	5	4	12	4
Bulgaria	20	16	11	10	12	10	3
Croatia	0	0	0	5	10	10	5
Czech Republic	20	20	20	16	10	10	6
Hungary	15	11	10	10	10	10	6
Macedonia	0	0	0	2	10	13	4
Montenegro	0	1	1	1	6	14	4
Poland	11	10	11	10	12	10	6
Romania	4	11	11	10	13	10	4
Serbia	1	1	1	0	7	10	7
Slovakia	20	20	20	20	12	10	9

Appendix Table 2: Number of years with data points by country and decade							
	**1950s**	**1960s**	**1970s**	**1980s**	**1990s**	**2000s**	**2010s**
Slovenia	0	0	0	10	10	10	6
**Central Asia**							
Armenia	0	0	0	11	10	15	6
Azerbaijan	0	0	0	11	10	17	5
Georgia	0	0	0	8	10	12	11
Kazakhstan	0	0	0	8	14	12	6
Kyrgyzstan	0	0	0	11	14	12	10
Mongolia	0	2	4	8	12	15	4
Tajikistan	0	0	0	8	11	14	5
Turkmenistan	0	0	0	7	9	1	2
Uzbekistan	0	0	0	7	11	12	0
**Central Latin America**							
Colombia	10	10	11	18	18	16	7
Costa Rica	9	10	12	13	11	11	8
El Salvador	10	10	11	12	13	16	7
Guatemala	10	10	10	11	13	14	9
Honduras	10	10	11	10	3	8	6
Mexico	10	10	11	12	13	15	9
Nicaragua	10	8	7	4	14	15	8
Panama	9	11	12	11	12	12	9
Venezuela	10	10	11	11	11	11	6
**Andean Latin America**							
Bolivia	8	2	3	3	4	9	0
Ecuador	6	10	12	13	16	13	10
Peru	10	9	11	14	14	18	13
**Caribbean**							
Antigua and Barbuda	10	8	9	6	12	10	4
The Bahamas	0	8	12	14	13	11	6
Barbados	10	10	10	10	6	11	6
Belize	10	10	10	9	13	12	9
Bermuda	10	11	10	10	10	11	8
Cuba	1	10	10	11	10	10	7
Dominica	10	8	10	10	10	10	7
Dominican Republic	10	10	12	13	11	15	6
Grenada	10	10	5	3	13	10	7
Guyana	10	3	6	3	11	14	5
Haiti	0	0	1	2	1	5	1
Jamaica	10	11	5	11	4	12	4
Puerto Rico	10	10	10	10	18	19	5
Saint Lucia	10	4	8	10	10	10	6
Saint Vincent and the Grenadines	7	5	7	8	7	10	7
Suriname	8	6	8	9	11	12	6
Trinidad and Tobago	10	10	11	11	10	14	0
Virgin Islands, U.S.	10	9	6	10	8	10	3
**Tropical Latin America**							
Brazil	0	1	7	11	19	21	7
Paraguay	10	10	12	11	14	13	7
**East Asia**							
China	0	0	2	7	26	45	13
North Korea	0	0	0	0	0	1	0
Taiwan	5	10	20	20	20	20	4
**Southeast Asia**							
Cambodia	0	0	0	0	4	4	2
Indonesia	0	1	3	3	16	19	6
Laos	0	0	0	0	1	1	2
Malaysia	8	10	8	4	11	16	0
Maldives	0	0	6	11	14	13	3
Mauritius	10	10	10	10	10	10	8
Myanmar	0	0	0	1	1	3	2
Philippines	9	10	13	19	15	15	5

Appendix Table 2: Number of years with data points by country and decade							
	1950s	1960s	1970s	1980s	1990s	2000s	2010s
Sri Lanka	10	11	12	11	14	16	1
Seychelles	7	10	11	10	11	11	5
Thailand	10	10	12	14	11	12	3
Timor-Leste	0	0	0	0	6	4	0
Vietnam	0	0	0	1	2	7	6
**Oceania**							
American Samoa	8	10	5	7	7	10	3
Federated States of Micronesia	0	0	1	0	2	2	0
Fiji	11	11	12	9	5	19	3
Guam	10	10	10	7	9	10	3
Kiribati	0	2	2	0	9	4	1
Marshall Islands	0	0	0	4	9	3	1
Northern Mariana Islands	0	0	0	1	2	10	3
Papua New Guinea	0	0	2	2	3	2	0
Samoa	6	12	6	2	0	2	1
Solomon Islands	0	0	2	0	1	2	0
Tonga	3	6	1	2	4	6	1
Vanuatu	0	1	0	0	0	2	1
**North Africa and Middle East**							
Afghanistan	0	0	1	0	0	4	2
Algeria	7	2	0	5	3	6	3
Bahrain	0	0	1	16	17	11	8
Egypt	15	15	11	12	20	14	8
Iran	0	0	5	9	7	13	3
Iraq	3	10	4	4	2	8	2
Jordan	6	10	11	3	4	8	5
Kuwait	0	8	11	12	10	10	7
Lebanon	0	0	0	0	2	3	0
Libya	0	0	6	1	2	7	0
Morocco	0	0	0	4	11	4	1
Palestine	0	0	0	0	3	12	6
Oman	0	0	1	2	2	9	5
Qatar	0	0	0	11	10	14	5
Saudi Arabia	0	0	0	1	3	13	4
Sudan	0	0	1	2	3	3	3
Syria	0	6	11	3	3	5	2
Tunisia	0	3	10	7	5	2	3
Turkey	0	0	4	11	13	15	8
United Arab Emirates	0	0	1	1	6	9	0
Yemen	0	0	1	0	3	3	1
**South Asia**							
Bangladesh	0	0	3	11	16	16	9
Bhutan	0	0	0	0	1	1	1
India	10	5	10	13	16	16	6
Nepal	0	0	2	1	3	4	4
Pakistan	0	1	6	8	14	15	1
**Southern Sub-Saharan Africa**							
Botswana	0	0	0	1	2	3	1
Lesotho	0	0	1	1	0	4	2
Namibia	0	0	0	0	2	4	2
South Africa	0	0	0	4	16	17	8
Swaziland	0	0	0	0	1	3	2
Zimbabwe	0	0	0	1	4	4	4
**Western Sub-Saharan Africa**							
Benin	0	0	0	2	2	2	3
Burkina Faso	0	0	0	2	7	4	2
Cameroon	0	0	1	1	2	3	1
Cape Verde	4	5	7	4	3	2	4
Chad	0	0	0	0	2	2	2
Cote d'Ivoire	0	0	1	6	3	1	2

Appendix Table 2: Number of years with data points by country and decade							
	1950s	1960s	1970s	1980s	1990s	2000s	2010s
The Gambia	0	0	1	0	1	2	2
Ghana	0	0	1	1	4	7	4
Guinea	2	0	0	0	3	1	1
Guinea-Bissau	0	0	0	0	0	2	2
Liberia	0	0	0	2	0	5	2
Mali	0	0	1	2	3	3	2
Mauritania	0	0	0	2	2	2	1
Niger	0	0	0	0	3	2	1
Nigeria	0	0	0	0	2	7	3
Sao Tome and Principe	4	8	5	4	1	3	0
Senegal	0	0	1	1	3	5	5
Sierra Leone	0	0	0	0	0	4	2
Togo	0	1	0	1	1	1	4
**Eastern Sub-Saharan Africa**							
Burundi	0	0	1	1	2	2	2
Comoros	1	0	0	1	1	1	1
Djibouti	0	0	0	0	1	2	0
Eritrea	0	0	0	0	1	1	0
Ethiopia	0	0	0	1	1	4	2
Kenya	0	1	3	3	4	6	1
Madagascar	4	7	3	0	4	5	1
Malawi	0	0	2	3	4	6	5
Mozambique	0	0	0	0	4	5	1
Rwanda	0	0	2	1	2	6	5
Somalia	0	0	0	0	1	1	0
South Sudan	0	0	0	0	0	1	1
Tanzania	0	1	0	1	6	6	2
Uganda	0	0	0	1	2	6	3
Zambia	0	0	0	1	3	6	3
**Central Sub-Saharan Africa**							
Angola	0	0	0	0	0	2	1
Central African Republic	0	0	1	1	1	2	1
Congo	0	0	1	0	0	3	2
Democratic Republic of the Congo	0	0	0	0	1	2	2
Equatorial Guinea	0	0	0	0	0	1	1
Gabon	0	0	0	0	0	1	1

Appendix Table 3: Total number of country-years used in HIV excess mortality age pattern estimation		
**Country**	**Period covered**	**Total number of years used**
Argentina	1997 - 2010	14
Brazil	1996 - 2010	15
Colombia	1997 - 2008	12
Dominican Republic	2001 - 2004	3
Spain	1999 - 2010	12
France	2000 - 2001	2
Haiti	1999 - 1999	1
Italy	2003 - 2007	2
Mexico	1998 - 2012	15
Peru	2000 - 2004	5
Thailand	1994 - 2007	12
United States	1999 - 2010	12
Venezuela	1997 - 2009	13
South Africa	1996 - 2009	14
Total		132

Appendix Table 4: GBD world population age standard		
**Age**	**Percent of population**	**Rounded**
0-6 days	0.0367429	0.04
7-27 days	0.1079651	0.11
28-364 days	1.6850246	1.68*
0-1 years	1.8297326	1.83
1-5 years	7.1822788	7.18
5-9 years	8.6928104	8.69
10-14 years	8.3952037	8.4
15-19 years	8.098376	8.1
20-24 years	7.8144767	7.81
25-29 years	7.559828	7.56
30-34 years	7.2485193	7.25
35-39 years	6.8558773	6.86
40-44 years	6.3842306	6.38
45-49 years	5.8490788	5.85
50-54 years	5.2709755	5.27
55-59 years	4.6780164	4.68
60-64 years	4.05754	4.06
65-69 years	3.3590683	3.36
70-74 years	2.6292656	2.63
75-79 years	1.8966813	1.9
80-84 years	1.2134801	1.21
85-89 years	0.644468	0.64
90-94 years	0.2576025	0.26
95-99 years	0.0700765	0.07
100+ years	0.0124135	0.01
* rounded down in order for rounded numbers to sum to 100 percent		

Appendix Table 5: Total number of site years by cause and source type for 2015, excluding studies on lower respiratory infection and diarrhoea etiologi									
**Cause**	**Sort order**	**level**	**Vital registration**	**Verbal autopsy**	**Surveillance**	**Survey/Census**	**Cancer registry**	**Sibling history**	**Police records**
All causes	1	0	10801	534	1066	5		57	
Communicable, maternal, neonatal, and nutritional diseases	2	1	10872	1445	2010	184		3065	
HIV/AIDS and tuberculosis	3	2	10697	784	750				
Tuberculosis	4	3	10691	768	361				
HIV/AIDS	5	3	10344	200	390				
HIV/AIDS - Tuberculosis	6	4	4749		7				
HIV/AIDS resulting in other diseases	7	4	8164		51				
Diarrhea, lower respiratory, and other common infectious diseases	8	2	10835	993	560				
Diarrheal diseases	9	3	10770	910	509				
Intestinal infectious diseases	10	3	10627	510					
Typhoid fever	11	4	9280						
Paratyphoid fever	12	4	8542						
Other intestinal infectious diseases	13	4	8787						
Lower respiratory infections	14	3	10766	854	560				
Upper respiratory infections	15	3	9276						
Otitis media	16	3	9467	79					
Meningitis	17	3	10658	633	546				
Pneumococcal meningitis	18	4	8140						
H influenzae type B meningitis	19	4	8141						
Meningococcal meningitis	20	4	9359						
Other meningitis	21	4	8168						
Encephalitis	22	3	10150	128					
Diphtheria	23	3	9950	1					
Whooping cough	24	3	10249	286					
Tetanus	25	3	10578	621	393				
Measles	26	3	10669	495	524				
Varicella and herpes zoster	27	3	9327	1					
Neglected tropical diseases and malaria	28	2	10720	728	282				
Malaria	29	3	9382	670	1				
Chagas disease	30	3	3538						
Leishmaniasis	31	3	9561	111					
Visceral leishmaniasis	32	4	9482	111					
African trypanosomiasis	33	3	8096	109					
Schistosomiasis	34	3	8928	32					
Cysticercosis	35	3	8934						
Cystic echinococcosis	36	3	9013						
Dengue	37	3	9606	110	1				
Yellow fever	38	3	8946	110					
Rabies	39	3	10135	447	267				
Intestinal nematode infections	40	3	9638	157					
Ascariasis	41	4	8776						
Ebola	42	3	24		13				
Other neglected tropical diseases	43	3	9839	43					
Maternal disorders	44	2	10716	887	1091	184		3065	
Maternal hemorrhage	45	3	9762	475	731	7		1	
Maternal sepsis and other maternal infections	46	3	9290	321	455	6		1	
Maternal hypertensive disorders	47	3	9967	400	728	7		1	
Maternal obstructed labor and uterine rupture	48	3	9708	321	566	6		1	
Maternal abortion, miscarriage, and ectopic pregnancy	49	3	10280	424	589	7		1	
Indirect maternal deaths	50	3	9137	365	747	7		1	
Late maternal deaths	51	3	4190		140				
Maternal deaths aggravated by HIV/AIDS	52	3	10715	887	1091	184		3065	
Other maternal disorders	53	3	9518	3	704	5		1	
Neonatal disorders	54	2	10584	813	546				
Neonatal preterm birth complications	55	3	9300	166	558				
Neonatal encephalopathy due to birth asphyxia and trauma	56	3	9157	158	558				
Neonatal sepsis and other neonatal infections	57	3	8718		546				
Hemolytic disease and other neonatal jaundice	58	3	9411						
Other neonatal disorders	59	3	8306		552				
Nutritional deficiencies	60	2	10423	574	525				
Protein-energy malnutrition	61	3	10119	612					
Iodine deficiency	62	3	1350						
Iron-deficiency anemia	63	3	8987	521	1				
Other nutritional deficiencies	64	3	8508						
Other communicable, maternal, neonatal, and nutritional diseases	65	2	10710	587	843				
Sexually transmitted diseases excluding HIV	66	3	10562	145	228				
Syphilis	67	4	10011		228				
Chlamydial infection	68	4	8080						
Gonococcal infection	69	4	9585						
Other sexually transmitted diseases	70	4	8771						
Hepatitis	71	3	10380	529					
Acute hepatitis A	72	4	8128						
Acute hepatitis B	73	4	8508						
Acute hepatitis C	74	4	8080						
Acute hepatitis E	75	4	8086						
Other infectious diseases	76	3	10435	493	793				
Non-communicable diseases	77	1	10824	1177	626		2454		
Neoplasms	78	2	10814	891	625		2454		
Lip and oral cavity cancer	79	3	10049	134			2399		
Nasopharynx cancer	80	3	10070				2354		
Other pharynx cancer	81	3	10050				2323		
Esophageal cancer	82	3	10408	132			2411		
Stomach cancer	83	3	10414				2432		
Colon and rectum cancer	84	3	10413	134			2432		
Liver cancer	85	3	10118				2419		
Gallbladder and biliary tract cancer	90	3	10090				2331		
Pancreatic cancer	91	3	9796				2418		
Larynx cancer	92	3	10390				2405		
Tracheal, bronchus, and lung cancer	93	3	10413	147			2413		
Malignant skin melanoma	94	3	10103				2305		
Non-melanoma skin cancer	95	3	9680				1230		
Non-melanoma skin cancer (squamous-cell carcinoma)	96	4	8526				1230		

Appendix Table 5: Total number of site years by cause and source type for 2015, excluding studies on lower respiratory infection and diarrhoea etiologi									
Cause	Sort order	level	Vital registration	Verbal autopsy	Surveillance	Survey/Census	Cancer registry	Sibling history	Police records
Breast cancer	97	3	10402	153	1		2417		
Cervical cancer	98	3	10406				2382		
Uterine cancer	99	3	10325				2373		
Ovarian cancer	100	3	9802				2371		
Prostate cancer	101	3	10356				2351		
Testicular cancer	102	3	8869	84			2274		
Kidney cancer	103	3	10095				2387		
Bladder cancer	104	3	10100				2426		
Brain and nervous system cancer	105	3	9801	1			2418		
Thyroid cancer	106	3	9713				2375		
Mesothelioma	107	3	9095				1293		
Hodgkin lymphoma	108	3	10078				2289		
Non-Hodgkin lymphoma	109	3	10102				2394		
Multiple myeloma	110	3	10086				2294		
Leukemia	111	3	10413		551		2396		
Acute lymphoid leukemia	113	4	8236				260		
Chronic lymphoid leukemia	114	4	8226				249		
Acute myeloid leukemia	116	4	8235				259		
Chronic myeloid leukemia	117	4	8234				259		
Other neoplasms	118	3	10419		67		2444		
Cardiovascular diseases	119	2	10446	963	1				
Rheumatic heart disease	120	3	10417	11					
Ischemic heart disease	121	3	10652	734					
Cerebrovascular disease	122	3	10660	691	1				
Ischemic stroke	123	4	9207						
Hemorrhagic stroke	124	4	9211						
Hypertensive heart disease	125	3	10039						
Cardiomyopathy and myocarditis	126	3	10020						
Atrial fibrillation and flutter	127	3	8104						
Aortic aneurysm	128	3	9215						
Peripheral vascular disease	129	3	8087						
Endocarditis	130	3	9274						
Other cardiovascular and circulatory diseases	131	3	10339	1					
Chronic respiratory diseases	132	2	10714	866	517				
Chronic obstructive pulmonary disease	133	3	8928						
Pneumoconiosis	134	3	8181						
Silicosis	135	4	8158						
Asbestosis	136	4	8158						
Coal workers pneumoconiosis	137	4	8141						
Other pneumoconiosis	138	4	8163						
Asthma	139	3	8174						
Interstitial lung disease and pulmonary sarcoidosis	140	3	8174						
Other chronic respiratory diseases	141	3	8543						
Cirrhosis and other chronic liver diseases	142	2	10676	589					
Digestive diseases	147	2	10395	730	546				
Peptic ulcer disease	148	3	10636						
Gastritis and duodenitis	149	3	9029						
Appendicitis	150	3	10600	1					
Paralytic ileus and intestinal obstruction	151	3	10074	109					
Inguinal, femoral, and abdominal hernia	152	3	9628	342					
Inflammatory bowel disease	153	3	8137						
Vascular intestinal disorders	154	3	8111						
Gallbladder and biliary diseases	155	3	10332						
Pancreatitis	156	3	8555						
Other digestive diseases	157	3	8977						
Neurological disorders	158	2	10406	416	553				
Alzheimer disease and other dementias	159	3	9922	1					
Parkinson disease	160	3	8969						
Epilepsy	161	3	10282	324					
Multiple sclerosis	162	3	7663						
Motor neuron disease	163	3	8122						
Other neurological disorders	164	3	8159						
Mental and substance use disorders	165	2	10301	87					
Schizophrenia	166	3	9800	2					
Alcohol use disorders	167	3	9396	74					
Drug use disorders	168	3	10166	71					
Opioid use disorders	169	4	8465						
Cocaine use disorders	170	4	8456						
Amphetamine use disorders	171	4	8449						
Other drug use disorders	172	4	8469						
Eating disorders	173	3	8061						
Anorexia nervosa	174	4	8061						
Bulimia nervosa	175	4	7901						
Diabetes, urogenital, blood, and endocrine diseases	176	2	10468	765					
Diabetes mellitus	177	3	10455	570					
Acute glomerulonephritis	178	3	9382						
Chronic kidney disease	179	3	10432	721					
Chronic kidney disease due to diabetes mellitus	180	4	8528						
Chronic kidney disease due to hypertension	181	4	8537						
Chronic kidney disease due to glomerulonephritis	182	4	8528						
Chronic kidney disease due to other causes	183	4	8526						
Urinary diseases and male infertility	184	3	10399	131					
Interstitial nephritis and urinary tract infections	185	4	10406	1					
Urolithiasis	186	4	10076						
Other urinary diseases	187	4	8882						
Gynecological diseases	188	3	9872	235					
Uterine fibroids	189	4	8125						
Polycystic ovarian syndrome	190	4	6890						
Endometriosis	191	4	7082						
Genital prolapse	192	4	7984						
Other gynecological diseases	193	4	8115						
Hemoglobinopathies and hemolytic anemias	194	3	9713	513					

Appendix Table 5: Total number of site years by cause and source type for 2015, excluding studies on lower respiratory infection and diarrhoea etiologi

Cause	Sort order	level	Vital registration	Verbal autopsy	Surveillance	Survey/Census	Cancer registry	Sibling history	Police records
Thalassemias	195	4	8885	174					
Sickle cell disorders	196	4	8503	176					
G6PD deficiency	197	4	1077						
Other hemoglobinopathies and hemolytic anemias	198	4	8495						
Endocrine, metabolic, blood, and immune disorders	199	3	10333	344					
Musculoskeletal disorders	200	2	9654	133					
Rheumatoid arthritis	201	3	9053						
Other musculoskeletal disorders	202	3	9220						
Other non-communicable diseases	203	2	10451	627	559				
Congenital anomalies	204	3	10448	579	559				
Neural tube defects	205	4	9551		548				
Congenital heart anomalies	206	4	10310	1	557				
Cleft lip and cleft palate	207	4	6418						
Down syndrome	208	4	8136		540				
Other chromosomal abnormalities	209	4	8136						
Other congenital anomalies	210	4	9331		481				
Skin and subcutaneous diseases	211	3	10367	150					
Cellulitis	212	4	8533	1					
Pyoderma	213	4	9279	1					
Decubitus ulcer	214	4	8116						
Other skin and subcutaneous diseases	215	4	8179						
Sudden infant death syndrome	216	3	7914						
Injuries	217	1	10672	915	562	39			
Transport injuries	218	2	10544	640	557	17			
Road injuries	219	3	9478			5			61
Pedestrian road injuries	220	4	8172						66
Cyclist road injuries	221	4	8164						41
Motorcyclist road injuries	222	4	8172			2			48
Motor vehicle road injuries	223	4	8172			7			59
Other road injuries	224	4	8164						27
Other transport injuries	225	3	8854			2			2
Unintentional injuries	226	2	10594	728	556	8			
Falls	227	3	10556	494	554	5			
Drowning	228	3	10525	592	553	4			
Fire, heat, and hot substances	229	3	10537	575		3			
Poisonings	230	3	10500	127	523	2			
Exposure to mechanical forces	231	3	10184	90		5			
Unintentional firearm injuries	232	4	10071	82		2			
Unintentional suffocation	233	4	8483	82	558	1			
Other exposure to mechanical forces	234	4	9277	86		5			
Adverse effects of medical treatment	235	3	10170			2			
Animal contact	236	3	9801	481		3			
Venomous animal contact	237	4	8915	144					
Non-venomous animal contact	238	4	8534						
Foreign body	239	3	9305	135		2			
Pulmonary aspiration and foreign body in airway	240	4	8549						
Foreign body in other body part	241	4	8443						
Environmental heat and cold exposure	242	3	9613	75		2			
Other unintentional injuries	243	3	9394	135		3			
Self-harm and interpersonal violence	244	2	10593	751	1	13			
Self-harm	245	3	10579	710		5			
Interpersonal violence	246	3	10539	577		11			1256
Assault by firearm	247	4	8161	86		5			
Assault by sharp object	248	4	8162	84		1			
Assault by other means	249	4	8164	85					
Forces of nature, war, and legal intervention	250	2	10410	93		9			
Exposure to forces of nature	251	3	8554	81		5			
Collective violence and legal intervention	252	3	10169	87		5			

Appendix Table 6: Causes of death data source list by geography, source type, and study site, with years, age range, and sample size, excluding sources on lower respiratory infection and diarrhoea etiologies						
**Location**	**Source name**	**Source type**	**Site**	**Years**	**Ages**	**Sample size**
China: Anhui	China National Disease Surveillance Points (DSP)	Surveillance		1991-2000, 2002	All Ages	26929
China: Anhui	China National Maternal and Child Health Surveillance System Maternal Mortality By Cause 2006-2012 - MCHS	Surveillance		1996-2005	15 - 49 years	MMR
China: Anhui	China National Maternal and Child Health Surveillance System Child Mortality Data By Cause 1996-2012 - MCHS	Surveillance		1996-2012	birth - 4 years	1679
China: Anhui	China National Notifiable Infectious Diseases Reporting Information System (NIDRIS)	Surveillance		2004-2014	All Ages	2485
China: Anhui	China National Maternal and Child Health Surveillance System Maternal Mortality By Cause 1996-2005 - MCHS	Surveillance		2006-2012	15 - 49 years	MMR
China: Anhui	China National Maternal and Child Health Surveillance System Maternal Mortality By Cause 2013 - MCHS	Surveillance		2013	15 - 49 years	MMR
China: Anhui	China National Maternal and Child Health Surveillance System Child Mortality By Cause 2013 - MCHS	Surveillance		2013	birth - 4 years	301
China: Anhui	China National Disease Surveillance Points (DSP)	Vital Registration		2004-2007, 2013-2014	All Ages	474546
China: Anhui	China Mortality Registration and Reporting System	Vital Registration		2008-2012	All Ages	486213
China: Beijing	China National Disease Surveillance Points (DSP)	Surveillance		1991-2002	All Ages	6917
China: Beijing	China National Maternal and Child Health Surveillance System Maternal Mortality By Cause 2006-2012 - MCHS	Surveillance		1996-1999, 2005	15 - 49 years	MMR
China: Beijing	China National Maternal and Child Health Surveillance System Child Mortality Data By Cause 1996-2012 - MCHS	Surveillance		1996-2012	birth - 4 years	486
China: Beijing	China National Notifiable Infectious Diseases Reporting Information System (NIDRIS)	Surveillance		2004-2014	All Ages	362
China: Beijing	China National Maternal and Child Health Surveillance System Maternal Mortality By Cause 1996-2005 - MCHS	Surveillance		2006-2012	15 - 49 years	MMR
China: Beijing	China National Maternal and Child Health Surveillance System Maternal Mortality By Cause 2013 - MCHS	Surveillance		2013	15 - 49 years	MMR
China: Beijing	China National Maternal and Child Health Surveillance System Child Mortality By Cause 2013 - MCHS	Surveillance		2013	birth - 4 years	113
China: Beijing	China National Disease Surveillance Points (DSP)	Vital Registration		2004-2007, 2013-2014	All Ages	188252
China: Beijing	China Mortality Registration and Reporting System	Vital Registration		2008-2012	All Ages	368058
China: Chongqing	China National Disease Surveillance Points (DSP)	Surveillance		1991-1998, 2000-2002	All Ages	6106
China: Chongqing	China National Maternal and Child Health Surveillance System Maternal Mortality By Cause 2006-2012 - MCHS	Surveillance		1996-2005	15 - 49 years	MMR
China: Chongqing	China Maternal and Child Mortality Data 1996-2012 - China CDC	Surveillance		1996-2012	15 - 49 years	MMR
China: Chongqing	China National Maternal and Child Health Surveillance System Child Mortality Data By Cause 1996-2012 - MCHS	Surveillance		1996-2012	birth - 4 years	812
China: Chongqing	China National Notifiable Infectious Diseases Reporting Information System (NIDRIS)	Surveillance		2004-2014	All Ages	5112
China: Chongqing	China National Maternal and Child Health Surveillance System Maternal Mortality By Cause 1996-2005 - MCHS	Surveillance		2006-2012	15 - 49 years	MMR
China: Chongqing	China National Maternal and Child Health Surveillance System Maternal Mortality By Cause 2013 - MCHS	Surveillance		2013	15 - 49 years	MMR
China: Chongqing	China National Maternal and Child Health Surveillance System Child Mortality By Cause 2013 - MCHS	Surveillance		2013	birth - 4 years	191
China: Chongqing	China National Disease Surveillance Points (DSP)	Vital Registration		2004-2007, 2013-2014	All Ages	454936
China: Chongqing	China Mortality Registration and Reporting System	Vital Registration		2008-2012	All Ages	479548
China: Fujian	China National Disease Surveillance Points (DSP)	Surveillance		1991-2002	All Ages	16949
China: Fujian	China National Maternal and Child Health Surveillance System Maternal Mortality By Cause 2006-2012 - MCHS	Surveillance		1996-2005	15 - 49 years	MMR
China: Fujian	China National Maternal and Child Health Surveillance System Child Mortality Data By Cause 1996-2012 - MCHS	Surveillance		1996-2012	birth - 4 years	1516

Appendix Table 6: Causes of death data source list by geography, source type, and study site, with years, age range, and sample size, excluding sources on lower respiratory infection and diarrhoea etiologies						
**Location**	**Source name**	**Source type**	**Site**	**Years**	**Ages**	**Sample size**
China: Fujian	China National Notifiable Infectious Diseases Reporting Information System (NIDRIS)	Surveillance		2004-2014	All Ages	1336
China: Fujian	China National Maternal and Child Health Surveillance System Maternal Mortality By Cause 1996-2005 - MCHS	Surveillance		2006-2012	15 - 49 years	MMR
China: Fujian	China National Maternal and Child Health Surveillance System Maternal Mortality By Cause 2013 - MCHS	Surveillance		2013	15 - 49 years	MMR
China: Fujian	China National Maternal and Child Health Surveillance System Child Mortality By Cause 2013 - MCHS	Surveillance		2013	birth - 4 years	245
China: Fujian	China National Disease Surveillance Points (DSP)	Vital Registration		2004-2007, 2013-2014	All Ages	190784
China: Fujian	China Mortality Registration and Reporting System	Vital Registration		2008-2012	All Ages	150451
China: Gansu	China National Disease Surveillance Points (DSP)	Surveillance		1991-2002	All Ages	12155
China: Gansu	China National Maternal and Child Health Surveillance System Maternal Mortality By Cause 2006-2012 - MCHS	Surveillance		1996-2005	15 - 49 years	MMR
China: Gansu	China National Maternal and Child Health Surveillance System Child Mortality Data By Cause 1996-2012 - MCHS	Surveillance		1996-2012	birth - 4 years	1615
China: Gansu	China National Notifiable Infectious Diseases Reporting Information System (NIDRIS)	Surveillance		2004-2014	All Ages	394
China: Gansu	China National Maternal and Child Health Surveillance System Maternal Mortality By Cause 1996-2005 - MCHS	Surveillance		2006-2012	15 - 49 years	MMR
China: Gansu	China National Maternal and Child Health Surveillance System Maternal Mortality By Cause 2013 - MCHS	Surveillance		2013	15 - 49 years	MMR
China: Gansu	China National Maternal and Child Health Surveillance System Child Mortality By Cause 2013 - MCHS	Surveillance		2013	birth - 4 years	234
China: Gansu	China National Disease Surveillance Points (DSP)	Vital Registration		2004-2007, 2013-2014	All Ages	209705
China: Gansu	China Mortality Registration and Reporting System	Vital Registration		2008-2012	All Ages	100216
China: Guangdong	China National Disease Surveillance Points (DSP)	Surveillance		1991-2002	All Ages	27664
China: Guangdong	China National Maternal and Child Health Surveillance System Maternal Mortality By Cause 2006-2012 - MCHS	Surveillance		1996-2002, 2004-2005	15 - 49 years	MMR
China: Guangdong	China National Maternal and Child Health Surveillance System Child Mortality Data By Cause 1996-2012 - MCHS	Surveillance		1996-2012	birth - 4 years	1439
China: Guangdong	China National Notifiable Infectious Diseases Reporting Information System (NIDRIS)	Surveillance		2004-2014	All Ages	12129
China: Guangdong	China National Maternal and Child Health Surveillance System Maternal Mortality By Cause 1996-2005 - MCHS	Surveillance		2006-2012	15 - 49 years	MMR
China: Guangdong	China National Maternal and Child Health Surveillance System Maternal Mortality By Cause 2013 - MCHS	Surveillance		2013	15 - 49 years	MMR
China: Guangdong	China National Maternal and Child Health Surveillance System Child Mortality By Cause 2013 - MCHS	Surveillance		2013	birth - 4 years	331
China: Guangdong	China National Disease Surveillance Points (DSP)	Vital Registration		2004-2007, 2013-2014	All Ages	505229
China: Guangdong	China Mortality Registration and Reporting System	Vital Registration		2008-2012	All Ages	529075
China: Guangxi	China National Disease Surveillance Points (DSP)	Surveillance		1991-2002	All Ages	23352
China: Guangxi	China National Maternal and Child Health Surveillance System Maternal Mortality By Cause 2006-2012 - MCHS	Surveillance		1996-2005	15 - 49 years	MMR
China: Guangxi	China National Maternal and Child Health Surveillance System Child Mortality Data By Cause 1996-2012 - MCHS	Surveillance		1996-2012	birth - 4 years	2147
China: Guangxi	China National Notifiable Infectious Diseases Reporting Information System (NIDRIS)	Surveillance		2004-2014	All Ages	28123
China: Guangxi	China National Maternal and Child Health Surveillance System Maternal Mortality By Cause 1996-2005 - MCHS	Surveillance		2006-2012	15 - 49 years	MMR
China: Guangxi	China National Maternal and Child Health Surveillance System Maternal Mortality By Cause 2013 - MCHS	Surveillance		2013	15 - 49 years	MMR

Appendix Table 6: Causes of death data source list by geography, source type, and study site, with years, age range, and sample size, excluding sources on lower respiratory infection and diarrhoea etiologies						
**Location**	**Source name**	**Source type**	**Site**	**Years**	**Ages**	**Sample size**
China: Guangxi	China National Maternal and Child Health Surveillance System Child Mortality By Cause 2013 - MCHS	Surveillance		2013	birth - 4 years	338
China: Guangxi	China National Disease Surveillance Points (DSP)	Vital Registration		2004-2007, 2013-2014	All Ages	331651
China: Guangxi	China Mortality Registration and Reporting System	Vital Registration		2008-2012	All Ages	192135
China: Guizhou	China National Disease Surveillance Points (DSP)	Surveillance		1991-2002	All Ages	27954
China: Guizhou	China National Maternal and Child Health Surveillance System Maternal Mortality By Cause 2006-2012 - MCHS	Surveillance		1996-2005	15 - 49 years	MMR
China: Guizhou	China National Maternal and Child Health Surveillance System Child Mortality Data By Cause 1996-2012 - MCHS	Surveillance		1996-2012	birth - 4 years	3381
China: Guizhou	China National Notifiable Infectious Diseases Reporting Information System (NIDRIS)	Surveillance		2004-2014	All Ages	8232
China: Guizhou	China National Maternal and Child Health Surveillance System Maternal Mortality By Cause 1996-2005 - MCHS	Surveillance		2006-2012	15 - 49 years	MMR
China: Guizhou	China National Maternal and Child Health Surveillance System Maternal Mortality By Cause 2013 - MCHS	Surveillance		2013	15 - 49 years	MMR
China: Guizhou	China National Maternal and Child Health Surveillance System Child Mortality By Cause 2013 - MCHS	Surveillance		2013	birth - 4 years	357
China: Guizhou	China National Disease Surveillance Points (DSP)	Vital Registration		2004-2007, 2013-2014	All Ages	154702
China: Guizhou	China Mortality Registration and Reporting System	Vital Registration		2008-2012	All Ages	58640
China: Hainan	China National Disease Surveillance Points (DSP)	Surveillance		1991-2002	All Ages	4055
China: Hainan	China National Maternal and Child Health Surveillance System Maternal Mortality By Cause 2006-2012 - MCHS	Surveillance		1996-2005	15 - 49 years	MMR
China: Hainan	China National Maternal and Child Health Surveillance System Child Mortality Data By Cause 1996-2012 - MCHS	Surveillance		1996-2012	birth - 4 years	1683
China: Hainan	China National Notifiable Infectious Diseases Reporting Information System (NIDRIS)	Surveillance		2004-2014	All Ages	977
China: Hainan	China National Maternal and Child Health Surveillance System Maternal Mortality By Cause 1996-2005 - MCHS	Surveillance		2006-2012	15 - 49 years	MMR
China: Hainan	China National Maternal and Child Health Surveillance System Maternal Mortality By Cause 2013 - MCHS	Surveillance		2013	15 - 49 years	MMR
China: Hainan	China National Maternal and Child Health Surveillance System Child Mortality By Cause 2013 - MCHS	Surveillance		2013	birth - 4 years	167
China: Hainan	China National Disease Surveillance Points (DSP)	Vital Registration		2004-2007, 2013-2014	All Ages	40858
China: Hainan	China Mortality Registration and Reporting System	Vital Registration		2008-2012	All Ages	14474
China: Hebei	China National Disease Surveillance Points (DSP)	Surveillance		1991-2002	All Ages	22032
China: Hebei	China National Maternal and Child Health Surveillance System Maternal Mortality By Cause 2006-2012 - MCHS	Surveillance		1996-2005	15 - 49 years	MMR
China: Hebei	China National Maternal and Child Health Surveillance System Child Mortality Data By Cause 1996-2012 - MCHS	Surveillance		1996-2012	birth - 4 years	1105
China: Hebei	China National Notifiable Infectious Diseases Reporting Information System (NIDRIS)	Surveillance		2004-2014	All Ages	1550
China: Hebei	China National Maternal and Child Health Surveillance System Maternal Mortality By Cause 1996-2005 - MCHS	Surveillance		2006-2012	15 - 49 years	MMR
China: Hebei	China National Maternal and Child Health Surveillance System Maternal Mortality By Cause 2013 - MCHS	Surveillance		2013	15 - 49 years	MMR
China: Hebei	China National Maternal and Child Health Surveillance System Child Mortality By Cause 2013 - MCHS	Surveillance		2013	birth - 4 years	199
China: Hebei	China National Disease Surveillance Points (DSP)	Vital Registration		2004-2007, 2013-2014	All Ages	467306
China: Hebei	China Mortality Registration and Reporting System	Vital Registration		2008-2012	All Ages	311617
China: Heilongjiang	China National Disease Surveillance Points (DSP)	Surveillance		1991-2002	All Ages	12285

Appendix Table 6: Causes of death data source list by geography, source type, and study site, with years, age range, and sample size, excluding sources on lower respiratory infection and diarrhoea etiologies						
**Location**	**Source name**	**Source type**	**Site**	**Years**	**Ages**	**Sample size**
China: Heilongjiang	China National Maternal and Child Health Surveillance System Child Mortality Data By Cause 1996-2012 - MCHS	Surveillance		1996-2012	birth - 4 years	930
China: Heilongjiang	China National Maternal and Child Health Surveillance System Maternal Mortality By Cause 2006-2012 - MCHS	Surveillance		1997-2002, 2004-2005	15 - 49 years	MMR
China: Heilongjiang	China National Notifiable Infectious Diseases Reporting Information System (NIDRIS)	Surveillance		2004-2014	All Ages	924
China: Heilongjiang	China National Maternal and Child Health Surveillance System Maternal Mortality By Cause 1996-2005 - MCHS	Surveillance		2006-2012	15 - 49 years	MMR
China: Heilongjiang	China National Maternal and Child Health Surveillance System Maternal Mortality By Cause 2013 - MCHS	Surveillance		2013	15 - 49 years	MMR
China: Heilongjiang	China National Maternal and Child Health Surveillance System Child Mortality By Cause 2013 - MCHS	Surveillance		2013	birth - 4 years	101
China: Heilongjiang	China National Disease Surveillance Points (DSP)	Vital Registration		2004-2007, 2013-2014	All Ages	524404
China: Heilongjiang	China Mortality Registration and Reporting System	Vital Registration		2008-2012	All Ages	979278
China: Henan	China National Disease Surveillance Points (DSP)	Surveillance		1991-2002	All Ages	35272
China: Henan	China National Maternal and Child Health Surveillance System Maternal Mortality By Cause 2006-2012 - MCHS	Surveillance		1996-2005	15 - 49 years	MMR
China: Henan	China National Maternal and Child Health Surveillance System Child Mortality Data By Cause 1996-2012 - MCHS	Surveillance		1996-2012	birth - 4 years	1850
China: Henan	China National Notifiable Infectious Diseases Reporting Information System (NIDRIS)	Surveillance		2004-2014	All Ages	14827
China: Henan	China National Maternal and Child Health Surveillance System Maternal Mortality By Cause 1996-2005 - MCHS	Surveillance		2006-2012	15 - 49 years	MMR
China: Henan	China National Maternal and Child Health Surveillance System Maternal Mortality By Cause 2013 - MCHS	Surveillance		2013	15 - 49 years	MMR
China: Henan	China National Maternal and Child Health Surveillance System Child Mortality By Cause 2013 - MCHS	Surveillance		2013	birth - 4 years	385
China: Henan	China National Disease Surveillance Points (DSP)	Vital Registration		2004-2007, 2013-2014	All Ages	902508
China: Henan	China Mortality Registration and Reporting System	Vital Registration		2008-2012	All Ages	680747
China: Hong Kong Special Administrative Region of China	United Nations Office on Drugs and Crime Global Study on Homicide 2011	Police Records	Hong Kong Special Administrative Region of China CTS/National police	1996	All Ages	Death Rate
China: Hong Kong Special Administrative Region of China	WHO Mortality Database	Vital Registration		1980-2013	All Ages	1118401
China: Hubei	China National Disease Surveillance Points (DSP)	Surveillance		1991-2002	All Ages	28706
China: Hubei	China National Maternal and Child Health Surveillance System Maternal Mortality By Cause 2006-2012 - MCHS	Surveillance		1996-2005	15 - 49 years	MMR
China: Hubei	China National Maternal and Child Health Surveillance System Child Mortality Data By Cause 1996-2012 - MCHS	Surveillance		1996-2012	birth - 4 years	1348
China: Hubei	China National Notifiable Infectious Diseases Reporting Information System (NIDRIS)	Surveillance		2004-2014	All Ages	3991
China: Hubei	China National Maternal and Child Health Surveillance System Maternal Mortality By Cause 1996-2005 - MCHS	Surveillance		2006-2012	15 - 49 years	MMR
China: Hubei	China National Maternal and Child Health Surveillance System Maternal Mortality By Cause 2013 - MCHS	Surveillance		2013	15 - 49 years	MMR
China: Hubei	China National Maternal and Child Health Surveillance System Child Mortality By Cause 2013 - MCHS	Surveillance		2013	birth - 4 years	248
China: Hubei	China National Disease Surveillance Points (DSP)	Vital Registration		2004-2007, 2013-2014	All Ages	535964
China: Hubei	China Mortality Registration and Reporting System	Vital Registration		2008-2012	All Ages	329913
China: Hunan	China National Disease Surveillance Points (DSP)	Surveillance		1991-2002	All Ages	48781
China: Hunan	China National Maternal and Child Health Surveillance System Maternal Mortality By Cause 2006-2012 - MCHS	Surveillance		1996-2005	15 - 49 years	MMR

Appendix Table 6: Causes of death data source list by geography, source type, and study site, with years, age range, and sample size, excluding sources on lower respiratory infection and diarrhoea etiologies						
**Location**	**Source name**	**Source type**	**Site**	**Years**	**Ages**	**Sample size**
China: Hunan	China Maternal and Child Mortality Data 1996-2012 - China CDC	Surveillance		1996-2012	15 - 49 years	MMR
China: Hunan	China National Maternal and Child Health Surveillance System Child Mortality Data By Cause 1996-2012 - MCHS	Surveillance		1996-2012	birth - 4 years	1683
China: Hunan	China National Notifiable Infectious Diseases Reporting Information System (NIDRIS)	Surveillance		2004-2014	All Ages	8441
China: Hunan	China National Maternal and Child Health Surveillance System Maternal Mortality By Cause 1996-2005 - MCHS	Surveillance		2006-2012	15 - 49 years	MMR
China: Hunan	China National Maternal and Child Health Surveillance System Maternal Mortality By Cause 2013 - MCHS	Surveillance		2013	15 - 49 years	MMR
China: Hunan	China National Maternal and Child Health Surveillance System Child Mortality By Cause 2013 - MCHS	Surveillance		2013	birth - 4 years	322
China: Hunan	China National Disease Surveillance Points (DSP)	Vital Registration		2004-2007, 2013-2014	All Ages	732456
China: Hunan	China Mortality Registration and Reporting System	Vital Registration		2008-2012	All Ages	1238659
China: Inner Mongolia	China National Disease Surveillance Points (DSP)	Surveillance		1991-2002	All Ages	2625
China: Inner Mongolia	China National Maternal and Child Health Surveillance System Maternal Mortality By Cause 2006-2012 - MCHS	Surveillance		1996-2004	15 - 49 years	MMR
China: Inner Mongolia	China National Maternal and Child Health Surveillance System Child Mortality Data By Cause 1996-2012 - MCHS	Surveillance		1996-2012	birth - 4 years	2107
China: Inner Mongolia	China National Notifiable Infectious Diseases Reporting Information System (NIDRIS)	Surveillance		2004-2014	All Ages	297
China: Inner Mongolia	China National Maternal and Child Health Surveillance System Maternal Mortality By Cause 1996-2005 - MCHS	Surveillance		2006-2012	15 - 49 years	MMR
China: Inner Mongolia	China National Maternal and Child Health Surveillance System Maternal Mortality By Cause 2013 - MCHS	Surveillance		2013	15 - 49 years	MMR
China: Inner Mongolia	China National Maternal and Child Health Surveillance System Child Mortality By Cause 2013 - MCHS	Surveillance		2013	birth - 4 years	552
China: Inner Mongolia	China National Disease Surveillance Points (DSP)	Vital Registration		2004-2007, 2013-2014	All Ages	212787
China: Inner Mongolia	China Mortality Registration and Reporting System	Vital Registration		2008-2012	All Ages	293934
China: Jiangsu	China National Disease Surveillance Points (DSP)	Surveillance		1991-2002	All Ages	41754
China: Jiangsu	China National Maternal and Child Health Surveillance System Maternal Mortality By Cause 2006-2012 - MCHS	Surveillance		1996-2005	15 - 49 years	MMR
China: Jiangsu	China National Maternal and Child Health Surveillance System Child Mortality Data By Cause 1996-2012 - MCHS	Surveillance		1996-2012	birth - 4 years	1255
China: Jiangsu	China National Notifiable Infectious Diseases Reporting Information System (NIDRIS)	Surveillance		2004-2014	All Ages	2685
China: Jiangsu	China National Maternal and Child Health Surveillance System Maternal Mortality By Cause 1996-2005 - MCHS	Surveillance		2006-2012	15 - 49 years	MMR
China: Jiangsu	China National Maternal and Child Health Surveillance System Maternal Mortality By Cause 2013 - MCHS	Surveillance		2013	15 - 49 years	MMR
China: Jiangsu	China National Maternal and Child Health Surveillance System Child Mortality By Cause 2013 - MCHS	Surveillance		2013	birth - 4 years	195
China: Jiangsu	China National Disease Surveillance Points (DSP)	Vital Registration		2004-2007, 2013-2014	All Ages	968850
China: Jiangsu	China Mortality Registration and Reporting System	Vital Registration		2008-2012	All Ages	1672122
China: Jiangxi	China National Disease Surveillance Points (DSP)	Surveillance		1991-2002	All Ages	16150
China: Jiangxi	China National Maternal and Child Health Surveillance System Maternal Mortality By Cause 2006-2012 - MCHS	Surveillance		1996-2005	15 - 49 years	MMR
China: Jiangxi	China National Maternal and Child Health Surveillance System Child Mortality Data By Cause 1996-2012 - MCHS	Surveillance		1996-2012	birth - 4 years	2338
China: Jiangxi	China National Notifiable Infectious Diseases Reporting Information System (NIDRIS)	Surveillance		2004-2014	All Ages	2768

Appendix Table 6: Causes of death data source list by geography, source type, and study site, with years, age range, and sample size, excluding sources on lower respiratory infection and diarrhoea etiologies

Location	Source name	Source type	Site	Years	Ages	Sample size
China: Jiangxi	China National Maternal and Child Health Surveillance System Maternal Mortality By Cause 1996-2005 - MCHS	Surveillance		2006-2012	15 - 49 years	MMR
China: Jiangxi	China National Maternal and Child Health Surveillance System Maternal Mortality By Cause 2013 - MCHS	Surveillance		2013	15 - 49 years	MMR
China: Jiangxi	China National Maternal and Child Health Surveillance System Child Mortality By Cause 2013 - MCHS	Surveillance		2013	birth - 4 years	248
China: Jiangxi	China National Disease Surveillance Points (DSP)	Vital Registration		2004-2007, 2013-2014	All Ages	281897
China: Jiangxi	China Mortality Registration and Reporting System	Vital Registration		2008-2012	All Ages	109159
China: Jilin	China National Disease Surveillance Points (DSP)	Surveillance		1991-2002	All Ages	11179
China: Jilin	China National Maternal and Child Health Surveillance System Maternal Mortality By Cause 2006-2012 - MCHS	Surveillance		1996-2001, 2003, 2005	15 - 49 years	MMR
China: Jilin	China National Maternal and Child Health Surveillance System Child Mortality Data By Cause 1996-2012 - MCHS	Surveillance		1996-2012	birth - 4 years	906
China: Jilin	China National Notifiable Infectious Diseases Reporting Information System (NIDRIS)	Surveillance		2004-2014	All Ages	750
China: Jilin	China National Maternal and Child Health Surveillance System Maternal Mortality By Cause 1996-2005 - MCHS	Surveillance		2006-2012	15 - 49 years	MMR
China: Jilin	China National Maternal and Child Health Surveillance System Maternal Mortality By Cause 2013 - MCHS	Surveillance		2013	15 - 49 years	MMR
China: Jilin	China National Maternal and Child Health Surveillance System Child Mortality By Cause 2013 - MCHS	Surveillance		2013	birth - 4 years	148
China: Jilin	China National Disease Surveillance Points (DSP)	Vital Registration		2004-2007, 2013-2014	All Ages	280673
China: Jilin	China Mortality Registration and Reporting System	Vital Registration		2008-2012	All Ages	259826
China: Liaoning	China National Disease Surveillance Points (DSP)	Surveillance		1991-2002	All Ages	15137
China: Liaoning	China National Maternal and Child Health Surveillance System Maternal Mortality By Cause 2006-2012 - MCHS	Surveillance		1996-2000, 2002, 2005	15 - 49 years	MMR
China: Liaoning	China National Maternal and Child Health Surveillance System Child Mortality Data By Cause 1996-2012 - MCHS	Surveillance		1996-2012	birth - 4 years	1122
China: Liaoning	China National Notifiable Infectious Diseases Reporting Information System (NIDRIS)	Surveillance		2004-2014	All Ages	1124
China: Liaoning	China National Maternal and Child Health Surveillance System Maternal Mortality By Cause 1996-2005 - MCHS	Surveillance		2006-2012	15 - 49 years	MMR
China: Liaoning	China National Maternal and Child Health Surveillance System Maternal Mortality By Cause 2013 - MCHS	Surveillance		2013	15 - 49 years	MMR
China: Liaoning	China National Maternal and Child Health Surveillance System Child Mortality By Cause 2013 - MCHS	Surveillance		2013	birth - 4 years	138
China: Liaoning	China National Disease Surveillance Points (DSP)	Vital Registration		2004-2007, 2013-2014	All Ages	556387
China: Liaoning	China Mortality Registration and Reporting System	Vital Registration		2008-2012	All Ages	840701
China: Macao Special Administrative Region of China	WHO Mortality Database	Vital Registration		1994	All Ages	1329
China: Ningxia	China National Disease Surveillance Points (DSP)	Surveillance		1991-2002	All Ages	2103
China: Ningxia	China National Maternal and Child Health Surveillance System Maternal Mortality By Cause 2006-2012 - MCHS	Surveillance		1996-2005	15 - 49 years	MMR
China: Ningxia	China National Maternal and Child Health Surveillance System Child Mortality Data By Cause 1996-2012 - MCHS	Surveillance		1996-2012	birth - 4 years	2223
China: Ningxia	China National Notifiable Infectious Diseases Reporting Information System (NIDRIS)	Surveillance		2004-2014	All Ages	156
China: Ningxia	China National Maternal and Child Health Surveillance System Maternal Mortality By Cause 1996-2005 - MCHS	Surveillance		2006-2012	15 - 49 years	MMR
China: Ningxia	China National Maternal and Child Health Surveillance System Maternal Mortality By Cause 2013 - MCHS	Surveillance		2013	15 - 49 years	MMR

Appendix Table 6: Causes of death data source list by geography, source type, and study site, with years, age range, and sample size, excluding sources on lower respiratory infection and diarrhoea etiologies						
**Location**	**Source name**	**Source type**	**Site**	**Years**	**Ages**	**Sample size**
China: Ningxia	China National Maternal and Child Health Surveillance System Child Mortality By Cause 2013 - MCHS	Surveillance		2013	birth - 4 years	290
China: Ningxia	China National Disease Surveillance Points (DSP)	Vital Registration		2004-2007, 2013-2014	All Ages	66009
China: Ningxia	China Mortality Registration and Reporting System	Vital Registration		2008-2012	All Ages	102318
China: Qinghai	China National Disease Surveillance Points (DSP)	Surveillance		1991-2002	All Ages	5051
China: Qinghai	China National Maternal and Child Health Surveillance System Maternal Mortality By Cause 2006-2012 - MCHS	Surveillance		1996-2005	15 - 49 years	MMR
China: Qinghai	China National Maternal and Child Health Surveillance System Child Mortality Data By Cause 1996-2012 - MCHS	Surveillance		1996-2012	birth - 4 years	2400
China: Qinghai	China National Notifiable Infectious Diseases Reporting Information System (NIDRIS)	Surveillance		2004, 2006-2014	All Ages	154
China: Qinghai	China National Maternal and Child Health Surveillance System Maternal Mortality By Cause 1996-2005 - MCHS	Surveillance		2006-2012	15 - 49 years	MMR
China: Qinghai	China National Maternal and Child Health Surveillance System Maternal Mortality By Cause 2013 - MCHS	Surveillance		2013	15 - 49 years	MMR
China: Qinghai	China National Maternal and Child Health Surveillance System Child Mortality By Cause 2013 - MCHS	Surveillance		2013	birth - 4 years	233
China: Qinghai	China National Disease Surveillance Points (DSP)	Vital Registration		2004-2007, 2013-2014	All Ages	49910
China: Qinghai	China Mortality Registration and Reporting System	Vital Registration		2008-2012	All Ages	78790
China: Shaanxi	China National Disease Surveillance Points (DSP)	Surveillance		1991-2002	All Ages	17071
China: Shaanxi	China National Maternal and Child Health Surveillance System Maternal Mortality By Cause 2006-2012 - MCHS	Surveillance		1996-2005	15 - 49 years	MMR
China: Shaanxi	China National Maternal and Child Health Surveillance System Child Mortality Data By Cause 1996-2012 - MCHS	Surveillance		1996-2012	birth - 4 years	1272
China: Shaanxi	China National Notifiable Infectious Diseases Reporting Information System (NIDRIS)	Surveillance		2004-2014	All Ages	1169
China: Shaanxi	China National Maternal and Child Health Surveillance System Maternal Mortality By Cause 1996-2005 - MCHS	Surveillance		2006-2012	15 - 49 years	MMR
China: Shaanxi	China National Maternal and Child Health Surveillance System Maternal Mortality By Cause 2013 - MCHS	Surveillance		2013	15 - 49 years	MMR
China: Shaanxi	China National Maternal and Child Health Surveillance System Child Mortality By Cause 2013 - MCHS	Surveillance		2013	birth - 4 years	170
China: Shaanxi	China National Disease Surveillance Points (DSP)	Vital Registration		2004-2007, 2013-2014	All Ages	216684
China: Shaanxi	China Mortality Registration and Reporting System	Vital Registration		2008-2012	All Ages	138787
China: Shandong	China National Disease Surveillance Points (DSP)	Surveillance		1991-2002	All Ages	47142
China: Shandong	China National Maternal and Child Health Surveillance System Maternal Mortality By Cause 2006-2012 - MCHS	Surveillance		1996-2005	15 - 49 years	MMR
China: Shandong	China National Maternal and Child Health Surveillance System Child Mortality Data By Cause 1996-2012 - MCHS	Surveillance		1996-2012	birth - 4 years	2045
China: Shandong	China National Notifiable Infectious Diseases Reporting Information System (NIDRIS)	Surveillance		2004-2014	All Ages	1949
China: Shandong	China National Maternal and Child Health Surveillance System Maternal Mortality By Cause 1996-2005 - MCHS	Surveillance		2006-2012	15 - 49 years	MMR
China: Shandong	China National Maternal and Child Health Surveillance System Maternal Mortality By Cause 2013 - MCHS	Surveillance		2013	15 - 49 years	MMR
China: Shandong	China National Maternal and Child Health Surveillance System Child Mortality By Cause 2013 - MCHS	Surveillance		2013	birth - 4 years	242
China: Shandong	China National Disease Surveillance Points (DSP)	Vital Registration		2004-2007, 2013-2014	All Ages	1350124
China: Shandong	China Mortality Registration and Reporting System	Vital Registration		2008-2012	All Ages	1603052
China: Shanghai	China National Disease Surveillance Points (DSP)	Surveillance		1991-2002	All Ages	10752

Appendix Table 6: Causes of death data source list by geography, source type, and study site, with years, age range, and sample size, excluding sources on lower respiratory infection and diarrhoea etiologies						
**Location**	**Source name**	**Source type**	**Site**	**Years**	**Ages**	**Sample size**
China: Shanghai	China National Maternal and Child Health Surveillance System Child Mortality Data By Cause 1996-2012 - MCHS	Surveillance		1996-2012	birth - 4 years	203
China: Shanghai	China National Maternal and Child Health Surveillance System Maternal Mortality By Cause 2006-2012 - MCHS	Surveillance		1998, 2002	15 - 49 years	MMR
China: Shanghai	China National Notifiable Infectious Diseases Reporting Information System (NIDRIS)	Surveillance		2004-2014	All Ages	550
China: Shanghai	China National Maternal and Child Health Surveillance System Maternal Mortality By Cause 1996-2005 - MCHS	Surveillance		2007-2012	15 - 49 years	MMR
China: Shanghai	China National Maternal and Child Health Surveillance System Maternal Mortality By Cause 2013 - MCHS	Surveillance		2013	15 - 49 years	MMR
China: Shanghai	China National Maternal and Child Health Surveillance System Child Mortality By Cause 2013 - MCHS	Surveillance		2013	birth - 4 years	57
China: Shanghai	China National Disease Surveillance Points (DSP)	Vital Registration		2004-2007, 2013-2014	All Ages	193785
China: Shanghai	China Mortality Registration and Reporting System	Vital Registration		2008-2012	All Ages	430556
China: Shanxi	China National Disease Surveillance Points (DSP)	Surveillance		1991-2002	All Ages	16557
China: Shanxi	China National Maternal and Child Health Surveillance System Maternal Mortality By Cause 2006-2012 - MCHS	Surveillance		1996-2005	15 - 49 years	MMR
China: Shanxi	China National Maternal and Child Health Surveillance System Child Mortality Data By Cause 1996-2012 - MCHS	Surveillance		1996-2012	birth - 4 years	1519
China: Shanxi	China National Notifiable Infectious Diseases Reporting Information System (NIDRIS)	Surveillance		2004-2014	All Ages	1357
China: Shanxi	China National Maternal and Child Health Surveillance System Maternal Mortality By Cause 1996-2005 - MCHS	Surveillance		2006-2012	15 - 49 years	MMR
China: Shanxi	China National Maternal and Child Health Surveillance System Maternal Mortality By Cause 2013 - MCHS	Surveillance		2013	15 - 49 years	MMR
China: Shanxi	China National Maternal and Child Health Surveillance System Child Mortality By Cause 2013 - MCHS	Surveillance		2013	birth - 4 years	262
China: Shanxi	China National Disease Surveillance Points (DSP)	Vital Registration		2004-2007, 2013-2014	All Ages	271193
China: Shanxi	China Mortality Registration and Reporting System	Vital Registration		2008-2012	All Ages	266229
China: Sichuan	China National Disease Surveillance Points (DSP)	Surveillance		1991-2002	All Ages	33618
China: Sichuan	China National Maternal and Child Health Surveillance System Maternal Mortality By Cause 2006-2012 - MCHS	Surveillance		1996-2005	15 - 49 years	MMR
China: Sichuan	China National Maternal and Child Health Surveillance System Child Mortality Data By Cause 1996-2012 - MCHS	Surveillance		1996-2012	birth - 4 years	2185
China: Sichuan	China National Notifiable Infectious Diseases Reporting Information System (NIDRIS)	Surveillance		2004-2014	All Ages	17761
China: Sichuan	China National Maternal and Child Health Surveillance System Maternal Mortality By Cause 1996-2005 - MCHS	Surveillance		2006-2012	15 - 49 years	MMR
China: Sichuan	China National Maternal and Child Health Surveillance System Maternal Mortality By Cause 2013 - MCHS	Surveillance		2013	15 - 49 years	MMR
China: Sichuan	China National Maternal and Child Health Surveillance System Child Mortality By Cause 2013 - MCHS	Surveillance		2013	birth - 4 years	211
China: Sichuan	China National Disease Surveillance Points (DSP)	Vital Registration		2004-2007, 2013-2014	All Ages	941026
China: Sichuan	China Mortality Registration and Reporting System	Vital Registration		2008-2012	All Ages	1130413
China: Tianjin	China National Disease Surveillance Points (DSP)	Surveillance		1991-1998	All Ages	4560
China: Tianjin	China National Maternal and Child Health Surveillance System Maternal Mortality By Cause 2006-2012 - MCHS	Surveillance		1996-1997, 1999, 2003-2005	15 - 49 years	MMR
China: Tianjin	China National Maternal and Child Health Surveillance System Child Mortality Data By Cause 1996-2012 - MCHS	Surveillance		1996-2012	birth - 4 years	548
China: Tianjin	China National Disease Surveillance Points (DSP)	Surveillance		2000-2002	5 years +	627
China: Tianjin	China National Notifiable Infectious Diseases Reporting Information System (NIDRIS)	Surveillance		2004-2014	All Ages	314

Appendix Table 6: Causes of death data source list by geography, source type, and study site, with years, age range, and sample size, excluding sources on lower respiratory infection and diarrhoea etiologies						
**Location**	**Source name**	**Source type**	**Site**	**Years**	**Ages**	**Sample size**
China: Tianjin	China National Maternal and Child Health Surveillance System Maternal Mortality By Cause 1996-2005 - MCHS	Surveillance		2006-2012	15 - 49 years	MMR
China: Tianjin	China National Maternal and Child Health Surveillance System Maternal Mortality By Cause 2013 - MCHS	Surveillance		2013	15 - 49 years	MMR
China: Tianjin	China National Maternal and Child Health Surveillance System Child Mortality By Cause 2013 - MCHS	Surveillance		2013	birth - 4 years	140
China: Tianjin	China National Disease Surveillance Points (DSP)	Vital Registration		2004-2007, 2013-2014	All Ages	179925
China: Tianjin	China Mortality Registration and Reporting System	Vital Registration		2008-2012	All Ages	325255
China: Tibet	China National Disease Surveillance Points (DSP)	Surveillance		1991-2002	All Ages	3965
China: Tibet	China Maternal and Child Mortality Data 1996-2012 - China CDC	Surveillance		1996-2012	15 - 49 years	MMR
China: Tibet	China National Maternal and Child Health Surveillance System Child Mortality Data By Cause 1996-2012 - MCHS	Surveillance		1996-2012	birth - 4 years	1617
China: Tibet	China National Maternal and Child Health Surveillance System Maternal Mortality By Cause 2006-2012 - MCHS	Surveillance		1997-2001, 2003-2005	15 - 49 years	MMR
China: Tibet	China National Maternal and Child Health Surveillance System Maternal Mortality By Cause 1996-2005 - MCHS	Surveillance		2006-2012	15 - 49 years	MMR
China: Tibet	China National Notifiable Infectious Diseases Reporting Information System (NIDRIS)	Surveillance		2006-2014	All Ages	44
China: Tibet	China National Maternal and Child Health Surveillance System Maternal Mortality By Cause 2013 - MCHS	Surveillance		2013	15 - 49 years	MMR
China: Tibet	China National Maternal and Child Health Surveillance System Child Mortality By Cause 2013 - MCHS	Surveillance		2013	birth - 4 years	301
China: Tibet	China National Disease Surveillance Points (DSP)	Vital Registration		2004-2007, 2013-2014	All Ages	10955
China: Tibet	China Mortality Registration and Reporting System	Vital Registration		2008-2012	All Ages	5645
China: Xinjiang	China National Disease Surveillance Points (DSP)	Surveillance		1991-2000, 2002	All Ages	5930
China: Xinjiang	China National Maternal and Child Health Surveillance System Maternal Mortality By Cause 2006-2012 - MCHS	Surveillance		1996-2005	15 - 49 years	MMR
China: Xinjiang	China National Maternal and Child Health Surveillance System Child Mortality Data By Cause 1996-2012 - MCHS	Surveillance		1996-2012	birth - 4 years	4762
China: Xinjiang	China National Notifiable Infectious Diseases Reporting Information System (NIDRIS)	Surveillance		2004-2014	All Ages	5970
China: Xinjiang	China National Maternal and Child Health Surveillance System Maternal Mortality By Cause 1996-2005 - MCHS	Surveillance		2006-2012	15 - 49 years	MMR
China: Xinjiang	China National Maternal and Child Health Surveillance System Maternal Mortality By Cause 2013 - MCHS	Surveillance		2013	15 - 49 years	MMR
China: Xinjiang	China National Maternal and Child Health Surveillance System Child Mortality By Cause 2013 - MCHS	Surveillance		2013	birth - 4 years	1314
China: Xinjiang	China National Disease Surveillance Points (DSP)	Vital Registration		2004-2007, 2013-2014	All Ages	104589
China: Xinjiang	China Mortality Registration and Reporting System	Vital Registration		2008-2012	All Ages	82471
China: Yunnan	China National Disease Surveillance Points (DSP)	Surveillance		1991-2002	All Ages	28419
China: Yunnan	China National Maternal and Child Health Surveillance System Maternal Mortality By Cause 2006-2012 - MCHS	Surveillance		1996-2005	15 - 49 years	MMR
China: Yunnan	China National Maternal and Child Health Surveillance System Child Mortality Data By Cause 1996-2012 - MCHS	Surveillance		1996-2012	birth - 4 years	3312
China: Yunnan	China National Notifiable Infectious Diseases Reporting Information System (NIDRIS)	Surveillance		2004-2014	All Ages	20608
China: Yunnan	China National Maternal and Child Health Surveillance System Maternal Mortality By Cause 1996-2005 - MCHS	Surveillance		2006-2012	15 - 49 years	MMR
China: Yunnan	China National Maternal and Child Health Surveillance System Maternal Mortality By Cause 2013 - MCHS	Surveillance		2013	15 - 49 years	MMR

**Appendix Table 6: Causes of death data source list by geography, source type, and study site, with years, age range, and sample size, excluding sources on lower respiratory infection and diarrhoea etiologies**						
**Location**	**Source name**	**Source type**	**Site**	**Years**	**Ages**	**Sample size**
China: Yunnan	China National Maternal and Child Health Surveillance System Child Mortality By Cause 2013 - MCHS	Surveillance		2013	birth - 4 years	786
China: Yunnan	China National Disease Surveillance Points (DSP)	Vital Registration		2004-2007, 2013-2014	All Ages	442057
China: Yunnan	China Mortality Registration and Reporting System	Vital Registration		2008-2012	All Ages	561260
China: Zhejiang	China National Disease Surveillance Points (DSP)	Surveillance		1991-2002	All Ages	27130
China: Zhejiang	China National Maternal and Child Health Surveillance System Maternal Mortality By Cause 2006-2012 - MCHS	Surveillance		1996-1997, 1999-2005	15 - 49 years	MMR
China: Zhejiang	China National Maternal and Child Health Surveillance System Child Mortality Data By Cause 1996-2012 - MCHS	Surveillance		1996-2012	birth - 4 years	788
China: Zhejiang	China National Notifiable Infectious Diseases Reporting Information System (NIDRIS)	Surveillance		2004-2014	All Ages	1533
China: Zhejiang	China National Maternal and Child Health Surveillance System Maternal Mortality By Cause 1996-2005 - MCHS	Surveillance		2007-2012	15 - 49 years	MMR
China: Zhejiang	China National Maternal and Child Health Surveillance System Maternal Mortality By Cause 2013 - MCHS	Surveillance		2013	15 - 49 years	MMR
China: Zhejiang	China National Maternal and Child Health Surveillance System Child Mortality By Cause 2013 - MCHS	Surveillance		2013	birth - 4 years	86
China: Zhejiang	China National Disease Surveillance Points (DSP)	Vital Registration		2004-2007, 2013-2014	All Ages	239871
China: Zhejiang	China Mortality Registration and Reporting System	Vital Registration		2008-2012	All Ages	193637
North Korea	Korea, North Population Census 2008	Survey/Census		2008	15 - 49 years	MMR
Taiwan	United Nations Office on Drugs and Crime Global Study on Homicide 2011	Police Records	Taiwan NSO	1995-2007, 2009	All Ages	Death Rate
Taiwan	Taiwan Annual Statistical Summary of Causes of Death 1994	Vital Registration		1994	All Ages	109985
Taiwan	Taiwan Statistics of Causes of Death	Vital Registration		1995-2006, 2008-2012	All Ages	2188481
Taiwan	Taiwan Vital Statistics - Deaths 2007	Vital Registration		2007	All Ages	138966
Cambodia	United Nations Office on Drugs and Crime Global Study on Homicide 2011	Police Records	Cambodia NGO (e)	1995-2005	All Ages	Death Rate
Cambodia	Cambodia Demographic and Health Survey - Maternal Mortality Data	Sibling History		1986-2014	15 - 49 years	36
Cambodia	Comparing two survey methods for estimating maternal and perinatal mortality in rural Cambodia	Sibling History	Pailin and Battambang	2004	15 - 49 years	MMR
Cambodia	Cambodia Socioeconomic Survey	Survey/Census		2004	All Ages	501
Cambodia	Cambodia Census 2008	Survey/Census		2008	birth - 79 years	CF
Cambodia	Community-based surveillance: a pilot study from rural Cambodia	Verbal Autopsy	Trang Ta Saen Boeng Reang Preah Rumkel Chan Mul Choam Pir Thnu [rural]	2001	All Ages	445
Indonesia	United Nations Surveys on Crime Trends and the Operations of Criminal Justice Systems 1970-2006	Police Records		1981-1984, 1986	All Ages	Death Rate
Indonesia	United Nations Office on Drugs and Crime Global Study on Homicide 2011	Police Records	Indonesia Interpol	2003-2004	All Ages	Death Rate
Indonesia	Global Status Report on Road Safety 2009	Police Records		2007	All Ages	Death Rate
Indonesia	Indonesia Demographic and Health Survey - Maternal Mortality Data	Sibling History		1980-2012	15 - 49 years	250
Indonesia	Indonesia National Socioeconomic Survey (SUSENAS)	Survey/Census	2007	2001	birth - 27 days	1
Indonesia	Indonesia National Socioeconomic Survey (SUSENAS)	Survey/Census	2004	2001-2004	All Ages	8093
Indonesia	Indonesia National Socioeconomic Survey (SUSENAS)	Survey/Census	2007	2004-2007	All Ages	16930
Indonesia	Reproductive Mortality in Two Developing Countries	Verbal Autopsy	rural Bali	1981	15 - 49 years	1215

Appendix Table 6: Causes of death data source list by geography, source type, and study site, with years, age range, and sample size, excluding sources on lower respiratory infection and diarrhoea etiologies

Location	Source name	Source type	Site	Years	Ages	Sample size
Indonesia	The incidence of diarrhoeal diseases and diarrhoeal diseases related mortality in rural swampy low-landarea of south Sumatra, Indonesia	Verbal Autopsy		1983	All Ages	68
Indonesia	The pattern of the causes of death in children in rural swampy area of South Sumatra, Indonesia	Verbal Autopsy		1984	birth - 364 days	29
Indonesia	Efficacy of vitamin A in reducing preschool child mortality in Nepal	Verbal Autopsy	Terai district of Sarlahi [rural]	1989	birth - 4 years	358
Indonesia	Care-seeking for fatal illnesses in young children in Indramayu, West Java, Indonesia	Verbal Autopsy	Indramayu [rural]	1991	birth - 4 years	141
Indonesia	Age- and cause-specific childhood mortality in Lombok, Indonesia, as a factor for determining the appropriateness of introducing Haemophilus influenzae type b and pneumococcal vaccines	Verbal Autopsy	Lombok	1997	birth - 27 days	60
Indonesia	A district-based audit of the causes and circumstances of maternal deaths in South Kalimantan, Indonesia	Verbal Autopsy	rural South Kalimantan - Banjar Barito Kuala Hulu Sangai Seletan	1997	15 - 49 years	MMR
Indonesia	Demographic Surveillance System (DSS)	Verbal Autopsy		2000-2002	All Ages	1948
Indonesia	Indonesia Sample Registration System 2012, Indonesia Cause of Death Survey 2010-2011, Indonesia Mortality Registration System Strengthening Project (IMRSSP), and Indonesia Basic Health Research 2007-2008	Verbal Autopsy	VA sites combined	2010	All Ages	23609
Indonesia	Indonesia Sample Registration System Verbal Autopsy Data 2014	Verbal Autopsy		2014	All Ages	41590
Laos	United Nations Office on Drugs and Crime Global Study on Homicide 2011	Police Records	Laos National police	2010	All Ages	Death Rate
Laos	Laos Census 1995	Survey/Census		1995	15 - 49 years	4007
Laos	Laos Census 2005	Survey/Census		2005	15 - 49 years	5308
Laos	The Lao People's Democratic Republic: maternal mortality and female mortality: determining causes of deaths	Verbal Autopsy		1989	15 - 49 years	380
Malaysia	United Nations Surveys on Crime Trends and the Operations of Criminal Justice Systems 1970-2006	Police Records		1981-1984, 1986, 1990-1994	All Ages	Death Rate
Malaysia	United Nations Office on Drugs and Crime Global Study on Homicide 2011	Police Records	Malaysia CTS	1995-1996	All Ages	Death Rate
Malaysia	World Health Survey (WHS)	Survey/Census		1997	All Ages	20
Malaysia	Malaysia - Peninsular Vital Statistics	Vital Registration	Peninsular Malaysia	1980-1982	All Ages	71086
Malaysia	WHO Mortality Database	Vital Registration		1997, 2000-2008	All Ages	617571
Malaysia	Malaysia - Peninsular Vital Statistics	Vital Registration		1998	All Ages	43585
Maldives	United Nations Surveys on Crime Trends and the Operations of Criminal Justice Systems 1970-2006	Police Records		1986-1990	All Ages	Death Rate
Maldives	United Nations Office on Drugs and Crime Global Study on Homicide 2011	Police Records	Maldives CTS	1995, 1997, 2001-2002, 2007	All Ages	Death Rate
Maldives	Global Status Report on Road Safety 2009	Police Records		2007	All Ages	Death Rate
Maldives	WHO Mortality Database	Vital Registration		2000-2005, 2007-2008, 2010-2011	All Ages	10597
Mauritius	United Nations Surveys on Crime Trends and the Operations of Criminal Justice Systems 1970-2006	Police Records		1987, 1989-1994	All Ages	Death Rate
Mauritius	United Nations Office on Drugs and Crime Global Study on Homicide 2011	Police Records	Mauritius CTS	1995-2003, 2007, 2009	All Ages	Death Rate
Mauritius	United Nations Office on Drugs and Crime Global Study on Homicide 2011	Police Records	Mauritius WHO-MDB	1998-2003, 2007	All Ages	Death Rate
Mauritius	WHO Mortality Database	Vital Registration		1980-2014	All Ages	268115
Myanmar	United Nations Office on Drugs and Crime Global Study on Homicide 2011	Police Records	Myanmar NSO	1995, 2000-2007	All Ages	Death Rate

Appendix Table 6: Causes of death data source list by geography, source type, and study site, with years, age range, and sample size, excluding sources on lower respiratory infection and diarrhoea etiologies

Location	Source name	Source type	Site	Years	Ages	Sample size
Myanmar	Cause of Death Verification Study in Myanmar	Verbal Autopsy	Pyinmana Township	2007	All Ages	1775
Myanmar	Effects of malaria volunteer training on coverage and timeliness of diagnosis: a cluster randomized controlled trial in Myanmar	Verbal Autopsy	Bago Daik-U KyaukTaGar Oktwin Taungoo and YeDarShay townships	2009	All Ages	61
Philippines	United Nations Office on Drugs and Crime Global Study on Homicide 2011	Police Records	Philippines CTS	2009	All Ages	Death Rate
Philippines	Philippines Demographic and Health Survey - Maternal Mortality Data	Sibling History		1980-1998	15 - 54 years	214
Philippines	Philippines Field Health Services Information System (FHSIS)	Surveillance		1993-1994, 1996-1997, 2000-2003, 2006-2007	All Ages	2485239
Philippines	Philippines Field Health Services Information System (FHSIS)	Surveillance	Capitol Region	1998	All Ages	48333
Philippines	World Health Survey (WHS)	Survey/Census		1997	All Ages	51
Philippines	Effect of not breastfeeding on the risk of diarrheal and respiratory mortality in children under 2 years of age in Metro Cebu, The Philippines	Verbal Autopsy	Cebu [urban]	1989	birth - 364 days	425
Philippines	A comparison of vital registration and reproductive-age mortality survey in Bukidnon, Philippines, 2008	Verbal Autopsy	Bukidnon	2008	15 - 49 years	402
Philippines	Philippines Vital Statistics - Deaths 1979-2000	Vital Registration		1980, 1982-1991	All Ages	3506013
Philippines	WHO Mortality Database	Vital Registration		1981, 1992-2003, 2008	All Ages	4967428
Philippines	Philippines Vital Statistics - Deaths 2001-2005	Vital Registration		2004-2005	All Ages	829184
Philippines	Philippines Vital Registration - Deaths 2006	Vital Registration		2006	All Ages	440892
Philippines	Philippines Vital Registration - Deaths 2007	Vital Registration		2007	All Ages	441784
Philippines	Philippines Vital Registration - Deaths 2009	Vital Registration		2009	All Ages	480725
Philippines	Philippines Vital Registration - Deaths 2010	Vital Registration		2010	All Ages	488162
Philippines	Philippines Vital Registration - Deaths 2011	Vital Registration		2011	All Ages	498320
Philippines	Philippines Vital Registration - Deaths 2012	Vital Registration		2012	All Ages	514644
Sri Lanka	United Nations Surveys on Crime Trends and the Operations of Criminal Justice Systems 1970-2006	Police Records		1980-1986, 1990	All Ages	Death Rate
Sri Lanka	United Nations Office on Drugs and Crime Global Study on Homicide 2011	Police Records	Sri Lanka WHO-MDB	1997-2003	All Ages	Death Rate
Sri Lanka	United Nations Office on Drugs and Crime Global Study on Homicide 2011	Police Records	Sri Lanka National police	2005-2007, 2009	All Ages	Death Rate
Sri Lanka	Sri Lanka Achievements in Maternal Health Report	Surveillance		1995-2008	15 - 49 years	MMR
Sri Lanka	World Health Survey (WHS)	Survey/Census		1997	All Ages	151
Sri Lanka	WHO Mortality Database	Vital Registration		1980-1989, 1991-1992, 1997-2003, 2006	All Ages	2070480
Sri Lanka	Sri Lanka Vital Registration System	Vital Registration		1993-1996, 2004-2005, 2009-2010	All Ages	928066
Seychelles	United Nations Surveys on Crime Trends and the Operations of Criminal Justice Systems 1970-2006	Police Records		1980, 1988-1990	All Ages	Death Rate
Seychelles	WHO Mortality Database	Vital Registration		1981-1982, 1985-1987, 2001-2012	All Ages	10187
Thailand	Global Status Report on Road Safety 2009	Police Records		2007	All Ages	Death Rate
Thailand	United Nations Office on Drugs and Crime Global Study on Homicide 2011	Police Records	Thailand National police	2009-2010	All Ages	Death Rate
Thailand	Thailand Burden of Disease and Injuries 1998-1999	Verbal Autopsy		1997-1998	All Ages	18991
Thailand	Risk factors for a five-year death in the interASIA-south cohort	Verbal Autopsy	Songkhla	2000	35 - 64 years	50
Thailand	Perinatal death pattern in the four districts of Thailand: findings from the Prospective Cohort Study of Thai Children (PCTC)	Verbal Autopsy	Panomtuan Kanchanaburi; Thepa Songkhla; Kranuan Khon Kaen; Muang Nan	2001	birth - 6 days	14

Appendix Table 6: Causes of death data source list by geography, source type, and study site, with years, age range, and sample size, excluding sources on lower respiratory infection and diarrhoea etiologies

Location	Source name	Source type	Site	Years	Ages	Sample size
Thailand	Thailand Verbal Autopsy Study 2005	Verbal Autopsy		2005	All Ages	12270
Thailand	WHO Mortality Database	Vital Registration		1980-1987, 1990-1992, 1994-2000, 2002-2006	All Ages	6933895
Thailand	Hospital Mortality Data 2002-2007	Vital Registration		2007	All Ages	393163
Thailand	Thailand Public Health Statistics	Vital Registration		2009-2011	All Ages	1207783
Timor-Leste	United Nations Office on Drugs and Crime Global Study on Homicide 2011	Police Records	Timor-Leste UN-PKO	2004-2007, 2009-2010	All Ages	Death Rate
Timor-Leste	Demographic and Health Survey (DHS)	Sibling History		1995-2009	15 - 49 years	15
Vietnam	United Nations Office on Drugs and Crime Global Study on Homicide 2011	Police Records	Vietnam CTS	2005-2006	All Ages	Death Rate
Vietnam	Survey on immunization, diarrhoeal disease and mortality in Quang Ninh Province, Vietnam	Survey/Census	Quang Ninh province [urban]	1987	birth - 364 days	103
Vietnam	Vietnam Population Change and Family Planning Survey	Survey/Census		2006-2007	All Ages	CF
Vietnam	Are there social inequities in child morbidity and mortality in rural Vietnam	Verbal Autopsy	1/8 districts in Thai Binh province in the Red River Delta [rural]	1987	birth - 4 years	124
Vietnam	Maternal mortality in Vietnam in 1994-95	Verbal Autopsy	Quang Ngai [urban]	1994	15 - 49 years	812
Vietnam	Maternal mortality in Vietnam in 1994-95	Verbal Autopsy	Song Be [rural]	1994	15 - 49 years	794
Vietnam	Maternal mortality in Vietnam in 1994-95	Verbal Autopsy	Vinh Phu [rural]	1994	15 - 49 years	1236
Vietnam	Applying verbal autopsy to determine cause of death in rural Vietnam	Verbal Autopsy	Bavi District (FilaBavi) [rural]	1999	All Ages	189
Vietnam	Socio-economic status inequality and major causes of death in adults: a 5-year follow-up study in rural Vietnam	Verbal Autopsy	Bavi District (FilaBavi) [rural]	2001	20 years +	1067
Vietnam	Mortality patterns in Vietnam, 2006: Findings from a national verbal autopsy survey	Verbal Autopsy		2006	All Ages	6804
Vietnam	Demographic Surveillance System (DSS)	Verbal Autopsy		2006-2008	All Ages	853
Vietnam	Mortality measures from sample-based surveillance: evidence of the epidemiological transition in Viet Nam, Unpublished data	Verbal Autopsy		2008	All Ages	9291
Vietnam	Vietnam Burden of Disease and Injury Study 2008	Verbal Autopsy		2008	All Ages	541133
Vietnam	The causes of deaths in Chililab between 2008-2010 based on verbal autopsy method	Verbal Autopsy	seven villages Chililabarea [rural]	2008-2010	All Ages	896
Vietnam	Mortality measures from sample-based surveillance: evidence of the epidemiological transition in Viet Nam	Verbal Autopsy		2009	All Ages	9920
Vietnam	Causes of neonatal death: results from NeoKIP community-based trial in Quang Ninh province, Vietnam	Verbal Autopsy	Quang Ninh [rural]	2009	birth - 27 days	233
Vietnam	Maternal and Antenatal Risk Factors for Stillbirths and Neonatal Mortality in Rural Bangladesh: A Case-Control Study	Verbal Autopsy	[urban]	2011	birth - 27 days	68
American Samoa	United States National Vital Statistics System (NVSS)	Vital Registration		1997-2012	All Ages	4176
Federated States of Micronesia	Global Status Report on Road Safety 2009	Police Records		2007	All Ages	Death Rate
Fiji	United Nations Surveys on Crime Trends and the Operations of Criminal Justice Systems 1970-2006	Police Records		1980-1986, 1990-1994	All Ages	Death Rate
Fiji	United Nations Office on Drugs and Crime Global Study on Homicide 2011	Police Records	Fiji Interpol	2003-2004	All Ages	Death Rate
Fiji	Global Status Report on Road Safety 2009	Police Records		2007	All Ages	Death Rate
Fiji	WHO Mortality Database	Vital Registration		1999, 2001-2009, 2011-2012	All Ages	71250
Guam	United Nations Office on Drugs and Crime Global Study on Homicide 2011	Police Records	Guam National police	2000-2007	All Ages	Death Rate
Guam	United States National Vital Statistics System (NVSS)	Vital Registration		1994-2012	All Ages	13652

Appendix Table 6: Causes of death data source list by geography, source type, and study site, with years, age range, and sample size, excluding sources on lower respiratory infection and diarrhoea etiologies

Location	Source name	Source type	Site	Years	Ages	Sample size
Kiribati	United Nations Surveys on Crime Trends and the Operations of Criminal Justice Systems 1970-2006	Police Records		1986	All Ages	Death Rate
Kiribati	Global Status Report on Road Safety 2009	Police Records		2007	All Ages	Death Rate
Kiribati	WHO Mortality Database	Vital Registration		1991-2001	All Ages	8332
Marshall Islands	United Nations Surveys on Crime Trends and the Operations of Criminal Justice Systems 1970-2006	Police Records		1991-1992, 1994	All Ages	Death Rate
Marshall Islands	Global Status Report on Road Safety 2009	Police Records		2007	All Ages	Death Rate
Northern Mariana Islands	United States National Vital Statistics System (NVSS)	Vital Registration		1998-2012	All Ages	2251
Papua New Guinea	United Nations Office on Drugs and Crime Global Study on Homicide 2011	Police Records	Papua New Guinea National police	2006-2007	All Ages	Death Rate
Papua New Guinea	Global Status Report on Road Safety 2009	Police Records		2007	All Ages	Death Rate
Papua New Guinea	Discrepancies between national maternal mortality data and international estimates: the experience of Papua New Guinea	Sibling history		1995, 2006	15 - 49 years	MMR
Papua New Guinea	Discrepancies between national maternal mortality data and international estimates: the experience of Papua New Guinea	Survey/Census		2009	15 - 49 years	MMR
Papua New Guinea	Mortality and morbidity from acute lower respiratory tract infections in Tari, Southern Highlands Province 1977-1983	Verbal Autopsy	.(Tari Basin)	1980	All Ages	2193
Papua New Guinea	Demography and causes of death among the Huli in the Tari Basin	Verbal Autopsy	Tari Basin [rural]	1980	All Ages	2312
Papua New Guinea	Mortality in a rural area of Madang Province, Papua New Guinea	Verbal Autopsy	Madang [rural]	1983	All Ages	407
Papua New Guinea	Mortality rates and the utilization of health services during terminal illness in the Asaro Valley, Eastern Highlands Province, Papua New Guinea	Verbal Autopsy	Asaro Valley	1985	All Ages	582
Papua New Guinea	WHO Mortality Database	Vital Registration		1980	All Ages	3559
Solomon Islands	United Nations Office on Drugs and Crime Global Study on Homicide 2011	Police Records	Solomon Islands CTS	2004-2007	All Ages	Death Rate
Tonga	United Nations Surveys on Crime Trends and the Operations of Criminal Justice Systems 1970-2006	Police Records		1980, 1986-1990	All Ages	Death Rate
Tonga	Tonga Vital Statistics - Deaths 2003	Vital Registration		2003	All Ages	1682
Vanuatu	United Nations Surveys on Crime Trends and the Operations of Criminal Justice Systems 1970-2006	Police Records		1987-1990	All Ages	Death Rate
Armenia	United Nations Surveys on Crime Trends and the Operations of Criminal Justice Systems 1970-2006	Police Records		1986-1988	All Ages	Death Rate
Armenia	Global Status Report on Road Safety 2009	Police Records		2007	All Ages	Death Rate
Armenia	WHO Mortality Database	Vital Registration		1981-1982, 1985-2003, 2006, 2008-2012	All Ages	658840
Azerbaijan	United Nations Office on Drugs and Crime Global Study on Homicide 2011	Police Records	Azerbaijan CTS	2007	All Ages	Death Rate
Azerbaijan	WHO Mortality Database	Vital Registration		1981-1982, 1985-2004, 2007	All Ages	1085713
Georgia	United Nations Surveys on Crime Trends and the Operations of Criminal Justice Systems 1970-2006	Police Records		1990-1994	All Ages	Death Rate
Georgia	United Nations Office on Drugs and Crime Global Study on Homicide 2011	Police Records	Georgia CTS/Transmonee	1995-2007, 2009-2010	All Ages	Death Rate
Georgia	United Nations Office on Drugs and Crime Global Study on Homicide 2011	Police Records	Georgia WHO-HFA	1996-2001	All Ages	Death Rate
Georgia	WHO Mortality Database	Vital Registration		1981-1982, 1985-1992, 1994-2001, 2004-2007, 2009-2014	All Ages	1235263
Kazakhstan	United Nations Office on Drugs and Crime Global Study on Homicide 2011	Police Records	Kazakhstan Transmonee	1995-1999	All Ages	Death Rate

Appendix Table 6: Causes of death data source list by geography, source type, and study site, with years, age range, and sample size, excluding sources on lower respiratory infection and diarrhoea etiologies

Location	Source name	Source type	Site	Years	Ages	Sample size
Kazakhstan	United Nations Office on Drugs and Crime Global Study on Homicide 2011	Police Records	Kazakhstan WHO-HFA	1995-1999	All Ages	Death Rate
Kazakhstan	WHO Mortality Database	Vital Registration		1981-1982, 1985-2006, 2008-2012	All Ages	4170695
Kyrgyzstan	United Nations Office on Drugs and Crime Global Study on Homicide 2011	Police Records	Kyrgyzstan CTS/Transmonee	1995, 1997, 2000-2007, 2009	All Ages	Death Rate
Kyrgyzstan	United Nations Office on Drugs and Crime Global Study on Homicide 2011	Police Records	Kyrgyzstan WHO-HFA	1995, 1997, 2000-2007, 2009	All Ages	Death Rate
Kyrgyzstan	WHO Mortality Database	Vital Registration		1981-1982, 1985-2013	All Ages	1055953
Mongolia	United Nations Office on Drugs and Crime Global Study on Homicide 2011	Police Records	Mongolia CTS	2004-2007, 2009	All Ages	Death Rate
Mongolia	Tracking Maternal Mortality Declines in Mongolia Between 1992 and 2007: The Importance of Collaboration	Vital Registration		1992-1993, 1995-2007	15 - 49 years	MMR
Mongolia	WHO Mortality Database	Vital Registration		1994	All Ages	14923
Mongolia	Mongolia Vital Statistics - Deaths 2004-2008	Vital Registration		2004-2008	All Ages	77294
Mongolia	Mongolia Vital Registration - Deaths 2010	Vital Registration		2010	All Ages	17230
Tajikistan	United Nations Office on Drugs and Crime Global Study on Homicide 2011	Police Records	Tajikistan WHO-HFA	2000-2001	All Ages	Death Rate
Tajikistan	United Nations Office on Drugs and Crime Global Study on Homicide 2011	Police Records	Tajikistan CTS	2006-2007, 2009	All Ages	Death Rate
Tajikistan	Tajikistan - Infant Mortality in Tajikistan: Two Studies Look at Risk Factors	Verbal Autopsy	Sugd province and the Region of Republican Subordination (RSS)	2001	birth - 364 days	96
Tajikistan	Tajikistan - Infant Mortality in Tajikistan: Two Studies Look at Risk Factors	Verbal Autopsy	Khatlon province Dushanbe city	2003	birth - 364 days	217
Tajikistan	WHO Mortality Database	Vital Registration		1981-1982, 1985-2005	All Ages	731888
Turkmenistan	United Nations Office on Drugs and Crime Global Study on Homicide 2011	Police Records	Turkmenistan Transmonee	1995-1997, 1999-2006	All Ages	Death Rate
Turkmenistan	WHO Mortality Database	Vital Registration		1981-1982, 1985-1998, 2013	All Ages	482159
Uzbekistan	United Nations Office on Drugs and Crime Global Study on Homicide 2011	Police Records	Uzbekistan WHO-HFA	1995-2005	All Ages	Death Rate
Uzbekistan	United Nations Office on Drugs and Crime Global Study on Homicide 2011	Police Records	Uzbekistan CTS/Transmonee	1995-2007	All Ages	Death Rate
Uzbekistan	WHO Mortality Database	Vital Registration		1981-1982, 1985-2005	All Ages	3100622
Albania	Global Status Report on Road Safety 2009	Police Records		2007	All Ages	Death Rate
Albania	WHO Mortality Database	Vital Registration		1987-1989, 1992-2009	All Ages	346602
Albania	Albania Vital Registration - Causes of Death 1993-2010	Vital Registration		2010	All Ages	380
Bosnia and Herzegovina	World Health Survey (WHS)	Survey/Census		1997	All Ages	51
Bosnia and Herzegovina	WHO Mortality Database	Vital Registration		1985-1991, 2011	All Ages	242187
Bulgaria	Global Status Report on Road Safety 2009	Police Records		2007	All Ages	Death Rate
Bulgaria	WHO Mortality Database	Vital Registration		1980-2012	All Ages	3610447
Croatia	Global Status Report on Road Safety 2009	Police Records		2007	All Ages	Death Rate
Croatia	WHO Mortality Database	Vital Registration		1985-2013	All Ages	1495702
Czech Republic	Global Status Report on Road Safety 2009	Police Records		2007	All Ages	Death Rate
Czech Republic	WHO Mortality Database	Vital Registration		1986-2013	All Ages	3188714
Hungary	Global Status Report on Road Safety 2009	Police Records		2007	All Ages	Death Rate
Hungary	WHO Mortality Database	Vital Registration		1980-2013	All Ages	4745330
Macedonia	WHO Mortality Database	Vital Registration		1991-2010	All Ages	346691
Montenegro	Global Status Report on Road Safety 2009	Police Records		2007	All Ages	Death Rate

Appendix Table 6: Causes of death data source list by geography, source type, and study site, with years, age range, and sample size, excluding sources on lower respiratory infection and diarrhoea etiologies						
**Location**	**Source name**	**Source type**	**Site**	**Years**	**Ages**	**Sample size**
Montenegro	WHO Mortality Database	Vital Registration		2000-2004, 2006, 2009	All Ages	57121
Poland	Global Status Report on Road Safety 2009	Police Records		2007	All Ages	Death Rate
Poland	WHO Mortality Database	Vital Registration		1980-1996, 1999-2013	All Ages	11957283
Romania	WHO Mortality Database	Vital Registration		1980-2012	All Ages	8415641
Serbia	Global Status Report on Road Safety 2009	Police Records		2007	All Ages	Death Rate
Serbia	WHO Mortality Database	Vital Registration		1998-2013	All Ages	1642937
Slovakia	Global Status Report on Road Safety 2009	Police Records		2007	All Ages	Death Rate
Slovakia	WHO Mortality Database	Vital Registration		1992-2010, 2012-2014	All Ages	1155411
Slovenia	Global Status Report on Road Safety 2009	Police Records		2007	All Ages	Death Rate
Slovenia	WHO Mortality Database	Vital Registration		1985-2010	All Ages	493028
Belarus	WHO Mortality Database	Vital Registration		1981-1982, 1985-1995, 1997-2003, 2007-2009, 2011	All Ages	2947092
Estonia	World Health Survey (WHS)	Survey/Census		1997	All Ages	6
Estonia	WHO Mortality Database	Vital Registration		1981-1982, 1985-2012	All Ages	549124
Latvia	WHO Mortality Database	Vital Registration		1980-2012	All Ages	983159
Lithuania	Global Status Report on Road Safety 2009	Police Records		2006	All Ages	Death Rate
Lithuania	WHO Mortality Database	Vital Registration		1981-1982, 1985-2013	All Ages	1269969
Moldova	WHO Mortality Database	Vital Registration		1981-1982, 1985-2013	All Ages	1331728
Russia	Russia Road Safety Performance 2010	Police Records		2004-2008	All Ages	Death Rate
Russia	WHO Mortality Database	Vital Registration		1980-1988	All Ages	13992472
Russia	Russia Mortality Rates by Region, Age, Sex, and Cause of Death 1989-1998	Vital Registration		1989-1998	All Ages	19274200
Russia	Russia Mortality Rates by Region, Age, Sex, and Cause of Death 1999-2005	Vital Registration		1999-2000	All Ages	4357944
Russia	Russia Vital Registration System	Vital Registration		2001-2014	All Ages	29485688
Ukraine	WHO Mortality Database	Vital Registration		1981-1982, 1985-2012	All Ages	20960298
Brunei	United Nations Office on Drugs and Crime Global Study on Homicide 2011	Police Records	Brunei WHO-MDB	1996-1998	All Ages	Death Rate
Brunei	United Nations Office on Drugs and Crime Global Study on Homicide 2011	Police Records	Brunei CTS	2003-2004, 2006	All Ages	Death Rate
Brunei	Global Status Report on Road Safety 2009	Police Records		2007	All Ages	Death Rate
Brunei	WHO Mortality Database	Vital Registration		1996-2013	All Ages	19294
Japan: Aichi	Japan Vital Registration System	Vital Registration		1980-2013	All Ages	1514522
Japan: Akita	Japan Vital Registration System	Vital Registration		1980-2013	All Ages	393161
Japan: Aomori	Japan Vital Registration System	Vital Registration		1980-2013	All Ages	436414
Japan: Chiba	Japan Vital Registration System	Vital Registration		1980-2013	All Ages	1222403
Japan: Ehime	Japan Vital Registration System	Vital Registration		1980-2013	All Ages	465619
Japan: Fukui	Japan Vital Registration System	Vital Registration		1980-2013	All Ages	235887
Japan: Fukuoka	Japan Vital Registration System	Vital Registration		1980-2013	All Ages	1285379
Japan: Fukushima	Japan Vital Registration System	Vital Registration		1980-2013	All Ages	622295
Japan: Gifu	Japan Vital Registration System	Vital Registration		1980-2013	All Ages	550630
Japan: Gunma	Japan Vital Registration System	Vital Registration		1980-2013	All Ages	538270
Japan: Hiroshima	Japan Vital Registration System	Vital Registration		1980-2013	All Ages	776972
Japan: Hokkaidō	Japan Vital Registration System	Vital Registration		1980-2013	All Ages	1454522

Appendix Table 6: Causes of death data source list by geography, source type, and study site, with years, age range, and sample size, excluding sources on lower respiratory infection and diarrhoea etiologies						
**Location**	**Source name**	**Source type**	**Site**	**Years**	**Ages**	**Sample size**
Japan: Hyōgo	Japan Vital Registration System	Vital Registration		1980-2013	All Ages	1423264
Japan: Ibaraki	Japan Vital Registration System	Vital Registration		1980-2013	All Ages	755182
Japan: Ishikawa	Japan Vital Registration System	Vital Registration		1980-2013	All Ages	317957
Japan: Iwate	Japan Vital Registration System	Vital Registration		1980-2013	All Ages	431161
Japan: Kagawa	Japan Vital Registration System	Vital Registration		1980-2013	All Ages	309212
Japan: Kagoshima	Japan Vital Registration System	Vital Registration		1980-2013	All Ages	587598
Japan: Kanagawa	Japan Vital Registration System	Vital Registration		1980-2013	All Ages	1653904
Japan: Kōchi	Japan Vital Registration System	Vital Registration		1980-2013	All Ages	282426
Japan: Kumamoto	Japan Vital Registration System	Vital Registration		1980-2013	All Ages	545404
Japan: Kyōto	Japan Vital Registration System	Vital Registration		1980-2013	All Ages	684103
Japan: Mie	Japan Vital Registration System	Vital Registration		1980-2013	All Ages	513813
Japan: Miyagi	Japan Vital Registration System	Vital Registration		1980-2013	All Ages	576512
Japan: Miyazaki	Japan Vital Registration System	Vital Registration		1980-2013	All Ages	339283
Japan: Nagano	Japan Vital Registration System	Vital Registration		1980-2013	All Ages	643193
Japan: Nagasaki	Japan Vital Registration System	Vital Registration		1980-2013	All Ages	462617
Japan: Nara	Japan Vital Registration System	Vital Registration		1980-2013	All Ages	347724
Japan: Niigata	Japan Vital Registration System	Vital Registration		1980-2013	All Ages	732819
Japan: Ôita	Japan Vital Registration System	Vital Registration		1980-2013	All Ages	377766
Japan: Okayama	Japan Vital Registration System	Vital Registration		1980-2013	All Ages	568267
Japan: Okinawa	Japan Vital Registration System	Vital Registration		1980-2013	All Ages	259591
Japan: Ōsaka	Japan Vital Registration System	Vital Registration		1980-2013	All Ages	2071326
Japan: Saga	Japan Vital Registration System	Vital Registration		1980-2013	All Ages	266168
Japan: Saitama	Japan Vital Registration System	Vital Registration		1980-2013	All Ages	1323196
Japan: Shiga	Japan Vital Registration System	Vital Registration		1980-2013	All Ages	311351
Japan: Shimane	Japan Vital Registration System	Vital Registration		1980-2013	All Ages	263473
Japan: Shizuoka	Japan Vital Registration System	Vital Registration		1980-2013	All Ages	936226
Japan: Tochigi	Japan Vital Registration System	Vital Registration		1980-2013	All Ages	524080
Japan: Tokushima	Japan Vital Registration System	Vital Registration		1980-2013	All Ages	265449
Japan: Tōkyō	Japan Vital Registration System	Vital Registration		1980-2013	All Ages	2754255
Japan: Tottori	Japan Vital Registration System	Vital Registration		1980-2013	All Ages	190114
Japan: Toyama	Japan Vital Registration System	Vital Registration		1980-2013	All Ages	329982
Japan: Wakayama	Japan Vital Registration System	Vital Registration		1980-2013	All Ages	345954
Japan: Yamagata	Japan Vital Registration System	Vital Registration		1980-2013	All Ages	397344
Japan: Yamaguchi	Japan Vital Registration System	Vital Registration		1980-2013	All Ages	499828
Japan: Yamanashi	Japan Vital Registration System	Vital Registration		1980-2013	All Ages	240729
South Korea	United Nations Office on Drugs and Crime Global Study on Homicide 2011	Police Records	South Korea WHO-MDB	1995-1997	All Ages	Death Rate
South Korea	United Nations Office on Drugs and Crime Global Study on Homicide 2011	Police Records	South Korea National police	2009	All Ages	Death Rate
South Korea	WHO Mortality Database	Vital Registration		1985-2013	All Ages	6703551
Singapore	Global Status Report on Road Safety 2009	Police Records		2007	All Ages	Death Rate
Singapore	WHO Mortality Database	Vital Registration		1980-2014	All Ages	511814

**Appendix Table 6: Causes of death data source list by geography, source type, and study site, with years, age range, and sample size, excluding sources on lower respiratory infection and diarrhoea etiologies**						
**Location**	**Source name**	**Source type**	**Site**	**Years**	**Ages**	**Sample size**
Australia	Australia Maternal Deaths	Surveillance		1997-1999	15 - 44 years	90
Australia	Australia Maternal Deaths	Surveillance		2006-2011	15 - 54 years	201
Australia	WHO Mortality Database	Vital Registration		1980-2004, 2006-2011	All Ages	3895616
Australia	Australia Vital Registration - Deaths 2005	Vital Registration		2005	All Ages	131323
New Zealand	New Zealand Annual Report of the Perinatal and Maternal Mortality Review Committee 2012	Surveillance		2006-2012	20 - 54 years	MMR
New Zealand	WHO Mortality Database	Vital Registration		1980-1987	All Ages	210732
New Zealand	New Zealand Mortality Collection	Vital Registration		1988-2012	All Ages	697936
Austria	WHO Mortality Database	Vital Registration		1980-2014	All Ages	2843518
Belgium	WHO Mortality Database	Vital Registration		1980-2012	All Ages	3504510
Cyprus	Global Status Report on Road Safety 2009	Police Records		2007	All Ages	Death Rate
Cyprus	WHO Mortality Database	Vital Registration		1999-2000, 2004-2012	All Ages	57807
Denmark	Maternal mortality in Denmark, 1985-1994	Surveillance		1985-1994	10 - 54 years	MMR
Denmark	Maternal deaths in Denmark 2002-2006	Surveillance		2002-2006	10 - 54 years	MMR
Denmark	WHO Mortality Database	Vital Registration		1980-2012	All Ages	1894591
Finland	Pregnancy-associated deaths in Finland 1987-1994--definition problems and benefits of record linkage	Surveillance		1987-1994	15 - 49 years	MMR
Finland	WHO Mortality Database	Vital Registration		1980-2013	All Ages	1652210
France	[Maternal mortality in France, 2007-2009]	Surveillance		2001-2009	10 - 54 years	MMR
France	WHO Mortality Database	Vital Registration		1980-2011	All Ages	17092446
Germany	WHO Mortality Database	Vital Registration		1980-2013	All Ages	29929755
Greece	WHO Mortality Database	Vital Registration		1980-2012	All Ages	3289100
Iceland	WHO Mortality Database	Vital Registration		1980-2009	All Ages	53285
Ireland	Ireland Confidential Maternal Death Enquiry Report 2009-2012	Surveillance		2009-2012	10 - 54 years	MMR
Ireland	WHO Mortality Database	Vital Registration		1980-2012	All Ages	1016803
Israel	Global Status Report on Road Safety 2009	Police Records		2007	All Ages	Death Rate
Israel	WHO Mortality Database	Vital Registration		1980-2013	All Ages	1167904
Italy	WHO Mortality Database	Vital Registration		1980-2003, 2006-2012	All Ages	17357051
Luxembourg	WHO Mortality Database	Vital Registration		1980-2013	All Ages	129291
Malta	Global Status Report on Road Safety 2009	Police Records		2007	All Ages	Death Rate
Malta	WHO Mortality Database	Vital Registration		1980-2014	All Ages	105362
Netherlands	WHO Mortality Database	Vital Registration		1980-2013	All Ages	4484563
Norway	Maternal Deaths in Norway 2005-2009	Surveillance		2005-2009	10 - 54 years	MMR
Norway	WHO Mortality Database	Vital Registration		1980-2013	All Ages	1472045
Portugal	WHO Mortality Database	Vital Registration		1980-2003, 2007-2013	All Ages	3170914
Portugal	Portugal Vital Statistics - Deaths 2005-2006	Vital Registration		2005-2006	All Ages	195324
Spain	WHO Mortality Database	Vital Registration		1980-2013	All Ages	11832353
Sweden: Stockholm	Sweden Cause of Death Register	Vital Registration		1980-2011	All Ages	435595
Sweden: Sweden except Stockholm	Sweden Cause of Death Register	Vital Registration		1980-2011	All Ages	2161827
Switzerland	Global Status Report on Road Safety 2009	Police Records		2006	All Ages	Death Rate
Switzerland	WHO Mortality Database	Vital Registration		1980-2013	All Ages	2094173

Appendix Table 6: Causes of death data source list by geography, source type, and study site, with years, age range, and sample size, excluding sources on lower respiratory infection and diarrhoea etiologies						
**Location**	**Source name**	**Source type**	**Site**	**Years**	**Ages**	**Sample size**
United Kingdom: England: East Midlands	United Kingdom - England and Wales Vital Registration System	Vital Registration		1981-2013	All Ages	1416488
United Kingdom: England: East of England	United Kingdom - England and Wales Vital Registration System	Vital Registration		1981-2013	All Ages	1736957
United Kingdom: England: Greater London	United Kingdom - England and Wales Vital Registration System	Vital Registration		1981-2013	All Ages	2058518
United Kingdom: England: North East England	United Kingdom - England and Wales Vital Registration System	Vital Registration		1981-2013	All Ages	975240
United Kingdom: England: North West England	United Kingdom - England and Wales Vital Registration System	Vital Registration		1981-2013	All Ages	2566893
United Kingdom: England: South East England	United Kingdom - England and Wales Vital Registration System	Vital Registration		1981-2013	All Ages	2652034
United Kingdom: England: South West England	United Kingdom - England and Wales Vital Registration System	Vital Registration		1981-2013	All Ages	1803292
United Kingdom: England: West Midlands	United Kingdom - England and Wales Vital Registration System	Vital Registration		1981-2013	All Ages	1812346
United Kingdom: England: Yorkshire and the Humber	United Kingdom - England and Wales Vital Registration System	Vital Registration		1981-2013	All Ages	1794013
Northern Ireland	WHO Mortality Database	Vital Registration		1980-2013	All Ages	516306
Scotland	WHO Mortality Database	Vital Registration		1980-2013	All Ages	2022934
Wales	United Kingdom - England and Wales Vital Registration System	Vital Registration		1981-2013	All Ages	1107500
Argentina	United Nations Surveys on Crime Trends and the Operations of Criminal Justice Systems 1970-2006	Police Records		1980	All Ages	Death Rate
Argentina	United Nations Office on Drugs and Crime Global Study on Homicide 2011	Police Records	Argentina CTS/Ministry of Justice	1997-1999, 2001-2005, 2007	All Ages	Death Rate
Argentina	United Nations Office on Drugs and Crime Global Study on Homicide 2011	Police Records	Argentina PAHO	1997-1999, 2001-2005, 2007	All Ages	Death Rate
Argentina	Maternal Mortality in Argentina: A Closer Look at Women Who Die Outside of the Health System	Verbal Autopsy	Chaco Formosa Mendoza San Luis and Tucuman	2002	10 - 49 years	252
Argentina	WHO Mortality Database	Vital Registration		1980-2013	All Ages	9337228
Chile	United Nations Office on Drugs and Crime Global Study on Homicide 2011	Police Records	Chile PAHO	2000-2004, 2007	All Ages	Death Rate
Chile	United Nations Office on Drugs and Crime Global Study on Homicide 2011	Police Records	Chile CTS	2007, 2009	All Ages	Death Rate
Chile	WHO Mortality Database	Vital Registration		1980-1984, 1986-2013	All Ages	2675596
Chile	Chile Vital Registration - Deaths 1985	Vital Registration		1985	All Ages	73490
Uruguay	United Nations Surveys on Crime Trends and the Operations of Criminal Justice Systems 1970-2006	Police Records		1980-1986	All Ages	Death Rate
Uruguay	United Nations Office on Drugs and Crime Global Study on Homicide 2011	Police Records	Uruguay PAHO	1995-2001, 2003-2004	All Ages	Death Rate
Uruguay	United Nations Office on Drugs and Crime Global Study on Homicide 2011	Police Records	Uruguay Ministry of Interior/SES	2000-2007, 2009-2010	All Ages	Death Rate
Uruguay	WHO Mortality Database	Vital Registration		1980-1990, 1993-2010, 2012-2013	All Ages	954956
Uruguay	Uruguay Vital Registration - Deaths 1991	Vital Registration		1991	All Ages	29784
Canada	United Nations Office on Drugs and Crime Global Study on Homicide 2011	Police Records	Canada PAHO	1995-2004	All Ages	Death Rate
Canada	United Nations Office on Drugs and Crime Global Study on Homicide 2011	Police Records	Canada NSO	1995-2007, 2009	All Ages	Death Rate
Canada	Global Status Report on Road Safety 2009	Police Records		2006	All Ages	Death Rate
Canada	Under-reporting of maternal mortality in Canada: a question of definition	Surveillance		1988-1992	10 - 49 years	MMR

Appendix Table 6: Causes of death data source list by geography, source type, and study site, with years, age range, and sample size, excluding sources on lower respiratory infection and diarrhoea etiologies						
**Location**	**Source name**	**Source type**	**Site**	**Years**	**Ages**	**Sample size**
Canada	Temporal trends in maternal mortality in Canada II: estimates based on hospitalization data	Surveillance		1996-2007	15 - 44 years	MMR
Canada	WHO Mortality Database	Vital Registration		1980-2011	All Ages	6637878
Greenland	Greenland Vital Registration System	Vital Registration		1995-2013	All Ages	8472
United States: Alabama	United States National Vital Statistics System (NVSS)	Vital Registration		1980-2013	All Ages	1449614
United States: Alaska	United States National Vital Statistics System (NVSS)	Vital Registration		1980-2013	All Ages	91653
United States: Arizona	United States National Vital Statistics System (NVSS)	Vital Registration		1980-2013	All Ages	1224260
United States: Arkansas	United States National Vital Statistics System (NVSS)	Vital Registration		1980-2013	All Ages	901469
United States: California	United States National Vital Statistics System (NVSS)	Vital Registration		1980-2013	All Ages	7521454
United States: Colorado	United States National Vital Statistics System (NVSS)	Vital Registration		1980-2013	All Ages	872357
United States: Connecticut	United States National Vital Statistics System (NVSS)	Vital Registration		1980-2013	All Ages	978102
United States: Delaware	United States National Vital Statistics System (NVSS)	Vital Registration		1980-2013	All Ages	219935
United States: District of Columbia	United States National Vital Statistics System (NVSS)	Vital Registration		1980-2013	All Ages	212433
United States: Florida	United States National Vital Statistics System (NVSS)	Vital Registration		1980-2013	All Ages	5067902
United States: Georgia	United States National Vital Statistics System (NVSS)	Vital Registration		1980-2013	All Ages	2006006
United States: Hawaii	United States National Vital Statistics System (NVSS)	Vital Registration		1980-2013	All Ages	263117
United States: Idaho	United States National Vital Statistics System (NVSS)	Vital Registration		1980-2013	All Ages	308606
United States: Illinois	United States National Vital Statistics System (NVSS)	Vital Registration		1980-2013	All Ages	3523943
United States: Indiana	United States National Vital Statistics System (NVSS)	Vital Registration		1980-2013	All Ages	1793847
United States: Iowa	United States National Vital Statistics System (NVSS)	Vital Registration		1980-2013	All Ages	940059
United States: Kansas	United States National Vital Statistics System (NVSS)	Vital Registration		1980-2013	All Ages	801258
United States: Kentucky	United States National Vital Statistics System (NVSS)	Vital Registration		1980-2013	All Ages	1286270
United States: Louisiana	United States National Vital Statistics System (NVSS)	Vital Registration		1980-2013	All Ages	1340546
United States: Maine	United States National Vital Statistics System (NVSS)	Vital Registration		1980-2013	All Ages	404267
United States: Maryland	United States National Vital Statistics System (NVSS)	Vital Registration		1980-2013	All Ages	1385010
United States: Massachusetts	United States National Vital Statistics System (NVSS)	Vital Registration		1980-2013	All Ages	1856538
United States: Michigan	United States National Vital Statistics System (NVSS)	Vital Registration		1980-2013	All Ages	2831389
United States: Minnesota	United States National Vital Statistics System (NVSS)	Vital Registration		1980-2013	All Ages	1242918
United States: Mississippi	United States National Vital Statistics System (NVSS)	Vital Registration		1980-2013	All Ages	912672
United States: Missouri	United States National Vital Statistics System (NVSS)	Vital Registration		1980-2013	All Ages	1805703
United States: Montana	United States National Vital Statistics System (NVSS)	Vital Registration		1980-2013	All Ages	263181
United States: Nebraska	United States National Vital Statistics System (NVSS)	Vital Registration		1980-2013	All Ages	511604
United States: Nevada	United States National Vital Statistics System (NVSS)	Vital Registration		1980-2013	All Ages	453887
United States: New Hampshire	United States National Vital Statistics System (NVSS)	Vital Registration		1980-2013	All Ages	314806
United States: New Jersey	United States National Vital Statistics System (NVSS)	Vital Registration		1980-2013	All Ages	2425071
United States: New Mexico	United States National Vital Statistics System (NVSS)	Vital Registration		1980-2013	All Ages	432559
United States: New York	United States National Vital Statistics System (NVSS)	Vital Registration		1980-2013	All Ages	5490758
United States: North Carolina	United States National Vital Statistics System (NVSS)	Vital Registration		1980-2013	All Ages	2226286
United States: North Dakota	United States National Vital Statistics System (NVSS)	Vital Registration		1980-2013	All Ages	196355
United States: Ohio	United States National Vital Statistics System (NVSS)	Vital Registration		1980-2013	All Ages	3541782
United States: Oklahoma	United States National Vital Statistics System (NVSS)	Vital Registration		1980-2013	All Ages	1122998
United States: Oregon	United States National Vital Statistics System (NVSS)	Vital Registration		1980-2013	All Ages	948643

Appendix Table 6: Causes of death data source list by geography, source type, and study site, with years, age range, and sample size, excluding sources on lower respiratory infection and diarrhoea etiologies						
**Location**	**Source name**	**Source type**	**Site**	**Years**	**Ages**	**Sample size**
United States: Pennsylvania	United States National Vital Statistics System (NVSS)	Vital Registration		1980-2013	All Ages	4283631
United States: Rhode Island	United States National Vital Statistics System (NVSS)	Vital Registration		1980-2013	All Ages	327496
United States: South Carolina	United States National Vital Statistics System (NVSS)	Vital Registration		1980-2013	All Ages	1153477
United States: South Dakota	United States National Vital Statistics System (NVSS)	Vital Registration		1980-2013	All Ages	231976
United States: Tennessee	United States National Vital Statistics System (NVSS)	Vital Registration		1980-2013	All Ages	1744645
United States: Texas	United States National Vital Statistics System (NVSS)	Vital Registration		1980-2013	All Ages	4785663
United States: Utah	United States National Vital Statistics System (NVSS)	Vital Registration		1980-2013	All Ages	391623
United States: Vermont	United States National Vital Statistics System (NVSS)	Vital Registration		1980-2013	All Ages	167459
United States: Virginia	United States National Vital Statistics System (NVSS)	Vital Registration		1980-2013	All Ages	1788452
United States: Washington	United States National Vital Statistics System (NVSS)	Vital Registration		1980-2013	All Ages	1403201
United States: West Virginia	United States National Vital Statistics System (NVSS)	Vital Registration		1980-2013	All Ages	694917
United States: Wisconsin	United States National Vital Statistics System (NVSS)	Vital Registration		1980-2013	All Ages	1517296
United States: Wyoming	United States National Vital Statistics System (NVSS)	Vital Registration		1980-2013	All Ages	126812
Antigua and Barbuda	United Nations Surveys on Crime Trends and the Operations of Criminal Justice Systems 1970-2006	Police Records		1985-1986	All Ages	Death Rate
Antigua and Barbuda	United Nations Office on Drugs and Crime Global Study on Homicide 2011	Police Records	Antigua and Barbuda PAHO	1995, 2001-2002, 2004	All Ages	Death Rate
Antigua and Barbuda	United Nations Office on Drugs and Crime Global Study on Homicide 2011	Police Records	Antigua and Barbuda OAS/National police	2001-2002, 2004, 2007, 2009-2010	All Ages	Death Rate
Antigua and Barbuda	WHO Mortality Database	Vital Registration		1983, 1985-1995, 2000-2009, 2012-2013	All Ages	10421
Antigua and Barbuda	Antigua and Barbuda Vital Statistics - Deaths 1996-1999	Vital Registration		1996-1999	All Ages	1841
The Bahamas	United Nations Surveys on Crime Trends and the Operations of Criminal Justice Systems 1970-2006	Police Records		1981-1986, 1990-1994	All Ages	Death Rate
The Bahamas	United Nations Office on Drugs and Crime Global Study on Homicide 2011	Police Records	The Bahamas PAHO	1995-2004	All Ages	Death Rate
The Bahamas	United Nations Office on Drugs and Crime Global Study on Homicide 2011	Police Records	The Bahamas OAS	2000-2004, 2006-2007, 2009-2010	All Ages	Death Rate
The Bahamas	WHO Mortality Database	Vital Registration		1980-1981, 1984-1985, 1987, 1993-2012	All Ages	41292
Barbados	United Nations Surveys on Crime Trends and the Operations of Criminal Justice Systems 1970-2006	Police Records		1986	All Ages	Death Rate
Barbados	United Nations Office on Drugs and Crime Global Study on Homicide 2011	Police Records	Barbados CTS/OAS	1998-1999, 2009-2010	All Ages	Death Rate
Barbados	Global Status Report on Road Safety 2009	Police Records		2007	All Ages	Death Rate
Barbados	WHO Mortality Database	Vital Registration		1980-1995, 2000-2012	All Ages	65172
Belize	United Nations Surveys on Crime Trends and the Operations of Criminal Justice Systems 1970-2006	Police Records		1980	All Ages	Death Rate
Belize	United Nations Office on Drugs and Crime Global Study on Homicide 2011	Police Records	Belize PAHO	1998-2004, 2006-2007	All Ages	Death Rate
Belize	United Nations Office on Drugs and Crime Global Study on Homicide 2011	Police Records	Belize CTS/OAS	2000-2004, 2006-2007, 2009-2010	All Ages	Death Rate
Belize	WHO Mortality Database	Vital Registration		1980-1984, 1986-1987, 1989-1991, 1993-2013	All Ages	34348
Bermuda	United Nations Surveys on Crime Trends and the Operations of Criminal Justice Systems 1970-2006	Police Records		1981, 1988, 1991, 1994	All Ages	Death Rate
Bermuda	United Nations Office on Drugs and Crime Global Study on Homicide 2011	Police Records	Bermuda National police	1998, 2002, 2010	All Ages	Death Rate
Bermuda	WHO Mortality Database	Vital Registration		1980, 1983-2013	All Ages	13435

Appendix Table 6: Causes of death data source list by geography, source type, and study site, with years, age range, and sample size, excluding sources on lower respiratory infection and diarrhoea etiologies

Location	Source name	Source type	Site	Years	Ages	Sample size
Cuba	United Nations Office on Drugs and Crime Global Study on Homicide 2011	Police Records	Cuba PAHO	1995-2007	All Ages	Death Rate
Cuba	WHO Mortality Database	Vital Registration		1980-2013	All Ages	2554520
Dominica	United Nations Surveys on Crime Trends and the Operations of Criminal Justice Systems 1970-2006	Police Records		1982-1983, 1985-1986	All Ages	Death Rate
Dominica	United Nations Office on Drugs and Crime Global Study on Homicide 2011	Police Records	Dominica PAHO	1999-2000, 2002-2004	All Ages	Death Rate
Dominica	United Nations Office on Drugs and Crime Global Study on Homicide 2011	Police Records	Dominica OAS	2000, 2002-2004, 2007, 2009-2010	All Ages	Death Rate
Dominica	WHO Mortality Database	Vital Registration		1980-2013	All Ages	17591
Dominican Republic	United Nations Office on Drugs and Crime Global Study on Homicide 2011	Police Records	Dominican Republic PAHO	1996-2000, 2002	All Ages	Death Rate
Dominican Republic	United Nations Office on Drugs and Crime Global Study on Homicide 2011	Police Records	Dominican Republic NGO (b)/SES	1996-2000, 2002, 2007, 2009-2010	All Ages	Death Rate
Dominican Republic	Estimating Maternal Mortality in Monseñor Nouel Province, Dominican Republic	Sibling History	Monsenor Nouel Province	1986	15 - 49 years	MMR
Dominican Republic	Dominican Republic Demographic and Health Survey - Maternal Mortality Data	Sibling History		1988-2007	15 - 54 years	247
Dominican Republic	Dominican Republic Maternal Health Surveillance System Data 1999-2009	Surveillance	nationwide	1999-2008	15 - 49 years	MMR
Dominican Republic	World Health Survey (WHS)	Survey/Census		1997	All Ages	51
Dominican Republic	WHO Mortality Database	Vital Registration		1980-1992, 1994-2001, 2003-2012	All Ages	805975
Grenada	United Nations Office on Drugs and Crime Global Study on Homicide 2011	Police Records	Grenada OAS	2000, 2002-2003, 2010	All Ages	Death Rate
Grenada	United Nations Office on Drugs and Crime Global Study on Homicide 2011	Police Records	Grenada PAHO	2002	All Ages	Death Rate
Grenada	WHO Mortality Database	Vital Registration		1984-1985, 1988-1996, 2001-2013	All Ages	18506
Guyana	United Nations Surveys on Crime Trends and the Operations of Criminal Justice Systems 1970-2006	Police Records		1991-1993	All Ages	Death Rate
Guyana	United Nations Office on Drugs and Crime Global Study on Homicide 2011	Police Records	Guyana PAHO	2002-2003	All Ages	Death Rate
Guyana	United Nations Office on Drugs and Crime Global Study on Homicide 2011	Police Records	Guyana OAS/NSO	2002-2003, 2009-2010	All Ages	Death Rate
Guyana	WHO Mortality Database	Vital Registration		1984, 1988-1999, 2001-2011	All Ages	116271
Guyana	Guyana Vital Registration - Deaths 2000 ICD10 - PAHO	Vital Registration		2000	All Ages	4808
Haiti	United Nations Office on Drugs and Crime Global Study on Homicide 2011	Police Records	Haiti PAHO	2001	All Ages	Death Rate
Haiti	United Nations Office on Drugs and Crime Global Study on Homicide 2011	Police Records	Haiti UN-PKO	2007, 2009-2010	All Ages	Death Rate
Haiti	Haiti Demographic and Health Survey - Maternal Mortality Data	Sibling History		1986-2005	15 - 49 years	455
Haiti	The utility of verbal autopsies for identifying HIV-1-related deaths in Haitian children	Verbal Autopsy	Cite Soleil [rural]	1989	birth - 364 days	244
Haiti	Survey on Infant mortality in Mirebalais, Haiti	Verbal Autopsy	Mirebalais [urban]	1994	birth - 364 days	148
Haiti	Assessing the causes of under-five mortality in the Albert Schweitzer Hospital service area of rural Haiti	Verbal Autopsy	service area of the Albert Schweitzer Hospital [rural]	1997	birth - 4 years	97
Haiti	WHO Mortality Database	Vital Registration		1981, 1999, 2002-2004	All Ages	81870
Jamaica	United Nations Surveys on Crime Trends and the Operations of Criminal Justice Systems 1970-2006	Police Records		1981-1986, 1990-1994	All Ages	Death Rate
Jamaica	United Nations Office on Drugs and Crime Global Study on Homicide 2011	Police Records	Jamaica CTS/National police	1995-2007, 2009-2010	All Ages	Death Rate

Appendix Table 6: Causes of death data source list by geography, source type, and study site, with years, age range, and sample size, excluding sources on lower respiratory infection and diarrhoea etiologies						
**Location**	**Source name**	**Source type**	**Site**	**Years**	**Ages**	**Sample size**
Jamaica	Jamaica Maternal Mortality Surveillance System 1998-2008	Surveillance		1998-2003, 2005, 2008	15 - 49 years	8196
Jamaica	Neonatal mortality determinants in Jamaica	Verbal Autopsy	Jamaica	1987	birth - 6 days	792
Jamaica	WHO Mortality Database	Vital Registration		1980-1991, 2000-2006, 2009-2011	All Ages	303890
Puerto Rico	United Nations Office on Drugs and Crime Global Study on Homicide 2011	Police Records	Puerto Rico PAHO	1995-2007	All Ages	Death Rate
Puerto Rico	United Nations Office on Drugs and Crime Global Study on Homicide 2011	Police Records	Puerto Rico National police	1995-2007, 2009-2010	All Ages	Death Rate
Puerto Rico	Global Status Report on Road Safety 2009	Police Records		2007	All Ages	Death Rate
Puerto Rico	WHO Mortality Database	Vital Registration		1980-1993	All Ages	336021
Puerto Rico	United States National Vital Statistics System (NVSS)	Vital Registration		1994-2012	All Ages	554833
Saint Lucia	United Nations Office on Drugs and Crime Global Study on Homicide 2011	Police Records	Saint Lucia PAHO	1995-1997, 1999-2004	All Ages	Death Rate
Saint Lucia	United Nations Office on Drugs and Crime Global Study on Homicide 2011	Police Records	Saint Lucia OAS	2000-2004, 2006-2007, 2009-2010	All Ages	Death Rate
Saint Lucia	WHO Mortality Database	Vital Registration		1980-1981, 1983, 1986-2006, 2008-2012	All Ages	27708
Saint Vincent and the Grenadines	United Nations Surveys on Crime Trends and the Operations of Criminal Justice Systems 1970-2006	Police Records		1981-1985	All Ages	Death Rate
Saint Vincent and the Grenadines	United Nations Office on Drugs and Crime Global Study on Homicide 2011	Police Records	Saint Vincent and the Grenadines PAHO	1995-1996, 1998-2004, 2007	All Ages	Death Rate
Saint Vincent and the Grenadines	United Nations Office on Drugs and Crime Global Study on Homicide 2011	Police Records	Saint Vincent and the Grenadines OAS/NGO (c)	1995-1996, 1998-2004, 2007, 2009-2010	All Ages	Death Rate
Saint Vincent and the Grenadines	WHO Mortality Database	Vital Registration		1982-1987, 1990, 1995-2013	All Ages	19827
Suriname	United Nations Surveys on Crime Trends and the Operations of Criminal Justice Systems 1970-2006	Police Records		1984-1986	All Ages	Death Rate
Suriname	United Nations Office on Drugs and Crime Global Study on Homicide 2011	Police Records	Suriname PAHO	2001	All Ages	Death Rate
Suriname	United Nations Office on Drugs and Crime Global Study on Homicide 2011	Police Records	Suriname OAS	2001, 2006-2007	All Ages	Death Rate
Suriname	WHO Mortality Database	Vital Registration		1980-1982, 1984-1992, 1995-2006, 2008-2012	All Ages	69493
Suriname	Suriname Vital Statistics - Deaths 2007	Vital Registration		2007	All Ages	2008
Trinidad and Tobago	United Nations Surveys on Crime Trends and the Operations of Criminal Justice Systems 1970-2006	Police Records		1981-1986	All Ages	Death Rate
Trinidad and Tobago	United Nations Office on Drugs and Crime Global Study on Homicide 2011	Police Records	Trinidad and Tobago PAHO	1995-1997, 2000-2002, 2004-2006	All Ages	Death Rate
Trinidad and Tobago	United Nations Office on Drugs and Crime Global Study on Homicide 2011	Police Records	Trinidad and Tobago OAS/National police	2000-2007, 2009-2010	All Ages	Death Rate
Trinidad and Tobago	WHO Mortality Database	Vital Registration		1980-2009	All Ages	266335
Virgin Islands, U.S.	United Nations Office on Drugs and Crime Global Study on Homicide 2011	Police Records	Virgin Islands: U.S. PAHO	1997-2007	All Ages	Death Rate
Virgin Islands, U.S.	WHO Mortality Database	Vital Registration		1980	All Ages	540
Virgin Islands, U.S.	United States National Vital Statistics System (NVSS)	Vital Registration		1994-2012	All Ages	13200
Bolivia	United Nations Office on Drugs and Crime Global Study on Homicide 2011	Police Records	Bolivia National police	2005-2007, 2009-2010	All Ages	Death Rate
Bolivia	Global Status Report on Road Safety 2009	Police Records		2007	All Ages	Death Rate
Bolivia	Bolivia Demographic and Health Survey - Maternal Mortality Data	Sibling History		1980-2008	15 - 34 years	35
Bolivia	Bolivia Census 2001	Survey/Census		2000-2001	15 - 49 years	22782
Bolivia	WHO Mortality Database	Vital Registration		2000-2003	All Ages	88639

Appendix Table 6: Causes of death data source list by geography, source type, and study site, with years, age range, and sample size, excluding sources on lower respiratory infection and diarrhoea etiologies						
**Location**	**Source name**	**Source type**	**Site**	**Years**	**Ages**	**Sample size**
Ecuador	United Nations Surveys on Crime Trends and the Operations of Criminal Justice Systems 1970-2006	Police Records		1991	All Ages	Death Rate
Ecuador	United Nations Office on Drugs and Crime Global Study on Homicide 2011	Police Records	Ecuador PAHO	1996, 1999, 2006-2007	All Ages	Death Rate
Ecuador	United Nations Office on Drugs and Crime Global Study on Homicide 2011	Police Records	Ecuador National police	2009	All Ages	Death Rate
Ecuador	Ecuador Reproductive Health Survey - Maternal Mortality Data	Sibling history		1980-2003	15 - 49 years	CF
Ecuador	WHO Mortality Database	Vital Registration		1980-2013	All Ages	1874738
Peru	United Nations Surveys on Crime Trends and the Operations of Criminal Justice Systems 1970-2006	Police Records		1981-1982, 1984-1985	All Ages	Death Rate
Peru	United Nations Office on Drugs and Crime Global Study on Homicide 2011	Police Records	Peru PAHO	1999-2007	All Ages	Death Rate
Peru	United Nations Office on Drugs and Crime Global Study on Homicide 2011	Police Records	Peru National police/SES	1999-2007, 2009	All Ages	Death Rate
Peru	Global Status Report on Road Safety 2009	Police Records		2007	All Ages	Death Rate
Peru	Peru Demographic and Health Survey - Maternal Mortality Data	Sibling History		1980-2012	15 - 49 years	26
Peru	Peru Maternal Mortality 2002-2011	Surveillance		2002-2011	10 - 54 years	MMR
Peru	WHO Mortality Database	Vital Registration		1980-1983, 1986-1992, 1994-2000, 2007-2013	All Ages	2234898
Peru	Peru Vital Statistics - Deaths 2001-2006	Vital Registration		2001-2006	All Ages	511527
Colombia	United Nations Surveys on Crime Trends and the Operations of Criminal Justice Systems 1970-2006	Police Records		1990-1991	All Ages	Death Rate
Colombia	United Nations Office on Drugs and Crime Global Study on Homicide 2011	Police Records	Colombia PAHO	1995-2007	All Ages	Death Rate
Colombia	United Nations Office on Drugs and Crime Global Study on Homicide 2011	Police Records	Colombia National police	1995-2007, 2009-2010	All Ages	Death Rate
Colombia	Colombia Vital Statistics - Deaths 2008	Vital Registration		1980-1996, 2008	All Ages	2791049
Colombia	WHO Mortality Database	Vital Registration		1997-2002, 2004-2006, 2009-2012	All Ages	2462450
Colombia	Colombia Vital Registration 1980-2005	Vital Registration		2003	All Ages	192120
Costa Rica	United Nations Surveys on Crime Trends and the Operations of Criminal Justice Systems 1970-2006	Police Records		1980, 1983-1985	All Ages	Death Rate
Costa Rica	United Nations Office on Drugs and Crime Global Study on Homicide 2011	Police Records	Costa Rica PAHO	1997-2007	All Ages	Death Rate
Costa Rica	United Nations Office on Drugs and Crime Global Study on Homicide 2011	Police Records	Costa Rica CTS/Ministry of Justice	1997-2007, 2009-2010	All Ages	Death Rate
Costa Rica	WHO Mortality Database	Vital Registration		1980-2013	All Ages	468707
El Salvador	United Nations Office on Drugs and Crime Global Study on Homicide 2011	Police Records	El Salvador PAHO	1995-2007	All Ages	Death Rate
El Salvador	United Nations Office on Drugs and Crime Global Study on Homicide 2011	Police Records	El Salvador National police	1995-2007, 2009-2010	All Ages	Death Rate
El Salvador	El Salvador Reproductive Health Survey - Maternal Mortality Data	Sibling history		1980-2002	15 - 49 years	CF
El Salvador	El Salvador Maternal Mortality Data 2006-2010	Surveillance	nationwide	2006-2010	15 - 49 years	MMR
El Salvador	International Integrated Public Use Microdata Series (IPUMS)	Survey/Census		2007	1 year +	3150
El Salvador	WHO Mortality Database	Vital Registration		1981-1984, 1990-1993, 1995-2012	All Ages	790067
Guatemala	United Nations Surveys on Crime Trends and the Operations of Criminal Justice Systems 1970-2006	Police Records		1993-1994	All Ages	Death Rate
Guatemala	United Nations Office on Drugs and Crime Global Study on Homicide 2011	Police Records	Guatemala PAHO	2001-2004, 2007	All Ages	Death Rate

Appendix Table 6: Causes of death data source list by geography, source type, and study site, with years, age range, and sample size, excluding sources on lower respiratory infection and diarrhoea etiologies						
**Location**	**Source name**	**Source type**	**Site**	**Years**	**Ages**	**Sample size**
Guatemala	United Nations Office on Drugs and Crime Global Study on Homicide 2011	Police Records	Guatemala CTS/National police	2001-2004, 2007, 2009-2010	All Ages	Death Rate
Guatemala	Guatemala Demographic and Health Survey - Maternal Mortality Data	Sibling History		1981-1995	15 - 49 years	122
Guatemala	Guatemala Baseline Maternal Mortality for the Year 2000	Surveillance		2000	10 - 49 years	MMR
Guatemala	World Health Survey (WHS)	Survey/Census		1997	All Ages	15
Guatemala	WHO Mortality Database	Vital Registration		1980-1981, 1984, 1986-2013	All Ages	2139664
Honduras	United Nations Surveys on Crime Trends and the Operations of Criminal Justice Systems 1970-2006	Police Records		1980-1986	All Ages	Death Rate
Honduras	United Nations Office on Drugs and Crime Global Study on Homicide 2011	Police Records	Honduras OCAVI/National police	1999-2007, 2009-2010	All Ages	Death Rate
Honduras	Reproductive Health Survey (RHS)	Sibling history		1981-1995	15 - 49 years	0
Honduras	Honduras Maternal Mortality Ratio Update 2010	Surveillance		1990, 1997, 2010	15 - 49 years	MMR
Honduras	Honduras Population and Housing Census 2001	Survey/Census		2000-2001	15 - 49 years	4082
Honduras	Mortality of Women of Reproductive Age and Maternal Mortality	Verbal Autopsy		1990	15 - 49 years	MMR
Honduras	Honduras Maternal Mortality Ratio Update 2010	Verbal Autopsy		1990, 1997, 2010	15 - 49 years	MMR
Honduras	WHO Mortality Database	Vital Registration		1980-1983, 1987-1990, 2008-2013	All Ages	169544
Mexico: Aguascalientes	Mexico Intentional Search and Reclassification of Maternal Deaths 2005	Surveillance		2005	15 - 39 years	MMR
Mexico: Aguascalientes	Mexico Intentional Search and Reclassification of Maternal Deaths 2006	Surveillance		2006	20 - 44 years	MMR
Mexico: Aguascalientes	Mexico Intentional Search and Reclassification of Maternal Deaths 2008	Surveillance		2008	15 - 49 years	MMR
Mexico: Aguascalientes	Mexico Intentional Search and Reclassification of Maternal Deaths 2009	Surveillance		2009	15 - 49 years	MMR
Mexico: Aguascalientes	Mexico Intentional Search and Reclassification of Maternal Deaths 2010	Surveillance		2010	15 - 39 years	MMR
Mexico: Aguascalientes	Mexico Intentional Search and Reclassification of Maternal Deaths 2011	Surveillance		2011	15 - 34 years	MMR
Mexico: Aguascalientes	Mexico Intentional Search and Reclassification of Maternal Deaths 2012	Surveillance		2012	15 - 39 years	MMR
Mexico: Aguascalientes	Mexico Intentional Search and Reclassification of Maternal Deaths 2013	Surveillance		2013	15 - 44 years	MMR
Mexico: Aguascalientes	Mexico Vital Registration System - Mortality	Vital Registration		1980-2008, 2010-2014	All Ages	127661
Mexico: Aguascalientes	Mexico Vital Statistics - Deaths 2009	Vital Registration		2009	All Ages	4709
Mexico: Baja California	Mexico Intentional Search and Reclassification of Maternal Deaths 2005	Surveillance		2005	15 - 39 years	MMR
Mexico: Baja California	Mexico Intentional Search and Reclassification of Maternal Deaths 2006	Surveillance		2006	10 - 44 years	MMR
Mexico: Baja California	Mexico Intentional Search and Reclassification of Maternal Deaths 2008	Surveillance		2008	10 - 44 years	MMR
Mexico: Baja California	Mexico Intentional Search and Reclassification of Maternal Deaths 2009	Surveillance		2009	10 - 44 years	MMR
Mexico: Baja California	Mexico Intentional Search and Reclassification of Maternal Deaths 2010	Surveillance		2010	15 - 44 years	MMR
Mexico: Baja California	Mexico Intentional Search and Reclassification of Maternal Deaths 2011	Surveillance		2011	15 - 39 years	MMR
Mexico: Baja California	Mexico Intentional Search and Reclassification of Maternal Deaths 2012	Surveillance		2012	15 - 39 years	MMR
Mexico: Baja California	Mexico Intentional Search and Reclassification of Maternal Deaths 2013	Surveillance		2013	15 - 39 years	MMR

Appendix Table 6: Causes of death data source list by geography, source type, and study site, with years, age range, and sample size, excluding sources on lower respiratory infection and diarrhoea etiologies						
**Location**	**Source name**	**Source type**	**Site**	**Years**	**Ages**	**Sample size**
Mexico: Baja California	Mexico Vital Registration System - Mortality	Vital Registration		1980-2008, 2010-2014	All Ages	339821
Mexico: Baja California	Mexico Vital Statistics - Deaths 2009	Vital Registration		2009	All Ages	14203
Mexico: Baja California Sur	Mexico Intentional Search and Reclassification of Maternal Deaths 2005	Surveillance		2005	20 - 39 years	MMR
Mexico: Baja California Sur	Mexico Intentional Search and Reclassification of Maternal Deaths 2006	Surveillance		2006	10 - 39 years	MMR
Mexico: Baja California Sur	Mexico Intentional Search and Reclassification of Maternal Deaths 2008	Surveillance		2008	15 - 39 years	MMR
Mexico: Baja California Sur	Mexico Intentional Search and Reclassification of Maternal Deaths 2009	Surveillance		2009	15 - 39 years	MMR
Mexico: Baja California Sur	Mexico Intentional Search and Reclassification of Maternal Deaths 2010	Surveillance		2010	25 - 39 years	MMR
Mexico: Baja California Sur	Mexico Intentional Search and Reclassification of Maternal Deaths 2011	Surveillance		2011	15 - 29 years	MMR
Mexico: Baja California Sur	Mexico Intentional Search and Reclassification of Maternal Deaths 2012	Surveillance		2012	25 - 29 years	MMR
Mexico: Baja California Sur	Mexico Intentional Search and Reclassification of Maternal Deaths 2013	Surveillance		2013	15 - 34 years	MMR
Mexico: Baja California Sur	Mexico Vital Registration System - Mortality	Vital Registration		1980-2008, 2010-2014	All Ages	54731
Mexico: Baja California Sur	Mexico Vital Statistics - Deaths 2009	Vital Registration		2009	All Ages	2338
Mexico: Campeche	Mexico Intentional Search and Reclassification of Maternal Deaths 2005	Surveillance		2005	15 - 39 years	MMR
Mexico: Campeche	Mexico Intentional Search and Reclassification of Maternal Deaths 2006	Surveillance		2006	10 - 44 years	MMR
Mexico: Campeche	Mexico Intentional Search and Reclassification of Maternal Deaths 2008	Surveillance		2008	15 - 39 years	MMR
Mexico: Campeche	Mexico Intentional Search and Reclassification of Maternal Deaths 2009	Surveillance		2009	15 - 39 years	MMR
Mexico: Campeche	Mexico Intentional Search and Reclassification of Maternal Deaths 2010	Surveillance		2010	10 - 34 years	MMR
Mexico: Campeche	Mexico Intentional Search and Reclassification of Maternal Deaths 2011	Surveillance		2011	20 - 44 years	MMR
Mexico: Campeche	Mexico Intentional Search and Reclassification of Maternal Deaths 2012	Surveillance		2012	15 - 39 years	MMR
Mexico: Campeche	Mexico Intentional Search and Reclassification of Maternal Deaths 2013	Surveillance		2013	15 - 44 years	MMR
Mexico: Campeche	Mexico Vital Registration System - Mortality	Vital Registration		1980-2008, 2010-2014	All Ages	88811
Mexico: Campeche	Mexico Vital Statistics - Deaths 2009	Vital Registration		2009	All Ages	3396
Mexico: Chiapas	Mexico Intentional Search and Reclassification of Maternal Deaths 2005	Surveillance		2005	15 - 49 years	MMR
Mexico: Chiapas	Mexico Intentional Search and Reclassification of Maternal Deaths 2006	Surveillance		2006	10 - 49 years	MMR
Mexico: Chiapas	Mexico Intentional Search and Reclassification of Maternal Deaths 2008	Surveillance		2008	10 - 49 years	MMR
Mexico: Chiapas	Mexico Intentional Search and Reclassification of Maternal Deaths 2009	Surveillance		2009	10 - 49 years	MMR
Mexico: Chiapas	Mexico Intentional Search and Reclassification of Maternal Deaths 2010	Surveillance		2010	15 - 49 years	MMR
Mexico: Chiapas	Mexico Intentional Search and Reclassification of Maternal Deaths 2011	Surveillance		2011	15 - 44 years	MMR
Mexico: Chiapas	Mexico Intentional Search and Reclassification of Maternal Deaths 2012	Surveillance		2012	10 - 49 years	MMR

Appendix Table 6: Causes of death data source list by geography, source type, and study site, with years, age range, and sample size, excluding sources on lower respiratory infection and diarrhoea etiologies						
**Location**	**Source name**	**Source type**	**Site**	**Years**	**Ages**	**Sample size**
Mexico: Chiapas	Mexico Intentional Search and Reclassification of Maternal Deaths 2013	Surveillance		2013	15 - 44 years	MMR
Mexico: Chiapas	Mexico Vital Registration System - Mortality	Vital Registration		1980-2008, 2010-2014	All Ages	530775
Mexico: Chiapas	Mexico Vital Statistics - Deaths 2009	Vital Registration		2009	All Ages	19107
Mexico: Chihuahua	Mexico Intentional Search and Reclassification of Maternal Deaths 2005	Surveillance		2005	10 - 49 years	MMR
Mexico: Chihuahua	Mexico Intentional Search and Reclassification of Maternal Deaths 2006	Surveillance		2006	10 - 44 years	MMR
Mexico: Chihuahua	Mexico Intentional Search and Reclassification of Maternal Deaths 2008	Surveillance		2008	15 - 44 years	MMR
Mexico: Chihuahua	Mexico Intentional Search and Reclassification of Maternal Deaths 2009	Surveillance		2009	15 - 44 years	MMR
Mexico: Chihuahua	Mexico Intentional Search and Reclassification of Maternal Deaths 2010	Surveillance		2010	15 - 44 years	MMR
Mexico: Chihuahua	Mexico Intentional Search and Reclassification of Maternal Deaths 2011	Surveillance		2011	10 - 44 years	MMR
Mexico: Chihuahua	Mexico Intentional Search and Reclassification of Maternal Deaths 2012	Surveillance		2012	15 - 44 years	MMR
Mexico: Chihuahua	Mexico Intentional Search and Reclassification of Maternal Deaths 2013	Surveillance		2013	10 - 44 years	MMR
Mexico: Chihuahua	Mexico Vital Registration System - Mortality	Vital Registration		1980-2008, 2010-2014	All Ages	519446
Mexico: Chihuahua	Mexico Vital Statistics - Deaths 2009	Vital Registration		2009	All Ages	20869
Mexico: Coahuila	Mexico Intentional Search and Reclassification of Maternal Deaths 2005	Surveillance		2005	15 - 39 years	MMR
Mexico: Coahuila	Mexico Intentional Search and Reclassification of Maternal Deaths 2006	Surveillance		2006	15 - 44 years	MMR
Mexico: Coahuila	Mexico Intentional Search and Reclassification of Maternal Deaths 2008	Surveillance		2008	15 - 39 years	MMR
Mexico: Coahuila	Mexico Intentional Search and Reclassification of Maternal Deaths 2009	Surveillance		2009	15 - 39 years	MMR
Mexico: Coahuila	Mexico Intentional Search and Reclassification of Maternal Deaths 2010	Surveillance		2010	15 - 44 years	MMR
Mexico: Coahuila	Mexico Intentional Search and Reclassification of Maternal Deaths 2011	Surveillance		2011	15 - 44 years	MMR
Mexico: Coahuila	Mexico Intentional Search and Reclassification of Maternal Deaths 2012	Surveillance		2012	15 - 39 years	MMR
Mexico: Coahuila	Mexico Intentional Search and Reclassification of Maternal Deaths 2013	Surveillance		2013	15 - 44 years	MMR
Mexico: Coahuila	Mexico Vital Registration System - Mortality	Vital Registration		1980-2008, 2010-2014	All Ages	350621
Mexico: Coahuila	Mexico Vital Statistics - Deaths 2009	Vital Registration		2009	All Ages	12746
Mexico: Colima	Mexico Intentional Search and Reclassification of Maternal Deaths 2005	Surveillance		2005	30 - 34 years	MMR
Mexico: Colima	Mexico Intentional Search and Reclassification of Maternal Deaths 2006	Surveillance		2006	15 - 34 years	MMR
Mexico: Colima	Mexico Intentional Search and Reclassification of Maternal Deaths 2008	Surveillance		2008	15 - 39 years	MMR
Mexico: Colima	Mexico Intentional Search and Reclassification of Maternal Deaths 2009	Surveillance		2009	15 - 39 years	MMR
Mexico: Colima	Mexico Intentional Search and Reclassification of Maternal Deaths 2011	Surveillance		2011	15 - 34 years	MMR
Mexico: Colima	Mexico Intentional Search and Reclassification of Maternal Deaths 2012	Surveillance		2012	25 - 49 years	MMR

Appendix Table 6: Causes of death data source list by geography, source type, and study site, with years, age range, and sample size, excluding sources on lower respiratory infection and diarrhoea etiologies						
**Location**	**Source name**	**Source type**	**Site**	**Years**	**Ages**	**Sample size**
Mexico: Colima	Mexico Intentional Search and Reclassification of Maternal Deaths 2013	Surveillance		2013	15 - 34 years	MMR
Mexico: Colima	Mexico Vital Registration System - Mortality	Vital Registration		1980-2008, 2010-2014	All Ages	83744
Mexico: Colima	Mexico Vital Statistics - Deaths 2009	Vital Registration		2009	All Ages	2904
Mexico: Distrito Federal	Mexico Intentional Search and Reclassification of Maternal Deaths 2005	Surveillance		2005	10 - 49 years	MMR
Mexico: Distrito Federal	Mexico Intentional Search and Reclassification of Maternal Deaths 2006	Surveillance		2006	15 - 44 years	MMR
Mexico: Distrito Federal	Mexico Intentional Search and Reclassification of Maternal Deaths 2008	Surveillance		2008	15 - 44 years	MMR
Mexico: Distrito Federal	Mexico Intentional Search and Reclassification of Maternal Deaths 2009	Surveillance		2009	15 - 44 years	MMR
Mexico: Distrito Federal	Mexico Intentional Search and Reclassification of Maternal Deaths 2010	Surveillance		2010	15 - 54 years	MMR
Mexico: Distrito Federal	Mexico Intentional Search and Reclassification of Maternal Deaths 2011	Surveillance		2011	15 - 44 years	MMR
Mexico: Distrito Federal	Mexico Intentional Search and Reclassification of Maternal Deaths 2012	Surveillance		2012	10 - 49 years	MMR
Mexico: Distrito Federal	Mexico Intentional Search and Reclassification of Maternal Deaths 2013	Surveillance		2013	15 - 49 years	MMR
Mexico: Distrito Federal	Mexico Vital Registration System - Mortality	Vital Registration		1980-2008, 2010-2014	All Ages	1560430
Mexico: Distrito Federal	Mexico Vital Statistics - Deaths 2009	Vital Registration		2009	All Ages	50416
Mexico: Durango	Mexico Intentional Search and Reclassification of Maternal Deaths 2005	Surveillance		2005	15 - 49 years	MMR
Mexico: Durango	Mexico Intentional Search and Reclassification of Maternal Deaths 2006	Surveillance		2006	20 - 44 years	MMR
Mexico: Durango	Mexico Intentional Search and Reclassification of Maternal Deaths 2008	Surveillance		2008	15 - 49 years	MMR
Mexico: Durango	Mexico Intentional Search and Reclassification of Maternal Deaths 2009	Surveillance		2009	15 - 49 years	MMR
Mexico: Durango	Mexico Intentional Search and Reclassification of Maternal Deaths 2010	Surveillance		2010	15 - 44 years	MMR
Mexico: Durango	Mexico Intentional Search and Reclassification of Maternal Deaths 2011	Surveillance		2011	20 - 49 years	MMR
Mexico: Durango	Mexico Intentional Search and Reclassification of Maternal Deaths 2012	Surveillance		2012	15 - 39 years	MMR
Mexico: Durango	Mexico Intentional Search and Reclassification of Maternal Deaths 2013	Surveillance		2013	10 - 44 years	MMR
Mexico: Durango	Mexico Vital Registration System - Mortality	Vital Registration		1980-2008, 2010-2014	All Ages	211601
Mexico: Durango	Mexico Vital Statistics - Deaths 2009	Vital Registration		2009	All Ages	8845
Mexico: Guanajuato	Mexico Intentional Search and Reclassification of Maternal Deaths 2005	Surveillance		2005	10 - 44 years	MMR
Mexico: Guanajuato	Mexico Intentional Search and Reclassification of Maternal Deaths 2006	Surveillance		2006	15 - 49 years	MMR
Mexico: Guanajuato	Mexico Intentional Search and Reclassification of Maternal Deaths 2008	Surveillance		2008	15 - 49 years	MMR
Mexico: Guanajuato	Mexico Intentional Search and Reclassification of Maternal Deaths 2009	Surveillance		2009	15 - 49 years	MMR
Mexico: Guanajuato	Mexico Intentional Search and Reclassification of Maternal Deaths 2010	Surveillance		2010	15 - 44 years	MMR
Mexico: Guanajuato	Mexico Intentional Search and Reclassification of Maternal Deaths 2011	Surveillance		2011	15 - 44 years	MMR

Appendix Table 6: Causes of death data source list by geography, source type, and study site, with years, age range, and sample size, excluding sources on lower respiratory infection and diarrhoea etiologies						
Location	Source name	Source type	Site	Years	Ages	Sample size
Mexico: Guanajuato	Mexico Intentional Search and Reclassification of Maternal Deaths 2012	Surveillance		2012	15 - 49 years	MMR
Mexico: Guanajuato	Mexico Intentional Search and Reclassification of Maternal Deaths 2013	Surveillance		2013	10 - 49 years	MMR
Mexico: Guanajuato	Mexico Vital Registration System - Mortality	Vital Registration		1980-2008, 2010-2014	All Ages	746731
Mexico: Guanajuato	Mexico Vital Statistics - Deaths 2009	Vital Registration		2009	All Ages	23814
Mexico: Guerrero	Mexico Intentional Search and Reclassification of Maternal Deaths 2005	Surveillance		2005	10 - 49 years	MMR
Mexico: Guerrero	Mexico Intentional Search and Reclassification of Maternal Deaths 2006	Surveillance		2006	15 - 49 years	MMR
Mexico: Guerrero	Mexico Intentional Search and Reclassification of Maternal Deaths 2008	Surveillance		2008	10 - 44 years	MMR
Mexico: Guerrero	Mexico Intentional Search and Reclassification of Maternal Deaths 2009	Surveillance		2009	10 - 44 years	MMR
Mexico: Guerrero	Mexico Intentional Search and Reclassification of Maternal Deaths 2010	Surveillance		2010	15 - 54 years	MMR
Mexico: Guerrero	Mexico Intentional Search and Reclassification of Maternal Deaths 2011	Surveillance		2011	15 - 54 years	MMR
Mexico: Guerrero	Mexico Intentional Search and Reclassification of Maternal Deaths 2012	Surveillance		2012	15 - 49 years	MMR
Mexico: Guerrero	Mexico Intentional Search and Reclassification of Maternal Deaths 2013	Surveillance		2013	15 - 49 years	MMR
Mexico: Guerrero	Characteristics associated with under-registration of children's deaths in the state of Guerrero, Mexico	Verbal Autopsy	Guerrero [rural]	1993	birth - 4 years	366
Mexico: Guerrero	Mexico Vital Registration System - Mortality	Vital Registration		1980-2008, 2010-2014	All Ages	379057
Mexico: Guerrero	Mexico Vital Statistics - Deaths 2009	Vital Registration		2009	All Ages	15486
Mexico: Hidalgo	Mexico Intentional Search and Reclassification of Maternal Deaths 2005	Surveillance		2005	15 - 49 years	MMR
Mexico: Hidalgo	Mexico Intentional Search and Reclassification of Maternal Deaths 2006	Surveillance		2006	15 - 44 years	MMR
Mexico: Hidalgo	Mexico Intentional Search and Reclassification of Maternal Deaths 2008	Surveillance		2008	15 - 49 years	MMR
Mexico: Hidalgo	Mexico Intentional Search and Reclassification of Maternal Deaths 2009	Surveillance		2009	15 - 49 years	MMR
Mexico: Hidalgo	Mexico Intentional Search and Reclassification of Maternal Deaths 2010	Surveillance		2010	15 - 39 years	MMR
Mexico: Hidalgo	Mexico Intentional Search and Reclassification of Maternal Deaths 2011	Surveillance		2011	15 - 44 years	MMR
Mexico: Hidalgo	Mexico Intentional Search and Reclassification of Maternal Deaths 2012	Surveillance		2012	15 - 44 years	MMR
Mexico: Hidalgo	Mexico Intentional Search and Reclassification of Maternal Deaths 2013	Surveillance		2013	15 - 44 years	MMR
Mexico: Hidalgo	Mexico Vital Registration System - Mortality	Vital Registration		1980-2008, 2010-2014	All Ages	355019
Mexico: Hidalgo	Mexico Vital Statistics - Deaths 2009	Vital Registration		2009	All Ages	12180
Mexico: Jalisco	Mexico Intentional Search and Reclassification of Maternal Deaths 2005	Surveillance		2005	15 - 44 years	MMR
Mexico: Jalisco	Mexico Intentional Search and Reclassification of Maternal Deaths 2006	Surveillance		2006	15 - 44 years	MMR
Mexico: Jalisco	Mexico Intentional Search and Reclassification of Maternal Deaths 2008	Surveillance		2008	15 - 49 years	MMR
Mexico: Jalisco	Mexico Intentional Search and Reclassification of Maternal Deaths 2009	Surveillance		2009	15 - 49 years	MMR

Appendix Table 6: Causes of death data source list by geography, source type, and study site, with years, age range, and sample size, excluding sources on lower respiratory infection and diarrhoea etiologies						
**Location**	**Source name**	**Source type**	**Site**	**Years**	**Ages**	**Sample size**
Mexico: Jalisco	Mexico Intentional Search and Reclassification of Maternal Deaths 2010	Surveillance		2010	15 - 44 years	MMR
Mexico: Jalisco	Mexico Intentional Search and Reclassification of Maternal Deaths 2011	Surveillance		2011	10 - 44 years	MMR
Mexico: Jalisco	Mexico Intentional Search and Reclassification of Maternal Deaths 2012	Surveillance		2012	15 - 44 years	MMR
Mexico: Jalisco	Mexico Intentional Search and Reclassification of Maternal Deaths 2013	Surveillance		2013	15 - 44 years	MMR
Mexico: Jalisco	Mexico Vital Registration System - Mortality	Vital Registration		1980-2008, 2010-2014	All Ages	1035213
Mexico: Jalisco	Mexico Vital Statistics - Deaths 2009	Vital Registration		2009	All Ages	35371
Mexico: México	Mexico Intentional Search and Reclassification of Maternal Deaths 2005	Surveillance		2005	10 - 49 years	MMR
Mexico: México	Mexico Intentional Search and Reclassification of Maternal Deaths 2006	Surveillance		2006	15 - 49 years	MMR
Mexico: México	Mexico Intentional Search and Reclassification of Maternal Deaths 2008	Surveillance		2008	15 - 49 years	MMR
Mexico: México	Mexico Intentional Search and Reclassification of Maternal Deaths 2009	Surveillance		2009	15 - 49 years	MMR
Mexico: México	Mexico Intentional Search and Reclassification of Maternal Deaths 2010	Surveillance		2010	10 - 49 years	MMR
Mexico: México	Mexico Intentional Search and Reclassification of Maternal Deaths 2011	Surveillance		2011	15 - 54 years	MMR
Mexico: México	Mexico Intentional Search and Reclassification of Maternal Deaths 2012	Surveillance		2012	15 - 54 years	MMR
Mexico: México	Mexico Intentional Search and Reclassification of Maternal Deaths 2013	Surveillance		2013	15 - 49 years	MMR
Mexico: México	Mexico Vital Registration System - Mortality	Vital Registration		1980-2008, 2010-2014	All Ages	1773658
Mexico: México	Mexico Vital Statistics - Deaths 2009	Vital Registration		2009	All Ages	61674
Mexico: Michoacán de Ocampo	Mexico Intentional Search and Reclassification of Maternal Deaths 2005	Surveillance		2005	10 - 49 years	MMR
Mexico: Michoacán de Ocampo	Mexico Intentional Search and Reclassification of Maternal Deaths 2006	Surveillance		2006	15 - 44 years	MMR
Mexico: Michoacán de Ocampo	Mexico Intentional Search and Reclassification of Maternal Deaths 2008	Surveillance		2008	15 - 44 years	MMR
Mexico: Michoacán de Ocampo	Mexico Intentional Search and Reclassification of Maternal Deaths 2009	Surveillance		2009	15 - 44 years	MMR
Mexico: Michoacán de Ocampo	Mexico Intentional Search and Reclassification of Maternal Deaths 2010	Surveillance		2010	15 - 49 years	MMR
Mexico: Michoacán de Ocampo	Mexico Intentional Search and Reclassification of Maternal Deaths 2011	Surveillance		2011	15 - 49 years	MMR
Mexico: Michoacán de Ocampo	Mexico Intentional Search and Reclassification of Maternal Deaths 2012	Surveillance		2012	15 - 44 years	MMR
Mexico: Michoacán de Ocampo	Mexico Intentional Search and Reclassification of Maternal Deaths 2013	Surveillance		2013	10 - 44 years	MMR
Mexico: Michoacán de Ocampo	Mexico Vital Registration System - Mortality	Vital Registration		1980-2008, 2010-2014	All Ages	633828
Mexico: Michoacán de Ocampo	Mexico Vital Statistics - Deaths 2009	Vital Registration		2009	All Ages	22039
Mexico: Morelos	Mexico Intentional Search and Reclassification of Maternal Deaths 2005	Surveillance		2005	15 - 49 years	MMR
Mexico: Morelos	Mexico Intentional Search and Reclassification of Maternal Deaths 2006	Surveillance		2006	10 - 44 years	MMR
Mexico: Morelos	Mexico Intentional Search and Reclassification of Maternal Deaths 2008	Surveillance		2008	15 - 44 years	MMR

Appendix Table 6: Causes of death data source list by geography, source type, and study site, with years, age range, and sample size, excluding sources on lower respiratory infection and diarrhoea etiologies						
**Location**	**Source name**	**Source type**	**Site**	**Years**	**Ages**	**Sample size**
Mexico: Morelos	Mexico Intentional Search and Reclassification of Maternal Deaths 2009	Surveillance		2009	15 - 44 years	MMR
Mexico: Morelos	Mexico Intentional Search and Reclassification of Maternal Deaths 2010	Surveillance		2010	15 - 44 years	MMR
Mexico: Morelos	Mexico Intentional Search and Reclassification of Maternal Deaths 2011	Surveillance		2011	10 - 44 years	MMR
Mexico: Morelos	Mexico Intentional Search and Reclassification of Maternal Deaths 2012	Surveillance		2012	15 - 44 years	MMR
Mexico: Morelos	Mexico Intentional Search and Reclassification of Maternal Deaths 2013	Surveillance		2013	15 - 39 years	MMR
Mexico: Morelos	Mexico Vital Registration System - Mortality	Vital Registration		1980-2008, 2010-2014	All Ages	229516
Mexico: Morelos	Mexico Vital Statistics - Deaths 2009	Vital Registration		2009	All Ages	8484
Mexico: Nayarit	Mexico Intentional Search and Reclassification of Maternal Deaths 2005	Surveillance		2005	15 - 49 years	MMR
Mexico: Nayarit	Mexico Intentional Search and Reclassification of Maternal Deaths 2006	Surveillance		2006	15 - 44 years	MMR
Mexico: Nayarit	Mexico Intentional Search and Reclassification of Maternal Deaths 2008	Surveillance		2008	10 - 44 years	MMR
Mexico: Nayarit	Mexico Intentional Search and Reclassification of Maternal Deaths 2009	Surveillance		2009	10 - 44 years	MMR
Mexico: Nayarit	Mexico Intentional Search and Reclassification of Maternal Deaths 2010	Surveillance		2010	15 - 34 years	MMR
Mexico: Nayarit	Mexico Intentional Search and Reclassification of Maternal Deaths 2011	Surveillance		2011	10 - 49 years	MMR
Mexico: Nayarit	Mexico Intentional Search and Reclassification of Maternal Deaths 2012	Surveillance		2012	15 - 44 years	MMR
Mexico: Nayarit	Mexico Intentional Search and Reclassification of Maternal Deaths 2013	Surveillance		2013	15 - 44 years	MMR
Mexico: Nayarit	Mexico Vital Registration System - Mortality	Vital Registration		1980-2008, 2010-2014	All Ages	140306
Mexico: Nayarit	Mexico Vital Statistics - Deaths 2009	Vital Registration		2009	All Ages	5076
Mexico: Nuevo León	Mexico Intentional Search and Reclassification of Maternal Deaths 2005	Surveillance		2005	15 - 44 years	MMR
Mexico: Nuevo León	Mexico Intentional Search and Reclassification of Maternal Deaths 2006	Surveillance		2006	15 - 39 years	MMR
Mexico: Nuevo León	Mexico Intentional Search and Reclassification of Maternal Deaths 2008	Surveillance		2008	15 - 44 years	MMR
Mexico: Nuevo León	Mexico Intentional Search and Reclassification of Maternal Deaths 2009	Surveillance		2009	15 - 44 years	MMR
Mexico: Nuevo León	Mexico Intentional Search and Reclassification of Maternal Deaths 2010	Surveillance		2010	15 - 39 years	MMR
Mexico: Nuevo León	Mexico Intentional Search and Reclassification of Maternal Deaths 2011	Surveillance		2011	15 - 39 years	MMR
Mexico: Nuevo León	Mexico Intentional Search and Reclassification of Maternal Deaths 2012	Surveillance		2012	15 - 44 years	MMR
Mexico: Nuevo León	Mexico Intentional Search and Reclassification of Maternal Deaths 2013	Surveillance		2013	20 - 44 years	MMR
Mexico: Nuevo León	Mexico Vital Registration System - Mortality	Vital Registration		1980-2008, 2010-2014	All Ages	521847
Mexico: Nuevo León	Mexico Vital Statistics - Deaths 2009	Vital Registration		2009	All Ages	20804
Mexico: Oaxaca	Mexico Intentional Search and Reclassification of Maternal Deaths 2005	Surveillance		2005	15 - 44 years	MMR
Mexico: Oaxaca	Mexico Intentional Search and Reclassification of Maternal Deaths 2006	Surveillance		2006	10 - 44 years	MMR

Appendix Table 6: Causes of death data source list by geography, source type, and study site, with years, age range, and sample size, excluding sources on lower respiratory infection and diarrhoea etiologies						
**Location**	**Source name**	**Source type**	**Site**	**Years**	**Ages**	**Sample size**
Mexico: Oaxaca	Mexico Intentional Search and Reclassification of Maternal Deaths 2008	Surveillance		2008	15 - 44 years	MMR
Mexico: Oaxaca	Mexico Intentional Search and Reclassification of Maternal Deaths 2009	Surveillance		2009	15 - 44 years	MMR
Mexico: Oaxaca	Mexico Intentional Search and Reclassification of Maternal Deaths 2010	Surveillance		2010	15 - 49 years	MMR
Mexico: Oaxaca	Mexico Intentional Search and Reclassification of Maternal Deaths 2011	Surveillance		2011	10 - 49 years	MMR
Mexico: Oaxaca	Mexico Intentional Search and Reclassification of Maternal Deaths 2012	Surveillance		2012	15 - 44 years	MMR
Mexico: Oaxaca	Mexico Intentional Search and Reclassification of Maternal Deaths 2013	Surveillance		2013	15 - 49 years	MMR
Mexico: Oaxaca	Mexico Vital Registration System - Mortality	Vital Registration		1980-2008, 2010-2014	All Ages	630599
Mexico: Oaxaca	Mexico Vital Statistics - Deaths 2009	Vital Registration		2009	All Ages	19076
Mexico: Puebla	Mexico Intentional Search and Reclassification of Maternal Deaths 2005	Surveillance		2005	15 - 44 years	MMR
Mexico: Puebla	Mexico Intentional Search and Reclassification of Maternal Deaths 2006	Surveillance		2006	15 - 49 years	MMR
Mexico: Puebla	Mexico Intentional Search and Reclassification of Maternal Deaths 2008	Surveillance		2008	15 - 49 years	MMR
Mexico: Puebla	Mexico Intentional Search and Reclassification of Maternal Deaths 2009	Surveillance		2009	15 - 49 years	MMR
Mexico: Puebla	Mexico Intentional Search and Reclassification of Maternal Deaths 2010	Surveillance		2010	10 - 44 years	MMR
Mexico: Puebla	Mexico Intentional Search and Reclassification of Maternal Deaths 2011	Surveillance		2011	15 - 44 years	MMR
Mexico: Puebla	Mexico Intentional Search and Reclassification of Maternal Deaths 2012	Surveillance		2012	15 - 44 years	MMR
Mexico: Puebla	Mexico Intentional Search and Reclassification of Maternal Deaths 2013	Surveillance		2013	15 - 54 years	MMR
Mexico: Puebla	Mexico Vital Registration System - Mortality	Vital Registration		1980-2008, 2010-2014	All Ages	905837
Mexico: Puebla	Mexico Vital Statistics - Deaths 2009	Vital Registration		2009	All Ages	28235
Mexico: Querétaro	Mexico Intentional Search and Reclassification of Maternal Deaths 2005	Surveillance		2005	15 - 44 years	MMR
Mexico: Querétaro	Mexico Intentional Search and Reclassification of Maternal Deaths 2006	Surveillance		2006	15 - 44 years	MMR
Mexico: Querétaro	Mexico Intentional Search and Reclassification of Maternal Deaths 2008	Surveillance		2008	15 - 44 years	MMR
Mexico: Querétaro	Mexico Intentional Search and Reclassification of Maternal Deaths 2009	Surveillance		2009	15 - 44 years	MMR
Mexico: Querétaro	Mexico Intentional Search and Reclassification of Maternal Deaths 2010	Surveillance		2010	15 - 44 years	MMR
Mexico: Querétaro	Mexico Intentional Search and Reclassification of Maternal Deaths 2011	Surveillance		2011	15 - 44 years	MMR
Mexico: Querétaro	Mexico Intentional Search and Reclassification of Maternal Deaths 2012	Surveillance		2012	15 - 44 years	MMR
Mexico: Querétaro	Mexico Intentional Search and Reclassification of Maternal Deaths 2013	Surveillance		2013	15 - 44 years	MMR
Mexico: Querétaro	Mexico Vital Registration System - Mortality	Vital Registration		1980-2008, 2010-2014	All Ages	199997
Mexico: Querétaro	Mexico Vital Statistics - Deaths 2009	Vital Registration		2009	All Ages	7124
Mexico: Quintana Roo	Mexico Intentional Search and Reclassification of Maternal Deaths 2005	Surveillance		2005	15 - 34 years	MMR

Appendix Table 6: Causes of death data source list by geography, source type, and study site, with years, age range, and sample size, excluding sources on lower respiratory infection and diarrhoea etiologies						
Location	Source name	Source type	Site	Years	Ages	Sample size
Mexico: Quintana Roo	Mexico Intentional Search and Reclassification of Maternal Deaths 2006	Surveillance		2006	15 - 34 years	MMR
Mexico: Quintana Roo	Mexico Intentional Search and Reclassification of Maternal Deaths 2008	Surveillance		2008	10 - 39 years	MMR
Mexico: Quintana Roo	Mexico Intentional Search and Reclassification of Maternal Deaths 2009	Surveillance		2009	10 - 39 years	MMR
Mexico: Quintana Roo	Mexico Intentional Search and Reclassification of Maternal Deaths 2010	Surveillance		2010	15 - 39 years	MMR
Mexico: Quintana Roo	Mexico Intentional Search and Reclassification of Maternal Deaths 2011	Surveillance		2011	15 - 49 years	MMR
Mexico: Quintana Roo	Mexico Intentional Search and Reclassification of Maternal Deaths 2012	Surveillance		2012	15 - 49 years	MMR
Mexico: Quintana Roo	Mexico Intentional Search and Reclassification of Maternal Deaths 2013	Surveillance		2013	15 - 49 years	MMR
Mexico: Quintana Roo	Mexico Vital Registration System - Mortality	Vital Registration		1980-2008, 2010-2014	All Ages	76691
Mexico: Quintana Roo	Mexico Vital Statistics - Deaths 2009	Vital Registration		2009	All Ages	3842
Mexico: San Luis Potosí	Mexico Intentional Search and Reclassification of Maternal Deaths 2005	Surveillance		2005	15 - 49 years	MMR
Mexico: San Luis Potosí	Mexico Intentional Search and Reclassification of Maternal Deaths 2006	Surveillance		2006	15 - 39 years	MMR
Mexico: San Luis Potosí	Mexico Intentional Search and Reclassification of Maternal Deaths 2008	Surveillance		2008	15 - 49 years	MMR
Mexico: San Luis Potosí	Mexico Intentional Search and Reclassification of Maternal Deaths 2009	Surveillance		2009	15 - 49 years	MMR
Mexico: San Luis Potosí	Mexico Intentional Search and Reclassification of Maternal Deaths 2010	Surveillance		2010	15 - 44 years	MMR
Mexico: San Luis Potosí	Mexico Intentional Search and Reclassification of Maternal Deaths 2011	Surveillance		2011	15 - 44 years	MMR
Mexico: San Luis Potosí	Mexico Intentional Search and Reclassification of Maternal Deaths 2012	Surveillance		2012	15 - 44 years	MMR
Mexico: San Luis Potosí	Mexico Intentional Search and Reclassification of Maternal Deaths 2013	Surveillance		2013	15 - 44 years	MMR
Mexico: San Luis Potosí	Mexico Vital Registration System - Mortality	Vital Registration		1980-2008, 2010-2014	All Ages	354459
Mexico: San Luis Potosí	Mexico Vital Statistics - Deaths 2009	Vital Registration		2009	All Ages	11443
Mexico: Sinaloa	Mexico Intentional Search and Reclassification of Maternal Deaths 2005	Surveillance		2005	15 - 44 years	MMR
Mexico: Sinaloa	Mexico Intentional Search and Reclassification of Maternal Deaths 2006	Surveillance		2006	15 - 39 years	MMR
Mexico: Sinaloa	Mexico Intentional Search and Reclassification of Maternal Deaths 2008	Surveillance		2008	15 - 39 years	MMR
Mexico: Sinaloa	Mexico Intentional Search and Reclassification of Maternal Deaths 2009	Surveillance		2009	15 - 39 years	MMR
Mexico: Sinaloa	Mexico Intentional Search and Reclassification of Maternal Deaths 2010	Surveillance		2010	10 - 44 years	MMR
Mexico: Sinaloa	Mexico Intentional Search and Reclassification of Maternal Deaths 2011	Surveillance		2011	10 - 44 years	MMR
Mexico: Sinaloa	Mexico Intentional Search and Reclassification of Maternal Deaths 2012	Surveillance		2012	15 - 39 years	MMR
Mexico: Sinaloa	Mexico Intentional Search and Reclassification of Maternal Deaths 2013	Surveillance		2013	15 - 39 years	MMR
Mexico: Sinaloa	Mexico Vital Registration System - Mortality	Vital Registration		1980-2008, 2010-2014	All Ages	338434
Mexico: Sinaloa	Mexico Vital Statistics - Deaths 2009	Vital Registration		2009	All Ages	12848

Appendix Table 6: Causes of death data source list by geography, source type, and study site, with years, age range, and sample size, excluding sources on lower respiratory infection and diarrhoea etiologies						
**Location**	**Source name**	**Source type**	**Site**	**Years**	**Ages**	**Sample size**
Mexico: Sonora	Mexico Intentional Search and Reclassification of Maternal Deaths 2005	Surveillance		2005	15 - 44 years	MMR
Mexico: Sonora	Mexico Intentional Search and Reclassification of Maternal Deaths 2006	Surveillance		2006	15 - 39 years	MMR
Mexico: Sonora	Mexico Intentional Search and Reclassification of Maternal Deaths 2008	Surveillance		2008	10 - 49 years	MMR
Mexico: Sonora	Mexico Intentional Search and Reclassification of Maternal Deaths 2009	Surveillance		2009	10 - 49 years	MMR
Mexico: Sonora	Mexico Intentional Search and Reclassification of Maternal Deaths 2010	Surveillance		2010	15 - 44 years	MMR
Mexico: Sonora	Mexico Intentional Search and Reclassification of Maternal Deaths 2011	Surveillance		2011	15 - 44 years	MMR
Mexico: Sonora	Mexico Intentional Search and Reclassification of Maternal Deaths 2012	Surveillance		2012	15 - 44 years	MMR
Mexico: Sonora	Mexico Intentional Search and Reclassification of Maternal Deaths 2013	Surveillance		2013	15 - 39 years	MMR
Mexico: Sonora	Mexico Vital Registration System - Mortality	Vital Registration		1980-2008, 2010-2014	All Ages	353281
Mexico: Sonora	Mexico Vital Statistics - Deaths 2009	Vital Registration		2009	All Ages	13045
Mexico: Tabasco	Mexico Intentional Search and Reclassification of Maternal Deaths 2005	Surveillance		2005	15 - 49 years	MMR
Mexico: Tabasco	Mexico Intentional Search and Reclassification of Maternal Deaths 2006	Surveillance		2006	15 - 39 years	MMR
Mexico: Tabasco	Mexico Intentional Search and Reclassification of Maternal Deaths 2008	Surveillance		2008	15 - 39 years	MMR
Mexico: Tabasco	Mexico Intentional Search and Reclassification of Maternal Deaths 2009	Surveillance		2009	15 - 39 years	MMR
Mexico: Tabasco	Mexico Intentional Search and Reclassification of Maternal Deaths 2010	Surveillance		2010	15 - 39 years	MMR
Mexico: Tabasco	Mexico Intentional Search and Reclassification of Maternal Deaths 2011	Surveillance		2011	15 - 39 years	MMR
Mexico: Tabasco	Mexico Intentional Search and Reclassification of Maternal Deaths 2012	Surveillance		2012	15 - 39 years	MMR
Mexico: Tabasco	Mexico Intentional Search and Reclassification of Maternal Deaths 2013	Surveillance		2013	15 - 49 years	MMR
Mexico: Tabasco	Mexico Vital Registration System - Mortality	Vital Registration		1980-2008, 2010-2014	All Ages	256008
Mexico: Tabasco	Mexico Vital Statistics - Deaths 2009	Vital Registration		2009	All Ages	9417
Mexico: Tamaulipas	Mexico Intentional Search and Reclassification of Maternal Deaths 2005	Surveillance		2005	15 - 39 years	MMR
Mexico: Tamaulipas	Mexico Intentional Search and Reclassification of Maternal Deaths 2006	Surveillance		2006	15 - 44 years	MMR
Mexico: Tamaulipas	Mexico Intentional Search and Reclassification of Maternal Deaths 2008	Surveillance		2008	15 - 39 years	MMR
Mexico: Tamaulipas	Mexico Intentional Search and Reclassification of Maternal Deaths 2009	Surveillance		2009	15 - 39 years	MMR
Mexico: Tamaulipas	Mexico Intentional Search and Reclassification of Maternal Deaths 2010	Surveillance		2010	15 - 44 years	MMR
Mexico: Tamaulipas	Mexico Intentional Search and Reclassification of Maternal Deaths 2011	Surveillance		2011	15 - 39 years	MMR
Mexico: Tamaulipas	Mexico Intentional Search and Reclassification of Maternal Deaths 2012	Surveillance		2012	15 - 39 years	MMR
Mexico: Tamaulipas	Mexico Intentional Search and Reclassification of Maternal Deaths 2013	Surveillance		2013	10 - 39 years	MMR
Mexico: Tamaulipas	Mexico Vital Registration System - Mortality	Vital Registration		1980-2008, 2010-2014	All Ages	401069

Appendix Table 6: Causes of death data source list by geography, source type, and study site, with years, age range, and sample size, excluding sources on lower respiratory infection and diarrhoea etiologies						
**Location**	**Source name**	**Source type**	**Site**	**Years**	**Ages**	**Sample size**
Mexico: Tamaulipas	Mexico Vital Statistics - Deaths 2009	Vital Registration		2009	All Ages	14800
Mexico: Tlaxcala	Mexico Intentional Search and Reclassification of Maternal Deaths 2005	Surveillance		2005	15 - 39 years	MMR
Mexico: Tlaxcala	Mexico Intentional Search and Reclassification of Maternal Deaths 2006	Surveillance		2006	15 - 39 years	MMR
Mexico: Tlaxcala	Mexico Intentional Search and Reclassification of Maternal Deaths 2008	Surveillance		2008	20 - 44 years	MMR
Mexico: Tlaxcala	Mexico Intentional Search and Reclassification of Maternal Deaths 2009	Surveillance		2009	20 - 44 years	MMR
Mexico: Tlaxcala	Mexico Intentional Search and Reclassification of Maternal Deaths 2010	Surveillance		2010	15 - 39 years	MMR
Mexico: Tlaxcala	Mexico Intentional Search and Reclassification of Maternal Deaths 2011	Surveillance		2011	15 - 44 years	MMR
Mexico: Tlaxcala	Mexico Intentional Search and Reclassification of Maternal Deaths 2012	Surveillance		2012	15 - 39 years	MMR
Mexico: Tlaxcala	Mexico Intentional Search and Reclassification of Maternal Deaths 2013	Surveillance		2013	20 - 39 years	MMR
Mexico: Tlaxcala	Mexico Vital Registration System - Mortality	Vital Registration		1980-2008, 2010-2014	All Ages	151008
Mexico: Tlaxcala	Mexico Vital Statistics - Deaths 2009	Vital Registration		2009	All Ages	5046
Mexico: Veracruz de Ignacio de la Llave	Mexico Intentional Search and Reclassification of Maternal Deaths 2005	Surveillance		2005	10 - 44 years	MMR
Mexico: Veracruz de Ignacio de la Llave	Mexico Intentional Search and Reclassification of Maternal Deaths 2006	Surveillance		2006	15 - 44 years	MMR
Mexico: Veracruz de Ignacio de la Llave	Mexico Intentional Search and Reclassification of Maternal Deaths 2008	Surveillance		2008	10 - 44 years	MMR
Mexico: Veracruz de Ignacio de la Llave	Mexico Intentional Search and Reclassification of Maternal Deaths 2009	Surveillance		2009	10 - 44 years	MMR
Mexico: Veracruz de Ignacio de la Llave	Mexico Intentional Search and Reclassification of Maternal Deaths 2010	Surveillance		2010	15 - 44 years	MMR
Mexico: Veracruz de Ignacio de la Llave	Mexico Intentional Search and Reclassification of Maternal Deaths 2011	Surveillance		2011	10 - 44 years	MMR
Mexico: Veracruz de Ignacio de la Llave	Mexico Intentional Search and Reclassification of Maternal Deaths 2012	Surveillance		2012	15 - 44 years	MMR
Mexico: Veracruz de Ignacio de la Llave	Mexico Intentional Search and Reclassification of Maternal Deaths 2013	Surveillance		2013	15 - 44 years	MMR
Mexico: Veracruz de Ignacio de la Llave	Mexico Vital Registration System - Mortality	Vital Registration		1980-2008, 2010-2014	All Ages	1115326
Mexico: Veracruz de Ignacio de la Llave	Mexico Vital Statistics - Deaths 2009	Vital Registration		2009	All Ages	40691
Mexico: Yucatán	Mexico Intentional Search and Reclassification of Maternal Deaths 2005	Surveillance		2005	15 - 54 years	MMR
Mexico: Yucatán	Mexico Intentional Search and Reclassification of Maternal Deaths 2006	Surveillance		2006	20 - 44 years	MMR
Mexico: Yucatán	Mexico Intentional Search and Reclassification of Maternal Deaths 2008	Surveillance		2008	15 - 44 years	MMR
Mexico: Yucatán	Mexico Intentional Search and Reclassification of Maternal Deaths 2009	Surveillance		2009	15 - 44 years	MMR
Mexico: Yucatán	Mexico Intentional Search and Reclassification of Maternal Deaths 2010	Surveillance		2010	15 - 44 years	MMR
Mexico: Yucatán	Mexico Intentional Search and Reclassification of Maternal Deaths 2011	Surveillance		2011	15 - 44 years	MMR
Mexico: Yucatán	Mexico Intentional Search and Reclassification of Maternal Deaths 2012	Surveillance		2012	15 - 44 years	MMR
Mexico: Yucatán	Mexico Intentional Search and Reclassification of Maternal Deaths 2013	Surveillance		2013	15 - 39 years	MMR

Appendix Table 6: Causes of death data source list by geography, source type, and study site, with years, age range, and sample size, excluding sources on lower respiratory infection and diarrhoea etiologies						
**Location**	**Source name**	**Source type**	**Site**	**Years**	**Ages**	**Sample size**
Mexico: Yucatán	Mexico Vital Registration System - Mortality	Vital Registration		1980-2008, 2010-2014	All Ages	276801
Mexico: Yucatán	Mexico Vital Statistics - Deaths 2009	Vital Registration		2009	All Ages	9768
Mexico: Zacatecas	Mexico Intentional Search and Reclassification of Maternal Deaths 2005	Surveillance		2005	15 - 39 years	MMR
Mexico: Zacatecas	Mexico Intentional Search and Reclassification of Maternal Deaths 2006	Surveillance		2006	20 - 44 years	MMR
Mexico: Zacatecas	Mexico Intentional Search and Reclassification of Maternal Deaths 2008	Surveillance		2008	15 - 49 years	MMR
Mexico: Zacatecas	Mexico Intentional Search and Reclassification of Maternal Deaths 2009	Surveillance		2009	15 - 49 years	MMR
Mexico: Zacatecas	Mexico Intentional Search and Reclassification of Maternal Deaths 2010	Surveillance		2010	15 - 39 years	MMR
Mexico: Zacatecas	Mexico Intentional Search and Reclassification of Maternal Deaths 2011	Surveillance		2011	20 - 34 years	MMR
Mexico: Zacatecas	Mexico Intentional Search and Reclassification of Maternal Deaths 2012	Surveillance		2012	20 - 44 years	MMR
Mexico: Zacatecas	Mexico Intentional Search and Reclassification of Maternal Deaths 2013	Surveillance		2013	15 - 39 years	MMR
Mexico: Zacatecas	Mexico Vital Registration System - Mortality	Vital Registration		1980-2008, 2010-2014	All Ages	217005
Mexico: Zacatecas	Mexico Vital Statistics - Deaths 2009	Vital Registration		2009	All Ages	7345
Nicaragua	United Nations Surveys on Crime Trends and the Operations of Criminal Justice Systems 1970-2006	Police Records		1990-1994	All Ages	Death Rate
Nicaragua	United Nations Office on Drugs and Crime Global Study on Homicide 2011	Police Records	Nicaragua National police	1995, 1997-2007, 2009-2010	All Ages	Death Rate
Nicaragua	United Nations Office on Drugs and Crime Global Study on Homicide 2011	Police Records	Nicaragua PAHO	1997-2006	All Ages	Death Rate
Nicaragua	Global Status Report on Road Safety 2009	Police Records		2007	All Ages	Death Rate
Nicaragua	Reproductive Health Survey (RHS)	Sibling history		1980-1991	15 - 49 years	0
Nicaragua	Nicaragua Population and Housing Census 2005	Survey/Census		2004-2005	15 - 49 years	5130
Nicaragua	Comparing progress toward the millennium development goal for under-five mortality in León and Cuatro Santos, Nicaragua, 1990-2008	Verbal Autopsy	Leon []	2005	birth - 4 years	59
Nicaragua	Comparing progress toward the millennium development goal for under-five mortality in León and Cuatro Santos, Nicaragua, 1990-2008	Verbal Autopsy	Cuatro Santos []	2006	birth - 4 years	39
Nicaragua	WHO Mortality Database	Vital Registration		1988-1994, 1996-2013	All Ages	386454
Panama	United Nations Surveys on Crime Trends and the Operations of Criminal Justice Systems 1970-2006	Police Records		1980	All Ages	Death Rate
Panama	United Nations Office on Drugs and Crime Global Study on Homicide 2011	Police Records	Panama National police	1995-2007, 2009-2010	All Ages	Death Rate
Panama	United Nations Office on Drugs and Crime Global Study on Homicide 2011	Police Records	Panama PAHO	1996-2007	All Ages	Death Rate
Panama	WHO Mortality Database	Vital Registration		1980-1989, 1996-2013	All Ages	339468
Venezuela	United Nations Office on Drugs and Crime Global Study on Homicide 2011	Police Records	Venezuela NGO (d)	1995-2007, 2009	All Ages	Death Rate
Venezuela	United Nations Office on Drugs and Crime Global Study on Homicide 2011	Police Records	Venezuela PAHO	1996-2007	All Ages	Death Rate
Venezuela	WHO Mortality Database	Vital Registration		1980-1983, 1985-1990, 1992-1994, 1996-2012	All Ages	3125382
Brazil: Acre	Brazil Demographic and Health Survey - Maternal Mortality Data	Sibling History		1992	30 - 34 years	1

Appendix Table 6: Causes of death data source list by geography, source type, and study site, with years, age range, and sample size, excluding sources on lower respiratory infection and diarrhoea etiologies						
**Location**	**Source name**	**Source type**	**Site**	**Years**	**Ages**	**Sample size**
Brazil: Acre	Brazil Demographic and Health Survey - Maternal Mortality Data	Sibling History		1995	35 - 39 years	1
Brazil: Acre	Brazil Mortality Information System (SIM)	Vital Registration		1980-2013	All Ages	78302
Brazil: Alagoas	Brazil Demographic and Health Survey - Maternal Mortality Data	Sibling History		1982	35 - 39 years	1
Brazil: Alagoas	Brazil Demographic and Health Survey - Maternal Mortality Data	Sibling History		1985	40 - 44 years	1
Brazil: Alagoas	Brazil Demographic and Health Survey - Maternal Mortality Data	Sibling History		1988	35 - 49 years	2
Brazil: Alagoas	Brazil Demographic and Health Survey - Maternal Mortality Data	Sibling History		1991-1992	20 - 29 years	2
Brazil: Alagoas	Brazil Demographic and Health Survey - Maternal Mortality Data	Sibling History		1994	45 - 49 years	1
Brazil: Alagoas	Brazil Mortality Information System (SIM)	Vital Registration		1980-2013	All Ages	549284
Brazil: Amapá	Brazil Demographic and Health Survey - Maternal Mortality Data	Sibling History		1991	40 - 44 years	1
Brazil: Amapá	Brazil Mortality Information System (SIM)	Vital Registration		1980-2013	All Ages	52503
Brazil: Amazonas	Brazil Demographic and Health Survey - Maternal Mortality Data	Sibling History		1983-1989	25 - 29 years	2
Brazil: Amazonas	Brazil Demographic and Health Survey - Maternal Mortality Data	Sibling History		1994	15 - 59 years	4
Brazil: Amazonas	Brazil Demographic and Health Survey - Maternal Mortality Data	Sibling History		1996	50 - 54 years	2
Brazil: Amazonas	Brazil Mortality Information System (SIM)	Vital Registration		1980-2013	All Ages	324158
Brazil: Bahia	Brazil Demographic and Health Survey - Maternal Mortality Data	Sibling History		1982-1984	20 - 24 years	3
Brazil: Bahia	Brazil Demographic and Health Survey - Maternal Mortality Data	Sibling History		1986-1995	35 - 44 years	2
Brazil: Bahia	Brazil Mortality Information System (SIM)	Vital Registration		1980-2013	All Ages	2009973
Brazil: Ceará	Brazil Demographic and Health Survey - Maternal Mortality Data	Sibling History		1983-1984	25 - 54 years	2
Brazil: Ceará	Brazil Demographic and Health Survey - Maternal Mortality Data	Sibling History		1986	15 - 59 years	2
Brazil: Ceará	Brazil Demographic and Health Survey - Maternal Mortality Data	Sibling History		1990	30 - 54 years	3
Brazil: Ceará	Brazil Demographic and Health Survey - Maternal Mortality Data	Sibling History		1992-1996	20 - 29 years	3
Brazil: Ceará	A study of infant mortality and causes of death in a rural north-east Brazilian community	Verbal Autopsy	Trairi county Ceara state [rural]	1985	birth - 364 days	266
Brazil: Ceará	Vigilancia de obitos infantis em sistemas locais de saude: avaliacao da autopsia verbal e das informacoes de agentes de saude	Verbal Autopsy	Quixada Ceara Icapui Ceara Jucas Ceara	1993	birth - 27 days	201
Brazil: Ceará	Surveillance of infant deaths in local health systems: assessment of verbal autopsy reports and of information gathered from health agents	Verbal Autopsy	Quixada Icapui Jucas [urban]	1993	birth - 364 days	215
Brazil: Ceará	Brazil Mortality Information System (SIM)	Vital Registration		1980-2013	All Ages	1093812
Brazil: Distrito Federal	Brazil Demographic and Health Survey - Maternal Mortality Data	Sibling History		1982	20 - 24 years	2
Brazil: Distrito Federal	Brazil Demographic and Health Survey - Maternal Mortality Data	Sibling History		1985	30 - 49 years	3
Brazil: Distrito Federal	Brazil Demographic and Health Survey - Maternal Mortality Data	Sibling History		1990-1991	35 - 39 years	1

Appendix Table 6: Causes of death data source list by geography, source type, and study site, with years, age range, and sample size, excluding sources on lower respiratory infection and diarrhoea etiologies						
**Location**	**Source name**	**Source type**	**Site**	**Years**	**Ages**	**Sample size**
Brazil: Distrito Federal	Brazil Demographic and Health Survey - Maternal Mortality Data	Sibling History		1993-1994	30 - 34 years	1
Brazil: Distrito Federal	Brazil Mortality Information System (SIM)	Vital Registration		1980-2013	All Ages	273179
Brazil: Espírito Santo	Brazil Demographic and Health Survey - Maternal Mortality Data	Sibling History		1983, 1993	25 - 29 years	2
Brazil: Espírito Santo	Brazil Demographic and Health Survey - Maternal Mortality Data	Sibling History		1991	35 - 39 years	2
Brazil: Espírito Santo	Brazil Demographic and Health Survey - Maternal Mortality Data	Sibling History		1995-1996	30 - 34 years	1
Brazil: Espírito Santo	Brazil Mortality Information System (SIM)	Vital Registration		1980-2013	All Ages	569271
Brazil: Goiás	Brazil Demographic and Health Survey - Maternal Mortality Data	Sibling History		1983, 1994-1995	45 - 49 years	3
Brazil: Goiás	Brazil Demographic and Health Survey - Maternal Mortality Data	Sibling History		1985	35 - 39 years	1
Brazil: Goiás	Brazil Demographic and Health Survey - Maternal Mortality Data	Sibling History		1987	20 - 29 years	2
Brazil: Goiás	Brazil Demographic and Health Survey - Maternal Mortality Data	Sibling History		1990	50 - 54 years	1
Brazil: Goiás	Brazil Demographic and Health Survey - Maternal Mortality Data	Sibling History		1992	30 - 59 years	3
Brazil: Goiás	Brazil Mortality Information System (SIM)	Vital Registration		1980-2013	All Ages	818954
Brazil: Maranhão	Brazil Demographic and Health Survey - Maternal Mortality Data	Sibling History		1982-1984	20 - 59 years	2
Brazil: Maranhão	Brazil Demographic and Health Survey - Maternal Mortality Data	Sibling History		1986	20 - 24 years	2
Brazil: Maranhão	Brazil Demographic and Health Survey - Maternal Mortality Data	Sibling History		1990-1995	20 - 34 years	3
Brazil: Maranhão	Morbimortalidade infantil por diarréia aguda em área metropolitana da região Nordeste do Brasil, 1986-1989	Verbal Autopsy		1986, 1989	birth - 4 years	124
Brazil: Maranhão	Brazil Mortality Information System (SIM)	Vital Registration		1980-2013	All Ages	598743
Brazil: Mato Grosso	Brazil Demographic and Health Survey - Maternal Mortality Data	Sibling History		1982	15 - 34 years	3
Brazil: Mato Grosso	Brazil Demographic and Health Survey - Maternal Mortality Data	Sibling History		1984-1985	30 - 34 years	2
Brazil: Mato Grosso	Brazil Demographic and Health Survey - Maternal Mortality Data	Sibling History		1989-1990	25 - 29 years	1
Brazil: Mato Grosso	Brazil Demographic and Health Survey - Maternal Mortality Data	Sibling History		1992-1996	25 - 54 years	2
Brazil: Mato Grosso	Brazil Mortality Information System (SIM)	Vital Registration		1980-2013	All Ages	328303
Brazil: Mato Grosso do Sul	Brazil Demographic and Health Survey - Maternal Mortality Data	Sibling History		1982, 1986, 1988-1989	20 - 24 years	3
Brazil: Mato Grosso do Sul	Brazil Demographic and Health Survey - Maternal Mortality Data	Sibling History		1991-1992	25 - 29 years	1
Brazil: Mato Grosso do Sul	Brazil Demographic and Health Survey - Maternal Mortality Data	Sibling History		1995	45 - 49 years	1
Brazil: Mato Grosso do Sul	Brazil Mortality Information System (SIM)	Vital Registration		1980-2013	All Ages	363422
Brazil: Minas Gerais	Brazil Demographic and Health Survey - Maternal Mortality Data	Sibling History		1982	15 - 44 years	6
Brazil: Minas Gerais	Brazil Demographic and Health Survey - Maternal Mortality Data	Sibling History		1984-1995	15 - 34 years	5
Brazil: Minas Gerais	Brazil Mortality Information System (SIM)	Vital Registration		1980-2013	All Ages	3436489
Brazil: Pará	Brazil Demographic and Health Survey - Maternal Mortality Data	Sibling History		1987-1988, 1991	20 - 24 years	3

Appendix Table 6: Causes of death data source list by geography, source type, and study site, with years, age range, and sample size, excluding sources on lower respiratory infection and diarrhoea etiologies						
**Location**	**Source name**	**Source type**	**Site**	**Years**	**Ages**	**Sample size**
Brazil: Pará	Brazil Demographic and Health Survey - Maternal Mortality Data	Sibling History		1993-1995	50 - 54 years	1
Brazil: Pará	Brazil Mortality Information System (SIM)	Vital Registration		1980-2013	All Ages	749632
Brazil: Paraíba	Brazil Demographic and Health Survey - Maternal Mortality Data	Sibling History		1984, 1989	40 - 44 years	2
Brazil: Paraíba	Brazil Demographic and Health Survey - Maternal Mortality Data	Sibling History		1992	20 - 39 years	2
Brazil: Paraíba	Brazil Demographic and Health Survey - Maternal Mortality Data	Sibling History		1994-1995	20 - 59 years	5
Brazil: Paraíba	Brazil Mortality Information System (SIM)	Vital Registration		1980-2013	All Ages	705399
Brazil: Paraná	Brazil Demographic and Health Survey - Maternal Mortality Data	Sibling History		1983-1986	30 - 34 years	1
Brazil: Paraná	Brazil Demographic and Health Survey - Maternal Mortality Data	Sibling History		1988	45 - 49 years	1
Brazil: Paraná	Brazil Demographic and Health Survey - Maternal Mortality Data	Sibling History		1990	25 - 49 years	2
Brazil: Paraná	Brazil Demographic and Health Survey - Maternal Mortality Data	Sibling History		1992-1995	40 - 49 years	2
Brazil: Paraná	Brazil Mortality Information System (SIM)	Vital Registration		1980-2013	All Ages	1852297
Brazil: Pernambuco	Brazil Demographic and Health Survey - Maternal Mortality Data	Sibling History		1982	15 - 19 years	1
Brazil: Pernambuco	Brazil Demographic and Health Survey - Maternal Mortality Data	Sibling History		1984	15 - 39 years	4
Brazil: Pernambuco	Brazil Demographic and Health Survey - Maternal Mortality Data	Sibling History		1986-1996	20 - 29 years	2
Brazil: Pernambuco	Brazil Mortality Information System (SIM)	Vital Registration		1980-2013	All Ages	1813364
Brazil: Piaui	Brazil Demographic and Health Survey - Maternal Mortality Data	Sibling History		1982, 1984, 1987	25 - 29 years	4
Brazil: Piaui	Brazil Demographic and Health Survey - Maternal Mortality Data	Sibling History		1993-1995	30 - 34 years	1
Brazil: Piaui	Brazil Mortality Information System (SIM)	Vital Registration		1980-2013	All Ages	362009
Brazil: Rio de Janeiro	Brazil Demographic and Health Survey - Maternal Mortality Data	Sibling History		1982	25 - 29 years	1
Brazil: Rio de Janeiro	Brazil Demographic and Health Survey - Maternal Mortality Data	Sibling History		1984	25 - 34 years	2
Brazil: Rio de Janeiro	Brazil Demographic and Health Survey - Maternal Mortality Data	Sibling History		1987-1988	25 - 54 years	2
Brazil: Rio de Janeiro	Brazil Demographic and Health Survey - Maternal Mortality Data	Sibling History		1990	25 - 59 years	4
Brazil: Rio de Janeiro	Brazil Demographic and Health Survey - Maternal Mortality Data	Sibling History		1992-1995	35 - 59 years	4
Brazil: Rio de Janeiro	Brazil Mortality Information System (SIM)	Vital Registration		1980-2013	All Ages	3793988
Brazil: Rio Grande do Norte	Brazil Demographic and Health Survey - Maternal Mortality Data	Sibling History		1982-1984	25 - 34 years	2
Brazil: Rio Grande do Norte	Brazil Demographic and Health Survey - Maternal Mortality Data	Sibling History		1988-1989	35 - 39 years	2
Brazil: Rio Grande do Norte	Brazil Demographic and Health Survey - Maternal Mortality Data	Sibling History		1994	25 - 54 years	3
Brazil: Rio Grande do Norte	Brazil Mortality Information System (SIM)	Vital Registration		1980-2013	All Ages	426252
Brazil: Rio Grande do Sul	Brazil Demographic and Health Survey - Maternal Mortality Data	Sibling History		1982-1989	45 - 49 years	1
Brazil: Rio Grande do Sul	Brazil Demographic and Health Survey - Maternal Mortality Data	Sibling History		1991-1996	15 - 44 years	5

Appendix Table 6: Causes of death data source list by geography, source type, and study site, with years, age range, and sample size, excluding sources on lower respiratory infection and diarrhoea etiologies

Location	Source name	Source type	Site	Years	Ages	Sample size
Brazil: Rio Grande do Sul	Early childhood mortality in a Brazilian cohort: the roles of birthweight and socioeconomic status	Verbal Autopsy	Pelotas [urban]	1984	1 - 4 years	29
Brazil: Rio Grande do Sul	Evidence for protection by breast-feeding against infant deaths from infectious diseases in Brazil	Verbal Autopsy	Porto Alegre and Pelota [urban]	1984	birth - 364 days	357
Brazil: Rio Grande do Sul	International differences in clinical patterns of diarrhoeal deaths: a comparison of children from Brazil, Senegal, Bangladesh, and India	Verbal Autopsy	Porto Alegre Pelotas Rio Grande do Sul state [mixed]	1985	birth - 4 years	1892
Brazil: Rio Grande do Sul	Infant mortality in two population-based cohorts in southern Brazil: trends and differentials	Verbal Autopsy	Pelotas [urban]	1993	birth - 364 days	111
Brazil: Rio Grande do Sul	Brazil Mortality Information System (SIM)	Vital Registration		1980-2013	All Ages	2218443
Brazil: Rondônia	Brazil Demographic and Health Survey - Maternal Mortality Data	Sibling History		1986	15 - 19 years	1
Brazil: Rondônia	Brazil Demographic and Health Survey - Maternal Mortality Data	Sibling History		1990-1993	40 - 44 years	1
Brazil: Rondônia	Brazil Demographic and Health Survey - Maternal Mortality Data	Sibling History		1995	50 - 54 years	1
Brazil: Rondônia	Brazil Mortality Information System (SIM)	Vital Registration		1980-2013	All Ages	185131
Brazil: Roraima	Brazil Demographic and Health Survey - Maternal Mortality Data	Sibling History		1985	20 - 39 years	2
Brazil: Roraima	Brazil Mortality Information System (SIM)	Vital Registration		1980-2013	All Ages	36643
Brazil: Santa Catarina	Brazil Demographic and Health Survey - Maternal Mortality Data	Sibling History		1982	25 - 29 years	1
Brazil: Santa Catarina	Brazil Demographic and Health Survey - Maternal Mortality Data	Sibling History		1986	15 - 19 years	1
Brazil: Santa Catarina	Brazil Demographic and Health Survey - Maternal Mortality Data	Sibling History		1995	20 - 59 years	2
Brazil: Santa Catarina	Brazil Mortality Information System (SIM)	Vital Registration		1980-2013	All Ages	911317
Brazil: São Paulo	Brazil Demographic and Health Survey - Maternal Mortality Data	Sibling History		1982-1996	30 - 34 years	1
Brazil: São Paulo	Brazil Mortality Information System (SIM)	Vital Registration		1980-2013	All Ages	7576675
Brazil: Sergipe	Brazil Demographic and Health Survey - Maternal Mortality Data	Sibling History		1993	45 - 49 years	1
Brazil: Sergipe	Brazil Mortality Information System (SIM)	Vital Registration		1980-2013	All Ages	306865
Brazil: Tocantins	Brazil Demographic and Health Survey - Maternal Mortality Data	Sibling History		1989	30 - 34 years	1
Brazil: Tocantins	Brazil Demographic and Health Survey - Maternal Mortality Data	Sibling History		1991	35 - 39 years	1
Brazil: Tocantins	Brazil Demographic and Health Survey - Maternal Mortality Data	Sibling History		1995	40 - 44 years	1
Brazil: Tocantins	Brazil Mortality Information System (SIM)	Vital Registration		1990-2013	All Ages	110064
Paraguay	United Nations Surveys on Crime Trends and the Operations of Criminal Justice Systems 1970-2006	Police Records		1992-1993	All Ages	Death Rate
Paraguay	United Nations Office on Drugs and Crime Global Study on Homicide 2011	Police Records	Paraguay PAHO	1995-2007	All Ages	Death Rate
Paraguay	United Nations Office on Drugs and Crime Global Study on Homicide 2011	Police Records	Paraguay OAS	2004-2007, 2009-2010	All Ages	Death Rate
Paraguay	Global Status Report on Road Safety 2009	Police Records		2007	All Ages	Death Rate
Paraguay	Reproductive Health Survey (RHS)	Sibling history		1980-1994	15 - 49 years	0
Paraguay	World Health Survey (WHS)	Survey/Census		1997	All Ages	94
Paraguay	Paraguay Population and Housing Census 2002	Survey/Census		2001-2002	15 - 49 years	5750
Paraguay	Paraguay Population and Housing Census 2002	Survey/Census	rural area	2002	All Ages	1292

Appendix Table 6: Causes of death data source list by geography, source type, and study site, with years, age range, and sample size, excluding sources on lower respiratory infection and diarrhoea etiologies						
**Location**	**Source name**	**Source type**	**Site**	**Years**	**Ages**	**Sample size**
Paraguay	Paraguay Population and Housing Census 2002	Survey/Census	urban area	2002	All Ages	1927
Paraguay	WHO Mortality Database	Vital Registration		1980-1991, 1994-2013	All Ages	578352
Afghanistan	Special Demographic and Health Survey (DHS)	Sibling History		1996-2010	15 - 54 years	708
Afghanistan	Where giving birth is a forecast of death: maternal mortality in four districts of Afghanistan, 1999-2002	Verbal Autopsy	Alisheng district Laghman province [periurban]	2000	15 - 49 years	52
Afghanistan	Where giving birth is a forecast of death: maternal mortality in four districts of Afghanistan, 1999-2002	Verbal Autopsy	Kabul City Kabul province [urban]	2000	15 - 49 years	122
Afghanistan	Where giving birth is a forecast of death: maternal mortality in four districts of Afghanistan, 1999-2002	Verbal Autopsy	Maywand Kandahar province [rural]	2000	15 - 49 years	62
Afghanistan	Where giving birth is a forecast of death: maternal mortality in four districts of Afghanistan, 1999-2002	Verbal Autopsy	Ragh Badakshan Province [rural]	2000	15 - 49 years	121
Afghanistan	Afghanistan - Badghis Nutrition and Health Survey 2002	Verbal Autopsy	Badghis province [rural]	2001	All Ages	79
Afghanistan	Afghanistan Centers for Disease Control and Prevention Country Report 2003	Verbal Autopsy		2002	birth - 4 years	1039
Afghanistan	Special Demographic and Health Survey (DHS)	Verbal Autopsy		2008	All Ages	3139
Algeria	United Nations Office on Drugs and Crime Global Study on Homicide 2011	Police Records	Algeria CTS	2003-2007	All Ages	Death Rate
Algeria	Algeria - Study of Causes of Death, TAHINA 2002	Surveillance		2002	All Ages	5879
Algeria	Algeria National Maternal Mortality Survey 1999	Survey/Census		1999	15 - 49 years	7757
Algeria	Hospital Mortality Data 2002-2007	Vital Registration		2005-2006	All Ages	83052
Bahrain	United Nations Surveys on Crime Trends and the Operations of Criminal Justice Systems 1970-2006	Police Records		1980	All Ages	Death Rate
Bahrain	United Nations Office on Drugs and Crime Global Study on Homicide 2011	Police Records	Bahrain WHO-MDB	1997	All Ages	Death Rate
Bahrain	Global Status Report on Road Safety 2009	Police Records		2007	All Ages	Death Rate
Bahrain	Bahrain Vital Registration - Deaths 1986	Vital Registration		1986	All Ages	4189
Bahrain	WHO Mortality Database	Vital Registration		1998, 2000-2013	All Ages	37782
Egypt	United Nations Surveys on Crime Trends and the Operations of Criminal Justice Systems 1970-2006	Police Records		1982-1994	All Ages	Death Rate
Egypt	United Nations Office on Drugs and Crime Global Study on Homicide 2011	Police Records	Egypt CTS	2009	All Ages	Death Rate
Egypt	Egypt National Maternal Mortality Surveillance System 2004-2006	Surveillance		2004, 2006	15 - 49 years	43845
Egypt	A comparison of two cause-of-death classification systems for deaths among women of reproductive age in Menoufia, Egypt	Verbal Autopsy	Menoufia	1981	15 - 49 years	2005
Egypt	Maternal mortality in Giza, Egypt: magnitude, causes, and prevention	Verbal Autopsy	Giza governorate	1985	15 - 49 years	841
Egypt	Diarrhoeal Disease, Oral Rehydration, and Childhood Mortality in Rural Egypt	Verbal Autopsy	12 villages in Menoufia Governorate [rural]	1990	birth - 4 years	95
Egypt	National maternal mortality ratio in Egypt halved between 1992-93 and 2000	Verbal Autopsy	1992-3 survey included 21 governorates. 2000 survey covered 27 governorates	1992, 2000	15 - 49 years	15845
Egypt	Indices and sociodemographic determinants of childhood mortality in rural Upper Egypt	Verbal Autopsy	6 sites in Upper Egypt Beni Suef [rural]	1994	birth - 4 years	199
Egypt	WHO Mortality Database	Vital Registration		1980, 1987, 1991-1992, 2000-2013	All Ages	7991848
Iran	United Nations Office on Drugs and Crime Global Study on Homicide 2011	Police Records	Iran NSO	2009	All Ages	Death Rate
Iran	Iran Maternal Mortality Report 2012-2013	Surveillance		2002-2012	15 - 49 years	MMR

Appendix Table 6: Causes of death data source list by geography, source type, and study site, with years, age range, and sample size, excluding sources on lower respiratory infection and diarrhoea etiologies						
Location	Source name	Source type	Site	Years	Ages	Sample size
Iran	Iran - National Estimates for Maternal Mortality: An Analysis Based on Reproductive Age Mortality Study (RAMOS) in Iran	Verbal Autopsy	national	1996	15 - 49 years	8363
Iran	An Epidemiological Study on Infant Mortality and Factors Affecting it in Rural Areas of Birjand, Iran	Verbal Autopsy	Birjand [rural]	2004	birth - 364 days	156
Iran	Opium Use and Risk of Mortality from Digestive Diseases: A Prospective Cohort Study	Verbal Autopsy	Northeastern Iran []	2006	40 - 44 years	3110
Iran	Gastroesophageal Reflux Disease and overall and Cause-specific Mortality: A Prospective Study of 50000 Individuals	Verbal Autopsy	Golestan provinceGolestan Cohort Study [rural]	2008	40 - 44 years	3107
Iran	WHO Mortality Database	Vital Registration		1980-1985, 1987	All Ages	519197
Iran	Iran Death Registration System	Vital Registration		1996-2001, 2007-2011	All Ages	2200688
Iran	Iran Vital Statistics - Deaths 2000-2006	Vital Registration		2002-2006	All Ages	1344247
Iraq	United Nations Surveys on Crime Trends and the Operations of Criminal Justice Systems 1970-2006	Police Records		1990-1994	All Ages	Death Rate
Iraq	Iraq Child and Maternal Mortality Survey 1999	Survey/Census		1992, 1997	15 - 49 years	1377
Iraq	WHO Mortality Database	Vital Registration		2008	All Ages	106245
Jordan	United Nations Surveys on Crime Trends and the Operations of Criminal Justice Systems 1970-2006	Police Records		1980-1986, 1990-1994	All Ages	Death Rate
Jordan	Jordan Demographic and Health Survey - Maternal Mortality Data	Sibling History		1983-1997	15 - 44 years	24
Jordan	Mortality and causes of death in Jordan 1995-96: assessment by verbal autopsy	Verbal Autopsy	national 100 clusters	1995	birth - 79 years	947
Jordan	Jordan National Maternal Mortality Study 2008	Verbal Autopsy	nationwide	2007	15 - 49 years	1406
Jordan	Jordan Vital Registration - Deaths 2004-2006	Vital Registration		2004-2006	All Ages	53078
Jordan	WHO Mortality Database	Vital Registration		2008-2011	All Ages	61753
Kuwait	United Nations Surveys on Crime Trends and the Operations of Criminal Justice Systems 1970-2006	Police Records		1980-1983, 1986-1989, 1992-1994	All Ages	Death Rate
Kuwait	United Nations Office on Drugs and Crime Global Study on Homicide 2011	Police Records	Kuwait WHO-MDB	1995-2002	All Ages	Death Rate
Kuwait	United Nations Office on Drugs and Crime Global Study on Homicide 2011	Police Records	Kuwait CTS	2004-2007, 2009	All Ages	Death Rate
Kuwait	WHO Mortality Database	Vital Registration		1980-1987, 1993-2013	All Ages	135797
Lebanon	United Nations Surveys on Crime Trends and the Operations of Criminal Justice Systems 1970-2006	Police Records		1986-1988, 1993-1994	All Ages	Death Rate
Lebanon	United Nations Office on Drugs and Crime Global Study on Homicide 2011	Police Records	Lebanon National police	2004-2007, 2009-2010	All Ages	Death Rate
Lebanon	Lebanon Reproductive Age Mortality Survey 2009	Surveillance		2008	15 - 49 years	MMR
Lebanon	Non-communicable disease mortality rates using the verbal autopsy in a cohort of middle aged and older populations in Beirut during wartime, 1983-93	Verbal Autopsy	Beirut [urban]	1988	50 years +	416
Lebanon	Facility-based audit of maternal mortality in Lebanon: A feasibility study	Verbal Autopsy	Beirut	2000	15 - 49 years	279
Libya	United Nations Office on Drugs and Crime Global Study on Homicide 2011	Police Records	Libya Interpol	2003	All Ages	Death Rate
Libya	Libya Vital Statistics	Vital Registration		2006-2008	All Ages	62293
Morocco	United Nations Surveys on Crime Trends and the Operations of Criminal Justice Systems 1970-2006	Police Records		1980, 1990-1994	All Ages	Death Rate
Morocco	United Nations Office on Drugs and Crime Global Study on Homicide 2011	Police Records	Morocco CTS	1999-2004, 2006-2007, 2009-2010	All Ages	Death Rate
Morocco	Morocco Demographic and Health Survey - Maternal Mortality Data	Sibling History		1980-2003	15 - 54 years	319

Appendix Table 6: Causes of death data source list by geography, source type, and study site, with years, age range, and sample size, excluding sources on lower respiratory infection and diarrhoea etiologies						
**Location**	**Source name**	**Source type**	**Site**	**Years**	**Ages**	**Sample size**
Morocco	Morocco Health in Figures	Surveillance		2001-2004, 2008	15 - 49 years	12006
Morocco	Implementing a maternal mortality surveillance system in Morocco - challenges and opportunities	Surveillance		2009	15 - 49 years	3814
Morocco	Pan Arab Project for Child Development (PAPCHILD)	Survey/Census		1992, 1997	15 - 49 years	3883
Morocco	World Health Survey (WHS)	Survey/Census		1997	All Ages	11
Morocco	Morocco National Survey on Causes and Circumstances of Infant and Child Deaths 1988-1989	Verbal Autopsy	Morocco [mixed]	1988	birth - 4 years	382
Morocco	Morocco Cause of Death Data 2005	Vital Registration		2005	All Ages	135975
Morocco	WHO Mortality Database	Vital Registration		2008-2012	All Ages	248409
Palestine	United Nations Office on Drugs and Crime Global Study on Homicide 2011	Police Records	Palestine CTS	1997-1999, 2003-2005	All Ages	Death Rate
Palestine	Palestine Report on Maternal Mortality 2008-2009	Surveillance	Gaza Strip	2008-2009	15 - 49 years	MMR
Palestine	Palestine Report on Maternal Mortality 2008-2009	Surveillance	West Bank	2009	15 - 49 years	MMR
Palestine	Maternal mortality among Palestinian women in the West Bank	Verbal Autopsy	West Bank - West Bank districts	2000	15 - 49 years	431
Palestine	Palestine - West Bank Vital Registration - Deaths 1997	Vital Registration	West Bank	1997	All Ages	4918
Palestine	Palestine - West Bank Vital Registration - Deaths 1998	Vital Registration	West Bank	1998	All Ages	4495
Palestine	Palestine - West Bank Vital Registration - Deaths 1999	Vital Registration	West Bank	1999	All Ages	5181
Palestine	Palestine - West Bank Vital Registration - Deaths 2000	Vital Registration	West Bank	2000	All Ages	5368
Palestine	Palestine - West Bank Vital Registration - Deaths 2001	Vital Registration	West Bank	2001	All Ages	5172
Palestine	Palestine - West Bank Vital Registration - Deaths 2002	Vital Registration	West Bank	2002	All Ages	5387
Palestine	Palestine - West Bank Vital Registration - Deaths 2003	Vital Registration	West Bank	2003	All Ages	5663
Palestine	Palestine - West Bank Vital Registration - Deaths 2004	Vital Registration	West Bank	2004	1 year +	5902
Palestine	Palestine - West Bank Vital Registration - Deaths 2007	Vital Registration	West Bank	2004-2007	15 years +	25
Palestine	Palestine - West Bank Vital Registration - Deaths 2008	Vital Registration	West Bank	2004-2007	1 year +	745
Palestine	Palestine - West Bank Vital Registration - Deaths 2009	Vital Registration	West Bank	2004-2007	1 year +	190
Palestine	Palestine - West Bank Vital Registration - Deaths 2005	Vital Registration	West Bank	2005	1 year +	5813
Palestine	WHO Mortality Database	Vital Registration		2008-2009, 2011-2014	All Ages	40723
Oman	United Nations Office on Drugs and Crime Global Study on Homicide 2011	Police Records	Oman CTS	2001-2002	All Ages	Death Rate
Oman	Hospital Mortality Data 2002-2007	Vital Registration		2005-2007	All Ages	17186
Oman	WHO Mortality Database	Vital Registration		2009-2010	All Ages	14098
Qatar	United Nations Surveys on Crime Trends and the Operations of Criminal Justice Systems 1970-2006	Police Records		1980-1994	All Ages	Death Rate
Qatar	United Nations Office on Drugs and Crime Global Study on Homicide 2011	Police Records	Qatar CTS	1998-2000, 2003, 2007	All Ages	Death Rate
Qatar	Qatar Vital Statistics Annual Bulletin	Vital Registration		1984-1985, 2000, 2002-2003	All Ages	5138
Qatar	WHO Mortality Database	Vital Registration		1995, 2001, 2004-2012	All Ages	18539
Saudi Arabia: 'Asir	Saudi Arabia Vital Registration System	Vital Registration		1999-2012	All Ages	41280
Saudi Arabia: Bahah	Saudi Arabia Vital Registration System	Vital Registration		1999-2012	All Ages	10114
Saudi Arabia: Eastern Province	Saudi Arabia Vital Registration System	Vital Registration		1999-2012	All Ages	67064
Saudi Arabia: Ha'il	Saudi Arabia Vital Registration System	Vital Registration		1999-2012	All Ages	14605
Saudi Arabia: Jawf	Saudi Arabia Vital Registration System	Vital Registration		1999-2012	All Ages	9548
Saudi Arabia: Jizan	Saudi Arabia Vital Registration System	Vital Registration		1999-2012	All Ages	24751
Saudi Arabia: Madinah	Saudi Arabia Vital Registration System	Vital Registration		1999-2012	All Ages	51867

Appendix Table 6: Causes of death data source list by geography, source type, and study site, with years, age range, and sample size, excluding sources on lower respiratory infection and diarrhoea etiologies

Location	Source name	Source type	Site	Years	Ages	Sample size
Saudi Arabia: Makkah	Saudi Arabia Vital Registration System	Vital Registration		1999-2012	All Ages	160203
Saudi Arabia: Najran	Saudi Arabia Vital Registration System	Vital Registration		1999-2012	All Ages	7892
Saudi Arabia: Northern Borders	Saudi Arabia Vital Registration System	Vital Registration		1999-2012	All Ages	6997
Saudi Arabia: Qassim	Saudi Arabia Vital Registration System	Vital Registration		1999-2012	All Ages	28133
Saudi Arabia: Riyadh	Saudi Arabia Vital Registration System	Vital Registration		1999-2000, 2002-2012	All Ages	91554
Saudi Arabia: Tabuk	Saudi Arabia Vital Registration System	Vital Registration		1999-2012	All Ages	16010
Sudan	United Nations Surveys on Crime Trends and the Operations of Criminal Justice Systems 1970-2006	Police Records		1990-1994	All Ages	Death Rate
Sudan	Global Status Report on Road Safety 2009	Police Records		2007	All Ages	Death Rate
Sudan	United Nations Office on Drugs and Crime Global Study on Homicide 2011	Police Records	Sudan CTS	2007	All Ages	Death Rate
Sudan	Demographic and Health Survey (DHS)	Sibling History		1980-1989	15 - 44 years	49
Sudan	Pan Arab Project for Family Health (PAPFAM)	Surveillance		2005	15 - 49 years	13792
Sudan	International Integrated Public Use Microdata Series (IPUMS)	Survey/Census		2008	15 - 54 years	1110
Sudan	Sudan Population and Housing Census 2008	Survey/Census	Khartoum	2008	All Ages	63032
Sudan	Sudan Population and Housing Census 2008	Survey/Census	North Sudan	2008	All Ages	446449
Syria	United Nations Surveys on Crime Trends and the Operations of Criminal Justice Systems 1970-2006	Police Records		1980-1994	All Ages	Death Rate
Syria	United Nations Office on Drugs and Crime Global Study on Homicide 2011	Police Records	Syria CTS	2007	All Ages	Death Rate
Syria	Pan Arab Project for Family Health (PAPFAM)	Surveillance		1993, 1998	15 - 49 years	823
Syria	Causes of death among Syrian children using verbal autopsy	Verbal Autopsy	Syria (40000 households nationally)	1995	birth - 4 years	660
Syria	Maternal mortality in Syria: causes, contributing factors and preventability	Verbal Autopsy		2003	15 - 49 years	967
Syria	WHO Mortality Database	Vital Registration		1980, 1984-1985, 2010	All Ages	204742
Syria	Syria Vital Statistics - Deaths 2005-2007	Vital Registration		2005-2007	All Ages	170578
Tunisia	United Nations Office on Drugs and Crime Global Study on Homicide 2011	Police Records	Tunisia CTS	1998-2002	All Ages	Death Rate
Tunisia	Global Status Report on Road Safety 2009	Police Records		2007	All Ages	Death Rate
Tunisia	Tunisia National Maternal Mortality Survey 2009-2010	Survey/Census		1993, 2000, 2002, 2004, 2006, 2008	15 - 49 years	MMR
Tunisia	World Health Survey (WHS)	Survey/Census		1997	All Ages	32
Tunisia	Tunisia National Statistics on Medical Causes of Death 2006	Vital Registration		2006	All Ages	9646
Tunisia	WHO Mortality Database	Vital Registration		2009, 2013	All Ages	42737
Turkey	Turkey Road Traffic Accident Statistics 2010	Police Records		2001-2010	All Ages	Death Rate
Turkey	Infant Mortality Rates in Narlidere District, Turkey (1999 to 2001): Trends in Rates and Risk Factors	Verbal Autopsy	Narlidere District Izmir	2000	birth - 364 days	69
Turkey	Prevalence of Asthma, Allergy, and Respiratory Symptoms in Hasançelebi/Hekimhan/Malatya in Eastern Turkey	Verbal Autopsy	Hasancelebi Hekimhan Malatya [rural]	2001	20 years +	173
Turkey	Turkey Verbal Autopsy Survey 2003	Verbal Autopsy		2002	All Ages	1084
Turkey	Identifying and verifying causes of death in Turkey: National verbal autopsy survey	Verbal Autopsy	Turkey (12000 households)	2003	All Ages	1056
Turkey	The Turkey national maternal mortality study	Verbal Autopsy		2005	15 - 49 years	6887
Turkey	The Turkey national maternal mortality study	Verbal Autopsy	Turkey	2005	15 - 49 years	6876

Appendix Table 6: Causes of death data source list by geography, source type, and study site, with years, age range, and sample size, excluding sources on lower respiratory infection and diarrhoea etiologies						
**Location**	**Source name**	**Source type**	**Site**	**Years**	**Ages**	**Sample size**
Turkey	Statistical Analysis of Death Causes (2005-2010) in Villages with High Arsenic Levels in Drinking Water Supplies of Simav Plain, Turkey	Verbal Autopsy	Simav Plain [rural]	2011	All Ages	402
Turkey	WHO Mortality Database	Vital Registration		1983-1984, 1987-2013	All Ages	5627972
United Arab Emirates	United Nations Surveys on Crime Trends and the Operations of Criminal Justice Systems 1970-2006	Police Records		1980	All Ages	Death Rate
United Arab Emirates	United Nations Office on Drugs and Crime Global Study on Homicide 2011	Police Records	United Arab Emirates CTS	2003-2006	All Ages	Death Rate
United Arab Emirates	United Arab Emirates Annual Statistical Report	Vital Registration		2006-2007	All Ages	16274
Yemen	United Nations Office on Drugs and Crime Global Study on Homicide 2011	Police Records	Yemen NSO	2003-2007, 2009	All Ages	Death Rate
Yemen	Demographic and Health Survey (DHS)	Sibling History		1992	15 - 49 years	404
Yemen	Pan Arab Project for Family Health (PAPFAM)	Surveillance		2002	15 - 49 years	2584
Bangladesh	United Nations Surveys on Crime Trends and the Operations of Criminal Justice Systems 1970-2006	Police Records		1980-1986	All Ages	Death Rate
Bangladesh	United Nations Office on Drugs and Crime Global Study on Homicide 2011	Police Records	Bangladesh National police	2000, 2004-2007, 2009-2010	All Ages	Death Rate
Bangladesh	Global Status Report on Road Safety 2009	Police Records		2006	All Ages	Death Rate
Bangladesh	Bangladesh Demographic and Health Survey - Maternal Mortality Data	Sibling History		1987-2001	15 - 59 years	5089
Bangladesh	Maternal mortality in rural Bangladesh: the Tangail District	Survey/Census	Gopalpur and Bhuapur [rural]	1982	15 - 49 years	146
Bangladesh	Bangladesh Maternal Mortality and Health Service Survey 2010	Survey/Census		2009	15 - 49 years	816
Bangladesh	Demographic Surveillance System (DSS)	Verbal Autopsy		1980-2012	20 - 69 years	1146
Bangladesh	Maternal mortality in rural Bangladesh: the Jamalpur District	Verbal Autopsy	Dhaka - Jamalpur District	1982	15 - 44 years	126
Bangladesh	Neo-natal mortality patterns in rural Bangladesh	Verbal Autopsy	Ghatail Upazila District of Tangail [rural]	1985	birth - 27 days	69
Bangladesh	The effect of maternal and child health and family planning services on mortality: Is prevention enough?	Verbal Autopsy	Chittagong - Matlab	1986	15 - 44 years	102
Bangladesh	International differences in clinical patterns of diarrhoeal deaths: a comparison of children from Brazil, Senegal, Bangladesh, and India	Verbal Autopsy	Matlab [rural]	1988	birth - 4 years	1567
Bangladesh	Levels, Trends and Causes of Mortality in Children Below 5 Years of Age in Bangladesh: Findings from a national survey	Verbal Autopsy	Dhaka Khulna Rajshahi and Chittagong	1990	birth - 4 years	806
Bangladesh	A comparison of sisterhood information on causes of maternal death with the registration causes of maternal death in Matlab, Bangladesh	Verbal Autopsy	rural Chittagong - Matlab	1991	15 - 49 years	683
Bangladesh	Causes of childhood deaths in Bangladesh: an update	Verbal Autopsy		1991, 1994	birth - 4 years	1497
Bangladesh	Bangladesh Verbal Autopsy Study 2015	Verbal Autopsy	Bagerhat-Baintola (Rural)	1992	65 - 69 years	1
Bangladesh	Neonatal mortality in rural Bangladesh: an exploratory study	Verbal Autopsy	Dhaka Chittagong Rajshahi and Khulna [rural]	1992	birth - 27 days	47
Bangladesh	Determinants of mortality among children in the urban slums of Dhaka city, Bangladesh	Verbal Autopsy	Dhaka City [urban]	1992	birth - 9 years	402
Bangladesh	Causes of childhood deaths in Bangladesh: results of a nationwide verbal autopsy study	Verbal Autopsy	BDHS [mixed]	1993	birth - 4 years	823
Bangladesh	Exclusive breastfeeding reduces acute respiratory infection and diarrhea deaths among infants in Dhaka slums	Verbal Autopsy	Dhaka slums [urban]	1994	birth - 364 days	178
Bangladesh	Bangladesh Verbal Autopsy Study 2015	Verbal Autopsy	Rajbari-Boalia (Rural)	2000	80 years +	1
Bangladesh	Demographic Surveillance System (DSS)	Verbal Autopsy	hsid	2000	All Ages	694

Appendix Table 6: Causes of death data source list by geography, source type, and study site, with years, age range, and sample size, excluding sources on lower respiratory infection and diarrhoea etiologies

Location	Source name	Source type	Site	Years	Ages	Sample size
Bangladesh	Elevated Manganese Concentrations in Drinking Water May Be Beneficial for Fetal Survival	Verbal Autopsy	MatlabKorle-Bu Teaching Hospital Mortuary [rural]	2002	birth - 27 days	33
Bangladesh	Bangladesh Verbal Autopsy Study 2015	Verbal Autopsy	Madaripur-Khalia (Rural)	2003	65 - 69 years	1
Bangladesh	Demographic and Health Survey (DHS)	Verbal Autopsy		2004	birth - 4 years	604
Bangladesh	Causes of early childhood deaths in urban Dhaka, Bangladesh	Verbal Autopsy	Dhaka City [urban]	2004	birth - 4 years	914
Bangladesh	Bangladesh Verbal Autopsy Study 2015	Verbal Autopsy	Narail-Kotakol (Rural)	2004	60 - 64 years	1
Bangladesh	Effects of vitamin A or beta carotene supplementation on pregnancy-related mortality and infant mortality in rural Bangladesh: a cluster randomized trial	Verbal Autopsy	Rangpur [rural]	2004	birth - 27 days	951
Bangladesh	Beyond pregnancy--the neglected burden of mortality in young women of reproductive age in Bangladesh: a prospective cohort study	Verbal Autopsy	northwest rural BangladeshKorle-Bu Teaching Hospital Mortuary [rural]	2004	15 - 44 years	1107
Bangladesh	Bangladesh Verbal Autopsy Study 2015	Verbal Autopsy	Rajbari-Chandani (Rural)	2005-2006	80 years +	1
Bangladesh	Bangladesh Verbal Autopsy Study 2015	Verbal Autopsy	Madaripur-Amgram (Rural)	2006	80 years +	2
Bangladesh	Bangladesh - Dhaka Causes of Maternal, Neonatal and Child Deaths: An Exploratory Study of Dhaka's Slum Dwellers	Verbal Autopsy	Dhaka slums [urban]	2006, 2008	birth - 4 years	376
Bangladesh	Verbal autopsy for neonatal mortality: a community-based study	Verbal Autopsy	Mallickbari and Hobirbari Valuaka Mymenhingh rural [rural]	2007	birth - 27 days	54
Bangladesh	Bangladesh Verbal Autopsy Study 2015	Verbal Autopsy	Pirojpur-Dirgha (Rural)	2007	45 - 49 years	1
Bangladesh	Demographic Surveillance System (DSS)	Verbal Autopsy	Matlab HDSS	2007-2010	All Ages	6028
Bangladesh	Causes of neonatal and maternal deaths in Dhaka slums: implications for service delivery	Verbal Autopsy	Dhaka slums [urban]	2008	birth - 27 days	260
Bangladesh	Bangladesh Verbal Autopsy Study 2015	Verbal Autopsy	Rajbari-Boalia (Rural)	2008	40 - 44 years	1
Bangladesh	Bangladesh Verbal Autopsy Study 2015	Verbal Autopsy	Habiganj-Adaur (Rural)	2009	80 years +	2
Bangladesh	Bangladesh Verbal Autopsy Study 2015	Verbal Autopsy	Joypurhat-Ayma Rasulpur (Rural)	2009	80 years +	1
Bangladesh	Bangladesh Verbal Autopsy Study 2015	Verbal Autopsy	Netrokona-Sandikona (Rural)	2009	40 - 44 years	1
Bangladesh	Bangladesh Verbal Autopsy Study 2015	Verbal Autopsy	Kurigram-Joymanirhat (Rural)	2009-2010	45 - 49 years	1
Bangladesh	Bangladesh Verbal Autopsy Study 2015	Verbal Autopsy	Bogra-Majhira (Rural)	2010	50 - 54 years	1
Bangladesh	Bangladesh Verbal Autopsy Study 2015	Verbal Autopsy	Cox's Bazar-Kakara (Rural)	2010	80 years +	1
Bangladesh	Bangladesh Verbal Autopsy Study 2015	Verbal Autopsy	Netrokona-Sunai (Rural)	2010	80 years +	1
Bangladesh	Bangladesh Verbal Autopsy Study 2015	Verbal Autopsy	Rangpur-Darshan (Rural)	2010	80 years +	1
Bangladesh	Bangladesh Verbal Autopsy Study 2015	Verbal Autopsy	Sylhet-Dayamir (Rural)	2010	80 years +	1
Bangladesh	Bangladesh Verbal Autopsy Study 2015	Verbal Autopsy	Sylhet-Upashahar (Urban)	2010	60 - 74 years	3
Bangladesh	Bangladesh Verbal Autopsy Study 2015	Verbal Autopsy	Habiganj-Dighalbak (Rural)	2010, 2013-2015	65 - 69 years	2
Bangladesh	Bangladesh Verbal Autopsy Study 2015	Verbal Autopsy	Noakhali-Bazra (Rural)	2010-2011	60 - 64 years	3
Bangladesh	Bangladesh Verbal Autopsy Study 2015	Verbal Autopsy	Barishal-Vatikhana (Urban)	2010-2012	35 - 39 years	1
Bangladesh	Maternal and Antenatal Risk Factors for Stillbirths and Neonatal Mortality in Rural Bangladesh: A Case-Control Study	Verbal Autopsy	[rural]	2011	birth - 27 days	112
Bangladesh	Bangladesh Verbal Autopsy Study 2015	Verbal Autopsy	Barguna-Badarkhali (Rural)	2011	65 - 69 years	1
Bangladesh	Bangladesh Verbal Autopsy Study 2015	Verbal Autopsy	Barguna-Belagi sadar (Rural)	2011	80 years +	1
Bangladesh	Bangladesh Verbal Autopsy Study 2015	Verbal Autopsy	Chuadanga-Uthali (Rural)	2011	80 years +	1
Bangladesh	Bangladesh Verbal Autopsy Study 2015	Verbal Autopsy	Cox's Bazar-Harbang (Rural)	2011	75 - 79 years	1
Bangladesh	Bangladesh Verbal Autopsy Study 2015	Verbal Autopsy	Cox's Bazar-Raja palong (Rural)	2011	20 - 24 years	1

Appendix Table 6: Causes of death data source list by geography, source type, and study site, with years, age range, and sample size, excluding sources on lower respiratory infection and diarrhoea etiologies						
**Location**	**Source name**	**Source type**	**Site**	**Years**	**Ages**	**Sample size**
Bangladesh	Bangladesh Verbal Autopsy Study 2015	Verbal Autopsy	Dhaka-Badda (Urban)	2011	45 - 74 years	2
Bangladesh	Bangladesh Verbal Autopsy Study 2015	Verbal Autopsy	Dhaka-Uattra (Urban)	2011	60 - 64 years	1
Bangladesh	Bangladesh Verbal Autopsy Study 2015	Verbal Autopsy	Khulna-Sonadanga (Urban)	2011	5 - 9 years	1
Bangladesh	Bangladesh Verbal Autopsy Study 2015	Verbal Autopsy	Kurigram-Belgachha (Rural)	2011	80 years +	1
Bangladesh	Bangladesh Verbal Autopsy Study 2015	Verbal Autopsy	Kurigram-Charbhurungamari (Rural)	2011	80 years +	1
Bangladesh	Bangladesh Verbal Autopsy Study 2015	Verbal Autopsy	Kushtia-Ambaria (Rural)	2011	75 - 79 years	1
Bangladesh	Bangladesh Verbal Autopsy Study 2015	Verbal Autopsy	Meherpur-Bagoan (Rural)	2011	65 - 69 years	1
Bangladesh	Bangladesh Verbal Autopsy Study 2015	Verbal Autopsy	Moulovibazar-Alinagar (Rural)	2011	15 - 19 years	1
Bangladesh	Bangladesh Verbal Autopsy Study 2015	Verbal Autopsy	Mymensingh-Balikhan (Rural)	2011	65 - 69 years	1
Bangladesh	Bangladesh Verbal Autopsy Study 2015	Verbal Autopsy	Naogaon-Sreemantapur (Rural)	2011	40 - 44 years	1
Bangladesh	Bangladesh Verbal Autopsy Study 2015	Verbal Autopsy	Panchagarh-Dhamor (Rural)	2011	80 years +	1
Bangladesh	Bangladesh Verbal Autopsy Study 2015	Verbal Autopsy	Rajbari-Boalia (Rural)	2011	20 - 24 years	1
Bangladesh	Bangladesh Verbal Autopsy Study 2015	Verbal Autopsy	Rajshahi-Mohanpur (Rural)	2011	40 - 44 years	1
Bangladesh	Bangladesh Verbal Autopsy Study 2015	Verbal Autopsy	Rangpur-Haragachh (Rural)	2011	45 - 49 years	1
Bangladesh	Bangladesh Verbal Autopsy Study 2015	Verbal Autopsy	Sariatpur-Kedarpur (Rural)	2011	70 - 74 years	1
Bangladesh	Bangladesh Verbal Autopsy Study 2015	Verbal Autopsy	Sherpur-Rani Shimul (Rural)	2011	65 - 69 years	1
Bangladesh	Bangladesh Verbal Autopsy Study 2015	Verbal Autopsy	Bogra-Amrul (Rural)	2011, 2013-2015	50 - 54 years	2
Bangladesh	Bangladesh - Chandpur and Comilla District Verbal Autopsy Study 2011-2014	Verbal Autopsy	Matlab HDSS	2011-2013	All Ages	4433
Bangladesh	Bangladesh Verbal Autopsy Study 2015	Verbal Autopsy	Dhaka-Sharulia (Urban)	2011-2015	20 - 74 years	5
Bangladesh	Bangladesh Verbal Autopsy Study 2015	Verbal Autopsy	Dhaka-Tejgaon (Urban)	2011-2015	60 - 64 years	1
Bangladesh	Bangladesh Verbal Autopsy Study 2015	Verbal Autopsy	Rajbari-Baliakandi (Rural)	2011-2015	20 - 24 years	1
Bangladesh	Bangladesh Verbal Autopsy Study 2015	Verbal Autopsy	Faridpur-Bhawal (Rural)	2012	75 - 79 years	1
Bangladesh	Bangladesh Verbal Autopsy Study 2015	Verbal Autopsy	Habiganj-Gazipur (Rural)	2012	45 - 49 years	1
Bangladesh	Bangladesh Verbal Autopsy Study 2015	Verbal Autopsy	Jatrabari-West Jurain (Urban)	2012	65 - 69 years	1
Bangladesh	Bangladesh Verbal Autopsy Study 2015	Verbal Autopsy	Jessore-Sundoli (Rural)	2012	75 - 79 years	1
Bangladesh	Bangladesh Verbal Autopsy Study 2015	Verbal Autopsy	Kishoregonj-Jirani (Rural)	2012	80 years +	1
Bangladesh	Bangladesh Verbal Autopsy Study 2015	Verbal Autopsy	Kushtia-Korsha (Rural)	2012	55 - 59 years	1
Bangladesh	Bangladesh Verbal Autopsy Study 2015	Verbal Autopsy	Mymensingh-Char Ishwardia (Rural)	2012	30 - 34 years	1
Bangladesh	Bangladesh Verbal Autopsy Study 2015	Verbal Autopsy	Mymensingh-Char Nilaxmia (Rural)	2012	70 - 74 years	1
Bangladesh	Bangladesh Verbal Autopsy Study 2015	Verbal Autopsy	Noakhali-Ramnarayanpur (Rural)	2012	70 - 74 years	1
Bangladesh	Bangladesh Verbal Autopsy Study 2015	Verbal Autopsy	Pirojpur-Gulishakhali (Rural)	2012	70 - 74 years	1
Bangladesh	Bangladesh Verbal Autopsy Study 2015	Verbal Autopsy	Rangpur-Rajendrapur (Rural)	2012	80 years +	2
Bangladesh	Bangladesh Verbal Autopsy Study 2015	Verbal Autopsy	Sylhet-Dayamir (Rural)	2012	55 - 59 years	1
Bangladesh	Bangladesh Verbal Autopsy Study 2015	Verbal Autopsy	Sylhet-Upashahar (Urban)	2012	70 - 74 years	1
Bangladesh	Bangladesh Verbal Autopsy Study 2015	Verbal Autopsy	Barishal-Shikarpur (Rural)	2012-2013	60 - 64 years	1
Bangladesh	Bangladesh Verbal Autopsy Study 2015	Verbal Autopsy	Dhaka-Lalbag (Urban)	2012-2013	80 years +	1
Bangladesh	Bangladesh Verbal Autopsy Study 2015	Verbal Autopsy	Dhaka-Uttar Khan (Urban)	2012-2013	25 - 29 years	1
Bangladesh	Bangladesh Verbal Autopsy Study 2015	Verbal Autopsy	Noakhali-Mir Warishpur (Rural)	2012-2013	70 - 74 years	1

Appendix Table 6: Causes of death data source list by geography, source type, and study site, with years, age range, and sample size, excluding sources on lower respiratory infection and diarrhoea etiologies

Location	Source name	Source type	Site	Years	Ages	Sample size
Bangladesh	Bangladesh Verbal Autopsy Study 2015	Verbal Autopsy	Chandpur-Dakshin Rupsa (Rural)	2012-2014	70 - 74 years	1
Bangladesh	Bangladesh Verbal Autopsy Study 2015	Verbal Autopsy	Cox's Bazar-Garjania (Rural)	2012-2015	50 years +	2
Bangladesh	Bangladesh Verbal Autopsy Study 2015	Verbal Autopsy	Faridpur-Parameshwardi (Rural)	2012-2015	80 years +	2
Bangladesh	Bangladesh Verbal Autopsy Study 2015	Verbal Autopsy	Kushtia-Alampur (Rural)	2012-2015	60 - 64 years	1
Bangladesh	Bangladesh Verbal Autopsy Study 2015	Verbal Autopsy	Dhaka-Kamrangirchar (Urban)	2013	80 years +	1
Bangladesh	Bangladesh Verbal Autopsy Study 2015	Verbal Autopsy	Dhaka-Uattra (Urban)	2013	80 years +	1
Bangladesh	Bangladesh Verbal Autopsy Study 2015	Verbal Autopsy	Kushtia-Gopogram (Rural)	2013	80 years +	1
Bangladesh	Bangladesh Verbal Autopsy Study 2015	Verbal Autopsy	Noakhali-Arjuntala=28 (Rural)	2013	80 years +	1
Bangladesh	Bangladesh Verbal Autopsy Study 2015	Verbal Autopsy	Panchagarh-Dhamor (Rural)	2013	25 - 29 years	1
Bangladesh	Bangladesh Verbal Autopsy Study 2015	Verbal Autopsy	Sylhet-Gilachhara (Rural)	2013	80 years +	1
Bangladesh	Bangladesh Verbal Autopsy Study 2015	Verbal Autopsy	Sylhet-Paschim Amura (Rural)	2013	20 - 24 years	1
Bangladesh	Bangladesh Verbal Autopsy Study 2015	Verbal Autopsy	Bogra-Kharna (Rural)	2013-2014	60 - 64 years	1
Bangladesh	Bangladesh Verbal Autopsy Study 2015	Verbal Autopsy	Cox's Bazar-Harbang (Rural)	2013-2014	80 years +	2
Bangladesh	Bangladesh Verbal Autopsy Study 2015	Verbal Autopsy	Netrokona-Sunai (Rural)	2013-2014	5 - 29 years	2
Bangladesh	Bangladesh Verbal Autopsy Study 2015	Verbal Autopsy	Bagerhat-Baintola (Rural)	2013-2015	70 - 74 years	1
Bangladesh	Bangladesh Verbal Autopsy Study 2015	Verbal Autopsy	Bagerhat-Gazalia (Rural)	2013-2015	80 years +	1
Bangladesh	Bangladesh Verbal Autopsy Study 2015	Verbal Autopsy	Barguna-Aylapatakata (Rural)	2013-2015	65 - 69 years	2
Bangladesh	Bangladesh Verbal Autopsy Study 2015	Verbal Autopsy	Barguna-Badarkhali (Rural)	2013-2015	25 - 29 years	1
Bangladesh	Bangladesh Verbal Autopsy Study 2015	Verbal Autopsy	Bhola-Illisha (Rural)	2013-2015	45 - 49 years	1
Bangladesh	Bangladesh Verbal Autopsy Study 2015	Verbal Autopsy	Bhola-Paschim Char Umed (Rural)	2013-2015	50 - 54 years	1
Bangladesh	Bangladesh Verbal Autopsy Study 2015	Verbal Autopsy	Bhola-Uttar Dighaldi (Rural)	2013-2015	80 years +	1
Bangladesh	Bangladesh Verbal Autopsy Study 2015	Verbal Autopsy	Chuadanga-Uthali (Rural)	2013-2015	60 - 64 years	1
Bangladesh	Bangladesh Verbal Autopsy Study 2015	Verbal Autopsy	Cox's Bazar-Raja palong (Rural)	2013-2015	25 - 29 years	1
Bangladesh	Bangladesh Verbal Autopsy Study 2015	Verbal Autopsy	Dhaka-Hajaribag (Urban)	2013-2015	55 - 59 years	1
Bangladesh	Bangladesh Verbal Autopsy Study 2015	Verbal Autopsy	Dhaka-Sutrapur (Urban)	2013-2015	70 - 74 years	1
Bangladesh	Bangladesh Verbal Autopsy Study 2015	Verbal Autopsy	Jamalpur-Nayanagar (Rural)	2013-2015	65 - 69 years	1
Bangladesh	Bangladesh Verbal Autopsy Study 2015	Verbal Autopsy	Jamalpur-Satpoa (Rural)	2013-2015	70 - 74 years	1
Bangladesh	Bangladesh Verbal Autopsy Study 2015	Verbal Autopsy	Kurigram-Charbhurungamari (Rural)	2013-2015	5 - 9 years	1
Bangladesh	Bangladesh Verbal Autopsy Study 2015	Verbal Autopsy	Mymensingh-Achargaon (Rural)	2013-2015	80 years +	1
Bangladesh	Bangladesh Verbal Autopsy Study 2015	Verbal Autopsy	Naogaon-Kalikapur (Rural)	2013-2015	35 - 39 years	1
Bangladesh	Bangladesh Verbal Autopsy Study 2015	Verbal Autopsy	Netrokona-Chhiram (Rural)	2013-2015	75 - 79 years	1
Bangladesh	Bangladesh Verbal Autopsy Study 2015	Verbal Autopsy	Netrokona-Sandikona (Rural)	2013-2015	70 - 74 years	1
Bangladesh	Bangladesh Verbal Autopsy Study 2015	Verbal Autopsy	Nilphamari-Sonaroy (Rural)	2013-2015	80 years +	1
Bangladesh	Bangladesh Verbal Autopsy Study 2015	Verbal Autopsy	Noakhali-Bazra (Rural)	2013-2015	80 years +	2
Bangladesh	Bangladesh Verbal Autopsy Study 2015	Verbal Autopsy	Noakhali-Gopalpur (Rural)	2013-2015	5 years +	2
Bangladesh	Bangladesh Verbal Autopsy Study 2015	Verbal Autopsy	Rajbari-Chandani (Rural)	2013-2015	65 - 69 years	1
Bangladesh	Bangladesh Verbal Autopsy Study 2015	Verbal Autopsy	Rajshahi-Kazihata (Urban)	2013-2015	80 years +	1
Bangladesh	Bangladesh Verbal Autopsy Study 2015	Verbal Autopsy	Rajshahi-Mohanpur (Rural)	2013-2015	70 - 74 years	1
Bangladesh	Bangladesh Verbal Autopsy Study 2015	Verbal Autopsy	Rangpur-Gopalpur (Rural)	2013-2015	75 - 79 years	1
Bangladesh	Bangladesh Verbal Autopsy Study 2015	Verbal Autopsy	Rangpur-Haragachh (Rural)	2013-2015	65 - 69 years	1

Appendix Table 6: Causes of death data source list by geography, source type, and study site, with years, age range, and sample size, excluding sources on lower respiratory infection and diarrhoea etiologies						
Location	Source name	Source type	Site	Years	Ages	Sample size
Bangladesh	Bangladesh Verbal Autopsy Study 2015	Verbal Autopsy	Barishal-Andhar Manik (Rural)	2014	60 - 64 years	1
Bangladesh	Bangladesh Verbal Autopsy Study 2015	Verbal Autopsy	Chandpur-Paschim Gupti (Rural)	2014	45 years +	3
Bangladesh	Bangladesh Verbal Autopsy Study 2015	Verbal Autopsy	Chittagong-Kelishahar (Rural)	2014	55 - 59 years	1
Bangladesh	Bangladesh Verbal Autopsy Study 2015	Verbal Autopsy	Cox's Bazar-Lemsikhali (Rural)	2014	80 years +	1
Bangladesh	Bangladesh Verbal Autopsy Study 2015	Verbal Autopsy	Dhaka-Gulshan (Urban)	2014	50 - 64 years	2
Bangladesh	Bangladesh Verbal Autopsy Study 2015	Verbal Autopsy	Dhaka-Shampur (Urban)	2014	25 - 34 years	3
Bangladesh	Bangladesh Verbal Autopsy Study 2015	Verbal Autopsy	Jatrabari-West Jurain (Urban)	2014	35 - 39 years	1
Bangladesh	Bangladesh Verbal Autopsy Study 2015	Verbal Autopsy	Madaripur-Bajitpur (Rural)	2014	60 - 64 years	1
Bangladesh	Bangladesh Verbal Autopsy Study 2015	Verbal Autopsy	Meherpur-Bagoan (Rural)	2014	70 - 79 years	2
Bangladesh	Bangladesh Verbal Autopsy Study 2015	Verbal Autopsy	Munshiganj-Hossaindi (Rural)	2014	55 - 59 years	1
Bangladesh	Bangladesh Verbal Autopsy Study 2015	Verbal Autopsy	Mymensingh-Char Ishwardia (Rural)	2014	25 - 29 years	1
Bangladesh	Bangladesh Verbal Autopsy Study 2015	Verbal Autopsy	Mymensingh-Galagaon (Rural)	2014	20 - 24 years	1
Bangladesh	Bangladesh Verbal Autopsy Study 2015	Verbal Autopsy	Narail-Kotakol (Rural)	2014	80 years +	1
Bangladesh	Bangladesh Verbal Autopsy Study 2015	Verbal Autopsy	Nilphamari-Boragari (Rural)	2014	25 years +	2
Bangladesh	Bangladesh Verbal Autopsy Study 2015	Verbal Autopsy	Nilphamari-Ketkibari (Rural)	2014	45 years +	4
Bangladesh	Bangladesh Verbal Autopsy Study 2015	Verbal Autopsy	Noakhali-Kabilpur (Rural)	2014	15 - 69 years	2
Bangladesh	Bangladesh Verbal Autopsy Study 2015	Verbal Autopsy	Rajbari-Bahadurpur (Rural)	2014	15 years +	2
Bangladesh	Bangladesh Verbal Autopsy Study 2015	Verbal Autopsy	Sylhet-Upashahar (Urban)	2014	10 - 59 years	3
Bangladesh	Bangladesh Verbal Autopsy Study 2015	Verbal Autopsy	Bagerhat-Chitalmari sadar (Rural)	2014-2015	65 years +	2
Bangladesh	Bangladesh Verbal Autopsy Study 2015	Verbal Autopsy	Barishal-Vatikhana (Urban)	2014-2015	50 years +	2
Bangladesh	Bangladesh Verbal Autopsy Study 2015	Verbal Autopsy	Bogra-Dhupchanchia (Rural)	2014-2015	60 - 64 years	1
Bangladesh	Bangladesh Verbal Autopsy Study 2015	Verbal Autopsy	Bogra-Goshaibari (Rural)	2014-2015	50 - 54 years	1
Bangladesh	Bangladesh Verbal Autopsy Study 2015	Verbal Autopsy	Bogra-Majhira (Rural)	2014-2015	45 - 49 years	1
Bangladesh	Bangladesh Verbal Autopsy Study 2015	Verbal Autopsy	Bogra-Nasratpur (Rural)	2014-2015	50 years +	4
Bangladesh	Bangladesh Verbal Autopsy Study 2015	Verbal Autopsy	Bogra-Shibganj (Rural)	2014-2015	50 - 59 years	3
Bangladesh	Bangladesh Verbal Autopsy Study 2015	Verbal Autopsy	Chandpur-Uttar Rupsa (Rural)	2014-2015	70 years +	3
Bangladesh	Bangladesh Verbal Autopsy Study 2015	Verbal Autopsy	Chuadanga-Jehala (Rural)	2014-2015	25 years +	6
Bangladesh	Bangladesh Verbal Autopsy Study 2015	Verbal Autopsy	Cox's Bazar-Ali Akbar Deli (Rural)	2014-2015	30 - 64 years	2
Bangladesh	Bangladesh Verbal Autopsy Study 2015	Verbal Autopsy	Cox's Bazar-Palong Khali (Rural)	2014-2015	45 years +	2
Bangladesh	Bangladesh Verbal Autopsy Study 2015	Verbal Autopsy	Dhaka-Dhanmondi (Urban)	2014-2015	40 - 44 years	1
Bangladesh	Bangladesh Verbal Autopsy Study 2015	Verbal Autopsy	Dhaka-Tongi (Urban)	2014-2015	50 years +	2
Bangladesh	Bangladesh Verbal Autopsy Study 2015	Verbal Autopsy	Faridpur-Bhawal (Rural)	2014-2015	65 - 69 years	1
Bangladesh	Bangladesh Verbal Autopsy Study 2015	Verbal Autopsy	Faridpur-Gunbaha (Rural)	2014-2015	25 years +	2
Bangladesh	Bangladesh Verbal Autopsy Study 2015	Verbal Autopsy	Faridpur-Kaijuri (Rural)	2014-2015	80 years +	1
Bangladesh	Bangladesh Verbal Autopsy Study 2015	Verbal Autopsy	Gaibandha-Mahimaganj (Rural)	2014-2015	70 - 79 years	2
Bangladesh	Bangladesh Verbal Autopsy Study 2015	Verbal Autopsy	Gazipur-Konabari (Urban)	2014-2015	50 - 54 years	1
Bangladesh	Bangladesh Verbal Autopsy Study 2015	Verbal Autopsy	Habiganj-Adaur (Rural)	2014-2015	75 - 79 years	1
Bangladesh	Bangladesh Verbal Autopsy Study 2015	Verbal Autopsy	Habiganj-Gazipur (Rural)	2014-2015	65 - 69 years	1
Bangladesh	Bangladesh Verbal Autopsy Study 2015	Verbal Autopsy	Habiganj-Pailarkandi (Rural)	2014-2015	65 - 69 years	1

Appendix Table 6: Causes of death data source list by geography, source type, and study site, with years, age range, and sample size, excluding sources on lower respiratory infection and diarrhoea etiologies						
**Location**	**Source name**	**Source type**	**Site**	**Years**	**Ages**	**Sample size**
Bangladesh	Bangladesh Verbal Autopsy Study 2015	Verbal Autopsy	Jamalpur-Titpalla (Rural)	2014-2015	75 - 79 years	1
Bangladesh	Bangladesh Verbal Autopsy Study 2015	Verbal Autopsy	Jessore-Arabpur (Rural)	2014-2015	20 - 24 years	1
Bangladesh	Bangladesh Verbal Autopsy Study 2015	Verbal Autopsy	Jessore-Ganganandapur (Rural)	2014-2015	50 years +	3
Bangladesh	Bangladesh Verbal Autopsy Study 2015	Verbal Autopsy	Jessore-Goga (Rural)	2014-2015	55 years +	2
Bangladesh	Bangladesh Verbal Autopsy Study 2015	Verbal Autopsy	Jessore-Sundoli (Rural)	2014-2015	80 years +	2
Bangladesh	Bangladesh Verbal Autopsy Study 2015	Verbal Autopsy	Jhalokati-Kusanghal (Rural)	2014-2015	80 years +	1
Bangladesh	Bangladesh Verbal Autopsy Study 2015	Verbal Autopsy	Jhenaidah-Ganna (Rural)	2014-2015	25 - 79 years	3
Bangladesh	Bangladesh Verbal Autopsy Study 2015	Verbal Autopsy	Jhenaidah-Maliat (Rural)	2014-2015	30 - 79 years	2
Bangladesh	Bangladesh Verbal Autopsy Study 2015	Verbal Autopsy	Joypurhat-Dogachhi (Rural)	2014-2015	65 - 69 years	1
Bangladesh	Bangladesh Verbal Autopsy Study 2015	Verbal Autopsy	Khulna-Dhamalia (Rural)	2014-2015	55 years +	2
Bangladesh	Bangladesh Verbal Autopsy Study 2015	Verbal Autopsy	Khulna-Sonadanga (Urban)	2014-2015	35 - 74 years	2
Bangladesh	Bangladesh Verbal Autopsy Study 2015	Verbal Autopsy	Kurigram-Belgachha (Rural)	2014-2015	5 years +	6
Bangladesh	Bangladesh Verbal Autopsy Study 2015	Verbal Autopsy	Kurigram-Joymanirhat (Rural)	2014-2015	80 years +	2
Bangladesh	Bangladesh Verbal Autopsy Study 2015	Verbal Autopsy	Magura-Rajapur (Rural)	2014-2015	50 years +	2
Bangladesh	Bangladesh Verbal Autopsy Study 2015	Verbal Autopsy	Manikganj-Saturia (Rural)	2014-2015	70 - 74 years	1
Bangladesh	Bangladesh Verbal Autopsy Study 2015	Verbal Autopsy	Manikganj-Ulail (Rural)	2014-2015	40 - 79 years	2
Bangladesh	Bangladesh Verbal Autopsy Study 2015	Verbal Autopsy	Mymensingh-Moazzempur (Rural)	2014-2015	80 years +	3
Bangladesh	Bangladesh Verbal Autopsy Study 2015	Verbal Autopsy	Mymensingh-Payari (Rural)	2014-2015	60 - 64 years	1
Bangladesh	Bangladesh Verbal Autopsy Study 2015	Verbal Autopsy	Mymensingh-Rambhadrapur (Rural)	2014-2015	55 years +	2
Bangladesh	Bangladesh Verbal Autopsy Study 2015	Verbal Autopsy	Naogaon-Badalgachhi (Rural)	2014-2015	60 years +	2
Bangladesh	Bangladesh Verbal Autopsy Study 2015	Verbal Autopsy	Naogaon-Sreemantapur (Rural)	2014-2015	45 - 69 years	3
Bangladesh	Bangladesh Verbal Autopsy Study 2015	Verbal Autopsy	Netrokona-Kailati (Rural)	2014-2015	20 years +	5
Bangladesh	Bangladesh Verbal Autopsy Study 2015	Verbal Autopsy	Nilphamari-Dimla (Rural)	2014-2015	10 - 74 years	3
Bangladesh	Bangladesh Verbal Autopsy Study 2015	Verbal Autopsy	Pabna-Karamja (Rural)	2014-2015	70 - 74 years	1
Bangladesh	Bangladesh Verbal Autopsy Study 2015	Verbal Autopsy	Panchagarh-Salbahan (Rural)	2014-2015	45 - 64 years	2
Bangladesh	Bangladesh Verbal Autopsy Study 2015	Verbal Autopsy	Pirojpur-Gulishakhali (Rural)	2014-2015	60 - 74 years	4
Bangladesh	Bangladesh Verbal Autopsy Study 2015	Verbal Autopsy	Rajbari-Boalia (Rural)	2014-2015	55 years +	5
Bangladesh	Bangladesh Verbal Autopsy Study 2015	Verbal Autopsy	Rajshahi-Kismatgankoir (Rural)	2014-2015	40 - 44 years	1
Bangladesh	Bangladesh Verbal Autopsy Study 2015	Verbal Autopsy	Rangpur-Darshan (Rural)	2014-2015	40 - 64 years	2
Bangladesh	Bangladesh Verbal Autopsy Study 2015	Verbal Autopsy	Rangpur-Rajendrapur (Rural)	2014-2015	60 years +	2
Bangladesh	Bangladesh Verbal Autopsy Study 2015	Verbal Autopsy	Satkhira-Dhandia (Rural)	2014-2015	70 - 74 years	1
Bangladesh	Bangladesh Verbal Autopsy Study 2015	Verbal Autopsy	Sherpur-Ganapaddi (Rural)	2014-2015	50 years +	4
Bangladesh	Bangladesh Verbal Autopsy Study 2015	Verbal Autopsy	Sherpur-Rani Shimul (Rural)	2014-2015	20 - 24 years	1
Bangladesh	Bangladesh Verbal Autopsy Study 2015	Verbal Autopsy	Sirajganj-Sardabad (Rural)	2014-2015	70 - 74 years	1
Bangladesh	Bangladesh Verbal Autopsy Study 2015	Verbal Autopsy	Sylhet-Boaljur (Rural)	2014-2015	5 years +	3
Bangladesh	Bangladesh Verbal Autopsy Study 2015	Verbal Autopsy	Thakurgaw-Lehemba (Rural)	2014-2015	80 years +	1
Bangladesh	Bangladesh Verbal Autopsy Study 2015	Verbal Autopsy	Barguna-Belagi sadar (Rural)	2015	55 - 79 years	2
Bangladesh	Bangladesh Verbal Autopsy Study 2015	Verbal Autopsy	Barishal-Bhasan Char (Rural)	2015	45 years +	3
Bangladesh	Bangladesh Verbal Autopsy Study 2015	Verbal Autopsy	Barishal-Shikarpur (Rural)	2015	70 - 74 years	1
Bangladesh	Bangladesh Verbal Autopsy Study 2015	Verbal Autopsy	Bogra-Mirzapur (Rural)	2015	60 - 64 years	1

Appendix Table 6: Causes of death data source list by geography, source type, and study site, with years, age range, and sample size, excluding sources on lower respiratory infection and diarrhoea etiologies						
**Location**	**Source name**	**Source type**	**Site**	**Years**	**Ages**	**Sample size**
Bangladesh	Bangladesh Verbal Autopsy Study 2015	Verbal Autopsy	Chittagong-Lalkhanbazar (Urban)	2015	55 - 59 years	1
Bangladesh	Bangladesh Verbal Autopsy Study 2015	Verbal Autopsy	Comilla-Mainamati (Rural)	2015	10 - 69 years	2
Bangladesh	Bangladesh Verbal Autopsy Study 2015	Verbal Autopsy	Cox's Bazar-Kaiyarbil (Rural)	2015	40 - 54 years	2
Bangladesh	Bangladesh Verbal Autopsy Study 2015	Verbal Autopsy	Feni-Dharampur (Rural)	2015	5 years +	4
Bangladesh	Bangladesh Verbal Autopsy Study 2015	Verbal Autopsy	Gaibandha-Naldanga (Rural)	2015	45 years +	3
Bangladesh	Bangladesh Verbal Autopsy Study 2015	Verbal Autopsy	Habiganj-Bahara (Rural)	2015	25 years +	4
Bangladesh	Bangladesh Verbal Autopsy Study 2015	Verbal Autopsy	Joypurhat-Ayma Rasulpur (Rural)	2015	60 - 64 years	1
Bangladesh	Bangladesh Verbal Autopsy Study 2015	Verbal Autopsy	Kishoregonj-Achmita (Rural)	2015	15 - 79 years	2
Bangladesh	Bangladesh Verbal Autopsy Study 2015	Verbal Autopsy	Kishoregonj-Jirani (Rural)	2015	60 - 79 years	2
Bangladesh	Bangladesh Verbal Autopsy Study 2015	Verbal Autopsy	Kurigram-Chakirpashar (Rural)	2015	70 years +	2
Bangladesh	Bangladesh Verbal Autopsy Study 2015	Verbal Autopsy	Kurigram-Kaliganj (Rural)	2015	55 - 79 years	2
Bangladesh	Bangladesh Verbal Autopsy Study 2015	Verbal Autopsy	Kushtia-Ambaria (Rural)	2015	80 years +	2
Bangladesh	Bangladesh Verbal Autopsy Study 2015	Verbal Autopsy	Kushtia-Barakhada (Rural)	2015	70 years +	3
Bangladesh	Bangladesh Verbal Autopsy Study 2015	Verbal Autopsy	Kushtia-Gopogram (Rural)	2015	50 - 54 years	1
Bangladesh	Bangladesh Verbal Autopsy Study 2015	Verbal Autopsy	Kushtia-Korsha (Rural)	2015	70 years +	2
Bangladesh	Bangladesh Verbal Autopsy Study 2015	Verbal Autopsy	Madaripur-Amgram (Rural)	2015	30 years +	3
Bangladesh	Bangladesh Verbal Autopsy Study 2015	Verbal Autopsy	Manikganj-Baliakhora (Rural)	2015	65 years +	6
Bangladesh	Bangladesh Verbal Autopsy Study 2015	Verbal Autopsy	Manikganj-Dighulia (Rural)	2015	65 - 69 years	1
Bangladesh	Bangladesh Verbal Autopsy Study 2015	Verbal Autopsy	Moulovibazar-Alinagar (Rural)	2015	55 - 59 years	1
Bangladesh	Bangladesh Verbal Autopsy Study 2015	Verbal Autopsy	Mymensingh-Bharadoba (Rural)	2015	40 years +	4
Bangladesh	Bangladesh Verbal Autopsy Study 2015	Verbal Autopsy	Mymensingh-Jatia (Rural)	2015	55 years +	4
Bangladesh	Bangladesh Verbal Autopsy Study 2015	Verbal Autopsy	Mymensingh-Kachina (Rural)	2015	50 years +	3
Bangladesh	Bangladesh Verbal Autopsy Study 2015	Verbal Autopsy	Noakhali-Musapur (Rural)	2015	60 - 79 years	3
Bangladesh	Bangladesh Verbal Autopsy Study 2015	Verbal Autopsy	Noakhali-Ramnarayanpur (Rural)	2015	55 - 59 years	1
Bangladesh	Bangladesh Verbal Autopsy Study 2015	Verbal Autopsy	Patuakhali-Kalaiya (Rural)	2015	5 years +	4
Bangladesh	Bangladesh Verbal Autopsy Study 2015	Verbal Autopsy	Pirojpur-Dirgha (Rural)	2015	15 - 79 years	2
Bangladesh	Bangladesh Verbal Autopsy Study 2015	Verbal Autopsy	Rangamati-Bhasanya Adam (Rural)	2015	50 years +	9
Bangladesh	Bangladesh Verbal Autopsy Study 2015	Verbal Autopsy	Rangpur-Pirganj (Rural)	2015	45 - 74 years	3
Bangladesh	Bangladesh Verbal Autopsy Study 2015	Verbal Autopsy	Sariatpur-Kedarpur (Rural)	2015	60 - 64 years	1
Bangladesh	Bangladesh Verbal Autopsy Study 2015	Verbal Autopsy	Satkhira-Shyamnagar (Rural)	2015	30 years +	4
Bangladesh	Bangladesh Verbal Autopsy Study 2015	Verbal Autopsy	Sherpur-Jhenaigati (Rural)	2015	55 years +	2
Bangladesh	Bangladesh Verbal Autopsy Study 2015	Verbal Autopsy	Sherpur-Marichpuran (Rural)	2015	75 years +	2
Bangladesh	Bangladesh Verbal Autopsy Study 2015	Verbal Autopsy	Sherpur-Rupnarayankura (Rural)	2015	25 - 29 years	1
Bangladesh	Bangladesh Verbal Autopsy Study 2015	Verbal Autopsy	Sirajganj-Shialkol (Rural)	2015	65 years +	2
Bangladesh	Bangladesh Verbal Autopsy Study 2015	Verbal Autopsy	Sylhet-Dayamir (Rural)	2015	45 years +	3
Bangladesh	Bangladesh Verbal Autopsy Study 2015	Verbal Autopsy	Sylhet-Gilachhara (Rural)	2015	20 - 64 years	2
Bangladesh	Bangladesh Verbal Autopsy Study 2015	Verbal Autopsy	Sylhet-Omarpur (Rural)	2015	45 - 49 years	1
Bhutan	United Nations Office on Drugs and Crime Global Study on Homicide 2011	Police Records	Bhutan NSO	2000-2007	All Ages	Death Rate
Bhutan	Bhutan Demographic Sample Survey 1984	Survey/Census		1984	15 - 49 years	MMR

Appendix Table 6: Causes of death data source list by geography, source type, and study site, with years, age range, and sample size, excluding sources on lower respiratory infection and diarrhoea etiologies

Location	Source name	Source type	Site	Years	Ages	Sample size
Bhutan	Bhutan Health Survey 1994	Survey/Census		1994	15 - 49 years	MMR
Bhutan	Bhutan Health Survey 2000	Survey/Census		2000	15 - 49 years	MMR
Bhutan	Bhutan Population and Housing Census 2005	Survey/Census		2004	All Ages	4498
India: Andhra Pradesh, Rural	India District Level Household Survey (DLHS)	Sibling history		2001, 2006	10 - 54 years	517
India: Andhra Pradesh, Rural	Demographic and Health Survey (DHS)	Survey/Census		1992, 1998	All Ages	781
India: Andhra Pradesh, Rural	India Survey of Causes of Death (SCD)	Verbal Autopsy		1980-1998	All Ages	36338
India: Andhra Pradesh, Rural	Levels and causes of maternal mortality in southern India	Verbal Autopsy	Anantapur district Andhra Pradesh [rural]	1984	15 - 49 years	867
India: Andhra Pradesh, Rural	Chronic diseases now a leading cause of death in rural India – mortality data from the Andhra Pradesh Rural Health Initiative	Verbal Autopsy		2003	birth - 79 years	1339
India: Andhra Pradesh, Rural	India - Andhra Pradesh Rural Health Initiative Mortality Surveillance 2003-2007	Verbal Autopsy	Godavari	2004-2007	All Ages	5786
India: Andhra Pradesh, Rural	India - Andhra Pradesh and Odisha Mortality Survey 2005-2006	Verbal Autopsy		2005	15 years +	248682
India: Andhra Pradesh, Rural	India Sample Registration System (SRS)	Verbal Autopsy		2005, 2011	All Ages	11157
India: Andhra Pradesh, Rural	Effects on the estimated cause-specific mortality fraction of providing physician reviewers with different formats of verbal autopsy data	Verbal Autopsy	45 villages East and West Godavari Andhra Pradesh [rural]	2006	All Ages	2814
India: Andhra Pradesh, Rural	Study of causes of deaths among the insured population of a north easternv district of Andhra Pradesh	Verbal Autopsy	Vizianagaram []	2009	birth - 44 years	141
India: Andhra Pradesh, Rural	India Vital Statistics	Vital Registration		1983-1984, 1986-1995	All Ages	1050116
India: Andhra Pradesh, Urban	India District Level Household Survey (DLHS)	Sibling history		2001, 2006	10 - 54 years	119
India: Andhra Pradesh, Urban	Demographic and Health Survey (DHS)	Survey/Census		1992, 1998	All Ages	218
India: Andhra Pradesh, Urban	Levels and causes of maternal mortality in southern India	Verbal Autopsy	Anantapur district Andhra Pradesh [periurban]	1984	15 - 49 years	206
India: Andhra Pradesh, Urban	India - Andhra Pradesh and Odisha Mortality Survey 2005-2006	Verbal Autopsy		2005	15 years +	52748
India: Andhra Pradesh, Urban	India Sample Registration System (SRS)	Verbal Autopsy		2005, 2011	All Ages	7991
India: Andhra Pradesh, Urban	India Vital Statistics	Vital Registration		1980-1984, 1986-1995	All Ages	955646
India: Andhra Pradesh, Urban	India Medical Certification of Cause of Death (MCCD)	Vital Registration		1990-2010	All Ages	729764
India: Arunāchal Pradesh, Rural	India District Level Household Survey (DLHS)	Sibling history		2001, 2006	10 - 54 years	243
India: Arunāchal Pradesh, Rural	Demographic and Health Survey (DHS)	Survey/Census		1992, 1998	All Ages	305
India: Arunāchal Pradesh, Rural	India Survey of Causes of Death (SCD)	Verbal Autopsy		1980-1998	All Ages	955
India: Arunāchal Pradesh, Rural	India Sample Registration System (SRS)	Verbal Autopsy		2005, 2011	All Ages	271
India: Arunāchal Pradesh, Rural	India Vital Statistics	Vital Registration		1986-1995	All Ages	10261
India: Arunāchal Pradesh, Urban	India District Level Household Survey (DLHS)	Sibling history		2001, 2006	10 - 54 years	35
India: Arunāchal Pradesh, Urban	Demographic and Health Survey (DHS)	Survey/Census		1992, 1998	All Ages	22
India: Arunāchal Pradesh, Urban	India Sample Registration System (SRS)	Verbal Autopsy		2005, 2011	All Ages	185
India: Arunāchal Pradesh, Urban	India Vital Statistics	Vital Registration		1980-1984, 1986-1995	All Ages	6973
India: Arunāchal Pradesh, Urban	India Medical Certification of Cause of Death (MCCD)	Vital Registration		1990-2010	All Ages	24978
India: Assam, Rural	India District Level Household Survey (DLHS)	Sibling history		2001, 2006	10 - 54 years	672
India: Assam, Rural	Demographic and Health Survey (DHS)	Survey/Census		1992, 1998	All Ages	671
India: Assam, Rural	India Survey of Causes of Death (SCD)	Verbal Autopsy		1980-1998	All Ages	7415
India: Assam, Rural	India SRS Maternal Mortality: Trends, Causes and Risk Factors 1997-2003	Verbal Autopsy		1999-2003	10 - 54 years	MMR

Appendix Table 6: Causes of death data source list by geography, source type, and study site, with years, age range, and sample size, excluding sources on lower respiratory infection and diarrhoea etiologies						
**Location**	**Source name**	**Source type**	**Site**	**Years**	**Ages**	**Sample size**
India: Assam, Rural	India Study on Causes of Death by Verbal Autopsy 2003	Verbal Autopsy		2003	All Ages	1382
India: Assam, Rural	India SRS Maternal Mortality Tables 2001-2009	Verbal Autopsy		2004, 2006-2009	10 - 54 years	MMR
India: Assam, Rural	India Sample Registration System (SRS)	Verbal Autopsy		2005, 2011	All Ages	10047
India: Assam, Rural	India SRS Special Bulletin on Maternal Mortality 2010-2012	Verbal Autopsy		2010	10 - 54 years	MMR
India: Assam, Rural	India SRS Maternal Mortality Ratio Bulletin 2011-2013	Verbal Autopsy		2011-2013	10 - 54 years	MMR
India: Assam, Urban	India District Level Household Survey (DLHS)	Sibling history		2001, 2006	10 - 54 years	80
India: Assam, Urban	Demographic and Health Survey (DHS)	Survey/Census		1992, 1998	All Ages	197
India: Assam, Urban	India SRS Maternal Mortality: Trends, Causes and Risk Factors 1997-2003	Verbal Autopsy		1999-2003	10 - 54 years	MMR
India: Assam, Urban	India Study on Causes of Death by Verbal Autopsy 2003	Verbal Autopsy		2003	All Ages	499
India: Assam, Urban	India SRS Maternal Mortality Tables 2001-2009	Verbal Autopsy		2004, 2006-2009	10 - 54 years	MMR
India: Assam, Urban	India Sample Registration System (SRS)	Verbal Autopsy		2005, 2011	All Ages	6842
India: Assam, Urban	India SRS Special Bulletin on Maternal Mortality 2010-2012	Verbal Autopsy		2010	10 - 54 years	MMR
India: Assam, Urban	India SRS Maternal Mortality Ratio Bulletin 2011-2013	Verbal Autopsy		2011-2013	10 - 54 years	MMR
India: Assam, Urban	India Vital Statistics	Vital Registration		1980-1984, 1986-1987, 1989	All Ages	18270
India: Assam, Urban	India Medical Certification of Cause of Death (MCCD)	Vital Registration		1990-2010	All Ages	273416
India: Bihār, Rural	India District Level Household Survey (DLHS)	Sibling history		2001, 2006	10 - 54 years	1421
India: Bihār, Rural	Demographic and Health Survey (DHS)	Survey/Census		1992, 1998	All Ages	1755
India: Bihār, Rural	India Survey of Causes of Death (SCD)	Verbal Autopsy		1980-1998	All Ages	34500
India: Bihār, Rural	India Study on Causes of Death by Verbal Autopsy 2003	Verbal Autopsy		2003	All Ages	3088
India: Bihār, Rural	India Sample Registration System (SRS)	Verbal Autopsy		2005, 2011	All Ages	14022
India: Bihār, Rural	India Vital Statistics	Vital Registration		1986-1995	All Ages	1890401
India: Bihār, Urban	India District Level Household Survey (DLHS)	Sibling history		2001, 2006	10 - 54 years	133
India: Bihār, Urban	Infant and early childhood mortality in urban slums under ICDS scheme - a prospective study	Survey/Census	Patna [urban]	1987	birth - 364 days	32
India: Bihār, Urban	Demographic and Health Survey (DHS)	Survey/Census		1992, 1998	All Ages	245
India: Bihār, Urban	India Study on Causes of Death by Verbal Autopsy 2003	Verbal Autopsy		2003	All Ages	224
India: Bihār, Urban	India Sample Registration System (SRS)	Verbal Autopsy		2005, 2011	All Ages	9395
India: Bihār, Urban	India Vital Statistics	Vital Registration		1986-1995	All Ages	415011
India: Bihār, Urban	India Medical Certification of Cause of Death (MCCD)	Vital Registration		1999-2010	All Ages	136875
India: Chhattīsgarh, Rural	India District Level Household Survey (DLHS)	Sibling history		2001, 2006	10 - 54 years	405
India: Chhattīsgarh, Rural	India Sample Registration System (SRS)	Verbal Autopsy		2005, 2011	All Ages	2742
India: Chhattīsgarh, Urban	India District Level Household Survey (DLHS)	Sibling history		2001, 2006	10 - 54 years	54
India: Chhattīsgarh, Urban	India Sample Registration System (SRS)	Verbal Autopsy		2005, 2011	All Ages	1737
India: Chhattīsgarh, Urban	India Medical Certification of Cause of Death (MCCD)	Vital Registration		1999-2010, 2012	All Ages	90916
India: Delhi, Rural	India District Level Household Survey (DLHS)	Sibling history		2001	10 - 29 years	2
India: Delhi, Rural	India District Level Household Survey (DLHS)	Sibling history		2006	10 - 54 years	8
India: Delhi, Rural	Demographic and Health Survey (DHS)	Survey/Census		1992, 1998	All Ages	27
India: Delhi, Rural	India Survey of Causes of Death (SCD)	Verbal Autopsy		1980-1998	All Ages	987
India: Delhi, Rural	India Sample Registration System (SRS)	Verbal Autopsy		2005, 2011	All Ages	2979
India: Delhi, Rural	India Vital Statistics	Vital Registration		1983-1984, 1986-1995	All Ages	23100

Appendix Table 6: Causes of death data source list by geography, source type, and study site, with years, age range, and sample size, excluding sources on lower respiratory infection and diarrhoea etiologies

Location	Source name	Source type	Site	Years	Ages	Sample size
India: Delhi, Urban	Road safety in India: Challenges and opportunities	Police Records	Delhi	2003	All Ages	Death Rate
India: Delhi, Urban	India District Level Household Survey (DLHS)	Sibling history		2001, 2006	10 - 54 years	198
India: Delhi, Urban	Demographic and Health Survey (DHS)	Survey/Census		1992, 1998	All Ages	533
India: Delhi, Urban	Pathways to infant mortality in urban slums of Delhi, India: implications for improving the quality of community- and hospital-based programmes	Verbal Autopsy	2 urban slums in Delhi [urban]	1995	birth - 364 days	162
India: Delhi, Urban	Changing profile of disease contributing to mortality in a resettlement colony of Delhi	Verbal Autopsy	Gokulpuri East Delhi [urban]	1999	All Ages	515
India: Delhi, Urban	Community based retrospective study of sex in infant mortality in India	Verbal Autopsy	Sunder Nagari Tahirpur Amar Colony Delhi [urban]	1999	birth - 364 days	442
India: Delhi, Urban	India Sample Registration System (SRS)	Verbal Autopsy		2005, 2011	All Ages	2035
India: Delhi, Urban	India Vital Statistics	Vital Registration		1980-1984, 1986-1995	All Ages	732772
India: Delhi, Urban	India Medical Certification of Cause of Death (MCCD)	Vital Registration		1990-2010	All Ages	986258
India: Delhi, Urban	India - Delhi Report on Medical Certification of Cause of Deaths 2011	Vital Registration		2011	All Ages	67728
India: Delhi, Urban	India - Delhi Medical Certification of Cause of Death Report 2012	Vital Registration		2012	All Ages	66941
India: Delhi, Urban	India - Delhi Medical Certification of Cause of Death Report 2013	Vital Registration		2013	All Ages	67281
India: Goa, Rural	India District Level Household Survey (DLHS)	Sibling history		2001	15 - 54 years	2
India: Goa, Rural	India District Level Household Survey (DLHS)	Sibling history		2006	30 - 54 years	7
India: Goa, Rural	Demographic and Health Survey (DHS)	Survey/Census		1992, 1998	All Ages	299
India: Goa, Rural	India Survey of Causes of Death (SCD)	Verbal Autopsy		1980-1998	All Ages	2481
India: Goa, Rural	India Sample Registration System (SRS)	Verbal Autopsy		2005, 2011	All Ages	175
India: Goa, Rural	India Vital Statistics	Vital Registration		1983, 1989-1995	All Ages	34417
India: Goa, Urban	India District Level Household Survey (DLHS)	Sibling history		2001	25 - 54 years	1
India: Goa, Urban	India District Level Household Survey (DLHS)	Sibling history		2006	10 - 54 years	10
India: Goa, Urban	Demographic and Health Survey (DHS)	Survey/Census		1992, 1998	All Ages	204
India: Goa, Urban	India Sample Registration System (SRS)	Verbal Autopsy		2005, 2011	All Ages	121
India: Goa, Urban	India Vital Statistics	Vital Registration		1980-1984, 1986-1987, 1989-1995	All Ages	71782
India: Goa, Urban	India Medical Certification of Cause of Death (MCCD)	Vital Registration		1990-2010	All Ages	207921
India: Gujarāt, Rural	India District Level Household Survey (DLHS)	Sibling history		2001, 2006	10 - 54 years	384
India: Gujarāt, Rural	Demographic and Health Survey (DHS)	Survey/Census		1992, 1998	All Ages	609
India: Gujarāt, Rural	India Survey of Causes of Death (SCD)	Verbal Autopsy		1980-1998	All Ages	36004
India: Gujarāt, Rural	India SRS Maternal Mortality: Trends, Causes and Risk Factors 1997-2003	Verbal Autopsy		1999-2003	10 - 54 years	MMR
India: Gujarāt, Rural	India SRS Maternal Mortality Tables 2001-2009	Verbal Autopsy		2004, 2006-2009	10 - 54 years	MMR
India: Gujarāt, Rural	India Sample Registration System (SRS)	Verbal Autopsy		2005, 2011	All Ages	7976
India: Gujarāt, Rural	Changing epidemiology of maternal mortality in rural India: time to reset strategies for MDG-5	Verbal Autopsy		2006	15 - 44 years	871
India: Gujarāt, Rural	Causes of neonatal deaths among tribal women in Gujarat, India	Verbal Autopsy	Jhagadia block Gujarat [Sewa Rural registry]	2008	birth - 27 days	105
India: Gujarāt, Rural	India SRS Special Bulletin on Maternal Mortality 2010-2012	Verbal Autopsy		2010	10 - 54 years	MMR
India: Gujarāt, Rural	India SRS Maternal Mortality Ratio Bulletin 2011-2013	Verbal Autopsy		2011-2013	10 - 54 years	MMR
India: Gujarāt, Rural	India Vital Statistics	Vital Registration		1983-1984, 1986-1995	All Ages	1161206
India: Gujarāt, Urban	India District Level Household Survey (DLHS)	Sibling history		2001, 2006	10 - 54 years	151

Appendix Table 6: Causes of death data source list by geography, source type, and study site, with years, age range, and sample size, excluding sources on lower respiratory infection and diarrhoea etiologies						
**Location**	**Source name**	**Source type**	**Site**	**Years**	**Ages**	**Sample size**
India: Gujarāt, Urban	Demographic and Health Survey (DHS)	Survey/Census		1992, 1998	All Ages	304
India: Gujarāt, Urban	India SRS Maternal Mortality: Trends, Causes and Risk Factors 1997-2003	Verbal Autopsy		1999-2003	10 - 54 years	MMR
India: Gujarāt, Urban	India SRS Maternal Mortality Tables 2001-2009	Verbal Autopsy		2004, 2006-2009	10 - 54 years	MMR
India: Gujarāt, Urban	India Sample Registration System (SRS)	Verbal Autopsy		2005, 2011	All Ages	5495
India: Gujarāt, Urban	India SRS Special Bulletin on Maternal Mortality 2010-2012	Verbal Autopsy		2010	10 - 54 years	MMR
India: Gujarāt, Urban	India SRS Maternal Mortality Ratio Bulletin 2011-2013	Verbal Autopsy		2011-2013	10 - 54 years	MMR
India: Gujarāt, Urban	India Vital Statistics	Vital Registration		1980-1984, 1986-1995	All Ages	1184199
India: Gujarāt, Urban	India Medical Certification of Cause of Death (MCCD)	Vital Registration		1990-2010	All Ages	455660
India: Haryāna, Rural	India District Level Household Survey (DLHS)	Sibling history		2001, 2006	10 - 54 years	219
India: Haryāna, Rural	Demographic and Health Survey (DHS)	Survey/Census		1992, 1998	All Ages	494
India: Haryāna, Rural	India Survey of Causes of Death (SCD)	Verbal Autopsy		1980-1998	All Ages	11861
India: Haryāna, Rural	Morbidity and mortality in diarrhea in rural Haryana Indian	Verbal Autopsy	69 villages in Haryana [rural]	1983	birth - 4 years	117
India: Haryāna, Rural	"Development" is not essential to reduce infant mortality rate in India: experience from the Ballabgarh project	Verbal Autopsy	Ballabgarh [rural]	1983, 1993	birth - 364 days	606
India: Haryāna, Rural	Socio-biological factors in underfive deaths in a rural area	Verbal Autopsy	20 villages Ballabgarh Haryana [rural]	1985	birth - 4 years	281
India: Haryāna, Rural	Maternal mortality inquiry in a rural community of north India	Verbal Autopsy	5 community development blocks in Ambala North India. There were 774 villages. [rural]	1985	15 - 44 years	257
India: Haryāna, Rural	International differences in clinical patterns of diarrhoeal deaths: a comparison of children from Brazil, Senegal, Bangladesh, and India	Verbal Autopsy	Ballabgarh Haryana state [rural]	1988	birth - 4 years	811
India: Haryāna, Rural	Management of Sick Children by Health Workers in Ballabgarh: Lessons for Implementation of IMCI in India	Verbal Autopsy	Ballabgarh [rural]	1998	birth - 364 days	102
India: Haryāna, Rural	India SRS Maternal Mortality: Trends, Causes and Risk Factors 1997-2003	Verbal Autopsy		1999-2003	10 - 54 years	MMR
India: Haryāna, Rural	Suicide an emerging public health problem: evidence from rural Harya na, India	Verbal Autopsy	28 villages Ballabgarh block Haryana [rural]	2002-2007, 2009	10 years +	3914
India: Haryāna, Rural	Causes of death in rural adult population of North India (2002-2007), using verbal autopsy tool	Verbal Autopsy	28 villages Ballabgarh block Haryana [rural]	2004	15 years +	2294
India: Haryāna, Rural	India SRS Maternal Mortality Tables 2001-2009	Verbal Autopsy		2004, 2006-2009	10 - 54 years	MMR
India: Haryāna, Rural	India Sample Registration System (SRS)	Verbal Autopsy		2005, 2011	All Ages	6779
India: Haryāna, Rural	Adult mortality surveillance by routine health workers using a short verbal autopsy tool in rural north India	Verbal Autopsy	28 villages Ballabgarh [rural]	2008	10 years +	510
India: Haryāna, Rural	Performance of cause-specific childhood mortality surveillance by health workers using a short verbal autopsy tool	Verbal Autopsy	28 villages Ballabgarh [rural]	2008	birth - 4 years	143
India: Haryāna, Rural	Demographic Surveillance System (DSS)	Verbal Autopsy		2009-2012	All Ages	2543
India: Haryāna, Rural	Using three delays model to understand the social factors responsible for neonatal deaths in rural Haryana, India	Verbal Autopsy		2010	birth - 27 days	50
India: Haryāna, Rural	India SRS Special Bulletin on Maternal Mortality 2010-2012	Verbal Autopsy		2010	10 - 54 years	MMR
India: Haryāna, Rural	India SRS Maternal Mortality Ratio Bulletin 2011-2013	Verbal Autopsy		2011-2013	10 - 54 years	MMR
India: Haryāna, Rural	India Vital Statistics	Vital Registration		1984, 1986-1995	All Ages	656274
India: Haryāna, Urban	India District Level Household Survey (DLHS)	Sibling history		2001, 2006	10 - 54 years	82
India: Haryāna, Urban	Demographic and Health Survey (DHS)	Survey/Census		1992, 1998	All Ages	187
India: Haryāna, Urban	India SRS Maternal Mortality: Trends, Causes and Risk Factors 1997-2003	Verbal Autopsy		1999-2003	10 - 54 years	MMR

Appendix Table 6: Causes of death data source list by geography, source type, and study site, with years, age range, and sample size, excluding sources on lower respiratory infection and diarrhoea etiologies						
**Location**	**Source name**	**Source type**	**Site**	**Years**	**Ages**	**Sample size**
India: Haryāna, Urban	India SRS Maternal Mortality Tables 2001-2009	Verbal Autopsy		2004, 2006-2009	10 - 54 years	MMR
India: Haryāna, Urban	India Sample Registration System (SRS)	Verbal Autopsy		2005, 2011	All Ages	4621
India: Haryāna, Urban	India SRS Special Bulletin on Maternal Mortality 2010-2012	Verbal Autopsy		2010	10 - 54 years	MMR
India: Haryāna, Urban	India SRS Maternal Mortality Ratio Bulletin 2011-2013	Verbal Autopsy		2011-2013	10 - 54 years	MMR
India: Haryāna, Urban	India Vital Statistics	Vital Registration		1980-1984, 1986-1995	All Ages	293130
India: Haryāna, Urban	India Medical Certification of Cause of Death (MCCD)	Vital Registration		1990-2010	All Ages	288207
India: Himachal Pradesh, Rural	India District Level Household Survey (DLHS)	Sibling history		2001, 2006	10 - 54 years	123
India: Himachal Pradesh, Rural	Demographic and Health Survey (DHS)	Survey/Census		1992, 1998	All Ages	456
India: Himachal Pradesh, Rural	India Survey of Causes of Death (SCD)	Verbal Autopsy		1980-1998	All Ages	5043
India: Himachal Pradesh, Rural	India Sample Registration System (SRS)	Verbal Autopsy		2005, 2011	All Ages	1698
India: Himachal Pradesh, Rural	India Vital Statistics	Vital Registration		1988-1989, 1991-1995	All Ages	108534
India: Himachal Pradesh, Urban	India District Level Household Survey (DLHS)	Sibling history		2001	10 - 54 years	2
India: Himachal Pradesh, Urban	India District Level Household Survey (DLHS)	Sibling history		2006	20 - 54 years	14
India: Himachal Pradesh, Urban	Demographic and Health Survey (DHS)	Survey/Census		1992, 1998	All Ages	151
India: Himachal Pradesh, Urban	India Sample Registration System (SRS)	Verbal Autopsy		2005, 2011	All Ages	1160
India: Himachal Pradesh, Urban	India Vital Statistics	Vital Registration		1980-1984, 1986-1989, 1991-1995	All Ages	22569
India: Himachal Pradesh, Urban	India Medical Certification of Cause of Death (MCCD)	Vital Registration		1990-2010	All Ages	41218
India: Jammu and Kashmīr, Rural	India District Level Household Survey (DLHS)	Sibling history		2001, 2006	10 - 54 years	231
India: Jammu and Kashmīr, Rural	Demographic and Health Survey (DHS)	Survey/Census		1992, 1998	All Ages	462
India: Jammu and Kashmīr, Rural	India Survey of Causes of Death (SCD)	Verbal Autopsy		1980-1998	All Ages	7535
India: Jammu and Kashmīr, Rural	India Sample Registration System (SRS)	Verbal Autopsy		2005, 2011	All Ages	2798
India: Jammu and Kashmīr, Rural	India Vital Statistics	Vital Registration		1983-1984, 1991-1993, 1995	All Ages	123347
India: Jammu and Kashmīr, Urban	India District Level Household Survey (DLHS)	Sibling history		2001	20 - 54 years	4
India: Jammu and Kashmīr, Urban	India District Level Household Survey (DLHS)	Sibling history		2006	15 - 54 years	55
India: Jammu and Kashmīr, Urban	Demographic and Health Survey (DHS)	Survey/Census		1992, 1998	All Ages	212
India: Jammu and Kashmīr, Urban	India Sample Registration System (SRS)	Verbal Autopsy		2005, 2011	All Ages	1911
India: Jammu and Kashmīr, Urban	India Vital Statistics	Vital Registration		1983-1984, 1991-1993, 1995	All Ages	27567
India: Jharkhand, Rural	India District Level Household Survey (DLHS)	Sibling history		2001, 2006	10 - 54 years	745
India: Jharkhand, Rural	India Sample Registration System (SRS)	Verbal Autopsy		2005, 2011	All Ages	4856
India: Jharkhand, Urban	India District Level Household Survey (DLHS)	Sibling history		2001, 2006	10 - 54 years	109
India: Jharkhand, Urban	India Sample Registration System (SRS)	Verbal Autopsy		2005, 2011	All Ages	3253
India: Jharkhand, Urban	India Medical Certification of Cause of Death (MCCD)	Vital Registration		1999-2010	All Ages	43390
India: Karnātaka, Rural	India District Level Household Survey (DLHS)	Sibling history		2001, 2006	10 - 54 years	553
India: Karnātaka, Rural	Demographic and Health Survey (DHS)	Survey/Census		1992, 1998	All Ages	703
India: Karnātaka, Rural	India Survey of Causes of Death (SCD)	Verbal Autopsy		1980-1998	All Ages	20184
India: Karnātaka, Rural	India SRS Maternal Mortality: Trends, Causes and Risk Factors 1997-2003	Verbal Autopsy		1999-2003	10 - 54 years	MMR
India: Karnātaka, Rural	India Sample Registration System (SRS)	Verbal Autopsy		2005, 2011	All Ages	14418
India: Karnātaka, Rural	India SRS Maternal Mortality Tables 2001-2009	Verbal Autopsy		2006-2009	10 - 54 years	MMR
India: Karnātaka, Rural	India SRS Special Bulletin on Maternal Mortality 2010-2012	Verbal Autopsy		2010	10 - 54 years	MMR

Appendix Table 6: Causes of death data source list by geography, source type, and study site, with years, age range, and sample size, excluding sources on lower respiratory infection and diarrhoea etiologies						
Location	Source name	Source type	Site	Years	Ages	Sample size
India: Karnātaka, Rural	Institutionalizing district level infant death review: an experience from southern India	Verbal Autopsy	Raichur district Karnataka [rural]	2011	birth - 364 days	443
India: Karnātaka, Rural	India SRS Maternal Mortality Ratio Bulletin 2011-2013	Verbal Autopsy		2011-2013	10 - 54 years	MMR
India: Karnātaka, Rural	India Vital Statistics	Vital Registration		1988-1989, 1991-1995	All Ages	931329
India: Karnātaka, Urban	India District Level Household Survey (DLHS)	Sibling history		2001, 2006	10 - 54 years	207
India: Karnātaka, Urban	Demographic and Health Survey (DHS)	Survey/Census		1992, 1998	All Ages	283
India: Karnātaka, Urban	India SRS Maternal Mortality: Trends, Causes and Risk Factors 1997-2003	Verbal Autopsy		1999-2003	10 - 54 years	MMR
India: Karnātaka, Urban	India Sample Registration System (SRS)	Verbal Autopsy		2005, 2011	All Ages	10300
India: Karnātaka, Urban	India SRS Maternal Mortality Tables 2001-2009	Verbal Autopsy		2006-2009	10 - 54 years	MMR
India: Karnātaka, Urban	India SRS Special Bulletin on Maternal Mortality 2010-2012	Verbal Autopsy		2010	10 - 54 years	MMR
India: Karnātaka, Urban	Institutionalizing district level infant death review: an experience from southern India	Verbal Autopsy	Dakshina Kannada district Karnataka [urban]	2011	birth - 364 days	118
India: Karnātaka, Urban	India SRS Maternal Mortality Ratio Bulletin 2011-2013	Verbal Autopsy		2011-2013	10 - 54 years	MMR
India: Karnātaka, Urban	India Vital Statistics	Vital Registration		1980-1984, 1986-1989, 1991-1995	All Ages	943107
India: Karnātaka, Urban	India Medical Certification of Cause of Death (MCCD)	Vital Registration		1990-2012	All Ages	1509021
India: Kerala, Rural	India District Level Household Survey (DLHS)	Sibling history		2001	10 - 54 years	38
India: Kerala, Rural	India District Level Household Survey (DLHS)	Sibling history		2006	15 - 54 years	73
India: Kerala, Rural	Demographic and Health Survey (DHS)	Survey/Census		1992, 1998	All Ages	481
India: Kerala, Rural	India Survey of Causes of Death (SCD)	Verbal Autopsy		1980-1998	All Ages	9384
India: Kerala, Rural	India SRS Maternal Mortality: Trends, Causes and Risk Factors 1997-2003	Verbal Autopsy		1999-2003	10 - 54 years	MMR
India: Kerala, Rural	All-cause mortality and cardiovascular mortality in Kerala state of India: results from a 5-year follow-up of 161,942 rural community dwelling adults	Verbal Autopsy		2004	25 years +	4248
India: Kerala, Rural	Risk of mortality among alcohol using adult males in a population-based cohort in Kerala, India: PROFILE study	Verbal Autopsy	rural block in KeralaPROLIFE [rural]	2004	20 years +	1859
India: Kerala, Rural	India SRS Maternal Mortality Tables 2001-2009	Verbal Autopsy		2004, 2006-2009	10 - 54 years	MMR
India: Kerala, Rural	India Sample Registration System (SRS)	Verbal Autopsy		2005, 2011	All Ages	6753
India: Kerala, Rural	India SRS Maternal Mortality Ratio Bulletin 2011-2013	Verbal Autopsy		2011-2013	10 - 54 years	MMR
India: Kerala, Rural	India Vital Statistics	Vital Registration		1991-1995	All Ages	442644
India: Kerala, Urban	India District Level Household Survey (DLHS)	Sibling history		2001	10 - 54 years	24
India: Kerala, Urban	India District Level Household Survey (DLHS)	Sibling history		2006	15 - 54 years	21
India: Kerala, Urban	Demographic and Health Survey (DHS)	Survey/Census		1992, 1998	All Ages	166
India: Kerala, Urban	India SRS Maternal Mortality: Trends, Causes and Risk Factors 1997-2003	Verbal Autopsy		1999-2003	10 - 54 years	MMR
India: Kerala, Urban	India SRS Maternal Mortality Tables 2001-2009	Verbal Autopsy		2004, 2006-2009	10 - 54 years	MMR
India: Kerala, Urban	India Sample Registration System (SRS)	Verbal Autopsy		2005, 2011	All Ages	4819
India: Kerala, Urban	India SRS Maternal Mortality Ratio Bulletin 2011-2013	Verbal Autopsy		2011-2013	10 - 54 years	MMR
India: Kerala, Urban	India Vital Statistics	Vital Registration		1980-1984, 1986-1987, 1989, 1991-1995	All Ages	231294
India: Kerala, Urban	India Medical Certification of Cause of Death (MCCD)	Vital Registration		1990-2010	All Ages	265607
India: Madhya Pradesh, Rural	India District Level Household Survey (DLHS)	Sibling history		2001, 2006	10 - 54 years	923
India: Madhya Pradesh, Rural	Demographic and Health Survey (DHS)	Survey/Census		1992, 1998	All Ages	1499
India: Madhya Pradesh, Rural	India Survey of Causes of Death (SCD)	Verbal Autopsy		1980-1998	All Ages	37058

Appendix Table 6: Causes of death data source list by geography, source type, and study site, with years, age range, and sample size, excluding sources on lower respiratory infection and diarrhoea etiologies						
**Location**	**Source name**	**Source type**	**Site**	**Years**	**Ages**	**Sample size**
India: Madhya Pradesh, Rural	Causes of Neonatal Mortality: A community based study using verbal autopsy tool	Verbal Autopsy	Jabalpur district [rural]	2005	birth - 27 days	69
India: Madhya Pradesh, Rural	India Sample Registration System (SRS)	Verbal Autopsy		2005, 2011	All Ages	7930
India: Madhya Pradesh, Rural	India Vital Statistics	Vital Registration		1983-1984, 1986-1987, 1989-1993, 1995	All Ages	2925800
India: Madhya Pradesh, Urban	India District Level Household Survey (DLHS)	Sibling history		2001, 2006	10 - 54 years	270
India: Madhya Pradesh, Urban	Demographic and Health Survey (DHS)	Survey/Census		1992, 1998	All Ages	418
India: Madhya Pradesh, Urban	India Sample Registration System (SRS)	Verbal Autopsy		2005, 2011	All Ages	5026
India: Madhya Pradesh, Urban	India Vital Statistics	Vital Registration		1980-1984, 1986-1987, 1989-1993, 1995	All Ages	752171
India: Madhya Pradesh, Urban	India Medical Certification of Cause of Death (MCCD)	Vital Registration		1990-2010	All Ages	448071
India: Mahārāshtra, Rural	India District Level Household Survey (DLHS)	Sibling history		2001, 2006	10 - 54 years	548
India: Mahārāshtra, Rural	Demographic and Health Survey (DHS)	Survey/Census		1992, 1998	All Ages	537
India: Mahārāshtra, Rural	India Survey of Causes of Death (SCD)	Verbal Autopsy		1980-1998, 2011	All Ages	109469
India: Mahārāshtra, Rural	Reduction in pneumonia mortality and total childhood mortality by means of community-based intervention trial in Gadchiroli, India	Verbal Autopsy	18 villages in Gadchiroli [rural]	1988	birth - 4 years	161
India: Mahārāshtra, Rural	A prospective cohort study on the survival experience of under five children in rural western India	Verbal Autopsy	45 villages Shirur Development Block Pune District in Maharashtra [rural]	1990	birth - 4 years	286
India: Mahārāshtra, Rural	A study of registration of deaths at primary health centre--with special reference to verbal autopsy method	Verbal Autopsy	Kunjirwadi Kawathe and Tisgaon PHC areas [rural]	1991	All Ages	73
India: Mahārāshtra, Rural	India SRS Maternal Mortality: Trends, Causes and Risk Factors 1997-2003	Verbal Autopsy		1999-2003	10 - 54 years	MMR
India: Mahārāshtra, Rural	India Study on Causes of Death by Verbal Autopsy 2003	Verbal Autopsy		2003	All Ages	899
India: Mahārāshtra, Rural	Global health and infant mortality: application of verbal autopsy tool to categorize infant deaths, ascertain their causes and identify the gaps in health management information system in India	Verbal Autopsy	Maharashtra RuralYavatmal district [rural]	2004	birth - 364 days	78
India: Mahārāshtra, Rural	India SRS Maternal Mortality Tables 2001-2009	Verbal Autopsy		2004, 2006-2009	10 - 54 years	MMR
India: Mahārāshtra, Rural	A community based cross sectional study on feasibility of lay interviewers in ascertaining causes of adult deaths by using verbal autopsy in rural Wardha	Verbal Autopsy	15 villages of Primary Health Centre Anji Wardha district Maharashtra [rural]	2005	15 years +	209
India: Mahārāshtra, Rural	India Sample Registration System (SRS)	Verbal Autopsy		2005, 2011	All Ages	15687
India: Mahārāshtra, Rural	Demographic Surveillance System (DSS)	Verbal Autopsy		2009-2010	All Ages	746
India: Mahārāshtra, Rural	India SRS Special Bulletin on Maternal Mortality 2010-2012	Verbal Autopsy		2010	10 - 54 years	MMR
India: Mahārāshtra, Rural	India SRS Maternal Mortality Ratio Bulletin 2011-2013	Verbal Autopsy		2011-2013	10 - 54 years	MMR
India: Mahārāshtra, Rural	India Vital Statistics	Vital Registration		1983-1984, 1986-1995	All Ages	2614645
India: Mahārāshtra, Urban	Road safety in India: Challenges and opportunities	Police Records	Mumbai	1996	All Ages	Death Rate
India: Mahārāshtra, Urban	India District Level Household Survey (DLHS)	Sibling history		2001, 2006	10 - 54 years	304
India: Mahārāshtra, Urban	Demographic and Health Survey (DHS)	Survey/Census		1992, 1998	All Ages	476
India: Mahārāshtra, Urban	India SRS Maternal Mortality: Trends, Causes and Risk Factors 1997-2003	Verbal Autopsy		1999-2003	10 - 54 years	MMR
India: Mahārāshtra, Urban	India Study on Causes of Death by Verbal Autopsy 2003	Verbal Autopsy		2003	All Ages	626
India: Mahārāshtra, Urban	India SRS Maternal Mortality Tables 2001-2009	Verbal Autopsy		2004, 2006-2009	10 - 54 years	MMR
India: Mahārāshtra, Urban	India Sample Registration System (SRS)	Verbal Autopsy		2005, 2011	All Ages	10839
India: Mahārāshtra, Urban	Stillbirths and newborn deaths in slum settlements in Mumbai, India: a prospective verbal autopsy study	Verbal Autopsy	48 slum areas Mumbai [urban]	2006	birth - 27 days	116

Appendix Table 6: Causes of death data source list by geography, source type, and study site, with years, age range, and sample size, excluding sources on lower respiratory infection and diarrhoea etiologies						
**Location**	**Source name**	**Source type**	**Site**	**Years**	**Ages**	**Sample size**
India: Mahārāshtra, Urban	India SRS Special Bulletin on Maternal Mortality 2010-2012	Verbal Autopsy		2010	10 - 54 years	MMR
India: Mahārāshtra, Urban	India SRS Maternal Mortality Ratio Bulletin 2011-2013	Verbal Autopsy		2011-2013	10 - 54 years	MMR
India: Mahārāshtra, Urban	India Vital Statistics	Vital Registration		1980-1984, 1986-1995	All Ages	2656890
India: Mahārāshtra, Urban	India Medical Certification of Cause of Death (MCCD)	Vital Registration		1990-2010	All Ages	3532297
India: Manipur, Rural	India District Level Household Survey (DLHS)	Sibling history		2001, 2006	10 - 54 years	130
India: Manipur, Rural	Demographic and Health Survey (DHS)	Survey/Census		1992, 1998	All Ages	183
India: Manipur, Rural	India Survey of Causes of Death (SCD)	Verbal Autopsy		1980-1998	All Ages	1111
India: Manipur, Rural	India Sample Registration System (SRS)	Verbal Autopsy		2005, 2011	All Ages	717
India: Manipur, Rural	India Vital Statistics	Vital Registration		1983-1984, 1986-1993, 1995	All Ages	2239
India: Manipur, Urban	India District Level Household Survey (DLHS)	Sibling history		2001, 2006	10 - 54 years	32
India: Manipur, Urban	Demographic and Health Survey (DHS)	Survey/Census		1992, 1998	All Ages	93
India: Manipur, Urban	India Sample Registration System (SRS)	Verbal Autopsy		2005, 2011	All Ages	489
India: Manipur, Urban	India Vital Statistics	Vital Registration		1980-1984, 1986-1993, 1995	All Ages	13995
India: Manipur, Urban	India Medical Certification of Cause of Death (MCCD)	Vital Registration		1990-2010	All Ages	35154
India: Meghālaya, Rural	India District Level Household Survey (DLHS)	Sibling history		2001, 2006	10 - 54 years	159
India: Meghālaya, Rural	Demographic and Health Survey (DHS)	Survey/Census		1992, 1998	All Ages	213
India: Meghālaya, Rural	India Survey of Causes of Death (SCD)	Verbal Autopsy		1980-1998	All Ages	3357
India: Meghālaya, Rural	India Sample Registration System (SRS)	Verbal Autopsy		2005, 2011	All Ages	577
India: Meghālaya, Rural	India Vital Statistics	Vital Registration		1983	birth - 4 years	20
India: Meghālaya, Urban	India District Level Household Survey (DLHS)	Sibling history		2001, 2006	10 - 54 years	25
India: Meghālaya, Urban	Demographic and Health Survey (DHS)	Survey/Census		1992, 1998	All Ages	51
India: Meghālaya, Urban	India Sample Registration System (SRS)	Verbal Autopsy		2005, 2011	All Ages	394
India: Meghālaya, Urban	India Vital Statistics	Vital Registration		1980-1984, 1986-1987, 1989	All Ages	6123
India: Meghālaya, Urban	India Medical Certification of Cause of Death (MCCD)	Vital Registration		1990-2010	All Ages	41750
India: Mizoram, Rural	India District Level Household Survey (DLHS)	Sibling history		2001, 2006	10 - 54 years	95
India: Mizoram, Rural	Demographic and Health Survey (DHS)	Survey/Census		1992, 1998	All Ages	78
India: Mizoram, Rural	India Sample Registration System (SRS)	Verbal Autopsy		2005, 2011	All Ages	227
India: Mizoram, Rural	India Vital Statistics	Vital Registration		1988-1990	All Ages	4294
India: Mizoram, Urban	India District Level Household Survey (DLHS)	Sibling history		2001, 2006	10 - 54 years	36
India: Mizoram, Urban	Demographic and Health Survey (DHS)	Survey/Census		1992, 1998	All Ages	83
India: Mizoram, Urban	India Sample Registration System (SRS)	Verbal Autopsy		2005, 2011	All Ages	155
India: Mizoram, Urban	India Vital Statistics	Vital Registration		1980-1984, 1986-1990	All Ages	6735
India: Mizoram, Urban	India Medical Certification of Cause of Death (MCCD)	Vital Registration		1990-2010	All Ages	34361
India: Nāgāland, Rural	India District Level Household Survey (DLHS)	Sibling history		2001	10 - 54 years	9
India: Nāgāland, Rural	Demographic and Health Survey (DHS)	Survey/Census		1992, 1998	All Ages	106
India: Nāgāland, Rural	India Survey of Causes of Death (SCD)	Verbal Autopsy		1980-1998	All Ages	1380
India: Nāgāland, Rural	India Sample Registration System (SRS)	Verbal Autopsy		2005, 2011	All Ages	366
India: Nāgāland, Rural	India Vital Statistics	Vital Registration		1983-1984, 1986, 1988-1989, 1991-1995	All Ages	18997
India: Nāgāland, Urban	India District Level Household Survey (DLHS)	Sibling history		2001	10 - 49 years	2
India: Nāgāland, Urban	Demographic and Health Survey (DHS)	Survey/Census		1992, 1998	All Ages	15

**Appendix Table 6: Causes of death data source list by geography, source type, and study site, with years, age range, and sample size, excluding sources on lower respiratory infection and diarrhoea etiologies**						
**Location**	**Source name**	**Source type**	**Site**	**Years**	**Ages**	**Sample size**
India: Nāgāland, Urban	India Sample Registration System (SRS)	Verbal Autopsy		2005, 2011	All Ages	250
India: Nāgāland, Urban	India Vital Statistics	Vital Registration		1980-1984, 1986-1989, 1991-1995	All Ages	8355
India: Nāgāland, Urban	India Medical Certification of Cause of Death (MCCD)	Vital Registration		1990-2010	All Ages	5180
India: Orissa, Rural	India District Level Household Survey (DLHS)	Sibling history		2001, 2006	10 - 54 years	619
India: Orissa, Rural	Demographic and Health Survey (DHS)	Survey/Census		1992, 1998	All Ages	1026
India: Orissa, Rural	India Survey of Causes of Death (SCD)	Verbal Autopsy		1980-1998	All Ages	22640
India: Orissa, Rural	Validity of causes of infant death by verbal autopsy	Verbal Autopsy	16 clusters in districts Cuttack and Koraput Orissa [rural]	1992	birth - 364 days	56
India: Orissa, Rural	India SRS Maternal Mortality: Trends, Causes and Risk Factors 1997-2003	Verbal Autopsy		1999-2003	10 - 54 years	MMR
India: Orissa, Rural	India SRS Maternal Mortality Tables 2001-2009	Verbal Autopsy		2004, 2006-2009	10 - 54 years	MMR
India: Orissa, Rural	India - Andhra Pradesh and Odisha Mortality Survey 2005-2006	Verbal Autopsy		2005	15 years +	258520
India: Orissa, Rural	India Sample Registration System (SRS)	Verbal Autopsy		2005, 2011	All Ages	7514
India: Orissa, Rural	India Vital Statistics	Vital Registration		1986-1992, 1995	All Ages	1303997
India: Orissa, Urban	India District Level Household Survey (DLHS)	Sibling history		2001, 2006	10 - 54 years	102
India: Orissa, Urban	Demographic and Health Survey (DHS)	Survey/Census		1992, 1998	All Ages	213
India: Orissa, Urban	Validity of causes of infant death by verbal autopsy	Verbal Autopsy	7 clusters in districts Cuttack and Koraput Orissa [urban]	1992	birth - 364 days	123
India: Orissa, Urban	India SRS Maternal Mortality: Trends, Causes and Risk Factors 1997-2003	Verbal Autopsy		1999-2003	10 - 54 years	MMR
India: Orissa, Urban	India SRS Maternal Mortality Tables 2001-2009	Verbal Autopsy		2004, 2006-2009	10 - 54 years	MMR
India: Orissa, Urban	India - Andhra Pradesh and Odisha Mortality Survey 2005-2006	Verbal Autopsy		2005	15 years +	23078
India: Orissa, Urban	India Sample Registration System (SRS)	Verbal Autopsy		2005, 2011	All Ages	5031
India: Orissa, Urban	India Vital Statistics	Vital Registration		1980-1984, 1986-1992, 1995	All Ages	323259
India: Orissa, Urban	India Medical Certification of Cause of Death (MCCD)	Vital Registration		1990-2010	All Ages	477741
India: Orissa, Urban	India - Odisha Medical Certification of Cause of Death Data 2009	Vital Registration		2009	All Ages	34752
India: Orissa, Urban	India - Odisha Medical Certification of Cause of Death Data 2010	Vital Registration		2010	All Ages	34744
India: Orissa, Urban	India - Odisha Medical Certification of Cause of Death Data 2011	Vital Registration		2011	All Ages	33968
India: Orissa, Urban	India - Odisha Medical Certification of Cause of Death Data 2012	Vital Registration		2012	All Ages	32559
India: Orissa, Urban	India - Odisha Medical Certification of Cause of Death Data 2013	Vital Registration		2013	All Ages	34165
India: Punjab, Rural	India District Level Household Survey (DLHS)	Sibling history		2001, 2006	10 - 54 years	273
India: Punjab, Rural	Demographic and Health Survey (DHS)	Survey/Census		1992, 1998	All Ages	457
India: Punjab, Rural	India Survey of Causes of Death (SCD)	Verbal Autopsy		1980-1998	All Ages	27000
India: Punjab, Rural	India SRS Maternal Mortality: Trends, Causes and Risk Factors 1997-2003	Verbal Autopsy		1999-2003	10 - 54 years	MMR
India: Punjab, Rural	India SRS Maternal Mortality Tables 2001-2009	Verbal Autopsy		2004, 2006-2009	10 - 54 years	MMR
India: Punjab, Rural	India Sample Registration System (SRS)	Verbal Autopsy		2005, 2011	All Ages	7288
India: Punjab, Rural	India SRS Special Bulletin on Maternal Mortality 2010-2012	Verbal Autopsy		2010	10 - 54 years	MMR
India: Punjab, Rural	India SRS Maternal Mortality Ratio Bulletin 2011-2013	Verbal Autopsy		2011-2013	10 - 54 years	MMR
India: Punjab, Rural	India Vital Statistics	Vital Registration		1983-1984, 1986-1988	All Ages	411345

Appendix Table 6: Causes of death data source list by geography, source type, and study site, with years, age range, and sample size, excluding sources on lower respiratory infection and diarrhoea etiologies

Location	Source name	Source type	Site	Years	Ages	Sample size
India: Punjab, Urban	India District Level Household Survey (DLHS)	Sibling history		2001, 2006	10 - 54 years	115
India: Punjab, Urban	Demographic and Health Survey (DHS)	Survey/Census		1992, 1998	All Ages	207
India: Punjab, Urban	India SRS Maternal Mortality: Trends, Causes and Risk Factors 1997-2003	Verbal Autopsy		1999-2003	10 - 54 years	MMR
India: Punjab, Urban	India SRS Maternal Mortality Tables 2001-2009	Verbal Autopsy		2004, 2006-2009	10 - 54 years	MMR
India: Punjab, Urban	India Sample Registration System (SRS)	Verbal Autopsy		2005, 2011	All Ages	4975
India: Punjab, Urban	India SRS Special Bulletin on Maternal Mortality 2010-2012	Verbal Autopsy		2010	10 - 54 years	MMR
India: Punjab, Urban	India SRS Maternal Mortality Ratio Bulletin 2011-2013	Verbal Autopsy		2011-2013	10 - 54 years	MMR
India: Punjab, Urban	India Vital Statistics	Vital Registration		1980-1984, 1986-1989	All Ages	167375
India: Punjab, Urban	India Medical Certification of Cause of Death (MCCD)	Vital Registration		1990-2010	All Ages	187363
India: Rājasthān, Rural	India District Level Household Survey (DLHS)	Sibling history		2001, 2006	10 - 54 years	569
India: Rājasthān, Rural	Demographic and Health Survey (DHS)	Survey/Census		1992, 1998	All Ages	1433
India: Rājasthān, Rural	India Survey of Causes of Death (SCD)	Verbal Autopsy		1980-1998	All Ages	28891
India: Rājasthān, Rural	India SRS Maternal Mortality: Trends, Causes and Risk Factors 1997-2003	Verbal Autopsy		1999-2003	10 - 54 years	MMR
India: Rājasthān, Rural	Pregnancy-related deaths in rural Rajasthan, India: exploring causes, context, and care-seeking through verbal autopsy	Verbal Autopsy	a block of southern Rajasthan [rural]	2002	15 - 49 years	156
India: Rājasthān, Rural	India Study on Causes of Death by Verbal Autopsy 2003	Verbal Autopsy		2003	All Ages	2480
India: Rājasthān, Rural	Maternal mortality ratio and predictors of maternal deaths in selected desert districts in rajasthan a community-based survey and case control study	Verbal Autopsy	411 villages in Rajasthan - districts of Bikaner - Barmer - Jaisalmer - Jodhpur	2003	15 - 49 years	MMR
India: Rājasthān, Rural	India SRS Maternal Mortality Tables 2001-2009	Verbal Autopsy		2004, 2006-2009	10 - 54 years	MMR
India: Rājasthān, Rural	India Sample Registration System (SRS)	Verbal Autopsy		2005, 2011	All Ages	6199
India: Rājasthān, Rural	India SRS Special Bulletin on Maternal Mortality 2010-2012	Verbal Autopsy		2010	10 - 54 years	MMR
India: Rājasthān, Rural	India SRS Maternal Mortality Ratio Bulletin 2011-2013	Verbal Autopsy		2011-2013	10 - 54 years	MMR
India: Rājasthān, Rural	India Vital Statistics	Vital Registration		1983-1984, 1986-1993	All Ages	599238
India: Rājasthān, Urban	Road safety in India: Challenges and opportunities	Police Records	Kota	2007	All Ages	Death Rate
India: Rājasthān, Urban	India District Level Household Survey (DLHS)	Sibling history		2001, 2006	10 - 54 years	143
India: Rājasthān, Urban	Demographic and Health Survey (DHS)	Survey/Census		1992, 1998	All Ages	338
India: Rājasthān, Urban	India SRS Maternal Mortality: Trends, Causes and Risk Factors 1997-2003	Verbal Autopsy		1999-2003	10 - 54 years	MMR
India: Rājasthān, Urban	India Study on Causes of Death by Verbal Autopsy 2003	Verbal Autopsy		2003	All Ages	660
India: Rājasthān, Urban	India SRS Maternal Mortality Tables 2001-2009	Verbal Autopsy		2004, 2006-2009	10 - 54 years	MMR
India: Rājasthān, Urban	India Sample Registration System (SRS)	Verbal Autopsy		2005, 2011	All Ages	3924
India: Rājasthān, Urban	India SRS Special Bulletin on Maternal Mortality 2010-2012	Verbal Autopsy		2010	10 - 54 years	MMR
India: Rājasthān, Urban	India SRS Maternal Mortality Ratio Bulletin 2011-2013	Verbal Autopsy		2011-2013	10 - 54 years	MMR
India: Rājasthān, Urban	India Vital Statistics	Vital Registration		1980-1984, 1986-1993	All Ages	423450
India: Rājasthān, Urban	India Medical Certification of Cause of Death (MCCD)	Vital Registration		1990-2010	All Ages	475192
India: Sikkim, Rural	India District Level Household Survey (DLHS)	Sibling history		2001, 2006	10 - 54 years	125
India: Sikkim, Rural	Demographic and Health Survey (DHS)	Survey/Census		1998	All Ages	132
India: Sikkim, Rural	India Sample Registration System (SRS)	Verbal Autopsy		2005, 2011	All Ages	117
India: Sikkim, Urban	India District Level Household Survey (DLHS)	Sibling history		2001	25 - 54 years	0

Appendix Table 6: Causes of death data source list by geography, source type, and study site, with years, age range, and sample size, excluding sources on lower respiratory infection and diarrhoea etiologies						
Location	Source name	Source type	Site	Years	Ages	Sample size
India: Sikkim, Urban	India District Level Household Survey (DLHS)	Sibling history		2006	30 - 54 years	4
India: Sikkim, Urban	Demographic and Health Survey (DHS)	Survey/Census		1998	All Ages	8
India: Sikkim, Urban	India Sample Registration System (SRS)	Verbal Autopsy		2005, 2011	All Ages	80
India: Sikkim, Urban	India Vital Statistics	Vital Registration		1980-1984, 1986-1987, 1989	All Ages	2960
India: Sikkim, Urban	India Medical Certification of Cause of Death (MCCD)	Vital Registration		1990-2010	All Ages	13858
India: Tamil Nādu, Rural	India District Level Household Survey (DLHS)	Sibling history		2001, 2006	10 - 54 years	2707
India: Tamil Nādu, Rural	Demographic and Health Survey (DHS)	Survey/Census		1992, 1998	All Ages	681
India: Tamil Nādu, Rural	India Survey of Causes of Death (SCD)	Verbal Autopsy		1980-1998	All Ages	31691
India: Tamil Nādu, Rural	Suicides in young people in rural southern India	Verbal Autopsy	Vellore [rural]	1996	10 - 19 years	427
India: Tamil Nādu, Rural	India SRS Maternal Mortality: Trends, Causes and Risk Factors 1997-2003	Verbal Autopsy		1999-2003	10 - 54 years	MMR
India: Tamil Nādu, Rural	India Study on Causes of Death by Verbal Autopsy 2003	Verbal Autopsy		2003	All Ages	1703
India: Tamil Nādu, Rural	India SRS Maternal Mortality Tables 2001-2009	Verbal Autopsy		2004, 2006-2009	10 - 54 years	MMR
India: Tamil Nādu, Rural	India Sample Registration System (SRS)	Verbal Autopsy		2005, 2011	All Ages	16709
India: Tamil Nādu, Rural	India SRS Special Bulletin on Maternal Mortality 2010-2012	Verbal Autopsy		2010	10 - 54 years	MMR
India: Tamil Nādu, Rural	India SRS Maternal Mortality Ratio Bulletin 2011-2013	Verbal Autopsy		2011-2013	10 - 54 years	MMR
India: Tamil Nādu, Rural	India Vital Statistics	Vital Registration		1983-1984, 1986-1993, 1995	All Ages	1707490
India: Tamil Nādu, Urban	India District Level Household Survey (DLHS)	Sibling history		2001, 2006	10 - 54 years	949
India: Tamil Nādu, Urban	Demographic and Health Survey (DHS)	Survey/Census		1992, 1998	All Ages	318
India: Tamil Nādu, Urban	Reduced mortality among children in southern India receiving a small weekly dose of vitamin A	Verbal Autopsy	Trichy Tamil Nadu [urban]	1988	28 days - 4 years	88
India: Tamil Nādu, Urban	Verbal autopsy of 48 000 adult deaths attributable to medical causes in Chennai (formerly Madras), India	Verbal Autopsy		1996	25 years +	47217
India: Tamil Nādu, Urban	Infant mortality in an urban slum	Verbal Autopsy	Vellore [urban]	1999	birth - 364 days	219
India: Tamil Nādu, Urban	India SRS Maternal Mortality: Trends, Causes and Risk Factors 1997-2003	Verbal Autopsy		1999-2003	10 - 54 years	MMR
India: Tamil Nādu, Urban	India Study on Causes of Death by Verbal Autopsy 2003	Verbal Autopsy		2003	All Ages	645
India: Tamil Nādu, Urban	India SRS Maternal Mortality Tables 2001-2009	Verbal Autopsy		2004, 2006-2009	10 - 54 years	MMR
India: Tamil Nādu, Urban	India Sample Registration System (SRS)	Verbal Autopsy		2005, 2011	All Ages	11931
India: Tamil Nādu, Urban	India SRS Special Bulletin on Maternal Mortality 2010-2012	Verbal Autopsy		2010	10 - 54 years	MMR
India: Tamil Nādu, Urban	India SRS Maternal Mortality Ratio Bulletin 2011-2013	Verbal Autopsy		2011-2013	10 - 54 years	MMR
India: Tamil Nādu, Urban	India Vital Statistics	Vital Registration		1980-1984, 1986-1993, 1995	All Ages	1920695
India: Tamil Nādu, Urban	India Medical Certification of Cause of Death (MCCD)	Vital Registration		1990-2010	All Ages	1831846
India: Telangana, Rural	Incidence of suicides in three villages of Khammam district of South India	Verbal Autopsy	Telangana RuralKhammam district [rural]	2009	15 years +	252
India: Tripura, Rural	India District Level Household Survey (DLHS)	Sibling history		2001, 2006	10 - 54 years	72
India: Tripura, Rural	Demographic and Health Survey (DHS)	Survey/Census		1992, 1998	All Ages	241
India: Tripura, Rural	India Survey of Causes of Death (SCD)	Verbal Autopsy		1980-1998	All Ages	1796
India: Tripura, Rural	India Sample Registration System (SRS)	Verbal Autopsy		2005, 2011	All Ages	972
India: Tripura, Rural	India Vital Statistics	Vital Registration		1986, 1989, 1991	All Ages	8149
India: Tripura, Urban	India District Level Household Survey (DLHS)	Sibling history		2001	45 - 49 years	0
India: Tripura, Urban	India District Level Household Survey (DLHS)	Sibling history		2006	15 - 54 years	5
India: Tripura, Urban	Demographic and Health Survey (DHS)	Survey/Census		1992, 1998	All Ages	53

Appendix Table 6: Causes of death data source list by geography, source type, and study site, with years, age range, and sample size, excluding sources on lower respiratory infection and diarrhoea etiologies						
**Location**	**Source name**	**Source type**	**Site**	**Years**	**Ages**	**Sample size**
India: Tripura, Urban	India Sample Registration System (SRS)	Verbal Autopsy		2005, 2011	All Ages	662
India: Tripura, Urban	India Vital Statistics	Vital Registration		1980-1984, 1986-1987, 1989, 1991	All Ages	33051
India: Tripura, Urban	India Medical Certification of Cause of Death (MCCD)	Vital Registration		1990-2010	All Ages	169080
India: Uttar Pradesh, Rural	India District Level Household Survey (DLHS)	Sibling history		2001, 2006	10 - 54 years	2401
India: Uttar Pradesh, Rural	Demographic and Health Survey (DHS)	Survey/Census		1992, 1998	All Ages	2779
India: Uttar Pradesh, Rural	India Survey of Causes of Death (SCD)	Verbal Autopsy		1980-1998	All Ages	65581
India: Uttar Pradesh, Rural	Maternal mortality in seven districts of Uttar Pradesh - an ICMR task force study	Verbal Autopsy	Uttar Pradesh Agra [rural]	1989	15 - 49 years	MMR
India: Uttar Pradesh, Rural	Maternal mortality in seven districts of Uttar Pradesh - an ICMR task force study	Verbal Autopsy	Uttar Pradesh Badaun [rural]	1989	15 - 49 years	MMR
India: Uttar Pradesh, Rural	Maternal mortality in seven districts of Uttar Pradesh - an ICMR task force study	Verbal Autopsy	Uttar Pradesh Basti [rural]	1989	15 - 49 years	MMR
India: Uttar Pradesh, Rural	Maternal mortality in seven districts of Uttar Pradesh - an ICMR task force study	Verbal Autopsy	Uttar Pradesh Farrukhabad [rural]	1989	15 - 49 years	MMR
India: Uttar Pradesh, Rural	Maternal mortality in seven districts of Uttar Pradesh - an ICMR task force study	Verbal Autopsy	Uttar Pradesh Ghaziabad [rural]	1989	15 - 49 years	MMR
India: Uttar Pradesh, Rural	Maternal mortality in seven districts of Uttar Pradesh - an ICMR task force study	Verbal Autopsy	Uttar Pradesh Gorakhpur [rural]	1989	15 - 49 years	MMR
India: Uttar Pradesh, Rural	Maternal mortality in seven districts of Uttar Pradesh - an ICMR task force study	Verbal Autopsy	Uttar Pradesh Varanasi [rural]	1989	15 - 49 years	MMR
India: Uttar Pradesh, Rural	Determinants of childhood mortality	Verbal Autopsy	Aligarh [rural]	2005	birth - 4 years	56
India: Uttar Pradesh, Rural	India Sample Registration System (SRS)	Verbal Autopsy		2005, 2011	All Ages	20255
India: Uttar Pradesh, Rural	Rates, timing and causes of neonatal deaths in rural India: implications for neonatal health programmes	Verbal Autopsy	Barabanki and Unnao Uttar Pradesh [rural]	2006	birth - 27 days	618
India: Uttar Pradesh, Urban	India District Level Household Survey (DLHS)	Sibling history		2001, 2006	10 - 54 years	559
India: Uttar Pradesh, Urban	Demographic and Health Survey (DHS)	Survey/Census		1992, 1998	All Ages	510
India: Uttar Pradesh, Urban	Neonatal morbidity and mortality in ICDS Urban Slums	Verbal Autopsy	Gorakhpur city [urban]	1983	birth - 27 days	50
India: Uttar Pradesh, Urban	Mortality patterns in under six children in I.C.D.S. urban slum	Verbal Autopsy	Gorakhpur city [urban]	1983	1 - 4 years	205
India: Uttar Pradesh, Urban	Perinatal mortality in urban slums in Lucknow	Verbal Autopsy	Lucknow [urban]	1992	birth - 6 days	57
India: Uttar Pradesh, Urban	Cause-specific mortality in under fives in the urban slums of Lucknow, north India	Verbal Autopsy	Lucknow [urban]	1994	birth - 4 years	1101
India: Uttar Pradesh, Urban	Singh's verbal autopsy questionnaire for the assessment of causes of death, social autopsy, tobacco autopsy and dietary autopsy, based on medical records and interview	Verbal Autopsy	Moradabad [urban]	2000	25 - 64 years	2222
India: Uttar Pradesh, Urban	Determinants of childhood mortality	Verbal Autopsy	Aligarh [urban]	2005	birth - 4 years	26
India: Uttar Pradesh, Urban	India Sample Registration System (SRS)	Verbal Autopsy		2005, 2011	All Ages	12846
India: Uttar Pradesh, Urban	Determinants of tetanus and sepsis among the last neonatal deaths at household level in a peri-urban area of India	Verbal Autopsy	Kanpur [periurban]	2008	birth - 27 days	109
India: Uttar Pradesh, Urban	India Vital Statistics	Vital Registration		1980-1984, 1986-1987, 1989	All Ages	13825
India: Uttar Pradesh, Urban	India Medical Certification of Cause of Death (MCCD)	Vital Registration		1990-2010	All Ages	45014
India: Uttarakhand, Rural	India District Level Household Survey (DLHS)	Sibling history		2001, 2006	10 - 54 years	163
India: Uttarakhand, Rural	India Sample Registration System (SRS)	Verbal Autopsy		2005, 2011	All Ages	2256
India: Uttarakhand, Urban	India District Level Household Survey (DLHS)	Sibling history		2001, 2006	10 - 54 years	36
India: Uttarakhand, Urban	India Sample Registration System (SRS)	Verbal Autopsy		2005, 2011	All Ages	1541
India: Uttarakhand, Urban	India Medical Certification of Cause of Death (MCCD)	Vital Registration		1999-2010	All Ages	35701
India: West Bengal, Rural	India District Level Household Survey (DLHS)	Sibling history		2001, 2006	10 - 54 years	482

Appendix Table 6: Causes of death data source list by geography, source type, and study site, with years, age range, and sample size, excluding sources on lower respiratory infection and diarrhoea etiologies						
Location	Source name	Source type	Site	Years	Ages	Sample size
India: West Bengal, Rural	Demographic and Health Survey (DHS)	Survey/Census		1992, 1998	All Ages	761
India: West Bengal, Rural	India Survey of Causes of Death (SCD)	Verbal Autopsy		1980-1998	All Ages	5252
India: West Bengal, Rural	India SRS Maternal Mortality: Trends, Causes and Risk Factors 1997-2003	Verbal Autopsy		1999-2003	10 - 54 years	MMR
India: West Bengal, Rural	India SRS Maternal Mortality Tables 2001-2009	Verbal Autopsy		2004, 2006-2009	10 - 54 years	MMR
India: West Bengal, Rural	India Sample Registration System (SRS)	Verbal Autopsy		2005, 2011	All Ages	12601
India: West Bengal, Rural	India SRS Special Bulletin on Maternal Mortality 2010-2012	Verbal Autopsy		2010	10 - 54 years	MMR
India: West Bengal, Rural	India SRS Maternal Mortality Ratio Bulletin 2011-2013	Verbal Autopsy		2011-2013	10 - 54 years	MMR
India: West Bengal, Urban	India District Level Household Survey (DLHS)	Sibling history		2001, 2006	10 - 54 years	127
India: West Bengal, Urban	Demographic and Health Survey (DHS)	Survey/Census		1992, 1998	All Ages	319
India: West Bengal, Urban	India SRS Maternal Mortality: Trends, Causes and Risk Factors 1997-2003	Verbal Autopsy		1999-2003	10 - 54 years	MMR
India: West Bengal, Urban	Use of verbal autopsy to determine mortality patterns in an urban slum in Kolkata, India	Verbal Autopsy	Kolkata ward 29 and 30 [urban]	2003	All Ages	88465
India: West Bengal, Urban	India SRS Maternal Mortality Tables 2001-2009	Verbal Autopsy		2004, 2006-2009	10 - 54 years	MMR
India: West Bengal, Urban	India Sample Registration System (SRS)	Verbal Autopsy		2005, 2011	All Ages	8435
India: West Bengal, Urban	India SRS Special Bulletin on Maternal Mortality 2010-2012	Verbal Autopsy		2010	10 - 54 years	MMR
India: West Bengal, Urban	India SRS Maternal Mortality Ratio Bulletin 2011-2013	Verbal Autopsy		2011-2013	10 - 54 years	MMR
India: West Bengal, Urban	India Medical Certification of Cause of Death (MCCD)	Vital Registration		1999-2010	All Ages	124617
India: The Six Minor Territories, Rural	India District Level Household Survey (DLHS)	Sibling history		2001, 2006	10 - 54 years	63
India: The Six Minor Territories, Rural	India Survey of Causes of Death (SCD)	Verbal Autopsy		1980-1998	All Ages	5890
India: The Six Minor Territories, Rural	India Vital Statistics	Vital Registration		1983-1984, 1986-1995	All Ages	30705
India: The Six Minor Territories, Urban	India District Level Household Survey (DLHS)	Sibling history		2001, 2006	15 - 54 years	64
India: The Six Minor Territories, Urban	India Vital Statistics	Vital Registration		1980-1984, 1986-1995	All Ages	89408
India: The Six Minor Territories, Urban	India Medical Certification of Cause of Death (MCCD)	Vital Registration		1990-2010	All Ages	139322
Nepal	United Nations Surveys on Crime Trends and the Operations of Criminal Justice Systems 1970-2006	Police Records		1980-1986	All Ages	Death Rate
Nepal	United Nations Office on Drugs and Crime Global Study on Homicide 2011	Police Records	Nepal NSO	1997-2007, 2009	All Ages	Death Rate
Nepal	Nepal Demographic and Health Survey - Maternal Mortality Data	Sibling History		1992-2005	15 - 44 years	50
Nepal	Field test results of the motherhood method to measure maternal mortality	Sibling History	Bara District	2004	15 - 49 years	MMR
Nepal	Nepal Population and Housing Census 2011	Survey/Census		2011	15 - 49 years	9654
Nepal	Impact of a pilot acute respiratory infection (ARI) control programme in a rural community of the hill region of Nepal	Verbal Autopsy	Talku Dundcchaur Chhaimale in Kathmandu Valley [rural]	1984	birth - 4 years	64
Nepal	Reduction in total under-five mortality in western Nepal through community-based antimicrobial treatment of pneumonia	Verbal Autopsy	Jumla district [rural]	1987	birth - 4 years	2101
Nepal	A multicentre study of perinatal mortality in Nepal	Verbal Autopsy	Jumla district [rural]	1989	birth - 27 days	123
Nepal	A multicentre study of perinatal mortality in Nepal	Verbal Autopsy	Lalitpur district [rural]	1989	birth - 27 days	25
Nepal	Nepal Maternal Mortality and Morbidity Study 2008-2009	Verbal Autopsy	Kailali Rupandehi Okhaldhunga Surkhet Jumloa Baglung Rasuwa Sunsari [rural]	1998, 2008	15 - 49 years	2137
Nepal	Evaluation of neonatal verbal autopsy using physician review versus algorithm-based cause-of-death assignment in rural Nepal	Verbal Autopsy	Sarlahi district [rural]	1999	birth - 27 days	176

**Appendix Table 6: Causes of death data source list by geography, source type, and study site, with years, age range, and sample size, excluding sources on lower respiratory infection and diarrhoea etiologies**						
**Location**	**Source name**	**Source type**	**Site**	**Years**	**Ages**	**Sample size**
Nepal	Effect of daily zinc supplementation on child mortality in southern Nepal: a community-based, cluster randomised, placebo-controlled trial	Verbal Autopsy	Sarlahi district [rural]	2003	28 days - 4 years	333
Nepal	Verbal autopsy to ascertain causes of neonatal deaths in a community setting: a study from Morang, Nepal	Verbal Autopsy	Morang district [rural]	2005	birth - 27 days	183
Nepal	Demographic and Health Survey (DHS)	Verbal Autopsy	National	2006	birth - 4 years	474
Nepal	Maternal and infant mortality in Mahottari district of Nepal	Verbal Autopsy	11 VDCsin Mahottari District	2007	15 - 49 years	MMR
Pakistan	United Nations Surveys on Crime Trends and the Operations of Criminal Justice Systems 1970-2006	Police Records		1980	All Ages	Death Rate
Pakistan	United Nations Office on Drugs and Crime Global Study on Homicide 2011	Police Records	Pakistan NSO	1996-2007, 2009	All Ages	Death Rate
Pakistan	Maternal mortality in rural community: a challenge for achieving millennium development goal	Surveillance	Matiary - Sindh Province	2007	15 - 49 years	MMR
Pakistan	Pakistan Demographic Survey	Survey/Census	A10	1993-1994	All Ages	3394
Pakistan	World Health Survey (WHS)	Survey/Census		1997	All Ages	20
Pakistan	Early child health in Lahore, Pakistan: X. Mortality	Verbal Autopsy	Lahore [periurban]	1985	birth - 364 days	58
Pakistan	Early child health in Lahore, Pakistan: X. Mortality	Verbal Autopsy	Lahore [rural]	1985	birth - 364 days	61
Pakistan	Early child health in Lahore, Pakistan: X. Mortality	Verbal Autopsy	Lahore [urban]	1985	birth - 364 days	76
Pakistan	Acute respiratory infections in children: a case management intervention in Abbottabad District, Pakistan	Verbal Autopsy	Abbottabad district [rural]	1985-1986	birth - 4 years	93
Pakistan	Cause-specific child mortality in a mountainous community in Pakistan by verbal autopsy	Verbal Autopsy	Oshikhandass [rural]	1989	birth - 4 years	54
Pakistan	Maternal mortality in different Pakistani sites: ratios, clinical causes and determiniants	Verbal Autopsy	Balochistan (Pishin Loralai Lasbela Khuzdar)	1990	15 - 49 years	MMR
Pakistan	Time to focus child survival programmes on the newborn: assessment of levels and causes of infant mortality in rural Pakistan	Verbal Autopsy	Balochistan (Pishin Loralai Lasbela Khuzdar) [rural]	1990	birth - 364 days	522
Pakistan	Maternal mortality in different Pakistani sites: ratios, clinical causes and determiniants	Verbal Autopsy	Karachi [urban]	1990	15 - 49 years	MMR
Pakistan	Maternal mortality in different Pakistani sites: ratios, clinical causes and determiniants	Verbal Autopsy	North West Frontier Province (Abottabad Mansehra) [rural]	1990	15 - 49 years	MMR
Pakistan	Time to focus child survival programmes on the newborn: assessment of levels and causes of infant mortality in rural Pakistan	Verbal Autopsy	North-West Frontier Province (Dera Ismail Khan Peshawar Hazara) [rural]	1992	birth - 364 days	398
Pakistan	Time to focus child survival programmes on the newborn: assessment of levels and causes of infant mortality in rural Pakistan	Verbal Autopsy	Federally Administered Tribal Areas [rural]	1994	birth - 364 days	221
Pakistan	Neonatal mortality, risk factors and causes: a prospective population-based cohort study in urban Pakistan	Verbal Autopsy	Latifabad	2004	All Ages	53
Pakistan	Demographic and Health Survey (DHS)	Verbal Autopsy		2006	10 - 49 years	1062
Pakistan	Demographic and Health Survey (DHS)	Verbal Autopsy		2006	birth - 4 years	3100
Pakistan	Incidence of pneumonia, bacteremia, and invasive pneumococcal disease in Pakistani children	Verbal Autopsy	Sindh near Karachi	2007	birth - 4 years	54
Pakistan	To determine the probable causes of death in an urban slum community of Pakistan among adults 18 years and above by verbal autopsy	Verbal Autopsy	Nurpur Shahan Islamabad [urban]	2010	15 years +	300
Angola	United Nations Office on Drugs and Crime Global Study on Homicide 2011	Police Records	Angola Interpol	2004	All Ages	Death Rate
Central African Republic	Demographic and Health Survey (DHS)	Sibling History		1980-1994	15 - 39 years	33
Congo	Congo, Rep. Demographic and Health Survey - Maternal Mortality Data	Sibling History		1991-2011	15 - 39 years	31

Appendix Table 6: Causes of death data source list by geography, source type, and study site, with years, age range, and sample size, excluding sources on lower respiratory infection and diarrhoea etiologies

Location	Source name	Source type	Site	Years	Ages	Sample size
Congo	Mortality Data for Ebola Outbreaks 1994-2014	Surveillance		2005	All Ages	CF
Democratic Republic of the Congo	United Nations Office on Drugs and Crime Global Study on Homicide 2011	Police Records	Democratic Republic of the Congo UN-PKO	2004-2007, 2009-2010	All Ages	Death Rate
Democratic Republic of the Congo	Global Status Report on Road Safety 2009	Police Records		2007	All Ages	Death Rate
Democratic Republic of the Congo	Demographic and Health Survey (DHS)	Sibling History		1993-2013	15 - 39 years	29
Democratic Republic of the Congo	Mortality Data for Ebola Outbreaks 1994-2014	Surveillance		1995, 2007-2008, 2012, 2014	All Ages	CF
Democratic Republic of the Congo	Mortality in the Democratic Republic of Congo: a nationwide survey	Survey/Census	East	2000	birth - 4 years	198
Democratic Republic of the Congo	Mortality in the Democratic Republic of Congo: a nationwide survey	Survey/Census	West	2000	birth - 4 years	104
Democratic Republic of the Congo	Mortality in the Democratic Republic of Congo: a nationwide survey	Survey/Census	East	2003	All Ages	3169
Democratic Republic of the Congo	Mortality in the Democratic Republic of Congo: a nationwide survey	Survey/Census	West	2003	All Ages	827
Democratic Republic of the Congo	Etude de la mortalité globale et de la mortalité liée au paludisme dans le Kivu montagneux, Zaïre	Verbal Autopsy	Katana (Kivu Zaire) [rural]	1986	All Ages	1248
Democratic Republic of the Congo	Influence of nutritional status on child mortality in rural Zaire	Verbal Autopsy	Bwamanda Health zone [rural]	1990	birth - 4 years	246
Democratic Republic of the Congo	Incidence and duration of severe wasting in two African populations	Verbal Autopsy	Dwamanda [rural]	1990	28 days - 4 years	215
Gabon	Gabon Demographic and Health Survey - Maternal Mortality Data	Sibling History		1986-2012	15 - 39 years	5
Burundi	United Nations Surveys on Crime Trends and the Operations of Criminal Justice Systems 1970-2006	Police Records		1981-1982, 1984-1986	All Ages	Death Rate
Burundi	Demographic and Health Survey (DHS)	Sibling History		1996-2010	15 - 54 years	310
Burundi	Mortality and morbidity at young ages in a stable hyperendemic malaria region, community Nyanza-Lac, Imbo South, Burundi	Verbal Autopsy	Nyanza-Lac district Imbo Sud [rural]	1990	birth - 4 years	160
Comoros	Global Status Report on Road Safety 2009	Police Records		2007	All Ages	Death Rate
Comoros	Demographic and Health Surveys	Sibling History		1998-2012	15 - 34 years	12
Djibouti	Pan Arab Project for Family Health (PAPFAM)	Surveillance		2003	15 - 49 years	123
Eritrea	Eritrea Demographic and Health Survey - Maternal Mortality Data	Sibling History		1981-1995	15 - 39 years	20
Ethiopia	United Nations Office on Drugs and Crime Global Study on Homicide 2011	Police Records	Ethiopia Interpol	2004	All Ages	Death Rate
Ethiopia	Global Status Report on Road Safety 2009	Police Records		2006	All Ages	Death Rate
Ethiopia	Ethiopia Demographic and Health Survey - Maternal Mortality Data	Sibling History		1986-2010	15 - 54 years	1099
Ethiopia	High maternal mortality in rural south-west Ethiopia: estimate by using the sisterhood method	Sibling History	rural villages of Bonke in Gamo Gofa	2011	15 - 49 years	2552
Ethiopia	Maternal mortality in Addis Ababa, Ethiopia	Survey/Census	Addis Ababa [urban]	1982	15 - 49 years	MMR
Ethiopia	World Health Survey (WHS)	Survey/Census		1997	All Ages	5
Ethiopia	Ethiopia Population and Housing Census 2007	Survey/Census		2007	10 - 49 years	108296
Ethiopia	The Butajira rural health project in Ethiopia: mortality pattern of the under fives	Verbal Autopsy	Southern Shoa Administrative region Butajira district [rural]	1987	birth - 4 years	646
Ethiopia	Community based study on maternal mortality in Jimma town, south western Ethiopia	Verbal Autopsy	Jimma Town	1990	15 - 49 years	100
Ethiopia	Patterns of childhood mortality in three districts of north Gondar Administrative Zone. A community based study using the verbal autopsy method	Verbal Autopsy	Three Woredas of North Gondar [mixed]	1992	birth - 4 years	368

Appendix Table 6: Causes of death data source list by geography, source type, and study site, with years, age range, and sample size, excluding sources on lower respiratory infection and diarrhoea etiologies						
**Location**	**Source name**	**Source type**	**Site**	**Years**	**Ages**	**Sample size**
Ethiopia	The use of simplified verbal autopsy in identifying causes of adult death in a predominantly rural population in Ethiopia	Verbal Autopsy	Meskan and Mareko Butajira (BRPH-DSS) [rural]	1997	15 - 49 years	515
Ethiopia	Demographic Surveillance System (DSS)	Verbal Autopsy	Butajira	2000	All Ages	200
Ethiopia	La vigilancia de entierros detectó una reducción significativa en las muertes relacionadas con el VIH en Addis Ababa, Etiopía	Verbal Autopsy	Addis Ababa [urban]	2001, 2003	10 years +	990
Ethiopia	Determinants of under-five mortality in Gilgel Gibe Field Research Center, Southwest Ethiopia	Verbal Autopsy	Gilgel Gibe Field Research Center Soutwest Ethiopia	2004	birth - 4 years	168
Ethiopia	Ethiopia - Deployment of artemetherlumefantrine with rapid diagnostic tests at community level, Raya Valley, Tigray, Ethiopia	Verbal Autopsy	Alamata	2006	All Ages	991
Ethiopia	Ethiopia - Deployment of artemetherlumefantrine with rapid diagnostic tests at community level, Raya Valley, Tigray, Ethiopia	Verbal Autopsy	Rata Azebo	2006	All Ages	1106
Ethiopia	Ethiopia - Addis Ababa Mortality Surveillance Program Data 2006-2009	Verbal Autopsy	Addis Ababa [Urban]	2006-2009	10 years +	3708
Ethiopia	Factors associated with place of death in Addis Ababa, Ethiopia	Verbal Autopsy	Addis Ababa [urban]	2008	15 years +	4776
Ethiopia	Maternal and Neonatal Mortality in South-West Ethiopia: Estimates and Socio-Economic Inequality	Verbal Autopsy	Gamo Gofa [rural]	2010	15 - 44 years	220
Ethiopia	Emerging chronic non-communicable diseases in rural communities of Northern Ethiopia: evidence using population-based verbal autopsy method in Kilite Awlaelo surveillance site	Verbal Autopsy	Kilite Awlaelo Surveillance Site [rural]	2010	All Ages	403
Ethiopia	Demographic Surveillance System (DSS)	Verbal Autopsy		2010-2011	All Ages	559
Ethiopia	Determinants and Causes of Neonatal Mortality in Jimma Zone, Southwest Ethiopia: A Multilevel Analysis of Prospective Follow Up Study	Verbal Autopsy	Jimma Zone []	2012	birth - 364 days	99
Kenya: Baringo	Kenya Demographic and Health Survey - Maternal Mortality Data	Sibling History		1996	15 - 19 years	1
Kenya: Baringo	Kenya Demographic and Health Survey - Maternal Mortality Data	Sibling History		2002-2003	50 - 54 years	2
Kenya: Baringo	Kenya Demographic and Health Survey - Maternal Mortality Data	Sibling History		2005	35 - 39 years	1
Kenya: Baringo	Kenya Population and Housing Census 2009	Survey/Census		2009	All Ages	176
Kenya: Bomet	Kenya Demographic and Health Survey - Maternal Mortality Data	Sibling History		1989, 1993-2003	30 - 34 years	3
Kenya: Bomet	Kenya Demographic and Health Survey - Maternal Mortality Data	Sibling History		2006-2008	20 - 39 years	6
Kenya: Bomet	Kenya Population and Housing Census 2009	Survey/Census		2009	All Ages	256
Kenya: Bungoma	Kenya Demographic and Health Survey - Maternal Mortality Data	Sibling History		1985-2008	25 - 49 years	2
Kenya: Bungoma	Kenya Population and Housing Census 2009	Survey/Census		2009	All Ages	401
Kenya: Busia	Kenya Demographic and Health Survey - Maternal Mortality Data	Sibling History		1984-1985, 1987	15 - 19 years	2
Kenya: Busia	Kenya Demographic and Health Survey - Maternal Mortality Data	Sibling History		1991-2008	25 - 34 years	5
Kenya: Busia	Kenya Population and Housing Census 2009	Survey/Census		2009	All Ages	263
Kenya: Elgeyo-Marakwet	Kenya Demographic and Health Survey - Maternal Mortality Data	Sibling History		1987	30 - 34 years	1
Kenya: Elgeyo-Marakwet	Kenya Demographic and Health Survey - Maternal Mortality Data	Sibling History		1992	15 - 19 years	3

Appendix Table 6: Causes of death data source list by geography, source type, and study site, with years, age range, and sample size, excluding sources on lower respiratory infection and diarrhoea etiologies						
**Location**	**Source name**	**Source type**	**Site**	**Years**	**Ages**	**Sample size**
Kenya: Elgeyo-Marakwet	Kenya Demographic and Health Survey - Maternal Mortality Data	Sibling History		1997-2001, 2005	35 - 39 years	2
Kenya: Elgeyo-Marakwet	Kenya Demographic and Health Survey - Maternal Mortality Data	Sibling History		2007	20 - 44 years	2
Kenya: Elgeyo-Marakwet	Kenya Population and Housing Census 2009	Survey/Census		2009	All Ages	89
Kenya: Embu	Kenya Demographic and Health Survey - Maternal Mortality Data	Sibling History		1989	15 - 19 years	2
Kenya: Embu	Kenya Demographic and Health Survey - Maternal Mortality Data	Sibling History		1993-2005	30 - 34 years	7
Kenya: Embu	Kenya Demographic and Health Survey - Maternal Mortality Data	Sibling History		2007	20 - 49 years	2
Kenya: Embu	Kenya Population and Housing Census 2009	Survey/Census		2009	All Ages	131
Kenya: Garissa	Kenya Demographic and Health Survey - Maternal Mortality Data	Sibling History		1989-1990	25 - 44 years	4
Kenya: Garissa	Kenya Demographic and Health Survey - Maternal Mortality Data	Sibling History		1992-2004	15 - 24 years	2
Kenya: Garissa	Kenya Demographic and Health Survey - Maternal Mortality Data	Sibling History		2007-2008	40 - 44 years	1
Kenya: Garissa	Kenya Population and Housing Census 2009	Survey/Census		2009	All Ages	215
Kenya: HomaBay	Kenya Demographic and Health Survey - Maternal Mortality Data	Sibling History		1990	20 - 24 years	1
Kenya: HomaBay	Kenya Demographic and Health Survey - Maternal Mortality Data	Sibling History		1993-1995	45 - 49 years	1
Kenya: HomaBay	Kenya Demographic and Health Survey - Maternal Mortality Data	Sibling History		1997-2008	15 - 39 years	5
Kenya: HomaBay	Kenya Population and Housing Census 2009	Survey/Census		2009	All Ages	499
Kenya: Isiolo	Kenya Demographic and Health Survey - Maternal Mortality Data	Sibling History		1998-2001	25 - 49 years	2
Kenya: Isiolo	Kenya Demographic and Health Survey - Maternal Mortality Data	Sibling History		2003	40 - 44 years	1
Kenya: Isiolo	Kenya Demographic and Health Survey - Maternal Mortality Data	Sibling History		2006-2008	40 - 49 years	2
Kenya: Isiolo	Kenya Population and Housing Census 2009	Survey/Census		2009	All Ages	38
Kenya: Kajiado	Kenya Demographic and Health Survey - Maternal Mortality Data	Sibling History		1985	15 - 19 years	1
Kenya: Kajiado	Kenya Demographic and Health Survey - Maternal Mortality Data	Sibling History		1991, 1993	30 - 34 years	3
Kenya: Kajiado	Kenya Demographic and Health Survey - Maternal Mortality Data	Sibling History		1996-2006	25 - 29 years	2
Kenya: Kajiado	Kenya Population and Housing Census 2009	Survey/Census		2009	All Ages	185
Kenya: Kakamega	Kenya Demographic and Health Survey - Maternal Mortality Data	Sibling History		1984-1985	25 - 29 years	4
Kenya: Kakamega	Kenya Demographic and Health Survey - Maternal Mortality Data	Sibling History		1987-2008	15 - 39 years	6
Kenya: Kakamega	Kenya Population and Housing Census 2009	Survey/Census		2009	All Ages	540
Kenya: Kericho	Kenya Demographic and Health Survey - Maternal Mortality Data	Sibling History		1984-1986	20 - 54 years	2
Kenya: Kericho	Kenya Demographic and Health Survey - Maternal Mortality Data	Sibling History		1989-2001	15 - 59 years	5
Kenya: Kericho	Kenya Demographic and Health Survey - Maternal Mortality Data	Sibling History		2003-2006	30 - 34 years	7
Kenya: Kericho	Kenya Demographic and Health Survey - Maternal Mortality Data	Sibling History		2008	45 - 49 years	1

Appendix Table 6: Causes of death data source list by geography, source type, and study site, with years, age range, and sample size, excluding sources on lower respiratory infection and diarrhoea etiologies						
**Location**	**Source name**	**Source type**	**Site**	**Years**	**Ages**	**Sample size**
Kenya: Kericho	Kenya Population and Housing Census 2009	Survey/Census		2009	All Ages	173
Kenya: Kericho	The burden of malaria mortality among African children in the year 2000	Verbal Autopsy	Brookebond Tea Estate	1997	birth - 4 years	325
Kenya: Kiambu	Kenya Demographic and Health Survey - Maternal Mortality Data	Sibling History		1987	40 - 44 years	3
Kenya: Kiambu	Kenya Demographic and Health Survey - Maternal Mortality Data	Sibling History		1990	20 - 39 years	12
Kenya: Kiambu	Kenya Demographic and Health Survey - Maternal Mortality Data	Sibling History		1992-2008	35 - 39 years	4
Kenya: Kiambu	Kenya Population and Housing Census 2009	Survey/Census		2009	All Ages	397
Kenya: Kilifi	Kenya Demographic and Health Survey - Maternal Mortality Data	Sibling History		1987-1989	15 - 19 years	1
Kenya: Kilifi	Kenya Demographic and Health Survey - Maternal Mortality Data	Sibling History		1991-2004	15 - 49 years	18
Kenya: Kilifi	Kenya Demographic and Health Survey - Maternal Mortality Data	Sibling History		2006-2008	20 - 39 years	5
Kenya: Kilifi	Kenya Population and Housing Census 2009	Survey/Census		2009	All Ages	291
Kenya: Kilifi	Demographic Surveillance System (DSS)	Verbal Autopsy		2008-2011	All Ages	4448
Kenya: Kirinyaga	Kenya Demographic and Health Survey - Maternal Mortality Data	Sibling History		1989	25 - 29 years	1
Kenya: Kirinyaga	Kenya Demographic and Health Survey - Maternal Mortality Data	Sibling History		1991-1993	20 - 24 years	2
Kenya: Kirinyaga	Kenya Demographic and Health Survey - Maternal Mortality Data	Sibling History		1995-2008	30 - 39 years	5
Kenya: Kirinyaga	Kenya Population and Housing Census 2009	Survey/Census		2009	All Ages	170
Kenya: Kisii	Kenya Demographic and Health Survey - Maternal Mortality Data	Sibling History		1984-2008	15 - 44 years	27
Kenya: Kisii	Kenya Population and Housing Census 2009	Survey/Census		2009	All Ages	371
Kenya: Kisumu	Kenya Demographic and Health Survey - Maternal Mortality Data	Sibling History		1984	20 - 24 years	2
Kenya: Kisumu	Kenya Demographic and Health Survey - Maternal Mortality Data	Sibling History		1986-1987	15 - 34 years	9
Kenya: Kisumu	Kenya Demographic and Health Survey - Maternal Mortality Data	Sibling History		1989-2008	30 - 34 years	1
Kenya: Kisumu	Kenya Population and Housing Census 2009	Survey/Census		2009	All Ages	534
Kenya: Kisumu	Demographic Surveillance System (DSS)	Verbal Autopsy		2002-2010	birth - 4 years	935
Kenya: Kitui	Kenya Demographic and Health Survey - Maternal Mortality Data	Sibling History		1990	30 - 34 years	1
Kenya: Kitui	Kenya Demographic and Health Survey - Maternal Mortality Data	Sibling History		1992-2004	15 - 19 years	1
Kenya: Kitui	Kenya Demographic and Health Survey - Maternal Mortality Data	Sibling History		2006-2007	35 - 39 years	1
Kenya: Kitui	Kenya Population and Housing Census 2009	Survey/Census		2009	All Ages	333
Kenya: Kwale	Kenya Demographic and Health Survey - Maternal Mortality Data	Sibling History		1984	15 - 19 years	4
Kenya: Kwale	Kenya Demographic and Health Survey - Maternal Mortality Data	Sibling History		1987	40 - 44 years	1
Kenya: Kwale	Kenya Demographic and Health Survey - Maternal Mortality Data	Sibling History		1990-1992	25 - 29 years	1
Kenya: Kwale	Kenya Demographic and Health Survey - Maternal Mortality Data	Sibling History		1994-2007	15 - 34 years	10
Kenya: Kwale	Kenya Population and Housing Census 2009	Survey/Census		2009	All Ages	170

Appendix Table 6: Causes of death data source list by geography, source type, and study site, with years, age range, and sample size, excluding sources on lower respiratory infection and diarrhoea etiologies						
**Location**	**Source name**	**Source type**	**Site**	**Years**	**Ages**	**Sample size**
Kenya: Laikipia	Kenya Demographic and Health Survey - Maternal Mortality Data	Sibling History		1989	20 - 24 years	1
Kenya: Laikipia	Kenya Demographic and Health Survey - Maternal Mortality Data	Sibling History		1992-1994	20 - 39 years	2
Kenya: Laikipia	Kenya Demographic and Health Survey - Maternal Mortality Data	Sibling History		1997	15 - 44 years	2
Kenya: Laikipia	Kenya Demographic and Health Survey - Maternal Mortality Data	Sibling History		1999	15 - 19 years	2
Kenya: Laikipia	Kenya Demographic and Health Survey - Maternal Mortality Data	Sibling History		2002-2006	40 - 44 years	2
Kenya: Laikipia	Kenya Population and Housing Census 2009	Survey/Census		2009	All Ages	112
Kenya: Lamu	Kenya Demographic and Health Survey - Maternal Mortality Data	Sibling History		1994	25 - 29 years	3
Kenya: Lamu	Kenya Demographic and Health Survey - Maternal Mortality Data	Sibling History		1997-1998	25 - 39 years	2
Kenya: Lamu	Kenya Demographic and Health Survey - Maternal Mortality Data	Sibling History		2001-2005	35 - 39 years	2
Kenya: Lamu	Kenya Demographic and Health Survey - Maternal Mortality Data	Sibling History		2007	20 - 24 years	2
Kenya: Lamu	Kenya Population and Housing Census 2009	Survey/Census		2009	All Ages	26
Kenya: Machakos	Kenya Demographic and Health Survey - Maternal Mortality Data	Sibling History		1984-1985	25 - 29 years	1
Kenya: Machakos	Kenya Demographic and Health Survey - Maternal Mortality Data	Sibling History		1987-1999	15 - 34 years	4
Kenya: Machakos	Kenya Demographic and Health Survey - Maternal Mortality Data	Sibling History		2001-2007	15 - 44 years	16
Kenya: Machakos	Kenya Population and Housing Census 2009	Survey/Census		2009	All Ages	383
Kenya: Makueni	Kenya Demographic and Health Survey - Maternal Mortality Data	Sibling History		1989	35 - 39 years	2
Kenya: Makueni	Kenya Demographic and Health Survey - Maternal Mortality Data	Sibling History		1991	25 - 29 years	1
Kenya: Makueni	Kenya Demographic and Health Survey - Maternal Mortality Data	Sibling History		1993	15 - 19 years	1
Kenya: Makueni	Kenya Demographic and Health Survey - Maternal Mortality Data	Sibling History		1997-2002	15 - 29 years	3
Kenya: Makueni	Kenya Demographic and Health Survey - Maternal Mortality Data	Sibling History		2006-2008	30 - 34 years	1
Kenya: Makueni	Kenya Population and Housing Census 2009	Survey/Census		2009	All Ages	288
Kenya: Mandera	Kenya Demographic and Health Survey - Maternal Mortality Data	Sibling History		1990	30 - 34 years	1
Kenya: Mandera	Kenya Demographic and Health Survey - Maternal Mortality Data	Sibling History		1996	15 - 29 years	2
Kenya: Mandera	Kenya Demographic and Health Survey - Maternal Mortality Data	Sibling History		1998-2008	25 - 39 years	6
Kenya: Mandera	Kenya Population and Housing Census 2009	Survey/Census		2009	All Ages	460
Kenya: Marsabit	Kenya Demographic and Health Survey - Maternal Mortality Data	Sibling History		1991	30 - 34 years	1
Kenya: Marsabit	Kenya Demographic and Health Survey - Maternal Mortality Data	Sibling History		2000	20 - 34 years	2
Kenya: Marsabit	Kenya Demographic and Health Survey - Maternal Mortality Data	Sibling History		2002	25 - 29 years	1
Kenya: Marsabit	Kenya Demographic and Health Survey - Maternal Mortality Data	Sibling History		2005-2006	20 - 24 years	1

Appendix Table 6: Causes of death data source list by geography, source type, and study site, with years, age range, and sample size, excluding sources on lower respiratory infection and diarrhoea etiologies						
**Location**	**Source name**	**Source type**	**Site**	**Years**	**Ages**	**Sample size**
Kenya: Marsabit	Kenya Demographic and Health Survey - Maternal Mortality Data	Sibling History		2008	15 - 19 years	1
Kenya: Marsabit	Kenya Population and Housing Census 2009	Survey/Census		2009	All Ages	91
Kenya: Meru	Kenya Demographic and Health Survey - Maternal Mortality Data	Sibling History		1985	15 - 29 years	5
Kenya: Meru	Kenya Demographic and Health Survey - Maternal Mortality Data	Sibling History		1987-2008	20 - 24 years	7
Kenya: Meru	Kenya Population and Housing Census 2009	Survey/Census		2009	All Ages	382
Kenya: Migori	Kenya Demographic and Health Survey - Maternal Mortality Data	Sibling History		1989	15 - 29 years	7
Kenya: Migori	Kenya Demographic and Health Survey - Maternal Mortality Data	Sibling History		1991-1992	25 - 29 years	1
Kenya: Migori	Kenya Demographic and Health Survey - Maternal Mortality Data	Sibling History		1994-2008	25 - 34 years	2
Kenya: Migori	Kenya Population and Housing Census 2009	Survey/Census		2009	All Ages	551
Kenya: Mombasa	Kenya Demographic and Health Survey - Maternal Mortality Data	Sibling History		1986-1987	15 - 39 years	19
Kenya: Mombasa	Kenya Demographic and Health Survey - Maternal Mortality Data	Sibling History		1989	20 - 54 years	3
Kenya: Mombasa	Kenya Demographic and Health Survey - Maternal Mortality Data	Sibling History		1991-2003	20 - 29 years	2
Kenya: Mombasa	Kenya Demographic and Health Survey - Maternal Mortality Data	Sibling History		2005-2007	35 - 59 years	3
Kenya: Mombasa	Kenya Population and Housing Census 2009	Survey/Census		2009	All Ages	248
Kenya: Murang’a	Kenya Demographic and Health Survey - Maternal Mortality Data	Sibling History		1984-1987	20 - 24 years	1
Kenya: Murang’a	Kenya Demographic and Health Survey - Maternal Mortality Data	Sibling History		1989-1990, 2008	30 - 34 years	4
Kenya: Murang’a	Kenya Demographic and Health Survey - Maternal Mortality Data	Sibling History		1992	20 - 39 years	18
Kenya: Murang’a	Kenya Demographic and Health Survey - Maternal Mortality Data	Sibling History		1994-2006	25 - 54 years	4
Kenya: Murang’a	Kenya Population and Housing Census 2009	Survey/Census		2009	All Ages	272
Kenya: Murang’a	Mortality patterns in a rural Kenyan community	Verbal Autopsy	Nginda Mathithi and Kamahuha Muranga district [rural]	1986	birth - 4 years	216
Kenya: Nairobi	Kenya Demographic and Health Survey - Maternal Mortality Data	Sibling History		1985-1987	15 - 19 years	1
Kenya: Nairobi	Kenya Demographic and Health Survey - Maternal Mortality Data	Sibling History		1989-2008	15 - 39 years	4
Kenya: Nairobi	Kenya Population and Housing Census 2009	Survey/Census		2009	All Ages	899
Kenya: Nairobi	Demographic Surveillance System (DSS)	Verbal Autopsy		2002-2012	All Ages	6840
Kenya: Nairobi	Verbal autopsy interpretation: a comparative analysis of the InterVA model versus physician review in determining causes of death in the Nairobi DSS	Verbal Autopsy		2005	All Ages	572
Kenya: Nakuru	Kenya Demographic and Health Survey - Maternal Mortality Data	Sibling History		1987	30 - 39 years	2
Kenya: Nakuru	Kenya Demographic and Health Survey - Maternal Mortality Data	Sibling History		1989-2006	25 - 29 years	2
Kenya: Nakuru	Kenya Population and Housing Census 2009	Survey/Census		2009	All Ages	544
Kenya: Nandi	Kenya Demographic and Health Survey - Maternal Mortality Data	Sibling History		1986-1989	35 - 39 years	2
Kenya: Nandi	Kenya Demographic and Health Survey - Maternal Mortality Data	Sibling History		1991-2005	15 - 29 years	5

Appendix Table 6: Causes of death data source list by geography, source type, and study site, with years, age range, and sample size, excluding sources on lower respiratory infection and diarrhoea etiologies						
Location	Source name	Source type	Site	Years	Ages	Sample size
Kenya: Nandi	Kenya Demographic and Health Survey - Maternal Mortality Data	Sibling History		2007	55 - 59 years	1
Kenya: Nandi	Kenya Population and Housing Census 2009	Survey/Census		2009	All Ages	204
Kenya: Narok	Kenya Demographic and Health Survey - Maternal Mortality Data	Sibling History		1987	40 - 44 years	2
Kenya: Narok	Kenya Demographic and Health Survey - Maternal Mortality Data	Sibling History		1994-1995	20 - 24 years	1
Kenya: Narok	Kenya Demographic and Health Survey - Maternal Mortality Data	Sibling History		1998-1999	15 - 39 years	2
Kenya: Narok	Kenya Demographic and Health Survey - Maternal Mortality Data	Sibling History		2002-2006	30 - 39 years	6
Kenya: Narok	Kenya Population and Housing Census 2009	Survey/Census		2009	All Ages	237
Kenya: Nyamira	Kenya Demographic and Health Survey - Maternal Mortality Data	Sibling History		1986	20 - 39 years	5
Kenya: Nyamira	Kenya Demographic and Health Survey - Maternal Mortality Data	Sibling History		1988-1991	15 - 19 years	1
Kenya: Nyamira	Kenya Demographic and Health Survey - Maternal Mortality Data	Sibling History		1993-2004	15 - 34 years	5
Kenya: Nyamira	Kenya Demographic and Health Survey - Maternal Mortality Data	Sibling History		2006-2008	30 - 39 years	2
Kenya: Nyamira	Kenya Population and Housing Census 2009	Survey/Census		2009	All Ages	178
Kenya: Nyandarua	Kenya Demographic and Health Survey - Maternal Mortality Data	Sibling History		1989-1990	20 - 24 years	1
Kenya: Nyandarua	Kenya Demographic and Health Survey - Maternal Mortality Data	Sibling History		1992-1993	25 - 29 years	4
Kenya: Nyandarua	Kenya Demographic and Health Survey - Maternal Mortality Data	Sibling History		1996, 1998-1999	20 - 39 years	5
Kenya: Nyandarua	Kenya Demographic and Health Survey - Maternal Mortality Data	Sibling History		2001-2004	25 - 39 years	7
Kenya: Nyandarua	Kenya Demographic and Health Survey - Maternal Mortality Data	Sibling History		2007-2008	35 - 39 years	1
Kenya: Nyandarua	Kenya Population and Housing Census 2009	Survey/Census		2009	All Ages	180
Kenya: Nyeri	Kenya Demographic and Health Survey - Maternal Mortality Data	Sibling History		1984-1987	30 - 34 years	2
Kenya: Nyeri	Kenya Demographic and Health Survey - Maternal Mortality Data	Sibling History		1989-2007	15 - 19 years	3
Kenya: Nyeri	Kenya Population and Housing Census 2009	Survey/Census		2009	All Ages	250
Kenya: Samburu	Kenya Demographic and Health Survey - Maternal Mortality Data	Sibling History		1992-1993, 2003-2007	20 - 24 years	3
Kenya: Samburu	Kenya Demographic and Health Survey - Maternal Mortality Data	Sibling History		1996	25 - 29 years	1
Kenya: Samburu	Kenya Demographic and Health Survey - Maternal Mortality Data	Sibling History		1998-2001	30 - 34 years	1
Kenya: Samburu	Kenya Population and Housing Census 2009	Survey/Census		2009	All Ages	93
Kenya: Siaya	Kenya Demographic and Health Survey - Maternal Mortality Data	Sibling History		1986	25 - 29 years	1
Kenya: Siaya	Kenya Demographic and Health Survey - Maternal Mortality Data	Sibling History		1988-1990	15 - 34 years	15
Kenya: Siaya	Kenya Demographic and Health Survey - Maternal Mortality Data	Sibling History		1992-2008	15 - 39 years	4
Kenya: Siaya	Kenya Population and Housing Census 2009	Survey/Census		2009	All Ages	633
Kenya: Siaya	A reversal in reductions of child mortality in western Kenya, 2003-2009	Verbal Autopsy	Nyanza Province Asembo and Gem (2003) [rural]	2003	28 days - 4 years	1234

Appendix Table 6: Causes of death data source list by geography, source type, and study site, with years, age range, and sample size, excluding sources on lower respiratory infection and diarrhoea etiologies						
**Location**	**Source name**	**Source type**	**Site**	**Years**	**Ages**	**Sample size**
Kenya: Siaya	Causes of deaths using verbal autopsy among adolescents and adults in rural western Kenya	Verbal Autopsy	Nyanza Province Asembo and Gem [rural]	2003	10 - 79 years	1816
Kenya: Siaya	A reversal in reductions of child mortality in western Kenya, 2003-2009	Verbal Autopsy	Nyanza Province Asembo and Gem (2004) [rural]	2004	28 days - 4 years	1203
Kenya: Siaya	A reversal in reductions of child mortality in western Kenya, 2003-2009	Verbal Autopsy	Nyanza Province Asembo and Gem (2005) [rural]	2005	28 days - 4 years	1045
Kenya: Siaya	A reversal in reductions of child mortality in western Kenya, 2003-2009	Verbal Autopsy	Nyanza Province Asembo and Gem (2006) [rural]	2006	28 days - 4 years	873
Kenya: Siaya	A reversal in reductions of child mortality in western Kenya, 2003-2009	Verbal Autopsy	Nyanza Province Asembo and Gem (2007) [rural]	2007	28 days - 4 years	865
Kenya: Siaya	A reversal in reductions of child mortality in western Kenya, 2003-2009	Verbal Autopsy	Nyanza Province Asembo and Gem (2008) [rural]	2008	28 days - 4 years	1702
Kenya: TaitaTaveta	Kenya Demographic and Health Survey - Maternal Mortality Data	Sibling History		1987-1990	25 - 29 years	1
Kenya: TaitaTaveta	Kenya Demographic and Health Survey - Maternal Mortality Data	Sibling History		1992-2008	15 - 34 years	11
Kenya: TaitaTaveta	Kenya Population and Housing Census 2009	Survey/Census		2009	All Ages	84
Kenya: TanaRiver	Kenya Demographic and Health Survey - Maternal Mortality Data	Sibling History		1989	15 - 19 years	1
Kenya: TanaRiver	Kenya Demographic and Health Survey - Maternal Mortality Data	Sibling History		1998	25 - 49 years	3
Kenya: TanaRiver	Kenya Demographic and Health Survey - Maternal Mortality Data	Sibling History		2000	35 - 39 years	1
Kenya: TanaRiver	Kenya Demographic and Health Survey - Maternal Mortality Data	Sibling History		2002-2007	30 - 34 years	1
Kenya: TanaRiver	Kenya Population and Housing Census 2009	Survey/Census		2009	All Ages	74
Kenya: TharakaNithi	Kenya Demographic and Health Survey - Maternal Mortality Data	Sibling History		2002-2003	25 - 29 years	1
Kenya: TharakaNithi	Kenya Demographic and Health Survey - Maternal Mortality Data	Sibling History		2006-2007	45 - 49 years	1
Kenya: TharakaNithi	Kenya Population and Housing Census 2009	Survey/Census		2009	All Ages	117
Kenya: TransNzoia	Kenya Demographic and Health Survey - Maternal Mortality Data	Sibling History		1987	15 - 19 years	1
Kenya: TransNzoia	Kenya Demographic and Health Survey - Maternal Mortality Data	Sibling History		1994-2005	20 - 34 years	4
Kenya: TransNzoia	Kenya Demographic and Health Survey - Maternal Mortality Data	Sibling History		2007-2008	40 - 44 years	1
Kenya: TransNzoia	Kenya Population and Housing Census 2009	Survey/Census		2009	All Ages	262
Kenya: Turkana	Kenya Demographic and Health Survey - Maternal Mortality Data	Sibling History		1992, 1998	15 - 19 years	4
Kenya: Turkana	Kenya Demographic and Health Survey - Maternal Mortality Data	Sibling History		1995-1996	35 - 39 years	1
Kenya: Turkana	Kenya Demographic and Health Survey - Maternal Mortality Data	Sibling History		2003	25 - 44 years	3
Kenya: Turkana	Kenya Demographic and Health Survey - Maternal Mortality Data	Sibling History		2005-2006	20 - 44 years	2
Kenya: Turkana	Kenya Population and Housing Census 2009	Survey/Census		2009	All Ages	518
Kenya: UasinGishu	Kenya Demographic and Health Survey - Maternal Mortality Data	Sibling History		1985-1987	30 - 34 years	3
Kenya: UasinGishu	Kenya Demographic and Health Survey - Maternal Mortality Data	Sibling History		1990-2008	25 - 34 years	4
Kenya: UasinGishu	Kenya Population and Housing Census 2009	Survey/Census		2009	All Ages	270

Appendix Table 6: Causes of death data source list by geography, source type, and study site, with years, age range, and sample size, excluding sources on lower respiratory infection and diarrhoea etiologies

Location	Source name	Source type	Site	Years	Ages	Sample size
Kenya: Vihiga	Kenya Demographic and Health Survey - Maternal Mortality Data	Sibling History		1992-1996	30 - 34 years	3
Kenya: Vihiga	Kenya Demographic and Health Survey - Maternal Mortality Data	Sibling History		1999-2003	30 - 39 years	2
Kenya: Vihiga	Kenya Demographic and Health Survey - Maternal Mortality Data	Sibling History		2005	40 - 44 years	2
Kenya: Vihiga	Kenya Demographic and Health Survey - Maternal Mortality Data	Sibling History		2007-2008	45 - 49 years	1
Kenya: Vihiga	Kenya Population and Housing Census 2009	Survey/Census		2009	All Ages	228
Kenya: Wajir	Kenya Demographic and Health Survey - Maternal Mortality Data	Sibling History		1990-2005	15 - 24 years	2
Kenya: Wajir	Kenya Population and Housing Census 2009	Survey/Census		2009	All Ages	194
Kenya: WestPokot	Kenya Demographic and Health Survey - Maternal Mortality Data	Sibling History		1991, 1993, 2000-2002	25 - 29 years	3
Kenya: WestPokot	Kenya Demographic and Health Survey - Maternal Mortality Data	Sibling History		1997, 2005	20 - 24 years	4
Kenya: WestPokot	Kenya Population and Housing Census 2009	Survey/Census		2009	All Ages	310
Madagascar	United Nations Surveys on Crime Trends and the Operations of Criminal Justice Systems 1970-2006	Police Records		1980, 1985-1994	All Ages	Death Rate
Madagascar	United Nations Office on Drugs and Crime Global Study on Homicide 2011	Police Records	Madagascar UNU	2000	All Ages	Death Rate
Madagascar	Madagascar Demographic and Health Survey - Maternal Mortality Data	Sibling History		1980-2008	15 - 49 years	76
Madagascar	Madagascar - Antananorivo Mortality Report 1984-1995	Vital Registration	Antananarivo	1984-1995	All Ages	79862
Malawi	United Nations Surveys on Crime Trends and the Operations of Criminal Justice Systems 1970-2006	Police Records		1981-1985	All Ages	Death Rate
Malawi	Malawi Demographic and Health Survey - Maternal Mortality Data	Sibling History		1980-2010	15 - 39 years	16
Malawi	International Integrated Public Use Microdata Series (IPUMS)	Survey/Census		2008	15 - 49 years	2306
Malawi	Malawi Population and Housing Census 2008	Survey/Census		2008	10 - 49 years	24826
Malawi	Malawi Population and Housing Census 2008	Survey/Census		2008	All Ages	14871
Malawi	Infant and second-year mortality in rural Malawi: causes and descriptive epidemiology	Verbal Autopsy	Mangochi Malaria Research Project [rural]	1988	birth - 4 years	683
Malawi	Estimation of AIDS adult mortality by verbal autopsy in rural Malawi	Verbal Autopsy	three rural areas [rural]	1999	15 - 44 years	100
Malawi	Demographic Surveillance System (DSS)	Verbal Autopsy		2003-2011	All Ages	2063
Malawi	Declining child mortality in northern Malawi despite high rates of infection with HIV	Verbal Autopsy	Karonga district [rural]	2004	birth - 14 years	342
Malawi	Adult mortality and probable cause of death in rural northern Malawi in the era of HIV treatment	Verbal Autopsy	Karonga district [rural]	2004-2008	15 years +	877
Malawi	Rates and causes of death in Chiradzulu District, Malawi, 2008: a key informant study	Verbal Autopsy	Chiradzulu District [rural]	2008	All Ages	50
Malawi	Measuring causes of adult mortality in rural northern Malawi over a decade of change	Verbal Autopsy	Karonga District [rural]	2009-2012	15 - 64 years	538
Mozambique	United Nations Office on Drugs and Crime Global Study on Homicide 2011	Police Records	Mozambique UN-CASA	1999-2003	All Ages	Death Rate
Mozambique	Global Status Report on Road Safety 2009	Police Records		2007	All Ages	Death Rate
Mozambique	United Nations Office on Drugs and Crime Global Study on Homicide 2011	Police Records	Mozambique GBI	2007	All Ages	Death Rate
Mozambique	Mozambique Demographic and Health Survey - Maternal Mortality Data	Sibling History		1983-2011	15 - 39 years	45

Appendix Table 6: Causes of death data source list by geography, source type, and study site, with years, age range, and sample size, excluding sources on lower respiratory infection and diarrhoea etiologies						
**Location**	**Source name**	**Source type**	**Site**	**Years**	**Ages**	**Sample size**
Mozambique	Mozambique Census 2007	Survey/Census		2002	10 - 49 years	53567
Mozambique	Mortality rates, prevalence of malnutrition, and prevalence of lost pregnancies among the drought-ravaged population of Tete Province, Mozambique	Survey/Census	Cahora Bassa Changara Districts Tete Province [rural]	2003	All Ages	214
Mozambique	Quality of registration of maternal deaths in Mozambique: a community-based study in rural and urban areas	Verbal Autopsy	Chingussura sub-center Beira city Sofala Province [periurban]	1996	15 - 49 years	22
Mozambique	Quality of registration of maternal deaths in Mozambique: a community-based study in rural and urban areas	Verbal Autopsy	Dondo sub-center Dondo district Sofala Province	1996	15 - 49 years	54
Mozambique	Quality of registration of maternal deaths in Mozambique: a community-based study in rural and urban areas	Verbal Autopsy	Mafambisse sub-center Dondo district Sofala Province	1996	15 - 49 years	38
Mozambique	Quality of registration of maternal deaths in Mozambique: a community-based study in rural and urban areas	Verbal Autopsy	Manga sub-center Beira city Sofala Province [periurban]	1996	15 - 49 years	17
Mozambique	Quality of registration of maternal deaths in Mozambique: a community-based study in rural and urban areas	Verbal Autopsy	Metuchira sub-center Nhamatanda District Sofala province	1996	15 - 49 years	21
Mozambique	Quality of registration of maternal deaths in Mozambique: a community-based study in rural and urban areas	Verbal Autopsy	Nhamatanda sub-center Nhamatanda district Sofala province	1996	15 - 49 years	32
Mozambique	Quality of registration of maternal deaths in Mozambique: a community-based study in rural and urban areas	Verbal Autopsy	Ponta-Gea and Baxia [urban]	1996	15 - 49 years	18
Mozambique	Quality of registration of maternal deaths in Mozambique: a community-based study in rural and urban areas	Verbal Autopsy	Siluvo sub-center Nhamatanda district Sofala province	1996	15 - 49 years	12
Mozambique	A 10 year study of the cause of death in children under 15 years in Manhiça, Mozambique	Verbal Autopsy	Manhica District [rural]	2001	birth - 14 years	3696
Mozambique	Demographic Surveillance System (DSS)	Verbal Autopsy	Manhica	2002	All Ages	1248
Mozambique	Mozambique National Survey on the Causes of Death 2007-2008	Verbal Autopsy		2007	All Ages	10080
Mozambique	Mozambique Main Causes of Reported Death Study 2001	Vital Registration	Maputo Beira Chimoio Nampula	2001	All Ages	22865
Rwanda	United Nations Surveys on Crime Trends and the Operations of Criminal Justice Systems 1970-2006	Police Records		1986-1990	All Ages	Death Rate
Rwanda	United Nations Office on Drugs and Crime Global Study on Homicide 2011	Police Records	Rwanda National police	2003-2007	All Ages	Death Rate
Rwanda	Global Status Report on Road Safety 2009	Police Records		2007	All Ages	Death Rate
Rwanda	Rwanda Demographic and Health Survey - Maternal Mortality Data	Sibling History		1986-2010	15 - 39 years	21
South Sudan	Mortality Data for Ebola Outbreaks 1994-2014	Surveillance		2004	All Ages	CF
South Sudan	International Integrated Public Use Microdata Series (IPUMS)	Survey/Census		2008	1 year +	23488
South Sudan	Sudan Population and Housing Census 2008	Survey/Census	South Sudan	2008	All Ages	11755
Tanzania	United Nations Office on Drugs and Crime Global Study on Homicide 2011	Police Records	Tanzania Interpol	2003-2004	All Ages	Death Rate
Tanzania	Tanzania Demographic and Health Survey - Maternal Mortality Data	Sibling History		1982-2009	15 - 44 years	36
Tanzania	Causes and risk factors for maternal mortality in rural Tanzania--case of Rufiji Health and Demographic Surveillance Site (HDSS)	Surveillance	Rufiji	2004	15 - 49 years	767
Tanzania	Risk factors for deaths in children under 5 years old in Bagamoyo district, Tanzania	Verbal Autopsy	Bagamoyo District [rural]	1986	birth - 4 years	610
Tanzania	Mortality of under-fives in a rural area of holoendemic malaria transmission	Verbal Autopsy	Muheza District (Mkusi Kilulu Enzi Tingeni Lunguza) [rural]	1992	7 days - 4 years	83
Tanzania	Retrospective follow-up of maternal deaths and their associated risk factors in a rural district of Tanzania	Verbal Autopsy	Bagamoyo District [rural]	1993	15 - 49 years	MMR

Appendix Table 6: Causes of death data source list by geography, source type, and study site, with years, age range, and sample size, excluding sources on lower respiratory infection and diarrhoea etiologies						
**Location**	**Source name**	**Source type**	**Site**	**Years**	**Ages**	**Sample size**
Tanzania	Community based studies on childhood mortality in a malaria holoendemic area on the Tanzanian coast	Verbal Autopsy	Bagamoyo District [rural]	1993	birth - 4 years	118
Tanzania	Avoidable stillbirths and neonatal deaths in rural Tanzania	Verbal Autopsy	Mbulu and Hanang districts [rural]	1995	birth - 27 days	76
Tanzania	The Policy Implications of Tanzania's Mortality Burden	Verbal Autopsy	Dar	1995, 1998, 2001	All Ages	7219
Tanzania	The Policy Implications of Tanzania's Mortality Burden	Verbal Autopsy	Hai	1995, 1998, 2001	All Ages	13560
Tanzania	The Policy Implications of Tanzania's Mortality Burden	Verbal Autopsy	Morogoro	1995, 1998, 2001	All Ages	15216
Tanzania	Verbal autopsy in establishing cause of perinatal death	Verbal Autopsy	Hai Northern Sentinel [rural]	1996	birth - 6 days	31
Tanzania	Effect of HIV infection on pregnancy-related mortality in sub-Saharan Africa: secondary analyses of pooled community-based data from the network for Analysing Longitudinal Population-based HIV/AIDS data on Africa (ALPHA)	Verbal Autopsy	Kisesa [all]	2000	15 - 49 years	157
Tanzania	Demographic Surveillance System (DSS)	Verbal Autopsy	Rufiji	2000	All Ages	3930
Tanzania	Demographic Surveillance System (DSS)	Verbal Autopsy	Ifakara	2001	All Ages	1713
Tanzania	Patterns of malaria related mortality based on verbal autopsy in Muleba District, north-western Tanzania	Verbal Autopsy	Ijumbi Nshambya Ikondo Bushemba Bunywambele Kibanga villages Muleba District [rural]	2001	birth - 69 years	423
Tanzania	Tanzania - Clustering of mortality among children under five years due to malaria at the Ifakara Demographic Surveillance Site	Verbal Autopsy	IFAKARA [rural]	2002-2005	birth - 4 years	643
Tanzania	Effect of zinc supplementation on mortality in children aged 1-48 months: a community-based randomised placebo-controlled trial	Verbal Autopsy	Pemba	2004	birth - 4 years	389
Tanzania	Risk factors for injury mortality in rural Tanzania: a secondary data analysis	Verbal Autopsy	Rufiji [rural]	2004	1 year +	4471
Tanzania	Muertes de adultos y el futuro: Un análisis causa-específico de las muertes de adultos de un estudio longitudinal en Tanzania rural 2003-2007	Verbal Autopsy	Kilombero & Ulanga districts rural [Ifakara HDSS]	2005	15 - 29 years	1130
Tanzania	Risk Factors and Causes of Adult Deaths in the Ifakara Health and Demographic Surveillance System Population, 2003-2007	Verbal Autopsy	Kilombero and Ulanga districts IFAKARA [rural]	2005	15 - 59 years	1139
Tanzania	The contribution of reduction in malaria as a cause of rapid decline of under-five mortality: evidence from the Rufiji Health and Demographic Surveillance System (HDSS) in rural Tanzania	Verbal Autopsy	Rufiji districtRufiji HDSS [rural]	2005	birth - 4 years	1292
Tanzania	The Role of Birth Order in Infant Mortality in Ifakara DSS Area in Rural Tanzania	Verbal Autopsy	25 villages of Kilombero and Ulanga districts IFAKARA [rural]	2005-2007	birth - 364 days	1295
Tanzania	Neonatal deaths in rural southern Tanzania: care-seeking and causes of death	Verbal Autopsy	Lindi and Mtwara regions [rural]	2007	birth - 27 days	219
Uganda	United Nations Office on Drugs and Crime Global Study on Homicide 2011	Police Records	Uganda CTS	2003-2004	All Ages	Death Rate
Uganda	Global Status Report on Road Safety 2009	Police Records		2006	All Ages	Death Rate
Uganda	Uganda Demographic and Health Survey - Maternal Mortality Data	Sibling History		1981-2011	15 - 59 years	3761
Uganda	Mortality Data for Ebola Outbreaks 1994-2014	Surveillance		2007, 2011	All Ages	CF
Uganda	Effect of HIV infection on pregnancy-related mortality in sub-Saharan Africa: secondary analyses of pooled community-based data from the network for Analysing Longitudinal Population-based HIV/AIDS data on Africa (ALPHA)	Verbal Autopsy	Masaka [all]	2000	15 - 49 years	117

Appendix Table 6: Causes of death data source list by geography, source type, and study site, with years, age range, and sample size, excluding sources on lower respiratory infection and diarrhoea etiologies						
**Location**	**Source name**	**Source type**	**Site**	**Years**	**Ages**	**Sample size**
Uganda	Effect of HIV infection on pregnancy-related mortality in sub-Saharan Africa: secondary analyses of pooled community-based data from the network for Analysing Longitudinal Population-based HIV/AIDS data on Africa (ALPHA)	Verbal Autopsy	Rakai [all]	2000	15 - 49 years	139
Uganda	Using the three delays model to understand why newborn babies die in eastern Uganda	Verbal Autopsy	Iganga and Mayuge Districts [rural]	2006	birth - 27 days	64
Uganda	Uganda Child Verbal Autopsy Study 2007	Verbal Autopsy	national	2006	birth - 4 years	530
Uganda	Using verbal autopsy to assess the prevalence of HIV infection among deaths in the ART period in rural Uganda: a prospective cohort study, 2006-2008	Verbal Autopsy	rural southwest Uganda [rural]	2007	10 years +	264
Uganda	Two year mortality and associated factors in a cohort of children from rural Uganda	Verbal Autopsy	Iganga and Mayuge districts [rural]	2009	1 - 4 years	46
Zambia	United Nations Surveys on Crime Trends and the Operations of Criminal Justice Systems 1970-2006	Police Records		1980, 1990-1994	All Ages	Death Rate
Zambia	United Nations Office on Drugs and Crime Global Study on Homicide 2011	Police Records	Zambia CTS	1998-1999	All Ages	Death Rate
Zambia	Global Status Report on Road Safety 2009	Police Records		2007	All Ages	Death Rate
Zambia	Zambia Demographic and Health Survey - Maternal Mortality Data	Sibling History		1982-2013	15 - 44 years	81
Zambia	Zambia Census of Population and Housing 2010	Survey/Census		2010	15 - 49 years	MMR
Zambia	Effect of training traditional birth attendants on neonatal mortality (Lufwanyama Neonatal Survival Project): randomised controlled study	Verbal Autopsy	Lafwanyama district Copperbelt province [rural]	2007	birth - 27 days	58
Zambia	Causes of stillbirth, neonatal death and early childhood death in rural Zambia by verbal autopsy assessments	Verbal Autopsy	4 communities Kafue district [rural]	2008	birth - 4 years	98
Zambia	Zambia Sample Vital Registration with Verbal Autopsy (SAVVY) Data 2010	Verbal Autopsy		2009-2010	All Ages	1043
Botswana	United Nations Surveys on Crime Trends and the Operations of Criminal Justice Systems 1970-2006	Police Records		1982, 1984-1990	All Ages	Death Rate
Botswana	United Nations Office on Drugs and Crime Global Study on Homicide 2011	Police Records	Botswana CTS	2005-2006, 2009	All Ages	Death Rate
Botswana	Botswana Stats Brief Maternal Mortality Ratio 2005-2009	Surveillance		2005-2009	15 - 49 years	MMR
Botswana	Botswana Stats Brief Maternal Mortality Ratio 2007-2011	Surveillance		2010-2011	15 - 49 years	MMR
Botswana	Botswana AIDS Impact Survey	Survey/Census		2003, 2007	All Ages	51831
Lesotho	United Nations Surveys on Crime Trends and the Operations of Criminal Justice Systems 1970-2006	Police Records		1986, 1988, 1990-1994	All Ages	Death Rate
Lesotho	United Nations Office on Drugs and Crime Global Study on Homicide 2011	Police Records	Lesotho CTS	2009	All Ages	Death Rate
Lesotho	Lesotho Demographic and Health Survey - Maternal Mortality Data	Sibling History		1990-2009	15 - 49 years	135
Lesotho	Lesotho Population and Housing Census 1996	Survey/Census		1995-1996	15 - 39 years	3480
Lesotho	Lesotho Population and Housing Census 2006	Survey/Census		2006	All Ages	44580
Namibia	United Nations Office on Drugs and Crime Global Study on Homicide 2011	Police Records	Namibia Interpol	2004	All Ages	Death Rate
Namibia	Namibia Demographic and Health Survey - Maternal Mortality Data	Sibling History		1980-2013	15 - 59 years	2799
South Africa: Eastern Cape	Demographic and Health Survey (DHS)	Sibling History		1984-1998	15 - 19 years	1
South Africa: Eastern Cape	South Africa Saving Mothers	Surveillance		1998-2013	10 - 54 years	2510
South Africa: Eastern Cape	South Africa Vital Registration System	Vital Registration		1997-2013	All Ages	1190671
South Africa: Free State	Demographic and Health Survey (DHS)	Sibling History		1985-1988	30 - 39 years	3

**Appendix Table 6: Causes of death data source list by geography, source type, and study site, with years, age range, and sample size, excluding sources on lower respiratory infection and diarrhoea etiologies**						
**Location**	**Source name**	**Source type**	**Site**	**Years**	**Ages**	**Sample size**
South Africa: Free State	Demographic and Health Survey (DHS)	Sibling History		1990-1998	15 - 49 years	9
South Africa: Free State	South Africa Saving Mothers	Surveillance		1998-2013	10 - 54 years	2015
South Africa: Free State	South Africa Vital Registration System	Vital Registration		1997-2013	All Ages	752205
South Africa: Gauteng	Demographic and Health Survey (DHS)	Sibling History		1984-1998	40 - 44 years	1
South Africa: Gauteng	South Africa Saving Mothers	Surveillance		1998-2013	10 - 54 years	3691
South Africa: Gauteng	South Africa Vital Registration System	Vital Registration		1997-2013	All Ages	1606722
South Africa: KwaZulu-Natal	Demographic and Health Survey (DHS)	Sibling History		1984-1998	15 - 19 years	1
South Africa: KwaZulu-Natal	South Africa Saving Mothers	Surveillance		1998-2013	10 - 54 years	4699
South Africa: KwaZulu-Natal	Demographic Surveillance System (DSS)	Verbal Autopsy		2000-2011	All Ages	9290
South Africa: KwaZulu-Natal	Mortality in women of reproductive age in rural South Africa	Verbal Autopsy	KwaZulu-Natal districtAfrica Center for Health and Population Studies [rural]	2005	15 - 49 years	3098
South Africa: KwaZulu-Natal	South Africa Vital Registration System	Vital Registration		1997-2013	All Ages	1878626
South Africa: Limpopo	Demographic and Health Survey (DHS)	Sibling History		1984-1987, 1989-1998	45 - 49 years	2
South Africa: Limpopo	South Africa Saving Mothers	Surveillance		1998-2013	10 - 54 years	2487
South Africa: Limpopo	South Africa Vital Registration System	Vital Registration		1997-2013	All Ages	768255
South Africa: Mpumalanga	Demographic and Health Survey (DHS)	Sibling History		1984-1987	15 - 59 years	6
South Africa: Mpumalanga	Demographic and Health Survey (DHS)	Sibling History		1989-1998	15 - 49 years	5
South Africa: Mpumalanga	South Africa Saving Mothers	Surveillance		1998-2013	10 - 54 years	1717
South Africa: Mpumalanga	Demographic Surveillance System (DSS)	Verbal Autopsy		1992-2011	All Ages	9437
South Africa: Mpumalanga	South Africa Vital Registration System	Vital Registration		1997-2013	All Ages	658825
South Africa: North-West	Demographic and Health Survey (DHS)	Sibling History		1984-1986	20 - 44 years	12
South Africa: North-West	Demographic and Health Survey (DHS)	Sibling History		1988-1998	15 - 19 years	5
South Africa: North-West	South Africa Saving Mothers	Surveillance		1998-2013	10 - 54 years	1632
South Africa: North-West	South Africa Vital Registration System	Vital Registration		1997-2013	All Ages	703503
South Africa: Northern Cape	Demographic and Health Survey (DHS)	Sibling History		1984-1990	15 - 39 years	2
South Africa: Northern Cape	Demographic and Health Survey (DHS)	Sibling History		1992-1998	25 - 44 years	5
South Africa: Northern Cape	South Africa Saving Mothers	Surveillance		1998-2013	10 - 54 years	636
South Africa: Northern Cape	South Africa Vital Registration System	Vital Registration		1997-2013	All Ages	221494
South Africa: Western Cape	Demographic and Health Survey (DHS)	Sibling History		1984-1988	25 - 29 years	1
South Africa: Western Cape	Demographic and Health Survey (DHS)	Sibling History		1990-1998	35 - 39 years	1
South Africa: Western Cape	South Africa Saving Mothers	Surveillance		1998-2013	10 - 54 years	1020
South Africa: Western Cape	South Africa Vital Registration System	Vital Registration		1997-2013	All Ages	712064
Swaziland	United Nations Surveys on Crime Trends and the Operations of Criminal Justice Systems 1970-2006	Police Records		1986-1994	All Ages	Death Rate
Swaziland	United Nations Office on Drugs and Crime Global Study on Homicide 2011	Police Records	Swaziland CTS	1995, 2003-2004	All Ages	Death Rate
Swaziland	Demographic and Health Survey (DHS)	Sibling History		1992-2006	15 - 34 years	9
Swaziland	Effect of HIV infection on pregnancy-related mortality in sub-Saharan Africa: secondary analyses of pooled community-based data from the network for Analysing Longitudinal Population-based HIV/AIDS data on Africa (ALPHA)	Verbal Autopsy	uMkhanyakude [all]	2000	15 - 49 years	59
Zimbabwe	United Nations Surveys on Crime Trends and the Operations of Criminal Justice Systems 1970-2006	Police Records		1986	All Ages	Death Rate

Appendix Table 6: Causes of death data source list by geography, source type, and study site, with years, age range, and sample size, excluding sources on lower respiratory infection and diarrhoea etiologies						
**Location**	**Source name**	**Source type**	**Site**	**Years**	**Ages**	**Sample size**
Zimbabwe	United Nations Office on Drugs and Crime Global Study on Homicide 2011	Police Records	Zimbabwe CTS	1995-2000, 2003-2007	All Ages	Death Rate
Zimbabwe	Global Status Report on Road Safety 2009	Police Records		2006	All Ages	Death Rate
Zimbabwe	Zimbabwe Demographic and Health Survey - Maternal Mortality Data	Sibling History		1980-2010	15 - 49 years	78
Zimbabwe	Zimbabwe Population and Housing Census 1992	Survey/Census		1991-1992	15 - 49 years	25982
Zimbabwe	World Health Survey (WHS)	Survey/Census		1997	All Ages	10
Zimbabwe	Zimbabwe Population and Housing Census 2002	Survey/Census		2001-2002	15 - 49 years	102674
Zimbabwe	Zimbabwe Population and Housing Census 2012	Survey/Census		2012	15 - 49 years	MMR
Zimbabwe	Effect of HIV infection on pregnancy-related mortality in sub-Saharan Africa: secondary analyses of pooled community-based data from the network for Analysing Longitudinal Population-based HIV/AIDS data on Africa (ALPHA)	Verbal Autopsy	Manicaland [all]	2000	15 - 49 years	206
Zimbabwe	WHO Mortality Database	Vital Registration		1990	All Ages	34817
Zimbabwe	Zimbabwe Vital Statistics - Deaths 1995	Vital Registration		1995	All Ages	75489
Zimbabwe	Zimbabwe Mortality Report 2007	Vital Registration		2007	All Ages	62232
Benin	Benin Demographic and Health Survey - Maternal Mortality Data	Sibling History		1982-2006	15 - 44 years	68
Benin	Incidence de décès de 0 à 1 an dans une cohorte de 802 enfants en milieu rural au sud du Bénin	Verbal Autopsy	Pahou Avlekete [rural]	1989	birth - 364 days	25
Burkina Faso	United Nations Office on Drugs and Crime Global Study on Homicide 2011	Police Records	Burkina Faso NSO	2002-2007	All Ages	Death Rate
Burkina Faso	Global Status Report on Road Safety 2009	Police Records		2007	All Ages	Death Rate
Burkina Faso	Burkina Faso Demographic and Health Survey - Maternal Mortality Data	Sibling History		1984-2010	15 - 24 years	10
Burkina Faso	The epidemiology of pregnancy outcomes in rural Burkina Faso	Sibling History	Ourgaye and Diapaga districts [rural]	2004	15 - 49 years	MMR
Burkina Faso	Burkina Faso - Pissila Analysis of Mortality in Childhood after Three Years of Observation 1985-1987	Survey/Census	8 villages Pissila Region [rural]	1986	birth - 364 days	247
Burkina Faso	Burkina Faso - Niangoloko Childhood Mortality 1986-1988	Survey/Census	Korogora Kitobama Dionouna Tiempagora Letiefesso Fornefesso Katierla Nafona Yendere Diefoula Boko villages Niangoloko region [rural]	1987	birth - 4 years	164
Burkina Faso	Burkina Faso Population and Housing Census 2006	Survey/Census		2006	15 - 49 years	MMR
Burkina Faso	Burkina Faso Health Statistical Yearbook 2006	Survey/Census		2006	15 - 49 years	MMR
Burkina Faso	The burden of malaria mortality among African children in the year 2000	Verbal Autopsy	Kongodjan area [rural]	1984	birth - 4 years	43
Burkina Faso	Measuring the local burden of disease. A study of years of life lost in sub-Saharan Africa	Verbal Autopsy	Nouna	1998	All Ages	1400
Burkina Faso	Demographic Surveillance System (DSS)	Verbal Autopsy		1998-2011	All Ages	7589
Burkina Faso	Demographic Surveillance System (DSS)	Verbal Autopsy	Nouna	2000	All Ages	2087
Burkina Faso	Patterns of age-specific mortality in children in endemic areas of sub-Saharan Africa	Verbal Autopsy	Kourweogo [rural]	2002	birth - 14 years	1013
Burkina Faso	Causas de la distribución de muerte con el modelo InterVA y la codificación de médicos en un área rural de Burkina Faso	Verbal Autopsy	Nouna [rural]	2002	All Ages	5649
Burkina Faso	Patterns of age-specific mortality in children in endemic areas of sub-Saharan Africa	Verbal Autopsy	Oubritenga [rural]	2002	birth - 14 years	1491
Burkina Faso	The epidemiology of pregnancy outcomes in rural Burkina Faso	Verbal Autopsy	Ourgaye and Diapaga districts [rural]	2004	15 - 49 years	MMR

Appendix Table 6: Causes of death data source list by geography, source type, and study site, with years, age range, and sample size, excluding sources on lower respiratory infection and diarrhoea etiologies						
Location	Source name	Source type	Site	Years	Ages	Sample size
Burkina Faso	The high burden of infant deaths in rural Burkina Faso: a prospective community-based cohort study	Verbal Autopsy	Banfora Health District 24 villages [rural]	2007	birth - 364 days	75
Burkina Faso	An improved method for physician-certified verbal autopsy reduces the rate of discrepancy: experiences in the Nouna Health and Demographic Surveillance Site (NHDSS), Burkina Faso	Verbal Autopsy	Kossi Province	2009	All Ages	1256
Burkina Faso	The Kaya HDSS, Burkina Faso: a platform for epidemiological studies and health programme evaluation	Verbal Autopsy	north central region [rural]	2009	birth - 14 years	203
Burkina Faso	The Kaya HDSS, Burkina Faso: a platform for epidemiological studies and health programme evaluation	Verbal Autopsy	north central region [urban]	2009	birth - 14 years	347
Cameroon	United Nations Office on Drugs and Crime Global Study on Homicide 2011	Police Records	Cameroon CTS	2006-2007	All Ages	Death Rate
Cameroon	Global Status Report on Road Safety 2009	Police Records		2007	All Ages	Death Rate
Cameroon	Cameroon Demographic and Health Survey - Maternal Mortality Data	Sibling History		1984-2011	15 - 54 years	197
Cape Verde	United Nations Office on Drugs and Crime Global Study on Homicide 2011	Police Records	Cape Verde UN-OCHA	2007	All Ages	Death Rate
Cape Verde	Deaths among women of reproductive age in Cape Verde: causes and avoidability	Verbal Autopsy	Santiago Santo Antao Sao Vicente	1992	15 - 49 years	97
Cape Verde	WHO Mortality Database	Vital Registration		1980, 2011-2012	All Ages	7565
Chad	Chad Demographic and Health Survey - Maternal Mortality Data	Sibling History		1982-2004	15 - 34 years	21
Cote d'Ivoire	United Nations Office on Drugs and Crime Global Study on Homicide 2011	Police Records	Cote d'Ivoire CTS	2007	All Ages	Death Rate
Cote d'Ivoire	Côte d'Ivoire Demographic and Health Survey - Maternal Mortality Data	Sibling History		1980-1994	15 - 39 years	9
Cote d'Ivoire	Côte d'Ivoire Demographic and Health Survey - Maternal Mortality Data	Sibling History		1997-2011	15 - 44 years	254
Cote d'Ivoire	Effet de l'observance des d'approvisionnement en eau et de la therapie par voie orale sur les diarrhees chez les enfants de moins de 5 de la Cote d'Ivoire	Verbal Autopsy	Grand-Alepe Ahoutoue Brofodoume Ahoue [rural]	1988, 1990, 1992	birth - 4 years	113
Cote d'Ivoire	Demographic Surveillance System (DSS)	Verbal Autopsy		2009-2011	All Ages	844
The Gambia	United Nations Office on Drugs and Crime Global Study on Homicide 2011	Police Records	The Gambia Interpol	2004	All Ages	Death Rate
The Gambia	Demographic and Health Surveys	Sibling History		1999-2012	15 - 44 years	57
The Gambia	A prospective survey of the outcome of pregnancy in a rural area of the Gambia	Verbal Autopsy	41 villages and hamlets near Farafenni in North Bank Division [rural]	1982	birth - 27 days	33
The Gambia	Deaths in infancy and early childhood in a well-vaccinated, rural, West African population	Verbal Autopsy	Villages and hamlets near the town of Farafenni [rural]	1982	birth - 9 years	184
The Gambia	Sustained protection against mortality and morbidity from malaria in rural Gambian children by chemoprophylaxis given by village health workers	Verbal Autopsy	41 villages and hamlets west of Farafenni North Bank [rural]	1982, 1986	28 days - 4 years	151
The Gambia	The effect of insecticide-treated bed nets on mortality of Gambian children	Verbal Autopsy	73 villages South Bank east of Soma (17 PHC and 56 non-PHC villages) [rural] - control	1988-1989	birth - 4 years	296
The Gambia	The effect of insecticide-treated bed nets on mortality of Gambian children	Verbal Autopsy	73 villages South Bank east of Soma (17 PHC and 56 non-PHC villages) [rural] - intervention	1989	birth - 4 years	57
The Gambia	Risk factors for mortality from acute lower respiratory tract infections in young Gambian children	Verbal Autopsy	Upper River Division [rural]	1989	birth - 4 years	273
The Gambia	Changes in the pattern of infant and childhood mortality in upper river division, The Gambia, from 1989 to 1993	Verbal Autopsy	Upper River Division eastern Gambia [rural]	1989-1993	birth - 4 years	3776

Appendix Table 6: Causes of death data source list by geography, source type, and study site, with years, age range, and sample size, excluding sources on lower respiratory infection and diarrhoea etiologies

Location	Source name	Source type	Site	Years	Ages	Sample size
The Gambia	Mortality and morbidity from malaria in Gambian children after introduction of an impregnated bednet programme	Verbal Autopsy	Primary Health Care (PHC) Program villages [rural] - control	1992	1 - 9 years	182
The Gambia	Mortality and morbidity from malaria in Gambian children after introduction of an impregnated bednet programme	Verbal Autopsy	Primary Health Care (PHC) Program villages [rural] - intervention	1992	1 - 9 years	124
The Gambia	Maternal mortality in rural Gambia: levels, causes and contributing factors	Verbal Autopsy	Rural areas around Farafenni North Bank Division [rural]	1995	15 - 49 years	74
The Gambia	Demographic Surveillance System (DSS)	Verbal Autopsy		1998-2007	All Ages	2983
The Gambia	Reaching millennium development goal 4 - the Gambia	Verbal Autopsy	42 villages around the town of Farafenni [rural]	1999, 2002, 2006	birth - 4 years	738
The Gambia	Maternal deaths in rural Gambia	Verbal Autopsy	Central and Upper River Divisions [rural]	2002	15 - 49 years	MMR
The Gambia	Preventive measures in infancy to reduce under-five mortality: a case-control study in The Gambia	Verbal Autopsy	Farafenni villages and urban	2006	birth - 4 years	141
Ghana	Global Status Report on Road Safety 2009	Police Records		2007	All Ages	Death Rate
Ghana	United Nations Office on Drugs and Crime Global Study on Homicide 2011	Police Records	Ghana National police	2009	All Ages	Death Rate
Ghana	Ghana Demographic and Health Survey - Maternal Mortality Data	Sibling History		1993-2007	15 - 54 years	97
Ghana	Maternal mortality among the Kassena-Nankana of northern Ghana	Verbal Autopsy	Northern Region - Kassena-Nankana District	1989	15 - 49 years	738
Ghana	Vitamin A supplementation in northern Ghana: effects on clinic attendances, hospital admissions, and child mortality	Verbal Autopsy	Kassena-Nankana District [rural]	1990	28 days - 9 years	495
Ghana	Impact of permethrin impregnated bednets on child mortality in Kassena-Nankana district, Ghana: a randomized controlled trial	Verbal Autopsy	Kassena-Nankana District [rural] - control	1994	28 days - 4 years	461
Ghana	Impact of permethrin impregnated bednets on child mortality in Kassena-Nankana district, Ghana: a randomized controlled trial	Verbal Autopsy	Kassena-Nankana District [rural] - intervention	1994	28 days - 4 years	396
Ghana	How many years of life could be saved if malaria were eliminated from a hyperendemic area of northern Ghana?	Verbal Autopsy	Navrongo Health Research Center Kasena-Nankana district [rural]	1995	5 years +	2870
Ghana	Trend and causes of neonatal mortality in the Kassena-Nankana district of northern Ghana, 1995-2002	Verbal Autopsy	Lassena-Nankana district [rural]	1998	birth - 27 days	1068
Ghana	Demographic Surveillance System (DSS)	Verbal Autopsy	Navrongo	2000	All Ages	8483
Ghana	Maternal mortality decline in the Kassena-Nankana district of northern Ghana	Verbal Autopsy	Kassena-Nankana District [rural]	2003	10 - 49 years	516
Ghana	Aetiology of stillbirths and neonatal deaths in rural Ghana: implications for health programming in developing countries	Verbal Autopsy	Kintampo Brong Ahafo region [rural]	2003	birth - 27 days	590
Ghana	Effect of early infant feeding practices on infection-specific neonatal mortality: an investigation of the causal links with observational data from rural Ghana	Verbal Autopsy	Kintampo Wenchi Techiman Nkoranza districts Brong Ahafo region [rural]	2003	birth - 27 days	140
Ghana	Effect of vitamin A supplementation on cause-specific mortality in women of reproductive age in Ghana: a secondary analysis from the ObaapaVitA trial	Verbal Autopsy	seven districts of the Brong Ahafo region [rural]	2004	15 - 44 years	1298
Ghana	Determinants of epidemiologic transition in rural Africa: the role of socioeconomic status and drinking water source	Verbal Autopsy	Garu-Tempane district [rural]	2004, 2007, 2010	All Ages	1253
Ghana	Demographic Surveillance System (DSS)	Verbal Autopsy		2004-2011	All Ages	15200
Ghana	Why are babies dying in the first month after birth? A 7-year study of neonatalmortality in northern Ghana	Verbal Autopsy	Kassena-Nankana District [rural]	2006	birth - 27 days	424
Ghana	Special Demographic and Health Survey (DHS)	Verbal Autopsy		2007	10 - 49 years	4203

**Appendix Table 6: Causes of death data source list by geography, source type, and study site, with years, age range, and sample size, excluding sources on lower respiratory infection and diarrhoea etiologies**						
**Location**	**Source name**	**Source type**	**Site**	**Years**	**Ages**	**Sample size**
Ghana	Verbal autopsy: an analysis of the common causes of childhood death in the Barekese sub-district of Ghana	Verbal Autopsy	Barekese sub-district Atwima Nwabiagya district Ashanti [rural]	2007	birth - 4 years	117
Ghana	Ghana - Accra Births and Deaths Registry - Deaths 2000-2007	Vital Registration		2000, 2007	All Ages	29437
Guinea	Guinea Demographic and Health Survey - Maternal Mortality Data	Sibling History		1985-2012	15 - 39 years	66
Guinea	Inferred Total Deaths Among Confirmed Ebola Cases 2014-2015	Surveillance		2014-2015	All Ages	187416
Guinea	Guinea - Mandiana Mortality Study 1998-1999	Verbal Autopsy	Mandiana [rural]	1998	birth - 4 years	330
Guinea-Bissau	Global Status Report on Road Safety 2009	Police Records		2007	All Ages	Death Rate
Guinea-Bissau	Persistent and acute diarrhoea as the leading causes of child mortality in urban Guniea Bissau	Verbal Autopsy	Bandim II [urban]	1989	28 days - 4 years	135
Guinea-Bissau	Maternal mortality in Guinea-Bissau: the use of verbal autopsy in a multi-ethnic population	Verbal Autopsy	Clusters of 100 women from each of the 5 northern regions of Guinea-Bissau	1992	15 - 49 years	339
Guinea-Bissau	BCG vaccination scar associated with better childhood survival in Guinea-Bissau	Verbal Autopsy	Bandim I Bandim II Belem Mindara	1995	28 days - 4 years	114
Liberia	United Nations Surveys on Crime Trends and the Operations of Criminal Justice Systems 1970-2006	Police Records		1982-1985	All Ages	Death Rate
Liberia	United Nations Office on Drugs and Crime Global Study on Homicide 2011	Police Records	Liberia UN-PKO	2007, 2009-2010	All Ages	Death Rate
Liberia	Liberia Demographic and Health Survey - Maternal Mortality Data	Sibling History		1992-2013	15 - 44 years	58
Liberia	Inferred Total Deaths Among Confirmed Ebola Cases 2014-2015	Surveillance		2014	All Ages	25704
Liberia	Infant and child mortality in two counties of Liberia: results of a survey in 1988 and trends since 1984	Verbal Autopsy	Bomi and Grand Cape Mount counties	1984, 1987	birth - 4 years	949
Liberia	Application of the verbal autopsy during a clinical trial	Verbal Autopsy	Liberian Agricultural Company (LAC) rubber plantation [rural]	1990	birth - 49 years	24
Mali	Mali Demographic and Health Survey - Maternal Mortality Data	Sibling History		1981-2006	15 - 44 years	96
Mali	High maternal mortality estimated by the sisterhood method in a rural area of Mali	Sibling History	20 villages in Kita	2007	15 - 49 years	1233
Mali	Mali Population and Housing Census 2009	Survey/Census		2009	15 - 49 years	MMR
Mali	Assessment of maternal mortality and late maternal mortality among a cohort of pregnant women in Bamako, Mali	Verbal Autopsy	Bankoni a district of Bamako [urban]	1990	15 - 49 years	MMR
Mali	Mali - Twelve Years of Urban Mortality in the Sahel. Levels, Trends, Seasons, and Causes of Mortality in Bamako, 1974-1985	Vital Registration	Bamako	1981, 1984	All Ages	24895
Mauritania	Global Status Report on Road Safety 2009	Police Records		2007	All Ages	Death Rate
Mauritania	Demographic and Health Survey (DHS)	Sibling History		1986-2000	20 - 44 years	11
Niger	Global Status Report on Road Safety 2009	Police Records		2007	All Ages	Death Rate
Niger	Niger Demographic and Health Survey - Maternal Mortality Data	Sibling History		1980-2012	15 - 49 years	540
Niger	Retrospective determination of whether famine existed in Niger, 2005: two stage cluster survey	Verbal Autopsy	Agadez Diffa Dosso Maradi Tahoua Tillaberi Zinder and Niamey	2005	birth - 4 years	5737
Nigeria	United Nations Surveys on Crime Trends and the Operations of Criminal Justice Systems 1970-2006	Police Records		1990-1994	All Ages	Death Rate
Nigeria	United Nations Office on Drugs and Crime Global Study on Homicide 2011	Police Records	Nigeria NGO (a)	1995-2007	All Ages	Death Rate

Appendix Table 6: Causes of death data source list by geography, source type, and study site, with years, age range, and sample size, excluding sources on lower respiratory infection and diarrhoea etiologies						
**Location**	**Source name**	**Source type**	**Site**	**Years**	**Ages**	**Sample size**
Nigeria	Nigeria Demographic and Health Survey - Maternal Mortality Data	Sibling History		1994-2013	15 - 44 years	42
Nigeria	Community Study of Maternal Mortality in South West Nigeria: How Applicable is the Sisterhood Method	Sibling History	Ibadan North	2005	15 - 49 years	1130
Nigeria	Community Study of Maternal Mortality in South West Nigeria: How Applicable is the Sisterhood Method	Sibling History	Ido	2005	15 - 49 years	194
Nigeria	The use of the sisterhood method for estimating maternal mortality ratio in Lagos state, Nigeria	Sibling History	Lagos State	2008	15 - 49 years	474
Nigeria	Childhood diarrhoea in rural Nigeria. I. Studies on prevalence, mortality and socio-environmental factors	Survey/Census	Akoko North Ondo State [rural]	1987	birth - 4 years	120
Nigeria	Infant and maternal deaths in rural south west Nigeria: a prospective study	Survey/Census	Lagun [rural]	1995	birth - 44 years	177
Nigeria	Community-based surveillance of paediatric deaths in Cross River State, Nigeria	Verbal Autopsy	Nko Cross River State [rural]	1991	birth - 14 years	475
Nigeria	The Idikan adult mortality study	Verbal Autopsy	Ibadan [urban]	1994	15 years +	60
Nigeria	Aetiology of maternal mortality using verbal autopsy at Sokoto, North-Western Nigeria	Verbal Autopsy	Sokoto [urban]	2010	15 - 49 years	58
Nigeria	Health & demographic surveillance system profile: the Nahuche Health and Demographic Surveillance System, Northern Nigeria (Nahuche HDSS)	Verbal Autopsy	Zamfara StateNahuche HDSS [rural]	2012	birth - 19 years	2100
Nigeria	Nigeria Report of Livebirths, Deaths and Stillbirths 1994-2007	Vital Registration		2007	All Ages	23882
Sao Tome and Principe	United Nations Surveys on Crime Trends and the Operations of Criminal Justice Systems 1970-2006	Police Records		1990-1994	All Ages	Death Rate
Sao Tome and Principe	Demographic and Health Survey (DHS)	Sibling History		1994-2008	15 - 39 years	3
Sao Tome and Principe	WHO Mortality Database	Vital Registration		1985	All Ages	3292
Senegal	United Nations Surveys on Crime Trends and the Operations of Criminal Justice Systems 1970-2006	Police Records		1980	All Ages	Death Rate
Senegal	United Nations Office on Drugs and Crime Global Study on Homicide 2011	Police Records	Senegal Interpol	2003-2004	All Ages	Death Rate
Senegal	Senegal Demographic and Health Survey - Maternal Mortality Data	Sibling History		1980-2010	15 - 39 years	36
Senegal	Misclassification of pregnancy-related deaths in adult mortality surveys: case study in Senegal	Surveillance	Bandafassi	2006	10 - 49 years	60
Senegal	International Integrated Public Use Microdata Series (IPUMS)	Survey/Census		2002	All Ages	10022
Senegal	Senegal - Risk of Death Associated with Different Nutritional States in Children of Preschool age: Study Conducted in Niakhar (Senegal) 1983-1986	Verbal Autopsy	Niakhar Fatick region [rural]	1984	28 days - 4 years	830
Senegal	Incidence and duration of severe wasting in two African populations	Verbal Autopsy	Niakhar [rural]	1984	28 days - 4 years	356
Senegal	International differences in clinical patterns of diarrhoeal deaths: a comparison of children from Brazil, Senegal, Bangladesh, and India	Verbal Autopsy	Niakhar [rural]	1986	birth - 4 years	1517
Senegal	Impact of chloroquine resistance on malaria mortality	Verbal Autopsy	Bandafassi [rural]	1986, 1992	birth - 4 years	1167
Senegal	Childhood mortality and probable causes of death using verbal autopsy in Niakhar, Senegal, 1989-2000	Verbal Autopsy	Niakhar Fatick [rural]	1990, 1993, 1996, 1999	birth - 14 years	3424
Senegal	Adult mortality in a rural area of Senegal: Non-communicable diseases have a large impact in Mlomp	Verbal Autopsy	Mlomp [rural]	1994	15 - 59 years	363
Senegal	Demographic Surveillance System (DSS)	Verbal Autopsy		2005-2010	All Ages	1732
Sierra Leone	United Nations Office on Drugs and Crime Global Study on Homicide 2011	Police Records	Sierra Leone CTS	2004-2007	All Ages	Death Rate
Sierra Leone	Global Status Report on Road Safety 2009	Police Records		2007	All Ages	Death Rate

Appendix Table 6: Causes of death data source list by geography, source type, and study site, with years, age range, and sample size, excluding sources on lower respiratory infection and diarrhoea etiologies						
**Location**	**Source name**	**Source type**	**Site**	**Years**	**Ages**	**Sample size**
Sierra Leone	Demographic and Health Survey (DHS)	Sibling History		1994-2013	25 - 44 years	4
Sierra Leone	Inferred Total Deaths Among Confirmed Ebola Cases 2014-2015	Surveillance		2014-2015	All Ages	92503
Sierra Leone	International Integrated Public Use Microdata Series (IPUMS)	Survey/Census		2004	All Ages	9070
Sierra Leone	Sierra Leone Census 2004	Survey/Census		2004	All Ages	99278
Sierra Leone	Female Health and Family Planning in Sierra Leone	Survey/Census		2012	10 - 49 years	232
Sierra Leone	Malaria in a rural area of Sierra Leone. I. Initial results	Verbal Autopsy	15 villages north of Bo [rural] [rural]	1990	birth - 9 years	37
Sierra Leone	Immunization coverage and child mortality in two rural districts of Sierra Leone	Verbal Autopsy	Western Area and Porto Loko	1993	birth - 4 years	559
Togo	Global Status Report on Road Safety 2009	Police Records		2007	All Ages	Death Rate
Togo	Togo Demographic and Health Survey - Maternal Mortality Data	Sibling History		1984-2013	15 - 49 years	97

Appendix Table 7: List of International Classification of Diseases (ICD) codes mapped to the Global Burden of Disease cause list for causes of death

Cause	ICD10	ICD9
Communicable, maternal, neonatal, and nutritional diseases	A00-A00.9, A01.0-A14, A15-A28.9, A30-A30.9, A32-A39.4, A39.8-A39.9, A48.1-A48.2, A48.4-A48.52, A49.1, A50-A58, A60-A60.9, A63-A63.8, A65-A65.0, A68-A70, A74, A74.8-A75.9, A77-A96.9, A98-A98.8, B00-B06.9, B10-B10.89, B15-B17.9, B19-B27.99, B29.4, B33-B33.1, B33.3-B33.8, B47-B48.8, B50-B53.8, B55.0, B56-B57.5, B60-B60.8, B63, B65-B67.99, B69-B72.0, B74.3-B75, B77-B77.9, B83-B83.8, B90-B92, B94.1-B94.2, B95-B95.5, D50.1-D50.8, D51-D52.0, D52.8-D53.9, D64.3, D86.81, E00-E02, E40-E46.9, E51-E61.9, E63-E64.0, E64.2-E64.9, F07.1, G00.0-G00.8, G03-G03.8, G04-G05.8, G14-G14.6, H70-H70.93, I00, I02, I02.9, I98.0-I98.1, J01-J01.91, J02.0, J03.0-J03.01, J04.0, J05-J05.0, J05.11, J09-J15.8, J16-J16.9, J20-J21.9, J36-J36.0, K67.0-K67.8, K74.7-K74.8, K93.0, M03.1, M12.1-M12.19, M49.0-M49.1, M73.0-M73.1, M89.6-M89.69, N70-N71.9, N73-N74.8, N96, N98-N98.9, O00-O07.9, O09-O16.9, O20-O26.93, O28-O36.93, O40-O48.1, O60-O77.9, O80-O92.79, O96-P04.2, P04.5-P05.9, P07-P15.9, P19-P22.9, P23.0-P23.4, P24-P29.9, P35-P37.2, P37.5-P39.9, P50-P61.9, P70, P70.3-P72.9, P74-P78.9, P80-P81.9, P83-P84, P90-P94.9, P96, P96.3-P96.4, P96.8-P96.89, R19.7	001-001.9, 002.0-030.9, 032-034.9, 036-036.40, 036.5, 036.8-037.9, 039-039.4, 039.8-040, 040.1-041.09, 042-066.9, 070-070.21, 070.3-070.31, 070.4-070.43, 070.49-070.53, 070.59-074.1, 074.20, 074.3-075.9, 078.4-078.7, 079-079.7, 080-083.9, 084.0-084.5, 084.7-084.9, 085.0, 086-088.9, 090-101.6, 104-104.9, 120-124.9, 125.4-125.9, 127-127.1, 128-129.0, 136-136.29, 137-139.0, 244.2, 260-263.9, 265-269.9, 280.1-280.8, 281.0-281.9, 320.0-320.89, 321-323.9, 381-383.9, 390-390.9, 392, 392.9, 461-461.9, 464.0, 464.01, 464.11-464.2, 464.21, 464.31-464.4, 464.8-464.9, 466-469, 470.0, 475-475.9, 476.9, 480-482.89, 483.0-483.9, 484.0-484.7, 487-489, 613-614.9, 630-636.92, 638-638.92, 640-679.14, 716.0-716.09, 730.4-730.6, 760-760.64, 760.8-768, 768.2-770, 770.1-775, 775.4-779.34, 779.6-779.89, 787.91
HIV/AIDS and tuberculosis	A10-A14, A15-A19.9, B20-B24.9, B90-B90.9, K67.3, K93.0, M49.0, P37.0	010-019.9, 042-044.9, 137-137.9, 138.0-138.9, 730.4-730.6
Tuberculosis	A10-A14, A15-A19.9, B90-B90.9, K67.3, K93.0, M49.0, P37.0	010-019.9, 137-137.9, 138.0-138.9, 730.4-730.6
HIV/AIDS	B20-B24.9	042-044.9
HIV/AIDS - Tuberculosis	B20.0	
HIV/AIDS resulting in other diseases	B20.1-B23.9, B24.0	042.0-042.9, 043.0-043.9, 044.1-044.9
Diarrhea, lower respiratory, and other common infectious diseases	A00-A00.9, A01.0-A09.9, A33-A37.91, A39-A39.4, A39.8-A39.9, A48.1, A70, A83-A87.9, B01-B02.9, B05-B05.9, B94.1, D86.81, F07.1, G00.0-G00.8, G03-G03.8, G04-G05.8, H70-H70.93, J01-J01.91, J04.0, J05-J05.0, J05.11, J09-J15.8, J16-J16.9, J20-J21.9, J36-J36.0, P23.0-P23.4, P35.8, R19.7	001-001.9, 002.0-009.9, 032-033.9, 036-036.40, 036.5, 036.8-037.9, 047-049.9, 052-053.9, 055-055.9, 062-064.9, 073.0-073.6, 139.0, 320.0-320.89, 321-323, 323.1, 323.4-323.9, 381-383.9, 461-461.9, 464.0, 464.01, 464.11-464.2, 464.21, 464.31-464.4, 464.8-464.9, 466-469, 470.0, 475-475.9, 476.9, 480-482.89, 483.0-483.9, 484.0-484.4, 484.6-484.7, 487-489, 771.3, 787.91
Diarrheal diseases	A00-A00.9, A02-A04.1, A04.3, A04.5-A07, A07.2-A07.4, A08-A09.9, R19.7	001-001.9, 003-006.9, 007.4-007.8, 008.01-008.02, 008.04, 008.2-009.9, 787.91
Intestinal infectious diseases	A01.0-A01.4, A04.2, A04.4, A07.0-A07.1, A07.8-A07.9	002.0-002.9, 007-007.3, 007.9-008.00, 008.03, 008.09-008.1
Typhoid fever	A01.0-A01.09	002.0
Paratyphoid fever	A01.1-A01.4	002.1-002.9
Other intestinal infectious diseases	A04.2, A04.4, A07.0-A07.1, A07.8-A07.9	007-007.3, 007.9-008.00, 008.03, 008.09-008.1
Lower respiratory infections	A48.1, A70, J09-J15.8, J16-J16.9, J20-J21.9, P23.0-P23.4	073.0-073.6, 466-469, 470.0, 480-482.89, 483.0-483.9, 484.1-484.2, 484.6-484.7, 487-489
Upper respiratory infections	J01-J01.91, J04.0, J05-J05.0, J05.11, J36-J36.0	461-461.9, 464.0, 464.01, 464.11-464.2, 464.21, 464.31-464.4, 464.8-464.9, 475-475.9, 476.9
Otitis media	H70-H70.93	381-383.9
Meningitis	A39-A39.4, A39.8-A39.9, A87-A87.9, D86.81, G00.0-G00.8, G03-G03.8	036-036.40, 036.5, 036.8-036.9, 047-049.9, 320.0-320.89, 321-322.9
Pneumococcal meningitis	G00.1	320.1
H influenzae type B meningitis	G00.0	320.0
Meningococcal meningitis	A39-A39.4, A39.8-A39.9	036-036.40, 036.5, 036.8-036.9
Other meningitis	A87-A87.9, D86.81, G00.2-G00.8, G03-G03.8	047-049.9, 320.2-320.89, 321-322.9
Encephalitis	A83-A86.4, B94.1, F07.1, G04-G05.8	062-064.9, 139.0, 323, 323.4-323.9
Diphtheria	A36-A36.9	032-032.9
Whooping cough	A37-A37.91	033-033.9, 484.3-484.4
Tetanus	A33-A35.0	037-037.9, 771.3
Measles	B05-B05.9	055-055.9, 323.1, 484.0
Varicella and herpes zoster	B01-B02.9, P35.8	052-053.9
Neglected tropical diseases and malaria	A30-A30.9, A68-A68.9, A69.2-A69.9, A75-A75.9, A77-A79.9, A82-A82.9, A90-A96.9, A98-A98.8, B33.0-B33.1, B50-B53.8, B55.0, B56-B57.5, B60-B60.8, B65-B67.99, B69-B72.0, B74.3-B75, B77-B77.9, B83-B83.8, B92, P37.1	030-030.9, 060-061.8, 065-066.9, 071-071.9, 080, 080.2-083.9, 084.0-084.5, 084.7-084.9, 085.0, 086-088.9, 120-124.9, 125.4-125.9, 127-127.1, 128-129.0
Malaria	B50-B53.8	084.0-084.5, 084.7-084.9
Chagas disease	B57-B57.5	086-086.2, 086.9
Leishmaniasis	B55.0	085.0
Visceral leishmaniasis	B55.0	085.0
African trypanosomiasis	B56-B56.9	086.3-086.5
Schistosomiasis	B65-B65.9	120-120.9
Cysticercosis	B69-B69.9	123.1
Cystic echinococcosis	B67-B67.4, B67.8-B67.99	122-122.4, 122.8-122.9
Dengue	A90-A91.9	061-061.8
Yellow fever	A95-A95.9	060-060.9
Rabies	A82-A82.9	071-071.9
Intestinal nematode infections	B77-B77.9	127.0
Ascariasis	B77-B77.9	127.0
Ebola	A98.4	
Other neglected tropical diseases	A68-A68.9, A69.2-A69.9, A75-A75.9, A77-A79.9, A92-A94.0, A96-A96.9, A98-A98.3, A98.5-A98.8, B33.0-B33.1, B60-B60.8, B67.5-B67.7, B70-B72.0, B74.3-B75, B83-B83.8, P37.1	065-066.9, 080, 080.2-083.9, 087-088.9, 122.5-122.7, 123-123.0, 123.2-124.9, 125.4-125.9, 127, 127.1, 128-129.0
Maternal disorders	N96, N98-N98.9, O00-O07.9, O09-O16.9, O20-O26.93, O28-O36.93, O40-O48.1, O60-O77.9, O80-O92.79, O96-O99.91	630-636.92, 638-638.92, 640-679.14
Maternal hemorrhage	O20-O20.9, O43.2-O43.239, O44-O46.93, O62-O62.9, O67-O67.9, O70, O72-O72.3	640-641.93, 661-661.93, 665, 666-666.9
Maternal sepsis and other maternal infections	O23-O23.93, O85-O86.89, O91-O91.23	659.3-659.33, 670-670.9
Maternal hypertensive disorders	O10-O16.9	642-642.94
Maternal obstructed labor and uterine rupture	O32-O33.9, O64-O66.9, O71-O71.9	652-653.93, 660-660.93, 665.0-665.34
Maternal abortion, miscarriage, and ectopic pregnancy	N96, O00-O07.9	630-636.92, 638-638.92, 646.3-646.33
Indirect maternal deaths	O24-O25.3, O98-O99.91	646-646.24, 646.4-649.9, 674-674.94
Late maternal deaths	O96-O97.9	
Maternal deaths aggravated by HIV/AIDS		
Other maternal disorders	N98-N98.9, O09-O09.93, O21-O22.93, O26-O26.93, O28-O31.8, O34-O36.93, O40-O43.199, O43.8-O43.93, O47-O48.1, O60-O61.9, O63-O63.9, O68-O69.9, O70.0-O70.9, O73-O77.9, O80-O84.9, O87-O90.9, O92-O92.79	643-645.23, 650-651.93, 654-659.23, 659.4-659.93, 662-664.94, 665.4-665.94, 667-669.94, 671-673.9, 675-679.14
Neonatal disorders	P00-P04.2, P04.5-P05.9, P07-P15.9, P19-P22.9, P24-P29.9, P36-P36.9, P38-P39.9, P50-P61.9, P70, P70.3-P72.9, P74-P78.9, P80-P81.9, P83-P84, P90-P94.9, P96, P96.3-P96.4, P96.8-P96.89	760-760.64, 760.8-768, 768.2-770, 770.1-771, 771.4-775, 775.4-779.34, 779.6-779.89
Neonatal preterm birth complications	P01.0-P01.1, P07-P07.39, P22-P22.9, P25-P28.9, P61.2, P77-P77.9	761.0-761.1, 765-765.9, 769-769.9, 770.2-770.9, 776.6, 777.5-777.6
Neonatal encephalopathy due to birth asphyxia and trauma	P01.7, P02-P03.9, P10-P15.9, P20-P21.9, P24-P24.9, P90-P91.9	761.7-763.9, 767-768, 768.2-768.9, 770.1-770.18, 772.1-772.9, 779.0-779.2
Neonatal sepsis and other neonatal infections	P36-P36.9, P38-P39.9	771.4-771.9
Hemolytic disease and other neonatal jaundice	P55-P59.9	773-774.9
Other neonatal disorders	P00-P01, P01.2-P01.6, P01.8-P01.9, P04-P04.2, P04.5-P05.9, P08-P09, P19-P19.9, P29-P29.9, P50-P54.9, P60-P61.1, P61.3-P61.9, P70, P70.3-P72.9, P74-P76.9, P78-P78.9, P80-P81.9, P83-P84, P92-P94.9, P96, P96.3-P96.4, P96.8-P96.89	760-760.64, 760.8-761, 761.2-761.6, 764-764.99, 766-766.9, 770, 771, 772-772.0, 775, 775.4-776.5, 776.7-777.4, 777.7-779, 779.3-779.34, 779.6-779.89
Nutritional deficiencies	D50.1-D50.8, D51-D52.0, D52.8-D53.9, D64.3, E00-E02, E40-E46.9, E51-E61.9, E63-E64.0, E64.2-E64.9, M12.1-M12.19	244.2, 260-263.9, 265-269.9, 280.1-280.8, 281.0-281.9, 716.0-716.09
Protein-energy malnutrition	E40-E46.9, E64.0	260-263.9
Iodine deficiency	E00-E02	244.2
Iron-deficiency anemia	D50.1-D50.8, D64.3	280.1-280.8
Other nutritional deficiencies	D51-D52.0, D52.8-D53.9, E51-E61.9, E63-E64, E64.2-E64.3, M12.1-M12.19	265-269.9, 281.0-281.9, 716.0-716.09
Other communicable, maternal, neonatal, and nutritional diseases	A20-A28.9, A32-A32.9, A38-A38.9, A48.2, A48.4-A48.52, A49.1, A50-A58, A60-A60.9, A63-A63.8, A65-A65.0, A69-A69.1, A74, A74.8-A74.9, A80-A81.9, A88-A89.9, B00-B00.9, B03-B04, B06-B06.9, B10-B10.89, B15-B17.9, B19-B19.9, B25-B27.99, B29.4, B33, B33.3-B33.8, B47-B48.8, B63, B91, B94.2, B95-B95.5, G14-G14.6, I00, I02, I02.9, I98.0-I98.1, J02.0, J03.0-J03.01, K67.0-K67.2, K67.8, K74.7-K74.8, M03.1, M49.1, M73.0-M73.1, M89.6-M89.69, N70-N71.9, N73-N74.8, P35-P35.3, P35.9, P37, P37.2, P37.5-P37.9	020-029, 034-034.9, 039-039.4, 039.8-040, 040.1-041.09, 045-046.9, 050-051.9, 054-054.9, 056-059.9, 070-070.21, 070.3-070.31, 070.4-070.43, 070.49-070.53, 070.59-070.9, 072-073, 073.7-074.1, 074.20, 074.3-075.9, 078.4-078.7, 079-079.7, 080.0, 090-101.6, 104-104.9, 136-136.29, 138, 139, 323.0-323.02, 323.2-323.3, 390-390.9, 392, 392.9, 484.5, 613-614.9, 771.0-771.2

Appendix Table 7: List of International Classification of Diseases (ICD) codes mapped to the Global Burden of Disease cause list for causes of death

Cause	ICD10	ICD9
Sexually transmitted diseases excluding HIV	A50-A58, A60-A60.9, A63-A63.8, B63, I98.0, K67.0-K67.2, M03.1, M73.0-M73.1, N70-N71.9, N73-N74.8	054.1, 090-099.9, 613-614.9
Syphilis	A50-A53.9, I98.0, K67.2, M03.1, M73.1	090-097.9
Chlamydial infection	A55-A56.8, K67.0	099, 099.1-099.6
Gonococcal infection	A54-A54.9, K67.1, M73.0	098-098.9
Other sexually transmitted diseases	A57-A58, A63-A63.8	099.0, 099.8-099.9
Hepatitis	B15-B17.9, B19-B19.9, B94.2, P35.3	070-070.21, 070.3-070.31, 070.4-070.43, 070.49-070.53, 070.59-070.9
Acute hepatitis A	B15-B15.9	070.0-070.1
Acute hepatitis B	B16-B17.0, B19.1-B19.11, P35.3	070.2-070.21, 070.3-070.31, 070.42, 070.52
Acute hepatitis C	B17.1-B17.11, B19.2-B19.21	070.41, 070.51, 070.7-070.71
Acute hepatitis E	B17.2	070.43, 070.53
Other infectious diseases	A20-A28.9, A32-A32.9, A38-A38.9, A48.2, A48.4-A48.52, A49.1, A65-A65.0, A69-A69.1, A74, A74.8-A74.9, A80-A81.9, A88-A89.9, B00-B00.9, B03-B04, B06-B06.9, B10-B10.89, B25-B27.99, B29.4, B33, B33.3-B33.8, B47-B48.8, B91, B95-B95.5, G14-G14.6, I00, I02, I02.9, I98.1, J02.0, J03.0-J03.01, K67.8, K74.7-K74.8, M49.1, M89.6-M89.69, P35-P35.2, P35.9, P37, P37.2, P37.5-P37.9	020-029, 034-034.9, 039-039.4, 039.8-040, 040.1-041.09, 045-046.9, 050-051.9, 054-054.0, 054.10-054.9, 056-059.9, 072-073, 073.7-074.1, 074.20, 074.3-075.9, 078.4-078.7, 079-079.7, 080.0, 100-101.6, 104-104.9, 136-136.29, 138, 139, 323.0-323.02, 323.2-323.3, 390-390.9, 392, 392.9, 484.5, 771.0-771.2
Non-communicable diseases	A39.5-A39.53, A46-A46.0, A66-A67.9, B18-B18.9, B33.2-B33.24, B86, C0-C13.9, C15-C25.9, C3-C34.92, C37-C38.8, C4-C41.9, C43-C45.9, C47-C54.9, C56-C57.8, C58-C58.0, C60-C63.8, C64-C67.9, C68.0-C68.8, C69-C75.8, C81-C86.6, C88-C96.9, D00.00-D00.2, D01.0-D01.3, D02.0-D02.3, D03-D06.9, D07.0-D07.2, D07.4-D07.5, D09.0, D09.2-D09.3, D09.8, D10.0-D10.7, D11-D12.9, D13.0-D13.7, D14.0-D14.32, D15-D16.9, D22-D27.9, D28.0-D28.7, D29.0-D29.8, D30.0-D30.8, D31-D36, D36.1-D36.7, D37.01-D37.5, D38.0-D38.5, D39.1-D39.2, D39.8, D40.0-D40.8, D41.0-D41.8, D42-D43.9, D44.0-D44.8, D45-D45.9, D47-D47.0, D47.2-D47.9, D48.0-D48.62, D49.2-D49.4, D49.6, D49.81, D52.1, D55-D58.9, D59.0-D59.3, D59.5-D59.6, D60-D61.9, D63.1, D64.0, D64.4, D66-D67, D68.0-D69.8, D70-D75.89, D76-D78.89, D86-D86.8, D86.82-D86.9, D89-D89.3, E03-E07.1, E09-E14.9, E15.0, E16.0-E16.9, E20-E34.8, E36-E36.8, E65-E68, E70-E85.29, E87.71, E88-E89.9, F00-F03.91, F06.2, F10-F16.99, F18-F29.9, F50.0-F50.5, G10-G13.8, G20-G26.0, G30-G31.9, G35-G37.9, G40-G41.9, G45-G46.8, G47.3-G47.39, G61-G61.9, G70-G73.7, G90-G90.9, G93.7, G95-G95.9, G97-G97.9, H05.0-H05.119, I01-I01.9, I02.0, I05-I09.9, I11-I13.9, I20-I25.9, I27.1, I28-I28.8, I30-I31.1, I31.8-I43.9, I47-I48.92, I51.0-I51.5, I60-I61.9, I62.0-I62.03, I63-I63.9, I65-I66.9, I67.0-I67.3, I67.5-I67.7, I68.0-I68.2, I69.0-I69.398, I70.2-I70.799, I71-I73.9, I77-I89.9, I91.9, I95.2-I95.3, I97-I98, I98.2, I98.9, J30-J35.9, J37-J47.9, J60-J63.8, J65-J68.9, J70-J70.9, J82, J84-J84.9, J91-J92.9, J95-J95.9, K20-K29.91, K31-K31.89, K35-K38.9, K40-K46.9, K50-K52.9, K55-K62.9, K63.5, K64-K64.9, K66.8, K67, K68-K68.9, K70-K70.9, K71.3-K71.51, K71.7, K72.1-K74.69, K74.9, K75.2-K77.8, K80-K83.9, K85-K86.9, K90-K91.9, K92.8-K92.89, K94-K95.89, L00-L05.92, L08-L08.9, L10-L14.0, L51-L51.9, L88-L89.95, L93-L93.2, L97-L98.499, M00-M03.0, M03.2-M03.6, M05-M09.8, M30-M36.8, M40-M43.19, M65-M65.08, M71.0-M71.19, M80-M82.8, M86.3-M86.49, M87-M87.19, M88-M89.09, M89.5-M89.59, M89.7-M89.9, N00-N08.8, N10-N12.9, N14-N16.8, N18-N18.9, N20-N23.0, N25-N32.0, N32.3-N32.4, N34-N34.3, N36-N36.9, N39-N39.2, N41-N41.9, N44-N44.04, N45-N45.9, N49-N49.9, N60-N60.99, N65-N65.1, N72-N72.0, N75-N77.8, N80-N81.9, N83-N83.9, N84.0-N84.1, N87-N87.9, N99-N99.9, P04.3-P04.49, P70.0-P70.2, P96.0-P96.2, P96.5, Q00-Q07.9, Q10.4-Q18.9, Q20-Q28.9, Q30-Q36, Q37-Q45.9, Q50-Q87.89, Q89-Q89.8, Q90-Q93.9, Q95-Q99.8, R50.2, R50.82-R50.83, R73-R73.9, R78.0-R78.5, R95, X45-X45.9	035-035.9, 036.41-036.43, 036.6, 070.22-070.23, 070.32-070.33, 070.44, 070.54, 074.2, 074.21-074.23, 102-103.9, 133-133.6, 135-135.9, 136.6, 140-148.9, 150-158.9, 160-164.9, 170-175.9, 180-183.8, 184.0-184.4, 184.8, 185-186.9, 187.1-187.8, 188-188.9, 189.0-189.8, 190-194.8, 200-208.92, 209.0-209.17, 209.21-209.27, 209.31-209.57, 209.61, 209.63-209.67, 210.0-210.9, 211.0-211.8, 212.0-212.8, 213-213.9, 217-220.9, 221.0-221.8, 222.0-222.8, 223.0-223.89, 224-228.9, 229.0, 229.8, 230.1-230.8, 231.0-231.2, 232-232.9, 233.0-233.2, 233.31-233.32, 233.4-233.5, 233.7, 234.0-234.8, 235.0, 235.4, 235.6-235.8, 236.0-236.2, 236.4-236.5, 236.7, 236.91-237.3, 237.5-237.9, 238.0-238.5, 239.2-239.4, 239.6, 240-243.9, 244.0-244.1, 244.3-244.8, 245-246.9, 250-259.9, 270-273.9, 275-276, 277-277.2, 277.4-277.9, 278.0-278.8, 282-284.9, 286-286.5, 286.7-289.7, 290-292.9, 294.1-295.95, 303-303.93, 304.0-304.83, 305-305.93, 307.1, 307.51, 307.54, 327.2-327.8, 330-331.2, 331.5-337.9, 340-341.9, 345-345.91, 349-349.8, 353.6-353.9, 356-356.9, 357.0-357.7, 358-359.9, 376.0-376.1, 391-391.9, 392.0, 393-398.99, 402-404.93, 410-414.9, 416.1, 417-417.9, 420-423, 423.1-425.9, 427-427.32, 427.6-427.89, 429.0-429.1, 430-435.9, 437.0-437.2, 437.4-437.8, 441-443.9, 446-457.9, 459, 459.1-459.39, 470, 470.9-474.9, 476-476.1, 477-479, 490-504.9, 506-506.9, 508-509, 515, 516-517.8, 518.6-518.7, 518.9, 519.0-519.4, 530-536.1, 536.4-536.49, 537-537.6, 537.8-537.84, 538-543.9, 550-553.6, 555-558.9, 560-560.39, 560.8-560.9, 562-562.13, 564-564.7, 565-566.9, 569.0-569.44, 569.5-569.71, 569.84-569.85, 571-571.9, 572.3-572.9, 573.0-573.4, 573.8-577.9, 579-583.9, 585-585.9, 588-590.9, 592-593.89, 594-599.69, 599.8-599.89, 601-602.9, 604-604.99, 608.2-608.24, 610-610.9, 617-618.9, 620-620.9, 621.4-621.9, 622.3-622.7, 629-629.81, 680-689, 694-695.59, 707-707.9, 710-711.99, 714-714.33, 714.8-714.9, 728.86, 728.88, 730.1-730.19, 732-732.9, 733.0-733.19, 740-749.04, 749.2-758.9, 759.0-759.89, 760.7-760.79, 775.0-775.3, 779.4-779.5, 780.57, 780.59, 780.62-780.63, 786.03, 787.1, 788.0, 790.2-790.22, 790.3, 798-798.0, E850-E850.29, E850.9-E854.39, E860-E860.19
Neoplasms	C0-C13.9, C15-C25.9, C3-C34.92, C37-C38.8, C4-C41.9, C43-C45.9, C47-C54.9, C56-C57.8, C58-C58.0, C60-C63.8, C64-C67.9, C68.0-C68.8, C69-C75.8, C81-C86.6, C88-C96.9, D00.00-D00.2, D01.0-D01.3, D02.0-D02.3, D03-D06.9, D07.0-D07.2, D07.4-D07.5, D09.0, D09.2-D09.3, D09.8, D10.0-D10.7, D11-D12.9, D13.0-D13.7, D14.0-D14.32, D15-D16.9, D22-D24.9, D26.0, D27-D27.9, D28.0-D28.1, D28.7, D29.0-D29.8, D30.0-D30.8, D31-D36, D36.1-D36.7, D37.01-D37.5, D38.0-D38.5, D39.1-D39.2, D39.8, D40.0-D40.8, D41.0-D41.8, D42-D43.9, D44.0-D44.8, D45-D45.9, D47-D47.0, D47.2-D47.9, D48.0-D48.62, D49.2-D49.4, D49.6, D49.81, K31.7, K62.0-K62.1, K63.5, N60-N60.99, N84.0-N84.1, N87-N87.9	140-148.9, 150-158.9, 160-164.9, 170-175.9, 180-183.8, 184.0-184.4, 184.8, 185-186.9, 187.1-187.8, 188-188.9, 189.0-189.8, 190-194.8, 200-208.92, 209.0-209.17, 209.21-209.27, 209.31-209.57, 209.61, 209.63-209.67, 210.0-210.9, 211.0-211.8, 212.0-212.8, 213-213.9, 217-217.8, 219.0, 220-220.9, 221.0-221.8, 222.0-222.8, 223.0-223.89, 224-228.9, 229.0, 229.8, 230.1-230.8, 231.0-231.2, 232-232.9, 233.0-233.2, 233.31-233.32, 233.4-233.5, 233.7, 234.0-234.8, 235.0, 235.4, 235.6-235.8, 236.1-236.2, 236.4-236.5, 236.7, 236.91-237.3, 237.5-237.9, 238.0-238.5, 239.2-239.4, 239.6, 569.0, 569.43-569.44, 569.84-569.85, 610-610.9
Lip and oral cavity cancer	C0-C08.9, D00.00-D00.07, D10.0-D10.5, D11-D11.9, D37.01-D37.04, D37.09	140-145.9, 210.0-210.6, 235.0
Nasopharynx cancer	C11-C11.9, D00.08, D10.6, D37.05	147-147.9, 210.7-210.9
Other pharynx cancer	C09-C10.9, C12-C13.9, D10.7	146-146.9, 148-148.9
Esophageal cancer	C15-C15.9, D00.1, D13.0	150-150.9, 211.0, 230.1
Stomach cancer	C16-C16.9, D00.2, D13.1, D37.1	151-151.9, 209.23, 209.63, 211.1, 230.2
Colon and rectum cancer	C18-C21.9, D01.0-D01.3, D12-D12.9, D37.3-D37.5	153-154.9, 209.1-209.17, 209.5-209.57, 211.3-211.4, 230.3-230.6
Liver cancer	C22-C22.9, D13.4	155-155.9, 211.5
Liver cancer due to hepatitis B		
Liver cancer due to hepatitis C		
Liver cancer due to alcohol use		
Liver cancer due to other causes		
Gallbladder and biliary tract cancer	C23-C24.9, D13.5	156-156.9, 209.25-209.27, 209.65-209.67
Pancreatic cancer	C25-C25.9, D13.6-D13.7	157-157.9, 211.6-211.7
Larynx cancer	C32-C32.9, D02.0, D14.1, D38.0	161-161.9, 212.1, 231.0, 235.6
Tracheal, bronchus, and lung cancer	C33-C34.92, D02.1-D02.3, D14.2-D14.32, D38.1	162-162.9, 209.21, 209.61, 212.2-212.3, 231.1-231.2, 235.7
Malignant skin melanoma	C43-C43.9, D03-D03.9, D22-D23.9, D48.5	172-172.9
Non-melanoma skin cancer	C44-C44.99, D04-D04.9, D49.2	173-173.99, 222.4, 232-232.9, 238.2
Non-melanoma skin cancer (squamous-cell carcinoma)	C44-C44.99, D04-D04.9, D49.2	173-173.99, 222.4, 232-232.9, 238.2
Breast cancer	C50-C50.929, D05-D05.92, D24-D24.9, D48.6-D48.62, D49.3, N60-N60.99	174-175.9, 217-217.8, 233.0, 238.3, 239.3, 610-610.9
Cervical cancer	C53-C53.9, D06-D06.9, D26.0	180-180.9, 219.0, 233.1
Uterine cancer	C54-C54.9, D07.0-D07.2, N87-N87.9	182-182.8, 233.2
Ovarian cancer	C56-C56.9, D27-D27.9, D39.1-D39.12	183-183.0, 220-220.9, 236.2
Prostate cancer	C61-C61.9, D07.5, D29.1, D40.0	185-185.9, 222.2, 236.5
Testicular cancer	C62-C62.92, D29.2-D29.8, D40.1-D40.8	186-186.9, 222.0, 222.3, 236.4
Kidney cancer	C64-C65.9, D30.0-D30.12, D41.0-D41.12	189.0-189.1, 209.24, 209.64, 223.0-223.1, 236.91
Bladder cancer	C67-C67.9, D09.0, D30.3, D41.4-D41.8, D49.4	188-188.9, 223.3, 233.7, 236.7, 239.4
Brain and nervous system cancer	C70-C72.9	191-192.9
Thyroid cancer	C73-C73.9, D09.3, D09.8, D34-D34.9, D44.0	193-193.9, 226-226.9
Mesothelioma	C45-C45.9	158.9, 163-163.9, 212.4
Hodgkin lymphoma	C81-C81.99	201-201.98
Non-Hodgkin lymphoma	C82-C86.6, C96-C96.9	200-200.9, 202-202.98
Multiple myeloma	C88-C90.9	203-203.9
Leukemia	C91-C95.92	204-208.92
Acute lymphoid leukemia	C91.0-C91.02	204.0-204.02
Chronic lymphoid leukemia	C91.1-C91.12	204.1-204.12
Acute myeloid leukemia	C92.0-C92.02, C92.3-C92.62, C93.0-C93.02, C94.0-C94.02, C94.2-C94.22, C94.4-C94.5	205.0-205.02, 205.3-205.32, 206.0-206.02, 207.0
Chronic myeloid leukemia	C92.1-C92.12	205.1-205.12, 206.1-206.12, 207.1
Other neoplasms	C17-C17.9, C3-C31.9, C37-C38.8, C4-C41.9, C47-C5, C51-C52.9, C57-C57.8, C58-C58.0, C60-C60.9, C63-C63.8, C66-C66.9, C68.0-C68.8, C69-C7, C74-C75.8, D07.4, D09.2-D09.22, D13.2-D13.39, D14.0, D15-D16.9, D28.0-D28.1, D28.7, D29.0, D30.2-D30.22, D30.4-D30.8, D31-D33.9, D35-D36, D36.1-D36.7, D37.2, D38.2-D38.5, D39.2, D39.8, D41.2-D41.3, D42-D43.9, D44.1-D44.8, D45-D45.9, D47-D47.0, D47.2-D47.9, D48.0-D48.4, D49.6, D49.81, K31.7, K62.0-K62.1, K63.5, N84.0-N84.1	152-152.9, 158-158.8, 160-160.9, 164-164.9, 170-171.9, 181-181.9, 182.9, 183.2-183.8, 184.0-184.4, 184.8, 187.1-187.8, 189.2-189.8, 190-190.9, 194-194.8, 209.0-209.03, 209.22, 209.31-209.43, 211.2, 211.8, 212.0, 212.5-212.8, 213-213.9, 221.0-221.8, 222.1, 222.8, 223.2, 223.8-223.89, 224-225.9, 227-228.9, 229.0, 229.8, 230.7-230.8, 233.31-233.32, 233.4-233.5, 234.0-234.8, 235.4, 235.8, 236.1, 236.99-237.3, 237.5-237.9, 238.0-238.1, 238.4-238.5, 239.2, 239.6, 569.0, 569.43-569.44, 569.84-569.85
Cardiovascular diseases	A39.5-A39.53, B33.2-B33.24, D86.85, G45-G46.8, I01-I01.9, I02.0, I05-I09.9, I11-I11.9, I20-I25.9, I28-I28.8, I30-I31.1, I31.8-I43.9, I47-I48.92, I51.0-I51.5, I60-I61.9, I62.0-I62.03, I63-I63.9, I65-I66.9, I67.0-I67.3, I67.5-I67.6, I68.0-I68.2, I69.0-I69.398, I70.2-I70.799, I71-I73.9, I77-I83.93, I86-I89.9, I91.9, I98	036.41-036.43, 036.6, 074.2, 074.21-074.23, 391-391.9, 392.0, 393-398.99, 402-402.91, 410-414.9, 417-417.9, 420-423, 423.1-425.9, 427-427.32, 427.6-427.89, 429.0-429.1, 430-435.9, 437.0-437.2, 437.5-437.8, 441-443.9, 447-454.9, 456, 456.3-457.9, 459, 459.1-459.39

Appendix Table 7: List of International Classification of Diseases (ICD) codes mapped to the Global Burden of Disease cause list for causes of death

Cause	ICD10	ICD9
Rheumatic heart disease	I01-I01.9, I02.0, I05-I09.9	391-391.9, 392.0, 393-398.99
Ischemic heart disease	I20-I25.9	410-414.9
Cerebrovascular disease	G45-G46.8, I60-I61.9, I62.0-I62.03, I63-I63.9, I65-I66.9, I67.0-I67.3, I67.5-I67.6, I68.1-I68.2, I69.0-I69.398	430-435.9, 437.0-437.2, 437.5-437.8
Ischemic stroke	G45-G46.8, I63-I63.9, I65-I66.9, I67.2-I67.3, I67.5-I67.6, I69.3-I69.398	433-435.9, 437.0-437.1, 437.5-437.8
Hemorrhagic stroke	I60-I61.9, I62.0-I62.03, I67.0-I67.1, I68.1-I68.2, I69.0-I69.298	430-432.9, 437.2
Hypertensive heart disease	I11-I11.9	402-402.91
Cardiomyopathy and myocarditis	A39.52, B33.2-B33.24, D86.85, I40-I43.9, I51.4-I51.5	036.43, 036.6, 074.23, 422-422.99, 425-425.9, 429.0-429.1
Atrial fibrillation and flutter	I48-I48.92	427.3-427.32
Aortic aneurysm	I71-I71.9	441-441.9
Peripheral vascular disease	I70.2-I70.799, I73-I73.9	443.0-443.9
Endocarditis	A39.51, I33-I33.9, I38-I39.9	036.42, 074.22, 421-421.9, 424.9-424.91
Other cardiovascular and circulatory diseases	A39.5-A39.50, A39.53, I28-I28.8, I30-I31.1, I31.8-I32.8, I34-I37.9, I47-I47.9, I51.0-I51.3, I68.0, I72-I72.9, I77-I83.93, I86-I89.9, I91.9, I98	036.41, 074.2, 074.21, 417-417.9, 420-420.99, 423, 423.1-424.8, 424.99, 427-427.2, 427.6-427.89, 442-443, 447-454.9, 456, 456.3-457.9, 459, 459.1-459.39
Chronic respiratory diseases	D86-D86.2, D86.89-D86.9, G47.3-G47.39, J30-J35.9, J37-J47.9, J60-J63.8, J65-J68.9, J70-J70.1, J70.8-J70.9, J82, J84-J84.9, J91-J92.9	135-135.9, 136.6, 327.2-327.8, 470, 470.9-474.9, 476-476.1, 477-479, 490-504.9, 506-506.9, 508-509, 515, 516-517.8, 518.6, 518.9, 519.1-519.4, 780.57, 786.03
Chronic obstructive pulmonary disease	J40-J44.9, J47-J47.9	490-492.9, 494-494.9, 496-499
Pneumoconiosis	J60-J63.8, J65-J65.0, J92.0	500-504.9
Silicosis	J62-J62.9	502-502.9, 503.0, 503.9
Asbestosis	J61-J61.0, J92.0	501
Coal workers pneumoconiosis	J60-J60.0	500-500.9, 501.0-501.9
Other pneumoconiosis	J63-J63.8, J65-J65.0	503, 503.1, 504-504.9
Asthma	J45-J46.9	493-493.92
Interstitial lung disease and pulmonary sarcoidosis	D86-D86.2, D86.89-D86.9, J84-J84.9	135-135.9, 136.6, 515, 516-516.9
Other chronic respiratory diseases	G47.3-G47.39, J30-J35.9, J37-J39.9, J66-J68.9, J70-J70.1, J70.8-J70.9, J82, J91-J92, J92.9	327.2-327.8, 470, 470.9-474.9, 476-476.1, 477-479, 495-495.9, 506-506.9, 508-509, 517-517.8, 518.6, 518.9, 519.1-519.4, 780.57, 786.03
Cirrhosis and other chronic liver diseases	B18-B18.9, I85-I85.9, I98.2, K70-K70.9, K71.3-K71.51, K71.7, K72.1-K74.69, K74.9, K75.8-K76.0, K76.6-K76.7, K76.9	070.22-070.23, 070.32-070.33, 070.44, 070.54, 456.0-456.21, 571-571.9, 572.3-572.9, 573.0-573.3, 573.8-573.9
Cirrhosis and other chronic liver diseases due to hepatitis B		
Cirrhosis and other chronic liver diseases due to hepatitis C		
Cirrhosis and other chronic liver diseases due to alcohol use		
Cirrhosis and other chronic liver diseases due to other causes		
Digestive diseases	I84-I84.9, K20-K29.91, K31-K31.6, K31.8-K31.89, K35-K38.9, K40-K42.9, K44-K46.9, K50-K52.9, K55-K62, K62.2-K62.6, K62.8-K62.9, K64-K64.9, K66.8, K67, K68-K68.9, K75.2-K75.4, K76.1-K76.5, K76.8-K76.89, K77-K77.8, K80-K83.9, K85-K86.9, K90-K90.9, K92.8-K92.89, M09.1	455-455.9, 530-536.1, 537-537.6, 537.8-537.84, 538, 540-543.9, 550-551.1, 551.3-552.1, 552.3-553.6, 555-558.9, 560-560.39, 560.8-560.9, 562-562.13, 564-564.1, 564.5-564.7, 565-566.9, 569.1-569.42, 569.5, 569.7-569.71, 573.4, 574-577.9, 579-579.2, 579.4-579.9, 787.1
Peptic ulcer disease	K25-K28.9, K31, K31.1-K31.6, K31.8, K31.82-K31.89	531-534.91
Gastritis and duodenitis	K29-K29.91	535-535.9
Appendicitis	K35-K37.9, K38.3-K38.9	540-542.9
Paralytic ileus and intestinal obstruction	K56-K56.9	560-560.39, 560.8-560.9
Inguinal, femoral, and abdominal hernia	K40-K42.9, K44-K46.9	550-551.1, 551.3-552.1, 552.3-553.03, 553.6
Inflammatory bowel disease	K50-K52.9, M09.1	555-556.9, 558-558.9, 569.5
Vascular intestinal disorders	K55-K55.9	557-557.9
Gallbladder and biliary diseases	K80-K83.9	574-576.9
Pancreatitis	K85-K86.9	577-577.9, 579.4
Other digestive diseases	I84-I84.9, K20-K24, K31.0, K31.81-K31.819, K38-K38.2, K57-K62, K62.2-K62.6, K62.8-K62.9, K64-K64.9, K66.8, K67, K68-K68.9, K75.2-K75.4, K76.1-K76.5, K76.8-K76.89, K77-K77.8, K90-K90.9, K92.8-K92.89	455-455.9, 530-530.9, 536-536.1, 537-537.6, 537.8-537.84, 538, 543-543.9, 553.1-553.3, 562-562.13, 564-564.1, 564.5-564.7, 565-566.9, 569.1-569.42, 569.7-569.71, 573.4, 579-579.2, 579.8-579.9, 787.1
Neurological disorders	F00-F03.91, G10-G13.8, G20-G21.0, G21.2-G24, G24.1-G25.0, G25.2-G25.3, G25.5, G25.8-G26.0, G30-G31.1, G31.8-G31.9, G35-G37.9, G40-G41.9, G61-G61.9, G70-G72, G72.2-G73.7, G90-G90.9, G95-G95.9, M33-M33.99	290-290.9, 294.1-294.9, 330-331.2, 331.5-337.9, 340-341.9, 345-345.91, 349, 349.2-349.8, 353.6-353.9, 356-356.9, 357.0-357.1, 357.3-357.4, 357.7, 358-359.9, 728.86, 728.88, 775.2
Alzheimer disease and other dementias	F00-F03.91, G30-G31.1, G31.8-G31.9	290-290.9, 294.1-294.9, 331-331.2
Parkinson disease	G20-G21.0, G21.2-G22.0	332-332.9
Epilepsy	G40-G41.9	345-345.91
Multiple sclerosis	G35-G35.9	340-340.9
Motor neuron disease	G12.2-G12.9	335-335.29, 335.8-335.9
Other neurological disorders	G10-G12.1, G13-G13.8, G23-G24, G24.1-G25.0, G25.2-G25.3, G25.5, G25.8-G26.0, G36-G37.9, G61-G61.9, G70-G72, G72.2-G73.7, G90-G90.9, G95-G95.9, M33-M33.99	330-330.9, 331.5-331.9, 333-334.9, 335.3, 336-337.9, 341-341.9, 349, 349.2-349.8, 353.6-353.9, 356-356.9, 357.0-357.1, 357.3-357.4, 357.7, 358-359.9, 728.86, 728.88, 775.2
Mental and substance use disorders	F06.2, F10-F16.99, F18-F29.9, F50.0-F50.5, G31.2, G72.1, P04.3-P04.49, P96.1, Q86.0, R78.0-R78.5, X45-X45.9	291-292.9, 295-295.95, 303-303.93, 304.0-304.83, 305-305.93, 307.1, 307.51, 307.54, 357.5, 760.7-760.79, 780.59, 790.3, E850-E850.29, E850.9-E854.39, E860-E860.19
Schizophrenia	F06.2, F20-F23.9, F25-F29.9	295-295.95
Alcohol use disorders	F10-F10.99, G31.2, G72.1, P04.3, Q86.0, R78.0, X45-X45.9	291-291.9, 303-303.93, 305.0-305.03, 357.5, 790.3, E860-E860.19
Drug use disorders	F11-F16.99, F18-F19.99, P04.4-P04.49, P96.1, R78.1-R78.5	292-292.9, 304.0-304.83, 305, 305.1-305.93, 760.7-760.79, E850-E850.29, E850.9-E854.39
Opioid use disorders	F11-F11.99, P96.1, R78.1	304.0-304.03, 305.5-305.53, E850.0-E850.29
Cocaine use disorders	F14-F14.99, R78.2	304.2-304.23, 305.6-305.63
Amphetamine use disorders	F15-F15.99	304.4-304.43, 305.7-305.73
Other drug use disorders	F13-F13.99, F16-F16.99, F18-F19.99, R78.3-R78.5	292-292.9, 304.1-304.13, 304.5-304.83, 305, 305.1-305.13, 305.3-305.43, 305.8-305.93
Eating disorders	F50.0-F50.5	307.1, 307.51, 307.54
Anorexia nervosa	F50.0-F50.1	307.1, 307.54
Bulimia nervosa	F50.2-F50.5	307.51
Diabetes, urogenital, blood, and endocrine diseases	D25-D26, D26.1-D26.9, D28.2, D52.1, D55-D58.9, D59.0-D59.3, D59.5-D59.6, D60-D61.9, D63.1, D64.0, D64.4, D66-D67, D68.0-D69.8, D70-D75.89, D76-D78.89, D86.8, D86.82-D86.84, D86.86-D86.87, D89-D89.3, E03-E07.1, E09-E14.9, E15.0, E16.0-E16.9, E20-E34.8, E36-E36.8, E65-E68, E70-E85.29, E87.71, E88-E89.9, G21.1-G21.19, G24.0-G24.09, G25.1, G25.4, G25.6-G25.79, G72.0, G93.7, G97-G97.9, I12-I13.9, I95.2-I95.3, I97-I97.9, I98.9, J70.2-J70.5, J95-J95.9, K43-K43.9, K62.7, K91-K91.9, K94-K95.89, M87.1-M87.19, N00-N08.8, N10-N12.9, N14-N16.8, N18-N18.9, N20-N23.0, N25-N32.0, N32.3-N32.4, N34-N34.3, N36-N36.9, N39-N39.2, N41-N41.9, N44-N44.04, N45-N45.9, N49-N49.9, N65-N65.1, N72-N72.0, N75-N77.8, N80-N81.9, N83-N83.9, N99-N99.9, P70.0-P70.2, P96.2, P96.5, R50.2, R50.82-R50.83, R73-R73.9	218-219, 219.1-219.9, 236.0, 240-243.9, 244.0-244.1, 244.3-244.8, 245-246.9, 250-259.9, 270-273.9, 275-276, 277-277.2, 277.4-277.9, 278.0-278.8, 282-284.9, 286-286.5, 286.7-289.7, 349.0-349.1, 357.2, 357.6, 403-404.93, 518.7, 519.0-519.09, 536.4-536.49, 539-539.9, 551.2-551.29, 552.2-552.29, 564.2-564.4, 569.6-569.69, 579.3, 580-583.9, 585-585.9, 588-590.9, 592-593.89, 594-599.69, 599.8-599.89, 601-602.9, 604-604.99, 608.2-608.24, 617-618.9, 620-620.9, 621.4-621.9, 622.3-622.7, 629-629.81, 775.0-775.1, 775.3, 779.4-779.5, 780.62-780.63, 788.0, 790.2-790.22
Diabetes mellitus	E10-E10.11, E10.3-E11.1, E11.3-E12.1, E12.3-E13.11, E13.3-E14.1, E14.3-E14.9, P70.0-P70.2, R73-R73.9	250-250.39, 250.5-250.99, 357.2, 775.0-775.1, 790.2-790.22
Acute glomerulonephritis	N00-N01.9	580-580.9
Chronic kidney disease	D63.1, E10.2-E10.29, E11.2-E11.29, E12.2, E13.2-E13.29, E14.2, I12-I13.9, N02-N08.8, N15.0, N18-N18.9	250.4-250.49, 403-404.93, 581-583.9, 585-585.9, 589-589.9
Chronic kidney disease due to diabetes mellitus	E10.2-E10.29, E11.2-E11.29, E12.2, E13.2-E13.29, E14.2	250.4-250.49
Chronic kidney disease due to hypertension	I12-I13.9	403-404.93
Chronic kidney disease due to glomerulonephritis	N03-N06.9	581-583.9
Chronic kidney disease due to other causes	N02-N02.9, N07-N08.8, N15.0	589-589.9
Urinary diseases and male infertility	N10-N12.9, N15, N15.1-N16.8, N20-N23.0, N25-N32.0, N32.3-N32.4, N34-N34.3, N36-N36.9, N39-N39.2, N41-N41.9, N44-N44.04, N45-N45.9, N49-N49.9	588-588.9, 590-590.9, 592-593.89, 594-596.81, 596.89-598.1, 598.8-599.69, 599.8-599.89, 601-602.9, 604-604.99, 608.2-608.24, 788.0
Interstitial nephritis and urinary tract infections	N10-N12.9, N15, N15.1-N16.8, N30-N30.91, N34-N34.3, N39.0-N39.2	590-590.9, 595-595.9, 597-597.9, 599.0
Urolithiasis	N20-N23.0	592-592.9, 594-594.9, 788.0
Other urinary diseases	N25-N29.8, N31-N32.0, N32.3-N32.4, N36-N36.9, N39, N41-N41.9, N44-N44.04, N45-N45.9, N49-N49.9	588-588.9, 593-593.89, 596-596.81, 596.89-596.9, 598-598.1, 598.8-599, 599.1-599.69, 599.8-599.89, 601-602.9, 604-604.99, 608.2-608.24
Gynecological diseases	D25-D26, D26.1-D26.9, D28.2, E28.2, N72-N72.0, N75-N77.8, N80-N81.9, N83-N83.9	218-219, 219.1-219.9, 236.0, 256.4, 617-618.9, 620-620.9, 621.4-621.9, 622.3-622.7, 629-629.81

Appendix Table 7: List of International Classification of Diseases (ICD) codes mapped to the Global Burden of Disease cause list for causes of death

Cause	ICD10	ICD9
Uterine fibroids	D25-D26, D26.1-D26.9, D28.2	218-219, 219.1-219.9, 236.0
Polycystic ovarian syndrome	E28.2	256.4
Endometriosis	N80-N80.9	617-617.9
Genital prolapse	N81-N81.9	618-618.9
Other gynecological diseases	N72-N72.0, N75-N77.8, N83-N83.9	620-620.9, 621.4-621.9, 622.3-622.7, 629-629.81
Hemoglobinopathies and hemolytic anemias	D55-D58.9, D59.1, D59.3, D59.5, D60-D61.9, D64.0, D64.4	282-284.9
Thalassemias	D56-D56.9	282.4-282.49
Sickle cell disorders	D57-D57.219, D57.4-D57.819	282.6-282.68
G6PD deficiency	D55-D55.2	282.2-282.3
Other hemoglobinopathies and hemolytic anemias	D55.3-D55.9, D58-D58.9, D59.1, D59.3, D59.5, D60-D61.9, D64.0, D64.4	282-282.1, 282.69-284.9
Endocrine, metabolic, blood, and immune disorders	D52.1, D59.0, D59.2, D59.6, D66-D67, D68.0-D69.8, D70-D75.89, D76-D78.89, D86.8, D86.82-D86.84, D86.86-D86.87, D89-D89.3, E03-E07.1, E09-E09.9, E15.0, E16.0-E16.9, E20-E28.1, E28.3-E34.8, E36-E36.8, E65-E68, E70-E85.29, E87.71, E88-E89.9, G21.1-G21.19, G24.0-G24.09, G25.1, G25.4, G25.6-G25.79, G72.0, G93.7, G97-G97.9, I95.2-I95.3, I97-I97.9, I98.9, J70.2-J70.5, J95-J95.9, K43-K43.9, K62.7, K91-K91.9, K94-K95.89, M87.1-M87.19, N14-N14.4, N65-N65.1, N99-N99.9, P96.2, P96.5, R50.2, R50.82-R50.83	240-243.9, 244.0-244.1, 244.3-244.8, 245-246.9, 251-256.39, 256.8-259.9, 270-273.9, 275-276, 277-277.2, 277.4-277.9, 278.0-278.8, 286-286.5, 286.7-289.7, 349.0-349.1, 357.6, 518.7, 519.0-519.09, 536.4-536.49, 539-539.9, 551.2-551.29, 552.2-552.29, 564.2-564.4, 569.6-569.69, 579.3, 596.82-596.83, 598.2, 775.3, 779.4-779.5, 780.62-780.63
Musculoskeletal disorders	I27.1, I67.7, L93-L93.2, M00-M03.0, M03.2-M03.6, M05-M09.0, M09.2-M09.8, M30-M32.9, M34-M36.8, M40-M43.19, M65-M65.08, M71.0-M71.19, M80-M82.8, M86.3-M86.49, M87-M87.09, M88-M89.09, M89.5-M89.59, M89.7-M89.9	416.1, 437.4, 446-446.9, 695.4-695.59, 710-711.99, 714-714.33, 714.8-714.9, 730.1-730.19, 732-732.9, 733.0-733.19
Rheumatoid arthritis	M05-M06.9, M08.0-M08.89	714-714.33, 714.8-714.9
Other musculoskeletal disorders	I27.1, I67.7, L93-L93.2, M00-M03.0, M03.2-M03.6, M07-M08, M08.9-M09.0, M09.2-M09.8, M30-M32.9, M34-M36.8, M40-M43.19, M65-M65.08, M71.0-M71.19, M80-M82.8, M86.3-M86.49, M87-M87.09, M88-M89.09, M89.5-M89.59, M89.7-M89.9	416.1, 437.4, 446-446.9, 695.4-695.59, 710-711.99, 730.1-730.19, 732-732.9, 733.0-733.19
Other non-communicable diseases	A46-A46.0, A66-A67.9, B86, D86.3, H05.0-H05.119, L00-L05.92, L08-L08.9, L10-L14.0, L51-L51.9, L88-L89.95, L97-L98.499, P96.0, Q00-Q07.9, Q10.4-Q18.9, Q20-Q28.9, Q30-Q36, Q37-Q45.9, Q50-Q86, Q86.1-Q87.89, Q89-Q89.8, Q90-Q93.9, Q95-Q99.8, R95	035-035.9, 102-103.9, 133-133.6, 376.0-376.1, 680-689, 694-695.3, 707-707.9, 740-749.04, 749.2-758.9, 759.0-759.89, 798-798.0
Congenital anomalies	P96.0, Q00-Q07.9, Q10.4-Q18.9, Q20-Q28.9, Q30-Q36, Q37-Q45.9, Q50-Q86, Q86.1-Q87.89, Q89-Q89.8, Q90-Q93.9, Q95-Q99.8	740-749.04, 749.2-758.9, 759.0-759.89
Neural tube defects	Q00-Q01.9, Q05-Q05.9	740-741.93, 742.0
Congenital heart anomalies	Q20-Q28.9	745-747.9
Cleft lip and cleft palate	Q35-Q36, Q37-Q37.9	749-749.04, 749.2-749.25
Down syndrome	Q90-Q90.9	758.0
Other chromosomal abnormalities	Q91-Q93.9, Q95-Q95.9, Q97-Q97.9, Q99-Q99.8	758, 758.1-758.6, 758.8-758.9
Other congenital anomalies	P96.0, Q02-Q04.9, Q06-Q07.9, Q10.4-Q18.9, Q30-Q34.9, Q38-Q45.9, Q50-Q86, Q86.1-Q87.89, Q89-Q89.8	742, 742.1-744.9, 748-748.9, 749.6-757.9, 759.0-759.89
Skin and subcutaneous diseases	A46-A46.0, A66-A67.9, B86, D86.3, L00-L05.92, L08-L08.9, L10-L14.0, L51-L51.9, L88-L89.95, L97-L98.499	035-035.9, 102-103.9, 133-133.6, 680-689, 694-695.3, 707-707.9
Cellulitis	L03-L03.91	681-682.9
Pyoderma	A46-A46.0, A66-A67.9, L00-L02.93, L04-L05.92, L08-L08.9, L88, L97-L98.499	035-035.9, 102-103.9, 680-680.9, 683-689
Decubitus ulcer	L89-L89.95	707-707.9
Other skin and subcutaneous diseases	D86.3, L10-L14.0, L51-L51.9	694-695.3
Sudden infant death syndrome	R95	798-798.0
Injuries	V00-V86.99, V87.2-V87.3, V88.2-V88.3, V90-V98.8, W00-W46.2, W49-W62.9, W64-W70.9, W73-W75.9, W77-W81.9, W83-W94.9, W97.9, W99-X06.9, X08-X39.9, X46-X47, X47.1-X47.8, X48-X48.9, X50-X54.9, X57-X58.9, X60-Y08.9, Y35-Y84.9, Y87.0-Y87.1, Y88-Y88.3, Y89.0-Y89.1	E800-E800.3, E801-E801.3, E802-E802.3, E803-E803.3, E804-E804.3, E805-E805.3, E806-E806.3, E807-E807.3, E810.0-E810.7, E811.0-E811.7, E812.0-E812.7, E813.0-E813.7, E814.0-E814.7, E815.0-E815.7, E816.0-E816.7, E817.0-E817.7, E818.0-E818.7, E819.0-E819.7, E820.0-E820.7, E821.0-E821.7, E822.0-E822.7, E823.0-E823.7, E824.0-E824.7, E825.0-E825.7, E826.0-E826.4, E827.0-E827.4, E828.0-E828.4, E829.0-E829.4, E830-E838.9, E840-E849.9, E850.3-E850.89, E854.8, E856-E857.09, E860.2-E869.99, E870-E876.9, E878-E879.9, E880-E886.99, E888-E928.89, E929.1-E929.5, E930-E979.9, E990-E999.1
Transport injuries	V00-V86.99, V87.2-V87.3, V88.2-V88.3, V90-V98.8	E800-E800.3, E801-E801.3, E802-E802.3, E803-E803.3, E804-E804.3, E805-E805.3, E806-E806.3, E807-E807.3, E810.0-E810.7, E811.0-E811.7, E812.0-E812.7, E813.0-E813.7, E814.0-E814.7, E815.0-E815.7, E816.0-E816.7, E817.0-E817.7, E818.0-E818.7, E819.0-E819.7, E820.0-E820.7, E821.0-E821.7, E822.0-E822.7, E823.0-E823.7, E824.0-E824.7, E825.0-E825.7, E826.0-E826.4, E827.0-E827.4, E828.0-E828.4, E829.0-E829.4, E830-E838.9, E840-E849.9, E929.1
Road injuries	V01-V04.99, V06-V80.929, V82-V82.9, V87.2-V87.3	E800.3, E801.3, E802.3, E803.3, E804.3, E805.3, E806.3, E807.3, E810.0-E810.6, E811.0-E811.7, E812.0-E812.7, E813.0-E813.7, E814.0-E814.7, E815.0-E815.7, E816.0-E816.7, E817.0-E817.7, E818.0-E818.7, E819.0-E819.7, E820.0-E820.6, E821.0-E821.6, E822.0-E822.7, E823.0-E823.7, E824.0-E824.7, E825.0-E825.7, E826.0-E826.1, E826.3-E826.4, E827.0, E827.3-E827.4, E828.0, E828.4, E829.0-E829.4
Pedestrian road injuries	V01-V04.99, V06-V09.9	E811.7, E812.7, E813.7, E814.7, E815.7, E816.7, E817.7, E818.7, E819.7, E822.7, E823.7, E824.7, E825.7, E826.0, E827.0, E828.0, E829.0
Cyclist road injuries	V10-V19.9	E800.3, E801.3, E802.3, E803.3, E804.3, E805.3, E806.3, E807.3, E810.6, E811.6, E812.6, E813.6, E814.6, E815.6, E816.6, E817.6, E818.6, E819.6, E820.6, E821.6, E822.6, E823.6, E824.6, E825.6, E826.1
Motorcyclist road injuries	V20-V29.9	E810.2-E810.3, E811.2-E811.3, E812.2-E812.3, E813.2-E813.3, E814.2-E814.3, E815.2-E815.3, E816.2-E816.3, E817.2-E817.3, E818.2-E818.3, E819.2-E819.3, E820.2-E820.3, E821.2-E821.3, E822.2-E822.3, E823.2-E823.3, E824.2-E824.3, E825.2-E825.3
Motor vehicle road injuries	V30-V79.9, V87.2-V87.3	E810.0-E810.1, E811.0-E811.1, E812.0-E812.1, E813.0-E813.1, E814.0-E814.1, E815.0-E815.1, E816.0-E816.1, E817.0-E817.1, E818.0-E818.1, E819.0-E819.1, E820.0-E820.1, E821.0-E821.1, E822.0-E822.1, E823.0-E823.1, E824.0-E824.1, E825.0-E825.1
Other road injuries	V80-V80.929, V82-V82.9	E810.4-E810.5, E811.4-E811.5, E812.4-E812.5, E813.4-E813.5, E814.4-E814.5, E815.4-E815.5, E816.4-E816.5, E817.4-E817.5, E818.4-E818.5, E819.4-E819.5, E820.4-E820.5, E821.4-E821.5, E822.4-E822.5, E823.4-E823.5, E824.4-E824.5, E825.4-E825.5, E826.3-E826.4, E827.3-E827.4, E828.4, E829.4
Other transport injuries	V00-V00.898, V05-V05.99, V81-V81.9, V83-V86.99, V88.2-V88.3, V90-V98.8	E800-E800.2, E801-E801.2, E802-E802.2, E803-E803.2, E804-E804.2, E805-E805.2, E806-E806.2, E807-E807.2, E810.7, E820.7, E821.7, E826.2, E827.2, E828.2, E830-E838.9, E840-E849.9, E929.1
Unintentional injuries	W00-W46.2, W49-W62.9, W64-W70.9, W73-W75.9, W77-W81.9, W83-W94.9, W97.9, W99-X06.9, X08-X32.9, X39-X39.9, X46-X47, X47.1-X47.8, X48-X48.9, X50-X54.9, X57-X58.9, Y38.9-Y84.9, Y88-Y88.3	E850.3-E850.89, E854.8, E856-E857.09, E860.2-E869.99, E870-E876.9, E878-E879.9, E880-E886.99, E888-E906.99, E910-E928.89, E929.2-E929.5, E930-E949.9
Falls	W00-W19.9	E880-E886.99, E888-E888.9, E929.3
Drowning	W65-W70.9, W73-W74.9	E910-E910.99
Fire, heat, and hot substances	X00-X06.9, X08-X19.9	E890-E899.09, E924-E924.99, E929.4
Poisonings	X46-X47, X47.1-X47.8, X48-X48.9	E850.3-E850.89, E854.8, E856-E857.09, E860.2-E869.99, E929.2
Exposure to mechanical forces	W20-W38.9, W40-W43.9, W45.0-W45.2, W46-W46.2, W49-W52, W75-W75.9	E913-E913.19, E916-E922.99, E928.1-E928.7
Unintentional firearm injuries	W32-W34.9	E922-E922.99, E928.7
Unintentional suffocation	W75-W75.9	E913-E913.19
Other exposure to mechanical forces	W20-W31.9, W35-W38.9, W40-W43.9, W45.0-W45.2, W46-W46.2, W49-W52	E916-E921.99, E928.1-E928.6
Adverse effects of medical treatment	Y38.9-Y84.9, Y88-Y88.3	E870-E876.9, E878-E879.9, E930-E949.9
Animal contact	W52.0-W62.9, W64-W64.9, X20-X29.9	E905-E906.99
Venomous animal contact	X20-X29.9	E905-E905.99
Non-venomous animal contact	W52.0-W62.9, W64-W64.9	E906-E906.99
Foreign body	W44-W45, W45.3-W45.9, W78-W80.9, W83-W84.9	E911-E912.09, E913.8-E915.09
Pulmonary aspiration and foreign body in airway	W78-W80.9, W83-W84.9	E911-E912.09, E913.8-E913.99
Foreign body in other body part	W44-W45, W45.3-W45.9	E914-E915.09
Environmental heat and cold exposure	W88-W94.9, W97.9, W99-W99.9, X30-X32.9, X39-X39.9	E900-E902.99, E926-E926.99, E929.5
Other unintentional injuries	W39-W39.9, W77-W77.9, W81-W81.9, W85-W87.9, X50-X54.9, X57-X58.9	E903-E904.99, E913.2-E913.39, E923-E923.99, E925-E925.99, E927-E928.09, E928.8-E928.89
Self-harm and interpersonal violence	X60-Y08.9, Y87.0-Y87.1	E950-E969
Self-harm	X60-X84.9, Y87.0	E950-E959

Appendix Table 7: List of International Classification of Diseases (ICD) codes mapped to the Global Burden of Disease cause list for causes of death

Cause	ICD10	ICD9
Interpersonal violence	X85-Y08.9, Y87.1	E960-E969
Assault by firearm	X93-X94.0, X94.3-X94.7, X94.9-X95.9, X96.5	E965-E965.4
Assault by sharp object	X99-X99.9	E966
Assault by other means	X85-X92.9, X94.1-X94.2, X94.8, X96-X96.4, X96.6-X98.9, Y00-Y08.9, Y87.1	E960-E964, E965.5-E965.9, E967-E969
Forces of nature, war, and legal intervention	X33-X38.9, Y35-Y38.893, Y89.0-Y89.1	E907-E909.9, E970-E979.9, E990-E999.1
Exposure to forces of nature	X33-X38.9	E907-E909.9
Collective violence and legal intervention	Y35-Y38.893, Y89.0-Y89.1	E970-E979.9, E990-E999.1
Garbage Code	A01, A14.9, A29, A31-A31.9, A40-A45.9, A47-A48.0, A48.3, A48.8-A49.02, A49.2-A49.9, A59-A59.9, A61-A62, A64-A64.0, A71-A73, A74.0, A76, A97, A99-A99.0, B07-B09, B11-B14, B28-B29, B30-B32.4, B34-B46.9, B49-B49.9, B54-B55, B55.1-B55.9, B58-B59.9, B61-B62, B64, B68-B68.9, B73-B74.2, B76-B76.9, B78-B82.9, B83.9-B85.4, B87-B89, B93-B94.0, B94.8-B94.9, B95.6-B99.9, C14-C14.9, C26-C29, C35-C36, C39-C39.9, C42, C46-C46.9, C55-C55.9, C57.9, C59-C6, C63.9, C68, C68.9, C75.9-C80.9, C87, C97-D00.0, D01, D01.4-D02, D02.4-D02.9, D07, D07.3-D07.39, D07.6-D09, D09.1-D09.19, D09.7, D09.9-D10, D10.9, D13, D13.9-D14, D14.4, D17-D21.9, D28, D28.9-D29, D29.9-D30, D30.9, D36.0, D36.9-D37.0, D37.6-D38, D38.6-D39.0, D39.7, D39.9-D40, D40.9-D41, D41.9, D44, D44.9, D46-D46.9, D47.1, D48, D48.7-D49.1, D49.5, D49.7-D49.8, D49.89-D50.0, D50.9, D54, D59, D59.4, D59.8-D59.9, D62-D63.0, D63.8-D64, D64.1-D64.2, D64.8-D65.9, D68, D69.9, D75.9, D79-D85, D87-D88, D89.8-D99, E07.8-E08.9, E15, E16, E17-E19, E34.9-E35.8, E37-E39, E47-E50.9, E62, E64.1, E69, E85.3-E87.70, E87.79-E87.99, E90-E998, F04-F06.1, F06.3-F07.0, F07.2-F09.9, F17-F17.9, F30-F50, F50.8-G00, G00.9-G02.8, G03.9, G06-G09.9, G15-G19, G27-G29, G32-G34, G38-G39, G42-G44.89, G47-G47.29, G47.4-G60.9, G62-G69, G74-G89.4, G91-G93.6, G93.8-G94.8, G96-G96.9, G98-H05, H05.12-H69.93, H71-H99, I00.0, I03-I04, I10-I10.9, I14-I19, I26-I27.0, I27.2-I27.9, I28.9-I29.9, I31.2-I31.4, I44-I46.9, I49-I51, I51.6-I59, I62, I62.1-I62.9, I64-I64.9, I67, I67.4, I67.8-I68, I68.8-I69, I69.4-I70.1, I70.8-I70.92, I74-I76, I90, I92-I95.1, I95.8-I96.9, I98.4-I98.8, I99-J00.0, J02, J02.8-J03, J03.8-J04, J04.1-J04.31, J05.1-J05.10, J06-J08, J15.9, J17-J19.6, J22-J29, J48-J59, J64-J64.9, J69-J69.9, J71-J81.9, J83, J85-J90.9, J93-J94.9, J96-K19, K30, K31.9-K34, K39, K47-K49, K53-K54, K63-K63.4, K63.8-K63.9, K65-K66.1, K66.9, K69, K71-K71.2, K71.6, K71.8-K72.01, K75-K75.1, K78-K79, K84, K87-K89, K92-K92.2, K92.9-K93, K93.1-K93.8, K96-K99, L06-L07, L09, L15-L50.9, L52-L87.9, L90-L92.9, L94-L96, L98.5-L99.8, M04, M10-M12.09, M12.2-M29, M37-M39, M43.2-M49, M49.2-M64, M65.1-M71, M71.2-M73, M73.8-M79.9, M83-M86.29, M86.5-M86.9, M87.2-M87.9, M89.1-M89.49, M90-M99.9, N09, N13-N13.9, N17-N17.9, N19-N19.9, N24, N32.1-N32.2, N32.8-N33.8, N35-N35.9, N37-N38, N39.3-N40.9, N42-N43.42, N44.1-N44.8, N46-N48.9, N50-N59, N61-N64.9, N66-N69, N78-N79, N82-N82.9, N84, N84.2-N86, N88-N95.9, N97-N97.9, O08-O08.9, O17-O19, O27, O37-O39, O49-O59, O78-O79, O93-O95.9, P06, P16-P18, P23, P23.5-P23.9, P30-P34.2, P37.3-P37.4, P40-P49, P62-P69, P73, P79, P82, P85-P89, P96.9-P99.9, Q08-Q10.3, Q19, Q29, Q36.0-Q36.9, Q46-Q49, Q88, Q89.9, Q94, Q99.9-R19.6, R19.8-R50.1, R50.8-R50.81, R50.84-R72.9, R74-R78, R78.6-R94.8, R95.0-T71.161, T71.163-U03, U05-U99, V87-V87.1, V87.4-V88.1, V88.4-V89.9, V99-V99.0, W47-W48, W63, W71-W72, W76-W76.9, W82, W95-W97, W98, X07, X40-X44.9, X47.0, X47.9, X49-X49.9, X55-X56, X59-X59.9, Y09-Y34.9, Y85-Y87, Y87.2, Y89, Y89.9	000-000.9, 002, 031-031.9, 038-038.9, 039.6, 040.0, 041.1-041.9, 067-069, 076-078.3, 078.8-078.9, 079.8-079.99, 084, 084.6, 085, 085.1-085.9, 089-089.9, 105-119, 125-125.3, 126-126.9, 127.2-127.9, 130-132.9, 133.8-134.9, 136.3-136.5, 136.8-136.9, 139.1-139.9, 149-149.9, 159-159.9, 165-169, 176-179.9, 183.9-184, 184.5, 184.9, 187, 187.9, 189, 189.9, 194.9-199.9, 209, 209.2-209.20, 209.29-209.30, 209.6-209.60, 209.62, 209.69-210, 211, 211.9-212, 212.9, 214-216.9, 221, 221.9-222, 222.9-223, 223.9, 229, 229.1, 229.9-230.0, 230.9-231, 231.8-231.9, 233, 233.3-233.30, 233.39, 233.6, 233.9-234, 234.9-235, 235.1-235.3, 235.5, 235.9-236, 236.3, 236.6, 236.9-236.90, 237.4, 238, 238.6-239.1, 239.5, 239.7-239.9, 244, 244.9, 247-249.91, 264-264.9, 274-274.9, 276.0-276.9, 277.3-277.39, 278, 279-280.0, 280.9-281, 285-285.9, 286.6, 289.8-289.9, 293-294.0, 296-302.9, 304, 304.9-304.93, 306-307.0, 307.2-307.50, 307.52-307.53, 307.59-320, 320.9, 324-327.19, 328-329, 331.3-331.4, 338-339.89, 342-344.9, 346-348.9, 349.81-353.5, 354-355.9, 357, 357.8-357.9, 360-376, 376.10-380.9, 384-389.9, 399-401.9, 405-409.4, 415-416.0, 416.2-416.9, 418-419.9, 423.0, 426-426.9, 427.4-427.5, 427.9-429, 429.2-429.9, 436-437, 437.3, 437.9-440.9, 444-445.89, 458-458.9, 459.0, 459.5-460.9, 462-464, 464.00, 464.1-464.10, 464.20, 464.3-464.30, 464.5-464.51, 465-465.9, 482.9-483, 484, 484.8-486.9, 505-505.9, 507-507.9, 510-514.9, 515.0-515.9, 518-518.53, 518.8-518.89, 519, 519.8-529.9, 536.2-536.3, 536.8-536.9, 537.7, 537.89-537.9, 544-549, 553.8-553.9, 559-559.0, 560.4-560.7, 561, 562.2-563, 564.8-564.9, 567-569, 569.49, 569.79-569.83, 569.86-570.9, 572-572.2, 573, 573.5, 578-578.9, 584-584.9, 586-587.9, 591-591.9, 593.9, 599.7-599.72, 599.9-600.91, 603-603.9, 605-608.1, 608.3-609, 611-612.1, 615-616.9, 619-619.9, 621-621.35, 622-622.2, 622.8-628.9, 629.89-629.9, 637-637.92, 639-639.9, 690-693.9, 695.8-706.9, 708-709.9, 712-713.8, 714.4, 715-716, 716.1-728.85, 728.87, 728.89-730.09, 730.2-730.39, 730.7-731.9, 733, 733.2-739.9, 749.1-749.14, 759, 759.9, 770.0, 779.9-780.56, 780.58, 780.6-780.61, 780.64-786.02, 786.04-787.04, 787.2-787.9, 787.99-788, 788.1-790.1, 790.29, 790.4-797.9, 798.1-E80, E800.8-E800.9, E801.8-E801.9, E802.8-E802.9, E803.8-E803.9, E804.8-E804.9, E805.8-E805.9, E806.8-E806.9, E807.8-E810, E810.8-E811, E811.8-E812, E812.8-E813, E813.8-E814, E814.8-E815, E815.8-E816, E816.8-E817, E817.8-E818, E818.8-E819, E819.8-E820, E820.8-E821, E821.8-E822, E822.8-E823, E823.8-E824, E824.8-E825, E825.8-E826, E826.8-E827, E827.8-E828, E828.8-E829, E829.8-E83, E839, E85, E855-E855.99, E858-E859, E87, E877, E88, E887-E887.09, E928.9-E929.0, E929.8-E929.9, E980-E989
Still Born	P95-P95.9	768.0-768.1

Appendix Table 8: Restrictions on age and sex by cause for GBD 2015			
**Cause Name**	**Minimum Age**	**Maximum Age**	**Sex Restriction**
HIV/AIDS and tuberculosis	28 days		
Tuberculosis	28 days		
HIV/AIDS	28 days		
HIV/AIDS - Tuberculosis	28 days		
HIV/AIDS resulting in other diseases	28 days		
Intestinal infectious diseases	28 days		
Typhoid fever	28 days		
Paratyphoid fever	28 days		
Other intestinal infectious diseases	28 days		
Diphtheria	28 days	59 years	
Whooping cough	28 days	59 years	
Measles	28 days	59 years	
Malaria	7 days		
Chagas disease	28 days		
Leishmaniasis	28 days		
Visceral leishmaniasis	28 days		
African trypanosomiasis	1 years		
Schistosomiasis	28 days		
Cystic echinococcosis	1 years		
Dengue	28 days		
Yellow fever	7 days		
Rabies	28 days		
Intestinal nematode infections	28 days		
Ascariasis	28 days		
Maternal disorders	10 years	54 years	Females Only
Maternal hemorrhage	10 years	54 years	Females Only
Maternal sepsis and other maternal infections	10 years	54 years	Females Only
Maternal hypertensive disorders	10 years	54 years	Females Only
Maternal obstructed labor and uterine rupture	10 years	54 years	Females Only
Maternal abortion, miscarriage, and ectopic pregnancy	10 years	54 years	Females Only
Indirect maternal deaths	10 years	54 years	Females Only
Late maternal deaths	10 years	54 years	Females Only
Maternal deaths aggravated by HIV/AIDS	10 years	54 years	Females Only
Other maternal disorders	10 years	54 years	Females Only
Neonatal disorders		4 years	
Neonatal preterm birth complications		4 years	
Neonatal encephalopathy due to birth asphyxia and trauma		4 years	
Neonatal sepsis and other neonatal infections		4 years	
Hemolytic disease and other neonatal jaundice		4 years	
Other neonatal disorders		4 years	
Nutritional deficiencies	28 days		
Protein-energy malnutrition	28 days		
Iodine deficiency	28 days		
Iron-deficiency anemia	28 days		
Other nutritional deficiencies	28 days		
Chlamydial infection	10 years		
Gonococcal infection	10 years		
Other sexually transmitted diseases	10 years		
Hepatitis	28 days		
Acute hepatitis A	28 days		
Acute hepatitis B	28 days		

Appendix Table 8: Restrictions on age and sex by cause for GBD 2015			
**Cause Name**	**Minimum Age**	**Maximum Age**	**Sex Restriction**
Acute hepatitis C	5 years		
Acute hepatitis E	1 years		
Lip and oral cavity cancer	15 years		
Nasopharynx cancer	5 years		
Other pharynx cancer	15 years		
Esophageal cancer	15 years		
Stomach cancer	15 years		
Colon and rectum cancer	15 years		
Liver cancer	5 years		
Liver cancer due to hepatitis B	5 years		
Liver cancer due to hepatitis C	5 years		
Liver cancer due to alcohol use	15 years		
Liver cancer due to other causes	5 years		
Gallbladder and biliary tract cancer	15 years		
Pancreatic cancer	15 years		
Larynx cancer	15 years		
Tracheal, bronchus, and lung cancer	15 years		
Malignant skin melanoma	15 years		
Non-melanoma skin cancer	15 years		
Non-melanoma skin cancer (squamous-cell carcinoma)	15 years		
Breast cancer	15 years		
Cervical cancer	15 years		Females Only
Uterine cancer	15 years		Females Only
Ovarian cancer	15 years		Females Only
Prostate cancer	15 years		Males Only
Testicular cancer	15 years		Males Only
Bladder cancer	15 years		
Thyroid cancer	10 years		
Mesothelioma	15 years		
Multiple myeloma	15 years		
Chronic lymphoid leukemia	15 years		
Chronic myeloid leukemia	15 years		
Rheumatic heart disease	1 years		
Ischemic heart disease	28 days		
Ischemic stroke	28 days		
Hypertensive heart disease	28 days		
Atrial fibrillation and flutter	30 years		
Aortic aneurysm	15 years		
Peripheral vascular disease	40 years		
Chronic obstructive pulmonary disease	28 days		
Pneumoconiosis	15 years		
Silicosis	15 years		
Asbestosis	15 years		
Coal workers pneumoconiosis	15 years		
Other pneumoconiosis	15 years		
Asthma	1 years		
Interstitial lung disease and pulmonary sarcoidosis	1 years		
Cirrhosis and other chronic liver diseases	1 years		
Cirrhosis and other chronic liver diseases due to hepatitis B	1 years		
Cirrhosis and other chronic liver diseases due to hepatitis C	1 years		
Cirrhosis and other chronic liver diseases due to alcohol use	15 years		

Appendix Table 8: Restrictions on age and sex by cause for GBD 2015			
**Cause Name**	**Minimum Age**	**Maximum Age**	**Sex Restriction**
Cirrhosis and other chronic liver diseases due to other causes	1 years		
Peptic ulcer disease	1 years		
Gastritis and duodenitis	1 years		
Appendicitis	1 years		
Inguinal, femoral, and abdominal hernia	1 years		
Inflammatory bowel disease	1 years		
Vascular intestinal disorders	1 years		
Gallbladder and biliary diseases	1 years		
Pancreatitis	1 years		
Other digestive diseases	1 years		
Neurological disorders	28 days		
Alzheimer disease and other dementias	40 years		
Parkinson disease	20 years		
Epilepsy	28 days		
Multiple sclerosis	20 years		
Other neurological disorders	28 days		
Schizophrenia	25 years		
Alcohol use disorders	15 years		
Eating disorders	5 years	59 years	
Anorexia nervosa	5 years	59 years	
Bulimia nervosa	5 years	59 years	
Acute glomerulonephritis	28 days		
Chronic kidney disease	28 days		
Chronic kidney disease due to diabetes mellitus	28 days		
Chronic kidney disease due to hypertension	28 days		
Chronic kidney disease due to glomerulonephritis	28 days		
Chronic kidney disease due to other causes	28 days		
Urolithiasis	5 years		
Gynecological diseases	15 years		Females Only
Uterine fibroids	15 years		Females Only
Polycystic ovarian syndrome	15 years	54 years	Females Only
Endometriosis	15 years	54 years	Females Only
Genital prolapse	15 years		Females Only
Other gynecological diseases	15 years		Females Only
Musculoskeletal disorders	5 years		
Rheumatoid arthritis	5 years		
Other musculoskeletal disorders	5 years		
Cleft lip and cleft palate		4 years	
Skin and subcutaneous diseases	28 days		
Cellulitis	28 days		
Pyoderma	28 days		
Decubitus ulcer	1 years		
Other skin and subcutaneous diseases	28 days		
Sudden infant death syndrome	7 days	364 days	
Self-harm	5 years		

Appendix Table 9: HIV-related garbage code redistribution packages		
**Package name**	**ICD9 codes**	**ICD10 codes**
Infectious 1	039, 039.0, 039.1, 039.2, 039.3, 039.4, 039.6, 039.8, 039.9, 113, 113.2, 113.4, 113.5, 113.6	A42, A42.0, A42.1, A42.2, A42.7, A42.8, A42.81, A42.82, A42.89, A42.9
Infectious 2	088.0, 088.2, 088.3, 088.5, 088.7	A44, A44.0, A44.1, A44.8, A44.9
Infectious 3	112, 112.0, 112.1, 112.2, 112.3, 112.4, 112.5, 112.6, 112.8, 112.81, 112.82, 112.83, 112.84, 112.85, 112.89, 112.9	B37, B37.0, B37.1, B37.2, B37.3, B37.4, B37.41, B37.42, B37.49, B37.5, B37.6, B37.7, B37.8, B37.81, B37.82, B37.83, B37.84, B37.89, B37.9
Infectious 4	114, 114.0, 114.1, 114.2, 114.3, 114.4, 114.5, 114.6, 114.9	B38, B38.0, B38.1, B38.2, B38.3, B38.4, B38.7, B38.8, B38.81, B38.89, B38.9
Infectious 5	115, 115.0, 115.00, 115.01, 115.02, 115.03, 115.04, 115.05, 115.09, 115.1, 115.10, 115.11, 115.12, 115.13, 115.14, 115.15, 115.19, 115.2, 115.3, 115.4, 115.5, 115.9, 115.90, 115.91, 115.92, 115.93, 115.94, 115.95, 115.99	B39, B39.0, B39.1, B39.2, B39.3, B39.4, B39.5, B39.9
Infectious 6	116, 116.0, 116.2, 116.3, 116.4, 116.5, 116.6, 116.9	B40, B40.0, B40.1, B40.2, B40.3, B40.7, B40.8, B40.81, B40.89, B40.9
Infectious 7	116.1	B41, B41.0, B41.7, B41.8, B41.9
Infectious 8	117.1	B42, B42.0, B42.1, B42.7, B42.8, B42.81, B42.82, B42.89, B42.9, B43, B43.0, B43.1, B43.2, B43.8, B43.9
Infectious 9	117.3	B44, B44.0, B44.1, B44.2, B44.7, B44.8, B44.81, B44.89, B44.9
Infectious 10	117.7	B46, B46.0, B46.1, B46.2, B46.3, B46.4, B46.5, B46.8, B46.9
Infectious 11	130, 130.0, 130.1, 130.2, 130.3, 130.4, 130.5, 130.6, 130.7, 130.8, 130.9	B58, B58.0, B58.00, B58.01, B58.09, B58.1, B58.2, B58.3, B58.8, B58.81, B58.82, B58.83, B58.89, B58.9
Infectious 12	136.3, 136.4, 136.5	B59, B59.0, B59.9
Infectious 13	117.5	B45, B45.0, B45.1, B45.2, B45.3, B45.7, B45.8, B45.9
Infectious 14	117.2	A43, A43.0, A43.1, A43.8, A43.9
Infectious 15	117, 117.0, 117.4, 117.6, 117.8, 117.9, 118, 118.0, 118.1, 118.2, 118.3, 118.4, 118.5, 118.6, 118.9	B49, B49.5, B49.9
Infectious 16	085.1, 085.2, 085.3, 085.4, 085.5	B55.1, B55.2
Infectious 17	031, 031.0, 031.1, 031.2, 031.8, 031.9	A31, A31.0, A31.1, A31.2, A31.8, A31.9
Immunodeficiency - antibody	279.0, 279.00, 279.01, 279.02, 279.03, 279.04, 279.05, 279.06, 279.09, 279.1	D80, D80.0, D80.1, D80.2, D80.3, D80.4, D80.5, D80.6, D80.7, D80.8, D80.9
Immunodeficiency - WBC	279.10, 279.11, 279.12, 279.13, 279.19, 279.2, 279.3, 279.4, 279.41, 279.49	D81, D81.0, D81.1, D81.2, D81.3, D81.4, D81.5, D81.6, D81.7, D81.8, D81.81, D81.810, D81.818, D81.819, D81.89, D81.9, D82, D82.0, D82.1, D82.2, D82.3, D82.4, D82.8, D82.9
Immunodeficiency - other	279, 279.5, 279.50, 279.51, 279.52, 279.53, 279.6, 279.8, 279.9	D83, D83.0, D83.1, D83.2, D83.8, D83.9, D84, D84.0, D84.1, D84.8, D84.9, D89.8, D89.81, D89.810, D89.811, D89.812, D89.813, D89.82, D89.89, D89.9
Kaposi's sarcoma	176, 176.0, 176.1, 176.2, 176.3, 176.4, 176.5, 176.8, 176.9	C46, C46.0, C46.1, C46.2, C46.3, C46.4, C46.5, C46.50, C46.51, C46.52, C46.7, C46.8, C46.9

Appendix Table 10: CODEm covariates used, level of covariate, and expected direction of covariate by cause, sex, and age						
**Cause**	**Sex**	**Age start**	**Age end**	**Model type**	**Direction**	**Covariate**
Tuberculosis	Male	Post Neonatal	80 plus	Global	-1	Education (years per capita)
Tuberculosis	Male	Post Neonatal	80 plus	Global	-1	Health System Access 2 (unitless)
Tuberculosis	Male	Post Neonatal	80 plus	Global	-1	LDI (I$ per capita)
Tuberculosis	Male	Post Neonatal	80 plus	Global	-1	Sociodemographic Status
Tuberculosis	Male	Post Neonatal	80 plus	Global	1	Alcohol (litres per capita)
Tuberculosis	Male	Post Neonatal	80 plus	Global	1	Diabetes Fasting Plasma Glucose (mmol/L)
Tuberculosis	Male	Post Neonatal	80 plus	Global	1	Indoor Air Pollution (All Cooking Fuels)
Tuberculosis	Male	Post Neonatal	80 plus	Global	1	Log-transformed SEV scalar: TB
Tuberculosis	Male	Post Neonatal	80 plus	Global	1	Malnutrition (proportion <2SD weight for age)
Tuberculosis	Male	Post Neonatal	80 plus	Global	1	Population Density (500-1000 ppl/sqkm, proportion)
Tuberculosis	Male	Post Neonatal	80 plus	Global	1	Population Density (over 1000 ppl/sqkm, proportion)
Tuberculosis	Male	Post Neonatal	80 plus	Global	1	Smoking Prevalence
Tuberculosis	Male	Post Neonatal	80 plus	Data Rich	-1	Education (years per capita)
Tuberculosis	Male	Post Neonatal	80 plus	Data Rich	-1	Health System Access 2 (unitless)
Tuberculosis	Male	Post Neonatal	80 plus	Data Rich	-1	LDI (I$ per capita)
Tuberculosis	Male	Post Neonatal	80 plus	Data Rich	-1	Sociodemographic Status
Tuberculosis	Male	Post Neonatal	80 plus	Data Rich	1	Alcohol (litres per capita)
Tuberculosis	Male	Post Neonatal	80 plus	Data Rich	1	Cumulative Cigarettes (5 Years)
Tuberculosis	Male	Post Neonatal	80 plus	Data Rich	1	Diabetes Fasting Plasma Glucose (mmol/L)
Tuberculosis	Male	Post Neonatal	80 plus	Data Rich	1	Indoor Air Pollution (All Cooking Fuels)
Tuberculosis	Male	Post Neonatal	80 plus	Data Rich	1	Log-transformed SEV scalar: TB
Tuberculosis	Male	Post Neonatal	80 plus	Data Rich	1	Malnutrition (proportion <2SD weight for age)
Tuberculosis	Male	Post Neonatal	80 plus	Data Rich	1	Population Density (500-1000 ppl/sqkm, proportion)
Tuberculosis	Male	Post Neonatal	80 plus	Data Rich	1	Population Density (over 1000 ppl/sqkm, proportion)
Tuberculosis	Male	Post Neonatal	80 plus	Data Rich	1	Smoking Prevalence
Tuberculosis	Female	Post Neonatal	80 plus	Global	-1	Education (years per capita)
Tuberculosis	Female	Post Neonatal	80 plus	Global	-1	Health System Access 2 (unitless)
Tuberculosis	Female	Post Neonatal	80 plus	Global	-1	LDI (I$ per capita)
Tuberculosis	Female	Post Neonatal	80 plus	Global	-1	Sociodemographic Status
Tuberculosis	Female	Post Neonatal	80 plus	Global	1	Alcohol (litres per capita)
Tuberculosis	Female	Post Neonatal	80 plus	Global	1	Diabetes Fasting Plasma Glucose (mmol/L)
Tuberculosis	Female	Post Neonatal	80 plus	Global	1	Indoor Air Pollution (All Cooking Fuels)
Tuberculosis	Female	Post Neonatal	80 plus	Global	1	Log-transformed SEV scalar: TB
Tuberculosis	Female	Post Neonatal	80 plus	Global	1	Malnutrition (proportion <2SD weight for age)
Tuberculosis	Female	Post Neonatal	80 plus	Global	1	Population Density (500-1000 ppl/sqkm, proportion)
Tuberculosis	Female	Post Neonatal	80 plus	Global	1	Population Density (over 1000 ppl/sqkm, proportion)
Tuberculosis	Female	Post Neonatal	80 plus	Global	1	Smoking Prevalence
Tuberculosis	Female	Post Neonatal	80 plus	Data Rich	-1	Education (years per capita)
Tuberculosis	Female	Post Neonatal	80 plus	Data Rich	-1	Health System Access 2 (unitless)
Tuberculosis	Female	Post Neonatal	80 plus	Data Rich	-1	LDI (I$ per capita)
Tuberculosis	Female	Post Neonatal	80 plus	Data Rich	-1	Sociodemographic Status
Tuberculosis	Female	Post Neonatal	80 plus	Data Rich	1	Alcohol (litres per capita)
Tuberculosis	Female	Post Neonatal	80 plus	Data Rich	1	Cumulative Cigarettes (5 Years)
Tuberculosis	Female	Post Neonatal	80 plus	Data Rich	1	Diabetes Fasting Plasma Glucose (mmol/L)

Appendix Table 10: CODEm covariates used, level of covariate, and expected direction of covariate by cause, sex, and age						
**Cause**	**Sex**	**Age start**	**Age end**	**Model type**	**Direction**	**Covariate**
Tuberculosis	Female	Post Neonatal	80 plus	Data Rich	1	Indoor Air Pollution (All Cooking Fuels)
Tuberculosis	Female	Post Neonatal	80 plus	Data Rich	1	Log-transformed SEV scalar: TB
Tuberculosis	Female	Post Neonatal	80 plus	Data Rich	1	Malnutrition (proportion <2SD weight for age)
Tuberculosis	Female	Post Neonatal	80 plus	Data Rich	1	Population Density (500-1000 ppl/sqkm, proportion)
Tuberculosis	Female	Post Neonatal	80 plus	Data Rich	1	Population Density (over 1000 ppl/sqkm, proportion)
Tuberculosis	Female	Post Neonatal	80 plus	Data Rich	1	Smoking Prevalence
Diarrheal diseases	Male	5 to 9	80 plus	Global	-1	Education (years per capita)
Diarrheal diseases	Male	Early Neonatal	1 to 4	Global	-1	Education (years per capita)
Diarrheal diseases	Male	5 to 9	80 plus	Global	-1	Health System Access (unitless)
Diarrheal diseases	Male	5 to 9	80 plus	Global	-1	Improved Water Source (proportion with access)
Diarrheal diseases	Male	Early Neonatal	1 to 4	Global	-1	Improved Water Source (proportion with access)
Diarrheal diseases	Male	5 to 9	80 plus	Global	-1	LDI (I$ per capita)
Diarrheal diseases	Male	Early Neonatal	1 to 4	Global	-1	LDI (I$ per capita)
Diarrheal diseases	Male	5 to 9	80 plus	Global	-1	Rotavirus coverage (proportion)
Diarrheal diseases	Male	Early Neonatal	1 to 4	Global	-1	Rotavirus coverage (proportion)
Diarrheal diseases	Male	5 to 9	80 plus	Global	-1	Sanitation (proportion with access)
Diarrheal diseases	Male	Early Neonatal	1 to 4	Global	-1	Sanitation (proportion with access)
Diarrheal diseases	Male	5 to 9	80 plus	Global	-1	Sociodemographic Status
Diarrheal diseases	Male	Early Neonatal	1 to 4	Global	-1	Sociodemographic Status
Diarrheal diseases	Male	5 to 9	80 plus	Global	0	Population Density (over 1000 ppl/sqkm, proportion)
Diarrheal diseases	Male	Early Neonatal	1 to 4	Global	0	Population Density (over 1000 ppl/sqkm, proportion)
Diarrheal diseases	Male	Early Neonatal	1 to 4	Global	0	Population Density (under 150 ppl/sqkm, proportion)
Diarrheal diseases	Male	5 to 9	80 plus	Global	0	SEV unsafe sanitation
Diarrheal diseases	Male	5 to 9	80 plus	Global	0	SEV unsafe water
Diarrheal diseases	Male	Early Neonatal	1 to 4	Global	1	Latitude Under 15 (proportion)
Diarrheal diseases	Male	5 to 9	80 plus	Global	1	Log-transformed SEV scalar: Diarrhea
Diarrheal diseases	Male	Early Neonatal	1 to 4	Global	1	Log-transformed SEV scalar: Diarrhea
Diarrheal diseases	Male	Early Neonatal	1 to 4	Global	1	Malnutrition (proportion <2SD weight for age)
Diarrheal diseases	Male	Early Neonatal	1 to 4	Global	1	SEV unsafe sanitation
Diarrheal diseases	Male	Early Neonatal	1 to 4	Global	1	SEV unsafe water
Diarrheal diseases	Male	5 to 9	80 plus	Data Rich	-1	Education (years per capita)
Diarrheal diseases	Male	Early Neonatal	1 to 4	Data Rich	-1	Education (years per capita)
Diarrheal diseases	Male	5 to 9	80 plus	Data Rich	-1	Health System Access (unitless)
Diarrheal diseases	Male	Early Neonatal	1 to 4	Data Rich	-1	Improved Water Source (proportion with access)
Diarrheal diseases	Male	5 to 9	80 plus	Data Rich	-1	LDI (I$ per capita)
Diarrheal diseases	Male	Early Neonatal	1 to 4	Data Rich	-1	LDI (I$ per capita)
Diarrheal diseases	Male	5 to 9	80 plus	Data Rich	-1	Rotavirus coverage (proportion)
Diarrheal diseases	Male	Early Neonatal	1 to 4	Data Rich	-1	Rotavirus coverage (proportion)
Diarrheal diseases	Male	Early Neonatal	1 to 4	Data Rich	-1	Sanitation (proportion with access)
Diarrheal diseases	Male	5 to 9	80 plus	Data Rich	-1	Sociodemographic Status
Diarrheal diseases	Male	Early Neonatal	1 to 4	Data Rich	-1	Sociodemographic Status
Diarrheal diseases	Male	5 to 9	80 plus	Data Rich	0	Log-transformed SEV scalar: Diarrhea
Diarrheal diseases	Male	5 to 9	80 plus	Data Rich	0	Population Density (over 1000 ppl/sqkm, proportion)
Diarrheal diseases	Male	Early Neonatal	1 to 4	Data Rich	0	Population Density (over 1000 ppl/sqkm, proportion)

Appendix Table 10: CODEm covariates used, level of covariate, and expected direction of covariate by cause, sex, and age						
**Cause**	**Sex**	**Age start**	**Age end**	**Model type**	**Direction**	**Covariate**
Diarrheal diseases	Male	Early Neonatal	1 to 4	Data Rich	0	Population Density (under 150 ppl/sqkm, proportion)
Diarrheal diseases	Male	5 to 9	80 plus	Data Rich	0	SEV unsafe sanitation
Diarrheal diseases	Male	5 to 9	80 plus	Data Rich	0	SEV unsafe water
Diarrheal diseases	Male	5 to 9	80 plus	Data Rich	1	Improved Water Source (proportion with access)
Diarrheal diseases	Male	Early Neonatal	1 to 4	Data Rich	1	Latitude Under 15 (proportion)
Diarrheal diseases	Male	Early Neonatal	1 to 4	Data Rich	1	Log-transformed SEV scalar: Diarrhea
Diarrheal diseases	Male	5 to 9	80 plus	Data Rich	1	Malnutrition (proportion <2SD weight for age)
Diarrheal diseases	Male	Early Neonatal	1 to 4	Data Rich	1	Malnutrition (proportion <2SD weight for age)
Diarrheal diseases	Male	Early Neonatal	1 to 4	Data Rich	1	SEV unsafe sanitation
Diarrheal diseases	Male	Early Neonatal	1 to 4	Data Rich	1	SEV unsafe water
Diarrheal diseases	Male	5 to 9	80 plus	Data Rich	1	Sanitation (proportion with access)
Diarrheal diseases	Female	5 to 9	80 plus	Global	-1	Education (years per capita)
Diarrheal diseases	Female	Early Neonatal	1 to 4	Global	-1	Education (years per capita)
Diarrheal diseases	Female	5 to 9	80 plus	Global	-1	Health System Access (unitless)
Diarrheal diseases	Female	5 to 9	80 plus	Global	-1	Improved Water Source (proportion with access)
Diarrheal diseases	Female	Early Neonatal	1 to 4	Global	-1	Improved Water Source (proportion with access)
Diarrheal diseases	Female	5 to 9	80 plus	Global	-1	LDI (I$ per capita)
Diarrheal diseases	Female	Early Neonatal	1 to 4	Global	-1	LDI (I$ per capita)
Diarrheal diseases	Female	5 to 9	80 plus	Global	-1	Rotavirus coverage (proportion)
Diarrheal diseases	Female	Early Neonatal	1 to 4	Global	-1	Rotavirus coverage (proportion)
Diarrheal diseases	Female	5 to 9	80 plus	Global	-1	Sanitation (proportion with access)
Diarrheal diseases	Female	Early Neonatal	1 to 4	Global	-1	Sanitation (proportion with access)
Diarrheal diseases	Female	5 to 9	80 plus	Global	-1	Sociodemographic Status
Diarrheal diseases	Female	Early Neonatal	1 to 4	Global	-1	Sociodemographic Status
Diarrheal diseases	Female	5 to 9	80 plus	Global	0	Population Density (over 1000 ppl/sqkm, proportion)
Diarrheal diseases	Female	Early Neonatal	1 to 4	Global	0	Population Density (over 1000 ppl/sqkm, proportion)
Diarrheal diseases	Female	Early Neonatal	1 to 4	Global	0	Population Density (under 150 ppl/sqkm, proportion)
Diarrheal diseases	Female	5 to 9	80 plus	Global	0	SEV unsafe sanitation
Diarrheal diseases	Female	5 to 9	80 plus	Global	0	SEV unsafe water
Diarrheal diseases	Female	Early Neonatal	1 to 4	Global	1	Latitude Under 15 (proportion)
Diarrheal diseases	Female	5 to 9	80 plus	Global	1	Log-transformed SEV scalar: Diarrhea
Diarrheal diseases	Female	Early Neonatal	1 to 4	Global	1	Log-transformed SEV scalar: Diarrhea
Diarrheal diseases	Female	Early Neonatal	1 to 4	Global	1	Malnutrition (proportion <2SD weight for age)
Diarrheal diseases	Female	Early Neonatal	1 to 4	Global	1	SEV unsafe sanitation
Diarrheal diseases	Female	Early Neonatal	1 to 4	Global	1	SEV unsafe water
Diarrheal diseases	Female	5 to 9	80 plus	Data Rich	-1	Education (years per capita)
Diarrheal diseases	Female	Early Neonatal	1 to 4	Data Rich	-1	Education (years per capita)
Diarrheal diseases	Female	5 to 9	80 plus	Data Rich	-1	Health System Access (unitless)
Diarrheal diseases	Female	Early Neonatal	1 to 4	Data Rich	-1	Improved Water Source (proportion with access)
Diarrheal diseases	Female	5 to 9	80 plus	Data Rich	-1	LDI (I$ per capita)
Diarrheal diseases	Female	Early Neonatal	1 to 4	Data Rich	-1	LDI (I$ per capita)
Diarrheal diseases	Female	5 to 9	80 plus	Data Rich	-1	Rotavirus coverage (proportion)
Diarrheal diseases	Female	Early Neonatal	1 to 4	Data Rich	-1	Rotavirus coverage (proportion)
Diarrheal diseases	Female	Early Neonatal	1 to 4	Data Rich	-1	Sanitation (proportion with access)

Appendix Table 10: CODEm covariates used, level of covariate, and expected direction of covariate by cause, sex, and age						
**Cause**	**Sex**	**Age start**	**Age end**	**Model type**	**Direction**	**Covariate**
Diarrheal diseases	Female	5 to 9	80 plus	Data Rich	-1	Sociodemographic Status
Diarrheal diseases	Female	Early Neonatal	1 to 4	Data Rich	-1	Sociodemographic Status
Diarrheal diseases	Female	5 to 9	80 plus	Data Rich	0	Log-transformed SEV scalar: Diarrhea
Diarrheal diseases	Female	5 to 9	80 plus	Data Rich	0	Population Density (over 1000 ppl/sqkm, proportion)
Diarrheal diseases	Female	Early Neonatal	1 to 4	Data Rich	0	Population Density (over 1000 ppl/sqkm, proportion)
Diarrheal diseases	Female	Early Neonatal	1 to 4	Data Rich	0	Population Density (under 150 ppl/sqkm, proportion)
Diarrheal diseases	Female	5 to 9	80 plus	Data Rich	0	SEV unsafe sanitation
Diarrheal diseases	Female	5 to 9	80 plus	Data Rich	0	SEV unsafe water
Diarrheal diseases	Female	5 to 9	80 plus	Data Rich	1	Improved Water Source (proportion with access)
Diarrheal diseases	Female	Early Neonatal	1 to 4	Data Rich	1	Latitude Under 15 (proportion)
Diarrheal diseases	Female	Early Neonatal	1 to 4	Data Rich	1	Log-transformed SEV scalar: Diarrhea
Diarrheal diseases	Female	5 to 9	80 plus	Data Rich	1	Malnutrition (proportion <2SD weight for age)
Diarrheal diseases	Female	Early Neonatal	1 to 4	Data Rich	1	Malnutrition (proportion <2SD weight for age)
Diarrheal diseases	Female	Early Neonatal	1 to 4	Data Rich	1	SEV unsafe sanitation
Diarrheal diseases	Female	Early Neonatal	1 to 4	Data Rich	1	SEV unsafe water
Diarrheal diseases	Female	5 to 9	80 plus	Data Rich	1	Sanitation (proportion with access)
Other intestinal infectious diseases	Male	Post Neonatal	80 plus	Global	-1	Education (years per capita)
Other intestinal infectious diseases	Male	Post Neonatal	80 plus	Global	-1	Health System Access (capped)
Other intestinal infectious diseases	Male	Post Neonatal	80 plus	Global	-1	Improved Water Source (proportion with access)
Other intestinal infectious diseases	Male	Post Neonatal	80 plus	Global	-1	LDI (I$ per capita)
Other intestinal infectious diseases	Male	Post Neonatal	80 plus	Global	-1	Sanitation (proportion with access)
Other intestinal infectious diseases	Male	Post Neonatal	80 plus	Global	-1	Sociodemographic Status
Other intestinal infectious diseases	Male	Post Neonatal	80 plus	Data Rich	-1	Education (years per capita)
Other intestinal infectious diseases	Male	Post Neonatal	80 plus	Data Rich	-1	Health System Access (capped)
Other intestinal infectious diseases	Male	Post Neonatal	80 plus	Data Rich	-1	Improved Water Source (proportion with access)
Other intestinal infectious diseases	Male	Post Neonatal	80 plus	Data Rich	-1	LDI (I$ per capita)
Other intestinal infectious diseases	Male	Post Neonatal	80 plus	Data Rich	-1	Sanitation (proportion with access)
Other intestinal infectious diseases	Male	Post Neonatal	80 plus	Data Rich	-1	Sociodemographic Status
Other intestinal infectious diseases	Female	Post Neonatal	80 plus	Global	-1	Education (years per capita)
Other intestinal infectious diseases	Female	Post Neonatal	80 plus	Global	-1	Health System Access (capped)
Other intestinal infectious diseases	Female	Post Neonatal	80 plus	Global	-1	Improved Water Source (proportion with access)
Other intestinal infectious diseases	Female	Post Neonatal	80 plus	Global	-1	LDI (I$ per capita)
Other intestinal infectious diseases	Female	Post Neonatal	80 plus	Global	-1	Sanitation (proportion with access)
Other intestinal infectious diseases	Female	Post Neonatal	80 plus	Global	-1	Sociodemographic Status
Other intestinal infectious diseases	Female	Post Neonatal	80 plus	Data Rich	-1	Education (years per capita)
Other intestinal infectious diseases	Female	Post Neonatal	80 plus	Data Rich	-1	Health System Access (capped)
Other intestinal infectious diseases	Female	Post Neonatal	80 plus	Data Rich	-1	Improved Water Source (proportion with access)
Other intestinal infectious diseases	Female	Post Neonatal	80 plus	Data Rich	-1	LDI (I$ per capita)
Other intestinal infectious diseases	Female	Post Neonatal	80 plus	Data Rich	-1	Sanitation (proportion with access)
Other intestinal infectious diseases	Female	Post Neonatal	80 plus	Data Rich	-1	Sociodemographic Status
Lower respiratory infections	Male	5 to 9	80 plus	Global	-1	DTP3 Coverage (proportion)
Lower respiratory infections	Male	Early Neonatal	1 to 4	Global	-1	DTP3 Coverage (proportion)
Lower respiratory infections	Male	5 to 9	80 plus	Global	-1	Education (years per capita)
Lower respiratory infections	Male	Early Neonatal	1 to 4	Global	-1	Education (years per capita)

Appendix Table 10: CODEm covariates used, level of covariate, and expected direction of covariate by cause, sex, and age						
**Cause**	**Sex**	**Age start**	**Age end**	**Model type**	**Direction**	**Covariate**
Lower respiratory infections	Male	5 to 9	80 plus	Global	-1	Health System Access 2 (unitless)
Lower respiratory infections	Male	Early Neonatal	1 to 4	Global	-1	Health System Access 2 (unitless)
Lower respiratory infections	Male	Early Neonatal	1 to 4	Global	-1	Hib3 Vaccine Coverage (proportion)
Lower respiratory infections	Male	5 to 9	80 plus	Global	-1	LDI (I$ per capita)
Lower respiratory infections	Male	Early Neonatal	1 to 4	Global	-1	LDI (I$ per capita)
Lower respiratory infections	Male	5 to 9	80 plus	Global	-1	Malnutrition (proportion <2SD weight for age)
Lower respiratory infections	Male	Early Neonatal	1 to 4	Global	-1	Malnutrition (proportion <2SD weight for age)
Lower respiratory infections	Male	5 to 9	80 plus	Global	-1	PCV3 Coverage (proportion)
Lower respiratory infections	Male	Early Neonatal	1 to 4	Global	-1	PCV3 Coverage (proportion)
Lower respiratory infections	Male	5 to 9	80 plus	Global	-1	Sociodemographic Status
Lower respiratory infections	Male	Early Neonatal	1 to 4	Global	-1	Sociodemographic Status
Lower respiratory infections	Male	5 to 9	80 plus	Global	0	SEV unsafe sanitation
Lower respiratory infections	Male	Early Neonatal	1 to 4	Global	0	SEV unsafe sanitation
Lower respiratory infections	Male	5 to 9	80 plus	Global	1	Indoor Air Pollution (All Cooking Fuels)
Lower respiratory infections	Male	Early Neonatal	1 to 4	Global	1	Indoor Air Pollution (All Cooking Fuels)
Lower respiratory infections	Male	5 to 9	80 plus	Global	1	Log-transformed SEV scalar: LRI
Lower respiratory infections	Male	Early Neonatal	1 to 4	Global	1	Log-transformed SEV scalar: LRI
Lower respiratory infections	Male	5 to 9	80 plus	Global	1	Outdoor Air Pollution (PM2.5)
Lower respiratory infections	Male	Early Neonatal	1 to 4	Global	1	Outdoor Air Pollution (PM2.5)
Lower respiratory infections	Male	5 to 9	80 plus	Global	1	Smoking Prevalence
Lower respiratory infections	Male	Early Neonatal	1 to 4	Global	1	Smoking Prevalence
Lower respiratory infections	Male	5 to 9	80 plus	Data Rich	-1	DTP3 Coverage (proportion)
Lower respiratory infections	Male	Early Neonatal	1 to 4	Data Rich	-1	DTP3 Coverage (proportion)
Lower respiratory infections	Male	5 to 9	80 plus	Data Rich	-1	Education (years per capita)
Lower respiratory infections	Male	Early Neonatal	1 to 4	Data Rich	-1	Education (years per capita)
Lower respiratory infections	Male	5 to 9	80 plus	Data Rich	-1	Health System Access 2 (unitless)
Lower respiratory infections	Male	Early Neonatal	1 to 4	Data Rich	-1	Health System Access 2 (unitless)
Lower respiratory infections	Male	Early Neonatal	1 to 4	Data Rich	-1	Hib3 Vaccine Coverage (proportion)
Lower respiratory infections	Male	5 to 9	80 plus	Data Rich	-1	LDI (I$ per capita)
Lower respiratory infections	Male	Early Neonatal	1 to 4	Data Rich	-1	LDI (I$ per capita)
Lower respiratory infections	Male	5 to 9	80 plus	Data Rich	-1	Malnutrition (proportion <2SD weight for age)
Lower respiratory infections	Male	Early Neonatal	1 to 4	Data Rich	-1	Malnutrition (proportion <2SD weight for age)
Lower respiratory infections	Male	5 to 9	80 plus	Data Rich	-1	PCV3 Coverage (proportion)
Lower respiratory infections	Male	Early Neonatal	1 to 4	Data Rich	-1	PCV3 Coverage (proportion)
Lower respiratory infections	Male	5 to 9	80 plus	Data Rich	-1	Sociodemographic Status
Lower respiratory infections	Male	Early Neonatal	1 to 4	Data Rich	-1	Sociodemographic Status
Lower respiratory infections	Male	5 to 9	80 plus	Data Rich	0	SEV unsafe sanitation
Lower respiratory infections	Male	Early Neonatal	1 to 4	Data Rich	0	SEV unsafe sanitation
Lower respiratory infections	Male	5 to 9	80 plus	Data Rich	1	Indoor Air Pollution (All Cooking Fuels)
Lower respiratory infections	Male	Early Neonatal	1 to 4	Data Rich	1	Indoor Air Pollution (All Cooking Fuels)
Lower respiratory infections	Male	5 to 9	80 plus	Data Rich	1	Log-transformed SEV scalar: LRI
Lower respiratory infections	Male	Early Neonatal	1 to 4	Data Rich	1	Log-transformed SEV scalar: LRI
Lower respiratory infections	Male	5 to 9	80 plus	Data Rich	1	Outdoor Air Pollution (PM2.5)
Lower respiratory infections	Male	Early Neonatal	1 to 4	Data Rich	1	Outdoor Air Pollution (PM2.5)

Appendix Table 10: CODEm covariates used, level of covariate, and expected direction of covariate by cause, sex, and age						
**Cause**	**Sex**	**Age start**	**Age end**	**Model type**	**Direction**	**Covariate**
Lower respiratory infections	Male	5 to 9	80 plus	Data Rich	1	Smoking Prevalence
Lower respiratory infections	Male	Early Neonatal	1 to 4	Data Rich	1	Smoking Prevalence
Lower respiratory infections	Female	5 to 9	80 plus	Global	-1	DTP3 Coverage (proportion)
Lower respiratory infections	Female	Early Neonatal	1 to 4	Global	-1	DTP3 Coverage (proportion)
Lower respiratory infections	Female	5 to 9	80 plus	Global	-1	Education (years per capita)
Lower respiratory infections	Female	Early Neonatal	1 to 4	Global	-1	Education (years per capita)
Lower respiratory infections	Female	5 to 9	80 plus	Global	-1	Health System Access 2 (unitless)
Lower respiratory infections	Female	Early Neonatal	1 to 4	Global	-1	Health System Access 2 (unitless)
Lower respiratory infections	Female	Early Neonatal	1 to 4	Global	-1	Hib3 Vaccine Coverage (proportion)
Lower respiratory infections	Female	5 to 9	80 plus	Global	-1	LDI (I$ per capita)
Lower respiratory infections	Female	Early Neonatal	1 to 4	Global	-1	LDI (I$ per capita)
Lower respiratory infections	Female	5 to 9	80 plus	Global	-1	Malnutrition (proportion <2SD weight for age)
Lower respiratory infections	Female	Early Neonatal	1 to 4	Global	-1	Malnutrition (proportion <2SD weight for age)
Lower respiratory infections	Female	5 to 9	80 plus	Global	-1	PCV3 Coverage (proportion)
Lower respiratory infections	Female	Early Neonatal	1 to 4	Global	-1	PCV3 Coverage (proportion)
Lower respiratory infections	Female	5 to 9	80 plus	Global	-1	Sociodemographic Status
Lower respiratory infections	Female	Early Neonatal	1 to 4	Global	-1	Sociodemographic Status
Lower respiratory infections	Female	5 to 9	80 plus	Global	0	SEV unsafe sanitation
Lower respiratory infections	Female	Early Neonatal	1 to 4	Global	0	SEV unsafe sanitation
Lower respiratory infections	Female	5 to 9	80 plus	Global	1	Indoor Air Pollution (All Cooking Fuels)
Lower respiratory infections	Female	Early Neonatal	1 to 4	Global	1	Indoor Air Pollution (All Cooking Fuels)
Lower respiratory infections	Female	5 to 9	80 plus	Global	1	Log-transformed SEV scalar: LRI
Lower respiratory infections	Female	Early Neonatal	1 to 4	Global	1	Log-transformed SEV scalar: LRI
Lower respiratory infections	Female	5 to 9	80 plus	Global	1	Outdoor Air Pollution (PM2.5)
Lower respiratory infections	Female	Early Neonatal	1 to 4	Global	1	Outdoor Air Pollution (PM2.5)
Lower respiratory infections	Female	5 to 9	80 plus	Global	1	Smoking Prevalence
Lower respiratory infections	Female	Early Neonatal	1 to 4	Global	1	Smoking Prevalence
Lower respiratory infections	Female	5 to 9	80 plus	Data Rich	-1	DTP3 Coverage (proportion)
Lower respiratory infections	Female	Early Neonatal	1 to 4	Data Rich	-1	DTP3 Coverage (proportion)
Lower respiratory infections	Female	5 to 9	80 plus	Data Rich	-1	Education (years per capita)
Lower respiratory infections	Female	Early Neonatal	1 to 4	Data Rich	-1	Education (years per capita)
Lower respiratory infections	Female	5 to 9	80 plus	Data Rich	-1	Health System Access 2 (unitless)
Lower respiratory infections	Female	Early Neonatal	1 to 4	Data Rich	-1	Health System Access 2 (unitless)
Lower respiratory infections	Female	Early Neonatal	1 to 4	Data Rich	-1	Hib3 Vaccine Coverage (proportion)
Lower respiratory infections	Female	5 to 9	80 plus	Data Rich	-1	LDI (I$ per capita)
Lower respiratory infections	Female	Early Neonatal	1 to 4	Data Rich	-1	LDI (I$ per capita)
Lower respiratory infections	Female	5 to 9	80 plus	Data Rich	-1	Malnutrition (proportion <2SD weight for age)
Lower respiratory infections	Female	Early Neonatal	1 to 4	Data Rich	-1	Malnutrition (proportion <2SD weight for age)
Lower respiratory infections	Female	5 to 9	80 plus	Data Rich	-1	PCV3 Coverage (proportion)
Lower respiratory infections	Female	Early Neonatal	1 to 4	Data Rich	-1	PCV3 Coverage (proportion)
Lower respiratory infections	Female	5 to 9	80 plus	Data Rich	-1	Sociodemographic Status
Lower respiratory infections	Female	Early Neonatal	1 to 4	Data Rich	-1	Sociodemographic Status
Lower respiratory infections	Female	5 to 9	80 plus	Data Rich	0	SEV unsafe sanitation
Lower respiratory infections	Female	Early Neonatal	1 to 4	Data Rich	0	SEV unsafe sanitation

Appendix Table 10: CODEm covariates used, level of covariate, and expected direction of covariate by cause, sex, and age						
**Cause**	**Sex**	**Age start**	**Age end**	**Model type**	**Direction**	**Covariate**
Lower respiratory infections	Female	5 to 9	80 plus	Data Rich	1	Indoor Air Pollution (All Cooking Fuels)
Lower respiratory infections	Female	Early Neonatal	1 to 4	Data Rich	1	Indoor Air Pollution (All Cooking Fuels)
Lower respiratory infections	Female	5 to 9	80 plus	Data Rich	1	Log-transformed SEV scalar: LRI
Lower respiratory infections	Female	Early Neonatal	1 to 4	Data Rich	1	Log-transformed SEV scalar: LRI
Lower respiratory infections	Female	5 to 9	80 plus	Data Rich	1	Outdoor Air Pollution (PM2.5)
Lower respiratory infections	Female	Early Neonatal	1 to 4	Data Rich	1	Outdoor Air Pollution (PM2.5)
Lower respiratory infections	Female	5 to 9	80 plus	Data Rich	1	Smoking Prevalence
Lower respiratory infections	Female	Early Neonatal	1 to 4	Data Rich	1	Smoking Prevalence
Otitis media	Male	Early Neonatal	80 plus	Global	-1	DTP3 Coverage (proportion)
Otitis media	Male	Early Neonatal	80 plus	Global	-1	Education (years per capita)
Otitis media	Male	Early Neonatal	80 plus	Global	-1	Health System Access 2 (unitless)
Otitis media	Male	Early Neonatal	80 plus	Global	-1	Improved Water Source (proportion with access)
Otitis media	Male	Early Neonatal	80 plus	Global	-1	LDI (I$ per capita)
Otitis media	Male	Early Neonatal	80 plus	Global	-1	Sanitation (proportion with access)
Otitis media	Male	Early Neonatal	80 plus	Global	1	Elevation Over 1500m (proportion)
Otitis media	Male	Early Neonatal	80 plus	Global	1	Indoor Air Pollution (All Cooking Fuels)
Otitis media	Male	Early Neonatal	80 plus	Global	1	Log-transformed SEV scalar: Otitis
Otitis media	Male	Early Neonatal	80 plus	Global	1	Malnutrition (proportion <2SD weight for age)
Otitis media	Male	Early Neonatal	80 plus	Global	1	Outdoor Air Pollution (PM2.5)
Otitis media	Male	Early Neonatal	80 plus	Global	1	Population Density (over 1000 ppl/sqkm, proportion)
Otitis media	Male	Early Neonatal	80 plus	Global	1	Rainfall (Quintiles 4-5)
Otitis media	Male	Early Neonatal	80 plus	Global	1	Smoking Prevalence
Otitis media	Male	Early Neonatal	80 plus	Data Rich	-1	DTP3 Coverage (proportion)
Otitis media	Male	Early Neonatal	80 plus	Data Rich	-1	Education (years per capita)
Otitis media	Male	Early Neonatal	80 plus	Data Rich	-1	Health System Access 2 (unitless)
Otitis media	Male	Early Neonatal	80 plus	Data Rich	-1	Improved Water Source (proportion with access)
Otitis media	Male	Early Neonatal	80 plus	Data Rich	-1	LDI (I$ per capita)
Otitis media	Male	Early Neonatal	80 plus	Data Rich	-1	Sanitation (proportion with access)
Otitis media	Male	Early Neonatal	80 plus	Data Rich	-1	Sociodemographic Status
Otitis media	Male	Early Neonatal	80 plus	Data Rich	1	Elevation Over 1500m (proportion)
Otitis media	Male	Early Neonatal	80 plus	Data Rich	1	Indoor Air Pollution (All Cooking Fuels)
Otitis media	Male	Early Neonatal	80 plus	Data Rich	1	Log-transformed SEV scalar: Otitis
Otitis media	Male	Early Neonatal	80 plus	Data Rich	1	Malnutrition (proportion <2SD weight for age)
Otitis media	Male	Early Neonatal	80 plus	Data Rich	1	Outdoor Air Pollution (PM2.5)
Otitis media	Male	Early Neonatal	80 plus	Data Rich	1	Population Density (over 1000 ppl/sqkm, proportion)
Otitis media	Male	Early Neonatal	80 plus	Data Rich	1	Rainfall (Quintiles 4-5)
Otitis media	Male	Early Neonatal	80 plus	Data Rich	1	Smoking Prevalence
Otitis media	Female	Early Neonatal	80 plus	Global	-1	DTP3 Coverage (proportion)
Otitis media	Female	Early Neonatal	80 plus	Global	-1	Education (years per capita)
Otitis media	Female	Early Neonatal	80 plus	Global	-1	Health System Access 2 (unitless)
Otitis media	Female	Early Neonatal	80 plus	Global	-1	Improved Water Source (proportion with access)
Otitis media	Female	Early Neonatal	80 plus	Global	-1	LDI (I$ per capita)
Otitis media	Female	Early Neonatal	80 plus	Global	-1	Sanitation (proportion with access)
Otitis media	Female	Early Neonatal	80 plus	Global	1	Elevation Over 1500m (proportion)

Appendix Table 10: CODEm covariates used, level of covariate, and expected direction of covariate by cause, sex, and age						
**Cause**	**Sex**	**Age start**	**Age end**	**Model type**	**Direction**	**Covariate**
Otitis media	Female	Early Neonatal	80 plus	Global	1	Indoor Air Pollution (All Cooking Fuels)
Otitis media	Female	Early Neonatal	80 plus	Global	1	Log-transformed SEV scalar: Otitis
Otitis media	Female	Early Neonatal	80 plus	Global	1	Malnutrition (proportion <2SD weight for age)
Otitis media	Female	Early Neonatal	80 plus	Global	1	Outdoor Air Pollution (PM2.5)
Otitis media	Female	Early Neonatal	80 plus	Global	1	Population Density (over 1000 ppl/sqkm, proportion)
Otitis media	Female	Early Neonatal	80 plus	Global	1	Rainfall (Quintiles 4-5)
Otitis media	Female	Early Neonatal	80 plus	Global	1	Smoking Prevalence
Otitis media	Female	Early Neonatal	80 plus	Data Rich	-1	DTP3 Coverage (proportion)
Otitis media	Female	Early Neonatal	80 plus	Data Rich	-1	Education (years per capita)
Otitis media	Female	Early Neonatal	80 plus	Data Rich	-1	Health System Access 2 (unitless)
Otitis media	Female	Early Neonatal	80 plus	Data Rich	-1	Improved Water Source (proportion with access)
Otitis media	Female	Early Neonatal	80 plus	Data Rich	-1	LDI (I$ per capita)
Otitis media	Female	Early Neonatal	80 plus	Data Rich	-1	Sanitation (proportion with access)
Otitis media	Female	Early Neonatal	80 plus	Data Rich	-1	Sociodemographic Status
Otitis media	Female	Early Neonatal	80 plus	Data Rich	1	Elevation Over 1500m (proportion)
Otitis media	Female	Early Neonatal	80 plus	Data Rich	1	Indoor Air Pollution (All Cooking Fuels)
Otitis media	Female	Early Neonatal	80 plus	Data Rich	1	Log-transformed SEV scalar: Otitis
Otitis media	Female	Early Neonatal	80 plus	Data Rich	1	Malnutrition (proportion <2SD weight for age)
Otitis media	Female	Early Neonatal	80 plus	Data Rich	1	Outdoor Air Pollution (PM2.5)
Otitis media	Female	Early Neonatal	80 plus	Data Rich	1	Population Density (over 1000 ppl/sqkm, proportion)
Otitis media	Female	Early Neonatal	80 plus	Data Rich	1	Rainfall (Quintiles 4-5)
Otitis media	Female	Early Neonatal	80 plus	Data Rich	1	Smoking Prevalence
Meningitis	Male	5 to 9	80 plus	Global	-1	DTP3 Coverage (proportion)
Meningitis	Male	Early Neonatal	1 to 4	Global	-1	DTP3 Coverage (proportion)
Meningitis	Male	5 to 9	80 plus	Global	-1	Health System Access 2 (unitless)
Meningitis	Male	Early Neonatal	1 to 4	Global	-1	Health System Access 2 (unitless)
Meningitis	Male	5 to 9	80 plus	Global	-1	Improved Water Source (proportion with access)
Meningitis	Male	Early Neonatal	1 to 4	Global	-1	Improved Water Source (proportion with access)
Meningitis	Male	5 to 9	80 plus	Global	-1	LDI (I$ per capita)
Meningitis	Male	Early Neonatal	1 to 4	Global	-1	LDI (I$ per capita)
Meningitis	Male	5 to 9	80 plus	Global	-1	Maternal education (years per capita)
Meningitis	Male	Early Neonatal	1 to 4	Global	-1	Maternal education (years per capita)
Meningitis	Male	5 to 9	80 plus	Global	-1	Proportion of total population covered by menafrivac initiative (meningitis meningococcal type A vaccine)
Meningitis	Male	Early Neonatal	1 to 4	Global	-1	Proportion of total population covered by menafrivac initiative (meningitis meningococcal type A vaccine)
Meningitis	Male	5 to 9	80 plus	Global	-1	Sanitation (proportion with access)
Meningitis	Male	Early Neonatal	1 to 4	Global	-1	Sanitation (proportion with access)
Meningitis	Male	5 to 9	80 plus	Global	-1	Sociodemographic Status
Meningitis	Male	Early Neonatal	1 to 4	Global	-1	Sociodemographic Status
Meningitis	Male	5 to 9	80 plus	Global	1	Malnutrition (proportion <2SD weight for age)
Meningitis	Male	Early Neonatal	1 to 4	Global	1	Malnutrition (proportion <2SD weight for age)
Meningitis	Male	5 to 9	80 plus	Global	1	meningitis belt (proportion)
Meningitis	Male	Early Neonatal	1 to 4	Global	1	meningitis belt (proportion)
Meningitis	Male	5 to 9	80 plus	Data Rich	-1	DTP3 Coverage (proportion)
Meningitis	Male	Early Neonatal	1 to 4	Data Rich	-1	DTP3 Coverage (proportion)

Appendix Table 10: CODEm covariates used, level of covariate, and expected direction of covariate by cause, sex, and age						
**Cause**	**Sex**	**Age start**	**Age end**	**Model type**	**Direction**	**Covariate**
Meningitis	Male	5 to 9	80 plus	Data Rich	-1	Health System Access 2 (unitless)
Meningitis	Male	Early Neonatal	1 to 4	Data Rich	-1	Health System Access 2 (unitless)
Meningitis	Male	5 to 9	80 plus	Data Rich	-1	Improved Water Source (proportion with access)
Meningitis	Male	Early Neonatal	1 to 4	Data Rich	-1	Improved Water Source (proportion with access)
Meningitis	Male	5 to 9	80 plus	Data Rich	-1	LDI (I$ per capita)
Meningitis	Male	Early Neonatal	1 to 4	Data Rich	-1	LDI (I$ per capita)
Meningitis	Male	5 to 9	80 plus	Data Rich	-1	Maternal education (years per capita)
Meningitis	Male	Early Neonatal	1 to 4	Data Rich	-1	Maternal education (years per capita)
Meningitis	Male	5 to 9	80 plus	Data Rich	-1	Proportion of total population covered by menafrivac initiative (meningitis meningococcal type A vaccine)
Meningitis	Male	Early Neonatal	1 to 4	Data Rich	-1	Proportion of total population covered by menafrivac initiative (meningitis meningococcal type A vaccine)
Meningitis	Male	5 to 9	80 plus	Data Rich	-1	Sanitation (proportion with access)
Meningitis	Male	Early Neonatal	1 to 4	Data Rich	-1	Sanitation (proportion with access)
Meningitis	Male	5 to 9	80 plus	Data Rich	-1	Sociodemographic Status
Meningitis	Male	Early Neonatal	1 to 4	Data Rich	-1	Sociodemographic Status
Meningitis	Male	5 to 9	80 plus	Data Rich	1	Malnutrition (proportion <2SD weight for age)
Meningitis	Male	Early Neonatal	1 to 4	Data Rich	1	Malnutrition (proportion <2SD weight for age)
Meningitis	Male	5 to 9	80 plus	Data Rich	1	meningitis belt (proportion)
Meningitis	Male	Early Neonatal	1 to 4	Data Rich	1	meningitis belt (proportion)
Meningitis	Female	5 to 9	80 plus	Global	-1	DTP3 Coverage (proportion)
Meningitis	Female	Early Neonatal	1 to 4	Global	-1	DTP3 Coverage (proportion)
Meningitis	Female	5 to 9	80 plus	Global	-1	Health System Access 2 (unitless)
Meningitis	Female	Early Neonatal	1 to 4	Global	-1	Health System Access 2 (unitless)
Meningitis	Female	5 to 9	80 plus	Global	-1	Improved Water Source (proportion with access)
Meningitis	Female	Early Neonatal	1 to 4	Global	-1	Improved Water Source (proportion with access)
Meningitis	Female	5 to 9	80 plus	Global	-1	LDI (I$ per capita)
Meningitis	Female	Early Neonatal	1 to 4	Global	-1	LDI (I$ per capita)
Meningitis	Female	5 to 9	80 plus	Global	-1	Maternal education (years per capita)
Meningitis	Female	Early Neonatal	1 to 4	Global	-1	Maternal education (years per capita)
Meningitis	Female	5 to 9	80 plus	Global	-1	Proportion of total population covered by menafrivac initiative (meningitis meningococcal type A vaccine)
Meningitis	Female	Early Neonatal	1 to 4	Global	-1	Proportion of total population covered by menafrivac initiative (meningitis meningococcal type A vaccine)
Meningitis	Female	5 to 9	80 plus	Global	-1	Sanitation (proportion with access)
Meningitis	Female	Early Neonatal	1 to 4	Global	-1	Sanitation (proportion with access)
Meningitis	Female	5 to 9	80 plus	Global	-1	Sociodemographic Status
Meningitis	Female	Early Neonatal	1 to 4	Global	-1	Sociodemographic Status
Meningitis	Female	5 to 9	80 plus	Global	1	Malnutrition (proportion <2SD weight for age)
Meningitis	Female	Early Neonatal	1 to 4	Global	1	Malnutrition (proportion <2SD weight for age)
Meningitis	Female	5 to 9	80 plus	Global	1	meningitis belt (proportion)
Meningitis	Female	Early Neonatal	1 to 4	Global	1	meningitis belt (proportion)
Meningitis	Female	5 to 9	80 plus	Data Rich	-1	DTP3 Coverage (proportion)
Meningitis	Female	Early Neonatal	1 to 4	Data Rich	-1	DTP3 Coverage (proportion)
Meningitis	Female	5 to 9	80 plus	Data Rich	-1	Health System Access 2 (unitless)
Meningitis	Female	Early Neonatal	1 to 4	Data Rich	-1	Health System Access 2 (unitless)
Meningitis	Female	5 to 9	80 plus	Data Rich	-1	Improved Water Source (proportion with access)
Meningitis	Female	Early Neonatal	1 to 4	Data Rich	-1	Improved Water Source (proportion with access)

Appendix Table 10: CODEm covariates used, level of covariate, and expected direction of covariate by cause, sex, and age						
**Cause**	**Sex**	**Age start**	**Age end**	**Model type**	**Direction**	**Covariate**
Meningitis	Female	5 to 9	80 plus	Data Rich	-1	LDI (I$ per capita)
Meningitis	Female	Early Neonatal	1 to 4	Data Rich	-1	LDI (I$ per capita)
Meningitis	Female	5 to 9	80 plus	Data Rich	-1	Maternal education (years per capita)
Meningitis	Female	Early Neonatal	1 to 4	Data Rich	-1	Maternal education (years per capita)
Meningitis	Female	5 to 9	80 plus	Data Rich	-1	Proportion of total population covered by menafrivac initiative (meningitis meningococcal type A vaccine)
Meningitis	Female	Early Neonatal	1 to 4	Data Rich	-1	Proportion of total population covered by menafrivac initiative (meningitis meningococcal type A vaccine)
Meningitis	Female	5 to 9	80 plus	Data Rich	-1	Sanitation (proportion with access)
Meningitis	Female	Early Neonatal	1 to 4	Data Rich	-1	Sanitation (proportion with access)
Meningitis	Female	5 to 9	80 plus	Data Rich	-1	Sociodemographic Status
Meningitis	Female	Early Neonatal	1 to 4	Data Rich	-1	Sociodemographic Status
Meningitis	Female	5 to 9	80 plus	Data Rich	1	Malnutrition (proportion <2SD weight for age)
Meningitis	Female	Early Neonatal	1 to 4	Data Rich	1	Malnutrition (proportion <2SD weight for age)
Meningitis	Female	5 to 9	80 plus	Data Rich	1	meningitis belt (proportion)
Meningitis	Female	Early Neonatal	1 to 4	Data Rich	1	meningitis belt (proportion)
Encephalitis	Male	Early Neonatal	80 plus	Global	-1	Health System Access 2 (unitless)
Encephalitis	Male	Early Neonatal	80 plus	Global	-1	Improved Water Source (proportion with access)
Encephalitis	Male	Early Neonatal	80 plus	Global	-1	In-Facility Delivery (proportion)
Encephalitis	Male	Early Neonatal	80 plus	Global	-1	LDI (I$ per capita)
Encephalitis	Male	Early Neonatal	80 plus	Global	-1	Maternal education (years per capita)
Encephalitis	Male	Early Neonatal	80 plus	Global	-1	Sanitation (proportion with access)
Encephalitis	Male	Early Neonatal	80 plus	Global	-1	Sociodemographic Status
Encephalitis	Male	Early Neonatal	80 plus	Global	1	Japanese encelphalitis endemic area (binary)
Encephalitis	Male	Early Neonatal	80 plus	Global	1	Malnutrition (proportion <2SD weight for age)
Encephalitis	Male	Early Neonatal	80 plus	Data Rich	-1	Health System Access 2 (unitless)
Encephalitis	Male	Early Neonatal	80 plus	Data Rich	-1	Improved Water Source (proportion with access)
Encephalitis	Male	Early Neonatal	80 plus	Data Rich	-1	In-Facility Delivery (proportion)
Encephalitis	Male	Early Neonatal	80 plus	Data Rich	-1	LDI (I$ per capita)
Encephalitis	Male	Early Neonatal	80 plus	Data Rich	-1	Maternal education (years per capita)
Encephalitis	Male	Early Neonatal	80 plus	Data Rich	-1	Sanitation (proportion with access)
Encephalitis	Male	Early Neonatal	80 plus	Data Rich	-1	Sociodemographic Status
Encephalitis	Male	Early Neonatal	80 plus	Data Rich	1	Japanese encelphalitis endemic area (binary)
Encephalitis	Male	Early Neonatal	80 plus	Data Rich	1	Malnutrition (proportion <2SD weight for age)
Encephalitis	Female	Early Neonatal	80 plus	Global	-1	Health System Access 2 (unitless)
Encephalitis	Female	Early Neonatal	80 plus	Global	-1	Improved Water Source (proportion with access)
Encephalitis	Female	Early Neonatal	80 plus	Global	-1	In-Facility Delivery (proportion)
Encephalitis	Female	Early Neonatal	80 plus	Global	-1	LDI (I$ per capita)
Encephalitis	Female	Early Neonatal	80 plus	Global	-1	Maternal education (years per capita)
Encephalitis	Female	Early Neonatal	80 plus	Global	-1	Sanitation (proportion with access)
Encephalitis	Female	Early Neonatal	80 plus	Global	-1	Sociodemographic Status
Encephalitis	Female	Early Neonatal	80 plus	Global	1	Japanese encelphalitis endemic area (binary)
Encephalitis	Female	Early Neonatal	80 plus	Global	1	Malnutrition (proportion <2SD weight for age)
Encephalitis	Female	Early Neonatal	80 plus	Data Rich	-1	Health System Access 2 (unitless)
Encephalitis	Female	Early Neonatal	80 plus	Data Rich	-1	Improved Water Source (proportion with access)
Encephalitis	Female	Early Neonatal	80 plus	Data Rich	-1	In-Facility Delivery (proportion)

Appendix Table 10: CODEm covariates used, level of covariate, and expected direction of covariate by cause, sex, and age						
**Cause**	**Sex**	**Age start**	**Age end**	**Model type**	**Direction**	**Covariate**
Encephalitis	Female	Early Neonatal	80 plus	Data Rich	-1	LDI (I$ per capita)
Encephalitis	Female	Early Neonatal	80 plus	Data Rich	-1	Maternal education (years per capita)
Encephalitis	Female	Early Neonatal	80 plus	Data Rich	-1	Sanitation (proportion with access)
Encephalitis	Female	Early Neonatal	80 plus	Data Rich	-1	Sociodemographic Status
Encephalitis	Female	Early Neonatal	80 plus	Data Rich	1	Japanese encelphalitis endemic area (binary)
Encephalitis	Female	Early Neonatal	80 plus	Data Rich	1	Malnutrition (proportion <2SD weight for age)
Tetanus	Male	1 to 4	80 plus	Global	-1	DTP3 Coverage (proportion)
Tetanus	Male	Early Neonatal	Post Neonatal	Global	-1	DTP3 Coverage (proportion)
Tetanus	Male	1 to 4	80 plus	Global	-1	Education (years per capita)
Tetanus	Male	Early Neonatal	Post Neonatal	Global	-1	Education (years per capita)
Tetanus	Male	1 to 4	80 plus	Global	-1	Health System Access 2 (unitless)
Tetanus	Male	Early Neonatal	Post Neonatal	Global	-1	Health System Access 2 (unitless)
Tetanus	Male	Early Neonatal	Post Neonatal	Global	-1	In-Facility Delivery (proportion)
Tetanus	Male	1 to 4	80 plus	Global	-1	LDI (I$ per capita)
Tetanus	Male	Early Neonatal	Post Neonatal	Global	-1	LDI (I$ per capita)
Tetanus	Male	1 to 4	80 plus	Global	-1	Sanitation (proportion with access)
Tetanus	Male	Early Neonatal	Post Neonatal	Global	-1	Skilled Birth Attendance (proportion)
Tetanus	Male	1 to 4	80 plus	Global	-1	Sociodemographic Status
Tetanus	Male	Early Neonatal	Post Neonatal	Global	-1	Sociodemographic Status
Tetanus	Male	Early Neonatal	Post Neonatal	Global	-1	Tetanus Toxoid Coverage Smooth (proportion)
Tetanus	Male	1 to 4	80 plus	Data Rich	-1	DTP3 Coverage (proportion)
Tetanus	Male	Early Neonatal	Post Neonatal	Data Rich	-1	DTP3 Coverage (proportion)
Tetanus	Male	1 to 4	80 plus	Data Rich	-1	Education (years per capita)
Tetanus	Male	Early Neonatal	Post Neonatal	Data Rich	-1	Education (years per capita)
Tetanus	Male	1 to 4	80 plus	Data Rich	-1	Health System Access 2 (unitless)
Tetanus	Male	Early Neonatal	Post Neonatal	Data Rich	-1	Health System Access 2 (unitless)
Tetanus	Male	Early Neonatal	Post Neonatal	Data Rich	-1	In-Facility Delivery (proportion)
Tetanus	Male	1 to 4	80 plus	Data Rich	-1	LDI (I$ per capita)
Tetanus	Male	Early Neonatal	Post Neonatal	Data Rich	-1	LDI (I$ per capita)
Tetanus	Male	1 to 4	80 plus	Data Rich	-1	Sanitation (proportion with access)
Tetanus	Male	Early Neonatal	Post Neonatal	Data Rich	-1	Skilled Birth Attendance (proportion)
Tetanus	Male	1 to 4	80 plus	Data Rich	-1	Sociodemographic Status
Tetanus	Male	Early Neonatal	Post Neonatal	Data Rich	-1	Sociodemographic Status
Tetanus	Male	Early Neonatal	Post Neonatal	Data Rich	-1	Tetanus Toxoid Coverage Smooth (proportion)
Tetanus	Female	1 to 4	80 plus	Global	-1	DTP3 Coverage (proportion)
Tetanus	Female	Early Neonatal	Post Neonatal	Global	-1	DTP3 Coverage (proportion)
Tetanus	Female	1 to 4	80 plus	Global	-1	Education (years per capita)
Tetanus	Female	Early Neonatal	Post Neonatal	Global	-1	Education (years per capita)
Tetanus	Female	1 to 4	80 plus	Global	-1	Health System Access 2 (unitless)
Tetanus	Female	Early Neonatal	Post Neonatal	Global	-1	Health System Access 2 (unitless)
Tetanus	Female	Early Neonatal	Post Neonatal	Global	-1	In-Facility Delivery (proportion)
Tetanus	Female	1 to 4	80 plus	Global	-1	LDI (I$ per capita)
Tetanus	Female	Early Neonatal	Post Neonatal	Global	-1	LDI (I$ per capita)
Tetanus	Female	1 to 4	80 plus	Global	-1	Sanitation (proportion with access)

Appendix Table 10: CODEm covariates used, level of covariate, and expected direction of covariate by cause, sex, and age						
**Cause**	**Sex**	**Age start**	**Age end**	**Model type**	**Direction**	**Covariate**
Tetanus	Female	Early Neonatal	Post Neonatal	Global	-1	Skilled Birth Attendance (proportion)
Tetanus	Female	1 to 4	80 plus	Global	-1	Sociodemographic Status
Tetanus	Female	Early Neonatal	Post Neonatal	Global	-1	Sociodemographic Status
Tetanus	Female	Early Neonatal	Post Neonatal	Global	-1	Tetanus Toxoid Coverage Smooth (proportion)
Tetanus	Female	1 to 4	80 plus	Data Rich	-1	DTP3 Coverage (proportion)
Tetanus	Female	Early Neonatal	Post Neonatal	Data Rich	-1	DTP3 Coverage (proportion)
Tetanus	Female	1 to 4	80 plus	Data Rich	-1	Education (years per capita)
Tetanus	Female	Early Neonatal	Post Neonatal	Data Rich	-1	Education (years per capita)
Tetanus	Female	1 to 4	80 plus	Data Rich	-1	Health System Access 2 (unitless)
Tetanus	Female	Early Neonatal	Post Neonatal	Data Rich	-1	Health System Access 2 (unitless)
Tetanus	Female	Early Neonatal	Post Neonatal	Data Rich	-1	In-Facility Delivery (proportion)
Tetanus	Female	1 to 4	80 plus	Data Rich	-1	LDI (I$ per capita)
Tetanus	Female	Early Neonatal	Post Neonatal	Data Rich	-1	LDI (I$ per capita)
Tetanus	Female	1 to 4	80 plus	Data Rich	-1	Sanitation (proportion with access)
Tetanus	Female	Early Neonatal	Post Neonatal	Data Rich	-1	Skilled Birth Attendance (proportion)
Tetanus	Female	1 to 4	80 plus	Data Rich	-1	Sociodemographic Status
Tetanus	Female	Early Neonatal	Post Neonatal	Data Rich	-1	Sociodemographic Status
Tetanus	Female	Early Neonatal	Post Neonatal	Data Rich	-1	Tetanus Toxoid Coverage Smooth (proportion)
Chagas disease	Male	Post Neonatal	80 plus	Default	-1	Education (years per capita)
Chagas disease	Male	Post Neonatal	80 plus	Default	-1	Health System Access (unitless)
Chagas disease	Male	Post Neonatal	80 plus	Default	-1	LDI (I$ per capita)
Chagas disease	Male	Post Neonatal	80 plus	Default	-1	Sanitation (proportion with access)
Chagas disease	Male	Post Neonatal	80 plus	Default	1	Chagas Population-at-Risk 2 (proportion)
Chagas disease	Male	Post Neonatal	80 plus	Default	1	chagasPrevPAHO
Chagas disease	Female	Post Neonatal	80 plus	Default	-1	Education (years per capita)
Chagas disease	Female	Post Neonatal	80 plus	Default	-1	Health System Access (unitless)
Chagas disease	Female	Post Neonatal	80 plus	Default	-1	LDI (I$ per capita)
Chagas disease	Female	Post Neonatal	80 plus	Default	-1	Sanitation (proportion with access)
Chagas disease	Female	Post Neonatal	80 plus	Default	1	Chagas Population-at-Risk 2 (proportion)
Chagas disease	Female	Post Neonatal	80 plus	Default	1	chagasPrevPAHO
Dengue	Male	Post Neonatal	80 plus	Global	0	Education (years per capita)
Dengue	Male	Post Neonatal	80 plus	Global	0	Health System Access (unitless)
Dengue	Male	Post Neonatal	80 plus	Global	0	LDI (I$ per capita)
Dengue	Male	Post Neonatal	80 plus	Global	0	Sociodemographic Status
Dengue	Male	Post Neonatal	80 plus	Global	1	Dengue anomalies (deviation from mean dengue incidence rate)
Dengue	Male	Post Neonatal	80 plus	Global	1	Dengue outbreaks (binary)
Dengue	Male	Post Neonatal	80 plus	Global	1	Elevation Under 100m (proportion)
Dengue	Male	Post Neonatal	80 plus	Global	1	Latitude Under 15 (proportion)
Dengue	Male	Post Neonatal	80 plus	Global	1	Population Density (over 1000 ppl/sqkm, proportion)
Dengue	Male	Post Neonatal	80 plus	Global	1	Population weighted probability of dengue transmission
Dengue	Male	Post Neonatal	80 plus	Global	1	Rainfall Quintile 4 (proportion)
Dengue	Male	Post Neonatal	80 plus	Global	1	Rainfall Quintile 5 (proportion)
Dengue	Male	Post Neonatal	80 plus	Data Rich	0	Education (years per capita)
Dengue	Male	Post Neonatal	80 plus	Data Rich	0	Health System Access (unitless)

Appendix Table 10: CODEm covariates used, level of covariate, and expected direction of covariate by cause, sex, and age						
**Cause**	**Sex**	**Age start**	**Age end**	**Model type**	**Direction**	**Covariate**
Dengue	Male	Post Neonatal	80 plus	Data Rich	0	LDI (I$ per capita)
Dengue	Male	Post Neonatal	80 plus	Data Rich	0	Sociodemographic Status
Dengue	Male	Post Neonatal	80 plus	Data Rich	1	Dengue anomalies (deviation from mean dengue incidence rate)
Dengue	Male	Post Neonatal	80 plus	Data Rich	1	Dengue outbreaks (binary)
Dengue	Male	Post Neonatal	80 plus	Data Rich	1	Elevation Under 100m (proportion)
Dengue	Male	Post Neonatal	80 plus	Data Rich	1	Latitude Under 15 (proportion)
Dengue	Male	Post Neonatal	80 plus	Data Rich	1	Population Density (over 1000 ppl/sqkm, proportion)
Dengue	Male	Post Neonatal	80 plus	Data Rich	1	Population weighted probability of dengue transmission
Dengue	Male	Post Neonatal	80 plus	Data Rich	1	Rainfall Quintile 4 (proportion)
Dengue	Male	Post Neonatal	80 plus	Data Rich	1	Rainfall Quintile 5 (proportion)
Dengue	Female	Post Neonatal	80 plus	Global	0	Education (years per capita)
Dengue	Female	Post Neonatal	80 plus	Global	0	Health System Access (unitless)
Dengue	Female	Post Neonatal	80 plus	Global	0	LDI (I$ per capita)
Dengue	Female	Post Neonatal	80 plus	Global	0	Sociodemographic Status
Dengue	Female	Post Neonatal	80 plus	Global	1	Dengue anomalies (deviation from mean dengue incidence rate)
Dengue	Female	Post Neonatal	80 plus	Global	1	Dengue outbreaks (binary)
Dengue	Female	Post Neonatal	80 plus	Global	1	Elevation Under 100m (proportion)
Dengue	Female	Post Neonatal	80 plus	Global	1	Latitude Under 15 (proportion)
Dengue	Female	Post Neonatal	80 plus	Global	1	Population Density (over 1000 ppl/sqkm, proportion)
Dengue	Female	Post Neonatal	80 plus	Global	1	Population weighted probability of dengue transmission
Dengue	Female	Post Neonatal	80 plus	Global	1	Rainfall Quintile 4 (proportion)
Dengue	Female	Post Neonatal	80 plus	Global	1	Rainfall Quintile 5 (proportion)
Dengue	Female	Post Neonatal	80 plus	Data Rich	0	Education (years per capita)
Dengue	Female	Post Neonatal	80 plus	Data Rich	0	Health System Access (unitless)
Dengue	Female	Post Neonatal	80 plus	Data Rich	0	LDI (I$ per capita)
Dengue	Female	Post Neonatal	80 plus	Data Rich	0	Sociodemographic Status
Dengue	Female	Post Neonatal	80 plus	Data Rich	1	Dengue anomalies (deviation from mean dengue incidence rate)
Dengue	Female	Post Neonatal	80 plus	Data Rich	1	Dengue outbreaks (binary)
Dengue	Female	Post Neonatal	80 plus	Data Rich	1	Elevation Under 100m (proportion)
Dengue	Female	Post Neonatal	80 plus	Data Rich	1	Latitude Under 15 (proportion)
Dengue	Female	Post Neonatal	80 plus	Data Rich	1	Population Density (over 1000 ppl/sqkm, proportion)
Dengue	Female	Post Neonatal	80 plus	Data Rich	1	Population weighted probability of dengue transmission
Dengue	Female	Post Neonatal	80 plus	Data Rich	1	Rainfall Quintile 4 (proportion)
Dengue	Female	Post Neonatal	80 plus	Data Rich	1	Rainfall Quintile 5 (proportion)
Rabies	Male	Post Neonatal	80 plus	Global	-1	Antenatal Care (4 visits) Coverage (proportion)
Rabies	Male	Post Neonatal	80 plus	Global	-1	Health System Access (unitless)
Rabies	Male	Post Neonatal	80 plus	Global	-1	In-Facility Delivery (proportion)
Rabies	Male	Post Neonatal	80 plus	Global	-1	Skilled Birth Attendance (proportion)
Rabies	Male	Post Neonatal	80 plus	Global	-1	Sociodemographic Status
Rabies	Male	Post Neonatal	80 plus	Global	0	Population Density (500-1000 ppl/sqkm, proportion)
Rabies	Male	Post Neonatal	80 plus	Global	0	Population Density (under 150 ppl/sqkm, proportion)
Rabies	Male	Post Neonatal	80 plus	Data Rich	-1	Antenatal Care (4 visits) Coverage (proportion)
Rabies	Male	Post Neonatal	80 plus	Data Rich	-1	Health System Access (unitless)
Rabies	Male	Post Neonatal	80 plus	Data Rich	-1	In-Facility Delivery (proportion)

Appendix Table 10: CODEm covariates used, level of covariate, and expected direction of covariate by cause, sex, and age						
**Cause**	**Sex**	**Age start**	**Age end**	**Model type**	**Direction**	**Covariate**
Rabies	Male	Post Neonatal	80 plus	Data Rich	-1	Skilled Birth Attendance (proportion)
Rabies	Male	Post Neonatal	80 plus	Data Rich	-1	Sociodemographic Status
Rabies	Male	Post Neonatal	80 plus	Data Rich	0	Population Density (500-1000 ppl/sqkm, proportion)
Rabies	Male	Post Neonatal	80 plus	Data Rich	0	Population Density (under 150 ppl/sqkm, proportion)
Rabies	Female	Post Neonatal	80 plus	Global	-1	Antenatal Care (4 visits) Coverage (proportion)
Rabies	Female	Post Neonatal	80 plus	Global	-1	Health System Access (unitless)
Rabies	Female	Post Neonatal	80 plus	Global	-1	In-Facility Delivery (proportion)
Rabies	Female	Post Neonatal	80 plus	Global	-1	Skilled Birth Attendance (proportion)
Rabies	Female	Post Neonatal	80 plus	Global	-1	Sociodemographic Status
Rabies	Female	Post Neonatal	80 plus	Global	0	Population Density (500-1000 ppl/sqkm, proportion)
Rabies	Female	Post Neonatal	80 plus	Global	0	Population Density (under 150 ppl/sqkm, proportion)
Rabies	Female	Post Neonatal	80 plus	Data Rich	-1	Antenatal Care (4 visits) Coverage (proportion)
Rabies	Female	Post Neonatal	80 plus	Data Rich	-1	Health System Access (unitless)
Rabies	Female	Post Neonatal	80 plus	Data Rich	-1	In-Facility Delivery (proportion)
Rabies	Female	Post Neonatal	80 plus	Data Rich	-1	Skilled Birth Attendance (proportion)
Rabies	Female	Post Neonatal	80 plus	Data Rich	-1	Sociodemographic Status
Rabies	Female	Post Neonatal	80 plus	Data Rich	0	Population Density (500-1000 ppl/sqkm, proportion)
Rabies	Female	Post Neonatal	80 plus	Data Rich	0	Population Density (under 150 ppl/sqkm, proportion)
Other neglected tropical diseases	Male	Early Neonatal	80 plus	Global	-1	Education (years per capita)
Other neglected tropical diseases	Male	Early Neonatal	80 plus	Global	-1	Health System Access 2 (unitless)
Other neglected tropical diseases	Male	Early Neonatal	80 plus	Global	-1	LDI (I$ per capita)
Other neglected tropical diseases	Male	Early Neonatal	80 plus	Global	-1	Sanitation (proportion with access)
Other neglected tropical diseases	Male	Early Neonatal	80 plus	Global	-1	Sociodemographic Status
Other neglected tropical diseases	Male	Early Neonatal	80 plus	Global	1	Latitude Under 15 (proportion)
Other neglected tropical diseases	Male	Early Neonatal	80 plus	Global	1	Rainfall Quintile 5 (proportion)
Other neglected tropical diseases	Male	Early Neonatal	80 plus	Data Rich	-1	Education (years per capita)
Other neglected tropical diseases	Male	Early Neonatal	80 plus	Data Rich	-1	Health System Access 2 (unitless)
Other neglected tropical diseases	Male	Early Neonatal	80 plus	Data Rich	-1	LDI (I$ per capita)
Other neglected tropical diseases	Male	Early Neonatal	80 plus	Data Rich	-1	Sanitation (proportion with access)
Other neglected tropical diseases	Male	Early Neonatal	80 plus	Data Rich	-1	Sociodemographic Status
Other neglected tropical diseases	Male	Early Neonatal	80 plus	Data Rich	1	Latitude Under 15 (proportion)
Other neglected tropical diseases	Male	Early Neonatal	80 plus	Data Rich	1	Rainfall Quintile 5 (proportion)
Other neglected tropical diseases	Female	Early Neonatal	80 plus	Global	-1	Education (years per capita)
Other neglected tropical diseases	Female	Early Neonatal	80 plus	Global	-1	Health System Access 2 (unitless)
Other neglected tropical diseases	Female	Early Neonatal	80 plus	Global	-1	LDI (I$ per capita)
Other neglected tropical diseases	Female	Early Neonatal	80 plus	Global	-1	Sanitation (proportion with access)
Other neglected tropical diseases	Female	Early Neonatal	80 plus	Global	-1	Sociodemographic Status
Other neglected tropical diseases	Female	Early Neonatal	80 plus	Global	1	Latitude Under 15 (proportion)
Other neglected tropical diseases	Female	Early Neonatal	80 plus	Global	1	Rainfall Quintile 5 (proportion)
Other neglected tropical diseases	Female	Early Neonatal	80 plus	Data Rich	-1	Education (years per capita)
Other neglected tropical diseases	Female	Early Neonatal	80 plus	Data Rich	-1	Health System Access 2 (unitless)
Other neglected tropical diseases	Female	Early Neonatal	80 plus	Data Rich	-1	LDI (I$ per capita)
Other neglected tropical diseases	Female	Early Neonatal	80 plus	Data Rich	-1	Sanitation (proportion with access)
Other neglected tropical diseases	Female	Early Neonatal	80 plus	Data Rich	-1	Sociodemographic Status

Appendix Table 10: CODEm covariates used, level of covariate, and expected direction of covariate by cause, sex, and age						
**Cause**	**Sex**	**Age start**	**Age end**	**Model type**	**Direction**	**Covariate**
Other neglected tropical diseases	Female	Early Neonatal	80 plus	Data Rich	1	Latitude Under 15 (proportion)
Other neglected tropical diseases	Female	Early Neonatal	80 plus	Data Rich	1	Rainfall Quintile 5 (proportion)
Neonatal disorders	Male	Early Neonatal	1 to 4	Global	-1	Health System Access 2 (unitless)
Neonatal disorders	Male	Early Neonatal	1 to 4	Global	-1	In-Facility Delivery (proportion)
Neonatal disorders	Male	Early Neonatal	1 to 4	Global	-1	Sociodemographic Status
Neonatal disorders	Male	Early Neonatal	1 to 4	Global	0	Education (years per capita)
Neonatal disorders	Male	Early Neonatal	1 to 4	Global	0	Indoor Air Pollution (All Cooking Fuels)
Neonatal disorders	Male	Early Neonatal	1 to 4	Global	0	LDI (I$ per capita)
Neonatal disorders	Male	Early Neonatal	1 to 4	Global	0	Malnutrition (proportion <2SD weight for age)
Neonatal disorders	Male	Early Neonatal	1 to 4	Global	1	Smoking Prevalence (Reproductive Age Standardised)
Neonatal disorders	Male	Early Neonatal	1 to 4	Global	1	Total Fertility Rate
Neonatal disorders	Male	Early Neonatal	1 to 4	Data Rich	-1	Health System Access 2 (unitless)
Neonatal disorders	Male	Early Neonatal	1 to 4	Data Rich	-1	In-Facility Delivery (proportion)
Neonatal disorders	Male	Early Neonatal	1 to 4	Data Rich	-1	Sociodemographic Status
Neonatal disorders	Male	Early Neonatal	1 to 4	Data Rich	0	Education (years per capita)
Neonatal disorders	Male	Early Neonatal	1 to 4	Data Rich	0	Indoor Air Pollution (All Cooking Fuels)
Neonatal disorders	Male	Early Neonatal	1 to 4	Data Rich	0	LDI (I$ per capita)
Neonatal disorders	Male	Early Neonatal	1 to 4	Data Rich	0	Malnutrition (proportion <2SD weight for age)
Neonatal disorders	Male	Early Neonatal	1 to 4	Data Rich	1	Smoking Prevalence (Reproductive Age Standardised)
Neonatal disorders	Male	Early Neonatal	1 to 4	Data Rich	1	Total Fertility Rate
Neonatal disorders	Female	Early Neonatal	1 to 4	Global	-1	Health System Access 2 (unitless)
Neonatal disorders	Female	Early Neonatal	1 to 4	Global	-1	In-Facility Delivery (proportion)
Neonatal disorders	Female	Early Neonatal	1 to 4	Global	-1	Sociodemographic Status
Neonatal disorders	Female	Early Neonatal	1 to 4	Global	0	Education (years per capita)
Neonatal disorders	Female	Early Neonatal	1 to 4	Global	0	Indoor Air Pollution (All Cooking Fuels)
Neonatal disorders	Female	Early Neonatal	1 to 4	Global	0	LDI (I$ per capita)
Neonatal disorders	Female	Early Neonatal	1 to 4	Global	0	Malnutrition (proportion <2SD weight for age)
Neonatal disorders	Female	Early Neonatal	1 to 4	Global	1	Smoking Prevalence (Reproductive Age Standardised)
Neonatal disorders	Female	Early Neonatal	1 to 4	Global	1	Total Fertility Rate
Neonatal disorders	Female	Early Neonatal	1 to 4	Data Rich	-1	Health System Access 2 (unitless)
Neonatal disorders	Female	Early Neonatal	1 to 4	Data Rich	-1	In-Facility Delivery (proportion)
Neonatal disorders	Female	Early Neonatal	1 to 4	Data Rich	-1	Sociodemographic Status
Neonatal disorders	Female	Early Neonatal	1 to 4	Data Rich	0	Education (years per capita)
Neonatal disorders	Female	Early Neonatal	1 to 4	Data Rich	0	Indoor Air Pollution (All Cooking Fuels)
Neonatal disorders	Female	Early Neonatal	1 to 4	Data Rich	0	LDI (I$ per capita)
Neonatal disorders	Female	Early Neonatal	1 to 4	Data Rich	0	Malnutrition (proportion <2SD weight for age)
Neonatal disorders	Female	Early Neonatal	1 to 4	Data Rich	1	Smoking Prevalence (Reproductive Age Standardised)
Neonatal disorders	Female	Early Neonatal	1 to 4	Data Rich	1	Total Fertility Rate
Neonatal preterm birth complications	Male	Early Neonatal	1 to 4	Global	-1	Health System Access 2 (unitless)
Neonatal preterm birth complications	Male	Early Neonatal	1 to 4	Global	-1	In-Facility Delivery (proportion)
Neonatal preterm birth complications	Male	Early Neonatal	1 to 4	Global	-1	Sociodemographic Status
Neonatal preterm birth complications	Male	Early Neonatal	1 to 4	Global	0	Education (years per capita)
Neonatal preterm birth complications	Male	Early Neonatal	1 to 4	Global	0	LDI (I$ per capita)
Neonatal preterm birth complications	Male	Early Neonatal	1 to 4	Global	1	Indoor Air Pollution (All Cooking Fuels)

Appendix Table 10: CODEm covariates used, level of covariate, and expected direction of covariate by cause, sex, and age						
**Cause**	**Sex**	**Age start**	**Age end**	**Model type**	**Direction**	**Covariate**
Neonatal preterm birth complications	Male	Early Neonatal	1 to 4	Global	1	Live Births 35+ (proportion)
Neonatal preterm birth complications	Male	Early Neonatal	1 to 4	Global	1	Malnutrition (proportion <2SD weight for age)
Neonatal preterm birth complications	Male	Early Neonatal	1 to 4	Global	1	Smoking Prevalence (Reproductive Age Standardised)
Neonatal preterm birth complications	Male	Early Neonatal	1 to 4	Global	1	Total Fertility Rate
Neonatal preterm birth complications	Male	Early Neonatal	1 to 4	Data Rich	-1	Health System Access 2 (unitless)
Neonatal preterm birth complications	Male	Early Neonatal	1 to 4	Data Rich	-1	Sociodemographic Status
Neonatal preterm birth complications	Male	Early Neonatal	1 to 4	Data Rich	0	Education (years per capita)
Neonatal preterm birth complications	Male	Early Neonatal	1 to 4	Data Rich	0	LDI (I$ per capita)
Neonatal preterm birth complications	Male	Early Neonatal	1 to 4	Data Rich	1	In-Facility Delivery (proportion)
Neonatal preterm birth complications	Male	Early Neonatal	1 to 4	Data Rich	1	Indoor Air Pollution (All Cooking Fuels)
Neonatal preterm birth complications	Male	Early Neonatal	1 to 4	Data Rich	1	Live Births 35+ (proportion)
Neonatal preterm birth complications	Male	Early Neonatal	1 to 4	Data Rich	1	Malnutrition (proportion <2SD weight for age)
Neonatal preterm birth complications	Male	Early Neonatal	1 to 4	Data Rich	1	Smoking Prevalence (Reproductive Age Standardised)
Neonatal preterm birth complications	Male	Early Neonatal	1 to 4	Data Rich	1	Total Fertility Rate
Neonatal preterm birth complications	Female	Early Neonatal	1 to 4	Global	-1	Health System Access 2 (unitless)
Neonatal preterm birth complications	Female	Early Neonatal	1 to 4	Global	-1	In-Facility Delivery (proportion)
Neonatal preterm birth complications	Female	Early Neonatal	1 to 4	Global	-1	Sociodemographic Status
Neonatal preterm birth complications	Female	Early Neonatal	1 to 4	Global	0	Education (years per capita)
Neonatal preterm birth complications	Female	Early Neonatal	1 to 4	Global	0	LDI (I$ per capita)
Neonatal preterm birth complications	Female	Early Neonatal	1 to 4	Global	1	Indoor Air Pollution (All Cooking Fuels)
Neonatal preterm birth complications	Female	Early Neonatal	1 to 4	Global	1	Live Births 35+ (proportion)
Neonatal preterm birth complications	Female	Early Neonatal	1 to 4	Global	1	Malnutrition (proportion <2SD weight for age)
Neonatal preterm birth complications	Female	Early Neonatal	1 to 4	Global	1	Smoking Prevalence (Reproductive Age Standardised)
Neonatal preterm birth complications	Female	Early Neonatal	1 to 4	Global	1	Total Fertility Rate
Neonatal preterm birth complications	Female	Early Neonatal	1 to 4	Data Rich	-1	Health System Access 2 (unitless)
Neonatal preterm birth complications	Female	Early Neonatal	1 to 4	Data Rich	-1	In-Facility Delivery (proportion)
Neonatal preterm birth complications	Female	Early Neonatal	1 to 4	Data Rich	-1	Sociodemographic Status
Neonatal preterm birth complications	Female	Early Neonatal	1 to 4	Data Rich	0	Education (years per capita)
Neonatal preterm birth complications	Female	Early Neonatal	1 to 4	Data Rich	0	LDI (I$ per capita)
Neonatal preterm birth complications	Female	Early Neonatal	1 to 4	Data Rich	1	Indoor Air Pollution (All Cooking Fuels)
Neonatal preterm birth complications	Female	Early Neonatal	1 to 4	Data Rich	1	Live Births 35+ (proportion)
Neonatal preterm birth complications	Female	Early Neonatal	1 to 4	Data Rich	1	Malnutrition (proportion <2SD weight for age)
Neonatal preterm birth complications	Female	Early Neonatal	1 to 4	Data Rich	1	Smoking Prevalence (Reproductive Age Standardised)
Neonatal preterm birth complications	Female	Early Neonatal	1 to 4	Data Rich	1	Total Fertility Rate
Neonatal encephalopathy due to birth asphyxia and trauma	Male	Early Neonatal	1 to 4	Global	-1	Education (years per capita)
Neonatal encephalopathy due to birth asphyxia and trauma	Male	Early Neonatal	1 to 4	Global	-1	Health System Access 2 (unitless)
Neonatal encephalopathy due to birth asphyxia and trauma	Male	Early Neonatal	1 to 4	Global	-1	In-Facility Delivery (proportion)
Neonatal encephalopathy due to birth asphyxia and trauma	Male	Early Neonatal	1 to 4	Global	-1	LDI (I$ per capita)
Neonatal encephalopathy due to birth asphyxia and trauma	Male	Early Neonatal	1 to 4	Global	-1	Sociodemographic Status
Neonatal encephalopathy due to birth asphyxia and trauma	Male	Early Neonatal	1 to 4	Global	1	Indoor Air Pollution (All Cooking Fuels)
Neonatal encephalopathy due to birth asphyxia and trauma	Male	Early Neonatal	1 to 4	Global	1	Malnutrition (proportion <2SD weight for age)
Neonatal encephalopathy due to birth asphyxia and trauma	Male	Early Neonatal	1 to 4	Global	1	Smoking Prevalence (Reproductive Age Standardised)
Neonatal encephalopathy due to birth asphyxia and trauma	Male	Early Neonatal	1 to 4	Global	1	Total Fertility Rate
Neonatal encephalopathy due to birth asphyxia and trauma	Male	Early Neonatal	1 to 4	Data Rich	-1	Education (years per capita)

Appendix Table 10: CODEm covariates used, level of covariate, and expected direction of covariate by cause, sex, and age						
**Cause**	**Sex**	**Age start**	**Age end**	**Model type**	**Direction**	**Covariate**
Neonatal encephalopathy due to birth asphyxia and trauma	Male	Early Neonatal	1 to 4	Data Rich	-1	Health System Access 2 (unitless)
Neonatal encephalopathy due to birth asphyxia and trauma	Male	Early Neonatal	1 to 4	Data Rich	-1	In-Facility Delivery (proportion)
Neonatal encephalopathy due to birth asphyxia and trauma	Male	Early Neonatal	1 to 4	Data Rich	-1	LDI (I$ per capita)
Neonatal encephalopathy due to birth asphyxia and trauma	Male	Early Neonatal	1 to 4	Data Rich	-1	Sociodemographic Status
Neonatal encephalopathy due to birth asphyxia and trauma	Male	Early Neonatal	1 to 4	Data Rich	1	Indoor Air Pollution (All Cooking Fuels)
Neonatal encephalopathy due to birth asphyxia and trauma	Male	Early Neonatal	1 to 4	Data Rich	1	Malnutrition (proportion <2SD weight for age)
Neonatal encephalopathy due to birth asphyxia and trauma	Male	Early Neonatal	1 to 4	Data Rich	1	Smoking Prevalence (Reproductive Age Standardised)
Neonatal encephalopathy due to birth asphyxia and trauma	Male	Early Neonatal	1 to 4	Data Rich	1	Total Fertility Rate
Neonatal encephalopathy due to birth asphyxia and trauma	Female	Early Neonatal	1 to 4	Global	-1	Education (years per capita)
Neonatal encephalopathy due to birth asphyxia and trauma	Female	Early Neonatal	1 to 4	Global	-1	Health System Access 2 (unitless)
Neonatal encephalopathy due to birth asphyxia and trauma	Female	Early Neonatal	1 to 4	Global	-1	In-Facility Delivery (proportion)
Neonatal encephalopathy due to birth asphyxia and trauma	Female	Early Neonatal	1 to 4	Global	-1	LDI (I$ per capita)
Neonatal encephalopathy due to birth asphyxia and trauma	Female	Early Neonatal	1 to 4	Global	-1	Sociodemographic Status
Neonatal encephalopathy due to birth asphyxia and trauma	Female	Early Neonatal	1 to 4	Global	1	Indoor Air Pollution (All Cooking Fuels)
Neonatal encephalopathy due to birth asphyxia and trauma	Female	Early Neonatal	1 to 4	Global	1	Malnutrition (proportion <2SD weight for age)
Neonatal encephalopathy due to birth asphyxia and trauma	Female	Early Neonatal	1 to 4	Global	1	Smoking Prevalence (Reproductive Age Standardised)
Neonatal encephalopathy due to birth asphyxia and trauma	Female	Early Neonatal	1 to 4	Global	1	Total Fertility Rate
Neonatal encephalopathy due to birth asphyxia and trauma	Female	Early Neonatal	1 to 4	Data Rich	-1	Education (years per capita)
Neonatal encephalopathy due to birth asphyxia and trauma	Female	Early Neonatal	1 to 4	Data Rich	-1	Health System Access 2 (unitless)
Neonatal encephalopathy due to birth asphyxia and trauma	Female	Early Neonatal	1 to 4	Data Rich	-1	In-Facility Delivery (proportion)
Neonatal encephalopathy due to birth asphyxia and trauma	Female	Early Neonatal	1 to 4	Data Rich	-1	LDI (I$ per capita)
Neonatal encephalopathy due to birth asphyxia and trauma	Female	Early Neonatal	1 to 4	Data Rich	-1	Sociodemographic Status
Neonatal encephalopathy due to birth asphyxia and trauma	Female	Early Neonatal	1 to 4	Data Rich	1	Indoor Air Pollution (All Cooking Fuels)
Neonatal encephalopathy due to birth asphyxia and trauma	Female	Early Neonatal	1 to 4	Data Rich	1	Malnutrition (proportion <2SD weight for age)
Neonatal encephalopathy due to birth asphyxia and trauma	Female	Early Neonatal	1 to 4	Data Rich	1	Smoking Prevalence (Reproductive Age Standardised)
Neonatal encephalopathy due to birth asphyxia and trauma	Female	Early Neonatal	1 to 4	Data Rich	1	Total Fertility Rate
Neonatal sepsis and other neonatal infections	Male	Early Neonatal	1 to 4	Global	-1	Education (years per capita)
Neonatal sepsis and other neonatal infections	Male	Early Neonatal	1 to 4	Global	-1	Health System Access 2 (unitless)
Neonatal sepsis and other neonatal infections	Male	Early Neonatal	1 to 4	Global	-1	In-Facility Delivery (proportion)
Neonatal sepsis and other neonatal infections	Male	Early Neonatal	1 to 4	Global	-1	LDI (I$ per capita)
Neonatal sepsis and other neonatal infections	Male	Early Neonatal	1 to 4	Global	-1	Sociodemographic Status
Neonatal sepsis and other neonatal infections	Male	Early Neonatal	1 to 4	Global	1	Indoor Air Pollution (All Cooking Fuels)
Neonatal sepsis and other neonatal infections	Male	Early Neonatal	1 to 4	Global	1	Malnutrition (proportion <2SD weight for age)
Neonatal sepsis and other neonatal infections	Male	Early Neonatal	1 to 4	Global	1	Smoking Prevalence (Reproductive Age Standardised)
Neonatal sepsis and other neonatal infections	Male	Early Neonatal	1 to 4	Global	1	Total Fertility Rate
Neonatal sepsis and other neonatal infections	Male	Early Neonatal	1 to 4	Data Rich	-1	Education (years per capita)
Neonatal sepsis and other neonatal infections	Male	Early Neonatal	1 to 4	Data Rich	-1	Health System Access 2 (unitless)
Neonatal sepsis and other neonatal infections	Male	Early Neonatal	1 to 4	Data Rich	-1	In-Facility Delivery (proportion)
Neonatal sepsis and other neonatal infections	Male	Early Neonatal	1 to 4	Data Rich	-1	LDI (I$ per capita)
Neonatal sepsis and other neonatal infections	Male	Early Neonatal	1 to 4	Data Rich	-1	Sociodemographic Status
Neonatal sepsis and other neonatal infections	Male	Early Neonatal	1 to 4	Data Rich	1	Indoor Air Pollution (All Cooking Fuels)
Neonatal sepsis and other neonatal infections	Male	Early Neonatal	1 to 4	Data Rich	1	Malnutrition (proportion <2SD weight for age)
Neonatal sepsis and other neonatal infections	Male	Early Neonatal	1 to 4	Data Rich	1	Smoking Prevalence (Reproductive Age Standardised)
Neonatal sepsis and other neonatal infections	Male	Early Neonatal	1 to 4	Data Rich	1	Total Fertility Rate

Appendix Table 10: CODEm covariates used, level of covariate, and expected direction of covariate by cause, sex, and age						
**Cause**	**Sex**	**Age start**	**Age end**	**Model type**	**Direction**	**Covariate**
Neonatal sepsis and other neonatal infections	Female	Early Neonatal	1 to 4	Global	-1	Education (years per capita)
Neonatal sepsis and other neonatal infections	Female	Early Neonatal	1 to 4	Global	-1	Health System Access 2 (unitless)
Neonatal sepsis and other neonatal infections	Female	Early Neonatal	1 to 4	Global	-1	In-Facility Delivery (proportion)
Neonatal sepsis and other neonatal infections	Female	Early Neonatal	1 to 4	Global	-1	LDI (I$ per capita)
Neonatal sepsis and other neonatal infections	Female	Early Neonatal	1 to 4	Global	-1	Sociodemographic Status
Neonatal sepsis and other neonatal infections	Female	Early Neonatal	1 to 4	Global	1	Indoor Air Pollution (All Cooking Fuels)
Neonatal sepsis and other neonatal infections	Female	Early Neonatal	1 to 4	Global	1	Malnutrition (proportion <2SD weight for age)
Neonatal sepsis and other neonatal infections	Female	Early Neonatal	1 to 4	Global	1	Smoking Prevalence (Reproductive Age Standardised)
Neonatal sepsis and other neonatal infections	Female	Early Neonatal	1 to 4	Global	1	Total Fertility Rate
Neonatal sepsis and other neonatal infections	Female	Early Neonatal	1 to 4	Data Rich	-1	Education (years per capita)
Neonatal sepsis and other neonatal infections	Female	Early Neonatal	1 to 4	Data Rich	-1	Health System Access 2 (unitless)
Neonatal sepsis and other neonatal infections	Female	Early Neonatal	1 to 4	Data Rich	-1	In-Facility Delivery (proportion)
Neonatal sepsis and other neonatal infections	Female	Early Neonatal	1 to 4	Data Rich	-1	LDI (I$ per capita)
Neonatal sepsis and other neonatal infections	Female	Early Neonatal	1 to 4	Data Rich	-1	Sociodemographic Status
Neonatal sepsis and other neonatal infections	Female	Early Neonatal	1 to 4	Data Rich	1	Indoor Air Pollution (All Cooking Fuels)
Neonatal sepsis and other neonatal infections	Female	Early Neonatal	1 to 4	Data Rich	1	Malnutrition (proportion <2SD weight for age)
Neonatal sepsis and other neonatal infections	Female	Early Neonatal	1 to 4	Data Rich	1	Smoking Prevalence (Reproductive Age Standardised)
Neonatal sepsis and other neonatal infections	Female	Early Neonatal	1 to 4	Data Rich	1	Total Fertility Rate
Hemolytic disease and other neonatal jaundice	Male	Early Neonatal	1 to 4	Global	-1	Antenatal Care (4 visits) Coverage (proportion)
Hemolytic disease and other neonatal jaundice	Male	Early Neonatal	1 to 4	Global	-1	Education (years per capita)
Hemolytic disease and other neonatal jaundice	Male	Early Neonatal	1 to 4	Global	-1	Health System Access 2 (unitless)
Hemolytic disease and other neonatal jaundice	Male	Early Neonatal	1 to 4	Global	-1	In-Facility Delivery (proportion)
Hemolytic disease and other neonatal jaundice	Male	Early Neonatal	1 to 4	Global	-1	LDI (I$ per capita)
Hemolytic disease and other neonatal jaundice	Male	Early Neonatal	1 to 4	Global	-1	Skilled Birth Attendance (proportion)
Hemolytic disease and other neonatal jaundice	Male	Early Neonatal	1 to 4	Global	-1	Sociodemographic Status
Hemolytic disease and other neonatal jaundice	Male	Early Neonatal	1 to 4	Global	1	Total Fertility Rate
Hemolytic disease and other neonatal jaundice	Male	Early Neonatal	1 to 4	Data Rich	-1	Antenatal Care (4 visits) Coverage (proportion)
Hemolytic disease and other neonatal jaundice	Male	Early Neonatal	1 to 4	Data Rich	-1	Education (years per capita)
Hemolytic disease and other neonatal jaundice	Male	Early Neonatal	1 to 4	Data Rich	-1	Health System Access 2 (unitless)
Hemolytic disease and other neonatal jaundice	Male	Early Neonatal	1 to 4	Data Rich	-1	In-Facility Delivery (proportion)
Hemolytic disease and other neonatal jaundice	Male	Early Neonatal	1 to 4	Data Rich	-1	LDI (I$ per capita)
Hemolytic disease and other neonatal jaundice	Male	Early Neonatal	1 to 4	Data Rich	-1	Skilled Birth Attendance (proportion)
Hemolytic disease and other neonatal jaundice	Male	Early Neonatal	1 to 4	Data Rich	-1	Sociodemographic Status
Hemolytic disease and other neonatal jaundice	Male	Early Neonatal	1 to 4	Data Rich	1	Total Fertility Rate
Hemolytic disease and other neonatal jaundice	Female	Early Neonatal	1 to 4	Global	-1	Antenatal Care (4 visits) Coverage (proportion)
Hemolytic disease and other neonatal jaundice	Female	Early Neonatal	1 to 4	Global	-1	Education (years per capita)
Hemolytic disease and other neonatal jaundice	Female	Early Neonatal	1 to 4	Global	-1	Health System Access 2 (unitless)
Hemolytic disease and other neonatal jaundice	Female	Early Neonatal	1 to 4	Global	-1	In-Facility Delivery (proportion)
Hemolytic disease and other neonatal jaundice	Female	Early Neonatal	1 to 4	Global	-1	LDI (I$ per capita)
Hemolytic disease and other neonatal jaundice	Female	Early Neonatal	1 to 4	Global	-1	Skilled Birth Attendance (proportion)
Hemolytic disease and other neonatal jaundice	Female	Early Neonatal	1 to 4	Global	-1	Sociodemographic Status
Hemolytic disease and other neonatal jaundice	Female	Early Neonatal	1 to 4	Global	1	Total Fertility Rate
Hemolytic disease and other neonatal jaundice	Female	Early Neonatal	1 to 4	Data Rich	-1	Antenatal Care (4 visits) Coverage (proportion)
Hemolytic disease and other neonatal jaundice	Female	Early Neonatal	1 to 4	Data Rich	-1	Education (years per capita)

Appendix Table 10: CODEm covariates used, level of covariate, and expected direction of covariate by cause, sex, and age						
**Cause**	**Sex**	**Age start**	**Age end**	**Model type**	**Direction**	**Covariate**
Hemolytic disease and other neonatal jaundice	Female	Early Neonatal	1 to 4	Data Rich	-1	Health System Access 2 (unitless)
Hemolytic disease and other neonatal jaundice	Female	Early Neonatal	1 to 4	Data Rich	-1	In-Facility Delivery (proportion)
Hemolytic disease and other neonatal jaundice	Female	Early Neonatal	1 to 4	Data Rich	-1	LDI (I$ per capita)
Hemolytic disease and other neonatal jaundice	Female	Early Neonatal	1 to 4	Data Rich	-1	Skilled Birth Attendance (proportion)
Hemolytic disease and other neonatal jaundice	Female	Early Neonatal	1 to 4	Data Rich	-1	Sociodemographic Status
Hemolytic disease and other neonatal jaundice	Female	Early Neonatal	1 to 4	Data Rich	1	Total Fertility Rate
Other neonatal disorders	Male	Early Neonatal	1 to 4	Global	-1	Education (years per capita)
Other neonatal disorders	Male	Early Neonatal	1 to 4	Global	-1	Health System Access 2 (unitless)
Other neonatal disorders	Male	Early Neonatal	1 to 4	Global	-1	Skilled Birth Attendance (proportion)
Other neonatal disorders	Male	Early Neonatal	1 to 4	Global	-1	Sociodemographic Status
Other neonatal disorders	Male	Early Neonatal	1 to 4	Global	0	LDI (I$ per capita)
Other neonatal disorders	Male	Early Neonatal	1 to 4	Global	1	Indoor Air Pollution (All Cooking Fuels)
Other neonatal disorders	Male	Early Neonatal	1 to 4	Global	1	Malnutrition (proportion <2SD weight for age)
Other neonatal disorders	Male	Early Neonatal	1 to 4	Global	1	Smoking Prevalence (Reproductive Age Standardised)
Other neonatal disorders	Male	Early Neonatal	1 to 4	Global	1	Total Fertility Rate
Other neonatal disorders	Male	Early Neonatal	1 to 4	Data Rich	-1	Education (years per capita)
Other neonatal disorders	Male	Early Neonatal	1 to 4	Data Rich	-1	Health System Access 2 (unitless)
Other neonatal disorders	Male	Early Neonatal	1 to 4	Data Rich	-1	Skilled Birth Attendance (proportion)
Other neonatal disorders	Male	Early Neonatal	1 to 4	Data Rich	-1	Sociodemographic Status
Other neonatal disorders	Male	Early Neonatal	1 to 4	Data Rich	0	LDI (I$ per capita)
Other neonatal disorders	Male	Early Neonatal	1 to 4	Data Rich	1	Indoor Air Pollution (All Cooking Fuels)
Other neonatal disorders	Male	Early Neonatal	1 to 4	Data Rich	1	Malnutrition (proportion <2SD weight for age)
Other neonatal disorders	Male	Early Neonatal	1 to 4	Data Rich	1	Smoking Prevalence (Reproductive Age Standardised)
Other neonatal disorders	Male	Early Neonatal	1 to 4	Data Rich	1	Total Fertility Rate
Other neonatal disorders	Female	Early Neonatal	1 to 4	Global	-1	Education (years per capita)
Other neonatal disorders	Female	Early Neonatal	1 to 4	Global	-1	Health System Access 2 (unitless)
Other neonatal disorders	Female	Early Neonatal	1 to 4	Global	-1	Skilled Birth Attendance (proportion)
Other neonatal disorders	Female	Early Neonatal	1 to 4	Global	-1	Sociodemographic Status
Other neonatal disorders	Female	Early Neonatal	1 to 4	Global	0	LDI (I$ per capita)
Other neonatal disorders	Female	Early Neonatal	1 to 4	Global	1	Indoor Air Pollution (All Cooking Fuels)
Other neonatal disorders	Female	Early Neonatal	1 to 4	Global	1	Malnutrition (proportion <2SD weight for age)
Other neonatal disorders	Female	Early Neonatal	1 to 4	Global	1	Smoking Prevalence (Reproductive Age Standardised)
Other neonatal disorders	Female	Early Neonatal	1 to 4	Global	1	Total Fertility Rate
Other neonatal disorders	Female	Early Neonatal	1 to 4	Data Rich	-1	Education (years per capita)
Other neonatal disorders	Female	Early Neonatal	1 to 4	Data Rich	-1	Health System Access 2 (unitless)
Other neonatal disorders	Female	Early Neonatal	1 to 4	Data Rich	-1	Skilled Birth Attendance (proportion)
Other neonatal disorders	Female	Early Neonatal	1 to 4	Data Rich	-1	Sociodemographic Status
Other neonatal disorders	Female	Early Neonatal	1 to 4	Data Rich	0	LDI (I$ per capita)
Other neonatal disorders	Female	Early Neonatal	1 to 4	Data Rich	1	Indoor Air Pollution (All Cooking Fuels)
Other neonatal disorders	Female	Early Neonatal	1 to 4	Data Rich	1	Malnutrition (proportion <2SD weight for age)
Other neonatal disorders	Female	Early Neonatal	1 to 4	Data Rich	1	Smoking Prevalence (Reproductive Age Standardised)
Other neonatal disorders	Female	Early Neonatal	1 to 4	Data Rich	1	Total Fertility Rate
Nutritional deficiencies	Male	Post Neonatal	80 plus	Global	-1	Education (years per capita)
Nutritional deficiencies	Male	Post Neonatal	80 plus	Global	-1	Health System Access 2 (unitless)

Appendix Table 10: CODEm covariates used, level of covariate, and expected direction of covariate by cause, sex, and age						
**Cause**	**Sex**	**Age start**	**Age end**	**Model type**	**Direction**	**Covariate**
Nutritional deficiencies	Male	Post Neonatal	80 plus	Global	-1	Improved Water Source (proportion with access)
Nutritional deficiencies	Male	Post Neonatal	80 plus	Global	-1	LDI (I$ per capita)
Nutritional deficiencies	Male	Post Neonatal	80 plus	Global	-1	Sanitation (proportion with access)
Nutritional deficiencies	Male	Post Neonatal	80 plus	Global	-1	Sociodemographic Status
Nutritional deficiencies	Male	Post Neonatal	80 plus	Global	-1	Total Calories (kcal per capita)
Nutritional deficiencies	Male	Post Neonatal	80 plus	Global	0	Rainfall Quintile 1 (proportion)
Nutritional deficiencies	Male	Post Neonatal	80 plus	Global	0	Rainfall Quintile 2 (proportion)
Nutritional deficiencies	Male	Post Neonatal	80 plus	Global	1	Age-Standardize Prevalence of Severe Anemia
Nutritional deficiencies	Male	Post Neonatal	80 plus	Global	1	Famine (binary)
Nutritional deficiencies	Male	Post Neonatal	80 plus	Global	1	Malnutrition (proportion <2SD weight for age)
Nutritional deficiencies	Male	Post Neonatal	80 plus	Global	1	Mortality Rate Due to War Shocks (per 1 person)
Nutritional deficiencies	Male	Post Neonatal	80 plus	Data Rich	-1	Education (years per capita)
Nutritional deficiencies	Male	Post Neonatal	80 plus	Data Rich	-1	Health System Access 2 (unitless)
Nutritional deficiencies	Male	Post Neonatal	80 plus	Data Rich	-1	Improved Water Source (proportion with access)
Nutritional deficiencies	Male	Post Neonatal	80 plus	Data Rich	-1	LDI (I$ per capita)
Nutritional deficiencies	Male	Post Neonatal	80 plus	Data Rich	-1	Sanitation (proportion with access)
Nutritional deficiencies	Male	Post Neonatal	80 plus	Data Rich	-1	Sociodemographic Status
Nutritional deficiencies	Male	Post Neonatal	80 plus	Data Rich	-1	Total Calories (kcal per capita)
Nutritional deficiencies	Male	Post Neonatal	80 plus	Data Rich	0	Rainfall Quintile 1 (proportion)
Nutritional deficiencies	Male	Post Neonatal	80 plus	Data Rich	0	Rainfall Quintile 2 (proportion)
Nutritional deficiencies	Male	Post Neonatal	80 plus	Data Rich	1	Age-Standardize Prevalence of Severe Anemia
Nutritional deficiencies	Male	Post Neonatal	80 plus	Data Rich	1	Famine (binary)
Nutritional deficiencies	Male	Post Neonatal	80 plus	Data Rich	1	Malnutrition (proportion <2SD weight for age)
Nutritional deficiencies	Male	Post Neonatal	80 plus	Data Rich	1	Mortality Rate Due to War Shocks (per 1 person)
Nutritional deficiencies	Female	Post Neonatal	80 plus	Global	-1	Education (years per capita)
Nutritional deficiencies	Female	Post Neonatal	80 plus	Global	-1	Health System Access 2 (unitless)
Nutritional deficiencies	Female	Post Neonatal	80 plus	Global	-1	Improved Water Source (proportion with access)
Nutritional deficiencies	Female	Post Neonatal	80 plus	Global	-1	LDI (I$ per capita)
Nutritional deficiencies	Female	Post Neonatal	80 plus	Global	-1	Sanitation (proportion with access)
Nutritional deficiencies	Female	Post Neonatal	80 plus	Global	-1	Sociodemographic Status
Nutritional deficiencies	Female	Post Neonatal	80 plus	Global	-1	Total Calories (kcal per capita)
Nutritional deficiencies	Female	Post Neonatal	80 plus	Global	0	Rainfall Quintile 1 (proportion)
Nutritional deficiencies	Female	Post Neonatal	80 plus	Global	0	Rainfall Quintile 2 (proportion)
Nutritional deficiencies	Female	Post Neonatal	80 plus	Global	1	Age-Standardize Prevalence of Severe Anemia
Nutritional deficiencies	Female	Post Neonatal	80 plus	Global	1	Famine (binary)
Nutritional deficiencies	Female	Post Neonatal	80 plus	Global	1	Malnutrition (proportion <2SD weight for age)
Nutritional deficiencies	Female	Post Neonatal	80 plus	Global	1	Mortality Rate Due to War Shocks (per 1 person)
Nutritional deficiencies	Female	Post Neonatal	80 plus	Data Rich	-1	Education (years per capita)
Nutritional deficiencies	Female	Post Neonatal	80 plus	Data Rich	-1	Health System Access 2 (unitless)
Nutritional deficiencies	Female	Post Neonatal	80 plus	Data Rich	-1	Improved Water Source (proportion with access)
Nutritional deficiencies	Female	Post Neonatal	80 plus	Data Rich	-1	LDI (I$ per capita)
Nutritional deficiencies	Female	Post Neonatal	80 plus	Data Rich	-1	Sanitation (proportion with access)
Nutritional deficiencies	Female	Post Neonatal	80 plus	Data Rich	-1	Sociodemographic Status
Nutritional deficiencies	Female	Post Neonatal	80 plus	Data Rich	-1	Total Calories (kcal per capita)

Appendix Table 10: CODEm covariates used, level of covariate, and expected direction of covariate by cause, sex, and age						
**Cause**	**Sex**	**Age start**	**Age end**	**Model type**	**Direction**	**Covariate**
Nutritional deficiencies	Female	Post Neonatal	80 plus	Data Rich	0	Rainfall Quintile 1 (proportion)
Nutritional deficiencies	Female	Post Neonatal	80 plus	Data Rich	0	Rainfall Quintile 2 (proportion)
Nutritional deficiencies	Female	Post Neonatal	80 plus	Data Rich	1	Age-Standardize Prevalence of Severe Anemia
Nutritional deficiencies	Female	Post Neonatal	80 plus	Data Rich	1	Famine (binary)
Nutritional deficiencies	Female	Post Neonatal	80 plus	Data Rich	1	Malnutrition (proportion <2SD weight for age)
Nutritional deficiencies	Female	Post Neonatal	80 plus	Data Rich	1	Mortality Rate Due to War Shocks (per 1 person)
Protein-energy malnutrition	Male	5 to 9	80 plus	Global	-1	Education (years per capita)
Protein-energy malnutrition	Male	Post Neonatal	1 to 4	Global	-1	Education (years per capita)
Protein-energy malnutrition	Male	5 to 9	80 plus	Global	-1	Health System Access 2 (unitless)
Protein-energy malnutrition	Male	Post Neonatal	1 to 4	Global	-1	Health System Access 2 (unitless)
Protein-energy malnutrition	Male	5 to 9	80 plus	Global	-1	Improved Water Source (proportion with access)
Protein-energy malnutrition	Male	Post Neonatal	1 to 4	Global	-1	Improved Water Source (proportion with access)
Protein-energy malnutrition	Male	5 to 9	80 plus	Global	-1	LDI (I$ per capita)
Protein-energy malnutrition	Male	Post Neonatal	1 to 4	Global	-1	LDI (I$ per capita)
Protein-energy malnutrition	Male	5 to 9	80 plus	Global	-1	Sanitation (proportion with access)
Protein-energy malnutrition	Male	Post Neonatal	1 to 4	Global	-1	Sanitation (proportion with access)
Protein-energy malnutrition	Male	5 to 9	80 plus	Global	-1	Sociodemographic Status
Protein-energy malnutrition	Male	Post Neonatal	1 to 4	Global	-1	Sociodemographic Status
Protein-energy malnutrition	Male	5 to 9	80 plus	Global	-1	Total Calories (kcal per capita)
Protein-energy malnutrition	Male	Post Neonatal	1 to 4	Global	-1	Total Calories (kcal per capita)
Protein-energy malnutrition	Male	5 to 9	80 plus	Global	0	Rainfall Quintile 1 (proportion)
Protein-energy malnutrition	Male	Post Neonatal	1 to 4	Global	0	Rainfall Quintile 1 (proportion)
Protein-energy malnutrition	Male	5 to 9	80 plus	Global	0	Rainfall Quintile 2 (proportion)
Protein-energy malnutrition	Male	Post Neonatal	1 to 4	Global	0	Rainfall Quintile 2 (proportion)
Protein-energy malnutrition	Male	5 to 9	80 plus	Global	1	Age-Standardize Prevalence of Severe Anemia
Protein-energy malnutrition	Male	Post Neonatal	1 to 4	Global	1	Age-Standardize Prevalence of Severe Anemia
Protein-energy malnutrition	Male	5 to 9	80 plus	Global	1	Famine (binary)
Protein-energy malnutrition	Male	Post Neonatal	1 to 4	Global	1	Famine (binary)
Protein-energy malnutrition	Male	5 to 9	80 plus	Global	1	Malnutrition (proportion <2SD weight for age)
Protein-energy malnutrition	Male	Post Neonatal	1 to 4	Global	1	Malnutrition (proportion <2SD weight for age)
Protein-energy malnutrition	Male	5 to 9	80 plus	Global	1	Mortality Rate Due to War Shocks (per 1 person)
Protein-energy malnutrition	Male	Post Neonatal	1 to 4	Global	1	Mortality Rate Due to War Shocks (per 1 person)
Protein-energy malnutrition	Male	5 to 9	80 plus	Data Rich	-1	Education (years per capita)
Protein-energy malnutrition	Male	Post Neonatal	1 to 4	Data Rich	-1	Education (years per capita)
Protein-energy malnutrition	Male	5 to 9	80 plus	Data Rich	-1	Health System Access 2 (unitless)
Protein-energy malnutrition	Male	Post Neonatal	1 to 4	Data Rich	-1	Health System Access 2 (unitless)
Protein-energy malnutrition	Male	5 to 9	80 plus	Data Rich	-1	Improved Water Source (proportion with access)
Protein-energy malnutrition	Male	Post Neonatal	1 to 4	Data Rich	-1	Improved Water Source (proportion with access)
Protein-energy malnutrition	Male	5 to 9	80 plus	Data Rich	-1	LDI (I$ per capita)
Protein-energy malnutrition	Male	Post Neonatal	1 to 4	Data Rich	-1	LDI (I$ per capita)
Protein-energy malnutrition	Male	5 to 9	80 plus	Data Rich	-1	Sanitation (proportion with access)
Protein-energy malnutrition	Male	Post Neonatal	1 to 4	Data Rich	-1	Sanitation (proportion with access)
Protein-energy malnutrition	Male	5 to 9	80 plus	Data Rich	-1	Sociodemographic Status
Protein-energy malnutrition	Male	Post Neonatal	1 to 4	Data Rich	-1	Sociodemographic Status

Appendix Table 10: CODEm covariates used, level of covariate, and expected direction of covariate by cause, sex, and age						
**Cause**	**Sex**	**Age start**	**Age end**	**Model type**	**Direction**	**Covariate**
Protein-energy malnutrition	Male	5 to 9	80 plus	Data Rich	-1	Total Calories (kcal per capita)
Protein-energy malnutrition	Male	Post Neonatal	1 to 4	Data Rich	-1	Total Calories (kcal per capita)
Protein-energy malnutrition	Male	5 to 9	80 plus	Data Rich	0	Rainfall Quintile 1 (proportion)
Protein-energy malnutrition	Male	Post Neonatal	1 to 4	Data Rich	0	Rainfall Quintile 1 (proportion)
Protein-energy malnutrition	Male	5 to 9	80 plus	Data Rich	0	Rainfall Quintile 2 (proportion)
Protein-energy malnutrition	Male	Post Neonatal	1 to 4	Data Rich	0	Rainfall Quintile 2 (proportion)
Protein-energy malnutrition	Male	5 to 9	80 plus	Data Rich	1	Age-Standardize Prevalence of Severe Anemia
Protein-energy malnutrition	Male	Post Neonatal	1 to 4	Data Rich	1	Age-Standardize Prevalence of Severe Anemia
Protein-energy malnutrition	Male	5 to 9	80 plus	Data Rich	1	Famine (binary)
Protein-energy malnutrition	Male	Post Neonatal	1 to 4	Data Rich	1	Famine (binary)
Protein-energy malnutrition	Male	5 to 9	80 plus	Data Rich	1	Malnutrition (proportion <2SD weight for age)
Protein-energy malnutrition	Male	Post Neonatal	1 to 4	Data Rich	1	Malnutrition (proportion <2SD weight for age)
Protein-energy malnutrition	Male	5 to 9	80 plus	Data Rich	1	Mortality Rate Due to War Shocks (per 1 person)
Protein-energy malnutrition	Male	Post Neonatal	1 to 4	Data Rich	1	Mortality Rate Due to War Shocks (per 1 person)
Protein-energy malnutrition	Female	5 to 9	80 plus	Global	-1	Education (years per capita)
Protein-energy malnutrition	Female	Post Neonatal	1 to 4	Global	-1	Education (years per capita)
Protein-energy malnutrition	Female	5 to 9	80 plus	Global	-1	Health System Access 2 (unitless)
Protein-energy malnutrition	Female	Post Neonatal	1 to 4	Global	-1	Health System Access 2 (unitless)
Protein-energy malnutrition	Female	5 to 9	80 plus	Global	-1	Improved Water Source (proportion with access)
Protein-energy malnutrition	Female	Post Neonatal	1 to 4	Global	-1	Improved Water Source (proportion with access)
Protein-energy malnutrition	Female	5 to 9	80 plus	Global	-1	LDI (I$ per capita)
Protein-energy malnutrition	Female	Post Neonatal	1 to 4	Global	-1	LDI (I$ per capita)
Protein-energy malnutrition	Female	5 to 9	80 plus	Global	-1	Sanitation (proportion with access)
Protein-energy malnutrition	Female	Post Neonatal	1 to 4	Global	-1	Sanitation (proportion with access)
Protein-energy malnutrition	Female	5 to 9	80 plus	Global	-1	Sociodemographic Status
Protein-energy malnutrition	Female	Post Neonatal	1 to 4	Global	-1	Sociodemographic Status
Protein-energy malnutrition	Female	5 to 9	80 plus	Global	-1	Total Calories (kcal per capita)
Protein-energy malnutrition	Female	Post Neonatal	1 to 4	Global	-1	Total Calories (kcal per capita)
Protein-energy malnutrition	Female	5 to 9	80 plus	Global	0	Rainfall Quintile 1 (proportion)
Protein-energy malnutrition	Female	Post Neonatal	1 to 4	Global	0	Rainfall Quintile 1 (proportion)
Protein-energy malnutrition	Female	5 to 9	80 plus	Global	0	Rainfall Quintile 2 (proportion)
Protein-energy malnutrition	Female	Post Neonatal	1 to 4	Global	0	Rainfall Quintile 2 (proportion)
Protein-energy malnutrition	Female	5 to 9	80 plus	Global	1	Age-Standardize Prevalence of Severe Anemia
Protein-energy malnutrition	Female	Post Neonatal	1 to 4	Global	1	Age-Standardize Prevalence of Severe Anemia
Protein-energy malnutrition	Female	5 to 9	80 plus	Global	1	Famine (binary)
Protein-energy malnutrition	Female	Post Neonatal	1 to 4	Global	1	Famine (binary)
Protein-energy malnutrition	Female	5 to 9	80 plus	Global	1	Malnutrition (proportion <2SD weight for age)
Protein-energy malnutrition	Female	Post Neonatal	1 to 4	Global	1	Malnutrition (proportion <2SD weight for age)
Protein-energy malnutrition	Female	5 to 9	80 plus	Global	1	Mortality Rate Due to War Shocks (per 1 person)
Protein-energy malnutrition	Female	Post Neonatal	1 to 4	Global	1	Mortality Rate Due to War Shocks (per 1 person)
Protein-energy malnutrition	Female	5 to 9	80 plus	Data Rich	-1	Education (years per capita)
Protein-energy malnutrition	Female	Post Neonatal	1 to 4	Data Rich	-1	Education (years per capita)
Protein-energy malnutrition	Female	5 to 9	80 plus	Data Rich	-1	Health System Access 2 (unitless)
Protein-energy malnutrition	Female	Post Neonatal	1 to 4	Data Rich	-1	Health System Access 2 (unitless)

Appendix Table 10: CODEm covariates used, level of covariate, and expected direction of covariate by cause, sex, and age						
**Cause**	**Sex**	**Age start**	**Age end**	**Model type**	**Direction**	**Covariate**
Protein-energy malnutrition	Female	5 to 9	80 plus	Data Rich	-1	Improved Water Source (proportion with access)
Protein-energy malnutrition	Female	Post Neonatal	1 to 4	Data Rich	-1	Improved Water Source (proportion with access)
Protein-energy malnutrition	Female	5 to 9	80 plus	Data Rich	-1	LDI (I$ per capita)
Protein-energy malnutrition	Female	Post Neonatal	1 to 4	Data Rich	-1	LDI (I$ per capita)
Protein-energy malnutrition	Female	5 to 9	80 plus	Data Rich	-1	Sanitation (proportion with access)
Protein-energy malnutrition	Female	Post Neonatal	1 to 4	Data Rich	-1	Sanitation (proportion with access)
Protein-energy malnutrition	Female	5 to 9	80 plus	Data Rich	-1	Sociodemographic Status
Protein-energy malnutrition	Female	Post Neonatal	1 to 4	Data Rich	-1	Sociodemographic Status
Protein-energy malnutrition	Female	5 to 9	80 plus	Data Rich	-1	Total Calories (kcal per capita)
Protein-energy malnutrition	Female	Post Neonatal	1 to 4	Data Rich	-1	Total Calories (kcal per capita)
Protein-energy malnutrition	Female	5 to 9	80 plus	Data Rich	0	Rainfall Quintile 1 (proportion)
Protein-energy malnutrition	Female	Post Neonatal	1 to 4	Data Rich	0	Rainfall Quintile 1 (proportion)
Protein-energy malnutrition	Female	5 to 9	80 plus	Data Rich	0	Rainfall Quintile 2 (proportion)
Protein-energy malnutrition	Female	Post Neonatal	1 to 4	Data Rich	0	Rainfall Quintile 2 (proportion)
Protein-energy malnutrition	Female	5 to 9	80 plus	Data Rich	1	Age-Standardize Prevalence of Severe Anemia
Protein-energy malnutrition	Female	Post Neonatal	1 to 4	Data Rich	1	Age-Standardize Prevalence of Severe Anemia
Protein-energy malnutrition	Female	5 to 9	80 plus	Data Rich	1	Famine (binary)
Protein-energy malnutrition	Female	Post Neonatal	1 to 4	Data Rich	1	Famine (binary)
Protein-energy malnutrition	Female	5 to 9	80 plus	Data Rich	1	Malnutrition (proportion <2SD weight for age)
Protein-energy malnutrition	Female	Post Neonatal	1 to 4	Data Rich	1	Malnutrition (proportion <2SD weight for age)
Protein-energy malnutrition	Female	5 to 9	80 plus	Data Rich	1	Mortality Rate Due to War Shocks (per 1 person)
Protein-energy malnutrition	Female	Post Neonatal	1 to 4	Data Rich	1	Mortality Rate Due to War Shocks (per 1 person)
Iron-deficiency anemia	Male	Post Neonatal	80 plus	Global	-1	Education (years per capita)
Iron-deficiency anemia	Male	Post Neonatal	80 plus	Global	-1	Health System Access 2 (unitless)
Iron-deficiency anemia	Male	Post Neonatal	80 plus	Global	-1	Improved Water Source (proportion with access)
Iron-deficiency anemia	Male	Post Neonatal	80 plus	Global	-1	LDI (I$ per capita)
Iron-deficiency anemia	Male	Post Neonatal	80 plus	Global	-1	Sanitation (proportion with access)
Iron-deficiency anemia	Male	Post Neonatal	80 plus	Global	-1	Sociodemographic Status
Iron-deficiency anemia	Male	Post Neonatal	80 plus	Global	-1	Total Calories (kcal per capita)
Iron-deficiency anemia	Male	Post Neonatal	80 plus	Global	0	Rainfall Quintile 1 (proportion)
Iron-deficiency anemia	Male	Post Neonatal	80 plus	Global	0	Rainfall Quintile 2 (proportion)
Iron-deficiency anemia	Male	Post Neonatal	80 plus	Global	1	Age-Standardize Prevalence of Severe Anemia
Iron-deficiency anemia	Male	Post Neonatal	80 plus	Global	1	Malnutrition (proportion <2SD weight for age)
Iron-deficiency anemia	Male	Post Neonatal	80 plus	Global	1	Malnutrition Shock (binary)
Iron-deficiency anemia	Male	Post Neonatal	80 plus	Global	1	Mortality Rate Due to War Shocks (per 1 person)
Iron-deficiency anemia	Male	Post Neonatal	80 plus	Data Rich	-1	Education (years per capita)
Iron-deficiency anemia	Male	Post Neonatal	80 plus	Data Rich	-1	Health System Access 2 (unitless)
Iron-deficiency anemia	Male	Post Neonatal	80 plus	Data Rich	-1	Improved Water Source (proportion with access)
Iron-deficiency anemia	Male	Post Neonatal	80 plus	Data Rich	-1	LDI (I$ per capita)
Iron-deficiency anemia	Male	Post Neonatal	80 plus	Data Rich	-1	Sanitation (proportion with access)
Iron-deficiency anemia	Male	Post Neonatal	80 plus	Data Rich	-1	Sociodemographic Status
Iron-deficiency anemia	Male	Post Neonatal	80 plus	Data Rich	-1	Total Calories (kcal per capita)
Iron-deficiency anemia	Male	Post Neonatal	80 plus	Data Rich	0	Rainfall Quintile 1 (proportion)
Iron-deficiency anemia	Male	Post Neonatal	80 plus	Data Rich	0	Rainfall Quintile 2 (proportion)

Appendix Table 10: CODEm covariates used, level of covariate, and expected direction of covariate by cause, sex, and age						
**Cause**	**Sex**	**Age start**	**Age end**	**Model type**	**Direction**	**Covariate**
Iron-deficiency anemia	Male	Post Neonatal	80 plus	Data Rich	1	Age-Standardize Prevalence of Severe Anemia
Iron-deficiency anemia	Male	Post Neonatal	80 plus	Data Rich	1	Malnutrition (proportion <2SD weight for age)
Iron-deficiency anemia	Male	Post Neonatal	80 plus	Data Rich	1	Malnutrition Shock (binary)
Iron-deficiency anemia	Male	Post Neonatal	80 plus	Data Rich	1	Mortality Rate Due to War Shocks (per 1 person)
Iron-deficiency anemia	Female	Post Neonatal	80 plus	Global	-1	Education (years per capita)
Iron-deficiency anemia	Female	Post Neonatal	80 plus	Global	-1	Health System Access 2 (unitless)
Iron-deficiency anemia	Female	Post Neonatal	80 plus	Global	-1	Improved Water Source (proportion with access)
Iron-deficiency anemia	Female	Post Neonatal	80 plus	Global	-1	LDI (I$ per capita)
Iron-deficiency anemia	Female	Post Neonatal	80 plus	Global	-1	Sanitation (proportion with access)
Iron-deficiency anemia	Female	Post Neonatal	80 plus	Global	-1	Sociodemographic Status
Iron-deficiency anemia	Female	Post Neonatal	80 plus	Global	-1	Total Calories (kcal per capita)
Iron-deficiency anemia	Female	Post Neonatal	80 plus	Global	0	Rainfall Quintile 1 (proportion)
Iron-deficiency anemia	Female	Post Neonatal	80 plus	Global	0	Rainfall Quintile 2 (proportion)
Iron-deficiency anemia	Female	Post Neonatal	80 plus	Global	1	Age-Standardize Prevalence of Severe Anemia
Iron-deficiency anemia	Female	Post Neonatal	80 plus	Global	1	Malnutrition (proportion <2SD weight for age)
Iron-deficiency anemia	Female	Post Neonatal	80 plus	Global	1	Malnutrition Shock (binary)
Iron-deficiency anemia	Female	Post Neonatal	80 plus	Global	1	Mortality Rate Due to War Shocks (per 1 person)
Iron-deficiency anemia	Female	Post Neonatal	80 plus	Data Rich	-1	Education (years per capita)
Iron-deficiency anemia	Female	Post Neonatal	80 plus	Data Rich	-1	Health System Access 2 (unitless)
Iron-deficiency anemia	Female	Post Neonatal	80 plus	Data Rich	-1	Improved Water Source (proportion with access)
Iron-deficiency anemia	Female	Post Neonatal	80 plus	Data Rich	-1	LDI (I$ per capita)
Iron-deficiency anemia	Female	Post Neonatal	80 plus	Data Rich	-1	Sanitation (proportion with access)
Iron-deficiency anemia	Female	Post Neonatal	80 plus	Data Rich	-1	Sociodemographic Status
Iron-deficiency anemia	Female	Post Neonatal	80 plus	Data Rich	-1	Total Calories (kcal per capita)
Iron-deficiency anemia	Female	Post Neonatal	80 plus	Data Rich	0	Rainfall Quintile 1 (proportion)
Iron-deficiency anemia	Female	Post Neonatal	80 plus	Data Rich	0	Rainfall Quintile 2 (proportion)
Iron-deficiency anemia	Female	Post Neonatal	80 plus	Data Rich	1	Age-Standardize Prevalence of Severe Anemia
Iron-deficiency anemia	Female	Post Neonatal	80 plus	Data Rich	1	Malnutrition (proportion <2SD weight for age)
Iron-deficiency anemia	Female	Post Neonatal	80 plus	Data Rich	1	Malnutrition Shock (binary)
Iron-deficiency anemia	Female	Post Neonatal	80 plus	Data Rich	1	Mortality Rate Due to War Shocks (per 1 person)
Other nutritional deficiencies	Male	Post Neonatal	80 plus	Global	-1	Education (years per capita)
Other nutritional deficiencies	Male	Post Neonatal	80 plus	Global	-1	Health System Access 2 (unitless)
Other nutritional deficiencies	Male	Post Neonatal	80 plus	Global	-1	Improved Water Source (proportion with access)
Other nutritional deficiencies	Male	Post Neonatal	80 plus	Global	-1	LDI (I$ per capita)
Other nutritional deficiencies	Male	Post Neonatal	80 plus	Global	-1	Sanitation (proportion with access)
Other nutritional deficiencies	Male	Post Neonatal	80 plus	Global	-1	Sociodemographic Status
Other nutritional deficiencies	Male	Post Neonatal	80 plus	Global	-1	Total Calories (kcal per capita)
Other nutritional deficiencies	Male	Post Neonatal	80 plus	Global	0	Rainfall Quintile 1 (proportion)
Other nutritional deficiencies	Male	Post Neonatal	80 plus	Global	0	Rainfall Quintile 2 (proportion)
Other nutritional deficiencies	Male	Post Neonatal	80 plus	Global	1	Age-Standardize Prevalence of Severe Anemia
Other nutritional deficiencies	Male	Post Neonatal	80 plus	Global	1	Famine (binary)
Other nutritional deficiencies	Male	Post Neonatal	80 plus	Global	1	Malnutrition (proportion <2SD weight for age)
Other nutritional deficiencies	Male	Post Neonatal	80 plus	Global	1	Mortality Rate Due to War Shocks (per 1 person)
Other nutritional deficiencies	Male	Post Neonatal	80 plus	Data Rich	-1	Education (years per capita)

Appendix Table 10: CODEm covariates used, level of covariate, and expected direction of covariate by cause, sex, and age						
**Cause**	**Sex**	**Age start**	**Age end**	**Model type**	**Direction**	**Covariate**
Other nutritional deficiencies	Male	Post Neonatal	80 plus	Data Rich	-1	Health System Access 2 (unitless)
Other nutritional deficiencies	Male	Post Neonatal	80 plus	Data Rich	-1	Improved Water Source (proportion with access)
Other nutritional deficiencies	Male	Post Neonatal	80 plus	Data Rich	-1	LDI (I$ per capita)
Other nutritional deficiencies	Male	Post Neonatal	80 plus	Data Rich	-1	Sanitation (proportion with access)
Other nutritional deficiencies	Male	Post Neonatal	80 plus	Data Rich	-1	Sociodemographic Status
Other nutritional deficiencies	Male	Post Neonatal	80 plus	Data Rich	-1	Total Calories (kcal per capita)
Other nutritional deficiencies	Male	Post Neonatal	80 plus	Data Rich	0	Rainfall Quintile 1 (proportion)
Other nutritional deficiencies	Male	Post Neonatal	80 plus	Data Rich	0	Rainfall Quintile 2 (proportion)
Other nutritional deficiencies	Male	Post Neonatal	80 plus	Data Rich	1	Age-Standardize Prevalence of Severe Anemia
Other nutritional deficiencies	Male	Post Neonatal	80 plus	Data Rich	1	Famine (binary)
Other nutritional deficiencies	Male	Post Neonatal	80 plus	Data Rich	1	Malnutrition (proportion <2SD weight for age)
Other nutritional deficiencies	Male	Post Neonatal	80 plus	Data Rich	1	Mortality Rate Due to War Shocks (per 1 person)
Other nutritional deficiencies	Female	Post Neonatal	80 plus	Global	-1	Education (years per capita)
Other nutritional deficiencies	Female	Post Neonatal	80 plus	Global	-1	Health System Access 2 (unitless)
Other nutritional deficiencies	Female	Post Neonatal	80 plus	Global	-1	Improved Water Source (proportion with access)
Other nutritional deficiencies	Female	Post Neonatal	80 plus	Global	-1	LDI (I$ per capita)
Other nutritional deficiencies	Female	Post Neonatal	80 plus	Global	-1	Sanitation (proportion with access)
Other nutritional deficiencies	Female	Post Neonatal	80 plus	Global	-1	Sociodemographic Status
Other nutritional deficiencies	Female	Post Neonatal	80 plus	Global	-1	Total Calories (kcal per capita)
Other nutritional deficiencies	Female	Post Neonatal	80 plus	Global	0	Rainfall Quintile 1 (proportion)
Other nutritional deficiencies	Female	Post Neonatal	80 plus	Global	0	Rainfall Quintile 2 (proportion)
Other nutritional deficiencies	Female	Post Neonatal	80 plus	Global	1	Age-Standardize Prevalence of Severe Anemia
Other nutritional deficiencies	Female	Post Neonatal	80 plus	Global	1	Famine (binary)
Other nutritional deficiencies	Female	Post Neonatal	80 plus	Global	1	Malnutrition (proportion <2SD weight for age)
Other nutritional deficiencies	Female	Post Neonatal	80 plus	Global	1	Mortality Rate Due to War Shocks (per 1 person)
Other nutritional deficiencies	Female	Post Neonatal	80 plus	Data Rich	-1	Education (years per capita)
Other nutritional deficiencies	Female	Post Neonatal	80 plus	Data Rich	-1	Health System Access 2 (unitless)
Other nutritional deficiencies	Female	Post Neonatal	80 plus	Data Rich	-1	Improved Water Source (proportion with access)
Other nutritional deficiencies	Female	Post Neonatal	80 plus	Data Rich	-1	LDI (I$ per capita)
Other nutritional deficiencies	Female	Post Neonatal	80 plus	Data Rich	-1	Sanitation (proportion with access)
Other nutritional deficiencies	Female	Post Neonatal	80 plus	Data Rich	-1	Sociodemographic Status
Other nutritional deficiencies	Female	Post Neonatal	80 plus	Data Rich	-1	Total Calories (kcal per capita)
Other nutritional deficiencies	Female	Post Neonatal	80 plus	Data Rich	0	Rainfall Quintile 1 (proportion)
Other nutritional deficiencies	Female	Post Neonatal	80 plus	Data Rich	0	Rainfall Quintile 2 (proportion)
Other nutritional deficiencies	Female	Post Neonatal	80 plus	Data Rich	1	Age-Standardize Prevalence of Severe Anemia
Other nutritional deficiencies	Female	Post Neonatal	80 plus	Data Rich	1	Famine (binary)
Other nutritional deficiencies	Female	Post Neonatal	80 plus	Data Rich	1	Malnutrition (proportion <2SD weight for age)
Other nutritional deficiencies	Female	Post Neonatal	80 plus	Data Rich	1	Mortality Rate Due to War Shocks (per 1 person)
Sexually transmitted diseases excluding HIV	Male	10 to 14	80 plus	Global	-1	Antenatal Care (1 visit) Coverage (proportion)
Sexually transmitted diseases excluding HIV	Male	10 to 14	80 plus	Global	-1	Antenatal Care (4 visits) Coverage (proportion)
Sexually transmitted diseases excluding HIV	Male	10 to 14	80 plus	Global	-1	Education (years per capita)
Sexually transmitted diseases excluding HIV	Male	10 to 14	80 plus	Global	-1	Health System Access (capped)
Sexually transmitted diseases excluding HIV	Male	10 to 14	80 plus	Global	-1	LDI (I$ per capita)
Sexually transmitted diseases excluding HIV	Male	10 to 14	80 plus	Global	-1	Legality of Abortion

Appendix Table 10: CODEm covariates used, level of covariate, and expected direction of covariate by cause, sex, and age						
**Cause**	**Sex**	**Age start**	**Age end**	**Model type**	**Direction**	**Covariate**
Sexually transmitted diseases excluding HIV	Male	10 to 14	80 plus	Global	1	Age-Specific Fertility Rate
Sexually transmitted diseases excluding HIV	Male	10 to 14	80 plus	Global	1	Syphilis prevalence (proportion)
Sexually transmitted diseases excluding HIV	Male	10 to 14	80 plus	Global	1	Total Fertility Rate
Sexually transmitted diseases excluding HIV	Male	10 to 14	80 plus	Data Rich	-1	Antenatal Care (1 visit) Coverage (proportion)
Sexually transmitted diseases excluding HIV	Male	10 to 14	80 plus	Data Rich	-1	Antenatal Care (4 visits) Coverage (proportion)
Sexually transmitted diseases excluding HIV	Male	10 to 14	80 plus	Data Rich	-1	Education (years per capita)
Sexually transmitted diseases excluding HIV	Male	10 to 14	80 plus	Data Rich	-1	Health System Access (capped)
Sexually transmitted diseases excluding HIV	Male	10 to 14	80 plus	Data Rich	-1	LDI (I$ per capita)
Sexually transmitted diseases excluding HIV	Male	10 to 14	80 plus	Data Rich	-1	Legality of Abortion
Sexually transmitted diseases excluding HIV	Male	10 to 14	80 plus	Data Rich	1	Age-Specific Fertility Rate
Sexually transmitted diseases excluding HIV	Male	10 to 14	80 plus	Data Rich	1	Syphilis prevalence (proportion)
Sexually transmitted diseases excluding HIV	Male	10 to 14	80 plus	Data Rich	1	Total Fertility Rate
Sexually transmitted diseases excluding HIV	Female	10 to 14	80 plus	Global	-1	Antenatal Care (1 visit) Coverage (proportion)
Sexually transmitted diseases excluding HIV	Female	10 to 14	80 plus	Global	-1	Antenatal Care (4 visits) Coverage (proportion)
Sexually transmitted diseases excluding HIV	Female	10 to 14	80 plus	Global	-1	Education (years per capita)
Sexually transmitted diseases excluding HIV	Female	10 to 14	80 plus	Global	-1	Health System Access (capped)
Sexually transmitted diseases excluding HIV	Female	10 to 14	80 plus	Global	-1	LDI (I$ per capita)
Sexually transmitted diseases excluding HIV	Female	10 to 14	80 plus	Global	-1	Legality of Abortion
Sexually transmitted diseases excluding HIV	Female	10 to 14	80 plus	Global	1	Age-Specific Fertility Rate
Sexually transmitted diseases excluding HIV	Female	10 to 14	80 plus	Global	1	Syphilis prevalence (proportion)
Sexually transmitted diseases excluding HIV	Female	10 to 14	80 plus	Global	1	Total Fertility Rate
Sexually transmitted diseases excluding HIV	Female	10 to 14	80 plus	Data Rich	-1	Antenatal Care (1 visit) Coverage (proportion)
Sexually transmitted diseases excluding HIV	Female	10 to 14	80 plus	Data Rich	-1	Antenatal Care (4 visits) Coverage (proportion)
Sexually transmitted diseases excluding HIV	Female	10 to 14	80 plus	Data Rich	-1	Education (years per capita)
Sexually transmitted diseases excluding HIV	Female	10 to 14	80 plus	Data Rich	-1	Health System Access (capped)
Sexually transmitted diseases excluding HIV	Female	10 to 14	80 plus	Data Rich	-1	LDI (I$ per capita)
Sexually transmitted diseases excluding HIV	Female	10 to 14	80 plus	Data Rich	-1	Legality of Abortion
Sexually transmitted diseases excluding HIV	Female	10 to 14	80 plus	Data Rich	1	Age-Specific Fertility Rate
Sexually transmitted diseases excluding HIV	Female	10 to 14	80 plus	Data Rich	1	Syphilis prevalence (proportion)
Sexually transmitted diseases excluding HIV	Female	10 to 14	80 plus	Data Rich	1	Total Fertility Rate
Hepatitis	Male	Post Neonatal	80 plus	Global	-1	Education (years per capita)
Hepatitis	Male	Post Neonatal	80 plus	Global	-1	Health System Access 2 (unitless)
Hepatitis	Male	Post Neonatal	80 plus	Global	-1	Improved Water Source (proportion with access)
Hepatitis	Male	Post Neonatal	80 plus	Global	-1	LDI (I$ per capita)
Hepatitis	Male	Post Neonatal	80 plus	Global	-1	Sanitation (proportion with access)
Hepatitis	Male	Post Neonatal	80 plus	Global	-1	Sociodemographic Status
Hepatitis	Male	Post Neonatal	80 plus	Global	1	Hepatitis B (HBsAg) Seroprevalence (GBD 2015)
Hepatitis	Male	Post Neonatal	80 plus	Global	1	Hepatitis C (IgG) Seroprevalence (GBD 2015)
Hepatitis	Male	Post Neonatal	80 plus	Global	1	Log-transformed SEV scalar: Hep
Hepatitis	Male	Post Neonatal	80 plus	Global	1	Seroprevalence of anti-HAV (IgG
Hepatitis	Male	Post Neonatal	80 plus	Global	1	Seroprevalence of anti-HEV (IgG)
Hepatitis	Male	Post Neonatal	80 plus	Data Rich	-1	Education (years per capita)
Hepatitis	Male	Post Neonatal	80 plus	Data Rich	-1	Health System Access 2 (unitless)
Hepatitis	Male	Post Neonatal	80 plus	Data Rich	-1	Improved Water Source (proportion with access)

Appendix Table 10: CODEm covariates used, level of covariate, and expected direction of covariate by cause, sex, and age						
**Cause**	**Sex**	**Age start**	**Age end**	**Model type**	**Direction**	**Covariate**
Hepatitis	Male	Post Neonatal	80 plus	Data Rich	-1	LDI (I$ per capita)
Hepatitis	Male	Post Neonatal	80 plus	Data Rich	-1	Sanitation (proportion with access)
Hepatitis	Male	Post Neonatal	80 plus	Data Rich	-1	Sociodemographic Status
Hepatitis	Male	Post Neonatal	80 plus	Data Rich	1	Hepatitis B (HBsAg) Seroprevalence (GBD 2015)
Hepatitis	Male	Post Neonatal	80 plus	Data Rich	1	Hepatitis C (IgG) Seroprevalence (GBD 2015)
Hepatitis	Male	Post Neonatal	80 plus	Data Rich	1	Log-transformed SEV scalar: Hep
Hepatitis	Male	Post Neonatal	80 plus	Data Rich	1	Seroprevalence of anti-HAV (IgG
Hepatitis	Male	Post Neonatal	80 plus	Data Rich	1	Seroprevalence of anti-HEV (IgG)
Hepatitis	Female	Post Neonatal	80 plus	Global	-1	Education (years per capita)
Hepatitis	Female	Post Neonatal	80 plus	Global	-1	Health System Access 2 (unitless)
Hepatitis	Female	Post Neonatal	80 plus	Global	-1	Improved Water Source (proportion with access)
Hepatitis	Female	Post Neonatal	80 plus	Global	-1	LDI (I$ per capita)
Hepatitis	Female	Post Neonatal	80 plus	Global	-1	Sanitation (proportion with access)
Hepatitis	Female	Post Neonatal	80 plus	Global	-1	Sociodemographic Status
Hepatitis	Female	Post Neonatal	80 plus	Global	1	Hepatitis B (HBsAg) Seroprevalence (GBD 2015)
Hepatitis	Female	Post Neonatal	80 plus	Global	1	Hepatitis C (IgG) Seroprevalence (GBD 2015)
Hepatitis	Female	Post Neonatal	80 plus	Global	1	Log-transformed SEV scalar: Hep
Hepatitis	Female	Post Neonatal	80 plus	Global	1	Seroprevalence of anti-HAV (IgG
Hepatitis	Female	Post Neonatal	80 plus	Global	1	Seroprevalence of anti-HEV (IgG)
Hepatitis	Female	Post Neonatal	80 plus	Data Rich	-1	Education (years per capita)
Hepatitis	Female	Post Neonatal	80 plus	Data Rich	-1	Health System Access 2 (unitless)
Hepatitis	Female	Post Neonatal	80 plus	Data Rich	-1	Improved Water Source (proportion with access)
Hepatitis	Female	Post Neonatal	80 plus	Data Rich	-1	LDI (I$ per capita)
Hepatitis	Female	Post Neonatal	80 plus	Data Rich	-1	Sanitation (proportion with access)
Hepatitis	Female	Post Neonatal	80 plus	Data Rich	-1	Sociodemographic Status
Hepatitis	Female	Post Neonatal	80 plus	Data Rich	1	Hepatitis B (HBsAg) Seroprevalence (GBD 2015)
Hepatitis	Female	Post Neonatal	80 plus	Data Rich	1	Hepatitis C (IgG) Seroprevalence (GBD 2015)
Hepatitis	Female	Post Neonatal	80 plus	Data Rich	1	Log-transformed SEV scalar: Hep
Hepatitis	Female	Post Neonatal	80 plus	Data Rich	1	Seroprevalence of anti-HAV (IgG
Hepatitis	Female	Post Neonatal	80 plus	Data Rich	1	Seroprevalence of anti-HEV (IgG)
Other infectious diseases	Male	Early Neonatal	80 plus	Global	-1	Antenatal Care (1 visit) Coverage (proportion)
Other infectious diseases	Male	Early Neonatal	80 plus	Global	-1	DTP3 Coverage (proportion)
Other infectious diseases	Male	Early Neonatal	80 plus	Global	-1	Improved Water Source (proportion with access)
Other infectious diseases	Male	Early Neonatal	80 plus	Global	-1	Latitude Over 45 (proportion)
Other infectious diseases	Male	Early Neonatal	80 plus	Global	-1	Sanitation (proportion with access)
Other infectious diseases	Male	Early Neonatal	80 plus	Global	-1	Sociodemographic Status
Other infectious diseases	Male	Early Neonatal	80 plus	Global	0	Latitude 15 to 30 (proportion)
Other infectious diseases	Male	Early Neonatal	80 plus	Global	0	Latitude 30 to 45 (proportion)
Other infectious diseases	Male	Early Neonatal	80 plus	Global	1	Latitude Under 15 (proportion)
Other infectious diseases	Male	Early Neonatal	80 plus	Global	1	Malnutrition (proportion <2SD weight for age)
Other infectious diseases	Male	Early Neonatal	80 plus	Data Rich	-1	Antenatal Care (1 visit) Coverage (proportion)
Other infectious diseases	Male	Early Neonatal	80 plus	Data Rich	-1	DTP3 Coverage (proportion)
Other infectious diseases	Male	Early Neonatal	80 plus	Data Rich	-1	Education (years per capita)
Other infectious diseases	Male	Early Neonatal	80 plus	Data Rich	-1	Health System Access (unitless)

Appendix Table 10: CODEm covariates used, level of covariate, and expected direction of covariate by cause, sex, and age						
**Cause**	**Sex**	**Age start**	**Age end**	**Model type**	**Direction**	**Covariate**
Other infectious diseases	Male	Early Neonatal	80 plus	Data Rich	-1	Improved Water Source (proportion with access)
Other infectious diseases	Male	Early Neonatal	80 plus	Data Rich	-1	LDI (I$ per capita)
Other infectious diseases	Male	Early Neonatal	80 plus	Data Rich	-1	Latitude Over 45 (proportion)
Other infectious diseases	Male	Early Neonatal	80 plus	Data Rich	-1	Measles Vaccine Coverage (proportion)
Other infectious diseases	Male	Early Neonatal	80 plus	Data Rich	-1	Rainfall Quintile 1 (proportion)
Other infectious diseases	Male	Early Neonatal	80 plus	Data Rich	-1	Sanitation (proportion with access)
Other infectious diseases	Male	Early Neonatal	80 plus	Data Rich	-1	Sociodemographic Status
Other infectious diseases	Male	Early Neonatal	80 plus	Data Rich	0	Latitude 15 to 30 (proportion)
Other infectious diseases	Male	Early Neonatal	80 plus	Data Rich	0	Latitude 30 to 45 (proportion)
Other infectious diseases	Male	Early Neonatal	80 plus	Data Rich	0	Rainfall Quintile 2 (proportion)
Other infectious diseases	Male	Early Neonatal	80 plus	Data Rich	0	Rainfall Quintile 3 (proportion)
Other infectious diseases	Male	Early Neonatal	80 plus	Data Rich	0	Rainfall Quintile 4 (proportion)
Other infectious diseases	Male	Early Neonatal	80 plus	Data Rich	1	Latitude Under 15 (proportion)
Other infectious diseases	Male	Early Neonatal	80 plus	Data Rich	1	Malnutrition (proportion <2SD weight for age)
Other infectious diseases	Male	Early Neonatal	80 plus	Data Rich	1	Rainfall Quintile 5 (proportion)
Other infectious diseases	Female	Early Neonatal	80 plus	Global	-1	Antenatal Care (1 visit) Coverage (proportion)
Other infectious diseases	Female	Early Neonatal	80 plus	Global	-1	DTP3 Coverage (proportion)
Other infectious diseases	Female	Early Neonatal	80 plus	Global	-1	Improved Water Source (proportion with access)
Other infectious diseases	Female	Early Neonatal	80 plus	Global	-1	Latitude Over 45 (proportion)
Other infectious diseases	Female	Early Neonatal	80 plus	Global	-1	Sanitation (proportion with access)
Other infectious diseases	Female	Early Neonatal	80 plus	Global	-1	Sociodemographic Status
Other infectious diseases	Female	Early Neonatal	80 plus	Global	0	Latitude 15 to 30 (proportion)
Other infectious diseases	Female	Early Neonatal	80 plus	Global	0	Latitude 30 to 45 (proportion)
Other infectious diseases	Female	Early Neonatal	80 plus	Global	1	Latitude Under 15 (proportion)
Other infectious diseases	Female	Early Neonatal	80 plus	Global	1	Malnutrition (proportion <2SD weight for age)
Other infectious diseases	Female	Early Neonatal	80 plus	Data Rich	-1	Antenatal Care (1 visit) Coverage (proportion)
Other infectious diseases	Female	Early Neonatal	80 plus	Data Rich	-1	DTP3 Coverage (proportion)
Other infectious diseases	Female	Early Neonatal	80 plus	Data Rich	-1	Education (years per capita)
Other infectious diseases	Female	Early Neonatal	80 plus	Data Rich	-1	Health System Access (unitless)
Other infectious diseases	Female	Early Neonatal	80 plus	Data Rich	-1	Improved Water Source (proportion with access)
Other infectious diseases	Female	Early Neonatal	80 plus	Data Rich	-1	LDI (I$ per capita)
Other infectious diseases	Female	Early Neonatal	80 plus	Data Rich	-1	Latitude Over 45 (proportion)
Other infectious diseases	Female	Early Neonatal	80 plus	Data Rich	-1	Measles Vaccine Coverage (proportion)
Other infectious diseases	Female	Early Neonatal	80 plus	Data Rich	-1	Rainfall Quintile 1 (proportion)
Other infectious diseases	Female	Early Neonatal	80 plus	Data Rich	-1	Sanitation (proportion with access)
Other infectious diseases	Female	Early Neonatal	80 plus	Data Rich	-1	Sociodemographic Status
Other infectious diseases	Female	Early Neonatal	80 plus	Data Rich	0	Latitude 15 to 30 (proportion)
Other infectious diseases	Female	Early Neonatal	80 plus	Data Rich	0	Latitude 30 to 45 (proportion)
Other infectious diseases	Female	Early Neonatal	80 plus	Data Rich	0	Rainfall Quintile 2 (proportion)
Other infectious diseases	Female	Early Neonatal	80 plus	Data Rich	0	Rainfall Quintile 3 (proportion)
Other infectious diseases	Female	Early Neonatal	80 plus	Data Rich	0	Rainfall Quintile 4 (proportion)
Other infectious diseases	Female	Early Neonatal	80 plus	Data Rich	1	Latitude Under 15 (proportion)
Other infectious diseases	Female	Early Neonatal	80 plus	Data Rich	1	Malnutrition (proportion <2SD weight for age)
Other infectious diseases	Female	Early Neonatal	80 plus	Data Rich	1	Rainfall Quintile 5 (proportion)

Appendix Table 10: CODEm covariates used, level of covariate, and expected direction of covariate by cause, sex, and age						
**Cause**	**Sex**	**Age start**	**Age end**	**Model type**	**Direction**	**Covariate**
Lip and oral cavity cancer	Male	15 to 19	80 plus	Global	-1	Education (years per capita)
Lip and oral cavity cancer	Male	15 to 19	80 plus	Global	-1	Fruits (kcal per capita)
Lip and oral cavity cancer	Male	15 to 19	80 plus	Global	-1	Health System Access 2 (unitless)
Lip and oral cavity cancer	Male	15 to 19	80 plus	Global	-1	LDI (I$ per capita)
Lip and oral cavity cancer	Male	15 to 19	80 plus	Global	-1	Vegetables (kcal per capita)
Lip and oral cavity cancer	Male	15 to 19	80 plus	Global	0	Sociodemographic Status
Lip and oral cavity cancer	Male	15 to 19	80 plus	Global	1	Alcohol (litres per capita)
Lip and oral cavity cancer	Male	15 to 19	80 plus	Global	1	Cumulative Cigarettes (10 Years)
Lip and oral cavity cancer	Male	15 to 19	80 plus	Global	1	Cumulative Cigarettes (20 Years)
Lip and oral cavity cancer	Male	15 to 19	80 plus	Global	1	Log-transformed SEV scalar: Mouth C
Lip and oral cavity cancer	Male	15 to 19	80 plus	Global	1	Red Meat (kcal per capita)
Lip and oral cavity cancer	Male	15 to 19	80 plus	Global	1	Smoking Prevalence
Lip and oral cavity cancer	Male	15 to 19	80 plus	Data Rich	-1	Education (years per capita)
Lip and oral cavity cancer	Male	15 to 19	80 plus	Data Rich	-1	Fruits (kcal per capita)
Lip and oral cavity cancer	Male	15 to 19	80 plus	Data Rich	-1	Health System Access 2 (unitless)
Lip and oral cavity cancer	Male	15 to 19	80 plus	Data Rich	-1	LDI (I$ per capita)
Lip and oral cavity cancer	Male	15 to 19	80 plus	Data Rich	-1	Vegetables (kcal per capita)
Lip and oral cavity cancer	Male	15 to 19	80 plus	Data Rich	0	Sociodemographic Status
Lip and oral cavity cancer	Male	15 to 19	80 plus	Data Rich	1	Alcohol (litres per capita)
Lip and oral cavity cancer	Male	15 to 19	80 plus	Data Rich	1	Cumulative Cigarettes (10 Years)
Lip and oral cavity cancer	Male	15 to 19	80 plus	Data Rich	1	Cumulative Cigarettes (20 Years)
Lip and oral cavity cancer	Male	15 to 19	80 plus	Data Rich	1	Log-transformed SEV scalar: Mouth C
Lip and oral cavity cancer	Male	15 to 19	80 plus	Data Rich	1	Red Meat (kcal per capita)
Lip and oral cavity cancer	Male	15 to 19	80 plus	Data Rich	1	Smoking Prevalence
Lip and oral cavity cancer	Female	15 to 19	80 plus	Global	-1	Education (years per capita)
Lip and oral cavity cancer	Female	15 to 19	80 plus	Global	-1	Fruits (kcal per capita)
Lip and oral cavity cancer	Female	15 to 19	80 plus	Global	-1	Health System Access 2 (unitless)
Lip and oral cavity cancer	Female	15 to 19	80 plus	Global	-1	LDI (I$ per capita)
Lip and oral cavity cancer	Female	15 to 19	80 plus	Global	-1	Vegetables (kcal per capita)
Lip and oral cavity cancer	Female	15 to 19	80 plus	Global	0	Sociodemographic Status
Lip and oral cavity cancer	Female	15 to 19	80 plus	Global	1	Alcohol (litres per capita)
Lip and oral cavity cancer	Female	15 to 19	80 plus	Global	1	Cumulative Cigarettes (10 Years)
Lip and oral cavity cancer	Female	15 to 19	80 plus	Global	1	Cumulative Cigarettes (20 Years)
Lip and oral cavity cancer	Female	15 to 19	80 plus	Global	1	Red Meat (kcal per capita)
Lip and oral cavity cancer	Female	15 to 19	80 plus	Global	1	Smoking Prevalence
Lip and oral cavity cancer	Female	15 to 19	80 plus	Data Rich	-1	Education (years per capita)
Lip and oral cavity cancer	Female	15 to 19	80 plus	Data Rich	-1	Fruits (kcal per capita)
Lip and oral cavity cancer	Female	15 to 19	80 plus	Data Rich	-1	Health System Access 2 (unitless)
Lip and oral cavity cancer	Female	15 to 19	80 plus	Data Rich	-1	LDI (I$ per capita)
Lip and oral cavity cancer	Female	15 to 19	80 plus	Data Rich	-1	Vegetables (kcal per capita)
Lip and oral cavity cancer	Female	15 to 19	80 plus	Data Rich	0	Sociodemographic Status
Lip and oral cavity cancer	Female	15 to 19	80 plus	Data Rich	1	Alcohol (litres per capita)
Lip and oral cavity cancer	Female	15 to 19	80 plus	Data Rich	1	Cumulative Cigarettes (10 Years)
Lip and oral cavity cancer	Female	15 to 19	80 plus	Data Rich	1	Cumulative Cigarettes (20 Years)

Appendix Table 10: CODEm covariates used, level of covariate, and expected direction of covariate by cause, sex, and age						
**Cause**	**Sex**	**Age start**	**Age end**	**Model type**	**Direction**	**Covariate**
Lip and oral cavity cancer	Female	15 to 19	80 plus	Data Rich	1	Red Meat (kcal per capita)
Lip and oral cavity cancer	Female	15 to 19	80 plus	Data Rich	1	Smoking Prevalence
Nasopharynx cancer	Male	5 to 9	80 plus	Global	-1	Education (years per capita)
Nasopharynx cancer	Male	5 to 9	80 plus	Global	-1	Fruits (kcal per capita)
Nasopharynx cancer	Male	5 to 9	80 plus	Global	-1	Health System Access 2 (unitless)
Nasopharynx cancer	Male	5 to 9	80 plus	Global	-1	LDI (I$ per capita)
Nasopharynx cancer	Male	5 to 9	80 plus	Global	-1	Vegetables (kcal per capita)
Nasopharynx cancer	Male	5 to 9	80 plus	Global	-1	Whole Grains (kcal per capita)
Nasopharynx cancer	Male	5 to 9	80 plus	Global	1	Alcohol (litres per capita)
Nasopharynx cancer	Male	5 to 9	80 plus	Global	1	Log-transformed SEV scalar: Nasoph C
Nasopharynx cancer	Male	5 to 9	80 plus	Global	1	Malnutrition (proportion <2SD weight for age)
Nasopharynx cancer	Male	5 to 9	80 plus	Global	1	Population Density (over 1000 ppl/sqkm, proportion)
Nasopharynx cancer	Male	5 to 9	80 plus	Global	1	Population Density (under 150 ppl/sqkm, proportion)
Nasopharynx cancer	Male	5 to 9	80 plus	Data Rich	-1	Education (years per capita)
Nasopharynx cancer	Male	5 to 9	80 plus	Data Rich	-1	Fruits (kcal per capita)
Nasopharynx cancer	Male	5 to 9	80 plus	Data Rich	-1	Health System Access 2 (unitless)
Nasopharynx cancer	Male	5 to 9	80 plus	Data Rich	-1	LDI (I$ per capita)
Nasopharynx cancer	Male	5 to 9	80 plus	Data Rich	-1	Vegetables (kcal per capita)
Nasopharynx cancer	Male	5 to 9	80 plus	Data Rich	-1	Whole Grains (kcal per capita)
Nasopharynx cancer	Male	5 to 9	80 plus	Data Rich	0	Sociodemographic Status
Nasopharynx cancer	Male	5 to 9	80 plus	Data Rich	1	Alcohol (litres per capita)
Nasopharynx cancer	Male	5 to 9	80 plus	Data Rich	1	Log-transformed SEV scalar: Nasoph C
Nasopharynx cancer	Male	5 to 9	80 plus	Data Rich	1	Malnutrition (proportion <2SD weight for age)
Nasopharynx cancer	Male	5 to 9	80 plus	Data Rich	1	Population Density (over 1000 ppl/sqkm, proportion)
Nasopharynx cancer	Male	5 to 9	80 plus	Data Rich	1	Population Density (under 150 ppl/sqkm, proportion)
Nasopharynx cancer	Female	5 to 9	80 plus	Global	-1	Education (years per capita)
Nasopharynx cancer	Female	5 to 9	80 plus	Global	-1	Fruits (kcal per capita)
Nasopharynx cancer	Female	5 to 9	80 plus	Global	-1	Health System Access 2 (unitless)
Nasopharynx cancer	Female	5 to 9	80 plus	Global	-1	LDI (I$ per capita)
Nasopharynx cancer	Female	5 to 9	80 plus	Global	-1	Vegetables (kcal per capita)
Nasopharynx cancer	Female	5 to 9	80 plus	Global	-1	Whole Grains (kcal per capita)
Nasopharynx cancer	Female	5 to 9	80 plus	Global	0	Sociodemographic Status
Nasopharynx cancer	Female	5 to 9	80 plus	Global	1	Alcohol (litres per capita)
Nasopharynx cancer	Female	5 to 9	80 plus	Global	1	Log-transformed SEV scalar: Nasoph C
Nasopharynx cancer	Female	5 to 9	80 plus	Global	1	Malnutrition (proportion <2SD weight for age)
Nasopharynx cancer	Female	5 to 9	80 plus	Global	1	Population Density (over 1000 ppl/sqkm, proportion)
Nasopharynx cancer	Female	5 to 9	80 plus	Global	1	Population Density (under 150 ppl/sqkm, proportion)
Nasopharynx cancer	Female	5 to 9	80 plus	Data Rich	-1	Education (years per capita)
Nasopharynx cancer	Female	5 to 9	80 plus	Data Rich	-1	Fruits (kcal per capita)
Nasopharynx cancer	Female	5 to 9	80 plus	Data Rich	-1	Health System Access 2 (unitless)
Nasopharynx cancer	Female	5 to 9	80 plus	Data Rich	-1	LDI (I$ per capita)
Nasopharynx cancer	Female	5 to 9	80 plus	Data Rich	-1	Vegetables (kcal per capita)
Nasopharynx cancer	Female	5 to 9	80 plus	Data Rich	-1	Whole Grains (kcal per capita)
Nasopharynx cancer	Female	5 to 9	80 plus	Data Rich	0	Sociodemographic Status

Appendix Table 10: CODEm covariates used, level of covariate, and expected direction of covariate by cause, sex, and age						
**Cause**	**Sex**	**Age start**	**Age end**	**Model type**	**Direction**	**Covariate**
Nasopharynx cancer	Female	5 to 9	80 plus	Data Rich	1	Alcohol (litres per capita)
Nasopharynx cancer	Female	5 to 9	80 plus	Data Rich	1	Log-transformed SEV scalar: Nasoph C
Nasopharynx cancer	Female	5 to 9	80 plus	Data Rich	1	Malnutrition (proportion <2SD weight for age)
Nasopharynx cancer	Female	5 to 9	80 plus	Data Rich	1	Population Density (over 1000 ppl/sqkm, proportion)
Nasopharynx cancer	Female	5 to 9	80 plus	Data Rich	1	Population Density (under 150 ppl/sqkm, proportion)
Other pharynx cancer	Male	15 to 19	80 plus	Global	-1	Education (years per capita)
Other pharynx cancer	Male	15 to 19	80 plus	Global	-1	Fruits (kcal per capita)
Other pharynx cancer	Male	15 to 19	80 plus	Global	-1	Health System Access (capped)
Other pharynx cancer	Male	15 to 19	80 plus	Global	-1	LDI (I$ per capita)
Other pharynx cancer	Male	15 to 19	80 plus	Global	-1	Vegetables (kcal per capita)
Other pharynx cancer	Male	15 to 19	80 plus	Global	-1	Whole Grains (kcal per capita)
Other pharynx cancer	Male	15 to 19	80 plus	Global	0	Sociodemographic Status
Other pharynx cancer	Male	15 to 19	80 plus	Global	1	Alcohol (litres per capita)
Other pharynx cancer	Male	15 to 19	80 plus	Global	1	Cumulative Cigarettes (5 Years)
Other pharynx cancer	Male	15 to 19	80 plus	Global	1	Log-transformed SEV scalar: Oth Phar C
Other pharynx cancer	Male	15 to 19	80 plus	Global	1	Malnutrition (proportion <2SD weight for age)
Other pharynx cancer	Male	15 to 19	80 plus	Global	1	Population Density (over 1000 ppl/sqkm, proportion)
Other pharynx cancer	Male	15 to 19	80 plus	Global	1	Population Density (under 150 ppl/sqkm, proportion)
Other pharynx cancer	Male	15 to 19	80 plus	Global	1	Smoking Prevalence
Other pharynx cancer	Male	15 to 19	80 plus	Data Rich	-1	Education (years per capita)
Other pharynx cancer	Male	15 to 19	80 plus	Data Rich	-1	Fruits (kcal per capita)
Other pharynx cancer	Male	15 to 19	80 plus	Data Rich	-1	Health System Access (capped)
Other pharynx cancer	Male	15 to 19	80 plus	Data Rich	-1	LDI (I$ per capita)
Other pharynx cancer	Male	15 to 19	80 plus	Data Rich	-1	Vegetables (kcal per capita)
Other pharynx cancer	Male	15 to 19	80 plus	Data Rich	-1	Whole Grains (kcal per capita)
Other pharynx cancer	Male	15 to 19	80 plus	Data Rich	0	Sociodemographic Status
Other pharynx cancer	Male	15 to 19	80 plus	Data Rich	1	Alcohol (litres per capita)
Other pharynx cancer	Male	15 to 19	80 plus	Data Rich	1	Cumulative Cigarettes (5 Years)
Other pharynx cancer	Male	15 to 19	80 plus	Data Rich	1	Log-transformed SEV scalar: Oth Phar C
Other pharynx cancer	Male	15 to 19	80 plus	Data Rich	1	Malnutrition (proportion <2SD weight for age)
Other pharynx cancer	Male	15 to 19	80 plus	Data Rich	1	Population Density (over 1000 ppl/sqkm, proportion)
Other pharynx cancer	Male	15 to 19	80 plus	Data Rich	1	Population Density (under 150 ppl/sqkm, proportion)
Other pharynx cancer	Male	15 to 19	80 plus	Data Rich	1	Smoking Prevalence
Other pharynx cancer	Female	15 to 19	80 plus	Global	-1	Education (years per capita)
Other pharynx cancer	Female	15 to 19	80 plus	Global	-1	Fruits (kcal per capita)
Other pharynx cancer	Female	15 to 19	80 plus	Global	-1	Health System Access (capped)
Other pharynx cancer	Female	15 to 19	80 plus	Global	-1	LDI (I$ per capita)
Other pharynx cancer	Female	15 to 19	80 plus	Global	-1	Vegetables (kcal per capita)
Other pharynx cancer	Female	15 to 19	80 plus	Global	-1	Whole Grains (kcal per capita)
Other pharynx cancer	Female	15 to 19	80 plus	Global	0	Sociodemographic Status
Other pharynx cancer	Female	15 to 19	80 plus	Global	1	Alcohol (litres per capita)
Other pharynx cancer	Female	15 to 19	80 plus	Global	1	Cumulative Cigarettes (5 Years)
Other pharynx cancer	Female	15 to 19	80 plus	Global	1	Log-transformed SEV scalar: Oth Phar C
Other pharynx cancer	Female	15 to 19	80 plus	Global	1	Malnutrition (proportion <2SD weight for age)

Appendix Table 10: CODEm covariates used, level of covariate, and expected direction of covariate by cause, sex, and age						
**Cause**	**Sex**	**Age start**	**Age end**	**Model type**	**Direction**	**Covariate**
Other pharynx cancer	Female	15 to 19	80 plus	Global	1	Population Density (over 1000 ppl/sqkm, proportion)
Other pharynx cancer	Female	15 to 19	80 plus	Global	1	Population Density (under 150 ppl/sqkm, proportion)
Other pharynx cancer	Female	15 to 19	80 plus	Global	1	Smoking Prevalence
Other pharynx cancer	Female	15 to 19	80 plus	Data Rich	-1	Education (years per capita)
Other pharynx cancer	Female	15 to 19	80 plus	Data Rich	-1	Fruits (kcal per capita)
Other pharynx cancer	Female	15 to 19	80 plus	Data Rich	-1	Health System Access (capped)
Other pharynx cancer	Female	15 to 19	80 plus	Data Rich	-1	LDI (I$ per capita)
Other pharynx cancer	Female	15 to 19	80 plus	Data Rich	-1	Vegetables (kcal per capita)
Other pharynx cancer	Female	15 to 19	80 plus	Data Rich	-1	Whole Grains (kcal per capita)
Other pharynx cancer	Female	15 to 19	80 plus	Data Rich	0	Sociodemographic Status
Other pharynx cancer	Female	15 to 19	80 plus	Data Rich	1	Alcohol (litres per capita)
Other pharynx cancer	Female	15 to 19	80 plus	Data Rich	1	Cumulative Cigarettes (5 Years)
Other pharynx cancer	Female	15 to 19	80 plus	Data Rich	1	Log-transformed SEV scalar: Oth Phar C
Other pharynx cancer	Female	15 to 19	80 plus	Data Rich	1	Malnutrition (proportion <2SD weight for age)
Other pharynx cancer	Female	15 to 19	80 plus	Data Rich	1	Population Density (over 1000 ppl/sqkm, proportion)
Other pharynx cancer	Female	15 to 19	80 plus	Data Rich	1	Population Density (under 150 ppl/sqkm, proportion)
Other pharynx cancer	Female	15 to 19	80 plus	Data Rich	1	Smoking Prevalence
Esophageal cancer	Male	15 to 19	80 plus	Global	-1	Education (years per capita)
Esophageal cancer	Male	15 to 19	80 plus	Global	-1	Fruits (kcal per capita)
Esophageal cancer	Male	15 to 19	80 plus	Global	-1	Improved Water Source (proportion with access)
Esophageal cancer	Male	15 to 19	80 plus	Global	-1	LDI (I$ per capita)
Esophageal cancer	Male	15 to 19	80 plus	Global	-1	Sanitation (proportion with access)
Esophageal cancer	Male	15 to 19	80 plus	Global	-1	Vegetables (kcal per capita)
Esophageal cancer	Male	15 to 19	80 plus	Global	0	Sociodemographic Status
Esophageal cancer	Male	15 to 19	80 plus	Global	1	Alcohol (litres per capita)
Esophageal cancer	Male	15 to 19	80 plus	Global	1	Indoor Air Pollution (All Cooking Fuels)
Esophageal cancer	Male	15 to 19	80 plus	Global	1	Indoor Air Pollution (Biomass Cooking)
Esophageal cancer	Male	15 to 19	80 plus	Global	1	Indoor Air Pollution (Coal Cooking)
Esophageal cancer	Male	15 to 19	80 plus	Global	1	Mean BMI
Esophageal cancer	Male	15 to 19	80 plus	Global	1	Smoking Prevalence
Esophageal cancer	Male	15 to 19	80 plus	Global	1	Tobacco (cigarettes per capita)
Esophageal cancer	Male	15 to 19	80 plus	Data Rich	-1	Education (years per capita)
Esophageal cancer	Male	15 to 19	80 plus	Data Rich	-1	Fruits (kcal per capita)
Esophageal cancer	Male	15 to 19	80 plus	Data Rich	-1	Improved Water Source (proportion with access)
Esophageal cancer	Male	15 to 19	80 plus	Data Rich	-1	LDI (I$ per capita)
Esophageal cancer	Male	15 to 19	80 plus	Data Rich	-1	Sanitation (proportion with access)
Esophageal cancer	Male	15 to 19	80 plus	Data Rich	-1	Vegetables (kcal per capita)
Esophageal cancer	Male	15 to 19	80 plus	Data Rich	0	Sociodemographic Status
Esophageal cancer	Male	15 to 19	80 plus	Data Rich	1	Alcohol (litres per capita)
Esophageal cancer	Male	15 to 19	80 plus	Data Rich	1	Indoor Air Pollution (All Cooking Fuels)
Esophageal cancer	Male	15 to 19	80 plus	Data Rich	1	Indoor Air Pollution (Biomass Cooking)
Esophageal cancer	Male	15 to 19	80 plus	Data Rich	1	Indoor Air Pollution (Coal Cooking)
Esophageal cancer	Male	15 to 19	80 plus	Data Rich	1	Mean BMI
Esophageal cancer	Male	15 to 19	80 plus	Data Rich	1	Smoking Prevalence

Appendix Table 10: CODEm covariates used, level of covariate, and expected direction of covariate by cause, sex, and age						
**Cause**	**Sex**	**Age start**	**Age end**	**Model type**	**Direction**	**Covariate**
Esophageal cancer	Male	15 to 19	80 plus	Data Rich	1	Tobacco (cigarettes per capita)
Esophageal cancer	Female	15 to 19	80 plus	Global	-1	Education (years per capita)
Esophageal cancer	Female	15 to 19	80 plus	Global	-1	Fruits (kcal per capita)
Esophageal cancer	Female	15 to 19	80 plus	Global	-1	Improved Water Source (proportion with access)
Esophageal cancer	Female	15 to 19	80 plus	Global	-1	LDI (I$ per capita)
Esophageal cancer	Female	15 to 19	80 plus	Global	-1	Sanitation (proportion with access)
Esophageal cancer	Female	15 to 19	80 plus	Global	-1	Vegetables (kcal per capita)
Esophageal cancer	Female	15 to 19	80 plus	Global	0	Sociodemographic Status
Esophageal cancer	Female	15 to 19	80 plus	Global	1	Alcohol (litres per capita)
Esophageal cancer	Female	15 to 19	80 plus	Global	1	Indoor Air Pollution (All Cooking Fuels)
Esophageal cancer	Female	15 to 19	80 plus	Global	1	Indoor Air Pollution (Biomass Cooking)
Esophageal cancer	Female	15 to 19	80 plus	Global	1	Indoor Air Pollution (Coal Cooking)
Esophageal cancer	Female	15 to 19	80 plus	Global	1	Mean BMI
Esophageal cancer	Female	15 to 19	80 plus	Global	1	Smoking Prevalence
Esophageal cancer	Female	15 to 19	80 plus	Global	1	Tobacco (cigarettes per capita)
Esophageal cancer	Female	15 to 19	80 plus	Data Rich	-1	Education (years per capita)
Esophageal cancer	Female	15 to 19	80 plus	Data Rich	-1	Fruits (kcal per capita)
Esophageal cancer	Female	15 to 19	80 plus	Data Rich	-1	Improved Water Source (proportion with access)
Esophageal cancer	Female	15 to 19	80 plus	Data Rich	-1	LDI (I$ per capita)
Esophageal cancer	Female	15 to 19	80 plus	Data Rich	-1	Sanitation (proportion with access)
Esophageal cancer	Female	15 to 19	80 plus	Data Rich	-1	Vegetables (kcal per capita)
Esophageal cancer	Female	15 to 19	80 plus	Data Rich	0	Sociodemographic Status
Esophageal cancer	Female	15 to 19	80 plus	Data Rich	1	Alcohol (litres per capita)
Esophageal cancer	Female	15 to 19	80 plus	Data Rich	1	Indoor Air Pollution (All Cooking Fuels)
Esophageal cancer	Female	15 to 19	80 plus	Data Rich	1	Indoor Air Pollution (Biomass Cooking)
Esophageal cancer	Female	15 to 19	80 plus	Data Rich	1	Indoor Air Pollution (Coal Cooking)
Esophageal cancer	Female	15 to 19	80 plus	Data Rich	1	Mean BMI
Esophageal cancer	Female	15 to 19	80 plus	Data Rich	1	Smoking Prevalence
Esophageal cancer	Female	15 to 19	80 plus	Data Rich	1	Tobacco (cigarettes per capita)
Stomach cancer	Male	15 to 19	80 plus	Global	-1	Fruits (kcal per capita)
Stomach cancer	Male	15 to 19	80 plus	Global	-1	Improved Water Source (proportion with access)
Stomach cancer	Male	15 to 19	80 plus	Global	-1	LDI (I$ per capita)
Stomach cancer	Male	15 to 19	80 plus	Global	-1	Sanitation (proportion with access)
Stomach cancer	Male	15 to 19	80 plus	Global	-1	Vegetables (kcal per capita)
Stomach cancer	Male	15 to 19	80 plus	Global	0	Sociodemographic Status
Stomach cancer	Male	15 to 19	80 plus	Global	1	Alcohol (litres per capita)
Stomach cancer	Male	15 to 19	80 plus	Global	1	Cumulative Cigarettes (10 Years)
Stomach cancer	Male	15 to 19	80 plus	Global	1	Cumulative Cigarettes (15 Years)
Stomach cancer	Male	15 to 19	80 plus	Global	1	Indoor Air Pollution (All Cooking Fuels)
Stomach cancer	Male	15 to 19	80 plus	Global	1	Indoor Air Pollution (Biomass Cooking)
Stomach cancer	Male	15 to 19	80 plus	Global	1	Indoor Air Pollution (Coal Cooking)
Stomach cancer	Male	15 to 19	80 plus	Global	1	Log-transformed SEV scalar: Stomach C
Stomach cancer	Male	15 to 19	80 plus	Global	1	Mean BMI
Stomach cancer	Male	15 to 19	80 plus	Global	1	Outdoor Air Pollution (PM2.5)

Appendix Table 10: CODEm covariates used, level of covariate, and expected direction of covariate by cause, sex, and age						
**Cause**	**Sex**	**Age start**	**Age end**	**Model type**	**Direction**	**Covariate**
Stomach cancer	Male	15 to 19	80 plus	Global	1	Smoking Prevalence
Stomach cancer	Male	15 to 19	80 plus	Global	1	Tobacco (cigarettes per capita)
Stomach cancer	Male	15 to 19	80 plus	Data Rich	-1	Fruits (kcal per capita)
Stomach cancer	Male	15 to 19	80 plus	Data Rich	-1	Improved Water Source (proportion with access)
Stomach cancer	Male	15 to 19	80 plus	Data Rich	-1	LDI (I$ per capita)
Stomach cancer	Male	15 to 19	80 plus	Data Rich	-1	Sanitation (proportion with access)
Stomach cancer	Male	15 to 19	80 plus	Data Rich	-1	Vegetables (kcal per capita)
Stomach cancer	Male	15 to 19	80 plus	Data Rich	0	Sociodemographic Status
Stomach cancer	Male	15 to 19	80 plus	Data Rich	1	Alcohol (litres per capita)
Stomach cancer	Male	15 to 19	80 plus	Data Rich	1	Cumulative Cigarettes (10 Years)
Stomach cancer	Male	15 to 19	80 plus	Data Rich	1	Cumulative Cigarettes (15 Years)
Stomach cancer	Male	15 to 19	80 plus	Data Rich	1	Indoor Air Pollution (All Cooking Fuels)
Stomach cancer	Male	15 to 19	80 plus	Data Rich	1	Indoor Air Pollution (Biomass Cooking)
Stomach cancer	Male	15 to 19	80 plus	Data Rich	1	Indoor Air Pollution (Coal Cooking)
Stomach cancer	Male	15 to 19	80 plus	Data Rich	1	Log-transformed SEV scalar: Stomach C
Stomach cancer	Male	15 to 19	80 plus	Data Rich	1	Mean BMI
Stomach cancer	Male	15 to 19	80 plus	Data Rich	1	Outdoor Air Pollution (PM2.5)
Stomach cancer	Male	15 to 19	80 plus	Data Rich	1	Smoking Prevalence
Stomach cancer	Male	15 to 19	80 plus	Data Rich	1	Tobacco (cigarettes per capita)
Stomach cancer	Female	15 to 19	80 plus	Global	-1	Education (years per capita)
Stomach cancer	Female	15 to 19	80 plus	Global	-1	Fruits (kcal per capita)
Stomach cancer	Female	15 to 19	80 plus	Global	-1	Improved Water Source (proportion with access)
Stomach cancer	Female	15 to 19	80 plus	Global	-1	LDI (I$ per capita)
Stomach cancer	Female	15 to 19	80 plus	Global	-1	Sanitation (proportion with access)
Stomach cancer	Female	15 to 19	80 plus	Global	-1	Vegetables (kcal per capita)
Stomach cancer	Female	15 to 19	80 plus	Global	0	Sociodemographic Status
Stomach cancer	Female	15 to 19	80 plus	Global	1	Alcohol (litres per capita)
Stomach cancer	Female	15 to 19	80 plus	Global	1	Cumulative Cigarettes (10 Years)
Stomach cancer	Female	15 to 19	80 plus	Global	1	Indoor Air Pollution (All Cooking Fuels)
Stomach cancer	Female	15 to 19	80 plus	Global	1	Indoor Air Pollution (Biomass Cooking)
Stomach cancer	Female	15 to 19	80 plus	Global	1	Indoor Air Pollution (Coal Cooking)
Stomach cancer	Female	15 to 19	80 plus	Global	1	Log-transformed SEV scalar: Stomach C
Stomach cancer	Female	15 to 19	80 plus	Global	1	Mean BMI
Stomach cancer	Female	15 to 19	80 plus	Global	1	Outdoor Air Pollution (PM2.5)
Stomach cancer	Female	15 to 19	80 plus	Global	1	Smoking Prevalence
Stomach cancer	Female	15 to 19	80 plus	Global	1	Tobacco (cigarettes per capita)
Stomach cancer	Female	15 to 19	80 plus	Data Rich	-1	Education (years per capita)
Stomach cancer	Female	15 to 19	80 plus	Data Rich	-1	Fruits (kcal per capita)
Stomach cancer	Female	15 to 19	80 plus	Data Rich	-1	Improved Water Source (proportion with access)
Stomach cancer	Female	15 to 19	80 plus	Data Rich	-1	LDI (I$ per capita)
Stomach cancer	Female	15 to 19	80 plus	Data Rich	-1	Sanitation (proportion with access)
Stomach cancer	Female	15 to 19	80 plus	Data Rich	-1	Vegetables (kcal per capita)
Stomach cancer	Female	15 to 19	80 plus	Data Rich	0	Sociodemographic Status
Stomach cancer	Female	15 to 19	80 plus	Data Rich	1	Alcohol (litres per capita)

Appendix Table 10: CODEm covariates used, level of covariate, and expected direction of covariate by cause, sex, and age						
**Cause**	**Sex**	**Age start**	**Age end**	**Model type**	**Direction**	**Covariate**
Stomach cancer	Female	15 to 19	80 plus	Data Rich	1	Cumulative Cigarettes (10 Years)
Stomach cancer	Female	15 to 19	80 plus	Data Rich	1	Indoor Air Pollution (All Cooking Fuels)
Stomach cancer	Female	15 to 19	80 plus	Data Rich	1	Indoor Air Pollution (Biomass Cooking)
Stomach cancer	Female	15 to 19	80 plus	Data Rich	1	Indoor Air Pollution (Coal Cooking)
Stomach cancer	Female	15 to 19	80 plus	Data Rich	1	Log-transformed SEV scalar: Stomach C
Stomach cancer	Female	15 to 19	80 plus	Data Rich	1	Mean BMI
Stomach cancer	Female	15 to 19	80 plus	Data Rich	1	Outdoor Air Pollution (PM2.5)
Stomach cancer	Female	15 to 19	80 plus	Data Rich	1	Smoking Prevalence
Stomach cancer	Female	15 to 19	80 plus	Data Rich	1	Tobacco (cigarettes per capita)
Colon and rectum cancer	Male	15 to 19	80 plus	Global	-1	Education (years per capita)
Colon and rectum cancer	Male	15 to 19	80 plus	Global	-1	Fruits (kcal per capita)
Colon and rectum cancer	Male	15 to 19	80 plus	Global	-1	Health System Access 2 (unitless)
Colon and rectum cancer	Male	15 to 19	80 plus	Global	-1	LDI (I$ per capita)
Colon and rectum cancer	Male	15 to 19	80 plus	Global	-1	Nuts & Seeds (kcal per capita)
Colon and rectum cancer	Male	15 to 19	80 plus	Global	-1	PUFA Omega 3 - Seafood (kcal per capita)
Colon and rectum cancer	Male	15 to 19	80 plus	Global	-1	Vegetables (kcal per capita)
Colon and rectum cancer	Male	15 to 19	80 plus	Global	-1	Whole Grains (kcal per capita)
Colon and rectum cancer	Male	15 to 19	80 plus	Global	-1	ln-Milk (kcal per capita)
Colon and rectum cancer	Male	15 to 19	80 plus	Global	1	Alcohol (litres per capita)
Colon and rectum cancer	Male	15 to 19	80 plus	Global	1	Diabetes Age-Standardised Prevalence (proportion)
Colon and rectum cancer	Male	15 to 19	80 plus	Global	1	Log-transformed SEV scalar: Colorect C
Colon and rectum cancer	Male	15 to 19	80 plus	Global	1	Mean BMI
Colon and rectum cancer	Male	15 to 19	80 plus	Global	1	Red Meat (kcal per capita)
Colon and rectum cancer	Male	15 to 19	80 plus	Global	1	Smoking Prevalence
Colon and rectum cancer	Male	15 to 19	80 plus	Global	1	Sociodemographic Status
Colon and rectum cancer	Male	15 to 19	80 plus	Global	1	Tobacco (cigarettes per capita)
Colon and rectum cancer	Male	15 to 19	80 plus	Data Rich	-1	Education (years per capita)
Colon and rectum cancer	Male	15 to 19	80 plus	Data Rich	-1	Fruits (kcal per capita)
Colon and rectum cancer	Male	15 to 19	80 plus	Data Rich	-1	Health System Access 2 (unitless)
Colon and rectum cancer	Male	15 to 19	80 plus	Data Rich	-1	LDI (I$ per capita)
Colon and rectum cancer	Male	15 to 19	80 plus	Data Rich	-1	Nuts & Seeds (kcal per capita)
Colon and rectum cancer	Male	15 to 19	80 plus	Data Rich	-1	PUFA Omega 3 - Seafood (kcal per capita)
Colon and rectum cancer	Male	15 to 19	80 plus	Data Rich	-1	Vegetables (kcal per capita)
Colon and rectum cancer	Male	15 to 19	80 plus	Data Rich	-1	Whole Grains (kcal per capita)
Colon and rectum cancer	Male	15 to 19	80 plus	Data Rich	-1	ln-Milk (kcal per capita)
Colon and rectum cancer	Male	15 to 19	80 plus	Data Rich	1	Alcohol (litres per capita)
Colon and rectum cancer	Male	15 to 19	80 plus	Data Rich	1	Diabetes Age-Standardised Prevalence (proportion)
Colon and rectum cancer	Male	15 to 19	80 plus	Data Rich	1	Log-transformed SEV scalar: Colorect C
Colon and rectum cancer	Male	15 to 19	80 plus	Data Rich	1	Mean BMI
Colon and rectum cancer	Male	15 to 19	80 plus	Data Rich	1	Red Meat (kcal per capita)
Colon and rectum cancer	Male	15 to 19	80 plus	Data Rich	1	Smoking Prevalence
Colon and rectum cancer	Male	15 to 19	80 plus	Data Rich	1	Sociodemographic Status
Colon and rectum cancer	Male	15 to 19	80 plus	Data Rich	1	Tobacco (cigarettes per capita)
Colon and rectum cancer	Female	15 to 19	80 plus	Global	-1	Education (years per capita)

Appendix Table 10: CODEm covariates used, level of covariate, and expected direction of covariate by cause, sex, and age						
Cause	Sex	Age start	Age end	Model type	Direction	Covariate
Colon and rectum cancer	Female	15 to 19	80 plus	Global	-1	Fruits (kcal per capita)
Colon and rectum cancer	Female	15 to 19	80 plus	Global	-1	Health System Access 2 (unitless)
Colon and rectum cancer	Female	15 to 19	80 plus	Global	-1	LDI (I$ per capita)
Colon and rectum cancer	Female	15 to 19	80 plus	Global	-1	Nuts & Seeds (kcal per capita)
Colon and rectum cancer	Female	15 to 19	80 plus	Global	-1	PUFA Omega 3 - Seafood (kcal per capita)
Colon and rectum cancer	Female	15 to 19	80 plus	Global	-1	Vegetables (kcal per capita)
Colon and rectum cancer	Female	15 to 19	80 plus	Global	-1	Whole Grains (kcal per capita)
Colon and rectum cancer	Female	15 to 19	80 plus	Global	-1	ln-Milk (kcal per capita)
Colon and rectum cancer	Female	15 to 19	80 plus	Global	1	Alcohol (litres per capita)
Colon and rectum cancer	Female	15 to 19	80 plus	Global	1	Diabetes Age-Standardised Prevalence (proportion)
Colon and rectum cancer	Female	15 to 19	80 plus	Global	1	Log-transformed SEV scalar: Colorect C
Colon and rectum cancer	Female	15 to 19	80 plus	Global	1	Mean BMI
Colon and rectum cancer	Female	15 to 19	80 plus	Global	1	Red Meat (kcal per capita)
Colon and rectum cancer	Female	15 to 19	80 plus	Global	1	Smoking Prevalence
Colon and rectum cancer	Female	15 to 19	80 plus	Global	1	Sociodemographic Status
Colon and rectum cancer	Female	15 to 19	80 plus	Global	1	Tobacco (cigarettes per capita)
Colon and rectum cancer	Female	15 to 19	80 plus	Data Rich	-1	Education (years per capita)
Colon and rectum cancer	Female	15 to 19	80 plus	Data Rich	-1	Fruits (kcal per capita)
Colon and rectum cancer	Female	15 to 19	80 plus	Data Rich	-1	Health System Access 2 (unitless)
Colon and rectum cancer	Female	15 to 19	80 plus	Data Rich	-1	LDI (I$ per capita)
Colon and rectum cancer	Female	15 to 19	80 plus	Data Rich	-1	Nuts & Seeds (kcal per capita)
Colon and rectum cancer	Female	15 to 19	80 plus	Data Rich	-1	PUFA Omega 3 - Seafood (kcal per capita)
Colon and rectum cancer	Female	15 to 19	80 plus	Data Rich	-1	Vegetables (kcal per capita)
Colon and rectum cancer	Female	15 to 19	80 plus	Data Rich	-1	Whole Grains (kcal per capita)
Colon and rectum cancer	Female	15 to 19	80 plus	Data Rich	-1	ln-Milk (kcal per capita)
Colon and rectum cancer	Female	15 to 19	80 plus	Data Rich	1	Alcohol (litres per capita)
Colon and rectum cancer	Female	15 to 19	80 plus	Data Rich	1	Diabetes Age-Standardised Prevalence (proportion)
Colon and rectum cancer	Female	15 to 19	80 plus	Data Rich	1	Log-transformed SEV scalar: Colorect C
Colon and rectum cancer	Female	15 to 19	80 plus	Data Rich	1	Mean BMI
Colon and rectum cancer	Female	15 to 19	80 plus	Data Rich	1	Red Meat (kcal per capita)
Colon and rectum cancer	Female	15 to 19	80 plus	Data Rich	1	Smoking Prevalence
Colon and rectum cancer	Female	15 to 19	80 plus	Data Rich	1	Sociodemographic Status
Colon and rectum cancer	Female	15 to 19	80 plus	Data Rich	1	Tobacco (cigarettes per capita)
Liver cancer	Male	5 to 9	80 plus	Global	-1	Education (years per capita)
Liver cancer	Male	5 to 9	80 plus	Global	-1	Health System Access 2 (unitless)
Liver cancer	Male	5 to 9	80 plus	Global	-1	LDI (I$ per capita)
Liver cancer	Male	5 to 9	80 plus	Global	0	Sociodemographic Status
Liver cancer	Male	5 to 9	80 plus	Global	1	Alcohol (litres per capita)
Liver cancer	Male	5 to 9	80 plus	Global	1	Animal Fats (kcal per capita)
Liver cancer	Male	5 to 9	80 plus	Global	1	Cumulative Cigarettes (15 Years)
Liver cancer	Male	5 to 9	80 plus	Global	1	Cumulative Cigarettes (20 Years)
Liver cancer	Male	5 to 9	80 plus	Global	1	Diabetes Age-Standardised Prevalence (proportion)
Liver cancer	Male	5 to 9	80 plus	Global	1	Hepatitis B Prevalence (proportion)
Liver cancer	Male	5 to 9	80 plus	Global	1	Hepatitis C Prevalence (proportion)

Appendix Table 10: CODEm covariates used, level of covariate, and expected direction of covariate by cause, sex, and age						
**Cause**	**Sex**	**Age start**	**Age end**	**Model type**	**Direction**	**Covariate**
Liver cancer	Male	5 to 9	80 plus	Global	1	Log-transformed SEV scalar: Liver C
Liver cancer	Male	5 to 9	80 plus	Global	1	Mean BMI
Liver cancer	Male	5 to 9	80 plus	Global	1	Red Meat (kcal per capita)
Liver cancer	Male	5 to 9	80 plus	Global	1	Tobacco (cigarettes per capita)
Liver cancer	Male	5 to 9	80 plus	Data Rich	-1	Education (years per capita)
Liver cancer	Male	5 to 9	80 plus	Data Rich	-1	Health System Access 2 (unitless)
Liver cancer	Male	5 to 9	80 plus	Data Rich	-1	LDI (I$ per capita)
Liver cancer	Male	5 to 9	80 plus	Data Rich	0	Sociodemographic Status
Liver cancer	Male	5 to 9	80 plus	Data Rich	1	Alcohol (litres per capita)
Liver cancer	Male	5 to 9	80 plus	Data Rich	1	Animal Fats (kcal per capita)
Liver cancer	Male	5 to 9	80 plus	Data Rich	1	Cumulative Cigarettes (15 Years)
Liver cancer	Male	5 to 9	80 plus	Data Rich	1	Cumulative Cigarettes (20 Years)
Liver cancer	Male	5 to 9	80 plus	Data Rich	1	Diabetes Age-Standardised Prevalence (proportion)
Liver cancer	Male	5 to 9	80 plus	Data Rich	1	Hepatitis B Prevalence (proportion)
Liver cancer	Male	5 to 9	80 plus	Data Rich	1	Hepatitis C Prevalence (proportion)
Liver cancer	Male	5 to 9	80 plus	Data Rich	1	Log-transformed SEV scalar: Liver C
Liver cancer	Male	5 to 9	80 plus	Data Rich	1	Mean BMI
Liver cancer	Male	5 to 9	80 plus	Data Rich	1	Red Meat (kcal per capita)
Liver cancer	Male	5 to 9	80 plus	Data Rich	1	Tobacco (cigarettes per capita)
Liver cancer	Female	5 to 9	80 plus	Global	-1	Education (years per capita)
Liver cancer	Female	5 to 9	80 plus	Global	-1	Health System Access 2 (unitless)
Liver cancer	Female	5 to 9	80 plus	Global	-1	LDI (I$ per capita)
Liver cancer	Female	5 to 9	80 plus	Global	0	Sociodemographic Status
Liver cancer	Female	5 to 9	80 plus	Global	1	Alcohol (litres per capita)
Liver cancer	Female	5 to 9	80 plus	Global	1	Animal Fats (kcal per capita)
Liver cancer	Female	5 to 9	80 plus	Global	1	Cumulative Cigarettes (15 Years)
Liver cancer	Female	5 to 9	80 plus	Global	1	Cumulative Cigarettes (20 Years)
Liver cancer	Female	5 to 9	80 plus	Global	1	Diabetes Age-Standardised Prevalence (proportion)
Liver cancer	Female	5 to 9	80 plus	Global	1	Hepatitis B Prevalence (proportion)
Liver cancer	Female	5 to 9	80 plus	Global	1	Hepatitis C Prevalence (proportion)
Liver cancer	Female	5 to 9	80 plus	Global	1	Log-transformed SEV scalar: Liver C
Liver cancer	Female	5 to 9	80 plus	Global	1	Mean BMI
Liver cancer	Female	5 to 9	80 plus	Global	1	Red Meat (kcal per capita)
Liver cancer	Female	5 to 9	80 plus	Global	1	Tobacco (cigarettes per capita)
Liver cancer	Female	5 to 9	80 plus	Data Rich	-1	Education (years per capita)
Liver cancer	Female	5 to 9	80 plus	Data Rich	-1	Health System Access 2 (unitless)
Liver cancer	Female	5 to 9	80 plus	Data Rich	-1	LDI (I$ per capita)
Liver cancer	Female	5 to 9	80 plus	Data Rich	0	Sociodemographic Status
Liver cancer	Female	5 to 9	80 plus	Data Rich	1	Alcohol (litres per capita)
Liver cancer	Female	5 to 9	80 plus	Data Rich	1	Animal Fats (kcal per capita)
Liver cancer	Female	5 to 9	80 plus	Data Rich	1	Cumulative Cigarettes (15 Years)
Liver cancer	Female	5 to 9	80 plus	Data Rich	1	Cumulative Cigarettes (20 Years)
Liver cancer	Female	5 to 9	80 plus	Data Rich	1	Diabetes Age-Standardised Prevalence (proportion)
Liver cancer	Female	5 to 9	80 plus	Data Rich	1	Hepatitis B Prevalence (proportion)

Appendix Table 10: CODEm covariates used, level of covariate, and expected direction of covariate by cause, sex, and age						
**Cause**	**Sex**	**Age start**	**Age end**	**Model type**	**Direction**	**Covariate**
Liver cancer	Female	5 to 9	80 plus	Data Rich	1	Hepatitis C Prevalence (proportion)
Liver cancer	Female	5 to 9	80 plus	Data Rich	1	Log-transformed SEV scalar: Liver C
Liver cancer	Female	5 to 9	80 plus	Data Rich	1	Mean BMI
Liver cancer	Female	5 to 9	80 plus	Data Rich	1	Red Meat (kcal per capita)
Liver cancer	Female	5 to 9	80 plus	Data Rich	1	Tobacco (cigarettes per capita)
Gallbladder and biliary tract cancer	Male	15 to 19	80 plus	Global	-1	Education (years per capita)
Gallbladder and biliary tract cancer	Male	15 to 19	80 plus	Global	-1	Fruits (kcal per capita)
Gallbladder and biliary tract cancer	Male	15 to 19	80 plus	Global	-1	Health System Access (capped)
Gallbladder and biliary tract cancer	Male	15 to 19	80 plus	Global	-1	LDI (I$ per capita)
Gallbladder and biliary tract cancer	Male	15 to 19	80 plus	Global	-1	Vegetables (kcal per capita)
Gallbladder and biliary tract cancer	Male	15 to 19	80 plus	Global	0	Sociodemographic Status
Gallbladder and biliary tract cancer	Male	15 to 19	80 plus	Global	1	Alcohol (litres per capita)
Gallbladder and biliary tract cancer	Male	15 to 19	80 plus	Global	1	Cumulative Cigarettes (10 Years)
Gallbladder and biliary tract cancer	Male	15 to 19	80 plus	Global	1	Cumulative Cigarettes (5 Years)
Gallbladder and biliary tract cancer	Male	15 to 19	80 plus	Global	1	Diabetes Age-Standardised Prevalence (proportion)
Gallbladder and biliary tract cancer	Male	15 to 19	80 plus	Global	1	Log-transformed SEV scalar: Gallblad C
Gallbladder and biliary tract cancer	Male	15 to 19	80 plus	Global	1	Mean BMI
Gallbladder and biliary tract cancer	Male	15 to 19	80 plus	Global	1	Smoking Prevalence
Gallbladder and biliary tract cancer	Male	15 to 19	80 plus	Global	1	Tobacco (cigarettes per capita)
Gallbladder and biliary tract cancer	Male	15 to 19	80 plus	Global	1	Total Calories (kcal per capita)
Gallbladder and biliary tract cancer	Male	15 to 19	80 plus	Data Rich	-1	Education (years per capita)
Gallbladder and biliary tract cancer	Male	15 to 19	80 plus	Data Rich	-1	Fruits (kcal per capita)
Gallbladder and biliary tract cancer	Male	15 to 19	80 plus	Data Rich	-1	Health System Access (capped)
Gallbladder and biliary tract cancer	Male	15 to 19	80 plus	Data Rich	-1	LDI (I$ per capita)
Gallbladder and biliary tract cancer	Male	15 to 19	80 plus	Data Rich	-1	Vegetables (kcal per capita)
Gallbladder and biliary tract cancer	Male	15 to 19	80 plus	Data Rich	0	Sociodemographic Status
Gallbladder and biliary tract cancer	Male	15 to 19	80 plus	Data Rich	1	Alcohol (litres per capita)
Gallbladder and biliary tract cancer	Male	15 to 19	80 plus	Data Rich	1	Cumulative Cigarettes (10 Years)
Gallbladder and biliary tract cancer	Male	15 to 19	80 plus	Data Rich	1	Cumulative Cigarettes (5 Years)
Gallbladder and biliary tract cancer	Male	15 to 19	80 plus	Data Rich	1	Diabetes Age-Standardised Prevalence (proportion)
Gallbladder and biliary tract cancer	Male	15 to 19	80 plus	Data Rich	1	Log-transformed SEV scalar: Gallblad C
Gallbladder and biliary tract cancer	Male	15 to 19	80 plus	Data Rich	1	Mean BMI
Gallbladder and biliary tract cancer	Male	15 to 19	80 plus	Data Rich	1	Smoking Prevalence
Gallbladder and biliary tract cancer	Male	15 to 19	80 plus	Data Rich	1	Tobacco (cigarettes per capita)
Gallbladder and biliary tract cancer	Male	15 to 19	80 plus	Data Rich	1	Total Calories (kcal per capita)
Gallbladder and biliary tract cancer	Female	15 to 19	80 plus	Global	-1	Education (years per capita)
Gallbladder and biliary tract cancer	Female	15 to 19	80 plus	Global	-1	Fruits (kcal per capita)
Gallbladder and biliary tract cancer	Female	15 to 19	80 plus	Global	-1	Health System Access (capped)
Gallbladder and biliary tract cancer	Female	15 to 19	80 plus	Global	-1	LDI (I$ per capita)
Gallbladder and biliary tract cancer	Female	15 to 19	80 plus	Global	-1	Vegetables (kcal per capita)
Gallbladder and biliary tract cancer	Female	15 to 19	80 plus	Global	0	Sociodemographic Status
Gallbladder and biliary tract cancer	Female	15 to 19	80 plus	Global	1	Alcohol (litres per capita)
Gallbladder and biliary tract cancer	Female	15 to 19	80 plus	Global	1	Cumulative Cigarettes (10 Years)
Gallbladder and biliary tract cancer	Female	15 to 19	80 plus	Global	1	Cumulative Cigarettes (5 Years)

Appendix Table 10: CODEm covariates used, level of covariate, and expected direction of covariate by cause, sex, and age						
**Cause**	**Sex**	**Age start**	**Age end**	**Model type**	**Direction**	**Covariate**
Gallbladder and biliary tract cancer	Female	15 to 19	80 plus	Global	1	Diabetes Age-Standardised Prevalence (proportion)
Gallbladder and biliary tract cancer	Female	15 to 19	80 plus	Global	1	Log-transformed SEV scalar: Gallblad C
Gallbladder and biliary tract cancer	Female	15 to 19	80 plus	Global	1	Mean BMI
Gallbladder and biliary tract cancer	Female	15 to 19	80 plus	Global	1	Smoking Prevalence
Gallbladder and biliary tract cancer	Female	15 to 19	80 plus	Global	1	Tobacco (cigarettes per capita)
Gallbladder and biliary tract cancer	Female	15 to 19	80 plus	Global	1	Total Calories (kcal per capita)
Gallbladder and biliary tract cancer	Female	15 to 19	80 plus	Data Rich	-1	Education (years per capita)
Gallbladder and biliary tract cancer	Female	15 to 19	80 plus	Data Rich	-1	Fruits (kcal per capita)
Gallbladder and biliary tract cancer	Female	15 to 19	80 plus	Data Rich	-1	Health System Access (capped)
Gallbladder and biliary tract cancer	Female	15 to 19	80 plus	Data Rich	-1	LDI (I$ per capita)
Gallbladder and biliary tract cancer	Female	15 to 19	80 plus	Data Rich	-1	Vegetables (kcal per capita)
Gallbladder and biliary tract cancer	Female	15 to 19	80 plus	Data Rich	0	Sociodemographic Status
Gallbladder and biliary tract cancer	Female	15 to 19	80 plus	Data Rich	1	Alcohol (litres per capita)
Gallbladder and biliary tract cancer	Female	15 to 19	80 plus	Data Rich	1	Cumulative Cigarettes (10 Years)
Gallbladder and biliary tract cancer	Female	15 to 19	80 plus	Data Rich	1	Cumulative Cigarettes (5 Years)
Gallbladder and biliary tract cancer	Female	15 to 19	80 plus	Data Rich	1	Diabetes Age-Standardised Prevalence (proportion)
Gallbladder and biliary tract cancer	Female	15 to 19	80 plus	Data Rich	1	Log-transformed SEV scalar: Gallblad C
Gallbladder and biliary tract cancer	Female	15 to 19	80 plus	Data Rich	1	Mean BMI
Gallbladder and biliary tract cancer	Female	15 to 19	80 plus	Data Rich	1	Smoking Prevalence
Gallbladder and biliary tract cancer	Female	15 to 19	80 plus	Data Rich	1	Tobacco (cigarettes per capita)
Gallbladder and biliary tract cancer	Female	15 to 19	80 plus	Data Rich	1	Total Calories (kcal per capita)
Pancreatic cancer	Male	15 to 19	80 plus	Global	-1	Education (years per capita)
Pancreatic cancer	Male	15 to 19	80 plus	Global	-1	Fruits (kcal per capita)
Pancreatic cancer	Male	15 to 19	80 plus	Global	-1	Health System Access (unitless)
Pancreatic cancer	Male	15 to 19	80 plus	Global	-1	LDI (I$ per capita)
Pancreatic cancer	Male	15 to 19	80 plus	Global	-1	Vegetables (kcal per capita)
Pancreatic cancer	Male	15 to 19	80 plus	Global	1	Alcohol (litres per capita)
Pancreatic cancer	Male	15 to 19	80 plus	Global	1	Animal Fats (kcal per capita)
Pancreatic cancer	Male	15 to 19	80 plus	Global	1	Cumulative Cigarettes (10 Years)
Pancreatic cancer	Male	15 to 19	80 plus	Global	1	Cumulative Cigarettes (20 Years)
Pancreatic cancer	Male	15 to 19	80 plus	Global	1	Cumulative Cigarettes (5 Years)
Pancreatic cancer	Male	15 to 19	80 plus	Global	1	Diabetes Age-Standardised Prevalence (proportion)
Pancreatic cancer	Male	15 to 19	80 plus	Global	1	Log-transformed SEV scalar: Pancreas C
Pancreatic cancer	Male	15 to 19	80 plus	Global	1	Mean BMI
Pancreatic cancer	Male	15 to 19	80 plus	Global	1	Red Meat (kcal per capita)
Pancreatic cancer	Male	15 to 19	80 plus	Global	1	Smoking Prevalence
Pancreatic cancer	Male	15 to 19	80 plus	Global	1	Sociodemographic Status
Pancreatic cancer	Male	15 to 19	80 plus	Global	1	Tobacco (cigarettes per capita)
Pancreatic cancer	Male	15 to 19	80 plus	Global	1	Total Calories (kcal per capita)
Pancreatic cancer	Male	15 to 19	80 plus	Data Rich	-1	Education (years per capita)
Pancreatic cancer	Male	15 to 19	80 plus	Data Rich	-1	Fruits (kcal per capita)
Pancreatic cancer	Male	15 to 19	80 plus	Data Rich	-1	Health System Access (unitless)
Pancreatic cancer	Male	15 to 19	80 plus	Data Rich	-1	LDI (I$ per capita)
Pancreatic cancer	Male	15 to 19	80 plus	Data Rich	-1	Vegetables (kcal per capita)

Appendix Table 10: CODEm covariates used, level of covariate, and expected direction of covariate by cause, sex, and age						
**Cause**	**Sex**	**Age start**	**Age end**	**Model type**	**Direction**	**Covariate**
Pancreatic cancer	Male	15 to 19	80 plus	Data Rich	1	Alcohol (litres per capita)
Pancreatic cancer	Male	15 to 19	80 plus	Data Rich	1	Animal Fats (kcal per capita)
Pancreatic cancer	Male	15 to 19	80 plus	Data Rich	1	Cumulative Cigarettes (10 Years)
Pancreatic cancer	Male	15 to 19	80 plus	Data Rich	1	Cumulative Cigarettes (20 Years)
Pancreatic cancer	Male	15 to 19	80 plus	Data Rich	1	Cumulative Cigarettes (5 Years)
Pancreatic cancer	Male	15 to 19	80 plus	Data Rich	1	Diabetes Age-Standardised Prevalence (proportion)
Pancreatic cancer	Male	15 to 19	80 plus	Data Rich	1	Log-transformed SEV scalar: Pancreas C
Pancreatic cancer	Male	15 to 19	80 plus	Data Rich	1	Mean BMI
Pancreatic cancer	Male	15 to 19	80 plus	Data Rich	1	Red Meat (kcal per capita)
Pancreatic cancer	Male	15 to 19	80 plus	Data Rich	1	Smoking Prevalence
Pancreatic cancer	Male	15 to 19	80 plus	Data Rich	1	Sociodemographic Status
Pancreatic cancer	Male	15 to 19	80 plus	Data Rich	1	Tobacco (cigarettes per capita)
Pancreatic cancer	Male	15 to 19	80 plus	Data Rich	1	Total Calories (kcal per capita)
Pancreatic cancer	Female	15 to 19	80 plus	Global	-1	Education (years per capita)
Pancreatic cancer	Female	15 to 19	80 plus	Global	-1	Fruits (kcal per capita)
Pancreatic cancer	Female	15 to 19	80 plus	Global	-1	Health System Access (unitless)
Pancreatic cancer	Female	15 to 19	80 plus	Global	-1	LDI (I$ per capita)
Pancreatic cancer	Female	15 to 19	80 plus	Global	-1	Vegetables (kcal per capita)
Pancreatic cancer	Female	15 to 19	80 plus	Global	1	Alcohol (litres per capita)
Pancreatic cancer	Female	15 to 19	80 plus	Global	1	Animal Fats (kcal per capita)
Pancreatic cancer	Female	15 to 19	80 plus	Global	1	Cumulative Cigarettes (10 Years)
Pancreatic cancer	Female	15 to 19	80 plus	Global	1	Cumulative Cigarettes (20 Years)
Pancreatic cancer	Female	15 to 19	80 plus	Global	1	Cumulative Cigarettes (5 Years)
Pancreatic cancer	Female	15 to 19	80 plus	Global	1	Diabetes Age-Standardised Prevalence (proportion)
Pancreatic cancer	Female	15 to 19	80 plus	Global	1	Log-transformed SEV scalar: Pancreas C
Pancreatic cancer	Female	15 to 19	80 plus	Global	1	Mean BMI
Pancreatic cancer	Female	15 to 19	80 plus	Global	1	Red Meat (kcal per capita)
Pancreatic cancer	Female	15 to 19	80 plus	Global	1	Smoking Prevalence
Pancreatic cancer	Female	15 to 19	80 plus	Global	1	Sociodemographic Status
Pancreatic cancer	Female	15 to 19	80 plus	Global	1	Tobacco (cigarettes per capita)
Pancreatic cancer	Female	15 to 19	80 plus	Global	1	Total Calories (kcal per capita)
Pancreatic cancer	Female	15 to 19	80 plus	Data Rich	-1	Education (years per capita)
Pancreatic cancer	Female	15 to 19	80 plus	Data Rich	-1	Fruits (kcal per capita)
Pancreatic cancer	Female	15 to 19	80 plus	Data Rich	-1	Health System Access (unitless)
Pancreatic cancer	Female	15 to 19	80 plus	Data Rich	-1	LDI (I$ per capita)
Pancreatic cancer	Female	15 to 19	80 plus	Data Rich	-1	Vegetables (kcal per capita)
Pancreatic cancer	Female	15 to 19	80 plus	Data Rich	1	Alcohol (litres per capita)
Pancreatic cancer	Female	15 to 19	80 plus	Data Rich	1	Animal Fats (kcal per capita)
Pancreatic cancer	Female	15 to 19	80 plus	Data Rich	1	Cumulative Cigarettes (10 Years)
Pancreatic cancer	Female	15 to 19	80 plus	Data Rich	1	Cumulative Cigarettes (20 Years)
Pancreatic cancer	Female	15 to 19	80 plus	Data Rich	1	Cumulative Cigarettes (5 Years)
Pancreatic cancer	Female	15 to 19	80 plus	Data Rich	1	Diabetes Age-Standardised Prevalence (proportion)
Pancreatic cancer	Female	15 to 19	80 plus	Data Rich	1	Log-transformed SEV scalar: Pancreas C
Pancreatic cancer	Female	15 to 19	80 plus	Data Rich	1	Mean BMI

Appendix Table 10: CODEm covariates used, level of covariate, and expected direction of covariate by cause, sex, and age						
**Cause**	**Sex**	**Age start**	**Age end**	**Model type**	**Direction**	**Covariate**
Pancreatic cancer	Female	15 to 19	80 plus	Data Rich	1	Red Meat (kcal per capita)
Pancreatic cancer	Female	15 to 19	80 plus	Data Rich	1	Smoking Prevalence
Pancreatic cancer	Female	15 to 19	80 plus	Data Rich	1	Sociodemographic Status
Pancreatic cancer	Female	15 to 19	80 plus	Data Rich	1	Tobacco (cigarettes per capita)
Pancreatic cancer	Female	15 to 19	80 plus	Data Rich	1	Total Calories (kcal per capita)
Larynx cancer	Male	15 to 19	80 plus	Global	-1	Education (years per capita)
Larynx cancer	Male	15 to 19	80 plus	Global	-1	Fruits (kcal per capita)
Larynx cancer	Male	15 to 19	80 plus	Global	-1	LDI (I$ per capita)
Larynx cancer	Male	15 to 19	80 plus	Global	-1	Vegetables (kcal per capita)
Larynx cancer	Male	15 to 19	80 plus	Global	0	Sociodemographic Status
Larynx cancer	Male	15 to 19	80 plus	Global	1	Alcohol (litres per capita)
Larynx cancer	Male	15 to 19	80 plus	Global	1	Cumulative Cigarettes (10 Years)
Larynx cancer	Male	15 to 19	80 plus	Global	1	Cumulative Cigarettes (15 Years)
Larynx cancer	Male	15 to 19	80 plus	Global	1	Cumulative Cigarettes (20 Years)
Larynx cancer	Male	15 to 19	80 plus	Global	1	Cumulative Cigarettes (5 Years)
Larynx cancer	Male	15 to 19	80 plus	Global	1	Log-transformed SEV scalar: Larynx C
Larynx cancer	Male	15 to 19	80 plus	Global	1	Population Density (over 1000 ppl/sqkm, proportion)
Larynx cancer	Male	15 to 19	80 plus	Global	1	Population Density (under 150 ppl/sqkm, proportion)
Larynx cancer	Male	15 to 19	80 plus	Global	1	Smoking Prevalence
Larynx cancer	Male	15 to 19	80 plus	Global	1	Tobacco (cigarettes per capita)
Larynx cancer	Male	15 to 19	80 plus	Data Rich	-1	Education (years per capita)
Larynx cancer	Male	15 to 19	80 plus	Data Rich	-1	Fruits (kcal per capita)
Larynx cancer	Male	15 to 19	80 plus	Data Rich	-1	LDI (I$ per capita)
Larynx cancer	Male	15 to 19	80 plus	Data Rich	-1	Vegetables (kcal per capita)
Larynx cancer	Male	15 to 19	80 plus	Data Rich	0	Sociodemographic Status
Larynx cancer	Male	15 to 19	80 plus	Data Rich	1	Alcohol (litres per capita)
Larynx cancer	Male	15 to 19	80 plus	Data Rich	1	Cumulative Cigarettes (10 Years)
Larynx cancer	Male	15 to 19	80 plus	Data Rich	1	Cumulative Cigarettes (15 Years)
Larynx cancer	Male	15 to 19	80 plus	Data Rich	1	Cumulative Cigarettes (20 Years)
Larynx cancer	Male	15 to 19	80 plus	Data Rich	1	Cumulative Cigarettes (5 Years)
Larynx cancer	Male	15 to 19	80 plus	Data Rich	1	Log-transformed SEV scalar: Larynx C
Larynx cancer	Male	15 to 19	80 plus	Data Rich	1	Population Density (over 1000 ppl/sqkm, proportion)
Larynx cancer	Male	15 to 19	80 plus	Data Rich	1	Population Density (under 150 ppl/sqkm, proportion)
Larynx cancer	Male	15 to 19	80 plus	Data Rich	1	Smoking Prevalence
Larynx cancer	Male	15 to 19	80 plus	Data Rich	1	Tobacco (cigarettes per capita)
Larynx cancer	Female	15 to 19	80 plus	Global	-1	Education (years per capita)
Larynx cancer	Female	15 to 19	80 plus	Global	-1	Fruits (kcal per capita)
Larynx cancer	Female	15 to 19	80 plus	Global	-1	LDI (I$ per capita)
Larynx cancer	Female	15 to 19	80 plus	Global	-1	Vegetables (kcal per capita)
Larynx cancer	Female	15 to 19	80 plus	Global	0	Sociodemographic Status
Larynx cancer	Female	15 to 19	80 plus	Global	1	Alcohol (litres per capita)
Larynx cancer	Female	15 to 19	80 plus	Global	1	Cumulative Cigarettes (10 Years)
Larynx cancer	Female	15 to 19	80 plus	Global	1	Cumulative Cigarettes (15 Years)
Larynx cancer	Female	15 to 19	80 plus	Global	1	Cumulative Cigarettes (20 Years)

Appendix Table 10: CODEm covariates used, level of covariate, and expected direction of covariate by cause, sex, and age						
**Cause**	**Sex**	**Age start**	**Age end**	**Model type**	**Direction**	**Covariate**
Larynx cancer	Female	15 to 19	80 plus	Global	1	Cumulative Cigarettes (5 Years)
Larynx cancer	Female	15 to 19	80 plus	Global	1	Log-transformed SEV scalar: Larynx C
Larynx cancer	Female	15 to 19	80 plus	Global	1	Population Density (over 1000 ppl/sqkm, proportion)
Larynx cancer	Female	15 to 19	80 plus	Global	1	Population Density (under 150 ppl/sqkm, proportion)
Larynx cancer	Female	15 to 19	80 plus	Global	1	Smoking Prevalence
Larynx cancer	Female	15 to 19	80 plus	Global	1	Tobacco (cigarettes per capita)
Larynx cancer	Female	15 to 19	80 plus	Data Rich	-1	Education (years per capita)
Larynx cancer	Female	15 to 19	80 plus	Data Rich	-1	Fruits (kcal per capita)
Larynx cancer	Female	15 to 19	80 plus	Data Rich	-1	LDI (I$ per capita)
Larynx cancer	Female	15 to 19	80 plus	Data Rich	-1	Vegetables (kcal per capita)
Larynx cancer	Female	15 to 19	80 plus	Data Rich	0	Sociodemographic Status
Larynx cancer	Female	15 to 19	80 plus	Data Rich	1	Alcohol (litres per capita)
Larynx cancer	Female	15 to 19	80 plus	Data Rich	1	Cumulative Cigarettes (10 Years)
Larynx cancer	Female	15 to 19	80 plus	Data Rich	1	Cumulative Cigarettes (15 Years)
Larynx cancer	Female	15 to 19	80 plus	Data Rich	1	Cumulative Cigarettes (20 Years)
Larynx cancer	Female	15 to 19	80 plus	Data Rich	1	Cumulative Cigarettes (5 Years)
Larynx cancer	Female	15 to 19	80 plus	Data Rich	1	Log-transformed SEV scalar: Larynx C
Larynx cancer	Female	15 to 19	80 plus	Data Rich	1	Population Density (over 1000 ppl/sqkm, proportion)
Larynx cancer	Female	15 to 19	80 plus	Data Rich	1	Population Density (under 150 ppl/sqkm, proportion)
Larynx cancer	Female	15 to 19	80 plus	Data Rich	1	Smoking Prevalence
Larynx cancer	Female	15 to 19	80 plus	Data Rich	1	Tobacco (cigarettes per capita)
Tracheal, bronchus, and lung cancer	Male	15 to 19	80 plus	Global	-1	Education (years per capita)
Tracheal, bronchus, and lung cancer	Male	15 to 19	80 plus	Global	-1	LDI (I$ per capita)
Tracheal, bronchus, and lung cancer	Male	15 to 19	80 plus	Global	0	Sociodemographic Status
Tracheal, bronchus, and lung cancer	Male	15 to 19	80 plus	Global	1	Cumulative Cigarettes (10 Years)
Tracheal, bronchus, and lung cancer	Male	15 to 19	80 plus	Global	1	Cumulative Cigarettes (15 Years)
Tracheal, bronchus, and lung cancer	Male	15 to 19	80 plus	Global	1	Cumulative Cigarettes (20 Years)
Tracheal, bronchus, and lung cancer	Male	15 to 19	80 plus	Global	1	Cumulative Cigarettes (5 Years)
Tracheal, bronchus, and lung cancer	Male	15 to 19	80 plus	Global	1	Indoor Air Pollution (All Cooking Fuels)
Tracheal, bronchus, and lung cancer	Male	15 to 19	80 plus	Global	1	Outdoor Air Pollution (PM2.5)
Tracheal, bronchus, and lung cancer	Male	15 to 19	80 plus	Global	1	Smoking Prevalence
Tracheal, bronchus, and lung cancer	Male	15 to 19	80 plus	Global	1	Tobacco (cigarettes per capita)
Tracheal, bronchus, and lung cancer	Male	15 to 19	80 plus	Data Rich	-1	Education (years per capita)
Tracheal, bronchus, and lung cancer	Male	15 to 19	80 plus	Data Rich	-1	LDI (I$ per capita)
Tracheal, bronchus, and lung cancer	Male	15 to 19	80 plus	Data Rich	0	Sociodemographic Status
Tracheal, bronchus, and lung cancer	Male	15 to 19	80 plus	Data Rich	1	Cumulative Cigarettes (10 Years)
Tracheal, bronchus, and lung cancer	Male	15 to 19	80 plus	Data Rich	1	Cumulative Cigarettes (15 Years)
Tracheal, bronchus, and lung cancer	Male	15 to 19	80 plus	Data Rich	1	Cumulative Cigarettes (20 Years)
Tracheal, bronchus, and lung cancer	Male	15 to 19	80 plus	Data Rich	1	Cumulative Cigarettes (5 Years)
Tracheal, bronchus, and lung cancer	Male	15 to 19	80 plus	Data Rich	1	Indoor Air Pollution (All Cooking Fuels)
Tracheal, bronchus, and lung cancer	Male	15 to 19	80 plus	Data Rich	1	Outdoor Air Pollution (PM2.5)
Tracheal, bronchus, and lung cancer	Male	15 to 19	80 plus	Data Rich	1	Smoking Prevalence
Tracheal, bronchus, and lung cancer	Male	15 to 19	80 plus	Data Rich	1	Tobacco (cigarettes per capita)
Tracheal, bronchus, and lung cancer	Female	15 to 19	80 plus	Global	-1	Education (years per capita)

Appendix Table 10: CODEm covariates used, level of covariate, and expected direction of covariate by cause, sex, and age						
**Cause**	**Sex**	**Age start**	**Age end**	**Model type**	**Direction**	**Covariate**
Tracheal, bronchus, and lung cancer	Female	15 to 19	80 plus	Global	-1	LDI (I$ per capita)
Tracheal, bronchus, and lung cancer	Female	15 to 19	80 plus	Global	0	Sociodemographic Status
Tracheal, bronchus, and lung cancer	Female	15 to 19	80 plus	Global	1	Cumulative Cigarettes (10 Years)
Tracheal, bronchus, and lung cancer	Female	15 to 19	80 plus	Global	1	Cumulative Cigarettes (15 Years)
Tracheal, bronchus, and lung cancer	Female	15 to 19	80 plus	Global	1	Cumulative Cigarettes (20 Years)
Tracheal, bronchus, and lung cancer	Female	15 to 19	80 plus	Global	1	Cumulative Cigarettes (5 Years)
Tracheal, bronchus, and lung cancer	Female	15 to 19	80 plus	Global	1	Indoor Air Pollution (All Cooking Fuels)
Tracheal, bronchus, and lung cancer	Female	15 to 19	80 plus	Global	1	Outdoor Air Pollution (PM2.5)
Tracheal, bronchus, and lung cancer	Female	15 to 19	80 plus	Global	1	Smoking Prevalence
Tracheal, bronchus, and lung cancer	Female	15 to 19	80 plus	Global	1	Tobacco (cigarettes per capita)
Tracheal, bronchus, and lung cancer	Female	15 to 19	80 plus	Data Rich	-1	Education (years per capita)
Tracheal, bronchus, and lung cancer	Female	15 to 19	80 plus	Data Rich	-1	LDI (I$ per capita)
Tracheal, bronchus, and lung cancer	Female	15 to 19	80 plus	Data Rich	0	Sociodemographic Status
Tracheal, bronchus, and lung cancer	Female	15 to 19	80 plus	Data Rich	1	Cumulative Cigarettes (10 Years)
Tracheal, bronchus, and lung cancer	Female	15 to 19	80 plus	Data Rich	1	Cumulative Cigarettes (15 Years)
Tracheal, bronchus, and lung cancer	Female	15 to 19	80 plus	Data Rich	1	Cumulative Cigarettes (20 Years)
Tracheal, bronchus, and lung cancer	Female	15 to 19	80 plus	Data Rich	1	Cumulative Cigarettes (5 Years)
Tracheal, bronchus, and lung cancer	Female	15 to 19	80 plus	Data Rich	1	Indoor Air Pollution (All Cooking Fuels)
Tracheal, bronchus, and lung cancer	Female	15 to 19	80 plus	Data Rich	1	Outdoor Air Pollution (PM2.5)
Tracheal, bronchus, and lung cancer	Female	15 to 19	80 plus	Data Rich	1	Smoking Prevalence
Tracheal, bronchus, and lung cancer	Female	15 to 19	80 plus	Data Rich	1	Tobacco (cigarettes per capita)
Malignant skin melanoma	Male	15 to 19	80 plus	Global	-1	Education (years per capita)
Malignant skin melanoma	Male	15 to 19	80 plus	Global	-1	Fruits (kcal per capita)
Malignant skin melanoma	Male	15 to 19	80 plus	Global	-1	LDI (I$ per capita)
Malignant skin melanoma	Male	15 to 19	80 plus	Global	-1	Latitude 30 to 45 (proportion)
Malignant skin melanoma	Male	15 to 19	80 plus	Global	-1	Latitude Over 45 (proportion)
Malignant skin melanoma	Male	15 to 19	80 plus	Global	-1	Vegetables (kcal per capita)
Malignant skin melanoma	Male	15 to 19	80 plus	Global	0	Latitude 15 to 30 (proportion)
Malignant skin melanoma	Male	15 to 19	80 plus	Global	0	Sociodemographic Status
Malignant skin melanoma	Male	15 to 19	80 plus	Global	1	Alcohol (litres per capita)
Malignant skin melanoma	Male	15 to 19	80 plus	Global	1	Animal Fats (kcal per capita)
Malignant skin melanoma	Male	15 to 19	80 plus	Global	1	Cumulative Cigarettes (20 Years)
Malignant skin melanoma	Male	15 to 19	80 plus	Global	1	Mean BMI
Malignant skin melanoma	Male	15 to 19	80 plus	Global	1	Smoking Prevalence
Malignant skin melanoma	Male	15 to 19	80 plus	Global	1	Tobacco (cigarettes per capita)
Malignant skin melanoma	Male	15 to 19	80 plus	Data Rich	-1	Education (years per capita)
Malignant skin melanoma	Male	15 to 19	80 plus	Data Rich	-1	Fruits (kcal per capita)
Malignant skin melanoma	Male	15 to 19	80 plus	Data Rich	-1	LDI (I$ per capita)
Malignant skin melanoma	Male	15 to 19	80 plus	Data Rich	-1	Latitude 30 to 45 (proportion)
Malignant skin melanoma	Male	15 to 19	80 plus	Data Rich	-1	Latitude Over 45 (proportion)
Malignant skin melanoma	Male	15 to 19	80 plus	Data Rich	-1	Vegetables (kcal per capita)
Malignant skin melanoma	Male	15 to 19	80 plus	Data Rich	0	Latitude 15 to 30 (proportion)
Malignant skin melanoma	Male	15 to 19	80 plus	Data Rich	0	Sociodemographic Status
Malignant skin melanoma	Male	15 to 19	80 plus	Data Rich	1	Alcohol (litres per capita)

Appendix Table 10: CODEm covariates used, level of covariate, and expected direction of covariate by cause, sex, and age						
**Cause**	**Sex**	**Age start**	**Age end**	**Model type**	**Direction**	**Covariate**
Malignant skin melanoma	Male	15 to 19	80 plus	Data Rich	1	Animal Fats (kcal per capita)
Malignant skin melanoma	Male	15 to 19	80 plus	Data Rich	1	Cumulative Cigarettes (20 Years)
Malignant skin melanoma	Male	15 to 19	80 plus	Data Rich	1	Mean BMI
Malignant skin melanoma	Male	15 to 19	80 plus	Data Rich	1	Smoking Prevalence
Malignant skin melanoma	Male	15 to 19	80 plus	Data Rich	1	Tobacco (cigarettes per capita)
Malignant skin melanoma	Female	15 to 19	80 plus	Global	-1	Education (years per capita)
Malignant skin melanoma	Female	15 to 19	80 plus	Global	-1	Fruits (kcal per capita)
Malignant skin melanoma	Female	15 to 19	80 plus	Global	-1	LDI (I$ per capita)
Malignant skin melanoma	Female	15 to 19	80 plus	Global	-1	Latitude 30 to 45 (proportion)
Malignant skin melanoma	Female	15 to 19	80 plus	Global	-1	Latitude Over 45 (proportion)
Malignant skin melanoma	Female	15 to 19	80 plus	Global	-1	Vegetables (kcal per capita)
Malignant skin melanoma	Female	15 to 19	80 plus	Global	0	Latitude 15 to 30 (proportion)
Malignant skin melanoma	Female	15 to 19	80 plus	Global	0	Sociodemographic Status
Malignant skin melanoma	Female	15 to 19	80 plus	Global	1	Alcohol (litres per capita)
Malignant skin melanoma	Female	15 to 19	80 plus	Global	1	Animal Fats (kcal per capita)
Malignant skin melanoma	Female	15 to 19	80 plus	Global	1	Cumulative Cigarettes (20 Years)
Malignant skin melanoma	Female	15 to 19	80 plus	Global	1	Mean BMI
Malignant skin melanoma	Female	15 to 19	80 plus	Global	1	Smoking Prevalence
Malignant skin melanoma	Female	15 to 19	80 plus	Global	1	Tobacco (cigarettes per capita)
Malignant skin melanoma	Female	15 to 19	80 plus	Data Rich	-1	Education (years per capita)
Malignant skin melanoma	Female	15 to 19	80 plus	Data Rich	-1	Fruits (kcal per capita)
Malignant skin melanoma	Female	15 to 19	80 plus	Data Rich	-1	LDI (I$ per capita)
Malignant skin melanoma	Female	15 to 19	80 plus	Data Rich	-1	Latitude 30 to 45 (proportion)
Malignant skin melanoma	Female	15 to 19	80 plus	Data Rich	-1	Latitude Over 45 (proportion)
Malignant skin melanoma	Female	15 to 19	80 plus	Data Rich	-1	Vegetables (kcal per capita)
Malignant skin melanoma	Female	15 to 19	80 plus	Data Rich	0	Latitude 15 to 30 (proportion)
Malignant skin melanoma	Female	15 to 19	80 plus	Data Rich	0	Sociodemographic Status
Malignant skin melanoma	Female	15 to 19	80 plus	Data Rich	1	Alcohol (litres per capita)
Malignant skin melanoma	Female	15 to 19	80 plus	Data Rich	1	Animal Fats (kcal per capita)
Malignant skin melanoma	Female	15 to 19	80 plus	Data Rich	1	Cumulative Cigarettes (20 Years)
Malignant skin melanoma	Female	15 to 19	80 plus	Data Rich	1	Mean BMI
Malignant skin melanoma	Female	15 to 19	80 plus	Data Rich	1	Smoking Prevalence
Malignant skin melanoma	Female	15 to 19	80 plus	Data Rich	1	Tobacco (cigarettes per capita)
Non-melanoma skin cancer	Male	15 to 19	80 plus	Global	-1	Education (years per capita)
Non-melanoma skin cancer	Male	15 to 19	80 plus	Global	-1	LDI (I$ per capita)
Non-melanoma skin cancer	Male	15 to 19	80 plus	Global	0	Average latitude
Non-melanoma skin cancer	Male	15 to 19	80 plus	Global	0	Sociodemographic Status
Non-melanoma skin cancer	Male	15 to 19	80 plus	Global	1	Cumulative Cigarettes (10 Years)
Non-melanoma skin cancer	Male	15 to 19	80 plus	Global	1	Cumulative Cigarettes (15 Years)
Non-melanoma skin cancer	Male	15 to 19	80 plus	Global	1	Cumulative Cigarettes (5 Years)
Non-melanoma skin cancer	Male	15 to 19	80 plus	Global	1	Health System Access (capped)
Non-melanoma skin cancer	Male	15 to 19	80 plus	Global	1	Smoking Prevalence
Non-melanoma skin cancer	Male	15 to 19	80 plus	Data Rich	-1	Education (years per capita)
Non-melanoma skin cancer	Male	15 to 19	80 plus	Data Rich	-1	LDI (I$ per capita)

Appendix Table 10: CODEm covariates used, level of covariate, and expected direction of covariate by cause, sex, and age						
**Cause**	**Sex**	**Age start**	**Age end**	**Model type**	**Direction**	**Covariate**
Non-melanoma skin cancer	Male	15 to 19	80 plus	Data Rich	0	Average latitude
Non-melanoma skin cancer	Male	15 to 19	80 plus	Data Rich	0	Sociodemographic Status
Non-melanoma skin cancer	Male	15 to 19	80 plus	Data Rich	1	Cumulative Cigarettes (10 Years)
Non-melanoma skin cancer	Male	15 to 19	80 plus	Data Rich	1	Cumulative Cigarettes (15 Years)
Non-melanoma skin cancer	Male	15 to 19	80 plus	Data Rich	1	Cumulative Cigarettes (5 Years)
Non-melanoma skin cancer	Male	15 to 19	80 plus	Data Rich	1	Health System Access (capped)
Non-melanoma skin cancer	Male	15 to 19	80 plus	Data Rich	1	Smoking Prevalence
Non-melanoma skin cancer	Female	15 to 19	80 plus	Global	-1	Education (years per capita)
Non-melanoma skin cancer	Female	15 to 19	80 plus	Global	-1	LDI (I$ per capita)
Non-melanoma skin cancer	Female	15 to 19	80 plus	Global	0	Average latitude
Non-melanoma skin cancer	Female	15 to 19	80 plus	Global	0	Sociodemographic Status
Non-melanoma skin cancer	Female	15 to 19	80 plus	Global	1	Cumulative Cigarettes (10 Years)
Non-melanoma skin cancer	Female	15 to 19	80 plus	Global	1	Cumulative Cigarettes (15 Years)
Non-melanoma skin cancer	Female	15 to 19	80 plus	Global	1	Cumulative Cigarettes (5 Years)
Non-melanoma skin cancer	Female	15 to 19	80 plus	Global	1	Health System Access (capped)
Non-melanoma skin cancer	Female	15 to 19	80 plus	Global	1	Smoking Prevalence
Non-melanoma skin cancer	Female	15 to 19	80 plus	Data Rich	-1	Education (years per capita)
Non-melanoma skin cancer	Female	15 to 19	80 plus	Data Rich	-1	LDI (I$ per capita)
Non-melanoma skin cancer	Female	15 to 19	80 plus	Data Rich	0	Average latitude
Non-melanoma skin cancer	Female	15 to 19	80 plus	Data Rich	0	Sociodemographic Status
Non-melanoma skin cancer	Female	15 to 19	80 plus	Data Rich	1	Cumulative Cigarettes (10 Years)
Non-melanoma skin cancer	Female	15 to 19	80 plus	Data Rich	1	Cumulative Cigarettes (15 Years)
Non-melanoma skin cancer	Female	15 to 19	80 plus	Data Rich	1	Cumulative Cigarettes (5 Years)
Non-melanoma skin cancer	Female	15 to 19	80 plus	Data Rich	1	Health System Access (capped)
Non-melanoma skin cancer	Female	15 to 19	80 plus	Data Rich	1	Smoking Prevalence
Breast cancer	Male	15 to 19	80 plus	Global	-1	Education (years per capita)
Breast cancer	Male	15 to 19	80 plus	Global	-1	Fertility (15-19 year olds)
Breast cancer	Male	15 to 19	80 plus	Global	-1	Fruits (kcal per capita)
Breast cancer	Male	15 to 19	80 plus	Global	-1	Health System Access 2 (unitless)
Breast cancer	Male	15 to 19	80 plus	Global	-1	LDI (I$ per capita)
Breast cancer	Male	15 to 19	80 plus	Global	-1	Latitude 30 to 45 (proportion)
Breast cancer	Male	15 to 19	80 plus	Global	-1	Latitude Over 45 (proportion)
Breast cancer	Male	15 to 19	80 plus	Global	-1	Total Fertility Rate
Breast cancer	Male	15 to 19	80 plus	Global	-1	Vegetables (kcal per capita)
Breast cancer	Male	15 to 19	80 plus	Global	0	Latitude 15 to 30 (proportion)
Breast cancer	Male	15 to 19	80 plus	Global	0	Sociodemographic Status
Breast cancer	Male	15 to 19	80 plus	Global	1	Alcohol (litres per capita)
Breast cancer	Male	15 to 19	80 plus	Global	1	Animal Fats (kcal per capita)
Breast cancer	Male	15 to 19	80 plus	Global	1	Cumulative Cigarettes (10 Years)
Breast cancer	Male	15 to 19	80 plus	Global	1	Log-transformed SEV scalar: Breast C
Breast cancer	Male	15 to 19	80 plus	Global	1	Mean BMI
Breast cancer	Male	15 to 19	80 plus	Data Rich	-1	Education (years per capita)
Breast cancer	Male	15 to 19	80 plus	Data Rich	-1	Fertility (15-19 year olds)
Breast cancer	Male	15 to 19	80 plus	Data Rich	-1	Fruits (kcal per capita)

Appendix Table 10: CODEm covariates used, level of covariate, and expected direction of covariate by cause, sex, and age						
**Cause**	**Sex**	**Age start**	**Age end**	**Model type**	**Direction**	**Covariate**
Breast cancer	Male	15 to 19	80 plus	Data Rich	-1	Health System Access 2 (unitless)
Breast cancer	Male	15 to 19	80 plus	Data Rich	-1	LDI (I$ per capita)
Breast cancer	Male	15 to 19	80 plus	Data Rich	-1	Latitude 30 to 45 (proportion)
Breast cancer	Male	15 to 19	80 plus	Data Rich	-1	Latitude Over 45 (proportion)
Breast cancer	Male	15 to 19	80 plus	Data Rich	-1	Total Fertility Rate
Breast cancer	Male	15 to 19	80 plus	Data Rich	-1	Vegetables (kcal per capita)
Breast cancer	Male	15 to 19	80 plus	Data Rich	0	Latitude 15 to 30 (proportion)
Breast cancer	Male	15 to 19	80 plus	Data Rich	0	Sociodemographic Status
Breast cancer	Male	15 to 19	80 plus	Data Rich	1	Alcohol (litres per capita)
Breast cancer	Male	15 to 19	80 plus	Data Rich	1	Animal Fats (kcal per capita)
Breast cancer	Male	15 to 19	80 plus	Data Rich	1	Cumulative Cigarettes (10 Years)
Breast cancer	Male	15 to 19	80 plus	Data Rich	1	Log-transformed SEV scalar: Breast C
Breast cancer	Male	15 to 19	80 plus	Data Rich	1	Mean BMI
Breast cancer	Female	15 to 19	80 plus	Global	-1	Education (years per capita)
Breast cancer	Female	15 to 19	80 plus	Global	-1	Fertility (15-19 year olds)
Breast cancer	Female	15 to 19	80 plus	Global	-1	Fruits (kcal per capita)
Breast cancer	Female	15 to 19	80 plus	Global	-1	Health System Access 2 (unitless)
Breast cancer	Female	15 to 19	80 plus	Global	-1	LDI (I$ per capita)
Breast cancer	Female	15 to 19	80 plus	Global	-1	Latitude 30 to 45 (proportion)
Breast cancer	Female	15 to 19	80 plus	Global	-1	Latitude Over 45 (proportion)
Breast cancer	Female	15 to 19	80 plus	Global	-1	Total Fertility Rate
Breast cancer	Female	15 to 19	80 plus	Global	-1	Vegetables (kcal per capita)
Breast cancer	Female	15 to 19	80 plus	Global	0	Latitude 15 to 30 (proportion)
Breast cancer	Female	15 to 19	80 plus	Global	0	Sociodemographic Status
Breast cancer	Female	15 to 19	80 plus	Global	1	Alcohol (litres per capita)
Breast cancer	Female	15 to 19	80 plus	Global	1	Animal Fats (kcal per capita)
Breast cancer	Female	15 to 19	80 plus	Global	1	Cumulative Cigarettes (10 Years)
Breast cancer	Female	15 to 19	80 plus	Global	1	Log-transformed SEV scalar: Breast C
Breast cancer	Female	15 to 19	80 plus	Global	1	Mean BMI
Breast cancer	Female	15 to 19	80 plus	Data Rich	-1	Education (years per capita)
Breast cancer	Female	15 to 19	80 plus	Data Rich	-1	Fertility (15-19 year olds)
Breast cancer	Female	15 to 19	80 plus	Data Rich	-1	Fruits (kcal per capita)
Breast cancer	Female	15 to 19	80 plus	Data Rich	-1	Health System Access 2 (unitless)
Breast cancer	Female	15 to 19	80 plus	Data Rich	-1	LDI (I$ per capita)
Breast cancer	Female	15 to 19	80 plus	Data Rich	-1	Latitude 30 to 45 (proportion)
Breast cancer	Female	15 to 19	80 plus	Data Rich	-1	Latitude Over 45 (proportion)
Breast cancer	Female	15 to 19	80 plus	Data Rich	-1	Total Fertility Rate
Breast cancer	Female	15 to 19	80 plus	Data Rich	-1	Vegetables (kcal per capita)
Breast cancer	Female	15 to 19	80 plus	Data Rich	0	Latitude 15 to 30 (proportion)
Breast cancer	Female	15 to 19	80 plus	Data Rich	0	Sociodemographic Status
Breast cancer	Female	15 to 19	80 plus	Data Rich	1	Alcohol (litres per capita)
Breast cancer	Female	15 to 19	80 plus	Data Rich	1	Animal Fats (kcal per capita)
Breast cancer	Female	15 to 19	80 plus	Data Rich	1	Cumulative Cigarettes (10 Years)
Breast cancer	Female	15 to 19	80 plus	Data Rich	1	Log-transformed SEV scalar: Breast C

Appendix Table 10: CODEm covariates used, level of covariate, and expected direction of covariate by cause, sex, and age						
**Cause**	**Sex**	**Age start**	**Age end**	**Model type**	**Direction**	**Covariate**
Breast cancer	Female	15 to 19	80 plus	Data Rich	1	Mean BMI
Cervical cancer	Female	15 to 19	80 plus	Global	-1	Education (years per capita)
Cervical cancer	Female	15 to 19	80 plus	Global	-1	Fruits (kcal per capita)
Cervical cancer	Female	15 to 19	80 plus	Global	-1	Health System Access 2 (unitless)
Cervical cancer	Female	15 to 19	80 plus	Global	-1	LDI (I$ per capita)
Cervical cancer	Female	15 to 19	80 plus	Global	-1	Sociodemographic Status
Cervical cancer	Female	15 to 19	80 plus	Global	-1	Vegetables (kcal per capita)
Cervical cancer	Female	15 to 19	80 plus	Global	1	Abortion On-Demand Illegal (binary)
Cervical cancer	Female	15 to 19	80 plus	Global	1	Cumulative Cigarettes (10 Years)
Cervical cancer	Female	15 to 19	80 plus	Global	1	Cumulative Cigarettes (15 Years)
Cervical cancer	Female	15 to 19	80 plus	Global	1	Cumulative Cigarettes (5 Years)
Cervical cancer	Female	15 to 19	80 plus	Global	1	Fertility (15-19 year olds)
Cervical cancer	Female	15 to 19	80 plus	Global	1	HIV age-standardized prevalence
Cervical cancer	Female	15 to 19	80 plus	Global	1	Smoking Prevalence
Cervical cancer	Female	15 to 19	80 plus	Global	1	Total Fertility Rate
Cervical cancer	Female	15 to 19	80 plus	Data Rich	-1	Education (years per capita)
Cervical cancer	Female	15 to 19	80 plus	Data Rich	-1	Fruits (kcal per capita)
Cervical cancer	Female	15 to 19	80 plus	Data Rich	-1	Health System Access 2 (unitless)
Cervical cancer	Female	15 to 19	80 plus	Data Rich	-1	LDI (I$ per capita)
Cervical cancer	Female	15 to 19	80 plus	Data Rich	-1	Sociodemographic Status
Cervical cancer	Female	15 to 19	80 plus	Data Rich	-1	Vegetables (kcal per capita)
Cervical cancer	Female	15 to 19	80 plus	Data Rich	1	Abortion On-Demand Illegal (binary)
Cervical cancer	Female	15 to 19	80 plus	Data Rich	1	Cumulative Cigarettes (10 Years)
Cervical cancer	Female	15 to 19	80 plus	Data Rich	1	Cumulative Cigarettes (15 Years)
Cervical cancer	Female	15 to 19	80 plus	Data Rich	1	Cumulative Cigarettes (5 Years)
Cervical cancer	Female	15 to 19	80 plus	Data Rich	1	Fertility (15-19 year olds)
Cervical cancer	Female	15 to 19	80 plus	Data Rich	1	HIV age-standardized prevalence
Cervical cancer	Female	15 to 19	80 plus	Data Rich	1	Smoking Prevalence
Cervical cancer	Female	15 to 19	80 plus	Data Rich	1	Total Fertility Rate
Uterine cancer	Female	15 to 19	80 plus	Global	-1	Education (years per capita)
Uterine cancer	Female	15 to 19	80 plus	Global	-1	Fruits (kcal per capita)
Uterine cancer	Female	15 to 19	80 plus	Global	-1	Health System Access (unitless)
Uterine cancer	Female	15 to 19	80 plus	Global	-1	LDI (I$ per capita)
Uterine cancer	Female	15 to 19	80 plus	Global	-1	Vegetables (kcal per capita)
Uterine cancer	Female	15 to 19	80 plus	Global	0	Sociodemographic Status
Uterine cancer	Female	15 to 19	80 plus	Global	0	Total Fertility Rate
Uterine cancer	Female	15 to 19	80 plus	Global	1	Cumulative Cigarettes (10 Years)
Uterine cancer	Female	15 to 19	80 plus	Global	1	Cumulative Cigarettes (5 Years)
Uterine cancer	Female	15 to 19	80 plus	Global	1	Diabetes Age-Standardised Prevalence (proportion)
Uterine cancer	Female	15 to 19	80 plus	Global	1	Log-transformed SEV scalar: Uterus C
Uterine cancer	Female	15 to 19	80 plus	Global	1	Mean BMI
Uterine cancer	Female	15 to 19	80 plus	Global	1	Smoking Prevalence
Uterine cancer	Female	15 to 19	80 plus	Global	1	Tobacco (cigarettes per capita)
Uterine cancer	Female	15 to 19	80 plus	Data Rich	-1	Education (years per capita)

Appendix Table 10: CODEm covariates used, level of covariate, and expected direction of covariate by cause, sex, and age						
**Cause**	**Sex**	**Age start**	**Age end**	**Model type**	**Direction**	**Covariate**
Uterine cancer	Female	15 to 19	80 plus	Data Rich	-1	Fruits (kcal per capita)
Uterine cancer	Female	15 to 19	80 plus	Data Rich	-1	Health System Access (unitless)
Uterine cancer	Female	15 to 19	80 plus	Data Rich	-1	LDI (I$ per capita)
Uterine cancer	Female	15 to 19	80 plus	Data Rich	-1	Vegetables (kcal per capita)
Uterine cancer	Female	15 to 19	80 plus	Data Rich	0	Sociodemographic Status
Uterine cancer	Female	15 to 19	80 plus	Data Rich	0	Total Fertility Rate
Uterine cancer	Female	15 to 19	80 plus	Data Rich	1	Cumulative Cigarettes (10 Years)
Uterine cancer	Female	15 to 19	80 plus	Data Rich	1	Cumulative Cigarettes (5 Years)
Uterine cancer	Female	15 to 19	80 plus	Data Rich	1	Diabetes Age-Standardised Prevalence (proportion)
Uterine cancer	Female	15 to 19	80 plus	Data Rich	1	Log-transformed SEV scalar: Uterus C
Uterine cancer	Female	15 to 19	80 plus	Data Rich	1	Mean BMI
Uterine cancer	Female	15 to 19	80 plus	Data Rich	1	Smoking Prevalence
Uterine cancer	Female	15 to 19	80 plus	Data Rich	1	Tobacco (cigarettes per capita)
Ovarian cancer	Female	15 to 19	80 plus	Global	-1	Contraception (Modern) Prevalence (proportion)
Ovarian cancer	Female	15 to 19	80 plus	Global	-1	Education (years per capita)
Ovarian cancer	Female	15 to 19	80 plus	Global	-1	Fruits (kcal per capita)
Ovarian cancer	Female	15 to 19	80 plus	Global	-1	LDI (I$ per capita)
Ovarian cancer	Female	15 to 19	80 plus	Global	-1	Latitude 30 to 45 (proportion)
Ovarian cancer	Female	15 to 19	80 plus	Global	-1	Latitude Over 45 (proportion)
Ovarian cancer	Female	15 to 19	80 plus	Global	-1	Vegetables (kcal per capita)
Ovarian cancer	Female	15 to 19	80 plus	Global	0	Latitude 15 to 30 (proportion)
Ovarian cancer	Female	15 to 19	80 plus	Global	0	Sociodemographic Status
Ovarian cancer	Female	15 to 19	80 plus	Global	0	Total Fertility Rate
Ovarian cancer	Female	15 to 19	80 plus	Global	1	Alcohol (litres per capita)
Ovarian cancer	Female	15 to 19	80 plus	Global	1	Animal Fats (kcal per capita)
Ovarian cancer	Female	15 to 19	80 plus	Global	1	Cumulative Cigarettes (20 Years)
Ovarian cancer	Female	15 to 19	80 plus	Global	1	Diabetes Age-Standardised Prevalence (proportion)
Ovarian cancer	Female	15 to 19	80 plus	Global	1	Log-transformed SEV scalar: Ovary C
Ovarian cancer	Female	15 to 19	80 plus	Global	1	Mean BMI
Ovarian cancer	Female	15 to 19	80 plus	Global	1	Smoking Prevalence
Ovarian cancer	Female	15 to 19	80 plus	Global	1	Tobacco (cigarettes per capita)
Ovarian cancer	Female	15 to 19	80 plus	Global	1	Total Calories (kcal per capita)
Ovarian cancer	Female	15 to 19	80 plus	Data Rich	-1	Contraception (Modern) Prevalence (proportion)
Ovarian cancer	Female	15 to 19	80 plus	Data Rich	-1	Education (years per capita)
Ovarian cancer	Female	15 to 19	80 plus	Data Rich	-1	Fruits (kcal per capita)
Ovarian cancer	Female	15 to 19	80 plus	Data Rich	-1	LDI (I$ per capita)
Ovarian cancer	Female	15 to 19	80 plus	Data Rich	-1	Latitude 30 to 45 (proportion)
Ovarian cancer	Female	15 to 19	80 plus	Data Rich	-1	Latitude Over 45 (proportion)
Ovarian cancer	Female	15 to 19	80 plus	Data Rich	-1	Vegetables (kcal per capita)
Ovarian cancer	Female	15 to 19	80 plus	Data Rich	0	Latitude 15 to 30 (proportion)
Ovarian cancer	Female	15 to 19	80 plus	Data Rich	0	Sociodemographic Status
Ovarian cancer	Female	15 to 19	80 plus	Data Rich	0	Total Fertility Rate
Ovarian cancer	Female	15 to 19	80 plus	Data Rich	1	Alcohol (litres per capita)
Ovarian cancer	Female	15 to 19	80 plus	Data Rich	1	Animal Fats (kcal per capita)

Appendix Table 10: CODEm covariates used, level of covariate, and expected direction of covariate by cause, sex, and age						
**Cause**	**Sex**	**Age start**	**Age end**	**Model type**	**Direction**	**Covariate**
Ovarian cancer	Female	15 to 19	80 plus	Data Rich	1	Cumulative Cigarettes (20 Years)
Ovarian cancer	Female	15 to 19	80 plus	Data Rich	1	Diabetes Age-Standardised Prevalence (proportion)
Ovarian cancer	Female	15 to 19	80 plus	Data Rich	1	Log-transformed SEV scalar: Ovary C
Ovarian cancer	Female	15 to 19	80 plus	Data Rich	1	Mean BMI
Ovarian cancer	Female	15 to 19	80 plus	Data Rich	1	Smoking Prevalence
Ovarian cancer	Female	15 to 19	80 plus	Data Rich	1	Tobacco (cigarettes per capita)
Ovarian cancer	Female	15 to 19	80 plus	Data Rich	1	Total Calories (kcal per capita)
Prostate cancer	Male	15 to 19	80 plus	Global	-1	Health System Access 2 (unitless)
Prostate cancer	Male	15 to 19	80 plus	Global	0	Education (years per capita)
Prostate cancer	Male	15 to 19	80 plus	Global	0	LDI (I$ per capita)
Prostate cancer	Male	15 to 19	80 plus	Global	0	Sociodemographic Status
Prostate cancer	Male	15 to 19	80 plus	Global	1	Animal Fats (kcal per capita)
Prostate cancer	Male	15 to 19	80 plus	Global	1	Log-transformed SEV scalar: Prostate C
Prostate cancer	Male	15 to 19	80 plus	Data Rich	-1	Health System Access 2 (unitless)
Prostate cancer	Male	15 to 19	80 plus	Data Rich	0	Education (years per capita)
Prostate cancer	Male	15 to 19	80 plus	Data Rich	0	LDI (I$ per capita)
Prostate cancer	Male	15 to 19	80 plus	Data Rich	0	Sociodemographic Status
Prostate cancer	Male	15 to 19	80 plus	Data Rich	1	Animal Fats (kcal per capita)
Prostate cancer	Male	15 to 19	80 plus	Data Rich	1	Log-transformed SEV scalar: Prostate C
Testicular cancer	Male	15 to 19	80 plus	Global	-1	Education (years per capita)
Testicular cancer	Male	15 to 19	80 plus	Global	-1	Fruits (kcal per capita)
Testicular cancer	Male	15 to 19	80 plus	Global	-1	Health System Access 2 (unitless)
Testicular cancer	Male	15 to 19	80 plus	Global	-1	LDI (I$ per capita)
Testicular cancer	Male	15 to 19	80 plus	Global	-1	Vegetables (kcal per capita)
Testicular cancer	Male	15 to 19	80 plus	Global	0	Sociodemographic Status
Testicular cancer	Male	15 to 19	80 plus	Global	1	Cumulative Cigarettes (10 Years)
Testicular cancer	Male	15 to 19	80 plus	Global	1	Cumulative Cigarettes (15 Years)
Testicular cancer	Male	15 to 19	80 plus	Global	1	Cumulative Cigarettes (5 Years)
Testicular cancer	Male	15 to 19	80 plus	Data Rich	-1	Education (years per capita)
Testicular cancer	Male	15 to 19	80 plus	Data Rich	-1	Fruits (kcal per capita)
Testicular cancer	Male	15 to 19	80 plus	Data Rich	-1	Health System Access 2 (unitless)
Testicular cancer	Male	15 to 19	80 plus	Data Rich	-1	LDI (I$ per capita)
Testicular cancer	Male	15 to 19	80 plus	Data Rich	-1	Vegetables (kcal per capita)
Testicular cancer	Male	15 to 19	80 plus	Data Rich	0	Sociodemographic Status
Testicular cancer	Male	15 to 19	80 plus	Data Rich	1	Cumulative Cigarettes (10 Years)
Testicular cancer	Male	15 to 19	80 plus	Data Rich	1	Cumulative Cigarettes (15 Years)
Testicular cancer	Male	15 to 19	80 plus	Data Rich	1	Cumulative Cigarettes (5 Years)
Kidney cancer	Male	Post Neonatal	80 plus	Global	-1	Education (years per capita)
Kidney cancer	Male	Post Neonatal	80 plus	Global	-1	Health System Access 2 (unitless)
Kidney cancer	Male	Post Neonatal	80 plus	Global	-1	LDI (I$ per capita)
Kidney cancer	Male	Post Neonatal	80 plus	Global	0	Total Fertility Rate
Kidney cancer	Male	Post Neonatal	80 plus	Global	1	Alcohol (litres per capita)
Kidney cancer	Male	Post Neonatal	80 plus	Global	1	Cumulative Cigarettes (10 Years)
Kidney cancer	Male	Post Neonatal	80 plus	Global	1	Cumulative Cigarettes (15 Years)

Appendix Table 10: CODEm covariates used, level of covariate, and expected direction of covariate by cause, sex, and age						
**Cause**	**Sex**	**Age start**	**Age end**	**Model type**	**Direction**	**Covariate**
Kidney cancer	Male	Post Neonatal	80 plus	Global	1	Cumulative Cigarettes (5 Years)
Kidney cancer	Male	Post Neonatal	80 plus	Global	1	Diabetes Age-Standardised Prevalence (proportion)
Kidney cancer	Male	Post Neonatal	80 plus	Global	1	Log-transformed SEV scalar: Kidney C
Kidney cancer	Male	Post Neonatal	80 plus	Global	1	Mean BMI
Kidney cancer	Male	Post Neonatal	80 plus	Global	1	Smoking Prevalence
Kidney cancer	Male	Post Neonatal	80 plus	Global	1	Sociodemographic Status
Kidney cancer	Male	Post Neonatal	80 plus	Global	1	Systolic Blood Pressure (mmHg)
Kidney cancer	Male	Post Neonatal	80 plus	Global	1	Total Calories (kcal per capita)
Kidney cancer	Male	Post Neonatal	80 plus	Data Rich	-1	Education (years per capita)
Kidney cancer	Male	Post Neonatal	80 plus	Data Rich	-1	Health System Access 2 (unitless)
Kidney cancer	Male	Post Neonatal	80 plus	Data Rich	-1	LDI (I$ per capita)
Kidney cancer	Male	Post Neonatal	80 plus	Data Rich	0	Total Fertility Rate
Kidney cancer	Male	Post Neonatal	80 plus	Data Rich	1	Alcohol (litres per capita)
Kidney cancer	Male	Post Neonatal	80 plus	Data Rich	1	Cumulative Cigarettes (10 Years)
Kidney cancer	Male	Post Neonatal	80 plus	Data Rich	1	Cumulative Cigarettes (15 Years)
Kidney cancer	Male	Post Neonatal	80 plus	Data Rich	1	Cumulative Cigarettes (5 Years)
Kidney cancer	Male	Post Neonatal	80 plus	Data Rich	1	Diabetes Age-Standardised Prevalence (proportion)
Kidney cancer	Male	Post Neonatal	80 plus	Data Rich	1	Log-transformed SEV scalar: Kidney C
Kidney cancer	Male	Post Neonatal	80 plus	Data Rich	1	Mean BMI
Kidney cancer	Male	Post Neonatal	80 plus	Data Rich	1	Smoking Prevalence
Kidney cancer	Male	Post Neonatal	80 plus	Data Rich	1	Sociodemographic Status
Kidney cancer	Male	Post Neonatal	80 plus	Data Rich	1	Systolic Blood Pressure (mmHg)
Kidney cancer	Male	Post Neonatal	80 plus	Data Rich	1	Total Calories (kcal per capita)
Kidney cancer	Female	Post Neonatal	80 plus	Global	-1	Education (years per capita)
Kidney cancer	Female	Post Neonatal	80 plus	Global	-1	Health System Access 2 (unitless)
Kidney cancer	Female	Post Neonatal	80 plus	Global	-1	LDI (I$ per capita)
Kidney cancer	Female	Post Neonatal	80 plus	Global	0	Total Fertility Rate
Kidney cancer	Female	Post Neonatal	80 plus	Global	1	Alcohol (litres per capita)
Kidney cancer	Female	Post Neonatal	80 plus	Global	1	Cumulative Cigarettes (10 Years)
Kidney cancer	Female	Post Neonatal	80 plus	Global	1	Cumulative Cigarettes (15 Years)
Kidney cancer	Female	Post Neonatal	80 plus	Global	1	Cumulative Cigarettes (5 Years)
Kidney cancer	Female	Post Neonatal	80 plus	Global	1	Diabetes Age-Standardised Prevalence (proportion)
Kidney cancer	Female	Post Neonatal	80 plus	Global	1	Log-transformed SEV scalar: Kidney C
Kidney cancer	Female	Post Neonatal	80 plus	Global	1	Mean BMI
Kidney cancer	Female	Post Neonatal	80 plus	Global	1	Smoking Prevalence
Kidney cancer	Female	Post Neonatal	80 plus	Global	1	Sociodemographic Status
Kidney cancer	Female	Post Neonatal	80 plus	Global	1	Systolic Blood Pressure (mmHg)
Kidney cancer	Female	Post Neonatal	80 plus	Global	1	Total Calories (kcal per capita)
Kidney cancer	Female	Post Neonatal	80 plus	Data Rich	-1	Education (years per capita)
Kidney cancer	Female	Post Neonatal	80 plus	Data Rich	-1	Health System Access 2 (unitless)
Kidney cancer	Female	Post Neonatal	80 plus	Data Rich	-1	LDI (I$ per capita)
Kidney cancer	Female	Post Neonatal	80 plus	Data Rich	0	Total Fertility Rate
Kidney cancer	Female	Post Neonatal	80 plus	Data Rich	1	Alcohol (litres per capita)
Kidney cancer	Female	Post Neonatal	80 plus	Data Rich	1	Cumulative Cigarettes (10 Years)

Appendix Table 10: CODEm covariates used, level of covariate, and expected direction of covariate by cause, sex, and age						
**Cause**	**Sex**	**Age start**	**Age end**	**Model type**	**Direction**	**Covariate**
Kidney cancer	Female	Post Neonatal	80 plus	Data Rich	1	Cumulative Cigarettes (15 Years)
Kidney cancer	Female	Post Neonatal	80 plus	Data Rich	1	Cumulative Cigarettes (5 Years)
Kidney cancer	Female	Post Neonatal	80 plus	Data Rich	1	Diabetes Age-Standardised Prevalence (proportion)
Kidney cancer	Female	Post Neonatal	80 plus	Data Rich	1	Log-transformed SEV scalar: Kidney C
Kidney cancer	Female	Post Neonatal	80 plus	Data Rich	1	Mean BMI
Kidney cancer	Female	Post Neonatal	80 plus	Data Rich	1	Smoking Prevalence
Kidney cancer	Female	Post Neonatal	80 plus	Data Rich	1	Sociodemographic Status
Kidney cancer	Female	Post Neonatal	80 plus	Data Rich	1	Systolic Blood Pressure (mmHg)
Kidney cancer	Female	Post Neonatal	80 plus	Data Rich	1	Total Calories (kcal per capita)
Bladder cancer	Male	15 to 19	80 plus	Global	-1	Education (years per capita)
Bladder cancer	Male	15 to 19	80 plus	Global	-1	Fruits (kcal per capita)
Bladder cancer	Male	15 to 19	80 plus	Global	-1	Health System Access 2 (unitless)
Bladder cancer	Male	15 to 19	80 plus	Global	-1	LDI (I$ per capita)
Bladder cancer	Male	15 to 19	80 plus	Global	-1	Vegetables (kcal per capita)
Bladder cancer	Male	15 to 19	80 plus	Global	0	Sociodemographic Status
Bladder cancer	Male	15 to 19	80 plus	Global	1	Alcohol (litres per capita)
Bladder cancer	Male	15 to 19	80 plus	Global	1	Cumulative Cigarettes (10 Years)
Bladder cancer	Male	15 to 19	80 plus	Global	1	Cumulative Cigarettes (15 Years)
Bladder cancer	Male	15 to 19	80 plus	Global	1	Cumulative Cigarettes (5 Years)
Bladder cancer	Male	15 to 19	80 plus	Global	1	Log-transformed SEV scalar: Bladder C
Bladder cancer	Male	15 to 19	80 plus	Global	1	Population Density (over 1000 ppl/sqkm, proportion)
Bladder cancer	Male	15 to 19	80 plus	Global	1	Population Density (under 150 ppl/sqkm, proportion)
Bladder cancer	Male	15 to 19	80 plus	Global	1	Smoking Prevalence
Bladder cancer	Male	15 to 19	80 plus	Data Rich	-1	Education (years per capita)
Bladder cancer	Male	15 to 19	80 plus	Data Rich	-1	Fruits (kcal per capita)
Bladder cancer	Male	15 to 19	80 plus	Data Rich	-1	Health System Access 2 (unitless)
Bladder cancer	Male	15 to 19	80 plus	Data Rich	-1	LDI (I$ per capita)
Bladder cancer	Male	15 to 19	80 plus	Data Rich	-1	Vegetables (kcal per capita)
Bladder cancer	Male	15 to 19	80 plus	Data Rich	0	Sociodemographic Status
Bladder cancer	Male	15 to 19	80 plus	Data Rich	1	Alcohol (litres per capita)
Bladder cancer	Male	15 to 19	80 plus	Data Rich	1	Cumulative Cigarettes (10 Years)
Bladder cancer	Male	15 to 19	80 plus	Data Rich	1	Cumulative Cigarettes (15 Years)
Bladder cancer	Male	15 to 19	80 plus	Data Rich	1	Cumulative Cigarettes (5 Years)
Bladder cancer	Male	15 to 19	80 plus	Data Rich	1	Log-transformed SEV scalar: Bladder C
Bladder cancer	Male	15 to 19	80 plus	Data Rich	1	Population Density (over 1000 ppl/sqkm, proportion)
Bladder cancer	Male	15 to 19	80 plus	Data Rich	1	Population Density (under 150 ppl/sqkm, proportion)
Bladder cancer	Male	15 to 19	80 plus	Data Rich	1	Smoking Prevalence
Bladder cancer	Female	15 to 19	80 plus	Global	-1	Education (years per capita)
Bladder cancer	Female	15 to 19	80 plus	Global	-1	Fruits (kcal per capita)
Bladder cancer	Female	15 to 19	80 plus	Global	-1	Health System Access 2 (unitless)
Bladder cancer	Female	15 to 19	80 plus	Global	-1	LDI (I$ per capita)
Bladder cancer	Female	15 to 19	80 plus	Global	-1	Vegetables (kcal per capita)
Bladder cancer	Female	15 to 19	80 plus	Global	0	Sociodemographic Status
Bladder cancer	Female	15 to 19	80 plus	Global	1	Alcohol (litres per capita)

Appendix Table 10: CODEm covariates used, level of covariate, and expected direction of covariate by cause, sex, and age						
**Cause**	**Sex**	**Age start**	**Age end**	**Model type**	**Direction**	**Covariate**
Bladder cancer	Female	15 to 19	80 plus	Global	1	Cumulative Cigarettes (10 Years)
Bladder cancer	Female	15 to 19	80 plus	Global	1	Cumulative Cigarettes (15 Years)
Bladder cancer	Female	15 to 19	80 plus	Global	1	Cumulative Cigarettes (5 Years)
Bladder cancer	Female	15 to 19	80 plus	Global	1	Log-transformed SEV scalar: Bladder C
Bladder cancer	Female	15 to 19	80 plus	Global	1	Population Density (over 1000 ppl/sqkm, proportion)
Bladder cancer	Female	15 to 19	80 plus	Global	1	Population Density (under 150 ppl/sqkm, proportion)
Bladder cancer	Female	15 to 19	80 plus	Global	1	Smoking Prevalence
Bladder cancer	Female	15 to 19	80 plus	Data Rich	-1	Education (years per capita)
Bladder cancer	Female	15 to 19	80 plus	Data Rich	-1	Fruits (kcal per capita)
Bladder cancer	Female	15 to 19	80 plus	Data Rich	-1	Health System Access 2 (unitless)
Bladder cancer	Female	15 to 19	80 plus	Data Rich	-1	LDI (I$ per capita)
Bladder cancer	Female	15 to 19	80 plus	Data Rich	-1	Vegetables (kcal per capita)
Bladder cancer	Female	15 to 19	80 plus	Data Rich	0	Sociodemographic Status
Bladder cancer	Female	15 to 19	80 plus	Data Rich	1	Alcohol (litres per capita)
Bladder cancer	Female	15 to 19	80 plus	Data Rich	1	Cumulative Cigarettes (10 Years)
Bladder cancer	Female	15 to 19	80 plus	Data Rich	1	Cumulative Cigarettes (15 Years)
Bladder cancer	Female	15 to 19	80 plus	Data Rich	1	Cumulative Cigarettes (5 Years)
Bladder cancer	Female	15 to 19	80 plus	Data Rich	1	Log-transformed SEV scalar: Bladder C
Bladder cancer	Female	15 to 19	80 plus	Data Rich	1	Population Density (over 1000 ppl/sqkm, proportion)
Bladder cancer	Female	15 to 19	80 plus	Data Rich	1	Population Density (under 150 ppl/sqkm, proportion)
Bladder cancer	Female	15 to 19	80 plus	Data Rich	1	Smoking Prevalence
Brain and nervous system cancer	Male	1 to 4	80 plus	Global	-1	Education (years per capita)
Brain and nervous system cancer	Male	1 to 4	80 plus	Global	-1	Fruits (kcal per capita)
Brain and nervous system cancer	Male	1 to 4	80 plus	Global	-1	Health System Access 2 (unitless)
Brain and nervous system cancer	Male	1 to 4	80 plus	Global	-1	LDI (I$ per capita)
Brain and nervous system cancer	Male	1 to 4	80 plus	Global	-1	Vegetables (kcal per capita)
Brain and nervous system cancer	Male	1 to 4	80 plus	Global	1	Alcohol (litres per capita)
Brain and nervous system cancer	Male	1 to 4	80 plus	Global	1	Animal Fats (kcal per capita)
Brain and nervous system cancer	Male	1 to 4	80 plus	Global	1	Cholesterol (total, mean per capita)
Brain and nervous system cancer	Male	1 to 4	80 plus	Global	1	Cumulative Cigarettes (10 Years)
Brain and nervous system cancer	Male	1 to 4	80 plus	Global	1	Cumulative Cigarettes (15 Years)
Brain and nervous system cancer	Male	1 to 4	80 plus	Global	1	Red Meat (kcal per capita)
Brain and nervous system cancer	Male	1 to 4	80 plus	Global	1	Smoking Prevalence
Brain and nervous system cancer	Male	1 to 4	80 plus	Global	1	Sociodemographic Status
Brain and nervous system cancer	Male	1 to 4	80 plus	Global	1	Systolic Blood Pressure (mmHg)
Brain and nervous system cancer	Male	1 to 4	80 plus	Data Rich	-1	Education (years per capita)
Brain and nervous system cancer	Male	1 to 4	80 plus	Data Rich	-1	Fruits (kcal per capita)
Brain and nervous system cancer	Male	1 to 4	80 plus	Data Rich	-1	Health System Access 2 (unitless)
Brain and nervous system cancer	Male	1 to 4	80 plus	Data Rich	-1	LDI (I$ per capita)
Brain and nervous system cancer	Male	1 to 4	80 plus	Data Rich	-1	Vegetables (kcal per capita)
Brain and nervous system cancer	Male	1 to 4	80 plus	Data Rich	1	Alcohol (litres per capita)
Brain and nervous system cancer	Male	1 to 4	80 plus	Data Rich	1	Animal Fats (kcal per capita)
Brain and nervous system cancer	Male	1 to 4	80 plus	Data Rich	1	Cholesterol (total, mean per capita)
Brain and nervous system cancer	Male	1 to 4	80 plus	Data Rich	1	Cumulative Cigarettes (10 Years)

Appendix Table 10: CODEm covariates used, level of covariate, and expected direction of covariate by cause, sex, and age						
**Cause**	**Sex**	**Age start**	**Age end**	**Model type**	**Direction**	**Covariate**
Brain and nervous system cancer	Male	1 to 4	80 plus	Data Rich	1	Cumulative Cigarettes (15 Years)
Brain and nervous system cancer	Male	1 to 4	80 plus	Data Rich	1	Red Meat (kcal per capita)
Brain and nervous system cancer	Male	1 to 4	80 plus	Data Rich	1	Smoking Prevalence
Brain and nervous system cancer	Male	1 to 4	80 plus	Data Rich	1	Sociodemographic Status
Brain and nervous system cancer	Male	1 to 4	80 plus	Data Rich	1	Systolic Blood Pressure (mmHg)
Brain and nervous system cancer	Female	1 to 4	80 plus	Global	-1	Education (years per capita)
Brain and nervous system cancer	Female	1 to 4	80 plus	Global	-1	Fruits (kcal per capita)
Brain and nervous system cancer	Female	1 to 4	80 plus	Global	-1	Health System Access 2 (unitless)
Brain and nervous system cancer	Female	1 to 4	80 plus	Global	-1	LDI (I$ per capita)
Brain and nervous system cancer	Female	1 to 4	80 plus	Global	-1	Vegetables (kcal per capita)
Brain and nervous system cancer	Female	1 to 4	80 plus	Global	1	Alcohol (litres per capita)
Brain and nervous system cancer	Female	1 to 4	80 plus	Global	1	Animal Fats (kcal per capita)
Brain and nervous system cancer	Female	1 to 4	80 plus	Global	1	Cholesterol (total, mean per capita)
Brain and nervous system cancer	Female	1 to 4	80 plus	Global	1	Cumulative Cigarettes (10 Years)
Brain and nervous system cancer	Female	1 to 4	80 plus	Global	1	Cumulative Cigarettes (15 Years)
Brain and nervous system cancer	Female	1 to 4	80 plus	Global	1	Red Meat (kcal per capita)
Brain and nervous system cancer	Female	1 to 4	80 plus	Global	1	Smoking Prevalence
Brain and nervous system cancer	Female	1 to 4	80 plus	Global	1	Sociodemographic Status
Brain and nervous system cancer	Female	1 to 4	80 plus	Global	1	Systolic Blood Pressure (mmHg)
Brain and nervous system cancer	Female	1 to 4	80 plus	Data Rich	-1	Education (years per capita)
Brain and nervous system cancer	Female	1 to 4	80 plus	Data Rich	-1	Fruits (kcal per capita)
Brain and nervous system cancer	Female	1 to 4	80 plus	Data Rich	-1	Health System Access 2 (unitless)
Brain and nervous system cancer	Female	1 to 4	80 plus	Data Rich	-1	LDI (I$ per capita)
Brain and nervous system cancer	Female	1 to 4	80 plus	Data Rich	-1	Vegetables (kcal per capita)
Brain and nervous system cancer	Female	1 to 4	80 plus	Data Rich	1	Alcohol (litres per capita)
Brain and nervous system cancer	Female	1 to 4	80 plus	Data Rich	1	Animal Fats (kcal per capita)
Brain and nervous system cancer	Female	1 to 4	80 plus	Data Rich	1	Cholesterol (total, mean per capita)
Brain and nervous system cancer	Female	1 to 4	80 plus	Data Rich	1	Cumulative Cigarettes (10 Years)
Brain and nervous system cancer	Female	1 to 4	80 plus	Data Rich	1	Cumulative Cigarettes (15 Years)
Brain and nervous system cancer	Female	1 to 4	80 plus	Data Rich	1	Red Meat (kcal per capita)
Brain and nervous system cancer	Female	1 to 4	80 plus	Data Rich	1	Smoking Prevalence
Brain and nervous system cancer	Female	1 to 4	80 plus	Data Rich	1	Sociodemographic Status
Brain and nervous system cancer	Female	1 to 4	80 plus	Data Rich	1	Systolic Blood Pressure (mmHg)
Thyroid cancer	Male	10 to 14	80 plus	Global	-1	Education (years per capita)
Thyroid cancer	Male	10 to 14	80 plus	Global	-1	Fruits (kcal per capita)
Thyroid cancer	Male	10 to 14	80 plus	Global	-1	Improved Water Source (proportion with access)
Thyroid cancer	Male	10 to 14	80 plus	Global	-1	LDI (I$ per capita)
Thyroid cancer	Male	10 to 14	80 plus	Global	-1	Sanitation (proportion with access)
Thyroid cancer	Male	10 to 14	80 plus	Global	-1	Vegetables (kcal per capita)
Thyroid cancer	Male	10 to 14	80 plus	Global	0	Sociodemographic Status
Thyroid cancer	Male	10 to 14	80 plus	Global	1	Alcohol (litres per capita)
Thyroid cancer	Male	10 to 14	80 plus	Global	1	Log-transformed SEV scalar: Thyroid C
Thyroid cancer	Male	10 to 14	80 plus	Global	1	Mean BMI
Thyroid cancer	Male	10 to 14	80 plus	Global	1	Red Meat (kcal per capita)

Appendix Table 10: CODEm covariates used, level of covariate, and expected direction of covariate by cause, sex, and age						
**Cause**	**Sex**	**Age start**	**Age end**	**Model type**	**Direction**	**Covariate**
Thyroid cancer	Male	10 to 14	80 plus	Global	2	Smoking Prevalence
Thyroid cancer	Male	10 to 14	80 plus	Global	2	Tobacco (cigarettes per capita)
Thyroid cancer	Male	10 to 14	80 plus	Data Rich	-1	Education (years per capita)
Thyroid cancer	Male	10 to 14	80 plus	Data Rich	-1	Fruits (kcal per capita)
Thyroid cancer	Male	10 to 14	80 plus	Data Rich	-1	Improved Water Source (proportion with access)
Thyroid cancer	Male	10 to 14	80 plus	Data Rich	-1	LDI (I$ per capita)
Thyroid cancer	Male	10 to 14	80 plus	Data Rich	-1	Sanitation (proportion with access)
Thyroid cancer	Male	10 to 14	80 plus	Data Rich	-1	Vegetables (kcal per capita)
Thyroid cancer	Male	10 to 14	80 plus	Data Rich	0	Sociodemographic Status
Thyroid cancer	Male	10 to 14	80 plus	Data Rich	1	Alcohol (litres per capita)
Thyroid cancer	Male	10 to 14	80 plus	Data Rich	1	Log-transformed SEV scalar: Thyroid C
Thyroid cancer	Male	10 to 14	80 plus	Data Rich	1	Mean BMI
Thyroid cancer	Male	10 to 14	80 plus	Data Rich	1	Red Meat (kcal per capita)
Thyroid cancer	Male	10 to 14	80 plus	Data Rich	2	Smoking Prevalence
Thyroid cancer	Male	10 to 14	80 plus	Data Rich	2	Tobacco (cigarettes per capita)
Thyroid cancer	Female	10 to 14	80 plus	Global	-1	Education (years per capita)
Thyroid cancer	Female	10 to 14	80 plus	Global	-1	Fruits (kcal per capita)
Thyroid cancer	Female	10 to 14	80 plus	Global	-1	Improved Water Source (proportion with access)
Thyroid cancer	Female	10 to 14	80 plus	Global	-1	LDI (I$ per capita)
Thyroid cancer	Female	10 to 14	80 plus	Global	-1	Sanitation (proportion with access)
Thyroid cancer	Female	10 to 14	80 plus	Global	-1	Vegetables (kcal per capita)
Thyroid cancer	Female	10 to 14	80 plus	Global	0	Sociodemographic Status
Thyroid cancer	Female	10 to 14	80 plus	Global	1	Alcohol (litres per capita)
Thyroid cancer	Female	10 to 14	80 plus	Global	1	Log-transformed SEV scalar: Thyroid C
Thyroid cancer	Female	10 to 14	80 plus	Global	1	Mean BMI
Thyroid cancer	Female	10 to 14	80 plus	Global	1	Red Meat (kcal per capita)
Thyroid cancer	Female	10 to 14	80 plus	Global	2	Smoking Prevalence
Thyroid cancer	Female	10 to 14	80 plus	Global	2	Tobacco (cigarettes per capita)
Thyroid cancer	Female	10 to 14	80 plus	Data Rich	-1	Education (years per capita)
Thyroid cancer	Female	10 to 14	80 plus	Data Rich	-1	Fruits (kcal per capita)
Thyroid cancer	Female	10 to 14	80 plus	Data Rich	-1	Improved Water Source (proportion with access)
Thyroid cancer	Female	10 to 14	80 plus	Data Rich	-1	LDI (I$ per capita)
Thyroid cancer	Female	10 to 14	80 plus	Data Rich	-1	Sanitation (proportion with access)
Thyroid cancer	Female	10 to 14	80 plus	Data Rich	-1	Vegetables (kcal per capita)
Thyroid cancer	Female	10 to 14	80 plus	Data Rich	0	Sociodemographic Status
Thyroid cancer	Female	10 to 14	80 plus	Data Rich	1	Alcohol (litres per capita)
Thyroid cancer	Female	10 to 14	80 plus	Data Rich	1	Log-transformed SEV scalar: Thyroid C
Thyroid cancer	Female	10 to 14	80 plus	Data Rich	1	Mean BMI
Thyroid cancer	Female	10 to 14	80 plus	Data Rich	1	Red Meat (kcal per capita)
Thyroid cancer	Female	10 to 14	80 plus	Data Rich	2	Smoking Prevalence
Thyroid cancer	Female	10 to 14	80 plus	Data Rich	2	Tobacco (cigarettes per capita)
Mesothelioma	Male	15 to 19	80 plus	Global	-1	Education (years per capita)
Mesothelioma	Male	15 to 19	80 plus	Global	-1	Health System Access 2 (unitless)
Mesothelioma	Male	15 to 19	80 plus	Global	-1	LDI (I$ per capita)

Appendix Table 10: CODEm covariates used, level of covariate, and expected direction of covariate by cause, sex, and age						
**Cause**	**Sex**	**Age start**	**Age end**	**Model type**	**Direction**	**Covariate**
Mesothelioma	Male	15 to 19	80 plus	Global	0	Sociodemographic Status
Mesothelioma	Male	15 to 19	80 plus	Global	1	Asbestos production (binary)
Mesothelioma	Male	15 to 19	80 plus	Global	1	Asbestos production (kg) per capita
Mesothelioma	Male	15 to 19	80 plus	Global	1	Cumulative Cigarettes (5 Years)
Mesothelioma	Male	15 to 19	80 plus	Global	1	Elevation 500 to 1500m (proportion)
Mesothelioma	Male	15 to 19	80 plus	Global	1	Elevation Over 1500m (proportion)
Mesothelioma	Male	15 to 19	80 plus	Global	1	Gold production (binary)
Mesothelioma	Male	15 to 19	80 plus	Global	1	Gold production (kg) per capita
Mesothelioma	Male	15 to 19	80 plus	Global	1	Indoor Air Pollution (Coal Cooking)
Mesothelioma	Male	15 to 19	80 plus	Global	1	Log-transformed SEV scalar: Mesothel
Mesothelioma	Male	15 to 19	80 plus	Global	1	Population Density (over 1000 ppl/sqkm, proportion)
Mesothelioma	Male	15 to 19	80 plus	Global	1	Population Over 65 (proportion)
Mesothelioma	Male	15 to 19	80 plus	Global	1	Smoking Prevalence
Mesothelioma	Male	15 to 19	80 plus	Data Rich	-1	Education (years per capita)
Mesothelioma	Male	15 to 19	80 plus	Data Rich	-1	Health System Access 2 (unitless)
Mesothelioma	Male	15 to 19	80 plus	Data Rich	-1	LDI (I$ per capita)
Mesothelioma	Male	15 to 19	80 plus	Data Rich	0	Sociodemographic Status
Mesothelioma	Male	15 to 19	80 plus	Data Rich	1	Asbestos production (binary)
Mesothelioma	Male	15 to 19	80 plus	Data Rich	1	Asbestos production (kg) per capita
Mesothelioma	Male	15 to 19	80 plus	Data Rich	1	Cumulative Cigarettes (5 Years)
Mesothelioma	Male	15 to 19	80 plus	Data Rich	1	Elevation 500 to 1500m (proportion)
Mesothelioma	Male	15 to 19	80 plus	Data Rich	1	Elevation Over 1500m (proportion)
Mesothelioma	Male	15 to 19	80 plus	Data Rich	1	Gold production (binary)
Mesothelioma	Male	15 to 19	80 plus	Data Rich	1	Gold production (kg) per capita
Mesothelioma	Male	15 to 19	80 plus	Data Rich	1	Indoor Air Pollution (Coal Cooking)
Mesothelioma	Male	15 to 19	80 plus	Data Rich	1	Log-transformed SEV scalar: Mesothel
Mesothelioma	Male	15 to 19	80 plus	Data Rich	1	Population Density (over 1000 ppl/sqkm, proportion)
Mesothelioma	Male	15 to 19	80 plus	Data Rich	1	Population Over 65 (proportion)
Mesothelioma	Male	15 to 19	80 plus	Data Rich	1	Smoking Prevalence
Mesothelioma	Female	15 to 19	80 plus	Global	-1	Education (years per capita)
Mesothelioma	Female	15 to 19	80 plus	Global	-1	Health System Access 2 (unitless)
Mesothelioma	Female	15 to 19	80 plus	Global	-1	LDI (I$ per capita)
Mesothelioma	Female	15 to 19	80 plus	Global	0	Sociodemographic Status
Mesothelioma	Female	15 to 19	80 plus	Global	1	Asbestos production (binary)
Mesothelioma	Female	15 to 19	80 plus	Global	1	Asbestos production (kg) per capita
Mesothelioma	Female	15 to 19	80 plus	Global	1	Cumulative Cigarettes (5 Years)
Mesothelioma	Female	15 to 19	80 plus	Global	1	Elevation 500 to 1500m (proportion)
Mesothelioma	Female	15 to 19	80 plus	Global	1	Elevation Over 1500m (proportion)
Mesothelioma	Female	15 to 19	80 plus	Global	1	Gold production (binary)
Mesothelioma	Female	15 to 19	80 plus	Global	1	Gold production (kg) per capita
Mesothelioma	Female	15 to 19	80 plus	Global	1	Indoor Air Pollution (Coal Cooking)
Mesothelioma	Female	15 to 19	80 plus	Global	1	Log-transformed SEV scalar: Mesothel
Mesothelioma	Female	15 to 19	80 plus	Global	1	Population Density (over 1000 ppl/sqkm, proportion)
Mesothelioma	Female	15 to 19	80 plus	Global	1	Population Over 65 (proportion)

Appendix Table 10: CODEm covariates used, level of covariate, and expected direction of covariate by cause, sex, and age						
**Cause**	**Sex**	**Age start**	**Age end**	**Model type**	**Direction**	**Covariate**
Mesothelioma	Female	15 to 19	80 plus	Global	1	Smoking Prevalence
Mesothelioma	Female	15 to 19	80 plus	Data Rich	-1	Education (years per capita)
Mesothelioma	Female	15 to 19	80 plus	Data Rich	-1	Health System Access 2 (unitless)
Mesothelioma	Female	15 to 19	80 plus	Data Rich	-1	LDI (I$ per capita)
Mesothelioma	Female	15 to 19	80 plus	Data Rich	0	Sociodemographic Status
Mesothelioma	Female	15 to 19	80 plus	Data Rich	1	Asbestos production (binary)
Mesothelioma	Female	15 to 19	80 plus	Data Rich	1	Asbestos production (kg) per capita
Mesothelioma	Female	15 to 19	80 plus	Data Rich	1	Cumulative Cigarettes (5 Years)
Mesothelioma	Female	15 to 19	80 plus	Data Rich	1	Elevation 500 to 1500m (proportion)
Mesothelioma	Female	15 to 19	80 plus	Data Rich	1	Elevation Over 1500m (proportion)
Mesothelioma	Female	15 to 19	80 plus	Data Rich	1	Gold production (binary)
Mesothelioma	Female	15 to 19	80 plus	Data Rich	1	Gold production (kg) per capita
Mesothelioma	Female	15 to 19	80 plus	Data Rich	1	Indoor Air Pollution (Coal Cooking)
Mesothelioma	Female	15 to 19	80 plus	Data Rich	1	Log-transformed SEV scalar: Mesothel
Mesothelioma	Female	15 to 19	80 plus	Data Rich	1	Population Density (over 1000 ppl/sqkm, proportion)
Mesothelioma	Female	15 to 19	80 plus	Data Rich	1	Population Over 65 (proportion)
Mesothelioma	Female	15 to 19	80 plus	Data Rich	1	Smoking Prevalence
Hodgkin lymphoma	Male	Post Neonatal	80 plus	Global	-1	Education (years per capita)
Hodgkin lymphoma	Male	Post Neonatal	80 plus	Global	-1	Health System Access 2 (unitless)
Hodgkin lymphoma	Male	Post Neonatal	80 plus	Global	-1	LDI (I$ per capita)
Hodgkin lymphoma	Male	Post Neonatal	80 plus	Global	0	Sociodemographic Status
Hodgkin lymphoma	Male	Post Neonatal	80 plus	Global	1	Latitude 15 to 30 (proportion)
Hodgkin lymphoma	Male	Post Neonatal	80 plus	Global	1	Latitude 30 to 45 (proportion)
Hodgkin lymphoma	Male	Post Neonatal	80 plus	Global	1	Latitude Over 45 (proportion)
Hodgkin lymphoma	Male	Post Neonatal	80 plus	Global	1	Latitude Under 15 (proportion)
Hodgkin lymphoma	Male	Post Neonatal	80 plus	Data Rich	-1	Education (years per capita)
Hodgkin lymphoma	Male	Post Neonatal	80 plus	Data Rich	-1	Health System Access 2 (unitless)
Hodgkin lymphoma	Male	Post Neonatal	80 plus	Data Rich	-1	LDI (I$ per capita)
Hodgkin lymphoma	Male	Post Neonatal	80 plus	Data Rich	0	Sociodemographic Status
Hodgkin lymphoma	Male	Post Neonatal	80 plus	Data Rich	1	Latitude 15 to 30 (proportion)
Hodgkin lymphoma	Male	Post Neonatal	80 plus	Data Rich	1	Latitude 30 to 45 (proportion)
Hodgkin lymphoma	Male	Post Neonatal	80 plus	Data Rich	1	Latitude Over 45 (proportion)
Hodgkin lymphoma	Male	Post Neonatal	80 plus	Data Rich	1	Latitude Under 15 (proportion)
Hodgkin lymphoma	Female	Post Neonatal	80 plus	Global	-1	Education (years per capita)
Hodgkin lymphoma	Female	Post Neonatal	80 plus	Global	-1	Health System Access 2 (unitless)
Hodgkin lymphoma	Female	Post Neonatal	80 plus	Global	-1	LDI (I$ per capita)
Hodgkin lymphoma	Female	Post Neonatal	80 plus	Global	-1	Latitude 30 to 45 (proportion)
Hodgkin lymphoma	Female	Post Neonatal	80 plus	Global	-1	Latitude Over 45 (proportion)
Hodgkin lymphoma	Female	Post Neonatal	80 plus	Global	0	Sociodemographic Status
Hodgkin lymphoma	Female	Post Neonatal	80 plus	Global	1	Latitude 15 to 30 (proportion)
Hodgkin lymphoma	Female	Post Neonatal	80 plus	Global	1	Latitude Under 15 (proportion)
Hodgkin lymphoma	Female	Post Neonatal	80 plus	Data Rich	-1	Education (years per capita)
Hodgkin lymphoma	Female	Post Neonatal	80 plus	Data Rich	-1	Health System Access 2 (unitless)
Hodgkin lymphoma	Female	Post Neonatal	80 plus	Data Rich	-1	LDI (I$ per capita)

Appendix Table 10: CODEm covariates used, level of covariate, and expected direction of covariate by cause, sex, and age						
**Cause**	**Sex**	**Age start**	**Age end**	**Model type**	**Direction**	**Covariate**
Hodgkin lymphoma	Female	Post Neonatal	80 plus	Data Rich	-1	Latitude 30 to 45 (proportion)
Hodgkin lymphoma	Female	Post Neonatal	80 plus	Data Rich	-1	Latitude Over 45 (proportion)
Hodgkin lymphoma	Female	Post Neonatal	80 plus	Data Rich	0	Sociodemographic Status
Hodgkin lymphoma	Female	Post Neonatal	80 plus	Data Rich	1	Latitude 15 to 30 (proportion)
Hodgkin lymphoma	Female	Post Neonatal	80 plus	Data Rich	1	Latitude Under 15 (proportion)
Non-Hodgkin lymphoma	Male	Post Neonatal	80 plus	Global	-1	Health System Access 2 (unitless)
Non-Hodgkin lymphoma	Male	Post Neonatal	80 plus	Global	-1	LDI (I$ per capita)
Non-Hodgkin lymphoma	Male	Post Neonatal	80 plus	Global	0	Sociodemographic Status
Non-Hodgkin lymphoma	Male	Post Neonatal	80 plus	Global	0	Total Fertility Rate
Non-Hodgkin lymphoma	Male	Post Neonatal	80 plus	Global	1	Alcohol (litres per capita)
Non-Hodgkin lymphoma	Male	Post Neonatal	80 plus	Global	1	Cumulative Cigarettes (10 Years)
Non-Hodgkin lymphoma	Male	Post Neonatal	80 plus	Global	1	Smoking Prevalence
Non-Hodgkin lymphoma	Male	Post Neonatal	80 plus	Data Rich	-1	Health System Access 2 (unitless)
Non-Hodgkin lymphoma	Male	Post Neonatal	80 plus	Data Rich	-1	LDI (I$ per capita)
Non-Hodgkin lymphoma	Male	Post Neonatal	80 plus	Data Rich	0	Sociodemographic Status
Non-Hodgkin lymphoma	Male	Post Neonatal	80 plus	Data Rich	0	Total Fertility Rate
Non-Hodgkin lymphoma	Male	Post Neonatal	80 plus	Data Rich	1	Alcohol (litres per capita)
Non-Hodgkin lymphoma	Male	Post Neonatal	80 plus	Data Rich	1	Cumulative Cigarettes (10 Years)
Non-Hodgkin lymphoma	Male	Post Neonatal	80 plus	Data Rich	1	Smoking Prevalence
Non-Hodgkin lymphoma	Female	Post Neonatal	80 plus	Global	-1	Health System Access 2 (unitless)
Non-Hodgkin lymphoma	Female	Post Neonatal	80 plus	Global	-1	LDI (I$ per capita)
Non-Hodgkin lymphoma	Female	Post Neonatal	80 plus	Global	0	Sociodemographic Status
Non-Hodgkin lymphoma	Female	Post Neonatal	80 plus	Global	0	Total Fertility Rate
Non-Hodgkin lymphoma	Female	Post Neonatal	80 plus	Global	1	Alcohol (litres per capita)
Non-Hodgkin lymphoma	Female	Post Neonatal	80 plus	Global	1	Cumulative Cigarettes (10 Years)
Non-Hodgkin lymphoma	Female	Post Neonatal	80 plus	Global	1	Smoking Prevalence
Non-Hodgkin lymphoma	Female	Post Neonatal	80 plus	Data Rich	-1	Health System Access 2 (unitless)
Non-Hodgkin lymphoma	Female	Post Neonatal	80 plus	Data Rich	-1	LDI (I$ per capita)
Non-Hodgkin lymphoma	Female	Post Neonatal	80 plus	Data Rich	0	Sociodemographic Status
Non-Hodgkin lymphoma	Female	Post Neonatal	80 plus	Data Rich	0	Total Fertility Rate
Non-Hodgkin lymphoma	Female	Post Neonatal	80 plus	Data Rich	1	Alcohol (litres per capita)
Non-Hodgkin lymphoma	Female	Post Neonatal	80 plus	Data Rich	1	Cumulative Cigarettes (10 Years)
Non-Hodgkin lymphoma	Female	Post Neonatal	80 plus	Data Rich	1	Smoking Prevalence
Multiple myeloma	Male	15 to 19	80 plus	Global	-1	Education (years per capita)
Multiple myeloma	Male	15 to 19	80 plus	Global	-1	Fruits (kcal per capita)
Multiple myeloma	Male	15 to 19	80 plus	Global	-1	Improved Water Source (proportion with access)
Multiple myeloma	Male	15 to 19	80 plus	Global	-1	LDI (I$ per capita)
Multiple myeloma	Male	15 to 19	80 plus	Global	-1	Sanitation (proportion with access)
Multiple myeloma	Male	15 to 19	80 plus	Global	-1	Vegetables (kcal per capita)
Multiple myeloma	Male	15 to 19	80 plus	Global	0	Sociodemographic Status
Multiple myeloma	Male	15 to 19	80 plus	Global	1	Alcohol (litres per capita)
Multiple myeloma	Male	15 to 19	80 plus	Global	1	Mean BMI
Multiple myeloma	Male	15 to 19	80 plus	Global	1	Red Meat (kcal per capita)
Multiple myeloma	Male	15 to 19	80 plus	Global	1	Smoking Prevalence

Appendix Table 10: CODEm covariates used, level of covariate, and expected direction of covariate by cause, sex, and age						
**Cause**	**Sex**	**Age start**	**Age end**	**Model type**	**Direction**	**Covariate**
Multiple myeloma	Male	15 to 19	80 plus	Global	1	Tobacco (cigarettes per capita)
Multiple myeloma	Male	15 to 19	80 plus	Data Rich	-1	Education (years per capita)
Multiple myeloma	Male	15 to 19	80 plus	Data Rich	-1	Fruits (kcal per capita)
Multiple myeloma	Male	15 to 19	80 plus	Data Rich	-1	Improved Water Source (proportion with access)
Multiple myeloma	Male	15 to 19	80 plus	Data Rich	-1	LDI (I$ per capita)
Multiple myeloma	Male	15 to 19	80 plus	Data Rich	-1	Sanitation (proportion with access)
Multiple myeloma	Male	15 to 19	80 plus	Data Rich	-1	Vegetables (kcal per capita)
Multiple myeloma	Male	15 to 19	80 plus	Data Rich	0	Sociodemographic Status
Multiple myeloma	Male	15 to 19	80 plus	Data Rich	1	Alcohol (litres per capita)
Multiple myeloma	Male	15 to 19	80 plus	Data Rich	1	Mean BMI
Multiple myeloma	Male	15 to 19	80 plus	Data Rich	1	Red Meat (kcal per capita)
Multiple myeloma	Male	15 to 19	80 plus	Data Rich	1	Smoking Prevalence
Multiple myeloma	Male	15 to 19	80 plus	Data Rich	1	Tobacco (cigarettes per capita)
Multiple myeloma	Female	15 to 19	80 plus	Global	-1	Education (years per capita)
Multiple myeloma	Female	15 to 19	80 plus	Global	-1	Fruits (kcal per capita)
Multiple myeloma	Female	15 to 19	80 plus	Global	-1	Improved Water Source (proportion with access)
Multiple myeloma	Female	15 to 19	80 plus	Global	-1	LDI (I$ per capita)
Multiple myeloma	Female	15 to 19	80 plus	Global	-1	Sanitation (proportion with access)
Multiple myeloma	Female	15 to 19	80 plus	Global	-1	Vegetables (kcal per capita)
Multiple myeloma	Female	15 to 19	80 plus	Global	0	Sociodemographic Status
Multiple myeloma	Female	15 to 19	80 plus	Global	1	Alcohol (litres per capita)
Multiple myeloma	Female	15 to 19	80 plus	Global	1	Mean BMI
Multiple myeloma	Female	15 to 19	80 plus	Global	1	Red Meat (kcal per capita)
Multiple myeloma	Female	15 to 19	80 plus	Global	1	Smoking Prevalence
Multiple myeloma	Female	15 to 19	80 plus	Global	1	Tobacco (cigarettes per capita)
Multiple myeloma	Female	15 to 19	80 plus	Data Rich	-1	Education (years per capita)
Multiple myeloma	Female	15 to 19	80 plus	Data Rich	-1	Fruits (kcal per capita)
Multiple myeloma	Female	15 to 19	80 plus	Data Rich	-1	Improved Water Source (proportion with access)
Multiple myeloma	Female	15 to 19	80 plus	Data Rich	-1	LDI (I$ per capita)
Multiple myeloma	Female	15 to 19	80 plus	Data Rich	-1	Sanitation (proportion with access)
Multiple myeloma	Female	15 to 19	80 plus	Data Rich	-1	Vegetables (kcal per capita)
Multiple myeloma	Female	15 to 19	80 plus	Data Rich	0	Sociodemographic Status
Multiple myeloma	Female	15 to 19	80 plus	Data Rich	1	Alcohol (litres per capita)
Multiple myeloma	Female	15 to 19	80 plus	Data Rich	1	Mean BMI
Multiple myeloma	Female	15 to 19	80 plus	Data Rich	1	Red Meat (kcal per capita)
Multiple myeloma	Female	15 to 19	80 plus	Data Rich	1	Smoking Prevalence
Multiple myeloma	Female	15 to 19	80 plus	Data Rich	1	Tobacco (cigarettes per capita)
Leukemia	Male	Post Neonatal	80 plus	Global	-1	Education (years per capita)
Leukemia	Male	Post Neonatal	80 plus	Global	-1	Health System Access 2 (unitless)
Leukemia	Male	Post Neonatal	80 plus	Global	-1	LDI (I$ per capita)
Leukemia	Male	Post Neonatal	80 plus	Global	1	Alcohol (litres per capita)
Leukemia	Male	Post Neonatal	80 plus	Global	1	Cumulative Cigarettes (10 Years)
Leukemia	Male	Post Neonatal	80 plus	Global	1	Cumulative Cigarettes (15 Years)
Leukemia	Male	Post Neonatal	80 plus	Global	1	Cumulative Cigarettes (5 Years)

Appendix Table 10: CODEm covariates used, level of covariate, and expected direction of covariate by cause, sex, and age

Cause	Sex	Age start	Age end	Model type	Direction	Covariate
Leukemia	Male	Post Neonatal	80 plus	Global	1	Log-transformed SEV scalar: Leukemia
Leukemia	Male	Post Neonatal	80 plus	Global	1	Smoking Prevalence
Leukemia	Male	Post Neonatal	80 plus	Global	1	Sociodemographic Status
Leukemia	Male	Post Neonatal	80 plus	Data Rich	-1	Education (years per capita)
Leukemia	Male	Post Neonatal	80 plus	Data Rich	-1	Health System Access 2 (unitless)
Leukemia	Male	Post Neonatal	80 plus	Data Rich	-1	LDI (I$ per capita)
Leukemia	Male	Post Neonatal	80 plus	Data Rich	1	Alcohol (litres per capita)
Leukemia	Male	Post Neonatal	80 plus	Data Rich	1	Cumulative Cigarettes (10 Years)
Leukemia	Male	Post Neonatal	80 plus	Data Rich	1	Cumulative Cigarettes (15 Years)
Leukemia	Male	Post Neonatal	80 plus	Data Rich	1	Cumulative Cigarettes (5 Years)
Leukemia	Male	Post Neonatal	80 plus	Data Rich	1	Log-transformed SEV scalar: Leukemia
Leukemia	Male	Post Neonatal	80 plus	Data Rich	1	Smoking Prevalence
Leukemia	Male	Post Neonatal	80 plus	Data Rich	1	Sociodemographic Status
Leukemia	Female	Post Neonatal	80 plus	Global	-1	Education (years per capita)
Leukemia	Female	Post Neonatal	80 plus	Global	-1	Health System Access 2 (unitless)
Leukemia	Female	Post Neonatal	80 plus	Global	-1	LDI (I$ per capita)
Leukemia	Female	Post Neonatal	80 plus	Global	0	Total Fertility Rate
Leukemia	Female	Post Neonatal	80 plus	Global	1	Alcohol (litres per capita)
Leukemia	Female	Post Neonatal	80 plus	Global	1	Cumulative Cigarettes (10 Years)
Leukemia	Female	Post Neonatal	80 plus	Global	1	Cumulative Cigarettes (15 Years)
Leukemia	Female	Post Neonatal	80 plus	Global	1	Log-transformed SEV scalar: Leukemia
Leukemia	Female	Post Neonatal	80 plus	Global	1	Smoking Prevalence
Leukemia	Female	Post Neonatal	80 plus	Global	1	Sociodemographic Status
Leukemia	Female	Post Neonatal	80 plus	Data Rich	-1	Education (years per capita)
Leukemia	Female	Post Neonatal	80 plus	Data Rich	-1	Health System Access 2 (unitless)
Leukemia	Female	Post Neonatal	80 plus	Data Rich	-1	LDI (I$ per capita)
Leukemia	Female	Post Neonatal	80 plus	Data Rich	0	Total Fertility Rate
Leukemia	Female	Post Neonatal	80 plus	Data Rich	1	Alcohol (litres per capita)
Leukemia	Female	Post Neonatal	80 plus	Data Rich	1	Cumulative Cigarettes (10 Years)
Leukemia	Female	Post Neonatal	80 plus	Data Rich	1	Cumulative Cigarettes (15 Years)
Leukemia	Female	Post Neonatal	80 plus	Data Rich	1	Log-transformed SEV scalar: Leukemia
Leukemia	Female	Post Neonatal	80 plus	Data Rich	1	Smoking Prevalence
Leukemia	Female	Post Neonatal	80 plus	Data Rich	1	Sociodemographic Status
Acute lymphoid leukemia	Male	Post Neonatal	80 plus	Global	-1	Education (years per capita)
Acute lymphoid leukemia	Male	Post Neonatal	80 plus	Global	-1	Health System Access 2 (unitless)
Acute lymphoid leukemia	Male	Post Neonatal	80 plus	Global	-1	LDI (I$ per capita)
Acute lymphoid leukemia	Male	Post Neonatal	80 plus	Global	1	Alcohol (litres per capita)
Acute lymphoid leukemia	Male	Post Neonatal	80 plus	Global	1	Cumulative Cigarettes (10 Years)
Acute lymphoid leukemia	Male	Post Neonatal	80 plus	Global	1	Cumulative Cigarettes (5 Years)
Acute lymphoid leukemia	Male	Post Neonatal	80 plus	Global	1	Log-transformed SEV scalar: Leukemia
Acute lymphoid leukemia	Male	Post Neonatal	80 plus	Global	1	Smoking Prevalence
Acute lymphoid leukemia	Male	Post Neonatal	80 plus	Global	1	Sociodemographic Status
Acute lymphoid leukemia	Male	Post Neonatal	80 plus	Data Rich	-1	Education (years per capita)
Acute lymphoid leukemia	Male	Post Neonatal	80 plus	Data Rich	-1	Health System Access 2 (unitless)

Appendix Table 10: CODEm covariates used, level of covariate, and expected direction of covariate by cause, sex, and age						
**Cause**	**Sex**	**Age start**	**Age end**	**Model type**	**Direction**	**Covariate**
Acute lymphoid leukemia	Male	Post Neonatal	80 plus	Data Rich	-1	LDI (I$ per capita)
Acute lymphoid leukemia	Male	Post Neonatal	80 plus	Data Rich	1	Alcohol (litres per capita)
Acute lymphoid leukemia	Male	Post Neonatal	80 plus	Data Rich	1	Cumulative Cigarettes (10 Years)
Acute lymphoid leukemia	Male	Post Neonatal	80 plus	Data Rich	1	Cumulative Cigarettes (5 Years)
Acute lymphoid leukemia	Male	Post Neonatal	80 plus	Data Rich	1	Log-transformed SEV scalar: Leukemia
Acute lymphoid leukemia	Male	Post Neonatal	80 plus	Data Rich	1	Smoking Prevalence
Acute lymphoid leukemia	Male	Post Neonatal	80 plus	Data Rich	1	Sociodemographic Status
Acute lymphoid leukemia	Female	Post Neonatal	80 plus	Global	-1	Education (years per capita)
Acute lymphoid leukemia	Female	Post Neonatal	80 plus	Global	-1	Health System Access 2 (unitless)
Acute lymphoid leukemia	Female	Post Neonatal	80 plus	Global	-1	LDI (I$ per capita)
Acute lymphoid leukemia	Female	Post Neonatal	80 plus	Global	1	Alcohol (litres per capita)
Acute lymphoid leukemia	Female	Post Neonatal	80 plus	Global	1	Cumulative Cigarettes (10 Years)
Acute lymphoid leukemia	Female	Post Neonatal	80 plus	Global	1	Cumulative Cigarettes (5 Years)
Acute lymphoid leukemia	Female	Post Neonatal	80 plus	Global	1	Log-transformed SEV scalar: Leukemia
Acute lymphoid leukemia	Female	Post Neonatal	80 plus	Global	1	Smoking Prevalence
Acute lymphoid leukemia	Female	Post Neonatal	80 plus	Global	1	Sociodemographic Status
Acute lymphoid leukemia	Female	Post Neonatal	80 plus	Data Rich	-1	Education (years per capita)
Acute lymphoid leukemia	Female	Post Neonatal	80 plus	Data Rich	-1	Health System Access 2 (unitless)
Acute lymphoid leukemia	Female	Post Neonatal	80 plus	Data Rich	-1	LDI (I$ per capita)
Acute lymphoid leukemia	Female	Post Neonatal	80 plus	Data Rich	1	Alcohol (litres per capita)
Acute lymphoid leukemia	Female	Post Neonatal	80 plus	Data Rich	1	Cumulative Cigarettes (10 Years)
Acute lymphoid leukemia	Female	Post Neonatal	80 plus	Data Rich	1	Cumulative Cigarettes (5 Years)
Acute lymphoid leukemia	Female	Post Neonatal	80 plus	Data Rich	1	Log-transformed SEV scalar: Leukemia
Acute lymphoid leukemia	Female	Post Neonatal	80 plus	Data Rich	1	Smoking Prevalence
Acute lymphoid leukemia	Female	Post Neonatal	80 plus	Data Rich	1	Sociodemographic Status
Chronic lymphoid leukemia	Male	15 to 19	80 plus	Global	-1	Education (years per capita)
Chronic lymphoid leukemia	Male	15 to 19	80 plus	Global	-1	Health System Access 2 (unitless)
Chronic lymphoid leukemia	Male	15 to 19	80 plus	Global	-1	LDI (I$ per capita)
Chronic lymphoid leukemia	Male	15 to 19	80 plus	Global	1	Alcohol (litres per capita)
Chronic lymphoid leukemia	Male	15 to 19	80 plus	Global	1	Cumulative Cigarettes (10 Years)
Chronic lymphoid leukemia	Male	15 to 19	80 plus	Global	1	Cumulative Cigarettes (5 Years)
Chronic lymphoid leukemia	Male	15 to 19	80 plus	Global	1	Log-transformed SEV scalar: Leukemia
Chronic lymphoid leukemia	Male	15 to 19	80 plus	Global	1	Smoking Prevalence
Chronic lymphoid leukemia	Male	15 to 19	80 plus	Global	1	Sociodemographic Status
Chronic lymphoid leukemia	Male	15 to 19	80 plus	Data Rich	-1	Education (years per capita)
Chronic lymphoid leukemia	Male	15 to 19	80 plus	Data Rich	-1	Health System Access 2 (unitless)
Chronic lymphoid leukemia	Male	15 to 19	80 plus	Data Rich	-1	LDI (I$ per capita)
Chronic lymphoid leukemia	Male	15 to 19	80 plus	Data Rich	1	Alcohol (litres per capita)
Chronic lymphoid leukemia	Male	15 to 19	80 plus	Data Rich	1	Cumulative Cigarettes (10 Years)
Chronic lymphoid leukemia	Male	15 to 19	80 plus	Data Rich	1	Cumulative Cigarettes (5 Years)
Chronic lymphoid leukemia	Male	15 to 19	80 plus	Data Rich	1	Log-transformed SEV scalar: Leukemia
Chronic lymphoid leukemia	Male	15 to 19	80 plus	Data Rich	1	Smoking Prevalence
Chronic lymphoid leukemia	Male	15 to 19	80 plus	Data Rich	1	Sociodemographic Status
Chronic lymphoid leukemia	Female	15 to 19	80 plus	Global	-1	Education (years per capita)

Appendix Table 10: CODEm covariates used, level of covariate, and expected direction of covariate by cause, sex, and age						
**Cause**	**Sex**	**Age start**	**Age end**	**Model type**	**Direction**	**Covariate**
Chronic lymphoid leukemia	Female	15 to 19	80 plus	Global	-1	Health System Access 2 (unitless)
Chronic lymphoid leukemia	Female	15 to 19	80 plus	Global	-1	LDI (I$ per capita)
Chronic lymphoid leukemia	Female	15 to 19	80 plus	Global	1	Alcohol (litres per capita)
Chronic lymphoid leukemia	Female	15 to 19	80 plus	Global	1	Cumulative Cigarettes (10 Years)
Chronic lymphoid leukemia	Female	15 to 19	80 plus	Global	1	Cumulative Cigarettes (5 Years)
Chronic lymphoid leukemia	Female	15 to 19	80 plus	Global	1	Log-transformed SEV scalar: Leukemia
Chronic lymphoid leukemia	Female	15 to 19	80 plus	Global	1	Smoking Prevalence
Chronic lymphoid leukemia	Female	15 to 19	80 plus	Global	1	Sociodemographic Status
Chronic lymphoid leukemia	Female	15 to 19	80 plus	Data Rich	-1	Education (years per capita)
Chronic lymphoid leukemia	Female	15 to 19	80 plus	Data Rich	-1	Health System Access 2 (unitless)
Chronic lymphoid leukemia	Female	15 to 19	80 plus	Data Rich	-1	LDI (I$ per capita)
Chronic lymphoid leukemia	Female	15 to 19	80 plus	Data Rich	1	Alcohol (litres per capita)
Chronic lymphoid leukemia	Female	15 to 19	80 plus	Data Rich	1	Cumulative Cigarettes (10 Years)
Chronic lymphoid leukemia	Female	15 to 19	80 plus	Data Rich	1	Cumulative Cigarettes (5 Years)
Chronic lymphoid leukemia	Female	15 to 19	80 plus	Data Rich	1	Log-transformed SEV scalar: Leukemia
Chronic lymphoid leukemia	Female	15 to 19	80 plus	Data Rich	1	Smoking Prevalence
Chronic lymphoid leukemia	Female	15 to 19	80 plus	Data Rich	1	Sociodemographic Status
Acute myeloid leukemia	Male	Post Neonatal	80 plus	Global	-1	Education (years per capita)
Acute myeloid leukemia	Male	Post Neonatal	80 plus	Global	-1	Health System Access 2 (unitless)
Acute myeloid leukemia	Male	Post Neonatal	80 plus	Global	-1	LDI (I$ per capita)
Acute myeloid leukemia	Male	Post Neonatal	80 plus	Global	1	Alcohol (litres per capita)
Acute myeloid leukemia	Male	Post Neonatal	80 plus	Global	1	Cumulative Cigarettes (10 Years)
Acute myeloid leukemia	Male	Post Neonatal	80 plus	Global	1	Cumulative Cigarettes (5 Years)
Acute myeloid leukemia	Male	Post Neonatal	80 plus	Global	1	Log-transformed SEV scalar: Leukemia
Acute myeloid leukemia	Male	Post Neonatal	80 plus	Global	1	Smoking Prevalence
Acute myeloid leukemia	Male	Post Neonatal	80 plus	Global	1	Sociodemographic Status
Acute myeloid leukemia	Male	Post Neonatal	80 plus	Global	1	Tobacco (cigarettes per capita)
Acute myeloid leukemia	Male	Post Neonatal	80 plus	Data Rich	-1	Education (years per capita)
Acute myeloid leukemia	Male	Post Neonatal	80 plus	Data Rich	-1	Health System Access 2 (unitless)
Acute myeloid leukemia	Male	Post Neonatal	80 plus	Data Rich	-1	LDI (I$ per capita)
Acute myeloid leukemia	Male	Post Neonatal	80 plus	Data Rich	1	Alcohol (litres per capita)
Acute myeloid leukemia	Male	Post Neonatal	80 plus	Data Rich	1	Cumulative Cigarettes (10 Years)
Acute myeloid leukemia	Male	Post Neonatal	80 plus	Data Rich	1	Cumulative Cigarettes (5 Years)
Acute myeloid leukemia	Male	Post Neonatal	80 plus	Data Rich	1	Log-transformed SEV scalar: Leukemia
Acute myeloid leukemia	Male	Post Neonatal	80 plus	Data Rich	1	Smoking Prevalence
Acute myeloid leukemia	Male	Post Neonatal	80 plus	Data Rich	1	Sociodemographic Status
Acute myeloid leukemia	Male	Post Neonatal	80 plus	Data Rich	1	Tobacco (cigarettes per capita)
Acute myeloid leukemia	Female	Post Neonatal	80 plus	Global	-1	Education (years per capita)
Acute myeloid leukemia	Female	Post Neonatal	80 plus	Global	-1	Health System Access 2 (unitless)
Acute myeloid leukemia	Female	Post Neonatal	80 plus	Global	-1	LDI (I$ per capita)
Acute myeloid leukemia	Female	Post Neonatal	80 plus	Global	1	Alcohol (litres per capita)
Acute myeloid leukemia	Female	Post Neonatal	80 plus	Global	1	Cumulative Cigarettes (10 Years)
Acute myeloid leukemia	Female	Post Neonatal	80 plus	Global	1	Cumulative Cigarettes (5 Years)
Acute myeloid leukemia	Female	Post Neonatal	80 plus	Global	1	Log-transformed SEV scalar: Leukemia

Appendix Table 10: CODEm covariates used, level of covariate, and expected direction of covariate by cause, sex, and age						
**Cause**	**Sex**	**Age start**	**Age end**	**Model type**	**Direction**	**Covariate**
Acute myeloid leukemia	Female	Post Neonatal	80 plus	Global	1	Smoking Prevalence
Acute myeloid leukemia	Female	Post Neonatal	80 plus	Global	1	Sociodemographic Status
Acute myeloid leukemia	Female	Post Neonatal	80 plus	Global	1	Tobacco (cigarettes per capita)
Acute myeloid leukemia	Female	Post Neonatal	80 plus	Data Rich	-1	Education (years per capita)
Acute myeloid leukemia	Female	Post Neonatal	80 plus	Data Rich	-1	Health System Access 2 (unitless)
Acute myeloid leukemia	Female	Post Neonatal	80 plus	Data Rich	-1	LDI (I$ per capita)
Acute myeloid leukemia	Female	Post Neonatal	80 plus	Data Rich	1	Alcohol (litres per capita)
Acute myeloid leukemia	Female	Post Neonatal	80 plus	Data Rich	1	Cumulative Cigarettes (10 Years)
Acute myeloid leukemia	Female	Post Neonatal	80 plus	Data Rich	1	Cumulative Cigarettes (5 Years)
Acute myeloid leukemia	Female	Post Neonatal	80 plus	Data Rich	1	Log-transformed SEV scalar: Leukemia
Acute myeloid leukemia	Female	Post Neonatal	80 plus	Data Rich	1	Smoking Prevalence
Acute myeloid leukemia	Female	Post Neonatal	80 plus	Data Rich	1	Sociodemographic Status
Acute myeloid leukemia	Female	Post Neonatal	80 plus	Data Rich	1	Tobacco (cigarettes per capita)
Chronic myeloid leukemia	Male	Post Neonatal	80 plus	Global	-1	Education (years per capita)
Chronic myeloid leukemia	Male	Post Neonatal	80 plus	Global	-1	LDI (I$ per capita)
Chronic myeloid leukemia	Male	Post Neonatal	80 plus	Global	1	Alcohol (litres per capita)
Chronic myeloid leukemia	Male	Post Neonatal	80 plus	Global	1	Cumulative Cigarettes (10 Years)
Chronic myeloid leukemia	Male	Post Neonatal	80 plus	Global	1	Cumulative Cigarettes (5 Years)
Chronic myeloid leukemia	Male	Post Neonatal	80 plus	Global	1	Health System Access 2 (unitless)
Chronic myeloid leukemia	Male	Post Neonatal	80 plus	Global	1	Log-transformed SEV scalar: Leukemia
Chronic myeloid leukemia	Male	Post Neonatal	80 plus	Global	1	Smoking Prevalence
Chronic myeloid leukemia	Male	Post Neonatal	80 plus	Global	1	Sociodemographic Status
Chronic myeloid leukemia	Male	Post Neonatal	80 plus	Data Rich	-1	Education (years per capita)
Chronic myeloid leukemia	Male	Post Neonatal	80 plus	Data Rich	-1	LDI (I$ per capita)
Chronic myeloid leukemia	Male	Post Neonatal	80 plus	Data Rich	1	Alcohol (litres per capita)
Chronic myeloid leukemia	Male	Post Neonatal	80 plus	Data Rich	1	Cumulative Cigarettes (10 Years)
Chronic myeloid leukemia	Male	Post Neonatal	80 plus	Data Rich	1	Cumulative Cigarettes (5 Years)
Chronic myeloid leukemia	Male	Post Neonatal	80 plus	Data Rich	1	Health System Access 2 (unitless)
Chronic myeloid leukemia	Male	Post Neonatal	80 plus	Data Rich	1	Log-transformed SEV scalar: Leukemia
Chronic myeloid leukemia	Male	Post Neonatal	80 plus	Data Rich	1	Smoking Prevalence
Chronic myeloid leukemia	Male	Post Neonatal	80 plus	Data Rich	1	Sociodemographic Status
Chronic myeloid leukemia	Female	Post Neonatal	80 plus	Global	-1	Education (years per capita)
Chronic myeloid leukemia	Female	Post Neonatal	80 plus	Global	-1	LDI (I$ per capita)
Chronic myeloid leukemia	Female	Post Neonatal	80 plus	Global	1	Alcohol (litres per capita)
Chronic myeloid leukemia	Female	Post Neonatal	80 plus	Global	1	Cumulative Cigarettes (10 Years)
Chronic myeloid leukemia	Female	Post Neonatal	80 plus	Global	1	Cumulative Cigarettes (5 Years)
Chronic myeloid leukemia	Female	Post Neonatal	80 plus	Global	1	Health System Access 2 (unitless)
Chronic myeloid leukemia	Female	Post Neonatal	80 plus	Global	1	Log-transformed SEV scalar: Leukemia
Chronic myeloid leukemia	Female	Post Neonatal	80 plus	Global	1	Smoking Prevalence
Chronic myeloid leukemia	Female	Post Neonatal	80 plus	Global	1	Sociodemographic Status
Chronic myeloid leukemia	Female	Post Neonatal	80 plus	Data Rich	-1	Education (years per capita)
Chronic myeloid leukemia	Female	Post Neonatal	80 plus	Data Rich	-1	LDI (I$ per capita)
Chronic myeloid leukemia	Female	Post Neonatal	80 plus	Data Rich	1	Alcohol (litres per capita)
Chronic myeloid leukemia	Female	Post Neonatal	80 plus	Data Rich	1	Cumulative Cigarettes (10 Years)

Appendix Table 10: CODEm covariates used, level of covariate, and expected direction of covariate by cause, sex, and age						
**Cause**	**Sex**	**Age start**	**Age end**	**Model type**	**Direction**	**Covariate**
Chronic myeloid leukemia	Female	Post Neonatal	80 plus	Data Rich	1	Cumulative Cigarettes (5 Years)
Chronic myeloid leukemia	Female	Post Neonatal	80 plus	Data Rich	1	Health System Access 2 (unitless)
Chronic myeloid leukemia	Female	Post Neonatal	80 plus	Data Rich	1	Log-transformed SEV scalar: Leukemia
Chronic myeloid leukemia	Female	Post Neonatal	80 plus	Data Rich	1	Smoking Prevalence
Chronic myeloid leukemia	Female	Post Neonatal	80 plus	Data Rich	1	Sociodemographic Status
Other neoplasms	Male	Early Neonatal	80 plus	Global	-1	Education (years per capita)
Other neoplasms	Male	Early Neonatal	80 plus	Global	-1	Fruits (kcal per capita)
Other neoplasms	Male	Early Neonatal	80 plus	Global	-1	Health System Access 2 (unitless)
Other neoplasms	Male	Early Neonatal	80 plus	Global	-1	LDI (I$ per capita)
Other neoplasms	Male	Early Neonatal	80 plus	Global	-1	Nuts & Seeds (kcal per capita)
Other neoplasms	Male	Early Neonatal	80 plus	Global	-1	PUFA Omega 3 - Seafood (kcal per capita)
Other neoplasms	Male	Early Neonatal	80 plus	Global	-1	Vegetables (kcal per capita)
Other neoplasms	Male	Early Neonatal	80 plus	Global	0	Sociodemographic Status
Other neoplasms	Male	Early Neonatal	80 plus	Global	1	Smoking Prevalence
Other neoplasms	Male	Early Neonatal	80 plus	Global	1	Tobacco (cigarettes per capita)
Other neoplasms	Male	Early Neonatal	80 plus	Data Rich	-1	Education (years per capita)
Other neoplasms	Male	Early Neonatal	80 plus	Data Rich	-1	Fruits (kcal per capita)
Other neoplasms	Male	Early Neonatal	80 plus	Data Rich	-1	Health System Access 2 (unitless)
Other neoplasms	Male	Early Neonatal	80 plus	Data Rich	-1	LDI (I$ per capita)
Other neoplasms	Male	Early Neonatal	80 plus	Data Rich	-1	Nuts & Seeds (kcal per capita)
Other neoplasms	Male	Early Neonatal	80 plus	Data Rich	-1	PUFA Omega 3 - Seafood (kcal per capita)
Other neoplasms	Male	Early Neonatal	80 plus	Data Rich	-1	Vegetables (kcal per capita)
Other neoplasms	Male	Early Neonatal	80 plus	Data Rich	0	Sociodemographic Status
Other neoplasms	Male	Early Neonatal	80 plus	Data Rich	1	Smoking Prevalence
Other neoplasms	Male	Early Neonatal	80 plus	Data Rich	1	Tobacco (cigarettes per capita)
Other neoplasms	Female	Early Neonatal	80 plus	Global	-1	Education (years per capita)
Other neoplasms	Female	Early Neonatal	80 plus	Global	-1	Fruits (kcal per capita)
Other neoplasms	Female	Early Neonatal	80 plus	Global	-1	Health System Access 2 (unitless)
Other neoplasms	Female	Early Neonatal	80 plus	Global	-1	LDI (I$ per capita)
Other neoplasms	Female	Early Neonatal	80 plus	Global	-1	Nuts & Seeds (kcal per capita)
Other neoplasms	Female	Early Neonatal	80 plus	Global	-1	PUFA Omega 3 - Seafood (kcal per capita)
Other neoplasms	Female	Early Neonatal	80 plus	Global	-1	Vegetables (kcal per capita)
Other neoplasms	Female	Early Neonatal	80 plus	Global	0	Sociodemographic Status
Other neoplasms	Female	Early Neonatal	80 plus	Global	1	Smoking Prevalence
Other neoplasms	Female	Early Neonatal	80 plus	Global	1	Tobacco (cigarettes per capita)
Other neoplasms	Female	Early Neonatal	80 plus	Data Rich	-1	Education (years per capita)
Other neoplasms	Female	Early Neonatal	80 plus	Data Rich	-1	Fruits (kcal per capita)
Other neoplasms	Female	Early Neonatal	80 plus	Data Rich	-1	Health System Access 2 (unitless)
Other neoplasms	Female	Early Neonatal	80 plus	Data Rich	-1	Nuts & Seeds (kcal per capita)
Other neoplasms	Female	Early Neonatal	80 plus	Data Rich	-1	PUFA Omega 3 - Seafood (kcal per capita)
Other neoplasms	Female	Early Neonatal	80 plus	Data Rich	-1	Vegetables (kcal per capita)
Other neoplasms	Female	Early Neonatal	80 plus	Data Rich	0	LDI (I$ per capita)
Other neoplasms	Female	Early Neonatal	80 plus	Data Rich	0	Sociodemographic Status
Other neoplasms	Female	Early Neonatal	80 plus	Data Rich	1	Smoking Prevalence

Appendix Table 10: CODEm covariates used, level of covariate, and expected direction of covariate by cause, sex, and age						
**Cause**	**Sex**	**Age start**	**Age end**	**Model type**	**Direction**	**Covariate**
Other neoplasms	Female	Early Neonatal	80 plus	Data Rich	1	Tobacco (cigarettes per capita)
Cardiovascular diseases	Male	Early Neonatal	80 plus	Global	-1	LDI (I$ per capita)
Cardiovascular diseases	Male	Early Neonatal	80 plus	Global	-1	omega 3 adjusted(g)
Cardiovascular diseases	Male	Early Neonatal	80 plus	Global	-1	vegetables adjusted(g)
Cardiovascular diseases	Male	Early Neonatal	80 plus	Global	0	Alcohol (litres per capita)
Cardiovascular diseases	Male	Early Neonatal	80 plus	Global	0	Sociodemographic Status
Cardiovascular diseases	Male	Early Neonatal	80 plus	Global	1	Animal Fats (kcal per capita)
Cardiovascular diseases	Male	Early Neonatal	80 plus	Global	1	Cholesterol (total, mean per capita)
Cardiovascular diseases	Male	Early Neonatal	80 plus	Global	1	Elevation Under 100m (proportion)
Cardiovascular diseases	Male	Early Neonatal	80 plus	Global	1	Smoking Prevalence
Cardiovascular diseases	Male	Early Neonatal	80 plus	Global	1	red meats adjusted(g)
Cardiovascular diseases	Male	Early Neonatal	80 plus	Data Rich	-1	LDI (I$ per capita)
Cardiovascular diseases	Male	Early Neonatal	80 plus	Data Rich	-1	omega 3 adjusted(g)
Cardiovascular diseases	Male	Early Neonatal	80 plus	Data Rich	-1	vegetables adjusted(g)
Cardiovascular diseases	Male	Early Neonatal	80 plus	Data Rich	0	Alcohol (litres per capita)
Cardiovascular diseases	Male	Early Neonatal	80 plus	Data Rich	0	Sociodemographic Status
Cardiovascular diseases	Male	Early Neonatal	80 plus	Data Rich	1	Animal Fats (kcal per capita)
Cardiovascular diseases	Male	Early Neonatal	80 plus	Data Rich	1	Cholesterol (total, mean per capita)
Cardiovascular diseases	Male	Early Neonatal	80 plus	Data Rich	1	Elevation Under 100m (proportion)
Cardiovascular diseases	Male	Early Neonatal	80 plus	Data Rich	1	Smoking Prevalence
Cardiovascular diseases	Male	Early Neonatal	80 plus	Data Rich	1	red meats adjusted(g)
Cardiovascular diseases	Female	Early Neonatal	80 plus	Global	-1	LDI (I$ per capita)
Cardiovascular diseases	Female	Early Neonatal	80 plus	Global	-1	omega 3 adjusted(g)
Cardiovascular diseases	Female	Early Neonatal	80 plus	Global	-1	vegetables adjusted(g)
Cardiovascular diseases	Female	Early Neonatal	80 plus	Global	0	Alcohol (litres per capita)
Cardiovascular diseases	Female	Early Neonatal	80 plus	Global	0	Sociodemographic Status
Cardiovascular diseases	Female	Early Neonatal	80 plus	Global	1	Animal Fats (kcal per capita)
Cardiovascular diseases	Female	Early Neonatal	80 plus	Global	1	Cholesterol (total, mean per capita)
Cardiovascular diseases	Female	Early Neonatal	80 plus	Global	1	Elevation Under 100m (proportion)
Cardiovascular diseases	Female	Early Neonatal	80 plus	Global	1	Smoking Prevalence
Cardiovascular diseases	Female	Early Neonatal	80 plus	Global	1	red meats adjusted(g)
Cardiovascular diseases	Female	Early Neonatal	80 plus	Data Rich	-1	LDI (I$ per capita)
Cardiovascular diseases	Female	Early Neonatal	80 plus	Data Rich	-1	omega 3 adjusted(g)
Cardiovascular diseases	Female	Early Neonatal	80 plus	Data Rich	-1	vegetables adjusted(g)
Cardiovascular diseases	Female	Early Neonatal	80 plus	Data Rich	0	Alcohol (litres per capita)
Cardiovascular diseases	Female	Early Neonatal	80 plus	Data Rich	0	Sociodemographic Status
Cardiovascular diseases	Female	Early Neonatal	80 plus	Data Rich	1	Animal Fats (kcal per capita)
Cardiovascular diseases	Female	Early Neonatal	80 plus	Data Rich	1	Cholesterol (total, mean per capita)
Cardiovascular diseases	Female	Early Neonatal	80 plus	Data Rich	1	Elevation Under 100m (proportion)
Cardiovascular diseases	Female	Early Neonatal	80 plus	Data Rich	1	Smoking Prevalence
Cardiovascular diseases	Female	Early Neonatal	80 plus	Data Rich	1	red meats adjusted(g)
Rheumatic heart disease	Male	1 to 4	80 plus	Global	-1	Education (years per capita)
Rheumatic heart disease	Male	1 to 4	80 plus	Global	-1	Health System Access 2 (unitless)
Rheumatic heart disease	Male	1 to 4	80 plus	Global	-1	Improved Water Source (proportion with access)

Appendix Table 10: CODEm covariates used, level of covariate, and expected direction of covariate by cause, sex, and age						
**Cause**	**Sex**	**Age start**	**Age end**	**Model type**	**Direction**	**Covariate**
Rheumatic heart disease	Male	1 to 4	80 plus	Global	-1	LDI (I$ per capita)
Rheumatic heart disease	Male	1 to 4	80 plus	Global	-1	Sanitation (proportion with access)
Rheumatic heart disease	Male	1 to 4	80 plus	Global	-1	Sociodemographic Status
Rheumatic heart disease	Male	1 to 4	80 plus	Global	1	Log-transformed SEV scalar: RHD
Rheumatic heart disease	Male	1 to 4	80 plus	Global	1	Malnutrition (proportion <2SD weight for age)
Rheumatic heart disease	Male	1 to 4	80 plus	Global	1	Population Under 30 (proportion)
Rheumatic heart disease	Male	1 to 4	80 plus	Data Rich	-1	Education (years per capita)
Rheumatic heart disease	Male	1 to 4	80 plus	Data Rich	-1	Health System Access 2 (unitless)
Rheumatic heart disease	Male	1 to 4	80 plus	Data Rich	-1	Improved Water Source (proportion with access)
Rheumatic heart disease	Male	1 to 4	80 plus	Data Rich	-1	LDI (I$ per capita)
Rheumatic heart disease	Male	1 to 4	80 plus	Data Rich	-1	Sanitation (proportion with access)
Rheumatic heart disease	Male	1 to 4	80 plus	Data Rich	-1	Sociodemographic Status
Rheumatic heart disease	Male	1 to 4	80 plus	Data Rich	1	Log-transformed SEV scalar: RHD
Rheumatic heart disease	Male	1 to 4	80 plus	Data Rich	1	Malnutrition (proportion <2SD weight for age)
Rheumatic heart disease	Male	1 to 4	80 plus	Data Rich	1	Population Under 30 (proportion)
Rheumatic heart disease	Female	1 to 4	80 plus	Global	-1	Education (years per capita)
Rheumatic heart disease	Female	1 to 4	80 plus	Global	-1	Health System Access 2 (unitless)
Rheumatic heart disease	Female	1 to 4	80 plus	Global	-1	Improved Water Source (proportion with access)
Rheumatic heart disease	Female	1 to 4	80 plus	Global	-1	LDI (I$ per capita)
Rheumatic heart disease	Female	1 to 4	80 plus	Global	-1	Sanitation (proportion with access)
Rheumatic heart disease	Female	1 to 4	80 plus	Global	-1	Sociodemographic Status
Rheumatic heart disease	Female	1 to 4	80 plus	Global	1	Log-transformed SEV scalar: RHD
Rheumatic heart disease	Female	1 to 4	80 plus	Global	1	Malnutrition (proportion <2SD weight for age)
Rheumatic heart disease	Female	1 to 4	80 plus	Global	1	Population Under 30 (proportion)
Rheumatic heart disease	Female	1 to 4	80 plus	Data Rich	-1	Education (years per capita)
Rheumatic heart disease	Female	1 to 4	80 plus	Data Rich	-1	Health System Access 2 (unitless)
Rheumatic heart disease	Female	1 to 4	80 plus	Data Rich	-1	Improved Water Source (proportion with access)
Rheumatic heart disease	Female	1 to 4	80 plus	Data Rich	-1	LDI (I$ per capita)
Rheumatic heart disease	Female	1 to 4	80 plus	Data Rich	-1	Sanitation (proportion with access)
Rheumatic heart disease	Female	1 to 4	80 plus	Data Rich	-1	Sociodemographic Status
Rheumatic heart disease	Female	1 to 4	80 plus	Data Rich	1	Log-transformed SEV scalar: RHD
Rheumatic heart disease	Female	1 to 4	80 plus	Data Rich	1	Malnutrition (proportion <2SD weight for age)
Rheumatic heart disease	Female	1 to 4	80 plus	Data Rich	1	Population Under 30 (proportion)
Ischemic heart disease	Male	Post Neonatal	80 plus	Global	-1	LDI (I$ per capita)
Ischemic heart disease	Male	Post Neonatal	80 plus	Global	-1	omega 3 adjusted(g)
Ischemic heart disease	Male	Post Neonatal	80 plus	Global	-1	pulses legumes adjusted(g)
Ischemic heart disease	Male	Post Neonatal	80 plus	Global	-1	vegetables adjusted(g)
Ischemic heart disease	Male	Post Neonatal	80 plus	Global	0	Alcohol (litres per capita)
Ischemic heart disease	Male	Post Neonatal	80 plus	Global	0	Sociodemographic Status
Ischemic heart disease	Male	Post Neonatal	80 plus	Global	1	Animal Fats (kcal per capita)
Ischemic heart disease	Male	Post Neonatal	80 plus	Global	1	Cholesterol (total, mean per capita)
Ischemic heart disease	Male	Post Neonatal	80 plus	Global	1	Elevation Under 100m (proportion)
Ischemic heart disease	Male	Post Neonatal	80 plus	Global	1	Log-transformed SEV scalar: IHD
Ischemic heart disease	Male	Post Neonatal	80 plus	Global	1	Smoking Prevalence

Appendix Table 10: CODEm covariates used, level of covariate, and expected direction of covariate by cause, sex, and age						
**Cause**	**Sex**	**Age start**	**Age end**	**Model type**	**Direction**	**Covariate**
Ischemic heart disease	Male	Post Neonatal	80 plus	Global	1	red meats adjusted(g)
Ischemic heart disease	Male	Post Neonatal	80 plus	Data Rich	-1	LDI (I$ per capita)
Ischemic heart disease	Male	Post Neonatal	80 plus	Data Rich	-1	omega 3 adjusted(g)
Ischemic heart disease	Male	Post Neonatal	80 plus	Data Rich	-1	pulses legumes adjusted(g)
Ischemic heart disease	Male	Post Neonatal	80 plus	Data Rich	-1	vegetables adjusted(g)
Ischemic heart disease	Male	Post Neonatal	80 plus	Data Rich	0	Alcohol (litres per capita)
Ischemic heart disease	Male	Post Neonatal	80 plus	Data Rich	0	Sociodemographic Status
Ischemic heart disease	Male	Post Neonatal	80 plus	Data Rich	1	Animal Fats (kcal per capita)
Ischemic heart disease	Male	Post Neonatal	80 plus	Data Rich	1	Cholesterol (total, mean per capita)
Ischemic heart disease	Male	Post Neonatal	80 plus	Data Rich	1	Elevation Under 100m (proportion)
Ischemic heart disease	Male	Post Neonatal	80 plus	Data Rich	1	Log-transformed SEV scalar: IHD
Ischemic heart disease	Male	Post Neonatal	80 plus	Data Rich	1	Smoking Prevalence
Ischemic heart disease	Male	Post Neonatal	80 plus	Data Rich	1	red meats adjusted(g)
Ischemic heart disease	Female	Post Neonatal	80 plus	Global	-1	LDI (I$ per capita)
Ischemic heart disease	Female	Post Neonatal	80 plus	Global	-1	omega 3 adjusted(g)
Ischemic heart disease	Female	Post Neonatal	80 plus	Global	-1	pulses legumes adjusted(g)
Ischemic heart disease	Female	Post Neonatal	80 plus	Global	-1	vegetables adjusted(g)
Ischemic heart disease	Female	Post Neonatal	80 plus	Global	0	Alcohol (litres per capita)
Ischemic heart disease	Female	Post Neonatal	80 plus	Global	0	Sociodemographic Status
Ischemic heart disease	Female	Post Neonatal	80 plus	Global	1	Animal Fats (kcal per capita)
Ischemic heart disease	Female	Post Neonatal	80 plus	Global	1	Cholesterol (total, mean per capita)
Ischemic heart disease	Female	Post Neonatal	80 plus	Global	1	Elevation Under 100m (proportion)
Ischemic heart disease	Female	Post Neonatal	80 plus	Global	1	Log-transformed SEV scalar: IHD
Ischemic heart disease	Female	Post Neonatal	80 plus	Global	1	Smoking Prevalence
Ischemic heart disease	Female	Post Neonatal	80 plus	Global	1	red meats adjusted(g)
Ischemic heart disease	Female	Post Neonatal	80 plus	Data Rich	-1	LDI (I$ per capita)
Ischemic heart disease	Female	Post Neonatal	80 plus	Data Rich	-1	omega 3 adjusted(g)
Ischemic heart disease	Female	Post Neonatal	80 plus	Data Rich	-1	pulses legumes adjusted(g)
Ischemic heart disease	Female	Post Neonatal	80 plus	Data Rich	-1	vegetables adjusted(g)
Ischemic heart disease	Female	Post Neonatal	80 plus	Data Rich	0	Alcohol (litres per capita)
Ischemic heart disease	Female	Post Neonatal	80 plus	Data Rich	0	Sociodemographic Status
Ischemic heart disease	Female	Post Neonatal	80 plus	Data Rich	1	Animal Fats (kcal per capita)
Ischemic heart disease	Female	Post Neonatal	80 plus	Data Rich	1	Cholesterol (total, mean per capita)
Ischemic heart disease	Female	Post Neonatal	80 plus	Data Rich	1	Elevation Under 100m (proportion)
Ischemic heart disease	Female	Post Neonatal	80 plus	Data Rich	1	Log-transformed SEV scalar: IHD
Ischemic heart disease	Female	Post Neonatal	80 plus	Data Rich	1	Smoking Prevalence
Ischemic heart disease	Female	Post Neonatal	80 plus	Data Rich	1	red meats adjusted(g)
Cerebrovascular disease	Male	Early Neonatal	80 plus	Global	-1	Education (years per capita)
Cerebrovascular disease	Male	Early Neonatal	80 plus	Global	-1	LDI (I$ per capita)
Cerebrovascular disease	Male	Early Neonatal	80 plus	Global	-1	fruits adjusted(g)
Cerebrovascular disease	Male	Early Neonatal	80 plus	Global	-1	vegetables adjusted(g)
Cerebrovascular disease	Male	Early Neonatal	80 plus	Global	-1	whole grains adjusted(g)
Cerebrovascular disease	Male	Early Neonatal	80 plus	Global	0	Alcohol (litres per capita)
Cerebrovascular disease	Male	Early Neonatal	80 plus	Global	0	Sociodemographic Status

Appendix Table 10: CODEm covariates used, level of covariate, and expected direction of covariate by cause, sex, and age						
**Cause**	**Sex**	**Age start**	**Age end**	**Model type**	**Direction**	**Covariate**
Cerebrovascular disease	Male	Early Neonatal	80 plus	Global	1	Cholesterol (total, mean per capita)
Cerebrovascular disease	Male	Early Neonatal	80 plus	Global	1	Cumulative Cigarettes (5 Years)
Cerebrovascular disease	Male	Early Neonatal	80 plus	Global	1	Diabetes Age-Standardised Prevalence (proportion)
Cerebrovascular disease	Male	Early Neonatal	80 plus	Global	1	Log-transformed SEV scalar: Stroke
Cerebrovascular disease	Male	Early Neonatal	80 plus	Global	1	Mean BMI
Cerebrovascular disease	Male	Early Neonatal	80 plus	Global	1	Systolic Blood Pressure (mmHg)
Cerebrovascular disease	Male	Early Neonatal	80 plus	Global	1	red meats adjusted(g)
Cerebrovascular disease	Male	Early Neonatal	80 plus	Data Rich	-1	Education (years per capita)
Cerebrovascular disease	Male	Early Neonatal	80 plus	Data Rich	-1	LDI (I$ per capita)
Cerebrovascular disease	Male	Early Neonatal	80 plus	Data Rich	-1	fruits adjusted(g)
Cerebrovascular disease	Male	Early Neonatal	80 plus	Data Rich	-1	vegetables adjusted(g)
Cerebrovascular disease	Male	Early Neonatal	80 plus	Data Rich	-1	whole grains adjusted(g)
Cerebrovascular disease	Male	Early Neonatal	80 plus	Data Rich	0	Alcohol (litres per capita)
Cerebrovascular disease	Male	Early Neonatal	80 plus	Data Rich	0	Sociodemographic Status
Cerebrovascular disease	Male	Early Neonatal	80 plus	Data Rich	1	Cholesterol (total, mean per capita)
Cerebrovascular disease	Male	Early Neonatal	80 plus	Data Rich	1	Cumulative Cigarettes (5 Years)
Cerebrovascular disease	Male	Early Neonatal	80 plus	Data Rich	1	Diabetes Age-Standardised Prevalence (proportion)
Cerebrovascular disease	Male	Early Neonatal	80 plus	Data Rich	1	Log-transformed SEV scalar: Stroke
Cerebrovascular disease	Male	Early Neonatal	80 plus	Data Rich	1	Mean BMI
Cerebrovascular disease	Male	Early Neonatal	80 plus	Data Rich	1	Systolic Blood Pressure (mmHg)
Cerebrovascular disease	Male	Early Neonatal	80 plus	Data Rich	1	red meats adjusted(g)
Cerebrovascular disease	Female	Early Neonatal	80 plus	Global	-1	Education (years per capita)
Cerebrovascular disease	Female	Early Neonatal	80 plus	Global	-1	LDI (I$ per capita)
Cerebrovascular disease	Female	Early Neonatal	80 plus	Global	-1	fruits adjusted(g)
Cerebrovascular disease	Female	Early Neonatal	80 plus	Global	-1	vegetables adjusted(g)
Cerebrovascular disease	Female	Early Neonatal	80 plus	Global	-1	whole grains adjusted(g)
Cerebrovascular disease	Female	Early Neonatal	80 plus	Global	0	Alcohol (litres per capita)
Cerebrovascular disease	Female	Early Neonatal	80 plus	Global	0	Sociodemographic Status
Cerebrovascular disease	Female	Early Neonatal	80 plus	Global	1	Cholesterol (total, mean per capita)
Cerebrovascular disease	Female	Early Neonatal	80 plus	Global	1	Cumulative Cigarettes (5 Years)
Cerebrovascular disease	Female	Early Neonatal	80 plus	Global	1	Diabetes Age-Standardised Prevalence (proportion)
Cerebrovascular disease	Female	Early Neonatal	80 plus	Global	1	Log-transformed SEV scalar: Stroke
Cerebrovascular disease	Female	Early Neonatal	80 plus	Global	1	Mean BMI
Cerebrovascular disease	Female	Early Neonatal	80 plus	Global	1	Systolic Blood Pressure (mmHg)
Cerebrovascular disease	Female	Early Neonatal	80 plus	Global	1	red meats adjusted(g)
Cerebrovascular disease	Female	Early Neonatal	80 plus	Data Rich	-1	Education (years per capita)
Cerebrovascular disease	Female	Early Neonatal	80 plus	Data Rich	-1	LDI (I$ per capita)
Cerebrovascular disease	Female	Early Neonatal	80 plus	Data Rich	-1	fruits adjusted(g)
Cerebrovascular disease	Female	Early Neonatal	80 plus	Data Rich	-1	vegetables adjusted(g)
Cerebrovascular disease	Female	Early Neonatal	80 plus	Data Rich	-1	whole grains adjusted(g)
Cerebrovascular disease	Female	Early Neonatal	80 plus	Data Rich	0	Alcohol (litres per capita)
Cerebrovascular disease	Female	Early Neonatal	80 plus	Data Rich	0	Sociodemographic Status
Cerebrovascular disease	Female	Early Neonatal	80 plus	Data Rich	1	Cholesterol (total, mean per capita)
Cerebrovascular disease	Female	Early Neonatal	80 plus	Data Rich	1	Cumulative Cigarettes (5 Years)

Appendix Table 10: CODEm covariates used, level of covariate, and expected direction of covariate by cause, sex, and age						
**Cause**	**Sex**	**Age start**	**Age end**	**Model type**	**Direction**	**Covariate**
Cerebrovascular disease	Female	Early Neonatal	80 plus	Data Rich	1	Diabetes Age-Standardised Prevalence (proportion)
Cerebrovascular disease	Female	Early Neonatal	80 plus	Data Rich	1	Log-transformed SEV scalar: Stroke
Cerebrovascular disease	Female	Early Neonatal	80 plus	Data Rich	1	Mean BMI
Cerebrovascular disease	Female	Early Neonatal	80 plus	Data Rich	1	Systolic Blood Pressure (mmHg)
Cerebrovascular disease	Female	Early Neonatal	80 plus	Data Rich	1	red meats adjusted(g)
Ischemic stroke	Male	Post Neonatal	80 plus	Global	-1	Education (years per capita)
Ischemic stroke	Male	Post Neonatal	80 plus	Global	-1	Health System Access 2 (unitless)
Ischemic stroke	Male	Post Neonatal	80 plus	Global	-1	LDI (I$ per capita)
Ischemic stroke	Male	Post Neonatal	80 plus	Global	-1	Nuts & Seeds (kcal per capita)
Ischemic stroke	Male	Post Neonatal	80 plus	Global	-1	PUFA Omega 3 - Seafood (kcal per capita)
Ischemic stroke	Male	Post Neonatal	80 plus	Global	-1	fruits adjusted(g)
Ischemic stroke	Male	Post Neonatal	80 plus	Global	-1	milk adjusted(g)
Ischemic stroke	Male	Post Neonatal	80 plus	Global	-1	nuts seeds adjusted(g)
Ischemic stroke	Male	Post Neonatal	80 plus	Global	-1	omega 3 adjusted(g)
Ischemic stroke	Male	Post Neonatal	80 plus	Global	-1	pulses legumes adjusted(g)
Ischemic stroke	Male	Post Neonatal	80 plus	Global	-1	vegetables adjusted(g)
Ischemic stroke	Male	Post Neonatal	80 plus	Global	0	Alcohol (litres per capita)
Ischemic stroke	Male	Post Neonatal	80 plus	Global	0	Sociodemographic Status
Ischemic stroke	Male	Post Neonatal	80 plus	Global	1	Animal Fats (kcal per capita)
Ischemic stroke	Male	Post Neonatal	80 plus	Global	1	Cholesterol (total, mean per capita)
Ischemic stroke	Male	Post Neonatal	80 plus	Global	1	Log-transformed SEV scalar: Isch Stroke
Ischemic stroke	Male	Post Neonatal	80 plus	Global	1	Smoking Prevalence
Ischemic stroke	Male	Post Neonatal	80 plus	Data Rich	-1	Education (years per capita)
Ischemic stroke	Male	Post Neonatal	80 plus	Data Rich	-1	Health System Access 2 (unitless)
Ischemic stroke	Male	Post Neonatal	80 plus	Data Rich	-1	LDI (I$ per capita)
Ischemic stroke	Male	Post Neonatal	80 plus	Data Rich	-1	Nuts & Seeds (kcal per capita)
Ischemic stroke	Male	Post Neonatal	80 plus	Data Rich	-1	PUFA Omega 3 - Seafood (kcal per capita)
Ischemic stroke	Male	Post Neonatal	80 plus	Data Rich	-1	fruits adjusted(g)
Ischemic stroke	Male	Post Neonatal	80 plus	Data Rich	-1	milk adjusted(g)
Ischemic stroke	Male	Post Neonatal	80 plus	Data Rich	-1	nuts seeds adjusted(g)
Ischemic stroke	Male	Post Neonatal	80 plus	Data Rich	-1	omega 3 adjusted(g)
Ischemic stroke	Male	Post Neonatal	80 plus	Data Rich	-1	pulses legumes adjusted(g)
Ischemic stroke	Male	Post Neonatal	80 plus	Data Rich	-1	vegetables adjusted(g)
Ischemic stroke	Male	Post Neonatal	80 plus	Data Rich	0	Alcohol (litres per capita)
Ischemic stroke	Male	Post Neonatal	80 plus	Data Rich	0	Sociodemographic Status
Ischemic stroke	Male	Post Neonatal	80 plus	Data Rich	1	Animal Fats (kcal per capita)
Ischemic stroke	Male	Post Neonatal	80 plus	Data Rich	1	Cholesterol (total, mean per capita)
Ischemic stroke	Male	Post Neonatal	80 plus	Data Rich	1	Log-transformed SEV scalar: Isch Stroke
Ischemic stroke	Male	Post Neonatal	80 plus	Data Rich	1	Smoking Prevalence
Ischemic stroke	Female	Post Neonatal	80 plus	Global	-1	Education (years per capita)
Ischemic stroke	Female	Post Neonatal	80 plus	Global	-1	Health System Access 2 (unitless)
Ischemic stroke	Female	Post Neonatal	80 plus	Global	-1	LDI (I$ per capita)
Ischemic stroke	Female	Post Neonatal	80 plus	Global	-1	Nuts & Seeds (kcal per capita)
Ischemic stroke	Female	Post Neonatal	80 plus	Global	-1	PUFA Omega 3 - Seafood (kcal per capita)

Appendix Table 10: CODEm covariates used, level of covariate, and expected direction of covariate by cause, sex, and age						
**Cause**	**Sex**	**Age start**	**Age end**	**Model type**	**Direction**	**Covariate**
Ischemic stroke	Female	Post Neonatal	80 plus	Global	-1	fruits adjusted(g)
Ischemic stroke	Female	Post Neonatal	80 plus	Global	-1	milk adjusted(g)
Ischemic stroke	Female	Post Neonatal	80 plus	Global	-1	nuts seeds adjusted(g)
Ischemic stroke	Female	Post Neonatal	80 plus	Global	-1	omega 3 adjusted(g)
Ischemic stroke	Female	Post Neonatal	80 plus	Global	-1	pulses legumes adjusted(g)
Ischemic stroke	Female	Post Neonatal	80 plus	Global	-1	vegetables adjusted(g)
Ischemic stroke	Female	Post Neonatal	80 plus	Global	0	Alcohol (litres per capita)
Ischemic stroke	Female	Post Neonatal	80 plus	Global	0	Sociodemographic Status
Ischemic stroke	Female	Post Neonatal	80 plus	Global	1	Animal Fats (kcal per capita)
Ischemic stroke	Female	Post Neonatal	80 plus	Global	1	Cholesterol (total, mean per capita)
Ischemic stroke	Female	Post Neonatal	80 plus	Global	1	Log-transformed SEV scalar: Isch Stroke
Ischemic stroke	Female	Post Neonatal	80 plus	Global	1	Smoking Prevalence
Ischemic stroke	Female	Post Neonatal	80 plus	Data Rich	-1	Education (years per capita)
Ischemic stroke	Female	Post Neonatal	80 plus	Data Rich	-1	Health System Access 2 (unitless)
Ischemic stroke	Female	Post Neonatal	80 plus	Data Rich	-1	LDI (I$ per capita)
Ischemic stroke	Female	Post Neonatal	80 plus	Data Rich	-1	Nuts & Seeds (kcal per capita)
Ischemic stroke	Female	Post Neonatal	80 plus	Data Rich	-1	PUFA Omega 3 - Seafood (kcal per capita)
Ischemic stroke	Female	Post Neonatal	80 plus	Data Rich	-1	fruits adjusted(g)
Ischemic stroke	Female	Post Neonatal	80 plus	Data Rich	-1	milk adjusted(g)
Ischemic stroke	Female	Post Neonatal	80 plus	Data Rich	-1	nuts seeds adjusted(g)
Ischemic stroke	Female	Post Neonatal	80 plus	Data Rich	-1	omega 3 adjusted(g)
Ischemic stroke	Female	Post Neonatal	80 plus	Data Rich	-1	pulses legumes adjusted(g)
Ischemic stroke	Female	Post Neonatal	80 plus	Data Rich	-1	vegetables adjusted(g)
Ischemic stroke	Female	Post Neonatal	80 plus	Data Rich	0	Alcohol (litres per capita)
Ischemic stroke	Female	Post Neonatal	80 plus	Data Rich	0	Sociodemographic Status
Ischemic stroke	Female	Post Neonatal	80 plus	Data Rich	1	Animal Fats (kcal per capita)
Ischemic stroke	Female	Post Neonatal	80 plus	Data Rich	1	Cholesterol (total, mean per capita)
Ischemic stroke	Female	Post Neonatal	80 plus	Data Rich	1	Log-transformed SEV scalar: Isch Stroke
Ischemic stroke	Female	Post Neonatal	80 plus	Data Rich	1	Smoking Prevalence
Hemorrhagic stroke	Male	Early Neonatal	80 plus	Global	-1	Education (years per capita)
Hemorrhagic stroke	Male	Early Neonatal	80 plus	Global	-1	LDI (I$ per capita)
Hemorrhagic stroke	Male	Early Neonatal	80 plus	Global	-1	milk adjusted(g)
Hemorrhagic stroke	Male	Early Neonatal	80 plus	Global	-1	nuts seeds adjusted(g)
Hemorrhagic stroke	Male	Early Neonatal	80 plus	Global	-1	omega 3 adjusted(g)
Hemorrhagic stroke	Male	Early Neonatal	80 plus	Global	-1	pufa adjusted(percent)
Hemorrhagic stroke	Male	Early Neonatal	80 plus	Global	-1	pulses legumes adjusted(g)
Hemorrhagic stroke	Male	Early Neonatal	80 plus	Global	-1	vegetables adjusted(g)
Hemorrhagic stroke	Male	Early Neonatal	80 plus	Global	0	Alcohol (litres per capita)
Hemorrhagic stroke	Male	Early Neonatal	80 plus	Global	0	Sociodemographic Status
Hemorrhagic stroke	Male	Early Neonatal	80 plus	Global	1	Log-transformed SEV scalar: Hem Stroke
Hemorrhagic stroke	Male	Early Neonatal	80 plus	Global	1	Smoking Prevalence
Hemorrhagic stroke	Male	Early Neonatal	80 plus	Data Rich	-1	Education (years per capita)
Hemorrhagic stroke	Male	Early Neonatal	80 plus	Data Rich	-1	LDI (I$ per capita)
Hemorrhagic stroke	Male	Early Neonatal	80 plus	Data Rich	-1	milk adjusted(g)

Appendix Table 10: CODEm covariates used, level of covariate, and expected direction of covariate by cause, sex, and age						
**Cause**	**Sex**	**Age start**	**Age end**	**Model type**	**Direction**	**Covariate**
Hemorrhagic stroke	Male	Early Neonatal	80 plus	Data Rich	-1	nuts seeds adjusted(g)
Hemorrhagic stroke	Male	Early Neonatal	80 plus	Data Rich	-1	omega 3 adjusted(g)
Hemorrhagic stroke	Male	Early Neonatal	80 plus	Data Rich	-1	pufa adjusted(percent)
Hemorrhagic stroke	Male	Early Neonatal	80 plus	Data Rich	-1	pulses legumes adjusted(g)
Hemorrhagic stroke	Male	Early Neonatal	80 plus	Data Rich	-1	vegetables adjusted(g)
Hemorrhagic stroke	Male	Early Neonatal	80 plus	Data Rich	0	Alcohol (litres per capita)
Hemorrhagic stroke	Male	Early Neonatal	80 plus	Data Rich	0	Sociodemographic Status
Hemorrhagic stroke	Male	Early Neonatal	80 plus	Data Rich	1	Log-transformed SEV scalar: Hem Stroke
Hemorrhagic stroke	Male	Early Neonatal	80 plus	Data Rich	1	Smoking Prevalence
Hemorrhagic stroke	Female	Early Neonatal	80 plus	Global	-1	Education (years per capita)
Hemorrhagic stroke	Female	Early Neonatal	80 plus	Global	-1	LDI (I$ per capita)
Hemorrhagic stroke	Female	Early Neonatal	80 plus	Global	-1	milk adjusted(g)
Hemorrhagic stroke	Female	Early Neonatal	80 plus	Global	-1	nuts seeds adjusted(g)
Hemorrhagic stroke	Female	Early Neonatal	80 plus	Global	-1	omega 3 adjusted(g)
Hemorrhagic stroke	Female	Early Neonatal	80 plus	Global	-1	pufa adjusted(percent)
Hemorrhagic stroke	Female	Early Neonatal	80 plus	Global	-1	pulses legumes adjusted(g)
Hemorrhagic stroke	Female	Early Neonatal	80 plus	Global	-1	vegetables adjusted(g)
Hemorrhagic stroke	Female	Early Neonatal	80 plus	Global	0	Alcohol (litres per capita)
Hemorrhagic stroke	Female	Early Neonatal	80 plus	Global	0	Sociodemographic Status
Hemorrhagic stroke	Female	Early Neonatal	80 plus	Global	1	Log-transformed SEV scalar: Hem Stroke
Hemorrhagic stroke	Female	Early Neonatal	80 plus	Global	1	Smoking Prevalence
Hemorrhagic stroke	Female	Early Neonatal	80 plus	Data Rich	-1	Education (years per capita)
Hemorrhagic stroke	Female	Early Neonatal	80 plus	Data Rich	-1	LDI (I$ per capita)
Hemorrhagic stroke	Female	Early Neonatal	80 plus	Data Rich	-1	milk adjusted(g)
Hemorrhagic stroke	Female	Early Neonatal	80 plus	Data Rich	-1	nuts seeds adjusted(g)
Hemorrhagic stroke	Female	Early Neonatal	80 plus	Data Rich	-1	omega 3 adjusted(g)
Hemorrhagic stroke	Female	Early Neonatal	80 plus	Data Rich	-1	pufa adjusted(percent)
Hemorrhagic stroke	Female	Early Neonatal	80 plus	Data Rich	-1	pulses legumes adjusted(g)
Hemorrhagic stroke	Female	Early Neonatal	80 plus	Data Rich	-1	vegetables adjusted(g)
Hemorrhagic stroke	Female	Early Neonatal	80 plus	Data Rich	0	Alcohol (litres per capita)
Hemorrhagic stroke	Female	Early Neonatal	80 plus	Data Rich	0	Sociodemographic Status
Hemorrhagic stroke	Female	Early Neonatal	80 plus	Data Rich	1	Log-transformed SEV scalar: Hem Stroke
Hemorrhagic stroke	Female	Early Neonatal	80 plus	Data Rich	1	Smoking Prevalence
Hypertensive heart disease	Male	Post Neonatal	80 plus	Global	-1	Education (years per capita)
Hypertensive heart disease	Male	Post Neonatal	80 plus	Global	-1	Elevation Under 100m (proportion)
Hypertensive heart disease	Male	Post Neonatal	80 plus	Global	-1	LDI (I$ per capita)
Hypertensive heart disease	Male	Post Neonatal	80 plus	Global	-1	Sociodemographic Status
Hypertensive heart disease	Male	Post Neonatal	80 plus	Global	-1	milk adjusted(g)
Hypertensive heart disease	Male	Post Neonatal	80 plus	Global	-1	nuts seeds adjusted(g)
Hypertensive heart disease	Male	Post Neonatal	80 plus	Global	-1	omega 3 adjusted(g)
Hypertensive heart disease	Male	Post Neonatal	80 plus	Global	-1	pulses legumes adjusted(g)
Hypertensive heart disease	Male	Post Neonatal	80 plus	Global	-1	vegetables adjusted(g)
Hypertensive heart disease	Male	Post Neonatal	80 plus	Global	0	Alcohol (litres per capita)
Hypertensive heart disease	Male	Post Neonatal	80 plus	Global	1	Cumulative Cigarettes (10 Years)

Appendix Table 10: CODEm covariates used, level of covariate, and expected direction of covariate by cause, sex, and age						
**Cause**	**Sex**	**Age start**	**Age end**	**Model type**	**Direction**	**Covariate**
Hypertensive heart disease	Male	Post Neonatal	80 plus	Global	1	Indoor Air Pollution (All Cooking Fuels)
Hypertensive heart disease	Male	Post Neonatal	80 plus	Global	1	Outdoor Air Pollution (PM2.5)
Hypertensive heart disease	Male	Post Neonatal	80 plus	Global	1	Systolic Blood Pressure (mmHg)
Hypertensive heart disease	Male	Post Neonatal	80 plus	Global	1	energy unadjusted(kcal)
Hypertensive heart disease	Male	Post Neonatal	80 plus	Data Rich	-1	Education (years per capita)
Hypertensive heart disease	Male	Post Neonatal	80 plus	Data Rich	-1	Elevation Under 100m (proportion)
Hypertensive heart disease	Male	Post Neonatal	80 plus	Data Rich	-1	LDI (I$ per capita)
Hypertensive heart disease	Male	Post Neonatal	80 plus	Data Rich	-1	Sociodemographic Status
Hypertensive heart disease	Male	Post Neonatal	80 plus	Data Rich	-1	milk adjusted(g)
Hypertensive heart disease	Male	Post Neonatal	80 plus	Data Rich	-1	nuts seeds adjusted(g)
Hypertensive heart disease	Male	Post Neonatal	80 plus	Data Rich	-1	omega 3 adjusted(g)
Hypertensive heart disease	Male	Post Neonatal	80 plus	Data Rich	-1	pulses legumes adjusted(g)
Hypertensive heart disease	Male	Post Neonatal	80 plus	Data Rich	-1	vegetables adjusted(g)
Hypertensive heart disease	Male	Post Neonatal	80 plus	Data Rich	0	Alcohol (litres per capita)
Hypertensive heart disease	Male	Post Neonatal	80 plus	Data Rich	1	Cumulative Cigarettes (10 Years)
Hypertensive heart disease	Male	Post Neonatal	80 plus	Data Rich	1	Indoor Air Pollution (All Cooking Fuels)
Hypertensive heart disease	Male	Post Neonatal	80 plus	Data Rich	1	Outdoor Air Pollution (PM2.5)
Hypertensive heart disease	Male	Post Neonatal	80 plus	Data Rich	1	Systolic Blood Pressure (mmHg)
Hypertensive heart disease	Male	Post Neonatal	80 plus	Data Rich	1	energy unadjusted(kcal)
Hypertensive heart disease	Female	Post Neonatal	80 plus	Global	-1	Education (years per capita)
Hypertensive heart disease	Female	Post Neonatal	80 plus	Global	-1	Elevation Under 100m (proportion)
Hypertensive heart disease	Female	Post Neonatal	80 plus	Global	-1	LDI (I$ per capita)
Hypertensive heart disease	Female	Post Neonatal	80 plus	Global	-1	Sociodemographic Status
Hypertensive heart disease	Female	Post Neonatal	80 plus	Global	-1	milk adjusted(g)
Hypertensive heart disease	Female	Post Neonatal	80 plus	Global	-1	nuts seeds adjusted(g)
Hypertensive heart disease	Female	Post Neonatal	80 plus	Global	-1	omega 3 adjusted(g)
Hypertensive heart disease	Female	Post Neonatal	80 plus	Global	-1	pulses legumes adjusted(g)
Hypertensive heart disease	Female	Post Neonatal	80 plus	Global	-1	vegetables adjusted(g)
Hypertensive heart disease	Female	Post Neonatal	80 plus	Global	0	Alcohol (litres per capita)
Hypertensive heart disease	Female	Post Neonatal	80 plus	Global	1	Cumulative Cigarettes (10 Years)
Hypertensive heart disease	Female	Post Neonatal	80 plus	Global	1	Indoor Air Pollution (All Cooking Fuels)
Hypertensive heart disease	Female	Post Neonatal	80 plus	Global	1	Outdoor Air Pollution (PM2.5)
Hypertensive heart disease	Female	Post Neonatal	80 plus	Global	1	Systolic Blood Pressure (mmHg)
Hypertensive heart disease	Female	Post Neonatal	80 plus	Global	1	energy unadjusted(kcal)
Hypertensive heart disease	Female	Post Neonatal	80 plus	Data Rich	-1	Education (years per capita)
Hypertensive heart disease	Female	Post Neonatal	80 plus	Data Rich	-1	Elevation Under 100m (proportion)
Hypertensive heart disease	Female	Post Neonatal	80 plus	Data Rich	-1	LDI (I$ per capita)
Hypertensive heart disease	Female	Post Neonatal	80 plus	Data Rich	-1	Sociodemographic Status
Hypertensive heart disease	Female	Post Neonatal	80 plus	Data Rich	-1	milk adjusted(g)
Hypertensive heart disease	Female	Post Neonatal	80 plus	Data Rich	-1	nuts seeds adjusted(g)
Hypertensive heart disease	Female	Post Neonatal	80 plus	Data Rich	-1	omega 3 adjusted(g)
Hypertensive heart disease	Female	Post Neonatal	80 plus	Data Rich	-1	pulses legumes adjusted(g)
Hypertensive heart disease	Female	Post Neonatal	80 plus	Data Rich	-1	vegetables adjusted(g)
Hypertensive heart disease	Female	Post Neonatal	80 plus	Data Rich	0	Alcohol (litres per capita)

Appendix Table 10: CODEm covariates used, level of covariate, and expected direction of covariate by cause, sex, and age						
**Cause**	**Sex**	**Age start**	**Age end**	**Model type**	**Direction**	**Covariate**
Hypertensive heart disease	Female	Post Neonatal	80 plus	Data Rich	1	Cumulative Cigarettes (10 Years)
Hypertensive heart disease	Female	Post Neonatal	80 plus	Data Rich	1	Indoor Air Pollution (All Cooking Fuels)
Hypertensive heart disease	Female	Post Neonatal	80 plus	Data Rich	1	Outdoor Air Pollution (PM2.5)
Hypertensive heart disease	Female	Post Neonatal	80 plus	Data Rich	1	Systolic Blood Pressure (mmHg)
Hypertensive heart disease	Female	Post Neonatal	80 plus	Data Rich	1	energy unadjusted(kcal)
Cardiomyopathy and myocarditis	Male	Early Neonatal	80 plus	Global	-1	Education (years per capita)
Cardiomyopathy and myocarditis	Male	Early Neonatal	80 plus	Global	-1	Health System Access 2 (unitless)
Cardiomyopathy and myocarditis	Male	Early Neonatal	80 plus	Global	-1	LDI (I$ per capita)
Cardiomyopathy and myocarditis	Male	Early Neonatal	80 plus	Global	0	Alcohol (litres per capita)
Cardiomyopathy and myocarditis	Male	Early Neonatal	80 plus	Global	0	Sociodemographic Status
Cardiomyopathy and myocarditis	Male	Early Neonatal	80 plus	Global	1	Diabetes Age-Standardised Prevalence (proportion)
Cardiomyopathy and myocarditis	Male	Early Neonatal	80 plus	Global	1	Log-transformed SEV scalar: CMP
Cardiomyopathy and myocarditis	Male	Early Neonatal	80 plus	Global	1	Systolic Blood Pressure (mmHg)
Cardiomyopathy and myocarditis	Male	Early Neonatal	80 plus	Data Rich	-1	Education (years per capita)
Cardiomyopathy and myocarditis	Male	Early Neonatal	80 plus	Data Rich	-1	Health System Access 2 (unitless)
Cardiomyopathy and myocarditis	Male	Early Neonatal	80 plus	Data Rich	-1	LDI (I$ per capita)
Cardiomyopathy and myocarditis	Male	Early Neonatal	80 plus	Data Rich	0	Alcohol (litres per capita)
Cardiomyopathy and myocarditis	Male	Early Neonatal	80 plus	Data Rich	0	Sociodemographic Status
Cardiomyopathy and myocarditis	Male	Early Neonatal	80 plus	Data Rich	1	Diabetes Age-Standardised Prevalence (proportion)
Cardiomyopathy and myocarditis	Male	Early Neonatal	80 plus	Data Rich	1	Log-transformed SEV scalar: CMP
Cardiomyopathy and myocarditis	Male	Early Neonatal	80 plus	Data Rich	1	Systolic Blood Pressure (mmHg)
Cardiomyopathy and myocarditis	Female	Early Neonatal	80 plus	Global	-1	Education (years per capita)
Cardiomyopathy and myocarditis	Female	Early Neonatal	80 plus	Global	-1	Health System Access 2 (unitless)
Cardiomyopathy and myocarditis	Female	Early Neonatal	80 plus	Global	-1	LDI (I$ per capita)
Cardiomyopathy and myocarditis	Female	Early Neonatal	80 plus	Global	0	Alcohol (litres per capita)
Cardiomyopathy and myocarditis	Female	Early Neonatal	80 plus	Global	0	Sociodemographic Status
Cardiomyopathy and myocarditis	Female	Early Neonatal	80 plus	Global	1	Diabetes Age-Standardised Prevalence (proportion)
Cardiomyopathy and myocarditis	Female	Early Neonatal	80 plus	Global	1	Log-transformed SEV scalar: CMP
Cardiomyopathy and myocarditis	Female	Early Neonatal	80 plus	Global	1	Systolic Blood Pressure (mmHg)
Cardiomyopathy and myocarditis	Female	Early Neonatal	80 plus	Data Rich	-1	Education (years per capita)
Cardiomyopathy and myocarditis	Female	Early Neonatal	80 plus	Data Rich	-1	Health System Access 2 (unitless)
Cardiomyopathy and myocarditis	Female	Early Neonatal	80 plus	Data Rich	-1	LDI (I$ per capita)
Cardiomyopathy and myocarditis	Female	Early Neonatal	80 plus	Data Rich	0	Alcohol (litres per capita)
Cardiomyopathy and myocarditis	Female	Early Neonatal	80 plus	Data Rich	0	Sociodemographic Status
Cardiomyopathy and myocarditis	Female	Early Neonatal	80 plus	Data Rich	1	Diabetes Age-Standardised Prevalence (proportion)
Cardiomyopathy and myocarditis	Female	Early Neonatal	80 plus	Data Rich	1	Log-transformed SEV scalar: CMP
Cardiomyopathy and myocarditis	Female	Early Neonatal	80 plus	Data Rich	1	Systolic Blood Pressure (mmHg)
Aortic aneurysm	Male	15 to 19	80 plus	Global	-1	Education (years per capita)
Aortic aneurysm	Male	15 to 19	80 plus	Global	-1	Health System Access 2 (unitless)
Aortic aneurysm	Male	15 to 19	80 plus	Global	-1	LDI (I$ per capita)
Aortic aneurysm	Male	15 to 19	80 plus	Global	-1	fruits adjusted(g)
Aortic aneurysm	Male	15 to 19	80 plus	Global	-1	vegetables adjusted(g)
Aortic aneurysm	Male	15 to 19	80 plus	Global	-1	whole grains adjusted(g)
Aortic aneurysm	Male	15 to 19	80 plus	Global	0	Alcohol (litres per capita)

Appendix Table 10: CODEm covariates used, level of covariate, and expected direction of covariate by cause, sex, and age						
**Cause**	**Sex**	**Age start**	**Age end**	**Model type**	**Direction**	**Covariate**
Aortic aneurysm	Male	15 to 19	80 plus	Global	0	Sociodemographic Status
Aortic aneurysm	Male	15 to 19	80 plus	Global	1	Cholesterol (total, mean per capita)
Aortic aneurysm	Male	15 to 19	80 plus	Global	1	Cumulative Cigarettes (5 Years)
Aortic aneurysm	Male	15 to 19	80 plus	Global	1	Diabetes Age-Standardised Prevalence (proportion)
Aortic aneurysm	Male	15 to 19	80 plus	Global	1	Indoor Air Pollution (All Cooking Fuels)
Aortic aneurysm	Male	15 to 19	80 plus	Global	1	Log-transformed SEV scalar: Aort An
Aortic aneurysm	Male	15 to 19	80 plus	Global	1	Mean BMI
Aortic aneurysm	Male	15 to 19	80 plus	Global	1	Outdoor Air Pollution (PM2.5)
Aortic aneurysm	Male	15 to 19	80 plus	Global	1	Systolic Blood Pressure (mmHg)
Aortic aneurysm	Male	15 to 19	80 plus	Global	1	red meats adjusted(g)
Aortic aneurysm	Male	15 to 19	80 plus	Data Rich	-1	Education (years per capita)
Aortic aneurysm	Male	15 to 19	80 plus	Data Rich	-1	Health System Access 2 (unitless)
Aortic aneurysm	Male	15 to 19	80 plus	Data Rich	-1	LDI (I$ per capita)
Aortic aneurysm	Male	15 to 19	80 plus	Data Rich	-1	fruits adjusted(g)
Aortic aneurysm	Male	15 to 19	80 plus	Data Rich	-1	vegetables adjusted(g)
Aortic aneurysm	Male	15 to 19	80 plus	Data Rich	-1	whole grains adjusted(g)
Aortic aneurysm	Male	15 to 19	80 plus	Data Rich	0	Alcohol (litres per capita)
Aortic aneurysm	Male	15 to 19	80 plus	Data Rich	0	Sociodemographic Status
Aortic aneurysm	Male	15 to 19	80 plus	Data Rich	1	Cholesterol (total, mean per capita)
Aortic aneurysm	Male	15 to 19	80 plus	Data Rich	1	Cumulative Cigarettes (5 Years)
Aortic aneurysm	Male	15 to 19	80 plus	Data Rich	1	Diabetes Age-Standardised Prevalence (proportion)
Aortic aneurysm	Male	15 to 19	80 plus	Data Rich	1	Indoor Air Pollution (All Cooking Fuels)
Aortic aneurysm	Male	15 to 19	80 plus	Data Rich	1	Log-transformed SEV scalar: Aort An
Aortic aneurysm	Male	15 to 19	80 plus	Data Rich	1	Mean BMI
Aortic aneurysm	Male	15 to 19	80 plus	Data Rich	1	Outdoor Air Pollution (PM2.5)
Aortic aneurysm	Male	15 to 19	80 plus	Data Rich	1	Systolic Blood Pressure (mmHg)
Aortic aneurysm	Male	15 to 19	80 plus	Data Rich	1	red meats adjusted(g)
Aortic aneurysm	Female	15 to 19	80 plus	Global	-1	Education (years per capita)
Aortic aneurysm	Female	15 to 19	80 plus	Global	-1	Health System Access 2 (unitless)
Aortic aneurysm	Female	15 to 19	80 plus	Global	-1	LDI (I$ per capita)
Aortic aneurysm	Female	15 to 19	80 plus	Global	-1	fruits adjusted(g)
Aortic aneurysm	Female	15 to 19	80 plus	Global	-1	vegetables adjusted(g)
Aortic aneurysm	Female	15 to 19	80 plus	Global	-1	whole grains adjusted(g)
Aortic aneurysm	Female	15 to 19	80 plus	Global	0	Alcohol (litres per capita)
Aortic aneurysm	Female	15 to 19	80 plus	Global	0	Sociodemographic Status
Aortic aneurysm	Female	15 to 19	80 plus	Global	1	Cholesterol (total, mean per capita)
Aortic aneurysm	Female	15 to 19	80 plus	Global	1	Cumulative Cigarettes (5 Years)
Aortic aneurysm	Female	15 to 19	80 plus	Global	1	Diabetes Age-Standardised Prevalence (proportion)
Aortic aneurysm	Female	15 to 19	80 plus	Global	1	Indoor Air Pollution (All Cooking Fuels)
Aortic aneurysm	Female	15 to 19	80 plus	Global	1	Log-transformed SEV scalar: Aort An
Aortic aneurysm	Female	15 to 19	80 plus	Global	1	Mean BMI
Aortic aneurysm	Female	15 to 19	80 plus	Global	1	Outdoor Air Pollution (PM2.5)
Aortic aneurysm	Female	15 to 19	80 plus	Global	1	Systolic Blood Pressure (mmHg)
Aortic aneurysm	Female	15 to 19	80 plus	Global	1	red meats adjusted(g)

Appendix Table 10: CODEm covariates used, level of covariate, and expected direction of covariate by cause, sex, and age						
Cause	Sex	Age start	Age end	Model type	Direction	Covariate
Aortic aneurysm	Female	15 to 19	80 plus	Data Rich	-1	Education (years per capita)
Aortic aneurysm	Female	15 to 19	80 plus	Data Rich	-1	Health System Access 2 (unitless)
Aortic aneurysm	Female	15 to 19	80 plus	Data Rich	-1	LDI (I$ per capita)
Aortic aneurysm	Female	15 to 19	80 plus	Data Rich	-1	fruits adjusted(g)
Aortic aneurysm	Female	15 to 19	80 plus	Data Rich	-1	vegetables adjusted(g)
Aortic aneurysm	Female	15 to 19	80 plus	Data Rich	-1	whole grains adjusted(g)
Aortic aneurysm	Female	15 to 19	80 plus	Data Rich	0	Alcohol (litres per capita)
Aortic aneurysm	Female	15 to 19	80 plus	Data Rich	0	Sociodemographic Status
Aortic aneurysm	Female	15 to 19	80 plus	Data Rich	1	Cholesterol (total, mean per capita)
Aortic aneurysm	Female	15 to 19	80 plus	Data Rich	1	Cumulative Cigarettes (5 Years)
Aortic aneurysm	Female	15 to 19	80 plus	Data Rich	1	Diabetes Age-Standardised Prevalence (proportion)
Aortic aneurysm	Female	15 to 19	80 plus	Data Rich	1	Indoor Air Pollution (All Cooking Fuels)
Aortic aneurysm	Female	15 to 19	80 plus	Data Rich	1	Log-transformed SEV scalar: Aort An
Aortic aneurysm	Female	15 to 19	80 plus	Data Rich	1	Mean BMI
Aortic aneurysm	Female	15 to 19	80 plus	Data Rich	1	Outdoor Air Pollution (PM2.5)
Aortic aneurysm	Female	15 to 19	80 plus	Data Rich	1	Systolic Blood Pressure (mmHg)
Aortic aneurysm	Female	15 to 19	80 plus	Data Rich	1	red meats adjusted(g)
Peripheral vascular disease	Male	40 to 44	80 plus	Global	-1	Education (years per capita)
Peripheral vascular disease	Male	40 to 44	80 plus	Global	-1	Health System Access 2 (unitless)
Peripheral vascular disease	Male	40 to 44	80 plus	Global	-1	LDI (I$ per capita)
Peripheral vascular disease	Male	40 to 44	80 plus	Global	-1	fruits adjusted(g)
Peripheral vascular disease	Male	40 to 44	80 plus	Global	-1	nuts seeds adjusted(g)
Peripheral vascular disease	Male	40 to 44	80 plus	Global	-1	omega 3 adjusted(g)
Peripheral vascular disease	Male	40 to 44	80 plus	Global	-1	pufa adjusted(percent)
Peripheral vascular disease	Male	40 to 44	80 plus	Global	-1	pulses legumes adjusted(g)
Peripheral vascular disease	Male	40 to 44	80 plus	Global	-1	vegetables adjusted(g)
Peripheral vascular disease	Male	40 to 44	80 plus	Global	-1	whole grains adjusted(g)
Peripheral vascular disease	Male	40 to 44	80 plus	Global	0	Alcohol (litres per capita)
Peripheral vascular disease	Male	40 to 44	80 plus	Global	0	Sociodemographic Status
Peripheral vascular disease	Male	40 to 44	80 plus	Global	1	Cholesterol (total, mean per capita)
Peripheral vascular disease	Male	40 to 44	80 plus	Global	1	Cumulative Cigarettes (10 Years)
Peripheral vascular disease	Male	40 to 44	80 plus	Global	1	Diabetes Age-Standardised Prevalence (proportion)
Peripheral vascular disease	Male	40 to 44	80 plus	Global	1	Indoor Air Pollution (All Cooking Fuels)
Peripheral vascular disease	Male	40 to 44	80 plus	Global	1	Log-transformed SEV scalar: PVD
Peripheral vascular disease	Male	40 to 44	80 plus	Global	1	Mean BMI
Peripheral vascular disease	Male	40 to 44	80 plus	Global	1	Outdoor Air Pollution (PM2.5)
Peripheral vascular disease	Male	40 to 44	80 plus	Global	1	Smoking Prevalence
Peripheral vascular disease	Male	40 to 44	80 plus	Global	1	Systolic Blood Pressure (mmHg)
Peripheral vascular disease	Male	40 to 44	80 plus	Global	1	Tobacco (cigarettes per capita)
Peripheral vascular disease	Male	40 to 44	80 plus	Global	1	energy unadjusted(kcal)
Peripheral vascular disease	Male	40 to 44	80 plus	Global	1	red meats adjusted(g)
Peripheral vascular disease	Male	40 to 44	80 plus	Data Rich	-1	Education (years per capita)
Peripheral vascular disease	Male	40 to 44	80 plus	Data Rich	-1	Health System Access 2 (unitless)
Peripheral vascular disease	Male	40 to 44	80 plus	Data Rich	-1	LDI (I$ per capita)

Appendix Table 10: CODEm covariates used, level of covariate, and expected direction of covariate by cause, sex, and age						
**Cause**	**Sex**	**Age start**	**Age end**	**Model type**	**Direction**	**Covariate**
Peripheral vascular disease	Male	40 to 44	80 plus	Data Rich	-1	fruits adjusted(g)
Peripheral vascular disease	Male	40 to 44	80 plus	Data Rich	-1	nuts seeds adjusted(g)
Peripheral vascular disease	Male	40 to 44	80 plus	Data Rich	-1	omega 3 adjusted(g)
Peripheral vascular disease	Male	40 to 44	80 plus	Data Rich	-1	pufa adjusted(percent)
Peripheral vascular disease	Male	40 to 44	80 plus	Data Rich	-1	pulses legumes adjusted(g)
Peripheral vascular disease	Male	40 to 44	80 plus	Data Rich	-1	vegetables adjusted(g)
Peripheral vascular disease	Male	40 to 44	80 plus	Data Rich	-1	whole grains adjusted(g)
Peripheral vascular disease	Male	40 to 44	80 plus	Data Rich	0	Alcohol (litres per capita)
Peripheral vascular disease	Male	40 to 44	80 plus	Data Rich	0	Sociodemographic Status
Peripheral vascular disease	Male	40 to 44	80 plus	Data Rich	1	Cholesterol (total, mean per capita)
Peripheral vascular disease	Male	40 to 44	80 plus	Data Rich	1	Cumulative Cigarettes (10 Years)
Peripheral vascular disease	Male	40 to 44	80 plus	Data Rich	1	Diabetes Age-Standardised Prevalence (proportion)
Peripheral vascular disease	Male	40 to 44	80 plus	Data Rich	1	Indoor Air Pollution (All Cooking Fuels)
Peripheral vascular disease	Male	40 to 44	80 plus	Data Rich	1	Log-transformed SEV scalar: PVD
Peripheral vascular disease	Male	40 to 44	80 plus	Data Rich	1	Mean BMI
Peripheral vascular disease	Male	40 to 44	80 plus	Data Rich	1	Outdoor Air Pollution (PM2.5)
Peripheral vascular disease	Male	40 to 44	80 plus	Data Rich	1	Smoking Prevalence
Peripheral vascular disease	Male	40 to 44	80 plus	Data Rich	1	Systolic Blood Pressure (mmHg)
Peripheral vascular disease	Male	40 to 44	80 plus	Data Rich	1	Tobacco (cigarettes per capita)
Peripheral vascular disease	Male	40 to 44	80 plus	Data Rich	1	energy unadjusted(kcal)
Peripheral vascular disease	Male	40 to 44	80 plus	Data Rich	1	red meats adjusted(g)
Peripheral vascular disease	Female	40 to 44	80 plus	Global	-1	Education (years per capita)
Peripheral vascular disease	Female	40 to 44	80 plus	Global	-1	Health System Access 2 (unitless)
Peripheral vascular disease	Female	40 to 44	80 plus	Global	-1	LDI (I$ per capita)
Peripheral vascular disease	Female	40 to 44	80 plus	Global	-1	fruits adjusted(g)
Peripheral vascular disease	Female	40 to 44	80 plus	Global	-1	nuts seeds adjusted(g)
Peripheral vascular disease	Female	40 to 44	80 plus	Global	-1	omega 3 adjusted(g)
Peripheral vascular disease	Female	40 to 44	80 plus	Global	-1	pufa adjusted(percent)
Peripheral vascular disease	Female	40 to 44	80 plus	Global	-1	pulses legumes adjusted(g)
Peripheral vascular disease	Female	40 to 44	80 plus	Global	-1	vegetables adjusted(g)
Peripheral vascular disease	Female	40 to 44	80 plus	Global	-1	whole grains adjusted(g)
Peripheral vascular disease	Female	40 to 44	80 plus	Global	0	Alcohol (litres per capita)
Peripheral vascular disease	Female	40 to 44	80 plus	Global	0	Sociodemographic Status
Peripheral vascular disease	Female	40 to 44	80 plus	Global	1	Cholesterol (total, mean per capita)
Peripheral vascular disease	Female	40 to 44	80 plus	Global	1	Cumulative Cigarettes (10 Years)
Peripheral vascular disease	Female	40 to 44	80 plus	Global	1	Diabetes Age-Standardised Prevalence (proportion)
Peripheral vascular disease	Female	40 to 44	80 plus	Global	1	Indoor Air Pollution (All Cooking Fuels)
Peripheral vascular disease	Female	40 to 44	80 plus	Global	1	Log-transformed SEV scalar: PVD
Peripheral vascular disease	Female	40 to 44	80 plus	Global	1	Mean BMI
Peripheral vascular disease	Female	40 to 44	80 plus	Global	1	Outdoor Air Pollution (PM2.5)
Peripheral vascular disease	Female	40 to 44	80 plus	Global	1	Smoking Prevalence
Peripheral vascular disease	Female	40 to 44	80 plus	Global	1	Systolic Blood Pressure (mmHg)
Peripheral vascular disease	Female	40 to 44	80 plus	Global	1	Tobacco (cigarettes per capita)
Peripheral vascular disease	Female	40 to 44	80 plus	Global	1	energy unadjusted(kcal)

Appendix Table 10: CODEm covariates used, level of covariate, and expected direction of covariate by cause, sex, and age						
Cause	Sex	Age start	Age end	Model type	Direction	Covariate
Peripheral vascular disease	Female	40 to 44	80 plus	Global	1	red meats adjusted(g)
Peripheral vascular disease	Female	40 to 44	80 plus	Data Rich	-1	Education (years per capita)
Peripheral vascular disease	Female	40 to 44	80 plus	Data Rich	-1	Health System Access 2 (unitless)
Peripheral vascular disease	Female	40 to 44	80 plus	Data Rich	-1	LDI (I$ per capita)
Peripheral vascular disease	Female	40 to 44	80 plus	Data Rich	-1	fruits adjusted(g)
Peripheral vascular disease	Female	40 to 44	80 plus	Data Rich	-1	nuts seeds adjusted(g)
Peripheral vascular disease	Female	40 to 44	80 plus	Data Rich	-1	omega 3 adjusted(g)
Peripheral vascular disease	Female	40 to 44	80 plus	Data Rich	-1	pufa adjusted(percent)
Peripheral vascular disease	Female	40 to 44	80 plus	Data Rich	-1	pulses legumes adjusted(g)
Peripheral vascular disease	Female	40 to 44	80 plus	Data Rich	-1	vegetables adjusted(g)
Peripheral vascular disease	Female	40 to 44	80 plus	Data Rich	-1	whole grains adjusted(g)
Peripheral vascular disease	Female	40 to 44	80 plus	Data Rich	0	Alcohol (litres per capita)
Peripheral vascular disease	Female	40 to 44	80 plus	Data Rich	0	Sociodemographic Status
Peripheral vascular disease	Female	40 to 44	80 plus	Data Rich	1	Cholesterol (total, mean per capita)
Peripheral vascular disease	Female	40 to 44	80 plus	Data Rich	1	Cumulative Cigarettes (10 Years)
Peripheral vascular disease	Female	40 to 44	80 plus	Data Rich	1	Diabetes Age-Standardised Prevalence (proportion)
Peripheral vascular disease	Female	40 to 44	80 plus	Data Rich	1	Indoor Air Pollution (All Cooking Fuels)
Peripheral vascular disease	Female	40 to 44	80 plus	Data Rich	1	Log-transformed SEV scalar: PVD
Peripheral vascular disease	Female	40 to 44	80 plus	Data Rich	1	Mean BMI
Peripheral vascular disease	Female	40 to 44	80 plus	Data Rich	1	Outdoor Air Pollution (PM2.5)
Peripheral vascular disease	Female	40 to 44	80 plus	Data Rich	1	Smoking Prevalence
Peripheral vascular disease	Female	40 to 44	80 plus	Data Rich	1	Systolic Blood Pressure (mmHg)
Peripheral vascular disease	Female	40 to 44	80 plus	Data Rich	1	Tobacco (cigarettes per capita)
Peripheral vascular disease	Female	40 to 44	80 plus	Data Rich	1	energy unadjusted(kcal)
Peripheral vascular disease	Female	40 to 44	80 plus	Data Rich	1	red meats adjusted(g)
Endocarditis	Male	Early Neonatal	80 plus	Global	-1	Education (years per capita)
Endocarditis	Male	Early Neonatal	80 plus	Global	-1	Health System Access 2 (unitless)
Endocarditis	Male	Early Neonatal	80 plus	Global	-1	Improved Water Source (proportion with access)
Endocarditis	Male	Early Neonatal	80 plus	Global	-1	LDI (I$ per capita)
Endocarditis	Male	Early Neonatal	80 plus	Global	-1	Sanitation (proportion with access)
Endocarditis	Male	Early Neonatal	80 plus	Global	0	Sociodemographic Status
Endocarditis	Male	Early Neonatal	80 plus	Global	1	Log-transformed SEV scalar: Endocar
Endocarditis	Male	Early Neonatal	80 plus	Data Rich	-1	Education (years per capita)
Endocarditis	Male	Early Neonatal	80 plus	Data Rich	-1	Health System Access 2 (unitless)
Endocarditis	Male	Early Neonatal	80 plus	Data Rich	-1	Improved Water Source (proportion with access)
Endocarditis	Male	Early Neonatal	80 plus	Data Rich	-1	LDI (I$ per capita)
Endocarditis	Male	Early Neonatal	80 plus	Data Rich	-1	Sanitation (proportion with access)
Endocarditis	Male	Early Neonatal	80 plus	Data Rich	0	Sociodemographic Status
Endocarditis	Male	Early Neonatal	80 plus	Data Rich	1	Log-transformed SEV scalar: Endocar
Endocarditis	Female	Early Neonatal	80 plus	Global	-1	Education (years per capita)
Endocarditis	Female	Early Neonatal	80 plus	Global	-1	Health System Access 2 (unitless)
Endocarditis	Female	Early Neonatal	80 plus	Global	-1	Improved Water Source (proportion with access)
Endocarditis	Female	Early Neonatal	80 plus	Global	-1	LDI (I$ per capita)
Endocarditis	Female	Early Neonatal	80 plus	Global	-1	Sanitation (proportion with access)

Appendix Table 10: CODEm covariates used, level of covariate, and expected direction of covariate by cause, sex, and age						
**Cause**	**Sex**	**Age start**	**Age end**	**Model type**	**Direction**	**Covariate**
Endocarditis	Female	Early Neonatal	80 plus	Global	0	Sociodemographic Status
Endocarditis	Female	Early Neonatal	80 plus	Global	1	Log-transformed SEV scalar: Endocar
Endocarditis	Female	Early Neonatal	80 plus	Data Rich	-1	Education (years per capita)
Endocarditis	Female	Early Neonatal	80 plus	Data Rich	-1	Health System Access 2 (unitless)
Endocarditis	Female	Early Neonatal	80 plus	Data Rich	-1	Improved Water Source (proportion with access)
Endocarditis	Female	Early Neonatal	80 plus	Data Rich	-1	LDI (I$ per capita)
Endocarditis	Female	Early Neonatal	80 plus	Data Rich	-1	Sanitation (proportion with access)
Endocarditis	Female	Early Neonatal	80 plus	Data Rich	0	Sociodemographic Status
Endocarditis	Female	Early Neonatal	80 plus	Data Rich	1	Log-transformed SEV scalar: Endocar
Other cardiovascular and circulatory diseases	Male	Early Neonatal	80 plus	Global	-1	Education (years per capita)
Other cardiovascular and circulatory diseases	Male	Early Neonatal	80 plus	Global	-1	Elevation 100 to 500m (proportion)
Other cardiovascular and circulatory diseases	Male	Early Neonatal	80 plus	Global	-1	Health System Access 2 (unitless)
Other cardiovascular and circulatory diseases	Male	Early Neonatal	80 plus	Global	-1	LDI (I$ per capita)
Other cardiovascular and circulatory diseases	Male	Early Neonatal	80 plus	Global	-1	Sociodemographic Status
Other cardiovascular and circulatory diseases	Male	Early Neonatal	80 plus	Global	-1	Vegetables (kcal per capita)
Other cardiovascular and circulatory diseases	Male	Early Neonatal	80 plus	Global	-1	energy unadjusted(kcal)
Other cardiovascular and circulatory diseases	Male	Early Neonatal	80 plus	Global	-1	fruits adjusted(g)
Other cardiovascular and circulatory diseases	Male	Early Neonatal	80 plus	Global	-1	milk adjusted(g)
Other cardiovascular and circulatory diseases	Male	Early Neonatal	80 plus	Global	-1	nuts seeds adjusted(g)
Other cardiovascular and circulatory diseases	Male	Early Neonatal	80 plus	Global	-1	omega 3 adjusted(g)
Other cardiovascular and circulatory diseases	Male	Early Neonatal	80 plus	Global	-1	pufa adjusted(percent)
Other cardiovascular and circulatory diseases	Male	Early Neonatal	80 plus	Global	-1	pulses legumes adjusted(g)
Other cardiovascular and circulatory diseases	Male	Early Neonatal	80 plus	Global	-1	vegetables adjusted(g)
Other cardiovascular and circulatory diseases	Male	Early Neonatal	80 plus	Global	-1	whole grains adjusted(g)
Other cardiovascular and circulatory diseases	Male	Early Neonatal	80 plus	Global	0	Alcohol (litres per capita)
Other cardiovascular and circulatory diseases	Male	Early Neonatal	80 plus	Global	1	Animal Fats (kcal per capita)
Other cardiovascular and circulatory diseases	Male	Early Neonatal	80 plus	Global	1	Cholesterol (total, mean per capita)
Other cardiovascular and circulatory diseases	Male	Early Neonatal	80 plus	Global	1	Cumulative Cigarettes (10 Years)
Other cardiovascular and circulatory diseases	Male	Early Neonatal	80 plus	Global	1	Diabetes Age-Standardised Prevalence (proportion)
Other cardiovascular and circulatory diseases	Male	Early Neonatal	80 plus	Global	1	Indoor Air Pollution (All Cooking Fuels)
Other cardiovascular and circulatory diseases	Male	Early Neonatal	80 plus	Global	1	Log-transformed SEV scalar: Oth Cardio
Other cardiovascular and circulatory diseases	Male	Early Neonatal	80 plus	Global	1	Mean BMI
Other cardiovascular and circulatory diseases	Male	Early Neonatal	80 plus	Global	1	Outdoor Air Pollution (PM2.5)
Other cardiovascular and circulatory diseases	Male	Early Neonatal	80 plus	Global	1	Smoking Prevalence
Other cardiovascular and circulatory diseases	Male	Early Neonatal	80 plus	Global	1	Systolic Blood Pressure (mmHg)
Other cardiovascular and circulatory diseases	Male	Early Neonatal	80 plus	Global	1	Tobacco (cigarettes per capita)
Other cardiovascular and circulatory diseases	Male	Early Neonatal	80 plus	Global	1	red meats adjusted(g)
Other cardiovascular and circulatory diseases	Male	Early Neonatal	80 plus	Data Rich	-1	Education (years per capita)
Other cardiovascular and circulatory diseases	Male	Early Neonatal	80 plus	Data Rich	-1	Elevation 100 to 500m (proportion)
Other cardiovascular and circulatory diseases	Male	Early Neonatal	80 plus	Data Rich	-1	Health System Access 2 (unitless)
Other cardiovascular and circulatory diseases	Male	Early Neonatal	80 plus	Data Rich	-1	LDI (I$ per capita)
Other cardiovascular and circulatory diseases	Male	Early Neonatal	80 plus	Data Rich	-1	Sociodemographic Status
Other cardiovascular and circulatory diseases	Male	Early Neonatal	80 plus	Data Rich	-1	energy unadjusted(kcal)
Other cardiovascular and circulatory diseases	Male	Early Neonatal	80 plus	Data Rich	-1	fruits adjusted(g)

Appendix Table 10: CODEm covariates used, level of covariate, and expected direction of covariate by cause, sex, and age						
**Cause**	**Sex**	**Age start**	**Age end**	**Model type**	**Direction**	**Covariate**
Other cardiovascular and circulatory diseases	Male	Early Neonatal	80 plus	Data Rich	-1	milk adjusted(g)
Other cardiovascular and circulatory diseases	Male	Early Neonatal	80 plus	Data Rich	-1	nuts seeds adjusted(g)
Other cardiovascular and circulatory diseases	Male	Early Neonatal	80 plus	Data Rich	-1	omega 3 adjusted(g)
Other cardiovascular and circulatory diseases	Male	Early Neonatal	80 plus	Data Rich	-1	pufa adjusted(percent)
Other cardiovascular and circulatory diseases	Male	Early Neonatal	80 plus	Data Rich	-1	pulses legumes adjusted(g)
Other cardiovascular and circulatory diseases	Male	Early Neonatal	80 plus	Data Rich	-1	vegetables adjusted(g)
Other cardiovascular and circulatory diseases	Male	Early Neonatal	80 plus	Data Rich	-1	whole grains adjusted(g)
Other cardiovascular and circulatory diseases	Male	Early Neonatal	80 plus	Data Rich	0	Alcohol (litres per capita)
Other cardiovascular and circulatory diseases	Male	Early Neonatal	80 plus	Data Rich	1	Animal Fats (kcal per capita)
Other cardiovascular and circulatory diseases	Male	Early Neonatal	80 plus	Data Rich	1	Cholesterol (total, mean per capita)
Other cardiovascular and circulatory diseases	Male	Early Neonatal	80 plus	Data Rich	1	Cumulative Cigarettes (10 Years)
Other cardiovascular and circulatory diseases	Male	Early Neonatal	80 plus	Data Rich	1	Diabetes Age-Standardised Prevalence (proportion)
Other cardiovascular and circulatory diseases	Male	Early Neonatal	80 plus	Data Rich	1	Indoor Air Pollution (All Cooking Fuels)
Other cardiovascular and circulatory diseases	Male	Early Neonatal	80 plus	Data Rich	1	Log-transformed SEV scalar: Oth Cardio
Other cardiovascular and circulatory diseases	Male	Early Neonatal	80 plus	Data Rich	1	Mean BMI
Other cardiovascular and circulatory diseases	Male	Early Neonatal	80 plus	Data Rich	1	Outdoor Air Pollution (PM2.5)
Other cardiovascular and circulatory diseases	Male	Early Neonatal	80 plus	Data Rich	1	Smoking Prevalence
Other cardiovascular and circulatory diseases	Male	Early Neonatal	80 plus	Data Rich	1	Systolic Blood Pressure (mmHg)
Other cardiovascular and circulatory diseases	Male	Early Neonatal	80 plus	Data Rich	1	Tobacco (cigarettes per capita)
Other cardiovascular and circulatory diseases	Male	Early Neonatal	80 plus	Data Rich	1	red meats adjusted(g)
Other cardiovascular and circulatory diseases	Female	Early Neonatal	80 plus	Global	-1	Education (years per capita)
Other cardiovascular and circulatory diseases	Female	Early Neonatal	80 plus	Global	-1	Elevation 100 to 500m (proportion)
Other cardiovascular and circulatory diseases	Female	Early Neonatal	80 plus	Global	-1	Health System Access 2 (unitless)
Other cardiovascular and circulatory diseases	Female	Early Neonatal	80 plus	Global	-1	LDI (I$ per capita)
Other cardiovascular and circulatory diseases	Female	Early Neonatal	80 plus	Global	-1	Sociodemographic Status
Other cardiovascular and circulatory diseases	Female	Early Neonatal	80 plus	Global	-1	Vegetables (kcal per capita)
Other cardiovascular and circulatory diseases	Female	Early Neonatal	80 plus	Global	-1	energy unadjusted(kcal)
Other cardiovascular and circulatory diseases	Female	Early Neonatal	80 plus	Global	-1	fruits adjusted(g)
Other cardiovascular and circulatory diseases	Female	Early Neonatal	80 plus	Global	-1	milk adjusted(g)
Other cardiovascular and circulatory diseases	Female	Early Neonatal	80 plus	Global	-1	nuts seeds adjusted(g)
Other cardiovascular and circulatory diseases	Female	Early Neonatal	80 plus	Global	-1	omega 3 adjusted(g)
Other cardiovascular and circulatory diseases	Female	Early Neonatal	80 plus	Global	-1	pufa adjusted(percent)
Other cardiovascular and circulatory diseases	Female	Early Neonatal	80 plus	Global	-1	pulses legumes adjusted(g)
Other cardiovascular and circulatory diseases	Female	Early Neonatal	80 plus	Global	-1	vegetables adjusted(g)
Other cardiovascular and circulatory diseases	Female	Early Neonatal	80 plus	Global	-1	whole grains adjusted(g)
Other cardiovascular and circulatory diseases	Female	Early Neonatal	80 plus	Global	0	Alcohol (litres per capita)
Other cardiovascular and circulatory diseases	Female	Early Neonatal	80 plus	Global	1	Animal Fats (kcal per capita)
Other cardiovascular and circulatory diseases	Female	Early Neonatal	80 plus	Global	1	Cholesterol (total, mean per capita)
Other cardiovascular and circulatory diseases	Female	Early Neonatal	80 plus	Global	1	Cumulative Cigarettes (10 Years)
Other cardiovascular and circulatory diseases	Female	Early Neonatal	80 plus	Global	1	Diabetes Age-Standardised Prevalence (proportion)
Other cardiovascular and circulatory diseases	Female	Early Neonatal	80 plus	Global	1	Indoor Air Pollution (All Cooking Fuels)
Other cardiovascular and circulatory diseases	Female	Early Neonatal	80 plus	Global	1	Log-transformed SEV scalar: Oth Cardio
Other cardiovascular and circulatory diseases	Female	Early Neonatal	80 plus	Global	1	Mean BMI
Other cardiovascular and circulatory diseases	Female	Early Neonatal	80 plus	Global	1	Outdoor Air Pollution (PM2.5)

Appendix Table 10: CODEm covariates used, level of covariate, and expected direction of covariate by cause, sex, and age						
**Cause**	**Sex**	**Age start**	**Age end**	**Model type**	**Direction**	**Covariate**
Other cardiovascular and circulatory diseases	Female	Early Neonatal	80 plus	Global	1	Smoking Prevalence
Other cardiovascular and circulatory diseases	Female	Early Neonatal	80 plus	Global	1	Systolic Blood Pressure (mmHg)
Other cardiovascular and circulatory diseases	Female	Early Neonatal	80 plus	Global	1	Tobacco (cigarettes per capita)
Other cardiovascular and circulatory diseases	Female	Early Neonatal	80 plus	Global	1	red meats adjusted(g)
Other cardiovascular and circulatory diseases	Female	Early Neonatal	80 plus	Data Rich	-1	Education (years per capita)
Other cardiovascular and circulatory diseases	Female	Early Neonatal	80 plus	Data Rich	-1	Elevation 100 to 500m (proportion)
Other cardiovascular and circulatory diseases	Female	Early Neonatal	80 plus	Data Rich	-1	Health System Access 2 (unitless)
Other cardiovascular and circulatory diseases	Female	Early Neonatal	80 plus	Data Rich	-1	LDI (I$ per capita)
Other cardiovascular and circulatory diseases	Female	Early Neonatal	80 plus	Data Rich	-1	Sociodemographic Status
Other cardiovascular and circulatory diseases	Female	Early Neonatal	80 plus	Data Rich	-1	energy unadjusted(kcal)
Other cardiovascular and circulatory diseases	Female	Early Neonatal	80 plus	Data Rich	-1	fruits adjusted(g)
Other cardiovascular and circulatory diseases	Female	Early Neonatal	80 plus	Data Rich	-1	milk adjusted(g)
Other cardiovascular and circulatory diseases	Female	Early Neonatal	80 plus	Data Rich	-1	nuts seeds adjusted(g)
Other cardiovascular and circulatory diseases	Female	Early Neonatal	80 plus	Data Rich	-1	omega 3 adjusted(g)
Other cardiovascular and circulatory diseases	Female	Early Neonatal	80 plus	Data Rich	-1	pufa adjusted(percent)
Other cardiovascular and circulatory diseases	Female	Early Neonatal	80 plus	Data Rich	-1	pulses legumes adjusted(g)
Other cardiovascular and circulatory diseases	Female	Early Neonatal	80 plus	Data Rich	-1	vegetables adjusted(g)
Other cardiovascular and circulatory diseases	Female	Early Neonatal	80 plus	Data Rich	-1	whole grains adjusted(g)
Other cardiovascular and circulatory diseases	Female	Early Neonatal	80 plus	Data Rich	0	Alcohol (litres per capita)
Other cardiovascular and circulatory diseases	Female	Early Neonatal	80 plus	Data Rich	1	Animal Fats (kcal per capita)
Other cardiovascular and circulatory diseases	Female	Early Neonatal	80 plus	Data Rich	1	Cholesterol (total, mean per capita)
Other cardiovascular and circulatory diseases	Female	Early Neonatal	80 plus	Data Rich	1	Cumulative Cigarettes (10 Years)
Other cardiovascular and circulatory diseases	Female	Early Neonatal	80 plus	Data Rich	1	Diabetes Age-Standardised Prevalence (proportion)
Other cardiovascular and circulatory diseases	Female	Early Neonatal	80 plus	Data Rich	1	Indoor Air Pollution (All Cooking Fuels)
Other cardiovascular and circulatory diseases	Female	Early Neonatal	80 plus	Data Rich	1	Log-transformed SEV scalar: Oth Cardio
Other cardiovascular and circulatory diseases	Female	Early Neonatal	80 plus	Data Rich	1	Mean BMI
Other cardiovascular and circulatory diseases	Female	Early Neonatal	80 plus	Data Rich	1	Outdoor Air Pollution (PM2.5)
Other cardiovascular and circulatory diseases	Female	Early Neonatal	80 plus	Data Rich	1	Smoking Prevalence
Other cardiovascular and circulatory diseases	Female	Early Neonatal	80 plus	Data Rich	1	Systolic Blood Pressure (mmHg)
Other cardiovascular and circulatory diseases	Female	Early Neonatal	80 plus	Data Rich	1	Tobacco (cigarettes per capita)
Other cardiovascular and circulatory diseases	Female	Early Neonatal	80 plus	Data Rich	1	red meats adjusted(g)
Chronic respiratory diseases	Male	Early Neonatal	80 plus	Global	-1	Education (years per capita)
Chronic respiratory diseases	Male	Early Neonatal	80 plus	Global	-1	Health System Access 2 (unitless)
Chronic respiratory diseases	Male	Early Neonatal	80 plus	Global	-1	LDI (I$ per capita)
Chronic respiratory diseases	Male	Early Neonatal	80 plus	Global	0	Sociodemographic Status
Chronic respiratory diseases	Male	Early Neonatal	80 plus	Global	1	Cumulative Cigarettes (5 Years)
Chronic respiratory diseases	Male	Early Neonatal	80 plus	Global	1	Elevation 500 to 1500m (proportion)
Chronic respiratory diseases	Male	Early Neonatal	80 plus	Global	1	Elevation Over 1500m (proportion)
Chronic respiratory diseases	Male	Early Neonatal	80 plus	Global	1	Indoor Air Pollution (Biomass Cooking)
Chronic respiratory diseases	Male	Early Neonatal	80 plus	Global	1	Indoor Air Pollution (Coal Cooking)
Chronic respiratory diseases	Male	Early Neonatal	80 plus	Global	1	Outdoor Air Pollution (PM2.5)
Chronic respiratory diseases	Male	Early Neonatal	80 plus	Global	1	Population Density (over 1000 ppl/sqkm, proportion)
Chronic respiratory diseases	Male	Early Neonatal	80 plus	Global	1	Smoking Prevalence
Chronic respiratory diseases	Male	Early Neonatal	80 plus	Data Rich	-1	Education (years per capita)

Appendix Table 10: CODEm covariates used, level of covariate, and expected direction of covariate by cause, sex, and age						
**Cause**	**Sex**	**Age start**	**Age end**	**Model type**	**Direction**	**Covariate**
Chronic respiratory diseases	Male	Early Neonatal	80 plus	Data Rich	-1	Health System Access 2 (unitless)
Chronic respiratory diseases	Male	Early Neonatal	80 plus	Data Rich	-1	LDI (I$ per capita)
Chronic respiratory diseases	Male	Early Neonatal	80 plus	Data Rich	0	Sociodemographic Status
Chronic respiratory diseases	Male	Early Neonatal	80 plus	Data Rich	1	Cumulative Cigarettes (10 Years)
Chronic respiratory diseases	Male	Early Neonatal	80 plus	Data Rich	1	Cumulative Cigarettes (5 Years)
Chronic respiratory diseases	Male	Early Neonatal	80 plus	Data Rich	1	Elevation 500 to 1500m (proportion)
Chronic respiratory diseases	Male	Early Neonatal	80 plus	Data Rich	1	Elevation Over 1500m (proportion)
Chronic respiratory diseases	Male	Early Neonatal	80 plus	Data Rich	1	Indoor Air Pollution (Biomass Cooking)
Chronic respiratory diseases	Male	Early Neonatal	80 plus	Data Rich	1	Indoor Air Pollution (Coal Cooking)
Chronic respiratory diseases	Male	Early Neonatal	80 plus	Data Rich	1	Log-transformed SEV scalar: Chr Resp
Chronic respiratory diseases	Male	Early Neonatal	80 plus	Data Rich	1	Outdoor Air Pollution (PM2.5)
Chronic respiratory diseases	Male	Early Neonatal	80 plus	Data Rich	1	Population Density (over 1000 ppl/sqkm, proportion)
Chronic respiratory diseases	Male	Early Neonatal	80 plus	Data Rich	1	Smoking Prevalence
Chronic respiratory diseases	Female	Early Neonatal	80 plus	Global	-1	Education (years per capita)
Chronic respiratory diseases	Female	Early Neonatal	80 plus	Global	-1	Health System Access 2 (unitless)
Chronic respiratory diseases	Female	Early Neonatal	80 plus	Global	-1	LDI (I$ per capita)
Chronic respiratory diseases	Female	Early Neonatal	80 plus	Global	0	Sociodemographic Status
Chronic respiratory diseases	Female	Early Neonatal	80 plus	Global	1	Cumulative Cigarettes (10 Years)
Chronic respiratory diseases	Female	Early Neonatal	80 plus	Global	1	Cumulative Cigarettes (5 Years)
Chronic respiratory diseases	Female	Early Neonatal	80 plus	Global	1	Elevation 500 to 1500m (proportion)
Chronic respiratory diseases	Female	Early Neonatal	80 plus	Global	1	Elevation Over 1500m (proportion)
Chronic respiratory diseases	Female	Early Neonatal	80 plus	Global	1	Indoor Air Pollution (Biomass Cooking)
Chronic respiratory diseases	Female	Early Neonatal	80 plus	Global	1	Indoor Air Pollution (Coal Cooking)
Chronic respiratory diseases	Female	Early Neonatal	80 plus	Global	1	Log-transformed SEV scalar: Chr Resp
Chronic respiratory diseases	Female	Early Neonatal	80 plus	Global	1	Outdoor Air Pollution (PM2.5)
Chronic respiratory diseases	Female	Early Neonatal	80 plus	Global	1	Population Density (over 1000 ppl/sqkm, proportion)
Chronic respiratory diseases	Female	Early Neonatal	80 plus	Global	1	Smoking Prevalence
Chronic respiratory diseases	Female	Early Neonatal	80 plus	Data Rich	-1	Education (years per capita)
Chronic respiratory diseases	Female	Early Neonatal	80 plus	Data Rich	-1	Health System Access 2 (unitless)
Chronic respiratory diseases	Female	Early Neonatal	80 plus	Data Rich	-1	LDI (I$ per capita)
Chronic respiratory diseases	Female	Early Neonatal	80 plus	Data Rich	0	Sociodemographic Status
Chronic respiratory diseases	Female	Early Neonatal	80 plus	Data Rich	1	Cumulative Cigarettes (10 Years)
Chronic respiratory diseases	Female	Early Neonatal	80 plus	Data Rich	1	Cumulative Cigarettes (5 Years)
Chronic respiratory diseases	Female	Early Neonatal	80 plus	Data Rich	1	Elevation 500 to 1500m (proportion)
Chronic respiratory diseases	Female	Early Neonatal	80 plus	Data Rich	1	Elevation Over 1500m (proportion)
Chronic respiratory diseases	Female	Early Neonatal	80 plus	Data Rich	1	Indoor Air Pollution (Biomass Cooking)
Chronic respiratory diseases	Female	Early Neonatal	80 plus	Data Rich	1	Indoor Air Pollution (Coal Cooking)
Chronic respiratory diseases	Female	Early Neonatal	80 plus	Data Rich	1	Log-transformed SEV scalar: Chr Resp
Chronic respiratory diseases	Female	Early Neonatal	80 plus	Data Rich	1	Outdoor Air Pollution (PM2.5)
Chronic respiratory diseases	Female	Early Neonatal	80 plus	Data Rich	1	Population Density (over 1000 ppl/sqkm, proportion)
Chronic respiratory diseases	Female	Early Neonatal	80 plus	Data Rich	1	Smoking Prevalence
Chronic obstructive pulmonary disease	Male	Post Neonatal	80 plus	Global	-1	Education (years per capita)
Chronic obstructive pulmonary disease	Male	Post Neonatal	80 plus	Global	-1	Health System Access 2 (unitless)
Chronic obstructive pulmonary disease	Male	Post Neonatal	80 plus	Global	-1	LDI (I$ per capita)

Appendix Table 10: CODEm covariates used, level of covariate, and expected direction of covariate by cause, sex, and age						
**Cause**	**Sex**	**Age start**	**Age end**	**Model type**	**Direction**	**Covariate**
Chronic obstructive pulmonary disease	Male	Post Neonatal	80 plus	Global	0	Sociodemographic Status
Chronic obstructive pulmonary disease	Male	Post Neonatal	80 plus	Global	1	Cumulative Cigarettes (10 Years)
Chronic obstructive pulmonary disease	Male	Post Neonatal	80 plus	Global	1	Cumulative Cigarettes (5 Years)
Chronic obstructive pulmonary disease	Male	Post Neonatal	80 plus	Global	1	Elevation Over 1500m (proportion)
Chronic obstructive pulmonary disease	Male	Post Neonatal	80 plus	Global	1	Indoor Air Pollution (Biomass Cooking)
Chronic obstructive pulmonary disease	Male	Post Neonatal	80 plus	Global	1	Indoor Air Pollution (Coal Cooking)
Chronic obstructive pulmonary disease	Male	Post Neonatal	80 plus	Global	1	Outdoor Air Pollution (PM2.5)
Chronic obstructive pulmonary disease	Male	Post Neonatal	80 plus	Global	1	Smoking Prevalence
Chronic obstructive pulmonary disease	Male	Post Neonatal	80 plus	Data Rich	-1	Education (years per capita)
Chronic obstructive pulmonary disease	Male	Post Neonatal	80 plus	Data Rich	-1	Health System Access 2 (unitless)
Chronic obstructive pulmonary disease	Male	Post Neonatal	80 plus	Data Rich	-1	LDI (I$ per capita)
Chronic obstructive pulmonary disease	Male	Post Neonatal	80 plus	Data Rich	0	Sociodemographic Status
Chronic obstructive pulmonary disease	Male	Post Neonatal	80 plus	Data Rich	1	Cumulative Cigarettes (10 Years)
Chronic obstructive pulmonary disease	Male	Post Neonatal	80 plus	Data Rich	1	Cumulative Cigarettes (5 Years)
Chronic obstructive pulmonary disease	Male	Post Neonatal	80 plus	Data Rich	1	Elevation Over 1500m (proportion)
Chronic obstructive pulmonary disease	Male	Post Neonatal	80 plus	Data Rich	1	Indoor Air Pollution (Biomass Cooking)
Chronic obstructive pulmonary disease	Male	Post Neonatal	80 plus	Data Rich	1	Indoor Air Pollution (Coal Cooking)
Chronic obstructive pulmonary disease	Male	Post Neonatal	80 plus	Data Rich	1	Outdoor Air Pollution (PM2.5)
Chronic obstructive pulmonary disease	Male	Post Neonatal	80 plus	Data Rich	1	Smoking Prevalence
Chronic obstructive pulmonary disease	Female	Post Neonatal	80 plus	Global	-1	Education (years per capita)
Chronic obstructive pulmonary disease	Female	Post Neonatal	80 plus	Global	-1	Health System Access 2 (unitless)
Chronic obstructive pulmonary disease	Female	Post Neonatal	80 plus	Global	-1	LDI (I$ per capita)
Chronic obstructive pulmonary disease	Female	Post Neonatal	80 plus	Global	0	Sociodemographic Status
Chronic obstructive pulmonary disease	Female	Post Neonatal	80 plus	Global	1	Cumulative Cigarettes (10 Years)
Chronic obstructive pulmonary disease	Female	Post Neonatal	80 plus	Global	1	Cumulative Cigarettes (5 Years)
Chronic obstructive pulmonary disease	Female	Post Neonatal	80 plus	Global	1	Elevation Over 1500m (proportion)
Chronic obstructive pulmonary disease	Female	Post Neonatal	80 plus	Global	1	Indoor Air Pollution (Biomass Cooking)
Chronic obstructive pulmonary disease	Female	Post Neonatal	80 plus	Global	1	Indoor Air Pollution (Coal Cooking)
Chronic obstructive pulmonary disease	Female	Post Neonatal	80 plus	Global	1	Outdoor Air Pollution (PM2.5)
Chronic obstructive pulmonary disease	Female	Post Neonatal	80 plus	Global	1	Smoking Prevalence
Chronic obstructive pulmonary disease	Female	Post Neonatal	80 plus	Data Rich	-1	Education (years per capita)
Chronic obstructive pulmonary disease	Female	Post Neonatal	80 plus	Data Rich	-1	Health System Access 2 (unitless)
Chronic obstructive pulmonary disease	Female	Post Neonatal	80 plus	Data Rich	-1	LDI (I$ per capita)
Chronic obstructive pulmonary disease	Female	Post Neonatal	80 plus	Data Rich	0	Sociodemographic Status
Chronic obstructive pulmonary disease	Female	Post Neonatal	80 plus	Data Rich	1	Cumulative Cigarettes (10 Years)
Chronic obstructive pulmonary disease	Female	Post Neonatal	80 plus	Data Rich	1	Cumulative Cigarettes (5 Years)
Chronic obstructive pulmonary disease	Female	Post Neonatal	80 plus	Data Rich	1	Elevation Over 1500m (proportion)
Chronic obstructive pulmonary disease	Female	Post Neonatal	80 plus	Data Rich	1	Indoor Air Pollution (Biomass Cooking)
Chronic obstructive pulmonary disease	Female	Post Neonatal	80 plus	Data Rich	1	Indoor Air Pollution (Coal Cooking)
Chronic obstructive pulmonary disease	Female	Post Neonatal	80 plus	Data Rich	1	Outdoor Air Pollution (PM2.5)
Chronic obstructive pulmonary disease	Female	Post Neonatal	80 plus	Data Rich	1	Smoking Prevalence
Pneumoconiosis	Male	1 to 4	80 plus	Global	-1	Education (years per capita)
Pneumoconiosis	Male	1 to 4	80 plus	Global	-1	Health System Access 2 (unitless)
Pneumoconiosis	Male	1 to 4	80 plus	Global	-1	LDI (I$ per capita)

Appendix Table 10: CODEm covariates used, level of covariate, and expected direction of covariate by cause, sex, and age						
**Cause**	**Sex**	**Age start**	**Age end**	**Model type**	**Direction**	**Covariate**
Pneumoconiosis	Male	1 to 4	80 plus	Global	-1	Sociodemographic Status
Pneumoconiosis	Male	1 to 4	80 plus	Global	1	Asbestos production (kg) per capita
Pneumoconiosis	Male	1 to 4	80 plus	Global	1	Coal Production (per capita)
Pneumoconiosis	Male	1 to 4	80 plus	Global	1	Coal Reserves (teragrams per capita)
Pneumoconiosis	Male	1 to 4	80 plus	Global	1	Cumulative Cigarettes (5 Years)
Pneumoconiosis	Male	1 to 4	80 plus	Global	1	Elevation 500 to 1500m (proportion)
Pneumoconiosis	Male	1 to 4	80 plus	Global	1	Elevation Over 1500m (proportion)
Pneumoconiosis	Male	1 to 4	80 plus	Global	1	Gold production (kg) per capita
Pneumoconiosis	Male	1 to 4	80 plus	Global	1	Log-transformed SEV scalar: Pneumocon
Pneumoconiosis	Male	1 to 4	80 plus	Global	1	Smoking Prevalence
Pneumoconiosis	Male	1 to 4	80 plus	Data Rich	-1	Education (years per capita)
Pneumoconiosis	Male	1 to 4	80 plus	Data Rich	-1	Health System Access 2 (unitless)
Pneumoconiosis	Male	1 to 4	80 plus	Data Rich	-1	LDI (I$ per capita)
Pneumoconiosis	Male	1 to 4	80 plus	Data Rich	-1	Sociodemographic Status
Pneumoconiosis	Male	1 to 4	80 plus	Data Rich	1	Asbestos production (kg) per capita
Pneumoconiosis	Male	1 to 4	80 plus	Data Rich	1	Coal Production (per capita)
Pneumoconiosis	Male	1 to 4	80 plus	Data Rich	1	Coal Reserves (teragrams per capita)
Pneumoconiosis	Male	1 to 4	80 plus	Data Rich	1	Cumulative Cigarettes (5 Years)
Pneumoconiosis	Male	1 to 4	80 plus	Data Rich	1	Elevation 500 to 1500m (proportion)
Pneumoconiosis	Male	1 to 4	80 plus	Data Rich	1	Elevation Over 1500m (proportion)
Pneumoconiosis	Male	1 to 4	80 plus	Data Rich	1	Gold production (kg) per capita
Pneumoconiosis	Male	1 to 4	80 plus	Data Rich	1	Log-transformed SEV scalar: Pneumocon
Pneumoconiosis	Male	1 to 4	80 plus	Data Rich	1	Smoking Prevalence
Pneumoconiosis	Female	1 to 4	80 plus	Global	-1	Education (years per capita)
Pneumoconiosis	Female	1 to 4	80 plus	Global	-1	Health System Access 2 (unitless)
Pneumoconiosis	Female	1 to 4	80 plus	Global	-1	LDI (I$ per capita)
Pneumoconiosis	Female	1 to 4	80 plus	Global	-1	Sociodemographic Status
Pneumoconiosis	Female	1 to 4	80 plus	Global	1	Asbestos production (kg) per capita
Pneumoconiosis	Female	1 to 4	80 plus	Global	1	Coal Production (per capita)
Pneumoconiosis	Female	1 to 4	80 plus	Global	1	Coal Reserves (teragrams per capita)
Pneumoconiosis	Female	1 to 4	80 plus	Global	1	Cumulative Cigarettes (5 Years)
Pneumoconiosis	Female	1 to 4	80 plus	Global	1	Elevation 500 to 1500m (proportion)
Pneumoconiosis	Female	1 to 4	80 plus	Global	1	Elevation Over 1500m (proportion)
Pneumoconiosis	Female	1 to 4	80 plus	Global	1	Gold production (kg) per capita
Pneumoconiosis	Female	1 to 4	80 plus	Global	1	Log-transformed SEV scalar: Pneumocon
Pneumoconiosis	Female	1 to 4	80 plus	Global	1	Smoking Prevalence
Pneumoconiosis	Female	1 to 4	80 plus	Data Rich	-1	Education (years per capita)
Pneumoconiosis	Female	1 to 4	80 plus	Data Rich	-1	Health System Access 2 (unitless)
Pneumoconiosis	Female	1 to 4	80 plus	Data Rich	-1	LDI (I$ per capita)
Pneumoconiosis	Female	1 to 4	80 plus	Data Rich	-1	Sociodemographic Status
Pneumoconiosis	Female	1 to 4	80 plus	Data Rich	1	Asbestos production (kg) per capita
Pneumoconiosis	Female	1 to 4	80 plus	Data Rich	1	Coal Production (per capita)
Pneumoconiosis	Female	1 to 4	80 plus	Data Rich	1	Coal Reserves (teragrams per capita)
Pneumoconiosis	Female	1 to 4	80 plus	Data Rich	1	Cumulative Cigarettes (5 Years)

Appendix Table 10: CODEm covariates used, level of covariate, and expected direction of covariate by cause, sex, and age						
**Cause**	**Sex**	**Age start**	**Age end**	**Model type**	**Direction**	**Covariate**
Pneumoconiosis	Female	1 to 4	80 plus	Data Rich	1	Elevation 500 to 1500m (proportion)
Pneumoconiosis	Female	1 to 4	80 plus	Data Rich	1	Elevation Over 1500m (proportion)
Pneumoconiosis	Female	1 to 4	80 plus	Data Rich	1	Gold production (kg) per capita
Pneumoconiosis	Female	1 to 4	80 plus	Data Rich	1	Log-transformed SEV scalar: Pneumocon
Pneumoconiosis	Female	1 to 4	80 plus	Data Rich	1	Smoking Prevalence
Silicosis	Male	Early Neonatal	80 plus	Global	-1	Education (years per capita)
Silicosis	Male	Early Neonatal	80 plus	Global	-1	Health System Access 2 (unitless)
Silicosis	Male	Early Neonatal	80 plus	Global	-1	LDI (I$ per capita)
Silicosis	Male	Early Neonatal	80 plus	Global	-1	Sociodemographic Status
Silicosis	Male	Early Neonatal	80 plus	Global	1	Asbestos production (binary)
Silicosis	Male	Early Neonatal	80 plus	Global	1	Asbestos production (kg) per capita
Silicosis	Male	Early Neonatal	80 plus	Global	1	Cumulative Cigarettes (5 Years)
Silicosis	Male	Early Neonatal	80 plus	Global	1	Elevation 500 to 1500m (proportion)
Silicosis	Male	Early Neonatal	80 plus	Global	1	Elevation Over 1500m (proportion)
Silicosis	Male	Early Neonatal	80 plus	Global	1	Gold production (binary)
Silicosis	Male	Early Neonatal	80 plus	Global	1	Gold production (kg) per capita
Silicosis	Male	Early Neonatal	80 plus	Global	1	Indoor Air Pollution (Coal Cooking)
Silicosis	Male	Early Neonatal	80 plus	Global	1	Log-transformed SEV scalar: Silicosis
Silicosis	Male	Early Neonatal	80 plus	Global	1	Population Density (over 1000 ppl/sqkm, proportion)
Silicosis	Male	Early Neonatal	80 plus	Global	1	Smoking Prevalence
Silicosis	Male	Early Neonatal	80 plus	Data Rich	-1	Education (years per capita)
Silicosis	Male	Early Neonatal	80 plus	Data Rich	-1	Health System Access 2 (unitless)
Silicosis	Male	Early Neonatal	80 plus	Data Rich	-1	LDI (I$ per capita)
Silicosis	Male	Early Neonatal	80 plus	Data Rich	-1	Sociodemographic Status
Silicosis	Male	Early Neonatal	80 plus	Data Rich	1	Asbestos production (binary)
Silicosis	Male	Early Neonatal	80 plus	Data Rich	1	Asbestos production (kg) per capita
Silicosis	Male	Early Neonatal	80 plus	Data Rich	1	Cumulative Cigarettes (5 Years)
Silicosis	Male	Early Neonatal	80 plus	Data Rich	1	Elevation 500 to 1500m (proportion)
Silicosis	Male	Early Neonatal	80 plus	Data Rich	1	Elevation Over 1500m (proportion)
Silicosis	Male	Early Neonatal	80 plus	Data Rich	1	Gold production (binary)
Silicosis	Male	Early Neonatal	80 plus	Data Rich	1	Gold production (kg) per capita
Silicosis	Male	Early Neonatal	80 plus	Data Rich	1	Indoor Air Pollution (Coal Cooking)
Silicosis	Male	Early Neonatal	80 plus	Data Rich	1	Log-transformed SEV scalar: Silicosis
Silicosis	Male	Early Neonatal	80 plus	Data Rich	1	Population Density (over 1000 ppl/sqkm, proportion)
Silicosis	Male	Early Neonatal	80 plus	Data Rich	1	Smoking Prevalence
Silicosis	Female	Early Neonatal	80 plus	Global	-1	Education (years per capita)
Silicosis	Female	Early Neonatal	80 plus	Global	-1	Health System Access 2 (unitless)
Silicosis	Female	Early Neonatal	80 plus	Global	-1	LDI (I$ per capita)
Silicosis	Female	Early Neonatal	80 plus	Global	-1	Sociodemographic Status
Silicosis	Female	Early Neonatal	80 plus	Global	1	Asbestos production (binary)
Silicosis	Female	Early Neonatal	80 plus	Global	1	Asbestos production (kg) per capita
Silicosis	Female	Early Neonatal	80 plus	Global	1	Cumulative Cigarettes (5 Years)
Silicosis	Female	Early Neonatal	80 plus	Global	1	Elevation 500 to 1500m (proportion)
Silicosis	Female	Early Neonatal	80 plus	Global	1	Elevation Over 1500m (proportion)

Appendix Table 10: CODEm covariates used, level of covariate, and expected direction of covariate by cause, sex, and age						
**Cause**	**Sex**	**Age start**	**Age end**	**Model type**	**Direction**	**Covariate**
Silicosis	Female	Early Neonatal	80 plus	Global	1	Gold production (binary)
Silicosis	Female	Early Neonatal	80 plus	Global	1	Gold production (kg) per capita
Silicosis	Female	Early Neonatal	80 plus	Global	1	Indoor Air Pollution (Coal Cooking)
Silicosis	Female	Early Neonatal	80 plus	Global	1	Log-transformed SEV scalar: Silicosis
Silicosis	Female	Early Neonatal	80 plus	Global	1	Population Density (over 1000 ppl/sqkm, proportion)
Silicosis	Female	Early Neonatal	80 plus	Global	1	Smoking Prevalence
Silicosis	Female	Early Neonatal	80 plus	Data Rich	-1	Education (years per capita)
Silicosis	Female	Early Neonatal	80 plus	Data Rich	-1	Health System Access 2 (unitless)
Silicosis	Female	Early Neonatal	80 plus	Data Rich	-1	LDI (I$ per capita)
Silicosis	Female	Early Neonatal	80 plus	Data Rich	-1	Sociodemographic Status
Silicosis	Female	Early Neonatal	80 plus	Data Rich	1	Asbestos production (binary)
Silicosis	Female	Early Neonatal	80 plus	Data Rich	1	Asbestos production (kg) per capita
Silicosis	Female	Early Neonatal	80 plus	Data Rich	1	Cumulative Cigarettes (5 Years)
Silicosis	Female	Early Neonatal	80 plus	Data Rich	1	Elevation 500 to 1500m (proportion)
Silicosis	Female	Early Neonatal	80 plus	Data Rich	1	Elevation Over 1500m (proportion)
Silicosis	Female	Early Neonatal	80 plus	Data Rich	1	Gold production (binary)
Silicosis	Female	Early Neonatal	80 plus	Data Rich	1	Gold production (kg) per capita
Silicosis	Female	Early Neonatal	80 plus	Data Rich	1	Indoor Air Pollution (Coal Cooking)
Silicosis	Female	Early Neonatal	80 plus	Data Rich	1	Log-transformed SEV scalar: Silicosis
Silicosis	Female	Early Neonatal	80 plus	Data Rich	1	Population Density (over 1000 ppl/sqkm, proportion)
Silicosis	Female	Early Neonatal	80 plus	Data Rich	1	Smoking Prevalence
Asbestosis	Male	Early Neonatal	80 plus	Global	-1	Education (years per capita)
Asbestosis	Male	Early Neonatal	80 plus	Global	-1	Health System Access 2 (unitless)
Asbestosis	Male	Early Neonatal	80 plus	Global	-1	LDI (I$ per capita)
Asbestosis	Male	Early Neonatal	80 plus	Global	0	Sociodemographic Status
Asbestosis	Male	Early Neonatal	80 plus	Global	1	Asbestos production (binary)
Asbestosis	Male	Early Neonatal	80 plus	Global	1	Asbestos production (kg) per capita
Asbestosis	Male	Early Neonatal	80 plus	Global	1	Cumulative Cigarettes (5 Years)
Asbestosis	Male	Early Neonatal	80 plus	Global	1	Elevation 500 to 1500m (proportion)
Asbestosis	Male	Early Neonatal	80 plus	Global	1	Elevation Over 1500m (proportion)
Asbestosis	Male	Early Neonatal	80 plus	Global	1	Gold production (binary)
Asbestosis	Male	Early Neonatal	80 plus	Global	1	Gold production (kg) per capita
Asbestosis	Male	Early Neonatal	80 plus	Global	1	Indoor Air Pollution (Coal Cooking)
Asbestosis	Male	Early Neonatal	80 plus	Global	1	Log-transformed SEV scalar: Asbestosis
Asbestosis	Male	Early Neonatal	80 plus	Global	1	Population Density (over 1000 ppl/sqkm, proportion)
Asbestosis	Male	Early Neonatal	80 plus	Global	1	Smoking Prevalence
Asbestosis	Male	Early Neonatal	80 plus	Data Rich	-1	Education (years per capita)
Asbestosis	Male	Early Neonatal	80 plus	Data Rich	-1	Health System Access 2 (unitless)
Asbestosis	Male	Early Neonatal	80 plus	Data Rich	-1	LDI (I$ per capita)
Asbestosis	Male	Early Neonatal	80 plus	Data Rich	0	Sociodemographic Status
Asbestosis	Male	Early Neonatal	80 plus	Data Rich	1	Asbestos production (binary)
Asbestosis	Male	Early Neonatal	80 plus	Data Rich	1	Asbestos production (kg) per capita
Asbestosis	Male	Early Neonatal	80 plus	Data Rich	1	Cumulative Cigarettes (5 Years)
Asbestosis	Male	Early Neonatal	80 plus	Data Rich	1	Elevation 500 to 1500m (proportion)

Appendix Table 10: CODEm covariates used, level of covariate, and expected direction of covariate by cause, sex, and age						
**Cause**	**Sex**	**Age start**	**Age end**	**Model type**	**Direction**	**Covariate**
Asbestosis	Male	Early Neonatal	80 plus	Data Rich	1	Elevation Over 1500m (proportion)
Asbestosis	Male	Early Neonatal	80 plus	Data Rich	1	Gold production (binary)
Asbestosis	Male	Early Neonatal	80 plus	Data Rich	1	Gold production (kg) per capita
Asbestosis	Male	Early Neonatal	80 plus	Data Rich	1	Indoor Air Pollution (Coal Cooking)
Asbestosis	Male	Early Neonatal	80 plus	Data Rich	1	Log-transformed SEV scalar: Asbestosis
Asbestosis	Male	Early Neonatal	80 plus	Data Rich	1	Population Density (over 1000 ppl/sqkm, proportion)
Asbestosis	Male	Early Neonatal	80 plus	Data Rich	1	Smoking Prevalence
Asbestosis	Female	Early Neonatal	80 plus	Global	-1	Education (years per capita)
Asbestosis	Female	Early Neonatal	80 plus	Global	-1	Health System Access 2 (unitless)
Asbestosis	Female	Early Neonatal	80 plus	Global	-1	LDI (I$ per capita)
Asbestosis	Female	Early Neonatal	80 plus	Global	0	Sociodemographic Status
Asbestosis	Female	Early Neonatal	80 plus	Global	1	Asbestos production (binary)
Asbestosis	Female	Early Neonatal	80 plus	Global	1	Asbestos production (kg) per capita
Asbestosis	Female	Early Neonatal	80 plus	Global	1	Cumulative Cigarettes (5 Years)
Asbestosis	Female	Early Neonatal	80 plus	Global	1	Elevation 500 to 1500m (proportion)
Asbestosis	Female	Early Neonatal	80 plus	Global	1	Elevation Over 1500m (proportion)
Asbestosis	Female	Early Neonatal	80 plus	Global	1	Gold production (binary)
Asbestosis	Female	Early Neonatal	80 plus	Global	1	Gold production (kg) per capita
Asbestosis	Female	Early Neonatal	80 plus	Global	1	Indoor Air Pollution (Coal Cooking)
Asbestosis	Female	Early Neonatal	80 plus	Global	1	Log-transformed SEV scalar: Asbestosis
Asbestosis	Female	Early Neonatal	80 plus	Global	1	Population Density (over 1000 ppl/sqkm, proportion)
Asbestosis	Female	Early Neonatal	80 plus	Global	1	Smoking Prevalence
Asbestosis	Female	Early Neonatal	80 plus	Data Rich	-1	Education (years per capita)
Asbestosis	Female	Early Neonatal	80 plus	Data Rich	-1	Health System Access 2 (unitless)
Asbestosis	Female	Early Neonatal	80 plus	Data Rich	-1	LDI (I$ per capita)
Asbestosis	Female	Early Neonatal	80 plus	Data Rich	0	Sociodemographic Status
Asbestosis	Female	Early Neonatal	80 plus	Data Rich	1	Asbestos production (binary)
Asbestosis	Female	Early Neonatal	80 plus	Data Rich	1	Asbestos production (kg) per capita
Asbestosis	Female	Early Neonatal	80 plus	Data Rich	1	Cumulative Cigarettes (5 Years)
Asbestosis	Female	Early Neonatal	80 plus	Data Rich	1	Elevation 500 to 1500m (proportion)
Asbestosis	Female	Early Neonatal	80 plus	Data Rich	1	Elevation Over 1500m (proportion)
Asbestosis	Female	Early Neonatal	80 plus	Data Rich	1	Gold production (binary)
Asbestosis	Female	Early Neonatal	80 plus	Data Rich	1	Gold production (kg) per capita
Asbestosis	Female	Early Neonatal	80 plus	Data Rich	1	Indoor Air Pollution (Coal Cooking)
Asbestosis	Female	Early Neonatal	80 plus	Data Rich	1	Log-transformed SEV scalar: Asbestosis
Asbestosis	Female	Early Neonatal	80 plus	Data Rich	1	Population Density (over 1000 ppl/sqkm, proportion)
Asbestosis	Female	Early Neonatal	80 plus	Data Rich	1	Smoking Prevalence
Coal workers pneumoconiosis	Male	Early Neonatal	80 plus	Global	-1	Education (years per capita)
Coal workers pneumoconiosis	Male	Early Neonatal	80 plus	Global	-1	Health System Access 2 (unitless)
Coal workers pneumoconiosis	Male	Early Neonatal	80 plus	Global	-1	LDI (I$ per capita)
Coal workers pneumoconiosis	Male	Early Neonatal	80 plus	Global	-1	Sociodemographic Status
Coal workers pneumoconiosis	Male	Early Neonatal	80 plus	Global	1	Asbestos production (binary)
Coal workers pneumoconiosis	Male	Early Neonatal	80 plus	Global	1	Asbestos production (kg) per capita
Coal workers pneumoconiosis	Male	Early Neonatal	80 plus	Global	1	Cumulative Cigarettes (5 Years)

Appendix Table 10: CODEm covariates used, level of covariate, and expected direction of covariate by cause, sex, and age						
**Cause**	**Sex**	**Age start**	**Age end**	**Model type**	**Direction**	**Covariate**
Coal workers pneumoconiosis	Male	Early Neonatal	80 plus	Global	1	Elevation 500 to 1500m (proportion)
Coal workers pneumoconiosis	Male	Early Neonatal	80 plus	Global	1	Elevation Over 1500m (proportion)
Coal workers pneumoconiosis	Male	Early Neonatal	80 plus	Global	1	Gold production (binary)
Coal workers pneumoconiosis	Male	Early Neonatal	80 plus	Global	1	Gold production (kg) per capita
Coal workers pneumoconiosis	Male	Early Neonatal	80 plus	Global	1	Indoor Air Pollution (Coal Cooking)
Coal workers pneumoconiosis	Male	Early Neonatal	80 plus	Global	1	Log-transformed SEV scalar: Coal W
Coal workers pneumoconiosis	Male	Early Neonatal	80 plus	Global	1	Population Density (over 1000 ppl/sqkm, proportion)
Coal workers pneumoconiosis	Male	Early Neonatal	80 plus	Global	1	Smoking Prevalence
Coal workers pneumoconiosis	Male	Early Neonatal	80 plus	Data Rich	-1	Education (years per capita)
Coal workers pneumoconiosis	Male	Early Neonatal	80 plus	Data Rich	-1	Health System Access 2 (unitless)
Coal workers pneumoconiosis	Male	Early Neonatal	80 plus	Data Rich	-1	LDI (I$ per capita)
Coal workers pneumoconiosis	Male	Early Neonatal	80 plus	Data Rich	-1	Sociodemographic Status
Coal workers pneumoconiosis	Male	Early Neonatal	80 plus	Data Rich	1	Asbestos production (binary)
Coal workers pneumoconiosis	Male	Early Neonatal	80 plus	Data Rich	1	Asbestos production (kg) per capita
Coal workers pneumoconiosis	Male	Early Neonatal	80 plus	Data Rich	1	Cumulative Cigarettes (5 Years)
Coal workers pneumoconiosis	Male	Early Neonatal	80 plus	Data Rich	1	Elevation 500 to 1500m (proportion)
Coal workers pneumoconiosis	Male	Early Neonatal	80 plus	Data Rich	1	Elevation Over 1500m (proportion)
Coal workers pneumoconiosis	Male	Early Neonatal	80 plus	Data Rich	1	Gold production (binary)
Coal workers pneumoconiosis	Male	Early Neonatal	80 plus	Data Rich	1	Gold production (kg) per capita
Coal workers pneumoconiosis	Male	Early Neonatal	80 plus	Data Rich	1	Indoor Air Pollution (Coal Cooking)
Coal workers pneumoconiosis	Male	Early Neonatal	80 plus	Data Rich	1	Log-transformed SEV scalar: Coal W
Coal workers pneumoconiosis	Male	Early Neonatal	80 plus	Data Rich	1	Population Density (over 1000 ppl/sqkm, proportion)
Coal workers pneumoconiosis	Male	Early Neonatal	80 plus	Data Rich	1	Smoking Prevalence
Coal workers pneumoconiosis	Female	Early Neonatal	80 plus	Global	-1	Education (years per capita)
Coal workers pneumoconiosis	Female	Early Neonatal	80 plus	Global	-1	Health System Access 2 (unitless)
Coal workers pneumoconiosis	Female	Early Neonatal	80 plus	Global	-1	LDI (I$ per capita)
Coal workers pneumoconiosis	Female	Early Neonatal	80 plus	Global	-1	Sociodemographic Status
Coal workers pneumoconiosis	Female	Early Neonatal	80 plus	Global	1	Asbestos production (binary)
Coal workers pneumoconiosis	Female	Early Neonatal	80 plus	Global	1	Asbestos production (kg) per capita
Coal workers pneumoconiosis	Female	Early Neonatal	80 plus	Global	1	Cumulative Cigarettes (5 Years)
Coal workers pneumoconiosis	Female	Early Neonatal	80 plus	Global	1	Elevation 500 to 1500m (proportion)
Coal workers pneumoconiosis	Female	Early Neonatal	80 plus	Global	1	Elevation Over 1500m (proportion)
Coal workers pneumoconiosis	Female	Early Neonatal	80 plus	Global	1	Gold production (binary)
Coal workers pneumoconiosis	Female	Early Neonatal	80 plus	Global	1	Gold production (kg) per capita
Coal workers pneumoconiosis	Female	Early Neonatal	80 plus	Global	1	Indoor Air Pollution (Coal Cooking)
Coal workers pneumoconiosis	Female	Early Neonatal	80 plus	Global	1	Log-transformed SEV scalar: Coal W
Coal workers pneumoconiosis	Female	Early Neonatal	80 plus	Global	1	Population Density (over 1000 ppl/sqkm, proportion)
Coal workers pneumoconiosis	Female	Early Neonatal	80 plus	Global	1	Smoking Prevalence
Coal workers pneumoconiosis	Female	Early Neonatal	80 plus	Data Rich	-1	Education (years per capita)
Coal workers pneumoconiosis	Female	Early Neonatal	80 plus	Data Rich	-1	Health System Access 2 (unitless)
Coal workers pneumoconiosis	Female	Early Neonatal	80 plus	Data Rich	-1	LDI (I$ per capita)
Coal workers pneumoconiosis	Female	Early Neonatal	80 plus	Data Rich	-1	Sociodemographic Status
Coal workers pneumoconiosis	Female	Early Neonatal	80 plus	Data Rich	1	Asbestos production (binary)
Coal workers pneumoconiosis	Female	Early Neonatal	80 plus	Data Rich	1	Asbestos production (kg) per capita

Appendix Table 10: CODEm covariates used, level of covariate, and expected direction of covariate by cause, sex, and age						
**Cause**	**Sex**	**Age start**	**Age end**	**Model type**	**Direction**	**Covariate**
Coal workers pneumoconiosis	Female	Early Neonatal	80 plus	Data Rich	1	Cumulative Cigarettes (5 Years)
Coal workers pneumoconiosis	Female	Early Neonatal	80 plus	Data Rich	1	Elevation 500 to 1500m (proportion)
Coal workers pneumoconiosis	Female	Early Neonatal	80 plus	Data Rich	1	Elevation Over 1500m (proportion)
Coal workers pneumoconiosis	Female	Early Neonatal	80 plus	Data Rich	1	Gold production (binary)
Coal workers pneumoconiosis	Female	Early Neonatal	80 plus	Data Rich	1	Gold production (kg) per capita
Coal workers pneumoconiosis	Female	Early Neonatal	80 plus	Data Rich	1	Indoor Air Pollution (Coal Cooking)
Coal workers pneumoconiosis	Female	Early Neonatal	80 plus	Data Rich	1	Log-transformed SEV scalar: Coal W
Coal workers pneumoconiosis	Female	Early Neonatal	80 plus	Data Rich	1	Population Density (over 1000 ppl/sqkm, proportion)
Coal workers pneumoconiosis	Female	Early Neonatal	80 plus	Data Rich	1	Smoking Prevalence
Other pneumoconiosis	Male	Early Neonatal	80 plus	Global	-1	Education (years per capita)
Other pneumoconiosis	Male	Early Neonatal	80 plus	Global	-1	Health System Access 2 (unitless)
Other pneumoconiosis	Male	Early Neonatal	80 plus	Global	-1	LDI (I$ per capita)
Other pneumoconiosis	Male	Early Neonatal	80 plus	Global	-1	Sociodemographic Status
Other pneumoconiosis	Male	Early Neonatal	80 plus	Global	1	Asbestos production (binary)
Other pneumoconiosis	Male	Early Neonatal	80 plus	Global	1	Asbestos production (kg) per capita
Other pneumoconiosis	Male	Early Neonatal	80 plus	Global	1	Cumulative Cigarettes (5 Years)
Other pneumoconiosis	Male	Early Neonatal	80 plus	Global	1	Elevation 500 to 1500m (proportion)
Other pneumoconiosis	Male	Early Neonatal	80 plus	Global	1	Elevation Over 1500m (proportion)
Other pneumoconiosis	Male	Early Neonatal	80 plus	Global	1	Gold production (binary)
Other pneumoconiosis	Male	Early Neonatal	80 plus	Global	1	Gold production (kg) per capita
Other pneumoconiosis	Male	Early Neonatal	80 plus	Global	1	Indoor Air Pollution (Coal Cooking)
Other pneumoconiosis	Male	Early Neonatal	80 plus	Global	1	Log-transformed SEV scalar: Oth Pneum
Other pneumoconiosis	Male	Early Neonatal	80 plus	Global	1	Population Density (over 1000 ppl/sqkm, proportion)
Other pneumoconiosis	Male	Early Neonatal	80 plus	Global	1	Smoking Prevalence
Other pneumoconiosis	Male	Early Neonatal	80 plus	Data Rich	-1	Education (years per capita)
Other pneumoconiosis	Male	Early Neonatal	80 plus	Data Rich	-1	Health System Access 2 (unitless)
Other pneumoconiosis	Male	Early Neonatal	80 plus	Data Rich	-1	LDI (I$ per capita)
Other pneumoconiosis	Male	Early Neonatal	80 plus	Data Rich	-1	Sociodemographic Status
Other pneumoconiosis	Male	Early Neonatal	80 plus	Data Rich	1	Asbestos production (binary)
Other pneumoconiosis	Male	Early Neonatal	80 plus	Data Rich	1	Asbestos production (kg) per capita
Other pneumoconiosis	Male	Early Neonatal	80 plus	Data Rich	1	Cumulative Cigarettes (5 Years)
Other pneumoconiosis	Male	Early Neonatal	80 plus	Data Rich	1	Elevation 500 to 1500m (proportion)
Other pneumoconiosis	Male	Early Neonatal	80 plus	Data Rich	1	Elevation Over 1500m (proportion)
Other pneumoconiosis	Male	Early Neonatal	80 plus	Data Rich	1	Gold production (binary)
Other pneumoconiosis	Male	Early Neonatal	80 plus	Data Rich	1	Gold production (kg) per capita
Other pneumoconiosis	Male	Early Neonatal	80 plus	Data Rich	1	Indoor Air Pollution (Coal Cooking)
Other pneumoconiosis	Male	Early Neonatal	80 plus	Data Rich	1	Log-transformed SEV scalar: Oth Pneum
Other pneumoconiosis	Male	Early Neonatal	80 plus	Data Rich	1	Population Density (over 1000 ppl/sqkm, proportion)
Other pneumoconiosis	Male	Early Neonatal	80 plus	Data Rich	1	Smoking Prevalence
Other pneumoconiosis	Female	Early Neonatal	80 plus	Global	-1	Education (years per capita)
Other pneumoconiosis	Female	Early Neonatal	80 plus	Global	-1	Health System Access 2 (unitless)
Other pneumoconiosis	Female	Early Neonatal	80 plus	Global	-1	LDI (I$ per capita)
Other pneumoconiosis	Female	Early Neonatal	80 plus	Global	-1	Sociodemographic Status
Other pneumoconiosis	Female	Early Neonatal	80 plus	Global	1	Asbestos production (binary)

Appendix Table 10: CODEm covariates used, level of covariate, and expected direction of covariate by cause, sex, and age						
Cause	Sex	Age start	Age end	Model type	Direction	Covariate
Other pneumoconiosis	Female	Early Neonatal	80 plus	Global	1	Asbestos production (kg) per capita
Other pneumoconiosis	Female	Early Neonatal	80 plus	Global	1	Cumulative Cigarettes (5 Years)
Other pneumoconiosis	Female	Early Neonatal	80 plus	Global	1	Elevation 500 to 1500m (proportion)
Other pneumoconiosis	Female	Early Neonatal	80 plus	Global	1	Elevation Over 1500m (proportion)
Other pneumoconiosis	Female	Early Neonatal	80 plus	Global	1	Gold production (binary)
Other pneumoconiosis	Female	Early Neonatal	80 plus	Global	1	Gold production (kg) per capita
Other pneumoconiosis	Female	Early Neonatal	80 plus	Global	1	Indoor Air Pollution (Coal Cooking)
Other pneumoconiosis	Female	Early Neonatal	80 plus	Global	1	Log-transformed SEV scalar: Oth Pneum
Other pneumoconiosis	Female	Early Neonatal	80 plus	Global	1	Population Density (over 1000 ppl/sqkm, proportion)
Other pneumoconiosis	Female	Early Neonatal	80 plus	Global	1	Smoking Prevalence
Other pneumoconiosis	Female	Early Neonatal	80 plus	Data Rich	-1	Education (years per capita)
Other pneumoconiosis	Female	Early Neonatal	80 plus	Data Rich	-1	Health System Access 2 (unitless)
Other pneumoconiosis	Female	Early Neonatal	80 plus	Data Rich	-1	LDI (I$ per capita)
Other pneumoconiosis	Female	Early Neonatal	80 plus	Data Rich	-1	Sociodemographic Status
Other pneumoconiosis	Female	Early Neonatal	80 plus	Data Rich	1	Asbestos production (binary)
Other pneumoconiosis	Female	Early Neonatal	80 plus	Data Rich	1	Asbestos production (kg) per capita
Other pneumoconiosis	Female	Early Neonatal	80 plus	Data Rich	1	Cumulative Cigarettes (5 Years)
Other pneumoconiosis	Female	Early Neonatal	80 plus	Data Rich	1	Elevation 500 to 1500m (proportion)
Other pneumoconiosis	Female	Early Neonatal	80 plus	Data Rich	1	Elevation Over 1500m (proportion)
Other pneumoconiosis	Female	Early Neonatal	80 plus	Data Rich	1	Gold production (binary)
Other pneumoconiosis	Female	Early Neonatal	80 plus	Data Rich	1	Gold production (kg) per capita
Other pneumoconiosis	Female	Early Neonatal	80 plus	Data Rich	1	Indoor Air Pollution (Coal Cooking)
Other pneumoconiosis	Female	Early Neonatal	80 plus	Data Rich	1	Log-transformed SEV scalar: Oth Pneum
Other pneumoconiosis	Female	Early Neonatal	80 plus	Data Rich	1	Population Density (over 1000 ppl/sqkm, proportion)
Other pneumoconiosis	Female	Early Neonatal	80 plus	Data Rich	1	Smoking Prevalence
Asthma	Male	1 to 4	80 plus	Global	-1	Education (years per capita)
Asthma	Male	1 to 4	80 plus	Global	-1	Health System Access 2 (unitless)
Asthma	Male	1 to 4	80 plus	Global	-1	LDI (I$ per capita)
Asthma	Male	1 to 4	80 plus	Global	-1	Sociodemographic Status
Asthma	Male	1 to 4	80 plus	Global	1	Cumulative Cigarettes (10 Years)
Asthma	Male	1 to 4	80 plus	Global	1	Cumulative Cigarettes (5 Years)
Asthma	Male	1 to 4	80 plus	Global	1	Indoor Air Pollution (Biomass Cooking)
Asthma	Male	1 to 4	80 plus	Global	1	Indoor Air Pollution (Coal Cooking)
Asthma	Male	1 to 4	80 plus	Global	1	Log-transformed SEV scalar: Asthma
Asthma	Male	1 to 4	80 plus	Global	1	Outdoor Air Pollution (PM2.5)
Asthma	Male	1 to 4	80 plus	Global	1	Smoking Prevalence
Asthma	Male	1 to 4	80 plus	Data Rich	-1	Education (years per capita)
Asthma	Male	1 to 4	80 plus	Data Rich	-1	Health System Access 2 (unitless)
Asthma	Male	1 to 4	80 plus	Data Rich	-1	LDI (I$ per capita)
Asthma	Male	1 to 4	80 plus	Data Rich	-1	Sociodemographic Status
Asthma	Male	1 to 4	80 plus	Data Rich	1	Cumulative Cigarettes (10 Years)
Asthma	Male	1 to 4	80 plus	Data Rich	1	Cumulative Cigarettes (5 Years)
Asthma	Male	1 to 4	80 plus	Data Rich	1	Indoor Air Pollution (Biomass Cooking)
Asthma	Male	1 to 4	80 plus	Data Rich	1	Indoor Air Pollution (Coal Cooking)

Appendix Table 10: CODEm covariates used, level of covariate, and expected direction of covariate by cause, sex, and age						
**Cause**	**Sex**	**Age start**	**Age end**	**Model type**	**Direction**	**Covariate**
Asthma	Male	1 to 4	80 plus	Data Rich	1	Log-transformed SEV scalar: Asthma
Asthma	Male	1 to 4	80 plus	Data Rich	1	Outdoor Air Pollution (PM2.5)
Asthma	Male	1 to 4	80 plus	Data Rich	1	Smoking Prevalence
Asthma	Female	1 to 4	80 plus	Global	-1	Education (years per capita)
Asthma	Female	1 to 4	80 plus	Global	-1	Health System Access 2 (unitless)
Asthma	Female	1 to 4	80 plus	Global	-1	LDI (I$ per capita)
Asthma	Female	1 to 4	80 plus	Global	-1	Sociodemographic Status
Asthma	Female	1 to 4	80 plus	Global	1	Cumulative Cigarettes (10 Years)
Asthma	Female	1 to 4	80 plus	Global	1	Cumulative Cigarettes (5 Years)
Asthma	Female	1 to 4	80 plus	Global	1	Indoor Air Pollution (Biomass Cooking)
Asthma	Female	1 to 4	80 plus	Global	1	Indoor Air Pollution (Coal Cooking)
Asthma	Female	1 to 4	80 plus	Global	1	Log-transformed SEV scalar: Asthma
Asthma	Female	1 to 4	80 plus	Global	1	Outdoor Air Pollution (PM2.5)
Asthma	Female	1 to 4	80 plus	Global	1	Smoking Prevalence
Asthma	Female	1 to 4	80 plus	Data Rich	-1	Education (years per capita)
Asthma	Female	1 to 4	80 plus	Data Rich	-1	Health System Access 2 (unitless)
Asthma	Female	1 to 4	80 plus	Data Rich	-1	LDI (I$ per capita)
Asthma	Female	1 to 4	80 plus	Data Rich	-1	Sociodemographic Status
Asthma	Female	1 to 4	80 plus	Data Rich	1	Cumulative Cigarettes (10 Years)
Asthma	Female	1 to 4	80 plus	Data Rich	1	Cumulative Cigarettes (5 Years)
Asthma	Female	1 to 4	80 plus	Data Rich	1	Indoor Air Pollution (Biomass Cooking)
Asthma	Female	1 to 4	80 plus	Data Rich	1	Indoor Air Pollution (Coal Cooking)
Asthma	Female	1 to 4	80 plus	Data Rich	1	Log-transformed SEV scalar: Asthma
Asthma	Female	1 to 4	80 plus	Data Rich	1	Outdoor Air Pollution (PM2.5)
Asthma	Female	1 to 4	80 plus	Data Rich	1	Smoking Prevalence
Interstitial lung disease and pulmonary sarcoidosis	Male	1 to 4	80 plus	Global	-1	Education (years per capita)
Interstitial lung disease and pulmonary sarcoidosis	Male	1 to 4	80 plus	Global	-1	Health System Access 2 (unitless)
Interstitial lung disease and pulmonary sarcoidosis	Male	1 to 4	80 plus	Global	-1	LDI (I$ per capita)
Interstitial lung disease and pulmonary sarcoidosis	Male	1 to 4	80 plus	Global	0	Sociodemographic Status
Interstitial lung disease and pulmonary sarcoidosis	Male	1 to 4	80 plus	Global	1	Cumulative Cigarettes (5 Years)
Interstitial lung disease and pulmonary sarcoidosis	Male	1 to 4	80 plus	Global	1	Elevation 500 to 1500m (proportion)
Interstitial lung disease and pulmonary sarcoidosis	Male	1 to 4	80 plus	Global	1	Elevation Over 1500m (proportion)
Interstitial lung disease and pulmonary sarcoidosis	Male	1 to 4	80 plus	Global	1	Indoor Air Pollution (Biomass Cooking)
Interstitial lung disease and pulmonary sarcoidosis	Male	1 to 4	80 plus	Global	1	Indoor Air Pollution (Coal Cooking)
Interstitial lung disease and pulmonary sarcoidosis	Male	1 to 4	80 plus	Global	1	Log-transformed SEV scalar: ILD
Interstitial lung disease and pulmonary sarcoidosis	Male	1 to 4	80 plus	Global	1	Outdoor Air Pollution (PM2.5)
Interstitial lung disease and pulmonary sarcoidosis	Male	1 to 4	80 plus	Global	1	Population Density (over 1000 ppl/sqkm, proportion)
Interstitial lung disease and pulmonary sarcoidosis	Male	1 to 4	80 plus	Global	1	Smoking Prevalence
Interstitial lung disease and pulmonary sarcoidosis	Male	1 to 4	80 plus	Data Rich	-1	Education (years per capita)
Interstitial lung disease and pulmonary sarcoidosis	Male	1 to 4	80 plus	Data Rich	-1	Health System Access 2 (unitless)
Interstitial lung disease and pulmonary sarcoidosis	Male	1 to 4	80 plus	Data Rich	-1	LDI (I$ per capita)
Interstitial lung disease and pulmonary sarcoidosis	Male	1 to 4	80 plus	Data Rich	0	Sociodemographic Status
Interstitial lung disease and pulmonary sarcoidosis	Male	1 to 4	80 plus	Data Rich	1	Cumulative Cigarettes (5 Years)
Interstitial lung disease and pulmonary sarcoidosis	Male	1 to 4	80 plus	Data Rich	1	Elevation 500 to 1500m (proportion)

Appendix Table 10: CODEm covariates used, level of covariate, and expected direction of covariate by cause, sex, and age						
**Cause**	**Sex**	**Age start**	**Age end**	**Model type**	**Direction**	**Covariate**
Interstitial lung disease and pulmonary sarcoidosis	Male	1 to 4	80 plus	Data Rich	1	Elevation Over 1500m (proportion)
Interstitial lung disease and pulmonary sarcoidosis	Male	1 to 4	80 plus	Data Rich	1	Indoor Air Pollution (Biomass Cooking)
Interstitial lung disease and pulmonary sarcoidosis	Male	1 to 4	80 plus	Data Rich	1	Indoor Air Pollution (Coal Cooking)
Interstitial lung disease and pulmonary sarcoidosis	Male	1 to 4	80 plus	Data Rich	1	Log-transformed SEV scalar: ILD
Interstitial lung disease and pulmonary sarcoidosis	Male	1 to 4	80 plus	Data Rich	1	Outdoor Air Pollution (PM2.5)
Interstitial lung disease and pulmonary sarcoidosis	Male	1 to 4	80 plus	Data Rich	1	Population Density (over 1000 ppl/sqkm, proportion)
Interstitial lung disease and pulmonary sarcoidosis	Male	1 to 4	80 plus	Data Rich	1	Smoking Prevalence
Interstitial lung disease and pulmonary sarcoidosis	Female	1 to 4	80 plus	Global	-1	Education (years per capita)
Interstitial lung disease and pulmonary sarcoidosis	Female	1 to 4	80 plus	Global	-1	Health System Access 2 (unitless)
Interstitial lung disease and pulmonary sarcoidosis	Female	1 to 4	80 plus	Global	-1	LDI (I$ per capita)
Interstitial lung disease and pulmonary sarcoidosis	Female	1 to 4	80 plus	Global	0	Sociodemographic Status
Interstitial lung disease and pulmonary sarcoidosis	Female	1 to 4	80 plus	Global	1	Cumulative Cigarettes (5 Years)
Interstitial lung disease and pulmonary sarcoidosis	Female	1 to 4	80 plus	Global	1	Elevation 500 to 1500m (proportion)
Interstitial lung disease and pulmonary sarcoidosis	Female	1 to 4	80 plus	Global	1	Elevation Over 1500m (proportion)
Interstitial lung disease and pulmonary sarcoidosis	Female	1 to 4	80 plus	Global	1	Indoor Air Pollution (Biomass Cooking)
Interstitial lung disease and pulmonary sarcoidosis	Female	1 to 4	80 plus	Global	1	Indoor Air Pollution (Coal Cooking)
Interstitial lung disease and pulmonary sarcoidosis	Female	1 to 4	80 plus	Global	1	Log-transformed SEV scalar: ILD
Interstitial lung disease and pulmonary sarcoidosis	Female	1 to 4	80 plus	Global	1	Outdoor Air Pollution (PM2.5)
Interstitial lung disease and pulmonary sarcoidosis	Female	1 to 4	80 plus	Global	1	Population Density (over 1000 ppl/sqkm, proportion)
Interstitial lung disease and pulmonary sarcoidosis	Female	1 to 4	80 plus	Global	1	Smoking Prevalence
Interstitial lung disease and pulmonary sarcoidosis	Female	1 to 4	80 plus	Data Rich	-1	Education (years per capita)
Interstitial lung disease and pulmonary sarcoidosis	Female	1 to 4	80 plus	Data Rich	-1	Health System Access 2 (unitless)
Interstitial lung disease and pulmonary sarcoidosis	Female	1 to 4	80 plus	Data Rich	-1	LDI (I$ per capita)
Interstitial lung disease and pulmonary sarcoidosis	Female	1 to 4	80 plus	Data Rich	0	Sociodemographic Status
Interstitial lung disease and pulmonary sarcoidosis	Female	1 to 4	80 plus	Data Rich	1	Cumulative Cigarettes (5 Years)
Interstitial lung disease and pulmonary sarcoidosis	Female	1 to 4	80 plus	Data Rich	1	Elevation 500 to 1500m (proportion)
Interstitial lung disease and pulmonary sarcoidosis	Female	1 to 4	80 plus	Data Rich	1	Elevation Over 1500m (proportion)
Interstitial lung disease and pulmonary sarcoidosis	Female	1 to 4	80 plus	Data Rich	1	Indoor Air Pollution (Biomass Cooking)
Interstitial lung disease and pulmonary sarcoidosis	Female	1 to 4	80 plus	Data Rich	1	Indoor Air Pollution (Coal Cooking)
Interstitial lung disease and pulmonary sarcoidosis	Female	1 to 4	80 plus	Data Rich	1	Log-transformed SEV scalar: ILD
Interstitial lung disease and pulmonary sarcoidosis	Female	1 to 4	80 plus	Data Rich	1	Outdoor Air Pollution (PM2.5)
Interstitial lung disease and pulmonary sarcoidosis	Female	1 to 4	80 plus	Data Rich	1	Population Density (over 1000 ppl/sqkm, proportion)
Interstitial lung disease and pulmonary sarcoidosis	Female	1 to 4	80 plus	Data Rich	1	Smoking Prevalence
Other chronic respiratory diseases	Male	Early Neonatal	80 plus	Global	-1	Education (years per capita)
Other chronic respiratory diseases	Male	Early Neonatal	80 plus	Global	-1	Health System Access 2 (unitless)
Other chronic respiratory diseases	Male	Early Neonatal	80 plus	Global	-1	LDI (I$ per capita)
Other chronic respiratory diseases	Male	Early Neonatal	80 plus	Global	-1	Sociodemographic Status
Other chronic respiratory diseases	Male	Early Neonatal	80 plus	Global	1	Cumulative Cigarettes (5 Years)
Other chronic respiratory diseases	Male	Early Neonatal	80 plus	Global	1	Elevation 500 to 1500m (proportion)
Other chronic respiratory diseases	Male	Early Neonatal	80 plus	Global	1	Elevation Over 1500m (proportion)
Other chronic respiratory diseases	Male	Early Neonatal	80 plus	Global	1	Indoor Air Pollution (Biomass Cooking)
Other chronic respiratory diseases	Male	Early Neonatal	80 plus	Global	1	Indoor Air Pollution (Coal Cooking)
Other chronic respiratory diseases	Male	Early Neonatal	80 plus	Global	1	Log-transformed SEV scalar: Oth Resp
Other chronic respiratory diseases	Male	Early Neonatal	80 plus	Global	1	Outdoor Air Pollution (PM2.5)

Appendix Table 10: CODEm covariates used, level of covariate, and expected direction of covariate by cause, sex, and age						
**Cause**	**Sex**	**Age start**	**Age end**	**Model type**	**Direction**	**Covariate**
Other chronic respiratory diseases	Male	Early Neonatal	80 plus	Global	1	Population Density (over 1000 ppl/sqkm, proportion)
Other chronic respiratory diseases	Male	Early Neonatal	80 plus	Global	1	Smoking Prevalence
Other chronic respiratory diseases	Male	Early Neonatal	80 plus	Data Rich	-1	Education (years per capita)
Other chronic respiratory diseases	Male	Early Neonatal	80 plus	Data Rich	-1	Health System Access 2 (unitless)
Other chronic respiratory diseases	Male	Early Neonatal	80 plus	Data Rich	-1	LDI (I$ per capita)
Other chronic respiratory diseases	Male	Early Neonatal	80 plus	Data Rich	-1	Sociodemographic Status
Other chronic respiratory diseases	Male	Early Neonatal	80 plus	Data Rich	1	Cumulative Cigarettes (5 Years)
Other chronic respiratory diseases	Male	Early Neonatal	80 plus	Data Rich	1	Elevation 500 to 1500m (proportion)
Other chronic respiratory diseases	Male	Early Neonatal	80 plus	Data Rich	1	Elevation Over 1500m (proportion)
Other chronic respiratory diseases	Male	Early Neonatal	80 plus	Data Rich	1	Indoor Air Pollution (Biomass Cooking)
Other chronic respiratory diseases	Male	Early Neonatal	80 plus	Data Rich	1	Indoor Air Pollution (Coal Cooking)
Other chronic respiratory diseases	Male	Early Neonatal	80 plus	Data Rich	1	Log-transformed SEV scalar: Oth Resp
Other chronic respiratory diseases	Male	Early Neonatal	80 plus	Data Rich	1	Outdoor Air Pollution (PM2.5)
Other chronic respiratory diseases	Male	Early Neonatal	80 plus	Data Rich	1	Population Density (over 1000 ppl/sqkm, proportion)
Other chronic respiratory diseases	Male	Early Neonatal	80 plus	Data Rich	1	Smoking Prevalence
Other chronic respiratory diseases	Female	Early Neonatal	80 plus	Global	-1	Education (years per capita)
Other chronic respiratory diseases	Female	Early Neonatal	80 plus	Global	-1	Health System Access 2 (unitless)
Other chronic respiratory diseases	Female	Early Neonatal	80 plus	Global	-1	LDI (I$ per capita)
Other chronic respiratory diseases	Female	Early Neonatal	80 plus	Global	-1	Sociodemographic Status
Other chronic respiratory diseases	Female	Early Neonatal	80 plus	Global	1	Cumulative Cigarettes (5 Years)
Other chronic respiratory diseases	Female	Early Neonatal	80 plus	Global	1	Elevation 500 to 1500m (proportion)
Other chronic respiratory diseases	Female	Early Neonatal	80 plus	Global	1	Elevation Over 1500m (proportion)
Other chronic respiratory diseases	Female	Early Neonatal	80 plus	Global	1	Indoor Air Pollution (Biomass Cooking)
Other chronic respiratory diseases	Female	Early Neonatal	80 plus	Global	1	Indoor Air Pollution (Coal Cooking)
Other chronic respiratory diseases	Female	Early Neonatal	80 plus	Global	1	Log-transformed SEV scalar: Oth Resp
Other chronic respiratory diseases	Female	Early Neonatal	80 plus	Global	1	Outdoor Air Pollution (PM2.5)
Other chronic respiratory diseases	Female	Early Neonatal	80 plus	Global	1	Population Density (over 1000 ppl/sqkm, proportion)
Other chronic respiratory diseases	Female	Early Neonatal	80 plus	Global	1	Smoking Prevalence
Other chronic respiratory diseases	Female	Early Neonatal	80 plus	Data Rich	-1	Education (years per capita)
Other chronic respiratory diseases	Female	Early Neonatal	80 plus	Data Rich	-1	Health System Access 2 (unitless)
Other chronic respiratory diseases	Female	Early Neonatal	80 plus	Data Rich	-1	LDI (I$ per capita)
Other chronic respiratory diseases	Female	Early Neonatal	80 plus	Data Rich	-1	Sociodemographic Status
Other chronic respiratory diseases	Female	Early Neonatal	80 plus	Data Rich	1	Cumulative Cigarettes (5 Years)
Other chronic respiratory diseases	Female	Early Neonatal	80 plus	Data Rich	1	Elevation 500 to 1500m (proportion)
Other chronic respiratory diseases	Female	Early Neonatal	80 plus	Data Rich	1	Elevation Over 1500m (proportion)
Other chronic respiratory diseases	Female	Early Neonatal	80 plus	Data Rich	1	Indoor Air Pollution (Biomass Cooking)
Other chronic respiratory diseases	Female	Early Neonatal	80 plus	Data Rich	1	Indoor Air Pollution (Coal Cooking)
Other chronic respiratory diseases	Female	Early Neonatal	80 plus	Data Rich	1	Log-transformed SEV scalar: Oth Resp
Other chronic respiratory diseases	Female	Early Neonatal	80 plus	Data Rich	1	Outdoor Air Pollution (PM2.5)
Other chronic respiratory diseases	Female	Early Neonatal	80 plus	Data Rich	1	Population Density (over 1000 ppl/sqkm, proportion)
Other chronic respiratory diseases	Female	Early Neonatal	80 plus	Data Rich	1	Smoking Prevalence
Cirrhosis and other chronic liver diseases	Male	1 to 4	80 plus	Global	-1	Education (years per capita)
Cirrhosis and other chronic liver diseases	Male	1 to 4	80 plus	Global	-1	Health System Access 2 (unitless)
Cirrhosis and other chronic liver diseases	Male	1 to 4	80 plus	Global	-1	LDI (I$ per capita)

Appendix Table 10: CODEm covariates used, level of covariate, and expected direction of covariate by cause, sex, and age						
**Cause**	**Sex**	**Age start**	**Age end**	**Model type**	**Direction**	**Covariate**
Cirrhosis and other chronic liver diseases	Male	1 to 4	80 plus	Global	0	Sociodemographic Status
Cirrhosis and other chronic liver diseases	Male	1 to 4	80 plus	Global	1	Alcohol (litres per capita)
Cirrhosis and other chronic liver diseases	Male	1 to 4	80 plus	Global	1	Diabetes Age-Standardised Prevalence (proportion)
Cirrhosis and other chronic liver diseases	Male	1 to 4	80 plus	Global	1	Hepatitis B (HBsAg) Seroprevalence (GBD 2015)
Cirrhosis and other chronic liver diseases	Male	1 to 4	80 plus	Global	1	Hepatitis C (IgG) Seroprevalence (GBD 2015)
Cirrhosis and other chronic liver diseases	Male	1 to 4	80 plus	Global	1	Mean BMI
Cirrhosis and other chronic liver diseases	Male	1 to 4	80 plus	Global	1	Schistosomiasis Prevalence (proportion)
Cirrhosis and other chronic liver diseases	Male	1 to 4	80 plus	Data Rich	-1	Education (years per capita)
Cirrhosis and other chronic liver diseases	Male	1 to 4	80 plus	Data Rich	-1	Health System Access 2 (unitless)
Cirrhosis and other chronic liver diseases	Male	1 to 4	80 plus	Data Rich	-1	LDI (I$ per capita)
Cirrhosis and other chronic liver diseases	Male	1 to 4	80 plus	Data Rich	0	Sociodemographic Status
Cirrhosis and other chronic liver diseases	Male	1 to 4	80 plus	Data Rich	1	Alcohol (litres per capita)
Cirrhosis and other chronic liver diseases	Male	1 to 4	80 plus	Data Rich	1	Diabetes Age-Standardised Prevalence (proportion)
Cirrhosis and other chronic liver diseases	Male	1 to 4	80 plus	Data Rich	1	Hepatitis B (HBsAg) Seroprevalence (GBD 2015)
Cirrhosis and other chronic liver diseases	Male	1 to 4	80 plus	Data Rich	1	Hepatitis C (IgG) Seroprevalence (GBD 2015)
Cirrhosis and other chronic liver diseases	Male	1 to 4	80 plus	Data Rich	1	Mean BMI
Cirrhosis and other chronic liver diseases	Male	1 to 4	80 plus	Data Rich	1	Schistosomiasis Prevalence (proportion)
Cirrhosis and other chronic liver diseases	Female	1 to 4	80 plus	Global	-1	Education (years per capita)
Cirrhosis and other chronic liver diseases	Female	1 to 4	80 plus	Global	-1	Health System Access 2 (unitless)
Cirrhosis and other chronic liver diseases	Female	1 to 4	80 plus	Global	-1	LDI (I$ per capita)
Cirrhosis and other chronic liver diseases	Female	1 to 4	80 plus	Global	0	Sociodemographic Status
Cirrhosis and other chronic liver diseases	Female	1 to 4	80 plus	Global	1	Alcohol (litres per capita)
Cirrhosis and other chronic liver diseases	Female	1 to 4	80 plus	Global	1	Diabetes Age-Standardised Prevalence (proportion)
Cirrhosis and other chronic liver diseases	Female	1 to 4	80 plus	Global	1	Hepatitis B (HBsAg) Seroprevalence (GBD 2015)
Cirrhosis and other chronic liver diseases	Female	1 to 4	80 plus	Global	1	Hepatitis C (IgG) Seroprevalence (GBD 2015)
Cirrhosis and other chronic liver diseases	Female	1 to 4	80 plus	Global	1	Mean BMI
Cirrhosis and other chronic liver diseases	Female	1 to 4	80 plus	Global	1	Schistosomiasis Prevalence (proportion)
Cirrhosis and other chronic liver diseases	Female	1 to 4	80 plus	Data Rich	-1	Education (years per capita)
Cirrhosis and other chronic liver diseases	Female	1 to 4	80 plus	Data Rich	-1	Health System Access 2 (unitless)
Cirrhosis and other chronic liver diseases	Female	1 to 4	80 plus	Data Rich	-1	LDI (I$ per capita)
Cirrhosis and other chronic liver diseases	Female	1 to 4	80 plus	Data Rich	0	Sociodemographic Status
Cirrhosis and other chronic liver diseases	Female	1 to 4	80 plus	Data Rich	1	Alcohol (litres per capita)
Cirrhosis and other chronic liver diseases	Female	1 to 4	80 plus	Data Rich	1	Diabetes Age-Standardised Prevalence (proportion)
Cirrhosis and other chronic liver diseases	Female	1 to 4	80 plus	Data Rich	1	Hepatitis B (HBsAg) Seroprevalence (GBD 2015)
Cirrhosis and other chronic liver diseases	Female	1 to 4	80 plus	Data Rich	1	Hepatitis C (IgG) Seroprevalence (GBD 2015)
Cirrhosis and other chronic liver diseases	Female	1 to 4	80 plus	Data Rich	1	Mean BMI
Cirrhosis and other chronic liver diseases	Female	1 to 4	80 plus	Data Rich	1	Schistosomiasis Prevalence (proportion)
Digestive diseases	Male	1 to 4	80 plus	Global	-1	Education (years per capita)
Digestive diseases	Male	1 to 4	80 plus	Global	-1	Health System Access (capped)
Digestive diseases	Male	1 to 4	80 plus	Global	-1	LDI (I$ per capita)
Digestive diseases	Male	1 to 4	80 plus	Global	-1	Sanitation (proportion with access)
Digestive diseases	Male	1 to 4	80 plus	Global	-1	Sociodemographic Status
Digestive diseases	Male	1 to 4	80 plus	Global	-1	fruits adjusted(g)
Digestive diseases	Male	1 to 4	80 plus	Global	1	Alcohol (litres per capita)

Appendix Table 10: CODEm covariates used, level of covariate, and expected direction of covariate by cause, sex, and age						
Cause	Sex	Age start	Age end	Model type	Direction	Covariate
Digestive diseases	Male	1 to 4	80 plus	Global	1	Cumulative Cigarettes (5 Years)
Digestive diseases	Male	1 to 4	80 plus	Global	1	Malnutrition (proportion <2SD weight for age)
Digestive diseases	Male	1 to 4	80 plus	Global	1	red meats adjusted(g)
Digestive diseases	Male	1 to 4	80 plus	Data Rich	-1	Education (years per capita)
Digestive diseases	Male	1 to 4	80 plus	Data Rich	-1	Health System Access (capped)
Digestive diseases	Male	1 to 4	80 plus	Data Rich	-1	LDI (I$ per capita)
Digestive diseases	Male	1 to 4	80 plus	Data Rich	-1	Sanitation (proportion with access)
Digestive diseases	Male	1 to 4	80 plus	Data Rich	-1	Sociodemographic Status
Digestive diseases	Male	1 to 4	80 plus	Data Rich	-1	fruits adjusted(g)
Digestive diseases	Male	1 to 4	80 plus	Data Rich	1	Alcohol (litres per capita)
Digestive diseases	Male	1 to 4	80 plus	Data Rich	1	Cumulative Cigarettes (5 Years)
Digestive diseases	Male	1 to 4	80 plus	Data Rich	1	Malnutrition (proportion <2SD weight for age)
Digestive diseases	Male	1 to 4	80 plus	Data Rich	1	red meats adjusted(g)
Digestive diseases	Female	1 to 4	80 plus	Global	-1	Education (years per capita)
Digestive diseases	Female	1 to 4	80 plus	Global	-1	Health System Access (capped)
Digestive diseases	Female	1 to 4	80 plus	Global	-1	LDI (I$ per capita)
Digestive diseases	Female	1 to 4	80 plus	Global	-1	Sanitation (proportion with access)
Digestive diseases	Female	1 to 4	80 plus	Global	-1	Sociodemographic Status
Digestive diseases	Female	1 to 4	80 plus	Global	-1	fruits adjusted(g)
Digestive diseases	Female	1 to 4	80 plus	Global	1	Alcohol (litres per capita)
Digestive diseases	Female	1 to 4	80 plus	Global	1	Cumulative Cigarettes (5 Years)
Digestive diseases	Female	1 to 4	80 plus	Global	1	Malnutrition (proportion <2SD weight for age)
Digestive diseases	Female	1 to 4	80 plus	Global	1	red meats adjusted(g)
Digestive diseases	Female	1 to 4	80 plus	Data Rich	-1	Education (years per capita)
Digestive diseases	Female	1 to 4	80 plus	Data Rich	-1	Health System Access (capped)
Digestive diseases	Female	1 to 4	80 plus	Data Rich	-1	LDI (I$ per capita)
Digestive diseases	Female	1 to 4	80 plus	Data Rich	-1	Sanitation (proportion with access)
Digestive diseases	Female	1 to 4	80 plus	Data Rich	-1	Sociodemographic Status
Digestive diseases	Female	1 to 4	80 plus	Data Rich	-1	fruits adjusted(g)
Digestive diseases	Female	1 to 4	80 plus	Data Rich	1	Alcohol (litres per capita)
Digestive diseases	Female	1 to 4	80 plus	Data Rich	1	Cumulative Cigarettes (5 Years)
Digestive diseases	Female	1 to 4	80 plus	Data Rich	1	Malnutrition (proportion <2SD weight for age)
Digestive diseases	Female	1 to 4	80 plus	Data Rich	1	red meats adjusted(g)
Peptic ulcer disease	Male	1 to 4	80 plus	Global	-1	Health System Access (capped)
Peptic ulcer disease	Male	1 to 4	80 plus	Global	-1	LDI (I$ per capita)
Peptic ulcer disease	Male	1 to 4	80 plus	Global	-1	Maternal education (years per capita)
Peptic ulcer disease	Male	1 to 4	80 plus	Global	-1	Sanitation (proportion with access)
Peptic ulcer disease	Male	1 to 4	80 plus	Global	-1	Sociodemographic Status
Peptic ulcer disease	Male	1 to 4	80 plus	Global	-1	vegetables adjusted(g)
Peptic ulcer disease	Male	1 to 4	80 plus	Global	1	Alcohol (litres per capita)
Peptic ulcer disease	Male	1 to 4	80 plus	Global	1	Cumulative Cigarettes (10 Years)
Peptic ulcer disease	Male	1 to 4	80 plus	Global	1	Cumulative Cigarettes (5 Years)
Peptic ulcer disease	Male	1 to 4	80 plus	Global	1	Smoking Prevalence
Peptic ulcer disease	Male	1 to 4	80 plus	Data Rich	-1	Health System Access (capped)

Appendix Table 10: CODEm covariates used, level of covariate, and expected direction of covariate by cause, sex, and age						
**Cause**	**Sex**	**Age start**	**Age end**	**Model type**	**Direction**	**Covariate**
Peptic ulcer disease	Male	1 to 4	80 plus	Data Rich	-1	LDI (I$ per capita)
Peptic ulcer disease	Male	1 to 4	80 plus	Data Rich	-1	Maternal education (years per capita)
Peptic ulcer disease	Male	1 to 4	80 plus	Data Rich	-1	Sanitation (proportion with access)
Peptic ulcer disease	Male	1 to 4	80 plus	Data Rich	-1	Sociodemographic Status
Peptic ulcer disease	Male	1 to 4	80 plus	Data Rich	-1	vegetables adjusted(g)
Peptic ulcer disease	Male	1 to 4	80 plus	Data Rich	1	Alcohol (litres per capita)
Peptic ulcer disease	Male	1 to 4	80 plus	Data Rich	1	Cumulative Cigarettes (10 Years)
Peptic ulcer disease	Male	1 to 4	80 plus	Data Rich	1	Cumulative Cigarettes (5 Years)
Peptic ulcer disease	Male	1 to 4	80 plus	Data Rich	1	Smoking Prevalence
Peptic ulcer disease	Female	1 to 4	80 plus	Global	-1	Health System Access (capped)
Peptic ulcer disease	Female	1 to 4	80 plus	Global	-1	LDI (I$ per capita)
Peptic ulcer disease	Female	1 to 4	80 plus	Global	-1	Maternal education (years per capita)
Peptic ulcer disease	Female	1 to 4	80 plus	Global	-1	Sanitation (proportion with access)
Peptic ulcer disease	Female	1 to 4	80 plus	Global	-1	Sociodemographic Status
Peptic ulcer disease	Female	1 to 4	80 plus	Global	-1	vegetables adjusted(g)
Peptic ulcer disease	Female	1 to 4	80 plus	Global	1	Alcohol (litres per capita)
Peptic ulcer disease	Female	1 to 4	80 plus	Global	1	Cumulative Cigarettes (10 Years)
Peptic ulcer disease	Female	1 to 4	80 plus	Global	1	Cumulative Cigarettes (5 Years)
Peptic ulcer disease	Female	1 to 4	80 plus	Global	1	Smoking Prevalence
Peptic ulcer disease	Female	1 to 4	80 plus	Data Rich	-1	Health System Access (capped)
Peptic ulcer disease	Female	1 to 4	80 plus	Data Rich	-1	LDI (I$ per capita)
Peptic ulcer disease	Female	1 to 4	80 plus	Data Rich	-1	Maternal education (years per capita)
Peptic ulcer disease	Female	1 to 4	80 plus	Data Rich	-1	Sanitation (proportion with access)
Peptic ulcer disease	Female	1 to 4	80 plus	Data Rich	-1	Sociodemographic Status
Peptic ulcer disease	Female	1 to 4	80 plus	Data Rich	-1	vegetables adjusted(g)
Peptic ulcer disease	Female	1 to 4	80 plus	Data Rich	1	Alcohol (litres per capita)
Peptic ulcer disease	Female	1 to 4	80 plus	Data Rich	1	Cumulative Cigarettes (10 Years)
Peptic ulcer disease	Female	1 to 4	80 plus	Data Rich	1	Cumulative Cigarettes (5 Years)
Peptic ulcer disease	Female	1 to 4	80 plus	Data Rich	1	Smoking Prevalence
Gastritis and duodenitis	Male	1 to 4	80 plus	Global	-1	Education (years per capita)
Gastritis and duodenitis	Male	1 to 4	80 plus	Global	-1	Health System Access (capped)
Gastritis and duodenitis	Male	1 to 4	80 plus	Global	-1	Improved Water Source (proportion with access)
Gastritis and duodenitis	Male	1 to 4	80 plus	Global	-1	LDI (I$ per capita)
Gastritis and duodenitis	Male	1 to 4	80 plus	Global	-1	Sanitation (proportion with access)
Gastritis and duodenitis	Male	1 to 4	80 plus	Global	-1	Sociodemographic Status
Gastritis and duodenitis	Male	1 to 4	80 plus	Global	0	vegetables adjusted(g)
Gastritis and duodenitis	Male	1 to 4	80 plus	Global	1	Alcohol (litres per capita)
Gastritis and duodenitis	Male	1 to 4	80 plus	Global	1	Cumulative Cigarettes (10 Years)
Gastritis and duodenitis	Male	1 to 4	80 plus	Global	1	Cumulative Cigarettes (5 Years)
Gastritis and duodenitis	Male	1 to 4	80 plus	Global	1	Smoking Prevalence
Gastritis and duodenitis	Male	1 to 4	80 plus	Data Rich	-1	Education (years per capita)
Gastritis and duodenitis	Male	1 to 4	80 plus	Data Rich	-1	Health System Access (capped)
Gastritis and duodenitis	Male	1 to 4	80 plus	Data Rich	-1	Improved Water Source (proportion with access)
Gastritis and duodenitis	Male	1 to 4	80 plus	Data Rich	-1	LDI (I$ per capita)

Appendix Table 10: CODEm covariates used, level of covariate, and expected direction of covariate by cause, sex, and age						
**Cause**	**Sex**	**Age start**	**Age end**	**Model type**	**Direction**	**Covariate**
Gastritis and duodenitis	Male	1 to 4	80 plus	Data Rich	-1	Sanitation (proportion with access)
Gastritis and duodenitis	Male	1 to 4	80 plus	Data Rich	-1	Sociodemographic Status
Gastritis and duodenitis	Male	1 to 4	80 plus	Data Rich	0	vegetables adjusted(g)
Gastritis and duodenitis	Male	1 to 4	80 plus	Data Rich	1	Alcohol (litres per capita)
Gastritis and duodenitis	Male	1 to 4	80 plus	Data Rich	1	Cumulative Cigarettes (10 Years)
Gastritis and duodenitis	Male	1 to 4	80 plus	Data Rich	1	Cumulative Cigarettes (5 Years)
Gastritis and duodenitis	Male	1 to 4	80 plus	Data Rich	1	Smoking Prevalence
Gastritis and duodenitis	Female	1 to 4	80 plus	Global	-1	Education (years per capita)
Gastritis and duodenitis	Female	1 to 4	80 plus	Global	-1	Health System Access (capped)
Gastritis and duodenitis	Female	1 to 4	80 plus	Global	-1	Improved Water Source (proportion with access)
Gastritis and duodenitis	Female	1 to 4	80 plus	Global	-1	LDI (I$ per capita)
Gastritis and duodenitis	Female	1 to 4	80 plus	Global	-1	Sanitation (proportion with access)
Gastritis and duodenitis	Female	1 to 4	80 plus	Global	-1	Sociodemographic Status
Gastritis and duodenitis	Female	1 to 4	80 plus	Global	0	vegetables adjusted(g)
Gastritis and duodenitis	Female	1 to 4	80 plus	Global	1	Alcohol (litres per capita)
Gastritis and duodenitis	Female	1 to 4	80 plus	Global	1	Cumulative Cigarettes (10 Years)
Gastritis and duodenitis	Female	1 to 4	80 plus	Global	1	Cumulative Cigarettes (5 Years)
Gastritis and duodenitis	Female	1 to 4	80 plus	Global	1	Smoking Prevalence
Gastritis and duodenitis	Female	1 to 4	80 plus	Data Rich	-1	Education (years per capita)
Gastritis and duodenitis	Female	1 to 4	80 plus	Data Rich	-1	Health System Access (capped)
Gastritis and duodenitis	Female	1 to 4	80 plus	Data Rich	-1	Improved Water Source (proportion with access)
Gastritis and duodenitis	Female	1 to 4	80 plus	Data Rich	-1	LDI (I$ per capita)
Gastritis and duodenitis	Female	1 to 4	80 plus	Data Rich	-1	Sanitation (proportion with access)
Gastritis and duodenitis	Female	1 to 4	80 plus	Data Rich	-1	Sociodemographic Status
Gastritis and duodenitis	Female	1 to 4	80 plus	Data Rich	0	vegetables adjusted(g)
Gastritis and duodenitis	Female	1 to 4	80 plus	Data Rich	1	Alcohol (litres per capita)
Gastritis and duodenitis	Female	1 to 4	80 plus	Data Rich	1	Cumulative Cigarettes (10 Years)
Gastritis and duodenitis	Female	1 to 4	80 plus	Data Rich	1	Cumulative Cigarettes (5 Years)
Gastritis and duodenitis	Female	1 to 4	80 plus	Data Rich	1	Smoking Prevalence
Appendicitis	Male	1 to 4	80 plus	Global	-1	Education (years per capita)
Appendicitis	Male	1 to 4	80 plus	Global	-1	Health System Access (capped)
Appendicitis	Male	1 to 4	80 plus	Global	-1	LDI (I$ per capita)
Appendicitis	Male	1 to 4	80 plus	Global	-1	Sociodemographic Status
Appendicitis	Male	1 to 4	80 plus	Global	-1	fruits adjusted(g)
Appendicitis	Male	1 to 4	80 plus	Global	-1	vegetables adjusted(g)
Appendicitis	Male	1 to 4	80 plus	Data Rich	-1	Education (years per capita)
Appendicitis	Male	1 to 4	80 plus	Data Rich	-1	Health System Access (capped)
Appendicitis	Male	1 to 4	80 plus	Data Rich	-1	LDI (I$ per capita)
Appendicitis	Male	1 to 4	80 plus	Data Rich	-1	Sociodemographic Status
Appendicitis	Male	1 to 4	80 plus	Data Rich	-1	fruits adjusted(g)
Appendicitis	Male	1 to 4	80 plus	Data Rich	-1	vegetables adjusted(g)
Appendicitis	Female	1 to 4	80 plus	Global	-1	Education (years per capita)
Appendicitis	Female	1 to 4	80 plus	Global	-1	Health System Access (capped)
Appendicitis	Female	1 to 4	80 plus	Global	-1	LDI (I$ per capita)

Appendix Table 10: CODEm covariates used, level of covariate, and expected direction of covariate by cause, sex, and age						
**Cause**	**Sex**	**Age start**	**Age end**	**Model type**	**Direction**	**Covariate**
Appendicitis	Female	1 to 4	80 plus	Global	-1	Sociodemographic Status
Appendicitis	Female	1 to 4	80 plus	Global	-1	fruits adjusted(g)
Appendicitis	Female	1 to 4	80 plus	Global	-1	vegetables adjusted(g)
Appendicitis	Female	1 to 4	80 plus	Data Rich	-1	Education (years per capita)
Appendicitis	Female	1 to 4	80 plus	Data Rich	-1	Health System Access (capped)
Appendicitis	Female	1 to 4	80 plus	Data Rich	-1	LDI (I$ per capita)
Appendicitis	Female	1 to 4	80 plus	Data Rich	-1	Sociodemographic Status
Appendicitis	Female	1 to 4	80 plus	Data Rich	-1	fruits adjusted(g)
Appendicitis	Female	1 to 4	80 plus	Data Rich	-1	vegetables adjusted(g)
Paralytic ileus and intestinal obstruction	Male	Early Neonatal	80 plus	Global	-1	Education (years per capita)
Paralytic ileus and intestinal obstruction	Male	Early Neonatal	80 plus	Global	-1	Health System Access (capped)
Paralytic ileus and intestinal obstruction	Male	Early Neonatal	80 plus	Global	-1	LDI (I$ per capita)
Paralytic ileus and intestinal obstruction	Male	Early Neonatal	80 plus	Global	-1	Sociodemographic Status
Paralytic ileus and intestinal obstruction	Male	Early Neonatal	80 plus	Global	-1	fruits adjusted(g)
Paralytic ileus and intestinal obstruction	Male	Early Neonatal	80 plus	Global	-1	vegetables adjusted(g)
Paralytic ileus and intestinal obstruction	Male	Early Neonatal	80 plus	Data Rich	-1	Education (years per capita)
Paralytic ileus and intestinal obstruction	Male	Early Neonatal	80 plus	Data Rich	-1	Health System Access (capped)
Paralytic ileus and intestinal obstruction	Male	Early Neonatal	80 plus	Data Rich	-1	LDI (I$ per capita)
Paralytic ileus and intestinal obstruction	Male	Early Neonatal	80 plus	Data Rich	-1	Sociodemographic Status
Paralytic ileus and intestinal obstruction	Male	Early Neonatal	80 plus	Data Rich	-1	fruits adjusted(g)
Paralytic ileus and intestinal obstruction	Male	Early Neonatal	80 plus	Data Rich	-1	vegetables adjusted(g)
Paralytic ileus and intestinal obstruction	Female	Early Neonatal	80 plus	Global	-1	Education (years per capita)
Paralytic ileus and intestinal obstruction	Female	Early Neonatal	80 plus	Global	-1	Health System Access (capped)
Paralytic ileus and intestinal obstruction	Female	Early Neonatal	80 plus	Global	-1	LDI (I$ per capita)
Paralytic ileus and intestinal obstruction	Female	Early Neonatal	80 plus	Global	-1	Sociodemographic Status
Paralytic ileus and intestinal obstruction	Female	Early Neonatal	80 plus	Global	-1	fruits adjusted(g)
Paralytic ileus and intestinal obstruction	Female	Early Neonatal	80 plus	Global	-1	vegetables adjusted(g)
Paralytic ileus and intestinal obstruction	Female	Early Neonatal	80 plus	Data Rich	-1	Education (years per capita)
Paralytic ileus and intestinal obstruction	Female	Early Neonatal	80 plus	Data Rich	-1	Health System Access (capped)
Paralytic ileus and intestinal obstruction	Female	Early Neonatal	80 plus	Data Rich	-1	LDI (I$ per capita)
Paralytic ileus and intestinal obstruction	Female	Early Neonatal	80 plus	Data Rich	-1	Sociodemographic Status
Paralytic ileus and intestinal obstruction	Female	Early Neonatal	80 plus	Data Rich	-1	fruits adjusted(g)
Paralytic ileus and intestinal obstruction	Female	Early Neonatal	80 plus	Data Rich	-1	vegetables adjusted(g)
Inguinal, femoral, and abdominal hernia	Male	1 to 4	80 plus	Global	-1	Education (years per capita)
Inguinal, femoral, and abdominal hernia	Male	1 to 4	80 plus	Global	-1	Health System Access (capped)
Inguinal, femoral, and abdominal hernia	Male	1 to 4	80 plus	Global	-1	LDI (I$ per capita)
Inguinal, femoral, and abdominal hernia	Male	1 to 4	80 plus	Global	0	Sociodemographic Status
Inguinal, femoral, and abdominal hernia	Male	1 to 4	80 plus	Data Rich	-1	Education (years per capita)
Inguinal, femoral, and abdominal hernia	Male	1 to 4	80 plus	Data Rich	-1	Health System Access (capped)
Inguinal, femoral, and abdominal hernia	Male	1 to 4	80 plus	Data Rich	-1	LDI (I$ per capita)
Inguinal, femoral, and abdominal hernia	Male	1 to 4	80 plus	Data Rich	0	Sociodemographic Status
Inguinal, femoral, and abdominal hernia	Female	1 to 4	80 plus	Global	-1	Education (years per capita)
Inguinal, femoral, and abdominal hernia	Female	1 to 4	80 plus	Global	-1	Health System Access (capped)
Inguinal, femoral, and abdominal hernia	Female	1 to 4	80 plus	Global	-1	LDI (I$ per capita)

Appendix Table 10: CODEm covariates used, level of covariate, and expected direction of covariate by cause, sex, and age						
**Cause**	**Sex**	**Age start**	**Age end**	**Model type**	**Direction**	**Covariate**
Inguinal, femoral, and abdominal hernia	Female	1 to 4	80 plus	Global	0	Sociodemographic Status
Inguinal, femoral, and abdominal hernia	Female	1 to 4	80 plus	Data Rich	-1	Education (years per capita)
Inguinal, femoral, and abdominal hernia	Female	1 to 4	80 plus	Data Rich	-1	Health System Access (capped)
Inguinal, femoral, and abdominal hernia	Female	1 to 4	80 plus	Data Rich	-1	LDI (I$ per capita)
Inguinal, femoral, and abdominal hernia	Female	1 to 4	80 plus	Data Rich	0	Sociodemographic Status
Inflammatory bowel disease	Male	1 to 4	80 plus	Global	-1	Education (years per capita)
Inflammatory bowel disease	Male	1 to 4	80 plus	Global	-1	Health System Access (capped)
Inflammatory bowel disease	Male	1 to 4	80 plus	Global	-1	Latitude 15 to 30 (proportion)
Inflammatory bowel disease	Male	1 to 4	80 plus	Global	-1	fruits adjusted(g)
Inflammatory bowel disease	Male	1 to 4	80 plus	Global	-1	vegetables adjusted(g)
Inflammatory bowel disease	Male	1 to 4	80 plus	Global	0	LDI (I$ per capita)
Inflammatory bowel disease	Male	1 to 4	80 plus	Global	0	Sociodemographic Status
Inflammatory bowel disease	Male	1 to 4	80 plus	Global	1	Animal Fats (kcal per capita)
Inflammatory bowel disease	Male	1 to 4	80 plus	Global	1	Latitude 30 to 45 (proportion)
Inflammatory bowel disease	Male	1 to 4	80 plus	Global	1	Latitude Over 45 (proportion)
Inflammatory bowel disease	Male	1 to 4	80 plus	Global	1	red meats adjusted(g)
Inflammatory bowel disease	Male	1 to 4	80 plus	Data Rich	-1	Education (years per capita)
Inflammatory bowel disease	Male	1 to 4	80 plus	Data Rich	-1	Health System Access (capped)
Inflammatory bowel disease	Male	1 to 4	80 plus	Data Rich	-1	Latitude 15 to 30 (proportion)
Inflammatory bowel disease	Male	1 to 4	80 plus	Data Rich	-1	fruits adjusted(g)
Inflammatory bowel disease	Male	1 to 4	80 plus	Data Rich	-1	vegetables adjusted(g)
Inflammatory bowel disease	Male	1 to 4	80 plus	Data Rich	0	LDI (I$ per capita)
Inflammatory bowel disease	Male	1 to 4	80 plus	Data Rich	0	Sociodemographic Status
Inflammatory bowel disease	Male	1 to 4	80 plus	Data Rich	1	Animal Fats (kcal per capita)
Inflammatory bowel disease	Male	1 to 4	80 plus	Data Rich	1	Latitude 30 to 45 (proportion)
Inflammatory bowel disease	Male	1 to 4	80 plus	Data Rich	1	Latitude Over 45 (proportion)
Inflammatory bowel disease	Male	1 to 4	80 plus	Data Rich	1	red meats adjusted(g)
Inflammatory bowel disease	Female	1 to 4	80 plus	Global	-1	Education (years per capita)
Inflammatory bowel disease	Female	1 to 4	80 plus	Global	-1	Health System Access (capped)
Inflammatory bowel disease	Female	1 to 4	80 plus	Global	-1	Latitude 15 to 30 (proportion)
Inflammatory bowel disease	Female	1 to 4	80 plus	Global	-1	fruits adjusted(g)
Inflammatory bowel disease	Female	1 to 4	80 plus	Global	-1	vegetables adjusted(g)
Inflammatory bowel disease	Female	1 to 4	80 plus	Global	0	LDI (I$ per capita)
Inflammatory bowel disease	Female	1 to 4	80 plus	Global	0	Sociodemographic Status
Inflammatory bowel disease	Female	1 to 4	80 plus	Global	1	Animal Fats (kcal per capita)
Inflammatory bowel disease	Female	1 to 4	80 plus	Global	1	Latitude 30 to 45 (proportion)
Inflammatory bowel disease	Female	1 to 4	80 plus	Global	1	Latitude Over 45 (proportion)
Inflammatory bowel disease	Female	1 to 4	80 plus	Global	1	red meats adjusted(g)
Inflammatory bowel disease	Female	1 to 4	80 plus	Data Rich	-1	Education (years per capita)
Inflammatory bowel disease	Female	1 to 4	80 plus	Data Rich	-1	Health System Access (capped)
Inflammatory bowel disease	Female	1 to 4	80 plus	Data Rich	-1	Latitude 15 to 30 (proportion)
Inflammatory bowel disease	Female	1 to 4	80 plus	Data Rich	-1	fruits adjusted(g)
Inflammatory bowel disease	Female	1 to 4	80 plus	Data Rich	-1	vegetables adjusted(g)
Inflammatory bowel disease	Female	1 to 4	80 plus	Data Rich	0	LDI (I$ per capita)

Appendix Table 10: CODEm covariates used, level of covariate, and expected direction of covariate by cause, sex, and age						
**Cause**	**Sex**	**Age start**	**Age end**	**Model type**	**Direction**	**Covariate**
Inflammatory bowel disease	Female	1 to 4	80 plus	Data Rich	0	Sociodemographic Status
Inflammatory bowel disease	Female	1 to 4	80 plus	Data Rich	1	Animal Fats (kcal per capita)
Inflammatory bowel disease	Female	1 to 4	80 plus	Data Rich	1	Latitude 30 to 45 (proportion)
Inflammatory bowel disease	Female	1 to 4	80 plus	Data Rich	1	Latitude Over 45 (proportion)
Inflammatory bowel disease	Female	1 to 4	80 plus	Data Rich	1	red meats adjusted(g)
Vascular intestinal disorders	Male	1 to 4	80 plus	Global	-1	Education (years per capita)
Vascular intestinal disorders	Male	1 to 4	80 plus	Global	-1	Health System Access (capped)
Vascular intestinal disorders	Male	1 to 4	80 plus	Global	-1	LDI (I$ per capita)
Vascular intestinal disorders	Male	1 to 4	80 plus	Global	-1	fruits adjusted(g)
Vascular intestinal disorders	Male	1 to 4	80 plus	Global	-1	vegetables adjusted(g)
Vascular intestinal disorders	Male	1 to 4	80 plus	Global	0	Sociodemographic Status
Vascular intestinal disorders	Male	1 to 4	80 plus	Global	1	Alcohol (litres per capita)
Vascular intestinal disorders	Male	1 to 4	80 plus	Global	1	Animal Fats (kcal per capita)
Vascular intestinal disorders	Male	1 to 4	80 plus	Global	1	Cholesterol (total, mean per capita)
Vascular intestinal disorders	Male	1 to 4	80 plus	Global	1	Diabetes Age-Standardised Prevalence (proportion)
Vascular intestinal disorders	Male	1 to 4	80 plus	Global	1	Diabetes Fasting Plasma Glucose (mmol/L)
Vascular intestinal disorders	Male	1 to 4	80 plus	Global	1	Latitude Over 45 (proportion)
Vascular intestinal disorders	Male	1 to 4	80 plus	Global	1	Systolic Blood Pressure (mmHg)
Vascular intestinal disorders	Male	1 to 4	80 plus	Data Rich	-1	Education (years per capita)
Vascular intestinal disorders	Male	1 to 4	80 plus	Data Rich	-1	Health System Access (capped)
Vascular intestinal disorders	Male	1 to 4	80 plus	Data Rich	-1	LDI (I$ per capita)
Vascular intestinal disorders	Male	1 to 4	80 plus	Data Rich	-1	fruits adjusted(g)
Vascular intestinal disorders	Male	1 to 4	80 plus	Data Rich	-1	vegetables adjusted(g)
Vascular intestinal disorders	Male	1 to 4	80 plus	Data Rich	0	Sociodemographic Status
Vascular intestinal disorders	Male	1 to 4	80 plus	Data Rich	1	Alcohol (litres per capita)
Vascular intestinal disorders	Male	1 to 4	80 plus	Data Rich	1	Animal Fats (kcal per capita)
Vascular intestinal disorders	Male	1 to 4	80 plus	Data Rich	1	Cholesterol (total, mean per capita)
Vascular intestinal disorders	Male	1 to 4	80 plus	Data Rich	1	Diabetes Age-Standardised Prevalence (proportion)
Vascular intestinal disorders	Male	1 to 4	80 plus	Data Rich	1	Diabetes Fasting Plasma Glucose (mmol/L)
Vascular intestinal disorders	Male	1 to 4	80 plus	Data Rich	1	Latitude Over 45 (proportion)
Vascular intestinal disorders	Male	1 to 4	80 plus	Data Rich	1	Systolic Blood Pressure (mmHg)
Vascular intestinal disorders	Female	1 to 4	80 plus	Global	-1	Education (years per capita)
Vascular intestinal disorders	Female	1 to 4	80 plus	Global	-1	Health System Access (capped)
Vascular intestinal disorders	Female	1 to 4	80 plus	Global	-1	LDI (I$ per capita)
Vascular intestinal disorders	Female	1 to 4	80 plus	Global	-1	fruits adjusted(g)
Vascular intestinal disorders	Female	1 to 4	80 plus	Global	-1	vegetables adjusted(g)
Vascular intestinal disorders	Female	1 to 4	80 plus	Global	0	Sociodemographic Status
Vascular intestinal disorders	Female	1 to 4	80 plus	Global	1	Alcohol (litres per capita)
Vascular intestinal disorders	Female	1 to 4	80 plus	Global	1	Animal Fats (kcal per capita)
Vascular intestinal disorders	Female	1 to 4	80 plus	Global	1	Cholesterol (total, mean per capita)
Vascular intestinal disorders	Female	1 to 4	80 plus	Global	1	Diabetes Age-Standardised Prevalence (proportion)
Vascular intestinal disorders	Female	1 to 4	80 plus	Global	1	Diabetes Fasting Plasma Glucose (mmol/L)
Vascular intestinal disorders	Female	1 to 4	80 plus	Global	1	Latitude Over 45 (proportion)
Vascular intestinal disorders	Female	1 to 4	80 plus	Global	1	Systolic Blood Pressure (mmHg)

Appendix Table 10: CODEm covariates used, level of covariate, and expected direction of covariate by cause, sex, and age

Cause	Sex	Age start	Age end	Model type	Direction	Covariate
Vascular intestinal disorders	Female	1 to 4	80 plus	Data Rich	-1	Education (years per capita)
Vascular intestinal disorders	Female	1 to 4	80 plus	Data Rich	-1	Health System Access (capped)
Vascular intestinal disorders	Female	1 to 4	80 plus	Data Rich	-1	LDI (I$ per capita)
Vascular intestinal disorders	Female	1 to 4	80 plus	Data Rich	-1	fruits adjusted(g)
Vascular intestinal disorders	Female	1 to 4	80 plus	Data Rich	-1	vegetables adjusted(g)
Vascular intestinal disorders	Female	1 to 4	80 plus	Data Rich	0	Sociodemographic Status
Vascular intestinal disorders	Female	1 to 4	80 plus	Data Rich	1	Alcohol (litres per capita)
Vascular intestinal disorders	Female	1 to 4	80 plus	Data Rich	1	Animal Fats (kcal per capita)
Vascular intestinal disorders	Female	1 to 4	80 plus	Data Rich	1	Cholesterol (total, mean per capita)
Vascular intestinal disorders	Female	1 to 4	80 plus	Data Rich	1	Diabetes Age-Standardised Prevalence (proportion)
Vascular intestinal disorders	Female	1 to 4	80 plus	Data Rich	1	Diabetes Fasting Plasma Glucose (mmol/L)
Vascular intestinal disorders	Female	1 to 4	80 plus	Data Rich	1	Latitude Over 45 (proportion)
Vascular intestinal disorders	Female	1 to 4	80 plus	Data Rich	1	Systolic Blood Pressure (mmHg)
Gallbladder and biliary diseases	Male	1 to 4	80 plus	Global	-1	Health System Access (capped)
Gallbladder and biliary diseases	Male	1 to 4	80 plus	Global	0	Education (years per capita)
Gallbladder and biliary diseases	Male	1 to 4	80 plus	Global	0	LDI (I$ per capita)
Gallbladder and biliary diseases	Male	1 to 4	80 plus	Global	0	Sociodemographic Status
Gallbladder and biliary diseases	Male	1 to 4	80 plus	Global	1	Alcohol (litres per capita)
Gallbladder and biliary diseases	Male	1 to 4	80 plus	Global	1	Animal Fats (kcal per capita)
Gallbladder and biliary diseases	Male	1 to 4	80 plus	Global	1	Mean BMI
Gallbladder and biliary diseases	Male	1 to 4	80 plus	Global	1	Population Over 65 (proportion)
Gallbladder and biliary diseases	Male	1 to 4	80 plus	Global	1	red meats adjusted(g)
Gallbladder and biliary diseases	Male	1 to 4	80 plus	Data Rich	-1	Health System Access (capped)
Gallbladder and biliary diseases	Male	1 to 4	80 plus	Data Rich	0	Education (years per capita)
Gallbladder and biliary diseases	Male	1 to 4	80 plus	Data Rich	0	LDI (I$ per capita)
Gallbladder and biliary diseases	Male	1 to 4	80 plus	Data Rich	0	Sociodemographic Status
Gallbladder and biliary diseases	Male	1 to 4	80 plus	Data Rich	1	Alcohol (litres per capita)
Gallbladder and biliary diseases	Male	1 to 4	80 plus	Data Rich	1	Animal Fats (kcal per capita)
Gallbladder and biliary diseases	Male	1 to 4	80 plus	Data Rich	1	Mean BMI
Gallbladder and biliary diseases	Male	1 to 4	80 plus	Data Rich	1	Population Over 65 (proportion)
Gallbladder and biliary diseases	Male	1 to 4	80 plus	Data Rich	1	red meats adjusted(g)
Gallbladder and biliary diseases	Female	1 to 4	80 plus	Global	-1	Health System Access (capped)
Gallbladder and biliary diseases	Female	1 to 4	80 plus	Global	0	Education (years per capita)
Gallbladder and biliary diseases	Female	1 to 4	80 plus	Global	0	LDI (I$ per capita)
Gallbladder and biliary diseases	Female	1 to 4	80 plus	Global	0	Sociodemographic Status
Gallbladder and biliary diseases	Female	1 to 4	80 plus	Global	1	Alcohol (litres per capita)
Gallbladder and biliary diseases	Female	1 to 4	80 plus	Global	1	Animal Fats (kcal per capita)
Gallbladder and biliary diseases	Female	1 to 4	80 plus	Global	1	Mean BMI
Gallbladder and biliary diseases	Female	1 to 4	80 plus	Global	1	Population Over 65 (proportion)
Gallbladder and biliary diseases	Female	1 to 4	80 plus	Global	1	red meats adjusted(g)
Gallbladder and biliary diseases	Female	1 to 4	80 plus	Data Rich	-1	Health System Access (capped)
Gallbladder and biliary diseases	Female	1 to 4	80 plus	Data Rich	0	Education (years per capita)
Gallbladder and biliary diseases	Female	1 to 4	80 plus	Data Rich	0	LDI (I$ per capita)
Gallbladder and biliary diseases	Female	1 to 4	80 plus	Data Rich	0	Sociodemographic Status

Appendix Table 10: CODEm covariates used, level of covariate, and expected direction of covariate by cause, sex, and age						
Cause	Sex	Age start	Age end	Model type	Direction	Covariate
Gallbladder and biliary diseases	Female	1 to 4	80 plus	Data Rich	1	Alcohol (litres per capita)
Gallbladder and biliary diseases	Female	1 to 4	80 plus	Data Rich	1	Animal Fats (kcal per capita)
Gallbladder and biliary diseases	Female	1 to 4	80 plus	Data Rich	1	Mean BMI
Gallbladder and biliary diseases	Female	1 to 4	80 plus	Data Rich	1	Population Over 65 (proportion)
Gallbladder and biliary diseases	Female	1 to 4	80 plus	Data Rich	1	red meats adjusted(g)
Pancreatitis	Male	1 to 4	80 plus	Global	-1	Education (years per capita)
Pancreatitis	Male	1 to 4	80 plus	Global	-1	Health System Access (capped)
Pancreatitis	Male	1 to 4	80 plus	Global	0	LDI (I$ per capita)
Pancreatitis	Male	1 to 4	80 plus	Global	0	Sociodemographic Status
Pancreatitis	Male	1 to 4	80 plus	Global	1	Alcohol (litres per capita)
Pancreatitis	Male	1 to 4	80 plus	Global	1	Log-transformed SEV scalar: Pancreatit
Pancreatitis	Male	1 to 4	80 plus	Global	1	Mean BMI
Pancreatitis	Male	1 to 4	80 plus	Data Rich	-1	Education (years per capita)
Pancreatitis	Male	1 to 4	80 plus	Data Rich	-1	Health System Access (capped)
Pancreatitis	Male	1 to 4	80 plus	Data Rich	0	LDI (I$ per capita)
Pancreatitis	Male	1 to 4	80 plus	Data Rich	0	Sociodemographic Status
Pancreatitis	Male	1 to 4	80 plus	Data Rich	1	Alcohol (litres per capita)
Pancreatitis	Male	1 to 4	80 plus	Data Rich	1	Log-transformed SEV scalar: Pancreatit
Pancreatitis	Male	1 to 4	80 plus	Data Rich	1	Mean BMI
Pancreatitis	Female	1 to 4	80 plus	Global	-1	Education (years per capita)
Pancreatitis	Female	1 to 4	80 plus	Global	-1	Health System Access (capped)
Pancreatitis	Female	1 to 4	80 plus	Global	0	LDI (I$ per capita)
Pancreatitis	Female	1 to 4	80 plus	Global	0	Sociodemographic Status
Pancreatitis	Female	1 to 4	80 plus	Global	1	Alcohol (litres per capita)
Pancreatitis	Female	1 to 4	80 plus	Global	1	Log-transformed SEV scalar: Pancreatit
Pancreatitis	Female	1 to 4	80 plus	Global	1	Mean BMI
Pancreatitis	Female	1 to 4	80 plus	Data Rich	-1	Education (years per capita)
Pancreatitis	Female	1 to 4	80 plus	Data Rich	-1	Health System Access (capped)
Pancreatitis	Female	1 to 4	80 plus	Data Rich	0	LDI (I$ per capita)
Pancreatitis	Female	1 to 4	80 plus	Data Rich	0	Sociodemographic Status
Pancreatitis	Female	1 to 4	80 plus	Data Rich	1	Alcohol (litres per capita)
Pancreatitis	Female	1 to 4	80 plus	Data Rich	1	Log-transformed SEV scalar: Pancreatit
Pancreatitis	Female	1 to 4	80 plus	Data Rich	1	Mean BMI
Other digestive diseases	Male	1 to 4	80 plus	Global	-1	Education (years per capita)
Other digestive diseases	Male	1 to 4	80 plus	Global	-1	Health System Access 2 (unitless)
Other digestive diseases	Male	1 to 4	80 plus	Global	-1	Improved Water Source (proportion with access)
Other digestive diseases	Male	1 to 4	80 plus	Global	-1	LDI (I$ per capita)
Other digestive diseases	Male	1 to 4	80 plus	Global	-1	Sanitation (proportion with access)
Other digestive diseases	Male	1 to 4	80 plus	Global	-1	fruits adjusted(g)
Other digestive diseases	Male	1 to 4	80 plus	Global	0	Sociodemographic Status
Other digestive diseases	Male	1 to 4	80 plus	Global	0	vegetables adjusted(g)
Other digestive diseases	Male	1 to 4	80 plus	Global	1	Alcohol (litres per capita)
Other digestive diseases	Male	1 to 4	80 plus	Global	1	Animal Fats (kcal per capita)
Other digestive diseases	Male	1 to 4	80 plus	Global	1	Cumulative Cigarettes (10 Years)

Appendix Table 10: CODEm covariates used, level of covariate, and expected direction of covariate by cause, sex, and age						
**Cause**	**Sex**	**Age start**	**Age end**	**Model type**	**Direction**	**Covariate**
Other digestive diseases	Male	1 to 4	80 plus	Global	1	Cumulative Cigarettes (5 Years)
Other digestive diseases	Male	1 to 4	80 plus	Global	1	Diabetes Age-Standardised Prevalence (proportion)
Other digestive diseases	Male	1 to 4	80 plus	Global	1	Malnutrition (proportion <2SD weight for age)
Other digestive diseases	Male	1 to 4	80 plus	Global	1	Mean BMI
Other digestive diseases	Male	1 to 4	80 plus	Global	1	Smoking Prevalence
Other digestive diseases	Male	1 to 4	80 plus	Global	1	red meats adjusted(g)
Other digestive diseases	Male	1 to 4	80 plus	Data Rich	-1	Education (years per capita)
Other digestive diseases	Male	1 to 4	80 plus	Data Rich	-1	Health System Access 2 (unitless)
Other digestive diseases	Male	1 to 4	80 plus	Data Rich	-1	Improved Water Source (proportion with access)
Other digestive diseases	Male	1 to 4	80 plus	Data Rich	-1	LDI (I$ per capita)
Other digestive diseases	Male	1 to 4	80 plus	Data Rich	-1	Sanitation (proportion with access)
Other digestive diseases	Male	1 to 4	80 plus	Data Rich	-1	fruits adjusted(g)
Other digestive diseases	Male	1 to 4	80 plus	Data Rich	0	Sociodemographic Status
Other digestive diseases	Male	1 to 4	80 plus	Data Rich	0	vegetables adjusted(g)
Other digestive diseases	Male	1 to 4	80 plus	Data Rich	1	Alcohol (litres per capita)
Other digestive diseases	Male	1 to 4	80 plus	Data Rich	1	Animal Fats (kcal per capita)
Other digestive diseases	Male	1 to 4	80 plus	Data Rich	1	Cumulative Cigarettes (10 Years)
Other digestive diseases	Male	1 to 4	80 plus	Data Rich	1	Cumulative Cigarettes (5 Years)
Other digestive diseases	Male	1 to 4	80 plus	Data Rich	1	Diabetes Age-Standardised Prevalence (proportion)
Other digestive diseases	Male	1 to 4	80 plus	Data Rich	1	Malnutrition (proportion <2SD weight for age)
Other digestive diseases	Male	1 to 4	80 plus	Data Rich	1	Mean BMI
Other digestive diseases	Male	1 to 4	80 plus	Data Rich	1	Smoking Prevalence
Other digestive diseases	Male	1 to 4	80 plus	Data Rich	1	red meats adjusted(g)
Other digestive diseases	Female	1 to 4	80 plus	Global	-1	Education (years per capita)
Other digestive diseases	Female	1 to 4	80 plus	Global	-1	Health System Access 2 (unitless)
Other digestive diseases	Female	1 to 4	80 plus	Global	-1	Improved Water Source (proportion with access)
Other digestive diseases	Female	1 to 4	80 plus	Global	-1	LDI (I$ per capita)
Other digestive diseases	Female	1 to 4	80 plus	Global	-1	Sanitation (proportion with access)
Other digestive diseases	Female	1 to 4	80 plus	Global	-1	fruits adjusted(g)
Other digestive diseases	Female	1 to 4	80 plus	Global	0	Sociodemographic Status
Other digestive diseases	Female	1 to 4	80 plus	Global	0	vegetables adjusted(g)
Other digestive diseases	Female	1 to 4	80 plus	Global	1	Alcohol (litres per capita)
Other digestive diseases	Female	1 to 4	80 plus	Global	1	Animal Fats (kcal per capita)
Other digestive diseases	Female	1 to 4	80 plus	Global	1	Cumulative Cigarettes (10 Years)
Other digestive diseases	Female	1 to 4	80 plus	Global	1	Cumulative Cigarettes (5 Years)
Other digestive diseases	Female	1 to 4	80 plus	Global	1	Diabetes Age-Standardised Prevalence (proportion)
Other digestive diseases	Female	1 to 4	80 plus	Global	1	Malnutrition (proportion <2SD weight for age)
Other digestive diseases	Female	1 to 4	80 plus	Global	1	Mean BMI
Other digestive diseases	Female	1 to 4	80 plus	Global	1	Smoking Prevalence
Other digestive diseases	Female	1 to 4	80 plus	Global	1	red meats adjusted(g)
Other digestive diseases	Female	1 to 4	80 plus	Data Rich	-1	Education (years per capita)
Other digestive diseases	Female	1 to 4	80 plus	Data Rich	-1	Health System Access 2 (unitless)
Other digestive diseases	Female	1 to 4	80 plus	Data Rich	-1	Improved Water Source (proportion with access)
Other digestive diseases	Female	1 to 4	80 plus	Data Rich	-1	LDI (I$ per capita)

Appendix Table 10: CODEm covariates used, level of covariate, and expected direction of covariate by cause, sex, and age						
Cause	Sex	Age start	Age end	Model type	Direction	Covariate
Other digestive diseases	Female	1 to 4	80 plus	Data Rich	-1	Sanitation (proportion with access)
Other digestive diseases	Female	1 to 4	80 plus	Data Rich	-1	fruits adjusted(g)
Other digestive diseases	Female	1 to 4	80 plus	Data Rich	0	Sociodemographic Status
Other digestive diseases	Female	1 to 4	80 plus	Data Rich	0	vegetables adjusted(g)
Other digestive diseases	Female	1 to 4	80 plus	Data Rich	1	Alcohol (litres per capita)
Other digestive diseases	Female	1 to 4	80 plus	Data Rich	1	Animal Fats (kcal per capita)
Other digestive diseases	Female	1 to 4	80 plus	Data Rich	1	Cumulative Cigarettes (10 Years)
Other digestive diseases	Female	1 to 4	80 plus	Data Rich	1	Cumulative Cigarettes (5 Years)
Other digestive diseases	Female	1 to 4	80 plus	Data Rich	1	Diabetes Age-Standardised Prevalence (proportion)
Other digestive diseases	Female	1 to 4	80 plus	Data Rich	1	Malnutrition (proportion <2SD weight for age)
Other digestive diseases	Female	1 to 4	80 plus	Data Rich	1	Mean BMI
Other digestive diseases	Female	1 to 4	80 plus	Data Rich	1	Smoking Prevalence
Other digestive diseases	Female	1 to 4	80 plus	Data Rich	1	red meats adjusted(g)
Parkinson disease	Male	20 to 24	80 plus	Global	-1	Education (years per capita)
Parkinson disease	Male	20 to 24	80 plus	Global	-1	Fruits (kcal per capita)
Parkinson disease	Male	20 to 24	80 plus	Global	-1	Health System Access (capped)
Parkinson disease	Male	20 to 24	80 plus	Global	0	Cumulative Cigarettes (10 Years)
Parkinson disease	Male	20 to 24	80 plus	Global	0	Cumulative Cigarettes (5 Years)
Parkinson disease	Male	20 to 24	80 plus	Global	0	Improved Water Source (proportion with access)
Parkinson disease	Male	20 to 24	80 plus	Global	0	LDI (I$ per capita)
Parkinson disease	Male	20 to 24	80 plus	Global	0	Sanitation (proportion with access)
Parkinson disease	Male	20 to 24	80 plus	Global	1	Absolute value of average latitude
Parkinson disease	Male	20 to 24	80 plus	Global	1	Cholesterol (total, mean per capita)
Parkinson disease	Male	20 to 24	80 plus	Global	1	Sociodemographic Status
Parkinson disease	Male	20 to 24	80 plus	Data Rich	-1	Education (years per capita)
Parkinson disease	Male	20 to 24	80 plus	Data Rich	-1	Fruits (kcal per capita)
Parkinson disease	Male	20 to 24	80 plus	Data Rich	-1	Health System Access (capped)
Parkinson disease	Male	20 to 24	80 plus	Data Rich	0	Cumulative Cigarettes (10 Years)
Parkinson disease	Male	20 to 24	80 plus	Data Rich	0	Cumulative Cigarettes (5 Years)
Parkinson disease	Male	20 to 24	80 plus	Data Rich	0	Improved Water Source (proportion with access)
Parkinson disease	Male	20 to 24	80 plus	Data Rich	0	LDI (I$ per capita)
Parkinson disease	Male	20 to 24	80 plus	Data Rich	0	Sanitation (proportion with access)
Parkinson disease	Male	20 to 24	80 plus	Data Rich	1	Absolute value of average latitude
Parkinson disease	Male	20 to 24	80 plus	Data Rich	1	Cholesterol (total, mean per capita)
Parkinson disease	Male	20 to 24	80 plus	Data Rich	1	Sociodemographic Status
Parkinson disease	Female	20 to 24	80 plus	Global	-1	Education (years per capita)
Parkinson disease	Female	20 to 24	80 plus	Global	-1	Fruits (kcal per capita)
Parkinson disease	Female	20 to 24	80 plus	Global	-1	Health System Access (capped)
Parkinson disease	Female	20 to 24	80 plus	Global	0	Cumulative Cigarettes (10 Years)
Parkinson disease	Female	20 to 24	80 plus	Global	0	Cumulative Cigarettes (5 Years)
Parkinson disease	Female	20 to 24	80 plus	Global	0	Improved Water Source (proportion with access)
Parkinson disease	Female	20 to 24	80 plus	Global	0	LDI (I$ per capita)
Parkinson disease	Female	20 to 24	80 plus	Global	0	Sanitation (proportion with access)
Parkinson disease	Female	20 to 24	80 plus	Global	1	Absolute value of average latitude

Appendix Table 10: CODEm covariates used, level of covariate, and expected direction of covariate by cause, sex, and age						
Cause	Sex	Age start	Age end	Model type	Direction	Covariate
Parkinson disease	Female	20 to 24	80 plus	Global	1	Cholesterol (total, mean per capita)
Parkinson disease	Female	20 to 24	80 plus	Global	1	Sociodemographic Status
Parkinson disease	Female	20 to 24	80 plus	Data Rich	-1	Education (years per capita)
Parkinson disease	Female	20 to 24	80 plus	Data Rich	-1	Fruits (kcal per capita)
Parkinson disease	Female	20 to 24	80 plus	Data Rich	-1	Health System Access (capped)
Parkinson disease	Female	20 to 24	80 plus	Data Rich	0	Cumulative Cigarettes (10 Years)
Parkinson disease	Female	20 to 24	80 plus	Data Rich	0	Cumulative Cigarettes (5 Years)
Parkinson disease	Female	20 to 24	80 plus	Data Rich	0	Improved Water Source (proportion with access)
Parkinson disease	Female	20 to 24	80 plus	Data Rich	0	LDI (I$ per capita)
Parkinson disease	Female	20 to 24	80 plus	Data Rich	0	Sanitation (proportion with access)
Parkinson disease	Female	20 to 24	80 plus	Data Rich	1	Absolute value of average latitude
Parkinson disease	Female	20 to 24	80 plus	Data Rich	1	Cholesterol (total, mean per capita)
Parkinson disease	Female	20 to 24	80 plus	Data Rich	1	Sociodemographic Status
Epilepsy	Male	Post Neonatal	80 plus	Global	-1	Education (years per capita)
Epilepsy	Male	Post Neonatal	80 plus	Global	-1	Health System Access (capped)
Epilepsy	Male	Post Neonatal	80 plus	Global	-1	LDI (I$ per capita)
Epilepsy	Male	Post Neonatal	80 plus	Global	-1	Sociodemographic Status
Epilepsy	Male	Post Neonatal	80 plus	Global	1	Cholesterol (total, mean per capita)
Epilepsy	Male	Post Neonatal	80 plus	Global	1	Cumulative Cigarettes (10 Years)
Epilepsy	Male	Post Neonatal	80 plus	Global	1	Cumulative Cigarettes (5 Years)
Epilepsy	Male	Post Neonatal	80 plus	Global	1	Log-transformed SEV scalar: Epilepsy
Epilepsy	Male	Post Neonatal	80 plus	Global	1	Mean BMI
Epilepsy	Male	Post Neonatal	80 plus	Global	1	Pig Meat (kg per capita)
Epilepsy	Male	Post Neonatal	80 plus	Global	1	Pigs (per capita)
Epilepsy	Male	Post Neonatal	80 plus	Global	1	Systolic Blood Pressure (mmHg)
Epilepsy	Male	Post Neonatal	80 plus	Data Rich	-1	Education (years per capita)
Epilepsy	Male	Post Neonatal	80 plus	Data Rich	-1	Health System Access (capped)
Epilepsy	Male	Post Neonatal	80 plus	Data Rich	-1	LDI (I$ per capita)
Epilepsy	Male	Post Neonatal	80 plus	Data Rich	-1	Sociodemographic Status
Epilepsy	Male	Post Neonatal	80 plus	Data Rich	1	Cholesterol (total, mean per capita)
Epilepsy	Male	Post Neonatal	80 plus	Data Rich	1	Cumulative Cigarettes (10 Years)
Epilepsy	Male	Post Neonatal	80 plus	Data Rich	1	Cumulative Cigarettes (5 Years)
Epilepsy	Male	Post Neonatal	80 plus	Data Rich	1	Log-transformed SEV scalar: Epilepsy
Epilepsy	Male	Post Neonatal	80 plus	Data Rich	1	Mean BMI
Epilepsy	Male	Post Neonatal	80 plus	Data Rich	1	Pig Meat (kg per capita)
Epilepsy	Male	Post Neonatal	80 plus	Data Rich	1	Pigs (per capita)
Epilepsy	Male	Post Neonatal	80 plus	Data Rich	1	Systolic Blood Pressure (mmHg)
Epilepsy	Female	Post Neonatal	80 plus	Global	-1	Education (years per capita)
Epilepsy	Female	Post Neonatal	80 plus	Global	-1	Health System Access (capped)
Epilepsy	Female	Post Neonatal	80 plus	Global	-1	LDI (I$ per capita)
Epilepsy	Female	Post Neonatal	80 plus	Global	-1	Sociodemographic Status
Epilepsy	Female	Post Neonatal	80 plus	Global	1	Cholesterol (total, mean per capita)
Epilepsy	Female	Post Neonatal	80 plus	Global	1	Cumulative Cigarettes (10 Years)
Epilepsy	Female	Post Neonatal	80 plus	Global	1	Cumulative Cigarettes (5 Years)

Appendix Table 10: CODEm covariates used, level of covariate, and expected direction of covariate by cause, sex, and age						
**Cause**	**Sex**	**Age start**	**Age end**	**Model type**	**Direction**	**Covariate**
Epilepsy	Female	Post Neonatal	80 plus	Global	1	Log-transformed SEV scalar: Epilepsy
Epilepsy	Female	Post Neonatal	80 plus	Global	1	Mean BMI
Epilepsy	Female	Post Neonatal	80 plus	Global	1	Pig Meat (kg per capita)
Epilepsy	Female	Post Neonatal	80 plus	Global	1	Pigs (per capita)
Epilepsy	Female	Post Neonatal	80 plus	Global	1	Systolic Blood Pressure (mmHg)
Epilepsy	Female	Post Neonatal	80 plus	Data Rich	-1	Education (years per capita)
Epilepsy	Female	Post Neonatal	80 plus	Data Rich	-1	Health System Access (capped)
Epilepsy	Female	Post Neonatal	80 plus	Data Rich	-1	LDI (I$ per capita)
Epilepsy	Female	Post Neonatal	80 plus	Data Rich	-1	Sociodemographic Status
Epilepsy	Female	Post Neonatal	80 plus	Data Rich	1	Cholesterol (total, mean per capita)
Epilepsy	Female	Post Neonatal	80 plus	Data Rich	1	Cumulative Cigarettes (10 Years)
Epilepsy	Female	Post Neonatal	80 plus	Data Rich	1	Cumulative Cigarettes (5 Years)
Epilepsy	Female	Post Neonatal	80 plus	Data Rich	1	Log-transformed SEV scalar: Epilepsy
Epilepsy	Female	Post Neonatal	80 plus	Data Rich	1	Mean BMI
Epilepsy	Female	Post Neonatal	80 plus	Data Rich	1	Pig Meat (kg per capita)
Epilepsy	Female	Post Neonatal	80 plus	Data Rich	1	Pigs (per capita)
Epilepsy	Female	Post Neonatal	80 plus	Data Rich	1	Systolic Blood Pressure (mmHg)
Multiple sclerosis	Male	20 to 24	80 plus	Global	-1	Education (years per capita)
Multiple sclerosis	Male	20 to 24	80 plus	Global	-1	Health System Access (capped)
Multiple sclerosis	Male	20 to 24	80 plus	Global	-1	LDI (I$ per capita)
Multiple sclerosis	Male	20 to 24	80 plus	Global	1	Absolute value of average latitude
Multiple sclerosis	Male	20 to 24	80 plus	Global	1	Animal Fats (kcal per capita)
Multiple sclerosis	Male	20 to 24	80 plus	Global	1	Cholesterol (total, mean per capita)
Multiple sclerosis	Male	20 to 24	80 plus	Global	1	Cumulative Cigarettes (10 Years)
Multiple sclerosis	Male	20 to 24	80 plus	Global	1	Cumulative Cigarettes (5 Years)
Multiple sclerosis	Male	20 to 24	80 plus	Global	1	Smoking Prevalence
Multiple sclerosis	Male	20 to 24	80 plus	Global	1	Sociodemographic Status
Multiple sclerosis	Male	20 to 24	80 plus	Data Rich	-1	Education (years per capita)
Multiple sclerosis	Male	20 to 24	80 plus	Data Rich	-1	Health System Access (capped)
Multiple sclerosis	Male	20 to 24	80 plus	Data Rich	-1	LDI (I$ per capita)
Multiple sclerosis	Male	20 to 24	80 plus	Data Rich	1	Absolute value of average latitude
Multiple sclerosis	Male	20 to 24	80 plus	Data Rich	1	Animal Fats (kcal per capita)
Multiple sclerosis	Male	20 to 24	80 plus	Data Rich	1	Cholesterol (total, mean per capita)
Multiple sclerosis	Male	20 to 24	80 plus	Data Rich	1	Cumulative Cigarettes (10 Years)
Multiple sclerosis	Male	20 to 24	80 plus	Data Rich	1	Cumulative Cigarettes (5 Years)
Multiple sclerosis	Male	20 to 24	80 plus	Data Rich	1	Smoking Prevalence
Multiple sclerosis	Male	20 to 24	80 plus	Data Rich	1	Sociodemographic Status
Multiple sclerosis	Female	20 to 24	80 plus	Global	-1	Education (years per capita)
Multiple sclerosis	Female	20 to 24	80 plus	Global	-1	Health System Access (capped)
Multiple sclerosis	Female	20 to 24	80 plus	Global	-1	LDI (I$ per capita)
Multiple sclerosis	Female	20 to 24	80 plus	Global	1	Absolute value of average latitude
Multiple sclerosis	Female	20 to 24	80 plus	Global	1	Animal Fats (kcal per capita)
Multiple sclerosis	Female	20 to 24	80 plus	Global	1	Cholesterol (total, mean per capita)
Multiple sclerosis	Female	20 to 24	80 plus	Global	1	Cumulative Cigarettes (10 Years)

Appendix Table 10: CODEm covariates used, level of covariate, and expected direction of covariate by cause, sex, and age

Cause	Sex	Age start	Age end	Model type	Direction	Covariate
Multiple sclerosis	Female	20 to 24	80 plus	Global	1	Cumulative Cigarettes (5 Years)
Multiple sclerosis	Female	20 to 24	80 plus	Global	1	Smoking Prevalence
Multiple sclerosis	Female	20 to 24	80 plus	Global	1	Sociodemographic Status
Multiple sclerosis	Female	20 to 24	80 plus	Data Rich	-1	Education (years per capita)
Multiple sclerosis	Female	20 to 24	80 plus	Data Rich	-1	Health System Access (capped)
Multiple sclerosis	Female	20 to 24	80 plus	Data Rich	-1	LDI (I$ per capita)
Multiple sclerosis	Female	20 to 24	80 plus	Data Rich	1	Absolute value of average latitude
Multiple sclerosis	Female	20 to 24	80 plus	Data Rich	1	Animal Fats (kcal per capita)
Multiple sclerosis	Female	20 to 24	80 plus	Data Rich	1	Cholesterol (total, mean per capita)
Multiple sclerosis	Female	20 to 24	80 plus	Data Rich	1	Cumulative Cigarettes (10 Years)
Multiple sclerosis	Female	20 to 24	80 plus	Data Rich	1	Cumulative Cigarettes (5 Years)
Multiple sclerosis	Female	20 to 24	80 plus	Data Rich	1	Smoking Prevalence
Multiple sclerosis	Female	20 to 24	80 plus	Data Rich	1	Sociodemographic Status
Motor neuron disease	Male	Early Neonatal	80 plus	Global	-1	Health System Access (capped)
Motor neuron disease	Male	Early Neonatal	80 plus	Global	0	Cholesterol (total, mean per capita)
Motor neuron disease	Male	Early Neonatal	80 plus	Global	0	Education (years per capita)
Motor neuron disease	Male	Early Neonatal	80 plus	Global	0	Fruits (kcal per capita)
Motor neuron disease	Male	Early Neonatal	80 plus	Global	0	Improved Water Source (proportion with access)
Motor neuron disease	Male	Early Neonatal	80 plus	Global	0	LDI (I$ per capita)
Motor neuron disease	Male	Early Neonatal	80 plus	Global	0	Sanitation (proportion with access)
Motor neuron disease	Male	Early Neonatal	80 plus	Global	0	Sociodemographic Status
Motor neuron disease	Male	Early Neonatal	80 plus	Global	1	Absolute value of average latitude
Motor neuron disease	Male	Early Neonatal	80 plus	Global	1	Asbestos production (kg) per capita
Motor neuron disease	Male	Early Neonatal	80 plus	Data Rich	-1	Health System Access (capped)
Motor neuron disease	Male	Early Neonatal	80 plus	Data Rich	0	Cholesterol (total, mean per capita)
Motor neuron disease	Male	Early Neonatal	80 plus	Data Rich	0	Education (years per capita)
Motor neuron disease	Male	Early Neonatal	80 plus	Data Rich	0	Fruits (kcal per capita)
Motor neuron disease	Male	Early Neonatal	80 plus	Data Rich	0	Improved Water Source (proportion with access)
Motor neuron disease	Male	Early Neonatal	80 plus	Data Rich	0	LDI (I$ per capita)
Motor neuron disease	Male	Early Neonatal	80 plus	Data Rich	0	Sanitation (proportion with access)
Motor neuron disease	Male	Early Neonatal	80 plus	Data Rich	0	Sociodemographic Status
Motor neuron disease	Male	Early Neonatal	80 plus	Data Rich	1	Absolute value of average latitude
Motor neuron disease	Male	Early Neonatal	80 plus	Data Rich	1	Asbestos production (kg) per capita
Motor neuron disease	Female	Early Neonatal	80 plus	Global	-1	Health System Access (capped)
Motor neuron disease	Female	Early Neonatal	80 plus	Global	0	Cholesterol (total, mean per capita)
Motor neuron disease	Female	Early Neonatal	80 plus	Global	0	Education (years per capita)
Motor neuron disease	Female	Early Neonatal	80 plus	Global	0	Fruits (kcal per capita)
Motor neuron disease	Female	Early Neonatal	80 plus	Global	0	Improved Water Source (proportion with access)
Motor neuron disease	Female	Early Neonatal	80 plus	Global	0	LDI (I$ per capita)
Motor neuron disease	Female	Early Neonatal	80 plus	Global	0	Sanitation (proportion with access)
Motor neuron disease	Female	Early Neonatal	80 plus	Global	0	Sociodemographic Status
Motor neuron disease	Female	Early Neonatal	80 plus	Global	1	Absolute value of average latitude
Motor neuron disease	Female	Early Neonatal	80 plus	Global	1	Asbestos production (kg) per capita
Motor neuron disease	Female	Early Neonatal	80 plus	Data Rich	-1	Health System Access (capped)

Appendix Table 10: CODEm covariates used, level of covariate, and expected direction of covariate by cause, sex, and age						
**Cause**	**Sex**	**Age start**	**Age end**	**Model type**	**Direction**	**Covariate**
Motor neuron disease	Female	Early Neonatal	80 plus	Data Rich	0	Cholesterol (total, mean per capita)
Motor neuron disease	Female	Early Neonatal	80 plus	Data Rich	0	Education (years per capita)
Motor neuron disease	Female	Early Neonatal	80 plus	Data Rich	0	Fruits (kcal per capita)
Motor neuron disease	Female	Early Neonatal	80 plus	Data Rich	0	Improved Water Source (proportion with access)
Motor neuron disease	Female	Early Neonatal	80 plus	Data Rich	0	LDI (I$ per capita)
Motor neuron disease	Female	Early Neonatal	80 plus	Data Rich	0	Sanitation (proportion with access)
Motor neuron disease	Female	Early Neonatal	80 plus	Data Rich	0	Sociodemographic Status
Motor neuron disease	Female	Early Neonatal	80 plus	Data Rich	1	Absolute value of average latitude
Motor neuron disease	Female	Early Neonatal	80 plus	Data Rich	1	Asbestos production (kg) per capita
Other neurological disorders	Male	Post Neonatal	80 plus	Global	-1	Education (years per capita)
Other neurological disorders	Male	Post Neonatal	80 plus	Global	-1	Fruits (kcal per capita)
Other neurological disorders	Male	Post Neonatal	80 plus	Global	-1	Health System Access 2 (unitless)
Other neurological disorders	Male	Post Neonatal	80 plus	Global	-1	LDI (I$ per capita)
Other neurological disorders	Male	Post Neonatal	80 plus	Global	0	Sociodemographic Status
Other neurological disorders	Male	Post Neonatal	80 plus	Global	1	Alcohol (litres per capita)
Other neurological disorders	Male	Post Neonatal	80 plus	Global	1	Animal Fats (kcal per capita)
Other neurological disorders	Male	Post Neonatal	80 plus	Global	1	Cholesterol (total, mean per capita)
Other neurological disorders	Male	Post Neonatal	80 plus	Global	1	Cumulative Cigarettes (10 Years)
Other neurological disorders	Male	Post Neonatal	80 plus	Global	1	Cumulative Cigarettes (5 Years)
Other neurological disorders	Male	Post Neonatal	80 plus	Global	1	Malnutrition (proportion <2SD weight for age)
Other neurological disorders	Male	Post Neonatal	80 plus	Global	1	Mean BMI
Other neurological disorders	Male	Post Neonatal	80 plus	Global	1	Pig Meat (kg per capita)
Other neurological disorders	Male	Post Neonatal	80 plus	Global	1	Population Density (over 1000 ppl/sqkm, proportion)
Other neurological disorders	Male	Post Neonatal	80 plus	Global	1	Red Meat (kcal per capita)
Other neurological disorders	Male	Post Neonatal	80 plus	Global	1	Smoking Prevalence
Other neurological disorders	Male	Post Neonatal	80 plus	Global	1	Systolic Blood Pressure (mmHg)
Other neurological disorders	Male	Post Neonatal	80 plus	Data Rich	-1	Education (years per capita)
Other neurological disorders	Male	Post Neonatal	80 plus	Data Rich	-1	Fruits (kcal per capita)
Other neurological disorders	Male	Post Neonatal	80 plus	Data Rich	-1	Health System Access 2 (unitless)
Other neurological disorders	Male	Post Neonatal	80 plus	Data Rich	-1	LDI (I$ per capita)
Other neurological disorders	Male	Post Neonatal	80 plus	Data Rich	0	Sociodemographic Status
Other neurological disorders	Male	Post Neonatal	80 plus	Data Rich	1	Alcohol (litres per capita)
Other neurological disorders	Male	Post Neonatal	80 plus	Data Rich	1	Animal Fats (kcal per capita)
Other neurological disorders	Male	Post Neonatal	80 plus	Data Rich	1	Cholesterol (total, mean per capita)
Other neurological disorders	Male	Post Neonatal	80 plus	Data Rich	1	Cumulative Cigarettes (10 Years)
Other neurological disorders	Male	Post Neonatal	80 plus	Data Rich	1	Cumulative Cigarettes (5 Years)
Other neurological disorders	Male	Post Neonatal	80 plus	Data Rich	1	Malnutrition (proportion <2SD weight for age)
Other neurological disorders	Male	Post Neonatal	80 plus	Data Rich	1	Mean BMI
Other neurological disorders	Male	Post Neonatal	80 plus	Data Rich	1	Pig Meat (kg per capita)
Other neurological disorders	Male	Post Neonatal	80 plus	Data Rich	1	Population Density (over 1000 ppl/sqkm, proportion)
Other neurological disorders	Male	Post Neonatal	80 plus	Data Rich	1	Red Meat (kcal per capita)
Other neurological disorders	Male	Post Neonatal	80 plus	Data Rich	1	Smoking Prevalence
Other neurological disorders	Male	Post Neonatal	80 plus	Data Rich	1	Systolic Blood Pressure (mmHg)
Other neurological disorders	Female	Post Neonatal	80 plus	Global	-1	Education (years per capita)

Appendix Table 10: CODEm covariates used, level of covariate, and expected direction of covariate by cause, sex, and age						
**Cause**	**Sex**	**Age start**	**Age end**	**Model type**	**Direction**	**Covariate**
Other neurological disorders	Female	Post Neonatal	80 plus	Global	-1	Fruits (kcal per capita)
Other neurological disorders	Female	Post Neonatal	80 plus	Global	-1	Health System Access 2 (unitless)
Other neurological disorders	Female	Post Neonatal	80 plus	Global	-1	LDI (I$ per capita)
Other neurological disorders	Female	Post Neonatal	80 plus	Global	0	Sociodemographic Status
Other neurological disorders	Female	Post Neonatal	80 plus	Global	1	Alcohol (litres per capita)
Other neurological disorders	Female	Post Neonatal	80 plus	Global	1	Animal Fats (kcal per capita)
Other neurological disorders	Female	Post Neonatal	80 plus	Global	1	Cholesterol (total, mean per capita)
Other neurological disorders	Female	Post Neonatal	80 plus	Global	1	Cumulative Cigarettes (10 Years)
Other neurological disorders	Female	Post Neonatal	80 plus	Global	1	Cumulative Cigarettes (5 Years)
Other neurological disorders	Female	Post Neonatal	80 plus	Global	1	Malnutrition (proportion <2SD weight for age)
Other neurological disorders	Female	Post Neonatal	80 plus	Global	1	Mean BMI
Other neurological disorders	Female	Post Neonatal	80 plus	Global	1	Pig Meat (kg per capita)
Other neurological disorders	Female	Post Neonatal	80 plus	Global	1	Population Density (over 1000 ppl/sqkm, proportion)
Other neurological disorders	Female	Post Neonatal	80 plus	Global	1	Red Meat (kcal per capita)
Other neurological disorders	Female	Post Neonatal	80 plus	Global	1	Smoking Prevalence
Other neurological disorders	Female	Post Neonatal	80 plus	Global	1	Systolic Blood Pressure (mmHg)
Other neurological disorders	Female	Post Neonatal	80 plus	Data Rich	-1	Education (years per capita)
Other neurological disorders	Female	Post Neonatal	80 plus	Data Rich	-1	Fruits (kcal per capita)
Other neurological disorders	Female	Post Neonatal	80 plus	Data Rich	-1	Health System Access 2 (unitless)
Other neurological disorders	Female	Post Neonatal	80 plus	Data Rich	-1	LDI (I$ per capita)
Other neurological disorders	Female	Post Neonatal	80 plus	Data Rich	0	Sociodemographic Status
Other neurological disorders	Female	Post Neonatal	80 plus	Data Rich	1	Alcohol (litres per capita)
Other neurological disorders	Female	Post Neonatal	80 plus	Data Rich	1	Animal Fats (kcal per capita)
Other neurological disorders	Female	Post Neonatal	80 plus	Data Rich	1	Cholesterol (total, mean per capita)
Other neurological disorders	Female	Post Neonatal	80 plus	Data Rich	1	Cumulative Cigarettes (10 Years)
Other neurological disorders	Female	Post Neonatal	80 plus	Data Rich	1	Cumulative Cigarettes (5 Years)
Other neurological disorders	Female	Post Neonatal	80 plus	Data Rich	1	Malnutrition (proportion <2SD weight for age)
Other neurological disorders	Female	Post Neonatal	80 plus	Data Rich	1	Mean BMI
Other neurological disorders	Female	Post Neonatal	80 plus	Data Rich	1	Pig Meat (kg per capita)
Other neurological disorders	Female	Post Neonatal	80 plus	Data Rich	1	Population Density (over 1000 ppl/sqkm, proportion)
Other neurological disorders	Female	Post Neonatal	80 plus	Data Rich	1	Red Meat (kcal per capita)
Other neurological disorders	Female	Post Neonatal	80 plus	Data Rich	1	Smoking Prevalence
Other neurological disorders	Female	Post Neonatal	80 plus	Data Rich	1	Systolic Blood Pressure (mmHg)
Schizophrenia	Male	25 to 29	80 plus	Global	0	Alcohol (litres per capita)
Schizophrenia	Male	25 to 29	80 plus	Global	0	Cumulative Cigarettes (20 Years)
Schizophrenia	Male	25 to 29	80 plus	Global	0	Education (years per capita)
Schizophrenia	Male	25 to 29	80 plus	Global	0	Health System Access 2 (unitless)
Schizophrenia	Male	25 to 29	80 plus	Global	0	LDI (I$ per capita)
Schizophrenia	Male	25 to 29	80 plus	Global	0	Smoking Prevalence
Schizophrenia	Male	25 to 29	80 plus	Global	0	Sociodemographic Status
Schizophrenia	Male	25 to 29	80 plus	Global	1	Malnutrition (proportion <2SD weight for age)
Schizophrenia	Male	25 to 29	80 plus	Data Rich	0	Alcohol (litres per capita)
Schizophrenia	Male	25 to 29	80 plus	Data Rich	0	Cumulative Cigarettes (20 Years)
Schizophrenia	Male	25 to 29	80 plus	Data Rich	0	Education (years per capita)

Appendix Table 10: CODEm covariates used, level of covariate, and expected direction of covariate by cause, sex, and age						
**Cause**	**Sex**	**Age start**	**Age end**	**Model type**	**Direction**	**Covariate**
Schizophrenia	Male	25 to 29	80 plus	Data Rich	0	Health System Access 2 (unitless)
Schizophrenia	Male	25 to 29	80 plus	Data Rich	0	LDI (I$ per capita)
Schizophrenia	Male	25 to 29	80 plus	Data Rich	0	Smoking Prevalence
Schizophrenia	Male	25 to 29	80 plus	Data Rich	0	Sociodemographic Status
Schizophrenia	Male	25 to 29	80 plus	Data Rich	1	Malnutrition (proportion <2SD weight for age)
Schizophrenia	Female	25 to 29	80 plus	Global	0	Alcohol (litres per capita)
Schizophrenia	Female	25 to 29	80 plus	Global	0	Cumulative Cigarettes (20 Years)
Schizophrenia	Female	25 to 29	80 plus	Global	0	Education (years per capita)
Schizophrenia	Female	25 to 29	80 plus	Global	0	Health System Access 2 (unitless)
Schizophrenia	Female	25 to 29	80 plus	Global	0	LDI (I$ per capita)
Schizophrenia	Female	25 to 29	80 plus	Global	0	Smoking Prevalence
Schizophrenia	Female	25 to 29	80 plus	Global	0	Sociodemographic Status
Schizophrenia	Female	25 to 29	80 plus	Global	1	Malnutrition (proportion <2SD weight for age)
Schizophrenia	Female	25 to 29	80 plus	Data Rich	0	Alcohol (litres per capita)
Schizophrenia	Female	25 to 29	80 plus	Data Rich	0	Cumulative Cigarettes (20 Years)
Schizophrenia	Female	25 to 29	80 plus	Data Rich	0	Education (years per capita)
Schizophrenia	Female	25 to 29	80 plus	Data Rich	0	Health System Access 2 (unitless)
Schizophrenia	Female	25 to 29	80 plus	Data Rich	0	LDI (I$ per capita)
Schizophrenia	Female	25 to 29	80 plus	Data Rich	0	Smoking Prevalence
Schizophrenia	Female	25 to 29	80 plus	Data Rich	0	Sociodemographic Status
Schizophrenia	Female	25 to 29	80 plus	Data Rich	1	Malnutrition (proportion <2SD weight for age)
Alcohol use disorders	Male	15 to 19	80 plus	Global	-1	Education (years per capita)
Alcohol use disorders	Male	15 to 19	80 plus	Global	-1	Health System Access 2 (unitless)
Alcohol use disorders	Male	15 to 19	80 plus	Global	-1	LDI (I$ per capita)
Alcohol use disorders	Male	15 to 19	80 plus	Global	-1	Religion (binary, >50% Muslim)
Alcohol use disorders	Male	15 to 19	80 plus	Global	0	Cumulative Cigarettes (10 Years)
Alcohol use disorders	Male	15 to 19	80 plus	Global	0	Smoking Prevalence
Alcohol use disorders	Male	15 to 19	80 plus	Global	0	Sociodemographic Status
Alcohol use disorders	Male	15 to 19	80 plus	Global	1	Alcohol (litres per capita)
Alcohol use disorders	Male	15 to 19	80 plus	Global	1	Prevalence of binge drinking
Alcohol use disorders	Male	15 to 19	80 plus	Data Rich	-1	Education (years per capita)
Alcohol use disorders	Male	15 to 19	80 plus	Data Rich	-1	Health System Access 2 (unitless)
Alcohol use disorders	Male	15 to 19	80 plus	Data Rich	-1	LDI (I$ per capita)
Alcohol use disorders	Male	15 to 19	80 plus	Data Rich	-1	Religion (binary, >50% Muslim)
Alcohol use disorders	Male	15 to 19	80 plus	Data Rich	0	Cumulative Cigarettes (10 Years)
Alcohol use disorders	Male	15 to 19	80 plus	Data Rich	0	Smoking Prevalence
Alcohol use disorders	Male	15 to 19	80 plus	Data Rich	0	Sociodemographic Status
Alcohol use disorders	Male	15 to 19	80 plus	Data Rich	1	Alcohol (litres per capita)
Alcohol use disorders	Male	15 to 19	80 plus	Data Rich	1	Prevalence of binge drinking
Alcohol use disorders	Female	15 to 19	80 plus	Global	-1	Education (years per capita)
Alcohol use disorders	Female	15 to 19	80 plus	Global	-1	Health System Access 2 (unitless)
Alcohol use disorders	Female	15 to 19	80 plus	Global	-1	LDI (I$ per capita)
Alcohol use disorders	Female	15 to 19	80 plus	Global	-1	Religion (binary, >50% Muslim)
Alcohol use disorders	Female	15 to 19	80 plus	Global	0	Cumulative Cigarettes (10 Years)

Appendix Table 10: CODEm covariates used, level of covariate, and expected direction of covariate by cause, sex, and age						
**Cause**	**Sex**	**Age start**	**Age end**	**Model type**	**Direction**	**Covariate**
Alcohol use disorders	Female	15 to 19	80 plus	Global	0	Smoking Prevalence
Alcohol use disorders	Female	15 to 19	80 plus	Global	0	Sociodemographic Status
Alcohol use disorders	Female	15 to 19	80 plus	Global	1	Alcohol (litres per capita)
Alcohol use disorders	Female	15 to 19	80 plus	Global	1	Prevalence of binge drinking
Alcohol use disorders	Female	15 to 19	80 plus	Data Rich	-1	Education (years per capita)
Alcohol use disorders	Female	15 to 19	80 plus	Data Rich	-1	Health System Access 2 (unitless)
Alcohol use disorders	Female	15 to 19	80 plus	Data Rich	-1	LDI (I$ per capita)
Alcohol use disorders	Female	15 to 19	80 plus	Data Rich	-1	Religion (binary, >50% Muslim)
Alcohol use disorders	Female	15 to 19	80 plus	Data Rich	0	Cumulative Cigarettes (10 Years)
Alcohol use disorders	Female	15 to 19	80 plus	Data Rich	0	Smoking Prevalence
Alcohol use disorders	Female	15 to 19	80 plus	Data Rich	0	Sociodemographic Status
Alcohol use disorders	Female	15 to 19	80 plus	Data Rich	1	Alcohol (litres per capita)
Alcohol use disorders	Female	15 to 19	80 plus	Data Rich	1	Prevalence of binge drinking
Drug use disorders	Male	15 to 19	80 plus	Global	0	Education (years per capita)
Drug use disorders	Male	Early Neonatal	Late Neonatal	Global	0	Education (years per capita)
Drug use disorders	Male	15 to 19	80 plus	Global	0	Health System Access 2 (unitless)
Drug use disorders	Male	Early Neonatal	Late Neonatal	Global	0	Health System Access 2 (unitless)
Drug use disorders	Male	15 to 19	80 plus	Global	0	LDI (I$ per capita)
Drug use disorders	Male	Early Neonatal	Late Neonatal	Global	0	LDI (I$ per capita)
Drug use disorders	Male	15 to 19	80 plus	Global	0	Sociodemographic Status
Drug use disorders	Male	Early Neonatal	Late Neonatal	Global	0	Sociodemographic Status
Drug use disorders	Male	15 to 19	80 plus	Global	1	Alcohol (litres per capita)
Drug use disorders	Male	Early Neonatal	Late Neonatal	Global	1	Alcohol (litres per capita)
Drug use disorders	Male	15 to 19	80 plus	Global	1	Cumulative Cigarettes (10 Years)
Drug use disorders	Male	Early Neonatal	Late Neonatal	Global	1	Cumulative Cigarettes (10 Years)
Drug use disorders	Male	15 to 19	80 plus	Global	1	Cumulative Cigarettes (5 Years)
Drug use disorders	Male	Early Neonatal	Late Neonatal	Global	1	Cumulative Cigarettes (5 Years)
Drug use disorders	Male	15 to 19	80 plus	Global	1	Opium Cultivation (binary)
Drug use disorders	Male	Early Neonatal	Late Neonatal	Global	1	Opium Cultivation (binary)
Drug use disorders	Male	15 to 19	80 plus	Global	1	Smoking Prevalence
Drug use disorders	Male	Early Neonatal	Late Neonatal	Global	1	Smoking Prevalence
Drug use disorders	Male	15 to 19	80 plus	Data Rich	0	Education (years per capita)
Drug use disorders	Male	Early Neonatal	Late Neonatal	Data Rich	0	Education (years per capita)
Drug use disorders	Male	15 to 19	80 plus	Data Rich	0	Health System Access 2 (unitless)
Drug use disorders	Male	Early Neonatal	Late Neonatal	Data Rich	0	Health System Access 2 (unitless)
Drug use disorders	Male	15 to 19	80 plus	Data Rich	0	LDI (I$ per capita)
Drug use disorders	Male	Early Neonatal	Late Neonatal	Data Rich	0	LDI (I$ per capita)
Drug use disorders	Male	15 to 19	80 plus	Data Rich	0	Sociodemographic Status
Drug use disorders	Male	Early Neonatal	Late Neonatal	Data Rich	0	Sociodemographic Status
Drug use disorders	Male	15 to 19	80 plus	Data Rich	1	Alcohol (litres per capita)
Drug use disorders	Male	Early Neonatal	Late Neonatal	Data Rich	1	Alcohol (litres per capita)
Drug use disorders	Male	15 to 19	80 plus	Data Rich	1	Cumulative Cigarettes (10 Years)
Drug use disorders	Male	Early Neonatal	Late Neonatal	Data Rich	1	Cumulative Cigarettes (10 Years)
Drug use disorders	Male	15 to 19	80 plus	Data Rich	1	Cumulative Cigarettes (5 Years)

Appendix Table 10: CODEm covariates used, level of covariate, and expected direction of covariate by cause, sex, and age						
**Cause**	**Sex**	**Age start**	**Age end**	**Model type**	**Direction**	**Covariate**
Drug use disorders	Male	Early Neonatal	Late Neonatal	Data Rich	1	Cumulative Cigarettes (5 Years)
Drug use disorders	Male	15 to 19	80 plus	Data Rich	1	Opium Cultivation (binary)
Drug use disorders	Male	Early Neonatal	Late Neonatal	Data Rich	1	Opium Cultivation (binary)
Drug use disorders	Male	15 to 19	80 plus	Data Rich	1	Smoking Prevalence
Drug use disorders	Male	Early Neonatal	Late Neonatal	Data Rich	1	Smoking Prevalence
Drug use disorders	Female	15 to 19	80 plus	Global	0	Education (years per capita)
Drug use disorders	Female	Early Neonatal	Late Neonatal	Global	0	Education (years per capita)
Drug use disorders	Female	15 to 19	80 plus	Global	0	Health System Access 2 (unitless)
Drug use disorders	Female	Early Neonatal	Late Neonatal	Global	0	Health System Access 2 (unitless)
Drug use disorders	Female	15 to 19	80 plus	Global	0	LDI (I$ per capita)
Drug use disorders	Female	Early Neonatal	Late Neonatal	Global	0	LDI (I$ per capita)
Drug use disorders	Female	15 to 19	80 plus	Global	0	Sociodemographic Status
Drug use disorders	Female	Early Neonatal	Late Neonatal	Global	0	Sociodemographic Status
Drug use disorders	Female	15 to 19	80 plus	Global	1	Alcohol (litres per capita)
Drug use disorders	Female	Early Neonatal	Late Neonatal	Global	1	Alcohol (litres per capita)
Drug use disorders	Female	15 to 19	80 plus	Global	1	Cumulative Cigarettes (10 Years)
Drug use disorders	Female	Early Neonatal	Late Neonatal	Global	1	Cumulative Cigarettes (10 Years)
Drug use disorders	Female	15 to 19	80 plus	Global	1	Cumulative Cigarettes (5 Years)
Drug use disorders	Female	Early Neonatal	Late Neonatal	Global	1	Cumulative Cigarettes (5 Years)
Drug use disorders	Female	15 to 19	80 plus	Global	1	Opium Cultivation (binary)
Drug use disorders	Female	Early Neonatal	Late Neonatal	Global	1	Opium Cultivation (binary)
Drug use disorders	Female	15 to 19	80 plus	Global	1	Smoking Prevalence
Drug use disorders	Female	Early Neonatal	Late Neonatal	Global	1	Smoking Prevalence
Drug use disorders	Female	15 to 19	80 plus	Data Rich	0	Education (years per capita)
Drug use disorders	Female	Early Neonatal	Late Neonatal	Data Rich	0	Education (years per capita)
Drug use disorders	Female	15 to 19	80 plus	Data Rich	0	Health System Access 2 (unitless)
Drug use disorders	Female	Early Neonatal	Late Neonatal	Data Rich	0	Health System Access 2 (unitless)
Drug use disorders	Female	15 to 19	80 plus	Data Rich	0	LDI (I$ per capita)
Drug use disorders	Female	Early Neonatal	Late Neonatal	Data Rich	0	LDI (I$ per capita)
Drug use disorders	Female	15 to 19	80 plus	Data Rich	0	Sociodemographic Status
Drug use disorders	Female	Early Neonatal	Late Neonatal	Data Rich	0	Sociodemographic Status
Drug use disorders	Female	15 to 19	80 plus	Data Rich	1	Alcohol (litres per capita)
Drug use disorders	Female	Early Neonatal	Late Neonatal	Data Rich	1	Alcohol (litres per capita)
Drug use disorders	Female	15 to 19	80 plus	Data Rich	1	Cumulative Cigarettes (10 Years)
Drug use disorders	Female	Early Neonatal	Late Neonatal	Data Rich	1	Cumulative Cigarettes (10 Years)
Drug use disorders	Female	15 to 19	80 plus	Data Rich	1	Cumulative Cigarettes (5 Years)
Drug use disorders	Female	Early Neonatal	Late Neonatal	Data Rich	1	Cumulative Cigarettes (5 Years)
Drug use disorders	Female	15 to 19	80 plus	Data Rich	1	Opium Cultivation (binary)
Drug use disorders	Female	Early Neonatal	Late Neonatal	Data Rich	1	Opium Cultivation (binary)
Drug use disorders	Female	15 to 19	80 plus	Data Rich	1	Smoking Prevalence
Drug use disorders	Female	Early Neonatal	Late Neonatal	Data Rich	1	Smoking Prevalence
Opioid use disorders	Male	15 to 19	80 plus	Global	0	Education (years per capita)
Opioid use disorders	Male	Early Neonatal	Late Neonatal	Global	0	Education (years per capita)
Opioid use disorders	Male	15 to 19	80 plus	Global	0	Health System Access 2 (unitless)

Appendix Table 10: CODEm covariates used, level of covariate, and expected direction of covariate by cause, sex, and age						
**Cause**	**Sex**	**Age start**	**Age end**	**Model type**	**Direction**	**Covariate**
Opioid use disorders	Male	Early Neonatal	Late Neonatal	Global	0	Health System Access 2 (unitless)
Opioid use disorders	Male	15 to 19	80 plus	Global	0	LDI (I$ per capita)
Opioid use disorders	Male	Early Neonatal	Late Neonatal	Global	0	LDI (I$ per capita)
Opioid use disorders	Male	15 to 19	80 plus	Global	0	Sociodemographic Status
Opioid use disorders	Male	Early Neonatal	Late Neonatal	Global	0	Sociodemographic Status
Opioid use disorders	Male	15 to 19	80 plus	Global	1	Alcohol (litres per capita)
Opioid use disorders	Male	Early Neonatal	Late Neonatal	Global	1	Alcohol (litres per capita)
Opioid use disorders	Male	15 to 19	80 plus	Global	1	Cumulative Cigarettes (10 Years)
Opioid use disorders	Male	Early Neonatal	Late Neonatal	Global	1	Cumulative Cigarettes (10 Years)
Opioid use disorders	Male	15 to 19	80 plus	Global	1	Cumulative Cigarettes (5 Years)
Opioid use disorders	Male	Early Neonatal	Late Neonatal	Global	1	Cumulative Cigarettes (5 Years)
Opioid use disorders	Male	15 to 19	80 plus	Global	1	Opium Cultivation (binary)
Opioid use disorders	Male	Early Neonatal	Late Neonatal	Global	1	Opium Cultivation (binary)
Opioid use disorders	Male	15 to 19	80 plus	Global	1	Smoking Prevalence
Opioid use disorders	Male	Early Neonatal	Late Neonatal	Global	1	Smoking Prevalence
Opioid use disorders	Male	15 to 19	80 plus	Data Rich	0	Education (years per capita)
Opioid use disorders	Male	Early Neonatal	Late Neonatal	Data Rich	0	Education (years per capita)
Opioid use disorders	Male	15 to 19	80 plus	Data Rich	0	Health System Access 2 (unitless)
Opioid use disorders	Male	Early Neonatal	Late Neonatal	Data Rich	0	Health System Access 2 (unitless)
Opioid use disorders	Male	15 to 19	80 plus	Data Rich	0	LDI (I$ per capita)
Opioid use disorders	Male	Early Neonatal	Late Neonatal	Data Rich	0	LDI (I$ per capita)
Opioid use disorders	Male	15 to 19	80 plus	Data Rich	0	Sociodemographic Status
Opioid use disorders	Male	Early Neonatal	Late Neonatal	Data Rich	0	Sociodemographic Status
Opioid use disorders	Male	15 to 19	80 plus	Data Rich	1	Alcohol (litres per capita)
Opioid use disorders	Male	Early Neonatal	Late Neonatal	Data Rich	1	Alcohol (litres per capita)
Opioid use disorders	Male	15 to 19	80 plus	Data Rich	1	Cumulative Cigarettes (10 Years)
Opioid use disorders	Male	Early Neonatal	Late Neonatal	Data Rich	1	Cumulative Cigarettes (10 Years)
Opioid use disorders	Male	15 to 19	80 plus	Data Rich	1	Cumulative Cigarettes (5 Years)
Opioid use disorders	Male	Early Neonatal	Late Neonatal	Data Rich	1	Cumulative Cigarettes (5 Years)
Opioid use disorders	Male	15 to 19	80 plus	Data Rich	1	Opium Cultivation (binary)
Opioid use disorders	Male	Early Neonatal	Late Neonatal	Data Rich	1	Opium Cultivation (binary)
Opioid use disorders	Male	15 to 19	80 plus	Data Rich	1	Smoking Prevalence
Opioid use disorders	Male	Early Neonatal	Late Neonatal	Data Rich	1	Smoking Prevalence
Opioid use disorders	Female	15 to 19	80 plus	Global	0	Education (years per capita)
Opioid use disorders	Female	Early Neonatal	Late Neonatal	Global	0	Education (years per capita)
Opioid use disorders	Female	15 to 19	80 plus	Global	0	Health System Access 2 (unitless)
Opioid use disorders	Female	Early Neonatal	Late Neonatal	Global	0	Health System Access 2 (unitless)
Opioid use disorders	Female	15 to 19	80 plus	Global	0	LDI (I$ per capita)
Opioid use disorders	Female	Early Neonatal	Late Neonatal	Global	0	LDI (I$ per capita)
Opioid use disorders	Female	15 to 19	80 plus	Global	0	Sociodemographic Status
Opioid use disorders	Female	Early Neonatal	Late Neonatal	Global	0	Sociodemographic Status
Opioid use disorders	Female	15 to 19	80 plus	Global	1	Alcohol (litres per capita)
Opioid use disorders	Female	Early Neonatal	Late Neonatal	Global	1	Alcohol (litres per capita)
Opioid use disorders	Female	15 to 19	80 plus	Global	1	Cumulative Cigarettes (10 Years)

Appendix Table 10: CODEm covariates used, level of covariate, and expected direction of covariate by cause, sex, and age						
**Cause**	**Sex**	**Age start**	**Age end**	**Model type**	**Direction**	**Covariate**
Opioid use disorders	Female	Early Neonatal	Late Neonatal	Global	1	Cumulative Cigarettes (10 Years)
Opioid use disorders	Female	15 to 19	80 plus	Global	1	Cumulative Cigarettes (5 Years)
Opioid use disorders	Female	Early Neonatal	Late Neonatal	Global	1	Cumulative Cigarettes (5 Years)
Opioid use disorders	Female	15 to 19	80 plus	Global	1	Opium Cultivation (binary)
Opioid use disorders	Female	Early Neonatal	Late Neonatal	Global	1	Opium Cultivation (binary)
Opioid use disorders	Female	15 to 19	80 plus	Global	1	Smoking Prevalence
Opioid use disorders	Female	Early Neonatal	Late Neonatal	Global	1	Smoking Prevalence
Opioid use disorders	Female	15 to 19	80 plus	Data Rich	0	Education (years per capita)
Opioid use disorders	Female	Early Neonatal	Late Neonatal	Data Rich	0	Education (years per capita)
Opioid use disorders	Female	15 to 19	80 plus	Data Rich	0	Health System Access 2 (unitless)
Opioid use disorders	Female	Early Neonatal	Late Neonatal	Data Rich	0	Health System Access 2 (unitless)
Opioid use disorders	Female	15 to 19	80 plus	Data Rich	0	LDI (I$ per capita)
Opioid use disorders	Female	Early Neonatal	Late Neonatal	Data Rich	0	LDI (I$ per capita)
Opioid use disorders	Female	15 to 19	80 plus	Data Rich	0	Sociodemographic Status
Opioid use disorders	Female	Early Neonatal	Late Neonatal	Data Rich	0	Sociodemographic Status
Opioid use disorders	Female	15 to 19	80 plus	Data Rich	1	Alcohol (litres per capita)
Opioid use disorders	Female	Early Neonatal	Late Neonatal	Data Rich	1	Alcohol (litres per capita)
Opioid use disorders	Female	15 to 19	80 plus	Data Rich	1	Cumulative Cigarettes (10 Years)
Opioid use disorders	Female	Early Neonatal	Late Neonatal	Data Rich	1	Cumulative Cigarettes (10 Years)
Opioid use disorders	Female	15 to 19	80 plus	Data Rich	1	Cumulative Cigarettes (5 Years)
Opioid use disorders	Female	Early Neonatal	Late Neonatal	Data Rich	1	Cumulative Cigarettes (5 Years)
Opioid use disorders	Female	15 to 19	80 plus	Data Rich	1	Opium Cultivation (binary)
Opioid use disorders	Female	Early Neonatal	Late Neonatal	Data Rich	1	Opium Cultivation (binary)
Opioid use disorders	Female	15 to 19	80 plus	Data Rich	1	Smoking Prevalence
Opioid use disorders	Female	Early Neonatal	Late Neonatal	Data Rich	1	Smoking Prevalence
Cocaine use disorders	Male	15 to 19	80 plus	Global	0	Education (years per capita)
Cocaine use disorders	Male	15 to 19	80 plus	Global	0	LDI (I$ per capita)
Cocaine use disorders	Male	15 to 19	80 plus	Global	1	Alcohol (litres per capita)
Cocaine use disorders	Male	15 to 19	80 plus	Global	1	Cumulative Cigarettes (10 Years)
Cocaine use disorders	Male	15 to 19	80 plus	Global	1	Cumulative Cigarettes (5 Years)
Cocaine use disorders	Male	15 to 19	80 plus	Global	1	Health System Access 2 (unitless)
Cocaine use disorders	Male	15 to 19	80 plus	Global	1	Smoking Prevalence
Cocaine use disorders	Male	15 to 19	80 plus	Global	1	Sociodemographic Status
Cocaine use disorders	Male	15 to 19	80 plus	Data Rich	0	Education (years per capita)
Cocaine use disorders	Male	15 to 19	80 plus	Data Rich	0	LDI (I$ per capita)
Cocaine use disorders	Male	15 to 19	80 plus	Data Rich	1	Alcohol (litres per capita)
Cocaine use disorders	Male	15 to 19	80 plus	Data Rich	1	Cumulative Cigarettes (10 Years)
Cocaine use disorders	Male	15 to 19	80 plus	Data Rich	1	Cumulative Cigarettes (5 Years)
Cocaine use disorders	Male	15 to 19	80 plus	Data Rich	1	Health System Access 2 (unitless)
Cocaine use disorders	Male	15 to 19	80 plus	Data Rich	1	Smoking Prevalence
Cocaine use disorders	Male	15 to 19	80 plus	Data Rich	1	Sociodemographic Status
Cocaine use disorders	Female	15 to 19	80 plus	Global	0	Education (years per capita)
Cocaine use disorders	Female	15 to 19	80 plus	Global	0	LDI (I$ per capita)
Cocaine use disorders	Female	15 to 19	80 plus	Global	1	Alcohol (litres per capita)

Appendix Table 10: CODEm covariates used, level of covariate, and expected direction of covariate by cause, sex, and age						
**Cause**	**Sex**	**Age start**	**Age end**	**Model type**	**Direction**	**Covariate**
Cocaine use disorders	Female	15 to 19	80 plus	Global	1	Cumulative Cigarettes (10 Years)
Cocaine use disorders	Female	15 to 19	80 plus	Global	1	Cumulative Cigarettes (5 Years)
Cocaine use disorders	Female	15 to 19	80 plus	Global	1	Health System Access 2 (unitless)
Cocaine use disorders	Female	15 to 19	80 plus	Global	1	Smoking Prevalence
Cocaine use disorders	Female	15 to 19	80 plus	Global	1	Sociodemographic Status
Cocaine use disorders	Female	15 to 19	80 plus	Data Rich	0	Education (years per capita)
Cocaine use disorders	Female	15 to 19	80 plus	Data Rich	0	LDI (I$ per capita)
Cocaine use disorders	Female	15 to 19	80 plus	Data Rich	1	Alcohol (litres per capita)
Cocaine use disorders	Female	15 to 19	80 plus	Data Rich	1	Cumulative Cigarettes (10 Years)
Cocaine use disorders	Female	15 to 19	80 plus	Data Rich	1	Cumulative Cigarettes (5 Years)
Cocaine use disorders	Female	15 to 19	80 plus	Data Rich	1	Health System Access 2 (unitless)
Cocaine use disorders	Female	15 to 19	80 plus	Data Rich	1	Smoking Prevalence
Cocaine use disorders	Female	15 to 19	80 plus	Data Rich	1	Sociodemographic Status
Amphetamine use disorders	Male	15 to 19	80 plus	Global	0	Education (years per capita)
Amphetamine use disorders	Male	15 to 19	80 plus	Global	0	Health System Access 2 (unitless)
Amphetamine use disorders	Male	15 to 19	80 plus	Global	0	LDI (I$ per capita)
Amphetamine use disorders	Male	15 to 19	80 plus	Global	1	Alcohol (litres per capita)
Amphetamine use disorders	Male	15 to 19	80 plus	Global	1	Cumulative Cigarettes (10 Years)
Amphetamine use disorders	Male	15 to 19	80 plus	Global	1	Cumulative Cigarettes (5 Years)
Amphetamine use disorders	Male	15 to 19	80 plus	Global	1	Smoking Prevalence
Amphetamine use disorders	Male	15 to 19	80 plus	Global	1	Sociodemographic Status
Amphetamine use disorders	Male	15 to 19	80 plus	Data Rich	0	Education (years per capita)
Amphetamine use disorders	Male	15 to 19	80 plus	Data Rich	0	Health System Access 2 (unitless)
Amphetamine use disorders	Male	15 to 19	80 plus	Data Rich	0	LDI (I$ per capita)
Amphetamine use disorders	Male	15 to 19	80 plus	Data Rich	1	Alcohol (litres per capita)
Amphetamine use disorders	Male	15 to 19	80 plus	Data Rich	1	Cumulative Cigarettes (10 Years)
Amphetamine use disorders	Male	15 to 19	80 plus	Data Rich	1	Cumulative Cigarettes (5 Years)
Amphetamine use disorders	Male	15 to 19	80 plus	Data Rich	1	Smoking Prevalence
Amphetamine use disorders	Male	15 to 19	80 plus	Data Rich	1	Sociodemographic Status
Amphetamine use disorders	Female	15 to 19	80 plus	Global	0	Education (years per capita)
Amphetamine use disorders	Female	15 to 19	80 plus	Global	0	Health System Access 2 (unitless)
Amphetamine use disorders	Female	15 to 19	80 plus	Global	0	LDI (I$ per capita)
Amphetamine use disorders	Female	15 to 19	80 plus	Global	1	Alcohol (litres per capita)
Amphetamine use disorders	Female	15 to 19	80 plus	Global	1	Cumulative Cigarettes (10 Years)
Amphetamine use disorders	Female	15 to 19	80 plus	Global	1	Cumulative Cigarettes (5 Years)
Amphetamine use disorders	Female	15 to 19	80 plus	Global	1	Smoking Prevalence
Amphetamine use disorders	Female	15 to 19	80 plus	Global	1	Sociodemographic Status
Amphetamine use disorders	Female	15 to 19	80 plus	Data Rich	0	Education (years per capita)
Amphetamine use disorders	Female	15 to 19	80 plus	Data Rich	0	Health System Access 2 (unitless)
Amphetamine use disorders	Female	15 to 19	80 plus	Data Rich	0	LDI (I$ per capita)
Amphetamine use disorders	Female	15 to 19	80 plus	Data Rich	1	Alcohol (litres per capita)
Amphetamine use disorders	Female	15 to 19	80 plus	Data Rich	1	Cumulative Cigarettes (10 Years)
Amphetamine use disorders	Female	15 to 19	80 plus	Data Rich	1	Cumulative Cigarettes (5 Years)
Amphetamine use disorders	Female	15 to 19	80 plus	Data Rich	1	Smoking Prevalence

Appendix Table 10: CODEm covariates used, level of covariate, and expected direction of covariate by cause, sex, and age						
**Cause**	**Sex**	**Age start**	**Age end**	**Model type**	**Direction**	**Covariate**
Amphetamine use disorders	Female	15 to 19	80 plus	Data Rich	1	Sociodemographic Status
Other drug use disorders	Male	15 to 19	80 plus	Global	0	Education (years per capita)
Other drug use disorders	Male	15 to 19	80 plus	Global	0	Health System Access 2 (unitless)
Other drug use disorders	Male	15 to 19	80 plus	Global	0	LDI (I$ per capita)
Other drug use disorders	Male	15 to 19	80 plus	Global	0	Sociodemographic Status
Other drug use disorders	Male	15 to 19	80 plus	Global	1	Alcohol (litres per capita)
Other drug use disorders	Male	15 to 19	80 plus	Global	1	Cumulative Cigarettes (10 Years)
Other drug use disorders	Male	15 to 19	80 plus	Global	1	Cumulative Cigarettes (5 Years)
Other drug use disorders	Male	15 to 19	80 plus	Global	1	Smoking Prevalence
Other drug use disorders	Male	15 to 19	80 plus	Data Rich	0	Education (years per capita)
Other drug use disorders	Male	15 to 19	80 plus	Data Rich	0	Health System Access 2 (unitless)
Other drug use disorders	Male	15 to 19	80 plus	Data Rich	0	LDI (I$ per capita)
Other drug use disorders	Male	15 to 19	80 plus	Data Rich	0	Sociodemographic Status
Other drug use disorders	Male	15 to 19	80 plus	Data Rich	1	Alcohol (litres per capita)
Other drug use disorders	Male	15 to 19	80 plus	Data Rich	1	Cumulative Cigarettes (10 Years)
Other drug use disorders	Male	15 to 19	80 plus	Data Rich	1	Cumulative Cigarettes (5 Years)
Other drug use disorders	Male	15 to 19	80 plus	Data Rich	1	Smoking Prevalence
Other drug use disorders	Female	15 to 19	80 plus	Global	0	Education (years per capita)
Other drug use disorders	Female	15 to 19	80 plus	Global	0	Health System Access 2 (unitless)
Other drug use disorders	Female	15 to 19	80 plus	Global	0	LDI (I$ per capita)
Other drug use disorders	Female	15 to 19	80 plus	Global	0	Sociodemographic Status
Other drug use disorders	Female	15 to 19	80 plus	Global	1	Alcohol (litres per capita)
Other drug use disorders	Female	15 to 19	80 plus	Global	1	Cumulative Cigarettes (10 Years)
Other drug use disorders	Female	15 to 19	80 plus	Global	1	Cumulative Cigarettes (5 Years)
Other drug use disorders	Female	15 to 19	80 plus	Global	1	Smoking Prevalence
Other drug use disorders	Female	15 to 19	80 plus	Data Rich	0	Education (years per capita)
Other drug use disorders	Female	15 to 19	80 plus	Data Rich	0	Health System Access 2 (unitless)
Other drug use disorders	Female	15 to 19	80 plus	Data Rich	0	LDI (I$ per capita)
Other drug use disorders	Female	15 to 19	80 plus	Data Rich	0	Sociodemographic Status
Other drug use disorders	Female	15 to 19	80 plus	Data Rich	1	Alcohol (litres per capita)
Other drug use disorders	Female	15 to 19	80 plus	Data Rich	1	Cumulative Cigarettes (10 Years)
Other drug use disorders	Female	15 to 19	80 plus	Data Rich	1	Cumulative Cigarettes (5 Years)
Other drug use disorders	Female	15 to 19	80 plus	Data Rich	1	Smoking Prevalence
Eating disorders	Male	5 to 9	45 to 49	Global	1	Sociodemographic Status
Eating disorders	Male	5 to 9	45 to 49	Data Rich	1	Sociodemographic Status
Eating disorders	Female	5 to 9	45 to 49	Global	1	Sociodemographic Status
Eating disorders	Female	5 to 9	45 to 49	Data Rich	1	Sociodemographic Status
Anorexia nervosa	Male	5 to 9	45 to 49	Global	1	Sociodemographic Status
Anorexia nervosa	Male	5 to 9	45 to 49	Data Rich	1	Sociodemographic Status
Anorexia nervosa	Female	5 to 9	45 to 49	Global	1	Sociodemographic Status
Anorexia nervosa	Female	5 to 9	45 to 49	Data Rich	1	Sociodemographic Status
Bulimia nervosa	Male	5 to 9	45 to 49	Global	1	Sociodemographic Status
Bulimia nervosa	Male	5 to 9	45 to 49	Data Rich	1	Sociodemographic Status
Bulimia nervosa	Female	5 to 9	45 to 49	Global	1	Sociodemographic Status

Appendix Table 10: CODEm covariates used, level of covariate, and expected direction of covariate by cause, sex, and age						
**Cause**	**Sex**	**Age start**	**Age end**	**Model type**	**Direction**	**Covariate**
Bulimia nervosa	Female	5 to 9	45 to 49	Data Rich	1	Sociodemographic Status
Diabetes mellitus	Male	15 to 19	80 plus	Global	-1	Education (years per capita)
Diabetes mellitus	Male	Early Neonatal	10 to 14	Global	-1	Education (years per capita)
Diabetes mellitus	Male	15 to 19	80 plus	Global	-1	Health System Access 2 (unitless)
Diabetes mellitus	Male	Early Neonatal	10 to 14	Global	-1	Health System Access 2 (unitless)
Diabetes mellitus	Male	15 to 19	80 plus	Global	-1	LDI (I$ per capita)
Diabetes mellitus	Male	Early Neonatal	10 to 14	Global	-1	LDI (I$ per capita)
Diabetes mellitus	Male	15 to 19	80 plus	Global	-1	fruits adjusted(g)
Diabetes mellitus	Male	15 to 19	80 plus	Global	-1	vegetables adjusted(g)
Diabetes mellitus	Male	15 to 19	80 plus	Global	-1	whole grains adjusted(g)
Diabetes mellitus	Male	15 to 19	80 plus	Global	0	Sociodemographic Status
Diabetes mellitus	Male	15 to 19	80 plus	Global	1	Animal Fats (kcal per capita)
Diabetes mellitus	Male	15 to 19	80 plus	Global	1	Cholesterol (total, mean per capita)
Diabetes mellitus	Male	15 to 19	80 plus	Global	1	Diabetes Age-Standardised Prevalence (proportion)
Diabetes mellitus	Male	15 to 19	80 plus	Global	1	Diabetes Fasting Plasma Glucose (mmol/L)
Diabetes mellitus	Male	Early Neonatal	10 to 14	Global	1	Malnutrition (proportion <2SD weight for age)
Diabetes mellitus	Male	15 to 19	80 plus	Global	1	Mean BMI
Diabetes mellitus	Male	Early Neonatal	10 to 14	Global	1	Sociodemographic Status
Diabetes mellitus	Male	15 to 19	80 plus	Global	1	Systolic Blood Pressure (mmHg)
Diabetes mellitus	Male	15 to 19	80 plus	Global	1	energy unadjusted(kcal)
Diabetes mellitus	Male	15 to 19	80 plus	Data Rich	-1	Education (years per capita)
Diabetes mellitus	Male	Early Neonatal	10 to 14	Data Rich	-1	Education (years per capita)
Diabetes mellitus	Male	15 to 19	80 plus	Data Rich	-1	Health System Access 2 (unitless)
Diabetes mellitus	Male	Early Neonatal	10 to 14	Data Rich	-1	Health System Access 2 (unitless)
Diabetes mellitus	Male	15 to 19	80 plus	Data Rich	-1	LDI (I$ per capita)
Diabetes mellitus	Male	Early Neonatal	10 to 14	Data Rich	-1	LDI (I$ per capita)
Diabetes mellitus	Male	15 to 19	80 plus	Data Rich	-1	fruits adjusted(g)
Diabetes mellitus	Male	15 to 19	80 plus	Data Rich	-1	vegetables adjusted(g)
Diabetes mellitus	Male	15 to 19	80 plus	Data Rich	-1	whole grains adjusted(g)
Diabetes mellitus	Male	15 to 19	80 plus	Data Rich	0	Sociodemographic Status
Diabetes mellitus	Male	15 to 19	80 plus	Data Rich	1	Animal Fats (kcal per capita)
Diabetes mellitus	Male	15 to 19	80 plus	Data Rich	1	Cholesterol (total, mean per capita)
Diabetes mellitus	Male	15 to 19	80 plus	Data Rich	1	Diabetes Age-Standardised Prevalence (proportion)
Diabetes mellitus	Male	15 to 19	80 plus	Data Rich	1	Diabetes Fasting Plasma Glucose (mmol/L)
Diabetes mellitus	Male	Early Neonatal	10 to 14	Data Rich	1	Malnutrition (proportion <2SD weight for age)
Diabetes mellitus	Male	15 to 19	80 plus	Data Rich	1	Mean BMI
Diabetes mellitus	Male	Early Neonatal	10 to 14	Data Rich	1	Sociodemographic Status
Diabetes mellitus	Male	15 to 19	80 plus	Data Rich	1	Systolic Blood Pressure (mmHg)
Diabetes mellitus	Male	15 to 19	80 plus	Data Rich	1	energy unadjusted(kcal)
Diabetes mellitus	Female	15 to 19	80 plus	Global	-1	Education (years per capita)
Diabetes mellitus	Female	Early Neonatal	10 to 14	Global	-1	Education (years per capita)
Diabetes mellitus	Female	15 to 19	80 plus	Global	-1	Health System Access 2 (unitless)
Diabetes mellitus	Female	Early Neonatal	10 to 14	Global	-1	Health System Access 2 (unitless)
Diabetes mellitus	Female	15 to 19	80 plus	Global	-1	LDI (I$ per capita)

Appendix Table 10: CODEm covariates used, level of covariate, and expected direction of covariate by cause, sex, and age						
**Cause**	**Sex**	**Age start**	**Age end**	**Model type**	**Direction**	**Covariate**
Diabetes mellitus	Female	Early Neonatal	10 to 14	Global	-1	LDI (I$ per capita)
Diabetes mellitus	Female	15 to 19	80 plus	Global	-1	fruits adjusted(g)
Diabetes mellitus	Female	15 to 19	80 plus	Global	-1	vegetables adjusted(g)
Diabetes mellitus	Female	15 to 19	80 plus	Global	-1	whole grains adjusted(g)
Diabetes mellitus	Female	15 to 19	80 plus	Global	0	Sociodemographic Status
Diabetes mellitus	Female	15 to 19	80 plus	Global	1	Animal Fats (kcal per capita)
Diabetes mellitus	Female	15 to 19	80 plus	Global	1	Cholesterol (total, mean per capita)
Diabetes mellitus	Female	15 to 19	80 plus	Global	1	Diabetes Age-Standardised Prevalence (proportion)
Diabetes mellitus	Female	15 to 19	80 plus	Global	1	Diabetes Fasting Plasma Glucose (mmol/L)
Diabetes mellitus	Female	Early Neonatal	10 to 14	Global	1	Malnutrition (proportion <2SD weight for age)
Diabetes mellitus	Female	15 to 19	80 plus	Global	1	Mean BMI
Diabetes mellitus	Female	Early Neonatal	10 to 14	Global	1	Sociodemographic Status
Diabetes mellitus	Female	15 to 19	80 plus	Global	1	Systolic Blood Pressure (mmHg)
Diabetes mellitus	Female	15 to 19	80 plus	Global	1	energy unadjusted(kcal)
Diabetes mellitus	Female	15 to 19	80 plus	Data Rich	-1	Education (years per capita)
Diabetes mellitus	Female	Early Neonatal	10 to 14	Data Rich	-1	Education (years per capita)
Diabetes mellitus	Female	15 to 19	80 plus	Data Rich	-1	Health System Access 2 (unitless)
Diabetes mellitus	Female	Early Neonatal	10 to 14	Data Rich	-1	Health System Access 2 (unitless)
Diabetes mellitus	Female	15 to 19	80 plus	Data Rich	-1	LDI (I$ per capita)
Diabetes mellitus	Female	Early Neonatal	10 to 14	Data Rich	-1	LDI (I$ per capita)
Diabetes mellitus	Female	15 to 19	80 plus	Data Rich	-1	fruits adjusted(g)
Diabetes mellitus	Female	15 to 19	80 plus	Data Rich	-1	vegetables adjusted(g)
Diabetes mellitus	Female	15 to 19	80 plus	Data Rich	-1	whole grains adjusted(g)
Diabetes mellitus	Female	15 to 19	80 plus	Data Rich	0	Sociodemographic Status
Diabetes mellitus	Female	15 to 19	80 plus	Data Rich	1	Animal Fats (kcal per capita)
Diabetes mellitus	Female	15 to 19	80 plus	Data Rich	1	Cholesterol (total, mean per capita)
Diabetes mellitus	Female	15 to 19	80 plus	Data Rich	1	Diabetes Age-Standardised Prevalence (proportion)
Diabetes mellitus	Female	15 to 19	80 plus	Data Rich	1	Diabetes Fasting Plasma Glucose (mmol/L)
Diabetes mellitus	Female	Early Neonatal	10 to 14	Data Rich	1	Malnutrition (proportion <2SD weight for age)
Diabetes mellitus	Female	15 to 19	80 plus	Data Rich	1	Mean BMI
Diabetes mellitus	Female	Early Neonatal	10 to 14	Data Rich	1	Sociodemographic Status
Diabetes mellitus	Female	15 to 19	80 plus	Data Rich	1	Systolic Blood Pressure (mmHg)
Diabetes mellitus	Female	15 to 19	80 plus	Data Rich	1	energy unadjusted(kcal)
Acute glomerulonephritis	Male	Post Neonatal	80 plus	Global	-1	Education (years per capita)
Acute glomerulonephritis	Male	Post Neonatal	80 plus	Global	-1	Health System Access (unitless)
Acute glomerulonephritis	Male	Post Neonatal	80 plus	Global	-1	Improved Water Source (proportion with access)
Acute glomerulonephritis	Male	Post Neonatal	80 plus	Global	-1	LDI (I$ per capita)
Acute glomerulonephritis	Male	Post Neonatal	80 plus	Global	-1	Sanitation (proportion with access)
Acute glomerulonephritis	Male	Post Neonatal	80 plus	Global	-1	Sociodemographic Status
Acute glomerulonephritis	Male	Post Neonatal	80 plus	Global	1	Diabetes Age-Standardised Prevalence (proportion)
Acute glomerulonephritis	Male	Post Neonatal	80 plus	Global	1	Systolic Blood Pressure (mmHg)
Acute glomerulonephritis	Male	Post Neonatal	80 plus	Data Rich	-1	Education (years per capita)
Acute glomerulonephritis	Male	Post Neonatal	80 plus	Data Rich	-1	Health System Access (unitless)
Acute glomerulonephritis	Male	Post Neonatal	80 plus	Data Rich	-1	Improved Water Source (proportion with access)

Appendix Table 10: CODEm covariates used, level of covariate, and expected direction of covariate by cause, sex, and age						
**Cause**	**Sex**	**Age start**	**Age end**	**Model type**	**Direction**	**Covariate**
Acute glomerulonephritis	Male	Post Neonatal	80 plus	Data Rich	-1	LDI (I$ per capita)
Acute glomerulonephritis	Male	Post Neonatal	80 plus	Data Rich	-1	Sanitation (proportion with access)
Acute glomerulonephritis	Male	Post Neonatal	80 plus	Data Rich	-1	Sociodemographic Status
Acute glomerulonephritis	Male	Post Neonatal	80 plus	Data Rich	1	Diabetes Age-Standardised Prevalence (proportion)
Acute glomerulonephritis	Male	Post Neonatal	80 plus	Data Rich	1	Systolic Blood Pressure (mmHg)
Acute glomerulonephritis	Female	Post Neonatal	80 plus	Global	-1	Education (years per capita)
Acute glomerulonephritis	Female	Post Neonatal	80 plus	Global	-1	Health System Access (unitless)
Acute glomerulonephritis	Female	Post Neonatal	80 plus	Global	-1	Improved Water Source (proportion with access)
Acute glomerulonephritis	Female	Post Neonatal	80 plus	Global	-1	LDI (I$ per capita)
Acute glomerulonephritis	Female	Post Neonatal	80 plus	Global	-1	Sanitation (proportion with access)
Acute glomerulonephritis	Female	Post Neonatal	80 plus	Global	-1	Sociodemographic Status
Acute glomerulonephritis	Female	Post Neonatal	80 plus	Global	1	Diabetes Age-Standardised Prevalence (proportion)
Acute glomerulonephritis	Female	Post Neonatal	80 plus	Global	1	Systolic Blood Pressure (mmHg)
Acute glomerulonephritis	Female	Post Neonatal	80 plus	Data Rich	-1	Education (years per capita)
Acute glomerulonephritis	Female	Post Neonatal	80 plus	Data Rich	-1	Health System Access (unitless)
Acute glomerulonephritis	Female	Post Neonatal	80 plus	Data Rich	-1	Improved Water Source (proportion with access)
Acute glomerulonephritis	Female	Post Neonatal	80 plus	Data Rich	-1	LDI (I$ per capita)
Acute glomerulonephritis	Female	Post Neonatal	80 plus	Data Rich	-1	Sanitation (proportion with access)
Acute glomerulonephritis	Female	Post Neonatal	80 plus	Data Rich	-1	Sociodemographic Status
Acute glomerulonephritis	Female	Post Neonatal	80 plus	Data Rich	1	Diabetes Age-Standardised Prevalence (proportion)
Acute glomerulonephritis	Female	Post Neonatal	80 plus	Data Rich	1	Systolic Blood Pressure (mmHg)
Chronic kidney disease	Male	Post Neonatal	80 plus	Global	-1	Education (years per capita)
Chronic kidney disease	Male	Post Neonatal	80 plus	Global	-1	Health System Access (unitless)
Chronic kidney disease	Male	Post Neonatal	80 plus	Global	-1	LDI (I$ per capita)
Chronic kidney disease	Male	Post Neonatal	80 plus	Global	0	Animal Fats (kcal per capita)
Chronic kidney disease	Male	Post Neonatal	80 plus	Global	0	Red Meat (kcal per capita)
Chronic kidney disease	Male	Post Neonatal	80 plus	Global	0	Sociodemographic Status
Chronic kidney disease	Male	Post Neonatal	80 plus	Global	0	Whole Grains (kcal per capita)
Chronic kidney disease	Male	Post Neonatal	80 plus	Global	1	Cholesterol (total, mean per capita)
Chronic kidney disease	Male	Post Neonatal	80 plus	Global	1	Diabetes Age-Standardised Prevalence (proportion)
Chronic kidney disease	Male	Post Neonatal	80 plus	Global	1	Diabetes Fasting Plasma Glucose (mmol/L)
Chronic kidney disease	Male	Post Neonatal	80 plus	Global	1	Mean BMI
Chronic kidney disease	Male	Post Neonatal	80 plus	Global	1	Systolic Blood Pressure (mmHg)
Chronic kidney disease	Male	Post Neonatal	80 plus	Global	1	Total Calories (kcal per capita)
Chronic kidney disease	Male	Post Neonatal	80 plus	Data Rich	-1	Education (years per capita)
Chronic kidney disease	Male	Post Neonatal	80 plus	Data Rich	-1	Health System Access (unitless)
Chronic kidney disease	Male	Post Neonatal	80 plus	Data Rich	-1	LDI (I$ per capita)
Chronic kidney disease	Male	Post Neonatal	80 plus	Data Rich	0	Animal Fats (kcal per capita)
Chronic kidney disease	Male	Post Neonatal	80 plus	Data Rich	0	Red Meat (kcal per capita)
Chronic kidney disease	Male	Post Neonatal	80 plus	Data Rich	0	Sociodemographic Status
Chronic kidney disease	Male	Post Neonatal	80 plus	Data Rich	0	Whole Grains (kcal per capita)
Chronic kidney disease	Male	Post Neonatal	80 plus	Data Rich	1	Cholesterol (total, mean per capita)
Chronic kidney disease	Male	Post Neonatal	80 plus	Data Rich	1	Diabetes Age-Standardised Prevalence (proportion)
Chronic kidney disease	Male	Post Neonatal	80 plus	Data Rich	1	Diabetes Fasting Plasma Glucose (mmol/L)

Appendix Table 10: CODEm covariates used, level of covariate, and expected direction of covariate by cause, sex, and age						
**Cause**	**Sex**	**Age start**	**Age end**	**Model type**	**Direction**	**Covariate**
Chronic kidney disease	Male	Post Neonatal	80 plus	Data Rich	1	Mean BMI
Chronic kidney disease	Male	Post Neonatal	80 plus	Data Rich	1	Systolic Blood Pressure (mmHg)
Chronic kidney disease	Male	Post Neonatal	80 plus	Data Rich	1	Total Calories (kcal per capita)
Chronic kidney disease	Female	Post Neonatal	80 plus	Global	-1	Education (years per capita)
Chronic kidney disease	Female	Post Neonatal	80 plus	Global	-1	Health System Access (unitless)
Chronic kidney disease	Female	Post Neonatal	80 plus	Global	-1	LDI (I$ per capita)
Chronic kidney disease	Female	Post Neonatal	80 plus	Global	0	Animal Fats (kcal per capita)
Chronic kidney disease	Female	Post Neonatal	80 plus	Global	0	Red Meat (kcal per capita)
Chronic kidney disease	Female	Post Neonatal	80 plus	Global	0	Sociodemographic Status
Chronic kidney disease	Female	Post Neonatal	80 plus	Global	0	Whole Grains (kcal per capita)
Chronic kidney disease	Female	Post Neonatal	80 plus	Global	1	Cholesterol (total, mean per capita)
Chronic kidney disease	Female	Post Neonatal	80 plus	Global	1	Diabetes Age-Standardised Prevalence (proportion)
Chronic kidney disease	Female	Post Neonatal	80 plus	Global	1	Diabetes Fasting Plasma Glucose (mmol/L)
Chronic kidney disease	Female	Post Neonatal	80 plus	Global	1	Mean BMI
Chronic kidney disease	Female	Post Neonatal	80 plus	Global	1	Systolic Blood Pressure (mmHg)
Chronic kidney disease	Female	Post Neonatal	80 plus	Global	1	Total Calories (kcal per capita)
Chronic kidney disease	Female	Post Neonatal	80 plus	Data Rich	-1	Education (years per capita)
Chronic kidney disease	Female	Post Neonatal	80 plus	Data Rich	-1	Health System Access (unitless)
Chronic kidney disease	Female	Post Neonatal	80 plus	Data Rich	-1	LDI (I$ per capita)
Chronic kidney disease	Female	Post Neonatal	80 plus	Data Rich	0	Animal Fats (kcal per capita)
Chronic kidney disease	Female	Post Neonatal	80 plus	Data Rich	0	Red Meat (kcal per capita)
Chronic kidney disease	Female	Post Neonatal	80 plus	Data Rich	0	Sociodemographic Status
Chronic kidney disease	Female	Post Neonatal	80 plus	Data Rich	0	Whole Grains (kcal per capita)
Chronic kidney disease	Female	Post Neonatal	80 plus	Data Rich	1	Cholesterol (total, mean per capita)
Chronic kidney disease	Female	Post Neonatal	80 plus	Data Rich	1	Diabetes Age-Standardised Prevalence (proportion)
Chronic kidney disease	Female	Post Neonatal	80 plus	Data Rich	1	Diabetes Fasting Plasma Glucose (mmol/L)
Chronic kidney disease	Female	Post Neonatal	80 plus	Data Rich	1	Mean BMI
Chronic kidney disease	Female	Post Neonatal	80 plus	Data Rich	1	Systolic Blood Pressure (mmHg)
Chronic kidney disease	Female	Post Neonatal	80 plus	Data Rich	1	Total Calories (kcal per capita)
Urinary diseases and male infertility	Male	Early Neonatal	80 plus	Global	-1	Education (years per capita)
Urinary diseases and male infertility	Male	Early Neonatal	80 plus	Global	-1	Health System Access 2 (unitless)
Urinary diseases and male infertility	Male	Early Neonatal	80 plus	Global	-1	LDI (I$ per capita)
Urinary diseases and male infertility	Male	Early Neonatal	80 plus	Global	0	Latitude 15 to 30 (proportion)
Urinary diseases and male infertility	Male	Early Neonatal	80 plus	Global	0	Latitude 30 to 45 (proportion)
Urinary diseases and male infertility	Male	Early Neonatal	80 plus	Global	0	Latitude Over 45 (proportion)
Urinary diseases and male infertility	Male	Early Neonatal	80 plus	Global	0	Latitude Under 15 (proportion)
Urinary diseases and male infertility	Male	Early Neonatal	80 plus	Global	0	Sociodemographic Status
Urinary diseases and male infertility	Male	Early Neonatal	80 plus	Global	1	Mean BMI
Urinary diseases and male infertility	Male	Early Neonatal	80 plus	Data Rich	-1	Education (years per capita)
Urinary diseases and male infertility	Male	Early Neonatal	80 plus	Data Rich	-1	Health System Access 2 (unitless)
Urinary diseases and male infertility	Male	Early Neonatal	80 plus	Data Rich	-1	LDI (I$ per capita)
Urinary diseases and male infertility	Male	Early Neonatal	80 plus	Data Rich	0	Latitude 15 to 30 (proportion)
Urinary diseases and male infertility	Male	Early Neonatal	80 plus	Data Rich	0	Latitude 30 to 45 (proportion)
Urinary diseases and male infertility	Male	Early Neonatal	80 plus	Data Rich	0	Latitude Over 45 (proportion)

Appendix Table 10: CODEm covariates used, level of covariate, and expected direction of covariate by cause, sex, and age						
**Cause**	**Sex**	**Age start**	**Age end**	**Model type**	**Direction**	**Covariate**
Urinary diseases and male infertility	Male	Early Neonatal	80 plus	Data Rich	0	Latitude Under 15 (proportion)
Urinary diseases and male infertility	Male	Early Neonatal	80 plus	Data Rich	0	Sociodemographic Status
Urinary diseases and male infertility	Male	Early Neonatal	80 plus	Data Rich	1	Mean BMI
Urinary diseases and male infertility	Female	Early Neonatal	80 plus	Global	-1	Education (years per capita)
Urinary diseases and male infertility	Female	Early Neonatal	80 plus	Global	-1	Health System Access 2 (unitless)
Urinary diseases and male infertility	Female	Early Neonatal	80 plus	Global	-1	LDI (I$ per capita)
Urinary diseases and male infertility	Female	Early Neonatal	80 plus	Global	0	Latitude 15 to 30 (proportion)
Urinary diseases and male infertility	Female	Early Neonatal	80 plus	Global	0	Latitude 30 to 45 (proportion)
Urinary diseases and male infertility	Female	Early Neonatal	80 plus	Global	0	Latitude Over 45 (proportion)
Urinary diseases and male infertility	Female	Early Neonatal	80 plus	Global	0	Latitude Under 15 (proportion)
Urinary diseases and male infertility	Female	Early Neonatal	80 plus	Global	0	Sociodemographic Status
Urinary diseases and male infertility	Female	Early Neonatal	80 plus	Global	1	Mean BMI
Urinary diseases and male infertility	Female	Early Neonatal	80 plus	Data Rich	-1	Education (years per capita)
Urinary diseases and male infertility	Female	Early Neonatal	80 plus	Data Rich	-1	Health System Access 2 (unitless)
Urinary diseases and male infertility	Female	Early Neonatal	80 plus	Data Rich	-1	LDI (I$ per capita)
Urinary diseases and male infertility	Female	Early Neonatal	80 plus	Data Rich	0	Latitude 15 to 30 (proportion)
Urinary diseases and male infertility	Female	Early Neonatal	80 plus	Data Rich	0	Latitude 30 to 45 (proportion)
Urinary diseases and male infertility	Female	Early Neonatal	80 plus	Data Rich	0	Latitude Over 45 (proportion)
Urinary diseases and male infertility	Female	Early Neonatal	80 plus	Data Rich	0	Latitude Under 15 (proportion)
Urinary diseases and male infertility	Female	Early Neonatal	80 plus	Data Rich	0	Sociodemographic Status
Urinary diseases and male infertility	Female	Early Neonatal	80 plus	Data Rich	1	Mean BMI
Interstitial nephritis and urinary tract infections	Male	Early Neonatal	80 plus	Global	-1	Education (years per capita)
Interstitial nephritis and urinary tract infections	Male	Early Neonatal	80 plus	Global	-1	Health System Access 2 (unitless)
Interstitial nephritis and urinary tract infections	Male	Early Neonatal	80 plus	Global	-1	LDI (I$ per capita)
Interstitial nephritis and urinary tract infections	Male	Early Neonatal	80 plus	Global	0	Sociodemographic Status
Interstitial nephritis and urinary tract infections	Male	Early Neonatal	80 plus	Global	1	Sanitation (proportion with access)
Interstitial nephritis and urinary tract infections	Male	Early Neonatal	80 plus	Data Rich	-1	Education (years per capita)
Interstitial nephritis and urinary tract infections	Male	Early Neonatal	80 plus	Data Rich	-1	Health System Access 2 (unitless)
Interstitial nephritis and urinary tract infections	Male	Early Neonatal	80 plus	Data Rich	-1	LDI (I$ per capita)
Interstitial nephritis and urinary tract infections	Male	Early Neonatal	80 plus	Data Rich	0	Sociodemographic Status
Interstitial nephritis and urinary tract infections	Male	Early Neonatal	80 plus	Data Rich	1	Sanitation (proportion with access)
Interstitial nephritis and urinary tract infections	Female	Early Neonatal	80 plus	Global	-1	Education (years per capita)
Interstitial nephritis and urinary tract infections	Female	Early Neonatal	80 plus	Global	-1	Health System Access 2 (unitless)
Interstitial nephritis and urinary tract infections	Female	Early Neonatal	80 plus	Global	-1	LDI (I$ per capita)
Interstitial nephritis and urinary tract infections	Female	Early Neonatal	80 plus	Global	0	Sociodemographic Status
Interstitial nephritis and urinary tract infections	Female	Early Neonatal	80 plus	Global	1	Sanitation (proportion with access)
Interstitial nephritis and urinary tract infections	Female	Early Neonatal	80 plus	Data Rich	-1	Education (years per capita)
Interstitial nephritis and urinary tract infections	Female	Early Neonatal	80 plus	Data Rich	-1	Health System Access 2 (unitless)
Interstitial nephritis and urinary tract infections	Female	Early Neonatal	80 plus	Data Rich	-1	LDI (I$ per capita)
Interstitial nephritis and urinary tract infections	Female	Early Neonatal	80 plus	Data Rich	0	Sociodemographic Status
Interstitial nephritis and urinary tract infections	Female	Early Neonatal	80 plus	Data Rich	1	Sanitation (proportion with access)
Urolithiasis	Male	5 to 9	80 plus	Global	-1	Education (years per capita)
Urolithiasis	Male	5 to 9	80 plus	Global	-1	Fruits (kcal per capita)
Urolithiasis	Male	5 to 9	80 plus	Global	-1	Health System Access (unitless)

Appendix Table 10: CODEm covariates used, level of covariate, and expected direction of covariate by cause, sex, and age						
**Cause**	**Sex**	**Age start**	**Age end**	**Model type**	**Direction**	**Covariate**
Urolithiasis	Male	5 to 9	80 plus	Global	-1	LDI (I$ per capita)
Urolithiasis	Male	5 to 9	80 plus	Global	-1	Vegetables (kcal per capita)
Urolithiasis	Male	5 to 9	80 plus	Global	0	Sociodemographic Status
Urolithiasis	Male	5 to 9	80 plus	Global	1	90th percentile climatic temperature in the given country-year
Urolithiasis	Male	5 to 9	80 plus	Global	1	Animal Fats (kcal per capita)
Urolithiasis	Male	5 to 9	80 plus	Global	1	Red Meat (kcal per capita)
Urolithiasis	Male	5 to 9	80 plus	Data Rich	-1	Education (years per capita)
Urolithiasis	Male	5 to 9	80 plus	Data Rich	-1	Fruits (kcal per capita)
Urolithiasis	Male	5 to 9	80 plus	Data Rich	-1	Health System Access (unitless)
Urolithiasis	Male	5 to 9	80 plus	Data Rich	-1	LDI (I$ per capita)
Urolithiasis	Male	5 to 9	80 plus	Data Rich	-1	Vegetables (kcal per capita)
Urolithiasis	Male	5 to 9	80 plus	Data Rich	0	Sociodemographic Status
Urolithiasis	Male	5 to 9	80 plus	Data Rich	1	90th percentile climatic temperature in the given country-year
Urolithiasis	Male	5 to 9	80 plus	Data Rich	1	Animal Fats (kcal per capita)
Urolithiasis	Male	5 to 9	80 plus	Data Rich	1	Red Meat (kcal per capita)
Urolithiasis	Female	5 to 9	80 plus	Global	-1	Education (years per capita)
Urolithiasis	Female	5 to 9	80 plus	Global	-1	Fruits (kcal per capita)
Urolithiasis	Female	5 to 9	80 plus	Global	-1	Health System Access (unitless)
Urolithiasis	Female	5 to 9	80 plus	Global	-1	LDI (I$ per capita)
Urolithiasis	Female	5 to 9	80 plus	Global	-1	Vegetables (kcal per capita)
Urolithiasis	Female	5 to 9	80 plus	Global	0	Sociodemographic Status
Urolithiasis	Female	5 to 9	80 plus	Global	1	90th percentile climatic temperature in the given country-year
Urolithiasis	Female	5 to 9	80 plus	Global	1	Animal Fats (kcal per capita)
Urolithiasis	Female	5 to 9	80 plus	Global	1	Red Meat (kcal per capita)
Urolithiasis	Female	5 to 9	80 plus	Data Rich	-1	Education (years per capita)
Urolithiasis	Female	5 to 9	80 plus	Data Rich	-1	Fruits (kcal per capita)
Urolithiasis	Female	5 to 9	80 plus	Data Rich	-1	Health System Access (unitless)
Urolithiasis	Female	5 to 9	80 plus	Data Rich	-1	LDI (I$ per capita)
Urolithiasis	Female	5 to 9	80 plus	Data Rich	-1	Vegetables (kcal per capita)
Urolithiasis	Female	5 to 9	80 plus	Data Rich	0	Sociodemographic Status
Urolithiasis	Female	5 to 9	80 plus	Data Rich	1	90th percentile climatic temperature in the given country-year
Urolithiasis	Female	5 to 9	80 plus	Data Rich	1	Animal Fats (kcal per capita)
Urolithiasis	Female	5 to 9	80 plus	Data Rich	1	Red Meat (kcal per capita)
Other urinary diseases	Male	Early Neonatal	80 plus	Global	-1	Education (years per capita)
Other urinary diseases	Male	Early Neonatal	80 plus	Global	-1	Health System Access 2 (unitless)
Other urinary diseases	Male	Early Neonatal	80 plus	Global	-1	LDI (I$ per capita)
Other urinary diseases	Male	Early Neonatal	80 plus	Global	0	Sociodemographic Status
Other urinary diseases	Male	Early Neonatal	80 plus	Global	1	Mean BMI
Other urinary diseases	Male	Early Neonatal	80 plus	Data Rich	-1	Education (years per capita)
Other urinary diseases	Male	Early Neonatal	80 plus	Data Rich	-1	Health System Access 2 (unitless)
Other urinary diseases	Male	Early Neonatal	80 plus	Data Rich	-1	LDI (I$ per capita)
Other urinary diseases	Male	Early Neonatal	80 plus	Data Rich	0	Sociodemographic Status
Other urinary diseases	Male	Early Neonatal	80 plus	Data Rich	1	Mean BMI
Other urinary diseases	Female	Early Neonatal	80 plus	Global	0	Sociodemographic Status

Appendix Table 10: CODEm covariates used, level of covariate, and expected direction of covariate by cause, sex, and age						
**Cause**	**Sex**	**Age start**	**Age end**	**Model type**	**Direction**	**Covariate**
Other urinary diseases	Female	Early Neonatal	80 plus	Global	1	Education (years per capita)
Other urinary diseases	Female	Early Neonatal	80 plus	Global	1	Health System Access 2 (unitless)
Other urinary diseases	Female	Early Neonatal	80 plus	Global	1	LDI (I$ per capita)
Other urinary diseases	Female	Early Neonatal	80 plus	Global	1	Mean BMI
Other urinary diseases	Female	Early Neonatal	80 plus	Data Rich	0	Sociodemographic Status
Other urinary diseases	Female	Early Neonatal	80 plus	Data Rich	1	Education (years per capita)
Other urinary diseases	Female	Early Neonatal	80 plus	Data Rich	1	Health System Access 2 (unitless)
Other urinary diseases	Female	Early Neonatal	80 plus	Data Rich	1	LDI (I$ per capita)
Other urinary diseases	Female	Early Neonatal	80 plus	Data Rich	1	Mean BMI
Gynecological diseases	Female	15 to 19	80 plus	Global	-1	Education (years per capita)
Gynecological diseases	Female	15 to 19	80 plus	Global	-1	Health System Access (unitless)
Gynecological diseases	Female	15 to 19	80 plus	Global	-1	LDI (I$ per capita)
Gynecological diseases	Female	15 to 19	80 plus	Global	-1	Skilled Birth Attendance (proportion)
Gynecological diseases	Female	15 to 19	80 plus	Global	-1	Sociodemographic Status
Gynecological diseases	Female	15 to 19	80 plus	Global	0	Smoking Prevalence
Gynecological diseases	Female	15 to 19	80 plus	Global	1	Live Births 35+ (proportion)
Gynecological diseases	Female	15 to 19	80 plus	Global	1	Total Fertility Rate
Gynecological diseases	Female	15 to 19	80 plus	Data Rich	-1	Education (years per capita)
Gynecological diseases	Female	15 to 19	80 plus	Data Rich	-1	Health System Access (unitless)
Gynecological diseases	Female	15 to 19	80 plus	Data Rich	-1	LDI (I$ per capita)
Gynecological diseases	Female	15 to 19	80 plus	Data Rich	-1	Skilled Birth Attendance (proportion)
Gynecological diseases	Female	15 to 19	80 plus	Data Rich	-1	Sociodemographic Status
Gynecological diseases	Female	15 to 19	80 plus	Data Rich	0	Smoking Prevalence
Gynecological diseases	Female	15 to 19	80 plus	Data Rich	1	Live Births 35+ (proportion)
Gynecological diseases	Female	15 to 19	80 plus	Data Rich	1	Total Fertility Rate
Uterine fibroids	Female	15 to 19	80 plus	Global	-1	Education (years per capita)
Uterine fibroids	Female	15 to 19	80 plus	Global	-1	Health System Access (unitless)
Uterine fibroids	Female	15 to 19	80 plus	Global	-1	LDI (I$ per capita)
Uterine fibroids	Female	15 to 19	80 plus	Global	-1	Skilled Birth Attendance (proportion)
Uterine fibroids	Female	15 to 19	80 plus	Global	-1	Sociodemographic Status
Uterine fibroids	Female	15 to 19	80 plus	Global	0	Smoking Prevalence
Uterine fibroids	Female	15 to 19	80 plus	Global	1	Live Births 35+ (proportion)
Uterine fibroids	Female	15 to 19	80 plus	Global	1	Total Fertility Rate
Uterine fibroids	Female	15 to 19	80 plus	Data Rich	-1	Education (years per capita)
Uterine fibroids	Female	15 to 19	80 plus	Data Rich	-1	Health System Access (unitless)
Uterine fibroids	Female	15 to 19	80 plus	Data Rich	-1	LDI (I$ per capita)
Uterine fibroids	Female	15 to 19	80 plus	Data Rich	-1	Skilled Birth Attendance (proportion)
Uterine fibroids	Female	15 to 19	80 plus	Data Rich	-1	Sociodemographic Status
Uterine fibroids	Female	15 to 19	80 plus	Data Rich	0	Smoking Prevalence
Uterine fibroids	Female	15 to 19	80 plus	Data Rich	1	Live Births 35+ (proportion)
Uterine fibroids	Female	15 to 19	80 plus	Data Rich	1	Total Fertility Rate
Polycystic ovarian syndrome	Female	15 to 19	50 to 54	Global	-1	Education (years per capita)
Polycystic ovarian syndrome	Female	15 to 19	50 to 54	Global	-1	Health System Access (unitless)
Polycystic ovarian syndrome	Female	15 to 19	50 to 54	Global	-1	LDI (I$ per capita)

Appendix Table 10: CODEm covariates used, level of covariate, and expected direction of covariate by cause, sex, and age						
**Cause**	**Sex**	**Age start**	**Age end**	**Model type**	**Direction**	**Covariate**
Polycystic ovarian syndrome	Female	15 to 19	50 to 54	Global	-1	Skilled Birth Attendance (proportion)
Polycystic ovarian syndrome	Female	15 to 19	50 to 54	Global	-1	Sociodemographic Status
Polycystic ovarian syndrome	Female	15 to 19	50 to 54	Global	0	Smoking Prevalence
Polycystic ovarian syndrome	Female	15 to 19	50 to 54	Global	1	Live Births 35+ (proportion)
Polycystic ovarian syndrome	Female	15 to 19	50 to 54	Global	1	Total Fertility Rate
Polycystic ovarian syndrome	Female	15 to 19	50 to 54	Data Rich	-1	Education (years per capita)
Polycystic ovarian syndrome	Female	15 to 19	50 to 54	Data Rich	-1	Health System Access (unitless)
Polycystic ovarian syndrome	Female	15 to 19	50 to 54	Data Rich	-1	LDI (I$ per capita)
Polycystic ovarian syndrome	Female	15 to 19	50 to 54	Data Rich	-1	Skilled Birth Attendance (proportion)
Polycystic ovarian syndrome	Female	15 to 19	50 to 54	Data Rich	-1	Sociodemographic Status
Polycystic ovarian syndrome	Female	15 to 19	50 to 54	Data Rich	0	Smoking Prevalence
Polycystic ovarian syndrome	Female	15 to 19	50 to 54	Data Rich	1	Live Births 35+ (proportion)
Polycystic ovarian syndrome	Female	15 to 19	50 to 54	Data Rich	1	Total Fertility Rate
Endometriosis	Female	15 to 19	50 to 54	Global	-1	Education (years per capita)
Endometriosis	Female	15 to 19	50 to 54	Global	-1	Health System Access (unitless)
Endometriosis	Female	15 to 19	50 to 54	Global	-1	LDI (I$ per capita)
Endometriosis	Female	15 to 19	50 to 54	Global	-1	Skilled Birth Attendance (proportion)
Endometriosis	Female	15 to 19	50 to 54	Global	-1	Sociodemographic Status
Endometriosis	Female	15 to 19	50 to 54	Global	0	Smoking Prevalence
Endometriosis	Female	15 to 19	50 to 54	Global	1	Live Births 35+ (proportion)
Endometriosis	Female	15 to 19	50 to 54	Global	1	Total Fertility Rate
Endometriosis	Female	15 to 19	50 to 54	Data Rich	-1	Education (years per capita)
Endometriosis	Female	15 to 19	50 to 54	Data Rich	-1	Health System Access (unitless)
Endometriosis	Female	15 to 19	50 to 54	Data Rich	-1	LDI (I$ per capita)
Endometriosis	Female	15 to 19	50 to 54	Data Rich	-1	Skilled Birth Attendance (proportion)
Endometriosis	Female	15 to 19	50 to 54	Data Rich	-1	Sociodemographic Status
Endometriosis	Female	15 to 19	50 to 54	Data Rich	0	Smoking Prevalence
Endometriosis	Female	15 to 19	50 to 54	Data Rich	1	Live Births 35+ (proportion)
Endometriosis	Female	15 to 19	50 to 54	Data Rich	1	Total Fertility Rate
Genital prolapse	Female	15 to 19	80 plus	Global	-1	Education (years per capita)
Genital prolapse	Female	15 to 19	80 plus	Global	-1	Health System Access (unitless)
Genital prolapse	Female	15 to 19	80 plus	Global	-1	LDI (I$ per capita)
Genital prolapse	Female	15 to 19	80 plus	Global	-1	Skilled Birth Attendance (proportion)
Genital prolapse	Female	15 to 19	80 plus	Global	-1	Sociodemographic Status
Genital prolapse	Female	15 to 19	80 plus	Global	0	Smoking Prevalence
Genital prolapse	Female	15 to 19	80 plus	Global	1	Live Births 35+ (proportion)
Genital prolapse	Female	15 to 19	80 plus	Global	1	Total Fertility Rate
Genital prolapse	Female	15 to 19	80 plus	Data Rich	-1	Education (years per capita)
Genital prolapse	Female	15 to 19	80 plus	Data Rich	-1	Health System Access (unitless)
Genital prolapse	Female	15 to 19	80 plus	Data Rich	-1	LDI (I$ per capita)
Genital prolapse	Female	15 to 19	80 plus	Data Rich	-1	Skilled Birth Attendance (proportion)
Genital prolapse	Female	15 to 19	80 plus	Data Rich	-1	Sociodemographic Status
Genital prolapse	Female	15 to 19	80 plus	Data Rich	0	Smoking Prevalence
Genital prolapse	Female	15 to 19	80 plus	Data Rich	1	Live Births 35+ (proportion)

Appendix Table 10: CODEm covariates used, level of covariate, and expected direction of covariate by cause, sex, and age						
**Cause**	**Sex**	**Age start**	**Age end**	**Model type**	**Direction**	**Covariate**
Genital prolapse	Female	15 to 19	80 plus	Data Rich	1	Total Fertility Rate
Other gynecological diseases	Female	15 to 19	80 plus	Global	-1	Education (years per capita)
Other gynecological diseases	Female	15 to 19	80 plus	Global	-1	Health System Access (unitless)
Other gynecological diseases	Female	15 to 19	80 plus	Global	-1	LDI (I$ per capita)
Other gynecological diseases	Female	15 to 19	80 plus	Global	-1	Skilled Birth Attendance (proportion)
Other gynecological diseases	Female	15 to 19	80 plus	Global	-1	Sociodemographic Status
Other gynecological diseases	Female	15 to 19	80 plus	Global	0	Smoking Prevalence
Other gynecological diseases	Female	15 to 19	80 plus	Global	1	Live Births 35+ (proportion)
Other gynecological diseases	Female	15 to 19	80 plus	Global	1	Total Fertility Rate
Other gynecological diseases	Female	15 to 19	80 plus	Data Rich	-1	Education (years per capita)
Other gynecological diseases	Female	15 to 19	80 plus	Data Rich	-1	Health System Access (unitless)
Other gynecological diseases	Female	15 to 19	80 plus	Data Rich	-1	LDI (I$ per capita)
Other gynecological diseases	Female	15 to 19	80 plus	Data Rich	-1	Skilled Birth Attendance (proportion)
Other gynecological diseases	Female	15 to 19	80 plus	Data Rich	-1	Sociodemographic Status
Other gynecological diseases	Female	15 to 19	80 plus	Data Rich	0	Smoking Prevalence
Other gynecological diseases	Female	15 to 19	80 plus	Data Rich	1	Live Births 35+ (proportion)
Other gynecological diseases	Female	15 to 19	80 plus	Data Rich	1	Total Fertility Rate
Hemoglobinopathies and hemolytic anemias	Male	Early Neonatal	80 plus	Global	-1	Education (years per capita)
Hemoglobinopathies and hemolytic anemias	Male	Early Neonatal	80 plus	Global	-1	Health System Access (capped)
Hemoglobinopathies and hemolytic anemias	Male	Early Neonatal	80 plus	Global	-1	LDI (I$ per capita)
Hemoglobinopathies and hemolytic anemias	Male	Early Neonatal	80 plus	Global	-1	Latitude Over 45 (proportion)
Hemoglobinopathies and hemolytic anemias	Male	Early Neonatal	80 plus	Global	-1	Sociodemographic Status
Hemoglobinopathies and hemolytic anemias	Male	Early Neonatal	80 plus	Global	0	Latitude 30 to 45 (proportion)
Hemoglobinopathies and hemolytic anemias	Male	Early Neonatal	80 plus	Global	1	Hemoglobinopathies Prevalence x Excess Mortality
Hemoglobinopathies and hemolytic anemias	Male	Early Neonatal	80 plus	Global	1	Hemoglobinopathies Prevalence x Excess Mortality (excluding G6PD deficiency)
Hemoglobinopathies and hemolytic anemias	Male	Early Neonatal	80 plus	Global	1	Latitude 15 to 30 (proportion)
Hemoglobinopathies and hemolytic anemias	Male	Early Neonatal	80 plus	Global	1	Latitude Under 15 (proportion)
Hemoglobinopathies and hemolytic anemias	Male	Early Neonatal	80 plus	Data Rich	-1	Education (years per capita)
Hemoglobinopathies and hemolytic anemias	Male	Early Neonatal	80 plus	Data Rich	-1	Health System Access (capped)
Hemoglobinopathies and hemolytic anemias	Male	Early Neonatal	80 plus	Data Rich	-1	LDI (I$ per capita)
Hemoglobinopathies and hemolytic anemias	Male	Early Neonatal	80 plus	Data Rich	-1	Latitude Over 45 (proportion)
Hemoglobinopathies and hemolytic anemias	Male	Early Neonatal	80 plus	Data Rich	-1	Sociodemographic Status
Hemoglobinopathies and hemolytic anemias	Male	Early Neonatal	80 plus	Data Rich	0	Latitude 30 to 45 (proportion)
Hemoglobinopathies and hemolytic anemias	Male	Early Neonatal	80 plus	Data Rich	1	Hemoglobinopathies Prevalence x Excess Mortality
Hemoglobinopathies and hemolytic anemias	Male	Early Neonatal	80 plus	Data Rich	1	Hemoglobinopathies Prevalence x Excess Mortality (excluding G6PD deficiency)
Hemoglobinopathies and hemolytic anemias	Male	Early Neonatal	80 plus	Data Rich	1	Latitude 15 to 30 (proportion)
Hemoglobinopathies and hemolytic anemias	Male	Early Neonatal	80 plus	Data Rich	1	Latitude Under 15 (proportion)
Hemoglobinopathies and hemolytic anemias	Female	Early Neonatal	80 plus	Global	-1	Education (years per capita)
Hemoglobinopathies and hemolytic anemias	Female	Early Neonatal	80 plus	Global	-1	Health System Access (capped)
Hemoglobinopathies and hemolytic anemias	Female	Early Neonatal	80 plus	Global	-1	LDI (I$ per capita)
Hemoglobinopathies and hemolytic anemias	Female	Early Neonatal	80 plus	Global	-1	Latitude Over 45 (proportion)
Hemoglobinopathies and hemolytic anemias	Female	Early Neonatal	80 plus	Global	-1	Sociodemographic Status
Hemoglobinopathies and hemolytic anemias	Female	Early Neonatal	80 plus	Global	0	Latitude 30 to 45 (proportion)
Hemoglobinopathies and hemolytic anemias	Female	Early Neonatal	80 plus	Global	1	Hemoglobinopathies Prevalence x Excess Mortality

Appendix Table 10: CODEm covariates used, level of covariate, and expected direction of covariate by cause, sex, and age						
Cause	Sex	Age start	Age end	Model type	Direction	Covariate
Hemoglobinopathies and hemolytic anemias	Female	Early Neonatal	80 plus	Global	1	Hemoglobinopathies Prevalence x Excess Mortality (excluding G6PD deficiency)
Hemoglobinopathies and hemolytic anemias	Female	Early Neonatal	80 plus	Global	1	Latitude 15 to 30 (proportion)
Hemoglobinopathies and hemolytic anemias	Female	Early Neonatal	80 plus	Global	1	Latitude Under 15 (proportion)
Hemoglobinopathies and hemolytic anemias	Female	Early Neonatal	80 plus	Data Rich	-1	Education (years per capita)
Hemoglobinopathies and hemolytic anemias	Female	Early Neonatal	80 plus	Data Rich	-1	Health System Access (capped)
Hemoglobinopathies and hemolytic anemias	Female	Early Neonatal	80 plus	Data Rich	-1	LDI (I$ per capita)
Hemoglobinopathies and hemolytic anemias	Female	Early Neonatal	80 plus	Data Rich	-1	Latitude Over 45 (proportion)
Hemoglobinopathies and hemolytic anemias	Female	Early Neonatal	80 plus	Data Rich	-1	Sociodemographic Status
Hemoglobinopathies and hemolytic anemias	Female	Early Neonatal	80 plus	Data Rich	0	Latitude 30 to 45 (proportion)
Hemoglobinopathies and hemolytic anemias	Female	Early Neonatal	80 plus	Data Rich	1	Hemoglobinopathies Prevalence x Excess Mortality
Hemoglobinopathies and hemolytic anemias	Female	Early Neonatal	80 plus	Data Rich	1	Hemoglobinopathies Prevalence x Excess Mortality (excluding G6PD deficiency)
Hemoglobinopathies and hemolytic anemias	Female	Early Neonatal	80 plus	Data Rich	1	Latitude 15 to 30 (proportion)
Hemoglobinopathies and hemolytic anemias	Female	Early Neonatal	80 plus	Data Rich	1	Latitude Under 15 (proportion)
Endocrine, metabolic, blood, and immune disorders	Male	Early Neonatal	80 plus	Global	-1	Education (years per capita)
Endocrine, metabolic, blood, and immune disorders	Male	Early Neonatal	80 plus	Global	-1	Health System Access 2 (unitless)
Endocrine, metabolic, blood, and immune disorders	Male	Early Neonatal	80 plus	Global	-1	LDI (I$ per capita)
Endocrine, metabolic, blood, and immune disorders	Male	Early Neonatal	80 plus	Global	0	Sociodemographic Status
Endocrine, metabolic, blood, and immune disorders	Male	Early Neonatal	80 plus	Global	1	Alcohol (litres per capita)
Endocrine, metabolic, blood, and immune disorders	Male	Early Neonatal	80 plus	Global	1	Animal Fats (kcal per capita)
Endocrine, metabolic, blood, and immune disorders	Male	Early Neonatal	80 plus	Global	1	Cholesterol (total, mean per capita)
Endocrine, metabolic, blood, and immune disorders	Male	Early Neonatal	80 plus	Global	1	Mean BMI
Endocrine, metabolic, blood, and immune disorders	Male	Early Neonatal	80 plus	Global	1	Total Calories (kcal per capita)
Endocrine, metabolic, blood, and immune disorders	Male	Early Neonatal	80 plus	Data Rich	-1	Education (years per capita)
Endocrine, metabolic, blood, and immune disorders	Male	Early Neonatal	80 plus	Data Rich	-1	Health System Access 2 (unitless)
Endocrine, metabolic, blood, and immune disorders	Male	Early Neonatal	80 plus	Data Rich	-1	LDI (I$ per capita)
Endocrine, metabolic, blood, and immune disorders	Male	Early Neonatal	80 plus	Data Rich	0	Sociodemographic Status
Endocrine, metabolic, blood, and immune disorders	Male	Early Neonatal	80 plus	Data Rich	1	Alcohol (litres per capita)
Endocrine, metabolic, blood, and immune disorders	Male	Early Neonatal	80 plus	Data Rich	1	Animal Fats (kcal per capita)
Endocrine, metabolic, blood, and immune disorders	Male	Early Neonatal	80 plus	Data Rich	1	Cholesterol (total, mean per capita)
Endocrine, metabolic, blood, and immune disorders	Male	Early Neonatal	80 plus	Data Rich	1	Mean BMI
Endocrine, metabolic, blood, and immune disorders	Male	Early Neonatal	80 plus	Data Rich	1	Total Calories (kcal per capita)
Endocrine, metabolic, blood, and immune disorders	Female	Early Neonatal	80 plus	Global	-1	Education (years per capita)
Endocrine, metabolic, blood, and immune disorders	Female	Early Neonatal	80 plus	Global	-1	Health System Access 2 (unitless)
Endocrine, metabolic, blood, and immune disorders	Female	Early Neonatal	80 plus	Global	-1	LDI (I$ per capita)
Endocrine, metabolic, blood, and immune disorders	Female	Early Neonatal	80 plus	Global	0	Sociodemographic Status
Endocrine, metabolic, blood, and immune disorders	Female	Early Neonatal	80 plus	Global	1	Alcohol (litres per capita)
Endocrine, metabolic, blood, and immune disorders	Female	Early Neonatal	80 plus	Global	1	Animal Fats (kcal per capita)
Endocrine, metabolic, blood, and immune disorders	Female	Early Neonatal	80 plus	Global	1	Cholesterol (total, mean per capita)
Endocrine, metabolic, blood, and immune disorders	Female	Early Neonatal	80 plus	Global	1	Mean BMI
Endocrine, metabolic, blood, and immune disorders	Female	Early Neonatal	80 plus	Global	1	Total Calories (kcal per capita)
Endocrine, metabolic, blood, and immune disorders	Female	Early Neonatal	80 plus	Data Rich	-1	Education (years per capita)
Endocrine, metabolic, blood, and immune disorders	Female	Early Neonatal	80 plus	Data Rich	-1	Health System Access 2 (unitless)
Endocrine, metabolic, blood, and immune disorders	Female	Early Neonatal	80 plus	Data Rich	-1	LDI (I$ per capita)
Endocrine, metabolic, blood, and immune disorders	Female	Early Neonatal	80 plus	Data Rich	0	Sociodemographic Status

Appendix Table 10: CODEm covariates used, level of covariate, and expected direction of covariate by cause, sex, and age						
**Cause**	**Sex**	**Age start**	**Age end**	**Model type**	**Direction**	**Covariate**
Endocrine, metabolic, blood, and immune disorders	Female	Early Neonatal	80 plus	Data Rich	1	Alcohol (litres per capita)
Endocrine, metabolic, blood, and immune disorders	Female	Early Neonatal	80 plus	Data Rich	1	Animal Fats (kcal per capita)
Endocrine, metabolic, blood, and immune disorders	Female	Early Neonatal	80 plus	Data Rich	1	Cholesterol (total, mean per capita)
Endocrine, metabolic, blood, and immune disorders	Female	Early Neonatal	80 plus	Data Rich	1	Mean BMI
Endocrine, metabolic, blood, and immune disorders	Female	Early Neonatal	80 plus	Data Rich	1	Total Calories (kcal per capita)
Musculoskeletal disorders	Male	5 to 9	80 plus	Global	-1	Health System Access 2 (unitless)
Musculoskeletal disorders	Male	5 to 9	80 plus	Global	0	Education (years per capita)
Musculoskeletal disorders	Male	5 to 9	80 plus	Global	0	LDI (I$ per capita)
Musculoskeletal disorders	Male	5 to 9	80 plus	Global	0	Sociodemographic Status
Musculoskeletal disorders	Male	5 to 9	80 plus	Global	0	Vegetables (kcal per capita)
Musculoskeletal disorders	Male	5 to 9	80 plus	Global	1	Alcohol (litres per capita)
Musculoskeletal disorders	Male	5 to 9	80 plus	Global	1	Cholesterol (total, mean per capita)
Musculoskeletal disorders	Male	5 to 9	80 plus	Global	1	Cumulative Cigarettes (10 Years)
Musculoskeletal disorders	Male	5 to 9	80 plus	Global	1	Cumulative Cigarettes (5 Years)
Musculoskeletal disorders	Male	5 to 9	80 plus	Global	1	Mean BMI
Musculoskeletal disorders	Male	5 to 9	80 plus	Global	1	Smoking Prevalence
Musculoskeletal disorders	Male	5 to 9	80 plus	Data Rich	-1	Health System Access 2 (unitless)
Musculoskeletal disorders	Male	5 to 9	80 plus	Data Rich	0	Education (years per capita)
Musculoskeletal disorders	Male	5 to 9	80 plus	Data Rich	0	LDI (I$ per capita)
Musculoskeletal disorders	Male	5 to 9	80 plus	Data Rich	0	Sociodemographic Status
Musculoskeletal disorders	Male	5 to 9	80 plus	Data Rich	0	Vegetables (kcal per capita)
Musculoskeletal disorders	Male	5 to 9	80 plus	Data Rich	1	Alcohol (litres per capita)
Musculoskeletal disorders	Male	5 to 9	80 plus	Data Rich	1	Cholesterol (total, mean per capita)
Musculoskeletal disorders	Male	5 to 9	80 plus	Data Rich	1	Cumulative Cigarettes (10 Years)
Musculoskeletal disorders	Male	5 to 9	80 plus	Data Rich	1	Cumulative Cigarettes (5 Years)
Musculoskeletal disorders	Male	5 to 9	80 plus	Data Rich	1	Mean BMI
Musculoskeletal disorders	Male	5 to 9	80 plus	Data Rich	1	Smoking Prevalence
Musculoskeletal disorders	Female	5 to 9	80 plus	Global	-1	Health System Access 2 (unitless)
Musculoskeletal disorders	Female	5 to 9	80 plus	Global	0	Education (years per capita)
Musculoskeletal disorders	Female	5 to 9	80 plus	Global	0	LDI (I$ per capita)
Musculoskeletal disorders	Female	5 to 9	80 plus	Global	0	Sociodemographic Status
Musculoskeletal disorders	Female	5 to 9	80 plus	Global	0	Vegetables (kcal per capita)
Musculoskeletal disorders	Female	5 to 9	80 plus	Global	1	Alcohol (litres per capita)
Musculoskeletal disorders	Female	5 to 9	80 plus	Global	1	Cholesterol (total, mean per capita)
Musculoskeletal disorders	Female	5 to 9	80 plus	Global	1	Cumulative Cigarettes (10 Years)
Musculoskeletal disorders	Female	5 to 9	80 plus	Global	1	Cumulative Cigarettes (5 Years)
Musculoskeletal disorders	Female	5 to 9	80 plus	Global	1	Mean BMI
Musculoskeletal disorders	Female	5 to 9	80 plus	Global	1	Smoking Prevalence
Musculoskeletal disorders	Female	5 to 9	80 plus	Data Rich	-1	Health System Access 2 (unitless)
Musculoskeletal disorders	Female	5 to 9	80 plus	Data Rich	0	Education (years per capita)
Musculoskeletal disorders	Female	5 to 9	80 plus	Data Rich	0	LDI (I$ per capita)
Musculoskeletal disorders	Female	5 to 9	80 plus	Data Rich	0	Sociodemographic Status
Musculoskeletal disorders	Female	5 to 9	80 plus	Data Rich	0	Vegetables (kcal per capita)
Musculoskeletal disorders	Female	5 to 9	80 plus	Data Rich	1	Alcohol (litres per capita)

Appendix Table 10: CODEm covariates used, level of covariate, and expected direction of covariate by cause, sex, and age						
**Cause**	**Sex**	**Age start**	**Age end**	**Model type**	**Direction**	**Covariate**
Musculoskeletal disorders	Female	5 to 9	80 plus	Data Rich	1	Cholesterol (total, mean per capita)
Musculoskeletal disorders	Female	5 to 9	80 plus	Data Rich	1	Cumulative Cigarettes (10 Years)
Musculoskeletal disorders	Female	5 to 9	80 plus	Data Rich	1	Cumulative Cigarettes (5 Years)
Musculoskeletal disorders	Female	5 to 9	80 plus	Data Rich	1	Mean BMI
Musculoskeletal disorders	Female	5 to 9	80 plus	Data Rich	1	Smoking Prevalence
Rheumatoid arthritis	Male	5 to 9	80 plus	Global	-1	Education (years per capita)
Rheumatoid arthritis	Male	5 to 9	80 plus	Global	-1	Health System Access 2 (unitless)
Rheumatoid arthritis	Male	5 to 9	80 plus	Global	-1	LDI (I$ per capita)
Rheumatoid arthritis	Male	5 to 9	80 plus	Global	0	Sociodemographic Status
Rheumatoid arthritis	Male	5 to 9	80 plus	Global	0	Vegetables (kcal per capita)
Rheumatoid arthritis	Male	5 to 9	80 plus	Global	1	Alcohol (litres per capita)
Rheumatoid arthritis	Male	5 to 9	80 plus	Global	1	Cholesterol (total, mean per capita)
Rheumatoid arthritis	Male	5 to 9	80 plus	Global	1	Cumulative Cigarettes (10 Years)
Rheumatoid arthritis	Male	5 to 9	80 plus	Global	1	Cumulative Cigarettes (5 Years)
Rheumatoid arthritis	Male	5 to 9	80 plus	Global	1	Mean BMI
Rheumatoid arthritis	Male	5 to 9	80 plus	Global	1	Smoking Prevalence
Rheumatoid arthritis	Male	5 to 9	80 plus	Data Rich	-1	Education (years per capita)
Rheumatoid arthritis	Male	5 to 9	80 plus	Data Rich	-1	Health System Access 2 (unitless)
Rheumatoid arthritis	Male	5 to 9	80 plus	Data Rich	-1	LDI (I$ per capita)
Rheumatoid arthritis	Male	5 to 9	80 plus	Data Rich	0	Sociodemographic Status
Rheumatoid arthritis	Male	5 to 9	80 plus	Data Rich	0	Vegetables (kcal per capita)
Rheumatoid arthritis	Male	5 to 9	80 plus	Data Rich	1	Alcohol (litres per capita)
Rheumatoid arthritis	Male	5 to 9	80 plus	Data Rich	1	Cholesterol (total, mean per capita)
Rheumatoid arthritis	Male	5 to 9	80 plus	Data Rich	1	Cumulative Cigarettes (10 Years)
Rheumatoid arthritis	Male	5 to 9	80 plus	Data Rich	1	Cumulative Cigarettes (5 Years)
Rheumatoid arthritis	Male	5 to 9	80 plus	Data Rich	1	Mean BMI
Rheumatoid arthritis	Male	5 to 9	80 plus	Data Rich	1	Smoking Prevalence
Rheumatoid arthritis	Female	5 to 9	80 plus	Global	-1	Education (years per capita)
Rheumatoid arthritis	Female	5 to 9	80 plus	Global	-1	Health System Access 2 (unitless)
Rheumatoid arthritis	Female	5 to 9	80 plus	Global	-1	LDI (I$ per capita)
Rheumatoid arthritis	Female	5 to 9	80 plus	Global	0	Sociodemographic Status
Rheumatoid arthritis	Female	5 to 9	80 plus	Global	0	Vegetables (kcal per capita)
Rheumatoid arthritis	Female	5 to 9	80 plus	Global	1	Alcohol (litres per capita)
Rheumatoid arthritis	Female	5 to 9	80 plus	Global	1	Cholesterol (total, mean per capita)
Rheumatoid arthritis	Female	5 to 9	80 plus	Global	1	Cumulative Cigarettes (10 Years)
Rheumatoid arthritis	Female	5 to 9	80 plus	Global	1	Cumulative Cigarettes (5 Years)
Rheumatoid arthritis	Female	5 to 9	80 plus	Global	1	Mean BMI
Rheumatoid arthritis	Female	5 to 9	80 plus	Global	1	Smoking Prevalence
Rheumatoid arthritis	Female	5 to 9	80 plus	Data Rich	-1	Education (years per capita)
Rheumatoid arthritis	Female	5 to 9	80 plus	Data Rich	-1	Health System Access 2 (unitless)
Rheumatoid arthritis	Female	5 to 9	80 plus	Data Rich	-1	LDI (I$ per capita)
Rheumatoid arthritis	Female	5 to 9	80 plus	Data Rich	0	Sociodemographic Status
Rheumatoid arthritis	Female	5 to 9	80 plus	Data Rich	0	Vegetables (kcal per capita)
Rheumatoid arthritis	Female	5 to 9	80 plus	Data Rich	1	Alcohol (litres per capita)

Appendix Table 10: CODEm covariates used, level of covariate, and expected direction of covariate by cause, sex, and age						
**Cause**	**Sex**	**Age start**	**Age end**	**Model type**	**Direction**	**Covariate**
Rheumatoid arthritis	Female	5 to 9	80 plus	Data Rich	1	Cholesterol (total, mean per capita)
Rheumatoid arthritis	Female	5 to 9	80 plus	Data Rich	1	Cumulative Cigarettes (10 Years)
Rheumatoid arthritis	Female	5 to 9	80 plus	Data Rich	1	Cumulative Cigarettes (5 Years)
Rheumatoid arthritis	Female	5 to 9	80 plus	Data Rich	1	Mean BMI
Rheumatoid arthritis	Female	5 to 9	80 plus	Data Rich	1	Smoking Prevalence
Other musculoskeletal disorders	Male	5 to 9	80 plus	Global	-1	Health System Access 2 (unitless)
Other musculoskeletal disorders	Male	5 to 9	80 plus	Global	0	Education (years per capita)
Other musculoskeletal disorders	Male	5 to 9	80 plus	Global	0	LDI (I$ per capita)
Other musculoskeletal disorders	Male	5 to 9	80 plus	Global	0	Sociodemographic Status
Other musculoskeletal disorders	Male	5 to 9	80 plus	Global	0	Vegetables (kcal per capita)
Other musculoskeletal disorders	Male	5 to 9	80 plus	Global	1	Alcohol (litres per capita)
Other musculoskeletal disorders	Male	5 to 9	80 plus	Global	1	Cholesterol (total, mean per capita)
Other musculoskeletal disorders	Male	5 to 9	80 plus	Global	1	Cumulative Cigarettes (10 Years)
Other musculoskeletal disorders	Male	5 to 9	80 plus	Global	1	Cumulative Cigarettes (5 Years)
Other musculoskeletal disorders	Male	5 to 9	80 plus	Global	1	Mean BMI
Other musculoskeletal disorders	Male	5 to 9	80 plus	Global	1	Smoking Prevalence
Other musculoskeletal disorders	Male	5 to 9	80 plus	Data Rich	-1	Health System Access 2 (unitless)
Other musculoskeletal disorders	Male	5 to 9	80 plus	Data Rich	0	Education (years per capita)
Other musculoskeletal disorders	Male	5 to 9	80 plus	Data Rich	0	LDI (I$ per capita)
Other musculoskeletal disorders	Male	5 to 9	80 plus	Data Rich	0	Sociodemographic Status
Other musculoskeletal disorders	Male	5 to 9	80 plus	Data Rich	0	Vegetables (kcal per capita)
Other musculoskeletal disorders	Male	5 to 9	80 plus	Data Rich	1	Alcohol (litres per capita)
Other musculoskeletal disorders	Male	5 to 9	80 plus	Data Rich	1	Cholesterol (total, mean per capita)
Other musculoskeletal disorders	Male	5 to 9	80 plus	Data Rich	1	Cumulative Cigarettes (10 Years)
Other musculoskeletal disorders	Male	5 to 9	80 plus	Data Rich	1	Cumulative Cigarettes (5 Years)
Other musculoskeletal disorders	Male	5 to 9	80 plus	Data Rich	1	Mean BMI
Other musculoskeletal disorders	Male	5 to 9	80 plus	Data Rich	1	Smoking Prevalence
Other musculoskeletal disorders	Female	5 to 9	80 plus	Global	-1	Health System Access 2 (unitless)
Other musculoskeletal disorders	Female	5 to 9	80 plus	Global	0	Education (years per capita)
Other musculoskeletal disorders	Female	5 to 9	80 plus	Global	0	LDI (I$ per capita)
Other musculoskeletal disorders	Female	5 to 9	80 plus	Global	0	Sociodemographic Status
Other musculoskeletal disorders	Female	5 to 9	80 plus	Global	0	Vegetables (kcal per capita)
Other musculoskeletal disorders	Female	5 to 9	80 plus	Global	1	Alcohol (litres per capita)
Other musculoskeletal disorders	Female	5 to 9	80 plus	Global	1	Cholesterol (total, mean per capita)
Other musculoskeletal disorders	Female	5 to 9	80 plus	Global	1	Cumulative Cigarettes (10 Years)
Other musculoskeletal disorders	Female	5 to 9	80 plus	Global	1	Cumulative Cigarettes (5 Years)
Other musculoskeletal disorders	Female	5 to 9	80 plus	Global	1	Mean BMI
Other musculoskeletal disorders	Female	5 to 9	80 plus	Global	1	Smoking Prevalence
Other musculoskeletal disorders	Female	5 to 9	80 plus	Data Rich	-1	Health System Access 2 (unitless)
Other musculoskeletal disorders	Female	5 to 9	80 plus	Data Rich	0	Education (years per capita)
Other musculoskeletal disorders	Female	5 to 9	80 plus	Data Rich	0	LDI (I$ per capita)
Other musculoskeletal disorders	Female	5 to 9	80 plus	Data Rich	0	Sociodemographic Status
Other musculoskeletal disorders	Female	5 to 9	80 plus	Data Rich	0	Vegetables (kcal per capita)
Other musculoskeletal disorders	Female	5 to 9	80 plus	Data Rich	1	Alcohol (litres per capita)

Appendix Table 10: CODEm covariates used, level of covariate, and expected direction of covariate by cause, sex, and age						
Cause	Sex	Age start	Age end	Model type	Direction	Covariate
Other musculoskeletal disorders	Female	5 to 9	80 plus	Data Rich	1	Cholesterol (total, mean per capita)
Other musculoskeletal disorders	Female	5 to 9	80 plus	Data Rich	1	Cumulative Cigarettes (10 Years)
Other musculoskeletal disorders	Female	5 to 9	80 plus	Data Rich	1	Cumulative Cigarettes (5 Years)
Other musculoskeletal disorders	Female	5 to 9	80 plus	Data Rich	1	Mean BMI
Other musculoskeletal disorders	Female	5 to 9	80 plus	Data Rich	1	Smoking Prevalence
Congenital anomalies	Male	Early Neonatal	80 plus	Global	-1	Education (years per capita)
Congenital anomalies	Male	Early Neonatal	80 plus	Global	-1	Fruits (kcal per capita)
Congenital anomalies	Male	Early Neonatal	80 plus	Global	-1	LDI (I$ per capita)
Congenital anomalies	Male	Early Neonatal	80 plus	Global	-1	Legality of Abortion
Congenital anomalies	Male	Early Neonatal	80 plus	Global	-1	Sociodemographic Status
Congenital anomalies	Male	Early Neonatal	80 plus	Global	-1	Vegetables (kcal per capita)
Congenital anomalies	Male	Early Neonatal	80 plus	Global	0	Health System Access (capped)
Congenital anomalies	Male	Early Neonatal	80 plus	Global	1	Alcohol (litres per capita)
Congenital anomalies	Male	Early Neonatal	80 plus	Global	1	Indoor Air Pollution (All Cooking Fuels)
Congenital anomalies	Male	Early Neonatal	80 plus	Global	1	Live Births 35+ (proportion)
Congenital anomalies	Male	Early Neonatal	80 plus	Global	1	Smoking Prevalence (Reproductive Age Standardised)
Congenital anomalies	Male	Early Neonatal	80 plus	Data Rich	-1	Education (years per capita)
Congenital anomalies	Male	Early Neonatal	80 plus	Data Rich	-1	Fruits (kcal per capita)
Congenital anomalies	Male	Early Neonatal	80 plus	Data Rich	-1	LDI (I$ per capita)
Congenital anomalies	Male	Early Neonatal	80 plus	Data Rich	-1	Legality of Abortion
Congenital anomalies	Male	Early Neonatal	80 plus	Data Rich	-1	Sociodemographic Status
Congenital anomalies	Male	Early Neonatal	80 plus	Data Rich	-1	Vegetables (kcal per capita)
Congenital anomalies	Male	Early Neonatal	80 plus	Data Rich	0	Health System Access (capped)
Congenital anomalies	Male	Early Neonatal	80 plus	Data Rich	1	Alcohol (litres per capita)
Congenital anomalies	Male	Early Neonatal	80 plus	Data Rich	1	Indoor Air Pollution (All Cooking Fuels)
Congenital anomalies	Male	Early Neonatal	80 plus	Data Rich	1	Live Births 35+ (proportion)
Congenital anomalies	Male	Early Neonatal	80 plus	Data Rich	1	Smoking Prevalence (Reproductive Age Standardised)
Congenital anomalies	Female	Early Neonatal	80 plus	Global	-1	Education (years per capita)
Congenital anomalies	Female	Early Neonatal	80 plus	Global	-1	Fruits (kcal per capita)
Congenital anomalies	Female	Early Neonatal	80 plus	Global	-1	LDI (I$ per capita)
Congenital anomalies	Female	Early Neonatal	80 plus	Global	-1	Legality of Abortion
Congenital anomalies	Female	Early Neonatal	80 plus	Global	-1	Sociodemographic Status
Congenital anomalies	Female	Early Neonatal	80 plus	Global	-1	Vegetables (kcal per capita)
Congenital anomalies	Female	Early Neonatal	80 plus	Global	0	Health System Access (capped)
Congenital anomalies	Female	Early Neonatal	80 plus	Global	1	Alcohol (litres per capita)
Congenital anomalies	Female	Early Neonatal	80 plus	Global	1	Indoor Air Pollution (All Cooking Fuels)
Congenital anomalies	Female	Early Neonatal	80 plus	Global	1	Live Births 35+ (proportion)
Congenital anomalies	Female	Early Neonatal	80 plus	Global	1	Smoking Prevalence (Reproductive Age Standardised)
Congenital anomalies	Female	Early Neonatal	80 plus	Data Rich	-1	Education (years per capita)
Congenital anomalies	Female	Early Neonatal	80 plus	Data Rich	-1	Fruits (kcal per capita)
Congenital anomalies	Female	Early Neonatal	80 plus	Data Rich	-1	LDI (I$ per capita)
Congenital anomalies	Female	Early Neonatal	80 plus	Data Rich	-1	Legality of Abortion
Congenital anomalies	Female	Early Neonatal	80 plus	Data Rich	-1	Sociodemographic Status
Congenital anomalies	Female	Early Neonatal	80 plus	Data Rich	-1	Vegetables (kcal per capita)

Appendix Table 10: CODEm covariates used, level of covariate, and expected direction of covariate by cause, sex, and age						
**Cause**	**Sex**	**Age start**	**Age end**	**Model type**	**Direction**	**Covariate**
Congenital anomalies	Female	Early Neonatal	80 plus	Data Rich	0	Health System Access (capped)
Congenital anomalies	Female	Early Neonatal	80 plus	Data Rich	1	Alcohol (litres per capita)
Congenital anomalies	Female	Early Neonatal	80 plus	Data Rich	1	Indoor Air Pollution (All Cooking Fuels)
Congenital anomalies	Female	Early Neonatal	80 plus	Data Rich	1	Live Births 35+ (proportion)
Congenital anomalies	Female	Early Neonatal	80 plus	Data Rich	1	Smoking Prevalence (Reproductive Age Standardised)
Neural tube defects	Male	Early Neonatal	80 plus	Global	-1	Education (years per capita)
Neural tube defects	Male	Early Neonatal	80 plus	Global	-1	Fruits (kcal per capita)
Neural tube defects	Male	Early Neonatal	80 plus	Global	-1	Health System Access 2 (unitless)
Neural tube defects	Male	Early Neonatal	80 plus	Global	-1	LDI (I$ per capita)
Neural tube defects	Male	Early Neonatal	80 plus	Global	-1	Sociodemographic Status
Neural tube defects	Male	Early Neonatal	80 plus	Global	-1	Vegetables (kcal per capita)
Neural tube defects	Male	Early Neonatal	80 plus	Global	1	Alcohol (litres per capita)
Neural tube defects	Male	Early Neonatal	80 plus	Global	1	Diabetes Age-Standardised Prevalence (proportion)
Neural tube defects	Male	Early Neonatal	80 plus	Global	1	Indoor Air Pollution (All Cooking Fuels)
Neural tube defects	Male	Early Neonatal	80 plus	Global	1	Smoking Prevalence (Reproductive Age Standardised)
Neural tube defects	Male	Early Neonatal	80 plus	Data Rich	-1	Education (years per capita)
Neural tube defects	Male	Early Neonatal	80 plus	Data Rich	-1	Fruits (kcal per capita)
Neural tube defects	Male	Early Neonatal	80 plus	Data Rich	-1	Health System Access 2 (unitless)
Neural tube defects	Male	Early Neonatal	80 plus	Data Rich	-1	LDI (I$ per capita)
Neural tube defects	Male	Early Neonatal	80 plus	Data Rich	-1	Sociodemographic Status
Neural tube defects	Male	Early Neonatal	80 plus	Data Rich	-1	Vegetables (kcal per capita)
Neural tube defects	Male	Early Neonatal	80 plus	Data Rich	1	Alcohol (litres per capita)
Neural tube defects	Male	Early Neonatal	80 plus	Data Rich	1	Diabetes Age-Standardised Prevalence (proportion)
Neural tube defects	Male	Early Neonatal	80 plus	Data Rich	1	Indoor Air Pollution (All Cooking Fuels)
Neural tube defects	Male	Early Neonatal	80 plus	Data Rich	1	Smoking Prevalence (Reproductive Age Standardised)
Neural tube defects	Female	Early Neonatal	80 plus	Global	-1	Education (years per capita)
Neural tube defects	Female	Early Neonatal	80 plus	Global	-1	Fruits (kcal per capita)
Neural tube defects	Female	Early Neonatal	80 plus	Global	-1	Health System Access 2 (unitless)
Neural tube defects	Female	Early Neonatal	80 plus	Global	-1	LDI (I$ per capita)
Neural tube defects	Female	Early Neonatal	80 plus	Global	-1	Sociodemographic Status
Neural tube defects	Female	Early Neonatal	80 plus	Global	-1	Vegetables (kcal per capita)
Neural tube defects	Female	Early Neonatal	80 plus	Global	1	Alcohol (litres per capita)
Neural tube defects	Female	Early Neonatal	80 plus	Global	1	Diabetes Age-Standardised Prevalence (proportion)
Neural tube defects	Female	Early Neonatal	80 plus	Global	1	Indoor Air Pollution (All Cooking Fuels)
Neural tube defects	Female	Early Neonatal	80 plus	Global	1	Smoking Prevalence (Reproductive Age Standardised)
Neural tube defects	Female	Early Neonatal	80 plus	Data Rich	-1	Education (years per capita)
Neural tube defects	Female	Early Neonatal	80 plus	Data Rich	-1	Fruits (kcal per capita)
Neural tube defects	Female	Early Neonatal	80 plus	Data Rich	-1	Health System Access 2 (unitless)
Neural tube defects	Female	Early Neonatal	80 plus	Data Rich	-1	LDI (I$ per capita)
Neural tube defects	Female	Early Neonatal	80 plus	Data Rich	-1	Sociodemographic Status
Neural tube defects	Female	Early Neonatal	80 plus	Data Rich	-1	Vegetables (kcal per capita)
Neural tube defects	Female	Early Neonatal	80 plus	Data Rich	1	Alcohol (litres per capita)
Neural tube defects	Female	Early Neonatal	80 plus	Data Rich	1	Diabetes Age-Standardised Prevalence (proportion)
Neural tube defects	Female	Early Neonatal	80 plus	Data Rich	1	Indoor Air Pollution (All Cooking Fuels)

Appendix Table 10: CODEm covariates used, level of covariate, and expected direction of covariate by cause, sex, and age						
**Cause**	**Sex**	**Age start**	**Age end**	**Model type**	**Direction**	**Covariate**
Neural tube defects	Female	Early Neonatal	80 plus	Data Rich	1	Smoking Prevalence (Reproductive Age Standardised)
Congenital heart anomalies	Male	Early Neonatal	80 plus	Global	-1	Education (years per capita)
Congenital heart anomalies	Male	Early Neonatal	80 plus	Global	-1	Legality of Abortion
Congenital heart anomalies	Male	Early Neonatal	80 plus	Global	-1	Measles Vaccine Coverage (proportion)
Congenital heart anomalies	Male	Early Neonatal	80 plus	Global	0	LDI (I$ per capita)
Congenital heart anomalies	Male	Early Neonatal	80 plus	Global	0	Sociodemographic Status
Congenital heart anomalies	Male	Early Neonatal	80 plus	Global	1	Alcohol (litres per capita)
Congenital heart anomalies	Male	Early Neonatal	80 plus	Global	1	Diabetes Age-Standardised Prevalence (proportion)
Congenital heart anomalies	Male	Early Neonatal	80 plus	Global	1	Indoor Air Pollution (All Cooking Fuels)
Congenital heart anomalies	Male	Early Neonatal	80 plus	Global	1	Live Births 35+ (proportion)
Congenital heart anomalies	Male	Early Neonatal	80 plus	Global	1	Smoking Prevalence (Reproductive Age Standardised)
Congenital heart anomalies	Male	Early Neonatal	80 plus	Data Rich	-1	Education (years per capita)
Congenital heart anomalies	Male	Early Neonatal	80 plus	Data Rich	-1	Legality of Abortion
Congenital heart anomalies	Male	Early Neonatal	80 plus	Data Rich	-1	Measles Vaccine Coverage (proportion)
Congenital heart anomalies	Male	Early Neonatal	80 plus	Data Rich	0	LDI (I$ per capita)
Congenital heart anomalies	Male	Early Neonatal	80 plus	Data Rich	0	Sociodemographic Status
Congenital heart anomalies	Male	Early Neonatal	80 plus	Data Rich	1	Alcohol (litres per capita)
Congenital heart anomalies	Male	Early Neonatal	80 plus	Data Rich	1	Diabetes Age-Standardised Prevalence (proportion)
Congenital heart anomalies	Male	Early Neonatal	80 plus	Data Rich	1	Indoor Air Pollution (All Cooking Fuels)
Congenital heart anomalies	Male	Early Neonatal	80 plus	Data Rich	1	Live Births 35+ (proportion)
Congenital heart anomalies	Male	Early Neonatal	80 plus	Data Rich	1	Smoking Prevalence (Reproductive Age Standardised)
Congenital heart anomalies	Female	Early Neonatal	80 plus	Global	-1	Education (years per capita)
Congenital heart anomalies	Female	Early Neonatal	80 plus	Global	-1	Legality of Abortion
Congenital heart anomalies	Female	Early Neonatal	80 plus	Global	-1	Measles Vaccine Coverage (proportion)
Congenital heart anomalies	Female	Early Neonatal	80 plus	Global	0	LDI (I$ per capita)
Congenital heart anomalies	Female	Early Neonatal	80 plus	Global	0	Sociodemographic Status
Congenital heart anomalies	Female	Early Neonatal	80 plus	Global	1	Alcohol (litres per capita)
Congenital heart anomalies	Female	Early Neonatal	80 plus	Global	1	Diabetes Age-Standardised Prevalence (proportion)
Congenital heart anomalies	Female	Early Neonatal	80 plus	Global	1	Indoor Air Pollution (All Cooking Fuels)
Congenital heart anomalies	Female	Early Neonatal	80 plus	Global	1	Live Births 35+ (proportion)
Congenital heart anomalies	Female	Early Neonatal	80 plus	Global	1	Smoking Prevalence (Reproductive Age Standardised)
Congenital heart anomalies	Female	Early Neonatal	80 plus	Data Rich	-1	Education (years per capita)
Congenital heart anomalies	Female	Early Neonatal	80 plus	Data Rich	-1	Legality of Abortion
Congenital heart anomalies	Female	Early Neonatal	80 plus	Data Rich	-1	Measles Vaccine Coverage (proportion)
Congenital heart anomalies	Female	Early Neonatal	80 plus	Data Rich	0	LDI (I$ per capita)
Congenital heart anomalies	Female	Early Neonatal	80 plus	Data Rich	0	Sociodemographic Status
Congenital heart anomalies	Female	Early Neonatal	80 plus	Data Rich	1	Alcohol (litres per capita)
Congenital heart anomalies	Female	Early Neonatal	80 plus	Data Rich	1	Diabetes Age-Standardised Prevalence (proportion)
Congenital heart anomalies	Female	Early Neonatal	80 plus	Data Rich	1	Indoor Air Pollution (All Cooking Fuels)
Congenital heart anomalies	Female	Early Neonatal	80 plus	Data Rich	1	Live Births 35+ (proportion)
Congenital heart anomalies	Female	Early Neonatal	80 plus	Data Rich	1	Smoking Prevalence (Reproductive Age Standardised)
Cleft lip and cleft palate	Male	Early Neonatal	1 to 4	Global	-1	Education (years per capita)
Cleft lip and cleft palate	Male	Early Neonatal	1 to 4	Global	-1	Fruits (kcal per capita)
Cleft lip and cleft palate	Male	Early Neonatal	1 to 4	Global	-1	Health System Access (capped)

Appendix Table 10: CODEm covariates used, level of covariate, and expected direction of covariate by cause, sex, and age						
**Cause**	**Sex**	**Age start**	**Age end**	**Model type**	**Direction**	**Covariate**
Cleft lip and cleft palate	Male	Early Neonatal	1 to 4	Global	-1	LDI (I$ per capita)
Cleft lip and cleft palate	Male	Early Neonatal	1 to 4	Global	-1	Legality of Abortion
Cleft lip and cleft palate	Male	Early Neonatal	1 to 4	Global	-1	Sociodemographic Status
Cleft lip and cleft palate	Male	Early Neonatal	1 to 4	Global	-1	Vegetables (kcal per capita)
Cleft lip and cleft palate	Male	Early Neonatal	1 to 4	Global	1	Alcohol (litres per capita)
Cleft lip and cleft palate	Male	Early Neonatal	1 to 4	Global	1	Diabetes Age-Standardised Prevalence (proportion)
Cleft lip and cleft palate	Male	Early Neonatal	1 to 4	Global	1	Indoor Air Pollution (All Cooking Fuels)
Cleft lip and cleft palate	Male	Early Neonatal	1 to 4	Global	1	Smoking Prevalence (Reproductive Age Standardised)
Cleft lip and cleft palate	Male	Early Neonatal	1 to 4	Data Rich	-1	Education (years per capita)
Cleft lip and cleft palate	Male	Early Neonatal	1 to 4	Data Rich	-1	Fruits (kcal per capita)
Cleft lip and cleft palate	Male	Early Neonatal	1 to 4	Data Rich	-1	Health System Access (capped)
Cleft lip and cleft palate	Male	Early Neonatal	1 to 4	Data Rich	-1	LDI (I$ per capita)
Cleft lip and cleft palate	Male	Early Neonatal	1 to 4	Data Rich	-1	Legality of Abortion
Cleft lip and cleft palate	Male	Early Neonatal	1 to 4	Data Rich	-1	Sociodemographic Status
Cleft lip and cleft palate	Male	Early Neonatal	1 to 4	Data Rich	-1	Vegetables (kcal per capita)
Cleft lip and cleft palate	Male	Early Neonatal	1 to 4	Data Rich	1	Alcohol (litres per capita)
Cleft lip and cleft palate	Male	Early Neonatal	1 to 4	Data Rich	1	Diabetes Age-Standardised Prevalence (proportion)
Cleft lip and cleft palate	Male	Early Neonatal	1 to 4	Data Rich	1	Indoor Air Pollution (All Cooking Fuels)
Cleft lip and cleft palate	Male	Early Neonatal	1 to 4	Data Rich	1	Smoking Prevalence (Reproductive Age Standardised)
Cleft lip and cleft palate	Female	Early Neonatal	1 to 4	Global	-1	Education (years per capita)
Cleft lip and cleft palate	Female	Early Neonatal	1 to 4	Global	-1	Fruits (kcal per capita)
Cleft lip and cleft palate	Female	Early Neonatal	1 to 4	Global	-1	Health System Access (capped)
Cleft lip and cleft palate	Female	Early Neonatal	1 to 4	Global	-1	LDI (I$ per capita)
Cleft lip and cleft palate	Female	Early Neonatal	1 to 4	Global	-1	Legality of Abortion
Cleft lip and cleft palate	Female	Early Neonatal	1 to 4	Global	-1	Sociodemographic Status
Cleft lip and cleft palate	Female	Early Neonatal	1 to 4	Global	-1	Vegetables (kcal per capita)
Cleft lip and cleft palate	Female	Early Neonatal	1 to 4	Global	1	Alcohol (litres per capita)
Cleft lip and cleft palate	Female	Early Neonatal	1 to 4	Global	1	Diabetes Age-Standardised Prevalence (proportion)
Cleft lip and cleft palate	Female	Early Neonatal	1 to 4	Global	1	Indoor Air Pollution (All Cooking Fuels)
Cleft lip and cleft palate	Female	Early Neonatal	1 to 4	Global	1	Smoking Prevalence (Reproductive Age Standardised)
Cleft lip and cleft palate	Female	Early Neonatal	1 to 4	Data Rich	-1	Education (years per capita)
Cleft lip and cleft palate	Female	Early Neonatal	1 to 4	Data Rich	-1	Fruits (kcal per capita)
Cleft lip and cleft palate	Female	Early Neonatal	1 to 4	Data Rich	-1	Health System Access (capped)
Cleft lip and cleft palate	Female	Early Neonatal	1 to 4	Data Rich	-1	LDI (I$ per capita)
Cleft lip and cleft palate	Female	Early Neonatal	1 to 4	Data Rich	-1	Legality of Abortion
Cleft lip and cleft palate	Female	Early Neonatal	1 to 4	Data Rich	-1	Sociodemographic Status
Cleft lip and cleft palate	Female	Early Neonatal	1 to 4	Data Rich	-1	Vegetables (kcal per capita)
Cleft lip and cleft palate	Female	Early Neonatal	1 to 4	Data Rich	1	Alcohol (litres per capita)
Cleft lip and cleft palate	Female	Early Neonatal	1 to 4	Data Rich	1	Diabetes Age-Standardised Prevalence (proportion)
Cleft lip and cleft palate	Female	Early Neonatal	1 to 4	Data Rich	1	Indoor Air Pollution (All Cooking Fuels)
Cleft lip and cleft palate	Female	Early Neonatal	1 to 4	Data Rich	1	Smoking Prevalence (Reproductive Age Standardised)
Down syndrome	Male	Early Neonatal	80 plus	Global	-1	Education (years per capita)
Down syndrome	Male	Early Neonatal	80 plus	Global	-1	Health System Access 2 (unitless)
Down syndrome	Male	Early Neonatal	80 plus	Global	-1	LDI (I$ per capita)

Appendix Table 10: CODEm covariates used, level of covariate, and expected direction of covariate by cause, sex, and age						
**Cause**	**Sex**	**Age start**	**Age end**	**Model type**	**Direction**	**Covariate**
Down syndrome	Male	Early Neonatal	80 plus	Global	-1	Legality of Abortion
Down syndrome	Male	Early Neonatal	80 plus	Global	-1	Measles Vaccine Coverage (proportion)
Down syndrome	Male	Early Neonatal	80 plus	Global	-1	Sociodemographic Status
Down syndrome	Male	Early Neonatal	80 plus	Global	1	Alcohol (litres per capita)
Down syndrome	Male	Early Neonatal	80 plus	Global	1	Indoor Air Pollution (All Cooking Fuels)
Down syndrome	Male	Early Neonatal	80 plus	Global	1	Live Births 35+ (proportion)
Down syndrome	Male	Early Neonatal	80 plus	Global	1	Live Births 40+ (proportion)
Down syndrome	Male	Early Neonatal	80 plus	Global	1	Smoking Prevalence (Reproductive Age Standardised)
Down syndrome	Male	Early Neonatal	80 plus	Data Rich	-1	Education (years per capita)
Down syndrome	Male	Early Neonatal	80 plus	Data Rich	-1	Health System Access 2 (unitless)
Down syndrome	Male	Early Neonatal	80 plus	Data Rich	-1	LDI (I$ per capita)
Down syndrome	Male	Early Neonatal	80 plus	Data Rich	-1	Legality of Abortion
Down syndrome	Male	Early Neonatal	80 plus	Data Rich	-1	Measles Vaccine Coverage (proportion)
Down syndrome	Male	Early Neonatal	80 plus	Data Rich	-1	Sociodemographic Status
Down syndrome	Male	Early Neonatal	80 plus	Data Rich	1	Alcohol (litres per capita)
Down syndrome	Male	Early Neonatal	80 plus	Data Rich	1	Indoor Air Pollution (All Cooking Fuels)
Down syndrome	Male	Early Neonatal	80 plus	Data Rich	1	Live Births 35+ (proportion)
Down syndrome	Male	Early Neonatal	80 plus	Data Rich	1	Live Births 40+ (proportion)
Down syndrome	Male	Early Neonatal	80 plus	Data Rich	1	Smoking Prevalence (Reproductive Age Standardised)
Down syndrome	Female	Early Neonatal	80 plus	Global	-1	Education (years per capita)
Down syndrome	Female	Early Neonatal	80 plus	Global	-1	Health System Access 2 (unitless)
Down syndrome	Female	Early Neonatal	80 plus	Global	-1	LDI (I$ per capita)
Down syndrome	Female	Early Neonatal	80 plus	Global	-1	Legality of Abortion
Down syndrome	Female	Early Neonatal	80 plus	Global	-1	Measles Vaccine Coverage (proportion)
Down syndrome	Female	Early Neonatal	80 plus	Global	-1	Sociodemographic Status
Down syndrome	Female	Early Neonatal	80 plus	Global	1	Alcohol (litres per capita)
Down syndrome	Female	Early Neonatal	80 plus	Global	1	Indoor Air Pollution (All Cooking Fuels)
Down syndrome	Female	Early Neonatal	80 plus	Global	1	Live Births 35+ (proportion)
Down syndrome	Female	Early Neonatal	80 plus	Global	1	Live Births 40+ (proportion)
Down syndrome	Female	Early Neonatal	80 plus	Global	1	Smoking Prevalence (Reproductive Age Standardised)
Down syndrome	Female	Early Neonatal	80 plus	Data Rich	-1	Education (years per capita)
Down syndrome	Female	Early Neonatal	80 plus	Data Rich	-1	Health System Access 2 (unitless)
Down syndrome	Female	Early Neonatal	80 plus	Data Rich	-1	LDI (I$ per capita)
Down syndrome	Female	Early Neonatal	80 plus	Data Rich	-1	Legality of Abortion
Down syndrome	Female	Early Neonatal	80 plus	Data Rich	-1	Measles Vaccine Coverage (proportion)
Down syndrome	Female	Early Neonatal	80 plus	Data Rich	-1	Sociodemographic Status
Down syndrome	Female	Early Neonatal	80 plus	Data Rich	1	Alcohol (litres per capita)
Down syndrome	Female	Early Neonatal	80 plus	Data Rich	1	Indoor Air Pollution (All Cooking Fuels)
Down syndrome	Female	Early Neonatal	80 plus	Data Rich	1	Live Births 35+ (proportion)
Down syndrome	Female	Early Neonatal	80 plus	Data Rich	1	Live Births 40+ (proportion)
Down syndrome	Female	Early Neonatal	80 plus	Data Rich	1	Smoking Prevalence (Reproductive Age Standardised)
Other chromosomal abnormalities	Male	Early Neonatal	80 plus	Global	-1	Education (years per capita)
Other chromosomal abnormalities	Male	Early Neonatal	80 plus	Global	-1	Health System Access 2 (unitless)
Other chromosomal abnormalities	Male	Early Neonatal	80 plus	Global	-1	LDI (I$ per capita)

Appendix Table 10: CODEm covariates used, level of covariate, and expected direction of covariate by cause, sex, and age						
**Cause**	**Sex**	**Age start**	**Age end**	**Model type**	**Direction**	**Covariate**
Other chromosomal abnormalities	Male	Early Neonatal	80 plus	Global	-1	Legality of Abortion
Other chromosomal abnormalities	Male	Early Neonatal	80 plus	Global	-1	Measles Vaccine Coverage (proportion)
Other chromosomal abnormalities	Male	Early Neonatal	80 plus	Global	0	Sociodemographic Status
Other chromosomal abnormalities	Male	Early Neonatal	80 plus	Global	1	Alcohol (litres per capita)
Other chromosomal abnormalities	Male	Early Neonatal	80 plus	Global	1	Indoor Air Pollution (All Cooking Fuels)
Other chromosomal abnormalities	Male	Early Neonatal	80 plus	Global	1	Live Births 35+ (proportion)
Other chromosomal abnormalities	Male	Early Neonatal	80 plus	Global	1	Live Births 40+ (proportion)
Other chromosomal abnormalities	Male	Early Neonatal	80 plus	Global	1	Smoking Prevalence (Reproductive Age Standardised)
Other chromosomal abnormalities	Male	Early Neonatal	80 plus	Data Rich	-1	Education (years per capita)
Other chromosomal abnormalities	Male	Early Neonatal	80 plus	Data Rich	-1	Health System Access 2 (unitless)
Other chromosomal abnormalities	Male	Early Neonatal	80 plus	Data Rich	-1	LDI (I$ per capita)
Other chromosomal abnormalities	Male	Early Neonatal	80 plus	Data Rich	-1	Legality of Abortion
Other chromosomal abnormalities	Male	Early Neonatal	80 plus	Data Rich	-1	Measles Vaccine Coverage (proportion)
Other chromosomal abnormalities	Male	Early Neonatal	80 plus	Data Rich	0	Sociodemographic Status
Other chromosomal abnormalities	Male	Early Neonatal	80 plus	Data Rich	1	Alcohol (litres per capita)
Other chromosomal abnormalities	Male	Early Neonatal	80 plus	Data Rich	1	Indoor Air Pollution (All Cooking Fuels)
Other chromosomal abnormalities	Male	Early Neonatal	80 plus	Data Rich	1	Live Births 35+ (proportion)
Other chromosomal abnormalities	Male	Early Neonatal	80 plus	Data Rich	1	Live Births 40+ (proportion)
Other chromosomal abnormalities	Male	Early Neonatal	80 plus	Data Rich	1	Smoking Prevalence (Reproductive Age Standardised)
Other chromosomal abnormalities	Female	Early Neonatal	80 plus	Global	-1	Education (years per capita)
Other chromosomal abnormalities	Female	Early Neonatal	80 plus	Global	-1	Health System Access 2 (unitless)
Other chromosomal abnormalities	Female	Early Neonatal	80 plus	Global	-1	LDI (I$ per capita)
Other chromosomal abnormalities	Female	Early Neonatal	80 plus	Global	-1	Legality of Abortion
Other chromosomal abnormalities	Female	Early Neonatal	80 plus	Global	-1	Measles Vaccine Coverage (proportion)
Other chromosomal abnormalities	Female	Early Neonatal	80 plus	Global	0	Sociodemographic Status
Other chromosomal abnormalities	Female	Early Neonatal	80 plus	Global	1	Alcohol (litres per capita)
Other chromosomal abnormalities	Female	Early Neonatal	80 plus	Global	1	Indoor Air Pollution (All Cooking Fuels)
Other chromosomal abnormalities	Female	Early Neonatal	80 plus	Global	1	Live Births 35+ (proportion)
Other chromosomal abnormalities	Female	Early Neonatal	80 plus	Global	1	Live Births 40+ (proportion)
Other chromosomal abnormalities	Female	Early Neonatal	80 plus	Global	1	Smoking Prevalence (Reproductive Age Standardised)
Other chromosomal abnormalities	Female	Early Neonatal	80 plus	Data Rich	-1	Education (years per capita)
Other chromosomal abnormalities	Female	Early Neonatal	80 plus	Data Rich	-1	Health System Access 2 (unitless)
Other chromosomal abnormalities	Female	Early Neonatal	80 plus	Data Rich	-1	LDI (I$ per capita)
Other chromosomal abnormalities	Female	Early Neonatal	80 plus	Data Rich	-1	Legality of Abortion
Other chromosomal abnormalities	Female	Early Neonatal	80 plus	Data Rich	-1	Measles Vaccine Coverage (proportion)
Other chromosomal abnormalities	Female	Early Neonatal	80 plus	Data Rich	0	Sociodemographic Status
Other chromosomal abnormalities	Female	Early Neonatal	80 plus	Data Rich	1	Alcohol (litres per capita)
Other chromosomal abnormalities	Female	Early Neonatal	80 plus	Data Rich	1	Indoor Air Pollution (All Cooking Fuels)
Other chromosomal abnormalities	Female	Early Neonatal	80 plus	Data Rich	1	Live Births 35+ (proportion)
Other chromosomal abnormalities	Female	Early Neonatal	80 plus	Data Rich	1	Live Births 40+ (proportion)
Other chromosomal abnormalities	Female	Early Neonatal	80 plus	Data Rich	1	Smoking Prevalence (Reproductive Age Standardised)
Other congenital anomalies	Male	Early Neonatal	80 plus	Global	-1	Education (years per capita)
Other congenital anomalies	Male	Early Neonatal	80 plus	Global	-1	Health System Access 2 (unitless)
Other congenital anomalies	Male	Early Neonatal	80 plus	Global	-1	Legality of Abortion

Appendix Table 10: CODEm covariates used, level of covariate, and expected direction of covariate by cause, sex, and age						
**Cause**	**Sex**	**Age start**	**Age end**	**Model type**	**Direction**	**Covariate**
Other congenital anomalies	Male	Early Neonatal	80 plus	Global	-1	Measles Vaccine Coverage (proportion)
Other congenital anomalies	Male	Early Neonatal	80 plus	Global	0	LDI (I$ per capita)
Other congenital anomalies	Male	Early Neonatal	80 plus	Global	0	Sociodemographic Status
Other congenital anomalies	Male	Early Neonatal	80 plus	Global	1	Alcohol (litres per capita)
Other congenital anomalies	Male	Early Neonatal	80 plus	Global	1	Diabetes Age-Standardised Prevalence (proportion)
Other congenital anomalies	Male	Early Neonatal	80 plus	Global	1	Indoor Air Pollution (All Cooking Fuels)
Other congenital anomalies	Male	Early Neonatal	80 plus	Global	1	Live Births 35+ (proportion)
Other congenital anomalies	Male	Early Neonatal	80 plus	Global	1	Smoking Prevalence (Reproductive Age Standardised)
Other congenital anomalies	Male	Early Neonatal	80 plus	Data Rich	-1	Education (years per capita)
Other congenital anomalies	Male	Early Neonatal	80 plus	Data Rich	-1	Health System Access 2 (unitless)
Other congenital anomalies	Male	Early Neonatal	80 plus	Data Rich	-1	Legality of Abortion
Other congenital anomalies	Male	Early Neonatal	80 plus	Data Rich	-1	Measles Vaccine Coverage (proportion)
Other congenital anomalies	Male	Early Neonatal	80 plus	Data Rich	0	LDI (I$ per capita)
Other congenital anomalies	Male	Early Neonatal	80 plus	Data Rich	0	Sociodemographic Status
Other congenital anomalies	Male	Early Neonatal	80 plus	Data Rich	1	Alcohol (litres per capita)
Other congenital anomalies	Male	Early Neonatal	80 plus	Data Rich	1	Diabetes Age-Standardised Prevalence (proportion)
Other congenital anomalies	Male	Early Neonatal	80 plus	Data Rich	1	Indoor Air Pollution (All Cooking Fuels)
Other congenital anomalies	Male	Early Neonatal	80 plus	Data Rich	1	Live Births 35+ (proportion)
Other congenital anomalies	Male	Early Neonatal	80 plus	Data Rich	1	Smoking Prevalence (Reproductive Age Standardised)
Other congenital anomalies	Female	Early Neonatal	80 plus	Global	-1	Education (years per capita)
Other congenital anomalies	Female	Early Neonatal	80 plus	Global	-1	Health System Access 2 (unitless)
Other congenital anomalies	Female	Early Neonatal	80 plus	Global	-1	Legality of Abortion
Other congenital anomalies	Female	Early Neonatal	80 plus	Global	-1	Measles Vaccine Coverage (proportion)
Other congenital anomalies	Female	Early Neonatal	80 plus	Global	0	LDI (I$ per capita)
Other congenital anomalies	Female	Early Neonatal	80 plus	Global	0	Sociodemographic Status
Other congenital anomalies	Female	Early Neonatal	80 plus	Global	1	Alcohol (litres per capita)
Other congenital anomalies	Female	Early Neonatal	80 plus	Global	1	Diabetes Age-Standardised Prevalence (proportion)
Other congenital anomalies	Female	Early Neonatal	80 plus	Global	1	Indoor Air Pollution (All Cooking Fuels)
Other congenital anomalies	Female	Early Neonatal	80 plus	Global	1	Live Births 35+ (proportion)
Other congenital anomalies	Female	Early Neonatal	80 plus	Global	1	Smoking Prevalence (Reproductive Age Standardised)
Other congenital anomalies	Female	Early Neonatal	80 plus	Data Rich	-1	Education (years per capita)
Other congenital anomalies	Female	Early Neonatal	80 plus	Data Rich	-1	Health System Access 2 (unitless)
Other congenital anomalies	Female	Early Neonatal	80 plus	Data Rich	-1	Legality of Abortion
Other congenital anomalies	Female	Early Neonatal	80 plus	Data Rich	-1	Measles Vaccine Coverage (proportion)
Other congenital anomalies	Female	Early Neonatal	80 plus	Data Rich	0	LDI (I$ per capita)
Other congenital anomalies	Female	Early Neonatal	80 plus	Data Rich	0	Sociodemographic Status
Other congenital anomalies	Female	Early Neonatal	80 plus	Data Rich	1	Alcohol (litres per capita)
Other congenital anomalies	Female	Early Neonatal	80 plus	Data Rich	1	Diabetes Age-Standardised Prevalence (proportion)
Other congenital anomalies	Female	Early Neonatal	80 plus	Data Rich	1	Indoor Air Pollution (All Cooking Fuels)
Other congenital anomalies	Female	Early Neonatal	80 plus	Data Rich	1	Live Births 35+ (proportion)
Other congenital anomalies	Female	Early Neonatal	80 plus	Data Rich	1	Smoking Prevalence (Reproductive Age Standardised)
Skin and subcutaneous diseases	Male	Post Neonatal	80 plus	Global	-1	Education (years per capita)
Skin and subcutaneous diseases	Male	Post Neonatal	80 plus	Global	-1	Health System Access 2 (unitless)
Skin and subcutaneous diseases	Male	Post Neonatal	80 plus	Global	-1	Improved Water Source (proportion with access)

Appendix Table 10: CODEm covariates used, level of covariate, and expected direction of covariate by cause, sex, and age						
**Cause**	**Sex**	**Age start**	**Age end**	**Model type**	**Direction**	**Covariate**
Skin and subcutaneous diseases	Male	Post Neonatal	80 plus	Global	-1	LDI (I$ per capita)
Skin and subcutaneous diseases	Male	Post Neonatal	80 plus	Global	0	Sociodemographic Status
Skin and subcutaneous diseases	Male	Post Neonatal	80 plus	Global	1	Alcohol (litres per capita)
Skin and subcutaneous diseases	Male	Post Neonatal	80 plus	Global	1	Cumulative Cigarettes (10 Years)
Skin and subcutaneous diseases	Male	Post Neonatal	80 plus	Global	1	Cumulative Cigarettes (5 Years)
Skin and subcutaneous diseases	Male	Post Neonatal	80 plus	Global	1	Malnutrition (proportion <2SD weight for age)
Skin and subcutaneous diseases	Male	Post Neonatal	80 plus	Global	1	SEV unsafe sanitation
Skin and subcutaneous diseases	Male	Post Neonatal	80 plus	Global	1	Smoking Prevalence
Skin and subcutaneous diseases	Male	Post Neonatal	80 plus	Data Rich	-1	Education (years per capita)
Skin and subcutaneous diseases	Male	Post Neonatal	80 plus	Data Rich	-1	Health System Access 2 (unitless)
Skin and subcutaneous diseases	Male	Post Neonatal	80 plus	Data Rich	-1	Improved Water Source (proportion with access)
Skin and subcutaneous diseases	Male	Post Neonatal	80 plus	Data Rich	-1	LDI (I$ per capita)
Skin and subcutaneous diseases	Male	Post Neonatal	80 plus	Data Rich	0	Sociodemographic Status
Skin and subcutaneous diseases	Male	Post Neonatal	80 plus	Data Rich	1	Alcohol (litres per capita)
Skin and subcutaneous diseases	Male	Post Neonatal	80 plus	Data Rich	1	Cumulative Cigarettes (10 Years)
Skin and subcutaneous diseases	Male	Post Neonatal	80 plus	Data Rich	1	Cumulative Cigarettes (5 Years)
Skin and subcutaneous diseases	Male	Post Neonatal	80 plus	Data Rich	1	Malnutrition (proportion <2SD weight for age)
Skin and subcutaneous diseases	Male	Post Neonatal	80 plus	Data Rich	1	SEV unsafe sanitation
Skin and subcutaneous diseases	Male	Post Neonatal	80 plus	Data Rich	1	Smoking Prevalence
Skin and subcutaneous diseases	Female	Post Neonatal	80 plus	Global	-1	Education (years per capita)
Skin and subcutaneous diseases	Female	Post Neonatal	80 plus	Global	-1	Health System Access 2 (unitless)
Skin and subcutaneous diseases	Female	Post Neonatal	80 plus	Global	-1	Improved Water Source (proportion with access)
Skin and subcutaneous diseases	Female	Post Neonatal	80 plus	Global	-1	LDI (I$ per capita)
Skin and subcutaneous diseases	Female	Post Neonatal	80 plus	Global	0	Sociodemographic Status
Skin and subcutaneous diseases	Female	Post Neonatal	80 plus	Global	1	Alcohol (litres per capita)
Skin and subcutaneous diseases	Female	Post Neonatal	80 plus	Global	1	Cumulative Cigarettes (10 Years)
Skin and subcutaneous diseases	Female	Post Neonatal	80 plus	Global	1	Cumulative Cigarettes (5 Years)
Skin and subcutaneous diseases	Female	Post Neonatal	80 plus	Global	1	Malnutrition (proportion <2SD weight for age)
Skin and subcutaneous diseases	Female	Post Neonatal	80 plus	Global	1	SEV unsafe sanitation
Skin and subcutaneous diseases	Female	Post Neonatal	80 plus	Global	1	Smoking Prevalence
Skin and subcutaneous diseases	Female	Post Neonatal	80 plus	Data Rich	-1	Education (years per capita)
Skin and subcutaneous diseases	Female	Post Neonatal	80 plus	Data Rich	-1	Health System Access 2 (unitless)
Skin and subcutaneous diseases	Female	Post Neonatal	80 plus	Data Rich	-1	Improved Water Source (proportion with access)
Skin and subcutaneous diseases	Female	Post Neonatal	80 plus	Data Rich	-1	LDI (I$ per capita)
Skin and subcutaneous diseases	Female	Post Neonatal	80 plus	Data Rich	0	Sociodemographic Status
Skin and subcutaneous diseases	Female	Post Neonatal	80 plus	Data Rich	1	Alcohol (litres per capita)
Skin and subcutaneous diseases	Female	Post Neonatal	80 plus	Data Rich	1	Cumulative Cigarettes (10 Years)
Skin and subcutaneous diseases	Female	Post Neonatal	80 plus	Data Rich	1	Cumulative Cigarettes (5 Years)
Skin and subcutaneous diseases	Female	Post Neonatal	80 plus	Data Rich	1	Malnutrition (proportion <2SD weight for age)
Skin and subcutaneous diseases	Female	Post Neonatal	80 plus	Data Rich	1	SEV unsafe sanitation
Skin and subcutaneous diseases	Female	Post Neonatal	80 plus	Data Rich	1	Smoking Prevalence
Cellulitis	Male	Post Neonatal	80 plus	Global	-1	Education (years per capita)
Cellulitis	Male	Post Neonatal	80 plus	Global	-1	Health System Access 2 (unitless)
Cellulitis	Male	Post Neonatal	80 plus	Global	-1	Improved Water Source (proportion with access)

Appendix Table 10: CODEm covariates used, level of covariate, and expected direction of covariate by cause, sex, and age						
**Cause**	**Sex**	**Age start**	**Age end**	**Model type**	**Direction**	**Covariate**
Cellulitis	Male	Post Neonatal	80 plus	Global	-1	LDI (I$ per capita)
Cellulitis	Male	Post Neonatal	80 plus	Global	-1	Sociodemographic Status
Cellulitis	Male	Post Neonatal	80 plus	Global	1	Alcohol (litres per capita)
Cellulitis	Male	Post Neonatal	80 plus	Global	1	Cumulative Cigarettes (10 Years)
Cellulitis	Male	Post Neonatal	80 plus	Global	1	Cumulative Cigarettes (5 Years)
Cellulitis	Male	Post Neonatal	80 plus	Global	1	Malnutrition (proportion <2SD weight for age)
Cellulitis	Male	Post Neonatal	80 plus	Global	1	SEV unsafe sanitation
Cellulitis	Male	Post Neonatal	80 plus	Global	1	Smoking Prevalence
Cellulitis	Male	Post Neonatal	80 plus	Data Rich	-1	Education (years per capita)
Cellulitis	Male	Post Neonatal	80 plus	Data Rich	-1	Health System Access 2 (unitless)
Cellulitis	Male	Post Neonatal	80 plus	Data Rich	-1	Improved Water Source (proportion with access)
Cellulitis	Male	Post Neonatal	80 plus	Data Rich	-1	LDI (I$ per capita)
Cellulitis	Male	Post Neonatal	80 plus	Data Rich	-1	Sociodemographic Status
Cellulitis	Male	Post Neonatal	80 plus	Data Rich	1	Alcohol (litres per capita)
Cellulitis	Male	Post Neonatal	80 plus	Data Rich	1	Cumulative Cigarettes (10 Years)
Cellulitis	Male	Post Neonatal	80 plus	Data Rich	1	Cumulative Cigarettes (5 Years)
Cellulitis	Male	Post Neonatal	80 plus	Data Rich	1	Malnutrition (proportion <2SD weight for age)
Cellulitis	Male	Post Neonatal	80 plus	Data Rich	1	SEV unsafe sanitation
Cellulitis	Male	Post Neonatal	80 plus	Data Rich	1	Smoking Prevalence
Cellulitis	Female	Post Neonatal	80 plus	Global	-1	Education (years per capita)
Cellulitis	Female	Post Neonatal	80 plus	Global	-1	Health System Access 2 (unitless)
Cellulitis	Female	Post Neonatal	80 plus	Global	-1	Improved Water Source (proportion with access)
Cellulitis	Female	Post Neonatal	80 plus	Global	-1	LDI (I$ per capita)
Cellulitis	Female	Post Neonatal	80 plus	Global	-1	Sociodemographic Status
Cellulitis	Female	Post Neonatal	80 plus	Global	1	Alcohol (litres per capita)
Cellulitis	Female	Post Neonatal	80 plus	Global	1	Cumulative Cigarettes (10 Years)
Cellulitis	Female	Post Neonatal	80 plus	Global	1	Cumulative Cigarettes (5 Years)
Cellulitis	Female	Post Neonatal	80 plus	Global	1	Malnutrition (proportion <2SD weight for age)
Cellulitis	Female	Post Neonatal	80 plus	Global	1	SEV unsafe sanitation
Cellulitis	Female	Post Neonatal	80 plus	Global	1	Smoking Prevalence
Cellulitis	Female	Post Neonatal	80 plus	Data Rich	-1	Education (years per capita)
Cellulitis	Female	Post Neonatal	80 plus	Data Rich	-1	Health System Access 2 (unitless)
Cellulitis	Female	Post Neonatal	80 plus	Data Rich	-1	Improved Water Source (proportion with access)
Cellulitis	Female	Post Neonatal	80 plus	Data Rich	-1	LDI (I$ per capita)
Cellulitis	Female	Post Neonatal	80 plus	Data Rich	-1	Sociodemographic Status
Cellulitis	Female	Post Neonatal	80 plus	Data Rich	1	Alcohol (litres per capita)
Cellulitis	Female	Post Neonatal	80 plus	Data Rich	1	Cumulative Cigarettes (10 Years)
Cellulitis	Female	Post Neonatal	80 plus	Data Rich	1	Cumulative Cigarettes (5 Years)
Cellulitis	Female	Post Neonatal	80 plus	Data Rich	1	Malnutrition (proportion <2SD weight for age)
Cellulitis	Female	Post Neonatal	80 plus	Data Rich	1	SEV unsafe sanitation
Cellulitis	Female	Post Neonatal	80 plus	Data Rich	1	Smoking Prevalence
Pyoderma	Male	Early Neonatal	80 plus	Global	-1	Education (years per capita)
Pyoderma	Male	Early Neonatal	80 plus	Global	-1	Improved Water Source (proportion with access)
Pyoderma	Male	Early Neonatal	80 plus	Global	-1	LDI (I$ per capita)

Appendix Table 10: CODEm covariates used, level of covariate, and expected direction of covariate by cause, sex, and age						
**Cause**	**Sex**	**Age start**	**Age end**	**Model type**	**Direction**	**Covariate**
Pyoderma	Male	Early Neonatal	80 plus	Global	0	Sociodemographic Status
Pyoderma	Male	Early Neonatal	80 plus	Global	1	Alcohol (litres per capita)
Pyoderma	Male	Early Neonatal	80 plus	Global	1	Cumulative Cigarettes (10 Years)
Pyoderma	Male	Early Neonatal	80 plus	Global	1	Cumulative Cigarettes (5 Years)
Pyoderma	Male	Early Neonatal	80 plus	Global	1	Health System Access 2 (unitless)
Pyoderma	Male	Early Neonatal	80 plus	Global	1	Malnutrition (proportion <2SD weight for age)
Pyoderma	Male	Early Neonatal	80 plus	Global	1	SEV unsafe sanitation
Pyoderma	Male	Early Neonatal	80 plus	Global	1	Smoking Prevalence
Pyoderma	Male	Early Neonatal	80 plus	Data Rich	-1	Education (years per capita)
Pyoderma	Male	Early Neonatal	80 plus	Data Rich	-1	Improved Water Source (proportion with access)
Pyoderma	Male	Early Neonatal	80 plus	Data Rich	-1	LDI (I$ per capita)
Pyoderma	Male	Early Neonatal	80 plus	Data Rich	0	Sociodemographic Status
Pyoderma	Male	Early Neonatal	80 plus	Data Rich	1	Alcohol (litres per capita)
Pyoderma	Male	Early Neonatal	80 plus	Data Rich	1	Cumulative Cigarettes (10 Years)
Pyoderma	Male	Early Neonatal	80 plus	Data Rich	1	Cumulative Cigarettes (5 Years)
Pyoderma	Male	Early Neonatal	80 plus	Data Rich	1	Health System Access 2 (unitless)
Pyoderma	Male	Early Neonatal	80 plus	Data Rich	1	Malnutrition (proportion <2SD weight for age)
Pyoderma	Male	Early Neonatal	80 plus	Data Rich	1	SEV unsafe sanitation
Pyoderma	Male	Early Neonatal	80 plus	Data Rich	1	Smoking Prevalence
Pyoderma	Female	Early Neonatal	80 plus	Global	-1	Education (years per capita)
Pyoderma	Female	Early Neonatal	80 plus	Global	-1	Improved Water Source (proportion with access)
Pyoderma	Female	Early Neonatal	80 plus	Global	-1	LDI (I$ per capita)
Pyoderma	Female	Early Neonatal	80 plus	Global	0	Sociodemographic Status
Pyoderma	Female	Early Neonatal	80 plus	Global	1	Alcohol (litres per capita)
Pyoderma	Female	Early Neonatal	80 plus	Global	1	Cumulative Cigarettes (10 Years)
Pyoderma	Female	Early Neonatal	80 plus	Global	1	Cumulative Cigarettes (5 Years)
Pyoderma	Female	Early Neonatal	80 plus	Global	1	Health System Access 2 (unitless)
Pyoderma	Female	Early Neonatal	80 plus	Global	1	Malnutrition (proportion <2SD weight for age)
Pyoderma	Female	Early Neonatal	80 plus	Global	1	SEV unsafe sanitation
Pyoderma	Female	Early Neonatal	80 plus	Global	1	Smoking Prevalence
Pyoderma	Female	Early Neonatal	80 plus	Data Rich	-1	Education (years per capita)
Pyoderma	Female	Early Neonatal	80 plus	Data Rich	-1	Improved Water Source (proportion with access)
Pyoderma	Female	Early Neonatal	80 plus	Data Rich	-1	LDI (I$ per capita)
Pyoderma	Female	Early Neonatal	80 plus	Data Rich	0	Sociodemographic Status
Pyoderma	Female	Early Neonatal	80 plus	Data Rich	1	Alcohol (litres per capita)
Pyoderma	Female	Early Neonatal	80 plus	Data Rich	1	Cumulative Cigarettes (10 Years)
Pyoderma	Female	Early Neonatal	80 plus	Data Rich	1	Cumulative Cigarettes (5 Years)
Pyoderma	Female	Early Neonatal	80 plus	Data Rich	1	Health System Access 2 (unitless)
Pyoderma	Female	Early Neonatal	80 plus	Data Rich	1	Malnutrition (proportion <2SD weight for age)
Pyoderma	Female	Early Neonatal	80 plus	Data Rich	1	SEV unsafe sanitation
Pyoderma	Female	Early Neonatal	80 plus	Data Rich	1	Smoking Prevalence
Decubitus ulcer	Male	1 to 4	80 plus	Global	-1	Education (years per capita)
Decubitus ulcer	Male	1 to 4	80 plus	Global	-1	Health System Access 2 (unitless)
Decubitus ulcer	Male	1 to 4	80 plus	Global	-1	Improved Water Source (proportion with access)

Appendix Table 10: CODEm covariates used, level of covariate, and expected direction of covariate by cause, sex, and age						
**Cause**	**Sex**	**Age start**	**Age end**	**Model type**	**Direction**	**Covariate**
Decubitus ulcer	Male	1 to 4	80 plus	Global	-1	LDI (I$ per capita)
Decubitus ulcer	Male	1 to 4	80 plus	Global	0	Sociodemographic Status
Decubitus ulcer	Male	1 to 4	80 plus	Global	1	Alcohol (litres per capita)
Decubitus ulcer	Male	1 to 4	80 plus	Global	1	Cumulative Cigarettes (10 Years)
Decubitus ulcer	Male	1 to 4	80 plus	Global	1	Cumulative Cigarettes (5 Years)
Decubitus ulcer	Male	1 to 4	80 plus	Global	1	Malnutrition (proportion <2SD weight for age)
Decubitus ulcer	Male	1 to 4	80 plus	Global	1	SEV unsafe sanitation
Decubitus ulcer	Male	1 to 4	80 plus	Global	1	Smoking Prevalence
Decubitus ulcer	Male	1 to 4	80 plus	Data Rich	-1	Education (years per capita)
Decubitus ulcer	Male	1 to 4	80 plus	Data Rich	-1	Health System Access 2 (unitless)
Decubitus ulcer	Male	1 to 4	80 plus	Data Rich	-1	Improved Water Source (proportion with access)
Decubitus ulcer	Male	1 to 4	80 plus	Data Rich	-1	LDI (I$ per capita)
Decubitus ulcer	Male	1 to 4	80 plus	Data Rich	0	Sociodemographic Status
Decubitus ulcer	Male	1 to 4	80 plus	Data Rich	1	Alcohol (litres per capita)
Decubitus ulcer	Male	1 to 4	80 plus	Data Rich	1	Cumulative Cigarettes (10 Years)
Decubitus ulcer	Male	1 to 4	80 plus	Data Rich	1	Cumulative Cigarettes (5 Years)
Decubitus ulcer	Male	1 to 4	80 plus	Data Rich	1	Malnutrition (proportion <2SD weight for age)
Decubitus ulcer	Male	1 to 4	80 plus	Data Rich	1	SEV unsafe sanitation
Decubitus ulcer	Male	1 to 4	80 plus	Data Rich	1	Smoking Prevalence
Decubitus ulcer	Female	1 to 4	80 plus	Global	-1	Education (years per capita)
Decubitus ulcer	Female	1 to 4	80 plus	Global	-1	Health System Access 2 (unitless)
Decubitus ulcer	Female	1 to 4	80 plus	Global	-1	Improved Water Source (proportion with access)
Decubitus ulcer	Female	1 to 4	80 plus	Global	-1	LDI (I$ per capita)
Decubitus ulcer	Female	1 to 4	80 plus	Global	0	Sociodemographic Status
Decubitus ulcer	Female	1 to 4	80 plus	Global	1	Alcohol (litres per capita)
Decubitus ulcer	Female	1 to 4	80 plus	Global	1	Cumulative Cigarettes (10 Years)
Decubitus ulcer	Female	1 to 4	80 plus	Global	1	Cumulative Cigarettes (5 Years)
Decubitus ulcer	Female	1 to 4	80 plus	Global	1	Malnutrition (proportion <2SD weight for age)
Decubitus ulcer	Female	1 to 4	80 plus	Global	1	SEV unsafe sanitation
Decubitus ulcer	Female	1 to 4	80 plus	Global	1	Smoking Prevalence
Decubitus ulcer	Female	1 to 4	80 plus	Data Rich	-1	Education (years per capita)
Decubitus ulcer	Female	1 to 4	80 plus	Data Rich	-1	Health System Access 2 (unitless)
Decubitus ulcer	Female	1 to 4	80 plus	Data Rich	-1	Improved Water Source (proportion with access)
Decubitus ulcer	Female	1 to 4	80 plus	Data Rich	-1	LDI (I$ per capita)
Decubitus ulcer	Female	1 to 4	80 plus	Data Rich	0	Sociodemographic Status
Decubitus ulcer	Female	1 to 4	80 plus	Data Rich	1	Alcohol (litres per capita)
Decubitus ulcer	Female	1 to 4	80 plus	Data Rich	1	Cumulative Cigarettes (10 Years)
Decubitus ulcer	Female	1 to 4	80 plus	Data Rich	1	Cumulative Cigarettes (5 Years)
Decubitus ulcer	Female	1 to 4	80 plus	Data Rich	1	Malnutrition (proportion <2SD weight for age)
Decubitus ulcer	Female	1 to 4	80 plus	Data Rich	1	SEV unsafe sanitation
Decubitus ulcer	Female	1 to 4	80 plus	Data Rich	1	Smoking Prevalence
Other skin and subcutaneous diseases	Male	Post Neonatal	80 plus	Global	-1	Education (years per capita)
Other skin and subcutaneous diseases	Male	Post Neonatal	80 plus	Global	-1	Health System Access 2 (unitless)
Other skin and subcutaneous diseases	Male	Post Neonatal	80 plus	Global	-1	Improved Water Source (proportion with access)

Appendix Table 10: CODEm covariates used, level of covariate, and expected direction of covariate by cause, sex, and age						
**Cause**	**Sex**	**Age start**	**Age end**	**Model type**	**Direction**	**Covariate**
Other skin and subcutaneous diseases	Male	Post Neonatal	80 plus	Global	-1	LDI (I$ per capita)
Other skin and subcutaneous diseases	Male	Post Neonatal	80 plus	Global	0	Sociodemographic Status
Other skin and subcutaneous diseases	Male	Post Neonatal	80 plus	Global	1	Alcohol (litres per capita)
Other skin and subcutaneous diseases	Male	Post Neonatal	80 plus	Global	1	Cumulative Cigarettes (10 Years)
Other skin and subcutaneous diseases	Male	Post Neonatal	80 plus	Global	1	Cumulative Cigarettes (5 Years)
Other skin and subcutaneous diseases	Male	Post Neonatal	80 plus	Global	1	Malnutrition (proportion <2SD weight for age)
Other skin and subcutaneous diseases	Male	Post Neonatal	80 plus	Global	1	SEV unsafe sanitation
Other skin and subcutaneous diseases	Male	Post Neonatal	80 plus	Global	1	Smoking Prevalence
Other skin and subcutaneous diseases	Male	Post Neonatal	80 plus	Data Rich	-1	Education (years per capita)
Other skin and subcutaneous diseases	Male	Post Neonatal	80 plus	Data Rich	-1	Health System Access 2 (unitless)
Other skin and subcutaneous diseases	Male	Post Neonatal	80 plus	Data Rich	-1	Improved Water Source (proportion with access)
Other skin and subcutaneous diseases	Male	Post Neonatal	80 plus	Data Rich	-1	LDI (I$ per capita)
Other skin and subcutaneous diseases	Male	Post Neonatal	80 plus	Data Rich	0	Sociodemographic Status
Other skin and subcutaneous diseases	Male	Post Neonatal	80 plus	Data Rich	1	Alcohol (litres per capita)
Other skin and subcutaneous diseases	Male	Post Neonatal	80 plus	Data Rich	1	Cumulative Cigarettes (10 Years)
Other skin and subcutaneous diseases	Male	Post Neonatal	80 plus	Data Rich	1	Cumulative Cigarettes (5 Years)
Other skin and subcutaneous diseases	Male	Post Neonatal	80 plus	Data Rich	1	Malnutrition (proportion <2SD weight for age)
Other skin and subcutaneous diseases	Male	Post Neonatal	80 plus	Data Rich	1	SEV unsafe sanitation
Other skin and subcutaneous diseases	Male	Post Neonatal	80 plus	Data Rich	1	Smoking Prevalence
Other skin and subcutaneous diseases	Female	Post Neonatal	80 plus	Global	-1	Education (years per capita)
Other skin and subcutaneous diseases	Female	Post Neonatal	80 plus	Global	-1	Health System Access 2 (unitless)
Other skin and subcutaneous diseases	Female	Post Neonatal	80 plus	Global	-1	Improved Water Source (proportion with access)
Other skin and subcutaneous diseases	Female	Post Neonatal	80 plus	Global	-1	LDI (I$ per capita)
Other skin and subcutaneous diseases	Female	Post Neonatal	80 plus	Global	0	Sociodemographic Status
Other skin and subcutaneous diseases	Female	Post Neonatal	80 plus	Global	1	Alcohol (litres per capita)
Other skin and subcutaneous diseases	Female	Post Neonatal	80 plus	Global	1	Cumulative Cigarettes (10 Years)
Other skin and subcutaneous diseases	Female	Post Neonatal	80 plus	Global	1	Cumulative Cigarettes (5 Years)
Other skin and subcutaneous diseases	Female	Post Neonatal	80 plus	Global	1	Malnutrition (proportion <2SD weight for age)
Other skin and subcutaneous diseases	Female	Post Neonatal	80 plus	Global	1	SEV unsafe sanitation
Other skin and subcutaneous diseases	Female	Post Neonatal	80 plus	Global	1	Smoking Prevalence
Other skin and subcutaneous diseases	Female	Post Neonatal	80 plus	Data Rich	-1	Education (years per capita)
Other skin and subcutaneous diseases	Female	Post Neonatal	80 plus	Data Rich	-1	Health System Access 2 (unitless)
Other skin and subcutaneous diseases	Female	Post Neonatal	80 plus	Data Rich	-1	Improved Water Source (proportion with access)
Other skin and subcutaneous diseases	Female	Post Neonatal	80 plus	Data Rich	-1	LDI (I$ per capita)
Other skin and subcutaneous diseases	Female	Post Neonatal	80 plus	Data Rich	0	Sociodemographic Status
Other skin and subcutaneous diseases	Female	Post Neonatal	80 plus	Data Rich	1	Alcohol (litres per capita)
Other skin and subcutaneous diseases	Female	Post Neonatal	80 plus	Data Rich	1	Cumulative Cigarettes (10 Years)
Other skin and subcutaneous diseases	Female	Post Neonatal	80 plus	Data Rich	1	Cumulative Cigarettes (5 Years)
Other skin and subcutaneous diseases	Female	Post Neonatal	80 plus	Data Rich	1	Malnutrition (proportion <2SD weight for age)
Other skin and subcutaneous diseases	Female	Post Neonatal	80 plus	Data Rich	1	SEV unsafe sanitation
Other skin and subcutaneous diseases	Female	Post Neonatal	80 plus	Data Rich	1	Smoking Prevalence
Sudden infant death syndrome	Male	Late Neonatal	Post Neonatal	Global	-1	Education (years per capita)
Sudden infant death syndrome	Male	Late Neonatal	Post Neonatal	Global	-1	Health System Access 2 (unitless)
Sudden infant death syndrome	Male	Late Neonatal	Post Neonatal	Global	-1	In-Facility Delivery (proportion)

Appendix Table 10: CODEm covariates used, level of covariate, and expected direction of covariate by cause, sex, and age						
**Cause**	**Sex**	**Age start**	**Age end**	**Model type**	**Direction**	**Covariate**
Sudden infant death syndrome	Male	Late Neonatal	Post Neonatal	Global	-1	Skilled Birth Attendance (proportion)
Sudden infant death syndrome	Male	Late Neonatal	Post Neonatal	Global	0	LDI (I$ per capita)
Sudden infant death syndrome	Male	Late Neonatal	Post Neonatal	Global	0	Sociodemographic Status
Sudden infant death syndrome	Male	Late Neonatal	Post Neonatal	Global	1	Indoor Air Pollution (All Cooking Fuels)
Sudden infant death syndrome	Male	Late Neonatal	Post Neonatal	Global	1	Malnutrition (proportion <2SD weight for age)
Sudden infant death syndrome	Male	Late Neonatal	Post Neonatal	Global	1	Smoking Prevalence (Reproductive Age Standardised)
Sudden infant death syndrome	Male	Late Neonatal	Post Neonatal	Global	1	Total Fertility Rate
Sudden infant death syndrome	Male	Late Neonatal	Post Neonatal	Data Rich	-1	Education (years per capita)
Sudden infant death syndrome	Male	Late Neonatal	Post Neonatal	Data Rich	-1	Health System Access 2 (unitless)
Sudden infant death syndrome	Male	Late Neonatal	Post Neonatal	Data Rich	-1	In-Facility Delivery (proportion)
Sudden infant death syndrome	Male	Late Neonatal	Post Neonatal	Data Rich	-1	Skilled Birth Attendance (proportion)
Sudden infant death syndrome	Male	Late Neonatal	Post Neonatal	Data Rich	0	LDI (I$ per capita)
Sudden infant death syndrome	Male	Late Neonatal	Post Neonatal	Data Rich	0	Sociodemographic Status
Sudden infant death syndrome	Male	Late Neonatal	Post Neonatal	Data Rich	1	Indoor Air Pollution (All Cooking Fuels)
Sudden infant death syndrome	Male	Late Neonatal	Post Neonatal	Data Rich	1	Malnutrition (proportion <2SD weight for age)
Sudden infant death syndrome	Male	Late Neonatal	Post Neonatal	Data Rich	1	Smoking Prevalence (Reproductive Age Standardised)
Sudden infant death syndrome	Male	Late Neonatal	Post Neonatal	Data Rich	1	Total Fertility Rate
Sudden infant death syndrome	Female	Late Neonatal	Post Neonatal	Global	-1	Education (years per capita)
Sudden infant death syndrome	Female	Late Neonatal	Post Neonatal	Global	-1	Health System Access 2 (unitless)
Sudden infant death syndrome	Female	Late Neonatal	Post Neonatal	Global	-1	In-Facility Delivery (proportion)
Sudden infant death syndrome	Female	Late Neonatal	Post Neonatal	Global	-1	Skilled Birth Attendance (proportion)
Sudden infant death syndrome	Female	Late Neonatal	Post Neonatal	Global	0	LDI (I$ per capita)
Sudden infant death syndrome	Female	Late Neonatal	Post Neonatal	Global	0	Sociodemographic Status
Sudden infant death syndrome	Female	Late Neonatal	Post Neonatal	Global	1	Indoor Air Pollution (All Cooking Fuels)
Sudden infant death syndrome	Female	Late Neonatal	Post Neonatal	Global	1	Malnutrition (proportion <2SD weight for age)
Sudden infant death syndrome	Female	Late Neonatal	Post Neonatal	Global	1	Smoking Prevalence (Reproductive Age Standardised)
Sudden infant death syndrome	Female	Late Neonatal	Post Neonatal	Global	1	Total Fertility Rate
Sudden infant death syndrome	Female	Late Neonatal	Post Neonatal	Data Rich	-1	Education (years per capita)
Sudden infant death syndrome	Female	Late Neonatal	Post Neonatal	Data Rich	-1	Health System Access 2 (unitless)
Sudden infant death syndrome	Female	Late Neonatal	Post Neonatal	Data Rich	-1	In-Facility Delivery (proportion)
Sudden infant death syndrome	Female	Late Neonatal	Post Neonatal	Data Rich	-1	Skilled Birth Attendance (proportion)
Sudden infant death syndrome	Female	Late Neonatal	Post Neonatal	Data Rich	0	LDI (I$ per capita)
Sudden infant death syndrome	Female	Late Neonatal	Post Neonatal	Data Rich	0	Sociodemographic Status
Sudden infant death syndrome	Female	Late Neonatal	Post Neonatal	Data Rich	1	Indoor Air Pollution (All Cooking Fuels)
Sudden infant death syndrome	Female	Late Neonatal	Post Neonatal	Data Rich	1	Malnutrition (proportion <2SD weight for age)
Sudden infant death syndrome	Female	Late Neonatal	Post Neonatal	Data Rich	1	Smoking Prevalence (Reproductive Age Standardised)
Sudden infant death syndrome	Female	Late Neonatal	Post Neonatal	Data Rich	1	Total Fertility Rate
Transport injuries	Male	Early Neonatal	80 plus	Global	-1	Education (years per capita)
Transport injuries	Male	Early Neonatal	80 plus	Global	-1	Health System Access 2 (unitless)
Transport injuries	Male	Early Neonatal	80 plus	Global	-1	Sociodemographic Status
Transport injuries	Male	Early Neonatal	80 plus	Global	0	LDI (I$ per capita)
Transport injuries	Male	Early Neonatal	80 plus	Global	0	Population Density (300-500 ppl/sqkm, proportion)
Transport injuries	Male	Early Neonatal	80 plus	Global	0	Population Density (500-1000 ppl/sqkm, proportion)
Transport injuries	Male	Early Neonatal	80 plus	Global	1	Alcohol (litres per capita)

Appendix Table 10: CODEm covariates used, level of covariate, and expected direction of covariate by cause, sex, and age						
**Cause**	**Sex**	**Age start**	**Age end**	**Model type**	**Direction**	**Covariate**
Transport injuries	Male	Early Neonatal	80 plus	Global	1	Rainfall Quintile 5 (proportion)
Transport injuries	Male	Early Neonatal	80 plus	Global	1	Vehicles - 2 wheels fraction (proportion)
Transport injuries	Male	Early Neonatal	80 plus	Global	1	Vehicles - 2+4 wheels (per capita)
Transport injuries	Male	Early Neonatal	80 plus	Data Rich	-1	Education (years per capita)
Transport injuries	Male	Early Neonatal	80 plus	Data Rich	-1	Health System Access 2 (unitless)
Transport injuries	Male	Early Neonatal	80 plus	Data Rich	-1	Sociodemographic Status
Transport injuries	Male	Early Neonatal	80 plus	Data Rich	0	LDI (I$ per capita)
Transport injuries	Male	Early Neonatal	80 plus	Data Rich	0	Population Density (300-500 ppl/sqkm, proportion)
Transport injuries	Male	Early Neonatal	80 plus	Data Rich	0	Population Density (500-1000 ppl/sqkm, proportion)
Transport injuries	Male	Early Neonatal	80 plus	Data Rich	1	Alcohol (litres per capita)
Transport injuries	Male	Early Neonatal	80 plus	Data Rich	1	Rainfall Quintile 5 (proportion)
Transport injuries	Male	Early Neonatal	80 plus	Data Rich	1	Vehicles - 2 wheels fraction (proportion)
Transport injuries	Male	Early Neonatal	80 plus	Data Rich	1	Vehicles - 2+4 wheels (per capita)
Transport injuries	Female	Early Neonatal	80 plus	Global	-1	Education (years per capita)
Transport injuries	Female	Early Neonatal	80 plus	Global	-1	Health System Access 2 (unitless)
Transport injuries	Female	Early Neonatal	80 plus	Global	-1	Sociodemographic Status
Transport injuries	Female	Early Neonatal	80 plus	Global	0	LDI (I$ per capita)
Transport injuries	Female	Early Neonatal	80 plus	Global	0	Population Density (300-500 ppl/sqkm, proportion)
Transport injuries	Female	Early Neonatal	80 plus	Global	0	Population Density (500-1000 ppl/sqkm, proportion)
Transport injuries	Female	Early Neonatal	80 plus	Global	1	Alcohol (litres per capita)
Transport injuries	Female	Early Neonatal	80 plus	Global	1	Rainfall Quintile 5 (proportion)
Transport injuries	Female	Early Neonatal	80 plus	Global	1	Vehicles - 2 wheels fraction (proportion)
Transport injuries	Female	Early Neonatal	80 plus	Global	1	Vehicles - 2+4 wheels (per capita)
Transport injuries	Female	Early Neonatal	80 plus	Data Rich	-1	Education (years per capita)
Transport injuries	Female	Early Neonatal	80 plus	Data Rich	-1	Health System Access 2 (unitless)
Transport injuries	Female	Early Neonatal	80 plus	Data Rich	-1	Sociodemographic Status
Transport injuries	Female	Early Neonatal	80 plus	Data Rich	0	LDI (I$ per capita)
Transport injuries	Female	Early Neonatal	80 plus	Data Rich	0	Population Density (300-500 ppl/sqkm, proportion)
Transport injuries	Female	Early Neonatal	80 plus	Data Rich	0	Population Density (500-1000 ppl/sqkm, proportion)
Transport injuries	Female	Early Neonatal	80 plus	Data Rich	1	Alcohol (litres per capita)
Transport injuries	Female	Early Neonatal	80 plus	Data Rich	1	Rainfall Quintile 5 (proportion)
Transport injuries	Female	Early Neonatal	80 plus	Data Rich	1	Vehicles - 2 wheels fraction (proportion)
Transport injuries	Female	Early Neonatal	80 plus	Data Rich	1	Vehicles - 2+4 wheels (per capita)
Road injuries	Male	Early Neonatal	80 plus	Global	-1	Education (years per capita)
Road injuries	Male	Early Neonatal	80 plus	Global	-1	Health System Access 2 (unitless)
Road injuries	Male	Early Neonatal	80 plus	Global	-1	Sociodemographic Status
Road injuries	Male	Early Neonatal	80 plus	Global	0	LDI (I$ per capita)
Road injuries	Male	Early Neonatal	80 plus	Global	0	Population Density (300-500 ppl/sqkm, proportion)
Road injuries	Male	Early Neonatal	80 plus	Global	0	Population Density (500-1000 ppl/sqkm, proportion)
Road injuries	Male	Early Neonatal	80 plus	Global	1	Alcohol (litres per capita)
Road injuries	Male	Early Neonatal	80 plus	Global	1	Log-transformed SEV scalar: Road Inj
Road injuries	Male	Early Neonatal	80 plus	Global	1	Population 15 to 30 (proportion)
Road injuries	Male	Early Neonatal	80 plus	Global	1	Vehicles - 2 wheels (per capita)
Road injuries	Male	Early Neonatal	80 plus	Global	1	Vehicles - 2 wheels fraction (proportion)

Appendix Table 10: CODEm covariates used, level of covariate, and expected direction of covariate by cause, sex, and age						
**Cause**	**Sex**	**Age start**	**Age end**	**Model type**	**Direction**	**Covariate**
Road injuries	Male	Early Neonatal	80 plus	Global	1	Vehicles - 2+4 wheels (per capita)
Road injuries	Male	Early Neonatal	80 plus	Global	1	Vehicles - 4 wheels (per capita)
Road injuries	Male	Early Neonatal	80 plus	Data Rich	-1	Education (years per capita)
Road injuries	Male	Early Neonatal	80 plus	Data Rich	-1	Health System Access 2 (unitless)
Road injuries	Male	Early Neonatal	80 plus	Data Rich	-1	Sociodemographic Status
Road injuries	Male	Early Neonatal	80 plus	Data Rich	0	LDI (I$ per capita)
Road injuries	Male	Early Neonatal	80 plus	Data Rich	0	Population Density (300-500 ppl/sqkm, proportion)
Road injuries	Male	Early Neonatal	80 plus	Data Rich	0	Population Density (500-1000 ppl/sqkm, proportion)
Road injuries	Male	Early Neonatal	80 plus	Data Rich	1	Alcohol (litres per capita)
Road injuries	Male	Early Neonatal	80 plus	Data Rich	1	Log-transformed SEV scalar: Road Inj
Road injuries	Male	Early Neonatal	80 plus	Data Rich	1	Population 15 to 30 (proportion)
Road injuries	Male	Early Neonatal	80 plus	Data Rich	1	Vehicles - 2 wheels (per capita)
Road injuries	Male	Early Neonatal	80 plus	Data Rich	1	Vehicles - 2 wheels fraction (proportion)
Road injuries	Male	Early Neonatal	80 plus	Data Rich	1	Vehicles - 2+4 wheels (per capita)
Road injuries	Male	Early Neonatal	80 plus	Data Rich	1	Vehicles - 4 wheels (per capita)
Road injuries	Female	Early Neonatal	80 plus	Global	-1	Education (years per capita)
Road injuries	Female	Early Neonatal	80 plus	Global	-1	Health System Access 2 (unitless)
Road injuries	Female	Early Neonatal	80 plus	Global	-1	Sociodemographic Status
Road injuries	Female	Early Neonatal	80 plus	Global	0	LDI (I$ per capita)
Road injuries	Female	Early Neonatal	80 plus	Global	0	Population Density (300-500 ppl/sqkm, proportion)
Road injuries	Female	Early Neonatal	80 plus	Global	0	Population Density (500-1000 ppl/sqkm, proportion)
Road injuries	Female	Early Neonatal	80 plus	Global	1	Alcohol (litres per capita)
Road injuries	Female	Early Neonatal	80 plus	Global	1	Log-transformed SEV scalar: Road Inj
Road injuries	Female	Early Neonatal	80 plus	Global	1	Population 15 to 30 (proportion)
Road injuries	Female	Early Neonatal	80 plus	Global	1	Vehicles - 2 wheels (per capita)
Road injuries	Female	Early Neonatal	80 plus	Global	1	Vehicles - 2 wheels fraction (proportion)
Road injuries	Female	Early Neonatal	80 plus	Global	1	Vehicles - 2+4 wheels (per capita)
Road injuries	Female	Early Neonatal	80 plus	Global	1	Vehicles - 4 wheels (per capita)
Road injuries	Female	Early Neonatal	80 plus	Data Rich	-1	Education (years per capita)
Road injuries	Female	Early Neonatal	80 plus	Data Rich	-1	Health System Access 2 (unitless)
Road injuries	Female	Early Neonatal	80 plus	Data Rich	-1	Sociodemographic Status
Road injuries	Female	Early Neonatal	80 plus	Data Rich	0	LDI (I$ per capita)
Road injuries	Female	Early Neonatal	80 plus	Data Rich	0	Population Density (300-500 ppl/sqkm, proportion)
Road injuries	Female	Early Neonatal	80 plus	Data Rich	0	Population Density (500-1000 ppl/sqkm, proportion)
Road injuries	Female	Early Neonatal	80 plus	Data Rich	1	Alcohol (litres per capita)
Road injuries	Female	Early Neonatal	80 plus	Data Rich	1	Log-transformed SEV scalar: Road Inj
Road injuries	Female	Early Neonatal	80 plus	Data Rich	1	Population 15 to 30 (proportion)
Road injuries	Female	Early Neonatal	80 plus	Data Rich	1	Vehicles - 2 wheels (per capita)
Road injuries	Female	Early Neonatal	80 plus	Data Rich	1	Vehicles - 2 wheels fraction (proportion)
Road injuries	Female	Early Neonatal	80 plus	Data Rich	1	Vehicles - 2+4 wheels (per capita)
Road injuries	Female	Early Neonatal	80 plus	Data Rich	1	Vehicles - 4 wheels (per capita)
Pedestrian road injuries	Male	Early Neonatal	80 plus	Global	-1	Education (years per capita)
Pedestrian road injuries	Male	Early Neonatal	80 plus	Global	-1	Health System Access 2 (unitless)
Pedestrian road injuries	Male	Early Neonatal	80 plus	Global	-1	Sociodemographic Status

Appendix Table 10: CODEm covariates used, level of covariate, and expected direction of covariate by cause, sex, and age						
**Cause**	**Sex**	**Age start**	**Age end**	**Model type**	**Direction**	**Covariate**
Pedestrian road injuries	Male	Early Neonatal	80 plus	Global	0	LDI (I$ per capita)
Pedestrian road injuries	Male	Early Neonatal	80 plus	Global	0	Population Density (300-500 ppl/sqkm, proportion)
Pedestrian road injuries	Male	Early Neonatal	80 plus	Global	0	Population Density (500-1000 ppl/sqkm, proportion)
Pedestrian road injuries	Male	Early Neonatal	80 plus	Global	1	Alcohol (litres per capita)
Pedestrian road injuries	Male	Early Neonatal	80 plus	Global	1	Log-transformed SEV scalar: Pedest
Pedestrian road injuries	Male	Early Neonatal	80 plus	Global	1	Rainfall Quintile 5 (proportion)
Pedestrian road injuries	Male	Early Neonatal	80 plus	Global	1	Vehicles - 2 wheels fraction (proportion)
Pedestrian road injuries	Male	Early Neonatal	80 plus	Global	1	Vehicles - 2+4 wheels (per capita)
Pedestrian road injuries	Male	Early Neonatal	80 plus	Data Rich	-1	Education (years per capita)
Pedestrian road injuries	Male	Early Neonatal	80 plus	Data Rich	-1	Health System Access 2 (unitless)
Pedestrian road injuries	Male	Early Neonatal	80 plus	Data Rich	-1	Sociodemographic Status
Pedestrian road injuries	Male	Early Neonatal	80 plus	Data Rich	0	LDI (I$ per capita)
Pedestrian road injuries	Male	Early Neonatal	80 plus	Data Rich	0	Population Density (300-500 ppl/sqkm, proportion)
Pedestrian road injuries	Male	Early Neonatal	80 plus	Data Rich	0	Population Density (500-1000 ppl/sqkm, proportion)
Pedestrian road injuries	Male	Early Neonatal	80 plus	Data Rich	1	Alcohol (litres per capita)
Pedestrian road injuries	Male	Early Neonatal	80 plus	Data Rich	1	Log-transformed SEV scalar: Pedest
Pedestrian road injuries	Male	Early Neonatal	80 plus	Data Rich	1	Rainfall Quintile 5 (proportion)
Pedestrian road injuries	Male	Early Neonatal	80 plus	Data Rich	1	Vehicles - 2 wheels fraction (proportion)
Pedestrian road injuries	Male	Early Neonatal	80 plus	Data Rich	1	Vehicles - 2+4 wheels (per capita)
Pedestrian road injuries	Female	Early Neonatal	80 plus	Global	-1	Education (years per capita)
Pedestrian road injuries	Female	Early Neonatal	80 plus	Global	-1	Health System Access 2 (unitless)
Pedestrian road injuries	Female	Early Neonatal	80 plus	Global	-1	Sociodemographic Status
Pedestrian road injuries	Female	Early Neonatal	80 plus	Global	0	LDI (I$ per capita)
Pedestrian road injuries	Female	Early Neonatal	80 plus	Global	0	Population Density (300-500 ppl/sqkm, proportion)
Pedestrian road injuries	Female	Early Neonatal	80 plus	Global	0	Population Density (500-1000 ppl/sqkm, proportion)
Pedestrian road injuries	Female	Early Neonatal	80 plus	Global	1	Alcohol (litres per capita)
Pedestrian road injuries	Female	Early Neonatal	80 plus	Global	1	Log-transformed SEV scalar: Pedest
Pedestrian road injuries	Female	Early Neonatal	80 plus	Global	1	Rainfall Quintile 5 (proportion)
Pedestrian road injuries	Female	Early Neonatal	80 plus	Global	1	Vehicles - 2 wheels fraction (proportion)
Pedestrian road injuries	Female	Early Neonatal	80 plus	Global	1	Vehicles - 2+4 wheels (per capita)
Pedestrian road injuries	Female	Early Neonatal	80 plus	Data Rich	-1	Education (years per capita)
Pedestrian road injuries	Female	Early Neonatal	80 plus	Data Rich	-1	Health System Access 2 (unitless)
Pedestrian road injuries	Female	Early Neonatal	80 plus	Data Rich	-1	Sociodemographic Status
Pedestrian road injuries	Female	Early Neonatal	80 plus	Data Rich	0	LDI (I$ per capita)
Pedestrian road injuries	Female	Early Neonatal	80 plus	Data Rich	0	Population Density (300-500 ppl/sqkm, proportion)
Pedestrian road injuries	Female	Early Neonatal	80 plus	Data Rich	0	Population Density (500-1000 ppl/sqkm, proportion)
Pedestrian road injuries	Female	Early Neonatal	80 plus	Data Rich	1	Alcohol (litres per capita)
Pedestrian road injuries	Female	Early Neonatal	80 plus	Data Rich	1	Log-transformed SEV scalar: Pedest
Pedestrian road injuries	Female	Early Neonatal	80 plus	Data Rich	1	Rainfall Quintile 5 (proportion)
Pedestrian road injuries	Female	Early Neonatal	80 plus	Data Rich	1	Vehicles - 2 wheels fraction (proportion)
Pedestrian road injuries	Female	Early Neonatal	80 plus	Data Rich	1	Vehicles - 2+4 wheels (per capita)
Cyclist road injuries	Male	1 to 4	80 plus	Global	-1	Education (years per capita)
Cyclist road injuries	Male	1 to 4	80 plus	Global	-1	Health System Access 2 (unitless)
Cyclist road injuries	Male	1 to 4	80 plus	Global	0	LDI (I$ per capita)

Appendix Table 10: CODEm covariates used, level of covariate, and expected direction of covariate by cause, sex, and age						
**Cause**	**Sex**	**Age start**	**Age end**	**Model type**	**Direction**	**Covariate**
Cyclist road injuries	Male	1 to 4	80 plus	Global	0	Population Density (300-500 ppl/sqkm, proportion)
Cyclist road injuries	Male	1 to 4	80 plus	Global	0	Population Density (500-1000 ppl/sqkm, proportion)
Cyclist road injuries	Male	1 to 4	80 plus	Global	0	Sociodemographic Status
Cyclist road injuries	Male	1 to 4	80 plus	Global	1	Alcohol (litres per capita)
Cyclist road injuries	Male	1 to 4	80 plus	Global	1	Log-transformed SEV scalar: Cyclist
Cyclist road injuries	Male	1 to 4	80 plus	Global	1	Vehicles - 2 wheels fraction (proportion)
Cyclist road injuries	Male	1 to 4	80 plus	Global	1	Vehicles - 2+4 wheels (per capita)
Cyclist road injuries	Male	1 to 4	80 plus	Data Rich	-1	Education (years per capita)
Cyclist road injuries	Male	1 to 4	80 plus	Data Rich	-1	Health System Access 2 (unitless)
Cyclist road injuries	Male	1 to 4	80 plus	Data Rich	0	LDI (I$ per capita)
Cyclist road injuries	Male	1 to 4	80 plus	Data Rich	0	Population Density (300-500 ppl/sqkm, proportion)
Cyclist road injuries	Male	1 to 4	80 plus	Data Rich	0	Population Density (500-1000 ppl/sqkm, proportion)
Cyclist road injuries	Male	1 to 4	80 plus	Data Rich	0	Sociodemographic Status
Cyclist road injuries	Male	1 to 4	80 plus	Data Rich	1	Alcohol (litres per capita)
Cyclist road injuries	Male	1 to 4	80 plus	Data Rich	1	Log-transformed SEV scalar: Cyclist
Cyclist road injuries	Male	1 to 4	80 plus	Data Rich	1	Vehicles - 2 wheels fraction (proportion)
Cyclist road injuries	Male	1 to 4	80 plus	Data Rich	1	Vehicles - 2+4 wheels (per capita)
Cyclist road injuries	Female	1 to 4	80 plus	Global	-1	Education (years per capita)
Cyclist road injuries	Female	1 to 4	80 plus	Global	-1	Health System Access 2 (unitless)
Cyclist road injuries	Female	1 to 4	80 plus	Global	0	LDI (I$ per capita)
Cyclist road injuries	Female	1 to 4	80 plus	Global	0	Population Density (300-500 ppl/sqkm, proportion)
Cyclist road injuries	Female	1 to 4	80 plus	Global	0	Population Density (500-1000 ppl/sqkm, proportion)
Cyclist road injuries	Female	1 to 4	80 plus	Global	0	Sociodemographic Status
Cyclist road injuries	Female	1 to 4	80 plus	Global	1	Alcohol (litres per capita)
Cyclist road injuries	Female	1 to 4	80 plus	Global	1	Log-transformed SEV scalar: Cyclist
Cyclist road injuries	Female	1 to 4	80 plus	Global	1	Vehicles - 2 wheels fraction (proportion)
Cyclist road injuries	Female	1 to 4	80 plus	Global	1	Vehicles - 2+4 wheels (per capita)
Cyclist road injuries	Female	1 to 4	80 plus	Data Rich	-1	Education (years per capita)
Cyclist road injuries	Female	1 to 4	80 plus	Data Rich	-1	Health System Access 2 (unitless)
Cyclist road injuries	Female	1 to 4	80 plus	Data Rich	0	LDI (I$ per capita)
Cyclist road injuries	Female	1 to 4	80 plus	Data Rich	0	Population Density (300-500 ppl/sqkm, proportion)
Cyclist road injuries	Female	1 to 4	80 plus	Data Rich	0	Population Density (500-1000 ppl/sqkm, proportion)
Cyclist road injuries	Female	1 to 4	80 plus	Data Rich	0	Sociodemographic Status
Cyclist road injuries	Female	1 to 4	80 plus	Data Rich	1	Alcohol (litres per capita)
Cyclist road injuries	Female	1 to 4	80 plus	Data Rich	1	Log-transformed SEV scalar: Cyclist
Cyclist road injuries	Female	1 to 4	80 plus	Data Rich	1	Vehicles - 2 wheels fraction (proportion)
Cyclist road injuries	Female	1 to 4	80 plus	Data Rich	1	Vehicles - 2+4 wheels (per capita)
Motorcyclist road injuries	Male	Early Neonatal	80 plus	Global	-1	Education (years per capita)
Motorcyclist road injuries	Male	Early Neonatal	80 plus	Global	-1	Health System Access 2 (unitless)
Motorcyclist road injuries	Male	Early Neonatal	80 plus	Global	0	LDI (I$ per capita)
Motorcyclist road injuries	Male	Early Neonatal	80 plus	Global	0	Population Density (300-500 ppl/sqkm, proportion)
Motorcyclist road injuries	Male	Early Neonatal	80 plus	Global	0	Population Density (500-1000 ppl/sqkm, proportion)
Motorcyclist road injuries	Male	Early Neonatal	80 plus	Global	0	Sociodemographic Status
Motorcyclist road injuries	Male	Early Neonatal	80 plus	Global	1	Alcohol (litres per capita)

Appendix Table 10: CODEm covariates used, level of covariate, and expected direction of covariate by cause, sex, and age						
**Cause**	**Sex**	**Age start**	**Age end**	**Model type**	**Direction**	**Covariate**
Motorcyclist road injuries	Male	Early Neonatal	80 plus	Global	1	Log-transformed SEV scalar: Mot Cyc
Motorcyclist road injuries	Male	Early Neonatal	80 plus	Global	1	Rainfall Quintile 5 (proportion)
Motorcyclist road injuries	Male	Early Neonatal	80 plus	Global	1	Vehicles - 2 wheels (per capita)
Motorcyclist road injuries	Male	Early Neonatal	80 plus	Data Rich	-1	Education (years per capita)
Motorcyclist road injuries	Male	Early Neonatal	80 plus	Data Rich	-1	Health System Access 2 (unitless)
Motorcyclist road injuries	Male	Early Neonatal	80 plus	Data Rich	0	LDI (I$ per capita)
Motorcyclist road injuries	Male	Early Neonatal	80 plus	Data Rich	0	Population Density (300-500 ppl/sqkm, proportion)
Motorcyclist road injuries	Male	Early Neonatal	80 plus	Data Rich	0	Population Density (500-1000 ppl/sqkm, proportion)
Motorcyclist road injuries	Male	Early Neonatal	80 plus	Data Rich	0	Sociodemographic Status
Motorcyclist road injuries	Male	Early Neonatal	80 plus	Data Rich	1	Alcohol (litres per capita)
Motorcyclist road injuries	Male	Early Neonatal	80 plus	Data Rich	1	Log-transformed SEV scalar: Mot Cyc
Motorcyclist road injuries	Male	Early Neonatal	80 plus	Data Rich	1	Rainfall Quintile 5 (proportion)
Motorcyclist road injuries	Male	Early Neonatal	80 plus	Data Rich	1	Vehicles - 2 wheels (per capita)
Motorcyclist road injuries	Female	Early Neonatal	80 plus	Global	-1	Education (years per capita)
Motorcyclist road injuries	Female	Early Neonatal	80 plus	Global	-1	Health System Access 2 (unitless)
Motorcyclist road injuries	Female	Early Neonatal	80 plus	Global	0	LDI (I$ per capita)
Motorcyclist road injuries	Female	Early Neonatal	80 plus	Global	0	Population Density (300-500 ppl/sqkm, proportion)
Motorcyclist road injuries	Female	Early Neonatal	80 plus	Global	0	Population Density (500-1000 ppl/sqkm, proportion)
Motorcyclist road injuries	Female	Early Neonatal	80 plus	Global	0	Sociodemographic Status
Motorcyclist road injuries	Female	Early Neonatal	80 plus	Global	1	Alcohol (litres per capita)
Motorcyclist road injuries	Female	Early Neonatal	80 plus	Global	1	Log-transformed SEV scalar: Mot Cyc
Motorcyclist road injuries	Female	Early Neonatal	80 plus	Global	1	Rainfall Quintile 5 (proportion)
Motorcyclist road injuries	Female	Early Neonatal	80 plus	Global	1	Vehicles - 2 wheels (per capita)
Motorcyclist road injuries	Female	Early Neonatal	80 plus	Data Rich	-1	Education (years per capita)
Motorcyclist road injuries	Female	Early Neonatal	80 plus	Data Rich	-1	Health System Access 2 (unitless)
Motorcyclist road injuries	Female	Early Neonatal	80 plus	Data Rich	0	LDI (I$ per capita)
Motorcyclist road injuries	Female	Early Neonatal	80 plus	Data Rich	0	Population Density (300-500 ppl/sqkm, proportion)
Motorcyclist road injuries	Female	Early Neonatal	80 plus	Data Rich	0	Population Density (500-1000 ppl/sqkm, proportion)
Motorcyclist road injuries	Female	Early Neonatal	80 plus	Data Rich	0	Sociodemographic Status
Motorcyclist road injuries	Female	Early Neonatal	80 plus	Data Rich	1	Alcohol (litres per capita)
Motorcyclist road injuries	Female	Early Neonatal	80 plus	Data Rich	1	Log-transformed SEV scalar: Mot Cyc
Motorcyclist road injuries	Female	Early Neonatal	80 plus	Data Rich	1	Rainfall Quintile 5 (proportion)
Motorcyclist road injuries	Female	Early Neonatal	80 plus	Data Rich	1	Vehicles - 2 wheels (per capita)
Motor vehicle road injuries	Male	Early Neonatal	80 plus	Global	-1	Education (years per capita)
Motor vehicle road injuries	Male	Early Neonatal	80 plus	Global	-1	Health System Access 2 (unitless)
Motor vehicle road injuries	Male	Early Neonatal	80 plus	Global	0	LDI (I$ per capita)
Motor vehicle road injuries	Male	Early Neonatal	80 plus	Global	0	Population Density (300-500 ppl/sqkm, proportion)
Motor vehicle road injuries	Male	Early Neonatal	80 plus	Global	0	Population Density (500-1000 ppl/sqkm, proportion)
Motor vehicle road injuries	Male	Early Neonatal	80 plus	Global	0	Sociodemographic Status
Motor vehicle road injuries	Male	Early Neonatal	80 plus	Global	1	Alcohol (litres per capita)
Motor vehicle road injuries	Male	Early Neonatal	80 plus	Global	1	Log-transformed SEV scalar: Mot Veh
Motor vehicle road injuries	Male	Early Neonatal	80 plus	Global	1	Rainfall Quintile 5 (proportion)
Motor vehicle road injuries	Male	Early Neonatal	80 plus	Global	1	Vehicles - 4 wheels (per capita)
Motor vehicle road injuries	Male	Early Neonatal	80 plus	Data Rich	-1	Education (years per capita)

Appendix Table 10: CODEm covariates used, level of covariate, and expected direction of covariate by cause, sex, and age						
**Cause**	**Sex**	**Age start**	**Age end**	**Model type**	**Direction**	**Covariate**
Motor vehicle road injuries	Male	Early Neonatal	80 plus	Data Rich	-1	Health System Access 2 (unitless)
Motor vehicle road injuries	Male	Early Neonatal	80 plus	Data Rich	0	LDI (I$ per capita)
Motor vehicle road injuries	Male	Early Neonatal	80 plus	Data Rich	0	Population Density (300-500 ppl/sqkm, proportion)
Motor vehicle road injuries	Male	Early Neonatal	80 plus	Data Rich	0	Population Density (500-1000 ppl/sqkm, proportion)
Motor vehicle road injuries	Male	Early Neonatal	80 plus	Data Rich	0	Sociodemographic Status
Motor vehicle road injuries	Male	Early Neonatal	80 plus	Data Rich	1	Alcohol (litres per capita)
Motor vehicle road injuries	Male	Early Neonatal	80 plus	Data Rich	1	Log-transformed SEV scalar: Mot Veh
Motor vehicle road injuries	Male	Early Neonatal	80 plus	Data Rich	1	Rainfall Quintile 5 (proportion)
Motor vehicle road injuries	Male	Early Neonatal	80 plus	Data Rich	1	Vehicles - 4 wheels (per capita)
Motor vehicle road injuries	Female	Early Neonatal	80 plus	Global	-1	Education (years per capita)
Motor vehicle road injuries	Female	Early Neonatal	80 plus	Global	-1	Health System Access 2 (unitless)
Motor vehicle road injuries	Female	Early Neonatal	80 plus	Global	0	LDI (I$ per capita)
Motor vehicle road injuries	Female	Early Neonatal	80 plus	Global	0	Population Density (300-500 ppl/sqkm, proportion)
Motor vehicle road injuries	Female	Early Neonatal	80 plus	Global	0	Population Density (500-1000 ppl/sqkm, proportion)
Motor vehicle road injuries	Female	Early Neonatal	80 plus	Global	0	Sociodemographic Status
Motor vehicle road injuries	Female	Early Neonatal	80 plus	Global	1	Alcohol (litres per capita)
Motor vehicle road injuries	Female	Early Neonatal	80 plus	Global	1	Log-transformed SEV scalar: Mot Veh
Motor vehicle road injuries	Female	Early Neonatal	80 plus	Global	1	Rainfall Quintile 5 (proportion)
Motor vehicle road injuries	Female	Early Neonatal	80 plus	Global	1	Vehicles - 4 wheels (per capita)
Motor vehicle road injuries	Female	Early Neonatal	80 plus	Data Rich	-1	Education (years per capita)
Motor vehicle road injuries	Female	Early Neonatal	80 plus	Data Rich	-1	Health System Access 2 (unitless)
Motor vehicle road injuries	Female	Early Neonatal	80 plus	Data Rich	0	LDI (I$ per capita)
Motor vehicle road injuries	Female	Early Neonatal	80 plus	Data Rich	0	Population Density (300-500 ppl/sqkm, proportion)
Motor vehicle road injuries	Female	Early Neonatal	80 plus	Data Rich	0	Population Density (500-1000 ppl/sqkm, proportion)
Motor vehicle road injuries	Female	Early Neonatal	80 plus	Data Rich	0	Sociodemographic Status
Motor vehicle road injuries	Female	Early Neonatal	80 plus	Data Rich	1	Alcohol (litres per capita)
Motor vehicle road injuries	Female	Early Neonatal	80 plus	Data Rich	1	Log-transformed SEV scalar: Mot Veh
Motor vehicle road injuries	Female	Early Neonatal	80 plus	Data Rich	1	Rainfall Quintile 5 (proportion)
Motor vehicle road injuries	Female	Early Neonatal	80 plus	Data Rich	1	Vehicles - 4 wheels (per capita)
Other road injuries	Male	Early Neonatal	80 plus	Global	-1	Health System Access 2 (unitless)
Other road injuries	Male	Early Neonatal	80 plus	Global	-1	Sociodemographic Status
Other road injuries	Male	Early Neonatal	80 plus	Global	0	LDI (I$ per capita)
Other road injuries	Male	Early Neonatal	80 plus	Global	1	Alcohol (litres per capita)
Other road injuries	Male	Early Neonatal	80 plus	Global	1	Log-transformed SEV scalar: Oth Road
Other road injuries	Male	Early Neonatal	80 plus	Global	1	Rainfall Quintile 5 (proportion)
Other road injuries	Male	Early Neonatal	80 plus	Global	1	Vehicles - 2 wheels fraction (proportion)
Other road injuries	Male	Early Neonatal	80 plus	Global	1	Vehicles - 2+4 wheels (per capita)
Other road injuries	Male	Early Neonatal	80 plus	Data Rich	-1	Health System Access 2 (unitless)
Other road injuries	Male	Early Neonatal	80 plus	Data Rich	-1	Sociodemographic Status
Other road injuries	Male	Early Neonatal	80 plus	Data Rich	0	LDI (I$ per capita)
Other road injuries	Male	Early Neonatal	80 plus	Data Rich	1	Alcohol (litres per capita)
Other road injuries	Male	Early Neonatal	80 plus	Data Rich	1	Log-transformed SEV scalar: Oth Road
Other road injuries	Male	Early Neonatal	80 plus	Data Rich	1	Rainfall Quintile 5 (proportion)
Other road injuries	Male	Early Neonatal	80 plus	Data Rich	1	Vehicles - 2 wheels fraction (proportion)

Appendix Table 10: CODEm covariates used, level of covariate, and expected direction of covariate by cause, sex, and age						
**Cause**	**Sex**	**Age start**	**Age end**	**Model type**	**Direction**	**Covariate**
Other road injuries	Male	Early Neonatal	80 plus	Data Rich	1	Vehicles - 2+4 wheels (per capita)
Other road injuries	Female	Early Neonatal	80 plus	Global	-1	Health System Access 2 (unitless)
Other road injuries	Female	Early Neonatal	80 plus	Global	-1	Sociodemographic Status
Other road injuries	Female	Early Neonatal	80 plus	Global	0	LDI (I$ per capita)
Other road injuries	Female	Early Neonatal	80 plus	Global	1	Alcohol (litres per capita)
Other road injuries	Female	Early Neonatal	80 plus	Global	1	Log-transformed SEV scalar: Oth Road
Other road injuries	Female	Early Neonatal	80 plus	Global	1	Rainfall Quintile 5 (proportion)
Other road injuries	Female	Early Neonatal	80 plus	Global	1	Vehicles - 2 wheels fraction (proportion)
Other road injuries	Female	Early Neonatal	80 plus	Global	1	Vehicles - 2+4 wheels (per capita)
Other road injuries	Female	Early Neonatal	80 plus	Data Rich	-1	Health System Access 2 (unitless)
Other road injuries	Female	Early Neonatal	80 plus	Data Rich	-1	Sociodemographic Status
Other road injuries	Female	Early Neonatal	80 plus	Data Rich	0	LDI (I$ per capita)
Other road injuries	Female	Early Neonatal	80 plus	Data Rich	1	Alcohol (litres per capita)
Other road injuries	Female	Early Neonatal	80 plus	Data Rich	1	Log-transformed SEV scalar: Oth Road
Other road injuries	Female	Early Neonatal	80 plus	Data Rich	1	Rainfall Quintile 5 (proportion)
Other road injuries	Female	Early Neonatal	80 plus	Data Rich	1	Vehicles - 2 wheels fraction (proportion)
Other road injuries	Female	Early Neonatal	80 plus	Data Rich	1	Vehicles - 2+4 wheels (per capita)
Other transport injuries	Male	Early Neonatal	80 plus	Global	-1	Education (years per capita)
Other transport injuries	Male	Early Neonatal	80 plus	Global	-1	Health System Access 2 (unitless)
Other transport injuries	Male	Early Neonatal	80 plus	Global	0	LDI (I$ per capita)
Other transport injuries	Male	Early Neonatal	80 plus	Global	0	Population Density (300-500 ppl/sqkm, proportion)
Other transport injuries	Male	Early Neonatal	80 plus	Global	0	Population Density (500-1000 ppl/sqkm, proportion)
Other transport injuries	Male	Early Neonatal	80 plus	Global	0	Sociodemographic Status
Other transport injuries	Male	Early Neonatal	80 plus	Global	1	Alcohol (litres per capita)
Other transport injuries	Male	Early Neonatal	80 plus	Global	1	Log-transformed SEV scalar: Oth Trans
Other transport injuries	Male	Early Neonatal	80 plus	Global	1	Rainfall Quintile 5 (proportion)
Other transport injuries	Male	Early Neonatal	80 plus	Global	1	Vehicles - 2 wheels fraction (proportion)
Other transport injuries	Male	Early Neonatal	80 plus	Global	1	Vehicles - 2+4 wheels (per capita)
Other transport injuries	Male	Early Neonatal	80 plus	Data Rich	-1	Education (years per capita)
Other transport injuries	Male	Early Neonatal	80 plus	Data Rich	-1	Health System Access 2 (unitless)
Other transport injuries	Male	Early Neonatal	80 plus	Data Rich	0	LDI (I$ per capita)
Other transport injuries	Male	Early Neonatal	80 plus	Data Rich	0	Population Density (300-500 ppl/sqkm, proportion)
Other transport injuries	Male	Early Neonatal	80 plus	Data Rich	0	Population Density (500-1000 ppl/sqkm, proportion)
Other transport injuries	Male	Early Neonatal	80 plus	Data Rich	0	Sociodemographic Status
Other transport injuries	Male	Early Neonatal	80 plus	Data Rich	1	Alcohol (litres per capita)
Other transport injuries	Male	Early Neonatal	80 plus	Data Rich	1	Log-transformed SEV scalar: Oth Trans
Other transport injuries	Male	Early Neonatal	80 plus	Data Rich	1	Rainfall Quintile 5 (proportion)
Other transport injuries	Male	Early Neonatal	80 plus	Data Rich	1	Vehicles - 2 wheels fraction (proportion)
Other transport injuries	Male	Early Neonatal	80 plus	Data Rich	1	Vehicles - 2+4 wheels (per capita)
Other transport injuries	Female	Early Neonatal	80 plus	Global	-1	Education (years per capita)
Other transport injuries	Female	Early Neonatal	80 plus	Global	-1	Health System Access 2 (unitless)
Other transport injuries	Female	Early Neonatal	80 plus	Global	0	LDI (I$ per capita)
Other transport injuries	Female	Early Neonatal	80 plus	Global	0	Population Density (300-500 ppl/sqkm, proportion)
Other transport injuries	Female	Early Neonatal	80 plus	Global	0	Population Density (500-1000 ppl/sqkm, proportion)

Appendix Table 10: CODEm covariates used, level of covariate, and expected direction of covariate by cause, sex, and age						
**Cause**	**Sex**	**Age start**	**Age end**	**Model type**	**Direction**	**Covariate**
Other transport injuries	Female	Early Neonatal	80 plus	Global	0	Sociodemographic Status
Other transport injuries	Female	Early Neonatal	80 plus	Global	1	Alcohol (litres per capita)
Other transport injuries	Female	Early Neonatal	80 plus	Global	1	Log-transformed SEV scalar: Oth Trans
Other transport injuries	Female	Early Neonatal	80 plus	Global	1	Rainfall Quintile 5 (proportion)
Other transport injuries	Female	Early Neonatal	80 plus	Global	1	Vehicles - 2 wheels fraction (proportion)
Other transport injuries	Female	Early Neonatal	80 plus	Global	1	Vehicles - 2+4 wheels (per capita)
Other transport injuries	Female	Early Neonatal	80 plus	Data Rich	-1	Education (years per capita)
Other transport injuries	Female	Early Neonatal	80 plus	Data Rich	-1	Health System Access 2 (unitless)
Other transport injuries	Female	Early Neonatal	80 plus	Data Rich	0	LDI (I$ per capita)
Other transport injuries	Female	Early Neonatal	80 plus	Data Rich	0	Population Density (300-500 ppl/sqkm, proportion)
Other transport injuries	Female	Early Neonatal	80 plus	Data Rich	0	Population Density (500-1000 ppl/sqkm, proportion)
Other transport injuries	Female	Early Neonatal	80 plus	Data Rich	0	Sociodemographic Status
Other transport injuries	Female	Early Neonatal	80 plus	Data Rich	1	Alcohol (litres per capita)
Other transport injuries	Female	Early Neonatal	80 plus	Data Rich	1	Log-transformed SEV scalar: Oth Trans
Other transport injuries	Female	Early Neonatal	80 plus	Data Rich	1	Rainfall Quintile 5 (proportion)
Other transport injuries	Female	Early Neonatal	80 plus	Data Rich	1	Vehicles - 2 wheels fraction (proportion)
Other transport injuries	Female	Early Neonatal	80 plus	Data Rich	1	Vehicles - 2+4 wheels (per capita)
Falls	Male	Early Neonatal	80 plus	Global	-1	Health System Access 2 (unitless)
Falls	Male	Early Neonatal	80 plus	Global	-1	ln-Milk (kcal per capita)
Falls	Male	Early Neonatal	80 plus	Global	0	LDI (I$ per capita)
Falls	Male	Early Neonatal	80 plus	Global	0	Sociodemographic Status
Falls	Male	Early Neonatal	80 plus	Global	1	Alcohol (litres per capita)
Falls	Male	Early Neonatal	80 plus	Global	1	Elevation Over 1500m (proportion)
Falls	Male	Early Neonatal	80 plus	Global	1	Log-transformed SEV scalar: Falls
Falls	Male	Early Neonatal	80 plus	Data Rich	-1	Health System Access 2 (unitless)
Falls	Male	Early Neonatal	80 plus	Data Rich	-1	ln-Milk (kcal per capita)
Falls	Male	Early Neonatal	80 plus	Data Rich	0	LDI (I$ per capita)
Falls	Male	Early Neonatal	80 plus	Data Rich	0	Sociodemographic Status
Falls	Male	Early Neonatal	80 plus	Data Rich	1	Alcohol (litres per capita)
Falls	Male	Early Neonatal	80 plus	Data Rich	1	Elevation Over 1500m (proportion)
Falls	Male	Early Neonatal	80 plus	Data Rich	1	Log-transformed SEV scalar: Falls
Falls	Female	Early Neonatal	80 plus	Global	-1	Health System Access 2 (unitless)
Falls	Female	Early Neonatal	80 plus	Global	-1	ln-Milk (kcal per capita)
Falls	Female	Early Neonatal	80 plus	Global	0	LDI (I$ per capita)
Falls	Female	Early Neonatal	80 plus	Global	0	Sociodemographic Status
Falls	Female	Early Neonatal	80 plus	Global	1	Alcohol (litres per capita)
Falls	Female	Early Neonatal	80 plus	Global	1	Elevation Over 1500m (proportion)
Falls	Female	Early Neonatal	80 plus	Global	1	Log-transformed SEV scalar: Falls
Falls	Female	Early Neonatal	80 plus	Data Rich	-1	Health System Access 2 (unitless)
Falls	Female	Early Neonatal	80 plus	Data Rich	-1	ln-Milk (kcal per capita)
Falls	Female	Early Neonatal	80 plus	Data Rich	0	LDI (I$ per capita)
Falls	Female	Early Neonatal	80 plus	Data Rich	0	Sociodemographic Status
Falls	Female	Early Neonatal	80 plus	Data Rich	1	Alcohol (litres per capita)
Falls	Female	Early Neonatal	80 plus	Data Rich	1	Elevation Over 1500m (proportion)

Appendix Table 10: CODEm covariates used, level of covariate, and expected direction of covariate by cause, sex, and age						
**Cause**	**Sex**	**Age start**	**Age end**	**Model type**	**Direction**	**Covariate**
Falls	Female	Early Neonatal	80 plus	Data Rich	1	Log-transformed SEV scalar: Falls
Drowning	Male	Early Neonatal	80 plus	Global	-1	Education (years per capita)
Drowning	Male	Early Neonatal	80 plus	Global	-1	Landlocked Nation (binary)
Drowning	Male	Early Neonatal	80 plus	Global	-1	Rainfall Quintile 1 (proportion)
Drowning	Male	Early Neonatal	80 plus	Global	-1	Sociodemographic Status
Drowning	Male	Early Neonatal	80 plus	Global	0	LDI (I$ per capita)
Drowning	Male	Early Neonatal	80 plus	Global	1	Alcohol (litres per capita)
Drowning	Male	Early Neonatal	80 plus	Global	1	Coastal Population within 10km (proportion)
Drowning	Male	Early Neonatal	80 plus	Global	1	Elevation Under 100m (proportion)
Drowning	Male	Early Neonatal	80 plus	Global	1	Log-transformed SEV scalar: Drown
Drowning	Male	Early Neonatal	80 plus	Global	1	Rainfall Quintile 5 (proportion)
Drowning	Male	Early Neonatal	80 plus	Data Rich	-1	Education (years per capita)
Drowning	Male	Early Neonatal	80 plus	Data Rich	-1	Landlocked Nation (binary)
Drowning	Male	Early Neonatal	80 plus	Data Rich	-1	Rainfall Quintile 1 (proportion)
Drowning	Male	Early Neonatal	80 plus	Data Rich	-1	Sociodemographic Status
Drowning	Male	Early Neonatal	80 plus	Data Rich	0	LDI (I$ per capita)
Drowning	Male	Early Neonatal	80 plus	Data Rich	1	Alcohol (litres per capita)
Drowning	Male	Early Neonatal	80 plus	Data Rich	1	Coastal Population within 10km (proportion)
Drowning	Male	Early Neonatal	80 plus	Data Rich	1	Elevation Under 100m (proportion)
Drowning	Male	Early Neonatal	80 plus	Data Rich	1	Log-transformed SEV scalar: Drown
Drowning	Male	Early Neonatal	80 plus	Data Rich	1	Rainfall Quintile 5 (proportion)
Drowning	Female	Early Neonatal	80 plus	Global	-1	Education (years per capita)
Drowning	Female	Early Neonatal	80 plus	Global	-1	Landlocked Nation (binary)
Drowning	Female	Early Neonatal	80 plus	Global	-1	Rainfall Quintile 1 (proportion)
Drowning	Female	Early Neonatal	80 plus	Global	-1	Sociodemographic Status
Drowning	Female	Early Neonatal	80 plus	Global	0	LDI (I$ per capita)
Drowning	Female	Early Neonatal	80 plus	Global	1	Alcohol (litres per capita)
Drowning	Female	Early Neonatal	80 plus	Global	1	Coastal Population within 10km (proportion)
Drowning	Female	Early Neonatal	80 plus	Global	1	Elevation Under 100m (proportion)
Drowning	Female	Early Neonatal	80 plus	Global	1	Log-transformed SEV scalar: Drown
Drowning	Female	Early Neonatal	80 plus	Global	1	Rainfall Quintile 5 (proportion)
Drowning	Female	Early Neonatal	80 plus	Data Rich	-1	Education (years per capita)
Drowning	Female	Early Neonatal	80 plus	Data Rich	-1	Landlocked Nation (binary)
Drowning	Female	Early Neonatal	80 plus	Data Rich	-1	Rainfall Quintile 1 (proportion)
Drowning	Female	Early Neonatal	80 plus	Data Rich	-1	Sociodemographic Status
Drowning	Female	Early Neonatal	80 plus	Data Rich	0	LDI (I$ per capita)
Drowning	Female	Early Neonatal	80 plus	Data Rich	1	Alcohol (litres per capita)
Drowning	Female	Early Neonatal	80 plus	Data Rich	1	Coastal Population within 10km (proportion)
Drowning	Female	Early Neonatal	80 plus	Data Rich	1	Elevation Under 100m (proportion)
Drowning	Female	Early Neonatal	80 plus	Data Rich	1	Log-transformed SEV scalar: Drown
Drowning	Female	Early Neonatal	80 plus	Data Rich	1	Rainfall Quintile 5 (proportion)
Fire, heat, and hot substances	Male	Early Neonatal	80 plus	Global	-1	Education (years per capita)
Fire, heat, and hot substances	Male	Early Neonatal	80 plus	Global	-1	Health System Access 2 (unitless)
Fire, heat, and hot substances	Male	Early Neonatal	80 plus	Global	-1	Sociodemographic Status

Appendix Table 10: CODEm covariates used, level of covariate, and expected direction of covariate by cause, sex, and age						
**Cause**	**Sex**	**Age start**	**Age end**	**Model type**	**Direction**	**Covariate**
Fire, heat, and hot substances	Male	Early Neonatal	80 plus	Global	0	LDI (I$ per capita)
Fire, heat, and hot substances	Male	Early Neonatal	80 plus	Global	0	Population Density (over 1000 ppl/sqkm, proportion)
Fire, heat, and hot substances	Male	Early Neonatal	80 plus	Global	1	Alcohol (litres per capita)
Fire, heat, and hot substances	Male	Early Neonatal	80 plus	Global	1	Indoor Air Pollution (Biomass Cooking)
Fire, heat, and hot substances	Male	Early Neonatal	80 plus	Global	1	Log-transformed SEV scalar: Fire
Fire, heat, and hot substances	Male	Early Neonatal	80 plus	Global	1	Tobacco (cigarettes per capita)
Fire, heat, and hot substances	Male	Early Neonatal	80 plus	Data Rich	-1	Education (years per capita)
Fire, heat, and hot substances	Male	Early Neonatal	80 plus	Data Rich	-1	Health System Access 2 (unitless)
Fire, heat, and hot substances	Male	Early Neonatal	80 plus	Data Rich	-1	Sociodemographic Status
Fire, heat, and hot substances	Male	Early Neonatal	80 plus	Data Rich	0	LDI (I$ per capita)
Fire, heat, and hot substances	Male	Early Neonatal	80 plus	Data Rich	0	Population Density (over 1000 ppl/sqkm, proportion)
Fire, heat, and hot substances	Male	Early Neonatal	80 plus	Data Rich	1	Alcohol (litres per capita)
Fire, heat, and hot substances	Male	Early Neonatal	80 plus	Data Rich	1	Indoor Air Pollution (Biomass Cooking)
Fire, heat, and hot substances	Male	Early Neonatal	80 plus	Data Rich	1	Log-transformed SEV scalar: Fire
Fire, heat, and hot substances	Male	Early Neonatal	80 plus	Data Rich	1	Tobacco (cigarettes per capita)
Fire, heat, and hot substances	Female	Early Neonatal	80 plus	Global	-1	Education (years per capita)
Fire, heat, and hot substances	Female	Early Neonatal	80 plus	Global	-1	Health System Access 2 (unitless)
Fire, heat, and hot substances	Female	Early Neonatal	80 plus	Global	-1	Sociodemographic Status
Fire, heat, and hot substances	Female	Early Neonatal	80 plus	Global	0	LDI (I$ per capita)
Fire, heat, and hot substances	Female	Early Neonatal	80 plus	Global	0	Population Density (over 1000 ppl/sqkm, proportion)
Fire, heat, and hot substances	Female	Early Neonatal	80 plus	Global	1	Alcohol (litres per capita)
Fire, heat, and hot substances	Female	Early Neonatal	80 plus	Global	1	Indoor Air Pollution (Biomass Cooking)
Fire, heat, and hot substances	Female	Early Neonatal	80 plus	Global	1	Log-transformed SEV scalar: Fire
Fire, heat, and hot substances	Female	Early Neonatal	80 plus	Global	1	Tobacco (cigarettes per capita)
Fire, heat, and hot substances	Female	Early Neonatal	80 plus	Data Rich	-1	Education (years per capita)
Fire, heat, and hot substances	Female	Early Neonatal	80 plus	Data Rich	-1	Health System Access 2 (unitless)
Fire, heat, and hot substances	Female	Early Neonatal	80 plus	Data Rich	-1	Sociodemographic Status
Fire, heat, and hot substances	Female	Early Neonatal	80 plus	Data Rich	0	LDI (I$ per capita)
Fire, heat, and hot substances	Female	Early Neonatal	80 plus	Data Rich	0	Population Density (over 1000 ppl/sqkm, proportion)
Fire, heat, and hot substances	Female	Early Neonatal	80 plus	Data Rich	1	Alcohol (litres per capita)
Fire, heat, and hot substances	Female	Early Neonatal	80 plus	Data Rich	1	Indoor Air Pollution (Biomass Cooking)
Fire, heat, and hot substances	Female	Early Neonatal	80 plus	Data Rich	1	Log-transformed SEV scalar: Fire
Fire, heat, and hot substances	Female	Early Neonatal	80 plus	Data Rich	1	Tobacco (cigarettes per capita)
Poisonings	Male	Early Neonatal	80 plus	Global	-1	Education (years per capita)
Poisonings	Male	Early Neonatal	80 plus	Global	-1	Health System Access 2 (unitless)
Poisonings	Male	Early Neonatal	80 plus	Global	-1	Sociodemographic Status
Poisonings	Male	Early Neonatal	80 plus	Global	0	LDI (I$ per capita)
Poisonings	Male	Early Neonatal	80 plus	Global	0	Population Density (over 1000 ppl/sqkm, proportion)
Poisonings	Male	Early Neonatal	80 plus	Global	0	Population Density (under 150 ppl/sqkm, proportion)
Poisonings	Male	Early Neonatal	80 plus	Global	1	Log-transformed SEV scalar: Poison
Poisonings	Male	Early Neonatal	80 plus	Global	1	Opium Cultivation (binary)
Poisonings	Male	Early Neonatal	80 plus	Data Rich	-1	Education (years per capita)
Poisonings	Male	Early Neonatal	80 plus	Data Rich	-1	Health System Access 2 (unitless)
Poisonings	Male	Early Neonatal	80 plus	Data Rich	-1	Sociodemographic Status

Appendix Table 10: CODEm covariates used, level of covariate, and expected direction of covariate by cause, sex, and age						
**Cause**	**Sex**	**Age start**	**Age end**	**Model type**	**Direction**	**Covariate**
Poisonings	Male	Early Neonatal	80 plus	Data Rich	0	LDI (I$ per capita)
Poisonings	Male	Early Neonatal	80 plus	Data Rich	0	Population Density (over 1000 ppl/sqkm, proportion)
Poisonings	Male	Early Neonatal	80 plus	Data Rich	0	Population Density (under 150 ppl/sqkm, proportion)
Poisonings	Male	Early Neonatal	80 plus	Data Rich	1	Log-transformed SEV scalar: Poison
Poisonings	Male	Early Neonatal	80 plus	Data Rich	1	Opium Cultivation (binary)
Poisonings	Female	Early Neonatal	80 plus	Global	-1	Education (years per capita)
Poisonings	Female	Early Neonatal	80 plus	Global	-1	Health System Access 2 (unitless)
Poisonings	Female	Early Neonatal	80 plus	Global	-1	Sociodemographic Status
Poisonings	Female	Early Neonatal	80 plus	Global	0	LDI (I$ per capita)
Poisonings	Female	Early Neonatal	80 plus	Global	0	Population Density (over 1000 ppl/sqkm, proportion)
Poisonings	Female	Early Neonatal	80 plus	Global	0	Population Density (under 150 ppl/sqkm, proportion)
Poisonings	Female	Early Neonatal	80 plus	Global	1	Log-transformed SEV scalar: Poison
Poisonings	Female	Early Neonatal	80 plus	Global	1	Opium Cultivation (binary)
Poisonings	Female	Early Neonatal	80 plus	Data Rich	-1	Education (years per capita)
Poisonings	Female	Early Neonatal	80 plus	Data Rich	-1	Health System Access 2 (unitless)
Poisonings	Female	Early Neonatal	80 plus	Data Rich	-1	Sociodemographic Status
Poisonings	Female	Early Neonatal	80 plus	Data Rich	0	LDI (I$ per capita)
Poisonings	Female	Early Neonatal	80 plus	Data Rich	0	Population Density (over 1000 ppl/sqkm, proportion)
Poisonings	Female	Early Neonatal	80 plus	Data Rich	0	Population Density (under 150 ppl/sqkm, proportion)
Poisonings	Female	Early Neonatal	80 plus	Data Rich	1	Log-transformed SEV scalar: Poison
Poisonings	Female	Early Neonatal	80 plus	Data Rich	1	Opium Cultivation (binary)
Unintentional firearm injuries	Male	Early Neonatal	80 plus	Global	-1	Education (years per capita)
Unintentional firearm injuries	Male	Early Neonatal	80 plus	Global	-1	Health System Access (unitless)
Unintentional firearm injuries	Male	Early Neonatal	80 plus	Global	-1	Sociodemographic Status
Unintentional firearm injuries	Male	Early Neonatal	80 plus	Global	0	LDI (I$ per capita)
Unintentional firearm injuries	Male	Early Neonatal	80 plus	Global	0	Population Density (over 1000 ppl/sqkm, proportion)
Unintentional firearm injuries	Male	Early Neonatal	80 plus	Global	0	Population Density (under 150 ppl/sqkm, proportion)
Unintentional firearm injuries	Male	Early Neonatal	80 plus	Global	1	Alcohol (litres per capita)
Unintentional firearm injuries	Male	Early Neonatal	80 plus	Global	1	Log-transformed SEV scalar: Mech Gun
Unintentional firearm injuries	Male	Early Neonatal	80 plus	Data Rich	-1	Education (years per capita)
Unintentional firearm injuries	Male	Early Neonatal	80 plus	Data Rich	-1	Health System Access (unitless)
Unintentional firearm injuries	Male	Early Neonatal	80 plus	Data Rich	-1	Sociodemographic Status
Unintentional firearm injuries	Male	Early Neonatal	80 plus	Data Rich	0	LDI (I$ per capita)
Unintentional firearm injuries	Male	Early Neonatal	80 plus	Data Rich	0	Population Density (over 1000 ppl/sqkm, proportion)
Unintentional firearm injuries	Male	Early Neonatal	80 plus	Data Rich	0	Population Density (under 150 ppl/sqkm, proportion)
Unintentional firearm injuries	Male	Early Neonatal	80 plus	Data Rich	1	Alcohol (litres per capita)
Unintentional firearm injuries	Male	Early Neonatal	80 plus	Data Rich	1	Log-transformed SEV scalar: Mech Gun
Unintentional firearm injuries	Female	Early Neonatal	80 plus	Global	-1	Education (years per capita)
Unintentional firearm injuries	Female	Early Neonatal	80 plus	Global	-1	Health System Access (unitless)
Unintentional firearm injuries	Female	Early Neonatal	80 plus	Global	-1	Sociodemographic Status
Unintentional firearm injuries	Female	Early Neonatal	80 plus	Global	0	LDI (I$ per capita)
Unintentional firearm injuries	Female	Early Neonatal	80 plus	Global	0	Population Density (over 1000 ppl/sqkm, proportion)
Unintentional firearm injuries	Female	Early Neonatal	80 plus	Global	0	Population Density (under 150 ppl/sqkm, proportion)
Unintentional firearm injuries	Female	Early Neonatal	80 plus	Global	1	Alcohol (litres per capita)

Appendix Table 10: CODEm covariates used, level of covariate, and expected direction of covariate by cause, sex, and age						
**Cause**	**Sex**	**Age start**	**Age end**	**Model type**	**Direction**	**Covariate**
Unintentional firearm injuries	Female	Early Neonatal	80 plus	Global	1	Log-transformed SEV scalar: Mech Gun
Unintentional firearm injuries	Female	Early Neonatal	80 plus	Data Rich	-1	Education (years per capita)
Unintentional firearm injuries	Female	Early Neonatal	80 plus	Data Rich	-1	Health System Access (unitless)
Unintentional firearm injuries	Female	Early Neonatal	80 plus	Data Rich	-1	Sociodemographic Status
Unintentional firearm injuries	Female	Early Neonatal	80 plus	Data Rich	0	LDI (I$ per capita)
Unintentional firearm injuries	Female	Early Neonatal	80 plus	Data Rich	0	Population Density (over 1000 ppl/sqkm, proportion)
Unintentional firearm injuries	Female	Early Neonatal	80 plus	Data Rich	0	Population Density (under 150 ppl/sqkm, proportion)
Unintentional firearm injuries	Female	Early Neonatal	80 plus	Data Rich	1	Alcohol (litres per capita)
Unintentional firearm injuries	Female	Early Neonatal	80 plus	Data Rich	1	Log-transformed SEV scalar: Mech Gun
Unintentional suffocation	Male	Early Neonatal	80 plus	Global	-1	Education (years per capita)
Unintentional suffocation	Male	Early Neonatal	80 plus	Global	-1	Health System Access 2 (unitless)
Unintentional suffocation	Male	Early Neonatal	80 plus	Global	0	LDI (I$ per capita)
Unintentional suffocation	Male	Early Neonatal	80 plus	Global	0	Population Density (over 1000 ppl/sqkm, proportion)
Unintentional suffocation	Male	Early Neonatal	80 plus	Global	0	Population Density (under 150 ppl/sqkm, proportion)
Unintentional suffocation	Male	Early Neonatal	80 plus	Global	0	Sociodemographic Status
Unintentional suffocation	Male	Early Neonatal	80 plus	Global	1	Alcohol (litres per capita)
Unintentional suffocation	Male	Early Neonatal	80 plus	Global	1	Log-transformed SEV scalar: Mech Suff
Unintentional suffocation	Male	Early Neonatal	80 plus	Data Rich	-1	Education (years per capita)
Unintentional suffocation	Male	Early Neonatal	80 plus	Data Rich	-1	Health System Access 2 (unitless)
Unintentional suffocation	Male	Early Neonatal	80 plus	Data Rich	0	LDI (I$ per capita)
Unintentional suffocation	Male	Early Neonatal	80 plus	Data Rich	0	Population Density (over 1000 ppl/sqkm, proportion)
Unintentional suffocation	Male	Early Neonatal	80 plus	Data Rich	0	Population Density (under 150 ppl/sqkm, proportion)
Unintentional suffocation	Male	Early Neonatal	80 plus	Data Rich	0	Sociodemographic Status
Unintentional suffocation	Male	Early Neonatal	80 plus	Data Rich	1	Alcohol (litres per capita)
Unintentional suffocation	Male	Early Neonatal	80 plus	Data Rich	1	Log-transformed SEV scalar: Mech Suff
Unintentional suffocation	Female	Early Neonatal	80 plus	Global	-1	Education (years per capita)
Unintentional suffocation	Female	Early Neonatal	80 plus	Global	-1	Health System Access 2 (unitless)
Unintentional suffocation	Female	Early Neonatal	80 plus	Global	0	LDI (I$ per capita)
Unintentional suffocation	Female	Early Neonatal	80 plus	Global	0	Population Density (over 1000 ppl/sqkm, proportion)
Unintentional suffocation	Female	Early Neonatal	80 plus	Global	0	Population Density (under 150 ppl/sqkm, proportion)
Unintentional suffocation	Female	Early Neonatal	80 plus	Global	0	Sociodemographic Status
Unintentional suffocation	Female	Early Neonatal	80 plus	Global	1	Alcohol (litres per capita)
Unintentional suffocation	Female	Early Neonatal	80 plus	Global	1	Log-transformed SEV scalar: Mech Suff
Unintentional suffocation	Female	Early Neonatal	80 plus	Data Rich	-1	Education (years per capita)
Unintentional suffocation	Female	Early Neonatal	80 plus	Data Rich	-1	Health System Access 2 (unitless)
Unintentional suffocation	Female	Early Neonatal	80 plus	Data Rich	0	LDI (I$ per capita)
Unintentional suffocation	Female	Early Neonatal	80 plus	Data Rich	0	Population Density (over 1000 ppl/sqkm, proportion)
Unintentional suffocation	Female	Early Neonatal	80 plus	Data Rich	0	Population Density (under 150 ppl/sqkm, proportion)
Unintentional suffocation	Female	Early Neonatal	80 plus	Data Rich	0	Sociodemographic Status
Unintentional suffocation	Female	Early Neonatal	80 plus	Data Rich	1	Alcohol (litres per capita)
Unintentional suffocation	Female	Early Neonatal	80 plus	Data Rich	1	Log-transformed SEV scalar: Mech Suff
Other exposure to mechanical forces	Male	Early Neonatal	80 plus	Global	-1	Education (years per capita)
Other exposure to mechanical forces	Male	Early Neonatal	80 plus	Global	-1	Health System Access (unitless)
Other exposure to mechanical forces	Male	Early Neonatal	80 plus	Global	-1	Sociodemographic Status

Appendix Table 10: CODEm covariates used, level of covariate, and expected direction of covariate by cause, sex, and age						
Cause	Sex	Age start	Age end	Model type	Direction	Covariate
Other exposure to mechanical forces	Male	Early Neonatal	80 plus	Global	0	LDI (I$ per capita)
Other exposure to mechanical forces	Male	Early Neonatal	80 plus	Global	0	Population Density (over 1000 ppl/sqkm, proportion)
Other exposure to mechanical forces	Male	Early Neonatal	80 plus	Global	0	Population Density (under 150 ppl/sqkm, proportion)
Other exposure to mechanical forces	Male	Early Neonatal	80 plus	Global	1	Alcohol (litres per capita)
Other exposure to mechanical forces	Male	Early Neonatal	80 plus	Global	1	Log-transformed SEV scalar: Oth Mech
Other exposure to mechanical forces	Male	Early Neonatal	80 plus	Data Rich	-1	Education (years per capita)
Other exposure to mechanical forces	Male	Early Neonatal	80 plus	Data Rich	-1	Health System Access (unitless)
Other exposure to mechanical forces	Male	Early Neonatal	80 plus	Data Rich	-1	Sociodemographic Status
Other exposure to mechanical forces	Male	Early Neonatal	80 plus	Data Rich	0	LDI (I$ per capita)
Other exposure to mechanical forces	Male	Early Neonatal	80 plus	Data Rich	0	Population Density (over 1000 ppl/sqkm, proportion)
Other exposure to mechanical forces	Male	Early Neonatal	80 plus	Data Rich	0	Population Density (under 150 ppl/sqkm, proportion)
Other exposure to mechanical forces	Male	Early Neonatal	80 plus	Data Rich	1	Alcohol (litres per capita)
Other exposure to mechanical forces	Male	Early Neonatal	80 plus	Data Rich	1	Log-transformed SEV scalar: Oth Mech
Other exposure to mechanical forces	Female	Early Neonatal	80 plus	Global	-1	Education (years per capita)
Other exposure to mechanical forces	Female	Early Neonatal	80 plus	Global	-1	Health System Access (unitless)
Other exposure to mechanical forces	Female	Early Neonatal	80 plus	Global	-1	Sociodemographic Status
Other exposure to mechanical forces	Female	Early Neonatal	80 plus	Global	0	LDI (I$ per capita)
Other exposure to mechanical forces	Female	Early Neonatal	80 plus	Global	0	Population Density (over 1000 ppl/sqkm, proportion)
Other exposure to mechanical forces	Female	Early Neonatal	80 plus	Global	0	Population Density (under 150 ppl/sqkm, proportion)
Other exposure to mechanical forces	Female	Early Neonatal	80 plus	Global	1	Alcohol (litres per capita)
Other exposure to mechanical forces	Female	Early Neonatal	80 plus	Global	1	Log-transformed SEV scalar: Oth Mech
Other exposure to mechanical forces	Female	Early Neonatal	80 plus	Data Rich	-1	Education (years per capita)
Other exposure to mechanical forces	Female	Early Neonatal	80 plus	Data Rich	-1	Health System Access (unitless)
Other exposure to mechanical forces	Female	Early Neonatal	80 plus	Data Rich	-1	Sociodemographic Status
Other exposure to mechanical forces	Female	Early Neonatal	80 plus	Data Rich	0	LDI (I$ per capita)
Other exposure to mechanical forces	Female	Early Neonatal	80 plus	Data Rich	0	Population Density (over 1000 ppl/sqkm, proportion)
Other exposure to mechanical forces	Female	Early Neonatal	80 plus	Data Rich	0	Population Density (under 150 ppl/sqkm, proportion)
Other exposure to mechanical forces	Female	Early Neonatal	80 plus	Data Rich	1	Alcohol (litres per capita)
Other exposure to mechanical forces	Female	Early Neonatal	80 plus	Data Rich	1	Log-transformed SEV scalar: Oth Mech
Adverse effects of medical treatment	Male	Early Neonatal	80 plus	Global	0	Health System Access 2 (unitless)
Adverse effects of medical treatment	Male	Early Neonatal	80 plus	Global	0	LDI (I$ per capita)
Adverse effects of medical treatment	Male	Early Neonatal	80 plus	Global	0	Sociodemographic Status
Adverse effects of medical treatment	Male	Early Neonatal	80 plus	Data Rich	0	Health System Access 2 (unitless)
Adverse effects of medical treatment	Male	Early Neonatal	80 plus	Data Rich	0	LDI (I$ per capita)
Adverse effects of medical treatment	Male	Early Neonatal	80 plus	Data Rich	0	Sociodemographic Status
Adverse effects of medical treatment	Female	Early Neonatal	80 plus	Global	0	Health System Access 2 (unitless)
Adverse effects of medical treatment	Female	Early Neonatal	80 plus	Global	0	LDI (I$ per capita)
Adverse effects of medical treatment	Female	Early Neonatal	80 plus	Global	0	Sociodemographic Status
Adverse effects of medical treatment	Female	Early Neonatal	80 plus	Data Rich	0	Health System Access 2 (unitless)
Adverse effects of medical treatment	Female	Early Neonatal	80 plus	Data Rich	0	LDI (I$ per capita)
Adverse effects of medical treatment	Female	Early Neonatal	80 plus	Data Rich	0	Sociodemographic Status
Animal contact	Male	Early Neonatal	80 plus	Global	-1	Education (years per capita)
Animal contact	Male	Early Neonatal	80 plus	Global	-1	Health System Access 2 (unitless)
Animal contact	Male	Early Neonatal	80 plus	Global	-1	Sociodemographic Status

Appendix Table 10: CODEm covariates used, level of covariate, and expected direction of covariate by cause, sex, and age						
**Cause**	**Sex**	**Age start**	**Age end**	**Model type**	**Direction**	**Covariate**
Animal contact	Male	Early Neonatal	80 plus	Global	0	Elevation Over 1500m (proportion)
Animal contact	Male	Early Neonatal	80 plus	Global	0	Elevation Under 100m (proportion)
Animal contact	Male	Early Neonatal	80 plus	Global	0	LDI (I$ per capita)
Animal contact	Male	Early Neonatal	80 plus	Global	0	Population Density (over 1000 ppl/sqkm, proportion)
Animal contact	Male	Early Neonatal	80 plus	Global	0	Population Density (under 150 ppl/sqkm, proportion)
Animal contact	Male	Early Neonatal	80 plus	Global	0	Vehicles - 4 wheels (per capita)
Animal contact	Male	Early Neonatal	80 plus	Global	1	Alcohol (litres per capita)
Animal contact	Male	Early Neonatal	80 plus	Global	1	Log-transformed SEV scalar: Animal
Animal contact	Male	Early Neonatal	80 plus	Global	1	Population 15 to 30 (proportion)
Animal contact	Male	Early Neonatal	80 plus	Global	1	Vehicles - 2 wheels (per capita)
Animal contact	Male	Early Neonatal	80 plus	Data Rich	-1	Education (years per capita)
Animal contact	Male	Early Neonatal	80 plus	Data Rich	-1	Health System Access 2 (unitless)
Animal contact	Male	Early Neonatal	80 plus	Data Rich	-1	Sociodemographic Status
Animal contact	Male	Early Neonatal	80 plus	Data Rich	0	Elevation Over 1500m (proportion)
Animal contact	Male	Early Neonatal	80 plus	Data Rich	0	Elevation Under 100m (proportion)
Animal contact	Male	Early Neonatal	80 plus	Data Rich	0	LDI (I$ per capita)
Animal contact	Male	Early Neonatal	80 plus	Data Rich	0	Population Density (over 1000 ppl/sqkm, proportion)
Animal contact	Male	Early Neonatal	80 plus	Data Rich	0	Population Density (under 150 ppl/sqkm, proportion)
Animal contact	Male	Early Neonatal	80 plus	Data Rich	0	Vehicles - 4 wheels (per capita)
Animal contact	Male	Early Neonatal	80 plus	Data Rich	1	Alcohol (litres per capita)
Animal contact	Male	Early Neonatal	80 plus	Data Rich	1	Log-transformed SEV scalar: Animal
Animal contact	Male	Early Neonatal	80 plus	Data Rich	1	Population 15 to 30 (proportion)
Animal contact	Male	Early Neonatal	80 plus	Data Rich	1	Vehicles - 2 wheels (per capita)
Animal contact	Female	Early Neonatal	80 plus	Global	-1	Education (years per capita)
Animal contact	Female	Early Neonatal	80 plus	Global	-1	Health System Access 2 (unitless)
Animal contact	Female	Early Neonatal	80 plus	Global	-1	Sociodemographic Status
Animal contact	Female	Early Neonatal	80 plus	Global	0	Elevation Over 1500m (proportion)
Animal contact	Female	Early Neonatal	80 plus	Global	0	Elevation Under 100m (proportion)
Animal contact	Female	Early Neonatal	80 plus	Global	0	LDI (I$ per capita)
Animal contact	Female	Early Neonatal	80 plus	Global	0	Population Density (over 1000 ppl/sqkm, proportion)
Animal contact	Female	Early Neonatal	80 plus	Global	0	Population Density (under 150 ppl/sqkm, proportion)
Animal contact	Female	Early Neonatal	80 plus	Global	0	Vehicles - 4 wheels (per capita)
Animal contact	Female	Early Neonatal	80 plus	Global	1	Alcohol (litres per capita)
Animal contact	Female	Early Neonatal	80 plus	Global	1	Log-transformed SEV scalar: Animal
Animal contact	Female	Early Neonatal	80 plus	Global	1	Population 15 to 30 (proportion)
Animal contact	Female	Early Neonatal	80 plus	Global	1	Vehicles - 2 wheels (per capita)
Animal contact	Female	Early Neonatal	80 plus	Data Rich	-1	Education (years per capita)
Animal contact	Female	Early Neonatal	80 plus	Data Rich	-1	Health System Access 2 (unitless)
Animal contact	Female	Early Neonatal	80 plus	Data Rich	-1	Sociodemographic Status
Animal contact	Female	Early Neonatal	80 plus	Data Rich	0	Elevation Over 1500m (proportion)
Animal contact	Female	Early Neonatal	80 plus	Data Rich	0	Elevation Under 100m (proportion)
Animal contact	Female	Early Neonatal	80 plus	Data Rich	0	LDI (I$ per capita)
Animal contact	Female	Early Neonatal	80 plus	Data Rich	0	Population Density (over 1000 ppl/sqkm, proportion)
Animal contact	Female	Early Neonatal	80 plus	Data Rich	0	Population Density (under 150 ppl/sqkm, proportion)

Appendix Table 10: CODEm covariates used, level of covariate, and expected direction of covariate by cause, sex, and age						
**Cause**	**Sex**	**Age start**	**Age end**	**Model type**	**Direction**	**Covariate**
Animal contact	Female	Early Neonatal	80 plus	Data Rich	0	Vehicles - 4 wheels (per capita)
Animal contact	Female	Early Neonatal	80 plus	Data Rich	1	Alcohol (litres per capita)
Animal contact	Female	Early Neonatal	80 plus	Data Rich	1	Log-transformed SEV scalar: Animal
Animal contact	Female	Early Neonatal	80 plus	Data Rich	1	Population 15 to 30 (proportion)
Animal contact	Female	Early Neonatal	80 plus	Data Rich	1	Vehicles - 2 wheels (per capita)
Venomous animal contact	Male	Early Neonatal	80 plus	Global	-1	Education (years per capita)
Venomous animal contact	Male	Early Neonatal	80 plus	Global	-1	Health System Access 2 (unitless)
Venomous animal contact	Male	Early Neonatal	80 plus	Global	-1	Sociodemographic Status
Venomous animal contact	Male	Early Neonatal	80 plus	Global	0	Elevation Over 1500m (proportion)
Venomous animal contact	Male	Early Neonatal	80 plus	Global	0	Elevation Under 100m (proportion)
Venomous animal contact	Male	Early Neonatal	80 plus	Global	0	LDI (I$ per capita)
Venomous animal contact	Male	Early Neonatal	80 plus	Global	0	Population Density (over 1000 ppl/sqkm, proportion)
Venomous animal contact	Male	Early Neonatal	80 plus	Global	0	Population Density (under 150 ppl/sqkm, proportion)
Venomous animal contact	Male	Early Neonatal	80 plus	Global	0	Vehicles - 4 wheels (per capita)
Venomous animal contact	Male	Early Neonatal	80 plus	Global	1	Alcohol (litres per capita)
Venomous animal contact	Male	Early Neonatal	80 plus	Global	1	Log-transformed SEV scalar: Venom
Venomous animal contact	Male	Early Neonatal	80 plus	Global	1	Vehicles - 2 wheels (per capita)
Venomous animal contact	Male	Early Neonatal	80 plus	Data Rich	-1	Education (years per capita)
Venomous animal contact	Male	Early Neonatal	80 plus	Data Rich	-1	Health System Access 2 (unitless)
Venomous animal contact	Male	Early Neonatal	80 plus	Data Rich	-1	Sociodemographic Status
Venomous animal contact	Male	Early Neonatal	80 plus	Data Rich	0	Elevation Over 1500m (proportion)
Venomous animal contact	Male	Early Neonatal	80 plus	Data Rich	0	Elevation Under 100m (proportion)
Venomous animal contact	Male	Early Neonatal	80 plus	Data Rich	0	LDI (I$ per capita)
Venomous animal contact	Male	Early Neonatal	80 plus	Data Rich	0	Population Density (over 1000 ppl/sqkm, proportion)
Venomous animal contact	Male	Early Neonatal	80 plus	Data Rich	0	Population Density (under 150 ppl/sqkm, proportion)
Venomous animal contact	Male	Early Neonatal	80 plus	Data Rich	0	Vehicles - 4 wheels (per capita)
Venomous animal contact	Male	Early Neonatal	80 plus	Data Rich	1	Alcohol (litres per capita)
Venomous animal contact	Male	Early Neonatal	80 plus	Data Rich	1	Log-transformed SEV scalar: Venom
Venomous animal contact	Male	Early Neonatal	80 plus	Data Rich	1	Vehicles - 2 wheels (per capita)
Venomous animal contact	Female	Early Neonatal	80 plus	Global	-1	Education (years per capita)
Venomous animal contact	Female	Early Neonatal	80 plus	Global	-1	Health System Access 2 (unitless)
Venomous animal contact	Female	Early Neonatal	80 plus	Global	-1	Sociodemographic Status
Venomous animal contact	Female	Early Neonatal	80 plus	Global	0	Elevation Over 1500m (proportion)
Venomous animal contact	Female	Early Neonatal	80 plus	Global	0	Elevation Under 100m (proportion)
Venomous animal contact	Female	Early Neonatal	80 plus	Global	0	LDI (I$ per capita)
Venomous animal contact	Female	Early Neonatal	80 plus	Global	0	Population Density (over 1000 ppl/sqkm, proportion)
Venomous animal contact	Female	Early Neonatal	80 plus	Global	0	Population Density (under 150 ppl/sqkm, proportion)
Venomous animal contact	Female	Early Neonatal	80 plus	Global	0	Vehicles - 4 wheels (per capita)
Venomous animal contact	Female	Early Neonatal	80 plus	Global	1	Alcohol (litres per capita)
Venomous animal contact	Female	Early Neonatal	80 plus	Global	1	Log-transformed SEV scalar: Venom
Venomous animal contact	Female	Early Neonatal	80 plus	Global	1	Vehicles - 2 wheels (per capita)
Venomous animal contact	Female	Early Neonatal	80 plus	Data Rich	-1	Education (years per capita)
Venomous animal contact	Female	Early Neonatal	80 plus	Data Rich	-1	Health System Access 2 (unitless)
Venomous animal contact	Female	Early Neonatal	80 plus	Data Rich	-1	Sociodemographic Status

Appendix Table 10: CODEm covariates used, level of covariate, and expected direction of covariate by cause, sex, and age						
**Cause**	**Sex**	**Age start**	**Age end**	**Model type**	**Direction**	**Covariate**
Venomous animal contact	Female	Early Neonatal	80 plus	Data Rich	0	Elevation Over 1500m (proportion)
Venomous animal contact	Female	Early Neonatal	80 plus	Data Rich	0	Elevation Under 100m (proportion)
Venomous animal contact	Female	Early Neonatal	80 plus	Data Rich	0	LDI (I$ per capita)
Venomous animal contact	Female	Early Neonatal	80 plus	Data Rich	0	Population Density (over 1000 ppl/sqkm, proportion)
Venomous animal contact	Female	Early Neonatal	80 plus	Data Rich	0	Population Density (under 150 ppl/sqkm, proportion)
Venomous animal contact	Female	Early Neonatal	80 plus	Data Rich	0	Vehicles - 4 wheels (per capita)
Venomous animal contact	Female	Early Neonatal	80 plus	Data Rich	1	Alcohol (litres per capita)
Venomous animal contact	Female	Early Neonatal	80 plus	Data Rich	1	Log-transformed SEV scalar: Venom
Venomous animal contact	Female	Early Neonatal	80 plus	Data Rich	1	Vehicles - 2 wheels (per capita)
Non-venomous animal contact	Male	Early Neonatal	80 plus	Global	-1	Education (years per capita)
Non-venomous animal contact	Male	Early Neonatal	80 plus	Global	-1	Health System Access 2 (unitless)
Non-venomous animal contact	Male	Early Neonatal	80 plus	Global	-1	Sociodemographic Status
Non-venomous animal contact	Male	Early Neonatal	80 plus	Global	0	Elevation Over 1500m (proportion)
Non-venomous animal contact	Male	Early Neonatal	80 plus	Global	0	Elevation Under 100m (proportion)
Non-venomous animal contact	Male	Early Neonatal	80 plus	Global	0	LDI (I$ per capita)
Non-venomous animal contact	Male	Early Neonatal	80 plus	Global	0	Population Density (over 1000 ppl/sqkm, proportion)
Non-venomous animal contact	Male	Early Neonatal	80 plus	Global	0	Population Density (under 150 ppl/sqkm, proportion)
Non-venomous animal contact	Male	Early Neonatal	80 plus	Global	0	Vehicles - 4 wheels (per capita)
Non-venomous animal contact	Male	Early Neonatal	80 plus	Global	1	Alcohol (litres per capita)
Non-venomous animal contact	Male	Early Neonatal	80 plus	Global	1	Log-transformed SEV scalar: Non Ven
Non-venomous animal contact	Male	Early Neonatal	80 plus	Global	1	Vehicles - 2 wheels (per capita)
Non-venomous animal contact	Male	Early Neonatal	80 plus	Data Rich	-1	Education (years per capita)
Non-venomous animal contact	Male	Early Neonatal	80 plus	Data Rich	-1	Health System Access 2 (unitless)
Non-venomous animal contact	Male	Early Neonatal	80 plus	Data Rich	-1	Sociodemographic Status
Non-venomous animal contact	Male	Early Neonatal	80 plus	Data Rich	0	Elevation Over 1500m (proportion)
Non-venomous animal contact	Male	Early Neonatal	80 plus	Data Rich	0	Elevation Under 100m (proportion)
Non-venomous animal contact	Male	Early Neonatal	80 plus	Data Rich	0	LDI (I$ per capita)
Non-venomous animal contact	Male	Early Neonatal	80 plus	Data Rich	0	Population Density (over 1000 ppl/sqkm, proportion)
Non-venomous animal contact	Male	Early Neonatal	80 plus	Data Rich	0	Population Density (under 150 ppl/sqkm, proportion)
Non-venomous animal contact	Male	Early Neonatal	80 plus	Data Rich	0	Vehicles - 4 wheels (per capita)
Non-venomous animal contact	Male	Early Neonatal	80 plus	Data Rich	1	Alcohol (litres per capita)
Non-venomous animal contact	Male	Early Neonatal	80 plus	Data Rich	1	Log-transformed SEV scalar: Non Ven
Non-venomous animal contact	Male	Early Neonatal	80 plus	Data Rich	1	Vehicles - 2 wheels (per capita)
Non-venomous animal contact	Female	Early Neonatal	80 plus	Global	-1	Education (years per capita)
Non-venomous animal contact	Female	Early Neonatal	80 plus	Global	-1	Health System Access 2 (unitless)
Non-venomous animal contact	Female	Early Neonatal	80 plus	Global	-1	Sociodemographic Status
Non-venomous animal contact	Female	Early Neonatal	80 plus	Global	0	Elevation Over 1500m (proportion)
Non-venomous animal contact	Female	Early Neonatal	80 plus	Global	0	Elevation Under 100m (proportion)
Non-venomous animal contact	Female	Early Neonatal	80 plus	Global	0	LDI (I$ per capita)
Non-venomous animal contact	Female	Early Neonatal	80 plus	Global	0	Population Density (over 1000 ppl/sqkm, proportion)
Non-venomous animal contact	Female	Early Neonatal	80 plus	Global	0	Population Density (under 150 ppl/sqkm, proportion)
Non-venomous animal contact	Female	Early Neonatal	80 plus	Global	0	Vehicles - 4 wheels (per capita)
Non-venomous animal contact	Female	Early Neonatal	80 plus	Global	1	Alcohol (litres per capita)
Non-venomous animal contact	Female	Early Neonatal	80 plus	Global	1	Log-transformed SEV scalar: Non Ven

Appendix Table 10: CODEm covariates used, level of covariate, and expected direction of covariate by cause, sex, and age						
**Cause**	**Sex**	**Age start**	**Age end**	**Model type**	**Direction**	**Covariate**
Non-venomous animal contact	Female	Early Neonatal	80 plus	Global	1	Vehicles - 2 wheels (per capita)
Non-venomous animal contact	Female	Early Neonatal	80 plus	Data Rich	-1	Education (years per capita)
Non-venomous animal contact	Female	Early Neonatal	80 plus	Data Rich	-1	Health System Access 2 (unitless)
Non-venomous animal contact	Female	Early Neonatal	80 plus	Data Rich	-1	Sociodemographic Status
Non-venomous animal contact	Female	Early Neonatal	80 plus	Data Rich	0	Elevation Over 1500m (proportion)
Non-venomous animal contact	Female	Early Neonatal	80 plus	Data Rich	0	Elevation Under 100m (proportion)
Non-venomous animal contact	Female	Early Neonatal	80 plus	Data Rich	0	LDI (I$ per capita)
Non-venomous animal contact	Female	Early Neonatal	80 plus	Data Rich	0	Population Density (over 1000 ppl/sqkm, proportion)
Non-venomous animal contact	Female	Early Neonatal	80 plus	Data Rich	0	Population Density (under 150 ppl/sqkm, proportion)
Non-venomous animal contact	Female	Early Neonatal	80 plus	Data Rich	0	Vehicles - 4 wheels (per capita)
Non-venomous animal contact	Female	Early Neonatal	80 plus	Data Rich	1	Alcohol (litres per capita)
Non-venomous animal contact	Female	Early Neonatal	80 plus	Data Rich	1	Log-transformed SEV scalar: Non Ven
Non-venomous animal contact	Female	Early Neonatal	80 plus	Data Rich	1	Vehicles - 2 wheels (per capita)
Pulmonary aspiration and foreign body in airway	Male	Early Neonatal	80 plus	Global	-1	Health System Access 2 (unitless)
Pulmonary aspiration and foreign body in airway	Male	Early Neonatal	80 plus	Global	0	LDI (I$ per capita)
Pulmonary aspiration and foreign body in airway	Male	Early Neonatal	80 plus	Global	0	Sociodemographic Status
Pulmonary aspiration and foreign body in airway	Male	Early Neonatal	80 plus	Global	1	Alcohol (litres per capita)
Pulmonary aspiration and foreign body in airway	Male	Early Neonatal	80 plus	Global	1	Log-transformed SEV scalar: F Body Asp
Pulmonary aspiration and foreign body in airway	Male	Early Neonatal	80 plus	Global	1	Mean BMI
Pulmonary aspiration and foreign body in airway	Male	Early Neonatal	80 plus	Data Rich	-1	Health System Access 2 (unitless)
Pulmonary aspiration and foreign body in airway	Male	Early Neonatal	80 plus	Data Rich	0	LDI (I$ per capita)
Pulmonary aspiration and foreign body in airway	Male	Early Neonatal	80 plus	Data Rich	0	Sociodemographic Status
Pulmonary aspiration and foreign body in airway	Male	Early Neonatal	80 plus	Data Rich	1	Alcohol (litres per capita)
Pulmonary aspiration and foreign body in airway	Male	Early Neonatal	80 plus	Data Rich	1	Log-transformed SEV scalar: F Body Asp
Pulmonary aspiration and foreign body in airway	Male	Early Neonatal	80 plus	Data Rich	1	Mean BMI
Pulmonary aspiration and foreign body in airway	Female	Early Neonatal	80 plus	Global	-1	Health System Access 2 (unitless)
Pulmonary aspiration and foreign body in airway	Female	Early Neonatal	80 plus	Global	0	LDI (I$ per capita)
Pulmonary aspiration and foreign body in airway	Female	Early Neonatal	80 plus	Global	0	Sociodemographic Status
Pulmonary aspiration and foreign body in airway	Female	Early Neonatal	80 plus	Global	1	Alcohol (litres per capita)
Pulmonary aspiration and foreign body in airway	Female	Early Neonatal	80 plus	Global	1	Log-transformed SEV scalar: F Body Asp
Pulmonary aspiration and foreign body in airway	Female	Early Neonatal	80 plus	Global	1	Mean BMI
Pulmonary aspiration and foreign body in airway	Female	Early Neonatal	80 plus	Data Rich	-1	Health System Access 2 (unitless)
Pulmonary aspiration and foreign body in airway	Female	Early Neonatal	80 plus	Data Rich	0	LDI (I$ per capita)
Pulmonary aspiration and foreign body in airway	Female	Early Neonatal	80 plus	Data Rich	0	Sociodemographic Status
Pulmonary aspiration and foreign body in airway	Female	Early Neonatal	80 plus	Data Rich	1	Alcohol (litres per capita)
Pulmonary aspiration and foreign body in airway	Female	Early Neonatal	80 plus	Data Rich	1	Log-transformed SEV scalar: F Body Asp
Pulmonary aspiration and foreign body in airway	Female	Early Neonatal	80 plus	Data Rich	1	Mean BMI
Foreign body in other body part	Male	Early Neonatal	80 plus	Global	-1	Education (years per capita)
Foreign body in other body part	Male	Early Neonatal	80 plus	Global	-1	Health System Access 2 (unitless)
Foreign body in other body part	Male	Early Neonatal	80 plus	Global	-1	Sociodemographic Status
Foreign body in other body part	Male	Early Neonatal	80 plus	Global	0	Elevation Over 1500m (proportion)
Foreign body in other body part	Male	Early Neonatal	80 plus	Global	0	Elevation Under 100m (proportion)
Foreign body in other body part	Male	Early Neonatal	80 plus	Global	0	LDI (I$ per capita)
Foreign body in other body part	Male	Early Neonatal	80 plus	Global	0	Population Density (over 1000 ppl/sqkm, proportion)

Appendix Table 10: CODEm covariates used, level of covariate, and expected direction of covariate by cause, sex, and age						
**Cause**	**Sex**	**Age start**	**Age end**	**Model type**	**Direction**	**Covariate**
Foreign body in other body part	Male	Early Neonatal	80 plus	Global	0	Population Density (under 150 ppl/sqkm, proportion)
Foreign body in other body part	Male	Early Neonatal	80 plus	Global	1	Alcohol (litres per capita)
Foreign body in other body part	Male	Early Neonatal	80 plus	Global	1	Log-transformed SEV scalar: Oth F Body
Foreign body in other body part	Male	Early Neonatal	80 plus	Data Rich	-1	Education (years per capita)
Foreign body in other body part	Male	Early Neonatal	80 plus	Data Rich	-1	Health System Access 2 (unitless)
Foreign body in other body part	Male	Early Neonatal	80 plus	Data Rich	-1	Sociodemographic Status
Foreign body in other body part	Male	Early Neonatal	80 plus	Data Rich	0	Elevation Over 1500m (proportion)
Foreign body in other body part	Male	Early Neonatal	80 plus	Data Rich	0	Elevation Under 100m (proportion)
Foreign body in other body part	Male	Early Neonatal	80 plus	Data Rich	0	LDI (I$ per capita)
Foreign body in other body part	Male	Early Neonatal	80 plus	Data Rich	0	Population Density (over 1000 ppl/sqkm, proportion)
Foreign body in other body part	Male	Early Neonatal	80 plus	Data Rich	0	Population Density (under 150 ppl/sqkm, proportion)
Foreign body in other body part	Male	Early Neonatal	80 plus	Data Rich	1	Alcohol (litres per capita)
Foreign body in other body part	Male	Early Neonatal	80 plus	Data Rich	1	Log-transformed SEV scalar: Oth F Body
Foreign body in other body part	Female	Early Neonatal	80 plus	Global	-1	Education (years per capita)
Foreign body in other body part	Female	Early Neonatal	80 plus	Global	-1	Health System Access 2 (unitless)
Foreign body in other body part	Female	Early Neonatal	80 plus	Global	-1	Sociodemographic Status
Foreign body in other body part	Female	Early Neonatal	80 plus	Global	0	Elevation Over 1500m (proportion)
Foreign body in other body part	Female	Early Neonatal	80 plus	Global	0	Elevation Under 100m (proportion)
Foreign body in other body part	Female	Early Neonatal	80 plus	Global	0	LDI (I$ per capita)
Foreign body in other body part	Female	Early Neonatal	80 plus	Global	0	Population Density (over 1000 ppl/sqkm, proportion)
Foreign body in other body part	Female	Early Neonatal	80 plus	Global	0	Population Density (under 150 ppl/sqkm, proportion)
Foreign body in other body part	Female	Early Neonatal	80 plus	Global	1	Alcohol (litres per capita)
Foreign body in other body part	Female	Early Neonatal	80 plus	Global	1	Log-transformed SEV scalar: Oth F Body
Foreign body in other body part	Female	Early Neonatal	80 plus	Data Rich	-1	Education (years per capita)
Foreign body in other body part	Female	Early Neonatal	80 plus	Data Rich	-1	Health System Access 2 (unitless)
Foreign body in other body part	Female	Early Neonatal	80 plus	Data Rich	-1	Sociodemographic Status
Foreign body in other body part	Female	Early Neonatal	80 plus	Data Rich	0	Elevation Over 1500m (proportion)
Foreign body in other body part	Female	Early Neonatal	80 plus	Data Rich	0	Elevation Under 100m (proportion)
Foreign body in other body part	Female	Early Neonatal	80 plus	Data Rich	0	LDI (I$ per capita)
Foreign body in other body part	Female	Early Neonatal	80 plus	Data Rich	0	Population Density (over 1000 ppl/sqkm, proportion)
Foreign body in other body part	Female	Early Neonatal	80 plus	Data Rich	0	Population Density (under 150 ppl/sqkm, proportion)
Foreign body in other body part	Female	Early Neonatal	80 plus	Data Rich	1	Alcohol (litres per capita)
Foreign body in other body part	Female	Early Neonatal	80 plus	Data Rich	1	Log-transformed SEV scalar: Oth F Body
Environmental heat and cold exposure	Male	Early Neonatal	80 plus	Global	-1	Education (years per capita)
Environmental heat and cold exposure	Male	Early Neonatal	80 plus	Global	-1	Health System Access 2 (unitless)
Environmental heat and cold exposure	Male	Early Neonatal	80 plus	Global	-1	Sociodemographic Status
Environmental heat and cold exposure	Male	Early Neonatal	80 plus	Global	0	90th percentile climatic temperature in the given country-year
Environmental heat and cold exposure	Male	Early Neonatal	80 plus	Global	0	Elevation 500 to 1500m (proportion)
Environmental heat and cold exposure	Male	Early Neonatal	80 plus	Global	0	Elevation Over 1500m (proportion)
Environmental heat and cold exposure	Male	Early Neonatal	80 plus	Global	0	LDI (I$ per capita)
Environmental heat and cold exposure	Male	Early Neonatal	80 plus	Global	0	Population Density (150-300 ppl/sqkm, proportion)
Environmental heat and cold exposure	Male	Early Neonatal	80 plus	Global	0	Population-weighted mean temperature
Environmental heat and cold exposure	Male	Early Neonatal	80 plus	Global	0	Rainfall (Quintiles 4-5)
Environmental heat and cold exposure	Male	Early Neonatal	80 plus	Global	0	Sanitation (proportion with access)

Appendix Table 10: CODEm covariates used, level of covariate, and expected direction of covariate by cause, sex, and age						
**Cause**	**Sex**	**Age start**	**Age end**	**Model type**	**Direction**	**Covariate**
Environmental heat and cold exposure	Male	Early Neonatal	80 plus	Data Rich	-1	Education (years per capita)
Environmental heat and cold exposure	Male	Early Neonatal	80 plus	Data Rich	-1	Health System Access 2 (unitless)
Environmental heat and cold exposure	Male	Early Neonatal	80 plus	Data Rich	-1	Sociodemographic Status
Environmental heat and cold exposure	Male	Early Neonatal	80 plus	Data Rich	0	90th percentile climatic temperature in the given country-year
Environmental heat and cold exposure	Male	Early Neonatal	80 plus	Data Rich	0	Elevation 500 to 1500m (proportion)
Environmental heat and cold exposure	Male	Early Neonatal	80 plus	Data Rich	0	Elevation Over 1500m (proportion)
Environmental heat and cold exposure	Male	Early Neonatal	80 plus	Data Rich	0	LDI (I$ per capita)
Environmental heat and cold exposure	Male	Early Neonatal	80 plus	Data Rich	0	Population Density (150-300 ppl/sqkm, proportion)
Environmental heat and cold exposure	Male	Early Neonatal	80 plus	Data Rich	0	Population-weighted mean temperature
Environmental heat and cold exposure	Male	Early Neonatal	80 plus	Data Rich	0	Rainfall (Quintiles 4-5)
Environmental heat and cold exposure	Male	Early Neonatal	80 plus	Data Rich	0	Sanitation (proportion with access)
Environmental heat and cold exposure	Female	Early Neonatal	80 plus	Global	-1	Education (years per capita)
Environmental heat and cold exposure	Female	Early Neonatal	80 plus	Global	-1	Health System Access 2 (unitless)
Environmental heat and cold exposure	Female	Early Neonatal	80 plus	Global	-1	Sociodemographic Status
Environmental heat and cold exposure	Female	Early Neonatal	80 plus	Global	0	90th percentile climatic temperature in the given country-year
Environmental heat and cold exposure	Female	Early Neonatal	80 plus	Global	0	Elevation 500 to 1500m (proportion)
Environmental heat and cold exposure	Female	Early Neonatal	80 plus	Global	0	Elevation Over 1500m (proportion)
Environmental heat and cold exposure	Female	Early Neonatal	80 plus	Global	0	LDI (I$ per capita)
Environmental heat and cold exposure	Female	Early Neonatal	80 plus	Global	0	Population Density (150-300 ppl/sqkm, proportion)
Environmental heat and cold exposure	Female	Early Neonatal	80 plus	Global	0	Population-weighted mean temperature
Environmental heat and cold exposure	Female	Early Neonatal	80 plus	Global	0	Rainfall (Quintiles 4-5)
Environmental heat and cold exposure	Female	Early Neonatal	80 plus	Global	0	Sanitation (proportion with access)
Environmental heat and cold exposure	Female	Early Neonatal	80 plus	Data Rich	-1	Education (years per capita)
Environmental heat and cold exposure	Female	Early Neonatal	80 plus	Data Rich	-1	Health System Access 2 (unitless)
Environmental heat and cold exposure	Female	Early Neonatal	80 plus	Data Rich	-1	Sociodemographic Status
Environmental heat and cold exposure	Female	Early Neonatal	80 plus	Data Rich	0	90th percentile climatic temperature in the given country-year
Environmental heat and cold exposure	Female	Early Neonatal	80 plus	Data Rich	0	Elevation 500 to 1500m (proportion)
Environmental heat and cold exposure	Female	Early Neonatal	80 plus	Data Rich	0	Elevation Over 1500m (proportion)
Environmental heat and cold exposure	Female	Early Neonatal	80 plus	Data Rich	0	LDI (I$ per capita)
Environmental heat and cold exposure	Female	Early Neonatal	80 plus	Data Rich	0	Population Density (150-300 ppl/sqkm, proportion)
Environmental heat and cold exposure	Female	Early Neonatal	80 plus	Data Rich	0	Population-weighted mean temperature
Environmental heat and cold exposure	Female	Early Neonatal	80 plus	Data Rich	0	Rainfall (Quintiles 4-5)
Environmental heat and cold exposure	Female	Early Neonatal	80 plus	Data Rich	0	Sanitation (proportion with access)
Other unintentional injuries	Male	Early Neonatal	80 plus	Global	-1	Education (years per capita)
Other unintentional injuries	Male	Early Neonatal	80 plus	Global	-1	Health System Access 2 (unitless)
Other unintentional injuries	Male	Early Neonatal	80 plus	Global	0	Elevation Over 1500m (proportion)
Other unintentional injuries	Male	Early Neonatal	80 plus	Global	0	Elevation Under 100m (proportion)
Other unintentional injuries	Male	Early Neonatal	80 plus	Global	0	LDI (I$ per capita)
Other unintentional injuries	Male	Early Neonatal	80 plus	Global	0	Population Density (over 1000 ppl/sqkm, proportion)
Other unintentional injuries	Male	Early Neonatal	80 plus	Global	0	Population Density (under 150 ppl/sqkm, proportion)
Other unintentional injuries	Male	Early Neonatal	80 plus	Global	0	Sociodemographic Status
Other unintentional injuries	Male	Early Neonatal	80 plus	Global	0	Vehicles - 4 wheels (per capita)
Other unintentional injuries	Male	Early Neonatal	80 plus	Global	1	Alcohol (litres per capita)
Other unintentional injuries	Male	Early Neonatal	80 plus	Global	1	Log-transformed SEV scalar: Oth Unint

Appendix Table 10: CODEm covariates used, level of covariate, and expected direction of covariate by cause, sex, and age						
Cause	Sex	Age start	Age end	Model type	Direction	Covariate
Other unintentional injuries	Male	Early Neonatal	80 plus	Global	1	Vehicles - 2 wheels (per capita)
Other unintentional injuries	Male	Early Neonatal	80 plus	Data Rich	-1	Education (years per capita)
Other unintentional injuries	Male	Early Neonatal	80 plus	Data Rich	-1	Health System Access 2 (unitless)
Other unintentional injuries	Male	Early Neonatal	80 plus	Data Rich	0	Elevation Over 1500m (proportion)
Other unintentional injuries	Male	Early Neonatal	80 plus	Data Rich	0	Elevation Under 100m (proportion)
Other unintentional injuries	Male	Early Neonatal	80 plus	Data Rich	0	LDI (I$ per capita)
Other unintentional injuries	Male	Early Neonatal	80 plus	Data Rich	0	Population Density (over 1000 ppl/sqkm, proportion)
Other unintentional injuries	Male	Early Neonatal	80 plus	Data Rich	0	Population Density (under 150 ppl/sqkm, proportion)
Other unintentional injuries	Male	Early Neonatal	80 plus	Data Rich	0	Sociodemographic Status
Other unintentional injuries	Male	Early Neonatal	80 plus	Data Rich	0	Vehicles - 4 wheels (per capita)
Other unintentional injuries	Male	Early Neonatal	80 plus	Data Rich	1	Alcohol (litres per capita)
Other unintentional injuries	Male	Early Neonatal	80 plus	Data Rich	1	Log-transformed SEV scalar: Oth Unint
Other unintentional injuries	Male	Early Neonatal	80 plus	Data Rich	1	Vehicles - 2 wheels (per capita)
Other unintentional injuries	Female	Early Neonatal	80 plus	Global	-1	Education (years per capita)
Other unintentional injuries	Female	Early Neonatal	80 plus	Global	-1	Health System Access 2 (unitless)
Other unintentional injuries	Female	Early Neonatal	80 plus	Global	0	Elevation Over 1500m (proportion)
Other unintentional injuries	Female	Early Neonatal	80 plus	Global	0	Elevation Under 100m (proportion)
Other unintentional injuries	Female	Early Neonatal	80 plus	Global	0	LDI (I$ per capita)
Other unintentional injuries	Female	Early Neonatal	80 plus	Global	0	Population Density (over 1000 ppl/sqkm, proportion)
Other unintentional injuries	Female	Early Neonatal	80 plus	Global	0	Population Density (under 150 ppl/sqkm, proportion)
Other unintentional injuries	Female	Early Neonatal	80 plus	Global	0	Sociodemographic Status
Other unintentional injuries	Female	Early Neonatal	80 plus	Global	0	Vehicles - 4 wheels (per capita)
Other unintentional injuries	Female	Early Neonatal	80 plus	Global	1	Alcohol (litres per capita)
Other unintentional injuries	Female	Early Neonatal	80 plus	Global	1	Log-transformed SEV scalar: Oth Unint
Other unintentional injuries	Female	Early Neonatal	80 plus	Global	1	Vehicles - 2 wheels (per capita)
Other unintentional injuries	Female	Early Neonatal	80 plus	Data Rich	-1	Education (years per capita)
Other unintentional injuries	Female	Early Neonatal	80 plus	Data Rich	-1	Health System Access 2 (unitless)
Other unintentional injuries	Female	Early Neonatal	80 plus	Data Rich	0	Elevation Over 1500m (proportion)
Other unintentional injuries	Female	Early Neonatal	80 plus	Data Rich	0	Elevation Under 100m (proportion)
Other unintentional injuries	Female	Early Neonatal	80 plus	Data Rich	0	LDI (I$ per capita)
Other unintentional injuries	Female	Early Neonatal	80 plus	Data Rich	0	Population Density (over 1000 ppl/sqkm, proportion)
Other unintentional injuries	Female	Early Neonatal	80 plus	Data Rich	0	Population Density (under 150 ppl/sqkm, proportion)
Other unintentional injuries	Female	Early Neonatal	80 plus	Data Rich	0	Sociodemographic Status
Other unintentional injuries	Female	Early Neonatal	80 plus	Data Rich	0	Vehicles - 4 wheels (per capita)
Other unintentional injuries	Female	Early Neonatal	80 plus	Data Rich	1	Alcohol (litres per capita)
Other unintentional injuries	Female	Early Neonatal	80 plus	Data Rich	1	Log-transformed SEV scalar: Oth Unint
Other unintentional injuries	Female	Early Neonatal	80 plus	Data Rich	1	Vehicles - 2 wheels (per capita)
Self-harm	Male	10 to 14	80 plus	Global	-1	Religion (binary, >50% Muslim)
Self-harm	Male	10 to 14	80 plus	Global	0	Education (years per capita)
Self-harm	Male	10 to 14	80 plus	Global	0	LDI (I$ per capita)
Self-harm	Male	10 to 14	80 plus	Global	0	Population Density (150-300 ppl/sqkm, proportion)
Self-harm	Male	10 to 14	80 plus	Global	0	Population Density (300-500 ppl/sqkm, proportion)
Self-harm	Male	10 to 14	80 plus	Global	0	Population Density (500-1000 ppl/sqkm, proportion)
Self-harm	Male	10 to 14	80 plus	Global	0	Population Density (over 1000 ppl/sqkm, proportion)

Appendix Table 10: CODEm covariates used, level of covariate, and expected direction of covariate by cause, sex, and age						
**Cause**	**Sex**	**Age start**	**Age end**	**Model type**	**Direction**	**Covariate**
Self-harm	Male	10 to 14	80 plus	Global	0	Population Density (under 150 ppl/sqkm, proportion)
Self-harm	Male	10 to 14	80 plus	Global	0	Sociodemographic Status
Self-harm	Male	10 to 14	80 plus	Global	1	Alcohol (litres per capita)
Self-harm	Male	10 to 14	80 plus	Global	1	Log-transformed SEV scalar: Self Harm
Self-harm	Male	10 to 14	80 plus	Global	1	Major disorder from dismod interpolated to be used as covariate
Self-harm	Male	10 to 14	80 plus	Global	1	Opium Cultivation (binary)
Self-harm	Male	10 to 14	80 plus	Data Rich	-1	Religion (binary, >50% Muslim)
Self-harm	Male	10 to 14	80 plus	Data Rich	0	Education (years per capita)
Self-harm	Male	10 to 14	80 plus	Data Rich	0	LDI (I$ per capita)
Self-harm	Male	10 to 14	80 plus	Data Rich	0	Population Density (150-300 ppl/sqkm, proportion)
Self-harm	Male	10 to 14	80 plus	Data Rich	0	Population Density (300-500 ppl/sqkm, proportion)
Self-harm	Male	10 to 14	80 plus	Data Rich	0	Population Density (500-1000 ppl/sqkm, proportion)
Self-harm	Male	10 to 14	80 plus	Data Rich	0	Population Density (over 1000 ppl/sqkm, proportion)
Self-harm	Male	10 to 14	80 plus	Data Rich	0	Population Density (under 150 ppl/sqkm, proportion)
Self-harm	Male	10 to 14	80 plus	Data Rich	0	Sociodemographic Status
Self-harm	Male	10 to 14	80 plus	Data Rich	1	Alcohol (litres per capita)
Self-harm	Male	10 to 14	80 plus	Data Rich	1	Log-transformed SEV scalar: Self Harm
Self-harm	Male	10 to 14	80 plus	Data Rich	1	Major disorder from dismod interpolated to be used as covariate
Self-harm	Male	10 to 14	80 plus	Data Rich	1	Opium Cultivation (binary)
Self-harm	Female	10 to 14	80 plus	Global	-1	Religion (binary, >50% Muslim)
Self-harm	Female	10 to 14	80 plus	Global	0	Education (years per capita)
Self-harm	Female	10 to 14	80 plus	Global	0	LDI (I$ per capita)
Self-harm	Female	10 to 14	80 plus	Global	0	Population Density (150-300 ppl/sqkm, proportion)
Self-harm	Female	10 to 14	80 plus	Global	0	Population Density (300-500 ppl/sqkm, proportion)
Self-harm	Female	10 to 14	80 plus	Global	0	Population Density (500-1000 ppl/sqkm, proportion)
Self-harm	Female	10 to 14	80 plus	Global	0	Population Density (over 1000 ppl/sqkm, proportion)
Self-harm	Female	10 to 14	80 plus	Global	0	Population Density (under 150 ppl/sqkm, proportion)
Self-harm	Female	10 to 14	80 plus	Global	0	Sociodemographic Status
Self-harm	Female	10 to 14	80 plus	Global	1	Alcohol (litres per capita)
Self-harm	Female	10 to 14	80 plus	Global	1	Log-transformed SEV scalar: Self Harm
Self-harm	Female	10 to 14	80 plus	Global	1	Major disorder from dismod interpolated to be used as covariate
Self-harm	Female	10 to 14	80 plus	Global	1	Opium Cultivation (binary)
Self-harm	Female	10 to 14	80 plus	Data Rich	-1	Religion (binary, >50% Muslim)
Self-harm	Female	10 to 14	80 plus	Data Rich	0	Education (years per capita)
Self-harm	Female	10 to 14	80 plus	Data Rich	0	LDI (I$ per capita)
Self-harm	Female	10 to 14	80 plus	Data Rich	0	Population Density (150-300 ppl/sqkm, proportion)
Self-harm	Female	10 to 14	80 plus	Data Rich	0	Population Density (300-500 ppl/sqkm, proportion)
Self-harm	Female	10 to 14	80 plus	Data Rich	0	Population Density (500-1000 ppl/sqkm, proportion)
Self-harm	Female	10 to 14	80 plus	Data Rich	0	Population Density (over 1000 ppl/sqkm, proportion)
Self-harm	Female	10 to 14	80 plus	Data Rich	0	Population Density (under 150 ppl/sqkm, proportion)
Self-harm	Female	10 to 14	80 plus	Data Rich	0	Sociodemographic Status
Self-harm	Female	10 to 14	80 plus	Data Rich	1	Alcohol (litres per capita)
Self-harm	Female	10 to 14	80 plus	Data Rich	1	Log-transformed SEV scalar: Self Harm
Self-harm	Female	10 to 14	80 plus	Data Rich	1	Major disorder from dismod interpolated to be used as covariate

Appendix Table 10: CODEm covariates used, level of covariate, and expected direction of covariate by cause, sex, and age						
**Cause**	**Sex**	**Age start**	**Age end**	**Model type**	**Direction**	**Covariate**
Self-harm	Female	10 to 14	80 plus	Data Rich	1	Opium Cultivation (binary)
Interpersonal violence	Male	Early Neonatal	80 plus	Global	-1	Health System Access 2 (unitless)
Interpersonal violence	Male	Early Neonatal	80 plus	Global	0	Education (years per capita)
Interpersonal violence	Male	Early Neonatal	80 plus	Global	0	LDI (I$ per capita)
Interpersonal violence	Male	Early Neonatal	80 plus	Global	0	Sociodemographic Status
Interpersonal violence	Male	Early Neonatal	80 plus	Global	1	Alcohol (litres per capita)
Interpersonal violence	Male	Early Neonatal	80 plus	Global	1	Log-transformed SEV scalar: Violence
Interpersonal violence	Male	Early Neonatal	80 plus	Global	1	Opium Cultivation (binary)
Interpersonal violence	Male	Early Neonatal	80 plus	Global	1	Population Density (over 1000 ppl/sqkm, proportion)
Interpersonal violence	Male	Early Neonatal	80 plus	Data Rich	-1	Health System Access 2 (unitless)
Interpersonal violence	Male	Early Neonatal	80 plus	Data Rich	0	Education (years per capita)
Interpersonal violence	Male	Early Neonatal	80 plus	Data Rich	0	LDI (I$ per capita)
Interpersonal violence	Male	Early Neonatal	80 plus	Data Rich	0	Sociodemographic Status
Interpersonal violence	Male	Early Neonatal	80 plus	Data Rich	1	Alcohol (litres per capita)
Interpersonal violence	Male	Early Neonatal	80 plus	Data Rich	1	Log-transformed SEV scalar: Violence
Interpersonal violence	Male	Early Neonatal	80 plus	Data Rich	1	Opium Cultivation (binary)
Interpersonal violence	Male	Early Neonatal	80 plus	Data Rich	1	Population Density (over 1000 ppl/sqkm, proportion)
Interpersonal violence	Female	Early Neonatal	80 plus	Global	-1	Health System Access 2 (unitless)
Interpersonal violence	Female	Early Neonatal	80 plus	Global	0	Education (years per capita)
Interpersonal violence	Female	Early Neonatal	80 plus	Global	0	LDI (I$ per capita)
Interpersonal violence	Female	Early Neonatal	80 plus	Global	0	Sociodemographic Status
Interpersonal violence	Female	Early Neonatal	80 plus	Global	1	Alcohol (litres per capita)
Interpersonal violence	Female	Early Neonatal	80 plus	Global	1	Log-transformed SEV scalar: Violence
Interpersonal violence	Female	Early Neonatal	80 plus	Global	1	Opium Cultivation (binary)
Interpersonal violence	Female	Early Neonatal	80 plus	Global	1	Population Density (over 1000 ppl/sqkm, proportion)
Interpersonal violence	Female	Early Neonatal	80 plus	Data Rich	-1	Health System Access 2 (unitless)
Interpersonal violence	Female	Early Neonatal	80 plus	Data Rich	0	Education (years per capita)
Interpersonal violence	Female	Early Neonatal	80 plus	Data Rich	0	LDI (I$ per capita)
Interpersonal violence	Female	Early Neonatal	80 plus	Data Rich	0	Sociodemographic Status
Interpersonal violence	Female	Early Neonatal	80 plus	Data Rich	1	Alcohol (litres per capita)
Interpersonal violence	Female	Early Neonatal	80 plus	Data Rich	1	Log-transformed SEV scalar: Violence
Interpersonal violence	Female	Early Neonatal	80 plus	Data Rich	1	Opium Cultivation (binary)
Interpersonal violence	Female	Early Neonatal	80 plus	Data Rich	1	Population Density (over 1000 ppl/sqkm, proportion)
Assault by firearm	Male	Early Neonatal	80 plus	Global	-1	Health System Access 2 (unitless)
Assault by firearm	Male	Early Neonatal	80 plus	Global	0	Education (years per capita)
Assault by firearm	Male	Early Neonatal	80 plus	Global	0	LDI (I$ per capita)
Assault by firearm	Male	Early Neonatal	80 plus	Global	0	Sociodemographic Status
Assault by firearm	Male	Early Neonatal	80 plus	Global	1	Alcohol (litres per capita)
Assault by firearm	Male	Early Neonatal	80 plus	Global	1	Log-transformed SEV scalar: Viol Gun
Assault by firearm	Male	Early Neonatal	80 plus	Global	1	Opium Cultivation (binary)
Assault by firearm	Male	Early Neonatal	80 plus	Global	1	Population Density (over 1000 ppl/sqkm, proportion)
Assault by firearm	Male	Early Neonatal	80 plus	Data Rich	-1	Health System Access 2 (unitless)
Assault by firearm	Male	Early Neonatal	80 plus	Data Rich	0	Education (years per capita)
Assault by firearm	Male	Early Neonatal	80 plus	Data Rich	0	LDI (I$ per capita)

Appendix Table 10: CODEm covariates used, level of covariate, and expected direction of covariate by cause, sex, and age						
**Cause**	**Sex**	**Age start**	**Age end**	**Model type**	**Direction**	**Covariate**
Assault by firearm	Male	Early Neonatal	80 plus	Data Rich	0	Sociodemographic Status
Assault by firearm	Male	Early Neonatal	80 plus	Data Rich	1	Alcohol (litres per capita)
Assault by firearm	Male	Early Neonatal	80 plus	Data Rich	1	Log-transformed SEV scalar: Viol Gun
Assault by firearm	Male	Early Neonatal	80 plus	Data Rich	1	Opium Cultivation (binary)
Assault by firearm	Male	Early Neonatal	80 plus	Data Rich	1	Population Density (over 1000 ppl/sqkm, proportion)
Assault by firearm	Female	Early Neonatal	80 plus	Global	-1	Health System Access 2 (unitless)
Assault by firearm	Female	Early Neonatal	80 plus	Global	0	Education (years per capita)
Assault by firearm	Female	Early Neonatal	80 plus	Global	0	LDI (I$ per capita)
Assault by firearm	Female	Early Neonatal	80 plus	Global	0	Sociodemographic Status
Assault by firearm	Female	Early Neonatal	80 plus	Global	1	Alcohol (litres per capita)
Assault by firearm	Female	Early Neonatal	80 plus	Global	1	Log-transformed SEV scalar: Viol Gun
Assault by firearm	Female	Early Neonatal	80 plus	Global	1	Opium Cultivation (binary)
Assault by firearm	Female	Early Neonatal	80 plus	Global	1	Population Density (over 1000 ppl/sqkm, proportion)
Assault by firearm	Female	Early Neonatal	80 plus	Data Rich	-1	Health System Access 2 (unitless)
Assault by firearm	Female	Early Neonatal	80 plus	Data Rich	0	Education (years per capita)
Assault by firearm	Female	Early Neonatal	80 plus	Data Rich	0	LDI (I$ per capita)
Assault by firearm	Female	Early Neonatal	80 plus	Data Rich	0	Sociodemographic Status
Assault by firearm	Female	Early Neonatal	80 plus	Data Rich	1	Alcohol (litres per capita)
Assault by firearm	Female	Early Neonatal	80 plus	Data Rich	1	Log-transformed SEV scalar: Viol Gun
Assault by firearm	Female	Early Neonatal	80 plus	Data Rich	1	Opium Cultivation (binary)
Assault by firearm	Female	Early Neonatal	80 plus	Data Rich	1	Population Density (over 1000 ppl/sqkm, proportion)
Assault by sharp object	Male	Early Neonatal	80 plus	Global	-1	Health System Access 2 (unitless)
Assault by sharp object	Male	Early Neonatal	80 plus	Global	0	Education (years per capita)
Assault by sharp object	Male	Early Neonatal	80 plus	Global	0	LDI (I$ per capita)
Assault by sharp object	Male	Early Neonatal	80 plus	Global	0	Sociodemographic Status
Assault by sharp object	Male	Early Neonatal	80 plus	Global	1	Alcohol (litres per capita)
Assault by sharp object	Male	Early Neonatal	80 plus	Global	1	Log-transformed SEV scalar: Viol Knife
Assault by sharp object	Male	Early Neonatal	80 plus	Global	1	Opium Cultivation (binary)
Assault by sharp object	Male	Early Neonatal	80 plus	Global	1	Population Density (over 1000 ppl/sqkm, proportion)
Assault by sharp object	Male	Early Neonatal	80 plus	Data Rich	-1	Health System Access 2 (unitless)
Assault by sharp object	Male	Early Neonatal	80 plus	Data Rich	0	Education (years per capita)
Assault by sharp object	Male	Early Neonatal	80 plus	Data Rich	0	LDI (I$ per capita)
Assault by sharp object	Male	Early Neonatal	80 plus	Data Rich	0	Sociodemographic Status
Assault by sharp object	Male	Early Neonatal	80 plus	Data Rich	1	Alcohol (litres per capita)
Assault by sharp object	Male	Early Neonatal	80 plus	Data Rich	1	Log-transformed SEV scalar: Viol Knife
Assault by sharp object	Male	Early Neonatal	80 plus	Data Rich	1	Opium Cultivation (binary)
Assault by sharp object	Male	Early Neonatal	80 plus	Data Rich	1	Population Density (over 1000 ppl/sqkm, proportion)
Assault by sharp object	Female	Early Neonatal	80 plus	Global	-1	Health System Access 2 (unitless)
Assault by sharp object	Female	Early Neonatal	80 plus	Global	0	Education (years per capita)
Assault by sharp object	Female	Early Neonatal	80 plus	Global	0	LDI (I$ per capita)
Assault by sharp object	Female	Early Neonatal	80 plus	Global	0	Sociodemographic Status
Assault by sharp object	Female	Early Neonatal	80 plus	Global	1	Alcohol (litres per capita)
Assault by sharp object	Female	Early Neonatal	80 plus	Global	1	Log-transformed SEV scalar: Viol Knife
Assault by sharp object	Female	Early Neonatal	80 plus	Global	1	Opium Cultivation (binary)

Appendix Table 10: CODEm covariates used, level of covariate, and expected direction of covariate by cause, sex, and age						
**Cause**	**Sex**	**Age start**	**Age end**	**Model type**	**Direction**	**Covariate**
Assault by sharp object	Female	Early Neonatal	80 plus	Global	1	Population Density (over 1000 ppl/sqkm, proportion)
Assault by sharp object	Female	Early Neonatal	80 plus	Data Rich	-1	Health System Access 2 (unitless)
Assault by sharp object	Female	Early Neonatal	80 plus	Data Rich	0	Education (years per capita)
Assault by sharp object	Female	Early Neonatal	80 plus	Data Rich	0	LDI (I$ per capita)
Assault by sharp object	Female	Early Neonatal	80 plus	Data Rich	0	Sociodemographic Status
Assault by sharp object	Female	Early Neonatal	80 plus	Data Rich	1	Alcohol (litres per capita)
Assault by sharp object	Female	Early Neonatal	80 plus	Data Rich	1	Log-transformed SEV scalar: Viol Knife
Assault by sharp object	Female	Early Neonatal	80 plus	Data Rich	1	Opium Cultivation (binary)
Assault by sharp object	Female	Early Neonatal	80 plus	Data Rich	1	Population Density (over 1000 ppl/sqkm, proportion)
Assault by other means	Male	Early Neonatal	80 plus	Global	-1	Health System Access 2 (unitless)
Assault by other means	Male	Early Neonatal	80 plus	Global	0	Education (years per capita)
Assault by other means	Male	Early Neonatal	80 plus	Global	0	LDI (I$ per capita)
Assault by other means	Male	Early Neonatal	80 plus	Global	0	Sociodemographic Status
Assault by other means	Male	Early Neonatal	80 plus	Global	1	Alcohol (litres per capita)
Assault by other means	Male	Early Neonatal	80 plus	Global	1	Log-transformed SEV scalar: Oth Viol
Assault by other means	Male	Early Neonatal	80 plus	Global	1	Opium Cultivation (binary)
Assault by other means	Male	Early Neonatal	80 plus	Global	1	Population Density (over 1000 ppl/sqkm, proportion)
Assault by other means	Male	Early Neonatal	80 plus	Data Rich	-1	Health System Access 2 (unitless)
Assault by other means	Male	Early Neonatal	80 plus	Data Rich	0	Education (years per capita)
Assault by other means	Male	Early Neonatal	80 plus	Data Rich	0	LDI (I$ per capita)
Assault by other means	Male	Early Neonatal	80 plus	Data Rich	0	Sociodemographic Status
Assault by other means	Male	Early Neonatal	80 plus	Data Rich	1	Alcohol (litres per capita)
Assault by other means	Male	Early Neonatal	80 plus	Data Rich	1	Log-transformed SEV scalar: Oth Viol
Assault by other means	Male	Early Neonatal	80 plus	Data Rich	1	Opium Cultivation (binary)
Assault by other means	Male	Early Neonatal	80 plus	Data Rich	1	Population Density (over 1000 ppl/sqkm, proportion)
Assault by other means	Female	Early Neonatal	80 plus	Global	-1	Health System Access 2 (unitless)
Assault by other means	Female	Early Neonatal	80 plus	Global	0	Education (years per capita)
Assault by other means	Female	Early Neonatal	80 plus	Global	0	LDI (I$ per capita)
Assault by other means	Female	Early Neonatal	80 plus	Global	0	Sociodemographic Status
Assault by other means	Female	Early Neonatal	80 plus	Global	1	Alcohol (litres per capita)
Assault by other means	Female	Early Neonatal	80 plus	Global	1	Log-transformed SEV scalar: Oth Viol
Assault by other means	Female	Early Neonatal	80 plus	Global	1	Opium Cultivation (binary)
Assault by other means	Female	Early Neonatal	80 plus	Global	1	Population Density (over 1000 ppl/sqkm, proportion)
Assault by other means	Female	Early Neonatal	80 plus	Data Rich	-1	Health System Access 2 (unitless)
Assault by other means	Female	Early Neonatal	80 plus	Data Rich	0	Education (years per capita)
Assault by other means	Female	Early Neonatal	80 plus	Data Rich	0	LDI (I$ per capita)
Assault by other means	Female	Early Neonatal	80 plus	Data Rich	0	Sociodemographic Status
Assault by other means	Female	Early Neonatal	80 plus	Data Rich	1	Alcohol (litres per capita)
Assault by other means	Female	Early Neonatal	80 plus	Data Rich	1	Log-transformed SEV scalar: Oth Viol
Assault by other means	Female	Early Neonatal	80 plus	Data Rich	1	Opium Cultivation (binary)
Assault by other means	Female	Early Neonatal	80 plus	Data Rich	1	Population Density (over 1000 ppl/sqkm, proportion)

Appendix Table 11: CODEm predictive validity results by cause, sex, age, and location

Cause	Sex	Age start	Age end	Predictive validity					
				RMSE in	RMSE out	Trend in	Trend out	Coverage in	Coverage out
Dengue [Global]	Female	28-364 days	80+ years	0·452281	0·761531	0·297494	0·313454	0·993533	0·974533
Dengue [Global]	Male	28-364 days	80+ years	0·495625	0·774469	0·297248	0·313193	0·993714	0·969925
Otitis media [Data Rich]	Male	0-6 days	80+ years	0·425322	0·647622	0·335928	0·396117	0·989064	0·984749
Otitis media [Data Rich]	Female	0-6 days	80+ years	0·363955	0·586986	0·286561	0·334672	0·997475	0·993569
Otitis media [Global]	Male	0-6 days	80+ years	0·500444	0·899948	0·37738	0·388461	0·995628	0·972198
Otitis media [Global]	Female	0-6 days	80+ years	0·439121	0·865089	0·329661	0·338007	0·996799	0·983102
Iron-deficiency anemia [Global]	Male	28-364 days	80+ years	0·382265	0·636393	0·215093	0·202262	0·996194	0·991176
Iron-deficiency anemia [Global]	Female	28-364 days	80+ years	0·352926	0·647189	0·216384	0·198062	0·996112	0·987725
Iron-deficiency anemia [Data Rich]	Male	28-364 days	80+ years	0·3828	0·537444	0·189702	0·167057	0·997806	0·99668
Iron-deficiency anemia [Data Rich]	Female	28-364 days	80+ years	0·362012	0·51194	0·185488	0·153208	0·998197	0·99761
Hemoglobinopathies and hemolytic anemias [Global]	Male	0-6 days	80+ years	0·295039	0·54107	0·203266	0·238676	0·999529	0·992076
Hemoglobinopathies and hemolytic anemias [Global]	Female	0-6 days	80+ years	0·269417	0·532989	0·193873	0·233429	0·999429	0·991611
Lymphoid leukemia [Global]	Female	28-364 days	80+ years	0·281087	0·40354	0·232408	0·223963	0·998657	0·991705
Lymphoid leukemia [Data Rich]	Female	28-364 days	80+ years	0·242542	0·301024	0·210857	0·202506	0·998442	0·996523
Other neonatal disorders [Global]	Female	0-6 days	1-4 years	0·475694	0·654773	0·430413	0·392056	0·998997	0·997866
Other neonatal disorders [Global]	Male	0-6 days	1-4 years	0·479061	0·699084	0·433597	0·415694	0·998773	0·997215
Unintentional injuries [Global]	Female	28-364 days	80+ years	0·30561	0·460008	0·241532	0·25266	0·99481	0·978433
Unintentional injuries [Global]	Male	28-364 days	80+ years	0·279336	0·405252	0·227477	0·228916	0·994132	0·973144
Bladder cancer [Data Rich]	Male	15-19 years	80+ years	0·230582	0·274245	0·192908	0·213744	0·997134	0·995606
Bladder cancer [Data Rich]	Female	15-19 years	80+ years	0·25352	0·304321	0·214221	0·241128	0·992256	0·99
Acute lymphoid leukemia [Global]	Male	28-364 days	80+ years	0·292059	0·422015	0·234129	0·233471	0·998427	0·994249
Acute lymphoid leukemia [Global]	Female	28-364 days	80+ years	0·28521	0·434977	0·230676	0·234298	0·999145	0·994973
Acute myeloid leukemia [Global]	Male	28-364 days	80+ years	0·301073	0·4242	0·209321	0·206793	0·998524	0·99007
Acute myeloid leukemia [Data Rich]	Female	28-364 days	80+ years	0·219708	0·297696	0·182087	0·172123	0·999088	0·997735
Acute myeloid leukemia [Global]	Female	28-364 days	80+ years	0·266001	0·436025	0·213681	0·212177	0·998629	0·989176
Chronic myeloid leukemia [Data Rich]	Male	28-364 days	80+ years	0·22562	0·318327	0·181669	0·177122	0·9997	0·999022
Chronic myeloid leukemia [Global]	Male	28-364 days	80+ years	0·290725	0·470004	0·232	0·239949	0·999373	0·993826
Chronic myeloid leukemia [Data Rich]	Female	28-364 days	80+ years	0·239071	0·339418	0·189041	0·171448	0·999641	0·998772
Chronic myeloid leukemia [Global]	Female	28-364 days	80+ years	0·310585	0·5115	0·242015	0·2381	0·999176	0·992972
Urinary diseases and male infertility [Global]	Male	0-6 days	80+ years	0·290118	0·518003	0·179799	0·187004	0·999147	0·988445
Urinary diseases and male infertility [Global]	Female	0-6 days	80+ years	0·254053	0·532144	0·166862	0·179556	0·999222	0·984855
Urinary diseases and male infertility [Data Rich]	Female	0-6 days	80+ years	0·201183	0·262259	0·144319	0·168446	0·999212	0·99861
Malignant skin melanoma [Data Rich]	Male	15-19 years	80+ years	0·262729	0·302005	0·201705	0·226216	0·998992	0·99836
Malignant skin melanoma [Data Rich]	Female	15-19 years	80+ years	0·232067	0·270683	0·191654	0·213212	0·998429	0·99734
Other chronic respiratory diseases [Global]	Female	0-6 days	80+ years	0·497277	0·756624	0·270321	0·271602	0·966188	0·958657
Interstitial lung disease and pulmonary sarcoidosis [Global]	Female	1-4 years	80+ years	0·265464	0·500448	0·177692	0·182211	0·999523	0·98887
Other neurological disorders [Data Rich]	Male	28-364 days	80+ years	0·196833	0·291023	0·155117	0·163341	0·999872	0·999686
Other neurological disorders [Data Rich]	Female	28-364 days	80+ years	0·203245	0·305705	0·160134	0·180026	0·999771	0·999414
Parkinson disease [Global]	Male	20-24 years	80+ years	0·263589	0·570326	0·161554	0·168037	0·999423	0·983429
Parkinson disease [Global]	Female	20-24 years	80+ years	0·283927	0·557216	0·174262	0·177905	0·998717	0·97701
Multiple sclerosis [Data Rich]	Male	20-24 years	80+ years	0·228368	0·286237	0·17729	0·195852	0·9998	0·999435
Multiple sclerosis [Data Rich]	Female	20-24 years	80+ years	0·233113	0·288984	0·182819	0·19636	0·999693	0·99918
Asbestosis [Global]	Male	0-6 days	80+ years	0·690404	1·40039	0·527837	0·536617	0·715742	0·734776
Asbestosis [Global]	Female	0-6 days	80+ years	0·856584	1·706	0·697113	0·707529	0·572145	0·575385
Other pneumoconiosis [Global]	Male	0-6 days	80+ years	0·295102	0·659377	0·202672	0·209191	0·983825	0·97901
Other pneumoconiosis [Global]	Female	0-6 days	80+ years	0·451955	0·922729	0·312399	0·336793	0·915565	0·88803
Other chronic respiratory diseases [Global]	Male	0-6 days	80+ years	0·398143	0·658407	0·230832	0·228993	0·999041	0·987399
Cardiomyopathy and myocarditis [Global]	Male	0-6 days	80+ years	0·250984	0·460313	0·157951	0·165789	0·999158	0·976814
Cardiomyopathy and myocarditis [Global]	Female	0-6 days	80+ years	0·23834	0·505763	0·160754	0·167175	0·999059	0·979949
Interstitial nephritis and urinary tract infections [Global]	Male	0-6 days	80+ years	0·319793	0·593701	0·195313	0·199719	0·999159	0·98587
Interstitial nephritis and urinary tract infections [Data Rich]	Female	0-6 days	80+ years	0·269274	0·32451	0·170773	0·192106	0·999055	0·998369
Interstitial nephritis and urinary tract infections [Global]	Female	0-6 days	80+ years	0·281593	0·549433	0·180862	0·190699	0·999152	0·98237
Malignant skin melanoma [Global]	Male	15-19 years	80+ years	0·313044	0·456674	0·238482	0·242633	0·998837	0·991342
Malignant skin melanoma [Global]	Female	15-19 years	80+ years	0·290713	0·426007	0·230092	0·220124	0·998305	0·99137
Prostate cancer [Global]	Male	15-19 years	80+ years	0·359223	0·437882	0·275425	0·269968	0·995649	0·985716
Interstitial nephritis and urinary tract infections [Data Rich]	Male	0-6 days	80+ years	0·270685	0·320129	0·168228	0·183862	0·999072	0·998286
Kidney cancer [Global]	Male	28-364 days	80+ years	0·314755	0·430669	0·26012	0·268004	0·998815	0·993162
Kidney cancer [Data Rich]	Male	28-364 days	80+ years	0·260868	0·3111	0·219707	0·246669	0·998029	0·9965
Kidney cancer [Global]	Female	28-364 days	80+ years	0·325223	0·462785	0·267593	0·282602	0·999027	0·992144
Kidney cancer [Data Rich]	Female	28-364 days	80+ years	0·277883	0·33386	0·23482	0·258935	0·998537	0·997156
Bladder cancer [Global]	Male	15-19 years	80+ years	0·302561	0·433254	0·243984	0·244194	0·997993	0·990885
Bladder cancer [Global]	Female	15-19 years	80+ years	0·332237	0·482368	0·267378	0·278164	0·994702	0·990326
Thyroid cancer [Global]	Male	10-14 years	80+ years	0·386704	0·490533	0·30511	0·298633	0·992931	0·989852
Thyroid cancer [Data Rich]	Male	10-14 years	80+ years	0·318125	0·384007	0·272302	0·293744	0·993035	0·990499
Thyroid cancer [Data Rich]	Female	10-14 years	80+ years	0·390965	0·469181	0·330885	0·344486	0·982842	0·978791
Mesothelioma [Global]	Male	15-19 years	80+ years	0·333464	0·541584	0·267427	0·268572	0·999223	0·993907
Mesothelioma [Data Rich]	Male	15-19 years	80+ years	0·301885	0·416756	0·259511	0·266787	0·998977	0·997521
Mesothelioma [Global]	Female	15-19 years	80+ years	0·334721	0·526524	0·263788	0·263569	0·99836	0·994058
Mesothelioma [Data Rich]	Female	15-19 years	80+ years	0·296068	0·409144	0·248659	0·215724	0·998023	0·996154
Non-Hodgkin lymphoma [Global]	Female	28-364 days	80+ years	0·291953	0·406723	0·218606	0·222847	0·999117	0·994385
Multiple myeloma [Data Rich]	Female	15-19 years	80+ years	0·239585	0·285912	0·199032	0·221908	0·998399	0·997601
Multiple myeloma [Data Rich]	Male	15-19 years	80+ years	0·234373	0·277252	0·194235	0·214818	0·997828	0·996446
Lymphoid leukemia [Data Rich]	Male	28-364 days	80+ years	0·259791	0·327872	0·223759	0·214664	0·997285	0·994384
Lymphoid leukemia [Global]	Male	28-364 days	80+ years	0·296545	0·414002	0·245704	0·239179	0·998038	0·989861
Chronic lymphoid leukemia [Global]	Female	15-19 years	80+ years	0·273828	0·448008	0·20869	0·209412	0·997754	0·98794
Chronic lymphoid leukemia [Data Rich]	Female	15-19 years	80+ years	0·208156	0·284473	0·170142	0·150967	0·99783	0·996844

Appendix Table 11: CODEm predictive validity results by cause, sex, age, and location

				Predictive validity					
Cause	Sex	Age start	Age end	RMSE in	RMSE out	Trend in	Trend out	Coverage in	Coverage out
Chronic lymphoid leukemia [Global]	Male	15-19 years	80+ years	0·324657	0·565171	0·246054	0·231123	0·993437	0·978918
Chronic lymphoid leukemia [Data Rich]	Male	15-19 years	80+ years	0·251717	0·375992	0·202832	0·165172	0·995506	0·993173
Myeloid leukemia [Global]	Male	28-364 days	80+ years	0·264107	0·367963	0·22826	0·235117	0·998559	0·995366
Myeloid leukemia [Data Rich]	Male	28-364 days	80+ years	0·241722	0·310425	0·21654	0·215431	0·998226	0·996441
Myeloid leukemia [Global]	Female	28-364 days	80+ years	0·262614	0·368953	0·221638	0·222605	0·998953	0·995737
Myeloid leukemia [Data Rich]	Female	28-364 days	80+ years	0·233866	0·300389	0·207117	0·211628	0·998643	0·997247
Pneumoconiosis [Global]	Male	1-4 years	80+ years	0·274768	0·713999	0·192624	0·191428	0·999018	0·975007
Pneumoconiosis [Global]	Female	1-4 years	80+ years	0·431497	0·782126	0·26325	0·285921	0·998566	0·975428
Silicosis [Global]	Female	0-6 days	80+ years	0·738095	1·42628	0·603635	0·605145	0·965418	0·940557
Silicosis [Global]	Male	0-6 days	80+ years	0·564803	1·27714	0·410848	0·377914	0·975789	0·942476
Coal workers pneumoconiosis [Global]	Female	0-6 days	80+ years	0·853552	1·40888	0·704702	0·738858	0·956795	0·941497
Coal workers pneumoconiosis [Global]	Male	0-6 days	80+ years	0·756784	1·56009	0·60667	0·605158	0·93227	0·89397
Interstitial lung disease and pulmonary sarcoidosis [Global]	Male	1-4 years	80+ years	0·310765	0·522206	0·181818	0·184781	0·999596	0·985892
Decubitus ulcer [Global]	Male	1-4 years	80+ years	0·608107	1·09648	0·330465	0·335455	0·861304	0·868183
Decubitus ulcer [Global]	Female	1-4 years	80+ years	0·54698	1·06626	0·37974	0·382958	0·831532	0·817362
Decubitus ulcer [Data Rich]	Female	1-4 years	80+ years	0·518654	0·709457	0·351469	0·397348	0·791189	0·788037
Urinary diseases and male infertility [Data Rich]	Male	0-6 days	80+ years	0·203483	0·259476	0·147471	0·168749	0·999281	0·998366
Decubitus ulcer [Data Rich]	Male	1-4 years	80+ years	0·365486	0·58608	0·260101	0·234412	0·828238	0·823708
Dengue [Data Rich]	Female	28-364 days	80+ years	0·97684	1·39386	0·760718	0·75561	0·986668	0·974323
Dengue [Data Rich]	Male	28-364 days	80+ years	0·927027	1·35168	0·732674	0·783196	0·993862	0·982456
Rabies [Data Rich]	Male	28-364 days	80+ years	0·768906	1·10687	0·620719	0·655008	0·988712	0·975754
Rabies [Data Rich]	Female	28-364 days	80+ years	0·968031	1·35806	0·81295	0·804398	0·98589	0·970312
Other intestinal infectious diseases [Global]	Male	28-364 days	80+ years	0·992589	1·6831	0·780051	0·822336	0·989752	0·948416
Other intestinal infectious diseases [Global]	Female	28-364 days	80+ years	0·999257	1·70492	0·76854	0·7996	0·990149	0·942503
Cocaine use disorders [Global]	Male	15-19 years	80+ years	0·493227	1·15637	0·327438	0·356076	0·999086	0·976314
Cocaine use disorders [Global]	Female	15-19 years	80+ years	0·57383	1·19213	0·391823	0·42643	0·997849	0·956477
Amphetamine use disorders [Global]	Male	15-19 years	80+ years	0·679792	1·40702	0·503698	0·5731	0·997402	0·945715
Amphetamine use disorders [Global]	Female	15-19 years	80+ years	0·622953	1·36037	0·454983	0·506928	0·99776	0·95143
Opioid use disorders [Global]	Male	15-19 years	80+ years	0·337551	0·746751	0·219074	0·226597	0·99925	0·985253
Opioid use disorders [Global]	Female	15-19 years	80+ years	0·373499	0·684078	0·230164	0·229015	0·999761	0·986357
Transport injuries [Global]	Male	0-6 days	80+ years	0·283889	0·45019	0·262422	0·256959	0·998844	0·981899
Transport injuries [Global]	Female	0-6 days	80+ years	0·29309	0·472199	0·262526	0·257035	0·998445	0·986108
Assault by firearm [Global]	Male	0-6 days	80+ years	0·391295	0·849985	0·270406	0·274143	0·993582	0·955928
Assault by firearm [Global]	Female	0-6 days	80+ years	0·370184	0·698896	0·251335	0·250881	0·996601	0·979617
Assault by sharp object [Global]	Male	0-6 days	80+ years	0·300224	0·599585	0·212204	0·218017	0·998704	0·980926
Assault by sharp object [Global]	Female	0-6 days	80+ years	0·31063	0·564817	0·229442	0·229475	0·998523	0·989687
Assault by other means [Global]	Male	0-6 days	80+ years	0·27301	0·500816	0·182102	0·186098	0·998943	0·989735
Assault by other means [Global]	Female	0-6 days	80+ years	0·271816	0·427785	0·177985	0·181265	0·999308	0·992072
Falls [Global]	Male	0-6 days	80+ years	0·343073	0·524578	0·303459	0·301548	0·998134	0·984439
Falls [Global]	Female	0-6 days	80+ years	0·379093	0·588289	0·340659	0·337178	0·997436	0·987849
Drowning [Global]	Male	0-6 days	80+ years	0·32393	0·560614	0·282109	0·268935	0·998002	0·983663
Drowning [Global]	Female	0-6 days	80+ years	0·366486	0·674595	0·299785	0·295072	0·998377	0·982983
Exposure to mechanical forces [Global]	Male	0-6 days	80+ years	0·260603	0·518572	0·186527	0·191416	0·998254	0·982418
Exposure to mechanical forces [Global]	Female	0-6 days	80+ years	0·289234	0·534082	0·211625	0·222254	0·998539	0·989168
Unintentional firearm injuries [Global]	Male	0-6 days	80+ years	0·380002	0·757248	0·249882	0·270237	0·998297	0·981973
Unintentional suffocation [Global]	Male	0-6 days	80+ years	0·444429	0·807109	0·320102	0·309778	0·984917	0·977693
Adverse effects of medical treatment [Global]	Male	0-6 days	80+ years	0·378485	0·596084	0·216268	0·216427	0·995999	0·975618
Adverse effects of medical treatment [Global]	Female	0-6 days	80+ years	0·363599	0·573081	0·227408	0·23447	0·996762	0·985618
Tracheal, bronchus, and lung cancer [Global]	Male	15-19 years	80+ years	0·228214	0·381209	0·181738	0·18932	0·99663	0·965859
Tracheal, bronchus, and lung cancer [Data Rich]	Male	15-19 years	80+ years	0·191288	0·229277	0·156429	0·176259	0·995026	0·991993
Tracheal, bronchus, and lung cancer [Global]	Female	15-19 years	80+ years	0·241892	0·389713	0·190336	0·197429	0·997265	0·980216
Tracheal, bronchus, and lung cancer [Data Rich]	Female	15-19 years	80+ years	0·194024	0·231102	0·157856	0·174011	0·995272	0·992137
Animal contact [Global]	Male	0-6 days	80+ years	0·345879	0·725646	0·227677	0·22053	0·998105	0·98202
Animal contact [Global]	Female	0-6 days	80+ years	0·362565	0·741864	0·242731	0·242933	0·998465	0·977259
Venomous animal contact [Global]	Male	0-6 days	80+ years	0·436457	0·876913	0·324968	0·332817	0·979237	0·951199
Venomous animal contact [Global]	Female	0-6 days	80+ years	0·467447	0·933198	0·353029	0·361668	0·967595	0·94657
Non-venomous animal contact [Global]	Male	0-6 days	80+ years	0·303467	0·615797	0·213451	0·223971	0·999141	0·989831
Non-venomous animal contact [Global]	Female	0-6 days	80+ years	0·311477	0·62753	0·222653	0·214792	0·99797	0·990292
Foreign body in other body part [Global]	Male	0-6 days	80+ years	0·346611	0·71392	0·205642	0·22207	0·993198	0·982294
Foreign body in other body part [Global]	Female	0-6 days	80+ years	0·362749	0·798859	0·221562	0·221464	0·992403	0·976916
Environmental heat and cold exposure [Global]	Male	0-6 days	80+ years	0·347843	0·626097	0·226784	0·224232	0·998337	0·985044
Environmental heat and cold exposure [Global]	Female	0-6 days	80+ years	0·440412	0·769469	0·270234	0·279483	0·997114	0·981817
Other unintentional injuries [Global]	Male	0-6 days	80+ years	0·311686	0·567538	0·214164	0·240918	0·997571	0·982679
Other unintentional injuries [Global]	Female	0-6 days	80+ years	0·369476	0·671033	0·259216	0·260934	0·997012	0·979021
Unintentional suffocation [Global]	Female	0-6 days	80+ years	0·4887	0·924078	0·274664	0·269942	0·950526	0·94733
Unintentional firearm injuries [Global]	Female	0-6 days	80+ years	0·412714	0·746066	0·269471	0·271899	0·997979	0·98489
Gynecological diseases [Global]	Female	15-19 years	80+ years	0·548792	0·91314	0·397222	0·422307	0·9909	0·961432
Gynecological diseases [Data Rich]	Female	15-19 years	80+ years	0·391579	0·49923	0·310246	0·347241	0·99596	0·992389
Pneumoconiosis [Data Rich]	Male	1-4 years	80+ years	0·193949	0·335803	0·142766	0·143396	0·999649	0·999111
Pneumoconiosis [Data Rich]	Female	1-4 years	80+ years	0·260064	0·485382	0·188654	0·223199	0·998849	0·997762
Silicosis [Data Rich]	Male	0-6 days	80+ years	0·451497	0·67509	0·342666	0·262594	0·975622	0·969533
Silicosis [Data Rich]	Female	0-6 days	80+ years	0·603085	0·876895	0·499035	0·564117	0·960555	0·952949
Asbestosis [Data Rich]	Female	0-6 days	80+ years	0·730226	1·16868	0·591485	0·660837	0·492349	0·485559
Asbestosis [Data Rich]	Male	0-6 days	80+ years	0·661634	1·04559	0·447296	0·427334	0·669254	0·666376
Coal workers pneumoconiosis [Data Rich]	Female	0-6 days	80+ years	0·749455	1·25291	0·62212	0·786242	0·943803	0·931269
Motorcyclist road injuries [Global]	Female	0-6 days	80+ years	0·314399	0·633195	0·21735	0·230157	0·99847	0·990342

Appendix Table 11: CODEm predictive validity results by cause, sex, age, and location

Cause	Sex	Age start	Age end	Predictive validity					
				RMSE in	RMSE out	Trend in	Trend out	Coverage in	Coverage out
Coal workers pneumoconiosis [Data Rich]	Male	0-6 days	80+ years	0·647527	0·973172	0·521585	0·430946	0·918164	0·907193
Other pneumoconiosis [Data Rich]	Male	0-6 days	80+ years	0·203749	0·370089	0·149022	0·147903	0·99952	0·998736
Other pneumoconiosis [Data Rich]	Female	0-6 days	80+ years	0·309645	0·565709	0·228759	0·24275	0·998098	0·996462
Interstitial lung disease and pulmonary sarcoidosis [Data Rich]	Male	1-4 years	80+ years	0·264526	0·383919	0·170394	0·171602	0·999788	0·999315
Interstitial lung disease and pulmonary sarcoidosis [Data Rich]	Female	1-4 years	80+ years	0·242468	0·374141	0·160849	0·164833	0·999865	0·999588
Other chronic respiratory diseases [Data Rich]	Male	0-6 days	80+ years	0·24603	0·380977	0·186108	0·196881	0·999229	0·99803
Other chronic respiratory diseases [Data Rich]	Female	0-6 days	80+ years	0·307943	0·430853	0·216755	0·211372	0·964427	0·96724
Parkinson disease [Data Rich]	Male	20-24 years	80+ years	0·204634	0·288402	0·143224	0·166153	0·999834	0·999305
Parkinson disease [Data Rich]	Female	20-24 years	80+ years	0·21426	0·292921	0·149066	0·157488	0·999609	0·998857
Multiple sclerosis [Global]	Male	20-24 years	80+ years	0·265594	0·445323	0·201367	0·20526	0·999444	0·994219
Multiple sclerosis [Global]	Female	20-24 years	80+ years	0·273732	0·45904	0·207274	0·204134	0·999434	0·991673
Other neurological disorders [Global]	Male	28-364 days	80+ years	0·229709	0·35993	0·175619	0·177893	0·999384	0·996783
Other neurological disorders [Global]	Female	28-364 days	80+ years	0·242752	0·397001	0·182939	0·1881	0·999286	0·994985
Uterine fibroids [Global]	Female	15-19 years	80+ years	0·831259	1·42167	0·581183	0·559292	0·954769	0·920437
Uterine fibroids [Data Rich]	Female	15-19 years	80+ years	0·610697	0·898121	0·481093	0·414885	0·808729	0·8004
Endometriosis [Global]	Female	15-19 years	50-54 years	1·2887	2·06984	1·08835	1·11301	0·974465	0·928213
Endometriosis [Data Rich]	Female	15-19 years	50-54 years	1·20897	1·7166	1·03484	1·08318	0·968283	0·939262
Genital prolapse [Global]	Female	15-19 years	80+ years	0·822904	1·49475	0·662316	0·664792	0·92441	0·903091
Genital prolapse [Data Rich]	Female	15-19 years	80+ years	0·72838	1·04514	0·613509	0·553516	0·921189	0·913021
Other gynecological diseases [Data Rich]	Female	15-19 years	80+ years	0·475032	0·606628	0·376061	0·34065	0·994097	0·988637
Other gynecological diseases [Global]	Female	15-19 years	80+ years	0·612702	1·02632	0·442508	0·450577	0·99154	0·968759
Interpersonal violence [Global]	Male	0-6 days	80+ years	0·391595	0·637331	0·290019	0·298479	0·995107	0·967457
Interpersonal violence [Global]	Female	0-6 days	80+ years	0·394438	0·611023	0·305606	0·33	0·996401	0·980251
Road injuries [Global]	Male	0-6 days	80+ years	0·198869	0·346068	0·141742	0·149475	0·999342	0·982767
Road injuries [Global]	Female	0-6 days	80+ years	0·223423	0·388485	0·159485	0·167295	0·999641	0·990614
Pedestrian road injuries [Global]	Male	0-6 days	80+ years	0·266094	0·488447	0·191059	0·205695	0·996248	0·977509
Pedestrian road injuries [Global]	Female	0-6 days	80+ years	0·256462	0·470118	0·187008	0·196327	0·998889	0·986837
Cyclist road injuries [Global]	Male	1-4 years	80+ years	0·237389	0·509128	0·170398	0·175508	0·999676	0·986986
Cyclist road injuries [Global]	Female	1-4 years	80+ years	0·266409	0·613297	0·195219	0·200679	0·999748	0·990056
Motorcyclist road injuries [Global]	Male	0-6 days	80+ years	0·303464	0·572642	0·174362	0·18031	0·999341	0·987466
Self-harm and interpersonal violence [Global]	Male	0-6 days	80+ years	0·29019	0·492338	0·201151	0·217769	0·996937	0·975039
Self-harm and interpersonal violence [Global]	Female	0-6 days	80+ years	0·309662	0·520317	0·219455	0·228596	0·997812	0·984819
Other road injuries [Global]	Male	0-6 days	80+ years	0·444017	0·952973	0·277641	0·284919	0·99625	0·973205
Other road injuries [Global]	Female	0-6 days	80+ years	0·392116	0·821406	0·276312	0·28834	0·995148	0·982153
Other drug use disorders [Global]	Male	15-19 years	80+ years	0·405841	0·862877	0·27429	0·292883	0·998244	0·978518
Other drug use disorders [Global]	Female	15-19 years	80+ years	0·373594	0·760292	0·237787	0·24736	0·999391	0·986255
Chronic obstructive pulmonary disease [Data Rich]	Female	28-364 days	80+ years	0·166288	0·320895	0·126212	0·131782	0·99989	0·999297
Chronic obstructive pulmonary disease [Data Rich]	Male	28-364 days	80+ years	0·161432	0·271297	0·127485	0·133559	0·999763	0·999052
Motor neuron disease [Global]	Male	0-6 days	80+ years	0·286544	0·53082	0·184346	0·17945	0·998616	0·984688
Motor neuron disease [Global]	Female	0-6 days	80+ years	0·372456	0·665962	0·242236	0·246536	0·99725	0·983782
Motor neuron disease [Data Rich]	Male	0-6 days	80+ years	0·223626	0·381155	0·15729	0·156351	0·999255	0·998484
Motor neuron disease [Data Rich]	Female	0-6 days	80+ years	0·313087	0·491644	0·222994	0·217376	0·997634	0·996171
Musculoskeletal disorders [Global]	Male	5-9 years	80+ years	0·261301	0·486206	0·186022	0·194614	0·99949	0·991262
Musculoskeletal disorders [Global]	Female	5-9 years	80+ years	0·277164	0·593832	0·182925	0·186858	0·999108	0·975233
Neural tube defects [Data Rich]	Male	0-6 days	80+ years	0·387562	0·610863	0·289679	0·274562	0·924318	0·919164
Neural tube defects [Data Rich]	Female	0-6 days	80+ years	0·407466	0·633727	0·30585	0·322645	0·903004	0·898146
Other neonatal disorders [Data Rich]	Female	0-6 days	1-4 years	0·256939	0·432072	0·191114	0·229032	0·999249	0·997859
Other neonatal disorders [Data Rich]	Male	0-6 days	1-4 years	0·251125	0·450355	0·18285	0·221078	0·999127	0·997866
Neural tube defects [Global]	Male	0-6 days	80+ years	0·49258	0·997166	0·373257	0·383187	0·930921	0·943851
Neural tube defects [Global]	Female	0-6 days	80+ years	0·546155	1·03276	0·397349	0·404458	0·912696	0·944348
Congenital heart anomalies [Data Rich]	Male	0-6 days	80+ years	0·176974	0·256403	0·137396	0·158236	0·999923	0·999645
Congenital heart anomalies [Data Rich]	Female	0-6 days	80+ years	0·176007	0·255777	0·136717	0·153607	0·999923	0·999709
Congenital heart anomalies [Global]	Male	0-6 days	80+ years	0·249756	0·434542	0·206677	0·195221	0·999584	0·994641
Congenital heart anomalies [Global]	Female	0-6 days	80+ years	0·265183	0·439025	0·224486	0·223761	0·999807	0·996273
Cleft lip and cleft palate [Data Rich]	Male	0-6 days	1-4 years	0·812448	1·40346	0·703222	0·843667	0·920007	0·882407
Cleft lip and cleft palate [Data Rich]	Female	0-6 days	1-4 years	0·90533	1·57201	0·785559	0·91006	0·911418	0·854464
Cleft lip and cleft palate [Global]	Male	0-6 days	1-4 years	0·90081	1·52796	0·761271	0·779874	0·924246	0·901679
Cleft lip and cleft palate [Global]	Female	0-6 days	1-4 years	1·027	1·64223	0·822165	0·830418	0·919947	0·87413
Down syndrome [Data Rich]	Male	0-6 days	80+ years	0·298413	0·5402	0·200183	0·224403	0·997676	0·997086
Down syndrome [Data Rich]	Female	0-6 days	80+ years	0·363128	0·567814	0·213364	0·238244	0·994304	0·993344
Down syndrome [Global]	Male	0-6 days	80+ years	0·359432	0·651886	0·23758	0·245175	0·997585	0·9918
Down syndrome [Global]	Female	0-6 days	80+ years	0·417238	0·69618	0·262274	0·265475	0·992965	0·992354
Other chromosomal abnormalities [Data Rich]	Male	0-6 days	80+ years	0·485854	1·0171	0·317982	0·308168	0·790971	0·789899
Other chromosomal abnormalities [Data Rich]	Female	0-6 days	80+ years	0·566907	1·01064	0·339849	0·326814	0·791833	0·789805
Alcohol use disorders [Global]	Male	15-19 years	80+ years	0·239194	0·612527	0·173572	0·182927	0·99907	0·94774
Alcohol use disorders [Global]	Female	15-19 years	80+ years	0·268869	0·696149	0·198983	0·204312	0·999053	0·967496
Drug use disorders [Global]	Male	15-19 years	80+ years	0·344071	0·744503	0·224297	0·236649	0·998165	0·958979
Drug use disorders [Global]	Female	15-19 years	80+ years	0·36368	0·708413	0·248862	0·270849	0·998016	0·97335
Alcohol use disorders [Data Rich]	Male	15-19 years	80+ years	0·199453	0·252674	0·150158	0·173813	0·999503	0·998353
Alcohol use disorders [Data Rich]	Female	15-19 years	80+ years	0·220602	0·277758	0·170917	0·199168	0·999524	0·998561
Polycystic ovarian syndrome [Data Rich]	Female	15-19 years	50-54 years	0·387245	0·771971	0·285356	0·315243	0·996029	0·984019
Hemoglobinopathies and hemolytic anemias [Data Rich]	Male	0-6 days	80+ years	0·17901	0·276795	0·139014	0·159451	0·999921	0·999749
Hemoglobinopathies and hemolytic anemias [Data Rich]	Female	0-6 days	80+ years	0·174756	0·264252	0·128765	0·146914	0·999976	0·999871
Aortic aneurysm [Global]	Male	15-19 years	80+ years	0·182199	0·363829	0·130304	0·135468	0·999783	0·97444
Aortic aneurysm [Global]	Female	15-19 years	80+ years	0·203481	0·393351	0·144398	0·145741	0·99965	0·987712
Ischemic stroke [Global]	Male	28-364 days	80+ years	0·197159	0·377334	0·135414	0·141827	0·999437	0·983448

Appendix Table 11: CODEm predictive validity results by cause, sex, age, and location

				Predictive validity					
Cause	Sex	Age start	Age end	RMSE in	RMSE out	Trend in	Trend out	Coverage in	Coverage out
Ischemic stroke [Global]	Female	28-364 days	80+ years	0·212798	0·381924	0·145229	0·149829	0·999326	0·987279
Polycystic ovarian syndrome [Global]	Female	15-19 years	50-54 years	0·41505	0·811963	0·298443	0·267791	0·997606	0·983386
Nasopharynx cancer [Global]	Male	5-9 years	80+ years	0·345324	0·592894	0·279172	0·292845	0·998248	0·987922
Nasopharynx cancer [Data Rich]	Male	5-9 years	80+ years	0·294965	0·355039	0·25459	0·284967	0·997242	0·99532
Nasopharynx cancer [Global]	Female	5-9 years	80+ years	0·430091	0·66779	0·358953	0·352335	0·983778	0·973491
Nasopharynx cancer [Data Rich]	Female	5-9 years	80+ years	0·39848	0·488929	0·3444	0·393536	0·978754	0·976441
Other pharynx cancer [Global]	Male	15-19 years	80+ years	0·296629	0·520968	0·231789	0·231996	0·996492	0·977258
Other pharynx cancer [Data Rich]	Male	15-19 years	80+ years	0·252717	0·313299	0·203227	0·219991	0·994838	0·992681
Other pharynx cancer [Data Rich]	Female	15-19 years	80+ years	0·311859	0·373677	0·268036	0·29697	0·986151	0·984534
Rheumatoid arthritis [Data Rich]	Male	5-9 years	80+ years	0·313346	0·423517	0·232025	0·236326	0·983007	0·9815
Rheumatoid arthritis [Data Rich]	Female	5-9 years	80+ years	0·320342	0·452763	0·237746	0·278044	0·991008	0·989767
Rheumatoid arthritis [Global]	Male	5-9 years	80+ years	0·396668	0·849016	0·273352	0·286974	0·980281	0·951991
Rheumatoid arthritis [Global]	Female	5-9 years	80+ years	0·443895	0·837289	0·271747	0·291144	0·99056	0·958771
Musculoskeletal disorders [Data Rich]	Male	5-9 years	80+ years	0·212992	0·275273	0·159907	0·178277	0·99975	0·999455
Musculoskeletal disorders [Data Rich]	Female	5-9 years	80+ years	0·211038	0·296461	0·15722	0·174727	0·999393	0·998761
Peripheral vascular disease [Global]	Male	40-44 years	80+ years	0·319626	0·665863	0·221345	0·22302	0·996146	0·962651
Peripheral vascular disease [Global]	Female	40-44 years	80+ years	0·346023	0·785803	0·23743	0·244099	0·992567	0·963198
Opioid use disorders [Global]	Male	0-6 days	7-27 days	0·296939	0·769561	0·0939752	0·109648	0·99994	0·98284
Opioid use disorders [Global]	Female	0-6 days	7-27 days	0·211257	0·466951	0·0750898	0·0860498	1	0·998582
Sudden infant death syndrome [Global]	Male	7-27 days	28-364 days	0·417649	0·803598	0·292182	0·307994	0·996311	0·966204
Sudden infant death syndrome [Data Rich]	Male	7-27 days	28-364 days	0·326999	0·529029	0·248444	0·310564	0·995568	0·98977
Sudden infant death syndrome [Global]	Female	7-27 days	28-364 days	0·422621	0·812252	0·317634	0·3287	0·995032	0·968395
Sudden infant death syndrome [Data Rich]	Female	7-27 days	28-364 days	0·361528	0·539902	0·285363	0·323151	0·994302	0·988993
Drug use disorders [Global]	Male	0-6 days	7-27 days	0·171187	0·432487	0·0635834	0·0632366	1	0·993335
Drug use disorders [Global]	Female	0-6 days	7-27 days	0·206583	0·465464	0·0695082	0·0793245	1	0·99757
Chagas disease [Global]	Male	28-364 days	80+ years	0·282802	0·318226	0·169846	0·153684	0·998575	0·997535
Chagas disease [Global]	Female	28-364 days	80+ years	0·286958	0·323409	0·17498	0·148181	0·998114	0·996921
Transport injuries [Data Rich]	Male	0-6 days	80+ years	0·15741	0·191834	0·122443	0·140986	0·999693	0·998854
Transport injuries [Data Rich]	Female	0-6 days	80+ years	0·174237	0·214549	0·136713	0·15687	0·999775	0·99935
Road injuries [Data Rich]	Male	0-6 days	80+ years	0·163131	0·205248	0·126672	0·147742	0·999653	0·998966
Road injuries [Data Rich]	Female	0-6 days	80+ years	0·181679	0·228234	0·142741	0·164664	0·999758	0·999421
Pedestrian road injuries [Data Rich]	Male	0-6 days	80+ years	0·188023	0·294711	0·14636	0·174722	0·999566	0·99884
Pedestrian road injuries [Data Rich]	Female	0-6 days	80+ years	0·208126	0·329966	0·159937	0·195258	0·999384	0·99859
Cyclist road injuries [Data Rich]	Male	1-4 years	80+ years	0·196334	0·319555	0·150443	0·172642	0·999788	0·999459
Cyclist road injuries [Data Rich]	Female	1-4 years	80+ years	0·227579	0·428564	0·175322	0·199114	0·999903	0·999619
Motorcyclist road injuries [Data Rich]	Male	0-6 days	80+ years	0·209768	0·399901	0·151674	0·172994	0·999926	0·999225
Motorcyclist road injuries [Data Rich]	Female	0-6 days	80+ years	0·260476	0·474804	0·194035	0·214778	0·999137	0·99801
Other road injuries [Data Rich]	Male	0-6 days	80+ years	0·330213	0·735164	0·241869	0·28014	0·995009	0·992723
Other road injuries [Data Rich]	Female	0-6 days	80+ years	0·352592	0·66904	0·25466	0·343641	0·993887	0·992041
Unintentional injuries [Data Rich]	Male	28-364 days	80+ years	0·162452	0·19904	0·13024	0·149903	0·999266	0·997978
Unintentional injuries [Data Rich]	Female	28-364 days	80+ years	0·169806	0·20912	0·135688	0·158122	0·999248	0·99851
Falls [Data Rich]	Male	0-6 days	80+ years	0·177672	0·216141	0·138375	0·159085	0·999416	0·999032
Falls [Data Rich]	Female	0-6 days	80+ years	0·18308	0·222138	0·139186	0·159124	0·99943	0·998825
Drowning [Data Rich]	Male	0-6 days	80+ years	0·180133	0·220055	0·140736	0·162489	0·999745	0·999317
Drowning [Data Rich]	Female	0-6 days	80+ years	0·185746	0·233418	0·143101	0·163721	0·999737	0·999469
Exposure to mechanical forces [Data Rich]	Male	0-6 days	80+ years	0·188444	0·253642	0·142124	0·161764	0·998984	0·997495
Exposure to mechanical forces [Data Rich]	Female	0-6 days	80+ years	0·194739	0·260763	0·144864	0·163647	0·998906	0·998025
Unintentional firearm injuries [Data Rich]	Female	0-6 days	80+ years	0·230928	0·347695	0·164147	0·194572	0·999534	0·99905
Unintentional suffocation [Data Rich]	Male	0-6 days	80+ years	0·242873	0·44347	0·162988	0·17855	0·991802	0·990978
Unintentional suffocation [Data Rich]	Female	0-6 days	80+ years	0·277323	0·636534	0·173587	0·175288	0·955424	0·956664
Adverse effects of medical treatment [Data Rich]	Male	0-6 days	80+ years	0·200035	0·256949	0·13673	0·152363	0·999156	0·998043
Adverse effects of medical treatment [Data Rich]	Female	0-6 days	80+ years	0·209257	0·271633	0·142904	0·158812	0·998835	0·997959
Animal contact [Data Rich]	Male	0-6 days	80+ years	0·237162	0·308002	0·178547	0·199961	0·999586	0·998888
Animal contact [Data Rich]	Female	0-6 days	80+ years	0·263211	0·339243	0·200392	0·222792	0·999532	0·999077
Venomous animal contact [Data Rich]	Male	0-6 days	80+ years	0·364098	0·558344	0·286484	0·342262	0·972486	0·970646
Venomous animal contact [Data Rich]	Female	0-6 days	80+ years	0·395624	0·555611	0·315982	0·365293	0·958659	0·95581
Non-venomous animal contact [Data Rich]	Male	0-6 days	80+ years	0·25033	0·403081	0·192417	0·20808	0·999655	0·99919
Non-venomous animal contact [Data Rich]	Female	0-6 days	80+ years	0·263601	0·415761	0·200437	0·226941	0·999079	0·998399
Foreign body [Data Rich]	Male	0-6 days	80+ years	0·200774	0·279853	0·152086	0·168147	0·99931	0·998462
Foreign body [Data Rich]	Female	0-6 days	80+ years	0·198711	0·278799	0·151055	0·162079	0·999539	0·998717
Schizophrenia [Data Rich]	Male	25-29 years	80+ years	0·328985	0·472815	0·247967	0·283454	0·999151	0·99769
Schizophrenia [Data Rich]	Female	25-29 years	80+ years	0·337086	0·508316	0·251975	0·273396	0·989902	0·987763
Pulmonary aspiration and foreign body in airway [Data Rich]	Male	0-6 days	80+ years	0·20459	0·357634	0·15276	0·167299	0·99945	0·998926
Pulmonary aspiration and foreign body in airway [Data Rich]	Female	0-6 days	80+ years	0·204002	0·366433	0·153622	0·164895	0·999622	0·999225
Foreign body in other body part [Data Rich]	Male	0-6 days	80+ years	0·234183	0·452419	0·161259	0·190524	0·993886	0·993221
Foreign body in other body part [Data Rich]	Female	0-6 days	80+ years	0·266355	0·55455	0·186921	0·226246	0·991322	0·990451
Eating disorders [Global]	Male	5-9 years	45-49 years	0·660347	0·897937	0·45233	0·460141	0·993138	0·978601
Environmental heat and cold exposure [Data Rich]	Male	0-6 days	80+ years	0·243058	0·325978	0·174697	0·19703	0·999041	0·99836
Environmental heat and cold exposure [Data Rich]	Female	0-6 days	80+ years	0·27808	0·410647	0·205568	0·227444	0·998627	0·997651
Eating disorders [Global]	Female	5-9 years	45-49 years	0·66036	0·990841	0·454899	0·454186	0·992646	0·978331
Other unintentional injuries [Data Rich]	Male	0-6 days	80+ years	0·215766	0·282631	0·164275	0·182113	0·998689	0·997259
Other unintentional injuries [Data Rich]	Female	0-6 days	80+ years	0·269426	0·340741	0·206861	0·230613	0·998481	0·997103
Self-harm and interpersonal violence [Data Rich]	Male	0-6 days	80+ years	0·178477	0·219018	0·13763	0·156179	0·998382	0·996633
Eating disorders [Data Rich]	Male	5-9 years	45-49 years	0·601725	0·804447	0·442624	0·467514	0·991924	0·988047
Self-harm and interpersonal violence [Data Rich]	Female	0-6 days	80+ years	0·192316	0·234291	0·150929	0·173732	0·998958	0·997959
Eating disorders [Data Rich]	Female	5-9 years	45-49 years	0·597074	0·707839	0·429007	0·410538	0·991878	0·987707

Appendix Table 11: CODEm predictive validity results by cause, sex, age, and location									
				Predictive validity					
Cause	Sex	Age start	Age end	RMSE in	RMSE out	Trend in	Trend out	Coverage in	Coverage out
Interpersonal violence [Data Rich]	Male	0-6 days	80+ years	0·231404	0·29184	0·194979	0·24591	0·998606	0·996242
Interpersonal violence [Data Rich]	Female	0-6 days	80+ years	0·239231	0·301795	0·209759	0·27027	0·999596	0·998168
Prostate cancer [Data Rich]	Male	15-19 years	80+ years	0·276319	0·322874	0·224801	0·257638	0·997904	0·996337
Assault by firearm [Data Rich]	Male	0-6 days	80+ years	0·307905	0·53833	0·228464	0·301595	0·996366	0·994029
Assault by firearm [Data Rich]	Female	0-6 days	80+ years	0·288122	0·450542	0·207892	0·235228	0·998476	0·99742
Assault by sharp object [Data Rich]	Male	0-6 days	80+ years	0·245345	0·410701	0·181978	0·225263	0·999104	0·99849
Assault by sharp object [Data Rich]	Female	0-6 days	80+ years	0·255448	0·400136	0·194843	0·229798	0·999028	0·998139
Testicular cancer [Global]	Male	15-19 years	80+ years	0·350363	0·516668	0·282368	0·275422	0·998652	0·993452
Assault by other means [Data Rich]	Male	0-6 days	80+ years	0·199405	0·334886	0·151412	0·178979	0·999236	0·998548
Assault by other means [Data Rich]	Female	0-6 days	80+ years	0·193929	0·289371	0·150287	0·174954	0·999448	0·998967
Testicular cancer [Data Rich]	Male	15-19 years	80+ years	0·290631	0·357037	0·24608	0·26489	0·998838	0·997797
Unintentional firearm injuries [Data Rich]	Male	0-6 days	80+ years	0·242201	0·355165	0·17219	0·189712	0·99846	0·996927
Thyroid cancer [Global]	Female	10-14 years	80+ years	0·44209	0·617842	0·354264	0·363152	0·985002	0·978881
Other infectious diseases [Data Rich]	Male	0-6 days	80+ years	0·247567	0·336213	0·184589	0·216661	0·998824	0·997851
Other infectious diseases [Data Rich]	Female	0-6 days	80+ years	0·275511	0·334582	0·187979	0·203087	0·999278	0·998756
Other neglected tropical diseases [Data Rich]	Male	0-6 days	80+ years	0·654855	0·83924	0·503456	0·546483	0·992488	0·985797
Other neglected tropical diseases [Data Rich]	Female	0-6 days	80+ years	0·683054	0·915875	0·546277	0·594521	0·989435	0·980714
Other neglected tropical diseases [Global]	Male	0-6 days	80+ years	0·718068	1·33354	0·530034	0·548114	0·991652	0·949882
Other neglected tropical diseases [Global]	Female	0-6 days	80+ years	0·757848	1·44572	0·591676	0·629764	0·98709	0·945003
Neonatal preterm birth complications [Global]	Male	0-6 days	1-4 years	0·407355	0·53129	0·354744	0·341522	0·998356	0·995542
Neonatal preterm birth complications [Global]	Female	0-6 days	1-4 years	0·38925	0·566973	0·348593	0·347034	0·99786	0·989276
Neonatal encephalopathy due to birth asphyxia and trauma [Global]	Male	0-6 days	1-4 years	0·419801	0·618165	0·367825	0·371312	0·998251	0·993386
Neonatal encephalopathy due to birth asphyxia and trauma [Global]	Female	0-6 days	1-4 years	0·440817	0·625125	0·397075	0·389844	0·998533	0·995445
Other urinary diseases [Global]	Female	0-6 days	80+ years	0·461401	0·797934	0·308214	0·317809	0·98679	0·980024
Non-melanoma skin cancer [Data Rich]	Female	15-19 years	80+ years	0·350231	0·447207	0·296085	0·303472	0·996521	0·991423
Liver cancer [Global]	Male	5-9 years	80+ years	0·290082	0·476742	0·225369	0·221722	0·996852	0·976022
Liver cancer [Data Rich]	Male	5-9 years	80+ years	0·258174	0·315295	0·211928	0·23142	0·9955	0·994049
Liver cancer [Global]	Female	5-9 years	80+ years	0·31063	0·451614	0·236595	0·228335	0·997464	0·982088
Liver cancer [Data Rich]	Female	5-9 years	80+ years	0·263012	0·319542	0·218949	0·24845	0·995481	0·993135
Gallbladder and biliary tract cancer [Global]	Female	15-19 years	80+ years	0·278276	0·430721	0·22467	0·220495	0·995848	0·978058
Gallbladder and biliary tract cancer [Data Rich]	Female	15-19 years	80+ years	0·238737	0·291368	0·200595	0·221274	0·994438	0·992405
Gallbladder and biliary tract cancer [Data Rich]	Male	15-19 years	80+ years	0·222406	0·26676	0·184651	0·202219	0·996042	0·994092
Pancreatic cancer [Global]	Male	15-19 years	80+ years	0·214577	0·31694	0·166964	0·169107	0·997982	0·992707
Pancreatic cancer [Data Rich]	Male	15-19 years	80+ years	0·191774	0·229202	0·156801	0·160509	0·996913	0·994679
Pancreatic cancer [Data Rich]	Female	15-19 years	80+ years	0·20446	0·243656	0·17005	0·177108	0·996902	0·99513
Pancreatic cancer [Global]	Female	15-19 years	80+ years	0·223429	0·332292	0·178003	0·188806	0·998268	0·992794
Non-melanoma skin cancer [Data Rich]	Male	15-19 years	80+ years	0·265502	0·335342	0·219139	0·228069	0·996862	0·992957
Breast cancer [Data Rich]	Male	15-19 years	80+ years	0·392169	0·472006	0·318287	0·364945	0·961352	0·95677
Breast cancer [Data Rich]	Female	15-19 years	80+ years	0·174401	0·203662	0·142425	0·158337	0·99768	0·996237
Uterine cancer [Data Rich]	Female	15-19 years	80+ years	0·258718	0·309653	0·21103	0·239673	0·997831	0·996836
Ovarian cancer [Data Rich]	Female	15-19 years	80+ years	0·204563	0·24867	0·16511	0·17473	0·99654	0·994685
Ovarian cancer [Global]	Female	15-19 years	80+ years	0·241635	0·355918	0·190981	0·18942	0·997766	0·98983
Brain and nervous system cancer [Data Rich]	Male	1-4 years	80+ years	0·209583	0·259042	0·174496	0·192362	0·994879	0·991676
Brain and nervous system cancer [Data Rich]	Female	1-4 years	80+ years	0·209267	0·260436	0·175812	0·189434	0·99639	0·993864
Urolithiasis [Data Rich]	Male	5-9 years	80+ years	0·42136	0·543893	0·292909	0·293224	0·888907	0·887035
Urolithiasis [Global]	Female	5-9 years	80+ years	0·636837	1·15705	0·382204	0·404913	0·916319	0·895508
Larynx cancer [Data Rich]	Male	15-19 years	80+ years	0·224619	0·268673	0·188732	0·213041	0·998271	0·997093
Larynx cancer [Data Rich]	Female	15-19 years	80+ years	0·329174	0·399058	0·278224	0·31529	0·98414	0·983187
Epilepsy [Data Rich]	Female	28-364 days	80+ years	0·188304	0·23526	0·148624	0·171069	0·999958	0·999698
Other musculoskeletal disorders [Data Rich]	Male	5-9 years	80+ years	0·231326	0·294792	0·168318	0·18166	0·999855	0·999602
Other musculoskeletal disorders [Data Rich]	Female	5-9 years	80+ years	0·235195	0·339522	0·170989	0·196284	0·999266	0·998358
Acute glomerulonephritis [Data Rich]	Male	28-364 days	80+ years	0·822721	1·07592	0·590428	0·610626	0·849045	0·84292
Acute glomerulonephritis [Global]	Male	28-364 days	80+ years	0·875901	1·55662	0·637618	0·66549	0·869347	0·810993
Gallbladder and biliary tract cancer [Global]	Male	15-19 years	80+ years	0·267968	0·401503	0·213534	0·207024	0·997764	0·988182
Larynx cancer [Global]	Male	15-19 years	80+ years	0·303352	0·455785	0·240214	0·243127	0·998699	0·986616
Larynx cancer [Global]	Female	15-19 years	80+ years	0·402518	0·615279	0·321927	0·331513	0·989312	0·983366
Non-melanoma skin cancer [Global]	Female	15-19 years	80+ years	0·532321	0·686865	0·448283	0·440105	0·99093	0·979477
Non-melanoma skin cancer [Global]	Male	15-19 years	80+ years	0·36644	0·494445	0·297165	0·298866	0·997408	0·989267
Rheumatic heart disease [Global]	Male	1-4 years	80+ years	0·20831	0·449328	0·138955	0·145013	0·999067	0·982077
Rheumatic heart disease [Global]	Female	1-4 years	80+ years	0·225209	0·478289	0·153975	0·155001	0·999099	0·979783
Breast cancer [Global]	Male	15-19 years	80+ years	0·503985	0·724397	0·39972	0·409287	0·959088	0·952042
Breast cancer [Global]	Female	15-19 years	80+ years	0·197399	0·28708	0·154618	0·156554	0·998474	0·987978
Uterine cancer [Global]	Female	15-19 years	80+ years	0·337756	0·489442	0·265703	0·267647	0·998676	0·989682
Epilepsy [Data Rich]	Male	28-364 days	80+ years	0·175537	0·218203	0·137103	0·155969	0·999891	0·99944
Brain and nervous system cancer [Global]	Female	1-4 years	80+ years	0·258146	0·366432	0·204082	0·20635	0·998287	0·992424
Brain and nervous system cancer [Global]	Male	1-4 years	80+ years	0·277284	0·359934	0·20194	0·198461	0·997279	0·990957
Non-Hodgkin lymphoma [Global]	Male	28-364 days	80+ years	0·28056	0·391563	0·207353	0·210342	0·999161	0·991927
Leukemia [Global]	Male	28-364 days	80+ years	0·296249	0·39042	0·255909	0·258479	0·997943	0·990752
Chronic respiratory diseases [Global]	Male	0-6 days	80+ years	0·295672	0·53992	0·253584	0·261745	0·996574	0·980577
Endocrine, metabolic, blood, and immune disorders [Global]	Male	0-6 days	80+ years	0·26449	0·499392	0·178086	0·18813	0·998653	0·980418
Tetanus [Global]	Male	1-4 years	80+ years	0·705943	1·25464	0·459249	0·472194	0·91302	0·895396
Urolithiasis [Data Rich]	Female	5-9 years	80+ years	0·442466	0·655227	0·328163	0·420879	0·899323	0·899758
Other congenital anomalies [Data Rich]	Female	0-6 days	80+ years	0·217836	0·36899	0·159721	0·191762	0·999513	0·998887
Other urinary diseases [Data Rich]	Female	0-6 days	80+ years	0·292608	0·458339	0·197808	0·187394	0·986596	0·985275
Endocrine, metabolic, blood, and immune disorders [Global]	Female	0-6 days	80+ years	0·258226	0·461869	0·174068	0·179602	0·999295	0·987311
Tetanus [Global]	Female	1-4 years	80+ years	0·856993	1·64741	0·618678	0·655857	0·847954	0·823808

Appendix Table 11: CODEm predictive validity results by cause, sex, age, and location

Cause	Sex	Age start	Age end	Predictive validity					
				RMSE in	RMSE out	Trend in	Trend out	Coverage in	Coverage out
Other urinary diseases [Global]	Male	0-6 days	80+ years	0·428578	0·689687	0·248937	0·261553	0·991368	0·987624
Other urinary diseases [Data Rich]	Male	0-6 days	80+ years	0·242164	0·382149	0·159439	0·182024	0·990852	0·989955
Acute glomerulonephritis [Data Rich]	Female	28-364 days	80+ years	0·844377	1·09459	0·641913	0·681432	0·850557	0·843456
Acute glomerulonephritis [Global]	Female	28-364 days	80+ years	0·958925	1·72173	0·668908	0·716961	0·874865	0·813605
Tetanus [Global]	Male	0-6 days	28-364 days	0·504263	0·880319	0·374387	0·374379	0·994881	0·977627
Tetanus [Global]	Female	0-6 days	28-364 days	0·526891	0·888634	0·413795	0·425975	0·992174	0·983909
Leukemia [Data Rich]	Male	28-364 days	80+ years	0·228271	0·269915	0·193251	0·219447	0·99711	0·994521
Leukemia [Global]	Female	28-364 days	80+ years	0·301008	0·385321	0·256331	0·252217	0·998254	0·992683
Leukemia [Data Rich]	Female	28-364 days	80+ years	0·218963	0·260716	0·184439	0·209557	0·997746	0·995751
Asthma [Global]	Male	1-4 years	80+ years	0·249964	0·549165	0·185781	0·185203	0·99935	0·983294
Asthma [Global]	Female	1-4 years	80+ years	0·243613	0·4898	0·174765	0·17916	0·999551	0·988788
Endocrine, metabolic, blood, and immune disorders [Data Rich]	Male	0-6 days	80+ years	0·207104	0·256623	0·154659	0·177137	0·999324	0·997709
Endocrine, metabolic, blood, and immune disorders [Data Rich]	Female	0-6 days	80+ years	0·20465	0·253625	0·155399	0·175053	0·999579	0·998314
Endocarditis [Global]	Male	0-6 days	80+ years	0·304961	0·501951	0·165243	0·17317	0·999702	0·992563
Endocarditis [Global]	Female	0-6 days	80+ years	0·280473	0·513765	0·155737	0·163437	0·999718	0·990103
Chronic respiratory diseases [Global]	Female	0-6 days	80+ years	0·265202	0·508385	0·202048	0·193623	0·996976	0·981862
Chronic respiratory diseases [Data Rich]	Female	0-6 days	80+ years	0·172898	0·215378	0·125891	0·141505	0·999325	0·998295
Chronic respiratory diseases [Data Rich]	Male	0-6 days	80+ years	0·171666	0·212611	0·126038	0·140777	0·999102	0·99785
Asthma [Data Rich]	Male	1-4 years	80+ years	0·20249	0·377089	0·147593	0·173461	0·999834	0·99963
Asthma [Data Rich]	Female	1-4 years	80+ years	0·201411	0·327502	0·151448	0·169342	0·999893	0·999649
Neonatal sepsis and other neonatal infections [Data Rich]	Male	0-6 days	1-4 years	0·528184	0·674495	0·307613	0·3199	0·997934	0·994946
Neonatal sepsis and other neonatal infections [Data Rich]	Female	0-6 days	1-4 years	0·70428	0·87147	0·429272	0·465388	0·992487	0·98704
Neonatal sepsis and other neonatal infections [Global]	Female	0-6 days	1-4 years	0·858853	1·20911	0·696661	0·679882	0·995571	0·997205
Diabetes mellitus [Global]	Male	0-6 days	10-14 years	0·248942	0·483544	0·120251	0·125927	0·999263	0·994511
Diabetes mellitus [Global]	Female	0-6 days	10-14 years	0·270583	0·563457	0·132897	0·129902	0·999763	0·993796
Diabetes mellitus [Data Rich]	Male	0-6 days	10-14 years	0·165575	0·219991	0·0995983	0·11732	0·999975	0·999975
Diabetes mellitus [Data Rich]	Female	0-6 days	10-14 years	0·181534	0·252258	0·112038	0·127451	0·999923	0·999855
Peptic ulcer disease [Data Rich]	Female	1-4 years	80+ years	0·195855	0·241085	0·147098	0·168628	0·999444	0·999117
Peptic ulcer disease [Data Rich]	Male	1-4 years	80+ years	0·174201	0·212275	0·129176	0·143836	0·998484	0·998316
Peptic ulcer disease [Global]	Male	1-4 years	80+ years	0·252182	0·490374	0·184911	0·178638	0·996488	0·978121
Peptic ulcer disease [Global]	Female	1-4 years	80+ years	0·259441	0·533905	0·18469	0·203035	0·998602	0·981457
Neonatal preterm birth complications [Data Rich]	Female	0-6 days	1-4 years	0·20901	0·360328	0·153207	0·183126	0·999502	0·998645
Neonatal preterm birth complications [Data Rich]	Male	0-6 days	1-4 years	0·220755	0·35867	0·155926	0·185553	0·999627	0·999489
Neonatal encephalopathy due to birth asphyxia and trauma [Data Rich]	Female	0-6 days	1-4 years	0·225605	0·391741	0·16581	0·19873	0·99862	0·997518
Sexually transmitted diseases excluding HIV [Data Rich]	Female	10-14 years	80+ years	0·349148	0·426432	0·261537	0·288072	0·976696	0·97544
Congenital anomalies [Data Rich]	Male	0-6 days	80+ years	0·191558	0·262907	0·142908	0·158741	0·999684	0·998984
Congenital anomalies [Data Rich]	Female	0-6 days	80+ years	0·186964	0·263646	0·13997	0·161853	0·999784	0·999402
Congenital anomalies [Global]	Male	0-6 days	80+ years	0·243644	0·406747	0·154749	0·159156	0·999333	0·991278
Congenital anomalies [Global]	Female	0-6 days	80+ years	0·246549	0·383237	0·151914	0·158348	0·999596	0·994099
Neonatal encephalopathy due to birth asphyxia and trauma [Data Rich]	Male	0-6 days	1-4 years	0·231806	0·414732	0·169311	0·198503	0·99854	0·997192
Hemolytic disease and other neonatal jaundice [Data Rich]	Male	0-6 days	1-4 years	0·742973	1·04125	0·620276	0·582328	0·980598	0·965655
Hemolytic disease and other neonatal jaundice [Data Rich]	Female	0-6 days	1-4 years	0·829482	1·14945	0·706925	0·676703	0·972598	0·960982
Other congenital anomalies [Data Rich]	Male	0-6 days	80+ years	0·23993	0·339576	0·166096	0·202723	0·999514	0·998939
Other congenital anomalies [Global]	Male	0-6 days	80+ years	0·270803	0·480195	0·211497	0·220352	0·999361	0·991571
Other congenital anomalies [Global]	Female	0-6 days	80+ years	0·266134	0·501704	0·201256	0·206637	0·999359	0·990673
Chronic kidney disease [Data Rich]	Female	28-364 days	80+ years	0·185879	0·22742	0·146103	0·166891	0·999184	0·998129
Chronic kidney disease [Global]	Female	28-364 days	80+ years	0·222734	0·40666	0·158481	0·163582	0·998411	0·975682
Chronic kidney disease [Data Rich]	Male	28-364 days	80+ years	0·180054	0·218044	0·141203	0·157289	0·999335	0·998454
Sexually transmitted diseases excluding HIV [Data Rich]	Male	10-14 years	80+ years	0·553312	0·676139	0·444756	0·477918	0·990744	0·982198
Diabetes mellitus [Global]	Male	15-19 years	80+ years	0·217538	0·439178	0·164509	0·157682	0·997353	0·964674
Diabetes mellitus [Global]	Female	15-19 years	80+ years	0·210116	0·430607	0·151106	0·157058	0·999131	0·969479
Diabetes mellitus [Data Rich]	Male	15-19 years	80+ years	0·171769	0·210543	0·134149	0·154189	0·999434	0·997943
Diabetes mellitus [Data Rich]	Female	15-19 years	80+ years	0·173436	0·214459	0·133848	0·153047	0·999316	0·997948
Chronic kidney disease [Global]	Male	28-364 days	80+ years	0·23198	0·391861	0·155572	0·156653	0·998288	0·976638
Cardiovascular diseases [Global]	Male	0-6 days	80+ years	0·169307	0·322962	0·118066	0·125409	0·998178	0·961493
Cardiovascular diseases [Global]	Female	0-6 days	80+ years	0·171362	0·313795	0·120172	0·122979	0·998588	0·975545
Cerebrovascular disease [Global]	Male	0-6 days	80+ years	0·198954	0·378368	0·135884	0·138689	0·998958	0·973586
Cerebrovascular disease [Global]	Female	0-6 days	80+ years	0·187137	0·382782	0·130865	0·134796	0·999261	0·979278
Hemorrhagic stroke [Global]	Male	0-6 days	80+ years	0·207991	0·375961	0·145933	0·152469	0·999247	0·984863
Hemorrhagic stroke [Global]	Female	0-6 days	80+ years	0·204726	0·372164	0·14328	0·146654	0·999392	0·988202
Hypertensive heart disease [Global]	Male	28-364 days	80+ years	0·261659	0·652723	0·155297	0·159703	0·958196	0·928237
Hypertensive heart disease [Global]	Female	28-364 days	80+ years	0·276519	0·618354	0·143769	0·145815	0·963531	0·943911
Ischemic heart disease [Global]	Male	28-364 days	80+ years	0·212464	0·411426	0·136687	0·143346	0·998111	0·948388
Ischemic heart disease [Global]	Female	28-364 days	80+ years	0·208296	0·440276	0·133356	0·139166	0·997648	0·967468
Other musculoskeletal disorders [Global]	Female	5-9 years	80+ years	0·310387	0·571592	0·197649	0·208472	0·998989	0·979197
Other musculoskeletal disorders [Global]	Male	5-9 years	80+ years	0·28484	0·449628	0·193563	0·191324	0·999534	0·994184
Skin and subcutaneous diseases [Global]	Male	28-364 days	80+ years	0·434128	0·773754	0·212619	0·2446	0·998567	0·976954
Skin and subcutaneous diseases [Global]	Female	28-364 days	80+ years	0·437465	0·801166	0·212616	0·222822	0·997049	0·966636
Skin and subcutaneous diseases [Data Rich]	Male	28-364 days	80+ years	0·355402	0·396546	0·180599	0·187337	0·998774	0·997626
Skin and subcutaneous diseases [Data Rich]	Female	28-364 days	80+ years	0·305836	0·35353	0·178772	0·190845	0·997527	0·996039
Lip and oral cavity cancer [Global]	Male	15-19 years	80+ years	0·264283	0·424968	0·211032	0·214489	0·99801	0·987116
Lip and oral cavity cancer [Data Rich]	Male	15-19 years	80+ years	0·218045	0·25946	0·183235	0·203019	0·998185	0·996814
Lip and oral cavity cancer [Global]	Female	15-19 years	80+ years	0·278991	0·424667	0·226078	0·230394	0·998813	0·992366
Lip and oral cavity cancer [Data Rich]	Female	15-19 years	80+ years	0·242025	0·287635	0·206022	0·232479	0·998552	0·997401
Esophageal cancer [Global]	Male	15-19 years	80+ years	0·263924	0·47238	0·209448	0·215339	0·995995	0·967151
Esophageal cancer [Data Rich]	Male	15-19 years	80+ years	0·216113	0·259867	0·180307	0·207895	0·997218	0·99528

Appendix Table 11: CODEm predictive validity results by cause, sex, age, and location									
				Predictive validity					
Cause	Sex	Age start	Age end	RMSE in	RMSE out	Trend in	Trend out	Coverage in	Coverage out
Esophageal cancer [Global]	Female	15-19 years	80+ years	0·304075	0·521657	0·239273	0·239344	0·995405	0·970769
Esophageal cancer [Data Rich]	Female	15-19 years	80+ years	0·234222	0·28035	0·195052	0·222812	0·997564	0·996041
Stomach cancer [Global]	Male	15-19 years	80+ years	0·206954	0·378518	0·163647	0·167061	0·997975	0·97272
Stomach cancer [Data Rich]	Male	15-19 years	80+ years	0·173986	0·205468	0·142241	0·155695	0·997167	0·994945
Stomach cancer [Global]	Female	15-19 years	80+ years	0·218389	0·363072	0·171477	0·169152	0·998307	0·97887
Stomach cancer [Data Rich]	Female	15-19 years	80+ years	0·184613	0·21712	0·152694	0·168233	0·996799	0·994778
Colon and rectum cancer [Global]	Male	15-19 years	80+ years	0·231669	0·319018	0·188826	0·191093	0·995324	0·982704
Colon and rectum cancer [Data Rich]	Male	15-19 years	80+ years	0·192877	0·227535	0·161784	0·181199	0·990844	0·986686
Colon and rectum cancer [Global]	Female	15-19 years	80+ years	0·231465	0·319431	0·188847	0·18982	0·995625	0·983714
Colon and rectum cancer [Data Rich]	Female	15-19 years	80+ years	0·196713	0·232876	0·167012	0·189957	0·990792	0·986932
Other neoplasms [Global]	Male	0-6 days	80+ years	0·313467	0·393575	0·250272	0·247976	0·997338	0·993388
Other neoplasms [Data Rich]	Male	0-6 days	80+ years	0·287791	0·343732	0·24545	0·282339	0·996592	0·993884
Other neoplasms [Global]	Female	0-6 days	80+ years	0·295042	0·397178	0·243107	0·251892	0·998521	0·996016
Other neoplasms [Data Rich]	Female	0-6 days	80+ years	0·2752	0·328283	0·235062	0·268303	0·998236	0·996055
Tuberculosis [Data Rich]	Male	28-364 days	80+ years	0·211407	0·262429	0·158322	0·179585	0·997017	0·996574
Tuberculosis [Data Rich]	Female	28-364 days	80+ years	0·238311	0·295622	0·18238	0·210855	0·998385	0·997841
Other cardiovascular and circulatory diseases [Global]	Male	0-6 days	80+ years	0·240368	0·484629	0·153867	0·157924	0·997411	0·985108
Other cardiovascular and circulatory diseases [Global]	Female	0-6 days	80+ years	0·25037	0·487232	0·159746	0·165778	0·997575	0·990026
Meningitis [Data Rich]	Female	0-6 days	1-4 years	0·209598	0·265216	0·163216	0·185298	0·999774	0·999384
Meningitis [Data Rich]	Male	0-6 days	1-4 years	0·21087	0·263655	0·162963	0·186937	0·99985	0·999186
Meningitis [Data Rich]	Male	5-9 years	80+ years	0·194416	0·235862	0·152382	0·171437	0·999598	0·999221
Meningitis [Data Rich]	Female	5-9 years	80+ years	0·198588	0·241136	0·156496	0·171924	0·999635	0·999145
Meningitis [Global]	Female	0-6 days	1-4 years	0·478859	0·659913	0·437851	0·420983	0·99871	0·995785
Meningitis [Global]	Male	0-6 days	1-4 years	0·490113	0·661961	0·451611	0·444092	0·99852	0·995987
Meningitis [Global]	Male	5-9 years	80+ years	0·283233	0·475565	0·199971	0·180462	0·998392	0·990799
Meningitis [Global]	Female	5-9 years	80+ years	0·278375	0·471974	0·210727	0·203868	0·998418	0·991174
Digestive diseases [Data Rich]	Female	1-4 years	80+ years	0·159657	0·192775	0·127111	0·146311	0·999938	0·999667
Digestive diseases [Data Rich]	Male	1-4 years	80+ years	0·15445	0·186012	0·12383	0·139952	0·999912	0·99955
Digestive diseases [Global]	Male	1-4 years	80+ years	0·21356	0·332161	0·171533	0·170629	0·99918	0·983681
Digestive diseases [Global]	Female	1-4 years	80+ years	0·225149	0·336988	0·196922	0·206501	0·999598	0·986916
Epilepsy [Global]	Male	28-364 days	80+ years	0·231456	0·457192	0·170253	0·178153	0·999598	0·988644
Epilepsy [Global]	Female	28-364 days	80+ years	0·251622	0·484595	0·187684	0·203495	0·999454	0·990392
Diarrheal diseases [Global]	Male	0-6 days	1-4 years	0·629232	0·905192	0·619909	0·655135	0·984972	0·971925
Diarrheal diseases [Global]	Female	0-6 days	1-4 years	0·640011	0·890755	0·625665	0·605561	0·985942	0·975753
Diarrheal diseases [Data Rich]	Male	0-6 days	1-4 years	0·260308	0·33367	0·189919	0·216302	0·99906	0·997783
Lower respiratory infections [Global]	Male	5-9 years	80+ years	0·287042	0·449983	0·229126	0·217209	0·994574	0·970305
Diarrheal diseases [Data Rich]	Female	0-6 days	1-4 years	0·264922	0·338532	0·19623	0·219421	0·99951	0·998077
Lower respiratory infections [Global]	Male	0-6 days	1-4 years	0·375726	0·528378	0·314334	0·332475	0·993932	0·983312
Lower respiratory infections [Global]	Female	0-6 days	1-4 years	0·446471	0·576516	0·39099	0·364012	0·992328	0·984348
Lower respiratory infections [Global]	Female	5-9 years	80+ years	0·311163	0·441977	0·262073	0·231978	0·993583	0·976055
Lower respiratory infections [Data Rich]	Male	0-6 days	1-4 years	0·258624	0·294767	0·192894	0·199509	0·999172	0·998821
Lower respiratory infections [Data Rich]	Female	0-6 days	1-4 years	0·277992	0·315998	0·208068	0·228479	0·999169	0·99883
Lower respiratory infections [Data Rich]	Male	5-9 years	80+ years	0·202729	0·23538	0·150603	0·163558	0·998718	0·997362
Lower respiratory infections [Data Rich]	Female	5-9 years	80+ years	0·203391	0·231352	0·153891	0·163046	0·999091	0·998197
Diarrheal diseases [Global]	Male	5-9 years	80+ years	0·315516	0·671702	0·21102	0·220453	0·998837	0·972869
Diarrheal diseases [Global]	Female	5-9 years	80+ years	0·325523	0·660551	0·217348	0·215835	0·998188	0·979055
Diarrheal diseases [Data Rich]	Male	5-9 years	80+ years	0·277202	0·339703	0·179774	0·200534	0·999626	0·99871
Diarrheal diseases [Data Rich]	Female	5-9 years	80+ years	0·251801	0·315704	0·175355	0·190742	0·999167	0·997921
Neonatal sepsis and other neonatal infections [Global]	Male	0-6 days	1-4 years	0·79296	1·16365	0·678902	0·678757	0·997859	0·996364
Cervical cancer [Global]	Female	15-19 years	80+ years	0·278576	0·410607	0·221742	0·223249	0·998283	0·985679
Cervical cancer [Data Rich]	Female	15-19 years	80+ years	0·211751	0·250798	0·17357	0·191575	0·997483	0·994811
Gastritis and duodenitis [Data Rich]	Male	1-4 years	80+ years	0·289875	0·582981	0·204764	0·212129	0·988561	0·986857
Gastritis and duodenitis [Data Rich]	Female	1-4 years	80+ years	0·330937	0·663577	0·236599	0·250947	0·982695	0·981106
Gastritis and duodenitis [Global]	Female	1-4 years	80+ years	0·478308	0·90286	0·302949	0·305291	0·98483	0·965214
Gastritis and duodenitis [Global]	Male	1-4 years	80+ years	0·375868	0·802617	0·256172	0·258818	0·990084	0·969651
Appendicitis [Data Rich]	Female	1-4 years	80+ years	0·245567	0·30029	0·194247	0·218738	0·999471	0·99881
Appendicitis [Data Rich]	Male	1-4 years	80+ years	0·224136	0·27277	0·176263	0·201586	0·999806	0·999199
Appendicitis [Global]	Male	1-4 years	80+ years	0·298285	0·529542	0·210927	0·231073	0·999003	0·990729
Appendicitis [Global]	Female	1-4 years	80+ years	0·312269	0·501027	0·226371	0·234019	0·998957	0·991668
Other digestive diseases [Data Rich]	Female	1-4 years	80+ years	0·187788	0·271664	0·144124	0·153374	0·999758	0·999571
Other digestive diseases [Data Rich]	Male	1-4 years	80+ years	0·176642	0·250597	0·135475	0·155106	1	0·999797
Other digestive diseases [Global]	Male	1-4 years	80+ years	0·253123	0·39836	0·168002	0·177113	0·999644	0·994528
Other digestive diseases [Global]	Female	1-4 years	80+ years	0·251109	0·453833	0·180941	0·194624	0·999141	0·994738
Hodgkin lymphoma [Global]	Male	28-364 days	80+ years	0·369664	0·537094	0·27558	0·27732	0·99861	0·99135
Hodgkin lymphoma [Global]	Female	28-364 days	80+ years	0·511433	0·710323	0·345017	0·347415	0·983266	0·976593
Anorexia nervosa [Global]	Male	5-9 years	45-49 years	0·676188	0·936886	0·466817	0·46792	0·991506	0·977399
Hodgkin lymphoma [Data Rich]	Female	28-364 days	80+ years	0·360903	0·459231	0·295331	0·329001	0·974471	0·97383
Anorexia nervosa [Global]	Female	5-9 years	45-49 years	0·689124	1·00558	0·465121	0·484152	0·992397	0·980226
Anorexia nervosa [Data Rich]	Male	5-9 years	45-49 years	0·611363	0·827428	0·451743	0·468925	0·99085	0·986131
Anorexia nervosa [Data Rich]	Female	5-9 years	45-49 years	0·607575	0·738699	0·438523	0·418384	0·991989	0·987068
Hodgkin lymphoma [Data Rich]	Male	28-364 days	80+ years	0·291003	0·374267	0·239089	0·262947	0·996633	0·995042
Encephalitis [Data Rich]	Female	0-6 days	80+ years	0·238036	0·310555	0·182385	0·195822	0·999749	0·999318
Encephalitis [Data Rich]	Male	0-6 days	80+ years	0·242173	0·309767	0·178017	0·197319	0·999871	0·99958
Encephalitis [Global]	Male	0-6 days	80+ years	0·294021	0·572937	0·213487	0·216659	0·999043	0·982793
Encephalitis [Global]	Female	0-6 days	80+ years	0·314949	0·561877	0·211558	0·207291	0·999105	0·984247
Cardiovascular diseases [Data Rich]	Male	0-6 days	80+ years	0·128789	0·157006	0·0977253	0·111164	0·999528	0·998792

Appendix Table 11: CODEm predictive validity results by cause, sex, age, and location

Cause	Sex	Age start	Age end	Predictive validity					
				RMSE in	RMSE out	Trend in	Trend out	Coverage in	Coverage out
Cardiovascular diseases [Data Rich]	Female	0-6 days	80+ years	0·13191	0·159791	0·100678	0·115697	0·999587	0·999022
Rheumatic heart disease [Data Rich]	Male	1-4 years	80+ years	0·154642	0·191258	0·116127	0·131276	0·999744	0·999166
Rheumatic heart disease [Data Rich]	Female	1-4 years	80+ years	0·167888	0·210923	0·127274	0·145119	0·999717	0·999285
Ischemic heart disease [Data Rich]	Male	28-364 days	80+ years	0·148811	0·185673	0·106221	0·123874	0·999568	0·999002
Ischemic heart disease [Data Rich]	Female	28-364 days	80+ years	0·156253	0·194679	0·112207	0·127082	0·998142	0·997551
Cerebrovascular disease [Data Rich]	Male	0-6 days	80+ years	0·148468	0·180354	0·111289	0·127271	0·999843	0·999328
Cerebrovascular disease [Data Rich]	Female	0-6 days	80+ years	0·14839	0·179681	0·111404	0·124484	0·999812	0·999444
Ischemic stroke [Data Rich]	Male	28-364 days	80+ years	0·162974	0·208628	0·122859	0·140799	0·999829	0·999542
Ischemic stroke [Data Rich]	Female	28-364 days	80+ years	0·176751	0·226582	0·138814	0·156014	0·999427	0·999145
Hemorrhagic stroke [Data Rich]	Male	0-6 days	80+ years	0·159354	0·206351	0·120562	0·138704	0·999531	0·998722
Hemorrhagic stroke [Data Rich]	Female	0-6 days	80+ years	0·159895	0·210713	0·121499	0·136864	0·999653	0·999104
Hypertensive heart disease [Data Rich]	Male	28-364 days	80+ years	0·20642	0·305453	0·122387	0·146038	0·940738	0·94004
Hypertensive heart disease [Data Rich]	Female	28-364 days	80+ years	0·18966	0·280052	0·112534	0·131474	0·947252	0·946682
Cardiomyopathy and myocarditis [Data Rich]	Male	0-6 days	80+ years	0·172748	0·251127	0·12851	0·151246	0·999407	0·998112
Cardiomyopathy and myocarditis [Data Rich]	Female	0-6 days	80+ years	0·168162	0·241965	0·124297	0·143526	0·99929	0·997977
Rabies [Global]	Male	28-364 days	80+ years	0·847679	1·7383	0·657715	0·692842	0·985947	0·909435
Rabies [Global]	Female	28-364 days	80+ years	1·03519	1·92995	0·815822	0·856311	0·983796	0·891839
Aortic aneurysm [Data Rich]	Male	15-19 years	80+ years	0·154626	0·241811	0·119887	0·133369	0·999707	0·999376
Aortic aneurysm [Data Rich]	Female	15-19 years	80+ years	0·165558	0·242339	0·12879	0·139556	0·999684	0·999434
Non-Hodgkin lymphoma [Data Rich]	Female	28-364 days	80+ years	0·24593	0·287152	0·189891	0·211923	0·998826	0·99803
Peripheral vascular disease [Data Rich]	Male	40-44 years	80+ years	0·274108	0·570584	0·199772	0·206832	0·996571	0·993439
Peripheral vascular disease [Data Rich]	Female	40-44 years	80+ years	0·283709	0·624997	0·20301	0·233713	0·994326	0·99139
Non-Hodgkin lymphoma [Data Rich]	Male	28-364 days	80+ years	0·241756	0·279973	0·180896	0·201326	0·998656	0·997671
Endocarditis [Data Rich]	Male	0-6 days	80+ years	0·18127	0·330453	0·131639	0·153831	0·99991	0·999673
Endocarditis [Data Rich]	Female	0-6 days	80+ years	0·172816	0·335074	0·125424	0·146561	0·999901	0·999684
Other cardiovascular and circulatory diseases [Data Rich]	Male	0-6 days	80+ years	0·167965	0·205341	0·123035	0·137883	0·998134	0·99649
Other cardiovascular and circulatory diseases [Data Rich]	Female	0-6 days	80+ years	0·166953	0·207281	0·121027	0·135104	0·998454	0·996831
Multiple myeloma [Global]	Female	15-19 years	80+ years	0·291795	0·437811	0·232924	0·238089	0·998433	0·994079
Multiple myeloma [Global]	Male	15-19 years	80+ years	0·29141	0·434029	0·233432	0·232682	0·998274	0·993662
Acute lymphoid leukemia [Data Rich]	Female	28-364 days	80+ years	0·224145	0·306577	0·187613	0·182849	0·999711	0·998635
Acute lymphoid leukemia [Data Rich]	Male	28-364 days	80+ years	0·232432	0·294645	0·193586	0·165457	0·999559	0·997808
Acute myeloid leukemia [Data Rich]	Male	28-364 days	80+ years	0·222903	0·297504	0·179173	0·170446	0·999055	0·997756
Nutritional deficiencies [Data Rich]	Male	28-364 days	80+ years	0·287957	0·356919	0·224006	0·257262	0·998454	0·996931
Nutritional deficiencies [Data Rich]	Female	28-364 days	80+ years	0·302854	0·371886	0·234974	0·266771	0·998176	0·996242
Nutritional deficiencies [Global]	Male	28-364 days	80+ years	0·446745	0·741584	0·385084	0·351559	0·997517	0·975916
Nutritional deficiencies [Global]	Female	28-364 days	80+ years	0·347798	0·679271	0·261051	0·281852	0·997554	0·979993
Protein-energy malnutrition [Global]	Male	28-364 days	1-4 years	0·393049	0·829268	0·288978	0·306836	0·995362	0·967327
Protein-energy malnutrition [Global]	Female	28-364 days	1-4 years	0·359054	0·826946	0·256126	0·277439	0·995869	0·966732
Protein-energy malnutrition [Data Rich]	Male	28-364 days	1-4 years	0·28764	0·413384	0·223883	0·236355	0·998012	0·995415
Protein-energy malnutrition [Data Rich]	Female	28-364 days	1-4 years	0·274686	0·410124	0·20662	0·228832	0·998621	0·995608
Protein-energy malnutrition [Global]	Male	5-9 years	80+ years	0·416663	0·877191	0·308952	0·317449	0·97512	0·951571
Protein-energy malnutrition [Global]	Female	5-9 years	80+ years	0·41455	0·915499	0·291363	0·310148	0·972486	0·940543
Protein-energy malnutrition [Data Rich]	Male	5-9 years	80+ years	0·363626	0·493479	0·278261	0·290284	0·969737	0·967988
Protein-energy malnutrition [Data Rich]	Female	5-9 years	80+ years	0·464366	0·557276	0·280241	0·302441	0·965152	0·963959
Other pharynx cancer [Global]	Female	15-19 years	80+ years	0·35216	0·539415	0·283676	0·289152	0·99096	0·984027
Hepatitis [Global]	Male	28-364 days	80+ years	0·389791	0·804471	0·279954	0·29963	0·998649	0·9654
Hepatitis [Global]	Female	28-364 days	80+ years	0·409244	0·785417	0·297423	0·306438	0·998383	0·970787
Hepatitis [Data Rich]	Male	28-364 days	80+ years	0·345241	0·467759	0·252615	0·275611	0·999633	0·998343
Hepatitis [Data Rich]	Female	28-364 days	80+ years	0·360704	0·479611	0·266419	0·292711	0·999524	0·997872
Cirrhosis and other chronic liver diseases [Global]	Male	1-4 years	80+ years	0·197443	0·437792	0·13986	0·141803	0·99945	0·955733
Cirrhosis and other chronic liver diseases [Global]	Female	1-4 years	80+ years	0·227061	0·447483	0·147538	0·152808	0·999689	0·972188
Cirrhosis and other chronic liver diseases [Data Rich]	Male	1-4 years	80+ years	0·169942	0·210325	0·127095	0·148426	0·999859	0·999331
Cirrhosis and other chronic liver diseases [Data Rich]	Female	1-4 years	80+ years	0·16852	0·206811	0·132009	0·151937	0·999911	0·999634
Sexually transmitted diseases excluding HIV [Global]	Male	10-14 years	80+ years	0·747028	1·25787	0·562003	0·607499	0·90862	0·90912
Sexually transmitted diseases excluding HIV [Global]	Female	10-14 years	80+ years	0·533124	0·783629	0·416827	0·384073	0·982706	0·974463
Paralytic ileus and intestinal obstruction [Data Rich]	Female	0-6 days	80+ years	0·245266	0·323878	0·171958	0·202057	0·999431	0·999196
Paralytic ileus and intestinal obstruction [Data Rich]	Male	0-6 days	80+ years	0·231533	0·298973	0·155815	0·170793	0·999945	0·999768
Paralytic ileus and intestinal obstruction [Global]	Male	0-6 days	80+ years	0·275141	0·481517	0·180579	0·18018	0·999465	0·988245
Paralytic ileus and intestinal obstruction [Global]	Female	0-6 days	80+ years	0·317291	0·525888	0·189343	0·200588	0·999155	0·991053
Inflammatory bowel disease [Data Rich]	Male	1-4 years	80+ years	0·238707	0·402211	0·174534	0·200338	0·999768	0·999231
Inflammatory bowel disease [Data Rich]	Female	1-4 years	80+ years	0·270577	0·455568	0·205051	0·246676	0·998859	0·997845
Inflammatory bowel disease [Global]	Female	1-4 years	80+ years	0·331179	0·582828	0·236738	0·246681	0·998232	0·988306
Inflammatory bowel disease [Global]	Male	1-4 years	80+ years	0·314979	0·572279	0·211475	0·21159	0·999346	0·990028
Gallbladder and biliary diseases [Data Rich]	Female	1-4 years	80+ years	0·216154	0·262167	0·16576	0·179555	0·999345	0·998466
Gallbladder and biliary diseases [Data Rich]	Male	1-4 years	80+ years	0·187988	0·231465	0·136002	0·15178	0·999815	0·99955
Gallbladder and biliary diseases [Global]	Male	1-4 years	80+ years	0·299531	0·506202	0·207202	0·218847	0·997903	0·987375
Gallbladder and biliary diseases [Global]	Female	1-4 years	80+ years	0·29401	0·499604	0·215492	0·223702	0·998064	0·988359
Pancreatitis [Data Rich]	Male	1-4 years	80+ years	0·213743	0·273859	0·152357	0·154195	0·999817	0·999238
Pancreatitis [Data Rich]	Female	1-4 years	80+ years	0·21213	0·264481	0·160885	0·163192	0·999526	0·999018
Pancreatitis [Global]	Female	1-4 years	80+ years	0·272855	0·48984	0·191205	0·196191	0·999046	0·990544
Pancreatitis [Global]	Male	1-4 years	80+ years	0·258021	0·493992	0·176007	0·175723	0·99929	0·987921
Self-harm [Data Rich]	Male	10-14 years	80+ years	0·169493	0·210637	0·131578	0·157327	0·999122	0·997806
Self-harm [Data Rich]	Female	10-14 years	80+ years	0·186507	0·229924	0·147491	0·168381	0·998981	0·998069
Self-harm [Global]	Male	10-14 years	80+ years	0·231596	0·530969	0·169919	0·176416	0·998491	0·967269
Self-harm [Global]	Female	10-14 years	80+ years	0·282112	0·586441	0·189594	0·194433	0·998415	0·97333
Vascular intestinal disorders [Data Rich]	Male	1-4 years	80+ years	0·226376	0·297727	0·166524	0·185016	0·999254	0·998599

Appendix Table 11: CODEm predictive validity results by cause, sex, age, and location									
				Predictive validity					
Cause	Sex	Age start	Age end	RMSE in	RMSE out	Trend in	Trend out	Coverage in	Coverage out
Vascular intestinal disorders [Data Rich]	Female	1-4 years	80+ years	0·302709	0·437618	0·237126	0·27905	0·997846	0·996199
Vascular intestinal disorders [Global]	Female	1-4 years	80+ years	0·336809	0·622238	0·243898	0·255409	0·997896	0·97966
Vascular intestinal disorders [Global]	Male	1-4 years	80+ years	0·273602	0·51832	0·185471	0·187851	0·998844	0·981979
Other intestinal infectious diseases [Data Rich]	Male	28-364 days	80+ years	0·849938	1·33793	0·68134	0·6654	0·994814	0·982096
Other intestinal infectious diseases [Data Rich]	Female	28-364 days	80+ years	0·866065	1·38311	0·704459	0·715279	0·991669	0·981203
Schizophrenia [Global]	Male	25-29 years	80+ years	0·410656	0·817724	0·298778	0·328747	0·998071	0·968228
Schizophrenia [Global]	Female	25-29 years	80+ years	0·435666	0·844652	0·324554	0·340493	0·99663	0·962595
Bulimia nervosa [Global]	Male	5-9 years	45-49 years	1·24174	1·52224	0·778907	0·773927	0·74696	0·874477
Bulimia nervosa [Global]	Female	5-9 years	45-49 years	1·4908	2·06527	0·947039	0·975532	0·804551	0·880664
Bulimia nervosa [Data Rich]	Male	5-9 years	45-49 years	0·995135	1·1258	0·748965	0·613634	0·977221	0·970577
Bulimia nervosa [Data Rich]	Female	5-9 years	45-49 years	1·2863	1·69218	0·906183	0·829909	0·980867	0·973485
Other nutritional deficiencies [Global]	Male	28-364 days	80+ years	0·39963	0·80588	0·28936	0·308623	0·971702	0·959621
Other nutritional deficiencies [Global]	Female	28-364 days	80+ years	0·523603	0·876513	0·305436	0·312428	0·969894	0·960787
Other nutritional deficiencies [Data Rich]	Male	28-364 days	80+ years	0·308784	0·525993	0·229308	0·257695	0·974574	0·972624
Other nutritional deficiencies [Data Rich]	Female	28-364 days	80+ years	0·324322	0·504118	0·242634	0·267201	0·972955	0·970879
Tuberculosis [Global]	Male	28-364 days	80+ years	0·351171	0·65737	0·237563	0·244588	0·996165	0·964623
Tuberculosis [Global]	Female	28-364 days	80+ years	0·407543	0·684353	0·305384	0·285322	0·996038	0·972464
Other chromosomal abnormalities [Global]	Male	0-6 days	80+ years	0·555838	1·23856	0·360116	0·351254	0·795613	0·811537
Other chromosomal abnormalities [Global]	Female	0-6 days	80+ years	0·551614	1·2223	0·368571	0·349653	0·796089	0·793422
Poisonings [Global]	Female	0-6 days	80+ years	0·40562	0·695221	0·347477	0·342542	0·998082	0·981669
Poisonings [Global]	Male	0-6 days	80+ years	0·425385	0·668781	0·372386	0·348576	0·998602	0·980538
Other exposure to mechanical forces [Data Rich]	Male	0-6 days	80+ years	0·177802	0·23645	0·135374	0·151503	0·999657	0·999125
Other exposure to mechanical forces [Global]	Male	0-6 days	80+ years	0·268158	0·468237	0·165544	0·166591	0·999418	0·992068
Other exposure to mechanical forces [Data Rich]	Female	0-6 days	80+ years	0·210717	0·27508	0·163324	0·185215	0·999748	0·999414
Other exposure to mechanical forces [Global]	Female	0-6 days	80+ years	0·280513	0·504001	0·191167	0·20147	0·9994	0·994472
Other transport injuries [Global]	Male	0-6 days	80+ years	0·299572	0·657955	0·204568	0·221147	0·998102	0·978056
Other transport injuries [Global]	Female	0-6 days	80+ years	0·343051	0·672291	0·24969	0·266889	0·998576	0·983956
Fire, heat, and hot substances [Global]	Male	0-6 days	80+ years	0·297138	0·555057	0·201519	0·215421	0·996976	0·985349
Fire, heat, and hot substances [Data Rich]	Female	0-6 days	80+ years	0·178866	0·21795	0·137678	0·152169	0·999587	0·999097
Fire, heat, and hot substances [Global]	Female	0-6 days	80+ years	0·329355	0·550967	0·200576	0·199456	0·996738	0·975327
Motor vehicle road injuries [Global]	Male	0-6 days	80+ years	0·256125	0·413546	0·156501	0·167159	0·998793	0·982647
Motor vehicle road injuries [Data Rich]	Female	0-6 days	80+ years	0·2055	0·312268	0·152528	0·174802	0·999392	0·998927
Motor vehicle road injuries [Global]	Female	0-6 days	80+ years	0·248694	0·437119	0·168686	0·175274	0·999188	0·991423
Motor vehicle road injuries [Data Rich]	Male	0-6 days	80+ years	0·194584	0·306149	0·136924	0·163675	0·999721	0·999274
Fire, heat, and hot substances [Data Rich]	Male	0-6 days	80+ years	0·179665	0·216958	0·140302	0·156649	0·99958	0·999183
Other transport injuries [Data Rich]	Female	0-6 days	80+ years	0·278647	0·365364	0·22004	0·26146	0·998686	0·997421
Other transport injuries [Data Rich]	Male	0-6 days	80+ years	0·232459	0·328819	0·171761	0·200061	0·998751	0·997558
Poisonings [Data Rich]	Male	0-6 days	80+ years	0·223922	0·274639	0·164063	0·184121	0·998576	0·99756
Poisonings [Data Rich]	Female	0-6 days	80+ years	0·203502	0·25589	0·150142	0·184139	0·998746	0·997905
Urolithiasis [Global]	Male	5-9 years	80+ years	0·634033	1·03335	0·363189	0·345785	0·90906	0·896325
Other infectious diseases [Global]	Male	0-6 days	80+ years	0·436824	0·703696	0·372887	0·380364	0·998012	0·98788
Other infectious diseases [Global]	Female	0-6 days	80+ years	0·404484	0·666774	0·352461	0·351163	0·99826	0·987921
Opioid use disorders [Data Rich]	Female	15-19 years	80+ years	0·249261	0·64034	0·181122	0·216088	0·999871	0·999433
Drug use disorders [Data Rich]	Female	15-19 years	80+ years	0·248363	0·348583	0·185059	0·226215	0·999359	0·998674
Drug use disorders [Data Rich]	Male	15-19 years	80+ years	0·256162	0·374162	0·181443	0·213387	0·998565	0·996579
Drug use disorders [Data Rich]	Male	0-6 days	7-27 days	0·125699	0·188416	0·0582317	0·0789605	1	0·999923
Drug use disorders [Data Rich]	Female	0-6 days	7-27 days	0·167167	0·291342	0·0571552	0·0867337	1	1
Opioid use disorders [Data Rich]	Male	15-19 years	80+ years	0·252296	0·664022	0·175473	0·212255	0·999671	0·998623
Opioid use disorders [Data Rich]	Male	0-6 days	7-27 days	0·220885	0·668543	0·0717221	0·117162	1	0·999751
Opioid use disorders [Data Rich]	Female	0-6 days	7-27 days	0·178574	0·49436	0·0603308	0·0787125	1	0·999972
Cocaine use disorders [Data Rich]	Male	15-19 years	80+ years	0·432421	1·0317	0·297133	0·400751	0·999215	0·998329
Cocaine use disorders [Data Rich]	Female	15-19 years	80+ years	0·44167	1·07188	0·33154	0·420939	0·997974	0·995546
Amphetamine use disorders [Data Rich]	Male	15-19 years	80+ years	0·666088	1·40622	0·490885	0·692138	0·997806	0·989087
Amphetamine use disorders [Data Rich]	Female	15-19 years	80+ years	0·543521	1·28717	0·412366	0·554011	0·997283	0·993797
Other drug use disorders [Data Rich]	Male	15-19 years	80+ years	0·350076	0·73773	0·245166	0·329733	0·998046	0·996034
Other drug use disorders [Data Rich]	Female	15-19 years	80+ years	0·276318	0·614065	0·195738	0·245738	0·999672	0·99895
Inguinal, femoral, and abdominal hernia [Data Rich]	Female	1-4 years	80+ years	0·266119	0·378788	0·165046	0·190263	0·999515	0·998892
Inguinal, femoral, and abdominal hernia [Data Rich]	Male	1-4 years	80+ years	0·244194	0·348262	0·15474	0·16721	0·999432	0·99891
Inguinal, femoral, and abdominal hernia [Global]	Male	1-4 years	80+ years	0·352325	0·56361	0·195181	0·200477	0·987658	0·985031
Inguinal, femoral, and abdominal hernia [Global]	Female	1-4 years	80+ years	0·405669	0·649066	0·21152	0·218621	0·965085	0·964823
Foreign body [Global]	Male	0-6 days	80+ years	0·247554	0·498481	0·168645	0·180559	0·99842	0·976161
Foreign body [Global]	Female	0-6 days	80+ years	0·250739	0·501313	0·171661	0·175892	0·998777	0·981552
Pulmonary aspiration and foreign body in airway [Global]	Female	0-6 days	80+ years	0·28528	0·521315	0·170286	0·176321	0·999622	0·986722
Pulmonary aspiration and foreign body in airway [Global]	Male	0-6 days	80+ years	0·272622	0·488325	0·167191	0·169241	0·999341	0·982436
Hemolytic disease and other neonatal jaundice [Global]	Female	0-6 days	1-4 years	0·961686	1·41027	0·805645	0·790481	0·986118	0·970519
Hemolytic disease and other neonatal jaundice [Global]	Male	0-6 days	1-4 years	0·818985	1·32544	0·669326	0·660534	0·985005	0·943838
Neonatal disorders [Global]	Female	0-6 days	1-4 years	0·372323	0·503852	0·340966	0·318839	0·997492	0·993728
Neonatal disorders [Data Rich]	Female	0-6 days	1-4 years	0·182086	0·240292	0·131319	0·157579	0·998552	0·997739
Neonatal disorders [Global]	Male	0-6 days	1-4 years	0·377325	0·495069	0·350862	0·332023	0·998148	0·994451
Neonatal disorders [Data Rich]	Male	0-6 days	1-4 years	0·178929	0·236307	0·129316	0·15385	0·998366	0·99772
Chronic obstructive pulmonary disease [Global]	Female	28-364 days	80+ years	0·210367	0·409778	0·142489	0·143877	0·999719	0·987232
Chronic obstructive pulmonary disease [Global]	Male	28-364 days	80+ years	0·199208	0·367817	0·144564	0·146433	0·999615	0·986572
Tetanus [Data Rich]	Male	1-4 years	80+ years	0·477838	0·626049	0·376792	0·445355	0·993303	0·988722
Tetanus [Data Rich]	Female	1-4 years	80+ years	0·711927	0·936873	0·586905	0·759172	0·986539	0·977636
Tetanus [Data Rich]	Male	0-6 days	28-364 days	0·325834	0·446585	0·230826	0·288571	0·99744	0·995266
Tetanus [Data Rich]	Female	0-6 days	28-364 days	0·325221	0·435176	0·241054	0·29489	0·995113	0·992831

Appendix Table 11: CODEm predictive validity results by cause, sex, age, and location

Cause	Sex	Age start	Age end	Predictive validity					
				RMSE in	RMSE out	Trend in	Trend out	Coverage in	Coverage out
Cellulitis [Global]	Male	28-364 days	80+ years	0·647528	0·991265	0·327955	0·314785	0·95777	0·949362
Cellulitis [Global]	Female	28-364 days	80+ years	0·56462	0·999479	0·381335	0·3964	0·939903	0·9297
Pyoderma [Global]	Male	0-6 days	80+ years	0·302043	0·642404	0·186472	0·196764	0·997313	0·988465
Pyoderma [Global]	Female	0-6 days	80+ years	0·320125	0·628437	0·197569	0·206477	0·993824	0·983398
Pyoderma [Data Rich]	Male	0-6 days	80+ years	0·224209	0·338661	0·152163	0·179769	0·997054	0·996441
Pyoderma [Data Rich]	Female	0-6 days	80+ years	0·244354	0·360529	0·166964	0·195222	0·991924	0·99144
Other skin and subcutaneous diseases [Global]	Male	28-364 days	80+ years	0·49163	0·823367	0·372127	0·371828	0·949544	0·949265
Other skin and subcutaneous diseases [Global]	Female	28-364 days	80+ years	0·508233	0·793385	0·366162	0·358275	0·939575	0·934646
Other skin and subcutaneous diseases [Data Rich]	Male	28-364 days	80+ years	0·391667	0·537225	0·299679	0·303829	0·959256	0·957097
Other skin and subcutaneous diseases [Data Rich]	Female	28-364 days	80+ years	0·436082	0·528737	0·301994	0·297398	0·951311	0·948737
Cellulitis [Data Rich]	Male	28-364 days	80+ years	0·385964	0·695383	0·257354	0·270248	0·953723	0·952762
Cellulitis [Data Rich]	Female	28-364 days	80+ years	0·462116	0·790565	0·328841	0·384048	0·933416	0·931072

Appendix Table 12: Comparison of GBD 2013 and GBD 2015 covariates and level of covariates in cause of death modeling								
			GBD 2013			GBD 2015		
Cause	Sex	Covariate	Level 1	Level 2	Level 3	Level 1	Level 2	Level 3
Tuberculosis	Male	Cumulative Cigarettes (5 Years)	X			X		
Tuberculosis	Male	Diabetes Fasting Plasma Glucose (mmol/L)	X			X		
Tuberculosis	Male	Health System Access 2 (unitless)	X			X		
Tuberculosis	Male	Indoor Air Pollution (All Cooking Fuels)	X			X		
Tuberculosis	Male	Log-transformed SEV scalar: TB				X		
Tuberculosis	Male	Malnutrition (proportion <2SD weight for age)	X			X		
Tuberculosis	Male	Smoking Prevalence	X			X		
Tuberculosis	Male	Alcohol (litres per capita)		X			X	
Tuberculosis	Male	Diabetes Fasting Plasma Glucose (mmol/L)	X				X	
Tuberculosis	Male	Health System Access 2 (unitless)	X				X	
Tuberculosis	Male	Indoor Air Pollution (All Cooking Fuels)	X				X	
Tuberculosis	Male	Malnutrition (proportion <2SD weight for age)	X				X	
Tuberculosis	Male	Population Density (500-1000 ppl/sqkm, proportion)		X			X	
Tuberculosis	Male	Population Density (over 1000 ppl/sqkm, proportion)		X			X	
Tuberculosis	Male	Smoking Prevalence	X				X	
Tuberculosis	Male	Education (years per capita)			X			X
Tuberculosis	Male	LDI (I$ per capita)			X			X
Tuberculosis	Male	Sociodemographic Status						X
Tuberculosis	Female	Cumulative Cigarettes (5 Years)	X			X		
Tuberculosis	Female	Diabetes Fasting Plasma Glucose (mmol/L)	X			X		
Tuberculosis	Female	Health System Access 2 (unitless)	X			X		
Tuberculosis	Female	Indoor Air Pollution (All Cooking Fuels)	X			X		
Tuberculosis	Female	Log-transformed SEV scalar: TB				X		
Tuberculosis	Female	Malnutrition (proportion <2SD weight for age)	X			X		
Tuberculosis	Female	Smoking Prevalence	X			X		
Tuberculosis	Female	Alcohol (litres per capita)		X			X	
Tuberculosis	Female	Diabetes Fasting Plasma Glucose (mmol/L)	X				X	
Tuberculosis	Female	Health System Access 2 (unitless)	X				X	
Tuberculosis	Female	Indoor Air Pollution (All Cooking Fuels)	X				X	
Tuberculosis	Female	Malnutrition (proportion <2SD weight for age)	X				X	
Tuberculosis	Female	Population Density (500-1000 ppl/sqkm, proportion)		X			X	
Tuberculosis	Female	Population Density (over 1000 ppl/sqkm, proportion)		X			X	
Tuberculosis	Female	Smoking Prevalence	X				X	
Tuberculosis	Female	Education (years per capita)			X			X
Tuberculosis	Female	LDI (I$ per capita)			X			X
Tuberculosis	Female	Sociodemographic Status						X
Diarrheal diseases	Male	Improved Water Source (proportion with access)	X			X		
Diarrheal diseases	Male	Log-transformed SEV scalar: Diarrhea				X		
Diarrheal diseases	Male	Rotavirus coverage (proportion)				X		
Diarrheal diseases	Male	SEV unsafe sanitation				X		
Diarrheal diseases	Male	SEV unsafe water				X		
Diarrheal diseases	Male	Sanitation (proportion with access)	X			X		
Diarrheal diseases	Male	Health System Access (unitless)					X	
Diarrheal diseases	Male	Malnutrition (proportion <2SD weight for age)		X			X	
Diarrheal diseases	Male	Education (years per capita)			X			X
Diarrheal diseases	Male	LDI (I$ per capita)			X			X
Diarrheal diseases	Male	Latitude Under 15 (proportion)			X			X
Diarrheal diseases	Male	Population Density (over 1000 ppl/sqkm, proportion)			X			X
Diarrheal diseases	Male	Population Density (under 150 ppl/sqkm, proportion)			X			X
Diarrheal diseases	Male	Sociodemographic Status						X
Diarrheal diseases	Male	Health System Access 2 (unitless)	X					
Diarrheal diseases	Male	Rotavirus Vaccine Introduced (binary)	X					
Diarrheal diseases	Female	Improved Water Source (proportion with access)	X			X		
Diarrheal diseases	Female	Log-transformed SEV scalar: Diarrhea				X		
Diarrheal diseases	Female	Rotavirus coverage (proportion)				X		
Diarrheal diseases	Female	SEV unsafe sanitation				X		
Diarrheal diseases	Female	SEV unsafe water				X		
Diarrheal diseases	Female	Sanitation (proportion with access)	X			X		
Diarrheal diseases	Female	Health System Access (unitless)					X	
Diarrheal diseases	Female	Malnutrition (proportion <2SD weight for age)		X			X	
Diarrheal diseases	Female	Education (years per capita)			X			X
Diarrheal diseases	Female	LDI (I$ per capita)			X			X
Diarrheal diseases	Female	Latitude Under 15 (proportion)			X			X
Diarrheal diseases	Female	Population Density (over 1000 ppl/sqkm, proportion)			X			X
Diarrheal diseases	Female	Population Density (under 150 ppl/sqkm, proportion)			X			X
Diarrheal diseases	Female	Sociodemographic Status						X
Diarrheal diseases	Female	Health System Access 2 (unitless)	X					
Diarrheal diseases	Female	Rotavirus Vaccine Introduced (binary)	X					
Other intestinal infectious diseases	Male	Improved Water Source (proportion with access)				X		
Other intestinal infectious diseases	Male	Sanitation (proportion with access)				X		
Other intestinal infectious diseases	Male	Health System Access (capped)					X	
Other intestinal infectious diseases	Male	LDI (I$ per capita)					X	
Other intestinal infectious diseases	Male	Education (years per capita)						X
Other intestinal infectious diseases	Male	Sociodemographic Status						X
Other intestinal infectious diseases	Female	Improved Water Source (proportion with access)				X		
Other intestinal infectious diseases	Female	Sanitation (proportion with access)				X		
Other intestinal infectious diseases	Female	Health System Access (capped)					X	
Other intestinal infectious diseases	Female	LDI (I$ per capita)					X	
Other intestinal infectious diseases	Female	Education (years per capita)						X
Other intestinal infectious diseases	Female	Sociodemographic Status						X
Lower respiratory infections	Male	Hib3 Vaccine Coverage (proportion)	X			X		
Lower respiratory infections	Male	Indoor Air Pollution (All Cooking Fuels)	X			X		
Lower respiratory infections	Male	Log-transformed SEV scalar: LRI				X		
Lower respiratory infections	Male	Malnutrition (proportion <2SD weight for age)	X			X		

Appendix Table 12: Comparison of GBD 2013 and GBD 2015 covariates and level of covariates in cause of death modeling

			GBD 2013			GBD 2015		
Lower respiratory infections	Male	PCV3 Coverage (proportion)	X			X		
Lower respiratory infections	Male	Smoking Prevalence	X			X		
Lower respiratory infections	Male	DTP3 Coverage (proportion)		X			X	
Lower respiratory infections	Male	Health System Access 2 (unitless)	X				X	
Lower respiratory infections	Male	Education (years per capita)			X			X
Lower respiratory infections	Male	LDI (I$ per capita)			X			X
Lower respiratory infections	Male	Outdoor Air Pollution (PM2.5)	X					X
Lower respiratory infections	Male	SEV unsafe sanitation						X
Lower respiratory infections	Male	Sociodemographic Status						X
Lower respiratory infections	Male	Elevation Over 1500m (proportion)		X				
Lower respiratory infections	Male	Improved Water Source (proportion with access)		X				
Lower respiratory infections	Male	Population Density (over 1000 ppl/sqkm, proportion)			X			
Lower respiratory infections	Male	Rainfall (Quintiles 4-5)		X				
Lower respiratory infections	Male	Sanitation (proportion with access)		X				
Lower respiratory infections	Female	Hib3 Vaccine Coverage (proportion)	X			X		
Lower respiratory infections	Female	Indoor Air Pollution (All Cooking Fuels)	X			X		
Lower respiratory infections	Female	Log-transformed SEV scalar: LRI				X		
Lower respiratory infections	Female	Malnutrition (proportion <2SD weight for age)	X			X		
Lower respiratory infections	Female	PCV3 Coverage (proportion)	X			X		
Lower respiratory infections	Female	Smoking Prevalence	X			X		
Lower respiratory infections	Female	DTP3 Coverage (proportion)		X			X	
Lower respiratory infections	Female	Health System Access 2 (unitless)	X				X	
Lower respiratory infections	Female	PCV3 Coverage (proportion)	X				X	
Lower respiratory infections	Female	Education (years per capita)			X			X
Lower respiratory infections	Female	LDI (I$ per capita)			X			X
Lower respiratory infections	Female	Outdoor Air Pollution (PM2.5)	X					X
Lower respiratory infections	Female	SEV unsafe sanitation						X
Lower respiratory infections	Female	Sociodemographic Status						X
Lower respiratory infections	Female	Elevation Over 1500m (proportion)		X				
Lower respiratory infections	Female	Improved Water Source (proportion with access)		X				
Lower respiratory infections	Female	Population Density (over 1000 ppl/sqkm, proportion)			X			
Lower respiratory infections	Female	Rainfall (Quintiles 4-5)		X				
Lower respiratory infections	Female	Sanitation (proportion with access)		X				
Otitis media	Male	Health System Access 2 (unitless)	X			X		
Otitis media	Male	Indoor Air Pollution (All Cooking Fuels)	X			X		
Otitis media	Male	Log-transformed SEV scalar: Otitis				X		
Otitis media	Male	Malnutrition (proportion <2SD weight for age)	X			X		
Otitis media	Male	Outdoor Air Pollution (PM2.5)	X			X		
Otitis media	Male	Smoking Prevalence	X			X		
Otitis media	Male	DTP3 Coverage (proportion)		X			X	
Otitis media	Male	Elevation Over 1500m (proportion)		X			X	
Otitis media	Male	Improved Water Source (proportion with access)		X			X	
Otitis media	Male	Rainfall (Quintiles 4-5)		X			X	
Otitis media	Male	Sanitation (proportion with access)		X			X	
Otitis media	Male	Education (years per capita)			X			X
Otitis media	Male	LDI (I$ per capita)			X			X
Otitis media	Male	Population Density (over 1000 ppl/sqkm, proportion)			X			X
Otitis media	Male	Sociodemographic Status						X
Otitis media	Female	Health System Access 2 (unitless)	X			X		
Otitis media	Female	Indoor Air Pollution (All Cooking Fuels)	X			X		
Otitis media	Female	Log-transformed SEV scalar: Otitis				X		
Otitis media	Female	Malnutrition (proportion <2SD weight for age)	X			X		
Otitis media	Female	Outdoor Air Pollution (PM2.5)	X			X		
Otitis media	Female	Smoking Prevalence	X			X		
Otitis media	Female	DTP3 Coverage (proportion)		X			X	
Otitis media	Female	Elevation Over 1500m (proportion)		X			X	
Otitis media	Female	Improved Water Source (proportion with access)		X			X	
Otitis media	Female	Rainfall (Quintiles 4-5)		X			X	
Otitis media	Female	Sanitation (proportion with access)		X			X	
Otitis media	Female	Education (years per capita)			X			X
Otitis media	Female	LDI (I$ per capita)			X			X
Otitis media	Female	Population Density (over 1000 ppl/sqkm, proportion)			X			X
Otitis media	Female	Sociodemographic Status						X
Meningitis	Male	meningitis belt (proportion)				X		
Meningitis	Male	Health System Access 2 (unitless)	X				X	
Meningitis	Male	Improved Water Source (proportion with access)			X		X	
Meningitis	Male	Malnutrition (proportion <2SD weight for age)	X				X	
Meningitis	Male	DTP3 Coverage (proportion)						X
Meningitis	Male	LDI (I$ per capita)			X			X
Meningitis	Male	Maternal education (years per capita)						X
Meningitis	Male	Proportion of total population covered by menafrivac initiative (meningitis meningococcal type A vaccine)						X
Meningitis	Male	Sanitation (proportion with access)			X			X
Meningitis	Male	Sociodemographic Status						X
Meningitis	Male	Education (years per capita)			X			
Meningitis	Male	Meningitis Belt (binary)	X					
Meningitis	Male	Population Density (over 1000 ppl/sqkm, proportion)		X				
Meningitis	Female	meningitis belt (proportion)				X		
Meningitis	Female	Health System Access 2 (unitless)	X				X	
Meningitis	Female	Improved Water Source (proportion with access)			X		X	
Meningitis	Female	Malnutrition (proportion <2SD weight for age)	X				X	
Meningitis	Female	DTP3 Coverage (proportion)						X
Meningitis	Female	LDI (I$ per capita)			X			X
Meningitis	Female	Maternal education (years per capita)						X
Meningitis	Female	Proportion of total population covered by menafrivac initiative (meningitis meningococcal type A vaccine)						X

Appendix Table 12: Comparison of GBD 2013 and GBD 2015 covariates and level of covariates in cause of death modeling								
			GBD 2013			GBD 2015		
Meningitis	Female	Sanitation (proportion with access)			X			X
Meningitis	Female	Sociodemographic Status						X
Meningitis	Female	Education (years per capita)			X			
Meningitis	Female	Meningitis Belt (binary)	X					
Meningitis	Female	Population Density (over 1000 ppl/sqkm, proportion)		X				
Encephalitis	Male	Health System Access 2 (unitless)	X			X		
Encephalitis	Male	Japanese encelphalitis endemic area (binary)				X		
Encephalitis	Male	Malnutrition (proportion <2SD weight for age)	X			X		
Encephalitis	Male	LDI (I$ per capita)			X		X	
Encephalitis	Male	Improved Water Source (proportion with access)			X			X
Encephalitis	Male	In-Facility Delivery (proportion)						X
Encephalitis	Male	Maternal education (years per capita)						X
Encephalitis	Male	Sanitation (proportion with access)			X			X
Encephalitis	Male	Sociodemographic Status						X
Encephalitis	Male	Education (years per capita)			X			
Encephalitis	Male	Meningitis Belt (binary)	X					
Encephalitis	Male	Population Density (over 1000 ppl/sqkm, proportion)		X				
Encephalitis	Female	Health System Access 2 (unitless)	X			X		
Encephalitis	Female	Japanese encelphalitis endemic area (binary)				X		
Encephalitis	Female	Malnutrition (proportion <2SD weight for age)	X			X		
Encephalitis	Female	LDI (I$ per capita)			X		X	
Encephalitis	Female	Improved Water Source (proportion with access)			X			X
Encephalitis	Female	In-Facility Delivery (proportion)						X
Encephalitis	Female	Maternal education (years per capita)						X
Encephalitis	Female	Sanitation (proportion with access)			X			X
Encephalitis	Female	Sociodemographic Status						X
Encephalitis	Female	Education (years per capita)			X			
Encephalitis	Female	Meningitis Belt (binary)	X					
Encephalitis	Female	Population Density (over 1000 ppl/sqkm, proportion)		X				
Tetanus	Male	DTP3 Coverage (proportion)	X			X		
Tetanus	Male	Health System Access 2 (unitless)	X			X		
Tetanus	Male	Tetanus Toxoid Coverage Smooth (proportion)	X			X		
Tetanus	Male	In-Facility Delivery (proportion)		X			X	
Tetanus	Male	Skilled Birth Attendance (proportion)		X			X	
Tetanus	Male	Education (years per capita)			X			X
Tetanus	Male	LDI (I$ per capita)			X			X
Tetanus	Male	Sanitation (proportion with access)			X			X
Tetanus	Male	Sociodemographic Status						X
Tetanus	Female	DTP3 Coverage (proportion)	X			X		
Tetanus	Female	Health System Access 2 (unitless)	X			X		
Tetanus	Female	Tetanus Toxoid Coverage Smooth (proportion)	X			X		
Tetanus	Female	In-Facility Delivery (proportion)		X			X	
Tetanus	Female	Skilled Birth Attendance (proportion)		X			X	
Tetanus	Female	Education (years per capita)			X			X
Tetanus	Female	LDI (I$ per capita)			X			X
Tetanus	Female	Sanitation (proportion with access)			X			X
Tetanus	Female	Sociodemographic Status						X
Malaria	Male	Health System Access (capped)	X			X		
Malaria	Male	ITN Coverage (proportion)	X			X		
Malaria	Male	Interaction of malaria ITN and log PFPR covariates	X			X		
Malaria	Male	Malaria Endemicity (40-100%)	X			X		
Malaria	Male	Malaria Endemicity (5-100%)	X			X		
Malaria	Male	Malaria Indoor Residual Spraying Coverage	X			X		
Malaria	Male	Malaria Lysenko PFPR (2 Highest Endemicity)	X			X		
Malaria	Male	Malaria Lysenko PFPR (3 Highest Endemicity)	X			X		
Malaria	Male	Malaria PFPR (rate)	X			X		
Malaria	Male	Malaria Population-At-Risk (proportion)	X			X		
Malaria	Male	Malaria Prevalence-Weighted Resistance	X			X		
Malaria	Male	Rainfall (Quintiles 2-5)	X			X		
Malaria	Male	Rainfall (Quintiles 3-5)	X			X		
Malaria	Male	Rainfall (Quintiles 4-5)	X			X		
Malaria	Male	Education (years per capita)			X			X
Malaria	Male	LDI (I$ per capita)			X			X
Malaria	Female	Health System Access (capped)	X			X		
Malaria	Female	ITN Coverage (proportion)	X			X		
Malaria	Female	Interaction of malaria ITN and log PFPR covariates	X			X		
Malaria	Female	Malaria Endemicity (40-100%)	X			X		
Malaria	Female	Malaria Endemicity (5-100%)	X			X		
Malaria	Female	Malaria Indoor Residual Spraying Coverage	X			X		
Malaria	Female	Malaria Lysenko PFPR (2 Highest Endemicity)	X			X		
Malaria	Female	Malaria Lysenko PFPR (3 Highest Endemicity)	X			X		
Malaria	Female	Malaria PFPR (rate)	X			X		
Malaria	Female	Malaria Population-At-Risk (proportion)	X			X		
Malaria	Female	Malaria Prevalence-Weighted Resistance	X			X		
Malaria	Female	Rainfall (Quintiles 2-5)	X			X		
Malaria	Female	Rainfall (Quintiles 3-5)	X			X		
Malaria	Female	Rainfall (Quintiles 4-5)	X			X		
Malaria	Female	Education (years per capita)			X			X
Malaria	Female	LDI (I$ per capita)			X			X
Chagas disease	Male	Chagas Population-at-Risk 2 (proportion)	X			X		
Chagas disease	Male	Health System Access (unitless)	X			X		
Chagas disease	Male	chagasPrevPAHO				X		
Chagas disease	Male	Sanitation (proportion with access)		X			X	
Chagas disease	Male	Education (years per capita)			X			X
Chagas disease	Male	LDI (I$ per capita)			X			X

Appendix Table 12: Comparison of GBD 2013 and GBD 2015 covariates and level of covariates in cause of death modeling

			GBD 2013			GBD 2015		
Chagas disease	Female	Chagas Population-at-Risk 2 (proportion)	X			X		
Chagas disease	Female	Health System Access (unitless)	X			X		
Chagas disease	Female	chagasPrevPAHO				X		
Chagas disease	Female	Sanitation (proportion with access)		X			X	
Chagas disease	Female	Education (years per capita)			X			X
Chagas disease	Female	LDI (I$ per capita)			X			X
Dengue	Male	Population Density (over 1000 ppl/sqkm, proportion)	X			X		
Dengue	Male	Population weighted probability of dengue transmission	X			X		
Dengue	Male	Dengue anomalies (deviation from mean dengue incidence rate)		X			X	
Dengue	Male	Dengue outbreaks (binary)		X			X	
Dengue	Male	Elevation Under 100m (proportion)		X			X	
Dengue	Male	Health System Access (unitless)		X			X	
Dengue	Male	Latitude Under 15 (proportion)		X			X	
Dengue	Male	Rainfall Quintile 4 (proportion)		X			X	
Dengue	Male	Rainfall Quintile 5 (proportion)		X			X	
Dengue	Male	Education (years per capita)			X			X
Dengue	Male	LDI (I$ per capita)			X			X
Dengue	Male	Sociodemographic Status						X
Dengue	Female	Population Density (over 1000 ppl/sqkm, proportion)	X			X		
Dengue	Female	Population weighted probability of dengue transmission	X			X		
Dengue	Female	Dengue anomalies (deviation from mean dengue incidence rate)		X			X	
Dengue	Female	Dengue outbreaks (binary)		X			X	
Dengue	Female	Elevation Under 100m (proportion)		X			X	
Dengue	Female	Health System Access (unitless)		X			X	
Dengue	Female	Latitude Under 15 (proportion)		X			X	
Dengue	Female	Rainfall Quintile 4 (proportion)		X			X	
Dengue	Female	Rainfall Quintile 5 (proportion)		X			X	
Dengue	Female	Education (years per capita)			X			X
Dengue	Female	LDI (I$ per capita)			X			X
Dengue	Female	Sociodemographic Status						X
Rabies	Male	Antenatal Care (4 visits) Coverage (proportion)				X		
Rabies	Male	Health System Access (unitless)	X			X		
Rabies	Male	In-Facility Delivery (proportion)				X		
Rabies	Male	Skilled Birth Attendance (proportion)					X	
Rabies	Male	Population Density (500-1000 ppl/sqkm, proportion)		X				X
Rabies	Male	Population Density (under 150 ppl/sqkm, proportion)						X
Rabies	Male	Sociodemographic Status						X
Rabies	Male	Population Density (300-500 ppl/sqkm, proportion)		X				
Rabies	Male	Population Density (over 1000 ppl/sqkm, proportion)		X				
Rabies	Female	Antenatal Care (4 visits) Coverage (proportion)				X		
Rabies	Female	Health System Access (unitless)	X			X		
Rabies	Female	In-Facility Delivery (proportion)				X		
Rabies	Female	Skilled Birth Attendance (proportion)					X	
Rabies	Female	Population Density (500-1000 ppl/sqkm, proportion)		X				X
Rabies	Female	Population Density (under 150 ppl/sqkm, proportion)						X
Rabies	Female	Sociodemographic Status						X
Rabies	Female	Population Density (300-500 ppl/sqkm, proportion)		X				
Rabies	Female	Population Density (over 1000 ppl/sqkm, proportion)		X				
Other neglected tropical diseases	Male	Health System Access 2 (unitless)	X			X		
Other neglected tropical diseases	Male	Latitude Under 15 (proportion)	X			X		
Other neglected tropical diseases	Male	Rainfall Quintile 5 (proportion)		X			X	
Other neglected tropical diseases	Male	Sanitation (proportion with access)		X			X	
Other neglected tropical diseases	Male	Education (years per capita)			X			X
Other neglected tropical diseases	Male	LDI (I$ per capita)			X			X
Other neglected tropical diseases	Male	Sociodemographic Status						X
Other neglected tropical diseases	Female	Health System Access 2 (unitless)	X			X		
Other neglected tropical diseases	Female	Latitude Under 15 (proportion)	X			X		
Other neglected tropical diseases	Female	Rainfall Quintile 5 (proportion)		X			X	
Other neglected tropical diseases	Female	Sanitation (proportion with access)		X			X	
Other neglected tropical diseases	Female	Education (years per capita)			X			X
Other neglected tropical diseases	Female	LDI (I$ per capita)			X			X
Other neglected tropical diseases	Female	Sociodemographic Status						X
Neonatal disorders	Male	Health System Access 2 (unitless)	X			X		
Neonatal disorders	Male	Indoor Air Pollution (All Cooking Fuels)	X			X		
Neonatal disorders	Male	Smoking Prevalence (Reproductive Age Standardised)	X			X		
Neonatal disorders	Male	Education (years per capita)		X			X	
Neonatal disorders	Male	In-Facility Delivery (proportion)					X	
Neonatal disorders	Male	LDI (I$ per capita)		X			X	
Neonatal disorders	Male	Malnutrition (proportion <2SD weight for age)		X			X	
Neonatal disorders	Male	Sociodemographic Status						X
Neonatal disorders	Male	Total Fertility Rate			X			X
Neonatal disorders	Male	Skilled Birth Attendance (proportion)		X				
Neonatal disorders	Female	Health System Access 2 (unitless)	X			X		
Neonatal disorders	Female	Indoor Air Pollution (All Cooking Fuels)	X			X		
Neonatal disorders	Female	Smoking Prevalence (Reproductive Age Standardised)	X			X		
Neonatal disorders	Female	Education (years per capita)		X			X	
Neonatal disorders	Female	In-Facility Delivery (proportion)					X	
Neonatal disorders	Female	LDI (I$ per capita)		X			X	
Neonatal disorders	Female	Malnutrition (proportion <2SD weight for age)		X			X	
Neonatal disorders	Female	Sociodemographic Status						X
Neonatal disorders	Female	Total Fertility Rate			X			X
Neonatal disorders	Female	Skilled Birth Attendance (proportion)		X				
Neonatal preterm birth complications	Male	Health System Access 2 (unitless)	X			X		
Neonatal preterm birth complications	Male	In-Facility Delivery (proportion)				X		
Neonatal preterm birth complications	Male	Indoor Air Pollution (All Cooking Fuels)	X			X		

Appendix Table 12: Comparison of GBD 2013 and GBD 2015 covariates and level of covariates in cause of death modeling

			GBD 2013			GBD 2015		
Neonatal preterm birth complications	Male	Smoking Prevalence (Reproductive Age Standardised)	X			X		
Neonatal preterm birth complications	Male	In-Facility Delivery (proportion)					X	
Neonatal preterm birth complications	Male	Live Births 35+ (proportion)					X	
Neonatal preterm birth complications	Male	Malnutrition (proportion <2SD weight for age)		X			X	
Neonatal preterm birth complications	Male	Education (years per capita)			X			X
Neonatal preterm birth complications	Male	LDI (I$ per capita)			X			X
Neonatal preterm birth complications	Male	Sociodemographic Status						X
Neonatal preterm birth complications	Male	Total Fertility Rate			X			X
Neonatal preterm birth complications	Male	Skilled Birth Attendance (proportion)		X				
Neonatal preterm birth complications	Female	Health System Access 2 (unitless)	X			X		
Neonatal preterm birth complications	Female	Indoor Air Pollution (All Cooking Fuels)	X			X		
Neonatal preterm birth complications	Female	Smoking Prevalence (Reproductive Age Standardised)	X			X		
Neonatal preterm birth complications	Female	In-Facility Delivery (proportion)					X	
Neonatal preterm birth complications	Female	Live Births 35+ (proportion)					X	
Neonatal preterm birth complications	Female	Malnutrition (proportion <2SD weight for age)		X			X	
Neonatal preterm birth complications	Female	Education (years per capita)			X			X
Neonatal preterm birth complications	Female	LDI (I$ per capita)			X			X
Neonatal preterm birth complications	Female	Sociodemographic Status						X
Neonatal preterm birth complications	Female	Total Fertility Rate			X			X
Neonatal preterm birth complications	Female	Skilled Birth Attendance (proportion)		X				
Neonatal encephalopathy due to birth asphyxia and trauma	Male	Health System Access 2 (unitless)	X			X		
[truncated: 742,799 more chars]
