# Supplementary figures and images for: Global, regional, and national life expectancy, all-cause mortality, and cause-specific mortality for 249 causes of death, 1980–2015: a systematic analysis for the Global Burden of Disease Study 2015
Source: Lancet. 2016 Oct 8;388(10053):1459–544. doi: 10.1016/S0140-6736(16)31012-1 (PMC5388903; doi:10.1016/S0140-6736(16)31012-1)

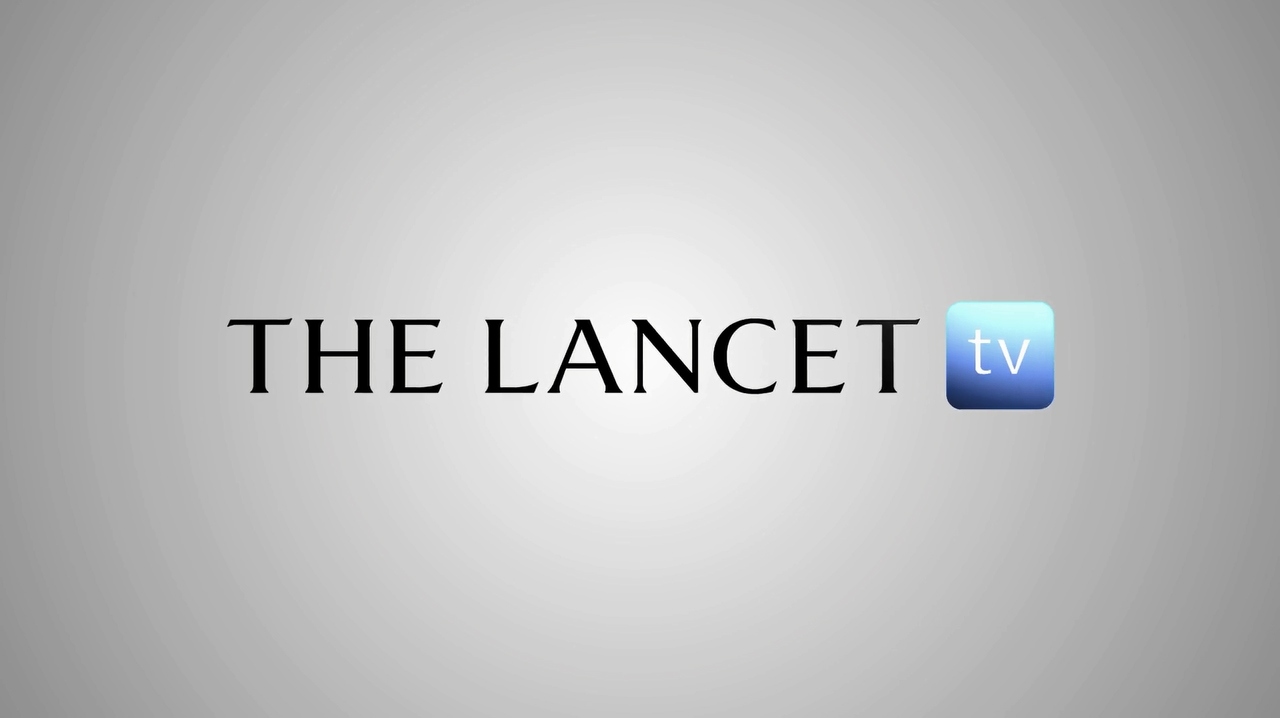

Supplement: Supplementary video — New results from the Global Burden of Disease Study 2015 examine causes of death and categorise regions according to the Socio-demographic Index, or ‘SDI’. [file mmc3.jpg]
