# Supplementary material for: Global, regional, and national life expectancy, all-cause mortality, and cause-specific mortality for 249 causes of death, 1980–2015: a systematic analysis for the Global Burden of Disease Study 2015
Source: Lancet. 2016 Oct 8;388(10053):1459–544. doi: 10.1016/S0140-6736(16)31012-1 (PMC5388903; doi:10.1016/S0140-6736(16)31012-1)
Supplement: Supplementary appendix [file mmc2.pdf]

THE LANCET

Supplementary appendix

This appendix formed part of the original submission and has been peer reviewed. We post it as supplied by the authors.

Supplement to: GBD 2015 Mortality and Causes of Death Collaborators. Global, regional, and national life expectancy, all-cause mortality, and cause-specific mortality for 249 causes of death, 1980–2015: a systematic analysis for the Global Burden of Disease Study 2015. *Lancet* 2016; **388:** 1459–544.

Supplementary Results for Global, regional, and national life expectancy, all-cause and cause-specific mortality for 249 causes of death, 1980–2015: a systematic analysis for the Global Burden of Disease Study 2015

This supplement provides additional figures and more detailed results for all-cause mortality, causes of death, and years of life lost.

Complete results from the Global Burden of Disease Study (GBD 2015) for mortality, causes of death and illness, and risk factors for 195 countries and territories from 1990-2015 are now measured in terabytes. These results are available in an interactive data downloading tool in the Global Health Data exchange (GHDx). The tool contains the complete set of results from all GBD 2015 papers; however, specialized tables from the papers will available as separate entries in the GHDx, as was made available in GBD 2013. Access the results and tables through the GHDx at http://ghdx.healthdata.org/global-burden-disease-study-2015.

Figures and Tables

SR Figure 1: Global comparison of GBD 2015 and UNAIDS estimates of deaths due to HIV/AIDS, 1980–2015 4

SR Figure 2: Comparison of GBD and United Nations Population Division estimates of life expectancy at birth in 2015 5

SR Figure 3: Global decomposition of changes in leading 30 causes of death due to population growth, population aging, and changes in age-specific mortality rates, 1980–2015 6

SR Figure 4: Global decomposition of changes in leading 30 causes of death due to population growth, population ageing, and changes in age-specific mortality rates, 2000–2015.......... 7

SR Table 1: Under-5 mortality (5q0) per 1,000 live births, adult mortality (45q15) per 1,000, life expectancy at birth (years), and life expectancy at age 50 (years), by geography, both sexes, 2005 and 2015 (95% uncertainty intervals).......... 8

SR Table 2: Under-5 mortality (5q0) per 1,000 live births, adult mortality (45q15) per 1,000, life expectancy at birth (years), and life expectancy at age 50 (years), by geography, females, 2005 and 2015 (95% uncertainty intervals).......... 14

SR Table 3: Under-5 mortality (5q0) per 1,000 live births, adult mortality (45q15) per 1,000, life expectancy at birth (years), and life expectancy at age 50 (years), by geography, males, 2005 and 2015 (95% uncertainty intervals).......... 20

SR Table 4: Regional deaths (thousands) by age and sex (with 95% uncertainty intervals) in 1990, 2005, and 2015 26

SR Table 5: Estimates of life expectancy at birth by geography (with 95% uncertainty intervals), both sexes, 1980–2015 ... 36

SR Table 6: Estimates of life expectancy at birth by geography (with 95% uncertainty intervals), females, 1980–2015 ... 40

SR Table 7: Estimates of life expectancy at birth by geography (with 95% uncertainty intervals), males, 1980–2015 ... 44

SR Table 8: Global deaths for all ages (thousands) and age-standardized rates (per 100,000), and percent change (with 95% uncertainty intervals), for females, 2005 and 2015 ... 48

SR Table 9: Global deaths for all ages (thousands) and age-standardized rates (per 100,000), and percent change (with 95% uncertainty intervals), for males, 2005 and 2015 ... 54

SR Table 10: Global deaths by cause and age group for females (with 95% uncertainty intervals), 1990, 1995, 2000, 2005, 2010, and 2015 ... 60

SR Table 11: Global deaths by cause and age group for males (with 95% uncertainty intervals), 1990, 1995, 2000, 2005, 2010, and 2015 ... 96

SR Table 12: Global deaths by cause and age group for both sexes combined (with 95% uncertainty intervals), 1990, 1995, 2000, 2005, 2010, and 2015 ... 132

SR Table 13: Global death rates (per 100,000) by cause and age group for females (with 95% uncertainty intervals), 1990, 1995, 2000, 2005, 2010, and 2015 ... 168

SR Table 14: Global death rates (per 100,000) by cause and age group for males (with 95% uncertainty intervals), 1990, 1995, 2000, 2005, 2010, and 201 ... 204

SR Table 15: Global death rates (per 100,000) by cause and age group for both sexes combined (with 95% uncertainty intervals), 1990, 1995, 2000, 2005, 2010, and 2015 ... 240

SR Table 16: Global YLLs by cause and age group for females (with 95% uncertainty intervals), 1990, 1995, 2000, 2005, 2010, and 2015 ... 276

SR Table 17: Global YLLs by cause and age group for males (with 95% uncertainty intervals), 1990, 1995, 2000, 2005, 2010, and 2015 ... 312

SR Table 18: Global YLLs by cause and age group for both sexes combined (with 95% uncertainty intervals), 1990, 1995, 2000, 2005, 2010, and 2015 ... 328

SR Table 19: Deaths by geography, cause, and age group for females (with 95% uncertainty intervals), 2015 ... 384

SR Table 20: Deaths by geography, cause, and age group for males (with 95% uncertainty intervals), 2015 ... 1509

SR Table 21: Deaths by geography, cause, and age group for both sexes combined (with 95% uncertainty intervals), 2015 ... 2634

SR Table 22: Counterfactual deaths and population attributable fractions for diarrhea and lower respiratory infection pathogens among children under 5 and all ages combined (with 95% uncertainty intervals), 1990 and 2015 ... 3984

SR Table 23: Comparison of GBD and Maternal and Child Epidemiology Estimation group (MCEE) estimated deaths for select causes for neonates and children under 5, 2015 3985

SR Table 24: Comparison of GBD and Maternal and Child Epidemiology Estimation group (MCEE) estimated child deaths for select causes, for the 10 countries with the highest number of deaths in children under 5 in 2015 3986

SR Table 25: Comparison of GBD and GLOBOCAN estimated cancer deaths by cause and sex, 2012 3990

SR Figure 1: Global comparison of GBD and UNAIDS estimates of deaths due to HIV/AIDS, 1980–2015

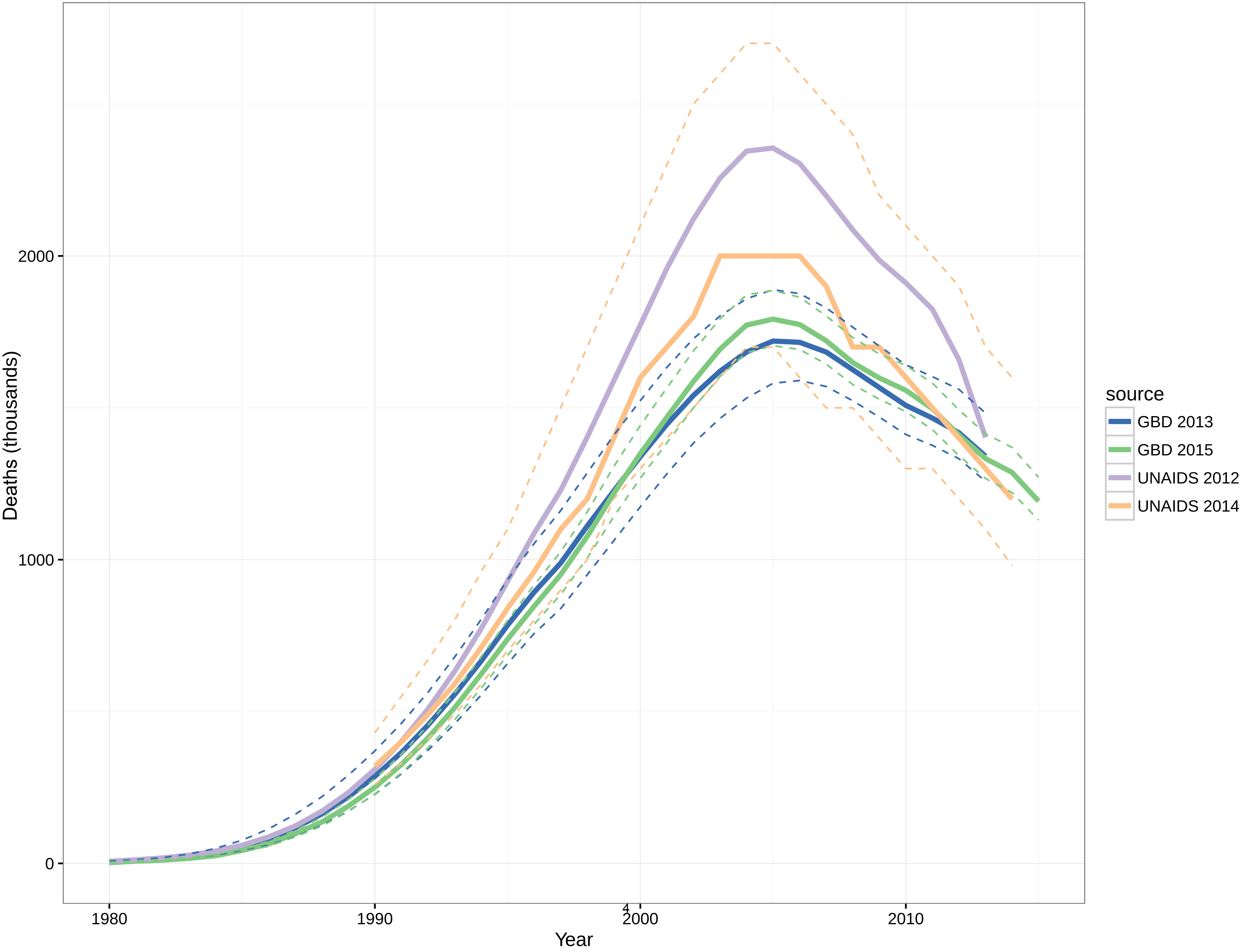

SR Figure 2: Comparison of life expectancy in 2015, IHME GBD 2015 and UNPOP 2015

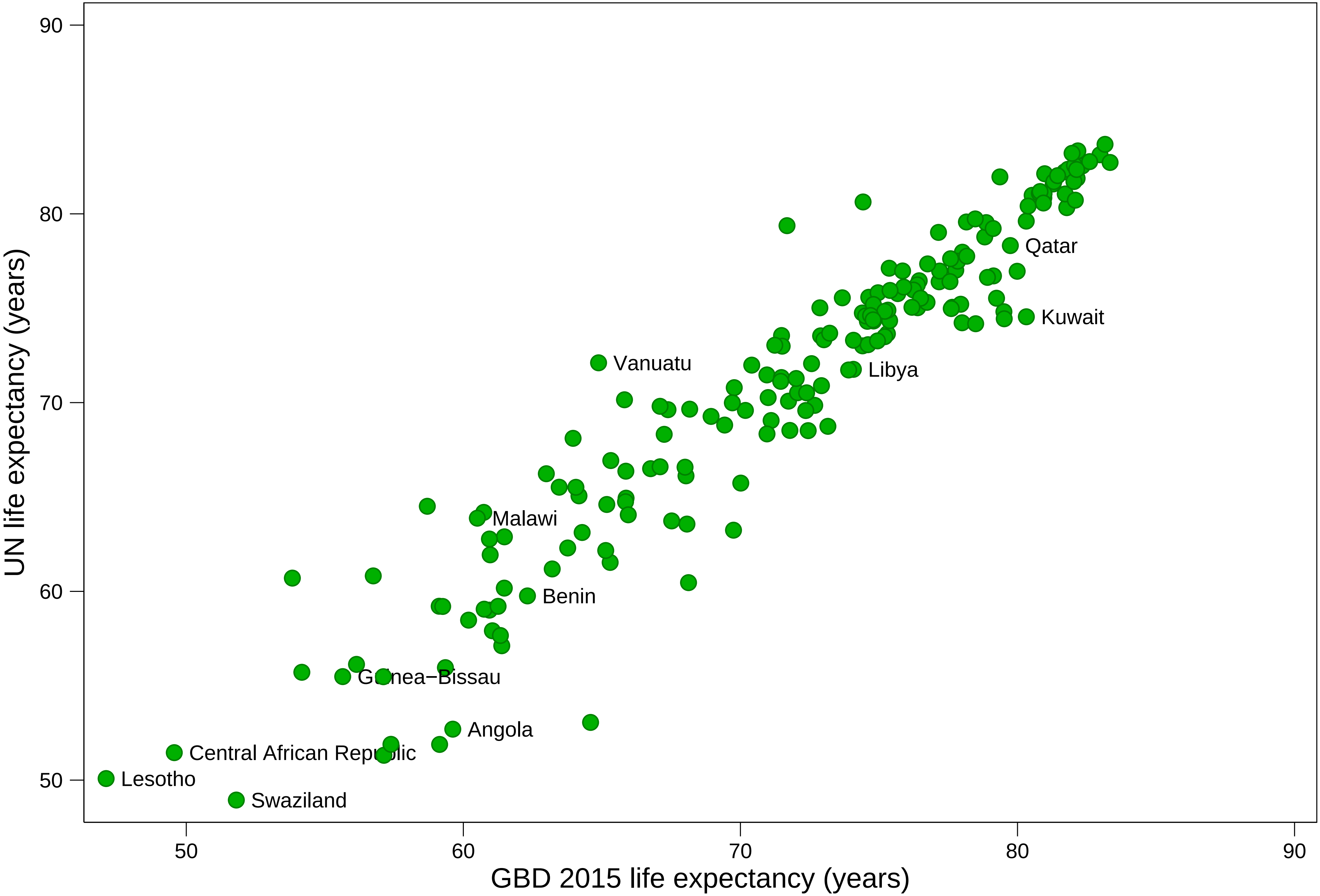

SR Figure 3. Global decomposition of changes in leading 30 causes of death due to population growth, population ageing, and changes in age-specific mortality rates, 1980 to 2015

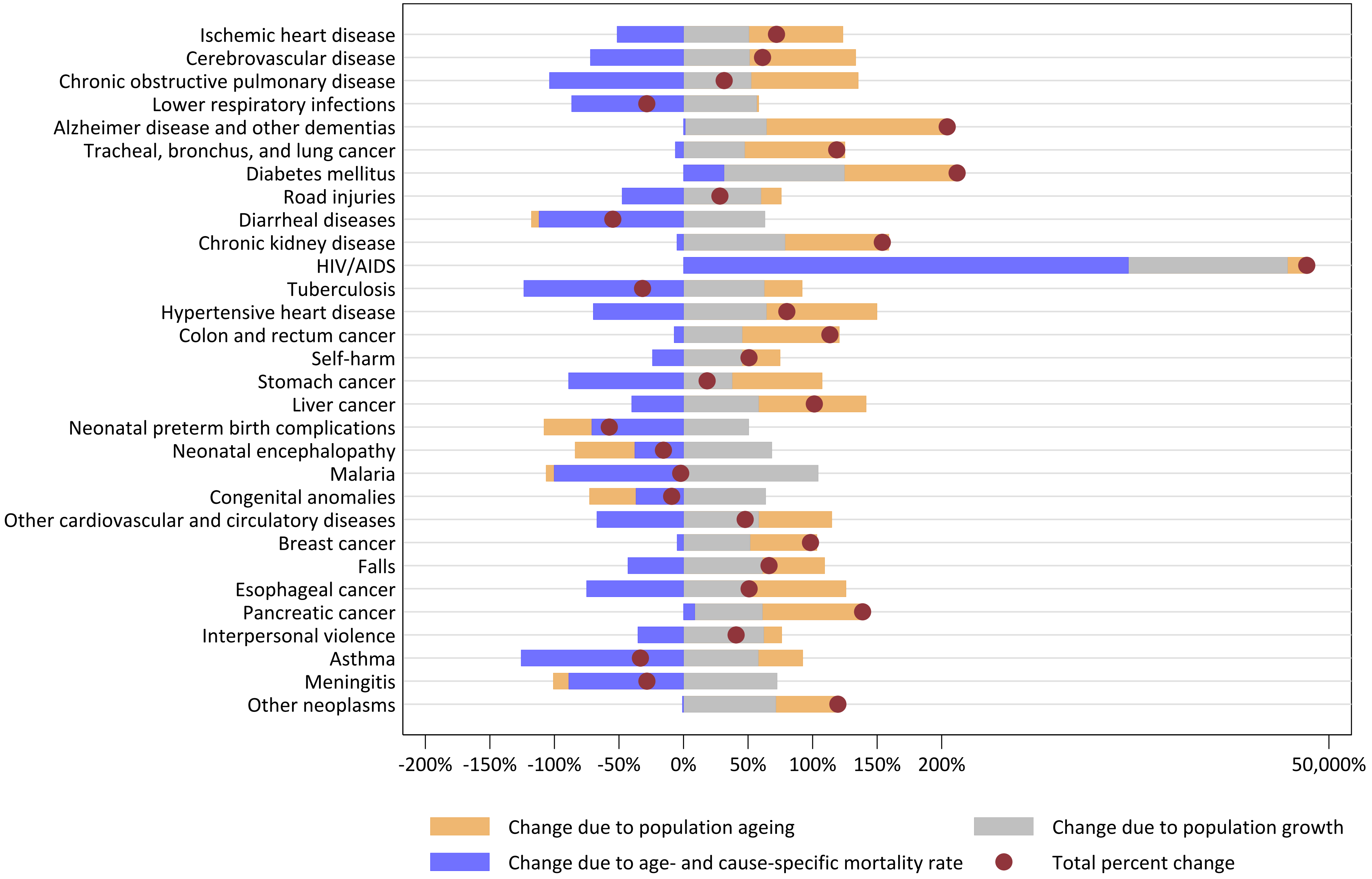

SR Figure 4. Global decomposition of changes in leading 30 causes of death due to population growth, population ageing, and changes in age-specific mortality rates, 2000 to 2015

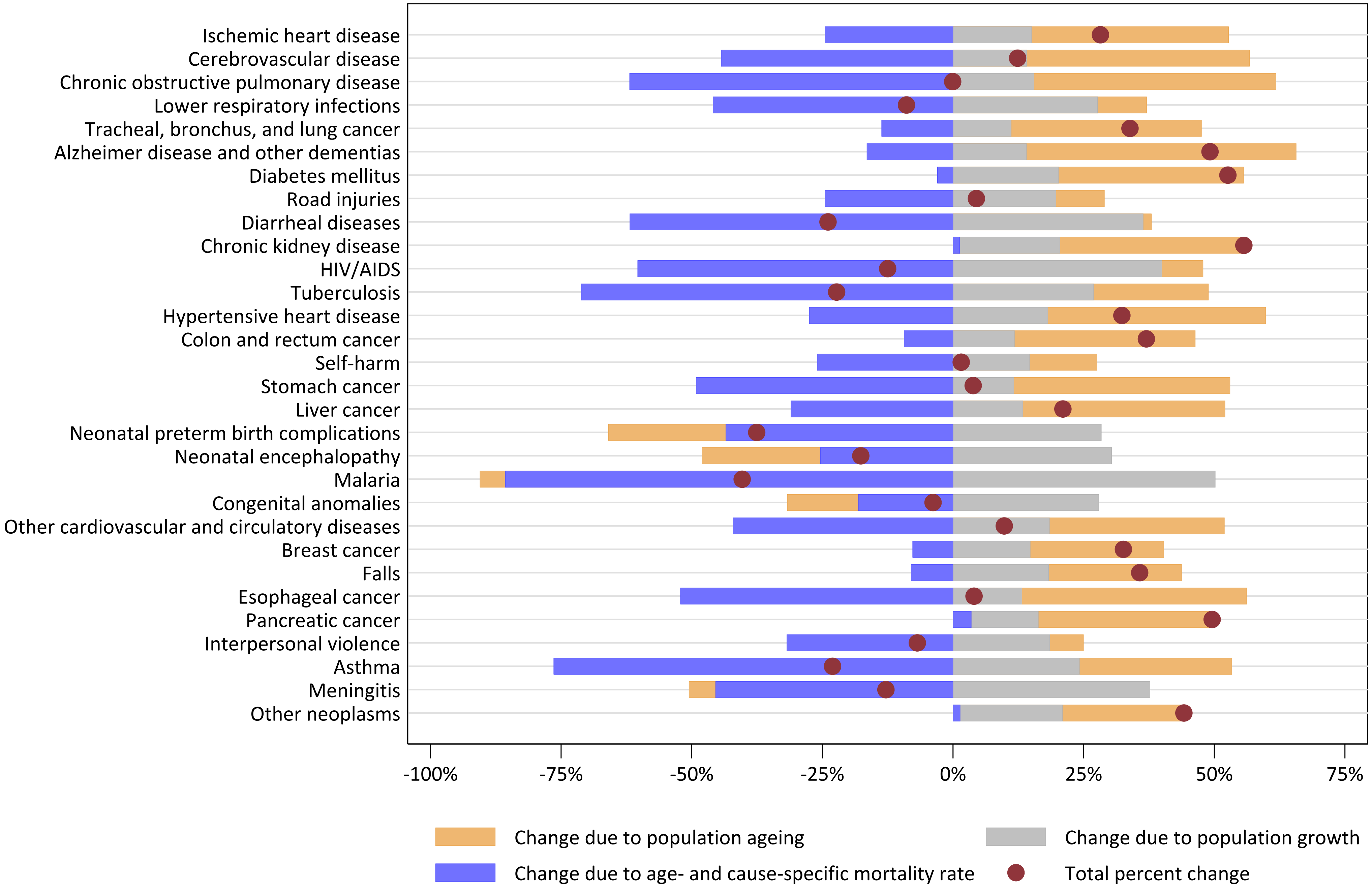

SR Table 1: Under-5 mortality (5q0) per 1,000 live births, adult mortality (45q15) per 1,000, life expectancy at birth (years), and life expectancy at age 50 (years), by geography, both sexes, 2005 and 2015 (95% uncertainty intervals)

Location	2005				2015			
	Under-5 mortality (5q0)	Adult mortality (45q15)	Life expectancy at birth	Life expectancy at age 50	Under-5 mortality (5q0)	Adult mortality (45q15)	Life expectancy at birth	Life expectancy at age 50
Global	60.0 (58.1-62.0)	184.8 (182.3-187.2)	68.2 (68.0-68.4)	27.0 (26.9-27.1)	41.4 (37.9-45.5)	151.5 (147.9-155.2)	71.8 (71.5-72.2)	28.7 (28.6-28.9)
High SDI	7.4 (7.3-7.5)	128.4 (127.9-128.9)	76.8 (76.7-76.8)	30.1 (30.1-30.1)	5.3 (5.0-5.6)	100.0 (99.0-101.1)	79.3 (79.2-79.4)	31.8 (31.8-31.9)
High-middle SDI	25.6 (24.1-27.0)	156.4 (153.5-159.3)	72.2 (72.0-72.4)	27.6 (27.5-27.8)	15.6 (13.9-17.7)	118.5 (115.1-122.3)	75.8 (75.5-76.1)	29.7 (29.5-29.9)
Middle SDI	37.9 (36.0-39.7)	178.2 (173.9-182.6)	69.2 (68.9-69.5)	25.7 (25.5-25.9)	23.4 (20.9-26.1)	149.1 (143.4-155.1)	72.5 (72.1-72.9)	27.5 (27.2-27.8)
Low-middle SDI	91.3 (88.5-94.3)	257.8 (248.5-267.2)	61.4 (60.9-61.9)	23.7 (23.4-24.0)	62.1 (57.5-67.1)	214.5 (203.2-226.6)	65.7 (65.0-66.4)	25.0 (24.6-25.4)
Low SDI	123.4 (119.8-127.2)	360.8 (342.7-381.4)	55.3 (54.5-56.1)	21.8 (21.2-22.4)	84.0 (76.0-93.2)	293.5 (264.0-328.7)	60.7 (59.1-62.3)	23.1 (22.0-24.2)
High-income	6.8 (6.7-6.8)	90.1 (89.9-90.4)	79.3 (79.3-79.3)	31.7 (31.7-31.7)	5.1 (4.8-5.4)	79.7 (79.4-80.1)	80.8 (80.8-80.8)	32.8 (32.8-32.9)
High-income North America	7.8 (7.7-7.9)	108.1 (107.7-108.7)	77.9 (77.9-78.0)	30.9 (30.9-30.9)	5.9 (5.7-6.1)	98.6 (98.0-99.2)	79.4 (79.4-79.5)	32.0 (31.9-32.0)
Canada	6.1 (5.9-6.2)	75.4 (75.0-75.8)	80.2 (80.2-80.3)	32.3 (32.3-32.3)	5.0 (4.7-5.4)	65.4 (63.9-66.9)	81.7 (81.6-81.9)	33.5 (33.4-33.6)
Greenland	18.6 (17.1-20.2)	219.0 (208.4-229.6)	69.1 (68.5-69.6)	24.6 (24.3-25.0)	14.9 (13.4-16.7)	170.0 (155.7-184.7)	72.1 (71.4-72.8)	26.6 (26.2-27.0)
United States	8.0 (7.9-8.1)	111.7 (111.3-112.4)	77.6 (77.6-77.7)	30.7 (30.7-30.7)	6.0 (5.8-6.2)	102.5 (101.8-103.2)	79.1 (79.1-79.2)	31.8 (31.7-31.8)
Australasia	5.9 (5.8-6.1)	68.0 (67.6-68.3)	80.9 (80.9-81.0)	33.0 (32.9-33.0)	4.2 (3.9-4.4)	60.3 (59.5-61.1)	82.2 (82.1-82.3)	33.9 (33.9-34.0)
Australia	5.8 (5.6-5.9)	66.1 (65.7-66.5)	81.2 (81.1-81.2)	33.2 (33.1-33.2)	3.9 (3.7-4.2)	59.2 (58.4-60.0)	82.3 (82.3-82.4)	34.0 (34.0-34.1)
New Zealand	6.5 (6.3-6.8)	77.4 (76.4-78.4)	79.8 (79.7-79.8)	32.0 (32.0-32.1)	5.3 (4.9-5.6)	65.8 (63.9-67.9)	81.4 (81.2-81.6)	33.4 (33.2-33.5)
High-income Asia Pacific	4.2 (4.1-4.2)	75.5 (74.8-76.0)	81.3 (81.3-81.3)	33.2 (33.2-33.3)	2.9 (2.7-3.2)	64.4 (63.1-65.7)	82.7 (82.6-82.8)	34.3 (34.2-34.4)
Brunei	8.8 (8.2-9.4)	105.7 (100.5-111.0)	76.7 (76.4-77.0)	29.6 (29.3-29.8)	8.9 (7.8-10.2)	97.9 (90.7-105.9)	77.1 (76.7-77.6)	29.8 (29.4-30.1)
Japan	3.7 (3.6-3.8)	69.8 (69.6-69.9)	82.0 (81.9-82.0)	33.7 (33.7-33.7)	2.7 (2.5-3.0)	60.3 (59.9-60.6)	83.2 (83.1-83.2)	34.7 (34.6-34.7)
Singapore	2.9 (2.7-3.0)	67.9 (66.7-69.2)	80.0 (79.9-80.2)	31.5 (31.3-31.6)	2.2 (2.0-2.4)	53.2 (50.8-55.6)	82.0 (81.7-82.2)	33.1 (32.9-33.3)
South Korea	5.3 (5.1-5.4)	90.3 (87.9-92.3)	78.7 (78.5-78.9)	31.0 (30.9-31.1)	3.2 (2.7-3.8)	72.9 (69.0-76.8)	81.0 (80.6-81.3)	32.8 (32.5-33.0)
Western Europe	4.9 (4.8-4.9)	80.2 (80.1-80.3)	79.9 (79.8-79.9)	31.8 (31.8-31.8)	3.6 (3.2-4.0)	67.5 (67.2-67.9)	81.5 (81.4-81.5)	33.1 (33.0-33.1)
Andorra	2.8 (2.3-3.4)	47.7 (45.3-50.2)	84.3 (84.0-84.5)	35.5 (35.3-35.8)	1.9 (1.6-2.4)	44.7 (42.2-47.7)	84.8 (84.5-85.1)	35.9 (35.7-36.2)
Austria	5.1 (4.8-5.4)	83.0 (82.4-83.7)	79.5 (79.5-79.6)	31.5 (31.5-31.5)	3.5 (3.1-4.0)	66.6 (65.6-67.7)	81.3 (81.2-81.4)	32.8 (32.7-32.9)
Belgium	4.9 (4.8-5.1)	88.4 (87.5-89.3)	79.1 (79.1-79.2)	31.2 (31.1-31.3)	3.8 (3.4-4.2)	77.0 (73.7-80.5)	80.5 (80.2-80.8)	32.3 (32.0-32.5)
Cyprus	6.0 (5.5-6.4)	84.6 (82.8-86.3)	79.0 (78.8-79.1)	31.2 (31.1-31.3)	4.8 (4.2-5.6)	60.1 (58.2-62.1)	81.8 (81.6-82.0)	33.4 (33.3-33.6)
Denmark	4.9 (4.6-5.2)	90.1 (89.2-91.0)	78.3 (78.2-78.4)	30.2 (30.2-30.3)	3.5 (3.0-4.2)	72.8 (70.8-75.0)	80.4 (80.2-80.6)	31.9 (31.7-32.1)
Finland	3.9 (3.6-4.3)	98.5 (97.3-99.6)	79.0 (79.0-79.1)	31.3 (31.2-31.4)	2.4 (1.9-3.0)	78.3 (75.7-80.8)	80.9 (80.7-81.2)	32.7 (32.5-32.9)
France	4.4 (4.2-4.5)	91.6 (91.3-91.9)	80.4 (80.4-80.4)	32.6 (32.6-32.6)	3.3 (2.6-4.2)	80.5 (78.8-82.1)	81.8 (81.7-82.0)	33.6 (33.5-33.8)
Germany	4.6 (4.4-4.7)	83.8 (83.5-84.1)	79.4 (79.4-79.4)	31.3 (31.2-31.3)	3.5 (3.0-4.2)	71.3 (70.4-72.2)	80.8 (80.7-80.9)	32.3 (32.2-32.4)
Greece	4.6 (4.4-4.8)	80.7 (80.0-81.5)	79.6 (79.5-79.6)	31.6 (31.6-31.7)	3.3 (3.0-3.6)	72.1 (69.9-74.4)	81.0 (80.7-81.2)	32.7 (32.5-32.8)
Iceland	3.1 (2.8-3.5)	61.8 (60.0-63.9)	81.7 (81.4-81.9)	33.1 (32.9-33.4)	2.0 (1.7-2.4)	50.8 (48.7-53.0)	83.3 (83.1-83.6)	34.6 (34.3-34.8)
Ireland	4.9 (4.7-5.2)	77.7 (76.4-79.0)	79.7 (79.6-80.0)	31.6 (31.5-31.9)	3.5 (3.2-3.9)	63.9 (61.6-66.1)	81.7 (81.4-82.1)	33.3 (32.9-33.7)
Israel	5.8 (5.6-6.1)	73.4 (72.3-74.6)	79.7 (79.6-79.8)	31.7 (31.7-31.8)	3.8 (3.5-4.2)	57.7 (55.9-59.7)	82.1 (81.8-82.2)	33.5 (33.3-33.7)
Italy	4.4 (4.2-4.5)	64.2 (64.0-64.4)	81.1 (81.1-81.1)	32.8 (32.8-32.8)	3.1 (2.6-3.8)	56.3 (55.3-57.3)	82.2 (82.1-82.3)	33.5 (33.4-33.6)
Luxembourg	3.5 (3.1-3.9)	78.3 (76.6-80.1)	80.0 (79.8-80.1)	31.7 (31.6-31.9)	2.6 (2.2-3.0)	60.6 (58.7-62.6)	82.2 (82.0-82.4)	33.5 (33.3-33.7)
Malta	6.8 (6.2-7.5)	67.2 (64.8-69.7)	80.7 (80.4-80.9)	32.7 (32.4-32.9)	6.3 (5.5-7.2)	55.7 (53.2-58.2)	82.1 (81.8-82.3)	33.8 (33.6-34.0)
Netherlands	5.5 (5.3-5.7)	72.2 (71.5-72.9)	79.6 (79.5-79.6)	31.3 (31.2-31.3)	3.9 (3.5-4.4)	60.6 (58.4-62.7)	81.3 (81.1-81.6)	32.7 (32.5-32.9)
Norway	3.9 (3.7-4.2)	71.9 (70.8-73.1)	80.2 (80.1-80.3)	32.0 (31.9-32.1)	2.7 (2.4-3.1)	57.3 (55.5-58.9)	82.0 (81.9-82.2)	33.4 (33.3-33.6)
Portugal	5.0 (4.8-5.1)	97.2 (96.4-98.0)	78.4 (78.4-78.5)	31.0 (30.9-31.0)	3.0 (2.8-3.2)	77.6 (76.1-79.1)	80.8 (80.7-80.9)	32.7 (32.6-32.8)
Spain	4.8 (4.6-4.9)	77.7 (77.5-78.0)	80.3 (80.2-80.3)	32.2 (32.2-32.3)	3.0 (2.6-3.4)	59.6 (58.9-60.4)	82.6 (82.5-82.7)	34.0 (33.9-34.1)
Sweden	3.4 (3.2-3.7)	65.8 (65.0-66.7)	80.6 (80.5-80.6)	32.1 (32.0-32.2)	2.6 (2.3-3.0)	54.7 (53.1-56.2)	82.1 (82.0-82.3)	33.5 (33.3-33.6)

SR Table 1: Under-5 mortality (5q0) per 1,000 live births, adult mortality (45q15) per 1,000, life expectancy at birth (years), and life expectancy at age 50 (years), by geography, both sexes, 2005 and 2015 (95% uncertainty intervals)

Location	2005				2015			
	Under-5 mortality (5q0)	Adult mortality (45q15)	Life expectancy at birth	Life expectancy at age 50	Under-5 mortality (5q0)	Adult mortality (45q15)	Life expectancy at birth	Life expectancy at age 50
Switzerland	4.9 (4.7-5.2)	65.2 (64.3-66.1)	81.3 (81.2-81.4)	33.0 (33.0-33.1)	4.0 (3.5-4.8)	52.3 (50.1-54.3)	83.0 (82.7-83.2)	34.4 (34.2-34.6)
United Kingdom	6.1 (5.9-6.2)	81.2 (80.9-81.5)	79.0 (79.0-79.0)	31.0 (31.0-31.1)	4.7 (4.3-5.1)	68.6 (67.9-69.2)	81.0 (80.9-81.0)	32.7 (32.6-32.8)
England	6.1 (5.9-6.2)	78.2 (77.9-78.4)	79.3 (79.2-79.3)	31.3 (31.2-31.3)	4.7 (4.3-5.1)	65.8 (65.2-66.5)	81.2 (81.1-81.3)	32.9 (32.8-33.0)
Northern Ireland	6.8 (6.2-7.4)	86.9 (85.4-88.6)	78.4 (78.2-78.5)	30.6 (30.5-30.7)	5.3 (4.2-6.7)	75.2 (72.3-78.6)	80.2 (79.8-80.4)	32.1 (31.8-32.3)
Scotland	6.3 (5.9-6.7)	105.1 (104.1-106.2)	77.0 (76.9-77.1)	29.5 (29.5-29.6)	4.4 (3.7-5.2)	88.4 (85.9-91.3)	79.2 (79.0-79.5)	31.4 (31.2-31.6)
Wales	5.2 (4.8-5.5)	86.3 (85.3-87.3)	78.5 (78.4-78.6)	30.5 (30.4-30.6)	4.4 (4.0-4.8)	76.0 (73.8-78.4)	80.1 (79.9-80.3)	31.9 (31.7-32.1)
Southern Latin America	14.8 (14.7-15.0)	114.5 (114.0-114.9)	76.0 (76.0-76.0)	29.4 (29.4-29.5)	11.3 (10.7-11.9)	104.1 (101.9-106.2)	77.3 (77.1-77.4)	30.3 (30.2-30.4)
Argentina	16.9 (16.7-17.0)	123.1 (122.5-123.7)	75.3 (75.2-75.3)	29.0 (28.9-29.0)	12.6 (12.1-13.1)	113.8 (111.2-116.3)	76.5 (76.3-76.6)	29.7 (29.5-29.8)
Chile	9.2 (9.0-9.3)	92.8 (91.8-93.7)	78.2 (78.1-78.3)	30.9 (30.9-31.0)	7.6 (7.2-8.1)	82.8 (78.2-87.6)	79.4 (79.0-79.8)	31.8 (31.5-32.1)
Uruguay	13.3 (12.6-14.0)	123.5 (121.9-125.0)	75.5 (75.4-75.6)	28.9 (28.8-29.0)	9.6 (7.4-12.4)	112.4 (109.1-116.0)	76.8 (76.5-77.1)	29.7 (29.5-29.9)
Central Europe, Eastern Europe, and Central Asia	21.8 (20.4-23.3)	246.5 (245.5-247.6)	68.2 (68.0-68.3)	24.5 (24.5-24.6)	13.9 (12.1-16.0)	177.3 (174.7-179.8)	72.7 (72.5-72.9)	27.4 (27.3-27.4)
Eastern Europe	14.2 (13.5-15.0)	307.7 (306.2-309.5)	66.0 (65.9-66.1)	23.1 (23.1-23.1)	8.7 (8.0-9.6)	212.2 (208.2-216.6)	71.3 (71.1-71.5)	26.4 (26.3-26.6)
Belarus	11.2 (9.1-13.6)	250.3 (245.7-255.1)	68.7 (68.4-68.9)	24.0 (23.9-24.2)	5.7 (4.3-7.6)	213.9 (204.9-222.8)	71.0 (70.5-71.5)	25.3 (24.9-25.6)
Estonia	7.6 (6.8-8.4)	189.9 (187.7-191.9)	72.5 (72.4-72.7)	26.7 (26.6-26.8)	3.3 (2.5-4.3)	114.2 (110.8-117.5)	77.8 (77.5-78.1)	30.1 (29.9-30.4)
Latvia	10.1 (9.2-11.1)	213.6 (211.6-215.3)	71.1 (71.0-71.2)	25.7 (25.7-25.8)	5.2 (3.6-7.3)	150.6 (146.2-155.1)	75.4 (75.1-75.7)	28.6 (28.3-28.8)
Lithuania	8.4 (7.8-8.9)	217.1 (215.8-218.3)	71.7 (71.6-71.7)	26.5 (26.5-26.6)	4.1 (3.4-4.9)	165.3 (162.1-168.1)	75.2 (75.0-75.4)	28.8 (28.7-28.9)
Moldova	15.6 (12.2-19.8)	214.0 (212.7-215.3)	69.5 (69.2-69.8)	24.1 (24.0-24.2)	10.7 (7.2-15.4)	149.2 (146.0-152.6)	73.9 (73.5-74.2)	27.3 (27.1-27.4)
Russia	14.3 (14.1-14.5)	327.0 (324.9-329.4)	65.1 (65.0-65.3)	22.7 (22.7-22.8)	8.7 (8.6-8.9)	219.9 (214.0-226.0)	71.0 (70.7-71.3)	26.4 (26.2-26.6)
Ukraine	15.4 (12.2-19.2)	277.6 (276.8-278.5)	67.1 (66.9-67.3)	23.5 (23.5-23.6)	9.6 (6.1-14.4)	200.7 (193.6-208.4)	71.5 (70.9-71.9)	26.2 (26.0-26.5)
Central Europe	10.3 (10.0-10.5)	143.2 (142.9-143.5)	74.2 (74.1-74.2)	27.3 (27.3-27.3)	5.9 (4.6-7.5)	113.9 (112.7-115.2)	77.0 (76.8-77.1)	29.3 (29.3-29.4)
Albania	21.1 (16.9-25.8)	99.0 (95.7-102.4)	76.2 (75.7-76.6)	30.1 (29.9-30.3)	12.2 (8.5-17.2)	83.8 (79.7-88.4)	78.0 (77.4-78.6)	30.8 (30.2-31.5)
Bosnia and Herzegovina	8.8 (8.2-9.3)	102.5 (101.6-103.5)	77.0 (76.9-77.2)	29.8 (29.7-30.0)	5.4 (4.6-6.2)	89.5 (86.9-92.2)	78.9 (78.7-79.2)	31.1 (30.9-31.3)
Bulgaria	13.2 (12.5-13.9)	156.2 (155.2-157.2)	72.6 (72.5-72.6)	26.1 (26.0-26.1)	8.7 (6.2-12.1)	133.7 (128.9-138.9)	74.8 (74.4-75.2)	27.6 (27.4-27.9)
Croatia	6.8 (6.5-7.2)	115.7 (114.5-116.9)	75.7 (75.6-75.7)	28.1 (28.0-28.2)	4.4 (3.9-5.0)	95.0 (91.9-98.2)	77.8 (77.6-78.1)	29.7 (29.5-29.9)
Czech Republic	4.3 (4.0-4.6)	112.3 (111.4-113.1)	76.2 (76.1-76.3)	28.3 (28.3-28.4)	2.5 (2.1-2.9)	84.6 (83.0-86.3)	78.8 (78.7-79.0)	30.4 (30.3-30.5)
Hungary	7.6 (7.1-8.0)	180.2 (179.0-181.4)	73.1 (73.0-73.1)	26.3 (26.2-26.4)	4.9 (3.5-6.9)	126.7 (122.5-131.1)	76.7 (76.4-77.1)	28.9 (28.7-29.2)
Macedonia	13.3 (12.3-14.4)	118.8 (117.3-120.3)	73.8 (73.7-74.0)	26.6 (26.5-26.7)	9.6 (6.4-14.0)	97.8 (94.0-101.4)	76.5 (76.0-77.0)	29.0 (28.6-29.4)
Montenegro	10.4 (9.2-11.6)	128.1 (126.0-130.1)	75.0 (74.8-75.2)	28.0 (27.9-28.2)	5.2 (3.8-7.0)	108.2 (102.6-113.0)	77.2 (76.8-77.6)	29.6 (29.2-30.0)
Poland	7.4 (7.1-7.6)	144.3 (143.8-144.7)	75.1 (75.0-75.1)	28.1 (28.0-28.1)	4.5 (3.2-6.3)	116.0 (114.1-118.1)	77.6 (77.4-77.8)	29.9 (29.8-30.0)
Romania	17.6 (17.0-18.1)	162.0 (161.2-162.7)	72.4 (72.3-72.5)	26.5 (26.4-26.5)	8.3 (6.3-11.0)	135.0 (130.5-139.6)	75.2 (74.9-75.5)	28.1 (27.9-28.3)
Serbia	10.2 (9.2-11.2)	137.2 (134.9-139.5)	73.2 (73.1-73.4)	26.1 (26.0-26.3)	7.7 (7.0-8.5)	112.5 (110.3-114.7)	76.2 (76.0-76.3)	28.8 (28.6-28.9)
Slovakia	8.6 (8.0-9.2)	139.2 (137.9-140.4)	74.3 (74.2-74.3)	27.1 (27.0-27.1)	6.0 (5.0-7.2)	101.9 (100.1-104.0)	77.6 (77.4-77.7)	29.7 (29.5-29.8)
Slovenia	4.3 (4.0-4.7)	106.6 (105.3-107.7)	77.6 (77.5-77.7)	29.8 (29.7-29.9)	2.6 (2.3-3.0)	71.7 (70.1-73.4)	80.9 (80.8-81.1)	32.4 (32.2-32.5)
Central Asia	40.8 (37.0-44.9)	210.6 (207.7-213.2)	67.5 (67.2-67.8)	24.5 (24.4-24.6)	25.6 (21.2-31.1)	168.5 (164.0-173.1)	71.3 (70.8-71.8)	26.7 (26.5-26.9)
Armenia	24.9 (21.1-29.0)	149.0 (142.8-154.6)	71.8 (71.3-72.2)	26.4 (26.2-26.6)	14.9 (11.6-19.0)	125.4 (117.6-132.8)	74.7 (74.2-75.2)	28.4 (28.1-28.7)
Azerbaijan	51.8 (45.5-58.7)	163.5 (155.8-171.7)	68.2 (67.6-68.8)	25.3 (24.9-25.6)	29.7 (23.1-37.7)	130.9 (123.5-139.1)	72.9 (72.1-73.7)	28.0 (27.4-28.4)
Georgia	28.0 (24.2-32.1)	151.6 (142.9-159.8)	71.8 (71.2-72.4)	26.8 (26.5-27.2)	17.4 (13.9-21.5)	157.0 (144.6-168.7)	72.9 (72.2-73.6)	27.3 (26.8-27.8)
Kazakhstan	28.5 (24.1-33.6)	286.4 (285.0-287.9)	65.3 (64.9-65.6)	23.0 (22.9-23.0)	17.3 (13.4-22.1)	208.3 (194.0-222.7)	70.2 (69.3-71.0)	25.7 (25.2-26.2)
Kyrgyzstan	42.3 (39.9-44.7)	231.0 (222.0-239.1)	66.5 (66.0-66.9)	23.6 (23.3-23.9)	29.0 (25.7-32.5)	190.6 (180.9-200.4)	69.8 (69.2-70.3)	25.5 (25.1-25.9)
Mongolia	47.6 (42.3-53.4)	290.4 (279.8-301.0)	63.6 (63.0-64.2)	21.9 (21.6-22.2)	28.1 (22.6-35.6)	246.2 (233.2-258.3)	67.1 (66.4-67.8)	23.7 (23.3-24.1)

SR Table 1: Under-5 mortality (5q0) per 1,000 live births, adult mortality (45q15) per 1,000, life expectancy at birth (years), and life expectancy at age 50 (years), by geography, both sexes, 2005 and 2015 (95% uncertainty intervals)

Location	2005				2015			
	Under-5 mortality (5q0)	Adult mortality (45q15)	Life expectancy at birth	Life expectancy at age 50	Under-5 mortality (5q0)	Adult mortality (45q15)	Life expectancy at birth	Life expectancy at age 50
Tajikistan	51.7 (46.2-57.8)	163.2 (156.7-170.4)	68.0 (67.4-68.5)	25.0 (24.7-25.3)	32.8 (26.3-40.5)	135.9 (128.8-143.7)	72.4 (71.7-73.0)	27.8 (27.4-28.1)
Turkmenistan	61.2 (46.1-79.0)	219.1 (213.9-224.4)	65.8 (64.5-66.9)	24.7 (24.3-25.0)	39.6 (29.4-52.3)	172.8 (169.4-176.0)	70.0 (69.0-70.8)	26.6 (26.4-26.9)
Uzbekistan	37.6 (30.2-45.9)	187.4 (179.7-195.1)	68.5 (67.7-69.2)	24.7 (24.4-25.1)	24.2 (18.8-30.8)	158.0 (149.3-166.5)	71.8 (71.1-72.5)	26.8 (26.4-27.2)
Latin America and Caribbean	26.7 (24.5-28.7)	143.4 (141.8-145.0)	73.4 (73.3-73.6)	28.9 (28.9-29.0)	18.3 (14.7-22.8)	128.7 (126.0-131.3)	75.2 (74.8-75.5)	29.8 (29.6-29.9)
Central Latin America	23.6 (21.4-25.7)	131.3 (130.5-132.3)	74.7 (74.6-74.9)	29.8 (29.8-29.9)	16.7 (13.4-20.9)	120.8 (118.2-123.4)	76.0 (75.7-76.4)	30.4 (30.2-30.5)
Colombia	21.9 (19.6-24.4)	113.6 (113.0-114.1)	75.1 (74.9-75.3)	30.1 (30.1-30.2)	15.8 (11.8-21.0)	89.2 (86.3-92.1)	78.0 (77.5-78.4)	31.9 (31.7-32.1)
Costa Rica	13.5 (11.2-16.6)	85.8 (84.9-86.8)	79.0 (78.7-79.2)	32.2 (32.2-32.3)	9.8 (7.1-13.2)	80.6 (77.1-84.1)	80.3 (79.9-80.7)	33.1 (32.9-33.4)
El Salvador	19.6 (16.0-23.4)	178.0 (172.1-184.2)	73.0 (72.6-73.5)	29.9 (29.7-30.1)	11.3 (8.0-15.7)	163.8 (152.1-176.5)	74.9 (74.1-75.8)	30.7 (30.3-31.2)
Guatemala	41.7 (37.8-45.9)	197.7 (195.7-199.6)	69.8 (69.5-70.1)	28.4 (28.3-28.5)	26.0 (21.2-31.8)	170.2 (151.4-191.5)	72.6 (71.3-73.9)	29.0 (28.1-29.9)
Honduras	30.3 (28.4-32.3)	164.6 (134.8-199.7)	71.2 (69.1-73.1)	27.1 (25.5-28.6)	22.2 (18.9-26.5)	147.1 (117.9-182.0)	73.0 (70.9-75.0)	27.9 (26.4-29.4)
Mexico	22.2 (19.0-25.8)	130.9 (130.5-131.3)	75.1 (74.9-75.4)	29.7 (29.7-29.7)	15.4 (11.7-20.0)	124.0 (123.1-124.8)	75.9 (75.5-76.1)	29.8 (29.7-29.8)
Nicaragua	26.4 (24.1-28.8)	119.0 (113.3-124.1)	76.6 (76.1-77.0)	31.6 (31.3-31.9)	15.4 (12.6-18.7)	110.6 (101.4-119.8)	78.0 (77.2-78.7)	31.9 (31.3-32.4)
Panama	21.2 (17.8-25.2)	103.6 (98.3-109.4)	77.2 (76.6-77.7)	31.8 (31.4-32.1)	17.6 (13.2-23.2)	99.4 (88.6-111.9)	78.2 (77.1-79.1)	32.5 (31.7-33.2)
Venezuela	18.4 (18.3-18.6)	139.5 (138.5-140.5)	74.6 (74.5-74.7)	29.7 (29.7-29.8)	16.9 (15.8-18.1)	141.6 (126.2-158.0)	74.8 (73.7-75.9)	29.9 (29.2-30.7)
Andean Latin America	31.6 (30.2-32.9)	114.6 (110.1-119.2)	74.9 (74.6-75.3)	30.2 (30.0-30.5)	19.2 (16.9-21.6)	96.9 (89.9-104.7)	77.2 (76.6-77.8)	31.1 (30.6-31.5)
Bolivia	51.3 (47.5-55.2)	151.7 (137.9-165.6)	70.2 (69.3-71.1)	27.5 (26.8-28.2)	29.4 (24.2-35.7)	135.2 (112.2-162.2)	73.2 (71.4-74.7)	28.4 (27.1-29.6)
Ecuador	27.8 (25.3-30.5)	132.8 (126.0-140.0)	74.1 (73.6-74.6)	29.4 (29.0-29.7)	17.7 (15.2-20.7)	116.4 (106.1-127.2)	75.9 (75.1-76.6)	29.9 (29.3-30.6)
Peru	25.2 (24.0-26.5)	94.4 (87.8-100.9)	77.2 (76.7-77.7)	31.7 (31.3-32.1)	15.8 (13.5-18.8)	75.5 (67.5-85.2)	79.5 (78.6-80.3)	32.7 (32.0-33.4)
Caribbean	45.0 (42.2-48.2)	165.7 (158.5-174.2)	70.9 (70.4-71.4)	28.5 (28.2-28.8)	31.9 (24.0-42.2)	145.3 (135.7-155.1)	73.1 (72.1-73.9)	29.1 (28.7-29.4)
Antigua and Barbuda	15.7 (12.0-20.2)	125.7 (119.0-131.7)	74.9 (74.2-75.5)	28.6 (28.1-29.2)	12.9 (8.8-18.4)	102.5 (92.7-112.3)	76.4 (75.6-77.4)	29.5 (28.8-30.5)
The Bahamas	20.5 (14.5-27.8)	177.8 (169.0-185.8)	71.7 (71.0-72.4)	27.5 (27.1-27.9)	16.2 (9.1-28.8)	151.7 (138.1-165.6)	73.7 (72.5-74.8)	28.4 (27.8-29.1)
Barbados	19.9 (14.2-27.1)	121.6 (117.6-125.6)	75.0 (74.4-75.6)	29.1 (28.8-29.5)	16.2 (9.1-27.3)	114.2 (104.7-125.0)	75.7 (74.6-76.6)	29.3 (28.6-30.1)
Belize	21.8 (17.4-27.4)	196.8 (187.9-205.5)	70.0 (69.4-70.7)	26.0 (25.6-26.4)	16.7 (10.5-26.6)	177.0 (162.9-191.2)	71.7 (70.7-72.7)	26.9 (26.3-27.5)
Bermuda	6.5 (4.7-8.6)	120.9 (113.7-127.2)	76.4 (75.9-76.9)	29.3 (29.0-29.7)	4.3 (2.8-6.4)	97.8 (88.5-107.6)	78.5 (77.7-79.2)	30.8 (30.3-31.3)
Cuba	7.0 (6.9-7.2)	107.4 (106.7-108.3)	77.1 (77.1-77.2)	29.7 (29.6-29.7)	6.1 (5.8-6.5)	95.0 (91.7-98.1)	78.2 (77.9-78.4)	30.4 (30.2-30.6)
Dominica	24.5 (18.6-32.1)	133.9 (127.1-142.2)	74.2 (73.4-74.9)	29.1 (28.6-29.5)	26.4 (17.1-39.7)	144.0 (132.0-158.1)	73.1 (71.7-74.4)	28.4 (27.5-29.2)
Dominican Republic	35.2 (32.1-38.6)	143.4 (135.2-153.0)	73.3 (72.8-73.8)	29.9 (29.6-30.2)	25.0 (20.5-30.4)	119.0 (113.5-127.9)	75.3 (74.7-75.8)	30.2 (29.9-30.3)
Grenada	18.4 (12.3-25.2)	200.0 (192.7-207.6)	70.3 (69.5-70.9)	25.8 (25.5-26.1)	15.9 (8.7-27.5)	181.4 (169.7-195.2)	71.5 (70.4-72.5)	26.5 (25.9-27.0)
Guyana	39.0 (35.3-42.9)	281.4 (267.1-297.3)	64.4 (63.7-65.2)	23.1 (22.6-23.6)	28.0 (22.0-35.4)	251.4 (227.6-279.5)	66.8 (65.5-68.0)	24.1 (23.4-24.9)
Haiti	88.6 (81.0-97.3)	305.5 (272.8-345.6)	60.0 (58.2-61.9)	23.6 (22.2-25.1)	58.4 (42.1-79.7)	253.8 (209.6-300.0)	64.3 (61.8-66.8)	24.2 (22.7-25.9)
Jamaica	20.5 (13.9-30.4)	129.4 (124.0-134.9)	75.4 (74.5-76.0)	30.4 (30.1-30.7)	18.1 (11.6-27.6)	127.7 (116.5-140.2)	75.0 (74.0-76.0)	29.3 (28.6-30.0)
Puerto Rico	9.6 (9.3-10.0)	126.8 (125.3-128.6)	76.6 (76.4-76.7)	30.3 (30.2-30.3)	7.0 (6.2-7.9)	106.1 (99.9-112.1)	78.5 (78.0-78.9)	31.5 (31.2-31.9)
Saint Lucia	21.9 (14.6-31.4)	144.9 (139.3-150.1)	73.1 (72.3-73.9)	27.9 (27.5-28.4)	17.7 (9.2-31.5)	128.0 (117.3-138.4)	74.8 (73.5-75.9)	29.0 (28.4-29.7)
Saint Vincent and the Grenadines	28.1 (21.2-36.8)	174.8 (169.0-181.1)	70.4 (69.7-71.1)	26.1 (25.8-26.6)	21.9 (13.8-33.6)	171.7 (160.5-183.1)	71.2 (70.2-72.2)	26.5 (25.9-27.0)
Suriname	41.1 (34.8-48.6)	178.5 (169.8-186.6)	69.9 (69.1-70.6)	27.1 (26.5-27.7)	31.1 (24.9-39.5)	160.0 (145.6-174.3)	72.0 (70.8-73.2)	28.1 (27.2-29.0)
Trinidad and Tobago	24.2 (19.0-30.6)	178.8 (176.3-181.4)	71.1 (70.7-71.5)	26.9 (26.7-27.0)	19.2 (11.9-30.0)	163.2 (149.8-177.9)	72.4 (71.3-73.4)	27.4 (26.7-28.0)
Virgin Islands, U.S.	10.8 (9.6-12.1)	143.5 (136.5-150.5)	74.4 (74.0-74.9)	28.3 (28.0-28.6)	8.7 (7.2-10.5)	148.0 (135.6-163.2)	74.4 (73.5-75.2)	28.1 (27.5-28.7)
Tropical Latin America	25.1 (21.4-28.9)	156.6 (153.7-159.7)	72.4 (72.1-72.8)	28.0 (27.8-28.1)	17.0 (13.4-21.4)	140.8 (135.4-146.2)	74.4 (73.9-74.9)	29.1 (28.8-29.4)
Brazil	25.1 (21.3-29.1)	157.2 (154.3-160.3)	72.4 (72.0-72.7)	28.0 (27.8-28.1)	16.9 (13.4-21.4)	141.0 (135.5-146.5)	74.4 (73.9-74.9)	29.1 (28.8-29.4)
Paraguay	25.2 (21.9-28.9)	132.4 (123.1-142.0)	74.0 (73.3-74.7)	28.9 (28.4-29.4)	17.9 (14.3-22.4)	133.1 (117.3-151.3)	74.4 (73.2-75.6)	28.7 (27.8-29.5)

SR Table 1: Under-5 mortality (5q0) per 1,000 live births, adult mortality (45q15) per 1,000, life expectancy at birth (years), and life expectancy at age 50 (years), by geography, both sexes, 2005 and 2015 (95% uncertainty intervals)

Location	2005				2015			
	Under-5 mortality (5q0)	Adult mortality (45q15)	Life expectancy at birth	Life expectancy at age 50	Under-5 mortality (5q0)	Adult mortality (45q15)	Life expectancy at birth	Life expectancy at age 50
Southeast Asia, East Asia, and Oceania	29.0 (26.7-31.5)	155.4 (150.8-160.2)	71.1 (70.8-71.4)	26.5 (26.3-26.7)	16.4 (13.7-19.6)	119.9 (114.2-126.2)	74.9 (74.5-75.4)	28.8 (28.5-29.1)
East Asia	23.4 (19.8-27.8)	144.5 (140.0-149.0)	72.0 (71.5-72.4)	26.6 (26.3-26.8)	12.4 (8.8-17.2)	102.3 (97.6-107.7)	76.2 (75.7-76.8)	29.3 (28.9-29.7)
China	23.4 (19.6-28.0)	144.5 (140.0-149.2)	71.9 (71.5-72.3)	26.5 (26.3-26.7)	12.3 (8.6-17.2)	101.3 (96.6-106.8)	76.2 (75.7-76.8)	29.3 (28.9-29.7)
North Korea	31.5 (15.9-55.0)	176.2 (144.2-212.1)	70.2 (67.7-72.5)	26.2 (24.9-27.6)	19.2 (9.0-36.3)	167.4 (132.1-210.8)	72.1 (69.7-74.2)	26.9 (25.5-28.4)
Taiwan	6.8 (6.5-7.1)	112.5 (111.5-113.4)	77.4 (77.3-77.5)	30.3 (30.2-30.4)	6.6 (4.2-10.3)	90.7 (78.2-104.0)	79.5 (78.4-80.6)	32.0 (31.2-32.9)
Southeast Asia	35.9 (34.8-36.9)	182.8 (170.8-195.6)	69.6 (68.9-70.3)	26.2 (25.8-26.8)	21.5 (19.2-24.1)	160.9 (145.4-177.0)	72.2 (71.3-73.1)	27.3 (26.7-28.0)
Cambodia	65.2 (61.9-68.9)	241.0 (225.6-256.1)	64.0 (63.2-64.8)	23.5 (23.0-24.1)	31.4 (27.2-36.0)	186.5 (159.9-216.6)	69.4 (68.0-70.9)	25.6 (24.6-26.7)
Indonesia	39.5 (37.6-41.3)	187.2 (171.5-202.6)	68.7 (67.9-69.6)	25.4 (24.9-26.1)	23.7 (20.4-27.5)	170.1 (140.3-202.2)	71.1 (69.5-73.0)	26.4 (25.3-27.8)
Laos	112.8 (103.1-123.3)	252.2 (231.7-274.8)	60.1 (58.8-61.2)	23.6 (22.8-24.3)	60.2 (45.8-77.7)	190.7 (157.0-227.3)	67.1 (64.9-69.1)	25.7 (24.3-27.1)
Malaysia	8.1 (7.9-8.2)	131.9 (131.2-132.6)	74.3 (74.2-74.3)	27.5 (27.4-27.5)	6.5 (5.5-7.7)	129.0 (114.7-144.7)	75.3 (74.3-76.4)	28.4 (27.6-29.2)
Maldives	24.4 (22.0-26.9)	87.8 (84.2-91.5)	76.9 (76.5-77.2)	30.9 (30.7-31.2)	12.4 (10.3-15.2)	66.1 (56.0-76.4)	80.0 (78.9-81.1)	32.5 (31.6-33.5)
Mauritius	16.1 (15.5-16.7)	159.8 (157.0-162.7)	72.4 (72.2-72.5)	26.3 (26.2-26.4)	13.4 (12.3-14.7)	138.0 (131.0-144.9)	74.5 (74.1-75.0)	28.1 (27.7-28.4)
Myanmar	61.6 (61.2-62.0)	260.6 (160.0-371.3)	63.4 (58.9-68.4)	23.0 (20.0-26.8)	32.4 (24.8-43.0)	214.2 (133.5-310.2)	68.0 (63.5-72.6)	24.8 (21.9-28.2)
Philippines	32.0 (30.3-33.8)	186.8 (186.3-187.4)	69.1 (68.9-69.2)	25.4 (25.4-25.4)	23.2 (20.5-26.3)	168.6 (156.4-181.5)	71.0 (70.2-71.7)	26.2 (25.7-26.8)
Sri Lanka	17.4 (17.0-17.8)	137.4 (134.6-140.0)	73.9 (73.7-74.0)	28.2 (28.1-28.3)	8.1 (6.2-10.5)	108.0 (85.0-134.4)	77.6 (75.8-79.5)	30.9 (29.5-32.3)
Seychelles	13.0 (11.5-14.6)	182.8 (174.1-191.9)	72.0 (71.6-72.4)	26.6 (26.4-26.8)	10.6 (8.6-13.2)	149.2 (130.8-168.7)	74.1 (73.0-75.2)	27.9 (27.2-28.8)
Thailand	10.9 (9.4-12.5)	177.8 (168.8-187.3)	73.1 (72.5-73.6)	28.7 (28.3-29.1)	5.6 (4.5-7.0)	153.8 (132.5-179.7)	74.7 (73.3-76.2)	28.6 (27.6-29.8)
Timor-Leste	69.9 (61.5-80.0)	169.7 (152.4-187.9)	67.5 (66.2-68.8)	26.7 (25.6-27.8)	38.7 (23.9-59.2)	133.2 (106.1-162.0)	72.4 (70.0-74.7)	28.5 (27.0-30.1)
Vietnam	21.6 (19.7-23.5)	143.9 (111.2-178.6)	73.3 (71.2-75.6)	28.0 (26.5-29.9)	13.4 (11.5-15.9)	124.2 (90.0-164.7)	75.4 (73.3-77.7)	29.1 (27.7-31.0)
Oceania	58.3 (40.4-79.9)	347.2 (256.2-450.4)	60.0 (56.1-64.0)	20.4 (18.1-23.0)	39.4 (24.1-61.8)	318.8 (237.3-434.9)	62.5 (58.0-66.4)	21.2 (18.5-23.6)
American Samoa	11.3 (10.1-12.6)	178.7 (158.6-201.0)	72.0 (70.8-73.1)	26.3 (25.4-27.1)	7.1 (5.7-8.8)	170.9 (143.8-201.1)	72.9 (71.1-74.6)	26.6 (25.3-27.9)
Federated States of Micronesia	17.6 (12.0-24.5)	248.3 (151.3-378.8)	67.6 (62.0-72.2)	23.5 (19.6-26.6)	11.5 (7.7-16.8)	232.6 (154.0-371.8)	68.9 (62.8-73.2)	24.1 (19.8-27.2)
Fiji	31.9 (23.6-41.9)	278.7 (253.5-307.3)	64.8 (63.4-66.0)	22.1 (21.3-22.8)	30.4 (18.2-48.6)	259.2 (225.0-298.9)	65.8 (63.7-67.8)	22.6 (21.5-23.8)
Guam	13.6 (12.3-15.0)	154.6 (138.6-173.3)	73.1 (72.1-73.9)	27.2 (26.5-27.7)	17.5 (13.6-22.3)	172.1 (142.6-203.9)	71.7 (70.1-73.2)	26.4 (25.4-27.4)
Kiribati	58.8 (46.0-74.0)	329.8 (292.8-369.4)	60.8 (58.9-62.6)	20.9 (20.0-21.8)	39.1 (21.3-67.1)	315.2 (271.5-359.3)	63.0 (60.6-65.3)	21.5 (20.4-22.7)
Marshall Islands	33.2 (23.4-44.9)	278.9 (243.2-312.0)	64.7 (63.2-66.4)	22.2 (21.3-23.3)	18.8 (10.6-31.0)	261.8 (221.1-300.7)	66.9 (65.1-68.9)	23.2 (21.9-24.4)
Northern Mariana Islands	8.3 (6.2-10.8)	118.7 (109.5-128.7)	75.9 (75.3-76.5)	28.9 (28.3-29.3)	6.0 (3.4-9.8)	125.3 (109.2-143.4)	76.2 (75.1-77.2)	29.0 (28.2-29.7)
Papua New Guinea	67.8 (45.9-95.1)	379.2 (248.4-519.2)	57.9 (52.8-63.2)	19.4 (16.4-23.1)	44.5 (26.8-70.5)	343.8 (230.7-494.5)	60.9 (55.3-66.1)	20.3 (16.8-23.8)
Samoa	12.8 (6.5-22.0)	177.4 (151.3-204.2)	71.8 (70.3-73.3)	26.2 (25.2-27.2)	8.7 (4.3-16.0)	160.1 (131.2-191.1)	73.2 (71.4-75.1)	26.9 (25.6-28.4)
Solomon Islands	31.0 (22.6-41.3)	348.7 (228.0-485.8)	61.8 (56.8-67.1)	20.2 (17.0-23.7)	19.8 (13.5-28.5)	319.8 (200.0-473.4)	64.0 (58.0-69.4)	21.1 (17.3-24.7)
Tonga	23.2 (14.7-34.6)	189.8 (172.2-208.5)	70.0 (68.8-71.2)	25.5 (24.8-26.1)	17.8 (10.5-28.8)	175.4 (144.7-215.3)	71.5 (69.4-73.5)	26.3 (25.2-27.5)
Vanuatu	40.3 (32.0-49.7)	308.6 (199.0-442.7)	62.7 (57.6-67.7)	21.2 (18.0-24.5)	27.0 (18.5-40.1)	284.8 (194.6-411.4)	64.9 (59.7-69.0)	22.1 (18.8-24.9)
North Africa and Middle East	44.1 (41.6-46.7)	158.0 (149.3-166.8)	70.5 (70.0-71.1)	27.2 (26.9-27.6)	30.2 (26.6-34.6)	148.3 (138.3-159.1)	72.4 (71.7-73.0)	28.2 (27.8-28.5)
North Africa and Middle East	44.1 (41.6-46.7)	158.0 (149.3-166.8)	70.5 (70.0-71.1)	27.2 (26.9-27.6)	30.2 (26.6-34.6)	148.3 (138.3-159.1)	72.4 (71.7-73.0)	28.2 (27.8-28.5)
Afghanistan	113.1 (103.8-122.5)	460.3 (378.9-548.4)	52.0 (49.2-55.0)	17.5 (15.8-19.4)	82.6 (69.5-98.0)	452.1 (376.4-533.4)	53.8 (50.7-56.9)	18.2 (16.6-20.1)
Algeria	30.9 (26.3-36.0)	109.0 (98.0-120.7)	74.8 (73.9-75.7)	29.7 (29.1-30.4)	22.7 (17.9-29.4)	97.3 (86.2-108.6)	76.4 (75.4-77.4)	30.4 (29.7-31.1)
Bahrain	9.8 (9.0-10.6)	109.4 (97.9-121.6)	76.1 (75.5-76.8)	29.0 (28.6-29.4)	6.6 (5.6-7.7)	79.9 (66.2-98.2)	79.1 (77.8-80.2)	31.3 (30.3-32.2)
Egypt	34.1 (30.7-37.3)	173.8 (173.2-174.4)	70.1 (69.9-70.4)	25.4 (25.4-25.4)	21.5 (16.3-28.3)	167.1 (162.7-171.3)	71.5 (70.9-72.0)	25.8 (25.6-26.0)
Iran	26.9 (20.3-35.0)	141.2 (117.3-168.2)	71.9 (70.3-73.7)	27.3 (26.0-28.5)	14.7 (10.8-19.5)	123.8 (99.6-154.2)	74.6 (72.9-76.3)	28.4 (27.2-29.6)
Iraq	38.8 (36.2-41.4)	247.3 (204.5-303.6)	65.7 (63.4-67.8)	23.6 (22.1-25.0)	27.3 (23.2-32.6)	233.3 (180.2-292.9)	67.4 (64.7-70.0)	24.7 (23.0-26.4)

SR Table 1: Under-5 mortality (5q0) per 1,000 live births, adult mortality (45q15) per 1,000, life expectancy at birth (years), and life expectancy at age 50 (years), by geography, both sexes, 2005 and 2015 (95% uncertainty intervals)

Location	2005				2015			
	Under-5 mortality (5q0)	Adult mortality (45q15)	Life expectancy at birth	Life expectancy at age 50	Under-5 mortality (5q0)	Adult mortality (45q15)	Life expectancy at birth	Life expectancy at age 50
Jordan	20.4 (19.3-21.5)	109.0 (102.0-116.2)	74.5 (74.0-75.1)	28.3 (27.8-28.8)	14.8 (12.9-17.1)	79.7 (71.4-90.1)	78.5 (77.6-79.3)	31.5 (30.7-32.1)
Kuwait	10.8 (10.1-11.5)	91.2 (88.9-93.4)	77.7 (77.5-77.9)	30.4 (30.2-30.6)	8.2 (6.5-10.2)	61.2 (52.6-72.1)	80.3 (79.3-81.2)	32.2 (31.4-32.9)
Lebanon	14.7 (11.1-19.1)	91.7 (74.1-113.7)	77.3 (75.6-78.8)	30.5 (29.0-31.7)	8.1 (5.9-11.3)	81.4 (59.3-106.6)	78.9 (76.9-80.9)	31.2 (29.7-32.8)
Libya	23.4 (18.9-28.4)	109.6 (101.9-117.9)	75.4 (74.7-76.0)	29.6 (29.2-30.0)	17.5 (13.2-22.6)	138.9 (114.4-164.6)	74.1 (72.5-75.6)	28.9 (27.9-29.9)
Morocco	35.2 (30.7-40.0)	130.3 (104.2-166.0)	72.7 (70.5-74.4)	28.3 (26.6-29.6)	22.4 (18.0-27.9)	120.3 (89.6-158.1)	74.6 (72.0-76.8)	28.9 (26.9-30.6)
Palestine	23.3 (20.7-26.2)	123.0 (111.5-135.7)	74.6 (73.7-75.3)	29.2 (28.4-29.7)	17.3 (13.5-21.8)	125.8 (97.1-157.1)	74.6 (72.8-76.6)	28.6 (27.2-30.2)
Oman	10.9 (10.4-11.4)	110.1 (99.5-121.9)	76.0 (75.2-76.8)	28.9 (28.3-29.5)	9.4 (8.1-10.8)	95.4 (78.6-113.2)	77.2 (75.9-78.7)	29.9 (28.9-31.1)
Qatar	13.3 (10.0-17.3)	104.6 (91.8-120.9)	76.3 (75.1-77.4)	29.4 (28.4-30.4)	8.6 (6.0-12.1)	71.9 (55.7-90.7)	79.7 (78.0-81.3)	32.0 (30.4-33.3)
Saudi Arabia	17.8 (13.3-23.8)	86.2 (82.3-90.0)	77.8 (77.2-78.3)	31.2 (31.0-31.5)	11.5 (8.3-16.3)	75.0 (68.2-83.2)	79.5 (78.7-80.3)	32.1 (31.5-32.8)
Sudan	80.0 (72.7-87.4)	209.6 (157.4-270.7)	64.6 (61.8-67.4)	25.0 (23.0-27.2)	56.0 (43.3-73.7)	189.9 (139.1-251.0)	67.5 (64.4-70.5)	25.8 (23.5-28.0)
Syria	16.2 (15.9-16.4)	110.0 (102.6-117.0)	75.5 (75.0-76.0)	28.9 (28.5-29.4)	22.4 (16.2-29.3)	240.7 (149.2-322.7)	68.2 (63.6-73.6)	28.3 (27.2-29.7)
Tunisia	20.9 (18.5-23.4)	99.6 (82.8-117.4)	76.2 (75.1-77.4)	30.1 (29.2-31.1)	13.8 (11.5-16.5)	89.2 (70.5-110.3)	77.6 (76.0-79.3)	30.7 (29.3-32.1)
Turkey	27.6 (23.8-32.1)	96.6 (91.2-102.5)	76.2 (75.7-76.7)	30.8 (30.5-31.1)	16.6 (12.3-22.2)	75.6 (71.9-79.6)	79.2 (78.7-79.8)	32.4 (32.0-32.8)
United Arab Emirates	8.9 (7.3-10.9)	130.6 (118.4-143.5)	75.2 (74.5-75.8)	28.5 (27.9-28.9)	5.5 (3.2-9.1)	133.1 (98.0-172.8)	75.4 (73.1-77.4)	28.3 (26.5-29.8)
Yemen	65.1 (61.7-68.7)	227.4 (125.6-351.7)	64.6 (59.4-70.1)	23.9 (20.1-28.2)	53.6 (45.9-63.2)	220.8 (133.2-348.4)	66.0 (60.2-70.9)	24.5 (20.5-28.3)
South Asia	71.6 (68.0-75.0)	232.8 (226.0-239.6)	63.8 (63.4-64.2)	24.0 (23.7-24.3)	50.0 (44.9-56.3)	197.4 (190.4-205.0)	67.4 (66.8-67.9)	25.2 (24.9-25.6)
South Asia	71.6 (68.0-75.0)	232.8 (226.0-239.6)	63.8 (63.4-64.2)	24.0 (23.7-24.3)	50.0 (44.9-56.3)	197.4 (190.4-205.0)	67.4 (66.8-67.9)	25.2 (24.9-25.6)
Bangladesh	65.4 (63.1-67.7)	180.2 (167.0-195.4)	67.1 (66.3-67.9)	25.9 (25.3-26.5)	39.0 (36.1-42.7)	161.5 (140.3-185.0)	70.4 (69.0-71.7)	26.8 (25.8-27.8)
Bhutan	66.7 (59.9-74.6)	147.6 (126.9-171.0)	69.3 (67.8-70.8)	28.1 (27.0-29.2)	44.1 (38.0-51.8)	124.1 (97.4-151.8)	72.7 (70.9-74.6)	29.1 (27.8-30.6)
India	70.5 (66.4-74.5)	238.1 (230.6-245.7)	63.6 (63.1-64.0)	23.8 (23.5-24.1)	48.9 (42.6-56.9)	200.9 (193.0-208.9)	67.2 (66.6-67.9)	25.1 (24.8-25.5)
Nepal	63.2 (60.3-66.0)	180.1 (161.5-200.2)	67.4 (66.3-68.5)	26.2 (25.3-27.1)	36.9 (33.6-40.8)	176.5 (147.1-209.4)	69.7 (67.8-71.5)	26.6 (25.2-28.0)
Pakistan	84.0 (80.4-87.8)	243.6 (216.8-271.8)	62.2 (60.9-63.5)	23.8 (22.9-24.8)	63.0 (57.3-69.4)	205.8 (175.8-237.6)	65.9 (64.3-67.4)	24.9 (23.8-26.0)
Sub-Saharan Africa	123.7 (120.9-126.8)	371.1 (356.6-388.9)	55.1 (54.4-55.8)	22.4 (21.9-23.0)	82.2 (75.8-89.6)	275.6 (252.3-303.3)	61.8 (60.4-63.1)	24.2 (23.3-25.1)
Southern Sub-Saharan Africa	79.5 (74.8-84.8)	548.6 (522.1-575.6)	50.8 (49.7-52.1)	20.3 (19.4-21.4)	46.4 (41.1-52.3)	359.4 (338.3-385.6)	60.4 (59.1-61.5)	23.6 (22.6-24.4)
Botswana	53.8 (45.3-63.3)	606.3 (358.5-888.9)	49.3 (39.1-62.2)	18.5 (10.6-27.5)	28.2 (22.1-36.6)	431.4 (236.0-784.3)	58.7 (45.8-69.2)	21.6 (12.1-28.9)
Lesotho	119.6 (112.7-126.8)	691.9 (614.8-759.7)	43.1 (40.3-46.1)	16.4 (14.6-18.4)	83.7 (70.3-99.4)	625.3 (485.6-759.9)	47.1 (42.3-52.9)	17.7 (13.8-22.5)
Namibia	64.3 (59.1-70.3)	520.5 (455.3-592.4)	53.1 (50.2-56.0)	19.9 (17.7-22.3)	40.5 (31.4-51.6)	304.9 (224.1-407.9)	64.2 (59.3-68.8)	25.3 (21.4-28.9)
South Africa	78.3 (70.0-86.3)	505.4 (477.8-533.4)	52.7 (51.3-54.2)	21.1 (20.0-22.3)	42.2 (35.8-49.7)	340.7 (324.9-359.3)	61.3 (60.2-62.3)	23.9 (23.1-24.7)
Swaziland	110.0 (103.0-117.3)	721.0 (636.6-799.5)	42.5 (39.2-46.0)	16.2 (13.6-19.0)	55.0 (45.0-66.6)	550.2 (403.6-698.8)	51.8 (46.3-58.6)	19.2 (14.7-25.2)
Zimbabwe	83.0 (79.6-86.5)	697.2 (624.7-755.8)	45.1 (42.4-48.3)	17.6 (15.3-20.1)	54.8 (48.1-63.3)	380.5 (291.1-484.5)	59.3 (54.9-64.0)	23.1 (19.3-27.2)
Western Sub-Saharan Africa	147.5 (143.7-151.2)	289.6 (268.1-312.0)	56.6 (55.6-57.7)	24.0 (23.2-24.8)	99.2 (92.0-107.8)	234.7 (204.7-279.0)	62.5 (60.4-64.2)	25.5 (23.9-26.6)
Benin	116.8 (111.7-122.0)	289.3 (215.9-366.5)	58.6 (55.4-62.0)	23.3 (20.6-25.9)	77.9 (69.5-88.3)	262.2 (138.1-427.5)	62.3 (55.9-68.9)	23.4 (18.9-28.3)
Burkina Faso	154.3 (147.9-160.5)	292.3 (245.7-343.9)	55.9 (53.6-58.0)	23.6 (21.6-25.3)	110.4 (100.1-123.0)	238.7 (140.2-370.2)	60.9 (55.6-66.2)	24.1 (20.1-28.1)
Cameroon	124.6 (118.3-131.0)	372.5 (318.4-433.6)	54.9 (52.5-57.5)	22.2 (20.0-24.5)	89.8 (77.9-103.4)	322.9 (222.5-451.8)	59.4 (53.9-64.5)	23.6 (19.3-27.5)
Cape Verde	37.7 (33.5-42.3)	194.4 (144.9-252.1)	69.3 (66.5-72.2)	26.5 (24.7-28.6)	28.2 (25.3-31.3)	145.1 (121.7-177.4)	72.9 (70.9-74.5)	28.5 (27.0-29.7)
Chad	167.9 (157.7-178.3)	343.2 (255.4-452.0)	53.0 (49.0-56.8)	22.4 (19.1-25.6)	130.5 (115.9-148.8)	293.1 (164.8-458.4)	57.4 (51.2-63.8)	23.3 (18.5-28.2)
Cote d'Ivoire	128.1 (121.5-134.6)	405.8 (349.7-463.6)	53.4 (51.1-55.7)	21.3 (19.1-23.4)	93.5 (83.7-105.7)	314.1 (212.3-449.2)	59.1 (54.1-64.3)	22.8 (18.8-26.8)
The Gambia	70.9 (66.8-74.9)	219.6 (180.3-261.8)	64.9 (62.9-67.1)	25.0 (23.5-26.7)	45.4 (40.9-50.3)	197.4 (135.7-276.1)	68.1 (64.3-71.7)	25.9 (23.1-28.6)
Ghana	85.4 (82.1-88.8)	290.5 (227.6-367.8)	60.8 (57.6-64.0)	23.5 (21.0-26.1)	55.5 (49.8-62.7)	237.2 (133.5-372.8)	65.3 (59.5-71.0)	24.6 (20.3-28.9)
Guinea	151.0 (144.3-158.1)	290.1 (243.7-337.7)	56.0 (54.0-58.1)	23.2 (21.6-25.0)	108.7 (97.1-121.4)	284.6 (191.4-398.2)	59.1 (54.6-63.7)	23.0 (19.7-26.6)

SR Table 1: Under-5 mortality (5q0) per 1,000 live births, adult mortality (45q15) per 1,000, life expectancy at birth (years), and life expectancy at age 50 (years), by geography, both sexes, 2005 and 2015 (95% uncertainty intervals)

Location	2005				2015			
	Under-5 mortality (5q0)	Adult mortality (45q15)	Life expectancy at birth	Life expectancy at age 50	Under-5 mortality (5q0)	Adult mortality (45q15)	Life expectancy at birth	Life expectancy at age 50
Guinea-Bissau	164.8 (150.2-180.2)	330.6 (129.3-693.0)	52.9 (41.7-63.3)	21.6 (13.6-29.8)	123.9 (108.1-143.8)	329.1 (139.8-746.4)	55.6 (42.3-65.8)	21.9 (12.7-29.6)
Liberia	119.9 (115.1-124.6)	289.0 (245.4-334.6)	58.4 (56.5-60.6)	23.7 (22.1-25.5)	71.4 (64.6-79.7)	256.6 (180.5-351.4)	63.2 (58.9-67.4)	24.3 (21.1-27.6)
Mali	167.5 (160.4-174.9)	245.3 (208.4-287.9)	56.9 (54.9-58.7)	24.9 (23.1-26.3)	130.2 (115.4-148.9)	228.7 (152.1-329.2)	60.2 (56.0-64.4)	25.1 (21.5-28.2)
Mauritania	83.0 (78.2-87.8)	172.8 (139.9-212.1)	66.3 (64.2-68.4)	26.7 (24.9-28.3)	55.0 (49.3-62.2)	153.1 (88.6-237.2)	69.7 (65.4-74.1)	27.5 (24.1-30.9)
Niger	175.9 (168.6-183.4)	253.7 (211.8-295.7)	55.9 (54.2-57.8)	24.4 (23.1-25.8)	112.8 (101.3-127.1)	237.2 (151.9-347.7)	61.0 (56.4-65.4)	24.4 (21.0-27.7)
Nigeria	161.5 (155.8-167.8)	266.7 (226.6-310.0)	56.8 (54.7-58.9)	25.1 (23.4-26.6)	103.8 (92.6-116.3)	194.1 (146.6-279.0)	64.6 (60.1-67.6)	27.7 (24.2-29.8)
Sao Tome and Principe	60.7 (55.3-66.2)	217.2 (172.1-272.3)	65.5 (63.1-67.9)	24.3 (22.5-26.1)	38.3 (33.3-44.1)	203.9 (107.2-321.8)	68.0 (62.7-73.5)	24.9 (21.2-29.0)
Senegal	92.0 (89.1-95.0)	242.3 (176.2-324.3)	62.2 (58.9-65.5)	24.1 (21.5-26.7)	52.3 (48.2-57.2)	236.9 (126.9-389.6)	65.3 (59.0-71.5)	24.0 (19.6-28.5)
Sierra Leone	181.5 (175.4-187.3)	328.5 (286.7-371.9)	52.3 (50.8-53.9)	21.7 (20.4-23.2)	122.4 (113.0-132.2)	307.3 (216.5-421.4)	57.1 (52.8-61.3)	22.7 (19.3-26.1)
Togo	110.4 (105.1-115.7)	347.4 (288.8-406.6)	56.8 (54.4-59.5)	22.7 (20.4-24.9)	78.0 (71.2-86.5)	287.2 (196.9-395.6)	61.5 (57.1-66.1)	23.7 (20.2-27.4)
Eastern Sub-Saharan Africa	104.4 (101.3-107.4)	398.0 (375.5-424.4)	55.3 (54.3-56.3)	21.8 (20.9-22.6)	67.3 (60.0-76.4)	284.9 (246.6-332.9)	62.4 (60.4-64.4)	23.8 (22.1-25.3)
Burundi	118.2 (109.0-127.5)	380.6 (324.2-443.0)	54.7 (52.4-57.0)	20.7 (18.8-22.5)	76.8 (57.3-104.3)	282.0 (169.6-417.7)	61.4 (55.9-67.1)	22.7 (18.9-27.0)
Comoros	68.8 (53.0-87.7)	195.3 (157.7-236.5)	66.0 (63.6-68.4)	25.3 (23.7-26.9)	44.0 (30.7-62.0)	194.9 (122.2-301.3)	68.1 (63.1-72.4)	25.4 (21.9-28.5)
Djibouti	89.6 (80.0-100.2)	285.6 (154.4-469.0)	60.7 (53.8-67.5)	23.5 (18.4-28.7)	65.5 (55.4-78.7)	254.0 (129.0-446.6)	63.8 (56.6-70.5)	24.1 (19.0-29.1)
Eritrea	81.7 (70.2-93.6)	305.2 (158.1-493.2)	59.9 (53.4-67.0)	21.9 (17.3-27.2)	67.1 (54.6-84.0)	308.8 (148.4-525.6)	60.7 (52.9-68.7)	21.5 (16.4-27.3)
Ethiopia	110.2 (104.9-115.2)	362.2 (315.0-407.1)	56.1 (54.4-57.9)	21.6 (20.2-23.2)	60.3 (53.7-68.5)	229.4 (139.1-349.9)	65.2 (60.0-70.1)	24.6 (20.8-28.3)
Kenya	71.2 (67.4-75.0)	422.6 (399.6-448.3)	57.2 (56.1-58.4)	23.3 (22.3-24.2)	50.8 (45.4-57.6)	262.5 (237.4-291.8)	65.1 (63.6-66.5)	25.3 (24.2-26.3)
Madagascar	83.6 (75.9-91.5)	272.2 (221.6-329.1)	61.2 (58.9-63.5)	22.5 (20.8-24.1)	64.2 (51.9-80.6)	254.5 (143.0-390.5)	63.5 (58.0-69.3)	23.1 (19.5-27.2)
Malawi	121.1 (113.1-128.8)	554.8 (489.9-615.3)	48.5 (46.0-51.2)	20.5 (18.0-22.8)	83.4 (70.2-98.4)	307.1 (229.5-418.3)	60.5 (55.7-64.8)	24.6 (20.5-28.0)
Mozambique	126.3 (121.4-131.1)	427.5 (359.8-496.6)	52.9 (50.1-56.1)	22.0 (19.3-24.9)	77.9 (70.4-87.3)	391.5 (281.8-521.5)	57.1 (51.9-62.7)	22.4 (18.2-27.0)
Rwanda	103.6 (96.9-110.9)	294.7 (252.7-344.1)	59.6 (57.4-61.8)	24.6 (22.6-26.3)	65.6 (53.5-81.8)	209.7 (132.5-333.8)	65.9 (60.3-70.6)	25.6 (21.4-29.0)
Somalia	139.1 (128.8-149.8)	388.7 (118.4-756.9)	52.1 (41.1-64.8)	19.2 (12.2-28.6)	112.2 (97.5-130.4)	379.8 (133.8-752.8)	54.2 (42.1-66.0)	19.6 (12.3-28.2)
South Sudan	122.0 (113.7-131.1)	341.7 (129.0-725.1)	55.3 (43.1-66.0)	21.2 (12.9-29.3)	107.0 (89.4-127.3)	344.0 (143.6-713.4)	56.1 (43.6-66.7)	21.4 (13.1-29.1)
Tanzania	96.0 (91.6-100.6)	376.7 (320.6-438.4)	57.1 (54.3-60.1)	23.6 (21.1-26.0)	60.1 (52.9-69.2)	261.3 (166.4-402.8)	64.1 (57.9-69.4)	24.8 (20.2-28.9)
Uganda	108.7 (104.6-112.9)	435.1 (382.9-489.3)	53.5 (51.5-55.6)	20.6 (18.7-22.7)	69.4 (62.8-77.4)	307.0 (194.0-440.2)	61.3 (56.0-67.2)	23.3 (19.4-28.1)
Zambia	108.6 (100.9-117.1)	602.0 (552.1-652.1)	47.1 (45.1-49.1)	17.6 (16.2-19.1)	65.9 (55.4-78.0)	412.0 (301.4-536.9)	56.7 (52.3-61.7)	20.6 (17.3-24.3)
Central Sub-Saharan Africa	126.5 (119.0-134.7)	348.8 (287.4-444.6)	55.5 (52.0-58.2)	21.9 (19.6-23.9)	85.6 (71.8-100.7)	305.2 (226.3-424.3)	60.0 (55.7-63.8)	22.5 (19.6-25.4)
Angola	123.3 (110.0-137.5)	341.7 (125.5-704.7)	55.2 (43.7-66.1)	20.9 (13.2-29.3)	78.7 (60.3-100.9)	306.6 (122.6-704.1)	59.6 (45.9-70.0)	21.9 (13.2-29.7)
Central African Republic	154.9 (135.3-175.7)	573.5 (395.3-740.4)	45.5 (40.5-52.7)	17.2 (13.4-22.8)	126.8 (98.0-165.8)	503.7 (310.8-699.0)	49.6 (43.3-57.6)	18.1 (13.9-24.2)
Congo	90.0 (84.0-96.4)	381.4 (327.9-438.6)	56.7 (54.2-59.1)	21.3 (19.6-23.3)	56.1 (44.4-70.1)	317.0 (210.4-450.8)	61.5 (56.0-66.7)	22.4 (18.6-26.3)
Democratic Republic of the Congo	128.8 (118.0-139.0)	320.9 (279.7-366.2)	56.4 (54.5-58.4)	22.7 (21.1-24.2)	88.0 (70.2-108.9)	281.3 (193.4-379.8)	60.8 (56.6-65.3)	23.1 (19.9-26.5)
Equatorial Guinea	133.3 (112.7-156.5)	313.7 (147.6-652.5)	56.0 (44.8-65.2)	22.9 (14.4-29.7)	87.5 (65.5-118.6)	271.3 (135.5-619.1)	61.0 (48.7-69.3)	24.1 (15.0-29.8)
Gabon	66.8 (62.6-71.0)	338.6 (284.6-396.6)	60.1 (57.7-62.6)	22.4 (20.5-24.3)	46.1 (40.2-52.7)	247.9 (171.1-369.3)	65.9 (60.4-70.2)	24.8 (20.9-28.2)

SR Table 2: Under-5 mortality (5q0) per 1,000 live births, adult mortality (45q15) per 1,000, life expectancy at birth (years), and life expectancy at age 50 (years), by geography, females, 2005 and 2015 (95% uncertainty intervals)

Location	2005				2015			
	Under-5 mortality (5q0)	Adult mortality (45q15)	Life expectancy at birth	Life expectancy at age 50	Under-5 mortality (5q0)	Adult mortality (45q15)	Life expectancy at birth	Life expectancy at age 50
Global	58.3 (56.4-60.4)	144.1 (141.3-147.1)	70.7 (70.5-71.0)	28.9 (28.8-29.0)	39.8 (36.4-43.8)	110.9 (107.2-115.1)	74.8 (74.4-75.2)	30.9 (30.7-31.1)
High SDI	6.7 (6.6-6.8)	79.8 (79.5-80.2)	80.3 (80.2-80.3)	32.5 (32.5-32.5)	4.8 (4.6-5.1)	64.6 (63.9-65.4)	82.3 (82.3-82.4)	34.1 (34.0-34.1)
High-middle SDI	23.9 (22.5-25.3)	110.3 (107.3-113.3)	75.3 (75.0-75.5)	29.7 (29.6-29.9)	14.5 (12.9-16.4)	77.5 (74.7-80.5)	79.1 (78.8-79.4)	32.2 (31.9-32.4)
Middle SDI	35.9 (34.2-37.7)	135.2 (130.5-139.7)	71.8 (71.5-72.2)	27.5 (27.2-27.8)	22.0 (19.6-24.6)	103.2 (97.6-109.5)	75.7 (75.2-76.2)	29.8 (29.4-30.1)
Low-middle SDI	90.1 (87.2-93.3)	228.8 (217.4-240.5)	62.6 (62.0-63.2)	24.5 (24.1-24.9)	60.3 (55.7-65.6)	174.1 (161.0-188.7)	67.7 (66.8-68.6)	26.2 (25.6-26.8)
Low SDI	117.3 (113.6-121.1)	343.2 (318.3-369.1)	56.2 (55.2-57.2)	22.0 (21.3-22.9)	79.1 (71.0-88.0)	262.1 (225.0-305.9)	62.3 (60.2-64.3)	23.8 (22.2-25.2)
High-income	6.1 (6.0-6.2)	62.5 (62.3-62.7)	82.1 (82.1-82.1)	33.9 (33.9-33.9)	4.6 (4.3-4.9)	56.5 (56.2-56.8)	83.4 (83.4-83.5)	35.0 (34.9-35.0)
High-income North America	7.0 (6.8-7.2)	78.9 (78.6-79.4)	80.4 (80.4-80.5)	32.7 (32.6-32.7)	5.4 (5.2-5.6)	73.2 (72.6-73.9)	81.8 (81.7-81.8)	33.7 (33.6-33.8)
Canada	5.5 (5.3-5.7)	56.8 (56.3-57.3)	82.5 (82.5-82.6)	34.1 (34.1-34.2)	4.6 (4.3-5.0)	50.1 (48.3-51.9)	83.9 (83.7-84.1)	35.3 (35.1-35.5)
Greenland	17.1 (15.6-18.8)	185.3 (171.7-199.3)	71.1 (70.2-71.9)	25.9 (25.3-26.5)	13.8 (12.3-15.4)	133.2 (119.1-150.4)	74.9 (73.8-75.7)	28.7 (27.8-29.2)
United States	7.1 (6.9-7.4)	81.4 (81.0-81.9)	80.2 (80.2-80.2)	32.5 (32.5-32.5)	5.5 (5.2-5.7)	75.9 (75.2-76.6)	81.5 (81.4-81.6)	33.5 (33.5-33.6)
Australasia	5.3 (5.1-5.5)	49.9 (49.5-50.3)	83.3 (83.2-83.3)	34.8 (34.8-34.8)	3.8 (3.6-4.1)	45.0 (44.1-45.8)	84.3 (84.2-84.4)	35.6 (35.5-35.7)
Australia	5.2 (5.0-5.5)	47.7 (47.2-48.2)	83.5 (83.5-83.6)	35.0 (35.0-35.1)	3.6 (3.4-3.9)	43.6 (42.6-44.5)	84.5 (84.4-84.6)	35.8 (35.7-35.9)
New Zealand	5.9 (5.5-6.2)	61.1 (59.8-62.4)	81.8 (81.7-81.9)	33.6 (33.5-33.7)	4.8 (4.4-5.2)	52.0 (49.9-54.4)	83.3 (83.0-83.5)	34.8 (34.6-35.0)
High-income Asia Pacific	3.9 (3.8-4.0)	47.2 (46.8-47.7)	84.7 (84.7-84.8)	36.2 (36.1-36.2)	2.7 (2.5-3.0)	40.8 (40.0-41.8)	86.0 (85.8-86.0)	37.2 (37.1-37.3)
Brunei	8.0 (7.5-8.7)	86.4 (80.2-92.1)	79.0 (78.5-79.3)	31.3 (31.0-31.6)	8.2 (7.1-9.3)	78.6 (70.2-87.3)	79.4 (78.8-79.9)	31.6 (31.2-32.0)
Japan	3.5 (3.3-3.6)	45.6 (45.5-45.8)	85.3 (85.3-85.4)	36.7 (36.7-36.7)	2.6 (2.4-2.8)	41.0 (40.6-41.3)	86.3 (86.3-86.4)	37.5 (37.5-37.6)
Singapore	2.7 (2.5-2.9)	49.7 (48.3-51.2)	82.2 (82.0-82.4)	33.3 (33.2-33.5)	2.1 (1.9-2.3)	38.7 (36.2-41.0)	84.0 (83.7-84.4)	34.9 (34.6-35.2)
South Korea	4.9 (4.7-5.1)	50.4 (48.9-51.9)	82.0 (81.8-82.1)	33.6 (33.5-33.8)	3.1 (2.6-3.6)	40.6 (38.0-43.5)	84.3 (83.9-84.6)	35.6 (35.3-35.9)
Western Europe	4.4 (4.3-4.5)	54.4 (54.3-54.5)	82.6 (82.6-82.6)	34.0 (34.0-34.1)	3.3 (2.9-3.7)	46.9 (46.5-47.3)	84.0 (83.9-84.0)	35.1 (35.1-35.2)
Andorra	2.6 (2.1-3.2)	25.5 (23.7-27.5)	87.9 (87.6-88.2)	38.7 (38.5-39.0)	1.8 (1.5-2.3)	24.5 (22.6-26.6)	88.4 (88.1-88.7)	39.1 (38.9-39.4)
Austria	4.6 (4.3-4.9)	54.8 (54.2-55.4)	82.2 (82.1-82.3)	33.6 (33.6-33.7)	3.2 (2.9-3.6)	45.9 (44.9-47.0)	83.7 (83.5-83.8)	34.8 (34.7-34.9)
Belgium	4.4 (4.2-4.6)	61.8 (60.7-62.7)	82.0 (81.9-82.1)	33.5 (33.4-33.6)	3.4 (3.1-3.8)	55.2 (51.5-58.7)	83.2 (82.8-83.6)	34.5 (34.2-34.8)
Cyprus	5.5 (5.1-6.0)	55.7 (54.2-57.3)	82.1 (82.0-82.3)	33.8 (33.6-33.9)	4.5 (3.9-5.2)	38.5 (36.7-40.5)	85.0 (84.8-85.3)	36.3 (36.1-36.5)
Denmark	4.4 (4.1-4.7)	68.6 (67.5-69.7)	80.5 (80.4-80.6)	32.0 (31.9-32.1)	3.2 (2.7-3.8)	55.4 (53.2-57.7)	82.4 (82.2-82.7)	33.6 (33.4-33.8)
Finland	3.6 (3.3-3.9)	59.6 (58.5-60.8)	82.4 (82.3-82.6)	34.0 (33.9-34.1)	2.2 (1.7-2.8)	49.2 (46.8-51.5)	83.9 (83.6-84.1)	35.1 (34.8-35.3)
France	3.9 (3.8-4.1)	56.7 (56.4-57.0)	83.9 (83.9-83.9)	35.5 (35.4-35.5)	3.0 (2.4-3.7)	50.1 (48.7-51.6)	85.2 (85.0-85.3)	36.4 (36.3-36.6)
Germany	4.1 (4.0-4.3)	57.0 (56.6-57.3)	82.1 (82.0-82.1)	33.4 (33.4-33.5)	3.2 (2.8-3.8)	49.9 (49.0-50.8)	83.2 (83.1-83.3)	34.4 (34.3-34.4)
Greece	4.4 (4.2-4.5)	47.6 (46.8-48.4)	82.3 (82.2-82.4)	33.7 (33.6-33.8)	3.2 (2.9-3.4)	44.2 (42.2-46.2)	83.5 (83.2-83.7)	34.6 (34.4-34.9)
Iceland	3.0 (2.6-3.3)	48.4 (46.2-50.7)	83.6 (83.3-84.1)	34.9 (34.5-35.3)	1.9 (1.6-2.3)	37.6 (35.2-40.0)	85.8 (85.4-86.3)	36.8 (36.4-37.3)
Ireland	4.5 (4.3-4.8)	58.3 (56.9-59.8)	81.8 (81.5-82.1)	33.3 (33.0-33.7)	3.3 (2.9-3.7)	46.4 (43.9-49.1)	84.3 (83.6-85.0)	35.6 (34.8-36.3)
Israel	5.6 (5.3-5.8)	51.8 (50.6-53.0)	81.7 (81.6-81.9)	33.2 (33.1-33.3)	3.7 (3.4-4.1)	41.6 (39.6-43.6)	83.8 (83.6-84.1)	34.9 (34.7-35.2)
Italy	4.1 (3.9-4.2)	44.2 (43.9-44.5)	83.7 (83.7-83.8)	35.0 (34.9-35.0)	2.9 (2.4-3.6)	39.9 (39.0-41.0)	84.6 (84.4-84.7)	35.6 (35.5-35.7)
Luxembourg	3.2 (2.8-3.5)	54.4 (52.4-56.3)	82.5 (82.3-82.7)	33.8 (33.6-34.0)	2.4 (2.1-2.7)	44.4 (42.0-46.4)	84.3 (84.0-84.6)	35.4 (35.1-35.7)
Malta	6.2 (5.7-6.9)	49.8 (46.7-52.9)	82.7 (82.3-83.1)	34.4 (34.0-34.7)	5.7 (5.1-6.5)	38.4 (35.7-41.1)	84.5 (84.2-84.8)	35.8 (35.6-36.2)
Netherlands	4.9 (4.7-5.1)	61.2 (60.3-62.1)	81.6 (81.5-81.7)	33.1 (33.1-33.2)	3.6 (3.2-4.0)	50.5 (47.8-53.3)	83.4 (83.1-83.7)	34.6 (34.3-34.9)
Norway	3.6 (3.3-3.8)	55.4 (53.9-56.7)	82.4 (82.3-82.6)	33.8 (33.7-34.0)	2.5 (2.2-2.8)	43.5 (41.4-45.5)	84.1 (83.8-84.3)	35.1 (34.9-35.3)
Portugal	4.5 (4.4-4.7)	57.2 (56.5-58.0)	81.7 (81.7-81.8)	33.4 (33.3-33.5)	2.8 (2.6-3.0)	45.2 (43.8-46.6)	83.9 (83.7-84.0)	35.1 (34.9-35.2)
Spain	4.4 (4.2-4.5)	45.0 (44.7-45.3)	83.6 (83.6-83.7)	35.0 (35.0-35.0)	2.8 (2.5-3.2)	38.5 (37.7-39.3)	85.3 (85.2-85.4)	36.3 (36.2-36.4)

SR Table 2: Under-5 mortality (5q0) per 1,000 live births, adult mortality (45q15) per 1,000, life expectancy at birth (years), and life expectancy at age 50 (years), by geography, females, 2005 and 2015 (95% uncertainty intervals)								
Location	2005				2015			
	Under-5 mortality (5q0)	Adult mortality (45q15)	Life expectancy at birth	Life expectancy at age 50	Under-5 mortality (5q0)	Adult mortality (45q15)	Life expectancy at birth	Life expectancy at age 50
Sweden	3.1 (2.9-3.3)	51.2 (50.2-52.1)	82.6 (82.5-82.7)	33.8 (33.7-33.9)	2.4 (2.1-2.7)	42.3 (40.5-44.2)	84.0 (83.7-84.2)	35.0 (34.8-35.2)
Switzerland	4.5 (4.2-4.7)	46.2 (45.1-47.3)	83.7 (83.6-83.8)	35.1 (35.0-35.2)	3.7 (3.1-4.3)	37.8 (35.6-39.9)	85.2 (84.9-85.5)	36.3 (36.0-36.5)
United Kingdom	5.4 (5.3-5.6)	62.1 (61.8-62.4)	81.1 (81.1-81.2)	32.8 (32.7-32.8)	4.3 (3.9-4.6)	53.5 (52.7-54.2)	82.8 (82.7-82.9)	34.2 (34.1-34.3)
England	5.4 (5.3-5.6)	60.1 (59.8-60.5)	81.4 (81.3-81.4)	33.0 (32.9-33.0)	4.3 (4.0-4.6)	51.6 (50.8-52.4)	83.0 (82.9-83.2)	34.4 (34.3-34.5)
Northern Ireland	6.3 (5.7-6.8)	64.4 (62.7-66.4)	80.8 (80.6-81.0)	32.5 (32.3-32.7)	5.0 (3.9-6.2)	57.0 (53.4-60.6)	82.3 (81.9-82.7)	33.8 (33.5-34.1)
Scotland	5.6 (5.3-6.0)	77.5 (76.1-78.7)	79.5 (79.4-79.6)	31.4 (31.3-31.5)	4.0 (3.3-4.7)	67.5 (63.7-71.0)	81.2 (80.9-81.6)	32.8 (32.5-33.2)
Wales	4.6 (4.4-4.9)	66.2 (65.0-67.5)	80.7 (80.5-80.8)	32.2 (32.1-32.4)	4.0 (3.6-4.4)	58.4 (55.9-61.1)	82.2 (81.9-82.5)	33.6 (33.3-33.8)
Southern Latin America	13.3 (12.9-13.7)	79.2 (78.7-79.7)	79.3 (79.2-79.3)	32.0 (31.9-32.0)	10.1 (9.5-10.8)	72.1 (70.0-74.4)	80.4 (80.2-80.6)	32.7 (32.6-32.9)
Argentina	15.1 (14.5-15.6)	86.0 (85.4-86.7)	78.7 (78.6-78.8)	31.7 (31.6-31.7)	11.2 (10.6-11.9)	79.5 (76.7-82.2)	79.8 (79.5-80.0)	32.3 (32.1-32.5)
Chile	8.3 (8.0-8.6)	62.6 (61.5-63.6)	81.0 (80.9-81.1)	32.9 (32.8-33.0)	7.0 (6.5-7.4)	56.2 (52.0-60.8)	82.0 (81.5-82.5)	33.8 (33.3-34.2)
Uruguay	11.8 (11.0-12.6)	85.6 (83.9-87.6)	79.2 (79.0-79.3)	31.8 (31.7-31.9)	8.5 (6.6-10.9)	75.0 (71.5-78.5)	80.5 (80.2-80.9)	32.7 (32.5-33.0)
Central Europe, Eastern Europe, and Central Asia	19.4 (18.1-20.8)	137.0 (136.2-137.7)	73.6 (73.5-73.7)	27.8 (27.8-27.9)	12.3 (10.7-14.2)	100.0 (97.9-102.1)	77.3 (77.1-77.5)	30.4 (30.2-30.5)
Eastern Europe	12.3 (11.7-13.1)	163.7 (162.8-164.8)	72.7 (72.6-72.8)	26.9 (26.9-26.9)	7.6 (7.0-8.4)	114.3 (111.1-117.9)	76.7 (76.4-76.9)	29.8 (29.6-29.9)
Belarus	9.7 (7.9-11.9)	127.9 (124.9-131.1)	74.9 (74.6-75.1)	27.9 (27.8-28.1)	5.1 (4.0-6.8)	104.5 (98.3-110.5)	77.1 (76.6-77.6)	29.5 (29.1-29.9)
Estonia	6.7 (6.0-7.5)	99.5 (96.7-101.7)	77.7 (77.5-77.8)	30.1 (30.0-30.2)	3.0 (2.3-3.9)	62.5 (59.6-65.8)	81.4 (81.0-81.9)	32.8 (32.4-33.3)
Latvia	8.9 (8.1-9.8)	113.9 (112.2-115.7)	76.6 (76.5-76.8)	29.4 (29.3-29.5)	4.7 (3.3-6.6)	83.9 (80.2-88.3)	79.7 (79.3-80.1)	31.7 (31.4-32.0)
Lithuania	7.4 (6.9-7.9)	109.1 (107.8-110.4)	77.7 (77.6-77.8)	30.4 (30.3-30.4)	3.7 (3.2-4.4)	84.6 (82.1-87.4)	80.4 (80.2-80.6)	32.3 (32.1-32.5)
Moldova	13.6 (10.7-17.4)	133.6 (132.0-135.3)	73.4 (73.1-73.7)	26.5 (26.4-26.6)	9.4 (6.4-13.5)	85.1 (82.0-88.4)	77.7 (77.3-78.1)	29.8 (29.6-30.1)
Russia	12.3 (11.9-12.8)	173.8 (172.5-175.1)	72.2 (72.1-72.3)	26.6 (26.6-26.7)	7.6 (7.3-7.9)	118.8 (114.2-123.6)	76.6 (76.2-76.9)	29.8 (29.5-30.0)
Ukraine	13.4 (10.6-16.8)	149.4 (148.4-150.3)	73.2 (72.9-73.4)	27.1 (27.0-27.1)	8.5 (5.5-12.5)	109.3 (104.0-114.9)	76.5 (75.9-76.9)	29.4 (29.1-29.7)
Central Europe	9.1 (8.9-9.4)	84.9 (84.5-85.2)	77.9 (77.9-77.9)	30.1 (30.1-30.1)	5.3 (4.3-6.7)	67.1 (66.1-68.2)	80.5 (80.3-80.6)	32.0 (32.0-32.1)
Albania	19.2 (15.5-23.5)	67.1 (64.3-70.3)	79.1 (78.6-79.5)	32.2 (31.9-32.5)	11.1 (7.9-15.6)	54.7 (50.7-58.5)	81.3 (80.7-81.9)	33.5 (33.0-34.0)
Bosnia and Herzegovina	7.7 (7.2-8.3)	68.4 (67.2-69.5)	79.9 (79.7-80.0)	31.9 (31.8-32.0)	4.9 (4.2-5.6)	58.1 (55.4-60.8)	81.8 (81.4-82.2)	33.4 (33.0-33.8)
Bulgaria	11.7 (11.0-12.4)	92.5 (91.2-93.6)	76.2 (76.1-76.3)	28.8 (28.8-28.9)	7.8 (5.7-10.7)	79.3 (74.9-83.9)	78.4 (77.9-78.8)	30.4 (30.1-30.8)
Croatia	6.2 (5.8-6.6)	65.6 (64.2-67.0)	79.2 (79.1-79.3)	30.8 (30.7-31.0)	4.1 (3.6-4.6)	57.2 (54.1-59.8)	80.9 (80.6-81.3)	32.2 (31.9-32.5)
Czech Republic	3.9 (3.6-4.1)	68.8 (68.0-69.7)	79.4 (79.3-79.5)	30.8 (30.8-30.9)	2.3 (2.0-2.7)	53.7 (52.1-55.3)	81.7 (81.5-81.8)	32.7 (32.6-32.9)
Hungary	6.8 (6.4-7.3)	106.9 (105.7-108.3)	77.2 (77.1-77.3)	29.5 (29.4-29.6)	4.5 (3.3-6.2)	81.1 (77.0-85.3)	79.9 (79.6-80.3)	31.5 (31.2-31.9)
Macedonia	12.1 (11.1-13.1)	80.9 (79.1-82.7)	76.4 (76.2-76.6)	28.6 (28.5-28.7)	8.8 (6.0-12.8)	66.0 (62.4-69.7)	79.1 (78.6-79.6)	31.1 (30.7-31.4)
Montenegro	9.3 (8.1-10.4)	88.6 (86.2-91.4)	78.0 (77.7-78.2)	30.4 (30.1-30.6)	4.8 (3.5-6.4)	71.4 (66.2-76.5)	80.2 (79.7-80.7)	31.9 (31.5-32.3)
Poland	6.6 (6.3-6.8)	79.4 (78.9-79.9)	79.4 (79.3-79.4)	31.2 (31.2-31.2)	4.1 (2.9-5.6)	63.0 (61.5-64.9)	81.6 (81.4-81.8)	33.0 (32.8-33.1)
Romania	15.6 (14.9-16.2)	98.9 (98.1-99.7)	76.1 (76.0-76.2)	29.1 (29.0-29.2)	7.5 (5.8-9.7)	77.5 (74.0-81.6)	79.0 (78.6-79.4)	31.0 (30.7-31.2)
Serbia	9.1 (8.2-10.0)	92.9 (90.4-95.6)	75.9 (75.7-76.2)	28.1 (27.9-28.3)	7.0 (6.3-7.7)	75.7 (73.4-78.0)	78.9 (78.7-79.0)	30.8 (30.6-30.9)
Slovakia	7.6 (7.1-8.2)	77.4 (76.2-78.6)	78.2 (78.1-78.3)	30.1 (30.0-30.1)	5.4 (4.6-6.4)	58.7 (56.7-60.8)	80.9 (80.7-81.1)	32.4 (32.2-32.6)
Slovenia	4.1 (3.7-4.4)	63.6 (62.3-65.0)	81.1 (80.9-81.2)	32.5 (32.4-32.7)	2.5 (2.2-2.9)	44.8 (43.1-46.6)	83.8 (83.6-84.0)	34.8 (34.6-35.0)
Central Asia	36.7 (33.2-40.5)	137.6 (134.7-140.4)	71.5 (71.1-71.8)	27.0 (26.8-27.1)	22.7 (18.6-27.6)	106.9 (102.9-111.4)	75.3 (74.8-75.8)	29.2 (29.0-29.5)
Armenia	22.0 (18.5-25.8)	93.5 (88.6-98.4)	75.3 (74.8-75.8)	28.6 (28.3-29.0)	13.2 (10.2-17.0)	76.1 (69.9-82.1)	78.4 (77.9-78.9)	30.9 (30.6-31.4)
Azerbaijan	47.2 (40.8-54.6)	110.4 (103.4-118.2)	71.4 (70.6-72.1)	27.3 (26.9-27.7)	26.5 (20.3-34.2)	84.5 (77.4-91.3)	76.3 (75.6-77.1)	30.2 (29.7-30.7)
Georgia	24.8 (21.2-29.1)	85.3 (79.1-92.3)	76.0 (75.4-76.6)	29.7 (29.2-30.2)	15.4 (12.2-19.0)	80.4 (71.8-89.9)	78.0 (77.3-78.7)	30.9 (30.3-31.5)
Kazakhstan	25.4 (21.3-30.2)	164.4 (162.5-166.1)	71.1 (70.7-71.4)	26.3 (26.2-26.4)	15.4 (11.9-19.7)	119.5 (108.4-131.6)	75.0 (74.1-75.9)	28.7 (28.0-29.3)

SR Table 2: Under-5 mortality (5q0) per 1,000 live births, adult mortality (45q15) per 1,000, life expectancy at birth (years), and life expectancy at age 50 (years), by geography, females, 2005 and 2015 (95% uncertainty intervals)

Location	2005				2015			
	Under-5 mortality (5q0)	Adult mortality (45q15)	Life expectancy at birth	Life expectancy at age 50	Under-5 mortality (5q0)	Adult mortality (45q15)	Life expectancy at birth	Life expectancy at age 50
Kyrgyzstan	38.3 (35.5-41.3)	147.0 (139.6-154.4)	70.7 (70.2-71.2)	26.2 (25.8-26.6)	25.9 (22.6-29.6)	114.2 (106.6-122.0)	74.1 (73.4-74.7)	28.3 (27.9-28.8)
Mongolia	43.6 (38.1-49.5)	202.3 (191.8-214.5)	67.7 (67.0-68.4)	24.2 (23.8-24.7)	25.2 (20.1-31.8)	154.3 (142.6-166.2)	71.9 (71.0-72.7)	26.5 (26.1-27.0)
Tajikistan	47.0 (41.3-53.3)	131.5 (123.0-140.0)	70.2 (69.4-70.9)	26.3 (25.9-26.8)	29.3 (23.4-36.5)	103.8 (96.3-113.1)	74.8 (73.9-75.5)	29.2 (28.7-29.7)
Turkmenistan	56.3 (41.4-74.2)	150.8 (144.8-155.9)	69.5 (68.2-70.7)	26.9 (26.5-27.3)	35.7 (26.1-48.0)	116.9 (113.5-120.5)	73.7 (72.7-74.5)	28.9 (28.6-29.2)
Uzbekistan	33.3 (26.6-41.9)	137.9 (129.2-146.1)	71.5 (70.7-72.3)	26.6 (26.2-27.1)	21.2 (16.3-27.2)	108.7 (99.9-118.2)	75.2 (74.3-76.0)	29.0 (28.4-29.6)
Latin America and Caribbean	24.1 (22.2-26.0)	101.4 (99.8-103.2)	76.5 (76.3-76.7)	30.7 (30.5-30.8)	16.5 (13.3-20.6)	87.8 (85.2-90.6)	78.3 (77.9-78.6)	31.5 (31.3-31.7)
Central Latin America	21.3 (19.4-23.3)	92.8 (91.7-94.0)	77.6 (77.4-77.8)	31.3 (31.2-31.4)	15.1 (12.1-18.7)	82.1 (79.6-84.6)	78.8 (78.4-79.2)	31.8 (31.6-32.0)
Colombia	19.9 (17.6-22.3)	73.5 (73.0-74.0)	78.3 (78.1-78.5)	31.7 (31.6-31.7)	14.4 (10.9-18.9)	57.9 (55.1-60.7)	80.8 (80.4-81.2)	33.5 (33.2-33.7)
Costa Rica	12.1 (10.0-14.9)	59.6 (58.5-60.8)	81.5 (81.2-81.7)	33.9 (33.8-34.0)	8.8 (6.5-11.7)	56.3 (52.8-59.9)	82.6 (82.1-83.0)	34.6 (34.3-35.0)
El Salvador	17.5 (14.3-20.9)	105.7 (100.7-110.3)	77.0 (76.6-77.4)	31.2 (30.9-31.5)	10.1 (7.3-14.0)	95.9 (85.7-105.9)	78.9 (78.1-79.8)	32.2 (31.6-32.9)
Guatemala	38.7 (34.8-42.9)	136.0 (133.7-138.2)	73.2 (72.8-73.5)	29.5 (29.3-29.7)	23.7 (19.4-29.0)	115.7 (94.6-140.8)	75.2 (73.5-77.0)	29.6 (28.3-31.1)
Honduras	28.3 (26.3-30.4)	150.6 (112.0-195.2)	72.0 (69.5-74.8)	27.3 (25.5-29.5)	20.6 (17.3-24.7)	131.4 (93.5-174.0)	74.0 (71.4-76.7)	28.2 (26.4-30.3)
Mexico	19.8 (16.9-23.1)	97.6 (97.2-98.0)	77.7 (77.5-77.9)	31.2 (31.2-31.2)	13.7 (10.5-17.7)	88.1 (87.1-89.0)	78.3 (78.0-78.6)	31.1 (31.1-31.2)
Nicaragua	23.6 (21.4-26.0)	84.3 (78.7-90.3)	79.3 (78.7-79.8)	33.1 (32.8-33.5)	13.7 (11.2-16.6)	73.9 (65.5-82.9)	80.8 (79.9-81.7)	33.6 (32.9-34.3)
Panama	19.4 (16.4-23.2)	73.1 (67.9-79.1)	79.7 (79.1-80.3)	33.3 (32.8-33.8)	16.1 (12.2-21.1)	67.7 (56.1-79.5)	81.0 (79.8-82.2)	34.2 (33.3-35.1)
Venezuela	16.6 (16.1-17.0)	89.2 (88.0-90.3)	78.2 (78.1-78.3)	31.5 (31.4-31.6)	15.2 (14.1-16.3)	82.1 (69.7-95.8)	79.2 (78.0-80.4)	32.2 (31.3-33.2)
Andean Latin America	29.2 (27.7-30.7)	93.2 (88.1-98.5)	76.8 (76.3-77.2)	31.3 (30.9-31.7)	17.6 (15.4-19.8)	75.6 (67.6-84.1)	79.0 (78.2-79.8)	32.1 (31.5-32.8)
Bolivia	48.0 (43.8-52.3)	136.6 (119.4-156.1)	71.4 (69.9-72.7)	28.1 (26.7-29.2)	26.8 (21.9-32.6)	119.2 (91.0-149.9)	74.2 (72.1-76.4)	28.7 (27.0-30.5)
Ecuador	25.9 (23.2-28.6)	96.3 (89.8-103.6)	76.9 (76.3-77.4)	31.0 (30.6-31.4)	16.5 (14.1-19.3)	82.0 (72.5-94.0)	78.6 (77.6-79.5)	31.6 (30.8-32.5)
Peru	23.0 (21.6-24.5)	78.8 (71.8-86.5)	78.8 (78.2-79.4)	32.6 (32.1-33.1)	14.4 (12.3-17.0)	59.7 (49.9-69.5)	81.1 (80.0-82.2)	33.7 (32.8-34.7)
Caribbean	41.6 (38.8-44.6)	143.1 (132.5-156.1)	72.7 (71.9-73.4)	29.7 (29.1-30.2)	29.1 (21.5-38.7)	118.9 (106.0-135.7)	75.2 (73.9-76.3)	30.4 (29.8-31.0)
Antigua and Barbuda	14.2 (11.0-18.2)	94.4 (87.0-101.5)	77.2 (76.5-78.0)	30.2 (29.6-30.8)	11.7 (8.1-16.5)	75.5 (65.4-87.4)	78.9 (78.1-79.8)	31.3 (30.7-32.3)
The Bahamas	18.6 (13.1-25.1)	132.8 (123.7-141.9)	74.4 (73.7-75.2)	28.8 (28.4-29.3)	14.6 (8.4-25.4)	109.7 (95.1-124.7)	76.4 (75.1-77.7)	29.9 (29.1-30.8)
Barbados	17.7 (12.6-24.2)	95.6 (91.4-100.4)	77.0 (76.2-77.7)	30.3 (29.6-30.8)	14.5 (8.2-24.2)	92.0 (79.3-105.6)	77.6 (76.2-78.7)	30.5 (29.7-31.3)
Belize	20.0 (15.9-25.4)	148.4 (139.0-157.9)	73.1 (72.4-73.8)	27.8 (27.3-28.2)	15.3 (9.6-24.5)	131.3 (117.6-144.8)	74.7 (73.7-75.8)	28.7 (28.0-29.5)
Bermuda	6.1 (4.5-8.0)	79.6 (72.0-87.1)	79.6 (79.1-80.2)	31.7 (31.3-32.1)	4.1 (2.7-6.0)	56.7 (48.7-66.0)	82.2 (81.2-83.3)	33.7 (32.9-34.6)
Cuba	6.3 (6.1-6.5)	86.2 (85.3-87.2)	79.1 (79.0-79.2)	31.2 (31.1-31.3)	5.5 (5.2-5.9)	70.7 (67.0-74.4)	80.6 (80.2-81.0)	32.3 (32.0-32.7)
Dominica	22.0 (16.7-28.9)	94.3 (86.9-103.0)	77.2 (76.3-77.9)	30.9 (30.3-31.5)	23.9 (15.4-35.9)	95.2 (82.8-108.3)	76.8 (75.5-78.1)	30.8 (29.8-31.6)
Dominican Republic	32.2 (28.9-35.4)	107.0 (99.7-116.1)	76.2 (75.5-76.6)	31.7 (31.1-31.9)	22.6 (18.5-27.6)	85.9 (83.2-91.1)	77.9 (77.4-78.3)	31.8 (31.5-31.9)
Grenada	16.5 (11.2-23.0)	149.0 (140.1-158.0)	73.4 (72.6-74.1)	27.6 (27.2-28.1)	14.3 (7.9-24.6)	138.0 (124.4-155.3)	74.3 (72.9-75.4)	28.1 (27.3-28.8)
Guyana	35.0 (31.2-38.8)	216.4 (201.4-231.0)	67.8 (67.0-68.7)	24.8 (24.3-25.4)	24.9 (19.4-31.7)	183.6 (160.0-211.4)	70.5 (69.0-71.9)	26.1 (25.1-27.0)
Haiti	82.6 (74.9-91.2)	314.1 (265.2-371.7)	59.9 (57.3-62.3)	23.1 (21.1-25.6)	53.7 (37.8-73.7)	247.1 (185.6-321.8)	64.7 (61.2-68.2)	23.9 (21.6-26.7)
Jamaica	18.5 (12.3-27.9)	111.0 (104.6-117.1)	76.7 (75.9-77.4)	30.9 (30.5-31.3)	16.3 (10.3-24.8)	101.1 (86.5-114.7)	77.0 (75.7-78.2)	30.4 (29.6-31.3)
Puerto Rico	8.6 (8.3-9.0)	74.7 (73.0-76.4)	80.2 (80.1-80.4)	32.5 (32.4-32.6)	6.4 (5.7-7.2)	63.3 (57.9-68.9)	81.8 (81.2-82.4)	33.6 (33.1-34.1)
Saint Lucia	19.7 (13.2-28.5)	102.1 (95.6-108.7)	76.1 (75.3-77.1)	29.7 (29.3-30.4)	15.9 (8.4-28.2)	95.8 (85.0-107.5)	77.1 (75.8-78.3)	30.2 (29.4-31.1)
Saint Vincent and the Grenadines	26.1 (19.5-34.3)	135.4 (127.7-144.0)	73.0 (72.2-73.7)	27.6 (27.1-28.1)	20.3 (12.7-31.1)	129.2 (117.3-142.7)	73.9 (72.8-75.0)	28.0 (27.4-28.7)
Suriname	37.6 (31.5-45.2)	133.1 (125.2-141.5)	72.7 (71.9-73.6)	28.7 (27.9-29.4)	28.2 (22.4-35.8)	111.9 (97.6-128.2)	75.3 (73.9-76.5)	30.1 (28.9-31.0)
Trinidad and Tobago	21.5 (17.0-27.3)	131.7 (128.9-134.5)	74.5 (74.1-74.9)	29.0 (28.9-29.2)	17.1 (10.6-26.6)	113.5 (100.5-127.7)	76.1 (74.9-77.2)	29.8 (29.0-30.6)
Virgin Islands, U.S.	9.9 (8.8-11.2)	92.3 (84.6-99.8)	78.0 (77.5-78.5)	30.6 (30.3-31.0)	8.0 (6.7-9.6)	87.5 (76.2-100.1)	78.7 (77.9-79.5)	31.1 (30.5-31.7)

SR Table 2: Under-5 mortality (5q0) per 1,000 live births, adult mortality (45q15) per 1,000, life expectancy at birth (years), and life expectancy at age 50 (years), by geography, females, 2005 and 2015 (95% uncertainty intervals)

Location	2005				2015			
	Under-5 mortality (5q0)	Adult mortality (45q15)	Life expectancy at birth	Life expectancy at age 50	Under-5 mortality (5q0)	Adult mortality (45q15)	Life expectancy at birth	Life expectancy at age 50
Tropical Latin America	22.4 (19.0-26.1)	103.0 (100.3-105.8)	76.2 (75.8-76.6)	30.2 (30.0-30.4)	15.2 (12.0-19.0)	90.4 (85.8-95.3)	78.2 (77.6-78.7)	31.4 (31.0-31.8)
Brazil	22.4 (18.8-26.2)	103.0 (100.2-105.9)	76.2 (75.8-76.6)	30.2 (30.0-30.4)	15.1 (11.9-19.0)	90.2 (85.7-95.3)	78.2 (77.6-78.7)	31.4 (31.0-31.8)
Paraguay	22.8 (19.5-26.2)	103.2 (93.7-112.8)	76.1 (75.2-77.0)	30.0 (29.3-30.8)	16.1 (12.8-20.1)	98.0 (80.4-117.7)	76.9 (75.6-78.4)	30.2 (29.2-31.4)
Southeast Asia, East Asia, and Oceania	26.5 (24.4-29.1)	114.1 (109.7-118.7)	74.0 (73.6-74.3)	28.3 (28.0-28.6)	14.9 (12.5-17.9)	79.3 (74.1-85.1)	78.4 (77.8-78.9)	31.4 (31.0-31.8)
East Asia	21.3 (17.8-25.6)	103.3 (99.0-107.9)	74.9 (74.5-75.4)	28.6 (28.3-28.9)	11.3 (8.1-15.5)	63.3 (59.5-67.4)	79.8 (79.2-80.5)	32.1 (31.6-32.7)
China	21.3 (17.7-26.0)	103.4 (99.0-107.9)	74.9 (74.4-75.4)	28.6 (28.2-28.9)	11.3 (7.9-15.6)	62.4 (58.5-66.8)	79.9 (79.2-80.6)	32.2 (31.7-32.8)
North Korea	29.2 (14.7-51.2)	136.6 (104.7-174.6)	72.6 (69.8-75.1)	27.5 (26.0-29.1)	17.6 (8.4-33.4)	121.0 (84.2-166.0)	74.9 (72.2-77.5)	28.6 (26.7-30.5)
Taiwan	6.4 (6.1-6.8)	66.3 (65.2-67.4)	80.7 (80.6-80.8)	32.6 (32.5-32.7)	6.2 (4.1-9.5)	53.1 (42.4-66.3)	82.5 (81.2-83.9)	34.2 (33.1-35.4)
Southeast Asia	32.5 (31.4-33.7)	141.1 (128.9-153.8)	72.2 (71.5-72.9)	27.6 (27.1-28.2)	19.2 (17.0-21.6)	115.8 (100.7-132.5)	75.3 (74.3-76.3)	29.3 (28.6-30.0)
Cambodia	59.1 (55.3-63.3)	199.2 (179.5-220.0)	66.2 (65.3-67.3)	24.4 (23.8-25.2)	27.6 (23.6-32.0)	139.7 (109.0-176.3)	72.4 (70.5-74.6)	27.2 (25.9-28.9)
Indonesia	35.1 (33.1-37.3)	165.7 (147.7-185.9)	70.1 (69.2-71.1)	25.9 (25.2-26.6)	20.8 (17.8-24.3)	138.1 (105.7-175.6)	73.3 (71.2-75.4)	27.6 (26.3-29.2)
Laos	105.1 (94.8-116.0)	215.4 (186.2-246.3)	62.1 (60.3-63.9)	24.5 (23.0-25.8)	55.0 (40.9-72.8)	152.8 (114.9-197.3)	69.5 (66.7-72.2)	26.9 (24.9-28.9)
Malaysia	7.3 (7.0-7.5)	90.6 (89.8-91.5)	76.3 (76.2-76.4)	28.5 (28.4-28.5)	5.9 (5.0-7.0)	82.4 (69.2-96.7)	78.6 (77.7-79.6)	30.7 (30.0-31.4)
Maldives	22.4 (20.1-24.8)	73.8 (69.0-78.4)	78.2 (77.8-78.7)	31.7 (31.4-32.1)	11.4 (9.5-13.8)	48.0 (38.2-58.1)	82.3 (81.0-83.5)	34.4 (33.3-35.4)
Mauritius	14.5 (13.7-15.1)	107.3 (104.1-110.8)	75.7 (75.4-75.9)	28.6 (28.4-28.8)	12.1 (11.0-13.3)	92.0 (85.6-99.7)	77.7 (77.1-78.2)	30.3 (29.8-30.8)
Myanmar	57.5 (54.9-60.1)	207.6 (122.9-304.3)	66.0 (61.8-70.8)	24.3 (21.4-28.2)	29.4 (22.3-39.3)	158.7 (95.7-237.1)	71.2 (66.9-75.3)	26.5 (23.7-29.6)
Philippines	29.1 (27.1-31.0)	124.9 (124.2-125.6)	73.0 (72.9-73.2)	28.2 (28.2-28.3)	20.9 (18.5-23.8)	117.7 (105.9-132.0)	74.4 (73.4-75.3)	28.7 (27.9-29.4)
Sri Lanka	16.1 (15.5-16.6)	76.0 (73.4-78.6)	78.0 (77.7-78.2)	31.0 (30.9-31.3)	7.6 (5.9-9.7)	59.7 (42.6-81.4)	81.2 (79.1-83.2)	33.3 (31.6-35.0)
Seychelles	12.1 (10.8-13.6)	114.8 (105.4-125.0)	75.9 (75.4-76.5)	29.1 (28.7-29.5)	10.0 (8.1-12.2)	91.6 (76.4-110.2)	77.9 (76.8-79.0)	30.5 (29.8-31.3)
Thailand	9.9 (8.6-11.5)	119.1 (111.4-127.0)	76.5 (75.9-77.1)	30.2 (29.8-30.6)	5.3 (4.3-6.5)	92.5 (75.4-115.2)	79.0 (77.5-80.5)	31.5 (30.4-32.6)
Timor-Leste	65.5 (56.6-75.3)	164.7 (139.6-191.1)	68.0 (66.2-69.9)	26.7 (25.2-28.1)	35.3 (21.4-55.1)	127.1 (92.2-167.8)	73.0 (70.0-75.7)	28.5 (26.3-30.4)
Vietnam	19.3 (17.5-21.3)	90.6 (62.7-125.5)	77.0 (74.4-79.2)	30.4 (28.3-32.3)	12.1 (10.2-14.4)	70.5 (43.3-104.4)	79.4 (77.0-82.2)	31.9 (29.9-34.4)
Oceania	54.7 (37.3-75.9)	325.6 (235.2-429.5)	60.9 (56.9-65.0)	20.7 (18.3-23.2)	36.3 (21.8-57.9)	293.3 (213.6-407.0)	63.7 (59.0-67.7)	21.6 (18.8-24.1)
American Samoa	10.3 (9.1-11.7)	136.6 (114.8-161.1)	74.6 (73.0-75.9)	28.0 (26.8-28.9)	6.6 (5.4-8.1)	138.0 (105.4-177.2)	74.8 (72.4-77.1)	27.9 (26.1-29.6)
Federated States of Micronesia	15.9 (10.8-22.0)	201.3 (119.3-316.3)	69.9 (64.4-74.5)	24.8 (20.9-28.0)	10.4 (7.1-15.0)	182.7 (116.3-304.1)	71.5 (65.3-75.8)	25.7 (21.3-28.9)
Fiji	28.9 (21.0-38.5)	232.3 (200.7-265.2)	67.1 (65.4-68.8)	23.4 (22.3-24.5)	27.6 (16.5-44.1)	215.2 (167.2-267.8)	68.1 (65.5-70.9)	24.0 (22.1-25.8)
Guam	12.3 (11.0-13.8)	105.5 (88.6-124.6)	76.5 (75.4-77.6)	29.5 (28.8-30.3)	15.8 (12.3-20.1)	116.2 (89.8-147.7)	75.3 (73.6-77.3)	28.8 (27.6-30.3)
Kiribati	55.1 (42.4-70.2)	233.6 (194.5-278.6)	64.9 (62.7-67.1)	23.2 (21.9-24.5)	36.1 (19.0-62.7)	214.9 (168.7-269.9)	67.4 (64.6-70.2)	24.0 (22.3-25.7)
Marshall Islands	30.4 (21.0-41.4)	235.9 (195.6-283.6)	66.8 (64.6-69.0)	23.3 (21.8-24.7)	17.0 (9.5-28.6)	208.1 (166.3-257.7)	69.5 (67.0-71.9)	24.5 (22.8-26.2)
Northern Mariana Islands	7.7 (5.8-9.9)	103.1 (91.8-114.4)	77.4 (76.7-78.1)	30.0 (29.5-30.5)	5.6 (3.3-9.0)	93.9 (77.2-111.5)	78.5 (77.2-79.6)	30.7 (29.8-31.6)
Papua New Guinea	63.8 (42.0-90.6)	364.2 (236.1-502.5)	58.5 (53.4-63.9)	19.4 (16.5-23.0)	41.2 (24.4-66.2)	323.7 (215.5-470.4)	61.8 (56.0-67.0)	20.5 (17.0-23.8)
Samoa	11.7 (6.2-20.3)	148.9 (115.9-183.3)	73.7 (71.7-75.8)	27.4 (25.9-29.0)	8.0 (4.2-14.4)	146.8 (111.1-185.2)	74.2 (71.8-76.6)	27.6 (25.8-29.4)
Solomon Islands	28.2 (20.4-38.3)	326.6 (210.4-460.8)	62.7 (57.5-68.2)	20.4 (17.3-24.1)	17.9 (12.4-25.8)	290.5 (178.0-440.0)	65.1 (59.2-70.8)	21.5 (17.8-25.3)
Tonga	21.0 (13.3-31.5)	147.0 (126.3-168.1)	72.7 (71.3-74.0)	27.1 (26.3-28.0)	16.1 (9.4-26.0)	125.8 (92.7-167.0)	74.7 (72.2-77.1)	28.4 (26.7-30.0)
Vanuatu	37.1 (28.6-46.9)	277.2 (174.2-404.3)	64.2 (59.0-69.2)	21.9 (18.5-25.1)	24.6 (16.7-36.9)	248.5 (165.5-369.5)	66.6 (61.2-70.9)	23.0 (19.5-25.9)
North Africa and Middle East	41.9 (39.3-44.5)	128.9 (118.0-140.8)	72.5 (71.8-73.2)	28.5 (28.0-29.0)	28.6 (25.0-32.9)	113.8 (103.6-125.7)	74.9 (74.1-75.7)	29.8 (29.3-30.2)
North Africa and Middle East	41.9 (39.3-44.5)	128.9 (118.0-140.8)	72.5 (71.8-73.2)	28.5 (28.0-29.0)	28.6 (25.0-32.9)	113.8 (103.6-125.7)	74.9 (74.1-75.7)	29.8 (29.3-30.2)
Afghanistan	110.9 (100.0-121.7)	477.8 (363.8-604.8)	51.4 (47.4-55.6)	16.9 (14.6-19.3)	80.5 (66.8-96.9)	442.3 (329.7-560.3)	54.5 (50.2-58.8)	17.7 (15.5-20.4)
Algeria	28.6 (24.0-33.6)	95.3 (82.0-109.0)	76.1 (74.9-77.1)	30.4 (29.5-31.2)	20.9 (16.4-27.3)	86.2 (72.6-102.1)	77.4 (76.0-78.5)	30.9 (29.8-31.7)

SR Table 2: Under-5 mortality (5q0) per 1,000 live births, adult mortality (45q15) per 1,000, life expectancy at birth (years), and life expectancy at age 50 (years), by geography, females, 2005 and 2015 (95% uncertainty intervals)

Location	2005				2015			
	Under-5 mortality (5q0)	Adult mortality (45q15)	Life expectancy at birth	Life expectancy at age 50	Under-5 mortality (5q0)	Adult mortality (45q15)	Life expectancy at birth	Life expectancy at age 50
Bahrain	9.5 (8.7-10.3)	90.8 (77.5-106.3)	78.1 (77.2-78.9)	30.6 (30.0-31.2)	6.5 (5.6-7.6)	69.3 (53.6-91.3)	80.3 (78.9-81.8)	32.3 (31.2-33.5)
Egypt	32.9 (29.6-36.4)	131.2 (130.5-131.8)	72.6 (72.4-72.9)	27.3 (27.2-27.3)	20.6 (15.6-27.3)	119.8 (115.3-124.3)	74.4 (73.9-74.9)	28.0 (27.8-28.3)
Iran	24.7 (18.4-32.5)	95.3 (74.5-121.4)	74.6 (72.5-76.5)	28.5 (27.0-30.0)	13.4 (9.8-17.8)	79.5 (56.5-110.0)	78.0 (75.9-80.0)	30.7 (29.0-32.3)
Iraq	36.1 (33.2-39.0)	199.7 (149.1-253.4)	68.2 (65.7-70.6)	24.7 (23.0-26.3)	25.3 (21.3-30.4)	174.7 (123.1-235.7)	70.7 (67.5-73.6)	26.1 (23.9-28.1)
Jordan	19.6 (18.4-20.8)	105.1 (95.0-116.9)	74.4 (73.5-75.3)	27.8 (27.1-28.6)	14.2 (12.4-16.5)	56.7 (47.8-66.8)	80.7 (79.7-81.8)	33.1 (32.2-34.1)
Kuwait	10.1 (9.4-10.8)	60.7 (57.6-63.5)	80.2 (79.9-80.5)	32.3 (32.0-32.5)	7.8 (6.2-9.7)	36.3 (29.6-44.2)	81.6 (80.5-82.7)	32.9 (31.9-33.9)
Lebanon	13.7 (10.4-17.7)	88.0 (64.5-118.6)	77.6 (75.8-79.4)	30.5 (29.1-31.9)	7.7 (5.7-10.4)	73.0 (45.3-106.1)	79.9 (77.8-82.3)	32.0 (30.5-34.0)
Libya	22.0 (17.7-26.8)	91.0 (81.1-100.7)	76.9 (76.1-77.6)	30.5 (29.9-31.1)	16.5 (12.4-21.3)	97.7 (79.8-119.5)	76.9 (75.3-78.2)	30.3 (29.2-31.4)
Morocco	32.8 (28.4-37.4)	116.4 (83.2-158.8)	73.8 (70.9-76.2)	28.7 (26.4-30.8)	20.7 (16.6-25.7)	103.3 (63.3-151.5)	75.8 (72.7-79.0)	29.5 (27.2-32.1)
Palestine	21.5 (18.9-24.3)	86.1 (75.2-96.3)	77.3 (76.6-78.1)	30.9 (30.2-31.5)	15.9 (12.4-20.3)	83.1 (60.3-113.8)	77.9 (75.8-79.8)	30.9 (29.2-32.4)
Oman	10.3 (9.7-10.9)	82.7 (72.4-95.2)	78.4 (77.4-79.3)	30.6 (29.8-31.4)	8.9 (7.7-10.2)	69.8 (55.1-88.3)	79.6 (78.3-81.1)	31.7 (30.6-33.0)
Qatar	12.4 (9.4-16.0)	80.6 (68.6-92.3)	78.3 (77.5-79.1)	30.9 (30.2-31.6)	8.2 (5.8-11.4)	57.6 (41.4-75.5)	81.2 (79.4-82.9)	33.1 (31.5-34.6)
Saudi Arabia	16.6 (12.4-22.3)	60.2 (57.1-63.3)	80.0 (79.4-80.6)	32.8 (32.4-33.2)	10.8 (7.8-14.9)	48.1 (42.9-54.8)	82.2 (81.3-83.1)	34.2 (33.4-35.0)
Sudan	74.3 (66.9-82.1)	185.8 (119.3-265.7)	66.1 (62.3-70.0)	25.6 (22.5-28.6)	51.4 (39.3-67.8)	161.6 (96.0-233.6)	69.3 (65.4-73.6)	26.5 (23.8-29.9)
Syria	15.0 (14.4-15.6)	83.9 (76.6-91.5)	77.4 (76.7-78.0)	30.3 (29.8-30.8)	22.1 (15.5-29.4)	126.8 (88.3-162.9)	74.8 (72.1-77.8)	30.4 (29.3-31.7)
Tunisia	19.7 (17.5-22.2)	68.3 (51.8-87.2)	79.0 (77.5-80.5)	32.1 (30.8-33.4)	13.1 (10.9-15.6)	57.2 (40.2-78.0)	80.7 (78.8-82.7)	33.1 (31.4-34.8)
Turkey	25.8 (22.0-30.1)	60.4 (55.8-65.4)	79.5 (78.9-80.1)	33.1 (32.7-33.6)	15.4 (11.4-20.5)	45.8 (42.8-49.1)	82.4 (81.8-82.9)	34.8 (34.4-35.2)
United Arab Emirates	8.4 (6.9-10.3)	106.6 (93.5-120.2)	77.3 (76.5-78.0)	30.1 (29.5-30.5)	5.3 (3.1-8.4)	101.3 (73.2-138.5)	78.0 (75.5-79.9)	30.4 (28.6-31.8)
Yemen	62.8 (58.7-66.8)	224.9 (123.1-349.4)	64.7 (59.6-70.4)	23.6 (20.0-28.2)	52.0 (43.6-61.5)	208.5 (121.6-337.9)	66.6 (60.8-71.6)	24.3 (20.4-28.4)
South Asia	73.4 (69.4-77.6)	193.4 (185.4-202.0)	65.2 (64.7-65.8)	25.1 (24.7-25.5)	50.5 (45.0-57.1)	151.1 (142.9-159.4)	69.6 (68.8-70.3)	26.8 (26.4-27.3)
South Asia	73.4 (69.4-77.6)	193.4 (185.4-202.0)	65.2 (64.7-65.8)	25.1 (24.7-25.5)	50.5 (45.0-57.1)	151.1 (142.9-159.4)	69.6 (68.8-70.3)	26.8 (26.4-27.3)
Bangladesh	63.9 (61.2-66.7)	153.7 (137.3-170.1)	68.7 (67.6-69.9)	27.0 (26.1-28.0)	37.3 (34.2-40.8)	128.8 (105.2-156.0)	72.5 (70.6-74.5)	28.1 (26.5-29.8)
Bhutan	67.5 (60.0-76.4)	134.6 (104.5-169.0)	70.2 (67.8-72.3)	28.7 (26.9-30.3)	43.9 (37.6-51.7)	104.4 (77.0-141.6)	74.3 (71.6-76.5)	30.3 (28.2-32.1)
India	73.2 (68.5-78.1)	195.9 (187.6-205.6)	65.0 (64.4-65.7)	25.0 (24.6-25.4)	50.1 (43.0-58.5)	151.0 (142.3-159.6)	69.5 (68.7-70.4)	26.8 (26.3-27.4)
Nepal	62.6 (59.3-66.0)	153.8 (129.8-182.3)	68.8 (67.0-70.6)	27.1 (25.6-28.5)	35.7 (32.1-39.7)	149.2 (112.4-191.8)	71.2 (68.8-73.9)	27.3 (25.3-29.5)
Pakistan	83.6 (79.3-88.2)	218.7 (186.4-257.5)	63.2 (61.4-65.0)	24.3 (22.8-25.7)	62.2 (56.2-69.3)	175.1 (137.5-216.3)	67.3 (65.2-69.8)	25.7 (24.0-27.7)
Sub-Saharan Africa	117.6 (114.5-120.9)	352.8 (335.0-373.5)	55.9 (55.1-56.7)	22.8 (22.1-23.4)	77.2 (71.1-84.6)	242.0 (213.7-275.8)	63.6 (61.9-65.2)	25.2 (23.9-26.4)
Southern Sub-Saharan Africa	74.9 (70.1-80.1)	514.8 (483.7-545.8)	51.8 (50.5-53.2)	21.4 (20.3-22.5)	42.8 (37.8-48.5)	304.6 (281.0-332.3)	63.2 (61.7-64.7)	25.7 (24.6-26.8)
Botswana	50.2 (41.4-59.8)	559.8 (318.7-862.7)	50.8 (39.4-64.3)	20.2 (11.6-29.4)	25.6 (20.0-33.0)	353.9 (181.1-721.3)	62.0 (47.7-72.3)	23.7 (13.5-30.7)
Lesotho	111.4 (103.7-119.2)	641.4 (553.3-729.9)	44.8 (41.3-48.6)	18.3 (15.6-20.3)	77.1 (64.2-92.4)	532.3 (365.5-721.3)	50.4 (43.6-58.5)	20.1 (14.5-26.0)
Namibia	60.5 (55.1-66.6)	469.9 (389.4-557.3)	55.1 (51.3-59.1)	21.8 (18.7-24.7)	37.4 (28.5-48.6)	224.6 (149.0-344.2)	68.4 (61.6-73.0)	28.1 (22.4-31.1)
South Africa	74.4 (65.7-82.7)	470.3 (437.7-501.4)	53.6 (52.2-55.4)	22.1 (20.8-23.4)	39.0 (32.8-46.7)	292.3 (272.6-316.2)	64.0 (62.5-65.2)	26.1 (25.0-27.1)
Swaziland	105.7 (97.8-113.7)	689.2 (593.1-782.7)	43.2 (39.3-47.4)	17.3 (14.4-19.9)	51.7 (41.7-63.3)	463.8 (292.6-680.0)	54.9 (46.7-63.9)	21.4 (15.0-28.3)
Zimbabwe	77.5 (73.0-82.2)	674.7 (596.4-746.4)	45.6 (42.4-49.3)	18.3 (15.5-20.4)	50.4 (43.5-58.6)	312.0 (217.9-443.3)	62.5 (56.3-68.3)	25.5 (20.1-30.3)
Western Sub-Saharan Africa	141.3 (137.0-145.9)	276.1 (250.2-304.7)	57.5 (56.1-58.8)	24.4 (23.2-25.4)	94.0 (86.8-102.6)	207.3 (169.4-269.1)	64.2 (60.9-66.5)	26.3 (23.6-28.0)
Benin	111.1 (105.3-117.2)	240.5 (162.0-343.4)	61.0 (56.4-65.3)	24.7 (20.8-28.0)	73.2 (64.9-83.5)	210.6 (88.8-432.9)	64.8 (55.8-72.4)	24.8 (18.3-30.8)
Burkina Faso	148.8 (141.4-156.8)	278.1 (217.4-353.7)	56.6 (53.5-59.7)	23.9 (20.8-26.2)	105.4 (94.8-118.8)	221.7 (103.5-411.6)	61.9 (54.3-68.9)	24.4 (18.8-29.9)
Cameroon	119.1 (111.8-126.3)	347.7 (278.2-421.5)	55.9 (52.8-59.5)	22.8 (19.9-25.7)	84.9 (72.7-98.3)	283.3 (158.3-457.8)	61.2 (54.0-68.5)	24.7 (18.8-30.0)
Cape Verde	34.3 (30.2-38.8)	93.3 (84.0-129.3)	76.0 (73.4-76.9)	31.1 (29.0-31.6)	25.4 (22.6-28.3)	93.8 (81.9-121.9)	76.6 (74.3-77.6)	30.7 (28.8-31.5)

SR Table 2: Under-5 mortality (5q0) per 1,000 live births, adult mortality (45q15) per 1,000, life expectancy at birth (years), and life expectancy at age 50 (years), by geography, females, 2005 and 2015 (95% uncertainty intervals)

Location	2005				2015			
	Under-5 mortality (5q0)	Adult mortality (45q15)	Life expectancy at birth	Life expectancy at age 50	Under-5 mortality (5q0)	Adult mortality (45q15)	Life expectancy at birth	Life expectancy at age 50
Chad	161.4 (150.1-174.1)	310.5 (196.0-452.1)	54.5 (49.3-60.0)	23.3 (18.9-27.6)	124.7 (109.2-143.0)	261.5 (114.4-512.2)	58.9 (49.4-67.6)	24.1 (17.0-30.8)
Cote d'Ivoire	119.4 (112.2-126.7)	372.7 (305.1-451.4)	54.8 (51.8-58.2)	21.9 (19.1-25.0)	86.3 (76.3-98.4)	267.4 (149.8-425.1)	61.4 (55.1-68.5)	23.9 (18.9-29.7)
The Gambia	67.4 (62.9-72.1)	198.9 (147.7-252.8)	66.2 (63.5-69.3)	25.6 (23.4-28.1)	42.3 (37.5-47.5)	173.0 (98.4-275.9)	69.6 (64.4-74.6)	26.6 (22.8-30.6)
Ghana	79.7 (75.6-83.5)	264.2 (194.1-348.4)	62.2 (58.4-66.3)	24.2 (21.1-27.7)	51.1 (45.7-58.1)	196.0 (95.0-370.2)	67.6 (59.6-74.2)	25.8 (20.0-31.1)
Guinea	144.4 (136.4-152.7)	275.8 (220.5-338.0)	56.9 (54.2-59.8)	23.5 (21.1-25.7)	103.0 (91.5-115.8)	257.1 (138.6-403.8)	60.5 (54.5-67.1)	23.6 (19.0-28.8)
Guinea-Bissau	158.5 (142.8-174.7)	322.6 (106.3-689.4)	53.4 (41.9-65.2)	21.8 (13.8-30.9)	118.2 (102.4-137.9)	313.9 (112.8-734.8)	56.4 (42.5-67.6)	22.2 (13.0-30.7)
Liberia	113.9 (108.0-120.1)	292.9 (235.2-356.2)	58.4 (55.8-61.5)	23.4 (20.9-26.0)	66.8 (59.7-75.3)	255.2 (150.5-396.7)	63.4 (57.5-69.4)	24.0 (19.5-28.8)
Mali	160.7 (152.3-169.4)	249.2 (199.8-309.2)	57.1 (54.2-59.6)	24.6 (22.2-26.3)	124.1 (108.6-142.4)	228.6 (121.7-374.9)	60.4 (54.4-66.7)	24.7 (19.7-29.5)
Mauritania	77.7 (72.7-83.2)	183.3 (138.2-243.0)	66.1 (63.1-68.9)	26.1 (23.5-28.2)	50.8 (45.0-57.5)	155.6 (73.8-277.6)	69.8 (63.7-75.5)	27.0 (22.4-31.8)
Niger	173.0 (164.2-182.9)	241.6 (189.8-297.8)	56.7 (53.9-59.4)	24.9 (22.7-26.8)	109.6 (97.4-124.0)	207.5 (112.1-340.9)	62.5 (56.7-68.2)	25.2 (20.6-29.7)
Nigeria	155.1 (147.6-163.2)	263.7 (213.4-319.6)	57.3 (54.4-59.9)	25.1 (22.7-27.1)	98.5 (86.8-111.5)	169.4 (110.6-289.9)	66.3 (59.5-70.3)	28.6 (23.2-31.5)
Sao Tome and Principe	57.7 (52.0-63.3)	203.2 (143.6-276.1)	66.2 (63.0-69.5)	24.4 (22.1-27.1)	35.6 (30.6-41.2)	189.3 (80.1-354.1)	68.8 (61.3-75.8)	25.1 (19.9-30.5)
Senegal	86.6 (83.1-90.6)	223.3 (138.4-330.0)	63.3 (58.7-68.2)	24.5 (20.9-28.3)	48.2 (44.1-53.1)	210.7 (86.6-406.3)	66.7 (58.3-74.4)	24.6 (18.7-30.6)
Sierra Leone	174.0 (165.3-183.7)	309.2 (258.3-366.9)	53.2 (51.2-55.5)	22.0 (19.9-24.0)	116.2 (106.5-126.7)	296.3 (184.7-452.3)	57.7 (52.0-63.3)	22.6 (18.1-27.2)
Togo	105.5 (99.6-112.1)	299.8 (234.3-371.0)	59.0 (55.8-62.5)	24.2 (21.2-26.8)	73.8 (66.8-82.4)	224.2 (131.2-362.2)	64.6 (58.5-70.5)	25.6 (20.6-30.4)
Eastern Sub-Saharan Africa	98.2 (95.2-101.4)	380.2 (352.1-411.2)	56.1 (54.8-57.3)	21.9 (20.9-23.1)	62.6 (55.3-71.4)	247.0 (198.0-307.4)	64.3 (61.4-67.1)	24.7 (22.5-26.9)
Burundi	112.7 (102.6-122.8)	366.8 (296.6-448.2)	55.3 (52.3-58.1)	20.5 (18.3-23.1)	72.4 (53.1-97.0)	253.1 (123.8-432.4)	62.7 (54.7-70.2)	23.1 (18.1-28.8)
Comoros	63.9 (48.5-82.1)	186.4 (136.4-243.3)	66.7 (63.7-70.0)	25.3 (23.1-27.8)	40.1 (27.2-56.2)	175.2 (84.9-293.2)	69.3 (63.4-75.4)	25.8 (21.6-30.4)
Djibouti	84.1 (74.3-93.9)	220.4 (100.1-467.2)	63.9 (53.9-71.7)	25.4 (17.8-31.5)	60.9 (51.1-72.8)	192.4 (92.8-424.2)	67.0 (57.0-74.0)	26.0 (18.7-31.5)
Eritrea	75.7 (64.5-88.0)	301.3 (128.1-538.0)	60.2 (51.5-69.8)	21.6 (16.1-29.0)	61.8 (49.5-78.6)	297.0 (103.0-571.5)	61.3 (51.8-71.8)	21.4 (15.4-29.1)
Ethiopia	102.8 (96.6-109.0)	364.4 (301.4-434.9)	56.1 (53.5-58.7)	21.1 (19.0-23.5)	55.2 (48.7-62.8)	201.7 (97.6-364.4)	66.8 (59.4-73.4)	25.3 (19.8-30.7)
Kenya	66.6 (62.6-70.9)	392.2 (369.0-420.4)	58.5 (57.1-59.9)	24.2 (23.0-25.4)	47.0 (41.2-53.6)	215.2 (188.5-246.7)	67.6 (65.8-69.3)	26.6 (25.2-28.1)
Madagascar	78.7 (71.0-87.1)	250.6 (189.7-318.6)	62.3 (59.4-65.2)	22.7 (20.6-25.2)	60.0 (47.8-75.1)	222.6 (98.6-396.3)	65.0 (57.8-72.3)	23.7 (18.8-29.2)
Malawi	114.2 (105.8-123.6)	528.6 (460.5-601.3)	49.3 (46.3-52.6)	20.9 (18.1-23.5)	77.8 (65.0-93.0)	254.7 (166.3-388.7)	63.0 (57.0-68.7)	25.8 (20.8-30.3)
Mozambique	120.6 (114.0-127.0)	384.4 (305.9-476.3)	54.8 (51.1-58.7)	23.6 (19.8-26.6)	73.4 (65.5-82.6)	327.7 (203.4-512.1)	59.9 (52.1-67.3)	24.2 (18.0-30.1)
Rwanda	96.7 (89.5-104.4)	271.7 (217.0-327.3)	61.0 (58.0-64.0)	25.2 (22.5-27.5)	60.5 (49.0-76.2)	179.7 (92.1-331.4)	67.7 (60.5-73.7)	26.5 (20.9-31.0)
Somalia	132.2 (120.5-143.7)	383.6 (93.6-755.5)	52.5 (41.1-66.6)	19.1 (12.4-29.3)	106.0 (91.8-125.1)	365.6 (98.7-748.0)	55.0 (42.4-67.9)	19.5 (12.5-28.9)
South Sudan	117.1 (107.2-127.2)	334.3 (105.2-715.1)	55.7 (43.1-67.5)	21.2 (13.2-30.1)	102.4 (84.0-122.5)	330.5 (115.6-707.0)	56.9 (43.9-68.7)	21.4 (13.3-30.3)
Tanzania	91.1 (85.9-96.3)	372.2 (304.2-456.3)	57.2 (53.5-60.8)	23.4 (20.1-26.2)	56.2 (48.9-65.0)	231.6 (118.3-429.4)	65.5 (57.0-72.6)	25.4 (19.0-31.2)
Uganda	101.0 (96.1-106.0)	389.0 (324.4-456.2)	55.3 (52.7-58.4)	21.3 (19.2-24.5)	63.6 (57.4-71.5)	241.6 (125.7-411.8)	64.3 (56.9-71.6)	24.9 (19.3-30.7)
Zambia	102.3 (94.1-110.9)	560.1 (490.6-632.3)	48.5 (45.7-51.3)	18.9 (16.9-20.7)	61.2 (50.7-73.0)	336.7 (206.4-491.3)	59.8 (53.6-67.0)	22.7 (18.1-28.4)
Central Sub-Saharan Africa	120.6 (112.1-130.2)	328.9 (257.4-433.6)	56.4 (52.6-59.7)	22.2 (19.7-24.7)	80.7 (67.3-95.4)	284.4 (184.3-412.8)	61.0 (56.0-66.2)	22.9 (19.6-26.8)
Angola	117.7 (103.6-132.6)	344.8 (105.3-707.6)	55.2 (43.5-67.9)	20.6 (13.3-30.2)	74.2 (56.2-95.9)	306.1 (99.9-704.1)	59.8 (45.9-71.6)	21.7 (13.3-30.7)
Central African Republic	148.8 (129.0-170.3)	502.7 (284.2-753.9)	47.6 (39.7-57.6)	18.8 (13.1-26.6)	121.2 (91.1-158.4)	435.6 (209.1-726.3)	51.7 (42.5-63.1)	19.6 (13.4-28.5)
Congo	85.3 (78.1-92.5)	406.8 (329.3-486.5)	55.6 (52.6-58.8)	20.1 (18.0-22.5)	52.4 (40.6-66.8)	349.0 (193.0-550.5)	60.0 (52.7-68.0)	21.0 (16.1-27.1)
Democratic Republic of the Congo	122.7 (111.5-134.7)	294.9 (240.2-355.0)	57.6 (55.1-60.6)	23.2 (20.9-25.6)	82.9 (65.5-103.1)	253.8 (146.8-380.3)	62.1 (56.8-68.1)	23.7 (19.7-28.6)
Equatorial Guinea	127.3 (106.8-150.9)	305.4 (116.1-649.6)	56.5 (44.8-67.7)	22.9 (14.4-31.3)	82.6 (60.7-112.2)	254.4 (102.7-599.9)	61.9 (48.9-71.6)	24.3 (15.4-31.5)
Gabon	62.4 (57.7-67.5)	306.0 (243.4-372.3)	61.3 (58.5-64.2)	21.9 (20.0-24.0)	42.4 (36.3-49.1)	189.2 (93.6-336.7)	68.5 (61.5-74.3)	25.5 (20.6-30.1)

SR Table 3: Under-5 mortality (5q0) per 1,000 live births, adult mortality (45q15) per 1,000, life expectancy at birth (years), and life expectancy at age 50 (years), by geography, males, 2005 and 2015 (95% uncertainty intervals)

Location	2005				2015			
	Under-5 mortality (5q0)	Adult mortality (45q15)	Life expectancy at birth	Life expectancy at age 50	Under-5 mortality (5q0)	Adult mortality (45q15)	Life expectancy at birth	Life expectancy at age 50
Global	61.6 (59.7-63.7)	223.5 (219.9-227.2)	65.7 (65.5-65.9)	25.1 (25.0-25.3)	42.9 (39.4-46.9)	190.1 (184.9-195.6)	69.0 (68.6-69.4)	26.6 (26.4-26.8)
High SDI	8.1 (8.0-8.2)	175.3 (174.6-176.1)	73.2 (73.2-73.3)	27.5 (27.4-27.5)	5.8 (5.5-6.1)	134.7 (132.9-136.5)	76.2 (76.1-76.3)	29.4 (29.3-29.4)
High-middle SDI	27.1 (25.6-28.6)	199.9 (195.6-205.0)	69.3 (69.0-69.5)	25.6 (25.4-25.7)	16.7 (14.8-18.8)	157.3 (151.6-164.0)	72.7 (72.3-73.1)	27.4 (27.0-27.7)
Middle SDI	39.6 (37.7-41.5)	218.2 (211.1-225.2)	66.8 (66.4-67.2)	24.1 (23.8-24.4)	24.7 (22.1-27.4)	191.9 (182.9-201.5)	69.5 (69.0-70.1)	25.4 (25.0-25.8)
Low-middle SDI	92.3 (89.5-95.3)	285.3 (273.1-297.9)	60.3 (59.7-60.9)	22.9 (22.5-23.3)	63.7 (59.1-68.8)	252.5 (237.7-270.2)	63.8 (63.0-64.6)	23.7 (23.2-24.3)
Low SDI	129.3 (125.3-133.4)	378.8 (355.1-404.0)	54.5 (53.5-55.4)	21.6 (20.8-22.3)	88.8 (80.6-98.4)	324.4 (280.2-376.5)	59.1 (56.9-61.3)	22.4 (20.8-23.9)
High-income	7.4 (7.3-7.5)	117.3 (117.0-117.7)	76.4 (76.3-76.4)	29.3 (29.3-29.3)	5.5 (5.1-5.9)	102.7 (102.1-103.3)	78.1 (78.1-78.2)	30.6 (30.5-30.6)
High-income North America	8.6 (8.4-8.8)	136.9 (136.3-137.8)	75.3 (75.3-75.4)	28.9 (28.9-28.9)	6.4 (6.2-6.7)	123.9 (122.9-124.9)	77.0 (77.0-77.1)	30.1 (30.1-30.2)
Canada	6.6 (6.4-6.8)	93.7 (93.1-94.4)	77.8 (77.8-77.9)	30.3 (30.2-30.3)	5.4 (5.1-5.8)	80.4 (78.0-83.0)	79.5 (79.3-79.7)	31.6 (31.4-31.8)
Greenland	20.1 (18.3-21.7)	242.8 (227.8-259.9)	67.4 (66.7-68.1)	23.6 (23.1-24.0)	16.1 (14.4-18.0)	198.5 (176.8-221.1)	70.0 (69.1-70.9)	25.1 (24.6-25.6)
United States	8.7 (8.5-8.9)	141.7 (141.1-142.8)	75.1 (75.0-75.1)	28.8 (28.8-28.8)	6.5 (6.3-6.8)	129.0 (127.9-130.1)	76.7 (76.7-76.8)	30.0 (29.9-30.0)
Australasia	6.5 (6.2-6.7)	85.8 (85.2-86.3)	78.6 (78.5-78.6)	31.1 (31.0-31.1)	4.5 (4.2-4.8)	75.6 (74.3-76.8)	80.1 (80.0-80.2)	32.2 (32.1-32.3)
Australia	6.3 (6.0-6.6)	84.1 (83.5-84.8)	78.8 (78.7-78.8)	31.2 (31.2-31.2)	4.3 (4.0-4.6)	74.7 (73.4-75.9)	80.2 (80.1-80.3)	32.2 (32.1-32.3)
New Zealand	7.1 (6.8-7.5)	94.0 (92.7-95.4)	77.6 (77.5-77.7)	30.3 (30.3-30.4)	5.7 (5.3-6.2)	80.0 (76.8-83.2)	79.6 (79.3-79.9)	31.9 (31.7-32.1)
High-income Asia Pacific	4.4 (4.3-4.5)	102.9 (101.8-104.0)	77.8 (77.7-77.8)	30.1 (30.0-30.1)	3.0 (2.8-3.3)	87.2 (85.0-89.6)	79.4 (79.2-79.5)	31.3 (31.2-31.4)
Brunei	9.5 (8.8-10.2)	123.3 (115.3-131.3)	74.8 (74.4-75.2)	28.0 (27.7-28.3)	9.6 (8.4-11.0)	115.5 (103.7-128.0)	75.1 (74.4-75.9)	28.2 (27.7-28.6)
Japan	4.0 (3.8-4.1)	93.3 (93.1-93.6)	78.5 (78.4-78.5)	30.5 (30.5-30.5)	2.9 (2.6-3.2)	79.0 (78.4-79.7)	79.9 (79.9-80.0)	31.7 (31.7-31.8)
Singapore	3.0 (2.8-3.2)	85.8 (83.8-87.9)	77.9 (77.7-78.0)	29.5 (29.4-29.7)	2.3 (2.0-2.5)	67.4 (63.7-71.3)	79.8 (79.5-80.2)	31.2 (30.8-31.5)
South Korea	5.6 (5.4-5.8)	128.6 (124.6-132.5)	75.2 (74.9-75.5)	28.0 (27.8-28.2)	3.4 (2.8-4.0)	104.2 (97.4-111.5)	77.5 (77.0-78.1)	29.7 (29.3-30.1)
Western Europe	5.3 (5.2-5.4)	105.5 (105.4-105.7)	77.0 (77.0-77.0)	29.4 (29.4-29.4)	3.9 (3.4-4.4)	87.9 (87.3-88.6)	78.9 (78.9-79.0)	30.8 (30.8-30.9)
Andorra	3.0 (2.4-3.7)	69.5 (64.9-74.3)	80.4 (80.0-80.8)	32.0 (31.7-32.3)	2.0 (1.6-2.5)	64.8 (59.9-69.7)	81.2 (80.8-81.6)	32.6 (32.3-32.9)
Austria	5.5 (5.2-5.9)	110.7 (109.7-111.7)	76.6 (76.6-76.7)	29.1 (29.0-29.1)	3.8 (3.4-4.3)	87.1 (85.5-88.9)	78.8 (78.7-78.9)	30.7 (30.5-30.8)
Belgium	5.4 (5.2-5.6)	114.1 (112.6-115.6)	76.2 (76.1-76.3)	28.7 (28.6-28.8)	4.2 (3.7-4.6)	98.3 (92.7-104.3)	77.8 (77.4-78.3)	29.9 (29.6-30.3)
Cyprus	6.4 (5.9-6.9)	113.5 (110.6-116.2)	75.9 (75.7-76.1)	28.7 (28.6-28.8)	5.1 (4.4-5.9)	82.5 (79.3-85.9)	78.7 (78.4-79.0)	30.7 (30.5-31.0)
Denmark	5.4 (5.1-5.7)	110.8 (109.4-112.1)	76.1 (76.0-76.2)	28.4 (28.3-28.5)	3.9 (3.3-4.5)	89.8 (86.3-93.0)	78.3 (78.0-78.6)	30.1 (29.9-30.4)
Finland	4.3 (3.9-4.6)	135.2 (133.4-137.3)	75.6 (75.4-75.7)	28.4 (28.3-28.5)	2.6 (2.0-3.3)	106.2 (101.5-110.8)	78.0 (77.6-78.3)	30.2 (30.0-30.5)
France	4.8 (4.6-4.9)	126.2 (125.7-126.7)	76.8 (76.8-76.8)	29.5 (29.5-29.6)	3.6 (2.8-4.5)	111.2 (108.4-114.2)	78.4 (78.2-78.6)	30.7 (30.5-30.8)
Germany	5.0 (4.8-5.2)	109.5 (109.1-109.9)	76.6 (76.5-76.6)	28.8 (28.8-28.8)	3.8 (3.2-4.6)	92.0 (90.6-93.5)	78.4 (78.2-78.5)	30.2 (30.1-30.3)
Greece	4.8 (4.6-5.1)	112.8 (111.6-114.1)	76.8 (76.7-76.9)	29.5 (29.4-29.5)	3.4 (3.1-3.8)	100.5 (96.6-104.7)	78.4 (78.1-78.7)	30.6 (30.3-30.8)
Iceland	3.3 (2.9-3.7)	74.4 (71.4-77.5)	79.7 (79.4-79.9)	31.4 (31.2-31.6)	2.1 (1.8-2.5)	64.3 (60.7-68.6)	80.9 (80.6-81.2)	32.4 (32.1-32.6)
Ireland	5.3 (5.0-5.6)	96.3 (94.2-98.5)	77.7 (77.4-77.9)	29.9 (29.7-30.1)	3.7 (3.3-4.2)	81.3 (77.4-85.0)	79.2 (78.9-79.5)	31.1 (30.8-31.3)
Israel	6.1 (5.9-6.4)	95.6 (93.9-97.7)	77.6 (77.5-77.8)	30.1 (30.0-30.2)	4.0 (3.6-4.4)	74.3 (71.1-77.2)	80.2 (79.9-80.5)	31.9 (31.7-32.2)
Italy	4.7 (4.5-4.9)	84.1 (83.7-84.4)	78.3 (78.3-78.3)	30.3 (30.3-30.3)	3.3 (2.7-4.1)	72.8 (71.3-74.4)	79.6 (79.5-79.8)	31.3 (31.2-31.4)
Luxembourg	3.8 (3.4-4.2)	100.8 (98.3-103.4)	77.2 (77.0-77.4)	29.4 (29.3-29.6)	2.8 (2.4-3.2)	76.1 (72.9-79.3)	79.9 (79.6-80.1)	31.4 (31.2-31.6)
Malta	7.4 (6.6-8.2)	84.0 (80.1-87.9)	78.3 (77.9-78.7)	30.6 (30.3-30.9)	6.7 (5.8-7.7)	72.3 (68.0-76.7)	79.7 (79.3-80.1)	31.6 (31.3-32.0)
Netherlands	6.0 (5.7-6.3)	82.9 (81.9-83.9)	77.3 (77.2-77.4)	29.2 (29.1-29.3)	4.2 (3.8-4.8)	70.5 (66.9-73.8)	79.2 (78.9-79.5)	30.7 (30.5-31.0)
Norway	4.2 (3.9-4.5)	87.7 (86.0-89.4)	77.8 (77.7-78.0)	30.0 (29.9-30.1)	2.9 (2.6-3.3)	70.4 (67.4-73.0)	79.9 (79.7-80.2)	31.6 (31.4-31.9)
Portugal	5.4 (5.2-5.6)	137.3 (135.9-138.7)	75.1 (75.0-75.1)	28.4 (28.3-28.4)	3.2 (3.0-3.4)	110.1 (107.6-112.7)	77.7 (77.5-77.8)	30.0 (29.9-30.2)
Spain	5.2 (5.0-5.4)	109.7 (109.2-110.1)	76.9 (76.9-77.0)	29.4 (29.3-29.4)	3.2 (2.8-3.6)	80.5 (79.3-81.8)	79.8 (79.7-80.0)	31.5 (31.4-31.6)

SR Table 3: Under-5 mortality (5q0) per 1,000 live births, adult mortality (45q15) per 1,000, life expectancy at birth (years), and life expectancy at age 50 (years), by geography, males, 2005 and 2015 (95% uncertainty intervals)								
	2005				2015			
Location	Under-5 mortality (5q0)	Adult mortality (45q15)	Life expectancy at birth	Life expectancy at age 50	Under-5 mortality (5q0)	Adult mortality (45q15)	Life expectancy at birth	Life expectancy at age 50
Sweden	3.7 (3.4-4.0)	79.9 (78.5-81.2)	78.4 (78.3-78.5)	30.2 (30.1-30.3)	2.8 (2.4-3.2)	66.5 (64.2-69.0)	80.2 (80.0-80.5)	31.8 (31.6-32.0)
Switzerland	5.4 (5.1-5.7)	83.7 (82.3-85.2)	78.7 (78.5-78.8)	30.8 (30.7-30.9)	4.4 (3.7-5.2)	66.3 (62.9-69.8)	80.7 (80.3-81.0)	32.3 (32.1-32.6)
United Kingdom	6.6 (6.5-6.8)	100.2 (99.8-100.6)	76.7 (76.7-76.8)	29.2 (29.2-29.2)	5.1 (4.7-5.6)	83.6 (82.5-84.6)	79.0 (78.9-79.1)	31.1 (31.0-31.2)
England	6.6 (6.5-6.8)	96.1 (95.7-96.5)	77.1 (77.0-77.1)	29.4 (29.4-29.4)	5.1 (4.7-5.6)	80.0 (78.9-81.0)	79.3 (79.2-79.4)	31.3 (31.3-31.4)
Northern Ireland	7.3 (6.5-8.0)	109.3 (106.9-112.2)	75.8 (75.6-76.0)	28.5 (28.4-28.7)	5.6 (4.4-7.2)	93.3 (88.2-98.3)	77.9 (77.5-78.4)	30.2 (29.9-30.6)
Scotland	7.0 (6.6-7.5)	132.9 (131.2-134.7)	74.4 (74.2-74.5)	27.5 (27.4-27.6)	4.8 (4.0-5.8)	109.6 (105.2-114.5)	77.2 (76.8-77.5)	29.8 (29.6-30.1)
Wales	5.6 (5.3-6.0)	106.4 (104.9-108.1)	76.2 (76.1-76.3)	28.7 (28.6-28.7)	4.8 (4.4-5.3)	93.6 (90.0-97.5)	78.0 (77.7-78.3)	30.2 (30.0-30.4)
Southern Latin America	16.3 (15.9-16.7)	149.8 (149.1-150.6)	72.6 (72.6-72.7)	26.7 (26.7-26.7)	12.4 (11.7-13.2)	136.1 (132.3-139.6)	74.0 (73.7-74.2)	27.6 (27.4-27.7)
Argentina	18.6 (18.0-19.1)	160.5 (159.5-161.3)	71.8 (71.7-71.8)	26.1 (26.0-26.1)	13.8 (13.2-14.6)	148.3 (144.3-152.5)	73.0 (72.8-73.3)	26.8 (26.6-27.0)
Chile	10.0 (9.6-10.3)	122.7 (121.2-124.1)	75.4 (75.3-75.5)	28.8 (28.7-28.9)	8.2 (7.7-8.8)	108.9 (101.0-117.3)	76.6 (75.9-77.2)	29.6 (29.1-30.1)
Uruguay	14.8 (13.9-15.7)	162.4 (159.8-164.6)	71.8 (71.6-72.0)	25.7 (25.6-25.8)	10.6 (8.0-13.8)	150.4 (144.9-156.7)	72.9 (72.5-73.4)	26.5 (26.2-26.8)
Central Europe, Eastern Europe, and Central Asia	24.1 (22.6-25.8)	351.5 (350.0-353.0)	62.9 (62.8-63.1)	20.9 (20.9-21.0)	15.4 (13.4-17.7)	254.9 (250.5-259.2)	67.9 (67.7-68.2)	23.9 (23.7-24.0)
Eastern Europe	16.0 (15.2-17.0)	442.3 (440.3-444.5)	59.8 (59.6-59.9)	18.9 (18.9-18.9)	9.7 (8.9-10.7)	311.1 (303.9-318.4)	65.7 (65.4-66.1)	22.4 (22.1-22.6)
Belarus	12.5 (10.3-15.4)	367.4 (359.1-374.7)	62.8 (62.4-63.1)	19.8 (19.6-20.0)	6.3 (4.7-8.4)	322.2 (306.1-338.4)	65.0 (64.3-65.7)	20.7 (20.2-21.2)
Estonia	8.4 (7.5-9.3)	283.1 (279.6-286.7)	67.1 (66.9-67.3)	22.7 (22.6-22.8)	3.6 (2.7-4.6)	169.4 (163.3-175.7)	73.4 (73.1-73.8)	26.6 (26.3-26.9)
Latvia	11.3 (10.1-12.4)	313.9 (311.1-316.9)	65.5 (65.3-65.6)	21.4 (21.4-21.5)	5.6 (3.9-8.1)	219.1 (211.6-226.2)	70.6 (70.2-71.0)	24.7 (24.4-25.0)
Lithuania	9.2 (8.6-9.9)	324.2 (322.0-326.3)	65.7 (65.6-65.8)	22.1 (22.1-22.2)	4.4 (3.7-5.3)	246.7 (240.9-251.3)	69.7 (69.5-70.0)	24.5 (24.3-24.7)
Moldova	17.5 (13.7-22.1)	296.4 (294.2-298.5)	65.5 (65.2-65.8)	21.4 (21.3-21.5)	11.9 (7.9-17.2)	215.4 (209.8-220.9)	69.9 (69.5-70.4)	24.3 (24.1-24.5)
Russia	16.1 (15.6-16.6)	467.5 (464.7-470.5)	58.7 (58.5-58.9)	18.4 (18.4-18.4)	9.8 (9.5-10.1)	321.4 (311.5-331.4)	65.3 (64.8-65.8)	22.3 (22.0-22.6)
Ukraine	17.3 (13.7-21.7)	402.2 (400.9-403.5)	61.4 (61.1-61.6)	19.5 (19.5-19.6)	10.6 (6.6-16.1)	294.9 (282.8-307.4)	66.3 (65.6-66.9)	22.4 (22.1-22.8)
Central Europe	11.3 (11.0-11.6)	200.0 (199.5-200.6)	70.5 (70.4-70.5)	24.4 (24.4-24.4)	6.4 (5.0-8.2)	159.5 (157.5-161.7)	73.4 (73.2-73.6)	26.4 (26.3-26.5)
Albania	22.9 (18.2-27.9)	128.4 (122.8-134.4)	73.5 (73.0-74.0)	28.2 (27.9-28.4)	13.1 (9.0-18.6)	113.2 (105.7-121.1)	75.0 (74.1-75.9)	28.3 (27.5-29.4)
Bosnia and Herzegovina	9.7 (9.1-10.4)	134.8 (133.3-136.3)	74.1 (73.9-74.2)	27.5 (27.4-27.7)	5.9 (5.0-6.8)	118.6 (114.5-122.6)	76.0 (75.7-76.3)	28.7 (28.5-29.0)
Bulgaria	14.6 (13.8-15.6)	218.3 (216.5-220.1)	69.1 (69.0-69.2)	23.3 (23.2-23.3)	9.6 (6.8-13.3)	184.7 (176.9-193.9)	71.4 (70.8-71.9)	24.8 (24.4-25.1)
Croatia	7.5 (7.0-8.0)	164.0 (162.1-165.9)	72.0 (71.9-72.1)	25.1 (25.0-25.2)	4.7 (4.1-5.4)	132.1 (126.8-137.4)	74.6 (74.3-75.0)	27.0 (26.7-27.3)
Czech Republic	4.7 (4.4-5.1)	154.4 (153.1-155.7)	73.0 (72.9-73.1)	25.6 (25.6-25.7)	2.7 (2.3-3.2)	114.0 (111.5-116.7)	75.9 (75.7-76.1)	27.9 (27.8-28.1)
Hungary	8.3 (7.8-8.8)	253.7 (251.8-255.5)	68.8 (68.7-68.9)	22.8 (22.7-22.9)	5.3 (3.7-7.4)	173.5 (165.9-181.2)	73.2 (72.8-73.7)	25.8 (25.5-26.2)
Macedonia	14.5 (13.3-15.7)	155.8 (153.7-158.1)	71.4 (71.2-71.5)	24.6 (24.5-24.7)	10.3 (6.8-15.1)	128.2 (121.9-134.3)	73.9 (73.3-74.5)	26.9 (26.5-27.4)
Montenegro	11.4 (10.0-12.8)	167.1 (163.7-170.7)	72.0 (71.8-72.2)	25.6 (25.4-25.7)	5.5 (4.0-7.6)	143.7 (135.0-152.6)	74.2 (73.6-74.9)	27.2 (26.6-27.9)
Poland	8.1 (7.8-8.5)	207.5 (206.8-208.3)	70.8 (70.7-70.8)	24.6 (24.6-24.7)	4.9 (3.4-6.9)	167.9 (164.6-171.1)	73.4 (73.2-73.7)	26.5 (26.4-26.7)
Romania	19.4 (18.7-20.1)	223.3 (222.1-224.7)	68.8 (68.7-68.9)	23.7 (23.7-23.8)	9.1 (6.8-12.1)	190.8 (182.4-198.7)	71.5 (71.0-72.0)	25.1 (24.8-25.5)
Serbia	11.3 (10.1-12.4)	180.5 (176.6-184.3)	70.6 (70.4-70.8)	24.1 (23.9-24.2)	8.4 (7.6-9.4)	148.8 (145.3-152.5)	73.5 (73.2-73.7)	26.7 (26.4-26.9)
Slovakia	9.5 (8.8-10.3)	199.9 (197.9-201.9)	70.3 (70.2-70.4)	23.8 (23.7-23.9)	6.5 (5.4-7.8)	144.0 (140.7-147.3)	74.0 (73.7-74.2)	26.6 (26.4-26.8)
Slovenia	4.6 (4.2-5.0)	146.5 (144.7-148.4)	73.9 (73.8-74.0)	26.7 (26.6-26.8)	2.8 (2.4-3.2)	96.8 (93.7-99.8)	77.9 (77.7-78.2)	29.7 (29.6-29.9)
Central Asia	44.6 (40.5-49.2)	284.3 (279.6-288.8)	63.6 (63.3-64.0)	21.9 (21.7-22.0)	28.3 (23.4-34.3)	231.2 (223.5-239.2)	67.4 (66.9-67.9)	23.9 (23.6-24.2)
Armenia	27.4 (23.2-32.0)	207.2 (196.7-216.7)	68.2 (67.6-68.8)	23.9 (23.7-24.2)	16.5 (12.7-21.2)	180.9 (168.0-194.6)	70.7 (70.0-71.5)	25.5 (25.0-25.9)
Azerbaijan	55.8 (49.0-63.2)	217.4 (203.8-231.3)	65.1 (64.3-65.9)	23.2 (22.7-23.7)	32.6 (25.3-41.3)	178.0 (164.0-191.9)	69.6 (68.5-70.6)	25.5 (24.6-26.2)
Georgia	30.8 (26.3-35.7)	221.8 (206.5-237.2)	67.4 (66.6-68.2)	23.7 (23.2-24.1)	19.2 (15.2-24.0)	235.5 (212.8-256.1)	67.9 (66.9-69.0)	23.5 (22.9-24.4)
Kazakhstan	31.4 (26.5-37.0)	404.8 (402.7-407.2)	59.8 (59.4-60.1)	19.3 (19.3-19.4)	19.1 (14.9-24.5)	296.9 (272.3-322.4)	65.3 (64.1-66.5)	22.2 (21.5-23.0)

SR Table 3: Under-5 mortality (5q0) per 1,000 live births, adult mortality (45q15) per 1,000, life expectancy at birth (years), and life expectancy at age 50 (years), by geography, males, 2005 and 2015 (95% uncertainty intervals)

Location	2005				2015			
	Under-5 mortality (5q0)	Adult mortality (45q15)	Life expectancy at birth	Life expectancy at age 50	Under-5 mortality (5q0)	Adult mortality (45q15)	Life expectancy at birth	Life expectancy at age 50
Kyrgyzstan	46.0 (43.0-49.2)	312.4 (297.0-326.5)	62.6 (62.0-63.2)	21.0 (20.6-21.4)	31.9 (28.3-35.8)	266.0 (249.5-282.9)	65.6 (64.8-66.4)	22.5 (21.9-23.0)
Mongolia	51.5 (45.8-57.7)	372.7 (354.9-390.7)	60.0 (59.2-60.7)	19.6 (19.2-20.1)	30.9 (25.1-39.0)	334.7 (315.0-354.3)	62.8 (61.9-63.6)	20.8 (20.3-21.3)
Tajikistan	56.1 (50.2-63.0)	194.3 (182.1-206.4)	65.9 (65.2-66.7)	23.8 (23.4-24.3)	36.2 (29.0-44.6)	167.3 (155.2-180.7)	70.1 (69.2-70.9)	26.4 (25.9-26.9)
Turkmenistan	65.8 (49.7-84.9)	287.0 (279.1-295.8)	62.1 (60.8-63.3)	22.3 (21.9-22.7)	43.3 (32.2-56.5)	230.1 (224.9-235.0)	66.3 (65.3-67.2)	24.1 (23.8-24.4)
Uzbekistan	41.6 (33.5-51.0)	238.3 (223.5-251.3)	65.5 (64.6-66.4)	22.7 (22.2-23.2)	27.1 (20.9-34.7)	209.0 (194.2-224.1)	68.4 (67.5-69.3)	24.4 (24.0-24.9)
Latin America and Caribbean	29.1 (26.7-31.3)	185.1 (182.5-187.7)	70.4 (70.2-70.7)	27.1 (27.0-27.3)	20.0 (16.0-25.0)	169.6 (165.0-174.2)	72.2 (71.7-72.6)	27.9 (27.7-28.1)
Central Latin America	25.8 (23.3-28.2)	169.8 (168.6-171.2)	71.9 (71.7-72.1)	28.2 (28.2-28.3)	18.3 (14.6-22.9)	159.7 (155.0-164.0)	73.3 (72.8-73.7)	28.8 (28.7-29.0)
Colombia	23.9 (21.2-26.7)	153.7 (152.9-154.6)	72.0 (71.8-72.2)	28.5 (28.4-28.5)	17.2 (12.7-22.9)	120.7 (116.0-125.5)	75.1 (74.6-75.6)	30.2 (30.0-30.5)
Costa Rica	14.9 (12.3-18.2)	111.4 (109.8-113.2)	76.6 (76.3-76.8)	30.6 (30.5-30.7)	10.7 (7.6-14.4)	104.4 (98.4-110.7)	78.1 (77.5-78.6)	31.6 (31.2-31.9)
El Salvador	21.6 (17.6-25.9)	257.6 (246.9-268.9)	68.8 (68.1-69.5)	28.4 (28.1-28.8)	12.4 (8.6-17.2)	241.3 (218.2-263.2)	70.6 (69.3-71.9)	28.9 (28.2-29.7)
Guatemala	44.5 (40.4-49.1)	263.6 (260.5-266.5)	66.4 (66.1-66.7)	27.2 (27.1-27.3)	28.1 (22.9-34.3)	228.9 (192.8-264.4)	69.8 (67.8-71.8)	28.3 (27.1-29.5)
Honduras	32.2 (30.1-34.4)	178.3 (136.4-230.6)	70.4 (67.2-72.9)	27.0 (24.6-28.7)	23.7 (20.3-28.3)	162.5 (120.5-217.7)	72.1 (68.7-74.7)	27.6 (25.3-29.5)
Mexico	24.5 (21.0-28.5)	164.0 (163.5-164.7)	72.5 (72.2-72.8)	28.1 (28.1-28.1)	16.9 (12.9-22.0)	160.0 (158.6-161.4)	73.4 (73.0-73.7)	28.4 (28.3-28.4)
Nicaragua	29.1 (26.6-31.8)	156.3 (147.0-166.3)	73.7 (73.1-74.4)	29.9 (29.5-30.3)	16.9 (13.9-20.7)	150.1 (131.5-168.6)	75.0 (73.8-76.3)	29.9 (29.1-30.8)
Panama	22.8 (19.2-27.2)	133.2 (123.8-142.8)	74.8 (74.0-75.5)	30.3 (29.7-30.8)	18.9 (14.2-25.0)	129.9 (111.4-151.6)	75.5 (73.9-76.9)	30.8 (29.6-31.8)
Venezuela	20.2 (19.7-20.7)	187.6 (186.0-189.2)	71.2 (71.1-71.3)	27.9 (27.8-27.9)	18.5 (17.2-19.9)	198.1 (170.7-226.8)	70.7 (69.0-72.4)	27.6 (26.6-28.7)
Andean Latin America	33.8 (32.3-35.4)	136.0 (128.7-143.6)	73.1 (72.6-73.6)	29.1 (28.7-29.4)	20.7 (18.4-23.4)	118.2 (106.8-131.2)	75.4 (74.5-76.3)	29.9 (29.2-30.5)
Bolivia	54.5 (50.3-59.0)	166.6 (146.8-187.8)	69.0 (67.9-70.1)	27.0 (26.3-27.7)	31.8 (26.2-38.5)	150.8 (116.3-191.5)	72.1 (69.6-74.2)	28.1 (26.0-29.6)
Ecuador	29.6 (27.0-32.5)	168.5 (156.6-180.3)	71.5 (70.8-72.2)	27.8 (27.3-28.2)	19.0 (16.3-22.1)	150.2 (133.1-168.7)	73.3 (72.1-74.4)	28.2 (27.2-29.1)
Peru	27.3 (25.8-28.8)	110.1 (99.6-120.7)	75.5 (74.7-76.4)	30.6 (30.0-31.2)	17.1 (14.5-20.4)	91.5 (77.9-109.8)	77.9 (76.4-79.1)	31.6 (30.4-32.6)
Caribbean	48.2 (45.1-51.6)	188.5 (179.1-198.7)	69.1 (68.5-69.7)	27.3 (27.0-27.6)	34.5 (26.2-45.1)	171.8 (158.5-186.4)	70.9 (69.9-71.9)	27.6 (27.3-28.1)
Antigua and Barbuda	17.2 (13.1-22.2)	157.4 (146.9-167.6)	72.4 (71.6-73.4)	27.0 (26.1-27.9)	14.0 (9.6-20.0)	131.6 (115.2-148.5)	73.8 (72.7-75.3)	27.5 (26.5-29.1)
The Bahamas	22.4 (15.7-30.4)	223.4 (209.3-237.9)	68.9 (67.9-69.9)	25.9 (25.3-26.5)	17.7 (9.8-30.9)	193.9 (173.3-214.5)	70.9 (69.4-72.3)	26.7 (25.9-27.7)
Barbados	22.0 (15.5-29.8)	148.8 (142.3-154.9)	72.9 (72.2-73.5)	27.8 (27.2-28.2)	17.9 (10.0-29.5)	137.9 (120.7-155.2)	73.6 (72.3-74.8)	27.9 (26.8-28.9)
Belize	23.6 (18.6-29.7)	242.1 (228.4-256.5)	67.3 (66.4-68.1)	24.4 (23.7-25.0)	18.0 (11.5-28.4)	221.0 (200.0-244.2)	69.1 (67.7-70.3)	25.3 (24.4-26.2)
Bermuda	6.8 (4.9-9.3)	161.4 (150.7-171.7)	73.3 (72.7-74.0)	27.1 (26.6-27.6)	4.5 (2.9-6.8)	138.5 (121.7-155.6)	75.0 (73.9-76.0)	28.0 (27.3-28.6)
Cuba	7.7 (7.5-8.0)	128.0 (126.7-129.4)	75.2 (75.1-75.3)	28.2 (28.1-28.2)	6.6 (6.3-7.1)	118.4 (113.5-123.3)	75.9 (75.5-76.2)	28.6 (28.3-28.9)
Dominica	26.8 (20.5-35.2)	172.8 (160.9-185.7)	71.4 (70.3-72.3)	27.4 (26.6-27.9)	28.9 (18.8-42.9)	191.5 (170.6-216.0)	69.7 (67.8-71.5)	26.1 (24.9-27.4)
Dominican Republic	38.1 (34.8-41.8)	178.8 (167.2-191.8)	70.8 (70.0-71.4)	28.4 (28.0-28.6)	27.3 (22.3-32.9)	151.8 (141.8-168.6)	72.8 (71.9-73.4)	28.7 (28.1-28.9)
Grenada	20.1 (13.4-27.8)	247.6 (236.5-258.6)	67.2 (66.4-67.9)	23.9 (23.5-24.2)	17.4 (9.4-29.7)	222.7 (203.8-243.2)	68.8 (67.5-70.0)	24.7 (24.0-25.4)
Guyana	42.7 (38.5-47.3)	341.7 (318.2-367.6)	61.3 (60.2-62.3)	21.4 (20.7-22.2)	30.9 (24.4-38.7)	312.9 (273.1-356.9)	63.5 (61.7-65.3)	22.3 (21.2-23.4)
Haiti	94.3 (86.0-103.8)	295.7 (251.3-344.8)	60.3 (58.2-62.8)	24.1 (22.7-26.1)	62.8 (46.0-84.6)	260.1 (199.5-326.3)	63.9 (60.6-67.2)	24.5 (22.6-26.6)
Jamaica	22.5 (15.2-33.2)	148.3 (139.4-157.4)	74.0 (73.0-74.8)	29.9 (29.5-30.4)	19.9 (12.7-30.0)	154.3 (136.4-173.9)	73.0 (71.7-74.3)	28.2 (27.1-29.2)
Puerto Rico	10.6 (10.1-11.0)	182.4 (179.7-185.3)	72.8 (72.7-73.0)	27.8 (27.7-27.9)	7.6 (6.7-8.6)	151.0 (140.7-161.2)	75.0 (74.4-75.7)	29.2 (28.7-29.7)
Saint Lucia	24.0 (16.2-34.0)	187.3 (179.3-195.1)	70.2 (69.2-71.1)	26.1 (25.5-26.8)	19.3 (10.0-34.0)	160.3 (142.3-177.9)	72.5 (71.0-73.9)	27.7 (26.8-28.6)
Saint Vincent and the Grenadines	30.0 (22.6-39.3)	210.8 (201.8-220.1)	68.0 (67.3-68.7)	24.7 (24.2-25.2)	23.5 (14.9-35.6)	210.2 (193.7-228.5)	68.8 (67.6-69.9)	25.0 (24.1-25.7)
Suriname	44.4 (37.9-52.2)	221.4 (207.8-235.4)	67.1 (66.1-68.2)	25.4 (24.7-26.3)	33.9 (27.1-42.6)	205.0 (181.4-230.2)	68.8 (67.1-70.5)	26.0 (24.7-27.3)
Trinidad and Tobago	26.8 (21.0-33.6)	223.3 (219.4-227.1)	67.8 (67.3-68.2)	24.6 (24.5-24.8)	21.3 (13.2-32.9)	210.8 (187.8-235.3)	68.9 (67.4-70.3)	24.9 (24.0-25.8)
Virgin Islands, U.S.	11.7 (10.3-13.3)	197.5 (185.9-209.5)	70.9 (70.3-71.5)	26.0 (25.5-26.4)	9.4 (7.6-11.4)	211.0 (188.4-238.0)	70.3 (68.9-71.6)	25.3 (24.3-26.3)

SR Table 3: Under-5 mortality (5q0) per 1,000 live births, adult mortality (45q15) per 1,000, life expectancy at birth (years), and life expectancy at age 50 (years), by geography, males, 2005 and 2015 (95% uncertainty intervals)								
	2005				2015			
**Location**	**Under-5 mortality (5q0)**	**Adult mortality (45q15)**	**Life expectancy at birth**	**Life expectancy at age 50**	**Under-5 mortality (5q0)**	**Adult mortality (45q15)**	**Life expectancy at birth**	**Life expectancy at age 50**
Tropical Latin America	27.6 (23.6-32.1)	209.7 (204.8-214.8)	68.8 (68.4-69.2)	25.7 (25.5-25.9)	18.7 (14.8-23.6)	190.7 (181.9-199.9)	70.8 (70.1-71.4)	26.7 (26.3-27.1)
Brazil	27.6 (23.4-32.2)	211.0 (206.0-216.2)	68.7 (68.3-69.1)	25.6 (25.4-25.8)	18.7 (14.8-23.6)	191.5 (182.5-201.0)	70.7 (70.0-71.3)	26.7 (26.3-27.1)
Paraguay	27.6 (24.0-31.5)	159.6 (145.1-174.9)	72.0 (71.1-72.9)	27.8 (27.2-28.3)	19.6 (15.6-24.5)	165.6 (137.4-196.1)	72.1 (70.2-73.8)	27.2 (25.9-28.4)
Southeast Asia, East Asia, and Oceania	31.2 (28.7-34.0)	193.8 (186.6-200.8)	68.5 (68.1-68.9)	24.7 (24.4-25.0)	17.7 (14.7-21.3)	157.8 (149.2-167.3)	71.9 (71.3-72.5)	26.5 (26.0-26.9)
East Asia	25.2 (21.1-30.0)	182.2 (174.8-190.2)	69.4 (68.9-69.9)	24.8 (24.4-25.1)	13.3 (9.4-18.7)	138.2 (129.7-148.0)	73.2 (72.4-73.9)	26.9 (26.4-27.4)
China	25.2 (20.8-30.1)	182.1 (174.6-190.2)	69.3 (68.8-69.9)	24.7 (24.4-25.0)	13.3 (9.1-18.9)	137.1 (128.2-147.2)	73.2 (72.4-73.9)	26.9 (26.3-27.4)
North Korea	33.8 (17.4-58.2)	215.2 (157.6-271.2)	67.5 (64.3-71.0)	24.4 (22.8-26.7)	20.7 (9.5-38.4)	211.5 (157.9-279.3)	68.9 (65.7-71.9)	24.7 (22.8-26.8)
Taiwan	7.2 (6.8-7.6)	156.1 (154.4-157.7)	74.5 (74.4-74.6)	28.3 (28.2-28.3)	6.9 (4.4-10.9)	127.4 (105.0-150.7)	76.6 (75.1-78.3)	29.9 (28.7-31.2)
Southeast Asia	39.1 (37.9-40.3)	223.6 (207.5-240.7)	67.1 (66.2-68.0)	24.8 (24.2-25.5)	23.7 (21.3-26.5)	204.7 (181.4-229.3)	69.2 (68.0-70.5)	25.2 (24.4-26.2)
Cambodia	70.8 (66.8-75.0)	289.7 (267.7-311.1)	61.5 (60.5-62.6)	22.5 (21.6-23.2)	35.0 (30.3-40.2)	238.6 (201.3-282.1)	66.2 (64.3-68.0)	23.6 (22.2-24.6)
Indonesia	43.6 (41.4-45.9)	207.9 (184.7-230.6)	67.4 (66.2-68.6)	25.0 (24.1-26.0)	26.5 (22.9-30.6)	200.4 (155.3-249.2)	69.0 (66.7-71.9)	25.2 (23.6-27.5)
Laos	120.2 (109.5-131.8)	290.1 (254.9-325.4)	58.1 (56.6-59.5)	22.6 (21.7-23.4)	65.1 (50.2-84.4)	229.1 (178.0-287.9)	64.7 (62.2-67.5)	24.4 (22.9-26.1)
Malaysia	8.8 (8.5-9.1)	171.0 (169.9-172.2)	72.3 (72.3-72.4)	26.6 (26.5-26.6)	7.0 (5.9-8.4)	174.9 (148.8-201.9)	72.4 (70.8-74.0)	26.4 (25.1-27.7)
Maldives	26.3 (23.6-29.1)	101.8 (96.0-108.1)	75.7 (75.2-76.2)	30.3 (29.9-30.7)	13.4 (11.1-16.3)	84.6 (68.5-103.7)	78.1 (76.4-79.6)	31.1 (29.6-32.4)
Mauritius	17.6 (16.9-18.5)	210.4 (205.7-215.4)	69.2 (68.9-69.4)	23.9 (23.7-24.1)	14.7 (13.4-16.2)	182.1 (170.8-192.5)	71.4 (70.8-72.1)	25.7 (25.3-26.2)
Myanmar	65.6 (63.0-68.1)	314.3 (198.8-435.5)	60.7 (56.3-65.9)	21.6 (18.5-25.3)	35.3 (27.1-46.9)	270.0 (173.5-381.9)	64.9 (60.3-69.9)	23.0 (20.1-26.7)
Philippines	34.9 (32.8-36.9)	246.0 (245.1-246.9)	65.4 (65.3-65.6)	22.6 (22.6-22.7)	25.4 (22.4-28.7)	216.9 (197.3-237.5)	67.7 (66.6-68.8)	23.8 (23.0-24.5)
Sri Lanka	18.7 (18.1-19.3)	197.8 (193.0-202.4)	70.1 (69.9-70.4)	25.6 (25.4-25.8)	8.5 (6.4-11.1)	157.7 (118.0-206.3)	74.1 (71.1-76.9)	28.3 (26.3-30.5)
Seychelles	13.8 (12.2-15.6)	241.6 (228.3-255.2)	68.2 (67.6-68.8)	24.0 (23.7-24.3)	11.3 (9.0-14.0)	201.3 (169.9-235.6)	70.5 (68.8-72.1)	25.3 (24.2-26.6)
Thailand	11.8 (10.2-13.7)	234.4 (218.0-250.1)	69.8 (68.8-70.8)	27.1 (26.4-27.8)	6.0 (4.8-7.4)	212.9 (172.4-256.8)	70.8 (68.8-73.2)	25.8 (24.3-27.8)
Timor-Leste	74.2 (65.0-85.0)	174.4 (151.1-200.3)	67.0 (65.3-68.6)	26.7 (25.3-28.0)	41.8 (26.0-62.9)	138.8 (101.5-187.6)	72.0 (68.9-75.1)	28.6 (26.5-30.8)
Vietnam	23.6 (21.4-25.7)	197.5 (137.1-258.3)	69.4 (66.4-72.9)	25.3 (23.1-28.1)	14.7 (12.6-17.3)	176.4 (119.5-252.7)	71.3 (67.8-74.8)	26.0 (23.9-28.7)
Oceania	61.7 (43.5-84.3)	367.8 (275.7-470.8)	59.0 (55.3-63.0)	20.1 (17.9-22.7)	42.2 (26.5-65.1)	343.0 (257.3-460.3)	61.4 (57.1-65.3)	20.8 (18.2-23.3)
American Samoa	12.3 (10.8-13.9)	216.1 (185.4-250.2)	69.6 (67.9-71.1)	24.6 (23.5-25.7)	7.6 (6.0-9.5)	201.5 (158.8-249.1)	70.9 (68.6-73.2)	25.2 (23.7-27.0)
Federated States of Micronesia	19.2 (13.2-26.6)	291.8 (181.3-434.8)	65.4 (60.0-70.2)	22.2 (18.4-25.1)	12.5 (8.4-18.1)	281.1 (189.0-434.2)	66.5 (60.4-70.8)	22.6 (18.5-25.4)
Fiji	34.7 (25.5-45.5)	320.8 (280.2-367.4)	62.7 (60.9-64.5)	20.8 (19.8-21.9)	33.1 (19.8-52.0)	299.3 (242.7-367.5)	63.7 (60.8-66.5)	21.3 (19.6-23.0)
Guam	14.7 (13.2-16.4)	199.3 (172.0-227.0)	70.1 (68.7-71.3)	25.1 (24.2-25.7)	19.0 (14.5-24.4)	221.8 (173.8-273.4)	68.5 (66.2-70.8)	24.2 (22.7-25.6)
Kiribati	62.3 (48.8-78.4)	421.4 (360.3-484.1)	56.9 (54.6-59.3)	18.5 (17.3-19.7)	41.9 (23.4-70.2)	410.9 (344.0-489.7)	58.9 (55.7-61.8)	18.9 (17.2-20.5)
Marshall Islands	36.0 (25.3-48.2)	318.6 (268.4-368.2)	62.7 (60.8-64.9)	21.2 (20.0-22.5)	20.5 (11.4-33.5)	310.7 (246.7-375.0)	64.6 (62.0-67.3)	21.8 (20.1-23.5)
Northern Mariana Islands	9.0 (6.6-11.9)	130.6 (117.2-145.2)	74.5 (73.6-75.4)	27.8 (27.1-28.4)	6.3 (3.5-10.6)	147.6 (124.1-174.0)	74.2 (72.7-75.5)	27.6 (26.5-28.5)
Papua New Guinea	71.5 (49.1-99.7)	393.8 (259.7-535.1)	57.2 (52.4-62.7)	19.4 (16.4-23.3)	47.5 (29.5-73.7)	362.8 (245.0-517.4)	60.1 (54.6-65.3)	20.2 (16.7-23.8)
Samoa	13.7 (6.9-23.8)	202.9 (163.2-246.3)	70.0 (67.9-72.0)	24.9 (23.6-26.3)	9.3 (4.4-17.3)	172.1 (132.7-218.3)	72.2 (69.7-74.5)	26.2 (24.5-27.9)
Solomon Islands	33.5 (24.5-44.7)	369.9 (245.1-508.6)	60.9 (56.1-65.9)	20.0 (16.8-23.3)	21.6 (14.5-30.6)	347.4 (221.3-500.7)	62.9 (57.0-68.1)	20.7 (17.0-24.0)
Tonga	25.3 (16.0-37.1)	233.5 (206.7-265.9)	67.3 (65.7-68.8)	23.7 (22.7-24.5)	19.4 (11.5-31.0)	223.8 (170.6-290.8)	68.4 (65.5-71.1)	24.2 (22.5-25.9)
Vanuatu	43.3 (34.5-53.3)	337.7 (220.9-477.8)	61.4 (56.4-66.4)	20.7 (17.5-24.0)	29.3 (20.3-42.9)	320.0 (221.8-452.8)	63.2 (58.3-67.3)	21.3 (18.1-24.0)
North Africa and Middle East	46.3 (43.7-49.1)	185.3 (172.7-197.2)	68.6 (68.0-69.3)	26.0 (25.6-26.5)	31.8 (28.0-36.3)	179.6 (164.3-195.5)	70.1 (69.2-70.9)	26.7 (26.2-27.1)
North Africa and Middle East	46.3 (43.7-49.1)	185.3 (172.7-197.2)	68.6 (68.0-69.3)	26.0 (25.6-26.5)	31.8 (28.0-36.3)	179.6 (164.3-195.5)	70.1 (69.2-70.9)	26.7 (26.2-27.1)
Afghanistan	115.3 (105.0-126.0)	439.5 (326.7-557.2)	52.7 (48.9-56.7)	18.2 (15.8-21.5)	84.7 (70.8-100.9)	458.0 (357.4-574.3)	53.3 (49.2-57.7)	18.8 (16.4-22.0)
Algeria	33.1 (28.1-38.6)	122.2 (106.3-139.2)	73.5 (72.3-74.8)	29.0 (28.2-29.9)	24.5 (19.3-31.3)	108.0 (91.5-125.6)	75.4 (74.1-76.8)	29.9 (29.0-30.9)

SR Table 3: Under-5 mortality (5q0) per 1,000 live births, adult mortality (45q15) per 1,000, life expectancy at birth (years), and life expectancy at age 50 (years), by geography, males, 2005 and 2015 (95% uncertainty intervals)

Location	2005				2015			
	Under-5 mortality (5q0)	Adult mortality (45q15)	Life expectancy at birth	Life expectancy at age 50	Under-5 mortality (5q0)	Adult mortality (45q15)	Life expectancy at birth	Life expectancy at age 50
Bahrain	10.1 (9.2-11.1)	120.3 (104.7-138.3)	74.6 (73.8-75.6)	27.8 (27.3-28.4)	6.7 (5.6-7.9)	85.7 (66.5-110.0)	78.2 (76.5-80.0)	30.5 (29.1-32.0)
Egypt	35.2 (31.5-38.5)	214.1 (213.2-214.9)	67.7 (67.4-67.9)	23.5 (23.4-23.5)	22.4 (16.8-29.4)	211.6 (204.4-218.4)	68.7 (68.1-69.3)	23.7 (23.4-23.9)
Iran	29.1 (22.2-37.7)	183.1 (146.2-226.5)	69.6 (67.2-72.0)	26.2 (24.6-27.8)	15.9 (11.7-21.3)	165.1 (126.1-219.2)	71.9 (69.2-74.2)	26.5 (24.7-28.3)
Iraq	41.4 (38.5-44.5)	292.3 (224.6-374.3)	63.3 (60.0-66.5)	22.5 (20.4-24.6)	29.2 (24.8-34.6)	288.6 (209.2-375.5)	64.3 (60.8-68.5)	23.2 (20.9-26.4)
Jordan	21.2 (19.8-22.4)	112.4 (102.4-123.3)	74.7 (73.9-75.4)	28.7 (28.1-29.4)	15.3 (13.3-17.8)	100.8 (86.1-117.9)	76.5 (75.1-77.6)	29.9 (28.7-30.8)
Kuwait	11.4 (10.6-12.2)	110.1 (106.8-113.3)	76.3 (76.0-76.5)	29.3 (29.1-29.5)	8.6 (6.7-10.9)	78.4 (64.7-95.6)	79.4 (77.9-80.7)	31.7 (30.6-32.7)
Lebanon	15.6 (11.7-20.4)	95.1 (67.6-127.6)	77.0 (74.2-79.6)	30.5 (28.1-32.5)	8.5 (6.1-12.1)	88.8 (57.9-126.7)	77.9 (75.1-80.9)	30.5 (28.2-32.9)
Libya	24.8 (20.1-30.0)	126.0 (113.9-139.4)	73.9 (73.1-74.8)	28.7 (28.2-29.3)	18.4 (13.8-23.9)	178.0 (138.4-217.2)	71.4 (68.9-73.8)	27.6 (26.0-29.0)
Morocco	37.5 (32.8-42.6)	144.2 (105.6-195.6)	71.6 (68.7-74.1)	27.8 (25.5-29.6)	24.1 (19.3-29.7)	137.3 (88.7-198.3)	73.3 (69.5-76.5)	28.3 (25.2-30.8)
Palestine	25.0 (22.2-28.3)	157.3 (140.0-178.0)	72.1 (70.5-73.1)	27.5 (26.0-28.2)	18.5 (14.4-23.5)	166.1 (119.3-222.1)	71.6 (68.7-74.5)	26.4 (24.4-28.7)
Oman	11.5 (10.8-12.1)	127.7 (111.5-146.0)	74.1 (72.9-75.2)	27.4 (26.6-28.3)	9.8 (8.4-11.3)	106.0 (84.8-131.0)	75.7 (74.1-77.7)	28.6 (27.4-30.3)
Qatar	14.2 (10.4-18.5)	112.4 (96.6-132.4)	75.2 (73.6-76.9)	28.7 (27.2-30.1)	9.1 (6.2-13.0)	76.2 (55.8-98.4)	79.1 (76.9-81.2)	31.5 (29.5-33.3)
Saudi Arabia	19.0 (14.1-25.4)	104.7 (98.5-110.8)	75.9 (75.3-76.6)	29.8 (29.5-30.2)	12.2 (8.7-17.3)	90.6 (80.5-102.3)	77.4 (76.3-78.5)	30.4 (29.5-31.3)
Sudan	85.5 (77.9-93.4)	232.1 (151.7-318.2)	63.1 (59.4-67.2)	24.5 (22.0-27.5)	60.4 (46.8-78.9)	216.8 (142.1-310.1)	65.8 (61.6-69.9)	25.1 (22.1-27.9)
Syria	17.2 (16.7-17.8)	133.8 (122.0-145.6)	73.9 (73.1-74.7)	27.7 (27.1-28.4)	22.8 (16.7-29.8)	337.5 (203.4-452.4)	62.6 (56.5-70.2)	26.4 (24.9-28.2)
Tunisia	22.1 (19.5-24.6)	130.8 (103.2-162.1)	73.7 (71.8-75.4)	28.3 (27.0-29.5)	14.5 (12.1-17.4)	120.8 (91.1-161.8)	74.6 (72.0-77.0)	28.3 (26.3-30.4)
Turkey	29.4 (25.2-34.2)	135.0 (125.5-146.3)	72.9 (72.3-73.6)	28.3 (27.9-28.7)	17.7 (13.0-23.5)	106.6 (99.7-113.6)	75.9 (75.2-76.5)	29.7 (29.2-30.2)
United Arab Emirates	9.4 (7.6-11.5)	137.9 (122.1-154.4)	74.2 (73.3-74.9)	27.7 (26.9-28.2)	5.7 (3.2-9.6)	141.0 (100.7-188.8)	74.5 (71.8-77.1)	27.6 (25.4-29.4)
Yemen	67.4 (63.6-71.3)	229.7 (127.6-353.5)	64.4 (59.2-69.9)	24.3 (20.4-28.3)	55.1 (47.5-64.5)	232.3 (143.7-361.2)	65.4 (59.7-70.2)	24.7 (20.6-28.3)
South Asia	69.9 (66.3-73.5)	268.3 (258.6-278.3)	62.4 (61.9-63.0)	22.9 (22.6-23.3)	49.5 (44.7-55.4)	239.4 (228.3-250.5)	65.4 (64.7-66.1)	23.8 (23.4-24.2)
South Asia	69.9 (66.3-73.5)	268.3 (258.6-278.3)	62.4 (61.9-63.0)	22.9 (22.6-23.3)	49.5 (44.7-55.4)	239.4 (228.3-250.5)	65.4 (64.7-66.1)	23.8 (23.4-24.2)
Bangladesh	66.8 (63.8-69.5)	204.4 (184.1-228.9)	65.7 (64.6-66.8)	25.0 (24.2-25.7)	40.7 (37.6-44.6)	192.1 (156.9-229.3)	68.5 (66.5-70.3)	25.7 (24.3-26.9)
Bhutan	65.8 (58.9-74.5)	157.7 (126.5-194.6)	68.7 (66.4-70.7)	27.7 (26.0-29.2)	44.3 (38.2-51.9)	139.3 (100.6-180.2)	71.5 (69.1-74.2)	28.3 (26.4-30.3)
India	68.1 (63.8-72.3)	276.0 (264.9-287.3)	62.2 (61.7-62.8)	22.6 (22.2-23.0)	47.8 (41.9-55.3)	245.6 (233.4-258.4)	65.2 (64.5-66.0)	23.5 (23.1-24.0)
Nepal	63.7 (60.6-67.3)	205.7 (174.2-240.0)	66.0 (64.4-67.7)	25.3 (24.2-26.7)	38.1 (34.5-42.1)	205.5 (154.8-261.9)	68.1 (65.2-70.9)	25.8 (23.8-28.0)
Pakistan	84.4 (80.0-88.5)	266.4 (225.3-309.4)	61.3 (59.6-63.2)	23.3 (22.3-24.6)	63.8 (58.2-70.0)	234.3 (191.1-284.3)	64.5 (62.2-66.5)	24.1 (22.7-25.5)
Sub-Saharan Africa	129.7 (126.3-132.9)	390.1 (371.1-410.3)	54.3 (53.5-55.1)	22.1 (21.4-22.7)	87.0 (80.4-94.3)	308.9 (277.2-346.9)	60.0 (58.4-61.6)	23.2 (22.1-24.3)
Southern Sub-Saharan Africa	84.0 (78.9-89.4)	584.5 (558.1-610.8)	49.9 (48.7-51.1)	19.2 (18.3-20.4)	49.8 (44.3-56.0)	416.4 (388.0-449.8)	57.5 (56.0-58.9)	21.2 (20.1-22.2)
Botswana	57.3 (48.6-67.0)	648.4 (391.8-908.0)	47.9 (38.8-60.2)	16.8 (9.5-25.6)	30.7 (24.1-39.6)	499.2 (276.2-838.4)	55.8 (44.0-66.6)	19.6 (10.7-27.1)
Lesotho	127.6 (119.7-136.8)	750.5 (664.9-821.9)	41.2 (38.7-44.6)	14.4 (12.3-17.7)	90.0 (76.0-106.6)	706.8 (508.9-871.6)	44.1 (38.6-51.8)	14.8 (10.5-21.6)
Namibia	67.9 (62.0-74.7)	573.7 (491.2-659.9)	51.1 (48.1-54.3)	18.0 (15.6-20.9)	43.6 (34.0-55.4)	384.8 (259.6-554.0)	60.0 (53.6-66.3)	22.3 (17.4-27.1)
South Africa	82.2 (73.7-90.2)	542.6 (513.8-570.4)	51.7 (50.4-53.1)	20.1 (19.1-21.3)	45.3 (38.6-52.4)	392.2 (367.6-418.4)	58.6 (57.3-59.9)	21.5 (20.6-22.5)
Swaziland	114.2 (106.2-123.3)	753.3 (653.6-840.6)	41.7 (38.4-45.8)	15.0 (11.9-19.5)	58.3 (47.8-70.7)	621.8 (415.4-823.8)	49.0 (42.3-58.3)	16.9 (11.5-24.8)
Zimbabwe	88.5 (83.9-92.9)	718.7 (636.1-784.8)	44.6 (42.0-48.4)	17.0 (14.1-21.2)	59.1 (52.2-67.4)	442.7 (312.0-586.4)	56.3 (51.1-62.6)	20.8 (16.8-26.1)
Western Sub-Saharan Africa	153.4 (148.9-157.7)	303.1 (272.9-335.5)	55.8 (54.5-57.2)	23.7 (22.6-24.8)	104.1 (96.7-112.6)	261.1 (220.0-321.7)	61.0 (58.2-63.0)	24.7 (22.7-26.1)
Benin	122.4 (115.9-128.4)	336.3 (221.7-450.6)	56.2 (52.0-61.3)	21.8 (18.3-25.5)	82.4 (73.3-93.4)	304.4 (137.4-556.3)	59.9 (51.4-68.3)	22.0 (16.1-28.0)
Burkina Faso	159.5 (151.2-167.3)	309.0 (236.2-383.8)	55.0 (52.2-58.3)	23.3 (21.2-25.9)	115.1 (104.2-127.4)	253.0 (138.4-452.9)	60.0 (52.6-66.0)	23.9 (18.0-28.4)
Cameroon	130.0 (122.6-137.7)	396.2 (315.9-480.7)	53.8 (50.7-57.2)	21.7 (18.7-24.4)	94.4 (82.0-108.4)	355.9 (221.6-549.4)	57.6 (50.4-64.2)	22.5 (16.9-27.4)
Cape Verde	41.0 (36.6-45.9)	301.2 (210.7-408.4)	63.2 (59.2-68.0)	22.2 (19.5-25.8)	30.9 (27.7-34.4)	201.3 (158.3-254.0)	69.1 (66.2-71.8)	25.9 (23.8-28.0)

SR Table 3: Under-5 mortality (5q0) per 1,000 live births, adult mortality (45q15) per 1,000, life expectancy at birth (years), and life expectancy at age 50 (years), by geography, males, 2005 and 2015 (95% uncertainty intervals)

Location	2005				2015			
	Under-5 mortality (5q0)	Adult mortality (45q15)	Life expectancy at birth	Life expectancy at age 50	Under-5 mortality (5q0)	Adult mortality (45q15)	Life expectancy at birth	Life expectancy at age 50
Chad	174.2 (162.4-186.3)	373.0 (235.7-526.2)	51.5 (46.5-57.5)	21.5 (17.1-26.4)	136.2 (120.7-155.1)	315.7 (157.0-559.3)	55.9 (47.6-63.6)	22.5 (16.1-28.2)
Cote d'Ivoire	136.5 (129.5-144.2)	430.4 (354.8-503.1)	52.2 (49.4-55.2)	20.9 (18.2-23.4)	100.5 (90.0-112.7)	349.0 (195.8-558.7)	57.2 (50.0-64.5)	21.9 (16.3-27.4)
The Gambia	74.3 (69.3-79.4)	238.4 (180.1-310.1)	63.8 (60.6-66.9)	24.5 (22.5-26.8)	48.3 (43.4-53.4)	221.3 (146.1-335.8)	66.7 (61.5-70.7)	25.3 (21.4-28.1)
Ghana	90.7 (86.6-95.0)	315.3 (226.5-426.6)	59.4 (55.1-63.5)	22.8 (19.3-26.1)	59.7 (53.6-67.0)	273.9 (146.9-486.2)	63.1 (55.0-69.8)	23.3 (17.7-28.1)
Guinea	157.5 (148.4-166.2)	303.7 (234.2-380.7)	55.2 (52.4-58.2)	22.9 (20.9-25.1)	114.3 (101.0-128.1)	307.2 (166.7-502.6)	57.8 (50.8-64.5)	22.5 (17.1-27.7)
Guinea-Bissau	171.0 (155.6-188.5)	338.9 (150.6-694.6)	52.3 (41.5-61.6)	21.5 (13.5-28.6)	129.4 (112.8-150.3)	344.6 (164.8-757.1)	54.9 (42.0-64.0)	21.7 (12.4-28.4)
Liberia	125.6 (119.9-131.6)	284.4 (228.7-350.3)	58.4 (55.8-61.1)	24.1 (22.2-26.2)	75.8 (68.5-83.9)	255.8 (170.8-380.4)	63.1 (57.6-67.7)	24.6 (20.5-28.0)
Mali	173.9 (165.4-183.1)	240.6 (190.5-302.7)	56.7 (54.2-59.2)	25.2 (23.2-27.3)	136.0 (120.8-154.5)	225.9 (150.3-364.2)	60.0 (54.2-64.3)	25.5 (21.1-28.6)
Mauritania	88.0 (82.5-93.4)	161.1 (116.7-213.4)	66.8 (63.9-69.7)	27.5 (25.2-29.9)	59.1 (52.8-66.7)	148.7 (65.5-270.2)	69.8 (63.7-75.8)	28.0 (23.8-32.7)
Niger	178.6 (169.7-187.6)	263.7 (209.4-333.8)	55.2 (52.5-57.7)	24.0 (21.9-25.8)	115.8 (104.2-130.2)	260.4 (144.8-434.3)	59.6 (52.9-65.4)	23.7 (18.5-27.9)
Nigeria	167.5 (159.9-175.3)	269.3 (215.2-330.4)	56.3 (53.7-59.0)	25.0 (22.9-27.4)	108.9 (97.3-122.2)	216.7 (167.2-333.1)	62.9 (57.4-65.8)	26.8 (22.7-28.4)
Sao Tome and Principe	63.6 (58.0-69.6)	231.5 (163.1-310.5)	64.7 (61.1-68.1)	24.2 (21.6-26.6)	40.9 (35.6-47.1)	215.0 (91.3-395.0)	67.2 (59.4-74.8)	24.8 (19.4-30.3)
Senegal	97.2 (93.3-101.0)	261.8 (168.9-391.9)	61.0 (55.8-65.5)	23.6 (19.6-26.9)	56.1 (51.7-61.1)	259.4 (136.2-515.9)	63.8 (54.4-70.6)	23.4 (16.9-28.4)
Sierra Leone	188.9 (179.8-197.5)	347.9 (290.9-410.4)	51.3 (49.2-53.5)	21.4 (19.8-23.1)	128.5 (117.6-139.5)	315.2 (193.3-472.4)	56.5 (51.0-62.4)	22.7 (18.0-27.3)
Togo	115.3 (108.5-122.2)	396.0 (308.6-485.4)	54.6 (51.3-58.2)	21.1 (18.1-24.1)	82.2 (74.5-91.1)	348.1 (199.3-525.7)	58.5 (52.3-65.7)	21.7 (17.1-27.2)
Eastern Sub-Saharan Africa	110.4 (107.0-113.8)	416.9 (388.3-446.8)	54.5 (53.3-55.7)	21.6 (20.6-22.5)	71.9 (64.4-81.4)	322.4 (266.5-390.8)	60.5 (57.7-63.2)	22.8 (20.7-24.7)
Burundi	123.5 (113.6-134.1)	393.0 (310.3-481.4)	54.2 (50.9-57.5)	20.9 (18.0-23.5)	81.1 (61.8-109.5)	305.5 (159.1-506.4)	60.1 (53.1-67.7)	22.3 (17.2-27.6)
Comoros	73.5 (57.2-93.1)	203.6 (149.5-259.7)	65.3 (62.3-68.6)	25.3 (23.6-27.6)	47.7 (33.6-66.5)	211.7 (108.2-366.9)	66.9 (60.0-73.4)	25.0 (20.2-29.6)
Djibouti	94.8 (84.3-107.0)	336.3 (157.6-625.4)	57.8 (47.9-66.5)	21.7 (14.8-28.0)	69.9 (58.7-84.8)	301.8 (142.5-612.4)	60.9 (50.3-69.1)	22.2 (14.9-28.3)
Eritrea	87.4 (75.7-100.7)	300.1 (145.7-582.8)	59.8 (49.8-67.6)	22.3 (15.6-28.1)	72.2 (59.1-89.1)	309.8 (135.3-574.8)	60.3 (50.9-69.2)	21.7 (15.5-28.0)
Ethiopia	117.3 (111.0-123.4)	359.2 (289.7-424.4)	56.0 (53.5-59.0)	22.2 (20.3-24.5)	65.1 (58.2-73.9)	253.7 (141.2-454.7)	63.6 (55.7-69.6)	23.9 (18.3-28.3)
Kenya	75.6 (71.4-79.8)	454.0 (424.8-486.4)	56.0 (54.6-57.2)	22.3 (21.2-23.4)	54.6 (49.1-61.3)	308.0 (272.7-347.4)	62.8 (61.1-64.6)	23.9 (22.6-25.2)
Madagascar	88.3 (79.9-97.1)	293.0 (224.2-375.1)	60.1 (56.8-63.0)	22.3 (19.7-24.2)	68.3 (55.6-84.7)	280.5 (124.8-486.5)	61.9 (54.5-69.9)	22.5 (17.2-28.3)
Malawi	127.8 (119.1-136.7)	580.3 (499.6-655.5)	47.7 (44.8-51.4)	20.0 (16.7-23.4)	88.8 (74.9-104.5)	353.1 (242.7-508.2)	58.2 (52.0-63.7)	23.2 (18.2-27.4)
Mozambique	131.9 (125.8-138.6)	474.1 (385.6-557.7)	50.9 (47.9-54.7)	20.3 (17.2-23.8)	82.4 (74.5-92.3)	454.1 (294.2-658.1)	54.3 (47.3-61.9)	20.3 (15.1-26.7)
Rwanda	110.3 (102.5-119.2)	324.0 (262.8-393.1)	58.1 (55.3-61.2)	23.7 (21.6-26.2)	70.7 (58.1-87.0)	243.2 (143.0-422.7)	63.7 (56.4-69.3)	24.5 (18.9-28.4)
Somalia	145.7 (133.8-157.9)	394.0 (141.7-760.8)	51.7 (41.0-63.1)	19.3 (12.0-28.0)	118.1 (102.4-137.0)	394.1 (161.4-759.2)	53.4 (41.9-64.1)	19.6 (12.0-27.6)
South Sudan	126.8 (117.6-136.4)	349.4 (151.2-729.0)	54.9 (43.1-64.3)	21.3 (12.8-28.4)	111.5 (93.0-132.7)	357.5 (168.5-723.3)	55.5 (43.2-64.9)	21.3 (12.9-28.2)
Tanzania	100.8 (95.3-106.3)	380.1 (306.1-467.7)	57.1 (53.5-60.8)	23.9 (20.9-26.9)	63.8 (56.3-72.9)	285.3 (168.9-481.9)	62.7 (54.8-68.5)	24.2 (18.2-28.1)
Uganda	116.2 (110.7-121.8)	481.0 (410.7-554.8)	51.7 (49.1-54.6)	19.8 (17.3-22.3)	75.0 (67.7-84.0)	363.2 (206.7-563.1)	58.4 (51.3-66.0)	21.6 (16.4-27.2)
Zambia	114.8 (106.5-123.4)	644.6 (577.2-708.1)	45.8 (43.6-48.3)	16.2 (14.5-18.8)	70.6 (59.3-83.5)	475.5 (304.2-640.6)	54.0 (48.5-61.1)	18.4 (14.6-23.8)
Central Sub-Saharan Africa	132.2 (123.4-141.1)	368.9 (300.3-462.3)	54.6 (51.2-57.4)	21.6 (19.3-23.7)	90.4 (76.5-106.1)	324.5 (219.7-455.6)	58.9 (54.0-63.9)	22.1 (18.5-25.7)
Angola	128.6 (114.4-144.5)	338.4 (145.3-699.1)	55.2 (44.1-64.7)	21.3 (13.3-28.6)	83.1 (64.0-106.0)	307.0 (144.0-705.2)	59.5 (46.1-68.6)	22.1 (13.2-28.5)
Central African Republic	160.7 (141.5-182.4)	622.4 (380.8-839.3)	43.6 (37.1-52.6)	15.6 (10.8-23.3)	132.2 (103.4-170.3)	545.9 (257.6-805.9)	47.5 (39.6-59.1)	16.5 (11.3-25.8)
Congo	94.7 (88.2-102.0)	352.7 (282.5-433.9)	58.1 (54.7-61.6)	23.1 (20.3-25.9)	59.8 (47.6-74.3)	277.3 (173.8-452.2)	63.4 (55.9-68.7)	24.3 (19.0-27.9)
Democratic Republic of the Congo	134.8 (123.2-146.1)	346.5 (284.4-412.4)	55.2 (52.6-57.9)	22.2 (20.3-24.1)	93.0 (74.8-114.7)	305.4 (180.1-458.6)	59.4 (53.2-65.6)	22.5 (18.0-27.1)
Equatorial Guinea	139.2 (116.8-162.8)	320.2 (168.2-651.7)	55.6 (44.5-63.4)	22.9 (14.5-28.4)	92.3 (69.1-124.0)	285.9 (159.8-625.9)	60.3 (48.5-67.4)	23.9 (15.1-28.4)
Gabon	71.0 (65.8-76.3)	367.6 (289.1-454.3)	59.3 (55.6-63.5)	23.2 (20.0-26.6)	49.7 (43.4-56.4)	296.4 (188.3-456.5)	63.6 (56.8-69.0)	24.2 (19.1-27.8)

SR Table 4: Regional deaths (thousands) by age and sex (with 95% uncertainty intervals) in 1990, 2005, and 2015

	1990			2005			2015		
Age (years)	Male	Female	Total	Male	Female	Total	Male	Female	Total
**East Asia**									
Early Neonatal	272.60 (260.80 - 284.20)	199.80 (192.00 - 208.20)	472.20 (453.40 - 492.00)	96.00 (91.20 - 100.60)	67.20 (64.00 - 70.40)	163.00 (155.20 - 171.00)	47.40 (42.20 - 53.20)	35.60 (32.00 - 39.60)	83.00 (74.60 - 92.60)
Late Neonatal	66.40 (62.00 - 70.80)	58.80 (54.80 - 63.20)	125.20 (117.20 - 133.40)	18.80 (18.00 - 19.60)	15.40 (14.40 - 16.20)	34.00 (32.40 - 35.80)	12.40 (11.60 - 13.40)	10.20 (9.40 - 11.00)	22.60 (21.00 - 24.20)
Post Neonatal	299.00 (278.60 - 321.20)	248.40 (226.40 - 272.80)	547.40 (506.80 - 594.80)	60.60 (56.40 - 64.60)	46.00 (42.00 - 50.20)	106.60 (98.80 - 114.80)	34.80 (32.00 - 37.60)	25.80 (23.40 - 28.40)	60.60 (55.40 - 66.00)
1 to 4	193.80 (170.80 - 223.60)	167.80 (148.00 - 191.00)	361.80 (319.80 - 412.80)	44.60 (40.20 - 49.80)	31.60 (28.60 - 34.80)	76.20 (69.00 - 84.80)	28.80 (25.40 - 32.20)	19.60 (17.60 - 21.80)	48.20 (42.80 - 54.20)
5 to 9	73.60 (70.20 - 77.00)	45.00 (43.00 - 47.20)	118.60 (113.20 - 123.80)	27.80 (26.80 - 28.80)	14.00 (13.60 - 14.60)	42.00 (40.40 - 43.40)	17.60 (16.40 - 18.80)	11.20 (10.60 - 11.80)	28.80 (27.00 - 30.60)
10 to 14	56.60 (54.00 - 58.80)	37.00 (35.40 - 38.80)	93.60 (90.40 - 96.80)	37.00 (35.80 - 38.00)	19.60 (19.00 - 20.20)	56.60 (55.20 - 58.20)	18.00 (17.00 - 19.00)	10.00 (9.40 - 10.40)	28.00 (26.60 - 29.40)
15 to 19	85.80 (79.40 - 92.00)	59.80 (55.60 - 64.20)	145.40 (138.20 - 153.40)	67.20 (64.20 - 70.20)	33.60 (32.40 - 35.00)	100.80 (97.60 - 104.20)	27.80 (25.80 - 30.00)	11.00 (10.20 - 11.60)	38.60 (36.60 - 41.00)
20 to 24	120.20 (111.00 - 129.60)	87.00 (80.60 - 94.00)	207.20 (196.80 - 219.20)	72.80 (69.40 - 76.00)	32.60 (31.20 - 34.00)	105.20 (101.80 - 108.80)	52.40 (48.80 - 56.40)	19.20 (18.20 - 20.40)	71.60 (68.00 - 76.00)
25 to 29	101.00 (93.40 - 108.60)	71.60 (66.20 - 77.60)	172.80 (164.00 - 182.40)	79.20 (75.80 - 82.60)	36.00 (34.40 - 37.60)	115.20 (111.60 - 119.00)	83.00 (77.60 - 89.00)	31.60 (29.60 - 33.60)	114.60 (109.00 - 120.80)
30 to 34	106.20 (98.20 - 114.00)	73.00 (67.40 - 78.80)	179.00 (170.00 - 188.80)	132.00 (126.40 - 137.80)	59.60 (56.80 - 62.40)	191.40 (185.40 - 197.80)	80.00 (74.80 - 85.80)	31.00 (29.20 - 33.00)	111.00 (105.80 - 117.20)
35 to 39	139.20 (127.40 - 150.80)	94.00 (85.40 - 103.20)	233.20 (219.20 - 248.20)	184.40 (176.00 - 193.60)	88.20 (83.40 - 93.20)	272.60 (263.00 - 282.60)	93.40 (86.60 - 101.00)	36.20 (33.60 - 39.40)	129.80 (122.60 - 138.00)
40 to 44	141.00 (128.80 - 153.40)	92.60 (84.80 - 100.60)	233.60 (219.80 - 248.20)	221.80 (211.60 - 232.80)	102.20 (97.40 - 107.40)	324.00 (312.80 - 335.80)	176.80 (164.00 - 192.00)	69.60 (64.80 - 75.00)	246.40 (233.20 - 262.00)
45 to 49	174.20 (159.20 - 189.80)	105.60 (97.00 - 114.20)	279.60 (263.20 - 297.00)	245.80 (234.00 - 258.80)	125.00 (119.40 - 131.20)	370.80 (357.80 - 384.40)	288.00 (268.00 - 311.00)	121.60 (113.80 - 129.80)	409.60 (389.00 - 433.40)
50 to 54	251.40 (231.20 - 272.60)	151.00 (139.80 - 162.60)	402.40 (380.20 - 426.40)	387.00 (369.60 - 406.20)	209.80 (201.00 - 219.20)	597.00 (577.00 - 618.00)	350.20 (326.40 - 377.20)	154.80 (145.60 - 164.80)	505.20 (480.60 - 531.80)
55 to 59	352.80 (326.20 - 379.80)	214.60 (199.00 - 231.00)	567.40 (537.60 - 598.80)	408.60 (389.80 - 428.80)	233.20 (223.20 - 243.80)	641.80 (620.60 - 663.20)	412.00 (384.60 - 442.60)	182.80 (171.00 - 195.20)	594.60 (565.60 - 627.00)
60 to 64	461.00 (429.80 - 492.80)	290.40 (269.20 - 311.80)	751.60 (715.60 - 790.00)	463.80 (444.00 - 483.80)	265.80 (254.60 - 277.40)	729.60 (707.40 - 752.00)	680.40 (639.00 - 725.40)	321.80 (301.20 - 344.00)	1,002.20 (955.40 - 1,053.60)
65 to 69	523.60 (490.20 - 557.40)	370.60 (343.40 - 397.60)	894.20 (853.20 - 938.00)	664.00 (637.60 - 691.60)	397.40 (381.00 - 414.40)	1,061.40 (1,029.60 - 1,093.00)	711.80 (669.00 - 757.40)	356.60 (333.80 - 381.40)	1,068.40 (1,018.60 - 1,121.20)
70 to 74	639.00 (600.40 - 678.00)	498.60 (462.40 - 535.40)	1,137.60 (1,086.40 - 1,189.60)	804.00 (774.00 - 835.60)	552.20 (529.60 - 575.80)	1,356.20 (1,317.80 - 1,395.00)	717.60 (677.40 - 760.60)	395.60 (371.80 - 420.60)	1,113.20 (1,067.40 - 1,163.80)
75 to 79	508.40 (479.40 - 537.00)	497.40 (463.20 - 532.00)	1,005.80 (962.60 - 1,048.80)	731.40 (705.80 - 757.00)	584.20 (562.00 - 607.40)	1,315.60 (1,281.20 - 1,350.00)	799.40 (759.40 - 843.20)	514.20 (484.80 - 545.20)	1,313.60 (1,263.80 - 1,366.80)
80 plus	448.00 (432.20 - 463.40)	706.20 (675.80 - 735.00)	1,154.20 (1,120.00 - 1,187.00)	991.00 (970.60 - 1,011.40)	1,188.40 (1,162.00 - 1,214.60)	2,179.60 (2,145.40 - 2,213.20)	1,400.20 (1,354.60 - 1,445.60)	1,410.00 (1,366.00 - 1,459.00)	2,810.20 (2,749.00 - 2,877.40)
**Southeast Asia**									
Early Neonatal	127.20 (120.00 - 134.00)	97.40 (92.80 - 101.60)	224.60 (213.40 - 235.60)	84.00 (79.80 - 88.00)	63.40 (61.00 - 65.80)	147.40 (140.60 - 153.40)	58.20 (52.80 - 63.80)	43.60 (40.00 - 47.40)	101.80 (92.80 - 111.20)
Late Neonatal	50.60 (48.80 - 52.60)	40.60 (38.80 - 42.40)	91.20 (88.00 - 94.40)	26.20 (25.00 - 27.20)	21.00 (20.20 - 22.00)	47.20 (45.20 - 49.00)	17.00 (15.60 - 18.40)	13.60 (12.40 - 14.80)	30.40 (28.00 - 33.20)
Post Neonatal	175.00 (164.40 - 185.80)	139.20 (126.40 - 152.40)	314.20 (291.20 - 338.20)	81.60 (77.00 - 85.80)	63.20 (57.40 - 68.60)	144.80 (135.00 - 154.00)	43.20 (38.40 - 48.40)	32.20 (28.00 - 37.20)	75.40 (66.80 - 85.40)
1 to 4	122.20 (105.40 - 142.00)	115.40 (101.80 - 131.00)	237.40 (206.60 - 272.80)	49.00 (42.40 - 56.80)	42.20 (37.00 - 47.80)	91.20 (79.40 - 104.40)	29.20 (24.80 - 34.20)	23.60 (20.20 - 27.40)	52.60 (45.00 - 61.20)
5 to 9	44.00 (42.40 - 45.80)	35.80 (34.60 - 37.00)	79.80 (77.40 - 82.20)	24.60 (23.60 - 26.20)	18.80 (17.80 - 19.80)	43.40 (42.00 - 45.40)	16.40 (15.20 - 18.00)	12.20 (11.40 - 13.80)	28.60 (26.60 - 31.60)
10 to 14	31.20 (30.40 - 32.00)	23.40 (22.80 - 24.20)	54.60 (53.40 - 56.00)	24.20 (23.40 - 25.60)	16.40 (15.60 - 17.20)	40.60 (39.40 - 42.40)	17.60 (16.40 - 19.60)	11.80 (11.00 - 13.40)	29.40 (27.60 - 32.40)
15 to 19	40.00 (38.00 - 42.20)	26.60 (25.00 - 28.20)	66.60 (63.60 - 69.60)	38.20 (35.40 - 40.80)	19.40 (18.20 - 20.60)	57.60 (54.00 - 60.80)	32.80 (28.00 - 37.40)	14.80 (13.20 - 16.20)	47.40 (42.00 - 52.40)
20 to 24	56.20 (52.80 - 59.60)	33.00 (30.80 - 35.20)	89.20 (84.60 - 93.80)	56.80 (52.00 - 61.00)	25.40 (23.40 - 27.40)	82.20 (76.20 - 87.80)	51.20 (43.80 - 59.00)	19.60 (17.20 - 22.40)	70.80 (62.00 - 79.20)
25 to 29	56.80 (53.20 - 60.80)	33.60 (31.20 - 36.80)	90.40 (85.40 - 96.20)	64.20 (59.00 - 69.60)	30.40 (27.80 - 33.60)	94.60 (87.60 - 102.40)	58.00 (50.20 - 67.60)	24.40 (20.60 - 29.20)	82.40 (72.40 - 93.80)
30 to 34	57.20 (53.20 - 61.60)	35.40 (32.60 - 39.00)	92.60 (87.00 - 99.20)	73.20 (67.60 - 79.40)	36.60 (33.20 - 40.80)	109.80 (101.80 - 119.20)	72.20 (63.80 - 84.20)	32.60 (27.20 - 39.20)	104.60 (93.40 - 119.00)
35 to 39	58.40 (54.00 - 63.00)	37.80 (34.80 - 42.60)	96.20 (89.80 - 104.60)	84.60 (78.40 - 92.40)	45.40 (41.20 - 50.80)	130.00 (121.00 - 141.20)	84.20 (74.40 - 97.20)	41.80 (34.80 - 50.40)	125.80 (112.00 - 142.20)
40 to 44	56.60 (52.00 - 61.20)	37.60 (34.60 - 41.80)	94.20 (87.80 - 102.60)	96.20 (88.60 - 105.20)	55.60 (50.40 - 61.60)	151.80 (140.80 - 165.20)	103.80 (91.00 - 119.40)	55.40 (46.40 - 65.60)	159.00 (141.40 - 178.80)
45 to 49	67.20 (61.60 - 73.00)	46.00 (42.40 - 50.80)	113.20 (105.20 - 122.80)	110.80 (101.20 - 121.80)	68.40 (61.80 - 75.60)	179.40 (165.80 - 195.20)	131.00 (114.60 - 150.00)	74.40 (63.40 - 86.60)	205.40 (182.20 - 229.60)
50 to 54	85.80 (78.80 - 93.40)	61.80 (56.60 - 68.60)	147.80 (137.00 - 160.40)	125.60 (114.20 - 138.00)	84.80 (76.20 - 93.40)	210.40 (193.40 - 228.80)	164.60 (142.80 - 187.20)	98.00 (84.60 - 112.80)	262.60 (233.60 - 291.60)
55 to 59	103.40 (95.20 - 112.80)	78.00 (70.60 - 86.60)	181.40 (167.60 - 196.00)	134.20 (123.00 - 145.60)	96.00 (87.40 - 104.00)	230.20 (213.80 - 246.80)	201.00 (174.40 - 225.20)	122.00 (106.80 - 138.60)	323.00 (289.00 - 356.00)
60 to 64	122.60 (113.80 - 132.60)	98.60 (89.80 - 108.80)	221.20 (205.00 - 238.20)	150.00 (137.40 - 162.00)	117.40 (108.20 - 126.20)	267.40 (249.40 - 285.20)	224.20 (196.40 - 248.00)	140.20 (124.20 - 156.00)	364.40 (329.60 - 398.20)
65 to 69	127.80 (119.60 - 137.00)	111.20 (101.20 - 123.00)	239.20 (222.20 - 257.20)	174.00 (159.40 - 187.20)	150.40 (138.80 - 160.80)	324.60 (303.20 - 344.60)	216.60 (191.40 - 236.40)	156.80 (141.00 - 171.80)	373.40 (341.60 - 402.80)
70 to 74	131.40 (124.00 - 139.60)	128.00 (118.00 - 140.00)	259.40 (243.20 - 276.60)	189.40 (173.40 - 203.00)	184.00 (172.00 - 196.00)	373.40 (350.80 - 394.60)	218.80 (193.00 - 237.80)	179.60 (163.00 - 195.60)	398.40 (365.40 - 427.20)
75 to 79	120.60 (115.00 - 127.40)	133.00 (123.80 - 144.00)	253.80 (240.40 - 268.60)	169.40 (155.00 - 181.20)	187.60 (176.40 - 200.20)	357.00 (336.20 - 376.60)	217.80 (193.00 - 234.20)	214.20 (195.20 - 230.60)	432.00 (400.00 - 460.00)
80 plus	149.20 (145.20 - 154.40)	226.00 (216.20 - 238.20)	375.00 (362.80 - 389.20)	256.60 (241.80 - 267.60)	392.40 (374.00 - 415.60)	648.80 (623.60 - 676.20)	374.00 (348.60 - 390.20)	522.60 (480.40 - 552.20)	896.60 (847.00 - 935.00)
**Oceania**									
Early Neonatal	1.60 (1.40 - 2.00)	1.40 (1.20 - 1.60)	3.20 (2.80 - 3.40)	2.00 (1.60 - 2.40)	1.60 (1.40 - 2.00)	3.60 (3.00 - 4.20)	1.80 (1.40 - 2.20)	1.40 (1.00 - 1.80)	3.20 (2.40 - 4.00)
Late Neonatal	0.60 (0.40 - 0.60)	0.60 (0.40 - 0.60)	1.00 (1.00 - 1.20)	0.60 (0.40 - 0.80)	0.60 (0.40 - 0.80)	1.20 (0.80 - 1.60)	0.40 (0.20 - 0.60)	0.40 (0.20 - 0.60)	0.80 (0.60 - 1.20)

Post Neonatal	3.80 (3.20 - 4.60)	3.00 (2.40 - 4.00)	7.00 (5.60 - 8.40)	3.80 (2.60 - 5.40)	3.00 (2.00 - 4.40)	6.80 (4.60 - 9.60)	2.60 (1.60 - 4.20)	2.00 (1.20 - 3.40)	4.80 (2.80 - 7.60)
1 to 4	2.80 (1.80 - 3.80)	2.40 (1.80 - 3.20)	5.20 (3.60 - 7.00)	2.40 (1.40 - 3.80)	2.00 (1.20 - 3.00)	4.40 (2.60 - 6.80)	1.40 (0.80 - 2.60)	1.20 (0.60 - 2.00)	2.60 (1.40 - 4.60)
5 to 9	0.60 (0.60 - 0.60)	0.60 (0.40 - 0.60)	1.00 (1.00 - 1.20)	0.60 (0.40 - 0.80)	0.60 (0.40 - 0.60)	1.20 (0.80 - 1.40)	0.60 (0.40 - 0.80)	0.40 (0.40 - 0.60)	1.00 (0.80 - 1.40)
10 to 14	0.40 (0.40 - 0.60)	0.40 (0.40 - 0.60)	0.80 (0.80 - 1.00)	0.60 (0.40 - 0.60)	0.40 (0.40 - 0.60)	1.00 (0.80 - 1.20)	0.60 (0.40 - 0.60)	0.40 (0.40 - 0.60)	1.00 (0.80 - 1.20)
15 to 19	0.80 (0.60 - 1.00)	0.60 (0.40 - 0.80)	1.40 (1.20 - 2.00)	1.00 (0.80 - 1.20)	0.60 (0.60 - 1.00)	1.60 (1.20 - 2.20)	1.00 (0.80 - 1.40)	0.80 (0.60 - 1.00)	1.80 (1.40 - 2.40)
20 to 24	1.20 (0.80 - 1.40)	0.80 (0.60 - 1.00)	1.80 (1.40 - 2.40)	1.40 (1.00 - 1.80)	1.00 (0.60 - 1.40)	2.40 (1.80 - 3.20)	1.60 (1.20 - 2.00)	1.00 (0.60 - 1.40)	2.40 (1.80 - 3.40)
25 to 29	1.20 (0.80 - 1.40)	0.80 (0.60 - 1.20)	2.00 (1.40 - 2.60)	1.60 (1.20 - 2.20)	1.20 (0.80 - 1.80)	3.00 (2.00 - 4.00)	1.60 (1.20 - 2.40)	1.20 (0.80 - 1.80)	2.80 (2.00 - 4.20)
30 to 34	1.20 (1.00 - 1.60)	1.00 (0.60 - 1.40)	2.20 (1.60 - 3.00)	2.00 (1.40 - 2.60)	1.60 (1.00 - 2.20)	3.40 (2.40 - 5.00)	2.00 (1.40 - 2.80)	1.40 (1.00 - 2.40)	3.40 (2.40 - 5.20)
35 to 39	1.40 (1.00 - 1.80)	1.20 (0.80 - 1.80)	2.60 (1.80 - 3.80)	2.00 (1.40 - 2.80)	1.80 (1.00 - 2.80)	3.80 (2.60 - 5.80)	2.40 (1.80 - 3.60)	2.00 (1.20 - 3.40)	4.40 (3.00 - 7.00)
40 to 44	1.60 (1.20 - 2.20)	1.20 (0.80 - 1.80)	2.80 (2.00 - 4.00)	2.40 (1.60 - 3.40)	2.00 (1.20 - 3.00)	4.40 (3.00 - 6.40)	3.00 (2.00 - 4.60)	2.40 (1.60 - 3.80)	5.40 (3.60 - 8.40)
45 to 49	1.80 (1.20 - 2.60)	1.40 (1.00 - 1.80)	3.20 (2.20 - 4.40)	2.80 (1.80 - 4.00)	2.20 (1.60 - 3.20)	5.00 (3.40 - 7.20)	3.20 (2.20 - 5.00)	2.60 (1.80 - 4.00)	6.00 (4.00 - 9.00)
50 to 54	2.00 (1.40 - 2.80)	1.60 (1.00 - 2.20)	3.60 (2.60 - 5.00)	3.00 (2.20 - 4.40)	2.60 (1.80 - 3.60)	5.80 (4.00 - 8.00)	3.80 (2.60 - 5.60)	3.20 (2.20 - 4.60)	7.00 (4.80 - 10.20)
55 to 59	2.20 (1.60 - 3.00)	1.80 (1.20 - 2.40)	4.00 (2.80 - 5.20)	3.20 (2.20 - 4.20)	2.80 (2.00 - 3.80)	6.00 (4.20 - 8.20)	4.20 (3.00 - 6.00)	3.80 (2.60 - 5.20)	7.80 (5.60 - 11.20)
60 to 64	2.40 (1.60 - 2.80)	2.00 (1.40 - 2.60)	4.20 (3.20 - 5.40)	3.40 (2.40 - 4.20)	3.20 (2.20 - 4.00)	6.40 (4.60 - 8.40)	4.40 (3.20 - 6.00)	4.20 (3.20 - 5.80)	8.60 (6.40 - 11.80)
65 to 69	2.20 (1.60 - 2.60)	2.20 (1.60 - 2.80)	4.40 (3.40 - 5.40)	3.20 (2.40 - 3.80)	3.40 (2.40 - 4.20)	6.40 (4.80 - 8.20)	4.20 (3.20 - 5.20)	4.40 (3.40 - 5.80)	8.40 (6.40 - 11.20)
70 to 74	1.80 (1.40 - 2.20)	2.20 (1.60 - 2.60)	4.00 (3.20 - 4.80)	2.80 (2.20 - 3.20)	3.40 (2.60 - 4.20)	6.20 (4.80 - 7.60)	3.80 (3.00 - 4.60)	4.60 (3.40 - 6.00)	8.20 (6.60 - 10.40)
75 to 79	1.40 (1.20 - 1.60)	1.80 (1.60 - 2.20)	3.40 (2.80 - 3.80)	2.20 (1.80 - 2.40)	3.00 (2.40 - 3.60)	5.20 (4.20 - 6.00)	3.00 (2.60 - 3.60)	4.20 (3.20 - 5.20)	7.20 (5.80 - 8.60)
80 plus	1.40 (1.20 - 1.40)	2.20 (2.00 - 2.40)	3.60 (3.20 - 3.80)	2.20 (2.00 - 2.40)	4.20 (3.60 - 4.40)	6.40 (5.60 - 6.80)	3.40 (3.00 - 3.60)	6.20 (5.40 - 6.80)	9.60 (8.40 - 10.40)
**Central Asia**									
Early Neonatal	21.00 (20.00 - 22.00)	15.60 (15.00 - 16.20)	36.60 (35.00 - 38.20)	14.00 (13.20 - 15.00)	10.20 (9.60 - 10.80)	24.20 (22.80 - 25.80)	12.20 (11.00 - 13.40)	8.80 (8.00 - 9.60)	21.00 (19.00 - 23.00)
Late Neonatal	5.60 (5.40 - 5.80)	4.80 (4.60 - 5.00)	10.40 (9.80 - 10.80)	3.20 (2.80 - 3.40)	2.60 (2.40 - 3.00)	5.80 (5.40 - 6.40)	2.60 (2.20 - 2.80)	2.20 (2.00 - 2.40)	4.60 (4.20 - 5.20)
Post Neonatal	23.80 (22.40 - 25.20)	19.20 (17.60 - 20.80)	43.00 (40.20 - 46.00)	12.00 (10.80 - 13.40)	9.40 (8.40 - 10.60)	21.40 (19.20 - 24.00)	8.20 (7.20 - 9.40)	6.20 (5.40 - 7.20)	14.40 (12.60 - 16.60)
1 to 4	15.60 (13.80 - 17.80)	13.40 (12.00 - 15.20)	29.20 (25.80 - 32.80)	6.80 (5.80 - 8.00)	5.60 (4.60 - 6.40)	12.40 (10.60 - 14.60)	5.20 (4.40 - 6.20)	4.00 (3.40 - 4.80)	9.20 (7.80 - 10.80)
5 to 9	4.00 (4.00 - 4.20)	2.80 (2.60 - 2.80)	6.80 (6.60 - 7.00)	3.20 (3.00 - 3.40)	2.20 (2.00 - 2.40)	5.40 (5.00 - 5.80)	2.20 (2.00 - 2.40)	1.40 (1.40 - 1.60)	3.60 (3.40 - 4.00)
10 to 14	3.00 (3.00 - 3.00)	1.80 (1.80 - 1.80)	4.80 (4.60 - 4.80)	3.60 (3.40 - 3.80)	2.20 (2.20 - 2.40)	5.80 (5.60 - 6.00)	2.00 (1.80 - 2.00)	1.20 (1.00 - 1.20)	3.00 (3.00 - 3.20)
15 to 19	3.80 (3.80 - 4.00)	2.20 (2.20 - 2.20)	6.00 (6.00 - 6.20)	4.60 (4.60 - 4.80)	2.40 (2.20 - 2.40)	7.00 (6.80 - 7.20)	3.80 (3.60 - 4.00)	1.60 (1.60 - 1.60)	5.20 (5.00 - 5.60)
20 to 24	5.60 (5.40 - 5.60)	3.00 (2.80 - 3.00)	8.40 (8.40 - 8.60)	7.20 (7.00 - 7.40)	3.20 (3.20 - 3.40)	10.40 (10.20 - 10.60)	7.20 (6.80 - 7.60)	2.60 (2.40 - 2.80)	9.80 (9.40 - 10.20)
25 to 29	7.40 (7.40 - 7.60)	3.40 (3.40 - 3.60)	11.00 (10.80 - 11.00)	9.00 (8.80 - 9.20)	3.60 (3.60 - 3.80)	12.60 (12.40 - 12.80)	10.00 (9.40 - 10.40)	3.60 (3.40 - 3.80)	13.40 (13.00 - 14.00)
30 to 34	8.40 (8.20 - 8.60)	3.80 (3.80 - 4.00)	12.20 (12.00 - 12.40)	10.60 (10.40 - 10.80)	4.40 (4.20 - 4.40)	15.00 (14.60 - 15.20)	10.60 (10.20 - 11.00)	3.80 (3.60 - 4.00)	14.40 (13.80 - 15.00)
35 to 39	8.40 (8.40 - 8.60)	4.00 (3.80 - 4.00)	12.40 (12.20 - 12.60)	12.00 (11.80 - 12.40)	5.20 (5.00 - 5.40)	17.20 (17.00 - 17.60)	11.00 (10.60 - 11.60)	4.40 (4.20 - 4.60)	15.60 (15.00 - 16.00)
40 to 44	8.40 (8.20 - 8.40)	4.00 (3.80 - 4.00)	12.20 (12.00 - 12.40)	16.80 (16.60 - 17.20)	7.40 (7.20 - 7.60)	24.20 (23.80 - 24.60)	13.60 (13.00 - 14.20)	5.80 (5.60 - 6.20)	19.40 (18.80 - 20.20)
45 to 49	9.20 (9.00 - 9.40)	4.40 (4.20 - 4.40)	13.60 (13.40 - 13.80)	20.60 (20.20 - 21.00)	9.40 (9.20 - 9.80)	30.00 (29.60 - 30.40)	17.00 (16.40 - 17.80)	8.00 (7.60 - 8.40)	25.00 (24.20 - 25.80)
50 to 54	19.60 (19.40 - 20.00)	10.00 (9.80 - 10.20)	29.60 (29.20 - 30.00)	22.40 (22.00 - 22.80)	11.20 (10.80 - 11.40)	33.60 (33.00 - 34.00)	26.40 (25.40 - 27.40)	13.20 (12.80 - 13.80)	39.60 (38.40 - 40.80)
55 to 59	20.40 (20.00 - 20.80)	11.40 (11.20 - 11.60)	31.80 (31.40 - 32.20)	20.80 (20.40 - 21.20)	11.80 (11.40 - 12.00)	32.60 (32.00 - 33.00)	29.80 (28.60 - 30.80)	16.40 (15.80 - 17.00)	46.00 (44.80 - 47.20)
60 to 64	28.60 (28.20 - 29.20)	18.80 (18.60 - 19.20)	47.60 (47.00 - 48.20)	21.20 (20.80 - 21.60)	13.20 (12.80 - 13.40)	34.40 (34.00 - 34.80)	32.00 (31.00 - 33.00)	19.60 (19.00 - 20.40)	51.60 (50.40 - 53.00)
65 to 69	19.60 (19.20 - 19.80)	19.40 (19.00 - 19.80)	39.00 (38.40 - 39.40)	39.00 (38.40 - 39.60)	28.20 (27.60 - 28.80)	67.20 (66.20 - 68.00)	24.20 (23.40 - 25.00)	17.00 (16.20 - 17.60)	41.20 (40.20 - 42.20)
70 to 74	16.00 (15.60 - 16.20)	20.40 (20.00 - 20.80)	36.40 (35.80 - 36.80)	35.80 (35.20 - 36.40)	31.40 (30.60 - 32.00)	67.20 (66.20 - 68.00)	24.60 (23.80 - 25.20)	20.40 (19.60 - 21.20)	44.80 (43.80 - 46.00)
75 to 79	19.80 (19.60 - 20.20)	29.40 (28.80 - 29.80)	49.20 (48.60 - 49.80)	35.60 (35.00 - 36.20)	40.00 (39.20 - 41.00)	75.60 (74.60 - 76.60)	37.60 (36.60 - 38.80)	39.20 (37.40 - 40.80)	76.80 (74.80 - 78.60)
80 plus	37.20 (36.80 - 37.60)	69.60 (68.80 - 70.40)	106.80 (105.80 - 107.60)	31.20 (30.80 - 31.60)	73.60 (72.40 - 74.60)	104.80 (103.60 - 105.80)	53.40 (52.40 - 54.40)	85.60 (82.80 - 87.60)	139.00 (136.20 - 141.20)
**Central Europe**									
Early Neonatal	8.80 (8.40 - 9.00)	6.00 (5.80 - 6.20)	14.80 (14.40 - 15.20)	2.60 (2.60 - 2.80)	2.00 (1.80 - 2.00)	4.60 (4.40 - 4.60)	1.40 (1.20 - 1.60)	1.00 (1.00 - 1.20)	2.40 (2.00 - 2.80)
Late Neonatal	2.40 (2.40 - 2.60)	2.00 (2.00 - 2.00)	4.40 (4.20 - 4.60)	1.00 (1.00 - 1.00)	0.80 (0.80 - 0.80)	1.80 (1.60 - 1.80)	0.60 (0.60 - 0.60)	0.40 (0.40 - 0.60)	1.00 (1.00 - 1.20)
Post Neonatal	7.20 (7.00 - 7.40)	5.80 (5.40 - 6.20)	13.00 (12.40 - 13.60)	2.20 (2.00 - 2.20)	1.80 (1.60 - 1.80)	4.00 (3.80 - 4.20)	1.20 (1.20 - 1.40)	1.00 (0.80 - 1.20)	2.20 (2.00 - 2.60)
1 to 4	3.20 (2.80 - 3.60)	2.60 (2.40 - 3.00)	5.80 (5.20 - 6.60)	1.00 (1.00 - 1.20)	0.80 (0.80 - 1.00)	1.80 (1.60 - 2.00)	0.60 (0.60 - 0.80)	0.40 (0.40 - 0.60)	1.00 (1.00 - 1.20)
5 to 9	2.00 (2.00 - 2.20)	1.40 (1.40 - 1.40)	3.40 (3.40 - 3.60)	0.80 (0.80 - 0.80)	0.60 (0.60 - 0.60)	1.20 (1.20 - 1.40)	0.40 (0.40 - 0.40)	0.40 (0.20 - 0.40)	0.80 (0.60 - 0.80)
10 to 14	2.00 (2.00 - 2.20)	1.20 (1.20 - 1.20)	3.20 (3.20 - 3.40)	1.00 (1.00 - 1.00)	0.60 (0.60 - 0.60)	1.60 (1.60 - 1.60)	0.60 (0.40 - 0.60)	0.40 (0.40 - 0.40)	0.80 (0.80 - 1.00)

15 to 19	4.60 (4.60 - 4.80)	1.80 (1.80 - 1.80)	6.60 (6.40 - 6.60)	3.00 (3.00 - 3.00)	1.20 (1.20 - 1.20)	4.20 (4.20 - 4.20)	1.80 (1.80 - 1.80)	0.80 (0.60 - 0.80)	2.40 (2.40 - 2.60)
20 to 24	6.60 (6.40 - 6.60)	2.00 (2.00 - 2.00)	8.60 (8.60 - 8.60)	4.80 (4.80 - 4.80)	1.40 (1.40 - 1.40)	6.20 (6.20 - 6.20)	3.20 (3.20 - 3.20)	1.00 (0.80 - 1.00)	4.20 (4.00 - 4.20)
25 to 29	7.20 (7.00 - 7.20)	2.40 (2.40 - 2.40)	9.60 (9.40 - 9.60)	5.40 (5.40 - 5.60)	1.80 (1.80 - 1.80)	7.20 (7.20 - 7.20)	4.20 (4.00 - 4.20)	1.20 (1.20 - 1.20)	5.40 (5.20 - 5.40)
30 to 34	11.20 (11.20 - 11.20)	4.00 (4.00 - 4.00)	15.20 (15.20 - 15.20)	6.60 (6.60 - 6.80)	2.40 (2.40 - 2.40)	9.00 (9.00 - 9.00)	5.60 (5.40 - 5.60)	1.80 (1.80 - 1.80)	7.40 (7.40 - 7.40)
35 to 39	16.80 (16.80 - 16.80)	6.40 (6.40 - 6.60)	23.20 (23.20 - 23.40)	9.60 (9.60 - 9.80)	3.80 (3.80 - 3.80)	13.40 (13.40 - 13.40)	8.20 (8.20 - 8.40)	3.20 (3.00 - 3.20)	11.40 (11.20 - 11.60)
40 to 44	22.00 (22.00 - 22.00)	9.00 (8.80 - 9.00)	31.00 (30.80 - 31.00)	15.60 (15.60 - 15.60)	6.20 (6.20 - 6.20)	21.80 (21.80 - 21.80)	12.00 (11.80 - 12.20)	4.80 (4.80 - 4.80)	16.80 (16.60 - 17.00)
45 to 49	26.40 (26.20 - 26.40)	10.80 (10.80 - 10.80)	37.00 (37.00 - 37.20)	30.40 (30.20 - 30.40)	12.40 (12.20 - 12.40)	42.80 (42.60 - 42.80)	19.40 (19.20 - 19.80)	8.00 (7.80 - 8.20)	27.40 (27.00 - 27.80)
50 to 54	41.20 (41.20 - 41.40)	17.60 (17.40 - 17.60)	58.80 (58.60 - 59.00)	47.20 (47.00 - 47.20)	19.60 (19.60 - 19.80)	66.80 (66.60 - 67.00)	32.80 (32.40 - 33.40)	13.80 (13.60 - 14.00)	46.60 (46.20 - 47.20)
55 to 59	60.20 (60.00 - 60.60)	27.60 (27.60 - 27.80)	88.00 (87.60 - 88.20)	56.40 (56.20 - 56.60)	25.20 (25.20 - 25.40)	81.60 (81.40 - 81.80)	54.60 (53.80 - 55.40)	24.20 (23.80 - 24.60)	78.80 (78.00 - 79.80)
60 to 64	79.00 (78.60 - 79.40)	42.80 (42.60 - 43.00)	121.80 (121.40 - 122.20)	58.20 (58.00 - 58.40)	29.20 (29.00 - 29.20)	87.40 (87.20 - 87.60)	74.00 (73.00 - 75.00)	36.00 (35.40 - 36.40)	110.00 (108.80 - 111.00)
65 to 69	88.20 (87.80 - 88.60)	62.80 (62.60 - 63.20)	151.00 (150.60 - 151.60)	79.40 (79.20 - 79.60)	48.20 (48.00 - 48.40)	127.60 (127.40 - 128.00)	69.80 (69.00 - 70.80)	40.00 (39.40 - 40.60)	109.80 (108.80 - 111.00)
70 to 74	63.80 (63.40 - 64.00)	57.40 (57.00 - 57.60)	121.20 (120.80 - 121.60)	98.80 (98.40 - 99.00)	77.20 (77.00 - 77.60)	176.00 (175.60 - 176.40)	72.60 (71.80 - 73.60)	53.40 (52.60 - 54.20)	126.20 (124.80 - 127.40)
75 to 79	96.20 (95.80 - 96.60)	107.40 (106.80 - 108.00)	203.60 (203.00 - 204.40)	104.40 (104.20 - 104.80)	112.00 (111.60 - 112.60)	216.40 (216.00 - 217.00)	93.20 (92.20 - 94.40)	92.00 (90.60 - 93.40)	185.20 (183.40 - 187.20)
80 plus	140.80 (140.20 - 141.20)	227.40 (226.60 - 228.60)	368.20 (367.40 - 369.60)	146.40 (146.20 - 146.60)	259.80 (259.00 - 260.40)	406.20 (405.40 - 407.00)	204.00 (202.40 - 205.80)	333.80 (330.60 - 337.20)	538.00 (534.20 - 541.80)
**Eastern Europe**									
Early Neonatal	15.20 (14.00 - 16.20)	10.40 (9.60 - 11.00)	25.40 (23.60 - 27.00)	6.80 (6.00 - 7.60)	4.60 (4.20 - 5.20)	11.60 (10.40 - 12.80)	4.40 (4.00 - 5.20)	3.20 (3.00 - 3.60)	7.80 (6.80 - 8.80)
Late Neonatal	4.00 (3.80 - 4.40)	3.20 (3.00 - 3.60)	7.40 (7.00 - 7.80)	2.20 (2.20 - 2.40)	1.80 (1.80 - 2.00)	4.20 (3.80 - 4.40)	1.80 (1.80 - 2.00)	1.60 (1.40 - 1.60)	3.40 (3.20 - 3.80)
Post Neonatal	10.80 (10.00 - 11.60)	7.80 (6.80 - 8.80)	18.60 (17.00 - 20.40)	5.00 (4.60 - 5.40)	3.60 (3.20 - 4.00)	8.60 (7.80 - 9.40)	3.80 (3.20 - 4.20)	2.80 (2.40 - 3.20)	6.40 (5.60 - 7.20)
1 to 4	7.60 (6.20 - 9.40)	5.80 (4.80 - 7.00)	13.40 (11.00 - 16.60)	3.40 (2.80 - 4.20)	2.40 (2.00 - 3.00)	5.80 (4.80 - 7.20)	2.60 (2.00 - 3.20)	1.80 (1.60 - 2.20)	4.40 (3.60 - 5.40)
5 to 9	6.20 (6.00 - 6.40)	3.40 (3.20 - 3.40)	9.60 (9.20 - 9.80)	2.40 (2.20 - 2.60)	1.40 (1.40 - 1.60)	4.00 (3.80 - 4.20)	1.60 (1.40 - 1.60)	1.00 (1.00 - 1.00)	2.60 (2.40 - 2.60)
10 to 14	4.80 (4.80 - 5.00)	2.40 (2.40 - 2.60)	7.40 (7.20 - 7.40)	3.20 (3.00 - 3.40)	1.80 (1.60 - 1.80)	4.80 (4.60 - 5.20)	1.40 (1.40 - 1.40)	0.80 (0.80 - 0.80)	2.20 (2.20 - 2.40)
15 to 19	11.60 (11.60 - 11.80)	4.60 (4.60 - 4.60)	16.20 (16.20 - 16.40)	14.20 (13.40 - 14.80)	5.40 (5.20 - 5.80)	19.60 (18.60 - 20.60)	4.40 (4.20 - 4.60)	1.80 (1.60 - 1.80)	6.20 (5.80 - 6.40)
20 to 24	18.40 (18.40 - 18.60)	4.80 (4.80 - 4.80)	23.40 (23.20 - 23.40)	32.20 (31.20 - 33.20)	8.40 (8.20 - 8.60)	40.60 (39.40 - 41.80)	17.20 (16.40 - 17.80)	4.60 (4.40 - 4.80)	21.60 (21.00 - 22.40)
25 to 29	27.60 (27.40 - 27.60)	6.60 (6.60 - 6.60)	34.20 (34.00 - 34.20)	45.40 (44.60 - 46.00)	11.80 (11.60 - 12.00)	57.20 (56.20 - 58.00)	33.80 (32.80 - 34.80)	9.40 (9.00 - 9.80)	43.20 (42.00 - 44.20)
30 to 34	38.40 (38.40 - 38.60)	9.80 (9.80 - 9.80)	48.20 (48.20 - 48.40)	55.60 (55.00 - 56.20)	15.40 (15.20 - 15.60)	71.00 (70.40 - 71.80)	43.20 (42.00 - 44.60)	13.00 (12.40 - 13.40)	56.20 (54.80 - 57.60)
35 to 39	46.60 (46.40 - 46.60)	13.60 (13.40 - 13.60)	60.00 (59.80 - 60.20)	65.00 (64.40 - 65.60)	19.20 (19.20 - 19.40)	84.20 (83.60 - 85.00)	52.40 (50.80 - 54.00)	16.60 (16.00 - 17.20)	69.00 (67.40 - 70.80)
40 to 44	52.60 (52.40 - 52.80)	17.40 (17.20 - 17.40)	70.00 (69.80 - 70.20)	104.80 (104.20 - 105.40)	31.60 (31.40 - 31.80)	136.20 (135.40 - 137.00)	49.60 (48.20 - 51.20)	17.00 (16.40 - 17.60)	66.60 (65.00 - 68.20)
45 to 49	51.80 (51.80 - 52.00)	19.20 (19.00 - 19.20)	71.00 (70.80 - 71.20)	147.20 (146.60 - 148.00)	47.60 (47.40 - 47.80)	194.80 (194.00 - 195.80)	72.00 (70.00 - 74.20)	26.20 (25.40 - 27.00)	98.20 (96.00 - 100.60)
50 to 54	115.80 (115.60 - 116.20)	46.40 (46.20 - 46.60)	162.20 (161.80 - 162.60)	173.80 (173.20 - 174.60)	63.60 (63.40 - 64.00)	237.40 (236.60 - 238.40)	121.80 (118.40 - 125.40)	47.00 (45.60 - 48.60)	168.80 (165.20 - 172.80)
55 to 59	116.80 (116.40 - 117.20)	54.60 (54.20 - 54.80)	171.40 (170.80 - 171.80)	169.20 (168.60 - 169.80)	75.60 (75.40 - 76.00)	244.80 (244.20 - 245.60)	149.20 (145.20 - 153.20)	68.20 (66.20 - 70.40)	217.40 (213.20 - 222.20)
60 to 64	178.40 (177.80 - 178.80)	104.40 (103.80 - 104.80)	282.60 (282.00 - 283.40)	137.60 (137.20 - 137.80)	72.20 (72.00 - 72.60)	209.80 (209.20 - 210.20)	212.20 (207.20 - 217.60)	106.20 (103.20 - 109.40)	318.40 (312.80 - 324.80)
65 to 69	122.80 (122.40 - 123.20)	126.00 (125.40 - 126.60)	249.00 (248.20 - 249.60)	248.60 (248.20 - 249.20)	169.20 (168.40 - 169.80)	417.80 (416.80 - 418.80)	78.60 (77.00 - 80.40)	53.40 (52.20 - 55.00)	132.00 (130.00 - 134.40)
70 to 74	97.20 (96.80 - 97.60)	131.60 (131.00 - 132.40)	228.80 (228.00 - 229.60)	197.00 (196.60 - 197.60)	184.40 (183.80 - 185.20)	381.60 (380.60 - 382.60)	240.20 (235.40 - 245.40)	226.00 (220.00 - 232.60)	466.20 (458.00 - 475.00)
75 to 79	131.20 (130.80 - 131.80)	241.20 (240.00 - 242.40)	372.40 (371.00 - 373.80)	217.20 (216.80 - 217.80)	312.40 (311.20 - 313.40)	529.60 (528.40 - 531.00)	130.60 (128.20 - 133.40)	163.80 (159.60 - 168.40)	294.60 (289.40 - 300.00)
80 plus	166.60 (166.20 - 167.00)	493.80 (492.00 - 495.60)	660.40 (658.60 - 662.40)	169.40 (169.20 - 169.80)	560.80 (559.00 - 562.40)	730.20 (728.40 - 732.00)	294.40 (290.60 - 298.40)	710.40 (698.00 - 724.60)	1,004.80 (991.40 - 1,019.40)
**High-income Asia Pacific**									
Early Neonatal	3.20 (2.60 - 4.00)	2.40 (2.00 - 2.80)	5.40 (4.60 - 6.80)	1.20 (1.00 - 1.20)	1.00 (0.80 - 1.00)	2.00 (2.00 - 2.20)	0.60 (0.60 - 0.80)	0.60 (0.60 - 0.60)	1.20 (1.20 - 1.40)
Late Neonatal	1.00 (1.00 - 1.20)	0.80 (0.80 - 1.00)	2.00 (1.80 - 2.20)	0.40 (0.40 - 0.40)	0.40 (0.40 - 0.40)	0.80 (0.80 - 0.80)	0.40 (0.20 - 0.40)	0.20 (0.20 - 0.20)	0.60 (0.60 - 0.60)
Post Neonatal	2.80 (2.40 - 3.20)	2.20 (2.00 - 2.60)	5.00 (4.20 - 5.60)	1.20 (1.20 - 1.20)	1.00 (1.00 - 1.00)	2.20 (2.00 - 2.20)	0.80 (0.80 - 0.80)	0.60 (0.60 - 0.80)	1.40 (1.40 - 1.60)
1 to 4	2.40 (2.00 - 3.00)	1.80 (1.60 - 2.20)	4.20 (3.60 - 5.00)	1.00 (1.00 - 1.20)	0.80 (0.80 - 0.80)	1.80 (1.60 - 2.00)	0.60 (0.60 - 0.60)	0.60 (0.40 - 0.60)	1.20 (1.00 - 1.20)
5 to 9	2.00 (1.80 - 2.20)	1.40 (1.20 - 1.40)	3.20 (2.80 - 3.80)	0.80 (0.80 - 0.80)	0.40 (0.40 - 0.60)	1.20 (1.20 - 1.20)	0.40 (0.40 - 0.40)	0.40 (0.20 - 0.40)	0.80 (0.60 - 0.80)
10 to 14	1.60 (1.60 - 1.80)	1.20 (1.00 - 1.20)	2.80 (2.60 - 3.00)	0.60 (0.60 - 0.60)	0.40 (0.40 - 0.40)	1.20 (1.20 - 1.20)	0.40 (0.40 - 0.60)	0.40 (0.40 - 0.40)	0.80 (0.80 - 0.80)
15 to 19	5.60 (5.40 - 5.60)	2.40 (2.40 - 2.40)	7.80 (7.80 - 8.00)	2.00 (2.00 - 2.00)	1.00 (1.00 - 1.00)	3.00 (3.00 - 3.00)	1.40 (1.40 - 1.60)	0.80 (0.80 - 0.80)	2.20 (2.20 - 2.20)
20 to 24	6.40 (6.40 - 6.60)	2.80 (2.80 - 3.00)	9.40 (9.20 - 9.40)	3.60 (3.40 - 3.60)	1.80 (1.60 - 1.80)	5.20 (5.20 - 5.20)	2.60 (2.60 - 2.80)	1.20 (1.20 - 1.20)	4.00 (3.80 - 4.00)
25 to 29	6.60 (6.60 - 6.60)	3.40 (3.20 - 3.40)	10.00 (9.80 - 10.00)	4.40 (4.40 - 4.40)	2.20 (2.20 - 2.20)	6.60 (6.60 - 6.60)	3.40 (3.20 - 3.40)	1.60 (1.60 - 1.60)	5.00 (4.80 - 5.00)
30 to 34	8.00 (7.80 - 8.00)	4.20 (4.20 - 4.20)	12.20 (12.00 - 12.20)	6.40 (6.20 - 6.40)	3.20 (3.20 - 3.20)	9.60 (9.40 - 9.60)	4.20 (4.20 - 4.40)	2.20 (2.20 - 2.40)	6.60 (6.40 - 6.60)

35 to 39	11.20 (11.00 - 11.20)	5.80 (5.60 - 5.80)	16.80 (16.80 - 17.00)	8.60 (8.60 - 8.80)	4.40 (4.20 - 4.40)	13.00 (12.80 - 13.20)	7.80 (7.60 - 8.00)	4.20 (4.00 - 4.20)	12.00 (11.80 - 12.20)
40 to 44	17.80 (17.60 - 18.00)	8.40 (8.40 - 8.40)	26.20 (26.00 - 26.40)	13.20 (13.00 - 13.40)	6.00 (5.80 - 6.00)	19.20 (19.00 - 19.40)	11.80 (11.40 - 12.00)	5.80 (5.60 - 6.00)	17.40 (17.20 - 17.80)
45 to 49	24.00 (23.80 - 24.20)	10.80 (10.80 - 11.00)	34.80 (34.60 - 35.00)	19.80 (19.40 - 20.20)	8.40 (8.40 - 8.60)	28.40 (28.00 - 28.60)	17.00 (16.60 - 17.60)	7.80 (7.60 - 8.00)	24.80 (24.20 - 25.40)
50 to 54	32.80 (32.60 - 33.00)	14.80 (14.80 - 15.00)	47.60 (47.40 - 48.00)	29.80 (29.40 - 30.00)	13.00 (13.00 - 13.20)	42.80 (42.40 - 43.20)	24.20 (23.60 - 25.00)	10.60 (10.40 - 10.80)	34.80 (34.00 - 35.80)
55 to 59	47.60 (47.40 - 47.80)	21.20 (21.00 - 21.20)	68.60 (68.40 - 69.00)	46.00 (45.80 - 46.40)	19.80 (19.60 - 20.00)	65.80 (65.60 - 66.20)	36.40 (35.40 - 37.40)	15.40 (15.20 - 15.80)	51.80 (50.80 - 52.80)
60 to 64	57.00 (56.80 - 57.20)	28.00 (27.80 - 28.20)	85.00 (84.80 - 85.40)	58.40 (58.00 - 58.80)	25.20 (25.00 - 25.20)	83.40 (83.00 - 83.80)	67.60 (66.60 - 68.60)	28.60 (28.40 - 29.00)	96.20 (95.20 - 97.20)
65 to 69	59.20 (59.00 - 59.40)	38.20 (38.00 - 38.40)	97.40 (97.00 - 97.80)	75.00 (74.60 - 75.60)	35.40 (35.20 - 35.60)	110.40 (109.80 - 111.00)	82.80 (81.60 - 84.00)	37.40 (36.80 - 37.80)	120.00 (118.80 - 121.40)
70 to 74	67.00 (66.80 - 67.20)	51.60 (51.40 - 51.80)	118.60 (118.20 - 119.00)	100.00 (99.40 - 100.60)	54.80 (54.40 - 55.20)	154.80 (154.20 - 155.40)	100.20 (98.80 - 101.60)	51.00 (50.20 - 52.00)	151.20 (149.60 - 153.00)
75 to 79	83.00 (82.80 - 83.20)	75.80 (75.40 - 76.20)	159.00 (158.40 - 159.40)	118.60 (118.20 - 119.20)	80.00 (79.40 - 80.60)	198.60 (198.00 - 199.40)	141.00 (139.40 - 142.80)	87.60 (86.20 - 89.00)	228.60 (226.40 - 230.80)
80 plus	149.60 (149.40 - 149.60)	216.60 (216.00 - 217.00)	366.00 (365.60 - 366.60)	249.20 (248.80 - 249.80)	371.40 (370.40 - 372.40)	620.60 (619.60 - 621.80)	411.00 (408.80 - 413.20)	582.40 (578.80 - 586.20)	993.20 (988.80 - 997.80)
**Australasia**									
Early Neonatal	0.80 (0.60 - 0.80)	0.60 (0.60 - 0.60)	1.40 (1.20 - 1.40)	0.40 (0.40 - 0.40)	0.40 (0.40 - 0.40)	0.80 (0.80 - 0.80)	0.40 (0.40 - 0.40)	0.20 (0.20 - 0.40)	0.60 (0.60 - 0.60)
Late Neonatal	0.20 (0.20 - 0.20)	0.20 (0.20 - 0.20)	0.20 (0.20 - 0.20)	0.20 (0.20 - 0.20)	0.00 (0.00 - 0.00)	0.20 (0.20 - 0.20)	0.00 (0.00 - 0.20)	0.00 (0.00 - 0.00)	0.20 (0.20 - 0.20)
Post Neonatal	0.60 (0.60 - 0.60)	0.40 (0.40 - 0.40)	1.00 (0.80 - 1.00)	0.40 (0.40 - 0.40)	0.20 (0.20 - 0.20)	0.60 (0.60 - 0.60)	0.20 (0.20 - 0.40)	0.20 (0.20 - 0.20)	0.40 (0.40 - 0.60)
1 to 4	0.40 (0.20 - 0.40)	0.20 (0.20 - 0.20)	0.60 (0.40 - 0.60)	0.20 (0.20 - 0.20)	0.20 (0.20 - 0.20)	0.40 (0.20 - 0.40)	0.20 (0.20 - 0.20)	0.20 (0.00 - 0.20)	0.20 (0.20 - 0.40)
5 to 9	0.20 (0.20 - 0.20)	0.20 (0.20 - 0.20)	0.20 (0.20 - 0.40)	0.20 (0.20 - 0.20)	0.00 (0.00 - 0.00)	0.20 (0.20 - 0.20)	0.00 (0.00 - 0.00)	0.00 (0.00 - 0.00)	0.20 (0.20 - 0.20)
10 to 14	0.20 (0.20 - 0.20)	0.20 (0.20 - 0.20)	0.40 (0.20 - 0.40)	0.20 (0.20 - 0.20)	0.00 (0.00 - 0.00)	0.20 (0.20 - 0.20)	0.00 (0.00 - 0.00)	0.00 (0.00 - 0.00)	0.20 (0.20 - 0.20)
15 to 19	0.80 (0.80 - 0.80)	0.40 (0.40 - 0.40)	1.20 (1.20 - 1.20)	0.60 (0.40 - 0.60)	0.20 (0.20 - 0.20)	0.80 (0.80 - 0.80)	0.40 (0.40 - 0.40)	0.20 (0.20 - 0.20)	0.80 (0.80 - 0.80)
20 to 24	1.20 (1.20 - 1.20)	0.40 (0.40 - 0.40)	1.60 (1.60 - 1.60)	0.80 (0.80 - 0.80)	0.20 (0.20 - 0.20)	1.00 (1.00 - 1.00)	0.80 (0.80 - 0.80)	0.20 (0.20 - 0.20)	1.00 (1.00 - 1.00)
25 to 29	1.20 (1.20 - 1.20)	0.40 (0.40 - 0.40)	1.60 (1.60 - 1.60)	0.80 (0.80 - 0.80)	0.20 (0.20 - 0.20)	1.00 (1.00 - 1.00)	0.80 (0.80 - 0.80)	0.40 (0.40 - 0.40)	1.20 (1.20 - 1.20)
30 to 34	1.20 (1.20 - 1.20)	0.40 (0.40 - 0.40)	1.60 (1.60 - 1.60)	1.00 (1.00 - 1.00)	0.40 (0.40 - 0.40)	1.40 (1.40 - 1.40)	0.80 (0.80 - 0.80)	0.40 (0.40 - 0.40)	1.20 (1.20 - 1.20)
35 to 39	1.20 (1.20 - 1.20)	0.60 (0.60 - 0.60)	1.80 (1.80 - 1.80)	1.00 (1.00 - 1.00)	0.60 (0.60 - 0.60)	1.60 (1.60 - 1.60)	1.20 (1.00 - 1.20)	0.60 (0.60 - 0.60)	1.80 (1.80 - 1.80)
40 to 44	1.60 (1.60 - 1.60)	0.80 (0.80 - 0.80)	2.40 (2.40 - 2.40)	1.60 (1.60 - 1.60)	1.00 (1.00 - 1.00)	2.40 (2.40 - 2.40)	1.40 (1.40 - 1.40)	0.80 (0.80 - 0.80)	2.20 (2.20 - 2.20)
45 to 49	2.00 (2.00 - 2.00)	1.20 (1.20 - 1.20)	3.00 (3.00 - 3.00)	2.20 (2.20 - 2.20)	1.40 (1.40 - 1.40)	3.40 (3.40 - 3.60)	2.20 (2.20 - 2.20)	1.40 (1.40 - 1.40)	3.60 (3.60 - 3.80)
50 to 54	2.80 (2.80 - 2.80)	1.60 (1.60 - 1.60)	4.40 (4.40 - 4.40)	2.80 (2.80 - 2.80)	1.80 (1.80 - 1.80)	4.60 (4.60 - 4.60)	3.00 (3.00 - 3.00)	2.00 (2.00 - 2.00)	5.00 (5.00 - 5.20)
55 to 59	4.20 (4.20 - 4.20)	2.20 (2.20 - 2.20)	6.40 (6.40 - 6.40)	4.20 (4.20 - 4.20)	2.60 (2.60 - 2.60)	6.60 (6.60 - 6.80)	4.00 (4.00 - 4.20)	2.60 (2.60 - 2.60)	6.80 (6.60 - 6.80)
60 to 64	7.00 (7.00 - 7.00)	3.80 (3.60 - 3.80)	10.80 (10.80 - 10.80)	5.20 (5.20 - 5.20)	3.20 (3.20 - 3.20)	8.40 (8.20 - 8.40)	7.00 (6.80 - 7.00)	4.40 (4.20 - 4.40)	11.20 (11.20 - 11.40)
65 to 69	9.80 (9.80 - 10.00)	5.60 (5.60 - 5.60)	15.60 (15.40 - 15.60)	6.80 (6.80 - 6.80)	4.00 (4.00 - 4.20)	10.80 (10.80 - 11.00)	8.00 (7.80 - 8.20)	4.80 (4.80 - 5.00)	12.80 (12.80 - 13.00)
70 to 74	11.00 (11.00 - 11.00)	7.60 (7.40 - 7.60)	18.60 (18.40 - 18.60)	9.00 (9.00 - 9.00)	5.80 (5.80 - 5.80)	14.80 (14.80 - 14.80)	9.80 (9.60 - 9.80)	6.20 (6.20 - 6.40)	16.00 (15.80 - 16.20)
75 to 79	12.80 (12.80 - 12.80)	10.40 (10.40 - 10.60)	23.20 (23.20 - 23.40)	13.00 (12.80 - 13.00)	9.40 (9.40 - 9.40)	22.40 (22.20 - 22.40)	11.80 (11.80 - 12.00)	8.20 (8.00 - 8.40)	20.00 (19.80 - 20.40)
80 plus	20.00 (20.00 - 20.20)	31.00 (30.80 - 31.20)	51.00 (50.80 - 51.20)	33.40 (33.20 - 33.40)	47.60 (47.20 - 47.80)	80.80 (80.60 - 81.20)	49.20 (48.80 - 49.80)	61.00 (60.20 - 61.80)	110.40 (109.60 - 111.20)
**Western Europe**									
Early Neonatal	10.40 (10.20 - 10.60)	7.60 (7.40 - 7.80)	18.00 (17.60 - 18.40)	4.80 (4.60 - 4.80)	3.80 (3.80 - 3.80)	8.60 (8.40 - 8.80)	3.40 (3.20 - 3.60)	2.60 (2.60 - 2.80)	6.00 (5.60 - 6.40)
Late Neonatal	3.00 (2.80 - 3.00)	2.40 (2.20 - 2.40)	5.20 (5.20 - 5.40)	1.80 (1.60 - 1.80)	1.40 (1.40 - 1.40)	3.00 (3.00 - 3.20)	1.20 (1.20 - 1.40)	1.00 (1.00 - 1.20)	2.20 (2.20 - 2.40)
Post Neonatal	7.00 (6.80 - 7.00)	5.20 (5.00 - 5.40)	12.20 (11.80 - 12.40)	3.40 (3.40 - 3.60)	2.80 (2.60 - 2.80)	6.20 (6.00 - 6.40)	2.80 (2.60 - 3.00)	2.20 (2.00 - 2.20)	5.00 (4.60 - 5.20)
1 to 4	4.00 (3.60 - 4.40)	3.00 (2.80 - 3.20)	7.00 (6.40 - 7.60)	2.00 (1.80 - 2.00)	1.60 (1.40 - 1.60)	3.40 (3.20 - 3.80)	1.40 (1.20 - 1.60)	1.20 (1.00 - 1.20)	2.60 (2.40 - 2.80)
5 to 9	2.80 (2.80 - 2.80)	2.00 (2.00 - 2.00)	4.80 (4.60 - 4.80)	1.40 (1.20 - 1.40)	1.00 (1.00 - 1.00)	2.20 (2.20 - 2.40)	1.00 (1.00 - 1.00)	0.80 (0.80 - 0.80)	1.80 (1.60 - 1.80)
10 to 14	3.00 (3.00 - 3.20)	2.00 (2.00 - 2.00)	5.00 (5.00 - 5.00)	1.60 (1.60 - 1.60)	1.20 (1.20 - 1.20)	2.80 (2.80 - 2.80)	1.20 (1.20 - 1.20)	0.80 (0.80 - 0.80)	2.00 (2.00 - 2.00)
15 to 19	11.40 (11.40 - 11.40)	4.20 (4.20 - 4.20)	15.60 (15.40 - 15.60)	6.20 (6.20 - 6.20)	2.60 (2.40 - 2.60)	8.60 (8.60 - 8.60)	4.00 (4.00 - 4.20)	1.80 (1.80 - 1.80)	5.80 (5.80 - 6.00)
20 to 24	18.40 (18.40 - 18.40)	5.60 (5.60 - 5.60)	24.00 (24.00 - 24.20)	9.80 (9.80 - 9.80)	3.20 (3.20 - 3.20)	13.00 (13.00 - 13.00)	7.40 (7.40 - 7.60)	2.60 (2.60 - 2.60)	10.00 (10.00 - 10.20)
25 to 29	19.80 (19.80 - 20.00)	6.80 (6.80 - 6.80)	26.60 (26.60 - 26.80)	10.60 (10.60 - 10.60)	3.80 (3.80 - 3.80)	14.40 (14.40 - 14.40)	8.40 (8.40 - 8.60)	3.20 (3.20 - 3.20)	11.60 (11.60 - 11.80)
30 to 34	21.00 (20.80 - 21.00)	8.40 (8.40 - 8.60)	29.40 (29.40 - 29.40)	13.60 (13.60 - 13.60)	5.80 (5.80 - 5.80)	19.40 (19.40 - 19.40)	10.40 (10.20 - 10.40)	4.60 (4.40 - 4.60)	14.80 (14.80 - 15.00)
35 to 39	24.60 (24.40 - 24.60)	11.80 (11.60 - 11.80)	36.20 (36.20 - 36.40)	20.40 (20.20 - 20.40)	10.00 (10.00 - 10.00)	30.40 (30.20 - 30.40)	14.40 (14.20 - 14.40)	7.20 (7.20 - 7.40)	21.60 (21.60 - 21.80)
40 to 44	34.60 (34.60 - 34.80)	17.60 (17.60 - 17.60)	52.40 (52.20 - 52.40)	31.40 (31.40 - 31.40)	16.60 (16.60 - 16.60)	48.00 (48.00 - 48.20)	25.40 (25.20 - 25.60)	13.80 (13.80 - 14.00)	39.20 (39.00 - 39.60)
45 to 49	47.20 (47.20 - 47.40)	24.40 (24.20 - 24.40)	71.60 (71.40 - 71.60)	46.40 (46.20 - 46.40)	25.20 (25.00 - 25.20)	71.40 (71.40 - 71.60)	45.00 (44.60 - 45.40)	25.60 (25.40 - 25.80)	70.60 (70.20 - 71.00)
50 to 54	75.40 (75.40 - 75.60)	37.20 (37.20 - 37.40)	112.60 (112.60 - 112.80)	67.20 (67.00 - 67.20)	35.40 (35.40 - 35.60)	102.60 (102.40 - 102.80)	65.00 (64.40 - 65.60)	36.20 (35.80 - 36.40)	101.20 (100.60 - 101.80)

55 to 59	113.20 (113.00 - 113.40)	54.60 (54.60 - 54.80)	167.80 (167.60 - 168.00)	96.80 (96.80 - 97.00)	50.40 (50.40 - 50.60)	147.40 (147.20 - 147.60)	86.60 (85.80 - 87.20)	47.00 (46.60 - 47.40)	133.60 (132.80 - 134.40)
60 to 64	167.60 (167.40 - 168.00)	86.80 (86.60 - 87.00)	254.60 (254.20 - 254.80)	123.40 (123.20 - 123.60)	64.60 (64.40 - 64.80)	188.00 (187.80 - 188.20)	146.00 (145.00 - 147.00)	78.40 (77.60 - 79.00)	224.40 (223.20 - 225.60)
65 to 69	224.00 (223.80 - 224.40)	140.20 (140.00 - 140.60)	364.40 (363.80 - 364.80)	178.40 (178.00 - 178.60)	98.40 (98.20 - 98.60)	276.60 (276.20 - 277.00)	155.00 (154.00 - 156.00)	86.20 (85.60 - 86.80)	241.20 (240.00 - 242.40)
70 to 74	219.20 (218.80 - 219.60)	165.80 (165.40 - 166.20)	385.00 (384.60 - 385.60)	236.80 (236.40 - 237.20)	150.20 (149.80 - 150.60)	387.20 (386.60 - 387.60)	237.40 (235.60 - 239.00)	145.00 (143.80 - 146.20)	382.40 (380.40 - 384.40)
75 to 79	319.60 (319.00 - 320.20)	310.80 (310.00 - 311.40)	630.20 (629.40 - 631.20)	304.20 (303.80 - 304.60)	248.40 (247.80 - 249.00)	552.60 (551.80 - 553.20)	297.60 (295.60 - 299.40)	217.00 (215.20 - 218.60)	514.60 (512.00 - 517.00)
80 plus	594.00 (593.40 - 594.80)	1,033.80 (1,032.20 - 1,035.60)	1,627.80 (1,626.20 - 1,629.60)	710.00 (709.00 - 710.60)	1,205.20 (1,203.20 - 1,207.20)	1,915.20 (1,913.20 - 1,917.20)	970.60 (966.20 - 975.20)	1,456.80 (1,448.60 - 1,464.60)	2,427.60 (2,418.20 - 2,436.60)
**Southern Latin America**									
Early Neonatal	6.60 (6.20 - 6.80)	4.80 (4.60 - 5.00)	11.40 (10.80 - 11.80)	3.60 (3.40 - 4.00)	2.80 (2.60 - 3.00)	6.40 (6.00 - 6.80)	2.60 (2.40 - 2.80)	2.00 (1.80 - 2.20)	4.60 (4.20 - 5.00)
Late Neonatal	1.60 (1.60 - 1.80)	1.40 (1.20 - 1.40)	3.00 (2.80 - 3.20)	1.20 (1.00 - 1.20)	0.80 (0.80 - 1.00)	2.00 (2.00 - 2.20)	1.00 (0.80 - 1.00)	0.80 (0.60 - 0.80)	1.60 (1.60 - 1.80)
Post Neonatal	5.00 (4.60 - 5.00)	4.00 (3.60 - 4.40)	9.00 (8.40 - 9.40)	2.60 (2.40 - 2.80)	2.00 (1.80 - 2.40)	4.60 (4.40 - 5.00)	2.00 (2.00 - 2.20)	1.60 (1.60 - 1.80)	3.80 (3.40 - 4.00)
1 to 4	2.00 (1.40 - 2.40)	1.60 (1.20 - 2.00)	3.60 (2.60 - 4.40)	1.20 (0.80 - 1.60)	1.00 (0.80 - 1.20)	2.20 (1.60 - 2.80)	1.00 (0.60 - 1.20)	0.80 (0.60 - 1.00)	1.60 (1.20 - 2.20)
5 to 9	1.00 (1.00 - 1.00)	0.60 (0.60 - 0.60)	1.60 (1.60 - 1.60)	0.60 (0.60 - 0.60)	0.60 (0.40 - 0.60)	1.20 (1.20 - 1.20)	0.60 (0.60 - 0.60)	0.40 (0.40 - 0.40)	1.00 (1.00 - 1.00)
10 to 14	1.00 (1.00 - 1.00)	0.60 (0.60 - 0.60)	1.60 (1.60 - 1.60)	0.80 (0.80 - 0.80)	0.60 (0.60 - 0.60)	1.40 (1.40 - 1.40)	0.60 (0.60 - 0.80)	0.40 (0.40 - 0.60)	1.20 (1.20 - 1.20)
15 to 19	2.00 (2.00 - 2.20)	1.00 (1.00 - 1.00)	3.00 (3.00 - 3.20)	2.20 (2.20 - 2.20)	1.00 (1.00 - 1.00)	3.20 (3.20 - 3.20)	2.00 (2.00 - 2.20)	1.00 (1.00 - 1.00)	3.00 (3.00 - 3.00)
20 to 24	2.80 (2.80 - 2.80)	1.20 (1.20 - 1.20)	4.00 (4.00 - 4.00)	3.00 (3.00 - 3.00)	1.00 (1.00 - 1.00)	4.00 (4.00 - 4.20)	3.00 (3.00 - 3.20)	1.00 (1.00 - 1.00)	4.20 (4.00 - 4.20)
25 to 29	3.00 (3.00 - 3.00)	1.40 (1.40 - 1.40)	4.20 (4.20 - 4.20)	3.20 (3.20 - 3.20)	1.20 (1.20 - 1.20)	4.40 (4.40 - 4.40)	3.20 (3.20 - 3.20)	1.20 (1.20 - 1.20)	4.40 (4.20 - 4.40)
30 to 34	3.20 (3.20 - 3.20)	1.60 (1.60 - 1.80)	5.00 (5.00 - 5.00)	3.20 (3.20 - 3.20)	1.40 (1.40 - 1.40)	4.80 (4.80 - 4.80)	3.40 (3.40 - 3.40)	1.60 (1.40 - 1.60)	4.80 (4.80 - 5.00)
35 to 39	4.00 (4.00 - 4.00)	2.40 (2.20 - 2.40)	6.40 (6.20 - 6.40)	3.80 (3.80 - 3.80)	2.00 (2.00 - 2.00)	5.60 (5.60 - 5.80)	4.20 (4.00 - 4.40)	2.20 (2.00 - 2.20)	6.40 (6.20 - 6.60)
40 to 44	5.60 (5.40 - 5.60)	3.00 (3.00 - 3.00)	8.60 (8.60 - 8.60)	5.00 (5.00 - 5.00)	2.80 (2.80 - 2.80)	7.80 (7.80 - 7.80)	5.20 (5.00 - 5.40)	2.80 (2.80 - 3.00)	8.00 (7.80 - 8.20)
45 to 49	7.60 (7.60 - 7.60)	4.00 (4.00 - 4.00)	11.60 (11.60 - 11.60)	7.20 (7.20 - 7.20)	4.20 (4.20 - 4.20)	11.40 (11.40 - 11.40)	7.40 (7.20 - 7.60)	4.20 (4.20 - 4.40)	11.80 (11.40 - 12.00)
50 to 54	10.40 (10.40 - 10.60)	5.20 (5.20 - 5.40)	15.80 (15.60 - 15.80)	10.40 (10.40 - 10.60)	6.00 (6.00 - 6.00)	16.40 (16.40 - 16.60)	11.00 (10.60 - 11.40)	6.20 (6.00 - 6.40)	17.20 (16.80 - 17.60)
55 to 59	14.80 (14.60 - 14.80)	7.40 (7.40 - 7.40)	22.20 (22.00 - 22.20)	14.20 (14.20 - 14.40)	7.80 (7.80 - 7.80)	22.00 (22.00 - 22.20)	15.60 (15.20 - 16.00)	8.80 (8.40 - 9.00)	24.40 (23.80 - 24.80)
60 to 64	19.60 (19.60 - 19.80)	10.40 (10.40 - 10.60)	30.20 (30.00 - 30.20)	18.20 (18.20 - 18.40)	10.20 (10.20 - 10.20)	28.40 (28.40 - 28.60)	21.20 (20.60 - 21.80)	12.20 (11.80 - 12.40)	33.20 (32.60 - 34.00)
65 to 69	22.60 (22.40 - 22.60)	13.40 (13.40 - 13.40)	36.00 (35.80 - 36.00)	21.80 (21.80 - 22.00)	13.00 (13.00 - 13.20)	34.80 (34.80 - 35.00)	25.80 (25.00 - 26.40)	15.40 (14.80 - 15.80)	41.00 (40.20 - 41.80)
70 to 74	24.00 (24.00 - 24.20)	17.40 (17.20 - 17.60)	41.40 (41.40 - 41.60)	27.40 (27.20 - 27.40)	18.60 (18.40 - 18.60)	45.80 (45.60 - 46.00)	29.20 (28.60 - 30.00)	19.60 (19.00 - 20.20)	48.80 (48.00 - 49.80)
75 to 79	25.20 (25.00 - 25.20)	23.20 (23.00 - 23.40)	48.40 (48.20 - 48.60)	30.40 (30.20 - 30.60)	26.20 (26.00 - 26.40)	56.60 (56.40 - 56.80)	31.00 (30.40 - 31.80)	25.80 (25.00 - 26.60)	56.80 (55.80 - 57.80)
80 plus	40.60 (40.60 - 40.80)	56.60 (56.40 - 56.80)	97.20 (97.00 - 97.60)	59.40 (59.20 - 59.60)	96.60 (96.20 - 97.00)	156.00 (155.60 - 156.40)	81.40 (80.20 - 82.60)	140.40 (138.00 - 143.20)	222.00 (219.00 - 224.80)
**High-income North America**									
Early Neonatal	12.40 (12.20 - 12.80)	9.60 (9.40 - 9.60)	22.00 (21.60 - 22.40)	9.00 (8.80 - 9.20)	7.00 (7.00 - 7.20)	16.00 (15.60 - 16.20)	6.40 (6.20 - 6.60)	5.20 (5.00 - 5.20)	11.60 (11.20 - 11.80)
Late Neonatal	2.60 (2.40 - 2.60)	2.00 (2.00 - 2.00)	4.60 (4.40 - 4.60)	2.00 (2.00 - 2.20)	1.60 (1.60 - 1.60)	3.80 (3.60 - 3.80)	1.60 (1.60 - 1.60)	1.40 (1.20 - 1.40)	3.00 (2.80 - 3.00)
Post Neonatal	8.00 (8.00 - 8.20)	6.00 (5.80 - 6.20)	14.20 (13.80 - 14.40)	5.60 (5.40 - 5.60)	4.20 (4.20 - 4.40)	9.80 (9.60 - 10.00)	4.20 (4.00 - 4.20)	3.20 (3.20 - 3.40)	7.40 (7.20 - 7.60)
1 to 4	4.40 (4.00 - 4.60)	3.20 (3.00 - 3.60)	7.60 (7.00 - 8.20)	3.20 (3.00 - 3.40)	2.40 (2.20 - 2.80)	5.60 (5.20 - 6.20)	2.40 (2.20 - 2.60)	1.80 (1.80 - 2.00)	4.20 (4.00 - 4.60)
5 to 9	2.60 (2.60 - 2.60)	1.80 (1.80 - 1.80)	4.40 (4.40 - 4.40)	1.60 (1.60 - 2.00)	1.40 (1.20 - 1.60)	3.00 (3.00 - 3.40)	1.40 (1.40 - 1.40)	1.00 (1.00 - 1.00)	2.40 (2.40 - 2.40)
10 to 14	3.20 (3.20 - 3.20)	1.80 (1.80 - 1.80)	5.00 (5.00 - 5.00)	2.40 (2.40 - 3.00)	1.60 (1.60 - 2.00)	4.20 (4.00 - 4.60)	1.80 (1.60 - 1.80)	1.20 (1.20 - 1.20)	3.00 (2.80 - 3.00)
15 to 19	12.20 (12.20 - 12.20)	4.40 (4.40 - 4.40)	16.40 (16.40 - 16.60)	10.60 (10.40 - 11.20)	4.20 (4.20 - 4.40)	14.80 (14.60 - 15.40)	7.80 (7.80 - 7.80)	3.20 (3.20 - 3.20)	11.00 (10.80 - 11.00)
20 to 24	17.40 (17.20 - 17.40)	5.20 (5.20 - 5.20)	22.60 (22.60 - 22.60)	16.60 (16.40 - 17.40)	5.20 (5.20 - 5.60)	22.00 (21.60 - 22.60)	15.00 (14.80 - 15.20)	5.00 (5.00 - 5.20)	20.00 (20.00 - 20.20)
25 to 29	20.80 (20.80 - 21.00)	7.20 (7.20 - 7.20)	28.20 (28.00 - 28.20)	15.20 (15.00 - 15.80)	5.80 (5.80 - 6.00)	21.00 (20.80 - 21.60)	16.00 (15.80 - 16.20)	6.20 (6.20 - 6.20)	22.20 (22.00 - 22.40)
30 to 34	26.00 (25.80 - 26.00)	10.00 (10.00 - 10.00)	36.00 (35.80 - 36.00)	16.20 (16.00 - 16.80)	7.80 (7.60 - 8.00)	23.80 (23.60 - 24.40)	16.60 (16.60 - 16.80)	8.00 (8.00 - 8.20)	24.80 (24.60 - 25.00)
35 to 39	29.40 (29.20 - 29.40)	12.40 (12.40 - 12.60)	41.80 (41.80 - 41.80)	21.80 (21.60 - 22.40)	12.20 (12.20 - 12.60)	34.00 (33.80 - 34.60)	17.80 (17.60 - 18.00)	10.40 (10.20 - 10.40)	28.20 (28.00 - 28.40)
40 to 44	32.60 (32.40 - 32.60)	16.20 (16.20 - 16.20)	48.80 (48.60 - 48.80)	35.40 (35.20 - 36.00)	21.40 (21.20 - 21.60)	56.80 (56.60 - 57.40)	26.20 (26.00 - 26.60)	16.80 (16.60 - 17.00)	43.00 (42.80 - 43.40)
45 to 49	36.20 (36.00 - 36.20)	20.40 (20.40 - 20.40)	56.40 (56.40 - 56.60)	52.80 (52.60 - 53.60)	32.20 (32.00 - 32.60)	85.00 (84.60 - 85.80)	45.60 (45.20 - 46.00)	29.40 (29.20 - 29.80)	75.00 (74.60 - 75.60)
50 to 54	45.60 (45.40 - 45.60)	27.40 (27.20 - 27.40)	73.00 (72.80 - 73.00)	69.60 (69.40 - 70.40)	42.00 (41.80 - 42.40)	111.60 (111.40 - 112.40)	77.20 (76.40 - 77.80)	48.80 (48.40 - 49.20)	126.00 (125.00 - 126.80)
55 to 59	66.40 (66.20 - 66.60)	40.60 (40.40 - 40.60)	107.00 (106.80 - 107.00)	85.40 (85.20 - 86.00)	54.40 (54.20 - 54.80)	139.80 (139.40 - 140.40)	101.60 (100.60 - 102.40)	64.00 (63.40 - 64.60)	165.60 (164.40 - 166.60)
60 to 64	103.00 (102.80 - 103.20)	65.40 (65.20 - 65.40)	168.40 (168.20 - 168.60)	95.60 (95.40 - 96.00)	65.60 (65.40 - 65.80)	161.00 (160.80 - 161.60)	138.00 (137.00 - 139.20)	90.80 (90.00 - 91.60)	229.00 (227.60 - 230.20)
65 to 69	140.80 (140.60 - 141.20)	96.40 (96.20 - 96.60)	237.20 (237.00 - 237.60)	109.40 (109.00 - 109.80)	80.40 (80.20 - 80.80)	189.80 (189.40 - 190.20)	139.40 (138.20 - 140.40)	100.40 (99.60 - 101.20)	239.80 (238.40 - 241.20)
70 to 74	163.00 (162.60 - 163.20)	123.00 (122.80 - 123.20)	286.00 (285.60 - 286.40)	137.20 (137.00 - 137.60)	110.60 (110.20 - 111.00)	247.80 (247.40 - 248.40)	144.40 (143.40 - 145.60)	112.40 (111.40 - 113.40)	256.80 (255.40 - 258.40)

75 to 79	173.80 (173.60 - 174.20)	156.00 (155.60 - 156.20)	329.80 (329.40 - 330.20)	176.40 (176.00 - 176.80)	162.00 (161.40 - 162.40)	338.40 (337.80 - 339.00)	158.60 (157.40 - 159.60)	137.20 (136.00 - 138.40)	295.60 (294.00 - 297.40)
80 plus	320.60 (320.20 - 320.80)	519.40 (519.00 - 519.80)	840.00 (839.40 - 840.40)	461.20 (460.80 - 462.00)	728.40 (727.60 - 729.60)	1,189.80 (1,188.80 - 1,191.00)	583.20 (581.40 - 585.20)	830.60 (827.40 - 833.80)	1,414.00 (1,410.20 - 1,417.60)
**Caribbean**									
Early Neonatal	9.00 (8.40 - 9.80)	6.40 (6.00 - 6.80)	15.40 (14.60 - 16.40)	6.60 (6.00 - 7.00)	4.60 (4.40 - 4.80)	11.20 (10.40 - 11.80)	5.20 (4.40 - 5.80)	3.60 (3.20 - 4.00)	8.80 (7.60 - 9.80)
Late Neonatal	3.80 (3.60 - 4.00)	3.40 (3.20 - 3.60)	7.00 (6.80 - 7.40)	2.20 (2.20 - 2.40)	2.00 (1.80 - 2.20)	4.40 (4.00 - 4.60)	1.60 (1.40 - 2.00)	1.40 (1.20 - 1.80)	3.00 (2.60 - 3.80)
Post Neonatal	11.80 (10.60 - 12.80)	10.20 (9.00 - 11.40)	21.80 (19.60 - 24.20)	7.00 (6.20 - 7.60)	6.00 (5.20 - 6.80)	13.00 (11.60 - 14.40)	4.40 (3.40 - 5.80)	3.80 (2.80 - 5.00)	8.20 (6.20 - 10.60)
1 to 4	9.20 (7.40 - 11.20)	8.60 (7.20 - 10.20)	17.80 (14.60 - 21.20)	4.60 (3.60 - 5.60)	4.00 (3.20 - 5.00)	8.60 (7.00 - 10.60)	2.60 (2.00 - 3.80)	2.20 (1.60 - 3.20)	5.00 (3.60 - 6.80)
5 to 9	2.20 (2.20 - 2.40)	1.80 (1.80 - 2.00)	4.00 (4.00 - 4.20)	1.80 (1.60 - 1.80)	1.40 (1.40 - 1.60)	3.20 (3.00 - 3.40)	1.20 (1.00 - 1.40)	1.00 (0.80 - 1.20)	2.20 (1.80 - 2.60)
10 to 14	1.60 (1.60 - 1.60)	1.20 (1.20 - 1.40)	2.80 (2.80 - 3.00)	1.60 (1.40 - 1.60)	1.20 (1.00 - 1.20)	2.60 (2.60 - 2.80)	1.20 (1.20 - 1.40)	1.00 (0.80 - 1.00)	2.20 (2.00 - 2.40)
15 to 19	2.20 (2.20 - 2.40)	1.60 (1.60 - 1.60)	3.80 (3.80 - 4.00)	2.20 (2.00 - 2.40)	1.40 (1.40 - 1.60)	3.60 (3.40 - 3.80)	2.20 (1.80 - 2.40)	1.20 (1.00 - 1.40)	3.40 (3.00 - 3.60)
20 to 24	3.40 (3.20 - 3.60)	2.20 (2.20 - 2.40)	5.80 (5.40 - 6.00)	3.20 (3.00 - 3.60)	2.20 (2.00 - 2.80)	5.40 (5.00 - 6.20)	3.20 (2.80 - 3.60)	1.80 (1.40 - 2.00)	4.80 (4.40 - 5.40)
25 to 29	4.00 (3.80 - 4.20)	2.60 (2.40 - 3.00)	6.60 (6.20 - 7.00)	3.80 (3.40 - 4.20)	3.00 (2.40 - 3.80)	6.80 (5.80 - 7.80)	3.80 (3.20 - 4.40)	2.40 (2.00 - 2.80)	6.00 (5.40 - 6.80)
30 to 34	4.00 (3.80 - 4.20)	2.60 (2.40 - 3.00)	6.60 (6.20 - 7.00)	4.60 (4.00 - 5.00)	3.60 (3.20 - 4.20)	8.20 (7.40 - 9.00)	4.20 (3.80 - 5.00)	2.80 (2.40 - 3.40)	7.00 (6.40 - 7.80)
35 to 39	4.20 (4.00 - 4.40)	3.00 (2.80 - 3.20)	7.00 (6.80 - 7.40)	5.40 (5.00 - 6.00)	4.20 (3.80 - 4.80)	9.80 (9.00 - 10.40)	4.40 (4.00 - 5.00)	3.00 (2.60 - 3.80)	7.60 (6.80 - 8.40)
40 to 44	4.20 (4.00 - 4.40)	3.20 (3.00 - 3.40)	7.40 (7.00 - 7.60)	6.40 (5.80 - 6.80)	4.60 (4.20 - 5.20)	11.00 (10.40 - 11.60)	5.40 (5.00 - 6.00)	3.80 (3.20 - 4.40)	9.20 (8.60 - 10.00)
45 to 49	5.00 (4.80 - 5.20)	3.80 (3.60 - 4.00)	8.80 (8.40 - 9.20)	6.60 (6.20 - 7.20)	5.00 (4.40 - 5.40)	11.60 (10.80 - 12.40)	7.20 (6.60 - 8.00)	5.00 (4.40 - 5.80)	12.20 (11.40 - 13.20)
50 to 54	5.80 (5.60 - 6.00)	5.00 (4.60 - 5.20)	10.80 (10.40 - 11.20)	7.60 (7.20 - 8.20)	5.80 (5.20 - 6.60)	13.40 (12.60 - 14.40)	9.60 (9.00 - 10.40)	7.00 (6.20 - 7.80)	16.60 (15.40 - 17.80)
55 to 59	6.80 (6.60 - 7.00)	5.80 (5.40 - 6.20)	12.60 (12.20 - 13.20)	8.60 (8.20 - 9.00)	6.80 (6.20 - 7.40)	15.40 (14.60 - 16.20)	10.80 (10.00 - 11.60)	8.20 (7.20 - 9.20)	19.00 (17.80 - 20.20)
60 to 64	8.80 (8.40 - 9.00)	7.60 (7.20 - 8.00)	16.40 (16.00 - 16.80)	10.60 (10.20 - 11.00)	8.60 (7.80 - 9.40)	19.20 (18.40 - 20.20)	13.80 (13.00 - 14.60)	10.80 (9.60 - 11.80)	24.60 (23.20 - 25.80)
65 to 69	10.40 (10.20 - 10.80)	9.40 (9.00 - 9.80)	19.80 (19.40 - 20.40)	12.60 (12.00 - 13.00)	10.80 (9.80 - 11.80)	23.40 (22.20 - 24.40)	15.20 (14.40 - 16.00)	12.20 (11.00 - 13.40)	27.40 (26.00 - 28.60)
70 to 74	12.60 (12.20 - 12.80)	11.40 (11.00 - 11.80)	24.00 (23.40 - 24.40)	14.40 (13.80 - 14.80)	13.00 (12.00 - 13.80)	27.40 (26.40 - 28.40)	17.60 (16.80 - 18.40)	15.00 (13.60 - 16.20)	32.60 (31.00 - 34.00)
75 to 79	13.40 (13.20 - 13.60)	12.80 (12.40 - 13.00)	26.20 (25.80 - 26.60)	15.80 (15.20 - 16.00)	14.80 (14.00 - 15.40)	30.60 (29.60 - 31.40)	19.80 (19.00 - 20.60)	18.00 (16.80 - 19.40)	37.80 (36.40 - 39.20)
80 plus	27.40 (27.20 - 27.60)	30.40 (30.00 - 30.60)	57.80 (57.40 - 58.20)	42.00 (41.60 - 42.40)	51.20 (50.00 - 52.00)	93.20 (92.00 - 94.20)	56.20 (55.20 - 57.20)	70.80 (69.00 - 72.80)	127.20 (125.00 - 129.20)
**Andean Latin America**									
Early Neonatal	11.00 (10.20 - 12.00)	8.60 (8.00 - 9.00)	19.60 (18.20 - 21.00)	6.60 (6.20 - 7.20)	5.20 (4.80 - 5.40)	11.80 (11.20 - 12.60)	4.40 (3.80 - 5.00)	3.40 (3.00 - 3.80)	7.80 (6.80 - 8.80)
Late Neonatal	5.60 (5.40 - 5.80)	4.80 (4.60 - 5.00)	10.40 (10.00 - 10.80)	2.40 (2.40 - 2.60)	2.00 (2.00 - 2.20)	4.40 (4.20 - 4.60)	1.60 (1.60 - 1.80)	1.40 (1.20 - 1.60)	3.00 (2.80 - 3.40)
Post Neonatal	19.20 (17.80 - 20.60)	16.60 (14.80 - 18.40)	35.80 (32.80 - 39.00)	7.00 (6.40 - 7.40)	6.00 (5.40 - 6.60)	12.80 (11.80 - 14.00)	3.80 (3.40 - 4.40)	3.20 (2.80 - 3.80)	7.20 (6.20 - 8.00)
1 to 4	13.40 (11.00 - 16.00)	13.20 (11.20 - 15.20)	26.60 (22.20 - 31.20)	4.40 (3.60 - 5.20)	3.80 (3.20 - 4.40)	8.20 (6.80 - 9.60)	2.80 (2.40 - 3.40)	2.20 (1.80 - 2.60)	5.00 (4.20 - 6.00)
5 to 9	3.40 (3.20 - 3.40)	2.60 (2.60 - 2.80)	6.00 (5.80 - 6.20)	1.80 (1.60 - 1.80)	1.20 (1.20 - 1.40)	3.00 (3.00 - 3.20)	1.20 (1.00 - 1.20)	0.80 (0.80 - 1.00)	2.00 (1.80 - 2.20)
10 to 14	2.40 (2.40 - 2.60)	1.80 (1.80 - 1.80)	4.20 (4.20 - 4.40)	1.60 (1.60 - 1.80)	1.20 (1.20 - 1.20)	2.80 (2.80 - 3.00)	1.20 (1.20 - 1.40)	0.80 (0.80 - 0.80)	2.00 (2.00 - 2.20)
15 to 19	2.80 (2.80 - 3.00)	1.80 (1.80 - 2.00)	4.60 (4.60 - 4.80)	2.20 (2.20 - 2.40)	1.40 (1.20 - 1.40)	3.60 (3.40 - 3.80)	2.00 (1.80 - 2.20)	1.00 (1.00 - 1.20)	3.00 (2.80 - 3.40)
20 to 24	3.80 (3.60 - 4.00)	2.20 (2.00 - 2.20)	6.00 (5.80 - 6.20)	3.20 (3.00 - 3.40)	1.60 (1.40 - 1.60)	4.60 (4.40 - 4.80)	3.00 (2.80 - 3.40)	1.20 (1.20 - 1.40)	4.20 (4.00 - 4.60)
25 to 29	3.80 (3.60 - 4.00)	2.20 (2.00 - 2.20)	5.80 (5.60 - 6.00)	3.40 (3.20 - 3.60)	1.60 (1.60 - 1.80)	5.00 (4.80 - 5.40)	3.40 (3.00 - 3.80)	1.40 (1.20 - 1.60)	4.80 (4.40 - 5.20)
30 to 34	3.60 (3.40 - 3.80)	2.20 (2.20 - 2.40)	5.80 (5.60 - 6.00)	3.60 (3.40 - 3.80)	1.80 (1.80 - 2.00)	5.40 (5.20 - 5.60)	3.60 (3.20 - 4.00)	1.60 (1.40 - 1.80)	5.20 (4.80 - 5.60)
35 to 39	3.80 (3.60 - 4.00)	2.60 (2.40 - 2.80)	6.40 (6.20 - 6.60)	3.80 (3.60 - 4.00)	2.20 (2.20 - 2.40)	6.00 (5.80 - 6.40)	4.00 (3.60 - 4.40)	2.00 (1.80 - 2.40)	6.00 (5.60 - 6.40)
40 to 44	3.80 (3.60 - 4.00)	2.60 (2.60 - 2.80)	6.40 (6.20 - 6.80)	4.20 (4.00 - 4.40)	2.80 (2.60 - 2.80)	6.80 (6.60 - 7.20)	4.40 (4.00 - 5.00)	2.60 (2.40 - 3.00)	7.00 (6.60 - 7.60)
45 to 49	4.20 (4.00 - 4.40)	3.00 (2.80 - 3.20)	7.20 (7.00 - 7.60)	4.80 (4.40 - 5.00)	3.40 (3.20 - 3.60)	8.20 (7.80 - 8.40)	5.20 (4.60 - 6.00)	3.60 (3.20 - 4.00)	8.80 (8.20 - 9.60)
50 to 54	4.80 (4.60 - 5.20)	3.60 (3.40 - 3.80)	8.40 (8.20 - 8.80)	5.60 (5.20 - 6.00)	4.40 (4.00 - 4.60)	10.00 (9.40 - 10.40)	6.60 (5.80 - 7.40)	4.60 (4.20 - 5.20)	11.20 (10.40 - 12.20)
55 to 59	5.60 (5.40 - 6.00)	4.40 (4.20 - 4.60)	10.00 (9.60 - 10.40)	6.20 (5.80 - 6.60)	4.80 (4.60 - 5.20)	11.00 (10.60 - 11.60)	7.80 (7.00 - 8.80)	6.00 (5.40 - 6.80)	13.80 (12.80 - 15.20)
60 to 64	6.80 (6.60 - 7.20)	5.60 (5.40 - 6.00)	12.60 (12.20 - 13.00)	7.80 (7.40 - 8.20)	6.40 (5.80 - 6.80)	14.20 (13.60 - 14.80)	10.20 (9.20 - 11.20)	8.00 (7.20 - 9.00)	18.20 (17.00 - 19.60)
65 to 69	7.60 (7.20 - 7.80)	6.60 (6.20 - 7.00)	14.20 (13.60 - 14.80)	9.20 (8.80 - 9.80)	7.60 (7.20 - 8.20)	17.00 (16.20 - 17.60)	11.40 (10.40 - 12.80)	9.20 (8.20 - 10.40)	20.80 (19.20 - 22.40)
70 to 74	8.60 (8.20 - 8.80)	8.00 (7.60 - 8.60)	16.60 (16.20 - 17.20)	11.00 (10.60 - 11.60)	9.80 (9.20 - 10.40)	20.80 (20.00 - 21.60)	13.80 (12.60 - 15.20)	11.80 (10.40 - 13.20)	25.60 (23.80 - 27.60)
75 to 79	8.80 (8.40 - 9.00)	8.80 (8.40 - 9.40)	17.60 (17.00 - 18.20)	11.80 (11.20 - 12.40)	12.20 (11.60 - 13.00)	24.00 (23.20 - 24.80)	15.20 (13.80 - 16.60)	14.20 (12.60 - 15.80)	29.20 (27.40 - 31.40)
80 plus	13.00 (12.60 - 13.20)	15.60 (15.20 - 16.20)	28.60 (28.00 - 29.20)	27.80 (27.20 - 28.60)	32.40 (31.20 - 33.40)	60.20 (59.00 - 61.40)	41.20 (39.00 - 43.40)	53.40 (49.60 - 57.00)	94.40 (90.20 - 99.20)
**Central Latin America**									
Early Neonatal	36.20 (35.20 - 37.20)	26.40 (25.80 - 27.00)	62.60 (61.00 - 64.20)	23.60 (22.60 - 24.60)	17.20 (16.60 - 17.80)	40.80 (39.20 - 42.40)	16.00 (14.80 - 17.40)	11.80 (11.00 - 12.80)	28.00 (25.80 - 30.40)

Late Neonatal	13.00 (12.60 - 13.20)	10.40 (10.20 - 10.60)	23.40 (22.80 - 24.00)	7.80 (7.40 - 8.00)	6.20 (6.00 - 6.40)	13.80 (13.40 - 14.40)	6.00 (5.60 - 6.40)	4.60 (4.40 - 5.00)	10.60 (10.20 - 11.20)
Post Neonatal	43.20 (41.80 - 44.80)	35.40 (33.60 - 37.20)	78.60 (75.60 - 81.80)	20.20 (19.20 - 21.20)	16.40 (15.40 - 17.40)	36.60 (34.60 - 38.60)	13.20 (12.40 - 14.20)	10.80 (9.80 - 11.60)	24.00 (22.20 - 26.00)
1 to 4	23.60 (21.60 - 25.60)	22.00 (20.40 - 23.60)	45.40 (42.00 - 49.00)	12.40 (11.20 - 13.40)	10.60 (9.80 - 11.60)	22.80 (21.00 - 25.00)	8.60 (7.80 - 9.40)	7.20 (6.40 - 7.80)	15.60 (14.20 - 17.20)
5 to 9	9.80 (9.60 - 9.80)	7.20 (7.00 - 7.40)	16.80 (16.60 - 17.20)	5.40 (5.40 - 5.60)	4.20 (4.00 - 4.20)	9.60 (9.40 - 10.00)	4.00 (3.80 - 4.20)	3.00 (2.80 - 3.20)	7.00 (6.60 - 7.40)
10 to 14	8.80 (8.60 - 8.80)	5.60 (5.60 - 5.80)	14.40 (14.20 - 14.60)	6.40 (6.40 - 6.60)	4.40 (4.20 - 4.40)	10.80 (10.60 - 11.00)	5.20 (5.00 - 5.40)	3.40 (3.40 - 3.60)	8.80 (8.40 - 9.00)
15 to 19	15.20 (15.00 - 15.40)	5.80 (5.80 - 6.00)	21.20 (21.00 - 21.40)	15.20 (15.00 - 15.40)	5.60 (5.60 - 5.80)	20.80 (20.60 - 21.20)	15.60 (14.80 - 16.20)	5.20 (5.00 - 5.40)	20.80 (20.20 - 21.40)
20 to 24	22.00 (21.80 - 22.20)	6.60 (6.60 - 6.80)	28.60 (28.40 - 29.00)	22.80 (22.60 - 23.20)	6.60 (6.40 - 6.60)	29.40 (29.20 - 29.60)	25.00 (24.00 - 25.80)	6.20 (6.00 - 6.40)	31.20 (30.00 - 32.00)
25 to 29	21.60 (21.40 - 21.80)	6.80 (6.60 - 6.80)	28.40 (28.20 - 28.60)	22.80 (22.60 - 23.00)	6.80 (6.60 - 7.00)	29.60 (29.40 - 30.00)	25.40 (24.40 - 26.20)	6.80 (6.40 - 7.00)	32.20 (31.20 - 33.00)
30 to 34	19.60 (19.40 - 19.60)	7.00 (7.00 - 7.20)	26.60 (26.60 - 26.80)	21.60 (21.40 - 21.80)	7.60 (7.40 - 7.60)	29.00 (28.80 - 29.40)	24.60 (23.80 - 25.40)	7.80 (7.40 - 8.20)	32.60 (31.60 - 33.40)
35 to 39	19.00 (18.80 - 19.00)	8.40 (8.40 - 8.60)	27.40 (27.20 - 27.40)	21.80 (21.60 - 22.00)	9.20 (9.00 - 9.40)	31.00 (30.80 - 31.20)	24.80 (24.00 - 25.40)	9.80 (9.40 - 10.20)	34.60 (33.60 - 35.40)
40 to 44	17.40 (17.20 - 17.40)	9.00 (8.80 - 9.00)	26.40 (26.20 - 26.40)	23.20 (23.00 - 23.40)	11.80 (11.60 - 12.00)	34.80 (34.60 - 35.20)	26.40 (25.60 - 27.20)	12.80 (12.20 - 13.20)	39.20 (38.20 - 40.00)
45 to 49	18.60 (18.60 - 18.60)	10.60 (10.60 - 10.80)	29.20 (29.20 - 29.40)	25.60 (25.40 - 25.80)	15.20 (15.00 - 15.40)	40.60 (40.40 - 41.00)	31.60 (30.60 - 32.60)	18.00 (17.60 - 18.60)	49.80 (48.60 - 50.80)
50 to 54	20.40 (20.40 - 20.60)	13.20 (13.20 - 13.20)	33.60 (33.60 - 33.80)	29.00 (28.60 - 29.20)	19.00 (18.80 - 19.20)	48.00 (47.60 - 48.20)	38.20 (36.80 - 39.20)	24.40 (23.60 - 25.20)	62.40 (61.00 - 63.80)
55 to 59	24.00 (23.80 - 24.00)	17.00 (16.80 - 17.00)	40.80 (40.80 - 41.00)	31.80 (31.60 - 32.00)	22.80 (22.60 - 23.00)	54.60 (54.20 - 55.00)	43.60 (42.20 - 44.80)	30.60 (29.80 - 31.40)	74.20 (72.60 - 75.60)
60 to 64	28.40 (28.20 - 28.40)	21.40 (21.20 - 21.60)	49.80 (49.60 - 49.80)	36.20 (36.00 - 36.60)	28.20 (27.80 - 28.40)	64.40 (64.00 - 64.80)	50.40 (49.20 - 51.60)	38.60 (37.60 - 39.40)	89.00 (87.40 - 90.40)
65 to 69	30.80 (30.80 - 31.00)	24.60 (24.40 - 24.80)	55.40 (55.20 - 55.60)	42.00 (41.60 - 42.40)	34.00 (33.60 - 34.40)	76.00 (75.40 - 76.60)	55.60 (54.20 - 56.80)	43.80 (42.60 - 45.00)	99.40 (97.60 - 101.20)
70 to 74	31.60 (31.40 - 31.60)	26.60 (26.40 - 26.80)	58.00 (57.80 - 58.20)	48.00 (47.60 - 48.40)	41.80 (41.40 - 42.20)	89.80 (89.20 - 90.40)	61.80 (60.40 - 63.20)	51.40 (50.00 - 52.80)	113.20 (111.20 - 115.20)
75 to 79	33.20 (33.00 - 33.20)	30.20 (30.00 - 30.40)	63.40 (63.20 - 63.60)	50.00 (49.60 - 50.60)	46.80 (46.20 - 47.20)	96.80 (96.20 - 97.60)	65.80 (64.40 - 67.20)	58.40 (56.80 - 60.00)	124.20 (122.00 - 126.40)
80 plus	67.20 (67.20 - 67.40)	79.80 (79.60 - 80.00)	147.00 (146.80 - 147.40)	107.80 (107.40 - 108.60)	135.60 (134.40 - 136.20)	243.40 (242.20 - 244.40)	178.80 (176.40 - 180.80)	233.20 (229.40 - 236.80)	412.00 (407.60 - 416.00)
**Tropical Latin America**									
Early Neonatal	34.60 (33.60 - 35.60)	26.40 (25.60 - 27.00)	61.00 (59.20 - 62.60)	21.00 (20.00 - 22.00)	15.40 (14.80 - 16.00)	36.40 (34.60 - 38.00)	12.60 (11.40 - 14.00)	9.40 (8.60 - 10.40)	22.00 (20.00 - 24.20)
Late Neonatal	11.20 (10.80 - 11.80)	11.60 (11.20 - 12.20)	23.00 (22.20 - 23.80)	5.60 (5.40 - 5.80)	5.60 (5.40 - 5.80)	11.20 (10.60 - 11.80)	3.80 (3.60 - 4.20)	3.80 (3.60 - 4.00)	7.60 (7.20 - 8.20)
Post Neonatal	47.60 (46.00 - 49.40)	39.00 (36.80 - 41.20)	86.60 (83.00 - 90.60)	18.00 (16.80 - 19.00)	13.80 (12.80 - 15.00)	31.80 (29.60 - 33.80)	10.00 (9.40 - 10.80)	7.80 (7.20 - 8.40)	17.80 (16.60 - 19.20)
1 to 4	14.60 (13.00 - 16.60)	11.00 (9.80 - 12.20)	25.60 (22.80 - 28.80)	6.00 (5.40 - 6.80)	4.00 (3.60 - 4.60)	10.20 (9.20 - 11.40)	3.80 (3.40 - 4.20)	2.40 (2.20 - 2.80)	6.20 (5.60 - 7.00)
5 to 9	7.40 (7.20 - 7.60)	5.20 (5.00 - 5.40)	12.60 (12.20 - 13.00)	4.40 (4.20 - 4.60)	3.20 (3.00 - 3.40)	7.60 (7.40 - 8.20)	2.80 (2.60 - 3.00)	2.00 (2.00 - 2.20)	4.80 (4.60 - 5.20)
10 to 14	7.60 (7.40 - 8.00)	4.60 (4.60 - 4.80)	12.40 (12.00 - 12.80)	5.40 (5.20 - 5.80)	3.40 (3.40 - 3.60)	8.80 (8.60 - 9.40)	4.40 (4.20 - 4.60)	2.80 (2.60 - 3.00)	7.20 (6.80 - 7.60)
15 to 19	12.80 (12.40 - 13.00)	4.60 (4.40 - 4.80)	17.40 (17.00 - 17.80)	16.00 (15.40 - 16.40)	4.40 (4.40 - 4.60)	20.40 (20.00 - 20.80)	14.20 (13.60 - 15.00)	3.80 (3.80 - 4.00)	18.20 (17.40 - 18.80)
20 to 24	18.80 (18.20 - 19.40)	5.80 (5.60 - 6.00)	24.40 (24.00 - 25.00)	23.80 (23.20 - 24.60)	5.60 (5.40 - 6.00)	29.60 (28.80 - 30.40)	20.60 (19.60 - 21.60)	4.60 (4.40 - 5.00)	25.20 (24.20 - 26.40)
25 to 29	20.60 (20.00 - 21.20)	6.80 (6.60 - 7.00)	27.40 (26.60 - 28.20)	23.20 (22.40 - 24.20)	6.80 (6.60 - 7.20)	30.20 (29.20 - 31.20)	23.00 (21.80 - 24.20)	6.40 (6.00 - 6.80)	29.20 (27.80 - 30.80)
30 to 34	21.40 (20.80 - 22.00)	8.00 (7.80 - 8.20)	29.40 (28.80 - 30.00)	23.00 (22.20 - 23.80)	8.00 (7.80 - 8.40)	31.00 (30.20 - 32.00)	26.20 (24.80 - 27.80)	8.60 (8.20 - 9.00)	34.80 (33.20 - 36.60)
35 to 39	22.00 (21.20 - 22.80)	9.60 (9.20 - 10.00)	31.60 (30.80 - 32.40)	25.60 (24.80 - 26.40)	10.60 (10.20 - 11.00)	36.20 (35.40 - 37.20)	28.00 (26.60 - 29.80)	11.00 (10.20 - 11.60)	39.00 (37.20 - 40.80)
40 to 44	23.20 (22.40 - 23.80)	11.40 (11.00 - 11.60)	34.40 (33.60 - 35.20)	30.40 (29.40 - 31.40)	14.40 (14.00 - 15.00)	44.80 (43.80 - 46.00)	30.80 (29.20 - 32.60)	14.20 (13.40 - 15.00)	45.00 (43.00 - 47.00)
45 to 49	24.80 (24.00 - 25.60)	13.20 (12.80 - 13.60)	38.20 (37.20 - 39.00)	35.00 (34.00 - 36.20)	18.60 (18.20 - 19.20)	53.80 (52.60 - 55.00)	37.20 (35.00 - 39.40)	19.40 (18.40 - 20.60)	56.60 (54.00 - 59.20)
50 to 54	29.80 (29.00 - 30.80)	17.00 (16.40 - 17.40)	46.80 (45.80 - 47.80)	40.00 (38.80 - 41.20)	22.60 (22.00 - 23.40)	62.80 (61.40 - 64.20)	49.00 (46.00 - 51.80)	27.40 (26.00 - 29.00)	76.40 (73.00 - 79.80)
55 to 59	33.20 (32.20 - 34.20)	20.20 (19.60 - 20.80)	53.40 (52.40 - 54.60)	43.60 (42.40 - 44.80)	26.40 (25.60 - 27.20)	69.80 (68.60 - 71.40)	56.20 (53.20 - 59.60)	33.60 (31.80 - 35.40)	89.80 (86.00 - 93.60)
60 to 64	39.40 (38.20 - 40.40)	26.00 (25.20 - 26.60)	65.20 (64.00 - 66.40)	47.20 (46.00 - 48.40)	31.00 (30.20 - 31.80)	78.20 (76.80 - 79.60)	65.60 (62.20 - 69.00)	42.00 (39.80 - 44.40)	107.40 (103.00 - 111.80)
65 to 69	41.80 (40.80 - 42.80)	29.40 (28.60 - 30.20)	71.20 (69.80 - 72.40)	55.40 (54.00 - 56.80)	39.80 (38.80 - 41.00)	95.20 (93.40 - 97.00)	70.80 (67.20 - 74.40)	49.00 (46.40 - 51.80)	119.80 (115.00 - 124.20)
70 to 74	42.80 (41.80 - 43.80)	34.40 (33.40 - 35.40)	77.20 (75.80 - 78.60)	58.60 (57.20 - 60.00)	46.40 (45.20 - 47.80)	105.00 (103.00 - 107.00)	73.20 (69.80 - 76.80)	55.60 (52.60 - 58.80)	128.80 (123.60 - 133.60)
75 to 79	41.60 (40.60 - 42.40)	38.60 (37.60 - 39.60)	80.20 (78.80 - 81.40)	60.40 (59.20 - 61.80)	55.20 (53.60 - 56.60)	115.60 (113.60 - 117.80)	80.60 (77.00 - 84.60)	69.80 (66.20 - 73.80)	150.40 (144.80 - 155.80)
80 plus	48.40 (47.60 - 49.00)	62.80 (61.80 - 64.00)	111.20 (110.00 - 112.40)	102.60 (101.20 - 104.00)	132.40 (130.20 - 134.60)	234.80 (232.40 - 237.60)	180.00 (175.40 - 185.00)	226.40 (219.60 - 233.20)	406.40 (397.00 - 415.20)
**North Africa and Middle East**									
Early Neonatal	136.20 (130.80 - 141.60)	93.00 (90.20 - 95.80)	229.20 (221.20 - 237.40)	97.20 (93.00 - 101.40)	67.00 (64.40 - 69.40)	164.00 (157.40 - 170.60)	80.40 (74.20 - 86.80)	56.80 (53.20 - 60.60)	137.20 (127.20 - 147.40)
Late Neonatal	58.40 (56.40 - 60.60)	51.20 (49.40 - 53.20)	109.60 (106.00 - 113.40)	33.20 (31.80 - 34.60)	29.60 (28.20 - 31.00)	62.60 (60.00 - 65.40)	26.80 (24.80 - 29.00)	23.80 (21.80 - 25.80)	50.40 (46.80 - 54.80)
Post Neonatal	162.20 (154.60 - 169.40)	159.00 (149.00 - 168.40)	321.20 (304.00 - 337.40)	84.60 (79.40 - 89.80)	82.40 (76.20 - 89.00)	167.00 (155.80 - 178.40)	61.40 (55.80 - 67.60)	60.40 (54.20 - 67.00)	121.80 (110.20 - 134.60)
1 to 4	114.60 (103.60 - 127.60)	117.80 (107.60 - 128.00)	232.20 (211.80 - 255.20)	59.80 (52.60 - 68.00)	56.80 (51.00 - 63.40)	116.60 (103.80 - 131.20)	49.60 (42.60 - 57.20)	45.00 (39.00 - 52.00)	94.40 (81.60 - 109.20)
5 to 9	36.40 (34.60 - 38.20)	28.00 (26.40 - 29.60)	64.60 (61.20 - 67.60)	21.20 (20.40 - 22.20)	16.40 (15.80 - 17.20)	37.80 (36.40 - 39.20)	19.60 (17.60 - 21.80)	15.20 (13.60 - 16.80)	34.80 (31.20 - 38.40)

10 to 14	26.20 (24.20 - 28.20)	18.20 (16.80 - 19.60)	44.40 (41.20 - 47.60)	18.40 (17.80 - 19.20)	13.20 (12.60 - 14.00)	31.80 (30.40 - 33.00)	18.40 (16.20 - 20.40)	13.00 (11.60 - 14.40)	31.20 (28.00 - 34.80)
15 to 19	27.60 (25.20 - 30.60)	16.80 (15.40 - 18.20)	44.40 (41.00 - 48.00)	32.40 (29.80 - 34.80)	17.80 (16.20 - 19.40)	50.20 (47.00 - 53.40)	41.20 (32.40 - 50.40)	17.80 (15.40 - 20.20)	59.00 (48.40 - 70.00)
20 to 24	32.40 (29.40 - 35.80)	18.60 (17.00 - 20.40)	51.00 (47.20 - 55.40)	43.20 (39.60 - 47.20)	21.20 (19.20 - 23.40)	64.60 (60.20 - 69.40)	59.40 (46.80 - 72.40)	21.40 (18.80 - 24.20)	80.80 (66.60 - 95.60)
25 to 29	31.40 (29.00 - 34.60)	19.00 (17.40 - 20.80)	50.60 (47.20 - 54.20)	41.00 (37.80 - 44.40)	21.80 (19.40 - 24.60)	63.00 (58.40 - 67.40)	59.00 (49.20 - 69.20)	24.40 (21.60 - 27.80)	83.40 (71.80 - 95.60)
30 to 34	31.20 (28.80 - 34.00)	19.80 (18.20 - 21.60)	51.00 (47.80 - 54.60)	39.40 (36.40 - 42.80)	22.40 (20.00 - 25.40)	62.00 (57.60 - 66.40)	58.80 (50.80 - 67.20)	28.20 (25.00 - 32.20)	87.00 (77.20 - 97.20)
35 to 39	33.60 (31.20 - 36.40)	22.60 (20.80 - 25.00)	56.40 (52.80 - 60.00)	41.80 (38.40 - 45.40)	26.80 (23.60 - 30.80)	68.60 (63.60 - 74.00)	60.00 (53.00 - 67.20)	33.40 (29.20 - 38.80)	93.40 (84.20 - 103.60)
40 to 44	33.00 (30.60 - 35.40)	23.00 (21.00 - 25.20)	55.80 (52.40 - 59.20)	48.60 (44.60 - 52.60)	30.80 (27.60 - 34.60)	79.40 (74.20 - 85.00)	65.20 (58.80 - 72.20)	36.80 (32.60 - 42.00)	102.00 (93.60 - 111.00)
45 to 49	37.80 (35.40 - 40.60)	25.60 (23.80 - 28.00)	63.60 (60.20 - 67.20)	60.00 (55.20 - 64.80)	38.00 (34.20 - 41.80)	97.80 (91.60 - 104.40)	78.80 (72.00 - 86.40)	45.80 (41.20 - 51.40)	124.80 (116.20 - 134.60)
50 to 54	49.80 (46.80 - 53.00)	34.40 (32.20 - 37.00)	84.20 (80.00 - 88.40)	74.60 (69.20 - 80.00)	50.80 (46.40 - 55.40)	125.40 (118.20 - 132.80)	97.40 (90.00 - 105.80)	61.20 (55.60 - 67.40)	158.60 (149.20 - 169.20)
55 to 59	63.40 (59.80 - 67.20)	43.40 (40.60 - 46.20)	106.80 (102.00 - 111.80)	78.20 (73.00 - 83.20)	56.80 (52.20 - 61.80)	135.00 (127.80 - 142.80)	113.00 (105.00 - 122.20)	74.40 (67.80 - 81.60)	187.40 (176.80 - 199.60)
60 to 64	77.80 (73.80 - 82.20)	57.60 (54.60 - 61.00)	135.40 (130.00 - 141.00)	90.40 (85.00 - 95.60)	69.60 (65.00 - 74.80)	160.00 (152.60 - 167.80)	135.60 (127.20 - 145.00)	95.00 (87.80 - 102.80)	230.60 (219.20 - 243.40)
65 to 69	85.00 (81.00 - 89.20)	67.00 (63.80 - 70.80)	152.00 (146.60 - 157.60)	108.80 (102.80 - 114.60)	87.00 (81.40 - 92.80)	195.80 (187.40 - 204.60)	134.80 (127.40 - 142.80)	103.40 (96.40 - 111.00)	238.20 (227.40 - 249.80)
70 to 74	80.80 (77.60 - 83.80)	73.80 (70.80 - 77.20)	154.40 (149.80 - 159.40)	130.20 (123.20 - 137.00)	111.20 (104.60 - 117.80)	241.40 (231.40 - 251.60)	139.60 (132.40 - 147.60)	115.40 (108.20 - 123.00)	255.20 (244.80 - 266.20)
75 to 79	89.80 (87.00 - 92.60)	81.00 (78.20 - 84.00)	170.60 (166.60 - 174.80)	129.80 (123.60 - 135.80)	118.00 (111.80 - 124.80)	247.80 (238.40 - 257.40)	148.40 (140.80 - 156.20)	128.20 (121.20 - 135.40)	276.60 (265.80 - 287.80)
80 plus	106.00 (104.00 - 107.80)	137.60 (134.40 - 140.80)	243.60 (239.80 - 247.40)	168.80 (163.00 - 174.40)	210.00 (203.00 - 217.20)	378.80 (369.20 - 388.40)	250.20 (240.80 - 258.40)	289.60 (279.60 - 300.40)	539.60 (525.80 - 553.60)
**South Asia**									
Early Neonatal	775.40 (747.20 - 800.40)	608.40 (587.80 - 627.00)	1,383.80 (1,336.00 - 1,426.00)	597.60 (579.20 - 615.80)	463.00 (450.00 - 475.80)	1,060.60 (1,030.00 - 1,091.00)	464.00 (445.80 - 482.20)	362.00 (348.40 - 374.60)	826.00 (795.00 - 856.40)
Late Neonatal	284.60 (278.20 - 291.00)	263.60 (258.40 - 269.60)	548.20 (539.40 - 557.60)	167.80 (162.80 - 173.40)	160.60 (156.60 - 165.00)	328.40 (320.20 - 337.00)	109.60 (103.40 - 115.80)	107.20 (101.80 - 112.80)	216.80 (205.40 - 228.00)
Post Neonatal	550.40 (532.00 - 567.20)	582.60 (552.60 - 615.60)	1,133.20 (1,086.20 - 1,180.20)	319.60 (309.00 - 329.80)	344.80 (327.00 - 363.80)	664.40 (637.00 - 692.40)	194.40 (183.80 - 205.60)	215.40 (200.60 - 230.20)	409.80 (385.20 - 434.20)
1 to 4	508.40 (465.40 - 554.20)	668.80 (621.60 - 720.20)	1,177.20 (1,088.40 - 1,274.40)	248.20 (227.00 - 273.20)	305.20 (280.60 - 334.20)	553.40 (507.80 - 606.40)	138.60 (124.40 - 153.80)	158.20 (142.40 - 175.80)	296.80 (267.20 - 329.20)
5 to 9	133.80 (132.20 - 135.60)	152.60 (150.40 - 154.60)	286.40 (283.40 - 289.20)	106.20 (103.00 - 109.80)	102.00 (99.20 - 105.60)	208.20 (202.80 - 215.00)	76.60 (73.40 - 80.00)	64.00 (61.00 - 67.00)	140.60 (134.80 - 146.60)
10 to 14	82.00 (79.80 - 84.40)	86.00 (83.40 - 89.00)	168.00 (164.60 - 171.80)	85.00 (81.00 - 92.40)	74.40 (71.00 - 78.40)	159.40 (152.80 - 169.60)	64.40 (61.60 - 68.40)	50.60 (48.20 - 53.20)	114.80 (110.40 - 120.40)
15 to 19	107.20 (101.80 - 112.60)	135.20 (129.00 - 141.80)	242.40 (234.40 - 250.80)	126.40 (120.60 - 132.20)	132.20 (126.40 - 138.20)	258.80 (249.40 - 267.40)	106.20 (100.80 - 112.00)	94.60 (89.00 - 100.00)	200.80 (192.80 - 208.80)
20 to 24	136.40 (129.80 - 143.80)	153.80 (146.00 - 162.00)	290.20 (280.00 - 300.60)	170.20 (162.80 - 178.20)	157.60 (150.00 - 165.60)	327.80 (316.00 - 339.40)	161.60 (153.00 - 171.20)	120.80 (113.60 - 128.00)	282.40 (270.60 - 294.20)
25 to 29	146.20 (139.40 - 153.00)	139.40 (132.40 - 147.40)	285.60 (276.20 - 296.20)	188.80 (180.20 - 197.20)	147.80 (139.60 - 155.00)	336.60 (323.60 - 349.20)	190.20 (180.80 - 200.60)	124.60 (117.00 - 132.20)	315.00 (302.20 - 328.00)
30 to 34	161.20 (153.40 - 169.20)	127.40 (120.80 - 134.60)	288.60 (278.40 - 298.80)	216.60 (207.60 - 226.20)	138.60 (132.00 - 145.60)	355.20 (343.00 - 368.20)	216.00 (204.80 - 227.80)	123.20 (116.20 - 130.60)	339.20 (325.60 - 353.20)
35 to 39	179.60 (170.20 - 189.60)	127.80 (120.20 - 136.60)	307.40 (295.20 - 320.20)	251.60 (241.00 - 262.80)	147.00 (139.60 - 155.60)	398.60 (385.00 - 413.00)	258.40 (244.00 - 273.20)	132.80 (124.00 - 141.60)	391.20 (374.00 - 409.20)
40 to 44	186.80 (176.40 - 197.80)	127.20 (120.40 - 135.00)	314.20 (301.60 - 327.00)	281.60 (269.00 - 294.80)	158.00 (150.80 - 167.00)	439.60 (424.40 - 455.60)	294.20 (277.60 - 311.60)	146.80 (137.80 - 156.20)	441.00 (422.20 - 461.80)
45 to 49	227.20 (214.40 - 240.40)	155.00 (147.20 - 164.00)	382.20 (367.20 - 398.00)	325.40 (310.20 - 340.40)	186.00 (177.60 - 196.20)	511.40 (493.40 - 530.40)	362.20 (341.60 - 383.40)	192.00 (180.40 - 203.80)	554.20 (530.60 - 579.60)
50 to 54	283.80 (268.00 - 300.40)	209.80 (199.40 - 220.80)	493.60 (474.20 - 513.00)	385.00 (367.40 - 403.40)	228.80 (218.60 - 240.80)	613.80 (592.80 - 635.80)	444.80 (420.80 - 469.20)	245.20 (230.60 - 260.60)	690.00 (662.20 - 719.60)
55 to 59	343.20 (325.80 - 361.20)	264.80 (251.80 - 278.60)	608.00 (586.20 - 630.40)	410.80 (393.60 - 429.00)	285.60 (272.60 - 300.40)	696.40 (673.20 - 719.60)	546.20 (519.20 - 574.80)	319.00 (300.00 - 339.20)	865.20 (832.40 - 901.20)
60 to 64	415.60 (395.40 - 436.00)	322.60 (307.60 - 338.60)	738.20 (712.60 - 763.60)	495.40 (475.60 - 515.60)	374.00 (357.80 - 392.00)	869.40 (841.80 - 896.20)	639.20 (609.00 - 670.00)	430.40 (405.80 - 455.40)	1,069.60 (1,031.40 - 1,110.40)
65 to 69	436.40 (416.60 - 455.40)	368.80 (352.60 - 386.20)	805.20 (779.20 - 831.60)	572.20 (551.00 - 594.00)	485.00 (464.20 - 507.80)	1,057.40 (1,026.20 - 1,090.40)	660.20 (631.00 - 690.40)	513.40 (483.80 - 543.40)	1,173.80 (1,134.00 - 1,217.00)
70 to 74	426.20 (408.40 - 444.00)	385.00 (368.60 - 402.60)	811.20 (786.40 - 836.40)	578.40 (557.80 - 599.20)	535.00 (512.20 - 558.60)	1,113.40 (1,081.60 - 1,146.60)	693.40 (664.40 - 724.20)	579.40 (546.60 - 612.40)	1,272.80 (1,230.20 - 1,317.80)
75 to 79	342.60 (329.60 - 355.00)	326.00 (313.60 - 339.80)	668.60 (649.80 - 687.20)	508.60 (492.40 - 524.80)	472.80 (454.00 - 492.80)	981.40 (956.20 - 1,007.20)	635.60 (611.40 - 661.00)	562.60 (533.00 - 592.80)	1,198.20 (1,161.00 - 1,237.20)
80 plus	366.00 (357.20 - 374.40)	422.80 (413.40 - 433.40)	788.80 (775.60 - 802.20)	657.80 (645.60 - 669.60)	729.40 (712.20 - 748.20)	1,387.20 (1,365.00 - 1,409.40)	1,020.40 (996.80 - 1,043.20)	1,051.60 (1,017.20 - 1,084.40)	2,071.80 (2,030.80 - 2,114.00)
**Central Sub-Saharan Africa**									
Early Neonatal	39.60 (34.60 - 44.80)	31.20 (28.00 - 34.60)	70.80 (62.80 - 79.20)	53.00 (46.60 - 59.40)	41.00 (37.00 - 44.80)	93.80 (83.40 - 104.00)	55.80 (48.60 - 63.80)	42.80 (38.00 - 47.80)	98.60 (86.80 - 111.40)
Late Neonatal	14.80 (13.80 - 15.80)	13.00 (11.80 - 14.00)	27.60 (25.60 - 29.80)	16.60 (15.40 - 17.60)	14.60 (13.40 - 16.00)	31.20 (29.00 - 33.60)	14.80 (12.40 - 17.40)	13.20 (11.00 - 15.60)	27.80 (23.20 - 32.80)
Post Neonatal	70.80 (62.20 - 79.80)	66.60 (56.00 - 78.20)	137.40 (118.00 - 157.40)	83.60 (74.00 - 93.80)	79.00 (67.00 - 92.40)	162.60 (140.80 - 184.80)	71.80 (58.80 - 86.20)	67.80 (53.80 - 83.40)	139.60 (113.00 - 168.40)
1 to 4	93.40 (77.40 - 109.80)	85.40 (73.40 - 98.00)	178.80 (151.60 - 207.80)	95.80 (77.00 - 114.80)	85.80 (71.60 - 100.40)	181.60 (148.60 - 215.00)	71.00 (52.60 - 93.80)	61.00 (45.60 - 80.00)	132.00 (98.60 - 173.20)
5 to 9	10.20 (9.40 - 11.00)	9.20 (7.80 - 10.00)	19.40 (17.80 - 20.80)	13.60 (12.60 - 14.60)	12.40 (11.20 - 13.40)	26.20 (24.20 - 27.80)	13.60 (11.60 - 15.60)	11.60 (9.80 - 13.80)	25.20 (21.80 - 29.20)
10 to 14	5.80 (4.80 - 6.60)	4.80 (3.80 - 6.00)	10.60 (9.20 - 12.40)	8.40 (7.80 - 9.40)	7.40 (6.20 - 8.80)	15.80 (14.20 - 18.20)	10.00 (8.80 - 11.60)	8.40 (6.80 - 10.60)	18.40 (15.80 - 21.80)
15 to 19	6.40 (4.40 - 9.00)	5.40 (3.60 - 7.40)	12.00 (8.80 - 16.00)	9.00 (7.20 - 12.00)	8.40 (6.40 - 11.20)	17.60 (14.00 - 23.00)	11.80 (8.20 - 16.40)	9.40 (6.00 - 13.60)	21.20 (15.80 - 28.40)
20 to 24	8.60 (5.60 - 12.20)	7.20 (4.80 - 10.00)	15.80 (11.60 - 21.40)	11.80 (9.00 - 16.00)	12.00 (9.00 - 16.40)	23.80 (18.80 - 31.60)	15.00 (9.60 - 21.40)	12.40 (7.80 - 19.00)	27.20 (19.60 - 38.40)
25 to 29	9.80 (6.60 - 13.60)	8.00 (5.20 - 11.80)	17.80 (13.00 - 24.20)	14.20 (10.80 - 18.60)	15.20 (11.40 - 21.40)	29.40 (23.00 - 39.20)	16.40 (10.80 - 23.20)	15.40 (10.20 - 24.40)	31.80 (22.80 - 46.00)

30 to 34	10.60 (7.20 - 14.80)	8.20 (5.40 - 12.20)	18.80 (13.80 - 26.00)	16.80 (13.20 - 21.80)	16.40 (12.60 - 22.40)	33.40 (27.00 - 44.00)	18.40 (12.20 - 26.20)	16.80 (11.20 - 26.20)	35.20 (25.20 - 50.80)
35 to 39	11.20 (7.40 - 16.00)	9.00 (5.60 - 14.20)	20.20 (14.40 - 29.40)	18.40 (14.40 - 24.40)	16.80 (12.80 - 25.00)	35.20 (28.00 - 48.60)	19.80 (12.80 - 29.00)	18.00 (11.60 - 31.80)	37.80 (26.80 - 57.60)
40 to 44	11.60 (7.60 - 16.80)	8.80 (5.80 - 13.60)	20.40 (14.60 - 28.80)	19.00 (14.80 - 25.40)	15.60 (11.60 - 23.00)	34.60 (27.40 - 48.00)	20.80 (13.40 - 32.00)	17.60 (11.20 - 29.20)	38.40 (27.00 - 58.80)
45 to 49	12.40 (7.80 - 18.40)	9.80 (6.40 - 15.00)	22.20 (15.40 - 31.80)	19.20 (14.60 - 26.60)	15.60 (11.20 - 22.60)	34.80 (26.80 - 48.80)	22.00 (13.60 - 35.00)	18.20 (11.00 - 29.40)	40.20 (27.80 - 62.00)
50 to 54	13.00 (8.20 - 19.40)	11.60 (7.40 - 17.40)	24.60 (17.00 - 35.20)	19.00 (14.00 - 26.60)	16.60 (11.80 - 24.00)	35.60 (26.80 - 50.00)	23.20 (14.20 - 37.40)	20.20 (11.40 - 32.00)	43.20 (29.20 - 64.80)
55 to 59	13.60 (8.60 - 20.00)	13.40 (8.80 - 20.00)	27.00 (19.00 - 37.60)	19.00 (14.20 - 25.60)	18.40 (13.20 - 25.60)	37.40 (28.60 - 50.40)	24.20 (14.60 - 38.20)	22.60 (12.80 - 35.20)	46.80 (31.60 - 69.40)
60 to 64	14.60 (9.80 - 20.80)	16.00 (10.60 - 22.80)	30.60 (22.60 - 41.00)	20.20 (15.60 - 26.40)	21.80 (15.80 - 29.20)	42.00 (33.00 - 54.40)	26.20 (16.40 - 39.20)	26.00 (15.20 - 38.80)	52.20 (36.00 - 74.00)
65 to 69	14.80 (10.20 - 20.40)	18.00 (12.20 - 25.20)	32.60 (24.40 - 42.80)	20.80 (16.40 - 26.40)	24.60 (18.00 - 32.20)	45.40 (36.00 - 57.40)	27.20 (17.80 - 39.00)	29.60 (17.60 - 42.60)	56.80 (40.60 - 77.40)
70 to 74	14.20 (10.20 - 18.80)	18.80 (13.40 - 25.20)	32.80 (25.60 - 41.40)	20.40 (16.80 - 24.60)	26.00 (19.80 - 33.00)	46.40 (37.80 - 56.60)	27.40 (19.00 - 37.40)	32.40 (20.60 - 45.80)	59.80 (44.00 - 78.40)
75 to 79	11.00 (8.40 - 14.00)	16.00 (12.20 - 20.60)	27.00 (22.00 - 33.00)	16.60 (14.20 - 19.40)	23.00 (18.00 - 28.40)	39.80 (33.20 - 46.80)	23.20 (17.20 - 30.60)	30.00 (20.00 - 40.80)	53.20 (40.20 - 67.60)
80 plus	10.20 (8.40 - 11.80)	17.60 (15.00 - 20.60)	28.00 (24.60 - 31.60)	16.80 (15.40 - 18.20)	27.80 (23.60 - 31.80)	44.60 (39.60 - 49.20)	25.40 (21.00 - 30.40)	39.40 (30.60 - 48.60)	64.80 (54.20 - 76.00)
**Eastern Sub-Saharan Africa**									
Early Neonatal	153.60 (143.20 - 164.00)	111.80 (105.40 - 117.60)	265.40 (248.40 - 281.40)	156.00 (147.60 - 164.40)	113.00 (108.00 - 117.60)	269.20 (255.80 - 282.00)	148.00 (141.60 - 155.20)	108.20 (104.20 - 112.40)	256.20 (245.80 - 267.20)
Late Neonatal	68.00 (66.60 - 69.40)	54.00 (52.00 - 56.00)	121.80 (119.00 - 124.60)	51.20 (50.00 - 52.60)	41.60 (40.20 - 43.00)	92.80 (90.60 - 95.40)	41.40 (39.40 - 43.60)	34.00 (32.40 - 35.80)	75.60 (72.00 - 79.40)
Post Neonatal	253.00 (238.40 - 266.80)	233.40 (216.00 - 251.40)	486.40 (454.20 - 517.80)	219.60 (209.20 - 230.00)	203.80 (190.40 - 217.00)	423.20 (399.40 - 447.00)	165.00 (156.80 - 174.40)	153.40 (144.00 - 163.20)	318.40 (301.20 - 337.80)
1 to 4	292.80 (266.60 - 319.20)	278.80 (259.40 - 300.00)	571.60 (526.80 - 618.00)	211.20 (191.80 - 231.00)	191.60 (176.80 - 207.00)	403.00 (368.20 - 437.40)	138.80 (125.60 - 153.80)	119.80 (108.80 - 131.60)	258.60 (235.00 - 284.80)
5 to 9	38.20 (36.20 - 41.20)	34.40 (32.60 - 37.20)	72.60 (69.40 - 77.40)	44.40 (43.00 - 45.80)	39.40 (38.20 - 40.60)	84.00 (81.80 - 86.00)	38.80 (37.00 - 40.60)	31.80 (30.20 - 33.60)	70.60 (67.60 - 73.60)
10 to 14	22.60 (20.80 - 25.80)	19.00 (17.40 - 21.20)	41.80 (38.60 - 46.40)	31.00 (30.00 - 32.00)	27.20 (26.00 - 28.60)	58.20 (56.20 - 60.40)	33.40 (31.60 - 35.20)	27.40 (25.20 - 30.00)	60.80 (57.60 - 64.40)
15 to 19	30.20 (22.80 - 42.80)	22.80 (20.20 - 26.20)	53.20 (43.80 - 67.00)	31.40 (28.40 - 34.80)	28.80 (26.80 - 31.20)	60.20 (55.80 - 65.20)	39.40 (33.00 - 47.20)	26.80 (21.80 - 32.20)	66.20 (57.40 - 76.20)
20 to 24	40.60 (30.20 - 58.20)	30.40 (27.20 - 34.40)	71.00 (58.80 - 90.20)	41.40 (37.00 - 46.40)	49.60 (43.80 - 55.60)	90.80 (82.60 - 99.80)	49.20 (39.60 - 61.00)	37.80 (30.80 - 46.20)	87.00 (74.80 - 102.80)
25 to 29	43.00 (34.40 - 56.60)	33.00 (29.40 - 37.20)	76.00 (65.20 - 91.40)	59.60 (53.00 - 66.60)	74.40 (65.60 - 84.20)	134.00 (121.20 - 148.60)	59.00 (48.00 - 72.40)	50.60 (41.60 - 62.00)	109.60 (94.60 - 129.80)
30 to 34	44.20 (37.40 - 55.00)	33.60 (29.80 - 38.00)	77.80 (69.00 - 90.00)	78.80 (71.20 - 86.40)	81.40 (74.20 - 89.00)	160.20 (148.40 - 173.20)	68.60 (56.40 - 84.00)	55.00 (45.20 - 67.20)	123.60 (107.60 - 144.40)
35 to 39	45.00 (38.60 - 53.60)	35.60 (31.20 - 41.20)	80.60 (71.80 - 91.60)	81.20 (74.60 - 88.20)	74.20 (67.40 - 82.20)	155.40 (143.60 - 168.80)	72.40 (59.20 - 88.40)	54.80 (43.80 - 70.60)	127.20 (109.00 - 150.40)
40 to 44	46.00 (39.80 - 54.20)	35.00 (30.80 - 40.20)	81.00 (72.40 - 92.00)	77.00 (71.00 - 83.40)	63.80 (58.00 - 70.40)	140.60 (131.00 - 152.00)	72.20 (58.00 - 90.60)	50.00 (39.40 - 64.80)	122.20 (103.60 - 147.00)
45 to 49	46.60 (40.40 - 53.40)	36.20 (31.40 - 41.80)	82.80 (74.20 - 93.20)	72.20 (65.40 - 80.20)	60.40 (54.00 - 67.60)	132.60 (121.80 - 146.00)	69.40 (54.60 - 89.40)	48.80 (37.20 - 64.40)	118.20 (97.80 - 144.40)
50 to 54	47.80 (41.40 - 55.00)	41.00 (36.20 - 46.80)	88.80 (80.00 - 99.80)	66.00 (58.80 - 74.00)	60.80 (53.20 - 69.20)	126.80 (115.40 - 139.60)	68.60 (53.20 - 89.00)	53.00 (39.60 - 71.40)	121.60 (99.40 - 149.20)
55 to 59	52.40 (45.80 - 59.60)	49.20 (43.40 - 55.60)	101.60 (91.80 - 113.20)	63.80 (56.00 - 72.20)	64.60 (55.80 - 73.40)	128.20 (115.40 - 142.20)	70.80 (54.40 - 92.60)	60.00 (43.60 - 80.60)	130.80 (106.20 - 161.40)
60 to 64	54.80 (48.40 - 61.20)	55.80 (50.20 - 61.80)	110.60 (101.00 - 121.00)	64.40 (56.80 - 72.60)	70.20 (61.00 - 79.20)	134.60 (121.60 - 149.00)	78.40 (61.80 - 100.80)	70.80 (53.00 - 91.80)	149.40 (123.20 - 180.80)
65 to 69	56.80 (50.80 - 62.60)	63.20 (57.20 - 69.60)	120.00 (110.40 - 131.00)	67.00 (59.60 - 74.80)	77.00 (66.80 - 86.80)	144.20 (130.80 - 158.60)	85.00 (67.80 - 108.00)	83.80 (62.60 - 108.60)	169.00 (140.00 - 204.60)
70 to 74	56.00 (51.00 - 60.80)	62.80 (57.40 - 68.40)	118.80 (110.20 - 127.40)	72.60 (66.00 - 79.60)	85.60 (75.80 - 95.20)	158.20 (145.40 - 171.60)	87.00 (70.80 - 108.00)	91.00 (69.80 - 116.80)	178.00 (150.40 - 212.60)
75 to 79	46.20 (42.20 - 49.40)	54.20 (50.20 - 58.20)	100.40 (94.20 - 106.60)	60.40 (55.40 - 65.20)	72.60 (65.00 - 79.60)	132.80 (123.20 - 142.60)	78.60 (65.40 - 95.40)	87.00 (68.80 - 108.80)	165.40 (142.40 - 194.60)
80 plus	41.40 (39.40 - 43.00)	59.40 (56.60 - 62.00)	100.80 (97.00 - 104.20)	72.20 (68.60 - 75.80)	97.60 (91.00 - 104.00)	169.80 (161.80 - 177.60)	108.80 (97.00 - 123.20)	134.40 (114.60 - 156.60)	243.00 (219.00 - 270.20)
**Southern Sub-Saharan Africa**									
Early Neonatal	18.40 (17.60 - 19.20)	14.00 (13.60 - 14.60)	32.40 (31.00 - 33.60)	19.40 (18.60 - 20.40)	14.80 (14.20 - 15.20)	34.20 (32.60 - 35.60)	16.40 (15.40 - 17.60)	12.40 (11.80 - 13.00)	28.80 (27.20 - 30.60)
Late Neonatal	5.80 (5.60 - 6.20)	5.20 (5.00 - 5.60)	11.20 (10.60 - 11.60)	4.40 (4.20 - 4.60)	4.40 (4.20 - 4.60)	8.80 (8.40 - 9.20)	4.00 (3.60 - 4.40)	3.80 (3.40 - 4.00)	7.80 (7.20 - 8.40)
Post Neonatal	23.00 (21.80 - 24.20)	20.20 (18.60 - 21.60)	43.20 (40.40 - 45.80)	29.00 (27.40 - 30.80)	26.40 (24.40 - 28.40)	55.60 (52.00 - 59.20)	15.40 (14.00 - 16.80)	13.60 (12.20 - 15.00)	29.00 (26.20 - 31.80)
1 to 4	17.80 (15.80 - 19.80)	16.80 (15.20 - 18.60)	34.60 (31.00 - 38.20)	20.00 (17.60 - 22.40)	17.60 (15.80 - 19.60)	37.60 (33.40 - 42.00)	9.60 (8.20 - 11.00)	8.20 (7.20 - 9.60)	17.80 (15.40 - 20.60)
5 to 9	5.00 (4.80 - 5.20)	3.80 (3.60 - 4.00)	8.80 (8.40 - 9.00)	8.60 (8.20 - 9.00)	7.80 (7.60 - 8.20)	16.60 (15.80 - 17.20)	7.00 (6.60 - 7.40)	6.20 (5.80 - 6.60)	13.20 (12.60 - 14.00)
10 to 14	3.20 (3.00 - 3.40)	2.60 (2.60 - 2.80)	6.00 (5.80 - 6.20)	6.00 (5.60 - 6.40)	5.60 (5.20 - 6.00)	11.60 (10.80 - 12.40)	8.20 (7.80 - 8.80)	7.40 (6.80 - 7.80)	15.60 (14.60 - 16.60)
15 to 19	5.40 (4.80 - 5.80)	3.60 (3.40 - 4.00)	9.00 (8.40 - 9.60)	8.60 (7.40 - 9.80)	8.40 (7.60 - 9.40)	17.00 (15.00 - 19.00)	8.40 (7.40 - 9.60)	6.20 (5.40 - 7.00)	14.60 (13.20 - 16.20)
20 to 24	9.40 (8.40 - 10.40)	5.40 (5.00 - 6.00)	14.80 (13.60 - 16.00)	18.20 (15.80 - 20.60)	24.40 (20.60 - 29.20)	42.60 (37.20 - 49.00)	16.20 (14.20 - 18.40)	14.20 (12.20 - 16.40)	30.40 (27.20 - 33.80)
25 to 29	10.00 (9.00 - 11.20)	6.20 (5.40 - 6.80)	16.20 (14.80 - 17.80)	36.80 (32.20 - 41.80)	47.00 (39.80 - 54.80)	83.80 (73.00 - 96.20)	27.20 (24.20 - 30.40)	24.80 (21.20 - 29.00)	52.00 (46.40 - 58.20)
30 to 34	10.00 (9.00 - 11.00)	6.60 (6.00 - 7.40)	16.60 (15.20 - 18.20)	47.00 (42.00 - 52.40)	49.60 (44.20 - 55.60)	96.60 (87.20 - 107.20)	32.20 (29.00 - 35.60)	26.20 (23.20 - 29.60)	58.40 (53.20 - 64.20)
35 to 39	9.60 (8.60 - 10.80)	6.60 (5.80 - 7.40)	16.20 (15.00 - 18.00)	46.40 (41.80 - 51.20)	44.00 (39.40 - 49.20)	90.40 (82.00 - 99.80)	32.20 (29.40 - 35.60)	24.60 (22.20 - 27.40)	56.80 (52.40 - 62.00)
40 to 44	9.00 (8.20 - 10.00)	6.00 (5.40 - 6.60)	15.00 (13.80 - 16.40)	42.40 (39.00 - 45.60)	35.60 (32.00 - 39.20)	78.00 (71.80 - 84.20)	28.20 (25.60 - 31.00)	20.40 (18.40 - 22.60)	48.40 (45.00 - 53.00)
45 to 49	9.80 (9.00 - 11.00)	5.80 (5.40 - 6.60)	15.80 (14.40 - 17.20)	37.80 (34.60 - 41.20)	29.40 (26.40 - 32.60)	67.20 (61.40 - 73.20)	25.40 (23.00 - 28.60)	18.80 (16.80 - 21.00)	44.20 (40.60 - 48.80)

50 to 54	10.20 (9.20 - 11.20)	6.20 (5.60 - 7.00)	16.40 (15.00 - 18.00)	31.80 (28.20 - 35.20)	24.60 (21.60 - 27.80)	56.40 (50.60 - 62.00)	25.40 (22.60 - 28.80)	18.20 (16.00 - 20.60)	43.60 (39.60 - 48.60)
55 to 59	11.40 (10.40 - 12.60)	8.20 (7.40 - 9.00)	19.60 (18.00 - 21.20)	28.00 (24.60 - 31.60)	20.40 (17.80 - 23.80)	48.40 (42.80 - 54.40)	26.40 (23.40 - 30.00)	16.60 (14.20 - 19.60)	43.00 (38.60 - 48.00)
60 to 64	12.20 (11.20 - 13.40)	13.20 (12.20 - 14.40)	25.40 (23.80 - 27.20)	28.40 (25.00 - 31.80)	25.80 (22.40 - 29.60)	54.40 (47.80 - 60.80)	32.40 (29.00 - 36.80)	19.80 (17.00 - 23.20)	52.20 (47.40 - 58.60)
65 to 69	15.00 (13.80 - 16.40)	18.60 (17.00 - 20.40)	33.60 (31.60 - 36.00)	23.80 (20.80 - 26.60)	28.40 (24.60 - 32.60)	52.20 (46.00 - 58.60)	27.80 (24.80 - 31.20)	20.20 (17.20 - 23.60)	47.80 (43.40 - 53.40)
70 to 74	13.40 (12.40 - 14.40)	17.20 (15.80 - 18.60)	30.40 (28.80 - 32.40)	22.00 (19.20 - 24.20)	35.00 (30.00 - 40.20)	57.00 (50.00 - 64.00)	26.60 (23.60 - 29.60)	26.60 (22.80 - 31.00)	53.20 (48.00 - 59.20)
75 to 79	17.80 (16.60 - 19.00)	25.80 (24.00 - 27.60)	43.60 (41.40 - 45.80)	19.20 (17.00 - 21.00)	34.20 (29.60 - 38.80)	53.20 (47.00 - 59.40)	20.40 (18.20 - 22.80)	30.00 (26.00 - 34.60)	50.40 (45.60 - 56.00)
80 plus	23.20 (22.40 - 24.20)	42.60 (40.80 - 44.20)	65.80 (64.00 - 67.80)	33.60 (31.60 - 35.40)	71.80 (66.80 - 77.00)	105.40 (98.80 - 111.80)	39.60 (37.20 - 42.00)	78.80 (73.40 - 85.20)	118.60 (112.20 - 125.80)
**Western Sub-Saharan Africa**									
Early Neonatal	183.20 (166.60 - 201.20)	129.80 (119.20 - 140.80)	313.00 (286.00 - 341.60)	224.60 (203.60 - 245.60)	157.40 (145.20 - 169.80)	382.20 (349.20 - 415.40)	222.00 (202.80 - 240.60)	155.60 (143.60 - 167.00)	377.60 (346.40 - 408.00)
Late Neonatal	70.60 (68.60 - 72.60)	56.80 (54.00 - 59.60)	127.40 (123.00 - 131.80)	73.60 (71.40 - 76.00)	60.60 (57.80 - 63.60)	134.00 (129.60 - 138.80)	62.00 (58.00 - 66.40)	51.60 (48.00 - 55.60)	113.60 (106.40 - 121.60)
Post Neonatal	236.80 (217.00 - 258.00)	219.20 (195.80 - 245.00)	456.00 (414.00 - 502.40)	262.20 (241.00 - 284.40)	244.80 (218.80 - 272.80)	507.00 (461.40 - 557.00)	216.80 (198.00 - 236.60)	203.80 (182.60 - 228.40)	420.40 (380.60 - 464.80)
1 to 4	407.20 (366.40 - 450.40)	390.00 (356.60 - 422.80)	797.20 (723.20 - 868.40)	395.20 (351.20 - 441.80)	376.40 (341.40 - 411.80)	771.60 (695.00 - 851.80)	291.20 (251.60 - 333.20)	270.60 (236.60 - 304.40)	561.80 (488.00 - 637.00)
5 to 9	44.60 (43.40 - 45.60)	37.40 (34.00 - 41.00)	82.00 (78.20 - 86.00)	52.60 (51.20 - 54.00)	44.20 (40.80 - 48.20)	96.80 (92.80 - 101.00)	50.80 (48.00 - 54.00)	40.40 (37.60 - 45.00)	91.00 (86.00 - 97.00)
10 to 14	22.60 (20.40 - 24.40)	17.60 (15.20 - 20.20)	40.20 (36.60 - 43.80)	30.00 (28.00 - 31.40)	23.40 (21.40 - 25.60)	53.40 (50.20 - 56.20)	33.60 (31.80 - 36.60)	25.20 (22.80 - 29.40)	59.00 (55.20 - 63.80)
15 to 19	19.60 (15.60 - 25.00)	18.00 (13.60 - 22.40)	37.60 (31.00 - 44.60)	27.20 (23.60 - 30.40)	24.80 (20.60 - 29.60)	52.00 (46.00 - 58.20)	33.40 (28.20 - 41.20)	23.40 (18.80 - 31.60)	56.60 (49.00 - 67.80)
20 to 24	23.80 (18.80 - 31.20)	21.80 (16.60 - 27.60)	45.60 (37.60 - 55.00)	35.40 (30.80 - 40.80)	38.20 (32.20 - 44.80)	73.80 (64.60 - 82.60)	41.80 (34.00 - 53.40)	32.20 (25.20 - 43.20)	74.00 (62.20 - 89.80)
25 to 29	24.80 (19.80 - 32.40)	22.20 (16.80 - 28.60)	47.00 (38.80 - 57.40)	45.00 (38.80 - 51.80)	51.00 (42.80 - 59.80)	96.00 (84.00 - 108.60)	48.80 (39.00 - 62.20)	41.80 (32.00 - 54.60)	90.60 (75.60 - 108.80)
30 to 34	25.20 (19.80 - 33.00)	22.20 (17.00 - 29.00)	47.40 (39.40 - 58.00)	53.80 (47.00 - 61.20)	54.60 (48.40 - 61.40)	108.40 (98.00 - 119.80)	56.40 (44.60 - 71.20)	47.00 (36.80 - 60.60)	103.40 (86.60 - 123.80)
35 to 39	26.40 (20.60 - 35.40)	23.60 (17.80 - 31.60)	50.20 (40.80 - 61.80)	56.00 (49.40 - 63.40)	51.60 (45.60 - 58.00)	107.60 (97.00 - 118.20)	61.00 (48.60 - 77.20)	48.20 (37.80 - 61.80)	109.20 (92.20 - 131.40)
40 to 44	28.40 (21.80 - 39.20)	24.20 (18.60 - 33.00)	52.80 (43.20 - 65.20)	56.40 (50.20 - 63.60)	46.80 (42.00 - 51.80)	103.00 (94.60 - 112.00)	61.60 (50.40 - 77.40)	45.60 (36.60 - 59.40)	107.20 (91.80 - 129.20)
45 to 49	31.80 (24.40 - 44.00)	27.40 (21.20 - 37.80)	59.40 (48.80 - 73.40)	55.40 (48.60 - 63.20)	44.80 (39.80 - 50.20)	100.20 (91.00 - 110.20)	61.00 (49.40 - 79.20)	44.40 (35.00 - 60.00)	105.20 (89.20 - 128.00)
50 to 54	35.20 (27.40 - 47.60)	32.00 (24.80 - 45.00)	67.20 (55.60 - 82.60)	54.20 (47.20 - 62.40)	46.60 (40.60 - 53.20)	100.80 (90.40 - 111.80)	63.00 (51.00 - 81.80)	47.60 (37.00 - 66.40)	110.60 (93.80 - 135.40)
55 to 59	38.00 (29.80 - 51.20)	37.60 (29.20 - 52.60)	75.60 (62.60 - 92.60)	55.80 (48.20 - 64.80)	51.60 (44.40 - 60.00)	107.40 (96.00 - 120.20)	64.60 (52.20 - 84.20)	52.40 (40.00 - 75.00)	117.00 (98.80 - 144.80)
60 to 64	44.20 (35.60 - 56.80)	46.80 (37.00 - 63.60)	91.20 (76.60 - 109.80)	62.60 (54.20 - 71.60)	61.60 (52.80 - 72.80)	124.00 (111.00 - 139.20)	70.80 (58.80 - 90.40)	61.40 (47.80 - 87.20)	132.20 (113.40 - 162.40)
65 to 69	46.40 (38.00 - 58.60)	53.20 (42.60 - 71.60)	99.60 (84.80 - 119.80)	64.00 (55.80 - 72.40)	68.20 (59.00 - 80.00)	132.20 (118.80 - 147.60)	75.00 (63.00 - 94.60)	69.20 (54.40 - 98.40)	144.20 (124.00 - 176.60)
70 to 74	46.20 (38.60 - 55.80)	56.20 (46.20 - 72.20)	102.40 (88.80 - 120.00)	63.60 (56.00 - 71.00)	71.80 (63.20 - 82.20)	135.20 (122.80 - 148.80)	76.40 (65.60 - 93.60)	75.60 (60.80 - 102.80)	152.00 (132.60 - 181.40)
75 to 79	35.00 (29.80 - 40.80)	45.20 (38.60 - 55.60)	80.20 (71.40 - 92.00)	51.20 (45.80 - 56.60)	60.60 (54.80 - 67.40)	111.80 (103.20 - 121.20)	61.60 (53.80 - 72.80)	67.00 (55.00 - 87.00)	128.60 (113.60 - 149.20)
80 plus	28.00 (25.00 - 30.80)	39.40 (35.40 - 45.00)	67.40 (62.40 - 73.60)	46.00 (42.60 - 49.00)	59.80 (55.80 - 64.00)	105.60 (100.20 - 111.40)	58.00 (52.80 - 64.60)	73.20 (63.40 - 86.80)	131.20 (120.00 - 145.20)

SR Table 5: Estimates of life expectancy at birth by geography (with 95% uncertainty intervals), both sexes, 1980- 2015

Location	1980	1981	1982	1983	1984	1985	1986	1987	1988	1989	1990	1991
Global	61.7 (61.4-61.9)	62.0 (61.7-62.3)	62.4 (62.0-62.7)	62.6 (62.3-62.9)	62.9 (62.6-63.3)	63.3 (63.0-63.6)	63.8 (63.5-64.1)	64.1 (63.8-64.4)	64.3 (64.0-64.6)	64.6 (64.3-64.8)	64.8 (64.5-65.0)	64.9 (64.6-65.2)
High SDI	72.4 (72.4-72.5)	72.8 (72.7-72.8)	73.1 (73.0-73.1)	73.2 (73.1-73.2)	73.3 (73.2-73.3)	73.6 (73.5-73.6)	74.0 (74.0-74.1)	74.2 (74.2-74.3)	74.4 (74.3-74.4)	74.5 (74.4-74.5)	74.6 (74.5-74.6)	74.7 (74.6-74.7)
High-middle SDI	66.4 (66.0-66.8)	66.6 (66.2-67.0)	67.0 (66.5-67.4)	67.4 (66.9-67.8)	67.7 (67.3-68.1)	68.1 (67.7-68.4)	68.5 (68.1-68.9)	68.7 (68.3-69.1)	68.8 (68.4-69.1)	69.0 (68.7-69.4)	69.1 (68.8-69.5)	69.4 (69.0-69.7)
Middle SDI	60.9 (60.3-61.5)	61.2 (60.6-61.8)	61.6 (61.1-62.2)	62.2 (61.6-62.7)	62.6 (62.1-63.2)	63.0 (62.4-63.5)	63.4 (62.8-63.9)	63.7 (63.1-64.2)	63.9 (63.3-64.4)	64.3 (63.7-64.8)	64.6 (64.1-65.1)	64.9 (64.4-65.4)
Low-middle SDI	53.5 (52.7-54.2)	53.9 (53.1-54.6)	54.3 (53.6-55.0)	54.7 (53.9-55.4)	55.1 (54.4-55.7)	55.4 (54.7-56.1)	55.9 (55.2-56.5)	56.3 (55.6-56.9)	56.6 (55.9-57.2)	56.9 (56.3-57.5)	57.2 (56.6-57.8)	57.2 (56.6-57.8)
Low SDI	48.1 (46.6-49.5)	48.3 (46.9-49.6)	48.6 (47.2-49.8)	47.9 (46.4-49.3)	47.9 (46.4-49.3)	49.1 (47.9-50.3)	49.6 (48.5-50.7)	50.1 (49.0-51.1)	50.5 (49.5-51.4)	50.8 (49.9-51.7)	50.6 (49.7-51.5)	51.0 (50.1-51.7)
High-income	73.7 (73.6-73.7)	74.0 (74.0-74.0)	74.3 (74.3-74.4)	74.5 (74.5-74.5)	74.8 (74.8-74.8)	75.0 (75.0-75.0)	75.2 (75.2-75.2)	75.5 (75.4-75.5)	75.6 (75.6-75.6)	75.8 (75.8-75.8)	76.0 (76.0-76.0)	76.2 (76.2-76.2)
High-income North America	73.9 (73.9-73.9)	74.2 (74.2-74.2)	74.6 (74.6-74.6)	74.7 (74.7-74.8)	74.9 (74.9-74.9)	74.9 (74.9-74.9)	75.0 (75.0-75.0)	75.1 (75.1-75.1)	75.2 (75.1-75.2)	75.4 (75.3-75.4)	75.6 (75.6-75.6)	75.8 (75.8-75.8)
Canada	75.1 (75.1-75.2)	75.5 (75.4-75.5)	75.8 (75.7-75.8)	76.1 (76.0-76.1)	76.3 (76.3-76.4)	76.4 (76.4-76.5)	76.6 (76.6-76.6)	76.8 (76.7-76.8)	77.0 (77.0-77.0)	77.2 (77.1-77.2)	77.4 (77.4-77.5)	77.6 (77.6-77.6)
Greenland	63.6 (63.2-64.2)	63.6 (63.1-64.2)	63.5 (63.0-64.1)	63.5 (62.9-64.0)	63.4 (62.8-63.9)	63.5 (63.0-64.0)	63.7 (63.2-64.2)	63.9 (63.3-64.4)	64.2 (63.6-64.8)	64.5 (63.9-65.1)	64.6 (64.0-65.3)	65.1 (64.5-65.7)
United States	73.8 (73.7-73.8)	74.1 (74.0-74.1)	74.4 (74.4-74.4)	74.6 (74.6-74.6)	74.7 (74.7-74.7)	74.7 (74.7-74.7)	74.8 (74.8-74.8)	74.9 (74.9-74.9)	74.9 (74.9-75.0)	75.1 (75.1-75.2)	75.4 (75.4-75.4)	75.5 (75.5-75.6)
Australasia	74.3 (74.2-74.3)	74.6 (74.5-74.6)	74.7 (74.6-74.7)	75.0 (75.0-75.1)	75.2 (75.2-75.3)	75.3 (75.3-75.4)	75.7 (75.7-75.8)	75.9 (75.8-75.9)	76.1 (76.1-76.1)	76.2 (76.2-76.3)	76.7 (76.7-76.8)	77.1 (77.1-77.2)
Australia	74.5 (74.5-74.6)	74.8 (74.7-74.8)	74.9 (74.8-74.9)	75.3 (75.2-75.3)	75.5 (75.4-75.5)	75.6 (75.5-75.6)	76.0 (76.0-76.1)	76.2 (76.1-76.2)	76.4 (76.3-76.4)	76.5 (76.4-76.5)	77.0 (76.9-77.0)	77.4 (77.4-77.4)
New Zealand	73.1 (73.0-73.2)	73.5 (73.4-73.6)	73.7 (73.6-73.8)	73.8 (73.7-73.9)	74.0 (73.9-74.1)	74.1 (74.0-74.2)	74.3 (74.2-74.4)	74.5 (74.4-74.6)	74.7 (74.6-74.8)	75.1 (75.0-75.2)	75.6 (75.5-75.7)	75.9 (75.8-76.0)
High-income Asia Pacific	74.2 (74.0-74.3)	74.6 (74.4-74.7)	75.0 (74.8-75.1)	75.1 (75.0-75.3)	75.5 (75.4-75.6)	75.9 (75.8-76.0)	76.3 (76.2-76.4)	76.7 (76.5-76.8)	76.9 (76.8-77.0)	77.2 (77.1-77.3)	77.4 (77.3-77.5)	77.7 (77.5-77.7)
Brunei	70.7 (70.2-71.3)	71.0 (70.5-71.5)	71.2 (70.7-71.7)	71.6 (71.1-72.0)	71.9 (71.5-72.3)	72.3 (71.9-72.7)	72.7 (72.3-73.0)	73.1 (72.7-73.4)	73.4 (73.1-73.8)	73.6 (73.3-74.0)	73.8 (73.4-74.2)	73.8 (73.5-74.2)
Japan	76.2 (76.2-76.2)	76.6 (76.6-76.6)	77.0 (76.9-77.0)	77.1 (77.1-77.1)	77.4 (77.4-77.4)	77.7 (77.7-77.8)	78.1 (78.1-78.1)	78.4 (78.4-78.5)	78.6 (78.5-78.6)	78.8 (78.8-78.9)	79.0 (79.0-79.0)	79.1 (79.1-79.2)
Singapore	71.5 (71.3-71.6)	71.6 (71.5-71.8)	71.8 (71.7-72.0)	72.2 (72.0-72.3)	72.5 (72.4-72.7)	73.0 (72.8-73.1)	73.4 (73.3-73.6)	73.8 (73.6-73.9)	74.0 (73.8-74.1)	74.3 (74.1-74.4)	74.9 (74.8-75.0)	75.6 (75.4-75.7)
South Korea	67.0 (66.5-67.4)	67.6 (67.1-68.0)	68.0 (67.5-68.3)	68.3 (67.9-68.7)	68.9 (68.5-69.2)	69.4 (69.0-69.7)	69.8 (69.5-70.1)	70.2 (69.9-70.5)	70.8 (70.4-71.1)	71.3 (70.9-71.5)	71.8 (71.4-72.0)	72.2 (71.9-72.5)
Western Europe	73.9 (73.9-74.0)	74.2 (74.2-74.3)	74.5 (74.5-74.5)	74.7 (74.7-74.7)	75.0 (75.0-75.1)	75.2 (75.2-75.2)	75.5 (75.5-75.5)	75.7 (75.7-75.7)	75.9 (75.9-75.9)	76.1 (76.1-76.1)	76.3 (76.3-76.3)	76.4 (76.4-76.4)
Andorra	80.6 (80.1-81.1)	80.6 (80.1-81.2)	80.6 (80.1-81.1)	80.7 (80.2-81.3)	80.9 (80.4-81.4)	81.0 (80.5-81.5)	81.1 (80.7-81.6)	81.3 (80.8-81.7)	81.4 (81.0-81.9)	81.6 (81.1-82.0)	81.7 (81.3-82.1)	81.8 (81.4-82.2)
Austria	72.7 (72.6-72.7)	73.0 (72.9-73.0)	73.0 (73.0-73.1)	73.2 (73.2-73.3)	73.7 (73.6-73.8)	74.1 (74.0-74.1)	74.5 (74.4-74.5)	74.9 (74.9-75.0)	75.4 (75.3-75.4)	75.5 (75.4-75.5)	75.7 (75.6-75.8)	75.8 (75.7-75.8)
Belgium	73.1 (73.0-73.2)	73.5 (73.4-73.6)	73.6 (73.5-73.7)	73.8 (73.7-73.8)	74.2 (74.1-74.3)	74.6 (74.5-74.6)	74.8 (74.7-74.9)	75.1 (75.0-75.2)	75.6 (75.5-75.6)	75.7 (75.6-75.8)	76.0 (75.9-76.1)	76.2 (76.1-76.3)
Cyprus	75.6 (75.5-75.8)	76.3 (76.2-76.5)	76.2 (76.1-76.4)	76.6 (76.4-76.7)	76.4 (76.2-76.5)	76.2 (76.0-76.4)	76.6 (76.4-76.8)	75.8 (75.7-75.9)	76.1 (75.9-76.3)	76.4 (76.2-76.6)	76.8 (76.6-77.0)	76.4 (76.3-76.6)
Denmark	74.2 (74.1-74.2)	74.4 (74.3-74.5)	74.7 (74.6-74.8)	74.6 (74.6-74.7)	74.8 (74.7-74.8)	74.7 (74.6-74.8)	74.8 (74.8-74.9)	74.8 (74.8-74.9)	75.1 (75.0-75.1)	75.0 (74.9-75.0)	75.1 (75.0-75.2)	75.4 (75.3-75.5)
Finland	73.6 (73.5-73.7)	74.0 (73.9-74.1)	74.3 (74.2-74.4)	74.5 (74.4-74.6)	74.5 (74.4-74.6)	74.6 (74.5-74.7)	74.7 (74.6-74.8)	74.7 (74.6-74.8)	74.8 (74.7-74.9)	75.0 (74.8-75.1)	75.1 (75.0-75.2)	75.4 (75.3-75.5)
France	74.6 (74.5-74.6)	74.8 (74.8-74.8)	75.0 (75.0-75.0)	75.1 (75.1-75.2)	75.5 (75.5-75.6)	75.7 (75.7-75.8)	76.0 (76.0-76.0)	76.5 (76.4-76.5)	76.7 (76.7-76.7)	76.9 (76.9-76.9)	77.2 (77.1-77.2)	77.3 (77.3-77.4)
Germany	73.1 (73.1-73.1)	73.3 (73.3-73.4)	73.6 (73.6-73.6)	74.0 (73.9-74.0)	74.4 (74.3-74.4)	74.6 (74.6-74.6)	74.8 (74.8-74.9)	75.1 (75.0-75.1)	75.2 (75.2-75.3)	75.4 (75.4-75.4)	75.4 (75.4-75.4)	75.6 (75.6-75.7)
Greece	75.3 (75.3-75.4)	75.6 (75.5-75.7)	75.7 (75.6-75.8)	75.7 (75.6-75.8)	75.9 (75.8-76.0)	75.9 (75.8-75.9)	76.3 (76.3-76.4)	76.3 (76.2-76.4)	76.7 (76.7-76.8)	76.9 (76.8-77.0)	77.0 (77.0-77.1)	77.1 (77.0-77.2)
Iceland	76.0 (75.7-76.3)	76.4 (76.1-76.7)	76.7 (76.4-76.9)	76.8 (76.6-77.0)	77.0 (76.8-77.2)	77.2 (76.9-77.5)	77.4 (77.1-77.7)	77.6 (77.4-77.9)	77.9 (77.6-78.2)	78.2 (77.9-78.3)	78.3 (78.0-78.4)	78.4 (78.2-78.6)
Ireland	73.6 (73.4-73.9)	74.1 (73.8-74.4)	74.4 (74.1-74.6)	74.4 (74.2-74.7)	74.7 (74.4-75.0)	75.0 (74.7-75.3)	75.3 (75.1-75.6)	75.6 (75.4-75.9)	75.9 (75.7-76.1)	75.9 (75.7-76.3)	76.3 (75.9-76.6)	76.7 (76.5-77.0)
Israel	73.9 (73.7-74.1)	74.1 (73.9-74.3)	74.5 (74.2-74.7)	74.3 (74.1-74.5)	74.5 (74.3-74.7)	74.9 (74.7-75.1)	75.0 (74.8-75.1)	75.3 (75.1-75.4)	75.6 (75.4-75.7)	76.2 (76.1-76.3)	76.7 (76.5-76.8)	76.5 (76.4-76.6)
Italy	74.1 (74.0-74.2)	74.5 (74.5-74.6)	74.9 (74.9-74.9)	74.9 (74.9-74.9)	75.4 (75.4-75.5)	75.7 (75.6-75.7)	76.0 (75.9-76.0)	76.3 (76.3-76.3)	76.6 (76.5-76.6)	76.8 (76.8-76.9)	77.0 (77.0-77.0)	77.0 (77.0-77.0)
Luxembourg	72.5 (72.3-72.7)	72.7 (72.5-72.9)	72.9 (72.8-73.1)	73.3 (73.1-73.5)	73.5 (73.3-73.7)	73.8 (73.6-74.0)	74.2 (74.0-74.3)	74.4 (74.2-74.5)	74.6 (74.4-74.8)	74.8 (74.7-75.0)	75.2 (75.0-75.4)	75.5 (75.4-75.7)
Malta	73.5 (73.2-73.7)	74.2 (74.0-74.5)	74.0 (73.8-74.3)	74.4 (74.2-74.7)	75.0 (74.8-75.3)	75.3 (75.1-75.5)	76.0 (75.8-76.3)	76.8 (76.5-77.0)	76.6 (76.3-76.9)	76.8 (76.5-77.0)	77.1 (76.8-77.4)	77.3 (77.0-77.6)
Netherlands	75.8 (75.7-75.9)	76.0 (75.9-76.1)	76.1 (76.0-76.1)	76.2 (76.2-76.3)	76.4 (76.3-76.4)	76.4 (76.3-76.5)	76.4 (76.3-76.4)	76.7 (76.7-76.8)	77.0 (77.0-77.1)	76.8 (76.8-76.9)	77.0 (77.0-77.1)	77.2 (77.1-77.3)
Norway	75.7 (75.5-75.8)	75.8 (75.7-76.0)	76.0 (75.9-76.1)	76.0 (75.9-76.1)	76.2 (76.1-76.4)	76.0 (75.9-76.2)	76.2 (76.1-76.3)	76.1 (76.0-76.2)	76.2 (76.1-76.3)	76.4 (76.3-76.6)	76.6 (76.5-76.8)	77.0 (76.9-77.2)
Portugal	71.7 (71.7-71.8)	71.9 (71.8-71.9)	72.6 (72.5-72.6)	72.6 (72.5-72.6)	72.8 (72.8-72.9)	73.1 (73.1-73.2)	73.4 (73.4-73.5)	73.8 (73.8-73.9)	73.9 (73.9-74.0)	74.3 (74.3-74.4)	74.2 (74.1-74.2)	74.2 (74.1-74.2)
Spain	75.4 (75.4-75.5)	75.7 (75.6-75.7)	76.1 (76.1-76.2)	76.1 (76.1-76.1)	76.3 (76.3-76.4)	76.5 (76.4-76.5)	76.6 (76.6-76.7)	76.7 (76.7-76.7)	76.8 (76.7-76.8)	76.8 (76.8-76.9)	76.9 (76.9-77.0)	77.0 (77.0-77.1)
Sweden	75.7 (75.6-75.8)	76.1 (76.0-76.2)	76.4 (76.3-76.4)	76.6 (76.6-76.7)	76.9 (76.8-76.9)	76.8 (76.7-76.9)	77.0 (76.9-77.1)	77.1 (77.0-77.2)	77.1 (77.0-77.2)	77.5 (77.4-77.6)	77.6 (77.5-77.7)	77.7 (77.6-77.8)
Switzerland	75.7 (75.6-75.8)	75.9 (75.8-76.0)	76.0 (75.9-76.1)	76.3 (76.2-76.4)	76.6 (76.6-76.8)	76.9 (76.8-77.0)	77.1 (77.0-77.2)	77.3 (77.2-77.4)	77.4 (77.3-77.5)	77.4 (77.3-77.6)	77.5 (77.4-77.6)	77.6 (77.5-77.7)
United Kingdom	73.5 (73.4-73.5)	73.8 (73.8-73.8)	74.0 (74.0-74.0)	74.3 (74.3-74.3)	74.6 (74.5-74.6)	74.7 (74.6-74.7)	74.9 (74.8-74.9)	75.1 (75.1-75.1)	75.3 (75.2-75.3)	75.5 (75.4-75.5)	75.7 (75.7-75.7)	76.0 (75.9-76.0)
England	73.7 (73.7-73.8)	74.1 (74.0-74.1)	74.3 (74.2-74.3)	74.6 (74.5-74.6)	74.8 (74.8-74.9)	74.9 (74.9-74.9)	75.1 (75.1-75.1)	75.3 (75.3-75.4)	75.5 (75.5-75.5)	75.7 (75.7-75.7)	75.9 (75.9-76.0)	76.2 (76.2-76.2)
Northern Ireland	71.9 (71.6-72.1)	71.8 (71.6-72.1)	72.2 (72.0-72.4)	72.7 (72.5-72.9)	73.2 (73.0-73.3)	73.4 (73.2-73.5)	73.6 (73.4-73.8)	73.9 (73.7-74.1)	74.1 (73.9-74.3)	74.6 (74.5-74.8)	74.9 (74.8-75.1)	75.3 (75.1-75.4)
Scotland	72.1 (72.0-72.2)	72.1 (72.0-72.2)	72.3 (72.2-72.4)	72.7 (72.6-72.8)	72.9 (72.8-73.0)	73.0 (72.9-73.1)	73.2 (73.2-73.3)	73.4 (73.4-73.5)	73.6 (73.5-73.7)	73.7 (73.6-73.8)	74.0 (73.9-74.1)	74.2 (74.1-74.3)
Wales	73.1 (72.9-73.2)	73.3 (73.2-73.4)	73.6 (73.5-73.8)	73.9 (73.8-74.0)	74.3 (74.2-74.4)	74.3 (74.2-74.4)	74.6 (74.5-74.6)	74.9 (74.8-74.9)	75.2 (75.1-75.3)	75.4 (75.3-75.5)	75.6 (75.6-75.7)	75.8 (75.7-75.9)
Southern Latin America	70.0 (69.9-70.1)	70.4 (70.3-70.5)	70.9 (70.9-71.0)	70.8 (70.8-70.9)	71.1 (71.1-71.2)	71.7 (71.5-71.9)	72.2 (72.1-72.2)	72.3 (72.3-72.4)	72.4 (72.4-72.4)	72.5 (72.5-72.5)	72.7 (72.6-72.7)	73.0 (72.9-73.0)
Argentina	69.9 (69.8-70.1)	70.1 (70.0-70.2)	70.7 (70.6-70.7)	70.6 (70.5-70.7)	70.9 (70.9-71.0)	71.6 (71.6-71.7)	71.9 (71.8-71.9)	72.0 (71.9-72.0)	72.1 (72.0-72.1)	72.3 (72.2-72.3)	72.5 (72.4-72.5)	72.7 (72.6-72.7)
Chile	70.0 (70.0-70.1)	71.0 (70.9-71.1)	71.4 (71.3-71.4)	71.3 (71.2-71.4)	71.5 (71.4-71.6)	71.8 (71.2-72.4)	72.9 (72.8-73.0)	73.3 (73.2-73.4)	73.3 (73.2-73.3)	72.9 (72.9-73.0)	73.1 (73.0-73.2)	74.1 (74.0-74.1)
Uruguay	70.5 (70.3-70.6)	71.0 (70.9-71.1)	71.5 (71.3-71.6)	71.7 (71.6-71.8)	71.8 (71.6-71.9)	71.9 (71.8-72.0)	72.1 (72.0-72.3)	72.3 (72.2-72.5)	72.6 (72.4-72.7)	72.8 (72.7-73.0)	73.0 (72.9-73.1)	72.6 (72.4-72.7)
Central Europe, Eastern Europe, and Central Asia	68.0 (67.8-68.1)	68.2 (68.1-68.3)	68.5 (68.4-68.6)	68.4 (68.3-68.5)	68.2 (68.1-68.2)	68.6 (68.5-68.6)	69.6 (69.5-69.6)	69.6 (69.5-69.6)	69.4 (69.3-69.5)	69.4 (69.3-69.4)	69.2 (69.1-69.3)	69.0 (68.9-69.0)
Eastern Europe	68.1 (67.9-68.2)	68.3 (68.1-68.4)	68.6 (68.5-68.7)	68.5 (68.4-68.5)	68.1 (68.0-68.1)	68.7 (68.6-68.8)	70.3 (70.2-70.3)	70.3 (70.2-70.3)	70.2 (70.1-70.2)	69.8 (69.7-69.9)	69.5 (69.4-69.6)	69.2 (69.2-69.3)
Belarus	70.6 (70.2-71.0)	70.7 (70.3-71.0)	70.7 (70.3-71.0)	70.6 (70.2-71.0)	70.3 (70.0-70.7)	70.6 (70.2-70.9)	71.4 (71.0-71.7)	71.3 (71.0-71.7)	71.2 (70.9-71.6)	70.8 (70.5-71.2)	70.5 (70.1-70.8)	70.1 (69.7-70.4)
Estonia	69.6 (69.4-69.7)	69.3 (69.1-69.4)	69.8 (69.6-69.9)	69.7 (69.6-69.9)	69.7 (69.5-69.8)	69.8 (69.7-70.0)	71.1 (70.9-71.2)	71.1 (71.0-71.2)	71.0 (70.9-71.2)	70.3 (70.2-70.5)	70.1 (69.9-70.2)	69.6 (69.4-69.7)
Latvia	69.1 (69.0-69.2)	69.0 (68.9-69.1)	69.6 (69.5-69.7)	69.4 (69.2-69.5)	69.4 (69.3-69.5)	69.7 (69.6-69.8)	71.0 (70.9-71.1)	71.0 (70.9-71.1)	71.0 (70.9-71.1)	70.2 (70.1-70.4)	69.6 (69.5-69.7)	69.1 (69.0-69.2)
Lithuania	70.9 (70.8-71.0)	70.7 (70.6-70.8)	70.9 (70.9-71.0)	71.0 (70.9-71.1)	70.6 (70.5-70.7)	70.9 (70.8-71.0)	72.4 (72.3-72.5)	72.1 (72.1-72.2)	72.2 (72.1-72.3)	71.6 (71.5-71.7)	71.5 (71.4-71.5)	70.5 (70.4-70.5)
Moldova	65.1 (64.3-65.8)	65.4 (64.7-66.0)	65.6 (65.0-66.1)	66.0 (65.4-66.5)	66.3 (65.7-66.8)	65.9 (65.4-66.3)	67.6 (67.1-67.9)	68.3 (67.9-68.7)	68.3 (67.9-68.7)	68.4 (68.0-68.7)	68.2 (67.8-68.5)	67.4 (67.0-67.7)
Russia	67.6 (67.5-67.6)	67.8 (67.8-67.9)	68.3 (68.3-68.3)	68.0 (67.9-68.0)	67.5 (67.5-67.5)	68.3 (68.2-68.3)	69.9 (69.9-70.0)	70.0 (69.9-70.0)	69.8 (69.8-69.9)	69.5 (69.5-69.5)	69.2 (69.2-69.3)	69.1 (69.1-69.2)
Ukraine	68.9 (68.4-69.4)	69.0 (68.5-69.4)	69.3 (68.8-69.7)	69.4 (69.0-69.8)	69.1 (68.8-69.4)	69.7 (69.4-70.0)	71.1 (70.7-71.3)	70.9 (70.6-71.2)	70.9 (70.6-71.1)	70.4 (70.1-70.7)	70.0 (69.7-70.3)	69.4 (69.1-69.7)
Central Europe	70.2 (70.1-70.3)	70.5 (70.4-70.6)	70.6 (70.5-70.7)	70.6 (70.6-70.7)	70.6 (70.5-70.7)	70.5 (70.4-70.6)	70.7 (70.7-70.8)	70.7 (70.7-70.8)	71.0 (70.9-71.0)	70.9 (70.9-71.0)	71.0 (70.9-71.0)	70.9 (70.8-70.9)
Albania	72.0 (70.8-73.0)	72.1 (71.0-73.0)	72.2 (71.3-73.1)	72.2 (71.4-73.0)	72.4 (71.7-73.1)	72.6 (72.0-73.2)	72.8 (72.3-73.4)	72.8 (72.2-73.3)	72.8 (72.3-73.2)	72.9 (72.4-73.3)	73.3 (72.8-73.7)	73.4 (73.0-73.8)
Bosnia and Herzegovina	70.4 (69.9-70.9)	70.7 (70.3-71.1)	70.9 (70.6-71.3)	71.2 (70.9-71.5)	71.4 (71.2-71.6)	71.2 (70.8-71.6)	71.5 (71.1-71.9)	71.8 (71.4-72.2)	72.2 (71.8-72.5)	72.4 (72.1-72.8)	72.7 (72.4-73.0)	72.7 (72.4-73.0)
Bulgaria	71.2 (71.1-71.3)	71.4 (71.3-71.5)	71.2 (71.2-71.3)	71.3 (71.2-71.4)	71.3 (71.2-71.3)	71.2 (71.1-71.3)	71.4 (71.3-71.5)	71.4 (71.3-71.5)	71.4 (71.3-71.5)	71.4 (71.3-71.5)	71.4 (71.3-71.5)	71.3 (71.2-71.4)
Croatia	70.9 (70.3-71.4)	71.1 (70.7-71.6)	71.3 (70.9-71.7)	71.5 (71.2-71.8)	71.6 (71.4-71.8)	71.7 (71.5-71.8)	71.8 (71.7-71.9)	71.9 (71.8-72.0)	72.1 (72.0-72.2)	72.3 (72.2-72.4)	72.6 (72.5-72.7)	71.9 (71.1-72.6)
Czech Republic	70.6 (70.6-70.7)	70.8 (70.7-70.8)	70.8 (70.7-70.9)	70.8 (70.8-70.9)	70.9 (70.9-71.0)	71.1 (71.1-71.2)	71.3 (71.2-71.4)	71.5 (71.4-71.6)	71.6 (71.6-71.7)	71.7 (71.6-71.8)	71.8 (71.7-71.8)	72.0 (71.9-72.1)
Hungary	69.2 (69.2-69.3)	69.3 (69.2-69.3)	69.2 (69.1-69.3)	69.1 (69.0-69.2)	69.0 (68.9-69.1)	69.0 (68.9-69.1)	69.2 (69.2-69.3)	69.6 (69.5-69.7)	69.8 (69.7-69.8)	69.6 (69.6-69.7)	69.5 (69.4-69.6)	69.2 (69.2-69.3)
Macedonia	70.6 (69.1-71.8)	70.7 (69.9-71.4)	70.7 (70.4-70.9)	71.0 (70.3-71.6)	71.3 (70.4-72.1)	71.6 (70.7-72.5)	71.7 (70.7-72.5)	71.6 (70.8-72.3)	71.4 (70.9-71.9)	71.5 (71.3-71.7)	71.7 (71.5-71.9)	71.7 (71.5-71.8)

Location	1992	1993	1994	1995	1996	1997	1998	1999	2000	2001	2002	2003
Global	65.1 (64.9-65.4)	65.2 (64.9-65.4)	65.1 (64.8-65.4)	65.5 (65.3-65.7)	65.8 (65.6-66.0)	66.0 (65.8-66.2)	66.3 (66.1-66.5)	66.4 (66.2-66.6)	66.6 (66.4-66.8)	66.8 (66.6-67.0)	67.1 (66.9-67.3)	67.4 (67.2-67.6)
High SDI	74.7 (74.7-74.8)	74.3 (74.3-74.4)	74.2 (74.2-74.2)	74.5 (74.5-74.5)	75.1 (75.0-75.1)	75.5 (75.5-75.6)	75.8 (75.8-75.8)	75.7 (75.6-75.7)	75.8 (75.7-75.8)	75.9 (75.9-75.9)	76.1 (76.0-76.1)	76.2 (76.2-76.2)
High-middle SDI	69.5 (69.2-69.9)	69.7 (69.3-70.0)	69.8 (69.5-70.1)	69.9 (69.6-70.2)	70.1 (69.9-70.3)	70.4 (70.1-70.6)	70.6 (70.4-70.8)	70.7 (70.5-70.9)	71.0 (70.8-71.2)	71.2 (71.0-71.4)	71.3 (71.1-71.5)	71.5 (71.3-71.7)
Middle SDI	65.2 (64.7-65.6)	65.5 (65.0-66.0)	65.9 (65.4-66.3)	66.1 (65.7-66.6)	66.5 (66.1-66.9)	66.8 (66.4-67.2)	67.0 (66.7-67.4)	67.3 (66.9-67.7)	67.6 (67.2-67.9)	67.7 (67.4-68.1)	68.0 (67.6-68.3)	68.5 (68.2-68.8)
Low-middle SDI	57.7 (57.2-58.3)	57.9 (57.4-58.5)	58.2 (57.7-58.7)	58.5 (57.9-59.0)	58.7 (58.2-59.2)	58.9 (58.4-59.4)	59.1 (58.7-59.6)	59.5 (59.0-59.9)	59.7 (59.2-60.2)	60.0 (59.5-60.5)	60.3 (59.8-60.8)	60.7 (60.2-61.2)
Low SDI	51.2 (50.4-51.9)	51.3 (50.4-52.0)	48.2 (45.7-50.5)	51.3 (50.5-52.0)	51.2 (50.5-51.9)	51.6 (50.9-52.2)	51.7 (51.0-52.4)	51.9 (51.2-52.6)	52.2 (51.5-53.0)	53.0 (52.2-53.7)	53.5 (52.7-54.2)	54.0 (53.3-54.7)
High-income	76.5 (76.4-76.5)	76.6 (76.6-76.6)	76.8 (76.8-76.8)	76.9 (76.9-77.0)	77.3 (77.2-77.3)	77.6 (77.6-77.6)	77.8 (77.8-77.8)	77.9 (77.9-77.9)	78.2 (78.2-78.2)	78.4 (78.3-78.4)	78.5 (78.5-78.6)	78.7 (78.7-78.7)
High-income North America	76.0 (76.0-76.0)	75.9 (75.9-75.9)	76.0 (76.0-76.0)	76.1 (76.1-76.1)	76.5 (76.5-76.5)	76.9 (76.8-76.9)	77.1 (77.0-77.1)	77.1 (77.1-77.1)	77.2 (77.2-77.2)	77.2 (77.2-77.2)	77.4 (77.3-77.4)	77.5 (77.5-77.5)
Canada	77.7 (77.7-77.8)	77.8 (77.8-77.8)	77.9 (77.9-78.0)	78.0 (78.0-78.1)	78.3 (78.2-78.3)	78.6 (78.5-78.6)	78.8 (78.7-78.8)	79.0 (78.9-79.0)	79.2 (79.2-79.3)	79.5 (79.4-79.5)	79.6 (79.6-79.7)	79.8 (79.8-79.9)
Greenland	65.5 (64.9-66.2)	65.9 (65.3-66.6)	66.3 (65.7-66.8)	66.5 (66.0-67.1)	66.8 (66.2-67.4)	66.9 (66.3-67.4)	67.2 (66.7-67.8)	67.5 (66.9-68.0)	67.8 (67.2-68.3)	68.0 (67.4-68.5)	68.3 (67.8-68.9)	68.5 (68.0-69.1)
United States	75.8 (75.7-75.8)	75.7 (75.7-75.7)	75.8 (75.8-75.8)	75.9 (75.8-75.9)	76.3 (76.2-76.3)	76.6 (76.6-76.7)	76.8 (76.8-76.9)	76.9 (76.8-76.9)	76.9 (76.9-76.9)	76.9 (76.9-77.0)	77.1 (77.1-77.1)	77.3 (77.2-77.3)
Australasia	77.3 (77.3-77.4)	77.7 (77.6-77.7)	77.8 (77.8-77.9)	78.1 (78.1-78.1)	78.3 (78.3-78.3)	78.6 (78.6-78.7)	79.0 (78.9-79.0)	79.2 (79.2-79.3)	79.5 (79.5-79.6)	79.8 (79.8-79.9)	80.1 (80.0-80.1)	80.4 (80.3-80.4)
Australia	77.6 (77.5-77.6)	77.9 (77.9-78.0)	78.1 (78.0-78.1)	78.3 (78.3-78.4)	78.5 (78.5-78.6)	78.8 (78.8-78.9)	79.2 (79.1-79.2)	79.4 (79.4-79.5)	79.8 (79.7-79.8)	80.1 (80.0-80.1)	80.3 (80.3-80.3)	80.6 (80.6-80.7)
New Zealand	76.2 (76.1-76.3)	76.5 (76.4-76.6)	76.7 (76.6-76.8)	77.0 (76.9-77.1)	77.3 (77.2-77.4)	77.6 (77.5-77.7)	78.0 (77.9-78.1)	78.2 (78.1-78.2)	78.4 (78.3-78.5)	78.6 (78.5-78.7)	78.9 (78.8-79.0)	79.1 (79.1-79.2)
High-income Asia Pacific	77.9 (77.8-77.9)	78.1 (78.0-78.2)	78.4 (78.4-78.5)	78.5 (78.4-78.6)	79.0 (78.9-79.1)	79.3 (79.2-79.3)	79.4 (79.4-79.5)	79.6 (79.6-79.7)	80.0 (80.0-80.1)	80.4 (80.3-80.4)	80.7 (80.6-80.7)	80.9 (80.8-80.9)
Brunei	74.0 (73.6-74.5)	74.2 (73.8-74.7)	74.4 (74.0-74.9)	74.7 (74.2-75.1)	74.9 (74.5-75.3)	75.2 (74.9-75.6)	75.6 (75.2-75.9)	75.8 (75.5-76.1)	75.9 (75.6-76.2)	76.1 (75.8-76.4)	76.3 (76.0-76.7)	76.5 (76.2-76.8)
Japan	79.3 (79.2-79.3)	79.4 (79.4-79.5)	79.8 (79.7-79.8)	79.7 (79.5-79.8)	80.2 (80.2-80.2)	80.4 (80.4-80.5)	80.5 (80.5-80.5)	80.6 (80.6-80.6)	81.0 (81.0-81.0)	81.3 (81.3-81.3)	81.6 (81.6-81.6)	81.7 (81.7-81.7)
Singapore	76.0 (75.9-76.2)	76.2 (76.1-76.4)	76.3 (76.2-76.5)	76.4 (76.3-76.6)	76.7 (76.6-76.9)	77.2 (77.1-77.3)	77.6 (77.5-77.7)	78.0 (77.8-78.1)	78.3 (78.2-78.5)	78.6 (78.5-78.8)	78.9 (78.8-79.1)	79.3 (79.2-79.5)
South Korea	72.7 (72.4-73.0)	73.1 (72.9-73.4)	73.6 (73.3-73.8)	74.0 (73.7-74.2)	74.5 (74.3-74.7)	74.9 (74.8-75.1)	75.4 (75.2-75.6)	75.8 (75.6-76.0)	76.3 (76.1-76.4)	76.7 (76.5-76.9)	77.1 (77.0-77.3)	77.7 (77.5-77.9)
Western Europe	76.7 (76.7-76.7)	76.9 (76.9-76.9)	77.1 (77.1-77.2)	77.3 (77.3-77.3)	77.6 (77.6-77.6)	77.9 (77.9-77.9)	78.1 (78.1-78.1)	78.3 (78.3-78.3)	78.5 (78.5-78.6)	78.8 (78.8-78.8)	79.0 (79.0-79.0)	79.2 (79.1-79.2)
Andorra	81.9 (81.5-82.3)	82.2 (81.8-82.5)	82.3 (82.0-82.7)	82.6 (82.2-82.9)	82.8 (82.5-83.1)	83.1 (82.8-83.4)	83.3 (83.0-83.6)	83.5 (83.2-83.8)	83.7 (83.4-84.0)	83.9 (83.6-84.2)	84.1 (83.9-84.4)	84.2 (83.9-84.4)
Austria	76.0 (75.9-76.1)	76.2 (76.2-76.3)	76.5 (76.5-76.6)	76.8 (76.8-76.9)	77.1 (77.0-77.1)	77.6 (77.5-77.6)	77.8 (77.7-77.8)	78.0 (77.9-78.0)	78.2 (78.1-78.2)	78.6 (78.5-78.6)	78.8 (78.8-78.9)	79.0 (78.9-79.0)
Belgium	76.3 (76.3-76.4)	76.5 (76.4-76.5)	76.6 (76.5-76.7)	76.9 (76.8-76.9)	77.2 (77.1-77.3)	77.4 (77.3-77.4)	77.5 (77.5-77.6)	77.7 (77.6-77.8)	77.8 (77.7-77.9)	78.0 (77.9-78.1)	78.2 (78.1-78.3)	78.5 (78.4-78.6)
Cyprus	76.7 (76.5-76.8)	76.8 (76.6-76.9)	76.4 (76.3-76.5)	76.7 (76.5-76.8)	77.1 (77.0-77.2)	77.1 (77.0-77.3)	77.1 (77.0-77.2)	77.5 (77.3-77.6)	77.7 (77.6-77.9)	78.5 (78.3-78.6)	78.7 (78.5-78.8)	79.0 (78.9-79.1)
Denmark	75.4 (75.4-75.5)	75.4 (75.4-75.5)	75.4 (75.3-75.5)	75.6 (75.5-75.6)	75.9 (75.8-76.0)	76.2 (76.1-76.3)	76.7 (76.6-76.8)	76.3 (76.2-76.4)	77.1 (77.0-77.2)	77.2 (77.1-77.3)	77.2 (77.2-77.3)	77.6 (77.5-77.6)
Finland	75.7 (75.6-75.8)	76.0 (75.9-76.1)	76.4 (76.3-76.5)	76.7 (76.6-76.8)	76.8 (76.7-76.9)	77.1 (77.0-77.2)	77.3 (77.2-77.4)	77.5 (77.4-77.6)	77.8 (77.7-77.9)	78.1 (78.0-78.2)	78.3 (78.2-78.4)	78.5 (78.4-78.6)
France	77.6 (77.5-77.6)	77.7 (77.7-77.7)	78.0 (78.0-78.0)	78.2 (78.1-78.2)	78.4 (78.3-78.4)	78.7 (78.7-78.7)	78.7 (78.7-78.7)	78.8 (78.8-78.8)	79.1 (79.1-79.1)	79.2 (79.2-79.3)	79.4 (79.4-79.5)	79.6 (79.6-79.6)
Germany	76.0 (75.9-76.0)	76.1 (76.1-76.2)	76.4 (76.4-76.4)	76.6 (76.6-76.7)	76.9 (76.9-76.9)	77.3 (77.2-77.3)	77.6 (77.6-77.6)	77.8 (77.8-77.9)	78.1 (78.1-78.1)	78.4 (78.4-78.4)	78.6 (78.6-78.6)	78.8 (78.8-78.8)
Greece	77.1 (77.1-77.2)	77.5 (77.4-77.5)	77.7 (77.6-77.7)	77.8 (77.7-77.9)	77.9 (77.8-78.0)	78.1 (78.1-78.2)	78.2 (78.1-78.3)	78.3 (78.2-78.3)	78.6 (78.5-78.6)	78.9 (78.8-79.0)	79.1 (79.0-79.2)	79.3 (79.2-79.3)
Iceland	78.6 (78.4-78.9)	78.8 (78.6-79.1)	79.1 (78.9-79.3)	78.9 (78.6-79.1)	79.4 (79.2-79.6)	79.6 (79.4-79.8)	79.9 (79.6-80.1)	80.2 (79.9-80.4)	80.5 (80.2-80.7)	80.8 (80.5-81.0)	81.0 (80.8-81.2)	81.3 (81.0-81.5)
Ireland	76.8 (76.5-77.1)	76.9 (76.6-77.1)	77.3 (76.9-77.6)	77.1 (76.9-77.4)	77.5 (77.2-77.9)	77.4 (77.1-77.8)	77.6 (77.3-78.0)	77.7 (77.4-78.1)	77.8 (77.5-78.2)	78.4 (78.1-78.7)	78.8 (78.5-79.0)	79.1 (78.9-79.3)
Israel	76.3 (76.2-76.4)	76.9 (76.8-77.0)	77.1 (77.0-77.2)	77.4 (77.3-77.5)	77.7 (77.6-77.8)	77.9 (77.8-78.0)	78.1 (78.0-78.3)	78.5 (78.4-78.6)	78.6 (78.5-78.7)	78.8 (78.6-78.9)	78.6 (78.5-78.8)	79.1 (78.9-79.3)
Italy	77.3 (77.3-77.3)	77.6 (77.6-77.6)	77.8 (77.8-77.9)	78.1 (78.0-78.1)	78.3 (78.3-78.4)	78.7 (78.7-78.7)	78.9 (78.9-78.9)	79.3 (79.2-79.3)	79.6 (79.5-79.6)	79.8 (79.8-79.9)	80.1 (80.1-80.1)	80.1 (80.1-80.2)
Luxembourg	75.8 (75.7-76.0)	76.2 (76.0-76.3)	76.5 (76.4-76.7)	76.9 (76.7-77.0)	77.1 (76.9-77.3)	77.4 (77.2-77.5)	77.7 (77.6-77.9)	78.1 (77.9-78.3)	78.5 (78.3-78.7)	78.7 (78.6-78.9)	79.0 (78.8-79.2)	79.1 (78.9-79.3)
Malta	77.5 (77.2-77.9)	77.9 (77.6-78.3)	78.2 (77.8-78.5)	78.4 (78.0-78.7)	78.5 (78.1-78.8)	78.8 (78.4-79.1)	78.9 (78.6-79.3)	79.0 (78.6-79.3)	79.2 (78.8-79.5)	79.4 (79.1-79.8)	79.8 (79.4-80.1)	80.1 (79.8-80.4)
Netherlands	77.3 (77.2-77.4)	77.2 (77.1-77.2)	77.5 (77.4-77.5)	77.6 (77.5-77.7)	77.6 (77.6-77.7)	77.9 (77.8-78.0)	78.1 (78.0-78.1)	78.0 (77.9-78.1)	78.1 (78.0-78.2)	78.3 (78.2-78.4)	78.5 (78.4-78.6)	78.7 (78.6-78.8)
Norway	77.1 (77.0-77.2)	77.5 (77.3-77.6)	77.7 (77.6-77.8)	77.9 (77.8-78.0)	78.2 (78.0-78.3)	78.3 (78.2-78.4)	78.3 (78.2-78.4)	78.5 (78.4-78.7)	78.6 (78.5-78.7)	78.9 (78.8-79.1)	79.1 (79.0-79.2)	79.5 (79.4-79.7)
Portugal	74.6 (74.5-74.6)	74.7 (74.7-74.8)	75.5 (75.5-75.6)	75.5 (75.4-75.5)	75.5 (75.4-75.5)	75.8 (75.8-75.9)	76.1 (76.1-76.2)	76.4 (76.3-76.4)	76.8 (76.7-76.8)	76.8 (76.7-76.9)	77.3 (77.2-77.3)	77.6 (77.5-77.6)
Spain	77.4 (77.3-77.4)	77.6 (77.6-77.6)	77.8 (77.8-77.8)	77.9 (77.9-77.9)	78.1 (78.0-78.1)	78.6 (78.6-78.7)	78.8 (78.8-78.9)	78.8 (78.8-78.8)	79.2 (79.1-79.2)	79.5 (79.4-79.5)	79.6 (79.6-79.6)	79.6 (79.6-79.7)
Sweden	78.1 (78.1-78.2)	78.3 (78.2-78.4)	78.5 (78.1-78.7)	78.9 (78.8-79.0)	79.1 (79.0-79.2)	79.4 (79.3-79.4)	79.5 (79.4-79.6)	79.5 (79.5-79.6)	79.7 (79.6-79.8)	79.8 (79.7-79.9)	80.0 (79.9-80.1)	80.2 (80.1-80.3)
Switzerland	77.9 (77.8-78.0)	78.3 (78.2-78.4)	78.4 (78.3-78.5)	78.5 (78.4-78.6)	79.1 (79.0-79.2)	79.2 (79.1-79.3)	79.5 (79.4-79.6)	79.8 (79.8-79.9)	79.8 (79.7-79.9)	80.2 (80.1-80.3)	80.4 (80.3-80.5)	80.6 (80.5-80.7)
United Kingdom	76.2 (76.2-76.3)	76.3 (76.3-76.4)	76.6 (76.6-76.7)	76.7 (76.7-76.7)	76.9 (76.9-76.9)	77.1 (77.1-77.2)	77.3 (77.3-77.3)	77.5 (77.5-77.5)	77.8 (77.8-77.8)	78.0 (78.0-78.0)	78.2 (78.2-78.2)	78.4 (78.4-78.4)
England	76.5 (76.4-76.5)	76.6 (76.5-76.6)	76.9 (76.8-76.9)	76.9 (76.9-77.0)	77.2 (77.1-77.2)	77.4 (77.4-77.4)	77.6 (77.5-77.6)	77.7 (77.7-77.8)	78.0 (78.0-78.1)	78.3 (78.2-78.3)	78.5 (78.4-78.5)	78.7 (78.6-78.7)
Northern Ireland	75.6 (75.5-75.8)	75.7 (75.6-75.9)	76.1 (75.9-76.3)	76.3 (76.1-76.4)	76.6 (76.4-76.7)	76.8 (76.7-77.0)	77.1 (76.9-77.2)	77.2 (77.0-77.3)	77.6 (77.4-77.7)	77.8 (77.6-77.9)	78.0 (77.8-78.1)	78.1 (77.9-78.2)
Scotland	74.5 (74.4-74.6)	74.6 (74.5-74.7)	74.9 (74.8-75.0)	74.9 (74.9-75.0)	75.1 (75.0-75.2)	75.4 (75.3-75.5)	75.5 (75.4-75.6)	75.6 (75.6-75.7)	75.8 (75.7-75.9)	76.1 (76.0-76.1)	76.2 (76.1-76.3)	76.4 (76.3-76.5)
Wales	76.1 (76.0-76.2)	76.2 (76.1-76.3)	76.4 (76.3-76.5)	76.5 (76.4-76.6)	76.6 (76.5-76.7)	76.8 (76.7-76.9)	77.0 (76.9-77.1)	77.1 (77.0-77.1)	77.4 (77.3-77.5)	77.6 (77.5-77.7)	77.8 (77.7-77.9)	77.9 (77.8-78.0)
Southern Latin America	73.3 (73.3-73.4)	73.6 (73.5-73.6)	73.8 (73.8-73.9)	73.9 (73.9-74.0)	74.1 (74.0-74.1)	74.2 (74.2-74.3)	74.5 (74.4-74.5)	74.6 (74.6-74.7)	75.0 (74.9-75.0)	75.1 (75.0-75.1)	75.2 (75.1-75.2)	75.3 (75.3-75.4)
Argentina	72.8 (72.7-72.8)	73.1 (73.0-73.1)	73.4 (73.3-73.4)	73.5 (73.4-73.5)	73.5 (73.5-73.6)	73.6 (73.6-73.7)	73.8 (73.7-73.8)	73.9 (73.8-73.9)	74.2 (74.2-74.2)	74.3 (74.3-74.3)	74.3 (74.3-74.4)	74.5 (74.5-74.6)
Chile	75.0 (74.9-75.0)	75.2 (75.1-75.3)	75.3 (75.2-75.4)	75.4 (75.3-75.5)	75.7 (75.6-75.8)	76.2 (76.1-76.3)	76.6 (76.5-76.7)	76.9 (76.8-77.0)	77.3 (77.2-77.4)	77.4 (77.3-77.5)	77.7 (77.6-77.8)	77.8 (77.8-77.9)
Uruguay	73.2 (73.1-73.4)	73.2 (73.1-73.4)	73.3 (73.2-73.5)	73.4 (73.2-73.5)	73.5 (73.4-73.6)	73.7 (73.6-73.9)	74.0 (73.9-74.1)	74.3 (74.1-74.4)	74.6 (74.5-74.8)	74.8 (74.7-74.9)	75.0 (74.8-75.1)	75.0 (74.9-75.1)
Central Europe, Eastern Europe, and Central Asia	68.3 (68.2-68.4)	67.1 (67.0-67.2)	66.3 (66.2-66.4)	66.4 (66.3-66.5)	67.1 (67.0-67.2)	67.8 (67.7-67.9)	68.2 (68.1-68.3)	67.8 (67.6-67.9)	67.6 (67.5-67.7)	67.7 (67.6-67.9)	67.8 (67.6-67.9)	67.8 (67.7-67.9)
Eastern Europe	68.3 (68.2-68.4)	66.2 (66.2-66.3)	65.0 (64.9-65.0)	65.2 (65.1-65.2)	66.2 (66.1-66.3)	67.2 (67.1-67.3)	67.6 (67.5-67.6)	66.5 (66.4-66.7)	66.0 (65.9-66.1)	66.0 (65.9-66.1)	65.8 (65.7-66.0)	65.8 (65.7-65.9)
Belarus	69.6 (69.3-70.0)	68.9 (68.6-69.2)	68.6 (68.3-68.9)	68.2 (67.9-68.5)	68.1 (67.8-68.5)	68.0 (67.7-68.3)	67.9 (67.6-68.2)	67.7 (67.4-68.1)	68.1 (67.8-68.4)	68.0 (67.7-68.3)	67.9 (67.6-68.1)	68.2 (67.9-68.5)
Estonia	69.3 (69.1-69.4)	68.2 (68.1-68.4)	66.3 (65.9-66.7)	67.7 (67.5-67.8)	69.8 (69.6-69.9)	70.0 (69.8-70.1)	69.5 (69.4-69.7)	70.2 (70.1-70.4)	70.4 (70.3-70.6)	70.0 (69.8-70.2)	70.9 (70.8-71.1)	71.5 (71.4-71.7)
Latvia	68.4 (68.2-68.5)	66.7 (66.6-66.9)	65.7 (65.6-65.8)	66.5 (66.4-66.7)	68.6 (68.5-68.7)	69.3 (69.2-69.5)	69.0 (68.9-69.2)	70.0 (69.8-70.1)	70.5 (70.4-70.6)	70.0 (69.8-70.1)	70.4 (70.2-70.5)	70.8 (70.7-70.9)
Lithuania	70.3 (70.2-70.4)	69.1 (69.0-69.2)	68.7 (68.6-68.7)	69.0 (68.9-69.1)	70.2 (70.1-70.3)	71.1 (71.0-71.2)	71.3 (71.3-71.4)	71.8 (71.7-71.9)	72.1 (72.0-72.1)	71.6 (71.5-71.7)	72.1 (72.0-72.1)	72.1 (72.1-72.2)
Moldova	67.6 (67.1-68.1)	67.4 (67.1-67.8)	65.8 (65.4-66.1)	65.4 (65.0-65.7)	66.1 (65.7-66.5)	67.9 (67.5-68.3)	68.5 (68.1-68.9)	68.4 (68.0-68.8)	68.6 (68.1-68.9)	69.2 (68.8-69.5)	69.6 (69.3-70.0)	69.9 (69.5-70.2)
Russia	68.0 (68.0-68.0)	65.2 (65.2-65.3)	63.8 (63.7-63.8)	64.4 (64.3-64.5)	65.7 (65.7-65.8)	67.0 (66.9-67.0)	67.2 (67.2-67.3)	65.9 (65.7-66.0)	65.1 (65.0-65.2)	65.1 (65.0-65.2)	64.9 (64.7-65.0)	64.7 (64.5-64.9)
Ukraine	68.8 (68.5-69.1)	68.3 (68.0-68.6)	67.5 (67.2-67.8)	66.5 (66.2-66.7)	66.7 (66.4-66.9)	67.3 (67.0-67.6)	68.1 (67.8-68.4)	67.7 (67.3-68.0)	67.3 (67.0-67.6)	67.5 (67.2-67.8)	67.5 (67.2-67.7)	67.7 (67.4-67.9)
Central Europe	70.9 (70.8-71.0)	71.1 (71.0-71.2)	71.2 (71.2-71.3)	71.2 (71.2-71.3)	71.6 (71.6-71.7)	71.8 (71.8-71.8)	72.1 (72.1-72.2)	72.4 (72.3-72.5)	72.9 (72.9-72.9)	73.2 (73.2-73.2)	73.5 (73.4-73.5)	73.7 (73.7-73.7)
Albania	73.6 (73.1-74.0)	73.6 (73.1-74.0)	73.6 (73.2-74.0)	73.7 (73.3-74.1)	73.8 (73.4-74.2)	74.1 (73.7-74.5)	74.2 (73.8-74.5)	74.2 (73.7-74.5)	74.7 (74.2-75.0)	75.1 (74.7-75.5)	75.5 (75.1-75.8)	75.7 (75.3-76.1)
Bosnia and Herzegovina	68.0 (66.6-69.4)	68.3 (66.7-70.0)	71.1 (69.3-72.8)	67.0 (65.8-68.4)	73.8 (73.6-74.0)	74.4 (74.2-74.6)	74.8 (74.7-75.0)	75.3 (75.2-75.4)	75.7 (75.6-75.9)	76.2 (76.1-76.4)	76.4 (76.3-76.6)	76.7 (76.5-76.8)
Bulgaria	71.2 (71.1-71.3)	71.1 (71.1-71.2)	70.9 (70.8-71.0)	71.0 (70.9-71.0)	70.9 (70.8-70.9)	70.6 (70.5-70.7)	71.0 (71.0-71.1)	71.4 (71.3-71.5)	71.6 (71.5-71.7)	71.8 (71.8-71.9)	72.1 (72.0-72.2)	72.3 (72.2-72.4)
Croatia	72.3 (71.7-72.9)	72.9 (72.6-73.2)	73.4 (73.2-73.5)	73.0 (72.5-73.4)	73.4 (73.3-73.5)	73.4 (73.3-73.5)	73.5 (73.4-73.6)	73.8 (73.7-73.9)	74.2 (74.1-74.3)	74.6 (74.5-74.7)	74.9 (74.8-75.0)	75.1 (75.0-75.2)
Czech Republic	72.3 (72.3-72.4)	72.7 (72.7-72.8)	73.0 (73.0-73.1)	73.4 (73.4-73.5)	73.8 (73.8-73.9)	74.2 (74.1-74.2)	74.5 (74.4-74.6)	74.7 (74.7-74.8)	75.0 (75.0-75.1)	75.3 (75.2-75.3)	75.4 (75.4-75.5)	75.6 (75.6-75.7)
Hungary	69.1 (69.1-69.2)	69.2 (69.2-69.3)	69.5 (69.5-69.6)	70.1 (70.0-70.1)	70.5 (70.5-70.6)	70.9 (70.8-71.0)	71.1 (71.0-71.2)	71.3 (71.2-71.4)	71.8 (71.7-71.8)	72.3 (72.2-72.3)	72.6 (72.5-72.6)	72.7 (72.6-72.8)
Macedonia	71.6 (71.4-71.7)	71.5 (71.3-71.7)	71.7 (71.6-71.8)	71.8 (71.6-71.9)	72.1 (71.9-72.2)	72.3 (72.1-72.4)	72.4 (72.3-72.5)	72.6 (72.5-72.8)	72.9 (72.8-73.0)	73.0 (72.8-73.2)	73.3 (73.1-73.4)	73.4 (73.3-73.5)

Location	2004	2005	2006	2007	2008	2009	2010	2011	2012	2013	2014	2015
Global	67.7 (67.5-68.0)	68.2 (68.0-68.4)	68.7 (68.4-68.9)	69.1 (68.9-69.3)	69.4 (69.2-69.6)	69.9 (69.7-70.1)	70.2 (69.9-70.4)	70.6 (70.4-70.9)	71.0 (70.7-71.3)	71.3 (71.0-71.6)	71.6 (71.2-71.9)	71.8 (71.5-72.2)
High SDI	76.6 (76.6-76.6)	76.8 (76.7-76.8)	77.3 (77.2-77.3)	77.6 (77.6-77.7)	77.9 (77.8-77.9)	78.2 (78.2-78.2)	78.4 (78.4-78.5)	78.7 (78.7-78.7)	79.0 (78.9-79.0)	79.1 (79.1-79.2)	79.2 (79.2-79.2)	79.3 (79.2-79.4)
High-middle SDI	71.8 (71.6-72.0)	72.2 (72.0-72.4)	72.6 (72.4-72.8)	72.9 (72.7-73.1)	73.2 (73.0-73.4)	73.6 (73.4-73.8)	74.0 (73.8-74.2)	74.5 (74.3-74.8)	75.0 (74.8-75.2)	75.4 (75.1-75.6)	75.6 (75.3-75.8)	75.8 (75.5-76.1)
Middle SDI	68.6 (68.3-69.0)	69.2 (68.9-69.5)	69.6 (69.3-69.9)	70.0 (69.8-70.3)	70.3 (70.0-70.6)	70.7 (70.4-71.0)	71.0 (70.7-71.3)	71.3 (71.0-71.6)	71.6 (71.3-71.9)	71.9 (71.6-72.3)	72.1 (71.8-72.5)	72.5 (72.1-72.9)
Low-middle SDI	61.1 (60.6-61.6)	61.4 (60.9-61.9)	62.0 (61.5-62.5)	62.4 (62.0-62.9)	62.7 (62.2-63.3)	63.4 (62.9-63.9)	63.4 (62.8-64.0)	64.2 (63.6-64.8)	64.5 (63.9-65.1)	64.9 (64.3-65.5)	65.3 (64.7-65.9)	65.7 (65.0-66.4)
Low SDI	54.7 (53.9-55.4)	55.3 (54.5-56.1)	56.0 (55.2-56.9)	56.7 (55.8-57.6)	57.4 (56.4-58.3)	58.0 (57.0-59.0)	58.6 (57.4-59.7)	59.1 (57.8-60.2)	59.5 (58.3-60.8)	59.9 (58.6-61.4)	60.3 (58.9-61.8)	60.7 (59.1-62.3)
High-income	79.1 (79.1-79.1)	79.3 (79.3-79.3)	79.5 (79.5-79.5)	79.7 (79.7-79.7)	79.9 (79.9-79.9)	80.1 (80.1-80.1)	80.3 (80.3-80.3)	80.4 (80.4-80.5)	80.6 (80.6-80.6)	80.7 (80.6-80.7)	80.7 (80.7-80.7)	80.8 (80.8-80.8)
High-income North America	77.9 (77.8-77.9)	77.9 (77.9-78.0)	78.1 (78.1-78.1)	78.4 (78.4-78.4)	78.6 (78.6-78.6)	78.8 (78.8-78.8)	79.1 (79.1-79.1)	79.2 (79.2-79.2)	79.3 (79.3-79.3)	79.3 (79.3-79.4)	79.4 (79.3-79.4)	79.4 (79.4-79.5)
Canada	80.1 (80.0-80.1)	80.2 (80.2-80.3)	80.5 (80.4-80.5)	80.6 (80.6-80.7)	80.8 (80.8-80.8)	81.1 (81.0-81.1)	81.3 (81.3-81.4)	81.6 (81.6-81.6)	81.6 (81.5-81.7)	81.6 (81.5-81.7)	81.7 (81.5-81.8)	81.7 (81.6-81.9)
Greenland	68.8 (68.2-69.3)	69.1 (68.5-69.6)	69.5 (69.0-70.1)	70.0 (69.5-70.4)	70.3 (69.8-70.8)	70.6 (70.0-71.1)	70.8 (70.3-71.3)	71.2 (70.7-71.7)	71.6 (71.0-72.1)	71.8 (71.2-72.4)	72.0 (71.3-72.6)	72.1 (71.4-72.8)
United States	77.6 (77.6-77.6)	77.6 (77.6-77.7)	77.8 (77.8-77.9)	78.1 (78.1-78.1)	78.3 (78.3-78.3)	78.5 (78.5-78.6)	78.8 (78.8-78.8)	78.9 (78.9-78.9)	79.0 (79.0-79.0)	79.1 (79.0-79.1)	79.1 (79.0-79.1)	79.1 (79.1-79.2)
Australasia	80.6 (80.6-80.7)	80.9 (80.9-81.0)	81.1 (81.1-81.2)	81.3 (81.2-81.3)	81.4 (81.4-81.4)	81.6 (81.5-81.6)	81.8 (81.8-81.8)	82.0 (81.9-82.0)	82.2 (82.1-82.2)	82.2 (82.2-82.3)	82.2 (82.1-82.2)	82.2 (82.1-82.3)
Australia	80.9 (80.9-80.9)	81.2 (81.1-81.2)	81.4 (81.3-81.4)	81.5 (81.4-81.5)	81.6 (81.6-81.7)	81.8 (81.8-81.8)	82.0 (82.0-82.1)	82.2 (82.2-82.3)	82.4 (82.3-82.4)	82.4 (82.3-82.4)	82.3 (82.3-82.4)	82.3 (82.3-82.4)
New Zealand	79.4 (79.3-79.5)	79.8 (79.7-79.8)	79.9 (79.8-80.0)	80.1 (80.0-80.2)	80.3 (80.2-80.4)	80.5 (80.4-80.6)	80.8 (80.7-80.8)	80.8 (80.7-80.9)	81.1 (81.0-81.2)	81.4 (81.2-81.5)	81.4 (81.2-81.6)	81.4 (81.2-81.6)
High-income Asia Pacific	81.2 (81.1-81.2)	81.3 (81.3-81.3)	81.6 (81.5-81.6)	81.8 (81.7-81.8)	81.9 (81.9-82.0)	82.2 (82.1-82.2)	82.2 (82.2-82.3)	82.2 (81.9-82.4)	82.5 (82.4-82.5)	82.6 (82.5-82.6)	82.6 (82.6-82.7)	82.7 (82.6-82.8)
Brunei	76.7 (76.3-76.9)	76.7 (76.4-77.0)	76.8 (76.5-77.1)	76.8 (76.5-77.1)	76.9 (76.6-77.2)	77.0 (76.7-77.3)	77.1 (76.8-77.3)	76.9 (76.6-77.2)	76.9 (76.5-77.2)	76.9 (76.6-77.3)	77.1 (76.7-77.5)	77.1 (76.7-77.6)
Japan	81.9 (81.9-81.9)	82.0 (81.9-82.0)	82.2 (82.2-82.2)	82.4 (82.3-82.4)	82.5 (82.5-82.5)	82.7 (82.7-82.7)	82.7 (82.7-82.8)	82.6 (82.2-82.8)	82.9 (82.9-83.0)	83.0 (83.0-83.0)	83.1 (83.1-83.1)	83.2 (83.1-83.2)
Singapore	79.8 (79.7-79.9)	80.0 (79.9-80.2)	80.2 (80.1-80.3)	80.4 (80.2-80.5)	80.6 (80.5-80.8)	81.0 (80.8-81.1)	81.3 (81.2-81.4)	81.5 (81.3-81.6)	81.6 (81.4-81.7)	81.7 (81.6-81.8)	81.9 (81.7-82.0)	82.0 (81.7-82.2)
South Korea	78.2 (78.0-78.4)	78.7 (78.5-78.9)	79.1 (78.9-79.3)	79.4 (79.3-79.6)	79.8 (79.6-80.0)	80.1 (79.9-80.3)	80.4 (80.2-80.5)	80.6 (80.4-80.8)	80.7 (80.6-80.9)	80.9 (80.7-81.1)	80.9 (80.7-81.2)	81.0 (80.6-81.3)
Western Europe	79.6 (79.6-79.6)	79.9 (79.8-79.9)	80.1 (80.1-80.1)	80.3 (80.3-80.3)	80.5 (80.5-80.5)	80.7 (80.7-80.7)	80.9 (80.9-80.9)	81.1 (81.1-81.1)	81.2 (81.2-81.3)	81.3 (81.3-81.4)	81.4 (81.4-81.4)	81.5 (81.4-81.5)
Andorra	84.1 (83.8-84.4)	84.3 (84.0-84.5)	84.4 (84.1-84.7)	84.5 (84.2-84.8)	84.6 (84.3-84.8)	84.6 (84.3-84.8)	84.7 (84.4-85.0)	84.7 (84.5-85.0)	84.7 (84.5-85.0)	84.8 (84.5-85.0)	84.8 (84.5-85.1)	84.8 (84.5-85.1)
Austria	79.4 (79.3-79.4)	79.5 (79.5-79.6)	80.0 (79.9-80.1)	80.1 (80.1-80.2)	80.4 (80.3-80.4)	80.3 (80.2-80.4)	80.5 (80.5-80.6)	80.7 (80.7-80.8)	80.9 (80.9-81.0)	81.1 (81.0-81.2)	81.2 (81.1-81.3)	81.3 (81.2-81.4)
Belgium	79.0 (78.9-79.0)	79.1 (79.1-79.2)	79.5 (79.4-79.6)	79.5 (79.4-79.6)	79.6 (79.5-79.7)	79.8 (79.7-79.9)	80.1 (80.0-80.1)	80.2 (80.1-80.2)	80.3 (80.2-80.4)	80.4 (80.2-80.5)	80.5 (80.2-80.7)	80.5 (80.2-80.8)
Cyprus	79.1 (78.9-79.2)	79.0 (78.8-79.1)	79.5 (79.3-79.6)	79.8 (79.6-79.9)	80.0 (79.8-80.1)	80.3 (80.1-80.4)	80.6 (80.4-80.7)	81.1 (80.9-81.3)	81.5 (81.4-81.7)	81.7 (81.5-81.8)	81.7 (81.5-81.9)	81.8 (81.6-82.0)
Denmark	77.8 (77.7-77.9)	78.3 (78.2-78.4)	78.3 (78.3-78.4)	78.4 (78.4-78.5)	78.9 (78.8-79.0)	79.1 (79.0-79.2)	79.4 (79.3-79.5)	79.9 (79.8-80.0)	80.3 (80.1-80.4)	80.3 (80.1-80.4)	80.3 (80.2-80.5)	80.4 (80.2-80.6)
Finland	78.8 (78.7-78.9)	79.0 (79.0-79.1)	79.2 (79.1-79.3)	79.4 (79.3-79.5)	79.6 (79.5-79.7)	79.8 (79.7-79.9)	80.0 (79.9-80.1)	80.3 (80.2-80.4)	80.5 (80.4-80.6)	80.8 (80.6-80.9)	80.9 (80.7-81.0)	80.9 (80.7-81.2)
France	80.2 (80.2-80.3)	80.4 (80.4-80.4)	80.7 (80.7-80.7)	81.0 (80.9-81.0)	81.1 (81.1-81.1)	81.2 (81.2-81.2)	81.4 (81.4-81.4)	81.6 (81.6-81.6)	81.6 (81.5-81.7)	81.7 (81.6-81.7)	81.7 (81.6-81.9)	81.8 (81.7-82.0)
Germany	79.2 (79.1-79.2)	79.4 (79.4-79.4)	79.7 (79.7-79.7)	79.9 (79.8-79.9)	80.0 (80.0-80.0)	80.1 (80.1-80.1)	80.3 (80.2-80.3)	80.5 (80.4-80.5)	80.6 (80.6-80.7)	80.7 (80.6-80.7)	80.7 (80.7-80.8)	80.8 (80.7-80.9)
Greece	79.4 (79.3-79.4)	79.6 (79.5-79.6)	80.0 (79.9-80.0)	79.8 (79.7-79.8)	80.2 (80.1-80.3)	80.2 (80.2-80.3)	80.4 (80.4-80.5)	80.5 (80.5-80.6)	80.6 (80.5-80.6)	81.0 (80.9-81.1)	81.0 (80.8-81.1)	81.0 (80.7-81.2)
Iceland	81.5 (81.2-81.7)	81.7 (81.4-81.9)	81.8 (81.6-82.1)	82.1 (81.9-82.4)	82.4 (82.1-82.7)	82.5 (82.3-82.8)	82.8 (82.5-83.1)	82.9 (82.7-83.2)	83.1 (82.8-83.3)	83.2 (83.0-83.5)	83.3 (83.0-83.5)	83.3 (83.1-83.6)
Ireland	79.3 (79.1-79.5)	79.7 (79.6-80.0)	79.8 (79.6-80.1)	80.1 (79.8-80.4)	80.1 (79.9-80.3)	80.3 (79.9-80.5)	81.0 (80.8-81.3)	80.6 (80.4-80.9)	81.1 (80.9-81.4)	81.5 (81.3-81.8)	81.6 (81.3-81.9)	81.7 (81.4-82.1)
Israel	79.5 (79.4-79.7)	79.7 (79.6-79.8)	79.6 (79.4-79.7)	80.3 (80.2-80.4)	80.6 (80.5-80.7)	81.0 (80.9-81.1)	81.6 (81.5-81.7)	81.6 (81.5-81.7)	82.1 (82.0-82.3)	82.0 (81.9-82.2)	81.3 (81.1-81.4)	82.1 (81.8-82.2)
Italy	80.9 (80.9-80.9)	81.1 (81.1-81.1)	81.2 (81.1-81.2)	81.3 (81.3-81.3)	81.5 (81.5-81.5)	81.6 (81.6-81.7)	81.9 (81.9-82.0)	82.0 (81.9-82.0)	82.0 (82.0-82.1)	82.1 (82.0-82.1)	82.1 (82.0-82.2)	82.2 (82.1-82.3)
Luxembourg	79.5 (79.4-79.7)	80.0 (79.8-80.1)	80.3 (80.2-80.5)	80.6 (80.5-80.8)	81.0 (80.8-81.1)	81.2 (81.0-81.3)	81.3 (81.2-81.5)	81.5 (81.3-81.6)	81.7 (81.6-81.9)	81.9 (81.7-82.1)	82.1 (81.9-82.3)	82.2 (82.0-82.4)
Malta	80.3 (80.1-80.6)	80.7 (80.4-80.9)	81.0 (80.7-81.3)	80.9 (80.6-81.2)	80.7 (80.5-81.0)	81.2 (81.0-81.4)	81.3 (81.1-81.5)	81.3 (81.1-81.6)	81.6 (81.3-81.8)	81.7 (81.5-82.0)	82.0 (81.7-82.2)	82.1 (81.8-82.3)
Netherlands	79.1 (79.1-79.2)	79.6 (79.5-79.6)	79.9 (79.8-79.9)	80.2 (80.2-80.3)	80.3 (80.3-80.4)	80.6 (80.6-80.7)	80.8 (80.7-80.9)	80.9 (80.8-81.0)	81.0 (80.9-81.1)	81.2 (81.1-81.3)	81.3 (81.1-81.4)	81.3 (81.1-81.6)
Norway	79.8 (79.7-79.9)	80.2 (80.1-80.3)	80.4 (80.3-80.5)	80.6 (80.5-80.7)	80.8 (80.7-80.9)	80.8 (80.7-81.0)	81.1 (81.0-81.2)	81.1 (81.0-81.2)	81.6 (81.5-81.7)	81.6 (81.5-81.7)	82.0 (81.9-82.1)	82.0 (81.9-82.2)
Portugal	78.2 (78.1-78.2)	78.4 (78.4-78.5)	79.0 (78.9-79.0)	79.2 (79.1-79.2)	79.4 (79.4-79.5)	79.6 (79.5-79.6)	79.9 (79.8-79.9)	80.2 (80.1-80.2)	80.4 (80.4-80.5)	80.7 (80.6-80.8)	80.7 (80.6-80.8)	80.8 (80.7-80.9)
Spain	80.0 (80.0-80.1)	80.3 (80.2-80.3)	80.7 (80.6-80.7)	80.8 (80.8-80.8)	81.1 (81.0-81.1)	81.5 (81.5-81.5)	81.8 (81.8-81.8)	82.0 (81.9-82.0)	82.2 (82.2-82.2)	82.5 (82.4-82.5)	82.5 (82.5-82.6)	82.6 (82.5-82.7)
Sweden	80.2 (80.1-80.2)	80.6 (80.5-80.6)	80.8 (80.7-80.9)	80.9 (80.9-81.0)	81.1 (81.0-81.2)	81.2 (81.2-81.3)	81.6 (81.5-81.7)	81.7 (81.6-81.8)	81.8 (81.7-81.9)	81.9 (81.8-82.0)	82.1 (82.0-82.2)	82.1 (82.0-82.3)
Switzerland	81.0 (80.9-81.1)	81.3 (81.2-81.4)	81.5 (81.4-81.6)	81.7 (81.6-81.8)	82.0 (81.9-82.1)	82.0 (81.9-82.1)	82.3 (82.2-82.4)	82.6 (82.5-82.7)	82.7 (82.6-82.8)	82.8 (82.7-83.0)	82.9 (82.7-83.1)	83.0 (82.7-83.2)
United Kingdom	78.7 (78.7-78.8)	79.0 (79.0-79.0)	79.2 (79.2-79.3)	79.5 (79.4-79.5)	79.7 (79.6-79.7)	80.0 (79.9-80.0)	80.2 (80.2-80.3)	80.5 (80.5-80.6)	80.7 (80.7-80.7)	80.8 (80.8-80.8)	80.9 (80.8-80.9)	81.0 (80.9-81.0)
England	79.0 (79.0-79.0)	79.3 (79.2-79.3)	79.5 (79.5-79.5)	79.8 (79.7-79.8)	80.0 (79.9-80.0)	80.3 (80.3-80.3)	80.5 (80.5-80.6)	80.8 (80.8-80.8)	81.0 (80.9-81.0)	81.1 (81.0-81.1)	81.1 (81.1-81.2)	81.2 (81.1-81.3)
Northern Ireland	78.3 (78.1-78.4)	78.4 (78.2-78.5)	78.4 (78.3-78.6)	78.5 (78.4-78.7)	78.7 (78.5-78.8)	78.9 (78.7-79.0)	79.1 (78.9-79.3)	79.6 (79.4-79.7)	79.8 (79.7-80.0)	80.0 (79.8-80.2)	80.1 (79.8-80.3)	80.2 (79.8-80.4)
Scotland	76.8 (76.7-76.9)	77.0 (76.9-77.1)	77.3 (77.2-77.3)	77.4 (77.3-77.5)	77.6 (77.5-77.7)	77.9 (77.9-78.0)	78.3 (78.2-78.3)	78.6 (78.5-78.7)	78.8 (78.7-78.9)	79.0 (78.9-79.2)	79.2 (79.0-79.3)	79.2 (79.0-79.5)
Wales	78.2 (78.1-78.3)	78.5 (78.4-78.6)	78.7 (78.6-78.8)	78.8 (78.7-78.9)	79.0 (79.0-79.1)	79.3 (79.2-79.4)	79.5 (79.4-79.6)	79.7 (79.6-79.8)	79.8 (79.7-79.9)	79.9 (79.8-80.1)	80.0 (79.8-80.2)	80.1 (79.9-80.3)
Southern Latin America	75.7 (75.7-75.8)	76.0 (76.0-76.0)	76.1 (76.0-76.1)	76.1 (76.0-76.1)	76.3 (76.2-76.3)	76.4 (76.3-76.4)	76.6 (76.5-76.6)	76.8 (76.7-76.8)	76.9 (76.8-76.9)	77.0 (76.9-77.0)	77.1 (77.0-77.2)	77.3 (77.1-77.4)
Argentina	75.0 (74.9-75.0)	75.3 (75.2-75.3)	75.3 (75.3-75.4)	75.2 (75.2-75.3)	75.4 (75.4-75.5)	75.6 (75.5-75.6)	75.8 (75.8-75.9)	76.0 (75.9-76.0)	76.1 (76.0-76.1)	76.2 (76.1-76.2)	76.3 (76.2-76.4)	76.5 (76.3-76.6)
Chile	78.0 (77.9-78.1)	78.2 (78.1-78.3)	78.2 (78.1-78.3)	78.3 (78.3-78.4)	78.5 (78.4-78.6)	78.5 (78.4-78.6)	78.5 (78.4-78.6)	78.9 (78.9-79.0)	79.1 (79.0-79.2)	79.1 (79.1-79.2)	79.2 (79.0-79.5)	79.4 (79.0-79.8)
Uruguay	75.3 (75.1-75.4)	75.5 (75.4-75.6)	75.7 (75.6-75.8)	75.7 (75.6-75.9)	76.0 (75.8-76.1)	76.2 (76.0-76.3)	76.2 (76.1-76.4)	76.3 (76.1-76.4)	76.3 (76.1-76.4)	76.4 (76.2-76.5)	76.6 (76.3-76.8)	76.8 (76.5-77.1)
Central Europe, Eastern Europe, and Central Asia	68.1 (68.0-68.2)	68.2 (68.0-68.3)	69.0 (68.9-69.1)	69.6 (69.4-69.7)	69.9 (69.7-70.0)	70.6 (70.5-70.7)	71.0 (70.8-71.1)	71.6 (71.5-71.7)	72.0 (71.9-72.1)	72.4 (72.2-72.5)	72.5 (72.3-72.6)	72.7 (72.5-72.9)
Eastern Europe	66.1 (65.9-66.2)	66.0 (65.9-66.1)	67.3 (67.2-67.3)	67.9 (67.8-67.9)	68.2 (68.1-68.2)	69.2 (69.2-69.3)	69.5 (69.5-69.6)	70.3 (70.3-70.4)	70.7 (70.7-70.8)	71.1 (71.0-71.1)	71.1 (71.1-71.2)	71.3 (71.1-71.5)
Belarus	68.5 (68.3-68.8)	68.7 (68.4-68.9)	69.1 (68.9-69.4)	69.7 (69.5-70.0)	70.0 (69.7-70.2)	70.1 (69.9-70.4)	70.2 (69.9-70.5)	70.3 (70.0-70.6)	70.4 (70.0-70.7)	70.6 (70.2-71.0)	70.7 (70.3-71.2)	71.0 (70.5-71.5)
Estonia	72.0 (71.9-72.2)	72.5 (72.4-72.7)	72.9 (72.8-73.1)	73.0 (72.9-73.1)	74.2 (74.0-74.3)	74.9 (74.8-75.1)	76.4 (76.2-76.5)	76.3 (76.1-76.5)	76.7 (76.5-76.8)	77.5 (77.3-77.7)	77.6 (77.4-77.9)	77.8 (77.5-78.1)
Latvia	71.2 (71.1-71.4)	71.1 (71.0-71.2)	70.8 (70.7-70.9)	71.3 (71.2-71.4)	72.4 (72.3-72.5)	73.3 (73.2-73.5)	73.9 (73.8-74.1)	74.6 (74.5-74.7)	75.1 (74.9-75.2)	75.0 (74.8-75.2)	75.2 (75.0-75.5)	75.4 (75.1-75.7)
Lithuania	72.2 (72.1-72.3)	71.7 (71.6-71.7)	71.5 (71.4-71.6)	71.4 (71.3-71.4)	72.1 (72.0-72.2)	73.6 (73.5-73.7)	73.9 (73.8-73.9)	74.3 (74.2-74.4)	74.7 (74.6-74.8)	74.9 (74.7-75.0)	75.0 (74.9-75.2)	75.2 (75.0-75.4)
Moldova	70.2 (69.9-70.5)	69.5 (69.2-69.8)	69.8 (69.5-70.1)	70.0 (69.7-70.3)	70.4 (70.1-70.6)	70.6 (70.4-70.9)	70.5 (70.2-70.7)	72.3 (72.0-72.5)	72.4 (72.1-72.7)	73.3 (73.0-73.6)	73.6 (73.3-73.9)	73.9 (73.5-74.2)
Russia	65.1 (64.9-65.2)	65.1 (65.0-65.3)	66.7 (66.7-66.7)	67.7 (67.6-67.7)	67.9 (67.9-68.0)	68.8 (68.7-68.8)	68.9 (68.9-69.0)	69.8 (69.8-69.8)	70.4 (70.3-70.4)	70.8 (70.8-70.8)	70.9 (70.9-70.9)	71.0 (70.7-71.3)
Ukraine	67.5 (67.2-67.7)	67.1 (66.9-67.3)	67.9 (67.6-68.1)	67.6 (67.4-67.8)	67.8 (67.6-68.0)	69.6 (69.4-69.8)	70.5 (70.3-70.7)	71.1 (70.9-71.3)	71.2 (71.0-71.4)	71.3 (71.1-71.5)	71.3 (70.9-71.6)	71.5 (70.9-71.9)
Central Europe	74.0 (73.9-74.0)	74.2 (74.1-74.2)	74.4 (74.4-74.5)	74.7 (74.6-74.7)	74.9 (74.9-74.9)	75.1 (75.1-75.2)	75.5 (75.5-75.5)	76.0 (75.9-76.0)	76.2 (76.2-76.2)	76.6 (76.5-76.6)	76.8 (76.7-76.9)	77.0 (76.8-77.1)
Albania	75.9 (75.5-76.3)	76.2 (75.7-76.6)	76.4 (76.0-76.8)	76.6 (76.2-77.0)	76.8 (76.2-77.2)	77.0 (76.4-77.5)	77.2 (76.5-77.7)	77.4 (76.6-78.0)	77.5 (76.8-78.2)	77.7 (77.0-78.3)	77.9 (77.1-78.5)	78.0 (77.4-78.6)
Bosnia and Herzegovina	76.9 (76.8-77.0)	77.0 (76.9-77.2)	77.1 (77.0-77.3)	77.3 (77.2-77.4)	77.5 (77.4-77.6)	77.7 (77.6-77.8)	77.9 (77.8-78.0)	78.2 (78.0-78.3)	78.4 (78.2-78.5)	78.6 (78.4-78.8)	78.7 (78.5-79.0)	78.9 (78.7-79.2)
Bulgaria	72.5 (72.4-72.5)	72.6 (72.5-72.6)	72.7 (72.6-72.8)	72.9 (72.9-73.0)	73.2 (73.1-73.3)	73.5 (73.4-73.6)	73.7 (73.7-73.8)	74.2 (74.1-74.3)	74.5 (74.4-74.6)	74.6 (74.4-74.8)	74.7 (74.4-75.0)	74.8 (74.4-75.2)
Croatia	75.3 (75.2-75.4)	75.7 (75.6-75.7)	75.9 (75.8-75.9)	76.0 (75.9-76.1)	76.2 (76.1-76.3)	76.4 (76.3-76.5)	76.7 (76.7-76.8)	77.1 (77.0-77.2)	77.4 (77.3-77.5)	77.6 (77.5-77.8)	77.8 (77.6-77.9)	77.8 (77.6-78.1)
Czech Republic	75.9 (75.8-76.0)	76.2 (76.1-76.3)	76.5 (76.5-76.6)	76.8 (76.8-76.9)	77.1 (77.1-77.2)	77.3 (77.2-77.3)	77.5 (77.4-77.6)	77.8 (77.7-77.9)	78.1 (78.0-78.1)	78.3 (78.3-78.4)	78.6 (78.5-78.7)	78.8 (78.7-79.0)
Hungary	72.9 (72.8-73.0)	73.1 (73.0-73.1)	73.3 (73.2-73.4)	73.6 (73.5-73.6)	73.9 (73.8-74.0)	74.3 (74.2-74.3)	74.6 (74.6-74.7)	75.0 (75.0-75.1)	75.5 (75.4-75.6)	76.1 (76.0-76.2)	76.5 (76.3-76.7)	76.7 (76.4-77.1)
Macedonia	73.6 (73.5-73.7)	73.8 (73.7-74.0)	74.1 (74.0-74.2)	74.3 (74.2-74.4)	74.5 (74.4-74.7)	74.7 (74.6-74.8)	75.2 (75.0-75.3)	75.6 (75.5-75.8)	75.8 (75.6-76.0)	76.0 (75.7-76.3)	76.2 (75.8-76.6)	76.5 (76.0-77.0)

SR Table 5: Estimates of life expectancy at birth by geography (with 95% uncertainty intervals), both sexes, 1980- 2015

Location	1980	1981	1982	1983	1984	1985	1986	1987	1988	1989	1990	1991
Montenegro	73.6 (72.4-74.5)	73.6 (72.5-74.6)	73.7 (72.7-74.6)	73.8 (72.8-74.6)	73.8 (72.8-74.7)	73.9 (72.9-74.7)	73.9 (73.0-74.7)	74.0 (73.2-74.8)	74.1 (73.3-74.9)	74.3 (73.6-75.0)	74.6 (73.9-75.2)	74.4 (73.8-75.0)
Poland	70.4 (70.4-70.5)	71.2 (71.1-71.2)	71.3 (71.2-71.3)	71.1 (71.1-71.1)	70.8 (70.8-70.9)	70.7 (70.6-70.7)	70.9 (70.9-71.0)	71.0 (71.0-71.0)	71.3 (71.3-71.3)	71.1 (71.0-71.1)	70.9 (70.9-71.0)	70.7 (70.6-70.7)
Romania	69.5 (69.5-69.6)	69.7 (69.7-69.8)	70.1 (70.1-70.2)	70.3 (70.2-70.4)	70.3 (70.2-70.3)	69.9 (69.8-70.0)	70.0 (70.0-70.1)	69.3 (69.3-69.4)	69.7 (69.6-69.7)	69.7 (69.6-69.9)	70.0 (69.9-70.0)	70.5 (70.4-70.5)
Serbia	71.5 (70.3-72.4)	71.6 (70.5-72.5)	71.7 (70.7-72.6)	71.8 (70.8-72.7)	71.9 (71.0-72.7)	72.1 (71.2-72.9)	72.2 (71.5-73.0)	72.4 (71.7-73.0)	72.5 (71.9-73.1)	72.5 (72.0-73.1)	72.5 (72.1-73.0)	71.4 (70.9-71.8)
Slovakia	70.5 (70.4-70.6)	70.6 (70.5-70.7)	70.8 (70.7-70.9)	70.6 (70.5-70.7)	70.7 (70.6-70.8)	70.8 (70.7-70.9)	70.9 (70.8-71.0)	71.0 (70.9-71.1)	71.1 (71.0-71.2)	71.0 (70.9-71.1)	71.0 (70.9-71.0)	71.1 (71.0-71.1)
Slovenia	70.5 (70.2-70.7)	70.7 (70.5-70.9)	70.9 (70.8-71.0)	71.0 (70.9-71.1)	71.4 (71.2-71.5)	71.8 (71.7-71.9)	72.2 (72.1-72.3)	72.3 (72.1-72.4)	72.8 (72.7-72.9)	73.2 (73.1-73.3)	73.7 (73.6-73.8)	73.6 (73.5-73.7)
Central Asia	65.3 (64.9-65.6)	65.6 (65.3-65.8)	65.9 (65.6-66.1)	66.0 (65.7-66.2)	66.1 (65.8-66.3)	66.4 (66.2-66.6)	66.9 (66.7-67.1)	67.1 (66.8-67.3)	66.2 (65.7-66.7)	67.1 (66.9-67.3)	67.1 (66.9-67.4)	66.9 (66.7-67.2)
Armenia	69.5 (68.8-70.2)	69.8 (69.1-70.4)	70.3 (69.6-70.8)	70.2 (69.6-70.8)	70.5 (70.0-71.1)	70.5 (70.0-71.0)	71.0 (70.5-71.5)	71.1 (70.7-71.6)	55.5 (48.9-63.7)	70.4 (70.0-70.9)	70.1 (69.6-70.5)	69.7 (69.3-70.2)
Azerbaijan	64.7 (63.6-65.8)	64.7 (63.7-65.7)	64.9 (64.0-65.8)	65.1 (64.2-65.9)	65.0 (64.1-65.7)	65.3 (64.5-66.1)	65.3 (64.6-66.1)	65.4 (64.6-66.0)	65.2 (64.5-65.9)	65.7 (65.0-66.3)	65.6 (64.8-66.2)	65.3 (64.6-66.0)
Georgia	69.1 (68.3-69.8)	69.3 (68.6-70.0)	69.5 (68.9-70.1)	69.6 (69.0-70.3)	69.7 (69.0-70.4)	69.7 (69.1-70.4)	69.8 (69.2-70.4)	69.8 (69.2-70.4)	69.9 (69.3-70.4)	69.8 (69.3-70.4)	69.9 (69.3-70.4)	69.9 (69.3-70.5)
Kazakhstan	65.7 (65.3-66.2)	66.2 (65.9-66.5)	66.7 (66.3-67.0)	66.7 (66.3-67.1)	66.6 (66.3-67.0)	67.2 (66.9-67.5)	68.4 (68.1-68.6)	68.6 (68.3-68.8)	68.3 (68.0-68.5)	68.0 (67.8-68.3)	68.0 (67.8-68.2)	67.6 (67.4-67.9)
Kyrgyzstan	64.1 (63.5-64.7)	64.4 (63.8-64.9)	64.6 (64.0-65.1)	64.8 (64.3-65.3)	65.0 (64.5-65.5)	65.3 (64.8-65.8)	65.6 (65.2-66.1)	65.8 (65.4-66.3)	65.9 (65.5-66.4)	66.0 (65.5-66.4)	66.0 (65.5-66.4)	65.9 (65.4-66.3)
Mongolia	59.0 (58.1-59.8)	59.3 (58.5-60.2)	59.6 (58.9-60.5)	60.0 (59.3-60.8)	60.4 (59.7-61.2)	60.8 (60.1-61.4)	61.3 (60.6-61.9)	61.7 (61.1-62.3)	61.9 (61.3-62.5)	62.2 (61.5-62.8)	62.3 (61.6-62.9)	62.3 (61.7-62.9)
Tajikistan	62.5 (61.3-63.5)	62.9 (61.8-63.8)	63.2 (62.3-64.1)	63.5 (62.6-64.3)	63.8 (63.0-64.7)	64.2 (63.3-65.1)	64.5 (63.6-65.3)	64.6 (63.8-65.4)	64.8 (64.1-65.6)	65.0 (64.3-65.8)	65.3 (64.6-65.9)	65.3 (64.6-66.0)
Turkmenistan	60.9 (59.2-62.4)	61.6 (59.9-62.8)	62.0 (60.6-63.2)	62.2 (60.8-63.3)	62.4 (61.2-63.5)	62.7 (61.6-63.8)	63.0 (61.9-64.0)	63.3 (62.3-64.3)	63.5 (62.5-64.5)	63.7 (62.8-64.6)	63.9 (62.9-64.8)	63.5 (62.6-64.4)
Uzbekistan	66.1 (65.4-66.8)	66.3 (65.7-67.0)	66.5 (65.8-67.2)	66.6 (65.9-67.2)	66.7 (66.1-67.3)	67.1 (66.4-67.6)	67.4 (66.7-67.9)	67.6 (67.0-68.1)	67.7 (67.2-68.3)	67.8 (67.2-68.3)	67.8 (67.2-68.3)	67.6 (67.1-68.1)
Latin America and Caribbean	65.9 (65.7-66.2)	66.3 (66.0-66.5)	66.6 (66.4-66.9)	67.0 (66.8-67.2)	67.3 (67.1-67.5)	67.4 (67.1-67.6)	68.0 (67.8-68.2)	68.3 (68.1-68.5)	68.7 (68.5-68.9)	69.0 (68.8-69.2)	69.3 (69.1-69.4)	69.5 (69.3-69.6)
Central Latin America	67.0 (66.8-67.2)	67.2 (67.0-67.4)	67.8 (67.6-68.1)	68.4 (68.2-68.6)	69.0 (68.8-69.2)	68.6 (68.3-68.9)	69.7 (69.5-69.9)	70.0 (69.8-70.2)	70.4 (70.2-70.5)	70.7 (70.5-70.8)	71.2 (71.0-71.3)	71.3 (71.1-71.5)
Colombia	69.1 (68.9-69.3)	69.5 (69.3-69.7)	70.0 (69.8-70.2)	70.3 (70.1-70.5)	70.7 (70.5-70.8)	68.1 (67.2-68.9)	70.8 (70.6-70.9)	70.8 (70.6-70.9)	70.9 (70.8-71.1)	71.1 (70.9-71.3)	71.5 (71.3-71.7)	71.1 (71.0-71.3)
Costa Rica	74.6 (74.3-74.9)	75.0 (74.8-75.3)	75.4 (75.2-75.7)	75.6 (75.3-75.9)	75.7 (75.5-76.0)	75.9 (75.6-76.1)	76.1 (75.8-76.3)	76.2 (75.9-76.4)	76.2 (76.0-76.5)	76.4 (76.2-76.7)	76.6 (76.3-76.8)	76.5 (76.3-76.8)
El Salvador	64.6 (63.8-65.3)	58.0 (55.7-60.6)	62.0 (60.8-63.3)	60.5 (58.9-62.4)	62.5 (61.3-63.8)	64.2 (63.3-65.2)	64.2 (63.2-65.4)	65.7 (65.0-66.6)	67.1 (66.5-67.7)	65.4 (64.9-66.1)	67.7 (67.2-68.3)	68.1 (67.5-68.7)
Guatemala	59.8 (59.1-60.5)	59.0 (57.5-60.5)	57.9 (55.7-60.2)	61.4 (60.9-61.8)	63.2 (62.8-63.5)	62.7 (62.4-63.1)	63.1 (62.7-63.5)	63.4 (63.1-63.7)	64.2 (63.8-64.5)	64.9 (64.6-65.3)	64.6 (64.3-64.9)	64.0 (63.8-64.3)
Honduras	67.3 (66.9-67.7)	67.8 (67.4-68.2)	68.1 (67.8-68.5)	68.5 (68.2-68.9)	68.9 (68.5-69.3)	69.1 (68.7-69.5)	69.4 (69.0-69.8)	69.8 (69.4-70.2)	70.0 (69.5-70.5)	70.1 (69.7-70.7)	70.3 (69.7-71.0)	70.3 (69.6-71.0)
Mexico	66.3 (65.9-66.6)	66.9 (66.6-67.2)	67.6 (67.3-67.9)	68.2 (67.9-68.5)	68.8 (68.5-69.1)	69.0 (68.6-69.3)	69.7 (69.4-70.0)	70.2 (69.9-70.4)	70.5 (70.2-70.8)	70.9 (70.7-71.2)	71.5 (71.2-71.8)	71.9 (71.6-72.2)
Nicaragua	67.2 (66.2-68.0)	67.5 (66.5-68.5)	67.9 (66.9-69.0)	65.7 (63.7-67.7)	65.0 (62.3-68.0)	65.4 (62.7-68.4)	66.9 (64.5-69.4)	66.7 (63.8-69.8)	69.7 (68.3-71.4)	72.4 (71.9-72.9)	73.3 (72.9-73.8)	73.7 (73.2-74.1)
Panama	73.2 (72.7-73.7)	73.5 (73.0-74.1)	73.8 (73.2-74.3)	73.9 (73.4-74.5)	74.1 (73.6-74.6)	74.2 (73.6-74.7)	74.3 (73.7-74.8)	74.4 (73.8-74.9)	74.4 (73.9-75.0)	74.1 (73.3-74.9)	74.9 (74.3-75.5)	75.0 (74.4-75.5)
Venezuela	69.9 (69.8-70.0)	70.4 (70.3-70.5)	70.6 (70.5-70.7)	71.2 (71.1-71.3)	71.6 (71.5-71.6)	71.9 (71.8-72.0)	72.4 (72.3-72.5)	72.2 (72.1-72.2)	72.3 (72.2-72.4)	72.3 (72.2-72.4)	72.5 (72.4-72.6)	72.4 (72.3-72.5)
Andean Latin America	63.2 (62.8-63.6)	63.6 (63.2-64.0)	64.0 (63.6-64.4)	64.2 (63.8-64.6)	64.7 (64.2-65.1)	65.3 (64.9-65.7)	65.9 (65.5-66.3)	66.1 (65.5-66.6)	67.0 (66.6-67.4)	67.4 (67.1-67.8)	67.9 (67.5-68.3)	68.3 (67.9-68.7)
Bolivia	56.4 (55.4-57.4)	56.9 (55.9-57.9)	57.3 (56.3-58.3)	57.5 (56.5-58.5)	58.3 (57.3-59.2)	58.9 (57.9-59.8)	59.5 (58.5-60.4)	59.9 (59.0-60.8)	60.4 (59.5-61.3)	61.0 (60.1-61.9)	61.6 (60.8-62.6)	62.2 (61.3-63.1)
Ecuador	67.5 (67.1-67.8)	68.1 (67.8-68.4)	68.6 (68.3-68.9)	68.9 (68.6-69.2)	69.4 (69.0-69.7)	69.8 (69.5-70.1)	70.1 (69.8-70.4)	68.8 (67.4-70.1)	70.7 (70.3-71.0)	71.0 (70.6-71.3)	71.2 (70.8-71.5)	71.4 (71.1-71.8)
Peru	63.8 (63.2-64.5)	64.1 (63.5-64.8)	64.4 (63.8-65.0)	64.6 (64.0-65.3)	64.9 (64.2-65.6)	65.7 (65.1-66.3)	66.4 (65.8-67.0)	67.1 (66.6-67.6)	67.8 (67.3-68.3)	68.2 (67.7-68.7)	68.7 (68.2-69.2)	69.2 (68.6-69.8)
Caribbean	65.7 (65.1-66.3)	66.1 (65.5-66.6)	66.4 (65.9-66.9)	66.6 (66.1-67.1)	66.8 (66.2-67.3)	66.8 (66.3-67.3)	67.0 (66.5-67.5)	67.2 (66.8-67.7)	67.5 (67.1-67.9)	67.6 (67.3-68.0)	67.8 (67.5-68.2)	68.0 (67.6-68.3)
Antigua and Barbuda	70.5 (69.6-71.3)	71.2 (70.4-72.0)	71.8 (71.1-72.6)	72.6 (71.8-73.4)	72.8 (72.1-73.5)	73.1 (72.4-73.8)	73.4 (72.7-74.0)	73.5 (72.9-74.2)	73.6 (73.0-74.3)	73.5 (72.9-74.2)	73.5 (72.8-74.2)	73.6 (72.9-74.2)
The Bahamas	66.3 (65.1-67.4)	66.6 (65.3-67.6)	66.8 (65.6-67.8)	67.1 (65.9-68.1)	67.3 (66.2-68.3)	66.3 (64.7-67.6)	67.4 (66.3-68.4)	67.7 (66.5-68.6)	68.0 (66.9-68.9)	68.3 (67.2-69.2)	68.5 (67.5-69.4)	68.9 (68.0-69.7)
Barbados	72.3 (71.2-73.2)	72.7 (71.6-73.5)	73.0 (71.8-73.8)	73.0 (71.9-73.8)	72.9 (71.9-73.8)	72.9 (71.9-73.7)	73.1 (72.1-73.9)	73.1 (72.1-73.9)	73.1 (72.2-73.9)	73.1 (72.1-73.9)	73.0 (72.1-73.8)	73.2 (72.4-74.0)
Belize	69.0 (68.2-69.9)	69.3 (68.4-70.2)	69.6 (68.7-70.4)	69.9 (69.1-70.7)	70.3 (69.4-71.0)	70.8 (70.0-71.5)	71.1 (70.3-71.8)	71.3 (70.5-72.0)	71.5 (70.8-72.1)	71.4 (70.7-72.1)	71.4 (70.6-72.0)	71.2 (70.4-72.0)
Bermuda	70.4 (69.9-70.9)	70.4 (69.9-70.9)	70.6 (70.1-71.1)	70.7 (70.2-71.2)	70.7 (70.2-71.2)	70.6 (70.1-71.1)	70.5 (70.0-71.0)	70.8 (70.3-71.3)	71.0 (70.6-71.5)	71.0 (70.5-71.4)	71.1 (70.6-71.5)	71.2 (70.7-71.7)
Cuba	74.1 (74.0-74.2)	74.3 (74.2-74.3)	74.5 (74.4-74.5)	74.5 (74.4-74.6)	74.5 (74.4-74.6)	74.4 (74.3-74.5)	74.4 (74.3-74.5)	74.4 (74.4-74.5)	74.5 (74.4-74.6)	74.6 (74.5-74.6)	74.7 (74.6-74.8)	74.8 (74.7-74.8)
Dominica	72.7 (71.8-73.5)	73.2 (72.4-74.0)	73.4 (72.6-74.1)	73.7 (72.9-74.4)	73.6 (72.7-74.3)	73.5 (72.7-74.2)	73.4 (72.6-74.1)	73.3 (72.5-74.0)	73.2 (72.4-73.9)	72.9 (72.1-73.6)	72.8 (72.0-73.5)	72.6 (71.8-73.4)
Dominican Republic	69.3 (68.8-69.8)	69.7 (69.2-70.2)	70.2 (69.7-70.7)	70.6 (70.1-71.0)	70.8 (70.3-71.2)	70.8 (70.4-71.3)	71.0 (70.5-71.4)	71.0 (70.6-71.5)	71.3 (70.9-71.8)	71.7 (71.3-72.2)	71.8 (71.4-72.3)	72.2 (71.7-72.6)
Grenada	67.8 (65.1-69.6)	68.0 (65.3-69.8)	68.2 (65.6-69.9)	68.3 (65.9-70.0)	68.4 (66.1-69.9)	68.6 (66.6-70.0)	68.8 (67.0-70.1)	69.1 (67.5-70.2)	69.3 (67.9-70.4)	69.6 (68.4-70.6)	69.9 (68.7-70.8)	70.2 (69.2-71.0)
Guyana	62.8 (61.9-63.6)	63.1 (62.2-63.9)	63.3 (62.5-64.2)	63.6 (62.8-64.4)	63.9 (63.1-64.7)	63.9 (63.2-64.7)	64.0 (63.3-64.7)	64.2 (63.5-64.8)	64.3 (63.7-64.9)	64.2 (63.5-64.8)	64.2 (63.6-64.8)	64.3 (63.7-64.9)
Haiti	50.2 (48.5-52.1)	51.0 (49.4-52.7)	51.6 (50.0-53.3)	52.0 (50.4-53.6)	52.3 (50.8-53.8)	52.6 (51.1-54.0)	52.9 (51.5-54.3)	53.4 (52.1-54.8)	53.9 (52.7-55.2)	54.0 (52.8-55.2)	54.3 (53.0-55.5)	54.4 (53.2-55.6)
Jamaica	71.6 (70.8-72.3)	72.1 (71.4-72.7)	72.3 (71.7-72.9)	72.5 (71.9-73.1)	72.7 (72.0-73.3)	72.9 (72.2-73.4)	73.2 (72.5-73.7)	73.6 (73.0-74.1)	74.0 (73.4-74.6)	74.5 (73.8-75.0)	74.8 (74.0-75.4)	74.9 (74.2-75.6)
Puerto Rico	73.7 (73.6-73.8)	73.9 (73.8-74.0)	74.2 (74.0-74.3)	74.4 (74.2-74.5)	74.4 (74.3-74.5)	73.8 (73.4-74.2)	74.3 (74.1-74.4)	73.9 (73.8-74.0)	73.7 (73.6-73.9)	73.7 (73.6-73.9)	73.8 (73.6-73.9)	73.8 (73.6-73.9)
Saint Lucia	67.4 (65.9-68.7)	68.3 (67.0-69.5)	68.7 (67.3-69.8)	69.1 (67.8-70.2)	69.3 (68.0-70.5)	69.4 (68.1-70.5)	69.5 (68.1-70.6)	69.6 (68.2-70.7)	68.9 (67.5-70.3)	70.0 (68.8-70.9)	70.2 (69.0-71.1)	70.4 (69.3-71.3)
Saint Vincent and the Grenadines	67.3 (65.4-68.8)	67.8 (66.1-69.2)	68.3 (66.7-69.7)	68.7 (67.1-70.0)	68.9 (67.4-70.1)	69.3 (67.9-70.4)	69.4 (68.1-70.5)	69.5 (68.3-70.6)	69.7 (68.4-70.7)	69.7 (68.5-70.8)	69.7 (68.5-70.7)	69.7 (68.7-70.6)
Suriname	65.4 (64.7-66.1)	65.9 (65.3-66.6)	66.4 (65.8-67.0)	67.1 (66.5-67.7)	67.6 (66.9-68.2)	68.1 (67.4-68.7)	68.2 (67.5-69.0)	68.6 (67.8-69.3)	68.7 (68.0-69.5)	68.2 (67.1-69.4)	69.7 (69.1-70.3)	69.9 (69.3-70.5)
Trinidad and Tobago	67.8 (67.5-68.1)	68.0 (67.7-68.3)	68.2 (67.9-68.5)	68.2 (67.9-68.5)	68.3 (68.0-68.6)	68.5 (68.1-68.8)	68.6 (68.3-68.9)	68.7 (68.4-69.0)	68.8 (68.5-69.1)	69.0 (68.7-69.3)	69.1 (68.8-69.4)	69.3 (68.9-69.6)
Virgin Islands, U.S.	70.4 (69.9-71.0)	70.9 (70.4-71.4)	71.4 (70.9-71.8)	71.9 (71.4-72.3)	72.2 (71.8-72.6)	72.5 (72.0-72.9)	72.7 (72.2-73.1)	72.8 (72.3-73.2)	72.9 (72.5-73.4)	73.0 (72.6-73.5)	73.1 (72.7-73.6)	73.2 (72.7-73.6)
Tropical Latin America	65.5 (65.0-66.0)	65.9 (65.5-66.4)	66.1 (65.7-66.6)	66.4 (65.9-66.8)	66.4 (66.0-66.9)	66.7 (66.3-67.1)	67.1 (66.7-67.5)	67.4 (67.0-67.8)	67.7 (67.3-68.1)	68.0 (67.6-68.3)	68.0 (67.7-68.4)	68.3 (67.9-68.6)
Brazil	65.3 (64.8-65.8)	65.8 (65.3-66.3)	66.0 (65.5-66.5)	66.2 (65.8-66.7)	66.3 (65.9-66.7)	66.5 (66.1-66.9)	66.9 (66.5-67.3)	67.2 (66.8-67.6)	67.6 (67.2-68.0)	67.8 (67.5-68.2)	67.9 (67.5-68.2)	68.1 (67.7-68.5)
Paraguay	71.8 (71.2-72.5)	72.0 (71.4-72.7)	72.3 (71.7-73.0)	72.4 (71.8-73.0)	72.5 (71.9-73.2)	72.8 (72.3-73.5)	73.2 (72.6-73.8)	73.6 (73.0-74.2)	73.9 (73.3-74.6)	74.0 (73.4-74.6)	74.2 (73.7-74.8)	74.2 (73.6-74.8)
Southeast Asia, East Asia, and Oceania	63.3 (62.5-64.0)	63.5 (62.7-64.3)	64.0 (63.2-64.7)	64.5 (63.7-65.2)	64.9 (64.1-65.6)	65.3 (64.5-65.9)	65.6 (64.8-66.3)	65.9 (65.1-66.6)	66.0 (65.1-66.6)	66.1 (65.3-66.7)	66.4 (65.7-67.0)	66.7 (65.9-67.3)
East Asia	64.1 (62.9-65.2)	64.3 (63.1-65.3)	64.8 (63.6-65.8)	65.3 (64.1-66.3)	65.7 (64.6-66.7)	66.0 (65.0-67.0)	66.3 (65.2-67.2)	66.6 (65.4-67.5)	66.5 (65.4-67.5)	66.5 (65.5-67.4)	66.8 (65.9-67.7)	67.1 (66.1-68.0)
China	64.0 (62.8-65.0)	64.1 (62.9-65.2)	64.7 (63.5-65.7)	65.1 (63.9-66.2)	65.6 (64.5-66.6)	65.9 (64.9-66.9)	66.1 (65.0-67.1)	66.4 (65.2-67.5)	66.4 (65.3-67.4)	66.4 (65.4-67.3)	66.7 (65.7-67.6)	67.0 (65.9-67.8)
North Korea	65.0 (57.7-69.9)	65.4 (58.7-70.2)	65.8 (59.8-70.4)	66.2 (60.0-70.8)	66.6 (60.8-71.0)	67.0 (61.3-71.1)	67.4 (62.0-71.4)	67.7 (62.9-71.6)	68.2 (63.5-71.9)	68.5 (64.2-72.2)	68.9 (64.7-72.4)	69.2 (64.9-72.6)
Taiwan	71.5 (71.4-71.6)	71.7 (71.6-71.8)	72.1 (72.0-72.2)	72.3 (72.2-72.3)	72.6 (72.6-72.7)	73.0 (72.9-73.1)	73.2 (73.1-73.3)	73.4 (73.3-73.5)	73.3 (73.2-73.4)	73.5 (73.4-73.5)	73.8 (73.7-73.8)	74.2 (74.1-74.3)
Southeast Asia	61.6 (60.6-62.5)	62.1 (61.1-62.9)	62.5 (61.5-63.3)	62.9 (62.0-63.7)	63.3 (62.4-64.1)	63.7 (62.8-64.4)	64.1 (63.3-64.9)	64.5 (63.7-65.2)	64.8 (64.1-65.5)	65.2 (64.5-65.9)	65.5 (64.8-66.2)	65.9 (65.1-66.6)
Cambodia	52.8 (50.5-55.3)	53.2 (51.0-55.6)	53.6 (51.6-55.9)	54.0 (52.1-56.3)	54.4 (52.5-56.5)	54.7 (52.9-56.7)	55.1 (53.3-56.9)	56.9 (55.8-58.0)	57.3 (56.3-58.4)	57.3 (56.4-58.3)	57.6 (56.7-58.5)	57.9 (57.1-58.8)
Indonesia	59.3 (58.3-60.4)	60.0 (59.1-61.0)	60.6 (59.7-61.5)	61.1 (60.3-61.9)	61.6 (60.8-62.4)	62.1 (61.3-62.8)	62.5 (61.8-63.2)	62.7 (62.1-63.4)	63.2 (62.5-63.8)	63.7 (63.0-64.3)	64.0 (63.4-64.7)	64.5 (63.8-65.1)
Laos	47.5 (44.2-50.9)	48.0 (45.0-51.2)	48.5 (45.5-51.5)	49.0 (46.1-51.8)	49.5 (46.8-52.4)	49.9 (47.4-52.7)	50.2 (47.9-52.8)	50.5 (48.3-53.1)	50.8 (48.7-53.2)	51.2 (49.1-53.4)	51.5 (49.5-53.7)	52.0 (49.9-54.1)
Malaysia	68.9 (68.5-69.2)	69.2 (68.9-69.6)	69.6 (69.3-69.9)	69.9 (69.7-70.1)	70.1 (70.0-70.2)	70.3 (70.3-70.4)	70.7 (70.6-70.8)	71.1 (71.0-71.2)	71.4 (71.2-71.6)	71.6 (71.5-71.8)	71.8 (71.7-71.9)	72.0 (72.0-72.1)
Maldives	58.3 (57.0-59.7)	59.2 (58.0-60.3)	60.0 (59.1-61.0)	60.8 (60.0-61.6)	61.5 (60.9-62.3)	62.4 (61.8-62.9)	63.1 (62.6-63.7)	63.9 (63.4-64.4)	64.6 (64.1-65.1)	65.3 (64.8-65.7)	66.0 (65.5-66.5)	66.7 (66.2-67.2)
Mauritius	65.8 (65.6-65.9)	66.4 (66.2-66.6)	66.9 (66.8-67.1)	67.2 (67.0-67.4)	67.4 (67.2-67.6)	67.6 (67.4-67.8)	68.0 (67.8-68.2)	68.4 (68.2-68.6)	68.7 (68.5-68.8)	69.0 (68.8-69.1)	69.3 (69.1-69.5)	69.6 (69.4-69.8)
Myanmar	54.3 (49.4-59.4)	54.6 (49.6-59.8)	54.9 (49.8-60.0)	55.3 (50.4-60.4)	55.7 (51.0-60.8)	56.0 (51.4-61.2)	56.4 (51.7-61.6)	56.7 (51.9-61.7)	56.8 (52.2-61.9)	57.3 (52.8-62.3)	57.6 (53.0-62.7)	57.8 (53.2-63.0)
Philippines	65.0 (64.8-65.3)	65.4 (65.1-65.7)	65.3 (65.1-65.6)	65.5 (65.2-65.7)	65.6 (65.4-65.9)	65.5 (65.3-65.7)	65.7 (65.5-65.9)	66.1 (65.8-66.2)	66.6 (66.4-66.8)	67.1 (66.9-67.2)	67.6 (67.4-67.7)	67.9 (67.7-68.2)

Location	1992	1993	1994	1995	1996	1997	1998	1999	2000	2001	2002	2003
Montenegro	74.2 (73.6-74.7)	73.9 (73.5-74.4)	73.7 (73.3-74.1)	73.5 (73.3-73.8)	73.4 (73.1-73.7)	73.3 (73.0-73.6)	73.1 (72.8-73.4)	73.0 (72.7-73.3)	73.3 (73.0-73.5)	73.5 (73.4-73.7)	74.0 (73.8-74.2)	74.5 (74.3-74.6)
Poland	71.1 (71.1-71.1)	71.7 (71.7-71.7)	71.7 (71.7-71.8)	71.9 (71.9-72.0)	72.4 (72.3-72.4)	72.7 (72.6-72.7)	73.0 (73.0-73.0)	73.1 (73.1-73.2)	73.8 (73.8-73.8)	74.2 (74.2-74.3)	74.6 (74.5-74.6)	74.9 (74.8-74.9)
Romania	70.0 (69.9-70.1)	69.8 (69.8-69.9)	69.5 (69.5-69.6)	69.4 (69.3-69.5)	69.1 (69.0-69.1)	69.1 (69.1-69.2)	69.8 (69.8-69.9)	70.6 (70.6-70.7)	71.1 (71.0-71.1)	71.1 (71.0-71.1)	71.2 (71.2-71.3)	71.6 (71.5-71.6)
Serbia	72.4 (72.1-72.7)	72.3 (72.0-72.5)	72.1 (72.0-72.2)	72.0 (71.9-72.0)	71.9 (71.9-72.0)	72.1 (72.0-72.2)	71.5 (71.0-72.0)	71.0 (70.0-72.0)	72.1 (71.9-72.3)	72.4 (72.2-72.6)	72.5 (72.3-72.7)	72.7 (72.5-72.9)
Slovakia	71.4 (71.3-71.5)	71.9 (71.8-72.0)	72.3 (72.2-72.4)	72.4 (72.3-72.5)	72.8 (72.7-72.9)	72.8 (72.7-72.9)	72.8 (72.7-72.9)	73.0 (72.9-73.1)	73.3 (73.2-73.4)	73.6 (73.5-73.7)	73.8 (73.7-73.9)	73.9 (73.8-74.0)
Slovenia	73.8 (73.7-73.9)	73.9 (73.8-73.9)	74.2 (74.1-74.3)	74.6 (74.5-74.7)	75.0 (74.9-75.1)	75.2 (75.1-75.3)	75.4 (75.3-75.5)	75.7 (75.6-75.8)	76.1 (76.0-76.2)	76.3 (76.2-76.4)	76.6 (76.5-76.7)	76.8 (76.7-76.9)
Central Asia	66.2 (65.9-66.5)	65.7 (65.4-66.0)	65.4 (65.1-65.7)	65.3 (65.0-65.6)	65.3 (65.0-65.6)	65.7 (65.4-65.9)	65.9 (65.6-66.2)	66.4 (66.1-66.6)	66.5 (66.2-66.8)	66.8 (66.5-67.1)	67.0 (66.7-67.3)	67.1 (66.8-67.4)
Armenia	68.8 (68.3-69.3)	68.3 (67.9-68.8)	68.6 (68.2-69.1)	69.1 (68.6-69.5)	69.4 (69.0-69.9)	70.0 (69.6-70.4)	70.5 (70.1-70.9)	70.8 (70.3-71.2)	71.3 (70.8-71.7)	71.4 (70.9-71.8)	71.6 (71.2-72.1)	71.5 (71.1-71.9)
Azerbaijan	63.7 (62.7-64.6)	64.0 (63.2-64.7)	63.5 (62.7-64.3)	64.6 (63.9-65.3)	65.0 (64.3-65.7)	65.3 (64.7-66.0)	65.6 (64.9-66.2)	65.9 (65.3-66.6)	66.5 (65.8-67.1)	67.2 (66.6-67.9)	67.6 (67.0-68.3)	67.6 (67.0-68.2)
Georgia	69.4 (68.7-70.1)	69.2 (68.4-70.0)	70.2 (69.6-70.9)	70.7 (70.2-71.3)	71.2 (70.7-71.8)	71.3 (70.8-71.9)	71.4 (70.8-72.0)	71.4 (70.8-72.0)	71.4 (70.8-72.0)	71.7 (71.0-72.3)	71.6 (71.0-72.2)	71.7 (71.0-72.2)
Kazakhstan	67.1 (66.9-67.4)	65.3 (64.8-65.8)	64.6 (64.1-65.1)	63.6 (63.1-64.1)	63.5 (62.9-64.0)	63.9 (63.3-64.4)	64.2 (63.7-64.8)	65.0 (64.6-65.3)	64.9 (64.6-65.2)	65.1 (64.8-65.5)	65.4 (65.1-65.8)	65.3 (65.0-65.7)
Kyrgyzstan	65.6 (65.1-66.0)	65.3 (64.8-65.8)	65.0 (64.6-65.5)	65.1 (64.6-65.5)	65.2 (64.7-65.6)	65.3 (64.9-65.8)	65.6 (65.1-66.0)	65.8 (65.3-66.2)	65.9 (65.5-66.3)	66.0 (65.5-66.4)	65.9 (65.4-66.3)	66.0 (65.5-66.4)
Mongolia	62.2 (61.5-62.8)	62.0 (61.4-62.6)	62.0 (61.4-62.6)	61.7 (61.2-62.4)	61.7 (61.2-62.4)	61.9 (61.3-62.5)	62.1 (61.5-62.6)	62.3 (61.7-62.8)	62.5 (61.8-63.0)	62.7 (62.1-63.2)	62.9 (62.3-63.5)	63.1 (62.5-63.6)
Tajikistan	62.6 (61.2-64.1)	64.2 (63.4-64.9)	64.1 (63.4-64.9)	64.1 (63.3-64.8)	63.3 (62.5-64.0)	64.8 (64.1-65.4)	64.9 (64.2-65.6)	65.5 (64.9-66.1)	65.8 (65.2-66.4)	66.1 (65.4-66.7)	66.5 (65.8-67.1)	66.9 (66.4-67.6)
Turkmenistan	63.5 (62.5-64.4)	63.5 (62.6-64.4)	63.4 (62.4-64.3)	63.3 (62.2-64.2)	63.2 (62.1-64.1)	63.2 (62.1-64.2)	63.3 (62.1-64.3)	63.5 (62.2-64.5)	63.7 (62.4-64.9)	64.1 (62.7-65.2)	64.4 (63.1-65.6)	64.8 (63.5-66.0)
Uzbekistan	67.3 (66.7-67.8)	67.0 (66.4-67.5)	66.7 (66.2-67.3)	66.6 (66.1-67.2)	66.7 (66.1-67.3)	66.9 (66.3-67.5)	67.1 (66.5-67.7)	67.4 (66.8-68.0)	67.6 (66.9-68.2)	67.8 (67.2-68.4)	67.9 (67.3-68.5)	68.1 (67.4-68.7)
Latin America and Caribbean	69.6 (69.5-69.8)	69.9 (69.7-70.0)	70.1 (70.0-70.3)	70.5 (70.3-70.6)	70.8 (70.6-70.9)	71.1 (70.9-71.3)	71.3 (71.1-71.5)	71.5 (71.3-71.7)	72.1 (71.9-72.3)	72.4 (72.2-72.6)	72.7 (72.5-72.8)	72.9 (72.7-73.1)
Central Latin America	71.4 (71.3-71.6)	71.6 (71.5-71.8)	71.9 (71.7-72.1)	72.3 (72.2-72.5)	72.7 (72.5-72.9)	73.0 (72.8-73.2)	72.9 (72.7-73.1)	73.1 (72.8-73.3)	73.8 (73.7-74.0)	74.0 (73.9-74.2)	74.2 (74.0-74.3)	74.3 (74.1-74.5)
Colombia	71.1 (71.0-71.3)	71.4 (71.2-71.5)	71.6 (71.4-71.8)	72.0 (71.8-72.2)	72.2 (72.1-72.4)	72.8 (72.6-72.9)	73.1 (72.9-73.3)	73.2 (73.0-73.3)	73.3 (73.2-73.5)	73.5 (73.3-73.6)	73.7 (73.5-73.9)	74.5 (74.3-74.6)
Costa Rica	76.6 (76.4-76.9)	76.6 (76.4-76.9)	76.7 (76.4-76.9)	76.8 (76.5-77.0)	76.8 (76.5-77.0)	77.0 (76.7-77.2)	77.2 (76.9-77.5)	77.5 (77.2-77.7)	77.7 (77.4-77.9)	78.0 (77.7-78.2)	78.2 (78.0-78.5)	78.4 (78.2-78.7)
El Salvador	68.9 (68.4-69.4)	69.3 (68.8-69.9)	69.6 (69.1-70.2)	70.0 (69.5-70.6)	70.3 (69.8-70.9)	70.8 (70.2-71.3)	71.0 (70.5-71.5)	71.6 (71.0-72.1)	71.9 (71.4-72.5)	71.7 (71.2-72.3)	72.5 (72.0-72.9)	72.6 (72.1-73.1)
Guatemala	63.8 (63.6-64.1)	64.2 (63.9-64.4)	64.6 (64.4-64.9)	66.9 (66.6-67.2)	68.1 (67.8-68.4)	67.7 (67.5-68.0)	67.7 (67.4-67.9)	68.8 (68.5-69.0)	68.8 (68.5-69.0)	69.3 (69.1-69.6)	70.0 (69.8-70.3)	70.1 (69.8-70.3)
Honduras	70.1 (69.3-70.9)	69.9 (69.1-70.8)	70.1 (69.1-71.1)	70.1 (68.9-71.1)	70.1 (68.9-71.3)	70.2 (68.8-71.4)	63.9 (61.1-66.8)	70.3 (68.8-71.8)	70.5 (68.7-72.0)	70.6 (68.7-72.2)	70.7 (68.7-72.4)	70.8 (68.8-72.6)
Mexico	72.1 (71.8-72.4)	72.4 (72.1-72.6)	72.7 (72.4-73.0)	73.0 (72.7-73.2)	73.3 (73.1-73.6)	73.6 (73.3-73.8)	73.7 (73.5-74.0)	74.1 (73.9-74.4)	74.6 (74.3-74.9)	74.9 (74.6-75.1)	74.9 (74.6-75.1)	74.9 (74.6-75.1)
Nicaragua	73.6 (73.1-74.1)	73.9 (73.4-74.4)	74.1 (73.6-74.6)	74.4 (73.8-74.9)	74.7 (74.2-75.2)	75.0 (74.6-75.5)	73.2 (72.2-74.1)	75.6 (75.2-76.1)	76.0 (75.6-76.4)	76.2 (75.7-76.6)	76.3 (75.8-76.7)	76.3 (75.9-76.8)
Panama	75.0 (74.5-75.5)	75.2 (74.6-75.7)	75.2 (74.6-75.7)	75.3 (74.7-75.9)	75.4 (74.8-76.0)	75.6 (75.0-76.1)	75.8 (75.3-76.4)	76.2 (75.7-76.7)	76.5 (76.0-77.0)	76.7 (76.2-77.2)	76.9 (76.3-77.3)	77.0 (76.4-77.5)
Venezuela	72.4 (72.3-72.5)	72.5 (72.4-72.6)	72.4 (72.3-72.4)	72.7 (72.5-72.8)	73.1 (73.0-73.1)	73.6 (73.5-73.7)	73.6 (73.5-73.7)	70.2 (68.7-71.7)	73.8 (73.7-73.8)	73.8 (73.7-73.8)	73.8 (73.7-73.9)	73.5 (73.5-73.6)
Andean Latin America	68.7 (68.3-69.0)	69.1 (68.7-69.4)	69.6 (69.2-69.9)	70.0 (69.7-70.4)	70.6 (70.2-70.9)	71.1 (70.7-71.4)	71.7 (71.3-72.0)	72.3 (71.9-72.6)	72.8 (72.4-73.1)	73.3 (72.9-73.6)	73.7 (73.4-74.0)	74.1 (73.8-74.5)
Bolivia	62.7 (61.8-63.5)	63.2 (62.4-64.0)	63.8 (63.0-64.6)	64.4 (63.6-65.2)	65.1 (64.3-65.9)	65.6 (64.9-66.4)	66.3 (65.6-67.0)	66.9 (66.3-67.7)	67.6 (66.9-68.3)	68.3 (67.6-69.0)	68.7 (68.0-69.5)	69.3 (68.5-70.0)
Ecuador	71.7 (71.3-72.0)	71.8 (71.4-72.2)	72.2 (71.8-72.5)	72.3 (72.0-72.7)	72.5 (72.1-72.9)	72.7 (72.3-73.1)	72.8 (72.4-73.2)	73.1 (72.7-73.5)	73.2 (72.7-73.6)	73.4 (72.9-73.9)	73.6 (73.1-74.0)	73.7 (73.2-74.1)
Peru	69.5 (69.0-70.1)	70.0 (69.5-70.5)	70.5 (70.0-71.0)	71.1 (70.5-71.5)	71.7 (71.2-72.2)	72.4 (71.8-72.9)	73.1 (72.7-73.6)	73.9 (73.4-74.4)	74.6 (74.0-75.1)	75.2 (74.7-75.7)	75.7 (75.3-76.2)	76.2 (75.7-76.7)
Caribbean	68.1 (67.8-68.5)	68.1 (67.7-68.5)	68.3 (67.9-68.7)	68.7 (68.3-69.1)	68.9 (68.5-69.3)	69.2 (68.8-69.6)	69.5 (69.1-69.9)	69.8 (69.4-70.2)	70.1 (69.7-70.6)	70.4 (69.9-70.8)	70.7 (70.2-71.1)	70.8 (70.3-71.2)
Antigua and Barbuda	73.5 (72.8-74.1)	73.4 (72.7-74.0)	73.4 (72.8-74.0)	73.5 (72.9-74.1)	73.5 (72.9-74.1)	73.9 (73.4-74.5)	74.1 (73.6-74.7)	74.0 (73.5-74.6)	73.8 (73.1-74.3)	74.1 (73.6-74.7)	74.3 (73.8-74.9)	74.5 (74.0-75.2)
The Bahamas	69.2 (68.3-70.0)	69.7 (68.9-70.4)	69.5 (68.7-70.2)	69.7 (68.9-70.3)	70.0 (69.4-70.7)	70.2 (69.5-70.8)	70.1 (69.4-70.8)	70.1 (69.4-70.8)	70.2 (69.5-70.9)	70.3 (69.6-71.1)	70.4 (69.6-71.1)	71.1 (70.2-71.9)
Barbados	73.2 (72.3-73.9)	73.1 (72.2-73.8)	73.0 (72.2-73.7)	73.3 (72.4-74.0)	73.7 (72.8-74.4)	73.9 (73.1-74.7)	74.1 (73.3-74.9)	74.2 (73.4-75.0)	74.0 (73.2-74.7)	74.4 (73.6-75.1)	74.6 (73.9-75.2)	74.8 (74.1-75.4)
Belize	71.1 (70.2-71.9)	70.8 (70.1-71.7)	70.6 (69.9-71.4)	70.5 (69.8-71.2)	70.4 (69.7-71.0)	69.7 (69.0-70.4)	69.5 (68.9-70.2)	69.3 (68.7-69.9)	69.0 (68.4-69.6)	68.8 (68.1-69.5)	69.3 (68.7-69.9)	69.6 (69.0-70.2)
Bermuda	71.1 (70.6-71.5)	71.0 (70.5-71.5)	71.4 (70.9-71.9)	72.5 (72.0-73.0)	72.5 (72.0-73.0)	73.7 (73.2-74.2)	74.3 (73.8-74.7)	74.4 (73.9-74.8)	74.1 (73.6-74.6)	75.1 (74.6-75.5)	75.4 (74.9-75.8)	75.7 (75.3-76.2)
Cuba	74.7 (74.7-74.8)	74.7 (74.6-74.7)	74.8 (74.8-74.9)	75.0 (75.0-75.1)	75.2 (75.1-75.2)	75.4 (75.3-75.4)	75.6 (75.5-75.7)	75.8 (75.7-75.9)	76.2 (76.1-76.2)	76.4 (76.4-76.5)	76.8 (76.7-76.9)	76.9 (76.9-77.0)
Dominica	72.6 (71.8-73.4)	72.6 (71.8-73.5)	72.7 (71.9-73.6)	72.8 (71.9-73.6)	72.8 (72.0-73.6)	73.0 (72.2-73.8)	72.7 (71.8-73.6)	73.4 (72.6-74.2)	73.7 (72.9-74.4)	73.9 (73.1-74.6)	74.0 (73.1-74.7)	74.1 (73.2-74.7)
Dominican Republic	72.5 (72.0-72.9)	72.5 (72.0-73.0)	72.5 (72.0-73.0)	72.6 (72.1-73.2)	72.6 (72.1-73.2)	72.9 (72.3-73.5)	72.9 (72.3-73.6)	73.2 (72.6-73.8)	73.3 (72.6-73.9)	73.4 (72.8-74.0)	73.5 (72.9-74.0)	73.5 (72.8-74.0)
Grenada	70.2 (69.2-70.9)	70.0 (69.2-70.7)	70.4 (69.6-71.0)	70.7 (70.0-71.4)	70.8 (70.2-71.4)	70.8 (70.1-71.3)	70.6 (70.0-71.2)	70.6 (69.9-71.2)	70.5 (69.8-71.1)	70.3 (69.6-71.0)	70.2 (69.5-70.8)	70.2 (69.5-70.8)
Guyana	64.5 (63.9-65.0)	64.4 (63.9-65.0)	64.5 (63.9-65.0)	64.4 (63.8-65.0)	64.3 (63.7-64.9)	64.3 (63.7-64.9)	64.3 (63.6-64.9)	64.3 (63.6-64.9)	64.2 (63.5-64.9)	63.9 (63.2-64.6)	63.5 (62.9-64.2)	63.7 (63.1-64.4)
Haiti	54.9 (53.6-56.2)	54.7 (53.3-56.0)	55.4 (54.0-56.8)	56.2 (54.9-57.6)	56.7 (55.4-58.2)	57.0 (55.7-58.5)	57.5 (56.2-59.1)	58.0 (56.6-59.6)	58.7 (57.3-60.3)	59.2 (57.7-60.8)	59.6 (58.0-61.2)	59.7 (58.1-61.3)
Jamaica	74.9 (74.1-75.6)	74.8 (74.1-75.6)	74.8 (74.0-75.5)	74.7 (73.9-75.4)	74.5 (73.8-75.2)	74.4 (73.7-75.1)	74.5 (73.7-75.1)	74.6 (73.9-75.2)	74.4 (73.7-74.9)	74.2 (73.5-74.8)	74.5 (73.8-75.1)	74.8 (74.1-75.4)
Puerto Rico	73.7 (73.6-73.9)	73.8 (73.7-73.9)	73.1 (73.0-73.3)	73.3 (73.2-73.5)	73.6 (73.5-73.7)	74.5 (74.4-74.7)	75.1 (75.0-75.2)	75.2 (75.1-75.4)	75.6 (75.5-75.7)	75.8 (75.6-75.9)	76.2 (76.1-76.3)	76.5 (76.4-76.6)
Saint Lucia	70.5 (69.6-71.4)	70.7 (69.9-71.5)	70.9 (70.1-71.6)	71.1 (70.4-71.7)	71.2 (70.5-71.8)	71.5 (70.8-72.0)	71.7 (71.0-72.3)	71.8 (71.2-72.4)	72.0 (71.3-72.6)	72.1 (71.5-72.7)	72.3 (71.6-72.9)	72.5 (71.8-73.1)
Saint Vincent and the Grenadines	69.7 (68.8-70.6)	69.7 (68.7-70.5)	69.6 (68.7-70.4)	69.6 (68.7-70.3)	69.4 (68.7-70.1)	69.7 (69.0-70.4)	69.8 (69.1-70.4)	69.6 (68.9-70.2)	69.1 (68.4-69.7)	69.7 (69.0-70.3)	69.7 (69.0-70.4)	70.1 (69.4-70.7)
Suriname	69.8 (69.2-70.4)	69.7 (69.1-70.3)	69.8 (69.2-70.4)	70.1 (69.4-70.7)	70.2 (69.6-70.9)	70.2 (69.6-70.9)	70.1 (69.4-70.8)	69.9 (69.2-70.5)	69.7 (69.0-70.3)	69.5 (68.7-70.1)	69.4 (68.7-70.1)	69.5 (68.8-70.3)
Trinidad and Tobago	69.2 (68.8-69.5)	69.3 (68.9-69.6)	69.2 (68.8-69.5)	69.3 (68.9-69.7)	69.3 (68.9-69.6)	69.4 (69.0-69.8)	69.6 (69.2-69.9)	69.3 (68.9-69.6)	69.5 (69.0-69.8)	69.8 (69.3-70.1)	69.9 (69.4-70.3)	70.4 (70.0-70.7)
Virgin Islands, U.S.	73.3 (72.8-73.7)	73.4 (72.9-73.8)	73.4 (72.9-73.8)	73.4 (72.9-73.8)	73.6 (73.1-74.0)	73.7 (73.2-74.1)	73.8 (73.3-74.2)	73.9 (73.5-74.3)	74.0 (73.6-74.4)	74.1 (73.7-74.5)	74.2 (73.8-74.6)	74.3 (73.9-74.7)
Tropical Latin America	68.4 (68.0-68.8)	68.7 (68.3-69.0)	68.9 (68.5-69.2)	69.1 (68.8-69.5)	69.4 (69.0-69.7)	69.7 (69.3-70.1)	70.0 (69.7-70.4)	70.4 (70.0-70.7)	70.8 (70.4-71.1)	71.2 (70.8-71.5)	71.5 (71.1-71.8)	71.8 (71.4-72.1)
Brazil	68.3 (67.9-68.7)	68.5 (68.2-68.9)	68.8 (68.4-69.1)	69.0 (68.7-69.4)	69.3 (68.9-69.6)	69.6 (69.2-70.0)	69.9 (69.6-70.3)	70.3 (69.9-70.6)	70.7 (70.3-71.1)	71.1 (70.7-71.4)	71.4 (71.0-71.7)	71.7 (71.4-72.0)
Paraguay	74.1 (73.5-74.8)	74.1 (73.4-74.8)	74.0 (73.4-74.7)	74.1 (73.5-74.8)	74.1 (73.5-74.8)	74.2 (73.5-74.8)	74.2 (73.6-74.8)	74.3 (73.7-74.8)	74.2 (73.6-74.8)	74.2 (73.6-74.8)	74.1 (73.5-74.7)	74.1 (73.4-74.7)
Southeast Asia, East Asia, and Oceania	66.9 (66.2-67.6)	67.3 (66.6-67.9)	67.6 (67.0-68.2)	67.8 (67.3-68.4)	68.2 (67.7-68.7)	68.5 (68.0-68.9)	68.7 (68.3-69.2)	69.0 (68.5-69.4)	69.2 (68.7-69.6)	69.3 (68.9-69.8)	69.6 (69.2-69.9)	70.2 (69.9-70.6)
East Asia	67.3 (66.4-68.2)	67.7 (66.7-68.5)	68.0 (67.1-68.9)	68.3 (67.4-69.1)	68.7 (67.9-69.4)	69.0 (68.3-69.6)	69.2 (68.6-69.9)	69.5 (68.9-70.1)	69.7 (69.1-70.3)	69.9 (69.2-70.5)	70.1 (69.4-70.6)	70.9 (70.4-71.4)
China	67.2 (66.2-68.1)	67.5 (66.6-68.4)	67.9 (67.0-68.8)	68.4 (67.5-69.1)	68.8 (68.0-69.5)	69.1 (68.4-69.7)	69.4 (68.7-69.9)	69.6 (69.0-70.2)	69.8 (69.2-70.4)	70.0 (69.4-70.5)	70.2 (69.6-70.7)	70.9 (70.4-71.3)
North Korea	69.4 (65.3-72.8)	69.6 (65.5-72.9)	69.7 (65.6-72.9)	58.6 (41.9-69.1)	58.7 (41.4-69.1)	58.8 (41.3-68.8)	58.9 (41.7-68.6)	59.0 (42.2-68.7)	59.1 (42.9-68.7)	59.2 (43.4-68.7)	59.3 (43.5-68.8)	69.9 (67.0-72.4)
Taiwan	74.3 (74.3-74.4)	74.5 (74.4-74.6)	74.6 (74.5-74.7)	74.6 (74.5-74.7)	74.7 (74.6-74.8)	75.2 (75.1-75.2)	75.4 (75.3-75.5)	75.3 (75.0-75.6)	76.0 (75.9-76.1)	76.4 (76.3-76.5)	76.9 (76.9-77.0)	77.3 (77.2-77.3)
Southeast Asia	66.2 (65.5-66.9)	66.5 (65.8-67.2)	66.7 (66.0-67.4)	67.0 (66.3-67.6)	67.2 (66.5-67.9)	67.4 (66.7-68.0)	67.6 (66.9-68.3)	67.9 (67.2-68.5)	68.1 (67.4-68.7)	68.4 (67.7-69.0)	68.7 (68.0-69.4)	69.0 (68.3-69.7)
Cambodia	58.2 (57.4-59.0)	58.1 (57.4-58.9)	58.0 (57.3-58.7)	58.1 (57.4-58.8)	58.3 (57.6-59.0)	58.7 (58.0-59.4)	59.0 (58.4-59.8)	59.5 (58.9-60.3)	60.0 (59.3-60.7)	60.8 (60.1-61.6)	61.6 (60.9-62.3)	62.4 (61.7-63.2)
Indonesia	64.9 (64.2-65.5)	65.3 (64.6-65.9)	65.7 (65.1-66.3)	66.1 (65.5-66.7)	66.4 (65.9-67.0)	66.7 (66.2-67.3)	67.0 (66.5-67.6)	67.2 (66.7-67.8)	67.5 (66.9-68.1)	67.8 (67.1-68.4)	68.0 (67.3-68.7)	68.2 (67.5-69.0)
Laos	52.4 (50.4-54.4)	52.8 (51.0-54.7)	53.2 (51.5-55.0)	53.6 (52.0-55.3)	54.0 (52.4-55.6)	54.4 (52.9-55.9)	54.9 (53.5-56.3)	55.5 (54.1-56.8)	56.1 (54.8-57.3)	56.6 (55.4-57.8)	57.6 (56.4-58.7)	58.4 (57.3-59.6)
Malaysia	72.2 (72.2-72.3)	72.4 (72.3-72.5)	72.4 (72.4-72.5)	72.4 (72.4-72.5)	72.5 (72.5-72.6)	72.8 (72.7-72.8)	73.0 (72.9-73.1)	73.1 (73.1-73.2)	73.3 (73.2-73.4)	73.6 (73.5-73.7)	73.8 (73.7-73.9)	73.9 (73.8-73.9)
Maldives	67.3 (66.8-67.8)	67.9 (67.5-68.5)	68.6 (68.1-69.2)	69.3 (68.8-69.9)	70.0 (69.5-70.6)	70.8 (70.3-71.4)	71.7 (71.2-72.2)	72.3 (71.8-72.8)	73.0 (72.6-73.4)	73.7 (73.3-74.1)	74.4 (74.0-74.8)	75.3 (74.9-75.7)
Mauritius	69.7 (69.6-69.9)	69.8 (69.7-70.0)	70.0 (69.8-70.2)	70.3 (70.1-70.5)	70.5 (70.3-70.7)	70.7 (70.5-70.9)	71.0 (70.8-71.2)	71.2 (71.1-71.4)	71.4 (71.2-71.6)	71.6 (71.5-71.8)	71.7 (71.5-71.9)	71.9 (71.7-72.1)
Myanmar	58.1 (53.7-63.2)	58.4 (54.0-63.5)	58.8 (54.3-63.7)	59.0 (54.7-64.0)	59.4 (55.0-64.4)	59.7 (55.3-64.7)	60.1 (55.6-65.1)	60.4 (56.0-65.5)	60.8 (56.4-65.8)	61.3 (56.9-66.4)	61.7 (57.3-67.0)	62.3 (58.0-67.6)
Philippines	68.2 (68.1-68.4)	68.3 (68.2-68.5)	68.6 (68.5-68.7)	68.8 (68.7-68.9)	68.9 (68.7-69.0)	69.0 (68.9-69.1)	69.0 (68.9-69.1)	69.2 (69.1-69.4)	69.1 (69.0-69.3)	69.0 (68.9-69.1)	69.0 (68.9-69.1)	69.1 (69.0-69.3)

Location	2004	2005	2006	2007	2008	2009	2010	2011	2012	2013	2014	2015
Montenegro	74.8 (74.6-75.0)	75.0 (74.8-75.2)	75.2 (75.0-75.4)	75.6 (75.3-75.8)	76.0 (75.8-76.2)	76.3 (76.1-76.5)	76.5 (76.3-76.8)	76.7 (76.4-77.0)	76.8 (76.5-77.2)	76.9 (76.5-77.3)	77.1 (76.7-77.5)	77.2 (76.8-77.6)
Poland	75.0 (74.9-75.0)	75.1 (75.0-75.1)	75.2 (75.1-75.2)	75.2 (75.2-75.3)	75.4 (75.4-75.5)	75.7 (75.7-75.8)	76.2 (76.2-76.3)	76.5 (76.5-76.6)	76.7 (76.7-76.8)	77.3 (77.2-77.3)	77.4 (77.3-77.5)	77.6 (77.4-77.8)
Romania	72.0 (72.0-72.1)	72.4 (72.3-72.5)	72.8 (72.8-72.9)	73.3 (73.2-73.3)	73.2 (73.2-73.3)	73.3 (73.3-73.4)	73.7 (73.6-73.7)	74.5 (74.5-74.6)	74.7 (74.6-74.7)	74.9 (74.7-75.0)	75.1 (74.8-75.3)	75.2 (74.9-75.5)
Serbia	72.9 (72.7-73.1)	73.2 (73.1-73.4)	73.6 (73.5-73.7)	74.0 (73.8-74.1)	74.3 (74.2-74.4)	74.6 (74.5-74.6)	74.8 (74.8-74.9)	75.1 (75.0-75.1)	75.4 (75.3-75.4)	75.7 (75.6-75.7)	75.9 (75.8-76.1)	76.2 (76.0-76.3)
Slovakia	74.2 (74.1-74.3)	74.3 (74.2-74.3)	74.5 (74.4-74.6)	74.6 (74.5-74.7)	74.9 (74.9-75.0)	75.2 (75.1-75.3)	75.7 (75.6-75.7)	76.1 (76.0-76.2)	76.5 (76.4-76.6)	77.0 (76.9-77.1)	77.4 (77.3-77.5)	77.6 (77.4-77.7)
Slovenia	77.2 (77.1-77.3)	77.6 (77.5-77.7)	77.9 (77.8-78.0)	78.2 (78.1-78.3)	78.7 (78.6-78.8)	79.1 (79.0-79.2)	79.5 (79.4-79.6)	79.9 (79.8-80.0)	80.1 (79.9-80.2)	80.3 (80.2-80.4)	80.8 (80.7-81.0)	80.9 (80.8-81.1)
Central Asia	67.3 (67.0-67.6)	67.5 (67.2-67.8)	67.8 (67.5-68.1)	68.2 (67.9-68.5)	68.7 (68.4-69.0)	69.3 (68.9-69.6)	69.6 (69.2-69.9)	70.0 (69.6-70.3)	70.5 (70.1-70.8)	70.8 (70.4-71.2)	71.1 (70.6-71.5)	71.3 (70.8-71.8)
Armenia	71.8 (71.4-72.2)	71.8 (71.3-72.2)	71.7 (71.3-72.1)	72.3 (71.8-72.7)	72.3 (71.8-72.7)	72.6 (72.2-73.1)	73.0 (72.6-73.4)	73.5 (73.0-73.9)	73.9 (73.5-74.4)	74.3 (73.8-74.7)	74.5 (74.0-75.0)	74.7 (74.2-75.2)
Azerbaijan	67.8 (67.2-68.5)	68.2 (67.6-68.8)	68.7 (68.0-69.3)	69.1 (68.4-69.8)	69.2 (68.5-69.9)	69.7 (69.0-70.4)	70.1 (69.3-70.8)	70.8 (70.0-71.6)	71.5 (70.7-72.3)	72.1 (71.3-72.9)	72.5 (71.7-73.3)	72.9 (72.1-73.7)
Georgia	71.5 (71.0-72.1)	71.8 (71.2-72.4)	71.9 (71.4-72.5)	72.1 (71.5-72.7)	71.8 (71.2-72.4)	72.1 (71.5-72.7)	72.2 (71.6-72.8)	72.2 (71.6-72.9)	72.4 (71.8-72.9)	72.5 (71.9-73.1)	72.6 (72.0-73.3)	72.9 (72.2-73.6)
Kazakhstan	65.3 (65.0-65.6)	65.3 (64.9-65.6)	65.3 (65.0-65.6)	65.8 (65.5-66.1)	67.1 (66.8-67.4)	68.2 (67.9-68.5)	68.5 (68.2-68.8)	69.1 (68.8-69.4)	69.7 (69.4-70.0)	70.0 (69.6-70.5)	70.2 (69.5-70.8)	70.2 (69.3-71.0)
Kyrgyzstan	66.2 (65.8-66.7)	66.5 (66.0-66.9)	66.7 (66.3-67.2)	67.0 (66.6-67.5)	67.3 (66.9-67.7)	67.7 (67.2-68.1)	67.9 (67.5-68.3)	68.5 (68.1-69.0)	69.0 (68.5-69.4)	69.4 (68.9-69.9)	69.6 (69.1-70.1)	69.8 (69.2-70.3)
Mongolia	63.4 (62.8-63.9)	63.6 (63.0-64.2)	64.0 (63.4-64.6)	64.4 (63.8-65.0)	64.8 (64.2-65.4)	65.0 (64.4-65.6)	65.2 (64.6-65.9)	65.6 (64.9-66.3)	66.0 (65.3-66.7)	66.4 (65.7-67.1)	66.8 (66.0-67.4)	67.1 (66.4-67.8)
Tajikistan	67.5 (66.9-68.0)	68.0 (67.4-68.5)	68.7 (68.1-69.3)	69.2 (68.7-69.8)	69.6 (69.0-70.2)	70.0 (69.4-70.6)	70.1 (69.6-70.7)	70.1 (69.5-70.7)	70.8 (70.2-71.4)	71.4 (70.8-72.1)	71.9 (71.2-72.6)	72.4 (71.7-73.0)
Turkmenistan	65.3 (63.9-66.4)	65.8 (64.5-66.9)	66.3 (65.0-67.4)	66.8 (65.5-67.9)	67.2 (66.0-68.2)	67.6 (66.5-68.6)	68.1 (66.9-69.0)	68.5 (67.4-69.4)	68.9 (67.9-69.7)	69.2 (68.2-70.0)	69.6 (68.7-70.4)	70.0 (69.0-70.8)
Uzbekistan	68.3 (67.6-68.9)	68.5 (67.7-69.2)	68.8 (68.1-69.6)	69.2 (68.4-70.0)	69.6 (68.8-70.4)	69.9 (69.1-70.6)	70.2 (69.5-70.9)	70.6 (69.8-71.3)	70.9 (70.2-71.6)	71.2 (70.5-71.9)	71.5 (70.8-72.2)	71.8 (71.1-72.5)
Latin America and Caribbean	73.2 (73.0-73.3)	73.4 (73.3-73.6)	73.7 (73.5-73.9)	74.0 (73.8-74.2)	74.1 (73.9-74.3)	74.2 (74.0-74.4)	73.2 (72.0-74.2)	74.6 (74.4-74.9)	74.8 (74.5-75.1)	74.9 (74.6-75.2)	75.0 (74.7-75.4)	75.2 (74.8-75.5)
Central Latin America	74.6 (74.4-74.8)	74.7 (74.6-74.9)	74.9 (74.8-75.1)	75.2 (75.0-75.4)	75.2 (75.0-75.4)	75.2 (74.9-75.4)	75.4 (75.1-75.6)	75.7 (75.4-75.9)	75.7 (75.5-76.0)	75.7 (75.4-75.9)	75.8 (75.5-76.1)	76.0 (75.7-76.4)
Colombia	74.7 (74.5-74.9)	75.1 (74.9-75.3)	75.3 (75.1-75.5)	75.9 (75.6-76.1)	76.2 (75.9-76.4)	76.2 (75.9-76.4)	76.4 (76.1-76.7)	77.0 (76.7-77.3)	77.3 (76.9-77.6)	77.5 (77.1-77.9)	77.8 (77.4-78.1)	78.0 (77.5-78.4)
Costa Rica	78.7 (78.4-78.9)	79.0 (78.7-79.2)	79.3 (79.0-79.5)	79.6 (79.3-79.8)	79.8 (79.5-80.0)	79.8 (79.6-80.0)	79.8 (79.6-80.0)	79.9 (79.7-80.1)	80.0 (79.7-80.2)	80.1 (79.8-80.3)	80.2 (79.9-80.5)	80.3 (79.9-80.7)
El Salvador	72.8 (72.3-73.3)	73.0 (72.6-73.5)	73.3 (72.8-73.7)	73.5 (73.0-73.9)	73.7 (73.2-74.2)	73.8 (73.3-74.3)	74.1 (73.5-74.6)	74.3 (73.7-74.9)	74.5 (73.8-75.1)	74.6 (73.9-75.3)	74.8 (74.0-75.5)	74.9 (74.1-75.8)
Guatemala	70.2 (69.9-70.5)	69.8 (69.5-70.1)	70.2 (69.9-70.5)	71.0 (70.6-71.3)	71.2 (70.8-71.5)	70.9 (70.6-71.3)	71.2 (70.8-71.5)	71.6 (71.2-72.0)	72.1 (71.6-72.4)	72.3 (71.9-72.7)	72.4 (71.7-73.2)	72.6 (71.3-73.9)
Honduras	71.0 (68.9-72.9)	71.2 (69.1-73.1)	71.4 (69.3-73.4)	71.6 (69.4-73.6)	71.8 (69.6-73.7)	71.9 (69.8-73.9)	72.0 (69.9-73.9)	72.2 (70.1-74.2)	72.3 (70.2-74.2)	72.6 (70.4-74.5)	72.8 (70.7-74.7)	73.0 (70.9-75.0)
Mexico	75.2 (74.9-75.4)	75.1 (74.9-75.4)	75.4 (75.1-75.6)	75.6 (75.3-75.9)	75.4 (75.1-75.6)	75.3 (75.0-75.5)	75.4 (75.1-75.7)	75.7 (75.4-76.0)	75.7 (75.4-76.0)	75.4 (75.1-75.7)	75.6 (75.3-75.9)	75.9 (75.5-76.1)
Nicaragua	76.4 (76.0-76.9)	76.6 (76.1-77.0)	76.7 (76.3-77.1)	76.8 (76.4-77.2)	77.0 (76.6-77.5)	77.1 (76.8-77.5)	77.2 (76.8-77.6)	77.4 (77.0-77.8)	77.5 (77.1-77.9)	77.6 (77.1-78.1)	77.8 (77.1-78.3)	78.0 (77.2-78.7)
Panama	77.1 (76.5-77.6)	77.2 (76.6-77.7)	77.2 (76.7-77.7)	77.3 (76.8-77.8)	77.3 (76.8-77.8)	77.3 (76.8-77.8)	77.3 (76.8-77.8)	77.4 (76.8-77.9)	77.5 (76.9-78.0)	77.6 (77.0-78.3)	77.9 (77.1-78.8)	78.2 (77.1-79.1)
Venezuela	74.3 (74.2-74.4)	74.6 (74.5-74.7)	74.5 (74.4-74.5)	74.4 (74.3-74.4)	74.2 (74.2-74.3)	74.4 (74.4-74.5)	74.9 (74.8-74.9)	74.8 (74.8-74.9)	74.6 (74.5-74.7)	74.7 (74.3-75.1)	74.8 (74.0-75.5)	74.8 (73.7-75.9)
Andean Latin America	74.5 (74.2-74.9)	74.9 (74.6-75.3)	75.3 (74.9-75.7)	75.5 (75.1-75.9)	75.8 (75.4-76.2)	76.0 (75.6-76.3)	76.1 (75.7-76.5)	76.3 (75.9-76.8)	76.6 (76.1-77.0)	76.8 (76.3-77.3)	77.0 (76.5-77.5)	77.2 (76.6-77.8)
Bolivia	69.8 (68.9-70.6)	70.2 (69.3-71.1)	70.6 (69.6-71.6)	70.9 (69.8-71.9)	71.2 (70.0-72.3)	71.6 (70.3-72.7)	71.8 (70.5-73.1)	72.1 (70.7-73.4)	72.4 (70.9-73.8)	72.7 (71.0-74.1)	72.9 (71.3-74.4)	73.2 (71.4-74.7)
Ecuador	73.9 (73.4-74.4)	74.1 (73.6-74.6)	74.3 (73.8-74.8)	74.5 (74.0-75.0)	74.7 (74.2-75.2)	74.9 (74.4-75.4)	75.1 (74.5-75.5)	75.3 (74.7-75.8)	75.5 (74.8-76.0)	75.6 (75.0-76.2)	75.8 (75.1-76.4)	75.9 (75.1-76.6)
Peru	76.7 (76.2-77.2)	77.2 (76.7-77.7)	77.7 (77.2-78.3)	77.9 (77.4-78.4)	78.2 (77.7-78.7)	78.2 (77.7-78.7)	78.4 (77.8-78.9)	78.5 (78.0-79.1)	78.7 (78.2-79.3)	79.1 (78.5-79.7)	79.3 (78.5-80.0)	79.5 (78.6-80.3)
Caribbean	70.4 (69.8-71.0)	70.9 (70.4-71.4)	71.1 (70.6-71.6)	71.3 (70.7-71.8)	71.4 (70.9-71.9)	71.7 (71.2-72.2)	58.7 (49.2-69.5)	72.1 (71.5-72.7)	72.3 (71.6-73.0)	72.6 (71.7-73.3)	72.8 (71.9-73.6)	73.1 (72.1-73.9)
Antigua and Barbuda	74.6 (74.0-75.3)	74.9 (74.2-75.5)	74.9 (74.2-75.6)	75.3 (74.6-76.0)	75.5 (74.8-76.2)	75.7 (75.0-76.4)	75.8 (75.1-76.5)	75.9 (75.2-76.7)	76.1 (75.3-76.8)	76.2 (75.4-77.0)	76.3 (75.5-77.2)	76.4 (75.6-77.4)
The Bahamas	71.7 (70.9-72.4)	71.7 (71.0-72.4)	72.0 (71.3-72.7)	71.8 (71.0-72.6)	72.2 (71.2-73.0)	72.7 (71.9-73.5)	72.7 (71.9-73.6)	72.6 (71.6-73.5)	72.7 (71.8-73.6)	72.9 (71.7-74.0)	73.5 (72.4-74.6)	73.7 (72.5-74.8)
Barbados	74.9 (74.3-75.5)	75.0 (74.4-75.6)	75.0 (74.4-75.6)	75.1 (74.5-75.7)	75.3 (74.6-75.9)	75.3 (74.6-75.8)	75.2 (74.6-75.8)	75.2 (74.5-75.8)	75.4 (74.6-76.2)	75.5 (74.6-76.3)	75.6 (74.6-76.5)	75.7 (74.6-76.6)
Belize	69.9 (69.2-70.6)	70.0 (69.4-70.7)	70.3 (69.7-70.9)	70.6 (70.0-71.2)	70.7 (70.1-71.3)	70.9 (70.3-71.4)	71.2 (70.7-71.7)	71.2 (70.7-71.8)	71.3 (70.7-71.9)	71.3 (70.6-72.0)	71.5 (70.6-72.3)	71.7 (70.7-72.7)
Bermuda	76.0 (75.6-76.5)	76.4 (75.9-76.9)	76.4 (75.9-76.9)	76.8 (76.3-77.2)	77.1 (76.7-77.6)	77.5 (77.0-77.9)	77.6 (77.2-78.1)	78.0 (77.6-78.5)	78.3 (77.8-78.9)	78.3 (77.7-78.8)	78.4 (77.7-79.0)	78.5 (77.7-79.2)
Cuba	77.0 (77.0-77.1)	77.1 (77.1-77.2)	77.3 (77.2-77.4)	77.5 (77.4-77.6)	77.6 (77.5-77.7)	77.7 (77.7-77.8)	77.8 (77.7-77.9)	77.9 (77.9-78.0)	78.0 (77.9-78.1)	78.0 (77.9-78.1)	78.1 (78.0-78.3)	78.2 (77.9-78.4)
Dominica	74.1 (73.3-74.8)	74.2 (73.4-74.9)	74.2 (73.4-74.9)	74.2 (73.3-74.9)	74.1 (73.2-74.9)	73.9 (73.0-74.7)	73.7 (72.7-74.5)	73.4 (72.3-74.3)	73.2 (72.1-74.3)	73.0 (71.8-74.1)	73.0 (71.7-74.2)	73.1 (71.7-74.4)
Dominican Republic	73.1 (72.5-73.7)	73.3 (72.8-73.8)	73.5 (73.0-73.9)	73.7 (73.2-74.1)	74.1 (73.6-74.5)	74.3 (73.8-74.7)	74.4 (73.9-74.9)	74.6 (74.1-75.0)	74.7 (74.2-75.2)	74.9 (74.4-75.4)	75.1 (74.5-75.6)	75.3 (74.7-75.8)
Grenada	69.3 (68.2-70.3)	70.3 (69.5-70.9)	70.5 (69.8-71.1)	70.7 (70.0-71.3)	70.8 (70.2-71.4)	70.9 (70.2-71.5)	71.0 (70.3-71.6)	71.0 (70.2-71.6)	71.1 (70.3-71.8)	71.2 (70.3-72.0)	71.4 (70.3-72.3)	71.5 (70.4-72.5)
Guyana	64.2 (63.4-64.9)	64.4 (63.7-65.2)	64.8 (64.1-65.5)	64.9 (64.2-65.6)	65.0 (64.3-65.8)	65.3 (64.5-66.1)	65.5 (64.6-66.3)	65.6 (64.7-66.6)	65.9 (64.9-66.9)	66.2 (65.0-67.3)	66.4 (65.2-67.6)	66.8 (65.5-68.0)
Haiti	58.6 (56.7-60.5)	60.0 (58.2-61.9)	60.3 (58.5-62.1)	60.8 (58.9-62.5)	61.0 (59.2-62.8)	61.7 (59.9-63.6)	32.2 (21.2-54.5)	62.4 (60.4-64.6)	62.9 (60.5-65.1)	63.4 (60.9-65.7)	63.8 (61.2-66.3)	64.3 (61.8-66.8)
Jamaica	75.0 (74.3-75.7)	75.4 (74.5-76.0)	75.5 (74.7-76.2)	75.3 (74.4-76.0)	75.1 (74.2-75.7)	74.8 (73.9-75.4)	74.8 (74.0-75.5)	74.6 (73.7-75.3)	74.7 (73.9-75.5)	74.8 (73.9-75.6)	74.8 (73.9-75.8)	75.0 (74.0-76.0)
Puerto Rico	76.5 (76.4-76.6)	76.6 (76.4-76.7)	76.8 (76.6-76.9)	76.9 (76.8-77.0)	77.0 (76.9-77.1)	77.1 (76.9-77.2)	77.1 (77.0-77.3)	77.4 (77.3-77.5)	77.7 (77.5-77.8)	77.9 (77.7-78.0)	78.2 (77.9-78.5)	78.5 (78.0-78.9)
Saint Lucia	72.8 (72.1-73.5)	73.1 (72.3-73.9)	73.4 (72.6-74.2)	73.7 (72.9-74.5)	74.1 (73.2-74.8)	74.3 (73.4-75.0)	74.5 (73.6-75.3)	74.6 (73.7-75.4)	74.7 (73.8-75.6)	74.7 (73.7-75.7)	74.8 (73.6-75.8)	74.8 (73.5-75.9)
Saint Vincent and the Grenadines	70.2 (69.5-70.8)	70.4 (69.7-71.1)	70.6 (69.9-71.3)	70.8 (70.1-71.4)	70.9 (70.1-71.6)	71.1 (70.3-71.8)	71.1 (70.3-71.8)	71.0 (70.2-71.8)	71.1 (70.2-71.9)	70.8 (69.9-71.7)	71.1 (70.1-72.0)	71.2 (70.2-72.2)
Suriname	69.7 (69.0-70.4)	69.9 (69.1-70.6)	70.0 (69.2-70.7)	70.1 (69.3-70.9)	70.2 (69.4-71.0)	70.5 (69.7-71.4)	70.7 (69.9-71.6)	71.0 (70.1-71.9)	71.3 (70.4-72.3)	71.6 (70.5-72.6)	71.8 (70.7-72.9)	72.0 (70.8-73.2)
Trinidad and Tobago	70.9 (70.5-71.3)	71.1 (70.7-71.5)	71.3 (70.8-71.7)	71.5 (71.0-72.0)	71.7 (71.2-72.2)	71.8 (71.2-72.3)	71.8 (71.1-72.3)	71.9 (71.2-72.6)	72.0 (71.2-72.8)	72.1 (71.2-73.0)	72.3 (71.3-73.2)	72.4 (71.3-73.4)
Virgin Islands, U.S.	74.3 (73.9-74.8)	74.4 (74.0-74.9)	74.5 (74.0-74.9)	74.5 (74.0-74.9)	74.4 (73.9-74.9)	74.3 (73.8-74.8)	74.3 (73.8-74.9)	74.4 (73.8-74.9)	74.4 (73.7-75.0)	74.4 (73.7-75.1)	74.4 (73.6-75.2)	74.4 (73.5-75.2)
Tropical Latin America	72.1 (71.7-72.4)	72.4 (72.1-72.8)	72.7 (72.4-73.1)	73.0 (72.6-73.3)	73.2 (72.9-73.6)	73.5 (73.1-73.8)	73.6 (73.2-74.0)	73.8 (73.5-74.2)	74.0 (73.6-74.4)	74.1 (73.7-74.5)	74.3 (73.8-74.7)	74.4 (73.9-74.9)
Brazil	72.0 (71.7-72.4)	72.4 (72.0-72.7)	72.7 (72.3-73.0)	73.0 (72.6-73.3)	73.2 (72.8-73.6)	73.4 (73.0-73.8)	73.6 (73.2-74.0)	73.8 (73.5-74.2)	74.0 (73.6-74.4)	74.1 (73.7-74.5)	74.3 (73.8-74.7)	74.4 (73.9-74.9)
Paraguay	73.8 (73.2-74.5)	74.0 (73.3-74.7)	74.0 (73.2-74.6)	74.0 (73.2-74.7)	74.0 (73.2-74.7)	74.0 (73.1-74.7)	74.0 (73.1-74.7)	74.0 (73.2-74.8)	74.1 (73.1-74.9)	74.1 (73.1-75.1)	74.3 (73.2-75.3)	74.4 (73.2-75.6)
Southeast Asia, East Asia, and Oceania	70.4 (70.0-70.8)	71.1 (70.8-71.4)	71.6 (71.3-72.0)	72.1 (71.8-72.4)	72.2 (71.8-72.5)	72.9 (72.5-73.2)	73.2 (72.9-73.5)	73.7 (73.3-74.0)	74.1 (73.7-74.4)	74.4 (74.0-74.8)	74.6 (74.2-75.0)	74.9 (74.5-75.4)
East Asia	71.5 (71.0-71.9)	72.0 (71.5-72.4)	72.6 (72.2-73.0)	73.1 (72.7-73.5)	73.4 (73.0-73.8)	73.9 (73.6-74.3)	74.3 (74.0-74.6)	74.9 (74.5-75.2)	75.3 (75.0-75.7)	75.7 (75.3-76.1)	75.9 (75.4-76.4)	76.2 (75.7-76.8)
China	71.4 (70.9-71.9)	71.9 (71.5-72.3)	72.6 (72.2-73.0)	73.1 (72.7-73.5)	73.4 (73.0-73.7)	73.9 (73.6-74.3)	74.3 (73.9-74.6)	74.9 (74.5-75.2)	75.3 (75.0-75.7)	75.7 (75.3-76.1)	75.9 (75.4-76.4)	76.2 (75.7-76.8)
North Korea	70.0 (67.3-72.5)	70.2 (67.7-72.5)	70.4 (68.1-72.5)	70.6 (68.5-72.5)	70.9 (68.9-72.6)	71.0 (69.1-72.8)	71.2 (69.3-73.1)	71.4 (69.4-73.3)	71.6 (69.5-73.6)	71.8 (69.5-73.9)	71.9 (69.5-74.1)	72.1 (69.7-74.2)
Taiwan	77.3 (77.2-77.4)	77.4 (77.3-77.5)	77.8 (77.7-77.9)	78.2 (78.1-78.3)	78.4 (78.3-78.5)	78.7 (78.5-78.8)	79.0 (78.9-79.1)	78.8 (78.7-78.9)	79.0 (78.9-79.1)	79.3 (78.8-79.7)	79.4 (78.6-80.3)	79.5 (78.4-80.6)
Southeast Asia	68.4 (67.4-69.4)	69.6 (68.9-70.3)	69.9 (69.2-70.6)	70.2 (69.5-70.9)	69.9 (69.1-70.7)	70.7 (69.9-71.4)	70.9 (70.2-71.7)	71.2 (70.4-72.0)	71.5 (70.7-72.3)	71.7 (70.8-72.5)	71.9 (71.1-72.8)	72.2 (71.3-73.1)
Cambodia	63.2 (62.5-63.9)	64.0 (63.2-64.8)	64.8 (64.0-65.5)	65.4 (64.7-66.2)	66.1 (65.2-66.9)	66.6 (65.7-67.4)	67.0 (66.0-67.9)	67.6 (66.5-68.6)	68.2 (67.1-69.3)	68.7 (67.4-69.9)	69.1 (67.7-70.5)	69.4 (68.0-70.9)
Indonesia	66.7 (65.2-68.3)	68.7 (67.9-69.6)	68.9 (68.0-69.8)	69.2 (68.3-70.2)	69.4 (68.5-70.5)	69.6 (68.6-70.8)	69.8 (68.8-71.1)	70.1 (69.0-71.4)	70.3 (69.1-71.9)	70.6 (69.2-72.3)	70.8 (69.4-72.6)	71.1 (69.5-73.0)
Laos	59.3 (58.2-60.4)	60.1 (58.8-61.2)	60.8 (59.7-62.0)	61.7 (60.5-62.9)	62.5 (61.2-63.7)	63.3 (62.0-64.6)	64.1 (62.7-65.5)	65.0 (63.5-66.5)	65.6 (64.0-67.1)	66.1 (64.3-67.8)	66.6 (64.7-68.6)	67.1 (64.9-69.1)
Malaysia	74.0 (74.0-74.1)	74.3 (74.2-74.3)	74.5 (74.4-74.5)	74.5 (74.5-74.6)	74.5 (74.4-74.5)	74.3 (74.3-74.4)	74.4 (74.3-74.6)	74.6 (74.2-74.9)	74.7 (74.1-75.3)	74.9 (74.1-75.6)	75.1 (74.1-76.0)	75.3 (74.3-76.4)
Maldives	74.7 (73.9-75.6)	76.9 (76.5-77.2)	77.5 (77.1-77.9)	78.1 (77.7-78.5)	78.5 (78.1-79.0)	78.9 (78.5-79.3)	79.2 (78.8-79.7)	79.6 (79.1-80.1)	79.8 (79.2-80.4)	79.9 (79.1-80.7)	80.0 (79.1-81.0)	80.0 (78.9-81.1)
Mauritius	72.1 (71.9-72.3)	72.4 (72.2-72.5)	72.5 (72.3-72.7)	72.7 (72.5-72.9)	72.9 (72.7-73.1)	73.2 (73.0-73.4)	73.4 (73.2-73.6)	73.7 (73.5-73.9)	74.1 (73.9-74.3)	74.2 (74.0-74.4)	74.3 (74.0-74.6)	74.5 (74.1-75.0)
Myanmar	62.8 (58.5-67.9)	63.4 (58.9-68.4)	64.0 (59.6-68.8)	64.6 (59.9-69.2)	59.4 (54.2-65.4)	65.6 (60.7-70.3)	66.0 (61.1-70.7)	66.4 (61.5-71.0)	66.8 (62.1-71.4)	67.2 (62.5-71.8)	67.6 (63.0-72.2)	68.0 (63.5-72.6)
Philippines	69.1 (69.0-69.3)	69.1 (68.9-69.2)	69.2 (69.0-69.3)	69.7 (69.5-69.8)	69.8 (69.7-70.0)	69.9 (69.7-70.0)	70.1 (69.9-70.2)	70.2 (70.0-70.3)	70.3 (70.1-70.5)	70.3 (70.0-70.6)	70.7 (70.2-71.2)	71.0 (70.2-71.7)

SR Table 5: Estimates of life expectancy at birth by geography (with 95% uncertainty intervals), both sexes, 1980- 2015

Location	1980	1981	1982	1983	1984	1985	1986	1987	1988	1989
Sri Lanka	69.7 (69.6-69.8)	70.6 (70.5-70.6)	70.2 (70.1-70.4)	70.1 (70.0-70.2)	69.6 (69.3-69.9)	70.0 (69.8-70.3)	70.6 (70.3-70.8)	70.6 (70.3-70.9)	70.7 (70.4-71.1)	70.4 (70.2-70.6)
Seychelles	69.8 (69.3-70.4)	70.2 (69.7-70.8)	70.3 (69.8-70.9)	70.3 (69.7-70.8)	70.0 (69.5-70.6)	69.7 (69.2-70.2)	69.4 (69.0-69.9)	69.3 (68.9-69.7)	69.2 (68.7-69.8)	69.1 (68.6-69.6)
Thailand	66.3 (65.8-66.9)	66.6 (66.1-67.2)	67.1 (66.5-67.6)	67.7 (67.2-68.3)	68.5 (68.0-69.1)	69.3 (68.8-69.9)	69.9 (69.4-70.4)	70.3 (69.8-70.9)	70.6 (70.1-71.2)	70.9 (70.3-71.4)
Timor-Leste	50.1 (47.6-52.5)	51.4 (48.9-53.9)	52.6 (50.4-55.1)	53.6 (51.3-56.0)	54.4 (52.1-56.8)	55.3 (53.1-57.6)	56.2 (54.1-58.5)	56.9 (54.9-59.1)	57.5 (55.4-59.6)	58.0 (56.0-60.0)
Vietnam	66.1 (63.4-68.5)	66.5 (64.0-68.8)	66.8 (64.5-69.2)	67.1 (64.9-69.4)	67.5 (65.5-69.7)	67.8 (65.9-69.9)	68.2 (66.3-70.1)	68.5 (66.7-70.2)	68.8 (67.2-70.3)	69.1 (67.5-70.5)
Oceania	57.9 (54.6-61.3)	57.9 (54.5-61.4)	58.0 (54.5-61.5)	58.1 (54.6-61.6)	58.2 (54.7-61.7)	58.2 (54.9-61.8)	58.3 (54.9-61.8)	58.4 (55.1-62.0)	58.5 (55.2-62.0)	58.6 (55.2-62.0)
American Samoa	69.5 (68.0-70.9)	69.6 (68.2-71.0)	69.7 (68.3-71.0)	69.7 (68.4-70.9)	69.7 (68.6-70.9)	69.9 (68.7-71.0)	69.9 (68.8-71.1)	70.0 (68.9-71.2)	70.0 (68.9-71.2)	70.2 (69.1-71.5)
Federated States of Micronesia	62.5 (57.6-67.6)	62.5 (57.7-67.8)	62.6 (58.0-68.1)	62.9 (58.2-68.1)	63.1 (58.3-68.4)	63.3 (58.5-68.6)	63.6 (58.8-68.9)	63.8 (59.1-69.2)	64.0 (59.2-69.3)	64.2 (59.3-69.5)
Fiji	64.3 (62.9-65.7)	64.3 (62.9-65.6)	64.6 (63.2-65.9)	64.8 (63.5-66.1)	65.1 (63.7-66.3)	65.0 (63.6-66.2)	65.0 (63.6-66.4)	65.1 (63.6-66.6)	65.1 (63.5-66.8)	65.0 (63.2-66.9)
Guam	71.0 (70.0-72.0)	71.4 (70.4-72.4)	71.7 (70.7-72.8)	72.1 (70.8-73.2)	72.3 (71.2-73.4)	72.6 (71.6-73.7)	72.8 (71.7-73.8)	72.8 (71.7-73.7)	72.8 (71.8-73.7)	72.7 (71.8-73.6)
Kiribati	57.6 (54.0-60.6)	57.5 (53.8-60.4)	57.4 (54.0-60.3)	57.3 (54.0-60.1)	57.3 (54.2-60.0)	57.3 (54.5-59.9)	57.3 (54.9-59.7)	57.4 (55.3-59.6)	57.6 (55.6-59.6)	57.8 (55.9-59.6)
Marshall Islands	63.3 (61.0-65.6)	63.6 (61.5-65.7)	63.7 (61.7-65.6)	63.8 (61.8-65.6)	63.9 (62.0-65.6)	64.0 (62.2-65.7)	64.2 (62.4-65.9)	64.6 (62.8-66.1)	64.6 (63.0-66.1)	64.7 (63.2-66.1)
Northern Mariana Islands	69.9 (67.8-71.8)	70.1 (67.9-71.9)	70.3 (68.2-72.1)	70.5 (68.5-72.3)	70.7 (68.9-72.4)	70.9 (69.1-72.6)	71.1 (69.4-72.7)	71.3 (69.7-72.8)	71.5 (70.0-72.9)	71.7 (70.2-73.1)
Papua New Guinea	55.4 (51.2-60.1)	55.4 (51.1-60.3)	55.5 (51.0-60.4)	55.5 (51.0-60.5)	55.5 (51.1-60.6)	55.6 (51.3-60.7)	55.7 (51.4-60.7)	55.8 (51.4-60.9)	55.9 (51.6-60.8)	56.0 (51.7-60.8)
Samoa	67.1 (65.3-68.8)	67.2 (65.3-69.1)	67.4 (65.4-69.3)	67.6 (65.5-69.6)	67.8 (65.6-69.8)	68.0 (65.8-70.1)	68.2 (66.0-70.4)	68.4 (66.1-70.6)	68.7 (66.3-70.9)	68.9 (66.6-71.1)
Solomon Islands	58.7 (54.3-63.7)	59.0 (54.5-63.9)	59.1 (54.4-64.1)	59.2 (54.4-64.4)	59.4 (54.5-64.4)	59.5 (54.5-64.4)	58.9 (54.0-63.7)	59.7 (54.9-64.6)	59.8 (55.0-64.6)	59.9 (55.0-65.0)
Tonga	66.8 (64.5-69.2)	67.0 (64.6-69.4)	67.1 (64.8-69.5)	67.3 (65.0-69.8)	67.7 (65.5-70.0)	68.0 (65.9-70.2)	68.3 (66.2-70.5)	68.6 (66.6-70.7)	68.8 (66.8-70.8)	69.0 (67.0-70.8)
Vanuatu	60.5 (56.0-65.1)	60.7 (56.1-65.3)	60.9 (56.1-65.5)	61.1 (56.3-65.7)	61.3 (56.3-65.9)	61.5 (56.4-66.1)	61.6 (56.6-66.2)	61.0 (56.3-65.4)	61.8 (57.0-66.3)	61.8 (57.0-66.3)
North Africa and Middle East	60.2 (59.6-60.8)	60.3 (59.5-61.0)	60.5 (59.6-61.4)	61.2 (59.9-62.5)	61.9 (60.8-63.1)	62.6 (61.6-63.5)	63.4 (62.5-64.1)	63.8 (63.0-64.6)	63.9 (62.8-65.1)	65.8 (65.4-66.3)
North Africa and Middle East	60.2 (59.6-60.8)	60.3 (59.5-61.0)	60.5 (59.6-61.4)	61.2 (59.9-62.5)	61.9 (60.8-63.1)	62.6 (61.6-63.5)	63.4 (62.5-64.1)	63.8 (63.0-64.6)	63.9 (62.8-65.1)	65.8 (65.4-66.3)
Afghanistan	42.5 (38.9-46.5)	43.2 (39.8-46.9)	42.8 (39.0-46.8)	41.0 (35.9-46.6)	38.6 (32.5-46.0)	40.2 (34.6-46.8)	41.0 (35.5-47.4)	43.0 (38.2-48.2)	45.1 (41.2-49.2)	48.7 (46.2-51.9)
Algeria	61.5 (60.6-62.5)	62.8 (61.9-63.6)	63.9 (63.1-64.8)	65.3 (64.5-66.1)	66.5 (65.8-67.3)	67.7 (66.9-68.4)	68.3 (67.5-69.0)	68.8 (68.1-69.6)	69.3 (68.5-70.1)	69.8 (68.9-70.6)
Bahrain	71.2 (69.7-72.7)	71.8 (70.6-73.2)	72.0 (70.9-73.3)	72.0 (70.9-73.2)	71.9 (70.9-73.2)	72.4 (71.3-73.5)	71.9 (70.9-73.0)	71.7 (70.8-72.9)	71.6 (70.6-72.8)	71.8 (70.8-72.9)
Egypt	58.8 (58.2-59.3)	59.0 (58.5-59.6)	59.6 (59.1-60.1)	60.2 (59.8-60.7)	61.1 (60.7-61.5)	60.9 (60.5-61.2)	61.6 (61.2-62.0)	62.1 (61.6-62.6)	64.0 (63.7-64.4)	64.5 (64.2-64.9)
Iran	63.1 (61.1-64.9)	61.9 (59.0-64.8)	62.8 (59.9-65.7)	63.8 (60.7-66.8)	65.5 (63.2-67.8)	66.3 (64.2-68.5)	66.2 (64.1-68.3)	66.3 (64.0-68.5)	66.1 (63.9-68.2)	68.0 (66.5-69.5)
Iraq	60.9 (57.3-64.5)	56.3 (49.8-63.4)	56.6 (50.5-63.9)	56.8 (50.5-63.9)	59.4 (54.7-64.7)	59.7 (55.1-64.6)	59.8 (55.3-64.7)	60.0 (55.6-64.9)	49.0 (39.3-62.6)	65.6 (63.6-67.6)
Jordan	65.9 (64.7-66.9)	66.4 (65.3-67.6)	66.9 (65.7-68.0)	67.5 (66.3-68.7)	68.3 (67.0-69.5)	68.9 (67.6-70.2)	69.4 (67.9-70.7)	70.0 (68.5-71.3)	70.7 (69.2-71.9)	71.1 (69.6-72.4)
Kuwait	72.8 (72.5-73.1)	73.4 (73.1-73.6)	73.3 (73.1-73.6)	74.0 (73.7-74.2)	74.5 (74.2-74.8)	74.9 (74.6-75.1)	75.7 (75.5-75.9)	76.3 (76.0-76.5)	76.5 (76.3-76.8)	77.1 (76.9-77.4)
Lebanon	52.4 (44.7-62.8)	52.6 (44.9-63.1)	44.7 (33.3-64.1)	63.8 (58.8-68.8)	64.0 (59.0-69.0)	64.4 (59.6-69.4)	64.6 (59.8-69.5)	54.0 (46.1-64.2)	54.1 (46.4-64.5)	69.9 (68.1-71.8)
Libya	70.9 (70.0-71.8)	71.1 (70.2-72.1)	71.5 (70.6-72.4)	71.8 (70.8-72.8)	72.0 (71.0-73.0)	72.4 (71.3-73.4)	72.6 (71.5-73.6)	69.6 (66.7-72.4)	72.9 (71.8-74.0)	73.0 (71.9-74.0)
Morocco	61.0 (59.5-62.4)	61.7 (60.2-63.2)	62.8 (61.4-64.3)	63.5 (62.1-64.9)	64.3 (62.9-65.6)	65.0 (63.6-66.3)	65.6 (64.2-66.9)	66.1 (64.7-67.4)	66.6 (65.3-67.9)	67.1 (65.8-68.3)
Palestine	68.8 (66.2-71.2)	69.2 (66.7-71.5)	42.8 (33.5-57.8)	70.2 (67.6-72.3)	70.6 (68.1-72.8)	71.1 (68.5-73.2)	71.4 (68.8-73.6)	71.3 (68.7-73.5)	71.4 (69.0-73.6)	71.6 (69.2-73.6)
Oman	64.8 (62.3-67.6)	65.8 (63.3-68.5)	66.8 (64.2-69.5)	67.7 (65.2-70.5)	68.6 (66.0-71.3)	69.4 (66.9-72.2)	70.1 (67.7-72.9)	70.7 (68.3-73.4)	71.1 (68.9-73.8)	71.6 (69.3-74.2)
Qatar	69.1 (67.2-70.9)	69.1 (67.5-70.5)	69.3 (67.9-70.6)	69.9 (68.6-71.4)	71.5 (69.9-72.9)	72.1 (70.8-73.3)	72.9 (71.7-74.0)	73.4 (72.3-74.5)	73.7 (72.7-74.8)	74.2 (73.2-75.2)
Saudi Arabia	71.3 (69.8-72.7)	71.8 (70.5-73.3)	72.2 (70.9-73.6)	72.5 (71.2-73.9)	72.8 (71.5-74.1)	73.0 (71.8-74.3)	73.3 (72.1-74.6)	73.5 (72.4-74.8)	73.8 (72.6-74.9)	74.0 (72.8-75.1)
Sudan	55.3 (53.5-56.9)	56.0 (54.3-57.7)	56.3 (54.6-57.9)	51.3 (46.3-55.6)	51.8 (46.2-56.1)	52.2 (46.7-56.5)	57.3 (55.4-59.1)	57.8 (55.9-59.6)	58.2 (56.2-60.2)	58.2 (56.2-60.4)
Syria	66.5 (65.4-67.4)	66.9 (65.9-67.9)	63.7 (60.9-66.7)	67.8 (66.8-68.9)	68.3 (67.2-69.4)	68.5 (67.3-69.6)	68.9 (67.6-70.0)	69.2 (67.9-70.4)	69.6 (68.3-70.8)	70.0 (68.6-71.2)
Tunisia	65.7 (65.0-66.4)	66.9 (66.2-67.6)	67.8 (67.1-68.5)	68.7 (68.0-69.4)	69.4 (68.7-70.1)	70.0 (69.3-70.7)	70.4 (69.7-71.1)	70.8 (70.1-71.4)	71.2 (70.5-71.8)	71.5 (70.8-72.1)
Turkey	62.8 (61.8-63.7)	63.4 (62.4-64.2)	64.1 (63.2-64.9)	64.7 (63.8-65.6)	64.9 (64.1-65.8)	65.8 (65.0-66.6)	66.4 (65.6-67.3)	66.9 (66.1-67.7)	67.4 (66.7-68.2)	67.5 (66.7-68.2)
United Arab Emirates	68.5 (65.2-71.5)	69.0 (65.7-71.8)	69.4 (66.4-72.1)	69.4 (66.5-72.1)	69.9 (67.0-72.5)	70.0 (67.3-72.6)	70.1 (67.6-72.7)	70.3 (67.8-72.7)	70.3 (68.1-72.6)	70.4 (68.1-72.6)
Yemen	52.4 (48.0-57.4)	53.2 (48.9-58.2)	53.5 (49.3-58.5)	54.8 (50.7-59.9)	55.5 (51.4-60.6)	56.1 (52.0-61.1)	54.1 (50.3-58.7)	57.1 (52.9-62.2)	57.6 (53.2-62.7)	58.0 (53.5-63.2)
South Asia	54.0 (53.4-54.4)	54.5 (54.0-55.0)	55.0 (54.6-55.5)	55.5 (55.0-56.0)	55.9 (55.5-56.4)	56.3 (55.9-56.8)	56.8 (56.4-57.3)	57.3 (56.8-57.7)	57.6 (57.2-58.1)	58.1 (57.7-58.5)
South Asia	54.0 (53.4-54.4)	54.5 (54.0-55.0)	55.0 (54.6-55.5)	55.5 (55.0-56.0)	55.9 (55.5-56.4)	56.3 (55.9-56.8)	56.8 (56.4-57.3)	57.3 (56.8-57.7)	57.6 (57.2-58.1)	58.1 (57.7-58.5)
Bangladesh	53.1 (51.9-54.5)	53.4 (52.3-54.7)	53.9 (52.8-55.2)	54.3 (53.3-55.5)	54.8 (53.8-55.9)	54.8 (53.9-55.9)	55.8 (54.9-56.9)	56.5 (55.5-57.5)	56.9 (56.0-57.8)	57.8 (56.9-58.8)
Bhutan	56.3 (53.9-58.8)	56.7 (54.3-59.3)	57.2 (54.9-59.6)	57.7 (55.4-60.1)	58.2 (56.0-60.6)	58.7 (56.6-61.0)	59.2 (57.0-61.4)	59.8 (57.6-62.0)	60.3 (58.2-62.4)	60.8 (58.8-62.9)
India	53.6 (53.0-54.1)	54.2 (53.6-54.7)	54.7 (54.2-55.3)	55.2 (54.7-55.7)	55.6 (55.1-56.2)	56.1 (55.6-56.6)	56.6 (56.0-57.1)	57.0 (56.5-57.5)	57.4 (56.9-57.9)	57.8 (57.3-58.3)
Nepal	52.9 (50.9-54.9)	53.3 (51.4-55.3)	53.9 (52.0-55.9)	54.4 (52.4-56.4)	54.9 (53.0-56.9)	55.5 (53.6-57.5)	56.0 (54.2-58.1)	56.6 (54.8-58.6)	57.1 (55.4-59.0)	57.8 (56.2-59.6)
Pakistan	59.7 (58.3-61.0)	60.1 (58.7-61.3)	60.5 (59.1-61.7)	60.8 (59.5-62.0)	61.2 (60.0-62.4)	61.4 (60.2-62.6)	61.6 (60.4-62.8)	61.7 (60.5-62.8)	61.8 (60.5-63.0)	61.9 (60.7-63.1)
Sub-Saharan Africa	51.5 (50.0-52.8)	51.7 (50.3-53.0)	52.0 (50.6-53.2)	51.7 (50.3-53.0)	51.9 (50.6-53.1)	52.6 (51.4-53.7)	52.9 (51.8-54.0)	53.2 (52.1-54.2)	53.4 (52.4-54.4)	53.5 (52.5-54.4)
Southern Sub-Saharan Africa	57.3 (55.9-58.7)	57.8 (56.4-59.1)	58.2 (56.9-59.4)	58.5 (57.4-59.7)	59.3 (58.2-60.3)	59.8 (58.9-60.7)	60.3 (59.4-61.2)	61.0 (60.1-61.8)	61.7 (60.9-62.4)	62.0 (61.2-62.8)
Botswana	59.5 (48.6-71.1)	59.9 (49.4-71.5)	60.3 (49.7-71.7)	60.7 (49.9-72.2)	61.1 (50.2-72.4)	61.5 (50.0-72.5)	61.8 (50.0-72.5)	62.2 (50.0-72.6)	62.4 (50.5-72.6)	62.5 (50.9-72.7)
Lesotho	58.5 (52.9-64.6)	58.7 (53.3-64.6)	58.9 (53.7-64.5)	59.1 (54.1-64.6)	59.3 (54.6-64.3)	59.5 (55.1-64.2)	59.8 (55.6-64.0)	60.0 (56.0-63.8)	60.2 (56.8-63.7)	60.4 (57.4-63.5)
Namibia	59.1 (56.8-61.7)	59.3 (57.1-61.8)	59.5 (57.3-61.7)	60.0 (57.9-62.1)	60.6 (58.6-62.6)	60.9 (59.1-62.9)	61.2 (59.6-63.2)	61.6 (59.9-63.5)	61.6 (60.0-63.4)	61.7 (60.2-63.4)
South Africa	56.6 (54.9-58.4)	57.0 (55.3-58.6)	57.3 (55.8-58.8)	57.7 (56.2-59.0)	58.4 (57.1-59.6)	58.9 (57.7-60.1)	59.4 (58.3-60.5)	60.1 (59.1-61.1)	61.0 (60.1-62.0)	61.6 (60.6-62.5)
Swaziland	56.8 (50.6-63.3)	57.2 (51.1-63.6)	57.6 (51.6-63.9)	57.0 (51.5-63.2)	58.3 (53.1-64.6)	58.9 (54.2-65.0)	59.3 (54.7-65.1)	59.8 (55.4-65.0)	60.2 (56.2-65.0)	60.5 (56.8-64.8)
Zimbabwe	58.8 (56.3-61.5)	59.9 (57.6-62.3)	60.6 (58.4-62.7)	61.3 (59.2-63.3)	62.0 (60.0-63.9)	62.8 (60.9-64.5)	63.6 (61.8-65.2)	64.0 (62.3-65.5)	64.0 (62.2-65.6)	63.7 (61.8-65.6)
Western Sub-Saharan Africa	51.9 (48.9-54.3)	52.2 (49.4-54.6)	52.6 (49.8-55.0)	52.9 (50.1-55.3)	53.1 (50.5-55.5)	53.5 (50.9-55.7)	53.6 (51.2-55.7)	53.7 (51.4-55.7)	54.0 (51.7-55.9)	54.1 (52.0-55.9)
Benin	50.4 (47.5-53.7)	50.9 (48.5-53.9)	51.6 (49.3-54.3)	52.3 (50.2-54.8)	52.8 (50.7-55.1)	53.5 (51.4-55.5)	54.2 (52.1-56.1)	55.0 (52.9-56.9)	55.6 (53.5-57.3)	56.0 (54.2-57.8)
Burkina Faso	49.0 (45.7-52.2)	49.6 (46.7-52.4)	50.1 (47.3-52.5)	50.4 (48.0-52.5)	51.0 (49.0-52.9)	51.3 (49.5-53.0)	51.3 (49.5-52.9)	51.2 (49.5-52.8)	51.1 (49.3-52.8)	51.4 (49.5-53.1)
Cameroon	55.1 (51.2-59.4)	55.7 (52.2-59.6)	56.2 (53.0-59.7)	56.7 (53.8-59.7)	57.0 (54.6-59.5)	57.5 (55.6-59.6)	57.6 (56.0-59.4)	58.1 (56.6-60.0)	58.3 (56.8-60.1)	58.4 (56.9-60.2)
Cape Verde	67.1 (66.4-67.8)	66.9 (66.3-67.5)	66.8 (66.3-67.3)	66.7 (66.3-67.1)	66.4 (66.0-66.8)	66.9 (66.5-67.4)	67.3 (66.8-67.9)	67.7 (67.1-68.3)	68.1 (67.5-68.7)	68.4 (67.8-69.0)
Chad	50.6 (47.5-53.2)	50.6 (47.7-53.1)	50.8 (48.3-53.0)	51.4 (49.2-53.4)	51.8 (49.7-53.8)	53.0 (50.9-54.8)	52.9 (50.8-54.7)	51.7 (49.1-54.1)	54.3 (52.5-56.1)	54.2 (52.4-56.1)
Cote d'Ivoire	56.1 (54.2-58.2)	56.6 (54.7-58.6)	56.7 (54.9-58.7)	56.9 (55.1-58.7)	56.8 (55.1-58.7)	57.0 (55.2-59.0)	56.9 (55.1-58.9)	56.7 (54.7-58.5)	56.2 (54.2-58.2)	55.8 (53.8-57.7)
The Gambia	58.4 (51.9-64.8)	56.4 (50.0-62.7)	59.0 (52.0-65.4)	59.3 (52.1-65.7)	59.6 (52.4-66.0)	59.9 (52.7-66.1)	60.2 (53.3-66.1)	60.4 (53.9-66.1)	60.7 (54.4-66.2)	61.0 (55.1-66.2)
Ghana	55.4 (49.6-61.2)	55.5 (49.6-61.3)	55.6 (49.8-61.1)	55.7 (50.3-61.4)	55.9 (50.6-61.7)	56.2 (51.1-62.0)	56.5 (51.6-62.1)	56.9 (52.2-62.0)	57.3 (52.9-62.0)	57.7 (53.5-61.8)
Guinea	48.0 (44.2-51.8)	48.3 (44.9-51.9)	48.7 (45.7-51.9)	48.9 (46.2-51.9)	49.4 (46.8-52.0)	49.8 (47.6-52.1)	50.3 (48.3-52.4)	50.6 (48.8-52.6)	50.9 (49.2-52.7)	51.4 (49.8-53.2)
Guinea-Bissau	49.9 (39.8-58.8)	49.9 (40.0-58.8)	50.0 (40.4-59.0)	50.2 (40.9-59.1)	50.4 (41.5-59.2)	50.5 (41.4-59.3)	50.7 (41.4-59.4)	50.9 (41.2-59.6)	51.1 (41.3-59.9)	51.3 (41.5-60.1)

Location	1990	1991	1992	1993	1994	1995	1996	1997	1998	1999	2000	2001	2002
Sri Lanka	70.5 (70.2-70.7)	71.3 (70.9-71.6)	72.0 (71.8-72.3)	72.9 (72.8-73.0)	72.8 (72.6-72.9)	71.7 (71.6-71.8)	71.3 (71.0-71.5)	71.4 (71.3-71.6)	72.0 (71.8-72.2)	72.4 (72.3-72.6)	72.4 (72.2-72.6)	73.3 (73.2-73.4)	73.7 (73.5-73.9)
Seychelles	69.1 (68.6-69.6)	69.1 (68.6-69.6)	69.2 (68.7-69.7)	69.3 (68.9-69.9)	69.6 (69.1-70.1)	69.8 (69.3-70.4)	69.9 (69.4-70.4)	70.1 (69.7-70.7)	70.4 (69.9-70.9)	70.7 (70.2-71.1)	70.9 (70.4-71.3)	71.1 (70.6-71.5)	71.3 (70.8-71.7)
Thailand	71.0 (70.5-71.5)	71.0 (70.4-71.6)	70.9 (70.3-71.4)	70.8 (70.2-71.3)	70.6 (70.0-71.2)	70.6 (70.0-71.2)	70.6 (69.9-71.2)	70.5 (69.8-71.2)	70.4 (69.8-71.1)	70.5 (69.9-71.2)	70.7 (70.1-71.4)	71.2 (70.5-71.8)	71.7 (71.0-72.3)
Timor-Leste	58.5 (56.5-60.6)	59.2 (57.1-61.0)	59.9 (58.0-61.7)	60.6 (58.9-62.3)	61.3 (59.7-63.0)	61.8 (60.3-63.4)	62.3 (60.9-63.8)	62.4 (61.0-63.8)	62.8 (61.4-64.2)	62.3 (60.7-63.9)	60.7 (57.9-63.5)	64.4 (63.1-65.8)	65.3 (64.1-66.6)
Vietnam	69.3 (67.7-70.7)	69.6 (67.7-71.0)	69.8 (68.0-71.4)	70.1 (68.1-71.8)	70.3 (68.4-72.1)	70.6 (68.8-72.4)	70.9 (69.1-72.6)	71.1 (69.5-72.7)	71.5 (70.0-73.0)	71.7 (70.2-73.4)	72.0 (70.5-73.7)	72.2 (70.7-74.0)	72.5 (70.8-74.4)
Oceania	58.6 (55.1-62.1)	58.6 (55.0-62.1)	58.8 (55.0-62.3)	58.9 (55.2-62.5)	59.1 (55.2-62.6)	59.2 (55.5-62.8)	59.3 (55.7-63.0)	59.4 (55.7-63.0)	59.0 (55.4-62.6)	59.6 (55.9-63.4)	59.7 (56.0-63.6)	59.7 (56.0-63.6)	59.7 (56.0-63.8)
American Samoa	70.2 (69.1-71.4)	70.2 (69.1-71.3)	70.2 (69.2-71.4)	70.5 (69.3-71.7)	70.5 (69.3-71.8)	70.6 (69.2-71.9)	70.7 (69.3-71.9)	70.8 (69.6-72.0)	71.0 (69.9-72.0)	71.4 (70.2-72.3)	71.7 (70.6-72.7)	71.7 (70.5-72.7)	71.7 (70.6-72.8)
Federated States of Micronesia	64.3 (59.4-69.9)	64.5 (59.5-70.0)	64.7 (59.8-70.1)	64.9 (60.2-70.4)	65.1 (60.5-70.5)	65.4 (60.8-70.4)	65.7 (60.8-70.4)	65.9 (60.8-70.5)	66.1 (61.1-70.7)	66.3 (61.5-70.9)	66.6 (61.8-71.2)	66.8 (61.9-71.4)	66.0 (61.2-70.5)
Fiji	65.1 (63.1-67.0)	65.1 (63.1-67.0)	65.0 (63.1-66.9)	64.8 (62.9-66.5)	64.7 (63.0-66.5)	64.5 (62.9-66.2)	64.3 (62.8-65.8)	64.1 (62.6-65.5)	64.2 (62.8-65.5)	64.1 (62.8-65.4)	64.2 (62.8-65.7)	64.4 (63.0-65.8)	64.5 (63.2-66.0)
Guam	72.6 (71.7-73.5)	72.4 (71.5-73.3)	72.4 (71.4-73.4)	72.5 (71.5-73.6)	72.7 (71.6-73.7)	73.0 (71.9-73.9)	73.2 (72.1-74.1)	69.4 (66.8-71.9)	73.4 (72.4-74.3)	73.5 (72.5-74.4)	73.6 (72.6-74.4)	73.6 (72.7-74.5)	73.6 (72.7-74.5)
Kiribati	58.0 (56.3-59.7)	58.2 (56.5-59.9)	58.4 (56.9-60.0)	58.4 (56.9-60.0)	58.7 (57.3-60.2)	58.9 (57.6-60.4)	59.2 (57.8-60.6)	59.4 (58.0-60.8)	59.5 (58.1-60.8)	59.6 (58.3-61.0)	59.8 (58.4-61.2)	60.1 (58.6-61.5)	60.2 (58.7-61.7)
Marshall Islands	64.6 (63.2-66.0)	64.8 (63.5-66.1)	64.9 (63.6-66.2)	65.1 (63.8-66.3)	65.3 (64.0-66.5)	65.2 (64.0-66.4)	65.3 (63.9-66.5)	65.1 (63.7-66.4)	65.0 (63.4-66.4)	64.8 (63.2-66.3)	64.7 (63.0-66.2)	64.7 (63.0-66.2)	64.7 (63.0-66.3)
Northern Mariana Islands	72.3 (70.8-73.6)	72.8 (71.4-73.9)	73.2 (71.8-74.3)	73.5 (72.4-74.7)	73.9 (72.7-75.0)	74.2 (73.2-75.2)	74.5 (73.5-75.4)	74.7 (73.9-75.6)	74.9 (74.1-75.6)	75.2 (74.5-75.9)	75.5 (74.9-76.2)	75.6 (74.9-76.2)	75.7 (75.0-76.3)
Papua New Guinea	56.0 (51.7-60.9)	56.0 (51.5-60.8)	56.2 (51.5-61.1)	56.4 (51.7-61.3)	56.7 (51.9-61.6)	56.9 (52.1-61.9)	57.0 (52.3-62.1)	57.2 (52.6-62.4)	56.5 (51.9-61.7)	57.4 (52.7-62.6)	57.5 (52.8-62.9)	57.6 (52.9-63.0)	57.6 (52.8-63.2)
Samoa	69.1 (66.7-71.3)	69.1 (66.6-71.3)	69.5 (67.0-71.6)	69.7 (67.3-71.8)	69.8 (67.5-72.1)	70.0 (67.8-72.2)	70.2 (67.9-72.3)	70.4 (68.2-72.4)	70.6 (68.5-72.6)	70.7 (68.7-72.6)	70.8 (68.8-72.7)	71.0 (69.1-72.8)	71.2 (69.5-72.9)
Solomon Islands	60.1 (55.1-65.2)	60.0 (55.1-65.3)	60.4 (55.6-65.6)	60.6 (55.9-65.8)	60.9 (56.2-66.0)	61.1 (56.4-66.1)	61.4 (56.6-66.3)	61.5 (56.7-66.5)	61.6 (56.9-66.5)	61.7 (56.9-66.7)	61.7 (56.9-66.7)	61.7 (57.0-66.6)	61.7 (56.9-66.8)
Tonga	69.1 (67.3-70.9)	69.3 (67.6-70.9)	69.4 (67.9-70.9)	69.5 (68.0-70.9)	69.5 (68.2-70.9)	69.6 (68.4-70.9)	69.7 (68.5-70.8)	69.7 (68.6-70.8)	69.7 (68.6-70.8)	69.6 (68.5-70.6)	69.6 (68.5-70.6)	69.6 (68.5-70.6)	69.7 (68.5-70.7)
Vanuatu	61.9 (57.1-66.6)	62.0 (57.0-66.8)	62.1 (56.9-67.0)	62.2 (56.8-67.1)	62.2 (56.9-67.2)	62.3 (56.8-67.2)	62.4 (57.0-67.2)	61.2 (56.1-65.8)	62.5 (57.2-67.2)	62.0 (56.8-66.7)	62.5 (57.4-67.6)	62.5 (57.6-67.7)	62.5 (57.8-67.7)
North Africa and Middle East	65.9 (65.4-66.4)	66.3 (65.8-66.7)	66.8 (66.4-67.3)	67.1 (66.6-67.5)	67.2 (66.8-67.7)	67.5 (67.1-68.0)	67.8 (67.3-68.3)	68.1 (67.7-68.6)	68.5 (68.0-68.9)	68.8 (68.3-69.3)	69.4 (68.9-69.9)	69.6 (69.2-70.1)	69.9 (69.4-70.4)
North Africa and Middle East	65.9 (65.4-66.4)	66.3 (65.8-66.7)	66.8 (66.4-67.3)	67.1 (66.6-67.5)	67.2 (66.8-67.7)	67.5 (67.1-68.0)	67.8 (67.3-68.3)	68.1 (67.7-68.6)	68.5 (68.0-68.9)	68.8 (68.3-69.3)	69.4 (68.9-69.9)	69.6 (69.2-70.1)	69.9 (69.4-70.4)
Afghanistan	49.5 (46.9-52.6)	49.3 (46.6-52.3)	49.4 (46.6-52.6)	49.5 (46.5-52.7)	48.9 (46.1-52.0)	49.4 (46.5-52.4)	49.7 (46.9-52.8)	49.5 (46.7-52.7)	48.6 (45.9-51.5)	50.0 (47.2-52.9)	50.1 (47.4-53.0)	50.4 (47.7-53.2)	51.0 (48.3-53.9)
Algeria	70.2 (69.3-71.0)	70.5 (69.6-71.4)	70.9 (70.0-71.8)	71.2 (70.3-72.1)	71.4 (70.4-72.3)	71.6 (70.7-72.5)	72.1 (71.2-73.0)	72.4 (71.4-73.2)	72.6 (71.7-73.4)	73.0 (72.1-73.8)	73.3 (72.4-74.1)	73.5 (72.6-74.3)	73.8 (72.9-74.7)
Bahrain	71.8 (70.8-72.9)	72.0 (70.9-73.0)	72.1 (71.1-73.1)	72.5 (71.4-73.5)	72.9 (71.7-73.9)	73.0 (72.0-74.0)	73.4 (72.5-74.1)	73.8 (73.0-74.5)	74.0 (73.3-74.7)	74.2 (73.5-74.9)	73.7 (72.9-74.6)	74.3 (73.5-75.2)	74.8 (74.2-75.6)
Egypt	65.2 (64.9-65.5)	65.4 (65.1-65.8)	66.1 (65.7-66.5)	66.4 (66.1-66.7)	66.7 (66.3-67.0)	67.4 (67.1-67.7)	67.9 (67.6-68.1)	68.2 (67.9-68.4)	68.6 (68.3-68.8)	69.0 (68.7-69.2)	69.7 (69.5-70.0)	69.7 (69.5-69.9)	69.8 (69.5-70.0)
Iran	66.0 (64.2-67.8)	67.8 (66.6-69.0)	68.5 (67.1-69.9)	69.1 (67.7-70.6)	69.6 (68.1-71.0)	69.9 (68.5-71.2)	69.8 (68.4-71.0)	70.3 (69.2-71.4)	70.8 (69.7-71.8)	71.3 (70.2-72.2)	71.4 (70.2-72.3)	71.3 (70.1-72.4)	71.3 (70.0-72.5)
Iraq	65.7 (63.6-67.7)	63.9 (61.9-65.9)	65.4 (63.4-67.3)	65.4 (63.5-67.3)	65.4 (63.5-67.2)	65.3 (63.4-67.1)	65.3 (63.4-67.1)	65.2 (63.3-67.0)	65.7 (63.8-67.4)	65.9 (64.0-67.7)	65.8 (63.9-67.6)	66.4 (64.4-68.3)	66.1 (64.0-68.0)
Jordan	71.5 (69.9-72.7)	71.9 (70.4-73.1)	72.2 (70.7-73.4)	72.2 (70.7-73.4)	72.4 (70.8-73.6)	72.5 (71.1-73.7)	72.6 (71.3-73.8)	72.8 (71.6-73.9)	73.0 (71.9-74.0)	73.2 (72.2-74.2)	73.4 (72.5-74.4)	73.6 (72.7-74.5)	73.8 (73.0-74.7)
Kuwait	77.2 (76.2-78.2)	64.4 (61.6-67.3)	80.0 (79.7-80.2)	78.7 (78.5-78.9)	77.6 (77.4-77.9)	76.5 (76.2-76.7)	76.0 (75.7-76.2)	76.2 (76.0-76.4)	76.3 (76.1-76.6)	77.3 (77.1-77.5)	77.7 (77.5-77.9)	77.6 (77.3-77.7)	78.2 (78.0-78.5)
Lebanon	69.3 (67.6-71.1)	71.9 (70.2-73.5)	72.2 (70.4-73.6)	72.5 (70.9-73.8)	73.0 (71.5-74.3)	73.4 (72.1-74.7)	74.0 (72.7-75.3)	74.4 (73.1-75.7)	74.9 (73.5-76.2)	75.6 (74.1-76.8)	75.9 (74.4-77.2)	76.3 (74.8-77.7)	76.6 (75.1-78.0)
Libya	73.4 (72.3-74.4)	73.7 (72.6-74.7)	73.8 (72.8-74.8)	74.2 (73.2-75.2)	74.4 (73.5-75.3)	74.6 (73.6-75.5)	74.6 (73.7-75.5)	74.8 (73.9-75.6)	74.8 (74.0-75.6)	74.9 (74.0-75.6)	74.8 (74.0-75.5)	75.0 (74.2-75.7)	75.0 (74.2-75.7)
Morocco	67.7 (66.4-68.9)	68.1 (66.8-69.3)	68.4 (67.1-69.7)	68.6 (67.4-70.0)	69.1 (67.9-70.5)	69.5 (68.3-70.9)	70.0 (68.7-71.5)	70.4 (69.0-71.7)	70.8 (69.3-72.2)	71.1 (69.6-72.5)	71.5 (69.9-72.9)	71.8 (70.1-73.3)	72.0 (70.3-73.6)
Palestine	71.8 (69.4-73.7)	72.0 (69.8-73.9)	72.4 (70.3-74.1)	72.8 (70.7-74.4)	73.3 (71.3-74.8)	73.7 (71.9-75.1)	74.0 (72.4-75.3)	74.2 (72.9-75.4)	74.5 (73.3-75.5)	74.7 (73.7-75.7)	74.4 (73.4-75.4)	74.7 (73.8-75.5)	74.4 (73.5-75.2)
Oman	72.1 (69.9-74.6)	72.5 (70.3-75.0)	72.9 (70.7-75.4)	73.3 (71.0-75.7)	73.6 (71.3-75.8)	73.9 (71.7-76.1)	74.2 (72.2-76.3)	74.5 (72.7-76.4)	74.8 (73.1-76.6)	75.1 (73.4-76.7)	75.2 (73.8-76.8)	75.4 (74.1-76.8)	75.4 (74.3-76.6)
Qatar	74.5 (73.4-75.6)	74.4 (73.2-75.5)	74.5 (73.3-75.7)	74.5 (73.3-75.5)	74.4 (73.4-75.4)	74.4 (73.3-75.4)	74.5 (73.3-75.5)	74.6 (73.5-75.6)	74.6 (73.5-75.7)	74.6 (73.6-75.7)	74.7 (73.7-75.7)	75.0 (73.9-76.1)	75.0 (74.0-76.1)
Saudi Arabia	73.9 (72.2-75.2)	74.3 (73.2-75.4)	74.6 (73.6-75.7)	74.9 (73.9-75.9)	75.1 (74.2-76.0)	75.5 (74.5-76.3)	75.8 (74.9-76.6)	76.0 (75.1-76.8)	76.3 (75.5-77.0)	76.6 (75.8-77.2)	76.8 (76.1-77.5)	77.1 (76.5-77.7)	77.2 (76.6-77.8)
Sudan	58.9 (56.7-61.1)	59.2 (57.0-61.5)	59.4 (57.1-61.9)	59.5 (57.1-61.8)	60.2 (58.0-62.7)	60.5 (58.1-63.0)	60.6 (58.1-63.1)	60.8 (58.2-63.5)	61.2 (58.6-63.8)	62.0 (59.4-64.7)	62.4 (59.7-65.1)	62.8 (60.0-65.5)	63.3 (60.5-66.1)
Syria	70.5 (69.0-71.7)	71.0 (69.6-72.3)	71.8 (70.2-73.0)	72.0 (70.4-73.3)	72.3 (70.6-73.7)	72.7 (70.9-74.0)	73.1 (71.4-74.4)	73.4 (71.8-74.7)	73.8 (72.2-75.0)	74.1 (72.7-75.2)	74.4 (73.1-75.5)	74.6 (73.5-75.6)	74.9 (73.9-75.8)
Tunisia	71.8 (71.1-72.4)	72.0 (71.4-72.7)	72.2 (71.5-72.8)	72.2 (71.6-72.8)	72.5 (71.9-73.2)	72.9 (72.2-73.6)	73.4 (72.7-74.1)	73.9 (73.1-74.7)	74.3 (73.5-75.1)	74.7 (73.9-75.6)	75.0 (74.1-75.9)	75.3 (74.4-76.2)	75.5 (74.6-76.6)
Turkey	68.1 (67.3-68.8)	68.5 (67.8-69.3)	69.2 (68.4-69.9)	69.7 (69.0-70.4)	69.8 (69.1-70.5)	70.0 (69.3-70.7)	70.6 (70.0-71.4)	71.2 (70.6-71.9)	72.0 (71.4-72.6)	71.5 (70.6-72.3)	73.8 (73.1-74.4)	74.4 (73.8-75.0)	75.1 (74.5-75.7)
United Arab Emirates	70.6 (68.4-72.6)	70.8 (68.9-72.7)	71.1 (69.3-72.8)	71.3 (69.7-72.8)	71.6 (70.1-72.9)	71.9 (70.6-73.1)	72.1 (71.0-73.2)	72.4 (71.3-73.5)	72.8 (71.7-73.8)	73.0 (72.1-74.0)	73.3 (72.4-74.3)	73.6 (72.7-74.5)	73.8 (73.0-74.6)
Yemen	58.5 (53.9-63.7)	58.9 (54.2-64.1)	59.3 (54.6-64.3)	59.6 (54.9-64.6)	59.7 (54.9-64.6)	60.3 (55.3-65.1)	60.7 (55.8-65.4)	61.1 (56.2-65.8)	61.5 (56.6-66.4)	62.0 (57.0-67.0)	62.4 (57.4-67.6)	62.8 (57.9-68.2)	63.3 (58.5-68.8)
South Asia	58.4 (58.0-58.9)	58.6 (58.2-59.0)	59.1 (58.8-59.6)	59.5 (59.1-59.9)	59.9 (59.5-60.3)	60.2 (59.8-60.6)	60.5 (60.1-61.0)	60.8 (60.5-61.3)	61.2 (60.8-61.6)	61.6 (61.2-62.0)	61.9 (61.6-62.4)	62.3 (61.9-62.8)	62.7 (62.3-63.1)
South Asia	58.4 (58.0-58.9)	58.6 (58.2-59.0)	59.1 (58.8-59.6)	59.5 (59.1-59.9)	59.9 (59.5-60.3)	60.2 (59.8-60.6)	60.5 (60.1-61.0)	60.8 (60.5-61.3)	61.2 (60.8-61.6)	61.6 (61.2-62.0)	61.9 (61.6-62.4)	62.3 (61.9-62.8)	62.7 (62.3-63.1)
Bangladesh	57.9 (57.0-58.9)	56.4 (54.9-58.0)	59.7 (58.6-60.8)	60.5 (59.4-61.5)	61.2 (60.1-62.2)	61.6 (60.6-62.7)	62.4 (61.4-63.5)	63.2 (62.2-64.3)	63.9 (63.0-64.9)	64.6 (63.7-65.7)	64.9 (64.0-65.9)	65.4 (64.5-66.4)	65.8 (65.0-66.8)
Bhutan	61.4 (59.4-63.4)	61.9 (59.9-63.9)	62.4 (60.5-64.2)	62.8 (61.0-64.7)	63.1 (61.3-64.9)	63.8 (62.0-65.6)	64.7 (63.0-66.4)	65.1 (63.4-66.8)	65.6 (64.1-67.3)	66.5 (64.9-68.1)	65.9 (64.3-67.4)	67.5 (65.8-68.9)	68.1 (66.5-69.5)
India	58.2 (57.7-58.7)	58.5 (58.0-59.0)	58.8 (58.3-59.3)	59.1 (58.7-59.7)	59.5 (59.0-60.0)	59.9 (59.4-60.4)	60.2 (59.7-60.7)	60.5 (60.0-60.9)	60.8 (60.4-61.3)	61.2 (60.7-61.6)	61.5 (61.1-62.0)	61.9 (61.4-62.4)	62.3 (61.9-62.8)
Nepal	58.4 (56.9-60.2)	59.1 (57.6-60.7)	60.0 (58.6-61.5)	60.2 (58.9-61.6)	61.0 (59.7-62.4)	61.7 (60.5-63.0)	62.5 (61.4-63.8)	63.4 (62.3-64.5)	63.9 (62.9-64.9)	64.6 (63.6-65.5)	65.2 (64.4-66.1)	65.9 (65.1-66.8)	65.9 (65.1-66.8)
Pakistan	62.1 (60.9-63.3)	62.2 (60.9-63.4)	62.1 (60.8-63.3)	62.0 (60.7-63.3)	61.9 (60.6-63.3)	61.8 (60.5-63.1)	61.9 (60.7-63.2)	61.8 (60.7-63.1)	62.0 (60.8-63.2)	62.1 (60.9-63.4)	62.3 (61.1-63.6)	62.5 (61.1-63.8)	62.6 (61.3-63.9)
Sub-Saharan Africa	53.4 (52.4-54.2)	53.6 (52.7-54.4)	53.6 (52.8-54.4)	53.6 (52.8-54.3)	51.8 (50.3-53.3)	53.5 (52.8-54.1)	53.3 (52.6-53.9)	53.2 (52.6-53.8)	53.2 (52.5-53.8)	53.1 (52.5-53.7)	53.2 (52.5-53.7)	53.5 (52.9-54.2)	53.7 (53.0-54.3)
Southern Sub-Saharan Africa	62.2 (61.4-63.0)	62.5 (61.6-63.3)	62.2 (61.3-63.0)	62.3 (61.3-63.2)	61.8 (60.7-62.7)	61.1 (60.1-62.1)	59.9 (58.8-60.9)	58.3 (57.2-59.3)	57.0 (55.8-58.1)	55.8 (54.6-56.9)	54.3 (53.1-55.6)	53.6 (52.3-54.9)	52.5 (51.2-53.8)
Botswana	62.3 (50.6-72.5)	62.0 (50.0-72.3)	61.2 (49.1-71.9)	60.1 (48.2-71.3)	58.6 (46.5-70.5)	56.8 (44.7-69.3)	54.8 (43.0-67.9)	52.9 (41.6-66.5)	50.9 (40.0-64.8)	49.2 (39.0-63.1)	47.6 (37.9-61.5)	46.5 (37.1-60.0)	45.6 (36.7-58.8)
Lesotho	60.5 (57.7-63.2)	60.6 (58.1-63.0)	60.4 (58.0-62.5)	60.1 (57.7-62.3)	59.2 (56.9-61.6)	58.7 (56.3-61.4)	57.9 (55.5-60.9)	56.6 (53.9-59.9)	54.6 (51.6-57.9)	52.9 (49.7-56.0)	50.7 (47.5-53.7)	48.9 (45.8-52.0)	47.0 (44.0-50.1)
Namibia	61.7 (60.1-63.3)	61.9 (60.4-63.6)	62.0 (60.5-63.7)	62.0 (60.3-63.7)	61.5 (59.6-63.2)	60.5 (58.5-62.3)	59.3 (57.3-61.3)	58.1 (55.9-60.2)	56.7 (54.5-59.0)	55.4 (52.9-57.9)	54.0 (51.4-56.7)	53.4 (50.6-56.2)	52.7 (49.8-55.6)
South Africa	62.0 (61.1-62.9)	62.5 (61.6-63.3)	62.4 (61.6-63.3)	63.0 (62.2-63.8)	62.8 (62.0-63.6)	62.7 (62.0-63.4)	61.6 (61.0-62.3)	60.0 (59.2-60.7)	58.9 (58.0-59.8)	57.9 (56.9-58.9)	56.4 (55.3-57.6)	55.9 (54.6-57.2)	54.8 (53.5-56.2)
Swaziland	60.7 (57.3-64.4)	60.7 (57.6-64.0)	61.0 (58.1-64.3)	61.3 (58.3-64.7)	60.7 (57.6-64.5)	59.1 (55.7-63.2)	57.1 (53.5-61.1)	55.8 (52.0-59.8)	53.5 (49.8-57.5)	51.4 (47.9-55.4)	48.8 (45.3-52.6)	46.6 (43.2-50.4)	45.1 (41.8-48.7)
Zimbabwe	63.0 (60.8-65.4)	62.7 (59.9-65.6)	61.4 (58.1-64.7)	59.8 (56.1-63.4)	58.2 (54.3-62.0)	56.0 (51.9-60.4)	54.4 (50.3-58.9)	52.8 (48.7-57.2)	50.9 (46.9-55.3)	49.3 (45.5-53.7)	47.9 (44.2-52.1)	47.0 (43.5-51.1)	45.9 (42.6-49.8)
Western Sub-Saharan Africa	54.2 (52.3-55.8)	54.4 (52.6-55.9)	54.4 (52.9-55.8)	54.5 (53.0-55.8)	54.6 (53.3-55.8)	54.7 (53.5-55.7)	54.7 (53.7-55.7)	54.8 (53.8-55.7)	54.7 (53.8-55.6)	54.9 (54.0-55.9)	55.0 (54.0-55.9)	55.1 (54.2-56.1)	55.2 (54.3-56.3)
Benin	56.1 (54.4-57.8)	56.3 (54.7-58.1)	56.6 (54.9-58.3)	56.9 (55.1-58.6)	56.8 (55.1-58.6)	56.7 (54.8-58.7)	56.6 (54.5-58.5)	56.9 (54.8-58.9)	57.0 (54.8-59.1)	57.1 (54.8-59.3)	57.2 (54.9-59.4)	57.4 (55.1-59.8)	57.7 (55.3-60.2)
Burkina Faso	51.4 (49.6-53.2)	51.4 (49.6-53.3)	51.3 (49.6-53.1)	51.3 (49.6-53.1)	51.3 (49.5-53.2)	51.3 (49.6-53.2)	51.5 (49.7-53.4)	51.6 (49.9-53.5)	51.8 (50.1-53.7)	52.2 (50.4-54.0)	52.6 (50.6-54.4)	53.2 (51.3-55.1)	53.8 (51.7-55.8)
Cameroon	58.4 (56.7-60.0)	58.2 (56.3-59.9)	57.9 (56.0-59.6)	57.4 (55.5-59.2)	57.0 (55.1-59.0)	56.5 (54.6-58.5)	56.2 (54.2-58.2)	55.5 (53.5-57.7)	55.0 (53.0-57.2)	54.7 (52.6-57.0)	54.3 (52.1-56.6)	54.2 (52.1-56.6)	54.2 (52.1-56.7)
Cape Verde	68.7 (68.2-69.1)	68.6 (68.0-69.1)	68.6 (67.7-69.3)	68.4 (67.2-69.6)	68.3 (66.6-69.9)	68.3 (66.1-70.3)	68.2 (65.7-70.6)	68.2 (65.5-70.8)	68.2 (65.3-71.3)	68.2 (65.1-71.3)	68.4 (65.2-71.7)	68.6 (65.3-71.8)	68.7 (65.4-72.0)
Chad	53.7 (51.9-55.5)	54.3 (52.5-55.9)	53.9 (52.1-55.6)	54.0 (52.2-55.8)	53.6 (51.8-55.6)	53.6 (51.7-55.7)	53.0 (51.0-55.2)	52.5 (50.5-54.7)	52.1 (50.0-54.2)	51.7 (49.7-54.0)	51.5 (49.2-54.0)	51.7 (49.3-54.5)	51.9 (49.1-54.9)
Cote d'Ivoire	55.3 (53.3-57.2)	54.9 (52.9-56.9)	54.4 (52.4-56.5)	53.7 (51.8-55.9)	53.2 (51.2-55.5)	52.5 (50.5-54.8)	52.3 (50.3-54.6)	52.3 (50.2-54.7)	52.2 (50.2-54.5)	52.2 (50.1-54.5)	52.0 (50.0-54.2)	52.1 (50.0-54.3)	52.3 (50.2-54.5)
The Gambia	61.3 (55.7-66.4)	61.5 (56.4-66.5)	61.5 (56.6-66.3)	62.0 (57.3-66.7)	62.3 (57.9-66.5)	62.6 (58.6-66.5)	62.8 (59.3-66.4)	63.1 (59.9-66.3)	63.4 (60.5-66.3)	63.4 (60.8-66.1)	63.6 (61.3-66.1)	63.9 (61.8-66.3)	63.8 (61.8-66.1)
Ghana	58.0 (54.1-61.8)	58.4 (54.9-61.8)	58.7 (55.7-61.6)	59.5 (56.6-62.2)	59.6 (57.0-62.0)	60.0 (57.6-62.4)	60.1 (57.6-62.7)	59.8 (57.3-62.5)	60.1 (57.9-62.8)	60.1 (57.6-62.6)	60.0 (57.6-62.6)	59.9 (57.4-62.5)	60.0 (57.3-62.8)
Guinea	52.0 (50.4-53.6)	52.3 (50.8-54.0)	52.5 (51.0-54.1)	53.0 (51.4-54.4)	53.1 (51.5-54.6)	53.4 (51.8-54.9)	53.8 (52.1-55.2)	54.0 (52.4-55.5)	54.0 (52.5-55.6)	54.0 (52.5-55.7)	54.2 (52.7-56.0)	54.4 (52.7-56.2)	54.7 (53.0-56.5)
Guinea-Bissau	51.5 (41.4-60.3)	51.7 (41.1-60.6)	51.8 (40.8-60.8)	52.0 (41.2-61.0)	52.2 (41.2-61.4)	52.3 (41.1-61.5)	52.6 (41.7-61.8)	52.8 (42.2-62.2)	51.7 (41.6-61.0)	52.5 (42.0-62.1)	52.8 (42.1-62.6)	52.7 (42.2-62.7)	52.7 (42.2-63.0)

Location	2003	2004	2005	2006	2007	2008	2009	2010	2011	2012	2013	2014	2015
Sri Lanka	74.0 (74.0-74.1)	69.4 (66.3-72.8)	73.9 (73.7-74.0)	73.9 (73.7-74.2)	74.4 (74.3-74.6)	74.0 (73.6-74.4)	74.1 (73.6-74.6)	75.0 (74.3-75.8)	76.4 (76.1-76.8)	76.8 (76.1-77.6)	77.1 (76.0-78.3)	77.4 (75.9-78.9)	77.6 (75.8-79.5)
Seychelles	71.5 (71.0-71.9)	71.7 (71.3-72.1)	72.0 (71.6-72.4)	72.3 (71.9-72.8)	72.6 (72.2-73.2)	72.9 (72.4-73.4)	73.0 (72.5-73.5)	73.1 (72.6-73.6)	73.4 (72.8-73.9)	73.7 (72.9-74.3)	73.8 (73.0-74.6)	74.0 (73.0-74.9)	74.1 (73.0-75.2)
Thailand	72.1 (71.4-72.8)	72.2 (71.6-72.9)	73.1 (72.5-73.6)	73.5 (72.9-74.0)	73.8 (73.2-74.3)	73.9 (73.4-74.5)	74.0 (73.5-74.6)	74.2 (73.6-74.7)	74.3 (73.6-75.0)	74.4 (73.5-75.3)	74.4 (73.4-75.6)	74.6 (73.4-76.0)	74.7 (73.3-76.2)
Timor-Leste	65.7 (64.4-67.0)	66.5 (65.2-67.8)	67.5 (66.2-68.8)	68.5 (67.1-69.8)	69.2 (67.9-70.6)	69.9 (68.5-71.4)	70.4 (68.8-72.0)	70.8 (69.0-72.6)	71.3 (69.2-73.2)	71.7 (69.5-73.8)	72.0 (69.7-74.2)	72.3 (69.9-74.4)	72.4 (70.0-74.7)
Vietnam	72.8 (70.9-74.7)	73.0 (71.1-75.2)	73.3 (71.2-75.6)	73.5 (71.4-75.8)	73.8 (71.4-76.1)	74.1 (71.6-76.4)	74.3 (71.8-76.6)	74.5 (72.0-76.8)	74.7 (72.3-77.0)	74.9 (72.5-77.2)	75.0 (72.8-77.3)	75.2 (73.1-77.7)	75.4 (73.3-77.7)
Oceania	59.8 (56.0-63.9)	59.9 (55.9-63.9)	60.0 (56.1-64.0)	60.1 (56.1-64.1)	60.2 (56.1-64.4)	60.5 (56.3-64.6)	60.7 (56.3-64.7)	61.0 (56.5-65.0)	61.2 (56.8-65.2)	61.5 (56.9-65.5)	61.9 (57.1-65.9)	62.2 (57.6-66.1)	62.5 (58.0-66.4)
American Samoa	71.7 (70.6-72.8)	71.8 (70.7-72.9)	72.0 (70.8-73.1)	72.2 (71.1-73.3)	72.4 (71.3-73.6)	72.7 (71.5-73.8)	71.3 (70.0-72.7)	72.8 (71.6-74.0)	72.6 (71.3-73.8)	72.6 (71.2-73.9)	72.7 (71.0-74.1)	72.8 (71.1-74.4)	72.9 (71.1-74.6)
Federated States of Micronesia	67.3 (62.2-72.0)	67.4 (62.2-72.1)	67.6 (62.0-72.2)	67.7 (62.0-72.4)	67.9 (62.1-72.4)	68.0 (62.1-72.5)	68.1 (62.3-72.7)	68.3 (62.4-72.8)	68.4 (62.3-72.9)	68.6 (62.3-73.0)	68.7 (62.5-73.0)	68.8 (62.6-73.1)	68.9 (62.8-73.2)
Fiji	64.6 (63.2-65.9)	64.7 (63.4-66.0)	64.8 (63.4-66.0)	64.8 (63.4-66.0)	64.9 (63.4-66.2)	64.9 (63.5-66.2)	64.9 (63.5-66.3)	65.2 (63.7-66.6)	65.3 (63.7-66.9)	65.4 (63.7-67.0)	65.6 (63.7-67.3)	65.7 (63.7-67.6)	65.8 (63.7-67.8)
Guam	73.5 (72.6-74.3)	73.3 (72.3-74.1)	73.1 (72.1-73.9)	72.7 (71.8-73.6)	72.4 (71.4-73.3)	72.1 (71.0-73.1)	71.8 (70.6-72.8)	71.6 (70.6-72.7)	71.5 (70.4-72.6)	71.5 (70.2-72.6)	71.6 (70.2-72.8)	71.6 (70.1-73.0)	71.7 (70.1-73.2)
Kiribati	60.4 (58.7-62.0)	60.6 (58.8-62.3)	60.8 (58.9-62.6)	61.0 (59.0-62.9)	61.2 (59.1-63.3)	61.5 (59.4-63.6)	61.7 (59.5-63.8)	61.9 (59.8-64.1)	62.1 (59.9-64.3)	62.3 (60.0-64.6)	62.6 (60.2-64.9)	62.8 (60.4-65.1)	63.0 (60.6-65.3)
Marshall Islands	64.7 (63.1-66.2)	64.7 (63.0-66.3)	64.7 (63.2-66.4)	64.7 (63.1-66.3)	65.0 (63.3-66.7)	65.3 (63.5-67.0)	65.5 (63.7-67.4)	65.8 (63.9-67.7)	66.0 (64.2-68.0)	66.2 (64.3-68.2)	66.5 (64.6-68.5)	66.7 (64.8-68.8)	66.9 (65.1-68.9)
Northern Mariana Islands	75.8 (75.2-76.4)	75.9 (75.3-76.5)	75.9 (75.3-76.5)	76.2 (75.5-76.8)	76.4 (75.7-77.0)	76.4 (75.7-77.0)	76.3 (75.5-77.0)	76.4 (75.6-77.1)	76.3 (75.4-77.1)	76.3 (75.3-77.1)	76.2 (75.2-77.1)	76.2 (75.1-77.1)	76.2 (75.1-77.2)
Papua New Guinea	57.7 (52.8-63.1)	57.7 (52.7-63.1)	57.9 (52.8-63.2)	58.0 (52.9-63.5)	58.2 (53.0-63.8)	58.6 (53.2-64.2)	58.8 (53.4-64.3)	59.1 (53.6-64.5)	59.4 (53.8-64.9)	59.7 (53.9-65.2)	60.2 (54.4-65.5)	60.5 (54.8-65.8)	60.9 (55.3-66.1)
Samoa	71.4 (69.8-73.0)	71.6 (70.0-73.1)	71.8 (70.3-73.3)	72.0 (70.6-73.4)	72.1 (70.7-73.6)	72.3 (71.0-73.8)	70.4 (68.9-71.8)	72.6 (71.2-74.0)	72.7 (71.3-74.2)	72.7 (71.3-74.2)	73.0 (71.5-74.6)	73.1 (71.4-74.9)	73.2 (71.4-75.1)
Solomon Islands	61.7 (56.8-66.7)	61.7 (56.8-66.9)	61.8 (56.8-67.1)	61.9 (56.8-67.2)	61.9 (56.7-67.1)	62.3 (57.0-67.6)	62.4 (57.1-67.7)	62.7 (57.5-68.1)	63.0 (57.6-68.5)	63.3 (57.9-68.8)	63.5 (57.9-69.1)	63.6 (57.7-69.1)	64.0 (58.0-69.4)
Tonga	69.6 (68.4-70.7)	69.8 (68.6-70.9)	70.0 (68.8-71.2)	70.1 (68.9-71.4)	70.2 (68.9-71.6)	70.3 (68.9-71.8)	68.6 (67.0-70.1)	70.7 (69.1-72.3)	70.8 (69.2-72.6)	71.0 (69.3-72.8)	71.2 (69.2-73.0)	71.3 (69.3-73.3)	71.5 (69.4-73.5)
Vanuatu	62.5 (57.9-67.7)	62.6 (57.7-67.7)	62.7 (57.6-67.7)	62.9 (57.9-67.8)	63.2 (57.7-67.9)	63.4 (57.9-68.1)	63.6 (58.0-68.3)	63.9 (58.3-68.5)	64.1 (58.5-68.5)	64.4 (58.9-68.7)	64.6 (59.3-68.9)	64.7 (59.4-68.9)	64.9 (59.7-69.0)
North Africa and Middle East	69.8 (69.3-70.3)	70.3 (69.7-70.8)	70.5 (70.0-71.1)	70.7 (70.2-71.3)	71.1 (70.5-71.6)	71.4 (70.8-72.0)	71.7 (71.1-72.3)	71.9 (71.4-72.5)	71.9 (71.3-72.5)	72.1 (71.5-72.7)	72.4 (71.8-73.0)	72.4 (71.8-73.1)	72.4 (71.7-73.0)
North Africa and Middle East	69.8 (69.3-70.3)	70.3 (69.7-70.8)	70.5 (70.0-71.1)	70.7 (70.2-71.3)	71.1 (70.5-71.6)	71.4 (70.8-72.0)	71.7 (71.1-72.3)	71.9 (71.4-72.5)	71.9 (71.3-72.5)	72.1 (71.5-72.7)	72.4 (71.8-73.0)	72.4 (71.8-73.1)	72.4 (71.7-73.0)
Afghanistan	51.4 (48.8-54.3)	51.8 (49.1-54.8)	52.0 (49.2-55.0)	52.1 (49.3-55.0)	52.4 (49.6-55.3)	52.8 (50.0-55.7)	53.3 (50.3-56.2)	53.6 (50.7-56.5)	54.0 (51.1-56.8)	54.4 (51.5-57.3)	54.8 (52.0-57.8)	54.9 (52.0-57.8)	53.8 (50.7-56.9)
Algeria	73.9 (73.0-74.8)	74.4 (73.6-75.3)	74.8 (73.9-75.7)	75.0 (74.2-75.9)	75.3 (74.4-76.1)	75.5 (74.8-76.3)	75.7 (75.0-76.5)	76.0 (75.3-76.8)	76.1 (75.4-76.9)	76.2 (75.4-77.0)	76.3 (75.4-77.1)	76.3 (75.4-77.2)	76.4 (75.4-77.4)
Bahrain	75.3 (74.7-76.0)	75.7 (75.0-76.3)	76.1 (75.5-76.8)	76.3 (75.7-76.9)	77.0 (76.4-77.8)	77.6 (77.0-78.4)	78.2 (77.4-79.0)	78.7 (77.9-79.4)	78.8 (78.1-79.6)	79.0 (78.2-79.8)	79.1 (78.1-80.0)	79.1 (78.0-80.2)	79.1 (77.8-80.2)
Egypt	69.8 (69.5-70.0)	69.9 (69.7-70.2)	70.1 (69.9-70.4)	70.1 (69.8-70.3)	70.3 (70.0-70.6)	70.2 (70.0-70.5)	70.1 (69.8-70.3)	70.1 (69.8-70.3)	70.4 (70.1-70.6)	70.5 (70.2-70.7)	71.0 (70.8-71.3)	71.3 (70.9-71.7)	71.5 (70.9-72.0)
Iran	70.1 (68.6-71.7)	71.5 (70.0-73.0)	71.9 (70.3-73.7)	72.4 (70.5-74.2)	72.8 (70.8-74.6)	73.1 (71.2-75.0)	73.4 (71.4-75.4)	73.7 (71.7-75.6)	74.1 (72.1-75.9)	74.3 (72.7-76.2)	74.5 (72.9-76.3)	74.6 (72.9-76.3)	74.6 (72.9-76.3)
Iraq	66.1 (63.9-68.1)	66.3 (64.0-68.4)	65.7 (63.4-67.8)	65.1 (62.6-67.5)	65.3 (62.7-67.7)	66.6 (64.1-68.9)	67.1 (64.6-69.4)	67.3 (64.8-69.8)	67.7 (65.2-70.2)	68.1 (65.6-70.5)	68.3 (65.7-70.8)	67.7 (65.2-70.2)	67.4 (64.7-70.0)
Jordan	74.0 (73.3-74.8)	74.1 (73.5-74.8)	74.5 (74.0-75.1)	75.5 (75.0-76.0)	76.3 (75.9-76.7)	76.9 (76.6-77.2)	77.5 (77.2-77.7)	77.9 (77.7-78.2)	78.1 (77.8-78.4)	78.2 (77.9-78.6)	78.3 (77.8-78.8)	78.4 (77.7-79.1)	78.5 (77.6-79.3)
Kuwait	78.5 (78.3-78.7)	78.1 (77.8-78.3)	77.7 (77.5-77.9)	77.7 (77.5-77.9)	77.7 (77.6-78.0)	77.3 (77.2-77.5)	77.4 (77.2-77.7)	78.5 (78.1-78.8)	79.0 (78.8-79.2)	79.1 (78.9-79.3)	79.7 (79.5-79.9)	80.2 (79.6-80.7)	80.3 (79.3-81.2)
Lebanon	76.9 (75.3-78.2)	77.1 (75.5-78.6)	77.3 (75.6-78.8)	77.4 (75.7-79.0)	77.5 (75.7-79.1)	77.8 (75.8-79.5)	77.9 (76.0-79.7)	78.1 (76.2-80.0)	76.6 (74.6-78.8)	78.5 (76.5-80.4)	78.6 (76.6-80.5)	78.7 (76.7-80.6)	78.9 (76.9-80.9)
Libya	75.1 (74.4-75.7)	75.2 (74.6-75.8)	75.4 (74.7-76.0)	75.5 (74.9-76.2)	75.5 (74.9-76.2)	75.6 (74.9-76.3)	75.7 (75.0-76.4)	75.9 (75.1-76.7)	60.5 (53.1-69.8)	75.5 (74.5-76.4)	75.8 (74.7-76.7)	75.0 (73.8-76.1)	74.1 (72.5-75.6)
Morocco	72.3 (70.4-73.9)	72.5 (70.4-74.1)	72.7 (70.5-74.4)	72.9 (70.7-74.7)	73.1 (70.8-75.0)	73.3 (71.0-75.3)	73.5 (71.2-75.5)	73.7 (71.4-75.8)	73.9 (71.4-76.0)	74.1 (71.5-76.2)	74.3 (71.8-76.5)	74.4 (71.9-76.7)	74.6 (72.0-76.8)
Palestine	74.4 (73.6-75.1)	74.4 (73.6-75.1)	74.6 (73.7-75.3)	74.4 (73.2-75.2)	74.3 (73.1-75.2)	74.1 (72.9-75.2)	73.8 (72.4-75.0)	74.3 (72.7-75.7)	74.2 (72.6-75.8)	74.2 (72.5-75.9)	74.4 (72.6-76.2)	74.5 (72.7-76.4)	74.6 (72.8-76.6)
Oman	75.6 (74.6-76.6)	75.8 (74.9-76.6)	76.0 (75.2-76.8)	76.0 (75.3-76.7)	76.0 (75.4-76.6)	76.2 (75.6-76.8)	76.2 (75.5-76.8)	76.1 (75.3-76.8)	76.3 (75.5-77.1)	76.6 (75.7-77.6)	76.8 (75.8-78.1)	77.0 (75.9-78.4)	77.2 (75.9-78.7)
Qatar	75.2 (74.3-76.3)	75.8 (74.7-76.8)	76.3 (75.1-77.4)	76.7 (75.5-77.8)	77.3 (76.2-78.4)	77.9 (76.9-78.9)	78.5 (77.5-79.5)	79.2 (78.2-80.2)	79.7 (78.7-80.7)	79.9 (78.8-81.0)	79.9 (78.6-81.1)	79.8 (78.2-81.2)	79.7 (78.0-81.3)
Saudi Arabia	77.4 (76.8-77.9)	77.5 (76.9-78.0)	77.8 (77.2-78.3)	77.9 (77.3-78.5)	78.2 (77.6-78.7)	78.3 (77.8-78.9)	78.5 (77.9-79.0)	78.7 (78.1-79.3)	78.9 (78.3-79.5)	79.2 (78.5-79.7)	79.3 (78.6-80.0)	79.4 (78.7-80.2)	79.5 (78.7-80.3)
Sudan	63.5 (60.7-66.3)	63.7 (60.8-66.5)	64.6 (61.8-67.4)	64.9 (62.0-67.8)	65.3 (62.5-68.2)	65.5 (62.8-68.5)	65.7 (63.2-68.7)	66.1 (63.5-69.1)	66.3 (63.6-69.3)	66.7 (63.8-69.7)	66.9 (63.9-69.9)	67.2 (64.2-70.3)	67.5 (64.4-70.5)
Syria	75.1 (74.3-75.9)	75.3 (74.6-76.0)	75.5 (75.0-76.0)	75.7 (75.3-76.1)	75.9 (75.5-76.3)	76.1 (75.8-76.5)	76.3 (76.0-76.7)	76.5 (76.1-77.0)	75.1 (74.0-76.3)	68.1 (63.4-73.5)	69.0 (64.9-73.7)	67.2 (60.8-74.5)	68.2 (63.6-73.6)
Tunisia	75.7 (74.7-76.8)	76.0 (74.9-77.1)	76.2 (75.1-77.4)	76.4 (75.2-77.7)	76.6 (75.4-77.9)	76.8 (75.5-78.2)	77.0 (75.5-78.3)	77.1 (75.6-78.6)	77.2 (75.6-78.7)	77.4 (75.8-78.9)	77.5 (75.9-79.1)	77.6 (76.0-79.2)	77.6 (76.0-79.3)
Turkey	75.1 (74.5-75.7)	75.8 (75.3-76.4)	76.2 (75.7-76.7)	76.7 (76.3-77.2)	77.4 (76.9-77.8)	77.8 (77.4-78.2)	78.5 (78.0-78.9)	78.8 (78.3-79.2)	78.8 (78.4-79.3)	79.1 (78.6-79.5)	78.8 (78.4-79.3)	79.1 (78.6-79.5)	79.2 (78.7-79.8)
United Arab Emirates	74.1 (73.4-74.8)	74.4 (73.8-75.0)	75.2 (74.5-75.8)	75.7 (74.9-76.3)	75.6 (74.8-76.3)	75.6 (74.6-76.6)	75.6 (74.4-76.6)	75.6 (74.1-76.8)	75.5 (73.9-76.9)	75.5 (73.7-77.0)	75.4 (73.4-77.2)	75.4 (73.3-77.4)	75.4 (73.1-77.4)
Yemen	63.7 (58.8-69.2)	64.2 (59.1-69.8)	64.6 (59.4-70.1)	65.0 (59.8-70.6)	65.2 (59.9-70.8)	65.7 (60.1-71.2)	66.2 (60.5-71.7)	66.6 (60.7-72.0)	66.6 (60.9-72.0)	66.7 (61.0-72.0)	67.1 (61.3-72.4)	67.1 (61.4-72.3)	66.0 (60.2-70.9)
South Asia	63.1 (62.7-63.5)	63.5 (63.1-63.9)	63.8 (63.4-64.2)	64.2 (63.8-64.7)	64.6 (64.2-65.0)	64.9 (64.5-65.4)	65.3 (64.8-65.7)	65.6 (65.1-66.1)	65.9 (65.4-66.4)	66.3 (65.8-66.8)	66.6 (66.1-67.1)	67.0 (66.5-67.5)	67.4 (66.8-67.9)
South Asia	63.1 (62.7-63.5)	63.5 (63.1-63.9)	63.8 (63.4-64.2)	64.2 (63.8-64.7)	64.6 (64.2-65.0)	64.9 (64.5-65.4)	65.3 (64.8-65.7)	65.6 (65.1-66.1)	65.9 (65.4-66.4)	66.3 (65.8-66.8)	66.6 (66.1-67.1)	67.0 (66.5-67.5)	67.4 (66.8-67.9)
Bangladesh	66.3 (65.5-67.2)	66.8 (66.0-67.6)	67.1 (66.3-67.9)	67.5 (66.7-68.2)	67.7 (66.9-68.5)	68.3 (67.4-69.1)	68.6 (67.7-69.4)	68.8 (67.9-69.7)	69.3 (68.2-70.3)	69.4 (68.3-70.5)	69.8 (68.6-70.9)	70.1 (68.8-71.3)	70.4 (69.0-71.7)
Bhutan	68.5 (66.8-69.9)	68.9 (67.4-70.4)	69.3 (67.8-70.8)	69.8 (68.2-71.3)	70.3 (68.6-71.7)	70.7 (69.1-72.2)	70.9 (69.4-72.5)	71.4 (69.7-73.0)	71.7 (70.0-73.3)	71.9 (70.2-73.7)	72.2 (70.4-73.9)	72.4 (70.7-74.2)	72.7 (70.9-74.6)
India	62.8 (62.3-63.3)	63.2 (62.7-63.7)	63.6 (63.1-64.0)	63.9 (63.5-64.4)	64.3 (63.8-64.8)	64.7 (64.2-65.2)	65.0 (64.5-65.6)	65.4 (64.8-65.9)	65.7 (65.1-66.3)	66.1 (65.5-66.7)	66.5 (65.8-67.1)	66.9 (66.2-67.5)	67.2 (66.6-67.9)
Nepal	66.8 (65.9-67.7)	67.0 (66.0-68.0)	67.4 (66.3-68.5)	67.8 (66.5-69.0)	68.1 (66.8-69.5)	68.4 (67.0-69.9)	68.7 (67.2-70.3)	69.0 (67.4-70.7)	69.3 (67.6-71.1)	69.7 (67.9-71.4)	69.9 (68.2-71.7)	70.2 (68.4-72.0)	69.7 (67.8-71.5)
Pakistan	62.8 (61.5-64.1)	63.1 (61.8-64.3)	62.2 (60.9-63.5)	63.7 (62.4-65.0)	63.8 (62.5-65.2)	64.1 (62.6-65.5)	64.3 (62.9-65.8)	64.5 (63.1-66.1)	64.9 (63.4-66.4)	65.1 (63.6-66.7)	65.4 (63.8-66.9)	65.6 (64.1-67.1)	65.9 (64.3-67.4)
Sub-Saharan Africa	54.0 (53.3-54.6)	54.5 (53.8-55.1)	55.1 (54.4-55.8)	55.7 (55.0-56.4)	56.5 (55.7-57.2)	57.3 (56.6-58.1)	58.1 (57.2-58.9)	58.8 (57.9-59.6)	59.5 (58.5-60.4)	60.2 (59.1-61.2)	60.8 (59.6-61.9)	61.2 (59.9-62.4)	61.8 (60.4-63.1)
Southern Sub-Saharan Africa	51.5 (50.3-52.8)	50.8 (49.6-52.1)	50.8 (49.7-52.1)	51.0 (49.8-52.1)	51.6 (50.5-52.7)	52.2 (51.1-53.3)	52.7 (51.6-53.9)	53.6 (52.4-54.8)	55.4 (54.3-56.5)	57.6 (56.6-58.6)	59.1 (58.0-60.1)	59.7 (58.5-60.8)	60.4 (59.1-61.5)
Botswana	45.7 (36.8-58.6)	46.9 (37.6-59.9)	49.3 (39.1-62.2)	51.2 (40.2-64.3)	52.4 (41.2-65.4)	53.2 (41.6-65.7)	54.3 (42.3-66.5)	55.6 (43.4-67.5)	56.5 (44.1-68.1)	56.5 (43.6-68.1)	56.9 (44.1-67.9)	57.3 (44.3-67.9)	58.7 (45.8-69.2)
Lesotho	45.4 (42.4-48.3)	44.2 (41.3-47.2)	43.1 (40.3-46.1)	43.1 (40.6-45.9)	43.3 (40.7-46.2)	44.5 (41.7-47.6)	45.5 (42.3-48.8)	46.4 (43.0-50.3)	46.7 (42.9-50.9)	46.1 (42.2-50.6)	45.6 (41.6-50.4)	45.4 (41.2-50.5)	47.1 (42.3-52.9)
Namibia	52.4 (49.4-55.5)	52.5 (49.5-55.5)	53.1 (50.2-56.0)	54.9 (52.1-57.8)	57.5 (54.6-60.5)	59.1 (56.0-62.5)	60.3 (57.0-63.8)	61.4 (57.9-65.0)	62.6 (59.1-66.0)	63.6 (59.9-67.4)	63.9 (59.9-68.0)	64.1 (59.7-68.5)	64.2 (59.3-68.8)
South Africa	53.7 (52.3-55.2)	52.8 (51.4-54.4)	52.7 (51.3-54.2)	52.5 (51.2-54.0)	53.0 (51.8-54.5)	53.4 (52.2-54.8)	53.9 (52.7-55.4)	54.9 (53.7-56.3)	56.6 (55.5-57.9)	59.0 (58.0-60.0)	60.7 (59.9-61.6)	61.2 (60.2-62.1)	61.3 (60.2-62.3)
Swaziland	44.0 (40.7-47.4)	43.0 (39.8-46.5)	42.5 (39.2-46.0)	43.1 (40.0-46.8)	44.3 (41.1-48.2)	45.1 (41.7-49.3)	45.9 (42.3-50.5)	46.4 (42.5-51.2)	48.0 (43.8-53.3)	49.1 (44.5-54.9)	49.4 (44.6-55.5)	49.8 (44.9-56.0)	51.8 (46.3-58.6)
Zimbabwe	45.3 (42.2-49.1)	44.7 (41.9-48.3)	45.1 (42.4-48.3)	45.5 (43.1-48.4)	46.4 (44.3-49.0)	47.3 (45.3-49.8)	48.0 (45.9-50.5)	49.1 (46.9-51.8)	51.6 (49.1-54.6)	54.2 (51.4-57.7)	55.7 (52.4-59.6)	57.0 (53.1-61.4)	59.3 (54.9-64.0)
Western Sub-Saharan Africa	55.6 (54.6-56.6)	56.1 (55.1-57.1)	56.6 (55.6-57.7)	57.2 (56.1-58.3)	57.9 (56.7-59.0)	58.6 (57.4-59.7)	59.4 (58.2-60.6)	60.0 (58.8-61.3)	60.6 (59.3-62.0)	61.1 (59.5-62.6)	61.6 (59.9-63.1)	61.9 (59.9-63.5)	62.5 (60.4-64.2)
Benin	57.9 (55.3-60.7)	58.2 (55.3-61.3)	58.6 (55.4-62.0)	58.9 (55.3-62.8)	59.2 (55.4-63.5)	59.7 (55.5-64.2)	60.4 (55.7-65.1)	60.8 (55.5-65.8)	61.1 (55.7-66.4)	61.4 (55.7-67.1)	61.7 (55.7-67.8)	62.0 (55.7-68.2)	62.3 (55.9-68.9)
Burkina Faso	54.5 (52.3-56.5)	55.1 (52.9-57.2)	55.9 (53.6-58.0)	56.6 (54.4-58.9)	57.4 (54.9-59.9)	58.0 (55.3-60.8)	58.5 (55.4-61.7)	59.0 (55.6-62.7)	59.5 (55.7-63.6)	59.9 (55.6-64.4)	60.3 (55.4-64.8)	60.6 (55.3-65.4)	60.9 (55.6-66.2)
Cameroon	54.3 (52.1-56.8)	54.4 (52.1-57.0)	54.9 (52.5-57.5)	55.4 (52.9-58.2)	55.7 (53.0-58.7)	56.6 (53.7-59.8)	57.3 (54.2-60.8)	57.8 (54.5-61.7)	58.1 (54.5-62.3)	58.5 (54.4-63.2)	59.0 (54.4-63.8)	59.1 (54.0-64.3)	59.4 (53.9-64.5)
Cape Verde	68.9 (65.5-72.2)	69.1 (66.0-72.2)	69.3 (66.5-72.2)	69.6 (67.1-72.2)	69.6 (67.6-71.7)	70.4 (68.7-71.9)	70.7 (69.6-71.8)	71.1 (70.3-71.8)	71.4 (71.0-71.9)	71.9 (71.4-72.3)	72.3 (71.5-73.3)	72.7 (71.3-74.0)	72.9 (70.9-74.5)
Chad	52.1 (49.0-55.3)	52.6 (48.9-56.0)	53.0 (49.0-56.8)	53.1 (48.8-57.3)	54.0 (49.3-58.7)	54.3 (49.4-59.4)	55.2 (50.2-60.5)	55.8 (50.5-61.4)	56.1 (50.6-62.1)	56.3 (50.5-62.6)	56.6 (50.7-63.0)	56.8 (50.8-63.1)	57.4 (51.2-63.8)
Cote d'Ivoire	52.6 (50.4-54.8)	52.8 (50.6-55.1)	53.4 (51.1-55.7)	54.1 (51.7-56.6)	54.9 (52.4-57.6)	55.4 (52.7-58.3)	56.0 (53.0-59.2)	56.6 (53.3-60.0)	57.0 (53.3-60.8)	57.5 (53.5-61.6)	58.1 (53.6-62.4)	58.5 (53.8-63.3)	59.1 (54.1-64.3)
The Gambia	64.4 (62.3-66.7)	64.7 (62.6-67.0)	64.9 (62.9-67.1)	65.2 (63.2-67.4)	65.3 (63.3-67.4)	65.7 (63.6-68.0)	66.0 (63.8-68.4)	66.5 (64.2-69.0)	67.1 (64.4-69.6)	67.5 (64.4-70.2)	67.8 (64.5-70.8)	68.0 (64.4-71.3)	68.1 (64.3-71.7)
Ghana	60.2 (57.5-63.0)	60.5 (57.6-63.5)	60.8 (57.6-64.0)	61.2 (57.7-64.7)	61.6 (57.8-65.6)	62.0 (57.8-66.2)	62.4 (58.0-67.1)	62.9 (57.9-67.8)	63.5 (58.1-68.5)	64.1 (58.7-69.3)	64.5 (58.9-69.8)	64.8 (58.9-70.3)	65.3 (59.5-71.0)
Guinea	55.1 (53.4-57.0)	55.6 (53.7-57.6)	56.0 (54.0-58.1)	56.4 (54.3-58.5)	56.8 (54.6-59.0)	57.1 (55.0-59.5)	57.5 (55.3-60.1)	57.9 (55.4-60.7)	58.2 (55.3-61.2)	58.5 (55.1-61.8)	58.8 (55.0-62.5)	58.6 (54.5-62.7)	59.1 (54.6-63.7)
Guinea-Bissau	52.8 (41.8-63.1)	52.8 (41.5-63.2)	52.9 (41.7-63.3)	53.0 (41.7-63.5)	53.2 (42.1-63.7)	53.6 (42.3-64.1)	53.9 (42.4-64.3)	54.3 (42.6-64.6)	54.5 (42.8-64.8)	54.8 (42.4-65.1)	55.1 (42.6-65.4)	55.3 (42.4-65.5)	55.6 (42.3-65.8)

SR Table 5: Estimates of life expectancy at birth by geography (with 95% uncertainty intervals), both sexes, 1980- 2015

Location	1980	1981	1982	1983	1984	1985	1986	1987	1988	1989	1990	1991
Liberia	50.3 (44.1-55.9)	50.5 (44.3-56.0)	50.7 (44.9-55.8)	50.9 (45.3-55.9)	51.0 (45.6-55.9)	51.1 (46.3-55.7)	51.2 (47.0-55.5)	51.3 (47.5-55.3)	51.6 (48.1-55.1)	51.8 (48.6-55.0)	48.6 (45.2-52.1)	51.5 (48.9-54.2)
Mali	45.1 (42.9-47.4)	45.8 (43.6-48.0)	46.5 (44.4-48.5)	47.1 (45.3-49.1)	47.7 (45.9-49.5)	48.2 (46.5-49.9)	49.1 (47.5-50.7)	49.4 (47.9-50.9)	49.7 (48.3-51.1)	50.0 (48.6-51.4)	50.0 (48.7-51.4)	50.5 (49.1-52.0)
Mauritania	56.3 (50.9-60.9)	56.7 (51.8-61.0)	57.0 (52.8-60.9)	57.4 (53.6-60.9)	57.7 (54.6-60.9)	58.0 (55.3-60.8)	58.9 (56.7-61.3)	59.3 (57.4-61.5)	59.9 (58.1-61.9)	59.6 (57.8-61.3)	59.5 (57.8-61.3)	60.2 (58.5-62.0)
Niger	43.9 (42.0-45.9)	43.5 (41.6-45.4)	43.8 (42.0-45.7)	43.8 (42.0-45.7)	43.9 (42.1-45.6)	44.2 (42.4-45.9)	44.5 (42.8-46.3)	45.0 (43.3-46.8)	45.7 (44.0-47.4)	46.4 (44.8-48.1)	47.2 (45.6-48.8)	47.9 (46.3-49.6)
Nigeria	52.6 (46.9-58.1)	52.9 (47.4-58.4)	53.3 (47.7-58.6)	53.5 (47.9-58.5)	53.6 (48.2-58.6)	53.8 (48.6-58.6)	53.9 (48.9-58.6)	53.9 (49.1-58.2)	53.9 (49.6-58.0)	54.0 (50.1-57.9)	54.1 (50.4-57.7)	54.3 (50.7-57.5)
Sao Tome and Principe	63.0 (62.1-63.8)	63.1 (62.2-64.0)	63.2 (62.3-64.0)	63.2 (62.4-64.0)	63.2 (62.5-63.9)	63.2 (62.5-63.9)	63.3 (62.5-64.0)	63.3 (62.5-64.2)	63.3 (62.2-64.4)	63.4 (62.0-64.7)	63.4 (61.8-64.9)	63.5 (61.8-65.2)
Senegal	52.3 (50.2-54.5)	52.9 (50.9-55.1)	53.7 (51.7-55.8)	54.9 (53.0-56.9)	55.9 (54.0-57.7)	56.2 (54.3-58.1)	56.4 (54.5-58.5)	56.8 (55.0-58.8)	57.2 (55.3-59.0)	57.4 (55.6-59.3)	57.8 (56.0-59.7)	58.0 (56.2-59.9)
Sierra Leone	48.2 (43.1-54.0)	48.5 (43.5-54.0)	48.8 (43.8-54.3)	49.1 (44.1-54.4)	49.4 (44.6-54.8)	49.8 (45.0-54.8)	50.1 (45.6-54.7)	50.4 (46.1-54.9)	50.8 (46.7-55.0)	51.2 (47.2-55.1)	51.6 (48.0-55.2)	51.4 (48.1-54.6)
Togo	56.1 (52.2-60.0)	56.4 (53.0-60.0)	56.7 (53.8-59.8)	56.8 (54.1-59.6)	56.7 (54.2-59.3)	57.0 (54.6-59.2)	57.2 (55.0-59.2)	57.0 (55.0-59.0)	57.5 (55.5-59.5)	57.6 (55.7-59.7)	57.8 (55.9-60.0)	57.8 (55.9-60.0)
Eastern Sub-Saharan Africa	49.7 (47.8-51.5)	49.7 (48.0-51.3)	49.9 (48.3-51.5)	48.9 (46.9-50.8)	48.9 (47.0-50.6)	50.2 (48.8-51.5)	50.6 (49.4-51.8)	50.9 (49.7-52.1)	51.2 (50.1-52.3)	51.2 (50.1-52.2)	50.9 (49.8-51.8)	51.1 (50.1-52.0)
Burundi	44.0 (38.8-50.0)	44.4 (39.1-50.4)	44.8 (39.3-51.0)	45.1 (39.8-51.1)	45.4 (40.4-51.5)	45.6 (40.8-51.7)	45.7 (40.9-51.8)	45.8 (41.0-51.6)	45.8 (41.2-51.4)	45.7 (41.3-51.1)	45.6 (41.5-51.0)	45.4 (41.6-50.5)
Comoros	53.5 (48.0-59.9)	53.9 (48.1-60.4)	54.2 (48.2-60.8)	54.4 (48.4-60.9)	54.9 (48.8-61.4)	55.2 (49.3-61.9)	55.6 (49.9-62.3)	55.9 (50.3-62.5)	56.4 (50.8-63.0)	56.6 (51.3-62.7)	57.1 (51.9-63.1)	57.4 (52.3-63.1)
Djibouti	60.9 (54.2-67.2)	60.7 (54.1-66.8)	60.8 (54.5-66.9)	60.7 (54.7-66.7)	60.7 (54.7-66.7)	60.7 (54.5-66.6)	60.7 (54.7-66.7)	60.8 (55.2-66.4)	60.9 (55.4-66.3)	60.9 (55.9-66.1)	61.0 (56.2-65.8)	60.7 (56.1-65.5)
Eritrea	47.4 (44.7-50.4)	47.9 (45.4-50.7)	48.4 (46.3-51.0)	48.6 (46.6-50.9)	48.8 (46.8-51.1)	48.9 (46.9-51.2)	49.6 (47.6-51.9)	50.2 (48.2-52.4)	50.9 (48.9-53.0)	51.5 (49.5-53.7)	52.4 (50.4-54.6)	53.4 (51.4-55.6)
Ethiopia	42.3 (38.8-46.6)	42.5 (39.4-46.4)	42.8 (39.7-46.2)	39.4 (34.5-44.0)	39.8 (35.1-44.1)	43.7 (41.5-46.2)	44.5 (42.3-46.9)	45.2 (43.2-47.4)	45.7 (43.8-47.7)	45.6 (43.8-47.6)	45.2 (43.3-47.1)	46.9 (45.3-48.6)
Kenya	62.3 (60.9-63.7)	62.7 (61.3-64.1)	63.0 (61.6-64.3)	63.3 (61.9-64.5)	63.3 (61.9-64.6)	63.4 (62.0-64.6)	63.3 (61.9-64.5)	62.9 (61.6-64.1)	62.9 (61.5-64.1)	62.3 (60.9-63.5)	61.8 (60.4-63.1)	61.1 (59.7-62.3)
Madagascar	55.7 (53.8-57.5)	55.5 (53.8-57.1)	54.9 (53.3-56.5)	54.7 (53.3-56.2)	54.6 (53.2-56.1)	54.4 (52.9-55.9)	54.0 (52.6-55.6)	54.1 (52.7-55.6)	54.7 (53.3-56.1)	55.4 (54.0-56.7)	56.0 (54.6-57.3)	56.2 (54.9-57.5)
Malawi	49.1 (47.3-51.1)	49.5 (47.7-51.3)	49.8 (48.1-51.6)	50.0 (48.3-51.9)	50.1 (48.4-51.9)	50.0 (48.4-51.7)	49.7 (48.0-51.3)	49.5 (47.8-51.2)	49.3 (47.5-51.0)	49.0 (47.0-50.9)	48.8 (46.7-50.9)	48.6 (46.3-50.8)
Mozambique	50.6 (47.3-54.3)	47.9 (44.2-51.4)	48.0 (44.6-51.1)	48.0 (45.0-51.2)	45.2 (41.5-49.1)	45.2 (41.5-48.9)	47.1 (43.6-50.3)	47.4 (43.8-50.6)	50.8 (49.0-52.6)	51.2 (49.6-52.8)	51.5 (49.8-53.0)	51.7 (50.1-53.2)
Rwanda	52.6 (47.9-57.1)	53.6 (49.0-57.9)	54.4 (50.1-58.4)	54.9 (50.8-58.7)	55.1 (51.5-58.6)	55.2 (51.9-58.5)	55.2 (52.5-58.3)	54.9 (52.4-57.6)	54.1 (52.0-56.6)	52.9 (50.7-55.1)	50.5 (48.5-52.7)	49.3 (47.3-51.5)
Somalia	48.9 (38.5-60.3)	48.9 (38.2-60.4)	48.8 (38.1-60.4)	48.7 (38.0-60.1)	48.7 (38.3-60.3)	48.9 (38.4-60.4)	48.8 (38.3-60.2)	48.8 (38.9-60.2)	46.2 (37.3-57.4)	49.1 (39.3-60.6)	48.8 (38.9-60.2)	47.4 (38.5-58.4)
South Sudan	49.2 (39.7-58.5)	49.3 (39.6-58.7)	49.4 (39.4-58.7)	49.5 (39.4-58.8)	49.7 (39.6-58.9)	49.8 (39.7-59.1)	49.9 (39.9-59.3)	50.2 (40.1-59.5)	50.4 (40.5-59.9)	50.7 (40.8-60.3)	50.9 (41.0-60.7)	51.0 (40.7-60.8)
Tanzania	56.6 (53.5-59.1)	56.8 (54.1-59.2)	56.9 (54.4-59.1)	57.1 (54.8-59.1)	57.0 (55.0-59.0)	57.0 (55.1-58.8)	56.8 (55.0-58.6)	56.5 (54.7-58.3)	56.1 (54.4-57.9)	55.6 (53.8-57.5)	55.2 (53.5-57.1)	55.1 (53.3-57.1)
Uganda	48.2 (45.2-51.3)	48.7 (45.7-51.6)	49.2 (46.5-51.8)	49.7 (47.0-52.1)	49.9 (47.4-52.3)	50.0 (47.5-52.3)	50.0 (47.7-52.1)	51.6 (49.9-53.4)	51.7 (49.9-53.6)	51.3 (49.2-53.4)	50.7 (48.5-53.3)	50.0 (47.7-52.8)
Zambia	57.2 (54.0-60.5)	57.2 (54.4-60.0)	56.9 (54.5-59.5)	56.6 (54.3-59.1)	56.0 (54.0-58.4)	55.2 (53.3-57.3)	54.5 (52.6-56.5)	54.0 (52.2-56.2)	53.8 (51.8-56.1)	52.9 (50.9-55.2)	51.9 (49.8-54.2)	50.7 (48.5-53.1)
Central Sub-Saharan Africa	51.6 (47.1-56.6)	51.8 (47.3-56.8)	52.1 (47.8-57.0)	52.3 (48.0-56.9)	52.7 (48.4-57.3)	53.0 (48.9-57.4)	53.1 (49.2-57.3)	53.1 (49.3-57.2)	53.0 (49.3-56.8)	52.9 (49.4-56.4)	52.7 (49.1-56.0)	52.8 (49.2-55.9)
Angola	47.8 (38.0-59.8)	47.9 (38.1-59.6)	48.0 (38.6-59.7)	48.0 (38.7-59.9)	48.2 (39.1-60.2)	48.3 (39.4-60.1)	48.3 (39.6-60.0)	48.3 (39.6-59.6)	48.2 (39.1-59.5)	48.6 (39.6-60.4)	48.6 (39.4-60.3)	49.3 (39.7-61.1)
Central African Republic	49.3 (46.6-52.0)	49.7 (47.3-52.3)	50.2 (47.9-52.7)	50.2 (47.9-52.6)	50.2 (47.9-52.5)	50.3 (48.1-52.4)	50.1 (48.1-52.1)	50.0 (48.1-52.1)	49.3 (47.5-51.3)	48.8 (46.9-50.7)	48.4 (46.3-50.5)	48.1 (45.8-50.7)
Congo	53.2 (47.1-60.6)	53.7 (47.8-60.9)	54.1 (48.5-60.9)	54.5 (49.0-60.9)	54.8 (49.6-60.9)	55.0 (50.2-60.8)	55.1 (50.7-60.2)	55.0 (51.1-59.7)	54.9 (51.3-59.1)	54.6 (51.4-58.5)	54.3 (51.5-57.5)	54.4 (51.8-57.2)
Democratic Republic of the Congo	53.0 (47.0-59.7)	53.2 (47.5-59.9)	53.5 (47.8-60.1)	53.9 (48.1-60.2)	54.4 (48.8-60.5)	54.8 (49.6-60.5)	55.0 (50.1-60.4)	55.0 (50.3-60.2)	55.0 (50.6-59.7)	54.8 (50.7-59.2)	54.5 (50.8-58.6)	54.4 (51.0-58.0)
Equatorial Guinea	49.2 (39.4-61.9)	49.5 (39.6-62.5)	49.7 (39.6-62.5)	49.8 (39.9-62.6)	49.8 (39.8-62.5)	49.7 (39.7-62.3)	49.7 (40.0-62.0)	49.5 (39.8-61.8)	49.4 (39.7-61.8)	49.2 (39.7-61.9)	48.9 (39.5-61.8)	48.7 (39.2-61.7)
Gabon	56.9 (51.8-61.9)	57.3 (52.5-62.1)	57.7 (53.3-62.2)	58.1 (54.3-62.1)	58.4 (55.0-62.1)	58.7 (55.7-62.2)	59.8 (57.0-62.9)	60.2 (57.7-63.0)	59.9 (57.6-62.5)	59.7 (57.6-62.1)	59.5 (57.5-61.6)	59.8 (58.0-61.9)

Location	1992	1993	1994	1995	1996	1997	1998	1999	2000	2001	2002	2003
Liberia	51.8 (49.5-54.3)	50.1 (47.5-52.6)	48.9 (45.7-52.1)	50.9 (48.6-53.2)	50.4 (47.7-53.1)	53.8 (51.8-55.6)	54.4 (52.4-56.2)	55.2 (53.3-57.1)	55.8 (53.8-57.8)	56.3 (54.3-58.3)	55.4 (53.3-57.6)	55.2 (52.8-57.7)
Mali	50.8 (49.5-52.2)	51.2 (49.8-52.6)	51.2 (49.8-52.6)	51.4 (50.0-52.7)	51.8 (50.4-53.0)	52.2 (50.8-53.6)	50.9 (48.6-53.0)	53.5 (52.0-55.0)	53.5 (51.9-55.0)	54.1 (52.6-55.8)	54.6 (53.0-56.4)	55.5 (53.6-57.3)
Mauritania	60.4 (58.8-62.2)	60.7 (58.9-62.4)	60.7 (58.9-62.4)	61.2 (59.3-62.9)	61.5 (59.6-63.1)	62.0 (60.2-63.7)	62.5 (60.8-64.2)	63.2 (61.4-65.0)	63.8 (62.0-65.7)	64.2 (62.3-66.1)	64.9 (62.9-66.9)	65.5 (63.4-67.6)
Niger	48.2 (46.6-49.9)	48.6 (46.9-50.3)	49.1 (47.3-50.7)	49.5 (47.7-51.1)	50.2 (48.5-51.8)	50.6 (48.9-52.2)	51.2 (49.4-52.8)	51.8 (50.1-53.5)	52.4 (50.8-54.0)	52.9 (51.3-54.4)	53.7 (52.1-55.3)	54.4 (52.8-56.1)
Nigeria	54.4 (51.2-57.3)	54.5 (51.6-57.1)	54.9 (52.3-57.3)	55.0 (52.6-57.2)	55.0 (52.8-57.1)	55.0 (53.0-57.1)	55.1 (53.1-57.0)	55.2 (53.3-57.1)	55.2 (53.3-57.1)	55.4 (53.5-57.3)	55.3 (53.4-57.2)	55.6 (53.8-57.6)
Sao Tome and Principe	63.6 (61.8-65.5)	63.7 (61.7-65.6)	64.0 (61.9-65.9)	64.1 (62.0-66.1)	63.9 (61.8-65.9)	63.9 (62.0-65.9)	64.0 (62.1-66.0)	64.4 (62.5-66.4)	64.6 (62.8-66.6)	64.9 (63.1-66.9)	65.0 (63.1-66.9)	65.3 (63.4-67.2)
Senegal	58.0 (56.1-60.0)	58.2 (56.2-60.2)	58.2 (56.2-60.1)	58.4 (56.5-60.4)	58.8 (56.8-60.7)	58.9 (56.8-60.8)	59.1 (57.1-61.0)	59.2 (57.1-61.2)	59.7 (57.5-61.7)	60.2 (58.0-62.3)	60.4 (58.0-62.8)	61.3 (58.5-63.9)
Sierra Leone	51.9 (49.1-54.8)	52.1 (49.6-54.8)	51.6 (49.3-54.0)	50.9 (48.7-53.2)	51.9 (49.8-53.9)	51.3 (49.3-53.3)	49.7 (47.3-52.0)	49.2 (46.4-51.9)	51.5 (49.6-53.3)	51.8 (50.2-53.5)	51.6 (50.1-53.3)	51.7 (50.3-53.4)
Togo	57.9 (56.0-60.1)	57.8 (55.9-60.0)	57.6 (55.6-59.8)	57.6 (55.5-59.8)	57.3 (55.1-59.6)	56.9 (54.6-59.4)	56.6 (54.2-59.0)	56.8 (54.4-59.2)	56.7 (54.4-59.3)	56.7 (54.4-59.2)	56.7 (54.4-59.3)	56.4 (54.1-59.1)
Eastern Sub-Saharan Africa	51.3 (50.3-52.1)	51.3 (50.3-52.1)	47.0 (43.7-50.2)	51.1 (50.2-52.0)	51.2 (50.4-52.1)	51.3 (50.5-52.1)	51.5 (50.7-52.3)	51.4 (50.5-52.2)	51.7 (50.8-52.6)	52.6 (51.7-53.5)	53.2 (52.2-54.1)	53.8 (52.8-54.7)
Burundi	45.3 (41.7-49.7)	45.1 (41.9-49.0)	45.0 (42.0-48.5)	44.5 (41.9-47.6)	44.3 (41.9-47.1)	45.0 (42.8-47.6)	45.5 (43.4-47.9)	46.3 (44.1-48.7)	46.7 (44.7-49.0)	48.4 (46.1-50.7)	49.8 (47.6-52.0)	51.3 (49.1-53.6)
Comoros	57.8 (53.0-62.9)	58.2 (53.7-62.8)	58.5 (54.5-62.8)	58.9 (55.3-62.8)	58.4 (55.1-61.7)	59.4 (56.4-62.5)	60.0 (57.4-62.8)	61.4 (58.9-64.0)	62.1 (59.7-64.5)	63.0 (60.6-65.4)	63.8 (61.5-66.1)	64.8 (62.4-67.0)
Djibouti	60.4 (55.8-65.1)	60.7 (55.5-65.7)	60.0 (54.5-65.0)	60.4 (54.1-66.1)	60.3 (53.9-66.5)	60.1 (53.7-66.7)	60.0 (53.1-66.8)	59.9 (52.5-67.0)	60.0 (52.8-67.2)	60.1 (53.4-67.2)	60.2 (53.1-67.1)	60.3 (53.2-67.3)
Eritrea	54.9 (52.8-57.2)	56.2 (53.8-58.8)	57.0 (54.4-60.0)	57.8 (54.7-61.0)	58.4 (55.0-62.3)	59.0 (55.2-63.5)	58.8 (54.7-63.8)	52.2 (46.5-59.1)	37.6 (29.1-50.4)	59.9 (54.3-66.1)	60.0 (54.0-66.4)	59.9 (53.6-66.6)
Ethiopia	47.8 (46.3-49.5)	48.4 (46.9-50.0)	48.8 (47.4-50.4)	49.2 (47.8-50.8)	50.0 (48.5-51.6)	50.6 (49.0-52.3)	51.1 (49.5-52.8)	50.6 (48.9-52.4)	52.1 (50.3-53.9)	52.7 (50.9-54.5)	53.6 (51.8-55.5)	54.3 (52.4-56.2)
Kenya	60.3 (59.0-61.6)	59.5 (58.1-60.8)	58.7 (57.4-60.0)	58.1 (56.8-59.4)	57.4 (56.1-58.7)	56.7 (55.4-58.0)	56.1 (54.8-57.4)	55.8 (54.6-57.1)	55.6 (54.4-56.8)	55.6 (54.4-56.9)	55.7 (54.5-56.9)	55.8 (54.6-57.0)
Madagascar	56.4 (55.2-57.7)	56.3 (55.0-57.7)	56.8 (55.4-58.1)	57.2 (55.7-58.6)	57.6 (56.1-59.0)	58.0 (56.3-59.4)	58.3 (56.7-59.8)	58.8 (57.2-60.4)	59.1 (57.5-60.7)	59.6 (57.9-61.1)	59.8 (58.1-61.4)	60.1 (58.1-61.9)
Malawi	48.3 (45.8-50.9)	48.0 (45.3-51.0)	47.4 (44.7-50.5)	46.9 (44.0-50.0)	46.3 (43.4-49.4)	45.8 (43.0-48.8)	45.3 (42.7-48.2)	45.1 (42.5-48.0)	45.4 (42.8-48.3)	45.9 (43.2-48.7)	46.4 (43.7-49.0)	47.0 (44.2-49.7)
Mozambique	52.1 (50.4-53.6)	52.3 (50.5-53.8)	52.6 (50.6-54.2)	52.7 (50.8-54.4)	52.6 (50.9-54.4)	52.5 (50.7-54.2)	52.6 (50.7-54.4)	52.6 (50.7-54.6)	52.3 (50.3-54.4)	52.8 (50.4-55.2)	52.7 (50.3-55.5)	52.9 (50.3-55.9)
Rwanda	48.0 (46.0-50.0)	46.7 (45.0-48.6)	13.2 (8.5-26.6)	43.8 (42.0-45.9)	44.6 (43.1-46.3)	44.0 (42.5-45.8)	45.6 (44.0-47.2)	47.2 (45.6-49.0)	49.2 (47.5-51.0)	51.0 (49.2-52.9)	53.5 (51.6-55.5)	55.5 (53.5-57.5)
Somalia	48.4 (39.0-59.7)	49.7 (40.0-61.4)	49.7 (40.2-61.4)	49.9 (40.2-61.7)	49.9 (40.2-61.7)	49.6 (40.0-61.4)	50.3 (40.5-62.6)	50.4 (40.6-62.8)	50.7 (40.8-63.2)	50.9 (40.6-63.3)	51.1 (40.5-63.7)	51.5 (40.9-64.2)
South Sudan	51.6 (41.1-61.7)	51.9 (41.3-62.1)	52.3 (41.7-62.5)	52.7 (41.8-62.8)	53.1 (42.1-63.3)	53.4 (42.8-63.7)	53.8 (42.6-64.0)	54.1 (43.0-64.5)	54.4 (43.3-64.8)	54.7 (43.0-65.1)	54.9 (43.6-65.3)	55.0 (43.4-65.5)
Tanzania	54.7 (52.9-56.8)	54.5 (52.7-56.5)	54.0 (52.1-56.1)	53.9 (51.9-56.0)	53.8 (51.7-56.1)	53.8 (51.7-56.3)	53.7 (51.5-56.3)	53.8 (51.6-56.3)	54.3 (52.0-56.7)	54.8 (52.5-57.4)	55.4 (52.9-57.9)	55.9 (53.2-58.6)
Uganda	49.6 (47.1-52.4)	49.0 (46.6-51.8)	48.5 (46.2-51.2)	48.3 (46.1-51.0)	48.2 (46.0-50.8)	48.5 (46.4-50.9)	48.7 (46.7-50.9)	48.9 (47.0-51.0)	49.1 (47.4-51.2)	49.7 (47.9-51.7)	50.3 (48.6-52.4)	51.2 (49.4-53.2)
Zambia	49.6 (47.3-51.9)	48.6 (46.3-50.8)	47.7 (45.3-50.0)	46.9 (44.6-49.2)	46.3 (43.9-48.6)	45.9 (43.7-48.2)	45.4 (43.2-47.7)	45.0 (42.8-47.3)	44.8 (42.8-47.1)	44.9 (42.7-47.1)	45.1 (43.1-47.3)	45.3 (43.4-47.5)
Central Sub-Saharan Africa	52.8 (49.4-55.9)	52.5 (49.2-55.4)	52.8 (49.4-55.5)	52.8 (49.4-55.5)	51.4 (48.2-53.9)	52.0 (48.9-54.7)	52.4 (49.3-55.0)	52.8 (49.7-55.4)	53.2 (49.9-55.8)	53.4 (50.1-55.9)	53.8 (50.4-56.3)	54.1 (50.9-56.7)
Angola	49.6 (39.3-60.9)	48.4 (38.7-59.2)	50.0 (39.7-61.2)	50.9 (40.2-62.6)	51.3 (40.6-62.9)	51.7 (41.3-63.2)	51.8 (41.5-63.1)	51.8 (41.8-63.1)	52.3 (42.1-63.5)	52.5 (42.1-63.5)	53.3 (42.6-64.5)	53.9 (42.8-65.2)
Central African Republic	48.0 (45.6-51.0)	47.5 (44.8-50.9)	47.2 (43.9-50.9)	46.7 (43.3-51.1)	46.3 (42.6-51.3)	45.9 (42.0-51.4)	45.7 (41.6-51.4)	45.5 (41.0-51.7)	45.3 (40.8-51.6)	45.2 (40.4-51.9)	45.2 (40.2-51.8)	45.2 (40.2-52.1)
Congo	54.4 (52.1-57.0)	53.5 (51.2-55.9)	53.2 (51.0-55.7)	52.6 (50.5-55.2)	52.2 (50.1-54.7)	46.3 (41.8-51.4)	49.9 (47.8-52.2)	51.6 (49.5-53.9)	52.5 (50.4-54.8)	53.5 (51.3-55.8)	54.3 (52.2-56.6)	55.0 (52.8-57.2)
Democratic Republic of the Congo	54.3 (51.3-57.6)	54.3 (51.7-57.3)	54.3 (51.8-56.9)	54.0 (51.7-56.4)	51.8 (49.7-53.9)	53.2 (51.2-55.3)	53.5 (51.5-55.6)	54.0 (52.0-56.0)	54.3 (52.3-56.3)	54.5 (52.5-56.4)	54.7 (52.7-56.5)	54.9 (53.1-56.8)
Equatorial Guinea	48.7 (39.0-61.6)	48.6 (38.8-61.5)	48.5 (38.9-61.2)	48.5 (38.7-61.0)	48.9 (39.1-61.0)	50.3 (40.5-62.1)	51.2 (41.3-62.6)	52.0 (41.8-63.1)	52.9 (42.5-63.7)	54.0 (43.4-64.4)	54.9 (43.9-64.9)	55.3 (44.1-65.1)
Gabon	60.2 (58.2-62.3)	60.1 (58.2-62.2)	59.9 (58.0-62.1)	59.8 (57.9-62.0)	59.6 (57.7-61.7)	59.9 (58.0-62.0)	60.0 (58.0-62.1)	59.7 (57.6-61.8)	59.3 (57.1-61.5)	59.0 (56.9-61.2)	59.4 (57.1-61.9)	59.4 (57.0-62.0)

Location	2004	2005	2006	2007	2008	2009	2010	2011	2012	2013	2014	2015
Liberia	57.9 (56.0-60.0)	58.4 (56.5-60.6)	58.8 (56.9-61.0)	59.3 (57.3-61.6)	59.9 (57.8-62.3)	60.3 (58.1-62.9)	60.8 (58.3-63.5)	61.5 (58.6-64.6)	62.3 (58.9-65.6)	62.9 (59.2-66.6)	61.8 (58.1-65.8)	63.2 (58.9-67.4)
Mali	56.2 (54.3-58.1)	56.9 (54.9-58.7)	57.4 (55.4-59.4)	58.0 (56.0-60.1)	58.5 (56.3-60.7)	58.9 (56.6-61.4)	59.2 (56.7-61.9)	59.6 (56.8-62.3)	59.8 (56.4-62.8)	59.8 (56.1-63.2)	60.0 (56.1-63.8)	60.2 (56.0-64.4)
Mauritania	65.9 (63.7-68.0)	66.3 (64.2-68.4)	67.0 (64.8-69.1)	67.5 (65.2-69.6)	67.9 (65.3-70.1)	68.2 (65.3-70.7)	68.6 (65.5-71.5)	68.8 (65.6-72.0)	69.1 (65.4-72.5)	69.3 (65.4-73.1)	69.6 (65.5-73.7)	69.7 (65.4-74.1)
Niger	55.2 (53.5-57.0)	55.9 (54.2-57.8)	56.6 (54.6-58.5)	57.3 (55.1-59.3)	58.0 (55.6-60.0)	58.6 (56.0-60.9)	59.2 (56.5-61.8)	59.6 (56.6-62.6)	60.0 (56.6-63.4)	60.4 (56.6-64.2)	60.7 (56.6-64.9)	61.0 (56.4-65.4)
Nigeria	56.1 (54.2-58.1)	56.8 (54.7-58.9)	57.4 (55.3-59.4)	58.3 (56.0-60.4)	59.2 (56.8-61.4)	60.3 (57.9-62.6)	61.2 (58.7-63.6)	62.0 (58.9-64.5)	62.6 (59.3-65.3)	63.3 (59.5-66.0)	63.7 (59.7-66.6)	64.6 (60.1-67.6)
Sao Tome and Principe	65.4 (63.2-67.4)	65.5 (63.1-67.9)	65.7 (63.0-68.2)	65.7 (62.6-68.7)	66.0 (62.8-69.1)	66.7 (63.0-70.0)	66.9 (62.9-70.6)	67.2 (62.8-71.3)	67.4 (62.7-72.0)	67.6 (62.5-72.5)	67.8 (62.8-73.0)	68.0 (62.7-73.5)
Senegal	61.7 (58.7-64.7)	62.2 (58.9-65.5)	62.5 (58.8-66.2)	63.0 (59.0-67.0)	63.5 (59.0-68.0)	63.9 (59.0-68.7)	64.2 (58.9-69.3)	64.4 (59.0-69.8)	64.6 (58.8-70.2)	64.8 (58.7-70.6)	65.0 (58.8-71.0)	65.3 (59.0-71.5)
Sierra Leone	52.0 (50.6-53.6)	52.3 (50.8-53.9)	52.7 (51.1-54.4)	53.0 (51.3-55.0)	53.6 (51.8-55.7)	54.2 (52.2-56.4)	55.0 (52.8-57.4)	55.6 (53.1-58.4)	56.4 (53.5-59.6)	57.1 (53.7-60.7)	55.2 (51.6-58.8)	57.1 (52.8-61.3)
Togo	56.8 (54.4-59.7)	56.8 (54.4-59.5)	57.5 (55.0-60.2)	57.5 (54.9-60.3)	57.5 (54.9-60.4)	58.0 (55.3-61.1)	58.7 (55.6-61.8)	59.6 (56.3-63.0)	60.3 (56.8-64.1)	60.7 (57.0-64.7)	61.1 (57.1-65.6)	61.5 (57.1-66.1)
Eastern Sub-Saharan Africa	54.5 (53.5-55.4)	55.3 (54.3-56.3)	56.3 (55.1-57.3)	57.3 (56.0-58.4)	58.3 (57.1-59.6)	59.1 (57.7-60.5)	59.8 (58.2-61.2)	60.3 (58.7-61.9)	60.9 (59.1-62.5)	61.4 (59.6-63.2)	61.9 (60.0-63.8)	62.4 (60.4-64.4)
Burundi	53.0 (50.7-55.3)	54.7 (52.4-57.0)	56.4 (54.0-58.9)	57.9 (55.4-60.6)	59.1 (56.3-62.0)	60.0 (56.9-63.3)	60.4 (56.9-64.2)	60.8 (56.8-64.9)	61.1 (56.7-65.8)	61.3 (56.5-66.4)	61.4 (56.2-66.9)	61.4 (55.9-67.1)
Comoros	65.5 (63.1-67.9)	66.0 (63.6-68.4)	66.3 (64.0-68.7)	66.6 (64.3-68.9)	67.1 (64.7-69.4)	66.7 (64.1-69.3)	67.7 (65.0-70.3)	67.2 (64.2-70.3)	67.6 (64.2-70.9)	67.8 (63.7-71.4)	68.0 (63.5-71.8)	68.1 (63.1-72.4)
Djibouti	60.4 (53.3-67.2)	60.7 (53.8-67.5)	60.7 (53.7-67.5)	61.5 (54.4-68.5)	61.8 (54.7-68.7)	62.1 (54.8-69.0)	62.3 (54.8-69.3)	62.5 (55.0-69.5)	62.8 (55.4-69.7)	63.1 (55.7-70.0)	63.1 (55.8-70.0)	63.8 (56.6-70.5)
Eritrea	60.0 (53.6-66.8)	59.9 (53.4-67.0)	60.0 (53.3-67.2)	60.1 (53.2-67.5)	60.1 (53.0-67.5)	60.1 (52.9-67.5)	60.1 (52.8-67.5)	60.2 (52.7-67.9)	60.3 (52.7-67.9)	60.4 (52.8-68.2)	60.6 (52.9-68.6)	60.7 (52.9-68.7)
Ethiopia	55.2 (53.4-57.0)	56.1 (54.4-57.9)	57.2 (55.3-59.3)	58.6 (56.5-60.9)	60.0 (57.6-62.6)	61.2 (58.3-64.3)	62.1 (58.8-65.8)	62.9 (59.4-66.9)	63.6 (59.7-67.9)	64.2 (59.8-68.7)	64.7 (59.9-69.3)	65.2 (60.0-70.1)
Kenya	56.2 (55.0-57.3)	57.2 (56.1-58.4)	58.4 (57.2-59.6)	59.8 (58.6-61.0)	60.8 (59.5-62.0)	61.9 (60.5-63.1)	62.9 (61.6-64.1)	63.7 (62.3-64.9)	64.3 (62.9-65.6)	64.8 (63.3-66.0)	65.0 (63.5-66.3)	65.1 (63.6-66.5)
Madagascar	60.6 (58.4-62.5)	61.2 (58.9-63.5)	61.7 (59.1-64.3)	62.0 (59.1-64.9)	62.2 (59.1-65.5)	62.3 (58.8-66.0)	62.4 (58.5-66.5)	62.6 (58.3-66.9)	62.8 (58.1-67.5)	63.0 (58.1-67.9)	63.3 (57.9-68.8)	63.5 (58.0-69.3)
Malawi	47.5 (44.9-50.3)	48.5 (46.0-51.2)	49.6 (47.2-52.3)	51.0 (48.7-53.6)	52.4 (50.1-55.1)	53.9 (51.5-56.6)	55.4 (52.8-58.1)	56.6 (53.7-59.7)	58.0 (54.6-61.4)	59.3 (55.6-63.1)	60.1 (55.9-64.1)	60.5 (55.7-64.8)
Mozambique	53.0 (50.2-56.1)	52.9 (50.1-56.1)	53.0 (50.3-56.1)	53.2 (50.6-56.3)	54.0 (51.2-57.3)	54.4 (51.3-58.0)	54.4 (51.1-58.5)	54.5 (51.0-58.8)	54.5 (50.8-59.0)	54.8 (50.7-59.5)	56.1 (51.5-61.2)	57.1 (51.9-62.7)
Rwanda	57.6 (55.5-59.6)	59.6 (57.4-61.8)	61.6 (59.3-64.1)	63.1 (60.6-65.8)	64.1 (61.2-67.0)	64.3 (61.2-67.3)	65.1 (61.8-68.4)	65.3 (61.6-68.8)	65.5 (61.3-69.2)	65.6 (60.9-69.8)	65.7 (60.6-70.1)	65.9 (60.3-70.6)
Somalia	51.6 (40.9-64.3)	52.1 (41.1-64.8)	52.2 (41.1-64.8)	52.4 (40.9-64.9)	52.6 (40.8-65.1)	52.8 (41.1-65.0)	51.6 (40.6-63.5)	52.0 (40.1-63.8)	53.4 (41.5-65.5)	54.1 (41.8-66.2)	54.3 (42.0-66.3)	54.2 (42.1-66.0)
South Sudan	55.2 (43.3-65.7)	55.3 (43.1-66.0)	55.4 (43.2-66.1)	55.5 (42.9-66.1)	55.6 (42.7-66.2)	55.8 (42.9-66.2)	56.0 (42.9-66.5)	55.9 (42.7-66.3)	56.0 (42.9-66.5)	56.0 (42.9-66.5)	56.1 (43.1-66.7)	56.1 (43.6-66.7)
Tanzania	56.5 (53.8-59.3)	57.1 (54.3-60.1)	57.9 (54.8-61.0)	59.1 (55.6-62.5)	60.4 (56.4-64.1)	60.8 (56.4-64.8)	61.4 (56.8-65.6)	61.7 (56.9-66.2)	61.9 (56.6-66.6)	62.7 (57.0-67.7)	63.3 (57.3-68.5)	64.1 (57.9-69.4)
Uganda	52.0 (50.2-54.2)	53.5 (51.5-55.6)	54.9 (52.7-57.2)	55.3 (53.0-57.9)	56.0 (53.5-58.8)	57.0 (54.2-60.4)	57.8 (54.5-61.5)	58.6 (55.0-62.8)	59.3 (55.3-63.9)	60.1 (55.7-65.2)	60.7 (55.9-66.4)	61.3 (56.0-67.2)
Zambia	46.3 (44.5-48.3)	47.1 (45.1-49.1)	47.9 (46.0-50.0)	49.0 (46.8-51.2)	51.1 (48.7-53.6)	52.3 (49.7-54.9)	53.1 (50.3-55.9)	53.7 (50.8-56.8)	54.7 (51.5-58.1)	55.6 (52.1-59.6)	56.3 (52.3-60.6)	56.7 (52.3-61.7)
Central Sub-Saharan Africa	54.9 (51.5-57.5)	55.5 (52.0-58.2)	56.0 (52.4-58.7)	56.4 (52.7-59.0)	56.8 (53.1-59.5)	57.3 (53.6-60.1)	57.8 (54.0-60.6)	58.2 (54.4-61.2)	58.5 (54.6-61.7)	58.9 (54.7-62.3)	59.4 (55.1-63.1)	60.0 (55.7-63.8)
Angola	54.5 (43.4-65.8)	55.2 (43.7-66.1)	55.7 (44.1-66.6)	56.2 (44.3-67.0)	56.7 (44.6-67.5)	57.1 (44.7-67.8)	57.6 (44.9-68.3)	58.1 (45.5-68.5)	58.5 (45.6-68.8)	58.8 (45.6-69.2)	59.2 (45.5-69.7)	59.6 (45.9-70.0)
Central African Republic	45.4 (40.3-52.3)	45.5 (40.5-52.7)	45.8 (40.7-52.8)	46.2 (40.8-53.4)	46.8 (41.1-54.1)	47.6 (41.6-54.9)	47.9 (41.9-55.3)	48.1 (42.1-55.6)	48.5 (42.3-56.1)	47.8 (41.7-55.4)	48.2 (42.2-55.7)	49.6 (43.3-57.6)
Congo	55.8 (53.6-58.1)	56.7 (54.2-59.1)	57.8 (55.3-60.3)	58.3 (55.6-60.9)	58.8 (55.9-61.7)	59.8 (56.6-62.9)	60.4 (57.0-63.6)	60.9 (57.2-64.5)	61.3 (57.0-65.3)	61.5 (56.7-65.8)	61.5 (56.3-66.2)	61.5 (56.0-66.7)
Democratic Republic of the Congo	55.9 (54.0-57.8)	56.4 (54.5-58.4)	56.8 (54.9-58.8)	57.1 (55.0-59.1)	57.5 (55.1-59.6)	57.9 (55.4-60.2)	58.4 (55.9-61.0)	58.8 (56.1-61.7)	59.1 (56.2-62.4)	59.6 (56.2-63.2)	60.1 (56.4-64.3)	60.8 (56.6-65.3)
Equatorial Guinea	55.9 (44.4-65.5)	56.0 (44.8-65.2)	56.8 (45.1-66.0)	57.1 (45.2-66.4)	57.5 (45.4-66.8)	58.0 (46.2-67.1)	58.6 (46.8-67.5)	58.7 (46.8-67.6)	59.4 (47.4-68.0)	60.5 (48.2-68.8)	61.0 (48.7-69.2)	61.0 (48.7-69.3)
Gabon	59.4 (56.8-62.0)	60.1 (57.7-62.6)	60.9 (58.4-63.4)	61.6 (59.0-64.1)	61.7 (58.9-64.5)	62.1 (59.0-65.0)	63.0 (59.7-66.1)	63.3 (59.6-66.8)	63.9 (59.7-67.6)	64.4 (59.8-68.1)	65.0 (60.0-69.0)	65.9 (60.4-70.2)

SR Table 6: Estimates of life expectancy at birth by geography (with 95% uncertainty intervals), females, 1980–2015

Location	1980	1981	1982	1983	1984	1985	1986	1987	1988	1989	1990	1991	1992	1993	1994	1995	1996
Global	63.7 (63.3-64.1)	64.1 (63.7-64.4)	64.5 (64.1-64.9)	64.7 (64.3-65.2)	65.1 (64.6-65.5)	65.5 (65.1-65.9)	66.0 (65.6-66.4)	66.3 (65.9-66.7)	66.5 (66.2-66.9)	66.9 (66.5-67.2)	67.1 (66.7-67.4)	67.3 (66.9-67.6)	67.5 (67.2-67.8)	67.6 (67.3-67.9)	67.7 (67.4-68.0)	68.0 (67.7-68.3)	68.3 (68.0-68.5)
High SDI	76.1 (76.1-76.2)	76.4 (76.3-76.5)	76.7 (76.6-76.7)	76.7 (76.7-76.8)	76.9 (76.8-76.9)	77.1 (77.0-77.1)	77.5 (77.4-77.5)	77.7 (77.6-77.7)	77.8 (77.8-77.9)	78.0 (77.9-78.0)	78.1 (78.1-78.2)	78.3 (78.2-78.3)	78.4 (78.3-78.4)	78.1 (78.1-78.2)	78.1 (78.1-78.1)	78.3 (78.3-78.4)	78.7 (78.7-78.8)
High-middle SDI	69.1 (68.5-69.6)	69.3 (68.8-69.9)	69.8 (69.2-70.3)	70.1 (69.6-70.6)	70.5 (70.0-71.0)	70.8 (70.3-71.3)	71.3 (70.8-71.8)	71.5 (71.0-72.0)	71.7 (71.2-72.2)	72.0 (71.5-72.4)	72.2 (71.7-72.6)	72.4 (72.0-72.8)	72.6 (72.2-73.0)	72.8 (72.4-73.2)	73.0 (72.7-73.4)	73.2 (72.9-73.5)	73.4 (73.1-73.7)
Middle SDI	62.4 (61.6-63.2)	62.8 (62.0-63.5)	63.3 (62.5-64.0)	63.8 (63.0-64.5)	64.3 (63.6-65.0)	64.7 (64.0-65.4)	65.1 (64.4-65.8)	65.5 (64.8-66.2)	65.8 (65.1-66.5)	66.2 (65.6-66.9)	66.6 (66.0-67.3)	67.0 (66.3-67.6)	67.3 (66.7-67.9)	67.7 (67.1-68.2)	68.1 (67.5-68.6)	68.4 (67.8-68.9)	68.8 (68.3-69.3)
Low-middle SDI	53.6 (52.5-54.6)	54.1 (53.0-55.0)	54.6 (53.5-55.5)	54.9 (53.9-55.8)	55.4 (54.4-56.3)	55.8 (54.8-56.7)	56.3 (55.4-57.2)	56.7 (55.8-57.5)	57.0 (56.2-57.8)	57.4 (56.6-58.2)	57.7 (56.9-58.5)	57.9 (57.1-58.6)	58.3 (57.6-59.0)	58.6 (57.9-59.2)	58.9 (58.2-59.6)	59.2 (58.5-59.8)	59.4 (58.8-60.1)
Low SDI	49.4 (47.2-51.3)	49.6 (47.5-51.4)	49.9 (48.0-51.6)	49.3 (47.2-51.2)	49.5 (47.5-51.3)	50.6 (49.0-52.0)	51.1 (49.7-52.4)	51.5 (50.2-52.8)	51.9 (50.6-53.0)	52.1 (50.9-53.2)	52.1 (50.9-53.2)	52.3 (51.2-53.4)	52.5 (51.4-53.5)	52.5 (51.6-53.5)	50.9 (49.2-52.4)	52.5 (51.6-53.3)	52.4 (51.5-53.3)
High-income	77.0 (77.0-77.1)	77.3 (77.3-77.4)	77.6 (77.6-77.7)	77.8 (77.8-77.8)	78.1 (78.1-78.1)	78.3 (78.2-78.3)	78.5 (78.5-78.5)	78.7 (78.7-78.7)	78.9 (78.9-78.9)	79.1 (79.1-79.1)	79.3 (79.3-79.3)	79.5 (79.5-79.5)	79.7 (79.7-79.8)	79.8 (79.8-79.9)	80.1 (80.0-80.1)	80.2 (80.1-80.2)	80.4 (80.4-80.4)
High-income North America	77.6 (77.6-77.7)	77.9 (77.9-77.9)	78.2 (78.1-78.2)	78.2 (78.2-78.3)	78.3 (78.3-78.4)	78.4 (78.4-78.4)	78.5 (78.5-78.5)	78.6 (78.5-78.6)	78.6 (78.6-78.6)	78.8 (78.8-78.8)	79.0 (79.0-79.1)	79.1 (79.1-79.2)	79.3 (79.3-79.4)	79.2 (79.2-79.3)	79.3 (79.3-79.3)	79.3 (79.3-79.3)	79.5 (79.5-79.5)
Canada	78.8 (78.7-78.8)	79.1 (79.0-79.1)	79.3 (79.2-79.3)	79.6 (79.5-79.6)	79.8 (79.7-79.8)	79.8 (79.7-79.9)	79.9 (79.9-80.0)	80.1 (80.0-80.1)	80.3 (80.2-80.3)	80.4 (80.4-80.5)	80.6 (80.6-80.7)	80.7 (80.7-80.8)	80.9 (80.8-80.9)	80.9 (80.8-80.9)	80.9 (80.8-81.0)	81.0 (80.9-81.1)	81.1 (81.1-81.2)
Greenland	66.3 (65.5-67.1)	66.3 (65.5-67.1)	66.2 (65.4-66.9)	65.9 (65.2-66.7)	65.7 (64.9-66.5)	66.1 (65.3-66.9)	66.6 (65.7-67.4)	67.0 (66.2-67.8)	67.4 (66.5-68.3)	67.8 (66.9-68.6)	67.9 (67.0-68.7)	68.3 (67.4-69.1)	68.6 (67.6-69.5)	68.8 (67.8-69.8)	69.0 (68.1-69.9)	69.0 (68.2-69.9)	69.0 (68.2-69.9)
United States	77.5 (77.5-77.6)	77.8 (77.8-77.8)	78.1 (78.0-78.1)	78.1 (78.1-78.2)	78.2 (78.2-78.2)	78.2 (78.2-78.3)	78.3 (78.3-78.4)	78.4 (78.4-78.4)	78.4 (78.4-78.5)	78.6 (78.6-78.7)	78.9 (78.8-78.9)	79.0 (78.9-79.0)	79.2 (79.1-79.2)	79.1 (79.0-79.1)	79.1 (79.1-79.1)	79.1 (79.1-79.1)	79.3 (79.3-79.3)
Australasia	77.8 (77.8-77.9)	78.1 (78.0-78.2)	78.2 (78.1-78.2)	78.4 (78.3-78.5)	78.5 (78.5-78.6)	78.5 (78.5-78.6)	78.9 (78.9-79.0)	79.1 (79.0-79.2)	79.3 (79.2-79.3)	79.4 (79.3-79.5)	79.8 (79.8-79.9)	80.2 (80.1-80.2)	80.3 (80.2-80.4)	80.6 (80.6-80.7)	80.7 (80.7-80.8)	81.0 (80.9-81.0)	81.1 (81.1-81.2)
Australia	78.2 (78.1-78.2)	78.4 (78.3-78.5)	78.4 (78.4-78.5)	78.7 (78.6-78.8)	78.8 (78.8-78.9)	78.8 (78.8-78.9)	79.3 (79.2-79.3)	79.4 (79.4-79.5)	79.6 (79.5-79.7)	79.7 (79.6-79.7)	80.1 (80.0-80.2)	80.4 (80.4-80.5)	80.6 (80.5-80.6)	80.9 (80.8-81.0)	81.0 (80.9-81.1)	81.2 (81.2-81.3)	81.4 (81.3-81.5)
New Zealand	76.3 (76.2-76.5)	76.6 (76.5-76.8)	76.9 (76.8-77.1)	76.9 (76.8-77.1)	77.1 (77.0-77.3)	77.2 (77.1-77.4)	77.4 (77.2-77.5)	77.6 (77.4-77.7)	77.7 (77.6-77.9)	78.1 (78.0-78.3)	78.6 (78.5-78.7)	78.8 (78.7-79.0)	79.0 (78.8-79.1)	79.2 (79.1-79.4)	79.5 (79.4-79.7)	79.7 (79.6-79.9)	79.9 (79.7-80.0)
High-income Asia Pacific	77.0 (76.8-77.1)	77.4 (77.3-77.6)	77.8 (77.7-78.0)	78.1 (78.0-78.2)	78.5 (78.4-78.6)	78.9 (78.8-79.0)	79.3 (79.2-79.4)	79.7 (79.6-79.8)	79.9 (79.8-80.0)	80.2 (80.1-80.3)	80.5 (80.4-80.6)	80.8 (80.7-80.9)	81.0 (81.0-81.1)	81.3 (81.3-81.4)	81.7 (81.6-81.8)	81.9 (81.7-82.0)	82.3 (82.3-82.4)
Brunei	72.4 (71.6-73.0)	72.6 (71.8-73.3)	72.7 (72.0-73.5)	73.2 (72.5-73.9)	73.8 (73.2-74.5)	74.3 (73.6-74.9)	74.8 (74.2-75.3)	75.1 (74.5-75.7)	75.5 (74.9-76.1)	75.6 (75.0-76.2)	75.7 (75.1-76.3)	75.8 (75.2-76.3)	75.9 (75.3-76.5)	76.2 (75.5-76.8)	76.4 (75.7-77.1)	76.8 (76.1-77.3)	77.1 (76.5-77.6)
Japan	78.8 (78.8-78.8)	79.2 (79.1-79.2)	79.6 (79.6-79.6)	79.8 (79.8-79.8)	80.2 (80.1-80.2)	80.5 (80.4-80.5)	80.9 (80.8-80.9)	81.2 (81.2-81.2)	81.4 (81.3-81.4)	81.6 (81.6-81.7)	81.8 (81.8-81.9)	82.0 (82.0-82.1)	82.2 (82.2-82.2)	82.4 (82.4-82.5)	82.8 (82.8-82.8)	82.8 (82.6-82.9)	83.3 (83.3-83.3)
Singapore	74.3 (74.1-74.6)	74.5 (74.3-74.7)	74.8 (74.6-75.0)	75.2 (75.0-75.4)	75.6 (75.4-75.8)	75.8 (75.6-76.1)	76.1 (75.9-76.3)	76.3 (76.1-76.5)	76.5 (76.3-76.7)	76.9 (76.7-77.1)	77.4 (77.2-77.7)	78.0 (77.8-78.2)	78.3 (78.1-78.5)	78.4 (78.2-78.6)	78.6 (78.3-78.7)	78.8 (78.5-79.0)	79.1 (78.8-79.3)
South Korea	70.6 (70.1-71.0)	71.2 (70.8-71.6)	71.7 (71.2-72.0)	72.0 (71.7-72.4)	72.6 (72.3-72.9)	73.1 (72.8-73.4)	73.6 (73.3-73.9)	74.0 (73.7-74.3)	74.6 (74.3-74.8)	75.1 (74.8-75.3)	75.6 (75.3-75.8)	76.1 (75.8-76.3)	76.6 (76.3-76.8)	77.0 (76.8-77.3)	77.5 (77.2-77.7)	77.9 (77.7-78.1)	78.4 (78.1-78.6)
Western Europe	77.2 (77.2-77.2)	77.5 (77.5-77.5)	77.7 (77.7-77.8)	77.9 (77.9-77.9)	78.3 (78.2-78.3)	78.4 (78.4-78.5)	78.6 (78.6-78.7)	78.9 (78.9-78.9)	79.1 (79.1-79.1)	79.3 (79.3-79.3)	79.5 (79.5-79.5)	79.7 (79.7-79.7)	79.9 (79.9-79.9)	80.1 (80.1-80.1)	80.3 (80.3-80.3)	80.5 (80.5-80.5)	80.7 (80.7-80.7)
Andorra	84.0 (83.4-84.5)	83.9 (83.4-84.5)	83.9 (83.4-84.4)	84.0 (83.5-84.5)	84.1 (83.6-84.6)	84.2 (83.8-84.7)	84.4 (83.9-84.8)	84.5 (84.1-84.9)	84.7 (84.3-85.1)	84.9 (84.5-85.2)	85.0 (84.7-85.4)	85.2 (84.8-85.6)	85.4 (85.1-85.8)	85.4 (85.1-85.8)	85.5 (85.1-85.8)	85.8 (85.5-86.2)	86.1 (85.8-86.5)
Austria	76.0 (76.0-76.1)	76.3 (76.2-76.4)	76.4 (76.3-76.5)	76.5 (76.4-76.6)	77.1 (77.0-77.2)	77.3 (77.2-77.4)	77.7 (77.6-77.8)	78.1 (78.0-78.2)	78.6 (78.5-78.7)	78.7 (78.6-78.8)	78.8 (78.7-78.9)	79.0 (78.9-79.1)	79.2 (79.1-79.3)	79.3 (79.2-79.4)	79.6 (79.5-79.7)	80.0 (79.9-80.1)	80.1 (80.0-80.2)
Belgium	76.6 (76.5-76.7)	76.9 (76.7-77.0)	76.9 (76.8-77.0)	77.0 (76.9-77.1)	77.6 (77.4-77.7)	78.0 (77.8-78.1)	78.1 (77.9-78.2)	78.5 (78.4-78.6)	78.9 (78.7-79.0)	79.0 (78.8-79.1)	79.2 (79.1-79.3)	79.5 (79.4-79.6)	79.6 (79.4-79.7)	79.8 (79.7-79.9)	79.9 (79.8-80.0)	80.1 (80.0-80.3)	80.4 (80.3-80.5)
Cyprus	77.4 (77.2-77.6)	78.4 (78.2-78.6)	77.8 (77.6-78.0)	78.6 (78.4-78.8)	78.3 (78.0-78.5)	78.5 (78.3-78.7)	79.1 (78.8-79.3)	78.7 (78.5-78.9)	79.2 (79.0-79.5)	78.9 (78.7-79.1)	79.5 (79.3-79.8)	79.7 (79.4-79.9)	79.7 (79.5-79.9)	80.2 (80.0-80.5)	79.5 (79.3-79.7)	79.5 (79.4-79.7)	80.2 (80.0-80.4)
Denmark	77.1 (77.0-77.3)	77.4 (77.3-77.5)	77.7 (77.6-77.8)	77.7 (77.5-77.8)	77.7 (77.6-77.8)	77.6 (77.5-77.7)	77.7 (77.6-77.8)	77.7 (77.6-77.8)	77.9 (77.7-78.0)	77.8 (77.7-77.9)	77.8 (77.7-77.9)	78.1 (78.0-78.2)	78.1 (78.0-78.2)	78.0 (77.9-78.1)	78.1 (77.9-78.2)	78.0 (77.9-78.1)	78.4 (78.3-78.5)
Finland	77.8 (77.7-78.0)	78.1 (77.9-78.2)	78.4 (78.2-78.5)	78.5 (78.4-78.7)	78.5 (78.4-78.6)	78.5 (78.4-78.7)	78.7 (78.6-78.8)	78.7 (78.6-78.8)	78.8 (78.7-78.9)	78.9 (78.8-79.0)	79.0 (78.9-79.2)	79.3 (79.1-79.4)	79.5 (79.3-79.6)	79.7 (79.5-79.8)	80.0 (79.8-80.1)	80.3 (80.1-80.4)	80.4 (80.3-80.5)
France	78.6 (78.6-78.7)	78.9 (78.8-78.9)	79.1 (79.1-79.1)	79.2 (79.2-79.2)	79.6 (79.6-79.7)	79.8 (79.8-79.9)	80.1 (80.1-80.1)	80.5 (80.5-80.5)	80.7 (80.7-80.8)	80.9 (80.9-81.0)	81.2 (81.2-81.3)	81.4 (81.4-81.5)	81.6 (81.6-81.7)	81.7 (81.7-81.8)	82.0 (82.0-82.1)	82.1 (82.1-82.2)	82.3 (82.2-82.3)
Germany	76.1 (76.1-76.2)	76.3 (76.3-76.4)	76.6 (76.6-76.7)	77.0 (76.9-77.0)	77.3 (77.3-77.4)	77.6 (77.5-77.6)	77.8 (77.7-77.8)	78.0 (78.0-78.1)	78.2 (78.2-78.3)	78.4 (78.3-78.4)	78.5 (78.4-78.5)	78.7 (78.7-78.8)	79.1 (79.0-79.1)	79.2 (79.2-79.3)	79.5 (79.5-79.5)	79.7 (79.7-79.8)	80.0 (79.9-80.0)
Greece	77.7 (77.6-77.8)	77.9 (77.8-78.0)	78.2 (78.0-78.3)	78.1 (78.0-78.2)	78.3 (78.2-78.5)	78.4 (78.3-78.5)	78.8 (78.7-78.9)	78.8 (78.7-79.0)	79.3 (79.1-79.4)	79.4 (79.3-79.5)	79.5 (79.4-79.6)	79.8 (79.7-79.9)	79.7 (79.6-79.8)	80.1 (80.0-80.2)	80.4 (80.3-80.5)	80.6 (80.5-80.7)	80.7 (80.6-80.8)
Iceland	79.1 (78.9-79.4)	79.4 (79.2-79.7)	79.6 (79.3-79.9)	79.8 (79.5-80.0)	79.9 (79.6-80.2)	80.0 (79.7-80.3)	80.1 (79.7-80.4)	80.1 (79.7-80.4)	80.2 (79.9-80.5)	80.3 (80.0-80.6)	80.4 (80.1-80.7)	80.6 (80.3-80.8)	80.7 (80.5-81.0)	80.9 (80.6-81.1)	81.0 (80.8-81.3)	80.9 (80.6-81.2)	81.3 (81.0-81.7)
Ireland	77.0 (76.5-77.4)	77.3 (76.8-77.8)	77.5 (77.1-77.9)	77.7 (77.3-78.1)	77.9 (77.5-78.2)	78.1 (77.8-78.4)	78.3 (77.9-78.6)	78.6 (78.3-78.9)	78.6 (78.3-78.9)	78.6 (78.2-79.1)	78.7 (78.2-79.2)	79.3 (79.0-79.7)	79.4 (79.1-79.7)	79.5 (79.2-79.8)	79.5 (79.2-79.8)	79.6 (79.3-79.8)	80.2 (79.8-80.6)
Israel	75.7 (75.5-75.9)	75.8 (75.6-76.0)	76.3 (76.1-76.5)	76.3 (76.1-76.5)	76.4 (76.2-76.6)	76.8 (76.6-77.0)	76.9 (76.7-77.1)	77.2 (77.0-77.4)	77.7 (77.5-77.9)	78.0 (77.8-78.2)	78.3 (78.1-78.4)	78.3 (78.1-78.4)	78.2 (78.1-78.4)	78.9 (78.7-79.0)	79.0 (78.9-79.2)	79.4 (79.2-79.6)	79.6 (79.5-79.8)
Italy	77.5 (77.4-77.6)	77.8 (77.8-77.9)	78.2 (78.1-78.2)	78.2 (78.1-78.2)	78.7 (78.6-78.7)	78.9 (78.9-79.0)	79.1 (79.1-79.2)	79.5 (79.5-79.6)	79.7 (79.7-79.8)	80.1 (80.0-80.1)	80.3 (80.2-80.3)	80.3 (80.3-80.4)	80.6 (80.6-80.6)	80.8 (80.8-80.8)	81.0 (81.0-81.1)	81.2 (81.2-81.3)	81.4 (81.4-81.5)
Luxembourg	76.1 (75.8-76.3)	76.3 (76.0-76.5)	76.5 (76.3-76.7)	76.8 (76.6-77.0)	77.0 (76.8-77.2)	77.3 (77.0-77.5)	77.7 (77.4-77.9)	77.9 (77.6-78.1)	78.0 (77.8-78.3)	78.2 (77.9-78.4)	78.5 (78.2-78.7)	78.8 (78.6-79.1)	79.1 (78.9-79.4)	79.4 (79.2-79.7)	79.8 (79.5-80.0)	80.1 (79.9-80.3)	80.4 (80.2-80.6)
Malta	76.6 (76.2-76.9)	77.4 (77.0-77.7)	76.8 (76.6-77.2)	77.2 (76.8-77.6)	77.8 (77.5-78.1)	78.1 (77.8-78.3)	78.7 (78.4-79.0)	79.3 (78.9-79.6)	79.3 (79.0-79.5)	79.4 (79.1-79.7)	79.6 (79.3-79.9)	79.8 (79.5-80.1)	79.9 (79.6-80.3)	80.3 (80.0-80.7)	80.6 (80.2-81.1)	80.9 (80.5-81.4)	80.9 (80.6-81.3)
Netherlands	79.2 (79.1-79.3)	79.3 (79.2-79.4)	79.4 (79.3-79.5)	79.5 (79.4-79.6)	79.7 (79.6-79.8)	79.7 (79.6-79.8)	79.6 (79.5-79.7)	79.9 (79.8-80.0)	80.2 (80.1-80.3)	79.9 (79.8-80.0)	80.2 (80.1-80.3)	80.1 (80.0-80.2)	80.2 (80.1-80.3)	80.1 (80.0-80.2)	80.2 (80.1-80.3)	80.4 (80.3-80.5)	80.4 (80.3-80.5)
Norway	79.2 (79.0-79.3)	79.2 (79.0-79.4)	79.6 (79.4-79.7)	79.4 (79.3-79.6)	79.7 (79.5-79.9)	79.5 (79.3-79.7)	79.6 (79.4-79.7)	79.6 (79.4-79.7)	79.5 (79.3-79.7)	79.8 (79.6-79.9)	79.9 (79.7-80.1)	80.1 (80.0-80.3)	80.2 (80.1-80.4)	80.5 (80.3-80.6)	80.5 (80.4-80.7)	80.9 (80.8-81.1)	80.9 (80.7-81.0)
Portugal	75.2 (75.1-75.3)	75.5 (75.4-75.6)	76.0 (76.0-76.1)	76.1 (76.0-76.2)	76.3 (76.3-76.4)	76.6 (76.5-76.7)	77.0 (76.9-77.0)	77.3 (77.2-77.4)	77.6 (77.5-77.7)	77.8 (77.8-77.9)	77.7 (77.7-77.8)	77.8 (77.8-77.9)	78.3 (78.3-78.4)	78.4 (78.3-78.4)	79.1 (79.1-79.2)	79.2 (79.1-79.3)	79.3 (79.2-79.4)
Spain	78.5 (78.4-78.5)	78.8 (78.7-78.8)	79.2 (79.2-79.3)	79.2 (79.1-79.2)	79.6 (79.5-79.6)	79.8 (79.7-79.8)	79.8 (79.8-79.9)	80.0 (79.9-80.0)	80.2 (80.1-80.2)	80.4 (80.3-80.4)	80.5 (80.5-80.6)	80.7 (80.7-80.7)	81.1 (81.0-81.1)	81.2 (81.2-81.3)	81.4 (81.4-81.5)	81.6 (81.6-81.7)	81.7 (81.7-81.8)
Sweden	78.8 (78.7-78.9)	79.3 (79.2-79.4)	79.4 (79.3-79.5)	79.7 (79.6-79.9)	79.9 (79.8-80.1)	79.8 (79.7-80.0)	80.1 (80.0-80.2)	80.1 (80.0-80.3)	80.0 (79.9-80.1)	80.5 (80.3-80.6)	80.4 (80.3-80.5)	80.5 (80.4-80.7)	80.8 (80.7-80.9)	81.0 (80.9-81.1)	81.1 (80.9-81.3)	81.5 (81.4-81.6)	81.5 (81.4-81.7)
Switzerland	78.9 (78.7-79.0)	79.2 (79.1-79.4)	79.3 (79.2-79.5)	79.7 (79.6-79.8)	79.9 (79.8-80.1)	80.1 (80.0-80.3)	80.4 (80.3-80.5)	80.7 (80.5-80.8)	80.7 (80.5-80.8)	80.8 (80.6-80.9)	80.9 (80.7-81.0)	81.0 (80.9-81.2)	81.3 (81.1-81.4)	81.4 (81.3-81.6)	81.6 (81.4-81.7)	81.6 (81.4-81.7)	82.0 (81.9-82.1)
United Kingdom	76.5 (76.4-76.6)	76.8 (76.7-76.8)	77.0 (76.9-77.0)	77.2 (77.2-77.3)	77.4 (77.4-77.5)	77.5 (77.5-77.6)	77.7 (77.7-77.7)	77.9 (77.8-77.9)	78.0 (78.0-78.1)	78.2 (78.2-78.2)	78.5 (78.4-78.5)	78.7 (78.6-78.7)	78.9 (78.9-78.9)	79.0 (78.9-79.0)	79.2 (79.2-79.2)	79.3 (79.2-79.3)	79.4 (79.4-79.5)
England	76.7 (76.6-76.8)	77.0 (77.0-77.1)	77.2 (77.2-77.3)	77.5 (77.4-77.5)	77.7 (77.6-77.7)	77.7 (77.7-77.8)	77.9 (77.9-78.0)	78.1 (78.1-78.1)	78.2 (78.2-78.3)	78.4 (78.4-78.4)	78.7 (78.6-78.7)	78.9 (78.8-78.9)	79.1 (79.1-79.1)	79.2 (79.1-79.2)	79.4 (79.4-79.4)	79.5 (79.4-79.5)	79.6 (79.6-79.7)
Northern Ireland	75.4 (75.1-75.6)	75.3 (75.1-75.6)	75.6 (75.4-75.8)	76.1 (75.8-76.3)	76.5 (76.2-76.7)	76.6 (76.4-76.8)	76.9 (76.6-77.1)	77.0 (76.8-77.3)	77.2 (76.9-77.4)	77.5 (77.3-77.8)	77.8 (77.6-78.1)	78.1 (77.9-78.3)	78.4 (78.2-78.6)	78.5 (78.2-78.7)	78.8 (78.6-79.0)	78.9 (78.7-79.1)	79.2 (79.0-79.4)
Scotland	75.2 (75.1-75.3)	75.2 (75.1-75.3)	75.4 (75.2-75.5)	75.7 (75.6-75.8)	75.9 (75.8-76.0)	76.0 (75.9-76.1)	76.2 (76.1-76.4)	76.4 (76.3-76.5)	76.5 (76.4-76.7)	76.6 (76.4-76.7)	76.8 (76.7-77.0)	77.1 (76.9-77.2)	77.3 (77.1-77.4)	77.3 (77.2-77.5)	77.7 (77.5-77.8)	77.7 (77.6-77.9)	77.9 (77.8-78.1)
Wales	75.9 (75.7-76.1)	76.2 (76.0-76.3)	76.5 (76.3-76.7)	76.8 (76.6-76.9)	77.1 (77.0-77.2)	77.1 (77.0-77.3)	77.4 (77.3-77.5)	77.7 (77.5-77.8)	78.0 (77.9-78.1)	78.1 (78.0-78.3)	78.4 (78.3-78.6)	78.6 (78.4-78.7)	78.8 (78.7-78.9)	78.9 (78.7-79.0)	79.1 (78.9-79.2)	79.1 (78.9-79.2)	79.2 (79.1-79.3)
Southern Latin America	73.5 (73.4-73.6)	74.0 (73.9-74.1)	74.6 (74.5-74.7)	74.6 (74.5-74.7)	74.8 (74.7-74.9)	75.3 (75.1-75.4)	75.6 (75.5-75.7)	75.8 (75.7-75.8)	75.9 (75.8-75.9)	76.1 (76.0-76.1)	76.3 (76.2-76.4)	76.6 (76.5-76.6)	76.9 (76.8-77.0)	77.2 (77.1-77.3)	77.5 (77.4-77.6)	77.6 (77.6-77.7)	77.8 (77.8-77.9)
Argentina	73.5 (73.4-73.7)	73.8 (73.7-73.9)	74.4 (74.3-74.5)	74.3 (74.2-74.4)	74.6 (74.5-74.7)	75.1 (75.0-75.2)	75.4 (75.3-75.5)	75.6 (75.5-75.7)	75.7 (75.6-75.8)	75.9 (75.8-76.0)	76.1 (76.0-76.2)	76.3 (76.2-76.4)	76.5 (76.4-76.6)	76.8 (76.7-76.9)	77.2 (77.1-77.2)	77.3 (77.2-77.4)	77.4 (77.3-77.5)
Chile	73.2 (73.0-73.3)	74.2 (74.0-74.3)	74.8 (74.7-74.9)	75.0 (74.9-75.2)	75.2 (75.1-75.3)	75.4 (75.1-75.7)	76.0 (75.8-76.1)	76.1 (76.0-76.3)	76.2 (76.1-76.4)	76.3 (76.2-76.4)	76.5 (76.4-76.7)	77.2 (77.0-77.3)	77.9 (77.7-78.0)	78.1 (78.0-78.3)	78.4 (78.2-78.5)	78.6 (78.5-78.7)	79.0 (78.8-79.1)
Uruguay	74.1 (73.9-74.3)	74.7 (74.5-74.9)	75.1 (74.9-75.3)	75.3 (75.1-75.5)	75.4 (75.2-75.6)	75.5 (75.3-75.7)	75.7 (75.5-75.9)	76.0 (75.8-76.2)	76.3 (76.1-76.5)	76.6 (76.4-76.8)	76.8 (76.6-77.0)	76.4 (76.2-76.6)	77.1 (76.9-77.2)	77.1 (77.0-77.3)	77.3 (77.2-77.5)	77.4 (77.2-77.6)	77.6 (77.4-77.7)
Central Europe, Eastern Europe, and Central Asia	72.4 (72.3-72.5)	72.6 (72.5-72.7)	72.9 (72.8-73.0)	72.8 (72.7-72.9)	72.6 (72.5-72.7)	72.8 (72.7-72.8)	73.5 (73.4-73.5)	73.5 (73.4-73.6)	73.4 (73.3-73.5)	73.6 (73.5-73.7)	73.6 (73.5-73.7)	73.5 (73.5-73.6)	73.2 (73.1-73.3)	72.3 (72.2-72.4)	71.8 (71.7-71.9)	71.9 (71.8-72.0)	72.4 (72.3-72.5)
Eastern Europe	73.2 (73.0-73.3)	73.4 (73.2-73.5)	73.7 (73.6-73.8)	73.5 (73.4-73.6)	73.1 (73.1-73.2)	73.4 (73.3-73.5)	74.4 (74.4-74.5)	74.5 (74.4-74.6)	74.5 (74.4-74.6)	74.5 (74.4-74.6)	74.4 (74.3-74.5)	74.3 (74.2-74.4)	73.9 (73.8-73.9)	72.4 (72.4-72.5)	71.6 (71.5-71.7)	71.8 (71.7-71.8)	72.5 (72.4-72.6)
Belarus	75.2 (74.8-75.6)	75.4 (75.0-75.8)	75.4 (75.0-75.8)	75.3 (74.9-75.6)	75.0 (74.6-75.4)	75.0 (74.7-75.4)	75.5 (75.2-75.9)	75.5 (75.2-75.8)	75.5 (75.2-75.9)	75.5 (75.1-75.8)	75.2 (74.8-75.5)	75.0 (74.6-75.3)	74.8 (74.4-75.1)	74.3 (74.0-74.6)	74.2 (73.8-74.5)	73.9 (73.6-74.2)	73.9 (73.6-74.2)
Estonia	74.4 (74.2-74.5)	74.1 (73.9-74.3)	74.6 (74.4-74.7)	74.6 (74.4-74.8)	74.3 (74.2-74.5)	74.4 (74.2-74.6)	75.3 (75.1-75.4)	75.5 (75.3-75.7)	75.3 (75.1-75.5)	74.5 (74.3-74.7)	75.1 (75.0-75.3)	74.8 (74.6-75.0)	74.9 (74.7-75.1)	74.2 (74.0-74.4)	72.7 (72.5-72.9)	74.1 (73.9-74.3)	75.5 (75.3-75.7)
Latvia	74.1 (73.9-74.2)	74.1 (73.9-74.3)	74.6 (74.4-74.7)	74.4 (74.2-74.5)	74.3 (74.1-74.4)	74.2 (74.1-74.4)	75.1 (75.0-75.3)	75.1 (74.9-75.2)	75.3 (75.1-75.4)	74.8 (74.6-74.9)	74.6 (74.4-74.8)	74.5 (74.4-74.7)	74.2 (74.1-74.4)	73.2 (73.1-73.4)	72.6 (72.4-72.8)	73.0 (72.9-73.2)	74.5 (74.3-74.6)
Lithuania	75.8 (75.7-75.9)	75.6 (75.5-75.7)	75.8 (75.7-76.0)	75.9 (75.7-76.0)	75.5 (75.3-75.6)	75.4 (75.3-75.6)	76.5 (76.4-76.6)	76.4 (76.3-76.5)	76.5 (76.3-76.6)	76.1 (76.0-76.3)	76.3 (76.2-76.5)	75.7 (75.6-75.8)	75.8 (75.7-75.9)	75.1 (74.9-75.2)	74.8 (74.7-74.9)	75.0 (74.9-75.1)	75.9 (75.8-76.0)
Moldova	68.4 (67.6-69.1)	68.7 (68.0-69.3)	69.0 (68.3-69.5)	69.2 (68.6-69.8)	69.4 (68.8-69.9)	69.0 (68.5-69.4)	70.5 (70.1-71.0)	71.2 (70.7-71.5)	71.4 (70.9-71.7)	71.6 (71.2-71.9)	71.6 (71.2-71.9)	70.7 (70.4-71.0)	71.3 (70.9-71.7)	70.8 (70.4-71.1)	69.6 (69.2-69.9)	69.3 (68.9-69.6)	69.9 (69.4-70.2)
Russia	73.0 (73.0-73.1)	73.3 (73.2-73.4)	73.6 (73.6-73.7)	73.3 (73.2-73.3)	72.9 (72.8-73.0)	73.2 (73.1-73.3)	74.3 (74.2-74.4)	74.4 (74.3-74.4)	74.4 (74.3-74.5)	74.4 (74.3-74.5)	74.4 (74.3-74.4)	74.4 (74.3-74.5)	73.8 (73.8-73.9)	72.0 (71.9-72.0)	70.9 (70.9-71.0)	71.4 (71.4-71.5)	72.4 (72.3-72.4)
Ukraine	73.4 (72.9-73.8)	73.5 (73.0-73.9)	73.7 (73.3-74.1)	73.9 (73.5-74.2)	73.6 (73.3-73.9)	73.8 (73.5-74.1)	74.8 (74.5-75.1)	74.7 (74.4-75.0)	74.7 (74.4-75.0)	74.7 (74.4-75.0)	74.5 (74.2-74.8)	74.1 (73.9-74.4)	73.9 (73.6-74.1)	73.4 (73.1-73.6)	72.8 (72.6-73.1)	72.2 (71.9-72.4)	72.4 (72.1-72.6)
Central Europe	73.6 (73.5-73.7)	73.9 (73.8-74.0)	74.1 (74.0-74.2)	74.1 (74.0-74.2)	74.2 (74.1-74.3)	74.2 (74.1-74.2)	74.4 (74.3-74.5)	74.4 (74.3-74.4)	74.6 (74.6-74.7)	74.7 (74.7-74.8)	74.9 (74.8-74.9)	74.9 (74.9-75.0)	75.0 (75.0-75.1)	75.2 (75.1-75.3)	75.3 (75.2-75.3)	75.4 (75.3-75.4)	75.6 (75.6-75.7)
Albania	74.6 (73.4-75.6)	74.8 (73.7-75.8)	75.0 (74.0-75.9)	75.1 (74.2-75.9)	75.4 (74.6-76.1)	75.7 (75.0-76.4)	76.1 (75.5-76.6)	76.1 (75.6-76.6)	76.0 (75.5-76.5)	76.0 (75.5-76.5)	76.7 (76.3-77.1)	77.0 (76.6-77.5)	77.2 (76.7-77.6)	77.2 (76.8-77.7)	77.3 (76.9-77.8)	77.5 (77.0-77.9)	77.7 (77.2-78.1)
Bosnia and Herzegovina	73.6 (73.1-74.1)	73.9 (73.4-74.3)	74.2 (73.8-74.6)	74.5 (74.2-74.8)	74.8 (74.5-75.1)	74.9 (74.6-75.2)	75.1 (74.8-75.4)	75.4 (75.1-75.7)	75.8 (75.5-76.1)	76.1 (75.8-76.4)	76.3 (76.1-76.6)	76.3 (76.1-76.6)	74.0 (73.3-74.8)	74.1 (73.3-75.0)	75.5 (74.6-76.3)	73.6 (72.9-74.3)	77.1 (76.8-77.4)
Bulgaria	74.0 (73.9-74.1)	74.2 (74.1-74.3)	74.2 (74.0-74.3)	74.4 (74.3-74.5)	74.4 (74.3-74.5)	74.5 (74.3-74.6)	74.7 (74.6-74.8)	74.7 (74.6-74.8)	74.7 (74.6-74.8)	74.8 (74.7-74.9)	74.8 (74.7-74.9)	74.7 (74.6-74.8)	74.8 (74.7-74.9)	75.0 (74.9-75.1)	74.8 (74.7-75.0)	74.8 (74.7-75.0)	74.6 (74.5-74.7)
Croatia	74.4 (73.8-75.1)	74.7 (74.1-75.3)	74.9 (74.4-75.4)	75.1 (74.7-75.5)	75.3 (75.0-75.6)	75.4 (75.2-75.6)	75.5 (75.3-75.6)	75.6 (75.4-75.7)	75.8 (75.7-75.9)	76.1 (75.9-76.2)	76.4 (76.3-76.6)	76.2 (75.8-76.6)	76.5 (76.2-76.8)	76.8 (76.6-77.0)	77.1 (77.0-77.3)	77.0 (76.7-77.2)	77.1 (77.0-77.3)
Czech Republic	74.2 (74.1-74.3)	74.3 (74.2-74.4)	74.4 (74.3-74.5)	74.4 (74.3-74.5)	74.5 (74.4-74.7)	74.7 (74.6-74.8)	74.9 (74.8-75.0)	75.1 (75.0-75.2)	75.3 (75.2-75.4)	75.4 (75.4-75.5)	75.6 (75.5-75.7)	75.8 (75.7-75.9)	76.1 (76.0-76.2)	76.3 (76.3-76.4)	76.6 (76.5-76.7)	77.0 (76.9-77.1)	77.3 (77.2-77.4)
Hungary	72.9 (72.8-73.0)	73.0 (72.9-73.1)	73.1 (72.9-73.2)	73.1 (73.0-73.2)	73.1 (73.0-73.2)	73.1 (73.0-73.2)	73.3 (73.2-73.4)	73.6 (73.5-73.7)	73.8 (73.7-74.0)	73.9 (73.8-74.0)	73.9 (73.8-74.0)	73.8 (73.7-73.9)	73.9 (73.8-74.0)	74.1 (74.0-74.2)	74.4 (74.3-74.5)	74.8 (74.7-74.9)	75.1 (75.0-75.2)
Macedonia	72.9 (71.5-74.1)	73.0 (72.1-73.7)	72.9 (72.6-73.3)	73.3 (72.7-73.9)	73.8 (72.8-74.6)	74.0 (73.0-74.9)	74.1 (73.1-75.1)	74.1 (73.2-74.8)	73.9 (73.4-74.4)	73.9 (73.7-74.2)	74.1 (73.9-74.3)	74.1 (73.9-74.3)	74.0 (73.8-74.2)	74.1 (73.9-74.3)	74.2 (74.0-74.4)	74.2 (74.0-74.4)	74.5 (74.3-74.7)

Location	1997	1998	1999	2000	2001	2002	2003	2004	2005	2006	2007	2008	2009	2010	2011	2012	2013	2014	2015
Global	68.5 (68.3-68.8)	68.8 (68.5-69.0)	68.9 (68.7-69.1)	69.1 (68.9-69.4)	69.4 (69.1-69.6)	69.6 (69.4-69.8)	70.0 (69.7-70.2)	70.3 (70.1-70.5)	70.7 (70.5-71.0)	71.2 (71.0-71.5)	71.7 (71.5-72.0)	72.1 (71.9-72.4)	72.6 (72.4-72.9)	72.9 (72.6-73.2)	73.4 (73.1-73.8)	73.9 (73.5-74.2)	74.2 (73.9-74.6)	74.5 (74.1-74.9)	74.8 (74.4-75.2)
High SDI	79.1 (79.1-79.1)	79.3 (79.3-79.3)	79.2 (79.2-79.3)	79.4 (79.3-79.4)	79.5 (79.5-79.5)	79.6 (79.6-79.6)	79.8 (79.7-79.8)	80.1 (80.1-80.1)	80.3 (80.2-80.3)	80.7 (80.6-80.7)	81.0 (80.9-81.0)	81.1 (81.1-81.2)	81.4 (81.4-81.4)	81.6 (81.6-81.6)	81.8 (81.8-81.9)	82.0 (82.0-82.0)	82.1 (82.1-82.2)	82.2 (82.2-82.3)	82.3 (82.3-82.4)
High-middle SDI	73.6 (73.4-73.9)	73.9 (73.6-74.1)	74.0 (73.7-74.2)	74.2 (74.0-74.4)	74.4 (74.1-74.6)	74.5 (74.3-74.7)	74.7 (74.4-74.9)	74.9 (74.7-75.2)	75.3 (75.0-75.5)	75.7 (75.4-75.9)	76.0 (75.8-76.3)	76.4 (76.1-76.6)	76.8 (76.5-77.0)	77.2 (77.0-77.5)	77.8 (77.5-78.0)	78.3 (78.0-78.5)	78.6 (78.4-78.9)	78.9 (78.6-79.1)	79.1 (78.8-79.4)
Middle SDI	69.1 (68.6-69.6)	69.4 (69.0-69.9)	69.7 (69.3-70.1)	70.0 (69.5-70.4)	70.2 (69.8-70.6)	70.5 (70.1-70.9)	71.0 (70.7-71.4)	71.3 (70.9-71.6)	71.8 (71.5-72.2)	72.3 (72.0-72.7)	72.8 (72.5-73.1)	73.1 (72.8-73.5)	73.6 (73.3-73.9)	73.9 (73.6-74.3)	74.3 (74.0-74.7)	74.7 (74.3-75.1)	75.1 (74.7-75.5)	75.3 (74.9-75.8)	75.7 (75.2-76.2)
Low-middle SDI	59.7 (59.1-60.3)	60.0 (59.4-60.6)	60.3 (59.7-60.9)	60.6 (60.0-61.2)	61.0 (60.4-61.5)	61.3 (60.7-61.9)	61.7 (61.1-62.3)	62.2 (61.5-62.8)	62.6 (62.0-63.2)	63.2 (62.6-63.8)	63.8 (63.2-64.4)	64.2 (63.6-64.9)	64.9 (64.3-65.6)	65.1 (64.4-65.8)	65.9 (65.2-66.6)	66.4 (65.6-67.2)	66.8 (66.0-67.6)	67.3 (66.4-68.1)	67.7 (66.8-68.6)
Low SDI	52.7 (51.8-53.6)	52.8 (52.0-53.7)	53.0 (52.2-53.9)	53.3 (52.4-54.2)	53.8 (52.9-54.7)	54.3 (53.3-55.2)	54.9 (53.9-55.8)	55.5 (54.5-56.5)	56.2 (55.2-57.2)	57.0 (55.9-58.0)	57.7 (56.6-58.8)	58.5 (57.3-59.7)	59.2 (57.9-60.5)	59.8 (58.4-61.2)	60.4 (58.9-61.8)	60.9 (59.3-62.4)	61.4 (59.6-63.0)	61.8 (59.9-63.6)	62.3 (60.2-64.3)
High-income	80.6 (80.6-80.7)	80.8 (80.8-80.8)	80.9 (80.9-80.9)	81.1 (81.1-81.1)	81.3 (81.3-81.3)	81.4 (81.4-81.5)	81.6 (81.6-81.6)	81.9 (81.9-81.9)	82.1 (82.1-82.1)	82.3 (82.3-82.3)	82.5 (82.5-82.5)	82.6 (82.6-82.6)	82.8 (82.8-82.8)	83.0 (83.0-83.0)	83.1 (83.0-83.1)	83.2 (83.2-83.2)	83.3 (83.2-83.3)	83.3 (83.3-83.4)	83.4 (83.4-83.5)
High-income North America	79.6 (79.6-79.7)	79.8 (79.8-79.8)	79.7 (79.7-79.8)	79.8 (79.8-79.8)	79.8 (79.7-79.8)	79.9 (79.9-79.9)	80.0 (80.0-80.1)	80.3 (80.3-80.4)	80.4 (80.4-80.5)	80.6 (80.6-80.6)	80.9 (80.8-80.9)	81.0 (81.0-81.1)	81.2 (81.2-81.2)	81.5 (81.4-81.5)	81.5 (81.5-81.5)	81.6 (81.6-81.6)	81.6 (81.6-81.7)	81.7 (81.6-81.7)	81.8 (81.7-81.8)
Canada	81.3 (81.2-81.3)	81.4 (81.4-81.5)	81.6 (81.6-81.7)	81.8 (81.7-81.8)	82.0 (81.9-82.0)	82.0 (82.0-82.1)	82.2 (82.2-82.3)	82.4 (82.4-82.5)	82.5 (82.5-82.6)	82.8 (82.7-82.8)	82.9 (82.8-83.0)	83.0 (83.0-83.1)	83.3 (83.2-83.3)	83.5 (83.4-83.5)	83.6 (83.6-83.7)	83.7 (83.6-83.7)	83.7 (83.6-83.8)	83.8 (83.6-84.0)	83.9 (83.7-84.1)
Greenland	68.9 (68.1-69.7)	69.2 (68.5-70.0)	69.5 (68.7-70.3)	69.8 (69.0-70.7)	69.9 (69.1-70.9)	70.3 (69.5-71.3)	70.6 (69.7-71.5)	70.8 (70.0-71.7)	71.1 (70.2-71.9)	71.5 (70.7-72.4)	72.0 (71.2-72.8)	72.3 (71.5-73.1)	72.6 (71.8-73.4)	73.1 (72.3-73.9)	73.7 (72.8-74.4)	74.2 (73.3-74.8)	74.5 (73.7-75.2)	74.8 (73.8-75.5)	74.9 (73.8-75.7)
United States	79.5 (79.5-79.5)	79.6 (79.6-79.6)	79.5 (79.5-79.6)	79.6 (79.6-79.6)	79.6 (79.5-79.6)	79.7 (79.7-79.7)	79.8 (79.8-79.8)	80.1 (80.1-80.2)	80.2 (80.2-80.2)	80.4 (80.4-80.4)	80.6 (80.6-80.7)	80.8 (80.8-80.8)	81.0 (80.9-81.0)	81.2 (81.2-81.3)	81.2 (81.2-81.3)	81.4 (81.3-81.4)	81.4 (81.4-81.4)	81.5 (81.4-81.5)	81.5 (81.4-81.6)
Australasia	81.4 (81.3-81.4)	81.7 (81.6-81.7)	81.9 (81.8-82.0)	82.1 (82.1-82.2)	82.4 (82.3-82.4)	82.5 (82.5-82.6)	82.8 (82.7-82.8)	83.0 (82.9-83.1)	83.3 (83.2-83.3)	83.4 (83.3-83.4)	83.5 (83.4-83.5)	83.6 (83.6-83.7)	83.8 (83.7-83.8)	84.0 (83.9-84.0)	84.1 (84.0-84.1)	84.2 (84.2-84.3)	84.3 (84.2-84.3)	84.3 (84.2-84.4)	84.3 (84.2-84.4)
Australia	81.6 (81.5-81.7)	81.9 (81.9-82.0)	82.2 (82.1-82.2)	82.4 (82.3-82.4)	82.7 (82.6-82.7)	82.8 (82.7-82.9)	83.1 (83.0-83.1)	83.3 (83.2-83.4)	83.5 (83.5-83.6)	83.7 (83.6-83.7)	83.8 (83.7-83.8)	83.9 (83.8-84.0)	84.0 (84.0-84.1)	84.2 (84.2-84.3)	84.4 (84.3-84.4)	84.5 (84.5-84.6)	84.5 (84.4-84.6)	84.5 (84.4-84.6)	84.5 (84.4-84.6)
New Zealand	80.2 (80.1-80.4)	80.5 (80.4-80.6)	80.6 (80.5-80.8)	80.9 (80.8-81.0)	80.9 (80.8-81.0)	81.1 (81.0-81.2)	81.4 (81.2-81.5)	81.5 (81.4-81.7)	81.8 (81.7-81.9)	81.9 (81.8-82.1)	82.1 (82.0-82.2)	82.3 (82.1-82.4)	82.4 (82.3-82.5)	82.6 (82.5-82.7)	82.6 (82.5-82.8)	82.9 (82.8-83.0)	83.1 (83.0-83.3)	83.2 (83.0-83.4)	83.3 (83.0-83.5)
High-income Asia Pacific	82.6 (82.6-82.7)	82.8 (82.8-82.9)	83.1 (83.0-83.1)	83.5 (83.4-83.5)	83.8 (83.8-83.9)	84.1 (84.1-84.2)	84.4 (84.3-84.4)	84.6 (84.5-84.6)	84.7 (84.7-84.8)	84.9 (84.9-85.0)	85.1 (85.1-85.2)	85.3 (85.3-85.3)	85.6 (85.5-85.6)	85.6 (85.6-85.7)	85.6 (85.4-85.8)	85.8 (85.7-85.8)	85.8 (85.7-85.9)	85.9 (85.8-86.0)	86.0 (85.8-86.0)
Brunei	77.5 (77.0-77.8)	77.8 (77.4-78.1)	77.9 (77.7-78.3)	78.1 (77.8-78.5)	78.3 (78.0-78.9)	78.6 (78.1-79.0)	78.8 (78.2-79.2)	78.8 (78.4-79.2)	79.0 (78.5-79.3)	79.0 (78.6-79.4)	79.1 (78.6-79.4)	79.2 (78.8-79.5)	79.2 (78.9-79.5)	79.3 (79.0-79.6)	79.2 (78.9-79.5)	79.1 (78.8-79.5)	79.2 (78.7-79.6)	79.3 (78.8-79.7)	79.4 (78.8-79.9)
Japan	83.6 (83.5-83.6)	83.7 (83.7-83.8)	83.9 (83.9-84.0)	84.3 (84.3-84.3)	84.6 (84.6-84.7)	84.9 (84.9-85.0)	85.1 (85.1-85.2)	85.3 (85.2-85.3)	85.3 (85.3-85.4)	85.5 (85.5-85.6)	85.7 (85.7-85.7)	85.8 (85.8-85.8)	86.1 (86.0-86.1)	86.1 (86.1-86.1)	86.0 (85.6-86.2)	86.2 (86.1-86.2)	86.2 (86.2-86.2)	86.3 (86.2-86.3)	86.3 (86.3-86.4)
Singapore	79.4 (79.2-79.6)	79.8 (79.6-80.0)	80.1 (79.9-80.3)	80.5 (80.3-80.7)	80.8 (80.7-81.1)	81.2 (81.0-81.4)	81.6 (81.4-81.8)	82.0 (81.8-82.2)	82.2 (82.0-82.4)	82.3 (82.2-82.5)	82.5 (82.3-82.7)	82.8 (82.6-83.0)	83.1 (83.0-83.3)	83.4 (83.2-83.6)	83.5 (83.3-83.7)	83.6 (83.4-83.7)	83.7 (83.5-83.9)	83.9 (83.7-84.1)	84.0 (83.7-84.4)
South Korea	78.8 (78.6-79.0)	79.1 (78.9-79.3)	79.5 (79.3-79.6)	79.9 (79.7-80.0)	80.2 (80.0-80.4)	80.6 (80.4-80.8)	81.1 (80.9-81.2)	81.5 (81.3-81.7)	82.0 (81.8-82.1)	82.3 (82.1-82.5)	82.7 (82.5-82.8)	83.0 (82.9-83.2)	83.4 (83.2-83.5)	83.6 (83.4-83.8)	83.8 (83.6-84.1)	84.0 (83.8-84.2)	84.2 (83.9-84.4)	84.2 (83.9-84.5)	84.3 (83.9-84.6)
Western Europe	81.0 (80.9-81.0)	81.1 (81.1-81.1)	81.3 (81.3-81.3)	81.5 (81.5-81.5)	81.7 (81.7-81.7)	81.9 (81.8-81.9)	82.0 (82.0-82.0)	82.4 (82.4-82.4)	82.6 (82.6-82.6)	82.8 (82.8-82.9)	83.0 (83.0-83.0)	83.1 (83.1-83.1)	83.3 (83.2-83.3)	83.5 (83.5-83.5)	83.6 (83.6-83.6)	83.7 (83.7-83.7)	83.8 (83.7-83.8)	83.9 (83.8-83.9)	84.0 (83.9-84.0)
Andorra	86.4 (86.1-86.8)	86.7 (86.3-87.0)	86.9 (86.6-87.2)	87.2 (86.9-87.5)	87.4 (87.1-87.7)	87.6 (87.3-87.9)	87.8 (87.5-88.1)	87.7 (87.4-88.0)	87.9 (87.6-88.2)	88.0 (87.7-88.3)	88.1 (87.8-88.4)	88.1 (87.8-88.4)	88.0 (87.8-88.3)	88.2 (87.9-88.4)	88.2 (87.9-88.5)	88.3 (88.0-88.5)	88.3 (88.0-88.6)	88.4 (88.1-88.7)	88.4 (88.1-88.7)
Austria	80.7 (80.6-80.8)	80.8 (80.7-80.9)	80.8 (80.8-80.9)	81.0 (80.9-81.1)	81.4 (81.4-81.5)	81.6 (81.6-81.7)	81.6 (81.5-81.7)	82.2 (82.1-82.2)	82.2 (82.1-82.3)	82.7 (82.6-82.7)	82.8 (82.7-82.8)	82.9 (82.9-83.0)	83.0 (82.9-83.0)	83.2 (83.1-83.3)	83.2 (83.1-83.3)	83.4 (83.3-83.5)	83.6 (83.5-83.7)	83.6 (83.4-83.7)	83.7 (83.5-83.8)
Belgium	80.5 (80.4-80.6)	80.5 (80.4-80.7)	80.9 (80.8-81.0)	80.9 (80.8-81.0)	81.0 (80.9-81.1)	81.2 (81.1-81.3)	81.4 (81.3-81.5)	81.8 (81.7-81.9)	82.0 (81.9-82.1)	82.3 (82.1-82.4)	82.1 (82.0-82.2)	82.3 (82.2-82.5)	82.4 (82.3-82.5)	82.6 (82.5-82.7)	82.6 (82.5-82.7)	82.8 (82.7-83.0)	83.0 (82.7-83.2)	83.1 (82.8-83.4)	83.2 (82.8-83.6)
Cyprus	80.1 (80.0-80.3)	80.4 (80.2-80.6)	80.6 (80.4-80.8)	80.8 (80.6-81.0)	81.7 (81.5-81.9)	81.7 (81.5-81.9)	81.9 (81.7-82.1)	82.2 (82.0-82.4)	82.1 (82.0-82.3)	82.7 (82.6-82.9)	83.3 (83.1-83.5)	83.1 (82.9-83.3)	83.7 (83.4-83.9)	83.9 (83.7-84.1)	84.2 (84.0-84.4)	84.7 (84.5-84.9)	84.9 (84.6-85.1)	85.0 (84.7-85.2)	85.0 (84.8-85.3)
Denmark	78.5 (78.4-78.6)	79.1 (79.0-79.2)	78.6 (78.5-78.8)	79.4 (79.2-79.5)	79.4 (79.3-79.5)	79.5 (79.4-79.6)	79.9 (79.7-80.0)	80.1 (80.0-80.2)	80.5 (80.4-80.6)	80.6 (80.5-80.7)	80.6 (80.5-80.8)	81.0 (80.8-81.1)	81.2 (81.1-81.3)	81.4 (81.3-81.6)	81.9 (81.8-82.1)	82.2 (82.0-82.3)	82.3 (82.1-82.5)	82.4 (82.1-82.6)	82.4 (82.2-82.7)
Finland	80.6 (80.5-80.7)	80.8 (80.7-81.0)	81.0 (80.9-81.1)	81.2 (81.1-81.3)	81.4 (81.3-81.6)	81.6 (81.5-81.7)	81.8 (81.6-81.9)	82.1 (82.0-82.3)	82.4 (82.3-82.6)	82.6 (82.5-82.7)	82.8 (82.6-82.9)	83.0 (82.8-83.1)	83.1 (83.0-83.3)	83.2 (83.1-83.4)	83.4 (83.2-83.5)	83.5 (83.3-83.6)	83.7 (83.5-83.8)	83.8 (83.6-84.0)	83.9 (83.6-84.1)
France	82.5 (82.5-82.5)	82.5 (82.4-82.5)	82.6 (82.5-82.6)	82.8 (82.8-82.8)	82.9 (82.9-83.0)	83.1 (83.0-83.1)	83.2 (83.1-83.2)	83.7 (83.6-83.7)	83.9 (83.9-83.9)	84.1 (84.1-84.2)	84.4 (84.3-84.4)	84.4 (84.3-84.4)	84.4 (84.4-84.5)	84.6 (84.6-84.7)	84.8 (84.8-84.9)	84.8 (84.8-84.9)	84.9 (84.8-85.0)	85.1 (84.9-85.2)	85.2 (85.0-85.3)
Germany	80.3 (80.2-80.3)	80.5 (80.5-80.6)	80.7 (80.7-80.8)	81.0 (81.0-81.0)	81.2 (81.2-81.2)	81.4 (81.3-81.4)	81.5 (81.5-81.6)	81.9 (81.8-81.9)	82.1 (82.0-82.1)	82.3 (82.2-82.3)	82.4 (82.4-82.5)	82.5 (82.5-82.5)	82.6 (82.6-82.6)	82.7 (82.7-82.8)	82.9 (82.8-82.9)	83.0 (83.0-83.1)	83.0 (83.0-83.1)	83.1 (83.0-83.2)	83.2 (83.1-83.3)
Greece	81.0 (80.9-81.1)	81.0 (80.9-81.1)	81.1 (81.0-81.2)	81.5 (81.4-81.6)	81.7 (81.7-81.8)	81.7 (81.7-81.8)	82.0 (81.9-82.1)	82.1 (82.0-82.2)	82.3 (82.2-82.4)	82.7 (82.6-82.8)	82.6 (82.5-82.7)	83.0 (82.9-83.1)	83.1 (83.0-83.1)	83.1 (83.0-83.2)	83.2 (83.1-83.3)	83.2 (83.1-83.3)	83.5 (83.4-83.6)	83.5 (83.3-83.7)	83.5 (83.2-83.7)
Iceland	81.6 (81.2-81.9)	81.8 (81.4-82.2)	82.0 (81.7-82.4)	82.3 (81.9-82.7)	82.6 (82.2-83.0)	82.9 (82.4-83.3)	83.1 (82.7-83.5)	83.4 (83.0-83.8)	83.6 (83.3-84.1)	83.9 (83.5-84.3)	84.3 (83.9-84.8)	84.7 (84.2-85.2)	84.9 (84.5-85.4)	85.3 (84.8-85.7)	85.3 (84.8-85.8)	85.5 (85.1-86.0)	85.7 (85.2-86.1)	85.7 (85.3-86.2)	85.8 (85.4-86.3)
Ireland	79.9 (79.5-80.3)	80.4 (80.0-80.7)	80.2 (79.8-80.6)	80.2 (79.8-80.5)	80.5 (80.3-80.8)	81.3 (80.9-81.6)	81.3 (81.0-81.7)	81.6 (81.3-81.9)	81.8 (81.5-82.1)	82.0 (81.6-82.3)	82.6 (82.3-83.0)	82.4 (82.0-82.8)	82.7 (82.3-83.0)	83.8 (83.3-84.2)	83.2 (82.8-83.6)	83.5 (83.1-83.9)	84.1 (83.6-84.5)	84.2 (83.6-84.7)	84.3 (83.6-85.0)
Israel	80.0 (79.8-80.1)	80.3 (80.2-80.5)	80.4 (80.3-80.6)	80.8 (80.6-81.0)	81.0 (80.8-81.1)	80.9 (80.8-81.1)	81.2 (81.1-81.4)	81.7 (81.6-81.9)	81.7 (81.6-81.9)	81.9 (81.7-82.0)	82.3 (82.1-82.4)	82.7 (82.6-82.9)	82.9 (82.8-83.1)	83.4 (83.3-83.6)	83.5 (83.3-83.7)	84.0 (83.8-84.1)	83.8 (83.6-84.0)	83.4 (83.2-83.6)	83.8 (83.6-84.1)
Italy	81.8 (81.7-81.8)	81.9 (81.8-81.9)	82.3 (82.2-82.3)	82.5 (82.4-82.5)	82.7 (82.7-82.8)	82.9 (82.8-82.9)	82.9 (82.9-83.0)	83.6 (83.6-83.6)	83.7 (83.7-83.8)	83.8 (83.8-83.9)	83.9 (83.9-84.0)	84.1 (84.0-84.1)	84.1 (84.0-84.1)	84.4 (84.3-84.4)	84.3 (84.3-84.4)	84.4 (84.3-84.4)	84.4 (84.4-84.5)	84.5 (84.4-84.6)	84.6 (84.4-84.7)
Luxembourg	80.6 (80.3-80.8)	80.9 (80.7-81.1)	81.2 (81.0-81.5)	81.6 (81.4-81.8)	81.7 (81.5-81.9)	81.8 (81.6-82.0)	81.7 (81.5-81.9)	82.1 (81.9-82.3)	82.5 (82.3-82.7)	82.8 (82.6-83.0)	83.1 (82.9-83.3)	83.3 (83.1-83.6)	83.5 (83.3-83.7)	83.6 (83.4-83.8)	83.6 (83.4-83.9)	83.8 (83.5-84.0)	84.0 (83.8-84.2)	84.2 (83.9-84.5)	84.3 (84.0-84.6)
Malta	81.5 (81.0-81.9)	81.8 (81.3-82.3)	81.7 (81.2-82.2)	81.8 (81.4-82.3)	82.1 (81.7-82.5)	82.2 (81.7-82.5)	82.4 (82.0-82.7)	82.4 (82.1-82.7)	82.7 (82.3-83.1)	83.1 (82.7-83.6)	83.1 (82.7-83.5)	82.8 (82.4-83.1)	83.5 (83.2-83.8)	83.6 (83.4-83.9)	83.6 (83.3-83.8)	83.9 (83.6-84.1)	84.0 (83.7-84.3)	84.3 (84.0-84.7)	84.5 (84.2-84.8)
Netherlands	80.5 (80.4-80.6)	80.7 (80.6-80.7)	80.5 (80.4-80.6)	80.6 (80.5-80.7)	80.7 (80.6-80.8)	80.7 (80.6-80.8)	80.9 (80.8-81.0)	81.3 (81.3-81.4)	81.6 (81.5-81.7)	81.9 (81.8-82.0)	82.3 (82.2-82.3)	82.3 (82.2-82.4)	82.6 (82.5-82.7)	82.7 (82.6-82.8)	82.7 (82.6-82.8)	82.8 (82.7-82.9)	83.2 (83.1-83.3)	83.3 (83.1-83.5)	83.4 (83.1-83.7)
Norway	81.0 (80.8-81.1)	81.1 (80.9-81.3)	81.3 (81.1-81.4)	81.3 (81.1-81.5)	81.5 (81.4-81.7)	81.7 (81.5-81.8)	82.0 (81.8-82.1)	82.1 (81.9-82.3)	82.4 (82.3-82.6)	82.7 (82.5-82.8)	82.7 (82.5-82.9)	83.0 (82.9-83.2)	83.1 (82.9-83.2)	83.1 (83.0-83.3)	83.2 (83.0-83.4)	83.7 (83.5-83.8)	83.7 (83.5-83.8)	84.0 (83.8-84.2)	84.1 (83.8-84.3)
Portugal	79.4 (79.3-79.5)	79.8 (79.7-79.9)	80.0 (79.9-80.1)	80.3 (80.2-80.4)	80.4 (80.3-80.5)	80.7 (80.7-80.8)	80.9 (80.8-81.0)	81.5 (81.4-81.6)	81.7 (81.7-81.8)	82.4 (82.3-82.5)	82.4 (82.3-82.5)	82.5 (82.4-82.6)	82.6 (82.5-82.7)	82.9 (82.8-83.0)	83.1 (83.1-83.2)	83.4 (83.3-83.4)	83.6 (83.5-83.7)	83.7 (83.6-83.9)	83.9 (83.7-84.0)
Spain	82.2 (82.1-82.2)	82.4 (82.3-82.4)	82.4 (82.3-82.4)	82.7 (82.6-82.7)	82.9 (82.9-82.9)	83.0 (83.0-83.1)	83.0 (83.0-83.0)	83.4 (83.4-83.5)	83.6 (83.6-83.7)	84.0 (83.9-84.0)	84.1 (84.0-84.1)	84.2 (84.1-84.2)	84.5 (84.5-84.6)	84.8 (84.8-84.9)	84.9 (84.8-84.9)	85.0 (85.0-85.1)	85.1 (85.1-85.2)	85.2 (85.1-85.3)	85.3 (85.2-85.4)
Sweden	81.8 (81.7-82.0)	81.9 (81.8-82.0)	81.9 (81.8-82.0)	81.9 (81.8-82.0)	82.1 (81.9-82.2)	82.2 (82.1-82.3)	82.4 (82.2-82.5)	82.3 (82.2-82.4)	82.6 (82.5-82.7)	82.8 (82.7-82.9)	83.0 (82.9-83.1)	83.1 (83.0-83.2)	83.2 (83.1-83.3)	83.6 (83.5-83.8)	83.6 (83.5-83.7)	83.6 (83.5-83.7)	83.7 (83.5-83.8)	83.9 (83.7-84.1)	84.0 (83.7-84.2)
Switzerland	82.1 (81.9-82.2)	82.4 (82.3-82.5)	82.5 (82.4-82.7)	82.6 (82.4-82.7)	82.9 (82.7-83.0)	82.9 (82.8-83.0)	83.1 (83.0-83.2)	83.5 (83.4-83.7)	83.7 (83.6-83.8)	83.7 (83.6-83.9)	83.9 (83.8-84.1)	84.2 (84.1-84.3)	84.3 (84.2-84.4)	84.4 (84.2-84.5)	84.7 (84.6-84.8)	84.7 (84.6-84.9)	85.0 (84.8-85.1)	85.1 (84.9-85.3)	85.2 (84.9-85.5)
United Kingdom	79.6 (79.6-79.6)	79.8 (79.7-79.8)	79.9 (79.9-79.9)	80.2 (80.1-80.2)	80.4 (80.3-80.4)	80.5 (80.5-80.6)	80.6 (80.6-80.7)	80.9 (80.9-81.0)	81.1 (81.1-81.2)	81.4 (81.3-81.4)	81.6 (81.5-81.6)	81.7 (81.7-81.8)	82.0 (82.0-82.1)	82.2 (82.2-82.2)	82.4 (82.4-82.5)	82.5 (82.5-82.6)	82.6 (82.5-82.7)	82.7 (82.6-82.8)	82.8 (82.7-82.9)
England	79.8 (79.8-79.8)	79.9 (79.9-80.0)	80.1 (80.1-80.1)	80.4 (80.4-80.4)	80.6 (80.5-80.6)	80.7 (80.7-80.8)	80.8 (80.8-80.9)	81.2 (81.1-81.2)	81.4 (81.3-81.4)	81.6 (81.6-81.7)	81.8 (81.8-81.9)	82.0 (81.9-82.0)	82.3 (82.3-82.3)	82.5 (82.4-82.5)	82.7 (82.6-82.7)	82.8 (82.7-82.8)	82.8 (82.8-82.9)	82.9 (82.9-83.0)	83.0 (82.9-83.2)
Northern Ireland	79.4 (79.2-79.6)	79.6 (79.3-79.8)	79.7 (79.5-79.9)	80.0 (79.8-80.2)	80.2 (80.0-80.4)	80.4 (80.2-80.6)	80.4 (80.2-80.6)	80.7 (80.5-80.9)	80.8 (80.6-81.0)	80.9 (80.7-81.1)	81.0 (80.7-81.2)	81.0 (80.8-81.2)	81.2 (81.0-81.4)	81.4 (81.2-81.6)	81.8 (81.6-82.0)	81.9 (81.7-82.2)	82.1 (81.8-82.4)	82.2 (81.9-82.5)	82.3 (81.9-82.7)
Scotland	78.1 (78.0-78.3)	78.2 (78.1-78.4)	78.4 (78.3-78.5)	78.6 (78.5-78.7)	78.8 (78.7-78.9)	78.9 (78.8-79.0)	79.0 (78.9-79.1)	79.3 (79.2-79.4)	79.5 (79.4-79.6)	79.7 (79.6-79.9)	79.9 (79.7-80.0)	80.0 (79.9-80.1)	80.3 (80.2-80.4)	80.5 (80.4-80.6)	80.7 (80.6-80.9)	80.8 (80.6-81.0)	80.9 (80.8-81.1)	81.1 (80.8-81.4)	81.2 (80.9-81.6)
Wales	79.3 (79.2-79.4)	79.4 (79.3-79.6)	79.5 (79.4-79.6)	79.8 (79.7-79.9)	79.9 (79.8-80.1)	80.1 (80.0-80.2)	80.2 (80.1-80.3)	80.4 (80.3-80.6)	80.7 (80.5-80.8)	80.9 (80.8-81.0)	81.0 (80.9-81.2)	81.2 (81.1-81.3)	81.4 (81.3-81.6)	81.6 (81.5-81.8)	81.8 (81.7-82.0)	81.8 (81.7-82.0)	81.9 (81.7-82.1)	82.0 (81.8-82.3)	82.2 (81.9-82.5)
Southern Latin America	78.0 (77.9-78.0)	78.1 (78.0-78.2)	78.2 (78.1-78.3)	78.5 (78.4-78.6)	78.6 (78.6-78.7)	78.7 (78.7-78.8)	78.8 (78.7-78.9)	79.1 (79.0-79.1)	79.3 (79.2-79.3)	79.4 (79.3-79.4)	79.4 (79.3-79.4)	79.6 (79.5-79.6)	79.6 (79.5-79.7)	79.7 (79.6-79.7)	79.9 (79.8-79.9)	80.0 (79.9-80.0)	80.1 (80.0-80.2)	80.2 (80.1-80.4)	80.4 (80.2-80.6)
Argentina	77.5 (77.4-77.6)	77.6 (77.5-77.7)	77.6 (77.5-77.7)	77.9 (77.8-78.0)	78.0 (77.9-78.1)	78.1 (78.0-78.2)	78.1 (78.0-78.2)	78.4 (78.4-78.5)	78.7 (78.6-78.8)	78.8 (78.7-78.9)	78.7 (78.6-78.8)	78.9 (78.8-79.0)	78.9 (78.8-79.0)	79.0 (79.0-79.1)	79.2 (79.1-79.2)	79.3 (79.2-79.4)	79.4 (79.3-79.5)	79.6 (79.4-79.7)	79.8 (79.5-80.0)
Chile	79.4 (79.2-79.5)	79.6 (79.5-79.7)	79.9 (79.7-80.0)	80.2 (80.1-80.4)	80.4 (80.2-80.5)	80.6 (80.5-80.7)	80.7 (80.6-80.8)	80.8 (80.7-80.9)	81.0 (80.9-81.1)	80.9 (80.8-81.1)	81.1 (81.0-81.2)	81.3 (81.2-81.5)	81.4 (81.3-81.5)	81.4 (81.2-81.5)	81.7 (81.6-81.8)	81.8 (81.7-81.9)	81.8 (81.7-81.9)	81.9 (81.6-82.2)	82.0 (81.5-82.5)
Uruguay	77.8 (77.6-77.9)	78.0 (77.8-78.2)	78.2 (78.1-78.4)	78.5 (78.4-78.7)	78.7 (78.5-78.8)	78.7 (78.5-78.9)	78.7 (78.5-78.9)	78.9 (78.7-79.1)	79.2 (79.0-79.3)	79.4 (79.2-79.5)	79.4 (79.2-79.5)	79.5 (79.4-79.7)	79.8 (79.6-79.9)	79.8 (79.6-80.0)	79.9 (79.7-80.1)	79.9 (79.7-80.1)	80.1 (79.8-80.3)	80.3 (80.0-80.6)	80.5 (80.2-80.9)
Central Europe, Eastern Europe, and Central Asia	72.9 (72.8-73.0)	73.2 (73.1-73.3)	73.0 (72.9-73.1)	73.0 (72.9-73.1)	73.1 (73.0-73.2)	73.2 (73.1-73.3)	73.3 (73.1-73.4)	73.6 (73.4-73.7)	73.6 (73.5-73.7)	74.3 (74.2-74.4)	74.7 (74.6-74.8)	75.0 (74.9-75.1)	75.5 (75.4-75.6)	75.8 (75.7-75.9)	76.3 (76.2-76.4)	76.6 (76.5-76.8)	76.9 (76.8-77.1)	77.1 (77.0-77.3)	77.3 (77.1-77.5)
Eastern Europe	73.1 (73.0-73.2)	73.4 (73.4-73.5)	72.8 (72.7-72.9)	72.6 (72.5-72.6)	72.6 (72.5-72.7)	72.5 (72.4-72.6)	72.4 (72.3-72.5)	72.7 (72.7-72.8)	72.7 (72.6-72.8)	73.6 (73.6-73.7)	74.1 (74.1-74.2)	74.3 (74.2-74.3)	75.0 (74.9-75.0)	75.2 (75.2-75.3)	75.8 (75.8-75.9)	76.1 (76.1-76.2)	76.4 (76.4-76.5)	76.5 (76.4-76.6)	76.7 (76.4-76.9)
Belarus	73.8 (73.5-74.1)	73.8 (73.5-74.1)	73.7 (73.5-74.0)	74.1 (73.8-74.4)	74.1 (73.8-74.4)	74.0 (73.7-74.3)	74.4 (74.1-74.6)	74.7 (74.4-74.9)	74.9 (74.6-75.1)	75.3 (75.0-75.6)	75.7 (75.5-76.0)	76.0 (75.8-76.3)	76.1 (75.9-76.4)	76.3 (76.0-76.6)	76.4 (76.1-76.7)	76.5 (76.1-76.8)	76.6 (76.3-77.0)	76.8 (76.4-77.3)	77.1 (76.6-77.6)
Estonia	75.8 (75.6-75.9)	75.3 (75.1-75.5)	75.8 (75.6-76.0)	75.7 (75.5-76.0)	75.5 (75.3-75.7)	76.5 (76.3-76.7)	76.8 (76.6-77.0)	77.5 (77.3-77.6)	77.7 (77.5-77.8)	78.2 (78.0-78.4)	78.4 (78.3-78.6)	79.0 (78.9-79.2)	79.7 (79.5-79.8)	81.0 (80.8-81.2)	80.6 (80.4-80.9)	81.1 (80.8-81.4)	81.1 (80.8-81.4)	81.3 (80.9-81.7)	81.4 (81.0-81.9)
Latvia	74.9 (74.7-75.0)	74.6 (74.5-74.8)	75.3 (75.2-75.5)	76.0 (75.8-76.1)	75.5 (75.3-75.7)	75.9 (75.8-76.1)	75.9 (75.7-76.0)	76.4 (76.2-76.5)	76.6 (76.5-76.8)	76.2 (76.0-76.3)	76.7 (76.6-76.8)	77.5 (77.4-77.7)	78.1 (78.0-78.3)	78.7 (78.5-78.8)	79.1 (78.9-79.3)	79.7 (79.5-79.9)	79.3 (79.1-79.5)	79.6 (79.3-79.8)	79.7 (79.3-80.1)
Lithuania	76.6 (76.5-76.7)	76.6 (76.5-76.8)	77.1 (77.0-77.2)	77.4 (77.3-77.5)	77.2 (77.1-77.3)	77.7 (77.6-77.8)	77.7 (77.6-77.8)	78.0 (77.9-78.1)	77.7 (77.6-77.8)	77.6 (77.5-77.7)	77.6 (77.5-77.8)	77.8 (77.7-77.9)	79.1 (79.0-79.2)	79.3 (79.2-79.4)	79.5 (79.4-79.7)	79.9 (79.8-80.1)	80.1 (79.9-80.2)	80.2 (80.0-80.4)	80.4 (80.2-80.6)
Moldova	71.4 (70.9-71.8)	72.1 (71.7-72.5)	72.1 (71.7-72.5)	72.1 (71.7-72.5)	72.7 (72.3-73.0)	73.1 (72.8-73.4)	73.3 (73.0-73.6)	73.9 (73.6-74.2)	73.4 (73.1-73.7)	73.6 (73.3-73.8)	73.8 (73.5-74.0)	74.2 (73.9-74.4)	74.5 (74.3-74.8)	74.5 (74.3-74.8)	76.2 (75.9-76.4)	76.3 (76.0-76.5)	77.0 (76.7-77.3)	77.4 (77.1-77.7)	77.7 (77.3-78.1)
Russia	73.1 (73.1-73.2)	73.4 (73.3-73.4)	72.4 (72.3-72.5)	72.1 (72.0-72.2)	72.0 (72.0-72.1)	71.9 (71.8-72.0)	71.8 (71.6-71.9)	72.1 (72.0-72.2)	72.2 (72.1-72.3)	73.3 (73.3-73.4)	74.0 (74.0-74.1)	74.2 (74.2-74.3)	74.8 (74.7-74.8)	74.9 (74.8-74.9)	75.5 (75.5-75.6)	76.0 (75.9-76.0)	76.3 (76.3-76.4)	76.4 (76.3-76.4)	76.6 (76.2-76.9)
Ukraine	72.8 (72.5-73.1)	73.5 (73.1-73.8)	73.2 (72.9-73.5)	73.1 (72.8-73.4)	73.3 (73.0-73.6)	73.3 (73.0-73.6)	73.4 (73.1-73.7)	73.4 (73.1-73.7)	73.2 (72.9-73.4)	73.8 (73.5-74.0)	73.6 (73.4-73.8)	73.7 (73.6-73.9)	74.8 (74.7-75.0)	75.5 (75.3-75.6)	76.0 (75.8-76.2)	76.1 (75.9-76.3)	76.2 (76.0-76.4)	76.3 (75.9-76.6)	76.5 (75.9-76.9)
Central Europe	75.8 (75.8-75.8)	76.1 (76.0-76.1)	76.3 (76.2-76.3)	76.6 (76.6-76.7)	76.9 (76.9-77.0)	77.2 (77.1-77.2)	77.4 (77.4-77.5)	77.7 (77.7-77.7)	77.9 (77.9-77.9)	78.2 (78.1-78.2)	78.4 (78.4-78.4)	78.7 (78.6-78.7)	78.8 (78.8-78.9)	79.2 (79.2-79.2)	79.6 (79.5-79.6)	79.8 (79.7-79.8)	80.1 (80.0-80.1)	80.3 (80.2-80.4)	80.5 (80.3-80.6)
Albania	77.8 (77.3-78.2)	77.7 (77.3-78.1)	77.6 (77.2-78.0)	78.0 (77.6-78.5)	78.4 (78.0-78.8)	78.7 (78.2-79.1)	78.8 (78.4-79.2)	78.9 (78.5-79.4)	79.1 (78.6-79.5)	79.3 (78.8-79.7)	79.5 (79.0-79.9)	79.6 (79.1-80.1)	79.9 (79.3-80.4)	80.1 (79.5-80.7)	80.4 (79.7-81.0)	80.6 (80.0-81.2)	80.9 (80.2-81.5)	81.1 (80.5-81.7)	81.3 (80.7-81.9)
Bosnia and Herzegovina	77.7 (77.5-78.0)	78.1 (78.0-78.3)	78.5 (78.3-78.7)	79.0 (78.8-79.1)	79.3 (79.2-79.5)	79.5 (79.3-79.6)	79.6 (79.5-79.8)	79.8 (79.6-79.9)	79.9 (79.7-80.0)	80.0 (79.8-80.1)	80.2 (80.0-80.3)	80.4 (80.2-80.5)	80.5 (80.4-80.7)	80.7 (80.6-80.9)	80.9 (80.8-81.1)	81.1 (80.9-81.4)	81.4 (81.1-81.6)	81.6 (81.3-81.9)	81.8 (81.4-82.2)
Bulgaria	74.3 (74.2-74.5)	74.8 (74.6-74.9)	74.9 (74.7-75.0)	75.1 (74.9-75.2)	75.4 (75.2-75.5)	75.6 (75.5-75.7)	75.9 (75.8-76.0)	76.1 (76.0-76.2)	76.2 (76.1-76.3)	76.4 (76.2-76.5)	76.6 (76.5-76.7)	76.9 (76.8-77.0)	77.1 (77.0-77.2)	77.3 (77.2-77.4)	77.7 (77.6-77.9)	78.0 (77.8-78.2)	78.1 (77.9-78.4)	78.2 (77.9-78.6)	78.4 (77.9-78.8)
Croatia	77.1 (77.0-77.2)	77.2 (77.0-77.3)	77.4 (77.3-77.5)	77.7 (77.6-77.9)	78.1 (77.9-78.2)	78.4 (78.2-78.5)	78.6 (78.5-78.8)	78.9 (78.8-79.1)	79.2 (79.1-79.3)	79.4 (79.3-79.6)	79.6 (79.5-79.7)	79.8 (79.7-79.9)	80.0 (79.8-80.1)	80.2 (80.0-80.3)	80.4 (80.3-80.6)	80.6 (80.5-80.8)	80.7 (80.6-80.9)	80.8 (80.6-81.1)	80.9 (80.6-81.3)
Czech Republic	77.6 (77.5-77.7)	77.9 (77.8-78.0)	78.1 (78.0-78.2)	78.3 (78.2-78.4)	78.5 (78.4-78.6)	78.7 (78.6-78.8)	78.9 (78.8-79.0)	79.1 (79.0-79.2)	79.4 (79.3-79.5)	79.7 (79.6-79.8)	80.0 (79.9-80.0)	80.2 (80.1-80.3)	80.4 (80.3-80.5)	80.6 (80.5-80.7)	80.8 (80.7-80.9)	81.0 (80.9-81.1)	81.3 (81.1-81.3)	81.5 (81.4-81.6)	81.7 (81.5-81.8)
Hungary	75.4 (75.3-75.5)	75.6 (75.5-75.7)	75.8 (75.6-75.9)	76.1 (76.0-76.2)	76.5 (76.4-76.6)	76.7 (76.6-76.8)	76.9 (76.8-77.0)	77.1 (77.0-77.2)	77.2 (77.1-77.3)	77.5 (77.4-77.6)	77.7 (77.6-77.8)	77.9 (77.8-78.0)	78.2 (78.1-78.3)	78.5 (78.4-78.6)	78.8 (78.6-78.9)	79.0 (78.9-79.1)	79.4 (79.2-79.5)	79.7 (79.5-80.0)	79.9 (79.6-80.3)
Macedonia	74.7 (74.6-74.9)	74.9 (74.7-75.1)	75.2 (75.0-75.4)	75.5 (75.3-75.6)	75.7 (75.5-75.8)	75.9 (75.7-76.0)	76.0 (75.8-76.2)	76.2 (76.0-76.4)	76.4 (76.2-76.6)	76.6 (76.5-76.8)	76.8 (76.7-77.0)	77.1 (76.9-77.2)	77.1 (76.9-77.2)	77.4 (77.3-77.6)	78.0 (77.8-78.2)	78.2 (78.0-78.5)	78.5 (78.1-78.8)	78.8 (78.4-79.2)	79.1 (78.6-79.6)

SR Table 6: Estimates of life expectancy at birth for each geography (with 95% uncertainty intervals), females, 1980–2015

Location	1980	1981	1982	1983	1984	1985	1986	1987	1988	1989	1990	1991	1992	1993	1994	1995	1996	1997	1998	1999	2000	2001	2002	2003	2004	2005	2006	2007	2008	2009	2010	2011	2012	2013	2014	2015
Montenegro	76.2 (75.0-77.3)	76.3 (75.1-77.4)	76.4 (75.3-77.4)	76.4 (75.4-77.4)	76.5 (75.5-77.5)	76.6 (75.6-77.5)	76.8 (75.8-77.6)	76.9 (76.0-77.7)	77.0 (76.2-77.8)	77.2 (76.5-77.9)	77.4 (76.8-78.1)	77.4 (76.7-78.0)	77.3 (76.6-77.9)	77.0 (76.4-77.6)	76.7 (76.2-77.2)	76.6 (76.2-77.0)	76.4 (76.1-76.7)	76.3 (75.9-76.7)	76.2 (75.8-76.6)	76.1 (75.8-76.5)	76.3 (76.0-76.6)	76.4 (76.1-76.7)	77.0 (76.7-77.2)	77.4 (77.2-77.6)	77.7 (77.5-78.0)	78.0 (77.7-78.2)	78.2 (77.9-78.4)	78.5 (78.2-78.8)	78.9 (78.6-79.1)	79.2 (78.9-79.4)	79.4 (79.1-79.7)	79.6 (79.2-79.9)	79.7 (79.3-80.1)	79.8 (79.4-80.3)	80.0 (79.6-80.5)	80.2 (79.7-80.7)
Poland	74.7 (74.6-74.7)	75.2 (75.1-75.2)	75.2 (75.1-75.2)	75.1 (75.0-75.2)	75.0 (74.9-75.0)	74.8 (74.8-74.9)	75.1 (75.1-75.2)	75.2 (75.1-75.2)	75.5 (75.5-75.6)	75.5 (75.4-75.5)	75.5 (75.4-75.6)	75.4 (75.3-75.4)	75.7 (75.7-75.8)	76.1 (76.1-76.2)	76.1 (76.1-76.2)	76.4 (76.4-76.5)	76.7 (76.7-76.8)	77.0 (77.0-77.1)	77.4 (77.3-77.4)	77.5 (77.5-77.6)	78.0 (77.9-78.0)	78.4 (78.3-78.4)	78.7 (78.7-78.8)	79.0 (79.0-79.1)	79.2 (79.2-79.3)	79.4 (79.3-79.4)	79.5 (79.4-79.5)	79.6 (79.5-79.6)	79.8 (79.7-79.8)	79.9 (79.9-80.0)	80.5 (80.4-80.5)	80.7 (80.7-80.8)	80.9 (80.8-80.9)	81.3 (81.2-81.4)	81.5 (81.3-81.6)	81.6 (81.4-81.8)
Romania	72.2 (72.1-72.3)	72.6 (72.5-72.7)	73.0 (72.9-73.1)	73.2 (73.1-73.3)	73.3 (73.2-73.3)	73.0 (72.9-73.1)	73.2 (73.1-73.3)	72.5 (72.4-72.6)	72.8 (72.7-72.9)	72.9 (72.8-73.0)	73.4 (73.3-73.5)	73.9 (73.9-74.0)	73.8 (73.7-73.9)	73.7 (73.6-73.8)	73.6 (73.5-73.7)	73.7 (73.6-73.8)	73.4 (73.3-73.5)	73.5 (73.4-73.6)	73.9 (73.8-74.0)	74.4 (74.3-74.5)	74.8 (74.7-74.9)	74.9 (74.8-75.0)	75.1 (75.0-75.2)	75.4 (75.3-75.5)	75.9 (75.8-76.0)	76.1 (76.0-76.2)	76.5 (76.5-76.6)	77.0 (76.9-77.1)	77.2 (77.1-77.2)	77.3 (77.2-77.4)	77.6 (77.6-77.7)	78.3 (78.2-78.4)	78.4 (78.3-78.5)	78.6 (78.4-78.8)	78.8 (78.5-79.1)	79.0 (78.6-79.4)
Serbia	74.2 (72.9-75.3)	74.3 (73.1-75.4)	74.5 (73.4-75.5)	74.6 (73.5-75.6)	74.7 (73.7-75.6)	74.8 (74.0-75.7)	75.0 (74.2-75.7)	75.2 (74.5-75.8)	75.3 (74.6-75.9)	75.4 (74.8-75.9)	75.3 (74.8-75.8)	74.7 (74.3-75.2)	75.2 (74.8-75.6)	75.0 (74.7-75.3)	74.8 (74.6-75.0)	74.6 (74.5-74.8)	74.7 (74.5-74.8)	74.8 (74.7-74.9)	74.5 (74.2-74.8)	74.3 (73.8-74.8)	74.8 (74.6-75.1)	75.0 (74.8-75.3)	75.1 (74.9-75.4)	75.3 (75.1-75.6)	75.6 (75.4-75.9)	75.9 (75.7-76.2)	76.3 (76.1-76.5)	76.6 (76.5-76.8)	77.0 (76.9-77.1)	77.3 (77.2-77.4)	77.5 (77.4-77.6)	77.8 (77.7-77.8)	78.1 (78.0-78.1)	78.3 (78.2-78.4)	78.6 (78.5-78.7)	78.9 (78.7-79.0)
Slovakia	74.4 (74.2-74.5)	74.6 (74.4-74.7)	74.8 (74.6-74.9)	74.6 (74.5-74.8)	74.8 (74.7-75.0)	74.8 (74.6-74.9)	74.9 (74.8-75.1)	75.1 (74.9-75.2)	75.3 (75.2-75.4)	75.4 (75.3-75.5)	75.4 (75.3-75.5)	75.5 (75.3-75.6)	75.8 (75.7-75.9)	76.1 (76.0-76.2)	76.4 (76.2-76.5)	76.5 (76.3-76.6)	76.8 (76.7-76.9)	76.8 (76.7-76.9)	77.0 (76.8-77.1)	77.1 (77.0-77.2)	77.4 (77.3-77.5)	77.6 (77.5-77.7)	77.8 (77.7-78.0)	77.9 (77.8-78.0)	78.1 (77.9-78.2)	78.2 (78.1-78.3)	78.4 (78.3-78.5)	78.5 (78.4-78.6)	78.9 (78.7-79.0)	79.0 (78.8-79.1)	79.3 (79.2-79.5)	79.7 (79.6-79.8)	80.0 (79.9-80.1)	80.4 (80.2-80.5)	80.8 (80.6-80.9)	80.9 (80.7-81.1)
Slovenia	74.6 (74.3-74.9)	74.8 (74.6-75.1)	75.0 (74.8-75.2)	75.0 (74.9-75.2)	75.3 (75.2-75.5)	75.8 (75.7-76.0)	76.1 (75.9-76.2)	76.3 (76.1-76.4)	76.8 (76.6-76.9)	77.0 (76.9-77.2)	77.5 (77.3-77.6)	77.4 (77.2-77.5)	77.6 (77.5-77.7)	77.7 (77.6-77.8)	77.9 (77.8-78.1)	78.3 (78.2-78.5)	78.6 (78.5-78.8)	78.9 (78.8-79.1)	79.1 (79.0-79.2)	79.3 (79.2-79.5)	79.8 (79.6-79.9)	80.1 (79.9-80.2)	80.4 (80.2-80.5)	80.6 (80.4-80.7)	80.8 (80.7-80.9)	81.1 (80.9-81.2)	81.5 (81.4-81.6)	81.7 (81.6-81.8)	82.1 (82.0-82.2)	82.3 (82.2-82.5)	82.6 (82.5-82.8)	82.9 (82.7-83.0)	83.0 (82.9-83.2)	83.3 (83.1-83.4)	83.7 (83.6-83.9)	83.8 (83.6-84.0)
Central Asia	68.8 (68.5-69.2)	69.2 (68.9-69.6)	69.6 (69.2-69.8)	69.6 (69.3-69.9)	69.6 (69.3-69.9)	69.8 (69.6-70.1)	70.2 (69.9-70.4)	70.4 (70.1-70.6)	69.4 (68.7-70.0)	70.6 (70.3-70.8)	70.7 (70.4-70.9)	70.6 (70.3-70.8)	70.0 (69.8-70.3)	69.6 (69.3-70.0)	69.4 (69.1-69.7)	69.3 (69.0-69.6)	69.4 (69.1-69.6)	69.6 (69.3-69.9)	69.9 (69.6-70.2)	70.2 (69.9-70.5)	70.4 (70.1-70.7)	70.7 (70.4-71.0)	70.9 (70.6-71.2)	71.0 (70.7-71.3)	71.2 (70.9-71.6)	71.5 (71.1-71.8)	71.8 (71.4-72.1)	72.2 (71.9-72.6)	72.6 (72.3-73.0)	73.1 (72.7-73.4)	73.4 (73.1-73.8)	73.9 (73.5-74.3)	74.4 (74.0-74.7)	74.7 (74.3-75.1)	75.0 (74.6-75.5)	75.3 (74.8-75.8)
Armenia	72.6 (71.8-73.4)	72.9 (72.2-73.7)	73.3 (72.6-74.0)	73.2 (72.5-73.8)	73.6 (73.0-74.2)	73.3 (72.7-73.9)	73.7 (73.2-74.3)	73.7 (73.1-74.3)	55.9 (48.5-65.4)	73.5 (73.0-74.1)	73.5 (73.0-74.0)	73.5 (72.9-74.0)	72.7 (72.1-73.2)	72.5 (72.0-73.1)	73.0 (72.5-73.6)	73.1 (72.5-73.6)	73.2 (72.6-73.7)	73.7 (73.1-74.2)	74.0 (73.4-74.5)	74.3 (73.8-74.8)	74.7 (74.2-75.1)	74.8 (74.3-75.3)	75.2 (74.7-75.7)	75.0 (74.5-75.5)	75.4 (74.8-75.8)	75.3 (74.8-75.8)	75.2 (74.7-75.7)	75.9 (75.4-76.4)	76.0 (75.4-76.5)	76.5 (75.9-77.0)	76.8 (76.3-77.3)	77.4 (77.0-77.8)	77.8 (77.3-78.2)	78.0 (77.6-78.5)	78.2 (77.7-78.8)	78.4 (77.9-78.9)
Azerbaijan	68.3 (67.1-69.5)	68.3 (67.2-69.3)	68.5 (67.5-69.5)	68.6 (67.7-69.5)	68.5 (67.6-69.3)	68.7 (67.9-69.6)	68.6 (67.8-69.4)	68.7 (67.9-69.5)	68.7 (67.9-69.4)	69.2 (68.5-70.0)	69.5 (68.7-70.2)	69.3 (68.6-70.1)	68.5 (67.7-69.3)	68.5 (67.7-69.2)	68.3 (67.5-69.0)	68.6 (67.8-69.3)	68.8 (68.0-69.5)	69.0 (68.2-69.7)	69.0 (68.3-69.8)	69.3 (68.6-70.1)	69.8 (69.0-70.4)	70.4 (69.7-71.1)	70.8 (70.0-71.5)	70.7 (69.9-71.4)	71.0 (70.2-71.7)	71.4 (70.6-72.1)	71.9 (71.1-72.7)	72.3 (71.5-73.0)	72.4 (71.6-73.1)	72.8 (71.9-73.6)	73.2 (72.4-73.9)	74.0 (73.2-74.8)	74.8 (74.0-75.6)	75.4 (74.6-76.2)	75.9 (75.1-76.7)	76.3 (75.6-77.1)
Georgia	73.0 (72.1-73.8)	73.2 (72.5-74.0)	73.4 (72.7-74.1)	73.5 (72.8-74.2)	73.5 (72.7-74.2)	73.4 (72.7-74.1)	73.5 (72.8-74.1)	73.5 (72.9-74.2)	73.6 (73.0-74.2)	73.6 (73.0-74.3)	73.7 (73.1-74.3)	73.8 (73.2-74.4)	73.7 (73.0-74.3)	73.6 (72.8-74.3)	74.1 (73.4-74.8)	74.6 (73.9-75.2)	75.0 (74.3-75.7)	75.2 (74.4-75.8)	75.3 (74.5-75.9)	75.2 (74.5-75.9)	75.2 (74.5-75.8)	75.6 (74.9-76.3)	75.7 (75.0-76.3)	75.6 (74.9-76.3)	75.5 (74.9-76.2)	76.0 (75.4-76.6)	76.5 (75.9-77.1)	76.8 (76.2-77.5)	76.8 (76.2-77.5)	77.0 (76.4-77.6)	77.1 (76.6-77.7)	77.3 (76.7-77.8)	77.5 (76.9-78.0)	77.7 (77.1-78.2)	77.8 (77.3-78.4)	78.0 (77.3-78.7)
Kazakhstan	70.4 (69.8-70.9)	71.0 (70.6-71.4)	71.6 (71.2-71.9)	71.6 (71.1-72.0)	71.3 (70.9-71.7)	71.6 (71.3-71.9)	72.3 (72.0-72.6)	72.6 (72.3-72.8)	72.4 (72.1-72.7)	72.3 (72.1-72.6)	72.4 (72.2-72.7)	72.2 (71.9-72.5)	71.8 (71.5-72.1)	70.5 (70.1-70.8)	69.8 (69.4-70.2)	69.3 (68.9-69.7)	69.3 (68.9-69.7)	69.6 (69.1-70.0)	69.9 (69.4-70.3)	70.3 (69.9-70.6)	70.5 (70.1-70.8)	70.7 (70.3-71.1)	71.0 (70.6-71.3)	71.0 (70.6-71.3)	71.1 (70.7-71.5)	71.1 (70.7-71.4)	71.2 (70.8-71.5)	71.6 (71.3-71.9)	72.4 (72.1-72.7)	73.1 (72.8-73.4)	73.4 (73.1-73.7)	74.0 (73.7-74.3)	74.5 (74.1-74.8)	74.7 (74.3-75.2)	74.9 (74.2-75.7)	75.0 (74.1-75.9)
Kyrgyzstan	67.9 (67.2-68.7)	68.3 (67.6-69.0)	68.5 (67.8-69.1)	68.7 (68.1-69.3)	68.8 (68.1-69.4)	68.9 (68.3-69.6)	69.2 (68.6-69.8)	69.3 (68.8-69.9)	69.5 (68.9-70.1)	69.6 (69.0-70.2)	69.8 (69.2-70.4)	69.8 (69.2-70.4)	69.5 (69.0-70.1)	69.3 (68.8-69.9)	69.1 (68.6-69.7)	69.3 (68.7-69.9)	69.4 (68.9-70.0)	69.6 (69.0-70.1)	69.8 (69.2-70.3)	70.0 (69.4-70.5)	70.1 (69.6-70.6)	70.3 (69.7-70.8)	70.1 (69.6-70.6)	70.2 (69.7-70.7)	70.5 (70.0-71.0)	70.7 (70.2-71.2)	70.9 (70.3-71.4)	71.1 (70.6-71.7)	71.4 (71.0-71.9)	71.8 (71.4-72.4)	72.2 (71.7-72.7)	72.8 (72.3-73.3)	73.2 (72.7-73.7)	73.7 (73.1-74.2)	73.9 (73.3-74.5)	74.1 (73.4-74.7)
Mongolia	60.7 (59.7-61.9)	61.1 (60.1-62.2)	61.5 (60.5-62.5)	61.9 (61.0-62.9)	62.4 (61.4-63.4)	62.9 (62.0-63.8)	63.4 (62.5-64.3)	63.9 (63.0-64.8)	64.2 (63.4-65.1)	64.5 (63.7-65.4)	64.7 (63.9-65.6)	64.7 (63.9-65.5)	64.6 (63.9-65.4)	64.5 (63.8-65.3)	64.6 (63.8-65.3)	64.5 (63.8-65.2)	64.5 (63.8-65.2)	64.7 (63.9-65.4)	64.9 (64.1-65.6)	65.1 (64.4-65.8)	65.5 (64.8-66.3)	66.0 (65.3-66.7)	66.5 (65.8-67.2)	66.9 (66.2-67.6)	67.3 (66.6-68.0)	67.7 (67.0-68.4)	68.2 (67.4-68.9)	68.7 (68.0-69.4)	69.1 (68.4-69.9)	69.4 (68.7-70.2)	69.7 (68.9-70.5)	70.2 (69.4-70.9)	70.6 (69.8-71.4)	71.1 (70.3-71.9)	71.5 (70.6-72.3)	71.9 (71.0-72.7)
Tajikistan	64.5 (63.3-65.7)	64.9 (63.8-66.0)	65.3 (64.2-66.4)	65.6 (64.6-66.7)	66.1 (65.1-67.1)	66.5 (65.5-67.5)	66.7 (65.8-67.7)	66.9 (66.0-67.9)	67.2 (66.3-68.1)	67.4 (66.6-68.3)	67.7 (66.9-68.5)	67.8 (67.0-68.7)	66.1 (65.0-67.2)	66.9 (66.1-67.7)	66.7 (65.9-67.5)	66.8 (65.9-67.7)	66.5 (65.6-67.3)	67.3 (66.5-68.1)	67.5 (66.7-68.3)	67.8 (67.1-68.6)	68.1 (67.4-68.8)	68.4 (67.5-69.2)	68.7 (68.0-69.5)	69.1 (68.4-69.8)	69.6 (68.9-70.3)	70.2 (69.4-70.9)	70.9 (70.2-71.6)	71.4 (70.7-72.0)	71.5 (70.8-72.2)	72.1 (71.4-72.8)	72.4 (71.7-73.1)	72.4 (71.7-73.1)	73.1 (72.4-73.8)	73.8 (73.1-74.5)	74.3 (73.5-75.1)	74.8 (73.9-75.5)
Turkmenistan	64.1 (62.4-65.7)	64.8 (63.2-66.2)	65.2 (63.7-66.5)	65.3 (63.9-66.5)	65.5 (64.1-66.7)	65.8 (64.6-66.9)	66.0 (64.9-67.0)	66.3 (65.3-67.3)	66.5 (65.4-67.5)	66.7 (65.7-67.7)	66.9 (65.9-67.9)	66.8 (65.8-67.7)	66.7 (65.8-67.6)	66.7 (65.8-67.7)	66.6 (65.6-67.6)	66.6 (65.5-67.6)	66.6 (65.4-67.6)	66.7 (65.5-67.7)	66.9 (65.7-67.9)	67.1 (65.8-68.2)	67.5 (66.1-68.6)	67.8 (66.4-69.1)	68.2 (66.8-69.5)	68.6 (67.2-69.9)	69.0 (67.7-70.3)	69.5 (68.2-70.7)	70.0 (68.7-71.2)	70.5 (69.2-71.7)	70.9 (69.7-72.0)	71.3 (70.1-72.3)	71.7 (70.6-72.7)	72.1 (71.1-73.0)	72.5 (71.4-73.3)	72.8 (71.7-73.6)	73.3 (72.3-74.1)	73.7 (72.7-74.5)
Uzbekistan	69.0 (68.1-69.8)	69.3 (68.4-70.1)	69.5 (68.7-70.3)	69.5 (68.7-70.3)	69.6 (68.9-70.4)	69.9 (69.2-70.6)	70.1 (69.4-70.8)	70.4 (69.7-71.1)	70.5 (69.9-71.2)	70.6 (69.9-71.3)	70.7 (70.0-71.3)	70.5 (69.8-71.1)	70.1 (69.5-70.8)	69.8 (69.2-70.5)	69.6 (68.9-70.3)	69.5 (68.8-70.2)	69.6 (68.9-70.3)	69.8 (69.1-70.5)	70.0 (69.3-70.7)	70.3 (69.6-71.0)	70.5 (69.8-71.3)	70.7 (70.1-71.4)	70.9 (70.2-71.6)	71.1 (70.3-71.8)	71.3 (70.5-72.1)	71.5 (70.7-72.3)	71.9 (71.0-72.7)	72.3 (71.4-73.1)	72.7 (71.8-73.5)	73.1 (72.2-73.9)	73.5 (72.6-74.3)	73.9 (73.0-74.7)	74.2 (73.4-75.1)	74.6 (73.8-75.4)	74.9 (74.0-75.8)	75.2 (74.3-76.0)
Latin America and Caribbean	68.9 (68.6-69.1)	69.3 (69.0-69.5)	69.7 (69.4-69.9)	70.0 (69.8-70.3)	70.3 (70.1-70.6)	70.4 (70.2-70.7)	71.1 (70.9-71.3)	71.4 (71.2-71.6)	71.9 (71.7-72.1)	72.2 (72.0-72.4)	72.5 (72.3-72.7)	72.8 (72.6-73.0)	72.9 (72.7-73.1)	73.2 (73.0-73.4)	73.4 (73.2-73.6)	73.7 (73.5-73.9)	74.0 (73.8-74.2)	74.3 (74.1-74.5)	74.5 (74.3-74.7)	74.8 (74.6-75.0)	75.3 (75.1-75.5)	75.6 (75.4-75.8)	75.8 (75.6-76.0)	76.0 (75.8-76.2)	76.3 (76.0-76.4)	76.5 (76.3-76.7)	76.8 (76.5-77.0)	77.0 (76.8-77.2)	77.2 (77.0-77.4)	77.3 (77.1-77.5)	76.5 (75.5-77.3)	77.7 (77.5-78.0)	77.8 (77.6-78.1)	77.9 (77.6-78.2)	78.1 (77.7-78.4)	78.3 (77.9-78.6)
Central Latin America	70.3 (70.1-70.5)	70.7 (70.4-70.9)	71.2 (71.0-71.5)	71.7 (71.4-71.9)	72.2 (72.0-72.4)	71.9 (71.6-72.2)	72.9 (72.7-73.1)	73.3 (73.1-73.4)	73.7 (73.5-73.9)	74.0 (73.8-74.2)	74.4 (74.3-74.6)	74.6 (74.5-74.8)	74.7 (74.6-74.9)	75.0 (74.8-75.1)	75.2 (75.0-75.3)	75.5 (75.3-75.7)	75.8 (75.7-76.0)	76.1 (75.9-76.2)	76.0 (75.9-76.2)	76.2 (75.9-76.4)	76.9 (76.7-77.1)	77.1 (76.9-77.2)	77.2 (77.0-77.3)	77.3 (77.1-77.4)	77.5 (77.3-77.7)	77.6 (77.4-77.8)	77.8 (77.6-78.0)	78.1 (77.9-78.3)	78.2 (78.0-78.4)	78.1 (77.9-78.3)	78.3 (78.1-78.5)	78.6 (78.3-78.8)	78.6 (78.3-78.8)	78.5 (78.2-78.7)	78.6 (78.3-78.9)	78.8 (78.4-79.2)
Colombia	71.6 (71.3-71.9)	72.2 (71.9-72.4)	72.7 (72.5-73.0)	73.1 (72.8-73.3)	73.5 (73.3-73.8)	71.6 (70.9-72.4)	74.1 (73.9-74.2)	74.2 (74.1-74.4)	74.6 (74.4-74.8)	74.8 (74.6-75.0)	75.3 (75.2-75.5)	75.3 (75.1-75.5)	75.3 (75.1-75.5)	75.4 (75.3-75.6)	75.5 (75.4-75.7)	75.9 (75.7-76.0)	76.1 (75.9-76.3)	76.5 (76.3-76.7)	76.8 (76.6-76.9)	76.9 (76.8-77.1)	77.3 (77.1-77.4)	77.4 (77.2-77.6)	77.7 (77.5-77.9)	77.9 (77.7-78.1)	78.1 (77.9-78.3)	78.3 (78.1-78.5)	78.4 (78.2-78.6)	78.9 (78.7-79.1)	79.2 (79.0-79.5)	79.4 (79.1-79.6)	79.4 (79.1-79.6)	79.8 (79.5-80.1)	80.0 (79.7-80.3)	80.3 (80.0-80.7)	80.6 (80.2-81.0)	80.8 (80.4-81.2)
Costa Rica	76.8 (76.5-77.1)	77.2 (76.9-77.5)	77.6 (77.3-77.8)	77.6 (77.4-77.9)	77.7 (77.5-78.0)	77.9 (77.7-78.2)	78.2 (77.9-78.4)	78.3 (78.0-78.5)	78.5 (78.2-78.7)	78.8 (78.5-79.0)	78.9 (78.7-79.2)	78.9 (78.7-79.2)	79.0 (78.8-79.3)	79.1 (78.8-79.3)	79.2 (78.9-79.5)	79.2 (79.0-79.5)	79.3 (79.0-79.5)	79.4 (79.2-79.7)	79.7 (79.4-79.9)	79.9 (79.7-80.2)	80.2 (79.9-80.4)	80.5 (80.2-80.7)	80.7 (80.5-81.0)	80.8 (80.6-81.1)	81.0 (80.8-81.3)	81.5 (81.2-81.7)	81.8 (81.5-82.0)	82.0 (81.7-82.2)	82.2 (81.9-82.4)	82.3 (82.1-82.5)	82.3 (82.0-82.5)	82.3 (82.1-82.5)	82.3 (82.1-82.6)	82.4 (82.1-82.6)	82.5 (82.1-82.8)	82.6 (82.1-83.0)
El Salvador	68.2 (67.3-68.9)	65.0 (63.6-66.6)	67.1 (66.1-68.1)	66.7 (65.6-67.8)	68.1 (67.3-69.0)	69.4 (68.7-70.2)	69.5 (68.7-70.4)	70.8 (70.1-71.4)	71.7 (71.1-72.3)	71.0 (70.4-71.6)	72.3 (71.7-72.9)	72.7 (72.0-73.2)	73.1 (72.6-73.7)	73.5 (72.9-74.0)	73.7 (73.2-74.2)	74.2 (73.6-74.7)	74.4 (73.9-75.0)	74.9 (74.4-75.4)	75.2 (74.7-75.8)	75.8 (75.3-76.3)	76.3 (75.8-76.8)	76.1 (75.6-76.7)	76.7 (76.2-77.2)	76.7 (76.3-77.2)	76.9 (76.4-77.3)	77.0 (76.6-77.4)	77.2 (76.8-77.7)	77.5 (77.0-77.9)	77.8 (77.3-78.2)	77.9 (77.4-78.4)	78.1 (77.6-78.7)	78.3 (77.7-79.0)	78.5 (77.8-79.2)	78.6 (77.9-79.4)	78.8 (78.0-79.6)	78.9 (78.1-79.8)
Guatemala	63.3 (62.8-63.8)	63.4 (62.4-64.3)	62.3 (60.9-63.6)	63.9 (63.4-64.4)	65.5 (65.1-65.9)	65.2 (64.8-65.7)	66.0 (65.4-66.5)	66.5 (66.0-67.0)	67.4 (66.8-67.8)	67.8 (67.4-68.3)	67.5 (67.1-67.9)	66.7 (66.2-67.1)	66.2 (65.9-66.5)	66.9 (66.5-67.2)	67.5 (67.2-67.9)	70.2 (69.8-70.5)	71.3 (70.9-71.7)	71.1 (70.7-71.5)	71.1 (70.7-71.4)	71.9 (71.6-72.3)	71.8 (71.5-72.1)	72.8 (72.4-73.1)	73.3 (72.9-73.6)	73.4 (73.1-73.7)	73.4 (73.1-73.7)	73.2 (72.8-73.5)	73.5 (73.2-73.9)	73.9 (73.5-74.3)	74.2 (73.7-74.6)	73.7 (73.3-74.1)	73.8 (73.4-74.2)	74.0 (73.6-74.4)	74.3 (73.9-74.8)	74.6 (74.2-75.1)	74.9 (73.9-76.0)	75.2 (73.5-77.0)
Honduras	69.8 (69.3-70.4)	70.2 (69.7-70.8)	70.5 (70.0-71.0)	70.9 (70.4-71.4)	71.2 (70.7-71.7)	71.4 (70.9-71.9)	71.5 (71.0-72.0)	71.9 (71.3-72.4)	72.0 (71.4-72.6)	72.0 (71.3-72.7)	72.1 (71.3-72.9)	71.9 (71.0-72.8)	71.5 (70.4-72.6)	71.3 (70.1-72.6)	71.4 (70.0-72.8)	71.0 (69.6-72.7)	71.0 (69.5-72.8)	71.0 (69.4-73.0)	66.2 (63.7-68.8)	71.1 (69.2-73.3)	71.2 (69.1-73.5)	71.3 (69.1-73.8)	71.4 (69.2-74.0)	71.6 (69.3-74.1)	71.8 (69.4-74.4)	72.0 (69.5-74.8)	72.2 (69.6-75.1)	72.4 (69.9-75.3)	72.6 (70.1-75.5)	72.8 (70.3-75.7)	72.9 (70.4-75.8)	73.1 (70.4-76.0)	73.2 (70.6-76.1)	73.5 (70.9-76.4)	73.7 (71.1-76.5)	74.0 (71.4-76.7)
Mexico	70.1 (69.7-70.5)	70.6 (70.2-71.0)	71.3 (71.0-71.7)	71.8 (71.4-72.1)	72.2 (71.8-72.5)	72.3 (71.9-72.7)	73.0 (72.7-73.3)	73.4 (73.1-73.7)	73.8 (73.5-74.1)	74.2 (73.9-74.5)	74.7 (74.4-75.0)	75.2 (74.9-75.5)	75.4 (75.1-75.7)	75.6 (75.3-75.8)	75.9 (75.6-76.1)	76.0 (75.7-76.3)	76.3 (76.1-76.6)	76.5 (76.2-76.8)	76.7 (76.4-76.9)	76.9 (76.7-77.2)	77.3 (77.1-77.6)	77.5 (77.3-77.8)	77.5 (77.3-77.8)	77.5 (77.3-77.8)	77.7 (77.5-78.0)	77.7 (77.5-77.9)	78.0 (77.7-78.2)	78.2 (77.9-78.4)	78.1 (77.9-78.4)	78.0 (77.7-78.2)	78.3 (78.0-78.5)	78.6 (78.3-78.8)	78.5 (78.2-78.7)	78.0 (77.7-78.3)	78.1 (77.8-78.3)	78.3 (78.0-78.6)
Nicaragua	69.2 (68.0-70.2)	69.6 (68.3-70.8)	70.1 (68.8-71.3)	69.4 (68.0-70.8)	69.7 (68.1-71.3)	70.4 (68.8-72.1)	71.6 (70.2-73.0)	72.1 (70.5-73.7)	73.9 (72.9-74.8)	75.4 (74.8-76.0)	76.0 (75.4-76.6)	76.3 (75.7-76.9)	76.3 (75.7-76.9)	76.6 (76.0-77.2)	76.8 (76.2-77.4)	77.1 (76.5-77.7)	77.4 (76.9-78.0)	77.8 (77.3-78.3)	76.3 (75.4-77.3)	78.3 (77.9-78.9)	78.6 (78.1-79.1)	78.8 (78.3-79.3)	79.0 (78.5-79.5)	79.0 (78.5-79.5)	79.1 (78.6-79.7)	79.3 (78.7-79.8)	79.4 (78.8-79.8)	79.5 (79.0-79.9)	79.7 (79.2-80.2)	79.7 (79.2-80.2)	79.8 (79.3-80.2)	79.9 (79.5-80.4)	80.1 (79.6-80.6)	80.3 (79.8-80.8)	80.5 (79.9-81.2)	80.8 (79.9-81.7)
Panama	75.6 (75.0-76.2)	76.0 (75.3-76.6)	76.3 (75.6-76.9)	76.3 (75.7-77.0)	76.5 (75.9-77.2)	76.7 (76.0-77.3)	76.8 (76.2-77.5)	77.0 (76.4-77.6)	77.1 (76.4-77.8)	77.1 (76.3-77.8)	77.5 (76.8-78.2)	77.7 (77.1-78.3)	77.9 (77.2-78.5)	78.1 (77.5-78.7)	78.2 (77.6-78.9)	78.4 (77.8-79.1)	78.4 (77.7-79.1)	78.4 (77.8-79.0)	78.6 (78.0-79.2)	78.8 (78.2-79.4)	79.2 (78.6-79.7)	79.4 (78.8-79.9)	79.5 (78.9-80.1)	79.5 (79.0-80.2)	79.6 (79.0-80.3)	79.7 (79.1-80.3)	79.9 (79.3-80.4)	80.1 (79.5-80.6)	80.2 (79.6-80.7)	80.3 (79.7-80.9)	80.4 (79.8-81.0)	80.5 (79.9-81.0)	80.5 (79.9-81.1)	80.5 (79.8-81.2)	80.7 (79.8-81.7)	81.0 (79.8-82.2)
Venezuela	72.7 (72.5-72.8)	73.1 (73.0-73.3)	73.4 (73.3-73.5)	74.0 (73.9-74.2)	74.2 (74.0-74.3)	74.3 (74.2-74.4)	74.9 (74.8-75.0)	74.7 (74.6-74.9)	75.0 (74.9-75.1)	75.2 (75.0-75.3)	75.2 (75.1-75.4)	75.2 (75.1-75.4)	75.4 (75.2-75.5)	75.8 (75.7-75.9)	75.5 (75.4-75.6)	75.8 (75.5-76.0)	76.0 (75.9-76.1)	76.4 (76.3-76.5)	76.7 (76.6-76.8)	74.0 (72.8-75.2)	77.4 (77.3-77.5)	77.5 (77.4-77.6)	77.1 (77.0-77.2)	77.3 (77.2-77.4)	78.0 (77.9-78.1)	78.2 (78.1-78.3)	78.4 (78.3-78.5)	78.6 (78.5-78.7)	78.6 (78.5-78.7)	78.7 (78.6-78.8)	78.8 (78.7-78.9)	78.8 (78.7-78.9)	78.8 (78.7-78.9)	79.0 (78.5-79.4)	79.1 (78.2-80.0)	79.2 (78.0-80.4)
Andean Latin America	64.5 (63.9-65.2)	64.9 (64.3-65.5)	65.4 (64.7-66.0)	65.8 (65.1-66.4)	66.3 (65.6-66.9)	66.9 (66.2-67.4)	67.5 (66.9-68.0)	67.8 (67.1-68.4)	68.7 (68.1-69.2)	69.2 (68.6-69.8)	69.7 (69.2-70.2)	70.2 (69.6-70.7)	70.5 (70.0-71.0)	70.8 (70.3-71.3)	71.3 (70.8-71.8)	71.8 (71.3-72.3)	72.4 (72.0-72.9)	73.0 (72.5-73.4)	73.6 (73.2-74.0)	74.2 (73.8-74.6)	74.7 (74.3-75.1)	75.2 (74.8-75.6)	75.6 (75.2-76.0)	76.0 (75.6-76.4)	76.4 (75.9-76.8)	76.8 (76.3-77.2)	77.2 (76.7-77.7)	77.4 (76.9-77.9)	77.7 (77.1-78.2)	77.8 (77.2-78.3)	78.0 (77.4-78.5)	78.1 (77.5-78.7)	78.3 (77.7-78.9)	78.6 (77.9-79.3)	78.8 (78.1-79.5)	79.0 (78.2-79.8)
Bolivia	57.7 (56.1-59.3)	58.1 (56.6-59.7)	58.4 (56.8-60.0)	58.7 (57.1-60.3)	59.3 (57.8-60.9)	60.0 (58.4-61.5)	60.5 (58.9-62.0)	60.9 (59.4-62.4)	61.4 (59.9-62.9)	62.0 (60.4-63.4)	62.6 (61.1-64.0)	63.0 (61.6-64.4)	63.4 (62.1-64.7)	63.9 (62.6-65.2)	64.5 (63.3-65.7)	65.2 (64.0-66.4)	65.9 (64.7-67.1)	66.6 (65.5-67.7)	67.4 (66.3-68.5)	68.2 (67.0-69.2)	68.9 (67.8-70.0)	69.6 (68.5-70.7)	70.0 (68.9-71.2)	70.6 (69.4-71.8)	71.0 (69.6-72.2)	71.4 (69.9-72.7)	71.8 (70.2-73.1)	72.0 (70.3-73.4)	72.3 (70.5-73.8)	72.6 (70.7-74.3)	72.9 (70.9-74.7)	73.1 (71.2-75.0)	73.4 (71.4-75.4)	73.7 (71.6-75.7)	73.9 (71.8-76.1)	74.2 (72.1-76.4)
Ecuador	69.3 (68.9-69.7)	70.2 (69.8-70.6)	70.8 (70.4-71.2)	71.2 (70.7-71.6)	71.6 (71.2-72.0)	72.1 (71.6-72.5)	72.4 (72.0-72.8)	71.5 (70.3-72.6)	73.1 (72.7-73.5)	73.4 (73.0-73.9)	73.7 (73.3-74.1)	74.0 (73.6-74.4)	74.3 (73.8-74.7)	74.4 (73.9-74.8)	74.7 (74.2-75.2)	74.9 (74.4-75.4)	75.0 (74.5-75.5)	75.2 (74.7-75.7)	75.4 (74.9-75.9)	75.7 (75.2-76.2)	75.8 (75.3-76.3)	76.1 (75.6-76.6)	76.2 (75.7-76.7)	76.4 (75.9-76.9)	76.6 (76.1-77.1)	76.9 (76.3-77.4)	77.0 (76.5-77.6)	77.2 (76.7-77.8)	77.4 (76.9-78.0)	77.6 (77.0-78.2)	77.7 (77.2-78.4)	77.9 (77.3-78.6)	78.1 (77.4-78.9)	78.3 (77.5-79.1)	78.4 (77.6-79.3)	78.6 (77.6-79.5)
Peru	64.9 (64.0-65.8)	65.1 (64.2-66.0)	65.6 (64.8-66.4)	66.0 (65.2-66.9)	66.5 (65.7-67.3)	67.1 (66.3-67.9)	67.8 (67.1-68.6)	68.7 (67.9-69.4)	69.4 (68.7-70.1)	70.1 (69.3-70.8)	70.6 (70.0-71.4)	71.1 (70.4-71.8)	71.4 (70.7-72.2)	71.9 (71.1-72.6)	72.3 (71.6-73.0)	72.9 (72.2-73.5)	73.6 (72.9-74.2)	74.3 (73.6-74.8)	75.1 (74.5-75.6)	75.8 (75.2-76.3)	76.4 (75.8-77.0)	76.9 (76.4-77.5)	77.4 (76.8-78.0)	77.8 (77.3-78.4)	78.3 (77.7-78.9)	78.8 (78.2-79.4)	79.4 (78.8-80.1)	79.7 (79.0-80.3)	79.9 (79.2-80.6)	79.9 (79.3-80.6)	80.0 (79.4-80.7)	80.1 (79.5-80.8)	80.3 (79.6-81.0)	80.6 (79.9-81.4)	80.8 (79.9-81.8)	81.1 (80.0-82.2)
Caribbean	67.4 (66.5-68.2)	67.8 (66.9-68.5)	68.1 (67.3-68.8)	68.3 (67.6-69.0)	68.5 (67.8-69.1)	68.6 (67.9-69.2)	68.7 (68.0-69.3)	69.0 (68.3-69.5)	69.2 (68.6-69.8)	69.4 (68.8-69.9)	69.6 (69.1-70.1)	69.8 (69.3-70.3)	70.0 (69.5-70.6)	70.1 (69.6-70.6)	70.2 (69.7-70.8)	70.7 (70.2-71.2)	70.9 (70.4-71.4)	71.2 (70.6-71.7)	71.4 (70.9-72.1)	71.7 (71.1-72.3)	72.0 (71.4-72.7)	72.3 (71.6-72.8)	72.5 (71.8-73.1)	72.6 (71.9-73.2)	72.3 (71.5-73.1)	72.7 (71.9-73.4)	72.9 (72.1-73.7)	73.2 (72.3-74.0)	73.4 (72.6-74.2)	73.7 (72.8-74.5)	63.1 (54.8-72.0)	74.1 (73.1-75.0)	74.3 (73.3-75.2)	74.6 (73.5-75.6)	74.9 (73.7-76.0)	75.2 (73.9-76.3)
Antigua and Barbuda	73.2 (72.3-74.0)	73.8 (72.9-74.8)	74.5 (73.6-75.4)	75.2 (74.3-76.0)	75.4 (74.6-76.2)	75.7 (75.0-76.6)	76.1 (75.3-77.0)	76.2 (75.4-77.2)	76.3 (75.5-77.2)	76.2 (75.5-77.1)	76.1 (75.4-77.0)	76.1 (75.4-76.9)	76.0 (75.3-76.7)	75.9 (75.2-76.7)	75.8 (75.1-76.6)	75.8 (75.2-76.6)	75.7 (75.1-76.5)	76.0 (75.3-76.8)	76.1 (75.5-76.9)	76.1 (75.4-76.8)	76.0 (75.4-76.7)	76.3 (75.7-77.1)	76.4 (75.8-77.3)	76.7 (76.1-77.6)	76.9 (76.2-77.7)	77.2 (76.5-78.0)	77.4 (76.7-78.2)	77.8 (77.1-78.5)	78.0 (77.4-78.6)	78.3 (77.6-78.9)	78.4 (77.7-79.0)	78.5 (77.8-79.1)	78.6 (77.9-79.2)	78.6 (77.9-79.3)	78.8 (78.0-79.6)	78.9 (78.1-79.8)
The Bahamas	69.3 (68.0-70.5)	69.5 (68.2-70.7)	69.8 (68.6-70.9)	70.1 (68.9-71.2)	70.3 (69.1-71.5)	70.1 (68.9-71.3)	70.7 (69.5-71.8)	71.0 (69.8-72.1)	71.4 (70.2-72.4)	71.6 (70.4-72.6)	71.8 (70.8-72.8)	72.2 (71.2-73.1)	72.5 (71.5-73.3)	72.9 (72.0-73.8)	72.8 (71.9-73.6)	73.0 (72.3-73.8)	73.5 (72.8-74.3)	73.7 (72.9-74.4)	73.5 (72.8-74.3)	73.4 (72.6-74.2)	73.4 (72.6-74.2)	73.5 (72.8-74.4)	73.5 (72.7-74.2)	74.0 (73.1-74.8)	74.5 (73.7-75.2)	74.4 (73.7-75.2)	74.7 (74.0-75.5)	74.7 (73.9-75.5)	74.6 (73.1-75.6)	75.2 (74.3-76.0)	75.0 (74.0-75.9)	75.0 (74.0-76.0)	75.3 (74.3-76.3)	75.3 (73.9-76.7)	76.2 (74.9-77.4)	76.4 (75.1-77.7)
Barbados	74.8 (73.7-75.7)	75.1 (74.0-76.0)	75.2 (74.1-76.1)	75.1 (74.1-76.0)	75.1 (74.1-76.0)	75.3 (74.3-76.2)	75.4 (74.5-76.3)	75.5 (74.5-76.3)	75.6 (74.6-76.4)	75.6 (74.7-76.5)	75.7 (74.8-76.5)	75.8 (75.0-76.6)	75.9 (75.0-76.7)	75.9 (75.0-76.7)	76.0 (75.1-76.7)	76.1 (75.2-76.9)	76.3 (75.3-77.1)	76.4 (75.5-77.3)	76.5 (75.6-77.4)	76.6 (75.6-77.5)	76.3 (75.4-77.2)	76.7 (75.8-77.5)	76.6 (75.8-77.5)	76.7 (75.8-77.5)	76.8 (75.9-77.6)	77.0 (76.2-77.7)	77.0 (76.2-77.7)	77.0 (76.2-77.7)	77.1 (76.3-77.8)	76.9 (76.2-77.7)	76.9 (76.2-77.7)	76.8 (76.0-77.6)	77.1 (76.1-78.0)	77.3 (76.3-78.2)	77.4 (76.3-78.4)	77.6 (76.2-78.7)
Belize	70.7 (69.7-71.8)	71.0 (70.0-72.0)	71.3 (70.2-72.3)	71.7 (70.6-72.7)	72.2 (71.2-73.2)	72.6 (71.6-73.6)	73.0 (72.0-74.0)	73.3 (72.3-74.2)	73.5 (72.5-74.4)	73.5 (72.5-74.4)	73.5 (72.5-74.4)	73.4 (72.5-74.3)	73.3 (72.4-74.2)	73.3 (72.5-74.3)	73.4 (72.6-74.3)	73.5 (72.8-74.3)	73.7 (72.9-74.5)	73.0 (72.3-73.7)	72.7 (72.0-73.4)	72.5 (71.8-73.2)	72.3 (71.5-73.0)	72.2 (71.4-72.9)	72.6 (71.9-73.3)	72.8 (72.2-73.5)	73.0 (72.3-73.6)	73.1 (72.4-73.8)	73.3 (72.7-73.9)	73.5 (72.9-74.1)	73.7 (73.1-74.3)	73.9 (73.3-74.5)	74.2 (73.6-74.8)	74.3 (73.6-74.9)	74.3 (73.6-75.0)	74.3 (73.5-75.1)	74.5 (73.6-75.4)	74.7 (73.7-75.8)
Bermuda	75.4 (74.7-76.1)	75.5 (74.8-76.1)	75.6 (75.0-76.3)	75.7 (75.1-76.4)	75.6 (75.0-76.2)	75.5 (74.8-76.1)	75.4 (74.7-76.0)	75.6 (74.9-76.2)	75.7 (75.0-76.3)	75.6 (75.0-76.3)	75.5 (74.9-76.1)	75.4 (74.7-76.1)	75.2 (74.5-75.8)	75.2 (74.6-75.8)	75.5 (74.8-76.1)	76.0 (75.4-76.7)	76.2 (75.5-76.9)	76.9 (76.2-77.5)	77.4 (76.7-78.0)	77.4 (76.8-78.1)	77.3 (76.6-78.0)	78.0 (77.3-78.6)	78.4 (77.7-79.0)	79.1 (78.5-79.7)	79.3 (78.8-79.9)	79.6 (79.1-80.2)	79.9 (79.4-80.4)	80.3 (79.7-80.8)	80.6 (80.0-81.2)	80.8 (80.2-81.4)	81.0 (80.4-81.7)	81.4 (80.8-82.2)	82.0 (81.2-82.8)	82.0 (81.1-82.9)	82.1 (81.2-83.2)	82.2 (81.2-83.3)
Cuba	76.4 (76.2-76.5)	76.4 (76.3-76.6)	76.6 (76.5-76.7)	76.6 (76.5-76.7)	76.6 (76.5-76.7)	76.5 (76.4-76.6)	76.5 (76.4-76.6)	76.6 (76.5-76.7)	76.7 (76.6-76.8)	76.8 (76.7-76.9)	76.9 (76.8-77.0)	77.0 (76.9-77.1)	77.0 (76.9-77.1)	77.0 (76.9-77.1)	77.1 (77.0-77.2)	77.3 (77.2-77.4)	77.4 (77.3-77.5)	77.6 (77.5-77.7)	77.8 (77.7-77.9)	78.0 (77.9-78.1)	78.3 (78.2-78.4)	78.5 (78.4-78.6)	78.8 (78.7-78.9)	79.0 (78.9-79.0)	79.0 (78.9-79.1)	79.1 (79.0-79.2)	79.3 (79.2-79.4)	79.4 (79.4-79.5)	79.6 (79.5-79.7)	79.7 (79.6-79.8)	79.8 (79.7-79.9)	80.0 (79.9-80.1)	80.2 (80.1-80.3)	80.3 (80.2-80.4)	80.5 (80.2-80.7)	80.6 (80.2-81.0)
Dominica	73.7 (72.6-74.7)	74.2 (73.2-75.2)	74.2 (73.2-75.1)	74.4 (73.5-75.4)	74.4 (73.5-75.4)	74.5 (73.6-75.6)	74.6 (73.7-75.6)	74.5 (73.7-75.5)	74.4 (73.6-75.3)	74.3 (73.4-75.1)	74.2 (73.3-75.1)	74.1 (73.2-74.9)	74.3 (73.4-75.1)	74.4 (73.6-75.3)	74.6 (73.8-75.5)	74.9 (74.0-75.8)	75.0 (74.1-76.0)	75.2 (74.4-76.2)	75.3 (74.4-76.3)	75.8 (74.8-76.8)	76.2 (75.2-77.1)	76.5 (75.5-77.4)	76.7 (75.7-77.5)	76.8 (75.8-77.6)	77.0 (76.0-77.8)	77.2 (76.3-77.9)	77.2 (76.3-78.0)	77.3 (76.4-78.1)	77.3 (76.5-78.2)	77.3 (76.4-78.2)	77.2 (76.2-78.1)	77.0 (75.9-77.9)	76.9 (75.7-77.9)	76.7 (75.5-77.8)	76.8 (75.4-77.9)	76.8 (75.5-78.1)
Dominican Republic	70.8 (70.3-71.4)	71.3 (70.8-71.9)	71.9 (71.4-72.5)	72.4 (71.8-72.9)	72.6 (72.0-73.1)	72.7 (72.1-73.2)	72.8 (72.3-73.4)	73.0 (72.4-73.5)	73.3 (72.7-73.9)	73.7 (73.2-74.3)	73.9 (73.3-74.5)	74.3 (73.7-74.9)	74.7 (74.1-75.2)	74.7 (74.2-75.2)	74.9 (74.4-75.4)	75.1 (74.6-75.7)	75.3 (74.7-75.8)	75.6 (75.0-76.1)	75.7 (75.2-76.3)	76.0 (75.4-76.6)	76.2 (75.6-76.8)	76.3 (75.7-76.8)	76.4 (75.8-76.9)	76.3 (75.7-76.8)	76.1 (75.4-76.6)	76.2 (75.5-76.6)	76.3 (75.6-76.8)	76.5 (76.0-77.0)	76.9 (76.3-77.3)	77.1 (76.6-77.5)	77.3 (76.8-77.7)	77.4 (76.9-77.8)	77.5 (77.1-77.9)	77.7 (77.2-78.1)	77.8 (77.3-78.2)	77.9 (77.4-78.3)
Grenada	70.1 (67.5-72.0)	70.4 (67.7-72.3)	70.6 (67.9-72.4)	70.7 (68.3-72.5)	70.8 (68.6-72.4)	71.0 (69.0-72.4)	71.3 (69.5-72.6)	71.5 (69.9-72.7)	71.8 (70.3-72.9)	72.0 (70.7-73.1)	72.2 (71.0-73.2)	72.7 (71.5-73.5)	72.9 (72.0-73.8)	73.1 (72.3-73.9)	73.3 (72.5-74.0)	73.4 (72.7-74.1)	73.6 (72.9-74.2)	73.6 (72.9-74.3)	73.6 (72.9-74.3)	73.7 (72.9-74.3)	73.6 (72.9-74.3)	73.5 (72.7-74.2)	73.3 (72.5-74.0)	73.3 (72.5-74.0)	72.6 (71.5-73.6)	73.4 (72.6-74.1)	73.5 (72.7-74.2)	73.6 (72.9-74.3)	73.7 (72.9-74.4)	73.8 (73.0-74.5)	73.7 (72.9-74.5)	73.8 (73.0-74.5)	73.8 (73.0-74.6)	74.0 (73.0-74.8)	74.1 (73.0-75.1)	74.3 (72.9-75.4)
Guyana	65.7 (64.8-66.7)	66.1 (65.2-67.1)	66.5 (65.5-67.5)	66.8 (65.9-67.8)	67.3 (66.3-68.2)	67.3 (66.3-68.2)	67.3 (66.4-68.2)	67.5 (66.6-68.3)	67.6 (66.7-68.4)	67.6 (66.8-68.3)	67.6 (66.9-68.2)	67.6 (66.9-68.2)	67.6 (66.9-68.3)	67.5 (66.8-68.2)	67.4 (66.6-68.1)	67.1 (66.4-67.8)	67.2 (66.4-67.9)	67.3 (66.4-68.1)	67.3 (66.4-68.1)	67.6 (66.7-68.4)	67.7 (66.8-68.6)	67.3 (66.5-68.2)	66.9 (66.0-67.8)	67.3 (66.5-68.1)	67.7 (66.9-68.6)	67.8 (67.0-68.7)	68.1 (67.3-69.0)	68.3 (67.4-69.1)	68.5 (67.6-69.4)	68.7 (67.8-69.7)	68.9 (67.9-69.9)	69.1 (68.0-70.1)	69.5 (68.2-70.5)	69.8 (68.5-71.0)	70.1 (68.7-71.4)	70.5 (69.0-71.9)
Haiti	50.8 (48.5-53.3)	51.6 (49.4-53.9)	52.2 (50.0-54.3)	52.6 (50.6-54.7)	52.8 (50.9-54.9)	53.2 (51.2-55.1)	53.3 (51.6-55.1)	53.8 (52.0-55.4)	54.1 (52.3-55.7)	54.2 (52.6-55.7)	54.4 (52.9-56.0)	54.6 (53.1-56.1)	55.1 (53.6-56.7)	55.2 (53.7-56.8)	55.4 (53.8-57.1)	56.3 (54.7-58.0)	56.8 (55.1-58.5)	57.2 (55.5-59.1)	57.6 (55.8-59.7)	58.0 (56.1-60.0)	58.7 (56.7-60.8)	59.1 (57.0-61.2)	59.4 (57.3-61.6)	59.5 (57.3-61.8)	58.7 (56.3-61.2)	59.9 (57.3-62.3)	60.2 (57.6-63.0)	60.8 (58.2-63.7)	61.2 (58.6-64.1)	61.8 (59.2-64.9)	37.2 (25.7-57.0)	62.6 (59.6-65.8)	63.0 (59.9-66.1)	63.7 (60.3-67.0)	64.2 (60.9-67.6)	64.7 (61.2-68.2)
Jamaica	73.1 (72.3-73.8)	73.4 (72.7-74.1)	73.6 (72.9-74.2)	73.8 (73.1-74.4)	74.0 (73.3-74.6)	74.2 (73.6-74.8)	74.5 (73.8-75.0)	74.9 (74.2-75.5)	75.2 (74.5-75.8)	75.5 (74.8-76.1)	75.8 (75.0-76.4)	75.9 (75.1-76.7)	75.9 (75.1-76.8)	75.9 (75.0-76.8)	75.8 (74.9-76.8)	75.8 (74.9-76.7)	75.7 (74.9-76.6)	75.7 (74.9-76.5)	75.8 (75.0-76.6)	76.0 (75.3-76.8)	75.9 (75.1-76.5)	75.8 (75.1-76.4)	76.1 (75.3-76.7)	76.3 (75.4-76.9)	76.4 (75.6-77.1)	76.7 (75.9-77.4)	77.0 (76.1-77.7)	76.9 (76.0-77.6)	76.7 (75.8-77.5)	76.6 (75.6-77.3)	76.5 (75.6-77.3)	76.4 (75.5-77.2)	76.5 (75.6-77.5)	76.7 (75.7-77.8)	76.9 (75.7-78.0)	77.0 (75.7-78.2)
Puerto Rico	77.3 (77.1-77.4)	77.4 (77.2-77.6)	77.6 (77.4-77.8)	77.7 (77.6-77.9)	77.9 (77.7-78.1)	77.5 (77.1-77.9)	78.0 (77.8-78.2)	78.0 (77.8-78.2)	77.9 (77.7-78.1)	78.1 (77.9-78.3)	78.1 (78.0-78.3)	78.3 (78.1-78.5)	78.4 (78.2-78.6)	78.5 (78.4-78.7)	78.0 (77.8-78.2)	78.2 (78.0-78.4)	78.2 (78.0-78.3)	78.7 (78.6-78.9)	79.2 (79.1-79.4)	79.2 (79.1-79.4)	79.5 (79.4-79.7)	79.6 (79.4-79.7)	79.9 (79.7-80.0)	80.2 (80.0-80.3)	80.1 (80.0-80.3)	80.2 (80.1-80.4)	80.4 (80.3-80.6)	80.6 (80.4-80.7)	80.7 (80.5-80.8)	80.7 (80.5-80.8)	80.7 (80.6-80.9)	81.0 (80.8-81.1)	81.1 (80.9-81.3)	81.3 (81.0-81.5)	81.5 (81.1-81.9)	81.8 (81.2-82.4)
Saint Lucia	69.4 (67.9-70.7)	70.1 (68.8-71.4)	70.5 (69.1-71.7)	71.0 (69.6-72.1)	71.3 (69.8-72.4)	71.4 (69.9-72.5)	71.5 (70.1-72.6)	71.6 (70.3-72.7)	71.2 (69.7-72.4)	72.1 (70.8-73.1)	72.3 (71.2-73.2)	72.5 (71.5-73.5)	72.8 (71.8-73.8)	73.1 (72.2-73.9)	73.3 (72.5-74.0)	73.6 (72.8-74.2)	73.7 (73.1-74.3)	74.0 (73.4-74.7)	74.4 (73.7-75.0)	74.6 (73.9-75.3)	74.9 (74.2-75.6)	75.2 (74.4-75.9)	75.4 (74.6-76.1)	75.7 (74.9-76.4)	76.0 (75.1-76.8)	76.1 (75.3-77.1)	76.4 (75.5-77.4)	76.5 (75.6-77.5)	76.7 (75.6-77.6)	76.9 (75.8-77.8)	76.9 (75.9-77.8)	76.8 (75.8-77.7)	76.8 (75.7-77.8)	76.9 (75.8-78.0)	77.0 (75.8-78.1)	77.1 (75.8-78.3)
Saint Vincent and the Grenadines	69.2 (67.2-70.8)	69.7 (67.9-71.2)	70.2 (68.5-71.6)	70.4 (68.7-71.8)	70.5 (69.0-71.9)	70.8 (69.4-72.1)	71.1 (69.6-72.3)	71.2 (69.8-72.4)	71.3 (70.0-72.5)	71.2 (70.0-72.4)	71.2 (70.0-72.4)	71.2 (70.0-72.2)	71.1 (70.1-72.2)	71.2 (70.1-72.2)	71.2 (70.2-72.2)	71.2 (70.3-72.2)	71.2 (70.3-72.1)	71.6 (70.7-72.5)	71.8 (70.9-72.6)	71.8 (70.9-72.5)	71.5 (70.7-72.3)	72.1 (71.3-72.8)	72.2 (71.4-72.9)	72.6 (71.9-73.3)	72.8 (72.0-73.5)	73.0 (72.2-73.7)	73.4 (72.6-74.1)	73.5 (72.7-74.2)	73.5 (72.6-74.2)	73.7 (72.9-74.5)	73.8 (73.0-74.6)	73.7 (72.8-74.5)	73.7 (72.8-74.6)	73.5 (72.5-74.4)	73.8 (72.7-74.7)	73.9 (72.8-75.0)
Suriname	67.8 (67.0-68.6)	68.1 (67.3-68.9)	68.7 (67.8-69.5)	69.3 (68.4-70.1)	69.8 (68.9-70.6)	70.3 (69.5-71.1)	70.6 (69.8-71.4)	71.0 (70.2-71.8)	71.3 (70.6-72.1)	71.3 (70.5-72.1)	72.1 (71.4-72.8)	72.4 (71.7-73.1)	72.3 (71.6-73.1)	72.2 (71.5-72.9)	72.3 (71.5-73.0)	72.6 (71.8-73.4)	72.8 (72.0-73.6)	72.8 (72.0-73.6)	72.6 (71.8-73.5)	72.4 (71.6-73.2)	72.2 (71.4-73.1)	72.1 (71.2-72.9)	72.1 (71.2-72.9)	72.3 (71.4-73.1)	72.5 (71.7-73.3)	72.7 (71.9-73.6)	72.9 (72.1-73.7)	73.1 (72.2-74.0)	73.3 (72.4-74.2)	73.7 (72.7-74.6)	74.0 (72.9-74.9)	74.3 (73.2-75.2)	74.6 (73.5-75.5)	74.8 (73.6-75.8)	75.1 (73.8-76.2)	75.3 (73.9-76.5)
Trinidad and Tobago	70.3 (70.0-70.7)	70.6 (70.3-70.9)	70.9 (70.5-71.2)	70.8 (70.5-71.2)	70.9 (70.6-71.3)	71.1 (70.8-71.4)	71.3 (70.9-71.6)	71.4 (71.0-71.7)	71.4 (71.1-71.8)	71.7 (71.3-72.0)	71.7 (71.4-72.1)	72.1 (71.7-72.4)	72.0 (71.6-72.3)	72.1 (71.7-72.4)	72.1 (71.6-72.4)	72.3 (71.9-72.7)	72.3 (71.9-72.6)	72.4 (72.0-72.7)	72.6 (72.2-73.0)	72.2 (71.8-72.6)	72.5 (72.0-72.9)	72.9 (72.4-73.3)	73.1 (72.7-73.5)	73.8 (73.3-74.1)	74.6 (74.1-75.0)	74.5 (74.1-74.9)	74.8 (74.3-75.2)	75.2 (74.7-75.6)	75.3 (74.7-75.7)	75.3 (74.7-75.9)	75.4 (74.7-76.1)	75.5 (74.8-76.3)	75.7 (74.8-76.5)	75.8 (74.8-76.7)	76.0 (74.8-77.0)	76.1 (74.9-77.2)
Virgin Islands, U.S.	73.8 (73.1-74.5)	74.3 (73.5-74.9)	74.6 (73.9-75.2)	75.0 (74.4-75.6)	75.4 (74.8-75.9)	75.5 (74.9-76.0)	75.8 (75.2-76.3)	75.9 (75.3-76.4)	76.1 (75.5-76.6)	76.3 (75.7-76.8)	76.5 (75.9-77.0)	76.6 (76.0-77.1)	76.7 (76.2-77.3)	76.8 (76.3-77.4)	76.9 (76.3-77.4)	76.9 (76.4-77.4)	77.1 (76.6-77.6)	77.2 (76.7-77.7)	77.3 (76.8-77.8)	77.4 (76.9-77.9)	77.5 (77.1-78.0)	77.6 (77.2-78.2)	77.7 (77.3-78.3)	77.8 (77.4-78.4)	77.9 (77.4-78.4)	78.0 (77.5-78.5)	78.1 (77.6-78.6)	78.3 (77.7-78.7)	78.4 (77.9-78.9)	78.4 (77.9-78.9)	78.5 (77.9-79.0)	78.6 (78.0-79.1)	78.6 (78.0-79.2)	78.7 (77.9-79.3)	78.7 (77.9-79.4)	78.7 (77.9-79.5)
Tropical Latin America	68.9 (68.3-69.5)	69.3 (68.8-69.8)	69.6 (69.0-70.1)	69.9 (69.3-70.4)	70.0 (69.5-70.4)	70.3 (69.8-70.7)	70.7 (70.2-71.2)	71.1 (70.6-71.5)	71.5 (71.0-71.9)	71.8 (71.4-72.3)	71.9 (71.5-72.3)	72.2 (71.8-72.6)	72.4 (72.0-72.8)	72.6 (72.2-73.0)	72.9 (72.5-73.3)	73.1 (72.7-73.5)	73.3 (72.9-73.7)	73.6 (73.2-74.0)	74.0 (73.5-74.4)	74.3 (73.9-74.7)	74.6 (74.3-75.0)	75.0 (74.6-75.4)	75.3 (74.9-75.7)	75.6 (75.2-75.9)	75.9 (75.5-76.3)	76.2 (75.8-76.6)	76.5 (76.1-76.8)	76.7 (76.4-77.1)	77.0 (76.6-77.4)	77.2 (76.8-77.6)	77.4 (77.0-77.8)	77.6 (77.2-77.9)	77.7 (77.3-78.1)	77.9 (77.4-78.3)	78.0 (77.6-78.5)	78.2 (77.6-78.7)
Brazil	68.8 (68.2-69.4)	69.2 (68.7-69.8)	69.5 (68.9-70.0)	69.8 (69.2-70.3)	69.9 (69.4-70.4)	70.2 (69.6-70.6)	70.6 (70.1-71.1)	71.0 (70.5-71.4)	71.4 (70.9-71.8)	71.7 (71.3-72.2)	71.8 (71.3-72.2)	72.1 (71.7-72.5)	72.3 (71.9-72.7)	72.6 (72.1-73.0)	72.8 (72.4-73.2)	73.0 (72.6-73.4)	73.2 (72.8-73.6)	73.6 (73.1-74.0)	73.9 (73.5-74.3)	74.2 (73.8-74.6)	74.6 (74.2-75.0)	75.0 (74.6-75.3)	75.3 (74.9-75.7)	75.6 (75.2-76.0)	75.9 (75.5-76.3)	76.2 (75.8-76.6)	76.5 (76.1-76.9)	76.8 (76.4-77.1)	77.0 (76.6-77.4)	77.2 (76.8-77.6)	77.4 (77.0-77.8)	77.6 (77.2-78.0)	77.8 (77.4-78.1)	77.9 (77.5-78.3)	78.1 (77.6-78.5)	78.2 (77.6-78.7)
Paraguay	73.0 (72.1-73.9)	73.1 (72.3-74.0)	73.5 (72.7-74.3)	73.5 (72.6-74.3)	73.6 (72.7-74.5)	74.0 (73.1-74.8)	74.4 (73.5-75.2)	74.8 (74.0-75.6)	75.2 (74.4-76.0)	75.4 (74.5-76.1)	75.6 (74.7-76.4)	75.5 (74.6-76.3)	75.4 (74.5-76.3)	75.5 (74.5-76.4)	75.5 (74.6-76.5)	75.8 (74.8-76.7)	76.0 (75.0-76.9)	76.2 (75.2-77.0)	76.3 (75.3-77.1)	76.4 (75.4-77.1)	76.3 (75.3-77.1)	76.3 (75.3-77.1)	76.2 (75.3-77.0)	76.2 (75.2-77.0)	76.1 (75.1-76.9)	76.1 (75.2-77.0)	76.1 (75.2-77.0)	76.2 (75.3-77.1)	76.2 (75.3-77.2)	76.2 (75.3-77.2)	76.3 (75.4-77.2)	76.4 (75.4-77.4)	76.4 (75.4-77.5)	76.5 (75.4-77.7)	76.8 (75.5-78.0)	76.9 (75.6-78.4)
Southeast Asia, East Asia, and Oceania	64.9 (64.0-65.9)	65.3 (64.4-66.2)	65.8 (64.8-66.7)	66.2 (65.3-67.2)	66.7 (65.8-67.6)	67.1 (66.3-68.0)	67.5 (66.7-68.3)	67.9 (67.0-68.7)	68.1 (67.2-68.8)	68.3 (67.4-69.1)	68.6 (67.9-69.4)	68.9 (68.1-69.7)	69.3 (68.5-70.0)	69.6 (68.8-70.4)	70.1 (69.4-70.8)	70.4 (69.7-71.0)	70.8 (70.2-71.3)	71.1 (70.6-71.7)	71.4 (70.9-72.0)	71.7 (71.1-72.2)	71.8 (71.3-72.4)	72.1 (71.6-72.6)	72.4 (71.9-72.8)	73.0 (72.6-73.4)	73.3 (72.8-73.7)	74.0 (73.6-74.3)	74.5 (74.2-74.9)	75.0 (74.7-75.4)	75.3 (74.9-75.6)	76.0 (75.6-76.3)	76.4 (76.0-76.8)	76.9 (76.5-77.3)	77.4 (77.0-77.8)	77.8 (77.4-78.2)	78.0 (77.6-78.5)	78.4 (77.8-78.9)
East Asia	65.7 (64.3-66.9)	65.9 (64.7-67.2)	66.5 (65.1-67.7)	66.9 (65.5-68.2)	67.4 (66.1-68.6)	67.8 (66.6-69.0)	68.2 (67.0-69.4)	68.5 (67.2-69.6)	68.6 (67.4-69.7)	68.7 (67.6-69.8)	69.0 (68.0-70.1)	69.3 (68.2-70.3)	69.6 (68.6-70.7)	70.1 (69.0-71.1)	70.6 (69.6-71.5)	70.8 (69.9-71.7)	71.3 (70.5-72.1)	71.7 (70.9-72.5)	72.0 (71.3-72.7)	72.3 (71.5-73.0)	72.5 (71.8-73.2)	72.7 (72.0-73.3)	73.0 (72.3-73.6)	73.9 (73.3-74.4)	74.4 (73.9-74.9)	74.9 (74.5-75.4)	75.6 (75.2-76.1)	76.2 (75.7-76.6)	76.6 (76.1-77.0)	77.2 (76.8-77.6)	77.7 (77.3-78.1)	78.3 (77.9-78.7)	78.8 (78.4-79.2)	79.2 (78.8-79.7)	79.4 (78.9-80.0)	79.8 (79.2-80.5)
China	65.5 (64.1-66.8)	65.8 (64.5-67.1)	66.3 (64.9-67.6)	66.8 (65.3-68.1)	67.2 (66.0-68.4)	67.7 (66.5-68.9)	68.0 (66.8-69.3)	68.4 (67.1-69.5)	68.5 (67.2-69.6)	68.6 (67.4-69.7)	68.9 (67.8-70.0)	69.2 (68.1-70.3)	69.5 (68.4-70.5)	69.9 (68.9-71.0)	70.4 (69.5-71.4)	70.9 (70.0-71.8)	71.5 (70.6-72.2)	71.9 (71.1-72.6)	72.2 (71.4-72.9)	72.5 (71.7-73.2)	72.6 (72.0-73.3)	72.8 (72.2-73.5)	73.2 (72.5-73.7)	73.8 (73.2-74.3)	74.4 (73.8-74.9)	74.9 (74.4-75.4)	75.6 (75.1-76.0)	76.2 (75.7-76.6)	76.6 (76.1-77.0)	77.2 (76.8-77.6)	77.7 (77.2-78.1)	78.3 (77.9-78.7)	78.8 (78.4-79.3)	79.3 (78.8-79.8)	79.5 (78.9-80.0)	79.9 (79.2-80.6)
North Korea	66.5 (59.1-72.0)	66.9 (59.6-72.1)	67.3 (60.4-72.3)	67.7 (60.8-72.9)	68.2 (61.6-73.1)	68.6 (62.4-73.4)	69.1 (63.3-73.7)	69.5 (63.8-73.9)	69.9 (64.8-74.2)	70.3 (65.1-74.6)	70.7 (65.7-74.8)	71.0 (66.5-75.1)	71.3 (66.7-75.3)	71.5 (67.0-75.3)	71.6 (67.2-75.4)	60.7 (43.8-71.5)	60.8 (42.9-71.8)	60.9 (43.3-71.5)	61.0 (43.5-71.1)	61.1 (44.2-71.1)	61.2 (44.4-71.1)	61.4 (44.5-71.1)	61.6 (44.5-71.2)	72.1 (68.8-75.2)	72.3 (69.4-75.2)	72.6 (69.8-75.1)	72.8 (70.4-75.0)	73.0 (70.9-75.1)	73.3 (71.4-75.3)	73.5 (71.6-75.6)	73.8 (71.7-76.0)	74.0 (71.7-76.4)	74.2 (71.7-76.7)	74.5 (71.9-76.9)	74.7 (72.1-77.2)	74.9 (72.2-77.5)
Taiwan	74.1 (73.9-74.2)	74.5 (74.3-74.6)	74.8 (74.7-74.9)	74.9 (74.8-75.0)	75.4 (75.2-75.5)	75.6 (75.5-75.8)	75.9 (75.8-76.0)	76.2 (76.0-76.3)	76.2 (76.0-76.3)	76.4 (76.3-76.5)	76.7 (76.6-76.8)	77.1 (77.0-77.2)	77.3 (77.2-77.4)	77.4 (77.3-77.6)	77.7 (77.6-77.8)	77.7 (77.6-77.8)	77.9 (77.8-78.0)	78.4 (78.3-78.5)	78.6 (78.5-78.7)	78.4 (78.2-78.7)	79.1 (79.0-79.2)	79.5 (79.4-79.6)	80.0 (79.9-80.1)	80.3 (80.2-80.4)	80.5 (80.4-80.6)	80.7 (80.6-80.8)	81.2 (81.0-81.3)	81.4 (81.3-81.5)	81.7 (81.6-81.8)	81.9 (81.7-82.0)	82.2 (82.1-82.3)	82.2 (82.1-82.3)	82.3 (82.1-82.4)	82.3 (81.8-82.9)	82.4 (81.4-83.4)	82.5 (81.2-83.9)
Southeast Asia	63.5 (62.3-64.7)	64.1 (63.0-65.2)	64.5 (63.4-65.6)	64.9 (63.8-65.9)	65.4 (64.3-66.4)	65.8 (64.8-66.7)	66.3 (65.3-67.2)	66.7 (65.7-67.5)	67.1 (66.2-67.9)	67.5 (66.7-68.3)	67.9 (67.0-68.7)	68.3 (67.4-69.1)	68.6 (67.8-69.4)	68.9 (68.0-69.7)	69.2 (68.3-69.9)	69.4 (68.6-70.1)	69.6 (68.8-70.3)	69.8 (69.1-70.5)	70.0 (69.3-70.7)	70.3 (69.6-70.9)	70.5 (69.8-71.2)	70.8 (70.1-71.5)	71.1 (70.4-71.8)	71.5 (70.8-72.2)	71.1 (70.3-72.0)	72.2 (71.5-72.9)	72.5 (71.8-73.2)	72.9 (72.2-73.6)	72.7 (71.9-73.5)	73.5 (72.7-74.2)	73.8 (73.0-74.6)	74.1 (73.3-75.0)	74.5 (73.6-75.3)	74.7 (73.8-75.6)	75.0 (74.1-76.0)	75.3 (74.3-76.3)
Cambodia	56.3 (53.8-59.5)	56.7 (54.3-59.7)	57.0 (54.8-59.9)	57.4 (55.4-60.1)	57.8 (55.9-60.0)	58.1 (56.2-60.0)	58.3 (56.6-60.1)	59.3 (57.8-60.9)	59.5 (58.1-60.9)	59.4 (58.1-60.8)	59.6 (58.5-60.9)	59.8 (58.7-61.0)	60.0 (58.9-61.2)	59.9 (58.9-61.0)	59.8 (58.8-60.7)	60.0 (59.0-60.9)	60.1 (59.1-61.2)	60.5 (59.6-61.6)	61.0 (60.0-62.0)	61.5 (60.5-62.5)	62.0 (61.1-63.0)	62.8 (61.8-63.8)	63.6 (62.7-64.5)	64.5 (63.6-65.5)	65.3 (64.4-66.3)	66.2 (65.3-67.3)	67.1 (66.2-68.2)	67.9 (67.0-69.0)	68.7 (67.7-69.8)	69.4 (68.3-70.6)	69.9 (68.8-71.2)	70.5 (69.2-71.9)	71.2 (69.6-72.6)	71.6 (70.0-73.3)	72.1 (70.3-74.0)	72.4 (70.5-74.6)
Indonesia	60.4 (58.8-62.3)	61.2 (59.8-62.9)	61.8 (60.5-63.2)	62.2 (61.0-63.5)	62.8 (61.7-64.1)	63.3 (62.2-64.4)	63.8 (62.7-64.9)	64.1 (63.0-65.2)	64.5 (63.5-65.6)	65.0 (64.0-66.2)	65.4 (64.4-66.6)	66.0 (64.9-67.1)	66.4 (65.4-67.5)	66.7 (65.7-67.8)	67.1 (66.1-68.0)	67.3 (66.4-68.3)	67.6 (66.6-68.5)	67.8 (66.9-68.6)	68.0 (67.2-68.9)	68.2 (67.4-69.1)	68.5 (67.7-69.4)	68.8 (67.9-69.7)	69.1 (68.2-70.1)	69.4 (68.5-70.4)	68.4 (67.1-69.7)	70.1 (69.2-71.1)	70.3 (69.4-71.4)	70.8 (69.7-71.9)	71.1 (70.0-72.3)	71.4 (70.3-72.7)	71.7 (70.5-73.1)	72.0 (70.7-73.6)	72.4 (70.8-73.9)	72.7 (71.0-74.4)	73.0 (71.2-74.8)	73.3 (71.2-75.4)
Laos	48.8 (45.0-52.9)	49.3 (45.7-53.3)	49.8 (46.1-53.6)	50.3 (46.9-54.2)	50.9 (47.5-54.6)	51.3 (48.0-54.9)	51.6 (48.4-55.1)	51.9 (48.7-55.5)	52.2 (49.0-55.8)	52.6 (49.6-56.1)	53.0 (50.1-56.5)	53.4 (50.6-56.7)	53.8 (51.1-56.9)	54.2 (51.8-57.1)	54.6 (52.4-57.2)	55.0 (52.9-57.4)	55.4 (53.4-57.6)	55.9 (54.1-57.9)	56.5 (54.7-58.5)	57.0 (55.3-58.8)	57.7 (56.0-59.4)	58.3 (56.6-60.0)	59.3 (57.7-61.0)	60.3 (58.7-62.0)	61.2 (59.6-63.0)	62.1 (60.3-63.9)	63.0 (61.4-64.8)	63.9 (62.2-65.7)	64.7 (63.1-66.6)	65.6 (63.9-67.6)	66.5 (64.6-68.5)	67.3 (65.3-69.5)	68.0 (65.7-70.2)	68.5 (66.1-70.9)	69.0 (66.3-71.5)	69.5 (66.7-72.2)
Malaysia	71.4 (70.9-71.8)	71.8 (71.3-72.2)	72.2 (71.8-72.5)	72.4 (72.2-72.6)	72.6 (72.5-72.7)	72.8 (72.7-72.9)	73.1 (73.0-73.2)	73.4 (73.2-73.6)	73.7 (73.5-73.9)	73.9 (73.7-74.1)	74.0 (73.9-74.2)	74.3 (74.2-74.4)	74.5 (74.4-74.6)	74.7 (74.6-74.8)	74.8 (74.7-74.8)	74.8 (74.8-74.9)	75.0 (74.9-75.1)	75.2 (75.1-75.3)	75.3 (75.3-75.4)	75.5 (75.4-75.6)	75.6 (75.5-75.7)	75.7 (75.7-75.8)	75.9 (75.8-76.0)	76.0 (75.9-76.1)	76.2 (76.1-76.2)	76.3 (76.2-76.4)	76.5 (76.4-76.5)	76.6 (76.5-76.6)	76.6 (76.5-76.6)	76.6 (76.5-76.7)	77.0 (76.7-77.2)	77.4 (77.0-77.8)	77.7 (77.1-78.3)	78.0 (77.3-78.8)	78.3 (77.5-79.2)	78.6 (77.7-79.6)
Maldives	56.7 (55.0-58.8)	57.6 (56.2-59.3)	58.5 (57.3-59.8)	59.3 (58.3-60.4)	60.1 (59.3-60.9)	61.2 (60.5-61.8)	62.1 (61.5-62.7)	63.0 (62.4-63.5)	63.8 (63.2-64.3)	64.6 (64.1-65.2)	65.4 (64.9-66.0)	66.2 (65.7-66.8)	67.0 (66.5-67.6)	68.0 (67.3-68.7)	69.0 (68.2-69.8)	69.9 (69.2-70.8)	70.9 (70.1-71.7)	71.8 (71.2-72.5)	72.6 (72.0-73.3)	73.5 (72.8-74.1)	74.3 (73.7-74.8)	75.1 (74.5-75.5)	75.9 (75.4-76.2)	76.7 (76.3-77.1)	76.6 (75.9-77.3)	78.2 (77.8-78.7)	78.8 (78.4-79.3)	79.5 (79.0-80.0)	80.2 (79.7-80.7)	80.7 (80.1-81.3)	81.3 (80.6-81.9)	81.9 (81.2-82.5)	82.1 (81.2-82.9)	82.2 (81.2-83.2)	82.2 (81.1-83.4)	82.3 (81.0-83.5)
Mauritius	69.9 (69.6-70.2)	70.2 (69.9-70.5)	70.7 (70.4-71.0)	70.9 (70.6-71.2)	71.1 (70.9-71.4)	71.5 (71.2-71.7)	71.8 (71.6-72.1)	72.3 (72.0-72.6)	72.7 (72.4-73.0)	73.1 (72.8-73.4)	73.4 (73.1-73.6)	73.5 (73.2-73.8)	73.6 (73.3-73.9)	73.7 (73.4-74.0)	74.0 (73.7-74.3)	74.3 (74.0-74.6)	74.6 (74.3-74.9)	74.8 (74.5-75.1)	74.8 (74.5-75.1)	74.8 (74.5-75.1)	74.8 (74.5-75.1)	75.0 (74.7-75.3)	75.0 (74.7-75.3)	75.2 (74.9-75.4)	75.4 (75.2-75.7)	75.7 (75.4-75.9)	75.9 (75.6-76.1)	76.2 (75.9-76.5)	76.5 (76.3-76.8)	76.7 (76.5-77.0)	77.0 (76.7-77.2)	77.2 (77.0-77.5)	77.4 (77.1-77.7)	77.5 (77.2-77.7)	77.5 (77.1-77.9)	77.7 (77.1-78.2)
Myanmar	56.1 (51.1-61.3)	56.5 (51.4-61.6)	56.8 (51.7-62.1)	57.2 (52.2-62.5)	57.6 (52.7-62.9)	57.9 (53.2-63.3)	58.3 (53.6-63.6)	58.7 (53.9-63.8)	58.8 (54.2-64.0)	59.2 (54.6-64.3)	59.5 (54.9-64.7)	59.8 (55.1-65.0)	60.1 (55.4-65.3)	60.4 (55.8-65.5)	60.8 (56.3-65.8)	61.2 (56.7-66.2)	61.5 (57.1-66.5)	61.9 (57.4-66.9)	62.3 (57.8-67.4)	62.7 (58.3-67.8)	63.2 (58.8-68.1)	63.7 (59.2-68.7)	64.2 (59.8-69.3)	64.8 (60.6-70.0)	65.4 (61.2-70.4)	66.0 (61.8-70.8)	66.7 (62.4-71.4)	67.3 (62.9-71.8)	63.2 (58.3-68.6)	68.5 (63.7-72.9)	69.0 (64.1-73.4)	69.5 (64.8-73.8)	69.9 (65.5-74.2)	70.4 (65.8-74.6)	70.8 (66.3-74.9)	71.2 (66.9-75.3)
Philippines	68.7 (68.4-69.0)	69.0 (68.7-69.3)	69.0 (68.6-69.2)	69.2 (68.9-69.5)	69.4 (69.1-69.7)	69.2 (69.0-69.5)	69.4 (69.2-69.7)	69.8 (69.6-70.1)	70.4 (70.2-70.6)	70.9 (70.7-71.1)	71.5 (71.3-71.7)	71.9 (71.7-72.2)	72.2 (72.0-72.4)	72.4 (72.2-72.6)	72.7 (72.5-72.9)	72.9 (72.7-73.1)	73.0 (72.8-73.2)	73.1 (73.0-73.3)	73.2 (73.0-73.4)	73.4 (73.2-73.5)	73.2 (73.0-73.4)	73.0 (72.9-73.2)	73.0 (72.8-73.1)	73.1 (73.0-73.3)	73.1 (73.0-73.3)	73.0 (72.9-73.2)	73.0 (72.9-73.2)	73.5 (73.3-73.6)	73.6 (73.4-73.8)	73.5 (73.3-73.7)	73.6 (73.4-73.7)	73.7 (73.5-73.8)	73.7 (73.5-73.9)	73.8 (73.4-74.1)	74.1 (73.5-74.7)	74.4 (73.4-75.3)

SR Table 6: Estimates of life expectancy at birth for each geography (with 95% uncertainty intervals), females, 1980–2015

Location	1980	1981	1982	1983	1984	1985	1986	1987	1988	1989	1990	1991
Sri Lanka	72.1 (71.9-72.2)	72.8 (72.7-72.9)	73.0 (72.8-73.2)	73.1 (72.9-73.2)	73.0 (72.8-73.2)	73.7 (73.5-73.9)	74.4 (74.2-74.6)	74.4 (74.2-74.6)	74.9 (74.7-75.2)	75.2 (75.0-75.4)	75.3 (75.1-75.6)	75.6 (75.4-75.8)
Seychelles	73.4 (72.5-74.3)	73.7 (72.9-74.7)	73.8 (73.0-74.7)	73.6 (72.7-74.4)	73.3 (72.6-74.1)	73.1 (72.5-73.7)	73.0 (72.4-73.5)	73.0 (72.5-73.5)	73.3 (72.7-73.8)	73.6 (73.1-74.2)	74.1 (73.6-74.7)	74.4 (73.9-75.2)
Thailand	69.2 (68.5-69.9)	69.5 (68.9-70.2)	70.0 (69.3-70.7)	70.6 (70.0-71.4)	71.5 (70.8-72.2)	72.4 (71.7-73.0)	73.0 (72.3-73.6)	73.4 (72.8-74.0)	73.8 (73.2-74.4)	74.1 (73.5-74.7)	74.4 (73.8-75.0)	74.6 (73.9-75.2)
Timor-Leste	50.4 (46.9-54.3)	51.7 (48.2-55.5)	52.9 (49.6-56.7)	53.9 (50.4-57.6)	54.7 (51.1-58.3)	55.6 (52.2-59.1)	56.5 (53.2-59.9)	57.3 (54.2-60.4)	57.8 (54.9-60.8)	58.4 (55.5-61.2)	58.9 (56.1-61.7)	59.6 (56.8-62.3)
Vietnam	68.6 (65.0-71.8)	69.1 (65.6-72.1)	69.4 (65.9-72.3)	69.8 (66.4-72.5)	70.2 (67.2-72.6)	70.5 (67.7-72.9)	70.9 (68.3-73.2)	71.3 (68.8-73.3)	71.6 (69.3-73.4)	72.0 (69.8-73.6)	72.3 (70.0-74.0)	72.6 (70.2-74.4)
Oceania	59.6 (56.1-63.0)	59.6 (56.1-63.0)	59.6 (56.0-63.2)	59.7 (56.0-63.2)	59.7 (56.1-63.3)	59.8 (56.2-63.5)	59.8 (56.2-63.4)	59.9 (56.4-63.5)	59.9 (56.3-63.5)	60.0 (56.3-63.4)	60.0 (56.2-63.5)	59.9 (56.1-63.5)
American Samoa	72.8 (71.0-74.5)	72.8 (71.1-74.5)	72.7 (71.1-74.4)	72.7 (71.1-74.2)	72.8 (71.1-74.0)	72.9 (71.3-74.1)	73.2 (71.7-74.4)	73.8 (72.5-75.0)	74.2 (72.9-75.3)	74.6 (73.3-75.8)	74.7 (73.5-75.9)	74.7 (73.5-76.0)
Federated States of Micronesia	65.2 (60.3-70.2)	65.2 (60.4-70.4)	65.4 (60.7-70.6)	65.6 (60.9-70.7)	65.8 (60.9-70.9)	66.1 (61.2-71.2)	66.3 (61.4-71.4)	66.5 (61.8-71.7)	66.7 (61.7-72.0)	66.9 (61.9-72.1)	67.0 (62.2-72.2)	67.2 (62.2-72.5)
Fiji	67.3 (65.6-68.8)	67.2 (65.6-68.8)	67.5 (65.8-69.1)	67.9 (66.2-69.5)	68.2 (66.4-69.8)	68.1 (66.3-69.8)	68.0 (66.0-69.9)	68.0 (65.9-70.1)	67.9 (65.5-70.2)	67.8 (65.2-70.3)	67.8 (65.2-70.6)	67.7 (65.0-70.4)
Guam	75.0 (73.9-76.0)	75.3 (74.3-76.4)	75.6 (74.5-76.8)	75.9 (74.6-77.2)	76.2 (74.8-77.5)	76.4 (75.1-77.7)	76.4 (75.2-77.6)	76.3 (75.1-77.4)	76.2 (75.0-77.3)	76.1 (75.0-77.3)	76.0 (74.9-77.2)	75.7 (74.6-76.9)
Kiribati	60.0 (56.1-63.7)	60.0 (56.1-63.6)	59.9 (56.2-63.4)	59.8 (56.3-63.2)	59.8 (56.3-63.1)	59.9 (56.7-62.9)	59.9 (57.1-62.7)	60.0 (57.4-62.6)	60.2 (57.7-62.7)	60.5 (58.2-62.7)	60.8 (58.7-63.0)	61.0 (59.1-63.1)
Marshall Islands	67.3 (64.6-69.9)	67.7 (65.3-70.0)	67.7 (65.5-70.0)	67.9 (65.7-70.1)	68.0 (65.7-70.1)	68.1 (65.9-70.1)	68.3 (66.3-70.3)	68.3 (66.3-70.1)	68.0 (66.1-69.8)	68.0 (66.1-69.6)	67.8 (66.0-69.5)	67.9 (66.1-69.5)
Northern Mariana Islands	70.4 (67.7-72.7)	70.7 (68.0-72.8)	70.9 (68.3-73.0)	71.1 (68.6-73.3)	71.3 (68.9-73.4)	71.6 (69.3-73.5)	71.8 (69.6-73.7)	72.1 (69.9-74.0)	72.3 (70.1-74.1)	72.6 (70.4-74.3)	73.1 (71.1-74.8)	73.6 (71.8-75.1)
Papua New Guinea	56.7 (52.3-61.4)	56.7 (52.2-61.3)	56.7 (52.1-61.4)	56.7 (52.1-61.5)	56.7 (52.2-61.6)	56.8 (52.3-61.7)	56.8 (52.5-61.8)	56.9 (52.4-61.8)	57.0 (52.4-61.8)	57.0 (52.5-61.8)	57.0 (52.4-61.8)	57.0 (52.4-61.7)
Samoa	70.6 (68.7-72.2)	70.9 (68.8-72.6)	71.1 (69.0-72.9)	71.3 (69.0-73.2)	71.5 (69.0-73.5)	71.7 (69.1-73.8)	71.9 (69.2-74.1)	72.1 (69.3-74.5)	72.2 (69.3-74.7)	72.4 (69.4-74.9)	72.5 (69.5-75.1)	72.5 (69.5-75.1)
Solomon Islands	60.2 (55.6-65.2)	60.4 (55.7-65.5)	60.5 (55.7-65.7)	60.7 (55.7-65.8)	60.8 (55.7-65.9)	60.8 (55.7-65.9)	60.4 (55.4-65.4)	61.0 (56.0-66.1)	61.1 (56.2-66.0)	61.2 (56.1-66.3)	61.3 (56.2-66.6)	61.3 (56.2-66.7)
Tonga	68.1 (64.6-70.8)	68.2 (64.8-70.9)	68.3 (64.9-71.1)	68.6 (65.2-71.4)	69.0 (65.6-71.8)	69.3 (66.2-72.1)	69.7 (66.6-72.4)	70.0 (66.9-72.6)	70.2 (67.2-72.7)	70.4 (67.7-72.9)	70.6 (68.0-72.9)	70.8 (68.5-72.9)
Vanuatu	62.6 (57.9-67.0)	62.8 (58.0-67.3)	62.9 (58.0-67.4)	63.1 (58.2-67.7)	63.3 (58.2-67.9)	63.5 (58.3-68.0)	63.6 (58.4-68.2)	63.1 (58.1-67.6)	63.7 (58.7-68.2)	63.7 (58.7-68.3)	63.7 (58.7-68.4)	63.8 (58.5-68.6)
North Africa and Middle East	62.7 (62.1-63.3)	63.1 (62.5-63.7)	63.5 (62.8-64.1)	64.0 (63.1-64.9)	64.7 (63.9-65.5)	65.2 (64.4-66.0)	66.0 (65.4-66.6)	66.5 (65.8-67.1)	66.8 (66.0-67.5)	67.9 (67.3-68.4)	68.0 (67.4-68.6)	68.4 (67.8-68.9)
North Africa and Middle East	62.7 (62.1-63.3)	63.1 (62.5-63.7)	63.5 (62.8-64.1)	64.0 (63.1-64.9)	64.7 (63.9-65.5)	65.2 (64.4-66.0)	66.0 (65.4-66.6)	66.5 (65.8-67.1)	66.8 (66.0-67.5)	67.9 (67.3-68.4)	68.0 (67.4-68.6)	68.4 (67.8-68.9)
Afghanistan	44.1 (41.1-47.3)	44.5 (41.6-47.6)	44.5 (41.4-47.7)	43.9 (40.3-47.7)	43.0 (38.8-47.7)	44.0 (40.0-48.5)	44.6 (40.6-49.0)	45.8 (42.0-49.7)	46.9 (43.2-50.9)	48.6 (45.0-52.6)	49.0 (45.4-53.1)	48.9 (45.3-53.0)
Algeria	62.8 (61.6-64.1)	63.9 (62.9-65.1)	65.1 (64.1-66.2)	66.5 (65.6-67.6)	67.9 (66.9-68.9)	69.2 (68.3-70.3)	70.1 (69.1-71.1)	70.5 (69.4-71.5)	70.9 (69.8-71.9)	71.3 (70.2-72.4)	71.7 (70.5-72.8)	72.1 (70.9-73.2)
Bahrain	73.6 (72.3-75.3)	74.4 (73.1-75.9)	74.3 (73.1-75.7)	74.2 (73.0-75.4)	74.2 (73.1-75.3)	74.5 (73.5-75.9)	73.8 (72.8-74.8)	73.5 (72.4-74.4)	73.2 (72.1-74.2)	73.0 (71.8-73.9)	72.7 (71.5-73.8)	72.5 (71.3-73.6)
Egypt	61.2 (60.4-61.8)	61.3 (60.6-62.0)	61.9 (61.3-62.5)	62.4 (61.9-63.0)	63.2 (62.7-63.7)	63.0 (62.5-63.5)	63.7 (63.2-64.1)	64.4 (63.9-64.8)	66.1 (65.6-66.5)	66.6 (66.2-67.0)	67.3 (67.0-67.7)	67.8 (67.4-68.2)
Iran	66.8 (64.9-68.5)	66.8 (64.7-68.8)	67.7 (65.6-69.7)	68.6 (66.6-70.5)	69.6 (67.9-71.3)	70.3 (68.5-72.0)	70.1 (68.4-71.9)	70.0 (68.3-71.9)	70.0 (68.2-72.0)	70.9 (69.2-72.6)	69.3 (67.5-71.3)	70.8 (69.3-72.3)
Iraq	64.9 (62.4-67.5)	62.7 (58.8-66.7)	62.8 (59.0-67.0)	62.8 (59.0-66.8)	64.1 (61.0-67.4)	64.2 (61.1-67.5)	64.3 (61.2-67.4)	64.4 (61.3-67.6)	58.6 (52.2-65.7)	67.1 (64.9-69.6)	67.3 (65.2-69.6)	66.4 (64.3-68.5)
Jordan	68.7 (67.2-70.0)	69.0 (67.5-70.5)	69.2 (67.6-70.8)	69.3 (67.8-71.0)	70.0 (68.4-71.7)	70.5 (68.9-72.2)	70.8 (69.2-72.5)	71.4 (69.8-73.0)	71.6 (70.0-73.2)	72.1 (70.5-73.7)	72.3 (70.7-74.0)	72.5 (70.7-74.1)
Kuwait	75.0 (74.4-75.3)	75.4 (74.8-75.7)	75.2 (74.6-75.6)	75.9 (75.4-76.4)	76.4 (75.9-76.8)	76.8 (76.5-77.1)	77.3 (77.1-77.6)	77.9 (77.6-78.2)	77.2 (77.0-77.6)	77.9 (77.5-78.3)	78.8 (78.0-79.4)	73.6 (72.1-75.1)
Lebanon	62.8 (57.4-69.2)	63.0 (57.8-69.4)	58.0 (48.7-69.2)	69.3 (65.6-72.8)	69.6 (65.9-73.1)	70.0 (66.4-73.4)	70.3 (66.8-73.6)	64.5 (59.0-70.5)	64.7 (59.3-70.7)	73.2 (70.8-75.4)	72.7 (70.4-74.8)	73.9 (71.8-76.0)
Libya	72.6 (71.5-73.8)	73.0 (71.9-74.2)	73.3 (72.1-74.6)	73.6 (72.4-74.9)	73.8 (72.5-75.1)	74.0 (72.8-75.4)	74.2 (72.9-75.6)	72.9 (71.2-74.7)	74.4 (73.1-75.8)	74.5 (73.1-75.8)	74.7 (73.3-76.0)	75.0 (73.6-76.2)
Morocco	62.7 (60.5-64.6)	63.3 (61.1-65.2)	64.5 (62.6-66.2)	65.1 (63.2-66.8)	66.0 (64.1-67.6)	66.7 (64.9-68.3)	67.2 (65.4-68.8)	67.8 (65.9-69.3)	68.2 (66.3-69.7)	68.7 (66.6-70.2)	69.2 (67.3-70.8)	69.5 (67.6-71.1)
Palestine	70.3 (66.6-73.6)	70.8 (67.1-73.9)	56.4 (48.9-66.6)	71.7 (68.0-74.9)	72.2 (68.5-75.3)	72.6 (69.0-75.7)	73.0 (69.4-76.0)	73.1 (69.8-76.0)	73.3 (70.0-76.1)	73.5 (70.3-76.2)	73.7 (70.5-76.4)	74.0 (70.9-76.6)
Oman	66.0 (62.4-69.3)	67.1 (63.5-70.4)	68.1 (64.6-71.3)	69.1 (65.7-72.2)	70.0 (66.7-73.0)	70.9 (67.6-73.9)	71.6 (68.1-74.5)	72.2 (68.8-75.2)	72.7 (69.3-75.7)	73.2 (69.8-76.2)	73.8 (70.5-76.6)	74.2 (70.9-77.0)
Qatar	74.2 (72.1-76.2)	74.2 (72.4-76.0)	73.1 (71.7-74.6)	73.5 (72.2-75.0)	73.9 (72.6-75.3)	73.6 (72.3-75.0)	73.8 (72.4-75.2)	74.1 (72.8-75.6)	74.0 (72.7-75.3)	74.5 (73.2-76.0)	74.8 (73.5-76.3)	74.7 (73.3-76.2)
Saudi Arabia	72.8 (71.1-74.7)	73.4 (71.7-75.2)	73.8 (72.2-75.6)	74.2 (72.6-75.9)	74.5 (72.9-76.2)	74.8 (73.2-76.5)	75.1 (73.6-76.7)	75.4 (73.9-77.0)	75.7 (74.2-77.1)	75.9 (74.5-77.3)	76.0 (74.5-77.4)	76.3 (75.0-77.7)
Sudan	56.7 (54.2-59.1)	57.5 (55.0-59.8)	57.7 (55.1-59.9)	52.9 (45.2-58.0)	53.6 (46.2-58.6)	53.8 (46.6-58.7)	58.9 (56.3-61.3)	59.3 (56.6-61.7)	59.5 (56.7-62.2)	59.8 (56.8-62.5)	60.2 (57.2-63.1)	60.6 (57.4-63.5)
Syria	68.8 (67.2-70.2)	69.1 (67.5-70.5)	67.7 (65.6-69.7)	69.6 (68.0-71.1)	70.0 (68.4-71.5)	70.1 (68.4-71.7)	70.5 (68.6-72.1)	70.9 (68.9-72.6)	71.3 (69.2-73.1)	71.7 (69.5-73.6)	72.2 (69.9-74.1)	72.7 (70.4-74.6)
Tunisia	67.8 (67.0-68.8)	68.9 (68.0-69.9)	69.9 (69.0-70.8)	70.7 (69.8-71.6)	71.4 (70.5-72.3)	72.0 (71.1-72.9)	72.5 (71.6-73.3)	72.9 (72.1-73.7)	73.4 (72.5-74.1)	73.7 (72.9-74.5)	74.1 (73.3-74.8)	74.4 (73.6-75.2)
Turkey	66.3 (65.2-67.4)	66.8 (65.7-67.9)	67.2 (66.2-68.2)	68.1 (67.1-69.2)	68.6 (67.7-69.6)	69.3 (68.4-70.3)	69.9 (69.1-70.9)	70.3 (69.5-71.3)	70.9 (70.1-71.8)	70.9 (70.1-71.8)	71.5 (70.8-72.3)	72.0 (71.2-72.8)
United Arab Emirates	69.2 (65.2-72.7)	69.7 (66.1-73.1)	70.2 (66.8-73.4)	70.4 (66.9-73.4)	70.7 (67.5-73.7)	71.0 (68.0-73.9)	71.2 (68.3-73.8)	71.4 (68.7-73.8)	71.5 (68.8-73.8)	71.6 (69.1-73.9)	71.8 (69.4-74.0)	72.2 (69.9-74.2)
Yemen	53.2 (48.8-58.4)	54.0 (49.7-59.2)	54.3 (50.1-59.5)	55.4 (51.2-60.6)	56.0 (51.9-61.3)	56.6 (52.4-61.8)	55.9 (51.9-60.9)	57.5 (53.2-62.6)	57.9 (53.5-63.2)	58.3 (53.8-63.6)	58.7 (54.0-64.2)	59.1 (54.3-64.5)
South Asia	53.7 (53.0-54.4)	54.3 (53.6-55.0)	54.9 (54.2-55.6)	55.5 (54.8-56.1)	56.0 (55.3-56.6)	56.4 (55.8-57.1)	57.0 (56.4-57.6)	57.5 (56.8-58.1)	57.9 (57.3-58.5)	58.4 (57.8-59.0)	58.8 (58.3-59.4)	59.1 (58.5-59.7)
South Asia	53.7 (53.0-54.4)	54.3 (53.6-55.0)	54.9 (54.2-55.6)	55.5 (54.8-56.1)	56.0 (55.3-56.6)	56.4 (55.8-57.1)	57.0 (56.4-57.6)	57.5 (56.8-58.1)	57.9 (57.3-58.5)	58.4 (57.8-59.0)	58.8 (58.3-59.4)	59.1 (58.5-59.7)
Bangladesh	52.9 (51.0-54.8)	53.2 (51.5-55.2)	53.8 (52.1-55.6)	54.3 (52.6-56.0)	54.9 (53.2-56.5)	55.0 (53.5-56.5)	56.1 (54.7-57.7)	56.7 (55.3-58.3)	57.3 (56.0-58.9)	58.4 (57.0-59.9)	58.4 (56.9-59.9)	57.7 (56.0-59.4)
Bhutan	55.4 (51.9-58.8)	55.9 (52.4-59.3)	56.4 (52.9-59.7)	56.9 (53.6-60.2)	57.5 (54.2-60.7)	58.0 (54.7-61.3)	58.5 (55.3-61.7)	59.1 (56.0-62.3)	59.7 (56.6-62.7)	60.2 (57.3-63.3)	60.8 (57.9-63.8)	61.3 (58.5-64.3)
India	53.3 (52.6-54.1)	54.0 (53.2-54.7)	54.6 (53.9-55.3)	55.1 (54.4-55.9)	55.6 (54.9-56.3)	56.1 (55.4-56.8)	56.7 (56.0-57.4)	57.1 (56.4-57.8)	57.6 (56.9-58.3)	58.1 (57.4-58.7)	58.5 (57.8-59.2)	58.9 (58.3-59.6)
Nepal	52.7 (49.9-55.6)	53.2 (50.4-56.1)	53.8 (51.0-56.8)	54.3 (51.5-57.3)	54.8 (52.1-57.9)	55.4 (52.8-58.5)	56.0 (53.5-59.1)	56.6 (54.0-59.7)	57.2 (54.6-60.2)	58.0 (55.4-60.8)	58.6 (56.2-61.4)	59.3 (56.9-62.0)
Pakistan	59.7 (57.6-61.5)	60.2 (58.1-62.0)	60.7 (58.5-62.5)	61.1 (59.0-63.0)	61.7 (59.6-63.5)	61.7 (59.7-63.5)	61.9 (59.8-63.7)	61.8 (59.8-63.7)	62.0 (60.1-63.8)	62.1 (60.2-63.9)	62.3 (60.4-64.1)	62.4 (60.5-64.2)
Sub-Saharan Africa	52.5 (50.4-54.4)	52.7 (50.7-54.5)	53.0 (51.1-54.8)	52.8 (50.8-54.6)	53.1 (51.0-54.8)	53.8 (52.0-55.3)	54.1 (52.4-55.5)	54.4 (52.7-55.7)	54.6 (53.1-55.9)	54.7 (53.3-55.9)	54.7 (53.4-55.9)	54.9 (53.7-56.0)
Southern Sub-Saharan Africa	58.8 (56.9-60.5)	59.2 (57.5-60.8)	59.6 (58.0-61.1)	60.0 (58.6-61.4)	60.7 (59.4-62.0)	61.5 (60.3-62.7)	61.9 (60.8-63.0)	62.4 (61.3-63.4)	63.6 (62.6-64.5)	63.9 (62.9-64.8)	64.4 (63.4-65.3)	64.8 (63.8-65.6)
Botswana	61.4 (50.3-72.9)	61.8 (50.9-73.4)	62.3 (51.4-73.7)	62.7 (51.7-74.1)	63.2 (51.9-74.4)	63.6 (51.8-74.5)	64.0 (51.8-74.6)	64.4 (52.0-74.7)	64.7 (52.7-74.8)	64.9 (53.1-74.9)	64.9 (53.0-74.9)	64.7 (52.4-74.8)
Lesotho	61.9 (53.1-70.2)	62.1 (53.6-70.1)	62.4 (54.4-69.9)	62.7 (55.1-69.7)	62.9 (55.9-69.2)	63.2 (56.8-69.0)	63.5 (57.6-69.0)	63.7 (58.8-68.8)	64.0 (59.7-68.7)	64.3 (60.2-68.4)	64.5 (61.0-68.2)	64.7 (61.4-68.0)
Namibia	61.2 (58.0-64.5)	61.6 (58.6-64.7)	62.0 (59.0-64.9)	62.7 (60.0-65.5)	63.4 (60.8-66.1)	63.9 (61.7-66.5)	64.3 (62.0-66.8)	64.7 (62.5-67.2)	64.7 (62.7-67.1)	64.8 (62.7-67.1)	64.9 (63.0-67.0)	65.1 (63.2-67.2)
South Africa	57.8 (55.5-60.0)	58.2 (56.1-60.2)	58.6 (56.7-60.4)	58.9 (57.2-60.6)	59.5 (58.1-61.1)	60.4 (59.0-61.9)	60.7 (59.4-62.2)	61.3 (60.0-62.6)	62.7 (61.5-63.9)	63.2 (61.9-64.4)	64.0 (62.7-65.1)	64.6 (63.5-65.7)
Swaziland	58.8 (49.8-67.9)	59.2 (50.1-68.3)	59.7 (50.8-68.5)	59.2 (51.4-67.6)	60.5 (53.2-68.7)	61.1 (54.2-68.9)	61.7 (55.3-69.2)	62.2 (56.2-69.2)	62.7 (57.2-69.2)	63.2 (58.0-69.0)	63.5 (58.9-68.9)	63.7 (59.6-68.4)
Zimbabwe	61.3 (57.5-64.6)	62.2 (58.7-65.2)	62.6 (59.6-65.3)	63.3 (60.7-65.7)	64.1 (61.6-66.4)	64.9 (62.7-67.2)	65.8 (63.9-68.2)	66.2 (64.4-68.4)	66.2 (64.4-68.3)	66.0 (64.2-68.2)	65.3 (63.4-67.6)	64.9 (62.4-67.9)
Western Sub-Saharan Africa	52.5 (48.1-56.1)	52.8 (48.5-56.4)	53.2 (49.0-56.7)	53.5 (49.4-57.0)	53.8 (49.9-57.1)	54.2 (50.2-57.3)	54.4 (50.6-57.4)	54.5 (51.0-57.4)	54.7 (51.3-57.4)	54.9 (51.7-57.4)	55.1 (52.2-57.4)	55.3 (52.7-57.4)
Benin	51.7 (47.7-56.0)	52.2 (48.7-56.3)	52.9 (49.7-56.5)	53.6 (50.6-57.1)	54.3 (51.5-57.4)	54.9 (52.2-57.7)	55.8 (53.0-58.4)	56.6 (53.9-59.3)	57.5 (55.0-60.1)	57.9 (55.5-60.4)	58.1 (55.6-60.3)	58.5 (56.1-60.6)
Burkina Faso	50.8 (46.1-55.6)	51.4 (47.1-55.6)	51.8 (48.2-55.6)	52.2 (49.0-55.5)	52.4 (49.6-55.2)	52.7 (50.1-55.2)	52.6 (50.2-54.9)	52.4 (50.0-54.7)	52.2 (49.9-54.5)	52.5 (50.1-54.9)	52.7 (50.3-55.1)	52.8 (50.5-55.2)
Cameroon	54.5 (49.5-60.2)	55.2 (50.4-60.3)	55.8 (51.4-60.7)	56.4 (52.7-60.7)	57.0 (53.8-60.6)	57.7 (55.0-60.9)	58.2 (55.8-61.0)	58.9 (56.6-61.2)	59.3 (57.1-61.6)	59.4 (57.3-61.7)	59.6 (57.5-61.9)	59.5 (57.5-61.9)
Cape Verde	68.9 (67.9-69.8)	69.1 (68.2-69.8)	69.2 (68.5-69.9)	69.3 (68.7-69.9)	69.2 (68.7-69.8)	69.8 (69.2-70.3)	70.3 (69.5-70.9)	70.8 (70.0-71.5)	71.3 (70.5-72.1)	71.8 (71.0-72.5)	72.2 (71.6-72.8)	72.4 (71.7-73.0)
Chad	52.4 (48.0-56.5)	52.5 (48.6-56.1)	52.8 (49.1-56.1)	53.3 (50.0-56.4)	53.7 (50.6-56.6)	54.2 (51.3-57.0)	54.4 (51.7-57.1)	53.8 (50.9-56.7)	55.1 (52.6-57.8)	55.0 (52.3-57.6)	54.8 (52.1-57.4)	55.2 (52.6-57.8)
Cote d'Ivoire	57.3 (54.4-60.5)	57.5 (54.9-60.6)	57.6 (54.9-60.6)	57.7 (55.0-60.6)	58.0 (55.4-60.9)	58.3 (55.8-61.2)	58.5 (55.9-61.3)	58.4 (55.9-61.1)	58.2 (55.7-60.8)	58.0 (55.7-60.6)	57.8 (55.6-60.4)	57.7 (55.5-60.2)
The Gambia	57.9 (48.2-67.4)	57.1 (48.3-66.2)	58.5 (49.3-68.0)	58.8 (49.7-68.3)	59.0 (50.0-68.3)	59.3 (50.2-68.3)	59.6 (50.3-68.5)	59.9 (51.0-68.8)	60.2 (51.4-68.8)	60.4 (51.3-69.0)	60.7 (52.2-69.0)	60.9 (53.2-68.7)
Ghana	55.8 (46.9-64.6)	56.0 (47.0-64.7)	56.1 (47.6-64.8)	56.2 (48.2-64.7)	56.4 (48.7-64.7)	56.7 (49.2-64.5)	57.0 (49.8-64.3)	57.4 (50.5-64.3)	57.8 (51.5-64.4)	58.2 (52.6-64.2)	58.6 (53.4-63.9)	59.0 (54.3-63.7)
Guinea	47.9 (42.7-53.3)	48.2 (43.6-53.3)	48.5 (44.2-53.2)	48.7 (44.7-52.9)	49.1 (45.4-52.8)	49.4 (46.1-52.8)	49.9 (46.8-53.0)	50.4 (47.6-53.3)	50.5 (48.1-53.3)	51.1 (48.9-53.7)	51.8 (49.6-54.3)	52.2 (50.1-54.5)
Guinea-Bissau	50.8 (40.6-60.8)	50.8 (40.7-60.8)	51.0 (41.1-60.8)	51.1 (41.6-61.0)	51.3 (42.1-61.2)	51.4 (42.1-61.3)	51.6 (42.0-61.5)	51.7 (41.8-61.8)	51.9 (41.9-61.9)	52.1 (42.0-62.1)	52.3 (41.9-62.4)	52.5 (41.6-62.6)

Location	1992	1993	1994	1995	1996	1997	1998	1999	2000	2001	2002	2003
Sri Lanka	75.9 (75.8-76.1)	76.6 (76.5-76.8)	76.6 (76.5-76.7)	76.2 (76.0-76.3)	75.9 (75.7-76.2)	76.2 (76.0-76.3)	76.7 (76.6-76.9)	77.1 (77.0-77.2)	77.3 (77.1-77.4)	78.0 (77.9-78.1)	78.3 (78.1-78.4)	78.4 (78.3-78.5)
Seychelles	74.8 (74.2-75.5)	75.0 (74.5-75.9)	75.3 (74.7-76.2)	75.6 (74.9-76.5)	75.5 (74.9-76.3)	75.4 (74.9-76.0)	75.4 (74.8-75.9)	75.5 (75.0-76.0)	75.5 (75.0-76.0)	75.6 (75.1-76.0)	75.6 (75.1-76.1)	75.6 (75.2-76.2)
Thailand	74.6 (74.0-75.2)	74.6 (74.0-75.3)	74.7 (74.1-75.3)	74.8 (74.1-75.4)	74.8 (74.1-75.5)	74.6 (74.0-75.4)	74.5 (73.9-75.2)	74.5 (73.8-75.1)	74.7 (74.0-75.3)	75.0 (74.3-75.6)	75.4 (74.7-76.0)	75.7 (75.0-76.4)
Timor-Leste	60.3 (57.7-62.8)	61.0 (58.5-63.4)	61.7 (59.3-64.0)	62.2 (59.9-64.4)	62.7 (60.5-64.7)	62.8 (60.8-64.6)	63.0 (61.0-64.8)	63.1 (60.9-64.9)	62.7 (60.5-64.8)	64.9 (63.0-66.8)	65.7 (63.9-67.5)	66.1 (64.4-67.9)
Vietnam	72.9 (70.5-74.7)	73.2 (70.7-75.1)	73.5 (71.0-75.5)	73.8 (71.4-75.9)	74.2 (71.9-76.0)	74.5 (72.3-76.1)	75.0 (72.9-76.4)	75.3 (73.5-76.7)	75.6 (73.9-77.1)	75.9 (74.0-77.6)	76.2 (74.0-78.0)	76.5 (74.1-78.4)
Oceania	60.1 (56.1-63.7)	60.2 (56.2-63.8)	60.3 (56.2-64.0)	60.4 (56.5-64.1)	60.5 (56.7-64.3)	60.5 (56.7-64.3)	60.2 (56.4-64.0)	60.7 (56.8-64.6)	60.7 (56.9-64.8)	60.7 (56.9-64.8)	60.7 (56.8-64.9)	60.8 (56.7-64.9)
American Samoa	74.4 (73.3-75.8)	74.3 (72.8-75.7)	74.2 (72.5-75.8)	74.0 (72.3-75.6)	73.9 (72.1-75.5)	73.9 (72.2-75.4)	74.1 (72.6-75.4)	74.3 (72.7-75.5)	74.3 (72.8-75.5)	74.2 (72.7-75.4)	74.1 (72.6-75.4)	74.2 (72.7-75.5)
Federated States of Micronesia	67.4 (62.4-72.6)	67.6 (62.7-72.8)	67.7 (63.0-72.8)	68.0 (63.2-72.9)	68.2 (63.2-72.9)	68.4 (63.2-72.9)	68.6 (63.5-73.0)	68.8 (63.9-73.2)	69.0 (64.2-73.4)	69.2 (64.2-73.7)	68.6 (63.7-73.0)	69.6 (64.4-74.2)
Fiji	67.6 (65.0-70.2)	67.4 (64.9-70.1)	67.3 (64.6-70.0)	67.1 (64.8-69.5)	66.9 (64.8-69.1)	66.6 (64.8-68.6)	66.7 (64.9-68.6)	66.6 (64.8-68.5)	66.7 (64.9-68.7)	66.8 (65.1-68.8)	66.9 (65.2-68.7)	67.0 (65.2-68.7)
Guam	75.6 (74.5-76.8)	75.7 (74.7-76.9)	75.8 (74.7-77.1)	76.1 (75.0-77.3)	76.3 (75.4-77.5)	74.9 (73.0-76.5)	76.6 (75.5-77.7)	76.7 (75.6-77.8)	76.8 (75.8-77.9)	76.9 (75.8-78.0)	77.0 (75.9-78.1)	76.9 (75.9-78.0)
Kiribati	61.6 (59.7-63.5)	61.7 (59.8-63.6)	62.1 (60.3-64.0)	62.4 (60.6-64.1)	62.8 (61.1-64.6)	63.1 (61.5-65.0)	63.4 (61.7-65.2)	63.5 (61.8-65.3)	63.8 (62.0-65.6)	64.0 (62.2-65.9)	64.2 (62.3-66.1)	64.5 (62.3-66.4)
Marshall Islands	68.0 (66.3-69.5)	68.0 (66.1-69.4)	68.0 (66.1-69.5)	67.8 (66.0-69.4)	67.6 (65.8-69.2)	67.5 (65.6-69.1)	67.4 (65.4-69.1)	67.2 (65.2-69.0)	67.0 (64.8-69.0)	67.0 (64.7-69.0)	66.9 (64.7-69.0)	66.9 (64.8-69.1)
Northern Mariana Islands	74.0 (72.4-75.4)	74.4 (72.9-75.7)	74.8 (73.4-76.0)	75.1 (73.8-76.3)	75.5 (74.3-76.5)	75.8 (74.7-76.7)	76.1 (75.2-77.0)	76.3 (75.4-77.1)	76.7 (75.9-77.5)	76.7 (75.9-77.5)	76.9 (76.1-77.7)	77.2 (76.4-77.9)
Papua New Guinea	57.2 (52.3-62.0)	57.4 (52.4-62.2)	57.6 (52.6-62.5)	57.8 (52.8-62.7)	57.9 (53.1-62.9)	58.0 (53.2-63.1)	57.5 (52.7-62.7)	58.2 (53.4-63.4)	58.3 (53.4-63.6)	58.3 (53.5-63.6)	58.3 (53.4-63.7)	58.4 (53.3-63.7)
Samoa	72.7 (69.8-75.3)	72.8 (69.8-75.5)	72.9 (69.7-75.6)	73.0 (69.9-75.6)	73.0 (70.1-75.6)	73.1 (70.3-75.7)	73.1 (70.4-75.7)	73.1 (70.5-75.6)	73.2 (70.6-75.5)	73.3 (70.8-75.6)	73.4 (70.9-75.7)	73.5 (71.2-75.7)
Solomon Islands	61.6 (56.6-66.8)	61.7 (56.9-67.0)	62.0 (57.2-67.2)	62.2 (57.3-67.4)	62.4 (57.5-67.6)	62.5 (57.7-67.7)	62.6 (57.7-67.8)	62.7 (57.7-67.8)	62.7 (57.7-67.9)	62.6 (57.8-67.8)	62.6 (57.7-67.8)	62.6 (57.6-67.8)
Tonga	71.0 (68.8-72.9)	71.0 (69.1-72.8)	71.1 (69.3-72.8)	71.2 (69.6-72.7)	71.3 (69.9-72.7)	71.4 (70.1-72.8)	71.3 (70.1-72.5)	71.3 (70.1-72.5)	71.3 (70.1-72.4)	71.6 (70.3-72.7)	71.8 (70.5-73.0)	72.0 (70.7-73.2)
Vanuatu	63.8 (58.4-68.7)	63.9 (58.3-68.9)	63.9 (58.4-69.0)	63.9 (58.3-68.9)	64.0 (58.4-68.9)	63.0 (57.7-67.9)	64.0 (58.6-68.9)	63.6 (58.3-68.4)	64.0 (58.8-69.1)	64.0 (59.0-69.1)	64.0 (59.1-69.1)	64.0 (59.2-69.2)
North Africa and Middle East	68.9 (68.3-69.4)	69.1 (68.5-69.7)	69.3 (68.6-69.9)	69.6 (69.0-70.2)	69.9 (69.3-70.5)	70.2 (69.5-70.8)	70.5 (69.9-71.1)	70.8 (70.1-71.3)	71.5 (70.8-72.1)	71.7 (71.1-72.3)	71.9 (71.3-72.5)	71.9 (71.2-72.5)
North Africa and Middle East	68.9 (68.3-69.4)	69.1 (68.5-69.7)	69.3 (68.6-69.9)	69.6 (69.0-70.2)	69.9 (69.3-70.5)	70.2 (69.5-70.8)	70.5 (69.9-71.1)	70.8 (70.1-71.3)	71.5 (70.8-72.1)	71.7 (71.1-72.3)	71.9 (71.3-72.5)	71.9 (71.2-72.5)
Afghanistan	49.1 (45.4-53.4)	49.1 (45.3-53.5)	48.8 (45.1-53.1)	49.1 (45.2-53.0)	49.2 (45.4-53.4)	49.2 (45.5-53.3)	48.8 (45.2-52.8)	49.6 (46.0-53.7)	49.7 (46.2-53.8)	49.9 (46.2-54.0)	50.3 (46.7-54.1)	50.8 (47.1-54.6)
Algeria	72.4 (71.3-73.6)	72.8 (71.5-73.9)	73.1 (71.8-74.2)	73.4 (72.1-74.4)	73.8 (72.5-74.7)	74.1 (72.8-75.1)	74.4 (73.2-75.4)	74.6 (73.4-75.7)	74.9 (73.7-75.9)	75.0 (73.9-76.1)	75.3 (74.1-76.4)	75.4 (74.2-76.4)
Bahrain	72.7 (71.6-73.8)	73.1 (71.8-74.3)	73.7 (72.4-74.9)	74.2 (73.1-75.2)	75.0 (73.9-75.9)	75.7 (74.7-76.6)	76.0 (75.0-76.8)	76.2 (75.2-77.1)	76.2 (75.3-77.2)	76.4 (75.5-77.3)	77.0 (76.2-77.9)	77.3 (76.5-78.1)
Egypt	68.4 (68.0-68.8)	68.6 (68.2-68.9)	68.9 (68.5-69.3)	69.7 (69.4-70.0)	70.1 (69.8-70.4)	70.4 (70.1-70.7)	70.8 (70.5-71.1)	71.3 (71.0-71.6)	72.2 (72.0-72.5)	72.3 (72.0-72.5)	72.4 (72.1-72.6)	72.4 (72.1-72.6)
Iran	71.5 (69.7-73.4)	72.2 (70.3-74.1)	72.7 (70.8-74.4)	73.0 (71.3-74.5)	73.1 (71.7-74.5)	73.3 (72.1-74.5)	73.7 (72.6-74.8)	74.0 (72.8-75.1)	74.2 (73.0-75.3)	74.3 (73.0-75.5)	74.2 (72.8-75.5)	73.2 (71.5-74.8)
Iraq	67.0 (64.9-69.0)	67.0 (65.0-69.0)	67.1 (65.2-69.0)	67.0 (65.2-68.8)	66.9 (65.1-68.7)	66.9 (65.0-68.7)	67.2 (65.4-69.1)	67.6 (65.7-69.4)	67.7 (65.7-69.5)	68.1 (66.2-70.0)	68.1 (66.0-70.1)	68.2 (65.9-70.2)
Jordan	72.9 (71.1-74.5)	72.7 (71.1-74.3)	72.9 (71.3-74.6)	72.8 (71.2-74.5)	72.6 (71.0-74.3)	72.8 (71.1-74.5)	72.9 (71.1-74.5)	73.0 (71.3-74.5)	73.1 (71.6-74.5)	73.3 (71.9-74.5)	73.5 (72.2-74.6)	73.6 (72.5-74.7)
Kuwait	81.3 (81.0-81.6)	80.2 (79.9-80.5)	79.1 (78.7-79.5)	77.9 (77.5-78.2)	77.3 (76.9-77.6)	77.5 (77.2-77.9)	77.7 (77.3-78.0)	78.7 (78.4-79.0)	79.0 (78.7-79.3)	78.5 (78.2-78.8)	79.3 (79.0-79.6)	80.1 (79.7-80.4)
Lebanon	73.9 (71.9-76.1)	73.9 (72.0-76.0)	74.4 (72.4-76.4)	74.4 (72.4-76.4)	75.0 (73.0-76.9)	75.4 (73.3-77.2)	75.6 (73.6-77.4)	76.4 (74.3-78.0)	76.5 (74.5-78.1)	76.8 (74.8-78.4)	77.1 (75.1-78.7)	77.1 (75.1-78.8)
Libya	75.1 (73.8-76.3)	75.4 (74.1-76.6)	75.6 (74.3-76.7)	75.7 (74.5-76.8)	75.8 (74.7-76.9)	76.0 (74.8-77.0)	76.0 (74.9-77.0)	76.1 (75.0-77.0)	76.1 (75.0-77.0)	76.3 (75.3-77.1)	76.4 (75.4-77.2)	76.5 (75.6-77.3)
Morocco	69.8 (67.9-71.5)	70.0 (68.1-71.8)	70.4 (68.3-72.2)	70.8 (68.7-72.6)	71.3 (69.2-73.0)	71.6 (69.5-73.3)	72.1 (69.9-73.8)	72.4 (70.0-74.1)	72.8 (70.3-74.5)	73.1 (70.5-74.9)	73.1 (70.6-75.1)	73.4 (70.7-75.5)
Palestine	74.3 (71.4-76.8)	74.7 (72.0-77.0)	75.2 (72.6-77.4)	75.6 (73.2-77.5)	75.9 (73.7-77.7)	76.2 (74.1-77.8)	76.5 (74.5-77.8)	76.8 (75.2-77.9)	76.8 (75.6-77.8)	77.1 (76.2-78.0)	77.1 (76.2-77.9)	77.1 (76.4-77.9)
Oman	74.6 (71.5-77.3)	75.1 (71.9-77.7)	75.4 (72.4-78.0)	75.7 (72.9-78.2)	76.1 (73.4-78.4)	76.4 (74.0-78.6)	76.8 (74.5-78.7)	77.1 (75.0-78.9)	77.4 (75.5-79.0)	77.6 (76.0-79.0)	77.8 (76.5-78.9)	78.0 (76.8-79.1)
Qatar	74.7 (73.4-76.3)	74.6 (73.3-75.8)	74.6 (73.4-75.9)	74.8 (73.4-76.0)	75.2 (73.9-76.5)	75.4 (74.2-76.8)	75.6 (74.3-76.9)	75.7 (74.4-76.9)	75.9 (74.8-77.1)	76.4 (75.2-77.5)	76.6 (75.5-77.8)	77.0 (76.0-78.0)
Saudi Arabia	76.6 (75.4-77.9)	76.9 (75.7-78.2)	77.2 (76.0-78.3)	77.5 (76.4-78.6)	77.8 (76.8-78.8)	78.1 (77.1-79.0)	78.4 (77.5-79.2)	78.6 (77.8-79.4)	78.8 (78.1-79.5)	79.1 (78.4-79.7)	79.2 (78.5-79.7)	79.4 (78.8-80.0)
Sudan	60.8 (57.4-63.9)	61.0 (57.6-64.1)	61.5 (58.1-64.7)	61.8 (58.3-65.1)	62.0 (58.5-65.4)	62.3 (58.7-65.8)	62.7 (59.1-66.3)	63.4 (59.8-67.1)	63.9 (60.1-67.5)	64.3 (60.5-68.1)	64.8 (61.1-68.7)	65.1 (61.6-69.0)
Syria	73.4 (70.9-75.3)	73.7 (71.2-75.6)	74.1 (71.6-76.0)	74.5 (72.2-76.4)	74.9 (72.7-76.8)	75.3 (73.2-77.1)	75.6 (73.7-77.3)	75.9 (74.2-77.4)	76.2 (74.7-77.6)	76.5 (75.1-77.7)	76.8 (75.5-77.8)	77.0 (75.9-77.8)
Tunisia	74.6 (73.8-75.4)	74.7 (73.9-75.6)	75.1 (74.3-76.0)	75.5 (74.7-76.5)	76.1 (75.2-77.1)	76.6 (75.7-77.6)	77.0 (76.1-78.1)	77.4 (76.3-78.5)	77.7 (76.5-78.9)	78.0 (76.8-79.3)	78.3 (77.0-79.6)	78.5 (77.1-79.9)
Turkey	72.7 (71.9-73.5)	73.2 (72.5-74.0)	73.4 (72.6-74.2)	73.6 (72.8-74.4)	74.3 (73.5-75.0)	74.9 (74.1-75.7)	75.5 (74.7-76.2)	74.9 (74.1-75.8)	77.3 (76.7-78.0)	78.1 (77.4-78.7)	78.6 (78.0-79.3)	78.4 (77.8-79.1)
United Arab Emirates	72.6 (70.5-74.4)	72.9 (71.0-74.7)	73.3 (71.5-74.9)	73.6 (72.0-75.1)	73.8 (72.4-75.2)	74.1 (72.9-75.3)	74.5 (73.4-75.6)	74.8 (73.7-75.8)	75.1 (74.1-76.1)	75.4 (74.5-76.3)	75.8 (74.9-76.7)	76.2 (75.4-77.0)
Yemen	59.4 (54.7-64.7)	59.8 (55.0-64.9)	60.0 (55.2-65.1)	60.4 (55.4-65.3)	60.8 (55.7-65.7)	61.2 (56.2-66.1)	61.6 (56.6-66.7)	62.0 (57.1-67.3)	62.5 (57.5-67.8)	62.9 (58.0-68.4)	63.3 (58.5-69.0)	63.8 (59.0-69.5)
South Asia	59.6 (59.1-60.2)	60.1 (59.5-60.7)	60.5 (60.0-61.1)	60.9 (60.4-61.5)	61.3 (60.8-61.9)	61.7 (61.2-62.3)	62.2 (61.6-62.8)	62.6 (62.1-63.2)	63.1 (62.5-63.7)	63.5 (62.9-64.1)	63.9 (63.4-64.5)	64.4 (63.9-65.0)
South Asia	59.6 (59.1-60.2)	60.1 (59.5-60.7)	60.5 (60.0-61.1)	60.9 (60.4-61.5)	61.3 (60.8-61.9)	61.7 (61.2-62.3)	62.2 (61.6-62.8)	62.6 (62.1-63.2)	63.1 (62.5-63.7)	63.5 (62.9-64.1)	63.9 (63.4-64.5)	64.4 (63.9-65.0)
Bangladesh	60.3 (58.8-61.9)	61.2 (59.7-62.8)	62.0 (60.5-63.6)	62.5 (61.0-64.1)	63.5 (62.1-65.1)	64.4 (62.8-65.9)	65.2 (63.8-66.7)	66.0 (64.5-67.4)	66.3 (64.9-67.7)	66.8 (65.4-68.2)	67.2 (65.9-68.6)	67.8 (66.5-69.0)
Bhutan	61.9 (59.0-64.8)	62.3 (59.5-65.2)	62.7 (59.9-65.5)	63.4 (60.7-66.1)	64.2 (61.7-66.9)	64.7 (62.2-67.2)	65.3 (62.9-67.7)	66.3 (63.9-68.6)	66.2 (63.9-68.4)	67.8 (65.5-70.0)	68.6 (66.4-70.8)	69.2 (66.9-71.4)
India	59.3 (58.6-60.0)	59.7 (59.1-60.4)	60.2 (59.5-60.8)	60.6 (59.9-61.3)	61.0 (60.3-61.6)	61.3 (60.7-62.0)	61.8 (61.1-62.4)	62.2 (61.6-62.9)	62.7 (62.1-63.3)	63.1 (62.5-63.8)	63.6 (63.0-64.3)	64.1 (63.5-64.8)
Nepal	60.3 (58.0-62.7)	60.6 (58.5-63.0)	61.5 (59.6-63.8)	62.5 (60.7-64.6)	63.4 (61.7-65.3)	64.7 (63.1-66.4)	65.4 (64.0-67.0)	65.9 (64.6-67.4)	66.9 (65.6-68.3)	67.4 (66.1-68.8)	67.7 (66.3-69.0)	68.3 (66.8-69.7)
Pakistan	62.2 (60.4-64.1)	62.1 (60.3-64.1)	62.0 (60.1-64.1)	61.8 (59.9-63.9)	62.1 (60.2-64.2)	62.1 (60.4-64.1)	62.4 (60.7-64.3)	62.5 (60.7-64.5)	62.7 (60.9-64.7)	62.9 (60.9-64.9)	63.1 (61.1-65.1)	63.3 (61.4-65.1)
Sub-Saharan Africa	54.9 (53.8-55.8)	54.9 (53.9-55.8)	54.0 (52.7-55.1)	54.7 (53.9-55.5)	54.5 (53.7-55.3)	54.4 (53.7-55.2)	54.3 (53.6-55.1)	54.2 (53.5-55.0)	54.2 (53.5-54.9)	54.4 (53.7-55.2)	54.6 (53.8-55.3)	54.8 (54.0-55.6)
Southern Sub-Saharan Africa	64.4 (63.5-65.3)	64.7 (63.8-65.6)	64.2 (63.3-65.2)	64.0 (63.0-65.1)	62.8 (61.7-63.9)	61.0 (59.9-62.1)	59.6 (58.3-60.7)	58.1 (56.7-59.4)	56.2 (54.9-57.6)	55.2 (53.8-56.7)	53.9 (52.4-55.4)	52.6 (51.2-54.2)
Botswana	64.1 (51.8-74.5)	63.0 (50.6-73.9)	61.6 (48.9-73.2)	59.8 (46.8-72.1)	57.8 (44.8-70.9)	55.6 (43.0-69.4)	53.4 (41.2-67.8)	51.3 (39.6-66.0)	49.4 (38.4-64.2)	47.9 (37.4-62.4)	46.8 (36.9-61.0)	46.7 (37.0-60.5)
Lesotho	64.5 (61.2-67.7)	64.1 (61.3-67.4)	63.4 (60.5-66.7)	62.8 (59.7-66.4)	62.2 (58.8-65.9)	61.3 (57.7-65.1)	58.8 (54.9-62.5)	56.7 (52.6-60.5)	53.7 (49.6-57.4)	51.7 (47.7-55.6)	49.5 (45.5-53.5)	47.4 (43.6-51.2)
Namibia	65.2 (63.2-67.0)	64.9 (62.9-66.8)	64.4 (62.1-66.4)	63.3 (61.0-65.6)	62.2 (59.7-64.9)	60.6 (58.0-63.4)	59.1 (56.3-62.1)	57.5 (54.7-60.7)	56.0 (53.1-59.6)	55.2 (51.9-58.9)	54.4 (50.9-58.2)	54.2 (50.5-58.3)
South Africa	64.5 (63.4-65.5)	65.4 (64.4-66.3)	65.2 (64.3-66.0)	65.6 (64.7-66.4)	64.5 (63.7-65.3)	62.8 (61.9-63.6)	61.6 (60.6-62.6)	60.3 (59.2-61.5)	58.5 (57.2-59.9)	57.7 (56.3-59.2)	56.3 (54.8-57.9)	54.9 (53.4-56.6)
Swaziland	64.0 (60.1-68.4)	64.3 (60.8-68.6)	64.1 (60.6-68.7)	62.6 (58.7-67.5)	61.4 (57.1-66.2)	59.7 (55.2-64.3)	57.3 (52.6-62.0)	54.4 (49.7-59.2)	51.1 (46.5-55.6)	48.7 (44.2-53.2)	46.5 (42.3-51.0)	45.3 (41.3-49.7)
Zimbabwe	63.6 (60.6-66.9)	62.1 (58.7-65.9)	60.7 (56.8-64.6)	58.7 (54.7-62.7)	56.8 (52.7-61.2)	55.1 (50.9-59.3)	52.7 (48.7-57.0)	50.7 (46.7-54.8)	49.1 (45.2-53.1)	47.9 (44.1-51.9)	46.6 (43.0-50.7)	45.8 (42.4-50.0)
Western Sub-Saharan Africa	55.4 (53.1-57.3)	55.5 (53.5-57.2)	55.6 (53.9-57.1)	55.6 (54.0-57.0)	55.6 (54.1-57.0)	55.6 (54.3-56.9)	55.6 (54.3-56.9)	55.8 (54.5-57.0)	55.9 (54.6-57.1)	56.0 (54.7-57.3)	56.1 (54.7-57.4)	56.5 (55.1-57.8)
Benin	58.7 (56.3-60.7)	59.0 (56.5-61.0)	59.0 (56.4-61.1)	58.8 (56.0-61.1)	58.7 (55.8-61.2)	59.0 (56.1-61.6)	59.2 (56.3-62.0)	59.3 (56.5-62.3)	59.4 (56.4-62.4)	59.7 (56.4-62.8)	60.0 (56.6-63.6)	60.3 (56.6-64.1)
Burkina Faso	52.6 (50.3-55.1)	52.6 (50.5-55.1)	52.6 (50.5-55.2)	52.7 (50.5-55.2)	52.9 (50.7-55.4)	53.0 (50.7-55.3)	53.1 (50.9-55.7)	53.4 (51.0-56.1)	53.8 (51.3-56.5)	54.4 (51.8-57.0)	54.9 (52.2-57.5)	55.4 (52.6-58.1)
Cameroon	59.4 (57.3-61.8)	59.1 (56.9-61.6)	58.6 (56.4-61.2)	58.2 (55.9-60.8)	57.8 (55.4-60.5)	57.1 (54.7-60.1)	56.4 (54.0-59.8)	56.0 (53.4-59.4)	55.4 (52.7-58.9)	55.3 (52.5-58.8)	55.2 (52.4-58.8)	55.2 (52.4-58.7)
Cape Verde	72.5 (71.4-73.5)	72.6 (71.1-74.1)	72.8 (70.7-74.8)	73.0 (70.5-75.3)	73.2 (70.4-75.6)	73.5 (70.3-75.7)	73.8 (70.4-76.0)	74.0 (70.3-76.1)	74.4 (70.6-76.4)	74.8 (70.9-76.5)	75.1 (71.3-76.6)	75.4 (71.9-76.7)
Chad	55.1 (52.5-57.6)	55.1 (52.6-57.6)	55.0 (52.5-57.5)	54.9 (52.3-57.6)	54.6 (52.0-57.6)	54.4 (51.7-57.6)	53.9 (50.9-57.3)	53.7 (50.6-57.3)	53.6 (50.2-57.3)	53.7 (50.2-57.7)	53.7 (49.9-58.1)	53.7 (49.4-58.5)
Cote d'Ivoire	57.5 (55.4-59.9)	56.9 (54.8-59.4)	56.5 (54.4-59.2)	55.9 (53.5-58.7)	55.5 (53.1-58.4)	55.3 (52.9-58.3)	54.9 (52.5-57.9)	54.4 (52.0-57.6)	54.1 (51.4-57.1)	53.8 (51.0-56.7)	53.9 (51.1-57.0)	54.0 (51.2-57.2)
The Gambia	61.1 (53.3-68.4)	61.5 (54.3-68.4)	61.7 (55.3-68.0)	62.0 (56.3-67.8)	62.3 (57.2-67.7)	62.6 (58.2-67.5)	63.0 (59.0-67.5)	63.2 (59.6-67.2)	63.9 (60.6-67.7)	64.4 (61.4-67.8)	64.5 (61.8-67.6)	65.4 (62.8-68.5)
Ghana	59.3 (55.4-63.7)	60.1 (56.5-64.1)	60.3 (56.9-63.9)	60.5 (57.3-63.8)	60.4 (57.4-63.7)	60.3 (57.4-63.7)	60.7 (57.8-64.1)	60.8 (57.8-64.3)	60.9 (57.7-64.3)	60.7 (57.5-64.4)	60.9 (57.7-64.7)	61.3 (58.0-65.2)
Guinea	52.6 (50.4-54.9)	53.1 (50.9-55.3)	53.3 (51.1-55.6)	53.8 (51.7-56.1)	54.3 (52.1-56.6)	54.6 (52.5-56.9)	54.7 (52.6-57.1)	54.8 (52.8-57.3)	55.1 (53.0-57.7)	55.2 (52.9-57.9)	55.4 (53.0-58.2)	55.9 (53.4-58.9)
Guinea-Bissau	52.6 (41.3-62.7)	52.8 (41.7-63.1)	53.0 (41.6-63.5)	53.1 (41.6-63.7)	53.3 (42.0-64.0)	53.5 (42.6-64.2)	53.0 (42.2-63.7)	53.3 (42.6-64.4)	53.4 (42.4-64.7)	53.4 (42.4-64.7)	53.4 (42.3-64.7)	53.4 (42.1-65.0)

Location	2004	2005	2006	2007	2008	2009	2010	2011	2012	2013	2014	2015
Sri Lanka	74.3 (71.6-77.2)	78.0 (77.7-78.2)	78.2 (77.9-78.4)	78.4 (78.3-78.6)	78.1 (77.9-78.4)	78.1 (77.8-78.4)	78.7 (78.3-79.1)	79.6 (79.2-80.1)	80.1 (79.2-81.0)	80.5 (79.2-81.8)	80.8 (79.1-82.5)	81.2 (79.1-83.2)
Seychelles	75.7 (75.2-76.3)	75.9 (75.4-76.5)	76.1 (75.6-76.9)	76.6 (75.9-77.2)	76.9 (76.3-77.5)	77.0 (76.3-77.6)	77.1 (76.4-77.7)	77.3 (76.6-78.0)	77.5 (76.6-78.2)	77.6 (76.7-78.5)	77.8 (76.7-78.7)	77.9 (76.8-79.0)
Thailand	75.9 (75.2-76.6)	76.5 (75.9-77.1)	76.8 (76.3-77.3)	77.2 (76.7-77.7)	77.5 (77.0-78.0)	77.7 (77.3-78.3)	78.0 (77.5-78.7)	78.2 (77.6-79.1)	78.5 (77.7-79.5)	78.7 (77.5-79.9)	78.8 (77.5-80.2)	79.0 (77.5-80.5)
Timor-Leste	67.1 (65.3-69.0)	68.0 (66.2-69.9)	69.2 (67.3-70.9)	69.8 (67.8-71.6)	70.4 (68.1-72.2)	70.8 (68.6-72.7)	71.3 (68.7-73.3)	71.7 (69.1-74.1)	72.2 (69.4-74.6)	72.5 (69.6-75.1)	72.8 (69.9-75.4)	73.0 (70.0-75.7)
Vietnam	76.7 (74.2-78.8)	77.0 (74.4-79.2)	77.2 (74.6-79.6)	77.5 (74.9-79.8)	77.8 (75.2-80.2)	78.0 (75.5-80.6)	78.3 (75.8-80.8)	78.5 (76.1-81.1)	78.8 (76.3-81.4)	79.0 (76.5-81.5)	79.2 (76.8-81.8)	79.4 (77.0-82.2)
Oceania	60.8 (56.7-65.0)	60.9 (56.9-65.0)	61.1 (56.9-65.1)	61.3 (56.9-65.5)	61.5 (57.0-65.7)	61.7 (57.1-65.8)	62.0 (57.4-66.1)	62.3 (57.6-66.5)	62.6 (57.8-66.7)	63.0 (58.1-67.1)	63.4 (58.6-67.4)	63.7 (59.0-67.7)
American Samoa	74.4 (72.8-75.7)	74.6 (73.0-75.9)	74.6 (73.1-75.9)	74.7 (73.1-76.0)	74.8 (73.3-76.2)	73.8 (72.1-75.4)	75.0 (73.4-76.5)	74.6 (72.8-76.3)	74.5 (72.6-76.2)	74.6 (72.4-76.5)	74.7 (72.4-76.8)	74.8 (72.4-77.1)
Federated States of Micronesia	69.8 (64.4-74.3)	69.9 (64.4-74.5)	70.1 (64.4-74.6)	70.2 (64.4-74.7)	70.4 (64.6-74.8)	70.5 (64.7-75.0)	70.7 (64.7-75.2)	70.9 (64.7-75.3)	71.1 (64.8-75.5)	71.3 (65.2-75.5)	71.4 (65.1-75.7)	71.5 (65.3-75.8)
Fiji	67.1 (65.4-68.7)	67.1 (65.4-68.8)	67.1 (65.5-68.8)	67.1 (65.3-68.9)	67.1 (65.3-69.0)	67.1 (65.3-69.0)	67.3 (65.5-69.3)	67.5 (65.5-69.5)	67.6 (65.4-69.7)	67.8 (65.5-70.2)	68.0 (65.5-70.5)	68.1 (65.5-70.9)
Guam	76.7 (75.6-77.9)	76.5 (75.4-77.6)	76.3 (75.1-77.4)	76.0 (75.0-77.1)	75.8 (74.8-77.0)	75.4 (74.4-76.6)	75.2 (74.1-76.3)	75.1 (73.8-76.3)	75.0 (73.7-76.3)	75.2 (73.7-76.6)	75.3 (73.6-76.9)	75.3 (73.6-77.3)
Kiribati	64.7 (62.5-66.7)	64.9 (62.7-67.1)	65.2 (62.8-67.4)	65.4 (62.9-67.7)	65.7 (63.2-68.0)	66.0 (63.4-68.4)	66.2 (63.6-68.8)	66.4 (63.8-69.1)	66.7 (63.8-69.5)	66.9 (64.2-69.7)	67.2 (64.3-69.9)	67.4 (64.6-70.2)
Marshall Islands	66.9 (64.6-69.0)	66.8 (64.6-69.0)	66.8 (64.6-69.0)	67.2 (64.9-69.3)	67.5 (65.1-69.8)	67.8 (65.3-70.1)	68.1 (65.6-70.4)	68.4 (65.9-70.7)	68.7 (66.1-71.1)	69.0 (66.5-71.4)	69.2 (66.8-71.7)	69.5 (67.0-71.9)
Northern Mariana Islands	77.3 (76.6-78.1)	77.4 (76.7-78.1)	77.6 (76.8-78.3)	77.9 (77.1-78.6)	78.1 (77.2-78.9)	78.2 (77.2-79.0)	78.3 (77.3-79.2)	78.3 (77.2-79.2)	78.4 (77.2-79.4)	78.4 (77.2-79.5)	78.4 (77.2-79.5)	78.5 (77.2-79.6)
Papua New Guinea	58.4 (53.2-63.8)	58.5 (53.4-63.9)	58.7 (53.6-64.2)	59.0 (53.6-64.5)	59.3 (53.7-64.9)	59.5 (53.9-65.0)	59.9 (54.2-65.3)	60.2 (54.4-65.7)	60.6 (54.7-66.0)	61.0 (55.1-66.3)	61.4 (55.6-66.7)	61.8 (56.0-67.0)
Samoa	73.6 (71.4-75.8)	73.7 (71.7-75.8)	73.8 (71.9-75.9)	73.8 (72.0-75.8)	73.9 (72.0-75.9)	72.3 (70.4-74.2)	73.8 (71.8-75.8)	73.7 (71.6-75.8)	73.7 (71.6-75.8)	74.0 (71.8-76.2)	74.1 (71.8-76.4)	74.2 (71.8-76.6)
Solomon Islands	62.6 (57.6-68.1)	62.7 (57.5-68.2)	62.9 (57.6-68.4)	62.9 (57.5-68.3)	63.3 (57.9-68.9)	63.4 (57.9-69.0)	63.7 (58.2-69.4)	64.0 (58.6-69.8)	64.3 (58.8-70.1)	64.6 (58.8-70.4)	64.8 (58.8-70.5)	65.1 (59.2-70.8)
Tonga	72.4 (71.1-73.6)	72.7 (71.3-74.0)	73.0 (71.5-74.4)	73.1 (71.5-74.7)	73.3 (71.6-75.0)	72.8 (71.0-74.6)	73.7 (71.7-75.6)	73.9 (71.8-75.9)	74.1 (71.9-76.3)	74.3 (72.0-76.6)	74.5 (72.1-76.9)	74.7 (72.2-77.1)
Vanuatu	64.1 (59.1-69.1)	64.2 (59.0-69.2)	64.4 (59.1-69.3)	64.7 (59.0-69.4)	64.9 (59.1-69.7)	65.2 (59.5-70.0)	65.5 (59.8-70.1)	65.8 (60.1-70.2)	66.0 (60.3-70.5)	66.3 (60.8-70.7)	66.5 (61.1-70.7)	66.6 (61.2-70.9)
North Africa and Middle East	72.3 (71.6-72.9)	72.5 (71.8-73.2)	72.8 (72.1-73.5)	73.1 (72.4-73.9)	73.4 (72.7-74.1)	73.7 (73.0-74.4)	73.9 (73.2-74.7)	74.2 (73.4-74.9)	74.4 (73.7-75.1)	74.7 (74.0-75.4)	74.9 (74.1-75.6)	74.9 (74.1-75.7)
North Africa and Middle East	72.3 (71.6-72.9)	72.5 (71.8-73.2)	72.8 (72.1-73.5)	73.1 (72.4-73.9)	73.4 (72.7-74.1)	73.7 (73.0-74.4)	73.9 (73.2-74.7)	74.2 (73.4-74.9)	74.4 (73.7-75.1)	74.7 (74.0-75.4)	74.9 (74.1-75.6)	74.9 (74.1-75.7)
Afghanistan	51.1 (47.2-55.2)	51.4 (47.4-55.6)	51.6 (47.6-56.0)	52.0 (47.9-56.4)	52.3 (48.3-56.6)	52.8 (48.8-57.0)	53.2 (49.3-57.5)	53.6 (49.5-58.0)	54.1 (50.0-58.4)	54.5 (50.4-58.9)	54.7 (50.5-59.1)	54.5 (50.2-58.8)
Algeria	75.8 (74.7-76.8)	76.1 (74.9-77.1)	76.3 (75.3-77.3)	76.5 (75.6-77.4)	76.7 (75.8-77.6)	76.9 (76.0-77.7)	77.1 (76.2-77.9)	77.2 (76.3-78.0)	77.2 (76.3-78.1)	77.3 (76.2-78.2)	77.4 (76.1-78.4)	77.4 (76.0-78.5)
Bahrain	77.6 (76.9-78.4)	78.1 (77.2-78.9)	78.4 (77.6-79.2)	79.0 (78.2-79.6)	79.3 (78.6-79.9)	79.5 (78.8-80.2)	79.6 (79.0-80.4)	79.8 (79.1-80.5)	79.9 (79.2-80.7)	80.1 (79.3-81.2)	80.2 (79.1-81.6)	80.3 (78.9-81.8)
Egypt	72.5 (72.3-72.8)	72.6 (72.4-72.9)	72.7 (72.4-72.9)	73.0 (72.7-73.2)	72.8 (72.6-73.1)	72.6 (72.4-72.9)	72.6 (72.3-72.8)	73.2 (72.9-73.4)	73.2 (72.9-73.4)	73.9 (73.6-74.1)	74.2 (73.8-74.6)	74.4 (73.9-74.9)
Iran	74.2 (72.4-75.9)	74.6 (72.5-76.5)	75.0 (72.7-76.9)	75.3 (73.1-77.5)	75.7 (73.3-77.8)	76.0 (73.5-78.2)	76.2 (73.8-78.5)	76.8 (74.5-79.0)	77.3 (75.0-79.4)	77.6 (75.4-79.6)	77.8 (75.7-79.8)	78.0 (75.9-80.0)
Iraq	68.4 (66.0-70.6)	68.2 (65.7-70.6)	68.1 (65.3-70.6)	68.3 (65.4-71.0)	69.1 (66.0-71.9)	69.4 (66.4-72.2)	69.7 (66.5-72.6)	70.1 (67.0-73.0)	70.4 (67.3-73.5)	70.7 (67.6-73.7)	70.6 (67.6-73.5)	70.7 (67.5-73.6)
Jordan	73.8 (72.7-74.8)	74.4 (73.5-75.3)	75.7 (74.9-76.4)	77.0 (76.4-77.5)	77.9 (77.5-78.4)	78.8 (78.4-79.2)	79.5 (79.2-79.9)	79.9 (79.5-80.3)	80.1 (79.7-80.6)	80.4 (79.7-81.0)	80.5 (79.7-81.4)	80.7 (79.7-81.8)
Kuwait	80.0 (79.7-80.4)	80.2 (79.9-80.5)	80.1 (79.9-80.5)	79.4 (79.1-79.8)	78.7 (78.4-79.0)	78.4 (78.1-78.7)	78.7 (78.3-79.1)	78.9 (78.6-79.2)	79.1 (78.8-79.4)	80.3 (80.0-80.6)	81.1 (80.4-81.8)	81.6 (80.5-82.7)
Lebanon	77.4 (75.4-79.1)	77.6 (75.8-79.4)	77.8 (76.0-79.6)	78.0 (76.2-79.8)	78.3 (76.4-80.1)	78.6 (76.8-80.3)	78.8 (76.9-80.8)	78.3 (76.3-80.4)	79.3 (77.3-81.6)	79.4 (77.4-81.8)	79.6 (77.6-82.0)	79.9 (77.8-82.3)
Libya	76.7 (75.8-77.5)	76.9 (76.1-77.6)	77.2 (76.3-77.9)	77.2 (76.1-77.9)	77.4 (76.4-78.1)	77.5 (76.5-78.3)	77.7 (76.6-78.5)	70.0 (65.7-74.8)	77.5 (76.1-78.5)	77.6 (76.2-78.8)	77.3 (75.8-78.5)	76.9 (75.3-78.2)
Morocco	73.5 (70.8-75.8)	73.8 (70.9-76.2)	74.0 (71.1-76.5)	74.2 (71.4-76.8)	74.5 (71.5-77.1)	74.7 (71.6-77.4)	74.9 (71.8-77.7)	75.1 (72.0-78.0)	75.3 (72.2-78.4)	75.5 (72.3-78.6)	75.6 (72.5-78.8)	75.8 (72.7-79.0)
Palestine	77.2 (76.5-77.9)	77.3 (76.6-78.1)	77.3 (76.3-78.1)	77.3 (76.0-78.2)	77.3 (75.8-78.3)	77.1 (75.5-78.3)	77.4 (75.6-78.7)	77.4 (75.4-78.7)	77.4 (75.4-78.9)	77.6 (75.5-79.2)	77.7 (75.6-79.5)	77.9 (75.8-79.8)
Oman	78.2 (77.1-79.2)	78.4 (77.4-79.3)	78.4 (77.5-79.3)	78.4 (77.5-79.2)	78.6 (77.8-79.4)	78.5 (77.6-79.2)	78.2 (77.3-79.0)	78.6 (77.7-79.5)	79.0 (78.1-80.1)	79.3 (78.2-80.5)	79.5 (78.2-80.8)	79.6 (78.3-81.1)
Qatar	77.6 (76.7-78.5)	78.3 (77.5-79.1)	78.9 (78.1-79.8)	79.5 (78.6-80.6)	80.0 (79.0-81.1)	80.5 (79.4-81.6)	80.9 (79.9-81.8)	81.0 (79.8-82.0)	80.9 (79.7-82.2)	81.0 (79.5-82.4)	81.1 (79.4-82.7)	81.2 (79.4-82.9)
Saudi Arabia	79.7 (79.1-80.2)	80.0 (79.4-80.6)	80.3 (79.7-80.8)	80.5 (79.9-81.1)	80.7 (80.2-81.3)	80.9 (80.3-81.5)	81.2 (80.5-81.8)	81.4 (80.8-82.1)	81.7 (81.0-82.4)	81.9 (81.1-82.7)	82.1 (81.2-82.9)	82.2 (81.3-83.1)
Sudan	65.5 (61.8-69.5)	66.1 (62.3-70.0)	66.4 (62.5-70.3)	66.8 (63.0-70.7)	67.1 (63.4-71.1)	67.4 (63.9-71.5)	67.8 (64.1-71.9)	68.0 (64.3-72.2)	68.3 (64.5-72.5)	68.6 (64.9-72.8)	68.9 (65.1-73.2)	69.3 (65.4-73.6)
Syria	77.2 (76.3-77.9)	77.4 (76.7-78.0)	77.6 (77.1-78.0)	77.8 (77.4-78.1)	78.1 (77.7-78.4)	78.3 (78.0-78.7)	78.5 (78.1-79.0)	77.9 (77.2-78.7)	74.6 (72.1-77.4)	75.1 (72.9-77.6)	74.2 (70.9-77.8)	74.8 (72.1-77.8)
Tunisia	78.7 (77.3-80.2)	79.0 (77.5-80.5)	79.2 (77.6-80.8)	79.4 (77.8-81.0)	79.6 (78.1-81.3)	79.8 (78.2-81.6)	79.9 (78.3-81.8)	80.1 (78.4-82.0)	80.3 (78.5-82.2)	80.5 (78.7-82.4)	80.6 (78.8-82.6)	80.7 (78.8-82.7)
Turkey	79.3 (78.6-80.0)	79.5 (78.9-80.1)	80.0 (79.4-80.5)	80.6 (80.1-81.1)	81.0 (80.6-81.4)	81.8 (81.3-82.2)	82.0 (81.5-82.4)	82.0 (81.5-82.4)	82.2 (81.7-82.6)	82.0 (81.5-82.4)	82.2 (81.7-82.6)	82.4 (81.8-82.9)
United Arab Emirates	76.7 (75.9-77.3)	77.3 (76.5-78.0)	77.8 (76.8-78.5)	77.9 (76.9-78.9)	77.9 (76.7-79.0)	78.0 (76.6-79.2)	78.0 (76.4-79.3)	78.0 (76.2-79.5)	78.0 (76.0-79.6)	78.0 (75.8-79.7)	78.0 (75.7-79.8)	78.0 (75.5-79.9)
Yemen	64.2 (59.2-70.0)	64.7 (59.6-70.4)	65.2 (60.0-70.9)	65.6 (60.3-71.2)	66.0 (60.5-71.6)	66.4 (60.6-72.1)	66.8 (60.9-72.3)	67.0 (61.2-72.5)	67.1 (61.4-72.5)	67.4 (61.6-72.8)	67.5 (61.9-72.8)	66.6 (60.8-71.6)
South Asia	64.9 (64.3-65.5)	65.2 (64.7-65.8)	65.8 (65.2-66.3)	66.2 (65.7-66.8)	66.7 (66.1-67.3)	67.1 (66.5-67.7)	67.4 (66.8-68.1)	67.9 (67.2-68.5)	68.3 (67.7-68.9)	68.7 (68.0-69.4)	69.1 (68.4-69.8)	69.6 (68.8-70.3)
South Asia	64.9 (64.3-65.5)	65.2 (64.7-65.8)	65.8 (65.2-66.3)	66.2 (65.7-66.8)	66.7 (66.1-67.3)	67.1 (66.5-67.7)	67.4 (66.8-68.1)	67.9 (67.2-68.5)	68.3 (67.7-68.9)	68.7 (68.0-69.4)	69.1 (68.4-69.8)	69.6 (68.8-70.3)
Bangladesh	68.3 (67.2-69.5)	68.7 (67.6-69.9)	69.2 (68.0-70.4)	69.6 (68.4-70.9)	70.1 (68.8-71.4)	70.4 (69.2-71.8)	70.6 (69.3-72.1)	71.1 (69.6-72.6)	71.4 (69.8-73.0)	71.8 (70.1-73.5)	72.2 (70.3-74.0)	72.5 (70.6-74.5)
Bhutan	69.7 (67.4-71.8)	70.2 (67.8-72.3)	70.8 (68.4-72.9)	71.3 (68.8-73.4)	71.9 (69.4-74.0)	72.4 (69.9-74.4)	72.9 (70.4-74.9)	73.2 (70.7-75.3)	73.5 (70.8-75.6)	73.7 (71.1-75.9)	74.0 (71.3-76.2)	74.3 (71.6-76.5)
India	64.6 (63.9-65.2)	65.0 (64.4-65.7)	65.5 (64.9-66.1)	66.0 (65.3-66.6)	66.4 (65.8-67.1)	66.9 (66.2-67.6)	67.3 (66.6-67.9)	67.7 (67.0-68.4)	68.2 (67.5-68.9)	68.6 (67.9-69.4)	69.1 (68.3-69.9)	69.5 (68.7-70.4)
Nepal	68.5 (66.8-70.0)	68.8 (67.0-70.6)	69.2 (67.2-71.0)	69.5 (67.5-71.5)	69.8 (67.7-71.9)	70.1 (67.9-72.4)	70.4 (68.1-72.8)	70.7 (68.3-73.2)	71.0 (68.6-73.6)	71.3 (68.9-74.0)	71.6 (69.1-74.3)	71.2 (68.8-73.9)
Pakistan	63.7 (61.8-65.5)	63.2 (61.4-65.0)	64.5 (62.7-66.4)	65.0 (63.1-67.0)	65.4 (63.4-67.4)	65.6 (63.8-67.8)	65.9 (64.1-68.1)	66.2 (64.3-68.5)	66.5 (64.5-68.9)	66.8 (64.8-69.1)	67.0 (65.0-69.4)	67.3 (65.2-69.8)
Sub-Saharan Africa	55.3 (54.4-56.0)	55.9 (55.1-56.7)	56.7 (55.8-57.5)	57.5 (56.6-58.5)	58.5 (57.6-59.5)	59.4 (58.4-60.4)	60.1 (59.0-61.2)	60.9 (59.6-62.1)	61.8 (60.3-63.1)	62.5 (61.0-63.9)	63.0 (61.3-64.5)	63.6 (61.9-65.2)
Southern Sub-Saharan Africa	51.7 (50.3-53.2)	51.8 (50.5-53.2)	52.0 (50.7-53.4)	52.9 (51.6-54.2)	53.8 (52.5-55.1)	54.4 (53.1-55.8)	55.4 (53.9-56.7)	57.6 (56.2-58.9)	60.5 (59.1-61.7)	62.3 (60.9-63.5)	62.8 (61.4-64.1)	63.2 (61.7-64.7)
Botswana	48.0 (37.8-61.9)	50.8 (39.4-64.3)	52.8 (40.7-66.3)	54.1 (41.5-67.3)	55.0 (42.3-67.7)	56.3 (43.3-68.6)	58.1 (44.6-70.2)	59.2 (45.4-71.0)	59.1 (45.1-70.8)	60.2 (46.0-71.1)	60.7 (46.6-71.2)	62.0 (47.7-72.3)
Lesotho	46.1 (42.4-50.0)	44.8 (41.3-48.6)	45.2 (41.5-48.9)	45.3 (41.5-49.2)	46.7 (42.8-50.9)	47.9 (43.4-52.6)	49.5 (44.3-54.9)	49.8 (44.7-55.7)	48.6 (43.3-54.9)	48.3 (42.7-54.9)	48.1 (42.4-55.2)	50.4 (43.6-58.5)
Namibia	54.4 (50.4-58.4)	55.1 (51.3-59.1)	57.1 (53.4-61.0)	60.0 (56.0-64.3)	61.9 (57.6-66.5)	63.2 (58.6-67.8)	64.5 (59.6-69.0)	65.8 (61.2-69.9)	67.1 (62.2-71.2)	68.1 (62.7-72.1)	68.4 (62.4-72.7)	68.4 (61.6-73.0)
South Africa	53.7 (52.3-55.6)	53.6 (52.2-55.4)	53.6 (52.2-55.3)	54.3 (53.0-55.9)	55.0 (53.6-56.5)	55.6 (54.1-57.1)	56.4 (55.0-58.0)	58.7 (57.4-60.0)	61.8 (60.7-62.9)	63.8 (62.7-64.9)	64.2 (62.9-65.3)	64.0 (62.5-65.2)
Swaziland	44.0 (39.9-48.3)	43.2 (39.3-47.4)	43.9 (40.0-48.2)	45.3 (40.9-49.6)	46.9 (41.9-52.1)	48.1 (42.6-54.0)	48.7 (42.8-55.1)	50.6 (44.3-57.8)	51.9 (45.2-59.6)	52.2 (45.0-60.4)	52.8 (45.1-61.3)	54.9 (46.7-63.9)
Zimbabwe	45.1 (41.8-49.2)	45.6 (42.4-49.3)	46.1 (42.9-49.7)	47.2 (44.2-50.7)	48.8 (45.9-52.1)	49.6 (46.5-53.1)	50.7 (47.3-54.4)	53.8 (50.1-58.0)	57.0 (52.5-61.9)	58.8 (53.8-64.1)	60.3 (54.9-65.9)	62.5 (56.3-68.3)
Western Sub-Saharan Africa	56.9 (55.6-58.2)	57.5 (56.1-58.8)	58.1 (56.7-59.5)	58.8 (57.3-60.3)	59.6 (57.9-61.2)	60.4 (58.7-62.1)	61.2 (59.3-63.0)	61.8 (59.6-63.8)	62.4 (59.9-64.5)	63.0 (60.2-65.3)	63.4 (60.3-65.7)	64.2 (60.9-66.5)
Benin	60.6 (56.6-64.8)	61.0 (56.4-65.3)	61.3 (56.2-65.9)	61.6 (56.0-66.8)	62.1 (56.0-67.8)	62.8 (55.8-69.0)	63.2 (56.3-69.8)	63.6 (56.7-70.6)	63.9 (56.6-71.1)	64.2 (56.5-71.4)	64.5 (56.3-71.8)	64.8 (55.8-72.4)
Burkina Faso	55.9 (53.0-58.9)	56.6 (53.5-59.7)	57.3 (54.0-60.4)	58.1 (54.7-61.5)	58.7 (54.9-62.4)	59.2 (55.0-63.3)	59.7 (55.1-64.2)	60.2 (54.9-65.1)	60.7 (54.7-66.2)	61.1 (54.7-67.2)	61.5 (54.6-68.2)	61.9 (54.3-68.9)
Cameroon	55.4 (52.5-58.8)	55.9 (52.8-59.5)	56.6 (53.4-60.3)	57.0 (53.7-60.7)	58.0 (54.2-61.9)	58.8 (54.7-63.1)	59.2 (54.8-64.1)	59.6 (54.5-65.0)	60.0 (54.2-66.0)	60.7 (54.4-67.5)	61.0 (54.3-68.0)	61.2 (54.0-68.5)
Cape Verde	75.7 (72.6-76.8)	76.0 (73.4-76.9)	76.3 (74.2-77.1)	76.4 (74.8-77.0)	76.8 (75.5-77.2)	77.0 (76.1-77.3)	77.1 (76.7-77.3)	77.1 (76.9-77.3)	77.1 (76.6-77.3)	76.9 (76.1-77.4)	76.8 (75.4-77.5)	76.6 (74.3-77.6)
Chad	54.1 (49.3-59.2)	54.5 (49.3-60.0)	54.8 (49.1-60.8)	55.5 (49.2-61.9)	55.8 (49.0-62.8)	56.6 (49.5-64.1)	57.2 (49.6-65.1)	57.5 (49.4-65.7)	57.7 (49.1-65.9)	58.0 (49.1-66.4)	58.3 (49.3-66.9)	58.9 (49.4-67.6)
Cote d'Ivoire	54.4 (51.4-57.6)	54.8 (51.8-58.2)	55.7 (52.7-59.3)	56.6 (53.4-60.4)	57.1 (53.6-61.2)	57.8 (54.0-62.1)	58.6 (54.0-63.3)	58.9 (53.7-64.1)	59.5 (53.8-65.2)	60.1 (54.2-66.2)	60.6 (54.5-67.3)	61.4 (55.1-68.5)
The Gambia	65.8 (63.1-68.9)	66.2 (63.5-69.3)	66.5 (63.9-69.7)	66.5 (63.7-69.6)	67.0 (64.0-70.0)	67.2 (64.0-70.4)	67.8 (64.4-71.3)	68.5 (64.9-72.1)	69.0 (65.2-73.0)	69.4 (65.2-73.5)	69.5 (64.8-74.1)	69.6 (64.4-74.6)
Ghana	61.8 (58.3-65.8)	62.2 (58.4-66.3)	62.7 (58.4-67.4)	63.2 (58.5-68.4)	63.7 (58.5-69.7)	64.2 (58.7-70.6)	64.8 (58.8-71.4)	65.4 (58.8-72.0)	66.2 (59.1-72.9)	66.7 (59.3-73.4)	67.0 (59.4-73.8)	67.6 (59.6-74.2)
Guinea	56.4 (53.8-59.3)	56.9 (54.2-59.8)	57.2 (54.5-60.2)	57.7 (54.9-60.9)	58.1 (55.1-61.5)	58.6 (55.4-62.3)	59.1 (55.6-63.2)	59.4 (55.6-64.0)	59.8 (55.6-64.8)	60.2 (55.3-65.7)	60.0 (54.6-65.9)	60.5 (54.5-67.1)
Guinea-Bissau	53.4 (41.9-65.1)	53.4 (41.9-65.2)	53.5 (42.0-65.3)	53.7 (42.4-65.5)	54.1 (42.3-65.9)	54.5 (42.8-66.2)	54.8 (42.8-66.4)	55.1 (42.8-66.7)	55.4 (42.4-67.0)	55.8 (42.9-67.2)	56.0 (42.7-67.3)	56.4 (42.5-67.6)

SR Table 6: Estimates of life expectancy at birth for each geography (with 95% uncertainty intervals), females, 1980–2015

Location	1980	1981	1982	1983	1984	1985	1986	1987	1988	1989	1990	1991
Liberia	50.4 (41.9-59.0)	50.6 (42.2-59.0)	50.8 (43.0-58.9)	51.0 (43.7-58.8)	51.1 (44.4-58.4)	51.2 (44.9-57.9)	51.4 (45.5-57.5)	51.5 (46.4-57.3)	51.7 (47.0-57.0)	52.0 (47.5-56.7)	50.5 (46.5-54.8)	52.0 (48.0-55.8)
Mali	45.8 (43.3-49.0)	46.5 (43.9-49.5)	47.3 (44.8-50.2)	47.8 (45.4-50.7)	48.2 (45.9-50.9)	48.8 (46.6-51.3)	49.6 (47.5-52.0)	49.8 (47.9-52.2)	50.0 (48.0-52.3)	50.1 (48.1-52.3)	50.1 (48.1-52.3)	50.5 (48.6-52.8)
Mauritania	57.3 (50.9-64.6)	57.7 (51.8-64.3)	58.0 (52.7-64.1)	58.4 (53.4-63.8)	58.7 (54.4-63.5)	59.0 (55.2-63.1)	59.6 (56.3-63.3)	60.0 (57.1-63.0)	60.6 (58.1-63.4)	60.1 (57.8-62.9)	60.1 (57.7-62.8)	60.6 (58.1-63.3)
Niger	45.1 (42.1-47.8)	44.6 (41.8-47.2)	45.1 (42.3-47.6)	44.9 (42.1-47.4)	45.0 (42.3-47.5)	45.2 (42.5-47.8)	45.5 (42.8-48.0)	46.1 (43.4-48.5)	46.7 (44.1-49.0)	47.6 (45.1-49.8)	48.1 (45.7-50.3)	48.6 (46.0-50.8)
Nigeria	52.8 (44.7-61.2)	53.2 (45.5-61.5)	53.6 (45.9-61.6)	53.9 (46.3-61.4)	54.1 (46.9-61.4)	54.3 (47.2-61.4)	54.4 (47.5-61.1)	54.4 (47.8-60.6)	54.5 (48.1-60.1)	54.5 (48.7-59.7)	54.7 (49.2-59.4)	54.8 (49.9-59.1)
Sao Tome and Principe	64.7 (63.4-65.8)	64.8 (63.4-66.0)	65.0 (63.5-66.2)	65.1 (63.8-66.2)	65.2 (64.1-66.2)	65.0 (63.9-66.0)	65.0 (63.9-66.0)	65.0 (63.7-66.2)	64.8 (63.1-66.2)	64.7 (62.5-66.5)	64.5 (61.9-66.8)	64.4 (61.6-67.0)
Senegal	54.0 (51.1-57.3)	54.4 (51.6-57.5)	55.3 (52.6-58.2)	56.4 (53.6-58.9)	57.5 (54.7-59.9)	57.7 (54.9-60.2)	57.8 (55.1-60.3)	58.4 (55.6-61.0)	58.7 (55.8-61.2)	59.0 (56.2-61.5)	59.3 (56.6-61.9)	59.4 (56.7-62.0)
Sierra Leone	50.9 (43.6-57.6)	51.1 (44.1-57.8)	51.5 (44.4-58.0)	51.8 (45.0-58.1)	52.2 (45.5-58.3)	52.7 (46.2-58.4)	53.1 (46.9-58.5)	53.5 (47.5-58.7)	53.9 (48.3-58.8)	54.3 (49.0-59.0)	54.8 (49.9-59.1)	54.8 (50.5-58.7)
Togo	57.3 (52.0-62.2)	57.6 (52.8-62.1)	57.8 (53.6-62.0)	57.9 (54.1-61.6)	57.6 (54.2-61.0)	58.0 (54.7-61.2)	58.1 (55.2-61.2)	57.9 (55.0-61.0)	58.4 (55.5-61.4)	58.4 (55.5-61.4)	58.6 (55.8-61.6)	58.7 (56.1-61.8)
Eastern Sub-Saharan Africa	51.0 (48.4-53.5)	51.1 (48.7-53.3)	51.3 (49.1-53.4)	50.4 (47.7-52.8)	50.4 (48.0-52.7)	51.6 (49.9-53.4)	52.0 (50.5-53.7)	52.4 (50.9-53.8)	52.6 (51.3-54.0)	52.6 (51.3-53.9)	52.5 (51.3-53.7)	52.6 (51.5-53.7)
Burundi	45.1 (37.9-54.4)	45.4 (37.9-55.1)	45.8 (38.3-55.5)	46.1 (38.7-55.6)	46.4 (39.1-56.0)	46.6 (39.4-56.2)	46.8 (39.8-56.1)	47.0 (39.9-55.9)	47.0 (40.2-55.4)	47.0 (40.6-55.0)	47.0 (40.8-54.5)	46.8 (41.0-54.0)
Comoros	55.0 (46.4-64.5)	55.4 (47.0-64.8)	55.8 (47.5-65.0)	56.0 (48.0-65.3)	56.5 (48.6-65.9)	56.9 (49.0-66.1)	57.3 (49.3-66.2)	57.7 (49.9-65.9)	58.2 (50.4-66.1)	58.4 (50.9-66.1)	58.9 (51.5-66.4)	59.2 (52.3-66.6)
Djibouti	63.8 (55.4-70.6)	63.6 (55.2-70.5)	63.7 (54.9-70.6)	63.6 (54.8-70.4)	63.6 (54.4-70.4)	63.6 (55.1-70.3)	63.7 (55.8-70.4)	63.8 (56.2-70.3)	63.9 (56.6-70.2)	64.0 (56.7-70.2)	64.1 (57.1-70.1)	64.0 (57.6-69.7)
Eritrea	49.5 (45.8-53.3)	50.0 (46.8-53.4)	50.6 (47.5-53.8)	50.8 (47.8-54.0)	50.7 (47.7-53.8)	50.9 (48.0-53.8)	51.5 (48.7-54.5)	52.0 (49.2-55.0)	52.4 (49.6-55.4)	52.7 (49.7-55.5)	53.5 (50.5-56.4)	54.3 (51.3-57.2)
Ethiopia	44.0 (38.3-50.1)	44.3 (39.0-49.8)	44.5 (39.7-49.5)	41.3 (34.5-47.6)	41.7 (35.1-47.5)	45.5 (41.6-49.2)	46.3 (42.7-49.8)	46.9 (43.7-50.1)	47.4 (44.5-50.4)	47.6 (44.9-50.2)	47.6 (45.2-50.0)	48.6 (46.3-50.9)
Kenya	62.8 (60.6-64.8)	63.2 (61.1-65.1)	63.6 (61.5-65.3)	63.9 (61.9-65.6)	63.8 (61.9-65.6)	64.0 (62.2-65.8)	63.9 (62.1-65.6)	63.6 (61.8-65.3)	63.7 (61.9-65.4)	63.2 (61.5-64.8)	62.9 (61.3-64.6)	62.4 (60.7-64.0)
Madagascar	56.7 (54.2-59.4)	56.4 (54.2-59.1)	56.0 (53.9-58.8)	55.5 (53.4-58.1)	55.4 (53.4-57.8)	55.1 (53.2-57.4)	54.7 (52.9-56.7)	54.9 (53.2-56.9)	55.4 (53.7-57.2)	56.3 (54.7-58.0)	57.0 (55.3-58.6)	57.2 (55.5-58.9)
Malawi	50.0 (47.4-52.6)	50.4 (47.8-52.9)	50.7 (48.2-53.2)	50.8 (48.4-53.2)	50.9 (48.5-53.4)	50.7 (48.4-53.0)	50.4 (48.3-52.6)	50.3 (48.3-52.5)	50.1 (48.1-52.3)	49.9 (47.8-52.2)	49.8 (47.7-52.2)	49.7 (47.4-52.3)
Mozambique	51.1 (46.8-56.6)	48.9 (44.1-54.5)	49.1 (44.8-53.8)	48.9 (44.7-53.4)	47.9 (43.7-51.7)	47.7 (43.8-51.4)	49.9 (46.9-53.0)	50.6 (47.7-53.5)	52.7 (49.9-55.2)	53.3 (50.8-55.7)	53.8 (51.4-56.0)	54.1 (51.8-56.3)
Rwanda	53.3 (47.1-59.6)	54.3 (48.5-60.3)	55.0 (49.6-60.7)	55.5 (50.6-60.9)	55.7 (51.4-60.4)	55.7 (51.7-59.9)	55.7 (52.0-59.6)	55.4 (52.0-59.1)	54.7 (51.6-57.8)	53.5 (50.7-56.3)	51.9 (49.3-54.4)	50.6 (48.0-53.1)
Somalia	49.3 (38.6-61.8)	49.3 (38.4-62.1)	49.3 (37.9-62.1)	49.2 (38.0-62.0)	49.2 (38.2-62.0)	49.2 (38.5-62.0)	49.2 (38.3-61.9)	49.3 (39.1-62.1)	48.2 (38.5-60.2)	49.6 (39.4-62.6)	49.6 (39.3-62.6)	49.0 (38.9-61.6)
South Sudan	49.7 (40.1-60.3)	49.9 (39.8-60.3)	50.0 (39.8-60.4)	50.1 (39.7-60.5)	50.2 (39.7-60.6)	50.3 (40.0-60.8)	50.4 (40.1-61.1)	50.7 (40.4-61.3)	50.9 (40.7-61.7)	51.1 (41.2-62.1)	51.4 (41.1-62.6)	51.6 (41.1-62.8)
Tanzania	58.0 (53.4-61.7)	58.3 (54.1-61.5)	58.3 (54.6-61.3)	58.5 (55.3-61.2)	58.2 (55.4-60.7)	58.1 (55.5-60.5)	57.9 (55.3-60.2)	57.5 (55.0-60.0)	57.3 (54.8-59.7)	56.8 (54.3-59.3)	56.3 (53.9-58.9)	56.2 (53.8-58.8)
Uganda	50.6 (47.0-54.9)	51.1 (47.8-54.9)	51.6 (48.8-55.0)	52.2 (49.4-55.1)	52.2 (49.7-54.9)	52.5 (50.1-55.1)	52.6 (50.4-55.1)	53.4 (51.5-55.8)	53.4 (51.5-55.9)	53.0 (51.1-55.6)	52.6 (50.5-55.4)	52.0 (49.8-54.9)
Zambia	58.7 (54.4-62.9)	58.6 (54.9-62.3)	58.2 (54.8-61.5)	57.7 (54.8-60.8)	56.9 (54.3-59.7)	56.1 (53.7-58.7)	55.2 (53.1-57.8)	54.7 (52.6-57.4)	54.4 (52.4-57.1)	53.5 (51.3-56.2)	52.6 (50.4-55.5)	51.6 (49.3-54.3)
Central Sub-Saharan Africa	52.8 (46.9-59.4)	53.1 (46.9-59.5)	53.3 (47.4-59.5)	53.6 (47.9-59.6)	54.0 (48.5-59.9)	54.3 (49.1-59.9)	54.5 (49.7-59.8)	54.5 (49.9-59.6)	54.4 (49.8-59.1)	54.3 (49.9-58.7)	54.1 (50.0-58.1)	54.2 (50.2-58.0)
Angola	50.3 (39.9-62.5)	50.4 (39.9-62.3)	50.5 (40.4-62.5)	50.4 (40.5-62.4)	50.5 (40.7-62.8)	50.7 (41.2-62.9)	50.6 (41.3-62.5)	50.5 (41.1-62.2)	50.2 (40.7-62.0)	50.3 (40.7-62.5)	50.2 (40.5-62.3)	50.7 (40.6-62.8)
Central African Republic	52.3 (47.6-57.3)	52.8 (48.6-57.4)	53.4 (49.5-57.6)	53.0 (49.5-56.8)	53.2 (50.0-56.5)	53.4 (50.3-56.5)	53.3 (50.5-56.1)	53.0 (50.5-55.8)	51.9 (49.5-54.3)	51.6 (49.1-54.2)	50.9 (48.3-53.8)	51.0 (48.1-54.3)
Congo	53.7 (45.5-63.7)	54.2 (46.3-63.9)	54.7 (47.1-64.0)	55.3 (47.7-63.5)	55.7 (48.4-63.3)	56.1 (49.3-63.2)	56.3 (49.9-63.2)	56.4 (50.4-62.8)	56.5 (51.0-62.5)	56.4 (51.5-61.9)	56.2 (51.9-60.9)	56.3 (52.5-60.4)
Democratic Republic of the Congo	53.5 (45.6-62.9)	53.7 (45.8-62.7)	54.0 (46.2-62.6)	54.4 (46.7-62.6)	55.0 (47.3-63.1)	55.4 (48.2-63.2)	55.8 (49.0-63.1)	55.8 (49.9-62.7)	55.8 (50.5-62.4)	55.7 (50.6-61.8)	55.5 (50.7-61.2)	55.4 (51.2-60.4)
Equatorial Guinea	51.1 (40.7-63.9)	51.4 (41.0-64.2)	51.7 (40.8-64.4)	51.8 (41.2-64.5)	51.8 (41.2-64.4)	51.7 (41.3-64.2)	51.6 (41.3-64.0)	51.4 (41.2-63.7)	51.2 (41.0-63.7)	50.9 (40.9-63.8)	50.5 (40.6-63.5)	50.3 (40.2-63.6)
Gabon	60.7 (54.3-67.5)	61.1 (55.2-67.4)	61.5 (55.7-67.2)	61.9 (56.5-67.0)	62.3 (57.4-66.9)	62.6 (58.2-67.0)	62.9 (59.0-67.0)	63.0 (59.3-66.7)	62.9 (59.6-66.0)	62.7 (59.7-65.7)	63.0 (60.2-66.0)	63.3 (60.5-66.3)

Location	1992	1993	1994	1995	1996	1997	1998	1999	2000	2001	2002	2003
Liberia	52.3 (48.7-55.8)	51.7 (48.6-54.9)	51.2 (48.1-54.3)	52.4 (49.5-55.4)	52.4 (49.4-55.3)	54.3 (51.5-57.0)	55.0 (52.4-57.7)	55.9 (53.3-58.6)	56.5 (53.8-59.2)	57.1 (54.5-59.8)	56.7 (54.1-59.6)	56.8 (54.1-59.8)
Mali	50.8 (48.9-53.0)	51.3 (49.4-53.6)	51.4 (49.5-53.6)	51.4 (49.5-53.5)	51.7 (49.9-53.7)	52.0 (50.2-54.1)	51.8 (49.7-53.9)	53.2 (51.3-55.3)	53.3 (51.3-55.7)	53.9 (51.9-56.4)	54.6 (52.5-57.0)	55.5 (53.0-58.0)
Mauritania	60.8 (58.3-63.6)	61.0 (58.3-63.8)	61.2 (58.4-64.0)	61.5 (58.9-64.2)	61.7 (59.2-64.3)	62.3 (60.0-65.0)	62.7 (60.5-65.3)	63.1 (60.8-65.8)	63.6 (61.1-66.2)	64.0 (61.2-66.7)	64.6 (61.8-67.4)	65.2 (62.3-68.0)
Niger	48.7 (46.1-50.9)	48.9 (46.3-51.1)	49.3 (46.6-51.6)	49.6 (47.0-51.8)	50.3 (47.7-52.5)	50.7 (48.2-53.0)	51.3 (48.9-53.6)	52.0 (49.6-54.2)	52.7 (50.3-54.9)	53.2 (50.8-55.4)	54.1 (51.7-56.4)	55.0 (52.4-57.4)
Nigeria	55.0 (50.5-58.9)	55.1 (51.1-58.7)	55.4 (51.8-58.6)	55.4 (52.1-58.3)	55.4 (52.4-58.2)	55.4 (52.5-58.2)	55.5 (52.9-58.2)	55.7 (53.1-58.1)	55.7 (53.2-58.2)	55.9 (53.3-58.5)	55.7 (53.1-58.5)	56.1 (53.5-58.9)
Sao Tome and Principe	64.3 (61.4-67.1)	64.3 (61.4-67.2)	64.3 (61.4-67.2)	64.4 (61.5-67.3)	64.1 (61.4-67.1)	64.0 (61.4-67.0)	64.2 (61.6-67.2)	64.5 (62.0-67.3)	64.8 (62.3-67.5)	65.3 (62.9-68.1)	65.5 (62.8-68.3)	66.0 (63.4-68.8)
Senegal	59.5 (56.9-62.2)	59.7 (57.1-62.5)	59.6 (56.8-62.5)	59.9 (57.1-62.8)	60.1 (57.4-63.0)	60.2 (57.5-63.2)	60.4 (57.7-63.4)	60.4 (57.8-63.6)	60.9 (58.2-64.1)	61.3 (58.6-64.6)	61.8 (58.8-65.3)	62.4 (58.8-66.3)
Sierra Leone	55.2 (51.4-58.6)	55.4 (52.1-58.4)	54.9 (51.9-57.8)	54.5 (51.6-57.1)	54.8 (52.3-57.4)	54.2 (51.6-56.6)	53.3 (50.8-55.7)	52.8 (50.2-55.2)	53.7 (51.3-55.9)	53.5 (51.3-55.7)	53.1 (50.8-55.3)	53.1 (50.9-55.1)
Togo	59.0 (56.3-62.0)	59.0 (56.4-62.1)	59.0 (56.3-62.1)	59.3 (56.4-62.5)	59.3 (56.1-62.6)	58.9 (55.7-62.5)	58.7 (55.5-62.3)	58.8 (55.3-62.3)	58.7 (55.4-62.3)	58.7 (55.4-62.4)	58.6 (55.2-62.2)	58.5 (55.3-62.1)
Eastern Sub-Saharan Africa	52.6 (51.5-53.7)	52.5 (51.4-53.6)	50.2 (47.9-52.2)	52.2 (51.1-53.2)	52.2 (51.2-53.2)	52.2 (51.2-53.2)	52.3 (51.3-53.3)	52.3 (51.2-53.2)	52.5 (51.5-53.5)	53.2 (52.1-54.3)	53.8 (52.6-54.9)	54.4 (53.2-55.6)
Burundi	46.7 (41.3-53.2)	46.5 (41.6-52.2)	46.3 (42.1-51.5)	46.0 (42.1-50.8)	45.7 (42.0-49.8)	46.1 (42.7-49.7)	46.4 (43.2-49.7)	46.9 (43.8-50.1)	47.4 (44.5-50.5)	48.8 (46.0-51.7)	50.2 (47.6-53.1)	51.8 (49.0-54.7)
Comoros	59.5 (53.1-66.6)	59.9 (53.9-66.5)	60.2 (54.7-66.4)	60.6 (55.7-66.1)	60.6 (56.2-65.4)	61.2 (57.3-65.5)	61.7 (58.3-65.4)	62.4 (59.3-66.0)	62.9 (59.9-66.2)	63.6 (60.5-66.7)	64.5 (61.5-67.5)	65.2 (62.3-68.2)
Djibouti	63.9 (57.3-69.6)	64.0 (57.4-70.0)	63.5 (56.4-69.6)	63.9 (56.0-70.3)	63.9 (55.5-70.4)	63.8 (55.5-70.6)	63.7 (55.3-70.6)	63.6 (55.0-70.8)	63.6 (54.6-71.1)	63.7 (54.6-71.2)	63.6 (54.4-71.3)	63.7 (54.2-71.5)
Eritrea	55.5 (52.5-58.5)	56.6 (53.4-60.2)	57.3 (53.7-61.7)	58.0 (53.9-63.1)	58.6 (54.0-64.2)	59.2 (54.0-65.2)	59.3 (53.7-65.9)	56.3 (50.3-63.6)	47.8 (40.0-57.7)	60.1 (52.7-68.1)	60.2 (52.5-68.7)	60.2 (52.3-69.2)
Ethiopia	49.2 (47.1-51.2)	49.5 (47.4-51.4)	49.6 (47.7-51.5)	49.8 (47.8-51.8)	50.3 (48.3-52.4)	50.8 (48.7-52.8)	51.2 (49.1-53.4)	51.0 (48.9-53.2)	51.8 (49.7-54.1)	52.5 (50.2-54.9)	53.4 (50.8-55.8)	54.2 (51.6-56.8)
Kenya	61.7 (60.1-63.3)	60.9 (59.3-62.5)	60.1 (58.4-61.6)	59.3 (57.7-61.0)	58.7 (57.1-60.3)	57.9 (56.3-59.5)	57.3 (55.8-58.8)	57.1 (55.6-58.6)	56.8 (55.3-58.2)	56.8 (55.3-58.3)	56.8 (55.3-58.2)	56.9 (55.5-58.3)
Madagascar	57.3 (55.5-59.0)	57.3 (55.5-59.0)	57.7 (56.0-59.5)	58.1 (56.2-60.1)	58.5 (56.5-60.6)	58.8 (56.7-60.7)	59.2 (57.1-61.2)	59.7 (57.5-61.9)	60.0 (57.7-62.1)	60.4 (58.1-62.7)	60.6 (58.3-63.1)	61.1 (58.6-63.7)
Malawi	49.4 (46.9-52.2)	49.1 (46.5-52.2)	48.4 (45.6-51.7)	47.8 (45.0-51.1)	47.1 (44.3-50.4)	46.6 (43.7-49.8)	46.1 (43.3-49.3)	46.0 (43.1-49.1)	46.2 (43.6-49.5)	46.6 (43.8-49.9)	47.0 (44.2-50.3)	47.6 (44.5-50.9)
Mozambique	54.3 (52.0-56.4)	54.5 (52.3-56.6)	54.6 (52.5-56.7)	54.7 (52.5-56.8)	54.4 (52.1-56.7)	54.1 (51.9-56.5)	54.3 (51.8-56.8)	54.3 (51.8-57.2)	54.2 (51.5-57.4)	54.7 (51.6-58.4)	54.7 (51.2-58.6)	54.9 (51.2-58.8)
Rwanda	49.8 (47.3-52.3)	48.9 (46.4-51.4)	19.7 (9.8-37.4)	47.0 (44.7-49.4)	47.3 (45.1-49.7)	47.1 (45.1-49.5)	48.2 (46.1-50.6)	49.3 (47.1-51.7)	51.1 (48.7-53.5)	52.8 (50.3-55.4)	55.0 (52.5-57.8)	56.9 (54.3-59.7)
Somalia	49.5 (39.5-61.9)	50.1 (40.0-63.1)	50.2 (40.4-63.2)	50.3 (40.3-63.4)	50.4 (40.3-63.4)	50.1 (40.1-63.1)	50.7 (40.5-64.2)	50.8 (40.8-64.5)	51.0 (41.0-64.8)	51.3 (40.7-65.1)	51.5 (40.5-65.5)	51.9 (40.8-66.0)
South Sudan	52.1 (41.3-63.4)	52.4 (41.6-63.8)	52.8 (41.9-64.3)	53.2 (41.9-64.7)	53.6 (42.3-65.1)	53.9 (42.8-65.6)	54.3 (42.7-65.8)	54.6 (43.2-66.2)	54.9 (43.5-66.6)	55.2 (43.3-66.8)	55.4 (44.0-67.1)	55.5 (43.7-67.3)
Tanzania	55.9 (53.5-58.5)	55.5 (53.2-58.2)	55.0 (52.6-57.7)	54.5 (52.2-57.5)	54.3 (51.8-57.2)	54.0 (51.4-56.9)	53.8 (51.1-56.7)	53.9 (51.2-57.0)	54.3 (51.6-57.6)	54.9 (51.9-58.1)	55.4 (52.3-58.7)	55.8 (52.5-59.1)
Uganda	51.5 (49.2-54.6)	51.0 (48.8-54.1)	50.6 (48.3-53.6)	50.5 (48.0-53.3)	50.4 (48.0-53.1)	50.6 (48.3-53.3)	50.7 (48.4-53.3)	50.9 (48.5-53.4)	51.0 (48.6-53.6)	51.5 (49.1-54.0)	52.1 (49.7-54.8)	52.9 (50.5-55.7)
Zambia	50.5 (48.3-53.2)	49.7 (47.4-52.2)	48.6 (46.4-51.1)	47.7 (45.4-50.2)	47.1 (44.8-49.7)	46.7 (44.2-49.4)	46.2 (43.7-48.8)	45.8 (43.3-48.6)	45.7 (43.2-48.5)	45.8 (42.9-48.6)	46.1 (43.3-48.9)	46.3 (43.6-49.2)
Central Sub-Saharan Africa	54.3 (50.4-58.1)	54.1 (50.4-57.7)	54.2 (50.6-57.7)	54.2 (50.7-57.6)	53.2 (49.9-56.4)	53.9 (50.6-57.2)	54.1 (50.6-57.2)	54.5 (51.0-57.7)	54.7 (51.0-57.9)	54.7 (51.2-57.8)	55.0 (51.4-58.2)	55.3 (51.6-58.6)
Angola	51.0 (40.2-63.2)	50.5 (39.9-62.2)	51.3 (40.5-63.3)	51.8 (40.7-64.3)	52.0 (40.8-64.4)	52.3 (41.5-64.8)	52.4 (41.9-65.1)	52.5 (42.0-65.2)	52.7 (42.2-65.5)	52.9 (42.2-65.6)	53.6 (42.6-66.2)	54.1 (42.9-66.9)
Central African Republic	51.1 (47.9-54.8)	50.1 (46.5-54.6)	49.8 (45.9-55.3)	49.4 (44.9-56.0)	48.9 (43.8-56.1)	48.5 (43.1-56.2)	48.2 (42.4-56.4)	48.0 (41.5-56.6)	47.8 (41.0-56.5)	47.6 (40.3-56.5)	47.5 (39.8-56.7)	47.5 (39.7-56.8)
Congo	56.5 (53.0-60.2)	55.7 (52.5-59.2)	54.8 (51.8-58.2)	54.0 (50.9-57.4)	53.1 (50.2-56.5)	50.3 (46.9-53.8)	51.8 (49.0-54.9)	52.5 (49.9-55.6)	52.9 (50.4-55.8)	53.7 (51.1-56.6)	54.3 (51.6-57.4)	54.8 (51.9-57.8)
Democratic Republic of the Congo	55.4 (51.5-59.9)	55.6 (51.8-59.5)	55.5 (52.3-59.0)	55.4 (52.3-58.7)	53.9 (51.0-56.9)	55.2 (52.3-58.1)	55.3 (52.7-58.2)	56.0 (53.4-58.9)	56.2 (53.6-59.2)	56.1 (53.5-59.1)	56.2 (53.7-59.2)	56.5 (54.0-59.4)
Equatorial Guinea	50.3 (39.9-63.6)	50.1 (39.5-63.4)	49.8 (39.6-62.9)	49.7 (39.6-62.8)	49.9 (39.8-62.8)	51.3 (41.1-63.8)	52.1 (42.3-64.4)	52.8 (42.4-65.0)	53.7 (43.1-65.7)	54.7 (43.7-66.5)	55.6 (44.3-67.0)	55.9 (44.3-67.2)
Gabon	63.7 (60.8-66.7)	63.6 (60.7-66.5)	63.1 (60.3-65.9)	63.3 (60.6-65.9)	62.5 (60.0-65.0)	62.4 (59.9-64.8)	62.0 (59.3-64.5)	61.1 (58.2-63.7)	60.7 (57.9-63.5)	60.1 (57.3-63.0)	60.5 (57.7-63.3)	60.3 (57.4-63.2)

Location	2004	2005	2006	2007	2008	2009	2010	2011	2012	2013	2014	2015
Liberia	58.1 (55.5-61.1)	58.4 (55.8-61.5)	58.8 (56.3-62.0)	59.4 (56.8-62.5)	59.8 (56.9-63.1)	60.2 (57.1-63.7)	60.8 (57.4-64.7)	61.6 (57.9-65.9)	62.3 (58.3-66.9)	62.9 (58.2-68.1)	62.1 (56.9-67.4)	63.4 (57.5-69.4)
Mali	56.3 (53.5-58.9)	57.1 (54.2-59.6)	57.6 (54.8-60.3)	58.2 (55.4-61.1)	58.8 (55.7-61.9)	59.3 (56.0-62.6)	59.5 (55.9-63.1)	59.8 (55.8-63.6)	60.1 (55.6-64.5)	60.1 (55.1-64.9)	60.3 (54.7-65.7)	60.4 (54.4-66.7)
Mauritania	65.6 (62.7-68.5)	66.1 (63.1-68.9)	66.7 (63.5-69.5)	67.2 (63.9-70.1)	67.6 (64.2-70.6)	68.0 (64.4-71.4)	68.4 (64.6-72.2)	68.7 (64.7-72.8)	68.9 (64.3-73.5)	69.2 (64.0-74.2)	69.6 (63.9-74.9)	69.8 (63.7-75.5)
Niger	55.8 (53.0-58.3)	56.7 (53.9-59.4)	57.4 (54.6-60.3)	58.2 (55.4-61.3)	59.0 (55.8-62.0)	59.7 (56.1-62.9)	60.4 (56.7-63.8)	60.9 (56.7-64.6)	61.3 (56.8-65.5)	61.7 (56.9-66.5)	62.1 (56.7-67.3)	62.5 (56.7-68.2)
Nigeria	56.6 (53.8-59.4)	57.3 (54.4-59.9)	58.0 (55.1-60.6)	58.8 (55.8-61.6)	59.8 (56.6-62.8)	61.0 (57.5-64.4)	62.1 (58.3-65.5)	63.0 (58.9-66.4)	63.7 (59.0-67.4)	64.6 (59.2-68.4)	65.2 (59.1-69.1)	66.3 (59.5-70.3)
Sao Tome and Principe	65.9 (63.1-68.9)	66.2 (63.0-69.5)	66.6 (63.0-70.3)	66.6 (62.5-70.5)	66.9 (62.5-71.2)	67.3 (62.7-72.1)	67.6 (62.2-72.9)	67.9 (62.3-73.5)	68.1 (62.1-74.1)	68.3 (62.0-74.7)	68.6 (61.7-75.2)	68.8 (61.3-75.8)
Senegal	62.9 (58.6-67.2)	63.3 (58.7-68.2)	63.7 (58.6-69.0)	64.2 (58.8-69.9)	64.8 (59.1-71.0)	65.1 (59.1-71.8)	65.5 (59.0-72.4)	65.7 (58.7-73.1)	66.0 (58.7-73.3)	66.2 (58.3-73.8)	66.4 (58.3-74.0)	66.7 (58.3-74.4)
Sierra Leone	53.1 (51.0-55.2)	53.2 (51.2-55.5)	53.6 (51.2-55.9)	53.7 (51.2-56.3)	54.2 (51.5-57.1)	54.6 (51.8-57.8)	55.2 (51.9-58.9)	55.8 (52.2-59.8)	56.6 (52.4-61.1)	57.4 (52.5-62.4)	55.7 (50.8-60.5)	57.7 (52.0-63.3)
Togo	59.0 (55.7-62.5)	59.0 (55.8-62.5)	59.7 (56.3-63.2)	59.7 (56.1-63.3)	59.9 (56.2-63.6)	60.4 (56.5-64.2)	61.3 (57.2-65.0)	62.2 (57.9-66.5)	63.0 (58.2-67.7)	63.7 (58.6-68.8)	64.3 (58.6-69.8)	64.6 (58.5-70.5)
Eastern Sub-Saharan Africa	55.1 (53.9-56.4)	56.1 (54.8-57.3)	57.2 (55.8-58.5)	58.3 (57.0-59.8)	59.6 (58.1-61.2)	60.6 (58.8-62.4)	61.3 (59.2-63.3)	62.0 (59.7-64.1)	62.6 (60.1-64.9)	63.2 (60.6-65.6)	63.8 (61.1-66.4)	64.3 (61.4-67.1)
Burundi	53.4 (50.5-56.2)	55.3 (52.3-58.1)	57.1 (54.0-60.1)	58.7 (55.3-62.2)	60.0 (56.2-64.1)	60.9 (56.6-65.6)	61.4 (56.3-66.8)	61.8 (56.4-67.7)	62.2 (56.2-68.4)	62.5 (55.8-69.1)	62.7 (55.4-69.8)	62.7 (54.7-70.2)
Comoros	66.1 (63.2-69.2)	66.7 (63.7-70.0)	67.2 (64.2-70.5)	67.8 (64.8-71.0)	68.1 (65.0-71.4)	68.2 (65.0-71.6)	68.6 (65.1-72.4)	68.5 (64.6-72.6)	68.8 (64.5-73.4)	69.0 (64.1-74.1)	69.2 (63.6-74.9)	69.3 (63.4-75.4)
Djibouti	63.6 (53.9-71.4)	63.9 (53.9-71.7)	64.1 (54.0-71.8)	64.6 (54.3-72.3)	64.9 (55.0-72.5)	65.2 (55.4-72.7)	65.5 (55.6-72.9)	65.7 (55.7-73.0)	65.9 (55.6-73.0)	66.2 (56.4-73.4)	66.3 (56.5-73.5)	67.0 (57.0-74.0)
Eritrea	60.2 (51.8-69.5)	60.2 (51.5-69.8)	60.3 (51.5-70.3)	60.4 (51.2-70.5)	60.5 (51.2-70.7)	60.6 (50.9-70.9)	60.6 (51.0-70.9)	60.7 (50.9-71.0)	60.8 (51.0-71.0)	60.9 (51.2-71.2)	61.1 (51.4-71.5)	61.3 (51.8-71.8)
Ethiopia	55.1 (52.5-57.6)	56.1 (53.5-58.7)	57.5 (54.7-60.4)	59.1 (56.1-62.3)	60.9 (57.5-64.7)	62.3 (58.3-66.6)	63.4 (58.6-68.2)	64.4 (59.2-69.7)	65.2 (59.4-70.9)	65.8 (59.2-72.1)	66.3 (59.2-72.8)	66.8 (59.4-73.4)
Kenya	57.3 (55.9-58.7)	58.5 (57.1-59.9)	59.9 (58.5-61.2)	61.4 (60.0-62.8)	62.4 (60.8-63.8)	63.6 (62.0-64.9)	64.7 (63.1-66.0)	65.5 (63.9-66.9)	66.8 (65.2-68.3)	67.3 (65.7-68.8)	67.5 (65.8-69.1)	67.6 (65.8-69.3)
Madagascar	61.6 (58.9-64.2)	62.3 (59.4-65.2)	62.9 (59.4-66.1)	63.2 (59.3-66.9)	63.5 (59.0-67.7)	63.6 (58.6-68.5)	63.8 (58.2-69.1)	64.0 (58.0-69.8)	64.2 (57.7-70.5)	64.5 (57.8-71.1)	64.8 (57.8-71.7)	65.0 (57.8-72.3)
Malawi	48.1 (45.2-51.4)	49.3 (46.3-52.6)	50.7 (47.9-54.0)	52.5 (49.7-55.6)	54.2 (51.2-57.5)	55.9 (52.7-59.4)	57.5 (53.9-61.3)	58.9 (54.8-63.2)	60.4 (55.8-65.0)	61.8 (56.9-66.8)	62.6 (57.1-67.9)	63.0 (57.0-68.7)
Mozambique	54.9 (51.0-58.8)	54.8 (51.1-58.7)	55.0 (51.1-58.9)	55.2 (51.4-59.3)	56.3 (52.3-60.6)	56.6 (52.2-61.4)	56.6 (52.0-61.7)	56.7 (51.7-62.3)	56.6 (50.9-62.4)	56.9 (50.8-63.3)	58.5 (51.7-65.4)	59.9 (52.1-67.3)
Rwanda	58.8 (56.1-61.8)	61.0 (58.0-64.0)	63.1 (60.1-66.5)	64.7 (61.4-68.4)	65.7 (61.9-69.8)	66.2 (61.9-70.8)	66.8 (62.3-71.7)	67.1 (62.0-72.2)	67.2 (61.5-72.7)	67.3 (61.1-73.0)	67.5 (60.6-73.3)	67.7 (60.5-73.7)
Somalia	52.1 (41.1-66.2)	52.5 (41.1-66.6)	52.7 (41.3-66.7)	53.0 (41.0-66.7)	53.2 (41.0-66.9)	53.5 (41.3-67.0)	52.4 (40.3-65.4)	52.8 (40.4-66.0)	54.2 (41.6-67.5)	54.6 (42.0-67.9)	54.9 (42.4-68.0)	55.0 (42.4-67.9)
South Sudan	55.6 (43.5-67.5)	55.7 (43.1-67.5)	55.8 (43.2-67.7)	55.9 (43.1-67.9)	56.1 (42.8-67.9)	56.2 (42.8-67.9)	56.4 (43.0-68.0)	56.4 (42.8-67.9)	56.5 (43.0-68.1)	56.6 (43.0-68.3)	56.7 (43.4-68.5)	56.9 (43.9-68.7)
Tanzania	56.5 (53.1-59.9)	57.2 (53.5-60.8)	58.1 (54.2-62.0)	59.5 (55.1-63.7)	61.0 (56.1-66.0)	61.5 (56.0-66.8)	62.2 (56.2-67.9)	62.9 (56.2-69.0)	63.0 (55.5-69.5)	64.1 (56.3-70.9)	64.8 (56.7-71.8)	65.5 (57.0-72.6)
Uganda	53.8 (51.4-56.6)	55.3 (52.7-58.4)	56.8 (54.0-60.2)	57.3 (54.0-61.0)	58.2 (54.5-62.5)	59.3 (55.3-64.0)	60.1 (55.6-65.3)	61.0 (55.9-66.6)	61.7 (56.2-67.9)	62.9 (56.9-69.5)	63.8 (57.2-70.6)	64.3 (56.9-71.6)
Zambia	47.5 (44.9-50.2)	48.5 (45.7-51.3)	49.4 (46.5-52.4)	50.6 (47.7-53.8)	53.2 (49.8-56.8)	54.6 (50.9-58.6)	55.6 (51.8-59.8)	56.3 (52.2-60.9)	57.4 (52.9-62.6)	58.4 (53.2-64.3)	59.2 (53.6-65.8)	59.8 (53.6-67.0)
Central Sub-Saharan Africa	55.9 (52.2-59.1)	56.4 (52.6-59.7)	56.8 (52.9-60.1)	57.0 (53.2-60.4)	57.5 (53.5-61.1)	58.0 (53.9-61.8)	58.6 (54.5-62.4)	59.0 (54.7-63.1)	59.4 (54.8-63.6)	59.9 (54.9-64.4)	60.4 (55.5-65.4)	61.0 (56.0-66.2)
Angola	54.6 (43.3-67.4)	55.2 (43.5-67.9)	55.6 (43.8-68.2)	56.2 (44.0-68.6)	56.7 (44.3-69.1)	57.1 (44.3-69.4)	57.6 (44.7-69.9)	58.1 (45.2-70.3)	58.5 (45.3-70.6)	58.8 (45.3-71.0)	59.3 (45.2-71.2)	59.8 (45.9-71.6)
Central African Republic	47.5 (39.8-57.0)	47.6 (39.7-57.6)	47.9 (39.9-58.3)	48.2 (39.9-59.0)	48.9 (40.3-60.1)	49.7 (40.7-61.0)	49.9 (41.1-61.5)	50.3 (41.2-61.5)	50.6 (41.5-61.7)	50.5 (41.6-61.5)	50.7 (41.9-61.9)	51.7 (42.5-63.1)
Congo	55.3 (52.4-58.3)	55.6 (52.6-58.8)	56.6 (53.4-60.0)	56.9 (53.5-60.6)	57.3 (53.7-61.3)	58.3 (54.4-62.6)	58.7 (54.4-63.4)	59.2 (54.4-64.3)	60.1 (54.5-65.7)	60.1 (53.9-66.4)	60.1 (53.3-67.4)	60.0 (52.7-68.0)
Democratic Republic of the Congo	57.2 (54.6-60.2)	57.6 (55.1-60.6)	57.9 (55.2-61.0)	58.0 (55.2-61.1)	58.4 (55.5-62.0)	58.9 (55.7-62.5)	59.5 (56.1-63.3)	59.9 (56.2-64.1)	60.3 (56.2-64.8)	60.8 (56.4-65.7)	61.5 (56.7-67.0)	62.1 (56.8-68.1)
Equatorial Guinea	56.3 (44.5-67.5)	56.5 (44.8-67.7)	57.0 (45.0-68.0)	57.3 (45.1-68.3)	57.7 (45.4-68.7)	58.1 (45.9-69.1)	58.7 (46.4-69.5)	58.9 (46.5-69.5)	59.7 (47.1-70.1)	61.0 (48.2-70.9)	61.8 (48.7-71.6)	61.9 (48.9-71.6)
Gabon	60.1 (57.2-63.0)	61.3 (58.5-64.2)	62.0 (59.3-64.8)	63.2 (60.3-66.1)	63.5 (60.3-66.8)	64.1 (60.6-67.7)	65.3 (61.5-69.3)	65.7 (61.4-70.2)	66.5 (61.8-71.3)	67.4 (61.8-72.7)	68.0 (61.6-73.5)	68.5 (61.5-74.3)

SR Table 7: Estimates of life expectancy at birth by geography, males, 1980–2015

Location	1980	1981	1982	1983	1984	1985	1986	1987	1988	1989	1990	1991	1992	1993	1994	1995	1996	1997
Global	59.6 (59.3-60.0)	59.9 (59.6-60.2)	60.3 (59.9-60.6)	60.5 (60.1-60.9)	60.8 (60.4-61.2)	61.2 (60.9-61.6)	61.7 (61.3-62.0)	62.0 (61.6-62.3)	62.1 (61.7-62.4)	62.4 (62.0-62.7)	62.5 (62.2-62.8)	62.6 (62.3-62.9)	62.8 (62.5-63.1)	62.8 (62.5-63.1)	62.6 (62.2-63.0)	63.1 (62.8-63.4)	63.4 (63.1-63.6)	63.7 (63.4-63.9)
High SDI	68.7 (68.6-68.7)	69.0 (68.9-69.1)	69.3 (69.3-69.4)	69.4 (69.4-69.5)	69.6 (69.5-69.6)	69.9 (69.8-70.0)	70.5 (70.4-70.5)	70.7 (70.6-70.7)	70.8 (70.7-70.8)	70.8 (70.8-70.9)	70.9 (70.9-71.0)	71.0 (70.9-71.0)	71.0 (70.9-71.0)	70.5 (70.5-70.6)	70.3 (70.3-70.4)	70.7 (70.6-70.7)	71.3 (71.3-71.4)	72.0 (71.9-72.0)
High-middle SDI	63.9 (63.3-64.4)	64.0 (63.4-64.6)	64.4 (63.8-64.9)	64.8 (64.2-65.3)	65.1 (64.5-65.6)	65.4 (64.9-65.9)	65.9 (65.3-66.3)	66.0 (65.5-66.5)	66.0 (65.5-66.5)	66.2 (65.7-66.7)	66.3 (65.8-66.7)	66.5 (66.0-66.9)	66.6 (66.1-67.0)	66.7 (66.3-67.1)	66.8 (66.4-67.2)	66.9 (66.5-67.2)	67.0 (66.7-67.4)	67.3 (67.0-67.6)
Middle SDI	59.5 (58.8-60.3)	59.7 (59.0-60.4)	60.1 (59.4-60.9)	60.6 (59.9-61.3)	61.1 (60.4-61.7)	61.4 (60.7-62.1)	61.7 (61.0-62.4)	62.0 (61.3-62.7)	62.0 (61.3-62.7)	62.5 (61.8-63.1)	62.8 (62.1-63.4)	63.0 (62.4-63.6)	63.2 (62.6-63.8)	63.5 (62.9-64.1)	63.8 (63.3-64.4)	64.1 (63.6-64.6)	64.4 (63.9-64.9)	64.7 (64.2-65.1)
Low-middle SDI	53.3 (52.4-54.1)	53.7 (52.7-54.5)	54.1 (53.2-54.9)	54.4 (53.5-55.2)	54.8 (53.9-55.6)	55.1 (54.2-55.9)	55.5 (54.6-56.3)	55.9 (55.0-56.7)	56.1 (55.3-56.9)	56.4 (55.7-57.3)	56.7 (55.9-57.5)	56.6 (55.8-57.4)	57.2 (56.4-57.9)	57.3 (56.6-58.1)	57.6 (56.9-58.3)	57.8 (57.1-58.5)	58.0 (57.3-58.7)	58.1 (57.5-58.8)
Low SDI	46.9 (44.9-48.7)	47.1 (45.1-48.8)	47.3 (45.5-49.0)	46.5 (44.5-48.4)	46.3 (44.2-48.3)	47.6 (46.1-49.2)	48.1 (46.6-49.6)	48.6 (47.3-50.0)	49.2 (48.0-50.4)	49.5 (48.4-50.7)	49.2 (48.1-50.3)	49.6 (48.6-50.6)	49.9 (48.9-50.9)	50.1 (49.1-51.0)	45.6 (41.5-49.6)	50.1 (49.2-51.0)	50.1 (49.2-50.9)	50.5 (49.6-51.3)
High-income	70.3 (70.2-70.3)	70.6 (70.6-70.6)	70.9 (70.9-71.0)	71.1 (71.1-71.1)	71.4 (71.4-71.4)	71.6 (71.6-71.6)	71.8 (71.8-71.9)	72.1 (72.1-72.1)	72.3 (72.2-72.3)	72.4 (72.4-72.5)	72.6 (72.6-72.7)	72.8 (72.8-72.8)	73.1 (73.1-73.1)	73.2 (73.2-73.3)	73.5 (73.4-73.5)	73.6 (73.6-73.6)	74.0 (74.0-74.0)	74.4 (74.4-74.4)
High-income North America	70.1 (70.1-70.2)	70.5 (70.5-70.5)	70.9 (70.9-71.0)	71.2 (71.1-71.2)	71.3 (71.3-71.4)	71.4 (71.3-71.4)	71.4 (71.4-71.5)	71.6 (71.6-71.6)	71.7 (71.6-71.7)	71.8 (71.8-71.9)	72.1 (72.1-72.1)	72.3 (72.3-72.3)	72.6 (72.5-72.6)	72.5 (72.5-72.6)	72.7 (72.6-72.7)	72.8 (72.8-72.9)	73.4 (73.4-73.4)	73.9 (73.9-74.0)
Canada	71.6 (71.6-71.7)	72.0 (72.0-72.1)	72.4 (72.3-72.4)	72.7 (72.6-72.7)	73.0 (72.9-73.1)	73.1 (73.0-73.2)	73.3 (73.2-73.4)	73.5 (73.5-73.6)	73.7 (73.7-73.8)	73.9 (73.9-74.0)	74.2 (74.2-74.3)	74.4 (74.4-74.5)	74.6 (74.5-74.7)	74.7 (74.6-74.7)	74.9 (74.8-75.0)	75.0 (75.0-75.1)	75.4 (75.3-75.4)	75.7 (75.7-75.8)
Greenland	61.5 (60.8-62.1)	61.5 (60.9-62.2)	61.4 (60.8-62.1)	61.5 (60.8-62.1)	61.4 (60.8-62.1)	61.4 (60.8-62.1)	61.4 (60.7-62.0)	61.4 (60.7-62.2)	61.7 (60.9-62.5)	62.0 (61.1-62.8)	62.1 (61.3-62.9)	62.6 (61.7-63.4)	63.1 (62.3-64.0)	63.7 (62.9-64.4)	64.1 (63.4-64.8)	64.5 (63.8-65.2)	65.0 (64.2-65.7)	65.2 (64.5-66.0)
United States	70.0 (70.0-70.0)	70.4 (70.3-70.4)	70.8 (70.7-70.8)	71.0 (71.0-71.0)	71.2 (71.1-71.2)	71.2 (71.1-71.2)	71.2 (71.2-71.3)	71.4 (71.3-71.4)	71.4 (71.4-71.5)	71.6 (71.6-71.6)	71.9 (71.9-71.9)	72.1 (72.1-72.1)	72.3 (72.3-72.4)	72.3 (72.3-72.3)	72.4 (72.4-72.5)	72.6 (72.6-72.6)	73.2 (73.1-73.2)	73.7 (73.7-73.8)
Australasia	70.9 (70.8-70.9)	71.2 (71.1-71.2)	71.3 (71.3-71.4)	71.8 (71.7-71.8)	72.0 (72.0-72.1)	72.2 (72.1-72.2)	72.6 (72.5-72.6)	72.7 (72.7-72.8)	73.0 (72.9-73.1)	73.1 (73.1-73.2)	73.7 (73.6-73.8)	74.2 (74.1-74.2)	74.4 (74.3-74.5)	74.7 (74.7-74.8)	74.9 (74.9-75.0)	75.2 (75.2-75.3)	75.5 (75.4-75.5)	75.9 (75.8-75.9)
Australia	71.1 (71.0-71.1)	71.3 (71.3-71.4)	71.5 (71.4-71.6)	72.0 (71.9-72.0)	72.2 (72.2-72.3)	72.4 (72.3-72.5)	72.8 (72.8-72.9)	73.0 (72.9-73.1)	73.3 (73.2-73.3)	73.4 (73.3-73.4)	73.9 (73.8-74.0)	74.4 (74.3-74.5)	74.6 (74.6-74.7)	75.0 (74.9-75.0)	75.1 (75.1-75.2)	75.5 (75.4-75.5)	75.6 (75.6-75.7)	76.0 (76.0-76.1)
New Zealand	70.0 (69.9-70.2)	70.4 (70.3-70.6)	70.6 (70.5-70.7)	70.8 (70.7-70.9)	71.0 (70.9-71.1)	71.0 (70.9-71.2)	71.2 (71.1-71.4)	71.5 (71.4-71.7)	71.8 (71.6-71.9)	72.1 (72.0-72.2)	72.6 (72.5-72.7)	73.0 (72.9-73.1)	73.3 (73.2-73.5)	73.7 (73.5-73.8)	73.9 (73.8-74.1)	74.3 (74.1-74.4)	74.6 (74.5-74.7)	75.0 (74.8-75.1)
High-income Asia Pacific	71.3 (71.1-71.4)	71.6 (71.5-71.8)	72.0 (71.8-72.1)	72.1 (71.9-72.2)	72.4 (72.3-72.6)	72.8 (72.7-72.9)	73.1 (73.0-73.3)	73.5 (73.4-73.6)	73.7 (73.6-73.8)	74.0 (73.9-74.1)	74.2 (74.1-74.3)	74.4 (74.3-74.5)	74.5 (74.4-74.6)	74.7 (74.6-74.8)	75.0 (74.9-75.1)	75.0 (74.8-75.2)	75.5 (75.4-75.6)	75.8 (75.7-75.9)
Brunei	69.5 (68.8-70.3)	69.7 (69.1-70.4)	70.0 (69.4-70.6)	70.3 (69.7-70.8)	70.5 (70.0-71.0)	70.9 (70.4-71.3)	71.1 (70.7-71.5)	71.4 (71.0-71.8)	71.8 (71.4-72.2)	72.0 (71.6-72.5)	72.2 (71.8-72.7)	72.2 (71.8-72.8)	72.4 (71.9-73.1)	72.6 (72.0-73.3)	72.8 (72.2-73.4)	73.0 (72.4-73.6)	73.1 (72.6-73.7)	73.4 (72.8-74.0)
Japan	73.5 (73.5-73.5)	73.8 (73.8-73.9)	74.2 (74.2-74.2)	74.2 (74.2-74.2)	74.5 (74.5-74.5)	74.8 (74.8-74.9)	75.2 (75.1-75.2)	75.5 (75.5-75.5)	75.6 (75.6-75.6)	75.9 (75.8-75.9)	75.9 (75.9-76.0)	76.1 (76.1-76.1)	76.1 (76.1-76.2)	76.3 (76.2-76.3)	76.5 (76.5-76.6)	76.4 (76.1-76.6)	76.9 (76.9-77.0)	77.1 (77.1-77.2)
Singapore	68.9 (68.7-69.1)	69.0 (68.8-69.2)	69.2 (69.0-69.4)	69.5 (69.3-69.6)	69.8 (69.6-70.0)	70.4 (70.2-70.6)	71.0 (70.8-71.2)	71.4 (71.3-71.6)	71.6 (71.4-71.8)	71.8 (71.6-72.0)	72.5 (72.3-72.7)	73.3 (73.1-73.5)	73.8 (73.6-74.0)	74.1 (73.9-74.3)	74.1 (73.9-74.3)	74.2 (74.0-74.4)	74.5 (74.3-74.7)	75.0 (74.8-75.2)
South Korea	63.5 (63.0-64.0)	64.0 (63.6-64.4)	64.3 (63.9-64.7)	64.7 (64.2-65.0)	65.1 (64.7-65.5)	65.6 (65.2-65.9)	66.1 (65.7-66.4)	66.4 (66.1-66.8)	67.0 (66.6-67.3)	67.4 (67.1-67.7)	67.9 (67.5-68.2)	68.3 (68.0-68.6)	68.8 (68.5-69.1)	69.2 (68.9-69.5)	69.6 (69.3-69.9)	70.0 (69.7-70.3)	70.5 (70.2-70.8)	71.0 (70.7-71.3)
Western Europe	70.6 (70.5-70.6)	70.9 (70.9-70.9)	71.2 (71.1-71.2)	71.3 (71.3-71.4)	71.7 (71.7-71.7)	71.9 (71.9-71.9)	72.1 (72.1-72.2)	72.4 (72.4-72.4)	72.6 (72.6-72.6)	72.8 (72.8-72.8)	72.9 (72.9-72.9)	73.0 (73.0-73.1)	73.3 (73.3-73.3)	73.6 (73.5-73.6)	73.8 (73.8-73.9)	74.0 (74.0-74.0)	74.3 (74.3-74.3)	74.7 (74.7-74.7)
Andorra	76.8 (76.0-77.6)	76.9 (76.1-77.7)	76.9 (76.1-77.7)	77.0 (76.2-77.8)	77.1 (76.4-77.9)	77.3 (76.5-78.0)	77.4 (76.7-78.1)	77.5 (76.8-78.3)	77.7 (77.0-78.4)	77.8 (77.1-78.5)	77.9 (77.3-78.5)	78.0 (77.4-78.6)	78.0 (77.4-78.6)	78.4 (77.8-79.0)	78.8 (78.2-79.2)	78.9 (78.4-79.4)	79.1 (78.6-79.6)	79.3 (78.8-79.8)
Austria	69.0 (68.9-69.0)	69.2 (69.2-69.3)	69.3 (69.3-69.4)	69.6 (69.5-69.7)	69.9 (69.8-70.0)	70.4 (70.4-70.5)	70.9 (70.8-71.0)	71.4 (71.3-71.4)	71.8 (71.7-71.9)	71.9 (71.8-72.0)	72.3 (72.2-72.3)	72.2 (72.2-72.3)	72.5 (72.4-72.6)	72.8 (72.7-72.9)	73.1 (73.0-73.2)	73.3 (73.3-73.4)	73.7 (73.6-73.8)	74.1 (74.0-74.2)
Belgium	69.7 (69.6-69.8)	70.1 (70.0-70.2)	70.3 (70.2-70.4)	70.5 (70.4-70.6)	70.8 (70.7-71.0)	71.2 (71.1-71.3)	71.5 (71.4-71.6)	71.7 (71.6-71.9)	72.2 (72.1-72.3)	72.4 (72.3-72.5)	72.7 (72.6-72.8)	72.7 (72.6-72.9)	73.0 (72.9-73.1)	73.1 (73.0-73.2)	73.3 (73.2-73.4)	73.5 (73.4-73.6)	73.9 (73.8-74.0)	74.1 (74.0-74.2)
Cyprus	73.9 (73.7-74.1)	74.3 (74.1-74.5)	74.7 (74.5-74.9)	74.6 (74.4-74.8)	74.5 (74.3-74.8)	74.0 (73.7-74.3)	74.3 (74.0-74.6)	73.1 (73.0-73.4)	73.3 (73.1-73.5)	74.0 (73.7-74.3)	74.2 (73.9-74.5)	73.5 (73.3-73.7)	73.9 (73.7-74.1)	73.7 (73.5-73.8)	73.6 (73.4-73.8)	74.0 (73.8-74.1)	74.2 (74.0-74.4)	74.3 (74.1-74.5)
Denmark	71.3 (71.1-71.4)	71.5 (71.4-71.6)	71.7 (71.6-71.8)	71.7 (71.6-71.8)	71.9 (71.8-72.0)	71.8 (71.7-71.9)	72.0 (71.9-72.1)	72.0 (71.9-72.1)	72.3 (72.2-72.4)	72.1 (72.0-72.2)	72.3 (72.2-72.4)	72.6 (72.5-72.7)	72.8 (72.7-72.9)	72.8 (72.7-73.0)	72.7 (72.6-72.8)	73.1 (72.9-73.2)	73.3 (73.2-73.4)	73.8 (73.7-73.9)
Finland	69.2 (69.1-69.4)	69.7 (69.6-69.8)	70.1 (69.9-70.2)	70.2 (70.0-70.3)	70.3 (70.2-70.4)	70.4 (70.2-70.5)	70.6 (70.5-70.7)	70.5 (70.4-70.6)	70.7 (70.6-70.9)	70.8 (70.7-71.0)	71.0 (70.9-71.2)	71.4 (71.2-71.5)	71.8 (71.7-71.9)	72.2 (72.1-72.4)	72.6 (72.5-72.7)	72.9 (72.8-73.0)	73.1 (73.0-73.2)	73.4 (73.3-73.5)
France	70.5 (70.5-70.6)	70.8 (70.7-70.8)	70.9 (70.9-71.0)	71.1 (71.1-71.2)	71.5 (71.4-71.5)	71.7 (71.6-71.7)	71.9 (71.9-72.0)	72.4 (72.4-72.5)	72.7 (72.6-72.7)	72.8 (72.8-72.9)	73.1 (73.0-73.1)	73.2 (73.2-73.3)	73.5 (73.4-73.5)	73.6 (73.6-73.7)	73.9 (73.9-73.9)	74.1 (74.1-74.2)	74.4 (74.4-74.5)	74.8 (74.8-74.9)
Germany	69.6 (69.6-69.7)	69.9 (69.8-69.9)	70.2 (70.1-70.2)	70.5 (70.5-70.6)	71.0 (70.9-71.0)	71.2 (71.2-71.2)	71.4 (71.4-71.5)	71.7 (71.6-71.7)	71.8 (71.8-71.9)	72.0 (72.0-72.0)	72.0 (72.0-72.0)	72.2 (72.1-72.2)	72.5 (72.5-72.5)	72.7 (72.7-72.7)	73.0 (72.9-73.0)	73.3 (73.2-73.3)	73.6 (73.5-73.6)	74.0 (73.9-74.0)
Greece	73.0 (72.9-73.1)	73.2 (73.1-73.4)	73.3 (73.2-73.4)	73.2 (73.1-73.3)	73.4 (73.3-73.5)	73.3 (73.2-73.4)	73.9 (73.8-74.0)	73.8 (73.7-73.9)	74.2 (74.1-74.3)	74.4 (74.3-74.5)	74.6 (74.5-74.7)	74.5 (74.4-74.6)	74.6 (74.5-74.7)	74.8 (74.7-74.9)	75.0 (74.9-75.1)	75.0 (75.0-75.1)	75.1 (75.0-75.2)	75.3 (75.2-75.4)
Iceland	73.1 (72.7-73.6)	73.6 (73.2-74.0)	73.9 (73.6-74.2)	74.1 (73.9-74.4)	74.3 (74.0-74.6)	74.5 (74.2-75.0)	74.9 (74.5-75.3)	75.2 (74.8-75.6)	75.6 (75.2-76.0)	76.0 (75.6-76.2)	76.1 (75.8-76.3)	76.2 (75.9-76.5)	76.5 (76.2-76.8)	76.8 (76.5-77.1)	77.1 (76.8-77.4)	76.8 (76.5-77.2)	77.5 (77.2-77.7)	77.7 (77.4-77.9)
Ireland	70.5 (70.3-70.9)	71.2 (70.8-71.7)	71.4 (71.2-71.7)	71.4 (71.2-71.7)	71.7 (71.4-72.1)	72.0 (71.6-72.5)	72.5 (72.2-72.9)	72.7 (72.4-73.1)	73.2 (72.9-73.6)	73.3 (73.0-73.7)	73.8 (73.3-74.3)	74.1 (73.8-74.6)	74.1 (73.8-74.7)	74.3 (73.9-74.7)	75.0 (74.5-75.5)	74.6 (74.3-75.2)	74.8 (74.4-75.4)	74.9 (74.5-75.5)
Israel	72.1 (71.8-72.4)	72.4 (72.1-72.7)	72.6 (72.3-72.9)	72.4 (72.1-72.7)	72.6 (72.3-72.9)	73.0 (72.7-73.3)	73.0 (72.7-73.2)	73.3 (73.1-73.6)	73.5 (73.2-73.7)	74.4 (74.2-74.6)	75.0 (74.8-75.2)	74.7 (74.5-74.8)	74.4 (74.2-74.5)	74.8 (74.7-75.0)	75.0 (74.9-75.2)	75.2 (75.1-75.4)	75.6 (75.4-75.8)	75.8 (75.6-75.9)
Italy	70.7 (70.5-70.8)	71.2 (71.2-71.3)	71.6 (71.5-71.6)	71.5 (71.5-71.6)	72.1 (72.1-72.2)	72.3 (72.3-72.4)	72.7 (72.7-72.8)	73.0 (73.0-73.0)	73.3 (73.2-73.3)	73.5 (73.5-73.6)	73.6 (73.6-73.7)	73.6 (73.6-73.6)	73.9 (73.9-73.9)	74.3 (74.3-74.3)	74.5 (74.4-74.5)	74.8 (74.7-74.8)	75.1 (75.1-75.1)	75.5 (75.5-75.5)
Luxembourg	69.0 (68.7-69.2)	69.1 (68.9-69.3)	69.4 (69.1-69.6)	69.7 (69.5-70.0)	70.0 (69.7-70.2)	70.2 (70.0-70.5)	70.6 (70.3-70.8)	70.8 (70.6-71.0)	71.1 (70.8-71.3)	71.4 (71.1-71.6)	71.8 (71.5-72.0)	72.1 (71.9-72.4)	72.4 (72.2-72.6)	72.8 (72.5-73.0)	73.2 (72.9-73.4)	73.5 (73.3-73.7)	73.7 (73.4-73.9)	74.0 (73.8-74.2)
Malta	70.5 (70.2-70.9)	71.2 (71.0-71.5)	71.2 (70.9-71.6)	71.7 (71.4-72.0)	72.3 (72.0-72.6)	72.6 (72.2-72.8)	73.3 (73.0-73.7)	74.2 (73.8-74.6)	74.0 (73.6-74.3)	74.1 (73.8-74.5)	74.5 (74.1-74.9)	74.8 (74.4-75.3)	75.1 (74.6-75.7)	75.4 (74.9-76.0)	75.6 (75.1-76.2)	75.8 (75.3-76.3)	75.9 (75.3-76.5)	76.0 (75.5-76.5)
Netherlands	72.4 (72.3-72.5)	72.7 (72.6-72.8)	72.7 (72.6-72.8)	73.0 (72.9-73.1)	73.0 (72.9-73.1)	73.1 (73.0-73.2)	73.1 (73.0-73.2)	73.5 (73.4-73.6)	73.8 (73.7-73.9)	73.7 (73.6-73.8)	73.8 (73.7-73.9)	74.1 (74.0-74.2)	74.2 (74.2-74.3)	74.1 (74.0-74.2)	74.6 (74.5-74.6)	74.7 (74.6-74.8)	74.8 (74.7-74.9)	75.2 (75.1-75.3)
Norway	72.4 (72.2-72.5)	72.6 (72.5-72.8)	72.6 (72.4-72.8)	72.7 (72.6-72.9)	72.9 (72.7-73.1)	72.7 (72.5-72.9)	72.9 (72.7-73.1)	72.8 (72.6-73.0)	73.0 (72.8-73.1)	73.2 (73.0-73.4)	73.4 (73.2-73.6)	74.0 (73.8-74.1)	74.1 (73.9-74.2)	74.5 (74.3-74.6)	74.9 (74.7-75.0)	74.9 (74.7-75.0)	75.4 (75.2-75.6)	75.6 (75.4-75.7)
Portugal	68.2 (68.1-68.3)	68.3 (68.2-68.4)	69.0 (68.9-69.1)	69.0 (68.9-69.1)	69.3 (69.2-69.3)	69.6 (69.5-69.7)	69.9 (69.8-70.0)	70.3 (70.2-70.4)	70.3 (70.2-70.4)	70.8 (70.7-70.8)	70.6 (70.5-70.7)	70.5 (70.5-70.6)	70.8 (70.8-70.9)	71.1 (71.1-71.2)	71.9 (71.8-72.0)	71.7 (71.7-71.8)	71.7 (71.6-71.8)	72.2 (72.2-72.3)
Spain	72.3 (72.2-72.3)	72.5 (72.4-72.5)	73.0 (72.9-73.0)	72.9 (72.9-73.0)	73.0 (73.0-73.1)	73.1 (73.0-73.1)	73.3 (73.3-73.4)	73.4 (73.3-73.4)	73.3 (73.3-73.4)	73.3 (73.2-73.3)	73.3 (73.3-73.4)	73.4 (73.4-73.4)	73.7 (73.6-73.7)	74.0 (73.9-74.0)	74.2 (74.2-74.2)	74.2 (74.2-74.3)	74.4 (74.4-74.5)	75.1 (75.1-75.2)
Sweden	72.8 (72.7-72.9)	73.1 (73.0-73.2)	73.4 (73.3-73.5)	73.7 (73.6-73.8)	73.9 (73.8-74.0)	73.9 (73.8-74.0)	74.0 (73.9-74.2)	74.1 (74.0-74.2)	74.3 (74.2-74.4)	74.7 (74.6-74.8)	74.9 (74.8-75.0)	74.9 (74.8-75.0)	75.5 (75.4-75.6)	75.6 (75.5-75.7)	75.8 (75.2-76.1)	76.3 (76.2-76.4)	76.7 (76.5-76.8)	76.8 (76.7-76.9)
Switzerland	72.3 (72.2-72.5)	72.5 (72.4-72.7)	72.6 (72.5-72.7)	72.8 (72.7-73.0)	73.3 (73.1-73.4)	73.5 (73.4-73.7)	73.7 (73.6-73.9)	73.8 (73.7-74.0)	74.0 (73.9-74.1)	74.0 (73.9-74.1)	74.0 (73.9-74.2)	74.0 (73.9-74.2)	74.4 (74.2-74.5)	74.9 (74.8-75.1)	75.1 (75.0-75.3)	75.3 (75.1-75.4)	76.0 (75.8-76.1)	76.2 (76.0-76.3)
United Kingdom	70.4 (70.3-70.4)	70.7 (70.7-70.8)	71.0 (70.9-71.0)	71.3 (71.3-71.3)	71.6 (71.5-71.6)	71.7 (71.7-71.7)	71.9 (71.9-71.9)	72.2 (72.1-72.2)	72.4 (72.3-72.4)	72.6 (72.6-72.7)	72.9 (72.8-72.9)	73.1 (73.1-73.2)	73.5 (73.4-73.5)	73.6 (73.6-73.6)	73.9 (73.9-74.0)	74.0 (74.0-74.1)	74.3 (74.3-74.3)	74.6 (74.5-74.6)
England	70.6 (70.6-70.7)	71.0 (71.0-71.1)	71.3 (71.2-71.3)	71.6 (71.5-71.6)	71.9 (71.8-71.9)	72.0 (71.9-72.0)	72.2 (72.1-72.2)	72.4 (72.4-72.5)	72.6 (72.6-72.7)	72.9 (72.8-72.9)	73.1 (73.1-73.1)	73.4 (73.3-73.4)	73.7 (73.7-73.7)	73.9 (73.8-73.9)	74.2 (74.1-74.2)	74.3 (74.3-74.3)	74.6 (74.5-74.6)	74.8 (74.8-74.9)
Northern Ireland	68.4 (68.1-68.7)	68.4 (68.1-68.8)	68.9 (68.5-69.2)	69.4 (69.1-69.7)	69.9 (69.6-70.1)	70.1 (69.8-70.3)	70.4 (70.1-70.6)	70.7 (70.4-71.0)	70.9 (70.6-71.2)	71.6 (71.4-71.8)	71.9 (71.7-72.1)	72.3 (72.1-72.5)	72.7 (72.5-72.9)	72.9 (72.7-73.1)	73.3 (73.1-73.5)	73.5 (73.3-73.7)	73.8 (73.7-74.0)	74.1 (73.9-74.3)
Scotland	68.9 (68.8-69.0)	68.9 (68.8-69.0)	69.1 (69.0-69.3)	69.6 (69.4-69.7)	69.7 (69.6-69.8)	69.9 (69.8-70.0)	70.1 (70.0-70.2)	70.3 (70.2-70.5)	70.6 (70.4-70.7)	70.7 (70.6-70.8)	71.0 (70.9-71.1)	71.2 (71.1-71.3)	71.5 (71.4-71.6)	71.6 (71.5-71.8)	72.0 (71.9-72.1)	72.0 (71.9-72.1)	72.2 (72.0-72.3)	72.5 (72.3-72.6)
Wales	70.1 (69.9-70.3)	70.3 (70.2-70.5)	70.7 (70.5-70.8)	70.9 (70.8-71.1)	71.3 (71.2-71.5)	71.3 (71.2-71.5)	71.6 (71.5-71.7)	71.9 (71.8-72.0)	72.3 (72.2-72.4)	72.5 (72.4-72.6)	72.7 (72.6-72.9)	72.9 (72.8-73.1)	73.2 (73.1-73.4)	73.4 (73.2-73.5)	73.7 (73.5-73.8)	73.7 (73.6-73.9)	73.9 (73.8-74.1)	74.2 (74.0-74.3)
Southern Latin America	66.7 (66.5-66.8)	66.9 (66.8-67.1)	67.4 (67.4-67.5)	67.3 (67.2-67.4)	67.6 (67.6-67.7)	68.3 (68.0-68.5)	68.8 (68.8-68.9)	69.0 (68.9-69.1)	69.0 (68.9-69.1)	69.0 (68.9-69.1)	69.2 (69.1-69.2)	69.5 (69.4-69.6)	69.8 (69.7-69.8)	70.1 (70.0-70.1)	70.2 (70.2-70.3)	70.3 (70.2-70.3)	70.4 (70.3-70.4)	70.6 (70.5-70.6)
Argentina	66.6 (66.3-66.8)	66.6 (66.4-66.8)	67.2 (67.1-67.3)	67.1 (66.9-67.2)	67.5 (67.4-67.6)	68.3 (68.2-68.4)	68.5 (68.4-68.6)	68.5 (68.5-68.6)	68.6 (68.5-68.7)	68.8 (68.7-68.9)	69.0 (68.9-69.0)	69.1 (69.0-69.2)	69.1 (69.0-69.2)	69.4 (69.3-69.5)	69.7 (69.6-69.8)	69.7 (69.7-69.8)	69.7 (69.6-69.8)	69.8 (69.8-69.9)
Chile	67.0 (66.8-67.1)	67.8 (67.7-67.9)	68.0 (67.9-68.1)	67.7 (67.6-67.8)	68.0 (67.8-68.1)	68.3 (67.5-69.1)	69.9 (69.7-70.0)	70.5 (70.3-70.6)	70.3 (70.2-70.4)	69.6 (69.5-69.8)	69.8 (69.7-69.9)	70.9 (70.8-71.1)	72.0 (71.9-72.1)	72.2 (72.0-72.3)	72.2 (72.1-72.3)	72.1 (72.0-72.3)	72.5 (72.3-72.6)	73.0 (72.9-73.1)
Uruguay	67.1 (66.9-67.2)	67.5 (67.4-67.7)	68.0 (67.8-68.2)	68.3 (68.1-68.5)	68.3 (68.1-68.5)	68.5 (68.3-68.6)	68.6 (68.4-68.8)	68.8 (68.7-69.0)	69.0 (68.8-69.1)	69.2 (69.0-69.4)	69.3 (69.2-69.5)	68.9 (68.7-69.1)	69.5 (69.3-69.6)	69.4 (69.2-69.6)	69.4 (69.3-69.6)	69.4 (69.3-69.6)	69.5 (69.4-69.7)	69.7 (69.5-69.9)
Central Europe, Eastern Europe, and Central Asia	63.2 (63.1-63.3)	63.5 (63.4-63.6)	63.8 (63.7-63.9)	63.7 (63.6-63.8)	63.5 (63.4-63.6)	64.1 (64.0-64.1)	65.3 (65.2-65.4)	65.3 (65.2-65.4)	65.1 (65.0-65.2)	64.9 (64.8-65.0)	64.6 (64.5-64.7)	64.3 (64.2-64.4)	63.4 (63.3-63.5)	62.0 (61.9-62.1)	61.1 (61.0-61.2)	61.2 (61.1-61.3)	62.0 (61.9-62.1)	62.9 (62.8-63.0)
Eastern Europe	62.3 (62.2-62.4)	62.5 (62.4-62.7)	63.0 (62.9-63.1)	62.9 (62.8-63.0)	62.5 (62.4-62.6)	63.5 (63.4-63.6)	65.4 (65.3-65.5)	65.4 (65.3-65.5)	65.2 (65.2-65.3)	64.7 (64.6-64.7)	64.2 (64.1-64.3)	63.9 (63.8-63.9)	62.6 (62.6-62.7)	60.3 (60.2-60.4)	58.9 (58.8-59.0)	59.1 (59.0-59.2)	60.2 (60.1-60.3)	61.4 (61.4-61.5)
Belarus	65.3 (64.8-65.8)	65.3 (64.9-65.8)	65.4 (64.9-65.9)	65.4 (65.0-65.9)	65.2 (64.7-65.6)	65.7 (65.2-66.1)	66.6 (66.1-67.0)	66.6 (66.1-67.0)	66.4 (66.0-66.8)	65.8 (65.4-66.3)	65.4 (65.0-65.8)	64.9 (64.5-65.3)	64.4 (63.9-64.8)	63.5 (63.1-64.0)	63.1 (62.7-63.5)	62.6 (62.2-63.0)	62.6 (62.1-63.0)	62.4 (62.0-62.8)
Estonia	64.3 (64.1-64.5)	64.1 (63.9-64.3)	64.6 (64.4-64.8)	64.4 (64.2-64.6)	64.6 (64.4-64.8)	64.8 (64.6-65.0)	66.3 (66.2-66.5)	66.2 (66.1-66.4)	66.2 (66.1-66.4)	65.7 (65.6-65.9)	64.8 (64.6-64.9)	64.2 (64.0-64.4)	63.6 (63.5-63.8)	62.4 (62.2-62.6)	60.3 (59.8-60.8)	61.5 (61.3-61.7)	64.0 (63.8-64.2)	64.1 (64.0-64.3)
Latvia	63.7 (63.6-63.9)	63.5 (63.4-63.7)	64.2 (64.1-64.4)	64.0 (63.8-64.1)	64.1 (64.0-64.3)	64.8 (64.6-64.9)	66.3 (66.2-66.5)	66.4 (66.3-66.6)	66.2 (66.1-66.4)	65.3 (65.1-65.4)	64.3 (64.1-64.4)	63.5 (63.3-63.6)	62.5 (62.4-62.7)	60.5 (60.4-60.7)	59.2 (59.1-59.4)	60.3 (60.2-60.5)	62.7 (62.6-62.9)	63.6 (63.5-63.8)
Lithuania	65.7 (65.6-65.8)	65.6 (65.5-65.8)	65.8 (65.7-66.0)	65.9 (65.8-66.0)	65.5 (65.4-65.7)	66.1 (66.0-66.2)	68.0 (67.9-68.1)	67.5 (67.4-67.7)	67.6 (67.5-67.7)	66.8 (66.7-66.9)	66.4 (66.3-66.5)	65.1 (65.0-65.3)	64.7 (64.6-64.8)	63.3 (63.2-63.4)	62.8 (62.6-62.9)	63.2 (63.1-63.4)	64.6 (64.5-64.7)	65.6 (65.4-65.7)
Moldova	61.6 (60.7-62.3)	61.8 (61.1-62.4)	62.0 (61.4-62.6)	62.6 (62.0-63.1)	62.9 (62.3-63.5)	62.6 (62.1-63.1)	64.3 (63.8-64.7)	65.1 (64.7-65.5)	65.0 (64.6-65.4)	65.0 (64.5-65.3)	64.5 (64.1-64.9)	63.9 (63.5-64.2)	63.8 (63.2-64.4)	63.9 (63.5-64.3)	61.9 (61.5-62.3)	61.5 (61.1-61.8)	62.3 (61.9-62.7)	64.3 (63.8-64.7)
Russia	61.5 (61.4-61.6)	61.8 (61.7-61.8)	62.3 (62.3-62.4)	62.1 (62.0-62.2)	61.6 (61.6-61.7)	62.8 (62.7-62.8)	64.9 (64.8-64.9)	64.9 (64.8-64.9)	64.7 (64.6-64.7)	64.1 (64.1-64.2)	63.8 (63.7-63.8)	63.6 (63.6-63.7)	62.1 (62.1-62.2)	59.0 (59.0-59.1)	57.4 (57.3-57.5)	58.1 (58.0-58.1)	59.6 (59.5-59.6)	61.0 (61.0-61.1)
Ukraine	63.8 (63.3-64.3)	63.9 (63.4-64.4)	64.2 (63.7-64.6)	64.4 (63.9-64.7)	64.1 (63.7-64.4)	65.0 (64.6-65.3)	66.7 (66.3-67.0)	66.5 (66.1-66.8)	66.4 (66.1-66.7)	65.7 (65.4-66.0)	65.1 (64.8-65.4)	64.4 (64.1-64.6)	63.6 (63.3-63.9)	63.1 (62.8-63.4)	62.2 (61.9-62.5)	60.9 (60.6-61.2)	61.1 (60.8-61.4)	61.9 (61.6-62.2)
Central Europe	66.8 (66.6-66.9)	67.1 (67.0-67.2)	67.2 (67.1-67.3)	67.2 (67.1-67.3)	67.1 (67.0-67.2)	67.0 (66.9-67.1)	67.2 (67.1-67.2)	67.2 (67.1-67.2)	67.4 (67.3-67.4)	67.3 (67.2-67.3)	67.2 (67.2-67.3)	67.0 (66.9-67.1)	66.9 (66.8-67.0)	67.2 (67.1-67.3)	67.3 (67.2-67.4)	67.2 (67.1-67.3)	67.8 (67.7-67.8)	67.9 (67.9-68.0)
Albania	69.5 (68.4-70.6)	69.6 (68.5-70.6)	69.7 (68.7-70.6)	69.7 (68.8-70.5)	69.8 (68.9-70.5)	69.9 (69.1-70.5)	70.0 (69.3-70.6)	69.9 (69.3-70.5)	69.9 (69.4-70.5)	70.1 (69.6-70.6)	70.2 (69.7-70.7)	70.2 (69.7-70.7)	70.3 (69.8-70.8)	70.2 (69.7-70.8)	70.2 (69.6-70.7)	70.2 (69.7-70.7)	70.3 (69.7-70.7)	70.7 (70.2-71.2)
Bosnia and Herzegovina	67.1 (66.6-67.7)	67.3 (66.9-67.8)	67.5 (67.2-67.9)	67.7 (67.4-68.0)	67.9 (67.7-68.1)	67.4 (66.9-68.0)	67.8 (67.3-68.4)	68.1 (67.6-68.6)	68.4 (68.0-68.9)	68.7 (68.2-69.1)	69.0 (68.6-69.4)	69.0 (68.7-69.4)	62.6 (60.8-64.6)	63.1 (60.9-65.4)	66.9 (64.5-69.3)	61.4 (59.8-63.1)	70.5 (70.2-70.7)	71.0 (70.8-71.2)
Bulgaria	68.6 (68.5-68.7)	68.8 (68.7-68.9)	68.5 (68.4-68.6)	68.5 (68.4-68.6)	68.4 (68.3-68.5)	68.2 (68.1-68.3)	68.4 (68.3-68.5)	68.4 (68.3-68.5)	68.4 (68.3-68.5)	68.3 (68.2-68.4)	68.2 (68.1-68.3)	68.1 (68.0-68.2)	67.9 (67.8-68.0)	67.6 (67.5-67.7)	67.3 (67.2-67.4)	67.4 (67.3-67.5)	67.4 (67.3-67.5)	67.2 (67.1-67.3)
Croatia	67.2 (66.5-67.9)	67.4 (66.8-68.0)	67.6 (67.1-68.1)	67.7 (67.3-68.1)	67.8 (67.6-68.1)	67.9 (67.7-68.0)	68.0 (67.8-68.1)	68.1 (67.9-68.2)	68.2 (68.1-68.3)	68.5 (68.3-68.6)	68.8 (68.6-68.9)	67.6 (66.5-68.6)	68.1 (67.3-68.9)	69.0 (68.6-69.4)	69.5 (69.3-69.7)	69.0 (68.2-69.6)	69.7 (69.5-69.8)	69.7 (69.6-69.9)
Czech Republic	67.1 (67.0-67.2)	67.2 (67.2-67.3)	67.3 (67.2-67.4)	67.3 (67.2-67.4)	67.4 (67.3-67.5)	67.5 (67.5-67.6)	67.7 (67.6-67.8)	67.9 (67.8-68.0)	68.0 (67.9-68.1)	68.0 (67.9-68.1)	68.0 (67.9-68.1)	68.2 (68.1-68.3)	68.6 (68.5-68.7)	69.1 (69.0-69.2)	69.5 (69.4-69.6)	69.9 (69.8-69.9)	70.3 (70.2-70.4)	70.6 (70.6-70.7)
Hungary	65.7 (65.6-65.8)	65.6 (65.5-65.7)	65.5 (65.4-65.6)	65.3 (65.2-65.4)	65.1 (65.0-65.2)	65.0 (64.9-65.1)	65.3 (65.2-65.4)	65.6 (65.5-65.7)	65.8 (65.7-65.9)	65.5 (65.4-65.6)	65.3 (65.2-65.4)	64.9 (64.8-65.0)	64.7 (64.6-64.8)	64.7 (64.6-64.8)	65.0 (64.9-65.1)	65.5 (65.4-65.6)	66.1 (66.0-66.2)	66.5 (66.4-66.6)
Macedonia	68.4 (66.9-69.7)	68.5 (67.7-69.3)	68.6 (68.2-68.9)	68.8 (68.1-69.4)	69.1 (68.1-69.9)	69.3 (68.3-70.2)	69.4 (68.4-70.3)	69.2 (68.4-70.0)	69.1 (68.6-69.6)	69.2 (68.9-69.4)	69.4 (69.2-69.6)	69.3 (69.1-69.5)	69.2 (69.1-69.4)	69.0 (68.7-69.3)	69.4 (69.2-69.6)	69.4 (69.3-69.6)	69.7 (69.6-69.9)	69.9 (69.7-70.0)

Location	1998	1999	2000	2001	2002	2003	2004	2005	2006	2007	2008	2009	2010	2011	2012	2013	2014	2015
Global	63.9 (63.6-64.1)	64.0 (63.7-64.2)	64.2 (64.0-64.4)	64.4 (64.2-64.7)	64.6 (64.4-64.9)	65.0 (64.8-65.2)	65.3 (65.1-65.5)	65.7 (65.5-65.9)	66.2 (65.9-66.4)	66.6 (66.3-66.8)	66.8 (66.6-67.1)	67.3 (67.0-67.6)	67.5 (67.2-67.8)	68.0 (67.7-68.3)	68.3 (68.0-68.6)	68.6 (68.2-68.9)	68.8 (68.4-69.1)	69.0 (68.6-69.4)
High SDI	72.2 (72.2-72.2)	72.1 (72.0-72.1)	72.1 (72.1-72.2)	72.3 (72.3-72.4)	72.5 (72.4-72.5)	72.7 (72.6-72.7)	73.1 (73.0-73.1)	73.2 (73.2-73.3)	73.8 (73.8-73.9)	74.3 (74.3-74.3)	74.5 (74.5-74.6)	75.0 (74.9-75.0)	75.2 (75.2-75.3)	75.6 (75.5-75.6)	75.9 (75.8-75.9)	76.1 (76.0-76.1)	76.1 (76.1-76.2)	76.2 (76.1-76.3)
High-middle SDI	67.6 (67.3-67.8)	67.7 (67.4-67.9)	68.0 (67.7-68.2)	68.2 (67.9-68.4)	68.3 (68.0-68.6)	68.6 (68.3-68.8)	68.8 (68.5-69.1)	69.3 (69.0-69.5)	69.7 (69.4-70.0)	70.0 (69.7-70.3)	70.3 (70.0-70.5)	70.7 (70.4-70.9)	71.1 (70.8-71.3)	71.5 (71.2-71.8)	72.0 (71.6-72.3)	72.3 (71.9-72.6)	72.5 (72.1-72.8)	72.7 (72.3-73.1)
Middle SDI	64.9 (64.4-65.3)	65.1 (64.6-65.6)	65.4 (64.9-65.8)	65.5 (65.0-66.0)	65.7 (65.2-66.1)	66.2 (65.8-66.6)	66.3 (65.8-66.7)	66.8 (66.4-67.2)	67.2 (66.8-67.6)	67.5 (67.1-67.9)	67.7 (67.3-68.1)	68.1 (67.7-68.5)	68.3 (67.9-68.7)	68.6 (68.1-69.0)	68.8 (68.4-69.3)	69.1 (68.6-69.6)	69.3 (68.7-69.8)	69.5 (69.0-70.1)
Low-middle SDI	58.3 (57.7-59.0)	58.6 (58.0-59.3)	58.8 (58.2-59.4)	59.1 (58.5-59.7)	59.4 (58.8-59.9)	59.7 (59.1-60.3)	60.0 (59.5-60.6)	60.3 (59.7-60.9)	60.8 (60.2-61.4)	61.2 (60.5-61.8)	61.3 (60.7-62.0)	62.0 (61.3-62.6)	61.9 (61.1-62.6)	62.6 (61.9-63.3)	62.9 (62.1-63.6)	63.2 (62.3-63.9)	63.5 (62.6-64.2)	63.8 (63.0-64.6)
Low SDI	50.6 (49.7-51.4)	50.8 (49.9-51.7)	51.2 (50.3-52.1)	52.1 (51.2-53.0)	52.6 (51.7-53.5)	53.2 (52.3-54.1)	53.8 (52.9-54.8)	54.5 (53.5-55.4)	55.1 (54.1-56.2)	55.7 (54.6-56.8)	56.3 (55.1-57.5)	56.9 (55.5-58.2)	57.3 (55.9-58.7)	57.8 (56.1-59.3)	58.2 (56.4-59.9)	58.5 (56.6-60.4)	58.8 (56.8-60.9)	59.1 (56.9-61.3)
High-income	74.6 (74.6-74.7)	74.8 (74.8-74.8)	75.1 (75.1-75.1)	75.3 (75.3-75.3)	75.5 (75.5-75.5)	75.7 (75.7-75.8)	76.2 (76.1-76.2)	76.4 (76.3-76.4)	76.6 (76.6-76.6)	76.8 (76.8-76.9)	77.1 (77.0-77.1)	77.3 (77.3-77.3)	77.6 (77.5-77.6)	77.7 (77.6-77.7)	77.9 (77.9-77.9)	78.0 (78.0-78.0)	78.0 (78.0-78.1)	78.1 (78.1-78.2)
High-income North America	74.2 (74.2-74.2)	74.3 (74.3-74.3)	74.5 (74.4-74.5)	74.5 (74.5-74.6)	74.7 (74.7-74.7)	74.9 (74.9-74.9)	75.3 (75.2-75.3)	75.3 (75.3-75.4)	75.5 (75.5-75.6)	75.8 (75.8-75.9)	76.1 (76.1-76.1)	76.4 (76.3-76.4)	76.7 (76.7-76.7)	76.8 (76.8-76.8)	76.9 (76.9-76.9)	77.0 (77.0-77.0)	77.0 (76.9-77.0)	77.0 (77.0-77.1)
Canada	76.0 (76.0-76.1)	76.2 (76.2-76.3)	76.6 (76.6-76.7)	76.9 (76.8-76.9)	77.1 (77.1-77.2)	77.3 (77.3-77.4)	77.6 (77.5-77.7)	77.8 (77.8-77.9)	78.1 (78.0-78.2)	78.3 (78.2-78.3)	78.5 (78.4-78.5)	78.8 (78.7-78.8)	79.1 (79.0-79.2)	79.4 (79.4-79.5)	79.4 (79.4-79.5)	79.4 (79.3-79.6)	79.5 (79.3-79.6)	79.5 (79.3-79.7)
Greenland	65.6 (64.9-66.3)	65.8 (65.2-66.5)	66.1 (65.5-66.8)	66.4 (65.7-67.0)	66.7 (66.0-67.3)	66.9 (66.2-67.6)	67.1 (66.4-67.8)	67.4 (66.7-68.1)	67.9 (67.1-68.6)	68.3 (67.6-69.0)	68.7 (68.0-69.3)	68.9 (68.3-69.6)	69.0 (68.4-69.7)	69.3 (68.6-70.0)	69.6 (68.8-70.3)	69.7 (68.9-70.5)	69.9 (69.0-70.7)	70.0 (69.1-70.9)
United States	74.0 (74.0-74.0)	74.1 (74.1-74.1)	74.2 (74.2-74.3)	74.3 (74.2-74.3)	74.4 (74.4-74.5)	74.7 (74.6-74.7)	75.0 (75.0-75.0)	75.1 (75.0-75.1)	75.3 (75.2-75.3)	75.6 (75.5-75.6)	75.8 (75.8-75.8)	76.1 (76.1-76.1)	76.4 (76.4-76.4)	76.5 (76.5-76.5)	76.6 (76.6-76.6)	76.7 (76.7-76.7)	76.7 (76.7-76.8)	76.7 (76.7-76.8)
Australasia	76.2 (76.1-76.3)	76.5 (76.4-76.6)	76.9 (76.8-76.9)	77.3 (77.2-77.3)	77.6 (77.5-77.6)	77.9 (77.9-78.0)	78.2 (78.2-78.3)	78.6 (78.5-78.6)	78.8 (78.8-78.9)	79.0 (79.0-79.1)	79.2 (79.1-79.2)	79.4 (79.3-79.4)	79.6 (79.6-79.7)	79.9 (79.8-79.9)	80.1 (80.0-80.1)	80.2 (80.1-80.2)	80.1 (80.0-80.2)	80.1 (80.0-80.2)
Australia	76.4 (76.3-76.4)	76.7 (76.6-76.7)	77.1 (77.0-77.1)	77.5 (77.4-77.5)	77.8 (77.7-77.8)	78.1 (78.1-78.2)	78.5 (78.4-78.5)	78.8 (78.7-78.8)	79.0 (79.0-79.1)	79.2 (79.1-79.3)	79.3 (79.3-79.4)	79.6 (79.5-79.6)	79.8 (79.8-79.9)	80.1 (80.0-80.1)	80.2 (80.2-80.3)	80.3 (80.2-80.3)	80.2 (80.1-80.3)	80.2 (80.1-80.3)
New Zealand	75.4 (75.2-75.5)	75.6 (75.5-75.7)	75.9 (75.8-76.0)	76.1 (76.0-76.3)	76.5 (76.4-76.6)	76.8 (76.7-76.9)	77.1 (77.0-77.3)	77.6 (77.5-77.7)	77.8 (77.7-77.9)	78.0 (77.9-78.2)	78.2 (78.1-78.3)	78.5 (78.4-78.6)	78.8 (78.7-78.9)	78.9 (78.7-79.0)	79.2 (79.1-79.3)	79.5 (79.4-79.7)	79.6 (79.4-79.8)	79.6 (79.3-79.9)
High-income Asia Pacific	75.9 (75.8-76.0)	76.1 (76.0-76.1)	76.5 (76.4-76.5)	76.8 (76.7-76.8)	77.1 (77.0-77.2)	77.3 (77.2-77.3)	77.6 (77.6-77.7)	77.8 (77.7-77.8)	78.1 (78.0-78.1)	78.3 (78.2-78.4)	78.5 (78.4-78.5)	78.7 (78.6-78.7)	78.8 (78.7-78.9)	78.7 (78.4-78.9)	79.1 (79.0-79.2)	79.3 (79.2-79.4)	79.3 (79.2-79.4)	79.4 (79.2-79.5)
Brunei	73.7 (73.1-74.2)	74.0 (73.5-74.4)	74.0 (73.7-74.4)	74.2 (73.8-74.6)	74.4 (74.0-74.8)	74.5 (74.1-74.9)	74.7 (74.3-75.1)	74.8 (74.4-75.2)	74.8 (74.4-75.2)	74.8 (74.4-75.2)	74.9 (74.4-75.3)	75.0 (74.5-75.4)	75.1 (74.6-75.4)	74.9 (74.4-75.3)	74.9 (74.4-75.3)	74.9 (74.4-75.5)	75.1 (74.5-75.7)	75.1 (74.4-75.9)
Japan	77.1 (77.1-77.1)	77.2 (77.1-77.2)	77.6 (77.5-77.6)	77.8 (77.8-77.8)	78.1 (78.1-78.1)	78.1 (78.1-78.2)	78.5 (78.4-78.5)	78.5 (78.4-78.5)	78.7 (78.7-78.8)	78.9 (78.9-79.0)	79.1 (79.1-79.1)	79.2 (79.2-79.3)	79.3 (79.3-79.4)	79.1 (78.6-79.4)	79.6 (79.6-79.7)	79.8 (79.8-79.8)	79.9 (79.8-79.9)	79.9 (79.9-80.0)
Singapore	75.4 (75.3-75.6)	75.8 (75.7-76.0)	76.2 (76.0-76.4)	76.4 (76.3-76.6)	76.7 (76.5-76.8)	77.1 (76.9-77.2)	77.6 (77.4-77.7)	77.9 (77.7-78.0)	78.0 (77.9-78.2)	78.1 (78.0-78.3)	78.4 (78.2-78.6)	78.7 (78.6-78.9)	79.1 (78.9-79.2)	79.3 (79.2-79.5)	79.5 (79.3-79.7)	79.6 (79.4-79.8)	79.7 (79.5-79.9)	79.8 (79.5-80.2)
South Korea	71.5 (71.3-71.8)	72.0 (71.8-72.3)	72.5 (72.3-72.7)	73.0 (72.8-73.2)	73.5 (73.3-73.8)	74.1 (73.8-74.4)	74.7 (74.4-75.0)	75.2 (74.9-75.5)	75.6 (75.4-75.9)	76.0 (75.7-76.2)	76.3 (76.1-76.6)	76.6 (76.4-76.9)	76.9 (76.6-77.1)	77.1 (76.8-77.4)	77.2 (77.0-77.5)	77.4 (77.1-77.8)	77.5 (77.0-77.9)	77.5 (77.0-78.1)
Western Europe	74.9 (74.9-74.9)	75.1 (75.1-75.2)	75.4 (75.4-75.5)	75.7 (75.7-75.7)	76.0 (76.0-76.0)	76.2 (76.2-76.2)	76.7 (76.7-76.7)	77.0 (77.0-77.0)	77.3 (77.3-77.3)	77.5 (77.5-77.5)	77.8 (77.7-77.8)	78.0 (78.0-78.0)	78.3 (78.3-78.3)	78.5 (78.5-78.5)	78.7 (78.7-78.7)	78.8 (78.8-78.9)	78.9 (78.8-78.9)	78.9 (78.9-79.0)
Andorra	79.5 (79.1-79.9)	79.7 (79.3-80.1)	79.9 (79.5-80.3)	80.1 (79.7-80.5)	80.3 (79.9-80.7)	80.3 (79.9-80.7)	80.2 (79.8-80.6)	80.4 (80.0-80.8)	80.5 (80.2-80.9)	80.6 (80.3-81.0)	80.8 (80.5-81.2)	80.9 (80.6-81.3)	81.1 (80.7-81.5)	81.1 (80.7-81.5)	81.1 (80.7-81.5)	81.1 (80.7-81.5)	81.2 (80.8-81.5)	81.2 (80.8-81.6)
Austria	74.4 (74.3-74.5)	74.8 (74.8-74.9)	75.0 (75.0-75.1)	75.5 (75.4-75.6)	75.8 (75.7-75.9)	76.1 (76.0-76.2)	76.3 (76.3-76.4)	76.6 (76.6-76.7)	77.1 (77.0-77.2)	77.3 (77.2-77.4)	77.6 (77.6-77.7)	77.5 (77.4-77.6)	77.7 (77.7-77.8)	78.1 (78.1-78.2)	78.3 (78.3-78.4)	78.5 (78.4-78.5)	78.7 (78.6-78.8)	78.8 (78.7-78.9)
Belgium	74.4 (74.3-74.5)	74.5 (74.3-74.6)	74.6 (74.5-74.7)	74.9 (74.8-75.0)	75.2 (75.0-75.3)	75.5 (75.4-75.6)	76.0 (75.9-76.1)	76.2 (76.1-76.3)	76.7 (76.6-76.8)	76.8 (76.7-76.9)	76.8 (76.7-76.9)	77.1 (77.0-77.2)	77.4 (77.3-77.5)	77.6 (77.5-77.7)	77.6 (77.5-77.8)	77.7 (77.4-77.9)	77.8 (77.4-78.1)	77.8 (77.4-78.3)
Cyprus	74.0 (73.8-74.2)	74.5 (74.3-74.7)	74.8 (74.6-75.1)	75.4 (75.2-75.6)	75.8 (75.6-76.0)	76.2 (76.0-76.3)	76.1 (75.9-76.3)	75.9 (75.7-76.1)	76.4 (76.2-76.6)	76.5 (76.3-76.7)	77.1 (76.9-77.2)	77.1 (76.9-77.3)	77.5 (77.3-77.7)	78.2 (77.9-78.4)	78.5 (78.2-78.8)	78.6 (78.3-78.9)	78.6 (78.4-78.9)	78.7 (78.4-79.0)
Denmark	74.2 (74.1-74.3)	73.9 (73.8-74.0)	74.7 (74.6-74.8)	75.0 (74.9-75.1)	75.0 (74.9-75.1)	75.2 (75.1-75.3)	75.5 (75.4-75.6)	76.1 (76.0-76.2)	76.0 (75.9-76.1)	76.2 (76.1-76.3)	76.8 (76.7-76.9)	77.0 (76.9-77.1)	77.3 (77.2-77.4)	77.8 (77.7-78.0)	78.3 (78.1-78.4)	78.3 (78.1-78.5)	78.3 (78.0-78.5)	78.3 (78.0-78.6)
Finland	73.6 (73.5-73.7)	73.9 (73.7-74.0)	74.3 (74.2-74.4)	74.6 (74.5-74.8)	74.9 (74.7-75.0)	75.1 (75.0-75.2)	75.3 (75.2-75.5)	75.6 (75.4-75.7)	75.7 (75.6-75.8)	75.9 (75.8-76.1)	76.2 (76.1-76.4)	76.5 (76.3-76.6)	76.8 (76.7-76.9)	77.2 (77.0-77.3)	77.4 (77.3-77.6)	77.8 (77.6-78.0)	77.9 (77.7-78.2)	78.0 (77.6-78.3)
France	74.9 (74.8-74.9)	75.0 (75.0-75.0)	75.3 (75.2-75.3)	75.5 (75.4-75.5)	75.7 (75.7-75.8)	76.0 (75.9-76.0)	76.6 (76.6-76.7)	76.8 (76.8-76.8)	77.2 (77.1-77.2)	77.4 (77.4-77.5)	77.7 (77.6-77.7)	77.8 (77.7-77.8)	78.0 (77.9-78.0)	78.3 (78.2-78.3)	78.2 (78.2-78.3)	78.3 (78.1-78.4)	78.3 (78.2-78.5)	78.4 (78.2-78.6)
Germany	74.4 (74.3-74.4)	74.7 (74.6-74.7)	75.0 (74.9-75.0)	75.3 (75.3-75.4)	75.6 (75.5-75.6)	75.8 (75.8-75.8)	76.2 (76.2-76.3)	76.6 (76.5-76.6)	76.9 (76.9-76.9)	77.1 (77.1-77.2)	77.3 (77.3-77.4)	77.5 (77.5-77.5)	77.7 (77.7-77.7)	78.0 (77.9-78.0)	78.2 (78.2-78.2)	78.3 (78.2-78.3)	78.3 (78.2-78.4)	78.4 (78.2-78.5)
Greece	75.5 (75.4-75.6)	75.5 (75.4-75.6)	75.7 (75.6-75.8)	76.1 (76.0-76.2)	76.4 (76.3-76.5)	76.6 (76.5-76.7)	76.7 (76.6-76.7)	76.8 (76.7-76.9)	77.3 (77.2-77.3)	77.0 (76.9-77.1)	77.5 (77.4-77.6)	77.4 (77.3-77.5)	77.8 (77.7-77.9)	77.8 (77.7-77.9)	77.9 (77.8-78.0)	78.5 (78.3-78.6)	78.4 (78.2-78.6)	78.4 (78.1-78.7)
Iceland	77.9 (77.6-78.2)	78.3 (77.9-78.7)	78.6 (78.2-78.9)	78.9 (78.6-79.2)	79.1 (78.9-79.4)	79.4 (79.2-79.6)	79.5 (79.3-79.7)	79.7 (79.4-79.9)	79.8 (79.6-80.1)	80.0 (79.7-80.3)	80.2 (79.9-80.5)	80.2 (80.0-80.5)	80.4 (80.2-80.7)	80.6 (80.3-80.9)	80.7 (80.4-81.0)	80.8 (80.5-81.1)	80.9 (80.6-81.2)	80.9 (80.6-81.2)
Ireland	74.9 (74.5-75.5)	75.2 (74.7-75.7)	75.4 (74.9-76.0)	76.2 (75.7-76.7)	76.3 (76.0-76.7)	76.8 (76.5-77.1)	77.0 (76.6-77.2)	77.7 (77.4-77.9)	77.6 (77.2-77.9)	77.6 (77.2-78.0)	77.8 (77.4-78.0)	77.8 (77.4-78.2)	78.3 (78.1-78.6)	78.1 (77.8-78.5)	78.8 (78.4-79.0)	79.0 (78.7-79.2)	79.1 (78.9-79.4)	79.2 (78.9-79.5)
Israel	75.9 (75.7-76.0)	76.4 (76.3-76.6)	76.3 (76.2-76.4)	76.4 (76.3-76.6)	76.2 (76.0-76.4)	76.8 (76.6-77.0)	77.2 (77.1-77.4)	77.6 (77.5-77.8)	77.2 (77.0-77.4)	78.2 (78.0-78.3)	78.4 (78.2-78.5)	79.0 (78.8-79.1)	79.6 (79.4-79.7)	79.6 (79.4-79.8)	80.2 (80.0-80.4)	80.1 (79.9-80.3)	79.1 (78.9-79.3)	80.2 (79.9-80.5)
Italy	75.7 (75.7-75.8)	76.1 (76.1-76.1)	76.5 (76.5-76.5)	76.8 (76.7-76.8)	77.1 (77.1-77.1)	77.2 (77.1-77.2)	78.0 (78.0-78.1)	78.3 (78.3-78.3)	78.3 (78.3-78.3)	78.5 (78.5-78.6)	78.8 (78.7-78.8)	79.0 (79.0-79.0)	79.3 (79.3-79.4)	79.4 (79.4-79.5)	79.5 (79.5-79.6)	79.5 (79.4-79.6)	79.6 (79.5-79.7)	79.6 (79.5-79.8)
Luxembourg	74.4 (74.2-74.6)	74.8 (74.6-75.0)	75.2 (75.0-75.4)	75.6 (75.4-75.8)	76.0 (75.8-76.2)	76.3 (76.1-76.5)	76.8 (76.6-77.0)	77.2 (77.0-77.4)	77.7 (77.5-77.9)	78.0 (77.9-78.2)	78.4 (78.2-78.6)	78.6 (78.5-78.9)	78.9 (78.7-79.1)	79.1 (78.9-79.3)	79.5 (79.3-79.7)	79.7 (79.4-79.9)	79.8 (79.5-80.0)	79.9 (79.6-80.1)
Malta	76.1 (75.7-76.5)	76.2 (75.7-76.7)	76.4 (76.0-76.9)	76.7 (76.3-77.2)	77.2 (76.7-77.6)	77.7 (77.2-78.1)	78.0 (77.6-78.4)	78.3 (77.9-78.7)	78.5 (78.1-78.9)	78.5 (78.1-78.8)	78.5 (78.2-78.8)	78.8 (78.4-79.0)	78.9 (78.6-79.2)	79.0 (78.7-79.3)	79.2 (78.9-79.5)	79.4 (79.0-79.7)	79.6 (79.2-79.9)	79.7 (79.3-80.1)
Netherlands	75.3 (75.2-75.4)	75.4 (75.3-75.5)	75.5 (75.4-75.6)	75.8 (75.7-75.9)	76.1 (76.0-76.2)	76.3 (76.2-76.4)	76.8 (76.7-76.9)	77.3 (77.2-77.4)	77.7 (77.6-77.8)	78.0 (78.0-78.1)	78.2 (78.1-78.3)	78.5 (78.5-78.6)	78.7 (78.6-78.8)	79.0 (78.9-79.1)	79.1 (79.0-79.1)	79.2 (79.0-79.3)	79.2 (78.9-79.4)	79.2 (78.9-79.5)
Norway	75.4 (75.3-75.6)	75.8 (75.6-75.9)	75.8 (75.7-76.0)	76.3 (76.1-76.4)	76.5 (76.4-76.7)	77.0 (76.9-77.2)	77.3 (77.2-77.5)	77.8 (77.7-78.0)	78.0 (77.9-78.2)	78.4 (78.3-78.5)	78.4 (78.3-78.6)	78.5 (78.4-78.7)	78.9 (78.8-79.1)	78.9 (78.8-79.1)	79.4 (79.3-79.6)	79.4 (79.3-79.6)	79.9 (79.8-80.1)	79.9 (79.7-80.2)
Portugal	72.5 (72.4-72.6)	72.8 (72.7-72.8)	73.2 (73.1-73.3)	73.2 (73.1-73.3)	73.8 (73.7-73.8)	74.2 (74.1-74.3)	74.7 (74.7-74.8)	75.1 (75.0-75.1)	75.5 (75.4-75.6)	75.9 (75.8-75.9)	76.2 (76.1-76.3)	76.5 (76.4-76.5)	76.7 (76.7-76.8)	77.1 (77.0-77.1)	77.4 (77.3-77.4)	77.6 (77.5-77.7)	77.6 (77.5-77.7)	77.7 (77.5-77.8)
Spain	75.3 (75.3-75.4)	75.3 (75.2-75.3)	75.7 (75.6-75.7)	76.0 (76.0-76.0)	76.2 (76.1-76.2)	76.2 (76.2-76.3)	76.7 (76.6-76.7)	76.9 (76.9-77.0)	77.4 (77.3-77.4)	77.6 (77.5-77.6)	77.9 (77.9-78.0)	78.4 (78.4-78.5)	78.8 (78.7-78.8)	79.0 (79.0-79.1)	79.3 (79.2-79.3)	79.7 (79.7-79.8)	79.8 (79.7-79.9)	79.8 (79.7-80.0)
Sweden	77.0 (76.9-77.2)	77.1 (77.0-77.2)	77.4 (77.3-77.5)	77.5 (77.4-77.6)	77.8 (77.6-77.9)	78.0 (77.9-78.1)	78.0 (77.9-78.1)	78.4 (78.3-78.5)	78.7 (78.6-78.8)	78.8 (78.7-78.9)	79.0 (78.9-79.1)	79.2 (79.1-79.3)	79.5 (79.4-79.6)	79.7 (79.6-79.8)	79.9 (79.8-80.0)	80.0 (79.9-80.2)	80.2 (80.1-80.4)	80.2 (80.0-80.5)
Switzerland	76.4 (76.3-76.5)	76.9 (76.8-77.1)	76.9 (76.8-77.1)	77.3 (77.2-77.5)	77.7 (77.6-77.8)	77.9 (77.8-78.1)	78.3 (78.2-78.5)	78.7 (78.5-78.8)	79.0 (78.9-79.1)	79.3 (79.1-79.4)	79.6 (79.5-79.7)	79.6 (79.5-79.7)	80.1 (79.9-80.2)	80.3 (80.2-80.5)	80.5 (80.4-80.7)	80.6 (80.4-80.7)	80.6 (80.4-80.9)	80.7 (80.3-81.0)
United Kingdom	74.8 (74.7-74.8)	75.0 (74.9-75.0)	75.3 (75.2-75.3)	75.6 (75.5-75.6)	75.8 (75.8-75.8)	76.0 (76.0-76.1)	76.4 (76.4-76.5)	76.7 (76.7-76.8)	77.0 (77.0-77.0)	77.3 (77.2-77.3)	77.5 (77.5-77.5)	77.8 (77.8-77.9)	78.2 (78.1-78.2)	78.5 (78.5-78.6)	78.8 (78.7-78.8)	78.9 (78.9-79.0)	79.0 (78.9-79.1)	79.0 (78.9-79.1)
England	75.0 (75.0-75.1)	75.3 (75.2-75.3)	75.6 (75.5-75.6)	75.9 (75.8-75.9)	76.1 (76.1-76.1)	76.4 (76.3-76.4)	76.7 (76.7-76.8)	77.1 (77.0-77.1)	77.3 (77.3-77.4)	77.6 (77.6-77.7)	77.9 (77.8-77.9)	78.2 (78.2-78.2)	78.5 (78.5-78.5)	78.9 (78.8-78.9)	79.1 (79.1-79.1)	79.2 (79.2-79.3)	79.3 (79.2-79.4)	79.3 (79.2-79.4)
Northern Ireland	74.4 (74.2-74.6)	74.5 (74.3-74.7)	74.9 (74.7-75.1)	75.2 (75.0-75.4)	75.4 (75.2-75.6)	75.5 (75.3-75.7)	75.7 (75.5-75.9)	75.8 (75.6-76.0)	75.9 (75.7-76.1)	76.0 (75.8-76.2)	76.2 (76.0-76.4)	76.4 (76.1-76.6)	76.7 (76.4-76.9)	77.2 (77.0-77.4)	77.6 (77.4-77.8)	77.9 (77.6-78.1)	77.9 (77.6-78.3)	77.9 (77.5-78.4)
Scotland	72.5 (72.4-72.7)	72.7 (72.6-72.8)	72.9 (72.8-73.0)	73.2 (73.1-73.3)	73.4 (73.3-73.5)	73.7 (73.5-73.8)	74.1 (74.0-74.2)	74.4 (74.2-74.5)	74.7 (74.5-74.8)	74.8 (74.7-74.9)	75.1 (75.0-75.2)	75.5 (75.3-75.6)	75.9 (75.8-76.0)	76.3 (76.2-76.5)	76.8 (76.6-76.9)	77.0 (76.9-77.2)	77.1 (76.9-77.4)	77.2 (76.8-77.5)
Wales	74.4 (74.3-74.5)	74.5 (74.4-74.6)	74.8 (74.7-75.0)	75.1 (75.0-75.2)	75.4 (75.2-75.5)	75.6 (75.4-75.7)	75.9 (75.7-76.0)	76.2 (76.1-76.3)	76.4 (76.3-76.6)	76.6 (76.4-76.7)	76.8 (76.7-77.0)	77.1 (76.9-77.2)	77.3 (77.2-77.5)	77.6 (77.5-77.7)	77.8 (77.6-77.9)	77.9 (77.7-78.1)	77.9 (77.7-78.2)	78.0 (77.7-78.3)
Southern Latin America	70.8 (70.8-70.9)	71.1 (71.0-71.1)	71.4 (71.4-71.5)	71.5 (71.4-71.6)	71.6 (71.6-71.7)	71.9 (71.8-71.9)	72.3 (72.3-72.4)	72.6 (72.6-72.7)	72.7 (72.7-72.8)	72.7 (72.6-72.7)	72.8 (72.8-72.9)	73.0 (73.0-73.1)	73.3 (73.2-73.4)	73.5 (73.5-73.6)	73.6 (73.6-73.7)	73.7 (73.6-73.8)	73.8 (73.7-74.0)	74.0 (73.7-74.2)
Argentina	70.0 (69.9-70.1)	70.2 (70.1-70.3)	70.5 (70.4-70.6)	70.6 (70.5-70.7)	70.6 (70.6-70.7)	70.9 (70.8-71.0)	71.4 (71.4-71.5)	71.8 (71.7-71.8)	71.8 (71.8-71.9)	71.7 (71.6-71.8)	71.9 (71.8-72.0)	72.2 (72.1-72.2)	72.5 (72.5-72.6)	72.6 (72.6-72.7)	72.7 (72.6-72.8)	72.8 (72.7-72.9)	72.9 (72.8-73.1)	73.0 (72.8-73.3)
Chile	73.5 (73.4-73.6)	73.9 (73.8-74.0)	74.3 (74.2-74.4)	74.4 (74.3-74.5)	74.7 (74.6-74.8)	74.9 (74.8-75.0)	75.1 (75.0-75.2)	75.4 (75.3-75.5)	75.4 (75.3-75.5)	75.5 (75.4-75.6)	75.6 (75.5-75.7)	75.5 (75.4-75.6)	75.6 (75.4-75.7)	76.1 (76.0-76.2)	76.3 (76.2-76.4)	76.3 (76.2-76.5)	76.4 (76.1-76.8)	76.6 (75.9-77.2)
Uruguay	70.0 (69.9-70.2)	70.3 (70.2-70.5)	70.7 (70.6-70.9)	71.0 (70.8-71.1)	71.2 (71.0-71.3)	71.2 (71.1-71.4)	71.5 (71.4-71.7)	71.8 (71.6-72.0)	72.0 (71.9-72.2)	72.0 (71.9-72.2)	72.2 (72.1-72.4)	72.4 (72.3-72.6)	72.5 (72.3-72.7)	72.5 (72.3-72.7)	72.5 (72.3-72.7)	72.6 (72.5-72.8)	72.8 (72.5-73.1)	72.9 (72.5-73.4)
Central Europe, Eastern Europe, and Central Asia	63.3 (63.2-63.4)	62.7 (62.6-62.8)	62.5 (62.4-62.6)	62.6 (62.5-62.7)	62.6 (62.5-62.7)	62.7 (62.5-62.8)	62.9 (62.7-63.0)	62.9 (62.8-63.1)	63.9 (63.8-64.0)	64.5 (64.4-64.6)	64.8 (64.7-64.9)	65.8 (65.6-65.9)	66.1 (66.0-66.2)	66.8 (66.7-67.0)	67.3 (67.1-67.4)	67.7 (67.5-67.8)	67.8 (67.6-67.9)	67.9 (67.7-68.2)
Eastern Europe	61.8 (61.8-61.9)	60.6 (60.5-60.8)	59.9 (59.8-60.0)	59.9 (59.8-60.0)	59.7 (59.6-59.9)	59.7 (59.5-59.8)	59.9 (59.7-60.0)	59.8 (59.6-59.9)	61.2 (61.1-61.3)	61.9 (61.9-62.0)	62.3 (62.2-62.3)	63.5 (63.4-63.5)	63.9 (63.8-63.9)	64.7 (64.6-64.8)	65.2 (65.1-65.2)	65.6 (65.5-65.6)	65.6 (65.5-65.7)	65.7 (65.4-66.1)
Belarus	62.2 (61.8-62.6)	62.1 (61.7-62.5)	62.4 (62.1-62.9)	62.2 (61.9-62.6)	62.1 (61.7-62.5)	62.4 (62.0-62.7)	62.7 (62.4-63.0)	62.8 (62.4-63.1)	63.2 (62.9-63.6)	63.9 (63.6-64.2)	64.1 (63.7-64.4)	64.3 (63.9-64.6)	64.3 (63.9-64.7)	64.4 (64.0-64.8)	64.5 (64.0-65.0)	64.7 (64.1-65.3)	64.8 (64.2-65.5)	65.0 (64.3-65.7)
Estonia	63.8 (63.6-63.9)	64.6 (64.4-64.7)	65.0 (64.8-65.2)	64.4 (64.2-64.6)	65.3 (65.1-65.4)	66.1 (65.9-66.3)	66.4 (66.2-66.6)	67.1 (66.9-67.3)	67.4 (67.3-67.6)	67.4 (67.2-67.5)	68.9 (68.7-69.1)	69.8 (69.6-69.9)	71.1 (71.0-71.3)	71.4 (71.3-71.6)	71.6 (71.4-71.8)	73.3 (73.1-73.5)	73.3 (73.0-73.6)	73.4 (73.1-73.8)
Latvia	63.4 (63.2-63.5)	64.5 (64.3-64.6)	64.9 (64.7-65.0)	64.4 (64.2-64.5)	64.7 (64.5-64.8)	65.6 (65.4-65.7)	65.9 (65.7-66.1)	65.5 (65.3-65.6)	65.4 (65.2-65.5)	65.8 (65.6-65.9)	67.1 (66.9-67.2)	68.2 (68.1-68.4)	68.9 (68.7-69.0)	69.7 (69.6-69.9)	70.0 (69.8-70.2)	70.2 (70.0-70.4)	70.5 (70.1-70.8)	70.6 (70.2-71.0)
Lithuania	65.9 (65.8-66.0)	66.3 (66.2-66.5)	66.6 (66.4-66.7)	66.0 (65.9-66.1)	66.3 (66.2-66.4)	66.5 (66.4-66.6)	66.4 (66.3-66.5)	65.7 (65.6-65.8)	65.5 (65.4-65.6)	65.2 (65.1-65.3)	66.4 (66.3-66.5)	67.9 (67.8-68.0)	68.2 (68.1-68.3)	68.9 (68.7-69.0)	69.3 (69.1-69.4)	69.3 (69.2-69.5)	69.5 (69.3-69.8)	69.7 (69.5-70.0)
Moldova	64.8 (64.3-65.2)	64.6 (64.2-65.0)	64.9 (64.4-65.3)	65.6 (65.2-65.9)	66.0 (65.6-66.4)	66.3 (65.9-66.6)	66.4 (66.0-66.6)	65.5 (65.2-65.8)	66.0 (65.7-66.3)	66.2 (65.9-66.5)	66.5 (66.2-66.8)	66.7 (66.4-66.9)	66.5 (66.2-66.7)	68.3 (68.1-68.6)	68.5 (68.2-68.8)	69.5 (69.1-69.8)	69.7 (69.3-70.1)	69.9 (69.5-70.4)
Russia	61.3 (61.3-61.4)	59.7 (59.6-59.9)	58.8 (58.7-58.9)	58.8 (58.7-58.9)	58.6 (58.4-58.7)	58.4 (58.2-58.6)	58.7 (58.5-58.9)	58.7 (58.5-58.9)	60.4 (60.4-60.5)	61.5 (61.5-61.6)	61.9 (61.8-61.9)	62.9 (62.8-62.9)	63.0 (63.0-63.1)	64.0 (64.0-64.1)	64.7 (64.6-64.7)	65.1 (65.1-65.2)	65.2 (65.2-65.3)	65.3 (64.8-65.8)
Ukraine	62.7 (62.4-63.0)	62.2 (61.9-62.5)	61.7 (61.3-62.0)	61.9 (61.5-62.2)	61.8 (61.5-62.1)	62.1 (61.8-62.3)	61.8 (61.5-62.0)	61.4 (61.1-61.6)	62.2 (62.0-62.4)	61.8 (61.6-62.0)	62.1 (61.9-62.3)	64.4 (64.2-64.6)	65.4 (65.2-65.6)	66.0 (65.8-66.2)	66.1 (65.9-66.3)	66.3 (66.0-66.5)	66.1 (65.6-66.5)	66.3 (65.6-66.9)
Central Europe	68.3 (68.2-68.4)	68.6 (68.5-68.7)	69.2 (69.2-69.3)	69.5 (69.5-69.6)	69.8 (69.7-69.8)	70.0 (70.0-70.1)	70.3 (70.2-70.3)	70.5 (70.4-70.5)	70.7 (70.7-70.8)	71.0 (70.9-71.0)	71.2 (71.1-71.2)	71.4 (71.4-71.5)	71.8 (71.8-71.9)	72.3 (72.3-72.4)	72.6 (72.6-72.7)	73.0 (73.0-73.1)	73.3 (73.1-73.4)	73.4 (73.2-73.6)
Albania	71.0 (70.5-71.4)	71.1 (70.6-71.5)	71.6 (71.2-72.1)	72.2 (71.7-72.7)	72.6 (72.1-73.1)	73.0 (72.4-73.4)	73.2 (72.7-73.7)	73.5 (73.0-74.0)	73.8 (73.2-74.3)	74.0 (73.4-74.5)	74.1 (73.5-74.7)	74.4 (73.6-75.0)	74.5 (73.6-75.2)	74.6 (73.7-75.4)	74.7 (73.7-75.6)	74.8 (73.9-75.7)	74.9 (74.0-75.8)	75.0 (74.1-75.9)
Bosnia and Herzegovina	71.5 (71.3-71.6)	72.0 (71.8-72.1)	72.4 (72.2-72.7)	73.1 (72.8-73.3)	73.3 (73.1-73.6)	73.7 (73.4-73.8)	73.9 (73.8-74.1)	74.1 (73.9-74.2)	74.2 (74.1-74.4)	74.4 (74.3-74.5)	74.6 (74.5-74.8)	74.8 (74.7-75.0)	75.0 (74.9-75.2)	75.3 (75.1-75.5)	75.6 (75.4-75.7)	75.7 (75.5-76.0)	75.9 (75.6-76.1)	76.0 (75.7-76.3)
Bulgaria	67.6 (67.5-67.7)	68.1 (67.9-68.2)	68.3 (68.1-68.4)	68.5 (68.4-68.6)	68.8 (68.7-68.9)	68.9 (68.8-69.0)	69.0 (68.9-69.1)	69.1 (69.0-69.2)	69.2 (69.1-69.3)	69.4 (69.3-69.6)	69.7 (69.6-69.8)	70.0 (69.9-70.1)	70.3 (70.2-70.4)	70.7 (70.6-70.8)	71.0 (70.9-71.2)	71.2 (70.9-71.4)	71.3 (70.9-71.7)	71.4 (70.8-71.9)
Croatia	69.8 (69.7-70.0)	70.1 (70.0-70.2)	70.5 (70.4-70.6)	71.0 (70.8-71.1)	71.3 (71.2-71.5)	71.6 (71.4-71.7)	71.7 (71.5-71.8)	72.0 (71.9-72.1)	72.2 (72.1-72.3)	72.3 (72.2-72.4)	72.6 (72.5-72.7)	72.8 (72.7-73.0)	73.2 (73.1-73.3)	73.7 (73.6-73.8)	74.1 (74.0-74.2)	74.4 (74.2-74.6)	74.6 (74.3-74.8)	74.6 (74.3-75.0)
Czech Republic	71.0 (70.9-71.1)	71.3 (71.3-71.4)	71.6 (71.6-71.7)	71.9 (71.8-72.0)	72.1 (72.0-72.2)	72.3 (72.2-72.4)	72.6 (72.6-72.7)	73.0 (72.9-73.1)	73.3 (73.2-73.4)	73.7 (73.6-73.8)	73.9 (73.9-74.0)	74.1 (74.0-74.2)	74.4 (74.3-74.5)	74.7 (74.6-74.8)	75.0 (74.9-75.1)	75.3 (75.2-75.4)	75.7 (75.6-75.8)	75.9 (75.7-76.1)
Hungary	66.7 (66.6-66.8)	67.0 (66.9-67.0)	67.5 (67.4-67.6)	68.0 (67.9-68.1)	68.3 (68.2-68.4)	68.5 (68.4-68.6)	68.7 (68.5-68.8)	68.8 (68.7-68.9)	69.1 (69.0-69.2)	69.4 (69.3-69.4)	69.8 (69.7-69.9)	70.2 (70.1-70.3)	70.6 (70.5-70.7)	71.1 (71.0-71.2)	71.7 (71.6-71.8)	72.4 (72.3-72.6)	72.9 (72.7-73.2)	73.2 (72.8-73.7)
Macedonia	70.0 (69.8-70.1)	70.2 (70.0-70.3)	70.4 (70.3-70.6)	70.4 (70.2-70.7)	70.8 (70.6-70.9)	70.9 (70.7-71.1)	71.1 (70.9-71.2)	71.4 (71.2-71.5)	71.6 (71.5-71.8)	71.8 (71.7-72.0)	72.1 (71.9-72.2)	72.3 (72.2-72.5)	72.9 (72.7-73.1)	73.3 (73.1-73.5)	73.4 (73.1-73.6)	73.5 (73.1-73.9)	73.7 (73.2-74.2)	73.9 (73.3-74.5)

SR Table 7: Estimates of life expectancy at birth by geography, males, 1980–2015

Location	1980	1981	1982	1983	1984	1985	1986	1987	1988	1989	1990	1991	1992	1993	1994	1995	1996	1997
Montenegro	70.5 (69.2-71.7)	70.6 (69.4-71.7)	70.7 (69.5-71.8)	70.8 (69.5-71.9)	70.8 (69.6-71.9)	70.8 (69.6-71.9)	70.8 (69.7-71.9)	70.9 (69.8-71.9)	71.0 (70.0-72.0)	71.3 (70.4-72.1)	71.6 (70.8-72.3)	71.4 (70.7-72.1)	71.1 (70.5-71.7)	70.8 (70.3-71.3)	70.7 (70.3-71.1)	70.5 (70.2-70.9)	70.4 (70.1-70.7)	70.2 (69.9-70.5)
Poland	66.2 (66.2-66.3)	67.2 (67.1-67.2)	67.3 (67.2-67.4)	67.1 (67.0-67.1)	66.7 (66.7-66.8)	66.6 (66.5-66.6)	66.8 (66.7-66.8)	66.9 (66.8-66.9)	67.2 (67.1-67.2)	66.8 (66.7-66.8)	66.5 (66.5-66.6)	66.2 (66.1-66.2)	66.7 (66.6-66.7)	67.4 (67.3-67.4)	67.4 (67.4-67.5)	67.6 (67.5-67.6)	68.1 (68.1-68.2)	68.3 (68.3-68.4)
Romania	66.8 (66.7-66.9)	66.8 (66.8-66.9)	67.3 (67.2-67.4)	67.5 (67.4-67.6)	67.3 (67.3-67.4)	66.9 (66.8-67.0)	67.0 (66.9-67.0)	66.3 (66.2-66.4)	66.6 (66.5-66.7)	66.6 (66.4-66.8)	66.7 (66.6-66.8)	67.1 (67.0-67.2)	66.4 (66.3-66.5)	66.1 (66.1-66.2)	65.7 (65.6-65.8)	65.4 (65.4-65.5)	65.1 (65.0-65.2)	65.1 (65.0-65.2)
Serbia	68.8 (67.4-70.0)	68.9 (67.6-70.1)	69.0 (67.8-70.1)	69.1 (67.9-70.2)	69.2 (68.0-70.2)	69.3 (68.3-70.3)	69.5 (68.4-70.4)	69.6 (68.7-70.5)	69.7 (68.8-70.5)	69.7 (69.0-70.5)	69.8 (69.2-70.4)	68.1 (67.4-68.7)	69.6 (69.3-70.0)	69.5 (69.3-69.8)	69.4 (69.2-69.6)	69.3 (69.2-69.4)	69.3 (69.2-69.3)	69.4 (69.2-69.5)
Slovakia	66.8 (66.7-67.0)	66.9 (66.8-67.0)	67.0 (66.9-67.1)	66.8 (66.7-67.0)	66.8 (66.7-66.9)	66.9 (66.8-67.1)	67.1 (67.0-67.2)	67.1 (67.0-67.2)	67.1 (67.0-67.2)	66.8 (66.7-66.9)	66.7 (66.6-66.8)	66.8 (66.7-66.9)	67.2 (67.1-67.3)	67.8 (67.7-67.9)	68.3 (68.1-68.4)	68.5 (68.3-68.6)	68.8 (68.7-68.9)	68.8 (68.7-68.9)
Slovenia	66.3 (65.9-66.6)	66.5 (66.2-66.8)	66.7 (66.5-66.9)	66.8 (66.7-67.0)	67.3 (67.1-67.4)	67.7 (67.6-67.8)	68.1 (68.0-68.3)	68.1 (68.0-68.2)	68.7 (68.6-68.9)	69.1 (69.0-69.3)	69.6 (69.5-69.7)	69.7 (69.5-69.8)	69.8 (69.7-70.0)	69.8 (69.7-69.9)	70.2 (70.1-70.4)	70.7 (70.6-70.8)	71.1 (71.0-71.2)	71.2 (71.1-71.4)
Central Asia	61.4 (61.0-61.7)	61.6 (61.3-61.9)	61.9 (61.6-62.2)	62.0 (61.7-62.3)	62.2 (61.9-62.5)	62.7 (62.4-62.9)	63.3 (63.0-63.5)	63.5 (63.2-63.8)	62.8 (62.3-63.2)	63.4 (63.1-63.7)	63.4 (63.1-63.6)	63.1 (62.8-63.4)	62.2 (61.9-62.6)	61.7 (61.4-62.1)	61.5 (61.2-61.8)	61.3 (61.0-61.7)	61.3 (61.0-61.6)	61.8 (61.4-62.1)
Armenia	66.1 (65.2-66.9)	66.4 (65.6-67.2)	66.9 (66.2-67.7)	67.0 (66.2-67.6)	67.2 (66.5-67.8)	67.4 (66.8-68.1)	68.0 (67.3-68.5)	68.4 (67.7-69.0)	54.9 (49.1-61.7)	67.1 (66.5-67.7)	66.5 (65.9-67.1)	65.9 (65.3-66.5)	64.9 (64.3-65.5)	64.1 (63.5-64.8)	64.2 (63.6-64.9)	65.0 (64.4-65.6)	65.5 (64.9-66.1)	66.2 (65.6-66.8)
Azerbaijan	60.7 (59.4-61.9)	60.6 (59.5-61.8)	61.0 (59.9-62.0)	61.1 (60.2-62.1)	61.1 (60.1-61.9)	61.5 (60.6-62.4)	61.7 (60.8-62.5)	61.7 (60.9-62.5)	61.5 (60.8-62.3)	61.9 (61.0-62.7)	61.8 (60.9-62.5)	61.4 (60.6-62.2)	59.2 (57.9-60.4)	59.7 (58.8-60.5)	59.1 (58.0-60.1)	60.7 (59.8-61.5)	61.4 (60.6-62.2)	61.8 (61.0-62.6)
Georgia	64.6 (63.6-65.8)	64.9 (63.9-65.9)	65.1 (64.2-66.0)	65.3 (64.4-66.3)	65.5 (64.6-66.4)	65.6 (64.7-66.5)	65.7 (64.8-66.6)	65.7 (64.9-66.6)	65.7 (64.9-66.6)	65.7 (64.9-66.6)	65.8 (64.8-66.6)	65.7 (64.7-66.6)	65.0 (64.0-66.0)	64.6 (63.5-65.8)	66.1 (65.2-67.0)	66.6 (65.7-67.5)	67.2 (66.4-68.0)	67.3 (66.5-68.1)
Kazakhstan	60.7 (60.2-61.3)	61.0 (60.7-61.4)	61.4 (61.1-61.7)	61.5 (61.1-61.9)	61.6 (61.2-62.0)	62.4 (62.2-62.7)	63.9 (63.6-64.2)	64.1 (63.8-64.4)	63.7 (63.5-64.0)	63.4 (63.1-63.6)	63.3 (63.0-63.5)	62.8 (62.6-63.1)	62.4 (62.1-62.6)	60.3 (59.6-60.9)	59.6 (59.0-60.2)	58.4 (57.7-59.0)	58.1 (57.4-58.8)	58.5 (57.8-59.2)
Kyrgyzstan	59.9 (59.1-60.7)	60.1 (59.4-60.9)	60.3 (59.6-61.0)	60.6 (59.9-61.3)	60.9 (60.2-61.6)	61.3 (60.6-62.0)	61.7 (61.1-62.4)	62.0 (61.3-62.7)	62.1 (61.4-62.7)	62.0 (61.4-62.7)	62.0 (61.4-62.6)	61.9 (61.2-62.5)	61.6 (60.9-62.2)	61.3 (60.7-62.0)	61.0 (60.4-61.7)	61.0 (60.4-61.7)	61.1 (60.5-61.8)	61.3 (60.7-61.9)
Mongolia	57.2 (56.3-58.3)	57.5 (56.6-58.5)	57.8 (57.0-58.8)	58.2 (57.3-59.1)	58.5 (57.7-59.4)	58.8 (58.0-59.6)	59.2 (58.4-60.0)	59.6 (58.8-60.3)	59.7 (59.0-60.4)	59.9 (59.1-60.7)	60.0 (59.2-60.8)	60.0 (59.2-60.8)	59.9 (59.0-60.7)	59.6 (58.8-60.4)	59.5 (58.7-60.3)	59.2 (58.4-59.9)	59.2 (58.4-59.9)	59.4 (58.6-60.1)
Tajikistan	60.3 (59.0-61.5)	60.7 (59.5-61.8)	61.0 (59.9-62.0)	61.2 (60.1-62.3)	61.5 (60.4-62.6)	61.8 (60.7-62.8)	62.1 (61.1-63.0)	62.2 (61.3-63.2)	62.4 (61.5-63.3)	62.6 (61.7-63.4)	62.7 (61.9-63.6)	62.8 (62.0-63.7)	59.3 (57.5-61.3)	61.5 (60.5-62.4)	61.6 (60.7-62.5)	61.5 (60.6-62.4)	60.2 (59.3-61.0)	62.3 (61.4-63.1)
Turkmenistan	57.5 (55.8-59.0)	58.1 (56.5-59.5)	58.6 (57.2-59.9)	58.8 (57.4-60.1)	59.1 (57.8-60.3)	59.5 (58.3-60.6)	59.8 (58.7-60.9)	60.1 (59.1-61.2)	60.3 (59.4-61.3)	60.5 (59.5-61.4)	60.7 (59.7-61.6)	60.2 (59.2-61.2)	60.2 (59.2-61.1)	60.3 (59.3-61.2)	60.1 (59.2-61.1)	59.9 (58.9-60.9)	59.8 (58.7-60.8)	59.7 (58.6-60.7)
Uzbekistan	63.0 (62.1-63.9)	63.1 (62.3-64.0)	63.2 (62.3-64.0)	63.4 (62.5-64.2)	63.6 (62.8-64.4)	64.0 (63.2-64.7)	64.3 (63.6-65.0)	64.6 (63.9-65.3)	64.7 (64.0-65.4)	64.8 (64.0-65.5)	64.8 (64.1-65.5)	64.6 (63.8-65.3)	64.3 (63.6-65.0)	64.0 (63.3-64.7)	63.8 (63.1-64.5)	63.7 (62.9-64.4)	63.8 (63.0-64.5)	64.0 (63.2-64.7)
Latin America and Caribbean	63.1 (62.8-63.4)	63.4 (63.1-63.7)	63.8 (63.5-64.0)	64.1 (63.9-64.4)	64.4 (64.2-64.7)	64.4 (64.2-64.7)	65.1 (64.9-65.3)	65.3 (65.1-65.5)	65.7 (65.5-65.9)	65.9 (65.7-66.1)	66.2 (66.0-66.4)	66.3 (66.1-66.5)	66.5 (66.3-66.7)	66.7 (66.5-66.9)	67.0 (66.8-67.2)	67.3 (67.1-67.5)	67.7 (67.4-67.8)	68.0 (67.8-68.2)
Central Latin America	63.9 (63.7-64.2)	64.0 (63.7-64.2)	64.6 (64.3-64.9)	65.3 (65.1-65.5)	65.9 (65.7-66.1)	65.5 (65.1-65.8)	66.6 (66.4-66.8)	66.8 (66.7-67.0)	67.2 (67.0-67.4)	67.5 (67.3-67.7)	68.1 (67.9-68.2)	68.1 (67.9-68.2)	68.2 (68.0-68.4)	68.4 (68.3-68.6)	68.7 (68.6-68.9)	69.2 (69.0-69.4)	69.6 (69.5-69.8)	70.0 (69.8-70.2)
Colombia	66.6 (66.4-66.9)	66.9 (66.6-67.1)	67.3 (67.1-67.6)	67.6 (67.4-67.8)	67.8 (67.6-68.0)	64.6 (63.7-65.6)	67.5 (67.3-67.7)	67.4 (67.2-67.5)	67.4 (67.2-67.6)	67.5 (67.3-67.7)	67.8 (67.6-68.0)	67.1 (66.9-67.3)	67.1 (67.0-67.3)	67.4 (67.3-67.6)	67.8 (67.6-68.0)	68.3 (68.1-68.4)	68.5 (68.3-68.7)	69.1 (69.0-69.3)
Costa Rica	72.6 (72.2-72.9)	73.0 (72.7-73.3)	73.4 (73.1-73.8)	73.6 (73.4-74.0)	73.8 (73.5-74.1)	73.9 (73.6-74.2)	74.1 (73.8-74.3)	74.1 (73.9-74.4)	74.1 (73.8-74.4)	74.2 (73.9-74.5)	74.3 (74.0-74.6)	74.2 (74.0-74.5)	74.3 (74.0-74.6)	74.3 (74.0-74.5)	74.3 (74.0-74.6)	74.4 (74.1-74.6)	74.4 (74.1-74.7)	74.6 (74.2-74.8)
El Salvador	61.1 (60.2-61.9)	51.8 (48.9-55.2)	57.2 (55.5-58.8)	54.9 (52.6-57.4)	57.4 (55.7-59.1)	59.3 (58.1-60.6)	59.2 (57.8-60.8)	60.9 (59.9-62.1)	62.7 (61.9-63.5)	60.2 (59.5-61.0)	63.2 (62.5-64.0)	63.7 (62.9-64.4)	64.7 (64.0-65.5)	65.2 (64.5-66.0)	65.5 (64.8-66.3)	65.8 (65.1-66.6)	66.2 (65.5-67.0)	66.6 (65.9-67.3)
Guatemala	56.6 (55.6-57.6)	55.1 (52.9-57.3)	54.1 (51.0-57.2)	59.1 (58.4-59.7)	61.0 (60.5-61.4)	60.4 (59.9-60.8)	60.4 (60.0-60.8)	60.5 (60.2-60.9)	61.2 (60.8-61.5)	62.2 (61.8-62.5)	61.9 (61.5-62.2)	61.4 (61.1-61.7)	61.4 (61.1-61.7)	61.5 (61.2-61.8)	61.7 (61.5-62.0)	63.6 (63.4-63.9)	64.9 (64.6-65.2)	64.4 (64.1-64.7)
Honduras	65.0 (64.5-65.5)	65.5 (65.0-66.1)	65.9 (65.3-66.4)	66.3 (65.8-66.8)	66.7 (66.1-67.2)	67.0 (66.4-67.5)	67.3 (66.7-67.9)	67.8 (67.1-68.3)	68.0 (67.4-68.6)	68.3 (67.6-69.1)	68.6 (67.8-69.5)	68.6 (67.7-69.5)	68.7 (67.6-69.6)	68.6 (67.3-69.6)	68.9 (67.4-70.1)	69.1 (67.2-70.4)	69.3 (67.2-70.7)	69.4 (67.2-71.0)
Mexico	62.8 (62.4-63.1)	63.4 (63.1-63.8)	64.0 (63.7-64.3)	64.9 (64.6-65.2)	65.5 (65.2-65.8)	65.7 (65.3-66.1)	66.5 (66.2-66.8)	67.0 (66.7-67.3)	67.3 (67.0-67.6)	67.8 (67.5-68.1)	68.4 (68.1-68.6)	68.7 (68.4-69.0)	68.9 (68.6-69.2)	69.2 (68.9-69.5)	69.6 (69.3-69.9)	70.0 (69.7-70.3)	70.4 (70.1-70.7)	70.6 (70.3-70.9)
Nicaragua	65.1 (63.7-66.4)	65.4 (63.8-66.8)	65.7 (64.2-67.3)	62.1 (59.2-65.1)	60.7 (56.9-64.7)	60.8 (56.9-65.0)	62.4 (59.1-66.0)	61.6 (57.8-66.0)	65.7 (63.7-67.9)	69.3 (68.6-70.0)	70.5 (69.8-71.2)	70.9 (70.2-71.6)	70.8 (70.1-71.5)	71.0 (70.3-71.7)	71.3 (70.5-72.0)	71.5 (70.8-72.3)	71.8 (71.1-72.6)	72.2 (71.5-72.8)
Panama	71.1 (70.3-71.8)	71.4 (70.7-72.1)	71.5 (70.8-72.3)	71.8 (71.1-72.4)	71.9 (71.2-72.6)	72.0 (71.2-72.6)	72.0 (71.2-72.6)	72.0 (71.3-72.7)	72.0 (71.2-72.8)	71.4 (70.2-72.5)	72.4 (71.6-73.2)	72.5 (71.7-73.3)	72.4 (71.6-73.2)	72.5 (71.7-73.2)	72.4 (71.6-73.2)	72.5 (71.8-73.3)	72.7 (71.9-73.5)	73.0 (72.2-73.7)
Venezuela	67.3 (67.2-67.4)	67.8 (67.7-67.9)	68.0 (67.8-68.1)	68.6 (68.5-68.7)	69.1 (69.0-69.2)	69.5 (69.4-69.7)	70.0 (69.9-70.1)	69.7 (69.6-69.9)	69.8 (69.7-69.9)	69.6 (69.5-69.7)	69.9 (69.8-70.1)	69.7 (69.6-69.9)	69.6 (69.5-69.7)	69.5 (69.4-69.6)	69.4 (69.3-69.5)	69.7 (69.5-70.0)	70.3 (70.2-70.4)	70.9 (70.8-71.0)
Andean Latin America	61.9 (61.3-62.5)	62.3 (61.7-62.9)	62.6 (62.0-63.2)	62.7 (62.0-63.2)	63.1 (62.4-63.7)	63.8 (63.2-64.3)	64.4 (63.8-64.9)	64.4 (63.7-65.1)	65.3 (64.8-65.8)	65.7 (65.2-66.1)	66.1 (65.6-66.6)	66.5 (66.0-67.0)	66.9 (66.4-67.4)	67.3 (66.8-67.8)	67.8 (67.4-68.3)	68.3 (67.8-68.8)	68.8 (68.3-69.2)	69.2 (68.8-69.7)
Bolivia	55.1 (53.8-56.5)	55.6 (54.3-57.0)	56.2 (54.8-57.5)	56.4 (55.1-57.7)	57.3 (56.0-58.6)	57.8 (56.6-59.0)	58.4 (57.2-59.6)	58.9 (57.8-60.1)	59.4 (58.3-60.5)	60.1 (59.0-61.2)	60.7 (59.6-61.8)	61.3 (60.2-62.3)	61.9 (60.9-63.0)	62.5 (61.4-63.5)	63.1 (62.1-64.2)	63.7 (62.6-64.7)	64.3 (63.2-65.2)	64.7 (63.7-65.7)
Ecuador	65.7 (65.2-66.1)	66.2 (65.7-66.6)	66.5 (66.1-67.0)	66.7 (66.3-67.2)	67.2 (66.8-67.7)	67.6 (67.1-68.1)	67.9 (67.4-68.4)	66.3 (64.6-67.9)	68.4 (67.9-68.9)	68.6 (68.1-69.1)	68.8 (68.3-69.3)	69.0 (68.5-69.5)	69.2 (68.7-69.7)	69.3 (68.8-69.8)	69.7 (69.2-70.2)	69.9 (69.3-70.4)	70.1 (69.5-70.6)	70.2 (69.7-70.8)
Peru	62.8 (61.9-63.7)	63.0 (62.3-64.0)	63.3 (62.4-64.2)	63.2 (62.2-64.1)	63.4 (62.3-64.4)	64.2 (63.4-65.1)	64.9 (64.2-65.8)	65.6 (64.8-66.3)	66.1 (65.3-66.9)	66.4 (65.6-67.1)	66.8 (66.1-67.6)	67.2 (66.4-68.0)	67.7 (66.9-68.5)	68.1 (67.3-68.9)	68.7 (67.9-69.4)	69.2 (68.5-70.0)	69.8 (69.1-70.5)	70.4 (69.7-71.2)
Caribbean	64.1 (63.4-64.7)	64.5 (63.8-65.1)	64.8 (64.1-65.3)	65.0 (64.4-65.6)	65.1 (64.5-65.7)	65.1 (64.6-65.7)	65.3 (64.8-65.9)	65.5 (65.0-66.0)	65.8 (65.3-66.3)	65.9 (65.5-66.4)	66.1 (65.7-66.5)	66.2 (65.8-66.6)	66.3 (65.9-66.7)	66.2 (65.7-66.6)	66.5 (66.0-66.9)	66.8 (66.3-67.2)	67.0 (66.6-67.4)	67.3 (66.8-67.8)
Antigua and Barbuda	67.5 (66.5-68.4)	68.1 (67.2-69.0)	68.8 (67.9-69.8)	69.6 (68.6-70.8)	69.8 (68.8-70.8)	70.2 (69.2-71.2)	70.3 (69.5-71.2)	70.6 (69.8-71.4)	70.8 (69.9-71.6)	70.7 (69.8-71.5)	70.8 (69.9-71.6)	70.9 (70.1-71.7)	70.8 (70.0-71.7)	70.8 (69.8-71.7)	70.9 (69.9-71.8)	71.1 (70.1-71.9)	71.2 (70.2-72.0)	71.8 (70.9-72.5)
The Bahamas	63.4 (62.1-64.6)	63.7 (62.3-64.9)	63.8 (62.5-65.0)	64.1 (62.9-65.3)	64.4 (63.2-65.6)	62.6 (60.8-64.4)	64.2 (62.9-65.4)	64.4 (63.1-65.5)	64.7 (63.5-65.8)	65.0 (63.8-66.0)	65.3 (64.1-66.3)	65.7 (64.6-66.7)	66.0 (64.9-66.9)	66.4 (65.5-67.3)	66.3 (65.3-67.2)	66.3 (65.4-67.2)	66.6 (65.7-67.5)	66.7 (65.8-67.6)
Barbados	69.5 (68.4-70.4)	70.0 (68.8-70.9)	70.4 (69.2-71.3)	70.5 (69.3-71.4)	70.4 (69.2-71.3)	70.1 (69.1-71.0)	70.4 (69.3-71.3)	70.4 (69.4-71.3)	70.3 (69.3-71.2)	70.2 (69.2-71.0)	70.0 (69.0-70.8)	70.3 (69.3-71.1)	70.1 (69.2-70.9)	70.0 (69.1-70.8)	69.8 (68.9-70.6)	70.2 (69.2-71.0)	70.8 (69.8-71.7)	71.2 (70.1-72.1)
Belize	67.5 (66.3-68.5)	67.7 (66.7-68.7)	68.0 (67.0-68.9)	68.2 (67.3-69.1)	68.4 (67.5-69.2)	69.1 (68.2-69.8)	69.3 (68.4-70.1)	69.4 (68.6-70.2)	69.6 (68.8-70.3)	69.5 (68.7-70.3)	69.4 (68.5-70.2)	69.2 (68.1-70.2)	69.0 (67.9-70.1)	68.6 (67.5-69.8)	68.2 (67.2-69.4)	67.9 (67.0-68.8)	67.5 (66.7-68.4)	66.7 (65.8-67.6)
Bermuda	66.2 (65.6-66.9)	66.3 (65.6-66.9)	66.4 (65.8-67.0)	66.5 (65.9-67.1)	66.6 (66.0-67.3)	66.6 (65.9-67.2)	66.5 (65.8-67.1)	66.8 (66.1-67.4)	67.1 (66.5-67.8)	67.0 (66.3-67.7)	67.2 (66.5-67.9)	67.5 (66.8-68.1)	67.4 (66.7-68.1)	67.3 (66.6-68.0)	67.8 (67.1-68.5)	69.3 (68.7-70.0)	69.1 (68.5-69.8)	70.7 (70.1-71.4)
Cuba	72.1 (72.0-72.2)	72.3 (72.2-72.4)	72.5 (72.4-72.6)	72.6 (72.5-72.7)	72.6 (72.5-72.7)	72.5 (72.4-72.6)	72.5 (72.4-72.6)	72.4 (72.3-72.5)	72.5 (72.4-72.6)	72.5 (72.4-72.6)	72.6 (72.5-72.7)	72.7 (72.6-72.8)	72.7 (72.6-72.8)	72.5 (72.4-72.6)	72.7 (72.6-72.8)	72.9 (72.8-73.0)	73.1 (73.0-73.2)	73.3 (73.2-73.4)
Dominica	71.7 (70.8-72.7)	72.3 (71.3-73.2)	72.6 (71.7-73.6)	72.9 (72.0-73.8)	72.7 (71.8-73.6)	72.5 (71.6-73.4)	72.3 (71.4-73.1)	72.1 (71.2-72.9)	71.9 (70.9-72.7)	71.6 (70.6-72.5)	71.4 (70.2-72.3)	71.2 (69.9-72.2)	71.0 (69.8-72.1)	70.9 (69.7-72.0)	70.8 (69.7-71.9)	70.8 (69.6-71.9)	70.7 (69.6-71.8)	70.9 (69.8-71.9)
Dominican Republic	67.9 (67.2-68.5)	68.2 (67.6-68.9)	68.6 (68.0-69.2)	69.0 (68.4-69.5)	69.2 (68.6-69.7)	69.1 (68.6-69.7)	69.3 (68.7-69.8)	69.2 (68.6-69.8)	69.6 (68.9-70.2)	69.9 (69.3-70.5)	69.9 (69.2-70.5)	70.2 (69.6-70.9)	70.5 (69.8-71.1)	70.4 (69.8-71.1)	70.3 (69.6-71.1)	70.4 (69.6-71.2)	70.3 (69.5-71.1)	70.5 (69.7-71.3)
Grenada	65.3 (62.5-67.2)	65.5 (62.7-67.4)	65.6 (63.0-67.5)	65.7 (63.3-67.5)	65.7 (63.5-67.4)	65.9 (63.9-67.5)	66.2 (64.4-67.6)	66.5 (64.8-67.8)	66.7 (65.2-67.9)	67.1 (65.7-68.2)	67.4 (66.2-68.5)	67.7 (66.5-68.6)	67.4 (66.4-68.3)	67.0 (66.1-67.8)	67.5 (66.6-68.2)	67.9 (67.1-68.6)	68.0 (67.2-68.7)	67.8 (67.1-68.6)
Guyana	60.1 (58.9-61.3)	60.3 (59.1-61.5)	60.5 (59.4-61.7)	60.7 (59.6-61.9)	60.9 (59.7-62.0)	61.0 (59.9-62.1)	61.1 (60.0-62.1)	61.2 (60.1-62.1)	61.3 (60.3-62.2)	61.1 (60.2-61.9)	61.1 (60.1-61.9)	61.3 (60.4-62.1)	61.6 (60.7-62.4)	61.7 (60.8-62.4)	61.8 (61.0-62.6)	62.0 (61.2-62.9)	61.7 (60.9-62.6)	61.7 (60.9-62.5)
Haiti	49.7 (47.5-52.0)	50.5 (48.3-52.8)	51.1 (48.8-53.3)	51.5 (49.4-53.5)	51.7 (49.8-53.8)	52.0 (50.1-54.0)	52.6 (50.8-54.5)	53.2 (51.6-55.1)	53.8 (52.3-55.6)	54.0 (52.6-55.7)	54.3 (52.9-55.9)	54.3 (52.9-55.9)	54.8 (53.3-56.4)	54.3 (52.6-55.9)	55.5 (53.9-57.2)	56.2 (54.7-58.0)	56.8 (55.2-58.5)	57.0 (55.3-58.8)
Jamaica	70.2 (69.3-70.9)	70.8 (70.1-71.4)	71.1 (70.4-71.7)	71.2 (70.6-71.9)	71.4 (70.7-72.0)	71.5 (70.8-72.2)	71.8 (71.1-72.5)	72.3 (71.6-72.9)	72.9 (72.1-73.5)	73.4 (72.6-74.1)	73.7 (72.9-74.5)	73.9 (73.0-74.6)	73.8 (73.0-74.6)	73.8 (72.9-74.6)	73.7 (72.8-74.5)	73.5 (72.6-74.4)	73.3 (72.4-74.2)	73.1 (72.2-74.0)
Puerto Rico	70.2 (70.1-70.4)	70.6 (70.4-70.7)	70.9 (70.7-71.1)	71.1 (70.9-71.3)	71.1 (70.9-71.2)	70.3 (69.7-70.8)	70.7 (70.5-70.8)	70.0 (69.8-70.2)	69.7 (69.6-69.9)	69.6 (69.4-69.7)	69.5 (69.3-69.7)	69.5 (69.2-69.6)	69.2 (69.0-69.4)	69.3 (69.1-69.5)	68.5 (68.2-68.7)	68.7 (68.5-68.9)	69.2 (69.0-69.4)	70.4 (70.3-70.6)
Saint Lucia	65.3 (63.8-66.7)	66.3 (64.9-67.6)	66.7 (65.2-68.0)	67.1 (65.7-68.5)	67.3 (65.9-68.6)	67.3 (65.9-68.6)	67.4 (66.1-68.6)	67.6 (66.1-68.8)	66.7 (65.0-68.3)	67.9 (66.6-69.1)	68.1 (66.8-69.3)	68.3 (67.0-69.3)	68.3 (67.3-69.3)	68.4 (67.5-69.4)	68.5 (67.6-69.4)	68.7 (67.8-69.5)	68.8 (68.0-69.6)	68.9 (68.2-69.6)
Saint Vincent and the Grenadines	65.1 (63.2-66.8)	65.7 (64.0-67.3)	66.3 (64.7-67.7)	66.8 (65.3-68.1)	67.1 (65.6-68.4)	67.6 (66.2-68.8)	67.7 (66.3-68.9)	67.8 (66.4-69.0)	68.0 (66.7-69.2)	68.1 (66.7-69.3)	68.1 (66.8-69.3)	68.2 (67.0-69.5)	68.2 (67.1-69.4)	68.1 (67.1-69.2)	68.0 (67.0-69.0)	67.9 (67.0-68.8)	67.7 (66.7-68.5)	67.9 (67.0-68.7)
Suriname	63.3 (62.5-64.1)	63.9 (63.1-64.7)	64.4 (63.6-65.1)	65.1 (64.3-65.8)	65.5 (64.7-66.3)	66.1 (65.2-66.8)	66.0 (65.0-67.0)	66.3 (65.3-67.3)	66.3 (65.3-67.3)	65.4 (63.7-67.1)	67.5 (66.7-68.2)	67.5 (66.7-68.3)	67.4 (66.6-68.2)	67.3 (66.5-68.1)	67.5 (66.7-68.2)	67.7 (66.9-68.5)	67.8 (67.0-68.5)	67.8 (67.0-68.5)
Trinidad and Tobago	65.3 (65.0-65.7)	65.5 (65.1-65.8)	65.6 (65.3-66.0)	65.6 (65.3-66.0)	65.7 (65.4-66.1)	65.9 (65.6-66.3)	66.1 (65.7-66.5)	66.2 (65.8-66.5)	66.3 (66.0-66.7)	66.5 (66.1-66.8)	66.6 (66.2-67.0)	66.6 (66.2-67.0)	66.5 (66.1-66.9)	66.6 (66.1-67.0)	66.4 (66.0-66.8)	66.5 (66.1-66.9)	66.4 (66.0-66.8)	66.6 (66.2-67.0)
Virgin Islands, U.S.	67.0 (66.4-67.8)	67.6 (66.9-68.4)	68.1 (67.4-68.8)	68.7 (68.0-69.3)	69.1 (68.4-69.7)	69.4 (68.8-70.0)	69.6 (69.0-70.3)	69.7 (69.1-70.4)	69.8 (69.1-70.4)	69.9 (69.2-70.5)	69.8 (69.2-70.5)	69.9 (69.2-70.5)	69.9 (69.2-70.5)	69.9 (69.3-70.5)	69.9 (69.2-70.5)	69.9 (69.2-70.5)	70.2 (69.5-70.7)	70.2 (69.5-70.7)
Tropical Latin America	62.3 (61.7-62.9)	62.8 (62.2-63.3)	62.9 (62.4-63.4)	63.1 (62.6-63.6)	63.1 (62.6-63.6)	63.3 (62.8-63.8)	63.6 (63.2-64.1)	63.9 (63.5-64.3)	64.2 (63.7-64.6)	64.3 (63.9-64.8)	64.4 (63.9-64.8)	64.5 (64.1-65.0)	64.7 (64.2-65.1)	64.9 (64.5-65.4)	65.1 (64.7-65.5)	65.4 (65.0-65.8)	65.7 (65.2-66.1)	66.0 (65.5-66.4)
Brazil	62.1 (61.5-62.7)	62.6 (62.0-63.1)	62.8 (62.2-63.3)	62.9 (62.4-63.4)	62.9 (62.4-63.4)	63.1 (62.6-63.6)	63.4 (63.0-63.9)	63.7 (63.3-64.2)	64.0 (63.5-64.4)	64.1 (63.7-64.6)	64.2 (63.7-64.6)	64.3 (63.9-64.8)	64.5 (64.0-64.9)	64.7 (64.3-65.2)	64.9 (64.5-65.4)	65.2 (64.8-65.6)	65.5 (65.0-65.9)	65.8 (65.4-66.3)
Paraguay	70.6 (69.6-71.4)	70.9 (70.0-71.7)	71.1 (70.2-72.0)	71.2 (70.3-72.1)	71.4 (70.5-72.3)	71.6 (70.8-72.5)	72.0 (71.1-72.8)	72.3 (71.5-73.1)	72.6 (71.7-73.4)	72.6 (71.7-73.4)	72.8 (71.9-73.7)	72.8 (72.0-73.8)	72.8 (71.9-73.7)	72.7 (71.8-73.5)	72.5 (71.7-73.4)	72.4 (71.6-73.2)	72.3 (71.5-73.0)	72.3 (71.5-73.0)
Southeast Asia, East Asia, and Oceania	61.7 (60.8-62.6)	61.9 (60.9-62.6)	62.3 (61.4-63.1)	62.8 (61.9-63.6)	63.2 (62.3-64.0)	63.5 (62.7-64.3)	63.7 (62.8-64.5)	64.0 (63.1-64.8)	64.0 (63.2-64.8)	64.0 (63.2-64.8)	64.3 (63.5-65.1)	64.6 (63.8-65.3)	64.8 (64.0-65.6)	65.1 (64.3-65.8)	65.3 (64.6-66.0)	65.6 (64.9-66.2)	65.9 (65.2-66.4)	66.1 (65.5-66.6)
East Asia	62.7 (61.3-63.9)	62.7 (61.3-63.9)	63.2 (61.9-64.4)	63.7 (62.4-64.9)	64.1 (62.8-65.2)	64.4 (63.2-65.5)	64.5 (63.3-65.6)	64.8 (63.5-65.9)	64.7 (63.5-65.7)	64.5 (63.4-65.6)	64.8 (63.7-65.9)	65.1 (64.0-66.1)	65.3 (64.2-66.3)	65.6 (64.5-66.5)	65.8 (64.8-66.8)	66.0 (65.1-66.9)	66.3 (65.5-67.1)	66.6 (65.8-67.3)
China	62.5 (61.2-63.8)	62.6 (61.2-63.8)	63.1 (61.8-64.3)	63.6 (62.3-64.8)	64.0 (62.7-65.1)	64.3 (63.1-65.4)	64.4 (63.2-65.5)	64.7 (63.4-65.8)	64.6 (63.4-65.6)	64.4 (63.3-65.5)	64.7 (63.6-65.8)	65.0 (63.8-66.0)	65.2 (64.0-66.2)	65.4 (64.4-66.4)	65.7 (64.7-66.7)	66.1 (65.2-67.0)	66.4 (65.6-67.2)	66.7 (65.9-67.4)
North Korea	63.1 (55.7-68.4)	63.4 (56.5-68.7)	63.8 (57.3-69.1)	64.2 (58.2-69.3)	64.5 (58.5-69.4)	64.9 (58.9-69.7)	65.3 (59.7-69.9)	65.6 (60.1-70.1)	65.9 (60.7-70.4)	66.3 (61.0-70.7)	66.6 (61.4-70.8)	66.9 (62.0-71.1)	67.1 (62.1-71.2)	67.2 (62.6-71.4)	67.3 (62.5-71.5)	56.2 (39.6-66.8)	56.3 (40.2-66.7)	56.3 (40.3-66.9)
Taiwan	69.4 (69.3-69.5)	69.5 (69.4-69.6)	69.9 (69.8-70.0)	70.1 (70.0-70.2)	70.4 (70.3-70.5)	70.9 (70.7-71.0)	71.0 (70.9-71.1)	71.1 (71.0-71.2)	70.9 (70.8-71.0)	71.0 (70.9-71.1)	71.4 (71.2-71.5)	71.8 (71.7-71.9)	71.9 (71.8-72.0)	72.0 (71.9-72.1)	72.1 (72.0-72.2)	72.0 (71.9-72.1)	72.1 (71.9-72.2)	72.5 (72.4-72.6)
Southeast Asia	59.7 (58.7-60.7)	60.1 (59.1-61.0)	60.4 (59.5-61.3)	60.9 (60.0-61.7)	61.2 (60.3-62.0)	61.6 (60.8-62.4)	62.0 (61.2-62.8)	62.3 (61.5-63.1)	62.6 (61.8-63.4)	63.0 (62.2-63.7)	63.3 (62.5-64.0)	63.6 (62.8-64.4)	63.8 (63.1-64.6)	64.1 (63.3-64.9)	64.4 (63.6-65.1)	64.6 (63.8-65.3)	64.9 (64.1-65.6)	65.0 (64.3-65.8)
Cambodia	49.3 (46.1-52.7)	49.7 (46.6-52.9)	50.1 (47.1-53.3)	50.5 (47.6-53.5)	51.0 (48.2-53.8)	51.4 (48.7-54.0)	51.7 (49.2-54.3)	54.4 (52.9-56.1)	55.2 (53.8-56.6)	55.1 (53.9-56.6)	55.6 (54.4-56.8)	56.0 (54.8-57.2)	56.3 (55.2-57.4)	56.3 (55.3-57.4)	56.2 (55.1-57.3)	56.2 (55.2-57.3)	56.4 (55.5-57.5)	56.7 (55.7-57.8)
Indonesia	58.3 (57.0-59.6)	58.9 (57.7-60.0)	59.4 (58.3-60.4)	60.0 (59.0-60.9)	60.4 (59.5-61.2)	60.9 (60.0-61.7)	61.3 (60.4-62.1)	61.4 (60.6-62.2)	61.9 (61.0-62.7)	62.3 (61.5-63.1)	62.6 (61.8-63.4)	63.0 (62.3-63.8)	63.3 (62.6-64.1)	63.8 (63.1-64.6)	64.3 (63.6-65.1)	64.9 (64.1-65.7)	65.4 (64.6-66.2)	65.7 (64.9-66.4)
Laos	46.2 (42.6-50.3)	46.7 (43.4-50.6)	47.2 (43.9-51.0)	47.7 (44.5-51.4)	48.2 (45.1-51.8)	48.6 (45.5-52.2)	48.9 (45.8-52.3)	49.2 (46.1-52.6)	49.4 (46.5-52.7)	49.9 (47.1-53.1)	50.1 (47.3-53.1)	50.6 (47.9-53.4)	51.0 (48.4-53.7)	51.4 (49.0-54.0)	51.8 (49.5-54.2)	52.2 (49.9-54.4)	52.6 (50.5-54.6)	53.0 (51.0-54.8)
Malaysia	66.6 (66.2-67.1)	67.0 (66.5-67.4)	67.3 (66.9-67.7)	67.6 (67.4-67.8)	67.8 (67.7-67.9)	68.1 (68.0-68.2)	68.5 (68.4-68.7)	69.0 (68.8-69.2)	69.3 (69.1-69.5)	69.6 (69.4-69.7)	69.8 (69.7-69.9)	70.0 (69.9-70.1)	70.2 (70.1-70.3)	70.3 (70.3-70.4)	70.3 (70.2-70.4)	70.2 (70.1-70.3)	70.3 (70.2-70.4)	70.6 (70.5-70.7)
Maldives	59.6 (57.9-61.2)	60.4 (58.9-61.9)	61.2 (59.9-62.5)	61.9 (60.8-63.0)	62.6 (61.8-63.5)	63.2 (62.5-64.1)	63.8 (63.1-64.6)	64.5 (63.9-65.2)	65.1 (64.5-65.8)	65.7 (65.0-66.4)	66.3 (65.7-67.0)	67.0 (66.3-67.6)	67.4 (66.7-68.0)	67.8 (67.2-68.5)	68.2 (67.5-69.0)	68.7 (68.0-69.4)	69.2 (68.5-69.9)	70.0 (69.3-70.7)
Mauritius	62.0 (61.8-62.2)	62.9 (62.7-63.1)	63.4 (63.2-63.6)	63.7 (63.5-63.9)	63.8 (63.6-64.1)	64.1 (63.8-64.3)	64.4 (64.1-64.6)	64.7 (64.5-65.0)	64.9 (64.7-65.2)	65.1 (64.9-65.4)	65.5 (65.2-65.7)	65.8 (65.6-66.1)	66.1 (65.8-66.3)	66.2 (66.0-66.4)	66.3 (66.1-66.6)	66.4 (66.2-66.7)	66.6 (66.4-66.9)	66.9 (66.7-67.2)
Myanmar	52.5 (47.7-57.5)	52.8 (47.9-57.6)	53.1 (48.2-58.0)	53.4 (48.6-58.5)	53.8 (49.2-58.8)	54.1 (49.5-59.2)	54.5 (49.8-59.6)	54.8 (50.2-59.7)	54.8 (50.2-59.9)	55.4 (50.9-60.4)	55.7 (51.2-60.7)	55.9 (51.3-61.0)	56.1 (51.7-61.2)	56.4 (52.0-61.4)	56.7 (52.4-61.7)	56.9 (52.6-61.8)	57.3 (52.9-62.3)	57.5 (53.2-62.5)
Philippines	61.7 (61.3-62.0)	62.1 (61.7-62.4)	62.0 (61.6-62.3)	62.0 (61.7-62.3)	62.1 (61.8-62.4)	62.0 (61.7-62.3)	62.2 (61.9-62.5)	62.6 (62.3-62.8)	63.0 (62.8-63.3)	63.5 (63.3-63.7)	63.9 (63.7-64.1)	64.2 (63.9-64.5)	64.6 (64.4-64.7)	64.6 (64.5-64.8)	64.8 (64.7-65.0)	65.0 (64.9-65.2)	65.1 (64.9-65.2)	65.2 (65.1-65.4)

Location	1998	1999	2000	2001	2002	2003	2004	2005	2006	2007	2008	2009	2010	2011	2012	2013	2014	2015
Montenegro	70.1 (69.7-70.4)	70.0 (69.7-70.3)	70.2 (70.0-70.5)	70.7 (70.4-70.9)	71.1 (70.9-71.3)	71.5 (71.3-71.7)	71.8 (71.6-72.0)	72.0 (71.8-72.2)	72.2 (72.0-72.5)	72.6 (72.4-72.9)	73.1 (72.8-73.3)	73.4 (73.1-73.7)	73.6 (73.3-74.1)	73.8 (73.4-74.2)	73.9 (73.5-74.4)	73.9 (73.4-74.4)	74.1 (73.6-74.7)	74.2 (73.6-74.9)
Poland	68.7 (68.6-68.7)	68.8 (68.7-68.8)	69.6 (69.6-69.7)	70.0 (70.0-70.1)	70.4 (70.3-70.4)	70.7 (70.6-70.7)	70.7 (70.6-70.7)	70.8 (70.7-70.8)	70.8 (70.8-70.9)	70.9 (70.8-70.9)	71.1 (71.1-71.2)	71.5 (71.4-71.5)	71.9 (71.9-72.0)	72.3 (72.2-72.3)	72.5 (72.5-72.6)	73.1 (73.0-73.2)	73.3 (73.2-73.4)	73.4 (73.2-73.7)
Romania	66.0 (66.0-66.1)	67.0 (67.0-67.1)	67.5 (67.4-67.6)	67.4 (67.3-67.5)	67.6 (67.5-67.6)	67.9 (67.8-68.0)	68.4 (68.3-68.4)	68.8 (68.7-68.9)	69.3 (69.2-69.3)	69.6 (69.6-69.7)	69.4 (69.4-69.5)	69.5 (69.4-69.6)	69.9 (69.8-70.0)	70.9 (70.8-70.9)	71.0 (70.9-71.1)	71.2 (71.0-71.4)	71.4 (71.0-71.7)	71.5 (71.0-72.0)
Serbia	68.5 (67.8-69.3)	67.8 (66.3-69.2)	69.5 (69.2-69.7)	69.8 (69.5-70.0)	69.9 (69.6-70.2)	70.1 (69.8-70.3)	70.3 (70.0-70.5)	70.6 (70.4-70.8)	70.9 (70.8-71.1)	71.3 (71.2-71.4)	71.6 (71.5-71.7)	71.9 (71.8-72.0)	72.2 (72.1-72.2)	72.4 (72.3-72.5)	72.7 (72.6-72.8)	73.0 (72.8-73.1)	73.2 (73.0-73.4)	73.5 (73.2-73.7)
Slovakia	68.7 (68.6-68.8)	68.9 (68.8-69.0)	69.1 (69.0-69.3)	69.5 (69.4-69.6)	69.7 (69.6-69.9)	69.9 (69.8-70.0)	70.2 (70.1-70.3)	70.3 (70.2-70.4)	70.5 (70.4-70.6)	70.7 (70.6-70.8)	71.0 (70.9-71.1)	71.4 (71.2-71.5)	71.8 (71.7-72.0)	72.4 (72.3-72.5)	72.9 (72.7-73.0)	73.4 (73.3-73.5)	73.8 (73.7-74.0)	74.0 (73.7-74.2)
Slovenia	71.5 (71.4-71.6)	71.8 (71.7-71.9)	72.2 (72.0-72.3)	72.3 (72.2-72.4)	72.7 (72.6-72.8)	72.8 (72.7-73.0)	73.4 (73.2-73.5)	73.9 (73.8-74.0)	74.1 (74.0-74.3)	74.7 (74.5-74.8)	75.3 (75.1-75.4)	75.8 (75.7-75.9)	76.2 (76.1-76.4)	76.7 (76.6-76.9)	77.0 (76.8-77.1)	77.2 (77.1-77.4)	77.8 (77.6-78.0)	77.9 (77.7-78.2)
Central Asia	62.1 (61.7-62.4)	62.6 (62.3-62.9)	62.7 (62.4-63.0)	63.0 (62.7-63.3)	63.2 (62.9-63.5)	63.3 (62.9-63.6)	63.4 (63.1-63.8)	63.6 (63.3-64.0)	63.9 (63.5-64.2)	64.3 (63.9-64.7)	64.9 (64.5-65.3)	65.5 (65.1-65.9)	65.7 (65.3-66.1)	66.2 (65.7-66.5)	66.6 (66.2-67.0)	66.9 (66.5-67.4)	67.2 (66.7-67.7)	67.4 (66.9-67.9)
Armenia	66.8 (66.1-67.4)	67.0 (66.4-67.7)	67.6 (67.0-68.2)	67.7 (67.1-68.3)	67.9 (67.3-68.5)	67.9 (67.4-68.5)	68.1 (67.6-68.7)	68.2 (67.6-68.8)	68.2 (67.7-68.8)	68.8 (68.2-69.4)	68.9 (68.3-69.4)	69.1 (68.6-69.6)	69.5 (68.9-70.0)	69.8 (69.2-70.4)	70.2 (69.5-70.8)	70.4 (69.8-71.1)	70.6 (69.9-71.3)	70.7 (70.0-71.5)
Azerbaijan	62.2 (61.4-63.0)	62.6 (61.7-63.4)	63.2 (62.4-64.0)	64.1 (63.3-64.8)	64.5 (63.8-65.3)	64.6 (63.8-65.3)	64.8 (64.0-65.6)	65.1 (64.3-65.9)	65.5 (64.7-66.3)	66.0 (65.2-66.8)	66.1 (65.2-66.9)	66.7 (65.9-67.6)	67.1 (66.1-67.9)	67.7 (66.7-68.6)	68.3 (67.2-69.3)	68.8 (67.7-69.8)	69.2 (68.1-70.2)	69.6 (68.5-70.6)
Georgia	67.3 (66.4-68.1)	67.3 (66.5-68.2)	67.4 (66.5-68.2)	67.5 (66.7-68.4)	67.4 (66.5-68.3)	67.5 (66.7-68.4)	67.4 (66.6-68.3)	67.4 (66.6-68.2)	67.3 (66.6-68.1)	67.4 (66.5-68.2)	66.8 (65.9-67.7)	67.3 (66.5-68.2)	67.4 (66.5-68.3)	67.3 (66.5-68.2)	67.4 (66.7-68.3)	67.5 (66.7-68.3)	67.6 (66.7-68.6)	67.9 (66.9-69.0)
Kazakhstan	58.9 (58.2-59.6)	59.8 (59.4-60.2)	59.6 (59.2-59.9)	59.8 (59.4-60.1)	60.1 (59.8-60.5)	59.9 (59.6-60.3)	59.8 (59.5-60.2)	59.8 (59.4-60.1)	59.8 (59.5-60.1)	60.3 (60.0-60.6)	62.0 (61.7-62.3)	63.3 (63.0-63.6)	63.6 (63.2-63.9)	64.2 (63.9-64.5)	64.9 (64.6-65.2)	65.2 (64.7-65.8)	65.3 (64.4-66.3)	65.3 (64.1-66.5)
Kyrgyzstan	61.5 (60.8-62.1)	61.8 (61.1-62.4)	61.9 (61.2-62.5)	62.0 (61.3-62.6)	61.9 (61.3-62.6)	62.0 (61.4-62.6)	62.3 (61.7-62.9)	62.6 (62.0-63.2)	62.8 (62.3-63.5)	63.2 (62.6-63.8)	63.3 (62.7-64.0)	63.7 (63.1-64.3)	63.8 (63.2-64.4)	64.4 (63.8-65.1)	64.8 (64.2-65.5)	65.2 (64.6-65.9)	65.4 (64.7-66.2)	65.6 (64.8-66.4)
Mongolia	59.5 (58.8-60.3)	59.7 (58.9-60.4)	59.6 (58.9-60.4)	59.6 (58.8-60.3)	59.6 (58.9-60.3)	59.7 (58.9-60.4)	59.8 (59.0-60.6)	60.0 (59.2-60.7)	60.3 (59.4-61.0)	60.6 (59.7-61.3)	60.8 (60.1-61.7)	61.0 (60.2-61.9)	61.2 (60.4-62.0)	61.5 (60.7-62.4)	61.8 (60.9-62.7)	62.1 (61.2-63.1)	62.5 (61.6-63.4)	62.8 (61.9-63.6)
Tajikistan	62.4 (61.6-63.3)	63.2 (62.4-64.0)	63.6 (62.8-64.3)	63.9 (63.1-64.6)	64.4 (63.6-65.2)	64.9 (64.2-65.6)	65.4 (64.7-66.1)	65.9 (65.2-66.7)	66.6 (65.9-67.4)	67.2 (66.5-68.0)	67.7 (67.0-68.4)	68.0 (67.3-68.7)	67.9 (67.2-68.7)	67.9 (67.2-68.7)	68.6 (67.8-69.3)	69.2 (68.4-70.1)	69.7 (68.8-70.6)	70.1 (69.2-70.9)
Turkmenistan	59.7 (58.5-60.8)	59.9 (58.6-61.0)	60.1 (58.7-61.3)	60.4 (59.0-61.6)	60.8 (59.4-62.0)	61.2 (59.8-62.3)	61.6 (60.3-62.8)	62.1 (60.8-63.3)	62.6 (61.4-63.7)	63.1 (61.9-64.2)	63.6 (62.4-64.6)	64.0 (62.9-65.0)	64.4 (63.4-65.4)	64.9 (63.8-65.8)	65.3 (64.2-66.2)	65.6 (64.6-66.5)	66.0 (65.0-66.8)	66.3 (65.3-67.2)
Uzbekistan	64.2 (63.4-65.0)	64.5 (63.7-65.2)	64.6 (63.9-65.4)	64.9 (64.1-65.6)	65.0 (64.2-65.8)	65.1 (64.2-65.9)	65.3 (64.4-66.2)	65.5 (64.6-66.4)	65.8 (64.9-66.7)	66.1 (65.2-67.0)	66.4 (65.5-67.4)	66.7 (65.8-67.7)	67.0 (66.1-67.9)	67.3 (66.3-68.2)	67.6 (66.6-68.4)	67.8 (66.9-68.7)	68.1 (67.2-69.0)	68.4 (67.5-69.3)
Latin America and Caribbean	68.1 (67.9-68.4)	68.4 (68.2-68.6)	69.0 (68.8-69.2)	69.3 (69.1-69.5)	69.6 (69.4-69.8)	69.8 (69.6-70.1)	70.1 (69.9-70.3)	70.4 (70.2-70.7)	70.7 (70.5-70.9)	71.0 (70.7-71.2)	71.0 (70.8-71.3)	71.2 (70.9-71.4)	69.9 (68.6-71.1)	71.6 (71.4-71.9)	71.8 (71.5-72.1)	71.9 (71.5-72.2)	72.1 (71.6-72.4)	72.2 (71.7-72.6)
Central Latin America	69.8 (69.6-70.0)	70.0 (69.7-70.3)	70.8 (70.6-70.9)	71.0 (70.8-71.2)	71.2 (71.0-71.3)	71.4 (71.2-71.6)	71.7 (71.6-71.9)	71.9 (71.7-72.1)	72.1 (71.9-72.3)	72.4 (72.1-72.6)	72.2 (72.0-72.4)	72.2 (72.0-72.5)	72.4 (72.2-72.7)	72.8 (72.5-73.0)	72.9 (72.6-73.2)	72.9 (72.6-73.2)	73.1 (72.8-73.5)	73.3 (72.8-73.7)
Colombia	69.5 (69.3-69.7)	69.5 (69.3-69.7)	69.5 (69.3-69.7)	69.7 (69.5-69.9)	69.8 (69.6-70.0)	71.1 (70.9-71.3)	71.4 (71.1-71.5)	72.0 (71.8-72.2)	72.3 (72.0-72.5)	72.8 (72.5-73.0)	73.1 (72.8-73.3)	73.0 (72.7-73.3)	73.4 (73.1-73.7)	74.1 (73.8-74.5)	74.5 (74.1-74.8)	74.7 (74.3-75.1)	74.9 (74.4-75.4)	75.1 (74.6-75.6)
Costa Rica	74.8 (74.5-75.1)	75.1 (74.8-75.4)	75.3 (75.0-75.5)	75.5 (75.2-75.8)	75.8 (75.6-76.1)	76.1 (75.8-76.4)	76.4 (76.1-76.6)	76.6 (76.3-76.8)	76.9 (76.6-77.1)	77.2 (77.0-77.5)	77.4 (77.2-77.7)	77.4 (77.2-77.7)	77.4 (77.1-77.6)	77.5 (77.3-77.8)	77.7 (77.4-78.0)	77.8 (77.5-78.1)	77.9 (77.5-78.3)	78.1 (77.5-78.6)
El Salvador	66.7 (65.9-67.4)	67.2 (66.5-67.9)	67.5 (66.7-68.2)	67.2 (66.4-67.9)	68.1 (67.3-68.8)	68.3 (67.6-69.0)	68.6 (67.8-69.2)	68.8 (68.1-69.5)	69.1 (68.4-69.7)	69.2 (68.5-69.9)	69.4 (68.7-70.1)	69.4 (68.6-70.1)	69.7 (68.9-70.6)	69.9 (69.0-70.8)	70.1 (69.1-71.1)	70.3 (69.2-71.3)	70.4 (69.2-71.6)	70.6 (69.3-71.9)
Guatemala	64.3 (64.0-64.5)	65.6 (65.3-65.9)	65.7 (65.4-66.0)	65.9 (65.6-66.2)	66.8 (66.5-67.1)	66.6 (66.3-66.9)	66.9 (66.5-67.2)	66.4 (66.1-66.7)	66.8 (66.5-67.2)	68.0 (67.6-68.4)	68.1 (67.7-68.5)	68.0 (67.6-68.4)	68.5 (68.1-68.8)	69.1 (68.7-69.4)	69.7 (69.3-70.1)	69.8 (69.4-70.2)	69.8 (68.7-70.9)	69.8 (67.8-71.8)
Honduras	61.7 (58.4-65.3)	69.6 (67.1-71.5)	69.7 (67.0-71.8)	69.8 (67.0-72.1)	70.0 (67.1-72.4)	70.1 (67.0-72.6)	70.2 (67.1-72.7)	70.4 (67.2-72.9)	70.6 (67.3-73.1)	70.8 (67.4-73.3)	70.9 (67.5-73.5)	71.1 (67.7-73.7)	71.2 (67.8-73.8)	71.4 (68.0-74.1)	71.4 (68.0-74.0)	71.7 (68.3-74.3)	71.9 (68.5-74.5)	72.1 (68.7-74.7)
Mexico	70.8 (70.6-71.1)	71.3 (71.1-71.6)	71.8 (71.6-72.1)	72.2 (71.9-72.5)	72.2 (71.9-72.5)	72.2 (72.0-72.5)	72.6 (72.3-72.8)	72.5 (72.2-72.8)	72.8 (72.5-73.1)	73.0 (72.7-73.3)	72.7 (72.3-72.9)	72.6 (72.3-72.9)	72.6 (72.3-72.9)	72.9 (72.5-73.1)	73.1 (72.7-73.3)	72.9 (72.5-73.2)	73.2 (72.9-73.5)	73.4 (73.0-73.7)
Nicaragua	69.9 (68.8-71.1)	72.8 (72.2-73.5)	73.2 (72.6-73.9)	73.4 (72.7-74.1)	73.5 (72.8-74.2)	73.6 (72.9-74.2)	73.6 (72.9-74.2)	73.7 (73.1-74.4)	73.9 (73.3-74.5)	74.0 (73.4-74.6)	74.3 (73.7-74.9)	74.4 (73.8-75.0)	74.5 (74.0-75.1)	74.8 (74.2-75.4)	74.8 (74.2-75.4)	74.7 (74.0-75.4)	74.9 (73.8-75.8)	75.0 (73.8-76.3)
Panama	73.3 (72.6-74.0)	73.7 (73.0-74.4)	74.0 (73.2-74.7)	74.2 (73.5-74.9)	74.4 (73.6-75.1)	74.5 (73.8-75.2)	74.7 (73.9-75.4)	74.8 (74.0-75.5)	74.7 (73.9-75.4)	74.7 (74.0-75.4)	74.5 (73.8-75.2)	74.5 (73.7-75.2)	74.5 (73.7-75.2)	74.5 (73.8-75.4)	74.7 (73.9-75.5)	75.0 (74.1-75.9)	75.3 (74.0-76.5)	75.5 (73.9-76.9)
Venezuela	70.6 (70.5-70.7)	66.6 (64.9-68.3)	70.4 (70.3-70.5)	70.3 (70.2-70.4)	70.6 (70.5-70.7)	70.0 (69.9-70.1)	70.8 (70.7-70.9)	71.2 (71.1-71.3)	70.8 (70.7-70.9)	70.4 (70.3-70.5)	70.2 (70.1-70.3)	70.4 (70.3-70.5)	71.1 (71.0-71.2)	71.1 (71.0-71.2)	70.6 (70.5-70.7)	70.7 (70.1-71.3)	70.7 (69.5-71.9)	70.7 (69.0-72.4)
Andean Latin America	69.8 (69.3-70.2)	70.3 (69.9-70.8)	70.9 (70.4-71.4)	71.4 (70.9-71.9)	71.9 (71.4-72.3)	72.3 (71.8-72.7)	72.7 (72.2-73.2)	73.1 (72.6-73.6)	73.4 (72.9-73.9)	73.6 (73.1-74.1)	73.9 (73.4-74.4)	74.1 (73.5-74.6)	74.3 (73.7-74.8)	74.5 (73.9-75.1)	74.8 (74.1-75.4)	75.0 (74.3-75.7)	75.2 (74.4-76.0)	75.4 (74.5-76.3)
Bolivia	65.2 (64.2-66.1)	65.7 (64.8-66.7)	66.3 (65.4-67.2)	67.0 (66.0-67.9)	67.4 (66.5-68.4)	68.0 (67.0-68.9)	68.5 (67.5-69.6)	69.0 (67.9-70.1)	69.4 (68.2-70.6)	69.8 (68.5-71.2)	70.2 (68.8-71.6)	70.5 (69.0-72.2)	70.8 (69.2-72.5)	71.1 (69.2-72.8)	71.4 (69.3-73.2)	71.6 (69.4-73.5)	71.9 (69.5-73.8)	72.1 (69.6-74.2)
Ecuador	70.4 (69.8-71.0)	70.6 (70.0-71.2)	70.7 (70.0-71.3)	70.9 (70.1-71.5)	71.0 (70.2-71.6)	71.2 (70.4-71.8)	71.3 (70.6-72.0)	71.5 (70.8-72.2)	71.7 (71.0-72.4)	71.9 (71.2-72.6)	72.2 (71.3-72.8)	72.4 (71.5-73.1)	72.5 (71.6-73.3)	72.8 (71.8-73.5)	73.0 (72.0-73.8)	73.1 (72.1-74.0)	73.2 (72.1-74.2)	73.3 (72.1-74.4)
Peru	71.2 (70.5-72.0)	72.0 (71.3-72.8)	72.7 (72.0-73.5)	73.4 (72.7-74.2)	74.1 (73.3-74.9)	74.6 (73.8-75.4)	75.1 (74.3-75.9)	75.5 (74.7-76.4)	75.9 (75.2-76.8)	76.1 (75.4-76.8)	76.4 (75.7-77.1)	76.5 (75.7-77.2)	76.6 (75.9-77.4)	76.9 (76.1-77.7)	77.1 (76.3-78.0)	77.5 (76.5-78.5)	77.7 (76.4-78.8)	77.9 (76.4-79.1)
Caribbean	67.6 (67.1-68.0)	67.9 (67.4-68.3)	68.2 (67.7-68.7)	68.6 (68.0-69.1)	68.8 (68.3-69.3)	68.9 (68.4-69.5)	68.5 (67.8-69.2)	69.1 (68.5-69.7)	69.2 (68.6-69.9)	69.4 (68.8-70.1)	69.5 (68.9-70.2)	69.8 (69.2-70.4)	54.8 (44.6-66.9)	70.2 (69.5-70.9)	70.3 (69.5-71.1)	70.5 (69.6-71.4)	70.7 (69.7-71.7)	70.9 (69.9-71.9)
Antigua and Barbuda	72.0 (71.2-72.8)	71.8 (71.0-72.6)	71.3 (70.4-72.2)	71.8 (71.0-72.6)	72.1 (71.3-72.9)	72.2 (71.4-73.0)	72.3 (71.5-73.1)	72.4 (71.6-73.4)	72.3 (71.5-73.3)	72.8 (71.8-73.7)	72.8 (71.9-73.9)	73.1 (72.1-74.0)	73.1 (72.1-74.2)	73.3 (72.2-74.3)	73.4 (72.4-74.6)	73.6 (72.5-74.9)	73.7 (72.6-75.1)	73.8 (72.7-75.3)
The Bahamas	66.7 (65.8-67.6)	66.8 (65.9-67.7)	66.9 (66.0-67.8)	67.1 (66.1-68.0)	67.2 (66.2-68.2)	68.1 (67.0-69.2)	68.8 (67.7-69.8)	68.9 (67.9-69.9)	69.2 (68.3-70.2)	68.8 (67.7-70.0)	69.7 (68.6-70.7)	70.1 (69.1-71.2)	70.4 (69.3-71.5)	70.1 (68.9-71.3)	70.0 (68.8-71.3)	70.3 (69.0-71.7)	70.8 (69.4-72.1)	70.9 (69.4-72.3)
Barbados	71.5 (70.5-72.3)	71.7 (70.7-72.6)	71.5 (70.5-72.3)	71.9 (71.1-72.7)	72.3 (71.6-73.0)	72.7 (72.0-73.4)	72.9 (72.2-73.5)	72.9 (72.2-73.5)	72.9 (72.1-73.5)	73.1 (72.3-73.8)	73.3 (72.5-74.0)	73.4 (72.6-74.1)	73.4 (72.5-74.1)	73.5 (72.5-74.2)	73.6 (72.5-74.5)	73.6 (72.5-74.5)	73.6 (72.4-74.7)	73.6 (72.3-74.8)
Belize	66.8 (66.0-67.6)	66.5 (65.7-67.3)	66.1 (65.3-66.9)	65.9 (65.0-66.8)	66.4 (65.6-67.3)	66.7 (65.9-67.6)	67.1 (66.2-68.0)	67.3 (66.4-68.1)	67.6 (66.8-68.4)	68.0 (67.1-68.7)	68.1 (67.3-68.9)	68.2 (67.5-69.0)	68.6 (67.9-69.3)	68.6 (67.9-69.3)	68.7 (67.8-69.4)	68.7 (67.6-69.5)	68.8 (67.6-69.8)	69.1 (67.7-70.3)
Bermuda	71.4 (70.7-72.0)	71.5 (70.9-72.2)	71.2 (70.5-71.9)	72.4 (71.7-73.0)	72.5 (71.9-73.2)	72.6 (72.0-73.2)	72.9 (72.3-73.5)	73.3 (72.7-74.0)	73.1 (72.4-73.8)	73.6 (72.9-74.2)	73.9 (73.2-74.6)	74.4 (73.7-74.9)	74.5 (73.8-75.1)	74.9 (74.2-75.5)	75.0 (74.2-75.7)	74.9 (74.0-75.7)	75.0 (74.0-75.9)	75.0 (73.9-76.0)
Cuba	73.5 (73.4-73.6)	73.7 (73.6-73.8)	74.1 (74.0-74.2)	74.5 (74.4-74.6)	74.8 (74.7-74.9)	75.0 (74.9-75.1)	75.1 (75.0-75.2)	75.2 (75.1-75.3)	75.4 (75.3-75.5)	75.6 (75.5-75.7)	75.7 (75.6-75.8)	75.8 (75.7-75.9)	75.9 (75.8-76.0)	76.0 (75.9-76.1)	75.9 (75.8-76.0)	75.9 (75.7-76.0)	75.9 (75.7-76.1)	75.9 (75.5-76.2)
Dominica	70.3 (68.9-71.4)	71.1 (70.1-72.0)	71.4 (70.3-72.2)	71.4 (70.4-72.3)	71.4 (70.2-72.2)	71.4 (70.3-72.2)	71.4 (70.2-72.2)	71.4 (70.3-72.3)	71.4 (70.3-72.3)	71.2 (70.0-72.1)	71.1 (69.8-72.0)	70.7 (69.5-71.8)	70.4 (69.1-71.5)	70.0 (68.7-71.3)	69.9 (68.4-71.3)	69.6 (68.0-71.2)	69.6 (68.0-71.3)	69.7 (67.8-71.5)
Dominican Republic	70.5 (69.6-71.2)	70.7 (69.9-71.4)	70.8 (69.9-71.5)	70.9 (70.0-71.6)	70.9 (70.1-71.5)	70.9 (70.1-71.5)	70.5 (69.7-71.2)	70.8 (70.0-71.4)	70.9 (70.2-71.5)	71.1 (70.5-71.6)	71.5 (70.8-72.0)	71.7 (71.0-72.2)	71.8 (71.1-72.4)	72.0 (71.3-72.5)	72.1 (71.3-72.7)	72.4 (71.5-72.9)	72.6 (71.7-73.2)	72.8 (71.9-73.4)
Grenada	67.6 (66.8-68.4)	67.5 (66.6-68.2)	67.4 (66.6-68.1)	67.2 (66.4-67.9)	67.1 (66.3-67.8)	67.1 (66.3-67.8)	66.1 (64.8-67.3)	67.2 (66.4-67.9)	67.5 (66.7-68.2)	67.8 (67.0-68.5)	68.0 (67.2-68.8)	68.1 (67.3-68.8)	68.3 (67.4-69.0)	68.3 (67.4-69.0)	68.4 (67.5-69.3)	68.5 (67.5-69.5)	68.7 (67.5-69.8)	68.8 (67.5-70.0)
Guyana	61.5 (60.7-62.4)	61.3 (60.5-62.2)	61.1 (60.2-62.0)	60.8 (60.0-61.8)	60.5 (59.6-61.5)	60.5 (59.6-61.5)	61.0 (59.9-62.0)	61.3 (60.2-62.3)	61.7 (60.7-62.7)	61.8 (60.8-62.9)	61.9 (60.8-63.0)	62.1 (61.0-63.3)	62.4 (61.1-63.6)	62.5 (61.2-63.9)	62.8 (61.3-64.3)	63.0 (61.4-64.7)	63.1 (61.4-64.9)	63.5 (61.7-65.3)
Haiti	57.6 (55.9-59.5)	58.2 (56.4-60.1)	58.8 (57.0-60.8)	59.4 (57.6-61.5)	59.8 (58.0-61.9)	59.9 (58.0-62.0)	58.5 (56.2-61.1)	60.3 (58.2-62.8)	60.5 (58.3-63.1)	60.9 (58.7-63.4)	61.0 (58.7-63.6)	61.7 (59.1-64.3)	28.3 (18.2-52.4)	62.4 (59.7-65.4)	62.7 (59.9-65.8)	63.2 (60.2-66.2)	63.5 (60.4-66.7)	63.9 (60.6-67.2)
Jamaica	73.1 (72.2-73.8)	73.1 (72.2-73.9)	72.9 (72.0-73.6)	72.7 (71.8-73.4)	73.0 (72.2-73.8)	73.3 (72.4-74.1)	73.6 (72.7-74.5)	74.0 (73.0-74.8)	74.0 (73.0-74.9)	73.7 (72.7-74.5)	73.4 (72.4-74.2)	73.0 (72.0-73.8)	73.2 (72.2-74.0)	72.7 (71.8-73.7)	72.9 (71.9-73.9)	72.9 (71.8-73.9)	72.9 (71.7-74.0)	73.0 (71.7-74.3)
Puerto Rico	71.0 (70.9-71.2)	71.3 (71.1-71.5)	71.7 (71.5-71.9)	72.0 (71.8-72.1)	72.4 (72.3-72.6)	72.7 (72.6-72.9)	72.8 (72.6-72.9)	72.8 (72.7-73.0)	73.0 (72.9-73.2)	73.2 (73.0-73.4)	73.2 (73.0-73.4)	73.4 (73.2-73.5)	73.5 (73.3-73.6)	73.7 (73.6-73.9)	74.1 (73.9-74.3)	74.4 (74.1-74.6)	74.7 (74.3-75.2)	75.0 (74.4-75.7)
Saint Lucia	69.1 (68.3-69.8)	69.1 (68.3-69.8)	69.2 (68.4-69.8)	69.2 (68.5-69.9)	69.3 (68.5-70.1)	69.5 (68.6-70.3)	69.8 (68.9-70.7)	70.2 (69.2-71.1)	70.5 (69.6-71.5)	71.0 (69.9-71.9)	71.5 (70.6-72.3)	71.8 (70.9-72.6)	72.2 (71.3-73.0)	72.5 (71.5-73.4)	72.7 (71.6-73.6)	72.6 (71.4-73.6)	72.5 (71.2-73.8)	72.5 (71.0-73.9)
Saint Vincent and the Grenadines	67.9 (67.0-68.6)	67.5 (66.7-68.2)	66.8 (66.0-67.6)	67.5 (66.7-68.2)	67.4 (66.6-68.1)	67.7 (66.9-68.4)	67.8 (67.0-68.5)	68.0 (67.3-68.7)	68.1 (67.4-68.8)	68.3 (67.5-69.0)	68.4 (67.6-69.3)	68.6 (67.8-69.5)	68.6 (67.7-69.4)	68.6 (67.7-69.5)	68.6 (67.6-69.5)	68.4 (67.4-69.4)	68.7 (67.6-69.7)	68.8 (67.6-69.9)
Suriname	67.7 (66.8-68.4)	67.5 (66.6-68.3)	67.3 (66.3-68.1)	67.0 (66.0-67.8)	66.9 (65.9-67.8)	66.9 (65.9-67.9)	67.0 (66.0-68.1)	67.1 (66.1-68.2)	67.1 (66.2-68.3)	67.2 (66.2-68.3)	67.1 (66.1-68.3)	67.4 (66.4-68.6)	67.6 (66.5-68.9)	67.9 (66.7-69.2)	68.2 (66.9-69.6)	68.4 (66.9-69.9)	68.6 (67.1-70.2)	68.8 (67.1-70.5)
Trinidad and Tobago	66.7 (66.3-67.1)	66.4 (66.0-66.8)	66.6 (66.1-67.0)	66.8 (66.4-67.2)	66.8 (66.3-67.2)	67.2 (66.7-67.6)	67.5 (67.0-67.9)	67.8 (67.3-68.2)	67.9 (67.3-68.3)	68.1 (67.5-68.5)	68.3 (67.7-68.8)	68.4 (67.8-69.0)	68.3 (67.6-69.0)	68.4 (67.6-69.2)	68.5 (67.7-69.5)	68.6 (67.5-69.8)	68.8 (67.4-70.0)	68.9 (67.4-70.3)
Virgin Islands, U.S.	70.3 (69.6-70.8)	70.3 (69.7-70.9)	70.4 (69.8-71.0)	70.6 (70.0-71.1)	70.7 (70.0-71.3)	70.8 (70.2-71.4)	70.8 (70.2-71.4)	70.9 (70.3-71.5)	70.9 (70.2-71.6)	70.8 (70.1-71.5)	70.6 (69.9-71.4)	70.4 (69.5-71.2)	70.4 (69.6-71.3)	70.4 (69.4-71.3)	70.3 (69.3-71.3)	70.4 (69.2-71.4)	70.4 (69.0-71.5)	70.3 (68.9-71.6)
Tropical Latin America	66.3 (65.8-66.8)	66.6 (66.1-67.1)	67.1 (66.6-67.5)	67.5 (67.0-67.9)	67.8 (67.3-68.2)	68.1 (67.6-68.5)	68.4 (68.0-68.8)	68.8 (68.4-69.2)	69.1 (68.7-69.5)	69.4 (68.9-69.8)	69.6 (69.2-70.0)	69.8 (69.4-70.2)	70.0 (69.5-70.4)	70.2 (69.8-70.7)	70.4 (70.0-70.8)	70.5 (70.0-71.0)	70.6 (70.1-71.2)	70.8 (70.1-71.4)
Brazil	66.2 (65.7-66.6)	66.5 (66.0-66.9)	66.9 (66.5-67.4)	67.4 (66.9-67.8)	67.7 (67.2-68.1)	68.0 (67.5-68.4)	68.3 (67.9-68.7)	68.7 (68.3-69.1)	69.0 (68.6-69.4)	69.3 (68.8-69.7)	69.5 (69.1-70.0)	69.8 (69.3-70.2)	69.9 (69.5-70.4)	70.2 (69.7-70.6)	70.4 (69.9-70.8)	70.5 (69.9-70.9)	70.6 (70.0-71.1)	70.7 (70.0-71.3)
Paraguay	72.2 (71.5-72.9)	72.2 (71.5-72.9)	72.2 (71.4-72.9)	72.2 (71.4-72.9)	72.1 (71.3-72.9)	72.1 (71.3-72.9)	71.7 (70.9-72.6)	72.0 (71.1-72.9)	72.0 (71.0-72.8)	72.0 (70.8-72.8)	71.9 (70.8-72.8)	71.8 (70.7-72.8)	71.8 (70.6-72.8)	71.9 (70.6-72.9)	71.9 (70.5-73.1)	71.9 (70.3-73.3)	72.0 (70.3-73.5)	72.1 (70.2-73.8)
Southeast Asia, East Asia, and Oceania	66.3 (65.8-66.9)	66.5 (66.0-67.1)	66.7 (66.2-67.3)	66.9 (66.4-67.4)	67.1 (66.6-67.5)	67.7 (67.3-68.2)	67.8 (67.3-68.3)	68.5 (68.1-68.9)	69.0 (68.6-69.5)	69.5 (69.1-69.9)	69.5 (69.0-69.9)	70.1 (69.7-70.5)	70.4 (69.9-70.8)	70.8 (70.3-71.2)	71.2 (70.7-71.7)	71.4 (70.9-72.0)	71.6 (71.0-72.2)	71.9 (71.3-72.5)
East Asia	66.8 (66.0-67.5)	67.0 (66.3-67.7)	67.3 (66.5-68.0)	67.4 (66.7-68.1)	67.6 (66.9-68.2)	68.4 (67.9-69.0)	68.9 (68.3-69.5)	69.4 (68.9-69.9)	70.0 (69.5-70.5)	70.5 (70.0-70.9)	70.6 (70.1-71.1)	71.1 (70.7-71.6)	71.5 (71.0-71.9)	71.9 (71.5-72.4)	72.4 (71.9-72.9)	72.7 (72.2-73.3)	72.9 (72.3-73.5)	73.2 (72.4-73.9)
China	66.9 (66.2-67.6)	67.2 (66.4-67.8)	67.4 (66.7-68.0)	67.5 (66.9-68.1)	67.7 (67.1-68.3)	68.3 (67.7-68.9)	68.8 (68.3-69.4)	69.3 (68.8-69.9)	70.0 (69.5-70.5)	70.4 (69.9-70.9)	70.6 (70.1-71.1)	71.1 (70.6-71.6)	71.4 (70.9-71.9)	71.9 (71.4-72.4)	72.4 (71.9-72.9)	72.7 (72.2-73.3)	72.9 (72.3-73.5)	73.2 (72.4-73.9)
North Korea	56.4 (39.9-66.6)	56.5 (40.0-66.5)	56.5 (40.3-66.3)	56.6 (40.9-66.3)	56.8 (41.8-66.3)	67.2 (63.6-71.2)	67.4 (64.0-71.2)	67.5 (64.3-71.0)	67.7 (64.8-70.9)	67.8 (65.2-70.7)	68.0 (65.6-70.5)	68.2 (65.7-70.7)	68.3 (65.8-71.0)	68.5 (65.7-71.2)	68.6 (65.7-71.4)	68.7 (65.8-71.7)	68.8 (65.7-71.8)	68.9 (65.7-71.9)
Taiwan	72.7 (72.6-72.8)	72.6 (72.3-72.9)	73.3 (73.2-73.4)	73.7 (73.6-73.8)	74.3 (74.2-74.4)	74.6 (74.5-74.7)	74.5 (74.4-74.6)	74.5 (74.4-74.6)	74.9 (74.8-75.0)	75.3 (75.2-75.4)	75.5 (75.4-75.6)	75.8 (75.6-75.9)	76.1 (76.0-76.2)	75.8 (75.7-75.9)	76.0 (75.9-76.1)	76.5 (75.8-77.1)	76.6 (75.4-77.8)	76.6 (75.1-78.3)
Southeast Asia	65.3 (64.5-66.0)	65.5 (64.7-66.2)	65.7 (64.9-66.5)	66.1 (65.2-66.8)	66.3 (65.5-67.1)	66.6 (65.8-67.4)	65.8 (64.6-67.0)	67.1 (66.2-68.0)	67.3 (66.5-68.3)	67.6 (66.7-68.6)	67.1 (66.1-68.2)	67.9 (67.0-69.0)	68.2 (67.1-69.2)	68.4 (67.3-69.5)	68.6 (67.5-69.8)	68.7 (67.6-69.9)	68.9 (67.8-70.2)	69.2 (68.0-70.5)
Cambodia	57.0 (56.1-58.0)	57.5 (56.6-58.5)	57.9 (57.0-58.9)	58.7 (57.7-59.7)	59.4 (58.4-60.4)	60.2 (59.1-61.2)	60.8 (59.8-61.9)	61.5 (60.5-62.6)	62.2 (61.1-63.3)	62.7 (61.6-63.8)	63.2 (62.1-64.3)	63.6 (62.4-64.8)	63.9 (62.6-65.2)	64.4 (63.0-65.9)	65.0 (63.5-66.6)	65.5 (63.8-67.1)	65.9 (64.0-67.6)	66.2 (64.3-68.0)
Indonesia	66.1 (65.3-66.8)	66.3 (65.4-67.1)	66.5 (65.6-67.3)	66.8 (65.8-67.7)	66.9 (65.9-67.9)	67.1 (66.0-68.2)	65.0 (63.2-67.1)	67.4 (66.2-68.6)	67.4 (66.2-68.9)	67.6 (66.3-69.2)	67.8 (66.3-69.4)	67.9 (66.3-69.7)	68.0 (66.3-69.9)	68.2 (66.4-70.3)	68.4 (66.4-70.7)	68.6 (66.4-71.1)	68.8 (66.5-71.5)	69.0 (66.7-71.9)
Laos	53.3 (51.5-55.0)	54.0 (52.3-55.6)	54.5 (52.9-56.0)	55.0 (53.4-56.5)	56.0 (54.5-57.4)	56.6 (55.1-58.1)	57.4 (55.9-58.7)	58.1 (56.6-59.5)	58.7 (57.2-60.0)	59.5 (58.1-60.8)	60.2 (58.8-61.7)	61.0 (59.5-62.4)	61.8 (60.2-63.4)	62.6 (60.9-64.5)	63.2 (61.3-65.2)	63.7 (61.6-66.0)	64.3 (61.9-66.8)	64.7 (62.2-67.5)
Malaysia	70.8 (70.7-70.9)	71.0 (70.9-71.1)	71.2 (71.1-71.3)	71.6 (71.5-71.7)	71.9 (71.8-72.0)	71.9 (71.8-72.0)	72.1 (72.0-72.2)	72.3 (72.3-72.4)	72.6 (72.5-72.7)	72.7 (72.6-72.7)	72.5 (72.4-72.6)	72.3 (72.2-72.4)	72.1 (71.8-72.4)	72.0 (71.4-72.6)	72.0 (71.1-72.8)	72.1 (70.8-73.1)	72.2 (70.7-73.6)	72.4 (70.8-74.0)
Maldives	70.8 (70.2-71.6)	71.3 (70.7-72.0)	72.0 (71.4-72.6)	72.5 (72.0-73.1)	73.2 (72.6-73.7)	74.1 (73.5-74.6)	73.1 (72.1-74.3)	75.7 (75.2-76.2)	76.3 (75.7-76.9)	76.8 (76.2-77.4)	77.1 (76.6-77.7)	77.4 (76.9-77.9)	77.5 (77.0-78.1)	77.8 (77.1-78.5)	77.9 (77.1-78.8)	78.0 (76.9-79.1)	78.0 (76.6-79.4)	78.1 (76.4-79.6)
Mauritius	67.4 (67.1-67.6)	67.9 (67.6-68.1)	68.2 (68.0-68.4)	68.4 (68.2-68.7)	68.6 (68.3-68.8)	68.7 (68.5-69.0)	69.0 (68.7-69.2)	69.2 (68.9-69.4)	69.3 (69.0-69.5)	69.4 (69.1-69.6)	69.5 (69.2-69.7)	69.7 (69.5-70.0)	69.9 (69.7-70.2)	70.4 (70.1-70.6)	70.8 (70.5-71.1)	71.0 (70.8-71.3)	71.2 (70.8-71.5)	71.4 (70.8-72.1)
Myanmar	57.9 (53.6-62.7)	58.2 (53.7-63.1)	58.5 (54.1-63.5)	58.9 (54.5-63.9)	59.3 (54.8-64.5)	59.8 (55.4-65.2)	60.2 (55.8-65.6)	60.7 (56.3-65.9)	61.3 (56.8-66.3)	61.8 (57.2-66.8)	55.8 (50.2-62.2)	62.8 (57.9-67.8)	63.1 (58.2-68.1)	63.4 (58.5-68.3)	63.7 (58.9-68.6)	64.2 (59.3-69.1)	64.6 (59.9-69.6)	64.9 (60.3-69.9)
Philippines	65.2 (65.1-65.3)	65.5 (65.3-65.6)	65.4 (65.3-65.5)	65.4 (65.2-65.5)	65.3 (65.2-65.5)	65.5 (65.3-65.6)	65.5 (65.3-65.6)	65.4 (65.3-65.6)	65.6 (65.4-65.8)	66.2 (66.0-66.3)	66.4 (66.2-66.5)	66.5 (66.3-66.6)	66.8 (66.6-67.0)	66.9 (66.7-67.1)	67.1 (66.9-67.3)	67.0 (66.7-67.4)	67.5 (66.8-68.1)	67.7 (66.6-68.8)

SR Table 7: Estimates of life expectancy at birth by geography, males, 1980–2015

Location	1980	1981	1982	1983	1984	1985	1986	1987	1988	1989	1990	1991	1992	1993	1994	1995	1996	1997	1998	1999	2000	2001	2002	2003	2004	2005	2006	2007	2008	2009	2010	2011	2012	2013	2014	2015
Sri Lanka	67.8 (67.6-67.9)	68.6 (68.5-68.8)	67.9 (67.7-68.1)	67.5 (67.4-67.7)	66.7 (66.3-67.1)	67.0 (66.6-67.3)	67.3 (66.9-67.7)	67.4 (67.0-67.9)	67.2 (66.7-67.7)	66.4 (66.2-66.6)	66.4 (66.0-66.7)	67.5 (67.0-68.0)	68.6 (68.3-69.0)	69.6 (69.4-69.8)	69.4 (69.2-69.6)	67.8 (67.6-67.9)	67.2 (66.8-67.5)	67.3 (67.1-67.6)	67.8 (67.6-68.1)	68.4 (68.2-68.5)	68.2 (67.8-68.5)	69.1 (69.0-69.3)	69.6 (69.3-70.0)	70.1 (70.0-70.2)	65.0 (61.6-68.9)	70.1 (69.9-70.4)	70.0 (69.7-70.4)	70.6 (70.5-70.8)	70.0 (69.4-70.6)	70.2 (69.6-71.0)	71.4 (70.3-72.5)	73.2 (72.7-73.7)	73.5 (72.4-74.6)	73.7 (72.0-75.4)	73.9 (71.6-76.2)	74.1 (71.1-76.9)
Seychelles	66.5 (65.7-67.2)	66.9 (66.2-67.6)	67.0 (66.4-67.7)	67.2 (66.4-67.8)	67.0 (66.2-67.7)	66.5 (65.8-67.3)	66.2 (65.6-66.9)	65.8 (65.3-66.4)	65.6 (64.8-66.3)	65.0 (64.3-65.7)	64.6 (63.9-65.2)	64.4 (63.6-65.1)	64.4 (63.6-65.0)	64.5 (63.8-65.1)	64.7 (64.0-65.3)	64.9 (64.2-65.5)	65.1 (64.4-65.8)	65.6 (64.9-66.3)	66.0 (65.3-66.6)	66.4 (65.8-67.0)	66.7 (66.1-67.3)	67.0 (66.4-67.6)	67.3 (66.7-67.8)	67.5 (67.0-68.1)	67.9 (67.3-68.5)	68.2 (67.6-68.8)	68.6 (68.0-69.2)	69.0 (68.4-69.7)	69.2 (68.6-69.9)	69.3 (68.6-70.0)	69.5 (68.8-70.2)	69.8 (69.0-70.6)	70.2 (69.1-71.1)	70.3 (69.1-71.4)	70.5 (69.0-71.8)	70.5 (68.8-72.1)
Thailand	63.6 (62.8-64.4)	63.8 (63.1-64.6)	64.3 (63.6-65.1)	65.0 (64.2-65.8)	65.7 (65.0-66.5)	66.4 (65.6-67.1)	67.0 (66.2-67.7)	67.3 (66.5-68.1)	67.6 (66.8-68.3)	67.7 (67.0-68.5)	67.7 (67.0-68.5)	67.6 (66.8-68.4)	67.4 (66.5-68.2)	67.1 (66.2-68.0)	66.8 (66.0-67.8)	66.8 (65.8-67.6)	66.7 (65.7-67.7)	66.6 (65.6-67.6)	66.7 (65.7-67.6)	66.8 (65.8-67.8)	67.1 (66.0-68.1)	67.5 (66.5-68.6)	68.1 (67.1-69.2)	68.6 (67.6-69.7)	68.7 (67.8-69.8)	69.8 (68.8-70.8)	70.2 (69.3-71.1)	70.5 (69.5-71.4)	70.6 (69.7-71.5)	70.5 (69.7-71.5)	70.6 (69.6-71.6)	70.6 (69.5-71.9)	70.6 (69.3-72.2)	70.6 (69.0-72.6)	70.7 (68.9-72.9)	70.8 (68.8-73.2)
Timor-Leste	49.8 (46.6-53.2)	51.1 (48.0-54.5)	52.4 (49.1-55.6)	53.4 (50.1-56.5)	54.2 (51.0-57.3)	55.1 (51.8-58.0)	56.0 (52.9-58.9)	56.7 (53.7-59.6)	57.2 (54.2-59.9)	57.6 (54.8-60.2)	58.2 (55.3-60.6)	58.8 (56.4-61.2)	59.5 (57.2-61.7)	60.2 (58.1-62.3)	60.9 (58.9-63.0)	61.4 (59.5-63.4)	61.9 (60.0-63.7)	62.1 (60.3-63.9)	62.6 (60.8-64.3)	61.6 (59.6-63.6)	58.8 (55.0-62.7)	64.0 (62.4-65.7)	65.0 (63.5-66.6)	65.4 (63.8-67.0)	66.1 (64.5-67.7)	67.0 (65.3-68.6)	67.8 (66.2-69.5)	68.6 (66.9-70.4)	69.5 (67.6-71.5)	70.0 (67.9-72.2)	70.4 (68.1-72.9)	70.8 (68.3-73.5)	71.3 (68.5-74.0)	71.6 (68.7-74.5)	71.8 (68.8-74.8)	72.0 (68.9-75.1)
Vietnam	63.5 (60.0-67.2)	63.8 (60.4-67.3)	64.1 (60.8-67.5)	64.4 (61.1-67.6)	64.7 (61.6-67.8)	65.0 (62.2-67.7)	65.3 (62.9-67.7)	65.5 (63.2-67.7)	65.8 (63.5-68.0)	66.0 (63.6-68.0)	66.3 (63.9-68.2)	66.4 (64.0-68.6)	66.7 (64.0-68.9)	66.9 (64.1-69.1)	67.1 (64.3-69.4)	67.3 (64.7-69.7)	67.5 (65.1-69.8)	67.5 (65.4-70.0)	67.8 (65.7-70.3)	68.0 (65.9-70.5)	68.2 (65.9-70.9)	68.5 (66.2-71.4)	68.7 (66.2-71.8)	69.0 (66.2-72.2)	69.2 (66.4-72.6)	69.4 (66.4-72.9)	69.7 (66.5-73.4)	70.0 (66.5-73.8)	70.2 (66.4-74.1)	70.4 (66.6-73.9)	70.6 (66.8-74.0)	70.7 (67.0-74.1)	70.9 (67.1-74.4)	71.0 (67.5-74.4)	71.1 (67.8-74.5)	71.3 (67.8-74.8)
Oceania	56.5 (53.3-59.8)	56.5 (53.3-59.9)	56.6 (53.3-60.0)	56.7 (53.3-60.1)	56.8 (53.6-60.2)	56.9 (53.7-60.4)	56.9 (53.8-60.3)	57.1 (53.9-60.5)	57.2 (54.1-60.7)	57.3 (54.1-60.8)	57.4 (54.1-60.8)	57.4 (54.0-60.9)	57.6 (54.0-61.1)	57.8 (54.2-61.3)	58.0 (54.4-61.5)	58.1 (54.5-61.7)	58.3 (54.7-61.9)	58.3 (54.8-61.9)	57.8 (54.4-61.4)	58.6 (55.0-62.3)	58.7 (55.1-62.5)	58.7 (55.2-62.6)	58.8 (55.2-62.7)	58.9 (55.1-62.8)	58.9 (55.1-62.9)	59.0 (55.3-63.0)	59.2 (55.3-63.1)	59.3 (55.3-63.4)	59.6 (55.5-63.6)	59.7 (55.5-63.7)	60.0 (55.7-64.0)	60.2 (55.9-64.2)	60.4 (55.9-64.4)	60.8 (56.4-64.7)	61.1 (56.7-64.9)	61.4 (57.1-65.3)
American Samoa	66.7 (64.3-68.8)	66.9 (64.9-68.9)	67.1 (65.4-69.0)	67.1 (65.4-69.0)	67.2 (65.4-69.0)	67.4 (65.6-69.1)	67.1 (65.4-68.9)	67.0 (65.3-68.7)	66.7 (65.1-68.5)	66.8 (65.1-68.5)	66.7 (65.1-68.3)	66.7 (65.2-68.3)	66.9 (65.4-68.6)	67.3 (65.5-69.2)	67.5 (65.5-69.5)	67.7 (65.5-69.6)	67.8 (65.9-69.8)	68.1 (66.2-69.8)	68.4 (66.7-69.9)	68.8 (67.2-70.3)	69.3 (67.6-70.6)	69.5 (67.8-70.9)	69.6 (68.0-71.0)	69.4 (67.8-70.9)	69.4 (67.9-71.0)	69.6 (67.9-71.1)	69.9 (68.3-71.5)	70.2 (68.7-71.8)	70.6 (68.8-72.2)	68.9 (67.0-70.9)	70.5 (68.8-72.4)	70.6 (68.8-72.4)	70.7 (68.9-72.5)	70.8 (68.7-72.8)	70.8 (68.7-73.0)	70.9 (68.6-73.2)
Federated States of Micronesia	60.1 (55.3-65.4)	60.1 (55.3-65.5)	60.2 (55.6-65.7)	60.4 (55.8-65.8)	60.7 (56.1-66.1)	60.9 (56.2-66.3)	61.1 (56.5-66.5)	61.3 (56.7-66.8)	61.5 (56.8-67.0)	61.7 (57.0-67.1)	61.9 (57.3-67.5)	62.1 (57.4-67.7)	62.3 (57.5-67.8)	62.6 (57.9-68.3)	62.8 (58.2-68.4)	63.1 (58.5-68.3)	63.4 (58.6-68.1)	63.6 (58.6-68.3)	63.8 (59.0-68.5)	64.1 (59.4-68.8)	64.3 (59.7-69.1)	64.6 (59.8-69.3)	63.6 (58.9-68.3)	65.1 (60.1-70.0)	65.2 (60.0-70.1)	65.4 (60.0-70.2)	65.5 (60.0-70.2)	65.6 (59.9-70.3)	65.7 (60.0-70.4)	65.9 (60.1-70.6)	66.0 (60.1-70.6)	66.2 (60.1-70.6)	66.3 (60.1-70.6)	66.4 (60.2-70.7)	66.4 (60.3-70.7)	66.5 (60.4-70.8)
Fiji	61.8 (59.9-63.8)	61.8 (60.1-63.7)	62.0 (60.3-63.9)	62.3 (60.4-63.9)	62.4 (60.6-64.1)	62.3 (60.4-63.9)	62.4 (60.5-64.2)	62.6 (60.5-64.5)	62.7 (60.2-64.8)	62.7 (60.1-65.1)	62.8 (60.0-65.4)	62.7 (59.9-65.3)	62.7 (60.0-65.3)	62.5 (60.0-65.0)	62.4 (59.9-65.0)	62.2 (60.0-64.7)	62.0 (60.1-64.3)	61.8 (59.9-63.9)	61.9 (60.2-63.8)	61.8 (60.0-63.6)	62.0 (60.2-63.8)	62.1 (60.2-63.9)	62.3 (60.4-64.2)	62.4 (60.6-64.2)	62.6 (60.7-64.3)	62.7 (60.9-64.5)	62.8 (60.9-64.5)	62.8 (61.0-64.6)	62.9 (61.0-64.7)	63.0 (61.0-64.9)	63.2 (61.3-65.2)	63.4 (61.4-65.4)	63.4 (61.1-65.5)	63.5 (60.8-65.9)	63.6 (60.8-66.1)	63.7 (60.8-66.5)
Guam	67.7 (66.3-69.1)	68.2 (66.7-69.6)	68.7 (67.1-70.1)	69.0 (67.3-70.6)	69.2 (67.5-70.9)	69.6 (68.0-71.0)	69.8 (68.3-71.1)	69.9 (68.4-71.3)	70.1 (68.5-71.4)	70.0 (68.5-71.3)	69.9 (68.4-71.1)	69.8 (68.4-71.1)	69.8 (68.3-71.2)	69.9 (68.2-71.5)	70.0 (68.4-71.5)	70.3 (68.8-71.7)	70.5 (68.9-71.9)	65.0 (60.9-69.1)	70.7 (69.2-71.9)	70.7 (69.3-72.0)	70.8 (69.3-72.0)	70.7 (69.4-72.0)	70.7 (69.3-71.9)	70.5 (69.0-71.7)	70.3 (68.9-71.5)	70.1 (68.7-71.3)	69.7 (68.2-70.9)	69.2 (67.7-70.5)	68.8 (67.2-70.3)	68.5 (66.9-70.0)	68.5 (66.9-69.9)	68.4 (66.8-69.9)	68.3 (66.5-70.1)	68.4 (66.5-70.4)	68.5 (66.2-70.5)	68.5 (66.2-70.8)
Kiribati	55.2 (51.4-58.8)	55.1 (51.4-58.8)	55.0 (51.5-58.6)	54.9 (51.3-58.4)	54.9 (51.6-58.2)	54.8 (51.8-58.0)	54.9 (52.0-57.9)	54.9 (52.3-57.6)	55.0 (52.6-57.5)	55.2 (52.9-57.6)	55.4 (53.3-57.6)	55.5 (53.5-57.5)	55.4 (53.6-57.4)	55.4 (53.6-57.3)	55.5 (53.7-57.5)	55.7 (53.9-57.6)	55.9 (54.0-57.8)	56.0 (54.1-57.9)	55.9 (54.0-57.8)	56.0 (54.2-58.0)	56.0 (54.1-58.0)	56.3 (54.4-58.3)	56.5 (54.5-58.5)	56.6 (54.6-58.8)	56.8 (54.5-59.0)	56.9 (54.6-59.3)	57.1 (54.8-59.6)	57.3 (54.8-60.0)	57.5 (54.9-60.2)	57.7 (54.9-60.3)	57.9 (55.0-60.6)	58.1 (55.2-60.8)	58.3 (55.2-61.0)	58.5 (55.3-61.2)	58.7 (55.5-61.4)	58.9 (55.7-61.8)
Marshall Islands	60.1 (57.4-62.9)	60.3 (57.8-62.8)	60.4 (57.8-62.7)	60.5 (58.0-62.8)	60.5 (58.2-62.8)	60.6 (58.3-62.8)	60.8 (58.6-62.8)	61.5 (59.3-63.5)	61.7 (59.7-63.5)	61.8 (60.0-63.6)	61.8 (60.0-63.6)	62.1 (60.3-63.8)	62.2 (60.4-63.9)	62.5 (60.7-64.2)	62.9 (61.2-64.5)	62.9 (61.3-64.5)	63.1 (61.3-64.7)	62.8 (61.0-64.5)	62.7 (60.9-64.5)	62.6 (60.7-64.5)	62.5 (60.5-64.5)	62.5 (60.5-64.6)	62.6 (60.6-64.6)	62.6 (60.6-64.7)	62.6 (60.7-64.8)	62.7 (60.8-64.9)	62.7 (60.6-64.8)	62.9 (60.7-65.1)	63.2 (60.9-65.5)	63.4 (61.1-65.8)	63.6 (61.3-66.1)	63.8 (61.5-66.2)	64.0 (61.6-66.5)	64.2 (61.7-66.8)	64.4 (61.9-67.1)	64.6 (62.0-67.3)
Northern Mariana Islands	69.5 (66.8-72.1)	69.6 (67.1-72.2)	69.8 (67.3-72.4)	69.9 (67.5-72.3)	70.0 (67.7-72.4)	70.2 (67.9-72.5)	70.3 (68.2-72.5)	70.5 (68.5-72.6)	70.7 (68.7-72.7)	70.9 (69.0-72.8)	71.5 (69.7-73.3)	71.9 (70.1-73.6)	72.3 (70.6-74.0)	72.7 (71.1-74.3)	73.0 (71.5-74.4)	73.2 (71.8-74.6)	73.4 (72.2-74.8)	73.6 (72.5-74.8)	73.7 (72.7-74.8)	74.0 (73.1-75.1)	74.3 (73.4-75.2)	74.4 (73.6-75.3)	74.4 (73.6-75.3)	74.5 (73.6-75.3)	74.5 (73.6-75.3)	74.5 (73.6-75.4)	74.8 (73.9-75.6)	74.9 (74.1-75.7)	74.8 (73.8-75.7)	74.6 (73.6-75.6)	74.6 (73.5-75.6)	74.5 (73.3-75.6)	74.4 (73.1-75.5)	74.3 (72.9-75.5)	74.2 (72.8-75.5)	74.2 (72.7-75.5)
Papua New Guinea	54.3 (50.1-59.1)	54.3 (50.1-59.2)	54.3 (50.1-59.5)	54.4 (50.1-59.6)	54.5 (50.3-59.7)	54.5 (50.4-59.7)	54.6 (50.5-59.8)	54.8 (50.6-60.0)	54.9 (50.9-60.0)	55.1 (51.0-60.1)	55.1 (50.9-60.1)	55.1 (50.8-60.1)	55.3 (50.8-60.4)	55.6 (51.0-60.6)	55.9 (51.2-61.0)	56.1 (51.5-61.3)	56.3 (51.7-61.5)	56.5 (51.9-61.7)	55.7 (51.2-60.7)	56.8 (52.1-62.1)	56.9 (52.3-62.3)	56.9 (52.3-62.4)	57.0 (52.3-62.6)	57.1 (52.2-62.7)	57.1 (52.2-62.5)	57.2 (52.4-62.7)	57.4 (52.5-63.1)	57.6 (52.5-63.3)	58.0 (52.8-63.7)	58.2 (52.9-63.8)	58.4 (53.0-64.0)	58.7 (53.2-64.3)	58.9 (53.3-64.5)	59.4 (53.7-64.8)	59.8 (54.2-65.0)	60.1 (54.6-65.3)
Samoa	63.9 (61.6-66.5)	64.0 (61.6-66.7)	64.1 (61.6-66.9)	64.3 (61.7-67.2)	64.5 (61.8-67.6)	64.8 (61.9-67.9)	65.0 (62.1-68.2)	65.3 (62.2-68.4)	65.5 (62.4-68.7)	65.8 (62.7-69.1)	66.1 (62.9-69.4)	66.1 (62.9-69.3)	66.6 (63.3-69.8)	66.8 (63.6-70.0)	67.1 (63.9-70.1)	67.3 (64.3-70.3)	67.6 (64.6-70.5)	67.9 (64.9-70.7)	68.2 (65.3-70.9)	68.4 (65.6-71.0)	68.7 (66.0-71.0)	68.9 (66.4-71.3)	69.2 (66.8-71.4)	69.5 (67.2-71.6)	69.8 (67.5-71.8)	70.0 (67.9-72.0)	70.2 (68.2-72.1)	70.5 (68.5-72.4)	70.8 (68.9-72.7)	68.6 (66.7-70.4)	71.4 (69.5-73.2)	71.8 (69.7-73.7)	71.7 (69.6-73.7)	72.0 (69.8-74.1)	72.1 (69.8-74.4)	72.2 (69.7-74.5)
Solomon Islands	57.4 (53.2-62.5)	57.7 (53.3-62.6)	57.9 (53.3-62.9)	58.0 (53.3-63.1)	58.2 (53.4-63.2)	58.3 (53.4-63.2)	57.5 (52.9-62.2)	58.6 (53.9-63.3)	58.7 (54.0-63.4)	58.8 (54.1-63.8)	59.0 (54.2-64.1)	58.8 (54.0-63.9)	59.3 (54.6-64.5)	59.6 (54.9-64.6)	59.9 (55.3-64.9)	60.2 (55.5-65.0)	60.4 (55.8-65.2)	60.6 (55.8-65.3)	60.7 (56.1-65.4)	60.8 (56.2-65.6)	60.9 (56.2-65.6)	60.9 (56.3-65.6)	60.8 (56.2-65.7)	60.8 (56.1-65.8)	60.9 (56.0-65.9)	60.9 (56.1-65.9)	61.1 (56.1-66.2)	61.0 (55.8-65.9)	61.4 (56.2-66.5)	61.5 (56.3-66.6)	61.8 (56.6-67.0)	62.0 (56.8-67.3)	62.2 (57.0-67.6)	62.4 (56.9-67.8)	62.5 (56.7-67.7)	62.9 (57.0-68.1)
Tonga	65.6 (62.5-69.3)	65.8 (62.6-69.7)	65.9 (62.9-69.9)	66.2 (63.2-70.1)	66.5 (63.4-70.3)	66.7 (63.8-70.4)	67.0 (64.1-70.6)	67.2 (64.5-70.7)	67.4 (64.8-70.6)	67.5 (65.1-70.5)	67.7 (65.3-70.4)	67.8 (65.5-70.2)	67.9 (65.7-70.0)	67.9 (66.0-69.9)	68.0 (66.2-69.8)	68.0 (66.3-69.7)	68.0 (66.5-69.6)	68.1 (66.7-69.5)	68.2 (66.8-69.5)	67.9 (66.6-69.1)	67.8 (66.5-69.0)	67.6 (66.3-68.9)	67.5 (66.1-68.7)	67.3 (65.8-68.6)	67.1 (65.6-68.6)	67.3 (65.7-68.8)	67.3 (65.7-69.0)	67.4 (65.6-69.1)	67.4 (65.5-69.3)	64.6 (62.7-66.5)	67.7 (65.5-69.8)	67.8 (65.5-70.1)	68.0 (65.5-70.4)	68.1 (65.5-70.6)	68.2 (65.4-70.9)	68.4 (65.5-71.1)
Vanuatu	58.9 (54.4-63.5)	59.1 (54.6-63.8)	59.3 (54.7-64.0)	59.4 (54.8-64.1)	59.6 (54.8-64.3)	59.8 (54.9-64.4)	60.0 (55.1-64.4)	59.2 (54.6-63.7)	60.2 (55.5-64.7)	60.3 (55.6-64.8)	60.4 (55.6-64.9)	60.5 (55.6-65.1)	60.6 (55.6-65.4)	60.7 (55.5-65.7)	60.8 (55.6-65.8)	60.9 (55.5-65.7)	61.0 (55.8-65.7)	59.6 (54.6-64.1)	61.1 (56.0-65.9)	60.5 (55.5-65.3)	61.2 (56.3-66.2)	61.2 (56.4-66.4)	61.2 (56.6-66.5)	61.2 (56.7-66.5)	61.3 (56.4-66.4)	61.4 (56.4-66.4)	61.6 (56.6-66.4)	61.8 (56.6-66.5)	62.0 (56.6-66.7)	62.2 (56.8-66.9)	62.4 (57.0-66.9)	62.6 (57.2-67.1)	62.8 (57.4-67.2)	63.0 (57.9-67.3)	63.1 (57.9-67.2)	63.2 (58.3-67.3)
North Africa and Middle East	57.8 (57.0-58.6)	57.7 (56.7-58.8)	57.8 (56.6-59.0)	58.7 (56.8-60.6)	59.4 (57.8-61.0)	60.1 (58.8-61.5)	60.9 (59.8-62.0)	61.4 (60.3-62.5)	61.3 (59.7-62.9)	63.9 (63.3-64.5)	64.0 (63.3-64.5)	64.2 (63.7-64.8)	64.9 (64.3-65.4)	65.1 (64.6-65.7)	65.3 (64.7-65.8)	65.6 (65.0-66.2)	65.9 (65.3-66.5)	66.2 (65.6-66.8)	66.5 (66.0-67.1)	66.9 (66.3-67.5)	67.4 (66.9-68.0)	67.6 (67.0-68.3)	67.9 (67.3-68.5)	67.8 (67.2-68.4)	68.3 (67.7-69.0)	68.6 (68.0-69.3)	68.8 (68.1-69.5)	69.1 (68.4-69.8)	69.5 (68.8-70.2)	69.8 (69.1-70.5)	70.0 (69.3-70.8)	69.8 (69.1-70.6)	70.0 (69.3-70.8)	70.2 (69.5-71.0)	70.2 (69.4-71.0)	70.1 (69.2-70.9)
North Africa and Middle East	57.8 (57.0-58.6)	57.7 (56.7-58.8)	57.8 (56.6-59.0)	58.7 (56.8-60.6)	59.4 (57.8-61.0)	60.1 (58.8-61.5)	60.9 (59.8-62.0)	61.4 (60.3-62.5)	61.3 (59.7-62.9)	63.9 (63.3-64.5)	64.0 (63.3-64.5)	64.2 (63.7-64.8)	64.9 (64.3-65.4)	65.1 (64.6-65.7)	65.3 (64.7-65.8)	65.6 (65.0-66.2)	65.9 (65.3-66.5)	66.2 (65.6-66.8)	66.5 (66.0-67.1)	66.9 (66.3-67.5)	67.4 (66.9-68.0)	67.6 (67.0-68.3)	67.9 (67.3-68.5)	67.8 (67.2-68.4)	68.3 (67.7-69.0)	68.6 (68.0-69.3)	68.8 (68.1-69.5)	69.1 (68.4-69.8)	69.5 (68.8-70.2)	69.8 (69.1-70.5)	70.0 (69.3-70.8)	69.8 (69.1-70.6)	70.0 (69.3-70.8)	70.2 (69.5-71.0)	70.2 (69.4-71.0)	70.1 (69.2-70.9)
Afghanistan	41.1 (36.3-46.7)	42.1 (37.5-47.3)	41.3 (36.2-47.2)	38.5 (32.1-46.5)	35.1 (27.9-45.7)	37.2 (30.2-46.7)	38.1 (31.2-47.4)	40.6 (34.4-48.8)	43.6 (38.4-49.9)	49.0 (45.1-52.9)	50.1 (46.1-54.1)	49.6 (45.8-53.6)	49.8 (45.9-54.0)	50.0 (46.1-54.1)	49.1 (45.3-53.2)	49.8 (45.9-54.2)	50.3 (46.5-54.8)	49.9 (46.4-54.4)	48.5 (45.1-52.7)	50.5 (46.8-54.7)	50.6 (47.0-54.7)	50.9 (47.4-55.0)	51.7 (48.1-55.8)	52.1 (48.4-56.2)	52.5 (48.8-56.5)	52.7 (48.9-56.7)	52.7 (49.2-56.8)	52.9 (49.2-56.9)	53.4 (49.9-57.3)	53.8 (50.5-57.9)	54.1 (50.6-58.2)	54.5 (50.9-58.6)	54.9 (51.2-59.1)	55.2 (51.5-59.4)	55.1 (51.4-59.2)	53.3 (49.2-57.7)
Algeria	60.3 (59.0-61.5)	61.6 (60.4-62.8)	62.7 (61.6-63.7)	64.0 (62.9-65.0)	65.1 (64.0-66.1)	66.1 (65.1-67.1)	66.5 (65.4-67.5)	67.1 (66.1-68.2)	67.8 (66.7-68.9)	68.3 (67.1-69.4)	68.7 (67.5-69.9)	69.0 (67.8-70.3)	69.3 (68.2-70.6)	69.6 (68.4-70.8)	69.7 (68.6-70.9)	69.9 (68.7-71.2)	70.5 (69.4-71.7)	70.7 (69.5-72.0)	70.8 (69.7-72.0)	71.4 (70.3-72.6)	71.7 (70.6-72.9)	72.0 (70.9-73.2)	72.4 (71.2-73.6)	72.5 (71.3-73.7)	73.1 (71.9-74.4)	73.5 (72.3-74.8)	73.8 (72.7-75.0)	74.1 (73.0-75.2)	74.4 (73.4-75.5)	74.6 (73.6-75.7)	75.0 (74.0-76.1)	75.1 (74.1-76.2)	75.1 (74.1-76.3)	75.2 (74.1-76.5)	75.3 (74.1-76.6)	75.4 (74.1-76.8)
Bahrain	69.6 (67.8-71.7)	70.2 (68.4-72.2)	70.5 (68.8-72.4)	70.4 (68.8-72.4)	70.3 (68.8-72.2)	70.9 (69.3-72.6)	70.6 (69.1-72.3)	70.5 (69.1-72.2)	70.5 (69.1-72.3)	70.9 (69.3-72.5)	71.1 (69.5-72.7)	71.6 (69.9-73.0)	71.7 (70.0-73.1)	72.1 (70.3-73.6)	72.3 (70.5-73.7)	72.1 (70.7-73.5)	72.1 (70.9-73.2)	72.3 (71.3-73.4)	72.5 (71.5-73.4)	72.7 (71.7-73.7)	71.9 (70.7-73.1)	72.8 (71.8-74.0)	73.2 (72.3-74.3)	73.8 (72.9-74.8)	74.2 (73.3-75.1)	74.6 (73.8-75.6)	74.8 (73.9-75.7)	75.6 (74.8-76.8)	76.4 (75.4-77.7)	77.2 (76.0-78.5)	77.9 (76.7-79.1)	78.1 (76.9-79.2)	78.3 (77.0-79.6)	78.3 (76.8-79.7)	78.3 (76.6-80.0)	78.2 (76.5-80.0)
Egypt	56.5 (55.9-57.0)	56.8 (56.3-57.4)	57.4 (56.8-57.9)	58.1 (57.6-58.6)	59.0 (58.6-59.4)	58.8 (58.4-59.2)	59.6 (59.1-60.0)	59.9 (59.3-60.5)	62.0 (61.6-62.3)	62.5 (62.1-62.8)	63.1 (62.7-63.4)	63.2 (62.7-63.6)	63.8 (63.3-64.3)	64.3 (64.0-64.6)	64.5 (64.1-64.8)	65.1 (64.8-65.4)	65.6 (65.4-65.9)	66.0 (65.7-66.2)	66.4 (66.1-66.6)	66.6 (66.4-66.9)	67.2 (67.0-67.5)	67.2 (67.0-67.5)	67.2 (67.0-67.5)	67.3 (67.0-67.5)	67.4 (67.2-67.7)	67.7 (67.4-67.9)	67.6 (67.3-67.8)	67.8 (67.5-68.0)	67.7 (67.5-68.0)	67.6 (67.3-67.9)	67.7 (67.4-67.9)	67.7 (67.5-68.0)	68.0 (67.7-68.2)	68.4 (68.1-68.6)	68.6 (68.2-69.0)	68.7 (68.1-69.3)
Iran	60.0 (57.5-62.4)	58.0 (54.0-61.8)	58.8 (55.0-62.8)	59.9 (55.8-64.1)	62.0 (58.9-65.2)	63.0 (60.2-65.9)	62.9 (60.2-65.9)	63.1 (60.1-66.1)	62.8 (60.0-65.8)	65.5 (63.4-67.5)	63.2 (60.8-65.5)	65.3 (63.4-67.1)	66.0 (64.1-68.0)	66.5 (64.5-68.7)	67.0 (64.9-69.1)	67.3 (65.2-69.3)	67.0 (65.1-68.7)	67.8 (66.1-69.2)	68.3 (66.7-69.6)	68.9 (67.3-70.2)	68.8 (67.2-70.2)	68.7 (67.1-70.3)	68.8 (67.2-70.5)	67.5 (65.4-69.7)	69.1 (67.0-71.3)	69.6 (67.2-72.0)	70.1 (67.4-72.7)	70.5 (67.6-73.3)	70.8 (67.9-73.7)	71.2 (68.4-74.2)	71.4 (68.6-74.4)	71.7 (69.1-74.6)	71.8 (69.3-74.5)	71.8 (69.4-74.5)	71.9 (69.2-74.4)	71.9 (69.2-74.2)
Iraq	57.4 (52.6-62.6)	51.1 (43.1-61.2)	51.5 (44.1-61.7)	51.8 (43.8-62.2)	55.3 (49.1-62.4)	55.7 (49.5-63.1)	55.9 (49.7-63.3)	56.1 (50.1-62.9)	41.8 (31.6-59.3)	64.2 (61.2-67.2)	64.3 (61.1-67.3)	61.7 (58.5-64.6)	63.9 (60.7-66.9)	63.9 (60.8-66.9)	63.7 (60.6-66.7)	63.6 (60.7-66.4)	63.8 (60.9-66.7)	63.5 (60.8-66.4)	64.1 (61.4-67.0)	64.3 (61.6-67.3)	64.0 (61.3-66.9)	64.7 (61.8-67.7)	64.2 (61.2-67.2)	64.1 (61.0-67.1)	64.3 (61.2-67.4)	63.3 (60.0-66.5)	62.3 (58.8-65.9)	62.4 (59.0-66.1)	64.3 (60.8-67.8)	64.8 (61.4-68.5)	65.1 (61.6-68.9)	65.5 (62.1-69.5)	65.8 (62.3-69.8)	65.9 (62.4-69.9)	64.9 (61.4-68.7)	64.3 (60.8-68.5)
Jordan	63.5 (61.8-64.9)	64.3 (62.5-65.9)	64.9 (63.1-66.7)	66.0 (64.1-67.8)	66.7 (64.9-68.6)	67.5 (65.7-69.5)	68.2 (66.3-70.1)	68.8 (66.8-70.7)	69.8 (67.5-71.5)	70.2 (67.8-72.0)	70.7 (68.3-72.6)	71.3 (69.0-73.2)	71.6 (69.3-73.5)	71.8 (69.4-73.7)	72.0 (69.3-73.8)	72.3 (69.7-74.1)	72.6 (70.3-74.4)	72.9 (71.0-74.6)	73.2 (71.4-74.8)	73.5 (71.8-75.0)	73.7 (72.2-75.2)	74.0 (72.6-75.3)	74.3 (73.0-75.5)	74.4 (73.3-75.5)	74.6 (73.7-75.5)	74.7 (73.9-75.4)	75.3 (74.7-76.0)	75.7 (75.2-76.2)	75.9 (75.4-76.4)	76.2 (75.8-76.6)	76.4 (76.0-76.8)	76.5 (76.1-76.9)	76.5 (75.9-77.0)	76.5 (75.7-77.2)	76.5 (75.4-77.5)	76.5 (75.1-77.6)
Kuwait	71.3 (71.0-71.6)	72.0 (71.8-72.3)	72.1 (71.8-72.3)	72.6 (72.3-72.9)	73.2 (72.9-73.6)	73.5 (73.2-73.8)	74.5 (74.2-74.8)	75.1 (74.8-75.4)	76.0 (75.8-76.3)	76.6 (76.3-76.9)	76.0 (74.6-77.4)	57.9 (54.6-61.7)	79.1 (78.8-79.3)	77.7 (77.4-78.0)	76.7 (76.4-77.0)	75.6 (75.3-75.9)	75.2 (74.9-75.5)	75.4 (75.1-75.7)	75.5 (75.3-75.8)	76.4 (76.2-76.7)	76.9 (76.7-77.2)	77.0 (76.7-77.2)	77.6 (77.4-77.9)	77.6 (77.4-77.9)	76.9 (76.6-77.2)	76.3 (76.0-76.5)	76.2 (76.0-76.5)	76.7 (76.5-77.0)	76.5 (76.2-76.7)	76.8 (76.5-77.2)	78.3 (77.7-78.6)	79.1 (78.9-79.3)	79.0 (78.8-79.3)	79.3 (79.1-79.5)	79.5 (78.7-80.2)	79.4 (77.9-80.7)
Lebanon	44.4 (36.0-57.7)	44.6 (36.2-57.7)	35.9 (25.8-59.2)	58.8 (52.2-66.2)	59.0 (52.5-66.5)	59.3 (52.8-66.6)	59.5 (53.0-66.8)	45.7 (37.1-58.9)	45.8 (37.2-59.1)	66.9 (64.2-69.3)	66.2 (63.6-68.5)	69.9 (67.3-72.1)	70.5 (67.9-72.4)	71.0 (68.5-72.8)	71.6 (69.2-73.3)	72.5 (70.6-74.2)	73.0 (71.2-74.7)	73.5 (71.8-75.3)	74.2 (72.3-76.0)	74.7 (72.7-76.6)	75.3 (73.3-77.3)	75.9 (73.7-77.9)	76.2 (73.9-78.4)	76.6 (74.2-78.9)	76.9 (74.3-79.3)	77.0 (74.2-79.6)	77.0 (74.1-79.7)	76.9 (74.0-79.7)	77.3 (74.3-80.1)	77.3 (74.4-80.2)	77.4 (74.4-80.4)	75.0 (71.8-78.3)	77.7 (74.7-80.7)	77.7 (74.7-80.6)	77.8 (75.0-80.7)	77.9 (75.1-80.9)
Libya	69.4 (68.2-70.5)	69.5 (68.3-70.7)	69.9 (68.6-71.2)	70.3 (68.9-71.6)	70.5 (69.1-71.8)	70.9 (69.4-72.3)	71.2 (69.6-72.7)	67.0 (63.3-70.8)	71.6 (70.1-73.1)	71.7 (70.1-73.2)	72.2 (70.7-73.8)	72.6 (71.1-74.1)	72.7 (71.2-74.2)	73.1 (71.7-74.6)	73.4 (72.0-74.9)	73.6 (72.2-74.9)	73.5 (72.2-74.8)	73.8 (72.5-75.0)	73.8 (72.6-75.0)	73.8 (72.7-74.9)	73.6 (72.6-74.8)	73.8 (72.8-74.8)	73.7 (72.8-74.7)	73.8 (72.9-74.7)	73.8 (72.9-74.7)	73.9 (73.1-74.8)	74.0 (73.1-74.8)	74.0 (73.1-74.9)	74.0 (73.1-74.9)	74.1 (73.0-75.1)	74.3 (73.1-75.4)	53.3 (44.7-65.9)	73.6 (72.1-75.0)	73.9 (72.3-75.5)	72.7 (70.9-74.4)	71.4 (68.9-73.8)
Morocco	59.3 (57.2-61.4)	60.1 (58.0-62.1)	61.1 (59.2-63.1)	61.9 (60.0-63.8)	62.6 (60.8-64.5)	63.3 (61.5-65.1)	63.9 (62.2-65.8)	64.4 (62.7-66.1)	65.0 (63.3-66.7)	65.5 (63.8-67.2)	66.2 (64.5-67.9)	66.6 (64.8-68.3)	67.0 (65.1-68.7)	67.2 (65.4-68.9)	67.8 (65.8-69.5)	68.1 (66.2-69.9)	68.7 (66.8-70.4)	69.0 (67.1-70.8)	69.4 (67.3-71.3)	69.7 (67.7-71.7)	70.1 (68.0-72.2)	70.5 (68.2-72.6)	70.9 (68.3-73.0)	71.2 (68.6-73.4)	71.3 (68.6-73.6)	71.6 (68.7-74.1)	71.8 (68.7-74.4)	72.0 (68.7-74.7)	72.2 (68.9-75.1)	72.4 (68.8-75.3)	72.5 (68.8-75.7)	72.7 (68.9-75.9)	72.8 (69.1-76.1)	73.0 (69.2-76.4)	73.2 (69.3-76.5)	73.3 (69.5-76.5)
Palestine	67.3 (64.1-70.6)	67.7 (64.5-70.9)	34.5 (26.1-51.6)	68.6 (65.3-71.6)	69.1 (65.7-72.1)	69.5 (66.0-72.5)	69.8 (66.2-72.7)	69.5 (65.9-72.4)	69.6 (66.2-72.5)	69.7 (66.3-72.6)	69.9 (66.5-72.6)	70.1 (66.8-72.7)	70.4 (67.3-73.0)	70.8 (67.7-73.3)	71.4 (68.4-73.8)	71.9 (69.0-74.1)	72.1 (69.7-74.2)	72.3 (70.3-74.1)	72.5 (71.0-74.2)	72.7 (71.3-74.2)	72.1 (70.7-73.5)	72.3 (71.1-73.5)	71.8 (70.5-73.0)	71.7 (70.5-72.9)	71.8 (70.5-72.8)	72.1 (70.5-73.1)	71.6 (69.7-72.8)	71.4 (69.4-72.8)	71.2 (69.2-72.8)	70.7 (68.7-72.6)	71.4 (69.2-73.4)	71.3 (69.0-73.5)	71.2 (68.7-73.6)	71.4 (68.7-74.0)	71.5 (68.7-74.3)	71.6 (68.7-74.5)
Oman	63.6 (60.0-67.2)	64.5 (60.9-68.2)	65.5 (61.8-69.0)	66.4 (62.7-70.0)	67.2 (63.4-70.9)	68.0 (64.3-71.7)	68.6 (65.2-72.3)	69.2 (65.9-72.8)	69.6 (66.3-73.2)	70.1 (66.8-73.5)	70.6 (67.3-74.0)	71.0 (67.6-74.4)	71.5 (67.9-74.6)	71.8 (68.2-74.9)	72.2 (68.5-75.1)	72.5 (69.0-75.2)	72.8 (69.6-75.4)	73.1 (70.2-75.5)	73.3 (70.7-75.7)	73.4 (71.0-75.7)	73.5 (71.4-75.7)	73.5 (71.6-75.5)	73.4 (71.8-75.3)	73.6 (72.2-75.1)	73.8 (72.6-75.1)	74.1 (72.9-75.2)	74.1 (73.1-75.1)	74.0 (73.2-75.0)	74.2 (73.4-75.1)	74.4 (73.4-75.3)	74.5 (73.4-75.5)	74.7 (73.5-75.8)	75.0 (73.8-76.4)	75.3 (73.9-76.9)	75.5 (74.0-77.3)	75.7 (74.1-77.7)
Qatar	66.7 (64.4-69.1)	66.7 (64.7-68.6)	67.5 (65.7-69.2)	68.2 (66.6-70.2)	70.3 (68.4-72.1)	71.4 (69.6-73.0)	72.4 (70.9-73.9)	73.1 (71.7-74.4)	73.6 (72.3-75.0)	74.1 (72.8-75.4)	74.4 (73.0-75.7)	74.3 (72.8-75.7)	74.4 (72.9-75.8)	74.4 (72.9-75.9)	74.3 (72.9-75.6)	74.1 (72.7-75.4)	74.1 (72.6-75.4)	74.1 (72.6-75.5)	74.1 (72.6-75.6)	74.1 (72.6-75.5)	74.1 (72.7-75.6)	74.2 (72.8-75.7)	74.1 (72.7-75.6)	74.3 (72.9-75.8)	74.8 (73.3-76.3)	75.2 (73.6-76.9)	75.7 (74.0-77.1)	76.3 (74.8-77.7)	77.0 (75.6-78.4)	77.7 (76.3-78.9)	78.5 (77.2-79.8)	79.2 (77.8-80.5)	79.5 (78.1-80.8)	79.4 (77.7-81.0)	79.3 (77.3-81.0)	79.1 (76.9-81.2)
Saudi Arabia	69.8 (68.0-71.6)	70.4 (68.7-72.1)	70.7 (69.1-72.4)	71.0 (69.4-72.7)	71.3 (69.6-72.9)	71.5 (69.9-73.1)	71.7 (70.1-73.3)	72.0 (70.4-73.5)	72.2 (70.6-73.7)	72.3 (70.8-73.7)	72.1 (69.7-73.8)	72.7 (71.2-74.0)	73.0 (71.6-74.3)	73.3 (72.0-74.6)	73.5 (72.3-74.7)	73.8 (72.6-75.0)	74.1 (73.0-75.2)	74.3 (73.3-75.4)	74.6 (73.7-75.5)	74.9 (74.0-75.7)	75.2 (74.3-75.9)	75.5 (74.7-76.2)	75.6 (74.9-76.3)	75.7 (75.0-76.3)	75.8 (75.0-76.4)	75.9 (75.3-76.6)	76.0 (75.3-76.7)	76.3 (75.6-76.9)	76.4 (75.8-77.1)	76.6 (75.9-77.2)	76.7 (76.0-77.4)	76.9 (76.2-77.7)	77.2 (76.4-78.0)	77.3 (76.4-78.2)	77.4 (76.4-78.4)	77.4 (76.3-78.5)
Sudan	54.0 (51.7-56.2)	54.6 (52.4-56.9)	55.0 (52.9-57.2)	49.9 (42.4-55.1)	50.2 (43.2-55.3)	50.7 (43.7-55.6)	55.8 (53.5-58.1)	56.3 (53.9-58.8)	56.9 (54.4-59.5)	56.8 (54.1-59.6)	57.5 (55.0-60.3)	57.9 (55.2-60.8)	58.0 (55.2-61.1)	58.0 (55.0-61.2)	59.0 (56.0-62.3)	59.2 (56.0-62.6)	59.2 (55.9-62.7)	59.4 (55.6-62.9)	59.7 (55.9-63.4)	60.6 (56.7-64.3)	60.9 (57.0-64.7)	61.4 (57.4-65.0)	61.8 (58.0-65.7)	61.9 (58.2-65.9)	62.1 (58.2-66.1)	63.1 (59.4-67.2)	63.3 (59.6-67.4)	63.7 (60.2-67.7)	64.0 (60.4-68.0)	64.1 (60.4-68.2)	64.5 (60.6-68.5)	64.6 (60.7-68.7)	65.0 (61.0-69.1)	65.2 (61.2-69.1)	65.5 (61.4-69.6)	65.8 (61.6-69.9)
Syria	64.4 (63.0-65.7)	64.9 (63.5-66.3)	60.2 (56.3-64.8)	66.2 (64.6-67.6)	66.7 (65.1-68.1)	67.0 (65.4-68.5)	67.3 (65.5-68.9)	67.7 (65.9-69.2)	68.1 (66.2-69.6)	68.5 (66.5-70.0)	68.9 (66.8-70.5)	69.5 (67.2-71.2)	70.2 (67.9-71.9)	70.4 (67.9-72.2)	70.7 (68.0-72.5)	71.1 (68.3-72.9)	71.4 (68.7-73.2)	71.7 (69.0-73.5)	72.1 (69.6-73.8)	72.4 (70.1-74.0)	72.7 (70.6-74.2)	72.9 (71.2-74.3)	73.1 (71.7-74.4)	73.4 (72.1-74.5)	73.6 (72.6-74.6)	73.9 (73.1-74.7)	74.0 (73.4-74.7)	74.2 (73.7-74.8)	74.4 (73.9-75.0)	74.5 (74.0-75.1)	74.7 (74.0-75.4)	72.5 (70.9-74.2)	62.6 (56.5-70.1)	63.7 (58.2-70.3)	61.3 (53.3-71.5)	62.6 (56.5-70.2)
Tunisia	63.9 (62.9-64.8)	65.2 (64.2-66.1)	66.1 (65.0-67.0)	66.9 (65.8-67.9)	67.5 (66.6-68.6)	68.1 (67.1-69.1)	68.4 (67.5-69.4)	68.8 (67.8-69.7)	69.2 (68.2-70.1)	69.4 (68.5-70.3)	69.7 (68.7-70.6)	69.9 (69.0-70.8)	70.0 (69.0-70.9)	70.0 (69.1-70.9)	70.2 (69.3-71.2)	70.6 (69.6-71.6)	71.0 (69.9-72.2)	71.5 (70.4-72.7)	71.9 (70.7-73.1)	72.2 (71.0-73.6)	72.5 (71.2-73.9)	72.8 (71.3-74.2)	73.0 (71.4-74.5)	73.1 (71.5-74.8)	73.4 (71.6-75.1)	73.7 (71.8-75.4)	73.8 (72.0-75.7)	74.0 (72.0-75.9)	74.2 (72.0-76.2)	74.3 (72.0-76.3)	74.4 (72.0-76.5)	74.4 (71.8-76.5)	74.6 (71.9-76.7)	74.7 (72.0-76.9)	74.7 (72.1-77.1)	74.6 (72.0-77.0)
Turkey	59.5 (58.2-60.7)	60.2 (58.9-61.4)	61.2 (59.9-62.4)	61.5 (60.3-62.7)	61.5 (60.4-62.7)	62.6 (61.4-63.7)	63.2 (62.0-64.4)	63.7 (62.6-64.9)	64.2 (63.0-65.4)	64.3 (63.2-65.4)	64.9 (63.8-66.0)	65.3 (64.3-66.4)	65.9 (64.9-66.8)	66.3 (65.4-67.3)	66.3 (65.5-67.3)	66.6 (65.7-67.6)	67.2 (66.3-68.2)	67.7 (66.9-68.6)	68.6 (67.8-69.5)	68.1 (67.0-69.2)	70.3 (69.5-71.1)	70.9 (70.1-71.7)	71.6 (70.8-72.5)	71.8 (71.0-72.6)	72.4 (71.7-73.2)	72.9 (72.3-73.6)	73.4 (72.8-74.0)	74.0 (73.5-74.5)	74.5 (74.0-75.0)	75.1 (74.6-75.6)	75.4 (74.9-75.8)	75.5 (75.0-76.0)	75.8 (75.2-76.2)	75.5 (75.0-76.0)	75.8 (75.2-76.3)	75.9 (75.2-76.5)
United Arab Emirates	68.2 (64.1-71.9)	68.6 (64.6-72.3)	69.0 (65.1-72.6)	68.9 (65.1-72.4)	69.4 (65.7-73.0)	69.5 (65.9-73.0)	69.5 (66.2-73.1)	69.6 (66.4-73.1)	69.6 (66.6-72.8)	69.7 (66.9-72.7)	69.8 (67.1-72.8)	70.0 (67.6-72.7)	70.2 (68.0-72.6)	70.4 (68.4-72.5)	70.6 (68.8-72.5)	70.9 (69.3-72.5)	71.2 (69.8-72.7)	71.4 (70.1-72.8)	71.8 (70.6-73.2)	72.1 (70.9-73.4)	72.3 (71.2-73.6)	72.6 (71.5-73.8)	72.8 (71.7-73.9)	73.0 (72.1-73.9)	73.2 (72.5-73.9)	74.2 (73.3-74.9)	74.7 (73.8-75.5)	74.7 (73.6-75.5)	74.7 (73.4-75.8)	74.6 (73.1-75.9)	74.6 (72.8-76.1)	74.6 (72.6-76.3)	74.6 (72.4-76.5)	74.6 (72.2-76.7)	74.5 (72.0-76.9)	74.5 (71.8-77.1)
Yemen	51.4 (47.1-56.4)	52.3 (48.0-57.2)	52.5 (48.4-57.3)	54.1 (50.0-59.1)	54.9 (50.9-59.8)	55.5 (51.5-60.4)	52.3 (48.7-56.5)	56.7 (52.4-61.6)	57.3 (52.9-62.2)	57.7 (53.3-62.7)	58.3 (53.7-63.3)	58.7 (54.1-63.6)	59.1 (54.4-64.0)	59.5 (54.8-64.4)	59.5 (54.6-64.2)	60.2 (55.2-64.8)	60.6 (55.7-65.1)	61.1 (56.1-65.6)	61.4 (56.4-66.1)	61.9 (57.0-66.7)	62.4 (57.4-67.3)	62.8 (57.8-67.9)	63.2 (58.3-68.6)	63.6 (58.7-69.0)	64.1 (59.1-69.5)	64.4 (59.2-69.9)	64.9 (59.6-70.3)	65.0 (59.6-70.5)	65.4 (59.9-70.9)	66.0 (60.3-71.4)	66.4 (60.6-71.7)	66.3 (60.6-71.6)	66.4 (60.6-71.6)	66.8 (61.1-72.1)	66.7 (61.0-71.9)	65.4 (59.7-70.2)
South Asia	54.2 (53.6-54.8)	54.7 (54.1-55.3)	55.1 (54.6-55.7)	55.5 (55.0-56.1)	55.9 (55.3-56.5)	56.3 (55.7-56.8)	56.7 (56.1-57.3)	57.1 (56.5-57.7)	57.4 (56.9-58.0)	57.8 (57.2-58.4)	58.1 (57.5-58.7)	58.1 (57.5-58.7)	58.7 (58.2-59.3)	59.0 (58.4-59.6)	59.3 (58.7-59.9)	59.6 (59.0-60.1)	59.8 (59.3-60.4)	60.1 (59.5-60.6)	60.3 (59.8-60.9)	60.6 (60.1-61.2)	60.9 (60.5-61.5)	61.2 (60.7-61.8)	61.6 (61.1-62.1)	61.9 (61.4-62.4)	62.3 (61.7-62.8)	62.4 (61.9-63.0)	62.9 (62.3-63.4)	63.1 (62.5-63.6)	63.4 (62.8-63.9)	63.7 (63.1-64.2)	63.9 (63.3-64.5)	64.2 (63.6-64.8)	64.5 (63.9-65.1)	64.8 (64.1-65.4)	65.1 (64.5-65.8)	65.4 (64.7-66.1)
South Asia	54.2 (53.6-54.8)	54.7 (54.1-55.3)	55.1 (54.6-55.7)	55.5 (55.0-56.1)	55.9 (55.3-56.5)	56.3 (55.7-56.8)	56.7 (56.1-57.3)	57.1 (56.5-57.7)	57.4 (56.9-58.0)	57.8 (57.2-58.4)	58.1 (57.5-58.7)	58.1 (57.5-58.7)	58.7 (58.2-59.3)	59.0 (58.4-59.6)	59.3 (58.7-59.9)	59.6 (59.0-60.1)	59.8 (59.3-60.4)	60.1 (59.5-60.6)	60.3 (59.8-60.9)	60.6 (60.1-61.2)	60.9 (60.5-61.5)	61.2 (60.7-61.8)	61.6 (61.1-62.1)	61.9 (61.4-62.4)	62.3 (61.7-62.8)	62.4 (61.9-63.0)	62.9 (62.3-63.4)	63.1 (62.5-63.6)	63.4 (62.8-63.9)	63.7 (63.1-64.2)	63.9 (63.3-64.5)	64.2 (63.6-64.8)	64.5 (63.9-65.1)	64.8 (64.1-65.4)	65.1 (64.5-65.8)	65.4 (64.7-66.1)
Bangladesh	53.3 (51.6-55.0)	53.6 (52.0-55.2)	54.0 (52.4-55.5)	54.3 (52.8-55.7)	54.7 (53.3-56.0)	54.6 (53.3-55.9)	55.6 (54.3-56.9)	56.2 (54.9-57.5)	56.4 (55.2-57.7)	57.3 (56.0-58.6)	57.5 (56.1-58.7)	55.2 (53.4-57.3)	59.1 (57.8-60.5)	59.8 (58.4-61.1)	60.4 (59.0-61.8)	60.8 (59.5-62.2)	61.4 (60.1-62.9)	62.2 (60.9-63.6)	62.8 (61.5-64.2)	63.5 (62.2-64.9)	63.7 (62.5-65.1)	64.2 (63.0-65.5)	64.6 (63.4-65.8)	65.0 (63.8-66.1)	65.4 (64.3-66.5)	65.7 (64.6-66.8)	65.9 (64.9-67.0)	66.1 (65.0-67.1)	66.6 (65.5-67.7)	67.0 (65.8-68.2)	67.2 (65.9-68.5)	67.6 (66.1-68.9)	67.7 (66.0-69.1)	67.9 (66.2-69.5)	68.2 (66.4-70.0)	68.5 (66.5-70.3)
Bhutan	57.1 (53.8-60.1)	57.5 (54.2-60.5)	57.9 (54.8-60.9)	58.4 (55.3-61.5)	58.9 (56.0-62.0)	59.4 (56.5-62.4)	59.9 (57.0-62.8)	60.4 (57.7-63.2)	60.9 (58.2-63.6)	61.4 (58.8-64.0)	61.9 (59.3-64.5)	62.4 (59.9-64.9)	62.8 (60.5-65.3)	63.3 (61.0-65.8)	63.5 (61.3-65.9)	64.2 (62.0-66.5)	65.2 (63.0-67.3)	65.5 (63.2-67.5)	65.9 (63.7-67.9)	66.6 (64.4-68.6)	65.6 (63.5-67.6)	67.2 (65.0-69.1)	67.7 (65.4-69.5)	67.9 (65.6-69.8)	68.3 (66.1-70.3)	68.7 (66.4-70.7)	69.1 (66.7-71.1)	69.4 (67.1-71.5)	69.8 (67.4-71.9)	69.8 (67.6-72.0)	70.3 (67.9-72.5)	70.6 (68.2-72.9)	70.8 (68.4-73.2)	71.0 (68.5-73.5)	71.2 (68.8-73.9)	71.5 (69.1-74.2)
India	53.9 (53.2-54.6)	54.4 (53.7-55.1)	54.9 (54.2-55.6)	55.3 (54.6-56.0)	55.7 (55.0-56.4)	56.1 (55.4-56.7)	56.5 (55.8-57.1)	56.8 (56.2-57.5)	57.2 (56.5-57.8)	57.5 (56.9-58.2)	57.8 (57.2-58.5)	58.1 (57.4-58.7)	58.3 (57.7-59.0)	58.6 (58.0-59.3)	58.9 (58.3-59.6)	59.2 (58.6-59.8)	59.5 (58.9-60.0)	59.7 (59.1-60.3)	59.9 (59.4-60.5)	60.2 (59.7-60.8)	60.5 (60.0-61.1)	60.8 (60.3-61.4)	61.2 (60.7-61.8)	61.5 (61.0-62.1)	61.9 (61.3-62.5)	62.2 (61.7-62.8)	62.5 (61.9-63.1)	62.8 (62.2-63.4)	63.1 (62.5-63.7)	63.4 (62.8-64.0)	63.7 (63.0-64.3)	64.0 (63.3-64.7)	64.2 (63.5-64.9)	64.5 (63.8-65.3)	64.9 (64.1-65.6)	65.2 (64.5-66.0)
Nepal	53.1 (50.5-55.7)	53.4 (51.0-56.0)	54.0 (51.5-56.6)	54.5 (51.9-57.0)	54.9 (52.4-57.4)	55.5 (52.9-58.0)	56.0 (53.4-58.6)	56.5 (54.1-59.1)	57.0 (54.6-59.3)	57.6 (55.4-59.9)	58.2 (56.0-60.4)	58.8 (56.7-60.9)	59.7 (57.8-61.7)	59.7 (57.9-61.7)	60.4 (58.7-62.3)	60.9 (59.1-62.6)	61.6 (59.9-63.2)	62.0 (60.6-63.5)	62.3 (61.1-63.8)	63.1 (62.0-64.4)	63.4 (62.4-64.7)	64.3 (63.3-65.5)	64.2 (63.0-65.4)	65.3 (64.0-66.7)	65.5 (64.1-67.0)	66.0 (64.4-67.7)	66.4 (64.6-68.2)	66.7 (64.8-68.7)	67.0 (64.9-69.1)	67.3 (65.1-69.6)	67.6 (65.3-69.9)	67.9 (65.5-70.3)	68.2 (65.6-70.7)	68.5 (65.8-71.2)	68.7 (65.9-71.6)	68.1 (65.2-70.9)
Pakistan	59.7 (58.1-61.4)	60.0 (58.4-61.7)	60.3 (58.7-61.9)	60.5 (59.0-62.1)	60.8 (59.3-62.4)	61.1 (59.6-62.7)	61.5 (59.8-63.1)	61.6 (59.9-63.1)	61.6 (59.9-63.2)	61.8 (60.0-63.4)	61.9 (60.2-63.6)	62.0 (60.3-63.7)	62.0 (60.3-63.7)	61.9 (60.3-63.7)	61.9 (60.2-63.7)	61.7 (60.0-63.6)	61.7 (60.0-63.5)	61.6 (59.8-63.3)	61.7 (59.9-63.4)	61.8 (60.0-63.6)	61.9 (60.1-63.8)	62.1 (60.2-64.0)	62.2 (60.4-64.2)	62.3 (60.6-64.3)	62.5 (60.8-64.4)	61.3 (59.6-63.2)	62.9 (61.0-64.7)	62.7 (60.7-64.6)	62.9 (60.9-64.9)	63.1 (61.0-65.2)	63.3 (61.2-65.4)	63.6 (61.5-65.8)	63.9 (61.7-65.9)	64.1 (61.8-66.2)	64.3 (62.0-66.4)	64.5 (62.2-66.5)
Sub-Saharan Africa	50.5 (48.6-52.1)	50.7 (48.8-52.2)	50.9 (49.3-52.4)	50.7 (48.8-52.3)	50.8 (49.0-52.3)	51.5 (49.9-52.8)	51.8 (50.3-53.0)	52.0 (50.7-53.2)	52.2 (50.9-53.4)	52.3 (51.0-53.4)	52.0 (50.8-53.1)	52.3 (51.1-53.3)	52.4 (51.2-53.3)	52.3 (51.3-53.3)	49.8 (47.2-52.2)	52.3 (51.5-53.1)	52.1 (51.3-52.9)	52.1 (51.3-52.8)	52.1 (51.3-52.7)	52.0 (51.3-52.7)	52.1 (51.4-52.8)	52.6 (51.9-53.4)	52.9 (52.2-53.6)	53.2 (52.5-53.9)	53.7 (52.9-54.4)	54.3 (53.5-55.1)	54.8 (54.0-55.7)	55.5 (54.6-56.4)	56.2 (55.2-57.2)	56.8 (55.8-57.8)	57.4 (56.4-58.5)	58.0 (56.8-59.2)	58.6 (57.2-59.9)	59.1 (57.7-60.5)	59.5 (58.0-61.0)	60.0 (58.4-61.6)
Southern Sub-Saharan Africa	55.9 (54.0-57.9)	56.3 (54.6-58.2)	56.8 (55.2-58.5)	57.1 (55.6-58.6)	57.9 (56.5-59.3)	58.2 (56.9-59.5)	58.8 (57.4-60.0)	59.6 (58.3-60.7)	59.8 (58.7-60.8)	60.2 (59.1-61.3)	60.1 (59.1-61.2)	60.2 (59.1-61.3)	60.0 (58.9-61.1)	59.9 (58.6-61.0)	59.3 (58.1-60.4)	58.3 (57.1-59.5)	57.1 (55.9-58.3)	55.6 (54.4-56.8)	54.4 (53.2-55.6)	53.5 (52.2-54.7)	52.4 (51.1-53.7)	51.9 (50.6-53.2)	51.1 (49.8-52.4)	50.4 (49.1-51.7)	49.9 (48.7-51.2)	49.9 (48.7-51.1)	49.8 (48.8-51.1)	50.3 (49.2-51.5)	50.5 (49.5-51.7)	51.0 (49.9-52.2)	51.9 (50.7-53.1)	53.2 (52.0-54.4)	54.8 (53.7-55.9)	56.1 (54.8-57.2)	56.6 (55.3-57.9)	57.5 (56.0-58.9)
Botswana	57.4 (46.9-69.1)	57.8 (47.6-69.3)	58.2 (48.0-69.6)	58.6 (48.1-69.8)	58.9 (48.2-70.1)	59.2 (48.2-70.2)	59.5 (48.1-70.2)	59.8 (48.1-70.2)	59.9 (48.5-70.1)	59.9 (48.5-70.1)	59.6 (48.5-69.9)	59.2 (47.8-69.6)	58.3 (46.9-69.1)	57.2 (45.8-68.4)	55.7 (44.4-67.5)	53.9 (42.9-66.3)	52.1 (41.4-65.1)	50.4 (40.2-63.6)	48.7 (38.9-62.1)	47.4 (37.9-60.7)	46.1 (37.2-59.1)	45.2 (36.6-58.1)	44.6 (36.2-56.9)	44.7 (36.5-56.8)	45.8 (37.2-57.9)	47.9 (38.8-60.2)	49.7 (39.6-62.1)	50.9 (40.3-63.4)	51.5 (41.1-63.7)	52.5 (41.6-64.5)	53.3 (42.0-65.0)	54.1 (42.4-65.5)	54.2 (42.1-65.6)	54.1 (42.5-65.1)	54.3 (42.9-65.1)	55.8 (44.0-66.6)
Lesotho	54.8 (47.6-63.5)	55.0 (48.0-63.4)	55.2 (48.3-63.3)	55.4 (48.5-63.3)	55.6 (48.8-62.9)	55.8 (49.4-62.8)	56.0 (50.2-62.4)	56.1 (51.1-61.7)	56.4 (51.8-61.4)	56.5 (52.4-61.1)	56.5 (52.8-60.5)	56.5 (53.2-60.2)	56.2 (53.3-59.6)	56.0 (53.2-59.3)	55.0 (52.2-58.3)	54.6 (51.7-58.0)	53.7 (50.8-57.5)	52.2 (49.2-56.1)	50.5 (47.6-54.5)	49.0 (46.1-52.8)	47.5 (44.5-51.1)	45.9 (43.1-49.6)	44.4 (41.7-47.9)	43.2 (40.6-46.6)	42.1 (39.5-45.4)	41.2 (38.7-44.6)	41.0 (38.5-44.3)	41.2 (38.5-44.7)	42.2 (39.0-46.1)	43.0 (39.3-47.4)	43.4 (39.3-48.4)	43.6 (39.2-49.3)	43.5 (38.9-49.6)	43.0 (38.2-49.7)	42.8 (37.8-50.0)	44.1 (38.6-51.8)
Namibia	57.0 (53.4-60.6)	57.0 (53.7-60.5)	57.0 (53.8-60.3)	57.3 (54.3-60.5)	57.7 (54.8-60.7)	57.9 (55.3-60.8)	58.2 (55.9-61.0)	58.6 (56.4-61.4)	58.5 (56.4-61.4)	58.6 (56.4-61.3)	58.5 (56.4-61.0)	58.8 (56.8-61.4)	58.9 (56.7-61.6)	59.1 (56.7-61.9)	58.6 (56.2-61.5)	57.7 (55.1-60.4)	56.4 (53.7-58.9)	55.6 (52.9-58.2)	54.4 (51.6-57.1)	53.3 (50.5-56.1)	52.1 (49.4-54.9)	51.6 (48.7-54.6)	50.9 (47.9-53.9)	50.6 (47.6-53.7)	50.5 (47.4-53.8)	51.1 (48.1-54.3)	52.7 (49.5-56.0)	54.9 (51.6-58.6)	56.3 (52.8-60.2)	57.4 (53.7-61.8)	58.3 (54.1-63.0)	59.3 (54.9-64.1)	60.1 (54.8-65.4)	59.8 (54.1-65.7)	59.9 (53.8-65.9)	60.0 (53.6-66.3)
South Africa	55.6 (53.3-58.1)	55.9 (53.7-58.2)	56.2 (54.2-58.3)	56.5 (54.6-58.4)	57.3 (55.6-59.1)	57.6 (56.0-59.2)	58.1 (56.7-59.6)	59.1 (57.7-60.4)	59.4 (58.1-60.7)	60.0 (58.7-61.2)	60.1 (58.9-61.3)	60.3 (59.2-61.5)	60.4 (59.4-61.6)	60.6 (59.6-61.7)	60.5 (59.5-61.4)	59.9 (59.0-60.7)	58.8 (58.0-59.6)	57.2 (56.4-58.0)	56.2 (55.4-57.1)	55.4 (54.5-56.4)	54.4 (53.3-55.5)	54.1 (52.9-55.4)	53.3 (52.0-54.7)	52.5 (51.1-53.9)	51.9 (50.5-53.3)	51.7 (50.4-53.1)	51.4 (50.1-52.9)	51.7 (50.5-53.1)	51.8 (50.7-53.2)	52.3 (51.0-53.6)	53.2 (52.0-54.6)	54.4 (53.3-55.7)	56.2 (55.2-57.2)	57.6 (56.6-58.7)	58.2 (57.1-59.3)	58.6 (57.3-59.9)
Swaziland	54.6 (46.9-63.9)	55.0 (47.4-64.2)	55.3 (47.9-64.3)	54.6 (47.5-63.1)	55.8 (48.9-64.0)	56.4 (49.7-64.2)	56.7 (50.1-64.1)	57.1 (50.8-63.9)	57.3 (51.4-63.6)	57.5 (52.2-63.3)	57.6 (52.9-62.9)	57.4 (53.2-62.4)	57.8 (53.8-62.5)	58.1 (54.3-62.8)	57.2 (53.4-62.2)	55.7 (51.9-61.0)	53.2 (49.6-58.3)	52.1 (48.4-57.2)	50.0 (46.4-55.0)	48.5 (45.0-53.3)	46.5 (43.2-51.1)	44.6 (41.2-48.8)	43.6 (40.3-47.8)	42.6 (39.4-46.8)	41.9 (38.7-46.1)	41.7 (38.4-45.8)	42.3 (38.7-46.6)	43.2 (39.4-48.2)	43.2 (39.0-48.5)	43.8 (39.4-49.9)	44.3 (39.5-51.0)	45.6 (40.3-53.1)	46.4 (40.7-54.5)	46.7 (40.7-54.9)	47.1 (40.9-55.4)	49.0 (42.3-58.3)
Zimbabwe	56.4 (52.7-60.4)	57.7 (54.3-61.4)	58.7 (55.6-61.9)	59.3 (56.6-62.2)	60.0 (57.3-62.6)	60.7 (58.2-63.3)	61.4 (58.9-63.7)	61.9 (59.4-64.2)	61.8 (59.1-64.3)	61.5 (58.7-64.3)	60.8 (57.6-64.2)	60.6 (56.9-64.2)	59.3 (55.1-63.2)	57.6 (53.1-62.0)	55.8 (51.2-60.6)	53.5 (48.9-58.6)	52.2 (47.8-57.3)	50.7 (46.3-55.9)	49.1 (44.9-54.5)	48.0 (44.0-53.3)	46.7 (43.0-51.8)	46.1 (42.5-50.9)	45.3 (41.8-49.8)	44.8 (41.6-49.2)	44.4 (41.5-48.5)	44.6 (42.0-48.4)	44.9 (42.5-48.3)	45.6 (43.3-48.8)	46.0 (43.7-49.4)	46.6 (44.1-49.8)	47.7 (45.0-51.6)	49.7 (46.9-54.0)	51.7 (48.4-56.5)	52.9 (49.2-57.8)	54.1 (49.5-59.7)	56.3 (51.1-62.6)
Western Sub-Saharan Africa	51.4 (47.5-53.9)	51.7 (47.7-54.2)	52.0 (48.0-54.5)	52.3 (48.5-54.7)	52.5 (48.9-54.9)	52.8 (49.4-55.1)	52.9 (49.7-55.3)	53.0 (49.9-55.3)	53.2 (50.3-55.4)	53.3 (50.5-55.5)	53.3 (50.7-55.5)	53.5 (51.0-55.6)	53.6 (51.3-55.4)	53.6 (51.7-55.3)	53.7 (52.0-55.2)	53.8 (52.2-55.3)	53.9 (52.4-55.1)	53.9 (52.5-55.1)	53.7 (52.4-55.0)	54.0 (52.6-55.2)	54.1 (52.8-55.3)	54.3 (53.1-55.5)	54.4 (53.2-55.7)	54.8 (53.5-56.1)	55.3 (54.0-56.6)	55.8 (54.5-57.2)	56.3 (54.9-57.7)	57.0 (55.5-58.5)	57.6 (56.0-59.1)	58.3 (56.6-59.9)	58.9 (57.1-60.5)	59.4 (57.4-61.1)	59.9 (57.7-61.7)	60.3 (57.9-62.1)	60.4 (57.9-62.3)	61.0 (58.2-63.0)
Benin	49.1 (45.1-53.2)	49.6 (45.9-53.5)	50.3 (46.9-53.7)	50.9 (47.8-54.0)	51.3 (48.3-54.2)	51.9 (49.2-54.6)	52.5 (50.0-55.1)	53.3 (50.7-55.8)	53.6 (51.0-56.1)	53.8 (51.4-56.3)	54.0 (51.7-56.6)	54.0 (51.7-56.8)	54.4 (52.0-57.2)	54.6 (52.3-57.4)	54.6 (52.3-57.3)	54.6 (52.1-57.4)	54.4 (51.8-57.4)	54.7 (52.0-57.7)	54.7 (51.9-57.8)	54.8 (51.8-57.8)	54.9 (51.7-58.0)	55.0 (51.9-58.3)	55.3 (51.9-58.9)	55.5 (51.9-59.3)	55.8 (52.1-60.2)	56.2 (52.0-61.3)	56.5 (51.8-62.2)	56.9 (51.5-63.0)	57.3 (51.5-64.2)	58.0 (52.0-65.2)	58.4 (51.9-66.1)	58.6 (51.8-66.7)	59.0 (51.5-67.1)	59.3 (51.7-67.5)	59.6 (51.8-67.9)	59.9 (51.4-68.3)
Burkina Faso	47.2 (43.1-51.6)	47.7 (43.8-51.7)	48.2 (44.8-51.7)	48.6 (45.6-51.8)	49.6 (46.9-52.3)	49.8 (47.4-52.2)	50.0 (47.5-52.2)	50.0 (47.5-52.2)	49.9 (47.6-52.2)	50.1 (47.7-52.6)	50.1 (47.6-52.6)	50.0 (47.5-52.6)	49.9 (47.5-52.7)	49.8 (47.3-52.6)	49.8 (47.3-52.5)	49.8 (47.4-52.4)	50.0 (47.7-52.3)	50.2 (47.9-52.3)	50.4 (48.2-52.6)	50.8 (48.7-53.1)	51.1 (49.0-53.5)	51.9 (49.5-54.3)	52.6 (49.9-55.2)	53.4 (50.6-56.2)	54.2 (51.4-57.2)	55.0 (52.2-58.3)	55.9 (52.9-59.5)	56.6 (53.4-60.4)	57.2 (53.8-61.3)	57.8 (53.9-62.2)	58.3 (53.8-63.3)	58.8 (53.4-64.0)	59.1 (53.1-64.6)	59.4 (52.9-65.0)	59.7 (52.7-65.5)	60.0 (52.6-66.0)
Cameroon	56.0 (50.7-60.9)	56.4 (51.7-60.8)	56.8 (52.7-60.8)	57.1 (53.8-60.7)	57.0 (54.3-60.3)	57.4 (54.9-60.2)	57.1 (54.9-59.6)	57.4 (55.3-59.7)	57.4 (55.2-59.6)	57.3 (55.2-59.7)	57.1 (54.9-59.5)	56.8 (54.5-59.1)	56.3 (54.0-58.8)	55.7 (53.2-58.3)	55.4 (52.9-58.1)	54.9 (52.4-57.6)	54.6 (52.0-57.3)	54.0 (51.5-56.8)	53.6 (51.0-56.4)	53.4 (50.8-56.3)	53.1 (50.4-56.0)	53.1 (50.5-56.2)	53.2 (50.6-56.5)	53.3 (50.4-56.6)	53.5 (50.5-56.7)	53.8 (50.7-57.2)	54.3 (50.9-57.8)	54.5 (51.0-58.4)	55.2 (51.5-59.5)	55.9 (51.8-60.6)	56.4 (51.7-61.7)	56.7 (51.8-62.5)	57.1 (51.7-63.1)	57.3 (51.4-63.6)	57.3 (50.9-63.8)	57.6 (50.4-64.2)
Cape Verde	65.0 (64.0-66.0)	64.5 (63.7-65.3)	64.0 (63.3-64.7)	63.7 (63.1-64.3)	63.2 (62.6-63.9)	63.8 (63.1-64.5)	64.1 (63.2-65.0)	64.4 (63.4-65.3)	64.6 (63.6-65.6)	64.8 (63.9-65.6)	64.8 (64.1-65.5)	64.5 (63.7-65.3)	64.3 (63.1-65.5)	64.0 (62.1-65.8)	63.7 (61.2-66.1)	63.4 (60.3-66.5)	63.2 (59.7-66.9)	63.0 (58.9-67.3)	62.8 (58.1-67.5)	62.5 (57.6-67.5)	62.7 (57.6-68.0)	62.7 (57.6-68.2)	62.7 (57.8-68.4)	62.8 (58.1-68.3)	63.0 (58.5-68.3)	63.2 (59.2-68.0)	63.6 (59.9-67.7)	63.6 (60.5-67.1)	64.5 (62.1-67.1)	65.1 (63.3-66.8)	65.6 (64.4-66.7)	66.2 (65.5-66.8)	66.9 (66.2-67.6)	67.7 (66.6-69.5)	68.6 (66.6-70.7)	69.1 (66.2-71.8)
Chad	48.7 (44.5-52.5)	48.8 (45.0-52.4)	48.9 (45.5-52.0)	49.4 (46.2-52.5)	50.0 (46.8-52.8)	51.7 (49.0-54.3)	51.4 (48.8-53.9)	49.6 (45.6-53.2)	53.5 (51.1-56.1)	53.4 (50.9-56.0)	52.6 (50.0-55.2)	53.4 (50.9-55.7)	52.7 (50.4-55.1)	52.9 (50.5-55.4)	52.3 (49.9-55.0)	52.3 (49.6-55.1)	51.4 (48.6-54.3)	50.7 (47.8-53.7)	50.3 (47.3-53.2)	49.9 (46.9-53.0)	49.5 (46.2-52.9)	49.8 (46.6-53.5)	50.1 (46.6-54.2)	50.6 (46.7-55.4)	51.1 (46.7-56.4)	51.5 (46.5-57.5)	51.5 (45.9-57.9)	52.6 (46.3-59.3)	52.9 (46.1-59.9)	53.8 (46.8-61.1)	54.3 (47.2-61.7)	54.6 (47.4-62.2)	54.9 (47.5-62.5)	55.2 (47.5-62.9)	55.3 (47.4-62.9)	55.9 (47.6-63.6)
Cote d'Ivoire	55.0 (52.0-58.1)	55.7 (52.9-58.4)	55.9 (53.3-58.6)	56.1 (53.6-58.8)	55.8 (53.2-58.5)	55.8 (53.1-58.4)	55.6 (52.9-58.4)	55.1 (52.3-58.1)	54.6 (51.7-57.4)	53.9 (51.2-56.7)	53.3 (50.7-56.1)	52.7 (50.1-55.7)	52.0 (49.3-55.0)	51.3 (48.6-54.4)	50.7 (47.9-53.6)	50.0 (47.3-53.0)	49.9 (47.3-52.9)	50.1 (47.6-53.1)	50.2 (47.8-53.2)	50.3 (47.9-53.4)	50.4 (48.0-53.4)	50.7 (48.1-53.7)	50.9 (48.3-53.9)	51.3 (48.7-54.2)	51.5 (48.8-54.5)	52.2 (49.4-55.2)	52.8 (49.8-56.1)	53.5 (50.2-57.1)	54.0 (50.4-58.1)	54.5 (50.4-59.2)	55.0 (50.4-60.3)	55.4 (50.1-61.2)	55.9 (50.1-62.2)	56.3 (50.1-63.1)	56.7 (50.1-63.7)	57.2 (50.0-64.5)
The Gambia	58.9 (50.3-64.3)	55.6 (47.9-62.0)	59.6 (51.1-64.9)	59.9 (51.6-65.2)	60.2 (52.0-65.3)	60.4 (52.5-65.6)	60.7 (52.9-65.8)	61.0 (53.3-66.0)	61.3 (53.5-66.2)	61.5 (54.2-66.4)	61.8 (54.7-66.5)	62.0 (55.4-66.7)	61.9 (55.8-66.4)	62.6 (57.0-66.9)	62.9 (57.6-67.0)	63.1 (58.2-67.1)	63.3 (59.0-67.2)	63.5 (59.8-67.3)	63.7 (60.2-67.4)	63.5 (60.4-67.2)	63.4 (60.4-66.9)	63.4 (60.3-66.7)	63.2 (60.3-66.5)	63.5 (60.6-66.7)	63.8 (60.6-67.0)	63.8 (60.6-66.9)	63.9 (60.8-67.0)	64.1 (61.0-67.0)	64.5 (61.5-67.6)	64.9 (61.8-68.2)	65.3 (62.0-68.8)	65.7 (62.2-69.3)	66.1 (62.2-69.9)	66.3 (61.8-70.2)	66.5 (61.6-70.5)	66.7 (61.5-70.7)
Ghana	55.0 (47.3-62.5)	55.1 (47.4-62.7)	55.2 (47.7-62.8)	55.3 (47.7-62.8)	55.4 (48.0-62.9)	55.7 (48.6-63.1)	56.1 (49.2-63.2)	56.4 (49.8-63.2)	56.8 (50.7-63.3)	57.2 (51.4-63.0)	57.6 (52.5-62.7)	57.9 (53.1-62.5)	58.2 (53.7-62.5)	59.0 (54.9-63.1)	58.9 (55.2-62.5)	59.6 (56.3-63.0)	59.8 (56.9-63.2)	59.4 (56.3-62.8)	59.6 (56.6-62.9)	59.3 (55.9-62.6)	59.3 (55.6-62.7)	59.1 (55.5-62.8)	59.1 (55.2-62.8)	59.1 (55.6-62.9)	59.2 (55.5-63.3)	59.4 (55.1-63.5)	59.7 (55.0-64.2)	60.0 (54.7-64.8)	60.3 (54.6-65.7)	60.7 (54.7-66.6)	61.1 (54.7-67.3)	61.6 (55.1-68.0)	62.0 (54.7-68.6)	62.3 (55.0-69.0)	62.6 (54.8-69.3)	63.1 (55.0-69.8)
Guinea	48.3 (43.3-53.0)	48.7 (44.1-53.2)	49.1 (45.0-53.3)	49.3 (45.7-53.0)	49.8 (46.7-53.1)	50.4 (47.5-53.5)	50.8 (48.1-53.6)	51.0 (48.6-53.8)	51.5 (49.2-54.1)	51.9 (49.7-54.3)	52.3 (50.2-54.6)	52.6 (50.5-54.7)	52.6 (50.5-54.7)	52.9 (50.9-55.0)	53.0 (51.0-54.9)	53.1 (51.1-55.0)	53.2 (51.2-55.2)	53.3 (51.4-55.2)	53.3 (51.3-55.3)	53.3 (51.3-55.5)	53.4 (51.3-55.8)	53.6 (51.4-56.1)	53.9 (51.7-56.4)	54.4 (52.0-57.0)	54.8 (52.2-57.6)	55.2 (52.4-58.2)	55.5 (52.7-58.6)	55.8 (53.0-58.9)	56.2 (53.3-59.5)	56.5 (53.4-59.9)	56.8 (53.2-60.4)	57.1 (52.9-61.1)	57.3 (52.6-62.0)	57.5 (52.1-63.2)	57.3 (51.1-63.5)	57.8 (50.8-64.5)
Guinea-Bissau	48.9 (39.0-57.1)	49.0 (39.3-57.2)	49.1 (39.6-57.2)	49.3 (40.3-57.3)	49.5 (40.7-57.4)	49.7 (40.7-57.6)	49.9 (40.8-57.8)	50.1 (40.8-58.0)	50.3 (40.9-58.2)	50.5 (41.0-58.4)	50.7 (41.1-58.7)	50.9 (40.7-58.9)	51.1 (40.6-59.1)	51.2 (40.7-59.3)	51.4 (40.7-59.6)	51.6 (40.8-59.7)	51.8 (41.4-60.1)	52.1 (41.9-60.4)	50.3 (40.6-58.6)	51.6 (41.3-60.0)	52.1 (41.6-60.9)	52.0 (41.8-61.0)	52.1 (42.0-61.1)	52.2 (41.5-61.3)	52.2 (41.5-61.4)	52.3 (41.5-61.6)	52.5 (41.7-61.8)	52.7 (42.0-61.9)	53.1 (42.1-62.4)	53.4 (42.5-62.6)	53.7 (42.5-63.0)	54.0 (42.5-63.2)	54.3 (42.4-63.4)	54.4 (42.5-63.6)	54.5 (42.2-63.7)	54.9 (42.0-64.0)

SR Table 7: Estimates of life expectancy at birth by geography, males, 1980–2015

Location	1980	1981	1982	1983	1984	1985	1986	1987	1988	1989	1990	1991
Liberia	50.2 (42.9-55.8)	50.5 (43.3-56.0)	50.6 (43.9-56.2)	50.8 (44.5-56.5)	50.9 (45.1-56.6)	51.0 (45.3-56.5)	51.1 (45.8-56.6)	51.2 (46.3-56.6)	51.4 (46.7-56.4)	51.7 (47.3-56.1)	46.7 (41.8-52.2)	51.0 (47.5-54.7)
Mali	44.5 (41.3-47.8)	45.1 (42.1-48.3)	45.7 (42.8-48.7)	46.5 (43.9-49.2)	47.1 (44.7-49.5)	47.6 (45.4-49.9)	48.6 (46.5-50.7)	49.0 (47.2-51.0)	49.5 (47.7-51.4)	49.9 (48.2-51.8)	50.1 (48.4-51.9)	50.6 (48.9-52.5)
Mauritania	55.3 (48.3-61.4)	55.7 (49.3-61.2)	56.1 (50.4-60.9)	56.4 (51.6-60.8)	56.7 (52.6-60.7)	57.1 (53.5-60.5)	58.2 (55.0-61.3)	58.6 (55.8-61.3)	59.2 (56.4-61.8)	59.1 (56.5-61.5)	58.9 (56.5-61.4)	59.9 (57.4-62.4)
Niger	42.8 (40.2-45.4)	42.4 (39.9-44.8)	42.6 (40.2-45.0)	42.8 (40.4-45.1)	42.7 (40.4-45.1)	43.2 (40.7-45.5)	43.5 (41.1-45.9)	44.0 (41.5-46.3)	44.6 (42.1-47.0)	45.3 (42.7-47.7)	46.1 (43.7-48.5)	47.2 (44.9-49.6)
Nigeria	52.4 (45.0-57.6)	52.7 (45.4-58.0)	53.0 (45.6-58.2)	53.1 (45.9-58.3)	53.2 (46.3-58.3)	53.4 (46.7-58.5)	53.4 (47.0-58.4)	53.4 (47.4-58.4)	53.4 (47.7-58.3)	53.5 (48.1-58.3)	53.6 (48.6-58.2)	53.8 (49.0-58.0)
Sao Tome and Principe	61.2 (60.2-62.4)	61.3 (60.2-62.5)	61.4 (60.2-62.5)	61.3 (60.2-62.4)	61.2 (60.3-62.2)	61.4 (60.5-62.3)	61.5 (60.6-62.6)	61.7 (60.5-63.0)	61.8 (60.3-63.4)	62.0 (60.2-64.0)	62.3 (60.2-64.4)	62.6 (60.3-65.0)
Senegal	50.7 (47.7-53.7)	51.5 (48.7-54.3)	52.3 (49.5-54.9)	53.4 (50.8-56.0)	54.3 (51.8-56.8)	54.6 (52.1-57.0)	55.0 (52.5-57.4)	55.3 (52.8-57.7)	55.7 (53.1-58.2)	55.9 (53.4-58.3)	56.2 (53.8-58.7)	56.6 (54.1-59.2)
Sierra Leone	45.9 (39.6-52.9)	46.1 (39.8-52.9)	46.4 (40.3-53.0)	46.6 (40.5-53.2)	46.9 (40.9-53.5)	47.2 (41.3-53.6)	47.5 (41.8-53.8)	47.7 (42.3-53.9)	48.1 (42.8-54.1)	48.4 (43.3-54.2)	48.8 (44.1-54.2)	48.3 (44.1-53.0)
Togo	54.9 (49.3-60.5)	55.3 (50.3-60.3)	55.5 (51.0-60.0)	55.7 (51.9-59.8)	55.9 (52.2-59.4)	55.9 (52.6-59.2)	56.3 (53.2-59.3)	56.2 (53.4-58.9)	56.6 (53.9-59.3)	56.8 (54.2-59.5)	56.9 (54.1-59.7)	56.9 (54.1-59.8)
Eastern Sub-Saharan Africa	48.4 (45.6-50.9)	48.4 (45.9-50.7)	48.6 (46.5-50.7)	47.6 (44.8-50.0)	47.4 (44.9-49.8)	48.7 (47.0-50.4)	49.1 (47.6-50.7)	49.6 (48.1-51.0)	49.9 (48.5-51.2)	49.8 (48.5-51.0)	49.2 (47.9-50.5)	49.7 (48.5-50.8)
Burundi	43.0 (36.7-51.9)	43.3 (37.1-52.5)	43.7 (37.4-53.1)	44.1 (37.6-53.4)	44.4 (37.8-53.8)	44.5 (37.9-53.7)	44.5 (38.1-53.4)	44.6 (38.3-53.2)	44.5 (38.5-52.5)	44.3 (38.4-51.8)	44.2 (38.6-51.3)	44.0 (38.9-50.4)
Comoros	52.1 (44.2-60.9)	52.4 (44.5-61.3)	52.7 (44.8-61.7)	52.8 (44.9-61.8)	53.3 (45.2-62.2)	53.5 (45.6-62.6)	53.9 (46.3-62.7)	54.2 (46.6-62.9)	54.8 (47.0-63.1)	54.9 (47.3-63.1)	55.4 (48.2-63.3)	55.7 (48.9-63.2)
Djibouti	58.2 (48.9-65.7)	58.0 (49.2-65.5)	58.1 (49.4-65.5)	58.0 (49.2-65.5)	58.0 (49.8-65.4)	57.9 (49.7-65.5)	58.0 (50.1-65.5)	58.0 (50.3-65.4)	58.0 (51.0-65.4)	58.0 (51.8-65.0)	58.1 (51.7-64.8)	57.6 (51.2-64.0)
Eritrea	45.5 (41.9-49.8)	45.9 (42.7-49.7)	46.2 (43.2-49.8)	46.5 (43.8-49.8)	46.9 (44.2-50.0)	46.9 (44.2-50.0)	47.7 (45.0-50.6)	48.5 (45.8-51.4)	49.4 (46.7-52.6)	50.3 (47.6-53.5)	51.5 (48.7-54.6)	52.6 (49.6-56.0)
Ethiopia	40.7 (35.5-46.3)	40.9 (36.1-46.2)	41.2 (36.8-46.1)	37.7 (31.1-43.4)	38.2 (31.6-43.7)	42.0 (39.0-45.4)	42.9 (40.1-46.0)	43.6 (41.0-46.2)	44.1 (41.5-46.8)	43.8 (41.5-46.5)	43.0 (40.3-45.9)	45.3 (43.2-47.8)
Kenya	61.9 (60.2-63.6)	62.3 (60.6-63.9)	62.5 (60.8-64.0)	62.7 (61.1-64.2)	62.9 (61.3-64.4)	62.8 (61.1-64.3)	62.7 (61.1-64.1)	62.2 (60.7-63.7)	62.1 (60.5-63.5)	61.4 (59.9-62.9)	60.8 (59.3-62.2)	59.8 (58.3-61.3)
Madagascar	54.7 (52.2-57.2)	54.6 (52.3-57.0)	53.8 (51.6-55.9)	54.0 (51.9-56.0)	53.8 (51.8-55.7)	53.7 (51.6-55.7)	53.4 (51.2-55.4)	53.3 (51.1-55.4)	53.9 (51.7-56.0)	54.5 (52.4-56.5)	54.9 (53.0-56.8)	55.2 (53.4-57.0)
Malawi	48.2 (45.8-50.7)	48.6 (46.4-51.1)	49.0 (46.6-51.4)	49.2 (46.9-51.7)	49.4 (47.1-51.8)	49.3 (47.1-51.6)	49.0 (46.8-51.3)	48.7 (46.4-51.2)	48.5 (46.0-51.0)	48.2 (45.4-51.0)	47.9 (44.8-51.2)	47.6 (44.3-51.2)
Mozambique	50.1 (45.3-54.8)	46.8 (42.0-51.7)	46.9 (42.1-51.2)	47.1 (42.8-51.2)	42.7 (37.0-48.6)	42.7 (37.0-48.4)	44.4 (39.0-49.6)	44.2 (38.6-49.3)	48.7 (46.3-51.1)	49.0 (46.7-51.2)	48.9 (46.6-51.2)	49.2 (46.9-51.3)
Rwanda	52.0 (45.5-57.9)	53.0 (46.7-58.7)	53.8 (47.8-59.2)	54.4 (48.9-59.4)	54.7 (49.9-59.2)	54.8 (50.4-58.8)	54.9 (51.0-58.9)	54.6 (50.8-58.4)	53.8 (50.3-57.2)	52.4 (49.4-55.6)	49.3 (46.3-52.3)	48.1 (45.2-51.0)
Somalia	48.6 (38.6-59.1)	48.6 (38.1-59.2)	48.4 (38.0-58.9)	48.3 (37.8-58.6)	48.4 (38.3-58.8)	48.6 (38.4-59.1)	48.5 (38.2-58.9)	48.5 (38.6-58.7)	44.3 (35.1-55.6)	48.7 (38.9-59.1)	48.2 (38.6-58.6)	45.9 (37.5-55.9)
South Sudan	48.6 (39.2-57.1)	48.8 (39.3-57.2)	48.8 (39.3-57.2)	49.0 (39.2-57.4)	49.2 (39.5-57.5)	49.3 (39.5-57.7)	49.5 (39.7-58.0)	49.7 (39.8-58.3)	50.0 (40.3-58.6)	50.2 (40.5-58.9)	50.4 (40.7-59.2)	50.4 (40.5-59.1)
Tanzania	55.1 (51.2-59.5)	55.3 (51.8-59.2)	55.5 (52.4-58.9)	55.8 (53.0-58.7)	55.8 (53.3-58.6)	55.9 (53.4-58.5)	55.7 (53.4-58.2)	55.5 (53.3-58.0)	54.9 (52.7-57.4)	54.4 (52.0-56.9)	54.1 (51.8-56.6)	53.9 (51.5-56.6)
Uganda	46.0 (41.5-50.5)	46.5 (42.3-50.9)	46.9 (42.9-50.9)	47.4 (43.2-51.0)	47.7 (43.9-51.2)	47.7 (43.9-51.2)	47.5 (44.1-51.0)	49.9 (47.3-52.6)	50.0 (47.3-52.9)	49.5 (46.7-52.8)	48.9 (45.8-52.6)	48.1 (44.9-51.9)
Zambia	55.7 (51.5-60.3)	55.7 (52.0-59.7)	55.6 (52.3-59.3)	55.4 (52.3-58.9)	55.0 (52.4-58.3)	54.4 (51.9-57.4)	53.7 (51.2-56.7)	53.4 (50.8-56.5)	53.3 (50.5-56.7)	52.4 (49.3-56.1)	51.4 (48.3-54.9)	50.0 (46.9-53.4)
Central Sub-Saharan Africa	50.3 (44.9-56.0)	50.5 (45.1-56.3)	50.8 (45.6-56.4)	51.0 (45.9-56.4)	51.4 (46.6-56.7)	51.6 (46.9-56.9)	51.8 (46.9-56.9)	51.7 (47.0-56.7)	51.6 (47.1-56.2)	51.5 (47.4-56.0)	51.3 (47.3-55.6)	51.4 (47.5-55.3)
Angola	45.4 (36.2-57.0)	45.5 (36.3-56.9)	45.6 (36.8-57.2)	45.7 (37.0-57.4)	45.9 (37.4-57.8)	46.0 (37.6-57.5)	46.2 (37.9-57.3)	46.3 (38.0-57.4)	46.2 (37.8-57.2)	46.9 (38.3-58.1)	47.0 (38.3-58.2)	48.0 (38.7-59.2)
Central African Republic	46.6 (43.3-50.3)	46.9 (44.0-50.5)	47.3 (44.5-50.7)	47.7 (44.8-50.8)	47.6 (44.7-50.4)	47.5 (44.7-50.2)	47.2 (44.5-50.0)	47.2 (44.7-50.1)	46.9 (44.3-49.8)	46.1 (43.6-49.0)	46.0 (43.4-49.3)	45.4 (42.5-49.1)
Congo	52.8 (44.7-62.3)	53.2 (45.1-62.6)	53.5 (46.1-62.5)	53.7 (46.6-62.5)	53.9 (47.1-62.3)	54.0 (47.5-62.0)	53.9 (47.7-61.3)	53.6 (47.9-60.6)	53.3 (48.3-59.2)	52.9 (48.5-58.3)	52.4 (48.5-57.0)	52.5 (48.9-56.7)
Democratic Republic of the Congo	52.6 (44.5-60.2)	52.8 (44.7-60.4)	53.1 (45.4-60.7)	53.4 (46.0-60.8)	53.9 (47.1-61.4)	54.2 (47.1-61.4)	54.4 (47.5-61.4)	54.3 (47.9-61.2)	54.1 (48.1-60.8)	53.9 (48.1-60.1)	53.5 (48.3-59.5)	53.3 (48.6-58.9)
Equatorial Guinea	47.4 (37.8-59.8)	47.6 (38.1-60.3)	47.8 (38.2-60.6)	48.0 (38.7-60.6)	48.0 (38.6-60.6)	47.9 (38.7-60.4)	47.9 (38.8-60.3)	47.8 (38.8-60.0)	47.8 (38.5-60.2)	47.6 (38.7-60.3)	47.4 (38.4-60.1)	47.3 (38.4-60.0)
Gabon	53.4 (47.3-60.3)	53.8 (47.8-60.1)	54.1 (48.7-59.7)	54.4 (49.6-59.5)	54.8 (50.5-59.6)	55.1 (51.0-59.6)	56.7 (52.9-60.5)	57.4 (53.9-60.9)	56.9 (53.7-60.3)	56.9 (53.9-60.2)	56.1 (53.3-59.1)	56.5 (53.9-59.7)

Location	1992	1993	1994	1995	1996	1997	1998	1999	2000	2001	2002	2003
Liberia	51.5 (48.4-54.9)	48.5 (44.6-52.4)	46.7 (41.7-52.0)	49.5 (45.9-53.2)	48.6 (44.4-52.4)	53.3 (50.7-56.0)	53.7 (51.2-56.4)	54.6 (52.2-57.2)	55.2 (52.8-57.8)	55.6 (53.2-58.2)	54.1 (51.1-57.3)	53.7 (50.1-57.6)
Mali	50.9 (49.2-52.8)	51.2 (49.4-53.0)	51.1 (49.3-52.9)	51.5 (49.7-53.4)	51.9 (50.2-53.8)	52.6 (50.8-54.5)	50.3 (46.5-53.6)	54.0 (52.2-55.9)	53.8 (51.8-55.9)	54.3 (52.4-56.7)	54.8 (52.5-57.2)	55.5 (53.2-58.0)
Mauritania	60.2 (57.7-62.6)	60.5 (57.9-62.9)	60.4 (57.8-62.8)	60.9 (58.4-63.4)	61.3 (59.0-63.9)	61.9 (59.6-64.4)	62.4 (60.1-64.8)	63.4 (61.1-65.9)	64.1 (61.7-66.7)	64.6 (62.0-67.3)	65.3 (62.6-68.1)	66.0 (63.1-68.7)
Niger	47.8 (45.4-50.1)	48.3 (46.0-50.6)	49.0 (46.7-51.4)	49.4 (47.2-51.9)	50.2 (47.9-52.6)	50.6 (48.3-52.8)	51.0 (48.7-53.3)	51.6 (49.3-53.8)	52.2 (50.0-54.4)	52.6 (50.6-54.6)	53.4 (51.3-55.5)	53.9 (51.8-56.0)
Nigeria	53.9 (49.7-57.9)	54.0 (50.2-57.8)	54.5 (51.1-58.0)	54.7 (51.6-57.9)	54.7 (51.9-57.6)	54.7 (51.9-57.4)	54.7 (51.9-57.3)	54.8 (52.1-57.5)	54.7 (52.2-57.4)	54.8 (52.3-57.5)	54.8 (52.4-57.5)	55.2 (52.9-57.9)
Sao Tome and Principe	62.9 (60.6-65.5)	63.1 (60.8-65.8)	63.7 (61.2-66.5)	63.8 (61.3-66.8)	63.8 (61.2-66.8)	63.9 (61.2-66.8)	63.8 (61.1-66.8)	64.4 (61.9-67.3)	64.6 (61.9-67.4)	64.5 (61.8-67.2)	64.5 (61.9-67.4)	64.6 (61.6-67.5)
Senegal	56.6 (54.1-59.0)	56.7 (54.1-59.1)	56.7 (54.0-59.2)	57.0 (54.1-59.6)	57.4 (54.5-60.3)	57.5 (54.5-60.2)	57.8 (54.8-60.4)	58.0 (54.9-60.6)	58.5 (55.4-61.3)	58.9 (55.6-61.8)	59.0 (55.6-62.1)	60.0 (56.1-63.5)
Sierra Leone	49.0 (45.1-53.1)	49.2 (45.6-52.9)	48.5 (45.4-52.0)	47.6 (44.2-51.1)	49.2 (46.4-52.1)	48.7 (46.0-51.6)	46.5 (42.8-50.2)	46.1 (41.7-50.1)	49.4 (46.9-51.9)	50.1 (47.7-52.6)	50.1 (47.9-52.5)	50.4 (48.4-52.7)
Togo	56.8 (54.0-59.9)	56.5 (53.7-59.7)	56.1 (53.3-59.4)	55.8 (52.7-59.1)	55.4 (52.3-58.6)	54.9 (51.9-58.1)	54.4 (51.4-57.8)	54.7 (51.6-58.1)	54.7 (51.6-58.3)	54.6 (51.6-58.2)	54.7 (51.6-58.4)	54.3 (51.1-58.0)
Eastern Sub-Saharan Africa	49.9 (48.7-51.0)	50.0 (48.9-51.1)	44.1 (38.7-49.3)	50.1 (48.9-51.1)	50.3 (49.3-51.3)	50.5 (49.5-51.5)	50.8 (49.7-51.8)	50.6 (49.5-51.7)	50.9 (49.8-52.0)	52.0 (50.9-53.0)	52.6 (51.5-53.7)	53.1 (52.0-54.3)
Burundi	43.9 (39.5-49.6)	43.8 (39.7-48.6)	43.7 (39.9-48.2)	43.0 (39.6-47.1)	43.0 (39.6-46.7)	43.9 (40.9-47.3)	44.7 (41.9-48.0)	45.8 (43.0-49.1)	46.2 (43.4-49.3)	48.0 (45.0-51.4)	49.4 (46.4-52.7)	50.9 (48.0-54.5)
Comoros	56.1 (49.6-63.1)	56.4 (50.3-63.1)	56.8 (51.1-63.1)	57.2 (51.8-62.9)	56.3 (51.8-61.1)	57.7 (53.3-62.1)	58.5 (54.4-62.1)	60.5 (56.5-63.9)	61.4 (57.6-64.6)	62.5 (59.3-65.8)	63.2 (60.1-66.2)	64.4 (61.5-67.3)
Djibouti	57.2 (50.8-63.7)	57.6 (50.9-64.7)	56.8 (49.3-64.1)	57.2 (48.7-65.0)	57.1 (48.2-65.3)	56.9 (47.6-65.4)	56.8 (46.5-65.5)	56.6 (46.7-65.5)	56.7 (47.1-65.7)	57.0 (47.4-65.8)	57.1 (47.8-66.0)	57.3 (47.4-66.1)
Eritrea	54.4 (51.0-58.0)	56.0 (52.3-59.8)	57.1 (52.7-61.1)	57.9 (52.9-62.4)	58.5 (52.8-63.4)	59.0 (52.6-64.5)	58.5 (51.9-64.3)	48.7 (40.1-58.7)	31.0 (21.6-52.2)	59.9 (51.7-66.8)	60.0 (51.4-67.1)	59.8 (50.6-67.3)
Ethiopia	46.6 (44.4-48.9)	47.4 (45.2-49.8)	48.2 (46.1-50.5)	48.7 (46.7-51.0)	49.8 (47.7-52.0)	50.4 (48.3-52.6)	51.2 (49.0-53.4)	50.3 (47.9-52.8)	52.4 (50.0-54.9)	53.0 (50.6-55.7)	53.9 (51.5-56.5)	54.5 (52.0-57.1)
Kenya	59.0 (57.5-60.6)	58.1 (56.5-59.6)	57.4 (55.8-58.9)	56.8 (55.3-58.4)	56.1 (54.7-57.7)	55.5 (54.1-57.0)	54.8 (53.5-56.3)	54.6 (53.3-56.0)	54.4 (53.1-55.7)	54.5 (53.2-55.8)	54.6 (53.3-55.8)	54.7 (53.4-56.0)
Madagascar	55.5 (53.7-57.2)	55.4 (53.5-57.3)	55.9 (53.9-57.7)	56.3 (54.3-58.2)	56.7 (54.6-58.6)	57.2 (55.0-59.2)	57.5 (55.3-59.7)	58.0 (55.7-60.3)	58.3 (56.0-60.7)	58.7 (56.2-61.1)	58.9 (56.3-61.3)	59.2 (56.2-61.6)
Malawi	47.3 (44.0-51.2)	47.1 (43.6-51.2)	46.5 (43.0-50.7)	46.1 (42.5-50.3)	45.5 (42.1-49.5)	45.0 (41.8-49.0)	44.5 (41.4-48.2)	44.4 (41.4-47.9)	44.7 (41.8-48.1)	45.2 (42.2-48.6)	45.7 (42.9-49.0)	46.4 (43.5-49.8)
Mozambique	49.7 (47.6-52.0)	49.9 (47.7-52.2)	50.4 (48.1-52.6)	50.7 (48.4-52.9)	50.7 (48.4-53.1)	50.7 (48.3-53.2)	50.8 (48.4-53.2)	50.7 (48.3-53.3)	50.4 (47.9-53.1)	50.7 (48.1-53.6)	50.7 (48.0-53.9)	50.9 (48.1-54.2)
Rwanda	46.2 (43.5-49.0)	44.5 (42.1-47.2)	11.8 (7.1-31.9)	40.8 (38.4-43.4)	42.0 (40.0-44.3)	41.0 (38.9-43.5)	43.1 (41.2-45.3)	45.0 (42.9-47.5)	47.2 (45.0-49.9)	49.0 (46.6-51.8)	51.9 (49.3-54.7)	53.9 (51.3-56.8)
Somalia	47.4 (38.5-57.9)	49.4 (40.0-60.0)	49.4 (40.1-60.0)	49.6 (40.2-60.4)	49.5 (40.2-60.4)	49.2 (39.8-60.0)	50.0 (40.3-61.1)	50.1 (40.7-61.3)	50.4 (40.8-61.8)	50.5 (40.6-61.9)	50.7 (40.6-62.1)	51.1 (40.9-62.7)
South Sudan	51.1 (40.9-60.1)	51.5 (41.2-60.5)	51.9 (41.5-60.9)	52.2 (41.8-61.4)	52.6 (41.9-61.7)	53.0 (42.5-62.1)	53.3 (42.6-62.6)	53.6 (42.6-63.0)	53.9 (43.0-63.2)	54.2 (43.1-63.5)	54.5 (43.1-63.8)	54.6 (43.3-64.0)
Tanzania	53.6 (51.2-56.4)	53.4 (50.9-56.3)	53.1 (50.5-56.1)	53.3 (50.7-56.3)	53.3 (50.6-56.3)	53.6 (50.8-56.7)	53.8 (50.9-57.0)	53.8 (51.0-57.0)	54.4 (51.6-57.6)	54.8 (52.1-58.1)	55.4 (52.5-58.7)	56.0 (52.9-59.6)
Uganda	47.7 (44.3-51.4)	47.1 (43.9-50.8)	46.6 (43.6-50.1)	46.3 (43.5-49.7)	46.1 (43.3-49.3)	46.4 (43.7-49.4)	46.7 (44.3-49.7)	47.0 (44.7-49.9)	47.3 (45.1-50.3)	47.9 (45.6-50.8)	48.6 (46.2-51.4)	49.4 (46.9-52.1)
Zambia	48.8 (45.8-52.0)	47.7 (44.7-50.7)	46.8 (44.0-49.7)	46.0 (43.2-49.0)	45.4 (42.7-48.5)	45.1 (42.3-48.0)	44.6 (42.1-47.5)	44.2 (41.8-47.2)	43.9 (41.7-46.7)	44.0 (41.7-46.6)	44.2 (41.9-46.8)	44.3 (42.2-46.9)
Central Sub-Saharan Africa	51.3 (47.7-55.1)	50.9 (47.4-54.4)	51.3 (47.9-54.6)	51.4 (48.1-54.4)	49.6 (46.4-52.4)	50.2 (47.1-53.1)	50.8 (47.7-53.4)	51.2 (48.1-54.0)	51.7 (48.5-54.4)	52.1 (48.9-54.7)	52.5 (49.5-55.3)	52.9 (49.8-55.8)
Angola	48.1 (38.5-58.9)	46.4 (37.5-56.7)	48.8 (39.0-59.3)	50.1 (39.8-61.0)	50.7 (40.4-61.6)	51.1 (41.0-61.9)	51.2 (41.4-61.4)	51.3 (41.5-61.3)	51.8 (41.9-61.7)	52.1 (42.0-61.9)	53.1 (42.6-62.9)	53.8 (43.1-63.5)
Central African Republic	45.2 (42.1-49.4)	45.1 (41.5-50.0)	44.7 (40.8-50.3)	44.3 (40.0-50.7)	43.9 (39.4-50.4)	43.5 (38.8-50.7)	43.3 (38.2-50.7)	43.2 (37.9-51.0)	43.1 (37.5-51.2)	43.0 (37.3-51.2)	43.1 (37.1-51.7)	43.2 (37.0-52.0)
Congo	52.4 (49.1-56.3)	51.4 (48.4-55.1)	51.6 (48.7-55.0)	51.3 (48.5-54.5)	51.3 (48.5-54.5)	42.9 (37.1-50.1)	48.0 (45.3-51.1)	50.7 (47.8-53.8)	52.1 (49.2-55.1)	53.4 (50.3-56.3)	54.4 (51.4-57.4)	55.4 (52.3-58.4)
Democratic Republic of the Congo	53.2 (49.0-58.2)	53.2 (49.6-57.3)	53.0 (49.5-56.8)	52.7 (49.5-56.2)	49.7 (46.8-52.6)	51.3 (48.5-54.2)	51.7 (49.0-54.3)	52.1 (49.4-54.7)	52.5 (49.9-55.2)	52.9 (50.2-55.4)	53.2 (50.5-55.5)	53.4 (50.8-55.8)
Equatorial Guinea	47.4 (38.3-59.9)	47.4 (38.1-59.7)	47.4 (38.1-59.6)	47.5 (38.0-59.4)	48.0 (38.4-59.6)	49.4 (39.8-60.4)	50.4 (40.5-60.9)	51.2 (41.4-61.4)	52.2 (42.0-61.9)	53.3 (42.9-62.6)	54.3 (43.7-63.0)	54.8 (44.0-63.3)
Gabon	56.8 (53.9-60.0)	56.7 (53.8-60.0)	56.8 (53.8-59.9)	56.5 (53.5-59.6)	56.8 (53.8-60.0)	57.5 (54.6-60.8)	58.0 (55.0-61.2)	58.3 (55.2-61.7)	58.1 (54.9-61.5)	58.1 (54.7-61.7)	58.6 (55.2-62.6)	58.9 (55.2-63.0)

Location	2004	2005	2006	2007	2008	2009	2010	2011	2012	2013	2014	2015
Liberia	57.7 (55.3-60.3)	58.4 (55.8-61.1)	58.8 (56.2-61.7)	59.4 (56.6-62.3)	60.0 (57.1-63.3)	60.5 (57.2-64.1)	60.9 (57.4-65.0)	61.5 (57.5-65.8)	62.3 (57.8-66.6)	62.8 (57.9-67.3)	61.7 (56.7-66.0)	63.1 (57.6-67.7)
Mali	56.2 (53.7-58.7)	56.7 (54.2-59.2)	57.3 (54.6-60.0)	57.8 (55.0-60.6)	58.3 (55.5-61.3)	58.7 (55.6-61.9)	58.9 (55.5-62.2)	59.4 (55.3-62.9)	59.6 (55.2-63.3)	59.6 (54.8-63.5)	59.9 (54.6-64.0)	60.0 (54.2-64.3)
Mauritania	66.3 (63.4-69.1)	66.8 (63.9-69.7)	67.5 (64.6-70.6)	67.9 (64.8-71.0)	68.4 (64.8-71.8)	68.7 (64.7-72.3)	69.0 (64.5-72.9)	69.1 (64.6-73.4)	69.4 (64.3-74.1)	69.5 (64.3-74.8)	69.7 (64.1-75.5)	69.8 (63.7-75.8)
Niger	54.6 (52.3-56.9)	55.2 (52.5-57.7)	55.8 (53.0-58.4)	56.4 (53.5-59.2)	57.0 (53.9-60.0)	57.5 (54.1-60.9)	58.0 (54.0-61.7)	58.4 (53.7-62.4)	58.8 (53.4-63.2)	59.1 (53.3-64.1)	59.5 (53.1-64.8)	59.6 (52.9-65.4)
Nigeria	55.7 (53.2-58.3)	56.3 (53.7-59.0)	56.9 (54.1-59.7)	57.8 (54.8-60.6)	58.6 (55.6-61.4)	59.6 (56.5-62.3)	60.4 (56.9-63.0)	61.0 (57.3-63.7)	61.6 (57.5-64.2)	62.0 (57.5-64.7)	62.3 (57.4-65.1)	62.9 (57.4-65.8)
Sao Tome and Principe	64.8 (61.6-67.9)	64.7 (61.1-68.1)	64.7 (60.8-68.4)	65.1 (60.7-69.2)	65.2 (60.6-69.7)	66.0 (60.9-71.1)	66.2 (60.9-71.9)	66.4 (60.6-72.6)	66.6 (60.2-73.3)	66.8 (60.0-73.7)	67.0 (59.9-74.2)	67.2 (59.4-74.8)
Senegal	60.5 (55.9-64.5)	61.0 (55.8-65.5)	61.3 (55.5-66.2)	61.7 (55.4-67.2)	62.2 (55.5-68.0)	62.5 (55.3-68.5)	62.8 (55.1-68.9)	63.0 (55.1-69.3)	63.2 (55.0-69.6)	63.4 (54.6-70.0)	63.6 (54.6-70.3)	63.8 (54.4-70.6)
Sierra Leone	51.0 (48.9-53.2)	51.3 (49.2-53.5)	51.9 (49.5-54.1)	52.4 (49.8-54.8)	53.1 (50.4-55.9)	53.8 (50.9-56.9)	54.7 (51.6-58.2)	55.4 (52.0-59.2)	56.1 (52.1-60.4)	56.7 (52.1-61.9)	54.7 (49.9-59.7)	56.5 (51.0-62.4)
Togo	54.7 (51.3-58.4)	54.6 (51.3-58.2)	55.2 (51.7-58.8)	55.3 (51.8-59.0)	55.1 (51.7-58.9)	55.6 (52.0-59.5)	56.1 (52.3-60.3)	57.0 (52.9-61.5)	57.6 (53.0-62.6)	57.8 (52.9-63.5)	58.1 (52.6-64.6)	58.5 (52.3-65.7)
Eastern Sub-Saharan Africa	53.8 (52.6-54.9)	54.5 (53.3-55.7)	55.3 (54.0-56.6)	56.2 (54.8-57.6)	57.0 (55.5-58.6)	57.7 (56.0-59.4)	58.3 (56.3-60.1)	58.7 (56.5-60.8)	59.2 (56.8-61.5)	59.7 (57.1-62.1)	60.1 (57.4-62.6)	60.5 (57.7-63.2)
Burundi	52.6 (49.3-56.1)	54.2 (50.9-57.5)	55.8 (52.5-59.3)	57.1 (53.6-60.9)	58.2 (54.2-62.5)	59.1 (54.7-64.0)	59.5 (54.7-64.8)	59.7 (54.5-65.6)	60.0 (54.1-66.4)	60.1 (53.6-66.9)	60.1 (53.4-67.4)	60.1 (53.1-67.7)
Comoros	64.9 (62.0-67.9)	65.3 (62.3-68.6)	65.5 (62.4-68.6)	65.5 (62.4-68.6)	66.2 (62.9-69.4)	65.3 (62.0-68.7)	66.8 (62.9-70.6)	66.0 (61.6-70.1)	66.5 (61.7-71.3)	66.6 (61.0-72.0)	66.8 (60.6-72.9)	66.9 (60.0-73.4)
Djibouti	57.4 (47.6-66.1)	57.8 (47.9-66.5)	57.7 (47.9-66.3)	58.7 (49.1-67.3)	59.0 (49.6-67.5)	59.3 (49.9-67.7)	59.5 (49.9-68.1)	59.7 (50.2-68.3)	60.0 (50.5-68.5)	60.3 (50.8-68.8)	60.2 (50.3-68.6)	60.9 (50.3-69.1)
Eritrea	59.9 (50.4-67.5)	59.8 (49.8-67.6)	59.8 (49.9-67.9)	59.9 (50.0-68.0)	59.8 (50.4-68.1)	59.8 (50.1-68.3)	59.8 (50.0-68.4)	59.8 (49.9-68.6)	59.9 (49.9-68.7)	60.0 (50.2-68.9)	60.2 (50.7-69.1)	60.3 (50.9-69.2)
Ethiopia	55.3 (52.8-58.0)	56.0 (53.5-59.0)	57.0 (54.3-60.2)	58.1 (55.2-61.7)	59.2 (55.6-63.1)	60.0 (55.7-64.5)	60.8 (55.8-66.0)	61.5 (55.8-67.1)	62.1 (55.6-67.9)	62.6 (55.5-68.5)	63.1 (55.7-69.0)	63.6 (55.7-69.6)
Kenya	55.0 (53.7-56.3)	56.0 (54.6-57.2)	57.0 (55.7-58.2)	58.3 (56.9-59.6)	59.2 (57.8-60.6)	60.2 (58.8-61.7)	61.2 (59.8-62.7)	61.9 (60.4-63.4)	62.0 (60.4-63.6)	62.3 (60.7-64.0)	62.6 (61.0-64.3)	62.8 (61.1-64.6)
Madagascar	59.6 (56.5-62.2)	60.1 (56.8-63.0)	60.6 (56.9-64.1)	60.9 (57.0-64.9)	61.0 (56.7-65.4)	61.0 (56.3-65.8)	61.1 (56.0-66.6)	61.2 (55.8-67.2)	61.3 (55.5-67.8)	61.5 (54.8-68.4)	61.7 (54.6-69.1)	61.9 (54.5-69.9)
Malawi	46.9 (43.9-50.5)	47.7 (44.8-51.4)	48.6 (45.7-52.2)	49.6 (46.6-53.2)	50.7 (47.8-54.3)	52.1 (48.9-55.6)	53.4 (49.9-57.1)	54.5 (50.6-58.7)	55.7 (51.2-60.4)	56.9 (52.1-62.0)	57.7 (52.3-62.9)	58.2 (52.0-63.7)
Mozambique	51.0 (48.0-54.5)	50.9 (47.9-54.7)	50.9 (47.8-54.9)	51.1 (47.8-55.1)	51.7 (48.2-56.3)	52.0 (48.2-57.2)	52.0 (47.9-57.7)	52.2 (47.6-58.5)	52.3 (47.1-58.9)	52.6 (46.6-59.6)	53.5 (47.2-61.0)	54.3 (47.3-61.9)
Rwanda	56.2 (53.5-59.1)	58.1 (55.3-61.2)	59.9 (56.8-63.2)	61.3 (57.7-64.8)	62.2 (58.1-66.0)	62.2 (57.5-66.2)	63.1 (57.8-67.5)	63.3 (57.7-68.0)	63.4 (57.2-68.3)	63.5 (56.8-68.8)	63.6 (56.4-69.0)	63.7 (56.4-69.3)
Somalia	51.2 (40.8-62.6)	51.7 (41.0-63.1)	51.7 (41.0-63.1)	51.8 (40.7-63.2)	52.0 (40.8-63.3)	52.2 (40.9-63.5)	50.8 (40.0-62.0)	51.3 (39.6-62.4)	52.7 (41.1-63.9)	53.5 (41.7-64.6)	53.7 (41.9-64.9)	53.4 (41.9-64.1)
South Sudan	54.7 (43.0-64.2)	54.9 (43.1-64.3)	55.0 (43.1-64.5)	55.1 (43.0-64.7)	55.2 (42.7-64.7)	55.4 (42.8-64.8)	55.6 (42.9-65.0)	55.4 (42.6-64.9)	55.5 (42.8-65.0)	55.4 (42.8-64.9)	55.5 (43.0-64.9)	55.5 (43.2-64.9)
Tanzania	56.6 (53.1-60.1)	57.1 (53.5-60.8)	57.7 (53.5-61.6)	58.7 (54.0-62.7)	59.8 (54.5-64.1)	60.2 (54.6-64.7)	60.6 (54.5-65.3)	60.6 (54.2-65.6)	60.8 (53.7-66.1)	61.4 (54.1-67.0)	61.8 (54.5-67.6)	62.7 (54.8-68.5)
Uganda	50.3 (47.7-53.0)	51.7 (49.1-54.6)	52.9 (50.1-56.0)	53.3 (50.3-56.7)	53.9 (50.4-57.6)	54.7 (50.9-59.3)	55.5 (51.0-60.8)	56.3 (51.2-62.2)	56.9 (51.2-63.6)	57.4 (51.2-64.5)	57.8 (51.2-65.2)	58.4 (51.3-66.0)
Zambia	45.1 (43.0-47.6)	45.8 (43.6-48.3)	46.5 (44.2-49.1)	47.4 (44.9-50.1)	49.1 (46.2-52.1)	50.1 (47.1-53.2)	50.8 (47.7-54.2)	51.3 (48.0-55.4)	52.3 (48.3-57.0)	53.1 (48.5-58.5)	53.6 (48.6-59.8)	54.0 (48.5-61.1)
Central Sub-Saharan Africa	53.9 (50.7-56.7)	54.6 (51.2-57.4)	55.2 (51.7-58.1)	55.7 (52.1-58.5)	56.1 (52.6-59.0)	56.5 (53.1-59.6)	57.0 (53.4-60.2)	57.4 (53.4-60.8)	57.7 (53.4-61.6)	57.9 (53.5-62.2)	58.4 (53.7-62.9)	58.9 (54.0-63.9)
Angola	54.5 (43.7-64.1)	55.2 (44.1-64.7)	55.8 (44.6-65.2)	56.3 (44.5-65.6)	56.8 (45.0-66.0)	57.2 (45.1-66.3)	57.7 (45.1-66.7)	58.1 (45.6-67.1)	58.5 (46.0-67.6)	58.8 (45.9-67.9)	59.1 (45.8-68.2)	59.5 (46.1-68.6)
Central African Republic	43.4 (37.0-52.0)	43.6 (37.1-52.6)	43.9 (37.6-53.3)	44.3 (37.8-54.0)	44.9 (38.2-55.0)	45.6 (38.7-56.5)	45.9 (38.9-57.3)	46.2 (39.0-57.3)	46.5 (39.2-57.9)	45.5 (38.1-56.0)	45.9 (38.3-56.9)	47.5 (39.6-59.1)
Congo	56.7 (53.6-59.8)	58.1 (54.7-61.6)	59.4 (55.9-63.2)	60.0 (56.5-64.0)	60.8 (57.0-65.1)	61.7 (57.4-66.2)	62.7 (58.0-67.0)	63.2 (58.0-67.7)	62.9 (57.1-67.6)	63.2 (56.9-68.1)	63.3 (56.3-68.4)	63.4 (55.9-68.7)
Democratic Republic of the Congo	54.6 (52.1-56.9)	55.2 (52.6-57.9)	55.8 (53.0-58.6)	56.3 (53.4-59.1)	56.6 (53.4-59.5)	56.9 (53.5-60.2)	57.4 (53.6-61.0)	57.8 (53.7-61.6)	58.0 (53.5-62.3)	58.4 (53.6-63.5)	58.8 (53.6-64.6)	59.4 (53.2-65.6)
Equatorial Guinea	55.5 (44.4-63.7)	55.6 (44.5-63.4)	56.6 (45.3-64.5)	57.0 (45.6-64.7)	57.3 (45.7-65.1)	57.8 (46.4-65.5)	58.4 (47.2-65.8)	58.6 (47.3-66.0)	59.1 (47.5-66.3)	60.1 (48.3-67.1)	60.3 (48.4-67.3)	60.3 (48.5-67.4)
Gabon	58.9 (55.2-63.0)	59.3 (55.6-63.5)	60.0 (56.2-64.0)	60.3 (56.3-64.4)	60.1 (55.8-64.3)	60.3 (55.7-64.6)	61.0 (56.3-65.2)	61.2 (56.1-65.7)	61.6 (56.1-66.4)	61.8 (56.0-66.8)	62.4 (56.3-67.7)	63.6 (56.8-69.0)

SR Table 8: Global deaths for all ages (thousands) and age-standardized rates (per 100,000), and percent change (with 95% uncertainty intervals), for females, 2005 and 2015

	All age deaths (thousands)			Age-standardized rate (per 100,000)		
	2005	2015	Percent change	2005	2015	Percent change
**All causes**	24,760·6 (24,482·8 to 25,051·7)	24,940·7 (24,501·0 to 25,451·1)	0·7 (-1·3 to 3·0)	878·1 (868·3 to 888·2)	703·4 (691·0 to 717·8)	-20·0 (-21·5 to -18·1)
**Communicable, maternal, neonatal, and nutritional diseases**	6,769·4 (6,583·6 to 6,935·2)	5,271·3 (5,079·0 to 5,455·9)	-22·1 (-24·5 to -19·5)	214·9 (208·8 to 220·2)	146·2 (140·8 to 151·4)	-32·0 (-34·1 to -29·7)
**HIV/AIDS and tuberculosis**	1,377·5 (1,278·3 to 1,488·5)	941·1 (856·3 to 1,034·2)	-31·8 (-34·8 to -28·4)	44·2 (40·6 to 47·8)	25·7 (23·3 to 28·3)	-41·9 (-44·5 to -39·1)
Tuberculosis	498·2 (409·4 to 602·0)	385·4 (305·2 to 470·2)	-22·8 (-29·0 to -15·7)	17·2 (14·1 to 20·7)	10·8 (8·5 to 13·1)	-37·7 (-42·6 to -31·9)
HIV/AIDS	879·3 (835·5 to 929·2)	555·8 (521·8 to 592·0)	-36·8 (-40·1 to -33·5)	26·9 (25·6 to 28·4)	14·9 (14·0 to 15·9)	-44·6 (-47·4 to -41·8)
HIV/AIDS - Tuberculosis	167·4 (132·9 to 190·2)	94·5 (71·1 to 109·4)	-43·8 (-47·7 to -38·1)	5·2 (4·1 to 5·9)	2·5 (1·9 to 2·9)	-51·2 (-54·6 to -46·2)
HIV/AIDS resulting in other diseases	711·9 (663·8 to 764·1)	461·3 (424·9 to 498·2)	-35·2 (-39·4 to -31·2)	21·7 (20·3 to 23·3)	12·4 (11·4 to 13·4)	-43·1 (-46·7 to -39·6)
**Diarrhea, lower respiratory, and other common infectious diseases**	2,841·9 (2,708·2 to 2,977·0)	2,354·2 (2,217·9 to 2,478·7)	-17·2 (-20·4 to -13·4)	94·0 (89·5 to 98·2)	66·1 (62·2 to 69·6)	-29·7 (-32·4 to -26·5)
Diarrheal diseases	854·6 (797·6 to 915·7)	638·6 (595·9 to 686·8)	-25·3 (-30·7 to -19·0)	28·2 (26·4 to 30·1)	18·0 (16·8 to 19·3)	-36·5 (-40·8 to -31·3)
Intestinal infectious diseases	98·1 (55·7 to 160·6)	82·6 (46·6 to 135·6)	-15·6 (-22·9 to -9·5)	2·9 (1·7 to 4·8)	2·3 (1·3 to 3·8)	-21·3 (-28·2 to -15·4)
Typhoid fever	81·2 (44·7 to 137·1)	68·7 (37·6 to 114·7)	-15·1 (-22·9 to -8·6)	2·4 (1·3 to 4·1)	1·9 (1·0 to 3·2)	-20·7 (-28·0 to -14·6)
Paratyphoid fever	15·9 (7·2 to 30·4)	13·5 (6·3 to 26·1)	-14·8 (-24·3 to -5·5)	0·5 (0·2 to 0·9)	0·4 (0·2 to 0·7)	-20·9 (-29·5 to -12·3)
Other intestinal infectious diseases	1·0 (0·2 to 2·0)	0·4 (0·1 to 0·8)	-61·0 (-76·1 to -27·2)	0·0 (0·0 to 0·1)	0·0 (0·0 to 0·0)	-64·9 (-78·5 to -37·1)
Lower respiratory infections	1,373·2 (1,245·7 to 1,450·8)	1,297·4 (1,157·0 to 1,387·9)	-5·6 (-9·8 to -0·8)	46·9 (42·4 to 49·6)	36·5 (32·5 to 39·1)	-22·2 (-25·6 to -18·4)
Upper respiratory infections	1·8 (1·5 to 2·1)	1·4 (1·2 to 1·6)	-21·0 (-35·6 to -2·9)	0·1 (0·1 to 0·1)	0·0 (0·0 to 0·0)	-34·7 (-46·5 to -19·3)
Otitis media	1·9 (1·7 to 2·3)	1·6 (1·4 to 2·0)	-15·1 (-30·8 to 9·1)	0·1 (0·1 to 0·1)	0·0 (0·0 to 0·1)	-26·1 (-41·5 to -5·5)
Meningitis	189·7 (160·1 to 220·8)	168·9 (142·5 to 207·7)	-11·6 (-22·2 to 5·7)	5·9 (5·0 to 6·9)	4·7 (4·0 to 5·8)	-21·4 (-30·5 to -6·3)
Pneumococcal meningitis	52·3 (43·1 to 64·2)	50·1 (40·5 to 64·6)	-5·2 (-16·7 to 13·6)	1·6 (1·4 to 2·0)	1·4 (1·1 to 1·8)	-15·8 (-25·8 to 1·0)
H influenzae type B meningitis	51·9 (41·3 to 65·2)	32·7 (25·3 to 43·7)	-37·4 (-47·1 to -22·4)	1·6 (1·3 to 2·0)	0·9 (0·7 to 1·2)	-43·1 (-51·9 to -29·4)
Meningococcal meningitis	34·0 (26·2 to 43·7)	32·3 (25·1 to 43·4)	-5·5 (-20·9 to 16·7)	1·1 (0·8 to 1·3)	0·9 (0·7 to 1·2)	-15·4 (-28·7 to 4·4)
Other meningitis	51·5 (43·4 to 61·3)	53·7 (44·8 to 64·9)	3·7 (-6·2 to 20·8)	1·6 (1·4 to 2·0)	1·5 (1·2 to 1·8)	-9·9 (-18·0 to 4·9)
Encephalitis	66·9 (58·6 to 78·8)	67·9 (59·5 to 83·9)	1·6 (-9·1 to 12·1)	2·1 (1·9 to 2·6)	1·9 (1·7 to 2·3)	-11·7 (-20·9 to -2·9)
Diphtheria	2·7 (1·3 to 6·0)	1·0 (0·4 to 2·8)	-64·9 (-88·9 to 39·2)	0·1 (0·0 to 0·2)	0·0 (0·0 to 0·1)	-67·5 (-89·7 to 29·4)
Whooping cough	54·7 (20·2 to 124·4)	32·4 (11·2 to 69·3)	-40·8 (-78·2 to 62·7)	1·6 (0·6 to 3·7)	0·9 (0·3 to 1·9)	-44·9 (-79·7 to 51·5)
Tetanus	47·3 (38·5 to 71·3)	22·6 (18·0 to 32·6)	-52·1 (-62·2 to -42·4)	1·5 (1·2 to 2·3)	0·6 (0·5 to 0·9)	-57·4 (-66·4 to -48·6)
Measles	145·9 (54·6 to 305·7)	36·4 (13·0 to 80·1)	-75·5 (-84·3 to -59·1)	4·4 (1·6 to 9·1)	1·0 (0·4 to 2·2)	-77·2 (-85·4 to -61·9)
Varicella and herpes zoster	5·0 (4·2 to 5·9)	3·3 (2·7 to 4·3)	-34·5 (-47·7 to -14·1)	0·2 (0·1 to 0·2)	0·1 (0·1 to 0·1)	-46·5 (-56·9 to -30·7)
**Neglected tropical diseases and malaria**	647·4 (537·6 to 757·0)	413·6 (326·4 to 499·4)	-36·2 (-44·8 to -27·2)	19·9 (16·5 to 23·2)	11·5 (9·0 to 13·8)	-42·4 (-50·1 to -34·3)
Malaria	592·5 (484·6 to 702·8)	368·2 (282·6 to 456·7)	-38·0 (-47·5 to -28·1)	18·1 (14·8 to 21·5)	10·2 (7·8 to 12·7)	-43·8 (-52·5 to -34·7)
Chagas disease	3·1 (3·0 to 3·3)	3·5 (3·2 to 3·8)	11·8 (1·5 to 23·4)	0·1 (0·1 to 0·1)	0·1 (0·1 to 0·1)	-13·9 (-21·8 to -5·0)
Leishmaniasis	6·1 (3·7 to 8·9)	6·3 (4·3 to 8·5)	5·2 (-11·2 to 24·5)	0·2 (0·1 to 0·3)	0·2 (0·1 to 0·2)	-5·9 (-20·2 to 11·1)
Visceral leishmaniasis	6·1 (3·7 to 8·9)	6·3 (4·3 to 8·5)	5·2 (-11·2 to 24·5)	0·2 (0·1 to 0·3)	0·2 (0·1 to 0·2)	-5·9 (-20·2 to 11·1)
African trypanosomiasis	6·6 (3·5 to 10·9)	1·6 (0·8 to 2·7)	-76·1 (-84·0 to -65·6)	0·2 (0·1 to 0·3)	0·0 (0·0 to 0·1)	-79·1 (-85·9 to -69·9)
Schistosomiasis	3·7 (3·1 to 4·3)	1·9 (1·6 to 2·3)	-48·3 (-57·9 to -35·6)	0·1 (0·1 to 0·2)	0·1 (0·0 to 0·1)	-59·0 (-66·9 to -48·6)
Cysticercosis	0·3 (0·3 to 0·4)	0·2 (0·1 to 0·2)	-39·8 (-46·3 to -29·6)	0·0 (0·0 to 0·0)	0·0 (0·0 to 0·0)	-50·1 (-55·6 to -41·6)
Cystic echinococcosis	0·9 (0·8 to 0·9)	0·6 (0·5 to 0·6)	-35·1 (-39·0 to -30·7)	0·0 (0·0 to 0·0)	0·0 (0·0 to 0·0)	-46·7 (-49·8 to -43·0)
Dengue	6·2 (4·2 to 8·0)	8·5 (5·3 to 10·8)	40·4 (-8·7 to 93·7)	0·2 (0·1 to 0·2)	0·2 (0·1 to 0·3)	26·1 (-17·3 to 72·3)
Yellow fever	1·7 (0·3 to 4·4)	1·3 (0·3 to 3·7)	-22·0 (-40·1 to 1·9)	0·1 (0·0 to 0·1)	0·0 (0·0 to 0·1)	-28·7 (-44·6 to -6·8)
Rabies	13·1 (10·6 to 15·8)	6·9 (5·0 to 8·9)	-47·2 (-56·3 to -37·4)	0·4 (0·3 to 0·5)	0·2 (0·1 to 0·2)	-54·4 (-62·1 to -46·2)
Intestinal nematode infections	1·9 (1·6 to 2·2)	1·3 (1·2 to 1·5)	-30·7 (-39·9 to -20·8)	0·1 (0·1 to 0·1)	0·0 (0·0 to 0·0)	-36·7 (-45·1 to -27·7)
Ascariasis	1·9 (1·6 to 2·2)	1·3 (1·2 to 1·5)	-30·7 (-39·9 to -20·8)	0·1 (0·1 to 0·1)	0·0 (0·0 to 0·0)	-36·7 (-45·1 to -27·7)
Ebola	0·0 (0·0 to 0·0)	2·7 (2·2 to 3·3)	81,018·8 (81,018·8 to 81,018·8)	0·0 (0·0 to 0·0)	0·1 (0·1 to 0·1)	66,488·3 (66,488·3 to 66,488·3)

SR Table 8: Global deaths for all ages (thousands) and age-standardized rates (per 100,000), and percent change (with 95% uncertainty intervals), for females, 2005 and 2015

	All age deaths (thousands)			Age-standardized rate (per 100,000)		
	2005	2015	Percent change	2005	2015	Percent change
Other neglected tropical diseases	11·3 (8·2 to 16·7)	10·5 (7·7 to 14·8)	-7·2 (-23·4 to 21·2)	0·4 (0·3 to 0·5)	0·3 (0·2 to 0·4)	-18·5 (-32·9 to 6·2)
**Maternal disorders**	350·8 (327·9 to 376·6)	275·3 (243·8 to 315·5)	-21·8 (-30·3 to -10·7)	10·3 (9·6 to 11·1)	7·3 (6·5 to 8·4)	-29·2 (-36·9 to -19·0)
Maternal hemorrhage	99·7 (87·6 to 113·0)	83·1 (67·0 to 101·5)	-16·7 (-28·8 to -3·2)	2·9 (2·6 to 3·3)	2·2 (1·8 to 2·7)	-24·9 (-35·7 to -12·6)
Maternal sepsis and other maternal infections	24·7 (20·4 to 29·9)	17·9 (13·4 to 23·9)	-28·6 (-41·7 to -10·8)	0·7 (0·6 to 0·9)	0·5 (0·4 to 0·6)	-35·6 (-47·4 to -19·6)
Maternal hypertensive disorders	63·7 (54·9 to 74·3)	46·9 (37·1 to 59·6)	-26·7 (-36·7 to -13·1)	1·9 (1·6 to 2·2)	1·3 (1·0 to 1·6)	-33·0 (-42·2 to -20·6)
Maternal obstructed labor and uterine rupture	26·9 (22·1 to 32·0)	23·1 (17·2 to 30·0)	-14·4 (-27·4 to 1·5)	0·8 (0·7 to 0·9)	0·6 (0·5 to 0·8)	-22·7 (-34·3 to -8·3)
Maternal abortion, miscarriage, and ectopic pregnancy	41·2 (34·7 to 49·6)	31·7 (24·6 to 39·7)	-23·2 (-33·9 to -11·1)	1·2 (1·0 to 1·5)	0·8 (0·7 to 1·1)	-30·6 (-40·1 to -19·6)
Indirect maternal deaths	38·2 (31·6 to 45·4)	30·8 (23·1 to 40·5)	-20·3 (-32·4 to -1·9)	1·1 (0·9 to 1·3)	0·8 (0·6 to 1·1)	-27·6 (-38·6 to -10·7)
Late maternal deaths	7·9 (5·3 to 11·5)	6·7 (4·3 to 10·0)	-15·4 (-26·2 to -1·1)	0·2 (0·2 to 0·3)	0·2 (0·1 to 0·3)	-23·3 (-33·1 to -10·3)
Maternal deaths aggravated by HIV/AIDS	2·8 (1·8 to 3·8)	2·3 (1·4 to 3·3)	-16·5 (-33·1 to 8·0)	0·1 (0·1 to 0·1)	0·1 (0·0 to 0·1)	-25·4 (-40·3 to -3·5)
Other maternal disorders	45·7 (39·0 to 53·9)	32·7 (26·3 to 40·5)	-28·7 (-37·1 to -18·3)	1·3 (1·1 to 1·6)	0·9 (0·7 to 1·1)	-35·5 (-43·1 to -26·0)
**Neonatal disorders**	1,165·7 (1,131·9 to 1,200·7)	953·9 (921·9 to 987·9)	-18·2 (-20·5 to -15·6)	34·0 (33·0 to 35·0)	26·3 (25·4 to 27·2)	-22·6 (-24·9 to -20·2)
Neonatal preterm birth complications	496·2 (449·9 to 553·1)	363·0 (320·1 to 409·5)	-26·8 (-34·4 to -18·9)	14·5 (13·1 to 16·1)	10·0 (8·8 to 11·3)	-30·8 (-37·9 to -23·3)
Neonatal encephalopathy due to birth asphyxia and trauma	382·3 (334·1 to 433·0)	328·4 (281·5 to 384·5)	-14·3 (-25·7 to -1·2)	11·1 (9·7 to 12·6)	9·1 (7·8 to 10·6)	-18·9 (-29·7 to -6·6)
Neonatal sepsis and other neonatal infections	129·1 (62·5 to 194·9)	131·5 (65·2 to 200·8)	3·2 (-29·2 to 41·9)	3·8 (1·8 to 5·7)	3·6 (1·8 to 5·5)	-2·5 (-33·1 to 34·0)
Hemolytic disease and other neonatal jaundice	32·7 (16·5 to 56·2)	22·5 (12·7 to 37·7)	-29·1 (-51·9 to 1·3)	1·0 (0·5 to 1·6)	0·6 (0·4 to 1·0)	-33·0 (-54·6 to -4·3)
Other neonatal disorders	125·4 (85·5 to 182·6)	108·6 (75·3 to 152·2)	-12·5 (-40·6 to 24·4)	3·7 (2·5 to 5·3)	3·0 (2·1 to 4·2)	-17·4 (-43·9 to 17·5)
**Nutritional deficiencies**	226·4 (204·8 to 251·7)	192·3 (174·3 to 214·9)	-15·1 (-23·8 to -5·1)	7·5 (6·8 to 8·2)	5·4 (4·9 to 6·0)	-27·9 (-35·0 to -19·6)
Protein-energy malnutrition	182·4 (161·4 to 208·3)	150·5 (132·9 to 171·9)	-17·5 (-27·0 to -6·3)	6·0 (5·3 to 6·8)	4·2 (3·7 to 4·8)	-29·4 (-37·2 to -20·2)
Iodine deficiency	1·2 (0·9 to 1·8)	1·2 (0·9 to 1·7)	-4·7 (-39·4 to 48·4)	0·0 (0·0 to 0·1)	0·0 (0·0 to 0·0)	-20·4 (-48·3 to 21·6)
Iron-deficiency anemia	25·7 (17·5 to 34·1)	27·0 (18·5 to 35·2)	4·8 (-10·7 to 23·7)	0·8 (0·6 to 1·1)	0·8 (0·5 to 1·0)	-11·4 (-24·3 to 4·4)
Other nutritional deficiencies	17·0 (9·3 to 29·1)	13·7 (9·0 to 18·6)	-16·6 (-42·6 to 12·5)	0·6 (0·3 to 1·0)	0·4 (0·3 to 0·5)	-32·8 (-53·5 to -11·3)
**Other communicable, maternal, neonatal, and nutritional diseases**	159·9 (131·5 to 196·0)	140·7 (115·4 to 169·3)	-12·0 (-18·9 to -4·4)	5·1 (4·2 to 6·2)	3·9 (3·2 to 4·7)	-22·9 (-28·8 to -16·5)
Sexually transmitted diseases excluding HIV	67·7 (41·1 to 102·7)	55·3 (33·7 to 84·2)	-18·1 (-26·3 to -9·8)	2·0 (1·2 to 3·1)	1·5 (0·9 to 2·3)	-24·4 (-32·0 to -16·7)
Syphilis	66·8 (40·3 to 101·8)	54·5 (32·8 to 83·4)	-18·3 (-26·8 to -9·8)	2·0 (1·2 to 3·0)	1·5 (0·9 to 2·3)	-24·4 (-32·1 to -16·6)
Chlamydial infection	0·2 (0·1 to 0·2)	0·2 (0·1 to 0·2)	-5·7 (-17·0 to 10·6)	0·0 (0·0 to 0·0)	0·0 (0·0 to 0·0)	-23·5 (-32·6 to -10·5)
Gonococcal infection	0·5 (0·4 to 0·6)	0·5 (0·3 to 0·5)	-5·7 (-17·2 to 10·6)	0·0 (0·0 to 0·0)	0·0 (0·0 to 0·0)	-23·5 (-32·8 to -10·4)
Other sexually transmitted diseases	0·2 (0·2 to 0·3)	0·2 (0·2 to 0·3)	-8·3 (-19·8 to 8·0)	0·0 (0·0 to 0·0)	0·0 (0·0 to 0·0)	-24·3 (-33·8 to -10·5)
Hepatitis	51·5 (49·0 to 54·5)	41·0 (38·6 to 43·8)	-20·4 (-25·1 to -14·8)	1·7 (1·6 to 1·8)	1·1 (1·1 to 1·2)	-32·9 (-36·9 to -28·2)
Acute hepatitis A	8·4 (5·3 to 11·3)	5·4 (3·4 to 7·6)	-35·8 (-48·0 to -22·8)	0·3 (0·2 to 0·3)	0·1 (0·1 to 0·2)	-40·8 (-51·9 to -28·9)
Hepatitis B	27·2 (23·2 to 31·0)	23·3 (19·9 to 26·8)	-14·4 (-22·2 to -5·4)	0·9 (0·8 to 1·1)	0·6 (0·6 to 0·7)	-31·2 (-37·6 to -24·3)
Hepatitis C	1·2 (0·3 to 2·7)	1·0 (0·2 to 2·3)	-16·1 (-34·7 to 9·2)	0·0 (0·0 to 0·1)	0·0 (0·0 to 0·1)	-34·5 (-49·0 to -14·2)
Acute hepatitis E	14·8 (11·0 to 19·6)	11·4 (8·1 to 15·3)	-23·3 (-34·4 to -11·6)	0·5 (0·3 to 0·6)	0·3 (0·2 to 0·4)	-32·1 (-41·8 to -21·8)
Other infectious diseases	40·7 (26·6 to 47·8)	44·4 (25·8 to 53·4)	7·9 (-8·0 to 31·2)	1·3 (0·9 to 1·6)	1·2 (0·7 to 1·5)	-8·5 (-21·8 to 10·5)
**Non-communicable diseases**	16,527·0 (16,292·0 to 16,818·6)	18,270·1 (17,918·5 to 18,683·9)	10·5 (8·3 to 13·0)	614·9 (606·5 to 625·1)	518·5 (508·5 to 530·1)	-15·7 (-17·4 to -13·8)
**Neoplasms**	3,238·5 (3,176·9 to 3,309·5)	3,684·8 (3,592·2 to 3,773·8)	13·7 (10·7 to 17·0)	118·2 (116·0 to 120·8)	104·0 (101·4 to 106·5)	-12·0 (-14·4 to -9·5)
Lip and oral cavity cancer	37·7 (36·4 to 39·1)	48·2 (46·0 to 50·6)	27·8 (22·0 to 34·5)	1·4 (1·3 to 1·4)	1·4 (1·3 to 1·4)	-2·2 (-6·6 to 2·9)
Nasopharynx cancer	17·0 (16·2 to 17·9)	17·3 (16·3 to 18·3)	2·0 (-5·3 to 8·8)	0·6 (0·6 to 0·6)	0·5 (0·5 to 0·5)	-19·2 (-24·9 to -13·8)
Other pharynx cancer	15·4 (14·3 to 16·3)	17·4 (16·1 to 18·7)	11·7 (4·3 to 24·7)	0·6 (0·5 to 0·6)	0·5 (0·5 to 0·5)	-14·1 (-20·0 to -4·3)
Esophageal cancer	147·7 (140·4 to 154·5)	121·3 (114·7 to 128·4)	-18·0 (-23·7 to -11·0)	5·5 (5·2 to 5·7)	3·5 (3·3 to 3·7)	-37·0 (-41·3 to -31·6)
Stomach cancer	299·4 (288·3 to 310·2)	283·8 (273·6 to 294·0)	-5·2 (-9·1 to -0·6)	11·1 (10·7 to 11·5)	8·1 (7·8 to 8·3)	-27·2 (-30·1 to -23·6)
Colon and rectum cancer	321·2 (313·9 to 329·2)	376·2 (363·2 to 390·8)	17·1 (13·5 to 20·9)	12·0 (11·7 to 12·3)	10·7 (10·3 to 11·1)	-10·8 (-13·6 to -8·0)

SR Table 8: Global deaths for all ages (thousands) and age-standardized rates (per 100,000), and percent change (with 95% uncertainty intervals), for females, 2005 and 2015

	All age deaths (thousands)			Age-standardized rate (per 100,000)		
	2005	2015	Percent change	2005	2015	Percent change
Liver cancer	225·2 (191·9 to 238·9)	233·8 (203·6 to 254·8)	2·8 (-3·0 to 16·2)	8·2 (7·0 to 8·7)	6·6 (5·8 to 7·2)	-20·2 (-24·6 to -10·0)
Liver cancer due to hepatitis B	66·8 (54·8 to 73·5)	64·1 (54·8 to 73·1)	-5·3 (-12·4 to 13·1)	2·4 (1·9 to 2·6)	1·8 (1·5 to 2·0)	-25·4 (-31·0 to -11·5)
Liver cancer due to hepatitis C	62·6 (55·3 to 67·1)	71·8 (63·5 to 77·0)	14·0 (9·5 to 23·2)	2·3 (2·1 to 2·5)	2·1 (1·8 to 2·2)	-12·8 (-16·2 to -5·9)
Liver cancer due to alcohol use	36·0 (30·6 to 39·5)	40·9 (35·2 to 45·9)	12·6 (5·7 to 28·7)	1·3 (1·1 to 1·5)	1·2 (1·0 to 1·3)	-13·2 (-18·5 to -0·9)
Liver cancer due to other causes	59·8 (49·7 to 65·2)	57·0 (49·4 to 62·8)	-5·6 (-11·6 to 8·5)	2·2 (1·8 to 2·4)	1·6 (1·4 to 1·8)	-26·7 (-31·3 to -15·9)
Gallbladder and biliary tract cancer	74·5 (71·5 to 77·3)	81·0 (72·9 to 87·2)	8·3 (0·1 to 16·6)	2·8 (2·7 to 2·9)	2·3 (2·1 to 2·5)	-17·6 (-24·0 to -11·3)
Pancreatic cancer	151·7 (148·7 to 154·6)	197·0 (190·6 to 202·9)	29·9 (25·4 to 33·9)	5·7 (5·6 to 5·8)	5·6 (5·4 to 5·8)	-1·0 (-4·5 to 2·0)
Larynx cancer	17·5 (16·1 to 18·4)	19·5 (18·6 to 20·4)	11·1 (5·2 to 19·0)	0·6 (0·6 to 0·7)	0·6 (0·5 to 0·6)	-14·3 (-18·8 to -8·2)
Tracheal, bronchus, and lung cancer	428·7 (417·3 to 441·0)	516·8 (497·4 to 538·2)	20·2 (15·1 to 27·2)	15·9 (15·5 to 16·4)	14·7 (14·2 to 15·3)	-7·7 (-11·6 to -2·3)
Malignant skin melanoma	22·1 (19·9 to 23·4)	27·5 (24·0 to 29·1)	24·3 (18·0 to 31·5)	0·8 (0·7 to 0·8)	0·8 (0·7 to 0·8)	-3·9 (-8·6 to 1·6)
Non-melanoma skin cancer	11·4 (11·0 to 11·9)	17·7 (16·6 to 18·7)	54·9 (45·0 to 65·4)	0·4 (0·4 to 0·4)	0·5 (0·5 to 0·5)	16·4 (8·9 to 24·2)
Non-melanoma skin cancer (squamous-cell carcinoma)	11·4 (11·0 to 11·9)	17·7 (16·6 to 18·7)	54·9 (45·0 to 65·4)	0·4 (0·4 to 0·4)	0·5 (0·5 to 0·5)	16·4 (8·9 to 24·2)
Breast cancer	433·7 (413·0 to 455·8)	523·5 (492·3 to 543·3)	21·1 (14·2 to 26·6)	15·6 (14·9 to 16·3)	14·6 (13·7 to 15·1)	-6·2 (-11·4 to -2·0)
Cervical cancer	225·4 (213·9 to 237·8)	238·6 (225·3 to 252·4)	5·7 (-0·5 to 13·8)	8·0 (7·6 to 8·4)	6·6 (6·2 to 7·0)	-17·4 (-22·2 to -11·0)
Uterine cancer	81·8 (78·5 to 85·4)	89·9 (86·1 to 94·3)	9·8 (3·8 to 17·4)	3·0 (2·9 to 3·1)	2·5 (2·4 to 2·7)	-15·7 (-20·4 to -10·0)
Ovarian cancer	133·8 (130·8 to 138·6)	161·1 (156·5 to 166·5)	20·4 (16·5 to 24·4)	4·9 (4·8 to 5·0)	4·5 (4·4 to 4·7)	-6·9 (-10·0 to -3·9)
Prostate cancer	-	-	-	-	-	-
Testicular cancer	-	-	-	-	-	-
Kidney cancer	38·3 (37·4 to 39·4)	47·8 (46·2 to 49·4)	24·8 (20·0 to 29·3)	1·4 (1·4 to 1·5)	1·4 (1·3 to 1·4)	-4·0 (-7·6 to -0·5)
Bladder cancer	41·9 (41·0 to 43·0)	51·2 (49·3 to 53·3)	22·0 (17·3 to 27·3)	1·6 (1·5 to 1·6)	1·5 (1·4 to 1·5)	-8·0 (-11·6 to -4·0)
Brain and nervous system cancer	85·0 (80·8 to 87·9)	101·6 (96·4 to 106·2)	19·9 (11·9 to 27·1)	3·0 (2·8 to 3·1)	2·8 (2·7 to 3·0)	-3·7 (-10·1 to 1·9)
Thyroid cancer	15·9 (15·0 to 17·8)	18·5 (17·5 to 20·0)	16·9 (8·9 to 22·5)	0·6 (0·6 to 0·7)	0·5 (0·5 to 0·6)	-10·2 (-16·3 to -5·9)
Mesothelioma	7·0 (6·8 to 7·2)	9·2 (8·8 to 9·6)	31·7 (25·7 to 37·7)	0·3 (0·2 to 0·3)	0·3 (0·3 to 0·3)	2·4 (-2·2 to 7·1)
Hodgkin lymphoma	9·8 (8·0 to 12·5)	8·8 (7·4 to 11·8)	-9·9 (-15·9 to -4·1)	0·3 (0·3 to 0·4)	0·2 (0·2 to 0·3)	-27·0 (-31·9 to -22·1)
Non-Hodgkin lymphoma	78·8 (68·1 to 86·4)	98·0 (81·9 to 103·7)	24·9 (12·6 to 30·5)	2·9 (2·5 to 3·1)	2·8 (2·3 to 2·9)	-2·8 (-12·0 to 1·3)
Multiple myeloma	38·9 (37·3 to 40·3)	48·9 (46·3 to 51·2)	25·8 (20·2 to 31·2)	1·4 (1·4 to 1·5)	1·4 (1·3 to 1·5)	-3·9 (-8·2 to 0·2)
Leukemia	132·8 (129·0 to 137·0)	149·2 (144·1 to 154·2)	12·4 (8·3 to 16·4)	4·6 (4·5 to 4·7)	4·2 (4·0 to 4·3)	-8·9 (-12·2 to -5·7)
Acute lymphoid leukemia	41·6 (39·3 to 46·3)	45·5 (43·2 to 49·1)	9·3 (3·5 to 15·6)	1·4 (1·3 to 1·5)	1·3 (1·2 to 1·4)	-6·9 (-11·8 to -1·8)
Chronic lymphoid leukemia	24·1 (22·5 to 25·7)	26·8 (25·5 to 28·3)	11·2 (5·4 to 18·2)	0·9 (0·8 to 0·9)	0·8 (0·7 to 0·8)	-14·5 (-18·8 to -9·2)
Acute myeloid leukemia	51·4 (49·0 to 53·2)	62·0 (59·3 to 64·4)	20·6 (15·9 to 25·7)	1·8 (1·7 to 1·8)	1·7 (1·7 to 1·8)	-2·6 (-6·3 to 1·4)
Chronic myeloid leukemia	15·7 (14·6 to 16·9)	14·9 (14·0 to 15·9)	-5·1 (-11·2 to 0·9)	0·6 (0·5 to 0·6)	0·4 (0·4 to 0·4)	-25·7 (-30·3 to -21·1)
Other neoplasms	146·0 (136·5 to 152·1)	181·1 (162·1 to 192·4)	23·9 (17·1 to 31·8)	5·1 (4·8 to 5·4)	5·1 (4·6 to 5·4)	-1·4 (-6·7 to 4·8)
**Cardiovascular diseases**	7,876·5 (7,741·6 to 8,017·1)	8,501·4 (8,301·4 to 8,722·7)	7·9 (5·6 to 10·5)	297·6 (292·5 to 302·9)	242·0 (236·4 to 248·3)	-18·7 (-20·4 to -16·7)
Rheumatic heart disease	192·1 (175·9 to 206·2)	172·2 (157·4 to 186·2)	-10·3 (-16·3 to -4·9)	6·9 (6·3 to 7·4)	4·8 (4·4 to 5·2)	-29·3 (-34·0 to -25·0)
Ischemic heart disease	3,574·2 (3,510·8 to 3,646·5)	4,035·9 (3,941·3 to 4,146·3)	12·9 (10·6 to 15·5)	135·9 (133·4 to 138·6)	115·0 (112·3 to 118·1)	-15·4 (-17·1 to -13·5)
Cerebrovascular disease	3,107·2 (3,047·0 to 3,177·6)	3,094·9 (3,001·4 to 3,201·1)	-0·5 (-3·0 to 2·7)	117·3 (115·0 to 119·9)	88·3 (85·6 to 91·3)	-24·8 (-26·7 to -22·3)
Ischemic stroke	1,512·6 (1,439·3 to 1,582·2)	1,550·6 (1,477·7 to 1,619·5)	2·5 (-1·3 to 6·2)	58·0 (55·1 to 60·6)	44·4 (42·3 to 46·3)	-23·5 (-26·3 to -20·7)
Hemorrhagic stroke	1,594·6 (1,530·9 to 1,680·2)	1,544·4 (1,472·2 to 1,644·3)	-3·2 (-7·5 to 1·5)	59·3 (56·9 to 62·5)	43·9 (41·9 to 46·8)	-25·9 (-29·2 to -22·5)
Hypertensive heart disease	426·3 (378·6 to 473·5)	517·0 (426·4 to 565·1)	22·2 (6·6 to 29·3)	16·1 (14·3 to 17·9)	14·7 (12·1 to 16·1)	-7·9 (-19·7 to -2·5)
Cardiomyopathy and myocarditis	141·7 (137·3 to 145·8)	154·9 (149·3 to 161·3)	9·3 (5·1 to 13·5)	5·2 (5·0 to 5·3)	4·3 (4·2 to 4·5)	-15·9 (-19·1 to -12·7)
Atrial fibrillation and flutter	82·3 (67·9 to 98·8)	109·1 (89·4 to 131·8)	32·4 (29·6 to 35·7)	3·2 (2·6 to 3·9)	3·1 (2·5 to 3·7)	-4·4 (-6·2 to -2·3)
Aortic aneurysm	48·1 (46·7 to 49·8)	61·4 (59·3 to 64·1)	27·9 (23·1 to 31·7)	1·8 (1·8 to 1·9)	1·7 (1·7 to 1·8)	-4·1 (-7·6 to -1·2)

SR Table 8: Global deaths for all ages (thousands) and age-standardized rates (per 100,000), and percent change (with 95% uncertainty intervals), for females, 2005 and 2015	All age deaths (thousands)			Age-standardized rate (per 100,000)		
	2005	2015	Percent change	2005	2015	Percent change
Peripheral vascular disease	19·5<br>(18·4 to 20·8)	26·8<br>(24·5 to 29·3)	37·3<br>(26·1 to 51·1)	0·8<br>(0·7 to 0·8)	0·8<br>(0·7 to 0·8)	0·0<br>(-8·2 to 10·0)
Endocarditis	38·5<br>(32·2 to 43·9)	46·1<br>(39·4 to 53·3)	20·0<br>(12·9 to 26·5)	1·4<br>(1·2 to 1·6)	1·3<br>(1·1 to 1·5)	-7·1<br>(-12·0 to -2·5)
Other cardiovascular and circulatory diseases	246·5<br>(238·8 to 255·9)	282·9<br>(269·3 to 297·5)	14·8<br>(9·5 to 20·2)	9·2<br>(8·9 to 9·5)	8·0<br>(7·6 to 8·4)	-12·8<br>(-16·9 to -8·7)
**Chronic respiratory diseases**	1,647·7<br>(1,591·2 to 1,705·9)	1,590·4<br>(1,506·9 to 1,669·5)	-3·6<br>(-8·5 to 2·0)	62·0<br>(59·9 to 64·2)	45·6<br>(43·2 to 47·9)	-26·6<br>(-30·3 to -22·3)
Chronic obstructive pulmonary disease	1,363·5<br>(1,275·8 to 1,436·6)	1,311·9<br>(1,239·1 to 1,387·1)	-3·8<br>(-10·8 to 3·4)	51·7<br>(48·4 to 54·5)	37·7<br>(35·6 to 39·9)	-27·1<br>(-32·3 to -21·7)
Pneumoconiosis	9·9<br>(6·8 to 13·3)	12·4<br>(8·4 to 15·7)	23·5<br>(8·1 to 47·9)	0·4<br>(0·3 to 0·5)	0·4<br>(0·2 to 0·4)	-6·9<br>(-18·2 to 11·2)
Silicosis	0·4<br>(0·3 to 0·6)	0·4<br>(0·3 to 0·5)	-4·0<br>(-21·4 to 19·7)	0·0<br>(0·0 to 0·0)	0·0<br>(0·0 to 0·0)	-26·6<br>(-39·7 to -8·7)
Asbestosis	0·5<br>(0·4 to 0·6)	0·6<br>(0·4 to 0·7)	14·7<br>(-1·5 to 32·3)	0·0<br>(0·0 to 0·0)	0·0<br>(0·0 to 0·0)	-10·6<br>(-22·7 to 2·7)
Coal workers pneumoconiosis	0·2<br>(0·1 to 0·2)	0·1<br>(0·1 to 0·2)	-25·5<br>(-45·7 to 12·2)	0·0<br>(0·0 to 0·0)	0·0<br>(0·0 to 0·0)	-42·1<br>(-57·7 to -12·1)
Other pneumoconiosis	8·8<br>(6·0 to 12·1)	11·3<br>(7·6 to 14·5)	26·4<br>(10·2 to 52·7)	0·3<br>(0·2 to 0·5)	0·3<br>(0·2 to 0·4)	-5·1<br>(-16·6 to 14·3)
Asthma	223·8<br>(164·2 to 287·7)	195·8<br>(169·3 to 224·2)	-13·2<br>(-27·3 to 10·1)	8·1<br>(5·9 to 10·5)	5·6<br>(4·8 to 6·4)	-31·9<br>(-43·3 to -13·5)
Interstitial lung disease and pulmonary sarcoidosis	35·6<br>(26·2 to 40·3)	54·0<br>(40·2 to 59·4)	52·3<br>(40·5 to 61·6)	1·3<br>(1·0 to 1·5)	1·5<br>(1·1 to 1·7)	15·9<br>(7·3 to 23·0)
Other chronic respiratory diseases	15·0<br>(10·7 to 20·3)	16·2<br>(11·1 to 20·1)	8·3<br>(-7·8 to 26·4)	0·5<br>(0·4 to 0·7)	0·5<br>(0·3 to 0·6)	-8·2<br>(-20·9 to 7·5)
**Cirrhosis and other chronic liver diseases**	409·9<br>(387·4 to 462·6)	431·0<br>(407·7 to 489·6)	5·0<br>(1·0 to 9·9)	14·6<br>(13·8 to 16·5)	12·1<br>(11·4 to 13·7)	-17·5<br>(-20·5 to -13·7)
Cirrhosis and other chronic liver diseases due to hepatitis B	105·2<br>(95·1 to 123·8)	107·5<br>(97·4 to 127·3)	1·9<br>(-2·7 to 8·1)	3·8<br>(3·4 to 4·5)	3·0<br>(2·7 to 3·6)	-20·3<br>(-23·9 to -15·6)
Cirrhosis and other chronic liver diseases due to hepatitis C	92·0<br>(85·0 to 102·5)	100·8<br>(92·4 to 111·0)	9·5<br>(5·5 to 13·8)	3·3<br>(3·1 to 3·7)	2·8<br>(2·6 to 3·1)	-15·0<br>(-18·0 to -11·7)
Cirrhosis and other chronic liver diseases due to alcohol use	93·1<br>(85·8 to 104·8)	98·7<br>(90·1 to 112·0)	5·7<br>(1·0 to 12·2)	3·3<br>(3·0 to 3·7)	2·7<br>(2·5 to 3·1)	-17·1<br>(-20·8 to -12·1)
Cirrhosis and other chronic liver diseases due to other causes	119·5<br>(111·1 to 138·1)	124·1<br>(114·8 to 143·3)	3·6<br>(-0·1 to 8·6)	4·2<br>(3·9 to 4·8)	3·5<br>(3·2 to 4·0)	-17·1<br>(-20·1 to -13·3)
**Digestive diseases**	516·5<br>(499·3 to 539·2)	543·9<br>(515·7 to 569·7)	5·4<br>(0·1 to 10·3)	18·8<br>(18·2 to 19·6)	15·4<br>(14·6 to 16·1)	-18·3<br>(-22·1 to -14·5)
Peptic ulcer disease	121·4<br>(109·5 to 133·9)	111·4<br>(102·6 to 120·2)	-9·0<br>(-15·3 to 1·7)	4·4<br>(4·0 to 4·9)	3·2<br>(2·9 to 3·4)	-29·3<br>(-34·2 to -21·0)
Gastritis and duodenitis	22·0<br>(12·3 to 30·2)	21·0<br>(13·9 to 29·6)	-4·1<br>(-18·4 to 28·2)	0·8<br>(0·5 to 1·1)	0·6<br>(0·4 to 0·8)	-25·7<br>(-37·0 to -1·2)
Appendicitis	24·7<br>(18·4 to 30·6)	23·0<br>(16·7 to 27·4)	-6·5<br>(-21·9 to 9·5)	0·8<br>(0·6 to 1·0)	0·6<br>(0·5 to 0·8)	-22·5<br>(-34·3 to -9·5)
Paralytic ileus and intestinal obstruction	108·7<br>(91·7 to 123·7)	118·0<br>(102·6 to 134·8)	8·4<br>(1·1 to 17·4)	3·9<br>(3·3 to 4·5)	3·3<br>(2·9 to 3·8)	-15·3<br>(-20·5 to -8·8)
Inguinal, femoral, and abdominal hernia	20·7<br>(13·8 to 24·4)	21·9<br>(14·4 to 25·3)	5·5<br>(-3·7 to 17·9)	0·8<br>(0·5 to 0·9)	0·6<br>(0·4 to 0·7)	-18·9<br>(-25·8 to -9·1)
Inflammatory bowel disease	23·3<br>(20·0 to 26·9)	24·3<br>(21·9 to 27·3)	4·9<br>(-6·4 to 14·8)	0·8<br>(0·7 to 1·0)	0·7<br>(0·6 to 0·8)	-18·9<br>(-26·4 to -11·6)
Vascular intestinal disorders	47·6<br>(42·8 to 52·6)	58·7<br>(53·1 to 65·7)	23·3<br>(16·8 to 30·3)	1·8<br>(1·6 to 2·0)	1·7<br>(1·5 to 1·9)	-7·1<br>(-12·0 to -2·2)
Gallbladder and biliary diseases	57·3<br>(53·3 to 61·5)	62·6<br>(57·8 to 67·5)	9·5<br>(2·2 to 15·7)	2·1<br>(2·0 to 2·3)	1·8<br>(1·6 to 1·9)	-16·2<br>(-21·5 to -11·5)
Pancreatitis	42·8<br>(38·8 to 46·7)	47·6<br>(43·1 to 51·3)	11·4<br>(3·9 to 19·2)	1·5<br>(1·4 to 1·7)	1·3<br>(1·2 to 1·4)	-12·5<br>(-18·3 to -6·6)
Other digestive diseases	48·1<br>(42·4 to 53·3)	55·4<br>(51·6 to 60·3)	15·0<br>(5·2 to 27·1)	1·8<br>(1·6 to 2·0)	1·6<br>(1·5 to 1·7)	-12·3<br>(-19·6 to -3·3)
**Neurological disorders**	997·7<br>(854·2 to 1,136·9)	1,324·6<br>(1,122·4 to 1,524·4)	32·7<br>(30·5 to 34·9)	38·5<br>(32·8 to 44·0)	37·4<br>(31·7 to 42·9)	-3·1<br>(-4·4 to -1·7)
Alzheimer disease and other dementias	872·5<br>(726·6 to 1,014·1)	1,175·9<br>(973·9 to 1,377·3)	34·7<br>(32·7 to 36·8)	34·2<br>(28·4 to 39·7)	33·2<br>(27·5 to 38·8)	-2·9<br>(-4·1 to -1·5)
Parkinson disease	37·2<br>(35·8 to 38·7)	50·7<br>(48·5 to 53·0)	36·4<br>(30·2 to 42·2)	1·4<br>(1·4 to 1·5)	1·5<br>(1·4 to 1·5)	1·3<br>(-3·3 to 5·5)
Epilepsy	46·1<br>(42·6 to 51·6)	47·4<br>(44·5 to 50·7)	4·8<br>(-9·2 to 13·8)	1·5<br>(1·4 to 1·6)	1·3<br>(1·2 to 1·4)	-9·6<br>(-21·2 to -1·9)
Multiple sclerosis	9·8<br>(8·4 to 11·1)	11·2<br>(9·8 to 12·1)	14·2<br>(5·4 to 21·4)	0·3<br>(0·3 to 0·4)	0·3<br>(0·3 to 0·3)	-10·0<br>(-16·5 to -4·5)
Motor neuron disease	12·4<br>(11·9 to 13·5)	15·5<br>(14·5 to 16·3)	26·0<br>(12·7 to 29·9)	0·5<br>(0·4 to 0·5)	0·4<br>(0·4 to 0·5)	-2·1<br>(-12·2 to 0·8)
Other neurological disorders	19·6<br>(18·9 to 22·5)	23·9<br>(22·6 to 26·6)	22·3<br>(15·9 to 27·0)	0·7<br>(0·7 to 0·8)	0·7<br>(0·6 to 0·7)	-1·8<br>(-6·4 to 1·9)
**Mental and substance use disorders**	70·7<br>(68·2 to 73·0)	76·3<br>(71·1 to 79·4)	8·2<br>(2·6 to 12·5)	2·4<br>(2·3 to 2·4)	2·1<br>(1·9 to 2·2)	-11·8<br>(-16·3 to -8·3)
Schizophrenia	9·3<br>(8·7 to 9·9)	7·8<br>(7·3 to 8·4)	-16·3<br>(-23·1 to -7·3)	0·3<br>(0·3 to 0·3)	0·2<br>(0·2 to 0·2)	-33·7<br>(-39·1 to -26·8)
Alcohol use disorders	26·7<br>(25·7 to 27·8)	21·0<br>(20·0 to 21·9)	-21·5<br>(-26·0 to -17·2)	0·9<br>(0·9 to 0·9)	0·6<br>(0·5 to 0·6)	-37·2<br>(-40·7 to -33·8)
Drug use disorders	34·1<br>(31·5 to 36·0)	47·0<br>(41·8 to 49·5)	37·9<br>(27·5 to 46·4)	1·1<br>(1·0 to 1·2)	1·3<br>(1·1 to 1·3)	15·1<br>(6·7 to 22·1)
Opioid use disorders	22·3<br>(19·7 to 23·7)	31·0<br>(26·5 to 33·1)	39·3<br>(28·7 to 48·5)	0·7<br>(0·6 to 0·8)	0·8<br>(0·7 to 0·9)	16·6<br>(7·9 to 24·3)
Cocaine use disorders	2·1<br>(1·9 to 2·3)	3·2<br>(2·7 to 3·7)	56·5<br>(29·5 to 75·1)	0·1<br>(0·1 to 0·1)	0·1<br>(0·1 to 0·1)	30·7<br>(8·2 to 46·1)

SR Table 8: Global deaths for all ages (thousands) and age-standardized rates (per 100,000), and percent change (with 95% uncertainty intervals), for females, 2005 and 2015	All age deaths (thousands)			Age-standardized rate (per 100,000)		
	2005	2015	Percent change	2005	2015	Percent change
Amphetamine use disorders	1·1 (1·0 to 1·5)	1·9 (1·6 to 2·3)	74·5 (37·8 to 103·7)	0·0 (0·0 to 0·0)	0·1 (0·0 to 0·1)	46·2 (16·4 to 70·3)
Other drug use disorders	8·6 (7·8 to 10·8)	10·8 (9·7 to 13·2)	25·6 (12·6 to 38·0)	0·3 (0·3 to 0·4)	0·3 (0·3 to 0·4)	4·2 (-6·2 to 14·0)
Eating disorders	0·5 (0·3 to 0·7)	0·5 (0·3 to 0·7)	8·6 (2·5 to 16·0)	0·0 (0·0 to 0·0)	0·0 (0·0 to 0·0)	-3·0 (-8·3 to 3·3)
Anorexia nervosa	0·4 (0·3 to 0·6)	0·4 (0·3 to 0·6)	6·7 (0·4 to 13·7)	0·0 (0·0 to 0·0)	0·0 (0·0 to 0·0)	-4·5 (-10·1 to 1·5)
Bulimia nervosa	0·0 (0·0 to 0·0)	0·0 (0·0 to 0·1)	35·4 (19·2 to 68·3)	0·0 (0·0 to 0·0)	0·0 (0·0 to 0·0)	18·2 (5·3 to 44·2)
**Diabetes, urogenital, blood, and endocrine diseases**	1,364·0 (1,329·8 to 1,398·1)	1,697·1 (1,647·1 to 1,755·0)	24·4 (20·4 to 28·5)	50·0 (48·8 to 51·2)	48·2 (46·8 to 49·8)	-3·7 (-6·6 to -0·6)
Diabetes mellitus	619·4 (598·7 to 639·1)	772·8 (736·2 to 809·5)	24·9 (19·4 to 30·2)	23·1 (22·4 to 23·9)	22·1 (21·0 to 23·1)	-4·4 (-8·7 to -0·4)
Acute glomerulonephritis	5·9 (4·6 to 6·8)	5·1 (3·3 to 6·2)	-11·0 (-34·0 to -0·8)	0·2 (0·2 to 0·2)	0·1 (0·1 to 0·2)	-28·8 (-47·3 to -20·3)
Chronic kidney disease	466·4 (451·9 to 482·1)	597·1 (576·7 to 620·3)	28·0 (23·7 to 32·8)	17·2 (16·6 to 17·7)	16·9 (16·3 to 17·6)	-1·5 (-4·8 to 2·2)
Chronic kidney disease due to diabetes mellitus	149·8 (143·4 to 156·5)	200·9 (191·6 to 210·9)	34·1 (30·1 to 38·9)	5·6 (5·3 to 5·8)	5·7 (5·5 to 6·0)	2·8 (-0·3 to 6·5)
Chronic kidney disease due to hypertension	209·6 (200·7 to 218·7)	275·7 (263·7 to 288·7)	31·5 (26·7 to 36·9)	7·8 (7·4 to 8·1)	7·8 (7·5 to 8·2)	0·4 (-3·1 to 4·6)
Chronic kidney disease due to glomerulonephritis	95·1 (90·2 to 100·2)	106·2 (99·5 to 113·6)	11·7 (6·3 to 17·0)	3·4 (3·2 to 3·6)	3·0 (2·8 to 3·2)	-12·6 (-16·8 to -8·5)
Chronic kidney disease due to other causes	11·9 (9·9 to 14·1)	14·3 (12·0 to 16·8)	19·9 (12·2 to 28·1)	0·4 (0·4 to 0·5)	0·4 (0·3 to 0·5)	-5·6 (-11·9 to 0·3)
Urinary diseases and male infertility	104·1 (99·5 to 110·5)	137·2 (130·6 to 143·3)	32·2 (25·2 to 37·8)	3·9 (3·7 to 4·1)	3·9 (3·7 to 4·0)	0·7 (-4·5 to 5·0)
Interstitial nephritis and urinary tract infections	85·4 (80·9 to 90·2)	112·9 (106·9 to 118·2)	32·4 (25·5 to 38·4)	3·2 (3·0 to 3·4)	3·2 (3·0 to 3·3)	0·5 (-4·9 to 4·9)
Urolithiasis	6·9 (5·1 to 7·9)	7·8 (6·4 to 9·2)	12·8 (3·4 to 28·4)	0·2 (0·2 to 0·3)	0·2 (0·2 to 0·3)	-12·3 (-19·6 to -0·9)
Other urinary diseases	11·8 (10·5 to 13·9)	16·6 (15·0 to 20·0)	40·7 (25·1 to 55·2)	0·4 (0·4 to 0·5)	0·5 (0·4 to 0·6)	9·7 (-2·5 to 20·7)
Gynecological diseases	7·6 (6·1 to 8·6)	7·9 (5·9 to 9·2)	2·5 (-10·1 to 24·5)	0·3 (0·2 to 0·3)	0·2 (0·2 to 0·3)	-17·4 (-27·5 to 0·0)
Uterine fibroids	2·1 (1·2 to 2·7)	2·3 (1·2 to 3·0)	9·1 (-12·5 to 34·7)	0·1 (0·0 to 0·1)	0·1 (0·0 to 0·1)	-13·3 (-30·5 to 6·3)
Polycystic ovarian syndrome	0·7 (0·2 to 1·3)	0·6 (0·2 to 1·0)	-17·2 (-37·0 to 10·1)	0·0 (0·0 to 0·0)	0·0 (0·0 to 0·0)	-30·2 (-46·9 to -7·8)
Endometriosis	0·0 (0·0 to 0·1)	0·1 (0·0 to 0·1)	22·4 (-0·6 to 59·4)	0·0 (0·0 to 0·0)	0·0 (0·0 to 0·0)	4·5 (-15·0 to 35·8)
Genital prolapse	1·1 (0·6 to 1·8)	0·9 (0·6 to 1·5)	-19·5 (-36·0 to 12·9)	0·0 (0·0 to 0·1)	0·0 (0·0 to 0·0)	-38·5 (-50·9 to -14·0)
Other gynecological diseases	3·6 (2·7 to 4·5)	4·0 (3·0 to 4·8)	9·2 (-5·8 to 34·4)	0·1 (0·1 to 0·2)	0·1 (0·1 to 0·1)	-10·0 (-22·8 to 10·2)
Hemoglobinopathies and hemolytic anemias	102·0 (90·9 to 126·7)	102·2 (88·6 to 131·3)	-0·4 (-18·6 to 23·2)	3·3 (3·0 to 4·1)	2·9 (2·5 to 3·7)	-15·0 (-29·5 to 4·9)
Thalassemias	10·0 (8·5 to 12·4)	8·5 (7·2 to 10·5)	-15·4 (-32·0 to 8·4)	0·3 (0·3 to 0·4)	0·2 (0·2 to 0·3)	-25·4 (-39·3 to -6·0)
Sickle cell disorders	49·5 (39·0 to 73·1)	50·1 (37·3 to 79·5)	-0·6 (-30·8 to 49·2)	1·5 (1·2 to 2·2)	1·4 (1·0 to 2·2)	-9·0 (-36·3 to 36·8)
G6PD deficiency	8·2 (7·2 to 9·2)	9·9 (8·6 to 11·2)	20·7 (11·8 to 31·5)	0·3 (0·2 to 0·3)	0·3 (0·2 to 0·3)	1·6 (-5·9 to 10·5)
Other hemoglobinopathies and hemolytic anemias	34·4 (33·0 to 35·9)	33·8 (30·9 to 36·5)	-0·8 (-10·9 to 5·6)	1·3 (1·2 to 1·3)	1·0 (0·9 to 1·0)	-22·8 (-30·7 to -17·8)
Endocrine, metabolic, blood, and immune disorders	58·5 (56·8 to 60·7)	74·8 (71·8 to 77·4)	28·1 (20·9 to 33·4)	2·1 (2·0 to 2·2)	2·1 (2·0 to 2·2)	1·1 (-4·4 to 5·3)
**Musculoskeletal disorders**	53·4 (47·8 to 57·4)	62·5 (56·8 to 67·5)	17·0 (7·8 to 24·3)	1·9 (1·7 to 2·0)	1·8 (1·6 to 1·9)	-8·3 (-15·2 to -2·8)
Rheumatoid arthritis	18·7 (16·5 to 22·0)	20·7 (18·3 to 25·2)	10·2 (0·8 to 23·1)	0·7 (0·6 to 0·8)	0·6 (0·5 to 0·7)	-15·6 (-22·8 to -5·6)
Other musculoskeletal disorders	34·7 (30·5 to 38·2)	41·8 (35·8 to 45·1)	20·8 (9·1 to 28·3)	1·2 (1·1 to 1·3)	1·2 (1·0 to 1·3)	-4·0 (-12·6 to 1·7)
**Other non-communicable diseases**	352·2 (284·6 to 435·3)	358·1 (306·5 to 409·9)	5·8 (-13·2 to 13·8)	10·7 (8·7 to 13·2)	9·9 (8·5 to 11·4)	-4·2 (-20·5 to 3·2)
Congenital anomalies	302·2 (238·8 to 384·2)	295·3 (253·1 to 339·2)	2·3 (-18·3 to 12·1)	9·0 (7·1 to 11·4)	8·2 (7·0 to 9·4)	-4·9 (-23·9 to 4·2)
Neural tube defects	39·4 (26·1 to 62·6)	31·0 (19·2 to 44·0)	-19·3 (-42·0 to 4·2)	1·2 (0·8 to 1·8)	0·9 (0·5 to 1·2)	-24·1 (-45·6 to -2·0)
Congenital heart anomalies	149·1 (105·8 to 205·4)	141·0 (113·6 to 166·7)	-1·7 (-24·4 to 13·8)	4·4 (3·1 to 6·1)	3·9 (3·1 to 4·6)	-8·3 (-29·5 to 6·0)
Cleft lip and cleft palate	1·3 (0·8 to 1·7)	0·7 (0·5 to 0·9)	-49·6 (-63·3 to -33·1)	0·0 (0·0 to 0·1)	0·0 (0·0 to 0·0)	-52·4 (-65·3 to -36·9)
Down syndrome	16·8 (5·9 to 33·3)	15·5 (7·7 to 25·6)	-2·7 (-37·6 to 41·1)	0·5 (0·2 to 1·0)	0·4 (0·2 to 0·7)	-11·6 (-42·6 to 27·0)
Other chromosomal abnormalities	13·4 (5·4 to 35·5)	15·3 (7·2 to 30·1)	23·6 (-21·7 to 53·1)	0·4 (0·2 to 1·0)	0·4 (0·2 to 0·8)	15·8 (-26·5 to 43·3)
Other congenital anomalies	82·1 (67·2 to 112·8)	91·9 (76·2 to 119·9)	12·1 (-5·6 to 33·4)	2·5 (2·0 to 3·4)	2·5 (2·1 to 3·3)	3·3 (-12·4 to 23·0)
Skin and subcutaneous diseases	40·5 (24·2 to 57·1)	53·8 (32·0 to 73·1)	32·6 (23·4 to 42·8)	1·5 (0·9 to 2·1)	1·5 (0·9 to 2·1)	1·7 (-5·4 to 10·3)
Cellulitis	6·4 (3·5 to 9·3)	8·4 (4·6 to 11·7)	33·0 (15·0 to 51·8)	0·2 (0·1 to 0·3)	0·2 (0·1 to 0·3)	2·5 (-11·3 to 16·6)

SR Table 8: Global deaths for all ages (thousands) and age-standardized rates (per 100,000), and percent change (with 95% uncertainty intervals), for females, 2005 and 2015

	All age deaths (thousands)			Age-standardized rate (per 100,000)		
	**2005**	**2015**	**Percent change**	**2005**	**2015**	**Percent change**
Pyoderma	17·2 (10·3 to 26·7)	23·8 (14·5 to 37·0)	38·7 (26·2 to 50·0)	0·6 (0·4 to 0·9)	0·7 (0·4 to 1·0)	10·0 (0·1 to 19·1)
Decubitus ulcer	15·1 (8·1 to 19·7)	19·1 (10·5 to 24·5)	26·7 (14·3 to 43·4)	0·6 (0·3 to 0·8)	0·5 (0·3 to 0·7)	-6·4 (-15·6 to 6·2)
Other skin and subcutaneous diseases	1·8 (1·1 to 2·6)	2·4 (1·5 to 3·4)	33·0 (19·4 to 45·2)	0·1 (0·0 to 0·1)	0·1 (0·0 to 0·1)	4·3 (-6·3 to 14·1)
Sudden infant death syndrome	9·5 (6·9 to 13·1)	9·0 (6·9 to 13·1)	-4·2 (-26·4 to 24·1)	0·3 (0·2 to 0·4)	0·2 (0·2 to 0·4)	-10·1 (-30·9 to 16·5)
**Injuries**	1,464·1 (1,309·9 to 1,533·9)	1,399·3 (1,246·4 to 1,484·8)	-4·6 (-9·6 to 1·8)	48·3 (43·3 to 50·4)	38·7 (34·5 to 41·1)	-19·9 (-24·0 to -14·7)
**Transport injuries**	383·7 (366·2 to 400·5)	357·6 (336·5 to 379·1)	-6·9 (-14·2 to 1·6)	12·5 (12·0 to 13·0)	9·9 (9·3 to 10·5)	-21·1 (-27·2 to -14·1)
Road injuries	362·4 (346·0 to 378·9)	337·3 (317·5 to 357·0)	-7·1 (-14·3 to 0·9)	11·8 (11·3 to 12·3)	9·3 (8·8 to 9·9)	-21·3 (-27·3 to -14·5)
Pedestrian road injuries	166·2 (157·4 to 175·4)	148·8 (139·1 to 160·0)	-10·6 (-18·2 to -1·0)	5·5 (5·2 to 5·8)	4·1 (3·9 to 4·4)	-25·1 (-31·4 to -17·1)
Cyclist road injuries	16·8 (15·2 to 18·2)	14·5 (13·2 to 15·9)	-13·8 (-23·6 to -1·8)	0·5 (0·5 to 0·6)	0·4 (0·4 to 0·4)	-27·0 (-35·3 to -16·8)
Motorcyclist road injuries	48·5 (44·8 to 52·5)	50·4 (44·5 to 56·7)	4·3 (-9·9 to 17·9)	1·6 (1·4 to 1·7)	1·4 (1·2 to 1·6)	-10·5 (-22·6 to 1·2)
Motor vehicle road injuries	126·8 (119·0 to 135·6)	119·4 (111·8 to 127·2)	-6·0 (-12·6 to 2·1)	4·1 (3·8 to 4·3)	3·3 (3·1 to 3·5)	-19·4 (-25·0 to -12·6)
Other road injuries	4·1 (3·6 to 4·6)	4·2 (3·7 to 4·6)	-0·4 (-11·8 to 17·5)	0·1 (0·1 to 0·1)	0·1 (0·1 to 0·1)	-15·1 (-24·7 to -0·2)
Other transport injuries	21·3 (18·8 to 24·1)	20·3 (18·2 to 24·7)	-5·0 (-15·5 to 10·5)	0·7 (0·6 to 0·8)	0·6 (0·5 to 0·7)	-19·8 (-28·5 to -7·2)
**Unintentional injuries**	680·8 (566·5 to 729·8)	668·9 (549·6 to 717·7)	-2·2 (-6·3 to 5·0)	22·9 (19·1 to 24·4)	18·7 (15·3 to 20·0)	-18·8 (-22·2 to -13·1)
Falls	171·4 (146·3 to 180·9)	211·5 (176·0 to 227·5)	23·6 (14·1 to 32·3)	6·3 (5·4 to 6·6)	6·0 (5·0 to 6·4)	-5·4 (-12·8 to 1·3)
Drowning	126·0 (71·6 to 140·9)	100·7 (60·6 to 119·7)	-20·6 (-26·4 to -5·9)	4·0 (2·3 to 4·4)	2·8 (1·7 to 3·3)	-30·1 (-35·1 to -17·4)
Fire, heat, and hot substances	100·0 (74·3 to 110·7)	87·3 (65·1 to 97·8)	-13·7 (-20·4 to 1·8)	3·2 (2·4 to 3·6)	2·4 (1·8 to 2·7)	-26·1 (-31·9 to -13·7)
Poisonings	39·3 (27·4 to 46·9)	34·5 (22·9 to 41·4)	-12·7 (-24·7 to 4·3)	1·3 (0·9 to 1·5)	1·0 (0·6 to 1·1)	-25·4 (-34·9 to -11·0)
Exposure to mechanical forces	54·7 (47·7 to 58·4)	55·4 (44·4 to 59·9)	1·9 (-8·8 to 7·8)	1·8 (1·6 to 1·9)	1·5 (1·2 to 1·7)	-13·9 (-22·5 to -9·1)
Unintentional firearm injuries	8·6 (5·8 to 9·6)	9·2 (6·4 to 10·4)	7·5 (1·1 to 13·9)	0·3 (0·2 to 0·3)	0·3 (0·2 to 0·3)	-13·1 (-18·1 to -8·0)
Unintentional suffocation	12·1 (8·8 to 13·7)	12·0 (8·1 to 13·7)	0·2 (-17·7 to 15·3)	0·4 (0·3 to 0·4)	0·3 (0·2 to 0·4)	-10·9 (-26·6 to 2·1)
Other exposure to mechanical forces	34·1 (30·0 to 36·3)	34·2 (27·2 to 37·2)	1·1 (-11·5 to 9·0)	1·1 (1·0 to 1·2)	1·0 (0·8 to 1·0)	-15·1 (-24·8 to -8·4)
Adverse effects of medical treatment	42·7 (31·4 to 51·1)	43·0 (31·7 to 50·1)	0·3 (-5·2 to 9·5)	1·5 (1·1 to 1·8)	1·2 (0·9 to 1·4)	-18·2 (-22·3 to -12·0)
Animal contact	44·6 (24·7 to 50·0)	39·0 (20·9 to 55·0)	-14·5 (-22·1 to 16·6)	1·4 (0·8 to 1·6)	1·1 (0·6 to 1·5)	-26·2 (-32·6 to 0·7)
Venomous animal contact	38·4 (20·2 to 43·9)	33·5 (16·7 to 48·8)	-15·2 (-23·7 to 17·1)	1·2 (0·6 to 1·4)	0·9 (0·5 to 1·3)	-26·8 (-33·9 to 1·2)
Non-venomous animal contact	6·1 (4·4 to 7·7)	5·5 (4·0 to 6·5)	-9·5 (-22·8 to 9·2)	0·2 (0·1 to 0·2)	0·2 (0·1 to 0·2)	-22·1 (-33·1 to -6·5)
Foreign body	56·4 (45·1 to 76·2)	61·0 (51·8 to 74·0)	10·5 (-4·8 to 19·7)	1·9 (1·5 to 2·5)	1·7 (1·4 to 2·1)	-8·5 (-19·0 to -1·9)
Pulmonary aspiration and foreign body in airway	45·3 (34·1 to 66·9)	49·9 (40·4 to 63·9)	14·7 (-6·2 to 23·0)	1·5 (1·2 to 2·2)	1·4 (1·1 to 1·8)	-5·5 (-20·0 to -0·2)
Foreign body in other body part	11·1 (7·5 to 15·5)	11·1 (8·2 to 13·6)	1·8 (-16·5 to 16·0)	0·4 (0·3 to 0·5)	0·3 (0·2 to 0·4)	-14·4 (-29·9 to -3·6)
Environmental heat and cold exposure	16·0 (11·3 to 17·8)	13·6 (9·5 to 15·7)	-14·7 (-21·2 to -8·5)	0·6 (0·4 to 0·6)	0·4 (0·3 to 0·4)	-32·2 (-37·2 to -27·3)
Other unintentional injuries	29·7 (27·9 to 31·9)	22·8 (21·2 to 24·5)	-23·3 (-29·5 to -16·7)	1·0 (0·9 to 1·0)	0·6 (0·6 to 0·7)	-35·1 (-40·3 to -29·2)
**Self-harm and interpersonal violence**	359·3 (281·0 to 377·7)	328·2 (268·7 to 358·8)	-8·7 (-16·6 to 2·5)	11·6 (9·1 to 12·2)	9·0 (7·3 to 9·8)	-22·8 (-29·4 to -13·4)
Self-harm	271·1 (196·5 to 287·5)	249·2 (190·0 to 277·0)	-8·2 (-17·1 to 9·1)	8·8 (6·4 to 9·4)	6·8 (5·2 to 7·6)	-22·9 (-30·4 to -8·6)
Interpersonal violence	88·3 (84·2 to 91·9)	79·0 (73·1 to 85·0)	-10·6 (-15·9 to -4·2)	2·8 (2·7 to 2·9)	2·2 (2·0 to 2·3)	-22·9 (-27·5 to -17·5)
Assault by firearm	20·3 (16·8 to 22·4)	21·1 (17·2 to 24·3)	3·7 (-3·4 to 14·4)	0·6 (0·5 to 0·7)	0·6 (0·5 to 0·7)	-9·4 (-15·6 to -0·2)
Assault by sharp object	21·0 (19·8 to 22·8)	17·2 (15·9 to 19·1)	-18·4 (-23·9 to -12·1)	0·7 (0·6 to 0·7)	0·5 (0·4 to 0·5)	-30·0 (-34·8 to -24·8)
Assault by other means	46·9 (44·1 to 50·2)	40·7 (36·8 to 45·2)	-13·6 (-19·6 to -5·9)	1·5 (1·4 to 1·6)	1·1 (1·0 to 1·2)	-25·6 (-30·8 to -19·1)
**Forces of nature, war, and legal intervention**	40·3 (26·1 to 54·5)	44·6 (26·0 to 63·3)	10·9 (-30·9 to 64·5)	1·3 (0·8 to 1·7)	1·2 (0·7 to 1·7)	-2·1 (-39·4 to 44·7)
Exposure to forces of nature	32·7 (19·1 to 46·1)	4·5 (2·7 to 6·3)	-86·1 (-87·4 to -84·7)	1·0 (0·6 to 1·4)	0·1 (0·1 to 0·2)	-87·7 (-88·9 to -86·4)
Collective violence and legal intervention	7·6 (5·6 to 11·0)	40·1 (22·5 to 58·1)	438·5 (240·9 to 555·8)	0·2 (0·2 to 0·4)	1·1 (0·6 to 1·6)	377·2 (188·4 to 483·7)

SR Table 9: Global deaths for all ages (thousands) and age-standardized rates (per 100,000), and percent change (with 95% uncertainty intervals), for males, 2005 and 2015

	All age deaths (thousands)			Age-standardized rate (per 100,000)		
	2005	2015	Percent change	2005	2015	Percent change
**All causes**	28,857·9 (28,506·9 to 29,210·7)	30,852·2 (30,259·0 to 31,461·3)	6·9 (4·8 to 9·1)	1,195·0 (1,180·9 to 1,209·1)	1,018·6 (1,000·4 to 1,037·1)	-14·8 (-16·4 to -13·1)
**Communicable, maternal, neonatal, and nutritional diseases**	7,254·5 (7,057·8 to 7,529·3)	5,992·3 (5,738·8 to 6,281·3)	-17·4 (-19·9 to -14·9)	240·3 (232·6 to 251·0)	174·4 (166·4 to 183·0)	-27·4 (-29·6 to -25·2)
**HIV/AIDS and tuberculosis**	1,762·1 (1,580·0 to 2,041·6)	1,364·0 (1,185·1 to 1,617·7)	-22·5 (-27·6 to -18·0)	60·1 (53·0 to 71·1)	38·7 (33·2 to 46·7)	-35·5 (-40·0 to -31·7)
Tuberculosis	849·4 (666·3 to 1,134·3)	727·2 (545·2 to 974·9)	-14·0 (-23·3 to -6·8)	32·2 (25·2 to 43·3)	21·9 (16·5 to 29·5)	-31·7 (-39·2 to -25·9)
HIV/AIDS	912·7 (869·1 to 973·2)	636·8 (596·4 to 689·9)	-30·2 (-34·0 to -25·7)	27·9 (26·6 to 29·9)	16·8 (15·7 to 18·2)	-39·8 (-43·0 to -35·9)
HIV/AIDS - Tuberculosis	184·4 (148·2 to 208·6)	117·2 (90·7 to 135·7)	-36·6 (-41·1 to -30·6)	5·7 (4·6 to 6·4)	3·1 (2·4 to 3·6)	-45·8 (-49·7 to -40·7)
HIV/AIDS resulting in other diseases	728·2 (682·6 to 788·3)	519·5 (476·5 to 572·9)	-28·7 (-33·6 to -23·6)	22·2 (20·8 to 24·1)	13·7 (12·6 to 15·1)	-38·3 (-42·4 to -33·9)
**Diarrhea, lower respiratory, and other common infectious diseases**	2,931·3 (2,777·0 to 3,089·7)	2,605·6 (2,424·2 to 2,756·5)	-11·1 (-15·1 to -7·0)	106·3 (100·6 to 112·3)	81·8 (75·6 to 86·5)	-23·1 (-26·2 to -19·8)
Diarrheal diseases	802·6 (747·8 to 862·3)	673·5 (615·6 to 733·9)	-16·2 (-23·5 to -7·7)	28·2 (26·5 to 30·1)	20·4 (18·6 to 22·3)	-27·7 (-34·0 to -20·5)
Intestinal infectious diseases	110·5 (62·2 to 181·7)	96·0 (54·0 to 158·9)	-13·2 (-19·3 to -6·9)	3·2 (1·8 to 5·2)	2·5 (1·4 to 4·2)	-19·3 (-24·9 to -13·5)
Typhoid fever	91·7 (49·9 to 154·3)	80·0 (43·8 to 133·1)	-12·8 (-19·3 to -6·2)	2·6 (1·4 to 4·4)	2·1 (1·2 to 3·5)	-18·8 (-24·6 to -12·6)
Paratyphoid fever	18·1 (8·3 to 34·4)	15·7 (7·3 to 30·3)	-13·1 (-22·6 to -3·6)	0·5 (0·2 to 1·0)	0·4 (0·2 to 0·8)	-19·5 (-28·2 to -10·9)
Other intestinal infectious diseases	0·8 (0·2 to 1·6)	0·3 (0·1 to 0·7)	-65·7 (-79·6 to -39·8)	0·0 (0·0 to 0·1)	0·0 (0·0 to 0·0)	-69·9 (-81·4 to -47·0)
Lower respiratory infections	1,455·3 (1,315·8 to 1,571·1)	1,439·3 (1,274·4 to 1,564·7)	-1·1 (-6·0 to 3·5)	58·0 (52·1 to 63·2)	47·9 (42·2 to 52·3)	-17·4 (-21·0 to -13·9)
Upper respiratory infections	2·0 (1·7 to 2·3)	1·7 (1·5 to 2·0)	-15·6 (-28·9 to 1·9)	0·1 (0·1 to 0·1)	0·1 (0·0 to 0·1)	-30·6 (-41·7 to -16·2)
Otitis media	2·0 (1·8 to 2·3)	1·6 (1·4 to 1·8)	-21·2 (-29·4 to -9·3)	0·1 (0·1 to 0·1)	0·0 (0·0 to 0·0)	-30·4 (-38·2 to -19·9)
Meningitis	217·9 (175·9 to 253·4)	210·4 (167·1 to 255·0)	-3·9 (-14·1 to 10·3)	6·7 (5·4 to 7·8)	5·8 (4·6 to 7·0)	-14·4 (-23·2 to -2·4)
Pneumococcal meningitis	59·8 (47·7 to 74·7)	62·8 (49·1 to 80·8)	4·5 (-6·7 to 20·2)	1·9 (1·5 to 2·3)	1·7 (1·4 to 2·2)	-7·6 (-17·3 to 5·5)
H influenzae type B meningitis	58·7 (44·6 to 73·3)	38·7 (29·4 to 50·0)	-34·1 (-43·4 to -22·7)	1·7 (1·3 to 2·2)	1·0 (0·8 to 1·4)	-39·6 (-48·0 to -29·5)
Meningococcal meningitis	40·3 (30·5 to 51·6)	41·0 (31·1 to 52·8)	1·6 (-13·7 to 22·5)	1·2 (0·9 to 1·5)	1·1 (0·8 to 1·4)	-9·4 (-21·9 to 8·9)
Other meningitis	59·2 (48·0 to 70·6)	67·8 (51·9 to 83·1)	14·3 (2·9 to 29·7)	1·9 (1·5 to 2·3)	1·9 (1·5 to 2·3)	-1·1 (-10·5 to 11·3)
Encephalitis	80·1 (74·0 to 87·3)	81·6 (75·2 to 88·7)	2·1 (-6·1 to 9·4)	2·7 (2·5 to 2·9)	2·4 (2·2 to 2·6)	-11·7 (-18·5 to -5·4)
Diphtheria	2·8 (1·3 to 7·3)	1·1 (0·5 to 3·0)	-59·9 (-89·1 to 38·8)	0·1 (0·0 to 0·2)	0·0 (0·0 to 0·1)	-62·9 (-89·9 to 26·6)
Whooping cough	44·9 (16·4 to 103·2)	26·3 (9·0 to 57·0)	-41·9 (-78·1 to 64·9)	1·2 (0·5 to 2·9)	0·7 (0·2 to 1·5)	-45·8 (-79·6 to 53·7)
Tetanus	60·7 (49·2 to 89·9)	34·1 (26·6 to 52·0)	-44·1 (-52·0 to -32·9)	1·8 (1·5 to 2·9)	0·9 (0·7 to 1·4)	-50·1 (-56·8 to -40·1)
Measles	147·8 (55·0 to 306·4)	37·0 (13·2 to 82·0)	-75·6 (-84·8 to -58·3)	4·1 (1·5 to 8·5)	1·0 (0·3 to 2·1)	-77·2 (-85·8 to -61·1)
Varicella and herpes zoster	4·6 (3·9 to 5·5)	3·1 (2·4 to 4·1)	-33·7 (-48·4 to -11·4)	0·2 (0·1 to 0·2)	0·1 (0·1 to 0·1)	-46·3 (-58·2 to -29·4)
**Neglected tropical diseases and malaria**	651·2 (542·0 to 763·0)	429·5 (343·5 to 521·5)	-34·1 (-43·5 to -24·4)	19·2 (16·1 to 22·4)	11·5 (9·2 to 14·0)	-40·1 (-48·6 to -31·5)
Malaria	574·5 (466·8 to 685·9)	362·2 (275·6 to 456·5)	-36·8 (-47·5 to -26·2)	16·8 (13·7 to 20·0)	9·6 (7·3 to 12·2)	-42·5 (-52·2 to -33·0)
Chagas disease	4·4 (4·1 to 4·6)	4·6 (4·2 to 5·0)	4·3 (-5·4 to 15·7)	0·2 (0·2 to 0·2)	0·2 (0·1 to 0·2)	-19·3 (-26·7 to -10·3)
Leishmaniasis	17·0 (10·8 to 24·5)	17·9 (12·6 to 24·4)	5·6 (-9·6 to 23·6)	0·5 (0·3 to 0·7)	0·5 (0·3 to 0·6)	-7·2 (-20·3 to 8·0)
Visceral leishmaniasis	17·0 (10·8 to 24·5)	17·9 (12·6 to 24·4)	5·6 (-9·6 to 23·6)	0·5 (0·3 to 0·7)	0·5 (0·3 to 0·6)	-7·2 (-20·3 to 8·0)
African trypanosomiasis	7·6 (4·0 to 12·6)	1·9 (1·0 to 3·3)	-74·8 (-82·2 to -64·1)	0·2 (0·1 to 0·4)	0·1 (0·0 to 0·1)	-78·0 (-84·3 to -68·6)
Schistosomiasis	4·1 (3·5 to 4·9)	2·5 (2·1 to 2·9)	-40·2 (-52·3 to -27·5)	0·2 (0·1 to 0·2)	0·1 (0·1 to 0·1)	-52·7 (-62·8 to -42·5)
Cysticercosis	0·3 (0·3 to 0·4)	0·2 (0·2 to 0·3)	-35·8 (-42·7 to -24·3)	0·0 (0·0 to 0·0)	0·0 (0·0 to 0·0)	-46·8 (-52·5 to -37·2)
Cystic echinococcosis	0·9 (0·8 to 0·9)	0·6 (0·6 to 0·7)	-30·2 (-34·0 to -26·2)	0·0 (0·0 to 0·0)	0·0 (0·0 to 0·0)	-42·6 (-45·8 to -39·5)
Dengue	6·2 (3·7 to 7·9)	9·8 (5·4 to 12·9)	60·5 (4·7 to 108·3)	0·2 (0·1 to 0·2)	0·3 (0·1 to 0·4)	45·1 (-4·6 to 85·6)
Yellow fever	4·6 (1·0 to 12·7)	3·8 (0·8 to 10·5)	-18·3 (-35·0 to 5·5)	0·1 (0·0 to 0·4)	0·1 (0·0 to 0·3)	-25·7 (-40·4 to -4·2)
Rabies	19·0 (16·1 to 21·5)	10·5 (8·8 to 12·7)	-44·9 (-52·3 to -35·5)	0·6 (0·5 to 0·7)	0·3 (0·2 to 0·3)	-52·1 (-58·4 to -44·3)
Intestinal nematode infections	1·9 (1·7 to 2·2)	1·4 (1·2 to 1·6)	-26·2 (-36·3 to -15·7)	0·1 (0·0 to 0·1)	0·0 (0·0 to 0·0)	-32·5 (-41·6 to -23·0)
Ascariasis	1·9 (1·7 to 2·2)	1·4 (1·2 to 1·6)	-26·2 (-36·3 to -15·7)	0·1 (0·0 to 0·1)	0·0 (0·0 to 0·0)	-32·5 (-41·6 to -23·0)
Ebola	0·0 (0·0 to 0·0)	2·8 (2·2 to 3·3)	20,569·2 (20,569·2 to 20,569·2)	0·0 (0·0 to 0·0)	0·1 (0·1 to 0·1)	18,485·8 (18,485·8 to 18,485·8)

SR Table 9: Global deaths for all ages (thousands) and age-standardized rates (per 100,000), and percent change (with 95% uncertainty intervals), for males, 2005 and 2015	All age deaths (thousands)			Age-standardized rate (per 100,000)		
	2005	2015	Percent change	2005	2015	Percent change
Other neglected tropical diseases	10·6 (4·4 to 14·2)	11·2 (4·1 to 15·0)	4·2 (-12·1 to 42·1)	0·3 (0·1 to 0·5)	0·3 (0·1 to 0·4)	-9·1 (-24·4 to 22·6)
**Maternal disorders**	-	-	-	-	-	-
Maternal hemorrhage	-	-	-	-	-	-
Maternal sepsis and other maternal infections	-	-	-	-	-	-
Maternal hypertensive disorders	-	-	-	-	-	-
Maternal obstructed labor and uterine rupture	-	-	-	-	-	-
Maternal abortion, miscarriage, and ectopic pregnancy	-	-	-	-	-	-
Indirect maternal deaths	-	-	-	-	-	-
Late maternal deaths	-	-	-	-	-	-
Maternal deaths aggravated by HIV/AIDS	-	-	-	-	-	-
Other maternal disorders	-	-	-	-	-	-
**Neonatal disorders**	1,487·8 (1,440·4 to 1,539·8)	1,209·3 (1,165·9 to 1,257·5)	-18·7 (-20·8 to -16·5)	40·4 (39·2 to 41·9)	31·2 (30·1 to 32·4)	-22·9 (-24·9 to -20·8)
Neonatal preterm birth complications	591·8 (534·0 to 686·3)	442·8 (390·0 to 517·2)	-25·2 (-32·9 to -16·9)	16·1 (14·5 to 18·7)	11·4 (10·1 to 13·3)	-29·0 (-36·4 to -21·2)
Neonatal encephalopathy due to birth asphyxia and trauma	500·5 (440·4 to 566·2)	412·0 (357·1 to 469·2)	-17·8 (-27·0 to -5·9)	13·6 (12·0 to 15·4)	10·6 (9·2 to 12·1)	-22·0 (-30·7 to -10·8)
Neonatal sepsis and other neonatal infections	223·2 (141·4 to 328·0)	220·2 (144·2 to 317·4)	-1·3 (-19·4 to 22·1)	6·1 (3·8 to 8·9)	5·7 (3·7 to 8·2)	-6·4 (-23·6 to 15·8)
Hemolytic disease and other neonatal jaundice	35·6 (23·7 to 60·3)	22·6 (15·5 to 35·8)	-35·8 (-49·8 to -19·2)	1·0 (0·6 to 1·6)	0·6 (0·4 to 0·9)	-39·2 (-52·4 to -23·5)
Other neonatal disorders	136·7 (93·9 to 179·5)	111·7 (81·3 to 145·5)	-18·4 (-38·1 to 11·5)	3·7 (2·6 to 4·9)	2·9 (2·1 to 3·8)	-22·7 (-41·4 to 5·5)
**Nutritional deficiencies**	234·4 (153·7 to 307·7)	213·4 (137·0 to 291·0)	-8·8 (-27·2 to 12·9)	8·1 (5·4 to 10·4)	6·4 (4·1 to 8·6)	-21·3 (-34·6 to -5·2)
Protein-energy malnutrition	197·3 (127·6 to 264·9)	172·7 (112·1 to 240·4)	-12·3 (-32·7 to 12·3)	6·7 (4·4 to 8·8)	5·2 (3·4 to 7·0)	-23·6 (-38·9 to -5·4)
Iodine deficiency	0·8 (0·5 to 1·4)	0·8 (0·5 to 1·3)	0·9 (-40·8 to 66·5)	0·0 (0·0 to 0·0)	0·0 (0·0 to 0·0)	-15·5 (-45·6 to 35·7)
Iron-deficiency anemia	22·6 (10·0 to 33·9)	27·2 (12·4 to 41·9)	19·5 (-0·6 to 47·5)	0·8 (0·3 to 1·2)	0·8 (0·4 to 1·2)	0·8 (-15·1 to 22·1)
Other nutritional deficiencies	13·7 (10·0 to 18·7)	12·7 (9·6 to 16·9)	-7·4 (-21·3 to 7·3)	0·5 (0·4 to 0·7)	0·4 (0·3 to 0·5)	-24·1 (-34·9 to -13·0)
**Other communicable, maternal, neonatal, and nutritional diseases**	187·8 (157·5 to 224·7)	170·6 (139·9 to 202·7)	-9·2 (-15·2 to -3·0)	6·1 (5·2 to 7·1)	4·9 (4·0 to 5·7)	-20·3 (-25·8 to -15·2)
Sexually transmitted diseases excluding HIV	67·8 (40·3 to 104·7)	52·7 (30·7 to 82·5)	-22·3 (-30·6 to -14·3)	1·9 (1·1 to 2·9)	1·4 (0·8 to 2·1)	-28·1 (-35·8 to -20·6)
Syphilis	67·3 (39·8 to 104·1)	52·3 (30·3 to 82·1)	-22·3 (-30·6 to -14·2)	1·9 (1·1 to 2·9)	1·4 (0·8 to 2·1)	-27·9 (-35·7 to -20·4)
Chlamydial infection	-	-	-	-	-	-
Gonococcal infection	0·4 (0·3 to 0·4)	0·3 (0·2 to 0·3)	-29·7 (-42·5 to -14·1)	0·0 (0·0 to 0·0)	0·0 (0·0 to 0·0)	-43·6 (-53·8 to -30·9)
Other sexually transmitted diseases	0·2 (0·1 to 0·2)	0·1 (0·1 to 0·1)	-30·0 (-42·7 to -14·2)	0·0 (0·0 to 0·0)	0·0 (0·0 to 0·0)	-43·5 (-53·8 to -30·7)
Hepatitis	71·5 (68·5 to 74·6)	64·7 (60·7 to 68·5)	-9·5 (-14·8 to -4·4)	2·5 (2·4 to 2·6)	1·9 (1·8 to 2·0)	-24·7 (-28·8 to -20·6)
Acute hepatitis A	8·6 (5·4 to 12·0)	5·8 (3·5 to 8·5)	-32·2 (-44·6 to -18·8)	0·2 (0·2 to 0·4)	0·2 (0·1 to 0·2)	-37·9 (-48·8 to -25·7)
Hepatitis B	44·2 (38·3 to 49·9)	42·1 (35·8 to 47·4)	-4·9 (-13·0 to 4·0)	1·6 (1·4 to 1·8)	1·2 (1·1 to 1·4)	-23·6 (-29·8 to -16·7)
Hepatitis C	1·6 (0·3 to 3·6)	1·5 (0·3 to 3·6)	-5·4 (-26·5 to 20·7)	0·1 (0·0 to 0·1)	0·0 (0·0 to 0·1)	-26·4 (-43·3 to -6·5)
Acute hepatitis E	17·1 (11·6 to 23·4)	15·4 (10·0 to 21·7)	-10·7 (-24·5 to 4·5)	0·6 (0·4 to 0·8)	0·4 (0·3 to 0·6)	-21·8 (-34·1 to -8·4)
Other infectious diseases	48·5 (31·2 to 57·9)	53·1 (32·2 to 63·9)	9·0 (-3·5 to 26·7)	1·7 (1·1 to 2·0)	1·6 (1·0 to 1·9)	-5·6 (-17·1 to 7·8)
**Non-communicable diseases**	18,308·6 (17,995·9 to 18,607·1)	21,534·1 (21,080·8 to 22,019·1)	17·6 (15·0 to 20·3)	844·8 (831·7 to 856·7)	749·9 (735·2 to 765·7)	-11·2 (-13·0 to -9·4)
**Neoplasms**	4,254·3 (4,163·7 to 4,355·7)	5,079·8 (4,939·7 to 5,231·4)	19·4 (16·2 to 22·9)	189·0 (185·1 to 193·4)	171·9 (167·3 to 177·1)	-9·0 (-11·3 to -6·6)
Lip and oral cavity cancer	72·5 (70·5 to 74·7)	97·8 (94·3 to 101·5)	34·8 (29·5 to 40·7)	3·1 (3·0 to 3·1)	3·1 (3·0 to 3·3)	2·9 (-1·1 to 7·1)
Nasopharynx cancer	38·8 (28·3 to 41·4)	45·6 (33·7 to 49·2)	17·7 (8·2 to 26·3)	1·5 (1·1 to 1·6)	1·4 (1·0 to 1·5)	-7·4 (-14·7 to -1·2)
Other pharynx cancer	36·5 (35·4 to 37·5)	47·0 (45·0 to 49·2)	28·9 (22·8 to 34·6)	1·5 (1·5 to 1·5)	1·5 (1·4 to 1·5)	-1·8 (-6·4 to 2·4)
Esophageal cancer	311·6 (298·9 to 324·3)	317·8 (302·0 to 335·3)	2·0 (-4·6 to 9·5)	13·6 (13·1 to 14·1)	10·5 (10·0 to 11·1)	-22·5 (-27·5 to -16·9)
Stomach cancer	525·0 (510·6 to 540·9)	535·1 (515·9 to 556·3)	1·9 (-2·7 to 6·5)	23·6 (23·0 to 24·2)	18·3 (17·7 to 19·0)	-22·4 (-25·7 to -19·2)
Colon and rectum cancer	354·3 (347·7 to 361·7)	455·8 (442·4 to 468·2)	28·7 (24·7 to 32·5)	16·5 (16·2 to 16·8)	15·9 (15·4 to 16·3)	-3·5 (-6·4 to -0·7)

SR Table 9: Global deaths for all ages (thousands) and age-standardized rates (per 100,000), and percent change (with 95% uncertainty intervals), for males, 2005 and 2015	All age deaths (thousands)			Age-standardized rate (per 100,000)		
	2005	2015	Percent change	2005	2015	Percent change
Liver cancer	501·5 (424·3 to 529·8)	576·7 (524·4 to 622·0)	14·5 (7·3 to 27·0)	20·4 (17·4 to 21·5)	18·2 (16·6 to 19·6)	-10·9 (-16·2 to -2·4)
Liver cancer due to hepatitis B	196·3 (162·2 to 211·0)	201·2 (180·9 to 223·2)	1·8 (-5·9 to 17·5)	7·4 (6·2 to 8·0)	6·0 (5·4 to 6·7)	-19·5 (-25·1 to -8·2)
Liver cancer due to hepatitis C	75·2 (67·7 to 80·4)	95·3 (86·3 to 102·3)	26·7 (21·1 to 32·9)	3·4 (3·1 to 3·6)	3·3 (3·0 to 3·5)	-3·8 (-7·8 to 0·4)
Liver cancer due to alcohol use	158·6 (133·9 to 170·6)	204·3 (185·5 to 224·0)	28·4 (19·6 to 41·6)	6·7 (5·7 to 7·2)	6·5 (5·9 to 7·2)	-2·0 (-8·3 to 7·4)
Liver cancer due to other causes	71·5 (58·3 to 77·6)	75·9 (68·6 to 84·9)	5·5 (-1·6 to 18·5)	2·9 (2·4 to 3·1)	2·4 (2·2 to 2·7)	-17·5 (-22·7 to -8·4)
Gallbladder and biliary tract cancer	50·0 (48·5 to 51·5)	59·5 (56·5 to 62·4)	18·9 (14·2 to 25·7)	2·4 (2·3 to 2·5)	2·1 (2·0 to 2·2)	-10·8 (-14·4 to -5·9)
Pancreatic cancer	162·9 (160·0 to 166·0)	214·6 (209·6 to 219·8)	31·8 (28·3 to 35·1)	7·4 (7·2 to 7·5)	7·3 (7·1 to 7·5)	-0·8 (-3·3 to 1·6)
Larynx cancer	75·6 (73·7 to 77·7)	86·4 (83·2 to 89·9)	14·4 (10·6 to 18·3)	3·3 (3·2 to 3·4)	2·8 (2·7 to 2·9)	-13·2 (-16·0 to -10·2)
Tracheal, bronchus, and lung cancer	1,005·8 (981·2 to 1,029·1)	1,205·7 (1,164·6 to 1,251·9)	19·8 (16·0 to 24·3)	45·1 (44·1 to 46·1)	41·0 (39·6 to 42·5)	-9·1 (-12·0 to -5·9)
Malignant skin melanoma	24·9 (16·9 to 35·5)	32·3 (21·3 to 44·8)	30·4 (19·8 to 37·5)	1·1 (0·7 to 1·5)	1·1 (0·7 to 1·5)	0·0 (-8·4 to 5·4)
Non-melanoma skin cancer	24·9 (24·3 to 25·6)	34·2 (32·8 to 35·5)	37·5 (31·6 to 42·7)	1·2 (1·2 to 1·2)	1·2 (1·2 to 1·3)	2·0 (-2·5 to 6·0)
Non-melanoma skin cancer (squamous-cell carcinoma)	24·9 (24·3 to 25·6)	34·2 (32·8 to 35·5)	37·5 (31·6 to 42·7)	1·2 (1·2 to 1·2)	1·2 (1·2 to 1·3)	2·0 (-2·5 to 6·0)
Breast cancer	6·1 (5·5 to 6·3)	10·1 (9·4 to 10·6)	66·1 (56·8 to 75·6)	0·3 (0·2 to 0·3)	0·3 (0·3 to 0·4)	22·6 (15·7 to 29·8)
Cervical cancer	-	-	-	-	-	-
Uterine cancer	-	-	-	-	-	-
Ovarian cancer	-	-	-	-	-	-
Prostate cancer	277·4 (230·8 to 348·9)	365·9 (303·5 to 459·6)	32·0 (28·2 to 35·4)	15·0 (12·4 to 18·8)	14·2 (11·8 to 17·9)	-4·7 (-7·5 to -2·4)
Testicular cancer	8·6 (8·3 to 9·1)	9·4 (8·8 to 9·9)	8·7 (1·3 to 14·4)	0·3 (0·3 to 0·3)	0·3 (0·3 to 0·3)	-9·4 (-15·5 to -4·7)
Kidney cancer	66·2 (64·5 to 68·3)	89·1 (85·9 to 92·8)	34·9 (29·1 to 39·7)	2·9 (2·9 to 3·0)	3·0 (2·9 to 3·1)	2·4 (-2·1 to 6·1)
Bladder cancer	108·7 (106·5 to 110·9)	136·8 (132·7 to 140·7)	26·0 (21·9 to 30·0)	5·5 (5·3 to 5·6)	5·1 (4·9 to 5·2)	-7·1 (-10·2 to -4·3)
Brain and nervous system cancer	105·4 (89·2 to 116·8)	127·2 (108·0 to 140·9)	21·3 (8·5 to 32·2)	4·0 (3·4 to 4·4)	3·9 (3·3 to 4·3)	-2·9 (-12·3 to 5·0)
Thyroid cancer	9·7 (8·7 to 10·5)	13·4 (11·1 to 14·2)	39·9 (22·5 to 48·2)	0·4 (0·4 to 0·5)	0·5 (0·4 to 0·5)	6·4 (-6·9 to 12·5)
Mesothelioma	16·2 (15·8 to 16·6)	23·2 (22·1 to 24·1)	43·2 (35·9 to 49·2)	0·7 (0·7 to 0·8)	0·8 (0·8 to 0·8)	9·5 (3·3 to 14·4)
Hodgkin lymphoma	15·7 (13·3 to 19·4)	15·1 (13·0 to 19·3)	-3·5 (-8·4 to 1·5)	0·6 (0·5 to 0·7)	0·5 (0·4 to 0·6)	-22·1 (-26·3 to -18·2)
Non-Hodgkin lymphoma	100·6 (86·8 to 108·4)	133·4 (108·8 to 142·7)	34·6 (15·0 to 40·8)	4·3 (3·7 to 4·6)	4·4 (3·5 to 4·7)	3·1 (-10·4 to 7·6)
Multiple myeloma	38·7 (37·8 to 39·7)	52·2 (50·6 to 53·8)	35·2 (30·0 to 40·0)	1·8 (1·7 to 1·8)	1·8 (1·7 to 1·9)	0·7 (-3·0 to 4·3)
Leukemia	170·7 (166·5 to 176·8)	204·3 (197·1 to 211·5)	19·7 (15·7 to 23·8)	6·8 (6·7 to 7·0)	6·6 (6·3 to 6·8)	-3·6 (-6·7 to -0·7)
Acute lymphoid leukemia	55·9 (48·8 to 63·9)	65·0 (56·7 to 72·3)	16·4 (8·1 to 25·4)	1·9 (1·7 to 2·2)	1·9 (1·7 to 2·1)	-0·1 (-7·2 to 7·7)
Chronic lymphoid leukemia	27·4 (26·0 to 29·8)	33·9 (31·6 to 38·0)	24·0 (14·8 to 31·5)	1·3 (1·3 to 1·5)	1·2 (1·2 to 1·4)	-7·4 (-13·6 to -1·7)
Acute myeloid leukemia	67·6 (59·4 to 76·0)	85·1 (75·9 to 94·7)	25·9 (20·1 to 32·0)	2·7 (2·4 to 3·0)	2·7 (2·5 to 3·0)	0·8 (-3·5 to 5·1)
Chronic myeloid leukemia	19·7 (18·2 to 22·1)	20·3 (18·6 to 22·9)	2·8 (-2·3 to 9·0)	0·8 (0·8 to 0·9)	0·7 (0·6 to 0·8)	-19·5 (-23·7 to -14·7)
Other neoplasms	146·2 (126·2 to 154·2)	191·1 (160·0 to 205·5)	31·3 (22·6 to 37·3)	5·9 (5·1 to 6·2)	6·1 (5·1 to 6·5)	4·1 (-3·7 to 8·8)
**Cardiovascular diseases**	8,057·2 (7,921·2 to 8,192·9)	9,419·6 (9,199·7 to 9,648·1)	16·9 (14·3 to 19·9)	383·7 (377·6 to 389·8)	334·6 (327·3 to 342·2)	-12·8 (-14·7 to -10·8)
Rheumatic heart disease	141·0 (133·5 to 152·5)	147·2 (136·9 to 157·7)	4·4 (-1·5 to 10·0)	5·9 (5·5 to 6·3)	4·8 (4·5 to 5·1)	-18·4 (-22·9 to -14·2)
Ischemic heart disease	4,074·2 (4,002·5 to 4,153·5)	4,881·0 (4,747·4 to 5,023·0)	19·7 (16·8 to 23·0)	193·8 (190·4 to 197·2)	172·8 (168·2 to 177·5)	-10·9 (-12·9 to -8·6)
Cerebrovascular disease	2,913·7 (2,837·3 to 2,983·1)	3,231·2 (3,128·6 to 3,346·1)	10·8 (7·1 to 14·6)	140·0 (136·4 to 143·4)	115·6 (112·2 to 119·5)	-17·5 (-20·1 to -14·8)
Ischemic stroke	1,248·2 (1,206·1 to 1,292·2)	1,427·4 (1,369·6 to 1,484·1)	14·3 (10·5 to 18·2)	64·6 (62·4 to 66·6)	54·0 (52·0 to 56·1)	-16·3 (-18·9 to -13·6)
Hemorrhagic stroke	1,665·6 (1,607·4 to 1,722·8)	1,803·8 (1,728·9 to 1,888·6)	8·2 (3·8 to 13·1)	75·4 (72·7 to 77·9)	61·6 (59·1 to 64·5)	-18·4 (-21·7 to -14·6)
Hypertensive heart disease	334·3 (319·4 to 361·4)	445·4 (419·5 to 472·0)	33·5 (24·6 to 40·7)	16·1 (15·3 to 17·4)	16·0 (15·1 to 17·0)	-0·1 (-6·9 to 5·5)
Cardiomyopathy and myocarditis	187·0 (174·6 to 195·9)	198·8 (185·7 to 211·9)	6·2 (1·3 to 11·4)	7·8 (7·3 to 8·1)	6·5 (6·1 to 6·9)	-16·1 (-19·8 to -11·9)
Atrial fibrillation and flutter	60·4 (49·3 to 73·7)	86·2 (69·4 to 105·2)	42·5 (39·0 to 46·1)	3·7 (3·0 to 4·6)	3·6 (2·9 to 4·4)	-2·7 (-4·8 to -0·7)
Aortic aneurysm	86·7 (83·9 to 90·0)	106·8 (103·2 to 109·8)	24·5 (16·2 to 28·2)	4·3 (4·1 to 4·4)	3·9 (3·7 to 4·0)	-8·4 (-14·0 to -5·5)

SR Table 9: Global deaths for all ages (thousands) and age-standardized rates (per 100,000), and percent change (with 95% uncertainty intervals), for males, 2005 and 2015

	All age deaths (thousands)			Age-standardized rate (per 100,000)		
	2005	2015	Percent change	2005	2015	Percent change
Peripheral vascular disease	18·6 (17·9 to 19·4)	25·7 (24·2 to 27·2)	37·7 (29·6 to 47·4)	1·0 (1·0 to 1·0)	1·0 (0·9 to 1·1)	-0·8 (-7·2 to 6·3)
Endocarditis	30·3 (25·1 to 34·9)	38·8 (32·1 to 45·1)	28·9 (19·6 to 34·8)	1·3 (1·1 to 1·5)	1·3 (1·1 to 1·5)	0·0 (-6·3 to 4·5)
Other cardiovascular and circulatory diseases	210·9 (205·0 to 217·2)	258·5 (246·7 to 270·9)	22·6 (17·1 to 28·9)	9·9 (9·7 to 10·3)	9·2 (8·8 to 9·6)	-7·6 (-11·5 to -3·0)
**Chronic respiratory diseases**	2,061·4 (2,004·5 to 2,117·3)	2,205·1 (2,119·9 to 2,293·1)	6·9 (2·5 to 11·5)	102·5 (99·8 to 105·1)	81·2 (78·2 to 84·5)	-20·7 (-24·1 to -17·5)
Chronic obstructive pulmonary disease	1,737·0 (1,673·0 to 1,796·2)	1,876·4 (1,800·6 to 1,958·5)	7·8 (3·1 to 13·8)	88·2 (85·1 to 91·0)	70·1 (67·4 to 73·0)	-20·6 (-24·0 to -16·5)
Pneumoconiosis	22·0 (20·2 to 23·6)	23·7 (21·5 to 25·6)	7·9 (-0·6 to 16·9)	1·1 (1·0 to 1·1)	0·9 (0·8 to 0·9)	-19·2 (-25·6 to -13·0)
Silicosis	9·8 (9·0 to 10·9)	10·1 (9·0 to 11·3)	2·1 (-9·4 to 16·3)	0·5 (0·4 to 0·5)	0·4 (0·3 to 0·4)	-22·9 (-31·7 to -12·6)
Asbestosis	2·3 (1·6 to 2·7)	3·0 (2·0 to 3·6)	31·0 (19·6 to 44·2)	0·1 (0·1 to 0·1)	0·1 (0·1 to 0·1)	-2·6 (-11·6 to 7·5)
Coal workers pneumoconiosis	2·5 (2·3 to 2·8)	2·3 (2·0 to 2·7)	-5·2 (-21·7 to 9·0)	0·1 (0·1 to 0·1)	0·1 (0·1 to 0·1)	-30·4 (-42·4 to -20·2)
Other pneumoconiosis	7·3 (6·3 to 8·3)	8·3 (7·0 to 9·3)	12·9 (4·9 to 21·0)	0·4 (0·3 to 0·4)	0·3 (0·3 to 0·3)	-16·0 (-22·2 to -10·0)
Asthma	226·1 (178·7 to 261·9)	201·3 (176·6 to 225·9)	-12·1 (-22·4 to 5·2)	9·8 (7·7 to 11·4)	6·7 (5·9 to 7·5)	-32·7 (-40·8 to -19·1)
Interstitial lung disease and pulmonary sarcoidosis	44·9 (31·7 to 55·9)	67·8 (48·0 to 78·8)	54·3 (30·7 to 64·3)	2·2 (1·6 to 2·7)	2·5 (1·7 to 2·9)	13·4 (-2·7 to 21·1)
Other chronic respiratory diseases	31·4 (20·5 to 38·0)	35·9 (23·7 to 42·6)	14·9 (1·5 to 26·5)	1·2 (0·8 to 1·4)	1·1 (0·7 to 1·3)	-5·7 (-14·7 to 2·3)
**Cirrhosis and other chronic liver diseases**	761·8 (731·0 to 797·4)	861·1 (817·1 to 907·6)	13·0 (8·7 to 17·5)	29·1 (28·0 to 30·4)	25·9 (24·6 to 27·2)	-11·1 (-14·3 to -7·7)
Cirrhosis and other chronic liver diseases due to hepatitis B	236·2 (218·2 to 256·2)	263·7 (240·7 to 286·9)	11·7 (6·6 to 16·7)	9·1 (8·4 to 10·0)	8·0 (7·4 to 8·7)	-12·2 (-16·0 to -8·2)
Cirrhosis and other chronic liver diseases due to hepatitis C	195·4 (181·1 to 209·9)	224·8 (206·7 to 243·3)	15·0 (11·1 to 19·2)	7·7 (7·1 to 8·2)	6·9 (6·3 to 7·4)	-10·5 (-13·4 to -7·3)
Cirrhosis and other chronic liver diseases due to alcohol use	217·0 (200·6 to 233·7)	249·2 (228·5 to 269·3)	14·8 (10·1 to 20·7)	8·0 (7·4 to 8·7)	7·3 (6·7 to 7·9)	-9·5 (-13·1 to -5·2)
Cirrhosis and other chronic liver diseases due to other causes	113·2 (104·7 to 122·1)	123·4 (113·0 to 134·0)	8·9 (4·9 to 13·1)	4·3 (4·0 to 4·6)	3·8 (3·5 to 4·1)	-12·4 (-15·6 to -9·2)
**Digestive diseases**	596·8 (572·0 to 666·4)	659·1 (620·5 to 723·9)	10·4 (4·7 to 17·3)	25·5 (24·5 to 28·5)	21·8 (20·6 to 24·0)	-14·4 (-18·7 to -9·3)
Peptic ulcer disease	172·9 (160·5 to 192·4)	156·1 (141·4 to 177·2)	-10·6 (-17·1 to 0·8)	7·4 (6·9 to 8·2)	5·1 (4·7 to 5·8)	-30·8 (-35·7 to -21·8)
Gastritis and duodenitis	28·0 (23·0 to 34·6)	28·6 (25·0 to 33·0)	1·3 (-10·6 to 19·9)	1·2 (1·0 to 1·5)	0·9 (0·8 to 1·1)	-21·6 (-31·0 to -7·6)
Appendicitis	25·2 (21·7 to 28·7)	27·2 (21·8 to 30·7)	7·6 (-3·4 to 21·6)	0·9 (0·8 to 1·1)	0·8 (0·7 to 0·9)	-12·1 (-21·1 to -0·6)
Paralytic ileus and intestinal obstruction	124·7 (111·1 to 160·3)	146·3 (129·0 to 185·8)	17·3 (9·2 to 26·5)	5·3 (4·7 to 6·8)	4·8 (4·3 to 6·1)	-8·4 (-14·8 to -1·5)
Inguinal, femoral, and abdominal hernia	35·9 (22·3 to 41·4)	37·9 (22·9 to 45·9)	5·0 (-5·5 to 19·6)	1·6 (1·0 to 1·8)	1·3 (0·8 to 1·5)	-19·2 (-27·6 to -8·2)
Inflammatory bowel disease	19·3 (16·5 to 21·4)	23·1 (20·4 to 25·8)	19·3 (12·2 to 31·0)	0·9 (0·7 to 0·9)	0·8 (0·7 to 0·9)	-9·1 (-14·7 to -0·9)
Vascular intestinal disorders	36·7 (34·2 to 39·9)	47·2 (43·0 to 52·3)	28·3 (21·9 to 35·5)	1·8 (1·7 to 1·9)	1·7 (1·6 to 1·9)	-4·3 (-9·2 to 1·1)
Gallbladder and biliary diseases	39·1 (35·6 to 44·9)	49·1 (44·1 to 55·9)	25·5 (17·1 to 33·9)	1·9 (1·7 to 2·1)	1·8 (1·6 to 2·0)	-5·3 (-11·8 to 0·5)
Pancreatitis	66·8 (62·5 to 73·1)	85·1 (77·7 to 95·5)	27·3 (18·8 to 37·6)	2·5 (2·4 to 2·7)	2·6 (2·3 to 2·9)	1·8 (-5·1 to 9·7)
Other digestive diseases	48·2 (39·7 to 66·5)	58·5 (45·4 to 73·5)	21·6 (4·5 to 35·4)	2·1 (1·8 to 2·9)	2·0 (1·6 to 2·5)	-6·4 (-19·1 to 3·4)
**Neurological disorders**	673·3 (594·8 to 754·5)	934·3 (810·0 to 1,054·3)	38·8 (35·6 to 41·7)	38·3 (33·2 to 43·5)	37·5 (32·2 to 42·8)	-1·9 (-3·4 to -0·4)
Alzheimer disease and other dementias	508·2 (428·8 to 587·9)	732·3 (610·4 to 853·3)	44·2 (41·1 to 46·8)	31·5 (26·5 to 36·7)	30·9 (25·6 to 36·1)	-2·0 (-3·4 to -0·6)
Parkinson disease	45·2 (43·4 to 47·8)	66·6 (63·8 to 69·5)	47·5 (40·6 to 54·5)	2·6 (2·5 to 2·7)	2·7 (2·6 to 2·8)	4·5 (-0·4 to 9·6)
Epilepsy	72·8 (68·3 to 77·2)	77·5 (73·2 to 82·4)	6·0 (-0·2 to 15·4)	2·3 (2·2 to 2·5)	2·2 (2·0 to 2·3)	-8·5 (-13·4 to -1·3)
Multiple sclerosis	6·7 (6·2 to 7·2)	7·7 (7·2 to 8·3)	16·8 (8·3 to 21·9)	0·3 (0·2 to 0·3)	0·2 (0·2 to 0·2)	-8·5 (-14·8 to -4·6)
Motor neuron disease	15·3 (14·7 to 16·0)	19·8 (18·8 to 20·8)	30·0 (21·4 to 33·9)	0·6 (0·6 to 0·7)	0·6 (0·6 to 0·7)	0·1 (-6·5 to 3·1)
Other neurological disorders	25·2 (24·3 to 26·2)	30·4 (29·2 to 31·6)	21·0 (14·2 to 24·9)	0·9 (0·9 to 1·0)	0·9 (0·9 to 1·0)	-0·1 (-5·2 to 3·0)
**Mental and substance use disorders**	235·3 (224·2 to 244·3)	248·7 (233·7 to 260·5)	5·8 (0·6 to 10·4)	8·0 (7·6 to 8·3)	7·0 (6·6 to 7·3)	-12·6 (-16·8 to -9·0)
Schizophrenia	9·7 (9·0 to 10·4)	9·1 (8·3 to 10·0)	-7·1 (-17·1 to 6·8)	0·4 (0·3 to 0·4)	0·3 (0·3 to 0·3)	-25·0 (-32·7 to -14·6)
Alcohol use disorders	130·6 (120·5 to 136·6)	116·5 (110·5 to 123·0)	-11·2 (-15·4 to -3·9)	4·5 (4·2 to 4·7)	3·3 (3·1 to 3·5)	-27·7 (-31·1 to -22·1)
Drug use disorders	94·7 (90·7 to 99·2)	122·9 (108·1 to 131·8)	30·2 (16·4 to 39·3)	3·1 (2·9 to 3·2)	3·4 (3·0 to 3·6)	10·7 (-0·7 to 18·1)
Opioid use disorders	71·9 (68·9 to 78·1)	91·1 (80·6 to 98·3)	27·2 (14·2 to 36·0)	2·3 (2·3 to 2·5)	2·5 (2·2 to 2·7)	8·6 (-2·8 to 15·8)
Cocaine use disorders	5·3 (3·0 to 5·8)	7·9 (5·2 to 8·9)	47·0 (28·7 to 92·5)	0·2 (0·1 to 0·2)	0·2 (0·1 to 0·2)	24·5 (9·5 to 63·8)

SR Table 9: Global deaths for all ages (thousands) and age-standardized rates (per 100,000), and percent change (with 95% uncertainty intervals), for males, 2005 and 2015

	All age deaths (thousands)			Age-standardized rate (per 100,000)		
	2005	2015	Percent change	2005	2015	Percent change
Amphetamine use disorders	6·2 (3·1 to 7·0)	10·3 (6·5 to 12·1)	66·3 (21·5 to 129·8)	0·2 (0·1 to 0·2)	0·3 (0·2 to 0·3)	41·4 (3·7 to 95·1)
Other drug use disorders	11·3 (10·6 to 12·9)	13·7 (12·4 to 15·5)	21·4 (9·8 to 32·9)	0·4 (0·4 to 0·4)	0·4 (0·3 to 0·4)	1·7 (-7·7 to 11·1)
Eating disorders	0·2 (0·1 to 0·2)	0·2 (0·1 to 0·3)	1·6 (-7·4 to 24·1)	0·0 (0·0 to 0·0)	0·0 (0·0 to 0·0)	-10·4 (-18·3 to 8·9)
Anorexia nervosa	0·2 (0·1 to 0·2)	0·2 (0·1 to 0·2)	-1·0 (-9·9 to 21·0)	0·0 (0·0 to 0·0)	0·0 (0·0 to 0·0)	-12·2 (-20·0 to 6·9)
Bulimia nervosa	0·0 (0·0 to 0·0)	0·0 (0·0 to 0·0)	26·8 (12·6 to 48·5)	0·0 (0·0 to 0·0)	0·0 (0·0 to 0·0)	6·8 (-5·1 to 24·3)
**Diabetes, urogenital, blood, and endocrine diseases**	1,271·3 (1,161·2 to 1,340·6)	1,712·2 (1,581·8 to 1,802·5)	34·8 (29·6 to 39·6)	56·6 (52·3 to 59·0)	58·4 (54·1 to 61·1)	3·1 (-0·2 to 6·3)
Diabetes mellitus	530·7 (514·8 to 546·7)	746·2 (716·9 to 775·1)	40·7 (34·4 to 45·8)	24·3 (23·6 to 25·0)	25·7 (24·7 to 26·6)	5·8 (1·0 to 9·5)
Acute glomerulonephritis	6·8 (5·1 to 7·8)	6·7 (4·3 to 8·0)	-1·7 (-20·8 to 12·2)	0·3 (0·2 to 0·3)	0·2 (0·1 to 0·3)	-20·9 (-37·0 to -10·2)
Chronic kidney disease	471·3 (389·9 to 499·3)	637·8 (526·0 to 677·0)	35·3 (28·8 to 41·8)	21·4 (18·0 to 22·7)	22·1 (18·3 to 23·3)	2·9 (-2·3 to 7·5)
Chronic kidney disease due to diabetes mellitus	149·6 (127·0 to 158·8)	216·8 (185·4 to 232·3)	44·8 (38·6 to 51·0)	6·8 (5·9 to 7·2)	7·4 (6·4 to 8·0)	9·0 (4·3 to 13·4)
Chronic kidney disease due to hypertension	198·9 (164·1 to 212·0)	273·8 (222·2 to 293·1)	37·8 (29·9 to 45·0)	9·3 (7·8 to 10·0)	9·7 (7·9 to 10·3)	3·5 (-2·5 to 8·6)
Chronic kidney disease due to glomerulonephritis	110·5 (87·6 to 118·4)	131·5 (104·3 to 144·6)	18·9 (12·4 to 25·5)	4·7 (3·8 to 5·0)	4·4 (3·5 to 4·8)	-6·6 (-12·0 to -1·5)
Chronic kidney disease due to other causes	12·3 (9·6 to 14·9)	15·7 (12·5 to 18·9)	27·6 (18·3 to 37·7)	0·5 (0·4 to 0·6)	0·5 (0·4 to 0·6)	0·0 (-6·7 to 7·2)
Urinary diseases and male infertility	97·0 (85·3 to 111·5)	124·4 (105·4 to 137·4)	29·9 (17·4 to 37·5)	4·7 (4·1 to 5·3)	4·5 (3·8 to 4·9)	-2·5 (-12·6 to 3·0)
Interstitial nephritis and urinary tract infections	64·4 (55·7 to 74·2)	83·5 (70·8 to 94·6)	30·2 (19·5 to 40·5)	3·2 (2·8 to 3·6)	3·1 (2·6 to 3·4)	-2·7 (-11·3 to 5·0)
Urolithiasis	8·0 (6·6 to 11·2)	8·3 (6·8 to 12·4)	2·4 (-10·0 to 20·8)	0·4 (0·3 to 0·5)	0·3 (0·2 to 0·4)	-21·5 (-31·3 to -7·2)
Other urinary diseases	24·6 (18·4 to 30·5)	32·6 (23·9 to 37·3)	34·0 (15·1 to 51·3)	1·1 (0·9 to 1·4)	1·1 (0·8 to 1·3)	0·7 (-13·8 to 13·5)
Gynecological diseases	-	-	-	-	-	-
Uterine fibroids	-	-	-	-	-	-
Polycystic ovarian syndrome	-	-	-	-	-	-
Endometriosis	-	-	-	-	-	-
Genital prolapse	-	-	-	-	-	-
Other gynecological diseases	-	-	-	-	-	-
Hemoglobinopathies and hemolytic anemias	113·4 (73·8 to 161·4)	124·6 (80·3 to 186·3)	9·0 (-10·0 to 34·6)	3·8 (2·6 to 5·3)	3·6 (2·4 to 5·3)	-6·7 (-20·2 to 13·5)
Thalassemias	9·7 (6·8 to 12·2)	8·3 (5·7 to 10·6)	-14·8 (-31·4 to 7·3)	0·3 (0·2 to 0·4)	0·3 (0·2 to 0·3)	-22·9 (-35·3 to -6·6)
Sickle cell disorders	58·8 (32·6 to 99·7)	64·7 (35·4 to 116·4)	8·3 (-23·5 to 54·1)	1·7 (1·0 to 2·8)	1·7 (1·0 to 3·1)	-0·8 (-28·6 to 40·4)
G6PD deficiency	19·3 (14·9 to 23·6)	23·1 (17·7 to 28·6)	19·3 (9·6 to 30·9)	0·6 (0·5 to 0·8)	0·6 (0·5 to 0·8)	0·7 (-8·1 to 10·4)
Other hemoglobinopathies and hemolytic anemias	25·6 (19·0 to 31·6)	28·5 (21·0 to 35·5)	12·1 (1·0 to 20·9)	1·2 (0·9 to 1·4)	1·0 (0·7 to 1·2)	-14·4 (-23·1 to -8·4)
Endocrine, metabolic, blood, and immune disorders	52·0 (50·2 to 53·9)	72·5 (70·0 to 75·8)	39·4 (33·7 to 44·6)	2·1 (2·1 to 2·2)	2·4 (2·3 to 2·4)	10·9 (6·6 to 15·1)
**Musculoskeletal disorders**	22·8 (20·2 to 23·7)	27·6 (24·5 to 29·1)	21·3 (15·2 to 27·6)	1·0 (0·9 to 1·1)	0·9 (0·8 to 1·0)	-6·9 (-11·6 to -2·5)
Rheumatoid arthritis	7·8 (6·2 to 8·7)	9·3 (7·7 to 10·5)	19·6 (11·4 to 29·4)	0·4 (0·3 to 0·4)	0·3 (0·3 to 0·4)	-9·7 (-15·9 to -2·5)
Other musculoskeletal disorders	15·0 (13·6 to 16·1)	18·3 (16·3 to 19·4)	22·2 (15·4 to 28·1)	0·7 (0·6 to 0·7)	0·6 (0·5 to 0·7)	-5·3 (-10·9 to -0·4)
**Other non-communicable diseases**	374·4 (310·0 to 455·8)	386·5 (335·7 to 434·9)	5·2 (-12·5 to 12·3)	11·1 (9·2 to 13·4)	10·5 (9·1 to 11·9)	-3·8 (-18·9 to 2·3)
Congenital anomalies	331·9 (268·5 to 413·7)	332·6 (285·3 to 370·1)	2·5 (-16·4 to 9·3)	9·4 (7·6 to 11·6)	8·7 (7·5 to 9·7)	-4·8 (-21·9 to 1·2)
Neural tube defects	37·1 (25·9 to 56·0)	33·6 (24·5 to 45·9)	-7·9 (-35·3 to 21·9)	1·0 (0·7 to 1·5)	0·9 (0·6 to 1·2)	-13·3 (-39·2 to 14·7)
Congenital heart anomalies	169·9 (137·2 to 209·7)	162·3 (138·1 to 184·2)	-2·9 (-20·9 to 7·4)	4·8 (3·8 to 5·9)	4·2 (3·6 to 4·8)	-9·6 (-26·1 to 0·0)
Cleft lip and cleft palate	1·9 (1·5 to 2·4)	0·7 (0·5 to 0·9)	-64·8 (-73·2 to -57·1)	0·1 (0·0 to 0·1)	0·0 (0·0 to 0·0)	-66·7 (-74·6 to -59·4)
Down syndrome	9·7 (6·9 to 17·1)	11·0 (8·0 to 15·5)	18·0 (-20·5 to 37·7)	0·3 (0·2 to 0·5)	0·3 (0·2 to 0·4)	3·7 (-28·2 to 20·4)
Other chromosomal abnormalities	7·2 (3·8 to 15·5)	7·4 (4·8 to 11·8)	12·7 (-27·3 to 35·7)	0·2 (0·1 to 0·4)	0·2 (0·1 to 0·3)	4·7 (-31·7 to 25·3)
Other congenital anomalies	106·2 (83·6 to 138·4)	117·5 (97·6 to 136·8)	12·5 (-8·0 to 25·9)	3·1 (2·4 to 3·9)	3·1 (2·6 to 3·6)	3·8 (-14·4 to 15·5)
Skin and subcutaneous diseases	31·1 (18·3 to 46·6)	43·8 (26·4 to 68·8)	39·9 (31·9 to 52·4)	1·4 (0·9 to 2·1)	1·5 (0·9 to 2·4)	5·4 (-0·5 to 16·3)
Cellulitis	6·2 (3·3 to 10·9)	8·5 (4·7 to 14·5)	37·8 (20·4 to 50·9)	0·3 (0·1 to 0·5)	0·3 (0·2 to 0·5)	6·7 (-8·0 to 16·1)

SR Table 9: Global deaths for all ages (thousands) and age-standardized rates (per 100,000), and percent change (with 95% uncertainty intervals), for males, 2005 and 2015

	All age deaths (thousands)			Age-standardized rate (per 100,000)		
	2005	2015	Percent change	2005	2015	Percent change
Pyoderma	13·6<br>(8·0 to 22·2)	20·2<br>(12·4 to 34·7)	48·3<br>(35·7 to 62·9)	0·6<br>(0·3 to 0·9)	0·7<br>(0·4 to 1·1)	16·0<br>(5·7 to 28·0)
Decubitus ulcer	10·0<br>(4·6 to 13·9)	13·2<br>(6·2 to 19·3)	32·1<br>(23·0 to 47·7)	0·5<br>(0·2 to 0·7)	0·5<br>(0·2 to 0·7)	-4·6<br>(-11·7 to 6·8)
Other skin and subcutaneous diseases	1·3<br>(0·8 to 2·1)	1·8<br>(1·1 to 3·0)	39·3<br>(25·0 to 56·0)	0·1<br>(0·0 to 0·1)	0·1<br>(0·0 to 0·1)	7·6<br>(-3·1 to 22·8)
Sudden infant death syndrome	11·3<br>(8·3 to 22·4)	10·1<br>(7·6 to 17·2)	-9·3<br>(-27·8 to 7·7)	0·3<br>(0·2 to 0·6)	0·3<br>(0·2 to 0·4)	-14·8<br>(-32·1 to 1·2)
**Injuries**	3,294·9<br>(3,120·8 to 3,380·9)	3,325·8<br>(3,109·9 to 3,457·8)	0·9<br>(-2·7 to 5·2)	110·0<br>(104·1 to 112·7)	94·3<br>(88·1 to 98·0)	-14·3<br>(-17·2 to -10·8)
**Transport injuries**	1,110·3<br>(1,069·8 to 1,157·2)	1,108·9<br>(1,043·8 to 1,170·7)	0·1<br>(-6·2 to 5·2)	36·1<br>(34·9 to 37·6)	30·8<br>(29·0 to 32·5)	-14·5<br>(-19·8 to -10·1)
Road injuries	1,030·1<br>(986·2 to 1,075·7)	1,024·4<br>(966·0 to 1,081·0)	-0·3<br>(-6·8 to 4·8)	33·5<br>(32·2 to 34·9)	28·5<br>(26·9 to 30·0)	-14·8<br>(-20·2 to -10·5)
Pedestrian road injuries	415·1<br>(384·2 to 462·6)	411·8<br>(373·3 to 463·5)	-0·6<br>(-9·3 to 6·5)	14·0<br>(12·9 to 15·6)	11·7<br>(10·7 to 13·2)	-16·1<br>(-23·2 to -10·1)
Cyclist road injuries	46·7<br>(42·5 to 51·7)	44·2<br>(39·9 to 49·1)	-5·3<br>(-14·1 to 4·6)	1·6<br>(1·4 to 1·8)	1·3<br>(1·2 to 1·4)	-20·3<br>(-27·7 to -12·2)
Motorcyclist road injuries	197·2<br>(167·9 to 214·3)	206·7<br>(179·7 to 238·7)	4·2<br>(-7·8 to 18·5)	6·1<br>(5·1 to 6·6)	5·6<br>(4·8 to 6·4)	-9·1<br>(-19·6 to 3·8)
Motor vehicle road injuries	356·3<br>(318·8 to 403·5)	344·8<br>(299·0 to 387·3)	-3·0<br>(-10·4 to 3·5)	11·4<br>(10·1 to 12·8)	9·5<br>(8·2 to 10·6)	-16·5<br>(-23·0 to -11·0)
Other road injuries	14·8<br>(8·9 to 18·3)	16·9<br>(9·9 to 20·5)	13·5<br>(-6·7 to 41·3)	0·5<br>(0·3 to 0·6)	0·5<br>(0·3 to 0·6)	-3·0<br>(-20·6 to 20·2)
Other transport injuries	80·2<br>(72·0 to 92·2)	84·6<br>(71·1 to 105·4)	5·3<br>(-5·4 to 17·3)	2·6<br>(2·4 to 3·1)	2·3<br>(2·0 to 2·9)	-11·1<br>(-20·0 to -1·1)
**Unintentional injuries**	1,206·5<br>(1,121·4 to 1,256·1)	1,169·9<br>(1,052·4 to 1,232·9)	-3·3<br>(-7·2 to 2·6)	42·0<br>(38·9 to 43·6)	34·7<br>(31·1 to 36·4)	-17·7<br>(-20·9 to -13·2)
Falls	264·7<br>(242·5 to 275·5)	315·7<br>(269·7 to 334·9)	19·6<br>(10·2 to 26·9)	10·9<br>(10·0 to 11·3)	10·3<br>(8·8 to 10·9)	-5·5<br>(-12·5 to 0·0)
Drowning	277·1<br>(266·2 to 289·6)	223·1<br>(212·8 to 237·7)	-19·5<br>(-24·1 to -15·1)	8·5<br>(8·2 to 8·9)	6·1<br>(5·9 to 6·5)	-28·1<br>(-31·9 to -24·2)
Fire, heat, and hot substances	95·3<br>(77·2 to 102·5)	88·7<br>(71·0 to 95·9)	-6·7<br>(-14·1 to 2·6)	3·3<br>(2·7 to 3·5)	2·6<br>(2·1 to 2·8)	-21·5<br>(-27·7 to -14·8)
Poisonings	61·4<br>(39·0 to 75·6)	51·8<br>(31·3 to 64·4)	-16·3<br>(-29·1 to 1·1)	2·0<br>(1·3 to 2·4)	1·5<br>(0·9 to 1·8)	-28·7<br>(-38·1 to -15·0)
Exposure to mechanical forces	147·9<br>(124·6 to 158·1)	145·2<br>(111·4 to 159·3)	-1·1<br>(-13·2 to 8·7)	4·8<br>(4·1 to 5·2)	4·1<br>(3·1 to 4·5)	-15·0<br>(-25·2 to -6·9)
Unintentional firearm injuries	24·1<br>(16·6 to 26·7)	22·8<br>(15·3 to 25·4)	-5·5<br>(-11·6 to 1·6)	0·8<br>(0·5 to 0·9)	0·6<br>(0·4 to 0·7)	-19·0<br>(-24·3 to -13·2)
Unintentional suffocation	22·9<br>(17·0 to 26·9)	23·6<br>(16·6 to 27·7)	3·0<br>(-11·9 to 19·8)	0·8<br>(0·6 to 0·9)	0·7<br>(0·5 to 0·8)	-8·6<br>(-20·9 to 4·6)
Other exposure to mechanical forces	100·9<br>(80·9 to 109·1)	98·8<br>(70·4 to 110·7)	-1·3<br>(-16·2 to 11·7)	3·3<br>(2·6 to 3·6)	2·8<br>(2·0 to 3·1)	-15·8<br>(-28·5 to -4·9)
Adverse effects of medical treatment	54·6<br>(39·5 to 61·2)	56·7<br>(41·3 to 64·3)	3·6<br>(-1·9 to 10·9)	2·0<br>(1·5 to 2·2)	1·7<br>(1·3 to 2·0)	-14·1<br>(-17·9 to -10·2)
Animal contact	59·8<br>(36·2 to 66·3)	55·0<br>(30·4 to 79·7)	-12·6<br>(-19·6 to 24·2)	2·0<br>(1·2 to 2·2)	1·6<br>(0·9 to 2·2)	-25·1<br>(-31·1 to 6·0)
Venomous animal contact	49·8<br>(28·5 to 56·5)	46·1<br>(23·8 to 69·7)	-12·2<br>(-20·6 to 26·4)	1·6<br>(0·9 to 1·9)	1·3<br>(0·7 to 1·9)	-24·9<br>(-32·0 to 8·2)
Non-venomous animal contact	10·0<br>(7·6 to 11·8)	8·9<br>(6·6 to 10·6)	-13·7<br>(-21·8 to 18·9)	0·3<br>(0·3 to 0·4)	0·3<br>(0·2 to 0·3)	-26·4<br>(-32·6 to 0·4)
Foreign body	89·2<br>(70·7 to 113·8)	90·6<br>(75·0 to 106·8)	2·8<br>(-7·8 to 11·4)	3·2<br>(2·5 to 3·9)	2·7<br>(2·2 to 3·2)	-13·1<br>(-20·9 to -7·4)
Pulmonary aspiration and foreign body in airway	71·4<br>(56·2 to 90·9)	74·1<br>(60·8 to 87·3)	5·8<br>(-6·5 to 12·9)	2·6<br>(2·0 to 3·2)	2·2<br>(1·8 to 2·6)	-11·1<br>(-19·4 to -6·2)
Foreign body in other body part	17·9<br>(10·0 to 31·8)	16·5<br>(10·8 to 22·4)	-4·5<br>(-28·8 to 12·2)	0·6<br>(0·3 to 1·1)	0·5<br>(0·3 to 0·6)	-17·5<br>(-39·7 to -3·7)
Environmental heat and cold exposure	37·4<br>(27·0 to 40·5)	31·5<br>(23·1 to 34·7)	-16·1<br>(-22·5 to -7·4)	1·4<br>(1·0 to 1·5)	0·9<br>(0·7 to 1·0)	-31·1<br>(-36·2 to -24·7)
Other unintentional injuries	119·1<br>(112·1 to 127·8)	111·4<br>(102·4 to 125·1)	-6·6<br>(-14·8 to 3·2)	3·9<br>(3·6 to 4·2)	3·1<br>(2·9 to 3·5)	-19·3<br>(-26·9 to -10·7)
**Self-harm and interpersonal violence**	893·6<br>(816·1 to 919·9)	908·5<br>(851·2 to 949·1)	1·9<br>(-3·1 to 5·8)	29·2<br>(26·6 to 30·1)	25·2<br>(23·6 to 26·3)	-13·7<br>(-18·0 to -10·5)
Self-harm	556·6<br>(491·9 to 574·6)	578·9<br>(533·9 to 609·2)	4·5<br>(-2·7 to 9·4)	19·0<br>(16·7 to 19·6)	16·4<br>(15·1 to 17·3)	-12·9<br>(-18·9 to -9·0)
Interpersonal violence	337·1<br>(308·1 to 349·4)	329·6<br>(301·2 to 352·4)	-2·4<br>(-5·7 to 2·4)	10·3<br>(9·4 to 10·6)	8·7<br>(8·0 to 9·3)	-15·0<br>(-18·0 to -11·0)
Assault by firearm	142·6<br>(124·2 to 148·2)	152·0<br>(131·4 to 161·1)	6·5<br>(2·0 to 11·8)	4·2<br>(3·7 to 4·4)	4·0<br>(3·4 to 4·2)	-5·9<br>(-10·0 to -1·1)
Assault by sharp object	83·4<br>(77·7 to 89·1)	72·3<br>(66·6 to 79·2)	-13·4<br>(-18·4 to -7·8)	2·5<br>(2·4 to 2·7)	1·9<br>(1·8 to 2·1)	-24·7<br>(-29·1 to -19·9)
Assault by other means	111·1<br>(97·8 to 120·3)	105·3<br>(89·0 to 117·2)	-5·5<br>(-11·5 to 3·1)	3·5<br>(3·1 to 3·8)	2·8<br>(2·4 to 3·2)	-19·0<br>(-23·8 to -11·9)
**Forces of nature, war, and legal intervention**	84·4<br>(56·2 to 111·9)	138·5<br>(72·1 to 201·0)	64·7<br>(-6·4 to 147·6)	2·6<br>(1·7 to 3·4)	3·6<br>(1·9 to 5·2)	40·9<br>(-20·2 to 112·4)
Exposure to forces of nature	58·2<br>(33·8 to 82·0)	7·3<br>(4·4 to 10·1)	-87·5<br>(-88·6 to -86·4)	1·8<br>(1·0 to 2·5)	0·2<br>(0·1 to 0·3)	-89·0<br>(-90·0 to -88·0)
Collective violence and legal intervention	26·2<br>(19·5 to 32·9)	131·2<br>(66·8 to 194·2)	401·6<br>(222·5 to 529·2)	0·8<br>(0·6 to 1·0)	3·4<br>(1·7 to 5·1)	343·0<br>(181·7 to 455·4)

SR Table 10: Global deaths by cause and age group for females (with 95% uncertainty intervals), 1990

Cause	All ages	0-5 years	5-14 years	15-49 years	50-69 years	70+ years
All causes	22,606,547 (22,283,429–22,965,448)	5,755,006 (5,697,520–5,817,699)	610,628 (600,793–620,636)	2,932,427 (2,842,072–3,032,898)	4,695,061 (4,577,179–4,820,256)	8,613,423 (8,487,310–8,734,598)
Communicable, maternal, neonatal, and nutritional diseases	7,722,547 (7,477,722–7,950,026)	4,855,928 (4,660,254–5,014,206)	345,505 (327,011–367,033)	1,065,175 (1,012,063–1,133,866)	644,474 (591,477–688,322)	811,463 (757,958–854,168)
HIV/AIDS and tuberculosis	671,282 (553,557–799,888)	47,834 (36,240–71,542)	16,132 (12,784–24,347)	251,402 (207,830–301,493)	218,271 (164,902–255,772)	137,640 (98,227–164,839)
Tuberculosis	599,698 (479,292–721,775)	30,264 (18,843–53,325)	15,334 (12,012–23,587)	204,273 (159,950–255,225)	212,693 (159,780–250,141)	137,133 (97,727–164,277)
HIV/AIDS	71,583 (64,498–80,569)	17,570 (16,163–19,397)	798 (694–925)	47,128 (41,637–54,449)	5,578 (4,766–6,637)	507 (417–630)
HIV/AIDS - Tuberculosis	21,427 (17,950–25,037)	5,885 (4,803–6,789)	228 (188–269)	13,545 (11,302–16,125)	1,638 (1,387–1,909)	129 (106–157)
HIV/AIDS resulting in other diseases	50,155 (43,940–58,549)	11,684 (10,302–13,505)	570 (484–683)	33,582 (28,445–40,624)	3,940 (3,229–4,926)	377 (302–489)
Diarrhea, lower respiratory, and other common infectious diseases	4,002,937 (3,781,971–4,230,165)	2,527,972 (2,356,020–2,714,989)	250,012 (223,619–279,784)	302,650 (277,886–334,155)	331,307 (306,937–357,737)	590,995 (552,006–624,950)
Diarrheal diseases	1,197,694 (1,089,061–1,294,065)	684,789 (589,088–777,862)	64,238 (55,070–72,112)	113,110 (106,082–121,035)	150,278 (140,720–160,033)	185,277 (173,523–195,921)
Intestinal infectious diseases	119,310 (66,977–194,901)	34,903 (18,653–60,193)	45,926 (26,204–74,863)	31,832 (16,574–53,811)	4,813 (2,475–8,476)	1,834 (1,117–2,857)
Typhoid fever	93,501 (50,151–157,670)	25,995 (12,678–46,553)	36,455 (19,958–59,747)	25,704 (13,076–44,581)	3,965 (1,926–7,124)	1,380 (736–2,287)
Paratyphoid fever	22,810 (10,509–44,677)	6,787 (2,836–13,819)	9,185 (4,277–18,052)	5,849 (2,518–11,488)	735 (296–1,501)	252 (106–494)
Other intestinal infectious diseases	2,997 (697–6,494)	2,120 (404–5,172)	285 (47–859)	278 (76–594)	112 (40–194)	201 (81–366)
Lower respiratory infections	1,681,126 (1,534,480–1,803,454)	1,050,585 (934,323–1,156,675)	46,605 (34,079–53,873)	82,678 (67,625–94,127)	129,621 (105,568–144,936)	371,635 (329,667–399,678)
Upper respiratory infections	2,180 (1,832–2,625)	937 (656–1,318)	43 (28–64)	348 (281–422)	402 (319–503)	447 (353–578)
Otitis media	3,218 (2,865–3,689)	1,313 (1,102–1,583)	296 (264–329)	690 (620–773)	488 (421–583)	429 (297–745)
Meningitis	242,161 (194,876–283,368)	151,355 (114,435–190,172)	22,701 (17,663–26,119)	33,165 (27,635–38,887)	20,581 (17,488–24,522)	14,356 (12,795–17,101)
Pneumococcal meningitis	60,378 (46,596–75,387)	32,816 (21,829–46,261)	7,117 (4,739–9,807)	10,704 (8,508–13,641)	5,739 (4,325–7,556)	4,001 (3,055–5,055)
H influenzae type B meningitis	75,003 (56,211–94,639)	56,780 (40,033–75,689)	6,489 (4,230–8,874)	5,828 (4,240–7,527)	3,430 (2,513–4,572)	2,472 (1,900–3,245)
Meningococcal meningitis	40,579 (29,673–54,040)	25,888 (16,532–38,193)	3,794 (2,418–5,387)	5,413 (3,911–6,845)	3,327 (2,406–4,441)	2,156 (1,625–2,897)
Other meningitis	66,200 (52,041–80,849)	35,870 (24,995–49,366)	5,300 (3,709–7,195)	11,218 (9,200–13,572)	8,083 (6,613–10,008)	5,726 (4,723–6,860)
Encephalitis	74,149 (55,829–90,530)	31,122 (20,591–39,470)	10,045 (7,486–12,594)	12,733 (10,379–15,373)	10,694 (8,310–13,390)	9,554 (7,492–11,686)
Diphtheria	7,756 (4,291–14,975)	6,241 (2,974–13,287)	883 (432–1,639)	467 (311–686)	164 (86–287)	0 (0–0)
Whooping cough	88,865 (30,885–210,042)	82,903 (28,755–193,726)	4,882 (1,732–11,977)	947 (288–2,568)	131 (42–345)	0 (0–0)
Tetanus	157,270 (134,736–196,775)	116,719 (97,145–139,250)	5,971 (4,408–11,267)	16,174 (12,825–29,755)	12,842 (10,320–25,316)	5,561 (4,356–10,961)
Measles	422,757 (171,058–847,135)	365,040 (148,691–722,162)	47,894 (18,619–95,932)	9,560 (2,848–24,012)	261 (74–740)	0 (0–0)
Varicella and herpes zoster	6,448 (5,566–7,564)	2,059 (1,507–2,835)	522 (372–738)	938 (777–1,137)	1,027 (805–1,323)	1,899 (1,418–2,553)
Neglected tropical diseases and malaria	556,933 (436,443–679,983)	379,151 (267,258–497,954)	48,969 (35,954–63,699)	67,537 (52,951–86,191)	44,257 (34,336–57,899)	17,017 (12,820–22,815)
Malaria	482,944 (363,118–603,310)	359,992 (247,194–482,356)	36,811 (24,238–51,287)	45,296 (32,434–63,242)	29,981 (20,738–42,847)	10,861 (6,979–16,538)
Chagas disease	3,438 (3,272–3,617)	5 (4–5)	7 (6–8)	761 (706–822)	1,535 (1,434–1,635)	1,128 (1,055–1,203)
Leishmaniasis	5,164 (2,785–8,060)	1,376 (737–2,207)	751 (370–1,323)	2,422 (1,235–3,945)	487 (294–705)	126 (93–161)
Visceral leishmaniasis	5,164 (2,785–8,060)	1,376 (737–2,207)	751 (370–1,323)	2,422 (1,235–3,945)	487 (294–705)	126 (93–161)
Cutaneous and mucocutaneous leishmaniasis	0 (0–0)	0 (0–0)	0 (0–0)	0 (0–0)	0 (0–0)	0 (0–0)
African trypanosomiasis	11,077 (5,736–18,361)	848 (402–1,451)	1,370 (692–2,322)	6,144 (3,138–10,074)	2,203 (1,097–3,807)	509 (253–868)
Schistosomiasis	6,614 (5,608–7,869)	142 (83–256)	197 (130–289)	1,860 (1,501–2,334)	2,904 (2,257–3,780)	1,509 (1,132–1,992)
Cysticercosis	402 (351–477)	12 (6–22)	18 (14–23)	215 (187–259)	116 (102–138)	37 (33–42)
Cystic echinococcosis	1,552 (1,431–1,692)	134 (105–165)	175 (152–200)	539 (487–602)	409 (376–447)	292 (272–314)
Lymphatic filariasis	0 (0–0)	0 (0–0)	0 (0–0)	0 (0–0)	0 (0–0)	0 (0–0)
Onchocerciasis	0 (0–0)	0 (0–0)	0 (0–0)	0 (0–0)	0 (0–0)	0 (0–0)
Trachoma	0 (0–0)	0 (0–0)	0 (0–0)	0 (0–0)	0 (0–0)	0 (0–0)
Dengue	3,312 (1,514–6,988)	1,489 (466–3,684)	872 (369–1,794)	605 (360–1,096)	230 (159–383)	113 (74–189)
Yellow fever	3,398 (674–9,300)	372 (71–1,112)	952 (190–2,689)	1,594 (324–4,569)	402 (79–1,137)	76 (15–213)
Rabies	23,067 (19,286–26,641)	4,642 (3,063–6,261)	5,993 (4,720–7,430)	6,400 (4,978–7,658)	4,539 (3,765–5,577)	1,491 (1,172–2,041)
Intestinal nematode infections	3,584 (2,964–4,204)	2,936 (2,390–3,486)	409 (333–487)	108 (78–139)	73 (50–102)	56 (37–78)
Ascariasis	3,584 (2,964–4,204)	2,936 (2,390–3,486)	409 (333–487)	108 (78–139)	73 (50–102)	56 (37–78)
Trichuriasis	0 (0–0)	0 (0–0)	0 (0–0)	0 (0–0)	0 (0–0)	0 (0–0)
Hookworm disease	0 (0–0)	0 (0–0)	0 (0–0)	0 (0–0)	0 (0–0)	0 (0–0)
Food-borne trematodiases	0 (0–0)	0 (0–0)	0 (0–0)	0 (0–0)	0 (0–0)	0 (0–0)
Leprosy	0 (0–0)	0 (0–0)	0 (0–0)	0 (0–0)	0 (0–0)	0 (0–0)
Ebola	0 (0–0)	0 (0–0)	0 (0–0)	0 (0–0)	0 (0–0)	0 (0–0)
Other neglected tropical diseases	12,376 (8,277–19,537)	7,197 (4,482–10,567)	1,405 (874–2,346)	1,586 (1,110–2,717)	1,372 (915–2,732)	814 (543–1,688)
Maternal disorders	390,184 (365,192–416,234)	0 (0–0)	2,071 (1,731–2,415)	386,751 (362,141–412,417)	1,361 (1,033–1,924)	0 (0–0)
Maternal hemorrhage	105,609 (88,193–125,580)	0 (0–0)	549 (372–775)	104,631 (87,403–124,615)	428 (267–701)	0 (0–0)
Maternal sepsis and other maternal infections	29,359 (22,545–37,159)	0 (0–0)	95 (61–143)	29,154 (22,345–36,924)	108 (68–169)	0 (0–0)
Maternal hypertensive disorders	50,555 (40,186–62,936)	0 (0–0)	343 (216–493)	50,076 (39,782–62,498)	134 (84–201)	0 (0–0)
Maternal obstructed labor and uterine rupture	23,969 (18,435–30,166)	0 (0–0)	71 (41–117)	23,846 (18,318–30,056)	50 (32–80)	0 (0–0)
Maternal abortion, miscarriage, and ectopic pregnancy	55,686 (43,937–70,428)	0 (0–0)	366 (224–550)	55,072 (43,397–69,751)	248 (159–366)	0 (0–0)

SR Table 10 - Global deaths by age and cause for females in 1990						
**Cause**	**All ages**	**0-5 years**	**5-14 years**	**15-49 years**	**50-69 years**	**70+ years**
Indirect maternal deaths	41,491 (31,611–53,295)	0 (0–0)	249 (150–389)	41,138 (31,330–52,932)	102 (59–183)	0 (0–0)
Late maternal deaths	8,460 (5,791–11,935)	0 (0–0)	51 (26–86)	8,372 (5,714–11,830)	36 (19–67)	0 (0–0)
Maternal deaths aggravated by HIV/AIDS	754 (432–1,094)	0 (0–0)	0 (0–0)	752 (432–1,092)	1 (0–3)	0 (0–0)
Other maternal disorders	74,298 (61,159–89,652)	0 (0–0)	343 (224–490)	73,705 (60,608–88,977)	249 (165–367)	0 (0–0)
Neonatal disorders	1,554,695 (1,499,832–1,615,808)	1,554,695 (1,499,832–1,615,808)	0 (0–0)	0 (0–0)	0 (0–0)	0 (0–0)
Neonatal preterm birth complications	822,740 (737,303–915,376)	822,740 (737,303–915,376)	0 (0–0)	0 (0–0)	0 (0–0)	0 (0–0)
Neonatal encephalopathy due to birth asphyxia and trauma	391,734 (329,853–457,074)	391,734 (329,853–457,074)	0 (0–0)	0 (0–0)	0 (0–0)	0 (0–0)
Neonatal sepsis and other neonatal infections	119,808 (56,534–182,953)	119,808 (56,534–182,953)	0 (0–0)	0 (0–0)	0 (0–0)	0 (0–0)
Hemolytic disease and other neonatal jaundice	58,312 (27,410–128,581)	58,312 (27,410–128,581)	0 (0–0)	0 (0–0)	0 (0–0)	0 (0–0)
Other neonatal disorders	162,100 (108,281–251,244)	162,100 (108,281–251,244)	0 (0–0)	0 (0–0)	0 (0–0)	0 (0–0)
Nutritional deficiencies	328,272 (281,730–376,536)	217,160 (174,603–261,731)	11,386 (9,646–13,083)	25,138 (22,799–27,696)	25,128 (22,772–27,488)	49,458 (45,212–53,368)
Protein-energy malnutrition	271,561 (223,651–317,948)	195,944 (154,532–240,495)	8,688 (6,921–10,423)	12,971 (8,574–16,659)	15,314 (10,786–18,428)	38,642 (30,940–43,948)
Iodine deficiency	1,654 (1,109–2,571)	827 (398–1,637)	75 (37–145)	213 (137–337)	257 (153–429)	281 (170–461)
Vitamin A deficiency	0 (0–0)	0 (0–0)	0 (0–0)	0 (0–0)	0 (0–0)	0 (0–0)
Iron-deficiency anemia	27,159 (16,987–35,416)	10,073 (3,863–15,147)	1,127 (739–1,797)	7,136 (5,097–8,943)	5,649 (4,096–7,270)	3,172 (2,087–5,160)
Other nutritional deficiencies	27,897 (10,104–66,082)	10,315 (1,691–31,654)	1,494 (472–2,890)	4,817 (1,623–9,497)	3,906 (1,542–8,747)	7,362 (4,171–13,557)
Other communicable, maternal, neonatal, and nutritional diseases	218,240 (175,663–273,621)	129,112 (91,421–179,354)	16,934 (13,314–21,626)	31,695 (29,471–34,347)	24,147 (21,460–26,038)	16,351 (14,312–17,702)
Sexually transmitted diseases excluding HIV	106,338 (65,074–160,199)	87,502 (49,687–136,845)	8,466 (4,897–13,103)	5,394 (3,496–6,625)	2,965 (1,390–3,891)	2,010 (1,163–2,613)
Syphilis	104,978 (63,833–158,747)	87,502 (49,687–136,845)	8,439 (4,872–13,081)	4,790 (3,103–5,885)	2,520 (1,181–3,306)	1,725 (999–2,242)
Chlamydial infection	247 (143–302)	0 (0–0)	6 (4–8)	112 (72–139)	75 (35–99)	51 (30–66)
Gonococcal infection	739 (421–919)	0 (0–0)	11 (7–15)	297 (191–366)	249 (117–329)	180 (103–235)
Trichomoniasis	0 (0–0)	0 (0–0)	0 (0–0)	0 (0–0)	0 (0–0)	0 (0–0)
Genital herpes	0 (0–0)	0 (0–0)	0 (0–0)	0 (0–0)	0 (0–0)	0 (0–0)
Other sexually transmitted diseases	373 (217–462)	0 (0–0)	8 (5–10)	194 (125–239)	119 (55–157)	52 (30–69)
Hepatitis	70,375 (65,623–75,574)	21,752 (18,707–24,465)	6,097 (5,308–6,924)	21,286 (19,521–23,406)	13,313 (12,418–14,300)	7,925 (7,481–8,420)
Acute hepatitis A	18,158 (12,343–23,844)	16,352 (11,545–20,319)	934 (263–2,035)	583 (151–1,359)	186 (40–483)	101 (23–256)
Hepatitis B	29,148 (24,595–33,694)	1,187 (382–2,870)	605 (315–989)	10,618 (8,416–12,933)	10,853 (9,304–12,290)	5,883 (4,816–6,831)
Hepatitis C	1,192 (262–2,752)	0 (0–0)	75 (14–208)	320 (63–750)	552 (118–1,265)	244 (49–590)
Acute hepatitis E	21,875 (16,140–28,125)	4,212 (1,675–7,928)	4,482 (3,374–5,553)	9,764 (7,608–12,154)	1,720 (792–3,082)	1,695 (868–2,722)
Other infectious diseases	41,526 (27,301–48,234)	19,857 (9,564–26,281)	2,370 (1,670–2,740)	5,014 (4,212–5,515)	7,869 (6,141–8,812)	6,415 (4,965–7,334)
Non-communicable diseases	13,396,422 (13,096,584–13,762,836)	596,399 (490,992–796,757)	121,983 (111,070–136,424)	1,301,717 (1,250,983–1,365,748)	3,813,958 (3,711,710–3,920,221)	7,562,362 (7,449,350–7,682,569)
Neoplasms	2,495,273 (2,434,861–2,555,158)	22,378 (18,888–27,793)	25,773 (23,800–27,898)	409,127 (393,281–428,847)	1,058,597 (1,029,710–1,086,242)	979,396 (958,734–999,739)
Lip and oral cavity cancer	26,792 (25,659–27,981)	0 (0–0)	0 (0–0)	4,124 (3,878–4,411)	11,884 (11,240–12,580)	10,783 (10,352–11,245)
Nasopharynx cancer	15,313 (14,067–16,549)	0 (0–0)	137 (118–159)	5,375 (4,851–5,998)	6,852 (6,242–7,427)	2,947 (2,693–3,214)
Other pharynx cancer	12,581 (11,334–13,316)	0 (0–0)	0 (0–0)	1,940 (1,673–2,176)	6,715 (6,002–7,158)	3,924 (3,611–4,179)
Esophageal cancer	124,808 (114,645–136,513)	0 (0–0)	0 (0–0)	11,517 (10,424–12,466)	60,591 (55,211–66,828)	52,700 (48,346–57,899)
Stomach cancer	289,238 (277,579–302,063)	0 (0–0)	0 (0–0)	39,587 (36,875–43,352)	115,662 (110,158–121,684)	133,988 (129,295–138,825)
Colon and rectum cancer	244,684 (239,136–250,655)	0 (0–0)	0 (0–0)	22,992 (21,837–24,380)	89,928 (87,672–92,429)	131,764 (128,155–135,736)
Liver cancer	166,482 (138,009–180,959)	0 (0–0)	628 (442–738)	29,390 (21,260–33,642)	77,543 (62,348–85,250)	58,919 (52,044–64,506)
Liver cancer due to hepatitis B	55,011 (42,978–61,849)	0 (0–0)	297 (204–352)	16,374 (11,533–19,010)	25,914 (20,124–29,711)	12,425 (10,171–14,264)
Liver cancer due to hepatitis C	42,558 (37,819–46,234)	0 (0–0)	19 (13–26)	3,129 (2,348–3,690)	18,550 (15,413–20,768)	20,859 (19,153–22,794)
Liver cancer due to alcohol use	25,297 (21,235–28,036)	0 (0–0)	0 (0–0)	1,736 (1,263–2,087)	13,577 (10,877–15,667)	9,983 (8,740–11,057)
Liver cancer due to other causes	43,614 (34,992–48,914)	0 (0–0)	311 (220–371)	8,149 (5,807–9,512)	19,502 (15,198–22,523)	15,651 (13,167–17,714)
Gallbladder and biliary tract cancer	65,597 (62,990–68,464)	0 (0–0)	0 (0–0)	4,410 (3,959–4,797)	26,144 (24,766–27,560)	35,042 (33,855–36,406)
Pancreatic cancer	105,983 (103,485–108,389)	0 (0–0)	0 (0–0)	6,317 (6,047–6,600)	42,052 (40,871–43,186)	57,613 (56,304–58,936)
Larynx cancer	16,309 (14,784–17,461)	0 (0–0)	0 (0–0)	2,470 (2,062–2,745)	7,833 (7,027–8,472)	6,005 (5,648–6,353)
Tracheal, bronchus, and lung cancer	274,174 (264,809–285,607)	0 (0–0)	0 (0–0)	28,238 (26,502–30,392)	129,305 (124,569–135,073)	116,630 (112,716–121,730)
Malignant skin melanoma	16,098 (14,781–17,985)	0 (0–0)	0 (0–0)	3,546 (3,211–3,838)	6,032 (5,540–6,703)	6,519 (6,101–7,455)
Non-melanoma skin cancer	7,781 (7,383–8,182)	0 (0–0)	0 (0–0)	958 (880–1,034)	2,235 (2,067–2,405)	4,587 (4,348–4,854)
Non-melanoma skin cancer (squamous-cell carcinoma)	7,781 (7,383–8,182)	0 (0–0)	0 (0–0)	958 (880–1,034)	2,235 (2,067–2,405)	4,587 (4,348–4,854)
Non-melanoma skin cancer (basal-cell carcinoma)	0 (0–0)	0 (0–0)	0 (0–0)	0 (0–0)	0 (0–0)	0 (0–0)
Breast cancer	324,867 (312,956–347,331)	0 (0–0)	0 (0–0)	72,910 (65,133–81,961)	151,391 (145,646–161,465)	100,564 (97,267–105,228)
Cervical cancer	182,923 (169,229–194,903)	0 (0–0)	0 (0–0)	48,907 (44,234–53,051)	88,812 (82,023–95,406)	45,203 (42,777–49,033)
Uterine cancer	65,057 (61,740–69,074)	0 (0–0)	0 (0–0)	8,495 (7,667–9,372)	32,309 (30,466–34,357)	24,251 (23,340–25,997)
Ovarian cancer	103,083 (100,637–105,812)	0 (0–0)	0 (0–0)	17,832 (17,078–19,101)	51,519 (50,219–52,945)	33,731 (32,679–34,714)
Prostate cancer	0 (0–0)	0 (0–0)	0 (0–0)	0 (0–0)	0 (0–0)	0 (0–0)
Testicular cancer	0 (0–0)	0 (0–0)	0 (0–0)	0 (0–0)	0 (0–0)	0 (0–0)

SR Table 10 - Global deaths by age and cause for females in 1990

Cause	All ages	0-5 years	5-14 years	15-49 years	50-69 years	70+ years
Kidney cancer	29,265<br>(28,543–30,024)	990<br>(750–1,441)	585<br>(531–645)	2,831<br>(2,733–2,936)	12,220<br>(11,802–12,681)	12,637<br>(12,178–13,105)
Bladder cancer	31,390<br>(30,538–32,391)	0<br>(0–0)	0<br>(0–0)	1,868<br>(1,776–1,979)	9,539<br>(9,203–9,912)	19,981<br>(19,358–20,684)
Brain and nervous system cancer	61,599<br>(55,291–65,620)	3,025<br>(2,295–3,646)	4,685<br>(3,606–5,248)	17,289<br>(14,460–19,000)	24,000<br>(22,440–25,176)	12,599<br>(12,013–13,151)
Thyroid cancer	13,576<br>(12,557–15,310)	0<br>(0–0)	45<br>(35–53)	1,359<br>(1,220–1,749)	5,315<br>(4,857–6,016)	6,856<br>(6,396–7,612)
Mesothelioma	6,181<br>(5,609–6,649)	0<br>(0–0)	0<br>(0–0)	1,247<br>(1,145–1,344)	2,400<br>(2,168–2,597)	2,534<br>(2,260–2,757)
Hodgkin lymphoma	12,727<br>(8,456–16,424)	367<br>(148–541)	424<br>(240–620)	4,802<br>(3,312–6,033)	4,177<br>(2,616–5,682)	2,956<br>(2,003–3,840)
Non-Hodgkin lymphoma	54,902<br>(50,864–62,189)	1,641<br>(978–2,872)	1,537<br>(1,209–1,997)	9,660<br>(8,459–11,743)	19,386<br>(17,383–21,902)	22,676<br>(20,965–27,098)
Multiple myeloma	26,311<br>(25,349–27,760)	0<br>(0–0)	0<br>(0–0)	2,084<br>(1,962–2,255)	11,263<br>(10,766–11,893)	12,963<br>(12,407–13,939)
Leukemia	113,012<br>(107,779–118,405)	9,618<br>(7,417–11,564)	12,704<br>(11,600–14,018)	32,290<br>(30,078–35,002)	29,022<br>(27,880–30,106)	29,376<br>(28,493–30,378)
Acute lymphoid leukemia	39,468<br>(36,419–45,293)	6,750<br>(5,343–8,567)	9,209<br>(8,321–10,560)	12,094<br>(11,038–14,670)	6,893<br>(6,361–7,534)	4,520<br>(4,202–4,826)
Chronic lymphoid leukemia	19,441<br>(17,528–22,048)	0<br>(0–0)	0<br>(0–0)	4,174<br>(2,983–5,402)	5,823<br>(5,294–6,755)	9,442<br>(8,932–10,076)
Acute myeloid leukemia	37,140<br>(34,607–39,135)	2,868<br>(1,452–3,626)	3,495<br>(2,861–3,946)	10,792<br>(10,003–11,788)	10,343<br>(9,870–10,826)	9,641<br>(9,243–10,070)
Chronic myeloid leukemia	16,961<br>(15,590–18,609)	0<br>(0–0)	0<br>(0–0)	5,228<br>(4,475–6,154)	5,961<br>(5,470–6,503)	5,771<br>(5,412–6,223)
Other neoplasms	104,526<br>(97,330–110,786)	6,736<br>(5,103–10,653)	5,023<br>(4,465–5,606)	26,677<br>(23,873–29,218)	34,455<br>(31,354–37,308)	31,634<br>(29,418–33,657)
Cardiovascular diseases	6,416,432<br>(6,280,620–6,556,326)	53,450<br>(47,095–59,845)	21,072<br>(19,269–22,905)	429,533<br>(410,532–450,708)	1,639,510<br>(1,594,103–1,688,589)	4,272,865<br>(4,178,437–4,361,832)
Rheumatic heart disease	205,400<br>(190,198–223,392)	4,041<br>(2,708–5,183)	5,716<br>(5,049–6,475)	54,700<br>(49,745–60,797)	71,833<br>(66,191–78,419)	69,108<br>(63,668–74,877)
Ischemic heart disease	2,867,385<br>(2,810,229–2,924,549)	3,229<br>(2,625–4,150)	1,523<br>(1,329–1,799)	137,723<br>(131,248–146,103)	678,787<br>(660,855–696,877)	2,046,120<br>(2,000,354–2,088,169)
Cerebrovascular disease	2,495,098<br>(2,413,066–2,603,357)	19,705<br>(17,056–23,630)	7,551<br>(6,746–8,670)	165,338<br>(156,531–176,275)	676,098<br>(648,930–711,842)	1,626,404<br>(1,567,481–1,689,575)
Ischemic stroke	1,211,343<br>(1,116,828–1,327,032)	984<br>(752–1,279)	523<br>(435–639)	26,361<br>(21,887–32,821)	229,793<br>(199,800–265,376)	953,680<br>(893,641–1,025,672)
Hemorrhagic stroke	1,283,755<br>(1,197,378–1,358,836)	18,720<br>(16,153–22,685)	7,028<br>(6,275–8,111)	138,976<br>(130,735–149,761)	446,305<br>(415,399–477,004)	672,724<br>(617,136–711,422)
Hypertensive heart disease	363,903<br>(293,828–417,641)	529<br>(349–700)	251<br>(198–307)	26,501<br>(21,110–30,186)	109,049<br>(86,026–126,448)	227,571<br>(184,095–264,696)
Cardiomyopathy and myocarditis	127,452<br>(123,064–131,740)	8,213<br>(6,627–9,783)	2,190<br>(1,972–2,474)	14,393<br>(13,492–15,221)	26,481<br>(25,269–27,784)	76,175<br>(73,273–79,160)
Atrial fibrillation and flutter	54,775<br>(44,765–65,894)	0<br>(0–0)	0<br>(0–0)	209<br>(155–274)	3,564<br>(2,713–4,785)	51,000<br>(41,047–62,178)
Aortic aneurysm	32,222<br>(30,918–34,115)	0<br>(0–0)	0<br>(0–0)	2,020<br>(1,900–2,338)	7,478<br>(7,134–8,145)	22,723<br>(21,713–23,841)
Peripheral vascular disease	7,659<br>(7,051–8,225)	0<br>(0–0)	0<br>(0–0)	87<br>(81–93)	949<br>(862–1,072)	6,622<br>(6,085–7,082)
Endocarditis	26,945<br>(22,971–32,867)	2,488<br>(1,217–4,139)	665<br>(453–963)	4,328<br>(3,332–5,861)	5,701<br>(4,745–7,034)	13,761<br>(12,412–15,770)
Other cardiovascular and circulatory diseases	235,588<br>(225,300–246,860)	15,241<br>(12,662–18,449)	3,174<br>(2,781–3,669)	24,230<br>(22,480–26,455)	59,566<br>(55,987–63,611)	133,375<br>(125,856–140,620)
Chronic respiratory diseases	1,639,340<br>(1,556,791–1,743,236)	61,216<br>(51,583–69,763)	9,584<br>(8,559–10,646)	101,867<br>(95,062–110,501)	477,812<br>(450,557–509,979)	988,860<br>(937,082–1,065,365)
Chronic obstructive pulmonary disease	1,326,464<br>(1,159,314–1,495,928)	29,130<br>(18,772–37,833)	2,535<br>(1,557–4,432)	57,976<br>(45,606–74,165)	371,730<br>(305,892–434,530)	865,092<br>(776,019–960,202)
Pneumoconiosis	7,082<br>(4,577–10,465)	0<br>(0–0)	0<br>(0–0)	577<br>(294–995)	1,776<br>(982–2,904)	4,728<br>(3,241–6,759)
Silicosis	370<br>(213–590)	0<br>(0–0)	0<br>(0–0)	48<br>(18–95)	91<br>(51–145)	230<br>(140–351)
Asbestosis	371<br>(198–560)	0<br>(0–0)	0<br>(0–0)	111<br>(62–175)	105<br>(58–159)	154<br>(73–245)
Coal workers pneumoconiosis	177<br>(57–398)	0<br>(0–0)	0<br>(0–0)	41<br>(7–100)	52<br>(16–118)	84<br>(32–182)
Other pneumoconiosis	6,162<br>(3,967–9,147)	0<br>(0–0)	0<br>(0–0)	375<br>(182–696)	1,527<br>(815–2,576)	4,259<br>(2,924–6,150)
Asthma	269,727<br>(145,408–400,558)	18,549<br>(9,285–25,200)	6,284<br>(4,048–7,752)	40,550<br>(23,795–51,632)	97,804<br>(46,893–152,307)	106,538<br>(57,273–178,601)
Interstitial lung disease and pulmonary sarcoidosis	16,223<br>(13,361–21,318)	197<br>(100–336)	120<br>(93–156)	1,531<br>(1,209–1,889)	5,045<br>(4,061–6,398)	9,329<br>(7,777–12,952)
Other chronic respiratory diseases	19,843<br>(13,407–30,561)	13,338<br>(8,541–21,237)	644<br>(344–1,444)	1,232<br>(747–2,271)	1,456<br>(999–2,214)	3,171<br>(2,423–4,266)
Cirrhosis and other chronic liver diseases	340,882<br>(318,807–384,967)	12,066<br>(8,173–17,999)	6,120<br>(5,181–7,961)	74,640<br>(69,403–85,243)	143,145<br>(132,435–161,945)	104,908<br>(96,136–113,599)
Cirrhosis and other chronic liver diseases due to hepatitis B	90,737<br>(81,889–104,840)	59<br>(38–91)	119<br>(95–159)	19,792<br>(17,607–23,067)	40,648<br>(35,710–47,625)	30,116<br>(26,954–34,849)
Cirrhosis and other chronic liver diseases due to hepatitis C	73,849<br>(67,379–80,731)	30<br>(18–51)	68<br>(52–92)	14,485<br>(12,822–16,794)	32,422<br>(28,685–37,017)	26,843<br>(23,090–29,649)
Cirrhosis and other chronic liver diseases due to alcohol use	73,852<br>(67,848–81,717)	0<br>(0–0)	0<br>(0–0)	19,170<br>(17,287–22,121)	36,551<br>(32,467–41,171)	18,130<br>(16,243–20,385)
Cirrhosis and other chronic liver diseases due to other causes	102,443<br>(93,985–119,495)	11,977<br>(8,111–17,865)	5,932<br>(5,002–7,711)	21,191<br>(19,173–24,527)	33,523<br>(29,619–38,676)	29,817<br>(26,914–32,910)
Digestive diseases	469,566<br>(445,301–497,761)	32,434<br>(20,193–54,547)	13,502<br>(11,958–14,891)	82,459<br>(76,232–87,751)	130,439<br>(123,507–137,383)	210,730<br>(202,778–218,645)
Peptic ulcer disease	136,389<br>(117,403–156,025)	4,470<br>(2,298–8,869)	3,145<br>(2,486–4,004)	26,831<br>(22,646–31,608)	41,866<br>(35,217–48,598)	60,074<br>(52,208–68,364)
Gastritis and duodenitis	21,345<br>(7,605–30,411)	845<br>(224–1,840)	440<br>(163–705)	4,160<br>(1,397–6,055)	6,633<br>(2,236–9,859)	9,265<br>(3,689–13,736)
Appendicitis	23,987<br>(18,886–31,101)	5,500<br>(2,336–12,224)	2,360<br>(1,876–2,791)	6,777<br>(5,843–7,731)	4,804<br>(4,272–5,592)	4,543<br>(4,079–5,250)
Paralytic ileus and intestinal obstruction	88,858<br>(68,947–103,445)	8,381<br>(3,528–16,809)	3,906<br>(2,439–4,738)	17,201<br>(11,264–20,010)	23,550<br>(17,954–26,912)	35,817<br>(30,605–40,903)
Inguinal, femoral, and abdominal hernia	17,152<br>(11,871–20,010)	751<br>(249–1,342)	430<br>(253–568)	2,015<br>(1,150–2,528)	5,319<br>(3,546–6,350)	8,635<br>(6,384–9,924)
Inflammatory bowel disease	18,858<br>(14,536–24,126)	2,810<br>(686–6,552)	434<br>(260–658)	3,168<br>(2,245–4,307)	4,081<br>(3,387–4,774)	8,364<br>(7,259–9,125)
Vascular intestinal disorders	30,865<br>(27,466–34,022)	154<br>(55–337)	57<br>(35–82)	1,459<br>(996–1,905)	7,061<br>(5,856–8,185)	22,133<br>(20,362–23,908)
Gallbladder and biliary diseases	52,896<br>(49,123–57,162)	2,498<br>(1,183–4,852)	867<br>(709–1,042)	7,078<br>(6,322–7,981)	15,681<br>(14,339–16,983)	26,770<br>(25,226–28,622)
Pancreatitis	28,681<br>(25,477–32,128)	294<br>(142–563)	346<br>(253–450)	7,539<br>(6,222–8,941)	10,022<br>(8,861–11,263)	10,478<br>(9,718–11,266)
Other digestive diseases	50,532<br>(40,650–61,236)	6,727<br>(3,114–11,148)	1,514<br>(1,150–1,862)	6,226<br>(4,499–7,728)	11,417<br>(8,746–14,541)	24,647<br>(20,730–29,238)
Neurological disorders	666,059<br>(570,774–759,606)	9,897<br>(7,469–14,865)	6,597<br>(5,804–7,678)	28,078<br>(25,651–32,142)	51,621<br>(43,716–59,615)	569,864<br>(475,815–660,706)
Alzheimer disease and other dementias	570,281<br>(474,228–664,060)	0<br>(0–0)	0<br>(0–0)	408<br>(260–593)	31,460<br>(23,750–39,525)	538,412<br>(444,058–629,734)
Parkinson disease	22,176<br>(21,303–23,122)	0<br>(0–0)	0<br>(0–0)	182<br>(163–203)	3,403<br>(3,250–3,592)	18,590<br>(17,802–19,430)
Epilepsy	41,888<br>(37,331–49,840)	7,060<br>(4,982–11,153)	5,472<br>(4,748–6,375)	20,526<br>(18,443–23,978)	5,241<br>(4,904–5,595)	3,587<br>(3,395–3,783)

SR Table 10 - Global deaths by age and cause for females in 1990						
**Cause**	**All ages**	**0-5 years**	**5-14 years**	**15-49 years**	**50-69 years**	**70+ years**
Multiple sclerosis	7,806 (6,552–9,380)	0 (0–0)	0 (0–0)	2,909 (2,209–3,805)	3,452 (2,993–4,016)	1,444 (1,259–1,599)
Motor neuron disease	8,384 (7,880–10,287)	902 (479–1,601)	57 (46–82)	707 (655–958)	3,738 (3,507–4,407)	2,978 (2,849–3,168)
Migraine	0 (0–0)	0 (0–0)	0 (0–0)	0 (0–0)	0 (0–0)	0 (0–0)
Tension-type headache	0 (0–0)	0 (0–0)	0 (0–0)	0 (0–0)	0 (0–0)	0 (0–0)
Medication overuse headache	0 (0–0)	0 (0–0)	0 (0–0)	0 (0–0)	0 (0–0)	0 (0–0)
Other neurological disorders	15,534 (14,836–18,582)	1,945 (1,630–4,190)	1,067 (946–1,563)	3,344 (3,144–4,117)	4,324 (4,145–4,477)	4,851 (4,582–5,070)
Mental and substance use disorders	41,961 (38,587–43,887)	11 (10–13)	20 (10–29)	22,351 (19,884–23,907)	13,320 (12,690–13,844)	6,257 (5,889–6,563)
Schizophrenia	7,555 (6,902–8,346)	0 (0–0)	0 (0–0)	3,056 (2,709–3,531)	2,469 (2,238–2,736)	2,029 (1,846–2,229)
Alcohol use disorders	15,666 (15,092–16,209)	0 (0–0)	0 (0–0)	6,660 (6,339–6,968)	6,930 (6,621–7,253)	2,075 (1,987–2,154)
Drug use disorders	18,411 (14,865–19,731)	11 (10–13)	0 (0–0)	12,327 (9,603–13,399)	3,920 (3,384–4,197)	2,152 (1,840–2,351)
Opioid use disorders	9,985 (8,973–12,026)	11 (10–13)	0 (0–0)	6,755 (5,801–8,360)	2,204 (1,990–2,508)	1,012 (875–1,269)
Cocaine use disorders	386 (243–453)	0 (0–0)	0 (0–0)	281 (172–337)	75 (50–88)	28 (18–35)
Amphetamine use disorders	341 (197–976)	0 (0–0)	0 (0–0)	262 (140–767)	59 (39–160)	20 (12–42)
Cannabis use disorders	0 (0–0)	0 (0–0)	0 (0–0)	0 (0–0)	0 (0–0)	0 (0–0)
Other drug use disorders	7,698 (4,695–8,904)	0 (0–0)	0 (0–0)	5,026 (2,967–6,030)	1,581 (1,039–1,869)	1,090 (707–1,338)
Depressive disorders	0 (0–0)	0 (0–0)	0 (0–0)	0 (0–0)	0 (0–0)	0 (0–0)
Major depressive disorder	0 (0–0)	0 (0–0)	0 (0–0)	0 (0–0)	0 (0–0)	0 (0–0)
Dysthymia	0 (0–0)	0 (0–0)	0 (0–0)	0 (0–0)	0 (0–0)	0 (0–0)
Bipolar disorder	0 (0–0)	0 (0–0)	0 (0–0)	0 (0–0)	0 (0–0)	0 (0–0)
Anxiety disorders	0 (0–0)	0 (0–0)	0 (0–0)	0 (0–0)	0 (0–0)	0 (0–0)
Eating disorders	328 (218–532)	0 (0–0)	20 (10–29)	307 (208–505)	0 (0–0)	0 (0–0)
Anorexia nervosa	318 (212–513)	0 (0–0)	19 (9–29)	299 (201–487)	0 (0–0)	0 (0–0)
Bulimia nervosa	9 (5–20)	0 (0–0)	0 (0–1)	8 (4–19)	0 (0–0)	0 (0–0)
Autistic spectrum disorders	0 (0–0)	0 (0–0)	0 (0–0)	0 (0–0)	0 (0–0)	0 (0–0)
Autism	0 (0–0)	0 (0–0)	0 (0–0)	0 (0–0)	0 (0–0)	0 (0–0)
Asperger syndrome and other autistic spectrum disorders	0 (0–0)	0 (0–0)	0 (0–0)	0 (0–0)	0 (0–0)	0 (0–0)
Attention-deficit/hyperactivity disorder	0 (0–0)	0 (0–0)	0 (0–0)	0 (0–0)	0 (0–0)	0 (0–0)
Conduct disorder	0 (0–0)	0 (0–0)	0 (0–0)	0 (0–0)	0 (0–0)	0 (0–0)
Idiopathic developmental intellectual disability	0 (0–0)	0 (0–0)	0 (0–0)	0 (0–0)	0 (0–0)	0 (0–0)
Other mental and substance use disorders	0 (0–0)	0 (0–0)	0 (0–0)	0 (0–0)	0 (0–0)	0 (0–0)
Diabetes, urogenital, blood, and endocrine diseases	886,716 (861,016–916,712)	70,056 (50,895–89,134)	17,872 (15,819–19,843)	121,701 (116,273–128,898)	280,323 (269,997–290,317)	396,762 (384,980–409,191)
Diabetes mellitus	370,595 (355,927–384,299)	2,190 (1,657–2,670)	1,696 (1,480–2,013)	32,691 (31,054–34,791)	145,232 (138,486–151,363)	188,784 (180,816–196,641)
Acute glomerulonephritis	7,244 (6,010–11,307)	497 (360–748)	380 (317–540)	2,237 (1,856–3,113)	2,060 (1,644–3,267)	2,068 (1,568–3,740)
Chronic kidney disease	296,226 (287,448–306,921)	11,634 (10,042–13,192)	5,546 (5,024–6,061)	48,335 (45,869–51,574)	91,650 (88,338–95,521)	139,059 (134,289–144,012)
Chronic kidney disease due to diabetes mellitus	90,152 (85,922–94,456)	1,386 (1,107–1,694)	456 (378–542)	10,936 (9,949–12,002)	33,766 (31,269–36,559)	43,606 (41,060–46,172)
Chronic kidney disease due to hypertension	129,968 (124,622–135,758)	3,841 (3,233–4,416)	1,612 (1,374–1,883)	17,808 (16,495–19,215)	38,471 (35,764–41,089)	68,234 (64,635–71,955)
Chronic kidney disease due to glomerulonephritis	67,026 (63,519–71,141)	4,745 (4,044–5,465)	2,603 (2,308–2,925)	17,157 (15,747–18,944)	18,308 (16,819–19,940)	24,211 (22,450–26,186)
Chronic kidney disease due to other causes	9,078 (7,617–10,594)	1,659 (1,326–2,059)	872 (698–1,073)	2,433 (1,681–3,250)	1,104 (705–1,644)	3,007 (2,527–3,742)
Urinary diseases and male infertility	67,855 (63,455–76,010)	4,129 (2,975–5,108)	969 (828–1,144)	9,809 (8,823–11,227)	18,614 (16,917–22,277)	34,333 (32,375–38,783)
Interstitial nephritis and urinary tract infections	55,129 (51,298–62,051)	3,265 (2,319–3,971)	646 (530–774)	7,415 (6,586–8,533)	14,257 (12,735–17,268)	29,544 (27,597–33,326)
Urolithiasis	5,766 (4,590–6,923)	0 (0–0)	135 (98–176)	1,108 (793–1,377)	2,207 (1,787–2,739)	2,314 (1,800–2,664)
Benign prostatic hyperplasia	0 (0–0)	0 (0–0)	0 (0–0)	0 (0–0)	0 (0–0)	0 (0–0)
Male infertility	0 (0–0)	0 (0–0)	0 (0–0)	0 (0–0)	0 (0–0)	0 (0–0)
Other urinary diseases	6,959 (5,963–8,626)	864 (512–1,351)	186 (142–247)	1,285 (1,053–1,687)	2,148 (1,728–2,911)	2,473 (2,122–3,055)
Gynecological diseases	6,572 (5,093–8,073)	0 (0–0)	0 (0–0)	3,239 (2,377–3,944)	1,971 (1,476–2,495)	1,361 (1,087–1,779)
Uterine fibroids	1,768 (955–2,470)	0 (0–0)	0 (0–0)	723 (403–1,013)	702 (366–1,008)	342 (175–578)
Polycystic ovarian syndrome	881 (211–1,471)	0 (0–0)	0 (0–0)	637 (130–1,091)	243 (80–382)	0 (0–0)
Female infertility	0 (0–0)	0 (0–0)	0 (0–0)	0 (0–0)	0 (0–0)	0 (0–0)
Endometriosis	43 (27–64)	0 (0–0)	0 (0–0)	37 (24–55)	5 (3–9)	0 (0–0)
Genital prolapse	1,036 (505–1,906)	0 (0–0)	0 (0–0)	124 (54–219)	412 (156–843)	499 (278–927)
Premenstrual syndrome	0 (0–0)	0 (0–0)	0 (0–0)	0 (0–0)	0 (0–0)	0 (0–0)
Other gynecological diseases	2,842 (2,055–3,848)	0 (0–0)	0 (0–0)	1,716 (1,225–2,432)	607 (395–933)	519 (365–811)
Hemoglobinopathies and hemolytic anemias	95,229 (76,386–114,009)	41,943 (24,967–59,839)	7,303 (5,739–8,584)	17,864 (15,672–19,906)	11,105 (10,574–11,743)	17,012 (15,448–18,384)
Thalassemias	14,923 (9,635–20,396)	10,149 (5,503–15,347)	2,565 (1,881–3,106)	1,277 (1,075–1,505)	32 (28–39)	899 (719–1,205)
Thalassemias trait	0 (0–0)	0 (0–0)	0 (0–0)	0 (0–0)	0 (0–0)	0 (0–0)
Sickle cell disorders	43,408 (29,824–58,988)	30,883 (18,066–46,540)	3,841 (2,983–4,686)	7,103 (5,860–8,511)	441 (369–550)	1,138 (983–1,314)
Sickle cell trait	0 (0–0)	0 (0–0)	0 (0–0)	0 (0–0)	0 (0–0)	0 (0–0)

SR Table 10 - Global deaths by age and cause for females in 1990						
Cause	All ages	0-5 years	5-14 years	15-49 years	50-69 years	70+ years
G6PD deficiency	6,083<br>(5,127–6,973)	403<br>(188–660)	367<br>(258–470)	3,674<br>(3,147–4,232)	1,358<br>(1,185–1,560)	279<br>(240–323)
G6PD trait	0<br>(0–0)	0<br>(0–0)	0<br>(0–0)	0<br>(0–0)	0<br>(0–0)	0<br>(0–0)
Other hemoglobinopathies and hemolytic anemias	30,814<br>(29,317–32,251)	508<br>(299–783)	529<br>(421–621)	5,808<br>(5,196–6,403)	9,273<br>(8,787–9,811)	14,694<br>(13,224–15,748)
Endocrine, metabolic, blood, and immune disorders	42,992<br>(40,805–45,279)	9,661<br>(7,675–11,663)	1,976<br>(1,796–2,158)	7,523<br>(7,167–7,888)	9,688<br>(9,143–10,380)	14,144<br>(13,451–14,985)
Musculoskeletal disorders	36,088<br>(32,515–41,774)	0<br>(0–0)	1,049<br>(801–1,410)	9,256<br>(7,424–12,736)	9,392<br>(8,488–10,631)	16,388<br>(14,838–17,427)
Rheumatoid arthritis	15,899<br>(13,222–18,271)	0<br>(0–0)	199<br>(114–302)	1,546<br>(974–2,200)	5,309<br>(4,422–6,193)	8,845<br>(7,530–9,688)
Osteoarthritis	0<br>(0–0)	0<br>(0–0)	0<br>(0–0)	0<br>(0–0)	0<br>(0–0)	0<br>(0–0)
Low back and neck pain	0<br>(0–0)	0<br>(0–0)	0<br>(0–0)	0<br>(0–0)	0<br>(0–0)	0<br>(0–0)
Low back pain	0<br>(0–0)	0<br>(0–0)	0<br>(0–0)	0<br>(0–0)	0<br>(0–0)	0<br>(0–0)
Neck pain	0<br>(0–0)	0<br>(0–0)	0<br>(0–0)	0<br>(0–0)	0<br>(0–0)	0<br>(0–0)
Gout	0<br>(0–0)	0<br>(0–0)	0<br>(0–0)	0<br>(0–0)	0<br>(0–0)	0<br>(0–0)
Other musculoskeletal disorders	20,188<br>(18,014–24,553)	0<br>(0–0)	850<br>(641–1,156)	7,710<br>(6,133–10,964)	4,083<br>(3,683–5,096)	7,543<br>(6,770–8,236)
Other non-communicable diseases	404,089<br>(282,833–614,501)	334,874<br>(227,199–529,987)	20,390<br>(14,146–29,943)	22,699<br>(17,648–29,798)	9,795<br>(7,918–13,341)	16,329<br>(12,034–22,434)
Congenital anomalies	367,551<br>(246,226–574,951)	318,912<br>(208,269–510,951)	19,919<br>(13,726–29,573)	20,320<br>(15,746–27,647)	5,095<br>(4,264–5,982)	3,303<br>(2,929–3,732)
Neural tube defects	67,752<br>(42,901–119,423)	64,268<br>(40,282–114,201)	1,934<br>(1,247–3,258)	1,115<br>(785–1,701)	280<br>(179–547)	154<br>(103–259)
Congenital heart anomalies	190,844<br>(117,764–302,987)	158,170<br>(93,200–260,000)	14,527<br>(9,414–21,811)	14,876<br>(11,032–20,350)	1,867<br>(1,554–2,315)	1,402<br>(1,219–1,698)
Cleft lip and cleft palate	2,644<br>(1,129–4,254)	2,644<br>(1,129–4,254)	0<br>(0–0)	0<br>(0–0)	0<br>(0–0)	0<br>(0–0)
Down syndrome	20,560<br>(4,603–50,109)	17,305<br>(2,643–45,147)	558<br>(180–1,243)	1,654<br>(922–2,836)	972<br>(703–1,453)	69<br>(47–102)
Turner syndrome	0<br>(0–0)	0<br>(0–0)	0<br>(0–0)	0<br>(0–0)	0<br>(0–0)	0<br>(0–0)
Klinefelter syndrome	0<br>(0–0)	0<br>(0–0)	0<br>(0–0)	0<br>(0–0)	0<br>(0–0)	0<br>(0–0)
Other chromosomal abnormalities	13,529<br>(4,172–49,487)	12,934<br>(3,746–48,424)	204<br>(91–588)	246<br>(186–359)	105<br>(68–140)	38<br>(28–47)
Other congenital anomalies	72,219<br>(48,541–126,197)	63,588<br>(41,221–114,867)	2,695<br>(1,887–4,364)	2,426<br>(1,908–3,279)	1,868<br>(1,398–2,241)	1,639<br>(1,358–1,880)
Skin and subcutaneous diseases	23,763<br>(15,773–36,956)	3,188<br>(1,468–5,763)	470<br>(240–736)	2,379<br>(1,342–3,955)	4,699<br>(2,911–8,237)	13,025<br>(8,766–19,334)
Dermatitis	0<br>(0–0)	0<br>(0–0)	0<br>(0–0)	0<br>(0–0)	0<br>(0–0)	0<br>(0–0)
Psoriasis	0<br>(0–0)	0<br>(0–0)	0<br>(0–0)	0<br>(0–0)	0<br>(0–0)	0<br>(0–0)
Cellulitis	3,753<br>(2,129–6,214)	364<br>(128–790)	85<br>(40–142)	508<br>(246–849)	1,189<br>(599–2,129)	1,605<br>(1,019–2,555)
Pyoderma	9,218<br>(5,519–16,042)	2,618<br>(1,136–4,913)	245<br>(124–427)	1,254<br>(662–2,218)	1,965<br>(1,194–3,911)	3,133<br>(2,082–5,408)
Scabies	0<br>(0–0)	0<br>(0–0)	0<br>(0–0)	0<br>(0–0)	0<br>(0–0)	0<br>(0–0)
Fungal skin diseases	0<br>(0–0)	0<br>(0–0)	0<br>(0–0)	0<br>(0–0)	0<br>(0–0)	0<br>(0–0)
Viral skin diseases	0<br>(0–0)	0<br>(0–0)	0<br>(0–0)	0<br>(0–0)	0<br>(0–0)	0<br>(0–0)
Acne vulgaris	0<br>(0–0)	0<br>(0–0)	0<br>(0–0)	0<br>(0–0)	0<br>(0–0)	0<br>(0–0)
Alopecia areata	0<br>(0–0)	0<br>(0–0)	0<br>(0–0)	0<br>(0–0)	0<br>(0–0)	0<br>(0–0)
Pruritus	0<br>(0–0)	0<br>(0–0)	0<br>(0–0)	0<br>(0–0)	0<br>(0–0)	0<br>(0–0)
Urticaria	0<br>(0–0)	0<br>(0–0)	0<br>(0–0)	0<br>(0–0)	0<br>(0–0)	0<br>(0–0)
Decubitus ulcer	9,538<br>(5,624–14,082)	49<br>(13–111)	78<br>(35–127)	392<br>(210–665)	1,290<br>(701–2,398)	7,727<br>(4,362–10,948)
Other skin and subcutaneous diseases	1,253<br>(848–2,147)	155<br>(65–409)	61<br>(27–99)	223<br>(126–371)	253<br>(168–488)	558<br>(382–867)
Sense organ diseases	0<br>(0–0)	0<br>(0–0)	0<br>(0–0)	0<br>(0–0)	0<br>(0–0)	0<br>(0–0)
Glaucoma	0<br>(0–0)	0<br>(0–0)	0<br>(0–0)	0<br>(0–0)	0<br>(0–0)	0<br>(0–0)
Cataract	0<br>(0–0)	0<br>(0–0)	0<br>(0–0)	0<br>(0–0)	0<br>(0–0)	0<br>(0–0)
Macular degeneration	0<br>(0–0)	0<br>(0–0)	0<br>(0–0)	0<br>(0–0)	0<br>(0–0)	0<br>(0–0)
Refraction and accommodation disorders	0<br>(0–0)	0<br>(0–0)	0<br>(0–0)	0<br>(0–0)	0<br>(0–0)	0<br>(0–0)
Age-related and other hearing loss	0<br>(0–0)	0<br>(0–0)	0<br>(0–0)	0<br>(0–0)	0<br>(0–0)	0<br>(0–0)
Other vision loss	0<br>(0–0)	0<br>(0–0)	0<br>(0–0)	0<br>(0–0)	0<br>(0–0)	0<br>(0–0)
Other sense organ diseases	0<br>(0–0)	0<br>(0–0)	0<br>(0–0)	0<br>(0–0)	0<br>(0–0)	0<br>(0–0)
Oral disorders	0<br>(0–0)	0<br>(0–0)	0<br>(0–0)	0<br>(0–0)	0<br>(0–0)	0<br>(0–0)
Deciduous caries	0<br>(0–0)	0<br>(0–0)	0<br>(0–0)	0<br>(0–0)	0<br>(0–0)	0<br>(0–0)
Permanent caries	0<br>(0–0)	0<br>(0–0)	0<br>(0–0)	0<br>(0–0)	0<br>(0–0)	0<br>(0–0)
Periodontal diseases	0<br>(0–0)	0<br>(0–0)	0<br>(0–0)	0<br>(0–0)	0<br>(0–0)	0<br>(0–0)
Edentulism and severe tooth loss	0<br>(0–0)	0<br>(0–0)	0<br>(0–0)	0<br>(0–0)	0<br>(0–0)	0<br>(0–0)
Other oral disorders	0<br>(0–0)	0<br>(0–0)	0<br>(0–0)	0<br>(0–0)	0<br>(0–0)	0<br>(0–0)
Sudden infant death syndrome	12,774<br>(9,166–18,026)	12,774<br>(9,166–18,026)	0<br>(0–0)	0<br>(0–0)	0<br>(0–0)	0<br>(0–0)
Injuries	1,487,577<br>(1,295,129–1,600,455)	302,678<br>(237,737–365,007)	143,138<br>(125,915–155,052)	565,534<br>(485,649–602,345)	236,628<br>(210,205–249,485)	239,597<br>(213,763–251,837)
Transport injuries	333,971<br>(307,626–356,218)	48,795<br>(35,417–60,780)	38,992<br>(35,216–42,583)	142,581<br>(134,596–150,538)	63,590<br>(60,806–66,459)	40,011<br>(38,653–41,467)
Road injuries	315,956<br>(292,580–337,541)	45,213<br>(33,189–56,250)	37,283<br>(33,762–40,682)	135,264<br>(127,916–143,520)	60,039<br>(57,339–63,069)	38,154<br>(36,799–39,578)
Pedestrian road injuries	162,953<br>(146,189–176,517)	23,843<br>(17,824–29,441)	21,432<br>(19,425–23,300)	59,581<br>(54,143–64,485)	33,920<br>(30,737–36,371)	24,176<br>(22,459–25,454)
Cyclist road injuries	12,625<br>(10,750–16,056)	1,082<br>(679–1,968)	1,727<br>(1,394–2,243)	5,814<br>(4,809–7,532)	2,856<br>(2,485–3,307)	1,144<br>(1,009–1,314)
Motorcyclist road injuries	28,738<br>(24,141–34,832)	4,149<br>(2,010–7,101)	2,673<br>(2,084–3,394)	15,238<br>(13,219–18,376)	4,736<br>(4,064–5,922)	1,940<br>(1,589–2,578)

SR Table 10 - Global deaths by age and cause for females in 1990						
Cause	All ages	0-5 years	5-14 years	15-49 years	50-69 years	70+ years
Motor vehicle road injuries	108,093 (99,262–119,064)	15,422 (10,676–20,170)	11,033 (9,588–12,740)	53,226 (49,662–58,743)	17,884 (16,560–20,225)	10,526 (9,752–11,903)
Other road injuries	3,545 (2,443–4,332)	715 (263–1,284)	416 (272–548)	1,404 (981–1,679)	642 (482–780)	367 (295–460)
Other transport injuries	18,014 (12,662–21,141)	3,581 (1,567–5,022)	1,709 (1,151–2,080)	7,316 (5,233–8,639)	3,550 (2,706–4,031)	1,856 (1,541–2,107)
Unintentional injuries	769,550 (644,030–843,747)	233,858 (174,855–287,965)	84,549 (68,497–93,638)	193,593 (161,554–210,726)	99,317 (86,411–108,470)	158,230 (140,758–168,405)
Falls	141,117 (121,436–152,325)	18,922 (8,328–27,133)	7,195 (5,767–8,212)	17,661 (15,722–19,443)	21,780 (19,806–23,400)	75,558 (68,910–80,688)
Drowning	184,897 (101,716–216,870)	81,249 (31,268–104,865)	38,058 (24,196–43,967)	39,318 (25,074–44,673)	14,254 (8,978–16,931)	12,016 (8,423–14,119)
Fire, heat, and hot substances	128,362 (77,818–146,924)	28,836 (11,763–37,872)	9,537 (5,821–11,139)	56,669 (33,457–64,940)	15,355 (11,038–16,890)	17,962 (14,214–19,378)
Poisonings	51,269 (36,012–63,268)	16,751 (6,679–28,753)	4,785 (4,112–5,963)	17,580 (14,676–20,432)	6,797 (5,442–7,949)	5,355 (4,694–6,097)
Exposure to mechanical forces	59,201 (51,046–70,868)	24,449 (17,463–33,682)	5,353 (4,634–6,951)	12,765 (11,566–15,212)	7,364 (6,539–9,400)	9,267 (7,244–11,146)
Unintentional firearm injuries	7,555 (5,467–8,695)	937 (480–1,415)	678 (466–853)	2,322 (1,588–2,754)	1,050 (714–1,236)	2,565 (2,115–2,827)
Unintentional suffocation	18,734 (13,151–25,234)	14,134 (8,989–20,459)	1,322 (944–2,014)	1,323 (983–1,848)	613 (463–892)	1,340 (1,055–1,791)
Other exposure to mechanical forces	32,911 (28,261–41,253)	9,377 (6,049–14,344)	3,352 (2,860–4,496)	9,119 (8,201–11,335)	5,700 (4,950–7,770)	5,361 (3,845–6,854)
Adverse effects of medical treatment	47,808 (34,674–60,337)	13,167 (6,006–19,715)	2,635 (1,634–3,506)	10,528 (7,115–13,703)	8,255 (6,272–9,973)	13,220 (10,096–15,045)
Animal contact	48,643 (31,094–55,637)	11,102 (6,020–14,254)	7,901 (4,990–9,286)	16,951 (10,989–19,413)	8,919 (5,795–10,195)	3,767 (2,540–4,362)
Venomous animal contact	40,971 (25,697–46,898)	8,180 (4,255–10,730)	6,666 (4,085–7,924)	15,281 (9,681–17,599)	7,904 (4,976–9,139)	2,937 (1,828–3,569)
Non-venomous animal contact	7,672 (5,190–10,214)	2,922 (1,490–4,220)	1,234 (819–1,806)	1,670 (1,173–2,317)	1,014 (723–1,325)	830 (670–1,008)
Foreign body	57,015 (37,850–87,537)	27,728 (14,795–54,842)	3,968 (2,466–6,101)	8,430 (5,557–13,502)	6,057 (4,361–9,131)	10,830 (8,711–14,152)
Pulmonary aspiration and foreign body in airway	41,561 (25,198–75,691)	22,775 (9,380–50,375)	2,663 (1,406–5,022)	4,096 (2,950–6,540)	3,906 (3,131–5,075)	8,119 (7,222–9,072)
Foreign body in eyes	0 (0–0)	0 (0–0)	0 (0–0)	0 (0–0)	0 (0–0)	0 (0–0)
Foreign body in other body part	15,454 (6,577–33,246)	4,953 (1,302–8,689)	1,305 (550–2,979)	4,333 (2,001–10,100)	2,151 (989–5,429)	2,710 (1,524–6,123)
Environmental heat and cold exposure	16,642 (10,383–25,051)	2,964 (1,236–4,175)	667 (425–1,166)	2,700 (1,931–4,013)	4,168 (2,421–7,285)	6,141 (3,772–9,777)
Other unintentional injuries	34,590 (30,379–40,758)	8,684 (6,363–10,830)	4,445 (3,651–5,350)	10,987 (9,824–13,024)	6,364 (5,578–7,659)	4,108 (3,601–4,852)
Self-harm and interpersonal violence	341,588 (252,418–372,201)	11,197 (9,470–12,723)	11,732 (9,362–12,861)	211,518 (150,057–232,542)	68,309 (51,720–74,593)	38,830 (29,907–42,250)
Self-harm	264,009 (175,146–293,200)	0 (0–0)	5,425 (3,066–6,312)	167,008 (103,283–187,522)	58,028 (41,366–64,057)	33,547 (24,637–36,909)
Interpersonal violence	77,578 (73,936–81,750)	11,197 (9,470–12,723)	6,306 (5,833–6,826)	44,510 (42,590–47,158)	10,281 (9,866–10,731)	5,282 (5,029–5,535)
Assault by firearm	15,235 (12,964–16,853)	928 (621–1,307)	1,009 (819–1,174)	10,499 (8,918–11,430)	1,948 (1,669–2,274)	850 (712–1,012)
Assault by sharp object	17,855 (16,426–19,737)	1,125 (666–1,717)	1,108 (947–1,295)	11,973 (10,905–13,441)	2,345 (2,144–2,617)	1,302 (1,188–1,447)
Assault by other means	44,487 (40,882–48,367)	9,143 (7,563–10,530)	4,188 (3,772–4,667)	22,037 (20,223–24,326)	5,988 (5,539–6,474)	3,129 (2,884–3,343)
Forces of nature, war, and legal intervention	42,467 (28,637–57,800)	8,826 (4,312–15,211)	7,864 (4,297–12,478)	17,840 (11,473–25,675)	5,410 (3,261–7,923)	2,524 (1,265–5,267)
Exposure to forces of nature	18,855 (9,264–28,426)	2,807 (1,293–4,332)	4,649 (2,144–7,173)	7,683 (3,937–11,518)	2,619 (1,287–3,921)	1,094 (586–1,612)
Collective violence and legal intervention	23,611 (13,877–36,586)	6,019 (2,105–12,298)	3,215 (989–7,004)	10,156 (4,868–17,260)	2,790 (1,172–4,931)	1,429 (376–3,905)

SR Table 10 - Global deaths by age and cause for females in 1995

Cause	All ages	0-5 years	5-14 years	15-49 years	50-69 years	70+ years
All causes	23,541,653 (23,246,790–23,869,437)	5,019,133 (4,968,602–5,076,407)	592,919 (584,473–601,479)	3,270,912 (3,187,272–3,361,459)	4,966,411 (4,861,490–5,078,897)	9,692,276 (9,579,950–9,803,747)
Communicable, maternal, neonatal, and nutritional diseases	7,344,858 (7,124,349–7,546,260)	4,254,911 (4,089,372–4,375,713)	332,900 (315,793–353,802)	1,203,987 (1,154,992–1,272,072)	662,143 (612,705–704,420)	890,914 (835,154–935,192)
HIV/AIDS and tuberculosis	847,176 (735,704–966,200)	75,245 (66,325–91,639)	19,206 (16,188–26,544)	388,040 (349,402–435,881)	226,715 (177,438–256,985)	137,968 (101,398–164,103)
Tuberculosis	574,378 (458,141–684,209)	24,931 (16,389–41,191)	14,219 (11,434–21,474)	196,560 (157,380–244,063)	202,960 (152,608–233,855)	135,705 (99,142–161,481)
HIV/AIDS	272,798 (251,089–297,193)	50,314 (47,614–53,373)	4,986 (4,553–5,537)	191,480 (173,266–212,600)	23,754 (20,644–27,510)	2,263 (1,871–2,715)
HIV/AIDS - Tuberculosis	65,583 (55,579–78,626)	14,968 (12,738–17,133)	1,430 (1,121–1,681)	42,059 (35,426–51,276)	6,518 (5,494–7,655)	606 (494–733)
HIV/AIDS resulting in other diseases	207,215 (185,424–232,961)	35,345 (32,215–39,299)	3,555 (3,124–4,129)	149,420 (130,752–170,023)	17,235 (14,327–20,799)	1,657 (1,324–2,088)
Diarrhea, lower respiratory, and other common infectious diseases	3,558,091 (3,378,148–3,747,206)	2,047,301 (1,904,629–2,205,602)	231,893 (208,632–259,298)	298,292 (275,894–329,369)	331,388 (307,480–353,355)	649,215 (605,912–687,267)
Diarrheal diseases	1,028,884 (942,731–1,114,361)	539,827 (460,610–616,083)	56,992 (49,785–63,880)	107,781 (101,268–115,000)	141,796 (133,994–150,994)	182,485 (170,340–194,623)
Intestinal infectious diseases	115,817 (65,041–191,172)	32,737 (17,537–57,211)	46,544 (27,032–74,616)	30,103 (15,968–51,189)	4,576 (2,345–8,096)	1,855 (1,116–2,898)
Typhoid fever	94,405 (51,296–158,962)	26,376 (13,344–46,727)	38,687 (21,409–62,792)	24,379 (12,787–42,076)	3,602 (1,728–6,495)	1,359 (722–2,221)
Paratyphoid fever	19,238 (8,771–37,298)	4,967 (2,090–10,425)	7,613 (3,477–14,581)	5,480 (2,333–10,843)	870 (347–1,773)	307 (129–601)
Other intestinal infectious diseases	2,174 (423–4,480)	1,394 (198–3,221)	243 (37–675)	244 (68–472)	102 (37–176)	189 (77–361)
Lower respiratory infections	1,574,251 (1,444,828–1,678,086)	869,152 (775,371–954,234)	45,899 (33,749–52,675)	88,304 (73,172–101,322)	139,801 (113,090–154,446)	431,092 (383,750–463,202)
Upper respiratory infections	2,076 (1,754–2,489)	801 (550–1,177)	42 (27–64)	352 (290–425)	405 (312–504)	475 (372–612)
Otitis media	2,929 (2,614–3,345)	1,059 (890–1,252)	297 (266–332)	699 (638–773)	443 (386–511)	428 (296–770)
Meningitis	225,010 (187,279–262,214)	130,967 (100,337–166,005)	22,348 (17,633–25,781)	34,669 (29,279–40,766)	21,462 (18,236–25,751)	15,562 (13,924–18,697)
Pneumococcal meningitis	57,467 (45,652–71,587)	28,799 (19,510–40,868)	7,058 (4,704–9,797)	11,234 (8,930–14,149)	5,998 (4,484–7,860)	4,377 (3,403–5,519)
H influenzae type B meningitis	67,928 (52,427–86,002)	49,307 (35,050–65,949)	6,377 (4,170–8,730)	6,080 (4,428–7,865)	3,565 (2,601–4,738)	2,597 (2,004–3,420)
Meningococcal meningitis	39,300 (29,490–52,175)	23,822 (15,512–36,166)	3,793 (2,433–5,362)	5,785 (4,240–7,245)	3,517 (2,565–4,590)	2,381 (1,805–3,177)
Other meningitis	60,313 (49,351–72,983)	29,037 (20,370–40,617)	5,119 (3,598–7,015)	11,568 (9,602–14,164)	8,381 (6,793–10,377)	6,206 (5,119–7,339)
Encephalitis	74,387 (58,136–87,356)	29,379 (20,107–36,354)	10,238 (7,969–12,456)	13,414 (11,254–16,105)	10,834 (8,723–13,293)	10,520 (8,490–12,688)
Diphtheria	5,497 (2,760–11,171)	4,341 (1,685–9,888)	672 (323–1,352)	359 (232–529)	123 (58–227)	0 (0–0)
Whooping cough	73,952 (26,542–169,556)	68,811 (24,755–156,451)	4,222 (1,462–10,155)	808 (263–2,117)	110 (35–286)	0 (0–0)
Tetanus	104,568 (91,146–140,208)	71,536 (61,655–83,238)	4,652 (3,624–9,065)	13,045 (10,517–23,680)	10,596 (8,544–21,608)	4,737 (3,662–9,208)
Measles	344,294 (130,902–686,687)	296,834 (113,706–590,293)	39,467 (14,626–79,286)	7,781 (2,254–20,285)	210 (57–589)	0 (0–0)
Varicella and herpes zoster	6,419 (5,497–7,614)	1,851 (1,346–2,519)	514 (357–719)	971 (798–1,162)	1,027 (793–1,341)	2,055 (1,553–2,765)
Neglected tropical diseases and malaria	607,917 (482,692–732,471)	409,006 (294,070–530,969)	52,038 (38,767–67,146)	77,288 (59,617–99,263)	49,442 (37,510–65,166)	20,141 (14,945–27,151)
Malaria	536,891 (410,667–665,007)	392,752 (276,757–516,707)	40,284 (27,148–55,112)	54,479 (37,915–76,648)	35,482 (24,116–51,404)	13,892 (8,860–20,981)
Chagas disease	3,349 (3,189–3,517)	3 (3–4)	5 (4–6)	645 (598–696)	1,465 (1,366–1,569)	1,229 (1,156–1,313)
Leishmaniasis	5,489 (3,056–8,415)	1,376 (760–2,136)	815 (422–1,390)	2,626 (1,410–4,184)	524 (329–764)	145 (109–185)
Visceral leishmaniasis	5,489 (3,056–8,415)	1,376 (760–2,136)	815 (422–1,390)	2,626 (1,410–4,184)	524 (329–764)	145 (109–185)
Cutaneous and mucocutaneous leishmaniasis	0 (0–0)	0 (0–0)	0 (0–0)	0 (0–0)	0 (0–0)	0 (0–0)
African trypanosomiasis	13,624 (7,122–22,416)	1,004 (476–1,689)	1,701 (864–2,832)	7,461 (3,910–12,256)	2,796 (1,396–4,708)	661 (330–1,139)
Schistosomiasis	5,609 (4,857–6,551)	99 (57–179)	155 (103–231)	1,554 (1,253–1,922)	2,446 (1,895–3,132)	1,354 (1,004–1,782)
Cysticercosis	405 (353–483)	10 (5–19)	17 (13–21)	218 (189–261)	118 (104–140)	40 (35–45)
Cystic echinococcosis	1,338 (1,244–1,440)	94 (73–117)	144 (125–164)	475 (435–522)	355 (329–383)	268 (251–284)
Lymphatic filariasis	0 (0–0)	0 (0–0)	0 (0–0)	0 (0–0)	0 (0–0)	0 (0–0)
Onchocerciasis	0 (0–0)	0 (0–0)	0 (0–0)	0 (0–0)	0 (0–0)	0 (0–0)
Trachoma	0 (0–0)	0 (0–0)	0 (0–0)	0 (0–0)	0 (0–0)	0 (0–0)
Dengue	3,747 (2,270–6,452)	1,522 (651–3,157)	1,016 (578–1,741)	743 (508–1,189)	303 (226–455)	160 (111–245)
Yellow fever	2,463 (496–6,810)	246 (50–730)	709 (154–1,910)	1,181 (229–3,238)	270 (53–746)	55 (11–159)
Rabies	21,369 (17,627–24,787)	3,779 (2,651–5,344)	5,618 (4,315–7,085)	6,155 (4,741–7,456)	4,325 (3,521–5,309)	1,491 (1,146–2,010)
Intestinal nematode infections	2,923 (2,443–3,409)	2,318 (1,870–2,760)	373 (310–449)	104 (77–136)	70 (48–96)	57 (38–80)
Ascariasis	2,923 (2,443–3,409)	2,318 (1,870–2,760)	373 (310–449)	104 (77–136)	70 (48–96)	57 (38–80)
Trichuriasis	0 (0–0)	0 (0–0)	0 (0–0)	0 (0–0)	0 (0–0)	0 (0–0)
Hookworm disease	0 (0–0)	0 (0–0)	0 (0–0)	0 (0–0)	0 (0–0)	0 (0–0)
Food-borne trematodiases	0 (0–0)	0 (0–0)	0 (0–0)	0 (0–0)	0 (0–0)	0 (0–0)
Leprosy	0 (0–0)	0 (0–0)	0 (0–0)	0 (0–0)	0 (0–0)	0 (0–0)
Ebola	225 (181–270)	8 (6–9)	8 (6–9)	145 (117–174)	59 (48–71)	3 (2–4)
Other neglected tropical diseases	10,480 (7,421–16,150)	5,789 (3,883–8,466)	1,187 (750–1,869)	1,497 (1,078–2,416)	1,223 (856–2,285)	782 (536–1,515)
Maternal disorders	385,437 (362,582–411,234)	0 (0–0)	1,954 (1,657–2,284)	382,274 (359,544–407,827)	1,208 (864–1,829)	0 (0–0)
Maternal hemorrhage	107,246 (89,529–127,035)	0 (0–0)	545 (373–762)	106,281 (88,782–126,002)	419 (247–722)	0 (0–0)
Maternal sepsis and other maternal infections	27,991 (21,845–35,329)	0 (0–0)	86 (55–131)	27,819 (21,658–35,159)	85 (51–135)	0 (0–0)
Maternal hypertensive disorders	52,677 (42,746–64,722)	0 (0–0)	346 (224–502)	52,241 (42,180–64,260)	89 (51–146)	0 (0–0)
Maternal obstructed labor and uterine rupture	25,039 (19,504–31,507)	0 (0–0)	68 (38–110)	24,923 (19,401–31,382)	48 (28–83)	0 (0–0)
Maternal abortion, miscarriage, and ectopic pregnancy	51,847 (41,080–65,498)	0 (0–0)	327 (202–502)	51,312 (40,545–64,990)	207 (131–323)	0 (0–0)

SR Table 10 - Global deaths by age and cause for females in 1995

Cause	All ages	0-5 years	5-14 years	15-49 years	50-69 years	70+ years
Indirect maternal deaths	37,794 (29,579–48,179)	0 (0–0)	230 (138–348)	37,453 (29,252–47,752)	110 (61–213)	0 (0–0)
Late maternal deaths	8,338 (5,547–12,206)	0 (0–0)	49 (24–84)	8,250 (5,460–12,098)	38 (19–74)	0 (0–0)
Maternal deaths aggravated by HIV/AIDS	1,884 (1,167–2,610)	0 (0–0)	0 (0–0)	1,879 (1,164–2,602)	5 (2–10)	0 (0–0)
Other maternal disorders	72,616 (60,278–86,722)	0 (0–0)	299 (197–432)	72,114 (59,797–86,220)	203 (126–324)	0 (0–0)
Neonatal disorders	1,404,261 (1,359,613–1,451,748)	1,404,261 (1,359,613–1,451,748)	0 (0–0)	0 (0–0)	0 (0–0)	0 (0–0)
Neonatal preterm birth complications	700,476 (627,873–777,072)	700,476 (627,873–777,072)	0 (0–0)	0 (0–0)	0 (0–0)	0 (0–0)
Neonatal encephalopathy due to birth asphyxia and trauma	386,173 (331,870–441,233)	386,173 (331,870–441,233)	0 (0–0)	0 (0–0)	0 (0–0)	0 (0–0)
Neonatal sepsis and other neonatal infections	122,784 (58,700–185,323)	122,784 (58,700–185,323)	0 (0–0)	0 (0–0)	0 (0–0)	0 (0–0)
Hemolytic disease and other neonatal jaundice	51,451 (24,686–106,328)	51,451 (24,686–106,328)	0 (0–0)	0 (0–0)	0 (0–0)	0 (0–0)
Other neonatal disorders	143,376 (98,465–221,387)	143,376 (98,465–221,387)	0 (0–0)	0 (0–0)	0 (0–0)	0 (0–0)
Nutritional deficiencies	337,051 (286,454–392,772)	202,983 (161,278–246,797)	11,867 (9,901–13,806)	27,263 (24,080–30,623)	29,423 (25,310–33,633)	65,514 (55,548–76,397)
Protein-energy malnutrition	283,820 (230,511–342,942)	186,035 (146,763–229,448)	9,300 (7,399–11,383)	15,182 (10,900–19,380)	19,254 (14,509–23,956)	54,046 (43,406–65,698)
Iodine deficiency	1,489 (1,007–2,224)	645 (303–1,253)	68 (34–136)	214 (136–353)	262 (149–468)	298 (173–498)
Vitamin A deficiency	0 (0–0)	0 (0–0)	0 (0–0)	0 (0–0)	0 (0–0)	0 (0–0)
Iron-deficiency anemia	27,547 (17,661–35,069)	9,092 (3,659–13,182)	1,166 (774–1,825)	7,350 (5,356–9,062)	6,217 (4,602–7,933)	3,720 (2,426–5,663)
Other nutritional deficiencies	24,194 (10,071–53,429)	7,209 (1,441–20,429)	1,331 (447–2,501)	4,515 (1,685–8,545)	3,689 (1,526–8,101)	7,447 (4,382–13,262)
Other communicable, maternal, neonatal, and nutritional diseases	204,923 (167,486–254,770)	116,113 (82,770–161,156)	15,940 (12,686–20,359)	30,828 (28,860–33,377)	23,965 (21,857–25,626)	18,074 (16,268–19,489)
Sexually transmitted diseases excluding HIV	96,025 (58,901–145,166)	79,321 (45,321–125,015)	7,530 (4,331–11,772)	4,814 (3,377–5,889)	2,461 (1,339–3,099)	1,896 (1,187–2,431)
Syphilis	94,822 (57,631–143,808)	79,321 (45,321–125,015)	7,507 (4,305–11,747)	4,273 (2,998–5,226)	2,091 (1,138–2,637)	1,628 (1,019–2,087)
Chlamydial infection	218 (139–261)	0 (0–0)	5 (4–7)	100 (69–123)	62 (33–79)	49 (31–62)
Gonococcal infection	655 (414–787)	0 (0–0)	10 (7–13)	266 (185–326)	208 (113–264)	170 (106–217)
Trichomoniasis	0 (0–0)	0 (0–0)	0 (0–0)	0 (0–0)	0 (0–0)	0 (0–0)
Genital herpes	0 (0–0)	0 (0–0)	0 (0–0)	0 (0–0)	0 (0–0)	0 (0–0)
Other sexually transmitted diseases	328 (210–396)	0 (0–0)	7 (5–9)	173 (120–212)	98 (53–124)	49 (30–63)
Hepatitis	69,325 (65,214–74,087)	18,587 (16,012–21,025)	6,224 (5,417–7,029)	21,188 (19,700–23,201)	13,861 (13,109–14,648)	9,462 (8,990–9,951)
Acute hepatitis A	16,158 (10,753–21,413)	14,109 (10,080–17,295)	959 (253–2,022)	642 (165–1,501)	263 (60–679)	182 (45–435)
Hepatitis B	30,855 (26,285–35,336)	978 (347–2,395)	610 (325–1,000)	10,895 (8,347–13,170)	11,264 (9,719–12,640)	7,106 (5,874–8,223)
Hepatitis C	1,311 (282–2,995)	0 (0–0)	76 (14–200)	334 (69–786)	594 (127–1,398)	306 (63–727)
Acute hepatitis E	20,999 (15,342–27,206)	3,499 (1,371–6,819)	4,578 (3,376–5,714)	9,316 (7,097–11,630)	1,738 (820–3,044)	1,866 (1,007–3,076)
Other infectious diseases	39,573 (27,154–46,555)	18,205 (9,103–24,442)	2,185 (1,621–2,533)	4,824 (4,327–5,239)	7,642 (6,158–8,478)	6,715 (5,408–7,677)
Non-communicable diseases	14,683,165 (14,418,801–15,026,881)	519,786 (432,247–673,817)	122,491 (112,409–136,091)	1,455,276 (1,411,068–1,525,793)	4,052,670 (3,967,953–4,147,084)	8,532,941 (8,427,151–8,648,233)
Neoplasms	2,745,148 (2,690,036–2,808,666)	19,616 (16,972–24,272)	26,423 (24,626–28,595)	465,333 (449,414–485,647)	1,106,505 (1,079,844–1,135,896)	1,127,269 (1,106,318–1,148,601)
Lip and oral cavity cancer	29,828 (28,746–31,059)	0 (0–0)	0 (0–0)	4,666 (4,415–4,986)	12,660 (12,061–13,312)	12,501 (12,015–13,030)
Nasopharynx cancer	15,906 (14,760–17,109)	0 (0–0)	132 (117–152)	5,834 (5,331–6,514)	6,727 (6,232–7,239)	3,212 (2,991–3,448)
Other pharynx cancer	13,657 (12,441–14,457)	0 (0–0)	0 (0–0)	2,138 (1,877–2,388)	7,009 (6,339–7,528)	4,509 (4,192–4,784)
Esophageal cancer	132,554 (124,787–140,654)	0 (0–0)	0 (0–0)	13,167 (12,155–14,222)	61,106 (57,062–65,473)	58,280 (54,909–62,050)
Stomach cancer	293,136 (283,182–303,586)	0 (0–0)	0 (0–0)	41,876 (39,075–44,945)	111,910 (107,487–116,367)	139,350 (135,344–143,947)
Colon and rectum cancer	272,986 (267,229–278,372)	0 (0–0)	0 (0–0)	27,058 (25,883–28,740)	95,395 (92,905–97,700)	150,531 (146,781–154,137)
Liver cancer	184,788 (155,382–197,879)	0 (0–0)	615 (449–704)	33,428 (24,828–37,369)	81,577 (67,157–88,139)	69,166 (61,745–75,263)
Liver cancer due to hepatitis B	59,569 (47,169–65,692)	0 (0–0)	292 (209–338)	18,686 (13,493–20,973)	26,346 (20,841–29,971)	14,243 (11,750–16,121)
Liver cancer due to hepatitis C	49,082 (43,897–53,052)	0 (0–0)	18 (13–24)	3,579 (2,739–4,163)	20,268 (17,220–22,508)	25,216 (23,446–27,447)
Liver cancer due to alcohol use	29,338 (24,847–32,335)	0 (0–0)	0 (0–0)	2,213 (1,650–2,594)	15,213 (12,354–17,249)	11,911 (10,491–13,173)
Liver cancer due to other causes	46,796 (38,334–51,730)	0 (0–0)	304 (225–352)	8,948 (6,556–10,268)	19,749 (15,758–22,523)	17,794 (15,195–19,761)
Gallbladder and biliary tract cancer	69,416 (66,807–72,124)	0 (0–0)	0 (0–0)	4,949 (4,483–5,345)	26,476 (25,047–27,801)	37,990 (36,873–39,294)
Pancreatic cancer	118,870 (116,399–121,502)	0 (0–0)	0 (0–0)	7,449 (7,166–7,755)	44,503 (43,446–45,606)	66,917 (65,365–68,532)
Larynx cancer	17,513 (15,799–18,566)	0 (0–0)	0 (0–0)	2,680 (2,247–2,961)	8,145 (7,243–8,764)	6,687 (6,272–7,062)
Tracheal, bronchus, and lung cancer	320,877 (312,981–330,294)	0 (0–0)	0 (0–0)	34,645 (32,860–36,899)	139,224 (135,252–143,923)	147,007 (143,231–151,011)
Malignant skin melanoma	18,414 (16,629–20,272)	0 (0–0)	0 (0–0)	4,108 (3,712–4,447)	6,499 (5,886–7,031)	7,805 (7,083–8,619)
Non-melanoma skin cancer	8,629 (8,287–8,997)	0 (0–0)	0 (0–0)	1,067 (989–1,149)	2,282 (2,145–2,414)	5,279 (5,041–5,526)
Non-melanoma skin cancer (squamous-cell carcinoma)	8,629 (8,287–8,997)	0 (0–0)	0 (0–0)	1,067 (989–1,149)	2,282 (2,145–2,414)	5,279 (5,041–5,526)
Non-melanoma skin cancer (basal-cell carcinoma)	0 (0–0)	0 (0–0)	0 (0–0)	0 (0–0)	0 (0–0)	0 (0–0)
Breast cancer	365,325 (350,832–385,563)	0 (0–0)	0 (0–0)	86,016 (79,078–94,781)	161,581 (155,831–171,051)	117,726 (113,540–122,088)
Cervical cancer	195,090 (183,554–205,850)	0 (0–0)	0 (0–0)	54,460 (50,163–59,035)	91,150 (85,304–96,689)	49,479 (47,477–52,363)
Uterine cancer	71,354 (67,957–75,529)	0 (0–0)	0 (0–0)	9,690 (8,878–10,604)	34,038 (32,080–36,371)	27,626 (26,607–29,115)
Ovarian cancer	113,712 (111,275–117,236)	0 (0–0)	0 (0–0)	20,791 (19,979–22,418)	53,324 (52,090–55,086)	39,596 (38,515–40,729)
Prostate cancer	0 (0–0)	0 (0–0)	0 (0–0)	0 (0–0)	0 (0–0)	0 (0–0)
Testicular cancer	0 (0–0)	0 (0–0)	0 (0–0)	0 (0–0)	0 (0–0)	0 (0–0)

SR Table 10 - Global deaths by age and cause for females in 1995						
Cause	All ages	0-5 years	5-14 years	15-49 years	50-69 years	70+ years
Kidney cancer	32,448<br>(31,693–33,324)	814<br>(616–1,205)	572<br>(516–639)	3,192<br>(3,061–3,336)	12,788<br>(12,448–13,166)	15,081<br>(14,588–15,584)
Bladder cancer	35,357<br>(34,487–36,380)	0<br>(0–0)	0<br>(0–0)	2,098<br>(1,978–2,221)	10,174<br>(9,826–10,548)	23,085<br>(22,437–23,835)
Brain and nervous system cancer	70,130<br>(65,077–73,731)	2,883<br>(2,265–3,328)	4,983<br>(4,067–5,484)	20,241<br>(17,944–21,794)	26,175<br>(25,106–27,201)	15,846<br>(15,215–16,438)
Thyroid cancer	14,745<br>(13,720–16,462)	0<br>(0–0)	43<br>(35–48)	1,540<br>(1,405–1,858)	5,551<br>(5,048–6,217)	7,609<br>(7,122–8,306)
Mesothelioma	6,398<br>(6,012–6,815)	0<br>(0–0)	0<br>(0–0)	1,322<br>(1,239–1,402)	2,382<br>(2,207–2,540)	2,694<br>(2,507–3,005)
Hodgkin lymphoma	12,378<br>(8,592–15,319)	280<br>(120–403)	396<br>(226–557)	4,728<br>(3,402–5,629)	3,929<br>(2,593–5,136)	3,043<br>(2,136–3,794)
Non-Hodgkin lymphoma	64,680<br>(56,624–71,978)	1,513<br>(953–2,533)	1,641<br>(1,306–2,052)	11,302<br>(9,735–13,514)	21,679<br>(18,943–24,251)	28,544<br>(24,758–31,183)
Multiple myeloma	30,983<br>(29,692–32,282)	0<br>(0–0)	0<br>(0–0)	2,523<br>(2,389–2,729)	12,512<br>(12,019–13,096)	15,946<br>(14,967–16,723)
Leukemia	120,579<br>(115,720–126,154)	7,990<br>(6,656–9,498)	12,872<br>(11,832–14,196)	34,562<br>(32,342–37,451)	30,591<br>(29,464–31,888)	34,562<br>(33,482–35,517)
Acute lymphoid leukemia	40,167<br>(37,482–46,465)	5,479<br>(4,598–7,012)	9,191<br>(8,393–10,573)	12,764<br>(11,741–15,521)	7,469<br>(6,925–8,223)	5,262<br>(4,931–5,610)
Chronic lymphoid leukemia	21,148<br>(19,325–23,312)	0<br>(0–0)	0<br>(0–0)	4,206<br>(3,160–5,162)	5,972<br>(5,385–6,733)	10,969<br>(10,443–11,589)
Acute myeloid leukemia	41,370<br>(38,984–43,135)	2,510<br>(1,491–3,050)	3,680<br>(3,063–4,102)	12,205<br>(11,455–13,082)	11,201<br>(10,749–11,682)	11,771<br>(11,339–12,216)
Chronic myeloid leukemia	17,892<br>(16,533–19,509)	0<br>(0–0)	0<br>(0–0)	5,385<br>(4,634–6,259)	5,948<br>(5,495–6,496)	6,559<br>(6,175–7,013)
Other neoplasms	115,382<br>(108,420–121,159)	6,133<br>(4,804–9,315)	5,165<br>(4,614–5,733)	29,790<br>(27,051–32,269)	37,106<br>(34,710–39,601)	37,185<br>(35,312–39,118)
Cardiovascular diseases	7,050,424<br>(6,937,297–7,184,526)	42,222<br>(37,973–46,321)	20,231<br>(18,642–21,893)	477,074<br>(459,672–498,978)	1,751,539<br>(1,714,019–1,792,899)	4,759,357<br>(4,673,893–4,848,513)
Rheumatic heart disease	203,266<br>(190,308–218,734)	3,070<br>(2,099–3,893)	5,487<br>(4,840–6,199)	54,425<br>(49,517–60,110)	68,587<br>(63,956–74,314)	71,694<br>(67,802–76,253)
Ischemic heart disease	3,159,262<br>(3,106,483–3,218,246)	2,630<br>(2,232–3,224)	1,546<br>(1,357–1,824)	158,362<br>(151,849–167,509)	733,441<br>(715,749–751,457)	2,263,280<br>(2,221,602–2,308,450)
Cerebrovascular disease	2,766,903<br>(2,700,942–2,854,415)	15,443<br>(13,575–18,067)	7,107<br>(6,414–8,085)	182,912<br>(174,802–193,855)	728,659<br>(707,040–758,281)	1,832,779<br>(1,785,819–1,887,212)
Ischemic stroke	1,343,167<br>(1,260,392–1,435,476)	771<br>(599–989)	478<br>(403–572)	28,782<br>(24,614–34,183)	248,327<br>(222,195–278,839)	1,064,806<br>(1,011,761–1,127,750)
Hemorrhagic stroke	1,423,736<br>(1,352,609–1,504,331)	14,672<br>(12,858–17,381)	6,629<br>(5,972–7,553)	154,129<br>(146,372–165,092)	480,331<br>(454,448–511,020)	767,972<br>(718,328–808,151)
Hypertensive heart disease	385,967<br>(321,464–429,157)	421<br>(289–559)	250<br>(200–306)	28,641<br>(23,143–32,756)	111,349<br>(90,795–125,561)	245,304<br>(203,884–276,338)
Cardiomyopathy and myocarditis	132,310<br>(127,711–136,653)	6,856<br>(5,634–8,041)	2,048<br>(1,870–2,267)	18,055<br>(17,021–19,028)	28,118<br>(26,858–29,355)	77,231<br>(74,197–80,289)
Atrial fibrillation and flutter	63,292<br>(51,955–76,175)	0<br>(0–0)	0<br>(0–0)	250<br>(186–326)	3,887<br>(2,958–5,213)	59,154<br>(47,817–71,877)
Aortic aneurysm	38,058<br>(36,972–39,644)	0<br>(0–0)	0<br>(0–0)	2,517<br>(2,352–2,862)	8,296<br>(8,027–8,852)	27,244<br>(26,291–28,254)
Peripheral vascular disease	10,603<br>(9,988–11,300)	0<br>(0–0)	0<br>(0–0)	121<br>(114–129)	1,189<br>(1,104–1,323)	9,292<br>(8,723–9,876)
Endocarditis	30,231<br>(25,930–36,842)	2,187<br>(1,109–3,487)	665<br>(457–937)	4,920<br>(3,756–6,542)	6,087<br>(5,094–7,620)	16,370<br>(14,915–18,727)
Other cardiovascular and circulatory diseases	260,529<br>(252,096–270,908)	11,610<br>(9,881–13,660)	3,125<br>(2,834–3,524)	26,867<br>(25,285–28,844)	61,922<br>(58,987–65,508)	157,004<br>(150,644–164,432)
Chronic respiratory diseases	1,760,325<br>(1,693,257–1,828,695)	48,065<br>(41,849–54,248)	9,255<br>(8,266–10,377)	106,633<br>(101,044–114,495)	487,977<br>(466,196–513,222)	1,108,394<br>(1,064,150–1,151,198)
Chronic obstructive pulmonary disease	1,436,773<br>(1,293,794–1,564,144)	21,925<br>(14,466–28,241)	2,327<br>(1,505–3,971)	59,951<br>(49,008–73,660)	378,921<br>(326,152–431,384)	973,647<br>(894,017–1,037,292)
Pneumoconiosis	8,490<br>(5,571–12,021)	0<br>(0–0)	0<br>(0–0)	718<br>(372–1,125)	2,081<br>(1,149–3,264)	5,690<br>(3,985–7,881)
Silicosis	393<br>(252–626)	0<br>(0–0)	0<br>(0–0)	51<br>(26–97)	91<br>(57–151)	250<br>(168–376)
Asbestosis	361<br>(237–481)	0<br>(0–0)	0<br>(0–0)	128<br>(74–187)	96<br>(63–128)	137<br>(89–185)
Coal workers pneumoconiosis	178<br>(94–333)	0<br>(0–0)	0<br>(0–0)	40<br>(15–83)	51<br>(26–98)	87<br>(50–153)
Other pneumoconiosis	7,556<br>(4,908–10,890)	0<br>(0–0)	0<br>(0–0)	498<br>(243–829)	1,842<br>(988–2,990)	5,215<br>(3,596–7,342)
Asthma	274,134<br>(159,400–398,730)	14,568<br>(7,911–19,469)	6,109<br>(4,127–7,577)	42,410<br>(26,804–52,270)	98,944<br>(51,026–149,594)	112,101<br>(65,325–183,100)
Interstitial lung disease and pulmonary sarcoidosis	21,525<br>(17,336–26,218)	196<br>(102–316)	139<br>(106–183)	1,993<br>(1,606–2,379)	6,272<br>(5,062–7,727)	12,924<br>(10,433–16,286)
Other chronic respiratory diseases	19,400<br>(13,206–28,975)	11,374<br>(7,473–17,891)	678<br>(360–1,471)	1,559<br>(881–2,626)	1,756<br>(1,134–2,622)	4,030<br>(2,959–5,300)
Cirrhosis and other chronic liver diseases	369,702<br>(348,061–414,669)	9,986<br>(7,134–14,619)	6,208<br>(5,294–8,023)	83,834<br>(78,337–95,302)	153,432<br>(142,369–172,253)	116,239<br>(108,292–127,045)
Cirrhosis and other chronic liver diseases due to hepatitis B	97,675<br>(88,612–112,575)	49<br>(34–75)	121<br>(96–161)	21,940<br>(19,545–25,780)	42,384<br>(37,051–49,670)	33,179<br>(29,677–38,758)
Cirrhosis and other chronic liver diseases due to hepatitis C	80,309<br>(73,960–87,957)	24<br>(16–41)	70<br>(54–95)	16,299<br>(14,393–18,761)	34,529<br>(30,473–39,372)	29,386<br>(26,237–32,423)
Cirrhosis and other chronic liver diseases due to alcohol use	82,214<br>(75,427–91,051)	0<br>(0–0)	0<br>(0–0)	22,124<br>(20,042–25,386)	39,892<br>(35,323–44,896)	20,197<br>(18,099–22,608)
Cirrhosis and other chronic liver diseases due to other causes	109,502<br>(101,200–126,708)	9,912<br>(7,083–14,503)	6,016<br>(5,130–7,768)	23,469<br>(21,172–27,232)	36,626<br>(32,523–42,087)	33,476<br>(30,534–36,960)
Digestive diseases	485,344<br>(463,934–509,958)	22,940<br>(14,962–41,678)	12,786<br>(11,514–14,122)	84,984<br>(80,405–90,979)	133,582<br>(127,675–140,727)	231,050<br>(223,630–239,275)
Peptic ulcer disease	141,087<br>(123,661–159,015)	3,497<br>(1,837–7,090)	3,124<br>(2,501–3,894)	27,473<br>(23,515–32,073)	42,719<br>(36,730–49,059)	64,273<br>(57,109–71,872)
Gastritis and duodenitis	21,259<br>(8,343–29,537)	604<br>(179–1,229)	404<br>(158–643)	4,016<br>(1,525–5,706)	6,405<br>(2,398–9,249)	9,829<br>(4,195–13,939)
Appendicitis	23,658<br>(18,607–30,471)	4,399<br>(1,823–10,370)	2,201<br>(1,737–2,595)	7,076<br>(5,822–8,077)	5,104<br>(4,373–5,834)	4,877<br>(4,316–5,574)
Paralytic ileus and intestinal obstruction	93,591<br>(74,831–106,261)	6,608<br>(2,986–12,851)	3,752<br>(2,445–4,518)	17,708<br>(12,352–20,314)	24,786<br>(19,245–28,664)	40,735<br>(35,240–46,827)
Inguinal, femoral, and abdominal hernia	18,502<br>(12,695–21,462)	553<br>(192–999)	455<br>(249–607)	2,270<br>(1,229–2,909)	5,758<br>(3,703–6,798)	9,463<br>(7,039–11,101)
Inflammatory bowel disease	19,888<br>(16,357–24,173)	1,928<br>(594–4,609)	425<br>(276–623)	3,482<br>(2,543–4,620)	4,369<br>(3,637–5,079)	9,682<br>(8,486–10,454)
Vascular intestinal disorders	35,548<br>(31,859–39,157)	114<br>(44–251)	55<br>(35–79)	1,653<br>(1,164–2,145)	7,677<br>(6,358–8,929)	26,045<br>(24,112–28,036)
Gallbladder and biliary diseases	51,866<br>(48,498–55,935)	1,639<br>(801–3,551)	789<br>(650–938)	6,903<br>(6,229–7,734)	14,991<br>(13,720–16,290)	27,542<br>(26,020–29,303)
Pancreatitis	32,125<br>(28,894–35,486)	190<br>(104–349)	334<br>(249–428)	8,492<br>(7,219–9,824)	10,956<br>(9,742–12,269)	12,151<br>(11,311–13,036)
Other digestive diseases	47,814<br>(40,130–55,772)	3,404<br>(1,841–6,280)	1,243<br>(977–1,525)	5,905<br>(4,486–7,099)	10,812<br>(8,572–13,282)	26,447<br>(23,154–30,190)
Neurological disorders	767,789<br>(660,014–877,465)	9,336<br>(7,308–13,507)	7,062<br>(6,269–8,197)	31,713<br>(29,351–35,894)	56,617<br>(47,965–65,545)	663,059<br>(555,206–768,904)
Alzheimer disease and other dementias	661,464<br>(549,866–770,422)	0<br>(0–0)	0<br>(0–0)	490<br>(309–708)	34,945<br>(26,524–43,973)	626,027<br>(518,002–732,240)
Parkinson disease	25,507<br>(24,510–26,570)	0<br>(0–0)	0<br>(0–0)	211<br>(190–233)	3,528<br>(3,354–3,730)	21,767<br>(20,859–22,764)
Epilepsy	45,507<br>(41,077–52,965)	6,788<br>(5,024–10,279)	5,881<br>(5,154–6,795)	22,734<br>(20,607–26,383)	5,722<br>(5,376–6,126)	4,380<br>(4,176–4,606)

SR Table 10 - Global deaths by age and cause for females in 1995

Cause	All ages	0-5 years	5-14 years	15-49 years	50-69 years	70+ years
Multiple sclerosis	8,954 (7,210–10,472)	0 (0–0)	0 (0–0)	3,514 (2,544–4,418)	3,669 (3,099–4,177)	1,771 (1,441–1,909)
Motor neuron disease	9,429 (8,979–10,927)	759 (430–1,192)	57 (46–78)	871 (812–1,116)	4,084 (3,879–4,717)	3,656 (3,516–3,845)
Migraine	0 (0–0)	0 (0–0)	0 (0–0)	0 (0–0)	0 (0–0)	0 (0–0)
Tension-type headache	0 (0–0)	0 (0–0)	0 (0–0)	0 (0–0)	0 (0–0)	0 (0–0)
Medication overuse headache	0 (0–0)	0 (0–0)	0 (0–0)	0 (0–0)	0 (0–0)	0 (0–0)
Other neurological disorders	16,937 (16,263–20,030)	1,799 (1,538–3,631)	1,124 (1,004–1,706)	3,891 (3,688–4,843)	4,666 (4,527–4,807)	5,455 (5,203–5,669)
Mental and substance use disorders	57,497 (54,226–59,597)	12 (10–14)	21 (10–30)	31,429 (28,859–33,154)	17,987 (17,175–18,748)	8,047 (7,680–8,351)
Schizophrenia	8,893 (8,198–9,696)	0 (0–0)	0 (0–0)	3,753 (3,356–4,252)	2,663 (2,447–2,906)	2,476 (2,307–2,677)
Alcohol use disorders	24,347 (23,372–25,415)	0 (0–0)	0 (0–0)	11,332 (10,691–12,042)	10,448 (9,887–11,103)	2,567 (2,473–2,662)
Drug use disorders	23,879 (20,287–25,258)	12 (10–14)	0 (0–0)	15,987 (13,138–17,161)	4,874 (4,338–5,203)	3,003 (2,699–3,194)
Opioid use disorders	13,880 (12,752–16,310)	12 (10–14)	0 (0–0)	9,384 (8,303–11,475)	2,938 (2,682–3,269)	1,544 (1,410–1,883)
Cocaine use disorders	570 (403–663)	0 (0–0)	0 (0–0)	422 (297–491)	106 (76–126)	41 (29–49)
Amphetamine use disorders	420 (289–967)	0 (0–0)	0 (0–0)	321 (212–753)	72 (53–165)	25 (18–43)
Cannabis use disorders	0 (0–0)	0 (0–0)	0 (0–0)	0 (0–0)	0 (0–0)	0 (0–0)
Other drug use disorders	9,007 (6,161–10,082)	0 (0–0)	0 (0–0)	5,859 (3,822–6,775)	1,756 (1,296–1,972)	1,391 (1,000–1,592)
Depressive disorders	0 (0–0)	0 (0–0)	0 (0–0)	0 (0–0)	0 (0–0)	0 (0–0)
Major depressive disorder	0 (0–0)	0 (0–0)	0 (0–0)	0 (0–0)	0 (0–0)	0 (0–0)
Dysthymia	0 (0–0)	0 (0–0)	0 (0–0)	0 (0–0)	0 (0–0)	0 (0–0)
Bipolar disorder	0 (0–0)	0 (0–0)	0 (0–0)	0 (0–0)	0 (0–0)	0 (0–0)
Anxiety disorders	0 (0–0)	0 (0–0)	0 (0–0)	0 (0–0)	0 (0–0)	0 (0–0)
Eating disorders	376 (245–602)	0 (0–0)	21 (10–30)	355 (233–572)	0 (0–0)	0 (0–0)
Anorexia nervosa	363 (236–573)	0 (0–0)	20 (10–29)	342 (224–545)	0 (0–0)	0 (0–0)
Bulimia nervosa	13 (7–30)	0 (0–0)	0 (0–1)	12 (7–29)	0 (0–0)	0 (0–0)
Autistic spectrum disorders	0 (0–0)	0 (0–0)	0 (0–0)	0 (0–0)	0 (0–0)	0 (0–0)
Autism	0 (0–0)	0 (0–0)	0 (0–0)	0 (0–0)	0 (0–0)	0 (0–0)
Asperger syndrome and other autistic spectrum disorders	0 (0–0)	0 (0–0)	0 (0–0)	0 (0–0)	0 (0–0)	0 (0–0)
Attention-deficit/hyperactivity disorder	0 (0–0)	0 (0–0)	0 (0–0)	0 (0–0)	0 (0–0)	0 (0–0)
Conduct disorder	0 (0–0)	0 (0–0)	0 (0–0)	0 (0–0)	0 (0–0)	0 (0–0)
Idiopathic developmental intellectual disability	0 (0–0)	0 (0–0)	0 (0–0)	0 (0–0)	0 (0–0)	0 (0–0)
Other mental and substance use disorders	0 (0–0)	0 (0–0)	0 (0–0)	0 (0–0)	0 (0–0)	0 (0–0)
Diabetes, urogenital, blood, and endocrine diseases	1,026,489 (1,001,465–1,058,458)	66,312 (50,968–86,822)	18,294 (16,401–20,178)	138,672 (133,946–145,523)	323,540 (313,857–333,506)	479,669 (467,713–492,040)
Diabetes mellitus	453,814 (441,077–466,664)	1,860 (1,388–2,169)	1,810 (1,564–2,063)	40,090 (38,300–42,330)	174,964 (168,940–180,894)	235,088 (227,669–242,178)
Acute glomerulonephritis	6,656 (5,718–9,578)	338 (265–505)	316 (274–450)	2,128 (1,771–2,748)	1,828 (1,492–2,694)	2,043 (1,664–3,446)
Chronic kidney disease	334,122 (325,008–343,946)	10,101 (8,809–11,338)	5,668 (5,156–6,190)	53,778 (51,467–56,869)	101,255 (98,011–104,497)	163,318 (158,609–168,522)
Chronic kidney disease due to diabetes mellitus	102,515 (97,774–107,000)	1,304 (1,048–1,578)	476 (395–570)	12,336 (11,256–13,506)	38,032 (35,114–41,180)	50,365 (47,525–53,402)
Chronic kidney disease due to hypertension	148,987 (142,877–154,972)	3,364 (2,863–3,850)	1,664 (1,428–1,936)	19,875 (18,467–21,433)	42,579 (39,496–45,507)	81,504 (77,475–85,587)
Chronic kidney disease due to glomerulonephritis	72,853 (69,014–76,731)	4,032 (3,426–4,635)	2,676 (2,377–3,005)	18,984 (17,537–20,747)	19,403 (17,862–21,051)	27,755 (25,622–30,047)
Chronic kidney disease due to other causes	9,766 (8,160–11,626)	1,400 (1,129–1,736)	851 (686–1,035)	2,581 (1,767–3,482)	1,240 (773–1,870)	3,692 (3,064–4,621)
Urinary diseases and male infertility	78,114 (73,817–85,615)	3,687 (2,777–4,464)	1,035 (904–1,206)	11,601 (10,672–12,922)	20,768 (19,145–23,787)	41,020 (38,823–45,609)
Interstitial nephritis and urinary tract infections	63,114 (59,268–69,541)	2,856 (2,117–3,467)	671 (568–793)	8,618 (7,848–9,640)	15,769 (14,333–18,259)	35,198 (33,207–39,360)
Urolithiasis	5,979 (4,598–6,983)	0 (0–0)	141 (102–182)	1,259 (895–1,512)	2,233 (1,728–2,765)	2,344 (1,794–2,675)
Benign prostatic hyperplasia	0 (0–0)	0 (0–0)	0 (0–0)	0 (0–0)	0 (0–0)	0 (0–0)
Male infertility	0 (0–0)	0 (0–0)	0 (0–0)	0 (0–0)	0 (0–0)	0 (0–0)
Other urinary diseases	9,020 (7,979–10,705)	830 (528–1,220)	222 (175–281)	1,724 (1,444–2,133)	2,765 (2,318–3,554)	3,477 (3,077–4,069)
Gynecological diseases	6,972 (5,368–8,293)	0 (0–0)	0 (0–0)	3,457 (2,563–4,144)	2,039 (1,561–2,498)	1,475 (1,204–1,899)
Uterine fibroids	1,840 (1,008–2,514)	0 (0–0)	0 (0–0)	768 (432–1,080)	707 (360–996)	364 (190–614)
Polycystic ovarian syndrome	902 (214–1,518)	0 (0–0)	0 (0–0)	653 (133–1,119)	249 (78–384)	0 (0–0)
Female infertility	0 (0–0)	0 (0–0)	0 (0–0)	0 (0–0)	0 (0–0)	0 (0–0)
Endometriosis	40 (27–56)	0 (0–0)	0 (0–0)	35 (24–49)	5 (3–8)	0 (0–0)
Genital prolapse	1,074 (523–1,906)	0 (0–0)	0 (0–0)	122 (48–213)	425 (165–840)	526 (283–946)
Premenstrual syndrome	0 (0–0)	0 (0–0)	0 (0–0)	0 (0–0)	0 (0–0)	0 (0–0)
Other gynecological diseases	3,113 (2,319–4,174)	0 (0–0)	0 (0–0)	1,877 (1,382–2,596)	651 (432–986)	584 (409–909)
Hemoglobinopathies and hemolytic anemias	100,746 (85,191–120,923)	42,559 (28,567–62,868)	7,534 (6,082–8,767)	19,045 (17,058–20,903)	11,962 (11,381–12,590)	19,643 (17,880–21,386)
Thalassemias	12,968 (9,096–17,521)	8,019 (4,664–12,258)	2,389 (1,769–2,837)	1,206 (1,024–1,406)	39 (35–47)	1,313 (1,066–1,713)
Thalassemias trait	0 (0–0)	0 (0–0)	0 (0–0)	0 (0–0)	0 (0–0)	0 (0–0)
Sickle cell disorders	48,107 (35,285–67,625)	33,862 (21,911–52,519)	4,367 (3,512–5,304)	7,769 (6,562–8,984)	567 (490–652)	1,540 (1,307–1,868)
Sickle cell trait	0 (0–0)	0 (0–0)	0 (0–0)	0 (0–0)	0 (0–0)	0 (0–0)

SR Table 10 - Global deaths by age and cause for females in 1995						
**Cause**	**All ages**	**0-5 years**	**5-14 years**	**15-49 years**	**50-69 years**	**70+ years**
G6PD deficiency	6,792 (5,870–7,770)	338 (175–565)	369 (266–474)	3,961 (3,424–4,567)	1,737 (1,512–1,991)	385 (330–445)
G6PD trait	0 (0–0)	0 (0–0)	0 (0–0)	0 (0–0)	0 (0–0)	0 (0–0)
Other hemoglobinopathies and hemolytic anemias	32,878 (31,218–34,488)	338 (219–503)	408 (325–476)	6,107 (5,529–6,693)	9,618 (9,101–10,170)	16,405 (14,799–17,649)
Endocrine, metabolic, blood, and immune disorders	46,062 (44,466–47,724)	7,764 (6,479–9,005)	1,928 (1,787–2,086)	8,569 (8,232–8,921)	10,720 (10,268–11,269)	17,079 (16,405–17,886)
Musculoskeletal disorders	42,068 (37,874–47,321)	0 (0–0)	1,147 (901–1,458)	11,081 (9,164–14,434)	10,438 (9,385–11,673)	19,400 (17,375–20,415)
Rheumatoid arthritis	17,037 (14,381–19,344)	0 (0–0)	195 (111–278)	1,716 (1,162–2,264)	5,535 (4,733–6,374)	9,590 (8,294–10,863)
Osteoarthritis	0 (0–0)	0 (0–0)	0 (0–0)	0 (0–0)	0 (0–0)	0 (0–0)
Low back and neck pain	0 (0–0)	0 (0–0)	0 (0–0)	0 (0–0)	0 (0–0)	0 (0–0)
Low back pain	0 (0–0)	0 (0–0)	0 (0–0)	0 (0–0)	0 (0–0)	0 (0–0)
Neck pain	0 (0–0)	0 (0–0)	0 (0–0)	0 (0–0)	0 (0–0)	0 (0–0)
Gout	0 (0–0)	0 (0–0)	0 (0–0)	0 (0–0)	0 (0–0)	0 (0–0)
Other musculoskeletal disorders	25,030 (22,455–28,841)	0 (0–0)	952 (745–1,231)	9,365 (7,648–12,601)	4,902 (4,379–5,846)	9,810 (8,338–10,454)
Other non-communicable diseases	378,365 (278,579–534,212)	301,282 (213,881–444,713)	21,058 (15,245–29,513)	24,518 (19,642–30,736)	11,050 (8,616–14,738)	20,454 (14,546–27,531)
Congenital anomalies	338,595 (240,601–493,195)	286,938 (200,579–428,670)	20,531 (14,762–28,960)	21,652 (17,176–27,783)	5,461 (4,568–6,256)	4,010 (3,506–4,419)
Neural tube defects	53,930 (35,668–92,297)	50,898 (33,226–88,112)	1,716 (1,139–2,707)	939 (696–1,408)	243 (158–482)	133 (92–225)
Congenital heart anomalies	175,183 (114,173–263,890)	140,974 (87,582–219,368)	14,918 (10,145–21,181)	15,753 (12,119–20,267)	1,928 (1,600–2,321)	1,608 (1,407–1,873)
Cleft lip and cleft palate	2,365 (1,132–3,401)	2,365 (1,132–3,401)	0 (0–0)	0 (0–0)	0 (0–0)	0 (0–0)
Down syndrome	18,460 (4,987–42,923)	14,999 (2,815–37,357)	565 (201–1,202)	1,796 (1,053–2,922)	1,021 (771–1,448)	76 (56–110)
Turner syndrome	0 (0–0)	0 (0–0)	0 (0–0)	0 (0–0)	0 (0–0)	0 (0–0)
Klinefelter syndrome	0 (0–0)	0 (0–0)	0 (0–0)	0 (0–0)	0 (0–0)	0 (0–0)
Other chromosomal abnormalities	13,259 (4,468–44,154)	12,616 (3,981–43,068)	219 (106–592)	273 (208–386)	112 (68–147)	38 (29–47)
Other congenital anomalies	75,394 (54,472–120,925)	65,083 (45,108–108,624)	3,112 (2,296–4,888)	2,889 (2,361–3,847)	2,156 (1,659–2,517)	2,153 (1,731–2,408)
Skin and subcutaneous diseases	28,703 (18,465–43,747)	3,278 (1,499–5,699)	526 (250–784)	2,866 (1,538–4,636)	5,589 (3,315–9,537)	16,443 (10,747–23,501)
Dermatitis	0 (0–0)	0 (0–0)	0 (0–0)	0 (0–0)	0 (0–0)	0 (0–0)
Psoriasis	0 (0–0)	0 (0–0)	0 (0–0)	0 (0–0)	0 (0–0)	0 (0–0)
Cellulitis	4,352 (2,387–6,901)	343 (107–749)	88 (37–144)	568 (266–920)	1,334 (649–2,287)	2,017 (1,228–3,045)
Pyoderma	11,586 (6,902–19,319)	2,730 (1,180–4,923)	287 (137–484)	1,613 (792–2,706)	2,575 (1,488–4,767)	4,379 (2,891–7,212)
Scabies	0 (0–0)	0 (0–0)	0 (0–0)	0 (0–0)	0 (0–0)	0 (0–0)
Fungal skin diseases	0 (0–0)	0 (0–0)	0 (0–0)	0 (0–0)	0 (0–0)	0 (0–0)
Viral skin diseases	0 (0–0)	0 (0–0)	0 (0–0)	0 (0–0)	0 (0–0)	0 (0–0)
Acne vulgaris	0 (0–0)	0 (0–0)	0 (0–0)	0 (0–0)	0 (0–0)	0 (0–0)
Alopecia areata	0 (0–0)	0 (0–0)	0 (0–0)	0 (0–0)	0 (0–0)	0 (0–0)
Pruritus	0 (0–0)	0 (0–0)	0 (0–0)	0 (0–0)	0 (0–0)	0 (0–0)
Urticaria	0 (0–0)	0 (0–0)	0 (0–0)	0 (0–0)	0 (0–0)	0 (0–0)
Decubitus ulcer	11,330 (6,587–16,350)	48 (13–106)	82 (34–135)	431 (230–703)	1,395 (738–2,527)	9,373 (5,276–13,289)
Other skin and subcutaneous diseases	1,433 (932–2,309)	155 (64–361)	68 (29–104)	252 (142–395)	283 (184–518)	673 (452–1,022)
Sense organ diseases	0 (0–0)	0 (0–0)	0 (0–0)	0 (0–0)	0 (0–0)	0 (0–0)
Glaucoma	0 (0–0)	0 (0–0)	0 (0–0)	0 (0–0)	0 (0–0)	0 (0–0)
Cataract	0 (0–0)	0 (0–0)	0 (0–0)	0 (0–0)	0 (0–0)	0 (0–0)
Macular degeneration	0 (0–0)	0 (0–0)	0 (0–0)	0 (0–0)	0 (0–0)	0 (0–0)
Refraction and accommodation disorders	0 (0–0)	0 (0–0)	0 (0–0)	0 (0–0)	0 (0–0)	0 (0–0)
Age-related and other hearing loss	0 (0–0)	0 (0–0)	0 (0–0)	0 (0–0)	0 (0–0)	0 (0–0)
Other vision loss	0 (0–0)	0 (0–0)	0 (0–0)	0 (0–0)	0 (0–0)	0 (0–0)
Other sense organ diseases	0 (0–0)	0 (0–0)	0 (0–0)	0 (0–0)	0 (0–0)	0 (0–0)
Oral disorders	0 (0–0)	0 (0–0)	0 (0–0)	0 (0–0)	0 (0–0)	0 (0–0)
Deciduous caries	0 (0–0)	0 (0–0)	0 (0–0)	0 (0–0)	0 (0–0)	0 (0–0)
Permanent caries	0 (0–0)	0 (0–0)	0 (0–0)	0 (0–0)	0 (0–0)	0 (0–0)
Periodontal diseases	0 (0–0)	0 (0–0)	0 (0–0)	0 (0–0)	0 (0–0)	0 (0–0)
Edentulism and severe tooth loss	0 (0–0)	0 (0–0)	0 (0–0)	0 (0–0)	0 (0–0)	0 (0–0)
Other oral disorders	0 (0–0)	0 (0–0)	0 (0–0)	0 (0–0)	0 (0–0)	0 (0–0)
Sudden infant death syndrome	11,066 (7,787–15,835)	11,066 (7,787–15,835)	0 (0–0)	0 (0–0)	0 (0–0)	0 (0–0)
Injuries	1,513,629 (1,307,493–1,611,777)	244,434 (191,198–294,433)	137,527 (119,344–148,529)	611,648 (520,663–645,583)	251,597 (221,432–262,814)	268,420 (239,347–280,681)
Transport injuries	350,698 (331,322–368,377)	39,041 (30,011–48,493)	39,474 (36,144–42,745)	157,516 (150,309–165,799)	68,402 (66,100–70,985)	46,262 (44,918–47,714)
Road injuries	331,637 (314,696–348,659)	35,977 (27,964–44,550)	37,631 (34,506–40,682)	149,429 (142,775–157,800)	64,536 (62,294–67,162)	44,062 (42,756–45,519)
Pedestrian road injuries	171,356 (158,705–181,203)	18,702 (14,910–22,294)	21,569 (19,782–23,279)	67,227 (62,529–71,851)	36,379 (33,909–38,587)	27,477 (26,075–28,812)
Cyclist road injuries	14,086 (12,296–16,949)	891 (570–1,548)	1,772 (1,489–2,218)	6,833 (5,865–8,407)	3,164 (2,814–3,584)	1,423 (1,267–1,605)
Motorcyclist road injuries	29,922 (26,475–35,073)	3,179 (1,819–4,954)	2,636 (2,146–3,263)	16,613 (14,812–19,378)	5,153 (4,511–6,347)	2,339 (1,959–2,982)

SR Table 10 - Global deaths by age and cause for females in 1995

Cause	All ages	0-5 years	5-14 years	15-49 years	50-69 years	70+ years
Motor vehicle road injuries	112,610 (104,838–123,654)	12,624 (9,354–16,486)	11,238 (9,969–12,906)	57,217 (53,783–64,133)	19,139 (17,831–21,180)	12,390 (11,498–13,766)
Other road injuries	3,661 (2,724–4,262)	578 (253–926)	415 (296–515)	1,537 (1,172–1,800)	699 (536–860)	431 (346–538)
Other transport injuries	19,060 (14,542–22,080)	3,063 (1,566–4,261)	1,843 (1,317–2,212)	8,086 (6,140–9,462)	3,866 (3,038–4,405)	2,200 (1,852–2,518)
Unintentional injuries	760,234 (631,141–830,041)	192,213 (142,186–238,072)	82,813 (65,807–91,397)	204,413 (168,423–219,839)	105,112 (90,644–112,140)	175,681 (155,935–185,517)
Falls	147,129 (130,770–156,402)	15,175 (7,823–22,056)	7,217 (5,862–8,213)	18,930 (17,318–20,639)	22,695 (20,906–24,080)	83,111 (76,279–88,259)
Drowning	168,860 (92,904–192,231)	64,113 (23,096–81,129)	36,213 (22,067–41,038)	39,933 (25,043–44,099)	14,956 (9,452–17,065)	13,645 (9,837–15,267)
Fire, heat, and hot substances	135,162 (81,793–153,498)	24,902 (11,224–32,063)	9,868 (5,879–11,505)	62,089 (34,785–71,315)	17,454 (12,256–19,292)	20,847 (16,163–22,583)
Poisonings	47,000 (34,483–55,455)	12,708 (5,304–20,268)	4,555 (3,930–5,344)	17,451 (14,424–19,425)	6,869 (5,503–7,717)	5,415 (4,687–5,991)
Exposure to mechanical forces	57,363 (51,453–64,662)	20,475 (15,428–25,599)	5,244 (4,596–6,647)	13,522 (12,559–15,280)	7,853 (7,156–9,378)	10,268 (7,963–12,033)
Unintentional firearm injuries	8,115 (5,598–9,045)	863 (438–1,286)	672 (442–814)	2,566 (1,645–2,919)	1,151 (729–1,317)	2,862 (2,323–3,128)
Unintentional suffocation	16,288 (11,407–19,527)	11,548 (6,666–14,401)	1,259 (938–1,894)	1,364 (1,085–1,736)	638 (495–847)	1,476 (1,162–1,851)
Other exposure to mechanical forces	32,959 (29,694–38,778)	8,063 (5,573–11,641)	3,312 (2,874–4,263)	9,591 (8,854–11,460)	6,063 (5,455–7,765)	5,929 (4,250–7,367)
Adverse effects of medical treatment	47,061 (33,985–58,131)	10,953 (5,014–15,939)	2,504 (1,502–3,300)	10,842 (7,238–13,972)	8,333 (6,235–9,976)	14,427 (11,037–16,231)
Animal contact	48,809 (30,060–55,516)	9,773 (5,354–12,484)	7,977 (4,852–9,354)	17,443 (11,071–19,679)	9,443 (5,710–10,695)	4,172 (2,672–4,757)
Venomous animal contact	41,029 (24,701–46,637)	7,121 (3,767–9,275)	6,700 (3,938–7,922)	15,622 (9,679–17,690)	8,341 (4,902–9,532)	3,243 (1,918–3,846)
Non-venomous animal contact	7,780 (5,214–10,186)	2,652 (1,338–3,812)	1,276 (831–1,793)	1,820 (1,310–2,389)	1,102 (777–1,395)	928 (737–1,110)
Foreign body	56,254 (39,780–82,430)	24,436 (13,439–46,614)	3,931 (2,521–6,025)	8,651 (6,179–12,279)	6,283 (4,804–8,530)	12,952 (10,969–15,470)
Pulmonary aspiration and foreign body in airway	42,318 (27,542–71,746)	20,643 (9,014–43,782)	2,729 (1,467–4,986)	4,535 (3,331–7,013)	4,249 (3,491–5,437)	10,161 (9,153–11,122)
Foreign body in eyes	0 (0–0)	0 (0–0)	0 (0–0)	0 (0–0)	0 (0–0)	0 (0–0)
Foreign body in other body part	13,935 (7,025–27,021)	3,792 (1,204–6,148)	1,202 (559–2,386)	4,115 (2,142–8,332)	2,034 (1,088–4,436)	2,790 (1,769–5,417)
Environmental heat and cold exposure	16,484 (10,893–22,276)	2,237 (935–3,007)	609 (382–950)	3,187 (2,493–4,232)	4,264 (2,817–6,354)	6,183 (3,986–8,881)
Other unintentional injuries	36,107 (32,770–41,128)	7,438 (5,642–9,050)	4,692 (3,886–5,475)	12,362 (11,295–14,167)	6,956 (6,243–7,993)	4,657 (4,125–5,343)
Self-harm and interpersonal violence	381,567 (282,986–407,852)	9,876 (8,580–10,857)	12,399 (9,887–13,382)	240,499 (171,419–259,307)	74,445 (56,390–79,171)	44,346 (33,948–47,316)
Self-harm	289,899 (190,744–315,050)	0 (0–0)	5,626 (3,215–6,392)	185,710 (116,865–203,455)	61,140 (43,392–65,699)	37,421 (27,300–40,351)
Interpersonal violence	91,668 (87,724–95,665)	9,876 (8,580–10,857)	6,772 (6,295–7,296)	54,789 (52,305–57,679)	13,304 (12,613–13,936)	6,925 (6,598–7,248)
Assault by firearm	18,642 (15,698–20,227)	942 (684–1,245)	1,210 (958–1,369)	12,999 (10,853–13,917)	2,390 (2,073–2,772)	1,100 (936–1,296)
Assault by sharp object	21,920 (20,464–24,311)	991 (675–1,372)	1,185 (1,029–1,394)	14,816 (13,717–16,377)	3,154 (2,908–3,446)	1,772 (1,618–1,962)
Assault by other means	51,105 (47,213–54,873)	7,942 (6,855–8,811)	4,377 (3,964–4,885)	26,974 (24,815–29,611)	7,759 (7,152–8,258)	4,051 (3,715–4,305)
Forces of nature, war, and legal intervention	21,128 (15,790–28,119)	3,303 (2,383–4,405)	2,840 (2,077–3,856)	9,218 (6,955–11,993)	3,636 (2,528–5,776)	2,129 (1,123–5,220)
Exposure to forces of nature	7,271 (4,240–12,287)	658 (394–1,077)	1,195 (716–2,038)	2,971 (1,796–5,066)	1,417 (683–3,413)	1,028 (394–3,255)
Collective violence and legal intervention	13,856 (10,814–17,837)	2,644 (1,845–3,713)	1,644 (1,166–2,237)	6,247 (4,861–7,782)	2,219 (1,729–2,763)	1,100 (678–3,576)

SR Table 10 - Global deaths by age and cause for females in 2000

Cause	All ages	0-5 years	5-14 years	15-49 years	50-69 years	70+ years
All causes	24,291,101 (23,993,189–24,583,543)	4,374,317 (4,327,683–4,421,874)	547,178 (537,286–557,678)	3,570,122 (3,484,665–3,657,088)	5,164,819 (5,063,022–5,268,947)	10,634,664 (10,533,217–10,744,732)
Communicable, maternal, neonatal, and nutritional diseases	7,120,059 (6,925,504–7,304,629)	3,710,467 (3,576,552–3,809,079)	309,218 (293,693–326,610)	1,435,134 (1,378,612–1,506,150)	694,440 (648,538–736,613)	970,797 (908,742–1,018,798)
HIV/AIDS and tuberculosis	1,152,768 (1,046,569–1,266,867)	102,573 (95,901–113,646)	30,239 (27,721–36,047)	627,762 (582,383–680,222)	250,880 (209,608–284,434)	141,312 (108,163–166,668)
Tuberculosis	544,418 (439,904–656,199)	20,277 (14,816–30,777)	12,554 (10,365–18,475)	186,122 (153,468–228,140)	190,475 (147,874–223,027)	134,988 (102,015–160,433)
HIV/AIDS	608,349 (568,338–649,670)	82,295 (79,287–85,567)	17,685 (16,557–18,951)	441,639 (405,248–477,304)	60,404 (54,515–67,732)	6,324 (5,480–7,299)
HIV/AIDS - Tuberculosis	129,189 (108,964–152,222)	22,902 (19,855–26,169)	4,723 (3,856–5,525)	83,310 (69,324–99,414)	16,458 (14,052–18,951)	1,795 (1,506–2,096)
HIV/AIDS resulting in other diseases	479,160 (437,810–522,655)	59,393 (55,183–64,244)	12,962 (11,723–14,487)	358,328 (323,100–394,017)	43,945 (37,954–51,249)	4,529 (3,797–5,424)
Diarrhea, lower respiratory, and other common infectious diseases	3,179,609 (3,024,911–3,338,617)	1,629,807 (1,507,405–1,756,550)	201,319 (180,655–224,594)	297,025 (274,464–328,449)	336,132 (310,302–358,392)	715,324 (665,789–755,662)
Diarrheal diseases	909,706 (837,229–982,633)	426,910 (363,634–491,864)	47,413 (41,751–52,977)	105,587 (98,694–113,538)	137,492 (129,813–146,154)	192,303 (180,292–205,427)
Intestinal infectious diseases	107,790 (60,498–177,752)	28,645 (15,224–50,597)	43,052 (24,736–69,650)	29,722 (15,685–50,844)	4,441 (2,314–7,761)	1,928 (1,136–3,013)
Typhoid fever	88,566 (48,080–148,566)	23,475 (12,068–42,802)	36,001 (20,041–57,693)	24,146 (12,577–41,895)	3,500 (1,691–6,252)	1,441 (772–2,382)
Paratyphoid fever	17,718 (8,063–34,183)	4,269 (1,776–8,676)	6,872 (3,179–12,922)	5,387 (2,309–10,733)	861 (349–1,767)	327 (143–638)
Other intestinal infectious diseases	1,505 (221–3,161)	900 (89–2,077)	178 (20–519)	187 (38–398)	80 (21–146)	159 (50–312)
Lower respiratory infections	1,470,905 (1,352,565–1,563,743)	697,796 (627,991–770,187)	41,543 (30,643–47,261)	93,371 (76,994–105,821)	151,050 (122,300–165,138)	487,144 (432,654–519,863)
Upper respiratory infections	1,929 (1,651–2,304)	657 (445–996)	37 (23–56)	339 (280–412)	397 (312–492)	497 (387–646)
Otitis media	2,261 (2,003–2,644)	810 (684–959)	253 (228–283)	528 (492–569)	291 (260–328)	378 (244–722)
Meningitis	208,363 (174,898–241,114)	113,287 (87,994–143,735)	20,773 (16,849–24,060)	35,564 (30,224–41,480)	21,954 (18,365–25,960)	16,784 (14,638–19,869)
Pneumococcal meningitis	55,109 (44,826–68,232)	25,572 (17,366–37,349)	6,805 (4,633–9,545)	11,781 (9,367–14,960)	6,185 (4,627–7,982)	4,765 (3,738–6,030)
H influenzae type B meningitis	61,231 (47,863–76,985)	42,683 (30,604–58,314)	5,865 (3,850–7,929)	6,237 (4,525–8,088)	3,636 (2,650–4,849)	2,808 (2,166–3,644)
Meningococcal meningitis	36,466 (27,704–47,475)	20,895 (13,646–31,044)	3,511 (2,262–4,952)	5,919 (4,382–7,583)	3,607 (2,627–4,717)	2,532 (1,907–3,352)
Other meningitis	55,556 (45,917–66,262)	24,136 (17,130–33,344)	4,590 (3,251–6,380)	11,626 (9,542–14,292)	8,524 (6,872–10,469)	6,678 (5,460–7,978)
Encephalitis	69,761 (58,264–81,820)	26,397 (18,729–32,058)	9,776 (7,811–11,628)	13,673 (11,891–16,690)	10,071 (8,408–13,599)	9,842 (8,270–13,419)
Diphtheria	4,703 (2,224–11,122)	3,693 (1,301–10,084)	564 (240–1,197)	331 (210–504)	114 (55–210)	0 (0–0)
Whooping cough	66,988 (22,491–149,623)	62,295 (20,930–138,402)	3,838 (1,333–9,259)	750 (238–2,003)	104 (33–274)	0 (0–0)
Tetanus	73,521 (62,433–105,603)	46,074 (38,710–54,814)	3,507 (2,590–6,805)	10,482 (8,165–20,428)	9,081 (6,922–18,909)	4,377 (3,353–8,501)
Measles	257,661 (96,489–505,267)	221,632 (83,674–430,498)	30,104 (10,951–60,305)	5,770 (1,736–13,690)	153 (44–379)	0 (0–0)
Varicella and herpes zoster	6,014 (5,175–6,949)	1,605 (1,181–2,195)	454 (314–646)	905 (742–1,109)	981 (758–1,286)	2,068 (1,553–2,694)
Neglected tropical diseases and malaria	647,641 (526,440–765,206)	435,113 (325,442–545,915)	51,690 (38,267–66,027)	84,861 (64,798–109,819)	52,696 (39,100–70,921)	23,278 (17,042–31,939)
Malaria	584,365 (463,559–700,339)	421,093 (311,310–532,232)	41,523 (27,970–55,960)	64,342 (44,637–90,314)	40,396 (27,184–58,475)	17,009 (10,901–25,548)
Chagas disease	3,183 (3,023–3,360)	2 (2–3)	4 (3–5)	495 (457–535)	1,301 (1,208–1,399)	1,379 (1,293–1,472)
Leishmaniasis	5,684 (3,293–8,516)	1,321 (759–2,043)	809 (433–1,354)	2,851 (1,636–4,390)	540 (346–770)	162 (123–207)
Visceral leishmaniasis	5,684 (3,293–8,516)	1,321 (759–2,043)	809 (433–1,354)	2,851 (1,636–4,390)	540 (346–770)	162 (123–207)
Cutaneous and mucocutaneous leishmaniasis	0 (0–0)	0 (0–0)	0 (0–0)	0 (0–0)	0 (0–0)	0 (0–0)
African trypanosomiasis	11,440 (6,110–19,431)	884 (450–1,496)	1,546 (836–2,534)	6,215 (3,270–10,798)	2,261 (1,171–3,955)	533 (279–909)
Schistosomiasis	4,712 (4,038–5,490)	65 (38–126)	112 (76–160)	1,254 (1,010–1,537)	2,048 (1,586–2,598)	1,231 (912–1,708)
Cysticercosis	375 (324–451)	8 (4–16)	14 (11–19)	200 (171–240)	112 (97–133)	39 (35–45)
Cystic echinococcosis	1,098 (1,028–1,172)	62 (49–78)	110 (95–124)	385 (355–417)	297 (277–318)	242 (229–256)
Lymphatic filariasis	0 (0–0)	0 (0–0)	0 (0–0)	0 (0–0)	0 (0–0)	0 (0–0)
Onchocerciasis	0 (0–0)	0 (0–0)	0 (0–0)	0 (0–0)	0 (0–0)	0 (0–0)
Trachoma	0 (0–0)	0 (0–0)	0 (0–0)	0 (0–0)	0 (0–0)	0 (0–0)
Dengue	4,031 (2,424–6,928)	1,465 (675–2,984)	1,079 (604–1,835)	898 (593–1,468)	366 (246–582)	222 (132–363)
Yellow fever	2,030 (419–5,750)	202 (37–609)	608 (126–1,712)	953 (199–2,693)	220 (44–631)	45 (9–129)
Rabies	17,801 (14,540–20,985)	2,889 (1,997–4,189)	4,425 (3,393–5,579)	5,395 (4,222–6,532)	3,687 (2,981–4,545)	1,403 (1,055–1,900)
Intestinal nematode infections	2,364 (2,001–2,733)	1,822 (1,496–2,166)	313 (259–371)	99 (73–133)	68 (47–96)	59 (40–84)
Ascariasis	2,364 (2,001–2,733)	1,822 (1,496–2,166)	313 (259–371)	99 (73–133)	68 (47–96)	59 (40–84)
Trichuriasis	0 (0–0)	0 (0–0)	0 (0–0)	0 (0–0)	0 (0–0)	0 (0–0)
Hookworm disease	0 (0–0)	0 (0–0)	0 (0–0)	0 (0–0)	0 (0–0)	0 (0–0)
Food-borne trematodiases	0 (0–0)	0 (0–0)	0 (0–0)	0 (0–0)	0 (0–0)	0 (0–0)
Leprosy	0 (0–0)	0 (0–0)	0 (0–0)	0 (0–0)	0 (0–0)	0 (0–0)
Ebola	111 (89–133)	14 (11–17)	9 (7–10)	65 (53–78)	20 (16–24)	1 (1–2)
Other neglected tropical diseases	10,442 (7,312–15,426)	5,280 (3,572–7,872)	1,133 (697–1,867)	1,704 (1,171–2,519)	1,375 (926–2,320)	947 (625–1,685)
Maternal disorders	374,320 (351,336–400,418)	0 (0–0)	1,755 (1,487–2,067)	371,245 (348,356–397,372)	1,319 (945–2,008)	0 (0–0)
Maternal hemorrhage	97,688 (83,444–112,931)	0 (0–0)	504 (357–683)	96,723 (82,503–112,048)	460 (268–852)	0 (0–0)
Maternal sepsis and other maternal infections	25,723 (20,767–31,336)	0 (0–0)	74 (50–109)	25,556 (20,607–31,168)	92 (54–157)	0 (0–0)
Maternal hypertensive disorders	63,153 (52,613–74,858)	0 (0–0)	343 (236–482)	62,709 (52,220–74,407)	100 (60–166)	0 (0–0)
Maternal obstructed labor and uterine rupture	29,438 (23,418–35,836)	0 (0–0)	56 (33–88)	29,327 (23,328–35,727)	54 (32–91)	0 (0–0)
Maternal abortion, miscarriage, and ectopic pregnancy	54,347 (44,507–66,598)	0 (0–0)	271 (176–400)	53,865 (44,033–66,080)	210 (132–330)	0 (0–0)

SR Table 10 - Global deaths by age and cause for females in 2000						
**Cause**	**All ages**	**0-5 years**	**5-14 years**	**15-49 years**	**50-69 years**	**70+ years**
Indirect maternal deaths	44,521 (35,640–54,126)	0 (0–0)	216 (133–326)	44,173 (35,314–53,762)	131 (72–241)	0 (0–0)
Late maternal deaths	8,728 (5,830–12,716)	0 (0–0)	46 (23–79)	8,637 (5,751–12,612)	44 (22–96)	0 (0–0)
Maternal deaths aggravated by HIV/AIDS	2,704 (1,695–3,646)	0 (0–0)	0 (0–1)	2,695 (1,688–3,633)	8 (4–15)	0 (0–0)
Other maternal disorders	48,014 (39,806–57,496)	0 (0–0)	241 (164–339)	47,558 (39,386–57,009)	215 (137–343)	0 (0–0)
Neonatal disorders	1,278,608 (1,237,616–1,318,977)	1,278,608 (1,237,616–1,318,977)	0 (0–0)	0 (0–0)	0 (0–0)	0 (0–0)
Neonatal preterm birth complications	591,014 (532,824–660,907)	591,014 (532,824–660,907)	0 (0–0)	0 (0–0)	0 (0–0)	0 (0–0)
Neonatal encephalopathy due to birth asphyxia and trauma	386,604 (336,963–438,380)	386,604 (336,963–438,380)	0 (0–0)	0 (0–0)	0 (0–0)	0 (0–0)
Neonatal sepsis and other neonatal infections	124,836 (60,443–189,385)	124,836 (60,443–189,385)	0 (0–0)	0 (0–0)	0 (0–0)	0 (0–0)
Hemolytic disease and other neonatal jaundice	40,503 (19,583–78,012)	40,503 (19,583–78,012)	0 (0–0)	0 (0–0)	0 (0–0)	0 (0–0)
Other neonatal disorders	135,649 (91,148–203,425)	135,649 (91,148–203,425)	0 (0–0)	0 (0–0)	0 (0–0)	0 (0–0)
Nutritional deficiencies	303,421 (258,873–355,341)	167,150 (137,385–204,412)	10,747 (9,072–12,572)	25,623 (22,741–29,010)	29,277 (25,127–33,871)	70,622 (59,593–83,250)
Protein-energy malnutrition	254,837 (211,511–305,678)	153,659 (124,764–189,542)	8,465 (6,837–10,311)	14,348 (10,843–18,144)	19,330 (14,622–24,154)	59,033 (47,633–71,488)
Iodine deficiency	1,372 (953–2,018)	528 (240–1,108)	61 (28–123)	207 (135–326)	256 (147–465)	318 (189–530)
Vitamin A deficiency	0 (0–0)	0 (0–0)	0 (0–0)	0 (0–0)	0 (0–0)	0 (0–0)
Iron-deficiency anemia	26,947 (18,053–35,109)	8,239 (3,483–11,650)	1,175 (830–1,793)	7,074 (5,257–8,623)	6,428 (4,869–8,091)	4,029 (2,843–5,934)
Other nutritional deficiencies	20,264 (9,562–41,132)	4,723 (1,281–12,106)	1,044 (392–1,917)	3,993 (1,686–7,351)	3,261 (1,433–6,985)	7,240 (4,386–12,361)
Other communicable, maternal, neonatal, and nutritional diseases	183,689 (151,013–224,524)	97,213 (68,596–134,469)	13,465 (10,631–17,144)	28,616 (27,012–30,767)	24,135 (22,191–25,551)	20,259 (18,089–21,619)
Sexually transmitted diseases excluding HIV	81,046 (50,084–121,416)	66,834 (38,575–104,504)	6,442 (3,702–9,978)	4,070 (3,172–4,808)	1,960 (1,267–2,358)	1,738 (1,237–2,060)
Syphilis	80,026 (49,217–120,393)	66,834 (38,575–104,504)	6,421 (3,681–9,956)	3,610 (2,814–4,261)	1,666 (1,077–2,004)	1,492 (1,062–1,767)
Chlamydial infection	185 (136–213)	0 (0–0)	4 (3–6)	85 (66–101)	49 (31–59)	45 (32–53)
Gonococcal infection	557 (404–638)	0 (0–0)	8 (6–11)	226 (176–269)	166 (107–201)	155 (110–185)
Trichomoniasis	0 (0–0)	0 (0–0)	0 (0–0)	0 (0–0)	0 (0–0)	0 (0–0)
Genital herpes	0 (0–0)	0 (0–0)	0 (0–0)	0 (0–0)	0 (0–0)	0 (0–0)
Other sexually transmitted diseases	276 (203–320)	0 (0–0)	6 (4–7)	147 (114–174)	78 (50–94)	44 (31–53)
Hepatitis	62,740 (59,444–66,494)	13,119 (11,341–14,938)	5,009 (4,352–5,694)	19,556 (18,339–21,226)	14,219 (13,482–15,011)	10,834 (10,258–11,425)
Acute hepatitis A	12,076 (7,901–16,391)	10,039 (7,214–12,445)	794 (209–1,689)	648 (161–1,528)	339 (79–793)	254 (60–580)
Hepatitis B	31,287 (26,734–35,497)	718 (236–1,846)	509 (256–852)	10,339 (8,221–12,396)	11,527 (9,889–12,906)	8,192 (6,870–9,414)
Hepatitis C	1,408 (311–3,196)	0 (0–0)	65 (12–177)	329 (63–782)	640 (144–1,464)	373 (79–894)
Acute hepatitis E	17,968 (13,219–23,398)	2,361 (919–4,662)	3,640 (2,712–4,614)	8,238 (6,234–10,471)	1,712 (782–3,025)	2,014 (1,069–3,200)
Other infectious diseases	39,903 (26,786–46,697)	17,258 (8,731–23,570)	2,013 (1,529–2,356)	4,989 (4,381–5,447)	7,954 (6,198–8,820)	7,686 (5,816–8,694)
Non-communicable diseases	15,691,456 (15,455,243–15,968,684)	465,139 (388,562–582,069)	115,179 (106,161–126,566)	1,531,587 (1,490,188–1,589,667)	4,210,166 (4,128,945–4,301,208)	9,369,384 (9,271,254–9,476,935)
Neoplasms	3,004,388 (2,944,469–3,068,506)	17,438 (15,236–21,260)	25,718 (24,110–27,456)	498,933 (481,854–518,537)	1,173,252 (1,145,847–1,200,803)	1,289,045 (1,263,663–1,312,073)
Lip and oral cavity cancer	33,387 (32,225–34,636)	0 (0–0)	0 (0–0)	5,262 (4,968–5,574)	13,702 (13,051–14,378)	14,423 (13,954–14,957)
Nasopharynx cancer	16,955 (15,987–18,030)	0 (0–0)	119 (107–135)	5,955 (5,498–6,498)	7,216 (6,774–7,692)	3,664 (3,454–3,893)
Other pharynx cancer	14,608 (13,379–15,491)	0 (0–0)	0 (0–0)	2,326 (2,053–2,599)	7,294 (6,627–7,799)	4,987 (4,661–5,263)
Esophageal cancer	143,873 (136,405–151,738)	0 (0–0)	0 (0–0)	14,203 (13,213–15,256)	64,117 (60,459–68,135)	65,552 (61,934–69,349)
Stomach cancer	295,824 (286,919–305,738)	0 (0–0)	0 (0–0)	40,486 (37,945–42,991)	108,804 (104,383–113,380)	146,533 (142,407–150,750)
Colon and rectum cancer	297,943 (291,846–303,965)	0 (0–0)	0 (0–0)	29,071 (27,821–30,614)	98,619 (96,264–101,122)	170,253 (166,255–174,179)
Liver cancer	208,859 (174,288–221,512)	0 (0–0)	578 (442–646)	35,393 (26,744–39,097)	90,077 (73,968–96,532)	82,809 (73,372–88,002)
Liver cancer due to hepatitis B	64,311 (51,346–70,635)	0 (0–0)	272 (202–312)	19,428 (14,400–21,703)	28,442 (22,622–31,883)	16,168 (13,402–18,165)
Liver cancer due to hepatitis C	56,813 (50,155–61,111)	0 (0–0)	16 (12–19)	3,924 (3,015–4,542)	22,153 (18,591–24,633)	30,719 (28,108–33,034)
Liver cancer due to alcohol use	33,397 (28,142–36,670)	0 (0–0)	0 (0–0)	2,448 (1,844–2,879)	16,765 (13,510–19,068)	14,183 (12,370–15,540)
Liver cancer due to other causes	54,338 (44,605–59,511)	0 (0–0)	289 (225–329)	9,593 (7,093–10,869)	22,716 (17,866–25,660)	21,738 (18,380–23,746)
Gallbladder and biliary tract cancer	70,840 (68,334–73,470)	0 (0–0)	0 (0–0)	5,153 (4,689–5,568)	25,560 (24,290–26,744)	40,126 (38,895–41,426)
Pancreatic cancer	134,260 (131,495–137,085)	0 (0–0)	0 (0–0)	8,479 (8,192–8,797)	48,134 (46,968–49,287)	77,646 (75,939–79,403)
Larynx cancer	17,668 (15,969–18,744)	0 (0–0)	0 (0–0)	2,680 (2,265–2,951)	8,015 (7,165–8,564)	6,972 (6,558–7,377)
Tracheal, bronchus, and lung cancer	373,870 (365,190–384,231)	0 (0–0)	0 (0–0)	38,667 (36,984–40,581)	154,080 (149,934–158,732)	181,122 (176,710–185,756)
Malignant skin melanoma	20,150 (18,230–21,823)	0 (0–0)	0 (0–0)	4,250 (3,953–4,657)	6,854 (6,196–7,387)	9,045 (8,091–9,750)
Non-melanoma skin cancer	9,802 (9,455–10,182)	0 (0–0)	0 (0–0)	1,123 (1,054–1,192)	2,495 (2,364–2,626)	6,183 (5,935–6,467)
Non-melanoma skin cancer (squamous-cell carcinoma)	9,802 (9,455–10,182)	0 (0–0)	0 (0–0)	1,123 (1,054–1,192)	2,495 (2,364–2,626)	6,183 (5,935–6,467)
Non-melanoma skin cancer (basal-cell carcinoma)	0 (0–0)	0 (0–0)	0 (0–0)	0 (0–0)	0 (0–0)	0 (0–0)
Breast cancer	400,550 (381,423–422,365)	0 (0–0)	0 (0–0)	94,088 (85,041–103,509)	173,693 (165,766–183,333)	132,768 (128,063–137,257)
Cervical cancer	210,709 (199,081–222,653)	0 (0–0)	0 (0–0)	59,223 (54,618–63,982)	96,861 (90,887–102,744)	54,623 (52,671–56,696)
Uterine cancer	77,669 (74,293–81,670)	0 (0–0)	0 (0–0)	10,533 (9,721–11,404)	35,916 (34,036–38,255)	31,219 (30,161–32,526)
Ovarian cancer	123,315 (120,397–127,865)	0 (0–0)	0 (0–0)	22,579 (21,676–24,501)	55,703 (54,325–57,894)	45,032 (43,878–46,255)
Prostate cancer	0 (0–0)	0 (0–0)	0 (0–0)	0 (0–0)	0 (0–0)	0 (0–0)
Testicular cancer	0 (0–0)	0 (0–0)	0 (0–0)	0 (0–0)	0 (0–0)	0 (0–0)

SR Table 10 - Global deaths by age and cause for females in 2000

Cause	All ages	0-5 years	5-14 years	15-49 years	50-69 years	70+ years
Kidney cancer	34,992 (34,121–35,964)	793 (616–1,166)	563 (511–631)	3,399 (3,261–3,556)	12,869 (12,521–13,233)	17,365 (16,887–17,899)
Bladder cancer	38,733 (37,795–39,716)	0 (0–0)	0 (0–0)	2,204 (2,077–2,312)	10,520 (10,178–10,873)	26,007 (25,320–26,780)
Brain and nervous system cancer	79,181 (73,004–82,291)	2,628 (2,188–3,011)	4,924 (4,198–5,364)	22,300 (19,899–23,511)	29,499 (27,778–30,617)	19,828 (18,670–20,558)
Thyroid cancer	15,151 (14,263–17,162)	0 (0–0)	42 (36–47)	1,519 (1,400–1,809)	5,444 (5,058–6,271)	8,145 (7,704–8,979)
Mesothelioma	6,747 (6,509–6,979)	0 (0–0)	0 (0–0)	1,347 (1,255–1,436)	2,415 (2,315–2,512)	2,984 (2,857–3,107)
Hodgkin lymphoma	11,436 (8,343–13,557)	194 (96–269)	336 (198–460)	4,291 (3,178–5,026)	3,590 (2,513–4,420)	3,023 (2,266–3,628)
Non-Hodgkin lymphoma	73,112 (62,769–81,411)	1,391 (922–2,347)	1,686 (1,342–2,049)	12,185 (10,609–14,408)	23,548 (20,342–26,300)	34,300 (28,247–36,633)
Multiple myeloma	35,651 (33,822–37,013)	0 (0–0)	0 (0–0)	2,951 (2,797–3,161)	13,785 (13,280–14,384)	18,914 (17,219–19,684)
Leukemia	128,717 (124,471–133,515)	6,746 (5,770–7,881)	12,257 (11,352–13,341)	35,928 (34,075–37,913)	32,851 (31,760–34,106)	40,933 (39,700–42,172)
Acute lymphoid leukemia	41,591 (39,252–46,923)	4,574 (3,882–5,872)	8,655 (7,980–9,812)	13,521 (12,639–15,828)	8,454 (7,911–9,339)	6,386 (6,010–6,798)
Chronic lymphoid leukemia	23,028 (21,206–24,894)	0 (0–0)	0 (0–0)	4,183 (3,286–4,931)	6,155 (5,548–6,845)	12,688 (12,137–13,274)
Acute myeloid leukemia	46,879 (44,261–48,801)	2,171 (1,459–2,634)	3,602 (3,018–3,989)	13,307 (12,267–14,193)	12,659 (12,161–13,161)	15,138 (14,608–15,722)
Chronic myeloid leukemia	17,217 (16,051–18,632)	0 (0–0)	0 (0–0)	4,916 (4,282–5,640)	5,581 (5,225–6,040)	6,719 (6,358–7,073)
Other neoplasms	130,374 (122,550–136,204)	5,684 (4,536–8,369)	5,208 (4,718–5,755)	33,324 (29,950–35,870)	41,578 (39,172–43,970)	44,577 (42,707–46,388)
Cardiovascular diseases	7,481,903 (7,363,395–7,613,399)	33,812 (30,668–37,041)	18,185 (16,850–19,660)	496,462 (480,124–517,421)	1,789,350 (1,750,147–1,831,615)	5,144,092 (5,056,857–5,233,876)
Rheumatic heart disease	204,223 (189,096–219,487)	2,453 (1,796–3,026)	5,034 (4,461–5,663)	52,580 (46,296–58,269)	66,636 (61,400–72,686)	77,517 (73,044–82,649)
Ischemic heart disease	3,319,643 (3,265,902–3,381,909)	1,970 (1,704–2,362)	1,409 (1,243–1,634)	167,773 (160,946–176,856)	745,774 (727,714–764,804)	2,402,715 (2,359,137–2,449,043)
Cerebrovascular disease	2,984,573 (2,922,607–3,058,969)	11,772 (10,457–13,809)	6,279 (5,728–7,019)	190,096 (181,898–200,356)	755,774 (734,501–780,972)	2,020,649 (1,978,840–2,067,230)
Ischemic stroke	1,427,485 (1,352,713–1,506,709)	582 (458–718)	401 (344–474)	29,532 (25,481–33,648)	249,432 (225,777–275,473)	1,147,535 (1,097,659–1,195,684)
Hemorrhagic stroke	1,557,088 (1,490,342–1,642,240)	11,190 (9,891–13,291)	5,878 (5,333–6,580)	160,563 (152,430–171,590)	506,341 (481,429–536,755)	873,114 (826,898–916,908)
Hypertensive heart disease	409,115 (352,840–453,020)	316 (227–427)	223 (181–278)	29,269 (23,685–33,684)	112,621 (94,369–125,839)	266,684 (229,272–299,120)
Cardiomyopathy and myocarditis	132,275 (128,375–136,128)	6,196 (5,203–7,075)	1,998 (1,840–2,196)	20,759 (19,605–21,870)	27,111 (26,100–28,261)	76,209 (73,433–78,899)
Atrial fibrillation and flutter	70,661 (58,198–84,703)	0 (0–0)	0 (0–0)	287 (212–376)	4,028 (3,065–5,417)	66,345 (53,889–80,287)
Aortic aneurysm	43,995 (42,830–45,452)	0 (0–0)	0 (0–0)	2,998 (2,780–3,379)	9,210 (8,896–9,731)	31,787 (30,874–32,679)
Peripheral vascular disease	15,045 (14,179–15,985)	0 (0–0)	0 (0–0)	158 (149–169)	1,509 (1,414–1,672)	13,377 (12,554–14,191)
Endocarditis	34,903 (29,058–40,349)	2,013 (1,031–3,025)	674 (449–884)	6,087 (4,317–7,404)	6,688 (5,454–8,180)	19,438 (17,601–21,759)
Other cardiovascular and circulatory diseases	267,466 (259,249–277,986)	9,088 (7,907–10,657)	2,565 (2,325–2,882)	26,449 (25,049–28,408)	59,994 (57,401–63,356)	169,368 (163,270–175,989)
Chronic respiratory diseases	1,771,466 (1,715,666–1,830,031)	34,187 (29,841–38,465)	7,817 (7,001–8,685)	101,825 (96,816–107,693)	473,797 (453,472–495,593)	1,153,837 (1,115,870–1,190,997)
Chronic obstructive pulmonary disease	1,452,001 (1,335,662–1,562,114)	14,851 (9,776–19,138)	1,801 (1,205–3,021)	54,944 (46,045–66,816)	368,398 (325,029–414,500)	1,012,005 (943,996–1,068,488)
Pneumoconiosis	9,222 (6,133–12,781)	0 (0–0)	0 (0–0)	729 (388–1,083)	2,147 (1,188–3,305)	6,345 (4,517–8,689)
Silicosis	388 (262–606)	0 (0–0)	0 (0–0)	43 (25–79)	92 (58–148)	253 (179–377)
Asbestosis	423 (292–543)	0 (0–0)	0 (0–0)	142 (82–197)	102 (66–132)	179 (137–223)
Coal workers pneumoconiosis	182 (108–271)	0 (0–0)	0 (0–0)	32 (16–51)	53 (29–80)	96 (62–140)
Other pneumoconiosis	8,227 (5,440–11,608)	0 (0–0)	0 (0–0)	512 (264–806)	1,899 (1,034–3,028)	5,816 (4,095–8,034)
Asthma	264,380 (167,404–380,068)	10,388 (6,403–13,643)	5,232 (3,809–6,377)	42,058 (29,056–51,182)	93,661 (53,648–138,185)	113,039 (71,444–180,915)
Interstitial lung disease and pulmonary sarcoidosis	28,817 (22,043–33,282)	180 (100–279)	143 (107–187)	2,443 (1,978–2,856)	7,764 (6,100–9,287)	18,284 (13,370–20,880)
Other chronic respiratory diseases	17,044 (11,788–24,396)	8,766 (5,930–13,226)	639 (342–1,302)	1,649 (959–2,660)	1,825 (1,233–2,609)	4,163 (3,112–5,408)
Cirrhosis and other chronic liver diseases	385,769 (362,506–432,302)	7,854 (5,778–11,552)	5,771 (5,001–7,308)	87,947 (82,053–99,689)	158,794 (147,151–178,071)	125,402 (118,341–138,713)
Cirrhosis and other chronic liver diseases due to hepatitis B	101,351 (91,753–117,518)	37 (26–58)	110 (87–144)	22,368 (19,687–26,221)	43,314 (37,795–51,084)	35,520 (31,562–41,100)
Cirrhosis and other chronic liver diseases due to hepatitis C	85,028 (78,421–94,015)	19 (12–31)	66 (52–88)	17,587 (15,508–20,255)	36,177 (31,850–41,177)	31,177 (28,389–34,747)
Cirrhosis and other chronic liver diseases due to alcohol use	86,261 (78,838–95,695)	0 (0–0)	0 (0–0)	23,261 (21,130–26,847)	41,057 (36,256–46,468)	21,942 (19,588–24,604)
Cirrhosis and other chronic liver diseases due to other causes	113,128 (104,947–130,693)	7,797 (5,738–11,455)	5,593 (4,845–7,076)	24,729 (22,262–28,494)	38,245 (34,016–44,112)	36,762 (33,630–41,237)
Digestive diseases	500,533 (481,666–526,058)	18,351 (11,052–36,477)	11,030 (9,899–12,287)	84,668 (79,845–89,473)	134,359 (128,887–139,998)	252,124 (244,874–259,724)
Peptic ulcer disease	131,332 (116,583–146,011)	2,658 (1,263–6,041)	2,254 (1,824–2,767)	23,863 (20,684–27,202)	39,065 (34,174–44,169)	63,490 (57,116–70,338)
Gastritis and duodenitis	21,416 (10,447–28,594)	491 (159–1,078)	352 (162–556)	3,754 (1,735–5,293)	6,240 (2,850–8,739)	10,577 (5,203–14,602)
Appendicitis	24,076 (18,884–30,517)	3,748 (1,496–9,269)	1,932 (1,572–2,281)	7,425 (6,005–8,562)	5,408 (4,464–6,136)	5,559 (4,760–6,310)
Paralytic ileus and intestinal obstruction	103,295 (85,803–118,232)	5,813 (2,910–11,129)	3,566 (2,596–4,321)	19,237 (14,087–22,208)	26,850 (21,481–31,394)	47,827 (41,123–54,722)
Inguinal, femoral, and abdominal hernia	19,963 (13,518–23,331)	460 (167–973)	448 (240–635)	2,622 (1,343–3,386)	5,975 (3,742–7,153)	10,457 (7,774–12,448)
Inflammatory bowel disease	21,187 (17,946–25,184)	1,493 (488–3,629)	387 (266–550)	3,794 (2,845–5,000)	4,503 (3,805–5,191)	11,007 (9,811–11,809)
Vascular intestinal disorders	41,531 (37,381–45,776)	101 (36–237)	54 (34–78)	1,927 (1,383–2,496)	8,535 (7,090–9,900)	30,912 (28,639–33,511)
Gallbladder and biliary diseases	54,335 (50,553–58,445)	1,368 (639–2,982)	811 (670–968)	7,205 (6,407–8,185)	15,140 (13,822–16,661)	29,809 (28,145–31,765)
Pancreatitis	36,526 (33,096–40,378)	144 (75–267)	337 (254–431)	9,452 (8,102–10,950)	12,159 (10,804–13,576)	14,432 (13,412–15,397)
Other digestive diseases	46,868 (39,904–53,235)	2,069 (889–4,692)	886 (678–1,095)	5,384 (4,167–6,397)	10,478 (8,533–12,567)	28,049 (24,998–31,196)
Neurological disorders	860,821 (737,692–982,125)	8,613 (6,907–12,120)	6,868 (6,094–7,961)	33,014 (30,546–37,092)	62,000 (52,630–71,726)	750,324 (630,379–867,618)
Alzheimer disease and other dementias	743,691 (618,177–865,516)	0 (0–0)	0 (0–0)	564 (355–814)	38,174 (28,798–47,811)	704,951 (584,392–823,205)
Parkinson disease	31,079 (29,879–32,380)	0 (0–0)	0 (0–0)	234 (211–256)	3,773 (3,548–3,992)	27,070 (26,033–28,268)
Epilepsy	47,420 (43,318–54,286)	6,307 (4,835–9,084)	5,701 (5,000–6,522)	23,451 (21,293–26,856)	6,399 (6,031–6,869)	5,560 (5,297–5,813)

SR Table 10 - Global deaths by age and cause for females in 2000						
**Cause**	**All ages**	**0-5 years**	**5-14 years**	**15-49 years**	**50-69 years**	**70+ years**
Multiple sclerosis	9,497 (7,873–10,895)	0 (0–0)	0 (0–0)	3,469 (2,772–4,247)	4,014 (3,345–4,503)	2,013 (1,608–2,167)
Motor neuron disease	10,842 (10,376–12,204)	628 (390–937)	56 (45–73)	992 (925–1,249)	4,507 (4,303–5,109)	4,658 (4,488–4,862)
Migraine	0 (0–0)	0 (0–0)	0 (0–0)	0 (0–0)	0 (0–0)	0 (0–0)
Tension-type headache	0 (0–0)	0 (0–0)	0 (0–0)	0 (0–0)	0 (0–0)	0 (0–0)
Medication overuse headache	0 (0–0)	0 (0–0)	0 (0–0)	0 (0–0)	0 (0–0)	0 (0–0)
Other neurological disorders	18,301 (17,614–21,476)	1,687 (1,451–3,209)	1,110 (990–1,714)	4,301 (4,077–5,428)	5,130 (4,988–5,278)	6,070 (5,818–6,309)
Mental and substance use disorders	62,618 (59,736–64,687)	14 (12–16)	22 (11–31)	33,243 (31,674–34,796)	19,263 (18,485–19,994)	10,073 (9,505–10,398)
Schizophrenia	9,697 (9,020–10,355)	0 (0–0)	0 (0–0)	3,768 (3,416–4,121)	3,054 (2,836–3,259)	2,874 (2,700–3,064)
Alcohol use disorders	25,069 (24,112–26,009)	0 (0–0)	0 (0–0)	11,325 (10,692–11,994)	10,608 (10,029–11,204)	3,134 (3,038–3,245)
Drug use disorders	27,424 (24,674–28,722)	14 (12–16)	0 (0–0)	17,746 (16,135–18,910)	5,600 (5,047–5,891)	4,064 (3,610–4,291)
Opioid use disorders	16,947 (15,379–19,005)	14 (12–16)	0 (0–0)	11,095 (9,940–12,747)	3,467 (3,120–3,751)	2,369 (2,163–2,621)
Cocaine use disorders	1,145 (971–1,288)	0 (0–0)	0 (0–0)	818 (706–922)	241 (199–275)	85 (62–97)
Amphetamine use disorders	624 (519–1,078)	0 (0–0)	0 (0–0)	444 (361–802)	129 (106–205)	50 (39–71)
Cannabis use disorders	0 (0–0)	0 (0–0)	0 (0–0)	0 (0–0)	0 (0–0)	0 (0–0)
Other drug use disorders	8,707 (7,038–9,855)	0 (0–0)	0 (0–0)	5,387 (4,272–6,396)	1,761 (1,438–1,931)	1,558 (1,224–1,723)
Depressive disorders	0 (0–0)	0 (0–0)	0 (0–0)	0 (0–0)	0 (0–0)	0 (0–0)
Major depressive disorder	0 (0–0)	0 (0–0)	0 (0–0)	0 (0–0)	0 (0–0)	0 (0–0)
Dysthymia	0 (0–0)	0 (0–0)	0 (0–0)	0 (0–0)	0 (0–0)	0 (0–0)
Bipolar disorder	0 (0–0)	0 (0–0)	0 (0–0)	0 (0–0)	0 (0–0)	0 (0–0)
Anxiety disorders	0 (0–0)	0 (0–0)	0 (0–0)	0 (0–0)	0 (0–0)	0 (0–0)
Eating disorders	426 (267–649)	0 (0–0)	22 (11–31)	403 (255–620)	0 (0–0)	0 (0–0)
Anorexia nervosa	402 (255–610)	0 (0–0)	21 (10–30)	381 (243–581)	0 (0–0)	0 (0–0)
Bulimia nervosa	23 (10–38)	0 (0–0)	0 (0–1)	22 (9–38)	0 (0–0)	0 (0–0)
Autistic spectrum disorders	0 (0–0)	0 (0–0)	0 (0–0)	0 (0–0)	0 (0–0)	0 (0–0)
Autism	0 (0–0)	0 (0–0)	0 (0–0)	0 (0–0)	0 (0–0)	0 (0–0)
Asperger syndrome and other autistic spectrum disorders	0 (0–0)	0 (0–0)	0 (0–0)	0 (0–0)	0 (0–0)	0 (0–0)
Attention-deficit/hyperactivity disorder	0 (0–0)	0 (0–0)	0 (0–0)	0 (0–0)	0 (0–0)	0 (0–0)
Conduct disorder	0 (0–0)	0 (0–0)	0 (0–0)	0 (0–0)	0 (0–0)	0 (0–0)
Idiopathic developmental intellectual disability	0 (0–0)	0 (0–0)	0 (0–0)	0 (0–0)	0 (0–0)	0 (0–0)
Other mental and substance use disorders	0 (0–0)	0 (0–0)	0 (0–0)	0 (0–0)	0 (0–0)	0 (0–0)
Diabetes, urogenital, blood, and endocrine diseases	1,210,989 (1,178,280–1,247,929)	66,596 (51,769–94,592)	17,765 (16,136–19,492)	156,243 (150,882–162,885)	374,834 (364,350–385,867)	595,549 (582,039–609,111)
Diabetes mellitus	548,119 (533,134–563,320)	1,517 (1,111–1,755)	1,813 (1,450–2,064)	47,168 (44,736–49,491)	203,188 (196,064–210,752)	294,430 (286,956–302,981)
Acute glomerulonephritis	6,628 (5,180–7,747)	269 (213–355)	284 (229–341)	1,933 (1,434–2,177)	1,890 (1,401–2,284)	2,250 (1,725–2,986)
Chronic kidney disease	399,491 (388,465–410,790)	8,805 (7,715–9,766)	5,548 (5,050–6,051)	61,083 (58,419–64,113)	120,874 (117,107–124,975)	203,178 (197,404–209,150)
Chronic kidney disease due to diabetes mellitus	124,226 (118,867–129,993)	1,236 (998–1,494)	495 (410–593)	13,812 (12,571–15,153)	46,266 (43,049–50,029)	62,416 (59,310–65,693)
Chronic kidney disease due to hypertension	177,442 (170,150–184,616)	3,014 (2,586–3,433)	1,666 (1,424–1,939)	23,083 (21,424–24,979)	48,914 (45,532–52,479)	100,763 (96,258–105,598)
Chronic kidney disease due to glomerulonephritis	86,945 (82,510–91,303)	3,369 (2,862–3,860)	2,568 (2,272–2,901)	21,303 (19,720–23,025)	24,275 (22,494–26,189)	35,428 (32,890–38,137)
Chronic kidney disease due to other causes	10,876 (9,040–12,858)	1,185 (955–1,449)	818 (663–996)	2,884 (1,951–3,895)	1,418 (888–2,128)	4,569 (3,744–5,788)
Urinary diseases and male infertility	89,900 (85,376–96,571)	3,195 (2,392–3,900)	997 (881–1,168)	12,845 (11,831–14,424)	21,712 (20,032–24,481)	51,149 (48,516–55,175)
Interstitial nephritis and urinary tract infections	72,936 (68,835–78,673)	2,488 (1,850–2,998)	646 (560–757)	9,453 (8,663–10,625)	16,206 (14,795–18,353)	44,141 (41,632–47,928)
Urolithiasis	6,315 (4,661–7,189)	0 (0–0)	133 (97–169)	1,336 (926–1,575)	2,228 (1,587–2,680)	2,617 (2,006–2,996)
Benign prostatic hyperplasia	0 (0–0)	0 (0–0)	0 (0–0)	0 (0–0)	0 (0–0)	0 (0–0)
Male infertility	0 (0–0)	0 (0–0)	0 (0–0)	0 (0–0)	0 (0–0)	0 (0–0)
Other urinary diseases	10,648 (9,395–12,309)	707 (446–1,073)	218 (170–278)	2,056 (1,739–2,512)	3,276 (2,811–4,006)	4,390 (3,877–5,133)
Gynecological diseases	7,527 (5,867–8,584)	0 (0–0)	0 (0–0)	3,576 (2,682–4,167)	2,228 (1,705–2,612)	1,722 (1,424–2,152)
Uterine fibroids	2,005 (1,127–2,659)	0 (0–0)	0 (0–0)	797 (466–1,096)	793 (426–1,081)	414 (211–664)
Polycystic ovarian syndrome	874 (208–1,482)	0 (0–0)	0 (0–0)	627 (128–1,100)	246 (78–391)	0 (0–0)
Female infertility	0 (0–0)	0 (0–0)	0 (0–0)	0 (0–0)	0 (0–0)	0 (0–0)
Endometriosis	42 (31–57)	0 (0–0)	0 (0–0)	35 (26–48)	7 (4–10)	0 (0–0)
Genital prolapse	1,157 (576–1,955)	0 (0–0)	0 (0–0)	108 (42–194)	445 (184–827)	603 (325–969)
Premenstrual syndrome	0 (0–0)	0 (0–0)	0 (0–0)	0 (0–0)	0 (0–0)	0 (0–0)
Other gynecological diseases	3,447 (2,536–4,451)	0 (0–0)	0 (0–0)	2,007 (1,491–2,707)	735 (483–1,044)	704 (484–1,058)
Hemoglobinopathies and hemolytic anemias	108,103 (93,637–136,591)	46,548 (32,760–74,543)	7,379 (6,198–8,372)	19,976 (18,015–21,841)	12,644 (12,073–13,262)	21,554 (20,122–23,370)
Thalassemias	11,626 (9,019–14,719)	6,637 (4,405–9,629)	2,092 (1,650–2,431)	1,218 (1,074–1,381)	47 (43–54)	1,629 (1,349–2,042)
Thalassemias trait	0 (0–0)	0 (0–0)	0 (0–0)	0 (0–0)	0 (0–0)	0 (0–0)
Sickle cell disorders	54,993 (41,530–83,484)	39,312 (26,042–68,267)	4,584 (3,758–5,501)	8,506 (7,222–9,736)	636 (543–718)	1,954 (1,659–2,345)
Sickle cell trait	0 (0–0)	0 (0–0)	0 (0–0)	0 (0–0)	0 (0–0)	0 (0–0)

SR Table 10 - Global deaths by age and cause for females in 2000						
**Cause**	**All ages**	**0-5 years**	**5-14 years**	**15-49 years**	**50-69 years**	**70+ years**
G6PD deficiency	7,658 (6,725–8,692)	324 (198–537)	370 (281–458)	4,403 (3,854–5,058)	2,051 (1,810–2,347)	508 (437–589)
G6PD trait	0 (0–0)	0 (0–0)	0 (0–0)	0 (0–0)	0 (0–0)	0 (0–0)
Other hemoglobinopathies and hemolytic anemias	33,825 (32,318–35,532)	274 (194–403)	331 (278–380)	5,847 (5,407–6,325)	9,909 (9,433–10,496)	17,461 (16,182–18,791)
Endocrine, metabolic, blood, and immune disorders	51,218 (49,649–53,119)	6,259 (5,365–7,203)	1,741 (1,627–1,870)	9,659 (9,316–10,040)	12,295 (11,843–12,727)	21,262 (20,568–22,035)
Musculoskeletal disorders	49,000 (43,696–53,582)	0 (0–0)	1,269 (999–1,555)	12,855 (11,202–15,275)	11,715 (10,672–13,131)	23,159 (20,814–24,345)
Rheumatoid arthritis	17,996 (15,515–21,031)	0 (0–0)	175 (100–252)	1,638 (1,168–2,090)	5,652 (4,884–6,643)	10,529 (9,425–12,265)
Osteoarthritis	0 (0–0)	0 (0–0)	0 (0–0)	0 (0–0)	0 (0–0)	0 (0–0)
Low back and neck pain	0 (0–0)	0 (0–0)	0 (0–0)	0 (0–0)	0 (0–0)	0 (0–0)
Low back pain	0 (0–0)	0 (0–0)	0 (0–0)	0 (0–0)	0 (0–0)	0 (0–0)
Neck pain	0 (0–0)	0 (0–0)	0 (0–0)	0 (0–0)	0 (0–0)	0 (0–0)
Gout	0 (0–0)	0 (0–0)	0 (0–0)	0 (0–0)	0 (0–0)	0 (0–0)
Other musculoskeletal disorders	31,003 (26,811–34,416)	0 (0–0)	1,094 (870–1,335)	11,217 (9,776–13,438)	6,063 (5,365–7,041)	12,629 (10,592–13,471)
Other non-communicable diseases	363,956 (277,963–480,430)	278,260 (204,666–385,416)	20,731 (15,563–27,119)	26,393 (21,153–31,159)	12,796 (9,687–16,528)	25,774 (17,586–33,272)
Congenital anomalies	318,941 (235,106–433,953)	264,938 (192,538–370,072)	20,154 (15,058–26,505)	22,906 (18,205–27,374)	6,092 (5,061–6,818)	4,850 (3,974–5,271)
Neural tube defects	45,192 (29,741–75,188)	42,867 (28,006–72,097)	1,322 (833–2,140)	730 (531–1,109)	179 (126–343)	92 (68–160)
Congenital heart anomalies	161,078 (108,612–235,273)	126,220 (81,157–192,713)	14,643 (10,343–19,610)	16,407 (12,713–19,642)	2,059 (1,689–2,400)	1,747 (1,495–2,009)
Cleft lip and cleft palate	2,092 (1,114–2,844)	2,092 (1,114–2,844)	0 (0–0)	0 (0–0)	0 (0–0)	0 (0–0)
Down syndrome	18,019 (5,413–39,125)	14,214 (3,003–33,716)	555 (218–1,065)	1,968 (1,168–3,058)	1,192 (900–1,508)	89 (65–122)
Turner syndrome	0 (0–0)	0 (0–0)	0 (0–0)	0 (0–0)	0 (0–0)	0 (0–0)
Klinefelter syndrome	0 (0–0)	0 (0–0)	0 (0–0)	0 (0–0)	0 (0–0)	0 (0–0)
Other chromosomal abnormalities	13,209 (4,820–40,011)	12,535 (4,305–38,819)	221 (109–565)	292 (219–403)	117 (71–153)	42 (32–51)
Other congenital anomalies	79,348 (61,153–116,480)	67,008 (50,057–102,676)	3,411 (2,618–5,115)	3,507 (2,912–4,480)	2,542 (1,958–2,914)	2,878 (2,115–3,189)
Skin and subcutaneous diseases	35,021 (21,473–50,309)	3,328 (1,601–5,765)	576 (270–822)	3,487 (1,767–5,244)	6,703 (3,820–10,935)	20,924 (13,235–28,589)
Dermatitis	0 (0–0)	0 (0–0)	0 (0–0)	0 (0–0)	0 (0–0)	0 (0–0)
Psoriasis	0 (0–0)	0 (0–0)	0 (0–0)	0 (0–0)	0 (0–0)	0 (0–0)
Cellulitis	5,232 (2,814–7,869)	334 (112–728)	94 (40–153)	660 (302–1,031)	1,544 (747–2,534)	2,597 (1,517–3,699)
Pyoderma	14,689 (8,532–22,845)	2,803 (1,309–4,915)	321 (152–519)	2,022 (963–3,199)	3,263 (1,843–5,713)	6,279 (4,063–9,559)
Scabies	0 (0–0)	0 (0–0)	0 (0–0)	0 (0–0)	0 (0–0)	0 (0–0)
Fungal skin diseases	0 (0–0)	0 (0–0)	0 (0–0)	0 (0–0)	0 (0–0)	0 (0–0)
Viral skin diseases	0 (0–0)	0 (0–0)	0 (0–0)	0 (0–0)	0 (0–0)	0 (0–0)
Acne vulgaris	0 (0–0)	0 (0–0)	0 (0–0)	0 (0–0)	0 (0–0)	0 (0–0)
Alopecia areata	0 (0–0)	0 (0–0)	0 (0–0)	0 (0–0)	0 (0–0)	0 (0–0)
Pruritus	0 (0–0)	0 (0–0)	0 (0–0)	0 (0–0)	0 (0–0)	0 (0–0)
Urticaria	0 (0–0)	0 (0–0)	0 (0–0)	0 (0–0)	0 (0–0)	0 (0–0)
Decubitus ulcer	13,480 (7,473–18,218)	49 (15–108)	85 (36–140)	521 (265–766)	1,586 (808–2,681)	11,238 (6,000–14,930)
Other skin and subcutaneous diseases	1,617 (1,048–2,398)	140 (64–292)	74 (31–112)	283 (156–424)	310 (195–537)	808 (534–1,171)
Sense organ diseases	0 (0–0)	0 (0–0)	0 (0–0)	0 (0–0)	0 (0–0)	0 (0–0)
Glaucoma	0 (0–0)	0 (0–0)	0 (0–0)	0 (0–0)	0 (0–0)	0 (0–0)
Cataract	0 (0–0)	0 (0–0)	0 (0–0)	0 (0–0)	0 (0–0)	0 (0–0)
Macular degeneration	0 (0–0)	0 (0–0)	0 (0–0)	0 (0–0)	0 (0–0)	0 (0–0)
Refraction and accommodation disorders	0 (0–0)	0 (0–0)	0 (0–0)	0 (0–0)	0 (0–0)	0 (0–0)
Age-related and other hearing loss	0 (0–0)	0 (0–0)	0 (0–0)	0 (0–0)	0 (0–0)	0 (0–0)
Other vision loss	0 (0–0)	0 (0–0)	0 (0–0)	0 (0–0)	0 (0–0)	0 (0–0)
Other sense organ diseases	0 (0–0)	0 (0–0)	0 (0–0)	0 (0–0)	0 (0–0)	0 (0–0)
Oral disorders	0 (0–0)	0 (0–0)	0 (0–0)	0 (0–0)	0 (0–0)	0 (0–0)
Deciduous caries	0 (0–0)	0 (0–0)	0 (0–0)	0 (0–0)	0 (0–0)	0 (0–0)
Permanent caries	0 (0–0)	0 (0–0)	0 (0–0)	0 (0–0)	0 (0–0)	0 (0–0)
Periodontal diseases	0 (0–0)	0 (0–0)	0 (0–0)	0 (0–0)	0 (0–0)	0 (0–0)
Edentulism and severe tooth loss	0 (0–0)	0 (0–0)	0 (0–0)	0 (0–0)	0 (0–0)	0 (0–0)
Other oral disorders	0 (0–0)	0 (0–0)	0 (0–0)	0 (0–0)	0 (0–0)	0 (0–0)
Sudden infant death syndrome	9,993 (7,099–14,194)	9,993 (7,099–14,194)	0 (0–0)	0 (0–0)	0 (0–0)	0 (0–0)
Injuries	1,479,585 (1,306,675–1,564,290)	198,710 (155,433–239,571)	122,779 (107,413–133,323)	603,399 (529,491–634,620)	260,212 (231,673–270,670)	294,482 (268,446–306,223)
Transport injuries	369,678 (353,096–387,341)	31,445 (24,850–38,858)	35,987 (33,168–38,868)	170,717 (162,887–179,392)	76,126 (73,332–78,823)	55,402 (53,766–57,187)
Road injuries	349,284 (334,204–366,550)	28,823 (23,065–35,228)	34,201 (31,614–36,940)	161,896 (154,450–170,356)	71,753 (69,060–74,686)	52,609 (51,024–54,440)
Pedestrian road injuries	171,499 (161,575–181,270)	13,902 (11,227–16,970)	18,458 (17,078–19,953)	69,630 (65,319–73,953)	38,291 (36,065–40,455)	31,217 (29,820–32,822)
Cyclist road injuries	15,962 (14,596–17,826)	675 (452–1,052)	1,685 (1,461–1,962)	8,114 (7,299–9,191)	3,746 (3,405–4,089)	1,739 (1,567–1,938)
Motorcyclist road injuries	38,589 (34,704–43,256)	3,043 (2,091–4,137)	2,935 (2,457–3,454)	21,206 (19,210–23,786)	7,364 (6,516–8,726)	4,040 (3,487–4,901)

SR Table 10 - Global deaths by age and cause for females in 2000						
**Cause**	**All ages**	**0-5 years**	**5-14 years**	**15-49 years**	**50-69 years**	**70+ years**
Motor vehicle road injuries	119,325<br>(111,671–129,804)	10,737<br>(8,270–13,790)	10,722<br>(9,627–12,016)	61,227<br>(57,670–68,149)	21,566<br>(19,860–23,263)	15,071<br>(13,848–16,156)
Other road injuries	3,907<br>(3,145–4,436)	465<br>(250–686)	400<br>(303–485)	1,717<br>(1,379–1,968)	784<br>(622–925)	540<br>(439–649)
Other transport injuries	20,393<br>(16,669–23,595)	2,621<br>(1,447–3,826)	1,785<br>(1,377–2,107)	8,820<br>(7,220–10,292)	4,372<br>(3,634–4,954)	2,793<br>(2,415–3,208)
Unintentional injuries	710,877<br>(598,291–770,855)	153,534<br>(113,298–190,181)	70,925<br>(56,623–78,160)	192,135<br>(159,830–205,976)	104,203<br>(89,599–109,961)	190,077<br>(170,981–199,140)
Falls	153,177<br>(138,511–161,782)	12,205<br>(7,208–17,572)	6,335<br>(5,389–7,122)	18,661<br>(17,446–20,255)	23,531<br>(22,035–24,857)	92,444<br>(85,138–97,983)
Drowning	147,969<br>(81,934–167,239)	48,800<br>(18,414–62,779)	30,120<br>(18,426–33,892)	38,192<br>(23,199–41,775)	15,248<br>(9,659–16,808)	15,606<br>(10,809–16,996)
Fire, heat, and hot substances	117,830<br>(80,022–131,555)	19,024<br>(10,198–24,335)	8,062<br>(5,261–9,353)	53,921<br>(34,046–60,959)	16,051<br>(11,864–17,621)	20,770<br>(16,841–22,464)
Poisonings	42,830<br>(31,000–51,091)	9,888<br>(4,175–17,245)	3,905<br>(3,326–4,505)	16,529<br>(13,059–17,748)	6,785<br>(5,215–7,356)	5,720<br>(4,816–6,204)
Exposure to mechanical forces	56,040<br>(50,207–60,228)	17,266<br>(13,175–20,497)	4,838<br>(4,241–5,922)	14,018<br>(12,813–15,253)	8,437<br>(7,736–9,639)	11,479<br>(8,708–12,985)
Unintentional firearm injuries	8,494<br>(5,743–9,407)	779<br>(401–1,181)	625<br>(399–756)	2,692<br>(1,660–3,078)	1,257<br>(752–1,423)	3,138<br>(2,472–3,406)
Unintentional suffocation	14,171<br>(9,945–16,406)	9,437<br>(5,651–11,424)	1,116<br>(853–1,671)	1,382<br>(1,091–1,626)	677<br>(538–835)	1,558<br>(1,219–1,835)
Other exposure to mechanical forces	33,374<br>(30,619–36,775)	7,049<br>(5,199–9,312)	3,096<br>(2,689–3,811)	9,943<br>(9,189–11,220)	6,502<br>(5,927–7,889)	6,782<br>(4,726–8,028)
Adverse effects of medical treatment	44,287<br>(32,069–53,674)	8,887<br>(4,290–12,628)	2,211<br>(1,341–2,842)	10,651<br>(6,984–13,589)	8,187<br>(6,037–9,838)	14,348<br>(11,487–16,231)
Animal contact	46,093<br>(27,557–51,926)	8,125<br>(4,408–10,431)	6,996<br>(4,126–8,258)	17,098<br>(10,428–19,123)	9,402<br>(5,441–10,575)	4,471<br>(2,812–5,007)
Venomous animal contact	39,030<br>(22,516–44,204)	5,913<br>(3,107–7,740)	5,909<br>(3,350–7,104)	15,350<br>(9,100–17,324)	8,344<br>(4,686–9,457)	3,512<br>(2,017–4,068)
Non-venomous animal contact	7,063<br>(4,920–9,099)	2,211<br>(1,169–3,228)	1,087<br>(739–1,485)	1,748<br>(1,274–2,208)	1,058<br>(741–1,321)	958<br>(771–1,127)
Foreign body	55,849<br>(41,964–78,816)	21,476<br>(12,482–39,866)	3,712<br>(2,470–5,613)	8,811<br>(6,643–11,259)	6,610<br>(5,282–8,107)	15,238<br>(13,584–17,056)
Pulmonary aspiration and foreign body in airway	43,394<br>(30,552–68,607)	18,734<br>(8,967–37,658)	2,664<br>(1,498–4,738)	4,980<br>(3,738–7,546)	4,666<br>(3,925–5,895)	12,348<br>(11,437–13,416)
Foreign body in eyes	0<br>(0–0)	0<br>(0–0)	0<br>(0–0)	0<br>(0–0)	0<br>(0–0)	0<br>(0–0)
Foreign body in other body part	12,454<br>(7,515–21,438)	2,742<br>(1,130–4,460)	1,048<br>(563–1,855)	3,830<br>(2,325–6,570)	1,943<br>(1,171–3,605)	2,890<br>(2,023–4,658)
Environmental heat and cold exposure	13,556<br>(8,379–16,973)	1,707<br>(733–2,304)	519<br>(302–761)	2,439<br>(1,809–3,265)	3,527<br>(2,073–4,701)	5,362<br>(3,256–6,918)
Other unintentional injuries	33,241<br>(30,510–36,726)	6,152<br>(4,796–7,570)	4,223<br>(3,525–4,864)	11,811<br>(10,834–13,069)	6,420<br>(5,847–7,128)	4,634<br>(4,179–5,166)
Self-harm and interpersonal violence	371,944<br>(287,028–392,604)	8,025<br>(7,224–8,699)	11,836<br>(9,661–12,737)	228,431<br>(172,597–243,205)	76,424<br>(59,269–80,842)	47,226<br>(37,644–49,851)
Self-harm	277,524<br>(196,824–297,231)	0<br>(0–0)	5,498<br>(3,427–6,202)	171,396<br>(116,953–185,173)	62,111<br>(45,220–66,254)	38,518<br>(29,118–41,008)
Interpersonal violence	94,420<br>(90,804–98,414)	8,025<br>(7,224–8,699)	6,338<br>(5,925–6,800)	57,034<br>(54,748–59,663)	14,313<br>(13,521–14,964)	8,708<br>(8,285–9,082)
Assault by firearm	20,799<br>(17,169–22,522)	887<br>(699–1,117)	1,202<br>(939–1,363)	14,532<br>(11,924–15,553)	2,714<br>(2,355–3,102)	1,462<br>(1,187–1,713)
Assault by sharp object	22,958<br>(21,306–24,946)	800<br>(612–1,015)	1,097<br>(961–1,259)	15,317<br>(14,113–16,668)	3,469<br>(3,188–3,755)	2,274<br>(2,081–2,527)
Assault by other means	50,662<br>(47,319–54,670)	6,337<br>(5,638–6,936)	4,038<br>(3,671–4,467)	27,184<br>(24,990–29,946)	8,129<br>(7,454–8,686)	4,972<br>(4,513–5,262)
Forces of nature, war, and legal intervention	27,085<br>(17,605–40,844)	5,705<br>(2,343–11,226)	4,029<br>(1,624–8,040)	12,115<br>(6,628–19,434)	3,458<br>(1,847–5,354)	1,775<br>(879–3,255)
Exposure to forces of nature	3,851<br>(2,565–5,442)	378<br>(201–608)	686<br>(397–1,038)	1,711<br>(1,103–2,625)	647<br>(409–917)	427<br>(268–622)
Collective violence and legal intervention	23,233<br>(14,124–36,205)	5,327<br>(2,007–10,883)	3,343<br>(1,070–7,283)	10,403<br>(5,007–17,635)	2,811<br>(1,290–4,654)	1,347<br>(520–2,731)

Cause	All ages	0-5 years	5-14 years	15-49 years	50-69 years	70+ years
**SR Table 10 - Global deaths by age and cause for females in 2005**						
All causes	24,760,564 (24,482,834–25,051,695)	3,743,206 (3,699,681–3,790,059)	479,725 (470,619–489,377)	3,672,778 (3,583,141–3,761,185)	5,365,290 (5,269,610–5,472,162)	11,499,563 (11,394,063–11,604,686)
Communicable, maternal, neonatal, and nutritional diseases	6,769,439 (6,583,588–6,935,235)	3,168,617 (3,066,833–3,245,265)	278,504 (266,001–292,761)	1,572,289 (1,510,243–1,641,361)	709,270 (664,568–752,757)	1,040,757 (969,801–1,089,679)
HIV/AIDS and tuberculosis	1,377,465 (1,278,320–1,488,475)	106,789 (102,093–114,597)	47,008 (44,447–51,613)	812,538 (762,824–865,289)	268,660 (231,876–307,216)	142,468 (112,183–170,132)
Tuberculosis	498,182 (409,397–601,978)	15,877 (12,234–22,979)	10,280 (8,576–14,460)	166,310 (139,979–204,063)	173,967 (135,762–209,513)	131,745 (101,842–160,009)
HIV/AIDS	879,283 (835,513–929,222)	90,912 (87,891–93,955)	36,728 (35,031–38,516)	646,227 (605,739–692,010)	94,692 (87,507–103,632)	10,722 (9,581–12,233)
HIV/AIDS - Tuberculosis	167,382 (132,933–190,247)	24,102 (19,406–26,952)	8,491 (6,696–9,746)	106,198 (82,663–121,707)	25,324 (21,054–28,283)	3,265 (2,779–3,707)
HIV/AIDS resulting in other diseases	711,900 (663,768–764,075)	66,809 (62,566–71,990)	28,237 (26,236–30,769)	540,028 (496,199–586,670)	69,367 (62,332–78,280)	7,457 (6,503–8,818)
Diarrhea, lower respiratory, and other common infectious diseases	2,841,852 (2,708,244–2,977,003)	1,266,094 (1,181,386–1,360,904)	164,111 (148,451–182,199)	280,628 (258,566–306,615)	338,971 (312,822–363,172)	792,046 (728,976–833,127)
Diarrheal diseases	854,636 (797,590–915,659)	374,402 (326,547–425,027)	38,179 (33,893–42,844)	96,532 (89,851–104,443)	132,769 (123,677–141,963)	212,754 (199,625–227,071)
Intestinal infectious diseases	98,070 (55,727–160,590)	24,661 (13,088–42,633)	38,375 (22,283–61,790)	28,539 (14,955–48,318)	4,431 (2,292–7,669)	2,060 (1,179–3,271)
Typhoid fever	81,189 (44,654–137,095)	20,465 (10,394–36,410)	32,320 (17,933–51,874)	23,326 (12,163–40,861)	3,508 (1,683–6,283)	1,569 (833–2,565)
Paratyphoid fever	15,873 (7,150–30,445)	3,629 (1,534–7,516)	5,940 (2,769–11,476)	5,083 (2,218–10,032)	863 (354–1,733)	356 (153–671)
Other intestinal infectious diseases	1,006 (153–2,007)	566 (63–1,297)	115 (13–304)	130 (24–284)	60 (12–119)	134 (38–272)
Lower respiratory infections	1,373,226 (1,245,666–1,450,795)	539,097 (492,810–588,783)	36,589 (26,595–42,226)	94,760 (76,459–104,387)	160,898 (129,170–175,609)	541,881 (473,141–574,832)
Upper respiratory infections	1,753 (1,503–2,068)	536 (343–785)	30 (19–46)	303 (248–368)	377 (297–473)	505 (393–638)
Otitis media	1,883 (1,670–2,263)	626 (537–737)	198 (178–221)	434 (409–464)	232 (209–263)	391 (246–772)
Meningitis	189,749 (160,147–220,823)	97,809 (76,365–126,697)	18,263 (14,756–21,248)	33,677 (28,743–39,392)	21,862 (18,292–26,218)	18,136 (15,580–21,524)
Pneumococcal meningitis	52,338 (43,136–64,217)	22,882 (15,557–33,147)	6,303 (4,439–8,736)	11,580 (9,127–14,492)	6,318 (4,807–8,310)	5,253 (4,125–6,663)
H influenzae type B meningitis	51,930 (41,284–65,236)	35,428 (25,588–48,155)	4,883 (3,146–6,689)	5,355 (3,893–6,904)	3,338 (2,464–4,494)	2,924 (2,265–3,779)
Meningococcal meningitis	34,010 (26,175–43,691)	18,785 (12,300–28,310)	3,253 (2,123–4,549)	5,689 (4,191–7,266)	3,602 (2,614–4,777)	2,678 (2,021–3,545)
Other meningitis	51,469 (43,445–61,308)	20,712 (14,808–28,489)	3,823 (2,672–5,403)	11,052 (8,940–13,525)	8,602 (7,010–10,659)	7,279 (5,961–8,855)
Encephalitis	66,881 (58,573–78,768)	23,564 (17,616–27,641)	8,450 (6,874–10,088)	13,772 (12,315–16,742)	10,283 (8,890–14,090)	10,810 (9,452–14,316)
Diphtheria	2,710 (1,261–6,007)	2,063 (702–5,363)	358 (150–822)	211 (131–330)	76 (35–162)	0 (0–0)
Whooping cough	54,665 (20,153–124,371)	50,769 (18,725–115,050)	3,183 (1,168–7,248)	620 (197–1,727)	92 (29–255)	0 (0–0)
Tetanus	47,341 (38,505–71,303)	26,597 (22,157–32,389)	2,318 (1,620–4,327)	7,684 (5,384–15,164)	7,052 (4,833–15,262)	3,689 (2,629–7,389)
Measles	145,919 (54,552–305,665)	124,661 (46,961–261,964)	17,798 (6,555–37,714)	3,367 (1,030–8,283)	92 (28–226)	0 (0–0)
Varicella and herpes zoster	5,015 (4,237–5,932)	1,305 (917–1,911)	365 (244–576)	723 (584–900)	802 (599–1,098)	1,818 (1,361–2,463)
Neglected tropical diseases and malaria	647,356 (537,584–756,953)	434,374 (340,371–525,925)	46,536 (33,920–60,314)	86,720 (65,730–113,979)	54,397 (39,972–74,841)	25,328 (18,797–34,817)
Malaria	592,544 (484,635–702,833)	421,919 (328,430–514,377)	38,045 (24,574–51,874)	69,647 (49,084–96,872)	43,843 (29,611–63,484)	19,087 (12,618–28,232)
Chagas disease	3,101 (2,950–3,269)	1 (1–2)	3 (2–4)	390 (362–421)	1,198 (1,118–1,281)	1,507 (1,410–1,616)
Leishmaniasis	6,070 (3,734–8,879)	1,264 (766–1,903)	841 (463–1,352)	3,185 (1,946–4,671)	591 (397–829)	187 (143–236)
Visceral leishmaniasis	6,070 (3,734–8,879)	1,264 (766–1,903)	841 (463–1,352)	3,185 (1,946–4,671)	591 (397–829)	187 (143–236)
Cutaneous and mucocutaneous leishmaniasis	0 (0–0)	0 (0–0)	0 (0–0)	0 (0–0)	0 (0–0)	0 (0–0)
African trypanosomiasis	6,625 (3,501–10,941)	512 (262–864)	1,026 (539–1,674)	3,565 (1,826–5,970)	1,215 (630–2,064)	306 (159–510)
Schistosomiasis	3,685 (3,079–4,309)	41 (25–74)	81 (55–120)	927 (747–1,138)	1,617 (1,248–2,083)	1,017 (741–1,400)
Cysticercosis	299 (255–369)	6 (3–12)	11 (8–16)	155 (131–192)	92 (79–113)	33 (29–38)
Cystic echinococcosis	888 (832–946)	41 (33–51)	77 (67–87)	301 (278–324)	253 (236–271)	214 (203–226)
Lymphatic filariasis	0 (0–0)	0 (0–0)	0 (0–0)	0 (0–0)	0 (0–0)	0 (0–0)
Onchocerciasis	0 (0–0)	0 (0–0)	0 (0–0)	0 (0–0)	0 (0–0)	0 (0–0)
Trachoma	0 (0–0)	0 (0–0)	0 (0–0)	0 (0–0)	0 (0–0)	0 (0–0)
Dengue	6,167 (4,161–8,035)	2,003 (1,138–3,132)	1,681 (1,051–2,410)	1,440 (1,109–1,775)	636 (430–775)	405 (234–518)
Yellow fever	1,686 (340–4,431)	163 (32–496)	537 (107–1,459)	773 (153–2,115)	176 (41–487)	34 (7–96)
Rabies	13,083 (10,638–15,764)	1,945 (1,395–2,793)	2,835 (2,126–3,695)	4,170 (3,141–5,068)	2,964 (2,389–3,597)	1,167 (909–1,521)
Intestinal nematode infections	1,889 (1,640–2,162)	1,415 (1,199–1,647)	255 (208–310)	90 (64–118)	66 (45–92)	62 (43–86)
Ascariasis	1,889 (1,640–2,162)	1,415 (1,199–1,647)	255 (208–310)	90 (64–118)	66 (45–92)	62 (43–86)
Trichuriasis	0 (0–0)	0 (0–0)	0 (0–0)	0 (0–0)	0 (0–0)	0 (0–0)
Hookworm disease	0 (0–0)	0 (0–0)	0 (0–0)	0 (0–0)	0 (0–0)	0 (0–0)
Food-borne trematodiases	0 (0–0)	0 (0–0)	0 (0–0)	0 (0–0)	0 (0–0)	0 (0–0)
Leprosy	0 (0–0)	0 (0–0)	0 (0–0)	0 (0–0)	0 (0–0)	0 (0–0)
Ebola	3 (2–4)	0 (0–0)	0 (0–0)	1 (1–2)	1 (1–2)	0 (0–0)
Other neglected tropical diseases	11,311 (8,211–16,708)	5,057 (3,509–7,495)	1,139 (712–1,909)	2,069 (1,443–3,244)	1,739 (1,208–2,815)	1,305 (850–2,019)
Maternal disorders	350,795 (327,923–376,619)	0 (0–0)	1,638 (1,366–1,976)	347,824 (325,104–373,357)	1,332 (988–1,819)	0 (0–0)
Maternal hemorrhage	99,684 (87,611–112,970)	0 (0–0)	504 (374–658)	98,734 (86,673–111,999)	445 (283–681)	0 (0–0)
Maternal sepsis and other maternal infections	24,745 (20,416–29,934)	0 (0–0)	70 (49–102)	24,576 (20,265–29,772)	97 (60–157)	0 (0–0)
Maternal hypertensive disorders	63,699 (54,939–74,261)	0 (0–0)	333 (235–460)	63,257 (54,505–73,730)	108 (68–164)	0 (0–0)
Maternal obstructed labor and uterine rupture	26,890 (22,083–32,009)	0 (0–0)	48 (31–75)	26,786 (21,995–31,890)	55 (34–86)	0 (0–0)
Maternal abortion, miscarriage, and ectopic pregnancy	41,188 (34,661–49,571)	0 (0–0)	229 (156–320)	40,745 (34,197–49,117)	213 (140–321)	0 (0–0)

SR Table 10 - Global deaths by age and cause for females in 2005						
**Cause**	**All ages**	**0-5 years**	**5-14 years**	**15-49 years**	**50-69 years**	**70+ years**
Indirect maternal deaths	38,216 (31,610–45,445)	0 (0–0)	204 (135–290)	37,873 (31,311–45,105)	138 (83–227)	0 (0–0)
Late maternal deaths	7,908 (5,294–11,519)	0 (0–0)	45 (22–79)	7,817 (5,196–11,420)	46 (25–83)	0 (0–0)
Maternal deaths aggravated by HIV/AIDS	2,755 (1,768–3,758)	0 (0–0)	2 (1–4)	2,743 (1,761–3,743)	9 (4–15)	0 (0–0)
Other maternal disorders	45,706 (39,019–53,913)	0 (0–0)	198 (142–268)	45,288 (38,570–53,538)	219 (145–324)	0 (0–0)
Neonatal disorders	1,165,703 (1,131,943–1,200,738)	1,165,703 (1,131,943–1,200,738)	0 (0–0)	0 (0–0)	0 (0–0)	0 (0–0)
Neonatal preterm birth complications	496,177 (449,916–553,068)	496,177 (449,916–553,068)	0 (0–0)	0 (0–0)	0 (0–0)	0 (0–0)
Neonatal encephalopathy due to birth asphyxia and trauma	382,314 (334,149–433,030)	382,314 (334,149–433,030)	0 (0–0)	0 (0–0)	0 (0–0)	0 (0–0)
Neonatal sepsis and other neonatal infections	129,086 (62,455–194,933)	129,086 (62,455–194,933)	0 (0–0)	0 (0–0)	0 (0–0)	0 (0–0)
Hemolytic disease and other neonatal jaundice	32,749 (16,519–56,223)	32,749 (16,519–56,223)	0 (0–0)	0 (0–0)	0 (0–0)	0 (0–0)
Other neonatal disorders	125,375 (85,488–182,606)	125,375 (85,488–182,606)	0 (0–0)	0 (0–0)	0 (0–0)	0 (0–0)
Nutritional deficiencies	226,364 (204,849–251,721)	114,636 (95,450–137,683)	8,325 (7,286–9,459)	19,395 (17,919–21,203)	23,231 (21,504–25,238)	60,774 (56,929–65,038)
Protein-energy malnutrition	182,388 (161,370–208,324)	103,765 (85,585–125,989)	6,315 (5,346–7,376)	9,602 (8,005–11,669)	13,923 (11,852–16,231)	48,782 (43,436–54,341)
Iodine deficiency	1,249 (887–1,824)	418 (183–887)	53 (22–107)	189 (119–293)	247 (151–423)	339 (208–551)
Vitamin A deficiency	0 (0–0)	0 (0–0)	0 (0–0)	0 (0–0)	0 (0–0)	0 (0–0)
Iron-deficiency anemia	25,725 (17,454–34,069)	7,598 (3,624–10,985)	1,219 (892–1,948)	6,385 (4,875–7,813)	6,207 (4,782–7,602)	4,314 (2,897–6,227)
Other nutritional deficiencies	17,001 (9,329–29,102)	2,854 (1,064–5,664)	736 (326–1,226)	3,218 (1,603–5,276)	2,854 (1,475–5,447)	7,338 (4,551–11,662)
Other communicable, maternal, neonatal, and nutritional diseases	159,901 (131,504–196,023)	81,019 (55,875–113,373)	10,883 (8,402–14,000)	25,183 (23,679–26,920)	22,677 (20,508–24,127)	20,138 (17,446–21,506)
Sexually transmitted diseases excluding HIV	67,696 (41,111–102,700)	55,524 (31,322–87,566)	5,522 (3,115–8,597)	3,388 (2,768–4,127)	1,630 (1,164–1,940)	1,630 (1,204–1,923)
Syphilis	66,820 (40,296–101,776)	55,524 (31,322–87,566)	5,506 (3,100–8,581)	3,003 (2,458–3,653)	1,385 (989–1,645)	1,399 (1,034–1,651)
Chlamydial infection	159 (125–183)	0 (0–0)	3 (3–4)	71 (58–87)	41 (29–49)	43 (32–50)
Gonococcal infection	481 (372–554)	0 (0–0)	6 (5–8)	189 (155–232)	139 (99–166)	145 (106–172)
Trichomoniasis	0 (0–0)	0 (0–0)	0 (0–0)	0 (0–0)	0 (0–0)	0 (0–0)
Genital herpes	0 (0–0)	0 (0–0)	0 (0–0)	0 (0–0)	0 (0–0)	0 (0–0)
Other sexually transmitted diseases	234 (185–271)	0 (0–0)	4 (3–6)	123 (100–151)	64 (45–77)	42 (30–50)
Hepatitis	51,539 (48,973–54,466)	8,782 (7,631–10,095)	3,594 (3,101–4,140)	16,715 (15,623–18,068)	12,560 (11,972–13,284)	9,885 (9,349–10,517)
Acute hepatitis A	8,355 (5,256–11,266)	6,730 (4,696–8,340)	609 (170–1,228)	565 (151–1,281)	270 (60–671)	179 (40–432)
Hepatitis B	27,180 (23,170–30,996)	488 (171–1,149)	387 (204–658)	8,880 (7,080–10,653)	10,154 (8,688–11,350)	7,269 (5,976–8,451)
Hepatitis C	1,198 (257–2,739)	0 (0–0)	50 (9–138)	275 (54–649)	545 (121–1,284)	325 (67–801)
Acute hepatitis E	14,805 (10,951–19,616)	1,563 (604–3,055)	2,547 (1,868–3,235)	6,993 (5,244–9,017)	1,589 (735–3,019)	2,111 (1,126–3,297)
Other infectious diseases	40,666 (26,588–47,775)	16,712 (8,129–22,707)	1,766 (1,387–2,101)	5,078 (4,345–5,636)	8,486 (6,359–9,559)	8,622 (6,141–9,780)
Non-communicable diseases	16,527,013 (16,291,964–16,818,632)	412,716 (359,223–494,306)	99,057 (91,418–107,932)	1,518,447 (1,477,244–1,572,475)	4,372,446 (4,286,148–4,469,085)	10,124,344 (10,016,190–10,244,100)
Neoplasms	3,238,515 (3,176,935–3,309,483)	15,943 (14,110–18,996)	22,431 (21,105–23,848)	502,078 (485,749–520,508)	1,268,531 (1,240,114–1,298,601)	1,429,531 (1,403,059–1,458,306)
Lip and oral cavity cancer	37,661 (36,369–39,141)	0 (0–0)	0 (0–0)	5,743 (5,411–6,128)	15,317 (14,621–16,127)	16,600 (16,027–17,217)
Nasopharynx cancer	17,000 (16,164–17,905)	0 (0–0)	95 (85–105)	5,426 (5,095–5,803)	7,616 (7,195–8,056)	3,862 (3,662–4,063)
Other pharynx cancer	15,385 (14,321–16,265)	0 (0–0)	0 (0–0)	2,373 (2,134–2,620)	7,624 (7,037–8,106)	5,386 (5,066–5,657)
Esophageal cancer	147,686 (140,437–154,541)	0 (0–0)	0 (0–0)	13,097 (12,252–13,991)	63,750 (60,282–67,261)	70,838 (67,475–74,240)
Stomach cancer	299,430 (288,253–310,187)	0 (0–0)	0 (0–0)	37,075 (34,616–39,093)	108,793 (103,685–113,349)	153,561 (149,082–158,203)
Colon and rectum cancer	321,215 (313,948–329,222)	0 (0–0)	0 (0–0)	29,508 (28,283–30,966)	105,825 (102,921–109,136)	185,881 (181,288–190,419)
Liver cancer	225,192 (191,926–238,877)	0 (0–0)	435 (359–485)	32,688 (26,075–35,780)	96,329 (79,921–103,051)	95,738 (84,212–100,897)
Liver cancer due to hepatitis B	66,834 (54,821–73,514)	0 (0–0)	197 (159–224)	17,516 (13,793–19,509)	30,659 (24,841–34,340)	18,461 (15,328–20,682)
Liver cancer due to hepatitis C	62,608 (55,277–67,084)	0 (0–0)	13 (11–16)	3,876 (3,021–4,454)	23,077 (19,357–25,561)	35,640 (32,401–38,081)
Liver cancer due to alcohol use	35,959 (30,573–39,472)	0 (0–0)	0 (0–0)	2,307 (1,834–2,688)	17,680 (14,497–20,025)	15,970 (13,861–17,541)
Liver cancer due to other causes	59,790 (49,653–65,226)	0 (0–0)	224 (186–253)	8,987 (7,112–10,147)	24,912 (20,227–27,987)	25,665 (21,601–28,093)
Gallbladder and biliary tract cancer	74,487 (71,528–77,265)	0 (0–0)	0 (0–0)	5,245 (4,801–5,665)	26,314 (24,932–27,624)	42,927 (41,539–44,357)
Pancreatic cancer	151,675 (148,698–154,577)	0 (0–0)	0 (0–0)	9,026 (8,724–9,360)	54,116 (52,858–55,342)	88,532 (86,519–90,552)
Larynx cancer	17,508 (16,063–18,410)	0 (0–0)	0 (0–0)	2,456 (2,134–2,689)	7,830 (7,124–8,314)	7,222 (6,739–7,598)
Tracheal, bronchus, and lung cancer	428,731 (417,302–441,040)	0 (0–0)	0 (0–0)	39,822 (38,082–41,713)	175,197 (170,062–180,214)	213,711 (208,260–220,343)
Malignant skin melanoma	22,058 (19,890–23,422)	0 (0–0)	0 (0–0)	4,269 (3,968–4,732)	7,692 (6,943–8,185)	10,096 (8,917–10,689)
Non-melanoma skin cancer	11,395 (10,967–11,863)	0 (0–0)	0 (0–0)	1,151 (1,086–1,221)	2,831 (2,685–2,977)	7,412 (7,113–7,751)
Non-melanoma skin cancer (squamous-cell carcinoma)	11,395 (10,967–11,863)	0 (0–0)	0 (0–0)	1,151 (1,086–1,221)	2,831 (2,685–2,977)	7,412 (7,113–7,751)
Non-melanoma skin cancer (basal-cell carcinoma)	0 (0–0)	0 (0–0)	0 (0–0)	0 (0–0)	0 (0–0)	0 (0–0)
Breast cancer	433,723 (412,993–455,761)	0 (0–0)	0 (0–0)	97,106 (88,735–105,295)	192,053 (183,211–201,543)	144,563 (138,767–149,438)
Cervical cancer	225,446 (213,892–237,838)	0 (0–0)	0 (0–0)	61,916 (57,409–66,764)	105,339 (99,083–111,630)	58,189 (56,295–60,352)
Uterine cancer	81,779 (78,501–85,395)	0 (0–0)	0 (0–0)	10,098 (9,395–10,844)	38,153 (36,315–40,210)	33,527 (32,355–34,921)
Ovarian cancer	133,821 (130,764–138,606)	0 (0–0)	0 (0–0)	23,527 (22,576–25,295)	61,290 (59,735–63,721)	49,003 (47,743–50,380)
Prostate cancer	0 (0–0)	0 (0–0)	0 (0–0)	0 (0–0)	0 (0–0)	0 (0–0)
Testicular cancer	0 (0–0)	0 (0–0)	0 (0–0)	0 (0–0)	0 (0–0)	0 (0–0)

SR Table 10 - Global deaths by age and cause for females in 2005

Cause	All ages	0-5 years	5-14 years	15-49 years	50-69 years	70+ years
Kidney cancer	38,334 (37,397–39,413)	828 (663–1,175)	568 (513–639)	3,627 (3,478–3,812)	13,749 (13,401–14,163)	19,560 (19,045–20,123)
Bladder cancer	41,947 (40,971–43,029)	0 (0–0)	0 (0–0)	2,201 (2,092–2,308)	11,152 (10,788–11,527)	28,593 (27,814–29,422)
Brain and nervous system cancer	84,982 (80,832–87,855)	2,292 (2,056–2,566)	4,230 (3,880–4,577)	22,415 (20,976–23,475)	32,922 (31,699–34,019)	23,121 (21,856–23,816)
Thyroid cancer	15,886 (14,985–17,836)	0 (0–0)	36 (33–39)	1,490 (1,381–1,725)	5,633 (5,247–6,420)	8,725 (8,302–9,689)
Mesothelioma	6,999 (6,755–7,242)	0 (0–0)	0 (0–0)	1,255 (1,168–1,345)	2,492 (2,403–2,583)	3,251 (3,138–3,374)
Hodgkin lymphoma	9,777 (8,026–12,482)	116 (76–147)	236 (161–295)	3,557 (2,858–4,554)	3,076 (2,483–4,075)	2,790 (2,310–3,487)
Non-Hodgkin lymphoma	78,765 (68,110–86,399)	1,236 (902–2,041)	1,619 (1,318–1,920)	12,488 (11,048–14,502)	25,366 (22,526–27,984)	38,054 (31,410–40,513)
Multiple myeloma	38,851 (37,338–40,297)	0 (0–0)	0 (0–0)	3,110 (2,957–3,341)	14,943 (14,382–15,585)	20,797 (19,147–21,578)
Leukemia	132,791 (129,018–136,954)	6,095 (5,261–7,123)	10,190 (9,519–11,115)	35,158 (33,596–36,754)	35,581 (34,517–36,803)	45,765 (44,342–47,273)
Acute lymphoid leukemia	41,622 (39,311–46,339)	4,062 (3,409–5,053)	7,130 (6,545–8,135)	13,439 (12,660–15,453)	9,460 (8,903–10,494)	7,529 (7,045–8,051)
Chronic lymphoid leukemia	24,077 (22,488–25,663)	0 (0–0)	0 (0–0)	3,824 (3,149–4,375)	6,531 (5,932–7,166)	13,721 (13,155–14,320)
Acute myeloid leukemia	51,412 (48,967–53,211)	2,033 (1,523–2,442)	3,060 (2,704–3,357)	13,672 (12,650–14,520)	14,455 (13,848–15,003)	18,191 (17,541–18,818)
Chronic myeloid leukemia	15,678 (14,578–16,919)	0 (0–0)	0 (0–0)	4,221 (3,658–4,820)	5,133 (4,771–5,588)	6,323 (6,001–6,664)
Other neoplasms	145,980 (136,512–152,128)	5,374 (4,476–7,490)	5,018 (4,549–5,497)	36,239 (30,798–38,948)	47,533 (44,577–49,744)	51,813 (49,119–53,627)
Cardiovascular diseases	7,876,475 (7,741,640–8,017,141)	26,973 (25,024–29,011)	14,833 (13,747–16,084)	487,217 (471,134–506,315)	1,843,253 (1,800,847–1,887,114)	5,504,197 (5,408,153–5,606,784)
Rheumatic heart disease	192,126 (175,884–206,176)	1,782 (1,335–2,149)	4,037 (3,591–4,568)	46,326 (40,414–50,842)	62,197 (56,727–67,696)	77,783 (73,154–82,782)
Ischemic heart disease	3,574,195 (3,510,823–3,646,527)	1,407 (1,231–1,642)	1,126 (994–1,281)	170,719 (163,849–179,758)	794,588 (775,044–815,081)	2,606,353 (2,557,704–2,660,378)
Cerebrovascular disease	3,107,174 (3,046,990–3,177,600)	9,088 (8,128–10,324)	5,106 (4,672–5,673)	182,939 (175,042–192,330)	766,497 (744,429–790,416)	2,143,542 (2,098,851–2,192,812)
Ischemic stroke	1,512,620 (1,439,301–1,582,165)	440 (351–551)	317 (269–367)	29,451 (25,713–33,026)	256,286 (235,152–278,880)	1,226,124 (1,175,051–1,271,994)
Hemorrhagic stroke	1,594,553 (1,530,901–1,680,163)	8,647 (7,733–9,897)	4,788 (4,365–5,315)	153,488 (145,307–163,538)	510,210 (486,915–539,460)	917,417 (877,484–966,771)
Hypertensive heart disease	426,256 (378,628–473,513)	245 (184–322)	191 (158–236)	28,502 (23,131–33,168)	112,195 (94,733–126,953)	285,121 (257,501–318,006)
Cardiomyopathy and myocarditis	141,726 (137,333–145,761)	5,750 (5,050–6,448)	1,809 (1,670–1,976)	24,771 (23,389–26,100)	30,061 (28,853–31,329)	79,332 (76,485–82,059)
Atrial fibrillation and flutter	82,342 (67,876–98,808)	0 (0–0)	0 (0–0)	309 (228–404)	4,340 (3,312–5,819)	77,692 (63,018–94,456)
Aortic aneurysm	48,100 (46,739–49,804)	0 (0–0)	0 (0–0)	3,165 (2,917–3,506)	10,081 (9,713–10,630)	34,854 (33,799–35,995)
Peripheral vascular disease	19,497 (18,355–20,793)	0 (0–0)	0 (0–0)	188 (177–202)	2,024 (1,891–2,206)	17,283 (16,210–18,474)
Endocarditis	38,528 (32,204–43,873)	1,870 (1,013–2,680)	667 (453–858)	6,704 (4,669–8,091)	7,262 (5,923–8,894)	22,024 (19,806–24,609)
Other cardiovascular and circulatory diseases	246,527 (238,849–255,939)	6,828 (6,008–7,915)	1,895 (1,711–2,132)	23,591 (22,279–25,305)	54,004 (51,637–57,057)	160,207 (154,200–166,871)
Chronic respiratory diseases	1,647,724 (1,591,248–1,705,922)	23,652 (21,040–26,308)	5,905 (5,327–6,565)	86,404 (82,249–91,132)	417,798 (399,400–437,249)	1,113,963 (1,074,001–1,151,759)
Chronic obstructive pulmonary disease	1,363,498 (1,275,786–1,436,598)	9,614 (6,575–12,060)	1,203 (832–1,821)	46,170 (39,912–53,873)	328,714 (300,492–357,307)	977,794 (921,103–1,024,130)
Pneumoconiosis	9,877 (6,832–13,303)	0 (0–0)	0 (0–0)	668 (378–951)	2,164 (1,233–3,217)	7,045 (5,154–9,385)
Silicosis	398 (272–570)	0 (0–0)	0 (0–0)	37 (22–56)	94 (57–138)	266 (191–381)
Asbestosis	500 (357–625)	0 (0–0)	0 (0–0)	141 (85–189)	114 (74–148)	244 (196–297)
Coal workers pneumoconiosis	177 (109–238)	0 (0–0)	0 (0–0)	24 (13–35)	52 (28–72)	100 (66–132)
Other pneumoconiosis	8,802 (6,032–12,054)	0 (0–0)	0 (0–0)	464 (256–696)	1,903 (1,064–2,902)	6,434 (4,673–8,621)
Asthma	223,751 (164,195–287,690)	7,108 (5,166–9,084)	4,006 (3,143–4,831)	35,350 (27,163–41,577)	75,914 (52,020–100,584)	101,371 (73,956–143,028)
Interstitial lung disease and pulmonary sarcoidosis	35,569 (26,238–40,294)	156 (94–233)	124 (96–155)	2,644 (2,185–3,064)	9,076 (7,187–10,742)	23,568 (16,424–26,485)
Other chronic respiratory diseases	15,026 (10,676–20,338)	6,772 (4,685–9,706)	571 (318–1,035)	1,569 (974–2,348)	1,928 (1,321–2,618)	4,184 (3,028–5,523)
Cirrhosis and other chronic liver diseases	409,900 (387,404–462,595)	6,164 (4,776–8,955)	4,956 (4,285–6,264)	91,466 (85,388–104,613)	170,425 (159,912–192,246)	136,887 (128,880–152,764)
Cirrhosis and other chronic liver diseases due to hepatitis B	105,237 (95,125–123,774)	29 (20–45)	94 (74–123)	22,187 (19,606–26,122)	44,565 (38,759–52,900)	38,360 (34,259–44,684)
Cirrhosis and other chronic liver diseases due to hepatitis C	91,985 (85,016–102,512)	15 (10–24)	58 (45–78)	18,482 (16,324–21,313)	39,088 (34,631–44,398)	34,340 (31,273–38,346)
Cirrhosis and other chronic liver diseases due to alcohol use	93,135 (85,833–104,823)	0 (0–0)	0 (0–0)	24,685 (22,552–28,554)	45,013 (40,176–51,134)	23,437 (20,870–26,514)
Cirrhosis and other chronic liver diseases due to other causes	119,541 (111,144–138,144)	6,120 (4,743–8,884)	4,803 (4,165–6,067)	26,111 (23,571–30,177)	41,757 (37,290–48,353)	40,749 (37,257–46,172)
Digestive diseases	516,475 (499,345–539,166)	16,084 (9,070–30,614)	8,912 (8,016–10,086)	80,292 (76,208–84,809)	135,075 (129,772–140,742)	276,110 (268,038–284,607)
Peptic ulcer disease	121,422 (109,500–133,898)	2,042 (933–4,901)	1,541 (1,219–1,922)	19,490 (16,983–22,089)	35,294 (31,188–39,479)	63,054 (57,706–69,225)
Gastritis and duodenitis	21,962 (12,339–30,170)	417 (140–880)	271 (149–419)	3,381 (1,852–4,847)	6,211 (3,284–8,710)	11,678 (6,847–15,866)
Appendicitis	24,719 (18,433–30,619)	3,364 (1,295–8,044)	1,669 (1,362–1,966)	7,454 (5,682–8,414)	5,768 (4,534–6,464)	6,461 (5,287–7,252)
Paralytic ileus and intestinal obstruction	108,734 (91,713–123,685)	5,361 (2,802–10,880)	2,999 (2,370–3,712)	18,637 (14,197–21,802)	27,377 (22,631–32,711)	54,358 (46,581–61,776)
Inguinal, femoral, and abdominal hernia	20,650 (13,772–24,379)	400 (133–1,026)	357 (196–514)	2,615 (1,337–3,418)	6,032 (3,759–7,294)	11,244 (8,281–13,425)
Inflammatory bowel disease	23,264 (19,968–26,937)	1,322 (521–3,189)	313 (225–441)	3,821 (2,973–4,937)	4,859 (4,252–5,509)	12,947 (11,200–13,874)
Vascular intestinal disorders	47,555 (42,754–52,620)	94 (35–228)	48 (31–69)	2,044 (1,475–2,683)	9,528 (7,957–11,194)	35,840 (33,103–38,885)
Gallbladder and biliary diseases	57,300 (53,303–61,528)	1,229 (540–2,730)	707 (599–852)	7,103 (6,361–7,990)	15,231 (13,815–16,663)	33,028 (31,226–35,101)
Pancreatitis	42,750 (38,776–46,664)	138 (71–263)	324 (253–412)	10,764 (9,392–12,151)	14,330 (12,803–15,832)	17,193 (15,946–18,399)
Other digestive diseases	48,114 (42,379–53,297)	1,712 (755–3,757)	678 (512–851)	4,978 (4,009–5,786)	10,441 (8,884–12,043)	30,303 (27,640–32,976)
Neurological disorders	997,655 (854,227–1,136,895)	7,922 (6,603–10,512)	5,927 (5,294–6,793)	31,779 (29,557–35,777)	69,043 (58,697–80,034)	882,983 (741,741–1,021,545)
Alzheimer disease and other dementias	872,534 (726,611–1,014,104)	0 (0–0)	0 (0–0)	615 (390–888)	42,408 (32,218–53,256)	829,510 (687,235–968,357)
Parkinson disease	37,222 (35,820–38,694)	0 (0–0)	0 (0–0)	248 (225–270)	4,190 (3,902–4,454)	32,783 (31,490–34,083)
Epilepsy	46,144 (42,614–51,583)	5,788 (4,689–7,704)	4,828 (4,264–5,492)	22,070 (20,170–25,165)	6,929 (6,520–7,436)	6,527 (6,216–6,846)

SR Table 10 - Global deaths by age and cause for females in 2005

Cause	All ages	0-5 years	5-14 years	15-49 years	50-69 years	70+ years
Multiple sclerosis	9,807 (8,439–11,078)	0 (0–0)	0 (0–0)	3,171 (2,902–3,851)	4,465 (3,730–4,913)	2,171 (1,681–2,330)
Motor neuron disease	12,355 (11,863–13,496)	536 (374–745)	50 (42–60)	1,085 (1,021–1,295)	5,228 (5,005–5,789)	5,454 (5,251–5,697)
Migraine	0 (0–0)	0 (0–0)	0 (0–0)	0 (0–0)	0 (0–0)	0 (0–0)
Tension-type headache	0 (0–0)	0 (0–0)	0 (0–0)	0 (0–0)	0 (0–0)	0 (0–0)
Medication overuse headache	0 (0–0)	0 (0–0)	0 (0–0)	0 (0–0)	0 (0–0)	0 (0–0)
Other neurological disorders	19,601 (18,940–22,475)	1,607 (1,413–2,812)	1,048 (935–1,574)	4,586 (4,352–5,716)	5,821 (5,655–5,994)	6,536 (6,281–6,805)
Mental and substance use disorders	70,650 (68,167–72,990)	16 (13–18)	23 (10–30)	35,585 (34,152–37,336)	22,752 (21,850–23,592)	12,273 (10,937–12,890)
Schizophrenia	9,331 (8,694–9,910)	0 (0–0)	0 (0–0)	3,069 (2,767–3,337)	3,131 (2,906–3,344)	3,130 (2,934–3,330)
Alcohol use disorders	26,744 (25,706–27,811)	0 (0–0)	0 (0–0)	11,555 (10,941–12,299)	11,760 (11,104–12,488)	3,428 (3,311–3,545)
Drug use disorders	34,123 (31,528–36,027)	16 (13–18)	0 (0–0)	20,532 (19,458–22,191)	7,860 (7,142–8,300)	5,714 (4,412–6,285)
Opioid use disorders	22,304 (19,655–23,689)	16 (13–18)	0 (0–0)	13,509 (12,174–14,736)	5,148 (4,382–5,507)	3,630 (2,683–4,020)
Cocaine use disorders	2,072 (1,902–2,308)	0 (0–0)	0 (0–0)	1,397 (1,288–1,551)	552 (498–616)	122 (96–145)
Amphetamine use disorders	1,112 (977–1,538)	0 (0–0)	0 (0–0)	742 (645–1,042)	274 (237–346)	96 (73–142)
Cannabis use disorders	0 (0–0)	0 (0–0)	0 (0–0)	0 (0–0)	0 (0–0)	0 (0–0)
Other drug use disorders	8,633 (7,753–10,801)	0 (0–0)	0 (0–0)	4,883 (4,155–6,420)	1,885 (1,700–2,328)	1,864 (1,456–2,270)
Depressive disorders	0 (0–0)	0 (0–0)	0 (0–0)	0 (0–0)	0 (0–0)	0 (0–0)
Major depressive disorder	0 (0–0)	0 (0–0)	0 (0–0)	0 (0–0)	0 (0–0)	0 (0–0)
Dysthymia	0 (0–0)	0 (0–0)	0 (0–0)	0 (0–0)	0 (0–0)	0 (0–0)
Bipolar disorder	0 (0–0)	0 (0–0)	0 (0–0)	0 (0–0)	0 (0–0)	0 (0–0)
Anxiety disorders	0 (0–0)	0 (0–0)	0 (0–0)	0 (0–0)	0 (0–0)	0 (0–0)
Eating disorders	452 (279–668)	0 (0–0)	23 (10–30)	428 (267–644)	0 (0–0)	0 (0–0)
Anorexia nervosa	421 (265–623)	0 (0–0)	22 (10–29)	399 (252–590)	0 (0–0)	0 (0–0)
Bulimia nervosa	30 (12–48)	0 (0–0)	0 (0–1)	29 (12–47)	0 (0–0)	0 (0–0)
Autistic spectrum disorders	0 (0–0)	0 (0–0)	0 (0–0)	0 (0–0)	0 (0–0)	0 (0–0)
Autism	0 (0–0)	0 (0–0)	0 (0–0)	0 (0–0)	0 (0–0)	0 (0–0)
Asperger syndrome and other autistic spectrum disorders	0 (0–0)	0 (0–0)	0 (0–0)	0 (0–0)	0 (0–0)	0 (0–0)
Attention-deficit/hyperactivity disorder	0 (0–0)	0 (0–0)	0 (0–0)	0 (0–0)	0 (0–0)	0 (0–0)
Conduct disorder	0 (0–0)	0 (0–0)	0 (0–0)	0 (0–0)	0 (0–0)	0 (0–0)
Idiopathic developmental intellectual disability	0 (0–0)	0 (0–0)	0 (0–0)	0 (0–0)	0 (0–0)	0 (0–0)
Other mental and substance use disorders	0 (0–0)	0 (0–0)	0 (0–0)	0 (0–0)	0 (0–0)	0 (0–0)
Diabetes, urogenital, blood, and endocrine diseases	1,363,975 (1,329,757–1,398,064)	55,520 (44,064–80,023)	15,774 (14,341–17,317)	162,799 (157,104–169,634)	417,904 (405,601–430,551)	711,975 (696,345–729,013)
Diabetes mellitus	619,443 (598,659–639,085)	1,184 (887–1,349)	1,599 (1,213–1,822)	50,420 (47,778–53,133)	222,848 (213,306–231,579)	343,391 (333,000–354,753)
Acute glomerulonephritis	5,874 (4,579–6,824)	192 (150–252)	191 (154–234)	1,501 (1,122–1,682)	1,764 (1,309–2,092)	2,225 (1,705–2,734)
Chronic kidney disease	466,356 (451,867–482,061)	7,157 (6,419–7,862)	4,687 (4,289–5,135)	63,454 (60,615–66,764)	138,908 (133,759–144,489)	252,147 (243,962–260,772)
Chronic kidney disease due to diabetes mellitus	149,795 (143,422–156,521)	1,099 (905–1,317)	461 (383–562)	14,845 (13,484–16,297)	55,281 (51,639–59,718)	78,107 (74,282–82,250)
Chronic kidney disease due to hypertension	209,625 (200,717–218,698)	2,480 (2,161–2,818)	1,463 (1,258–1,689)	24,703 (22,833–26,709)	55,575 (51,648–59,450)	125,403 (119,442–131,652)
Chronic kidney disease due to glomerulonephritis	95,054 (90,210–100,247)	2,605 (2,243–3,006)	2,048 (1,785–2,334)	20,992 (19,558–22,803)	26,469 (24,398–28,648)	42,938 (39,834–46,171)
Chronic kidney disease due to other causes	11,880 (9,923–14,141)	973 (798–1,183)	713 (569–872)	2,913 (1,957–4,005)	1,581 (1,001–2,362)	5,698 (4,747–7,056)
Urinary diseases and male infertility	104,085 (99,548–110,531)	2,731 (2,092–3,295)	888 (787–1,036)	13,657 (12,746–15,216)	24,016 (22,448–26,445)	62,791 (59,634–66,173)
Interstitial nephritis and urinary tract infections	85,359 (80,930–90,185)	2,147 (1,606–2,571)	576 (500–662)	10,178 (9,465–11,243)	17,825 (16,520–19,596)	54,632 (51,246–57,814)
Urolithiasis	6,881 (5,068–7,879)	0 (0–0)	113 (88–146)	1,286 (899–1,545)	2,389 (1,660–2,828)	3,092 (2,392–3,593)
Benign prostatic hyperplasia	0 (0–0)	0 (0–0)	0 (0–0)	0 (0–0)	0 (0–0)	0 (0–0)
Male infertility	0 (0–0)	0 (0–0)	0 (0–0)	0 (0–0)	0 (0–0)	0 (0–0)
Other urinary diseases	11,844 (10,455–13,891)	584 (378–879)	198 (154–257)	2,192 (1,858–2,681)	3,801 (3,267–4,585)	5,066 (4,480–6,152)
Gynecological diseases	7,640 (6,090–8,606)	0 (0–0)	0 (0–0)	3,409 (2,693–3,903)	2,298 (1,775–2,659)	1,932 (1,556–2,330)
Uterine fibroids	2,122 (1,233–2,702)	0 (0–0)	0 (0–0)	788 (479–1,026)	868 (496–1,110)	466 (237–679)
Polycystic ovarian syndrome	747 (192–1,251)	0 (0–0)	0 (0–0)	528 (116–924)	219 (74–355)	0 (0–0)
Female infertility	0 (0–0)	0 (0–0)	0 (0–0)	0 (0–0)	0 (0–0)	0 (0–0)
Endometriosis	44 (34–60)	0 (0–0)	0 (0–0)	36 (27–50)	8 (5–12)	0 (0–0)
Genital prolapse	1,104 (569–1,766)	0 (0–0)	0 (0–0)	79 (35–145)	402 (179–722)	621 (345–958)
Premenstrual syndrome	0 (0–0)	0 (0–0)	0 (0–0)	0 (0–0)	0 (0–0)	0 (0–0)
Other gynecological diseases	3,621 (2,678–4,530)	0 (0–0)	0 (0–0)	1,977 (1,498–2,555)	799 (536–1,074)	843 (577–1,179)
Hemoglobinopathies and hemolytic anemias	102,041 (90,941–126,719)	38,898 (28,415–63,660)	6,879 (5,886–7,903)	19,607 (17,847–21,363)	13,385 (12,823–14,059)	23,271 (22,014–24,760)
Thalassemias	9,987 (8,452–12,380)	5,193 (3,828–7,504)	1,688 (1,391–1,945)	1,152 (1,024–1,299)	64 (59–71)	1,888 (1,597–2,312)
Thalassemias trait	0 (0–0)	0 (0–0)	0 (0–0)	0 (0–0)	0 (0–0)	0 (0–0)
Sickle cell disorders	49,482 (38,978–73,117)	33,268 (23,012–57,571)	4,605 (3,769–5,589)	8,517 (7,201–9,831)	727 (632–818)	2,362 (2,006–2,763)
Sickle cell trait	0 (0–0)	0 (0–0)	0 (0–0)	0 (0–0)	0 (0–0)	0 (0–0)

SR Table 10 - Global deaths by age and cause for females in 2005						
Cause	All ages	0-5 years	5-14 years	15-49 years	50-69 years	70+ years
G6PD deficiency	8,156 (7,234–9,207)	266 (175–441)	368 (288–454)	4,508 (3,983–5,151)	2,428 (2,136–2,773)	585 (504–676)
G6PD trait	0 (0–0)	0 (0–0)	0 (0–0)	0 (0–0)	0 (0–0)	0 (0–0)
Other hemoglobinopathies and hemolytic anemias	34,415 (33,006–35,881)	169 (130–241)	216 (184–247)	5,428 (5,034–5,874)	10,164 (9,678–10,723)	18,435 (17,208–19,431)
Endocrine, metabolic, blood, and immune disorders	58,531 (56,785–60,721)	5,356 (4,718–6,120)	1,528 (1,432–1,637)	10,747 (10,248–11,307)	14,684 (14,253–15,192)	26,214 (25,333–27,164)
Musculoskeletal disorders	53,423 (47,838–57,435)	0 (0–0)	1,177 (962–1,374)	13,324 (11,852–15,180)	12,959 (11,893–14,543)	25,962 (23,245–27,199)
Rheumatoid arthritis	18,733 (16,492–21,960)	0 (0–0)	146 (85–211)	1,517 (1,146–1,856)	5,819 (4,999–6,932)	11,249 (10,236–12,985)
Osteoarthritis	0 (0–0)	0 (0–0)	0 (0–0)	0 (0–0)	0 (0–0)	0 (0–0)
Low back and neck pain	0 (0–0)	0 (0–0)	0 (0–0)	0 (0–0)	0 (0–0)	0 (0–0)
Low back pain	0 (0–0)	0 (0–0)	0 (0–0)	0 (0–0)	0 (0–0)	0 (0–0)
Neck pain	0 (0–0)	0 (0–0)	0 (0–0)	0 (0–0)	0 (0–0)	0 (0–0)
Gout	0 (0–0)	0 (0–0)	0 (0–0)	0 (0–0)	0 (0–0)	0 (0–0)
Other musculoskeletal disorders	34,689 (30,504–38,188)	0 (0–0)	1,030 (863–1,205)	11,806 (10,381–13,600)	7,139 (6,380–8,275)	14,712 (12,365–15,750)
Other non-communicable diseases	352,207 (284,550–435,316)	260,430 (205,725–336,932)	19,116 (15,114–23,549)	27,498 (21,922–31,092)	14,703 (10,821–18,561)	30,458 (20,813–38,005)
Congenital anomalies	302,224 (238,813–384,174)	247,673 (193,961–323,917)	18,540 (14,633–22,908)	23,577 (18,876–26,651)	6,895 (5,633–7,521)	5,537 (4,410–5,973)
Neural tube defects	39,427 (26,070–62,590)	37,477 (24,681–60,372)	1,087 (658–1,741)	629 (464–909)	153 (113–261)	78 (61–117)
Congenital heart anomalies	149,109 (105,762–205,449)	115,371 (78,769–166,324)	13,160 (9,761–16,840)	16,547 (12,840–18,836)	2,204 (1,764–2,504)	1,825 (1,509–2,052)
Cleft lip and cleft palate	1,344 (786–1,715)	1,344 (786–1,715)	0 (0–0)	0 (0–0)	0 (0–0)	0 (0–0)
Down syndrome	16,825 (5,931–33,320)	12,808 (3,307–27,942)	512 (227–913)	2,014 (1,243–2,955)	1,391 (1,022–1,665)	98 (69–130)
Turner syndrome	0 (0–0)	0 (0–0)	0 (0–0)	0 (0–0)	0 (0–0)	0 (0–0)
Klinefelter syndrome	0 (0–0)	0 (0–0)	0 (0–0)	0 (0–0)	0 (0–0)	0 (0–0)
Other chromosomal abnormalities	13,422 (5,365–35,546)	12,721 (4,810–34,518)	221 (111–510)	302 (226–392)	129 (75–169)	46 (35–56)
Other congenital anomalies	82,095 (67,152–112,771)	67,950 (53,682–96,065)	3,558 (2,792–5,131)	4,082 (3,400–5,117)	3,015 (2,374–3,386)	3,487 (2,546–3,827)
Skin and subcutaneous diseases	40,508 (24,157–57,103)	3,281 (1,658–5,568)	575 (276–846)	3,921 (2,025–5,927)	7,808 (4,465–12,238)	24,921 (15,660–32,720)
Dermatitis	0 (0–0)	0 (0–0)	0 (0–0)	0 (0–0)	0 (0–0)	0 (0–0)
Psoriasis	0 (0–0)	0 (0–0)	0 (0–0)	0 (0–0)	0 (0–0)	0 (0–0)
Cellulitis	6,401 (3,485–9,321)	323 (115–713)	95 (41–151)	801 (362–1,205)	1,878 (858–2,894)	3,303 (1,850–4,582)
Pyoderma	17,244 (10,289–26,686)	2,777 (1,348–4,959)	320 (155–511)	2,241 (1,086–3,588)	3,863 (2,237–6,713)	8,040 (5,252–11,797)
Scabies	0 (0–0)	0 (0–0)	0 (0–0)	0 (0–0)	0 (0–0)	0 (0–0)
Fungal skin diseases	0 (0–0)	0 (0–0)	0 (0–0)	0 (0–0)	0 (0–0)	0 (0–0)
Viral skin diseases	0 (0–0)	0 (0–0)	0 (0–0)	0 (0–0)	0 (0–0)	0 (0–0)
Acne vulgaris	0 (0–0)	0 (0–0)	0 (0–0)	0 (0–0)	0 (0–0)	0 (0–0)
Alopecia areata	0 (0–0)	0 (0–0)	0 (0–0)	0 (0–0)	0 (0–0)	0 (0–0)
Pruritus	0 (0–0)	0 (0–0)	0 (0–0)	0 (0–0)	0 (0–0)	0 (0–0)
Urticaria	0 (0–0)	0 (0–0)	0 (0–0)	0 (0–0)	0 (0–0)	0 (0–0)
Decubitus ulcer	15,083 (8,098–19,658)	50 (16–102)	83 (35–138)	566 (269–788)	1,723 (853–2,690)	12,659 (6,733–16,123)
Other skin and subcutaneous diseases	1,778 (1,117–2,578)	129 (61–256)	76 (32–119)	311 (167–453)	342 (215–552)	918 (615–1,312)
Sense organ diseases	0 (0–0)	0 (0–0)	0 (0–0)	0 (0–0)	0 (0–0)	0 (0–0)
Glaucoma	0 (0–0)	0 (0–0)	0 (0–0)	0 (0–0)	0 (0–0)	0 (0–0)
Cataract	0 (0–0)	0 (0–0)	0 (0–0)	0 (0–0)	0 (0–0)	0 (0–0)
Macular degeneration	0 (0–0)	0 (0–0)	0 (0–0)	0 (0–0)	0 (0–0)	0 (0–0)
Refraction and accommodation disorders	0 (0–0)	0 (0–0)	0 (0–0)	0 (0–0)	0 (0–0)	0 (0–0)
Age-related and other hearing loss	0 (0–0)	0 (0–0)	0 (0–0)	0 (0–0)	0 (0–0)	0 (0–0)
Other vision loss	0 (0–0)	0 (0–0)	0 (0–0)	0 (0–0)	0 (0–0)	0 (0–0)
Other sense organ diseases	0 (0–0)	0 (0–0)	0 (0–0)	0 (0–0)	0 (0–0)	0 (0–0)
Oral disorders	0 (0–0)	0 (0–0)	0 (0–0)	0 (0–0)	0 (0–0)	0 (0–0)
Deciduous caries	0 (0–0)	0 (0–0)	0 (0–0)	0 (0–0)	0 (0–0)	0 (0–0)
Permanent caries	0 (0–0)	0 (0–0)	0 (0–0)	0 (0–0)	0 (0–0)	0 (0–0)
Periodontal diseases	0 (0–0)	0 (0–0)	0 (0–0)	0 (0–0)	0 (0–0)	0 (0–0)
Edentulism and severe tooth loss	0 (0–0)	0 (0–0)	0 (0–0)	0 (0–0)	0 (0–0)	0 (0–0)
Other oral disorders	0 (0–0)	0 (0–0)	0 (0–0)	0 (0–0)	0 (0–0)	0 (0–0)
Sudden infant death syndrome	9,474 (6,861–13,145)	9,474 (6,861–13,145)	0 (0–0)	0 (0–0)	0 (0–0)	0 (0–0)
Injuries	1,464,111 (1,309,878–1,533,890)	161,871 (127,550–193,122)	102,163 (90,581–110,036)	582,041 (520,430–606,914)	283,573 (253,507–294,384)	334,461 (299,876–347,682)
Transport injuries	383,724 (366,209–400,510)	27,589 (22,512–33,374)	29,684 (27,663–31,813)	174,850 (166,378–183,469)	87,450 (83,955–90,996)	64,150 (61,801–66,609)
Road injuries	362,425 (345,953–378,881)	25,352 (20,926–30,680)	28,187 (26,285–30,217)	165,709 (157,607–174,454)	82,391 (79,070–85,995)	60,784 (58,424–63,316)
Pedestrian road injuries	166,208 (157,428–175,358)	11,294 (9,461–13,495)	13,831 (12,929–14,962)	65,903 (62,023–70,114)	41,199 (38,959–43,978)	33,979 (32,341–36,137)
Cyclist road injuries	16,777 (15,247–18,210)	553 (399–777)	1,348 (1,201–1,508)	8,408 (7,614–9,208)	4,526 (4,106–4,955)	1,939 (1,728–2,144)
Motorcyclist road injuries	48,503 (44,842–52,491)	3,424 (2,648–4,336)	2,969 (2,606–3,357)	25,353 (23,495–27,291)	10,392 (9,372–11,705)	6,362 (5,674–7,376)

SR Table 10 - Global deaths by age and cause for females in 2005

Cause	All ages	0-5 years	5-14 years	15-49 years	50-69 years	70+ years
Motor vehicle road injuries	126,788 (118,989–135,593)	9,664 (7,577–12,297)	9,678 (8,781–10,654)	64,224 (60,348–70,206)	25,368 (23,271–27,050)	17,852 (16,029–19,006)
Other road injuries	4,147 (3,609–4,572)	415 (258–587)	359 (292–432)	1,818 (1,582–2,016)	903 (762–1,029)	650 (558–752)
Other transport injuries	21,299 (18,763–24,107)	2,236 (1,369–3,152)	1,497 (1,243–1,754)	9,140 (8,048–10,416)	5,058 (4,429–5,794)	3,365 (3,019–3,918)
Unintentional injuries	680,792 (566,541–729,795)	123,008 (90,508–150,640)	55,448 (44,673–60,794)	178,577 (147,386–191,114)	108,398 (91,919–114,241)	215,358 (185,935–225,465)
Falls	171,370 (146,338–180,919)	10,987 (6,625–15,279)	5,427 (4,690–6,023)	18,763 (17,296–20,197)	25,608 (23,191–26,988)	110,583 (94,396–116,855)
Drowning	126,016 (71,579–140,940)	36,295 (14,912–45,804)	21,830 (13,704–24,725)	35,103 (20,756–38,866)	15,497 (9,606–16,976)	17,288 (11,481–18,752)
Fire, heat, and hot substances	99,971 (74,344–110,726)	14,709 (9,165–18,136)	6,009 (4,465–7,014)	44,324 (30,918–49,918)	14,653 (11,327–16,169)	20,272 (16,610–21,865)
Poisonings	39,341 (27,391–46,889)	8,124 (3,377–14,595)	2,829 (2,392–3,230)	15,187 (11,444–16,229)	6,971 (5,061–7,471)	6,228 (4,923–6,600)
Exposure to mechanical forces	54,746 (47,686–58,355)	13,858 (11,484–16,033)	4,300 (3,825–5,002)	14,467 (12,513–15,419)	9,259 (8,365–10,092)	12,860 (9,366–14,148)
Unintentional firearm injuries	8,619 (5,844–9,614)	710 (377–1,083)	564 (366–689)	2,693 (1,689–3,132)	1,330 (805–1,498)	3,319 (2,574–3,606)
Unintentional suffocation	12,050 (8,838–13,742)	7,070 (4,752–8,443)	1,004 (778–1,428)	1,417 (1,110–1,587)	801 (623–921)	1,755 (1,259–2,081)
Other exposure to mechanical forces	34,076 (30,035–36,326)	6,077 (4,820–7,443)	2,730 (2,419–3,131)	10,355 (9,030–11,092)	7,127 (6,581–8,099)	7,785 (5,179–8,827)
Adverse effects of medical treatment	42,655 (31,429–51,095)	7,125 (3,948–9,810)	1,855 (1,162–2,378)	10,065 (6,690–12,550)	8,418 (6,248–9,894)	15,190 (12,180–17,150)
Animal contact	44,585 (24,729–49,989)	6,725 (3,605–8,295)	5,808 (3,330–6,992)	17,240 (9,339–19,784)	9,752 (5,253–11,040)	5,057 (2,910–5,640)
Venomous animal contact	38,440 (20,223–43,871)	4,967 (2,511–6,284)	4,948 (2,696–6,035)	15,704 (8,214–18,230)	8,741 (4,530–10,047)	4,077 (2,126–4,619)
Non-venomous animal contact	6,145 (4,399–7,711)	1,758 (1,001–2,478)	859 (605–1,149)	1,536 (1,134–1,913)	1,010 (709–1,218)	980 (781–1,126)
Foreign body	56,432 (45,083–76,178)	18,843 (11,593–33,761)	3,418 (2,372–5,037)	9,042 (7,209–11,161)	7,400 (6,227–8,411)	17,727 (15,951–18,953)
Pulmonary aspiration and foreign body in airway	45,294 (34,062–66,887)	16,817 (8,856–32,167)	2,519 (1,542–4,213)	5,560 (4,314–7,985)	5,593 (4,786–6,836)	14,802 (13,538–16,037)
Foreign body in eyes	0 (0–0)	0 (0–0)	0 (0–0)	0 (0–0)	0 (0–0)	0 (0–0)
Foreign body in other body part	11,138 (7,477–15,502)	2,025 (1,032–3,358)	898 (525–1,378)	3,481 (2,332–4,749)	1,806 (1,225–2,685)	2,924 (2,236–3,888)
Environmental heat and cold exposure	15,969 (11,300–17,837)	1,310 (611–1,697)	448 (271–589)	3,567 (2,973–4,022)	4,922 (3,552–5,651)	5,721 (3,706–6,771)
Other unintentional injuries	29,703 (27,853–31,858)	5,027 (4,164–6,047)	3,519 (3,081–4,001)	10,814 (9,957–11,761)	5,912 (5,440–6,426)	4,428 (4,088–4,790)
Self-harm and interpersonal violence	359,320 (281,034–377,679)	6,034 (5,509–6,587)	9,310 (7,957–9,974)	210,680 (164,138–222,927)	81,730 (63,767–86,302)	51,565 (40,066–54,546)
Self-harm	271,058 (196,458–287,537)	0 (0–0)	4,252 (2,929–4,762)	157,319 (113,512–168,139)	67,087 (48,742–71,345)	42,399 (31,172–45,330)
Interpersonal violence	88,262 (84,191–91,923)	6,034 (5,509–6,587)	5,058 (4,702–5,415)	53,361 (50,545–55,895)	14,642 (13,736–15,368)	9,166 (8,652–9,608)
Assault by firearm	20,325 (16,807–22,404)	824 (660–999)	1,145 (890–1,293)	14,001 (11,504–15,258)	2,755 (2,365–3,243)	1,598 (1,242–1,927)
Assault by sharp object	21,010 (19,771–22,838)	582 (492–678)	802 (716–908)	13,876 (12,909–15,110)	3,485 (3,257–3,800)	2,263 (2,102–2,587)
Assault by other means	46,926 (44,054–50,171)	4,627 (4,173–5,095)	3,110 (2,866–3,458)	25,483 (23,669–27,916)	8,401 (7,671–8,987)	5,303 (4,786–5,647)
Forces of nature, war, and legal intervention	40,273 (26,119–54,454)	5,239 (3,424–7,069)	7,719 (4,665–10,829)	17,932 (11,590–23,995)	5,994 (3,915–8,063)	3,387 (2,054–5,826)
Exposure to forces of nature	32,668 (19,106–46,069)	3,809 (2,165–5,458)	6,824 (3,847–9,928)	14,389 (8,336–20,346)	4,901 (2,930–6,837)	2,743 (1,639–3,987)
Collective violence and legal intervention	7,604 (5,600–10,968)	1,429 (1,001–1,889)	894 (623–1,209)	3,543 (2,635–4,551)	1,093 (840–1,407)	643 (351–3,048)

SR Table 10 - Global deaths by age and cause for females in 2010						
**Cause**	**All ages**	**0-5 years**	**5-14 years**	**15-49 years**	**50-69 years**	**70+ years**
All causes	24,524,648 (24,193,837–24,848,797)	3,202,366 (3,150,721–3,256,040)	439,508 (422,816–458,331)	3,398,973 (3,296,539–3,500,598)	5,273,346 (5,152,283–5,389,242)	12,210,452 (12,086,102–12,331,646)
Communicable, maternal, neonatal, and nutritional diseases	6,003,441 (5,833,198–6,170,642)	2,653,659 (2,574,105–2,724,856)	244,038 (232,466–257,629)	1,380,152 (1,314,703–1,450,020)	645,929 (601,919–689,533)	1,079,661 (996,545–1,133,918)
HIV/AIDS and tuberculosis	1,209,749 (1,122,029–1,309,496)	80,623 (76,610–86,444)	56,803 (54,290–60,684)	710,604 (666,657–762,146)	232,067 (199,300–265,486)	129,651 (100,167–154,648)
Tuberculosis	433,376 (353,237–521,172)	13,145 (10,409–18,368)	8,436 (7,015–11,601)	139,786 (117,079–170,700)	150,901 (118,077–181,251)	121,105 (92,337–146,010)
HIV/AIDS	776,373 (737,734–819,615)	67,477 (64,766–70,441)	48,366 (46,541–50,185)	570,817 (535,318–609,761)	81,165 (74,688–88,499)	8,546 (7,589–9,882)
HIV/AIDS - Tuberculosis	137,094 (104,283–153,263)	16,760 (12,783–18,927)	9,651 (7,117–11,075)	87,272 (64,859–98,311)	20,898 (16,861–23,079)	2,510 (2,128–2,887)
HIV/AIDS resulting in other diseases	639,278 (597,267–687,826)	50,717 (47,348–55,205)	38,714 (36,284–41,650)	483,545 (446,443–525,246)	60,266 (54,194–67,995)	6,035 (5,271–7,317)
Diarrhea, lower respiratory, and other common infectious diseases	2,537,641 (2,405,889–2,653,636)	986,670 (920,997–1,057,161)	134,725 (121,406–150,352)	250,457 (227,149–276,517)	323,036 (293,503–348,651)	842,751 (771,608–885,597)
Diarrheal diseases	748,293 (701,969–799,332)	314,061 (279,071–353,960)	29,881 (26,096–33,801)	78,846 (72,107–87,095)	115,340 (106,292–125,564)	210,163 (196,049–225,266)
Intestinal infectious diseases	86,911 (49,284–142,409)	20,882 (10,938–37,140)	33,089 (19,186–53,918)	26,525 (14,004–45,220)	4,337 (2,216–7,547)	2,075 (1,178–3,252)
Typhoid fever	72,096 (38,918–120,770)	17,426 (8,836–31,621)	27,857 (15,601–44,945)	21,730 (11,366–38,353)	3,468 (1,686–6,125)	1,612 (852–2,642)
Paratyphoid fever	14,176 (6,710–27,330)	3,119 (1,302–6,493)	5,160 (2,411–9,965)	4,713 (2,056–9,334)	827 (351–1,671)	355 (149–674)
Other intestinal infectious diseases	638 (132–1,260)	336 (50–774)	71 (9–185)	81 (18–192)	41 (10–86)	107 (38–239)
Lower respiratory infections	1,312,065 (1,179,837–1,390,032)	426,444 (387,564–468,221)	34,435 (24,480–41,062)	91,644 (72,550–103,507)	165,198 (130,914–183,042)	594,341 (515,845–630,762)
Upper respiratory infections	1,514 (1,286–1,774)	431 (285–655)	24 (15–38)	252 (207–310)	330 (260–416)	474 (376–592)
Otitis media	1,668 (1,467–1,996)	542 (467–631)	160 (144–178)	377 (357–402)	202 (182–227)	385 (248–706)
Meningitis	178,978 (151,900–210,176)	89,951 (68,823–118,336)	16,778 (13,892–19,984)	31,248 (25,870–36,609)	21,579 (17,745–25,813)	19,422 (16,266–22,418)
Pneumococcal meningitis	53,980 (44,086–66,999)	24,184 (16,509–34,530)	6,322 (4,509–8,612)	11,285 (8,890–14,031)	6,453 (4,814–8,406)	5,734 (4,464–7,138)
H influenzae type B meningitis	38,010 (29,790–48,839)	25,269 (18,068–35,511)	3,473 (2,277–4,934)	3,936 (2,893–5,126)	2,673 (1,978–3,576)	2,658 (2,048–3,382)
Meningococcal meningitis	34,718 (26,655–45,959)	19,453 (12,718–30,101)	3,360 (2,215–4,780)	5,415 (4,065–7,011)	3,605 (2,631–4,791)	2,882 (2,200–3,765)
Other meningitis	52,270 (43,585–61,797)	21,043 (15,145–29,391)	3,621 (2,522–5,071)	10,610 (8,547–12,924)	8,847 (7,075–10,801)	8,146 (6,605–9,777)
Encephalitis	65,948 (58,302–80,494)	22,198 (17,315–25,881)	7,808 (6,395–9,731)	13,632 (12,128–17,007)	10,551 (9,266–14,403)	11,757 (10,434–15,485)
Diphtheria	1,536 (653–4,011)	1,179 (339–3,573)	211 (75–525)	104 (68–162)	40 (21–76)	0 (0–0)
Whooping cough	41,386 (16,439–89,400)	38,414 (15,191–82,037)	2,481 (968–5,349)	424 (150–1,028)	65 (23–159)	0 (0–0)
Tetanus	29,576 (23,967–43,875)	15,633 (12,809–19,232)	1,417 (992–2,470)	5,157 (3,616–9,601)	4,751 (3,376–9,797)	2,617 (1,977–4,968)
Measles	65,780 (24,557–142,668)	55,888 (20,918–120,012)	8,135 (2,820–18,275)	1,707 (553–4,359)	48 (15–122)	0 (0–0)
Varicella and herpes zoster	3,981 (3,318–4,980)	1,041 (681–1,696)	300 (181–510)	535 (421–705)	590 (421–816)	1,514 (1,097–2,164)
Neglected tropical diseases and malaria	528,297 (442,198–614,406)	351,047 (276,716–426,010)	34,013 (23,566–47,963)	72,651 (56,558–96,763)	47,168 (35,407–63,992)	23,418 (17,959–31,492)
Malaria	479,860 (392,650–565,119)	339,497 (263,864–414,741)	26,749 (16,323–40,772)	58,485 (42,189–82,865)	38,081 (26,370–54,545)	17,046 (11,636–24,883)
Chagas disease	3,140 (2,979–3,326)	1 (1–1)	2 (2–3)	322 (296–351)	1,136 (1,058–1,219)	1,677 (1,560–1,804)
Leishmaniasis	6,189 (4,079–8,544)	1,317 (844–1,878)	832 (497–1,304)	3,217 (2,091–4,584)	612 (426–831)	208 (162–260)
Visceral leishmaniasis	6,189 (4,079–8,544)	1,317 (844–1,878)	832 (497–1,304)	3,217 (2,091–4,584)	612 (426–831)	208 (162–260)
Cutaneous and mucocutaneous leishmaniasis	0 (0–0)	0 (0–0)	0 (0–0)	0 (0–0)	0 (0–0)	0 (0–0)
African trypanosomiasis	2,810 (1,457–4,546)	219 (112–362)	486 (259–804)	1,462 (738–2,388)	515 (249–869)	127 (64–209)
Schistosomiasis	2,659 (2,244–3,103)	27 (17–47)	58 (40–83)	645 (514–797)	1,160 (902–1,501)	767 (569–1,008)
Cysticercosis	222 (183–286)	5 (2–9)	9 (6–13)	113 (92–146)	69 (57–88)	25 (21–30)
Cystic echinococcosis	696 (651–748)	30 (25–37)	57 (49–65)	226 (209–249)	200 (186–217)	180 (170–190)
Lymphatic filariasis	0 (0–0)	0 (0–0)	0 (0–0)	0 (0–0)	0 (0–0)	0 (0–0)
Onchocerciasis	0 (0–0)	0 (0–0)	0 (0–0)	0 (0–0)	0 (0–0)	0 (0–0)
Trachoma	0 (0–0)	0 (0–0)	0 (0–0)	0 (0–0)	0 (0–0)	0 (0–0)
Dengue	8,112 (5,187–9,973)	2,321 (1,349–3,287)	2,070 (1,207–2,880)	2,028 (1,430–2,486)	1,003 (592–1,274)	688 (355–889)
Yellow fever	1,583 (325–4,473)	161 (31–478)	554 (117–1,584)	694 (142–2,022)	143 (28–413)	29 (5–85)
Rabies	8,956 (7,138–10,944)	1,358 (945–2,075)	1,804 (1,274–2,395)	2,889 (2,076–3,676)	2,038 (1,592–2,487)	864 (679–1,093)
Intestinal nematode infections	1,576 (1,378–1,787)	1,156 (995–1,338)	213 (169–259)	79 (58–105)	62 (41–87)	64 (45–88)
Ascariasis	1,576 (1,378–1,787)	1,156 (995–1,338)	213 (169–259)	79 (58–105)	62 (41–87)	64 (45–88)
Trichuriasis	0 (0–0)	0 (0–0)	0 (0–0)	0 (0–0)	0 (0–0)	0 (0–0)
Hookworm disease	0 (0–0)	0 (0–0)	0 (0–0)	0 (0–0)	0 (0–0)	0 (0–0)
Food-borne trematodiases	0 (0–0)	0 (0–0)	0 (0–0)	0 (0–0)	0 (0–0)	0 (0–0)
Leprosy	0 (0–0)	0 (0–0)	0 (0–0)	0 (0–0)	0 (0–0)	0 (0–0)
Ebola	0 (0–0)	0 (0–0)	0 (0–0)	0 (0–0)	0 (0–0)	0 (0–0)
Other neglected tropical diseases	12,489 (8,678–18,837)	4,949 (3,462–7,248)	1,174 (693–1,990)	2,484 (1,571–4,051)	2,142 (1,241–3,570)	1,738 (1,021–2,853)
Maternal disorders	309,794 (283,378–340,510)	0 (0–0)	1,545 (1,279–1,888)	307,087 (281,040–337,459)	1,161 (853–1,610)	0 (0–0)
Maternal hemorrhage	88,487 (75,415–102,678)	0 (0–0)	470 (332–617)	87,648 (74,654–101,861)	368 (230–600)	0 (0–0)
Maternal sepsis and other maternal infections	23,208 (18,933–28,526)	0 (0–0)	68 (47–100)	23,050 (18,805–28,356)	88 (51–153)	0 (0–0)
Maternal hypertensive disorders	54,657 (45,860–64,952)	0 (0–0)	334 (232–461)	54,230 (45,432–64,490)	93 (58–144)	0 (0–0)
Maternal obstructed labor and uterine rupture	26,651 (21,761–32,430)	0 (0–0)	45 (28–68)	26,555 (21,663–32,315)	50 (30–80)	0 (0–0)
Maternal abortion, miscarriage, and ectopic pregnancy	38,142 (31,342–45,922)	0 (0–0)	199 (132–291)	37,751 (31,001–45,542)	191 (124–284)	0 (0–0)

SR Table 10 - Global deaths by age and cause for females in 2010						
**Cause**	**All ages**	**0-5 years**	**5-14 years**	**15-49 years**	**50-69 years**	**70+ years**
Indirect maternal deaths	34,002 (27,192–41,477)	0 (0–0)	207 (138–304)	33,670 (26,880–41,121)	123 (77–200)	0 (0–0)
Late maternal deaths	7,302 (4,824–10,563)	0 (0–0)	42 (20–77)	7,217 (4,752–10,441)	42 (23–73)	0 (0–0)
Maternal deaths aggravated by HIV/AIDS	2,493 (1,581–3,440)	0 (0–0)	3 (1–4)	2,479 (1,574–3,422)	10 (5–17)	0 (0–0)
Other maternal disorders	34,848 (29,561–41,490)	0 (0–0)	173 (122–241)	34,481 (29,169–41,149)	193 (129–281)	0 (0–0)
Neonatal disorders	1,065,391 (1,034,418–1,099,002)	1,065,391 (1,034,418–1,099,002)	0 (0–0)	0 (0–0)	0 (0–0)	0 (0–0)
Neonatal preterm birth complications	428,883 (385,218–476,491)	428,883 (385,218–476,491)	0 (0–0)	0 (0–0)	0 (0–0)	0 (0–0)
Neonatal encephalopathy due to birth asphyxia and trauma	361,476 (314,984–416,087)	361,476 (314,984–416,087)	0 (0–0)	0 (0–0)	0 (0–0)	0 (0–0)
Neonatal sepsis and other neonatal infections	132,265 (64,867–201,341)	132,265 (64,867–201,341)	0 (0–0)	0 (0–0)	0 (0–0)	0 (0–0)
Hemolytic disease and other neonatal jaundice	27,243 (14,738–45,490)	27,243 (14,738–45,490)	0 (0–0)	0 (0–0)	0 (0–0)	0 (0–0)
Other neonatal disorders	115,522 (81,597–161,287)	115,522 (81,597–161,287)	0 (0–0)	0 (0–0)	0 (0–0)	0 (0–0)
Nutritional deficiencies	207,839 (188,222–234,016)	99,462 (82,195–120,684)	7,475 (6,526–8,571)	16,776 (15,166–19,271)	21,248 (19,199–24,362)	62,875 (58,601–68,640)
Protein-energy malnutrition	166,495 (147,812–190,994)	90,124 (73,940–111,326)	5,627 (4,679–6,559)	8,045 (6,838–9,777)	12,477 (10,779–14,703)	50,221 (45,472–55,542)
Iodine deficiency	1,182 (838–1,723)	368 (154–784)	46 (20–96)	172 (111–271)	233 (142–373)	361 (226–566)
Vitamin A deficiency	0 (0–0)	0 (0–0)	0 (0–0)	0 (0–0)	0 (0–0)	0 (0–0)
Iron-deficiency anemia	25,615 (17,070–34,247)	7,200 (3,612–11,062)	1,285 (924–2,028)	6,035 (4,680–7,519)	6,141 (4,508–7,731)	4,953 (2,944–6,918)
Other nutritional deficiencies	14,545 (8,983–20,536)	1,769 (837–2,863)	516 (291–767)	2,524 (1,461–3,586)	2,396 (1,396–3,682)	7,339 (4,758–10,669)
Other communicable, maternal, neonatal, and nutritional diseases	144,726 (118,332–175,742)	70,463 (48,757–98,219)	9,476 (7,207–12,518)	22,574 (21,130–24,275)	21,247 (18,410–22,793)	20,964 (16,457–22,674)
Sexually transmitted diseases excluding HIV	58,675 (35,734–90,144)	47,521 (26,545–76,059)	5,029 (2,823–7,984)	3,013 (2,417–3,664)	1,477 (1,036–1,800)	1,633 (1,182–1,998)
Syphilis	57,867 (34,831–89,278)	47,521 (26,545–76,059)	5,015 (2,810–7,970)	2,670 (2,144–3,249)	1,256 (881–1,530)	1,402 (1,015–1,714)
Chlamydial infection	148 (112–173)	0 (0–0)	3 (2–4)	63 (50–77)	37 (26–45)	43 (32–52)
Gonococcal infection	446 (336–520)	0 (0–0)	6 (4–7)	169 (135–205)	125 (87–152)	145 (104–178)
Trichomoniasis	0 (0–0)	0 (0–0)	0 (0–0)	0 (0–0)	0 (0–0)	0 (0–0)
Genital herpes	0 (0–0)	0 (0–0)	0 (0–0)	0 (0–0)	0 (0–0)	0 (0–0)
Other sexually transmitted diseases	214 (163–250)	0 (0–0)	4 (3–5)	109 (87–133)	58 (41–71)	41 (29–52)
Hepatitis	43,735 (41,387–46,400)	6,674 (5,846–7,623)	2,801 (2,396–3,278)	14,476 (13,461–15,818)	10,911 (10,312–11,655)	8,871 (8,331–9,438)
Acute hepatitis A	6,420 (4,045–8,855)	5,096 (3,612–6,398)	506 (145–1,041)	496 (121–1,231)	202 (43–506)	119 (27–300)
Hepatitis B	23,630 (20,046–27,052)	401 (133–977)	316 (168–526)	7,758 (6,162–9,378)	8,790 (7,437–9,928)	6,364 (4,989–7,517)
Hepatitis C	1,003 (224–2,331)	0 (0–0)	42 (7–112)	235 (48–566)	452 (104–1,089)	272 (51–656)
Acute hepatitis E	12,681 (9,226–16,891)	1,177 (450–2,288)	1,936 (1,405–2,483)	5,986 (4,463–7,741)	1,466 (690–2,716)	2,114 (1,089–3,478)
Other infectious diseases	42,315 (25,798–49,835)	16,267 (7,915–22,769)	1,645 (1,282–2,017)	5,084 (4,063–5,706)	8,857 (6,117–10,079)	10,459 (6,149–11,990)
Non-communicable diseases	17,050,863 (16,797,445–17,348,761)	397,275 (352,982–456,697)	93,589 (86,656–101,565)	1,462,336 (1,415,942–1,516,507)	4,336,480 (4,239,375–4,437,751)	10,761,181 (10,647,427–10,897,507)
Neoplasms	3,412,374 (3,349,780–3,474,368)	16,221 (14,502–19,073)	22,520 (21,173–23,953)	494,915 (478,518–512,748)	1,314,113 (1,285,465–1,343,526)	1,564,603 (1,533,100–1,595,952)
Lip and oral cavity cancer	42,832 (41,245–44,680)	0 (0–0)	0 (0–0)	6,308 (5,894–6,795)	17,086 (16,244–18,033)	19,437 (18,738–20,242)
Nasopharynx cancer	16,903 (16,197–17,694)	0 (0–0)	91 (82–100)	4,878 (4,620–5,174)	7,692 (7,329–8,083)	4,241 (4,032–4,474)
Other pharynx cancer	16,338 (15,514–17,184)	0 (0–0)	0 (0–0)	2,399 (2,213–2,625)	8,006 (7,561–8,504)	5,931 (5,679–6,223)
Esophageal cancer	127,544 (122,119–133,233)	0 (0–0)	0 (0–0)	10,950 (10,257–11,790)	52,546 (49,893–55,526)	64,046 (61,050–67,066)
Stomach cancer	283,567 (276,310–291,205)	0 (0–0)	0 (0–0)	32,019 (30,367–33,503)	98,785 (95,382–102,265)	152,762 (148,689–157,147)
Colon and rectum cancer	342,955 (334,514–351,908)	0 (0–0)	0 (0–0)	29,358 (28,231–30,615)	109,685 (106,579–113,038)	203,910 (198,418–209,977)
Liver cancer	225,893 (195,990–240,468)	0 (0–0)	382 (331–440)	28,725 (24,251–31,408)	93,588 (80,255–100,169)	103,196 (90,839–109,608)
Liver cancer due to hepatitis B	63,371 (54,027–69,687)	0 (0–0)	171 (146–197)	15,123 (12,732–16,819)	28,776 (24,441–32,476)	19,299 (16,003–21,630)
Liver cancer due to hepatitis C	66,947 (59,369–71,312)	0 (0–0)	13 (10–15)	3,764 (2,962–4,298)	23,328 (19,688–25,680)	39,840 (36,022–42,475)
Liver cancer due to alcohol use	38,201 (33,038–41,622)	0 (0–0)	0 (0–0)	2,228 (1,846–2,585)	18,302 (15,430–20,688)	17,669 (15,411–19,311)
Liver cancer due to other causes	57,373 (48,843–62,241)	0 (0–0)	198 (169–228)	7,608 (6,336–8,466)	23,180 (19,446–25,784)	26,385 (22,657–28,829)
Gallbladder and biliary tract cancer	77,983 (74,258–80,950)	0 (0–0)	0 (0–0)	5,205 (4,756–5,556)	26,663 (25,133–27,847)	46,114 (44,074–47,928)
Pancreatic cancer	173,178 (169,659–176,773)	0 (0–0)	0 (0–0)	9,627 (9,334–9,927)	60,592 (59,252–61,944)	102,958 (100,453–105,619)
Larynx cancer	17,840 (16,741–18,664)	0 (0–0)	0 (0–0)	2,357 (2,115–2,556)	7,818 (7,311–8,240)	7,664 (7,255–8,078)
Tracheal, bronchus, and lung cancer	472,184 (460,688–483,501)	0 (0–0)	0 (0–0)	39,071 (37,807–40,518)	191,083 (186,424–195,788)	242,030 (235,265–248,808)
Malignant skin melanoma	24,684 (21,809–25,774)	0 (0–0)	0 (0–0)	4,290 (4,003–4,742)	8,510 (7,637–8,939)	11,883 (10,078–12,615)
Non-melanoma skin cancer	14,237 (13,692–14,847)	0 (0–0)	0 (0–0)	1,306 (1,238–1,385)	3,392 (3,210–3,582)	9,537 (9,093–10,044)
Non-melanoma skin cancer (squamous-cell carcinoma)	14,237 (13,692–14,847)	0 (0–0)	0 (0–0)	1,306 (1,238–1,385)	3,392 (3,210–3,582)	9,537 (9,093–10,044)
Non-melanoma skin cancer (basal-cell carcinoma)	0 (0–0)	0 (0–0)	0 (0–0)	0 (0–0)	0 (0–0)	0 (0–0)
Breast cancer	471,147 (446,576–487,370)	0 (0–0)	0 (0–0)	99,952 (92,480–105,712)	208,499 (197,234–216,652)	162,695 (153,348–168,140)
Cervical cancer	228,147 (215,835–238,407)	0 (0–0)	0 (0–0)	60,438 (56,287–64,594)	106,785 (99,988–112,654)	60,924 (58,855–63,067)
Uterine cancer	85,230 (81,990–88,203)	0 (0–0)	0 (0–0)	9,755 (9,278–10,309)	38,940 (37,271–40,780)	36,535 (34,725–37,876)
Ovarian cancer	144,674 (141,172–149,479)	0 (0–0)	0 (0–0)	24,503 (23,494–26,015)	66,179 (64,462–68,462)	53,991 (52,564–55,490)
Prostate cancer	0 (0–0)	0 (0–0)	0 (0–0)	0 (0–0)	0 (0–0)	0 (0–0)
Testicular cancer	0 (0–0)	0 (0–0)	0 (0–0)	0 (0–0)	0 (0–0)	0 (0–0)

SR Table 10 - Global deaths by age and cause for females in 2010

Cause	All ages	0-5 years	5-14 years	15-49 years	50-69 years	70+ years
Kidney cancer	42,698 (41,641–43,867)	868 (705–1,209)	625 (560–707)	3,772 (3,647–3,936)	14,760 (14,379–15,180)	22,671 (21,990–23,402)
Bladder cancer	45,594 (44,341–46,938)	0 (0–0)	0 (0–0)	2,205 (2,113–2,306)	11,689 (11,276–12,118)	31,698 (30,708–32,717)
Brain and nervous system cancer	92,183 (89,624–95,099)	2,315 (2,091–2,631)	4,412 (4,066–4,782)	22,623 (21,681–23,600)	35,870 (34,848–37,204)	26,961 (25,441–27,812)
Thyroid cancer	16,882 (15,967–18,492)	0 (0–0)	36 (33–39)	1,474 (1,359–1,668)	5,798 (5,359–6,469)	9,572 (9,074–10,545)
Mesothelioma	8,397 (8,129–8,668)	0 (0–0)	0 (0–0)	1,300 (1,216–1,388)	3,007 (2,820–3,130)	4,089 (3,932–4,251)
Hodgkin lymphoma	8,793 (7,472–11,796)	90 (63–119)	197 (140–255)	3,051 (2,475–4,111)	2,749 (2,325–3,924)	2,704 (2,319–3,472)
Non-Hodgkin lymphoma	87,223 (74,158–92,932)	1,300 (940–2,004)	1,750 (1,374–1,999)	13,201 (11,478–14,618)	27,879 (24,671–30,345)	43,092 (34,453–45,946)
Multiple myeloma	42,751 (40,971–44,271)	0 (0–0)	0 (0–0)	3,361 (3,205–3,564)	16,253 (15,626–16,996)	23,136 (21,151–24,120)
Leukemia	140,085 (136,095–144,762)	6,075 (5,302–7,109)	9,764 (9,106–10,681)	34,276 (32,987–35,858)	37,826 (36,680–39,118)	52,143 (50,308–53,799)
Acute lymphoid leukemia	43,647 (41,435–48,045)	3,997 (3,417–5,006)	6,794 (6,224–7,677)	13,392 (12,665–15,111)	10,535 (9,939–11,504)	8,927 (8,369–9,482)
Chronic lymphoid leukemia	25,285 (24,049–26,689)	0 (0–0)	0 (0–0)	3,447 (3,012–3,822)	6,657 (6,202–7,198)	15,179 (14,511–15,907)
Acute myeloid leukemia	56,497 (54,137–58,391)	2,077 (1,646–2,509)	2,969 (2,701–3,239)	13,792 (13,025–14,488)	15,971 (15,282–16,574)	21,686 (20,865–22,468)
Chronic myeloid leukemia	14,655 (13,649–15,723)	0 (0–0)	0 (0–0)	3,644 (3,164–4,148)	4,661 (4,334–5,043)	6,349 (5,994–6,743)
Other neoplasms	162,421 (146,579–169,301)	5,570 (4,739–7,437)	5,259 (4,736–5,776)	38,499 (32,600–41,856)	52,419 (48,349–55,112)	60,670 (53,521–62,849)
Cardiovascular diseases	8,033,516 (7,884,415–8,186,554)	24,481 (22,914–26,317)	13,140 (12,142–14,375)	460,606 (444,675–481,257)	1,763,086 (1,718,350–1,815,159)	5,772,201 (5,661,021–5,888,323)
Rheumatic heart disease	180,542 (166,467–193,735)	1,453 (1,136–1,734)	3,381 (2,977–3,897)	41,520 (36,091–45,365)	56,963 (52,016–61,570)	77,223 (73,217–82,105)
Ischemic heart disease	3,751,398 (3,675,226–3,839,281)	1,164 (1,016–1,340)	929 (811–1,075)	166,527 (159,923–175,182)	784,946 (763,836–810,281)	2,797,830 (2,740,519–2,861,188)
Cerebrovascular disease	3,014,518 (2,944,637–3,090,366)	7,692 (6,904–8,676)	4,456 (4,088–4,934)	167,555 (160,568–176,744)	698,532 (675,574–721,997)	2,136,281 (2,087,192–2,187,808)
Ischemic stroke	1,495,454 (1,424,950–1,563,148)	365 (294–448)	266 (225–311)	27,056 (23,419–30,731)	229,969 (209,417–250,649)	1,237,796 (1,184,726–1,283,866)
Hemorrhagic stroke	1,519,064 (1,447,027–1,607,890)	7,327 (6,560–8,293)	4,189 (3,832–4,654)	140,498 (133,215–149,686)	468,563 (444,555–496,678)	898,484 (852,103–960,042)
Hypertensive heart disease	473,132 (401,150–525,950)	229 (174–291)	180 (147–220)	28,712 (24,015–32,207)	114,918 (97,888–129,877)	329,091 (280,840–364,817)
Cardiomyopathy and myocarditis	145,545 (141,061–150,463)	5,737 (5,061–6,344)	1,751 (1,631–1,900)	22,761 (21,391–23,905)	28,991 (27,926–30,161)	86,303 (83,050–89,726)
Atrial fibrillation and flutter	94,283 (77,562–113,814)	0 (0–0)	0 (0–0)	326 (241–425)	4,531 (3,501–5,978)	89,425 (72,713–109,055)
Aortic aneurysm	53,038 (51,458–54,886)	0 (0–0)	0 (0–0)	3,369 (3,169–3,660)	10,885 (10,563–11,362)	38,783 (37,433–40,156)
Peripheral vascular disease	22,895 (21,343–24,478)	0 (0–0)	0 (0–0)	197 (185–209)	2,253 (2,119–2,439)	20,444 (18,972–21,994)
Endocarditis	42,161 (35,196–47,834)	1,838 (1,019–2,517)	666 (459–829)	6,998 (4,864–8,217)	7,759 (6,389–9,503)	24,897 (22,178–27,959)
Other cardiovascular and circulatory diseases	256,000 (246,218–266,516)	6,364 (5,686–7,175)	1,774 (1,614–1,967)	22,638 (21,332–24,283)	53,303 (49,846–56,582)	171,919 (164,512–179,658)
Chronic respiratory diseases	1,530,362 (1,474,374–1,584,177)	19,346 (17,448–21,392)	4,986 (4,491–5,547)	77,049 (72,850–81,706)	376,075 (358,644–394,362)	1,052,904 (1,011,662–1,091,087)
Chronic obstructive pulmonary disease	1,258,406 (1,198,723–1,314,796)	7,563 (5,229–9,389)	960 (704–1,310)	40,484 (36,224–45,261)	294,948 (276,179–314,591)	914,449 (873,994–954,256)
Pneumoconiosis	11,366 (7,863–14,500)	0 (0–0)	0 (0–0)	681 (379–932)	2,341 (1,323–3,270)	8,344 (6,106–10,696)
Silicosis	372 (257–517)	0 (0–0)	0 (0–0)	28 (16–37)	87 (54–120)	256 (186–363)
Asbestosis	525 (385–628)	0 (0–0)	0 (0–0)	151 (92–197)	118 (77–148)	255 (215–296)
Coal workers pneumoconiosis	131 (76–183)	0 (0–0)	0 (0–0)	13 (6–18)	37 (18–57)	80 (50–115)
Other pneumoconiosis	10,336 (7,130–13,293)	0 (0–0)	0 (0–0)	487 (262–686)	2,098 (1,170–3,007)	7,750 (5,620–10,035)
Asthma	200,054 (165,103–232,264)	5,570 (4,351–6,928)	3,292 (2,720–3,964)	31,127 (25,945–35,932)	65,415 (51,878–77,295)	94,647 (77,918–113,064)
Interstitial lung disease and pulmonary sarcoidosis	44,458 (32,776–49,805)	161 (102–244)	129 (102–157)	3,005 (2,552–3,450)	11,101 (8,975–12,853)	30,060 (20,509–33,662)
Other chronic respiratory diseases	16,076 (10,898–20,788)	6,050 (4,298–8,297)	603 (350–947)	1,750 (1,057–2,422)	2,268 (1,504–2,983)	5,403 (3,478–7,087)
Cirrhosis and other chronic liver diseases	416,014 (395,706–472,457)	5,272 (4,224–7,624)	4,326 (3,745–5,582)	86,429 (80,894–100,671)	172,895 (163,053–195,529)	147,090 (139,323–163,618)
Cirrhosis and other chronic liver diseases due to hepatitis B	104,089 (94,133–123,284)	24 (17–38)	80 (63–106)	20,572 (18,076–24,770)	43,176 (37,532–52,036)	40,235 (35,794–47,037)
Cirrhosis and other chronic liver diseases due to hepatitis C	95,997 (88,586–105,864)	12 (8–21)	51 (39–70)	17,896 (15,912–20,777)	40,650 (35,984–45,886)	37,385 (34,016–41,603)
Cirrhosis and other chronic liver diseases due to alcohol use	94,688 (87,429–107,147)	0 (0–0)	0 (0–0)	23,316 (21,143–27,449)	46,344 (41,415–53,023)	25,027 (22,262–28,424)
Cirrhosis and other chronic liver diseases due to other causes	121,238 (112,718–140,445)	5,235 (4,194–7,564)	4,193 (3,635–5,411)	24,643 (22,222–28,872)	42,724 (37,828–49,403)	44,441 (40,734–50,057)
Digestive diseases	518,422 (499,041–540,557)	14,138 (7,848–26,927)	7,587 (6,782–8,624)	74,490 (70,340–78,987)	131,284 (125,456–137,202)	290,921 (282,011–300,849)
Peptic ulcer disease	110,958 (102,837–121,278)	1,645 (739–3,989)	1,220 (965–1,504)	16,316 (14,534–18,354)	31,197 (28,258–34,456)	60,579 (56,652–65,999)
Gastritis and duodenitis	20,951 (13,436–29,758)	348 (132–708)	224 (135–363)	3,003 (1,894–4,394)	5,729 (3,490–8,212)	11,645 (7,560–16,405)
Appendicitis	23,754 (17,421–28,748)	2,924 (1,087–6,899)	1,414 (1,169–1,655)	6,900 (5,174–7,855)	5,733 (4,298–6,461)	6,780 (5,361–7,594)
Paralytic ileus and intestinal obstruction	111,612 (95,483–128,152)	4,885 (2,674–9,234)	2,576 (2,130–3,232)	17,681 (14,121–20,737)	27,176 (22,698–32,519)	59,292 (51,037–66,941)
Inguinal, femoral, and abdominal hernia	20,978 (13,753–24,808)	317 (115–837)	289 (170–414)	2,480 (1,241–3,313)	5,999 (3,579–7,316)	11,891 (8,520–13,925)
Inflammatory bowel disease	23,337 (20,730–26,334)	1,209 (498–2,893)	269 (199–373)	3,690 (2,921–4,637)	4,961 (4,412–5,451)	13,204 (11,960–14,052)
Vascular intestinal disorders	52,161 (47,059–58,181)	85 (34–207)	43 (29–62)	2,106 (1,560–2,785)	10,024 (8,499–11,899)	39,902 (36,788–43,797)
Gallbladder and biliary diseases	58,906 (55,144–63,037)	1,058 (466–2,205)	636 (547–757)	6,782 (6,067–7,592)	14,765 (13,435–16,073)	35,662 (33,780–38,210)
Pancreatitis	45,022 (41,034–48,718)	121 (60–244)	289 (234–361)	10,757 (9,383–12,006)	14,917 (13,317–16,432)	18,936 (17,525–20,307)
Other digestive diseases	50,740 (46,508–55,119)	1,542 (731–3,436)	623 (489–769)	4,769 (4,009–5,448)	10,778 (9,605–12,020)	33,026 (30,742–35,583)
Neurological disorders	1,146,642 (976,533–1,314,643)	7,838 (6,815–9,891)	5,581 (4,948–6,330)	31,291 (29,359–34,370)	75,954 (64,730–87,954)	1,025,976 (860,357–1,189,367)
Alzheimer disease and other dementias	1,010,976 (840,234–1,178,867)	0 (0–0)	0 (0–0)	657 (416–959)	46,752 (35,328–58,621)	963,566 (797,179–1,128,126)
Parkinson disease	43,614 (41,960–45,380)	0 (0–0)	0 (0–0)	257 (236–278)	4,543 (4,265–4,830)	38,813 (37,266–40,388)
Epilepsy	46,275 (43,437–49,801)	5,690 (4,816–7,050)	4,448 (3,867–5,113)	21,413 (19,804–23,378)	7,239 (6,878–7,659)	7,483 (7,115–7,832)

SR Table 10 - Global deaths by age and cause for females in 2010						
**Cause**	**All ages**	**0-5 years**	**5-14 years**	**15-49 years**	**50-69 years**	**70+ years**
Multiple sclerosis	10,324 (8,968–11,291)	0 (0–0)	0 (0–0)	3,014 (2,766–3,668)	4,895 (4,093–5,263)	2,413 (1,805–2,586)
Motor neuron disease	13,912 (13,420–14,649)	506 (367–659)	48 (41–55)	1,160 (1,098–1,294)	5,993 (5,760–6,412)	6,203 (5,965–6,460)
Migraine	0 (0–0)	0 (0–0)	0 (0–0)	0 (0–0)	0 (0–0)	0 (0–0)
Tension-type headache	0 (0–0)	0 (0–0)	0 (0–0)	0 (0–0)	0 (0–0)	0 (0–0)
Medication overuse headache	0 (0–0)	0 (0–0)	0 (0–0)	0 (0–0)	0 (0–0)	0 (0–0)
Other neurological disorders	21,550 (20,778–24,384)	1,651 (1,459–2,750)	1,084 (961–1,607)	4,788 (4,551–5,888)	6,530 (6,344–6,742)	7,496 (7,196–7,832)
Mental and substance use disorders	71,327 (67,339–73,709)	18 (16–20)	23 (10–31)	33,585 (32,255–35,905)	23,294 (21,834–24,111)	14,405 (11,771–15,405)
Schizophrenia	7,788 (7,456–8,166)	0 (0–0)	0 (0–0)	2,199 (2,064–2,349)	2,697 (2,567–2,839)	2,891 (2,733–3,048)
Alcohol use disorders	22,125 (21,412–22,902)	0 (0–0)	0 (0–0)	8,465 (8,067–8,900)	10,055 (9,584–10,555)	3,603 (3,467–3,731)
Drug use disorders	40,938 (36,932–43,084)	18 (16–20)	0 (0–0)	22,468 (21,296–24,588)	10,541 (9,042–11,135)	7,910 (5,295–8,871)
Opioid use disorders	27,144 (23,149–28,839)	18 (16–20)	0 (0–0)	14,934 (13,481–16,202)	6,907 (5,540–7,404)	5,284 (3,256–5,982)
Cocaine use disorders	2,750 (2,536–3,075)	0 (0–0)	0 (0–0)	1,688 (1,555–1,894)	900 (810–1,014)	161 (133–210)
Amphetamine use disorders	1,484 (1,300–1,884)	0 (0–0)	0 (0–0)	921 (812–1,165)	420 (360–523)	142 (102–205)
Cannabis use disorders	0 (0–0)	0 (0–0)	0 (0–0)	0 (0–0)	0 (0–0)	0 (0–0)
Other drug use disorders	9,559 (8,469–12,125)	0 (0–0)	0 (0–0)	4,924 (4,108–6,581)	2,312 (2,065–2,868)	2,322 (1,664–3,071)
Depressive disorders	0 (0–0)	0 (0–0)	0 (0–0)	0 (0–0)	0 (0–0)	0 (0–0)
Major depressive disorder	0 (0–0)	0 (0–0)	0 (0–0)	0 (0–0)	0 (0–0)	0 (0–0)
Dysthymia	0 (0–0)	0 (0–0)	0 (0–0)	0 (0–0)	0 (0–0)	0 (0–0)
Bipolar disorder	0 (0–0)	0 (0–0)	0 (0–0)	0 (0–0)	0 (0–0)	0 (0–0)
Anxiety disorders	0 (0–0)	0 (0–0)	0 (0–0)	0 (0–0)	0 (0–0)	0 (0–0)
Eating disorders	474 (295–686)	0 (0–0)	23 (10–31)	451 (285–658)	0 (0–0)	0 (0–0)
Anorexia nervosa	438 (275–630)	0 (0–0)	22 (10–29)	415 (265–602)	0 (0–0)	0 (0–0)
Bulimia nervosa	36 (16–57)	0 (0–0)	0 (0–1)	35 (15–56)	0 (0–0)	0 (0–0)
Autistic spectrum disorders	0 (0–0)	0 (0–0)	0 (0–0)	0 (0–0)	0 (0–0)	0 (0–0)
Autism	0 (0–0)	0 (0–0)	0 (0–0)	0 (0–0)	0 (0–0)	0 (0–0)
Asperger syndrome and other autistic spectrum disorders	0 (0–0)	0 (0–0)	0 (0–0)	0 (0–0)	0 (0–0)	0 (0–0)
Attention-deficit/hyperactivity disorder	0 (0–0)	0 (0–0)	0 (0–0)	0 (0–0)	0 (0–0)	0 (0–0)
Conduct disorder	0 (0–0)	0 (0–0)	0 (0–0)	0 (0–0)	0 (0–0)	0 (0–0)
Idiopathic developmental intellectual disability	0 (0–0)	0 (0–0)	0 (0–0)	0 (0–0)	0 (0–0)	0 (0–0)
Other mental and substance use disorders	0 (0–0)	0 (0–0)	0 (0–0)	0 (0–0)	0 (0–0)	0 (0–0)
Diabetes, urogenital, blood, and endocrine diseases	1,512,192 (1,470,047–1,559,153)	53,861 (41,460–79,952)	15,338 (13,863–17,090)	163,030 (156,505–171,620)	450,040 (434,894–467,199)	829,922 (808,099–853,702)
Diabetes mellitus	673,280 (647,342–698,190)	994 (767–1,134)	1,399 (1,043–1,599)	50,915 (48,207–53,934)	234,078 (223,158–244,857)	385,891 (370,819–400,303)
Acute glomerulonephritis	5,242 (3,763–6,036)	147 (114–191)	153 (120–179)	1,203 (838–1,362)	1,573 (1,050–1,858)	2,164 (1,510–2,569)
Chronic kidney disease	535,870 (518,701–555,707)	6,525 (5,938–7,158)	4,310 (3,893–4,789)	63,494 (60,702–67,059)	154,496 (148,679–161,231)	307,043 (296,829–318,830)
Chronic kidney disease due to diabetes mellitus	178,420 (170,931–187,177)	1,099 (921–1,298)	463 (386–560)	15,774 (14,256–17,290)	64,458 (60,294–69,388)	96,622 (91,659–101,966)
Chronic kidney disease due to hypertension	242,629 (231,785–253,286)	2,275 (2,008–2,580)	1,369 (1,166–1,601)	25,577 (23,640–27,648)	61,304 (56,935–65,753)	152,102 (144,747–159,939)
Chronic kidney disease due to glomerulonephritis	101,602 (95,862–107,829)	2,283 (1,973–2,626)	1,832 (1,578–2,117)	19,277 (17,802–21,241)	27,025 (24,765–29,656)	51,182 (47,593–55,112)
Chronic kidney disease due to other causes	13,217 (11,150–15,581)	866 (710–1,044)	644 (507–800)	2,865 (1,907–3,963)	1,706 (1,063–2,547)	7,135 (5,993–8,813)
Urinary diseases and male infertility	119,867 (114,124–126,369)	2,664 (2,101–3,180)	888 (785–1,018)	14,116 (13,164–15,812)	26,615 (24,939–29,199)	75,583 (70,324–78,837)
Interstitial nephritis and urinary tract infections	98,162 (92,806–103,418)	2,100 (1,613–2,507)	574 (498–665)	10,395 (9,653–11,511)	19,611 (18,254–21,413)	65,481 (60,593–68,760)
Urolithiasis	7,041 (5,665–8,192)	0 (0–0)	103 (86–130)	1,171 (900–1,411)	2,294 (1,701–2,743)	3,472 (2,932–4,218)
Benign prostatic hyperplasia	0 (0–0)	0 (0–0)	0 (0–0)	0 (0–0)	0 (0–0)	0 (0–0)
Male infertility	0 (0–0)	0 (0–0)	0 (0–0)	0 (0–0)	0 (0–0)	0 (0–0)
Other urinary diseases	14,663 (13,204–17,218)	563 (380–819)	209 (167–271)	2,549 (2,217–3,106)	4,710 (4,110–5,649)	6,629 (5,886–8,136)
Gynecological diseases	7,701 (5,727–8,736)	0 (0–0)	0 (0–0)	3,236 (2,439–3,722)	2,343 (1,659–2,701)	2,121 (1,609–2,469)
Uterine fibroids	2,255 (1,214–2,884)	0 (0–0)	0 (0–0)	763 (427–993)	943 (507–1,200)	548 (263–757)
Polycystic ovarian syndrome	650 (193–1,083)	0 (0–0)	0 (0–0)	448 (113–768)	201 (76–324)	0 (0–0)
Female infertility	0 (0–0)	0 (0–0)	0 (0–0)	0 (0–0)	0 (0–0)	0 (0–0)
Endometriosis	52 (41–77)	0 (0–0)	0 (0–0)	43 (34–65)	8 (6–13)	0 (0–0)
Genital prolapse	964 (549–1,594)	0 (0–0)	0 (0–0)	57 (27–104)	330 (158–607)	576 (343–897)
Premenstrual syndrome	0 (0–0)	0 (0–0)	0 (0–0)	0 (0–0)	0 (0–0)	0 (0–0)
Other gynecological diseases	3,778 (2,800–4,569)	0 (0–0)	0 (0–0)	1,922 (1,459–2,358)	858 (598–1,082)	996 (720–1,263)
Hemoglobinopathies and hemolytic anemias	103,445 (90,807–128,488)	38,201 (26,146–64,280)	7,078 (6,096–8,416)	19,027 (17,468–20,736)	13,926 (13,215–14,727)	25,212 (23,788–26,356)
Thalassemias	9,136 (7,815–11,151)	4,386 (3,271–6,409)	1,460 (1,254–1,650)	1,032 (915–1,178)	65 (60–71)	2,191 (1,893–2,603)
Thalassemias trait	0 (0–0)	0 (0–0)	0 (0–0)	0 (0–0)	0 (0–0)	0 (0–0)
Sickle cell disorders	50,303 (38,335–75,073)	33,425 (21,805–58,508)	5,063 (4,104–6,495)	8,239 (7,120–9,504)	776 (679–879)	2,798 (2,388–3,271)
Sickle cell trait	0 (0–0)	0 (0–0)	0 (0–0)	0 (0–0)	0 (0–0)	0 (0–0)

SR Table 10 - Global deaths by age and cause for females in 2010						
Cause	All ages	0-5 years	5-14 years	15-49 years	50-69 years	70+ years
G6PD deficiency	8,692 (7,737–9,799)	247 (165–386)	356 (286–431)	4,628 (4,092–5,281)	2,771 (2,439–3,177)	688 (596–791)
G6PD trait	0 (0–0)	0 (0–0)	0 (0–0)	0 (0–0)	0 (0–0)	0 (0–0)
Other hemoglobinopathies and hemolytic anemias	35,312 (33,530–36,901)	142 (108–204)	198 (172–227)	5,125 (4,732–5,550)	10,313 (9,749–10,938)	19,533 (18,204–20,456)
Endocrine, metabolic, blood, and immune disorders	66,783 (65,134–68,648)	5,328 (4,842–5,896)	1,507 (1,427–1,590)	11,035 (10,665–11,457)	17,006 (16,507–17,511)	31,905 (30,806–33,101)
Musculoskeletal disorders	55,965 (51,354–60,561)	0 (0–0)	1,168 (969–1,346)	13,155 (11,799–14,860)	13,562 (12,596–15,255)	28,079 (25,484–29,529)
Rheumatoid arthritis	18,959 (16,756–22,481)	0 (0–0)	135 (81–192)	1,384 (1,052–1,759)	5,688 (4,939–6,823)	11,750 (10,502–13,900)
Osteoarthritis	0 (0–0)	0 (0–0)	0 (0–0)	0 (0–0)	0 (0–0)	0 (0–0)
Low back and neck pain	0 (0–0)	0 (0–0)	0 (0–0)	0 (0–0)	0 (0–0)	0 (0–0)
Low back pain	0 (0–0)	0 (0–0)	0 (0–0)	0 (0–0)	0 (0–0)	0 (0–0)
Neck pain	0 (0–0)	0 (0–0)	0 (0–0)	0 (0–0)	0 (0–0)	0 (0–0)
Gout	0 (0–0)	0 (0–0)	0 (0–0)	0 (0–0)	0 (0–0)	0 (0–0)
Other musculoskeletal disorders	37,006 (32,828–39,811)	0 (0–0)	1,032 (874–1,202)	11,770 (10,494–13,276)	7,874 (6,983–8,963)	16,328 (13,345–17,466)
Other non-communicable diseases	354,032 (300,697–415,654)	256,085 (215,083–312,269)	18,916 (15,715–22,549)	27,782 (22,753–31,252)	16,171 (11,906–20,535)	35,075 (23,668–43,053)
Congenital anomalies	298,931 (251,271–358,285)	243,294 (203,414–299,537)	18,323 (15,256–22,007)	23,716 (19,580–26,544)	7,494 (6,060–8,088)	6,102 (4,721–6,648)
Neural tube defects	33,933 (21,988–50,668)	32,096 (20,589–48,162)	1,034 (600–1,628)	582 (419–825)	147 (109–238)	72 (57–100)
Congenital heart anomalies	147,263 (112,136–188,752)	114,255 (85,204–153,596)	12,639 (9,900–15,227)	16,242 (12,783–18,286)	2,251 (1,800–2,547)	1,875 (1,526–2,085)
Cleft lip and cleft palate	870 (556–1,110)	870 (556–1,110)	0 (0–0)	0 (0–0)	0 (0–0)	0 (0–0)
Down syndrome	15,475 (6,609–28,741)	11,297 (3,602–23,781)	485 (254–791)	2,054 (1,387–2,792)	1,530 (1,114–1,782)	107 (72–137)
Turner syndrome	0 (0–0)	0 (0–0)	0 (0–0)	0 (0–0)	0 (0–0)	0 (0–0)
Klinefelter syndrome	0 (0–0)	0 (0–0)	0 (0–0)	0 (0–0)	0 (0–0)	0 (0–0)
Other chromosomal abnormalities	14,222 (6,164–32,676)	13,464 (5,555–31,650)	244 (125–496)	321 (237–391)	140 (79–184)	52 (38–62)
Other congenital anomalies	87,166 (72,124–116,679)	71,310 (57,289–98,870)	3,920 (3,221–5,540)	4,515 (3,811–5,626)	3,425 (2,665–3,837)	3,994 (2,921–4,386)
Skin and subcutaneous diseases	45,858 (27,464–62,909)	3,549 (1,777–5,973)	592 (288–881)	4,065 (2,168–6,392)	8,676 (5,004–13,464)	28,973 (17,916–37,527)
Dermatitis	0 (0–0)	0 (0–0)	0 (0–0)	0 (0–0)	0 (0–0)	0 (0–0)
Psoriasis	0 (0–0)	0 (0–0)	0 (0–0)	0 (0–0)	0 (0–0)	0 (0–0)
Cellulitis	7,442 (3,993–10,507)	338 (123–748)	96 (42–153)	851 (396–1,256)	2,092 (1,010–3,081)	4,062 (2,219–5,441)
Pyoderma	20,088 (12,383–31,177)	3,015 (1,461–5,285)	330 (165–538)	2,329 (1,186–3,859)	4,436 (2,618–7,610)	9,976 (6,555–14,441)
Scabies	0 (0–0)	0 (0–0)	0 (0–0)	0 (0–0)	0 (0–0)	0 (0–0)
Fungal skin diseases	0 (0–0)	0 (0–0)	0 (0–0)	0 (0–0)	0 (0–0)	0 (0–0)
Viral skin diseases	0 (0–0)	0 (0–0)	0 (0–0)	0 (0–0)	0 (0–0)	0 (0–0)
Acne vulgaris	0 (0–0)	0 (0–0)	0 (0–0)	0 (0–0)	0 (0–0)	0 (0–0)
Alopecia areata	0 (0–0)	0 (0–0)	0 (0–0)	0 (0–0)	0 (0–0)	0 (0–0)
Pruritus	0 (0–0)	0 (0–0)	0 (0–0)	0 (0–0)	0 (0–0)	0 (0–0)
Urticaria	0 (0–0)	0 (0–0)	0 (0–0)	0 (0–0)	0 (0–0)	0 (0–0)
Decubitus ulcer	16,324 (9,184–21,132)	58 (19–119)	84 (37–133)	553 (271–776)	1,770 (897–2,687)	13,857 (7,590–17,532)
Other skin and subcutaneous diseases	2,003 (1,238–2,936)	137 (66–259)	81 (34–126)	331 (178–495)	377 (230–595)	1,076 (707–1,554)
Sense organ diseases	0 (0–0)	0 (0–0)	0 (0–0)	0 (0–0)	0 (0–0)	0 (0–0)
Glaucoma	0 (0–0)	0 (0–0)	0 (0–0)	0 (0–0)	0 (0–0)	0 (0–0)
Cataract	0 (0–0)	0 (0–0)	0 (0–0)	0 (0–0)	0 (0–0)	0 (0–0)
Macular degeneration	0 (0–0)	0 (0–0)	0 (0–0)	0 (0–0)	0 (0–0)	0 (0–0)
Refraction and accommodation disorders	0 (0–0)	0 (0–0)	0 (0–0)	0 (0–0)	0 (0–0)	0 (0–0)
Age-related and other hearing loss	0 (0–0)	0 (0–0)	0 (0–0)	0 (0–0)	0 (0–0)	0 (0–0)
Other vision loss	0 (0–0)	0 (0–0)	0 (0–0)	0 (0–0)	0 (0–0)	0 (0–0)
Other sense organ diseases	0 (0–0)	0 (0–0)	0 (0–0)	0 (0–0)	0 (0–0)	0 (0–0)
Oral disorders	0 (0–0)	0 (0–0)	0 (0–0)	0 (0–0)	0 (0–0)	0 (0–0)
Deciduous caries	0 (0–0)	0 (0–0)	0 (0–0)	0 (0–0)	0 (0–0)	0 (0–0)
Permanent caries	0 (0–0)	0 (0–0)	0 (0–0)	0 (0–0)	0 (0–0)	0 (0–0)
Periodontal diseases	0 (0–0)	0 (0–0)	0 (0–0)	0 (0–0)	0 (0–0)	0 (0–0)
Edentulism and severe tooth loss	0 (0–0)	0 (0–0)	0 (0–0)	0 (0–0)	0 (0–0)	0 (0–0)
Other oral disorders	0 (0–0)	0 (0–0)	0 (0–0)	0 (0–0)	0 (0–0)	0 (0–0)
Sudden infant death syndrome	9,241 (6,854–13,047)	9,241 (6,854–13,047)	0 (0–0)	0 (0–0)	0 (0–0)	0 (0–0)
Injuries	1,470,344 (1,303,470–1,574,285)	151,431 (122,084–176,142)	101,880 (85,506–118,541)	556,485 (496,405–599,627)	290,937 (260,255–308,992)	369,610 (328,370–386,269)
Transport injuries	367,612 (355,711–381,350)	25,310 (21,527–30,094)	26,070 (24,534–27,919)	159,247 (153,363–166,137)	89,267 (86,088–92,540)	67,716 (65,281–70,172)
Road injuries	347,229 (335,784–360,440)	23,287 (19,814–27,734)	24,775 (23,300–26,470)	150,818 (145,154–156,929)	84,176 (81,132–87,408)	64,170 (61,829–66,549)
Pedestrian road injuries	156,179 (150,146–163,787)	10,348 (8,790–12,278)	11,851 (11,165–12,734)	58,534 (55,725–61,554)	40,735 (38,959–43,272)	34,709 (33,149–36,846)
Cyclist road injuries	15,255 (14,317–16,257)	526 (394–723)	1,158 (1,049–1,293)	7,134 (6,661–7,653)	4,479 (4,157–4,853)	1,957 (1,763–2,169)
Motorcyclist road injuries	51,569 (47,789–55,959)	3,008 (2,415–3,777)	2,856 (2,515–3,269)	25,699 (23,826–27,854)	12,081 (10,975–13,485)	7,922 (7,050–8,997)

SR Table 10 - Global deaths by age and cause for females in 2010						
Cause	All ages	0-5 years	5-14 years	15-49 years	50-69 years	70+ years
Motor vehicle road injuries	120,023 (112,218–126,509)	9,044 (7,416–11,324)	8,579 (7,868–9,476)	57,650 (54,490–62,553)	25,909 (23,194–27,329)	18,840 (16,724–19,894)
Other road injuries	4,201 (3,815–4,599)	360 (250–514)	329 (281–390)	1,800 (1,627–1,984)	971 (843–1,107)	741 (631–848)
Other transport injuries	20,383 (18,152–24,182)	2,022 (1,360–2,868)	1,294 (1,095–1,527)	8,428 (7,582–9,845)	5,091 (4,475–6,074)	3,546 (3,194–4,279)
Unintentional injuries	662,750 (540,309–704,421)	108,699 (80,284–130,744)	48,215 (39,233–53,059)	159,817 (128,051–171,691)	105,913 (88,268–113,072)	240,104 (202,264–251,713)
Falls	191,030 (158,803–201,551)	10,844 (6,419–15,518)	5,388 (4,546–6,162)	18,664 (16,794–20,158)	26,822 (23,841–28,345)	129,309 (107,289–136,802)
Drowning	110,680 (65,659–124,175)	28,812 (13,482–36,087)	18,146 (11,880–21,060)	30,515 (17,878–34,994)	14,924 (9,011–16,912)	18,281 (12,210–20,006)
Fire, heat, and hot substances	88,420 (65,537–99,196)	12,751 (8,885–15,233)	4,966 (3,894–5,927)	37,439 (25,819–43,594)	13,199 (10,099–14,910)	20,062 (16,246–22,233)
Poisonings	36,450 (24,420–43,979)	7,980 (3,081–14,463)	2,410 (1,955–2,769)	13,068 (9,650–14,007)	6,578 (4,705–7,056)	6,412 (5,030–6,818)
Exposure to mechanical forces	55,483 (45,142–59,578)	12,982 (10,268–14,923)	4,054 (3,622–4,551)	14,280 (11,522–15,312)	9,748 (8,427–10,424)	14,417 (10,018–15,786)
Unintentional firearm injuries	8,747 (6,045–9,758)	706 (386–1,023)	536 (349–667)	2,610 (1,630–3,012)	1,342 (832–1,512)	3,553 (2,767–3,854)
Unintentional suffocation	12,240 (8,426–13,947)	6,800 (4,259–8,251)	995 (760–1,350)	1,421 (1,064–1,568)	935 (640–1,058)	2,087 (1,284–2,523)
Other exposure to mechanical forces	34,495 (27,811–37,081)	5,475 (4,566–6,458)	2,522 (2,225–2,841)	10,249 (8,057–11,054)	7,470 (6,524–8,070)	8,776 (5,477–9,836)
Adverse effects of medical treatment	41,996 (30,614–49,330)	6,340 (3,720–8,485)	1,703 (1,090–2,152)	9,298 (6,169–11,351)	8,346 (6,141–9,767)	16,307 (13,015–18,362)
Animal contact	41,992 (21,901–51,415)	5,826 (3,071–7,119)	4,941 (2,727–6,404)	16,172 (8,155–20,408)	9,627 (4,899–11,920)	5,425 (2,975–6,345)
Venomous animal contact	36,324 (17,856–45,697)	4,263 (2,101–5,436)	4,168 (2,170–5,621)	14,786 (7,136–18,976)	8,680 (4,200–10,874)	4,425 (2,165–5,308)
Non-venomous animal contact	5,668 (4,039–6,773)	1,562 (944–2,065)	772 (553–992)	1,386 (1,013–1,699)	947 (661–1,105)	1,000 (806–1,135)
Foreign body	58,152 (47,973–74,566)	18,083 (11,620–30,121)	3,385 (2,464–4,693)	8,829 (7,270–10,828)	7,697 (6,624–8,692)	20,156 (18,183–21,498)
Pulmonary aspiration and foreign body in airway	47,297 (37,246–64,967)	16,169 (9,073–28,391)	2,502 (1,640–3,923)	5,593 (4,459–7,764)	5,965 (5,091–7,066)	17,066 (15,545–18,388)
Foreign body in eyes	0 (0–0)	0 (0–0)	0 (0–0)	0 (0–0)	0 (0–0)	0 (0–0)
Foreign body in other body part	10,855 (7,607–13,402)	1,914 (1,045–3,201)	882 (534–1,234)	3,236 (2,267–3,969)	1,732 (1,223–2,207)	3,090 (2,451–3,679)
Environmental heat and cold exposure	14,310 (10,183–16,260)	1,092 (517–1,399)	401 (239–514)	2,856 (2,257–3,212)	4,260 (3,063–4,995)	5,700 (3,884–6,676)
Other unintentional injuries	24,231 (22,671–25,999)	3,984 (3,313–4,772)	2,817 (2,438–3,252)	8,691 (7,997–9,551)	4,707 (4,346–5,107)	4,031 (3,716–4,406)
Self-harm and interpersonal violence	336,153 (272,044–356,127)	5,322 (4,732–6,062)	8,074 (7,102–8,793)	190,608 (153,441–203,060)	79,555 (63,116–84,505)	52,591 (42,564–55,613)
Self-harm	255,384 (191,335–273,158)	0 (0–0)	3,567 (2,634–4,083)	142,985 (106,284–154,309)	65,878 (49,253–70,468)	42,954 (33,037–45,817)
Interpersonal violence	80,768 (76,936–84,845)	5,322 (4,732–6,062)	4,507 (4,141–4,886)	47,623 (44,772–50,370)	13,677 (12,738–14,577)	9,636 (9,052–10,182)
Assault by firearm	20,764 (17,140–23,243)	853 (658–1,060)	1,118 (878–1,282)	13,945 (11,449–15,379)	2,987 (2,601–3,561)	1,860 (1,403–2,295)
Assault by sharp object	17,724 (16,731–19,536)	501 (416–600)	667 (593–760)	11,284 (10,530–12,408)	2,996 (2,818–3,315)	2,273 (2,100–2,686)
Assault by other means	42,278 (38,612–45,752)	3,967 (3,513–4,529)	2,721 (2,448–3,023)	22,394 (20,399–24,657)	7,694 (6,879–8,447)	5,502 (4,842–5,951)
Forces of nature, war, and legal intervention	103,828 (28,651–186,340)	12,099 (3,844–21,044)	19,519 (4,763–35,767)	46,811 (12,997–83,964)	16,199 (4,588–28,942)	9,197 (2,553–16,587)
Exposure to forces of nature	94,743 (20,028–177,514)	10,318 (2,072–19,346)	18,464 (3,759–34,734)	42,617 (8,939–79,892)	14,783 (3,256–27,415)	8,560 (1,996–15,947)
Collective violence and legal intervention	9,085 (7,164–11,658)	1,781 (1,301–2,326)	1,055 (792–1,374)	4,194 (3,280–5,231)	1,416 (1,048–1,816)	637 (395–2,156)

SR Table 10 - Global deaths by age and cause for females in 2015

Cause	All ages	0-5 years	5-14 years	15-49 years	50-69 years	70+ years
All causes	24,940,690 (24,500,996–25,451,138)	2,697,033 (2,626,311–2,769,638)	372,563 (363,092–384,072)	3,072,209 (2,946,612–3,215,811)	5,572,272 (5,411,134–5,759,018)	13,226,611 (13,041,066–13,417,652)
Communicable, maternal, neonatal, and nutritional diseases	5,271,301 (5,078,971–5,455,889)	2,182,251 (2,113,490–2,254,038)	207,117 (195,833–220,514)	1,142,072 (1,070,540–1,226,773)	607,955 (556,370–660,009)	1,131,905 (1,039,707–1,192,859)
HIV/AIDS and tuberculosis	941,144 (856,323–1,034,187)	53,994 (50,509–59,158)	48,641 (46,403–52,047)	527,834 (491,124–571,225)	194,593 (164,579–223,507)	116,080 (88,333–139,927)
Tuberculosis	385,368 (305,223–470,234)	10,481 (7,782–14,862)	6,739 (5,440–9,596)	119,958 (98,675–148,433)	137,284 (105,901–165,212)	110,904 (83,314–134,828)
HIV/AIDS	555,775 (521,830–592,019)	43,513 (41,230–45,876)	41,902 (40,148–43,545)	407,875 (376,306–440,737)	57,308 (52,933–63,089)	5,176 (4,587–6,094)
HIV/AIDS - Tuberculosis	94,492 (71,083–109,403)	8,884 (6,652–10,187)	7,465 (5,435–8,971)	64,832 (48,315–75,793)	12,170 (9,847–13,766)	1,139 (960–1,297)
HIV/AIDS resulting in other diseases	461,282 (424,892–498,208)	34,628 (32,176–37,740)	34,436 (32,037–36,944)	343,042 (310,667–377,672)	45,138 (40,803–51,002)	4,036 (3,504–4,917)
Diarrhea, lower respiratory, and other common infectious diseases	2,354,237 (2,217,862–2,478,746)	774,118 (720,869–836,237)	117,347 (105,012–131,976)	235,328 (211,075–264,809)	325,178 (291,782–355,931)	902,264 (819,864–956,617)
Diarrheal diseases	638,635 (595,872–686,828)	241,591 (210,827–277,391)	23,507 (20,087–27,154)	68,114 (60,967–76,924)	105,443 (95,695–116,753)	199,979 (185,446–214,599)
Intestinal infectious diseases	82,597 (46,612–135,610)	19,564 (10,398–34,373)	30,603 (17,680–50,135)	25,701 (13,752–44,453)	4,565 (2,311–8,007)	2,162 (1,215–3,453)
Typhoid fever	68,735 (37,607–114,740)	16,445 (8,299–29,713)	25,818 (14,427–41,811)	21,100 (11,092–36,915)	3,664 (1,789–6,491)	1,706 (904–2,778)
Paratyphoid fever	13,478 (6,295–26,125)	2,929 (1,233–6,089)	4,744 (2,190–9,261)	4,551 (2,008–9,011)	874 (360–1,765)	379 (158–732)
Other intestinal infectious diseases	383 (103–805)	189 (37–444)	41 (6–108)	49 (13–124)	27 (8–66)	75 (25–182)
Lower respiratory infections	1,297,428 (1,156,952–1,387,934)	336,728 (306,217–372,579)	32,315 (22,486–39,472)	90,523 (71,319–104,924)	176,136 (140,012–198,327)	661,724 (576,426–709,940)
Upper respiratory infections	1,387 (1,198–1,632)	359 (232–557)	20 (12–33)	223 (180–280)	315 (249–399)	467 (369–591)
Otitis media	1,613 (1,375–2,046)	470 (398–564)	139 (122–158)	354 (330–383)	211 (187–241)	437 (269–855)
Meningitis	168,850 (142,483–207,679)	80,269 (60,903–114,157)	15,422 (12,782–18,712)	30,531 (25,289–36,173)	22,321 (17,925–26,487)	20,304 (16,710–23,209)
Pneumococcal meningitis	50,069 (40,467–64,631)	21,464 (14,235–33,295)	5,740 (3,974–7,980)	10,640 (8,303–13,522)	6,439 (4,734–8,371)	5,784 (4,530–7,241)
H influenzae type B meningitis	32,735 (25,330–43,702)	20,849 (14,224–30,685)	2,940 (1,870–4,272)	3,597 (2,627–4,766)	2,652 (1,917–3,572)	2,695 (2,045–3,504)
Meningococcal meningitis	32,348 (25,053–43,398)	17,318 (11,286–27,890)	3,142 (2,010–4,540)	5,266 (3,898–6,993)	3,652 (2,625–4,868)	2,968 (2,228–3,884)
Other meningitis	53,696 (44,803–64,930)	20,637 (14,354–29,712)	3,598 (2,469–5,111)	11,027 (8,752–13,709)	9,577 (7,583–11,813)	8,856 (6,987–10,621)
Encephalitis	67,890 (59,530–83,865)	21,377 (17,192–25,538)	7,358 (5,854–9,320)	13,949 (12,020–17,549)	11,625 (10,144–15,698)	13,579 (11,992–16,894)
Diphtheria	1,027 (427–2,823)	780 (214–2,590)	144 (50–414)	72 (48–110)	30 (16–54)	0 (0–0)
Whooping cough	32,426 (11,202–69,318)	30,138 (10,516–63,965)	1,929 (661–4,300)	310 (101–742)	47 (16–117)	0 (0–0)
Tetanus	22,613 (17,962–32,635)	11,037 (8,793–14,077)	1,041 (688–1,672)	4,278 (3,075–7,612)	3,992 (2,819–7,781)	2,263 (1,658–3,776)
Measles	36,444 (12,969–80,092)	30,949 (10,995–66,809)	4,619 (1,594–10,537)	852 (271–2,283)	23 (7–60)	0 (0–0)
Varicella and herpes zoster	3,322 (2,657–4,303)	850 (506–1,468)	244 (136–468)	416 (310–568)	464 (335–695)	1,345 (861–1,966)
Neglected tropical diseases and malaria	413,626 (326,392–499,360)	253,144 (183,872–327,032)	24,725 (16,271–36,886)	69,056 (51,767–94,756)	44,348 (32,907–61,712)	22,351 (17,116–30,129)
Malaria	368,242 (282,568–456,696)	242,897 (172,937–315,792)	18,492 (10,176–30,870)	55,126 (37,971–81,005)	35,815 (24,550–53,274)	15,910 (10,562–23,424)
Chagas disease	3,473 (3,172–3,814)	1 (1–1)	2 (1–2)	288 (254–327)	1,206 (1,076–1,358)	1,975 (1,787–2,194)
Leishmaniasis	6,330 (4,286–8,537)	1,394 (918–1,955)	782 (487–1,189)	3,248 (2,141–4,510)	664 (471–887)	240 (187–301)
Visceral leishmaniasis	6,330 (4,286–8,537)	1,394 (918–1,955)	782 (487–1,189)	3,248 (2,141–4,510)	664 (471–887)	240 (187–301)
Cutaneous and mucocutaneous leishmaniasis	0 (0–0)	0 (0–0)	0 (0–0)	0 (0–0)	0 (0–0)	0 (0–0)
African trypanosomiasis	1,587 (771–2,665)	134 (66–229)	296 (155–475)	819 (369–1,464)	270 (119–482)	66 (31–113)
Schistosomiasis	1,902 (1,623–2,260)	17 (10–31)	40 (26–59)	464 (369–596)	833 (652–1,056)	546 (433–683)
Cysticercosis	182 (141–245)	4 (2–7)	7 (4–11)	91 (69–126)	58 (45–77)	20 (17–26)
Cystic echinococcosis	576 (535–628)	22 (18–27)	42 (36–49)	179 (162–203)	175 (161–190)	156 (146–167)
Lymphatic filariasis	0 (0–0)	0 (0–0)	0 (0–0)	0 (0–0)	0 (0–0)	0 (0–0)
Onchocerciasis	0 (0–0)	0 (0–0)	0 (0–0)	0 (0–0)	0 (0–0)	0 (0–0)
Trachoma	0 (0–0)	0 (0–0)	0 (0–0)	0 (0–0)	0 (0–0)	0 (0–0)
Dengue	8,536 (5,347–10,815)	2,357 (1,215–3,570)	1,933 (1,142–3,104)	2,252 (1,578–2,931)	1,189 (728–1,524)	803 (416–1,032)
Yellow fever	1,320 (273–3,725)	135 (26–407)	464 (93–1,328)	582 (119–1,698)	113 (23–328)	23 (4–70)
Rabies	6,897 (4,995–8,934)	997 (640–1,627)	1,269 (865–1,875)	2,201 (1,467–3,116)	1,621 (1,158–2,047)	806 (573–1,014)
Intestinal nematode infections	1,307 (1,151–1,498)	929 (797–1,090)	179 (139–227)	72 (50–99)	63 (42–88)	62 (44–84)
Ascariasis	1,307 (1,151–1,498)	929 (797–1,090)	179 (139–227)	72 (50–99)	63 (42–88)	62 (44–84)
Trichuriasis	0 (0–0)	0 (0–0)	0 (0–0)	0 (0–0)	0 (0–0)	0 (0–0)
Hookworm disease	0 (0–0)	0 (0–0)	0 (0–0)	0 (0–0)	0 (0–0)	0 (0–0)
Food-borne trematodiases	0 (0–0)	0 (0–0)	0 (0–0)	0 (0–0)	0 (0–0)	0 (0–0)
Leprosy	0 (0–0)	0 (0–0)	0 (0–0)	0 (0–0)	0 (0–0)	0 (0–0)
Ebola	2,722 (2,195–3,263)	203 (164–244)	300 (242–360)	1,710 (1,379–2,049)	396 (319–475)	111 (90–134)
Other neglected tropical diseases	10,545 (7,722–14,784)	4,047 (2,792–5,966)	914 (548–1,504)	2,016 (1,378–3,156)	1,940 (1,207–3,127)	1,627 (1,006–2,535)
Maternal disorders	275,287 (243,757–315,490)	0 (0–0)	1,343 (1,104–1,640)	272,857 (241,723–312,860)	1,087 (761–1,631)	0 (0–0)
Maternal hemorrhage	83,125 (66,977–101,492)	0 (0–0)	416 (285–581)	82,367 (66,300–100,624)	340 (195–599)	0 (0–0)
Maternal sepsis and other maternal infections	17,894 (13,368–23,853)	0 (0–0)	56 (34–90)	17,753 (13,225–23,687)	83 (48–146)	0 (0–0)
Maternal hypertensive disorders	46,907 (37,085–59,639)	0 (0–0)	293 (186–443)	46,526 (36,691–59,130)	87 (53–147)	0 (0–0)
Maternal obstructed labor and uterine rupture	23,149 (17,209–29,965)	0 (0–0)	37 (20–64)	23,066 (17,140–29,892)	45 (26–76)	0 (0–0)
Maternal abortion, miscarriage, and ectopic pregnancy	31,658 (24,585–39,740)	0 (0–0)	168 (96–268)	31,316 (24,252–39,393)	173 (106–273)	0 (0–0)

SR Table 10 - Global deaths by age and cause for females in 2015

Cause	All ages	0-5 years	5-14 years	15-49 years	50-69 years	70+ years
Indirect maternal deaths	30,786 (23,136–40,519)	0 (0–0)	185 (111–286)	30,481 (22,851–40,187)	118 (69–208)	0 (0–0)
Late maternal deaths	6,711 (4,334–9,995)	0 (0–0)	37 (17–67)	6,634 (4,270–9,909)	39 (21–74)	0 (0–0)
Maternal deaths aggravated by HIV/AIDS	2,321 (1,394–3,336)	0 (0–0)	2 (1–4)	2,305 (1,386–3,318)	12 (6–25)	0 (0–0)
Other maternal disorders	32,733 (26,256–40,506)	0 (0–0)	145 (91–216)	32,404 (25,957–40,154)	184 (119–290)	0 (0–0)
Neonatal disorders	953,942 (921,873–987,899)	953,942 (921,873–987,899)	0 (0–0)	0 (0–0)	0 (0–0)	0 (0–0)
Neonatal preterm birth complications	362,978 (320,146–409,506)	362,978 (320,146–409,506)	0 (0–0)	0 (0–0)	0 (0–0)	0 (0–0)
Neonatal encephalopathy due to birth asphyxia and trauma	328,410 (281,510–384,470)	328,410 (281,510–384,470)	0 (0–0)	0 (0–0)	0 (0–0)	0 (0–0)
Neonatal sepsis and other neonatal infections	131,463 (65,181–200,823)	131,463 (65,181–200,823)	0 (0–0)	0 (0–0)	0 (0–0)	0 (0–0)
Hemolytic disease and other neonatal jaundice	22,507 (12,724–37,738)	22,507 (12,724–37,738)	0 (0–0)	0 (0–0)	0 (0–0)	0 (0–0)
Other neonatal disorders	108,582 (75,288–152,162)	108,582 (75,288–152,162)	0 (0–0)	0 (0–0)	0 (0–0)	0 (0–0)
Nutritional deficiencies	192,333 (174,291–214,920)	81,667 (67,537–99,144)	6,568 (5,657–7,573)	16,032 (14,097–18,753)	21,151 (18,710–24,758)	66,913 (61,725–74,442)
Protein-energy malnutrition	150,480 (132,905–171,896)	72,965 (59,096–90,213)	4,786 (3,940–5,672)	7,163 (5,997–8,879)	12,085 (10,339–14,593)	53,480 (47,987–60,153)
Iodine deficiency	1,189 (855–1,698)	326 (129–721)	42 (17–86)	170 (106–277)	251 (152–416)	399 (243–636)
Vitamin A deficiency	0 (0–0)	0 (0–0)	0 (0–0)	0 (0–0)	0 (0–0)	0 (0–0)
Iron-deficiency anemia	26,988 (18,548–35,245)	7,121 (3,562–10,846)	1,344 (958–2,078)	6,456 (4,953–8,247)	6,579 (4,828–8,473)	5,487 (3,320–7,463)
Other nutritional deficiencies	13,674 (9,045–18,561)	1,254 (690–1,992)	395 (243–570)	2,241 (1,467–3,064)	2,235 (1,404–3,209)	7,547 (5,170–10,685)
Other communicable, maternal, neonatal, and nutritional diseases	140,729 (115,370–169,274)	65,383 (45,134–91,750)	8,491 (6,381–11,271)	20,963 (19,279–22,718)	21,596 (18,764–23,553)	24,294 (17,045–27,557)
Sexually transmitted diseases excluding HIV	55,338 (33,672–84,156)	44,455 (24,522–71,013)	4,670 (2,649–7,324)	2,929 (2,322–3,649)	1,529 (1,062–1,882)	1,753 (1,260–2,169)
Syphilis	54,515 (32,832–83,352)	44,455 (24,522–71,013)	4,657 (2,633–7,312)	2,594 (2,058–3,234)	1,300 (904–1,603)	1,506 (1,083–1,862)
Chlamydial infection	151 (114–177)	0 (0–0)	3 (2–4)	62 (49–76)	38 (26–47)	47 (34–57)
Gonococcal infection	456 (340–535)	0 (0–0)	5 (4–7)	165 (130–205)	129 (89–159)	155 (111–193)
Trichomoniasis	0 (0–0)	0 (0–0)	0 (0–0)	0 (0–0)	0 (0–0)	0 (0–0)
Genital herpes	0 (0–0)	0 (0–0)	0 (0–0)	0 (0–0)	0 (0–0)	0 (0–0)
Other sexually transmitted diseases	216 (162–257)	0 (0–0)	3 (3–5)	106 (84–132)	60 (41–75)	44 (31–56)
Hepatitis	41,038 (38,552–43,822)	5,328 (4,631–6,174)	2,311 (1,960–2,737)	13,154 (12,036–14,482)	11,060 (10,332–11,967)	9,183 (8,566–9,911)
Acute hepatitis A	5,360 (3,416–7,605)	4,110 (2,914–5,182)	441 (131–899)	471 (113–1,133)	216 (44–546)	120 (27–299)
Hepatitis B	23,318 (19,921–26,839)	335 (109–800)	278 (144–474)	7,287 (5,641–8,883)	8,906 (7,504–10,102)	6,509 (5,237–7,786)
Hepatitis C	1,004 (206–2,335)	0 (0–0)	37 (7–101)	226 (44–539)	453 (96–1,052)	286 (56–728)
Acute hepatitis E	11,354 (8,147–15,276)	883 (314–1,748)	1,553 (1,092–2,033)	5,168 (3,767–6,696)	1,483 (677–2,685)	2,265 (1,209–3,599)
Other infectious diseases	44,352 (25,820–53,379)	15,598 (7,686–22,925)	1,509 (1,155–1,895)	4,879 (3,867–5,634)	9,006 (6,215–10,542)	13,357 (6,370–16,416)
Non-communicable diseases	18,270,124 (17,918,489–18,683,914)	378,243 (338,598–423,316)	87,589 (80,414–95,308)	1,438,989 (1,374,452–1,510,603)	4,667,804 (4,532,497–4,831,800)	11,697,497 (11,529,683–11,892,074)
Neoplasms	3,684,801 (3,592,175–3,773,780)	16,523 (14,700–18,837)	22,276 (20,509–24,077)	499,586 (477,327–522,855)	1,446,648 (1,403,907–1,492,351)	1,699,766 (1,658,958–1,741,014)
Lip and oral cavity cancer	48,189 (46,046–50,589)	0 (0–0)	0 (0–0)	6,734 (6,154–7,335)	19,273 (18,158–20,467)	22,180 (21,257–23,314)
Nasopharynx cancer	17,326 (16,285–18,274)	0 (0–0)	88 (78–99)	4,536 (4,234–4,864)	8,229 (7,689–8,750)	4,472 (4,177–4,769)
Other pharynx cancer	17,365 (16,130–18,727)	0 (0–0)	0 (0–0)	2,403 (2,173–2,643)	8,577 (7,933–9,271)	6,384 (5,951–6,908)
Esophageal cancer	121,267 (114,690–128,390)	0 (0–0)	0 (0–0)	10,141 (9,370–11,179)	50,805 (47,437–54,891)	60,320 (57,016–64,002)
Stomach cancer	283,815 (273,598–294,032)	0 (0–0)	0 (0–0)	28,794 (27,025–30,478)	100,283 (95,738–104,795)	154,737 (149,575–160,331)
Colon and rectum cancer	376,240 (363,199–390,834)	0 (0–0)	0 (0–0)	29,699 (28,349–31,302)	121,155 (116,710–125,714)	225,386 (216,247–236,014)
Liver cancer	233,802 (203,623–254,788)	0 (0–0)	326 (285–391)	26,531 (22,172–29,988)	98,342 (84,510–109,317)	108,602 (96,265–116,108)
Liver cancer due to hepatitis B	64,091 (54,837–73,126)	0 (0–0)	143 (122–175)	13,901 (11,612–16,162)	30,064 (25,705–34,934)	19,981 (16,970–22,556)
Liver cancer due to hepatitis C	71,768 (63,545–76,971)	0 (0–0)	12 (9–15)	3,664 (2,896–4,258)	24,717 (20,760–27,496)	43,373 (39,271–46,418)
Liver cancer due to alcohol use	40,913 (35,185–45,880)	0 (0–0)	0 (0–0)	2,196 (1,794–2,621)	20,044 (16,778–23,114)	18,671 (16,242–20,634)
Liver cancer due to other causes	57,029 (49,431–62,819)	0 (0–0)	170 (145–205)	6,768 (5,587–7,724)	23,515 (19,668–27,041)	26,575 (23,053–29,217)
Gallbladder and biliary tract cancer	80,980 (72,925–87,195)	0 (0–0)	0 (0–0)	4,995 (4,403–5,418)	27,674 (25,148–29,794)	48,310 (43,151–52,291)
Pancreatic cancer	196,952 (190,553–202,904)	0 (0–0)	0 (0–0)	10,233 (9,701–10,784)	70,178 (67,878–72,751)	116,540 (112,450–120,341)
Larynx cancer	19,487 (18,578–20,438)	0 (0–0)	0 (0–0)	2,415 (2,224–2,614)	8,644 (8,196–9,106)	8,427 (8,017–8,942)
Tracheal, bronchus, and lung cancer	516,783 (497,445–538,181)	0 (0–0)	0 (0–0)	38,700 (36,478–41,260)	214,385 (204,260–225,811)	263,697 (254,179–273,474)
Malignant skin melanoma	27,468 (24,017–29,111)	0 (0–0)	0 (0–0)	4,355 (4,035–4,747)	9,599 (8,536–10,280)	13,513 (11,361–14,574)
Non-melanoma skin cancer	17,655 (16,566–18,737)	0 (0–0)	0 (0–0)	1,416 (1,306–1,535)	4,150 (3,851–4,494)	12,088 (11,224–12,877)
Non-melanoma skin cancer (squamous-cell carcinoma)	17,655 (16,566–18,737)	0 (0–0)	0 (0–0)	1,416 (1,306–1,535)	4,150 (3,851–4,494)	12,088 (11,224–12,877)
Non-melanoma skin cancer (basal-cell carcinoma)	0 (0–0)	0 (0–0)	0 (0–0)	0 (0–0)	0 (0–0)	0 (0–0)
Breast cancer	523,487 (492,250–543,275)	0 (0–0)	0 (0–0)	105,011 (96,924–111,211)	236,553 (222,087–246,997)	181,922 (170,325–188,957)
Cervical cancer	238,620 (225,292–252,397)	0 (0–0)	0 (0–0)	59,413 (54,937–63,630)	114,019 (106,466–122,003)	65,188 (62,487–68,200)
Uterine cancer	89,946 (86,102–94,346)	0 (0–0)	0 (0–0)	9,307 (8,765–9,908)	41,479 (39,352–44,035)	39,159 (37,064–41,150)
Ovarian cancer	161,137 (156,506–166,503)	0 (0–0)	0 (0–0)	26,084 (24,658–27,532)	75,500 (73,014–78,467)	59,552 (57,601–61,637)
Prostate cancer	0 (0–0)	0 (0–0)	0 (0–0)	0 (0–0)	0 (0–0)	0 (0–0)
Testicular cancer	0 (0–0)	0 (0–0)	0 (0–0)	0 (0–0)	0 (0–0)	0 (0–0)

SR Table 10 - Global deaths by age and cause for females in 2015

Cause	All ages	0-5 years	5-14 years	15-49 years	50-69 years	70+ years
Kidney cancer	47,805 (46,213–49,365)	863 (714–1,140)	655 (571–757)	3,954 (3,774–4,160)	16,843 (16,228–17,470)	25,488 (24,383–26,573)
Bladder cancer	51,222 (49,258–53,309)	0 (0–0)	0 (0–0)	2,307 (2,186–2,450)	13,130 (12,497–13,851)	35,784 (34,257–37,379)
Brain and nervous system cancer	101,599 (96,426–106,246)	2,400 (2,112–2,754)	4,513 (4,002–4,986)	23,416 (21,783–25,035)	41,486 (39,373–43,886)	29,782 (28,152–30,985)
Thyroid cancer	18,488 (17,459–19,992)	0 (0–0)	36 (32–41)	1,465 (1,326–1,622)	6,440 (5,881–7,083)	10,545 (9,923–11,453)
Mesothelioma	9,212 (8,847–9,581)	0 (0–0)	0 (0–0)	1,282 (1,186–1,380)	3,376 (3,193–3,558)	4,553 (4,317–4,778)
Hodgkin lymphoma	8,801 (7,379–11,828)	80 (57–103)	179 (128–231)	2,854 (2,297–3,821)	2,882 (2,421–4,061)	2,804 (2,372–3,585)
Non-Hodgkin lymphoma	97,963 (81,927–103,662)	1,321 (959–1,887)	1,843 (1,373–2,201)	13,998 (11,934–15,188)	32,298 (28,076–35,035)	48,501 (38,327–52,209)
Multiple myeloma	48,878 (46,261–51,165)	0 (0–0)	0 (0–0)	3,720 (3,449–4,030)	19,079 (18,010–20,263)	26,078 (23,858–27,467)
Leukemia	149,163 (144,127–154,241)	6,139 (5,272–7,283)	9,283 (8,472–10,237)	33,847 (32,202–35,502)	42,607 (40,793–44,375)	57,285 (54,764–59,782)
Acute lymphoid leukemia	45,484 (43,166–49,084)	3,967 (3,367–4,842)	6,346 (5,690–7,086)	12,903 (12,112–14,136)	12,238 (11,476–13,126)	10,029 (9,327–10,698)
Chronic lymphoid leukemia	26,794 (25,476–28,299)	0 (0–0)	0 (0–0)	3,190 (2,862–3,505)	7,192 (6,764–7,734)	16,411 (15,476–17,465)
Acute myeloid leukemia	62,012 (59,254–64,433)	2,171 (1,807–2,627)	2,937 (2,650–3,273)	14,375 (13,432–15,173)	18,352 (17,302–19,162)	24,175 (23,020–25,287)
Chronic myeloid leukemia	14,870 (13,956–15,895)	0 (0–0)	0 (0–0)	3,377 (2,958–3,846)	4,822 (4,527–5,120)	6,670 (6,168–7,210)
Other neoplasms	181,138 (162,127–192,439)	5,718 (4,893–7,172)	5,349 (4,646–6,047)	41,962 (34,369–48,589)	59,650 (54,600–63,537)	68,457 (60,052–71,935)
Cardiovascular diseases	8,501,409 (8,301,354–8,722,665)	20,397 (18,841–22,352)	11,317 (10,166–12,722)	441,749 (417,539–468,486)	1,849,716 (1,785,561–1,926,363)	6,178,227 (6,032,217–6,331,678)
Rheumatic heart disease	172,217 (157,416–186,249)	1,102 (861–1,329)	2,799 (2,391–3,275)	37,009 (32,038–41,048)	55,307 (50,004–60,642)	75,997 (71,270–81,787)
Ischemic heart disease	4,035,935 (3,941,318–4,146,339)	918 (800–1,073)	796 (662–943)	162,874 (153,909–172,867)	834,503 (805,544–869,383)	3,036,842 (2,964,485–3,114,208)
Cerebrovascular disease	3,094,935 (3,001,358–3,201,113)	6,230 (5,604–7,049)	3,776 (3,384–4,338)	157,761 (148,119–169,398)	716,974 (685,072–753,523)	2,210,192 (2,147,339–2,279,167)
Ischemic stroke	1,550,557 (1,477,734–1,619,513)	295 (232–374)	216 (175–259)	25,454 (22,095–29,356)	234,128 (215,293–255,460)	1,290,461 (1,235,058–1,339,233)
Hemorrhagic stroke	1,544,378 (1,472,185–1,644,306)	5,935 (5,318–6,732)	3,559 (3,195–4,099)	132,306 (123,672–142,799)	482,846 (453,156–518,388)	919,731 (876,807–988,255)
Hypertensive heart disease	516,978 (426,400–565,131)	189 (148–244)	165 (135–206)	28,686 (24,299–32,117)	124,200 (104,461–139,644)	363,736 (295,224–395,975)
Cardiomyopathy and myocarditis	154,934 (149,307–161,298)	4,764 (4,265–5,414)	1,503 (1,386–1,656)	21,009 (19,714–22,336)	30,754 (29,390–32,361)	96,902 (92,629–101,176)
Atrial fibrillation and flutter	109,116 (89,388–131,762)	0 (0–0)	0 (0–0)	340 (250–444)	5,202 (4,005–6,879)	103,573 (84,104–126,022)
Aortic aneurysm	61,435 (59,264–64,076)	0 (0–0)	0 (0–0)	3,561 (3,341–3,788)	12,532 (12,103–12,955)	45,341 (43,408–47,584)
Peripheral vascular disease	26,825 (24,548–29,327)	0 (0–0)	0 (0–0)	209 (192–226)	2,598 (2,399–2,837)	24,017 (21,906–26,321)
Endocarditis	46,136 (39,416–53,283)	1,711 (1,014–2,266)	651 (457–802)	7,267 (5,161–8,460)	8,764 (7,238–10,875)	27,741 (24,975–31,561)
Other cardiovascular and circulatory diseases	282,893 (269,296–297,547)	5,478 (4,879–6,197)	1,625 (1,444–1,843)	23,029 (21,150–25,168)	58,877 (54,549–64,098)	193,882 (184,885–203,111)
Chronic respiratory diseases	1,590,401 (1,506,896–1,669,505)	15,942 (14,114–18,809)	4,312 (3,820–4,886)	72,279 (67,873–77,300)	393,730 (371,798–417,944)	1,104,136 (1,038,912–1,158,894)
Chronic obstructive pulmonary disease	1,311,924 (1,239,119–1,387,107)	6,075 (4,354–7,932)	808 (607–1,072)	37,821 (34,257–41,887)	309,965 (289,741–334,597)	957,252 (896,377–1,009,993)
Pneumoconiosis	12,369 (8,419–15,727)	0 (0–0)	0 (0–0)	673 (372–907)	2,580 (1,446–3,598)	9,115 (6,538–11,463)
Silicosis	382 (259–533)	0 (0–0)	0 (0–0)	25 (13–33)	95 (57–127)	261 (186–372)
Asbestosis	571 (408–679)	0 (0–0)	0 (0–0)	152 (90–198)	133 (82–171)	285 (230–336)
Coal workers pneumoconiosis	136 (76–191)	0 (0–0)	0 (0–0)	11 (5–16)	38 (19–59)	85 (52–122)
Other pneumoconiosis	11,279 (7,633–14,472)	0 (0–0)	0 (0–0)	484 (262–683)	2,312 (1,273–3,258)	8,482 (6,033–10,740)
Asthma	195,844 (169,293–224,225)	4,507 (3,569–5,575)	2,800 (2,303–3,388)	28,698 (24,369–33,033)	64,921 (54,450–75,383)	94,916 (82,204–111,033)
Interstitial lung disease and pulmonary sarcoidosis	54,026 (40,151–59,403)	157 (106–253)	127 (104–153)	3,325 (2,897–3,735)	13,689 (11,404–15,369)	36,726 (25,048–40,871)
Other chronic respiratory diseases	16,237 (11,148–20,055)	5,201 (3,821–6,857)	575 (362–834)	1,761 (1,088–2,311)	2,573 (1,742–3,240)	6,126 (3,909–7,796)
Cirrhosis and other chronic liver diseases	431,025 (407,697–489,571)	4,477 (3,572–6,289)	3,830 (3,255–4,981)	82,923 (76,904–96,924)	182,351 (170,426–207,362)	157,443 (148,661–174,106)
Cirrhosis and other chronic liver diseases due to hepatitis B	107,454 (97,384–127,296)	21 (15–32)	71 (54–96)	19,848 (17,196–24,060)	45,343 (39,540–55,025)	42,169 (37,643–49,382)
Cirrhosis and other chronic liver diseases due to hepatitis C	100,813 (92,363–111,040)	11 (7–18)	46 (34–63)	17,454 (15,343–20,581)	42,893 (37,734–48,601)	40,408 (36,802–44,827)
Cirrhosis and other chronic liver diseases due to alcohol use	98,684 (90,066–111,994)	0 (0–0)	0 (0–0)	22,574 (20,183–26,806)	49,070 (43,581–56,193)	27,040 (24,040–30,700)
Cirrhosis and other chronic liver diseases due to other causes	124,072 (114,830–143,343)	4,445 (3,549–6,242)	3,712 (3,161–4,815)	23,046 (20,645–27,638)	45,043 (39,664–52,588)	47,825 (43,803–53,518)
Digestive diseases	543,875 (515,703–569,714)	12,130 (6,709–25,570)	6,525 (5,749–7,485)	70,795 (65,877–75,909)	138,419 (129,390–146,429)	316,004 (301,370–329,072)
Peptic ulcer disease	111,353 (102,567–120,232)	1,283 (560–3,189)	1,026 (808–1,261)	14,849 (13,305–16,620)	31,336 (28,526–34,163)	62,857 (58,186–67,676)
Gastritis and duodenitis	21,040 (13,941–29,616)	295 (117–613)	190 (118–318)	2,784 (1,837–4,111)	5,804 (3,607–8,295)	11,964 (8,073–16,608)
Appendicitis	22,968 (16,721–27,406)	2,361 (908–5,948)	1,171 (987–1,389)	6,313 (4,692–7,189)	5,918 (4,331–6,712)	7,202 (5,580–8,195)
Paralytic ileus and intestinal obstruction	117,989 (102,625–134,814)	4,460 (2,421–8,906)	2,273 (1,891–2,869)	17,125 (14,203–20,387)	28,944 (24,472–34,923)	65,185 (56,494–73,795)
Inguinal, femoral, and abdominal hernia	21,887 (14,421–25,308)	244 (102–604)	242 (148–325)	2,342 (1,159–3,068)	6,397 (3,697–7,800)	12,659 (9,070–14,814)
Inflammatory bowel disease	24,278 (21,920–27,290)	1,084 (465–2,402)	236 (174–320)	3,680 (2,964–4,550)	5,371 (4,842–5,838)	13,905 (12,794–15,112)
Vascular intestinal disorders	58,678 (53,117–65,714)	75 (32–177)	37 (26–54)	2,180 (1,669–2,900)	11,246 (9,684–13,568)	45,137 (41,475–49,548)
Gallbladder and biliary diseases	62,629 (57,751–67,504)	842 (386–1,802)	542 (455–656)	6,475 (5,762–7,229)	15,509 (14,020–16,940)	39,260 (36,585–42,643)
Pancreatitis	47,601 (43,148–51,326)	94 (51–175)	240 (194–303)	10,273 (8,973–11,347)	16,026 (14,327–17,521)	20,966 (19,230–22,772)
Other digestive diseases	55,448 (51,621–60,289)	1,386 (709–2,948)	563 (459–682)	4,768 (4,145–5,480)	11,864 (10,755–12,987)	36,864 (34,270–40,194)
Neurological disorders	1,324,602 (1,122,417–1,524,389)	7,467 (6,611–8,844)	5,231 (4,521–6,022)	32,094 (29,810–34,622)	87,552 (74,051–102,121)	1,192,256 (997,853–1,386,880)
Alzheimer disease and other dementias	1,175,893 (973,930–1,377,324)	0 (0–0)	0 (0–0)	679 (427–992)	54,587 (40,878–68,913)	1,120,626 (924,932–1,316,519)
Parkinson disease	50,740 (48,523–53,007)	0 (0–0)	0 (0–0)	272 (251–293)	5,256 (4,988–5,578)	45,211 (43,111–47,311)
Epilepsy	47,435 (44,530–50,685)	5,380 (4,728–6,129)	4,073 (3,416–4,806)	21,815 (20,005–23,847)	7,951 (7,508–8,444)	8,214 (7,778–8,702)

SR Table 10 - Global deaths by age and cause for females in 2015						
**Cause**	**All ages**	**0-5 years**	**5-14 years**	**15-49 years**	**50-69 years**	**70+ years**
Multiple sclerosis	11,179 (9,769–12,108)	0 (0–0)	0 (0–0)	3,097 (2,805–3,778)	5,434 (4,539–5,830)	2,648 (1,941–2,867)
Motor neuron disease	15,462 (14,462–16,262)	449 (333–561)	43 (36–49)	1,210 (1,138–1,318)	6,861 (6,476–7,238)	6,895 (6,376–7,265)
Migraine	0 (0–0)	0 (0–0)	0 (0–0)	0 (0–0)	0 (0–0)	0 (0–0)
Tension-type headache	0 (0–0)	0 (0–0)	0 (0–0)	0 (0–0)	0 (0–0)	0 (0–0)
Medication overuse headache	0 (0–0)	0 (0–0)	0 (0–0)	0 (0–0)	0 (0–0)	0 (0–0)
Other neurological disorders	23,900 (22,624–26,552)	1,646 (1,418–2,581)	1,115 (954–1,590)	5,019 (4,658–6,037)	7,460 (7,140–7,748)	8,659 (8,183–9,156)
Mental and substance use disorders	76,287 (71,065–79,354)	17 (15–19)	22 (10–30)	33,942 (31,760–37,070)	25,879 (23,737–27,106)	16,424 (13,248–17,693)
Schizophrenia	7,825 (7,329–8,430)	0 (0–0)	0 (0–0)	1,955 (1,771–2,161)	2,848 (2,633–3,094)	3,021 (2,815–3,247)
Alcohol use disorders	20,985 (20,048–21,902)	0 (0–0)	0 (0–0)	7,292 (6,851–7,754)	9,885 (9,340–10,466)	3,807 (3,648–3,956)
Drug use disorders	46,985 (41,775–49,489)	17 (15–19)	0 (0–0)	24,226 (22,089–26,992)	13,145 (11,017–13,997)	9,595 (6,363–10,772)
Opioid use disorders	31,029 (26,474–33,104)	17 (15–19)	0 (0–0)	16,051 (14,420–17,965)	8,515 (6,762–9,252)	6,444 (3,983–7,320)
Cocaine use disorders	3,200 (2,662–3,702)	0 (0–0)	0 (0–0)	1,850 (1,563–2,119)	1,138 (910–1,339)	210 (161–281)
Amphetamine use disorders	1,923 (1,620–2,321)	0 (0–0)	0 (0–0)	1,129 (952–1,349)	597 (500–733)	197 (147–270)
Cannabis use disorders	0 (0–0)	0 (0–0)	0 (0–0)	0 (0–0)	0 (0–0)	0 (0–0)
Other drug use disorders	10,831 (9,720–13,244)	0 (0–0)	0 (0–0)	5,195 (4,572–6,691)	2,893 (2,538–3,466)	2,743 (1,905–3,626)
Depressive disorders	0 (0–0)	0 (0–0)	0 (0–0)	0 (0–0)	0 (0–0)	0 (0–0)
Major depressive disorder	0 (0–0)	0 (0–0)	0 (0–0)	0 (0–0)	0 (0–0)	0 (0–0)
Dysthymia	0 (0–0)	0 (0–0)	0 (0–0)	0 (0–0)	0 (0–0)	0 (0–0)
Bipolar disorder	0 (0–0)	0 (0–0)	0 (0–0)	0 (0–0)	0 (0–0)	0 (0–0)
Anxiety disorders	0 (0–0)	0 (0–0)	0 (0–0)	0 (0–0)	0 (0–0)	0 (0–0)
Eating disorders	491 (310–711)	0 (0–0)	22 (10–30)	468 (298–691)	0 (0–0)	0 (0–0)
Anorexia nervosa	449 (284–649)	0 (0–0)	21 (9–29)	427 (273–623)	0 (0–0)	0 (0–0)
Bulimia nervosa	41 (19–69)	0 (0–0)	1 (0–1)	40 (19–68)	0 (0–0)	0 (0–0)
Autistic spectrum disorders	0 (0–0)	0 (0–0)	0 (0–0)	0 (0–0)	0 (0–0)	0 (0–0)
Autism	0 (0–0)	0 (0–0)	0 (0–0)	0 (0–0)	0 (0–0)	0 (0–0)
Asperger syndrome and other autistic spectrum disorders	0 (0–0)	0 (0–0)	0 (0–0)	0 (0–0)	0 (0–0)	0 (0–0)
Attention-deficit/hyperactivity disorder	0 (0–0)	0 (0–0)	0 (0–0)	0 (0–0)	0 (0–0)	0 (0–0)
Conduct disorder	0 (0–0)	0 (0–0)	0 (0–0)	0 (0–0)	0 (0–0)	0 (0–0)
Idiopathic developmental intellectual disability	0 (0–0)	0 (0–0)	0 (0–0)	0 (0–0)	0 (0–0)	0 (0–0)
Other mental and substance use disorders	0 (0–0)	0 (0–0)	0 (0–0)	0 (0–0)	0 (0–0)	0 (0–0)
Diabetes, urogenital, blood, and endocrine diseases	1,697,106 (1,647,062–1,755,042)	50,221 (37,315–77,845)	14,447 (12,983–16,341)	163,974 (154,867–174,496)	509,327 (488,252–534,227)	959,135 (930,577–990,439)
Diabetes mellitus	772,786 (736,231–809,514)	824 (627–962)	1,223 (923–1,405)	52,833 (48,811–57,404)	268,265 (252,329–284,416)	449,640 (429,534–470,893)
Acute glomerulonephritis	5,109 (3,319–6,199)	135 (93–181)	131 (100–157)	999 (649–1,175)	1,635 (972–2,035)	2,208 (1,372–2,733)
Chronic kidney disease	597,089 (576,653–620,330)	5,787 (5,187–6,434)	3,902 (3,429–4,444)	62,552 (58,988–66,941)	172,197 (164,910–180,000)	352,649 (339,499–365,908)
Chronic kidney disease due to diabetes mellitus	200,922 (191,619–210,876)	1,039 (871–1,227)	452 (369–556)	15,979 (14,263–17,699)	73,139 (68,488–78,677)	110,311 (104,582–116,787)
Chronic kidney disease due to hypertension	275,739 (263,723–288,736)	2,016 (1,742–2,329)	1,256 (1,046–1,508)	26,193 (23,932–28,682)	69,167 (63,958–74,723)	177,104 (167,491–186,082)
Chronic kidney disease due to glomerulonephritis	106,174 (99,534–113,635)	1,982 (1,678–2,326)	1,625 (1,355–1,937)	17,586 (15,758–19,776)	27,989 (25,302–31,029)	56,991 (52,749–61,446)
Chronic kidney disease due to other causes	14,252 (12,028–16,835)	749 (608–917)	567 (439–716)	2,792 (1,826–3,989)	1,902 (1,200–2,812)	8,241 (6,905–10,182)
Urinary diseases and male infertility	137,226 (130,648–143,345)	2,471 (2,061–2,893)	852 (749–1,006)	14,553 (13,507–16,408)	30,608 (28,643–33,334)	88,740 (83,023–92,850)
Interstitial nephritis and urinary tract infections	112,857 (106,887–118,193)	1,996 (1,645–2,393)	558 (482–654)	10,785 (10,004–12,099)	22,666 (21,049–24,681)	76,850 (71,102–80,764)
Urolithiasis	7,806 (6,362–9,193)	0 (0–0)	98 (82–122)	1,164 (902–1,389)	2,544 (1,922–2,981)	3,999 (3,327–4,820)
Benign prostatic hyperplasia	0 (0–0)	0 (0–0)	0 (0–0)	0 (0–0)	0 (0–0)	0 (0–0)
Male infertility	0 (0–0)	0 (0–0)	0 (0–0)	0 (0–0)	0 (0–0)	0 (0–0)
Other urinary diseases	16,562 (14,967–20,044)	475 (341–661)	195 (162–262)	2,603 (2,274–3,185)	5,397 (4,721–6,516)	7,890 (7,026–9,633)
Gynecological diseases	7,863 (5,875–9,181)	0 (0–0)	0 (0–0)	3,224 (2,438–3,855)	2,417 (1,725–2,855)	2,220 (1,652–2,615)
Uterine fibroids	2,324 (1,209–3,034)	0 (0–0)	0 (0–0)	743 (401–992)	1,001 (521–1,283)	579 (270–800)
Polycystic ovarian syndrome	614 (193–1,017)	0 (0–0)	0 (0–0)	418 (114–712)	195 (80–324)	0 (0–0)
Female infertility	0 (0–0)	0 (0–0)	0 (0–0)	0 (0–0)	0 (0–0)	0 (0–0)
Endometriosis	55 (42–83)	0 (0–0)	0 (0–0)	45 (34–68)	9 (6–14)	0 (0–0)
Genital prolapse	888 (551–1,465)	0 (0–0)	0 (0–0)	50 (24–88)	295 (160–549)	542 (348–846)
Premenstrual syndrome	0 (0–0)	0 (0–0)	0 (0–0)	0 (0–0)	0 (0–0)	0 (0–0)
Other gynecological diseases	3,981 (2,998–4,833)	0 (0–0)	0 (0–0)	1,966 (1,518–2,442)	916 (648–1,160)	1,099 (813–1,373)
Hemoglobinopathies and hemolytic anemias	102,232 (88,606–131,290)	35,910 (23,165–64,750)	6,904 (5,874–8,680)	18,611 (16,769–20,668)	14,561 (13,400–15,580)	26,245 (23,816–28,477)
Thalassemias	8,491 (7,236–10,481)	3,652 (2,600–5,497)	1,230 (1,068–1,401)	987 (852–1,168)	82 (72–93)	2,537 (2,209–2,932)
Thalassemias trait	0 (0–0)	0 (0–0)	0 (0–0)	0 (0–0)	0 (0–0)	0 (0–0)
Sickle cell disorders	50,074 (37,306–79,549)	31,932 (19,566–60,574)	5,202 (4,197–7,052)	8,605 (7,361–10,358)	967 (814–1,166)	3,366 (2,883–3,894)
Sickle cell trait	0 (0–0)	0 (0–0)	0 (0–0)	0 (0–0)	0 (0–0)	0 (0–0)

SR Table 10 - Global deaths by age and cause for females in 2015

Cause	All ages	0-5 years	5-14 years	15-49 years	50-69 years	70+ years
G6PD deficiency	9,865 (8,626–11,150)	210 (138–333)	329 (264–398)	4,958 (4,263–5,706)	3,503 (3,020–4,011)	863 (740–1,003)
G6PD trait	0 (0–0)	0 (0–0)	0 (0–0)	0 (0–0)	0 (0–0)	0 (0–0)
Other hemoglobinopathies and hemolytic anemias	33,801 (30,885–36,531)	115 (85–174)	140 (121–163)	4,059 (3,678–4,505)	10,008 (9,262–10,790)	19,477 (17,464–21,324)
Endocrine, metabolic, blood, and immune disorders	74,797 (71,820–77,401)	5,092 (4,629–5,602)	1,433 (1,334–1,555)	11,199 (10,684–11,789)	19,641 (18,814–20,493)	37,430 (35,680–39,314)
Musculoskeletal disorders	62,498 (56,777–67,453)	0 (0–0)	1,108 (939–1,274)	13,218 (11,766–14,742)	15,666 (14,438–17,465)	32,504 (28,938–34,638)
Rheumatoid arthritis	20,716 (18,254–25,172)	0 (0–0)	118 (76–168)	1,312 (980–1,751)	6,270 (5,391–7,737)	13,014 (11,585–15,867)
Osteoarthritis	0 (0–0)	0 (0–0)	0 (0–0)	0 (0–0)	0 (0–0)	0 (0–0)
Low back and neck pain	0 (0–0)	0 (0–0)	0 (0–0)	0 (0–0)	0 (0–0)	0 (0–0)
Low back pain	0 (0–0)	0 (0–0)	0 (0–0)	0 (0–0)	0 (0–0)	0 (0–0)
Neck pain	0 (0–0)	0 (0–0)	0 (0–0)	0 (0–0)	0 (0–0)	0 (0–0)
Gout	0 (0–0)	0 (0–0)	0 (0–0)	0 (0–0)	0 (0–0)	0 (0–0)
Other musculoskeletal disorders	41,782 (35,794–45,127)	0 (0–0)	990 (843–1,147)	11,905 (10,541–13,317)	9,396 (8,202–10,551)	19,489 (15,162–21,336)
Other non-communicable diseases	358,106 (306,522–409,917)	251,056 (218,937–290,194)	18,517 (15,501–22,471)	28,423 (22,987–32,646)	18,512 (13,413–23,710)	41,596 (27,815–50,403)
Congenital anomalies	295,292 (253,082–339,211)	238,266 (206,797–277,376)	17,905 (14,985–21,836)	23,966 (19,269–27,858)	8,352 (6,597–9,240)	6,801 (5,093–7,514)
Neural tube defects	30,990 (19,153–43,999)	29,159 (17,822–41,954)	1,006 (570–1,566)	592 (405–851)	158 (113–243)	74 (56–104)
Congenital heart anomalies	140,953 (113,634–166,680)	108,651 (86,371–133,028)	11,948 (9,397–14,147)	15,935 (12,373–18,462)	2,403 (1,877–2,724)	2,014 (1,606–2,250)
Cleft lip and cleft palate	666 (455–880)	666 (455–880)	0 (0–0)	0 (0–0)	0 (0–0)	0 (0–0)
Down syndrome	15,499 (7,742–25,565)	11,086 (4,295–20,466)	471 (292–698)	2,151 (1,560–2,762)	1,676 (1,229–1,943)	113 (76–140)
Turner syndrome	0 (0–0)	0 (0–0)	0 (0–0)	0 (0–0)	0 (0–0)	0 (0–0)
Klinefelter syndrome	0 (0–0)	0 (0–0)	0 (0–0)	0 (0–0)	0 (0–0)	0 (0–0)
Other chromosomal abnormalities	15,288 (7,205–30,094)	14,449 (6,570–29,038)	267 (142–474)	353 (255–429)	158 (87–213)	58 (42–71)
Other congenital anomalies	91,893 (76,213–119,862)	74,252 (59,930–100,748)	4,211 (3,475–5,846)	4,933 (4,119–6,070)	3,955 (3,037–4,498)	4,540 (3,263–5,043)
Skin and subcutaneous diseases	53,774 (31,995–73,105)	3,750 (1,900–6,344)	611 (298–917)	4,457 (2,392–7,000)	10,159 (5,738–15,370)	34,794 (21,181–44,269)
Dermatitis	0 (0–0)	0 (0–0)	0 (0–0)	0 (0–0)	0 (0–0)	0 (0–0)
Psoriasis	0 (0–0)	0 (0–0)	0 (0–0)	0 (0–0)	0 (0–0)	0 (0–0)
Cellulitis	8,446 (4,632–11,676)	349 (134–776)	98 (43–156)	902 (417–1,364)	2,357 (1,188–3,496)	4,738 (2,546–6,388)
Pyoderma	23,818 (14,500–36,960)	3,188 (1,585–5,517)	339 (167–545)	2,611 (1,346–4,320)	5,346 (3,106–9,040)	12,332 (8,024–17,587)
Scabies	0 (0–0)	0 (0–0)	0 (0–0)	0 (0–0)	0 (0–0)	0 (0–0)
Fungal skin diseases	0 (0–0)	0 (0–0)	0 (0–0)	0 (0–0)	0 (0–0)	0 (0–0)
Viral skin diseases	0 (0–0)	0 (0–0)	0 (0–0)	0 (0–0)	0 (0–0)	0 (0–0)
Acne vulgaris	0 (0–0)	0 (0–0)	0 (0–0)	0 (0–0)	0 (0–0)	0 (0–0)
Alopecia areata	0 (0–0)	0 (0–0)	0 (0–0)	0 (0–0)	0 (0–0)	0 (0–0)
Pruritus	0 (0–0)	0 (0–0)	0 (0–0)	0 (0–0)	0 (0–0)	0 (0–0)
Urticaria	0 (0–0)	0 (0–0)	0 (0–0)	0 (0–0)	0 (0–0)	0 (0–0)
Decubitus ulcer	19,148 (10,546–24,464)	66 (22–139)	87 (38–139)	580 (287–839)	2,004 (1,039–3,041)	16,408 (8,915–20,292)
Other skin and subcutaneous diseases	2,361 (1,462–3,409)	145 (68–275)	86 (38–138)	363 (197–550)	450 (266–710)	1,314 (845–1,857)
Sense organ diseases	0 (0–0)	0 (0–0)	0 (0–0)	0 (0–0)	0 (0–0)	0 (0–0)
Glaucoma	0 (0–0)	0 (0–0)	0 (0–0)	0 (0–0)	0 (0–0)	0 (0–0)
Cataract	0 (0–0)	0 (0–0)	0 (0–0)	0 (0–0)	0 (0–0)	0 (0–0)
Macular degeneration	0 (0–0)	0 (0–0)	0 (0–0)	0 (0–0)	0 (0–0)	0 (0–0)
Refraction and accommodation disorders	0 (0–0)	0 (0–0)	0 (0–0)	0 (0–0)	0 (0–0)	0 (0–0)
Age-related and other hearing loss	0 (0–0)	0 (0–0)	0 (0–0)	0 (0–0)	0 (0–0)	0 (0–0)
Other vision loss	0 (0–0)	0 (0–0)	0 (0–0)	0 (0–0)	0 (0–0)	0 (0–0)
Other sense organ diseases	0 (0–0)	0 (0–0)	0 (0–0)	0 (0–0)	0 (0–0)	0 (0–0)
Oral disorders	0 (0–0)	0 (0–0)	0 (0–0)	0 (0–0)	0 (0–0)	0 (0–0)
Deciduous caries	0 (0–0)	0 (0–0)	0 (0–0)	0 (0–0)	0 (0–0)	0 (0–0)
Permanent caries	0 (0–0)	0 (0–0)	0 (0–0)	0 (0–0)	0 (0–0)	0 (0–0)
Periodontal diseases	0 (0–0)	0 (0–0)	0 (0–0)	0 (0–0)	0 (0–0)	0 (0–0)
Edentulism and severe tooth loss	0 (0–0)	0 (0–0)	0 (0–0)	0 (0–0)	0 (0–0)	0 (0–0)
Other oral disorders	0 (0–0)	0 (0–0)	0 (0–0)	0 (0–0)	0 (0–0)	0 (0–0)
Sudden infant death syndrome	9,039 (6,869–13,084)	9,039 (6,869–13,084)	0 (0–0)	0 (0–0)	0 (0–0)	0 (0–0)
Injuries	1,399,264 (1,246,352–1,484,818)	136,538 (111,878–155,519)	77,856 (70,251–85,934)	491,147 (439,194–526,723)	296,513 (264,965–318,137)	397,208 (353,513–415,670)
Transport injuries	357,622 (336,530–379,077)	22,728 (18,742–28,016)	22,692 (20,883–24,863)	145,441 (136,006–156,038)	94,868 (88,432–101,243)	71,891 (67,351–76,133)
Road injuries	337,280 (317,485–357,009)	20,870 (17,290–25,617)	21,523 (19,783–23,635)	137,364 (128,550–147,051)	89,421 (83,373–95,291)	68,100 (63,778–72,111)
Pedestrian road injuries	148,837 (139,081–160,020)	9,167 (7,612–11,189)	9,910 (9,054–10,927)	51,156 (47,318–55,582)	42,422 (39,058–46,234)	36,181 (33,627–39,103)
Cyclist road injuries	14,475 (13,171–15,892)	488 (372–676)	999 (894–1,128)	6,220 (5,597–6,924)	4,721 (4,206–5,267)	2,044 (1,812–2,298)
Motorcyclist road injuries	50,410 (44,536–56,712)	2,650 (2,040–3,596)	2,521 (2,135–3,076)	23,735 (20,948–26,769)	12,977 (11,322–14,847)	8,525 (7,267–9,986)

SR Table 10 - Global deaths by age and cause for females in 2015						
Cause	All ages	0-5 years	5-14 years	15-49 years	50-69 years	70+ years
Motor vehicle road injuries	119,402 (111,786–127,223)	8,236 (6,612–10,438)	7,800 (7,119–8,718)	54,531 (51,257–59,511)	28,274 (25,362–30,427)	20,558 (18,095–22,104)
Other road injuries	4,154 (3,687–4,640)	326 (243–450)	291 (245–350)	1,720 (1,502–1,963)	1,026 (866–1,208)	789 (660–915)
Other transport injuries	20,342 (18,192–24,673)	1,858 (1,303–2,696)	1,168 (996–1,400)	8,077 (7,177–9,910)	5,447 (4,737–6,941)	3,790 (3,319–4,807)
Unintentional injuries	668,851 (549,593–717,688)	96,518 (72,132–113,766)	42,449 (34,993–48,124)	149,252 (118,599–164,842)	112,488 (93,228–123,556)	268,142 (226,399–282,766)
Falls	211,496 (175,952–227,465)	10,947 (6,146–15,948)	5,184 (4,219–6,160)	18,345 (16,029–20,673)	30,076 (26,341–32,820)	146,942 (122,328–157,889)
Drowning	100,658 (60,602–119,693)	22,665 (11,240–29,972)	15,027 (9,941–18,383)	27,789 (15,781–34,650)	15,596 (9,359–19,037)	19,579 (13,079–21,961)
Fire, heat, and hot substances	87,252 (65,067–97,798)	11,564 (8,269–13,886)	4,561 (3,559–5,582)	35,529 (24,354–41,236)	13,919 (10,396–15,839)	21,677 (17,352–23,750)
Poisonings	34,538 (22,885–41,445)	7,546 (2,708–13,607)	2,012 (1,618–2,324)	11,430 (8,429–12,433)	6,855 (4,790–7,526)	6,693 (5,280–7,185)
Exposure to mechanical forces	55,426 (44,362–59,927)	11,841 (9,323–13,516)	3,724 (3,315–4,287)	13,524 (10,869–14,771)	10,416 (8,776–11,265)	15,919 (10,927–17,671)
Unintentional firearm injuries	9,246 (6,402–10,404)	696 (388–948)	519 (336–657)	2,622 (1,661–3,103)	1,443 (897–1,665)	3,964 (3,085–4,332)
Unintentional suffocation	11,999 (8,091–13,662)	6,212 (3,843–7,510)	939 (719–1,273)	1,365 (1,031–1,538)	1,067 (679–1,238)	2,414 (1,404–2,955)
Other exposure to mechanical forces	34,180 (27,154–37,168)	4,933 (4,279–5,800)	2,265 (1,992–2,634)	9,536 (7,478–10,455)	7,906 (6,766–8,626)	9,539 (5,853–10,795)
Adverse effects of medical treatment	43,042 (31,655–50,060)	5,559 (3,380–7,475)	1,547 (997–1,915)	9,025 (6,005–10,694)	8,883 (6,530–10,391)	18,026 (14,360–20,212)
Animal contact	38,987 (20,888–55,020)	4,968 (2,658–6,601)	4,245 (2,402–5,937)	14,578 (7,513–21,268)	9,651 (4,914–13,541)	5,542 (3,077–7,062)
Venomous animal contact	33,450 (16,743–48,809)	3,573 (1,804–5,098)	3,530 (1,901–5,311)	13,217 (6,470–19,618)	8,662 (4,217–12,422)	4,466 (2,177–5,813)
Non-venomous animal contact	5,537 (3,966–6,491)	1,395 (861–1,782)	715 (510–922)	1,361 (1,000–1,687)	988 (706–1,183)	1,076 (865–1,230)
Foreign body	61,014 (51,821–74,014)	17,143 (11,581–26,385)	3,337 (2,483–4,406)	8,938 (7,497–10,794)	8,468 (7,295–9,464)	23,127 (21,051–24,658)
Pulmonary aspiration and foreign body in airway	49,891 (40,429–63,871)	15,285 (9,312–24,754)	2,467 (1,736–3,584)	5,735 (4,648–7,574)	6,622 (5,672–7,621)	19,778 (17,994–21,244)
Foreign body in eyes	0 (0–0)	0 (0–0)	0 (0–0)	0 (0–0)	0 (0–0)	0 (0–0)
Foreign body in other body part	11,123 (8,173–13,553)	1,857 (1,065–2,949)	869 (543–1,225)	3,202 (2,331–3,985)	1,845 (1,344–2,385)	3,348 (2,699–3,987)
Environmental heat and cold exposure	13,647 (9,482–15,697)	959 (450–1,216)	367 (213–471)	2,374 (1,781–2,724)	4,041 (2,835–4,863)	5,903 (4,073–6,901)
Other unintentional injuries	22,786 (21,192–24,515)	3,321 (2,796–4,029)	2,440 (2,080–2,893)	7,715 (6,942–8,581)	4,578 (4,188–4,991)	4,731 (4,296–5,189)
Self-harm and interpersonal violence	328,208 (268,741–358,756)	4,681 (3,894–5,858)	7,067 (6,122–8,073)	177,925 (143,355–197,199)	83,625 (66,609–91,638)	54,909 (46,390–58,869)
Self-harm	249,229 (190,023–276,952)	0 (0–0)	3,085 (2,370–3,722)	132,483 (99,707–150,004)	69,194 (52,367–76,824)	44,465 (36,048–48,249)
Interpersonal violence	78,979 (73,056–85,036)	4,681 (3,894–5,858)	3,981 (3,512–4,520)	45,442 (41,879–49,130)	14,430 (13,341–15,726)	10,444 (9,606–11,118)
Assault by firearm	21,147 (17,175–24,312)	794 (587–1,085)	1,023 (793–1,219)	13,841 (11,155–15,792)	3,346 (2,862–4,050)	2,141 (1,627–2,656)
Assault by sharp object	17,155 (15,887–19,135)	454 (357–595)	590 (502–692)	10,480 (9,627–11,644)	3,143 (2,908–3,531)	2,486 (2,255–2,971)
Assault by other means	40,675 (36,775–45,200)	3,432 (2,826–4,334)	2,366 (2,057–2,723)	21,120 (18,696–23,749)	7,940 (7,071–8,878)	5,816 (5,033–6,332)
Forces of nature, war, and legal intervention	44,581 (25,955–63,252)	12,609 (8,080–17,182)	5,647 (2,968–8,263)	18,528 (10,028–26,985)	5,530 (3,220–8,026)	2,265 (1,358–3,241)
Exposure to forces of nature	4,531 (2,719–6,254)	408 (245–573)	832 (489–1,175)	2,022 (1,196–2,809)	813 (481–1,124)	453 (275–655)
Collective violence and legal intervention	40,050 (22,477–58,058)	12,201 (7,727–16,685)	4,814 (2,273–7,287)	16,506 (8,128–24,767)	4,716 (2,452–7,038)	1,811 (989–2,745)

SR Table 11: Global deaths by cause and age group for males (with 95% uncertainty intervals), 1990						
Cause	All ages	0-5 years	5-14 years	15-49 years	50-69 years	70+ years
All causes	25,314,405 (24,927,084–25,693,574)	6,365,122 (6,301,929–6,431,490)	719,886 (710,187–729,378)	4,441,030 (4,318,682–4,569,803)	6,692,703 (6,534,406–6,843,238)	7,095,662 (6,996,248–7,191,228)
Communicable, maternal, neonatal, and nutritional diseases	8,182,493 (7,931,026–8,503,642)	5,323,333 (5,130,163–5,468,520)	330,372 (310,282–354,415)	982,707 (919,973–1,065,328)	828,893 (757,478–931,247)	717,186 (669,203–789,868)
HIV/AIDS and tuberculosis	1,121,133 (926,626–1,418,538)	51,712 (39,153–81,200)	15,047 (10,824–22,369)	476,476 (404,202–570,219)	387,772 (301,934–499,185)	190,125 (147,352–266,295)
Tuberculosis	942,042 (741,893–1,234,604)	33,680 (21,119–63,195)	14,142 (9,923–21,502)	340,443 (266,002–434,351)	365,610 (279,145–476,424)	188,166 (145,610–264,374)
HIV/AIDS	179,090 (162,941–198,513)	18,032 (16,588–19,918)	904 (796–1,034)	136,033 (122,995–151,817)	22,161 (19,650–25,305)	1,959 (1,621–2,370)
HIV/AIDS - Tuberculosis	44,072 (37,653–51,730)	6,009 (4,921–6,927)	251 (212–293)	31,174 (26,568–37,095)	6,037 (5,154–6,978)	598 (495–724)
HIV/AIDS resulting in other diseases	135,018 (119,913–153,719)	12,022 (10,620–13,915)	652 (561–766)	104,858 (92,133–120,079)	16,124 (13,984–18,994)	1,360 (1,081–1,710)
Diarrhea, lower respiratory, and other common infectious diseases	3,993,133 (3,801,410–4,233,320)	2,588,866 (2,419,508–2,780,937)	236,778 (209,731–268,559)	353,938 (321,271–390,630)	346,991 (320,462–380,993)	466,559 (437,160–503,739)
Diarrheal diseases	1,122,853 (1,014,123–1,224,302)	702,824 (600,904–798,487)	54,441 (47,057–61,495)	110,786 (101,031–122,840)	124,925 (114,717–136,313)	129,874 (120,101–140,896)
Intestinal infectious diseases	127,064 (70,584–208,079)	36,458 (19,512–63,654)	47,142 (27,071–74,824)	37,230 (19,649–62,860)	4,631 (2,372–8,143)	1,600 (949–2,478)
Typhoid fever	99,763 (53,828–166,967)	27,294 (13,494–49,947)	37,432 (20,336–60,544)	30,094 (15,813–52,063)	3,754 (1,837–6,871)	1,188 (643–1,945)
Paratyphoid fever	24,368 (11,310–46,120)	7,072 (3,019–14,365)	9,503 (4,489–18,240)	6,852 (2,972–13,188)	719 (286–1,493)	220 (93–437)
Other intestinal infectious diseases	2,932 (1,167–6,788)	2,092 (670–5,699)	206 (68–576)	283 (117–473)	157 (61–267)	192 (73–355)
Lower respiratory infections	1,713,634 (1,579,967–1,838,126)	1,074,719 (962,162–1,175,617)	45,953 (34,333–53,025)	116,256 (94,800–132,718)	167,883 (142,861–193,301)	308,822 (276,772–342,655)
Upper respiratory infections	2,357 (2,026–2,768)	967 (668–1,348)	48 (31–70)	471 (394–562)	501 (398–614)	368 (293–461)
Otitis media	3,746 (3,218–4,345)	2,094 (1,698–2,630)	528 (461–591)	652 (578–719)	312 (270–356)	159 (134–191)
Meningitis	260,013 (201,964–307,057)	167,087 (120,440–210,733)	21,449 (13,996–25,390)	39,578 (28,019–46,775)	21,161 (15,624–26,881)	10,736 (8,150–14,347)
Pneumococcal meningitis	63,100 (47,355–79,630)	35,178 (22,905–49,862)	6,558 (4,169–9,140)	12,606 (8,821–16,206)	5,794 (4,069–8,015)	2,962 (2,134–4,107)
H influenzae type B meningitis	79,965 (58,496–102,107)	61,834 (42,652–82,715)	6,000 (3,664–8,476)	6,813 (4,619–9,117)	3,484 (2,349–4,748)	1,832 (1,338–2,557)
Meningococcal meningitis	45,762 (32,641–60,740)	30,359 (19,119–44,500)	3,889 (2,281–5,677)	6,539 (4,477–8,780)	3,433 (2,341–4,688)	1,540 (1,109–2,128)
Other meningitis	71,185 (54,596–87,182)	39,715 (26,947–53,682)	5,001 (3,021–6,932)	13,618 (9,113–16,540)	8,448 (6,010–10,802)	4,401 (3,228–5,633)
Encephalitis	80,681 (71,303–91,321)	31,214 (26,036–36,717)	9,836 (8,301–11,468)	18,116 (16,187–20,506)	12,343 (10,849–14,339)	9,170 (7,956–10,727)
Diphtheria	7,349 (4,178–13,678)	5,765 (2,780–11,954)	859 (430–1,523)	540 (371–742)	184 (100–323)	0 (0–0)
Whooping cough	71,876 (25,341–171,027)	67,141 (23,645–159,369)	3,710 (1,314–8,914)	900 (288–2,399)	124 (36–353)	0 (0–0)
Tetanus	179,751 (154,452–218,406)	134,332 (116,149–153,853)	6,768 (4,089–11,747)	20,442 (11,351–33,251)	13,617 (8,275–26,238)	4,590 (2,829–8,802)
Measles	417,920 (165,450–826,196)	364,159 (144,667–713,406)	45,497 (17,381–92,217)	7,952 (2,325–20,609)	311 (86–826)	0 (0–0)
Varicella and herpes zoster	5,884 (5,042–6,871)	2,101 (1,566–2,822)	541 (389–776)	1,009 (842–1,228)	993 (759–1,263)	1,236 (928–1,651)
Neglected tropical diseases and malaria	551,537 (429,220–684,784)	359,526 (241,889–488,664)	53,191 (39,373–70,511)	86,637 (69,462–107,317)	39,572 (32,974–48,553)	12,609 (10,192–15,970)
Malaria	456,068 (337,769–587,254)	341,378 (224,087–472,255)	37,058 (22,817–53,642)	48,416 (35,739–65,493)	22,234 (16,339–30,183)	6,981 (4,684–10,187)
Chagas disease	5,646 (5,336–5,948)	6 (5–7)	13 (11–15)	1,573 (1,448–1,704)	2,679 (2,486–2,876)	1,373 (1,271–1,492)
Leishmaniasis	14,087 (8,315–22,110)	2,725 (1,460–4,359)	1,513 (779–2,649)	7,950 (4,544–12,611)	1,642 (1,057–2,406)	254 (186–328)
Visceral leishmaniasis	14,087 (8,315–22,110)	2,725 (1,460–4,359)	1,513 (779–2,649)	7,950 (4,544–12,611)	1,642 (1,057–2,406)	254 (186–328)
Cutaneous and mucocutaneous leishmaniasis	0 (0–0)	0 (0–0)	0 (0–0)	0 (0–0)	0 (0–0)	0 (0–0)
African trypanosomiasis	11,938 (5,909–20,023)	855 (399–1,500)	1,430 (727–2,369)	7,707 (3,755–12,985)	1,616 (747–2,824)	328 (156–558)
Schistosomiasis	6,921 (5,935–8,038)	142 (82–250)	233 (160–337)	2,525 (2,074–3,128)	2,869 (2,182–3,629)	1,150 (873–1,513)
Cysticercosis	423 (379–483)	9 (4–16)	17 (13–21)	266 (239–304)	104 (93–119)	25 (22–28)
Cystic echinococcosis	1,385 (1,290–1,494)	93 (71–115)	151 (132–172)	555 (507–608)	399 (371–432)	185 (173–198)
Lymphatic filariasis	0 (0–0)	0 (0–0)	0 (0–0)	0 (0–0)	0 (0–0)	0 (0–0)
Onchocerciasis	0 (0–0)	0 (0–0)	0 (0–0)	0 (0–0)	0 (0–0)	0 (0–0)
Trachoma	0 (0–0)	0 (0–0)	0 (0–0)	0 (0–0)	0 (0–0)	0 (0–0)
Dengue	3,149 (1,685–6,143)	1,421 (435–3,336)	755 (419–1,374)	680 (471–1,122)	192 (122–321)	99 (64–171)
Yellow fever	9,503 (2,093–25,736)	903 (182–2,678)	2,445 (526–6,840)	5,234 (1,124–14,304)	787 (159–2,343)	132 (26–399)
Rabies	28,890 (25,524–31,531)	4,052 (2,796–5,690)	7,992 (6,649–9,420)	9,781 (8,374–10,892)	5,636 (4,640–6,345)	1,426 (1,114–1,672)
Intestinal nematode infections	3,370 (2,805–3,923)	2,638 (2,117–3,150)	446 (371–528)	148 (115–185)	88 (62–118)	48 (31–69)
Ascariasis	3,370 (2,805–3,923)	2,638 (2,117–3,150)	446 (371–528)	148 (115–185)	88 (62–118)	48 (31–69)
Trichuriasis	0 (0–0)	0 (0–0)	0 (0–0)	0 (0–0)	0 (0–0)	0 (0–0)
Hookworm disease	0 (0–0)	0 (0–0)	0 (0–0)	0 (0–0)	0 (0–0)	0 (0–0)
Food-borne trematodiases	0 (0–0)	0 (0–0)	0 (0–0)	0 (0–0)	0 (0–0)	0 (0–0)
Leprosy	0 (0–0)	0 (0–0)	0 (0–0)	0 (0–0)	0 (0–0)	0 (0–0)
Ebola	0 (0–0)	0 (0–0)	0 (0–0)	0 (0–0)	0 (0–0)	0 (0–0)
Other neglected tropical diseases	10,151 (5,050–15,569)	5,299 (2,473–7,385)	1,133 (554–1,957)	1,795 (909–3,182)	1,320 (649–2,307)	602 (336–1,114)
Maternal disorders	0 (0–0)	0 (0–0)	0 (0–0)	0 (0–0)	0 (0–0)	0 (0–0)
Maternal hemorrhage	0 (0–0)	0 (0–0)	0 (0–0)	0 (0–0)	0 (0–0)	0 (0–0)
Maternal sepsis and other maternal infections	0 (0–0)	0 (0–0)	0 (0–0)	0 (0–0)	0 (0–0)	0 (0–0)
Maternal hypertensive disorders	0 (0–0)	0 (0–0)	0 (0–0)	0 (0–0)	0 (0–0)	0 (0–0)
Maternal obstructed labor and uterine rupture	0 (0–0)	0 (0–0)	0 (0–0)	0 (0–0)	0 (0–0)	0 (0–0)
Maternal abortion, miscarriage, and ectopic pregnancy	0 (0–0)	0 (0–0)	0 (0–0)	0 (0–0)	0 (0–0)	0 (0–0)

SR Table 11 - Global deaths by age and cause for males in 1990						
Cause	All ages	0-5 years	5-14 years	15-49 years	50-69 years	70+ years
Indirect maternal deaths	0 (0–0)	0 (0–0)	0 (0–0)	0 (0–0)	0 (0–0)	0 (0–0)
Late maternal deaths	0 (0–0)	0 (0–0)	0 (0–0)	0 (0–0)	0 (0–0)	0 (0–0)
Maternal deaths aggravated by HIV/AIDS	0 (0–0)	0 (0–0)	0 (0–0)	0 (0–0)	0 (0–0)	0 (0–0)
Other maternal disorders	0 (0–0)	0 (0–0)	0 (0–0)	0 (0–0)	0 (0–0)	0 (0–0)
Neonatal disorders	1,961,093 (1,896,363–2,032,569)	1,961,093 (1,896,363–2,032,569)	0 (0–0)	0 (0–0)	0 (0–0)	0 (0–0)
Neonatal preterm birth complications	972,470 (875,305–1,081,518)	972,470 (875,305–1,081,518)	0 (0–0)	0 (0–0)	0 (0–0)	0 (0–0)
Neonatal encephalopathy due to birth asphyxia and trauma	523,588 (433,027–617,029)	523,588 (433,027–617,029)	0 (0–0)	0 (0–0)	0 (0–0)	0 (0–0)
Neonatal sepsis and other neonatal infections	209,488 (130,655–324,443)	209,488 (130,655–324,443)	0 (0–0)	0 (0–0)	0 (0–0)	0 (0–0)
Hemolytic disease and other neonatal jaundice	66,341 (42,794–123,354)	66,341 (42,794–123,354)	0 (0–0)	0 (0–0)	0 (0–0)	0 (0–0)
Other neonatal disorders	189,204 (128,530–255,227)	189,204 (128,530–255,227)	0 (0–0)	0 (0–0)	0 (0–0)	0 (0–0)
Nutritional deficiencies	307,312 (188,875–395,869)	221,042 (129,100–305,356)	10,441 (7,156–13,472)	21,339 (14,055–27,335)	22,401 (15,496–28,906)	32,087 (21,845–39,109)
Protein-energy malnutrition	269,765 (168,188–354,493)	206,208 (122,559–287,532)	8,569 (5,835–10,775)	13,174 (9,079–16,810)	15,965 (11,585–20,642)	25,847 (17,536–31,502)
Iodine deficiency	1,046 (629–1,909)	518 (179–1,266)	46 (20–91)	185 (108–332)	171 (98–309)	124 (72–224)
Vitamin A deficiency	0 (0–0)	0 (0–0)	0 (0–0)	0 (0–0)	0 (0–0)	0 (0–0)
Iron-deficiency anemia	19,073 (7,276–27,929)	7,587 (1,716–12,524)	815 (351–1,501)	4,537 (2,228–7,017)	3,746 (1,726–5,610)	2,386 (959–3,506)
Other nutritional deficiencies	17,427 (11,190–28,631)	6,728 (3,335–12,969)	1,010 (659–1,667)	3,441 (2,181–5,198)	2,517 (1,649–4,237)	3,728 (2,813–5,573)
Other communicable, maternal, neonatal, and nutritional diseases	248,283 (201,654–307,372)	141,091 (99,822–194,531)	14,914 (12,024–18,837)	44,316 (41,341–47,195)	32,156 (28,088–34,488)	15,804 (13,294–17,036)
Sexually transmitted diseases excluding HIV	107,799 (65,812–164,633)	92,850 (53,131–146,200)	6,734 (3,890–10,463)	3,506 (1,705–4,413)	3,037 (1,398–3,959)	1,671 (889–2,230)
Syphilis	106,773 (64,893–163,557)	92,850 (53,131–146,200)	6,707 (3,861–10,436)	2,991 (1,455–3,768)	2,753 (1,267–3,591)	1,470 (782–1,961)
Chlamydial infection	0 (0–0)	0 (0–0)	0 (0–0)	0 (0–0)	0 (0–0)	0 (0–0)
Gonococcal infection	709 (354–879)	0 (0–0)	15 (9–22)	365 (178–464)	175 (80–228)	154 (81–205)
Trichomoniasis	0 (0–0)	0 (0–0)	0 (0–0)	0 (0–0)	0 (0–0)	0 (0–0)
Genital herpes	0 (0–0)	0 (0–0)	0 (0–0)	0 (0–0)	0 (0–0)	0 (0–0)
Other sexually transmitted diseases	316 (157–393)	0 (0–0)	11 (7–16)	149 (71–189)	108 (49–141)	47 (25–63)
Hepatitis	85,827 (80,802–91,284)	20,529 (17,313–23,392)	5,447 (4,800–6,088)	32,350 (30,316–35,114)	19,364 (18,175–20,724)	8,135 (7,702–8,615)
Acute hepatitis A	17,722 (12,333–22,864)	15,773 (11,578–19,316)	808 (235–1,687)	796 (192–1,899)	256 (52–690)	87 (18–239)
Hepatitis B	45,086 (38,687–51,564)	1,157 (402–2,656)	675 (358–1,110)	21,135 (17,389–24,699)	15,994 (13,853–18,074)	6,122 (4,995–7,097)
Hepatitis C	1,561 (339–3,632)	0 (0–0)	78 (15–202)	530 (107–1,269)	725 (154–1,720)	226 (45–558)
Acute hepatitis E	21,456 (14,963–28,962)	3,597 (1,435–6,794)	3,885 (2,969–4,774)	9,887 (6,828–13,499)	2,387 (1,163–4,224)	1,698 (857–2,813)
Other infectious diseases	54,655 (32,127–69,019)	27,712 (10,789–41,509)	2,731 (1,822–3,242)	8,460 (6,580–9,638)	9,754 (6,468–11,303)	5,997 (4,101–6,931)
Non-communicable diseases	14,191,856 (13,843,562–14,565,385)	636,255 (532,102–821,968)	135,523 (125,026–145,521)	1,887,913 (1,818,591–1,962,256)	5,386,213 (5,242,037–5,532,083)	6,145,949 (6,040,599–6,241,005)
Neoplasms	3,184,028 (3,091,063–3,275,555)	28,428 (22,080–40,837)	33,704 (30,642–36,656)	439,494 (412,344–458,787)	1,536,241 (1,488,456–1,580,430)	1,146,159 (1,114,512–1,180,983)
Lip and oral cavity cancer	50,965 (48,960–53,421)	0 (0–0)	0 (0–0)	8,866 (8,418–9,321)	28,440 (27,105–30,008)	13,658 (13,079–14,307)
Nasopharynx cancer	30,175 (21,872–33,255)	0 (0–0)	284 (175–370)	9,076 (5,982–10,346)	15,101 (11,037–16,622)	5,713 (4,538–6,175)
Other pharynx cancer	26,484 (25,488–27,374)	0 (0–0)	0 (0–0)	4,131 (3,943–4,306)	16,622 (15,950–17,249)	5,730 (5,519–5,960)
Esophageal cancer	232,029 (214,124–252,549)	0 (0–0)	0 (0–0)	24,441 (22,163–26,990)	132,462 (121,823–144,434)	75,126 (69,353–82,650)
Stomach cancer	465,159 (446,346–484,685)	0 (0–0)	0 (0–0)	52,674 (49,703–56,059)	236,784 (225,985–247,483)	175,699 (169,819–181,868)
Colon and rectum cancer	243,174 (237,053–250,404)	0 (0–0)	0 (0–0)	26,385 (25,163–27,702)	107,168 (104,225–110,684)	109,621 (106,965–112,728)
Liver cancer	351,892 (294,167–385,458)	0 (0–0)	993 (673–1,238)	83,729 (61,023–94,220)	187,263 (157,346–206,738)	79,905 (73,602–87,696)
Liver cancer due to hepatitis B	147,212 (118,369–165,229)	0 (0–0)	639 (429–791)	51,758 (37,728–58,936)	73,055 (59,719–82,503)	21,759 (18,994–25,177)
Liver cancer due to hepatitis C	50,461 (45,691–55,488)	0 (0–0)	20 (14–29)	5,141 (3,964–5,992)	27,276 (23,872–30,495)	18,022 (16,443–20,163)
Liver cancer due to alcohol use	103,890 (88,407–115,814)	0 (0–0)	0 (0–0)	12,462 (9,098–14,486)	62,532 (52,832–70,727)	28,895 (25,841–32,031)
Liver cancer due to other causes	50,327 (40,528–56,640)	0 (0–0)	333 (230–421)	14,366 (10,183–16,514)	24,399 (20,158–28,141)	11,228 (9,877–12,834)
Gallbladder and biliary tract cancer	38,221 (37,031–39,453)	0 (0–0)	0 (0–0)	3,177 (2,953–3,406)	16,869 (16,270–17,476)	18,174 (17,667–18,733)
Pancreatic cancer	114,404 (111,453–117,163)	0 (0–0)	0 (0–0)	10,957 (10,501–11,454)	58,144 (56,480–59,842)	45,302 (44,219–46,376)
Larynx cancer	67,174 (65,297–69,129)	0 (0–0)	0 (0–0)	7,628 (7,320–7,953)	39,643 (38,418–40,895)	19,902 (19,226–20,576)
Tracheal, bronchus, and lung cancer	748,671 (727,370–774,206)	0 (0–0)	0 (0–0)	63,118 (60,104–66,292)	416,572 (404,292–430,669)	268,980 (261,396–279,329)
Malignant skin melanoma	16,527 (11,504–24,832)	0 (0–0)	0 (0–0)	4,308 (2,884–5,899)	7,330 (5,044–11,045)	4,888 (3,460–8,066)
Non-melanoma skin cancer	17,657 (17,079–18,225)	0 (0–0)	0 (0–0)	2,085 (1,986–2,195)	7,160 (6,898–7,420)	8,411 (8,141–8,712)
Non-melanoma skin cancer (squamous-cell carcinoma)	17,657 (17,079–18,225)	0 (0–0)	0 (0–0)	2,085 (1,986–2,195)	7,160 (6,898–7,420)	8,411 (8,141–8,712)
Non-melanoma skin cancer (basal-cell carcinoma)	0 (0–0)	0 (0–0)	0 (0–0)	0 (0–0)	0 (0–0)	0 (0–0)
Breast cancer	3,393 (3,127–3,548)	0 (0–0)	0 (0–0)	409 (386–436)	1,635 (1,465–1,718)	1,347 (1,236–1,427)
Cervical cancer	0 (0–0)	0 (0–0)	0 (0–0)	0 (0–0)	0 (0–0)	0 (0–0)
Uterine cancer	0 (0–0)	0 (0–0)	0 (0–0)	0 (0–0)	0 (0–0)	0 (0–0)
Ovarian cancer	0 (0–0)	0 (0–0)	0 (0–0)	0 (0–0)	0 (0–0)	0 (0–0)
Prostate cancer	191,896 (154,039–236,472)	0 (0–0)	0 (0–0)	1,710 (1,337–2,059)	50,045 (39,928–62,341)	140,140 (110,095–174,143)
Testicular cancer	8,607 (8,154–9,301)	0 (0–0)	0 (0–0)	6,058 (5,714–6,567)	1,572 (1,482–1,702)	977 (923–1,057)

SR Table 11 - Global deaths by age and cause for males in 1990

Cause	All ages	0-5 years	5-14 years	15-49 years	50-69 years	70+ years
Kidney cancer	43,703 (42,522–45,305)	1,563 (842–2,396)	520 (462–594)	4,615 (4,417–4,896)	21,516 (20,727–22,522)	15,488 (14,947–16,058)
Bladder cancer	81,208 (78,462–83,903)	0 (0–0)	0 (0–0)	3,838 (3,640–4,041)	31,755 (30,433–33,165)	45,615 (44,163–47,158)
Brain and nervous system cancer	78,416 (65,857–97,428)	4,293 (2,459–9,081)	5,998 (4,250–8,567)	23,942 (19,385–29,036)	31,794 (27,569–36,817)	12,387 (11,243–15,138)
Thyroid cancer	6,988 (6,372–7,621)	0 (0–0)	38 (30–43)	972 (865–1,066)	3,276 (2,928–3,575)	2,700 (2,476–2,979)
Mesothelioma	12,031 (10,967–12,932)	0 (0–0)	0 (0–0)	2,080 (1,735–2,240)	5,843 (5,342–6,246)	4,108 (3,774–4,665)
Hodgkin lymphoma	19,169 (13,752–25,525)	747 (378–925)	1,078 (655–1,370)	6,985 (5,048–9,152)	7,158 (5,025–9,711)	3,198 (2,401–4,415)
Non-Hodgkin lymphoma	68,759 (62,604–80,601)	2,300 (1,238–4,261)	2,789 (2,155–3,697)	15,333 (13,532–17,594)	26,709 (23,744–30,709)	21,626 (19,685–26,332)
Multiple myeloma	24,795 (24,017–25,841)	0 (0–0)	0 (0–0)	2,609 (2,487–2,786)	11,514 (11,078–12,071)	10,672 (10,339–11,057)
Leukemia	138,958 (134,201–144,991)	11,554 (9,860–13,555)	16,528 (15,283–17,773)	42,038 (39,870–44,689)	37,546 (36,270–38,984)	31,291 (30,447–32,186)
Acute lymphoid leukemia	51,004 (43,107–60,588)	7,545 (5,406–10,431)	11,709 (9,947–13,770)	17,564 (14,559–21,143)	9,620 (8,105–11,389)	4,563 (3,928–5,367)
Chronic lymphoid leukemia	20,133 (17,777–22,572)	0 (0–0)	0 (0–0)	3,088 (2,394–3,968)	7,037 (6,177–8,009)	10,008 (8,894–10,739)
Acute myeloid leukemia	48,459 (40,409–57,143)	4,009 (1,841–5,862)	4,818 (2,985–6,279)	14,684 (11,918–17,980)	14,313 (12,523–16,526)	10,633 (9,598–12,540)
Chronic myeloid leukemia	19,361 (17,297–21,817)	0 (0–0)	0 (0–0)	6,701 (5,729–7,940)	6,574 (5,804–7,473)	6,085 (5,564–6,633)
Other neoplasms	103,560 (92,013–111,442)	7,967 (4,624–14,044)	5,473 (4,364–6,102)	28,319 (23,345–31,017)	36,308 (31,075–40,525)	25,490 (23,226–29,233)
Cardiovascular diseases	6,173,282 (6,033,788–6,311,367)	63,384 (56,553–70,818)	23,143 (21,368–25,510)	689,867 (664,083–721,092)	2,344,750 (2,279,293–2,410,051)	3,052,135 (2,992,703–3,106,766)
Rheumatic heart disease	142,141 (134,082–153,454)	3,748 (2,954–4,765)	5,347 (4,792–5,957)	41,580 (38,513–45,779)	50,319 (46,822–54,627)	41,145 (38,920–43,813)
Ischemic heart disease	3,116,581 (3,050,314–3,182,633)	4,236 (3,433–5,266)	1,907 (1,664–2,222)	337,144 (324,024–351,405)	1,224,044 (1,192,198–1,255,937)	1,549,248 (1,518,159–1,577,764)
Cerebrovascular disease	2,138,728 (2,068,622–2,212,634)	25,883 (22,890–29,510)	9,099 (8,177–10,114)	207,936 (198,569–221,678)	805,116 (772,034–839,853)	1,090,692 (1,058,618–1,127,963)
Ischemic stroke	907,615 (869,633–948,607)	1,016 (810–1,299)	576 (497–687)	33,006 (30,050–36,442)	282,421 (265,642–299,758)	590,594 (569,646–614,453)
Hemorrhagic stroke	1,231,113 (1,174,265–1,285,540)	24,866 (22,011–28,323)	8,523 (7,646–9,484)	174,930 (166,489–186,451)	522,694 (495,692–549,443)	500,098 (476,632–521,701)
Hypertensive heart disease	296,932 (278,848–341,158)	561 (424–683)	288 (243–341)	33,884 (31,212–38,570)	115,622 (107,452–133,744)	146,575 (137,023–168,588)
Cardiomyopathy and myocarditis	141,849 (132,656–150,468)	9,312 (6,970–11,719)	2,400 (2,043–2,902)	31,532 (29,197–34,456)	48,917 (44,956–52,141)	49,686 (46,918–52,157)
Atrial fibrillation and flutter	37,272 (30,374–45,238)	0 (0–0)	0 (0–0)	386 (299–491)	4,380 (3,416–5,774)	32,505 (25,792–40,477)
Aortic aneurysm	63,464 (60,838–66,832)	0 (0–0)	0 (0–0)	5,115 (4,684–5,713)	22,627 (21,486–24,118)	35,721 (34,400–37,213)
Peripheral vascular disease	8,180 (7,717–8,710)	0 (0–0)	0 (0–0)	156 (143–170)	2,196 (2,046–2,393)	5,827 (5,490–6,194)
Endocarditis	19,735 (16,209–24,925)	2,842 (1,204–5,010)	539 (353–818)	4,653 (3,824–6,086)	5,540 (4,549–6,698)	6,158 (5,343–7,399)
Other cardiovascular and circulatory diseases	208,395 (199,519–218,431)	16,799 (14,617–20,952)	3,560 (3,206–4,193)	27,475 (25,800–29,574)	65,986 (62,612–69,426)	94,574 (89,890–98,995)
Chronic respiratory diseases	1,879,591 (1,798,621–1,955,586)	63,917 (54,789–74,626)	9,825 (8,742–10,779)	121,309 (113,911–131,103)	651,935 (617,343–686,789)	1,032,603 (990,104–1,070,238)
Chronic obstructive pulmonary disease	1,522,421 (1,376,526–1,644,809)	27,458 (19,498–36,688)	2,123 (1,625–2,805)	72,024 (60,081–83,164)	520,728 (457,818–575,855)	900,085 (827,805–958,343)
Pneumoconiosis	21,155 (18,089–24,262)	0 (0–0)	0 (0–0)	2,360 (1,916–2,876)	8,025 (6,722–9,285)	10,769 (9,262–12,364)
Silicosis	10,414 (8,309–13,001)	0 (0–0)	0 (0–0)	1,290 (881–1,745)	4,288 (3,380–5,410)	4,834 (3,898–5,986)
Asbestosis	1,206 (861–1,628)	0 (0–0)	0 (0–0)	185 (122–257)	426 (281–607)	594 (426–786)
Coal workers pneumoconiosis	3,087 (2,561–4,238)	0 (0–0)	0 (0–0)	154 (96–324)	1,071 (866–1,490)	1,862 (1,563–2,386)
Other pneumoconiosis	6,446 (5,447–7,703)	0 (0–0)	0 (0–0)	730 (548–960)	2,238 (1,802–2,790)	3,477 (2,921–4,031)
Asthma	278,708 (177,164–373,352)	18,095 (12,134–24,095)	6,670 (5,224–7,803)	41,131 (28,884–50,309)	109,373 (65,828–151,263)	103,437 (60,998–145,220)
Interstitial lung disease and pulmonary sarcoidosis	21,975 (16,888–32,176)	184 (72–601)	111 (67–245)	1,833 (1,305–3,074)	7,972 (5,788–12,434)	11,873 (9,309–17,176)
Other chronic respiratory diseases	35,331 (22,569–45,618)	18,177 (10,999–26,530)	920 (487–1,421)	3,959 (2,427–5,222)	5,835 (3,667–7,778)	6,438 (4,529–8,384)
Cirrhosis and other chronic liver diseases	589,654 (564,972–646,399)	13,040 (9,896–15,899)	6,650 (5,861–7,596)	188,995 (179,172–209,054)	273,303 (261,408–302,279)	107,664 (103,056–112,032)
Cirrhosis and other chronic liver diseases due to hepatitis B	187,919 (172,901–211,944)	138 (100–181)	279 (226–336)	62,032 (55,885–69,259)	89,112 (79,819–102,302)	36,355 (32,990–40,450)
Cirrhosis and other chronic liver diseases due to hepatitis C	145,700 (135,041–157,768)	74 (53–98)	151 (119–186)	43,302 (38,610–49,615)	70,449 (63,070–78,850)	31,722 (28,105–34,638)
Cirrhosis and other chronic liver diseases due to alcohol use	162,912 (151,576–177,807)	0 (0–0)	0 (0–0)	58,153 (53,123–64,511)	81,575 (73,648–91,380)	23,182 (20,714–25,873)
Cirrhosis and other chronic liver diseases due to other causes	93,123 (85,699–103,841)	12,827 (9,737–15,654)	6,219 (5,464–7,121)	25,507 (22,866–28,807)	32,165 (28,145–37,441)	16,403 (14,800–18,117)
Digestive diseases	539,582 (503,533–575,244)	25,806 (19,849–32,564)	14,722 (12,954–16,357)	142,057 (128,985–152,793)	188,466 (175,108–202,701)	168,529 (159,178–182,530)
Peptic ulcer disease	192,383 (176,099–212,934)	2,890 (1,628–5,463)	3,035 (2,454–3,760)	50,252 (44,925–56,214)	75,711 (68,795–83,465)	60,493 (55,220–67,736)
Gastritis and duodenitis	27,712 (19,535–35,576)	1,110 (532–1,956)	464 (313–741)	7,585 (5,329–9,894)	10,416 (7,219–13,820)	8,136 (5,705–10,967)
Appendicitis	22,255 (19,307–27,367)	2,693 (1,739–3,979)	2,390 (1,955–2,994)	7,926 (6,692–10,045)	5,505 (4,671–7,229)	3,738 (3,215–4,679)
Paralytic ileus and intestinal obstruction	105,710 (82,675–123,070)	7,338 (3,798–11,416)	4,830 (3,386–5,863)	30,134 (21,316–35,001)	34,451 (25,865–40,191)	28,956 (24,853–36,089)
Inguinal, femoral, and abdominal hernia	29,919 (18,432–36,180)	1,522 (380–2,764)	741 (359–954)	7,123 (3,742–8,705)	9,767 (6,335–12,809)	10,763 (7,170–12,939)
Inflammatory bowel disease	14,988 (11,874–17,416)	1,329 (579–3,358)	396 (256–637)	3,460 (2,627–4,073)	4,807 (3,884–5,614)	4,993 (4,126–5,769)
Vascular intestinal disorders	24,226 (22,266–26,572)	735 (364–1,470)	122 (93–159)	2,132 (1,893–2,381)	8,535 (7,854–9,351)	12,701 (11,880–13,730)
Gallbladder and biliary diseases	33,650 (29,413–37,086)	858 (479–1,465)	511 (387–717)	6,063 (4,882–7,016)	11,277 (9,479–12,449)	14,939 (13,570–16,199)
Pancreatitis	39,448 (36,285–44,025)	239 (134–406)	253 (200–334)	17,912 (16,052–20,325)	13,574 (12,439–15,252)	7,468 (6,868–8,175)
Other digestive diseases	49,286 (36,092–63,856)	7,088 (4,165–10,572)	1,974 (1,374–2,622)	9,466 (6,667–13,675)	14,420 (10,323–19,833)	16,336 (12,360–20,633)
Neurological disorders	440,735 (388,980–493,943)	15,740 (9,512–20,832)	8,728 (7,699–9,705)	43,948 (41,742–45,993)	56,651 (48,699–64,610)	315,665 (267,961–363,066)
Alzheimer disease and other dementias	316,770 (265,858–368,075)	0 (0–0)	0 (0–0)	438 (272–648)	31,809 (23,945–39,700)	284,522 (236,606–332,209)
Parkinson disease	24,894 (23,389–27,270)	0 (0–0)	0 (0–0)	254 (225–293)	4,474 (4,112–5,063)	20,165 (18,980–21,916)
Epilepsy	63,631 (56,928–69,286)	11,716 (6,781–15,916)	7,007 (6,147–7,824)	33,034 (31,115–34,938)	8,417 (7,925–8,925)	3,454 (3,267–3,681)

SR Table 11 - Global deaths by age and cause for males in 1990

Cause	All ages	0-5 years	5-14 years	15-49 years	50-69 years	70+ years
Multiple sclerosis	5,720 (4,870–6,276)	0 (0–0)	0 (0–0)	2,118 (1,689–2,411)	2,669 (2,327–2,885)	931 (781–988)
Motor neuron disease	9,638 (9,210–10,460)	839 (530–1,106)	78 (69–91)	1,436 (1,334–1,670)	4,563 (4,333–5,081)	2,720 (2,616–2,881)
Migraine	0 (0–0)	0 (0–0)	0 (0–0)	0 (0–0)	0 (0–0)	0 (0–0)
Tension-type headache	0 (0–0)	0 (0–0)	0 (0–0)	0 (0–0)	0 (0–0)	0 (0–0)
Medication overuse headache	0 (0–0)	0 (0–0)	0 (0–0)	0 (0–0)	0 (0–0)	0 (0–0)
Other neurological disorders	20,092 (19,226–22,121)	3,196 (1,928–4,653)	1,642 (1,394–1,965)	6,666 (6,078–7,462)	4,717 (4,297–5,291)	3,869 (3,588–4,221)
Mental and substance use disorders	138,117 (130,524–142,915)	18 (15–24)	12 (5–18)	84,394 (77,613–87,855)	43,981 (42,406–45,719)	9,710 (9,266–10,104)
Schizophrenia	7,981 (6,728–8,816)	0 (0–0)	0 (0–0)	4,085 (3,266–4,623)	2,646 (2,318–2,910)	1,249 (1,044–1,390)
Alcohol use disorders	79,858 (76,742–82,986)	0 (0–0)	0 (0–0)	41,714 (39,831–43,767)	32,188 (30,895–33,622)	5,956 (5,740–6,223)
Drug use disorders	50,171 (42,514–52,859)	18 (15–24)	0 (0–0)	38,500 (31,761–40,668)	9,146 (8,241–9,828)	2,505 (2,244–2,752)
Opioid use disorders	38,543 (33,040–40,619)	18 (15–24)	0 (0–0)	29,820 (24,963–31,675)	6,965 (6,301–7,544)	1,738 (1,547–1,915)
Cocaine use disorders	1,395 (1,128–1,800)	0 (0–0)	0 (0–0)	1,150 (944–1,486)	213 (157–263)	32 (24–43)
Amphetamine use disorders	931 (773–1,307)	0 (0–0)	0 (0–0)	740 (615–1,047)	162 (130–241)	29 (22–38)
Cannabis use disorders	0 (0–0)	0 (0–0)	0 (0–0)	0 (0–0)	0 (0–0)	0 (0–0)
Other drug use disorders	9,300 (6,592–10,310)	0 (0–0)	0 (0–0)	6,789 (4,614–7,535)	1,805 (1,431–2,060)	704 (566–836)
Depressive disorders	0 (0–0)	0 (0–0)	0 (0–0)	0 (0–0)	0 (0–0)	0 (0–0)
Major depressive disorder	0 (0–0)	0 (0–0)	0 (0–0)	0 (0–0)	0 (0–0)	0 (0–0)
Dysthymia	0 (0–0)	0 (0–0)	0 (0–0)	0 (0–0)	0 (0–0)	0 (0–0)
Bipolar disorder	0 (0–0)	0 (0–0)	0 (0–0)	0 (0–0)	0 (0–0)	0 (0–0)
Anxiety disorders	0 (0–0)	0 (0–0)	0 (0–0)	0 (0–0)	0 (0–0)	0 (0–0)
Eating disorders	106 (59–139)	0 (0–0)	12 (5–18)	93 (53–124)	0 (0–0)	0 (0–0)
Anorexia nervosa	96 (54–128)	0 (0–0)	11 (5–17)	85 (48–115)	0 (0–0)	0 (0–0)
Bulimia nervosa	9 (4–12)	0 (0–0)	0 (0–1)	8 (4–11)	0 (0–0)	0 (0–0)
Autistic spectrum disorders	0 (0–0)	0 (0–0)	0 (0–0)	0 (0–0)	0 (0–0)	0 (0–0)
Autism	0 (0–0)	0 (0–0)	0 (0–0)	0 (0–0)	0 (0–0)	0 (0–0)
Asperger syndrome and other autistic spectrum disorders	0 (0–0)	0 (0–0)	0 (0–0)	0 (0–0)	0 (0–0)	0 (0–0)
Attention-deficit/hyperactivity disorder	0 (0–0)	0 (0–0)	0 (0–0)	0 (0–0)	0 (0–0)	0 (0–0)
Conduct disorder	0 (0–0)	0 (0–0)	0 (0–0)	0 (0–0)	0 (0–0)	0 (0–0)
Idiopathic developmental intellectual disability	0 (0–0)	0 (0–0)	0 (0–0)	0 (0–0)	0 (0–0)	0 (0–0)
Other mental and substance use disorders	0 (0–0)	0 (0–0)	0 (0–0)	0 (0–0)	0 (0–0)	0 (0–0)
Diabetes, urogenital, blood, and endocrine diseases	815,039 (750,771–860,823)	79,881 (53,965–103,569)	18,573 (15,962–20,501)	145,043 (128,712–156,459)	275,309 (256,957–292,285)	296,230 (280,741–313,599)
Diabetes mellitus	298,032 (288,379–308,267)	2,178 (1,781–2,486)	1,315 (1,115–1,535)	38,878 (37,186–40,894)	132,674 (127,439–138,213)	122,985 (119,159–126,995)
Acute glomerulonephritis	7,863 (5,865–9,303)	540 (379–700)	400 (309–484)	2,521 (1,680–2,993)	2,550 (1,900–3,167)	1,849 (1,339–2,304)
Chronic kidney disease	296,694 (254,355–329,994)	11,387 (8,003–13,388)	5,607 (4,169–6,384)	63,490 (49,770–69,157)	98,633 (84,637–113,442)	117,576 (107,297–131,883)
Chronic kidney disease due to diabetes mellitus	89,037 (79,060–98,477)	1,042 (774–1,321)	395 (303–489)	13,889 (11,203–15,639)	35,430 (30,771–40,007)	38,278 (35,180–42,223)
Chronic kidney disease due to hypertension	124,204 (106,866–138,502)	3,774 (2,694–4,595)	1,560 (1,156–1,858)	23,811 (18,454–26,545)	40,152 (34,200–46,375)	54,904 (49,750–61,567)
Chronic kidney disease due to glomerulonephritis	74,223 (60,965–84,971)	4,857 (3,404–5,787)	2,672 (1,956–3,074)	23,014 (17,939–25,726)	21,738 (18,079–25,770)	21,940 (19,086–25,774)
Chronic kidney disease due to other causes	9,229 (7,191–11,156)	1,713 (1,033–2,293)	978 (672–1,278)	2,774 (1,932–3,783)	1,311 (816–2,006)	2,452 (2,026–3,064)
Urinary diseases and male infertility	74,289 (62,740–95,713)	6,542 (4,110–8,720)	1,218 (995–1,584)	11,691 (9,225–15,940)	22,099 (17,084–30,862)	32,737 (27,890–43,692)
Interstitial nephritis and urinary tract infections	47,149 (38,902–60,235)	5,164 (2,969–6,907)	722 (555–905)	6,894 (5,199–9,419)	12,549 (9,310–17,443)	21,818 (17,937–28,279)
Urolithiasis	9,718 (5,876–12,545)	0 (0–0)	204 (128–280)	1,922 (1,106–2,514)	3,802 (2,275–4,999)	3,789 (2,326–4,955)
Benign prostatic hyperplasia	0 (0–0)	0 (0–0)	0 (0–0)	0 (0–0)	0 (0–0)	0 (0–0)
Male infertility	0 (0–0)	0 (0–0)	0 (0–0)	0 (0–0)	0 (0–0)	0 (0–0)
Other urinary diseases	17,421 (12,852–27,965)	1,378 (820–2,609)	292 (209–484)	2,874 (1,894–5,082)	5,747 (3,670–10,070)	7,129 (5,086–11,319)
Gynecological diseases	0 (0–0)	0 (0–0)	0 (0–0)	0 (0–0)	0 (0–0)	0 (0–0)
Uterine fibroids	0 (0–0)	0 (0–0)	0 (0–0)	0 (0–0)	0 (0–0)	0 (0–0)
Polycystic ovarian syndrome	0 (0–0)	0 (0–0)	0 (0–0)	0 (0–0)	0 (0–0)	0 (0–0)
Female infertility	0 (0–0)	0 (0–0)	0 (0–0)	0 (0–0)	0 (0–0)	0 (0–0)
Endometriosis	0 (0–0)	0 (0–0)	0 (0–0)	0 (0–0)	0 (0–0)	0 (0–0)
Genital prolapse	0 (0–0)	0 (0–0)	0 (0–0)	0 (0–0)	0 (0–0)	0 (0–0)
Premenstrual syndrome	0 (0–0)	0 (0–0)	0 (0–0)	0 (0–0)	0 (0–0)	0 (0–0)
Other gynecological diseases	0 (0–0)	0 (0–0)	0 (0–0)	0 (0–0)	0 (0–0)	0 (0–0)
Hemoglobinopathies and hemolytic anemias	99,952 (70,087–123,579)	48,321 (25,349–69,797)	7,820 (6,214–9,917)	20,663 (15,809–25,646)	11,006 (8,452–13,911)	12,141 (9,395–15,149)
Thalassemias	13,620 (9,032–18,424)	9,553 (5,413–14,154)	2,471 (1,883–3,151)	875 (648–1,215)	26 (23–30)	692 (418–1,090)
Thalassemias trait	0 (0–0)	0 (0–0)	0 (0–0)	0 (0–0)	0 (0–0)	0 (0–0)
Sickle cell disorders	49,237 (28,400–70,211)	37,164 (18,854–57,648)	3,979 (2,866–5,006)	7,014 (4,766–9,455)	268 (205–332)	811 (583–1,137)
Sickle cell trait	0 (0–0)	0 (0–0)	0 (0–0)	0 (0–0)	0 (0–0)	0 (0–0)

SR Table 11 - Global deaths by age and cause for males in 1990						
Cause	All ages	0-5 years	5-14 years	15-49 years	50-69 years	70+ years
G6PD deficiency	12,970 (10,806–17,035)	1,053 (454–1,861)	942 (687–1,380)	7,301 (5,944–9,453)	3,094 (2,388–4,018)	577 (434–768)
G6PD trait	0 (0–0)	0 (0–0)	0 (0–0)	0 (0–0)	0 (0–0)	0 (0–0)
Other hemoglobinopathies and hemolytic anemias	24,124 (19,123–29,329)	549 (295–879)	426 (330–577)	5,472 (4,342–6,711)	7,617 (5,769–9,670)	10,059 (7,943–12,379)
Endocrine, metabolic, blood, and immune disorders	38,205 (36,677–40,110)	10,911 (9,184–12,852)	2,210 (2,061–2,366)	7,798 (7,264–8,436)	8,344 (7,713–9,109)	8,940 (8,398–9,533)
Musculoskeletal disorders	16,722 (14,037–17,830)	0 (0–0)	805 (641–976)	3,935 (3,616–4,361)	5,213 (4,230–5,665)	6,768 (5,346–7,307)
Rheumatoid arthritis	6,074 (4,391–6,692)	0 (0–0)	126 (85–190)	655 (484–750)	2,309 (1,677–2,574)	2,982 (2,174–3,306)
Osteoarthritis	0 (0–0)	0 (0–0)	0 (0–0)	0 (0–0)	0 (0–0)	0 (0–0)
Low back and neck pain	0 (0–0)	0 (0–0)	0 (0–0)	0 (0–0)	0 (0–0)	0 (0–0)
Low back pain	0 (0–0)	0 (0–0)	0 (0–0)	0 (0–0)	0 (0–0)	0 (0–0)
Neck pain	0 (0–0)	0 (0–0)	0 (0–0)	0 (0–0)	0 (0–0)	0 (0–0)
Gout	0 (0–0)	0 (0–0)	0 (0–0)	0 (0–0)	0 (0–0)	0 (0–0)
Other musculoskeletal disorders	10,648 (9,582–11,416)	0 (0–0)	678 (544–846)	3,279 (3,040–3,723)	2,903 (2,506–3,180)	3,786 (3,024–4,140)
Other non-communicable diseases	415,089 (314,407–600,910)	346,024 (251,923–519,881)	19,356 (14,394–25,079)	28,865 (25,883–34,765)	10,360 (8,194–12,866)	10,481 (8,016–13,941)
Congenital anomalies	381,078 (280,160–556,139)	328,034 (235,459–492,788)	18,919 (13,963–24,579)	25,766 (23,081–31,392)	5,259 (4,825–6,571)	3,099 (2,850–3,841)
Neural tube defects	71,153 (49,008–115,491)	66,896 (45,370–109,942)	2,070 (1,436–2,927)	1,623 (1,174–2,372)	345 (219–628)	219 (122–444)
Congenital heart anomalies	197,562 (141,453–291,603)	162,247 (111,845–250,856)	13,436 (9,715–17,725)	18,799 (16,482–22,372)	1,922 (1,627–2,303)	1,156 (958–1,453)
Cleft lip and cleft palate	3,054 (2,079–4,936)	3,054 (2,079–4,936)	0 (0–0)	0 (0–0)	0 (0–0)	0 (0–0)
Down syndrome	9,113 (5,315–22,444)	6,285 (2,694–18,949)	387 (235–821)	1,500 (1,108–2,516)	897 (642–1,403)	42 (30–65)
Turner syndrome	0 (0–0)	0 (0–0)	0 (0–0)	0 (0–0)	0 (0–0)	0 (0–0)
Klinefelter syndrome	0 (0–0)	0 (0–0)	0 (0–0)	0 (0–0)	0 (0–0)	0 (0–0)
Other chromosomal abnormalities	7,298 (3,020–21,445)	6,660 (2,375–20,622)	140 (86–267)	338 (245–478)	119 (82–167)	39 (27–53)
Other congenital anomalies	92,896 (66,339–136,696)	82,890 (57,738–125,488)	2,883 (2,133–3,917)	3,505 (2,832–4,312)	1,975 (1,639–2,446)	1,641 (1,414–2,047)
Skin and subcutaneous diseases	18,553 (11,563–28,606)	2,534 (1,104–5,028)	436 (254–781)	3,099 (1,871–5,194)	5,101 (2,872–7,450)	7,382 (4,817–10,594)
Dermatitis	0 (0–0)	0 (0–0)	0 (0–0)	0 (0–0)	0 (0–0)	0 (0–0)
Psoriasis	0 (0–0)	0 (0–0)	0 (0–0)	0 (0–0)	0 (0–0)	0 (0–0)
Cellulitis	3,888 (2,170–7,962)	408 (153–935)	94 (57–182)	899 (482–1,972)	1,412 (700–2,999)	1,073 (674–2,297)
Pyoderma	7,058 (4,251–12,271)	1,949 (820–3,853)	221 (117–413)	1,384 (805–2,436)	1,606 (1,042–2,734)	1,897 (1,299–2,991)
Scabies	0 (0–0)	0 (0–0)	0 (0–0)	0 (0–0)	0 (0–0)	0 (0–0)
Fungal skin diseases	0 (0–0)	0 (0–0)	0 (0–0)	0 (0–0)	0 (0–0)	0 (0–0)
Viral skin diseases	0 (0–0)	0 (0–0)	0 (0–0)	0 (0–0)	0 (0–0)	0 (0–0)
Acne vulgaris	0 (0–0)	0 (0–0)	0 (0–0)	0 (0–0)	0 (0–0)	0 (0–0)
Alopecia areata	0 (0–0)	0 (0–0)	0 (0–0)	0 (0–0)	0 (0–0)	0 (0–0)
Pruritus	0 (0–0)	0 (0–0)	0 (0–0)	0 (0–0)	0 (0–0)	0 (0–0)
Urticaria	0 (0–0)	0 (0–0)	0 (0–0)	0 (0–0)	0 (0–0)	0 (0–0)
Decubitus ulcer	6,674 (3,493–9,207)	41 (17–81)	70 (35–126)	605 (302–1,004)	1,847 (660–3,425)	4,109 (2,368–5,632)
Other skin and subcutaneous diseases	932 (564–1,470)	134 (59–284)	50 (29–97)	210 (124–393)	234 (116–494)	302 (188–458)
Sense organ diseases	0 (0–0)	0 (0–0)	0 (0–0)	0 (0–0)	0 (0–0)	0 (0–0)
Glaucoma	0 (0–0)	0 (0–0)	0 (0–0)	0 (0–0)	0 (0–0)	0 (0–0)
Cataract	0 (0–0)	0 (0–0)	0 (0–0)	0 (0–0)	0 (0–0)	0 (0–0)
Macular degeneration	0 (0–0)	0 (0–0)	0 (0–0)	0 (0–0)	0 (0–0)	0 (0–0)
Refraction and accommodation disorders	0 (0–0)	0 (0–0)	0 (0–0)	0 (0–0)	0 (0–0)	0 (0–0)
Age-related and other hearing loss	0 (0–0)	0 (0–0)	0 (0–0)	0 (0–0)	0 (0–0)	0 (0–0)
Other vision loss	0 (0–0)	0 (0–0)	0 (0–0)	0 (0–0)	0 (0–0)	0 (0–0)
Other sense organ diseases	0 (0–0)	0 (0–0)	0 (0–0)	0 (0–0)	0 (0–0)	0 (0–0)
Oral disorders	0 (0–0)	0 (0–0)	0 (0–0)	0 (0–0)	0 (0–0)	0 (0–0)
Deciduous caries	0 (0–0)	0 (0–0)	0 (0–0)	0 (0–0)	0 (0–0)	0 (0–0)
Permanent caries	0 (0–0)	0 (0–0)	0 (0–0)	0 (0–0)	0 (0–0)	0 (0–0)
Periodontal diseases	0 (0–0)	0 (0–0)	0 (0–0)	0 (0–0)	0 (0–0)	0 (0–0)
Edentulism and severe tooth loss	0 (0–0)	0 (0–0)	0 (0–0)	0 (0–0)	0 (0–0)	0 (0–0)
Other oral disorders	0 (0–0)	0 (0–0)	0 (0–0)	0 (0–0)	0 (0–0)	0 (0–0)
Sudden infant death syndrome	15,456 (11,517–38,425)	15,456 (11,517–38,425)	0 (0–0)	0 (0–0)	0 (0–0)	0 (0–0)
Injuries	2,940,055 (2,801,381–3,054,653)	405,533 (324,647–468,059)	253,990 (237,330–270,621)	1,570,409 (1,508,220–1,631,772)	477,596 (453,254–499,060)	232,526 (217,404–244,439)
Transport injuries	874,838 (830,918–913,261)	65,081 (47,479–81,292)	70,181 (64,220–75,155)	535,881 (511,155–560,107)	146,178 (138,276–154,164)	57,515 (54,738–60,714)
Road injuries	821,083 (776,959–860,064)	60,128 (43,892–75,282)	66,973 (61,292–71,824)	504,920 (482,100–527,528)	134,840 (127,152–142,154)	54,220 (51,538–57,138)
Pedestrian road injuries	355,742 (296,770–392,921)	34,217 (25,674–43,365)	38,690 (34,240–42,723)	185,096 (150,741–207,133)	67,440 (55,814–74,634)	30,296 (25,982–33,065)
Cyclist road injuries	33,264 (28,188–39,121)	1,579 (814–2,507)	3,961 (3,231–4,800)	16,757 (13,743–20,144)	7,736 (6,681–9,088)	3,229 (2,894–3,610)
Motorcyclist road injuries	122,660 (96,010–156,029)	5,030 (2,233–8,858)	5,253 (3,475–7,326)	93,797 (74,072–117,990)	14,562 (10,363–19,899)	4,015 (2,742–5,623)

SR Table 11 - Global deaths by age and cause for males in 1990

Cause	All ages	0-5 years	5-14 years	15-49 years	50-69 years	70+ years
Motor vehicle road injuries	300,141<br>(266,950–353,975)	18,268<br>(11,610–25,238)	18,131<br>(15,529–22,186)	204,209<br>(183,100–241,576)	43,571<br>(37,093–54,384)	15,960<br>(13,502–19,889)
Other road injuries	9,274<br>(5,448–12,424)	1,031<br>(295–2,446)	935<br>(469–1,465)	5,059<br>(2,926–6,815)	1,530<br>(1,003–2,117)	717<br>(485–1,084)
Other transport injuries	53,755<br>(44,266–60,787)	4,953<br>(2,603–6,783)	3,207<br>(2,421–3,743)	30,961<br>(25,831–34,579)	11,338<br>(9,302–13,042)	3,295<br>(2,709–3,759)
Unintentional injuries	1,270,043<br>(1,187,021–1,353,692)	319,267<br>(254,140–376,892)	156,742<br>(145,161–169,028)	482,380<br>(456,823–514,032)	189,249<br>(177,356–202,931)	122,403<br>(112,649–134,008)
Falls	211,542<br>(193,142–225,407)	20,708<br>(11,373–29,076)	12,480<br>(10,211–14,340)	75,220<br>(69,310–79,802)	51,922<br>(47,428–55,243)	51,209<br>(46,896–54,304)
Drowning	384,606<br>(352,117–418,012)	125,288<br>(101,915–148,635)	88,913<br>(82,070–95,803)	129,483<br>(118,249–140,005)	30,103<br>(27,588–32,914)	10,818<br>(9,900–11,890)
Fire, heat, and hot substances	103,424<br>(74,293–111,546)	25,223<br>(17,614–30,693)	7,350<br>(5,494–8,308)	43,655<br>(29,301–47,310)	15,795<br>(12,480–16,775)	11,400<br>(9,267–11,966)
Poisonings	87,210<br>(53,165–108,796)	26,548<br>(7,602–47,282)	6,308<br>(4,623–8,328)	34,491<br>(25,143–41,863)	14,831<br>(10,861–17,831)	5,029<br>(3,697–6,229)
Exposure to mechanical forces	143,411<br>(119,035–176,681)	32,483<br>(21,320–44,107)	11,680<br>(9,211–15,606)	68,708<br>(59,245–85,410)	21,059<br>(18,329–26,787)	9,479<br>(8,148–11,031)
Unintentional firearm injuries	25,501<br>(18,444–29,197)	2,910<br>(1,490–4,140)	2,277<br>(1,780–2,993)	15,270<br>(11,095–17,529)	3,112<br>(2,262–3,657)	1,930<br>(1,560–2,261)
Unintentional suffocation	27,613<br>(19,693–36,934)	20,004<br>(12,858–28,302)	2,420<br>(1,867–3,703)	2,869<br>(2,431–3,605)	823<br>(557–1,022)	1,495<br>(1,221–1,703)
Other exposure to mechanical forces	90,295<br>(71,478–120,138)	9,568<br>(5,053–15,296)	6,981<br>(4,968–10,006)	50,568<br>(41,205–67,260)	17,124<br>(14,354–23,060)	6,052<br>(5,046–7,551)
Adverse effects of medical treatment	54,868<br>(41,112–69,565)	16,784<br>(7,160–25,363)	3,497<br>(2,267–4,694)	14,960<br>(11,347–18,656)	10,218<br>(8,141–12,396)	9,407<br>(7,498–11,092)
Animal contact	64,130<br>(44,857–72,357)	12,178<br>(5,884–15,904)	10,027<br>(6,611–11,830)	25,144<br>(17,389–28,361)	12,147<br>(8,554–13,873)	4,632<br>(3,628–5,458)
Venomous animal contact	50,174<br>(34,027–57,836)	8,411<br>(4,054–10,938)	7,957<br>(5,097–9,562)	20,707<br>(13,822–24,163)	9,598<br>(6,591–11,479)	3,498<br>(2,447–4,354)
Non-venomous animal contact	13,955<br>(9,019–17,867)	3,766<br>(1,666–5,447)	2,069<br>(1,237–2,740)	4,436<br>(2,962–5,458)	2,548<br>(1,733–3,161)	1,134<br>(785–1,430)
Foreign body	95,716<br>(66,846–153,104)	45,205<br>(24,003–69,119)	7,388<br>(5,075–11,933)	22,484<br>(16,443–54,975)	11,181<br>(8,987–24,234)	9,456<br>(7,652–19,121)
Pulmonary aspiration and foreign body in airway	67,185<br>(46,643–95,306)	33,772<br>(15,262–56,265)	4,873<br>(3,160–6,961)	12,818<br>(10,632–15,193)	8,311<br>(6,896–9,530)	7,408<br>(6,507–8,249)
Foreign body in eyes	0<br>(0–0)	0<br>(0–0)	0<br>(0–0)	0<br>(0–0)	0<br>(0–0)	0<br>(0–0)
Foreign body in other body part	28,531<br>(12,905–99,636)	11,433<br>(4,536–20,955)	2,514<br>(1,051–8,129)	9,665<br>(4,097–42,360)	2,869<br>(1,333–15,376)	2,047<br>(941–11,168)
Environmental heat and cold exposure	30,979<br>(22,308–39,195)	4,327<br>(1,808–5,673)	1,100<br>(733–1,498)	12,334<br>(9,059–15,487)	8,649<br>(6,535–12,159)	4,566<br>(3,123–6,262)
Other unintentional injuries	94,153<br>(85,735–101,758)	10,516<br>(7,170–13,005)	7,996<br>(6,943–9,122)	55,896<br>(50,642–60,934)	13,341<br>(12,075–14,773)	6,402<br>(5,421–7,626)
Self-harm and interpersonal violence	689,937<br>(628,679–716,840)	12,762<br>(9,976–14,845)	16,032<br>(13,769–17,115)	478,809<br>(437,688–498,360)	132,134<br>(120,201–138,398)	50,197<br>(44,546–52,909)
Self-harm	421,802<br>(364,280–445,491)	0<br>(0–0)	6,337<br>(4,396–7,183)	270,586<br>(232,332–286,450)	101,399<br>(89,415–106,989)	43,479<br>(37,449–46,069)
Interpersonal violence	268,134<br>(254,844–280,112)	12,762<br>(9,976–14,845)	9,695<br>(8,919–10,537)	208,223<br>(198,527–217,067)	30,735<br>(28,943–33,078)	6,718<br>(6,301–7,581)
Assault by firearm	102,938<br>(97,963–111,841)	1,153<br>(828–1,678)	2,058<br>(1,856–2,384)	88,315<br>(84,134–95,239)	9,804<br>(8,804–11,272)	1,606<br>(1,438–1,809)
Assault by sharp object	68,432<br>(61,030–74,932)	1,695<br>(1,091–2,551)	1,876<br>(1,548–2,268)	55,902<br>(49,633–61,471)	7,392<br>(6,449–8,183)	1,565<br>(1,395–1,717)
Assault by other means	96,762<br>(83,157–108,300)	9,912<br>(7,350–11,890)	5,760<br>(4,860–6,603)	64,004<br>(54,787–71,095)	13,538<br>(12,411–15,498)	3,546<br>(3,245–4,254)
Forces of nature, war, and legal intervention	105,235<br>(71,430–143,893)	8,422<br>(4,208–14,277)	11,033<br>(6,021–17,557)	73,338<br>(44,570–108,154)	10,032<br>(5,804–15,223)	2,409<br>(1,435–3,599)
Exposure to forces of nature	32,048<br>(15,435–48,247)	2,990<br>(1,364–4,609)	6,509<br>(2,973–9,955)	17,107<br>(8,254–25,823)	4,143<br>(1,981–6,276)	1,298<br>(635–1,964)
Collective violence and legal intervention	73,187<br>(43,132–107,859)	5,431<br>(1,901–11,199)	4,523<br>(1,495–9,669)	56,230<br>(28,282–89,114)	5,889<br>(2,514–10,753)	1,111<br>(461–2,085)

SR Table 11 - Global deaths by age and cause for males in 1995

Cause	All ages	0-5 years	5-14 years	15-49 years	50-69 years	70+ years
All causes	26,846,748 (26,501,026–27,197,274)	5,608,069 (5,551,154–5,667,732)	707,989 (699,621–716,432)	5,227,088 (5,115,471–5,343,500)	7,285,502 (7,150,066–7,422,622)	8,018,099 (7,923,314–8,110,029)
Communicable, maternal, neonatal, and nutritional diseases	7,897,757 (7,677,987–8,224,916)	4,713,569 (4,564,077–4,837,486)	321,589 (302,012–342,942)	1,219,400 (1,153,893–1,311,094)	865,439 (794,367–963,720)	777,758 (731,948–847,657)
HIV/AIDS and tuberculosis	1,385,029 (1,199,102–1,675,996)	79,405 (69,367–99,736)	18,187 (14,799–24,772)	687,466 (612,759–778,527)	407,896 (332,236–511,805)	192,073 (150,174–267,556)
Tuberculosis	919,614 (728,320–1,210,730)	27,818 (18,526–49,349)	12,947 (9,745–19,491)	344,728 (270,074–437,317)	348,116 (269,928–452,450)	186,004 (144,182–261,790)
HIV/AIDS	465,415 (432,530–510,051)	51,587 (48,762–54,780)	5,240 (4,809–5,803)	342,738 (315,529–378,836)	59,780 (53,896–68,454)	6,068 (5,233–7,693)
HIV/AIDS - Tuberculosis	97,305 (83,139–114,576)	15,480 (13,218–17,675)	1,477 (1,164–1,730)	64,042 (54,429–76,556)	14,576 (12,350–17,254)	1,728 (1,427–2,079)
HIV/AIDS resulting in other diseases	368,109 (336,084–408,932)	36,106 (32,859–40,061)	3,763 (3,334–4,346)	278,696 (252,226–309,984)	45,204 (39,754–53,336)	4,339 (3,615–5,657)
Diarrhea, lower respiratory, and other common infectious diseases	3,567,800 (3,376,101–3,763,236)	2,111,728 (1,963,071–2,264,198)	222,445 (197,567–252,125)	364,436 (333,761–403,028)	354,966 (331,344–395,442)	514,222 (481,977–559,273)
Diarrheal diseases	956,886 (875,008–1,041,612)	556,476 (480,211–631,740)	48,009 (41,783–54,137)	107,243 (98,780–117,647)	117,020 (107,938–127,337)	128,136 (119,266–138,437)
Intestinal infectious diseases	125,179 (70,271–202,692)	35,254 (18,875–62,111)	48,635 (28,291–76,748)	35,240 (18,563–60,928)	4,439 (2,302–7,832)	1,609 (935–2,532)
Typhoid fever	101,440 (54,974–171,012)	28,269 (14,650–51,379)	40,076 (22,465–64,736)	28,456 (14,854–49,488)	3,460 (1,665–6,239)	1,176 (637–1,957)
Paratyphoid fever	21,743 (10,082–41,725)	5,712 (2,360–11,459)	8,393 (3,832–16,146)	6,519 (2,822–12,630)	846 (349–1,713)	270 (117–528)
Other intestinal infectious diseases	1,995 (713–4,425)	1,272 (352–3,370)	165 (50–440)	264 (122–449)	131 (57–217)	162 (62–298)
Lower respiratory infections	1,611,905 (1,496,043–1,741,411)	893,467 (802,398–977,785)	45,129 (33,558–51,245)	131,274 (110,665–151,427)	185,068 (159,483–216,308)	356,964 (321,029–397,975)
Upper respiratory infections	2,277 (1,945–2,735)	829 (571–1,220)	46 (31–67)	498 (417–593)	506 (403–629)	395 (317–490)
Otitis media	2,982 (2,608–3,378)	1,435 (1,172–1,739)	462 (403–516)	619 (555–684)	305 (265–351)	159 (132–197)
Meningitis	247,541 (198,832–291,862)	148,333 (109,887–186,505)	21,761 (14,483–25,694)	43,209 (30,926–51,170)	22,311 (16,718–27,981)	11,924 (9,046–15,457)
Pneumococcal meningitis	61,837 (48,038–77,645)	31,884 (21,430–46,270)	6,665 (4,285–9,249)	13,815 (9,872–17,621)	6,147 (4,309–8,442)	3,323 (2,416–4,561)
H influenzae type B meningitis	74,342 (55,008–93,569)	55,250 (38,554–74,086)	6,057 (3,752–8,563)	7,407 (4,997–9,947)	3,642 (2,500–4,999)	1,984 (1,466–2,806)
Meningococcal meningitis	44,859 (32,782–58,098)	28,258 (18,136–40,977)	4,009 (2,403–5,895)	7,182 (5,007–9,622)	3,679 (2,540–5,064)	1,729 (1,255–2,369)
Other meningitis	66,502 (52,528–80,383)	32,939 (23,009–44,614)	5,029 (3,138–6,895)	14,804 (10,053–17,699)	8,841 (6,313–11,236)	4,887 (3,614–6,226)
Encephalitis	81,378 (73,092–90,933)	29,414 (24,977–34,514)	10,090 (8,576–11,500)	19,622 (17,669–21,996)	12,539 (11,183–14,272)	9,710 (8,622–11,192)
Diphtheria	5,541 (2,898–11,596)	4,315 (1,854–10,339)	679 (324–1,414)	410 (286–595)	135 (72–243)	0 (0–0)
Whooping cough	60,394 (21,217–140,994)	56,180 (19,739–130,857)	3,308 (1,151–8,099)	801 (267–2,093)	103 (32–283)	0 (0–0)
Tetanus	122,282 (105,862–156,176)	83,882 (74,031–95,366)	5,419 (3,533–9,706)	17,717 (10,624–30,687)	11,285 (7,413–22,341)	3,977 (2,589–8,081)
Measles	345,517 (130,088–696,165)	300,174 (114,699–599,712)	38,349 (13,959–78,277)	6,743 (1,940–17,754)	249 (70–691)	0 (0–0)
Varicella and herpes zoster	5,913 (5,107–6,894)	1,963 (1,423–2,672)	551 (390–765)	1,054 (878–1,275)	999 (757–1,281)	1,344 (1,024–1,788)
Neglected tropical diseases and malaria	600,047 (472,559–730,078)	391,876 (273,659–521,850)	55,687 (41,618–72,070)	95,688 (77,083–117,553)	42,243 (34,329–51,809)	14,550 (11,528–18,694)
Malaria	507,727 (384,446–636,634)	376,163 (256,966–504,961)	40,102 (25,529–56,438)	57,001 (41,484–77,196)	25,672 (18,870–34,640)	8,787 (5,906–12,871)
Chagas disease	5,239 (4,961–5,554)	5 (4–8)	9 (7–10)	1,304 (1,199–1,421)	2,484 (2,316–2,680)	1,435 (1,326–1,568)
Leishmaniasis	14,959 (8,782–22,882)	2,579 (1,416–3,994)	1,647 (881–2,854)	8,688 (5,041–13,754)	1,749 (1,138–2,518)	294 (215–381)
Visceral leishmaniasis	14,959 (8,782–22,882)	2,579 (1,416–3,994)	1,647 (881–2,854)	8,688 (5,041–13,754)	1,749 (1,138–2,518)	294 (215–381)
Cutaneous and mucocutaneous leishmaniasis	0 (0–0)	0 (0–0)	0 (0–0)	0 (0–0)	0 (0–0)	0 (0–0)
African trypanosomiasis	14,806 (7,448–24,435)	1,015 (490–1,784)	1,800 (922–2,977)	9,554 (4,857–16,033)	2,025 (946–3,507)	411 (199–687)
Schistosomiasis	5,889 (5,031–6,874)	99 (55–177)	186 (122–281)	2,108 (1,722–2,576)	2,423 (1,873–3,111)	1,071 (783–1,457)
Cysticercosis	430 (385–492)	8 (4–14)	15 (12–19)	275 (247–314)	104 (93–119)	26 (23–30)
Cystic echinococcosis	1,249 (1,174–1,333)	67 (51–84)	126 (111–142)	527 (489–567)	357 (335–383)	170 (161–180)
Lymphatic filariasis	0 (0–0)	0 (0–0)	0 (0–0)	0 (0–0)	0 (0–0)	0 (0–0)
Onchocerciasis	0 (0–0)	0 (0–0)	0 (0–0)	0 (0–0)	0 (0–0)	0 (0–0)
Trachoma	0 (0–0)	0 (0–0)	0 (0–0)	0 (0–0)	0 (0–0)	0 (0–0)
Dengue	3,660 (2,277–5,770)	1,445 (585–2,703)	954 (583–1,586)	874 (644–1,396)	250 (163–377)	135 (91–210)
Yellow fever	6,692 (1,444–18,114)	599 (118–1,686)	1,770 (396–4,844)	3,691 (813–10,296)	534 (104–1,515)	97 (19–278)
Rabies	27,448 (24,358–30,296)	3,419 (2,384–4,755)	7,680 (6,393–9,072)	9,568 (8,130–10,773)	5,314 (4,463–6,058)	1,464 (1,153–1,730)
Intestinal nematode infections	2,820 (2,362–3,292)	2,129 (1,722–2,540)	410 (346–483)	147 (114–184)	84 (62–111)	48 (31–67)
Ascariasis	2,820 (2,362–3,292)	2,129 (1,722–2,540)	410 (346–483)	147 (114–184)	84 (62–111)	48 (31–67)
Trichuriasis	0 (0–0)	0 (0–0)	0 (0–0)	0 (0–0)	0 (0–0)	0 (0–0)
Hookworm disease	0 (0–0)	0 (0–0)	0 (0–0)	0 (0–0)	0 (0–0)	0 (0–0)
Food-borne trematodiases	0 (0–0)	0 (0–0)	0 (0–0)	0 (0–0)	0 (0–0)	0 (0–0)
Leprosy	0 (0–0)	0 (0–0)	0 (0–0)	0 (0–0)	0 (0–0)	0 (0–0)
Ebola	209 (168–250)	8 (7–10)	6 (5–8)	149 (120–178)	43 (35–52)	0 (0–0)
Other neglected tropical diseases	8,913 (4,829–13,395)	4,334 (2,182–6,031)	975 (518–1,578)	1,797 (988–2,990)	1,198 (679–1,989)	607 (367–1,063)
Maternal disorders	0 (0–0)	0 (0–0)	0 (0–0)	0 (0–0)	0 (0–0)	0 (0–0)
Maternal hemorrhage	0 (0–0)	0 (0–0)	0 (0–0)	0 (0–0)	0 (0–0)	0 (0–0)
Maternal sepsis and other maternal infections	0 (0–0)	0 (0–0)	0 (0–0)	0 (0–0)	0 (0–0)	0 (0–0)
Maternal hypertensive disorders	0 (0–0)	0 (0–0)	0 (0–0)	0 (0–0)	0 (0–0)	0 (0–0)
Maternal obstructed labor and uterine rupture	0 (0–0)	0 (0–0)	0 (0–0)	0 (0–0)	0 (0–0)	0 (0–0)
Maternal abortion, miscarriage, and ectopic pregnancy	0 (0–0)	0 (0–0)	0 (0–0)	0 (0–0)	0 (0–0)	0 (0–0)

SR Table 11 - Global deaths by age and cause for males in 1995

Cause	All ages	0-5 years	5-14 years	15-49 years	50-69 years	70+ years
Indirect maternal deaths	0 (0–0)	0 (0–0)	0 (0–0)	0 (0–0)	0 (0–0)	0 (0–0)
Late maternal deaths	0 (0–0)	0 (0–0)	0 (0–0)	0 (0–0)	0 (0–0)	0 (0–0)
Maternal deaths aggravated by HIV/AIDS	0 (0–0)	0 (0–0)	0 (0–0)	0 (0–0)	0 (0–0)	0 (0–0)
Other maternal disorders	0 (0–0)	0 (0–0)	0 (0–0)	0 (0–0)	0 (0–0)	0 (0–0)
Neonatal disorders	1,783,540 (1,724,386–1,845,505)	1,783,540 (1,724,386–1,845,505)	0 (0–0)	0 (0–0)	0 (0–0)	0 (0–0)
Neonatal preterm birth complications	833,095 (749,827–931,116)	833,095 (749,827–931,116)	0 (0–0)	0 (0–0)	0 (0–0)	0 (0–0)
Neonatal encephalopathy due to birth asphyxia and trauma	509,508 (432,245–590,167)	509,508 (432,245–590,167)	0 (0–0)	0 (0–0)	0 (0–0)	0 (0–0)
Neonatal sepsis and other neonatal infections	215,121 (133,555–322,818)	215,121 (133,555–322,818)	0 (0–0)	0 (0–0)	0 (0–0)	0 (0–0)
Hemolytic disease and other neonatal jaundice	58,423 (38,361–107,240)	58,423 (38,361–107,240)	0 (0–0)	0 (0–0)	0 (0–0)	0 (0–0)
Other neonatal disorders	167,390 (113,224–224,649)	167,390 (113,224–224,649)	0 (0–0)	0 (0–0)	0 (0–0)	0 (0–0)
Nutritional deficiencies	325,701 (225,071–422,612)	221,525 (141,459–306,925)	11,244 (8,564–14,934)	25,795 (19,440–32,123)	27,554 (20,732–35,341)	39,580 (28,160–47,822)
Protein-energy malnutrition	289,322 (197,059–380,658)	208,945 (132,410–292,473)	9,438 (7,044–12,137)	17,197 (12,439–21,995)	20,885 (15,504–26,828)	32,856 (23,348–39,741)
Iodine deficiency	931 (556–1,641)	400 (133–998)	42 (18–86)	187 (113–322)	170 (93–311)	131 (75–249)
Vitamin A deficiency	0 (0–0)	0 (0–0)	0 (0–0)	0 (0–0)	0 (0–0)	0 (0–0)
Iron-deficiency anemia	19,874 (8,037–29,987)	7,286 (1,797–12,119)	846 (371–1,646)	4,937 (2,497–7,560)	4,037 (1,932–6,181)	2,766 (1,109–4,137)
Other nutritional deficiencies	15,572 (10,439–25,085)	4,892 (2,565–9,040)	917 (608–1,497)	3,473 (2,311–5,068)	2,461 (1,674–4,094)	3,826 (2,928–5,591)
Other communicable, maternal, neonatal, and nutritional diseases	235,637 (193,724–288,703)	125,492 (87,682–174,675)	14,024 (11,409–17,601)	46,012 (43,409–48,583)	32,777 (29,053–34,781)	17,331 (15,017–18,545)
Sexually transmitted diseases excluding HIV	99,079 (60,410–150,532)	84,673 (48,545–131,940)	6,116 (3,531–9,436)	3,553 (1,733–4,477)	2,968 (1,408–3,814)	1,767 (901–2,371)
Syphilis	98,042 (59,336–149,384)	84,673 (48,545–131,940)	6,090 (3,506–9,413)	3,032 (1,478–3,812)	2,690 (1,277–3,456)	1,554 (792–2,086)
Chlamydial infection	0 (0–0)	0 (0–0)	0 (0–0)	0 (0–0)	0 (0–0)	0 (0–0)
Gonococcal infection	719 (360–889)	0 (0–0)	14 (9–21)	370 (182–466)	171 (81–220)	162 (83–218)
Trichomoniasis	0 (0–0)	0 (0–0)	0 (0–0)	0 (0–0)	0 (0–0)	0 (0–0)
Genital herpes	0 (0–0)	0 (0–0)	0 (0–0)	0 (0–0)	0 (0–0)	0 (0–0)
Other sexually transmitted diseases	317 (158–390)	0 (0–0)	10 (6–15)	150 (73–190)	105 (50–135)	50 (25–67)
Hepatitis	86,829 (82,669–91,563)	17,482 (14,842–19,848)	5,425 (4,788–6,022)	34,088 (32,265–36,717)	20,387 (19,407–21,548)	9,446 (8,983–9,905)
Acute hepatitis A	15,897 (10,731–20,823)	13,583 (9,989–16,444)	799 (231–1,733)	986 (241–2,396)	379 (89–888)	148 (34–354)
Hepatitis B	48,238 (41,897–54,590)	955 (327–2,245)	672 (355–1,158)	22,603 (18,529–26,279)	16,869 (14,670–18,858)	7,138 (5,991–8,194)
Hepatitis C	1,717 (369–3,956)	0 (0–0)	77 (15–195)	586 (117–1,385)	775 (165–1,811)	277 (57–684)
Acute hepatitis E	20,975 (14,470–28,814)	2,943 (1,170–5,591)	3,876 (2,913–4,762)	9,912 (6,825–13,737)	2,362 (1,134–4,103)	1,881 (1,010–3,000)
Other infectious diseases	49,729 (31,977–62,493)	23,336 (10,170–35,213)	2,482 (1,711–2,966)	8,370 (6,867–9,438)	9,421 (6,652–10,789)	6,117 (4,473–7,006)
Non-communicable diseases	15,802,035 (15,475,074–16,129,182)	565,853 (475,037–705,126)	138,010 (128,106–146,924)	2,235,727 (2,167,494–2,313,128)	5,887,773 (5,761,888–6,017,274)	6,974,670 (6,875,712–7,071,016)
Neoplasms	3,549,608 (3,455,893–3,630,356)	25,834 (20,538–36,199)	35,579 (32,528–38,311)	514,883 (487,410–534,560)	1,648,876 (1,607,414–1,686,775)	1,324,434 (1,287,128–1,360,413)
Lip and oral cavity cancer	58,476 (56,672–60,342)	0 (0–0)	0 (0–0)	10,893 (10,410–11,395)	31,606 (30,516–32,699)	15,976 (15,426–16,570)
Nasopharynx cancer	33,056 (24,256–35,893)	0 (0–0)	286 (182–353)	10,747 (7,132–12,003)	15,666 (11,838–17,011)	6,356 (5,202–6,776)
Other pharynx cancer	29,611 (28,632–30,522)	0 (0–0)	0 (0–0)	4,972 (4,761–5,186)	17,994 (17,316–18,653)	6,644 (6,390–6,891)
Esophageal cancer	254,358 (242,126–267,486)	0 (0–0)	0 (0–0)	28,823 (26,917–30,904)	140,210 (132,517–147,913)	85,325 (81,624–89,475)
Stomach cancer	482,498 (468,073–498,234)	0 (0–0)	0 (0–0)	57,970 (55,323–61,062)	237,906 (229,668–247,319)	186,621 (181,897–191,664)
Colon and rectum cancer	281,621 (275,809–287,509)	0 (0–0)	0 (0–0)	31,839 (30,712–33,263)	121,443 (118,692–124,467)	128,338 (125,887–130,986)
Liver cancer	401,071 (332,885–426,642)	0 (0–0)	1,067 (735–1,287)	102,873 (74,644–112,604)	202,357 (169,550–215,782)	94,772 (87,958–101,433)
Liver cancer due to hepatitis B	164,352 (130,453–178,774)	0 (0–0)	692 (470–840)	63,180 (45,727–69,890)	75,772 (62,037–83,653)	24,706 (21,914–27,898)
Liver cancer due to hepatitis C	58,883 (53,466–63,533)	0 (0–0)	20 (15–27)	6,156 (4,750–7,042)	30,402 (26,877–33,649)	22,304 (20,570–24,380)
Liver cancer due to alcohol use	123,037 (103,970–133,135)	0 (0–0)	0 (0–0)	16,747 (12,296–18,983)	71,412 (60,269–79,121)	34,877 (31,639–38,017)
Liver cancer due to other causes	54,797 (44,503–60,084)	0 (0–0)	354 (246–429)	16,789 (12,032–18,941)	24,769 (20,494–27,857)	12,883 (11,496–14,492)
Gallbladder and biliary tract cancer	41,409 (40,293–42,538)	0 (0–0)	0 (0–0)	3,536 (3,353–3,755)	17,676 (17,097–18,232)	20,196 (19,644–20,902)
Pancreatic cancer	128,551 (125,881–131,119)	0 (0–0)	0 (0–0)	13,273 (12,851–13,775)	63,351 (61,855–64,855)	51,925 (50,845–53,097)
Larynx cancer	73,296 (71,455–75,169)	0 (0–0)	0 (0–0)	8,986 (8,639–9,354)	41,905 (40,697–43,159)	22,403 (21,809–23,021)
Tracheal, bronchus, and lung cancer	841,298 (824,206–859,107)	0 (0–0)	0 (0–0)	76,828 (73,985–80,182)	447,448 (437,783–457,591)	317,022 (310,246–323,715)
Malignant skin melanoma	19,590 (13,345–28,449)	0 (0–0)	0 (0–0)	5,147 (3,519–7,141)	8,309 (5,628–12,212)	6,133 (4,095–9,454)
Non-melanoma skin cancer	19,325 (18,815–19,886)	0 (0–0)	0 (0–0)	2,304 (2,210–2,411)	7,525 (7,295–7,768)	9,495 (9,201–9,796)
Non-melanoma skin cancer (squamous-cell carcinoma)	19,325 (18,815–19,886)	0 (0–0)	0 (0–0)	2,304 (2,210–2,411)	7,525 (7,295–7,768)	9,495 (9,201–9,796)
Non-melanoma skin cancer (basal-cell carcinoma)	0 (0–0)	0 (0–0)	0 (0–0)	0 (0–0)	0 (0–0)	0 (0–0)
Breast cancer	4,074 (3,580–4,246)	0 (0–0)	0 (0–0)	527 (490–560)	1,902 (1,641–1,994)	1,644 (1,443–1,725)
Cervical cancer	0 (0–0)	0 (0–0)	0 (0–0)	0 (0–0)	0 (0–0)	0 (0–0)
Uterine cancer	0 (0–0)	0 (0–0)	0 (0–0)	0 (0–0)	0 (0–0)	0 (0–0)
Ovarian cancer	0 (0–0)	0 (0–0)	0 (0–0)	0 (0–0)	0 (0–0)	0 (0–0)
Prostate cancer	224,864 (179,861–274,673)	0 (0–0)	0 (0–0)	2,114 (1,651–2,567)	57,332 (46,360–71,275)	165,418 (129,301–203,003)
Testicular cancer	9,106 (8,631–9,725)	0 (0–0)	0 (0–0)	6,390 (5,990–6,875)	1,641 (1,559–1,760)	1,073 (1,031–1,132)

SR Table 11 - Global deaths by age and cause for males in 1995

Cause	All ages	0-5 years	5-14 years	15-49 years	50-69 years	70+ years
Kidney cancer	50,958 (49,643–52,639)	1,398 (736–2,206)	524 (469–588)	5,659 (5,469–5,927)	24,468 (23,836–25,192)	18,907 (18,382–19,458)
Bladder cancer	91,465 (89,516–93,408)	0 (0–0)	0 (0–0)	4,502 (4,304–4,706)	34,609 (33,697–35,458)	52,353 (51,064–53,670)
Brain and nervous system cancer	88,905 (75,300–105,781)	3,957 (2,474–7,433)	6,549 (4,766–8,829)	28,175 (23,219–33,033)	35,055 (30,292–39,742)	15,166 (13,662–17,466)
Thyroid cancer	7,896 (7,242–8,685)	0 (0–0)	39 (31–43)	1,153 (1,031–1,268)	3,594 (3,267–3,952)	3,109 (2,863–3,445)
Mesothelioma	12,979 (12,199–13,704)	0 (0–0)	0 (0–0)	2,090 (1,842–2,223)	6,055 (5,658–6,363)	4,833 (4,542–5,277)
Hodgkin lymphoma	18,932 (14,098–23,663)	556 (294–670)	1,004 (621–1,251)	7,089 (5,389–8,699)	6,937 (5,133–8,735)	3,344 (2,561–4,325)
Non-Hodgkin lymphoma	80,107 (70,526–90,009)	2,155 (1,246–3,767)	2,961 (2,365–3,775)	17,925 (15,781–20,392)	30,170 (26,515–33,626)	26,894 (23,088–30,420)
Multiple myeloma	29,773 (29,061–30,661)	0 (0–0)	0 (0–0)	3,248 (3,102–3,429)	13,152 (12,768–13,611)	13,372 (13,026–13,755)
Leukemia	151,114 (146,462–157,389)	10,344 (9,008–11,859)	17,369 (16,171–18,642)	45,778 (43,813–48,528)	40,589 (39,352–42,071)	37,031 (36,099–38,051)
Acute lymphoid leukemia	53,625 (46,362–62,964)	6,692 (5,038–8,809)	12,216 (10,566–14,343)	18,903 (16,007–22,503)	10,451 (9,007–12,151)	5,361 (4,700–6,240)
Chronic lymphoid leukemia	22,358 (20,272–24,570)	0 (0–0)	0 (0–0)	3,271 (2,642–3,960)	7,399 (6,682–8,280)	11,687 (10,566–12,417)
Acute myeloid leukemia	54,124 (46,063–62,883)	3,652 (1,899–5,217)	5,153 (3,325–6,506)	16,413 (13,525–19,583)	15,858 (14,158–18,086)	13,047 (11,981–14,894)
Chronic myeloid leukemia	21,006 (19,205–23,622)	0 (0–0)	0 (0–0)	7,190 (6,325–8,429)	6,880 (6,222–7,757)	6,935 (6,438–7,480)
Other neoplasms	115,260 (103,217–123,608)	7,421 (4,539–12,890)	5,775 (4,657–6,401)	32,029 (26,815–34,494)	39,962 (35,302–44,285)	30,071 (27,895–34,078)
Cardiovascular diseases	6,914,163 (6,784,801–7,044,546)	51,866 (46,983–56,559)	22,603 (21,060–24,367)	827,553 (801,985–860,407)	2,603,395 (2,547,546–2,657,098)	3,408,745 (3,348,822–3,464,436)
Rheumatic heart disease	144,438 (137,110–154,808)	3,046 (2,402–3,734)	5,164 (4,673–5,673)	43,443 (40,721–47,661)	49,526 (46,553–53,520)	43,257 (41,083–45,852)
Ischemic heart disease	3,476,508 (3,412,388–3,541,587)	3,646 (3,061–4,369)	1,973 (1,749–2,247)	409,907 (396,910–426,224)	1,361,124 (1,329,156–1,390,856)	1,699,855 (1,670,903–1,726,946)
Cerebrovascular disease	2,429,978 (2,361,109–2,501,078)	20,974 (18,726–23,452)	8,721 (7,995–9,460)	246,153 (236,024–258,654)	908,003 (877,720–940,498)	1,246,125 (1,214,027–1,280,379)
Ischemic stroke	1,022,667 (988,411–1,059,673)	790 (646–995)	546 (478–643)	39,340 (36,372–42,858)	322,148 (307,621–338,382)	659,841 (639,770–681,314)
Hemorrhagic stroke	1,407,310 (1,356,554–1,459,560)	20,183 (18,063–22,513)	8,174 (7,519–8,885)	206,812 (198,184–217,558)	585,855 (562,541–609,566)	586,284 (565,277–609,415)
Hypertensive heart disease	317,712 (300,343–352,803)	471 (356–572)	296 (246–352)	37,900 (35,053–42,210)	119,058 (111,509–132,772)	159,985 (150,654–177,547)
Cardiomyopathy and myocarditis	164,292 (154,849–173,118)	7,977 (6,102–9,533)	2,320 (2,036–2,847)	44,856 (41,852–48,142)	56,105 (52,760–59,664)	53,032 (50,071–55,495)
Atrial fibrillation and flutter	43,463 (35,324–52,926)	0 (0–0)	0 (0–0)	470 (366–594)	4,907 (3,794–6,517)	38,085 (30,187–47,379)
Aortic aneurysm	73,173 (70,669–76,177)	0 (0–0)	0 (0–0)	6,430 (6,005–6,996)	24,844 (23,850–26,104)	41,898 (40,589–43,353)
Peripheral vascular disease	10,531 (10,090–10,964)	0 (0–0)	0 (0–0)	209 (195–223)	2,583 (2,447–2,738)	7,738 (7,384–8,080)
Endocarditis	22,775 (18,910–27,682)	2,620 (1,152–4,224)	566 (374–788)	5,689 (4,670–7,331)	6,205 (5,242–7,469)	7,693 (6,786–9,137)
Other cardiovascular and circulatory diseases	231,288 (223,565–240,130)	13,127 (11,632–15,489)	3,560 (3,254–4,012)	32,492 (30,898–34,275)	71,035 (68,056–74,372)	111,072 (107,020–115,264)
Chronic respiratory diseases	2,064,299 (1,999,068–2,137,477)	51,585 (44,123–60,218)	9,574 (8,625–10,482)	137,004 (130,709–146,254)	689,374 (662,923–720,839)	1,176,760 (1,141,770–1,212,193)
Chronic obstructive pulmonary disease	1,698,557 (1,582,331–1,811,402)	21,011 (15,020–28,099)	1,989 (1,546–2,603)	83,017 (73,726–93,125)	556,823 (507,922–605,134)	1,035,714 (978,084–1,091,369)
Pneumoconiosis	22,907 (20,156–25,576)	0 (0–0)	0 (0–0)	2,908 (2,433–3,428)	8,117 (6,933–9,226)	11,880 (10,423–13,338)
Silicosis	11,292 (9,722–13,398)	0 (0–0)	0 (0–0)	1,594 (1,261–2,024)	4,322 (3,606–5,261)	5,375 (4,659–6,370)
Asbestosis	1,479 (1,073–1,842)	0 (0–0)	0 (0–0)	220 (170–265)	474 (316–652)	783 (562–966)
Coal workers pneumoconiosis	3,041 (2,606–3,801)	0 (0–0)	0 (0–0)	216 (156–354)	928 (767–1,191)	1,897 (1,643–2,251)
Other pneumoconiosis	7,094 (5,995–8,114)	0 (0–0)	0 (0–0)	877 (687–1,053)	2,392 (1,949–2,894)	3,824 (3,224–4,339)
Asthma	279,161 (188,180–357,629)	14,674 (10,233–19,173)	6,526 (5,251–7,569)	43,402 (32,098–52,059)	108,363 (70,005–144,337)	106,193 (67,903–141,884)
Interstitial lung disease and pulmonary sarcoidosis	28,296 (21,308–39,495)	172 (75–509)	126 (80–258)	2,409 (1,756–3,786)	9,816 (7,155–14,541)	15,770 (12,103–20,884)
Other chronic respiratory diseases	35,376 (23,021–44,255)	15,726 (9,655–22,606)	931 (504–1,411)	5,265 (3,165–6,568)	6,253 (4,018–7,887)	7,200 (5,094–9,110)
Cirrhosis and other chronic liver diseases	650,007 (625,398–708,821)	10,799 (8,556–13,156)	6,644 (5,936–7,669)	220,023 (209,742–241,855)	293,250 (281,757–320,397)	119,289 (114,511–124,633)
Cirrhosis and other chronic liver diseases due to hepatitis B	206,704 (191,240–233,023)	114 (84–148)	280 (227–338)	71,447 (64,613–80,445)	94,249 (84,691–108,097)	40,612 (36,935–45,821)
Cirrhosis and other chronic liver diseases due to hepatitis C	161,053 (149,539–173,409)	61 (45–80)	151 (118–186)	50,279 (44,890–56,704)	75,775 (68,009–84,323)	34,786 (31,617–38,032)
Cirrhosis and other chronic liver diseases due to alcohol use	182,632 (169,740–197,667)	0 (0–0)	0 (0–0)	69,119 (63,000–76,600)	88,041 (79,427–98,189)	25,470 (22,809–28,538)
Cirrhosis and other chronic liver diseases due to other causes	99,616 (92,030–110,475)	10,623 (8,416–12,938)	6,213 (5,547–7,141)	29,176 (26,225–33,037)	35,184 (30,824–40,489)	18,419 (16,676–20,425)
Digestive diseases	564,524 (532,991–603,519)	19,422 (14,982–24,797)	13,915 (12,348–15,506)	153,924 (141,548–163,820)	194,275 (182,297–208,624)	182,985 (174,940–199,372)
Peptic ulcer disease	199,094 (183,220–218,689)	2,300 (1,342–4,131)	2,961 (2,429–3,605)	53,758 (48,742–59,385)	76,558 (69,790–83,431)	63,515 (59,220–70,443)
Gastritis and duodenitis	27,989 (20,873–35,212)	886 (449–1,547)	433 (293–676)	7,771 (5,655–10,007)	10,224 (7,492–13,125)	8,673 (6,501–11,322)
Appendicitis	22,167 (19,296–26,832)	1,986 (1,310–2,979)	2,132 (1,767–2,621)	8,367 (7,217–10,359)	5,678 (4,895–7,065)	4,001 (3,515–4,874)
Paralytic ileus and intestinal obstruction	110,053 (90,508–130,644)	5,936 (3,610–9,126)	4,622 (3,528–5,672)	31,473 (23,922–36,334)	35,619 (28,450–42,342)	32,401 (28,459–40,966)
Inguinal, femoral, and abdominal hernia	32,014 (19,780–37,694)	1,222 (342–2,087)	726 (350–927)	7,795 (4,008–9,433)	10,390 (6,652–12,888)	11,879 (7,765–14,028)
Inflammatory bowel disease	17,135 (13,784–19,401)	1,106 (537–2,553)	416 (263–638)	4,260 (3,263–4,951)	5,537 (4,530–6,387)	5,813 (4,873–6,620)
Vascular intestinal disorders	27,771 (25,625–30,175)	641 (312–1,313)	120 (93–158)	2,542 (2,290–2,788)	9,702 (8,959–10,560)	14,764 (13,762–15,926)
Gallbladder and biliary diseases	33,654 (30,085–37,079)	596 (354–984)	473 (372–638)	5,859 (4,988–6,900)	10,928 (9,676–12,112)	15,797 (14,514–17,218)
Pancreatitis	46,965 (43,667–51,967)	183 (114–300)	252 (206–322)	22,341 (20,206–24,847)	15,431 (14,366–17,119)	8,756 (8,111–9,536)
Other digestive diseases	47,677 (36,501–63,648)	4,562 (2,939–6,709)	1,775 (1,244–2,355)	9,753 (7,271–14,141)	14,204 (10,627–19,343)	17,380 (13,894–21,845)
Neurological disorders	506,781 (447,852–566,837)	13,995 (8,850–18,352)	8,994 (7,973–9,987)	49,774 (47,756–51,826)	63,116 (54,152–71,902)	370,900 (315,604–426,423)
Alzheimer disease and other dementias	369,887 (311,252–428,111)	0 (0–0)	0 (0–0)	529 (328–783)	35,844 (26,838–44,478)	333,513 (277,925–388,331)
Parkinson disease	29,474 (28,094–31,894)	0 (0–0)	0 (0–0)	299 (275–335)	4,828 (4,559–5,371)	24,346 (23,134–26,080)
Epilepsy	67,998 (62,110–73,101)	10,404 (6,397–13,983)	7,186 (6,356–8,015)	37,056 (35,135–39,174)	9,239 (8,799–9,725)	4,110 (3,927–4,325)

SR Table 11 - Global deaths by age and cause for males in 1995

Cause	All ages	0-5 years	5-14 years	15-49 years	50-69 years	70+ years
Multiple sclerosis	6,467 (5,479–7,100)	0 (0–0)	0 (0–0)	2,524 (1,977–2,873)	2,841 (2,475–3,064)	1,100 (888–1,163)
Motor neuron disease	11,098 (10,664–11,779)	722 (465–954)	83 (72–97)	1,796 (1,689–2,013)	5,146 (4,916–5,600)	3,349 (3,221–3,511)
Migraine	0 (0–0)	0 (0–0)	0 (0–0)	0 (0–0)	0 (0–0)	0 (0–0)
Tension-type headache	0 (0–0)	0 (0–0)	0 (0–0)	0 (0–0)	0 (0–0)	0 (0–0)
Medication overuse headache	0 (0–0)	0 (0–0)	0 (0–0)	0 (0–0)	0 (0–0)	0 (0–0)
Other neurological disorders	21,865 (21,054–23,452)	2,877 (1,823–4,056)	1,724 (1,458–2,022)	7,568 (7,043–8,243)	5,215 (4,840–5,736)	4,479 (4,200–4,822)
Mental and substance use disorders	193,648 (176,315–201,601)	16 (13–21)	14 (5–20)	123,372 (112,230–129,161)	58,070 (54,615–60,809)	12,174 (11,690–12,624)
Schizophrenia	9,342 (8,426–10,109)	0 (0–0)	0 (0–0)	5,102 (4,445–5,626)	2,829 (2,570–3,042)	1,410 (1,296–1,526)
Alcohol use disorders	114,538 (106,122–120,289)	0 (0–0)	0 (0–0)	64,243 (58,344–68,161)	43,106 (39,852–45,502)	7,189 (6,898–7,462)
Drug use disorders	69,636 (62,163–73,588)	16 (13–21)	0 (0–0)	53,910 (47,236–57,178)	12,134 (11,341–13,076)	3,575 (3,326–3,845)
Opioid use disorders	54,132 (48,756–57,357)	16 (13–21)	0 (0–0)	42,286 (37,480–45,023)	9,300 (8,615–10,104)	2,529 (2,320–2,751)
Cocaine use disorders	2,137 (1,855–2,920)	0 (0–0)	0 (0–0)	1,764 (1,521–2,427)	323 (273–433)	49 (41–72)
Amphetamine use disorders	1,423 (1,174–2,074)	0 (0–0)	0 (0–0)	1,128 (923–1,634)	249 (191–368)	45 (35–56)
Cannabis use disorders	0 (0–0)	0 (0–0)	0 (0–0)	0 (0–0)	0 (0–0)	0 (0–0)
Other drug use disorders	11,943 (9,363–12,943)	0 (0–0)	0 (0–0)	8,731 (6,576–9,567)	2,261 (1,984–2,483)	951 (816–1,067)
Depressive disorders	0 (0–0)	0 (0–0)	0 (0–0)	0 (0–0)	0 (0–0)	0 (0–0)
Major depressive disorder	0 (0–0)	0 (0–0)	0 (0–0)	0 (0–0)	0 (0–0)	0 (0–0)
Dysthymia	0 (0–0)	0 (0–0)	0 (0–0)	0 (0–0)	0 (0–0)	0 (0–0)
Bipolar disorder	0 (0–0)	0 (0–0)	0 (0–0)	0 (0–0)	0 (0–0)	0 (0–0)
Anxiety disorders	0 (0–0)	0 (0–0)	0 (0–0)	0 (0–0)	0 (0–0)	0 (0–0)
Eating disorders	131 (71–171)	0 (0–0)	14 (5–20)	117 (65–153)	0 (0–0)	0 (0–0)
Anorexia nervosa	118 (65–154)	0 (0–0)	13 (5–19)	105 (58–138)	0 (0–0)	0 (0–0)
Bulimia nervosa	12 (6–17)	0 (0–0)	0 (0–1)	11 (5–16)	0 (0–0)	0 (0–0)
Autistic spectrum disorders	0 (0–0)	0 (0–0)	0 (0–0)	0 (0–0)	0 (0–0)	0 (0–0)
Autism	0 (0–0)	0 (0–0)	0 (0–0)	0 (0–0)	0 (0–0)	0 (0–0)
Asperger syndrome and other autistic spectrum disorders	0 (0–0)	0 (0–0)	0 (0–0)	0 (0–0)	0 (0–0)	0 (0–0)
Attention-deficit/hyperactivity disorder	0 (0–0)	0 (0–0)	0 (0–0)	0 (0–0)	0 (0–0)	0 (0–0)
Conduct disorder	0 (0–0)	0 (0–0)	0 (0–0)	0 (0–0)	0 (0–0)	0 (0–0)
Idiopathic developmental intellectual disability	0 (0–0)	0 (0–0)	0 (0–0)	0 (0–0)	0 (0–0)	0 (0–0)
Other mental and substance use disorders	0 (0–0)	0 (0–0)	0 (0–0)	0 (0–0)	0 (0–0)	0 (0–0)
Diabetes, urogenital, blood, and endocrine diseases	946,064 (872,261–997,367)	76,904 (51,763–102,991)	19,006 (16,326–21,105)	171,792 (154,796–182,964)	319,692 (299,958–335,404)	358,667 (340,445–376,457)
Diabetes mellitus	371,111 (360,577–382,092)	1,874 (1,493–2,112)	1,406 (1,134–1,612)	49,222 (47,264–51,510)	161,207 (155,533–166,671)	157,400 (153,062–161,829)
Acute glomerulonephritis	7,686 (5,620–8,576)	353 (267–448)	363 (279–423)	2,621 (1,674–2,946)	2,441 (1,867–2,841)	1,906 (1,362–2,261)
Chronic kidney disease	338,085 (289,557–376,115)	9,787 (7,065–11,304)	5,646 (4,168–6,299)	74,208 (59,120–80,611)	110,542 (94,389–125,780)	137,900 (123,938–153,508)
Chronic kidney disease due to diabetes mellitus	102,449 (91,001–113,117)	934 (716–1,184)	400 (310–494)	16,261 (13,217–18,255)	40,653 (35,257–45,864)	44,199 (40,435–48,334)
Chronic kidney disease due to hypertension	142,879 (122,691–160,252)	3,268 (2,395–3,940)	1,563 (1,171–1,860)	27,591 (21,639–30,327)	44,744 (38,002–50,610)	65,712 (59,407–73,325)
Chronic kidney disease due to glomerulonephritis	82,738 (68,895–92,385)	4,154 (2,914–4,901)	2,729 (2,035–3,117)	27,193 (21,715–29,967)	23,661 (19,921–28,024)	24,999 (21,918–28,987)
Chronic kidney disease due to other causes	10,017 (7,831–12,090)	1,430 (946–1,854)	952 (677–1,230)	3,161 (2,174–4,349)	1,483 (907–2,281)	2,989 (2,465–3,757)
Urinary diseases and male infertility	81,297 (70,263–100,413)	5,881 (3,772–7,696)	1,248 (1,031–1,560)	13,470 (11,090–17,173)	23,903 (19,245–31,138)	36,792 (32,071–46,552)
Interstitial nephritis and urinary tract infections	52,367 (43,786–64,265)	4,636 (2,814–6,126)	747 (573–916)	8,156 (6,472–10,391)	13,973 (10,822–18,119)	24,852 (20,716–31,039)
Urolithiasis	9,457 (6,527–11,703)	0 (0–0)	191 (131–263)	1,977 (1,291–2,526)	3,635 (2,475–4,576)	3,653 (2,471–4,662)
Benign prostatic hyperplasia	0 (0–0)	0 (0–0)	0 (0–0)	0 (0–0)	0 (0–0)	0 (0–0)
Male infertility	0 (0–0)	0 (0–0)	0 (0–0)	0 (0–0)	0 (0–0)	0 (0–0)
Other urinary diseases	19,472 (15,018–28,416)	1,244 (743–2,224)	309 (237–467)	3,336 (2,436–5,250)	6,294 (4,402–9,888)	8,286 (6,238–12,104)
Gynecological diseases	0 (0–0)	0 (0–0)	0 (0–0)	0 (0–0)	0 (0–0)	0 (0–0)
Uterine fibroids	0 (0–0)	0 (0–0)	0 (0–0)	0 (0–0)	0 (0–0)	0 (0–0)
Polycystic ovarian syndrome	0 (0–0)	0 (0–0)	0 (0–0)	0 (0–0)	0 (0–0)	0 (0–0)
Female infertility	0 (0–0)	0 (0–0)	0 (0–0)	0 (0–0)	0 (0–0)	0 (0–0)
Endometriosis	0 (0–0)	0 (0–0)	0 (0–0)	0 (0–0)	0 (0–0)	0 (0–0)
Genital prolapse	0 (0–0)	0 (0–0)	0 (0–0)	0 (0–0)	0 (0–0)	0 (0–0)
Premenstrual syndrome	0 (0–0)	0 (0–0)	0 (0–0)	0 (0–0)	0 (0–0)	0 (0–0)
Other gynecological diseases	0 (0–0)	0 (0–0)	0 (0–0)	0 (0–0)	0 (0–0)	0 (0–0)
Hemoglobinopathies and hemolytic anemias	106,468 (71,812–138,640)	49,698 (26,488–76,333)	8,021 (6,380–9,955)	22,892 (17,420–27,630)	11,925 (8,987–14,744)	13,930 (10,598–17,435)
Thalassemias	11,610 (8,080–15,202)	7,335 (4,395–10,814)	2,381 (1,824–2,968)	946 (687–1,281)	30 (27–34)	917 (528–1,422)
Thalassemias trait	0 (0–0)	0 (0–0)	0 (0–0)	0 (0–0)	0 (0–0)	0 (0–0)
Sickle cell disorders	54,503 (30,769–82,787)	41,092 (20,613–68,690)	4,315 (3,157–5,443)	7,806 (5,529–9,949)	299 (232–365)	990 (715–1,384)
Sickle cell trait	0 (0–0)	0 (0–0)	0 (0–0)	0 (0–0)	0 (0–0)	0 (0–0)

SR Table 11 - Global deaths by age and cause for males in 1995						
Cause	All ages	0-5 years	5-14 years	15-49 years	50-69 years	70+ years
G6PD deficiency	14,737 (11,941–18,582)	921 (432–1,649)	934 (684–1,339)	8,577 (6,875–10,876)	3,568 (2,729–4,518)	736 (547–968)
G6PD trait	0 (0–0)	0 (0–0)	0 (0–0)	0 (0–0)	0 (0–0)	0 (0–0)
Other hemoglobinopathies and hemolytic anemias	25,616 (19,900–31,281)	349 (196–578)	389 (307–520)	5,563 (4,340–6,763)	8,026 (5,956–10,150)	11,286 (8,809–13,893)
Endocrine, metabolic, blood, and immune disorders	41,415 (40,019–42,953)	9,309 (8,126–10,585)	2,318 (2,180–2,452)	9,377 (8,883–9,932)	9,671 (9,121–10,313)	10,737 (10,200–11,283)
Musculoskeletal disorders	19,075 (16,110–20,126)	0 (0–0)	849 (689–1,012)	4,693 (4,276–5,084)	5,723 (4,653–6,140)	7,807 (6,262–8,354)
Rheumatoid arthritis	6,542 (4,759–7,194)	0 (0–0)	122 (85–186)	752 (556–857)	2,438 (1,755–2,692)	3,228 (2,403–3,613)
Osteoarthritis	0 (0–0)	0 (0–0)	0 (0–0)	0 (0–0)	0 (0–0)	0 (0–0)
Low back and neck pain	0 (0–0)	0 (0–0)	0 (0–0)	0 (0–0)	0 (0–0)	0 (0–0)
Low back pain	0 (0–0)	0 (0–0)	0 (0–0)	0 (0–0)	0 (0–0)	0 (0–0)
Neck pain	0 (0–0)	0 (0–0)	0 (0–0)	0 (0–0)	0 (0–0)	0 (0–0)
Gout	0 (0–0)	0 (0–0)	0 (0–0)	0 (0–0)	0 (0–0)	0 (0–0)
Other musculoskeletal disorders	12,532 (11,184–13,271)	0 (0–0)	727 (592–888)	3,941 (3,678–4,353)	3,284 (2,841–3,539)	4,579 (3,645–4,958)
Other non-communicable diseases	393,852 (306,669–525,051)	315,418 (235,190–441,315)	20,827 (15,870–25,843)	32,704 (29,120–38,620)	11,998 (9,081–15,241)	12,903 (9,809–17,175)
Congenital anomalies	358,467 (273,484–485,712)	299,724 (221,603–422,381)	20,344 (15,314–25,319)	28,918 (26,018–34,582)	5,739 (5,302–7,136)	3,739 (3,452–4,578)
Neural tube defects	56,812 (40,582–87,449)	52,830 (37,140–82,615)	1,987 (1,477–2,637)	1,483 (1,184–2,067)	311 (219–534)	198 (123–393)
Congenital heart anomalies	187,533 (137,980–257,379)	148,524 (104,867–214,673)	14,449 (10,749–18,142)	21,231 (18,839–25,046)	2,014 (1,745–2,419)	1,312 (1,096–1,666)
Cleft lip and cleft palate	2,723 (1,902–3,928)	2,723 (1,902–3,928)	0 (0–0)	0 (0–0)	0 (0–0)	0 (0–0)
Down syndrome	9,136 (5,716–20,295)	6,044 (2,782–16,589)	411 (256–830)	1,665 (1,291–2,694)	965 (722–1,483)	48 (36–73)
Turner syndrome	0 (0–0)	0 (0–0)	0 (0–0)	0 (0–0)	0 (0–0)	0 (0–0)
Klinefelter syndrome	0 (0–0)	0 (0–0)	0 (0–0)	0 (0–0)	0 (0–0)	0 (0–0)
Other chromosomal abnormalities	7,006 (3,177–18,814)	6,316 (2,503–17,978)	150 (98–262)	377 (267–531)	124 (85–185)	39 (27–55)
Other congenital anomalies	95,255 (69,990–134,667)	83,285 (59,527–121,825)	3,345 (2,496–4,404)	4,160 (3,436–5,023)	2,323 (1,952–2,831)	2,140 (1,902–2,532)
Skin and subcutaneous diseases	22,134 (13,545–33,751)	2,443 (1,130–4,631)	482 (281–871)	3,786 (2,241–6,295)	6,258 (3,286–9,316)	9,164 (5,945–13,138)
Dermatitis	0 (0–0)	0 (0–0)	0 (0–0)	0 (0–0)	0 (0–0)	0 (0–0)
Psoriasis	0 (0–0)	0 (0–0)	0 (0–0)	0 (0–0)	0 (0–0)	0 (0–0)
Cellulitis	4,513 (2,456–8,575)	381 (141–835)	97 (57–187)	1,008 (533–2,165)	1,686 (785–3,374)	1,338 (833–2,713)
Pyoderma	8,672 (5,257–15,117)	1,894 (862–3,872)	252 (138–476)	1,857 (1,062–3,204)	2,121 (1,303–3,528)	2,545 (1,749–4,091)
Scabies	0 (0–0)	0 (0–0)	0 (0–0)	0 (0–0)	0 (0–0)	0 (0–0)
Fungal skin diseases	0 (0–0)	0 (0–0)	0 (0–0)	0 (0–0)	0 (0–0)	0 (0–0)
Viral skin diseases	0 (0–0)	0 (0–0)	0 (0–0)	0 (0–0)	0 (0–0)	0 (0–0)
Acne vulgaris	0 (0–0)	0 (0–0)	0 (0–0)	0 (0–0)	0 (0–0)	0 (0–0)
Alopecia areata	0 (0–0)	0 (0–0)	0 (0–0)	0 (0–0)	0 (0–0)	0 (0–0)
Pruritus	0 (0–0)	0 (0–0)	0 (0–0)	0 (0–0)	0 (0–0)	0 (0–0)
Urticaria	0 (0–0)	0 (0–0)	0 (0–0)	0 (0–0)	0 (0–0)	0 (0–0)
Decubitus ulcer	7,893 (3,942–11,171)	42 (18–73)	74 (37–136)	677 (334–1,119)	2,174 (719–4,310)	4,923 (2,790–6,857)
Other skin and subcutaneous diseases	1,055 (626–1,686)	124 (59–253)	57 (33–108)	241 (141–452)	275 (125–639)	355 (222–553)
Sense organ diseases	0 (0–0)	0 (0–0)	0 (0–0)	0 (0–0)	0 (0–0)	0 (0–0)
Glaucoma	0 (0–0)	0 (0–0)	0 (0–0)	0 (0–0)	0 (0–0)	0 (0–0)
Cataract	0 (0–0)	0 (0–0)	0 (0–0)	0 (0–0)	0 (0–0)	0 (0–0)
Macular degeneration	0 (0–0)	0 (0–0)	0 (0–0)	0 (0–0)	0 (0–0)	0 (0–0)
Refraction and accommodation disorders	0 (0–0)	0 (0–0)	0 (0–0)	0 (0–0)	0 (0–0)	0 (0–0)
Age-related and other hearing loss	0 (0–0)	0 (0–0)	0 (0–0)	0 (0–0)	0 (0–0)	0 (0–0)
Other vision loss	0 (0–0)	0 (0–0)	0 (0–0)	0 (0–0)	0 (0–0)	0 (0–0)
Other sense organ diseases	0 (0–0)	0 (0–0)	0 (0–0)	0 (0–0)	0 (0–0)	0 (0–0)
Oral disorders	0 (0–0)	0 (0–0)	0 (0–0)	0 (0–0)	0 (0–0)	0 (0–0)
Deciduous caries	0 (0–0)	0 (0–0)	0 (0–0)	0 (0–0)	0 (0–0)	0 (0–0)
Permanent caries	0 (0–0)	0 (0–0)	0 (0–0)	0 (0–0)	0 (0–0)	0 (0–0)
Periodontal diseases	0 (0–0)	0 (0–0)	0 (0–0)	0 (0–0)	0 (0–0)	0 (0–0)
Edentulism and severe tooth loss	0 (0–0)	0 (0–0)	0 (0–0)	0 (0–0)	0 (0–0)	0 (0–0)
Other oral disorders	0 (0–0)	0 (0–0)	0 (0–0)	0 (0–0)	0 (0–0)	0 (0–0)
Sudden infant death syndrome	13,249 (9,512–31,830)	13,249 (9,512–31,830)	0 (0–0)	0 (0–0)	0 (0–0)	0 (0–0)
Injuries	3,146,955 (2,992,649–3,248,534)	328,646 (266,930–377,414)	248,390 (232,998–263,571)	1,771,960 (1,697,880–1,823,443)	532,289 (505,372–548,934)	265,669 (247,633–275,846)
Transport injuries	961,931 (922,089–1,004,059)	54,568 (41,337–66,058)	72,500 (66,367–77,354)	606,092 (582,686–630,437)	161,842 (154,677–169,192)	66,927 (64,326–69,663)
Road injuries	900,881 (860,969–942,249)	50,400 (38,193–61,391)	68,957 (63,174–73,594)	569,768 (547,274–594,919)	148,840 (141,321–156,242)	62,914 (60,342–65,626)
Pedestrian road injuries	398,874 (347,708–434,537)	27,800 (22,264–34,224)	40,043 (35,883–43,934)	219,595 (188,596–241,912)	76,050 (65,579–82,655)	35,383 (31,105–38,212)
Cyclist road injuries	37,717 (32,976–43,644)	1,321 (762–2,004)	4,007 (3,325–4,786)	20,003 (17,095–23,645)	8,793 (7,772–10,288)	3,590 (3,239–4,083)
Motorcyclist road injuries	133,642 (107,007–167,049)	3,996 (2,063–6,368)	5,306 (3,649–7,282)	104,037 (83,590–129,184)	15,665 (11,769–20,646)	4,637 (3,347–6,255)

SR Table 11 - Global deaths by age and cause for males in 1995

Cause	All ages	0-5 years	5-14 years	15-49 years	50-69 years	70+ years
Motor vehicle road injuries	319,854 (288,613–377,820)	16,292 (10,806–20,714)	18,546 (16,215–22,508)	220,021 (199,118–260,660)	46,567 (40,328–56,820)	18,426 (15,660–22,267)
Other road injuries	10,791 (6,476–14,094)	989 (309–2,457)	1,052 (546–1,561)	6,109 (3,667–8,032)	1,764 (1,241–2,375)	875 (630–1,304)
Other transport injuries	61,050 (52,281–68,405)	4,167 (2,455–5,366)	3,542 (2,798–4,099)	36,324 (31,242–40,536)	13,001 (11,052–15,056)	4,013 (3,446–4,684)
Unintentional injuries	1,285,641 (1,210,840–1,351,755)	259,442 (206,509–303,598)	154,310 (143,783–164,817)	527,806 (502,599–551,791)	205,201 (192,618–215,048)	138,880 (127,392–147,143)
Falls	232,330 (214,545–244,408)	17,439 (11,137–23,650)	13,025 (10,784–14,748)	86,345 (79,966–90,689)	56,773 (52,355–59,760)	58,747 (53,899–61,823)
Drowning	362,368 (340,984–385,237)	95,544 (80,198–112,183)	86,793 (81,317–91,993)	134,940 (128,443–143,348)	32,751 (31,149–34,732)	12,338 (11,714–13,060)
Fire, heat, and hot substances	111,532 (82,532–119,832)	21,595 (15,339–25,744)	7,452 (5,577–8,422)	50,654 (35,589–54,974)	18,865 (15,337–20,138)	12,964 (10,606–13,645)
Poisonings	81,688 (52,622–98,999)	19,884 (6,229–35,850)	5,901 (4,245–7,477)	35,891 (26,741–42,121)	14,919 (10,981–17,001)	5,091 (3,663–6,007)
Exposure to mechanical forces	145,344 (128,903–168,878)	27,956 (20,504–35,605)	11,175 (9,360–14,851)	73,913 (67,011–87,860)	21,671 (19,517–26,443)	10,627 (9,102–11,924)
Unintentional firearm injuries	25,504 (18,209–28,837)	2,283 (1,263–3,373)	2,067 (1,577–2,727)	15,953 (11,256–17,776)	3,067 (2,260–3,478)	2,131 (1,696–2,389)
Unintentional suffocation	25,815 (19,424–32,582)	17,426 (11,317–23,704)	2,361 (1,891–3,683)	3,270 (2,849–4,029)	900 (594–1,067)	1,857 (1,521–2,039)
Other exposure to mechanical forces	94,024 (80,997–118,331)	8,245 (4,820–12,131)	6,746 (5,235–9,242)	54,689 (48,443–68,435)	17,703 (15,727–22,459)	6,638 (5,508–7,780)
Adverse effects of medical treatment	56,371 (41,005–67,569)	15,114 (6,141–21,240)	3,509 (2,179–4,406)	16,649 (12,202–19,797)	10,650 (8,275–12,481)	10,448 (8,194–11,883)
Animal contact	64,330 (41,924–72,021)	10,545 (5,128–13,708)	9,899 (6,203–11,520)	26,143 (17,367–29,192)	12,608 (8,270–14,152)	5,134 (3,755–5,879)
Venomous animal contact	50,389 (32,240–57,023)	7,234 (3,490–9,356)	7,839 (4,704–9,216)	21,460 (13,866–24,464)	9,965 (6,325–11,604)	3,888 (2,736–4,671)
Non-venomous animal contact	13,941 (9,657–17,117)	3,311 (1,482–4,757)	2,060 (1,321–2,627)	4,682 (3,399–5,588)	2,642 (1,955–3,172)	1,245 (937–1,529)
Foreign body	92,348 (67,635–131,252)	38,792 (21,952–58,486)	7,140 (5,138–10,697)	23,442 (18,186–45,073)	11,770 (9,824–19,887)	11,203 (9,188–18,092)
Pulmonary aspiration and foreign body in airway	67,261 (48,921–91,796)	30,015 (14,217–48,643)	4,784 (3,143–6,784)	14,224 (11,894–16,881)	9,089 (7,639–10,376)	9,148 (7,727–10,048)
Foreign body in eyes	0 (0–0)	0 (0–0)	0 (0–0)	0 (0–0)	0 (0–0)	0 (0–0)
Foreign body in other body part	25,086 (12,606–71,882)	8,777 (3,778–15,219)	2,356 (1,124–6,056)	9,217 (4,411–31,485)	2,680 (1,415–10,735)	2,054 (1,044–8,662)
Environmental heat and cold exposure	34,678 (25,870–40,157)	3,446 (1,394–4,565)	1,091 (682–1,406)	15,289 (11,667–17,810)	9,980 (7,969–12,682)	4,870 (3,382–6,082)
Other unintentional injuries	104,647 (97,125–111,502)	9,123 (6,807–11,263)	8,321 (7,322–9,353)	64,537 (59,851–69,501)	15,209 (14,024–16,547)	7,454 (6,463–8,654)
Self-harm and interpersonal violence	841,612 (757,486–870,269)	11,480 (9,021–12,801)	17,542 (14,553–18,653)	595,563 (537,023–616,524)	158,805 (144,456–164,684)	58,220 (51,214–60,494)
Self-harm	509,053 (439,233–530,749)	0 (0–0)	6,809 (4,725–7,574)	332,968 (284,651–348,718)	119,364 (105,000–124,436)	49,910 (42,686–52,075)
Interpersonal violence	332,559 (308,924–344,994)	11,480 (9,021–12,801)	10,732 (9,525–11,494)	262,595 (245,708–272,808)	39,441 (36,922–41,394)	8,309 (7,888–8,932)
Assault by firearm	125,165 (117,064–131,947)	1,204 (933–1,594)	2,453 (2,255–2,727)	108,421 (101,683–113,804)	11,178 (10,097–12,216)	1,908 (1,701–2,088)
Assault by sharp object	86,493 (79,095–92,028)	1,401 (938–2,034)	2,041 (1,655–2,479)	71,189 (65,130–76,150)	9,874 (8,865–10,787)	1,986 (1,800–2,145)
Assault by other means	120,899 (106,173–130,286)	8,874 (6,849–10,149)	6,237 (5,187–6,920)	82,984 (72,416–90,052)	18,388 (16,650–20,169)	4,413 (4,070–4,966)
Forces of nature, war, and legal intervention	57,770 (44,826–70,801)	3,154 (2,278–4,161)	4,037 (2,999–5,505)	42,497 (32,888–52,276)	6,440 (4,542–9,750)	1,640 (1,051–3,171)
Exposure to forces of nature	11,865 (7,029–19,652)	727 (432–1,216)	1,685 (1,048–2,855)	6,577 (3,857–11,284)	2,064 (1,008–5,005)	810 (345–2,324)
Collective violence and legal intervention	45,904 (35,793–55,643)	2,426 (1,697–3,293)	2,351 (1,655–3,117)	35,919 (27,795–43,840)	4,375 (3,396–5,342)	830 (644–1,030)

SR Table 11 - Global deaths by age and cause for males in 2000						
Cause	All ages	0-5 years	5-14 years	15-49 years	50-69 years	70+ years
All causes	27,852,062 (27,517,468–28,179,760)	4,928,179 (4,877,583–4,978,873)	676,161 (667,138–687,634)	5,697,411 (5,580,238–5,809,808)	7,526,488 (7,396,576–7,649,242)	9,023,820 (8,928,784–9,115,898)
Communicable, maternal, neonatal, and nutritional diseases	7,633,208 (7,435,007–7,941,360)	4,153,465 (4,028,822–4,261,534)	307,510 (291,074–327,381)	1,430,169 (1,356,374–1,529,196)	902,003 (834,318–1,006,260)	840,059 (791,675–918,382)
HIV/AIDS and tuberculosis	1,631,960 (1,447,776–1,907,030)	107,548 (99,898–120,019)	29,647 (26,860–35,341)	872,213 (792,849–967,051)	426,427 (356,283–531,091)	196,124 (156,398–271,442)
Tuberculosis	891,070 (705,956–1,174,117)	22,803 (16,014–36,054)	11,554 (8,776–16,965)	345,357 (270,043–439,910)	326,563 (256,710–432,052)	184,790 (145,277–259,667)
HIV/AIDS	740,889 (697,060–800,164)	84,744 (81,631–88,103)	18,092 (16,938–19,399)	526,855 (489,863–574,388)	99,863 (91,672–113,408)	11,334 (10,164–13,819)
HIV/AIDS - Tuberculosis	154,472 (131,582–180,045)	23,885 (20,716–27,222)	4,934 (4,032–5,744)	97,764 (82,582–115,297)	24,696 (20,899–28,379)	3,191 (2,723–3,698)
HIV/AIDS resulting in other diseases	586,417 (542,075–641,573)	60,859 (56,614–65,893)	13,158 (11,897–14,712)	429,090 (391,574–471,486)	75,167 (67,158–87,228)	8,142 (7,108–10,332)
Diarrhea, lower respiratory, and other common infectious diseases	3,216,532 (3,049,003–3,409,083)	1,698,872 (1,581,403–1,825,840)	199,926 (178,099–227,554)	381,878 (349,621–420,571)	369,290 (340,707–406,321)	566,564 (526,478–618,698)
Diarrheal diseases	837,842 (771,764–907,405)	441,461 (382,208–504,864)	41,563 (36,440–46,740)	109,363 (100,659–120,908)	113,997 (105,259–124,028)	131,456 (122,221–141,443)
Intestinal infectious diseases	119,227 (67,046–195,551)	31,662 (16,324–55,109)	46,892 (27,538–74,270)	34,676 (18,243–59,211)	4,322 (2,211–7,608)	1,673 (957–2,630)
Typhoid fever	97,962 (53,506–164,261)	26,060 (12,956–46,448)	39,071 (22,262–62,076)	28,201 (14,626–49,362)	3,374 (1,612–6,019)	1,253 (675–2,062)
Paratyphoid fever	20,012 (9,156–37,874)	4,853 (2,018–9,620)	7,702 (3,544–14,593)	6,298 (2,636–12,361)	859 (354–1,760)	299 (126–583)
Other intestinal infectious diseases	1,252 (364–2,611)	748 (177–1,864)	118 (30–287)	176 (64–342)	88 (32–169)	120 (38–242)
Lower respiratory infections	1,527,430 (1,404,298–1,650,868)	729,987 (663,330–799,690)	41,815 (31,914–47,475)	146,738 (122,379–166,028)	203,963 (171,898–233,187)	404,926 (361,798–450,769)
Upper respiratory infections	2,156 (1,872–2,541)	693 (484–1,020)	42 (27–63)	504 (421–604)	497 (398–612)	417 (335–523)
Otitis media	2,465 (2,150–2,794)	1,014 (827–1,221)	390 (343–436)	589 (524–668)	303 (259–361)	167 (135–218)
Meningitis	232,789 (188,205–274,323)	129,376 (98,124–163,532)	21,149 (14,507–25,282)	45,916 (33,287–55,934)	23,131 (17,445–29,144)	13,215 (10,062–17,182)
Pneumococcal meningitis	60,826 (47,491–77,073)	28,792 (19,370–41,758)	6,738 (4,454–9,349)	15,072 (10,793–19,246)	6,485 (4,559–8,887)	3,737 (2,707–5,135)
H influenzae type B meningitis	68,028 (51,262–85,081)	48,457 (33,797–64,391)	5,798 (3,650–8,067)	7,819 (5,313–10,341)	3,750 (2,580–5,113)	2,201 (1,616–3,102)
Meningococcal meningitis	42,035 (30,981–54,848)	24,830 (16,260–36,592)	3,874 (2,376–5,618)	7,640 (5,407–10,186)	3,810 (2,675–5,267)	1,879 (1,371–2,596)
Other meningitis	61,898 (49,962–73,699)	27,295 (19,332–36,549)	4,737 (3,079–6,576)	15,384 (10,705–18,744)	9,085 (6,593–11,753)	5,396 (4,053–6,854)
Encephalitis	79,895 (72,477–88,034)	27,104 (22,838–31,582)	10,152 (8,690–11,698)	21,148 (19,212–23,368)	11,920 (10,756–13,279)	9,569 (8,616–10,878)
Diphtheria	4,852 (2,301–11,612)	3,719 (1,286–10,237)	600 (277–1,363)	405 (273–590)	126 (66–232)	0 (0–0)
Whooping cough	55,000 (18,161–123,133)	51,090 (16,850–114,239)	3,039 (1,037–6,955)	772 (253–2,001)	97 (30–269)	0 (0–0)
Tetanus	88,851 (75,040–122,955)	55,350 (47,588–65,092)	4,330 (3,030–8,233)	15,620 (10,248–29,500)	9,805 (6,571–21,096)	3,746 (2,409–8,052)
Measles	260,480 (99,329–510,132)	225,711 (87,287–440,010)	29,453 (10,386–59,734)	5,129 (1,484–12,201)	185 (52–450)	0 (0–0)
Varicella and herpes zoster	5,539 (4,747–6,559)	1,700 (1,222–2,441)	496 (347–710)	1,012 (836–1,235)	938 (724–1,216)	1,392 (1,041–1,831)
Neglected tropical diseases and malaria	642,631 (521,708–776,450)	419,990 (304,687–540,366)	55,471 (41,354–71,320)	105,505 (84,313–130,459)	44,842 (36,215–56,088)	16,822 (13,175–22,165)
Malaria	557,755 (439,324–691,894)	406,403 (291,210–527,494)	41,521 (26,864–58,025)	69,163 (50,217–94,413)	29,699 (21,563–40,744)	10,967 (7,400–15,950)
Chagas disease	4,738 (4,477–5,009)	3 (2–3)	5 (5–6)	975 (896–1,056)	2,215 (2,059–2,382)	1,538 (1,416–1,670)
Leishmaniasis	15,958 (9,696–23,611)	2,468 (1,429–3,676)	1,671 (941–2,815)	9,578 (5,699–14,424)	1,899 (1,245–2,695)	340 (251–435)
Visceral leishmaniasis	15,958 (9,696–23,611)	2,468 (1,429–3,676)	1,671 (941–2,815)	9,578 (5,699–14,424)	1,899 (1,245–2,695)	340 (251–435)
Cutaneous and mucocutaneous leishmaniasis	0 (0–0)	0 (0–0)	0 (0–0)	0 (0–0)	0 (0–0)	0 (0–0)
African trypanosomiasis	12,754 (6,896–21,267)	875 (456–1,452)	1,616 (875–2,602)	8,267 (4,470–14,026)	1,660 (854–2,881)	332 (171–554)
Schistosomiasis	5,031 (4,355–5,814)	66 (38–121)	135 (91–197)	1,784 (1,471–2,172)	2,093 (1,602–2,756)	952 (685–1,342)
Cysticercosis	400 (357–459)	6 (3–12)	13 (10–17)	254 (226–293)	99 (88–113)	26 (23–29)
Cystic echinococcosis	1,058 (1,000–1,124)	46 (36–57)	100 (88–112)	451 (422–486)	300 (281–320)	159 (151–169)
Lymphatic filariasis	0 (0–0)	0 (0–0)	0 (0–0)	0 (0–0)	0 (0–0)	0 (0–0)
Onchocerciasis	0 (0–0)	0 (0–0)	0 (0–0)	0 (0–0)	0 (0–0)	0 (0–0)
Trachoma	0 (0–0)	0 (0–0)	0 (0–0)	0 (0–0)	0 (0–0)	0 (0–0)
Dengue	3,738 (2,292–6,155)	1,313 (555–2,590)	942 (551–1,519)	1,047 (682–1,701)	281 (168–443)	153 (100–236)
Yellow fever	5,584 (1,163–14,995)	489 (94–1,455)	1,488 (302–4,133)	3,096 (649–8,670)	430 (89–1,213)	79 (16–234)
Rabies	23,768 (20,723–26,651)	2,696 (1,870–3,863)	6,657 (5,387–8,091)	8,426 (7,176–9,619)	4,586 (3,827–5,329)	1,400 (1,068–1,712)
Intestinal nematode infections	2,352 (1,990–2,723)	1,720 (1,396–2,042)	347 (288–413)	147 (115–185)	84 (62–112)	52 (34–73)
Ascariasis	2,352 (1,990–2,723)	1,720 (1,396–2,042)	347 (288–413)	147 (115–185)	84 (62–112)	52 (34–73)
Trichuriasis	0 (0–0)	0 (0–0)	0 (0–0)	0 (0–0)	0 (0–0)	0 (0–0)
Hookworm disease	0 (0–0)	0 (0–0)	0 (0–0)	0 (0–0)	0 (0–0)	0 (0–0)
Food-borne trematodiases	0 (0–0)	0 (0–0)	0 (0–0)	0 (0–0)	0 (0–0)	0 (0–0)
Leprosy	0 (0–0)	0 (0–0)	0 (0–0)	0 (0–0)	0 (0–0)	0 (0–0)
Ebola	114 (92–136)	14 (11–17)	8 (7–10)	67 (54–80)	21 (17–26)	1 (1–2)
Other neglected tropical diseases	9,376 (4,698–14,069)	3,886 (1,922–5,494)	960 (465–1,469)	2,242 (1,020–3,570)	1,470 (698–2,329)	816 (407–1,361)
Maternal disorders	0 (0–0)	0 (0–0)	0 (0–0)	0 (0–0)	0 (0–0)	0 (0–0)
Maternal hemorrhage	0 (0–0)	0 (0–0)	0 (0–0)	0 (0–0)	0 (0–0)	0 (0–0)
Maternal sepsis and other maternal infections	0 (0–0)	0 (0–0)	0 (0–0)	0 (0–0)	0 (0–0)	0 (0–0)
Maternal hypertensive disorders	0 (0–0)	0 (0–0)	0 (0–0)	0 (0–0)	0 (0–0)	0 (0–0)
Maternal obstructed labor and uterine rupture	0 (0–0)	0 (0–0)	0 (0–0)	0 (0–0)	0 (0–0)	0 (0–0)
Maternal abortion, miscarriage, and ectopic pregnancy	0 (0–0)	0 (0–0)	0 (0–0)	0 (0–0)	0 (0–0)	0 (0–0)

SR Table 11 - Global deaths by age and cause for males in 2000						
**Cause**	**All ages**	**0-5 years**	**5-14 years**	**15-49 years**	**50-69 years**	**70+ years**
Indirect maternal deaths	0 (0–0)	0 (0–0)	0 (0–0)	0 (0–0)	0 (0–0)	0 (0–0)
Late maternal deaths	0 (0–0)	0 (0–0)	0 (0–0)	0 (0–0)	0 (0–0)	0 (0–0)
Maternal deaths aggravated by HIV/AIDS	0 (0–0)	0 (0–0)	0 (0–0)	0 (0–0)	0 (0–0)	0 (0–0)
Other maternal disorders	0 (0–0)	0 (0–0)	0 (0–0)	0 (0–0)	0 (0–0)	0 (0–0)
Neonatal disorders	1,630,897 (1,577,823–1,688,677)	1,630,897 (1,577,823–1,688,677)	0 (0–0)	0 (0–0)	0 (0–0)	0 (0–0)
Neonatal preterm birth complications	700,765 (634,774–798,215)	700,765 (634,774–798,215)	0 (0–0)	0 (0–0)	0 (0–0)	0 (0–0)
Neonatal encephalopathy due to birth asphyxia and trauma	514,453 (442,437–585,897)	514,453 (442,437–585,897)	0 (0–0)	0 (0–0)	0 (0–0)	0 (0–0)
Neonatal sepsis and other neonatal infections	217,743 (137,286–322,558)	217,743 (137,286–322,558)	0 (0–0)	0 (0–0)	0 (0–0)	0 (0–0)
Hemolytic disease and other neonatal jaundice	44,633 (29,103–80,126)	44,633 (29,103–80,126)	0 (0–0)	0 (0–0)	0 (0–0)	0 (0–0)
Other neonatal disorders	153,300 (102,585–205,422)	153,300 (102,585–205,422)	0 (0–0)	0 (0–0)	0 (0–0)	0 (0–0)
Nutritional deficiencies	299,143 (212,540–388,952)	192,015 (128,047–271,049)	10,495 (8,222–14,147)	26,098 (20,201–34,093)	28,393 (21,249–37,570)	42,141 (30,403–51,821)
Protein-energy malnutrition	263,140 (184,527–346,996)	180,693 (118,842–255,824)	8,751 (6,666–11,577)	17,116 (12,240–22,378)	21,548 (15,328–28,577)	35,031 (25,250–43,122)
Iodine deficiency	853 (540–1,432)	332 (113–819)	35 (16–67)	183 (112–304)	165 (96–291)	137 (83–235)
Vitamin A deficiency	0 (0–0)	0 (0–0)	0 (0–0)	0 (0–0)	0 (0–0)	0 (0–0)
Iron-deficiency anemia	20,950 (8,993–32,258)	7,339 (1,910–12,435)	890 (408–1,756)	5,309 (2,879–8,609)	4,300 (2,152–6,667)	3,110 (1,270–4,675)
Other nutritional deficiencies	14,198 (9,837–21,835)	3,649 (1,946–6,445)	818 (558–1,286)	3,489 (2,427–5,110)	2,379 (1,650–3,821)	3,861 (2,974–5,507)
Other communicable, maternal, neonatal, and nutritional diseases	212,042 (176,877–256,196)	104,141 (72,280–144,899)	11,970 (9,694–15,043)	44,474 (42,294–46,620)	33,049 (30,175–34,675)	18,406 (16,451–19,524)
Sexually transmitted diseases excluding HIV	82,131 (49,561–125,489)	71,485 (40,914–111,693)	5,169 (3,022–7,882)	2,405 (1,663–2,920)	1,911 (1,272–2,385)	1,160 (846–1,454)
Syphilis	81,445 (48,842–124,777)	71,485 (40,914–111,693)	5,150 (3,004–7,864)	2,055 (1,419–2,503)	1,733 (1,153–2,162)	1,020 (744–1,278)
Chlamydial infection	0 (0–0)	0 (0–0)	0 (0–0)	0 (0–0)	0 (0–0)	0 (0–0)
Gonococcal infection	474 (336–555)	0 (0–0)	10 (7–14)	246 (169–298)	110 (73–136)	107 (77–134)
Trichomoniasis	0 (0–0)	0 (0–0)	0 (0–0)	0 (0–0)	0 (0–0)	0 (0–0)
Genital herpes	0 (0–0)	0 (0–0)	0 (0–0)	0 (0–0)	0 (0–0)	0 (0–0)
Other sexually transmitted diseases	211 (145–246)	0 (0–0)	7 (5–10)	102 (70–124)	68 (45–85)	32 (24–41)
Hepatitis	82,023 (78,581–85,788)	12,324 (10,520–14,161)	4,568 (4,033–5,099)	33,401 (31,763–35,572)	21,385 (20,471–22,380)	10,342 (9,897–10,822)
Acute hepatitis A	12,015 (7,834–16,565)	9,571 (6,963–11,857)	688 (192–1,470)	1,043 (259–2,392)	521 (118–1,193)	190 (43–446)
Hepatitis B	49,355 (43,015–55,736)	707 (229–1,676)	587 (315–958)	22,542 (18,776–25,956)	17,666 (15,454–19,698)	7,852 (6,597–8,994)
Hepatitis C	1,808 (381–4,091)	0 (0–0)	67 (13–178)	601 (130–1,366)	829 (176–1,900)	309 (65–767)
Acute hepatitis E	18,843 (12,870–25,941)	2,045 (795–3,848)	3,224 (2,438–3,969)	9,214 (6,208–12,727)	2,368 (1,153–4,155)	1,990 (1,047–3,185)
Other infectious diseases	47,888 (31,695–57,916)	20,332 (9,505–29,614)	2,232 (1,591–2,692)	8,667 (7,053–9,722)	9,752 (6,880–11,047)	6,903 (5,014–7,821)
Non-communicable diseases	17,008,750 (16,694,139–17,288,724)	511,170 (431,537–628,935)	134,909 (125,707–143,206)	2,420,044 (2,351,257–2,484,129)	6,063,513 (5,944,893–6,173,284)	7,879,112 (7,781,545–7,968,541)
Neoplasms	3,892,712 (3,799,262–3,973,313)	22,679 (18,265–31,085)	36,032 (33,152–38,558)	555,612 (526,146–574,395)	1,710,363 (1,668,715–1,746,704)	1,568,025 (1,526,467–1,606,186)
Lip and oral cavity cancer	64,342 (62,627–66,115)	0 (0–0)	0 (0–0)	12,216 (11,712–12,764)	33,132 (32,159–34,096)	18,993 (18,379–19,606)
Nasopharynx cancer	36,498 (26,499–39,154)	0 (0–0)	274 (193–333)	11,740 (7,700–12,901)	16,972 (12,559–18,258)	7,511 (6,051–7,945)
Other pharynx cancer	32,690 (31,766–33,648)	0 (0–0)	0 (0–0)	5,392 (5,182–5,599)	19,209 (18,609–19,853)	8,088 (7,792–8,363)
Esophageal cancer	280,544 (268,718–292,590)	0 (0–0)	0 (0–0)	31,341 (29,476–33,267)	147,838 (140,599–155,254)	101,364 (97,172–105,612)
Stomach cancer	497,065 (483,594–511,120)	0 (0–0)	0 (0–0)	58,060 (55,712–60,698)	230,479 (222,993–237,888)	208,526 (203,496–213,683)
Colon and rectum cancer	313,775 (307,949–319,434)	0 (0–0)	0 (0–0)	34,548 (33,400–35,900)	127,120 (124,420–129,635)	152,105 (149,182–155,077)
Liver cancer	464,690 (378,074–490,486)	0 (0–0)	1,120 (785–1,312)	116,798 (85,121–126,412)	226,584 (187,980–239,172)	120,186 (106,815–125,915)
Liver cancer due to hepatitis B	186,425 (147,132–201,069)	0 (0–0)	737 (508–879)	70,982 (51,474–77,388)	84,680 (68,513–92,763)	30,024 (25,231–33,433)
Liver cancer due to hepatitis C	68,306 (60,875–73,253)	0 (0–0)	20 (15–24)	6,953 (5,316–7,923)	32,636 (28,576–35,855)	28,696 (26,470–30,918)
Liver cancer due to alcohol use	144,320 (119,638–154,968)	0 (0–0)	0 (0–0)	19,544 (14,300–22,057)	80,197 (66,124–87,635)	44,578 (39,090–48,092)
Liver cancer due to other causes	65,637 (51,888–71,365)	0 (0–0)	363 (256–427)	19,317 (13,821–21,610)	29,069 (23,393–32,432)	16,886 (14,494–18,632)
Gallbladder and biliary tract cancer	44,545 (43,413–45,693)	0 (0–0)	0 (0–0)	3,711 (3,512–3,932)	17,758 (17,209–18,259)	23,075 (22,437–23,804)
Pancreatic cancer	143,142 (140,584–145,704)	0 (0–0)	0 (0–0)	14,671 (14,241–15,162)	66,990 (65,615–68,355)	61,480 (60,195–62,870)
Larynx cancer	73,701 (71,823–75,686)	0 (0–0)	0 (0–0)	8,677 (8,344–9,039)	39,735 (38,633–40,895)	25,289 (24,590–25,972)
Tracheal, bronchus, and lung cancer	919,285 (902,316–935,845)	0 (0–0)	0 (0–0)	82,404 (79,443–85,705)	452,484 (443,877–461,883)	384,395 (377,276–391,718)
Malignant skin melanoma	21,920 (14,998–31,664)	0 (0–0)	0 (0–0)	5,380 (3,833–7,689)	8,989 (6,122–13,058)	7,551 (4,872–11,133)
Non-melanoma skin cancer	21,967 (21,431–22,593)	0 (0–0)	0 (0–0)	2,461 (2,366–2,577)	8,157 (7,922–8,414)	11,348 (11,039–11,672)
Non-melanoma skin cancer (squamous-cell carcinoma)	21,967 (21,431–22,593)	0 (0–0)	0 (0–0)	2,461 (2,366–2,577)	8,157 (7,922–8,414)	11,348 (11,039–11,672)
Non-melanoma skin cancer (basal-cell carcinoma)	0 (0–0)	0 (0–0)	0 (0–0)	0 (0–0)	0 (0–0)	0 (0–0)
Breast cancer	5,078 (4,417–5,262)	0 (0–0)	0 (0–0)	685 (639–725)	2,289 (1,993–2,385)	2,102 (1,800–2,196)
Cervical cancer	0 (0–0)	0 (0–0)	0 (0–0)	0 (0–0)	0 (0–0)	0 (0–0)
Uterine cancer	0 (0–0)	0 (0–0)	0 (0–0)	0 (0–0)	0 (0–0)	0 (0–0)
Ovarian cancer	0 (0–0)	0 (0–0)	0 (0–0)	0 (0–0)	0 (0–0)	0 (0–0)
Prostate cancer	250,852 (204,109–310,647)	0 (0–0)	0 (0–0)	2,398 (1,888–2,925)	60,371 (48,853–74,266)	188,082 (151,101–234,657)
Testicular cancer	8,884 (8,513–9,352)	0 (0–0)	0 (0–0)	6,203 (5,880–6,584)	1,538 (1,476–1,619)	1,142 (1,097–1,190)

SR Table 11 - Global deaths by age and cause for males in 2000						
Cause	All ages	0-5 years	5-14 years	15-49 years	50-69 years	70+ years
Kidney cancer	57,371 (56,067–59,214)	1,407 (734–2,248)	533 (483–594)	6,340 (6,132–6,615)	25,996 (25,348–26,691)	23,093 (22,491–23,747)
Bladder cancer	99,871 (97,798–101,916)	0 (0–0)	0 (0–0)	4,818 (4,642–5,008)	34,640 (33,819–35,422)	60,411 (58,947–61,911)
Brain and nervous system cancer	98,578 (81,382–111,426)	3,520 (2,334–6,010)	6,797 (5,005–8,570)	30,966 (24,780–34,991)	38,326 (32,383–42,153)	18,967 (16,281–20,449)
Thyroid cancer	8,628 (7,918–9,538)	0 (0–0)	43 (35–48)	1,243 (1,096–1,362)	3,723 (3,377–4,101)	3,617 (3,384–4,060)
Mesothelioma	14,620 (14,054–15,080)	0 (0–0)	0 (0–0)	2,051 (1,903–2,155)	6,405 (6,130–6,628)	6,162 (5,923–6,423)
Hodgkin lymphoma	17,845 (13,602–21,184)	390 (227–469)	862 (545–1,027)	6,635 (5,139–7,860)	6,406 (4,901–7,628)	3,550 (2,773–4,338)
Non-Hodgkin lymphoma	90,603 (78,366–98,867)	2,047 (1,230–3,518)	3,074 (2,539–3,677)	19,442 (17,341–22,345)	32,928 (28,808–35,955)	33,109 (27,061–35,371)
Multiple myeloma	34,261 (33,420–35,298)	0 (0–0)	0 (0–0)	3,742 (3,588–3,945)	14,327 (13,952–14,831)	16,192 (15,732–16,650)
Leukemia	162,976 (158,662–168,563)	8,442 (7,383–9,729)	17,364 (16,281–18,552)	48,390 (46,585–50,785)	43,633 (42,523–44,951)	45,145 (44,026–46,418)
Acute lymphoid leukemia	56,220 (49,075–64,926)	5,368 (4,260–6,945)	12,202 (10,687–14,168)	20,298 (17,467–23,840)	11,641 (10,038–13,429)	6,710 (5,914–7,749)
Chronic lymphoid leukemia	24,626 (22,994–26,719)	0 (0–0)	0 (0–0)	3,400 (2,903–4,056)	7,592 (6,970–8,365)	13,634 (12,828–14,441)
Acute myeloid leukemia	61,238 (52,905–69,659)	3,074 (1,760–4,264)	5,161 (3,440–6,401)	17,829 (14,607–20,766)	17,922 (16,008–20,063)	17,249 (15,780–18,932)
Chronic myeloid leukemia	20,891 (19,263–23,420)	0 (0–0)	0 (0–0)	6,862 (6,115–7,965)	6,477 (5,891–7,312)	7,551 (7,061–8,139)
Other neoplasms	128,969 (115,029–137,683)	6,870 (4,455–11,633)	5,960 (4,947–6,595)	35,287 (29,645–37,968)	44,321 (39,374–47,897)	36,529 (33,744–40,474)
Cardiovascular diseases	7,418,544 (7,297,526–7,545,083)	41,988 (38,566–45,261)	21,125 (19,819–22,481)	897,583 (871,929–929,282)	2,662,319 (2,609,720–2,712,850)	3,795,527 (3,738,229–3,851,285)
Rheumatic heart disease	144,790 (136,910–156,584)	2,546 (2,104–3,073)	4,937 (4,498–5,428)	43,478 (40,389–47,648)	48,168 (45,176–52,829)	45,658 (43,322–48,515)
Ischemic heart disease	3,689,690 (3,623,971–3,753,333)	2,910 (2,500–3,393)	1,959 (1,753–2,200)	444,766 (431,460–463,689)	1,377,433 (1,348,351–1,405,851)	1,862,621 (1,831,411–1,891,476)
Cerebrovascular disease	2,689,987 (2,619,826–2,761,408)	16,348 (14,759–18,110)	7,993 (7,412–8,585)	274,537 (264,120–286,821)	957,324 (923,412–987,263)	1,433,782 (1,398,642–1,468,122)
Ischemic stroke	1,115,071 (1,077,610–1,154,195)	598 (493–748)	496 (440–574)	43,732 (40,434–47,984)	326,841 (311,813–342,739)	743,401 (721,732–765,967)
Hemorrhagic stroke	1,574,916 (1,522,669–1,627,840)	15,749 (14,235–17,449)	7,496 (6,958–8,053)	230,805 (221,607–241,195)	630,483 (606,891–653,840)	690,380 (667,347–714,004)
Hypertensive heart disease	325,176 (309,194–356,188)	380 (289–465)	285 (240–337)	38,828 (35,987–42,601)	115,152 (108,676–127,128)	170,530 (161,296–186,493)
Cardiomyopathy and myocarditis	166,047 (156,618–175,026)	7,034 (5,241–8,137)	2,263 (1,980–2,674)	47,819 (44,522–51,302)	54,395 (51,358–58,106)	54,535 (51,581–57,206)
Atrial fibrillation and flutter	49,981 (40,747–60,936)	0 (0–0)	0 (0–0)	532 (409–678)	5,216 (4,038–6,897)	44,231 (35,136–55,014)
Aortic aneurysm	81,142 (78,509–84,366)	0 (0–0)	0 (0–0)	7,611 (7,154–8,229)	26,194 (25,114–27,487)	47,336 (45,879–48,915)
Peripheral vascular disease	14,106 (13,514–14,660)	0 (0–0)	0 (0–0)	287 (270–303)	3,337 (3,180–3,481)	10,480 (9,994–10,905)
Endocarditis	26,756 (22,037–30,985)	2,357 (1,131–3,586)	587 (395–756)	6,987 (5,439–8,345)	7,110 (5,930–8,351)	9,713 (8,502–11,238)
Other cardiovascular and circulatory diseases	230,864 (224,309–238,359)	10,411 (9,256–12,115)	3,098 (2,845–3,446)	32,734 (31,190–34,330)	67,984 (65,500–70,723)	116,635 (112,921–120,866)
Chronic respiratory diseases	2,124,701 (2,062,895–2,185,795)	38,654 (32,491–45,864)	8,572 (7,746–9,428)	141,696 (135,556–150,137)	667,373 (641,506–692,802)	1,268,404 (1,233,449–1,301,815)
Chronic obstructive pulmonary disease	1,768,206 (1,677,951–1,866,756)	14,928 (10,387–20,784)	1,643 (1,289–2,110)	86,014 (78,332–95,412)	543,084 (505,822–585,323)	1,122,534 (1,074,331–1,173,647)
Pneumoconiosis	22,686 (20,559–24,721)	0 (0–0)	0 (0–0)	2,774 (2,331–3,187)	7,232 (6,342–8,080)	12,679 (11,457–13,833)
Silicosis	10,773 (9,715–12,305)	0 (0–0)	0 (0–0)	1,498 (1,242–1,811)	3,678 (3,176–4,330)	5,596 (5,090–6,266)
Asbestosis	1,937 (1,323–2,352)	0 (0–0)	0 (0–0)	237 (193–276)	520 (355–717)	1,179 (750–1,393)
Coal workers pneumoconiosis	2,740 (2,417–3,141)	0 (0–0)	0 (0–0)	231 (180–298)	769 (643–932)	1,739 (1,554–1,973)
Other pneumoconiosis	7,235 (6,131–8,210)	0 (0–0)	0 (0–0)	807 (665–926)	2,263 (1,823–2,705)	4,164 (3,506–4,705)
Asthma	264,199 (189,395–326,489)	11,009 (7,716–14,624)	5,922 (4,847–6,874)	44,569 (34,494–52,931)	98,878 (67,566–126,793)	103,819 (72,429–133,396)
Interstitial lung disease and pulmonary sarcoidosis	36,682 (26,380–48,157)	156 (76–427)	127 (83–252)	2,993 (2,155–4,488)	11,764 (8,386–16,601)	21,640 (15,390–26,602)
Other chronic respiratory diseases	32,927 (21,516–40,485)	12,560 (7,860–18,152)	877 (477–1,347)	5,344 (3,197–6,622)	6,414 (3,984–7,895)	7,730 (5,393–9,514)
Cirrhosis and other chronic liver diseases	697,109 (672,938–738,734)	8,617 (7,011–10,589)	6,462 (5,809–7,264)	240,991 (230,136–257,331)	309,250 (297,646–331,798)	131,786 (127,392–137,796)
Cirrhosis and other chronic liver diseases due to hepatitis B	219,093 (203,061–239,605)	89 (66–118)	270 (219–326)	76,416 (69,390–85,039)	97,480 (87,462–110,195)	44,836 (40,813–49,459)
Cirrhosis and other chronic liver diseases due to hepatitis C	176,020 (163,678–188,657)	48 (36–64)	147 (116–181)	56,490 (50,586–62,842)	81,519 (73,184–89,920)	37,813 (34,635–41,624)
Cirrhosis and other chronic liver diseases due to alcohol use	197,196 (183,775–212,971)	0 (0–0)	0 (0–0)	76,086 (69,481–83,597)	92,750 (83,576–102,795)	28,360 (25,399–31,818)
Cirrhosis and other chronic liver diseases due to other causes	104,799 (97,117–113,837)	8,479 (6,897–10,414)	6,044 (5,431–6,804)	31,998 (28,843–35,565)	37,500 (32,933–42,665)	20,775 (18,719–23,040)
Digestive diseases	576,626 (549,785–630,706)	14,489 (10,953–19,272)	12,284 (11,038–14,168)	158,976 (148,301–170,018)	191,753 (182,629–210,980)	199,123 (191,488–220,867)
Peptic ulcer disease	187,280 (173,175–205,657)	1,616 (940–2,894)	2,322 (1,929–2,820)	50,468 (45,705–55,414)	70,005 (64,555–76,537)	62,867 (58,425–70,036)
Gastritis and duodenitis	27,551 (21,404–34,586)	744 (370–1,415)	363 (250–561)	7,519 (5,639–9,527)	9,708 (7,484–12,249)	9,216 (7,304–11,793)
Appendicitis	22,839 (20,248–27,400)	1,464 (971–2,210)	1,862 (1,583–2,265)	8,980 (7,870–10,948)	5,961 (5,180–7,349)	4,570 (4,004–5,475)
Paralytic ileus and intestinal obstruction	118,139 (102,700–146,066)	4,799 (3,095–7,412)	4,420 (3,642–5,657)	33,924 (27,589–41,182)	37,201 (31,725–46,487)	37,792 (33,460–48,022)
Inguinal, femoral, and abdominal hernia	34,885 (21,603–40,517)	953 (299–1,555)	681 (336–878)	8,757 (4,490–10,511)	11,004 (7,019–13,616)	13,488 (8,722–15,884)
Inflammatory bowel disease	17,551 (14,738–19,660)	854 (462–1,783)	393 (257–568)	4,399 (3,499–5,096)	5,148 (4,403–5,880)	6,755 (5,819–7,596)
Vascular intestinal disorders	31,609 (29,386–34,217)	538 (274–1,088)	117 (92–151)	2,918 (2,623–3,231)	10,320 (9,561–11,388)	17,714 (16,563–19,184)
Gallbladder and biliary diseases	36,032 (32,668–40,382)	472 (273–775)	480 (376–632)	6,588 (5,642–7,781)	10,890 (9,766–12,326)	17,600 (16,244–19,524)
Pancreatitis	54,275 (50,956–59,577)	145 (93–235)	264 (219–334)	25,740 (23,793–28,417)	17,384 (16,113–19,008)	10,740 (9,948–11,762)
Other digestive diseases	46,461 (37,355–63,955)	2,900 (1,785–4,559)	1,378 (1,023–1,965)	9,678 (7,391–14,245)	14,126 (10,918–19,738)	18,376 (15,661–23,906)
Neurological disorders	572,012 (504,909–639,442)	12,472 (8,208–16,024)	9,021 (8,046–9,930)	52,552 (50,477–54,714)	69,277 (59,624–78,847)	428,688 (365,522–490,804)
Alzheimer disease and other dementias	421,712 (354,928–487,713)	0 (0–0)	0 (0–0)	640 (399–939)	39,110 (29,304–48,675)	381,961 (318,866–444,383)
Parkinson disease	36,593 (35,109–39,089)	0 (0–0)	0 (0–0)	345 (324–387)	5,293 (5,043–5,843)	30,954 (29,596–32,731)
Epilepsy	70,678 (65,309–75,373)	9,334 (5,929–12,305)	7,164 (6,364–7,919)	38,750 (36,766–40,889)	10,371 (9,929–10,809)	5,058 (4,862–5,276)

SR Table 11 - Global deaths by age and cause for males in 2000						
**Cause**	**All ages**	**0-5 years**	**5-14 years**	**15-49 years**	**50-69 years**	**70+ years**
Multiple sclerosis	6,680 (5,928–7,168)	0 (0–0)	0 (0–0)	2,475 (2,177–2,744)	2,982 (2,655–3,165)	1,222 (1,022–1,282)
Motor neuron disease	12,958 (12,469–13,670)	604 (418–794)	89 (76–102)	2,094 (1,965–2,320)	5,772 (5,522–6,189)	4,397 (4,242–4,575)
Migraine	0 (0–0)	0 (0–0)	0 (0–0)	0 (0–0)	0 (0–0)	0 (0–0)
Tension-type headache	0 (0–0)	0 (0–0)	0 (0–0)	0 (0–0)	0 (0–0)	0 (0–0)
Medication overuse headache	0 (0–0)	0 (0–0)	0 (0–0)	0 (0–0)	0 (0–0)	0 (0–0)
Other neurological disorders	23,398 (22,568–24,585)	2,544 (1,721–3,433)	1,767 (1,485–2,039)	8,246 (7,739–8,897)	5,746 (5,393–6,237)	5,093 (4,792–5,462)
Mental and substance use disorders	212,810 (195,663–219,254)	15 (12–19)	15 (6–22)	133,533 (122,821–138,293)	63,685 (57,842–66,139)	15,560 (14,735–16,095)
Schizophrenia	10,378 (9,241–11,186)	0 (0–0)	0 (0–0)	5,419 (4,688–5,921)	3,307 (2,955–3,557)	1,651 (1,519–1,770)
Alcohol use disorders	119,815 (111,811–124,803)	0 (0–0)	0 (0–0)	65,228 (61,078–68,694)	45,778 (42,176–47,915)	8,809 (8,413–9,149)
Drug use disorders	82,458 (74,465–85,468)	15 (12–19)	0 (0–0)	62,743 (56,421–65,365)	14,599 (13,243–15,386)	5,099 (4,669–5,430)
Opioid use disorders	64,086 (57,522–66,611)	15 (12–19)	0 (0–0)	49,056 (43,909–51,269)	11,111 (10,139–11,788)	3,903 (3,529–4,170)
Cocaine use disorders	3,514 (2,386–3,963)	0 (0–0)	0 (0–0)	2,827 (1,905–3,215)	598 (393–700)	88 (70–112)
Amphetamine use disorders	2,751 (1,727–3,080)	0 (0–0)	0 (0–0)	2,130 (1,342–2,381)	516 (317–596)	104 (65–119)
Cannabis use disorders	0 (0–0)	0 (0–0)	0 (0–0)	0 (0–0)	0 (0–0)	0 (0–0)
Other drug use disorders	12,104 (10,839–12,961)	0 (0–0)	0 (0–0)	8,729 (7,602–9,331)	2,373 (2,127–2,600)	1,002 (887–1,156)
Depressive disorders	0 (0–0)	0 (0–0)	0 (0–0)	0 (0–0)	0 (0–0)	0 (0–0)
Major depressive disorder	0 (0–0)	0 (0–0)	0 (0–0)	0 (0–0)	0 (0–0)	0 (0–0)
Dysthymia	0 (0–0)	0 (0–0)	0 (0–0)	0 (0–0)	0 (0–0)	0 (0–0)
Bipolar disorder	0 (0–0)	0 (0–0)	0 (0–0)	0 (0–0)	0 (0–0)	0 (0–0)
Anxiety disorders	0 (0–0)	0 (0–0)	0 (0–0)	0 (0–0)	0 (0–0)	0 (0–0)
Eating disorders	157 (80–197)	0 (0–0)	15 (6–22)	142 (73–177)	0 (0–0)	0 (0–0)
Anorexia nervosa	142 (72–175)	0 (0–0)	14 (5–21)	127 (64–157)	0 (0–0)	0 (0–0)
Bulimia nervosa	15 (7–21)	0 (0–0)	0 (0–1)	14 (7–20)	0 (0–0)	0 (0–0)
Autistic spectrum disorders	0 (0–0)	0 (0–0)	0 (0–0)	0 (0–0)	0 (0–0)	0 (0–0)
Autism	0 (0–0)	0 (0–0)	0 (0–0)	0 (0–0)	0 (0–0)	0 (0–0)
Asperger syndrome and other autistic spectrum disorders	0 (0–0)	0 (0–0)	0 (0–0)	0 (0–0)	0 (0–0)	0 (0–0)
Attention-deficit/hyperactivity disorder	0 (0–0)	0 (0–0)	0 (0–0)	0 (0–0)	0 (0–0)	0 (0–0)
Conduct disorder	0 (0–0)	0 (0–0)	0 (0–0)	0 (0–0)	0 (0–0)	0 (0–0)
Idiopathic developmental intellectual disability	0 (0–0)	0 (0–0)	0 (0–0)	0 (0–0)	0 (0–0)	0 (0–0)
Other mental and substance use disorders	0 (0–0)	0 (0–0)	0 (0–0)	0 (0–0)	0 (0–0)	0 (0–0)
Diabetes, urogenital, blood, and endocrine diseases	1,110,086 (1,018,013–1,175,365)	77,419 (50,070–116,050)	19,239 (16,459–21,259)	197,094 (176,413–209,105)	369,545 (343,885–384,497)	446,787 (422,935–464,968)
Diabetes mellitus	455,181 (442,341–468,134)	1,538 (1,220–1,712)	1,443 (1,114–1,618)	59,338 (56,914–62,048)	189,697 (183,755–196,001)	203,162 (197,576–209,110)
Acute glomerulonephritis	7,698 (5,351–8,641)	266 (190–343)	362 (243–409)	2,450 (1,540–2,715)	2,456 (1,797–2,875)	2,162 (1,444–2,497)
Chronic kidney disease	399,740 (336,375–433,139)	8,535 (6,211–9,821)	5,710 (4,233–6,300)	85,545 (66,124–91,588)	129,736 (107,679–142,381)	170,211 (149,643–185,546)
Chronic kidney disease due to diabetes mellitus	122,106 (105,163–130,387)	854 (669–1,072)	420 (329–517)	18,570 (14,901–20,911)	48,328 (41,061–53,032)	53,932 (48,216–57,819)
Chronic kidney disease due to hypertension	166,311 (139,963–180,148)	2,953 (2,203–3,490)	1,633 (1,224–1,914)	31,998 (24,369–35,231)	50,248 (41,312–55,060)	79,477 (69,960–86,323)
Chronic kidney disease due to glomerulonephritis	100,292 (81,380–109,919)	3,520 (2,520–4,150)	2,750 (2,007–3,155)	31,364 (23,998–34,238)	29,471 (23,889–33,642)	33,186 (28,491–37,600)
Chronic kidney disease due to other causes	11,029 (8,639–13,332)	1,207 (831–1,556)	905 (646–1,131)	3,612 (2,450–4,979)	1,688 (1,034–2,525)	3,615 (2,949–4,524)
Urinary diseases and male infertility	86,835 (76,873–105,318)	5,153 (3,463–6,836)	1,206 (1,012–1,517)	14,094 (12,084–17,466)	23,699 (20,015–29,692)	42,681 (37,951–51,821)
Interstitial nephritis and urinary tract infections	57,241 (48,962–67,756)	4,097 (2,640–5,477)	742 (578–912)	8,559 (7,034–10,654)	13,953 (11,108–17,522)	29,887 (25,307–35,834)
Urolithiasis	8,245 (6,561–11,190)	0 (0–0)	154 (121–248)	1,673 (1,298–2,445)	2,971 (2,326–4,024)	3,445 (2,659–4,600)
Benign prostatic hyperplasia	0 (0–0)	0 (0–0)	0 (0–0)	0 (0–0)	0 (0–0)	0 (0–0)
Male infertility	0 (0–0)	0 (0–0)	0 (0–0)	0 (0–0)	0 (0–0)	0 (0–0)
Other urinary diseases	21,348 (16,748–29,010)	1,056 (617–1,915)	308 (239–447)	3,861 (2,970–5,503)	6,774 (5,078–9,718)	9,348 (7,195–12,537)
Gynecological diseases	0 (0–0)	0 (0–0)	0 (0–0)	0 (0–0)	0 (0–0)	0 (0–0)
Uterine fibroids	0 (0–0)	0 (0–0)	0 (0–0)	0 (0–0)	0 (0–0)	0 (0–0)
Polycystic ovarian syndrome	0 (0–0)	0 (0–0)	0 (0–0)	0 (0–0)	0 (0–0)	0 (0–0)
Female infertility	0 (0–0)	0 (0–0)	0 (0–0)	0 (0–0)	0 (0–0)	0 (0–0)
Endometriosis	0 (0–0)	0 (0–0)	0 (0–0)	0 (0–0)	0 (0–0)	0 (0–0)
Genital prolapse	0 (0–0)	0 (0–0)	0 (0–0)	0 (0–0)	0 (0–0)	0 (0–0)
Premenstrual syndrome	0 (0–0)	0 (0–0)	0 (0–0)	0 (0–0)	0 (0–0)	0 (0–0)
Other gynecological diseases	0 (0–0)	0 (0–0)	0 (0–0)	0 (0–0)	0 (0–0)	0 (0–0)
Hemoglobinopathies and hemolytic anemias	115,865 (75,097–164,060)	54,132 (27,642–93,106)	8,287 (6,389–10,013)	25,271 (18,689–30,407)	12,693 (9,318–15,610)	15,481 (11,399–19,680)
Thalassemias	10,963 (7,725–13,916)	6,368 (4,046–9,249)	2,321 (1,735–2,713)	1,008 (703–1,429)	35 (31–39)	1,229 (706–1,901)
Thalassemias trait	0 (0–0)	0 (0–0)	0 (0–0)	0 (0–0)	0 (0–0)	0 (0–0)
Sickle cell disorders	62,037 (33,797–103,650)	46,554 (21,982–86,302)	4,628 (3,401–5,957)	9,140 (6,429–11,568)	341 (272–429)	1,371 (933–1,915)
Sickle cell trait	0 (0–0)	0 (0–0)	0 (0–0)	0 (0–0)	0 (0–0)	0 (0–0)

SR Table 11 - Global deaths by age and cause for males in 2000

Cause	All ages	0-5 years	5-14 years	15-49 years	50-69 years	70+ years
G6PD deficiency	17,374 (13,621–21,583)	916 (501–1,529)	1,004 (771–1,343)	10,006 (7,746–12,413)	4,439 (3,340–5,520)	1,008 (747–1,327)
G6PD trait	0 (0–0)	0 (0–0)	0 (0–0)	0 (0–0)	0 (0–0)	0 (0–0)
Other hemoglobinopathies and hemolytic anemias	25,491 (19,260–31,548)	293 (179–457)	332 (267–414)	5,115 (3,768–6,448)	7,877 (5,674–9,998)	11,872 (8,973–14,809)
Endocrine, metabolic, blood, and immune disorders	44,764 (43,245–46,216)	7,792 (6,964–8,751)	2,229 (2,109–2,359)	10,393 (9,914–10,924)	11,260 (10,683–11,904)	13,088 (12,525–13,690)
Musculoskeletal disorders	21,083 (18,236–22,010)	0 (0–0)	870 (726–1,024)	5,220 (4,690–5,597)	6,047 (5,134–6,403)	8,944 (7,336–9,445)
Rheumatoid arthritis	7,109 (5,573–8,060)	0 (0–0)	119 (85–181)	775 (628–947)	2,550 (1,961–2,801)	3,664 (2,861–4,203)
Osteoarthritis	0 (0–0)	0 (0–0)	0 (0–0)	0 (0–0)	0 (0–0)	0 (0–0)
Low back and neck pain	0 (0–0)	0 (0–0)	0 (0–0)	0 (0–0)	0 (0–0)	0 (0–0)
Low back pain	0 (0–0)	0 (0–0)	0 (0–0)	0 (0–0)	0 (0–0)	0 (0–0)
Neck pain	0 (0–0)	0 (0–0)	0 (0–0)	0 (0–0)	0 (0–0)	0 (0–0)
Gout	0 (0–0)	0 (0–0)	0 (0–0)	0 (0–0)	0 (0–0)	0 (0–0)
Other musculoskeletal disorders	13,973 (12,519–14,778)	0 (0–0)	751 (630–901)	4,444 (4,045–4,826)	3,497 (3,069–3,776)	5,280 (4,354–5,677)
Other non-communicable diseases	383,052 (303,470–501,359)	294,822 (226,312–406,592)	21,285 (16,520–26,001)	36,782 (31,962–42,747)	13,896 (10,471–16,855)	16,264 (11,897–21,120)
Congenital anomalies	344,232 (268,652–460,230)	280,261 (211,868–391,659)	20,751 (15,859–25,608)	32,009 (27,739–37,188)	6,636 (6,025–7,945)	4,572 (4,187–5,410)
Neural tube defects	44,907 (31,915–71,138)	41,729 (29,111–67,396)	1,547 (1,130–2,022)	1,224 (1,009–1,599)	257 (198–379)	148 (105–260)
Congenital heart anomalies	179,890 (137,547–240,608)	138,317 (101,757–194,861)	14,795 (11,137–18,407)	23,108 (19,984–27,019)	2,174 (1,883–2,581)	1,494 (1,255–1,914)
Cleft lip and cleft palate	2,538 (1,869–3,404)	2,538 (1,869–3,404)	0 (0–0)	0 (0–0)	0 (0–0)	0 (0–0)
Down syndrome	9,339 (6,286–18,769)	5,702 (2,984–14,192)	434 (275–794)	1,956 (1,563–2,930)	1,185 (969–1,635)	60 (48–85)
Turner syndrome	0 (0–0)	0 (0–0)	0 (0–0)	0 (0–0)	0 (0–0)	0 (0–0)
Klinefelter syndrome	0 (0–0)	0 (0–0)	0 (0–0)	0 (0–0)	0 (0–0)	0 (0–0)
Other chromosomal abnormalities	7,048 (3,418–17,242)	6,291 (2,675–16,353)	152 (106–254)	426 (284–596)	132 (90–193)	45 (32–65)
Other congenital anomalies	100,508 (75,846–139,497)	85,682 (63,228–123,490)	3,821 (2,893–4,948)	5,292 (4,254–6,227)	2,887 (2,363–3,380)	2,824 (2,435–3,189)
Skin and subcutaneous diseases	26,672 (15,695–39,806)	2,413 (1,196–4,666)	533 (305–919)	4,773 (2,669–7,545)	7,259 (3,827–9,982)	11,692 (7,207–16,345)
Dermatitis	0 (0–0)	0 (0–0)	0 (0–0)	0 (0–0)	0 (0–0)	0 (0–0)
Psoriasis	0 (0–0)	0 (0–0)	0 (0–0)	0 (0–0)	0 (0–0)	0 (0–0)
Cellulitis	5,242 (2,813–9,593)	363 (141–727)	106 (58–188)	1,172 (583–2,350)	1,878 (898–3,500)	1,721 (1,058–3,210)
Pyoderma	11,261 (6,568–18,500)	1,886 (927–3,636)	283 (155–527)	2,519 (1,305–4,006)	2,885 (1,627–4,511)	3,686 (2,386–5,773)
Scabies	0 (0–0)	0 (0–0)	0 (0–0)	0 (0–0)	0 (0–0)	0 (0–0)
Fungal skin diseases	0 (0–0)	0 (0–0)	0 (0–0)	0 (0–0)	0 (0–0)	0 (0–0)
Viral skin diseases	0 (0–0)	0 (0–0)	0 (0–0)	0 (0–0)	0 (0–0)	0 (0–0)
Acne vulgaris	0 (0–0)	0 (0–0)	0 (0–0)	0 (0–0)	0 (0–0)	0 (0–0)
Alopecia areata	0 (0–0)	0 (0–0)	0 (0–0)	0 (0–0)	0 (0–0)	0 (0–0)
Pruritus	0 (0–0)	0 (0–0)	0 (0–0)	0 (0–0)	0 (0–0)	0 (0–0)
Urticaria	0 (0–0)	0 (0–0)	0 (0–0)	0 (0–0)	0 (0–0)	0 (0–0)
Decubitus ulcer	8,980 (4,238–12,233)	43 (19–76)	79 (39–138)	800 (361–1,238)	2,208 (763–3,723)	5,847 (3,053–8,157)
Other skin and subcutaneous diseases	1,188 (708–1,829)	119 (61–222)	64 (36–113)	280 (158–489)	286 (140–555)	436 (277–670)
Sense organ diseases	0 (0–0)	0 (0–0)	0 (0–0)	0 (0–0)	0 (0–0)	0 (0–0)
Glaucoma	0 (0–0)	0 (0–0)	0 (0–0)	0 (0–0)	0 (0–0)	0 (0–0)
Cataract	0 (0–0)	0 (0–0)	0 (0–0)	0 (0–0)	0 (0–0)	0 (0–0)
Macular degeneration	0 (0–0)	0 (0–0)	0 (0–0)	0 (0–0)	0 (0–0)	0 (0–0)
Refraction and accommodation disorders	0 (0–0)	0 (0–0)	0 (0–0)	0 (0–0)	0 (0–0)	0 (0–0)
Age-related and other hearing loss	0 (0–0)	0 (0–0)	0 (0–0)	0 (0–0)	0 (0–0)	0 (0–0)
Other vision loss	0 (0–0)	0 (0–0)	0 (0–0)	0 (0–0)	0 (0–0)	0 (0–0)
Other sense organ diseases	0 (0–0)	0 (0–0)	0 (0–0)	0 (0–0)	0 (0–0)	0 (0–0)
Oral disorders	0 (0–0)	0 (0–0)	0 (0–0)	0 (0–0)	0 (0–0)	0 (0–0)
Deciduous caries	0 (0–0)	0 (0–0)	0 (0–0)	0 (0–0)	0 (0–0)	0 (0–0)
Permanent caries	0 (0–0)	0 (0–0)	0 (0–0)	0 (0–0)	0 (0–0)	0 (0–0)
Periodontal diseases	0 (0–0)	0 (0–0)	0 (0–0)	0 (0–0)	0 (0–0)	0 (0–0)
Edentulism and severe tooth loss	0 (0–0)	0 (0–0)	0 (0–0)	0 (0–0)	0 (0–0)	0 (0–0)
Other oral disorders	0 (0–0)	0 (0–0)	0 (0–0)	0 (0–0)	0 (0–0)	0 (0–0)
Sudden infant death syndrome	12,147 (8,639–26,648)	12,147 (8,639–26,648)	0 (0–0)	0 (0–0)	0 (0–0)	0 (0–0)
Injuries	3,210,102 (3,073,447–3,299,739)	263,542 (216,150–305,550)	233,741 (220,379–247,285)	1,847,198 (1,776,200–1,904,061)	560,971 (535,641–576,552)	304,648 (287,708–314,710)
Transport injuries	1,024,324 (987,877–1,061,834)	43,260 (34,673–52,547)	69,327 (64,298–73,683)	653,036 (630,540–680,270)	178,597 (171,768–184,821)	80,103 (77,115–83,267)
Road injuries	953,915 (918,969–989,618)	39,882 (31,856–48,911)	65,647 (60,820–69,862)	610,301 (588,360–636,442)	163,302 (156,226–169,438)	74,780 (71,784–77,843)
Pedestrian road injuries	408,414 (368,249–447,942)	20,686 (16,999–25,475)	36,415 (33,187–39,887)	230,283 (206,036–254,578)	80,254 (71,957–87,837)	40,773 (37,079–45,182)
Cyclist road injuries	42,556 (38,725–47,871)	1,065 (692–1,497)	4,077 (3,511–4,671)	22,920 (20,565–26,240)	10,201 (9,308–11,412)	4,292 (3,961–4,891)
Motorcyclist road injuries	160,561 (131,431–184,741)	3,869 (2,425–5,294)	6,042 (4,438–7,544)	123,681 (103,001–141,627)	20,225 (15,552–24,357)	6,742 (4,541–8,824)

SR Table 11 - Global deaths by age and cause for males in 2000

Cause	All ages	0-5 years	5-14 years	15-49 years	50-69 years	70+ years
Motor vehicle road injuries	329,609<br>(297,839–381,496)	13,342<br>(10,253–17,457)	17,970<br>(15,744–21,462)	225,933<br>(207,030–264,541)	50,514<br>(43,409–59,030)	21,849<br>(18,428–25,836)
Other road injuries	12,772<br>(7,505–16,173)	918<br>(320–2,167)	1,140<br>(602–1,601)	7,483<br>(4,194–9,516)	2,107<br>(1,471–2,765)	1,122<br>(850–1,568)
Other transport injuries	70,408<br>(63,067–78,995)	3,377<br>(2,258–4,417)	3,679<br>(3,118–4,197)	42,734<br>(38,325–47,666)	15,294<br>(13,434–17,663)	5,322<br>(4,736–6,135)
Unintentional injuries	1,223,410<br>(1,159,984–1,277,124)	205,354<br>(165,239–241,889)	140,602<br>(131,726–149,498)	514,215<br>(491,904–536,886)	206,135<br>(195,572–214,286)	157,101<br>(145,952–164,676)
Falls	238,221<br>(226,996–249,313)	14,288<br>(10,102–18,981)	11,930<br>(10,554–13,376)	84,113<br>(80,180–92,103)	59,183<br>(56,145–62,410)	68,705<br>(63,797–71,993)
Drowning	324,543<br>(309,987–340,013)	70,041<br>(58,983–82,098)	78,235<br>(74,090–82,416)	128,393<br>(123,890–134,962)	33,567<br>(32,305–34,953)	14,305<br>(13,853–14,871)
Fire, heat, and hot substances	102,785<br>(81,748–110,163)	17,191<br>(12,261–20,703)	6,503<br>(5,063–7,357)	46,830<br>(36,427–50,009)	18,873<br>(16,002–19,997)	13,387<br>(11,588–14,337)
Poisonings	72,956<br>(46,785–90,830)	15,003<br>(4,942–31,044)	5,208<br>(3,683–6,482)	33,319<br>(24,070–38,437)	14,073<br>(10,087–15,771)	5,350<br>(3,718–6,141)
Exposure to mechanical forces	144,300<br>(130,138–163,204)	23,929<br>(18,206–29,322)	10,346<br>(8,904–13,235)	75,203<br>(68,531–86,980)	22,566<br>(20,445–26,157)	12,254<br>(10,416–13,426)
Unintentional firearm injuries	24,839<br>(17,241–27,374)	1,917<br>(1,100–2,879)	1,735<br>(1,297–2,297)	15,734<br>(10,652–17,474)	3,114<br>(2,213–3,494)	2,338<br>(1,795–2,584)
Unintentional suffocation	24,064<br>(18,380–28,908)	14,795<br>(9,487–19,190)	2,232<br>(1,806–3,445)	3,683<br>(3,236–4,494)	971<br>(664–1,135)	2,380<br>(1,987–2,561)
Other exposure to mechanical forces	95,395<br>(84,090–115,176)	7,216<br>(4,593–9,986)	6,378<br>(5,238–8,401)	55,785<br>(49,864–67,669)	18,481<br>(16,816–22,018)	7,534<br>(6,184–8,563)
Adverse effects of medical treatment	54,985<br>(39,335–63,780)	13,078<br>(5,486–18,310)	3,278<br>(2,006–4,009)	17,445<br>(12,230–20,175)	10,410<br>(7,974–12,109)	10,772<br>(8,575–12,314)
Animal contact	61,236<br>(38,976–67,966)	8,487<br>(4,266–11,025)	9,045<br>(5,517–10,552)	25,698<br>(16,490–28,395)	12,441<br>(7,834–13,892)	5,564<br>(3,939–6,346)
Venomous animal contact	48,904<br>(29,994–54,415)	5,793<br>(2,939–7,382)	7,271<br>(4,314–8,534)	21,528<br>(13,378–23,963)	10,045<br>(6,123–11,508)	4,265<br>(2,925–4,983)
Non-venomous animal contact	12,332<br>(8,877–14,868)	2,693<br>(1,281–3,925)	1,773<br>(1,168–2,231)	4,169<br>(3,091–4,961)	2,396<br>(1,749–2,900)	1,298<br>(1,019–1,592)
Foreign body	88,233<br>(67,573–118,540)	32,490<br>(19,029–48,283)	6,538<br>(4,783–9,284)	23,863<br>(18,858–37,999)	12,332<br>(10,280–17,366)	13,009<br>(10,532–17,601)
Pulmonary aspiration and foreign body in airway	66,733<br>(50,823–88,885)	26,326<br>(13,626–41,809)	4,500<br>(3,059–6,341)	15,130<br>(12,706–18,130)	9,832<br>(8,319–11,195)	10,942<br>(8,833–11,892)
Foreign body in eyes	0<br>(0–0)	0<br>(0–0)	0<br>(0–0)	0<br>(0–0)	0<br>(0–0)	0<br>(0–0)
Foreign body in other body part	21,500<br>(11,162–50,773)	6,163<br>(2,590–10,602)	2,038<br>(1,010–4,064)	8,732<br>(4,550–23,428)	2,499<br>(1,422–7,454)	2,066<br>(1,176–6,205)
Environmental heat and cold exposure	28,054<br>(19,647–35,457)	2,667<br>(1,110–3,591)	993<br>(580–1,236)	11,606<br>(8,390–15,650)	7,957<br>(5,908–11,259)	4,828<br>(3,353–5,663)
Other unintentional injuries	108,092<br>(101,357–115,548)	8,177<br>(6,261–9,981)	8,521<br>(7,459–9,622)	67,740<br>(63,233–73,296)	14,729<br>(13,609–15,976)	8,923<br>(7,643–10,629)
Self-harm and interpersonal violence	881,573<br>(794,779–905,675)	9,561<br>(7,750–10,490)	18,087<br>(14,897–19,267)	617,905<br>(556,407–636,111)	169,719<br>(154,525–174,715)	66,300<br>(59,103–68,796)
Self-harm	538,749<br>(467,420–557,164)	0<br>(0–0)	7,539<br>(5,430–8,343)	347,369<br>(299,748–360,808)	127,446<br>(113,202–131,746)	56,393<br>(49,421–58,713)
Interpersonal violence	342,824<br>(312,198–354,656)	9,561<br>(7,750–10,490)	10,548<br>(9,044–11,218)	270,536<br>(246,816–279,899)	42,272<br>(39,125–44,328)	9,906<br>(9,341–10,456)
Assault by firearm	138,701<br>(123,503–144,586)	1,172<br>(926–1,423)	2,361<br>(2,103–2,557)	120,224<br>(107,006–124,970)	12,631<br>(11,258–13,526)	2,310<br>(2,061–2,493)
Assault by sharp object	87,432<br>(79,492–92,429)	1,093<br>(790–1,502)	2,063<br>(1,578–2,462)	71,069<br>(65,030–75,076)	10,760<br>(9,796–11,651)	2,445<br>(2,220–2,628)
Assault by other means	116,690<br>(103,785–126,763)	7,295<br>(5,914–8,216)	6,123<br>(5,085–6,701)	79,241<br>(69,999–86,868)	18,880<br>(17,267–20,486)	5,150<br>(4,741–5,635)
Forces of nature, war, and legal intervention	80,794<br>(46,984–117,387)	5,366<br>(2,188–10,405)	5,723<br>(2,311–11,508)	62,040<br>(31,074–98,375)	6,518<br>(3,524–10,814)	1,144<br>(802–1,551)
Exposure to forces of nature	6,228<br>(4,118–8,655)	424<br>(223–752)	970<br>(581–1,565)	3,590<br>(2,359–5,114)	901<br>(571–1,301)	341<br>(210–503)
Collective violence and legal intervention	74,565<br>(41,485–111,142)	4,942<br>(1,814–9,948)	4,753<br>(1,514–10,393)	58,450<br>(27,718–94,659)	5,616<br>(2,713–9,937)	802<br>(512–1,187)

SR Table 11 - Global deaths by age and cause for males in 2005

Cause	All ages	0-5 years	5-14 years	15-49 years	50-69 years	70+ years
All causes	28,857,905 (28,506,905–29,210,677)	4,253,440 (4,208,413–4,301,145)	593,363 (584,891–603,903)	5,931,491 (5,814,145–6,051,401)	8,010,209 (7,869,930–8,149,430)	10,069,401 (9,958,503–10,181,033)
Communicable, maternal, neonatal, and nutritional diseases	7,254,451 (7,057,817–7,529,254)	3,577,744 (3,490,294–3,652,915)	290,221 (275,377–306,654)	1,529,264 (1,449,453–1,625,515)	938,434 (862,626–1,034,534)	918,786 (856,489–993,347)
HIV/AIDS and tuberculosis	1,762,066 (1,579,991–2,041,557)	112,044 (106,441–122,315)	47,433 (44,575–52,735)	965,220 (888,816–1,059,086)	441,611 (374,228–548,358)	195,756 (156,630–269,631)
Tuberculosis	849,415 (666,312–1,134,336)	17,991 (13,497–28,214)	9,750 (7,736–14,930)	327,549 (254,597–417,477)	312,723 (243,533–418,079)	181,400 (142,066–255,741)
HIV/AIDS	912,650 (869,054–973,198)	94,053 (91,030–97,181)	37,683 (35,932–39,556)	637,670 (599,006–682,870)	128,887 (119,601–143,866)	14,355 (13,092–16,411)
HIV/AIDS - Tuberculosis	184,406 (148,229–208,638)	25,266 (20,393–28,255)	9,070 (7,177–10,415)	114,401 (91,135–130,809)	31,543 (26,109–35,400)	4,125 (3,556–4,644)
HIV/AIDS resulting in other diseases	728,244 (682,628–788,316)	68,786 (64,362–73,978)	28,613 (26,548–31,242)	523,269 (483,483–569,354)	97,343 (88,496–111,586)	10,230 (9,126–12,197)
Diarrhea, lower respiratory, and other common infectious diseases	2,931,256 (2,776,986–3,089,672)	1,332,030 (1,238,659–1,430,578)	171,919 (154,561–193,260)	389,328 (356,339–425,876)	393,963 (357,972–427,179)	644,016 (588,570–692,188)
Diarrheal diseases	802,578 (747,750–862,296)	385,233 (338,111–439,377)	37,274 (32,877–41,981)	111,986 (103,433–122,501)	118,707 (110,616–127,757)	149,375 (140,131–158,771)
Intestinal infectious diseases	110,529 (62,225–181,745)	28,015 (14,750–48,240)	43,224 (25,447–69,217)	33,118 (17,282–57,535)	4,400 (2,251–7,725)	1,771 (986–2,801)
Typhoid fever	91,700 (49,853–154,344)	23,380 (11,979–40,941)	36,317 (20,677–58,505)	27,174 (14,107–47,386)	3,468 (1,694–6,146)	1,359 (734–2,261)
Paratyphoid fever	18,052 (8,331–34,357)	4,193 (1,727–8,657)	6,830 (3,151–13,015)	5,831 (2,484–11,738)	873 (356–1,778)	324 (138–623)
Other intestinal infectious diseases	776 (222–1,635)	440 (104–1,105)	76 (17–196)	112 (37–239)	59 (21–125)	86 (28–197)
Lower respiratory infections	1,455,301 (1,315,787–1,571,111)	575,824 (529,339–628,421)	37,359 (28,319–42,455)	155,768 (130,083–175,758)	223,590 (186,258–252,218)	462,758 (407,754–511,517)
Upper respiratory infections	2,003 (1,743–2,300)	553 (385–809)	34 (22–53)	485 (406–576)	494 (395–601)	435 (348–539)
Otitis media	1,999 (1,763–2,259)	749 (616–898)	295 (260–330)	515 (457–588)	269 (228–324)	168 (135–224)
Meningitis	217,940 (175,883–253,401)	112,201 (86,038–141,536)	19,613 (14,014–23,451)	47,156 (34,692–58,248)	24,428 (18,558–31,245)	14,541 (11,404–19,095)
Pneumococcal meningitis	59,808 (47,737–74,710)	25,937 (17,673–37,141)	6,670 (4,468–9,197)	16,004 (11,508–20,634)	7,017 (4,925–9,588)	4,177 (3,053–5,725)
H influenzae type B meningitis	58,681 (44,604–73,325)	40,276 (28,235–53,962)	5,131 (3,285–7,262)	7,261 (5,016–9,571)	3,675 (2,532–5,076)	2,335 (1,716–3,269)
Meningococcal meningitis	40,274 (30,481–51,609)	22,323 (14,624–32,074)	3,764 (2,347–5,424)	8,078 (5,697–10,902)	4,051 (2,867–5,466)	2,056 (1,507–2,783)
Other meningitis	59,177 (48,010–70,588)	23,663 (16,817–32,270)	4,046 (2,691–5,911)	15,811 (11,273–19,570)	9,684 (7,158–12,651)	5,971 (4,527–7,729)
Encephalitis	80,134 (73,993–87,311)	24,570 (21,384–28,306)	9,714 (8,373–11,168)	22,585 (20,853–24,895)	12,761 (11,635–14,137)	10,502 (9,598–11,779)
Diphtheria	2,845 (1,266–7,272)	2,071 (624–6,524)	401 (156–970)	281 (188–446)	89 (43–182)	0 (0–0)
Whooping cough	44,890 (16,432–103,174)	41,600 (15,215–94,563)	2,538 (946–6,110)	661 (229–1,808)	89 (28–260)	0 (0–0)
Tetanus	60,681 (49,183–89,862)	32,921 (27,807–39,531)	3,357 (2,331–6,775)	12,927 (9,109–26,260)	8,240 (5,743–17,896)	3,233 (2,234–7,159)
Measles	147,776 (55,044–306,384)	126,932 (47,544–258,442)	17,717 (6,445–38,174)	3,008 (940–7,236)	117 (36–287)	0 (0–0)
Varicella and herpes zoster	4,575 (3,872–5,527)	1,355 (943–1,932)	386 (251–606)	831 (662–1,054)	771 (584–1,059)	1,229 (880–1,708)
Neglected tropical diseases and malaria	651,177 (542,004–763,025)	419,740 (321,780–520,008)	52,276 (38,317–68,433)	111,590 (88,546–141,316)	48,250 (38,879–61,375)	19,319 (15,149–25,361)
Malaria	574,469 (466,770–685,877)	407,313 (308,706–508,318)	40,302 (25,691–56,989)	79,068 (57,333–108,669)	34,360 (24,983–47,559)	13,423 (9,331–19,389)
Chagas disease	4,373 (4,129–4,634)	2 (1–2)	4 (4–5)	762 (699–829)	2,028 (1,882–2,180)	1,576 (1,455–1,719)
Leishmaniasis	17,008 (10,840–24,464)	2,421 (1,513–3,519)	1,694 (1,007–2,694)	10,413 (6,499–15,175)	2,097 (1,424–2,909)	381 (288–483)
Visceral leishmaniasis	17,008 (10,840–24,464)	2,421 (1,513–3,519)	1,694 (1,007–2,694)	10,413 (6,499–15,175)	2,097 (1,424–2,909)	381 (288–483)
Cutaneous and mucocutaneous leishmaniasis	0 (0–0)	0 (0–0)	0 (0–0)	0 (0–0)	0 (0–0)	0 (0–0)
African trypanosomiasis	7,622 (4,009–12,554)	507 (261–858)	1,039 (553–1,712)	4,947 (2,546–8,272)	926 (474–1,554)	202 (107–330)
Schistosomiasis	4,143 (3,524–4,943)	41 (24–71)	98 (68–138)	1,401 (1,132–1,727)	1,776 (1,347–2,433)	825 (611–1,148)
Cysticercosis	320 (280–377)	5 (2–9)	10 (7–14)	200 (173–238)	82 (71–96)	21 (19–25)
Cystic echinococcosis	885 (835–938)	31 (25–38)	68 (60–76)	375 (351–403)	262 (246–280)	146 (138–154)
Lymphatic filariasis	0 (0–0)	0 (0–0)	0 (0–0)	0 (0–0)	0 (0–0)	0 (0–0)
Onchocerciasis	0 (0–0)	0 (0–0)	0 (0–0)	0 (0–0)	0 (0–0)	0 (0–0)
Trachoma	0 (0–0)	0 (0–0)	0 (0–0)	0 (0–0)	0 (0–0)	0 (0–0)
Dengue	6,177 (3,697–7,862)	1,942 (937–3,094)	1,552 (974–2,181)	1,852 (1,153–2,356)	536 (303–679)	293 (178–387)
Yellow fever	4,644 (996–12,661)	397 (75–1,173)	1,326 (278–3,717)	2,518 (546–6,890)	341 (77–940)	60 (12–185)
Rabies	18,992 (16,116–21,506)	2,026 (1,423–2,869)	4,835 (3,848–6,022)	7,035 (5,920–8,046)	3,877 (3,220–4,551)	1,217 (948–1,496)
Intestinal nematode infections	1,924 (1,683–2,190)	1,360 (1,152–1,600)	282 (230–339)	144 (113–181)	83 (60–111)	53 (36–71)
Ascariasis	1,924 (1,683–2,190)	1,360 (1,152–1,600)	282 (230–339)	144 (113–181)	83 (60–111)	53 (36–71)
Trichuriasis	0 (0–0)	0 (0–0)	0 (0–0)	0 (0–0)	0 (0–0)	0 (0–0)
Hookworm disease	0 (0–0)	0 (0–0)	0 (0–0)	0 (0–0)	0 (0–0)	0 (0–0)
Food-borne trematodiases	0 (0–0)	0 (0–0)	0 (0–0)	0 (0–0)	0 (0–0)	0 (0–0)
Leprosy	0 (0–0)	0 (0–0)	0 (0–0)	0 (0–0)	0 (0–0)	0 (0–0)
Ebola	13 (10–16)	0 (0–0)	0 (0–0)	13 (10–16)	0 (0–0)	0 (0–0)
Other neglected tropical diseases	10,600 (4,401–14,153)	3,690 (1,743–5,138)	1,060 (425–1,492)	2,855 (1,011–3,984)	1,877 (727–2,589)	1,117 (454–1,600)
Maternal disorders	0 (0–0)	0 (0–0)	0 (0–0)	0 (0–0)	0 (0–0)	0 (0–0)
Maternal hemorrhage	0 (0–0)	0 (0–0)	0 (0–0)	0 (0–0)	0 (0–0)	0 (0–0)
Maternal sepsis and other maternal infections	0 (0–0)	0 (0–0)	0 (0–0)	0 (0–0)	0 (0–0)	0 (0–0)
Maternal hypertensive disorders	0 (0–0)	0 (0–0)	0 (0–0)	0 (0–0)	0 (0–0)	0 (0–0)
Maternal obstructed labor and uterine rupture	0 (0–0)	0 (0–0)	0 (0–0)	0 (0–0)	0 (0–0)	0 (0–0)
Maternal abortion, miscarriage, and ectopic pregnancy	0 (0–0)	0 (0–0)	0 (0–0)	0 (0–0)	0 (0–0)	0 (0–0)

SR Table 11 - Global deaths by age and cause for males in 2005						
Cause	All ages	0-5 years	5-14 years	15-49 years	50-69 years	70+ years
Indirect maternal deaths	0 (0–0)	0 (0–0)	0 (0–0)	0 (0–0)	0 (0–0)	0 (0–0)
Late maternal deaths	0 (0–0)	0 (0–0)	0 (0–0)	0 (0–0)	0 (0–0)	0 (0–0)
Maternal deaths aggravated by HIV/AIDS	0 (0–0)	0 (0–0)	0 (0–0)	0 (0–0)	0 (0–0)	0 (0–0)
Other maternal disorders	0 (0–0)	0 (0–0)	0 (0–0)	0 (0–0)	0 (0–0)	0 (0–0)
Neonatal disorders	1,487,766 (1,440,391–1,539,752)	1,487,766 (1,440,391–1,539,752)	0 (0–0)	0 (0–0)	0 (0–0)	0 (0–0)
Neonatal preterm birth complications	591,836 (533,980–686,315)	591,836 (533,980–686,315)	0 (0–0)	0 (0–0)	0 (0–0)	0 (0–0)
Neonatal encephalopathy due to birth asphyxia and trauma	500,467 (440,405–566,213)	500,467 (440,405–566,213)	0 (0–0)	0 (0–0)	0 (0–0)	0 (0–0)
Neonatal sepsis and other neonatal infections	223,213 (141,390–328,001)	223,213 (141,390–328,001)	0 (0–0)	0 (0–0)	0 (0–0)	0 (0–0)
Hemolytic disease and other neonatal jaundice	35,576 (23,740–60,281)	35,576 (23,740–60,281)	0 (0–0)	0 (0–0)	0 (0–0)	0 (0–0)
Other neonatal disorders	136,674 (93,875–179,471)	136,674 (93,875–179,471)	0 (0–0)	0 (0–0)	0 (0–0)	0 (0–0)
Nutritional deficiencies	234,431 (153,707–307,701)	140,234 (86,141–201,876)	8,512 (6,295–12,330)	21,977 (15,615–31,432)	22,774 (16,442–32,376)	40,931 (27,995–51,664)
Protein-energy malnutrition	197,313 (127,643–264,879)	129,696 (79,161–187,651)	6,784 (4,854–9,797)	12,514 (8,868–17,126)	15,454 (11,385–22,015)	32,864 (22,508–41,409)
Iodine deficiency	812 (511–1,415)	281 (91–774)	32 (14–64)	178 (104–304)	169 (97–302)	149 (88–248)
Vitamin A deficiency	0 (0–0)	0 (0–0)	0 (0–0)	0 (0–0)	0 (0–0)	0 (0–0)
Iron-deficiency anemia	22,645 (9,960–33,945)	7,605 (2,188–12,617)	986 (455–1,887)	5,735 (3,218–9,707)	4,707 (2,362–7,509)	3,610 (1,465–5,222)
Other nutritional deficiencies	13,659 (10,003–18,733)	2,651 (1,470–4,253)	709 (503–1,072)	3,548 (2,518–4,960)	2,443 (1,777–3,540)	4,306 (3,327–5,669)
Other communicable, maternal, neonatal, and nutritional diseases	187,752 (157,491–224,687)	85,927 (59,020–119,538)	10,078 (8,065–12,716)	41,147 (38,331–43,285)	31,835 (28,294–33,746)	18,762 (16,388–19,956)
Sexually transmitted diseases excluding HIV	67,778 (40,299–104,707)	59,156 (33,724–93,117)	4,427 (2,490–6,885)	1,861 (1,328–2,293)	1,458 (984–1,878)	875 (637–1,172)
Syphilis	67,252 (39,807–104,137)	59,156 (33,724–93,117)	4,412 (2,478–6,870)	1,590 (1,135–1,959)	1,322 (893–1,704)	770 (561–1,030)
Chlamydial infection	0 (0–0)	0 (0–0)	0 (0–0)	0 (0–0)	0 (0–0)	0 (0–0)
Gonococcal infection	364 (273–433)	0 (0–0)	8 (6–11)	191 (136–236)	83 (56–106)	80 (58–108)
Trichomoniasis	0 (0–0)	0 (0–0)	0 (0–0)	0 (0–0)	0 (0–0)	0 (0–0)
Genital herpes	0 (0–0)	0 (0–0)	0 (0–0)	0 (0–0)	0 (0–0)	0 (0–0)
Other sexually transmitted diseases	162 (118–193)	0 (0–0)	6 (4–8)	79 (55–97)	52 (35–67)	24 (18–33)
Hepatitis	71,506 (68,509–74,640)	8,334 (7,148–9,786)	3,688 (3,232–4,145)	30,008 (28,503–31,679)	19,595 (18,711–20,536)	9,880 (9,458–10,350)
Acute hepatitis A	8,562 (5,389–12,011)	6,461 (4,695–8,058)	590 (168–1,263)	912 (210–2,072)	447 (108–1,070)	150 (33–383)
Hepatitis B	44,211 (38,255–49,943)	490 (166–1,213)	478 (251–815)	19,799 (16,460–22,957)	16,094 (13,883–17,948)	7,348 (6,070–8,505)
Hepatitis C	1,591 (342–3,640)	0 (0–0)	56 (11–145)	527 (101–1,238)	724 (151–1,760)	283 (55–681)
Acute hepatitis E	17,141 (11,556–23,436)	1,382 (538–2,675)	2,563 (1,882–3,172)	8,768 (6,051–11,957)	2,328 (1,136–4,053)	2,098 (1,149–3,332)
Other infectious diseases	48,466 (31,216–57,875)	18,437 (8,902–26,138)	1,962 (1,401–2,395)	9,277 (7,075–10,234)	10,782 (7,328–12,154)	8,006 (5,645–9,052)
Non-communicable diseases	18,308,566 (17,995,933–18,607,057)	459,182 (397,814–542,192)	115,623 (108,051–123,109)	2,508,034 (2,436,777–2,578,502)	6,432,520 (6,310,149–6,550,589)	8,793,205 (8,680,419–8,900,641)
Neoplasms	4,254,263 (4,163,673–4,355,686)	20,703 (17,101–27,396)	29,992 (28,139–31,983)	560,460 (537,693–579,464)	1,843,383 (1,801,257–1,884,056)	1,799,723 (1,760,398–1,847,775)
Lip and oral cavity cancer	72,490 (70,531–74,654)	0 (0–0)	0 (0–0)	13,424 (12,844–14,151)	37,106 (36,051–38,255)	21,959 (21,325–22,560)
Nasopharynx cancer	38,798 (28,305–41,440)	0 (0–0)	225 (185–266)	11,731 (7,999–12,709)	18,565 (13,590–19,853)	8,275 (6,573–8,785)
Other pharynx cancer	36,485 (35,401–37,481)	0 (0–0)	0 (0–0)	5,530 (5,296–5,774)	21,636 (20,953–22,307)	9,317 (9,001–9,621)
Esophageal cancer	311,575 (298,862–324,322)	0 (0–0)	0 (0–0)	30,803 (29,160–32,702)	161,616 (154,546–168,756)	119,156 (114,393–124,110)
Stomach cancer	525,028 (510,574–540,913)	0 (0–0)	0 (0–0)	54,907 (52,678–57,401)	237,458 (229,653–246,021)	232,662 (226,993–238,402)
Colon and rectum cancer	354,318 (347,745–361,672)	0 (0–0)	0 (0–0)	37,416 (36,114–39,148)	140,353 (137,428–143,440)	176,548 (173,285–180,126)
Liver cancer	501,529 (424,280–529,795)	0 (0–0)	826 (677–943)	114,139 (90,075–123,276)	245,454 (208,348–259,509)	141,108 (125,039–147,728)
Liver cancer due to hepatitis B	196,283 (162,249–211,018)	0 (0–0)	532 (426–626)	68,830 (54,274–75,103)	92,166 (77,024–101,022)	34,754 (28,962–38,397)
Liver cancer due to hepatitis C	75,159 (67,659–80,360)	0 (0–0)	17 (13–21)	7,086 (5,599–8,040)	33,893 (29,630–37,191)	34,163 (31,592–36,660)
Liver cancer due to alcohol use	158,585 (133,941–170,592)	0 (0–0)	0 (0–0)	19,327 (15,251–21,574)	87,236 (74,210–95,314)	52,022 (45,459–56,216)
Liver cancer due to other causes	71,500 (58,304–77,581)	0 (0–0)	276 (222–316)	18,895 (14,615–21,321)	32,158 (26,604–35,870)	20,169 (17,209–22,224)
Gallbladder and biliary tract cancer	49,976 (48,452–51,503)	0 (0–0)	0 (0–0)	3,942 (3,694–4,181)	19,116 (18,385–19,738)	26,916 (26,200–27,713)
Pancreatic cancer	162,886 (159,989–166,027)	0 (0–0)	0 (0–0)	15,724 (15,284–16,299)	75,195 (73,723–76,754)	71,966 (70,550–73,485)
Larynx cancer	75,611 (73,675–77,697)	0 (0–0)	0 (0–0)	8,190 (7,859–8,579)	40,382 (39,278–41,605)	27,038 (26,254–27,834)
Tracheal, bronchus, and lung cancer	1,005,802 (981,221–1,029,138)	0 (0–0)	0 (0–0)	82,019 (78,902–85,514)	482,019 (468,894–493,567)	441,763 (432,153–451,255)
Malignant skin melanoma	24,926 (16,880–35,545)	0 (0–0)	0 (0–0)	5,604 (4,139–8,247)	10,310 (6,948–14,856)	9,012 (5,545–12,583)
Non-melanoma skin cancer	24,880 (24,252–25,575)	0 (0–0)	0 (0–0)	2,527 (2,428–2,648)	8,856 (8,587–9,139)	13,496 (13,119–13,911)
Non-melanoma skin cancer (squamous-cell carcinoma)	24,880 (24,252–25,575)	0 (0–0)	0 (0–0)	2,527 (2,428–2,648)	8,856 (8,587–9,139)	13,496 (13,119–13,911)
Non-melanoma skin cancer (basal-cell carcinoma)	0 (0–0)	0 (0–0)	0 (0–0)	0 (0–0)	0 (0–0)	0 (0–0)
Breast cancer	6,093 (5,470–6,320)	0 (0–0)	0 (0–0)	788 (745–840)	2,811 (2,543–2,939)	2,493 (2,188–2,603)
Cervical cancer	0 (0–0)	0 (0–0)	0 (0–0)	0 (0–0)	0 (0–0)	0 (0–0)
Uterine cancer	0 (0–0)	0 (0–0)	0 (0–0)	0 (0–0)	0 (0–0)	0 (0–0)
Ovarian cancer	0 (0–0)	0 (0–0)	0 (0–0)	0 (0–0)	0 (0–0)	0 (0–0)
Prostate cancer	277,382 (230,800–348,922)	0 (0–0)	0 (0–0)	2,585 (2,031–3,183)	64,645 (52,858–80,448)	210,150 (173,390–267,444)
Testicular cancer	8,641 (8,268–9,083)	0 (0–0)	0 (0–0)	5,927 (5,616–6,319)	1,527 (1,464–1,592)	1,186 (1,136–1,238)

SR Table 11 - Global deaths by age and cause for males in 2005

Cause	All ages	0-5 years	5-14 years	15-49 years	50-69 years	70+ years
Kidney cancer	66,194 (64,495–68,304)	1,503 (798–2,445)	543 (493–609)	7,177 (6,917–7,519)	29,504 (28,777–30,357)	27,466 (26,745–28,168)
Bladder cancer	108,660 (106,478–110,864)	0 (0–0)	0 (0–0)	4,903 (4,680–5,153)	35,818 (34,941–36,662)	67,938 (66,473–69,435)
Brain and nervous system cancer	105,424 (89,154–116,848)	3,036 (2,289–4,885)	5,519 (4,463–6,647)	31,841 (26,121–35,851)	42,643 (36,630–47,275)	22,382 (18,661–23,887)
Thyroid cancer	9,661 (8,691–10,468)	0 (0–0)	39 (34–43)	1,290 (1,132–1,396)	4,103 (3,659–4,444)	4,228 (3,847–4,625)
Mesothelioma	16,194 (15,764–16,646)	0 (0–0)	0 (0–0)	1,989 (1,893–2,097)	6,803 (6,578–7,024)	7,401 (7,166–7,652)
Hodgkin lymphoma	15,655 (13,275–19,412)	240 (177–307)	560 (411–689)	5,698 (4,827–7,099)	5,779 (4,905–7,161)	3,375 (2,908–4,064)
Non-Hodgkin lymphoma	100,556 (86,790–108,431)	1,856 (1,236–3,193)	2,868 (2,461–3,323)	20,765 (18,638–23,803)	36,677 (32,459–40,162)	38,387 (31,094–40,918)
Multiple myeloma	38,652 (37,757–39,704)	0 (0–0)	0 (0–0)	4,082 (3,914–4,325)	15,950 (15,502–16,482)	18,619 (18,104–19,099)
Leukemia	170,672 (166,530–176,757)	7,586 (6,716–8,834)	13,864 (13,026–14,775)	48,621 (46,946–50,949)	47,820 (46,588–49,287)	52,779 (51,498–54,188)
Acute lymphoid leukemia	55,864 (48,822–63,901)	4,766 (3,899–6,129)	9,774 (8,678–11,261)	20,703 (17,753–24,017)	12,822 (11,013–14,639)	7,797 (6,917–8,848)
Chronic lymphoid leukemia	27,429 (25,983–29,849)	0 (0–0)	0 (0–0)	3,374 (2,998–4,032)	8,361 (7,817–9,247)	15,692 (14,967–17,031)
Acute myeloid leukemia	67,635 (59,425–76,001)	2,820 (1,867–3,815)	4,089 (2,989–5,147)	18,511 (15,436–21,546)	20,540 (18,427–22,986)	21,673 (19,429–23,298)
Chronic myeloid leukemia	19,743 (18,168–22,098)	0 (0–0)	0 (0–0)	6,031 (5,341–6,983)	6,096 (5,508–6,910)	7,615 (7,125–8,330)
Other neoplasms	146,163 (126,177–154,224)	6,479 (4,481–10,513)	5,544 (4,756–6,199)	38,824 (32,079–41,867)	51,225 (44,051–54,083)	44,090 (39,543–47,013)
Cardiovascular diseases	8,057,234 (7,921,158–8,192,896)	33,444 (31,303–35,707)	17,094 (16,025–18,129)	943,849 (916,461–978,036)	2,832,457 (2,775,909–2,887,904)	4,230,387 (4,161,815–4,295,448)
Rheumatic heart disease	141,034 (133,474–152,464)	1,972 (1,648–2,326)	4,071 (3,689–4,505)	41,040 (38,116–45,135)	46,577 (43,578–51,069)	47,372 (44,958–50,674)
Ischemic heart disease	4,074,209 (4,002,473–4,153,495)	2,160 (1,882–2,490)	1,634 (1,463–1,817)	475,337 (461,156–496,409)	1,498,613 (1,466,547–1,533,050)	2,096,463 (2,058,383–2,132,476)
Cerebrovascular disease	2,913,723 (2,837,276–2,983,084)	12,781 (11,633–14,004)	6,556 (6,087–7,036)	282,708 (271,822–294,440)	1,001,752 (970,671–1,032,791)	1,609,924 (1,569,569–1,646,964)
Ischemic stroke	1,248,168 (1,206,081–1,292,182)	469 (384–586)	396 (349–459)	46,794 (43,311–51,367)	349,587 (332,802–366,910)	850,920 (824,287–875,066)
Hemorrhagic stroke	1,665,555 (1,607,397–1,722,758)	12,312 (11,223–13,493)	6,159 (5,723–6,611)	235,914 (226,162–246,062)	652,164 (626,722–678,271)	759,004 (732,752–783,347)
Hypertensive heart disease	334,270 (319,350–361,394)	305 (241–373)	243 (202–292)	39,272 (36,299–42,839)	114,469 (108,157–125,468)	179,979 (170,934–195,039)
Cardiomyopathy and myocarditis	187,042 (174,590–195,910)	6,229 (4,463–7,077)	1,888 (1,649–2,141)	58,254 (53,818–61,952)	62,474 (59,055–67,509)	58,195 (54,255–60,797)
Atrial fibrillation and flutter	60,449 (49,328–73,697)	0 (0–0)	0 (0–0)	568 (436–726)	5,685 (4,437–7,516)	54,196 (43,062–67,027)
Aortic aneurysm	86,680 (83,936–90,024)	0 (0–0)	0 (0–0)	8,512 (7,998–9,199)	27,838 (26,753–29,194)	50,329 (48,768–51,962)
Peripheral vascular disease	18,635 (17,933–19,368)	0 (0–0)	0 (0–0)	393 (363–424)	4,920 (4,649–5,190)	13,321 (12,745–13,913)
Endocarditis	30,284 (25,141–34,926)	2,081 (1,142–2,990)	554 (386–702)	7,889 (6,087–9,221)	8,085 (6,780–9,532)	11,673 (10,263–13,527)
Other cardiovascular and circulatory diseases	210,903 (204,964–217,159)	7,913 (7,061–9,146)	2,145 (1,975–2,394)	29,872 (28,418–31,521)	62,039 (59,730–64,594)	108,932 (105,556–112,692)
Chronic respiratory diseases	2,061,371 (2,004,487–2,117,273)	28,162 (23,870–33,317)	7,019 (6,310–7,714)	131,828 (125,779–139,200)	613,734 (590,071–637,697)	1,280,626 (1,249,324–1,314,016)
Chronic obstructive pulmonary disease	1,736,981 (1,672,964–1,796,232)	10,309 (7,002–14,675)	1,194 (965–1,479)	81,048 (75,228–87,560)	504,525 (479,490–530,269)	1,139,904 (1,104,217–1,175,227)
Pneumoconiosis	21,976 (20,243–23,597)	0 (0–0)	0 (0–0)	2,421 (2,118–2,748)	6,592 (5,856–7,244)	12,962 (11,952–13,895)
Silicosis	9,841 (8,958–10,927)	0 (0–0)	0 (0–0)	1,259 (1,093–1,487)	3,188 (2,806–3,666)	5,392 (4,938–5,910)
Asbestosis	2,302 (1,567–2,679)	0 (0–0)	0 (0–0)	242 (199–282)	566 (409–733)	1,493 (907–1,715)
Coal workers pneumoconiosis	2,505 (2,264–2,794)	0 (0–0)	0 (0–0)	194 (159–244)	707 (616–830)	1,603 (1,450–1,783)
Other pneumoconiosis	7,326 (6,292–8,288)	0 (0–0)	0 (0–0)	724 (615–828)	2,129 (1,724–2,524)	4,471 (3,818–5,040)
Asthma	226,148 (178,747–261,896)	7,811 (5,873–9,806)	4,875 (4,071–5,636)	39,573 (32,026–46,750)	81,990 (62,879–97,219)	91,896 (71,957–108,003)
Interstitial lung disease and pulmonary sarcoidosis	44,853 (31,733–55,921)	142 (77–329)	113 (77–207)	3,316 (2,435–4,692)	13,637 (9,862–18,049)	27,644 (18,811–32,987)
Other chronic respiratory diseases	31,411 (20,472–38,044)	9,898 (6,444–14,391)	837 (469–1,226)	5,468 (3,346–6,800)	6,988 (4,257–8,366)	8,219 (5,434–9,890)
Cirrhosis and other chronic liver diseases	761,839 (731,031–797,373)	6,821 (5,697–8,356)	5,791 (5,167–6,614)	256,250 (241,996–269,245)	344,505 (327,924–360,690)	148,470 (142,570–154,138)
Cirrhosis and other chronic liver diseases due to hepatitis B	236,183 (218,200–256,249)	70 (52–93)	237 (189–291)	79,397 (71,630–88,129)	105,937 (94,335–119,162)	50,540 (45,957–55,367)
Cirrhosis and other chronic liver diseases due to hepatitis C	195,441 (181,096–209,864)	37 (28–49)	131 (102–165)	60,238 (53,787–67,466)	92,185 (82,433–101,266)	42,847 (39,094–46,973)
Cirrhosis and other chronic liver diseases due to alcohol use	216,974 (200,577–233,730)	0 (0–0)	0 (0–0)	81,893 (74,564–89,775)	103,879 (92,903–114,607)	31,201 (27,919–34,927)
Cirrhosis and other chronic liver diseases due to other causes	113,240 (104,712–122,148)	6,713 (5,612–8,224)	5,421 (4,828–6,154)	34,720 (30,974–38,656)	42,503 (37,235–47,811)	23,880 (21,454–26,374)
Digestive diseases	596,771 (571,983–666,440)	11,725 (8,840–15,688)	10,355 (9,306–12,358)	159,114 (149,682–173,832)	195,830 (186,799–222,004)	219,744 (212,070–244,521)
Peptic ulcer disease	172,900 (160,526–192,356)	1,097 (646–1,964)	1,621 (1,371–1,973)	43,779 (39,906–48,732)	63,978 (58,927–71,160)	62,424 (58,210–69,397)
Gastritis and duodenitis	27,963 (22,964–34,606)	662 (329–1,217)	280 (194–431)	7,030 (5,476–8,893)	9,754 (7,977–12,151)	10,234 (8,659–12,686)
Appendicitis	25,163 (21,741–28,655)	1,216 (796–1,826)	1,662 (1,378–1,995)	9,985 (8,474–11,473)	6,809 (5,859–7,893)	5,489 (4,778–6,346)
Paralytic ileus and intestinal obstruction	124,663 (111,073–160,297)	3,982 (2,654–5,900)	3,998 (3,378–5,342)	34,187 (29,390–42,890)	38,747 (33,891–50,989)	43,746 (39,017–56,148)
Inguinal, femoral, and abdominal hernia	35,904 (22,296–41,376)	742 (270–1,282)	586 (313–766)	8,594 (4,629–10,475)	11,273 (7,288–13,813)	14,706 (9,425–17,217)
Inflammatory bowel disease	19,302 (16,473–21,382)	777 (432–1,461)	331 (230–456)	4,541 (3,635–5,254)	5,558 (4,840–6,404)	8,092 (6,973–8,944)
Vascular intestinal disorders	36,696 (34,156–39,860)	424 (221–841)	105 (81–134)	3,259 (2,905–3,657)	11,763 (10,868–13,145)	21,142 (19,776–23,159)
Gallbladder and biliary diseases	39,143 (35,610–44,932)	398 (232–633)	432 (339–567)	6,829 (5,904–8,198)	11,324 (10,125–13,310)	20,158 (18,751–22,394)
Pancreatitis	66,816 (62,471–73,133)	130 (87–198)	261 (218–325)	31,207 (28,456–34,385)	21,765 (20,263–23,910)	13,452 (12,448–14,829)
Other digestive diseases	48,217 (39,678–66,508)	2,293 (1,398–3,635)	1,075 (811–1,592)	9,697 (7,604–14,144)	14,854 (11,810–20,912)	20,296 (17,734–26,570)
Neurological disorders	673,320 (594,825–754,499)	11,166 (7,892–13,993)	7,643 (6,882–8,423)	54,899 (52,554–57,375)	78,472 (67,876–89,005)	521,138 (444,696–596,603)
Alzheimer disease and other dementias	508,223 (428,839–587,910)	0 (0–0)	0 (0–0)	658 (407–966)	43,764 (33,142–54,435)	463,799 (387,147–540,204)
Parkinson disease	45,182 (43,359–47,839)	0 (0–0)	0 (0–0)	385 (361–437)	5,968 (5,720–6,575)	38,829 (37,238–40,794)
Epilepsy	72,815 (68,303–77,234)	8,400 (5,704–10,842)	6,005 (5,412–6,643)	40,328 (38,211–42,515)	12,082 (11,563–12,620)	5,999 (5,786–6,223)

SR Table 11 - Global deaths by age and cause for males in 2005

Cause	All ages	0-5 years	5-14 years	15-49 years	50-69 years	70+ years
Multiple sclerosis	6,669 (6,165–7,157)	0 (0–0)	0 (0–0)	2,259 (2,153–2,547)	3,147 (2,842–3,341)	1,262 (1,093–1,316)
Motor neuron disease	15,254 (14,694–16,039)	533 (416–682)	71 (64–78)	2,384 (2,233–2,615)	6,855 (6,579–7,253)	5,409 (5,221–5,626)
Migraine	0 (0–0)	0 (0–0)	0 (0–0)	0 (0–0)	0 (0–0)	0 (0–0)
Tension-type headache	0 (0–0)	0 (0–0)	0 (0–0)	0 (0–0)	0 (0–0)	0 (0–0)
Medication overuse headache	0 (0–0)	0 (0–0)	0 (0–0)	0 (0–0)	0 (0–0)	0 (0–0)
Other neurological disorders	25,185 (24,340–26,167)	2,242 (1,651–2,867)	1,567 (1,357–1,772)	8,883 (8,391–9,529)	6,653 (6,300–7,129)	5,838 (5,523–6,233)
Mental and substance use disorders	235,263 (224,238–244,275)	13 (10–17)	13 (6–18)	141,092 (134,700–147,155)	75,126 (69,008–78,329)	19,017 (17,547–19,674)
Schizophrenia	9,742 (9,010–10,415)	0 (0–0)	0 (0–0)	4,541 (4,117–4,932)	3,399 (3,146–3,651)	1,802 (1,691–1,910)
Alcohol use disorders	130,636 (120,527–136,551)	0 (0–0)	0 (0–0)	67,814 (63,070–71,594)	52,522 (48,181–55,323)	10,299 (9,535–10,698)
Drug use disorders	94,715 (90,667–99,240)	13 (10–17)	0 (0–0)	68,582 (65,471–72,308)	19,203 (17,980–20,389)	6,915 (6,031–7,289)
Opioid use disorders	71,926 (68,944–78,132)	13 (10–17)	0 (0–0)	52,248 (49,777–57,221)	14,202 (13,323–15,497)	5,462 (4,732–5,797)
Cocaine use disorders	5,319 (3,016–5,840)	0 (0–0)	0 (0–0)	4,007 (2,268–4,404)	1,176 (628–1,338)	135 (95–152)
Amphetamine use disorders	6,158 (3,106–7,049)	0 (0–0)	0 (0–0)	4,578 (2,288–5,279)	1,338 (656–1,598)	241 (139–284)
Cannabis use disorders	0 (0–0)	0 (0–0)	0 (0–0)	0 (0–0)	0 (0–0)	0 (0–0)
Other drug use disorders	11,311 (10,564–12,919)	0 (0–0)	0 (0–0)	7,748 (7,200–8,885)	2,485 (2,290–2,881)	1,076 (955–1,204)
Depressive disorders	0 (0–0)	0 (0–0)	0 (0–0)	0 (0–0)	0 (0–0)	0 (0–0)
Major depressive disorder	0 (0–0)	0 (0–0)	0 (0–0)	0 (0–0)	0 (0–0)	0 (0–0)
Dysthymia	0 (0–0)	0 (0–0)	0 (0–0)	0 (0–0)	0 (0–0)	0 (0–0)
Bipolar disorder	0 (0–0)	0 (0–0)	0 (0–0)	0 (0–0)	0 (0–0)	0 (0–0)
Anxiety disorders	0 (0–0)	0 (0–0)	0 (0–0)	0 (0–0)	0 (0–0)	0 (0–0)
Eating disorders	167 (87–212)	0 (0–0)	13 (6–18)	154 (80–197)	0 (0–0)	0 (0–0)
Anorexia nervosa	150 (77–187)	0 (0–0)	12 (5–17)	137 (70–172)	0 (0–0)	0 (0–0)
Bulimia nervosa	17 (9–25)	0 (0–0)	0 (0–1)	16 (8–24)	0 (0–0)	0 (0–0)
Autistic spectrum disorders	0 (0–0)	0 (0–0)	0 (0–0)	0 (0–0)	0 (0–0)	0 (0–0)
Autism	0 (0–0)	0 (0–0)	0 (0–0)	0 (0–0)	0 (0–0)	0 (0–0)
Asperger syndrome and other autistic spectrum disorders	0 (0–0)	0 (0–0)	0 (0–0)	0 (0–0)	0 (0–0)	0 (0–0)
Attention-deficit/hyperactivity disorder	0 (0–0)	0 (0–0)	0 (0–0)	0 (0–0)	0 (0–0)	0 (0–0)
Conduct disorder	0 (0–0)	0 (0–0)	0 (0–0)	0 (0–0)	0 (0–0)	0 (0–0)
Idiopathic developmental intellectual disability	0 (0–0)	0 (0–0)	0 (0–0)	0 (0–0)	0 (0–0)	0 (0–0)
Other mental and substance use disorders	0 (0–0)	0 (0–0)	0 (0–0)	0 (0–0)	0 (0–0)	0 (0–0)
Diabetes, urogenital, blood, and endocrine diseases	1,271,303 (1,161,226–1,340,593)	68,133 (44,399–105,747)	17,693 (14,854–20,057)	214,782 (191,329–227,306)	426,655 (395,201–444,227)	544,038 (512,406–563,096)
Diabetes mellitus	530,740 (514,774–546,709)	1,215 (963–1,348)	1,291 (1,018–1,424)	65,625 (62,678–68,747)	217,939 (210,085–225,757)	244,669 (237,513–251,974)
Acute glomerulonephritis	6,799 (5,108–7,759)	177 (128–231)	218 (158–249)	1,986 (1,369–2,209)	2,258 (1,773–2,691)	2,159 (1,525–2,516)
Chronic kidney disease	471,307 (389,864–499,302)	7,203 (5,486–8,123)	5,066 (3,802–5,594)	93,580 (70,700–100,286)	152,730 (123,437–162,999)	212,727 (183,605–224,388)
Chronic kidney disease due to diabetes mellitus	149,575 (127,022–158,837)	756 (598–932)	398 (306–498)	21,031 (16,581–23,695)	59,007 (49,799–64,829)	68,381 (60,580–73,250)
Chronic kidney disease due to hypertension	198,881 (164,102–211,968)	2,520 (1,968–2,982)	1,492 (1,125–1,766)	35,912 (26,992–39,353)	59,106 (47,199–64,663)	99,850 (85,697–106,215)
Chronic kidney disease due to glomerulonephritis	110,524 (87,637–118,423)	2,902 (2,138–3,406)	2,343 (1,725–2,714)	32,718 (24,629–36,236)	32,700 (25,482–36,256)	39,859 (33,020–43,876)
Chronic kidney disease due to other causes	12,326 (9,576–14,895)	1,024 (734–1,303)	831 (608–1,034)	3,917 (2,579–5,479)	1,917 (1,180–2,925)	4,636 (3,822–5,630)
Urinary diseases and male infertility	97,020 (85,311–111,482)	4,441 (3,144–5,827)	1,070 (899–1,349)	14,932 (12,593–17,551)	25,576 (21,529–29,862)	50,999 (45,620–58,210)
Interstitial nephritis and urinary tract infections	64,421 (55,718–74,186)	3,514 (2,400–4,691)	663 (536–833)	9,055 (7,353–10,851)	15,008 (12,340–17,895)	36,179 (31,673–41,372)
Urolithiasis	8,030 (6,606–11,195)	0 (0–0)	129 (100–205)	1,523 (1,269–2,323)	2,798 (2,233–3,998)	3,578 (2,854–4,830)
Benign prostatic hyperplasia	0 (0–0)	0 (0–0)	0 (0–0)	0 (0–0)	0 (0–0)	0 (0–0)
Male infertility	0 (0–0)	0 (0–0)	0 (0–0)	0 (0–0)	0 (0–0)	0 (0–0)
Other urinary diseases	24,569 (18,410–30,460)	926 (540–1,567)	277 (213–381)	4,352 (3,110–5,626)	7,770 (5,569–10,028)	11,241 (8,607–14,182)
Gynecological diseases	0 (0–0)	0 (0–0)	0 (0–0)	0 (0–0)	0 (0–0)	0 (0–0)
Uterine fibroids	0 (0–0)	0 (0–0)	0 (0–0)	0 (0–0)	0 (0–0)	0 (0–0)
Polycystic ovarian syndrome	0 (0–0)	0 (0–0)	0 (0–0)	0 (0–0)	0 (0–0)	0 (0–0)
Female infertility	0 (0–0)	0 (0–0)	0 (0–0)	0 (0–0)	0 (0–0)	0 (0–0)
Endometriosis	0 (0–0)	0 (0–0)	0 (0–0)	0 (0–0)	0 (0–0)	0 (0–0)
Genital prolapse	0 (0–0)	0 (0–0)	0 (0–0)	0 (0–0)	0 (0–0)	0 (0–0)
Premenstrual syndrome	0 (0–0)	0 (0–0)	0 (0–0)	0 (0–0)	0 (0–0)	0 (0–0)
Other gynecological diseases	0 (0–0)	0 (0–0)	0 (0–0)	0 (0–0)	0 (0–0)	0 (0–0)
Hemoglobinopathies and hemolytic anemias	113,418 (73,819–161,446)	48,159 (24,529–86,595)	8,095 (6,030–10,005)	26,515 (19,670–32,073)	13,707 (10,058–16,718)	16,941 (12,419–21,234)
Thalassemias	9,698 (6,766–12,213)	5,287 (3,597–7,708)	1,906 (1,426–2,189)	1,023 (714–1,480)	41 (36–46)	1,439 (870–2,199)
Thalassemias trait	0 (0–0)	0 (0–0)	0 (0–0)	0 (0–0)	0 (0–0)	0 (0–0)
Sickle cell disorders	58,819 (32,557–99,693)	41,855 (19,890–81,156)	4,990 (3,589–6,631)	9,860 (7,225–12,489)	402 (324–509)	1,710 (1,163–2,324)
Sickle cell trait	0 (0–0)	0 (0–0)	0 (0–0)	0 (0–0)	0 (0–0)	0 (0–0)

SR Table 11 - Global deaths by age and cause for males in 2005

Cause	All ages	0-5 years	5-14 years	15-49 years	50-69 years	70+ years
G6PD deficiency	19,344 (14,895–23,621)	821 (509–1,299)	977 (769–1,297)	11,019 (8,423–13,641)	5,323 (4,068–6,500)	1,202 (888–1,566)
G6PD trait	0 (0–0)	0 (0–0)	0 (0–0)	0 (0–0)	0 (0–0)	0 (0–0)
Other hemoglobinopathies and hemolytic anemias	25,556 (18,985–31,616)	194 (131–305)	221 (180–271)	4,611 (3,269–5,913)	7,939 (5,685–9,995)	12,589 (9,433–15,482)
Endocrine, metabolic, blood, and immune disorders	52,016 (50,160–53,851)	6,936 (6,398–7,590)	1,952 (1,820–2,096)	12,142 (11,603–12,741)	14,443 (13,834–15,108)	16,541 (15,924–17,226)
Musculoskeletal disorders	22,799 (20,192–23,745)	0 (0–0)	742 (645–858)	5,390 (4,997–5,936)	6,496 (5,779–6,853)	10,170 (8,385–10,657)
Rheumatoid arthritis	7,792 (6,246–8,680)	0 (0–0)	100 (77–131)	804 (647–962)	2,710 (2,148–2,967)	4,176 (3,308–4,737)
Osteoarthritis	0 (0–0)	0 (0–0)	0 (0–0)	0 (0–0)	0 (0–0)	0 (0–0)
Low back and neck pain	0 (0–0)	0 (0–0)	0 (0–0)	0 (0–0)	0 (0–0)	0 (0–0)
Low back pain	0 (0–0)	0 (0–0)	0 (0–0)	0 (0–0)	0 (0–0)	0 (0–0)
Neck pain	0 (0–0)	0 (0–0)	0 (0–0)	0 (0–0)	0 (0–0)	0 (0–0)
Gout	0 (0–0)	0 (0–0)	0 (0–0)	0 (0–0)	0 (0–0)	0 (0–0)
Other musculoskeletal disorders	15,006 (13,646–16,064)	0 (0–0)	641 (556–756)	4,586 (4,274–5,123)	3,785 (3,443–4,211)	5,993 (5,057–6,405)
Other non-communicable diseases	374,387 (309,973–455,761)	279,001 (225,484–359,818)	19,276 (15,449–22,524)	40,363 (34,141–45,829)	15,859 (11,991–19,591)	19,886 (14,169–25,782)
Congenital anomalies	331,941 (268,528–413,700)	265,423 (213,514–343,954)	18,767 (14,947–21,964)	34,699 (29,440–39,649)	7,736 (6,760–8,884)	5,314 (4,801–6,211)
Neural tube defects	37,051 (25,949–55,965)	34,992 (24,291–53,947)	925 (627–1,285)	850 (663–1,141)	185 (141–260)	96 (66–160)
Congenital heart anomalies	169,861 (137,241–209,740)	127,659 (101,038–164,691)	13,300 (10,247–15,671)	24,807 (20,891–28,290)	2,438 (2,104–2,846)	1,655 (1,421–2,072)
Cleft lip and cleft palate	1,947 (1,540–2,400)	1,947 (1,540–2,400)	0 (0–0)	0 (0–0)	0 (0–0)	0 (0–0)
Down syndrome	9,733 (6,873–17,053)	5,631 (3,226–12,039)	428 (284–712)	2,145 (1,757–3,047)	1,457 (1,237–1,861)	70 (58–92)
Turner syndrome	0 (0–0)	0 (0–0)	0 (0–0)	0 (0–0)	0 (0–0)	0 (0–0)
Klinefelter syndrome	0 (0–0)	0 (0–0)	0 (0–0)	0 (0–0)	0 (0–0)	0 (0–0)
Other chromosomal abnormalities	7,178 (3,845–15,461)	6,375 (3,061–14,546)	144 (101–223)	461 (298–625)	145 (96–207)	50 (37–70)
Other congenital anomalies	106,169 (83,589–138,351)	88,816 (68,518–120,019)	3,968 (3,074–4,922)	6,432 (5,093–7,434)	3,509 (2,805–3,988)	3,441 (2,933–3,860)
Skin and subcutaneous diseases	31,125 (18,315–46,599)	2,256 (1,277–4,411)	509 (296–891)	5,664 (3,036–8,727)	8,122 (4,530–11,942)	14,571 (8,771–20,401)
Dermatitis	0 (0–0)	0 (0–0)	0 (0–0)	0 (0–0)	0 (0–0)	0 (0–0)
Psoriasis	0 (0–0)	0 (0–0)	0 (0–0)	0 (0–0)	0 (0–0)	0 (0–0)
Cellulitis	6,204 (3,290–10,938)	326 (139–674)	104 (55–183)	1,431 (646–2,516)	2,127 (1,024–3,776)	2,214 (1,339–3,856)
Pyoderma	13,616 (7,964–22,211)	1,769 (987–3,567)	263 (155–508)	3,015 (1,541–4,860)	3,588 (1,998–5,731)	4,978 (3,094–7,665)
Scabies	0 (0–0)	0 (0–0)	0 (0–0)	0 (0–0)	0 (0–0)	0 (0–0)
Fungal skin diseases	0 (0–0)	0 (0–0)	0 (0–0)	0 (0–0)	0 (0–0)	0 (0–0)
Viral skin diseases	0 (0–0)	0 (0–0)	0 (0–0)	0 (0–0)	0 (0–0)	0 (0–0)
Acne vulgaris	0 (0–0)	0 (0–0)	0 (0–0)	0 (0–0)	0 (0–0)	0 (0–0)
Alopecia areata	0 (0–0)	0 (0–0)	0 (0–0)	0 (0–0)	0 (0–0)	0 (0–0)
Pruritus	0 (0–0)	0 (0–0)	0 (0–0)	0 (0–0)	0 (0–0)	0 (0–0)
Urticaria	0 (0–0)	0 (0–0)	0 (0–0)	0 (0–0)	0 (0–0)	0 (0–0)
Decubitus ulcer	9,974 (4,591–13,921)	45 (21–78)	75 (37–126)	895 (387–1,340)	2,114 (830–2,970)	6,843 (3,350–9,422)
Other skin and subcutaneous diseases	1,330 (797–2,055)	115 (62–197)	66 (36–116)	322 (178–546)	291 (158–478)	535 (332–811)
Sense organ diseases	0 (0–0)	0 (0–0)	0 (0–0)	0 (0–0)	0 (0–0)	0 (0–0)
Glaucoma	0 (0–0)	0 (0–0)	0 (0–0)	0 (0–0)	0 (0–0)	0 (0–0)
Cataract	0 (0–0)	0 (0–0)	0 (0–0)	0 (0–0)	0 (0–0)	0 (0–0)
Macular degeneration	0 (0–0)	0 (0–0)	0 (0–0)	0 (0–0)	0 (0–0)	0 (0–0)
Refraction and accommodation disorders	0 (0–0)	0 (0–0)	0 (0–0)	0 (0–0)	0 (0–0)	0 (0–0)
Age-related and other hearing loss	0 (0–0)	0 (0–0)	0 (0–0)	0 (0–0)	0 (0–0)	0 (0–0)
Other vision loss	0 (0–0)	0 (0–0)	0 (0–0)	0 (0–0)	0 (0–0)	0 (0–0)
Other sense organ diseases	0 (0–0)	0 (0–0)	0 (0–0)	0 (0–0)	0 (0–0)	0 (0–0)
Oral disorders	0 (0–0)	0 (0–0)	0 (0–0)	0 (0–0)	0 (0–0)	0 (0–0)
Deciduous caries	0 (0–0)	0 (0–0)	0 (0–0)	0 (0–0)	0 (0–0)	0 (0–0)
Permanent caries	0 (0–0)	0 (0–0)	0 (0–0)	0 (0–0)	0 (0–0)	0 (0–0)
Periodontal diseases	0 (0–0)	0 (0–0)	0 (0–0)	0 (0–0)	0 (0–0)	0 (0–0)
Edentulism and severe tooth loss	0 (0–0)	0 (0–0)	0 (0–0)	0 (0–0)	0 (0–0)	0 (0–0)
Other oral disorders	0 (0–0)	0 (0–0)	0 (0–0)	0 (0–0)	0 (0–0)	0 (0–0)
Sudden infant death syndrome	11,320 (8,282–22,395)	11,320 (8,282–22,395)	0 (0–0)	0 (0–0)	0 (0–0)	0 (0–0)
Injuries	3,294,888 (3,120,843–3,380,923)	216,513 (183,583–247,113)	187,518 (175,639–197,359)	1,894,192 (1,816,858–1,948,916)	639,253 (603,365–655,489)	357,409 (331,116–367,004)
Transport injuries	1,110,343 (1,069,768–1,157,198)	37,004 (30,627–44,173)	55,618 (51,985–59,110)	707,026 (678,039–740,055)	215,746 (206,287–224,289)	94,947 (91,529–98,234)
Road injuries	1,030,096 (986,185–1,075,682)	34,104 (28,010–40,923)	52,524 (49,052–55,917)	658,139 (626,934–690,395)	197,010 (187,873–205,367)	88,318 (84,978–91,595)
Pedestrian road injuries	415,145 (384,213–462,600)	16,187 (13,578–19,849)	26,157 (23,861–28,965)	234,453 (214,960–261,514)	91,822 (84,710–102,846)	46,524 (43,211–52,658)
Cyclist road injuries	46,651 (42,511–51,744)	881 (643–1,170)	3,136 (2,788–3,505)	24,466 (22,046–27,177)	13,024 (11,783–14,511)	5,141 (4,678–5,759)
Motorcyclist road injuries	197,213 (167,899–214,330)	4,210 (2,873–5,401)	6,023 (4,706–7,110)	147,843 (128,613–161,632)	29,376 (23,031–33,345)	9,759 (6,426–12,382)

SR Table 11 - Global deaths by age and cause for males in 2005

Cause	All ages	0-5 years	5-14 years	15-49 years	50-69 years	70+ years
Motor vehicle road injuries	356,288<br>(318,830–403,450)	11,899<br>(9,427–14,830)	16,014<br>(13,963–18,877)	242,717<br>(221,613–275,647)	60,168<br>(50,610–68,196)	25,489<br>(21,544–28,721)
Other road injuries	14,797<br>(8,850–18,305)	925<br>(364–2,016)	1,192<br>(627–1,630)	8,658<br>(4,871–10,788)	2,617<br>(1,812–3,336)	1,403<br>(1,066–1,929)
Other transport injuries	80,247<br>(72,025–92,199)	2,900<br>(2,093–3,674)	3,093<br>(2,781–3,488)	48,887<br>(43,830–56,168)	18,735<br>(16,657–22,298)	6,629<br>(5,890–7,865)
Unintentional injuries	1,206,511<br>(1,121,384–1,256,115)	166,314<br>(136,945–194,580)	106,484<br>(99,109–113,294)	521,397<br>(489,507–540,993)	228,664<br>(210,945–237,005)	183,650<br>(165,957–190,418)
Falls	264,704<br>(242,524–275,454)	12,827<br>(9,419–16,447)	10,405<br>(9,226–11,487)	88,473<br>(82,855–93,969)	67,752<br>(62,315–70,750)	85,245<br>(75,783–89,029)
Drowning	277,052<br>(266,244–289,589)	52,454<br>(45,032–61,662)	52,417<br>(49,425–55,246)	121,075<br>(116,658–127,003)	34,897<br>(33,621–36,226)	16,206<br>(15,705–16,739)
Fire, heat, and hot substances	95,265<br>(77,220–102,473)	13,982<br>(9,946–17,071)	5,374<br>(4,140–6,099)	42,942<br>(34,623–45,966)	18,920<br>(16,195–20,145)	14,045<br>(12,096–14,792)
Poisonings	61,414<br>(39,006–75,571)	12,216<br>(3,805–24,290)	3,461<br>(2,331–4,278)	27,563<br>(20,119–33,148)	12,591<br>(8,829–14,224)	5,581<br>(3,801–6,240)
Exposure to mechanical forces	147,895<br>(124,560–158,109)	20,520<br>(15,239–25,220)	9,066<br>(7,968–10,840)	79,154<br>(64,845–84,785)	25,351<br>(21,281–27,485)	13,802<br>(11,325–14,792)
Unintentional firearm injuries	24,104<br>(16,577–26,670)	1,733<br>(961–2,609)	1,441<br>(1,083–1,942)	15,348<br>(10,405–17,189)	3,165<br>(2,310–3,531)	2,416<br>(1,899–2,670)
Unintentional suffocation	22,892<br>(17,010–26,937)	12,509<br>(7,643–16,138)	2,099<br>(1,699–3,068)	4,246<br>(3,613–4,988)	1,166<br>(799–1,358)	2,869<br>(2,330–3,100)
Other exposure to mechanical forces	100,898<br>(80,934–109,073)	6,277<br>(4,439–8,132)	5,525<br>(4,644–6,687)	59,559<br>(47,185–64,894)	21,020<br>(17,335–22,932)	8,516<br>(6,594–9,352)
Adverse effects of medical treatment	54,635<br>(39,489–61,196)	10,810<br>(5,252–14,650)	2,862<br>(1,818–3,488)	18,055<br>(12,409–20,887)	11,079<br>(8,448–12,789)	11,827<br>(9,340–13,495)
Animal contact	59,790<br>(36,151–66,251)	6,756<br>(3,700–8,452)	8,003<br>(4,682–9,346)	26,058<br>(15,648–29,697)	12,918<br>(7,679–14,398)	6,053<br>(4,020–6,709)
Venomous animal contact	49,752<br>(28,490–56,514)	4,740<br>(2,429–5,965)	6,658<br>(3,740–7,981)	22,738<br>(12,883–26,467)	10,817<br>(6,117–12,304)	4,797<br>(2,993–5,420)
Non-venomous animal contact	10,037<br>(7,609–11,758)	2,015<br>(1,064–2,817)	1,345<br>(914–1,676)	3,320<br>(2,622–3,903)	2,100<br>(1,582–2,496)	1,255<br>(1,010–1,514)
Foreign body	89,232<br>(70,711–113,782)	27,224<br>(16,530–39,215)	5,800<br>(4,256–7,895)	25,782<br>(20,476–33,555)	15,178<br>(12,253–18,147)	15,246<br>(12,062–17,684)
Pulmonary aspiration and foreign body in airway	71,353<br>(56,231–90,878)	23,053<br>(13,299–35,310)	4,164<br>(2,947–5,820)	17,874<br>(14,805–21,223)	12,892<br>(10,191–14,621)	13,368<br>(10,220–14,493)
Foreign body in eyes	0<br>(0–0)	0<br>(0–0)	0<br>(0–0)	0<br>(0–0)	0<br>(0–0)	0<br>(0–0)
Foreign body in other body part	17,878<br>(9,995–31,770)	4,170<br>(1,494–6,764)	1,635<br>(827–2,668)	7,908<br>(4,563–14,969)	2,285<br>(1,476–4,832)	1,877<br>(1,334–3,921)
Environmental heat and cold exposure	37,404<br>(27,047–40,475)	2,016<br>(957–2,650)	841<br>(500–1,028)	16,149<br>(11,813–17,854)	12,906<br>(8,899–14,219)	5,489<br>(3,825–6,105)
Other unintentional injuries	119,116<br>(112,062–127,813)	7,504<br>(6,035–9,072)	8,249<br>(7,222–9,341)	76,141<br>(70,734–82,913)	17,068<br>(15,944–18,370)	10,151<br>(8,478–12,653)
Self-harm and interpersonal violence	893,639<br>(816,111–919,943)	7,619<br>(6,728–8,375)	14,294<br>(12,559–15,189)	612,371<br>(561,564–632,619)	184,496<br>(167,900–190,171)	74,857<br>(65,990–77,591)
Self-harm	556,574<br>(491,881–574,604)	0<br>(0–0)	5,905<br>(4,644–6,492)	346,493<br>(306,709–359,379)	139,973<br>(124,206–144,632)	64,202<br>(55,298–66,824)
Interpersonal violence	337,065<br>(308,076–349,391)	7,619<br>(6,728–8,375)	8,389<br>(7,685–8,912)	265,878<br>(240,005–276,136)	44,522<br>(41,334–46,785)	10,654<br>(10,138–11,232)
Assault by firearm	142,606<br>(124,180–148,153)	1,141<br>(893–1,303)	2,388<br>(1,996–2,574)	122,872<br>(106,949–127,772)	13,706<br>(12,283–14,659)	2,498<br>(2,295–2,709)
Assault by sharp object	83,383<br>(77,670–89,120)	790<br>(634–1,023)	1,356<br>(1,176–1,558)	67,496<br>(62,638–72,271)	11,235<br>(10,369–12,188)	2,504<br>(2,309–2,719)
Assault by other means	111,075<br>(97,815–120,334)	5,688<br>(5,008–6,369)	4,644<br>(4,067–5,172)	75,509<br>(66,106–83,265)	19,580<br>(17,785–21,115)	5,652<br>(5,144–6,094)
Forces of nature, war, and legal intervention	84,394<br>(56,196–111,868)	5,574<br>(3,544–7,564)	11,121<br>(6,629–15,577)	53,396<br>(36,890–69,491)	10,346<br>(6,835–13,814)	3,954<br>(2,463–5,422)
Exposure to forces of nature	58,151<br>(33,797–82,021)	4,241<br>(2,343–6,090)	9,820<br>(5,579–14,039)	32,644<br>(18,889–46,403)	8,012<br>(4,697–11,238)	3,431<br>(2,006–4,875)
Collective violence and legal intervention	26,242<br>(19,492–32,894)	1,332<br>(924–1,745)	1,300<br>(941–1,686)	20,751<br>(15,272–26,021)	2,334<br>(1,747–2,938)	523<br>(384–684)

SR Table 11 - Global deaths by age and cause for males in 2010

Cause	All ages	0-5 years	5-14 years	15-49 years	50-69 years	70+ years
All causes	29,488,225<br>(29,074,627–29,898,175)	3,673,465<br>(3,617,366–3,730,946)	558,755<br>(537,261–582,759)	5,767,033<br>(5,611,878–5,924,203)	8,303,969<br>(8,150,199–8,452,668)	11,185,001<br>(11,063,985–11,300,226)
Communicable, maternal, neonatal, and nutritional diseases	6,585,685<br>(6,376,310–6,828,782)	3,029,977<br>(2,956,503–3,098,500)	266,657<br>(253,447–282,497)	1,377,118<br>(1,292,794–1,466,441)	911,527<br>(831,205–991,504)	1,000,404<br>(920,370–1,064,203)
HIV/AIDS and tuberculosis	1,558,650<br>(1,386,295–1,828,596)	84,513<br>(80,198–93,726)	57,916<br>(55,300–62,966)	832,455<br>(761,439–916,803)	398,827<br>(335,032–499,277)	184,938<br>(145,910–258,786)
Tuberculosis	777,448<br>(599,951–1,043,012)	14,614<br>(11,234–23,523)	8,095<br>(6,358–12,578)	293,539<br>(222,567–378,823)	287,599<br>(222,165–386,352)	173,599<br>(133,309–246,588)
HIV/AIDS	781,201<br>(743,023–831,189)	69,898<br>(67,040–72,921)	49,821<br>(47,913–51,727)	538,915<br>(505,741–581,353)	111,227<br>(103,163–123,438)	11,338<br>(10,335–12,781)
HIV/AIDS - Tuberculosis	154,635<br>(120,553–173,981)	17,482<br>(13,378–19,691)	10,606<br>(7,884–12,132)	97,291<br>(74,858–110,504)	26,223<br>(21,492–29,500)	3,031<br>(2,630–3,447)
HIV/AIDS resulting in other diseases	626,566<br>(584,727–679,050)	52,416<br>(49,044–56,911)	39,214<br>(36,709–42,302)	441,624<br>(407,082–482,429)	85,003<br>(77,495–96,773)	8,306<br>(7,446–9,585)
Diarrhea, lower respiratory, and other common infectious diseases	2,728,382<br>(2,554,499–2,871,410)	1,051,423<br>(977,223–1,126,553)	150,050<br>(134,404–168,950)	382,812<br>(345,456–422,721)	413,218<br>(365,222–447,684)	730,876<br>(655,625–778,184)
Diarrheal diseases	753,246<br>(703,368–805,560)	329,552<br>(293,813–372,519)	33,116<br>(29,586–36,773)	108,131<br>(98,290–119,735)	119,133<br>(107,897–130,849)	163,313<br>(148,246–178,951)
Intestinal infectious diseases	99,911<br>(56,661–164,840)	23,917<br>(12,960–41,318)	38,691<br>(22,775–62,390)	31,068<br>(16,070–53,584)	4,425<br>(2,261–7,654)	1,809<br>(1,012–2,893)
Typhoid fever	83,132<br>(45,540–140,695)	20,086<br>(10,402–35,394)	32,517<br>(18,464–52,745)	25,571<br>(13,041–44,942)	3,532<br>(1,727–6,217)	1,424<br>(762–2,338)
Paratyphoid fever	16,316<br>(7,594–31,633)	3,591<br>(1,500–7,350)	6,126<br>(2,822–11,857)	5,425<br>(2,288–10,850)	853<br>(347–1,701)	320<br>(136–605)
Other intestinal infectious diseases	462<br>(154–1,042)	240<br>(66–576)	46<br>(11–118)	71<br>(28–175)	39<br>(15–90)	64<br>(27–151)
Lower respiratory infections	1,429,251<br>(1,273,392–1,544,714)	460,034<br>(423,165–501,033)	36,176<br>(27,265–41,822)	158,050<br>(127,763–176,886)	241,638<br>(198,172–268,237)	533,350<br>(464,413–585,910)
Upper respiratory infections	1,819<br>(1,602–2,112)	459<br>(314–674)	29<br>(19–46)	439<br>(361–531)	458<br>(372–571)	432<br>(348–536)
Otitis media	1,721<br>(1,541–1,930)	638<br>(535–769)	248<br>(224–277)	444<br>(398–506)	227<br>(195–267)	161<br>(129–206)
Meningitis	214,712<br>(171,794–251,897)	103,736<br>(79,555–133,806)	19,513<br>(14,179–23,806)	48,609<br>(36,082–61,486)	26,325<br>(19,969–34,084)	16,527<br>(12,773–21,370)
Pneumococcal meningitis	64,431<br>(50,927–80,509)	27,438<br>(18,585–39,143)	7,195<br>(4,877–9,848)	17,245<br>(12,372–22,668)	7,736<br>(5,528–10,585)	4,816<br>(3,537–6,566)
H influenzae type B meningitis	44,231<br>(33,800–56,798)	28,598<br>(19,755–40,016)	4,036<br>(2,534–5,844)	5,993<br>(4,282–8,087)	3,319<br>(2,325–4,619)	2,284<br>(1,699–3,207)
Meningococcal meningitis	42,689<br>(32,572–54,012)	23,182<br>(15,212–34,001)	4,147<br>(2,627–6,205)	8,582<br>(6,049–11,744)	4,425<br>(3,127–6,049)	2,351<br>(1,714–3,127)
Other meningitis	63,359<br>(49,979–76,340)	24,518<br>(17,509–33,936)	4,133<br>(2,696–6,047)	16,788<br>(11,943–21,065)	10,842<br>(7,907–14,010)	7,075<br>(5,304–8,975)
Encephalitis	80,827<br>(74,921–87,576)	22,952<br>(20,261–25,908)	9,319<br>(7,969–10,776)	23,101<br>(21,293–25,374)	13,745<br>(12,513–15,180)	11,708<br>(10,728–13,022)
Diphtheria	1,758<br>(776–4,645)	1,280<br>(374–4,222)	259<br>(88–756)	166<br>(108–262)	52<br>(29–96)	0<br>(0–0)
Whooping cough	33,735<br>(13,141–72,693)	31,234<br>(12,164–67,136)	1,961<br>(750–4,402)	473<br>(177–1,147)	65<br>(22–169)	0<br>(0–0)
Tetanus	42,041<br>(34,223–65,262)	20,236<br>(16,809–24,679)	2,489<br>(1,706–4,822)	10,272<br>(6,977–20,673)	6,503<br>(4,625–13,860)	2,539<br>(1,834–5,758)
Measles	65,694<br>(24,298–142,786)	56,285<br>(20,975–122,094)	7,932<br>(2,730–17,881)	1,411<br>(440–3,568)	66<br>(21–166)	0<br>(0–0)
Varicella and herpes zoster	3,661<br>(2,969–4,648)	1,095<br>(704–1,784)	312<br>(190–530)	644<br>(496–840)	576<br>(414–819)	1,032<br>(722–1,537)
Neglected tropical diseases and malaria	542,338<br>(455,759–638,796)	337,371<br>(255,865–422,618)	41,460<br>(29,030–57,359)	100,291<br>(79,179–127,759)	44,393<br>(35,409–55,905)	18,821<br>(15,011–24,359)
Malaria	472,540<br>(384,452–572,169)	325,664<br>(243,832–411,579)	31,136<br>(19,212–46,607)	71,322<br>(51,493–98,338)	31,745<br>(22,941–43,416)	12,671<br>(8,905–18,088)
Chagas disease	4,250<br>(3,989–4,522)	1<br>(1–1)	3<br>(3–4)	642<br>(587–703)	1,931<br>(1,774–2,095)	1,671<br>(1,535–1,824)
Leishmaniasis	17,406<br>(11,996–24,052)	2,390<br>(1,593–3,375)	1,699<br>(1,087–2,560)	10,698<br>(7,203–15,065)	2,193<br>(1,560–2,974)	424<br>(330–533)
Visceral leishmaniasis	17,406<br>(11,996–24,052)	2,390<br>(1,593–3,375)	1,699<br>(1,087–2,560)	10,698<br>(7,203–15,065)	2,193<br>(1,560–2,974)	424<br>(330–533)
Cutaneous and mucocutaneous leishmaniasis	0<br>(0–0)	0<br>(0–0)	0<br>(0–0)	0<br>(0–0)	0<br>(0–0)	0<br>(0–0)
African trypanosomiasis	3,380<br>(1,733–5,596)	212<br>(110–353)	485<br>(260–782)	2,197<br>(1,097–3,718)	400<br>(198–679)	84<br>(43–139)
Schistosomiasis	3,241<br>(2,752–3,816)	28<br>(17–51)	71<br>(48–103)	1,080<br>(892–1,309)	1,390<br>(1,076–1,805)	670<br>(507–889)
Cysticercosis	248<br>(207–316)	4<br>(2–7)	8<br>(5–12)	155<br>(129–199)	63<br>(54–78)	16<br>(14–19)
Cystic echinococcosis	725<br>(679–775)	23<br>(19–28)	51<br>(45–57)	301<br>(278–326)	219<br>(204–234)	128<br>(121–136)
Lymphatic filariasis	0<br>(0–0)	0<br>(0–0)	0<br>(0–0)	0<br>(0–0)	0<br>(0–0)	0<br>(0–0)
Onchocerciasis	0<br>(0–0)	0<br>(0–0)	0<br>(0–0)	0<br>(0–0)	0<br>(0–0)	0<br>(0–0)
Trachoma	0<br>(0–0)	0<br>(0–0)	0<br>(0–0)	0<br>(0–0)	0<br>(0–0)	0<br>(0–0)
Dengue	8,743<br>(5,113–11,177)	2,349<br>(1,188–3,519)	2,021<br>(1,159–2,924)	2,832<br>(1,633–3,571)	966<br>(495–1,290)	573<br>(284–793)
Yellow fever	4,459<br>(911–12,637)	398<br>(76–1,205)	1,351<br>(272–3,705)	2,358<br>(471–6,608)	298<br>(59–856)	53<br>(9–153)
Rabies	13,328<br>(11,554–15,213)	1,524<br>(1,095–2,143)	3,217<br>(2,577–4,043)	4,981<br>(4,259–5,699)	2,683<br>(2,284–3,146)	921<br>(745–1,094)
Intestinal nematode infections	1,652<br>(1,457–1,868)	1,127<br>(958–1,317)	244<br>(199–294)	137<br>(107–173)	84<br>(60–113)	57<br>(38–79)
Ascariasis	1,652<br>(1,457–1,868)	1,127<br>(958–1,317)	244<br>(199–294)	137<br>(107–173)	84<br>(60–113)	57<br>(38–79)
Trichuriasis	0<br>(0–0)	0<br>(0–0)	0<br>(0–0)	0<br>(0–0)	0<br>(0–0)	0<br>(0–0)
Hookworm disease	0<br>(0–0)	0<br>(0–0)	0<br>(0–0)	0<br>(0–0)	0<br>(0–0)	0<br>(0–0)
Food-borne trematodiases	0<br>(0–0)	0<br>(0–0)	0<br>(0–0)	0<br>(0–0)	0<br>(0–0)	0<br>(0–0)
Leprosy	0<br>(0–0)	0<br>(0–0)	0<br>(0–0)	0<br>(0–0)	0<br>(0–0)	0<br>(0–0)
Ebola	0<br>(0–0)	0<br>(0–0)	0<br>(0–0)	0<br>(0–0)	0<br>(0–0)	0<br>(0–0)
Other neglected tropical diseases	12,360<br>(4,239–16,665)	3,644<br>(1,572–5,299)	1,168<br>(366–1,644)	3,583<br>(1,012–4,829)	2,415<br>(744–3,228)	1,547<br>(490–2,148)
Maternal disorders	0<br>(0–0)	0<br>(0–0)	0<br>(0–0)	0<br>(0–0)	0<br>(0–0)	0<br>(0–0)
Maternal hemorrhage	0<br>(0–0)	0<br>(0–0)	0<br>(0–0)	0<br>(0–0)	0<br>(0–0)	0<br>(0–0)
Maternal sepsis and other maternal infections	0<br>(0–0)	0<br>(0–0)	0<br>(0–0)	0<br>(0–0)	0<br>(0–0)	0<br>(0–0)
Maternal hypertensive disorders	0<br>(0–0)	0<br>(0–0)	0<br>(0–0)	0<br>(0–0)	0<br>(0–0)	0<br>(0–0)
Maternal obstructed labor and uterine rupture	0<br>(0–0)	0<br>(0–0)	0<br>(0–0)	0<br>(0–0)	0<br>(0–0)	0<br>(0–0)
Maternal abortion, miscarriage, and ectopic pregnancy	0<br>(0–0)	0<br>(0–0)	0<br>(0–0)	0<br>(0–0)	0<br>(0–0)	0<br>(0–0)

SR Table 11 - Global deaths by age and cause for males in 2010						
**Cause**	**All ages**	**0-5 years**	**5-14 years**	**15-49 years**	**50-69 years**	**70+ years**
Indirect maternal deaths	0<br>(0–0)	0<br>(0–0)	0<br>(0–0)	0<br>(0–0)	0<br>(0–0)	0<br>(0–0)
Late maternal deaths	0<br>(0–0)	0<br>(0–0)	0<br>(0–0)	0<br>(0–0)	0<br>(0–0)	0<br>(0–0)
Maternal deaths aggravated by HIV/AIDS	0<br>(0–0)	0<br>(0–0)	0<br>(0–0)	0<br>(0–0)	0<br>(0–0)	0<br>(0–0)
Other maternal disorders	0<br>(0–0)	0<br>(0–0)	0<br>(0–0)	0<br>(0–0)	0<br>(0–0)	0<br>(0–0)
Neonatal disorders	1,353,949<br>(1,311,935–1,404,789)	1,353,949<br>(1,311,935–1,404,789)	0<br>(0–0)	0<br>(0–0)	0<br>(0–0)	0<br>(0–0)
Neonatal preterm birth complications	517,312<br>(465,890–600,946)	517,312<br>(465,890–600,946)	0<br>(0–0)	0<br>(0–0)	0<br>(0–0)	0<br>(0–0)
Neonatal encephalopathy due to birth asphyxia and trauma	462,178<br>(403,347–523,965)	462,178<br>(403,347–523,965)	0<br>(0–0)	0<br>(0–0)	0<br>(0–0)	0<br>(0–0)
Neonatal sepsis and other neonatal infections	225,452<br>(143,952–325,185)	225,452<br>(143,952–325,185)	0<br>(0–0)	0<br>(0–0)	0<br>(0–0)	0<br>(0–0)
Hemolytic disease and other neonatal jaundice	28,514<br>(19,336–46,051)	28,514<br>(19,336–46,051)	0<br>(0–0)	0<br>(0–0)	0<br>(0–0)	0<br>(0–0)
Other neonatal disorders	120,490<br>(89,151–154,434)	120,490<br>(89,151–154,434)	0<br>(0–0)	0<br>(0–0)	0<br>(0–0)	0<br>(0–0)
Nutritional deficiencies	228,562<br>(151,695–299,711)	129,383<br>(80,341–184,822)	8,239<br>(6,155–12,309)	22,042<br>(15,721–31,825)	23,443<br>(16,742–32,959)	45,453<br>(30,232–56,586)
Protein-energy malnutrition	189,357<br>(126,355–252,194)	119,202<br>(73,515–174,471)	6,528<br>(4,752–9,621)	12,052<br>(8,607–16,790)	15,397<br>(11,453–22,208)	36,175<br>(24,169–45,313)
Iodine deficiency	802<br>(506–1,394)	249<br>(76–709)	30<br>(13–66)	176<br>(101–312)	179<br>(105–343)	166<br>(96–292)
Vitamin A deficiency	0<br>(0–0)	0<br>(0–0)	0<br>(0–0)	0<br>(0–0)	0<br>(0–0)	0<br>(0–0)
Iron-deficiency anemia	25,166<br>(11,034–37,819)	7,914<br>(2,299–14,139)	1,086<br>(513–2,078)	6,397<br>(3,692–10,777)	5,396<br>(2,675–8,524)	4,371<br>(1,661–6,227)
Other nutritional deficiencies	13,236<br>(9,755–17,307)	2,017<br>(1,039–3,226)	594<br>(417–925)	3,415<br>(2,377–4,746)	2,469<br>(1,852–3,350)	4,739<br>(3,550–6,044)
Other communicable, maternal, neonatal, and nutritional diseases	173,802<br>(144,087–208,710)	73,336<br>(50,047–104,237)	8,989<br>(7,159–11,374)	39,516<br>(35,441–41,838)	31,644<br>(26,726–33,930)	20,315<br>(16,463–22,039)
Sexually transmitted diseases excluding HIV	57,363<br>(33,590–89,411)	49,870<br>(28,019–79,406)	3,983<br>(2,264–6,312)	1,572<br>(1,031–1,961)	1,219<br>(781–1,602)	717<br>(502–973)
Syphilis	56,921<br>(33,155–89,012)	49,870<br>(28,019–79,406)	3,971<br>(2,252–6,300)	1,341<br>(880–1,673)	1,106<br>(708–1,453)	631<br>(441–856)
Chlamydial infection	0<br>(0–0)	0<br>(0–0)	0<br>(0–0)	0<br>(0–0)	0<br>(0–0)	0<br>(0–0)
Gonococcal infection	306<br>(208–374)	0<br>(0–0)	6<br>(4–9)	163<br>(106–207)	69<br>(45–90)	66<br>(46–89)
Trichomoniasis	0<br>(0–0)	0<br>(0–0)	0<br>(0–0)	0<br>(0–0)	0<br>(0–0)	0<br>(0–0)
Genital herpes	0<br>(0–0)	0<br>(0–0)	0<br>(0–0)	0<br>(0–0)	0<br>(0–0)	0<br>(0–0)
Other sexually transmitted diseases	136<br>(92–166)	0<br>(0–0)	5<br>(3–7)	67<br>(43–84)	43<br>(27–57)	20<br>(14–27)
Hepatitis	64,932<br>(61,817–67,723)	6,346<br>(5,491–7,239)	3,147<br>(2,762–3,536)	27,824<br>(25,987–29,432)	18,106<br>(17,132–19,029)	9,506<br>(9,046–10,046)
Acute hepatitis A	6,690<br>(4,254–9,729)	4,890<br>(3,662–6,056)	525<br>(134–1,108)	812<br>(178–1,939)	345<br>(78–903)	116<br>(23–306)
Hepatitis B	40,787<br>(34,950–45,905)	396<br>(132–952)	421<br>(225–700)	18,269<br>(15,049–21,141)	14,819<br>(12,641–16,508)	6,880<br>(5,518–8,129)
Hepatitis C	1,429<br>(304–3,306)	0<br>(0–0)	51<br>(9–133)	476<br>(99–1,123)	642<br>(134–1,565)	259<br>(51–631)
Acute hepatitis E	16,024<br>(10,949–22,226)	1,059<br>(413–2,020)	2,148<br>(1,572–2,701)	8,266<br>(5,627–11,358)	2,298<br>(1,104–4,033)	2,250<br>(1,178–3,618)
Other infectious diseases	51,506<br>(31,647–61,557)	17,119<br>(8,540–25,259)	1,859<br>(1,322–2,316)	10,119<br>(7,031–11,316)	12,317<br>(7,825–14,158)	10,090<br>(6,341–11,651)
Non-communicable diseases	19,558,041<br>(19,224,429–19,913,976)	443,548<br>(393,849–502,576)	110,835<br>(104,017–117,504)	2,512,278<br>(2,430,070–2,600,050)	6,712,081<br>(6,575,200–6,857,209)	9,779,296<br>(9,654,281–9,910,300)
Neoplasms	4,590,741<br>(4,489,704–4,700,578)	21,634<br>(17,979–28,393)	29,423<br>(27,707–31,290)	556,529<br>(535,469–577,534)	1,947,069<br>(1,901,825–1,995,823)	2,036,085<br>(1,985,883–2,095,900)
Lip and oral cavity cancer	85,241<br>(82,956–87,990)	0<br>(0–0)	0<br>(0–0)	14,795<br>(14,088–15,657)	43,129<br>(41,866–44,507)	27,316<br>(26,528–28,154)
Nasopharynx cancer	42,010<br>(30,950–44,464)	0<br>(0–0)	218<br>(184–251)	11,563<br>(8,292–12,461)	20,499<br>(14,945–21,831)	9,729<br>(7,578–10,326)
Other pharynx cancer	41,554<br>(40,377–42,798)	0<br>(0–0)	0<br>(0–0)	5,739<br>(5,481–6,083)	24,560<br>(23,835–25,324)	11,254<br>(10,884–11,621)
Esophageal cancer	301,994<br>(290,379–313,456)	0<br>(0–0)	0<br>(0–0)	27,829<br>(26,349–29,461)	154,269<br>(148,186–160,831)	119,895<br>(115,282–124,849)
Stomach cancer	514,495<br>(501,001–529,500)	0<br>(0–0)	0<br>(0–0)	48,758<br>(46,952–50,940)	224,555<br>(217,491–232,656)	241,181<br>(235,624–247,087)
Colon and rectum cancer	399,299<br>(391,122–408,578)	0<br>(0–0)	0<br>(0–0)	39,980<br>(38,491–41,881)	154,419<br>(150,972–158,098)	204,898<br>(200,298–209,654)
Liver cancer	531,222<br>(479,530–558,263)	0<br>(0–0)	739<br>(667–849)	109,626<br>(97,216–117,191)	259,955<br>(235,566–274,108)	160,900<br>(145,228–168,131)
Liver cancer due to hepatitis B	198,119<br>(176,862–213,679)	0<br>(0–0)	471<br>(415–558)	65,078<br>(57,702–70,560)	94,194<br>(83,495–103,648)	38,375<br>(33,171–42,522)
Liver cancer due to hepatitis C	83,517<br>(75,748–89,624)	0<br>(0–0)	16<br>(13–20)	7,089<br>(5,910–8,053)	36,406<br>(32,053–40,141)	40,004<br>(37,158–43,009)
Liver cancer due to alcohol use	177,004<br>(158,668–191,927)	0<br>(0–0)	0<br>(0–0)	20,364<br>(17,753–22,684)	96,406<br>(87,062–106,058)	60,233<br>(53,709–64,999)
Liver cancer due to other causes	72,581<br>(63,757–79,496)	0<br>(0–0)	251<br>(219–286)	17,093<br>(14,866–19,163)	32,948<br>(28,969–37,244)	22,287<br>(19,405–24,516)
Gallbladder and biliary tract cancer	55,063<br>(52,931–56,736)	0<br>(0–0)	0<br>(0–0)	3,962<br>(3,686–4,202)	20,246<br>(19,378–20,982)	30,854<br>(29,683–31,779)
Pancreatic cancer	187,367<br>(184,000–191,229)	0<br>(0–0)	0<br>(0–0)	16,570<br>(16,066–17,229)	85,206<br>(83,488–87,043)	85,590<br>(83,680–87,491)
Larynx cancer	78,764<br>(76,480–81,599)	0<br>(0–0)	0<br>(0–0)	7,773<br>(7,416–8,202)	41,378<br>(40,099–42,900)	29,613<br>(28,673–30,683)
Tracheal, bronchus, and lung cancer	1,090,444<br>(1,064,974–1,118,556)	0<br>(0–0)	0<br>(0–0)	78,593<br>(75,298–82,576)	511,106<br>(497,671–525,672)	500,744<br>(489,531–512,547)
Malignant skin melanoma	28,407<br>(18,695–39,356)	0<br>(0–0)	0<br>(0–0)	5,740<br>(4,277–8,183)	11,745<br>(7,892–16,890)	10,921<br>(6,461–14,318)
Non-melanoma skin cancer	29,339<br>(28,504–30,254)	0<br>(0–0)	0<br>(0–0)	2,691<br>(2,575–2,822)	10,072<br>(9,740–10,423)	16,574<br>(16,046–17,136)
Non-melanoma skin cancer (squamous-cell carcinoma)	29,339<br>(28,504–30,254)	0<br>(0–0)	0<br>(0–0)	2,691<br>(2,575–2,822)	10,072<br>(9,740–10,423)	16,574<br>(16,046–17,136)
Non-melanoma skin cancer (basal-cell carcinoma)	0<br>(0–0)	0<br>(0–0)	0<br>(0–0)	0<br>(0–0)	0<br>(0–0)	0<br>(0–0)
Breast cancer	8,335<br>(7,686–8,641)	0<br>(0–0)	0<br>(0–0)	1,039<br>(976–1,103)	4,063<br>(3,806–4,247)	3,231<br>(2,883–3,379)
Cervical cancer	0<br>(0–0)	0<br>(0–0)	0<br>(0–0)	0<br>(0–0)	0<br>(0–0)	0<br>(0–0)
Uterine cancer	0<br>(0–0)	0<br>(0–0)	0<br>(0–0)	0<br>(0–0)	0<br>(0–0)	0<br>(0–0)
Ovarian cancer	0<br>(0–0)	0<br>(0–0)	0<br>(0–0)	0<br>(0–0)	0<br>(0–0)	0<br>(0–0)
Prostate cancer	312,911<br>(260,321–393,480)	0<br>(0–0)	0<br>(0–0)	2,743<br>(2,159–3,337)	69,502<br>(56,420–86,806)	240,665<br>(199,132–308,043)
Testicular cancer	8,889<br>(8,505–9,370)	0<br>(0–0)	0<br>(0–0)	5,974<br>(5,676–6,395)	1,592<br>(1,519–1,669)	1,322<br>(1,261–1,387)

SR Table 11 - Global deaths by age and cause for males in 2010						
Cause	All ages	0-5 years	5-14 years	15-49 years	50-69 years	70+ years
Kidney cancer	77,417 (75,384–79,763)	1,742 (898–2,980)	606 (544–687)	7,840 (7,549–8,172)	33,566 (32,713–34,521)	33,661 (32,740–34,573)
Bladder cancer	120,396 (117,552–123,204)	0 (0–0)	0 (0–0)	5,010 (4,796–5,275)	37,568 (36,627–38,493)	77,818 (75,851–79,886)
Brain and nervous system cancer	114,804 (98,026–126,163)	2,976 (2,394–4,423)	5,446 (4,416–6,205)	32,576 (27,486–36,589)	47,447 (41,218–52,692)	26,356 (21,238–28,077)
Thyroid cancer	11,639 (9,735–12,193)	0 (0–0)	40 (34–45)	1,450 (1,175–1,542)	4,782 (4,040–5,054)	5,364 (4,448–5,635)
Mesothelioma	19,451 (18,883–20,021)	0 (0–0)	0 (0–0)	2,163 (2,070–2,281)	7,813 (7,565–8,060)	9,474 (9,126–9,829)
Hodgkin lymphoma	14,776 (12,933–19,064)	187 (142–253)	453 (348–594)	5,108 (4,435–6,597)	5,591 (4,905–7,233)	3,435 (3,039–4,302)
Non-Hodgkin lymphoma	116,537 (96,693–123,088)	1,935 (1,325–3,122)	3,035 (2,588–3,400)	22,946 (20,000–25,274)	42,961 (36,150–45,819)	45,657 (35,454–49,065)
Multiple myeloma	44,301 (43,265–45,338)	0 (0–0)	0 (0–0)	4,570 (4,390–4,801)	18,059 (17,565–18,531)	21,671 (21,031–22,292)
Leukemia	186,444 (181,521–192,720)	8,077 (7,086–9,352)	13,241 (12,388–14,163)	49,073 (47,234–51,659)	53,015 (51,506–54,559)	63,036 (61,215–64,727)
Acute lymphoid leukemia	60,599 (53,289–67,720)	5,063 (4,189–6,341)	9,448 (8,452–10,701)	21,397 (18,530–24,260)	14,891 (12,600–16,672)	9,798 (8,363–10,879)
Chronic lymphoid leukemia	30,568 (29,000–33,959)	0 (0–0)	0 (0–0)	3,257 (2,937–4,060)	8,939 (8,380–9,880)	18,371 (17,418–20,559)
Acute myeloid leukemia	75,960 (67,759–84,716)	3,013 (2,097–3,962)	3,792 (2,871–4,868)	19,010 (16,030–22,295)	23,409 (21,019–26,101)	26,734 (23,469–28,688)
Chronic myeloid leukemia	19,314 (17,794–21,879)	0 (0–0)	0 (0–0)	5,408 (4,795–6,344)	5,775 (5,244–6,566)	8,131 (7,578–9,091)
Other neoplasms	169,630 (142,448–180,656)	6,715 (4,770–10,641)	5,640 (4,819–6,301)	42,405 (34,341–46,401)	59,958 (49,798–63,971)	54,909 (46,338–58,020)
Cardiovascular diseases	8,607,169 (8,445,650–8,786,275)	29,979 (28,302–31,940)	15,639 (14,632–16,680)	947,830 (918,458–988,309)	2,923,538 (2,855,176–2,993,049)	4,690,180 (4,609,857–4,769,857)
Rheumatic heart disease	146,148 (137,742–156,564)	1,760 (1,490–2,094)	3,588 (3,218–4,015)	41,380 (38,344–45,443)	47,899 (44,656–51,835)	51,519 (48,684–54,572)
Ischemic heart disease	4,414,882 (4,321,288–4,516,181)	1,779 (1,564–2,065)	1,417 (1,263–1,588)	480,595 (463,849–505,104)	1,572,280 (1,532,614–1,615,798)	2,358,809 (2,311,272–2,407,105)
Cerebrovascular disease	3,016,721 (2,936,879–3,099,678)	10,851 (9,953–12,010)	5,982 (5,535–6,438)	283,436 (271,506–296,211)	997,594 (963,227–1,031,808)	1,718,856 (1,676,620–1,764,066)
Ischemic stroke	1,321,145 (1,273,686–1,368,182)	384 (317–479)	339 (297–392)	45,968 (42,149–50,631)	345,773 (327,192–363,892)	928,679 (898,753–958,502)
Hemorrhagic stroke	1,695,576 (1,630,257–1,755,295)	10,467 (9,608–11,587)	5,642 (5,220–6,070)	237,467 (227,045–248,236)	651,821 (623,070–679,398)	790,177 (761,258–821,669)
Hypertensive heart disease	393,714 (374,018–422,234)	271 (218–337)	232 (190–286)	42,459 (39,016–46,308)	127,356 (119,736–137,887)	223,393 (211,810–238,762)
Cardiomyopathy and myocarditis	187,219 (173,707–195,937)	6,102 (4,243–6,931)	1,826 (1,541–2,010)	51,357 (46,520–54,494)	62,619 (59,544–68,617)	65,314 (60,390–68,424)
Atrial fibrillation and flutter	71,941 (58,293–87,495)	0 (0–0)	0 (0–0)	596 (456–761)	6,146 (4,854–7,974)	65,199 (51,604–80,515)
Aortic aneurysm	93,606 (91,044–96,366)	0 (0–0)	0 (0–0)	9,256 (8,788–9,845)	29,733 (28,722–30,826)	54,617 (53,186–56,083)
Peripheral vascular disease	21,823 (20,899–22,707)	0 (0–0)	0 (0–0)	366 (343–392)	5,386 (5,127–5,648)	16,070 (15,295–16,858)
Endocarditis	34,493 (28,455–39,287)	1,966 (1,165–2,686)	557 (401–691)	8,559 (6,500–9,843)	9,207 (7,688–11,000)	14,202 (12,246–16,158)
Other cardiovascular and circulatory diseases	226,617 (218,821–235,670)	7,248 (6,480–8,591)	2,035 (1,881–2,230)	29,823 (28,146–31,801)	65,313 (62,383–68,432)	122,197 (117,740–126,907)
Chronic respiratory diseases	2,026,939 (1,965,917–2,092,754)	23,132 (19,463–27,642)	6,209 (5,609–6,911)	126,128 (119,325–134,147)	579,790 (555,811–605,294)	1,291,678 (1,253,120–1,332,431)
Chronic obstructive pulmonary disease	1,713,075 (1,654,522–1,771,939)	8,080 (5,504–11,786)	1,034 (875–1,236)	77,962 (72,333–83,403)	476,449 (453,786–498,784)	1,149,549 (1,110,595–1,189,563)
Pneumoconiosis	22,267 (20,288–23,995)	0 (0–0)	0 (0–0)	2,217 (1,949–2,501)	6,343 (5,625–7,038)	13,707 (12,576–14,742)
Silicosis	9,633 (8,816–10,722)	0 (0–0)	0 (0–0)	1,124 (986–1,305)	3,022 (2,683–3,453)	5,485 (5,049–6,017)
Asbestosis	2,705 (1,782–3,177)	0 (0–0)	0 (0–0)	250 (209–293)	608 (461–795)	1,847 (1,067–2,129)
Coal workers pneumoconiosis	2,292 (2,041–2,593)	0 (0–0)	0 (0–0)	180 (143–236)	632 (535–762)	1,479 (1,334–1,664)
Other pneumoconiosis	7,636 (6,390–8,693)	0 (0–0)	0 (0–0)	662 (557–764)	2,079 (1,658–2,525)	4,894 (4,111–5,570)
Asthma	202,037 (174,716–226,271)	6,146 (4,799–7,889)	4,166 (3,609–4,878)	35,967 (30,701–42,218)	72,424 (61,301–82,545)	83,331 (71,486–93,918)
Interstitial lung disease and pulmonary sarcoidosis	55,925 (39,143–67,018)	146 (88–279)	119 (86–197)	3,829 (2,889–5,095)	16,529 (12,322–20,816)	35,301 (23,308–41,301)
Other chronic respiratory diseases	33,631 (21,867–40,619)	8,759 (5,997–12,431)	888 (527–1,251)	6,150 (3,689–7,583)	8,044 (4,809–9,577)	9,788 (6,170–11,632)
Cirrhosis and other chronic liver diseases	810,608 (770,273–845,775)	5,876 (4,936–7,201)	5,367 (4,740–6,222)	258,871 (244,047–275,511)	373,855 (353,067–389,526)	166,637 (159,449–173,071)
Cirrhosis and other chronic liver diseases due to hepatitis B	248,468 (228,975–269,733)	62 (46–82)	222 (175–276)	79,594 (71,490–89,098)	112,216 (99,878–126,673)	56,373 (51,160–61,897)
Cirrhosis and other chronic liver diseases due to hepatitis C	210,136 (193,323–226,349)	31 (23–41)	118 (91–151)	60,904 (54,341–68,168)	101,023 (89,690–111,623)	48,057 (43,549–52,611)
Cirrhosis and other chronic liver diseases due to alcohol use	233,595 (215,957–251,628)	0 (0–0)	0 (0–0)	83,881 (76,510–92,203)	114,514 (102,084–126,522)	35,199 (31,478–39,449)
Cirrhosis and other chronic liver diseases due to other causes	118,408 (109,326–127,690)	5,782 (4,859–7,079)	5,026 (4,428–5,817)	34,490 (30,622–38,538)	46,101 (40,353–52,151)	27,007 (24,269–29,643)
Digestive diseases	617,936 (591,248–687,366)	10,221 (7,860–13,991)	9,278 (8,351–11,151)	155,759 (144,247–170,962)	201,463 (191,650–228,564)	241,214 (232,765–269,614)
Peptic ulcer disease	158,760 (146,889–178,828)	821 (506–1,447)	1,253 (1,049–1,573)	37,386 (33,838–42,674)	58,358 (53,623–65,482)	60,939 (56,830–68,334)
Gastritis and duodenitis	27,724 (23,791–33,351)	616 (298–1,105)	229 (164–352)	6,502 (5,245–8,144)	9,653 (8,254–11,721)	10,722 (9,423–12,778)
Appendicitis	26,059 (22,004–29,376)	1,021 (677–1,484)	1,480 (1,219–1,773)	10,049 (8,292–11,357)	7,332 (6,228–8,443)	6,176 (5,355–7,071)
Paralytic ileus and intestinal obstruction	134,088 (119,846–171,918)	3,586 (2,498–5,297)	3,736 (3,144–5,016)	34,802 (29,828–44,521)	41,424 (36,640–54,651)	50,538 (44,823–63,611)
Inguinal, femoral, and abdominal hernia	36,894 (22,386–43,445)	613 (236–1,047)	506 (272–662)	8,278 (4,312–10,550)	11,528 (7,212–13,973)	15,968 (10,131–18,683)
Inflammatory bowel disease	20,754 (18,270–23,184)	724 (421–1,282)	308 (227–415)	4,640 (3,827–5,372)	6,066 (5,371–6,979)	9,014 (8,065–10,049)
Vascular intestinal disorders	41,189 (38,438–45,678)	382 (203–758)	100 (77–131)	3,370 (2,968–3,950)	12,744 (11,770–14,526)	24,592 (23,210–27,144)
Gallbladder and biliary diseases	43,511 (40,008–50,219)	352 (212–545)	410 (323–523)	7,051 (6,125–8,402)	12,152 (11,003–14,572)	23,543 (22,131–26,397)
Pancreatitis	76,007 (70,634–84,671)	116 (80–172)	254 (212–323)	33,862 (30,903–37,836)	25,554 (23,734–28,644)	16,220 (15,115–18,211)
Other digestive diseases	52,947 (42,150–69,093)	1,986 (1,252–3,106)	998 (754–1,432)	9,816 (7,320–13,466)	16,648 (12,359–21,715)	23,497 (19,993–29,441)
Neurological disorders	795,267 (698,138–894,877)	10,679 (8,035–13,197)	7,082 (6,436–7,737)	55,885 (53,394–59,113)	87,765 (76,157–99,658)	633,854 (538,521–729,077)
Alzheimer disease and other dementias	613,293 (514,405–711,035)	0 (0–0)	0 (0–0)	733 (454–1,077)	48,532 (36,710–60,160)	564,027 (468,501–660,810)
Parkinson disease	55,329 (53,312–57,892)	0 (0–0)	0 (0–0)	428 (401–480)	6,872 (6,607–7,503)	48,028 (46,207–50,112)
Epilepsy	74,437 (70,550–78,722)	7,993 (5,849–10,113)	5,491 (4,932–6,076)	40,715 (38,433–43,355)	13,252 (12,598–14,004)	6,984 (6,737–7,265)

SR Table 11 - Global deaths by age and cause for males in 2010

Cause	All ages	0-5 years	5-14 years	15-49 years	50-69 years	70+ years
Multiple sclerosis	7,064 (6,610–7,620)	0 (0–0)	0 (0–0)	2,198 (2,075–2,559)	3,449 (3,128–3,669)	1,416 (1,213–1,494)
Motor neuron disease	17,479 (16,875–18,343)	495 (409–602)	66 (62–72)	2,566 (2,405–2,791)	7,981 (7,690–8,374)	6,369 (6,146–6,628)
Migraine	0 (0–0)	0 (0–0)	0 (0–0)	0 (0–0)	0 (0–0)	0 (0–0)
Tension-type headache	0 (0–0)	0 (0–0)	0 (0–0)	0 (0–0)	0 (0–0)	0 (0–0)
Medication overuse headache	0 (0–0)	0 (0–0)	0 (0–0)	0 (0–0)	0 (0–0)	0 (0–0)
Other neurological disorders	27,672 (26,792–28,695)	2,200 (1,686–2,704)	1,523 (1,329–1,722)	9,242 (8,747–9,896)	7,677 (7,307–8,155)	7,028 (6,679–7,434)
Mental and substance use disorders	231,468 (221,584–239,970)	12 (9–15)	12 (6–17)	131,378 (126,253–138,207)	77,255 (72,763–79,959)	22,809 (19,859–23,670)
Schizophrenia	8,543 (8,124–9,026)	0 (0–0)	0 (0–0)	3,553 (3,336–3,820)	3,191 (3,029–3,370)	1,798 (1,698–1,902)
Alcohol use disorders	117,097 (112,820–121,732)	0 (0–0)	0 (0–0)	55,931 (53,445–58,738)	50,010 (47,183–52,185)	11,155 (10,150–11,633)
Drug use disorders	105,659 (99,076–110,954)	12 (9–15)	0 (0–0)	71,737 (67,471–76,925)	24,054 (21,647–25,454)	9,855 (7,691–10,519)
Opioid use disorders	79,548 (74,331–84,201)	12 (9–15)	0 (0–0)	53,974 (50,557–59,176)	17,589 (15,981–18,674)	7,971 (6,095–8,542)
Cocaine use disorders	6,319 (3,887–6,881)	0 (0–0)	0 (0–0)	4,487 (2,869–4,929)	1,649 (888–1,836)	182 (140–218)
Amphetamine use disorders	7,846 (4,564–8,720)	0 (0–0)	0 (0–0)	5,544 (3,203–6,253)	1,921 (1,113–2,269)	380 (223–444)
Cannabis use disorders	0 (0–0)	0 (0–0)	0 (0–0)	0 (0–0)	0 (0–0)	0 (0–0)
Other drug use disorders	11,944 (11,095–13,482)	0 (0–0)	0 (0–0)	7,731 (7,166–8,958)	2,892 (2,662–3,293)	1,320 (1,134–1,476)
Depressive disorders	0 (0–0)	0 (0–0)	0 (0–0)	0 (0–0)	0 (0–0)	0 (0–0)
Major depressive disorder	0 (0–0)	0 (0–0)	0 (0–0)	0 (0–0)	0 (0–0)	0 (0–0)
Dysthymia	0 (0–0)	0 (0–0)	0 (0–0)	0 (0–0)	0 (0–0)	0 (0–0)
Bipolar disorder	0 (0–0)	0 (0–0)	0 (0–0)	0 (0–0)	0 (0–0)	0 (0–0)
Anxiety disorders	0 (0–0)	0 (0–0)	0 (0–0)	0 (0–0)	0 (0–0)	0 (0–0)
Eating disorders	168 (96–234)	0 (0–0)	12 (6–17)	155 (89–217)	0 (0–0)	0 (0–0)
Anorexia nervosa	148 (83–205)	0 (0–0)	11 (5–16)	136 (77–190)	0 (0–0)	0 (0–0)
Bulimia nervosa	19 (10–30)	0 (0–0)	0 (0–1)	18 (10–29)	0 (0–0)	0 (0–0)
Autistic spectrum disorders	0 (0–0)	0 (0–0)	0 (0–0)	0 (0–0)	0 (0–0)	0 (0–0)
Autism	0 (0–0)	0 (0–0)	0 (0–0)	0 (0–0)	0 (0–0)	0 (0–0)
Asperger syndrome and other autistic spectrum disorders	0 (0–0)	0 (0–0)	0 (0–0)	0 (0–0)	0 (0–0)	0 (0–0)
Attention-deficit/hyperactivity disorder	0 (0–0)	0 (0–0)	0 (0–0)	0 (0–0)	0 (0–0)	0 (0–0)
Conduct disorder	0 (0–0)	0 (0–0)	0 (0–0)	0 (0–0)	0 (0–0)	0 (0–0)
Idiopathic developmental intellectual disability	0 (0–0)	0 (0–0)	0 (0–0)	0 (0–0)	0 (0–0)	0 (0–0)
Other mental and substance use disorders	0 (0–0)	0 (0–0)	0 (0–0)	0 (0–0)	0 (0–0)	0 (0–0)
Diabetes, urogenital, blood, and endocrine diseases	1,473,710 (1,347,031–1,548,597)	66,824 (43,219–110,134)	17,780 (14,807–20,482)	231,857 (208,631–247,159)	496,079 (459,303–515,971)	661,167 (618,849–683,654)
Diabetes mellitus	616,721 (598,270–637,209)	1,037 (845–1,148)	1,169 (930–1,298)	71,386 (67,826–75,018)	251,027 (241,839–260,702)	292,100 (283,410–301,585)
Acute glomerulonephritis	6,511 (4,631–7,525)	132 (96–166)	172 (126–197)	1,720 (1,100–1,941)	2,189 (1,598–2,574)	2,296 (1,531–2,709)
Chronic kidney disease	559,814 (459,627–586,757)	6,631 (5,179–7,574)	4,881 (3,771–5,395)	101,046 (77,344–108,615)	181,458 (145,792–191,889)	265,795 (226,808–276,780)
Chronic kidney disease due to diabetes mellitus	186,767 (159,552–198,373)	732 (572–908)	407 (316–511)	24,151 (19,197–27,390)	73,756 (62,167–80,600)	87,719 (77,503–93,084)
Chronic kidney disease due to hypertension	234,956 (190,893–249,397)	2,325 (1,840–2,729)	1,439 (1,113–1,723)	39,336 (29,595–43,578)	69,119 (54,430–76,125)	122,735 (103,112–130,167)
Chronic kidney disease due to glomerulonephritis	123,977 (97,633–133,647)	2,656 (2,002–3,162)	2,242 (1,696–2,605)	33,451 (25,703–37,170)	36,336 (27,431–40,398)	49,289 (39,672–54,320)
Chronic kidney disease due to other causes	14,113 (11,008–16,965)	916 (675–1,147)	792 (586–990)	4,106 (2,723–5,811)	2,246 (1,387–3,459)	6,050 (4,893–7,254)
Urinary diseases and male infertility	109,584 (93,691–122,581)	4,268 (3,115–5,592)	1,043 (884–1,314)	15,656 (12,708–18,603)	28,094 (23,227–31,815)	60,520 (53,135–67,355)
Interstitial nephritis and urinary tract infections	72,764 (62,410–82,247)	3,386 (2,436–4,577)	646 (527–812)	9,292 (7,398–11,181)	16,217 (13,331–18,986)	43,221 (37,543–48,623)
Urolithiasis	7,669 (6,531–11,738)	0 (0–0)	115 (90–185)	1,344 (1,111–2,261)	2,543 (2,130–4,043)	3,665 (3,030–5,329)
Benign prostatic hyperplasia	0 (0–0)	0 (0–0)	0 (0–0)	0 (0–0)	0 (0–0)	0 (0–0)
Male infertility	0 (0–0)	0 (0–0)	0 (0–0)	0 (0–0)	0 (0–0)	0 (0–0)
Other urinary diseases	29,150 (21,185–33,781)	882 (509–1,397)	281 (208–366)	5,020 (3,423–5,987)	9,333 (6,420–11,133)	13,633 (10,281–16,680)
Gynecological diseases	0 (0–0)	0 (0–0)	0 (0–0)	0 (0–0)	0 (0–0)	0 (0–0)
Uterine fibroids	0 (0–0)	0 (0–0)	0 (0–0)	0 (0–0)	0 (0–0)	0 (0–0)
Polycystic ovarian syndrome	0 (0–0)	0 (0–0)	0 (0–0)	0 (0–0)	0 (0–0)	0 (0–0)
Female infertility	0 (0–0)	0 (0–0)	0 (0–0)	0 (0–0)	0 (0–0)	0 (0–0)
Endometriosis	0 (0–0)	0 (0–0)	0 (0–0)	0 (0–0)	0 (0–0)	0 (0–0)
Genital prolapse	0 (0–0)	0 (0–0)	0 (0–0)	0 (0–0)	0 (0–0)	0 (0–0)
Premenstrual syndrome	0 (0–0)	0 (0–0)	0 (0–0)	0 (0–0)	0 (0–0)	0 (0–0)
Other gynecological diseases	0 (0–0)	0 (0–0)	0 (0–0)	0 (0–0)	0 (0–0)	0 (0–0)
Hemoglobinopathies and hemolytic anemias	119,004 (76,853–177,081)	47,612 (23,847–91,452)	8,528 (6,285–11,000)	28,379 (21,234–35,195)	15,268 (11,042–18,562)	19,215 (13,866–23,700)
Thalassemias	8,938 (6,099–11,272)	4,510 (3,010–6,257)	1,636 (1,233–1,900)	964 (650–1,393)	44 (38–49)	1,782 (1,054–2,597)
Thalassemias trait	0 (0–0)	0 (0–0)	0 (0–0)	0 (0–0)	0 (0–0)	0 (0–0)
Sickle cell disorders	61,514 (34,433–108,170)	42,157 (19,609–85,592)	5,726 (3,970–7,923)	10,964 (8,055–14,026)	489 (393–605)	2,176 (1,463–2,901)
Sickle cell trait	0 (0–0)	0 (0–0)	0 (0–0)	0 (0–0)	0 (0–0)	0 (0–0)

SR Table 11 - Global deaths by age and cause for males in 2010

Cause	All ages	0-5 years	5-14 years	15-49 years	50-69 years	70+ years
G6PD deficiency	21,093 (16,000–25,496)	780 (517–1,224)	966 (769–1,267)	11,670 (8,857–14,339)	6,242 (4,635–7,668)	1,433 (1,048–1,827)
G6PD trait	0 (0–0)	0 (0–0)	0 (0–0)	0 (0–0)	0 (0–0)	0 (0–0)
Other hemoglobinopathies and hemolytic anemias	27,458 (19,959–33,735)	163 (118–234)	198 (158–248)	4,780 (3,369–6,180)	8,491 (5,987–10,635)	13,823 (10,197–16,692)
Endocrine, metabolic, blood, and immune disorders	62,074 (60,117–64,329)	7,141 (6,567–7,799)	1,984 (1,821–2,171)	13,668 (13,021–14,485)	18,040 (17,413–18,781)	21,239 (20,563–21,993)
Musculoskeletal disorders	24,517 (21,922–25,678)	0 (0–0)	711 (628–818)	5,404 (5,038–6,026)	6,905 (6,401–7,383)	11,495 (9,535–12,082)
Rheumatoid arthritis	8,323 (6,864–9,287)	0 (0–0)	95 (75–118)	789 (642–936)	2,780 (2,345–3,037)	4,657 (3,718–5,271)
Osteoarthritis	0 (0–0)	0 (0–0)	0 (0–0)	0 (0–0)	0 (0–0)	0 (0–0)
Low back and neck pain	0 (0–0)	0 (0–0)	0 (0–0)	0 (0–0)	0 (0–0)	0 (0–0)
Low back pain	0 (0–0)	0 (0–0)	0 (0–0)	0 (0–0)	0 (0–0)	0 (0–0)
Neck pain	0 (0–0)	0 (0–0)	0 (0–0)	0 (0–0)	0 (0–0)	0 (0–0)
Gout	0 (0–0)	0 (0–0)	0 (0–0)	0 (0–0)	0 (0–0)	0 (0–0)
Other musculoskeletal disorders	16,194 (14,725–17,284)	0 (0–0)	616 (543–716)	4,615 (4,310–5,201)	4,124 (3,803–4,596)	6,837 (5,737–7,307)
Other non-communicable diseases	379,671 (325,867–437,211)	275,177 (231,747–331,732)	19,329 (15,951–21,663)	42,632 (36,210–49,536)	18,357 (13,845–23,461)	24,173 (17,074–32,760)
Congenital anomalies	332,262 (278,774–387,460)	262,131 (219,125–316,658)	18,810 (15,419–21,079)	36,251 (30,885–41,967)	8,860 (7,540–9,968)	6,209 (5,504–7,120)
Neural tube defects	34,750 (24,890–50,382)	32,864 (23,220–48,671)	853 (564–1,224)	782 (585–1,095)	169 (130–234)	79 (57–124)
Congenital heart anomalies	167,120 (140,722–196,307)	124,130 (103,076–151,058)	13,092 (10,546–14,847)	25,469 (21,775–29,370)	2,622 (2,239–3,020)	1,805 (1,578–2,228)
Cleft lip and cleft palate	935 (770–1,189)	935 (770–1,189)	0 (0–0)	0 (0–0)	0 (0–0)	0 (0–0)
Down syndrome	10,528 (7,330–16,419)	6,058 (3,317–10,876)	433 (299–654)	2,276 (1,902–3,155)	1,680 (1,361–2,040)	79 (66–100)
Turner syndrome	0 (0–0)	0 (0–0)	0 (0–0)	0 (0–0)	0 (0–0)	0 (0–0)
Klinefelter syndrome	0 (0–0)	0 (0–0)	0 (0–0)	0 (0–0)	0 (0–0)	0 (0–0)
Other chromosomal abnormalities	7,232 (4,185–13,246)	6,383 (3,366–12,327)	146 (106–214)	486 (309–669)	159 (104–223)	56 (40–76)
Other congenital anomalies	111,694 (90,829–137,988)	91,759 (72,793–117,652)	4,283 (3,428–5,185)	7,235 (5,855–8,367)	4,227 (3,370–4,782)	4,188 (3,549–4,681)
Skin and subcutaneous diseases	36,686 (21,977–57,173)	2,324 (1,396–4,647)	519 (310–951)	6,381 (3,497–10,342)	9,497 (5,508–14,660)	17,963 (11,011–26,292)
Dermatitis	0 (0–0)	0 (0–0)	0 (0–0)	0 (0–0)	0 (0–0)	0 (0–0)
Psoriasis	0 (0–0)	0 (0–0)	0 (0–0)	0 (0–0)	0 (0–0)	0 (0–0)
Cellulitis	7,340 (3,994–12,322)	316 (142–673)	102 (55–184)	1,607 (730–2,797)	2,465 (1,236–4,222)	2,848 (1,698–4,844)
Pyoderma	16,495 (9,977–28,193)	1,838 (1,095–3,841)	270 (168–541)	3,472 (1,859–5,896)	4,471 (2,588–7,482)	6,441 (4,009–10,440)
Scabies	0 (0–0)	0 (0–0)	0 (0–0)	0 (0–0)	0 (0–0)	0 (0–0)
Fungal skin diseases	0 (0–0)	0 (0–0)	0 (0–0)	0 (0–0)	0 (0–0)	0 (0–0)
Viral skin diseases	0 (0–0)	0 (0–0)	0 (0–0)	0 (0–0)	0 (0–0)	0 (0–0)
Acne vulgaris	0 (0–0)	0 (0–0)	0 (0–0)	0 (0–0)	0 (0–0)	0 (0–0)
Alopecia areata	0 (0–0)	0 (0–0)	0 (0–0)	0 (0–0)	0 (0–0)	0 (0–0)
Pruritus	0 (0–0)	0 (0–0)	0 (0–0)	0 (0–0)	0 (0–0)	0 (0–0)
Urticaria	0 (0–0)	0 (0–0)	0 (0–0)	0 (0–0)	0 (0–0)	0 (0–0)
Decubitus ulcer	11,308 (5,198–16,077)	51 (25–91)	75 (39–131)	933 (418–1,455)	2,230 (913–3,265)	8,016 (3,825–11,167)
Other skin and subcutaneous diseases	1,542 (942–2,428)	117 (64–211)	70 (40–126)	368 (207–655)	330 (186–514)	656 (412–1,020)
Sense organ diseases	0 (0–0)	0 (0–0)	0 (0–0)	0 (0–0)	0 (0–0)	0 (0–0)
Glaucoma	0 (0–0)	0 (0–0)	0 (0–0)	0 (0–0)	0 (0–0)	0 (0–0)
Cataract	0 (0–0)	0 (0–0)	0 (0–0)	0 (0–0)	0 (0–0)	0 (0–0)
Macular degeneration	0 (0–0)	0 (0–0)	0 (0–0)	0 (0–0)	0 (0–0)	0 (0–0)
Refraction and accommodation disorders	0 (0–0)	0 (0–0)	0 (0–0)	0 (0–0)	0 (0–0)	0 (0–0)
Age-related and other hearing loss	0 (0–0)	0 (0–0)	0 (0–0)	0 (0–0)	0 (0–0)	0 (0–0)
Other vision loss	0 (0–0)	0 (0–0)	0 (0–0)	0 (0–0)	0 (0–0)	0 (0–0)
Other sense organ diseases	0 (0–0)	0 (0–0)	0 (0–0)	0 (0–0)	0 (0–0)	0 (0–0)
Oral disorders	0 (0–0)	0 (0–0)	0 (0–0)	0 (0–0)	0 (0–0)	0 (0–0)
Deciduous caries	0 (0–0)	0 (0–0)	0 (0–0)	0 (0–0)	0 (0–0)	0 (0–0)
Permanent caries	0 (0–0)	0 (0–0)	0 (0–0)	0 (0–0)	0 (0–0)	0 (0–0)
Periodontal diseases	0 (0–0)	0 (0–0)	0 (0–0)	0 (0–0)	0 (0–0)	0 (0–0)
Edentulism and severe tooth loss	0 (0–0)	0 (0–0)	0 (0–0)	0 (0–0)	0 (0–0)	0 (0–0)
Other oral disorders	0 (0–0)	0 (0–0)	0 (0–0)	0 (0–0)	0 (0–0)	0 (0–0)
Sudden infant death syndrome	10,722 (8,006–19,488)	10,722 (8,006–19,488)	0 (0–0)	0 (0–0)	0 (0–0)	0 (0–0)
Injuries	3,344,499 (3,135,924–3,500,816)	199,939 (171,736–227,155)	181,263 (159,001–205,476)	1,877,636 (1,776,504–1,972,504)	680,360 (630,931–708,335)	405,299 (373,608–419,256)
Transport injuries	1,100,946 (1,055,690–1,147,502)	33,763 (28,715–39,349)	48,596 (45,333–51,627)	680,880 (651,092–713,268)	233,530 (223,664–242,684)	104,175 (99,829–108,259)
Road injuries	1,020,761 (973,240–1,065,920)	31,260 (26,475–36,623)	45,969 (42,881–48,856)	633,106 (601,836–664,009)	213,626 (202,521–222,501)	96,798 (92,665–100,805)
Pedestrian road injuries	410,915 (370,835–455,634)	14,824 (12,596–17,550)	22,147 (20,234–24,437)	224,876 (201,874–248,793)	98,601 (91,252–110,559)	50,465 (47,441–57,054)
Cyclist road injuries	44,079 (40,433–47,609)	801 (615–1,050)	2,599 (2,312–2,898)	21,874 (19,186–23,927)	13,360 (12,389–14,722)	5,444 (5,023–6,114)
Motorcyclist road injuries	208,479 (177,149–235,990)	3,728 (2,647–4,843)	5,826 (4,685–7,016)	150,824 (133,753–173,652)	35,979 (28,548–40,763)	12,120 (8,008–14,803)

SR Table 11 - Global deaths by age and cause for males in 2010

Cause	All ages	0-5 years	5-14 years	15-49 years	50-69 years	70+ years
Motor vehicle road injuries	341,396 (296,924–385,466)	11,029 (8,522–13,691)	14,180 (11,935–16,494)	226,312 (201,278–257,807)	62,743 (51,285–69,549)	27,130 (22,686–29,595)
Other road injuries	15,890 (9,199–19,285)	876 (340–1,846)	1,215 (613–1,675)	9,219 (4,964–11,502)	2,941 (1,987–3,692)	1,637 (1,231–2,288)
Other transport injuries	80,184 (69,947–97,327)	2,503 (1,893–3,182)	2,627 (2,296–2,985)	47,773 (41,535–58,072)	19,903 (16,974–24,980)	7,377 (6,395–9,131)
Unintentional injuries	1,174,602 (1,067,962–1,222,868)	146,976 (121,643–170,650)	92,614 (86,001–98,172)	489,408 (451,720–514,006)	234,845 (211,382–246,248)	210,756 (187,444–219,711)
Falls	290,661 (254,934–303,653)	12,572 (8,883–16,042)	10,435 (8,932–11,515)	90,141 (79,753–96,056)	74,355 (65,772–77,656)	103,156 (89,191–107,793)
Drowning	245,305 (235,527–258,257)	42,737 (36,680–50,498)	42,468 (39,936–45,204)	107,798 (103,438–114,095)	34,503 (33,165–36,069)	17,798 (17,146–18,463)
Fire, heat, and hot substances	88,001 (71,596–94,776)	12,357 (9,080–14,784)	4,846 (3,753–5,444)	38,193 (29,965–41,955)	17,786 (14,968–19,038)	14,818 (12,749–15,784)
Poisonings	54,144 (34,031–67,137)	11,548 (3,271–22,258)	2,857 (1,876–3,456)	22,947 (17,025–28,586)	11,238 (7,784–12,928)	5,552 (3,846–6,298)
Exposure to mechanical forces	147,668 (115,591–159,158)	18,872 (13,345–23,313)	8,434 (7,558–9,592)	77,229 (58,921–83,364)	27,395 (20,808–29,696)	15,735 (12,068–17,036)
Unintentional firearm injuries	23,224 (16,013–25,802)	1,698 (904–2,468)	1,317 (975–1,719)	14,476 (9,788–16,297)	3,175 (2,255–3,582)	2,555 (1,989–2,830)
Unintentional suffocation	23,717 (16,798–28,099)	11,546 (6,869–15,239)	2,128 (1,756–2,889)	4,945 (3,809–5,466)	1,584 (910–1,876)	3,512 (2,678–3,839)
Other exposure to mechanical forces	100,726 (73,776–109,240)	5,627 (4,136–7,095)	4,988 (3,972–5,826)	57,807 (41,489–63,145)	22,635 (16,691–24,705)	9,667 (6,910–10,760)
Adverse effects of medical treatment	54,795 (39,774–61,918)	9,804 (5,192–12,951)	2,696 (1,759–3,255)	17,722 (12,189–20,922)	11,543 (8,789–13,409)	13,027 (10,340–14,832)
Animal contact	56,834 (33,038–71,451)	5,670 (3,074–7,041)	7,031 (4,002–8,917)	24,901 (14,162–32,794)	12,921 (7,480–15,993)	6,309 (4,064–7,502)
Venomous animal contact	47,795 (26,160–62,509)	3,935 (1,973–5,470)	5,838 (3,158–7,850)	21,975 (11,798–29,584)	11,001 (6,000–13,859)	5,045 (3,024–6,112)
Non-venomous animal contact	9,038 (7,012–10,328)	1,734 (990–2,264)	1,193 (845–1,459)	2,926 (2,373–3,482)	1,919 (1,495–2,370)	1,264 (1,035–1,590)
Foreign body	89,429 (72,005–110,041)	25,032 (15,843–34,599)	5,577 (4,208–7,272)	25,164 (20,454–31,241)	16,040 (13,034–18,525)	17,614 (13,930–19,697)
Pulmonary aspiration and foreign body in airway	72,817 (58,497–89,470)	21,449 (13,313–31,163)	4,059 (2,986–5,415)	17,786 (14,845–21,053)	13,849 (11,119–15,550)	15,673 (11,942–16,906)
Foreign body in eyes	0 (0–0)	0 (0–0)	0 (0–0)	0 (0–0)	0 (0–0)	0 (0–0)
Foreign body in other body part	16,611 (10,256–24,314)	3,583 (1,449–5,499)	1,517 (840–2,285)	7,377 (4,573–11,848)	2,191 (1,491–3,565)	1,941 (1,498–3,140)
Environmental heat and cold exposure	33,221 (24,187–36,010)	1,754 (853–2,319)	776 (444–958)	13,029 (9,899–14,640)	11,832 (8,248–13,229)	5,829 (4,043–6,469)
Other unintentional injuries	114,540 (106,262–125,716)	6,626 (5,386–8,119)	7,490 (6,460–8,734)	72,280 (66,072–81,057)	17,227 (15,925–18,649)	10,914 (8,819–14,204)
Self-harm and interpersonal violence	881,448 (814,339–912,310)	6,610 (5,767–7,700)	12,674 (11,545–13,483)	592,566 (545,845–615,065)	187,985 (173,674–194,800)	81,611 (74,289–84,789)
Self-harm	552,837 (502,628–574,894)	0 (0–0)	5,055 (4,311–5,570)	333,331 (301,293–348,616)	144,429 (131,755–150,285)	70,020 (62,804–72,918)
Interpersonal violence	328,611 (298,658–341,786)	6,610 (5,767–7,700)	7,619 (6,940–8,202)	259,235 (233,835–270,369)	43,556 (40,444–46,226)	11,590 (10,898–12,298)
Assault by firearm	151,534 (131,195–157,810)	1,129 (871–1,322)	2,441 (1,970–2,626)	129,796 (112,169–134,956)	15,294 (13,652–16,613)	2,873 (2,645–3,209)
Assault by sharp object	72,908 (67,785–78,822)	646 (518–854)	1,072 (942–1,247)	58,441 (54,094–63,435)	10,212 (9,512–11,187)	2,534 (2,351–2,773)
Assault by other means	104,168 (91,129–112,605)	4,834 (4,196–5,668)	4,105 (3,664–4,581)	70,997 (60,221–78,045)	18,049 (16,095–19,702)	6,182 (5,525–6,675)
Forces of nature, war, and legal intervention	187,501 (62,642–321,455)	12,588 (3,940–22,093)	27,377 (6,687–50,013)	114,781 (42,936–191,761)	23,998 (7,660–41,622)	8,756 (2,367–15,637)
Exposure to forces of nature	155,912 (32,632–291,957)	10,919 (2,188–20,542)	25,835 (5,222–48,546)	90,022 (18,884–167,986)	21,021 (4,786–38,603)	8,112 (1,763–14,962)
Collective violence and legal intervention	31,589 (24,958–37,973)	1,669 (1,223–2,186)	1,541 (1,164–1,977)	24,758 (19,273–30,084)	2,977 (2,228–3,741)	643 (477–843)

SR Table 11 - Global deaths by age and cause for males in 2015

Cause	All ages	0-5 years	5-14 years	15-49 years	50-69 years	70+ years
All causes	30,852,193 (30,259,042–31,461,340)	3,123,815 (3,046,310–3,201,443)	481,648 (472,218–492,530)	5,603,998 (5,417,543–5,811,103)	9,172,781 (8,937,374–9,411,577)	12,469,949 (12,296,166–12,651,539)
Communicable, maternal, neonatal, and nutritional diseases	5,992,331 (5,738,840–6,281,332)	2,521,108 (2,443,143–2,604,189)	232,385 (219,183–248,416)	1,243,087 (1,155,800–1,342,670)	917,170 (830,191–1,006,316)	1,078,578 (983,380–1,148,660)
HIV/AIDS and tuberculosis	1,364,028 (1,185,065–1,617,735)	57,188 (53,106–65,376)	49,933 (47,666–54,355)	705,711 (633,197–787,182)	375,159 (305,954–474,072)	176,034 (133,687–244,062)
Tuberculosis	727,238 (545,161–974,906)	11,819 (8,569–19,488)	6,592 (5,233–10,787)	266,512 (196,672–347,428)	276,200 (205,707–374,703)	166,112 (123,591–233,838)
HIV/AIDS	636,790 (596,440–689,943)	45,369 (43,048–47,794)	43,341 (41,506–45,037)	439,198 (404,636–483,282)	98,958 (90,609–113,253)	9,921 (8,961–11,363)
HIV/AIDS - Tuberculosis	117,243 (90,737–135,656)	9,346 (7,002–10,695)	8,351 (6,135–9,962)	78,235 (60,186–91,371)	19,286 (15,615–22,275)	2,023 (1,671–2,342)
HIV/AIDS resulting in other diseases	519,546 (476,540–572,906)	36,022 (33,468–39,094)	34,990 (32,474–37,615)	360,963 (324,471–402,788)	79,672 (71,859–92,551)	7,898 (7,003–9,152)
Diarrhea, lower respiratory, and other common infectious diseases	2,605,595 (2,424,164–2,756,454)	834,430 (773,398–898,771)	135,957 (121,585–153,097)	381,614 (342,780–425,898)	441,586 (386,799–481,500)	812,006 (724,247–865,010)
Diarrheal diseases	673,493 (615,645–733,863)	257,297 (223,385–295,300)	28,129 (24,698–31,613)	103,437 (90,877–118,197)	118,277 (102,901–135,256)	166,350 (146,966–190,060)
Intestinal infectious diseases	95,950 (54,011–158,942)	22,594 (12,175–39,289)	36,141 (21,034–58,140)	30,622 (15,849–52,940)	4,695 (2,379–8,222)	1,895 (1,062–3,045)
Typhoid fever	80,019 (43,794–133,128)	19,076 (10,027–33,707)	30,416 (17,056–49,404)	25,253 (12,875–44,054)	3,761 (1,794–6,626)	1,511 (807–2,503)
Paratyphoid fever	15,672 (7,287–30,340)	3,392 (1,421–6,941)	5,699 (2,598–11,132)	5,327 (2,238–10,615)	909 (366–1,823)	343 (147–654)
Other intestinal infectious diseases	258 (109–682)	126 (39–308)	26 (7–65)	41 (17–124)	24 (9–70)	40 (17–113)
Lower respiratory infections	1,439,286 (1,274,448–1,564,710)	367,189 (332,162–405,068)	34,607 (25,752–40,593)	161,738 (130,164–184,013)	267,126 (219,591–301,051)	608,623 (530,399–672,213)
Upper respiratory infections	1,693 (1,471–1,959)	371 (252–559)	25 (15–40)	408 (337–497)	455 (370–559)	431 (340–537)
Otitis media	1,581 (1,431–1,757)	546 (448–683)	216 (194–241)	419 (378–473)	229 (198–267)	169 (130–225)
Meningitis	210,369 (167,117–254,967)	92,788 (70,076–128,687)	19,259 (14,008–24,965)	51,134 (37,335–64,985)	29,026 (21,745–37,827)	18,160 (14,109–23,785)
Pneumococcal meningitis	62,815 (49,083–80,836)	24,592 (16,204–37,362)	7,096 (4,751–9,838)	17,649 (12,916–23,868)	8,330 (5,982–11,685)	5,146 (3,804–7,106)
H influenzae type B meningitis	38,717 (29,382–50,010)	23,246 (15,719–33,507)	3,640 (2,197–5,519)	5,868 (4,076–8,124)	3,509 (2,387–4,955)	2,452 (1,771–3,519)
Meningococcal meningitis	40,993 (31,056–52,828)	20,656 (13,644–31,506)	4,102 (2,559–6,161)	8,920 (6,209–12,518)	4,766 (3,289–6,633)	2,546 (1,860–3,418)
Other meningitis	67,843 (51,884–83,099)	24,293 (17,231–35,604)	4,419 (2,861–6,415)	18,695 (13,237–24,126)	12,419 (8,903–16,269)	8,015 (5,935–10,183)
Encephalitis	81,626 (75,175–88,714)	21,517 (18,633–24,504)	8,965 (7,539–10,535)	22,858 (20,821–25,122)	15,154 (13,710–16,780)	13,130 (11,919–14,735)
Diphtheria	1,122 (493–2,958)	779 (222–2,406)	180 (63–543)	121 (82–189)	41 (24–71)	0 (0–0)
Whooping cough	26,290 (9,040–56,951)	24,354 (8,432–52,487)	1,531 (520–3,368)	356 (122–850)	47 (16–120)	0 (0–0)
Tetanus	34,128 (26,610–51,960)	14,459 (11,741–18,003)	2,051 (1,328–3,685)	9,249 (5,588–17,252)	6,035 (4,087–11,767)	2,331 (1,519–4,800)
Measles	37,000 (13,206–81,974)	31,638 (11,264–69,389)	4,583 (1,584–10,817)	746 (239–1,967)	32 (10–85)	0 (0–0)
Varicella and herpes zoster	3,052 (2,384–4,094)	892 (547–1,585)	264 (152–496)	518 (380–734)	464 (331–654)	912 (601–1,410)
Neglected tropical diseases and malaria	429,473 (343,545–521,536)	242,272 (171,076–320,433)	30,833 (20,837–46,409)	95,635 (73,109–125,508)	42,263 (32,492–55,404)	18,468 (14,378–23,851)
Malaria	362,247 (275,634–456,544)	231,248 (159,059–309,066)	21,587 (11,959–37,113)	67,360 (45,400–96,325)	29,845 (20,017–42,630)	12,206 (8,243–17,707)
Chagas disease	4,573 (4,174–5,027)	1 (1–1)	2 (2–3)	593 (521–677)	2,064 (1,842–2,309)	1,911 (1,699–2,151)
Leishmaniasis	17,871 (12,587–24,368)	2,389 (1,644–3,326)	1,659 (1,078–2,423)	10,956 (7,513–15,187)	2,387 (1,716–3,216)	478 (376–594)
Visceral leishmaniasis	17,871 (12,587–24,368)	2,389 (1,644–3,326)	1,659 (1,078–2,423)	10,956 (7,513–15,187)	2,387 (1,716–3,216)	478 (376–594)
Cutaneous and mucocutaneous leishmaniasis	0 (0–0)	0 (0–0)	0 (0–0)	0 (0–0)	0 (0–0)	0 (0–0)
African trypanosomiasis	1,927 (952–3,338)	126 (61–219)	288 (153–464)	1,247 (543–2,247)	219 (96–402)	46 (22–78)
Schistosomiasis	2,462 (2,087–2,872)	19 (11–33)	49 (33–72)	826 (674–1,014)	1,059 (832–1,339)	507 (403–647)
Cysticercosis	208 (164–285)	3 (1–6)	6 (4–10)	130 (101–180)	53 (43–69)	14 (11–17)
Cystic echinococcosis	618 (573–667)	17 (14–21)	39 (34–44)	250 (227–277)	197 (182–212)	113 (107–121)
Lymphatic filariasis	0 (0–0)	0 (0–0)	0 (0–0)	0 (0–0)	0 (0–0)	0 (0–0)
Onchocerciasis	0 (0–0)	0 (0–0)	0 (0–0)	0 (0–0)	0 (0–0)	0 (0–0)
Trachoma	0 (0–0)	0 (0–0)	0 (0–0)	0 (0–0)	0 (0–0)	0 (0–0)
Dengue	9,825 (5,368–12,914)	2,564 (1,144–4,190)	2,069 (1,126–3,262)	3,359 (1,801–4,456)	1,188 (605–1,617)	643 (336–852)
Yellow fever	3,819 (776–10,543)	324 (62–922)	1,169 (243–3,410)	2,032 (421–5,853)	250 (51–722)	43 (8–129)
Rabies	10,478 (8,793–12,672)	1,280 (845–1,961)	2,478 (1,885–3,299)	3,835 (3,204–4,620)	2,085 (1,772–2,508)	799 (653–973)
Intestinal nematode infections	1,420 (1,232–1,625)	927 (768–1,105)	212 (169–264)	131 (100–167)	89 (65–116)	59 (40–81)
Ascariasis	1,420 (1,232–1,625)	927 (768–1,105)	212 (169–264)	131 (100–167)	89 (65–116)	59 (40–81)
Trichuriasis	0 (0–0)	0 (0–0)	0 (0–0)	0 (0–0)	0 (0–0)	0 (0–0)
Hookworm disease	0 (0–0)	0 (0–0)	0 (0–0)	0 (0–0)	0 (0–0)	0 (0–0)
Food-borne trematodiases	0 (0–0)	0 (0–0)	0 (0–0)	0 (0–0)	0 (0–0)	0 (0–0)
Leprosy	0 (0–0)	0 (0–0)	0 (0–0)	0 (0–0)	0 (0–0)	0 (0–0)
Ebola	2,775 (2,238–3,325)	211 (170–253)	289 (233–347)	1,733 (1,398–2,077)	439 (354–526)	100 (81–120)
Other neglected tropical diseases	11,244 (4,102–15,023)	3,158 (1,420–4,945)	980 (350–1,428)	3,179 (989–4,174)	2,382 (811–3,092)	1,544 (535–2,012)
Maternal disorders	0 (0–0)	0 (0–0)	0 (0–0)	0 (0–0)	0 (0–0)	0 (0–0)
Maternal hemorrhage	0 (0–0)	0 (0–0)	0 (0–0)	0 (0–0)	0 (0–0)	0 (0–0)
Maternal sepsis and other maternal infections	0 (0–0)	0 (0–0)	0 (0–0)	0 (0–0)	0 (0–0)	0 (0–0)
Maternal hypertensive disorders	0 (0–0)	0 (0–0)	0 (0–0)	0 (0–0)	0 (0–0)	0 (0–0)
Maternal obstructed labor and uterine rupture	0 (0–0)	0 (0–0)	0 (0–0)	0 (0–0)	0 (0–0)	0 (0–0)
Maternal abortion, miscarriage, and ectopic pregnancy	0 (0–0)	0 (0–0)	0 (0–0)	0 (0–0)	0 (0–0)	0 (0–0)

SR Table 11 - Global deaths by age and cause for males in 2015						
Cause	All ages	0-5 years	5-14 years	15-49 years	50-69 years	70+ years
Indirect maternal deaths	0 (0–0)	0 (0–0)	0 (0–0)	0 (0–0)	0 (0–0)	0 (0–0)
Late maternal deaths	0 (0–0)	0 (0–0)	0 (0–0)	0 (0–0)	0 (0–0)	0 (0–0)
Maternal deaths aggravated by HIV/AIDS	0 (0–0)	0 (0–0)	0 (0–0)	0 (0–0)	0 (0–0)	0 (0–0)
Other maternal disorders	0 (0–0)	0 (0–0)	0 (0–0)	0 (0–0)	0 (0–0)	0 (0–0)
Neonatal disorders	1,209,262 (1,165,896–1,257,482)	1,209,262 (1,165,896–1,257,482)	0 (0–0)	0 (0–0)	0 (0–0)	0 (0–0)
Neonatal preterm birth complications	442,800 (389,990–517,234)	442,800 (389,990–517,234)	0 (0–0)	0 (0–0)	0 (0–0)	0 (0–0)
Neonatal encephalopathy due to birth asphyxia and trauma	412,013 (357,099–469,179)	412,013 (357,099–469,179)	0 (0–0)	0 (0–0)	0 (0–0)	0 (0–0)
Neonatal sepsis and other neonatal infections	220,203 (144,176–317,424)	220,203 (144,176–317,424)	0 (0–0)	0 (0–0)	0 (0–0)	0 (0–0)
Hemolytic disease and other neonatal jaundice	22,580 (15,470–35,755)	22,580 (15,470–35,755)	0 (0–0)	0 (0–0)	0 (0–0)	0 (0–0)
Other neonatal disorders	111,664 (81,264–145,526)	111,664 (81,264–145,526)	0 (0–0)	0 (0–0)	0 (0–0)	0 (0–0)
Nutritional deficiencies	213,400 (136,977–290,959)	111,155 (68,109–160,954)	7,459 (5,546–11,407)	21,803 (15,927–33,254)	24,286 (17,390–35,167)	48,694 (32,837–60,095)
Protein-energy malnutrition	172,692 (112,087–240,387)	101,449 (60,635–149,360)	5,787 (4,183–9,032)	11,308 (8,049–16,670)	15,563 (11,204–22,802)	38,583 (26,169–48,303)
Iodine deficiency	808 (525–1,317)	214 (61–612)	27 (11–62)	185 (110–337)	196 (112–342)	184 (107–340)
Vitamin A deficiency	0 (0–0)	0 (0–0)	0 (0–0)	0 (0–0)	0 (0–0)	0 (0–0)
Iron-deficiency anemia	27,243 (12,402–41,854)	7,949 (2,315–14,244)	1,162 (585–2,229)	7,202 (4,163–12,309)	6,040 (3,031–9,394)	4,889 (1,918–7,021)
Other nutritional deficiencies	12,656 (9,560–16,932)	1,542 (779–2,562)	483 (325–783)	3,107 (2,193–4,364)	2,486 (1,906–3,570)	5,036 (3,835–6,375)
Other communicable, maternal, neonatal, and nutritional diseases	170,569 (139,923–202,749)	66,798 (44,294–95,271)	8,201 (6,488–10,366)	38,322 (33,969–41,252)	33,873 (28,325–36,855)	23,373 (17,790–26,086)
Sexually transmitted diseases excluding HIV	52,700 (30,667–82,520)	46,046 (25,742–73,778)	3,726 (2,138–5,846)	1,331 (822–1,702)	1,037 (639–1,392)	558 (391–773)
Syphilis	52,329 (30,341–82,133)	46,046 (25,742–73,778)	3,716 (2,129–5,835)	1,134 (700–1,448)	941 (579–1,263)	491 (344–680)
Chlamydial infection	0 (0–0)	0 (0–0)	0 (0–0)	0 (0–0)	0 (0–0)	0 (0–0)
Gonococcal infection	256 (170–317)	0 (0–0)	5 (4–8)	140 (87–181)	58 (36–78)	51 (35–70)
Trichomoniasis	0 (0–0)	0 (0–0)	0 (0–0)	0 (0–0)	0 (0–0)	0 (0–0)
Genital herpes	0 (0–0)	0 (0–0)	0 (0–0)	0 (0–0)	0 (0–0)	0 (0–0)
Other sexually transmitted diseases	114 (75–140)	0 (0–0)	4 (2–5)	56 (34–73)	37 (22–49)	15 (11–22)
Hepatitis	64,729 (60,689–68,471)	5,188 (4,467–5,927)	2,761 (2,397–3,148)	26,927 (24,754–29,063)	19,457 (18,275–20,701)	10,394 (9,770–11,068)
Acute hepatitis A	5,800 (3,474–8,513)	4,003 (2,835–5,005)	479 (131–984)	801 (185–1,915)	391 (87–962)	125 (25–332)
Hepatitis B	42,051 (35,829–47,362)	343 (115–816)	379 (197–635)	17,896 (14,605–20,910)	15,952 (13,546–17,902)	7,479 (5,958–8,772)
Hepatitis C	1,511 (324–3,583)	0 (0–0)	47 (9–128)	476 (100–1,130)	705 (140–1,741)	282 (61–662)
Acute hepatitis E	15,366 (9,972–21,652)	841 (321–1,654)	1,855 (1,325–2,352)	7,752 (4,856–11,039)	2,408 (1,171–4,317)	2,507 (1,329–3,983)
Other infectious diseases	53,139 (32,229–63,852)	15,563 (7,758–23,750)	1,713 (1,227–2,189)	10,062 (7,065–11,456)	13,379 (8,499–15,708)	12,420 (7,005–15,010)
Non-communicable diseases	21,534,072 (21,080,820–22,019,072)	426,275 (378,745–474,280)	105,886 (98,873–112,977)	2,536,272 (2,432,247–2,643,831)	7,522,573 (7,331,109–7,728,665)	10,943,065 (10,774,780–11,126,707)
Neoplasms	5,079,806 (4,939,703–5,231,378)	22,155 (18,411–28,265)	29,119 (26,973–31,058)	561,754 (534,344–589,711)	2,200,906 (2,134,167–2,274,206)	2,265,870 (2,203,957–2,338,705)
Lip and oral cavity cancer	97,771 (94,328–101,473)	0 (0–0)	0 (0–0)	15,846 (14,920–16,841)	49,997 (48,094–52,049)	31,928 (30,727–33,256)
Nasopharynx cancer	45,623 (33,703–49,157)	0 (0–0)	211 (179–241)	11,401 (8,311–12,693)	23,194 (16,995–25,193)	10,815 (8,394–11,665)
Other pharynx cancer	46,991 (45,016–49,185)	0 (0–0)	0 (0–0)	6,029 (5,683–6,447)	28,151 (26,878–29,584)	12,810 (12,256–13,415)
Esophageal cancer	317,757 (301,960–335,327)	0 (0–0)	0 (0–0)	27,204 (25,559–29,041)	166,179 (156,831–176,775)	124,372 (118,296–131,092)
Stomach cancer	535,107 (515,941–556,283)	0 (0–0)	0 (0–0)	44,975 (42,573–47,755)	239,126 (228,239–250,625)	251,005 (242,919–259,000)
Colon and rectum cancer	455,807 (442,417–468,248)	0 (0–0)	0 (0–0)	42,183 (40,290–44,439)	178,373 (172,452–184,046)	235,251 (227,616–242,035)
Liver cancer	576,680 (524,352–621,990)	0 (0–0)	689 (611–843)	107,127 (96,398–119,266)	294,149 (268,188–321,449)	174,714 (157,091–184,432)
Liver cancer due to hepatitis B	201,200 (180,855–223,209)	0 (0–0)	436 (377–551)	61,606 (54,991–68,927)	99,922 (88,495–112,717)	39,235 (34,286–43,935)
Liver cancer due to hepatitis C	95,293 (86,349–102,278)	0 (0–0)	16 (13–21)	7,463 (6,317–8,599)	42,313 (36,970–47,222)	45,500 (42,089–49,046)
Liver cancer due to alcohol use	204,333 (185,527–223,972)	0 (0–0)	0 (0–0)	21,951 (19,217–25,508)	115,914 (103,837–129,058)	66,467 (59,428–71,976)
Liver cancer due to other causes	75,852 (68,561–84,909)	0 (0–0)	236 (205–283)	16,105 (14,081–18,321)	35,998 (31,648–41,685)	23,510 (20,666–26,089)
Gallbladder and biliary tract cancer	59,517 (56,489–62,391)	0 (0–0)	0 (0–0)	3,884 (3,554–4,143)	21,918 (20,624–23,203)	33,714 (31,937–35,303)
Pancreatic cancer	214,646 (209,609–219,754)	0 (0–0)	0 (0–0)	17,572 (16,850–18,442)	99,222 (96,493–101,935)	97,850 (95,219–100,503)
Larynx cancer	86,447 (83,187–89,857)	0 (0–0)	0 (0–0)	7,817 (7,411–8,300)	46,095 (44,373–47,905)	32,535 (31,259–33,929)
Tracheal, bronchus, and lung cancer	1,205,705 (1,164,610–1,251,918)	0 (0–0)	0 (0–0)	76,849 (72,962–81,653)	579,103 (556,813–603,202)	549,753 (531,610–568,031)
Malignant skin melanoma	32,312 (21,291–44,784)	0 (0–0)	0 (0–0)	5,991 (4,497–8,572)	13,526 (9,126–19,754)	12,794 (7,594–16,752)
Non-melanoma skin cancer	34,198 (32,774–35,499)	0 (0–0)	0 (0–0)	2,778 (2,610–2,961)	12,030 (11,460–12,559)	19,389 (18,540–20,219)
Non-melanoma skin cancer (squamous-cell carcinoma)	34,198 (32,774–35,499)	0 (0–0)	0 (0–0)	2,778 (2,610–2,961)	12,030 (11,460–12,559)	19,389 (18,540–20,219)
Non-melanoma skin cancer (basal-cell carcinoma)	0 (0–0)	0 (0–0)	0 (0–0)	0 (0–0)	0 (0–0)	0 (0–0)
Breast cancer	10,110 (9,393–10,629)	0 (0–0)	0 (0–0)	1,187 (1,100–1,287)	5,123 (4,762–5,466)	3,799 (3,436–4,053)
Cervical cancer	0 (0–0)	0 (0–0)	0 (0–0)	0 (0–0)	0 (0–0)	0 (0–0)
Uterine cancer	0 (0–0)	0 (0–0)	0 (0–0)	0 (0–0)	0 (0–0)	0 (0–0)
Ovarian cancer	0 (0–0)	0 (0–0)	0 (0–0)	0 (0–0)	0 (0–0)	0 (0–0)
Prostate cancer	365,933 (303,491–459,613)	0 (0–0)	0 (0–0)	2,960 (2,280–3,602)	80,971 (65,116–101,436)	282,001 (233,048–358,457)
Testicular cancer	9,366 (8,825–9,910)	0 (0–0)	0 (0–0)	6,110 (5,733–6,580)	1,777 (1,655–1,899)	1,478 (1,381–1,581)

SR Table 11 - Global deaths by age and cause for males in 2015						
**Cause**	**All ages**	**0-5 years**	**5-14 years**	**15-49 years**	**50-69 years**	**70+ years**
Kidney cancer	89,128 (85,929–92,800)	1,811 (998–3,139)	642 (566–744)	8,346 (7,934–8,817)	39,042 (37,592–40,590)	39,286 (37,736–40,955)
Bladder cancer	136,795 (132,658–140,711)	0 (0–0)	0 (0–0)	5,346 (5,056–5,684)	42,761 (41,280–44,170)	88,688 (85,572–91,717)
Brain and nervous system cancer	127,169 (107,968–140,907)	2,967 (2,435–3,936)	5,403 (4,165–6,151)	33,921 (28,417–38,519)	55,333 (47,646–61,756)	29,544 (23,559–31,831)
Thyroid cancer	13,383 (11,078–14,214)	0 (0–0)	42 (35–47)	1,546 (1,224–1,676)	5,525 (4,645–5,919)	6,268 (5,072–6,679)
Mesothelioma	23,160 (22,103–24,118)	0 (0–0)	0 (0–0)	2,259 (2,130–2,398)	9,109 (8,747–9,473)	11,791 (10,933–12,488)
Hodgkin lymphoma	15,114 (12,997–19,280)	153 (123–208)	391 (302–512)	4,915 (4,156–6,366)	6,021 (5,176–7,765)	3,632 (3,176–4,470)
Non-Hodgkin lymphoma	133,441 (108,765–142,662)	1,951 (1,405–2,996)	3,161 (2,618–3,632)	24,777 (21,270–27,129)	50,936 (42,402–54,918)	52,613 (40,312–57,037)
Multiple myeloma	52,235 (50,626–53,800)	0 (0–0)	0 (0–0)	5,175 (4,915–5,436)	21,759 (21,066–22,472)	25,301 (24,349–26,225)
Leukemia	204,293 (197,075–211,514)	8,423 (7,281–9,774)	12,892 (11,693–14,029)	50,060 (47,798–52,774)	61,326 (59,208–63,516)	71,590 (68,870–74,125)
Acute lymphoid leukemia	64,986 (56,718–72,279)	5,209 (4,322–6,441)	9,166 (8,123–10,311)	21,667 (18,656–24,483)	17,699 (14,627–19,629)	11,243 (9,516–12,386)
Chronic lymphoid leukemia	33,928 (31,639–38,023)	0 (0–0)	0 (0–0)	3,109 (2,715–3,747)	10,069 (9,240–11,154)	20,749 (19,374–23,555)
Acute myeloid leukemia	85,068 (75,919–94,749)	3,213 (2,331–4,161)	3,725 (2,891–4,805)	20,110 (16,975–23,426)	27,368 (24,612–30,550)	30,650 (27,120–33,037)
Chronic myeloid leukemia	20,309 (18,576–22,901)	0 (0–0)	0 (0–0)	5,172 (4,528–6,045)	6,190 (5,536–6,958)	8,946 (8,275–9,984)
Other neoplasms	191,104 (159,976–205,545)	6,848 (5,014–10,457)	5,685 (4,872–6,459)	45,483 (36,653–50,804)	70,149 (58,012–75,215)	62,937 (53,050–66,987)
Cardiovascular diseases	9,419,637 (9,199,719–9,648,088)	24,636 (22,815–26,856)	13,947 (12,691–15,196)	946,477 (903,930–995,056)	3,241,267 (3,146,117–3,337,808)	5,193,307 (5,090,764–5,297,785)
Rheumatic heart disease	147,224 (136,892–157,705)	1,389 (1,176–1,647)	3,082 (2,662–3,503)	39,549 (36,037–43,309)	49,574 (45,494–53,754)	53,627 (50,425–57,085)
Ischemic heart disease	4,881,028 (4,747,380–5,022,975)	1,448 (1,236–1,716)	1,290 (1,096–1,475)	487,034 (464,295–515,834)	1,750,682 (1,692,062–1,811,529)	2,640,572 (2,574,215–2,703,714)
Cerebrovascular disease	3,231,199 (3,128,644–3,346,127)	8,711 (7,990–9,814)	5,271 (4,747–5,776)	276,754 (261,544–291,590)	1,094,893 (1,050,881–1,140,428)	1,845,569 (1,794,227–1,906,724)
Ischemic stroke	1,427,423 (1,369,627–1,484,115)	297 (241–366)	286 (241–333)	44,559 (40,201–49,071)	378,205 (355,076–399,858)	1,004,073 (969,100–1,038,473)
Hemorrhagic stroke	1,803,776 (1,728,915–1,888,569)	8,413 (7,729–9,481)	4,984 (4,494–5,456)	232,194 (218,384–245,045)	716,687 (681,745–755,794)	841,496 (806,434–882,866)
Hypertensive heart disease	445,446 (419,492–471,996)	220 (177–278)	212 (172–269)	43,350 (39,390–47,545)	144,532 (134,526–154,512)	257,131 (242,592–272,797)
Cardiomyopathy and myocarditis	198,790 (185,662–211,910)	5,041 (3,624–5,775)	1,637 (1,399–1,859)	48,300 (44,022–52,110)	68,261 (64,129–76,175)	75,548 (69,655–79,903)
Atrial fibrillation and flutter	86,189 (69,422–105,187)	0 (0–0)	0 (0–0)	623 (477–796)	7,080 (5,585–9,201)	78,485 (62,054–97,371)
Aortic aneurysm	106,769 (103,208–109,809)	0 (0–0)	0 (0–0)	9,574 (9,148–10,129)	33,816 (32,674–34,862)	63,377 (61,079–65,355)
Peripheral vascular disease	25,685 (24,234–27,242)	0 (0–0)	0 (0–0)	367 (340–394)	5,943 (5,588–6,323)	19,374 (18,085–20,680)
Endocarditis	38,767 (32,134–45,111)	1,775 (1,155–2,349)	550 (405–673)	9,221 (6,983–10,846)	10,776 (8,977–12,863)	16,443 (14,221–18,847)
Other cardiovascular and circulatory diseases	258,537 (246,673–270,931)	6,050 (5,366–7,360)	1,903 (1,699–2,134)	31,701 (29,175–34,460)	75,705 (71,483–80,048)	143,176 (137,360–149,763)
Chronic respiratory diseases	2,205,060 (2,119,874–2,293,084)	19,322 (16,032–23,880)	5,495 (4,890–6,178)	124,086 (116,405–132,206)	640,942 (608,673–675,447)	1,415,212 (1,362,872–1,469,074)
Chronic obstructive pulmonary disease	1,876,402 (1,800,621–1,958,537)	6,519 (4,486–9,484)	905 (766–1,068)	76,966 (70,665–82,702)	530,078 (501,195–562,837)	1,261,932 (1,213,993–1,313,285)
Pneumoconiosis	23,700 (21,537–25,634)	0 (0–0)	0 (0–0)	2,066 (1,829–2,337)	6,990 (6,213–7,720)	14,643 (13,355–15,837)
Silicosis	10,058 (8,998–11,305)	0 (0–0)	0 (0–0)	1,014 (860–1,220)	3,380 (2,929–3,971)	5,663 (5,103–6,294)
Asbestosis	3,025 (1,961–3,563)	0 (0–0)	0 (0–0)	257 (218–303)	696 (529–892)	2,071 (1,185–2,435)
Coal workers pneumoconiosis	2,349 (2,045–2,722)	0 (0–0)	0 (0–0)	168 (125–229)	672 (556–808)	1,508 (1,328–1,733)
Other pneumoconiosis	8,267 (7,014–9,320)	0 (0–0)	0 (0–0)	626 (530–722)	2,241 (1,793–2,692)	5,399 (4,612–6,058)
Asthma	201,269 (176,642–225,889)	4,975 (3,854–6,389)	3,589 (3,134–4,210)	34,090 (29,230–40,057)	74,093 (63,996–84,734)	84,519 (74,267–94,688)
Interstitial lung disease and pulmonary sarcoidosis	67,801 (47,972–78,830)	143 (95–254)	119 (89–179)	4,298 (3,319–5,386)	20,352 (15,679–24,463)	42,887 (28,524–49,068)
Other chronic respiratory diseases	35,886 (23,736–42,644)	7,683 (5,529–10,753)	880 (561–1,184)	6,664 (4,014–8,170)	9,428 (5,694–11,343)	11,229 (7,125–13,344)
Cirrhosis and other chronic liver diseases	861,111 (817,106–907,634)	5,114 (4,262–6,277)	4,933 (4,252–5,874)	257,169 (240,759–276,495)	409,796 (386,884–430,148)	184,097 (174,756–192,055)
Cirrhosis and other chronic liver diseases due to hepatitis B	263,680 (240,694–286,867)	54 (40–73)	204 (156–258)	78,440 (69,747–88,396)	123,135 (108,793–139,216)	61,846 (56,225–67,993)
Cirrhosis and other chronic liver diseases due to hepatitis C	224,787 (206,745–243,324)	27 (20–36)	108 (82–142)	60,887 (54,099–68,843)	110,301 (97,770–122,195)	53,462 (48,085–58,319)
Cirrhosis and other chronic liver diseases due to alcohol use	249,241 (228,481–269,290)	0 (0–0)	0 (0–0)	84,368 (76,481–93,919)	125,860 (112,269–139,114)	39,012 (34,823–43,728)
Cirrhosis and other chronic liver diseases due to other causes	123,401 (113,043–133,968)	5,032 (4,202–6,187)	4,620 (3,986–5,489)	33,472 (29,584–37,698)	50,499 (44,019–57,366)	29,776 (26,802–32,806)
Digestive diseases	659,131 (620,487–723,912)	8,579 (6,631–11,747)	8,289 (7,361–10,046)	155,145 (140,556–171,075)	219,667 (204,643–244,496)	267,450 (254,417–293,649)
Peptic ulcer disease	156,121 (141,353–177,200)	601 (385–1,007)	999 (831–1,256)	33,923 (29,605–40,429)	58,464 (52,767–66,676)	62,131 (56,495–69,263)
Gastritis and duodenitis	28,621 (24,965–32,974)	518 (268–977)	190 (139–283)	6,257 (5,205–7,551)	10,255 (8,902–11,927)	11,400 (10,053–13,129)
Appendicitis	27,164 (21,764–30,685)	819 (556–1,243)	1,285 (1,048–1,532)	10,055 (7,701–11,595)	8,076 (6,535–9,347)	6,926 (5,784–7,870)
Paralytic ileus and intestinal obstruction	146,331 (128,964–185,785)	3,162 (2,288–4,348)	3,508 (3,006–4,705)	35,591 (30,271–45,453)	46,441 (40,382–60,378)	57,627 (50,991–71,537)
Inguinal, femoral, and abdominal hernia	37,933 (22,853–45,860)	480 (208–816)	427 (231–562)	7,778 (4,019–10,383)	12,181 (7,335–15,203)	17,065 (10,715–20,423)
Inflammatory bowel disease	23,074 (20,352–25,815)	649 (396–1,109)	287 (218–379)	4,945 (4,109–5,756)	7,003 (6,144–7,999)	10,187 (9,238–11,361)
Vascular intestinal disorders	47,163 (43,018–52,306)	317 (177–590)	90 (70–119)	3,538 (3,026–4,306)	14,589 (13,148–16,769)	28,628 (26,509–31,388)
Gallbladder and biliary diseases	49,101 (44,073–55,874)	298 (188–443)	384 (302–479)	7,278 (6,271–8,488)	13,883 (12,328–16,435)	27,257 (24,740–30,244)
Pancreatitis	85,113 (77,686–95,455)	98 (69–137)	228 (186–305)	35,838 (31,778–40,968)	29,857 (27,335–33,883)	19,090 (17,598–21,487)
Other digestive diseases	58,506 (45,392–73,543)	1,633 (1,016–2,554)	885 (644–1,235)	9,938 (7,134–13,152)	18,914 (13,638–24,292)	27,133 (22,663–33,015)
Neurological disorders	934,311 (809,977–1,054,268)	9,802 (7,889–11,912)	6,544 (5,926–7,143)	58,287 (54,825–62,479)	102,140 (87,913–116,071)	757,537 (639,754–874,736)
Alzheimer disease and other dementias	732,259 (610,417–853,335)	0 (0–0)	0 (0–0)	774 (479–1,147)	56,778 (42,677–70,677)	674,706 (556,275–792,872)
Parkinson disease	66,625 (63,778–69,459)	0 (0–0)	0 (0–0)	483 (453–529)	8,361 (8,005–8,918)	57,779 (55,200–60,240)
Epilepsy	77,503 (73,220–82,436)	7,292 (5,749–9,124)	4,997 (4,468–5,605)	42,494 (39,459–46,098)	14,850 (13,956–15,963)	7,868 (7,549–8,206)

SR Table 11 - Global deaths by age and cause for males in 2015						
**Cause**	**All ages**	**0-5 years**	**5-14 years**	**15-49 years**	**50-69 years**	**70+ years**
Multiple sclerosis	7,744 (7,194–8,325)	0 (0–0)	0 (0–0)	2,269 (2,094–2,659)	3,885 (3,553–4,163)	1,588 (1,365–1,685)
Motor neuron disease	19,764 (18,757–20,771)	437 (366–528)	58 (54–65)	2,717 (2,547–2,968)	9,271 (8,823–9,755)	7,280 (6,834–7,611)
Migraine	0 (0–0)	0 (0–0)	0 (0–0)	0 (0–0)	0 (0–0)	0 (0–0)
Tension-type headache	0 (0–0)	0 (0–0)	0 (0–0)	0 (0–0)	0 (0–0)	0 (0–0)
Medication overuse headache	0 (0–0)	0 (0–0)	0 (0–0)	0 (0–0)	0 (0–0)	0 (0–0)
Other neurological disorders	30,423 (29,197–31,601)	2,081 (1,665–2,494)	1,487 (1,315–1,671)	9,547 (8,911–10,228)	8,992 (8,459–9,515)	8,313 (7,854–8,742)
Mental and substance use disorders	248,661 (233,710–260,498)	9 (8–11)	11 (6–16)	135,717 (126,664–145,243)	86,277 (80,985–90,173)	26,645 (23,012–27,850)
Schizophrenia	9,083 (8,319–9,969)	0 (0–0)	0 (0–0)	3,485 (3,093–3,971)	3,601 (3,308–3,943)	1,996 (1,856–2,151)
Alcohol use disorders	116,514 (110,450–122,978)	0 (0–0)	0 (0–0)	52,174 (49,259–55,634)	52,205 (48,956–55,468)	12,134 (11,128–12,815)
Drug use disorders	122,887 (108,135–131,844)	9 (8–11)	0 (0–0)	79,892 (70,085–87,458)	30,470 (26,170–32,712)	12,514 (9,627–13,426)
Opioid use disorders	91,069 (80,558–98,255)	9 (8–11)	0 (0–0)	59,177 (52,095–65,545)	21,817 (18,752–23,646)	10,065 (7,592–10,877)
Cocaine use disorders	7,866 (5,237–8,900)	0 (0–0)	0 (0–0)	5,390 (3,661–6,236)	2,217 (1,388–2,543)	257 (198–330)
Amphetamine use disorders	10,256 (6,502–12,135)	0 (0–0)	0 (0–0)	6,923 (4,352–8,282)	2,796 (1,773–3,393)	537 (346–660)
Cannabis use disorders	0 (0–0)	0 (0–0)	0 (0–0)	0 (0–0)	0 (0–0)	0 (0–0)
Other drug use disorders	13,695 (12,369–15,459)	0 (0–0)	0 (0–0)	8,401 (7,520–9,817)	3,639 (3,259–4,111)	1,654 (1,425–1,866)
Depressive disorders	0 (0–0)	0 (0–0)	0 (0–0)	0 (0–0)	0 (0–0)	0 (0–0)
Major depressive disorder	0 (0–0)	0 (0–0)	0 (0–0)	0 (0–0)	0 (0–0)	0 (0–0)
Dysthymia	0 (0–0)	0 (0–0)	0 (0–0)	0 (0–0)	0 (0–0)	0 (0–0)
Bipolar disorder	0 (0–0)	0 (0–0)	0 (0–0)	0 (0–0)	0 (0–0)	0 (0–0)
Anxiety disorders	0 (0–0)	0 (0–0)	0 (0–0)	0 (0–0)	0 (0–0)	0 (0–0)
Eating disorders	176 (104–253)	0 (0–0)	11 (6–16)	164 (97–237)	0 (0–0)	0 (0–0)
Anorexia nervosa	154 (89–220)	0 (0–0)	11 (5–15)	143 (83–204)	0 (0–0)	0 (0–0)
Bulimia nervosa	22 (12–35)	0 (0–0)	0 (0–1)	21 (12–34)	0 (0–0)	0 (0–0)
Autistic spectrum disorders	0 (0–0)	0 (0–0)	0 (0–0)	0 (0–0)	0 (0–0)	0 (0–0)
Autism	0 (0–0)	0 (0–0)	0 (0–0)	0 (0–0)	0 (0–0)	0 (0–0)
Asperger syndrome and other autistic spectrum disorders	0 (0–0)	0 (0–0)	0 (0–0)	0 (0–0)	0 (0–0)	0 (0–0)
Attention-deficit/hyperactivity disorder	0 (0–0)	0 (0–0)	0 (0–0)	0 (0–0)	0 (0–0)	0 (0–0)
Conduct disorder	0 (0–0)	0 (0–0)	0 (0–0)	0 (0–0)	0 (0–0)	0 (0–0)
Idiopathic developmental intellectual disability	0 (0–0)	0 (0–0)	0 (0–0)	0 (0–0)	0 (0–0)	0 (0–0)
Other mental and substance use disorders	0 (0–0)	0 (0–0)	0 (0–0)	0 (0–0)	0 (0–0)	0 (0–0)
Diabetes, urogenital, blood, and endocrine diseases	1,712,241 (1,581,761–1,802,483)	65,815 (40,897–114,849)	17,456 (14,490–20,984)	246,669 (222,351–266,801)	591,908 (551,374–621,087)	790,391 (738,175–820,365)
Diabetes mellitus	746,179 (716,925–775,084)	876 (703–973)	1,053 (854–1,165)	79,280 (74,463–85,106)	306,893 (292,641–321,253)	358,075 (344,717–371,401)
Acute glomerulonephritis	6,688 (4,295–8,033)	120 (80–155)	145 (104–171)	1,502 (886–1,779)	2,414 (1,607–2,938)	2,505 (1,493–3,074)
Chronic kidney disease	637,842 (525,974–676,999)	5,876 (4,685–7,094)	4,489 (3,538–5,160)	104,239 (80,774–113,818)	211,620 (170,239–226,034)	311,616 (264,582–327,517)
Chronic kidney disease due to diabetes mellitus	216,828 (185,423–232,341)	680 (528–890)	397 (303–506)	25,494 (20,371–29,123)	87,072 (73,313–95,620)	103,183 (90,678–110,697)
Chronic kidney disease due to hypertension	273,778 (222,189–293,113)	2,059 (1,641–2,489)	1,335 (1,046–1,637)	41,993 (31,830–47,388)	82,143 (64,510–90,897)	146,246 (123,044–156,396)
Chronic kidney disease due to glomerulonephritis	131,499 (104,308–144,564)	2,348 (1,856–2,980)	2,039 (1,562–2,426)	32,449 (25,239–36,923)	39,704 (30,096–44,923)	54,958 (44,293–60,604)
Chronic kidney disease due to other causes	15,735 (12,462–18,933)	788 (597–995)	717 (543–916)	4,301 (2,795–6,176)	2,699 (1,620–4,087)	7,229 (5,836–8,737)
Urinary diseases and male infertility	124,423 (105,367–137,354)	3,997 (3,053–5,324)	995 (841–1,264)	16,361 (13,520–19,634)	32,297 (26,510–36,429)	70,772 (61,408–77,146)
Interstitial nephritis and urinary tract infections	83,520 (70,778–94,624)	3,223 (2,469–4,353)	628 (519–806)	9,831 (7,938–12,230)	18,840 (15,327–22,136)	50,996 (43,629–56,497)
Urolithiasis	8,264 (6,823–12,422)	0 (0–0)	108 (85–167)	1,294 (1,053–2,173)	2,786 (2,249–4,327)	4,075 (3,259–5,719)
Benign prostatic hyperplasia	0 (0–0)	0 (0–0)	0 (0–0)	0 (0–0)	0 (0–0)	0 (0–0)
Male infertility	0 (0–0)	0 (0–0)	0 (0–0)	0 (0–0)	0 (0–0)	0 (0–0)
Other urinary diseases	32,638 (23,902–37,288)	773 (457–1,184)	259 (191–336)	5,235 (3,539–6,255)	10,669 (7,389–12,429)	15,700 (11,889–18,968)
Gynecological diseases	0 (0–0)	0 (0–0)	0 (0–0)	0 (0–0)	0 (0–0)	0 (0–0)
Uterine fibroids	0 (0–0)	0 (0–0)	0 (0–0)	0 (0–0)	0 (0–0)	0 (0–0)
Polycystic ovarian syndrome	0 (0–0)	0 (0–0)	0 (0–0)	0 (0–0)	0 (0–0)	0 (0–0)
Female infertility	0 (0–0)	0 (0–0)	0 (0–0)	0 (0–0)	0 (0–0)	0 (0–0)
Endometriosis	0 (0–0)	0 (0–0)	0 (0–0)	0 (0–0)	0 (0–0)	0 (0–0)
Genital prolapse	0 (0–0)	0 (0–0)	0 (0–0)	0 (0–0)	0 (0–0)	0 (0–0)
Premenstrual syndrome	0 (0–0)	0 (0–0)	0 (0–0)	0 (0–0)	0 (0–0)	0 (0–0)
Other gynecological diseases	0 (0–0)	0 (0–0)	0 (0–0)	0 (0–0)	0 (0–0)	0 (0–0)
Hemoglobinopathies and hemolytic anemias	124,638 (80,303–186,255)	47,611 (22,658–96,211)	8,739 (6,305–11,969)	30,189 (22,567–38,542)	17,005 (12,400–21,412)	21,092 (15,649–25,960)
Thalassemias	8,330 (5,660–10,631)	3,905 (2,579–5,623)	1,451 (1,093–1,715)	897 (615–1,321)	49 (41–57)	2,026 (1,301–2,867)
Thalassemias trait	0 (0–0)	0 (0–0)	0 (0–0)	0 (0–0)	0 (0–0)	0 (0–0)
Sickle cell disorders	64,678 (35,384–116,359)	42,914 (19,217–90,921)	6,268 (4,386–9,256)	12,284 (9,197–16,487)	589 (483–745)	2,621 (1,779–3,498)
Sickle cell trait	0 (0–0)	0 (0–0)	0 (0–0)	0 (0–0)	0 (0–0)	0 (0–0)

SR Table 11 - Global deaths by age and cause for males in 2015						
**Cause**	**All ages**	**0-5 years**	**5-14 years**	**15-49 years**	**50-69 years**	**70+ years**
G6PD deficiency	23,121<br>(17,665–28,557)	623<br>(416–960)	815<br>(642–1,073)	12,629<br>(9,619–15,580)	7,268<br>(5,463–8,982)	1,783<br>(1,303–2,254)
G6PD trait	0<br>(0–0)	0<br>(0–0)	0<br>(0–0)	0<br>(0–0)	0<br>(0–0)	0<br>(0–0)
Other hemoglobinopathies and hemolytic anemias	28,508<br>(20,989–35,518)	167<br>(119–253)	204<br>(163–271)	4,377<br>(3,116–6,062)	9,097<br>(6,437–11,595)	14,661<br>(11,051–17,691)
Endocrine, metabolic, blood, and immune disorders	72,468<br>(69,972–75,799)	7,333<br>(6,377–8,501)	2,032<br>(1,804–2,351)	15,096<br>(14,150–16,399)	21,676<br>(20,900–22,651)	26,329<br>(25,246–27,434)
Musculoskeletal disorders	27,634<br>(24,529–29,080)	0<br>(0–0)	680<br>(612–779)	5,626<br>(5,228–6,228)	7,987<br>(7,298–8,501)	13,339<br>(10,884–14,198)
Rheumatoid arthritis	9,319<br>(7,667–10,533)	0<br>(0–0)	89<br>(70–108)	779<br>(632–937)	3,168<br>(2,683–3,537)	5,281<br>(4,221–6,121)
Osteoarthritis	0<br>(0–0)	0<br>(0–0)	0<br>(0–0)	0<br>(0–0)	0<br>(0–0)	0<br>(0–0)
Low back and neck pain	0<br>(0–0)	0<br>(0–0)	0<br>(0–0)	0<br>(0–0)	0<br>(0–0)	0<br>(0–0)
Low back pain	0<br>(0–0)	0<br>(0–0)	0<br>(0–0)	0<br>(0–0)	0<br>(0–0)	0<br>(0–0)
Neck pain	0<br>(0–0)	0<br>(0–0)	0<br>(0–0)	0<br>(0–0)	0<br>(0–0)	0<br>(0–0)
Gout	0<br>(0–0)	0<br>(0–0)	0<br>(0–0)	0<br>(0–0)	0<br>(0–0)	0<br>(0–0)
Other musculoskeletal disorders	18,314<br>(16,295–19,440)	0<br>(0–0)	590<br>(529–684)	4,847<br>(4,490–5,455)	4,818<br>(4,387–5,275)	8,057<br>(6,458–8,703)
Other non-communicable diseases	386,466<br>(335,687–434,944)	270,830<br>(232,395–303,976)	19,408<br>(15,600–21,928)	45,337<br>(37,710–53,521)	21,679<br>(16,075–28,129)	29,211<br>(20,536–40,076)
Congenital anomalies	332,557<br>(285,304–370,111)	258,318<br>(220,205–290,681)	18,875<br>(15,038–21,313)	38,027<br>(32,375–44,505)	10,217<br>(8,577–11,523)	7,119<br>(6,214–8,105)
Neural tube defects	33,612<br>(24,499–45,920)	31,694<br>(22,853–44,051)	841<br>(558–1,181)	813<br>(591–1,169)	183<br>(144–247)	79<br>(60–118)
Congenital heart anomalies	162,328<br>(138,112–184,217)	118,413<br>(100,034–136,504)	12,832<br>(10,031–14,595)	26,208<br>(22,199–30,735)	2,903<br>(2,495–3,361)	1,970<br>(1,736–2,399)
Cleft lip and cleft palate	681<br>(549–881)	681<br>(549–881)	0<br>(0–0)	0<br>(0–0)	0<br>(0–0)	0<br>(0–0)
Down syndrome	10,999<br>(7,960–15,453)	6,155<br>(3,700–9,640)	446<br>(315–636)	2,416<br>(1,986–3,218)	1,891<br>(1,475–2,240)	88<br>(71–109)
Turner syndrome	0<br>(0–0)	0<br>(0–0)	0<br>(0–0)	0<br>(0–0)	0<br>(0–0)	0<br>(0–0)
Klinefelter syndrome	0<br>(0–0)	0<br>(0–0)	0<br>(0–0)	0<br>(0–0)	0<br>(0–0)	0<br>(0–0)
Other chromosomal abnormalities	7,442<br>(4,779–11,772)	6,518<br>(3,891–10,837)	156<br>(117–223)	526<br>(319–758)	178<br>(112–244)	62<br>(43–83)
Other congenital anomalies	117,492<br>(97,575–136,788)	94,854<br>(78,117–112,543)	4,597<br>(3,681–5,530)	8,061<br>(6,482–9,359)	5,060<br>(3,997–5,711)	4,918<br>(4,167–5,469)
Skin and subcutaneous diseases	43,795<br>(26,427–68,833)	2,398<br>(1,501–4,817)	532<br>(321–984)	7,310<br>(4,047–12,277)	11,461<br>(6,628–18,344)	22,092<br>(13,556–32,805)
Dermatitis	0<br>(0–0)	0<br>(0–0)	0<br>(0–0)	0<br>(0–0)	0<br>(0–0)	0<br>(0–0)
Psoriasis	0<br>(0–0)	0<br>(0–0)	0<br>(0–0)	0<br>(0–0)	0<br>(0–0)	0<br>(0–0)
Cellulitis	8,472<br>(4,705–14,524)	309<br>(140–640)	101<br>(54–187)	1,764<br>(835–3,135)	2,868<br>(1,478–4,978)	3,428<br>(2,092–5,702)
Pyoderma	20,232<br>(12,449–34,708)	1,906<br>(1,190–3,953)	276<br>(175–543)	4,132<br>(2,216–7,102)	5,673<br>(3,332–9,723)	8,243<br>(5,148–13,556)
Scabies	0<br>(0–0)	0<br>(0–0)	0<br>(0–0)	0<br>(0–0)	0<br>(0–0)	0<br>(0–0)
Fungal skin diseases	0<br>(0–0)	0<br>(0–0)	0<br>(0–0)	0<br>(0–0)	0<br>(0–0)	0<br>(0–0)
Viral skin diseases	0<br>(0–0)	0<br>(0–0)	0<br>(0–0)	0<br>(0–0)	0<br>(0–0)	0<br>(0–0)
Acne vulgaris	0<br>(0–0)	0<br>(0–0)	0<br>(0–0)	0<br>(0–0)	0<br>(0–0)	0<br>(0–0)
Alopecia areata	0<br>(0–0)	0<br>(0–0)	0<br>(0–0)	0<br>(0–0)	0<br>(0–0)	0<br>(0–0)
Pruritus	0<br>(0–0)	0<br>(0–0)	0<br>(0–0)	0<br>(0–0)	0<br>(0–0)	0<br>(0–0)
Urticaria	0<br>(0–0)	0<br>(0–0)	0<br>(0–0)	0<br>(0–0)	0<br>(0–0)	0<br>(0–0)
Decubitus ulcer	13,240<br>(6,189–19,324)	59<br>(30–113)	78<br>(42–141)	986<br>(448–1,592)	2,520<br>(1,084–3,855)	9,595<br>(4,563–13,589)
Other skin and subcutaneous diseases	1,849<br>(1,144–3,033)	122<br>(69–235)	76<br>(43–141)	426<br>(241–771)	398<br>(234–605)	825<br>(515–1,280)
Sense organ diseases	0<br>(0–0)	0<br>(0–0)	0<br>(0–0)	0<br>(0–0)	0<br>(0–0)	0<br>(0–0)
Glaucoma	0<br>(0–0)	0<br>(0–0)	0<br>(0–0)	0<br>(0–0)	0<br>(0–0)	0<br>(0–0)
Cataract	0<br>(0–0)	0<br>(0–0)	0<br>(0–0)	0<br>(0–0)	0<br>(0–0)	0<br>(0–0)
Macular degeneration	0<br>(0–0)	0<br>(0–0)	0<br>(0–0)	0<br>(0–0)	0<br>(0–0)	0<br>(0–0)
Refraction and accommodation disorders	0<br>(0–0)	0<br>(0–0)	0<br>(0–0)	0<br>(0–0)	0<br>(0–0)	0<br>(0–0)
Age-related and other hearing loss	0<br>(0–0)	0<br>(0–0)	0<br>(0–0)	0<br>(0–0)	0<br>(0–0)	0<br>(0–0)
Other vision loss	0<br>(0–0)	0<br>(0–0)	0<br>(0–0)	0<br>(0–0)	0<br>(0–0)	0<br>(0–0)
Other sense organ diseases	0<br>(0–0)	0<br>(0–0)	0<br>(0–0)	0<br>(0–0)	0<br>(0–0)	0<br>(0–0)
Oral disorders	0<br>(0–0)	0<br>(0–0)	0<br>(0–0)	0<br>(0–0)	0<br>(0–0)	0<br>(0–0)
Deciduous caries	0<br>(0–0)	0<br>(0–0)	0<br>(0–0)	0<br>(0–0)	0<br>(0–0)	0<br>(0–0)
Permanent caries	0<br>(0–0)	0<br>(0–0)	0<br>(0–0)	0<br>(0–0)	0<br>(0–0)	0<br>(0–0)
Periodontal diseases	0<br>(0–0)	0<br>(0–0)	0<br>(0–0)	0<br>(0–0)	0<br>(0–0)	0<br>(0–0)
Edentulism and severe tooth loss	0<br>(0–0)	0<br>(0–0)	0<br>(0–0)	0<br>(0–0)	0<br>(0–0)	0<br>(0–0)
Other oral disorders	0<br>(0–0)	0<br>(0–0)	0<br>(0–0)	0<br>(0–0)	0<br>(0–0)	0<br>(0–0)
Sudden infant death syndrome	10,113<br>(7,592–17,233)	10,113<br>(7,592–17,233)	0<br>(0–0)	0<br>(0–0)	0<br>(0–0)	0<br>(0–0)
Injuries	3,325,789<br>(3,109,875–3,457,825)	176,431<br>(151,144–199,086)	143,376<br>(133,946–151,940)	1,824,637<br>(1,712,565–1,913,141)	733,038<br>(677,306–767,800)	448,305<br>(414,982–465,720)
Transport injuries	1,108,934<br>(1,043,835–1,170,738)	31,004<br>(25,835–36,433)	43,313<br>(39,723–47,072)	658,956<br>(619,229–699,626)	260,228<br>(243,013–276,782)	115,431<br>(108,523–121,970)
Road injuries	1,024,378<br>(965,972–1,081,016)	28,651<br>(23,895–33,766)	40,906<br>(37,595–44,489)	610,185<br>(570,484–648,040)	237,554<br>(220,912–252,482)	107,080<br>(100,402–113,140)
Pedestrian road injuries	411,778<br>(373,293–463,516)	13,302<br>(11,105–15,863)	18,973<br>(17,158–21,079)	214,470<br>(188,703–239,793)	109,347<br>(99,936–124,993)	55,684<br>(51,221–64,327)
Cyclist road injuries	44,201<br>(39,929–49,115)	721<br>(558–919)	2,245<br>(1,990–2,545)	20,536<br>(17,838–22,878)	14,776<br>(13,379–16,758)	5,922<br>(5,363–6,763)
Motorcyclist road injuries	206,673<br>(179,698–238,728)	3,493<br>(2,409–4,879)	5,351<br>(4,224–6,662)	143,938<br>(127,374–170,452)	40,287<br>(31,681–46,104)	13,602<br>(8,955–16,288)

SR Table 11 - Global deaths by age and cause for males in 2015						
Cause	All ages	0-5 years	5-14 years	15-49 years	50-69 years	70+ years
Motor vehicle road injuries	344,794 (298,981–387,315)	10,307 (8,021–12,647)	13,159 (11,034–15,169)	221,562 (197,148–250,499)	69,757 (56,776–77,501)	30,007 (25,113–33,107)
Other road injuries	16,930 (9,912–20,529)	826 (338–1,620)	1,177 (607–1,653)	9,677 (5,374–12,247)	3,384 (2,237–4,311)	1,863 (1,397–2,637)
Other transport injuries	84,555 (71,110–105,377)	2,353 (1,759–3,013)	2,406 (2,068–2,760)	48,770 (40,971–60,711)	22,674 (18,726–29,347)	8,350 (6,970–10,814)
Unintentional injuries	1,169,894 (1,052,358–1,232,886)	127,803 (105,595–147,805)	80,350 (74,782–86,163)	469,283 (426,007–505,680)	254,068 (225,503–273,600)	238,387 (210,805–250,998)
Falls	315,747 (269,651–334,877)	12,396 (8,432–15,953)	10,246 (8,664–11,707)	89,999 (78,079–97,246)	84,261 (72,445–90,284)	118,842 (101,557–126,149)
Drowning	223,105 (212,831–237,691)	33,423 (28,310–39,963)	33,731 (31,145–36,720)	100,376 (95,359–108,688)	36,421 (34,727–38,971)	19,152 (18,349–20,171)
Fire, heat, and hot substances	88,735 (70,994–95,930)	10,921 (8,355–13,027)	4,569 (3,609–5,150)	37,834 (28,756–41,643)	18,959 (15,542–20,904)	16,450 (13,985–17,680)
Poisonings	51,814 (31,327–64,389)	10,866 (2,880–21,778)	2,327 (1,582–2,862)	20,993 (15,269–26,137)	11,852 (8,076–13,717)	5,775 (3,925–6,463)
Exposure to mechanical forces	145,220 (111,386–159,294)	16,909 (11,810–20,831)	7,835 (6,977–9,129)	73,346 (54,840–80,910)	29,571 (21,487–33,469)	17,558 (13,087–19,504)
Unintentional firearm injuries	22,796 (15,311–25,445)	1,559 (837–2,215)	1,218 (880–1,554)	13,967 (9,251–15,845)	3,331 (2,319–3,690)	2,719 (2,108–2,973)
Unintentional suffocation	23,634 (16,633–27,672)	10,235 (6,052–13,712)	2,048 (1,703–2,760)	5,205 (3,924–5,819)	1,955 (1,017–2,367)	4,188 (3,129–4,645)
Other exposure to mechanical forces	98,789 (70,366–110,677)	5,113 (3,763–6,254)	4,567 (3,590–5,448)	54,173 (38,295–60,996)	24,283 (16,926–27,919)	10,651 (7,230–12,299)
Adverse effects of medical treatment	56,712 (41,285–64,273)	8,696 (5,146–11,473)	2,519 (1,686–3,035)	18,086 (12,171–21,403)	12,781 (9,678–14,940)	14,628 (11,573–16,497)
Animal contact	55,031 (30,407–79,710)	4,886 (2,643–7,203)	6,336 (3,545–9,419)	23,733 (12,657–36,189)	13,443 (7,485–18,972)	6,631 (4,124–8,727)
Venomous animal contact	46,139 (23,833–69,693)	3,350 (1,648–5,675)	5,245 (2,760–8,330)	20,865 (10,352–32,683)	11,408 (5,941–16,492)	5,269 (3,035–7,088)
Non-venomous animal contact	8,891 (6,639–10,551)	1,535 (944–1,959)	1,091 (770–1,324)	2,868 (2,286–3,665)	2,035 (1,541–2,702)	1,361 (1,109–1,840)
Foreign body	90,592 (75,022–106,768)	22,429 (15,230–29,761)	5,310 (4,138–6,630)	25,422 (20,757–31,300)	17,319 (14,191–19,669)	20,110 (16,191–22,182)
Pulmonary aspiration and foreign body in airway	74,138 (60,750–87,310)	19,309 (12,926–26,431)	3,888 (2,956–4,984)	17,933 (14,948–21,093)	15,000 (12,302–16,684)	18,006 (14,067–19,359)
Foreign body in eyes	0 (0–0)	0 (0–0)	0 (0–0)	0 (0–0)	0 (0–0)	0 (0–0)
Foreign body in other body part	16,454 (10,839–22,369)	3,120 (1,472–4,555)	1,422 (838–2,024)	7,489 (4,595–11,275)	2,318 (1,555–3,451)	2,104 (1,634–2,990)
Environmental heat and cold exposure	31,519 (23,059–34,674)	1,555 (763–2,042)	733 (404–943)	11,476 (8,443–13,114)	11,529 (8,422–13,280)	6,224 (4,281–6,961)
Other unintentional injuries	111,414 (102,429–125,087)	5,720 (4,642–7,068)	6,739 (5,719–7,991)	68,014 (61,348–79,022)	17,928 (16,350–19,862)	13,012 (10,404–16,921)
Self-harm and interpersonal violence	908,482 (851,157–949,119)	5,834 (4,838–7,235)	11,448 (10,471–12,513)	591,637 (550,113–620,594)	207,501 (193,669–217,590)	92,061 (85,478–96,911)
Self-harm	578,879 (533,925–609,206)	0 (0–0)	4,699 (4,042–5,322)	335,092 (307,461–354,360)	160,007 (148,057–168,616)	79,079 (72,096–83,470)
Interpersonal violence	329,603 (301,240–352,370)	5,834 (4,838–7,235)	6,748 (6,091–7,555)	256,544 (230,642–272,713)	47,493 (43,787–51,940)	12,981 (11,997–14,003)
Assault by firearm	151,999 (131,387–161,076)	1,017 (750–1,289)	2,179 (1,828–2,413)	128,141 (109,159–135,594)	17,260 (15,476–19,168)	3,399 (3,038–3,908)
Assault by sharp object	72,339 (66,551–79,200)	561 (436–772)	895 (779–1,039)	56,953 (52,080–62,613)	11,063 (10,185–12,429)	2,866 (2,626–3,162)
Assault by other means	105,263 (88,991–117,241)	4,254 (3,494–5,332)	3,673 (3,240–4,259)	71,450 (59,107–80,060)	19,169 (17,016–21,510)	6,715 (5,993–7,377)
Forces of nature, war, and legal intervention	138,478 (72,118–201,033)	11,788 (7,551–16,197)	8,265 (4,534–11,906)	104,759 (52,855–155,181)	11,239 (5,933–16,321)	2,425 (1,420–3,379)
Exposure to forces of nature	7,256 (4,435–10,136)	448 (265–638)	1,184 (703–1,694)	3,972 (2,372–5,543)	1,185 (723–1,662)	465 (282–649)
Collective violence and legal intervention	131,222 (66,767–194,150)	11,339 (7,210–15,660)	7,080 (3,397–10,682)	100,787 (49,555–150,078)	10,054 (4,956–14,897)	1,959 (990–2,868)

SR Table 12: Global deaths by cause and age group for both sexes combined (with 95% uncertainty intervals), 1990

Cause	All ages	0-5 years	5-14 years	15-49 years	50-69 years	70+ years
All causes	47,920,952 (47,422,084–48,454,663)	12,120,128 (12,010,665–12,240,141)	1,330,515 (1,314,157–1,345,827)	7,373,458 (7,215,451–7,549,882)	11,387,764 (11,187,811–11,596,081)	15,709,086 (15,556,409–15,861,032)
Communicable, maternal, neonatal, and nutritional diseases	15,905,040 (15,505,263–16,331,151)	10,179,261 (9,873,602–10,456,969)	675,878 (639,170–718,182)	2,047,883 (1,957,100–2,162,248)	1,473,367 (1,372,725–1,588,572)	1,528,649 (1,450,809–1,613,246)
HIV/AIDS and tuberculosis	1,792,415 (1,558,987–2,131,615)	99,547 (77,432–146,333)	31,179 (25,417–43,958)	727,878 (641,710–834,241)	606,043 (499,728–731,050)	327,765 (266,150–420,995)
Tuberculosis	1,541,741 (1,304,838–1,876,228)	63,944 (41,643–111,227)	29,477 (23,720–42,422)	544,716 (455,447–652,371)	578,303 (472,928–702,774)	325,299 (263,498–418,438)
HIV/AIDS	250,673 (228,668–278,132)	35,602 (32,752–39,316)	1,702 (1,489–1,959)	183,161 (164,954–205,579)	27,740 (24,590–31,676)	2,466 (2,053–2,950)
HIV/AIDS - Tuberculosis	65,500 (55,444–76,563)	11,895 (9,723–13,713)	479 (401–563)	44,720 (37,978–53,000)	7,675 (6,596–8,846)	728 (607–871)
HIV/AIDS resulting in other diseases	185,173 (164,266–211,890)	23,707 (20,926–27,401)	1,222 (1,045–1,449)	138,441 (120,893–160,336)	20,064 (17,368–23,615)	1,737 (1,416–2,153)
Diarrhea, lower respiratory, and other common infectious diseases	7,996,071 (7,629,671–8,421,266)	5,116,838 (4,813,156–5,484,409)	486,790 (434,743–548,053)	656,588 (610,101–718,095)	678,298 (644,299–722,660)	1,057,555 (1,008,071–1,102,758)
Diarrheal diseases	2,320,548 (2,115,293–2,495,198)	1,387,614 (1,204,766–1,553,535)	118,679 (103,236–131,848)	223,897 (212,148–237,840)	275,204 (260,542–290,431)	315,151 (299,522–332,100)
Intestinal infectious diseases	246,374 (136,888–404,648)	71,361 (38,370–123,682)	93,069 (53,075–150,173)	69,063 (36,684–116,531)	9,445 (4,858–16,631)	3,435 (2,053–5,318)
Typhoid fever	193,264 (104,340–324,822)	53,289 (26,164–96,020)	73,887 (40,111–120,989)	55,798 (28,866–97,384)	7,720 (3,727–13,981)	2,568 (1,389–4,213)
Paratyphoid fever	47,179 (22,058–89,503)	13,860 (5,884–28,304)	18,689 (8,801–36,419)	12,702 (5,396–24,906)	1,454 (582–2,995)	472 (200–941)
Other intestinal infectious diseases	5,930 (2,346–11,608)	4,212 (1,363–9,287)	492 (163–1,182)	562 (276–936)	269 (138–427)	393 (201–679)
Lower respiratory infections	3,394,760 (3,151,259–3,613,441)	2,125,305 (1,908,454–2,311,784)	92,559 (71,889–105,267)	198,935 (171,104–217,309)	297,504 (261,594–322,333)	680,457 (626,100–719,044)
Upper respiratory infections	4,537 (3,997–5,190)	1,904 (1,453–2,486)	91 (65–127)	820 (710–947)	903 (767–1,062)	816 (682–986)
Otitis media	6,965 (6,306–7,730)	3,408 (2,887–4,006)	824 (740–904)	1,342 (1,245–1,453)	800 (720–903)	588 (451–894)
Meningitis	502,174 (412,531–573,478)	318,443 (245,478–384,536)	44,151 (35,351–50,568)	72,743 (59,899–82,836)	41,742 (35,262–48,786)	25,093 (21,625–29,814)
Pneumococcal meningitis	123,478 (97,648–152,890)	67,994 (45,832–93,581)	13,675 (9,236–18,487)	23,310 (18,205–28,939)	11,534 (8,767–14,859)	6,963 (5,297–8,876)
H influenzae type B meningitis	154,968 (119,276–191,673)	118,614 (85,713–154,175)	12,490 (8,292–17,124)	12,642 (9,255–16,338)	6,915 (5,099–9,191)	4,305 (3,347–5,591)
Meningococcal meningitis	86,342 (65,362–113,263)	56,248 (37,472–81,821)	7,683 (5,014–10,813)	11,953 (8,947–15,130)	6,760 (4,885–8,864)	3,696 (2,799–4,879)
Other meningitis	137,385 (110,871–165,273)	75,586 (54,574–100,041)	10,301 (7,284–14,043)	24,836 (19,386–29,229)	16,532 (13,238–19,982)	10,128 (8,308–12,071)
Encephalitis	154,831 (134,325–174,376)	62,337 (51,039–73,075)	19,881 (16,598–23,326)	30,849 (27,372–34,699)	23,038 (19,764–26,636)	18,724 (15,898–21,333)
Diphtheria	15,106 (9,547–24,121)	12,006 (6,765–20,441)	1,742 (988–2,784)	1,007 (753–1,321)	349 (227–544)	0 (0–0)
Whooping cough	160,741 (56,654–383,760)	150,045 (52,714–356,469)	8,592 (3,074–20,985)	1,848 (571–4,995)	255 (79–699)	0 (0–0)
Tetanus	337,021 (304,312–401,573)	251,051 (223,899–284,126)	12,739 (9,996–20,396)	36,617 (28,704–57,451)	26,459 (21,647–46,222)	10,152 (8,253–17,935)
Measles	840,677 (337,343–1,670,077)	729,199 (295,378–1,430,012)	93,391 (35,749–188,131)	17,513 (5,212–44,383)	573 (160–1,547)	0 (0–0)
Varicella and herpes zoster	12,332 (11,121–14,055)	4,161 (3,323–5,216)	1,064 (836–1,366)	1,948 (1,701–2,239)	2,021 (1,680–2,416)	3,135 (2,568–3,910)
Neglected tropical diseases and malaria	1,108,471 (868,374–1,356,075)	738,678 (510,394–986,548)	102,161 (75,866–134,035)	154,175 (125,479–188,520)	83,830 (67,729–104,812)	29,626 (23,228–38,746)
Malaria	939,013 (700,771–1,190,222)	701,371 (473,421–953,514)	73,870 (48,097–105,643)	93,713 (69,225–126,521)	52,216 (37,642–72,022)	17,842 (11,716–26,538)
Chagas disease	9,085 (8,748–9,443)	12 (10–13)	20 (18–23)	2,335 (2,201–2,482)	4,215 (4,005–4,426)	2,501 (2,381–2,635)
Leishmaniasis	19,252 (11,146–29,887)	4,101 (2,181–6,507)	2,265 (1,145–3,958)	10,373 (5,919–16,383)	2,130 (1,371–3,096)	381 (278–490)
Visceral leishmaniasis	19,252 (11,146–29,887)	4,101 (2,181–6,507)	2,265 (1,145–3,958)	10,373 (5,919–16,383)	2,130 (1,371–3,096)	381 (278–490)
Cutaneous and mucocutaneous leishmaniasis	0 (0–0)	0 (0–0)	0 (0–0)	0 (0–0)	0 (0–0)	0 (0–0)
African trypanosomiasis	23,016 (11,647–38,069)	1,704 (797–2,948)	2,801 (1,432–4,667)	13,851 (6,987–23,033)	3,820 (1,889–6,594)	838 (418–1,415)
Schistosomiasis	13,535 (12,079–15,337)	284 (180–449)	431 (323–572)	4,386 (3,774–5,226)	5,773 (4,785–6,993)	2,659 (2,143–3,235)
Cysticercosis	825 (732–958)	21 (11–38)	35 (28–44)	482 (427–561)	221 (198–258)	63 (56–71)
Cystic echinococcosis	2,937 (2,740–3,160)	227 (177–279)	327 (285–372)	1,095 (1,009–1,192)	809 (751–871)	478 (447–508)
Lymphatic filariasis	0 (0–0)	0 (0–0)	0 (0–0)	0 (0–0)	0 (0–0)	0 (0–0)
Onchocerciasis	0 (0–0)	0 (0–0)	0 (0–0)	0 (0–0)	0 (0–0)	0 (0–0)
Trachoma	0 (0–0)	0 (0–0)	0 (0–0)	0 (0–0)	0 (0–0)	0 (0–0)
Dengue	6,462 (3,571–10,568)	2,910 (1,142–5,530)	1,627 (856–2,768)	1,286 (889–1,841)	423 (306–590)	213 (152–305)
Yellow fever	12,901 (2,826–34,787)	1,276 (259–3,713)	3,398 (733–9,320)	6,828 (1,504–18,225)	1,190 (248–3,504)	208 (42–614)
Rabies	51,957 (46,104–57,340)	8,694 (6,151–11,151)	13,986 (11,646–16,113)	16,182 (14,126–18,247)	10,175 (8,801–11,598)	2,917 (2,398–3,649)
Intestinal nematode infections	6,955 (5,793–8,040)	5,575 (4,536–6,564)	856 (729–988)	256 (208–311)	162 (125–203)	104 (77–136)
Ascariasis	6,955 (5,793–8,040)	5,575 (4,536–6,564)	856 (729–988)	256 (208–311)	162 (125–203)	104 (77–136)
Trichuriasis	0 (0–0)	0 (0–0)	0 (0–0)	0 (0–0)	0 (0–0)	0 (0–0)
Hookworm disease	0 (0–0)	0 (0–0)	0 (0–0)	0 (0–0)	0 (0–0)	0 (0–0)
Food-borne trematodiases	0 (0–0)	0 (0–0)	0 (0–0)	0 (0–0)	0 (0–0)	0 (0–0)
Leprosy	0 (0–0)	0 (0–0)	0 (0–0)	0 (0–0)	0 (0–0)	0 (0–0)
Ebola	0 (0–0)	0 (0–0)	0 (0–0)	0 (0–0)	0 (0–0)	0 (0–0)
Other neglected tropical diseases	22,527 (15,703–30,489)	12,497 (8,344–16,505)	2,539 (1,691–3,805)	3,382 (2,237–5,303)	2,692 (1,723–4,220)	1,416 (943–2,477)
Maternal disorders	390,184 (365,192–416,234)	0 (0–0)	2,071 (1,731–2,415)	386,751 (362,141–412,417)	1,361 (1,033–1,924)	0 (0–0)
Maternal hemorrhage	105,609 (88,193–125,580)	0 (0–0)	549 (372–775)	104,631 (87,403–124,615)	428 (267–701)	0 (0–0)
Maternal sepsis and other maternal infections	29,359 (22,545–37,159)	0 (0–0)	95 (61–143)	29,154 (22,345–36,924)	108 (68–169)	0 (0–0)
Maternal hypertensive disorders	50,555 (40,186–62,936)	0 (0–0)	343 (216–493)	50,076 (39,782–62,498)	134 (84–201)	0 (0–0)
Maternal obstructed labor and uterine rupture	23,969 (18,435–30,166)	0 (0–0)	71 (41–117)	23,846 (18,318–30,056)	50 (32–80)	0 (0–0)
Maternal abortion, miscarriage, and ectopic pregnancy	55,686 (43,937–70,428)	0 (0–0)	366 (224–550)	55,072 (43,397–69,751)	248 (159–366)	0 (0–0)

SR Table 12 - Global deaths by age and cause for both sexes in 1990						
**Cause**	**All ages**	**0-5 years**	**5-14 years**	**15-49 years**	**50-69 years**	**70+ years**
Indirect maternal deaths	41,491 (31,611–53,295)	0 (0–0)	249 (150–389)	41,138 (31,330–52,932)	102 (59–183)	0 (0–0)
Late maternal deaths	8,460 (5,791–11,935)	0 (0–0)	51 (26–86)	8,372 (5,714–11,830)	36 (19–67)	0 (0–0)
Maternal deaths aggravated by HIV/AIDS	754 (432–1,094)	0 (0–0)	0 (0–0)	752 (432–1,092)	1 (0–3)	0 (0–0)
Other maternal disorders	74,298 (61,159–89,652)	0 (0–0)	343 (224–490)	73,705 (60,608–88,977)	249 (165–367)	0 (0–0)
Neonatal disorders	3,515,789 (3,420,303–3,624,470)	3,515,789 (3,420,303–3,624,470)	0 (0–0)	0 (0–0)	0 (0–0)	0 (0–0)
Neonatal preterm birth complications	1,795,211 (1,658,046–1,945,923)	1,795,211 (1,658,046–1,945,923)	0 (0–0)	0 (0–0)	0 (0–0)	0 (0–0)
Neonatal encephalopathy due to birth asphyxia and trauma	915,323 (787,962–1,045,953)	915,323 (787,962–1,045,953)	0 (0–0)	0 (0–0)	0 (0–0)	0 (0–0)
Neonatal sepsis and other neonatal infections	329,296 (227,567–462,474)	329,296 (227,567–462,474)	0 (0–0)	0 (0–0)	0 (0–0)	0 (0–0)
Hemolytic disease and other neonatal jaundice	124,653 (75,327–225,701)	124,653 (75,327–225,701)	0 (0–0)	0 (0–0)	0 (0–0)	0 (0–0)
Other neonatal disorders	351,304 (255,141–465,743)	351,304 (255,141–465,743)	0 (0–0)	0 (0–0)	0 (0–0)	0 (0–0)
Nutritional deficiencies	635,584 (513,862–753,023)	438,203 (335,953–550,460)	21,827 (18,176–25,800)	46,478 (40,071–52,589)	47,529 (41,165–53,909)	81,546 (72,813–87,510)
Protein-energy malnutrition	541,326 (438,107–650,716)	402,153 (307,626–509,668)	17,257 (14,061–20,596)	26,146 (21,228–31,212)	31,280 (26,109–36,713)	64,489 (56,330–70,765)
Iodine deficiency	2,700 (1,950–4,098)	1,345 (705–2,600)	121 (67–209)	399 (281–576)	428 (288–667)	405 (271–603)
Vitamin A deficiency	0 (0–0)	0 (0–0)	0 (0–0)	0 (0–0)	0 (0–0)	0 (0–0)
Iron-deficiency anemia	46,232 (29,040–61,186)	17,660 (7,088–25,530)	1,943 (1,344–3,021)	11,673 (8,233–14,863)	9,395 (6,805–12,118)	5,559 (3,625–7,762)
Other nutritional deficiencies	45,324 (26,458–89,877)	17,044 (7,359–41,207)	2,505 (1,418–4,228)	8,259 (4,948–13,724)	6,424 (3,901–11,881)	11,091 (7,954–18,115)
Other communicable, maternal, neonatal, and nutritional diseases	466,523 (382,021–577,516)	270,204 (192,790–372,417)	31,848 (25,353–40,429)	76,011 (72,052–79,874)	56,304 (49,921–59,311)	32,156 (28,029–34,144)
Sexually transmitted diseases excluding HIV	214,138 (131,039–324,249)	180,353 (103,080–282,800)	15,200 (8,811–23,500)	8,900 (5,902–10,438)	6,002 (3,087–7,366)	3,682 (2,241–4,644)
Syphilis	211,751 (128,999–321,740)	180,353 (103,080–282,800)	15,147 (8,748–23,450)	7,781 (5,185–9,125)	5,273 (2,723–6,453)	3,195 (1,942–4,030)
Chlamydial infection	247 (143–302)	0 (0–0)	6 (4–8)	112 (72–139)	75 (35–99)	51 (30–66)
Gonococcal infection	1,449 (864–1,705)	0 (0–0)	27 (19–35)	662 (417–783)	425 (220–525)	334 (202–422)
Trichomoniasis	0 (0–0)	0 (0–0)	0 (0–0)	0 (0–0)	0 (0–0)	0 (0–0)
Genital herpes	0 (0–0)	0 (0–0)	0 (0–0)	0 (0–0)	0 (0–0)	0 (0–0)
Other sexually transmitted diseases	690 (419–807)	0 (0–0)	19 (13–25)	343 (224–406)	227 (116–279)	99 (59–126)
Hepatitis	156,203 (147,635–164,962)	42,281 (36,210–47,282)	11,545 (10,227–12,805)	53,636 (50,792–57,598)	32,678 (31,120–34,588)	16,061 (15,374–16,812)
Acute hepatitis A	35,881 (24,846–46,902)	32,126 (23,699–39,283)	1,742 (550–3,578)	1,379 (356–3,085)	443 (105–1,060)	189 (44–461)
Hepatitis B	74,234 (64,038–84,189)	2,344 (886–5,267)	1,280 (746–2,011)	31,754 (26,310–36,942)	26,847 (23,655–29,920)	12,006 (10,064–13,723)
Hepatitis C	2,753 (602–6,266)	0 (0–0)	154 (31–396)	850 (180–1,996)	1,278 (275–2,874)	470 (100–1,128)
Acute hepatitis E	43,332 (31,446–56,387)	7,810 (3,235–14,288)	8,367 (6,426–10,210)	19,651 (15,197–24,606)	4,108 (2,075–7,266)	3,394 (1,807–5,396)
Other infectious diseases	96,182 (64,330–114,334)	47,569 (25,799–63,385)	5,102 (3,799–5,822)	13,474 (11,038–14,867)	17,623 (13,013–19,509)	12,412 (9,459–13,673)
Non-communicable diseases	27,588,279 (27,085,305–28,191,734)	1,232,655 (1,053,757–1,564,268)	257,507 (237,462–277,299)	3,189,630 (3,107,061–3,297,388)	9,200,172 (9,020,286–9,394,994)	13,708,312 (13,546,224–13,866,363)
Neoplasms	5,679,301 (5,565,628–5,790,440)	50,807 (42,923–65,987)	59,478 (55,296–63,685)	848,622 (819,466–878,344)	2,594,838 (2,535,931–2,650,668)	2,125,555 (2,085,505–2,166,186)
Lip and oral cavity cancer	77,758 (75,531–80,300)	0 (0–0)	0 (0–0)	12,990 (12,481–13,525)	40,325 (38,872–41,899)	24,442 (23,815–25,117)
Nasopharynx cancer	45,489 (37,642–48,950)	0 (0–0)	421 (319–518)	14,452 (11,733–16,011)	21,954 (18,052–23,630)	8,660 (7,417–9,182)
Other pharynx cancer	39,066 (37,381–40,324)	0 (0–0)	0 (0–0)	6,072 (5,751–6,360)	23,338 (22,317–24,137)	9,655 (9,234–10,042)
Esophageal cancer	356,838 (336,343–381,256)	0 (0–0)	0 (0–0)	35,958 (33,595–38,606)	193,053 (181,270–206,223)	127,826 (120,586–137,673)
Stomach cancer	754,397 (731,105–778,281)	0 (0–0)	0 (0–0)	92,262 (88,125–98,208)	352,446 (339,773–365,628)	309,688 (301,943–317,849)
Colon and rectum cancer	487,859 (479,468–496,478)	0 (0–0)	0 (0–0)	49,377 (47,525–51,559)	197,096 (193,271–201,073)	241,385 (236,383–246,372)
Liver cancer	518,374 (445,647–557,607)	0 (0–0)	1,622 (1,234–1,864)	113,119 (85,132–125,320)	264,807 (228,403–285,617)	138,825 (127,903–148,046)
Liver cancer due to hepatitis B	202,224 (164,986–222,067)	0 (0–0)	936 (689–1,087)	68,133 (50,891–76,184)	98,970 (82,710–110,226)	34,184 (30,033–38,395)
Liver cancer due to hepatitis C	93,020 (85,177–100,266)	0 (0–0)	40 (31–51)	8,271 (6,643–9,493)	45,826 (40,504–50,293)	38,881 (35,993–42,248)
Liver cancer due to alcohol use	129,187 (112,286–142,061)	0 (0–0)	0 (0–0)	14,199 (10,617–16,386)	76,109 (65,129–85,504)	38,879 (35,141–42,493)
Liver cancer due to other causes	93,941 (78,846–102,980)	0 (0–0)	645 (505–751)	22,515 (16,895–25,611)	43,901 (36,751–49,501)	26,879 (23,661–29,799)
Gallbladder and biliary tract cancer	103,819 (100,608–107,193)	0 (0–0)	0 (0–0)	7,588 (7,011–8,053)	43,013 (41,363–44,692)	53,217 (51,800–54,780)
Pancreatic cancer	220,387 (216,610–224,567)	0 (0–0)	0 (0–0)	17,275 (16,761–17,925)	100,196 (98,179–102,437)	102,916 (101,092–104,799)
Larynx cancer	83,484 (80,897–85,994)	0 (0–0)	0 (0–0)	10,099 (9,550–10,606)	47,477 (45,974–48,957)	25,907 (25,087–26,727)
Tracheal, bronchus, and lung cancer	1,022,845 (999,983–1,053,324)	0 (0–0)	0 (0–0)	91,356 (87,746–95,573)	545,877 (532,764–562,612)	385,611 (376,340–398,977)
Malignant skin melanoma	32,625 (27,035–41,728)	0 (0–0)	0 (0–0)	7,855 (6,418–9,525)	13,362 (10,859–17,236)	11,407 (9,754–14,940)
Non-melanoma skin cancer	25,439 (24,671–26,216)	0 (0–0)	0 (0–0)	3,043 (2,914–3,189)	9,396 (9,050–9,734)	12,999 (12,620–13,444)
Non-melanoma skin cancer (squamous-cell carcinoma)	25,439 (24,671–26,216)	0 (0–0)	0 (0–0)	3,043 (2,914–3,189)	9,396 (9,050–9,734)	12,999 (12,620–13,444)
Non-melanoma skin cancer (basal-cell carcinoma)	0 (0–0)	0 (0–0)	0 (0–0)	0 (0–0)	0 (0–0)	0 (0–0)
Breast cancer	328,260 (316,336–350,689)	0 (0–0)	0 (0–0)	73,319 (65,566–82,383)	153,027 (147,322–163,086)	101,912 (98,584–106,581)
Cervical cancer	182,923 (169,229–194,903)	0 (0–0)	0 (0–0)	48,907 (44,234–53,051)	88,812 (82,023–95,406)	45,203 (42,777–49,033)
Uterine cancer	65,057 (61,740–69,074)	0 (0–0)	0 (0–0)	8,495 (7,667–9,372)	32,309 (30,466–34,357)	24,251 (23,340–25,997)
Ovarian cancer	103,083 (100,637–105,812)	0 (0–0)	0 (0–0)	17,832 (17,078–19,101)	51,519 (50,219–52,945)	33,731 (32,679–34,714)
Prostate cancer	191,896 (154,039–236,472)	0 (0–0)	0 (0–0)	1,710 (1,337–2,059)	50,045 (39,928–62,341)	140,140 (110,095–174,143)
Testicular cancer	8,607 (8,154–9,301)	0 (0–0)	0 (0–0)	6,058 (5,714–6,567)	1,572 (1,482–1,702)	977 (923–1,057)

SR Table 12 - Global deaths by age and cause for both sexes in 1990						
**Cause**	**All ages**	**0-5 years**	**5-14 years**	**15-49 years**	**50-69 years**	**70+ years**
Kidney cancer	72,968 (71,624–74,681)	2,553 (1,654–3,619)	1,106 (1,009–1,208)	7,446 (7,202–7,748)	33,736 (32,812–34,846)	28,125 (27,347–28,909)
Bladder cancer	112,598 (109,651–115,754)	0 (0–0)	0 (0–0)	5,706 (5,485–5,959)	41,294 (39,897–42,797)	65,597 (63,918–67,451)
Brain and nervous system cancer	140,015 (127,351–156,207)	7,319 (5,357–12,362)	10,684 (8,950–13,015)	41,231 (36,546–45,585)	55,794 (51,300–59,738)	24,986 (23,718–27,670)
Thyroid cancer	20,565 (19,484–22,688)	0 (0–0)	83 (67–93)	2,332 (2,182–2,745)	8,592 (8,071–9,410)	9,557 (9,038–10,430)
Mesothelioma	18,213 (17,021–19,204)	0 (0–0)	0 (0–0)	3,327 (2,983–3,525)	8,243 (7,688–8,667)	6,642 (6,205–7,273)
Hodgkin lymphoma	31,896 (23,475–41,474)	1,115 (565–1,379)	1,502 (958–1,958)	11,788 (8,989–15,008)	11,336 (8,107–15,072)	6,154 (4,617–8,045)
Non-Hodgkin lymphoma	123,662 (115,786–140,861)	3,942 (2,301–6,595)	4,327 (3,477–5,474)	24,993 (22,628–28,903)	46,095 (42,097–51,778)	44,303 (41,192–52,076)
Multiple myeloma	51,107 (49,603–53,238)	0 (0–0)	0 (0–0)	4,694 (4,505–4,983)	22,777 (22,024–23,731)	23,635 (22,917–24,583)
Leukemia	251,971 (244,524–261,881)	21,173 (18,366–24,411)	29,233 (27,176–31,462)	74,328 (71,113–79,158)	66,568 (64,627–68,473)	60,667 (59,344–62,076)
Acute lymphoid leukemia	90,472 (82,098–102,910)	14,295 (11,609–18,023)	20,919 (18,843–23,757)	29,658 (26,572–34,468)	16,514 (14,987–18,500)	9,084 (8,440–10,015)
Chronic lymphoid leukemia	39,575 (36,642–42,101)	0 (0–0)	0 (0–0)	7,263 (5,773–8,324)	12,861 (11,792–13,784)	19,450 (18,292–20,326)
Acute myeloid leukemia	85,600 (76,923–94,217)	6,877 (4,521–8,699)	8,313 (6,336–9,790)	25,476 (22,336–28,619)	24,656 (22,671–27,012)	20,275 (19,128–22,351)
Chronic myeloid leukemia	36,322 (33,295–39,476)	0 (0–0)	0 (0–0)	11,929 (10,345–13,458)	12,535 (11,477–13,621)	11,857 (11,158–12,543)
Other neoplasms	208,087 (194,334–217,761)	14,703 (10,991–21,221)	10,497 (9,308–11,484)	54,997 (48,422–59,088)	70,763 (63,960–75,061)	57,124 (54,104–60,611)
Cardiovascular diseases	12,589,715 (12,383,444–12,797,498)	116,835 (104,456–129,378)	44,215 (40,739–48,080)	1,119,401 (1,088,216–1,163,262)	3,984,260 (3,901,473–4,066,487)	7,325,001 (7,201,359–7,442,154)
Rheumatic heart disease	347,542 (328,308–367,073)	7,790 (6,314–9,323)	11,063 (10,077–12,019)	96,281 (89,624–103,304)	122,152 (115,092–129,908)	110,253 (103,879–116,507)
Ischemic heart disease	5,983,967 (5,889,836–6,082,402)	7,466 (6,183–9,296)	3,430 (3,046–3,962)	474,868 (461,698–492,615)	1,902,832 (1,863,964–1,940,498)	3,595,369 (3,530,160–3,657,684)
Cerebrovascular disease	4,633,827 (4,511,983–4,779,391)	45,589 (40,564–52,913)	16,650 (15,174–18,748)	373,275 (360,470–390,579)	1,481,214 (1,433,012–1,534,275)	2,717,097 (2,642,474–2,798,517)
Ischemic stroke	2,118,958 (2,017,544–2,235,761)	2,001 (1,696–2,464)	1,099 (981–1,279)	59,368 (54,287–66,082)	512,214 (478,914–548,370)	1,544,274 (1,478,610–1,624,826)
Hemorrhagic stroke	2,514,868 (2,409,946–2,606,304)	43,587 (38,770–50,827)	15,551 (14,166–17,540)	313,906 (302,101–328,281)	968,999 (922,789–1,006,847)	1,172,822 (1,111,994–1,219,640)
Hypertensive heart disease	660,836 (593,921–742,125)	1,090 (824–1,327)	540 (467–619)	60,386 (55,523–66,418)	224,671 (201,249–252,339)	374,147 (326,897–420,097)
Cardiomyopathy and myocarditis	269,302 (259,746–279,753)	17,525 (14,185–20,771)	4,590 (4,110–5,216)	45,925 (43,460–49,176)	75,398 (71,468–78,859)	125,862 (122,033–129,790)
Atrial fibrillation and flutter	92,047 (75,422–111,271)	0 (0–0)	0 (0–0)	596 (455–757)	7,944 (6,142–10,515)	83,505 (66,862–102,622)
Aortic aneurysm	95,687 (92,328–100,135)	0 (0–0)	0 (0–0)	7,136 (6,662–7,783)	30,105 (28,793–31,891)	58,445 (56,468–60,681)
Peripheral vascular disease	15,840 (14,941–16,677)	0 (0–0)	0 (0–0)	244 (229–259)	3,145 (2,956–3,374)	12,450 (11,714–13,091)
Endocarditis	46,680 (41,369–54,186)	5,330 (3,423–7,750)	1,205 (954–1,568)	8,982 (7,844–10,994)	11,241 (9,860–12,691)	19,919 (18,099–22,237)
Other cardiovascular and circulatory diseases	443,984 (429,972–460,038)	32,041 (28,073–39,088)	6,734 (6,089–7,779)	51,705 (49,120–54,973)	125,552 (120,274–131,099)	227,949 (218,645–236,986)
Chronic respiratory diseases	3,518,932 (3,409,539–3,642,535)	125,133 (108,719–140,596)	19,409 (17,446–21,173)	223,177 (213,447–236,999)	1,129,747 (1,088,327–1,175,382)	2,021,464 (1,956,524–2,094,772)
Chronic obstructive pulmonary disease	2,848,885 (2,676,578–3,020,422)	56,588 (42,812–70,354)	4,658 (3,413–6,532)	130,001 (115,192–147,660)	892,458 (821,508–971,188)	1,765,178 (1,671,796–1,848,458)
Pneumoconiosis	28,237 (24,178–32,361)	0 (0–0)	0 (0–0)	2,937 (2,367–3,511)	9,801 (8,283–11,332)	15,498 (13,247–17,742)
Silicosis	10,784 (8,747–13,281)	0 (0–0)	0 (0–0)	1,339 (930–1,803)	4,380 (3,494–5,500)	5,065 (4,162–6,183)
Asbestosis	1,577 (1,207–1,977)	0 (0–0)	0 (0–0)	296 (210–388)	531 (384–716)	749 (558–950)
Coal workers pneumoconiosis	3,265 (2,784–4,357)	0 (0–0)	0 (0–0)	195 (111–349)	1,123 (934–1,535)	1,946 (1,674–2,462)
Other pneumoconiosis	12,608 (9,836–15,967)	0 (0–0)	0 (0–0)	1,105 (792–1,483)	3,766 (2,817–4,939)	7,736 (6,217–9,753)
Asthma	548,435 (349,918–688,749)	36,645 (25,508–46,945)	12,955 (10,083–15,218)	81,681 (58,763–97,548)	207,177 (125,042–267,633)	209,976 (127,634–272,827)
Interstitial lung disease and pulmonary sarcoidosis	38,199 (31,783–50,165)	382 (215–755)	231 (181–360)	3,365 (2,667–4,547)	13,017 (10,452–17,597)	21,202 (17,948–27,994)
Other chronic respiratory diseases	55,174 (39,298–71,490)	31,516 (21,608–44,620)	1,564 (941–2,695)	5,191 (3,491–6,795)	7,292 (4,994–9,177)	9,609 (7,474–11,594)
Cirrhosis and other chronic liver diseases	930,537 (899,292–1,005,336)	25,107 (18,732–32,352)	12,771 (11,246–14,959)	263,636 (251,804–288,029)	416,449 (399,830–451,439)	212,573 (202,015–222,168)
Cirrhosis and other chronic liver diseases due to hepatitis B	278,656 (258,288–310,373)	197 (141–259)	399 (324–485)	81,825 (74,447–92,185)	129,761 (116,511–147,421)	66,472 (60,670–73,014)
Cirrhosis and other chronic liver diseases due to hepatitis C	219,549 (204,598–236,286)	104 (75–140)	220 (172–270)	57,788 (51,743–65,504)	102,871 (92,428–114,760)	58,565 (52,271–63,931)
Cirrhosis and other chronic liver diseases due to alcohol use	236,764 (220,803–254,972)	0 (0–0)	0 (0–0)	77,323 (71,048–85,298)	118,127 (106,791–130,803)	41,313 (37,244–45,812)
Cirrhosis and other chronic liver diseases due to other causes	195,566 (181,406–217,563)	24,805 (18,499–32,023)	12,152 (10,685–14,256)	46,699 (42,513–52,273)	65,689 (58,421–74,880)	46,220 (42,047–50,678)
Digestive diseases	1,009,149 (960,111–1,052,910)	58,241 (42,794–81,613)	28,225 (25,302–30,945)	224,517 (208,629–236,663)	318,905 (301,696–337,453)	379,259 (364,745–397,142)
Peptic ulcer disease	328,773 (302,895–360,463)	7,361 (4,271–12,763)	6,181 (5,269–7,408)	77,084 (70,741–85,657)	117,578 (107,045–130,174)	120,568 (109,262–134,270)
Gastritis and duodenitis	49,057 (36,296–63,567)	1,955 (1,039–3,101)	904 (605–1,268)	11,745 (8,743–15,121)	17,049 (12,846–22,396)	17,401 (12,540–23,209)
Appendicitis	46,243 (40,588–54,366)	8,194 (4,417–15,199)	4,751 (3,947–5,532)	14,704 (13,367–16,805)	10,310 (9,359–12,017)	8,282 (7,563–9,523)
Paralytic ileus and intestinal obstruction	194,569 (165,871–216,508)	15,719 (9,558–25,718)	8,736 (6,796–10,272)	47,336 (36,360–53,048)	58,001 (49,005–64,792)	64,774 (58,728–73,573)
Inguinal, femoral, and abdominal hernia	47,071 (33,994–54,245)	2,274 (804–3,787)	1,172 (721–1,458)	9,139 (5,593–10,997)	15,086 (11,007–17,941)	19,398 (14,410–22,228)
Inflammatory bowel disease	33,846 (28,338–40,509)	4,140 (1,534–9,165)	830 (591–1,257)	6,629 (5,247–8,175)	8,888 (7,541–10,168)	13,358 (11,808–14,644)
Vascular intestinal disorders	55,092 (51,145–59,543)	889 (519–1,581)	179 (144–226)	3,591 (3,063–4,111)	15,596 (14,150–17,242)	34,834 (32,764–37,334)
Gallbladder and biliary diseases	86,547 (81,064–92,241)	3,356 (1,875–5,808)	1,379 (1,168–1,668)	13,141 (11,873–14,519)	26,959 (24,948–28,798)	41,710 (39,769–44,298)
Pancreatitis	68,130 (63,002–75,171)	533 (306–898)	600 (474–754)	25,452 (23,080–28,624)	23,597 (21,779–25,879)	17,946 (16,941–19,294)
Other digestive diseases	99,818 (82,555–117,781)	13,815 (8,097–19,350)	3,489 (2,784–4,348)	15,692 (12,228–20,069)	25,837 (20,576–31,949)	40,983 (34,884–47,464)
Neurological disorders	1,106,794 (964,049–1,250,409)	25,638 (17,360–34,457)	15,326 (13,713–16,981)	72,027 (68,966–76,488)	108,273 (92,362–123,921)	885,529 (747,965–1,021,604)
Alzheimer disease and other dementias	887,052 (741,868–1,030,006)	0 (0–0)	0 (0–0)	847 (530–1,243)	63,270 (47,445–79,024)	822,934 (684,635–958,569)
Parkinson disease	47,070 (45,395–49,978)	0 (0–0)	0 (0–0)	436 (403–484)	7,877 (7,547–8,604)	38,756 (37,323–40,819)
Epilepsy	105,519 (95,266–116,995)	18,777 (12,082–25,951)	12,480 (11,101–13,836)	53,560 (50,546–57,864)	13,658 (13,175–14,139)	7,042 (6,792–7,316)

SR Table 12 - Global deaths by age and cause for both sexes in 1990						
**Cause**	**All ages**	**0-5 years**	**5-14 years**	**15-49 years**	**50-69 years**	**70+ years**
Multiple sclerosis	13,526 (11,705–15,582)	0 (0–0)	0 (0–0)	5,027 (3,997–6,213)	6,122 (5,517–6,854)	2,375 (2,097–2,557)
Motor neuron disease	18,022 (17,300–20,000)	1,742 (1,034–2,260)	136 (118–161)	2,143 (2,004–2,518)	8,301 (7,910–9,210)	5,698 (5,515–5,998)
Migraine	0 (0–0)	0 (0–0)	0 (0–0)	0 (0–0)	0 (0–0)	0 (0–0)
Tension-type headache	0 (0–0)	0 (0–0)	0 (0–0)	0 (0–0)	0 (0–0)	0 (0–0)
Medication overuse headache	0 (0–0)	0 (0–0)	0 (0–0)	0 (0–0)	0 (0–0)	0 (0–0)
Other neurological disorders	35,626 (34,325–38,625)	5,142 (3,681–7,989)	2,710 (2,403–3,298)	10,010 (9,319–10,815)	9,042 (8,524–9,707)	8,720 (8,280–9,214)
Mental and substance use disorders	180,078 (170,131–185,086)	30 (25–36)	32 (17–44)	106,746 (98,157–110,770)	57,301 (55,530–59,144)	15,968 (15,402–16,498)
Schizophrenia	15,536 (13,968–16,734)	0 (0–0)	0 (0–0)	7,141 (6,204–7,907)	5,115 (4,655–5,510)	3,279 (3,016–3,523)
Alcohol use disorders	95,525 (92,201–98,797)	0 (0–0)	0 (0–0)	48,375 (46,373–50,564)	39,118 (37,707–40,677)	8,031 (7,788–8,316)
Drug use disorders	68,582 (57,903–71,483)	30 (25–36)	0 (0–0)	50,827 (42,135–53,343)	13,067 (11,736–13,829)	4,657 (4,199–5,019)
Opioid use disorders	48,528 (42,809–51,020)	30 (25–36)	0 (0–0)	36,576 (31,155–38,912)	9,170 (8,485–9,810)	2,751 (2,530–3,070)
Cocaine use disorders	1,782 (1,456–2,103)	0 (0–0)	0 (0–0)	1,432 (1,167–1,698)	288 (228–339)	61 (46–73)
Amphetamine use disorders	1,273 (1,047–1,966)	0 (0–0)	0 (0–0)	1,003 (819–1,548)	221 (176–342)	49 (37–74)
Cannabis use disorders	0 (0–0)	0 (0–0)	0 (0–0)	0 (0–0)	0 (0–0)	0 (0–0)
Other drug use disorders	16,998 (11,801–18,741)	0 (0–0)	0 (0–0)	11,815 (8,050–13,093)	3,387 (2,520–3,818)	1,795 (1,332–2,119)
Depressive disorders	0 (0–0)	0 (0–0)	0 (0–0)	0 (0–0)	0 (0–0)	0 (0–0)
Major depressive disorder	0 (0–0)	0 (0–0)	0 (0–0)	0 (0–0)	0 (0–0)	0 (0–0)
Dysthymia	0 (0–0)	0 (0–0)	0 (0–0)	0 (0–0)	0 (0–0)	0 (0–0)
Bipolar disorder	0 (0–0)	0 (0–0)	0 (0–0)	0 (0–0)	0 (0–0)	0 (0–0)
Anxiety disorders	0 (0–0)	0 (0–0)	0 (0–0)	0 (0–0)	0 (0–0)	0 (0–0)
Eating disorders	434 (301–633)	0 (0–0)	32 (17–44)	401 (284–598)	0 (0–0)	0 (0–0)
Anorexia nervosa	415 (289–606)	0 (0–0)	30 (16–41)	384 (271–570)	0 (0–0)	0 (0–0)
Bulimia nervosa	18 (11–28)	0 (0–0)	1 (0–2)	17 (10–27)	0 (0–0)	0 (0–0)
Autistic spectrum disorders	0 (0–0)	0 (0–0)	0 (0–0)	0 (0–0)	0 (0–0)	0 (0–0)
Autism	0 (0–0)	0 (0–0)	0 (0–0)	0 (0–0)	0 (0–0)	0 (0–0)
Asperger syndrome and other autistic spectrum disorders	0 (0–0)	0 (0–0)	0 (0–0)	0 (0–0)	0 (0–0)	0 (0–0)
Attention-deficit/hyperactivity disorder	0 (0–0)	0 (0–0)	0 (0–0)	0 (0–0)	0 (0–0)	0 (0–0)
Conduct disorder	0 (0–0)	0 (0–0)	0 (0–0)	0 (0–0)	0 (0–0)	0 (0–0)
Idiopathic developmental intellectual disability	0 (0–0)	0 (0–0)	0 (0–0)	0 (0–0)	0 (0–0)	0 (0–0)
Other mental and substance use disorders	0 (0–0)	0 (0–0)	0 (0–0)	0 (0–0)	0 (0–0)	0 (0–0)
Diabetes, urogenital, blood, and endocrine diseases	1,701,755 (1,645,755–1,762,088)	149,938 (117,955–183,372)	36,446 (32,685–39,952)	266,745 (253,573–280,758)	555,633 (536,556–577,751)	692,993 (673,424–715,552)
Diabetes mellitus	668,628 (651,699–685,183)	4,369 (3,612–4,974)	3,012 (2,683–3,482)	71,570 (69,168–74,559)	277,906 (269,807–286,291)	311,769 (303,099–320,205)
Acute glomerulonephritis	15,107 (12,786–20,852)	1,037 (784–1,387)	781 (683–1,027)	4,759 (3,911–5,982)	4,611 (3,757–6,401)	3,917 (3,207–6,108)
Chronic kidney disease	592,921 (556,105–631,250)	23,021 (19,190–26,315)	11,153 (9,674–12,293)	111,825 (100,799–118,925)	190,283 (177,989–206,410)	256,636 (247,610–273,751)
Chronic kidney disease due to diabetes mellitus	179,189 (168,686–190,863)	2,429 (1,941–2,950)	852 (702–1,030)	24,825 (22,181–27,434)	69,197 (63,425–75,932)	81,884 (77,148–87,962)
Chronic kidney disease due to hypertension	254,172 (239,341–271,273)	7,616 (6,249–8,837)	3,173 (2,662–3,690)	41,620 (36,928–45,013)	78,623 (72,560–85,480)	123,138 (116,691–131,611)
Chronic kidney disease due to glomerulonephritis	141,250 (129,694–153,283)	9,602 (7,832–11,060)	5,275 (4,550–5,912)	40,172 (36,142–43,927)	40,047 (36,233–44,650)	46,152 (42,351–50,466)
Chronic kidney disease due to other causes	18,307 (15,316–21,491)	3,373 (2,574–4,277)	1,851 (1,438–2,321)	5,207 (3,682–6,885)	2,415 (1,539–3,602)	5,459 (4,586–6,678)
Urinary diseases and male infertility	142,145 (128,018–165,672)	10,672 (8,135–13,285)	2,188 (1,867–2,598)	21,500 (18,513–26,401)	40,714 (34,979–50,617)	67,070 (60,861–78,060)
Interstitial nephritis and urinary tract infections	102,279 (91,675–116,809)	8,429 (6,429–10,448)	1,369 (1,158–1,605)	14,309 (12,218–17,264)	26,807 (22,707–32,521)	51,363 (46,158–58,776)
Urolithiasis	15,484 (11,445–18,447)	0 (0–0)	339 (250–422)	3,030 (2,168–3,670)	6,010 (4,411–7,311)	6,103 (4,424–7,347)
Benign prostatic hyperplasia	0 (0–0)	0 (0–0)	0 (0–0)	0 (0–0)	0 (0–0)	0 (0–0)
Male infertility	0 (0–0)	0 (0–0)	0 (0–0)	0 (0–0)	0 (0–0)	0 (0–0)
Other urinary diseases	24,381 (19,160–35,440)	2,242 (1,487–3,556)	479 (360–679)	4,159 (3,052–6,527)	7,896 (5,643–12,700)	9,603 (7,403–14,146)
Gynecological diseases	6,572 (5,093–8,073)	0 (0–0)	0 (0–0)	3,239 (2,377–3,944)	1,971 (1,476–2,495)	1,361 (1,087–1,779)
Uterine fibroids	1,768 (955–2,470)	0 (0–0)	0 (0–0)	723 (403–1,013)	702 (366–1,008)	342 (175–578)
Polycystic ovarian syndrome	881 (211–1,471)	0 (0–0)	0 (0–0)	637 (130–1,091)	243 (80–382)	0 (0–0)
Female infertility	0 (0–0)	0 (0–0)	0 (0–0)	0 (0–0)	0 (0–0)	0 (0–0)
Endometriosis	43 (27–64)	0 (0–0)	0 (0–0)	37 (24–55)	5 (3–9)	0 (0–0)
Genital prolapse	1,036 (505–1,906)	0 (0–0)	0 (0–0)	124 (54–219)	412 (156–843)	499 (278–927)
Premenstrual syndrome	0 (0–0)	0 (0–0)	0 (0–0)	0 (0–0)	0 (0–0)	0 (0–0)
Other gynecological diseases	2,842 (2,055–3,848)	0 (0–0)	0 (0–0)	1,716 (1,225–2,432)	607 (395–933)	519 (365–811)
Hemoglobinopathies and hemolytic anemias	195,182 (164,156–225,027)	90,264 (63,127–119,428)	15,123 (12,464–18,151)	38,527 (34,214–44,254)	22,112 (19,679–25,125)	29,153 (25,734–33,262)
Thalassemias	28,543 (20,732–37,028)	19,702 (12,534–27,928)	5,037 (4,008–6,117)	2,152 (1,867–2,529)	58 (53–68)	1,592 (1,217–2,240)
Thalassemias trait	0 (0–0)	0 (0–0)	0 (0–0)	0 (0–0)	0 (0–0)	0 (0–0)
Sickle cell disorders	92,645 (69,715–119,583)	68,047 (46,695–94,770)	7,820 (6,198–9,387)	14,117 (11,816–16,898)	709 (616–823)	1,950 (1,633–2,376)
Sickle cell trait	0 (0–0)	0 (0–0)	0 (0–0)	0 (0–0)	0 (0–0)	0 (0–0)

SR Table 12 - Global deaths by age and cause for both sexes in 1990						
**Cause**	**All ages**	**0-5 years**	**5-14 years**	**15-49 years**	**50-69 years**	**70+ years**
G6PD deficiency	19,053 (16,499–23,562)	1,457 (700–2,355)	1,310 (961–1,794)	10,975 (9,592–13,470)	4,452 (3,659–5,462)	857 (695–1,078)
G6PD trait	0 (0–0)	0 (0–0)	0 (0–0)	0 (0–0)	0 (0–0)	0 (0–0)
Other hemoglobinopathies and hemolytic anemias	54,938 (49,367–60,782)	1,057 (658–1,528)	955 (775–1,149)	11,280 (10,246–12,771)	16,891 (15,047–19,251)	24,753 (21,853–27,952)
Endocrine, metabolic, blood, and immune disorders	81,198 (78,205–84,514)	20,572 (17,131–23,834)	4,186 (3,900–4,462)	15,321 (14,566–16,120)	18,032 (16,980–19,248)	23,084 (21,961–24,334)
Musculoskeletal disorders	52,811 (48,855–58,102)	0 (0–0)	1,855 (1,490–2,326)	13,192 (11,385–16,536)	14,606 (13,269–15,743)	23,157 (20,632–24,341)
Rheumatoid arthritis	21,974 (17,991–24,297)	0 (0–0)	326 (215–435)	2,202 (1,542–2,864)	7,618 (6,317–8,461)	11,827 (9,942–12,681)
Osteoarthritis	0 (0–0)	0 (0–0)	0 (0–0)	0 (0–0)	0 (0–0)	0 (0–0)
Low back and neck pain	0 (0–0)	0 (0–0)	0 (0–0)	0 (0–0)	0 (0–0)	0 (0–0)
Low back pain	0 (0–0)	0 (0–0)	0 (0–0)	0 (0–0)	0 (0–0)	0 (0–0)
Neck pain	0 (0–0)	0 (0–0)	0 (0–0)	0 (0–0)	0 (0–0)	0 (0–0)
Gout	0 (0–0)	0 (0–0)	0 (0–0)	0 (0–0)	0 (0–0)	0 (0–0)
Other musculoskeletal disorders	30,837 (28,724–35,044)	0 (0–0)	1,529 (1,222–1,961)	10,990 (9,430–14,489)	6,987 (6,554–7,859)	11,330 (10,027–12,021)
Other non-communicable diseases	819,178 (632,035–1,171,903)	680,899 (504,771–1,012,692)	39,746 (30,598–52,207)	51,565 (46,267–62,901)	20,155 (17,201–24,740)	26,811 (21,462–33,730)
Congenital anomalies	748,630 (560,340–1,084,669)	646,946 (471,042–959,203)	38,839 (29,690–51,081)	46,086 (40,630–57,073)	10,354 (9,514–12,092)	6,402 (5,977–7,282)
Neural tube defects	138,906 (97,356–223,133)	131,164 (90,816–212,369)	4,004 (2,961–5,925)	2,738 (2,136–3,721)	625 (439–937)	373 (245–629)
Congenital heart anomalies	388,407 (284,785–545,429)	320,417 (226,863–466,050)	27,964 (20,714–36,468)	33,675 (29,427–41,860)	3,790 (3,342–4,343)	2,558 (2,277–2,970)
Cleft lip and cleft palate	5,698 (3,688–8,194)	5,698 (3,688–8,194)	0 (0–0)	0 (0–0)	0 (0–0)	0 (0–0)
Down syndrome	29,674 (15,663–58,161)	23,591 (10,606–50,031)	946 (548–1,685)	3,155 (2,179–4,771)	1,869 (1,364–2,684)	111 (79–157)
Turner syndrome	0 (0–0)	0 (0–0)	0 (0–0)	0 (0–0)	0 (0–0)	0 (0–0)
Klinefelter syndrome	0 (0–0)	0 (0–0)	0 (0–0)	0 (0–0)	0 (0–0)	0 (0–0)
Other chromosomal abnormalities	20,827 (7,699–67,429)	19,595 (6,634–65,472)	344 (194–837)	585 (473–767)	225 (156–284)	77 (58–98)
Other congenital anomalies	165,115 (128,482–244,057)	146,479 (111,956–222,853)	5,579 (4,286–7,777)	5,932 (4,967–7,171)	3,844 (3,275–4,351)	3,280 (2,989–3,675)
Skin and subcutaneous diseases	42,317 (30,653–57,535)	5,722 (3,216–9,246)	907 (589–1,355)	5,479 (3,832–7,991)	9,800 (6,741–14,208)	20,408 (14,568–27,371)
Dermatitis	0 (0–0)	0 (0–0)	0 (0–0)	0 (0–0)	0 (0–0)	0 (0–0)
Psoriasis	0 (0–0)	0 (0–0)	0 (0–0)	0 (0–0)	0 (0–0)	0 (0–0)
Cellulitis	7,641 (5,133–12,227)	772 (362–1,434)	179 (112–267)	1,408 (880–2,509)	2,601 (1,565–4,401)	2,678 (1,885–4,332)
Pyoderma	16,276 (11,555–25,128)	4,568 (2,489–7,694)	466 (290–760)	2,639 (1,751–3,954)	3,572 (2,589–5,852)	5,031 (3,764–7,557)
Scabies	0 (0–0)	0 (0–0)	0 (0–0)	0 (0–0)	0 (0–0)	0 (0–0)
Fungal skin diseases	0 (0–0)	0 (0–0)	0 (0–0)	0 (0–0)	0 (0–0)	0 (0–0)
Viral skin diseases	0 (0–0)	0 (0–0)	0 (0–0)	0 (0–0)	0 (0–0)	0 (0–0)
Acne vulgaris	0 (0–0)	0 (0–0)	0 (0–0)	0 (0–0)	0 (0–0)	0 (0–0)
Alopecia areata	0 (0–0)	0 (0–0)	0 (0–0)	0 (0–0)	0 (0–0)	0 (0–0)
Pruritus	0 (0–0)	0 (0–0)	0 (0–0)	0 (0–0)	0 (0–0)	0 (0–0)
Urticaria	0 (0–0)	0 (0–0)	0 (0–0)	0 (0–0)	0 (0–0)	0 (0–0)
Decubitus ulcer	16,213 (10,021–20,943)	91 (38–160)	148 (82–219)	997 (590–1,473)	3,138 (1,641–4,828)	11,836 (7,526–15,344)
Other skin and subcutaneous diseases	2,186 (1,624–3,261)	290 (180–568)	112 (68–163)	433 (296–669)	487 (318–775)	861 (652–1,188)
Sense organ diseases	0 (0–0)	0 (0–0)	0 (0–0)	0 (0–0)	0 (0–0)	0 (0–0)
Glaucoma	0 (0–0)	0 (0–0)	0 (0–0)	0 (0–0)	0 (0–0)	0 (0–0)
Cataract	0 (0–0)	0 (0–0)	0 (0–0)	0 (0–0)	0 (0–0)	0 (0–0)
Macular degeneration	0 (0–0)	0 (0–0)	0 (0–0)	0 (0–0)	0 (0–0)	0 (0–0)
Refraction and accommodation disorders	0 (0–0)	0 (0–0)	0 (0–0)	0 (0–0)	0 (0–0)	0 (0–0)
Age-related and other hearing loss	0 (0–0)	0 (0–0)	0 (0–0)	0 (0–0)	0 (0–0)	0 (0–0)
Other vision loss	0 (0–0)	0 (0–0)	0 (0–0)	0 (0–0)	0 (0–0)	0 (0–0)
Other sense organ diseases	0 (0–0)	0 (0–0)	0 (0–0)	0 (0–0)	0 (0–0)	0 (0–0)
Oral disorders	0 (0–0)	0 (0–0)	0 (0–0)	0 (0–0)	0 (0–0)	0 (0–0)
Deciduous caries	0 (0–0)	0 (0–0)	0 (0–0)	0 (0–0)	0 (0–0)	0 (0–0)
Permanent caries	0 (0–0)	0 (0–0)	0 (0–0)	0 (0–0)	0 (0–0)	0 (0–0)
Periodontal diseases	0 (0–0)	0 (0–0)	0 (0–0)	0 (0–0)	0 (0–0)	0 (0–0)
Edentulism and severe tooth loss	0 (0–0)	0 (0–0)	0 (0–0)	0 (0–0)	0 (0–0)	0 (0–0)
Other oral disorders	0 (0–0)	0 (0–0)	0 (0–0)	0 (0–0)	0 (0–0)	0 (0–0)
Sudden infant death syndrome	28,230 (22,632–53,425)	28,230 (22,632–53,425)	0 (0–0)	0 (0–0)	0 (0–0)	0 (0–0)
Injuries	4,427,633 (4,143,725–4,617,554)	708,211 (571,385–823,374)	397,129 (368,809–422,656)	2,135,944 (2,018,568–2,212,618)	714,224 (669,080–741,002)	472,123 (433,885–494,491)
Transport injuries	1,208,810 (1,153,429–1,259,972)	113,877 (86,986–137,124)	109,173 (100,909–117,014)	678,463 (653,322–704,127)	209,769 (200,879–218,232)	97,526 (94,207–100,851)
Road injuries	1,137,039 (1,085,205–1,186,580)	105,342 (80,056–126,862)	104,256 (96,470–111,562)	640,185 (616,794–665,427)	194,880 (186,304–202,915)	92,375 (89,219–95,485)
Pedestrian road injuries	518,695 (450,460–560,011)	58,061 (46,083–69,716)	60,123 (54,942–65,222)	244,677 (207,746–267,273)	101,360 (87,963–109,552)	54,473 (49,260–57,733)
Cyclist road injuries	45,889 (40,635–52,471)	2,662 (1,764–3,952)	5,689 (4,843–6,751)	22,571 (19,338–26,240)	10,592 (9,375–12,093)	4,373 (4,000–4,814)
Motorcyclist road injuries	151,398 (124,134–183,876)	9,180 (4,937–14,110)	7,927 (5,830–9,976)	109,036 (89,040–133,102)	19,298 (14,978–24,866)	5,955 (4,540–7,869)

SR Table 12 - Global deaths by age and cause for both sexes in 1990						
Cause	All ages	0-5 years	5-14 years	15-49 years	50-69 years	70+ years
Motor vehicle road injuries	408,235 (375,182–465,781)	33,691 (23,573–42,670)	29,164 (26,134–34,003)	257,435 (236,159–296,344)	61,455 (54,975–73,260)	26,487 (23,908–30,930)
Other road injuries	12,820 (8,261–16,019)	1,746 (623–3,188)	1,351 (824–1,910)	6,464 (4,120–8,283)	2,172 (1,528–2,737)	1,085 (798–1,430)
Other transport injuries	71,770 (60,383–78,674)	8,534 (4,839–10,880)	4,917 (3,730–5,612)	38,278 (32,834–42,128)	14,888 (12,533–16,557)	5,151 (4,475–5,724)
Unintentional injuries	2,039,594 (1,852,502–2,158,495)	553,125 (440,295–650,961)	241,292 (221,139–258,089)	675,973 (626,782–712,612)	288,567 (266,910–307,082)	280,634 (254,346–297,171)
Falls	352,660 (323,908–372,440)	39,630 (28,518–55,019)	19,675 (17,098–22,187)	92,882 (85,951–97,979)	73,702 (68,264–77,754)	126,768 (117,255–133,708)
Drowning	569,504 (484,043–623,166)	206,538 (153,151–247,956)	126,971 (112,663–136,939)	168,801 (155,783–179,845)	44,357 (39,176–47,759)	22,835 (19,331–25,103)
Fire, heat, and hot substances	231,787 (177,849–254,715)	54,060 (37,070–65,611)	16,888 (12,952–18,894)	100,324 (71,915–110,528)	31,150 (25,079–33,089)	29,363 (24,471–31,084)
Poisonings	138,479 (96,061–163,579)	43,300 (16,934–67,790)	11,093 (9,456–13,759)	52,072 (42,162–61,358)	21,628 (16,888–25,541)	10,385 (8,607–12,099)
Exposure to mechanical forces	202,613 (173,271–241,884)	56,933 (39,894–74,188)	17,034 (14,038–22,007)	81,474 (71,937–99,336)	28,424 (25,733–34,706)	18,746 (15,740–21,528)
Unintentional firearm injuries	33,057 (25,132–36,382)	3,848 (2,219–5,215)	2,956 (2,386–3,671)	17,593 (13,208–19,891)	4,163 (3,075–4,784)	4,496 (3,805–4,921)
Unintentional suffocation	46,348 (34,053–59,921)	34,139 (23,068–45,934)	3,743 (2,812–5,501)	4,193 (3,508–5,033)	1,436 (1,084–1,810)	2,835 (2,340–3,395)
Other exposure to mechanical forces	123,207 (103,149–156,182)	18,945 (11,981–27,831)	10,334 (8,157–13,800)	59,688 (50,183–77,329)	22,824 (20,266–29,599)	11,414 (9,137–13,752)
Adverse effects of medical treatment	102,677 (80,470–122,281)	29,952 (13,660–42,245)	6,133 (4,083–7,674)	25,488 (19,916–30,082)	18,474 (14,966–21,115)	22,627 (18,205–25,509)
Animal contact	112,773 (82,631–126,026)	23,280 (15,385–29,599)	17,928 (12,828–20,694)	42,096 (30,441–46,434)	21,067 (15,408–23,530)	8,400 (6,654–9,571)
Venomous animal contact	91,145 (65,233–102,613)	16,592 (10,554–21,156)	14,624 (10,257–17,052)	35,989 (25,333–40,288)	17,503 (12,525–20,136)	6,435 (4,878–7,682)
Non-venomous animal contact	21,628 (15,806–25,960)	6,688 (3,815–8,934)	3,304 (2,266–4,117)	6,107 (4,460–7,150)	3,563 (2,617–4,208)	1,964 (1,545–2,290)
Foreign body	152,732 (107,698–213,735)	72,934 (39,829–114,800)	11,357 (7,804–16,348)	30,914 (22,620–66,578)	17,238 (13,917–30,464)	20,286 (17,295–31,049)
Pulmonary aspiration and foreign body in airway	108,746 (73,155–158,476)	56,548 (25,408–96,914)	7,536 (4,733–11,137)	16,915 (14,122–20,482)	12,217 (10,500–13,797)	15,528 (13,822–16,735)
Foreign body in eyes	0 (0–0)	0 (0–0)	0 (0–0)	0 (0–0)	0 (0–0)	0 (0–0)
Foreign body in other body part	43,985 (21,908–125,071)	16,386 (6,735–27,502)	3,820 (1,753–9,650)	13,999 (6,420–50,358)	5,020 (2,448–17,843)	4,758 (2,734–15,141)
Environmental heat and cold exposure	47,621 (34,791–62,427)	7,292 (3,443–9,285)	1,767 (1,301–2,443)	15,035 (11,589–18,474)	12,817 (9,549–17,993)	10,708 (7,081–15,579)
Other unintentional injuries	128,743 (117,835–139,531)	19,201 (14,691–22,737)	12,441 (10,749–14,091)	66,883 (61,116–72,551)	19,705 (17,769–21,646)	10,511 (9,239–12,067)
Self-harm and interpersonal violence	1,031,525 (915,738–1,080,082)	23,959 (20,476–26,980)	27,765 (24,145–29,608)	690,327 (610,343–724,181)	200,444 (177,673–210,435)	89,028 (77,208–93,655)
Self-harm	685,812 (569,898–732,336)	0 (0–0)	11,763 (8,749–13,223)	437,594 (357,423–468,059)	159,427 (135,414–168,938)	77,026 (64,687–81,622)
Interpersonal violence	345,713 (332,350–357,438)	23,959 (20,476–26,980)	16,001 (14,982–17,211)	252,733 (243,703–263,034)	41,017 (39,262–42,944)	12,001 (11,516–12,877)
Assault by firearm	118,174 (112,555–127,015)	2,081 (1,564–2,728)	3,067 (2,789–3,449)	98,814 (94,565–105,759)	11,752 (10,751–13,151)	2,457 (2,223–2,691)
Assault by sharp object	86,288 (78,615–92,957)	2,821 (1,958–3,875)	2,984 (2,616–3,455)	67,876 (61,538–73,090)	9,737 (8,756–10,594)	2,868 (2,659–3,102)
Assault by other means	141,250 (127,293–153,735)	19,056 (15,822–21,867)	9,949 (8,914–10,984)	86,042 (76,932–93,769)	19,526 (18,213–21,565)	6,676 (6,261–7,429)
Forces of nature, war, and legal intervention	147,703 (107,351–193,866)	17,248 (9,614–26,721)	18,897 (11,246–27,823)	91,179 (61,018–126,625)	15,443 (9,961–21,710)	4,934 (2,862–7,574)
Exposure to forces of nature	50,904 (24,937–76,395)	5,797 (2,719–8,983)	11,158 (5,088–17,061)	24,791 (12,271–37,069)	6,762 (3,293–10,160)	2,393 (1,216–3,532)
Collective violence and legal intervention	96,799 (64,537–134,310)	11,451 (4,961–20,391)	7,738 (2,946–14,091)	66,387 (37,727–98,870)	8,680 (4,634–13,827)	2,540 (942–5,270)

SR Table 12 - Global deaths by age and cause for both sexes in 1995						
**Cause**	**All ages**	**0-5 years**	**5-14 years**	**15-49 years**	**50-69 years**	**70+ years**
All causes	50,388,402<br>(49,890,765–50,889,720)	10,627,202<br>(10,533,914–10,729,244)	1,300,909<br>(1,287,542–1,314,752)	8,498,000<br>(8,347,892–8,664,981)	12,251,913<br>(12,068,134–12,442,638)	17,710,375<br>(17,560,127–17,866,353)
Communicable, maternal, neonatal, and nutritional diseases	15,242,615<br>(14,885,504–15,636,903)	8,968,481<br>(8,716,618–9,188,688)	654,489<br>(618,681–695,516)	2,423,387<br>(2,332,691–2,555,860)	1,527,583<br>(1,438,906–1,628,846)	1,668,673<br>(1,596,195–1,761,347)
HIV/AIDS and tuberculosis	2,232,206<br>(2,005,845–2,558,968)	154,651<br>(136,942–187,852)	37,393<br>(32,170–49,017)	1,075,506<br>(991,171–1,182,160)	634,611<br>(532,744–748,466)	330,042<br>(275,186–417,718)
Tuberculosis	1,493,992<br>(1,259,752–1,803,802)	52,749<br>(36,663–86,838)	27,167<br>(22,220–38,722)	541,288<br>(448,942–647,552)	551,077<br>(449,404–666,624)	321,710<br>(266,753–409,333)
HIV/AIDS	738,213<br>(684,984–800,930)	101,901<br>(96,423–108,193)	10,226<br>(9,375–11,341)	534,218<br>(490,898–587,613)	83,534<br>(75,162–94,216)	8,332<br>(7,258–10,029)
HIV/AIDS - Tuberculosis	162,888<br>(139,045–192,857)	30,449<br>(25,950–34,821)	2,907<br>(2,286–3,412)	106,101<br>(89,773–127,662)	21,094<br>(17,934–24,794)	2,334<br>(1,946–2,780)
HIV/AIDS resulting in other diseases	575,325<br>(522,072–635,501)	71,452<br>(65,105–79,376)	7,319<br>(6,461–8,479)	428,116<br>(383,090–478,229)	62,439<br>(54,949–73,140)	5,997<br>(5,052–7,469)
Diarrhea, lower respiratory, and other common infectious diseases	7,125,891<br>(6,789,626–7,482,347)	4,159,030<br>(3,888,388–4,444,638)	454,339<br>(407,380–509,547)	662,729<br>(620,157–723,598)	686,354<br>(651,532–730,524)	1,163,438<br>(1,114,221–1,219,195)
Diarrheal diseases	1,985,771<br>(1,829,076–2,136,447)	1,096,304<br>(949,598–1,230,483)	105,002<br>(91,979–117,493)	215,024<br>(203,547–228,726)	258,817<br>(246,529–273,723)	310,622<br>(294,570–327,577)
Intestinal infectious diseases	240,996<br>(135,378–395,730)	67,992<br>(36,045–116,262)	95,179<br>(55,250–151,196)	65,344<br>(34,850–111,586)	9,015<br>(4,703–15,860)	3,465<br>(2,065–5,449)
Typhoid fever	195,845<br>(106,470–328,818)	54,645<br>(27,681–97,762)	78,764<br>(43,741–127,080)	52,835<br>(27,635–91,495)	7,063<br>(3,392–12,758)	2,535<br>(1,364–4,189)
Paratyphoid fever	40,981<br>(18,728–79,984)	10,679<br>(4,481–21,661)	16,006<br>(7,431–30,383)	11,999<br>(5,131–23,571)	1,717<br>(703–3,503)	577<br>(249–1,125)
Other intestinal infectious diseases	4,169<br>(1,540–7,772)	2,666<br>(714–5,798)	408<br>(127–963)	508<br>(251–836)	234<br>(117–361)	351<br>(179–623)
Lower respiratory infections	3,186,157<br>(2,972,390–3,371,488)	1,762,620<br>(1,596,263–1,916,238)	91,029<br>(71,102–102,383)	219,579<br>(192,668–245,270)	324,870<br>(288,863–353,540)	788,057<br>(730,792–838,710)
Upper respiratory infections	4,353<br>(3,814–4,966)	1,630<br>(1,217–2,187)	88<br>(62–120)	850<br>(736–974)	911<br>(773–1,077)	870<br>(735–1,052)
Otitis media	5,912<br>(5,385–6,509)	2,495<br>(2,128–2,877)	760<br>(683–830)	1,319<br>(1,231–1,427)	748<br>(677–826)	588<br>(448–925)
Meningitis	472,551<br>(396,441–536,179)	279,300<br>(220,103–338,410)	44,110<br>(35,896–50,766)	77,878<br>(64,530–90,318)	43,774<br>(37,018–51,679)	27,487<br>(23,753–32,932)
Pneumococcal meningitis	119,304<br>(96,270–145,504)	60,684<br>(41,765–83,663)	13,723<br>(9,295–18,800)	25,049<br>(19,760–31,264)	12,146<br>(9,150–15,703)	7,700<br>(5,906–9,866)
H influenzae type B meningitis	142,270<br>(111,348–176,126)	104,557<br>(76,440–136,222)	12,434<br>(8,412–17,097)	13,488<br>(9,934–17,465)	7,207<br>(5,299–9,572)	4,582<br>(3,547–5,982)
Meningococcal meningitis	84,160<br>(64,376–108,463)	52,081<br>(34,865–74,681)	7,802<br>(5,033–11,008)	12,967<br>(9,888–16,359)	7,197<br>(5,131–9,343)	4,111<br>(3,120–5,393)
Other meningitis	126,816<br>(106,134–149,524)	61,977<br>(45,884–81,903)	10,149<br>(7,188–13,730)	26,372<br>(20,715–31,230)	17,222<br>(13,860–20,851)	11,093<br>(9,102–13,276)
Encephalitis	155,765<br>(137,526–172,976)	58,794<br>(48,908–67,799)	20,329<br>(17,307–23,242)	33,036<br>(29,790–37,101)	23,374<br>(20,530–26,702)	20,231<br>(17,632–22,851)
Diphtheria	11,039<br>(6,491–19,262)	8,657<br>(4,347–16,781)	1,352<br>(788–2,287)	770<br>(566–1,037)	259<br>(157–406)	0<br>(0–0)
Whooping cough	134,347<br>(48,000–312,381)	124,992<br>(44,731–290,067)	7,530<br>(2,641–18,282)	1,610<br>(523–4,239)	214<br>(68–566)	0<br>(0–0)
Tetanus	226,850<br>(205,546–280,459)	155,418<br>(141,292–171,354)	10,072<br>(7,987–17,219)	30,763<br>(25,050–49,723)	21,881<br>(18,062–38,651)	8,714<br>(7,112–15,524)
Measles	689,811<br>(259,358–1,375,404)	597,008<br>(226,765–1,185,126)	77,817<br>(28,651–156,444)	14,525<br>(4,145–36,861)	460<br>(127–1,281)	0<br>(0–0)
Varicella and herpes zoster	12,333<br>(11,074–13,960)	3,814<br>(3,034–4,717)	1,065<br>(831–1,383)	2,026<br>(1,776–2,323)	2,027<br>(1,685–2,449)	3,399<br>(2,746–4,232)
Neglected tropical diseases and malaria	1,207,964<br>(961,752–1,459,914)	800,882<br>(568,285–1,052,298)	107,725<br>(81,100–137,471)	172,977<br>(138,993–212,173)	91,686<br>(72,859–116,157)	34,692<br>(26,675–46,062)
Malaria	1,044,619<br>(803,094–1,296,007)	768,916<br>(536,701–1,019,624)	80,387<br>(53,222–110,834)	111,481<br>(80,176–152,654)	61,155<br>(43,027–85,363)	22,679<br>(14,729–33,416)
Chagas disease	8,589<br>(8,275–8,929)	9<br>(7–12)	14<br>(13–16)	1,950<br>(1,842–2,071)	3,950<br>(3,760–4,164)	2,664<br>(2,523–2,819)
Leishmaniasis	20,449<br>(12,145–31,060)	3,955<br>(2,187–6,125)	2,463<br>(1,304–4,239)	11,315<br>(6,494–17,495)	2,274<br>(1,462–3,229)	440<br>(327–565)
Visceral leishmaniasis	20,449<br>(12,145–31,060)	3,955<br>(2,187–6,125)	2,463<br>(1,304–4,239)	11,315<br>(6,494–17,495)	2,274<br>(1,462–3,229)	440<br>(327–565)
Cutaneous and mucocutaneous leishmaniasis	0<br>(0–0)	0<br>(0–0)	0<br>(0–0)	0<br>(0–0)	0<br>(0–0)	0<br>(0–0)
African trypanosomiasis	28,431<br>(14,319–46,178)	2,019<br>(976–3,503)	3,501<br>(1,797–5,819)	17,015<br>(8,740–27,980)	4,821<br>(2,384–8,149)	1,072<br>(527–1,788)
Schistosomiasis	11,498<br>(10,342–12,959)	198<br>(125–312)	341<br>(249–465)	3,662<br>(3,153–4,265)	4,870<br>(4,034–5,849)	2,425<br>(1,952–3,000)
Cysticercosis	835<br>(740–970)	19<br>(10–34)	32<br>(25–41)	493<br>(437–573)	223<br>(199–257)	66<br>(59–75)
Cystic echinococcosis	2,588<br>(2,437–2,751)	161<br>(125–200)	271<br>(238–305)	1,002<br>(935–1,081)	713<br>(670–760)	438<br>(414–461)
Lymphatic filariasis	0<br>(0–0)	0<br>(0–0)	0<br>(0–0)	0<br>(0–0)	0<br>(0–0)	0<br>(0–0)
Onchocerciasis	0<br>(0–0)	0<br>(0–0)	0<br>(0–0)	0<br>(0–0)	0<br>(0–0)	0<br>(0–0)
Trachoma	0<br>(0–0)	0<br>(0–0)	0<br>(0–0)	0<br>(0–0)	0<br>(0–0)	0<br>(0–0)
Dengue	7,407<br>(4,958–10,684)	2,967<br>(1,465–5,034)	1,971<br>(1,262–2,875)	1,618<br>(1,220–2,230)	553<br>(425–714)	296<br>(220–392)
Yellow fever	9,155<br>(1,922–25,215)	846<br>(169–2,338)	2,479<br>(561–6,726)	4,872<br>(1,033–13,388)	804<br>(158–2,300)	152<br>(30–434)
Rabies	48,817<br>(43,609–54,109)	7,199<br>(5,261–9,443)	13,298<br>(10,983–15,525)	15,724<br>(13,714–17,327)	9,639<br>(8,308–10,780)	2,956<br>(2,437–3,581)
Intestinal nematode infections	5,743<br>(4,842–6,585)	4,447<br>(3,610–5,204)	784<br>(676–905)	251<br>(206–305)	154<br>(121–193)	105<br>(79–137)
Ascariasis	5,743<br>(4,842–6,585)	4,447<br>(3,610–5,204)	784<br>(676–905)	251<br>(206–305)	154<br>(121–193)	105<br>(79–137)
Trichuriasis	0<br>(0–0)	0<br>(0–0)	0<br>(0–0)	0<br>(0–0)	0<br>(0–0)	0<br>(0–0)
Hookworm disease	0<br>(0–0)	0<br>(0–0)	0<br>(0–0)	0<br>(0–0)	0<br>(0–0)	0<br>(0–0)
Food-borne trematodiases	0<br>(0–0)	0<br>(0–0)	0<br>(0–0)	0<br>(0–0)	0<br>(0–0)	0<br>(0–0)
Leprosy	0<br>(0–0)	0<br>(0–0)	0<br>(0–0)	0<br>(0–0)	0<br>(0–0)	0<br>(0–0)
Ebola	434<br>(350–520)	17<br>(13–20)	15<br>(12–18)	294<br>(237–353)	103<br>(83–124)	4<br>(3–4)
Other neglected tropical diseases	19,393<br>(13,890–26,299)	10,124<br>(7,051–13,222)	2,163<br>(1,488–3,039)	3,294<br>(2,254–4,858)	2,421<br>(1,650–3,665)	1,389<br>(968–2,283)
Maternal disorders	385,437<br>(362,582–411,234)	0<br>(0–0)	1,954<br>(1,657–2,284)	382,274<br>(359,544–407,827)	1,208<br>(864–1,829)	0<br>(0–0)
Maternal hemorrhage	107,246<br>(89,529–127,035)	0<br>(0–0)	545<br>(373–762)	106,281<br>(88,782–126,002)	419<br>(247–722)	0<br>(0–0)
Maternal sepsis and other maternal infections	27,991<br>(21,845–35,329)	0<br>(0–0)	86<br>(55–131)	27,819<br>(21,658–35,159)	85<br>(51–135)	0<br>(0–0)
Maternal hypertensive disorders	52,677<br>(42,746–64,722)	0<br>(0–0)	346<br>(224–502)	52,241<br>(42,180–64,260)	89<br>(51–146)	0<br>(0–0)
Maternal obstructed labor and uterine rupture	25,039<br>(19,504–31,507)	0<br>(0–0)	68<br>(38–110)	24,923<br>(19,401–31,382)	48<br>(28–83)	0<br>(0–0)
Maternal abortion, miscarriage, and ectopic pregnancy	51,847<br>(41,080–65,498)	0<br>(0–0)	327<br>(202–502)	51,312<br>(40,545–64,990)	207<br>(131–323)	0<br>(0–0)

SR Table 12 - Global deaths by age and cause for both sexes in 1995

Cause	All ages	0-5 years	5-14 years	15-49 years	50-69 years	70+ years
Indirect maternal deaths	37,794 (29,579–48,179)	0 (0–0)	230 (138–348)	37,453 (29,252–47,752)	110 (61–213)	0 (0–0)
Late maternal deaths	8,338 (5,547–12,206)	0 (0–0)	49 (24–84)	8,250 (5,460–12,098)	38 (19–74)	0 (0–0)
Maternal deaths aggravated by HIV/AIDS	1,884 (1,167–2,610)	0 (0–0)	0 (0–0)	1,879 (1,164–2,602)	5 (2–10)	0 (0–0)
Other maternal disorders	72,616 (60,278–86,722)	0 (0–0)	299 (197–432)	72,114 (59,797–86,220)	203 (126–324)	0 (0–0)
Neonatal disorders	3,187,802 (3,103,266–3,285,996)	3,187,802 (3,103,266–3,285,996)	0 (0–0)	0 (0–0)	0 (0–0)	0 (0–0)
Neonatal preterm birth complications	1,533,571 (1,419,648–1,681,428)	1,533,571 (1,419,648–1,681,428)	0 (0–0)	0 (0–0)	0 (0–0)	0 (0–0)
Neonatal encephalopathy due to birth asphyxia and trauma	895,681 (782,301–1,007,497)	895,681 (782,301–1,007,497)	0 (0–0)	0 (0–0)	0 (0–0)	0 (0–0)
Neonatal sepsis and other neonatal infections	337,905 (237,788–464,283)	337,905 (237,788–464,283)	0 (0–0)	0 (0–0)	0 (0–0)	0 (0–0)
Hemolytic disease and other neonatal jaundice	109,875 (67,004–198,298)	109,875 (67,004–198,298)	0 (0–0)	0 (0–0)	0 (0–0)	0 (0–0)
Other neonatal disorders	310,767 (227,559–415,606)	310,767 (227,559–415,606)	0 (0–0)	0 (0–0)	0 (0–0)	0 (0–0)
Nutritional deficiencies	662,752 (528,349–798,351)	424,508 (320,984–536,238)	23,111 (18,991–27,461)	53,058 (45,140–61,374)	56,978 (47,743–67,286)	105,094 (90,400–120,505)
Protein-energy malnutrition	573,143 (450,124–706,874)	394,980 (296,860–508,803)	18,738 (14,980–22,836)	32,380 (25,755–40,413)	40,140 (31,797–49,962)	86,903 (72,652–102,958)
Iodine deficiency	2,420 (1,766–3,382)	1,045 (549–1,954)	111 (64–199)	401 (282–604)	432 (282–680)	429 (287–642)
Vitamin A deficiency	0 (0–0)	0 (0–0)	0 (0–0)	0 (0–0)	0 (0–0)	0 (0–0)
Iron-deficiency anemia	47,422 (30,289–63,851)	16,379 (7,339–23,329)	2,013 (1,377–3,127)	12,288 (8,749–15,714)	10,254 (7,405–13,010)	6,487 (4,198–8,902)
Other nutritional deficiencies	39,766 (24,645–72,720)	12,102 (5,461–27,177)	2,249 (1,296–3,687)	7,988 (5,011–12,728)	6,151 (3,869–11,008)	11,274 (8,181–17,835)
Other communicable, maternal, neonatal, and nutritional diseases	440,561 (363,209–543,494)	241,606 (171,215–335,140)	29,965 (24,244–37,940)	76,840 (73,579–80,291)	56,743 (51,377–59,340)	35,406 (31,783–37,300)
Sexually transmitted diseases excluding HIV	195,104 (118,586–296,409)	163,995 (93,537–255,983)	13,646 (7,862–21,157)	8,368 (5,844–9,755)	5,429 (3,010–6,562)	3,664 (2,290–4,535)
Syphilis	192,864 (116,246–293,884)	163,995 (93,537–255,983)	13,597 (7,807–21,108)	7,306 (5,128–8,500)	4,782 (2,635–5,785)	3,182 (1,988–3,940)
Chlamydial infection	218 (139–261)	0 (0–0)	5 (4–7)	100 (69–123)	62 (33–79)	49 (31–62)
Gonococcal infection	1,375 (859–1,619)	0 (0–0)	25 (17–32)	636 (411–752)	380 (214–459)	332 (207–413)
Trichomoniasis	0 (0–0)	0 (0–0)	0 (0–0)	0 (0–0)	0 (0–0)	0 (0–0)
Genital herpes	0 (0–0)	0 (0–0)	0 (0–0)	0 (0–0)	0 (0–0)	0 (0–0)
Other sexually transmitted diseases	646 (412–751)	0 (0–0)	18 (12–23)	324 (222–378)	204 (113–246)	99 (61–124)
Hepatitis	156,154 (148,978–164,434)	36,069 (31,499–40,139)	11,650 (10,352–12,845)	55,277 (52,532–59,424)	34,249 (32,976–35,900)	18,908 (18,231–19,592)
Acute hepatitis A	32,056 (21,486–41,966)	27,693 (20,200–33,333)	1,759 (546–3,686)	1,629 (412–3,853)	642 (156–1,454)	331 (85–763)
Hepatitis B	79,094 (68,174–89,436)	1,933 (742–4,229)	1,282 (741–2,053)	33,498 (27,940–38,792)	28,134 (24,790–31,129)	14,244 (12,078–16,174)
Hepatitis C	3,029 (661–6,844)	0 (0–0)	153 (30–410)	920 (184–2,208)	1,370 (296–3,013)	584 (123–1,387)
Acute hepatitis E	41,974 (30,428–55,504)	6,442 (2,580–12,113)	8,454 (6,450–10,256)	19,228 (14,447–24,872)	4,101 (2,086–7,167)	3,747 (2,052–5,915)
Other infectious diseases	89,302 (63,007–105,475)	41,541 (23,501–55,788)	4,667 (3,641–5,361)	13,195 (11,390–14,363)	17,064 (13,226–18,707)	12,833 (10,212–14,035)
Non-communicable diseases	30,485,201 (30,046,033–31,001,882)	1,085,639 (934,184–1,322,110)	260,501 (241,691–279,778)	3,691,003 (3,610,127–3,796,443)	9,940,444 (9,779,401–10,117,103)	15,507,612 (15,359,280–15,663,579)
Neoplasms	6,294,757 (6,186,886–6,405,924)	45,450 (38,961–57,865)	62,002 (57,900–66,101)	980,217 (951,880–1,009,966)	2,755,381 (2,705,322–2,807,836)	2,451,704 (2,402,473–2,495,538)
Lip and oral cavity cancer	88,305 (86,190–90,509)	0 (0–0)	0 (0–0)	15,559 (15,013–16,176)	44,267 (43,053–45,575)	28,478 (27,758–29,167)
Nasopharynx cancer	48,962 (40,720–52,053)	0 (0–0)	418 (321–499)	16,581 (13,260–17,992)	22,393 (18,652–23,859)	9,568 (8,247–10,100)
Other pharynx cancer	43,269 (41,809–44,490)	0 (0–0)	0 (0–0)	7,110 (6,832–7,458)	25,004 (24,059–25,821)	11,154 (10,717–11,578)
Esophageal cancer	386,913 (373,123–402,178)	0 (0–0)	0 (0–0)	41,990 (39,817–44,437)	201,316 (193,502–210,227)	143,606 (138,408–149,269)
Stomach cancer	775,635 (757,848–796,166)	0 (0–0)	0 (0–0)	99,847 (96,128–105,296)	349,816 (340,386–360,734)	325,971 (319,298–332,997)
Colon and rectum cancer	554,607 (546,092–562,314)	0 (0–0)	0 (0–0)	58,898 (57,161–61,471)	216,839 (213,002–220,246)	278,869 (273,904–283,716)
Liver cancer	585,860 (506,675–618,621)	0 (0–0)	1,683 (1,278–1,918)	136,301 (103,469–147,394)	283,935 (246,118–299,777)	163,939 (151,096–173,992)
Liver cancer due to hepatitis B	223,922 (185,884–241,434)	0 (0–0)	984 (733–1,134)	81,866 (61,738–89,492)	102,119 (85,661–112,343)	38,950 (34,439–43,355)
Liver cancer due to hepatitis C	107,966 (99,486–115,573)	0 (0–0)	39 (31–48)	9,735 (7,863–11,085)	50,671 (45,269–55,469)	47,520 (44,403–51,310)
Liver cancer due to alcohol use	152,376 (132,315–164,149)	0 (0–0)	0 (0–0)	18,961 (14,215–21,411)	86,626 (74,010–95,634)	46,789 (42,748–50,725)
Liver cancer due to other causes	101,594 (86,479–110,183)	0 (0–0)	658 (515–750)	25,738 (19,526–28,756)	44,518 (37,615–49,509)	30,678 (27,315–33,811)
Gallbladder and biliary tract cancer	110,826 (107,538–114,216)	0 (0–0)	0 (0–0)	8,486 (7,888–8,941)	44,153 (42,380–45,734)	58,186 (56,788–59,735)
Pancreatic cancer	247,422 (243,927–251,194)	0 (0–0)	0 (0–0)	20,723 (20,209–21,363)	107,855 (106,093–109,704)	118,843 (116,734–120,975)
Larynx cancer	90,810 (88,486–93,057)	0 (0–0)	0 (0–0)	11,667 (11,211–12,182)	50,051 (48,608–51,467)	29,091 (28,309–29,827)
Tracheal, bronchus, and lung cancer	1,162,176 (1,143,362–1,183,815)	0 (0–0)	0 (0–0)	111,473 (108,067–115,875)	586,672 (575,933–598,180)	464,030 (456,376–472,747)
Malignant skin melanoma	38,004 (31,665–48,026)	0 (0–0)	0 (0–0)	9,256 (7,722–11,303)	14,809 (12,132–18,917)	13,939 (11,733–17,668)
Non-melanoma skin cancer	27,954 (27,272–28,646)	0 (0–0)	0 (0–0)	3,372 (3,256–3,526)	9,807 (9,521–10,105)	14,774 (14,366–15,172)
Non-melanoma skin cancer (squamous-cell carcinoma)	27,954 (27,272–28,646)	0 (0–0)	0 (0–0)	3,372 (3,256–3,526)	9,807 (9,521–10,105)	14,774 (14,366–15,172)
Non-melanoma skin cancer (basal-cell carcinoma)	0 (0–0)	0 (0–0)	0 (0–0)	0 (0–0)	0 (0–0)	0 (0–0)
Breast cancer	369,400 (354,781–389,651)	0 (0–0)	0 (0–0)	86,544 (79,599–95,330)	163,484 (157,690–172,852)	119,371 (115,083–123,644)
Cervical cancer	195,090 (183,554–205,850)	0 (0–0)	0 (0–0)	54,460 (50,163–59,035)	91,150 (85,304–96,689)	49,479 (47,477–52,363)
Uterine cancer	71,354 (67,957–75,529)	0 (0–0)	0 (0–0)	9,690 (8,878–10,604)	34,038 (32,080–36,371)	27,626 (26,607–29,115)
Ovarian cancer	113,712 (111,275–117,236)	0 (0–0)	0 (0–0)	20,791 (19,979–22,418)	53,324 (52,090–55,086)	39,596 (38,515–40,729)
Prostate cancer	224,864 (179,861–274,673)	0 (0–0)	0 (0–0)	2,114 (1,651–2,567)	57,332 (46,360–71,275)	165,418 (129,301–203,003)
Testicular cancer	9,106 (8,631–9,725)	0 (0–0)	0 (0–0)	6,390 (5,990–6,875)	1,641 (1,559–1,760)	1,073 (1,031–1,132)

SR Table 12 - Global deaths by age and cause for both sexes in 1995						
Cause	All ages	0-5 years	5-14 years	15-49 years	50-69 years	70+ years
Kidney cancer	83,407 (81,731–85,505)	2,213 (1,394–3,219)	1,096 (1,006–1,204)	8,851 (8,604–9,171)	37,256 (36,514–38,115)	33,988 (33,238–34,730)
Bladder cancer	126,823 (124,657–129,116)	0 (0–0)	0 (0–0)	6,600 (6,350–6,853)	44,783 (43,836–45,700)	75,438 (73,961–77,058)
Brain and nervous system cancer	159,035 (145,563–173,997)	6,841 (5,291–10,565)	11,533 (9,736–13,585)	48,417 (43,282–52,371)	61,230 (56,358–65,358)	31,013 (29,353–33,395)
Thyroid cancer	22,641 (21,458–24,453)	0 (0–0)	82 (68–90)	2,694 (2,540–3,068)	9,146 (8,616–9,943)	10,718 (10,152–11,580)
Mesothelioma	19,378 (18,491–20,134)	0 (0–0)	0 (0–0)	3,412 (3,152–3,594)	8,437 (8,005–8,780)	7,527 (7,159–7,982)
Hodgkin lymphoma	31,311 (24,040–38,356)	836 (452–1,015)	1,401 (905–1,755)	11,817 (9,304–14,188)	10,866 (8,160–13,590)	6,388 (4,905–7,958)
Non-Hodgkin lymphoma	144,788 (131,345–158,007)	3,668 (2,271–5,883)	4,603 (3,777–5,610)	29,227 (25,968–33,162)	51,849 (46,396–56,284)	55,439 (49,920–60,004)
Multiple myeloma	60,756 (59,176–62,507)	0 (0–0)	0 (0–0)	5,772 (5,546–6,079)	25,665 (24,933–26,498)	29,319 (28,107–30,231)
Leukemia	271,694 (264,779–283,002)	18,335 (16,131–20,801)	30,241 (28,250–32,481)	80,340 (77,262–85,485)	71,181 (69,389–73,885)	71,594 (70,001–73,177)
Acute lymphoid leukemia	93,793 (86,225–106,506)	12,172 (10,145–15,053)	21,407 (19,408–24,374)	31,668 (28,655–37,149)	17,920 (16,486–20,206)	10,623 (9,960–11,611)
Chronic lymphoid leukemia	43,507 (40,763–45,701)	0 (0–0)	0 (0–0)	7,477 (6,226–8,324)	13,372 (12,450–14,272)	22,657 (21,455–23,533)
Acute myeloid leukemia	95,494 (86,929–103,821)	6,163 (4,256–7,680)	8,833 (6,847–10,240)	28,618 (25,370–31,769)	27,059 (25,238–29,396)	24,818 (23,610–26,854)
Chronic myeloid leukemia	38,899 (36,241–41,867)	0 (0–0)	0 (0–0)	12,575 (11,140–13,975)	12,828 (11,942–13,837)	13,495 (12,806–14,195)
Other neoplasms	230,642 (216,987–241,412)	13,555 (10,286–19,471)	10,941 (9,819–11,861)	61,819 (55,610–65,709)	77,068 (71,084–81,118)	67,257 (64,487–71,241)
Cardiovascular diseases	13,964,588 (13,783,665–14,178,452)	94,088 (85,266–102,009)	42,835 (39,950–46,092)	1,304,627 (1,271,470–1,349,205)	4,354,934 (4,286,348–4,427,716)	8,168,102 (8,051,772–8,294,037)
Rheumatic heart disease	347,704 (331,013–364,386)	6,117 (5,031–7,202)	10,652 (9,715–11,619)	97,869 (91,322–105,159)	118,114 (112,169–124,475)	114,952 (110,073–119,718)
Ischemic heart disease	6,635,771 (6,552,463–6,732,848)	6,277 (5,376–7,466)	3,520 (3,133–4,047)	568,270 (553,593–589,703)	2,094,566 (2,059,520–2,129,110)	3,963,135 (3,902,463–4,025,281)
Cerebrovascular disease	5,196,881 (5,099,048–5,328,759)	36,418 (32,824–41,266)	15,829 (14,632–17,451)	429,065 (416,068–448,027)	1,636,662 (1,600,007–1,681,818)	3,078,905 (3,011,576–3,154,815)
Ischemic stroke	2,365,834 (2,279,534–2,460,808)	1,562 (1,344–1,868)	1,025 (934–1,167)	68,123 (63,585–73,733)	570,475 (542,368–599,978)	1,724,648 (1,666,025–1,790,264)
Hemorrhagic stroke	2,831,046 (2,738,566–2,923,086)	34,856 (31,371–39,634)	14,804 (13,677–16,373)	360,942 (348,972–376,163)	1,066,186 (1,028,972–1,102,585)	1,354,257 (1,294,264–1,401,772)
Hypertensive heart disease	703,680 (639,321–772,177)	893 (672–1,087)	546 (473–628)	66,542 (61,669–72,795)	230,407 (208,848–253,593)	405,289 (360,009–444,666)
Cardiomyopathy and myocarditis	296,603 (287,421–306,954)	14,834 (12,276–17,005)	4,369 (3,986–4,965)	62,911 (59,570–66,495)	84,223 (80,609–88,135)	130,263 (125,942–134,307)
Atrial fibrillation and flutter	106,756 (87,297–129,169)	0 (0–0)	0 (0–0)	720 (552–913)	8,794 (6,786–11,733)	97,240 (77,903–119,037)
Aortic aneurysm	111,231 (108,063–114,949)	0 (0–0)	0 (0–0)	8,947 (8,463–9,548)	33,141 (31,994–34,582)	69,142 (67,261–71,210)
Peripheral vascular disease	21,134 (20,255–21,988)	0 (0–0)	0 (0–0)	330 (314–347)	3,773 (3,609–3,959)	17,030 (16,272–17,770)
Endocarditis	53,006 (47,500–61,004)	4,808 (3,216–6,600)	1,231 (985–1,532)	10,609 (9,236–12,824)	12,292 (10,882–14,043)	24,064 (21,995–26,785)
Other cardiovascular and circulatory diseases	491,818 (478,636–505,441)	24,737 (22,092–29,049)	6,685 (6,155–7,475)	59,360 (57,045–62,460)	132,957 (128,598–138,255)	268,077 (260,144–276,899)
Chronic respiratory diseases	3,824,624 (3,733,995–3,925,289)	99,650 (88,260–111,846)	18,829 (17,010–20,577)	243,637 (234,794–258,200)	1,177,351 (1,143,563–1,219,428)	2,285,154 (2,228,684–2,341,274)
Chronic obstructive pulmonary disease	3,135,330 (2,984,955–3,300,000)	42,937 (33,200–53,273)	4,316 (3,236–5,983)	142,969 (128,813–159,025)	935,745 (876,053–1,010,171)	2,009,362 (1,925,065–2,087,370)
Pneumoconiosis	31,398 (27,256–36,447)	0 (0–0)	0 (0–0)	3,627 (2,966–4,302)	10,199 (8,617–12,034)	17,571 (15,191–20,206)
Silicosis	11,686 (10,121–13,848)	0 (0–0)	0 (0–0)	1,645 (1,303–2,084)	4,414 (3,704–5,344)	5,626 (4,906–6,628)
Asbestosis	1,840 (1,460–2,227)	0 (0–0)	0 (0–0)	349 (276–424)	571 (420–752)	920 (696–1,110)
Coal workers pneumoconiosis	3,220 (2,764–3,967)	0 (0–0)	0 (0–0)	256 (177–386)	979 (810–1,237)	1,984 (1,729–2,337)
Other pneumoconiosis	14,650 (11,687–18,460)	0 (0–0)	0 (0–0)	1,375 (1,047–1,750)	4,235 (3,253–5,542)	9,039 (7,306–11,336)
Asthma	553,296 (373,012–675,271)	29,243 (21,046–36,976)	12,636 (10,108–14,781)	85,813 (64,695–101,596)	207,308 (133,064–261,146)	218,294 (141,256–272,946)
Interstitial lung disease and pulmonary sarcoidosis	49,822 (40,661–61,107)	368 (218–684)	266 (207–396)	4,402 (3,580–5,681)	16,089 (12,989–20,769)	28,695 (23,698–34,311)
Other chronic respiratory diseases	54,777 (39,257–69,669)	27,101 (18,911–37,852)	1,610 (987–2,744)	6,825 (4,451–8,653)	8,009 (5,564–9,733)	11,230 (8,661–13,290)
Cirrhosis and other chronic liver diseases	1,019,709 (987,126–1,095,620)	20,785 (16,147–26,609)	12,853 (11,370–15,197)	303,858 (291,687–329,276)	446,682 (431,283–479,168)	235,529 (226,147–247,809)
Cirrhosis and other chronic liver diseases due to hepatitis B	304,380 (282,685–336,199)	164 (122–213)	402 (326–491)	93,388 (85,163–104,889)	136,633 (122,930–153,819)	73,792 (67,412–81,729)
Cirrhosis and other chronic liver diseases due to hepatitis C	241,363 (225,664–260,252)	85 (63–114)	221 (174–271)	66,578 (59,727–74,978)	110,304 (98,935–122,454)	64,172 (58,830–69,853)
Cirrhosis and other chronic liver diseases due to alcohol use	264,846 (247,301–283,845)	0 (0–0)	0 (0–0)	91,244 (83,979–100,947)	127,933 (115,323–141,683)	45,668 (41,140–50,817)
Cirrhosis and other chronic liver diseases due to other causes	209,119 (195,496–231,509)	20,536 (15,949–26,307)	12,229 (10,813–14,474)	52,646 (47,933–59,016)	71,811 (63,992–81,143)	51,896 (47,639–56,900)
Digestive diseases	1,049,868 (1,011,536–1,096,984)	42,363 (31,978–60,833)	26,702 (24,059–29,384)	238,908 (226,374–251,294)	327,857 (313,586–346,306)	414,036 (401,118–435,277)
Peptic ulcer disease	340,182 (317,428–369,984)	5,798 (3,374–10,029)	6,085 (5,242–7,179)	81,232 (75,580–88,520)	119,277 (110,230–129,641)	127,788 (117,659–140,328)
Gastritis and duodenitis	49,249 (37,435–62,875)	1,490 (804–2,286)	837 (571–1,172)	11,787 (9,058–14,960)	16,629 (12,846–21,552)	18,503 (13,901–24,151)
Appendicitis	45,825 (40,234–53,561)	6,385 (3,454–12,675)	4,333 (3,621–4,965)	15,444 (14,042–17,210)	10,782 (9,858–12,140)	8,879 (8,170–10,076)
Paralytic ileus and intestinal obstruction	203,644 (178,834–230,195)	12,544 (7,946–19,458)	8,374 (6,760–9,745)	49,182 (39,865–54,973)	60,406 (52,852–68,042)	73,137 (67,008–83,929)
Inguinal, femoral, and abdominal hernia	50,517 (35,875–57,958)	1,775 (672–2,755)	1,182 (710–1,470)	10,066 (5,956–12,123)	16,149 (11,577–18,925)	21,343 (16,204–24,181)
Inflammatory bowel disease	37,024 (31,679–42,554)	3,034 (1,320–6,439)	842 (615–1,228)	7,743 (6,225–9,260)	9,907 (8,329–11,177)	15,496 (13,818–16,767)
Vascular intestinal disorders	63,320 (59,501–68,316)	756 (444–1,379)	176 (144–220)	4,196 (3,676–4,778)	17,379 (15,980–19,281)	40,810 (38,715–43,479)
Gallbladder and biliary diseases	85,521 (81,283–91,330)	2,236 (1,287–4,047)	1,262 (1,082–1,496)	12,763 (11,821–14,087)	25,919 (24,306–27,745)	43,339 (41,406–46,038)
Pancreatitis	79,091 (74,316–85,826)	374 (239–590)	587 (473–720)	30,833 (28,452–33,937)	26,388 (24,678–28,720)	20,907 (19,783–22,337)
Other digestive diseases	95,491 (81,769–111,639)	7,967 (5,344–11,416)	3,018 (2,483–3,712)	15,659 (12,728–20,487)	25,017 (20,686–30,529)	43,828 (38,670–49,266)
Neurological disorders	1,274,570 (1,109,309–1,440,063)	23,331 (16,513–30,657)	16,057 (14,446–17,814)	81,487 (78,290–86,485)	119,734 (102,057–137,323)	1,033,959 (871,403–1,194,820)
Alzheimer disease and other dementias	1,031,351 (860,792–1,197,146)	0 (0–0)	0 (0–0)	1,020 (633–1,497)	70,790 (53,351–88,345)	959,541 (795,649–1,120,776)
Parkinson disease	54,981 (53,306–58,002)	0 (0–0)	0 (0–0)	510 (476–558)	8,357 (8,046–9,003)	46,113 (44,590–48,348)
Epilepsy	113,505 (104,351–124,794)	17,193 (11,646–23,122)	13,067 (11,709–14,439)	59,791 (56,560–64,802)	14,962 (14,475–15,482)	8,491 (8,204–8,802)

SR Table 12 - Global deaths by age and cause for both sexes in 1995						
**Cause**	**All ages**	**0-5 years**	**5-14 years**	**15-49 years**	**50-69 years**	**70+ years**
Multiple sclerosis	15,421 (12,992–17,467)	0 (0–0)	0 (0–0)	6,038 (4,678–7,253)	6,510 (5,811–7,204)	2,871 (2,416–3,053)
Motor neuron disease	20,528 (19,829–22,221)	1,482 (911–1,868)	141 (120–162)	2,668 (2,519–3,026)	9,230 (8,857–10,003)	7,005 (6,800–7,286)
Migraine	0 (0–0)	0 (0–0)	0 (0–0)	0 (0–0)	0 (0–0)	0 (0–0)
Tension-type headache	0 (0–0)	0 (0–0)	0 (0–0)	0 (0–0)	0 (0–0)	0 (0–0)
Medication overuse headache	0 (0–0)	0 (0–0)	0 (0–0)	0 (0–0)	0 (0–0)	0 (0–0)
Other neurological disorders	38,803 (37,589–42,106)	4,676 (3,446–7,074)	2,848 (2,512–3,530)	11,459 (10,835–12,277)	9,882 (9,463–10,437)	9,935 (9,506–10,380)
Mental and substance use disorders	251,146 (231,698–259,804)	29 (24–34)	35 (18–47)	154,801 (141,863–161,073)	76,057 (72,130–78,987)	20,222 (19,581–20,812)
Schizophrenia	18,235 (16,756–19,385)	0 (0–0)	0 (0–0)	8,855 (7,864–9,645)	5,493 (5,101–5,825)	3,886 (3,665–4,117)
Alcohol use disorders	138,886 (130,330–144,812)	0 (0–0)	0 (0–0)	75,575 (69,633–79,561)	53,554 (50,234–55,969)	9,756 (9,435–10,069)
Drug use disorders	93,515 (83,857–98,094)	29 (24–34)	0 (0–0)	69,898 (61,404–73,739)	17,009 (16,066–17,915)	6,579 (6,188–6,917)
Opioid use disorders	68,013 (62,719–72,659)	29 (24–34)	0 (0–0)	51,670 (46,475–55,449)	12,239 (11,496–13,156)	4,074 (3,832–4,433)
Cocaine use disorders	2,708 (2,375–3,451)	0 (0–0)	0 (0–0)	2,186 (1,910–2,768)	430 (369–540)	91 (75–114)
Amphetamine use disorders	1,843 (1,540–2,489)	0 (0–0)	0 (0–0)	1,449 (1,194–1,973)	321 (262–435)	71 (59–92)
Cannabis use disorders	0 (0–0)	0 (0–0)	0 (0–0)	0 (0–0)	0 (0–0)	0 (0–0)
Other drug use disorders	20,951 (15,908–22,784)	0 (0–0)	0 (0–0)	14,591 (10,840–15,989)	4,017 (3,356–4,330)	2,342 (1,898–2,595)
Depressive disorders	0 (0–0)	0 (0–0)	0 (0–0)	0 (0–0)	0 (0–0)	0 (0–0)
Major depressive disorder	0 (0–0)	0 (0–0)	0 (0–0)	0 (0–0)	0 (0–0)	0 (0–0)
Dysthymia	0 (0–0)	0 (0–0)	0 (0–0)	0 (0–0)	0 (0–0)	0 (0–0)
Bipolar disorder	0 (0–0)	0 (0–0)	0 (0–0)	0 (0–0)	0 (0–0)	0 (0–0)
Anxiety disorders	0 (0–0)	0 (0–0)	0 (0–0)	0 (0–0)	0 (0–0)	0 (0–0)
Eating disorders	507 (347–709)	0 (0–0)	35 (18–47)	472 (326–676)	0 (0–0)	0 (0–0)
Anorexia nervosa	481 (327–673)	0 (0–0)	33 (18–45)	448 (309–639)	0 (0–0)	0 (0–0)
Bulimia nervosa	26 (16–40)	0 (0–0)	1 (0–2)	24 (15–38)	0 (0–0)	0 (0–0)
Autistic spectrum disorders	0 (0–0)	0 (0–0)	0 (0–0)	0 (0–0)	0 (0–0)	0 (0–0)
Autism	0 (0–0)	0 (0–0)	0 (0–0)	0 (0–0)	0 (0–0)	0 (0–0)
Asperger syndrome and other autistic spectrum disorders	0 (0–0)	0 (0–0)	0 (0–0)	0 (0–0)	0 (0–0)	0 (0–0)
Attention-deficit/hyperactivity disorder	0 (0–0)	0 (0–0)	0 (0–0)	0 (0–0)	0 (0–0)	0 (0–0)
Conduct disorder	0 (0–0)	0 (0–0)	0 (0–0)	0 (0–0)	0 (0–0)	0 (0–0)
Idiopathic developmental intellectual disability	0 (0–0)	0 (0–0)	0 (0–0)	0 (0–0)	0 (0–0)	0 (0–0)
Other mental and substance use disorders	0 (0–0)	0 (0–0)	0 (0–0)	0 (0–0)	0 (0–0)	0 (0–0)
Diabetes, urogenital, blood, and endocrine diseases	1,972,553 (1,907,077–2,034,218)	143,217 (114,341–180,174)	37,301 (33,726–40,769)	310,465 (296,484–323,607)	643,232 (624,032–663,141)	838,336 (817,368–862,733)
Diabetes mellitus	824,925 (808,120–841,650)	3,734 (3,076–4,210)	3,217 (2,784–3,607)	89,313 (86,381–92,935)	336,171 (327,958–344,534)	392,488 (383,538–401,101)
Acute glomerulonephritis	14,342 (11,882–18,383)	691 (557–935)	680 (610–876)	4,750 (3,707–5,492)	4,270 (3,510–5,576)	3,949 (3,238–5,679)
Chronic kidney disease	672,208 (630,417–716,343)	19,889 (16,771–22,365)	11,314 (9,842–12,356)	127,987 (114,577–135,482)	211,798 (196,797–228,598)	301,218 (289,459–320,096)
Chronic kidney disease due to diabetes mellitus	204,964 (193,145–217,818)	2,238 (1,803–2,703)	876 (720–1,062)	28,597 (25,501–31,698)	78,685 (71,970–86,605)	94,564 (89,154–101,440)
Chronic kidney disease due to hypertension	291,867 (274,352–311,851)	6,632 (5,522–7,676)	3,227 (2,714–3,733)	47,467 (42,085–51,243)	87,323 (80,273–94,961)	147,216 (139,318–156,928)
Chronic kidney disease due to glomerulonephritis	155,592 (142,446–167,393)	8,187 (6,714–9,462)	5,406 (4,660–6,031)	46,178 (41,307–49,974)	43,065 (38,838–47,732)	52,755 (48,161–58,147)
Chronic kidney disease due to other causes	19,784 (16,498–23,353)	2,831 (2,169–3,527)	1,803 (1,430–2,230)	5,743 (4,029–7,649)	2,723 (1,696–4,070)	6,681 (5,581–8,333)
Urinary diseases and male infertility	159,411 (146,234–180,825)	9,569 (7,398–11,732)	2,284 (1,989–2,655)	25,071 (22,265–29,360)	44,671 (39,704–52,863)	77,813 (71,472–88,535)
Interstitial nephritis and urinary tract infections	115,482 (105,247–128,974)	7,493 (5,773–9,361)	1,419 (1,221–1,639)	16,774 (14,882–19,531)	29,743 (25,840–34,758)	60,050 (54,726–67,660)
Urolithiasis	15,437 (11,795–17,822)	0 (0–0)	332 (257–406)	3,237 (2,429–3,853)	5,869 (4,535–6,841)	5,998 (4,535–6,912)
Benign prostatic hyperplasia	0 (0–0)	0 (0–0)	0 (0–0)	0 (0–0)	0 (0–0)	0 (0–0)
Male infertility	0 (0–0)	0 (0–0)	0 (0–0)	0 (0–0)	0 (0–0)	0 (0–0)
Other urinary diseases	28,492 (23,669–38,309)	2,075 (1,389–3,178)	532 (423–707)	5,060 (4,009–7,017)	9,059 (7,018–13,102)	11,764 (9,562–16,003)
Gynecological diseases	6,972 (5,368–8,293)	0 (0–0)	0 (0–0)	3,457 (2,563–4,144)	2,039 (1,561–2,498)	1,475 (1,204–1,899)
Uterine fibroids	1,840 (1,008–2,514)	0 (0–0)	0 (0–0)	768 (432–1,080)	707 (360–996)	364 (190–614)
Polycystic ovarian syndrome	902 (214–1,518)	0 (0–0)	0 (0–0)	653 (133–1,119)	249 (78–384)	0 (0–0)
Female infertility	0 (0–0)	0 (0–0)	0 (0–0)	0 (0–0)	0 (0–0)	0 (0–0)
Endometriosis	40 (27–56)	0 (0–0)	0 (0–0)	35 (24–49)	5 (3–8)	0 (0–0)
Genital prolapse	1,074 (523–1,906)	0 (0–0)	0 (0–0)	122 (48–213)	425 (165–840)	526 (283–946)
Premenstrual syndrome	0 (0–0)	0 (0–0)	0 (0–0)	0 (0–0)	0 (0–0)	0 (0–0)
Other gynecological diseases	3,113 (2,319–4,174)	0 (0–0)	0 (0–0)	1,877 (1,382–2,596)	651 (432–986)	584 (409–909)
Hemoglobinopathies and hemolytic anemias	207,214 (171,844–243,714)	92,258 (65,702–127,572)	15,556 (13,063–18,251)	41,938 (37,525–47,215)	23,887 (21,061–26,974)	33,574 (29,332–38,459)
Thalassemias	24,578 (18,812–30,995)	15,354 (9,782–21,655)	4,770 (3,856–5,751)	2,152 (1,869–2,525)	69 (64–78)	2,230 (1,723–3,080)
Thalassemias trait	0 (0–0)	0 (0–0)	0 (0–0)	0 (0–0)	0 (0–0)	0 (0–0)
Sickle cell disorders	102,611 (76,161–137,926)	74,954 (50,930–108,635)	8,683 (7,028–10,388)	15,576 (13,509–17,732)	866 (776–971)	2,530 (2,104–3,170)
Sickle cell trait	0 (0–0)	0 (0–0)	0 (0–0)	0 (0–0)	0 (0–0)	0 (0–0)

SR Table 12 - Global deaths by age and cause for both sexes in 1995						
Cause	All ages	0-5 years	5-14 years	15-49 years	50-69 years	70+ years
G6PD deficiency	21,529 (18,840–26,030)	1,260 (663–2,057)	1,304 (969–1,748)	12,538 (11,007–15,162)	5,305 (4,367–6,396)	1,121 (912–1,408)
G6PD trait	0 (0–0)	0 (0–0)	0 (0–0)	0 (0–0)	0 (0–0)	0 (0–0)
Other hemoglobinopathies and hemolytic anemias	58,495 (52,342–64,867)	688 (446–1,006)	797 (651–961)	11,670 (10,601–12,977)	17,645 (15,642–20,129)	27,691 (24,351–31,273)
Endocrine, metabolic, blood, and immune disorders	87,478 (85,041–89,932)	17,073 (14,750–19,223)	4,247 (4,016–4,473)	17,946 (17,318–18,582)	20,392 (19,506–21,426)	27,817 (26,724–28,975)
Musculoskeletal disorders	61,143 (56,111–66,290)	0 (0–0)	1,997 (1,635–2,423)	15,775 (13,841–19,060)	16,162 (14,660–17,272)	27,208 (24,011–28,387)
Rheumatoid arthritis	23,579 (19,931–25,777)	0 (0–0)	317 (219–414)	2,468 (1,835–3,033)	7,974 (6,772–8,820)	12,818 (11,039–13,811)
Osteoarthritis	0 (0–0)	0 (0–0)	0 (0–0)	0 (0–0)	0 (0–0)	0 (0–0)
Low back and neck pain	0 (0–0)	0 (0–0)	0 (0–0)	0 (0–0)	0 (0–0)	0 (0–0)
Low back pain	0 (0–0)	0 (0–0)	0 (0–0)	0 (0–0)	0 (0–0)	0 (0–0)
Neck pain	0 (0–0)	0 (0–0)	0 (0–0)	0 (0–0)	0 (0–0)	0 (0–0)
Gout	0 (0–0)	0 (0–0)	0 (0–0)	0 (0–0)	0 (0–0)	0 (0–0)
Other musculoskeletal disorders	37,563 (34,372–41,702)	0 (0–0)	1,679 (1,375–2,051)	13,306 (11,564–16,674)	8,187 (7,586–9,071)	14,389 (12,238–15,161)
Other non-communicable diseases	772,217 (617,268–1,020,672)	616,701 (476,268–847,463)	41,885 (33,338–52,338)	57,223 (51,315–67,006)	23,049 (19,060–27,595)	33,357 (26,196–41,626)
Congenital anomalies	697,062 (540,892–936,006)	586,663 (442,839–813,646)	40,876 (32,498–50,868)	50,571 (45,354–60,280)	11,201 (10,253–12,819)	7,750 (7,239–8,674)
Neural tube defects	110,742 (81,906–168,428)	103,729 (75,600–159,537)	3,704 (2,871–5,044)	2,422 (2,002–3,066)	554 (415–793)	332 (233–543)
Congenital heart anomalies	362,716 (277,670–472,550)	289,498 (214,321–393,466)	29,368 (22,509–36,235)	36,984 (32,781–44,111)	3,943 (3,523–4,511)	2,921 (2,623–3,366)
Cleft lip and cleft palate	5,088 (3,493–6,682)	5,088 (3,493–6,682)	0 (0–0)	0 (0–0)	0 (0–0)	0 (0–0)
Down syndrome	27,596 (15,918–51,565)	21,044 (10,522–43,088)	977 (608–1,620)	3,462 (2,525–4,962)	1,986 (1,525–2,752)	125 (95–172)
Turner syndrome	0 (0–0)	0 (0–0)	0 (0–0)	0 (0–0)	0 (0–0)	0 (0–0)
Klinefelter syndrome	0 (0–0)	0 (0–0)	0 (0–0)	0 (0–0)	0 (0–0)	0 (0–0)
Other chromosomal abnormalities	20,266 (8,264–59,841)	18,932 (7,077–57,904)	369 (222–829)	650 (512–841)	236 (159–300)	77 (61–98)
Other congenital anomalies	170,650 (137,687–239,298)	148,369 (118,021–212,765)	6,457 (5,095–8,610)	7,050 (6,065–8,384)	4,479 (3,879–4,986)	4,293 (3,867–4,628)
Skin and subcutaneous diseases	50,838 (35,920–67,461)	5,721 (3,223–9,254)	1,009 (639–1,487)	6,652 (4,531–9,574)	11,847 (7,750–16,117)	25,607 (18,241–33,786)
Dermatitis	0 (0–0)	0 (0–0)	0 (0–0)	0 (0–0)	0 (0–0)	0 (0–0)
Psoriasis	0 (0–0)	0 (0–0)	0 (0–0)	0 (0–0)	0 (0–0)	0 (0–0)
Cellulitis	8,865 (5,773–13,556)	725 (320–1,295)	185 (110–275)	1,577 (955–2,791)	3,020 (1,747–4,792)	3,355 (2,323–5,163)
Pyoderma	20,259 (14,163–30,714)	4,625 (2,589–7,841)	540 (330–862)	3,471 (2,241–5,057)	4,697 (3,319–7,252)	6,925 (5,185–10,222)
Scabies	0 (0–0)	0 (0–0)	0 (0–0)	0 (0–0)	0 (0–0)	0 (0–0)
Fungal skin diseases	0 (0–0)	0 (0–0)	0 (0–0)	0 (0–0)	0 (0–0)	0 (0–0)
Viral skin diseases	0 (0–0)	0 (0–0)	0 (0–0)	0 (0–0)	0 (0–0)	0 (0–0)
Acne vulgaris	0 (0–0)	0 (0–0)	0 (0–0)	0 (0–0)	0 (0–0)	0 (0–0)
Alopecia areata	0 (0–0)	0 (0–0)	0 (0–0)	0 (0–0)	0 (0–0)	0 (0–0)
Pruritus	0 (0–0)	0 (0–0)	0 (0–0)	0 (0–0)	0 (0–0)	0 (0–0)
Urticaria	0 (0–0)	0 (0–0)	0 (0–0)	0 (0–0)	0 (0–0)	0 (0–0)
Decubitus ulcer	19,223 (11,680–24,603)	91 (39–157)	156 (85–229)	1,109 (646–1,589)	3,570 (1,757–5,842)	14,296 (8,891–18,377)
Other skin and subcutaneous diseases	2,489 (1,786–3,511)	280 (171–513)	126 (73–183)	494 (330–750)	558 (356–919)	1,029 (760–1,395)
Sense organ diseases	0 (0–0)	0 (0–0)	0 (0–0)	0 (0–0)	0 (0–0)	0 (0–0)
Glaucoma	0 (0–0)	0 (0–0)	0 (0–0)	0 (0–0)	0 (0–0)	0 (0–0)
Cataract	0 (0–0)	0 (0–0)	0 (0–0)	0 (0–0)	0 (0–0)	0 (0–0)
Macular degeneration	0 (0–0)	0 (0–0)	0 (0–0)	0 (0–0)	0 (0–0)	0 (0–0)
Refraction and accommodation disorders	0 (0–0)	0 (0–0)	0 (0–0)	0 (0–0)	0 (0–0)	0 (0–0)
Age-related and other hearing loss	0 (0–0)	0 (0–0)	0 (0–0)	0 (0–0)	0 (0–0)	0 (0–0)
Other vision loss	0 (0–0)	0 (0–0)	0 (0–0)	0 (0–0)	0 (0–0)	0 (0–0)
Other sense organ diseases	0 (0–0)	0 (0–0)	0 (0–0)	0 (0–0)	0 (0–0)	0 (0–0)
Oral disorders	0 (0–0)	0 (0–0)	0 (0–0)	0 (0–0)	0 (0–0)	0 (0–0)
Deciduous caries	0 (0–0)	0 (0–0)	0 (0–0)	0 (0–0)	0 (0–0)	0 (0–0)
Permanent caries	0 (0–0)	0 (0–0)	0 (0–0)	0 (0–0)	0 (0–0)	0 (0–0)
Periodontal diseases	0 (0–0)	0 (0–0)	0 (0–0)	0 (0–0)	0 (0–0)	0 (0–0)
Edentulism and severe tooth loss	0 (0–0)	0 (0–0)	0 (0–0)	0 (0–0)	0 (0–0)	0 (0–0)
Other oral disorders	0 (0–0)	0 (0–0)	0 (0–0)	0 (0–0)	0 (0–0)	0 (0–0)
Sudden infant death syndrome	24,316 (19,443–45,346)	24,316 (19,443–45,346)	0 (0–0)	0 (0–0)	0 (0–0)	0 (0–0)
Injuries	4,660,584 (4,335,808–4,839,607)	573,081 (470,025–666,795)	385,918 (358,566–408,614)	2,383,609 (2,250,558–2,448,846)	783,886 (733,111–805,901)	534,089 (491,202–553,975)
Transport injuries	1,312,629 (1,267,628–1,361,806)	93,609 (74,082–110,322)	111,974 (103,912–119,323)	763,609 (738,397–792,809)	230,245 (222,126–238,249)	113,190 (109,923–116,443)
Road injuries	1,232,518 (1,188,814–1,283,094)	86,377 (68,406–102,052)	106,588 (99,101–113,383)	719,197 (694,823–749,561)	213,377 (205,190–221,576)	106,976 (103,803–110,084)
Pedestrian road injuries	570,231 (513,236–609,295)	46,503 (38,757–54,632)	61,613 (56,663–66,154)	286,823 (254,311–309,951)	112,429 (101,039–119,496)	62,861 (58,101–66,138)
Cyclist road injuries	51,803 (46,640–58,335)	2,213 (1,552–3,111)	5,780 (4,977–6,680)	26,837 (23,629–30,731)	11,958 (10,798–13,465)	5,014 (4,623–5,516)
Motorcyclist road injuries	163,564 (136,775–196,384)	7,175 (4,423–10,121)	7,942 (6,085–9,831)	120,650 (99,944–145,689)	20,819 (16,871–26,077)	6,976 (5,604–8,854)

SR Table 12 - Global deaths by age and cause for both sexes in 1995						
**Cause**	**All ages**	**0-5 years**	**5-14 years**	**15-49 years**	**50-69 years**	**70+ years**
Motor vehicle road injuries	432,465 (398,239–496,335)	28,916 (21,353–34,967)	29,785 (26,792–34,720)	277,239 (256,470–318,994)	65,706 (59,138–76,378)	30,817 (27,882–35,066)
Other road injuries	14,453 (9,931–17,878)	1,567 (648–3,133)	1,467 (935–2,000)	7,647 (5,201–9,666)	2,463 (1,847–3,083)	1,306 (1,014–1,717)
Other transport injuries	80,110 (70,051–87,338)	7,231 (4,484–9,001)	5,385 (4,263–6,059)	44,411 (39,420–48,469)	16,868 (14,789–18,997)	6,213 (5,561–6,969)
Unintentional injuries	2,045,876 (1,859,024–2,155,337)	451,656 (363,316–531,133)	237,124 (216,569–253,370)	732,220 (678,015–761,731)	310,313 (285,667–323,104)	314,561 (284,136–329,447)
Falls	379,459 (351,400–395,969)	32,615 (24,060–43,411)	20,242 (17,712–22,567)	105,275 (98,449–109,861)	79,468 (74,033–83,051)	141,858 (132,016–148,659)
Drowning	531,229 (454,562–569,156)	159,657 (117,014–189,207)	123,006 (108,835–131,558)	174,873 (160,683–183,754)	47,708 (42,318–50,733)	25,984 (22,127–27,722)
Fire, heat, and hot substances	246,694 (189,045–269,983)	46,498 (32,778–55,913)	17,320 (13,231–19,365)	112,743 (81,233–124,430)	36,319 (29,315–38,778)	33,812 (28,144–35,782)
Poisonings	128,689 (91,567–148,564)	32,592 (13,421–51,513)	10,457 (8,664–12,430)	53,343 (42,806–59,856)	21,789 (17,035–24,370)	10,506 (8,687–11,845)
Exposure to mechanical forces	202,708 (183,613–229,181)	48,431 (37,002–58,425)	16,419 (14,173–20,984)	87,435 (80,508–102,307)	29,525 (27,443–34,470)	20,895 (17,377–23,375)
Unintentional firearm injuries	33,619 (25,211–36,352)	3,147 (1,941–4,336)	2,740 (2,182–3,378)	18,519 (13,321–20,406)	4,219 (3,096–4,679)	4,993 (4,179–5,433)
Unintentional suffocation	42,103 (32,488–50,322)	28,975 (19,217–36,158)	3,620 (2,846–5,400)	4,634 (4,034–5,402)	1,539 (1,167–1,782)	3,333 (2,733–3,807)
Other exposure to mechanical forces	126,984 (113,208–153,739)	16,309 (11,076–22,485)	10,059 (8,394–13,120)	64,280 (58,100–79,241)	23,766 (21,880–28,975)	12,568 (10,025–14,513)
Adverse effects of medical treatment	103,433 (78,925–119,340)	26,067 (11,513–35,270)	6,013 (3,825–7,339)	27,491 (20,783–31,337)	18,984 (15,339–21,324)	24,875 (19,754–27,617)
Animal contact	113,140 (79,529–125,752)	20,318 (13,536–25,863)	17,877 (12,126–20,534)	43,586 (30,679–47,645)	22,051 (15,431–24,441)	9,306 (6,946–10,432)
Venomous animal contact	91,419 (62,568–101,288)	14,355 (9,209–18,180)	14,540 (9,612–16,796)	37,083 (25,374–41,120)	18,307 (12,307–20,668)	7,132 (5,067–8,340)
Non-venomous animal contact	21,721 (16,586–25,787)	5,963 (3,467–7,986)	3,336 (2,393–4,105)	6,502 (5,098–7,495)	3,744 (2,906–4,353)	2,174 (1,787–2,490)
Foreign body	148,602 (111,437–193,504)	63,228 (36,422–98,828)	11,071 (7,934–14,894)	32,093 (24,704–56,349)	18,054 (15,202–26,478)	24,155 (20,703–31,864)
Pulmonary aspiration and foreign body in airway	109,580 (79,037–152,960)	50,658 (24,318–85,030)	7,513 (4,944–11,000)	18,760 (15,885–22,426)	13,338 (11,683–14,981)	19,309 (16,792–20,623)
Foreign body in eyes	0 (0–0)	0 (0–0)	0 (0–0)	0 (0–0)	0 (0–0)	0 (0–0)
Foreign body in other body part	39,022 (21,448–90,405)	12,569 (5,866–20,484)	3,558 (1,846–7,622)	13,332 (6,846–38,577)	4,715 (2,627–12,966)	4,845 (3,128–12,273)
Environmental heat and cold exposure	51,162 (39,032–61,052)	5,684 (2,613–7,174)	1,701 (1,183–2,234)	18,477 (14,829–21,350)	14,245 (11,242–17,976)	11,054 (7,681–14,528)
Other unintentional injuries	140,754 (131,403–150,295)	16,561 (13,000–19,648)	13,013 (11,409–14,533)	76,900 (71,892–82,396)	22,166 (20,393–23,916)	12,112 (10,846–13,637)
Self-harm and interpersonal violence	1,223,180 (1,080,112–1,266,557)	21,357 (18,221–23,402)	29,941 (25,555–31,643)	836,063 (734,812–867,440)	233,250 (206,145–241,941)	102,567 (88,330–106,935)
Self-harm	798,952 (665,095–836,672)	0 (0–0)	12,436 (9,140–13,722)	518,679 (429,106–545,201)	180,505 (153,864–188,324)	87,332 (72,954–91,642)
Interpersonal violence	424,227 (398,125–436,082)	21,357 (18,221–23,402)	17,504 (16,128–18,537)	317,384 (298,539–327,272)	52,745 (49,868–54,817)	15,234 (14,645–15,927)
Assault by firearm	143,808 (135,099–151,145)	2,146 (1,728–2,641)	3,663 (3,318–3,975)	121,420 (114,479–127,026)	13,568 (12,520–14,633)	3,009 (2,743–3,260)
Assault by sharp object	108,414 (100,266–114,184)	2,393 (1,808–3,145)	3,227 (2,776–3,708)	86,005 (79,467–91,041)	13,029 (11,921–13,946)	3,759 (3,524–4,009)
Assault by other means	172,004 (155,481–182,396)	16,817 (14,250–18,633)	10,614 (9,559–11,631)	109,958 (98,184–117,681)	26,147 (23,994–27,934)	8,465 (7,950–9,035)
Forces of nature, war, and legal intervention	78,899 (61,090–96,601)	6,457 (4,943–8,205)	6,877 (5,205–8,795)	51,716 (40,369–63,020)	10,077 (7,328–14,071)	3,770 (2,258–7,164)
Exposure to forces of nature	19,137 (11,736–29,204)	1,386 (912–2,075)	2,880 (1,860–4,260)	9,549 (5,844–15,012)	3,482 (1,778–7,087)	1,839 (806–4,512)
Collective violence and legal intervention	59,761 (47,418–71,568)	5,071 (3,792–6,528)	3,996 (2,998–5,076)	42,166 (33,098–50,898)	6,595 (5,158–8,009)	1,931 (1,331–4,555)

SR Table 12 - Global deaths by age and cause for both sexes in 2000						
**Cause**	**All ages**	**0-5 years**	**5-14 years**	**15-49 years**	**50-69 years**	**70+ years**
All causes	52,143,163 (51,673,019–52,592,849)	9,302,496 (9,217,834–9,386,325)	1,223,339 (1,207,620–1,241,388)	9,267,534 (9,117,583–9,427,691)	12,691,308 (12,519,083–12,849,596)	19,658,485 (19,520,290–19,801,255)
Communicable, maternal, neonatal, and nutritional diseases	14,753,267 (14,430,319–15,145,049)	7,863,933 (7,659,732–8,043,850)	616,729 (585,417–653,282)	2,865,304 (2,760,813–3,002,619)	1,596,444 (1,504,467–1,694,667)	1,810,857 (1,732,168–1,903,315)
HIV/AIDS and tuberculosis	2,784,728 (2,565,968–3,111,683)	210,122 (196,503–230,401)	59,886 (55,210–69,592)	1,499,975 (1,399,550–1,621,808)	677,307 (595,440–792,307)	337,437 (282,698–420,054)
Tuberculosis	1,435,489 (1,214,440–1,745,495)	43,081 (31,757–63,407)	24,109 (20,201–33,453)	531,480 (444,076–637,656)	517,039 (430,775–629,912)	319,779 (264,886–402,579)
HIV/AIDS	1,349,239 (1,267,302–1,442,122)	167,040 (160,945–173,580)	35,777 (33,489–38,363)	968,494 (898,735–1,044,917)	160,267 (147,105–176,723)	17,658 (15,949–20,700)
HIV/AIDS - Tuberculosis	283,661 (241,480–333,315)	46,787 (40,578–53,391)	9,657 (7,881–11,276)	181,075 (152,378–215,057)	41,154 (35,235–47,153)	4,987 (4,286–5,683)
HIV/AIDS resulting in other diseases	1,065,577 (981,677–1,153,742)	120,253 (111,805–130,043)	26,120 (23,622–29,199)	787,419 (715,177–859,512)	119,113 (106,280–135,583)	12,671 (11,202–15,490)
Diarrhea, lower respiratory, and other common infectious diseases	6,396,141 (6,109,293–6,699,668)	3,328,679 (3,109,827–3,564,820)	401,245 (360,757–451,833)	678,904 (635,567–742,500)	705,423 (667,911–745,111)	1,281,888 (1,217,844–1,346,670)
Diarrheal diseases	1,747,549 (1,622,933–1,857,675)	868,372 (760,437–976,412)	88,976 (79,181–98,818)	214,950 (203,098–230,177)	251,489 (239,163–266,343)	323,760 (308,263–342,762)
Intestinal infectious diseases	227,017 (128,178–373,267)	60,307 (31,463–104,812)	89,945 (52,513–143,029)	64,398 (33,849–110,088)	8,764 (4,555–15,371)	3,601 (2,108–5,627)
Typhoid fever	186,528 (101,500–313,929)	49,536 (24,880–89,196)	75,072 (42,358–120,983)	52,348 (27,267–90,491)	6,875 (3,291–12,381)	2,695 (1,440–4,429)
Paratyphoid fever	37,731 (17,185–72,391)	9,122 (3,841–18,343)	14,575 (6,721–27,566)	11,686 (4,933–23,071)	1,720 (705–3,438)	627 (269–1,225)
Other intestinal infectious diseases	2,757 (956–5,077)	1,648 (385–3,491)	296 (78–684)	364 (158–651)	168 (78–285)	279 (124–532)
Lower respiratory infections	2,998,336 (2,814,678–3,157,192)	1,427,783 (1,299,412–1,553,452)	83,358 (66,643–92,927)	240,109 (209,581–263,840)	355,013 (312,673–380,939)	892,071 (822,097–944,959)
Upper respiratory infections	4,086 (3,627–4,652)	1,351 (1,002–1,842)	79 (56–111)	843 (734–971)	895 (758–1,045)	915 (771–1,091)
Otitis media	4,727 (4,272–5,236)	1,825 (1,574–2,106)	644 (579–705)	1,117 (1,042–1,200)	594 (537–661)	545 (403–882)
Meningitis	441,153 (377,172–495,932)	242,664 (193,203–292,597)	41,922 (34,723–48,366)	81,481 (68,733–94,658)	45,086 (38,376–53,306)	29,999 (26,003–35,763)
Pneumococcal meningitis	115,936 (94,952–140,844)	54,364 (37,939–76,623)	13,544 (9,360–18,287)	26,853 (21,168–33,328)	12,670 (9,619–16,415)	8,503 (6,546–10,855)
H influenzae type B meningitis	129,260 (102,308–158,319)	91,141 (66,828–118,088)	11,664 (7,876–15,775)	14,056 (10,329–18,105)	7,387 (5,447–9,803)	5,010 (3,865–6,518)
Meningococcal meningitis	78,502 (61,040–99,968)	45,725 (31,179–65,927)	7,386 (4,861–10,416)	13,560 (10,320–17,205)	7,418 (5,409–9,738)	4,411 (3,339–5,767)
Other meningitis	117,454 (100,100–136,348)	51,432 (37,876–67,122)	9,327 (6,630–12,745)	27,010 (21,462–32,097)	17,609 (14,255–21,687)	12,074 (9,887–14,358)
Encephalitis	149,656 (134,959–165,779)	53,502 (44,583–61,328)	19,928 (17,169–22,611)	34,822 (31,880–38,995)	21,991 (19,586–25,976)	19,411 (17,157–23,319)
Diphtheria	9,555 (5,451–19,404)	7,413 (3,522–16,974)	1,165 (619–2,141)	736 (540–986)	240 (144–393)	0 (0–0)
Whooping cough	121,988 (41,080–272,088)	113,385 (37,931–252,507)	6,878 (2,379–16,389)	1,523 (487–3,951)	202 (62–532)	0 (0–0)
Tetanus	162,373 (142,731–213,476)	101,424 (90,085–115,991)	7,837 (6,037–13,598)	26,102 (21,073–45,638)	18,886 (14,636–35,557)	8,123 (6,302–14,876)
Measles	518,142 (199,749–1,014,121)	447,344 (173,963–865,079)	59,558 (21,332–119,718)	10,900 (3,271–25,677)	339 (97–825)	0 (0–0)
Varicella and herpes zoster	11,554 (10,337–12,974)	3,305 (2,597–4,286)	950 (731–1,243)	1,917 (1,662–2,216)	1,919 (1,587–2,369)	3,460 (2,825–4,202)
Neglected tropical diseases and malaria	1,290,272 (1,053,016–1,525,520)	855,104 (633,267–1,083,817)	107,161 (79,795–136,771)	190,366 (150,671–238,854)	97,539 (76,012–126,733)	40,101 (30,452–53,867)
Malaria	1,142,120 (907,792–1,384,054)	827,497 (605,556–1,058,270)	83,044 (55,514–112,854)	133,505 (95,317–183,315)	70,096 (49,171–98,348)	27,976 (18,347–41,273)
Chagas disease	7,921 (7,587–8,242)	5 (5–6)	10 (9–11)	1,470 (1,380–1,561)	3,516 (3,332–3,714)	2,917 (2,765–3,074)
Leishmaniasis	21,643 (13,135–32,337)	3,789 (2,177–5,756)	2,481 (1,403–4,135)	12,429 (7,431–18,920)	2,439 (1,602–3,417)	503 (381–640)
Visceral leishmaniasis	21,643 (13,135–32,337)	3,789 (2,177–5,756)	2,481 (1,403–4,135)	12,429 (7,431–18,920)	2,439 (1,602–3,417)	503 (381–640)
Cutaneous and mucocutaneous leishmaniasis	0 (0–0)	0 (0–0)	0 (0–0)	0 (0–0)	0 (0–0)	0 (0–0)
African trypanosomiasis	24,195 (12,980–40,165)	1,760 (894–2,968)	3,162 (1,722–5,142)	14,483 (7,722–24,353)	3,922 (2,041–6,706)	866 (456–1,451)
Schistosomiasis	9,743 (8,732–10,895)	131 (84–214)	247 (186–329)	3,039 (2,623–3,518)	4,141 (3,454–4,926)	2,183 (1,735–2,756)
Cysticercosis	775 (685–905)	15 (8–29)	27 (21–36)	454 (400–534)	211 (187–244)	66 (58–75)
Cystic echinococcosis	2,157 (2,039–2,276)	108 (85–134)	210 (185–236)	837 (784–892)	597 (564–636)	402 (382–423)
Lymphatic filariasis	0 (0–0)	0 (0–0)	0 (0–0)	0 (0–0)	0 (0–0)	0 (0–0)
Onchocerciasis	0 (0–0)	0 (0–0)	0 (0–0)	0 (0–0)	0 (0–0)	0 (0–0)
Trachoma	0 (0–0)	0 (0–0)	0 (0–0)	0 (0–0)	0 (0–0)	0 (0–0)
Dengue	7,770 (5,161–11,101)	2,778 (1,434–4,730)	2,022 (1,308–2,984)	1,946 (1,427–2,695)	647 (463–901)	375 (261–516)
Yellow fever	7,614 (1,600–20,777)	692 (130–1,992)	2,097 (416–5,807)	4,049 (849–11,516)	650 (134–1,818)	124 (25–348)
Rabies	41,569 (36,827–46,260)	5,586 (4,146–7,648)	11,083 (9,159–13,237)	13,822 (12,000–15,169)	8,274 (7,007–9,355)	2,803 (2,251–3,367)
Intestinal nematode infections	4,716 (4,019–5,376)	3,543 (2,935–4,142)	660 (563–764)	247 (204–299)	153 (119–193)	111 (84–146)
Ascariasis	4,716 (4,019–5,376)	3,543 (2,935–4,142)	660 (563–764)	247 (204–299)	153 (119–193)	111 (84–146)
Trichuriasis	0 (0–0)	0 (0–0)	0 (0–0)	0 (0–0)	0 (0–0)	0 (0–0)
Hookworm disease	0 (0–0)	0 (0–0)	0 (0–0)	0 (0–0)	0 (0–0)	0 (0–0)
Food-borne trematodiases	0 (0–0)	0 (0–0)	0 (0–0)	0 (0–0)	0 (0–0)	0 (0–0)
Leprosy	0 (0–0)	0 (0–0)	0 (0–0)	0 (0–0)	0 (0–0)	0 (0–0)
Ebola	225 (181–270)	28 (22–34)	17 (14–21)	133 (107–159)	42 (34–50)	3 (3–4)
Other neglected tropical diseases	19,818 (13,582–26,345)	9,166 (6,337–12,053)	2,094 (1,395–2,946)	3,946 (2,452–5,545)	2,845 (1,773–4,191)	1,764 (1,107–2,759)
Maternal disorders	374,320 (351,336–400,418)	0 (0–0)	1,755 (1,487–2,067)	371,245 (348,356–397,372)	1,319 (945–2,008)	0 (0–0)
Maternal hemorrhage	97,688 (83,444–112,931)	0 (0–0)	504 (357–683)	96,723 (82,503–112,048)	460 (268–852)	0 (0–0)
Maternal sepsis and other maternal infections	25,723 (20,767–31,336)	0 (0–0)	74 (50–109)	25,556 (20,607–31,168)	92 (54–157)	0 (0–0)
Maternal hypertensive disorders	63,153 (52,613–74,858)	0 (0–0)	343 (236–482)	62,709 (52,220–74,407)	100 (60–166)	0 (0–0)
Maternal obstructed labor and uterine rupture	29,438 (23,418–35,836)	0 (0–0)	56 (33–88)	29,327 (23,328–35,727)	54 (32–91)	0 (0–0)
Maternal abortion, miscarriage, and ectopic pregnancy	54,347 (44,507–66,598)	0 (0–0)	271 (176–400)	53,865 (44,033–66,080)	210 (132–330)	0 (0–0)

SR Table 12 - Global deaths by age and cause for both sexes in 2000

Cause	All ages	0-5 years	5-14 years	15-49 years	50-69 years	70+ years
Indirect maternal deaths	44,521 (35,640–54,126)	0 (0–0)	216 (133–326)	44,173 (35,314–53,762)	131 (72–241)	0 (0–0)
Late maternal deaths	8,728 (5,830–12,716)	0 (0–0)	46 (23–79)	8,637 (5,751–12,612)	44 (22–96)	0 (0–0)
Maternal deaths aggravated by HIV/AIDS	2,704 (1,695–3,646)	0 (0–0)	0 (0–1)	2,695 (1,688–3,633)	8 (4–15)	0 (0–0)
Other maternal disorders	48,014 (39,806–57,496)	0 (0–0)	241 (164–339)	47,558 (39,386–57,009)	215 (137–343)	0 (0–0)
Neonatal disorders	2,909,505 (2,828,628–2,994,936)	2,909,505 (2,828,628–2,994,936)	0 (0–0)	0 (0–0)	0 (0–0)	0 (0–0)
Neonatal preterm birth complications	1,291,780 (1,195,593–1,430,552)	1,291,780 (1,195,593–1,430,552)	0 (0–0)	0 (0–0)	0 (0–0)	0 (0–0)
Neonatal encephalopathy due to birth asphyxia and trauma	901,057 (802,358–1,002,844)	901,057 (802,358–1,002,844)	0 (0–0)	0 (0–0)	0 (0–0)	0 (0–0)
Neonatal sepsis and other neonatal infections	342,580 (243,682–467,795)	342,580 (243,682–467,795)	0 (0–0)	0 (0–0)	0 (0–0)	0 (0–0)
Hemolytic disease and other neonatal jaundice	85,137 (51,311–145,924)	85,137 (51,311–145,924)	0 (0–0)	0 (0–0)	0 (0–0)	0 (0–0)
Other neonatal disorders	288,949 (210,719–382,670)	288,949 (210,719–382,670)	0 (0–0)	0 (0–0)	0 (0–0)	0 (0–0)
Nutritional deficiencies	602,565 (489,234–730,249)	359,165 (274,789–457,954)	21,243 (17,605–25,727)	51,721 (43,683–60,951)	57,670 (47,451–69,466)	112,763 (96,432–129,876)
Protein-energy malnutrition	517,978 (413,946–639,926)	334,352 (254,660–430,699)	17,216 (13,944–21,017)	31,464 (24,788–39,997)	40,878 (31,655–51,864)	94,065 (78,417–111,422)
Iodine deficiency	2,226 (1,673–3,055)	860 (429–1,585)	96 (54–168)	390 (278–562)	422 (275–680)	456 (305–694)
Vitamin A deficiency	0 (0–0)	0 (0–0)	0 (0–0)	0 (0–0)	0 (0–0)	0 (0–0)
Iron-deficiency anemia	47,897 (31,450–64,719)	15,579 (7,541–22,320)	2,066 (1,465–3,242)	12,383 (9,245–15,946)	10,728 (7,956–13,653)	7,139 (4,739–9,748)
Other nutritional deficiencies	34,463 (22,617–57,886)	8,373 (4,206–16,704)	1,863 (1,150–2,956)	7,483 (4,964–11,330)	5,641 (3,683–9,628)	11,101 (8,210–16,871)
Other communicable, maternal, neonatal, and nutritional diseases	395,732 (329,959–482,007)	201,355 (142,008–279,608)	25,436 (20,306–32,232)	73,090 (70,401–76,253)	57,184 (52,831–59,484)	38,666 (34,997–40,386)
Sexually transmitted diseases excluding HIV	163,177 (99,577–247,455)	138,319 (80,460–216,200)	11,611 (6,744–17,879)	6,475 (5,218–7,307)	3,872 (2,732–4,525)	2,898 (2,247–3,373)
Syphilis	161,471 (98,176–245,668)	138,319 (80,460–216,200)	11,572 (6,701–17,835)	5,666 (4,574–6,402)	3,399 (2,400–3,974)	2,512 (1,952–2,923)
Chlamydial infection	185 (136–213)	0 (0–0)	4 (3–6)	85 (66–101)	49 (31–59)	45 (32–53)
Gonococcal infection	1,032 (797–1,144)	0 (0–0)	19 (15–24)	473 (380–535)	276 (194–323)	262 (203–306)
Trichomoniasis	0 (0–0)	0 (0–0)	0 (0–0)	0 (0–0)	0 (0–0)	0 (0–0)
Genital herpes	0 (0–0)	0 (0–0)	0 (0–0)	0 (0–0)	0 (0–0)	0 (0–0)
Other sexually transmitted diseases	488 (378–543)	0 (0–0)	14 (11–17)	249 (201–281)	146 (102–171)	77 (59–91)
Hepatitis	144,763 (139,423–150,759)	25,444 (22,201–28,577)	9,578 (8,516–10,700)	52,958 (50,814–56,336)	35,605 (34,334–36,950)	21,177 (20,401–21,914)
Acute hepatitis A	24,091 (15,957–33,100)	19,611 (14,471–23,867)	1,482 (431–3,082)	1,692 (428–3,843)	860 (194–1,917)	444 (110–989)
Hepatitis B	80,643 (70,691–90,462)	1,426 (511–3,427)	1,096 (616–1,729)	32,881 (27,719–37,824)	29,193 (25,836–32,116)	16,044 (13,780–18,098)
Hepatitis C	3,216 (708–7,260)	0 (0–0)	132 (25–359)	931 (189–2,131)	1,469 (334–3,336)	683 (142–1,630)
Acute hepatitis E	36,811 (26,146–48,987)	4,406 (1,775–8,408)	6,865 (5,210–8,421)	17,453 (13,007–22,400)	4,081 (2,004–7,169)	4,005 (2,258–6,207)
Other infectious diseases	87,791 (62,115–100,964)	37,591 (21,077–49,163)	4,246 (3,395–4,907)	13,656 (11,842–14,788)	17,707 (13,660–19,314)	14,589 (11,332–15,960)
Non-communicable diseases	32,700,207 (32,315,576–33,128,729)	976,309 (840,517–1,168,846)	250,089 (233,294–266,549)	3,951,631 (3,874,697–4,039,757)	10,273,679 (10,134,245–10,423,514)	17,248,496 (17,113,586–17,389,818)
Neoplasms	6,897,101 (6,783,370–7,003,387)	40,118 (34,492–50,634)	61,751 (58,116–65,339)	1,054,545 (1,019,864–1,081,923)	2,883,615 (2,829,942–2,928,486)	2,857,071 (2,805,347–2,900,333)
Lip and oral cavity cancer	97,730 (95,472–100,032)	0 (0–0)	0 (0–0)	17,478 (16,852–18,172)	46,835 (45,656–48,088)	33,416 (32,616–34,213)
Nasopharynx cancer	53,454 (43,627–56,493)	0 (0–0)	394 (317–462)	17,695 (13,872–19,123)	24,189 (19,800–25,629)	11,175 (9,584–11,655)
Other pharynx cancer	47,299 (45,764–48,452)	0 (0–0)	0 (0–0)	7,718 (7,396–8,092)	26,504 (25,611–27,234)	13,075 (12,618–13,477)
Esophageal cancer	424,417 (408,824–439,018)	0 (0–0)	0 (0–0)	45,545 (43,243–47,754)	211,955 (203,293–220,028)	166,916 (161,137–172,685)
Stomach cancer	792,890 (776,110–811,054)	0 (0–0)	0 (0–0)	98,547 (95,003–102,771)	339,283 (330,655–348,269)	355,059 (348,319–362,283)
Colon and rectum cancer	611,718 (602,479–620,483)	0 (0–0)	0 (0–0)	63,619 (61,806–66,186)	225,739 (221,856–229,367)	322,359 (316,523–327,897)
Liver cancer	673,550 (572,578–704,454)	0 (0–0)	1,699 (1,308–1,907)	152,192 (115,401–162,499)	316,662 (267,565–332,369)	202,996 (183,872–211,502)
Liver cancer due to hepatitis B	250,736 (202,840–268,342)	0 (0–0)	1,010 (756–1,147)	90,410 (67,987–97,712)	113,123 (93,439–123,244)	46,193 (39,515–51,196)
Liver cancer due to hepatitis C	125,120 (114,445–133,299)	0 (0–0)	36 (29–43)	10,878 (8,784–12,388)	54,789 (48,390–60,122)	59,416 (55,431–63,410)
Liver cancer due to alcohol use	177,718 (150,218–190,603)	0 (0–0)	0 (0–0)	21,992 (16,449–24,614)	96,963 (81,177–106,319)	58,762 (52,216–63,395)
Liver cancer due to other causes	119,975 (101,609–129,823)	0 (0–0)	653 (519–734)	28,911 (21,827–32,029)	51,786 (43,467–57,622)	38,624 (33,799–42,128)
Gallbladder and biliary tract cancer	115,385 (112,235–118,607)	0 (0–0)	0 (0–0)	8,864 (8,272–9,331)	43,319 (41,776–44,724)	63,201 (61,720–64,847)
Pancreatic cancer	277,403 (273,503–281,186)	0 (0–0)	0 (0–0)	23,151 (22,595–23,725)	115,124 (113,314–117,034)	139,127 (136,835–141,397)
Larynx cancer	91,370 (88,951–93,816)	0 (0–0)	0 (0–0)	11,357 (10,875–11,894)	47,751 (46,345–49,126)	32,261 (31,420–33,019)
Tracheal, bronchus, and lung cancer	1,293,155 (1,274,180–1,314,153)	0 (0–0)	0 (0–0)	121,072 (117,579–125,094)	606,564 (596,955–616,903)	565,518 (556,754–574,908)
Malignant skin melanoma	42,071 (35,010–52,700)	0 (0–0)	0 (0–0)	9,630 (8,145–12,072)	15,843 (12,910–20,059)	16,596 (13,718–20,644)
Non-melanoma skin cancer	31,769 (31,102–32,484)	0 (0–0)	0 (0–0)	3,585 (3,470–3,728)	10,652 (10,377–10,948)	17,532 (17,091–17,955)
Non-melanoma skin cancer (squamous-cell carcinoma)	31,769 (31,102–32,484)	0 (0–0)	0 (0–0)	3,585 (3,470–3,728)	10,652 (10,377–10,948)	17,532 (17,091–17,955)
Non-melanoma skin cancer (basal-cell carcinoma)	0 (0–0)	0 (0–0)	0 (0–0)	0 (0–0)	0 (0–0)	0 (0–0)
Breast cancer	405,629 (385,815–427,486)	0 (0–0)	0 (0–0)	94,773 (85,755–104,212)	175,983 (167,820–185,653)	134,871 (129,923–139,411)
Cervical cancer	210,709 (199,081–222,653)	0 (0–0)	0 (0–0)	59,223 (54,618–63,982)	96,861 (90,887–102,744)	54,623 (52,671–56,696)
Uterine cancer	77,669 (74,293–81,670)	0 (0–0)	0 (0–0)	10,533 (9,721–11,404)	35,916 (34,036–38,255)	31,219 (30,161–32,526)
Ovarian cancer	123,315 (120,397–127,865)	0 (0–0)	0 (0–0)	22,579 (21,676–24,501)	55,703 (54,325–57,894)	45,032 (43,878–46,255)
Prostate cancer	250,852 (204,109–310,647)	0 (0–0)	0 (0–0)	2,398 (1,888–2,925)	60,371 (48,853–74,266)	188,082 (151,101–234,657)
Testicular cancer	8,884 (8,513–9,352)	0 (0–0)	0 (0–0)	6,203 (5,880–6,584)	1,538 (1,476–1,619)	1,142 (1,097–1,190)

SR Table 12 - Global deaths by age and cause for both sexes in 2000						
**Cause**	**All ages**	**0-5 years**	**5-14 years**	**15-49 years**	**50-69 years**	**70+ years**
Kidney cancer	92,363 (90,655–94,586)	2,201 (1,399–3,246)	1,097 (1,013–1,202)	9,740 (9,485–10,076)	38,866 (38,074–39,694)	40,458 (39,610–41,340)
Bladder cancer	138,604 (136,220–140,876)	0 (0–0)	0 (0–0)	7,023 (6,800–7,270)	45,161 (44,300–46,033)	86,419 (84,638–88,161)
Brain and nervous system cancer	177,760 (159,900–189,012)	6,149 (4,918–8,655)	11,721 (9,877–13,408)	53,267 (46,791–57,251)	67,825 (61,550–70,989)	38,796 (35,644–40,386)
Thyroid cancer	23,779 (22,721–25,954)	0 (0–0)	86 (73–93)	2,762 (2,621–3,126)	9,167 (8,705–10,065)	11,763 (11,219–12,827)
Mesothelioma	21,367 (20,737–21,909)	0 (0–0)	0 (0–0)	3,399 (3,218–3,553)	8,821 (8,521–9,066)	9,147 (8,868–9,447)
Hodgkin lymphoma	29,282 (23,421–34,135)	585 (356–691)	1,198 (802–1,445)	10,927 (8,879–12,554)	9,996 (7,893–11,810)	6,573 (5,284–7,734)
Non-Hodgkin lymphoma	163,716 (145,507–177,321)	3,439 (2,221–5,463)	4,761 (3,995–5,598)	31,627 (28,506–35,278)	56,477 (50,313–60,623)	67,410 (57,611–71,042)
Multiple myeloma	69,912 (67,750–71,911)	0 (0–0)	0 (0–0)	6,693 (6,446–7,050)	28,112 (27,401–29,030)	35,106 (33,311–36,123)
Leukemia	291,693 (285,096–300,802)	15,188 (13,447–17,381)	29,622 (27,941–31,511)	84,319 (81,608–88,051)	76,484 (74,735–78,654)	86,079 (84,123–88,079)
Acute lymphoid leukemia	97,812 (90,260–109,201)	9,942 (8,453–12,291)	20,857 (19,175–23,328)	33,819 (30,787–38,490)	20,095 (18,404–22,375)	13,096 (12,299–14,256)
Chronic lymphoid leukemia	47,654 (45,177–49,922)	0 (0–0)	0 (0–0)	7,583 (6,509–8,326)	13,747 (12,911–14,543)	26,322 (25,320–27,256)
Acute myeloid leukemia	108,118 (99,542–116,055)	5,245 (3,876–6,468)	8,764 (6,847–10,028)	31,136 (27,608–33,972)	30,582 (28,706–32,819)	32,388 (30,888–34,258)
Chronic myeloid leukemia	38,108 (35,802–40,804)	0 (0–0)	0 (0–0)	11,778 (10,619–13,112)	12,058 (11,279–12,953)	14,270 (13,616–14,965)
Other neoplasms	259,343 (243,496–270,525)	12,554 (9,777–17,871)	11,169 (10,122–12,130)	68,612 (62,117–72,636)	85,899 (79,428–89,659)	81,107 (77,060–85,219)
Cardiovascular diseases	14,900,447 (14,720,981–15,093,916)	75,801 (69,739–81,690)	39,310 (36,801–42,044)	1,394,046 (1,363,605–1,437,452)	4,451,669 (4,389,387–4,520,536)	8,939,619 (8,817,135–9,057,858)
Rheumatic heart disease	349,013 (330,210–366,386)	5,000 (4,173–5,870)	9,972 (9,106–10,913)	96,059 (88,149–102,966)	114,805 (107,941–121,350)	123,176 (117,863–128,170)
Ischemic heart disease	7,009,334 (6,923,541–7,106,800)	4,881 (4,267–5,691)	3,368 (3,033–3,789)	612,540 (597,347–634,772)	2,123,207 (2,092,081–2,164,291)	4,265,336 (4,202,633–4,329,623)
Cerebrovascular disease	5,674,560 (5,580,214–5,784,495)	28,121 (25,400–31,478)	14,273 (13,305–15,515)	464,634 (451,938–481,108)	1,713,099 (1,677,560–1,750,561)	3,454,431 (3,395,149–3,518,530)
Ischemic stroke	2,542,556 (2,458,212–2,616,926)	1,181 (1,017–1,387)	897 (819–999)	73,265 (68,787–78,437)	576,274 (549,571–601,888)	1,890,937 (1,834,015–1,944,860)
Hemorrhagic stroke	3,132,004 (3,043,423–3,224,998)	26,940 (24,298–30,313)	13,375 (12,458–14,563)	391,369 (378,981–405,461)	1,136,824 (1,101,937–1,171,847)	1,563,494 (1,510,583–1,614,014)
Hypertensive heart disease	734,292 (678,522–795,817)	696 (545–859)	508 (441–594)	68,097 (63,761–74,906)	227,773 (209,628–250,493)	437,214 (399,208–475,203)
Cardiomyopathy and myocarditis	298,322 (288,042–308,628)	13,230 (11,213–14,884)	4,261 (3,916–4,750)	68,578 (65,244–72,319)	81,506 (78,259–85,541)	130,744 (126,576–134,661)
Atrial fibrillation and flutter	120,642 (99,114–145,049)	0 (0–0)	0 (0–0)	820 (624–1,044)	9,245 (7,109–12,312)	110,577 (89,270–135,223)
Aortic aneurysm	125,138 (122,009–128,775)	0 (0–0)	0 (0–0)	10,609 (10,084–11,207)	35,405 (34,299–36,702)	79,123 (77,159–81,109)
Peripheral vascular disease	29,151 (28,052–30,236)	0 (0–0)	0 (0–0)	446 (427–465)	4,847 (4,673–5,038)	23,857 (22,882–24,854)
Endocarditis	61,660 (53,964–68,152)	4,371 (3,010–5,825)	1,262 (980–1,490)	13,075 (10,657–14,947)	13,799 (11,979–15,590)	29,152 (26,500–31,649)
Other cardiovascular and circulatory diseases	498,331 (486,483–512,186)	19,499 (17,402–22,654)	5,663 (5,260–6,263)	59,184 (56,947–62,343)	127,979 (124,104–132,565)	286,004 (278,268–294,679)
Chronic respiratory diseases	3,896,167 (3,813,086–3,982,997)	72,841 (64,354–82,196)	16,390 (14,818–17,892)	243,521 (235,586–255,570)	1,141,170 (1,109,959–1,175,938)	2,422,242 (2,367,804–2,474,492)
Chronic obstructive pulmonary disease	3,220,207 (3,084,768–3,378,186)	29,780 (22,392–37,596)	3,444 (2,638–4,653)	140,958 (128,427–155,948)	911,482 (861,648–978,395)	2,134,540 (2,056,271–2,213,659)
Pneumoconiosis	31,909 (28,075–36,669)	0 (0–0)	0 (0–0)	3,504 (2,875–4,114)	9,379 (7,950–11,145)	19,025 (16,816–21,807)
Silicosis	11,162 (10,081–12,685)	0 (0–0)	0 (0–0)	1,541 (1,284–1,885)	3,770 (3,267–4,448)	5,849 (5,337–6,519)
Asbestosis	2,361 (1,730–2,798)	0 (0–0)	0 (0–0)	379 (313–454)	622 (448–833)	1,359 (926–1,569)
Coal workers pneumoconiosis	2,922 (2,574–3,338)	0 (0–0)	0 (0–0)	263 (203–335)	823 (688–990)	1,836 (1,643–2,067)
Other pneumoconiosis	15,462 (12,553–19,213)	0 (0–0)	0 (0–0)	1,319 (1,022–1,633)	4,163 (3,210–5,435)	9,980 (8,231–12,337)
Asthma	528,580 (379,697–627,829)	21,397 (15,884–27,098)	11,155 (9,196–13,014)	86,628 (68,900–102,128)	192,539 (132,920–236,773)	216,858 (152,573–263,311)
Interstitial lung disease and pulmonary sarcoidosis	65,499 (51,015–76,997)	336 (203–591)	271 (207–390)	5,437 (4,350–6,820)	19,528 (15,449–24,306)	39,924 (30,773–45,486)
Other chronic respiratory diseases	49,972 (36,105–61,940)	21,326 (14,999–29,191)	1,517 (939–2,478)	6,993 (4,637–8,819)	8,240 (5,671–9,807)	11,893 (9,220–13,951)
Cirrhosis and other chronic liver diseases	1,082,879 (1,047,356–1,148,533)	16,472 (13,071–21,000)	12,233 (10,907–14,222)	328,938 (315,668–350,276)	468,045 (450,957–493,967)	257,188 (247,949–272,917)
Cirrhosis and other chronic liver diseases due to hepatitis B	320,444 (297,005–350,385)	127 (95–168)	381 (309–464)	98,784 (89,742–110,251)	140,795 (126,507–157,893)	80,356 (72,998–89,281)
Cirrhosis and other chronic liver diseases due to hepatitis C	261,048 (243,475–280,202)	68 (50–90)	214 (167–262)	74,077 (66,404–82,616)	117,697 (105,477–129,555)	68,990 (63,417–75,743)
Cirrhosis and other chronic liver diseases due to alcohol use	283,458 (264,454–304,340)	0 (0–0)	0 (0–0)	99,348 (91,486–109,518)	133,807 (120,794–148,007)	50,302 (45,150–55,833)
Cirrhosis and other chronic liver diseases due to other causes	217,928 (203,838–240,170)	16,277 (12,909–20,761)	11,638 (10,382–13,538)	56,728 (51,506–62,761)	75,746 (67,332–85,214)	57,538 (52,816–63,406)
Digestive diseases	1,077,160 (1,042,308–1,135,478)	32,840 (23,912–51,915)	23,314 (21,086–26,260)	243,644 (232,300–255,660)	326,113 (315,101–346,958)	451,247 (438,472–476,845)
Peptic ulcer disease	318,612 (298,228–344,743)	4,274 (2,431–7,929)	4,576 (3,985–5,333)	74,331 (68,732–80,397)	109,071 (101,177–118,891)	126,357 (117,123–138,333)
Gastritis and duodenitis	48,968 (38,804–60,727)	1,235 (642–2,015)	715 (497–1,001)	11,273 (9,012–14,148)	15,949 (12,725–20,070)	19,794 (15,353–24,843)
Appendicitis	46,915 (41,956–54,683)	5,213 (2,702–10,818)	3,795 (3,295–4,332)	16,406 (14,814–18,269)	11,370 (10,451–12,734)	10,129 (9,346–11,288)
Paralytic ileus and intestinal obstruction	221,434 (197,659–253,211)	10,613 (7,007–16,839)	7,987 (6,753–9,532)	53,161 (45,737–61,203)	64,052 (57,353–73,923)	85,619 (78,510–98,518)
Inguinal, femoral, and abdominal hernia	54,848 (38,537–62,436)	1,413 (559–2,247)	1,129 (666–1,403)	11,379 (6,621–13,527)	16,980 (11,874–19,749)	23,945 (18,234–26,918)
Inflammatory bowel disease	38,739 (34,120–43,835)	2,348 (1,123–5,016)	780 (604–1,087)	8,194 (6,761–9,807)	9,652 (8,479–10,861)	17,763 (16,061–19,001)
Vascular intestinal disorders	73,140 (68,665–78,688)	639 (379–1,133)	171 (141–213)	4,845 (4,243–5,483)	18,856 (17,322–20,783)	48,627 (46,135–51,885)
Gallbladder and biliary diseases	90,368 (85,616–96,337)	1,840 (1,055–3,418)	1,291 (1,104–1,517)	13,794 (12,697–15,194)	26,030 (24,288–28,131)	47,410 (45,256–50,359)
Pancreatitis	90,802 (85,338–97,580)	290 (186–471)	601 (492–732)	35,193 (32,591–38,364)	29,543 (27,600–31,883)	25,172 (23,806–26,774)
Other digestive diseases	93,330 (81,776–112,812)	4,969 (3,126–8,022)	2,265 (1,867–2,984)	15,063 (12,450–19,986)	24,605 (20,798–30,543)	46,426 (42,069–52,699)
Neurological disorders	1,432,834 (1,244,107–1,617,549)	21,086 (15,433–27,226)	15,889 (14,443–17,636)	85,567 (82,147–90,965)	131,277 (112,610–150,134)	1,179,013 (997,165–1,361,094)
Alzheimer disease and other dementias	1,165,403 (972,817–1,352,263)	0 (0–0)	0 (0–0)	1,205 (752–1,758)	77,284 (58,574–96,591)	1,086,912 (903,239–1,269,859)
Parkinson disease	67,672 (65,796–70,640)	0 (0–0)	0 (0–0)	579 (543–632)	9,067 (8,712–9,873)	58,025 (56,362–60,329)
Epilepsy	118,099 (109,845–128,063)	15,641 (11,008–20,589)	12,865 (11,636–14,195)	62,201 (58,970–66,904)	16,770 (16,239–17,378)	10,619 (10,314–10,933)

SR Table 12 - Global deaths by age and cause for both sexes in 2000						
**Cause**	**All ages**	**0-5 years**	**5-14 years**	**15-49 years**	**50-69 years**	**70+ years**
Multiple sclerosis	16,177 (14,175–17,946)	0 (0–0)	0 (0–0)	5,945 (5,036–6,944)	6,996 (6,217–7,627)	3,236 (2,695–3,421)
Motor neuron disease	23,800 (23,062–25,280)	1,232 (822–1,517)	145 (124–162)	3,086 (2,926–3,411)	10,280 (9,908–11,014)	9,055 (8,811–9,346)
Migraine	0 (0–0)	0 (0–0)	0 (0–0)	0 (0–0)	0 (0–0)	0 (0–0)
Tension-type headache	0 (0–0)	0 (0–0)	0 (0–0)	0 (0–0)	0 (0–0)	0 (0–0)
Medication overuse headache	0 (0–0)	0 (0–0)	0 (0–0)	0 (0–0)	0 (0–0)	0 (0–0)
Other neurological disorders	41,699 (40,448–44,961)	4,231 (3,257–6,164)	2,878 (2,523–3,565)	12,548 (11,899–13,582)	10,877 (10,452–11,405)	11,164 (10,714–11,640)
Mental and substance use disorders	275,428 (254,945–282,158)	29 (25–34)	38 (20–51)	166,777 (155,159–171,753)	82,949 (76,977–85,498)	25,633 (24,479–26,277)
Schizophrenia	20,076 (18,739–21,102)	0 (0–0)	0 (0–0)	9,187 (8,191–9,852)	6,362 (5,934–6,679)	4,526 (4,290–4,755)
Alcohol use disorders	144,885 (136,910–149,930)	0 (0–0)	0 (0–0)	76,553 (72,083–79,987)	56,387 (53,112–58,663)	11,944 (11,535–12,313)
Drug use disorders	109,883 (100,544–113,340)	29 (25–34)	0 (0–0)	80,489 (73,208–83,489)	20,200 (18,595–21,046)	9,163 (8,391–9,561)
Opioid use disorders	81,034 (74,223–84,321)	29 (25–34)	0 (0–0)	60,151 (54,839–62,977)	14,578 (13,327–15,310)	6,273 (5,804–6,595)
Cocaine use disorders	4,660 (3,441–5,134)	0 (0–0)	0 (0–0)	3,645 (2,656–4,011)	840 (626–936)	174 (141–198)
Amphetamine use disorders	3,376 (2,336–3,899)	0 (0–0)	0 (0–0)	2,575 (1,783–2,967)	646 (443–756)	154 (115–178)
Cannabis use disorders	0 (0–0)	0 (0–0)	0 (0–0)	0 (0–0)	0 (0–0)	0 (0–0)
Other drug use disorders	20,812 (18,449–22,325)	0 (0–0)	0 (0–0)	14,117 (12,506–15,451)	4,134 (3,706–4,425)	2,560 (2,203–2,786)
Depressive disorders	0 (0–0)	0 (0–0)	0 (0–0)	0 (0–0)	0 (0–0)	0 (0–0)
Major depressive disorder	0 (0–0)	0 (0–0)	0 (0–0)	0 (0–0)	0 (0–0)	0 (0–0)
Dysthymia	0 (0–0)	0 (0–0)	0 (0–0)	0 (0–0)	0 (0–0)	0 (0–0)
Bipolar disorder	0 (0–0)	0 (0–0)	0 (0–0)	0 (0–0)	0 (0–0)	0 (0–0)
Anxiety disorders	0 (0–0)	0 (0–0)	0 (0–0)	0 (0–0)	0 (0–0)	0 (0–0)
Eating disorders	584 (379–771)	0 (0–0)	38 (20–51)	545 (359–728)	0 (0–0)	0 (0–0)
Anorexia nervosa	545 (355–723)	0 (0–0)	36 (19–48)	508 (335–689)	0 (0–0)	0 (0–0)
Bulimia nervosa	39 (21–52)	0 (0–0)	1 (0–3)	37 (20–50)	0 (0–0)	0 (0–0)
Autistic spectrum disorders	0 (0–0)	0 (0–0)	0 (0–0)	0 (0–0)	0 (0–0)	0 (0–0)
Autism	0 (0–0)	0 (0–0)	0 (0–0)	0 (0–0)	0 (0–0)	0 (0–0)
Asperger syndrome and other autistic spectrum disorders	0 (0–0)	0 (0–0)	0 (0–0)	0 (0–0)	0 (0–0)	0 (0–0)
Attention-deficit/hyperactivity disorder	0 (0–0)	0 (0–0)	0 (0–0)	0 (0–0)	0 (0–0)	0 (0–0)
Conduct disorder	0 (0–0)	0 (0–0)	0 (0–0)	0 (0–0)	0 (0–0)	0 (0–0)
Idiopathic developmental intellectual disability	0 (0–0)	0 (0–0)	0 (0–0)	0 (0–0)	0 (0–0)	0 (0–0)
Other mental and substance use disorders	0 (0–0)	0 (0–0)	0 (0–0)	0 (0–0)	0 (0–0)	0 (0–0)
Diabetes, urogenital, blood, and endocrine diseases	2,321,075 (2,229,637–2,405,661)	144,016 (107,996–193,206)	37,004 (33,566–40,392)	353,337 (334,377–367,045)	744,380 (719,805–765,080)	1,042,336 (1,017,326–1,067,167)
Diabetes mellitus	1,003,300 (983,793–1,024,623)	3,056 (2,515–3,404)	3,257 (2,634–3,600)	106,506 (102,984–110,249)	392,885 (382,875–403,505)	497,593 (487,708–508,598)
Acute glomerulonephritis	14,327 (10,833–16,653)	535 (432–659)	646 (519–744)	4,384 (3,152–4,783)	4,347 (3,292–5,123)	4,413 (3,313–5,454)
Chronic kidney disease	799,231 (740,442–832,938)	17,341 (14,773–19,406)	11,258 (9,830–12,260)	146,629 (128,707–153,908)	250,611 (229,596–265,578)	373,389 (354,850–391,960)
Chronic kidney disease due to diabetes mellitus	246,332 (230,694–259,317)	2,090 (1,705–2,534)	915 (759–1,110)	32,382 (28,524–35,909)	94,594 (86,463–102,965)	116,349 (109,689–123,168)
Chronic kidney disease due to hypertension	343,754 (319,417–361,142)	5,968 (5,031–6,847)	3,299 (2,768–3,837)	55,082 (48,332–59,347)	99,163 (90,705–106,273)	180,240 (170,546–190,405)
Chronic kidney disease due to glomerulonephritis	187,238 (169,390–199,167)	6,889 (5,704–7,924)	5,319 (4,569–5,969)	52,668 (46,263–56,990)	53,747 (48,228–58,663)	68,614 (63,181–74,695)
Chronic kidney disease due to other causes	21,905 (18,197–25,846)	2,392 (1,868–2,941)	1,724 (1,370–2,093)	6,496 (4,478–8,751)	3,106 (1,936–4,652)	8,185 (6,756–10,235)
Urinary diseases and male infertility	176,736 (164,132–194,533)	8,348 (6,454–10,241)	2,204 (1,955–2,569)	26,940 (24,377–30,858)	45,411 (40,859–51,941)	93,830 (87,428–103,554)
Interstitial nephritis and urinary tract infections	130,177 (119,967–142,086)	6,585 (5,078–8,211)	1,389 (1,208–1,592)	18,013 (16,271–20,495)	30,160 (26,604–34,462)	74,029 (68,014–80,870)
Urolithiasis	14,560 (12,025–17,274)	0 (0–0)	287 (239–373)	3,010 (2,459–3,687)	5,200 (4,267–6,159)	6,062 (4,962–7,136)
Benign prostatic hyperplasia	0 (0–0)	0 (0–0)	0 (0–0)	0 (0–0)	0 (0–0)	0 (0–0)
Male infertility	0 (0–0)	0 (0–0)	0 (0–0)	0 (0–0)	0 (0–0)	0 (0–0)
Other urinary diseases	31,997 (27,395–40,545)	1,763 (1,134–2,778)	526 (428–676)	5,917 (4,865–7,710)	10,050 (8,194–13,292)	13,738 (11,560–17,422)
Gynecological diseases	7,527 (5,867–8,584)	0 (0–0)	0 (0–0)	3,576 (2,682–4,167)	2,228 (1,705–2,612)	1,722 (1,424–2,152)
Uterine fibroids	2,005 (1,127–2,659)	0 (0–0)	0 (0–0)	797 (466–1,096)	793 (426–1,081)	414 (211–664)
Polycystic ovarian syndrome	874 (208–1,482)	0 (0–0)	0 (0–0)	627 (128–1,100)	246 (78–391)	0 (0–0)
Female infertility	0 (0–0)	0 (0–0)	0 (0–0)	0 (0–0)	0 (0–0)	0 (0–0)
Endometriosis	42 (31–57)	0 (0–0)	0 (0–0)	35 (26–48)	7 (4–10)	0 (0–0)
Genital prolapse	1,157 (576–1,955)	0 (0–0)	0 (0–0)	108 (42–194)	445 (184–827)	603 (325–969)
Premenstrual syndrome	0 (0–0)	0 (0–0)	0 (0–0)	0 (0–0)	0 (0–0)	0 (0–0)
Other gynecological diseases	3,447 (2,536–4,451)	0 (0–0)	0 (0–0)	2,007 (1,491–2,707)	735 (483–1,044)	704 (484–1,058)
Hemoglobinopathies and hemolytic anemias	223,969 (179,051–282,179)	100,681 (67,402–149,586)	15,666 (13,457–18,112)	45,247 (39,838–50,471)	25,338 (22,204–28,412)	37,036 (32,125–42,478)
Thalassemias	22,589 (18,779–27,062)	13,006 (9,289–17,562)	4,413 (3,777–5,065)	2,227 (1,897–2,657)	82 (76–90)	2,858 (2,211–3,837)
Thalassemias trait	0 (0–0)	0 (0–0)	0 (0–0)	0 (0–0)	0 (0–0)	0 (0–0)
Sickle cell disorders	117,030 (82,034–168,499)	85,866 (54,067–137,507)	9,213 (7,520–11,074)	17,647 (15,208–20,020)	978 (875–1,091)	3,326 (2,726–4,184)
Sickle cell trait	0 (0–0)	0 (0–0)	0 (0–0)	0 (0–0)	0 (0–0)	0 (0–0)

SR Table 12 - Global deaths by age and cause for both sexes in 2000						
Cause	All ages	0-5 years	5-14 years	15-49 years	50-69 years	70+ years
G6PD deficiency	25,033 (21,564–29,448)	1,240 (726–1,923)	1,375 (1,065–1,761)	14,409 (12,404–17,109)	6,490 (5,391–7,757)	1,517 (1,224–1,899)
G6PD trait	0 (0–0)	0 (0–0)	0 (0–0)	0 (0–0)	0 (0–0)	0 (0–0)
Other hemoglobinopathies and hemolytic anemias	59,316 (52,447–66,210)	567 (404–786)	664 (563–773)	10,963 (9,705–12,276)	17,787 (15,664–20,210)	29,334 (25,795–33,162)
Endocrine, metabolic, blood, and immune disorders	95,983 (93,811–98,504)	14,052 (12,522–15,616)	3,971 (3,775–4,173)	20,052 (19,427–20,704)	23,556 (22,709–24,386)	34,350 (33,265–35,454)
Musculoskeletal disorders	70,083 (63,657–74,989)	0 (0–0)	2,140 (1,806–2,520)	18,076 (16,368–20,556)	17,763 (16,286–18,995)	32,104 (28,939–33,398)
Rheumatoid arthritis	25,106 (21,815–27,497)	0 (0–0)	295 (207–388)	2,413 (1,927–2,901)	8,202 (7,159–9,046)	14,194 (12,614–15,711)
Osteoarthritis	0 (0–0)	0 (0–0)	0 (0–0)	0 (0–0)	0 (0–0)	0 (0–0)
Low back and neck pain	0 (0–0)	0 (0–0)	0 (0–0)	0 (0–0)	0 (0–0)	0 (0–0)
Low back pain	0 (0–0)	0 (0–0)	0 (0–0)	0 (0–0)	0 (0–0)	0 (0–0)
Neck pain	0 (0–0)	0 (0–0)	0 (0–0)	0 (0–0)	0 (0–0)	0 (0–0)
Gout	0 (0–0)	0 (0–0)	0 (0–0)	0 (0–0)	0 (0–0)	0 (0–0)
Other musculoskeletal disorders	44,977 (40,494–48,359)	0 (0–0)	1,845 (1,563–2,189)	15,662 (14,202–17,950)	9,560 (8,898–10,529)	17,909 (15,247–18,792)
Other non-communicable diseases	747,008 (613,587–953,291)	573,082 (454,335–766,811)	42,016 (34,034–50,695)	63,176 (55,907–71,266)	26,693 (21,453–31,054)	42,039 (31,581–50,217)
Congenital anomalies	663,173 (531,722–867,517)	545,200 (426,859–736,577)	40,905 (32,897–49,326)	54,915 (49,056–62,292)	12,729 (11,518–13,818)	9,422 (8,509–10,303)
Neural tube defects	90,099 (66,345–134,042)	84,597 (61,802–127,424)	2,869 (2,149–3,875)	1,955 (1,629–2,400)	437 (348–593)	240 (183–363)
Congenital heart anomalies	340,969 (269,598–424,447)	264,538 (202,243–341,628)	29,439 (23,257–35,046)	39,515 (34,835–44,834)	4,234 (3,788–4,706)	3,241 (2,906–3,738)
Cleft lip and cleft palate	4,630 (3,416–5,797)	4,630 (3,416–5,797)	0 (0–0)	0 (0–0)	0 (0–0)	0 (0–0)
Down syndrome	27,359 (17,174–47,240)	19,916 (10,471–38,315)	990 (666–1,518)	3,924 (3,018–5,159)	2,378 (1,989–2,915)	149 (121–188)
Turner syndrome	0 (0–0)	0 (0–0)	0 (0–0)	0 (0–0)	0 (0–0)	0 (0–0)
Klinefelter syndrome	0 (0–0)	0 (0–0)	0 (0–0)	0 (0–0)	0 (0–0)	0 (0–0)
Other chromosomal abnormalities	20,257 (8,861–55,309)	18,826 (7,523–53,258)	373 (231–791)	718 (545–899)	250 (168–316)	88 (70–111)
Other congenital anomalies	179,856 (147,601–235,277)	152,690 (123,454–205,416)	7,232 (5,821–9,330)	8,800 (7,624–10,141)	5,430 (4,626–5,943)	5,702 (4,816–6,100)
Skin and subcutaneous diseases	61,693 (41,873–79,276)	5,741 (3,426–8,935)	1,110 (678–1,584)	8,261 (5,317–11,300)	13,963 (8,947–18,322)	32,616 (22,312–40,780)
Dermatitis	0 (0–0)	0 (0–0)	0 (0–0)	0 (0–0)	0 (0–0)	0 (0–0)
Psoriasis	0 (0–0)	0 (0–0)	0 (0–0)	0 (0–0)	0 (0–0)	0 (0–0)
Cellulitis	10,474 (6,669–15,303)	698 (323–1,172)	201 (115–285)	1,833 (1,069–3,101)	3,422 (1,957–5,094)	4,319 (2,880–6,253)
Pyoderma	25,951 (17,639–36,820)	4,690 (2,786–7,583)	604 (372–917)	4,541 (2,750–6,262)	6,149 (4,112–8,934)	9,966 (7,155–13,674)
Scabies	0 (0–0)	0 (0–0)	0 (0–0)	0 (0–0)	0 (0–0)	0 (0–0)
Fungal skin diseases	0 (0–0)	0 (0–0)	0 (0–0)	0 (0–0)	0 (0–0)	0 (0–0)
Viral skin diseases	0 (0–0)	0 (0–0)	0 (0–0)	0 (0–0)	0 (0–0)	0 (0–0)
Acne vulgaris	0 (0–0)	0 (0–0)	0 (0–0)	0 (0–0)	0 (0–0)	0 (0–0)
Alopecia areata	0 (0–0)	0 (0–0)	0 (0–0)	0 (0–0)	0 (0–0)	0 (0–0)
Pruritus	0 (0–0)	0 (0–0)	0 (0–0)	0 (0–0)	0 (0–0)	0 (0–0)
Urticaria	0 (0–0)	0 (0–0)	0 (0–0)	0 (0–0)	0 (0–0)	0 (0–0)
Decubitus ulcer	22,461 (12,957–27,865)	92 (41–156)	165 (87–234)	1,322 (717–1,790)	3,794 (1,906–5,583)	17,085 (10,193–21,197)
Other skin and subcutaneous diseases	2,805 (1,970–3,788)	260 (159–458)	139 (79–194)	563 (366–828)	596 (389–897)	1,245 (900–1,653)
Sense organ diseases	0 (0–0)	0 (0–0)	0 (0–0)	0 (0–0)	0 (0–0)	0 (0–0)
Glaucoma	0 (0–0)	0 (0–0)	0 (0–0)	0 (0–0)	0 (0–0)	0 (0–0)
Cataract	0 (0–0)	0 (0–0)	0 (0–0)	0 (0–0)	0 (0–0)	0 (0–0)
Macular degeneration	0 (0–0)	0 (0–0)	0 (0–0)	0 (0–0)	0 (0–0)	0 (0–0)
Refraction and accommodation disorders	0 (0–0)	0 (0–0)	0 (0–0)	0 (0–0)	0 (0–0)	0 (0–0)
Age-related and other hearing loss	0 (0–0)	0 (0–0)	0 (0–0)	0 (0–0)	0 (0–0)	0 (0–0)
Other vision loss	0 (0–0)	0 (0–0)	0 (0–0)	0 (0–0)	0 (0–0)	0 (0–0)
Other sense organ diseases	0 (0–0)	0 (0–0)	0 (0–0)	0 (0–0)	0 (0–0)	0 (0–0)
Oral disorders	0 (0–0)	0 (0–0)	0 (0–0)	0 (0–0)	0 (0–0)	0 (0–0)
Deciduous caries	0 (0–0)	0 (0–0)	0 (0–0)	0 (0–0)	0 (0–0)	0 (0–0)
Permanent caries	0 (0–0)	0 (0–0)	0 (0–0)	0 (0–0)	0 (0–0)	0 (0–0)
Periodontal diseases	0 (0–0)	0 (0–0)	0 (0–0)	0 (0–0)	0 (0–0)	0 (0–0)
Edentulism and severe tooth loss	0 (0–0)	0 (0–0)	0 (0–0)	0 (0–0)	0 (0–0)	0 (0–0)
Other oral disorders	0 (0–0)	0 (0–0)	0 (0–0)	0 (0–0)	0 (0–0)	0 (0–0)
Sudden infant death syndrome	22,141 (17,828–38,459)	22,141 (17,828–38,459)	0 (0–0)	0 (0–0)	0 (0–0)	0 (0–0)
Injuries	4,689,688 (4,406,159–4,843,190)	462,253 (379,705–539,243)	356,521 (331,480–377,678)	2,450,598 (2,329,553–2,520,249)	821,184 (773,529–842,274)	599,131 (559,567–617,655)
Transport injuries	1,394,002 (1,351,567–1,439,114)	74,705 (61,571–88,353)	105,314 (98,234–111,661)	823,753 (799,330–855,279)	254,723 (247,092–261,852)	135,505 (131,779–139,289)
Road injuries	1,303,200 (1,258,864–1,347,824)	68,706 (56,855–81,260)	99,849 (93,280–105,842)	772,198 (746,088–802,473)	235,056 (227,300–242,164)	127,389 (123,860–131,039)
Pedestrian road injuries	579,914 (533,888–620,973)	34,589 (29,069–40,683)	54,873 (50,882–58,973)	299,914 (273,565–325,381)	118,545 (109,043–126,872)	71,991 (67,653–77,136)
Cyclist road injuries	58,519 (54,018–64,056)	1,740 (1,271–2,321)	5,763 (5,095–6,407)	31,034 (28,516–34,368)	13,948 (12,995–15,304)	6,031 (5,607–6,684)
Motorcyclist road injuries	199,151 (169,677–222,553)	6,913 (4,945–8,783)	8,978 (7,311–10,470)	144,887 (124,138–162,499)	27,589 (22,864–32,291)	10,782 (8,640–13,332)

SR Table 12 - Global deaths by age and cause for both sexes in 2000

Cause	All ages	0-5 years	5-14 years	15-49 years	50-69 years	70+ years
Motor vehicle road injuries	448,935 (417,050–506,510)	24,079 (19,396–29,457)	28,693 (25,983–32,883)	287,160 (267,955–329,474)	72,081 (64,814–81,202)	36,920 (33,096–41,176)
Other road injuries	16,679 (11,427–20,159)	1,383 (669–2,653)	1,540 (989–2,016)	9,200 (5,909–11,248)	2,891 (2,224–3,517)	1,663 (1,345–2,130)
Other transport injuries	90,802 (82,951–99,403)	5,998 (4,004–7,765)	5,465 (4,647–6,067)	51,555 (47,230–56,527)	19,667 (17,628–22,313)	8,115 (7,389–9,149)
Unintentional injuries	1,934,287 (1,771,918–2,027,558)	358,889 (288,934–422,833)	211,528 (193,383–225,573)	706,351 (659,642–731,581)	310,338 (287,909–322,118)	347,178 (317,910–361,160)
Falls	391,398 (373,788–405,807)	26,493 (20,316–34,287)	18,265 (16,487–20,144)	102,775 (98,550–110,901)	82,714 (79,013–86,119)	161,149 (151,063–168,192)
Drowning	472,512 (404,950–500,703)	118,842 (86,225–140,296)	108,356 (96,133–115,139)	166,586 (152,587–173,036)	48,816 (43,313–51,035)	29,911 (25,202–31,439)
Fire, heat, and hot substances	220,616 (176,281–237,931)	36,216 (26,764–43,535)	14,566 (11,410–16,319)	100,751 (77,439–109,373)	34,924 (29,106–37,099)	34,157 (29,404–36,201)
Poisonings	115,786 (82,867–134,337)	24,892 (10,781–43,166)	9,114 (7,480–10,501)	49,849 (38,937–54,345)	20,859 (15,848–22,883)	11,071 (8,950–12,149)
Exposure to mechanical forces	200,340 (184,383–219,607)	41,195 (32,637–48,181)	15,185 (13,310–18,835)	89,222 (82,281–101,489)	31,003 (28,835–34,813)	23,734 (19,394–25,923)
Unintentional firearm injuries	33,334 (24,118–35,931)	2,697 (1,739–3,757)	2,361 (1,862–2,884)	18,427 (12,791–20,366)	4,371 (3,096–4,853)	5,477 (4,490–5,905)
Unintentional suffocation	38,235 (29,514–43,722)	24,232 (15,653–29,045)	3,348 (2,697–4,926)	5,066 (4,502–5,861)	1,648 (1,282–1,855)	3,939 (3,259–4,306)
Other exposure to mechanical forces	128,770 (117,179–149,161)	14,265 (10,285–18,397)	9,474 (8,155–11,855)	65,729 (59,725–78,160)	24,983 (23,242–29,131)	14,317 (11,222–16,024)
Adverse effects of medical treatment	99,272 (75,060–112,290)	21,966 (10,116–29,570)	5,490 (3,541–6,690)	28,096 (20,613–31,410)	18,597 (14,885–20,821)	25,121 (20,993–27,839)
Animal contact	107,330 (74,505–118,404)	16,612 (10,672–21,146)	16,041 (10,884–18,502)	42,796 (29,962–46,556)	21,844 (14,625–24,202)	10,035 (7,252–11,179)
Venomous animal contact	87,934 (59,287–96,953)	11,707 (7,238–14,675)	13,180 (8,718–15,302)	36,878 (25,045–40,311)	18,389 (12,002–20,594)	7,778 (5,370–8,913)
Non-venomous animal contact	19,395 (15,022–22,859)	4,905 (2,936–6,692)	2,860 (2,075–3,494)	5,917 (4,783–6,802)	3,454 (2,651–4,042)	2,257 (1,883–2,575)
Foreign body	144,082 (112,895–180,759)	53,966 (32,263–82,925)	10,251 (7,602–13,426)	32,674 (26,137–47,274)	18,942 (16,288–24,012)	28,248 (24,429–33,021)
Pulmonary aspiration and foreign body in airway	110,127 (84,049–147,323)	45,061 (23,499–73,423)	7,164 (4,882–10,282)	20,111 (17,155–23,756)	14,498 (12,737–16,159)	23,291 (20,006–24,786)
Foreign body in eyes	0 (0–0)	0 (0–0)	0 (0–0)	0 (0–0)	0 (0–0)	0 (0–0)
Foreign body in other body part	33,955 (19,483–64,882)	8,905 (4,224–14,313)	3,086 (1,666–5,509)	12,562 (7,091–28,262)	4,443 (2,750–9,589)	4,956 (3,536–9,377)
Environmental heat and cold exposure	41,610 (30,410–48,555)	4,374 (2,091–5,570)	1,513 (1,002–1,912)	14,046 (10,798–18,203)	11,485 (8,529–14,405)	10,191 (6,981–12,246)
Other unintentional injuries	141,334 (133,145–149,898)	14,330 (11,404–17,041)	12,744 (11,139–14,145)	79,551 (74,872–85,407)	21,150 (19,703–22,643)	13,557 (12,128–15,440)
Self-harm and interpersonal violence	1,253,518 (1,114,478–1,287,422)	17,586 (15,170–19,012)	29,924 (25,286–31,666)	846,337 (751,646–870,916)	246,144 (219,064–253,336)	113,526 (100,635–117,617)
Self-harm	816,273 (701,759–844,642)	0 (0–0)	13,037 (9,878–14,293)	518,766 (442,509–539,040)	189,557 (164,961–196,248)	94,911 (82,460–98,745)
Interpersonal violence	437,244 (399,563–450,090)	17,586 (15,170–19,012)	16,886 (15,329–17,783)	327,570 (297,970–337,920)	56,586 (52,943–58,579)	18,615 (17,714–19,239)
Assault by firearm	159,501 (143,442–165,806)	2,060 (1,736–2,416)	3,564 (3,109–3,817)	134,757 (120,838–139,850)	15,346 (13,957–16,264)	3,772 (3,339–4,106)
Assault by sharp object	110,390 (100,623–116,244)	1,893 (1,462–2,367)	3,160 (2,656–3,599)	86,387 (78,806–90,843)	14,229 (13,217–15,159)	4,719 (4,421–5,017)
Assault by other means	167,353 (151,353–178,026)	13,632 (11,939–14,842)	10,161 (9,087–11,028)	106,425 (95,023–114,726)	27,009 (25,073–28,670)	10,123 (9,474–10,651)
Forces of nature, war, and legal intervention	107,879 (72,376–146,997)	11,072 (5,247–19,145)	9,753 (4,514–16,981)	74,156 (42,851–109,148)	9,977 (6,058–14,433)	2,919 (1,811–4,421)
Exposure to forces of nature	10,079 (7,011–13,320)	802 (473–1,216)	1,656 (1,075–2,381)	5,301 (3,578–7,219)	1,549 (1,030–2,119)	769 (498–1,062)
Collective violence and legal intervention	97,799 (63,149–135,955)	10,269 (4,442–18,427)	8,096 (3,006–15,115)	68,854 (38,243–103,772)	8,427 (4,706–12,969)	2,150 (1,146–3,513)

SR Table 12 - Global deaths by age and cause for both sexes in 2005

Cause	All ages	0-5 years	5-14 years	15-49 years	50-69 years	70+ years
All causes	53,618,470<br>(53,139,843–54,075,769)	7,996,646<br>(7,921,473–8,081,732)	1,073,089<br>(1,057,859–1,089,583)	9,604,269<br>(9,447,380–9,769,778)	13,375,500<br>(13,199,899–13,552,772)	21,568,964<br>(21,413,533–21,721,063)
Communicable, maternal, neonatal, and nutritional diseases	14,023,890<br>(13,734,842–14,335,279)	6,746,361<br>(6,599,367–6,880,633)	568,725<br>(542,743–598,565)	3,101,554<br>(2,985,665–3,229,604)	1,647,705<br>(1,551,380–1,737,965)	1,959,543<br>(1,860,891–2,034,178)
HIV/AIDS and tuberculosis	3,139,531<br>(2,938,605–3,469,781)	218,834<br>(209,705–234,674)	94,442<br>(89,670–102,765)	1,777,758<br>(1,678,388–1,896,809)	710,271<br>(634,917–824,665)	338,224<br>(289,611–421,470)
Tuberculosis	1,347,597<br>(1,152,871–1,658,703)	33,868<br>(26,985–47,960)	20,030<br>(17,117–27,312)	493,860<br>(415,191–594,020)	486,691<br>(410,900–593,547)	313,146<br>(264,116–395,645)
HIV/AIDS	1,791,933<br>(1,703,843–1,886,555)	184,965<br>(178,948–191,027)	74,412<br>(70,962–78,087)	1,283,897<br>(1,206,815–1,370,650)	223,579<br>(209,637–242,354)	25,078<br>(23,076–27,755)
HIV/AIDS - Tuberculosis	351,789<br>(281,694–400,397)	49,369<br>(39,812–55,179)	17,561<br>(13,881–20,156)	220,599<br>(174,273–253,203)	56,867<br>(47,507–63,681)	7,391<br>(6,405–8,209)
HIV/AIDS resulting in other diseases	1,440,144<br>(1,349,645–1,546,735)	135,595<br>(126,997–145,968)	56,850<br>(52,817–62,032)	1,063,298<br>(979,567–1,150,884)	166,711<br>(152,778–187,183)	17,687<br>(15,970–20,351)
Diarrhea, lower respiratory, and other common infectious diseases	5,773,109<br>(5,548,331–6,004,783)	2,598,124<br>(2,440,464–2,779,514)	336,030<br>(304,252–375,309)	669,956<br>(625,450–728,787)	732,934<br>(685,601–769,783)	1,436,062<br>(1,343,007–1,489,074)
Diarrheal diseases	1,657,214<br>(1,564,972–1,756,053)	759,635<br>(679,701–848,883)	75,453<br>(67,394–83,849)	208,518<br>(196,586–224,161)	251,476<br>(238,478–265,948)	362,129<br>(345,333–380,430)
Intestinal infectious diseases	208,600<br>(117,992–344,102)	52,676<br>(27,953–90,582)	81,600<br>(47,778–130,544)	61,657<br>(32,306–105,194)	8,832<br>(4,535–15,461)	3,831<br>(2,172–6,096)
Typhoid fever	172,890<br>(94,552–293,193)	43,845<br>(22,617–77,337)	68,637<br>(38,779–110,478)	50,500<br>(26,274–88,068)	6,976<br>(3,364–12,336)	2,929<br>(1,578–4,832)
Paratyphoid fever	33,926<br>(15,553–65,076)	7,823<br>(3,286–16,146)	12,770<br>(6,006–24,343)	10,914<br>(4,685–21,611)	1,736<br>(708–3,528)	681<br>(291–1,300)
Other intestinal infectious diseases	1,783<br>(633–3,253)	1,007<br>(256–2,123)	192<br>(54–424)	242<br>(92–451)	119<br>(50–215)	221<br>(89–432)
Lower respiratory infections	2,828,528<br>(2,628,624–2,965,849)	1,114,921<br>(1,032,799–1,196,344)	73,948<br>(58,839–83,401)	250,529<br>(215,687–272,131)	384,489<br>(333,287–410,668)	1,004,640<br>(911,495–1,051,321)
Upper respiratory infections	3,757<br>(3,349–4,228)	1,089<br>(811–1,460)	65<br>(45–91)	788<br>(681–908)	872<br>(740–1,016)	941<br>(794–1,110)
Otitis media	3,882<br>(3,524–4,344)	1,375<br>(1,201–1,571)	493<br>(447–541)	950<br>(888–1,025)	502<br>(453–563)	559<br>(405–942)
Meningitis	407,690<br>(351,086–457,104)	210,010<br>(168,211–254,015)	37,877<br>(31,279–43,906)	80,833<br>(68,072–94,756)	46,291<br>(39,853–55,370)	32,677<br>(28,762–38,967)
Pneumococcal meningitis	112,146<br>(93,316–135,170)	48,820<br>(34,662–69,329)	12,974<br>(9,159–17,245)	27,584<br>(21,745–34,081)	13,336<br>(10,120–17,517)	9,430<br>(7,298–12,038)
H influenzae type B meningitis	110,611<br>(88,291–135,759)	75,705<br>(55,488–98,924)	10,015<br>(6,680–13,631)	12,617<br>(9,280–16,263)	7,014<br>(5,148–9,250)	5,259<br>(4,112–6,833)
Meningococcal meningitis	74,284<br>(58,684–91,827)	41,109<br>(28,100–57,478)	7,017<br>(4,637–9,824)	13,768<br>(10,488–17,598)	7,653<br>(5,630–10,070)	4,735<br>(3,588–6,144)
Other meningitis	110,647<br>(94,870–128,596)	44,376<br>(32,775–59,335)	7,869<br>(5,496–11,067)	26,863<br>(21,726–32,207)	18,286<br>(14,883–22,884)	13,251<br>(10,922–15,704)
Encephalitis	147,015<br>(135,328–163,017)	48,134<br>(41,388–54,333)	18,164<br>(15,672–20,720)	36,358<br>(33,804–40,359)	23,044<br>(21,047–27,353)	21,312<br>(19,456–24,970)
Diphtheria	5,555<br>(3,027–11,015)	4,135<br>(1,718–9,628)	760<br>(389–1,482)	493<br>(344–684)	166<br>(98–290)	0<br>(0–0)
Whooping cough	99,555<br>(36,769–226,264)	92,369<br>(34,150–208,239)	5,722<br>(2,134–13,498)	1,281<br>(432–3,608)	182<br>(57–514)	0<br>(0–0)
Tetanus	108,022<br>(90,882–151,128)	59,518<br>(52,139–69,119)	5,676<br>(4,204–9,981)	20,611<br>(15,456–37,477)	15,293<br>(11,029–29,261)	6,922<br>(5,168–12,778)
Measles	293,695<br>(110,607–611,432)	251,593<br>(95,010–520,427)	35,515<br>(12,877–75,172)	6,376<br>(1,991–15,305)	209<br>(66–516)	0<br>(0–0)
Varicella and herpes zoster	9,590<br>(8,501–10,953)	2,661<br>(2,043–3,457)	752<br>(542–1,038)	1,555<br>(1,336–1,825)	1,574<br>(1,277–1,947)	3,047<br>(2,465–3,918)
Neglected tropical diseases and malaria	1,298,534<br>(1,082,739–1,509,065)	854,114<br>(658,822–1,048,858)	98,813<br>(72,278–128,039)	198,311<br>(156,008–254,904)	102,647<br>(79,273–135,008)	44,648<br>(34,215–59,803)
Malaria	1,167,013<br>(952,148–1,378,087)	829,233<br>(634,041–1,025,113)	78,348<br>(51,025–108,142)	148,716<br>(107,128–205,694)	78,204<br>(54,838–110,616)	32,511<br>(22,058–47,658)
Chagas disease	7,475<br>(7,181–7,795)	4<br>(3–4)	8<br>(7–8)	1,153<br>(1,082–1,228)	3,226<br>(3,055–3,413)	3,083<br>(2,920–3,256)
Leishmaniasis	23,078<br>(14,757–33,217)	3,685<br>(2,275–5,369)	2,536<br>(1,505–4,044)	13,599<br>(8,508–19,840)	2,688<br>(1,839–3,664)	569<br>(436–716)
Visceral leishmaniasis	23,078<br>(14,757–33,217)	3,685<br>(2,275–5,369)	2,536<br>(1,505–4,044)	13,599<br>(8,508–19,840)	2,688<br>(1,839–3,664)	569<br>(436–716)
Cutaneous and mucocutaneous leishmaniasis	0<br>(0–0)	0<br>(0–0)	0<br>(0–0)	0<br>(0–0)	0<br>(0–0)	0<br>(0–0)
African trypanosomiasis	14,248<br>(7,566–23,140)	1,019<br>(521–1,717)	2,066<br>(1,103–3,382)	8,513<br>(4,485–14,064)	2,141<br>(1,127–3,530)	508<br>(271–832)
Schistosomiasis	7,829<br>(6,971–8,919)	83<br>(55–125)	179<br>(135–239)	2,329<br>(2,003–2,738)	3,394<br>(2,763–4,171)	1,842<br>(1,472–2,342)
Cysticercosis	619<br>(536–744)	11<br>(6–21)	21<br>(16–30)	356<br>(307–430)	174<br>(151–208)	55<br>(48–64)
Cystic echinococcosis	1,773<br>(1,681–1,874)	73<br>(59–89)	145<br>(128–163)	676<br>(633–722)	516<br>(486–547)	361<br>(343–378)
Lymphatic filariasis	0<br>(0–0)	0<br>(0–0)	0<br>(0–0)	0<br>(0–0)	0<br>(0–0)	0<br>(0–0)
Onchocerciasis	0<br>(0–0)	0<br>(0–0)	0<br>(0–0)	0<br>(0–0)	0<br>(0–0)	0<br>(0–0)
Trachoma	0<br>(0–0)	0<br>(0–0)	0<br>(0–0)	0<br>(0–0)	0<br>(0–0)	0<br>(0–0)
Dengue	12,344<br>(8,616–15,057)	3,946<br>(2,344–5,704)	3,234<br>(2,141–4,177)	3,293<br>(2,518–3,882)	1,172<br>(801–1,377)	698<br>(470–860)
Yellow fever	6,331<br>(1,330–16,876)	561<br>(110–1,661)	1,863<br>(396–5,089)	3,292<br>(696–8,984)	518<br>(119–1,420)	95<br>(19–273)
Rabies	32,075<br>(28,025–36,129)	3,971<br>(3,008–5,351)	7,671<br>(6,279–9,262)	11,206<br>(9,735–12,570)	6,841<br>(5,900–7,801)	2,384<br>(1,935–2,842)
Intestinal nematode infections	3,814<br>(3,345–4,296)	2,776<br>(2,378–3,200)	538<br>(455–629)	234<br>(187–282)	150<br>(116–188)	115<br>(88–147)
Ascariasis	3,814<br>(3,345–4,296)	2,776<br>(2,378–3,200)	538<br>(455–629)	234<br>(187–282)	150<br>(116–188)	115<br>(88–147)
Trichuriasis	0<br>(0–0)	0<br>(0–0)	0<br>(0–0)	0<br>(0–0)	0<br>(0–0)	0<br>(0–0)
Hookworm disease	0<br>(0–0)	0<br>(0–0)	0<br>(0–0)	0<br>(0–0)	0<br>(0–0)	0<br>(0–0)
Food-borne trematodiases	0<br>(0–0)	0<br>(0–0)	0<br>(0–0)	0<br>(0–0)	0<br>(0–0)	0<br>(0–0)
Leprosy	0<br>(0–0)	0<br>(0–0)	0<br>(0–0)	0<br>(0–0)	0<br>(0–0)	0<br>(0–0)
Ebola	16<br>(13–20)	0<br>(0–0)	0<br>(0–0)	15<br>(12–18)	1<br>(1–2)	0<br>(0–0)
Other neglected tropical diseases	21,912<br>(13,984–27,719)	8,748<br>(6,048–11,511)	2,199<br>(1,353–3,019)	4,924<br>(2,762–6,259)	3,617<br>(2,092–4,754)	2,422<br>(1,407–3,260)
Maternal disorders	350,795<br>(327,923–376,619)	0<br>(0–0)	1,638<br>(1,366–1,976)	347,824<br>(325,104–373,357)	1,332<br>(988–1,819)	0<br>(0–0)
Maternal hemorrhage	99,684<br>(87,611–112,970)	0<br>(0–0)	504<br>(374–658)	98,734<br>(86,673–111,999)	445<br>(283–681)	0<br>(0–0)
Maternal sepsis and other maternal infections	24,745<br>(20,416–29,934)	0<br>(0–0)	70<br>(49–102)	24,576<br>(20,265–29,772)	97<br>(60–157)	0<br>(0–0)
Maternal hypertensive disorders	63,699<br>(54,939–74,261)	0<br>(0–0)	333<br>(235–460)	63,257<br>(54,505–73,730)	108<br>(68–164)	0<br>(0–0)
Maternal obstructed labor and uterine rupture	26,890<br>(22,083–32,009)	0<br>(0–0)	48<br>(31–75)	26,786<br>(21,995–31,890)	55<br>(34–86)	0<br>(0–0)
Maternal abortion, miscarriage, and ectopic pregnancy	41,188<br>(34,661–49,571)	0<br>(0–0)	229<br>(156–320)	40,745<br>(34,197–49,117)	213<br>(140–321)	0<br>(0–0)

SR Table 12 - Global deaths by age and cause for both sexes in 2005						
**Cause**	**All ages**	**0-5 years**	**5-14 years**	**15-49 years**	**50-69 years**	**70+ years**
Indirect maternal deaths	38,216<br>(31,610–45,445)	0<br>(0–0)	204<br>(135–290)	37,873<br>(31,311–45,105)	138<br>(83–227)	0<br>(0–0)
Late maternal deaths	7,908<br>(5,294–11,519)	0<br>(0–0)	45<br>(22–79)	7,817<br>(5,196–11,420)	46<br>(25–83)	0<br>(0–0)
Maternal deaths aggravated by HIV/AIDS	2,755<br>(1,768–3,758)	0<br>(0–0)	2<br>(1–4)	2,743<br>(1,761–3,743)	9<br>(4–15)	0<br>(0–0)
Other maternal disorders	45,706<br>(39,019–53,913)	0<br>(0–0)	198<br>(142–268)	45,288<br>(38,570–53,538)	219<br>(145–324)	0<br>(0–0)
Neonatal disorders	2,653,470<br>(2,583,390–2,728,085)	2,653,470<br>(2,583,390–2,728,085)	0<br>(0–0)	0<br>(0–0)	0<br>(0–0)	0<br>(0–0)
Neonatal preterm birth complications	1,088,013<br>(1,010,853–1,217,743)	1,088,013<br>(1,010,853–1,217,743)	0<br>(0–0)	0<br>(0–0)	0<br>(0–0)	0<br>(0–0)
Neonatal encephalopathy due to birth asphyxia and trauma	882,781<br>(800,697–974,347)	882,781<br>(800,697–974,347)	0<br>(0–0)	0<br>(0–0)	0<br>(0–0)	0<br>(0–0)
Neonatal sepsis and other neonatal infections	352,299<br>(252,004–465,710)	352,299<br>(252,004–465,710)	0<br>(0–0)	0<br>(0–0)	0<br>(0–0)	0<br>(0–0)
Hemolytic disease and other neonatal jaundice	68,326<br>(42,878–106,101)	68,326<br>(42,878–106,101)	0<br>(0–0)	0<br>(0–0)	0<br>(0–0)	0<br>(0–0)
Other neonatal disorders	262,049<br>(190,283–342,985)	262,049<br>(190,283–342,985)	0<br>(0–0)	0<br>(0–0)	0<br>(0–0)	0<br>(0–0)
Nutritional deficiencies	460,795<br>(380,340–541,579)	254,871<br>(195,975–326,173)	16,838<br>(14,247–21,003)	41,373<br>(35,292–51,073)	46,006<br>(39,249–56,033)	101,706<br>(88,806–113,825)
Protein-energy malnutrition	379,702<br>(314,338–457,087)	233,461<br>(180,241–301,318)	13,099<br>(11,036–16,358)	22,116<br>(18,659–27,500)	29,377<br>(25,227–36,417)	81,647<br>(71,590–91,917)
Iodine deficiency	2,061<br>(1,569–2,887)	700<br>(352–1,376)	85<br>(44–152)	368<br>(257–529)	417<br>(286–653)	489<br>(333–736)
Vitamin A deficiency	0<br>(0–0)	0<br>(0–0)	0<br>(0–0)	0<br>(0–0)	0<br>(0–0)	0<br>(0–0)
Iron-deficiency anemia	48,370<br>(31,949–63,106)	15,203<br>(7,735–21,261)	2,205<br>(1,561–3,423)	12,121<br>(8,951–16,294)	10,914<br>(7,739–13,910)	7,924<br>(5,109–10,335)
Other nutritional deficiencies	30,661<br>(21,731–43,705)	5,505<br>(3,204–8,306)	1,446<br>(952–2,047)	6,767<br>(4,893–9,312)	5,297<br>(3,745–7,996)	11,645<br>(8,656–16,388)
Other communicable, maternal, neonatal, and nutritional diseases	347,653<br>(291,150–419,818)	166,947<br>(115,564–232,936)	20,961<br>(16,478–26,659)	66,330<br>(63,490–69,124)	54,512<br>(49,369–57,128)	38,901<br>(34,517–40,809)
Sexually transmitted diseases excluding HIV	135,474<br>(81,161–207,167)	114,680<br>(65,012–180,221)	9,949<br>(5,608–15,380)	5,250<br>(4,471–6,039)	3,088<br>(2,332–3,609)	2,506<br>(2,059–2,912)
Syphilis	134,072<br>(79,901–205,663)	114,680<br>(65,012–180,221)	9,919<br>(5,579–15,347)	4,594<br>(3,908–5,290)	2,708<br>(2,050–3,172)	2,169<br>(1,784–2,521)
Chlamydial infection	159<br>(125–183)	0<br>(0–0)	3<br>(3–4)	71<br>(58–87)	41<br>(29–49)	43<br>(32–50)
Gonococcal infection	845<br>(691–949)	0<br>(0–0)	15<br>(12–19)	381<br>(319–435)	222<br>(168–260)	226<br>(185–263)
Trichomoniasis	0<br>(0–0)	0<br>(0–0)	0<br>(0–0)	0<br>(0–0)	0<br>(0–0)	0<br>(0–0)
Genital herpes	0<br>(0–0)	0<br>(0–0)	0<br>(0–0)	0<br>(0–0)	0<br>(0–0)	0<br>(0–0)
Other sexually transmitted diseases	396<br>(325–446)	0<br>(0–0)	10<br>(8–13)	202<br>(171–231)	116<br>(88–136)	66<br>(54–78)
Hepatitis	123,045<br>(118,587–127,579)	17,116<br>(14,919–19,291)	7,283<br>(6,428–8,115)	46,724<br>(44,697–49,230)	32,155<br>(31,062–33,308)	19,766<br>(19,016–20,551)
Acute hepatitis A	16,917<br>(10,918–23,080)	13,191<br>(9,807–16,111)	1,200<br>(371–2,418)	1,477<br>(390–3,259)	717<br>(181–1,677)	330<br>(71–792)
Hepatitis B	71,391<br>(62,032–80,141)	978<br>(368–2,238)	865<br>(501–1,428)	28,680<br>(23,997–33,256)	26,249<br>(22,849–28,835)	14,617<br>(12,397–16,665)
Hepatitis C	2,790<br>(599–6,432)	0<br>(0–0)	106<br>(21–276)	803<br>(160–1,841)	1,270<br>(275–3,003)	609<br>(124–1,440)
Acute hepatitis E	31,946<br>(22,995–42,329)	2,945<br>(1,175–5,674)	5,110<br>(3,861–6,323)	15,762<br>(11,600–20,403)	3,918<br>(1,951–6,899)	4,209<br>(2,383–6,454)
Other infectious diseases	89,133<br>(61,285–102,346)	35,150<br>(18,843–46,058)	3,729<br>(3,006–4,308)	14,356<br>(11,999–15,512)	19,268<br>(14,384–21,259)	16,628<br>(12,316–18,291)
Non-communicable diseases	34,835,579<br>(34,441,292–35,277,067)	871,898<br>(772,739–1,002,618)	214,681<br>(200,860–229,762)	4,026,481<br>(3,942,062–4,130,817)	10,804,967<br>(10,649,359–10,963,554)	18,917,549<br>(18,764,901–19,083,800)
Neoplasms	7,492,779<br>(7,378,397–7,616,532)	36,646<br>(31,562–45,440)	52,423<br>(49,765–55,546)	1,062,539<br>(1,036,814–1,091,186)	3,111,914<br>(3,059,388–3,163,669)	3,229,255<br>(3,177,895–3,285,767)
Lip and oral cavity cancer	110,152<br>(107,648–112,861)	0<br>(0–0)	0<br>(0–0)	19,167<br>(18,467–20,033)	52,423<br>(51,044–53,850)	38,560<br>(37,685–39,440)
Nasopharynx cancer	55,799<br>(45,890–58,827)	0<br>(0–0)	321<br>(283–365)	17,158<br>(13,629–18,327)	26,182<br>(21,405–27,623)	12,137<br>(10,410–12,719)
Other pharynx cancer	51,870<br>(50,468–53,239)	0<br>(0–0)	0<br>(0–0)	7,904<br>(7,590–8,309)	29,261<br>(28,453–30,082)	14,703<br>(14,260–15,119)
Esophageal cancer	459,262<br>(445,127–474,167)	0<br>(0–0)	0<br>(0–0)	43,900<br>(42,052–46,100)	225,366<br>(217,175–233,508)	189,995<br>(184,468–196,333)
Stomach cancer	824,459<br>(806,777–843,036)	0<br>(0–0)	0<br>(0–0)	91,983<br>(88,785–95,682)	346,252<br>(337,090–356,131)	386,223<br>(378,383–393,776)
Colon and rectum cancer	675,534<br>(664,900–688,214)	0<br>(0–0)	0<br>(0–0)	66,924<br>(64,927–69,666)	246,179<br>(241,794–251,176)	362,430<br>(356,002–368,802)
Liver cancer	726,721<br>(635,999–762,191)	0<br>(0–0)	1,262<br>(1,078–1,392)	146,827<br>(118,858–155,828)	341,783<br>(296,284–359,152)	236,847<br>(215,072–246,847)
Liver cancer due to hepatitis B	263,118<br>(224,398–282,523)	0<br>(0–0)	730<br>(617–828)	86,346<br>(70,558–92,916)	122,825<br>(105,356–133,798)	53,215<br>(45,561–58,610)
Liver cancer due to hepatitis C	137,767<br>(126,054–146,321)	0<br>(0–0)	30<br>(25–36)	10,962<br>(9,024–12,363)	56,970<br>(50,544–62,451)	69,803<br>(65,096–74,183)
Liver cancer due to alcohol use	194,545<br>(168,773–208,275)	0<br>(0–0)	0<br>(0–0)	21,635<br>(17,283–24,069)	104,917<br>(90,183–115,005)	67,992<br>(60,200–73,387)
Liver cancer due to other causes	131,290<br>(114,117–141,578)	0<br>(0–0)	501<br>(436–558)	27,882<br>(22,644–30,720)	57,070<br>(49,397–63,030)	45,835<br>(40,315–49,714)
Gallbladder and biliary tract cancer	124,464<br>(120,791–127,793)	0<br>(0–0)	0<br>(0–0)	9,188<br>(8,610–9,658)	45,431<br>(43,699–46,904)	69,844<br>(68,169–71,654)
Pancreatic cancer	314,561<br>(310,129–319,024)	0<br>(0–0)	0<br>(0–0)	24,751<br>(24,167–25,405)	129,311<br>(127,369–131,293)	160,498<br>(157,791–163,268)
Larynx cancer	93,120<br>(90,924–95,742)	0<br>(0–0)	0<br>(0–0)	10,646<br>(10,249–11,181)	48,212<br>(46,965–49,616)	34,260<br>(33,353–35,200)
Tracheal, bronchus, and lung cancer	1,434,534<br>(1,406,520–1,463,497)	0<br>(0–0)	0<br>(0–0)	121,841<br>(117,912–126,096)	657,217<br>(642,161–670,235)	655,475<br>(642,635–667,402)
Malignant skin melanoma	46,984<br>(38,710–58,576)	0<br>(0–0)	0<br>(0–0)	9,873<br>(8,529–12,593)	18,002<br>(14,563–22,927)	19,108<br>(15,508–23,238)
Non-melanoma skin cancer	36,276<br>(35,444–37,139)	0<br>(0–0)	0<br>(0–0)	3,678<br>(3,561–3,825)	11,687<br>(11,381–12,016)	20,909<br>(20,382–21,474)
Non-melanoma skin cancer (squamous-cell carcinoma)	36,276<br>(35,444–37,139)	0<br>(0–0)	0<br>(0–0)	3,678<br>(3,561–3,825)	11,687<br>(11,381–12,016)	20,909<br>(20,382–21,474)
Non-melanoma skin cancer (basal-cell carcinoma)	0<br>(0–0)	0<br>(0–0)	0<br>(0–0)	0<br>(0–0)	0<br>(0–0)	0<br>(0–0)
Breast cancer	439,816<br>(418,824–461,889)	0<br>(0–0)	0<br>(0–0)	97,895<br>(89,527–106,107)	194,864<br>(185,950–204,379)	147,056<br>(141,027–151,813)
Cervical cancer	225,446<br>(213,892–237,838)	0<br>(0–0)	0<br>(0–0)	61,916<br>(57,409–66,764)	105,339<br>(99,083–111,630)	58,189<br>(56,295–60,352)
Uterine cancer	81,779<br>(78,501–85,395)	0<br>(0–0)	0<br>(0–0)	10,098<br>(9,395–10,844)	38,153<br>(36,315–40,210)	33,527<br>(32,355–34,921)
Ovarian cancer	133,821<br>(130,764–138,606)	0<br>(0–0)	0<br>(0–0)	23,527<br>(22,576–25,295)	61,290<br>(59,735–63,721)	49,003<br>(47,743–50,380)
Prostate cancer	277,382<br>(230,800–348,922)	0<br>(0–0)	0<br>(0–0)	2,585<br>(2,031–3,183)	64,645<br>(52,858–80,448)	210,150<br>(173,390–267,444)
Testicular cancer	8,641<br>(8,268–9,083)	0<br>(0–0)	0<br>(0–0)	5,927<br>(5,616–6,319)	1,527<br>(1,464–1,592)	1,186<br>(1,136–1,238)

SR Table 12 - Global deaths by age and cause for both sexes in 2005

Cause	All ages	0-5 years	5-14 years	15-49 years	50-69 years	70+ years
Kidney cancer	104,529 (102,410–106,941)	2,332 (1,506–3,387)	1,111 (1,025–1,215)	10,804 (10,502–11,238)	43,253 (42,426–44,192)	47,027 (46,066–48,017)
Bladder cancer	150,607 (147,929–153,227)	0 (0–0)	0 (0–0)	7,104 (6,842–7,416)	46,971 (45,997–47,945)	96,531 (94,612–98,410)
Brain and nervous system cancer	190,407 (173,302–201,173)	5,328 (4,508–7,196)	9,750 (8,646–10,950)	54,257 (48,453–58,221)	75,566 (69,111–79,237)	45,504 (40,935–47,169)
Thyroid cancer	25,547 (24,441–27,588)	0 (0–0)	76 (68–81)	2,780 (2,627–3,114)	9,736 (9,215–10,572)	12,954 (12,408–14,003)
Mesothelioma	23,193 (22,668–23,751)	0 (0–0)	0 (0–0)	3,244 (3,091–3,406)	9,295 (9,044–9,534)	10,653 (10,381–10,948)
Hodgkin lymphoma	25,432 (22,659–29,682)	356 (268–422)	796 (624–929)	9,255 (8,205–10,892)	8,856 (7,930–10,398)	6,166 (5,491–7,220)
Non-Hodgkin lymphoma	179,321 (160,613–191,578)	3,092 (2,193–4,814)	4,487 (3,911–5,136)	33,254 (30,507–36,997)	62,044 (56,503–66,168)	76,441 (64,567–80,523)
Multiple myeloma	77,503 (75,641–79,713)	0 (0–0)	0 (0–0)	7,192 (6,923–7,577)	30,894 (30,168–31,948)	39,417 (37,838–40,524)
Leukemia	303,464 (297,296–311,845)	13,682 (12,236–15,604)	24,054 (22,729–25,687)	83,780 (81,440–86,772)	83,401 (81,578–85,596)	98,544 (96,385–101,016)
Acute lymphoid leukemia	97,487 (90,445–108,008)	8,828 (7,656–10,702)	16,904 (15,653–18,821)	34,143 (31,073–38,341)	22,283 (20,441–24,635)	15,326 (14,368–16,606)
Chronic lymphoid leukemia	51,506 (49,341–53,902)	0 (0–0)	0 (0–0)	7,199 (6,402–7,843)	14,893 (14,112–15,842)	29,413 (28,386–30,863)
Acute myeloid leukemia	119,048 (110,464–126,733)	4,853 (3,827–5,847)	7,149 (5,947–8,219)	32,184 (28,747–35,005)	34,995 (32,893–37,341)	39,865 (37,192–41,726)
Chronic myeloid leukemia	35,421 (33,439–38,161)	0 (0–0)	0 (0–0)	10,253 (9,299–11,492)	11,229 (10,526–12,092)	13,938 (13,336–14,728)
Other neoplasms	292,143 (270,852–302,569)	11,854 (9,608–16,373)	10,562 (9,632–11,431)	75,064 (66,650–79,512)	98,758 (89,984–102,834)	95,904 (90,270–99,282)
Cardiovascular diseases	15,933,709 (15,732,097–16,161,601)	60,417 (56,612–64,561)	31,928 (29,959–34,097)	1,431,067 (1,398,049–1,477,899)	4,675,710 (4,607,018–4,753,406)	9,734,585 (9,600,729–9,876,584)
Rheumatic heart disease	333,160 (313,063–349,502)	3,754 (3,193–4,353)	8,109 (7,433–9,019)	87,366 (80,067–93,596)	108,775 (101,389–114,540)	125,155 (119,305–130,482)
Ischemic heart disease	7,648,404 (7,551,450–7,774,303)	3,568 (3,158–4,070)	2,760 (2,473–3,080)	646,056 (629,905–671,523)	2,293,201 (2,257,496–2,341,117)	4,702,816 (4,630,440–4,782,487)
Cerebrovascular disease	6,020,898 (5,920,166–6,127,146)	21,869 (20,121–24,057)	11,662 (10,873–12,588)	465,648 (450,930–481,907)	1,768,249 (1,729,457–1,805,644)	3,753,467 (3,690,404–3,820,956)
Ischemic stroke	2,760,789 (2,682,694–2,837,510)	909 (791–1,070)	713 (652–789)	76,245 (71,639–81,470)	605,874 (579,819–631,523)	2,077,045 (2,018,415–2,131,245)
Hemorrhagic stroke	3,260,108 (3,169,222–3,359,838)	20,959 (19,266–23,122)	10,948 (10,184–11,829)	389,402 (375,918–404,284)	1,162,375 (1,126,305–1,198,122)	1,676,422 (1,628,996–1,732,973)
Hypertensive heart disease	760,527 (711,911–823,744)	551 (442–668)	435 (375–512)	67,775 (63,182–74,548)	226,665 (210,763–248,798)	465,101 (438,351–506,393)
Cardiomyopathy and myocarditis	328,769 (315,771–338,750)	11,979 (10,332–13,242)	3,698 (3,425–4,073)	83,026 (78,914–86,898)	92,536 (88,781–97,816)	137,527 (132,828–141,516)
Atrial fibrillation and flutter	142,792 (117,291–172,048)	0 (0–0)	0 (0–0)	877 (663–1,125)	10,025 (7,757–13,303)	131,889 (106,194–161,282)
Aortic aneurysm	134,780 (131,659–138,337)	0 (0–0)	0 (0–0)	11,677 (11,140–12,313)	37,919 (36,804–39,258)	85,183 (83,120–87,209)
Peripheral vascular disease	38,133 (36,726–39,640)	0 (0–0)	0 (0–0)	582 (549–616)	6,945 (6,638–7,249)	30,604 (29,294–31,983)
Endocarditis	68,813 (60,357–74,714)	3,951 (2,826–5,026)	1,221 (950–1,427)	14,593 (11,648–16,340)	15,348 (13,383–17,323)	33,697 (30,577–36,386)
Other cardiovascular and circulatory diseases	457,430 (446,711–470,615)	14,742 (13,354–16,711)	4,040 (3,746–4,476)	53,463 (51,234–56,238)	116,043 (112,240–120,275)	269,140 (261,455–277,345)
Chronic respiratory diseases	3,709,095 (3,631,623–3,795,956)	51,814 (46,086–58,167)	12,925 (11,759–14,124)	218,232 (211,097–229,225)	1,031,532 (1,003,142–1,061,679)	2,394,590 (2,343,623–2,448,143)
Chronic obstructive pulmonary disease	3,100,480 (2,997,681–3,194,886)	19,924 (15,001–25,171)	2,397 (1,888–3,069)	127,219 (117,483–136,522)	833,240 (796,605–871,646)	2,117,698 (2,052,935–2,173,077)
Pneumoconiosis	31,853 (28,432–36,310)	0 (0–0)	0 (0–0)	3,089 (2,687–3,594)	8,756 (7,553–10,282)	20,007 (17,872–22,642)
Silicosis	10,239 (9,356–11,363)	0 (0–0)	0 (0–0)	1,297 (1,129–1,528)	3,282 (2,895–3,773)	5,659 (5,211–6,178)
Asbestosis	2,802 (2,013–3,203)	0 (0–0)	0 (0–0)	383 (327–452)	681 (513–856)	1,737 (1,134–1,959)
Coal workers pneumoconiosis	2,683 (2,433–2,990)	0 (0–0)	0 (0–0)	219 (180–270)	759 (660–882)	1,704 (1,547–1,887)
Other pneumoconiosis	16,128 (13,343–19,672)	0 (0–0)	0 (0–0)	1,189 (955–1,462)	4,032 (3,163–5,255)	10,906 (9,158–13,226)
Asthma	449,899 (362,084–518,043)	14,920 (11,851–18,061)	8,881 (7,595–10,337)	74,924 (63,240–86,557)	157,905 (123,447–186,049)	193,267 (154,321–223,830)
Interstitial lung disease and pulmonary sarcoidosis	80,423 (61,218–92,814)	298 (194–471)	237 (187–327)	5,960 (4,903–7,245)	22,713 (18,208–27,125)	51,212 (37,143–58,039)
Other chronic respiratory diseases	46,438 (33,856–55,646)	16,670 (12,049–22,094)	1,408 (894–2,151)	7,037 (4,793–8,707)	8,916 (6,028–10,409)	12,404 (9,348–14,550)
Cirrhosis and other chronic liver diseases	1,171,740 (1,131,890–1,236,650)	12,985 (10,733–16,595)	10,747 (9,561–12,481)	347,717 (332,044–368,409)	514,930 (496,267–543,169)	285,358 (275,399–303,602)
Cirrhosis and other chronic liver diseases due to hepatitis B	341,420 (316,249–374,143)	99 (74–131)	332 (266–407)	101,585 (92,314–112,737)	150,503 (134,354–169,873)	88,900 (80,802–98,822)
Cirrhosis and other chronic liver diseases due to hepatitis C	287,426 (268,075–307,823)	52 (38–70)	190 (150–236)	78,720 (70,293–87,749)	131,274 (117,794–144,291)	77,188 (70,976–84,506)
Cirrhosis and other chronic liver diseases due to alcohol use	310,110 (289,424–333,408)	0 (0–0)	0 (0–0)	106,578 (97,747–116,490)	148,892 (134,833–163,937)	54,638 (49,111–60,813)
Cirrhosis and other chronic liver diseases due to other causes	232,782 (217,637–254,618)	12,834 (10,602–16,405)	10,225 (9,120–11,868)	60,832 (55,149–67,170)	84,261 (75,066–94,390)	64,630 (59,179–71,202)
Digestive diseases	1,113,247 (1,081,140–1,186,715)	27,810 (19,777–42,935)	19,267 (17,362–22,172)	239,407 (228,574–256,404)	330,906 (319,676–359,915)	495,855 (483,359–524,344)
Peptic ulcer disease	294,322 (278,048–321,995)	3,139 (1,722–6,210)	3,162 (2,724–3,801)	63,269 (59,203–68,960)	99,272 (92,994–109,267)	125,478 (117,877–137,330)
Gastritis and duodenitis	49,925 (40,193–60,881)	1,080 (578–1,823)	551 (385–797)	10,412 (8,432–13,177)	15,966 (12,981–19,782)	21,913 (17,536–26,610)
Appendicitis	49,883 (42,860–56,386)	4,580 (2,306–9,402)	3,332 (2,921–3,818)	17,440 (14,878–19,123)	12,578 (10,979–13,891)	11,951 (10,614–13,142)
Paralytic ileus and intestinal obstruction	233,397 (210,700–272,013)	9,344 (6,131–15,259)	6,998 (5,993–8,633)	52,824 (46,451–62,576)	66,124 (59,818–78,727)	98,104 (90,205–112,717)
Inguinal, femoral, and abdominal hernia	56,554 (39,958–63,819)	1,142 (472–1,945)	943 (579–1,197)	11,210 (6,565–13,466)	17,306 (12,084–20,013)	25,950 (19,472–28,936)
Inflammatory bowel disease	42,566 (38,284–47,340)	2,099 (1,143–3,987)	645 (509–854)	8,363 (7,029–9,859)	10,418 (9,342–11,523)	21,039 (18,687–22,370)
Vascular intestinal disorders	84,252 (78,962–91,241)	519 (313–904)	154 (126–193)	5,303 (4,647–6,070)	21,291 (19,517–23,672)	56,983 (53,811–60,989)
Gallbladder and biliary diseases	96,444 (91,143–103,079)	1,627 (910–3,055)	1,140 (986–1,342)	13,932 (12,814–15,418)	26,555 (24,755–28,856)	53,187 (50,875–56,718)
Pancreatitis	109,567 (103,025–117,238)	268 (176–437)	585 (488–707)	41,971 (38,797–45,646)	36,095 (33,740–38,662)	30,645 (28,871–32,433)
Other digestive diseases	96,331 (86,542–116,223)	4,006 (2,567–6,287)	1,753 (1,451–2,364)	14,676 (12,497–19,310)	25,295 (21,925–31,552)	50,599 (46,616–57,680)
Neurological disorders	1,670,975 (1,445,940–1,889,842)	19,088 (14,729–23,705)	13,570 (12,343–14,855)	86,679 (83,136–92,676)	147,515 (126,687–168,375)	1,404,122 (1,189,673–1,619,619)
Alzheimer disease and other dementias	1,380,757 (1,152,695–1,599,388)	0 (0–0)	0 (0–0)	1,274 (795–1,854)	86,173 (65,478–107,222)	1,293,310 (1,077,335–1,509,623)
Parkinson disease	82,405 (80,023–85,588)	0 (0–0)	0 (0–0)	634 (595–697)	10,158 (9,723–10,954)	71,612 (69,445–74,211)
Epilepsy	118,960 (111,925–127,594)	14,188 (10,637–17,947)	10,833 (9,792–11,903)	62,398 (58,951–66,906)	19,012 (18,366–19,733)	12,527 (12,140–12,921)

SR Table 12 - Global deaths by age and cause for both sexes in 2005						
**Cause**	**All ages**	**0-5 years**	**5-14 years**	**15-49 years**	**50-69 years**	**70+ years**
Multiple sclerosis	16,477 (14,914–18,082)	0 (0–0)	0 (0–0)	5,431 (5,145–6,321)	7,612 (6,764–8,188)	3,433 (2,848–3,617)
Motor neuron disease	27,609 (26,794–28,843)	1,070 (805–1,297)	121 (109–132)	3,470 (3,300–3,747)	12,083 (11,675–12,760)	10,863 (10,571–11,207)
Migraine	0 (0–0)	0 (0–0)	0 (0–0)	0 (0–0)	0 (0–0)	0 (0–0)
Tension-type headache	0 (0–0)	0 (0–0)	0 (0–0)	0 (0–0)	0 (0–0)	0 (0–0)
Medication overuse headache	0 (0–0)	0 (0–0)	0 (0–0)	0 (0–0)	0 (0–0)	0 (0–0)
Other neurological disorders	44,786 (43,552–47,849)	3,850 (3,133–5,386)	2,615 (2,324–3,218)	13,470 (12,852–14,593)	12,475 (12,061–12,999)	12,374 (11,937–12,866)
Mental and substance use disorders	305,914 (293,523–314,889)	29 (25–34)	36 (19–48)	176,678 (169,842–183,068)	97,878 (91,334–101,202)	31,291 (28,695–32,229)
Schizophrenia	19,073 (17,944–20,015)	0 (0–0)	0 (0–0)	7,610 (7,047–8,151)	6,530 (6,149–6,864)	4,932 (4,682–5,158)
Alcohol use disorders	157,381 (147,438–163,277)	0 (0–0)	0 (0–0)	79,370 (74,312–83,199)	64,283 (60,075–67,196)	13,727 (12,982–14,150)
Drug use disorders	128,838 (123,958–133,560)	29 (25–34)	0 (0–0)	89,114 (85,728–93,073)	27,064 (25,474–28,187)	12,630 (10,631–13,361)
Opioid use disorders	94,231 (90,547–99,710)	29 (25–34)	0 (0–0)	65,757 (62,818–71,096)	19,350 (18,327–20,361)	9,093 (7,585–9,656)
Cocaine use disorders	7,392 (5,026–7,949)	0 (0–0)	0 (0–0)	5,405 (3,646–5,844)	1,729 (1,165–1,897)	257 (204–287)
Amphetamine use disorders	7,271 (4,252–8,192)	0 (0–0)	0 (0–0)	5,320 (3,056–6,084)	1,612 (940–1,868)	337 (226–393)
Cannabis use disorders	0 (0–0)	0 (0–0)	0 (0–0)	0 (0–0)	0 (0–0)	0 (0–0)
Other drug use disorders	19,944 (18,624–22,759)	0 (0–0)	0 (0–0)	12,631 (11,636–14,764)	4,371 (4,076–5,003)	2,941 (2,518–3,361)
Depressive disorders	0 (0–0)	0 (0–0)	0 (0–0)	0 (0–0)	0 (0–0)	0 (0–0)
Major depressive disorder	0 (0–0)	0 (0–0)	0 (0–0)	0 (0–0)	0 (0–0)	0 (0–0)
Dysthymia	0 (0–0)	0 (0–0)	0 (0–0)	0 (0–0)	0 (0–0)	0 (0–0)
Bipolar disorder	0 (0–0)	0 (0–0)	0 (0–0)	0 (0–0)	0 (0–0)	0 (0–0)
Anxiety disorders	0 (0–0)	0 (0–0)	0 (0–0)	0 (0–0)	0 (0–0)	0 (0–0)
Eating disorders	619 (399–810)	0 (0–0)	36 (19–48)	583 (379–768)	0 (0–0)	0 (0–0)
Anorexia nervosa	571 (369–747)	0 (0–0)	35 (18–46)	536 (351–712)	0 (0–0)	0 (0–0)
Bulimia nervosa	48 (26–64)	0 (0–0)	1 (0–2)	46 (25–62)	0 (0–0)	0 (0–0)
Autistic spectrum disorders	0 (0–0)	0 (0–0)	0 (0–0)	0 (0–0)	0 (0–0)	0 (0–0)
Autism	0 (0–0)	0 (0–0)	0 (0–0)	0 (0–0)	0 (0–0)	0 (0–0)
Asperger syndrome and other autistic spectrum disorders	0 (0–0)	0 (0–0)	0 (0–0)	0 (0–0)	0 (0–0)	0 (0–0)
Attention-deficit/hyperactivity disorder	0 (0–0)	0 (0–0)	0 (0–0)	0 (0–0)	0 (0–0)	0 (0–0)
Conduct disorder	0 (0–0)	0 (0–0)	0 (0–0)	0 (0–0)	0 (0–0)	0 (0–0)
Idiopathic developmental intellectual disability	0 (0–0)	0 (0–0)	0 (0–0)	0 (0–0)	0 (0–0)	0 (0–0)
Other mental and substance use disorders	0 (0–0)	0 (0–0)	0 (0–0)	0 (0–0)	0 (0–0)	0 (0–0)
Diabetes, urogenital, blood, and endocrine diseases	2,635,278 (2,534,503–2,716,025)	123,653 (93,693–171,784)	33,468 (30,290–36,786)	377,582 (356,572–393,610)	844,560 (815,414–866,964)	1,256,014 (1,221,996–1,285,413)
Diabetes mellitus	1,150,184 (1,120,858–1,176,764)	2,399 (1,971–2,643)	2,890 (2,280–3,182)	116,045 (111,628–120,565)	440,787 (427,236–452,895)	588,060 (573,887–601,926)
Acute glomerulonephritis	12,674 (9,902–14,369)	369 (295–463)	409 (331–477)	3,488 (2,624–3,801)	4,022 (3,177–4,572)	4,385 (3,347–5,117)
Chronic kidney disease	937,664 (866,150–970,756)	14,361 (12,491–15,866)	9,753 (8,559–10,580)	157,035 (136,103–164,459)	291,638 (264,292–304,694)	464,875 (440,388–481,544)
Chronic kidney disease due to diabetes mellitus	299,371 (278,673–314,167)	1,855 (1,538–2,232)	860 (709–1,053)	35,877 (31,264–39,772)	114,288 (104,349–123,569)	146,488 (138,058–154,622)
Chronic kidney disease due to hypertension	408,507 (377,138–427,617)	5,000 (4,292–5,687)	2,955 (2,490–3,421)	60,615 (52,501–65,435)	114,681 (103,553–123,081)	225,254 (212,659–237,084)
Chronic kidney disease due to glomerulonephritis	205,578 (184,873–217,881)	5,507 (4,651–6,324)	4,391 (3,761–4,970)	53,711 (46,588–58,572)	59,169 (51,994–64,341)	82,797 (75,633–89,918)
Chronic kidney disease due to other causes	24,207 (20,150–28,610)	1,997 (1,588–2,436)	1,545 (1,220–1,890)	6,830 (4,668–9,405)	3,498 (2,189–5,254)	10,334 (8,658–12,628)
Urinary diseases and male infertility	201,106 (188,247–215,662)	7,172 (5,584–8,822)	1,959 (1,745–2,297)	28,590 (26,010–31,877)	49,593 (44,915–55,024)	113,791 (106,763–121,755)
Interstitial nephritis and urinary tract infections	149,781 (139,264–160,936)	5,661 (4,445–7,064)	1,240 (1,082–1,444)	19,233 (17,425–21,636)	32,833 (29,596–36,489)	90,811 (84,334–96,974)
Urolithiasis	14,912 (12,474–18,071)	0 (0–0)	243 (203–321)	2,810 (2,341–3,575)	5,187 (4,243–6,220)	6,671 (5,603–7,974)
Benign prostatic hyperplasia	0 (0–0)	0 (0–0)	0 (0–0)	0 (0–0)	0 (0–0)	0 (0–0)
Male infertility	0 (0–0)	0 (0–0)	0 (0–0)	0 (0–0)	0 (0–0)	0 (0–0)
Other urinary diseases	36,413 (30,826–42,697)	1,510 (986–2,304)	476 (388–591)	6,545 (5,306–7,897)	11,572 (9,394–14,127)	16,308 (13,910–19,624)
Gynecological diseases	7,640 (6,090–8,606)	0 (0–0)	0 (0–0)	3,409 (2,693–3,903)	2,298 (1,775–2,659)	1,932 (1,556–2,330)
Uterine fibroids	2,122 (1,233–2,702)	0 (0–0)	0 (0–0)	788 (479–1,026)	868 (496–1,110)	466 (237–679)
Polycystic ovarian syndrome	747 (192–1,251)	0 (0–0)	0 (0–0)	528 (116–924)	219 (74–355)	0 (0–0)
Female infertility	0 (0–0)	0 (0–0)	0 (0–0)	0 (0–0)	0 (0–0)	0 (0–0)
Endometriosis	44 (34–60)	0 (0–0)	0 (0–0)	36 (27–50)	8 (5–12)	0 (0–0)
Genital prolapse	1,104 (569–1,766)	0 (0–0)	0 (0–0)	79 (35–145)	402 (179–722)	621 (345–958)
Premenstrual syndrome	0 (0–0)	0 (0–0)	0 (0–0)	0 (0–0)	0 (0–0)	0 (0–0)
Other gynecological diseases	3,621 (2,678–4,530)	0 (0–0)	0 (0–0)	1,977 (1,498–2,555)	799 (536–1,074)	843 (577–1,179)
Hemoglobinopathies and hemolytic anemias	215,460 (173,451–273,956)	87,057 (58,167–136,302)	14,974 (12,813–17,542)	46,122 (40,246–52,193)	27,092 (23,808–30,098)	40,213 (35,301–45,297)
Thalassemias	19,685 (16,530–23,337)	10,481 (8,004–14,067)	3,594 (3,168–4,059)	2,176 (1,805–2,657)	105 (98–115)	3,327 (2,604–4,345)
Thalassemias trait	0 (0–0)	0 (0–0)	0 (0–0)	0 (0–0)	0 (0–0)	0 (0–0)
Sickle cell disorders	108,301 (78,476–159,818)	75,123 (47,862–124,842)	9,595 (7,777–11,864)	18,378 (16,017–21,143)	1,130 (1,019–1,273)	4,073 (3,374–5,010)
Sickle cell trait	0 (0–0)	0 (0–0)	0 (0–0)	0 (0–0)	0 (0–0)	0 (0–0)

SR Table 12 - Global deaths by age and cause for both sexes in 2005						
Cause	All ages	0-5 years	5-14 years	15-49 years	50-69 years	70+ years
G6PD deficiency	27,501 (23,535–32,095)	1,088 (719–1,630)	1,345 (1,078–1,719)	15,527 (13,176–18,466)	7,752 (6,478–9,112)	1,787 (1,449–2,221)
G6PD trait	0 (0–0)	0 (0–0)	0 (0–0)	0 (0–0)	0 (0–0)	0 (0–0)
Other hemoglobinopathies and hemolytic anemias	59,971 (53,174–66,508)	363 (279–502)	437 (380–502)	10,040 (8,815–11,383)	18,104 (15,982–20,265)	31,025 (27,366–34,497)
Endocrine, metabolic, blood, and immune disorders	110,548 (108,085–113,472)	12,292 (11,277–13,436)	3,481 (3,296–3,670)	22,890 (22,157–23,717)	29,127 (28,271–30,029)	42,756 (41,494–44,037)
Musculoskeletal disorders	76,223 (69,159–80,498)	0 (0–0)	1,920 (1,683–2,174)	18,714 (17,218–20,836)	19,455 (18,299–21,301)	36,132 (32,827–37,512)
Rheumatoid arthritis	26,526 (23,215–29,254)	0 (0–0)	247 (177–319)	2,321 (1,884–2,688)	8,530 (7,514–9,460)	15,426 (13,677–17,070)
Osteoarthritis	0 (0–0)	0 (0–0)	0 (0–0)	0 (0–0)	0 (0–0)	0 (0–0)
Low back and neck pain	0 (0–0)	0 (0–0)	0 (0–0)	0 (0–0)	0 (0–0)	0 (0–0)
Low back pain	0 (0–0)	0 (0–0)	0 (0–0)	0 (0–0)	0 (0–0)	0 (0–0)
Neck pain	0 (0–0)	0 (0–0)	0 (0–0)	0 (0–0)	0 (0–0)	0 (0–0)
Gout	0 (0–0)	0 (0–0)	0 (0–0)	0 (0–0)	0 (0–0)	0 (0–0)
Other musculoskeletal disorders	49,696 (45,531–53,117)	0 (0–0)	1,672 (1,469–1,915)	16,393 (15,154–18,315)	10,924 (10,279–12,225)	20,706 (17,690–21,725)
Other non-communicable diseases	726,594 (626,790–869,322)	539,431 (453,591–674,732)	38,392 (32,500–44,255)	67,862 (59,050–74,192)	30,562 (24,363–35,531)	50,345 (37,435–58,846)
Congenital anomalies	634,165 (535,864–773,401)	513,097 (425,377–646,654)	37,308 (31,432–43,077)	58,276 (51,297–63,752)	14,631 (12,949–15,504)	10,851 (9,569–11,649)
Neural tube defects	76,479 (56,721–107,029)	72,469 (53,283–102,004)	2,013 (1,388–2,810)	1,480 (1,193–1,818)	339 (275–441)	175 (137–245)
Congenital heart anomalies	318,970 (267,431–378,146)	243,030 (199,425–297,178)	26,460 (21,787–30,717)	41,355 (35,995–45,360)	4,643 (4,096–5,053)	3,480 (3,101–3,948)
Cleft lip and cleft palate	3,292 (2,622–3,826)	3,292 (2,622–3,826)	0 (0–0)	0 (0–0)	0 (0–0)	0 (0–0)
Down syndrome	26,558 (17,856–42,680)	18,439 (10,490–33,152)	941 (675–1,347)	4,160 (3,369–5,255)	2,848 (2,488–3,233)	168 (140–205)
Turner syndrome	0 (0–0)	0 (0–0)	0 (0–0)	0 (0–0)	0 (0–0)	0 (0–0)
Klinefelter syndrome	0 (0–0)	0 (0–0)	0 (0–0)	0 (0–0)	0 (0–0)	0 (0–0)
Other chromosomal abnormalities	20,600 (9,839–49,278)	19,097 (8,503–47,278)	366 (225–726)	764 (560–935)	274 (177–340)	97 (76–118)
Other congenital anomalies	188,264 (158,701–231,897)	156,767 (129,992–199,465)	7,527 (6,260–9,271)	10,515 (9,042–11,844)	6,525 (5,519–7,124)	6,928 (5,739–7,414)
Skin and subcutaneous diseases	71,633 (48,142–90,433)	5,538 (3,549–8,799)	1,084 (681–1,539)	9,585 (5,989–12,922)	15,930 (10,448–21,162)	39,493 (26,951–48,023)
Dermatitis	0 (0–0)	0 (0–0)	0 (0–0)	0 (0–0)	0 (0–0)	0 (0–0)
Psoriasis	0 (0–0)	0 (0–0)	0 (0–0)	0 (0–0)	0 (0–0)	0 (0–0)
Cellulitis	12,605 (7,750–17,429)	650 (330–1,082)	199 (113–274)	2,232 (1,189–3,391)	4,005 (2,253–5,724)	5,517 (3,551–7,525)
Pyoderma	30,860 (21,267–43,479)	4,547 (2,909–7,455)	583 (373–909)	5,257 (3,173–7,305)	7,452 (5,009–10,764)	13,019 (9,359–17,569)
Scabies	0 (0–0)	0 (0–0)	0 (0–0)	0 (0–0)	0 (0–0)	0 (0–0)
Fungal skin diseases	0 (0–0)	0 (0–0)	0 (0–0)	0 (0–0)	0 (0–0)	0 (0–0)
Viral skin diseases	0 (0–0)	0 (0–0)	0 (0–0)	0 (0–0)	0 (0–0)	0 (0–0)
Acne vulgaris	0 (0–0)	0 (0–0)	0 (0–0)	0 (0–0)	0 (0–0)	0 (0–0)
Alopecia areata	0 (0–0)	0 (0–0)	0 (0–0)	0 (0–0)	0 (0–0)	0 (0–0)
Pruritus	0 (0–0)	0 (0–0)	0 (0–0)	0 (0–0)	0 (0–0)	0 (0–0)
Urticaria	0 (0–0)	0 (0–0)	0 (0–0)	0 (0–0)	0 (0–0)	0 (0–0)
Decubitus ulcer	25,058 (14,502–30,724)	95 (46–159)	159 (85–226)	1,462 (756–1,916)	3,838 (2,033–5,107)	19,502 (11,532–23,467)
Other skin and subcutaneous diseases	3,109 (2,170–4,196)	245 (152–404)	143 (81–200)	633 (400–897)	633 (426–893)	1,453 (1,037–1,917)
Sense organ diseases	0 (0–0)	0 (0–0)	0 (0–0)	0 (0–0)	0 (0–0)	0 (0–0)
Glaucoma	0 (0–0)	0 (0–0)	0 (0–0)	0 (0–0)	0 (0–0)	0 (0–0)
Cataract	0 (0–0)	0 (0–0)	0 (0–0)	0 (0–0)	0 (0–0)	0 (0–0)
Macular degeneration	0 (0–0)	0 (0–0)	0 (0–0)	0 (0–0)	0 (0–0)	0 (0–0)
Refraction and accommodation disorders	0 (0–0)	0 (0–0)	0 (0–0)	0 (0–0)	0 (0–0)	0 (0–0)
Age-related and other hearing loss	0 (0–0)	0 (0–0)	0 (0–0)	0 (0–0)	0 (0–0)	0 (0–0)
Other vision loss	0 (0–0)	0 (0–0)	0 (0–0)	0 (0–0)	0 (0–0)	0 (0–0)
Other sense organ diseases	0 (0–0)	0 (0–0)	0 (0–0)	0 (0–0)	0 (0–0)	0 (0–0)
Oral disorders	0 (0–0)	0 (0–0)	0 (0–0)	0 (0–0)	0 (0–0)	0 (0–0)
Deciduous caries	0 (0–0)	0 (0–0)	0 (0–0)	0 (0–0)	0 (0–0)	0 (0–0)
Permanent caries	0 (0–0)	0 (0–0)	0 (0–0)	0 (0–0)	0 (0–0)	0 (0–0)
Periodontal diseases	0 (0–0)	0 (0–0)	0 (0–0)	0 (0–0)	0 (0–0)	0 (0–0)
Edentulism and severe tooth loss	0 (0–0)	0 (0–0)	0 (0–0)	0 (0–0)	0 (0–0)	0 (0–0)
Other oral disorders	0 (0–0)	0 (0–0)	0 (0–0)	0 (0–0)	0 (0–0)	0 (0–0)
Sudden infant death syndrome	20,795 (16,929–33,711)	20,795 (16,929–33,711)	0 (0–0)	0 (0–0)	0 (0–0)	0 (0–0)
Injuries	4,758,999 (4,451,381–4,893,087)	378,385 (319,071–432,514)	289,682 (269,048–306,629)	2,476,233 (2,350,935–2,542,146)	922,827 (863,587–944,438)	691,871 (634,083–711,192)
Transport injuries	1,494,068 (1,444,827–1,550,605)	64,594 (55,493–74,242)	85,303 (80,115–90,389)	881,876 (848,920–920,338)	303,196 (292,826–312,724)	159,098 (154,518–163,563)
Road injuries	1,392,521 (1,341,022–1,445,987)	59,456 (51,435–68,174)	80,712 (75,674–85,542)	823,848 (791,264–860,113)	279,401 (268,637–289,056)	149,102 (144,412–153,428)
Pedestrian road injuries	581,353 (546,292–631,317)	27,481 (23,834–31,685)	39,989 (37,152–43,341)	300,357 (280,739–327,947)	133,021 (124,708–145,248)	80,503 (76,213–87,818)
Cyclist road injuries	63,428 (58,658–68,940)	1,434 (1,108–1,801)	4,485 (4,058–4,894)	32,874 (30,217–35,816)	17,551 (16,100–19,200)	7,081 (6,508–7,770)
Motorcyclist road injuries	245,716 (215,166–263,819)	7,635 (6,131–9,153)	8,992 (7,509–10,223)	173,197 (153,548–186,874)	39,769 (33,787–44,202)	16,121 (12,954–18,904)

SR Table 12 - Global deaths by age and cause for both sexes in 2005						
**Cause**	**All ages**	**0-5 years**	**5-14 years**	**15-49 years**	**50-69 years**	**70+ years**
Motor vehicle road injuries	483,077 (445,885–536,583)	21,564 (18,190–25,623)	25,692 (23,451–28,819)	306,941 (286,012–344,852)	85,537 (76,194–93,340)	43,341 (38,592–47,172)
Other road injuries	18,945 (13,117–22,543)	1,340 (718–2,454)	1,551 (987–1,992)	10,477 (6,721–12,682)	3,521 (2,707–4,287)	2,054 (1,720–2,595)
Other transport injuries	101,546 (93,202–113,975)	5,137 (3,765–6,486)	4,590 (4,135–5,061)	58,028 (52,957–65,683)	23,794 (21,511–27,860)	9,995 (9,184–11,537)
Unintentional injuries	1,887,303 (1,700,962–1,969,640)	289,323 (233,685–337,853)	161,933 (147,448–173,158)	699,975 (645,955–725,411)	337,063 (305,085–349,157)	399,008 (355,146–413,463)
Falls	436,075 (402,277–451,202)	23,814 (17,862–30,069)	15,833 (14,291–17,227)	107,237 (101,567–113,289)	93,361 (87,202–96,522)	195,829 (174,167–204,026)
Drowning	403,068 (349,161–424,692)	88,750 (65,798–103,281)	74,248 (65,796–79,069)	156,179 (142,169–161,877)	50,395 (44,457–52,524)	33,495 (27,738–35,077)
Fire, heat, and hot substances	195,236 (161,404–208,999)	28,691 (22,403–33,477)	11,384 (9,432–12,840)	87,267 (70,172–94,259)	33,574 (28,711–35,628)	34,318 (29,802–36,264)
Poisonings	100,755 (71,392–117,406)	20,340 (8,353–35,626)	6,291 (5,081–7,157)	42,751 (33,378–47,249)	19,562 (14,675–20,959)	11,809 (9,252–12,553)
Exposure to mechanical forces	202,641 (175,377–214,018)	34,378 (27,682–40,088)	13,366 (11,892–15,655)	93,622 (78,174–99,608)	34,611 (30,075–36,779)	26,662 (21,030–28,636)
Unintentional firearm injuries	32,724 (23,815–35,648)	2,444 (1,577–3,429)	2,005 (1,591–2,501)	18,042 (12,516–19,960)	4,496 (3,309–4,964)	5,735 (4,728–6,137)
Unintentional suffocation	34,942 (27,146–39,393)	19,579 (13,137–23,830)	3,104 (2,532–4,371)	5,664 (4,901–6,345)	1,968 (1,494–2,184)	4,625 (3,669–5,071)
Other exposure to mechanical forces	134,975 (113,895–144,263)	12,355 (9,682–15,025)	8,256 (7,308–9,600)	69,915 (57,020–75,379)	28,147 (24,480–30,550)	16,301 (12,150–17,831)
Adverse effects of medical treatment	97,291 (74,071–107,795)	17,936 (9,500–23,576)	4,718 (3,235–5,684)	28,120 (20,469–31,300)	19,498 (15,556–21,788)	27,017 (22,475–29,852)
Animal contact	104,376 (67,090–114,938)	13,482 (8,413–16,568)	13,812 (8,980–16,083)	43,299 (27,444–48,072)	22,670 (14,376–25,189)	11,110 (7,558–12,164)
Venomous animal contact	88,192 (54,062–98,640)	9,708 (5,707–11,854)	11,607 (7,240–13,741)	38,442 (23,185–43,432)	19,559 (11,876–21,970)	8,874 (5,661–9,880)
Non-venomous animal contact	16,183 (13,091–18,663)	3,774 (2,390–4,986)	2,204 (1,649–2,702)	4,857 (4,007–5,551)	3,111 (2,460–3,544)	2,236 (1,897–2,520)
Foreign body	145,664 (118,707–175,931)	46,067 (28,838–69,059)	9,219 (6,981–11,770)	34,825 (28,840–42,431)	22,578 (18,940–25,680)	32,973 (28,038–36,001)
Pulmonary aspiration and foreign body in airway	116,648 (92,988–148,083)	39,871 (22,997–62,058)	6,684 (4,800–9,350)	23,435 (19,979–27,234)	18,486 (15,085–20,446)	28,170 (23,676–30,019)
Foreign body in eyes	0 (0–0)	0 (0–0)	0 (0–0)	0 (0–0)	0 (0–0)	0 (0–0)
Foreign body in other body part	29,016 (18,067–43,176)	6,196 (2,687–9,817)	2,534 (1,438–3,793)	11,390 (7,130–19,374)	4,092 (2,848–6,435)	4,802 (3,749–6,966)
Environmental heat and cold exposure	53,373 (39,901–57,243)	3,327 (1,803–4,211)	1,289 (877–1,535)	19,716 (14,977–21,561)	17,828 (12,774–19,508)	11,211 (7,883–12,621)
Other unintentional injuries	148,819 (141,447–157,713)	12,532 (10,550–14,642)	11,769 (10,531–13,155)	86,955 (81,902–93,705)	22,981 (21,534–24,422)	14,580 (12,797–17,095)
Self-harm and interpersonal violence	1,252,960 (1,125,626–1,288,753)	13,653 (12,443–14,749)	23,604 (21,096–24,951)	823,051 (740,915–848,387)	266,226 (239,090–274,130)	126,422 (110,662–131,035)
Self-harm	827,632 (725,352–855,524)	0 (0–0)	10,157 (8,197–11,069)	503,812 (443,854–522,603)	207,061 (180,934–214,253)	106,601 (91,332–111,057)
Interpersonal violence	425,327 (388,730–439,626)	13,653 (12,443–14,749)	13,447 (12,399–14,235)	319,239 (291,125–330,991)	59,164 (55,473–61,708)	19,821 (18,922–20,555)
Assault by firearm	162,932 (142,773–168,798)	1,965 (1,657–2,216)	3,533 (2,983–3,768)	136,873 (119,396–141,989)	16,461 (14,773–17,455)	4,096 (3,627–4,510)
Assault by sharp object	104,393 (97,491–110,653)	1,372 (1,165–1,638)	2,159 (1,959–2,409)	81,372 (75,685–86,379)	14,720 (13,922–15,757)	4,768 (4,529–5,200)
Assault by other means	158,001 (143,071–167,507)	10,315 (9,424–11,224)	7,754 (7,182–8,553)	100,993 (90,277–109,204)	27,982 (25,845–29,624)	10,955 (10,245–11,476)
Forces of nature, war, and legal intervention	124,667 (82,532–166,514)	10,813 (7,002–14,603)	18,841 (11,384–26,327)	71,329 (48,391–93,303)	16,341 (10,705–21,812)	7,341 (4,495–10,679)
Exposure to forces of nature	90,820 (53,038–127,993)	8,051 (4,558–11,547)	16,645 (9,438–23,890)	47,034 (27,312–66,219)	12,914 (7,684–17,935)	6,174 (3,626–8,659)
Collective violence and legal intervention	33,847 (25,478–42,967)	2,762 (1,972–3,552)	2,195 (1,602–2,849)	24,294 (18,003–30,611)	3,427 (2,645–4,286)	1,166 (749–3,718)

SR Table 12 - Global deaths by age and cause for both sexes in 2010

Cause	All ages	0-5 years	5-14 years	15-49 years	50-69 years	70+ years
All causes	54,012,874 (53,422,311–54,583,600)	6,875,832 (6,777,537–6,980,747)	998,264 (961,813–1,039,044)	9,166,007 (8,960,302–9,371,216)	13,577,316 (13,377,386–13,783,902)	23,395,454 (23,214,768–23,567,921)
Communicable, maternal, neonatal, and nutritional diseases	12,589,126 (12,295,253–12,869,371)	5,683,637 (5,559,943–5,811,539)	510,696 (486,291–539,027)	2,757,270 (2,638,834–2,889,221)	1,557,456 (1,455,713–1,637,012)	2,080,065 (1,962,701–2,148,745)
HIV/AIDS and tuberculosis	2,768,400 (2,557,478–3,056,579)	165,136 (157,667–177,051)	114,719 (110,073–121,996)	1,543,059 (1,449,345–1,652,024)	630,894 (557,052–738,312)	314,589 (263,103–390,690)
Tuberculosis	1,210,825 (1,018,167–1,497,206)	27,760 (22,880–39,803)	16,532 (14,104–22,944)	433,326 (354,426–527,556)	438,501 (365,370–547,453)	294,704 (243,224–371,041)
HIV/AIDS	1,557,574 (1,487,459–1,641,018)	137,376 (131,881–143,312)	98,187 (94,458–101,956)	1,109,732 (1,044,899–1,185,295)	192,393 (180,533–208,161)	19,884 (18,311–22,079)
HIV/AIDS - Tuberculosis	291,730 (224,519–327,643)	34,243 (26,167–38,625)	20,257 (15,001–23,214)	184,563 (139,725–208,381)	47,122 (38,218–52,527)	5,542 (4,802–6,177)
HIV/AIDS resulting in other diseases	1,265,844 (1,187,962–1,361,315)	103,133 (96,388–112,039)	77,929 (72,979–83,941)	925,169 (856,996–1,000,330)	145,270 (133,758–162,262)	14,341 (13,019–16,265)
Diarrhea, lower respiratory, and other common infectious diseases	5,266,023 (5,022,721–5,470,514)	2,038,094 (1,919,400–2,161,192)	284,776 (257,362–317,682)	633,270 (585,347–689,334)	736,255 (678,479–778,962)	1,573,628 (1,455,318–1,634,426)
Diarrheal diseases	1,501,539 (1,427,849–1,581,211)	643,613 (585,711–707,510)	62,998 (56,594–69,503)	186,977 (174,643–201,698)	234,473 (220,200–249,603)	373,476 (351,851–395,258)
Intestinal infectious diseases	186,822 (105,910–306,540)	44,800 (23,666–77,888)	71,781 (42,236–115,886)	57,594 (30,267–99,079)	8,762 (4,523–15,229)	3,884 (2,208–6,188)
Typhoid fever	155,228 (84,631–263,346)	37,512 (19,164–66,949)	60,375 (34,386–97,729)	47,301 (24,681–83,176)	7,001 (3,398–12,235)	3,037 (1,618–4,977)
Paratyphoid fever	30,493 (14,455–58,774)	6,710 (2,817–13,917)	11,286 (5,263–21,467)	10,139 (4,338–20,103)	1,680 (688–3,346)	675 (287–1,276)
Other intestinal infectious diseases	1,100 (419–2,013)	576 (171–1,148)	118 (35–255)	153 (63–311)	80 (36–159)	171 (77–335)
Lower respiratory infections	2,741,316 (2,513,026–2,872,150)	886,478 (826,640–949,912)	70,611 (55,315–80,645)	249,695 (209,794–271,109)	406,837 (345,349–436,374)	1,127,692 (1,018,534–1,179,607)
Upper respiratory infections	3,334 (2,974–3,751)	891 (666–1,177)	54 (37–78)	691 (599–805)	789 (671–920)	906 (772–1,065)
Otitis media	3,389 (3,107–3,786)	1,180 (1,047–1,348)	409 (371–450)	822 (767–891)	430 (390–481)	547 (405–877)
Meningitis	393,691 (335,308–444,127)	193,688 (154,328–239,352)	36,291 (30,466–42,681)	79,857 (66,875–95,453)	47,904 (40,605–57,471)	35,950 (31,166–42,730)
Pneumococcal meningitis	118,411 (98,850–143,758)	51,622 (36,481–72,056)	13,518 (9,736–17,982)	28,531 (22,621–35,611)	14,189 (10,643–18,241)	10,550 (8,184–13,203)
H influenzae type B meningitis	82,241 (65,792–103,087)	53,867 (39,193–74,731)	7,509 (4,983–10,751)	9,929 (7,559–13,055)	5,992 (4,409–7,985)	4,943 (3,883–6,483)
Meningococcal meningitis	77,408 (61,431–96,164)	42,636 (29,152–60,333)	7,508 (5,013–10,721)	13,998 (10,706–18,126)	8,031 (5,921–10,482)	5,233 (4,042–6,720)
Other meningitis	115,629 (97,818–135,547)	45,562 (33,891–61,833)	7,755 (5,476–10,812)	27,398 (22,030–33,014)	19,689 (15,778–24,718)	15,222 (12,516–18,012)
Encephalitis	146,775 (135,781–162,315)	45,150 (39,484–50,456)	17,128 (14,694–19,806)	36,734 (34,155–40,936)	24,296 (22,351–28,317)	23,465 (21,701–27,429)
Diphtheria	3,294 (1,678–7,124)	2,460 (957–6,250)	470 (213–1,113)	271 (193–383)	93 (58–152)	0 (0–0)
Whooping cough	75,121 (30,013–163,025)	69,649 (27,834–149,534)	4,443 (1,724–9,669)	897 (332–2,188)	130 (46–327)	0 (0–0)
Tetanus	71,618 (60,948–103,335)	35,870 (31,157–42,106)	3,906 (2,887–6,698)	15,429 (11,776–27,937)	11,255 (8,415–21,985)	5,156 (4,018–9,545)
Measles	131,474 (49,416–286,567)	112,173 (42,540–243,311)	16,068 (5,549–35,959)	3,118 (997–7,951)	114 (36–291)	0 (0–0)
Varicella and herpes zoster	7,643 (6,678–9,104)	2,136 (1,541–3,060)	613 (425–888)	1,179 (990–1,436)	1,166 (910–1,489)	2,546 (2,015–3,350)
Neglected tropical diseases and malaria	1,070,636 (898,927–1,245,127)	688,418 (538,226–842,580)	75,474 (53,588–104,620)	172,942 (136,353–223,046)	91,561 (71,544–119,570)	42,239 (33,395–55,121)
Malaria	952,401 (777,885–1,128,391)	665,162 (513,330–820,684)	57,886 (35,832–86,799)	129,808 (94,136–180,774)	69,826 (49,794–97,484)	29,717 (20,786–42,718)
Chagas disease	7,391 (7,076–7,707)	3 (2–3)	6 (5–7)	964 (903–1,030)	3,067 (2,897–3,243)	3,349 (3,156–3,549)
Leishmaniasis	23,595 (16,239–32,355)	3,708 (2,442–5,179)	2,532 (1,590–3,830)	13,916 (9,393–19,448)	2,805 (2,011–3,777)	633 (499–786)
Visceral leishmaniasis	23,595 (16,239–32,355)	3,708 (2,442–5,179)	2,532 (1,590–3,830)	13,916 (9,393–19,448)	2,805 (2,011–3,777)	633 (499–786)
Cutaneous and mucocutaneous leishmaniasis	0 (0–0)	0 (0–0)	0 (0–0)	0 (0–0)	0 (0–0)	0 (0–0)
African trypanosomiasis	6,190 (3,242–9,802)	431 (222–712)	971 (526–1,587)	3,660 (1,901–5,942)	915 (467–1,487)	211 (111–346)
Schistosomiasis	5,901 (5,224–6,619)	56 (38–85)	129 (96–171)	1,725 (1,482–2,007)	2,551 (2,099–3,093)	1,437 (1,158–1,755)
Cysticercosis	471 (393–602)	9 (4–17)	17 (12–25)	268 (223–348)	133 (112–166)	42 (36–49)
Cystic echinococcosis	1,421 (1,342–1,506)	54 (44–65)	109 (95–122)	528 (492–570)	420 (395–448)	309 (294–325)
Lymphatic filariasis	0 (0–0)	0 (0–0)	0 (0–0)	0 (0–0)	0 (0–0)	0 (0–0)
Onchocerciasis	0 (0–0)	0 (0–0)	0 (0–0)	0 (0–0)	0 (0–0)	0 (0–0)
Trachoma	0 (0–0)	0 (0–0)	0 (0–0)	0 (0–0)	0 (0–0)	0 (0–0)
Dengue	16,856 (10,980–20,355)	4,670 (2,760–6,331)	4,092 (2,583–5,231)	4,861 (3,392–5,814)	1,970 (1,218–2,444)	1,261 (736–1,570)
Yellow fever	6,042 (1,237–16,920)	559 (109–1,618)	1,905 (393–5,336)	3,052 (619–8,529)	441 (88–1,248)	82 (16–241)
Rabies	22,284 (19,514–25,665)	2,883 (2,189–4,027)	5,021 (4,029–6,137)	7,871 (6,853–9,029)	4,722 (4,088–5,393)	1,786 (1,485–2,092)
Intestinal nematode infections	3,228 (2,897–3,620)	2,284 (1,998–2,631)	458 (382–536)	217 (176–262)	147 (112–187)	121 (94–157)
Ascariasis	3,228 (2,897–3,620)	2,284 (1,998–2,631)	458 (382–536)	217 (176–262)	147 (112–187)	121 (94–157)
Trichuriasis	0 (0–0)	0 (0–0)	0 (0–0)	0 (0–0)	0 (0–0)	0 (0–0)
Hookworm disease	0 (0–0)	0 (0–0)	0 (0–0)	0 (0–0)	0 (0–0)	0 (0–0)
Food-borne trematodiases	0 (0–0)	0 (0–0)	0 (0–0)	0 (0–0)	0 (0–0)	0 (0–0)
Leprosy	0 (0–0)	0 (0–0)	0 (0–0)	0 (0–0)	0 (0–0)	0 (0–0)
Ebola	0 (0–0)	0 (0–0)	0 (0–0)	0 (0–0)	0 (0–0)	0 (0–0)
Other neglected tropical diseases	24,849 (14,474–31,602)	8,594 (5,808–11,202)	2,342 (1,307–3,234)	6,068 (2,903–7,944)	4,558 (2,350–6,123)	3,285 (1,851–4,460)
Maternal disorders	309,794 (283,378–340,510)	0 (0–0)	1,545 (1,279–1,888)	307,087 (281,040–337,459)	1,161 (853–1,610)	0 (0–0)
Maternal hemorrhage	88,487 (75,415–102,678)	0 (0–0)	470 (332–617)	87,648 (74,654–101,861)	368 (230–600)	0 (0–0)
Maternal sepsis and other maternal infections	23,208 (18,933–28,526)	0 (0–0)	68 (47–100)	23,050 (18,805–28,356)	88 (51–153)	0 (0–0)
Maternal hypertensive disorders	54,657 (45,860–64,952)	0 (0–0)	334 (232–461)	54,230 (45,432–64,490)	93 (58–144)	0 (0–0)
Maternal obstructed labor and uterine rupture	26,651 (21,761–32,430)	0 (0–0)	45 (28–68)	26,555 (21,663–32,315)	50 (30–80)	0 (0–0)
Maternal abortion, miscarriage, and ectopic pregnancy	38,142 (31,342–45,922)	0 (0–0)	199 (132–291)	37,751 (31,001–45,542)	191 (124–284)	0 (0–0)

SR Table 12 - Global deaths by age and cause for both sexes in 2010

Cause	All ages	0-5 years	5-14 years	15-49 years	50-69 years	70+ years
Indirect maternal deaths	34,002 (27,192–41,477)	0 (0–0)	207 (138–304)	33,670 (26,880–41,121)	123 (77–200)	0 (0–0)
Late maternal deaths	7,302 (4,824–10,563)	0 (0–0)	42 (20–77)	7,217 (4,752–10,441)	42 (23–73)	0 (0–0)
Maternal deaths aggravated by HIV/AIDS	2,493 (1,581–3,440)	0 (0–0)	3 (1–4)	2,479 (1,574–3,422)	10 (5–17)	0 (0–0)
Other maternal disorders	34,848 (29,561–41,490)	0 (0–0)	173 (122–241)	34,481 (29,169–41,149)	193 (129–281)	0 (0–0)
Neonatal disorders	2,419,340 (2,355,841–2,490,002)	2,419,340 (2,355,841–2,490,002)	0 (0–0)	0 (0–0)	0 (0–0)	0 (0–0)
Neonatal preterm birth complications	946,196 (874,957–1,051,016)	946,196 (874,957–1,051,016)	0 (0–0)	0 (0–0)	0 (0–0)	0 (0–0)
Neonatal encephalopathy due to birth asphyxia and trauma	823,655 (751,291–907,792)	823,655 (751,291–907,792)	0 (0–0)	0 (0–0)	0 (0–0)	0 (0–0)
Neonatal sepsis and other neonatal infections	357,718 (252,772–467,111)	357,718 (252,772–467,111)	0 (0–0)	0 (0–0)	0 (0–0)	0 (0–0)
Hemolytic disease and other neonatal jaundice	55,757 (35,933–84,036)	55,757 (35,933–84,036)	0 (0–0)	0 (0–0)	0 (0–0)	0 (0–0)
Other neonatal disorders	236,013 (181,086–298,224)	236,013 (181,086–298,224)	0 (0–0)	0 (0–0)	0 (0–0)	0 (0–0)
Nutritional deficiencies	436,402 (365,380–514,477)	228,846 (175,839–287,635)	15,714 (13,334–20,050)	38,819 (32,263–49,315)	44,692 (37,357–55,975)	108,329 (92,922–122,475)
Protein-energy malnutrition	355,852 (292,324–426,245)	209,326 (159,726–264,962)	12,155 (10,114–15,416)	20,097 (16,667–25,435)	27,875 (23,768–35,720)	86,396 (75,262–97,936)
Iodine deficiency	1,985 (1,508–2,772)	617 (288–1,214)	76 (39–144)	349 (244–521)	413 (282–634)	527 (367–791)
Vitamin A deficiency	0 (0–0)	0 (0–0)	0 (0–0)	0 (0–0)	0 (0–0)	0 (0–0)
Iron-deficiency anemia	50,782 (32,530–67,047)	15,115 (7,899–22,165)	2,371 (1,660–3,659)	12,432 (9,054–17,584)	11,537 (7,875–14,958)	9,325 (5,563–12,012)
Other nutritional deficiencies	27,782 (21,158–35,417)	3,786 (2,390–5,390)	1,110 (767–1,482)	5,939 (4,414–7,403)	4,866 (3,653–6,406)	12,078 (9,321–15,555)
Other communicable, maternal, neonatal, and nutritional diseases	318,528 (264,403–383,390)	143,800 (100,100–200,346)	18,465 (14,497–23,899)	62,091 (58,058–64,958)	52,891 (46,257–55,948)	41,279 (33,465–43,902)
Sexually transmitted diseases excluding HIV	116,039 (69,724–179,772)	97,391 (54,745–156,511)	9,012 (5,083–14,222)	4,585 (3,672–5,312)	2,697 (1,950–3,191)	2,351 (1,826–2,775)
Syphilis	114,788 (68,363–178,546)	97,391 (54,745–156,511)	8,987 (5,057–14,197)	4,011 (3,217–4,650)	2,363 (1,706–2,802)	2,033 (1,581–2,398)
Chlamydial infection	148 (112–173)	0 (0–0)	3 (2–4)	63 (50–77)	37 (26–45)	43 (32–52)
Gonococcal infection	752 (586–855)	0 (0–0)	12 (10–16)	333 (263–389)	194 (141–229)	211 (163–250)
Trichomoniasis	0 (0–0)	0 (0–0)	0 (0–0)	0 (0–0)	0 (0–0)	0 (0–0)
Genital herpes	0 (0–0)	0 (0–0)	0 (0–0)	0 (0–0)	0 (0–0)	0 (0–0)
Other sexually transmitted diseases	350 (274–399)	0 (0–0)	9 (7–11)	176 (140–204)	102 (73–120)	62 (47–73)
Hepatitis	108,667 (104,667–112,973)	13,021 (11,568–14,584)	5,948 (5,287–6,663)	42,301 (40,173–44,396)	29,018 (27,852–30,205)	18,377 (17,636–19,165)
Acute hepatitis A	13,110 (8,341–18,538)	9,986 (7,430–12,169)	1,032 (286–2,125)	1,308 (320–3,052)	548 (130–1,376)	235 (54–570)
Hepatitis B	64,418 (55,633–72,669)	797 (285–1,778)	737 (409–1,173)	26,027 (21,640–30,154)	23,610 (20,410–26,063)	13,245 (10,811–15,387)
Hepatitis C	2,432 (535–5,606)	0 (0–0)	93 (18–246)	712 (144–1,704)	1,095 (233–2,593)	531 (109–1,254)
Acute hepatitis E	28,706 (20,305–38,502)	2,237 (910–4,263)	4,085 (3,038–5,094)	14,253 (10,569–18,629)	3,764 (1,882–6,600)	4,365 (2,441–6,896)
Other infectious diseases	93,821 (61,231–106,820)	33,387 (17,486–43,626)	3,504 (2,779–4,111)	15,204 (11,928–16,617)	21,175 (14,832–23,550)	20,550 (13,156–22,950)
Non-communicable diseases	36,608,904 (36,151,875–37,142,487)	840,823 (768,434–930,696)	204,425 (191,199–218,108)	3,974,615 (3,876,278–4,100,983)	11,048,562 (10,866,446–11,237,862)	20,540,478 (20,364,421–20,758,195)
Neoplasms	8,003,116 (7,876,488–8,133,322)	37,855 (32,856–46,361)	51,943 (49,189–54,876)	1,051,445 (1,023,587–1,081,347)	3,261,182 (3,204,244–3,316,266)	3,600,688 (3,538,590–3,666,124)
Lip and oral cavity cancer	128,073 (125,032–131,446)	0 (0–0)	0 (0–0)	21,104 (20,203–22,178)	60,215 (58,556–61,999)	46,754 (45,596–47,966)
Nasopharynx cancer	58,914 (48,198–61,570)	0 (0–0)	309 (278–347)	16,442 (13,347–17,419)	28,191 (22,763–29,662)	13,971 (11,766–14,621)
Other pharynx cancer	57,893 (56,416–59,734)	0 (0–0)	0 (0–0)	8,139 (7,802–8,609)	32,567 (31,694–33,559)	17,186 (16,703–17,675)
Esophageal cancer	429,538 (416,900–441,683)	0 (0–0)	0 (0–0)	38,779 (37,161–40,563)	206,816 (200,246–213,826)	183,942 (178,303–189,119)
Stomach cancer	798,063 (781,244–814,661)	0 (0–0)	0 (0–0)	80,777 (77,982–83,795)	323,341 (314,890–331,842)	393,944 (386,428–401,712)
Colon and rectum cancer	742,254 (728,795–758,226)	0 (0–0)	0 (0–0)	69,339 (67,287–72,278)	264,105 (259,079–269,902)	408,809 (399,997–417,697)
Liver cancer	757,115 (691,592–793,636)	0 (0–0)	1,122 (1,032–1,270)	138,351 (124,266–147,211)	353,544 (322,924–372,073)	264,096 (243,249–275,614)
Liver cancer due to hepatitis B	261,490 (236,455–279,894)	0 (0–0)	642 (574–743)	80,202 (72,618–86,401)	122,971 (110,267–135,105)	57,675 (50,530–63,769)
Liver cancer due to hepatitis C	150,464 (138,408–159,926)	0 (0–0)	29 (24–36)	10,853 (9,202–12,229)	59,735 (52,886–65,553)	79,845 (74,436–84,728)
Liver cancer due to alcohol use	215,205 (195,061–232,270)	0 (0–0)	0 (0–0)	22,593 (19,771–25,037)	114,709 (103,520–125,690)	77,902 (69,971–83,720)
Liver cancer due to other causes	129,954 (116,840–140,078)	0 (0–0)	450 (401–509)	24,702 (21,793–27,133)	56,129 (50,056–62,378)	48,673 (43,438–52,983)
Gallbladder and biliary tract cancer	133,046 (128,214–136,734)	0 (0–0)	0 (0–0)	9,167 (8,605–9,637)	46,910 (44,939–48,374)	76,969 (74,493–79,169)
Pancreatic cancer	360,546 (355,417–366,683)	0 (0–0)	0 (0–0)	26,198 (25,600–26,974)	145,798 (143,467–148,319)	188,549 (185,017–192,482)
Larynx cancer	96,604 (94,193–99,626)	0 (0–0)	0 (0–0)	10,130 (9,718–10,674)	49,196 (47,824–50,891)	37,277 (36,298–38,502)
Tracheal, bronchus, and lung cancer	1,562,629 (1,534,378–1,593,792)	0 (0–0)	0 (0–0)	117,664 (113,979–122,019)	702,189 (688,179–716,878)	742,774 (728,577–758,345)
Malignant skin melanoma	53,092 (42,770–64,745)	0 (0–0)	0 (0–0)	10,030 (8,564–12,626)	20,256 (16,156–25,541)	22,804 (17,792–26,819)
Non-melanoma skin cancer	43,576 (42,529–44,748)	0 (0–0)	0 (0–0)	3,998 (3,854–4,182)	13,465 (13,058–13,883)	26,112 (25,378–26,916)
Non-melanoma skin cancer (squamous-cell carcinoma)	43,576 (42,529–44,748)	0 (0–0)	0 (0–0)	3,998 (3,854–4,182)	13,465 (13,058–13,883)	26,112 (25,378–26,916)
Non-melanoma skin cancer (basal-cell carcinoma)	0 (0–0)	0 (0–0)	0 (0–0)	0 (0–0)	0 (0–0)	0 (0–0)
Breast cancer	479,482 (454,930–495,620)	0 (0–0)	0 (0–0)	100,992 (93,466–106,770)	212,563 (201,139–220,762)	165,927 (156,412–171,424)
Cervical cancer	228,147 (215,835–238,407)	0 (0–0)	0 (0–0)	60,438 (56,287–64,594)	106,785 (99,988–112,654)	60,924 (58,855–63,067)
Uterine cancer	85,230 (81,990–88,203)	0 (0–0)	0 (0–0)	9,755 (9,278–10,309)	38,940 (37,271–40,780)	36,535 (34,725–37,876)
Ovarian cancer	144,674 (141,172–149,479)	0 (0–0)	0 (0–0)	24,503 (23,494–26,015)	66,179 (64,462–68,462)	53,991 (52,564–55,490)
Prostate cancer	312,911 (260,321–393,480)	0 (0–0)	0 (0–0)	2,743 (2,159–3,337)	69,502 (56,420–86,806)	240,665 (199,132–308,043)
Testicular cancer	8,889 (8,505–9,370)	0 (0–0)	0 (0–0)	5,974 (5,676–6,395)	1,592 (1,519–1,669)	1,322 (1,261–1,387)

SR Table 12 - Global deaths by age and cause for both sexes in 2010

Cause	All ages	0-5 years	5-14 years	15-49 years	50-69 years	70+ years
Kidney cancer	120,115 (117,606–123,066)	2,611 (1,649–3,906)	1,232 (1,125–1,363)	11,612 (11,258–12,035)	48,326 (47,366–49,385)	56,332 (55,038–57,587)
Bladder cancer	165,991 (162,629–169,323)	0 (0–0)	0 (0–0)	7,216 (6,970–7,531)	49,257 (48,163–50,390)	109,517 (106,983–112,158)
Brain and nervous system cancer	206,988 (189,485–218,585)	5,292 (4,632–6,900)	9,858 (8,761–10,783)	55,200 (50,116–59,444)	83,318 (76,884–88,687)	53,318 (46,572–55,261)
Thyroid cancer	28,521 (26,357–29,805)	0 (0–0)	77 (68–82)	2,925 (2,641–3,127)	10,580 (9,708–11,217)	14,937 (13,904–15,630)
Mesothelioma	27,849 (27,214–28,513)	0 (0–0)	0 (0–0)	3,463 (3,319–3,627)	10,821 (10,539–11,091)	13,563 (13,169–13,968)
Hodgkin lymphoma	23,570 (21,707–28,675)	277 (224–345)	651 (537–788)	8,159 (7,376–10,037)	8,341 (7,699–10,316)	6,140 (5,636–7,336)
Non-Hodgkin lymphoma	203,761 (175,607–213,657)	3,236 (2,349–4,873)	4,786 (4,130–5,241)	36,147 (32,371–38,593)	70,840 (62,577–74,029)	88,749 (71,881–94,245)
Multiple myeloma	87,053 (84,773–89,119)	0 (0–0)	0 (0–0)	7,931 (7,685–8,246)	34,313 (33,393–35,395)	44,807 (42,600–46,007)
Leukemia	326,529 (319,695–335,390)	14,152 (12,681–16,159)	23,005 (21,637–24,641)	83,350 (80,930–86,657)	90,841 (89,067–92,918)	115,180 (112,175–117,844)
Acute lymphoid leukemia	104,246 (96,691–113,199)	9,061 (7,957–10,763)	16,242 (15,034–17,867)	34,789 (31,814–38,125)	25,426 (23,047–27,462)	18,726 (17,188–19,939)
Chronic lymphoid leukemia	55,854 (53,783–59,187)	0 (0–0)	0 (0–0)	6,705 (6,116–7,426)	15,597 (14,868–16,577)	33,551 (32,259–35,696)
Acute myeloid leukemia	132,458 (123,205–141,153)	5,090 (4,031–6,144)	6,762 (5,832–7,855)	32,802 (29,664–35,941)	39,381 (37,018–42,078)	48,421 (44,742–50,430)
Chronic myeloid leukemia	33,969 (32,187–36,752)	0 (0–0)	0 (0–0)	9,052 (8,259–10,236)	10,436 (9,818–11,282)	14,480 (13,824–15,502)
Other neoplasms	332,051 (300,745–346,229)	12,286 (10,088–16,857)	10,900 (9,876–11,803)	80,905 (70,341–86,964)	112,377 (100,233–117,462)	115,580 (103,773–120,028)
Cardiovascular diseases	16,640,686 (16,402,567–16,932,282)	54,461 (51,629–57,848)	28,780 (26,839–31,045)	1,408,437 (1,371,741–1,467,132)	4,686,625 (4,604,078–4,783,766)	10,462,381 (10,297,419–10,633,827)
Rheumatic heart disease	326,691 (307,327–342,806)	3,214 (2,794–3,718)	6,969 (6,299–7,853)	82,900 (76,010–89,324)	104,863 (97,463–110,701)	128,743 (123,221–134,203)
Ischemic heart disease	8,166,280 (8,041,397–8,331,900)	2,943 (2,624–3,413)	2,346 (2,097–2,640)	647,123 (627,987–677,627)	2,357,226 (2,308,759–2,415,422)	5,156,640 (5,065,447–5,251,852)
Cerebrovascular disease	6,031,240 (5,911,599–6,163,240)	18,544 (17,142–20,355)	10,438 (9,749–11,302)	450,992 (436,043–470,171)	1,696,127 (1,653,628–1,739,464)	3,855,137 (3,780,964–3,934,049)
Ischemic stroke	2,816,600 (2,726,480–2,901,578)	750 (653–889)	606 (552–682)	73,025 (68,044–78,987)	575,742 (547,955–603,338)	2,166,475 (2,096,836–2,224,185)
Hemorrhagic stroke	3,214,640 (3,118,991–3,328,887)	17,794 (16,453–19,529)	9,831 (9,175–10,637)	377,966 (364,911–395,384)	1,120,384 (1,083,748–1,159,915)	1,688,662 (1,634,714–1,761,900)
Hypertensive heart disease	866,846 (798,268–938,557)	501 (421–605)	413 (353–488)	71,171 (66,318–77,020)	242,274 (225,017–264,940)	552,485 (497,480–597,808)
Cardiomyopathy and myocarditis	332,765 (319,758–343,786)	11,840 (10,009–12,936)	3,577 (3,328–3,875)	74,118 (69,603–77,851)	91,611 (88,309–98,030)	151,617 (146,235–156,613)
Atrial fibrillation and flutter	166,224 (136,291–200,382)	0 (0–0)	0 (0–0)	922 (700–1,180)	10,678 (8,359–13,948)	154,624 (124,309–189,299)
Aortic aneurysm	146,645 (143,557–149,901)	0 (0–0)	0 (0–0)	12,625 (12,122–13,215)	40,619 (39,617–41,754)	93,400 (91,220–95,702)
Peripheral vascular disease	44,719 (42,945–46,686)	0 (0–0)	0 (0–0)	563 (535–593)	7,640 (7,332–7,957)	36,515 (34,819–38,323)
Endocarditis	76,654 (67,034–82,775)	3,804 (2,760–4,742)	1,224 (956–1,409)	15,557 (12,234–17,116)	16,967 (14,848–19,152)	39,100 (35,435–41,878)
Other cardiovascular and circulatory diseases	482,618 (468,479–499,135)	13,613 (12,433–15,334)	3,810 (3,543–4,137)	52,461 (50,177–55,178)	118,616 (113,654–123,550)	294,116 (284,613–304,625)
Chronic respiratory diseases	3,557,302 (3,472,198–3,644,981)	42,479 (37,895–47,630)	11,195 (10,198–12,314)	203,177 (194,907–213,494)	955,866 (925,251–985,825)	2,344,583 (2,286,030–2,402,659)
Chronic obstructive pulmonary disease	2,971,482 (2,889,012–3,051,544)	15,644 (11,750–19,886)	1,994 (1,642–2,407)	118,446 (110,679–125,362)	771,397 (741,599–799,882)	2,063,998 (2,007,736–2,122,056)
Pneumoconiosis	33,634 (29,964–37,823)	0 (0–0)	0 (0–0)	2,898 (2,531–3,359)	8,684 (7,474–10,079)	22,051 (19,673–24,702)
Silicosis	10,005 (9,174–11,111)	0 (0–0)	0 (0–0)	1,153 (1,016–1,331)	3,110 (2,764–3,541)	5,742 (5,307–6,293)
Asbestosis	3,231 (2,268–3,732)	0 (0–0)	0 (0–0)	401 (344–476)	726 (559–923)	2,103 (1,317–2,393)
Coal workers pneumoconiosis	2,424 (2,178–2,723)	0 (0–0)	0 (0–0)	194 (158–247)	669 (579–802)	1,560 (1,419–1,750)
Other pneumoconiosis	17,973 (14,925–21,379)	0 (0–0)	0 (0–0)	1,149 (937–1,409)	4,178 (3,328–5,259)	12,645 (10,642–15,031)
Asthma	402,092 (359,412–446,328)	11,717 (9,732–14,084)	7,459 (6,488–8,694)	67,095 (59,649–77,509)	137,840 (121,142–154,824)	177,979 (158,046–196,361)
Interstitial lung disease and pulmonary sarcoidosis	100,384 (75,855–113,959)	307 (209–447)	249 (200–322)	6,835 (5,707–8,040)	27,630 (22,595–32,108)	65,361 (46,539–73,840)
Other chronic respiratory diseases	49,708 (35,525–59,025)	14,809 (11,106–18,944)	1,492 (978–2,082)	7,901 (5,237–9,666)	10,313 (6,790–12,049)	15,191 (10,644–17,983)
Cirrhosis and other chronic liver diseases	1,226,623 (1,184,442–1,297,585)	11,149 (9,369–14,196)	9,694 (8,580–11,532)	345,301 (328,997–368,158)	546,750 (526,920–575,741)	313,727 (303,515–332,480)
Cirrhosis and other chronic liver diseases due to hepatitis B	352,558 (326,168–388,236)	86 (65–116)	303 (239–378)	100,167 (90,229–112,110)	155,392 (138,507–175,610)	96,608 (87,645–107,777)
Cirrhosis and other chronic liver diseases due to hepatitis C	306,133 (284,669–328,200)	44 (33–60)	170 (132–214)	78,801 (70,630–88,126)	141,673 (126,984–155,923)	85,443 (78,579–93,789)
Cirrhosis and other chronic liver diseases due to alcohol use	328,284 (305,851–352,007)	0 (0–0)	0 (0–0)	107,198 (98,413–117,298)	160,859 (144,573–176,967)	60,226 (54,187–66,818)
Cirrhosis and other chronic liver diseases due to other causes	239,647 (223,707–262,099)	11,017 (9,259–14,028)	9,220 (8,173–11,021)	59,134 (53,491–65,848)	88,825 (78,971–99,919)	71,449 (65,488–78,768)
Digestive diseases	1,136,359 (1,100,099–1,207,978)	24,360 (17,115–38,587)	16,866 (15,198–19,527)	230,249 (217,634–245,997)	332,747 (320,077–363,153)	532,136 (518,310–563,527)
Peptic ulcer disease	269,718 (255,758–295,807)	2,466 (1,329–4,919)	2,473 (2,130–2,987)	53,703 (49,659–59,456)	89,556 (84,500–99,525)	121,518 (115,885–133,283)
Gastritis and duodenitis	48,675 (40,813–59,193)	965 (535–1,620)	454 (330–664)	9,506 (7,888–12,098)	15,382 (13,028–19,222)	22,368 (18,558–27,441)
Appendicitis	49,814 (41,588–55,646)	3,945 (1,964–7,990)	2,895 (2,542–3,300)	16,949 (14,077–18,546)	13,066 (11,205–14,373)	12,957 (11,372–14,129)
Paralytic ileus and intestinal obstruction	245,700 (224,771–288,389)	8,471 (5,703–13,378)	6,313 (5,457–7,843)	52,484 (46,848–63,261)	68,601 (62,580–82,386)	109,830 (100,539–124,949)
Inguinal, femoral, and abdominal hernia	57,872 (39,941–66,516)	930 (434–1,567)	795 (491–1,019)	10,758 (6,191–13,564)	17,527 (11,816–20,606)	27,860 (20,499–31,091)
Inflammatory bowel disease	44,091 (40,392–48,137)	1,934 (1,070–3,880)	578 (456–751)	8,330 (7,116–9,656)	11,028 (10,069–12,035)	22,218 (20,258–23,535)
Vascular intestinal disorders	93,350 (87,277–101,326)	467 (282–835)	143 (116–180)	5,476 (4,766–6,280)	22,768 (20,835–25,371)	64,494 (60,823–69,879)
Gallbladder and biliary diseases	102,418 (97,703–109,758)	1,410 (772–2,587)	1,047 (915–1,227)	13,834 (12,698–15,277)	26,918 (25,205–29,387)	59,206 (56,786–63,478)
Pancreatitis	121,029 (113,375–129,831)	237 (158–368)	543 (467–656)	44,619 (40,764–49,081)	40,471 (37,786–43,542)	35,156 (33,224–37,521)
Other digestive diseases	103,687 (93,376–120,523)	3,529 (2,334–5,651)	1,621 (1,365–2,149)	14,586 (12,267–18,355)	27,426 (23,287–32,590)	56,524 (52,793–63,704)
Neurological disorders	1,941,909 (1,670,977–2,206,519)	18,518 (15,107–22,462)	12,663 (11,547–13,784)	87,176 (83,731–92,913)	163,720 (140,927–186,444)	1,659,831 (1,401,166–1,920,455)
Alzheimer disease and other dementias	1,624,270 (1,353,001–1,891,694)	0 (0–0)	0 (0–0)	1,390 (873–2,036)	95,285 (72,071–118,511)	1,527,594 (1,268,080–1,790,906)
Parkinson disease	98,943 (96,028–102,255)	0 (0–0)	0 (0–0)	685 (651–750)	11,415 (11,002–12,138)	86,841 (84,111–89,678)
Epilepsy	120,712 (114,887–127,717)	13,684 (10,857–16,535)	9,940 (8,951–10,873)	62,128 (58,980–66,069)	20,491 (19,784–21,365)	14,467 (14,009–14,917)

SR Table 12 - Global deaths by age and cause for both sexes in 2010

Cause	All ages	0-5 years	5-14 years	15-49 years	50-69 years	70+ years
Multiple sclerosis	17,388<br>(15,997–18,584)	0<br>(0–0)	0<br>(0–0)	5,213<br>(4,871–6,167)	8,344<br>(7,380–8,788)	3,829<br>(3,092–4,037)
Motor neuron disease	31,392<br>(30,560–32,556)	1,002<br>(790–1,179)	114<br>(105–123)	3,727<br>(3,558–3,975)	13,974<br>(13,547–14,532)	12,572<br>(12,222–12,964)
Migraine	0<br>(0–0)	0<br>(0–0)	0<br>(0–0)	0<br>(0–0)	0<br>(0–0)	0<br>(0–0)
Tension-type headache	0<br>(0–0)	0<br>(0–0)	0<br>(0–0)	0<br>(0–0)	0<br>(0–0)	0<br>(0–0)
Medication overuse headache	0<br>(0–0)	0<br>(0–0)	0<br>(0–0)	0<br>(0–0)	0<br>(0–0)	0<br>(0–0)
Other neurological disorders	49,223<br>(48,046–52,404)	3,852<br>(3,204–5,225)	2,607<br>(2,320–3,234)	14,031<br>(13,441–15,220)	14,207<br>(13,799–14,700)	14,524<br>(14,067–15,029)
Mental and substance use disorders	302,796<br>(291,862–311,234)	30<br>(26–34)	36<br>(19–47)	164,963<br>(159,518–172,735)	100,550<br>(95,066–103,494)	37,215<br>(32,049–38,646)
Schizophrenia	16,332<br>(15,774–16,914)	0<br>(0–0)	0<br>(0–0)	5,753<br>(5,483–6,056)	5,888<br>(5,671–6,112)	4,690<br>(4,479–4,882)
Alcohol use disorders	139,222<br>(134,725–143,851)	0<br>(0–0)	0<br>(0–0)	64,397<br>(61,812–67,230)	60,066<br>(57,155–62,342)	14,759<br>(13,752–15,247)
Drug use disorders	146,598<br>(137,976–152,222)	30<br>(26–34)	0<br>(0–0)	94,206<br>(89,522–100,966)	34,595<br>(31,089–36,110)	17,766<br>(13,168–19,051)
Opioid use disorders	106,693<br>(99,958–111,540)	30<br>(26–34)	0<br>(0–0)	68,909<br>(64,731–74,380)	24,496<br>(21,802–25,747)	13,256<br>(9,572–14,245)
Cocaine use disorders	9,069<br>(6,710–9,684)	0<br>(0–0)	0<br>(0–0)	6,175<br>(4,531–6,643)	2,550<br>(1,787–2,757)	343<br>(291–401)
Amphetamine use disorders	9,330<br>(6,058–10,280)	0<br>(0–0)	0<br>(0–0)	6,465<br>(4,142–7,208)	2,342<br>(1,518–2,692)	522<br>(345–618)
Cannabis use disorders	0<br>(0–0)	0<br>(0–0)	0<br>(0–0)	0<br>(0–0)	0<br>(0–0)	0<br>(0–0)
Other drug use disorders	21,504<br>(20,053–24,526)	0<br>(0–0)	0<br>(0–0)	12,656<br>(11,672–14,905)	5,205<br>(4,855–5,848)	3,642<br>(2,890–4,407)
Depressive disorders	0<br>(0–0)	0<br>(0–0)	0<br>(0–0)	0<br>(0–0)	0<br>(0–0)	0<br>(0–0)
Major depressive disorder	0<br>(0–0)	0<br>(0–0)	0<br>(0–0)	0<br>(0–0)	0<br>(0–0)	0<br>(0–0)
Dysthymia	0<br>(0–0)	0<br>(0–0)	0<br>(0–0)	0<br>(0–0)	0<br>(0–0)	0<br>(0–0)
Bipolar disorder	0<br>(0–0)	0<br>(0–0)	0<br>(0–0)	0<br>(0–0)	0<br>(0–0)	0<br>(0–0)
Anxiety disorders	0<br>(0–0)	0<br>(0–0)	0<br>(0–0)	0<br>(0–0)	0<br>(0–0)	0<br>(0–0)
Eating disorders	643<br>(426–853)	0<br>(0–0)	36<br>(19–47)	606<br>(405–817)	0<br>(0–0)	0<br>(0–0)
Anorexia nervosa	587<br>(387–778)	0<br>(0–0)	34<br>(18–44)	552<br>(368–746)	0<br>(0–0)	0<br>(0–0)
Bulimia nervosa	56<br>(32–78)	0<br>(0–0)	1<br>(0–2)	54<br>(31–76)	0<br>(0–0)	0<br>(0–0)
Autistic spectrum disorders	0<br>(0–0)	0<br>(0–0)	0<br>(0–0)	0<br>(0–0)	0<br>(0–0)	0<br>(0–0)
Autism	0<br>(0–0)	0<br>(0–0)	0<br>(0–0)	0<br>(0–0)	0<br>(0–0)	0<br>(0–0)
Asperger syndrome and other autistic spectrum disorders	0<br>(0–0)	0<br>(0–0)	0<br>(0–0)	0<br>(0–0)	0<br>(0–0)	0<br>(0–0)
Attention-deficit/hyperactivity disorder	0<br>(0–0)	0<br>(0–0)	0<br>(0–0)	0<br>(0–0)	0<br>(0–0)	0<br>(0–0)
Conduct disorder	0<br>(0–0)	0<br>(0–0)	0<br>(0–0)	0<br>(0–0)	0<br>(0–0)	0<br>(0–0)
Idiopathic developmental intellectual disability	0<br>(0–0)	0<br>(0–0)	0<br>(0–0)	0<br>(0–0)	0<br>(0–0)	0<br>(0–0)
Other mental and substance use disorders	0<br>(0–0)	0<br>(0–0)	0<br>(0–0)	0<br>(0–0)	0<br>(0–0)	0<br>(0–0)
Diabetes, urogenital, blood, and endocrine diseases	2,985,902<br>(2,871,244–3,077,166)	120,685<br>(90,940–173,776)	33,118<br>(29,711–36,923)	394,888<br>(372,514–413,159)	946,119<br>(912,332–973,754)	1,491,090<br>(1,451,232–1,526,730)
Diabetes mellitus	1,290,002<br>(1,254,218–1,326,999)	2,031<br>(1,691–2,230)	2,569<br>(2,032–2,831)	122,301<br>(117,534–128,213)	485,106<br>(469,690–501,137)	677,992<br>(658,638–697,892)
Acute glomerulonephritis	11,754<br>(8,537–12,874)	280<br>(224–340)	326<br>(257–366)	2,923<br>(2,024–3,185)	3,763<br>(2,759–4,162)	4,461<br>(3,195–4,962)
Chronic kidney disease	1,095,685<br>(1,012,651–1,134,133)	13,156<br>(11,790–14,452)	9,192<br>(8,183–10,020)	164,541<br>(142,956–173,768)	335,955<br>(303,904–350,271)	572,839<br>(540,242–593,384)
Chronic kidney disease due to diabetes mellitus	365,187<br>(341,038–384,172)	1,832<br>(1,541–2,159)	870<br>(725–1,062)	39,926<br>(34,959–44,512)	138,215<br>(126,913–148,936)	184,342<br>(173,713–194,121)
Chronic kidney disease due to hypertension	477,585<br>(439,291–497,906)	4,601<br>(4,008–5,247)	2,808<br>(2,356–3,300)	64,914<br>(56,169–70,441)	130,423<br>(116,819–140,604)	274,838<br>(257,902–288,401)
Chronic kidney disease due to glomerulonephritis	225,580<br>(202,330–240,741)	4,940<br>(4,209–5,659)	4,075<br>(3,519–4,613)	52,729<br>(45,654–57,757)	63,362<br>(54,960–69,644)	100,472<br>(91,064–108,592)
Chronic kidney disease due to other causes	27,331<br>(22,717–32,220)	1,783<br>(1,430–2,169)	1,437<br>(1,143–1,758)	6,971<br>(4,717–9,620)	3,953<br>(2,459–5,958)	13,186<br>(11,127–16,010)
Urinary diseases and male infertility	229,452<br>(213,989–244,792)	6,933<br>(5,471–8,517)	1,931<br>(1,717–2,262)	29,773<br>(26,810–33,090)	54,710<br>(49,523–59,803)	136,103<br>(126,984–144,418)
Interstitial nephritis and urinary tract infections	170,927<br>(158,247–182,785)	5,487<br>(4,328–6,869)	1,221<br>(1,069–1,432)	19,687<br>(17,601–22,284)	35,828<br>(32,473–39,574)	108,702<br>(99,850–115,336)
Urolithiasis	14,710<br>(13,083–19,144)	0<br>(0–0)	219<br>(185–296)	2,515<br>(2,156–3,476)	4,837<br>(4,108–6,265)	7,138<br>(6,399–9,033)
Benign prostatic hyperplasia	0<br>(0–0)	0<br>(0–0)	0<br>(0–0)	0<br>(0–0)	0<br>(0–0)	0<br>(0–0)
Male infertility	0<br>(0–0)	0<br>(0–0)	0<br>(0–0)	0<br>(0–0)	0<br>(0–0)	0<br>(0–0)
Other urinary diseases	43,813<br>(36,489–49,318)	1,446<br>(951–2,092)	490<br>(415–599)	7,569<br>(6,012–8,835)	14,044<br>(11,172–16,189)	20,262<br>(17,149–23,791)
Gynecological diseases	7,701<br>(5,727–8,736)	0<br>(0–0)	0<br>(0–0)	3,236<br>(2,439–3,722)	2,343<br>(1,659–2,701)	2,121<br>(1,609–2,469)
Uterine fibroids	2,255<br>(1,214–2,884)	0<br>(0–0)	0<br>(0–0)	763<br>(427–993)	943<br>(507–1,200)	548<br>(263–757)
Polycystic ovarian syndrome	650<br>(193–1,083)	0<br>(0–0)	0<br>(0–0)	448<br>(113–768)	201<br>(76–324)	0<br>(0–0)
Female infertility	0<br>(0–0)	0<br>(0–0)	0<br>(0–0)	0<br>(0–0)	0<br>(0–0)	0<br>(0–0)
Endometriosis	52<br>(41–77)	0<br>(0–0)	0<br>(0–0)	43<br>(34–65)	8<br>(6–13)	0<br>(0–0)
Genital prolapse	964<br>(549–1,594)	0<br>(0–0)	0<br>(0–0)	57<br>(27–104)	330<br>(158–607)	576<br>(343–897)
Premenstrual syndrome	0<br>(0–0)	0<br>(0–0)	0<br>(0–0)	0<br>(0–0)	0<br>(0–0)	0<br>(0–0)
Other gynecological diseases	3,778<br>(2,800–4,569)	0<br>(0–0)	0<br>(0–0)	1,922<br>(1,459–2,358)	858<br>(598–1,082)	996<br>(720–1,263)
Hemoglobinopathies and hemolytic anemias	222,449<br>(176,494–288,524)	85,813<br>(56,477–138,996)	15,606<br>(12,892–19,031)	47,406<br>(40,945–54,283)	29,194<br>(25,449–32,695)	44,428<br>(38,928–49,538)
Thalassemias	18,074<br>(14,881–21,534)	8,896<br>(6,939–11,967)	3,096<br>(2,739–3,450)	1,997<br>(1,639–2,499)	109<br>(101–119)	3,974<br>(3,133–5,030)
Thalassemias trait	0<br>(0–0)	0<br>(0–0)	0<br>(0–0)	0<br>(0–0)	0<br>(0–0)	0<br>(0–0)
Sickle cell disorders	111,818<br>(78,825–168,948)	75,583<br>(47,632–128,679)	10,789<br>(8,430–14,002)	19,204<br>(16,527–22,420)	1,265<br>(1,145–1,419)	4,975<br>(4,156–6,012)
Sickle cell trait	0<br>(0–0)	0<br>(0–0)	0<br>(0–0)	0<br>(0–0)	0<br>(0–0)	0<br>(0–0)

SR Table 12 - Global deaths by age and cause for both sexes in 2010						
**Cause**	**All ages**	**0-5 years**	**5-14 years**	**15-49 years**	**50-69 years**	**70+ years**
G6PD deficiency	29,786<br>(25,216–34,901)	1,028<br>(704–1,491)	1,323<br>(1,080–1,669)	16,299<br>(13,710–19,113)	9,014<br>(7,484–10,697)	2,121<br>(1,729–2,596)
G6PD trait	0<br>(0–0)	0<br>(0–0)	0<br>(0–0)	0<br>(0–0)	0<br>(0–0)	0<br>(0–0)
Other hemoglobinopathies and hemolytic anemias	62,770<br>(55,174–69,730)	305<br>(241–412)	396<br>(349–458)	9,906<br>(8,489–11,398)	18,804<br>(16,369–21,069)	33,356<br>(29,245–36,737)
Endocrine, metabolic, blood, and immune disorders	128,857<br>(126,207–131,860)	12,469<br>(11,653–13,364)	3,492<br>(3,283–3,714)	24,704<br>(23,868–25,686)	35,046<br>(34,193–36,024)	53,144<br>(51,624–54,706)
Musculoskeletal disorders	80,483<br>(74,744–84,464)	0<br>(0–0)	1,880<br>(1,670–2,105)	18,559<br>(17,527–20,518)	20,468<br>(19,568–22,502)	39,574<br>(35,767–41,244)
Rheumatoid arthritis	27,282<br>(24,593–30,714)	0<br>(0–0)	230<br>(168–293)	2,174<br>(1,777–2,595)	8,468<br>(7,490–9,622)	16,408<br>(14,731–18,477)
Osteoarthritis	0<br>(0–0)	0<br>(0–0)	0<br>(0–0)	0<br>(0–0)	0<br>(0–0)	0<br>(0–0)
Low back and neck pain	0<br>(0–0)	0<br>(0–0)	0<br>(0–0)	0<br>(0–0)	0<br>(0–0)	0<br>(0–0)
Low back pain	0<br>(0–0)	0<br>(0–0)	0<br>(0–0)	0<br>(0–0)	0<br>(0–0)	0<br>(0–0)
Neck pain	0<br>(0–0)	0<br>(0–0)	0<br>(0–0)	0<br>(0–0)	0<br>(0–0)	0<br>(0–0)
Gout	0<br>(0–0)	0<br>(0–0)	0<br>(0–0)	0<br>(0–0)	0<br>(0–0)	0<br>(0–0)
Other musculoskeletal disorders	53,200<br>(48,957–56,079)	0<br>(0–0)	1,649<br>(1,461–1,859)	16,385<br>(15,231–18,165)	11,999<br>(11,347–13,259)	23,166<br>(19,298–24,459)
Other non-communicable diseases	733,703<br>(653,532–831,175)	531,263<br>(470,633–622,577)	38,246<br>(33,516–42,623)	70,415<br>(61,145–76,788)	34,529<br>(27,517–40,502)	59,248<br>(44,208–69,175)
Congenital anomalies	631,194<br>(557,615–723,794)	505,425<br>(442,181–596,216)	37,133<br>(32,402–41,496)	59,967<br>(53,141–65,636)	16,354<br>(14,170–17,340)	12,311<br>(10,634–13,168)
Neural tube defects	68,684<br>(50,957–91,533)	64,961<br>(47,781–87,609)	1,888<br>(1,288–2,637)	1,365<br>(1,080–1,706)	317<br>(261–403)	152<br>(124–202)
Congenital heart anomalies	314,384<br>(273,281–358,211)	238,385<br>(203,902–280,207)	25,732<br>(21,892–28,789)	41,711<br>(36,482–45,504)	4,873<br>(4,244–5,296)	3,680<br>(3,258–4,112)
Cleft lip and cleft palate	1,805<br>(1,490–2,176)	1,805<br>(1,490–2,176)	0<br>(0–0)	0<br>(0–0)	0<br>(0–0)	0<br>(0–0)
Down syndrome	26,003<br>(18,550–38,942)	17,355<br>(10,810–29,277)	918<br>(707–1,228)	4,331<br>(3,659–5,176)	3,210<br>(2,620–3,551)	187<br>(151–218)
Turner syndrome	0<br>(0–0)	0<br>(0–0)	0<br>(0–0)	0<br>(0–0)	0<br>(0–0)	0<br>(0–0)
Klinefelter syndrome	0<br>(0–0)	0<br>(0–0)	0<br>(0–0)	0<br>(0–0)	0<br>(0–0)	0<br>(0–0)
Other chromosomal abnormalities	21,455<br>(11,121–45,105)	19,847<br>(9,609–43,112)	390<br>(245–688)	808<br>(578–995)	299<br>(187–364)	108<br>(83–131)
Other congenital anomalies	198,861<br>(173,288–237,801)	163,070<br>(139,533–200,445)	8,203<br>(7,008–9,959)	11,750<br>(10,295–13,153)	7,653<br>(6,503–8,348)	8,183<br>(6,744–8,795)
Skin and subcutaneous diseases	82,545<br>(56,204–107,340)	5,874<br>(3,851–9,241)	1,112<br>(717–1,622)	10,447<br>(6,581–14,708)	18,174<br>(12,095–24,825)	46,936<br>(32,494–57,423)
Dermatitis	0<br>(0–0)	0<br>(0–0)	0<br>(0–0)	0<br>(0–0)	0<br>(0–0)	0<br>(0–0)
Psoriasis	0<br>(0–0)	0<br>(0–0)	0<br>(0–0)	0<br>(0–0)	0<br>(0–0)	0<br>(0–0)
Cellulitis	14,782<br>(9,130–20,086)	655<br>(351–1,136)	198<br>(114–284)	2,459<br>(1,299–3,663)	4,557<br>(2,623–6,395)	6,911<br>(4,481–9,084)
Pyoderma	36,584<br>(25,959–51,890)	4,854<br>(3,183–7,766)	601<br>(398–951)	5,801<br>(3,680–8,554)	8,907<br>(6,196–13,101)	16,418<br>(12,027–22,146)
Scabies	0<br>(0–0)	0<br>(0–0)	0<br>(0–0)	0<br>(0–0)	0<br>(0–0)	0<br>(0–0)
Fungal skin diseases	0<br>(0–0)	0<br>(0–0)	0<br>(0–0)	0<br>(0–0)	0<br>(0–0)	0<br>(0–0)
Viral skin diseases	0<br>(0–0)	0<br>(0–0)	0<br>(0–0)	0<br>(0–0)	0<br>(0–0)	0<br>(0–0)
Acne vulgaris	0<br>(0–0)	0<br>(0–0)	0<br>(0–0)	0<br>(0–0)	0<br>(0–0)	0<br>(0–0)
Alopecia areata	0<br>(0–0)	0<br>(0–0)	0<br>(0–0)	0<br>(0–0)	0<br>(0–0)	0<br>(0–0)
Pruritus	0<br>(0–0)	0<br>(0–0)	0<br>(0–0)	0<br>(0–0)	0<br>(0–0)	0<br>(0–0)
Urticaria	0<br>(0–0)	0<br>(0–0)	0<br>(0–0)	0<br>(0–0)	0<br>(0–0)	0<br>(0–0)
Decubitus ulcer	27,632<br>(16,163–34,142)	109<br>(55–185)	160<br>(91–230)	1,486<br>(780–2,009)	4,001<br>(2,173–5,401)	21,874<br>(12,823–26,269)
Other skin and subcutaneous diseases	3,546<br>(2,524–4,839)	254<br>(160–413)	151<br>(88–219)	699<br>(444–1,024)	707<br>(481–997)	1,732<br>(1,260–2,306)
Sense organ diseases	0<br>(0–0)	0<br>(0–0)	0<br>(0–0)	0<br>(0–0)	0<br>(0–0)	0<br>(0–0)
Glaucoma	0<br>(0–0)	0<br>(0–0)	0<br>(0–0)	0<br>(0–0)	0<br>(0–0)	0<br>(0–0)
Cataract	0<br>(0–0)	0<br>(0–0)	0<br>(0–0)	0<br>(0–0)	0<br>(0–0)	0<br>(0–0)
Macular degeneration	0<br>(0–0)	0<br>(0–0)	0<br>(0–0)	0<br>(0–0)	0<br>(0–0)	0<br>(0–0)
Refraction and accommodation disorders	0<br>(0–0)	0<br>(0–0)	0<br>(0–0)	0<br>(0–0)	0<br>(0–0)	0<br>(0–0)
Age-related and other hearing loss	0<br>(0–0)	0<br>(0–0)	0<br>(0–0)	0<br>(0–0)	0<br>(0–0)	0<br>(0–0)
Other vision loss	0<br>(0–0)	0<br>(0–0)	0<br>(0–0)	0<br>(0–0)	0<br>(0–0)	0<br>(0–0)
Other sense organ diseases	0<br>(0–0)	0<br>(0–0)	0<br>(0–0)	0<br>(0–0)	0<br>(0–0)	0<br>(0–0)
Oral disorders	0<br>(0–0)	0<br>(0–0)	0<br>(0–0)	0<br>(0–0)	0<br>(0–0)	0<br>(0–0)
Deciduous caries	0<br>(0–0)	0<br>(0–0)	0<br>(0–0)	0<br>(0–0)	0<br>(0–0)	0<br>(0–0)
Permanent caries	0<br>(0–0)	0<br>(0–0)	0<br>(0–0)	0<br>(0–0)	0<br>(0–0)	0<br>(0–0)
Periodontal diseases	0<br>(0–0)	0<br>(0–0)	0<br>(0–0)	0<br>(0–0)	0<br>(0–0)	0<br>(0–0)
Edentulism and severe tooth loss	0<br>(0–0)	0<br>(0–0)	0<br>(0–0)	0<br>(0–0)	0<br>(0–0)	0<br>(0–0)
Other oral disorders	0<br>(0–0)	0<br>(0–0)	0<br>(0–0)	0<br>(0–0)	0<br>(0–0)	0<br>(0–0)
Sudden infant death syndrome	19,963<br>(16,329–29,518)	19,963<br>(16,329–29,518)	0<br>(0–0)	0<br>(0–0)	0<br>(0–0)	0<br>(0–0)
Injuries	4,814,843<br>(4,468,644–5,058,093)	351,371<br>(296,563–397,784)	283,143<br>(244,617–323,075)	2,434,121<br>(2,289,398–2,567,713)	971,297<br>(898,148–1,013,673)	774,910<br>(706,659–802,104)
Transport injuries	1,468,559<br>(1,422,857–1,521,435)	59,073<br>(52,174–67,127)	74,666<br>(70,158–78,940)	840,127<br>(810,054–875,476)	322,798<br>(311,294–333,289)	171,892<br>(166,000–177,480)
Road injuries	1,367,991<br>(1,315,202–1,418,788)	54,548<br>(48,231–61,779)	70,744<br>(66,452–74,788)	783,925<br>(748,717–818,258)	297,803<br>(286,553–307,900)	160,969<br>(155,468–166,360)
Pedestrian road injuries	567,094<br>(533,645–613,517)	25,172<br>(22,189–28,450)	33,999<br>(31,829–36,586)	283,411<br>(260,461–307,621)	139,336<br>(131,499–152,447)	85,174<br>(81,598–92,982)
Cyclist road injuries	59,334<br>(55,850–63,683)	1,327<br>(1,056–1,625)	3,757<br>(3,424–4,082)	29,008<br>(26,558–31,227)	17,839<br>(16,768–19,320)	7,401<br>(6,906–8,206)
Motorcyclist road injuries	260,049<br>(231,473–288,449)	6,737<br>(5,452–8,024)	8,683<br>(7,409–10,066)	176,523<br>(159,478–199,875)	48,061<br>(40,709–53,125)	20,042<br>(16,368–22,881)

SR Table 12 - Global deaths by age and cause for both sexes in 2010						
**Cause**	**All ages**	**0-5 years**	**5-14 years**	**15-49 years**	**50-69 years**	**70+ years**
Motor vehicle road injuries	461,419 (417,167–506,248)	20,073 (17,059–23,521)	22,759 (20,541–25,315)	283,962 (260,110–316,136)	88,652 (77,148–94,935)	45,971 (40,556–48,764)
Other road injuries	20,092 (13,457–23,434)	1,236 (692–2,228)	1,544 (941–2,000)	11,019 (6,722–13,246)	3,912 (2,871–4,687)	2,378 (1,979–3,056)
Other transport injuries	100,568 (90,247–118,696)	4,525 (3,533–5,684)	3,921 (3,517–4,350)	56,201 (49,856–66,734)	24,995 (21,951–30,603)	10,923 (9,779–13,185)
Unintentional injuries	1,837,352 (1,633,384–1,914,359)	255,676 (206,483–293,511)	140,830 (127,904–150,466)	649,225 (587,896–679,455)	340,759 (302,359–356,863)	450,860 (396,032–468,586)
Falls	481,691 (429,858–500,398)	23,417 (16,618–28,880)	15,823 (13,966–17,325)	108,806 (98,383–114,936)	101,178 (90,997–105,213)	232,466 (202,653–242,481)
Drowning	355,986 (310,987–374,685)	71,549 (54,965–83,076)	60,615 (54,629–64,755)	138,313 (126,581–144,300)	49,427 (43,449–52,022)	36,079 (30,084–38,080)
Fire, heat, and hot substances	176,422 (145,318–189,563)	25,108 (20,048–28,736)	9,813 (8,176–11,169)	75,632 (60,254–83,015)	30,986 (25,971–33,114)	34,881 (30,195–37,265)
Poisonings	90,595 (63,082–105,143)	19,528 (7,354–32,508)	5,268 (4,175–5,909)	36,015 (28,004–40,258)	17,817 (13,303–19,150)	11,965 (9,493–12,705)
Exposure to mechanical forces	203,151 (163,449–216,369)	31,855 (24,375–37,209)	12,488 (11,325–13,844)	91,510 (71,646–97,949)	37,144 (29,894–39,632)	30,152 (22,558–32,430)
Unintentional firearm injuries	31,972 (23,278–34,893)	2,404 (1,483–3,287)	1,853 (1,437–2,260)	17,087 (11,950–19,011)	4,518 (3,289–4,971)	6,108 (5,016–6,528)
Unintentional suffocation	35,958 (26,290–41,004)	18,347 (11,675–22,697)	3,124 (2,624–4,102)	6,366 (5,159–6,895)	2,520 (1,630–2,866)	5,599 (4,041–6,226)
Other exposure to mechanical forces	135,221 (105,307–144,760)	11,103 (9,201–13,120)	7,511 (6,459–8,436)	68,056 (50,486–73,729)	30,106 (24,043–32,258)	18,443 (12,827–20,159)
Adverse effects of medical treatment	96,791 (74,774–105,871)	16,145 (9,346–20,882)	4,400 (3,087–5,178)	27,021 (19,425–30,608)	19,890 (15,738–22,491)	29,334 (24,494–32,476)
Animal contact	98,827 (60,109–121,393)	11,496 (6,819–13,805)	11,972 (7,411–15,097)	41,074 (24,327–52,542)	22,548 (13,565–27,479)	11,735 (7,582–13,628)
Venomous animal contact	84,120 (48,331–106,744)	8,199 (4,488–10,724)	10,007 (5,871–13,237)	36,761 (20,606–47,636)	19,682 (11,183–24,172)	9,470 (5,655–11,117)
Non-venomous animal contact	14,707 (11,860–16,530)	3,297 (2,137–4,158)	1,965 (1,504–2,370)	4,312 (3,545–4,979)	2,866 (2,289–3,365)	2,265 (1,944–2,603)
Foreign body	147,581 (124,218–172,019)	43,116 (28,168–61,093)	8,962 (6,928–11,145)	33,993 (28,619–39,618)	23,738 (20,022–26,213)	37,770 (32,086–40,537)
Pulmonary aspiration and foreign body in airway	120,115 (98,657–145,698)	37,619 (23,185–55,753)	6,562 (4,885–8,783)	23,379 (20,027–26,578)	19,814 (16,527–21,565)	32,739 (27,449–34,720)
Foreign body in eyes	0 (0–0)	0 (0–0)	0 (0–0)	0 (0–0)	0 (0–0)	0 (0–0)
Foreign body in other body part	27,466 (18,769–36,531)	5,497 (2,674–8,371)	2,399 (1,449–3,277)	10,614 (7,108–15,097)	3,923 (2,961–5,462)	5,031 (4,161–6,495)
Environmental heat and cold exposure	47,531 (35,464–51,277)	2,846 (1,630–3,583)	1,177 (779–1,406)	15,885 (12,313–17,441)	16,092 (11,454–17,887)	11,529 (8,248–12,877)
Other unintentional injuries	138,772 (130,084–150,363)	10,611 (8,921–12,631)	10,307 (9,092–11,673)	80,972 (74,611–89,763)	21,934 (20,613–23,405)	14,946 (12,847–18,313)
Self-harm and interpersonal violence	1,217,601 (1,106,598–1,255,671)	11,932 (10,723–13,531)	20,749 (18,838–22,060)	783,175 (710,086–810,069)	267,541 (243,128–276,441)	134,202 (120,710–139,086)
Self-harm	808,222 (720,727–838,187)	0 (0–0)	8,622 (7,290–9,385)	476,316 (424,665–495,648)	210,307 (187,317–218,737)	112,975 (99,142–117,483)
Interpersonal violence	409,379 (371,383–425,403)	11,932 (10,723–13,531)	12,127 (11,247–12,961)	306,858 (277,292–319,504)	57,234 (53,828–60,170)	21,226 (20,100–22,131)
Assault by firearm	172,299 (148,412–180,082)	1,982 (1,628–2,286)	3,559 (2,894–3,815)	143,741 (123,789–149,725)	18,281 (16,591–19,733)	4,733 (4,164–5,298)
Assault by sharp object	90,632 (85,429–97,024)	1,148 (975–1,422)	1,740 (1,592–1,945)	69,725 (64,940–75,023)	13,209 (12,512–14,297)	4,808 (4,563–5,322)
Assault by other means	146,447 (131,988–156,508)	8,801 (7,902–10,025)	6,826 (6,281–7,480)	93,391 (82,227–101,392)	25,743 (23,208–27,560)	11,684 (10,730–12,371)
Forces of nature, war, and legal intervention	291,330 (90,881–508,738)	24,688 (7,607–43,097)	46,897 (11,464–85,810)	161,592 (55,756–275,113)	40,198 (12,262–70,748)	17,953 (4,850–32,184)
Exposure to forces of nature	250,655 (52,997–469,802)	21,237 (4,354–39,905)	44,300 (9,005–83,291)	132,639 (27,999–247,892)	35,804 (8,177–66,155)	16,672 (3,750–31,053)
Collective violence and legal intervention	40,675 (32,174–49,339)	3,450 (2,615–4,353)	2,597 (2,037–3,214)	28,952 (22,833–35,019)	4,393 (3,322–5,546)	1,281 (898–2,754)

SR Table 12 - Global deaths by age and cause for both sexes in 2015						
**Cause**	**All ages**	**0-5 years**	**5-14 years**	**15-49 years**	**50-69 years**	**70+ years**
All causes	55,792,884 (54,984,081–56,640,301)	5,820,849 (5,672,972–5,965,164)	854,212 (837,379–873,323)	8,676,208 (8,415,004–8,926,243)	14,745,053 (14,435,282–15,069,368)	25,696,560 (25,438,332–25,972,635)
Communicable, maternal, neonatal, and nutritional diseases	11,263,632 (10,922,654–11,594,531)	4,703,359 (4,569,849–4,845,505)	439,502 (417,125–467,992)	2,385,160 (2,257,411–2,525,949)	1,525,125 (1,405,874–1,612,494)	2,210,484 (2,066,678–2,290,295)
HIV/AIDS and tuberculosis	2,305,173 (2,092,671–2,577,958)	111,183 (104,195–122,743)	98,575 (94,400–105,507)	1,233,545 (1,145,491–1,326,666)	569,753 (489,826–672,505)	292,115 (240,180–359,003)
Tuberculosis	1,112,606 (909,768–1,392,788)	22,300 (17,112–32,519)	13,331 (11,005–18,959)	386,471 (312,210–471,755)	413,485 (332,963–519,493)	277,016 (225,867–344,499)
HIV/AIDS	1,192,566 (1,130,790–1,270,291)	88,882 (84,253–93,731)	85,243 (81,659–88,570)	847,073 (791,097–910,622)	156,267 (145,438–173,143)	15,098 (13,807–16,920)
HIV/AIDS - Tuberculosis	211,736 (161,856–245,041)	18,231 (13,655–20,870)	15,816 (11,575–18,932)	143,068 (108,200–167,272)	31,456 (25,541–35,753)	3,163 (2,609–3,615)
HIV/AIDS resulting in other diseases	980,829 (914,654–1,063,620)	70,650 (65,683–76,845)	69,427 (64,508–74,621)	704,005 (646,360–768,653)	124,811 (114,605–140,876)	11,934 (10,760–13,534)
Diarrhea, lower respiratory, and other common infectious diseases	4,959,833 (4,711,609–5,179,403)	1,608,549 (1,509,976–1,720,569)	253,304 (227,899–282,758)	616,943 (563,033–678,396)	766,765 (698,364–820,649)	1,714,270 (1,586,436–1,790,059)
Diarrheal diseases	1,312,128 (1,233,574–1,391,253)	498,888 (447,449–557,643)	51,637 (45,815–57,482)	171,551 (156,805–188,342)	223,721 (205,093–243,373)	366,329 (338,251–393,937)
Intestinal infectious diseases	178,547 (100,881–293,731)	42,159 (22,517–73,174)	66,745 (38,395–108,245)	56,323 (29,640–96,734)	9,261 (4,722–16,403)	4,057 (2,271–6,429)
Typhoid fever	148,754 (81,867–249,677)	35,522 (18,369–63,375)	56,234 (31,607–91,318)	46,354 (24,206–80,978)	7,425 (3,577–13,097)	3,218 (1,709–5,279)
Paratyphoid fever	29,150 (13,742–56,335)	6,321 (2,633–13,053)	10,443 (4,805–20,100)	9,878 (4,262–19,446)	1,784 (727–3,591)	723 (305–1,387)
Other intestinal infectious diseases	642 (281–1,216)	315 (103–649)	67 (20–148)	91 (37–207)	51 (22–118)	116 (47–243)
Lower respiratory infections	2,736,714 (2,500,318–2,860,842)	703,917 (651,385–763,038)	66,922 (51,459–78,163)	252,262 (213,100–277,125)	443,262 (380,088–478,991)	1,270,348 (1,154,367–1,337,429)
Upper respiratory infections	3,080 (2,766–3,464)	731 (533–1,010)	46 (31–68)	632 (541–737)	770 (655–915)	899 (762–1,050)
Otitis media	3,194 (2,888–3,665)	1,016 (888–1,188)	356 (319–396)	774 (721–838)	440 (400–490)	606 (425–1,025)
Meningitis	379,220 (322,683–444,691)	173,058 (137,061–228,928)	34,682 (28,705–41,414)	81,666 (67,855–97,385)	51,347 (43,267–61,974)	38,464 (32,920–45,609)
Pneumococcal meningitis	112,885 (93,385–141,847)	46,057 (31,195–69,576)	12,836 (8,948–17,420)	28,290 (22,051–35,800)	14,770 (11,023–19,240)	10,930 (8,539–13,705)
H influenzae type B meningitis	71,453 (56,668–91,785)	44,095 (31,052–64,498)	6,581 (4,238–9,541)	9,465 (7,032–12,629)	6,162 (4,411–8,194)	5,147 (3,942–6,839)
Meningococcal meningitis	73,341 (57,993–93,221)	37,975 (26,310–56,404)	7,245 (4,596–10,643)	14,186 (10,790–18,751)	8,419 (6,171–10,992)	5,514 (4,180–7,113)
Other meningitis	121,540 (101,806–144,151)	44,930 (32,767–63,854)	8,018 (5,566–11,189)	29,723 (23,618–36,127)	21,996 (17,497–27,216)	16,871 (13,885–20,099)
Encephalitis	149,516 (137,602–167,003)	42,895 (37,029–48,640)	16,323 (13,590–19,024)	36,808 (34,011–41,182)	26,779 (24,617–30,976)	26,710 (24,656–30,241)
Diphtheria	2,149 (1,109–4,712)	1,560 (573–4,028)	324 (145–739)	194 (139–276)	71 (45–112)	0 (0–0)
Whooping cough	58,717 (20,259–126,615)	54,492 (18,827–117,022)	3,461 (1,176–7,615)	667 (224–1,560)	95 (31–230)	0 (0–0)
Tetanus	56,742 (48,199–80,041)	25,497 (21,784–30,943)	3,093 (2,240–5,174)	13,528 (10,578–23,533)	10,028 (7,726–18,608)	4,595 (3,608–7,961)
Measles	73,445 (26,147–161,441)	62,588 (22,372–135,835)	9,202 (3,181–21,429)	1,598 (512–4,196)	55 (18–152)	0 (0–0)
Varicella and herpes zoster	6,375 (5,381–7,815)	1,743 (1,202–2,577)	508 (331–822)	934 (750–1,197)	929 (731–1,238)	2,258 (1,664–3,084)
Neglected tropical diseases and malaria	843,099 (669,948–1,019,691)	495,417 (353,474–641,393)	55,558 (38,720–83,559)	164,691 (126,576–217,730)	86,611 (67,096–113,713)	40,820 (32,173–53,471)
Malaria	730,490 (555,810–904,014)	474,146 (333,275–623,744)	40,080 (22,874–68,418)	122,486 (86,076–174,610)	65,660 (46,547–94,079)	28,116 (19,384–40,570)
Chagas disease	8,047 (7,539–8,640)	2 (2–2)	4 (4–5)	881 (800–978)	3,271 (3,021–3,568)	3,886 (3,592–4,225)
Leishmaniasis	24,202 (17,085–32,525)	3,784 (2,595–5,234)	2,441 (1,586–3,547)	14,205 (9,857–19,434)	3,051 (2,212–4,060)	719 (574–885)
Visceral leishmaniasis	24,202 (17,085–32,525)	3,784 (2,595–5,234)	2,441 (1,586–3,547)	14,205 (9,857–19,434)	3,051 (2,212–4,060)	719 (574–885)
Cutaneous and mucocutaneous leishmaniasis	0 (0–0)	0 (0–0)	0 (0–0)	0 (0–0)	0 (0–0)	0 (0–0)
African trypanosomiasis	3,514 (1,788–5,662)	261 (129–448)	584 (313–942)	2,066 (990–3,444)	490 (231–836)	112 (57–182)
Schistosomiasis	4,365 (3,831–4,927)	37 (24–56)	90 (66–120)	1,290 (1,089–1,528)	1,892 (1,578–2,240)	1,054 (878–1,256)
Cysticercosis	390 (310–522)	7 (3–13)	14 (9–22)	222 (175–301)	111 (90–144)	34 (29–43)
Cystic echinococcosis	1,195 (1,119–1,278)	40 (33–49)	81 (71–93)	429 (394–470)	372 (347–398)	270 (255–286)
Lymphatic filariasis	0 (0–0)	0 (0–0)	0 (0–0)	0 (0–0)	0 (0–0)	0 (0–0)
Onchocerciasis	0 (0–0)	0 (0–0)	0 (0–0)	0 (0–0)	0 (0–0)	0 (0–0)
Trachoma	0 (0–0)	0 (0–0)	0 (0–0)	0 (0–0)	0 (0–0)	0 (0–0)
Dengue	18,361 (11,825–22,722)	4,921 (2,643–7,198)	4,002 (2,504–5,677)	5,612 (3,941–6,933)	2,377 (1,527–2,940)	1,447 (912–1,802)
Yellow fever	5,139 (1,053–14,181)	459 (87–1,292)	1,633 (330–4,739)	2,615 (555–7,436)	364 (75–1,045)	66 (14–195)
Rabies	17,375 (14,788–20,580)	2,278 (1,633–3,207)	3,747 (2,920–4,631)	6,036 (5,092–7,230)	3,706 (3,175–4,331)	1,605 (1,295–1,907)
Intestinal nematode infections	2,727 (2,428–3,072)	1,856 (1,605–2,160)	391 (324–465)	204 (161–251)	152 (118–191)	121 (94–151)
Ascariasis	2,727 (2,428–3,072)	1,856 (1,605–2,160)	391 (324–465)	204 (161–251)	152 (118–191)	121 (94–151)
Trichuriasis	0 (0–0)	0 (0–0)	0 (0–0)	0 (0–0)	0 (0–0)	0 (0–0)
Hookworm disease	0 (0–0)	0 (0–0)	0 (0–0)	0 (0–0)	0 (0–0)	0 (0–0)
Food-borne trematodiases	0 (0–0)	0 (0–0)	0 (0–0)	0 (0–0)	0 (0–0)	0 (0–0)
Leprosy	0 (0–0)	0 (0–0)	0 (0–0)	0 (0–0)	0 (0–0)	0 (0–0)
Ebola	5,497 (4,434–6,588)	415 (335–497)	590 (476–707)	3,443 (2,777–4,127)	835 (673–1,001)	212 (171–254)
Other neglected tropical diseases	21,790 (12,701–27,595)	7,205 (4,803–10,348)	1,894 (1,049–2,618)	5,196 (2,557–6,590)	4,323 (2,296–5,626)	3,171 (1,755–4,192)
Maternal disorders	275,287 (243,757–315,490)	0 (0–0)	1,343 (1,104–1,640)	272,857 (241,723–312,860)	1,087 (761–1,631)	0 (0–0)
Maternal hemorrhage	83,125 (66,977–101,492)	0 (0–0)	416 (285–581)	82,367 (66,300–100,624)	340 (195–599)	0 (0–0)
Maternal sepsis and other maternal infections	17,894 (13,368–23,853)	0 (0–0)	56 (34–90)	17,753 (13,225–23,687)	83 (48–146)	0 (0–0)
Maternal hypertensive disorders	46,907 (37,085–59,639)	0 (0–0)	293 (186–443)	46,526 (36,691–59,130)	87 (53–147)	0 (0–0)
Maternal obstructed labor and uterine rupture	23,149 (17,209–29,965)	0 (0–0)	37 (20–64)	23,066 (17,140–29,892)	45 (26–76)	0 (0–0)
Maternal abortion, miscarriage, and ectopic pregnancy	31,658 (24,585–39,740)	0 (0–0)	168 (96–268)	31,316 (24,252–39,393)	173 (106–273)	0 (0–0)

SR Table 12 - Global deaths by age and cause for both sexes in 2015						
Cause	All ages	0-5 years	5-14 years	15-49 years	50-69 years	70+ years
Indirect maternal deaths	30,786 (23,136–40,519)	0 (0–0)	185 (111–286)	30,481 (22,851–40,187)	118 (69–208)	0 (0–0)
Late maternal deaths	6,711 (4,334–9,995)	0 (0–0)	37 (17–67)	6,634 (4,270–9,909)	39 (21–74)	0 (0–0)
Maternal deaths aggravated by HIV/AIDS	2,321 (1,394–3,336)	0 (0–0)	2 (1–4)	2,305 (1,386–3,318)	12 (6–25)	0 (0–0)
Other maternal disorders	32,733 (26,256–40,506)	0 (0–0)	145 (91–216)	32,404 (25,957–40,154)	184 (119–290)	0 (0–0)
Neonatal disorders	2,163,205 (2,095,120–2,232,454)	2,163,205 (2,095,120–2,232,454)	0 (0–0)	0 (0–0)	0 (0–0)	0 (0–0)
Neonatal preterm birth complications	805,778 (736,182–898,643)	805,778 (736,182–898,643)	0 (0–0)	0 (0–0)	0 (0–0)	0 (0–0)
Neonatal encephalopathy due to birth asphyxia and trauma	740,424 (667,637–829,229)	740,424 (667,637–829,229)	0 (0–0)	0 (0–0)	0 (0–0)	0 (0–0)
Neonatal sepsis and other neonatal infections	351,667 (249,234–459,090)	351,667 (249,234–459,090)	0 (0–0)	0 (0–0)	0 (0–0)	0 (0–0)
Hemolytic disease and other neonatal jaundice	45,088 (30,111–67,315)	45,088 (30,111–67,315)	0 (0–0)	0 (0–0)	0 (0–0)	0 (0–0)
Other neonatal disorders	220,247 (167,601–276,792)	220,247 (167,601–276,792)	0 (0–0)	0 (0–0)	0 (0–0)	0 (0–0)
Nutritional deficiencies	405,733 (331,727–495,588)	192,823 (147,242–248,109)	14,027 (11,662–18,398)	37,836 (31,523–49,937)	45,437 (38,014–58,552)	115,608 (99,922–132,664)
Protein-energy malnutrition	323,173 (264,894–400,846)	174,414 (130,506–226,545)	10,573 (8,636–13,904)	18,472 (15,181–24,482)	27,648 (23,101–36,436)	92,063 (80,152–105,706)
Iodine deficiency	1,998 (1,525–2,716)	541 (257–1,044)	69 (36–127)	355 (247–539)	448 (301–663)	583 (394–869)
Vitamin A deficiency	0 (0–0)	0 (0–0)	0 (0–0)	0 (0–0)	0 (0–0)	0 (0–0)
Iron-deficiency anemia	54,231 (35,054–72,921)	15,070 (7,690–22,620)	2,506 (1,816–3,885)	13,658 (9,906–19,318)	12,619 (8,800–16,718)	10,377 (6,216–13,451)
Other nutritional deficiencies	26,330 (20,695–33,560)	2,796 (1,861–4,022)	878 (619–1,184)	5,349 (4,074–6,713)	4,721 (3,669–6,143)	12,583 (9,829–16,265)
Other communicable, maternal, neonatal, and nutritional diseases	311,299 (257,897–372,726)	132,181 (89,393–186,677)	16,692 (12,945–21,560)	59,285 (54,461–62,905)	55,470 (48,277–59,251)	47,668 (35,499–52,195)
Sexually transmitted diseases excluding HIV	108,039 (64,596–165,717)	90,502 (50,623–144,530)	8,396 (4,767–13,137)	4,261 (3,322–5,003)	2,566 (1,789–3,113)	2,312 (1,723–2,782)
Syphilis	106,844 (63,408–164,580)	90,502 (50,623–144,530)	8,373 (4,744–13,113)	3,729 (2,913–4,382)	2,241 (1,560–2,721)	1,997 (1,491–2,401)
Chlamydial infection	151 (114–177)	0 (0–0)	3 (2–4)	62 (49–76)	38 (26–47)	47 (34–57)
Gonococcal infection	713 (535–821)	0 (0–0)	11 (9–14)	306 (233–361)	188 (132–225)	207 (153–250)
Trichomoniasis	0 (0–0)	0 (0–0)	0 (0–0)	0 (0–0)	0 (0–0)	0 (0–0)
Genital herpes	0 (0–0)	0 (0–0)	0 (0–0)	0 (0–0)	0 (0–0)	0 (0–0)
Other sexually transmitted diseases	330 (247–379)	0 (0–0)	8 (6–10)	163 (126–191)	97 (68–118)	60 (44–73)
Hepatitis	105,767 (100,722–110,779)	10,517 (9,384–11,767)	5,072 (4,427–5,721)	40,082 (37,634–42,478)	30,517 (29,067–32,128)	19,577 (18,684–20,595)
Acute hepatitis A	11,161 (6,939–15,932)	8,113 (6,013–9,930)	920 (277–1,821)	1,273 (314–2,887)	608 (147–1,416)	245 (56–608)
Hepatitis B	65,369 (56,447–73,666)	678 (244–1,568)	657 (377–1,057)	25,183 (20,764–29,274)	24,858 (21,413–27,641)	13,989 (11,512–16,220)
Hepatitis C	2,516 (523–5,934)	0 (0–0)	85 (16–228)	703 (144–1,678)	1,158 (242–2,746)	568 (119–1,346)
Acute hepatitis E	26,720 (18,488–36,554)	1,724 (688–3,336)	3,409 (2,485–4,310)	12,921 (9,162–17,172)	3,892 (1,886–6,954)	4,773 (2,626–7,363)
Other infectious diseases	97,492 (61,338–112,919)	31,162 (16,242–42,352)	3,222 (2,502–3,833)	14,942 (11,704–16,578)	22,386 (15,945–25,207)	25,778 (14,098–30,013)
Non-communicable diseases	39,804,197 (39,210,814–40,452,236)	804,519 (733,815–868,906)	193,475 (180,427–207,408)	3,975,261 (3,848,962–4,119,528)	12,190,377 (11,938,854–12,466,469)	22,640,562 (22,400,434–22,919,488)
Neoplasms	8,764,608 (8,591,071–8,945,568)	38,679 (33,457–46,415)	51,395 (47,770–54,868)	1,061,341 (1,024,135–1,099,806)	3,647,554 (3,565,207–3,734,676)	3,965,636 (3,885,638–4,053,363)
Lip and oral cavity cancer	145,960 (141,582–150,560)	0 (0–0)	0 (0–0)	22,581 (21,449–23,908)	69,270 (66,944–71,711)	54,109 (52,435–56,024)
Nasopharynx cancer	62,950 (51,094–66,974)	0 (0–0)	299 (265–333)	15,938 (13,076–17,269)	31,424 (25,134–33,660)	15,287 (12,856–16,219)
Other pharynx cancer	64,357 (61,641–67,130)	0 (0–0)	0 (0–0)	8,433 (7,938–8,992)	36,728 (35,135–38,317)	19,195 (18,344–20,064)
Esophageal cancer	439,025 (422,615–456,866)	0 (0–0)	0 (0–0)	37,346 (35,454–39,524)	216,985 (206,989–227,468)	184,692 (177,766–191,693)
Stomach cancer	818,923 (795,488–843,710)	0 (0–0)	0 (0–0)	73,770 (70,533–77,410)	339,410 (326,846–352,172)	405,742 (395,225–415,640)
Colon and rectum cancer	832,048 (811,655–854,532)	0 (0–0)	0 (0–0)	71,882 (69,398–74,946)	299,528 (292,745–307,291)	460,637 (446,838–475,851)
Liver cancer	810,482 (749,717–862,770)	0 (0–0)	1,015 (925–1,192)	133,658 (121,454–145,361)	392,491 (361,947–421,586)	283,316 (262,478–296,858)
Liver cancer due to hepatitis B	265,292 (241,021–290,521)	0 (0–0)	579 (511–709)	75,508 (68,312–83,759)	129,986 (116,048–144,819)	59,217 (52,370–65,953)
Liver cancer due to hepatitis C	167,062 (153,918–177,891)	0 (0–0)	29 (23–35)	11,128 (9,488–12,674)	67,031 (59,162–73,846)	88,874 (82,829–94,532)
Liver cancer due to alcohol use	245,246 (225,149–266,579)	0 (0–0)	0 (0–0)	24,147 (21,397–27,756)	135,959 (122,183–150,417)	85,139 (76,866–91,825)
Liver cancer due to other causes	132,881 (119,765–144,391)	0 (0–0)	407 (361–469)	22,874 (20,231–25,564)	59,514 (52,752–67,101)	50,085 (44,987–54,378)
Gallbladder and biliary tract cancer	140,497 (131,445–147,231)	0 (0–0)	0 (0–0)	8,879 (8,188–9,419)	49,593 (46,538–51,981)	82,025 (76,401–86,192)
Pancreatic cancer	411,598 (403,618–420,700)	0 (0–0)	0 (0–0)	27,806 (26,824–29,006)	169,401 (165,719–173,303)	214,391 (209,339–219,645)
Larynx cancer	105,935 (102,689–109,488)	0 (0–0)	0 (0–0)	10,233 (9,781–10,831)	54,739 (52,904–56,639)	40,962 (39,590–42,445)
Tracheal, bronchus, and lung cancer	1,722,489 (1,673,741–1,772,655)	0 (0–0)	0 (0–0)	115,550 (110,989–120,961)	793,488 (768,243–820,529)	813,451 (788,440–836,387)
Malignant skin melanoma	59,781 (47,601–72,670)	0 (0–0)	0 (0–0)	10,347 (8,788–12,972)	23,126 (18,402–29,159)	26,307 (20,596–31,024)
Non-melanoma skin cancer	51,854 (49,861–53,798)	0 (0–0)	0 (0–0)	4,194 (3,972–4,442)	16,181 (15,512–16,866)	31,478 (30,163–32,714)
Non-melanoma skin cancer (squamous-cell carcinoma)	51,854 (49,861–53,798)	0 (0–0)	0 (0–0)	4,194 (3,972–4,442)	16,181 (15,512–16,866)	31,478 (30,163–32,714)
Non-melanoma skin cancer (basal-cell carcinoma)	0 (0–0)	0 (0–0)	0 (0–0)	0 (0–0)	0 (0–0)	0 (0–0)
Breast cancer	533,598 (502,185–553,104)	0 (0–0)	0 (0–0)	106,199 (98,073–112,368)	241,676 (227,148–252,007)	185,721 (174,149–192,800)
Cervical cancer	238,620 (225,292–252,397)	0 (0–0)	0 (0–0)	59,413 (54,937–63,630)	114,019 (106,466–122,003)	65,188 (62,487–68,200)
Uterine cancer	89,946 (86,102–94,346)	0 (0–0)	0 (0–0)	9,307 (8,765–9,908)	41,479 (39,352–44,035)	39,159 (37,064–41,150)
Ovarian cancer	161,137 (156,506–166,503)	0 (0–0)	0 (0–0)	26,084 (24,658–27,532)	75,500 (73,014–78,467)	59,552 (57,601–61,637)
Prostate cancer	365,933 (303,491–459,613)	0 (0–0)	0 (0–0)	2,960 (2,280–3,602)	80,971 (65,116–101,436)	282,001 (233,048–358,457)
Testicular cancer	9,366 (8,825–9,910)	0 (0–0)	0 (0–0)	6,110 (5,733–6,580)	1,777 (1,655–1,899)	1,478 (1,381–1,581)

SR Table 12 - Global deaths by age and cause for both sexes in 2015

Cause	All ages	0-5 years	5-14 years	15-49 years	50-69 years	70+ years
Kidney cancer	136,933 (132,959–141,256)	2,674 (1,750–4,064)	1,297 (1,164–1,451)	12,301 (11,812–12,837)	55,885 (54,292–57,585)	64,774 (62,557–66,907)
Bladder cancer	188,018 (182,839–192,743)	0 (0–0)	0 (0–0)	7,653 (7,328–8,043)	55,891 (54,139–57,528)	124,473 (120,714–128,096)
Brain and nervous system cancer	228,769 (209,520–244,694)	5,367 (4,666–6,501)	9,917 (8,548–10,876)	57,337 (52,278–62,651)	96,819 (89,584–104,310)	59,327 (51,998–62,090)
Thyroid cancer	31,872 (28,935–33,198)	0 (0–0)	78 (69–87)	3,012 (2,667–3,166)	11,966 (10,901–12,580)	16,814 (15,389–17,578)
Mesothelioma	32,372 (31,213–33,370)	0 (0–0)	0 (0–0)	3,541 (3,354–3,732)	12,486 (12,073–12,860)	16,344 (15,447–17,091)
Hodgkin lymphoma	23,915 (21,786–28,961)	234 (189–287)	570 (474–684)	7,770 (6,926–9,466)	8,903 (8,148–11,056)	6,436 (5,923–7,668)
Non-Hodgkin lymphoma	231,405 (195,677–243,842)	3,272 (2,466–4,696)	5,005 (4,121–5,678)	38,776 (33,886–41,317)	83,235 (72,347–87,746)	101,115 (81,389–108,343)
Multiple myeloma	101,113 (97,664–104,086)	0 (0–0)	0 (0–0)	8,895 (8,501–9,339)	40,838 (39,357–42,249)	51,379 (48,925–53,234)
Leukemia	353,456 (344,569–363,088)	14,562 (12,900–16,519)	22,175 (20,325–23,893)	83,908 (80,665–87,294)	103,934 (101,072–106,799)	128,876 (124,923–132,549)
Acute lymphoid leukemia	110,471 (101,226–118,362)	9,177 (7,977–10,665)	15,512 (14,038–17,024)	34,571 (31,234–37,605)	29,937 (26,720–32,107)	21,272 (19,329–22,641)
Chronic lymphoid leukemia	60,723 (57,941–64,551)	0 (0–0)	0 (0–0)	6,300 (5,779–6,917)	17,262 (16,291–18,292)	37,160 (35,411–39,952)
Acute myeloid leukemia	147,081 (137,308–156,953)	5,385 (4,379–6,483)	6,662 (5,834–7,869)	34,486 (31,161–37,789)	45,721 (42,721–48,889)	54,826 (50,867–57,646)
Chronic myeloid leukemia	35,179 (33,401–37,711)	0 (0–0)	0 (0–0)	8,550 (7,879–9,547)	11,012 (10,336–11,810)	15,616 (14,826–16,778)
Other neoplasms	372,243 (335,855–392,120)	12,567 (10,353–16,905)	11,035 (9,799–12,086)	87,445 (75,254–96,541)	129,799 (115,079–136,789)	131,395 (118,767–136,977)
Cardiovascular diseases	17,921,046 (17,590,537–18,276,848)	45,034 (41,889–49,145)	25,265 (22,992–27,815)	1,388,227 (1,336,203–1,451,979)	5,090,983 (4,967,573–5,220,607)	11,371,535 (11,159,587–11,577,908)
Rheumatic heart disease	319,441 (297,273–337,285)	2,492 (2,105–2,897)	5,882 (5,116–6,760)	76,559 (69,365–82,652)	104,882 (96,968–111,637)	129,624 (122,861–136,414)
Ischemic heart disease	8,916,964 (8,751,616–9,108,849)	2,366 (2,076–2,793)	2,086 (1,769–2,399)	649,909 (623,642–681,663)	2,585,186 (2,514,537–2,656,195)	5,677,415 (5,562,541–5,788,359)
Cerebrovascular disease	6,326,135 (6,175,215–6,492,925)	14,942 (13,781–16,744)	9,047 (8,265–9,959)	434,515 (415,265–455,870)	1,811,868 (1,751,429–1,874,042)	4,055,762 (3,962,376–4,156,545)
Ischemic stroke	2,977,980 (2,880,779–3,068,755)	593 (493–703)	503 (437–574)	70,014 (63,719–76,199)	612,333 (577,120–645,007)	2,294,534 (2,227,124–2,357,765)
Hemorrhagic stroke	3,348,155 (3,240,907–3,500,093)	14,348 (13,230–16,063)	8,544 (7,801–9,409)	364,500 (347,507–383,390)	1,199,534 (1,154,244–1,251,509)	1,761,227 (1,703,998–1,844,323)
Hypertensive heart disease	962,425 (873,583–1,024,508)	410 (348–507)	378 (321–461)	72,036 (66,789–78,002)	268,732 (249,790–289,015)	620,867 (549,344–661,777)
Cardiomyopathy and myocarditis	353,725 (339,522–370,624)	9,806 (8,490–10,784)	3,141 (2,907–3,488)	69,310 (64,934–73,796)	99,016 (94,521–107,755)	172,450 (165,273–179,562)
Atrial fibrillation and flutter	195,305 (159,518–236,176)	0 (0–0)	0 (0–0)	964 (728–1,231)	12,282 (9,639–16,102)	182,058 (146,200–223,121)
Aortic aneurysm	168,204 (163,552–172,770)	0 (0–0)	0 (0–0)	13,136 (12,602–13,746)	46,348 (45,074–47,520)	108,719 (105,230–112,343)
Peripheral vascular disease	52,510 (49,729–55,729)	0 (0–0)	0 (0–0)	576 (545–610)	8,542 (8,091–8,999)	43,391 (40,848–46,222)
Endocarditis	84,903 (74,684–93,024)	3,486 (2,615–4,230)	1,201 (949–1,374)	16,488 (13,077–18,266)	19,540 (17,075–22,470)	44,185 (40,404–47,856)
Other cardiovascular and circulatory diseases	541,430 (521,719–561,156)	11,529 (10,504–13,424)	3,528 (3,186–3,945)	54,730 (51,550–58,427)	134,582 (127,843–141,537)	337,059 (325,083–349,487)
Chronic respiratory diseases	3,795,462 (3,683,858–3,910,380)	35,265 (31,217–40,567)	9,807 (8,883–10,877)	196,366 (187,075–206,498)	1,034,673 (993,568–1,074,303)	2,519,349 (2,440,572–2,595,610)
Chronic obstructive pulmonary disease	3,188,326 (3,083,807–3,292,534)	12,595 (9,630–16,101)	1,714 (1,445–2,031)	114,787 (106,769–122,059)	840,044 (801,291–876,886)	2,219,184 (2,147,430–2,293,514)
Pneumoconiosis	36,069 (31,481–40,742)	0 (0–0)	0 (0–0)	2,739 (2,396–3,156)	9,571 (8,269–11,071)	23,759 (20,769–26,826)
Silicosis	10,440 (9,373–11,679)	0 (0–0)	0 (0–0)	1,039 (888–1,243)	3,476 (3,021–4,068)	5,924 (5,349–6,566)
Asbestosis	3,597 (2,473–4,156)	0 (0–0)	0 (0–0)	409 (342–483)	829 (637–1,032)	2,357 (1,438–2,718)
Coal workers pneumoconiosis	2,486 (2,187–2,853)	0 (0–0)	0 (0–0)	180 (138–238)	711 (594–849)	1,594 (1,421–1,811)
Other pneumoconiosis	19,546 (15,809–23,154)	0 (0–0)	0 (0–0)	1,110 (896–1,358)	4,553 (3,557–5,726)	13,882 (11,301–16,208)
Asthma	397,113 (362,953–438,706)	9,483 (7,838–11,534)	6,390 (5,593–7,515)	62,789 (56,491–72,368)	139,014 (125,324–155,403)	179,436 (163,100–197,650)
Interstitial lung disease and pulmonary sarcoidosis	121,827 (94,142–135,154)	301 (217–420)	246 (206–302)	7,623 (6,513–8,723)	34,041 (28,469–38,461)	79,613 (57,212–88,344)
Other chronic respiratory diseases	52,124 (38,036–60,963)	12,885 (9,918–16,343)	1,455 (1,009–1,914)	8,425 (5,686–10,155)	12,001 (7,965–14,099)	17,355 (12,024–20,447)
Cirrhosis and other chronic liver diseases	1,292,137 (1,239,944–1,371,720)	9,591 (8,094–12,040)	8,763 (7,639–10,616)	340,092 (321,532–366,086)	592,148 (567,529–626,409)	341,541 (330,437–361,884)
Cirrhosis and other chronic liver diseases due to hepatitis B	371,135 (341,646–409,972)	75 (56–100)	275 (213–350)	98,288 (87,785–110,489)	168,479 (149,715–189,952)	104,016 (94,119–115,771)
Cirrhosis and other chronic liver diseases due to hepatitis C	325,601 (301,785–349,885)	38 (28–52)	154 (118–199)	78,342 (69,833–88,221)	153,195 (137,134–168,947)	93,870 (86,546–102,833)
Cirrhosis and other chronic liver diseases due to alcohol use	347,926 (322,692–374,564)	0 (0–0)	0 (0–0)	106,942 (97,361–118,073)	174,930 (157,731–193,521)	66,052 (59,528–73,399)
Cirrhosis and other chronic liver diseases due to other causes	247,473 (230,310–272,229)	9,477 (8,000–11,909)	8,333 (7,278–10,087)	56,518 (50,917–63,732)	95,542 (84,738–107,401)	77,601 (71,055–85,299)
Digestive diseases	1,203,007 (1,150,475–1,270,031)	20,709 (14,578–34,119)	14,814 (13,210–17,282)	225,940 (209,565–243,444)	358,086 (340,104–382,126)	583,455 (561,684–610,990)
Peptic ulcer disease	267,475 (249,363–290,008)	1,885 (1,040–3,787)	2,026 (1,731–2,499)	48,772 (43,944–55,062)	89,800 (83,477–98,558)	124,989 (117,520–133,783)
Gastritis and duodenitis	49,661 (41,838–60,119)	814 (446–1,539)	381 (288–549)	9,042 (7,552–11,177)	16,059 (13,658–19,724)	23,364 (19,267–28,369)
Appendicitis	50,132 (41,154–55,996)	3,181 (1,642–6,779)	2,456 (2,136–2,837)	16,369 (13,126–18,294)	13,994 (11,567–15,394)	14,129 (11,969–15,524)
Paralytic ileus and intestinal obstruction	264,320 (242,380–307,364)	7,623 (5,324–12,462)	5,782 (5,009–7,190)	52,716 (46,534–62,931)	75,385 (68,645–90,534)	122,813 (113,599–139,847)
Inguinal, femoral, and abdominal hernia	59,820 (41,098–69,245)	724 (379–1,202)	669 (426–830)	10,121 (5,718–13,135)	18,579 (12,292–22,195)	29,724 (22,189–33,534)
Inflammatory bowel disease	47,352 (43,469–51,737)	1,733 (1,004–3,306)	524 (411–670)	8,625 (7,488–9,841)	12,375 (11,332–13,385)	24,093 (22,247–25,813)
Vascular intestinal disorders	105,842 (98,999–114,341)	393 (246–659)	128 (103–164)	5,718 (4,982–6,522)	25,835 (23,707–28,586)	73,766 (69,359–79,375)
Gallbladder and biliary diseases	111,731 (105,278–119,977)	1,140 (657–2,151)	926 (803–1,088)	13,753 (12,455–15,221)	29,392 (27,465–31,834)	66,517 (62,915–71,413)
Pancreatitis	132,715 (122,871–143,966)	192 (136–284)	468 (401–584)	46,112 (41,603–51,477)	45,883 (42,360–50,037)	40,057 (37,664–43,215)
Other digestive diseases	113,954 (101,945–131,107)	3,020 (2,010–4,843)	1,449 (1,214–1,876)	14,707 (11,995–18,096)	30,779 (25,714–36,270)	63,998 (59,878–71,681)
Neurological disorders	2,258,914 (1,937,845–2,574,428)	17,269 (14,692–20,142)	11,776 (10,676–12,869)	90,382 (86,024–95,962)	189,692 (162,182–218,029)	1,949,794 (1,641,699–2,260,765)
Alzheimer disease and other dementias	1,908,153 (1,586,694–2,229,136)	0 (0–0)	0 (0–0)	1,453 (903–2,147)	111,366 (83,087–139,575)	1,795,333 (1,484,809–2,109,553)
Parkinson disease	117,365 (113,908–121,264)	0 (0–0)	0 (0–0)	756 (720–813)	13,617 (13,191–14,200)	102,991 (99,673–106,544)
Epilepsy	124,939 (119,259–131,044)	12,672 (10,649–14,917)	9,071 (8,109–10,105)	64,310 (60,622–68,476)	22,801 (21,803–24,009)	16,083 (15,528–16,665)

SR Table 12 - Global deaths by age and cause for both sexes in 2015

Cause	All ages	0-5 years	5-14 years	15-49 years	50-69 years	70+ years
Multiple sclerosis	18,923 (17,306–20,037)	0 (0–0)	0 (0–0)	5,367 (4,940–6,357)	9,319 (8,359–9,830)	4,237 (3,427–4,509)
Motor neuron disease	35,226 (33,948–36,736)	886 (719–1,029)	102 (93–112)	3,927 (3,741–4,219)	16,133 (15,603–16,833)	14,176 (13,561–14,719)
Migraine	0 (0–0)	0 (0–0)	0 (0–0)	0 (0–0)	0 (0–0)	0 (0–0)
Tension-type headache	0 (0–0)	0 (0–0)	0 (0–0)	0 (0–0)	0 (0–0)	0 (0–0)
Medication overuse headache	0 (0–0)	0 (0–0)	0 (0–0)	0 (0–0)	0 (0–0)	0 (0–0)
Other neurological disorders	54,324 (52,981–57,481)	3,728 (3,148–4,942)	2,602 (2,303–3,209)	14,566 (13,903–15,736)	16,453 (15,966–16,956)	16,972 (16,370–17,601)
Mental and substance use disorders	324,949 (308,614–337,353)	27 (25–30)	34 (19–44)	169,660 (160,226–180,478)	112,156 (105,373–116,380)	43,070 (36,653–44,949)
Schizophrenia	16,908 (15,898–18,012)	0 (0–0)	0 (0–0)	5,441 (4,976–5,998)	6,449 (6,035–6,930)	5,017 (4,764–5,294)
Alcohol use disorders	137,500 (131,470–143,981)	0 (0–0)	0 (0–0)	59,466 (56,541–62,912)	62,091 (58,934–65,299)	15,942 (14,984–16,661)
Drug use disorders	169,872 (152,084–179,208)	27 (25–30)	0 (0–0)	104,119 (92,644–112,869)	43,615 (38,149–46,119)	22,109 (16,407–23,755)
Opioid use disorders	122,098 (109,547–129,679)	27 (25–30)	0 (0–0)	75,228 (66,818–82,068)	30,332 (26,347–32,334)	16,509 (11,939–17,827)
Cocaine use disorders	11,066 (8,508–12,173)	0 (0–0)	0 (0–0)	7,241 (5,565–8,135)	3,356 (2,439–3,729)	467 (394–552)
Amphetamine use disorders	12,180 (8,411–14,157)	0 (0–0)	0 (0–0)	8,052 (5,502–9,519)	3,393 (2,360–4,047)	734 (517–865)
Cannabis use disorders	0 (0–0)	0 (0–0)	0 (0–0)	0 (0–0)	0 (0–0)	0 (0–0)
Other drug use disorders	24,527 (22,727–27,341)	0 (0–0)	0 (0–0)	13,597 (12,449–15,817)	6,532 (5,996–7,243)	4,397 (3,434–5,282)
Depressive disorders	0 (0–0)	0 (0–0)	0 (0–0)	0 (0–0)	0 (0–0)	0 (0–0)
Major depressive disorder	0 (0–0)	0 (0–0)	0 (0–0)	0 (0–0)	0 (0–0)	0 (0–0)
Dysthymia	0 (0–0)	0 (0–0)	0 (0–0)	0 (0–0)	0 (0–0)	0 (0–0)
Bipolar disorder	0 (0–0)	0 (0–0)	0 (0–0)	0 (0–0)	0 (0–0)	0 (0–0)
Anxiety disorders	0 (0–0)	0 (0–0)	0 (0–0)	0 (0–0)	0 (0–0)	0 (0–0)
Eating disorders	667 (451–902)	0 (0–0)	34 (19–44)	633 (433–867)	0 (0–0)	0 (0–0)
Anorexia nervosa	603 (403–810)	0 (0–0)	32 (18–41)	570 (386–772)	0 (0–0)	0 (0–0)
Bulimia nervosa	64 (38–93)	0 (0–0)	1 (0–3)	62 (38–90)	0 (0–0)	0 (0–0)
Autistic spectrum disorders	0 (0–0)	0 (0–0)	0 (0–0)	0 (0–0)	0 (0–0)	0 (0–0)
Autism	0 (0–0)	0 (0–0)	0 (0–0)	0 (0–0)	0 (0–0)	0 (0–0)
Asperger syndrome and other autistic spectrum disorders	0 (0–0)	0 (0–0)	0 (0–0)	0 (0–0)	0 (0–0)	0 (0–0)
Attention-deficit/hyperactivity disorder	0 (0–0)	0 (0–0)	0 (0–0)	0 (0–0)	0 (0–0)	0 (0–0)
Conduct disorder	0 (0–0)	0 (0–0)	0 (0–0)	0 (0–0)	0 (0–0)	0 (0–0)
Idiopathic developmental intellectual disability	0 (0–0)	0 (0–0)	0 (0–0)	0 (0–0)	0 (0–0)	0 (0–0)
Other mental and substance use disorders	0 (0–0)	0 (0–0)	0 (0–0)	0 (0–0)	0 (0–0)	0 (0–0)
Diabetes, urogenital, blood, and endocrine diseases	3,409,347 (3,287,536–3,516,521)	116,036 (82,924–181,412)	31,903 (28,299–36,339)	410,643 (389,376–434,548)	1,101,236 (1,061,449–1,138,664)	1,749,527 (1,698,142–1,800,258)
Diabetes mellitus	1,518,966 (1,470,271–1,575,951)	1,700 (1,404–1,878)	2,277 (1,843–2,503)	132,113 (125,687–140,907)	575,158 (553,285–600,258)	807,715 (782,131–834,648)
Acute glomerulonephritis	11,798 (7,799–13,347)	255 (180–306)	276 (210–313)	2,501 (1,615–2,816)	4,049 (2,667–4,630)	4,714 (3,028–5,354)
Chronic kidney disease	1,234,931 (1,131,670–1,282,433)	11,663 (10,445–13,070)	8,392 (7,420–9,405)	166,791 (146,953–178,325)	383,818 (346,171–401,941)	664,266 (621,612–687,520)
Chronic kidney disease due to diabetes mellitus	417,750 (388,736–441,427)	1,719 (1,443–2,048)	849 (694–1,048)	41,474 (36,191–46,428)	160,211 (146,459–173,068)	213,494 (200,395–226,720)
Chronic kidney disease due to hypertension	549,518 (501,581–575,641)	4,075 (3,555–4,709)	2,592 (2,158–3,111)	68,187 (59,582–74,718)	151,311 (133,513–163,665)	323,350 (300,249–339,426)
Chronic kidney disease due to glomerulonephritis	237,674 (212,596–255,893)	4,330 (3,716–5,024)	3,664 (3,109–4,250)	50,035 (43,078–55,965)	67,693 (58,275–74,778)	111,950 (101,229–120,825)
Chronic kidney disease due to other causes	29,987 (25,003–35,213)	1,537 (1,235–1,870)	1,284 (1,012–1,610)	7,093 (4,713–9,937)	4,601 (2,870–6,829)	15,470 (13,085–18,750)
Urinary diseases and male infertility	261,650 (243,196–277,559)	6,469 (5,237–8,053)	1,847 (1,631–2,205)	30,915 (27,945–34,980)	62,905 (56,810–68,078)	159,512 (147,662–168,437)
Interstitial nephritis and urinary tract infections	196,378 (181,499–211,027)	5,220 (4,234–6,500)	1,186 (1,032–1,402)	20,617 (18,533–23,837)	41,507 (37,388–45,899)	127,846 (117,498–135,041)
Urolithiasis	16,070 (14,045–20,321)	0 (0–0)	206 (174–279)	2,458 (2,127–3,411)	5,330 (4,480–6,801)	8,075 (7,049–10,044)
Benign prostatic hyperplasia	0 (0–0)	0 (0–0)	0 (0–0)	0 (0–0)	0 (0–0)	0 (0–0)
Male infertility	0 (0–0)	0 (0–0)	0 (0–0)	0 (0–0)	0 (0–0)	0 (0–0)
Other urinary diseases	49,201 (41,213–55,424)	1,248 (852–1,717)	454 (388–563)	7,839 (6,196–8,943)	16,067 (12,936–18,141)	23,590 (20,027–27,545)
Gynecological diseases	7,863 (5,875–9,181)	0 (0–0)	0 (0–0)	3,224 (2,438–3,855)	2,417 (1,725–2,855)	2,220 (1,652–2,615)
Uterine fibroids	2,324 (1,209–3,034)	0 (0–0)	0 (0–0)	743 (401–992)	1,001 (521–1,283)	579 (270–800)
Polycystic ovarian syndrome	614 (193–1,017)	0 (0–0)	0 (0–0)	418 (114–712)	195 (80–324)	0 (0–0)
Female infertility	0 (0–0)	0 (0–0)	0 (0–0)	0 (0–0)	0 (0–0)	0 (0–0)
Endometriosis	55 (42–83)	0 (0–0)	0 (0–0)	45 (34–68)	9 (6–14)	0 (0–0)
Genital prolapse	888 (551–1,465)	0 (0–0)	0 (0–0)	50 (24–88)	295 (160–549)	542 (348–846)
Premenstrual syndrome	0 (0–0)	0 (0–0)	0 (0–0)	0 (0–0)	0 (0–0)	0 (0–0)
Other gynecological diseases	3,981 (2,998–4,833)	0 (0–0)	0 (0–0)	1,966 (1,518–2,442)	916 (648–1,160)	1,099 (813–1,373)
Hemoglobinopathies and hemolytic anemias	226,871 (177,198–306,189)	83,521 (50,435–148,839)	15,643 (12,667–19,928)	48,800 (42,213–57,160)	31,567 (27,281–36,405)	47,337 (41,532–53,877)
Thalassemias	16,821 (13,926–20,202)	7,557 (5,681–10,282)	2,682 (2,351–3,034)	1,885 (1,532–2,391)	132 (121–147)	4,563 (3,793–5,621)
Thalassemias trait	0 (0–0)	0 (0–0)	0 (0–0)	0 (0–0)	0 (0–0)	0 (0–0)
Sickle cell disorders	114,752 (78,342–183,235)	74,847 (43,296–138,711)	11,470 (8,826–15,468)	20,889 (17,798–25,425)	1,557 (1,374–1,807)	5,987 (5,032–7,115)
Sickle cell trait	0 (0–0)	0 (0–0)	0 (0–0)	0 (0–0)	0 (0–0)	0 (0–0)

SR Table 12 - Global deaths by age and cause for both sexes in 2015						
Cause	All ages	0-5 years	5-14 years	15-49 years	50-69 years	70+ years
G6PD deficiency	32,986 (27,969–38,881)	834 (581–1,240)	1,145 (927–1,439)	17,588 (14,863–20,914)	10,772 (9,016–12,765)	2,646 (2,158–3,201)
G6PD trait	0 (0–0)	0 (0–0)	0 (0–0)	0 (0–0)	0 (0–0)	0 (0–0)
Other hemoglobinopathies and hemolytic anemias	62,309 (54,670–71,051)	282 (216–395)	345 (295–418)	8,437 (7,134–10,263)	19,106 (16,559–22,080)	34,139 (29,989–38,466)
Endocrine, metabolic, blood, and immune disorders	147,265 (142,548–151,681)	12,426 (11,210–13,795)	3,466 (3,162–3,839)	26,295 (25,038–27,907)	41,317 (40,062–42,677)	63,759 (61,333–66,386)
Musculoskeletal disorders	90,133 (82,508–94,644)	0 (0–0)	1,789 (1,600–1,988)	18,845 (17,715–20,463)	23,654 (22,497–25,602)	45,844 (39,944–48,307)
Rheumatoid arthritis	30,035 (26,952–34,583)	0 (0–0)	207 (160–260)	2,092 (1,720–2,606)	9,438 (8,330–11,134)	18,296 (16,500–20,739)
Osteoarthritis	0 (0–0)	0 (0–0)	0 (0–0)	0 (0–0)	0 (0–0)	0 (0–0)
Low back and neck pain	0 (0–0)	0 (0–0)	0 (0–0)	0 (0–0)	0 (0–0)	0 (0–0)
Low back pain	0 (0–0)	0 (0–0)	0 (0–0)	0 (0–0)	0 (0–0)	0 (0–0)
Neck pain	0 (0–0)	0 (0–0)	0 (0–0)	0 (0–0)	0 (0–0)	0 (0–0)
Gout	0 (0–0)	0 (0–0)	0 (0–0)	0 (0–0)	0 (0–0)	0 (0–0)
Other musculoskeletal disorders	60,097 (53,078–63,479)	0 (0–0)	1,581 (1,415–1,773)	16,752 (15,518–18,459)	14,215 (13,159–15,490)	27,547 (21,911–29,704)
Other non-communicable diseases	744,572 (667,799–811,584)	521,886 (469,898–580,443)	37,925 (33,024–42,681)	73,760 (63,575–81,769)	40,191 (31,918–48,175)	70,808 (52,242–82,863)
Congenital anomalies	627,849 (567,309–694,356)	496,584 (444,357–554,583)	36,781 (32,007–41,264)	61,993 (54,285–68,714)	18,569 (15,766–19,889)	13,920 (11,747–14,907)
Neural tube defects	64,602 (47,925–83,371)	60,853 (44,966–79,201)	1,848 (1,287–2,508)	1,405 (1,111–1,812)	341 (282–444)	153 (127–198)
Congenital heart anomalies	303,282 (268,772–335,386)	227,065 (199,885–256,237)	24,781 (20,909–27,887)	42,143 (36,377–46,872)	5,306 (4,592–5,778)	3,984 (3,497–4,422)
Cleft lip and cleft palate	1,348 (1,110–1,658)	1,348 (1,110–1,658)	0 (0–0)	0 (0–0)	0 (0–0)	0 (0–0)
Down syndrome	26,498 (19,109–36,887)	17,241 (10,943–26,785)	917 (717–1,180)	4,568 (3,849–5,366)	3,568 (2,807–3,920)	202 (161–232)
Turner syndrome	0 (0–0)	0 (0–0)	0 (0–0)	0 (0–0)	0 (0–0)	0 (0–0)
Klinefelter syndrome	0 (0–0)	0 (0–0)	0 (0–0)	0 (0–0)	0 (0–0)	0 (0–0)
Other chromosomal abnormalities	22,730 (12,877–40,540)	20,968 (11,270–38,567)	424 (278–679)	880 (590–1,115)	336 (203–409)	121 (92–144)
Other congenital anomalies	209,386 (188,918–241,331)	169,107 (150,053–199,611)	8,809 (7,791–10,654)	12,994 (11,372–14,587)	9,016 (7,602–9,891)	9,458 (7,724–10,234)
Skin and subcutaneous diseases	97,569 (66,629–128,827)	6,149 (4,142–9,724)	1,144 (741–1,709)	11,767 (7,447–16,928)	21,621 (14,477–30,358)	56,887 (39,041–70,710)
Dermatitis	0 (0–0)	0 (0–0)	0 (0–0)	0 (0–0)	0 (0–0)	0 (0–0)
Psoriasis	0 (0–0)	0 (0–0)	0 (0–0)	0 (0–0)	0 (0–0)	0 (0–0)
Cellulitis	16,918 (10,449–22,994)	659 (378–1,152)	199 (117–294)	2,666 (1,475–4,041)	5,226 (3,017–7,366)	8,167 (5,283–10,661)
Pyoderma	44,050 (31,110–62,834)	5,094 (3,415–8,108)	615 (413–982)	6,744 (4,306–10,281)	11,019 (7,628–16,322)	20,576 (14,944–28,386)
Scabies	0 (0–0)	0 (0–0)	0 (0–0)	0 (0–0)	0 (0–0)	0 (0–0)
Fungal skin diseases	0 (0–0)	0 (0–0)	0 (0–0)	0 (0–0)	0 (0–0)	0 (0–0)
Viral skin diseases	0 (0–0)	0 (0–0)	0 (0–0)	0 (0–0)	0 (0–0)	0 (0–0)
Acne vulgaris	0 (0–0)	0 (0–0)	0 (0–0)	0 (0–0)	0 (0–0)	0 (0–0)
Alopecia areata	0 (0–0)	0 (0–0)	0 (0–0)	0 (0–0)	0 (0–0)	0 (0–0)
Pruritus	0 (0–0)	0 (0–0)	0 (0–0)	0 (0–0)	0 (0–0)	0 (0–0)
Urticaria	0 (0–0)	0 (0–0)	0 (0–0)	0 (0–0)	0 (0–0)	0 (0–0)
Decubitus ulcer	32,388 (19,263–40,009)	126 (65–214)	165 (96–238)	1,567 (835–2,164)	4,524 (2,523–6,241)	26,004 (15,555–31,050)
Other skin and subcutaneous diseases	4,211 (2,968–5,758)	268 (167–432)	163 (95–242)	789 (509–1,186)	849 (581–1,213)	2,139 (1,534–2,880)
Sense organ diseases	0 (0–0)	0 (0–0)	0 (0–0)	0 (0–0)	0 (0–0)	0 (0–0)
Glaucoma	0 (0–0)	0 (0–0)	0 (0–0)	0 (0–0)	0 (0–0)	0 (0–0)
Cataract	0 (0–0)	0 (0–0)	0 (0–0)	0 (0–0)	0 (0–0)	0 (0–0)
Macular degeneration	0 (0–0)	0 (0–0)	0 (0–0)	0 (0–0)	0 (0–0)	0 (0–0)
Refraction and accommodation disorders	0 (0–0)	0 (0–0)	0 (0–0)	0 (0–0)	0 (0–0)	0 (0–0)
Age-related and other hearing loss	0 (0–0)	0 (0–0)	0 (0–0)	0 (0–0)	0 (0–0)	0 (0–0)
Other vision loss	0 (0–0)	0 (0–0)	0 (0–0)	0 (0–0)	0 (0–0)	0 (0–0)
Other sense organ diseases	0 (0–0)	0 (0–0)	0 (0–0)	0 (0–0)	0 (0–0)	0 (0–0)
Oral disorders	0 (0–0)	0 (0–0)	0 (0–0)	0 (0–0)	0 (0–0)	0 (0–0)
Deciduous caries	0 (0–0)	0 (0–0)	0 (0–0)	0 (0–0)	0 (0–0)	0 (0–0)
Permanent caries	0 (0–0)	0 (0–0)	0 (0–0)	0 (0–0)	0 (0–0)	0 (0–0)
Periodontal diseases	0 (0–0)	0 (0–0)	0 (0–0)	0 (0–0)	0 (0–0)	0 (0–0)
Edentulism and severe tooth loss	0 (0–0)	0 (0–0)	0 (0–0)	0 (0–0)	0 (0–0)	0 (0–0)
Other oral disorders	0 (0–0)	0 (0–0)	0 (0–0)	0 (0–0)	0 (0–0)	0 (0–0)
Sudden infant death syndrome	19,153 (15,860–27,482)	19,153 (15,860–27,482)	0 (0–0)	0 (0–0)	0 (0–0)	0 (0–0)
Injuries	4,725,054 (4,398,513–4,905,180)	312,969 (265,152–348,421)	221,233 (205,408–236,618)	2,315,785 (2,168,883–2,417,865)	1,029,551 (954,727–1,076,634)	845,513 (773,365–874,034)
Transport injuries	1,466,557 (1,394,757–1,536,454)	53,733 (46,687–61,407)	66,005 (61,114–70,941)	804,397 (760,904–848,854)	355,097 (336,005–374,053)	187,322 (177,422–196,586)
Road injuries	1,361,658 (1,294,033–1,428,141)	49,521 (43,016–56,786)	62,430 (57,839–67,152)	747,549 (705,252–789,239)	326,975 (307,877–344,472)	175,181 (165,575–183,644)
Pedestrian road injuries	560,616 (525,755–617,078)	22,469 (19,557–25,755)	28,883 (26,531–31,548)	265,626 (239,455–291,999)	151,770 (140,994–169,339)	91,865 (86,055–102,172)
Cyclist road injuries	58,676 (54,352–64,412)	1,209 (989–1,486)	3,244 (2,947–3,592)	26,757 (24,216–29,381)	19,497 (17,757–21,789)	7,967 (7,291–9,003)
Motorcyclist road injuries	257,083 (230,605–290,895)	6,144 (4,863–7,898)	7,873 (6,564–9,417)	167,673 (151,075–195,385)	53,264 (45,128–59,630)	22,128 (18,196–25,048)

SR Table 12 - Global deaths by age and cause for both sexes in 2015						
**Cause**	**All ages**	**0-5 years**	**5-14 years**	**15-49 years**	**50-69 years**	**70+ years**
Motor vehicle road injuries	464,196 (417,794–508,345)	18,544 (15,598–21,836)	20,959 (18,728–23,369)	276,094 (252,176–306,629)	98,032 (85,155–106,122)	50,565 (44,943–54,661)
Other road injuries	21,084 (14,045–24,768)	1,152 (648–1,997)	1,469 (902–1,932)	11,398 (7,048–13,954)	4,411 (3,269–5,371)	2,653 (2,208–3,470)
Other transport injuries	104,898 (90,458–127,161)	4,212 (3,397–5,220)	3,575 (3,143–4,020)	56,847 (48,476–69,429)	28,121 (23,826–35,644)	12,141 (10,537–15,104)
Unintentional injuries	1,838,745 (1,634,601–1,939,145)	224,322 (180,259–256,610)	122,799 (111,334–132,898)	618,536 (553,054–665,676)	366,557 (322,376–395,772)	506,529 (445,749–529,526)
Falls	527,243 (467,795–554,525)	23,344 (15,184–30,322)	15,431 (13,112–17,383)	108,344 (95,768–115,713)	114,338 (100,743–121,551)	265,784 (233,733–280,176)
Drowning	323,763 (285,807–347,480)	56,088 (44,376–65,699)	48,759 (44,248–53,695)	128,165 (116,240–136,690)	52,018 (45,567–56,104)	38,732 (32,202–41,223)
Fire, heat, and hot substances	175,988 (145,147–189,551)	22,485 (17,938–26,194)	9,131 (7,560–10,411)	73,363 (57,548–80,790)	32,879 (27,416–35,731)	38,128 (33,039–40,518)
Poisonings	86,352 (58,623–100,990)	18,412 (6,405–31,515)	4,339 (3,494–4,969)	32,424 (24,970–36,225)	18,708 (13,522–20,627)	12,468 (9,834–13,454)
Exposure to mechanical forces	200,647 (157,601–216,704)	28,750 (22,043–33,576)	11,559 (10,489–12,974)	86,870 (67,118–94,868)	39,988 (31,068–43,943)	33,477 (24,547–36,445)
Unintentional firearm injuries	32,043 (23,315–35,072)	2,255 (1,375–2,991)	1,738 (1,321–2,077)	16,589 (11,416–18,608)	4,774 (3,434–5,208)	6,683 (5,545–7,165)
Unintentional suffocation	35,633 (25,552–40,296)	16,448 (10,571–20,579)	2,987 (2,574–3,828)	6,570 (5,225–7,177)	3,023 (1,775–3,517)	6,603 (4,594–7,427)
Other exposure to mechanical forces	132,970 (101,090–145,114)	10,046 (8,558–11,599)	6,833 (5,817–7,866)	63,710 (47,077–70,796)	32,189 (24,583–35,825)	20,190 (13,853–22,423)
Adverse effects of medical treatment	99,755 (77,255–109,034)	14,256 (9,132–18,027)	4,067 (2,910–4,777)	27,112 (19,292–30,993)	21,664 (17,119–24,532)	32,654 (27,284–36,012)
Animal contact	94,019 (55,764–132,269)	9,854 (5,858–13,424)	10,582 (6,486–14,957)	38,312 (21,783–56,566)	23,095 (13,502–32,140)	12,173 (7,780–15,671)
Venomous animal contact	79,589 (44,176–115,922)	6,923 (3,814–10,669)	8,775 (5,085–13,196)	34,082 (18,347–51,091)	20,071 (11,082–28,221)	9,736 (5,655–12,688)
Non-venomous animal contact	14,429 (11,432–16,373)	2,931 (1,989–3,572)	1,806 (1,380–2,183)	4,229 (3,411–5,082)	3,023 (2,350–3,813)	2,437 (2,057–2,995)
Foreign body	151,607 (132,621–169,823)	39,573 (27,653–53,206)	8,648 (6,870–10,437)	34,361 (29,326–39,572)	25,787 (22,244–28,236)	43,237 (37,256–46,188)
Pulmonary aspiration and foreign body in airway	124,029 (105,461–143,237)	34,594 (23,291–47,948)	6,356 (4,935–8,095)	23,669 (20,437–26,577)	21,623 (18,402–23,348)	37,785 (32,130–40,004)
Foreign body in eyes	0 (0–0)	0 (0–0)	0 (0–0)	0 (0–0)	0 (0–0)	0 (0–0)
Foreign body in other body part	27,578 (20,319–34,413)	4,978 (2,732–7,067)	2,291 (1,472–2,963)	10,691 (7,489–14,499)	4,164 (3,197–5,436)	5,452 (4,574–6,642)
Environmental heat and cold exposure	45,166 (33,466–49,464)	2,515 (1,438–3,153)	1,101 (699–1,340)	13,851 (10,374–15,423)	15,570 (11,462–17,706)	12,127 (8,674–13,572)
Other unintentional injuries	134,201 (124,827–147,818)	9,042 (7,713–10,647)	9,179 (7,995–10,558)	75,729 (68,929–86,736)	22,506 (20,899–24,454)	17,743 (15,069–21,668)
Self-harm and interpersonal violence	1,236,690 (1,130,115–1,287,886)	10,515 (8,886–12,769)	18,515 (16,687–20,252)	769,563 (700,262–803,286)	291,126 (267,095–304,482)	146,970 (134,695–153,722)
Self-harm	828,108 (745,844–868,688)	0 (0–0)	7,785 (6,712–8,735)	467,576 (420,839–493,065)	229,202 (205,946–241,124)	123,544 (112,214–129,999)
Interpersonal violence	408,582 (370,473–431,478)	10,515 (8,886–12,769)	10,730 (9,789–11,934)	301,986 (269,913–318,804)	61,923 (57,429–67,236)	23,425 (22,097–24,714)
Assault by firearm	173,147 (149,270–183,162)	1,812 (1,453–2,266)	3,203 (2,624–3,533)	141,982 (120,805–149,900)	20,606 (18,648–22,780)	5,541 (4,948–6,319)
Assault by sharp object	89,495 (83,332–97,252)	1,016 (820–1,302)	1,486 (1,338–1,670)	67,433 (62,278–73,484)	14,206 (13,317–15,679)	5,352 (5,006–5,938)
Assault by other means	145,939 (129,556–159,708)	7,687 (6,487–9,367)	6,040 (5,406–6,900)	92,570 (81,610–101,748)	27,110 (24,441–29,596)	12,531 (11,411–13,419)
Forces of nature, war, and legal intervention	183,060 (99,951–263,786)	24,397 (15,653–32,957)	13,912 (7,705–19,983)	123,288 (62,815–181,844)	16,770 (9,228–24,129)	4,690 (2,802–6,714)
Exposure to forces of nature	11,787 (7,160–16,368)	857 (518–1,205)	2,016 (1,206–2,858)	5,994 (3,614–8,344)	1,998 (1,215–2,786)	919 (552–1,308)
Collective violence and legal intervention	171,273 (88,084–251,133)	23,540 (14,874–31,872)	11,895 (5,689–17,719)	117,294 (57,756–175,043)	14,771 (7,365–21,931)	3,771 (2,023–5,610)

SR Table 13: Global death rates (per 100,000) by cause and age group for females (with 95% uncertainty intervals), 1990						
**Cause**	**All ages**	**0-5 years**	**5-14 years**	**15-49 years**	**50-69 years**	**70+ years**
All causes	857.4 (845.1–871.0)	1854.7 (1836.2–1874.9)	113.3 (111.5–115.2)	222.3 (215.4–229.9)	1347.3 (1313.5–1383.3)	7192.9 (7087.6–7294.1)
Communicable, maternal, neonatal, and nutritional diseases	292.9 (283.6–301.5)	1565.0 (1501.9–1616.0)	64.1 (60.7–68.1)	80.7 (76.7–85.9)	184.9 (169.7–197.5)	677.6 (633.0–713.3)
HIV/AIDS and tuberculosis	25.5 (21.0–30.3)	15.4 (11.7–23.1)	3.0 (2.4–4.5)	19.1 (15.8–22.9)	62.6 (47.3–73.4)	114.9 (82.0–137.7)
Tuberculosis	22.7 (18.2–27.4)	9.8 (6.1–17.2)	2.8 (2.2–4.4)	15.5 (12.1–19.3)	61.0 (45.9–71.8)	114.5 (81.6–137.2)
HIV/AIDS	2.7 (2.4–3.1)	5.7 (5.2–6.3)	0.1 (0.1–0.2)	3.6 (3.2–4.1)	1.6 (1.4–1.9)	0.4 (0.3–0.5)
HIV/AIDS - Tuberculosis	0.8 (0.7–0.9)	1.9 (1.5–2.2)	0.0 (0.0–0.0)	1.0 (0.9–1.2)	0.5 (0.4–0.5)	0.1 (0.1–0.1)
HIV/AIDS resulting in other diseases	1.9 (1.7–2.2)	3.8 (3.3–4.4)	0.1 (0.1–0.1)	2.5 (2.2–3.1)	1.1 (0.9–1.4)	0.3 (0.3–0.4)
Diarrhea, lower respiratory, and other common infectious diseases	151.8 (143.4–160.4)	814.7 (759.3–875.0)	46.4 (41.5–51.9)	22.9 (21.1–25.3)	95.1 (88.1–102.7)	493.5 (461.0–521.9)
Diarrheal diseases	45.4 (41.3–49.1)	220.7 (189.9–250.7)	11.9 (10.2–13.4)	8.6 (8.0–9.2)	43.1 (40.4–45.9)	154.7 (144.9–163.6)
Intestinal infectious diseases	4.5 (2.5–7.4)	11.2 (6.0–19.4)	8.5 (4.9–13.9)	2.4 (1.3–4.1)	1.4 (0.7–2.4)	1.5 (0.9–2.4)
Typhoid fever	3.5 (1.9–6.0)	8.4 (4.1–15.0)	6.8 (3.7–11.1)	1.9 (1.0–3.4)	1.1 (0.6–2.0)	1.2 (0.6–1.9)
Paratyphoid fever	0.9 (0.4–1.7)	2.2 (0.9–4.5)	1.7 (0.8–3.4)	0.4 (0.2–0.9)	0.2 (0.1–0.4)	0.2 (0.1–0.4)
Other intestinal infectious diseases	0.1 (0.0–0.2)	0.7 (0.1–1.7)	0.1 (0.0–0.2)	0.0 (0.0–0.0)	0.0 (0.0–0.1)	0.2 (0.1–0.3)
Lower respiratory infections	63.8 (58.2–68.4)	338.6 (301.1–372.8)	8.7 (6.3–10.0)	6.3 (5.1–7.1)	37.2 (30.3–41.6)	310.3 (275.3–333.8)
Upper respiratory infections	0.1 (0.1–0.1)	0.3 (0.2–0.4)	0.0 (0.0–0.0)	0.0 (0.0–0.0)	0.1 (0.1–0.1)	0.4 (0.3–0.5)
Otitis media	0.1 (0.1–0.1)	0.4 (0.4–0.5)	0.1 (0.0–0.1)	0.1 (0.0–0.1)	0.1 (0.1–0.2)	0.4 (0.2–0.6)
Meningitis	9.2 (7.4–10.7)	48.8 (36.9–61.3)	4.2 (3.3–4.8)	2.5 (2.1–2.9)	5.9 (5.0–7.0)	12.0 (10.7–14.3)
Pneumococcal meningitis	2.3 (1.8–2.9)	10.6 (7.0–14.9)	1.3 (0.9–1.8)	0.8 (0.6–1.0)	1.6 (1.2–2.2)	3.3 (2.6–4.2)
H influenzae type B meningitis	2.8 (2.1–3.6)	18.3 (12.9–24.4)	1.2 (0.8–1.6)	0.4 (0.3–0.6)	1.0 (0.7–1.3)	2.1 (1.6–2.7)
Meningococcal meningitis	1.5 (1.1–2.0)	8.3 (5.3–12.3)	0.7 (0.4–1.0)	0.4 (0.3–0.5)	1.0 (0.7–1.3)	1.8 (1.4–2.4)
Other meningitis	2.5 (2.0–3.1)	11.6 (8.1–15.9)	1.0 (0.7–1.3)	0.9 (0.7–1.0)	2.3 (1.9–2.9)	4.8 (3.9–5.7)
Encephalitis	2.8 (2.1–3.4)	10.0 (6.6–12.7)	1.9 (1.4–2.3)	1.0 (0.8–1.2)	3.1 (2.4–3.8)	8.0 (6.3–9.8)
Diphtheria	0.3 (0.2–0.6)	2.0 (1.0–4.3)	0.2 (0.1–0.3)	0.0 (0.0–0.1)	0.0 (0.0–0.1)	0 (0–0)
Whooping cough	3.4 (1.2–8.0)	26.7 (9.3–62.4)	0.9 (0.3–2.2)	0.1 (0.0–0.2)	0.0 (0.0–0.1)	0 (0–0)
Tetanus	6.0 (5.1–7.5)	37.6 (31.3–44.9)	1.1 (0.8–2.1)	1.2 (1.0–2.3)	3.7 (3.0–7.3)	4.6 (3.6–9.2)
Measles	16.0 (6.5–32.1)	117.6 (47.9–232.7)	8.9 (3.5–17.8)	0.7 (0.2–1.8)	0.1 (0.0–0.2)	0 (0–0)
Varicella and herpes zoster	0.2 (0.2–0.3)	0.7 (0.5–0.9)	0.1 (0.1–0.1)	0.1 (0.1–0.1)	0.3 (0.2–0.4)	1.6 (1.2–2.1)
Neglected tropical diseases and malaria	21.1 (16.6–25.8)	122.2 (86.1–160.5)	9.1 (6.7–11.8)	5.1 (4.0–6.5)	12.7 (9.9–16.6)	14.2 (10.7–19.1)
Malaria	18.3 (13.8–22.9)	116.0 (79.7–155.5)	6.8 (4.5–9.5)	3.4 (2.5–4.8)	8.6 (6.0–12.3)	9.1 (5.8–13.8)
Chagas disease	0.1 (0.1–0.1)	0.0 (0.0–0.0)	0.0 (0.0–0.0)	0.1 (0.1–0.1)	0.4 (0.4–0.5)	0.9 (0.9–1.0)
Leishmaniasis	0.2 (0.1–0.3)	0.4 (0.2–0.7)	0.1 (0.1–0.2)	0.2 (0.1–0.3)	0.1 (0.1–0.2)	0.1 (0.1–0.1)
Visceral leishmaniasis	0.2 (0.1–0.3)	0.4 (0.2–0.7)	0.1 (0.1–0.2)	0.2 (0.1–0.3)	0.1 (0.1–0.2)	0.1 (0.1–0.1)
Cutaneous and mucocutaneous leishmaniasis	0 (0–0)	0 (0–0)	0 (0–0)	0 (0–0)	0 (0–0)	0 (0–0)
African trypanosomiasis	0.4 (0.2–0.7)	0.3 (0.1–0.5)	0.3 (0.1–0.4)	0.5 (0.2–0.8)	0.6 (0.3–1.1)	0.4 (0.2–0.7)
Schistosomiasis	0.3 (0.2–0.3)	0.0 (0.0–0.1)	0.0 (0.0–0.1)	0.1 (0.1–0.2)	0.8 (0.6–1.1)	1.3 (0.9–1.7)
Cysticercosis	0.0 (0.0–0.0)	0.0 (0.0–0.0)	0.0 (0.0–0.0)	0.0 (0.0–0.0)	0.0 (0.0–0.0)	0.0 (0.0–0.0)
Cystic echinococcosis	0.1 (0.1–0.1)	0.0 (0.0–0.1)	0.0 (0.0–0.0)	0.0 (0.0–0.0)	0.1 (0.1–0.1)	0.2 (0.2–0.3)
Lymphatic filariasis	0 (0–0)	0 (0–0)	0 (0–0)	0 (0–0)	0 (0–0)	0 (0–0)
Onchocerciasis	0 (0–0)	0 (0–0)	0 (0–0)	0 (0–0)	0 (0–0)	0 (0–0)
Trachoma	0 (0–0)	0 (0–0)	0 (0–0)	0 (0–0)	0 (0–0)	0 (0–0)
Dengue	0.1 (0.1–0.3)	0.5 (0.2–1.2)	0.2 (0.1–0.3)	0.0 (0.0–0.1)	0.1 (0.0–0.1)	0.1 (0.1–0.2)
Yellow fever	0.1 (0.0–0.4)	0.1 (0.0–0.4)	0.2 (0.0–0.5)	0.1 (0.0–0.3)	0.1 (0.0–0.3)	0.1 (0.0–0.2)
Rabies	0.9 (0.7–1.0)	1.5 (1.0–2.0)	1.1 (0.9–1.4)	0.5 (0.4–0.6)	1.3 (1.1–1.6)	1.2 (1.0–1.7)
Intestinal nematode infections	0.1 (0.1–0.2)	0.9 (0.8–1.1)	0.1 (0.1–0.1)	0.0 (0.0–0.0)	0.0 (0.0–0.0)	0.0 (0.0–0.1)
Ascariasis	0.1 (0.1–0.2)	0.9 (0.8–1.1)	0.1 (0.1–0.1)	0.0 (0.0–0.0)	0.0 (0.0–0.0)	0.0 (0.0–0.1)
Trichuriasis	0 (0–0)	0 (0–0)	0 (0–0)	0 (0–0)	0 (0–0)	0 (0–0)
Hookworm disease	0 (0–0)	0 (0–0)	0 (0–0)	0 (0–0)	0 (0–0)	0 (0–0)
Food-borne trematodiases	0 (0–0)	0 (0–0)	0 (0–0)	0 (0–0)	0 (0–0)	0 (0–0)
Leprosy	0 (0–0)	0 (0–0)	0 (0–0)	0 (0–0)	0 (0–0)	0 (0–0)
Ebola	0 (0–0)	0 (0–0)	0 (0–0)	0 (0–0)	0 (0–0)	0 (0–0)
Other neglected tropical diseases	0.5 (0.3–0.7)	2.3 (1.4–3.4)	0.3 (0.2–0.4)	0.1 (0.1–0.2)	0.4 (0.3–0.8)	0.7 (0.5–1.4)
Maternal disorders	14.8 (13.9–15.8)	0 (0–0)	0.4 (0.3–0.4)	29.3 (27.4–31.3)	0.4 (0.3–0.6)	0 (0–0)
Maternal hemorrhage	4.0 (3.3–4.8)	0 (0–0)	0.1 (0.1–0.1)	7.9 (6.6–9.4)	0.1 (0.1–0.2)	0 (0–0)
Maternal sepsis and other maternal infections	1.1 (0.9–1.4)	0 (0–0)	0.0 (0.0–0.0)	2.2 (1.7–2.8)	0.0 (0.0–0.0)	0 (0–0)
Maternal hypertensive disorders	1.9 (1.5–2.4)	0 (0–0)	0.1 (0.0–0.1)	3.8 (3.0–4.7)	0.0 (0.0–0.1)	0 (0–0)
Maternal obstructed labor and uterine rupture	0.9 (0.7–1.1)	0 (0–0)	0.0 (0.0–0.0)	1.8 (1.4–2.3)	0.0 (0.0–0.0)	0 (0–0)
Maternal abortion, miscarriage, and ectopic pregnancy	2.1 (1.7–2.7)	0 (0–0)	0.1 (0.0–0.1)	4.2 (3.3–5.3)	0.1 (0.0–0.1)	0 (0–0)

SR Table 13 - Global death rates (per 100,000) by age and cause for females in 1990						
Cause	All ages	0-5 years	5-14 years	15-49 years	50-69 years	70+ years
Indirect maternal deaths	1.6 (1.2–2.0)	0 (0–0)	0.0 (0.0–0.1)	3.1 (2.4–4.0)	0.0 (0.0–0.1)	0 (0–0)
Late maternal deaths	0.3 (0.2–0.5)	0 (0–0)	0.0 (0.0–0.0)	0.6 (0.4–0.9)	0.0 (0.0–0.0)	0 (0–0)
Maternal deaths aggravated by HIV/AIDS	0.0 (0.0–0.0)	0 (0–0)	0.0 (0.0–0.0)	0.1 (0.0–0.1)	0.0 (0.0–0.0)	0 (0–0)
Other maternal disorders	2.8 (2.3–3.4)	0 (0–0)	0.1 (0.0–0.1)	5.6 (4.6–6.7)	0.1 (0.0–0.1)	0 (0–0)
Neonatal disorders	59.0 (56.9–61.3)	501.0 (483.4–520.7)	0 (0–0)	0 (0–0)	0 (0–0)	0 (0–0)
Neonatal preterm birth complications	31.2 (28.0–34.7)	265.2 (237.6–295.0)	0 (0–0)	0 (0–0)	0 (0–0)	0 (0–0)
Neonatal encephalopathy due to birth asphyxia and trauma	14.9 (12.5–17.3)	126.2 (106.3–147.3)	0 (0–0)	0 (0–0)	0 (0–0)	0 (0–0)
Neonatal sepsis and other neonatal infections	4.5 (2.1–6.9)	38.6 (18.2–59.0)	0 (0–0)	0 (0–0)	0 (0–0)	0 (0–0)
Hemolytic disease and other neonatal jaundice	2.2 (1.0–4.9)	18.8 (8.8–41.4)	0 (0–0)	0 (0–0)	0 (0–0)	0 (0–0)
Other neonatal disorders	6.1 (4.1–9.5)	52.2 (34.9–81.0)	0 (0–0)	0 (0–0)	0 (0–0)	0 (0–0)
Nutritional deficiencies	12.5 (10.7–14.3)	70.0 (56.3–84.4)	2.1 (1.8–2.4)	1.9 (1.7–2.1)	7.2 (6.5–7.9)	41.3 (37.8–44.6)
Protein-energy malnutrition	10.3 (8.5–12.1)	63.1 (49.8–77.5)	1.6 (1.3–1.9)	1.0 (0.6–1.3)	4.4 (3.1–5.3)	32.3 (25.8–36.7)
Iodine deficiency	0.1 (0.0–0.1)	0.3 (0.1–0.5)	0.0 (0.0–0.0)	0.0 (0.0–0.0)	0.1 (0.0–0.1)	0.2 (0.1–0.4)
Vitamin A deficiency	0 (0–0)	0 (0–0)	0 (0–0)	0 (0–0)	0 (0–0)	0 (0–0)
Iron-deficiency anemia	1.0 (0.6–1.3)	3.2 (1.2–4.9)	0.2 (0.1–0.3)	0.5 (0.4–0.7)	1.6 (1.2–2.1)	2.6 (1.7–4.3)
Other nutritional deficiencies	1.1 (0.4–2.5)	3.3 (0.5–10.2)	0.3 (0.1–0.5)	0.4 (0.1–0.7)	1.1 (0.4–2.5)	6.1 (3.5–11.3)
Other communicable, maternal, neonatal, and nutritional diseases	8.3 (6.7–10.4)	41.6 (29.5–57.8)	3.1 (2.5–4.0)	2.4 (2.2–2.6)	6.9 (6.2–7.5)	13.7 (12.0–14.8)
Sexually transmitted diseases excluding HIV	4.0 (2.5–6.1)	28.2 (16.0–44.1)	1.6 (0.9–2.4)	0.4 (0.3–0.5)	0.9 (0.4–1.1)	1.7 (1.0–2.2)
Syphilis	4.0 (2.4–6.0)	28.2 (16.0–44.1)	1.6 (0.9–2.4)	0.4 (0.2–0.4)	0.7 (0.3–0.9)	1.4 (0.8–1.9)
Chlamydial infection	0.0 (0.0–0.0)	0 (0–0)	0.0 (0.0–0.0)	0.0 (0.0–0.0)	0.0 (0.0–0.0)	0.0 (0.0–0.1)
Gonococcal infection	0.0 (0.0–0.0)	0 (0–0)	0.0 (0.0–0.0)	0.0 (0.0–0.0)	0.1 (0.0–0.1)	0.2 (0.1–0.2)
Trichomoniasis	0 (0–0)	0 (0–0)	0 (0–0)	0 (0–0)	0 (0–0)	0 (0–0)
Genital herpes	0 (0–0)	0 (0–0)	0 (0–0)	0 (0–0)	0 (0–0)	0 (0–0)
Other sexually transmitted diseases	0.0 (0.0–0.0)	0 (0–0)	0.0 (0.0–0.0)	0.0 (0.0–0.0)	0.0 (0.0–0.0)	0.0 (0.0–0.1)
Hepatitis	2.7 (2.5–2.9)	7.0 (6.0–7.9)	1.1 (1.0–1.3)	1.6 (1.5–1.8)	3.8 (3.6–4.1)	6.6 (6.2–7.0)
Acute hepatitis A	0.7 (0.5–0.9)	5.3 (3.7–6.5)	0.2 (0.0–0.4)	0.0 (0.0–0.1)	0.1 (0.0–0.1)	0.1 (0.0–0.2)
Hepatitis B	1.1 (0.9–1.3)	0.4 (0.1–0.9)	0.1 (0.1–0.2)	0.8 (0.6–1.0)	3.1 (2.7–3.5)	4.9 (4.0–5.7)
Hepatitis C	0.0 (0.0–0.1)	0 (0–0)	0.0 (0.0–0.0)	0.0 (0.0–0.1)	0.2 (0.0–0.4)	0.2 (0.0–0.5)
Acute hepatitis E	0.8 (0.6–1.1)	1.4 (0.5–2.6)	0.8 (0.6–1.0)	0.7 (0.6–0.9)	0.5 (0.2–0.9)	1.4 (0.7–2.3)
Other infectious diseases	1.6 (1.0–1.8)	6.4 (3.1–8.5)	0.4 (0.3–0.5)	0.4 (0.3–0.4)	2.3 (1.8–2.5)	5.4 (4.1–6.1)
Non-communicable diseases	508.1 (496.7–522.0)	192.2 (158.2–256.8)	22.6 (20.6–25.3)	98.7 (94.8–103.5)	1094.5 (1065.1–1125.0)	6315.2 (6220.8–6415.5)
Neoplasms	94.6 (92.3–96.9)	7.2 (6.1–9.0)	4.8 (4.4–5.2)	31.0 (29.8–32.5)	303.8 (295.5–311.7)	817.9 (800.6–834.9)
Lip and oral cavity cancer	1.0 (1.0–1.1)	0 (0–0)	0 (0–0)	0.3 (0.3–0.3)	3.4 (3.2–3.6)	9.0 (8.6–9.4)
Nasopharynx cancer	0.6 (0.5–0.6)	0 (0–0)	0.0 (0.0–0.0)	0.4 (0.4–0.5)	2.0 (1.8–2.1)	2.5 (2.2–2.7)
Other pharynx cancer	0.5 (0.4–0.5)	0 (0–0)	0 (0–0)	0.1 (0.1–0.2)	1.9 (1.7–2.1)	3.3 (3.0–3.5)
Esophageal cancer	4.7 (4.3–5.2)	0 (0–0)	0 (0–0)	0.9 (0.8–0.9)	17.4 (15.8–19.2)	44.0 (40.4–48.4)
Stomach cancer	11.0 (10.5–11.5)	0 (0–0)	0 (0–0)	3.0 (2.8–3.3)	33.2 (31.6–34.9)	111.9 (108.0–115.9)
Colon and rectum cancer	9.3 (9.1–9.5)	0 (0–0)	0 (0–0)	1.7 (1.7–1.8)	25.8 (25.2–26.5)	110.0 (107.0–113.4)
Liver cancer	6.3 (5.2–6.9)	0 (0–0)	0.1 (0.1–0.1)	2.2 (1.6–2.5)	22.3 (17.9–24.5)	49.2 (43.5–53.9)
Liver cancer due to hepatitis B	2.1 (1.6–2.3)	0 (0–0)	0.1 (0.0–0.1)	1.2 (0.9–1.4)	7.4 (5.8–8.5)	10.4 (8.5–11.9)
Liver cancer due to hepatitis C	1.6 (1.4–1.8)	0 (0–0)	0.0 (0.0–0.0)	0.2 (0.2–0.3)	5.3 (4.4–6.0)	17.4 (16.0–19.0)
Liver cancer due to alcohol use	1.0 (0.8–1.1)	0 (0–0)	0 (0–0)	0.1 (0.1–0.2)	3.9 (3.1–4.5)	8.3 (7.3–9.2)
Liver cancer due to other causes	1.7 (1.3–1.9)	0 (0–0)	0.1 (0.0–0.1)	0.6 (0.4–0.7)	5.6 (4.4–6.5)	13.1 (11.0–14.8)
Gallbladder and biliary tract cancer	2.5 (2.4–2.6)	0 (0–0)	0 (0–0)	0.3 (0.3–0.4)	7.5 (7.1–7.9)	29.3 (28.3–30.4)
Pancreatic cancer	4.0 (3.9–4.1)	0 (0–0)	0 (0–0)	0.5 (0.5–0.5)	12.1 (11.7–12.4)	48.1 (47.0–49.2)
Larynx cancer	0.6 (0.6–0.7)	0 (0–0)	0 (0–0)	0.2 (0.2–0.2)	2.2 (2.0–2.4)	5.0 (4.7–5.3)
Tracheal, bronchus, and lung cancer	10.4 (10.0–10.8)	0 (0–0)	0 (0–0)	2.1 (2.0–2.3)	37.1 (35.7–38.8)	97.4 (94.1–101.7)
Malignant skin melanoma	0.6 (0.6–0.7)	0 (0–0)	0 (0–0)	0.3 (0.2–0.3)	1.7 (1.6–1.9)	5.4 (5.1–6.2)
Non-melanoma skin cancer	0.3 (0.3–0.3)	0 (0–0)	0 (0–0)	0.1 (0.1–0.1)	0.6 (0.6–0.7)	3.8 (3.6–4.1)
Non-melanoma skin cancer (squamous-cell carcinoma)	0.3 (0.3–0.3)	0 (0–0)	0 (0–0)	0.1 (0.1–0.1)	0.6 (0.6–0.7)	3.8 (3.6–4.1)
Non-melanoma skin cancer (basal-cell carcinoma)	0 (0–0)	0 (0–0)	0 (0–0)	0 (0–0)	0 (0–0)	0 (0–0)
Breast cancer	12.3 (11.9–13.2)	0 (0–0)	0 (0–0)	5.5 (4.9–6.2)	43.4 (41.8–46.3)	84.0 (81.2–87.9)
Cervical cancer	6.9 (6.4–7.4)	0 (0–0)	0 (0–0)	3.7 (3.4–4.0)	25.5 (23.5–27.4)	37.7 (35.7–40.9)
Uterine cancer	2.5 (2.3–2.6)	0 (0–0)	0 (0–0)	0.6 (0.6–0.7)	9.3 (8.7–9.9)	20.3 (19.5–21.7)
Ovarian cancer	3.9 (3.8–4.0)	0 (0–0)	0 (0–0)	1.4 (1.3–1.4)	14.8 (14.4–15.2)	28.2 (27.3–29.0)
Prostate cancer	0 (0–0)	0 (0–0)	0 (0–0)	0 (0–0)	0 (0–0)	0 (0–0)
Testicular cancer	0 (0–0)	0 (0–0)	0 (0–0)	0 (0–0)	0 (0–0)	0 (0–0)

SR Table 13 - Global death rates (per 100,000) by age and cause for females in 1990

Cause	All ages	0-5 years	5-14 years	15-49 years	50-69 years	70+ years
Kidney cancer	1.1 (1.1–1.1)	0.3 (0.2–0.5)	0.1 (0.1–0.1)	0.2 (0.2–0.2)	3.5 (3.4–3.6)	10.6 (10.2–10.9)
Bladder cancer	1.2 (1.2–1.2)	0 (0–0)	0 (0–0)	0.1 (0.1–0.2)	2.7 (2.6–2.8)	16.7 (16.2–17.3)
Brain and nervous system cancer	2.3 (2.1–2.5)	1.0 (0.7–1.2)	0.9 (0.7–1.0)	1.3 (1.1–1.4)	6.9 (6.4–7.2)	10.5 (10.0–11.0)
Thyroid cancer	0.5 (0.5–0.6)	0 (0–0)	0.0 (0.0–0.0)	0.1 (0.1–0.1)	1.5 (1.4–1.7)	5.7 (5.3–6.4)
Mesothelioma	0.2 (0.2–0.3)	0 (0–0)	0 (0–0)	0.1 (0.1–0.1)	0.7 (0.6–0.7)	2.1 (1.9–2.3)
Hodgkin lymphoma	0.5 (0.3–0.6)	0.1 (0.0–0.2)	0.1 (0.0–0.1)	0.4 (0.3–0.5)	1.2 (0.8–1.6)	2.5 (1.7–3.2)
Non-Hodgkin lymphoma	2.1 (1.9–2.4)	0.5 (0.3–0.9)	0.3 (0.2–0.4)	0.7 (0.6–0.9)	5.6 (5.0–6.3)	18.9 (17.5–22.6)
Multiple myeloma	1.0 (1.0–1.1)	0 (0–0)	0 (0–0)	0.2 (0.1–0.2)	3.2 (3.1–3.4)	10.8 (10.4–11.6)
Leukemia	4.3 (4.1–4.5)	3.1 (2.4–3.7)	2.4 (2.2–2.6)	2.4 (2.3–2.7)	8.3 (8.0–8.6)	24.5 (23.8–25.4)
Acute lymphoid leukemia	1.5 (1.4–1.7)	2.2 (1.7–2.8)	1.7 (1.5–2.0)	0.9 (0.8–1.1)	2.0 (1.8–2.2)	3.8 (3.5–4.0)
Chronic lymphoid leukemia	0.7 (0.7–0.8)	0 (0–0)	0 (0–0)	0.3 (0.2–0.4)	1.7 (1.5–1.9)	7.9 (7.5–8.4)
Acute myeloid leukemia	1.4 (1.3–1.5)	0.9 (0.5–1.2)	0.6 (0.5–0.7)	0.8 (0.8–0.9)	3.0 (2.8–3.1)	8.1 (7.7–8.4)
Chronic myeloid leukemia	0.6 (0.6–0.7)	0 (0–0)	0 (0–0)	0.4 (0.3–0.5)	1.7 (1.6–1.9)	4.8 (4.5–5.2)
Other neoplasms	4.0 (3.7–4.2)	2.2 (1.6–3.4)	0.9 (0.8–1.0)	2.0 (1.8–2.2)	9.9 (9.0–10.7)	26.4 (24.6–28.1)
Cardiovascular diseases	243.4 (238.2–248.7)	17.2 (15.2–19.3)	3.9 (3.6–4.3)	32.6 (31.1–34.2)	470.5 (457.5–484.6)	3568.2 (3489.3–3642.5)
Rheumatic heart disease	7.8 (7.2–8.5)	1.3 (0.9–1.7)	1.1 (0.9–1.2)	4.1 (3.8–4.6)	20.6 (19.0–22.5)	57.7 (53.2–62.5)
Ischemic heart disease	108.8 (106.6–110.9)	1.0 (0.8–1.3)	0.3 (0.2–0.3)	10.4 (9.9–11.1)	194.8 (189.6–200.0)	1708.7 (1670.5–1743.8)
Cerebrovascular disease	94.6 (91.5–98.7)	6.4 (5.5–7.6)	1.4 (1.3–1.6)	12.5 (11.9–13.4)	194.0 (186.2–204.3)	1358.2 (1309.0–1410.9)
Ischemic stroke	45.9 (42.4–50.3)	0.3 (0.2–0.4)	0.1 (0.1–0.1)	2.0 (1.7–2.5)	65.9 (57.3–76.2)	796.4 (746.3–856.5)
Hemorrhagic stroke	48.7 (45.4–51.5)	6.0 (5.2–7.3)	1.3 (1.2–1.5)	10.5 (9.9–11.4)	128.1 (119.2–136.9)	561.8 (515.4–594.1)
Hypertensive heart disease	13.8 (11.1–15.8)	0.2 (0.1–0.2)	0.0 (0.0–0.1)	2.0 (1.6–2.3)	31.3 (24.7–36.3)	190.0 (153.7–221.0)
Cardiomyopathy and myocarditis	4.8 (4.7–5.0)	2.6 (2.1–3.2)	0.4 (0.4–0.5)	1.1 (1.0–1.2)	7.6 (7.3–8.0)	63.6 (61.2–66.1)
Atrial fibrillation and flutter	2.1 (1.7–2.5)	0 (0–0)	0 (0–0)	0.0 (0.0–0.0)	1.0 (0.8–1.4)	42.6 (34.3–51.9)
Aortic aneurysm	1.2 (1.2–1.3)	0 (0–0)	0 (0–0)	0.2 (0.1–0.2)	2.1 (2.0–2.3)	19.0 (18.1–19.9)
Peripheral vascular disease	0.3 (0.3–0.3)	0 (0–0)	0 (0–0)	0.0 (0.0–0.0)	0.3 (0.2–0.3)	5.5 (5.1–5.9)
Endocarditis	1.0 (0.9–1.2)	0.8 (0.4–1.3)	0.1 (0.1–0.2)	0.3 (0.3–0.4)	1.6 (1.4–2.0)	11.5 (10.4–13.2)
Other cardiovascular and circulatory diseases	8.9 (8.5–9.4)	4.9 (4.1–5.9)	0.6 (0.5–0.7)	1.8 (1.7–2.0)	17.1 (16.1–18.3)	111.4 (105.1–117.4)
Chronic respiratory diseases	62.2 (59.0–66.1)	19.7 (16.6–22.5)	1.8 (1.6–2.0)	7.7 (7.2–8.4)	137.1 (129.3–146.3)	825.8 (782.5–889.7)
Chronic obstructive pulmonary disease	50.3 (44.0–56.7)	9.4 (6.1–12.2)	0.5 (0.3–0.8)	4.4 (3.5–5.6)	106.7 (87.8–124.7)	722.4 (648.0–801.8)
Pneumoconiosis	0.3 (0.2–0.4)	0 (0–0)	0 (0–0)	0.0 (0.0–0.1)	0.5 (0.3–0.8)	3.9 (2.7–5.6)
Silicosis	0.0 (0.0–0.0)	0 (0–0)	0 (0–0)	0.0 (0.0–0.0)	0.0 (0.0–0.0)	0.2 (0.1–0.3)
Asbestosis	0.0 (0.0–0.0)	0 (0–0)	0 (0–0)	0.0 (0.0–0.0)	0.0 (0.0–0.0)	0.1 (0.1–0.2)
Coal workers pneumoconiosis	0.0 (0.0–0.0)	0 (0–0)	0 (0–0)	0.0 (0.0–0.0)	0.0 (0.0–0.0)	0.1 (0.0–0.2)
Other pneumoconiosis	0.2 (0.2–0.3)	0 (0–0)	0 (0–0)	0.0 (0.0–0.1)	0.4 (0.2–0.7)	3.6 (2.4–5.1)
Asthma	10.2 (5.5–15.2)	6.0 (3.0–8.1)	1.2 (0.8–1.4)	3.1 (1.8–3.9)	28.1 (13.5–43.7)	89.0 (47.8–149.1)
Interstitial lung disease and pulmonary sarcoidosis	0.6 (0.5–0.8)	0.1 (0.0–0.1)	0.0 (0.0–0.0)	0.1 (0.1–0.1)	1.4 (1.2–1.8)	7.8 (6.5–10.8)
Other chronic respiratory diseases	0.8 (0.5–1.2)	4.3 (2.8–6.8)	0.1 (0.1–0.3)	0.1 (0.1–0.2)	0.4 (0.3–0.6)	2.6 (2.0–3.6)
Cirrhosis and other chronic liver diseases	12.9 (12.1–14.6)	3.9 (2.6–5.8)	1.1 (1.0–1.5)	5.7 (5.3–6.5)	41.1 (38.0–46.5)	87.6 (80.3–94.9)
Cirrhosis and other chronic liver diseases due to hepatitis B	3.4 (3.1–4.0)	0.0 (0.0–0.0)	0.0 (0.0–0.0)	1.5 (1.3–1.7)	11.7 (10.2–13.7)	25.2 (22.5–29.1)
Cirrhosis and other chronic liver diseases due to hepatitis C	2.8 (2.6–3.1)	0.0 (0.0–0.0)	0.0 (0.0–0.0)	1.1 (1.0–1.3)	9.3 (8.2–10.6)	22.4 (19.3–24.8)
Cirrhosis and other chronic liver diseases due to alcohol use	2.8 (2.6–3.1)	0 (0–0)	0 (0–0)	1.5 (1.3–1.7)	10.5 (9.3–11.8)	15.1 (13.6–17.0)
Cirrhosis and other chronic liver diseases due to other causes	3.9 (3.6–4.5)	3.9 (2.6–5.8)	1.1 (0.9–1.4)	1.6 (1.5–1.9)	9.6 (8.5–11.1)	24.9 (22.5–27.5)
Digestive diseases	17.8 (16.9–18.9)	10.5 (6.5–17.6)	2.5 (2.2–2.8)	6.2 (5.8–6.7)	37.4 (35.4–39.4)	176.0 (169.3–182.6)
Peptic ulcer disease	5.2 (4.5–5.9)	1.4 (0.7–2.9)	0.6 (0.5–0.7)	2.0 (1.7–2.4)	12.0 (10.1–13.9)	50.2 (43.6–57.1)
Gastritis and duodenitis	0.8 (0.3–1.2)	0.3 (0.1–0.6)	0.1 (0.0–0.1)	0.3 (0.1–0.5)	1.9 (0.6–2.8)	7.7 (3.1–11.5)
Appendicitis	0.9 (0.7–1.2)	1.8 (0.8–3.9)	0.4 (0.3–0.5)	0.5 (0.4–0.6)	1.4 (1.2–1.6)	3.8 (3.4–4.4)
Paralytic ileus and intestinal obstruction	3.4 (2.6–3.9)	2.7 (1.1–5.4)	0.7 (0.5–0.9)	1.3 (0.9–1.5)	6.8 (5.2–7.7)	29.9 (25.6–34.2)
Inguinal, femoral, and abdominal hernia	0.7 (0.5–0.8)	0.2 (0.1–0.4)	0.1 (0.0–0.1)	0.2 (0.1–0.2)	1.5 (1.0–1.8)	7.2 (5.3–8.3)
Inflammatory bowel disease	0.7 (0.6–0.9)	0.9 (0.2–2.1)	0.1 (0.0–0.1)	0.2 (0.2–0.3)	1.2 (1.0–1.4)	7.0 (6.1–7.6)
Vascular intestinal disorders	1.2 (1.0–1.3)	0.0 (0.0–0.1)	0.0 (0.0–0.0)	0.1 (0.1–0.1)	2.0 (1.7–2.3)	18.5 (17.0–20.0)
Gallbladder and biliary diseases	2.0 (1.9–2.2)	0.8 (0.4–1.6)	0.2 (0.1–0.2)	0.5 (0.5–0.6)	4.5 (4.1–4.9)	22.4 (21.1–23.9)
Pancreatitis	1.1 (1.0–1.2)	0.1 (0.0–0.2)	0.1 (0.0–0.1)	0.6 (0.5–0.7)	2.9 (2.5–3.2)	8.8 (8.1–9.4)
Other digestive diseases	1.9 (1.5–2.3)	2.2 (1.0–3.6)	0.3 (0.2–0.3)	0.5 (0.3–0.6)	3.3 (2.5–4.2)	20.6 (17.3–24.4)
Neurological disorders	25.3 (21.6–28.8)	3.2 (2.4–4.8)	1.2 (1.1–1.4)	2.1 (1.9–2.4)	14.8 (12.5–17.1)	475.9 (397.3–551.7)
Alzheimer disease and other dementias	21.6 (18.0–25.2)	0 (0–0)	0 (0–0)	0.0 (0.0–0.0)	9.0 (6.8–11.3)	449.6 (370.8–525.9)
Parkinson disease	0.8 (0.8–0.9)	0 (0–0)	0 (0–0)	0.0 (0.0–0.0)	1.0 (0.9–1.0)	15.5 (14.9–16.2)
Epilepsy	1.6 (1.4–1.9)	2.3 (1.6–3.6)	1.0 (0.9–1.2)	1.6 (1.4–1.8)	1.5 (1.4–1.6)	3.0 (2.8–3.2)

SR Table 13 - Global death rates (per 100,000) by age and cause for females in 1990						
**Cause**	**All ages**	**0-5 years**	**5-14 years**	**15-49 years**	**50-69 years**	**70+ years**
Multiple sclerosis	0.3 (0.2–0.4)	0 (0–0)	0 (0–0)	0.2 (0.2–0.3)	1.0 (0.9–1.2)	1.2 (1.1–1.3)
Motor neuron disease	0.3 (0.3–0.4)	0.3 (0.2–0.5)	0.0 (0.0–0.0)	0.1 (0.0–0.1)	1.1 (1.0–1.3)	2.5 (2.4–2.6)
Migraine	0 (0–0)	0 (0–0)	0 (0–0)	0 (0–0)	0 (0–0)	0 (0–0)
Tension-type headache	0 (0–0)	0 (0–0)	0 (0–0)	0 (0–0)	0 (0–0)	0 (0–0)
Medication overuse headache	0 (0–0)	0 (0–0)	0 (0–0)	0 (0–0)	0 (0–0)	0 (0–0)
Other neurological disorders	0.6 (0.6–0.7)	0.6 (0.5–1.4)	0.2 (0.2–0.3)	0.3 (0.2–0.3)	1.2 (1.2–1.3)	4.1 (3.8–4.2)
Mental and substance use disorders	1.6 (1.5–1.7)	0.0 (0.0–0.0)	0.0 (0.0–0.0)	1.7 (1.5–1.8)	3.8 (3.6–4.0)	5.2 (4.9–5.5)
Schizophrenia	0.3 (0.3–0.3)	0 (0–0)	0 (0–0)	0.2 (0.2–0.3)	0.7 (0.6–0.8)	1.7 (1.5–1.9)
Alcohol use disorders	0.6 (0.6–0.6)	0 (0–0)	0 (0–0)	0.5 (0.5–0.5)	2.0 (1.9–2.1)	1.7 (1.7–1.8)
Drug use disorders	0.7 (0.6–0.7)	0.0 (0.0–0.0)	0 (0–0)	0.9 (0.7–1.0)	1.1 (1.0–1.2)	1.8 (1.5–2.0)
Opioid use disorders	0.4 (0.3–0.5)	0.0 (0.0–0.0)	0 (0–0)	0.5 (0.4–0.6)	0.6 (0.6–0.7)	0.8 (0.7–1.1)
Cocaine use disorders	0.0 (0.0–0.0)	0 (0–0)	0 (0–0)	0.0 (0.0–0.0)	0.0 (0.0–0.0)	0.0 (0.0–0.0)
Amphetamine use disorders	0.0 (0.0–0.0)	0 (0–0)	0 (0–0)	0.0 (0.0–0.1)	0.0 (0.0–0.0)	0.0 (0.0–0.0)
Cannabis use disorders	0 (0–0)	0 (0–0)	0 (0–0)	0 (0–0)	0 (0–0)	0 (0–0)
Other drug use disorders	0.3 (0.2–0.3)	0 (0–0)	0 (0–0)	0.4 (0.2–0.5)	0.5 (0.3–0.5)	0.9 (0.6–1.1)
Depressive disorders	0 (0–0)	0 (0–0)	0 (0–0)	0 (0–0)	0 (0–0)	0 (0–0)
Major depressive disorder	0 (0–0)	0 (0–0)	0 (0–0)	0 (0–0)	0 (0–0)	0 (0–0)
Dysthymia	0 (0–0)	0 (0–0)	0 (0–0)	0 (0–0)	0 (0–0)	0 (0–0)
Bipolar disorder	0 (0–0)	0 (0–0)	0 (0–0)	0 (0–0)	0 (0–0)	0 (0–0)
Anxiety disorders	0 (0–0)	0 (0–0)	0 (0–0)	0 (0–0)	0 (0–0)	0 (0–0)
Eating disorders	0.0 (0.0–0.0)	0 (0–0)	0.0 (0.0–0.0)	0.0 (0.0–0.0)	0 (0–0)	0 (0–0)
Anorexia nervosa	0.0 (0.0–0.0)	0 (0–0)	0.0 (0.0–0.0)	0.0 (0.0–0.0)	0 (0–0)	0 (0–0)
Bulimia nervosa	0.0 (0.0–0.0)	0 (0–0)	0.0 (0.0–0.0)	0.0 (0.0–0.0)	0 (0–0)	0 (0–0)
Autistic spectrum disorders	0 (0–0)	0 (0–0)	0 (0–0)	0 (0–0)	0 (0–0)	0 (0–0)
Autism	0 (0–0)	0 (0–0)	0 (0–0)	0 (0–0)	0 (0–0)	0 (0–0)
Asperger syndrome and other autistic spectrum disorders	0 (0–0)	0 (0–0)	0 (0–0)	0 (0–0)	0 (0–0)	0 (0–0)
Attention-deficit/hyperactivity disorder	0 (0–0)	0 (0–0)	0 (0–0)	0 (0–0)	0 (0–0)	0 (0–0)
Conduct disorder	0 (0–0)	0 (0–0)	0 (0–0)	0 (0–0)	0 (0–0)	0 (0–0)
Idiopathic developmental intellectual disability	0 (0–0)	0 (0–0)	0 (0–0)	0 (0–0)	0 (0–0)	0 (0–0)
Other mental and substance use disorders	0 (0–0)	0 (0–0)	0 (0–0)	0 (0–0)	0 (0–0)	0 (0–0)
Diabetes, urogenital, blood, and endocrine diseases	33.6 (32.7–34.8)	22.6 (16.4–28.7)	3.3 (2.9–3.7)	9.2 (8.8–9.8)	80.4 (77.5–83.3)	331.3 (321.5–341.7)
Diabetes mellitus	14.1 (13.5–14.6)	0.7 (0.5–0.9)	0.3 (0.3–0.4)	2.5 (2.4–2.6)	41.7 (39.7–43.4)	157.6 (151.0–164.2)
Acute glomerulonephritis	0.3 (0.2–0.4)	0.2 (0.1–0.2)	0.1 (0.1–0.1)	0.2 (0.1–0.2)	0.6 (0.5–0.9)	1.7 (1.3–3.1)
Chronic kidney disease	11.2 (10.9–11.6)	3.7 (3.2–4.3)	1.0 (0.9–1.1)	3.7 (3.5–3.9)	26.3 (25.4–27.4)	116.1 (112.1–120.3)
Chronic kidney disease due to diabetes mellitus	3.4 (3.3–3.6)	0.4 (0.4–0.5)	0.1 (0.1–0.1)	0.8 (0.8–0.9)	9.7 (9.0–10.5)	36.4 (34.3–38.6)
Chronic kidney disease due to hypertension	4.9 (4.7–5.1)	1.2 (1.0–1.4)	0.3 (0.3–0.3)	1.3 (1.3–1.5)	11.0 (10.3–11.8)	57.0 (54.0–60.1)
Chronic kidney disease due to glomerulonephritis	2.5 (2.4–2.7)	1.5 (1.3–1.8)	0.5 (0.4–0.5)	1.3 (1.2–1.4)	5.3 (4.8–5.7)	20.2 (18.7–21.9)
Chronic kidney disease due to other causes	0.3 (0.3–0.4)	0.5 (0.4–0.7)	0.2 (0.1–0.2)	0.2 (0.1–0.2)	0.3 (0.2–0.5)	2.5 (2.1–3.1)
Urinary diseases and male infertility	2.6 (2.4–2.9)	1.3 (1.0–1.6)	0.2 (0.2–0.2)	0.7 (0.7–0.9)	5.3 (4.9–6.4)	28.7 (27.0–32.4)
Interstitial nephritis and urinary tract infections	2.1 (1.9–2.4)	1.1 (0.7–1.3)	0.1 (0.1–0.1)	0.6 (0.5–0.6)	4.1 (3.7–5.0)	24.7 (23.0–27.8)
Urolithiasis	0.2 (0.2–0.3)	0 (0–0)	0.0 (0.0–0.0)	0.1 (0.1–0.1)	0.6 (0.5–0.8)	1.9 (1.5–2.2)
Benign prostatic hyperplasia	0 (0–0)	0 (0–0)	0 (0–0)	0 (0–0)	0 (0–0)	0 (0–0)
Male infertility	0 (0–0)	0 (0–0)	0 (0–0)	0 (0–0)	0 (0–0)	0 (0–0)
Other urinary diseases	0.3 (0.2–0.3)	0.3 (0.2–0.4)	0.0 (0.0–0.0)	0.1 (0.1–0.1)	0.6 (0.5–0.8)	2.1 (1.8–2.6)
Gynecological diseases	0.2 (0.2–0.3)	0 (0–0)	0 (0–0)	0.2 (0.2–0.3)	0.6 (0.4–0.7)	1.1 (0.9–1.5)
Uterine fibroids	0.1 (0.0–0.1)	0 (0–0)	0 (0–0)	0.1 (0.0–0.1)	0.2 (0.1–0.3)	0.3 (0.1–0.5)
Polycystic ovarian syndrome	0.0 (0.0–0.1)	0 (0–0)	0 (0–0)	0.0 (0.0–0.1)	0.1 (0.0–0.1)	0 (0–0)
Female infertility	0 (0–0)	0 (0–0)	0 (0–0)	0 (0–0)	0 (0–0)	0 (0–0)
Endometriosis	0.0 (0.0–0.0)	0 (0–0)	0 (0–0)	0.0 (0.0–0.0)	0.0 (0.0–0.0)	0 (0–0)
Genital prolapse	0.0 (0.0–0.1)	0 (0–0)	0 (0–0)	0.0 (0.0–0.0)	0.1 (0.0–0.2)	0.4 (0.2–0.8)
Premenstrual syndrome	0 (0–0)	0 (0–0)	0 (0–0)	0 (0–0)	0 (0–0)	0 (0–0)
Other gynecological diseases	0.1 (0.1–0.1)	0 (0–0)	0 (0–0)	0.1 (0.1–0.2)	0.2 (0.1–0.3)	0.4 (0.3–0.7)
Hemoglobinopathies and hemolytic anemias	3.6 (2.9–4.3)	13.5 (8.0–19.3)	1.4 (1.1–1.6)	1.4 (1.2–1.5)	3.2 (3.0–3.4)	14.2 (12.9–15.4)
Thalassemias	0.6 (0.4–0.8)	3.3 (1.8–4.9)	0.5 (0.3–0.6)	0.1 (0.1–0.1)	0.0 (0.0–0.0)	0.8 (0.6–1.0)
Thalassemias trait	0 (0–0)	0 (0–0)	0 (0–0)	0 (0–0)	0 (0–0)	0 (0–0)
Sickle cell disorders	1.6 (1.1–2.2)	10.0 (5.8–15.0)	0.7 (0.6–0.9)	0.5 (0.4–0.6)	0.1 (0.1–0.2)	1.0 (0.8–1.1)
Sickle cell trait	0 (0–0)	0 (0–0)	0 (0–0)	0 (0–0)	0 (0–0)	0 (0–0)

SR Table 13 - Global death rates (per 100,000) by age and cause for females in 1990						
**Cause**	**All ages**	**0-5 years**	**5-14 years**	**15-49 years**	**50-69 years**	**70+ years**
G6PD deficiency	0.2 (0.2–0.3)	0.1 (0.1–0.2)	0.1 (0.0–0.1)	0.3 (0.2–0.3)	0.4 (0.3–0.4)	0.2 (0.2–0.3)
G6PD trait	0 (0–0)	0 (0–0)	0 (0–0)	0 (0–0)	0 (0–0)	0 (0–0)
Other hemoglobinopathies and hemolytic anemias	1.2 (1.1–1.2)	0.2 (0.1–0.3)	0.1 (0.1–0.1)	0.4 (0.4–0.5)	2.7 (2.5–2.8)	12.3 (11.0–13.2)
Endocrine, metabolic, blood, and immune disorders	1.6 (1.5–1.7)	3.1 (2.5–3.8)	0.4 (0.3–0.4)	0.6 (0.5–0.6)	2.8 (2.6–3.0)	11.8 (11.2–12.5)
Musculoskeletal disorders	1.4 (1.2–1.6)	0 (0–0)	0.2 (0.1–0.3)	0.7 (0.6–1.0)	2.7 (2.4–3.1)	13.7 (12.4–14.6)
Rheumatoid arthritis	0.6 (0.5–0.7)	0 (0–0)	0.0 (0.0–0.1)	0.1 (0.1–0.2)	1.5 (1.3–1.8)	7.4 (6.3–8.1)
Osteoarthritis	0 (0–0)	0 (0–0)	0 (0–0)	0 (0–0)	0 (0–0)	0 (0–0)
Low back and neck pain	0 (0–0)	0 (0–0)	0 (0–0)	0 (0–0)	0 (0–0)	0 (0–0)
Low back pain	0 (0–0)	0 (0–0)	0 (0–0)	0 (0–0)	0 (0–0)	0 (0–0)
Neck pain	0 (0–0)	0 (0–0)	0 (0–0)	0 (0–0)	0 (0–0)	0 (0–0)
Gout	0 (0–0)	0 (0–0)	0 (0–0)	0 (0–0)	0 (0–0)	0 (0–0)
Other musculoskeletal disorders	0.8 (0.7–0.9)	0 (0–0)	0.2 (0.1–0.2)	0.6 (0.5–0.8)	1.2 (1.1–1.5)	6.3 (5.7–6.9)
Other non-communicable diseases	15.3 (10.7–23.3)	107.9 (73.2–170.8)	3.8 (2.6–5.6)	1.7 (1.3–2.3)	2.8 (2.3–3.8)	13.6 (10.1–18.7)
Congenital anomalies	13.9 (9.3–21.8)	102.8 (67.1–164.7)	3.7 (2.5–5.5)	1.5 (1.2–2.1)	1.5 (1.2–1.7)	2.8 (2.4–3.1)
Neural tube defects	2.6 (1.6–4.5)	20.7 (13.0–36.8)	0.4 (0.2–0.6)	0.1 (0.1–0.1)	0.1 (0.1–0.2)	0.1 (0.1–0.2)
Congenital heart anomalies	7.2 (4.5–11.5)	51.0 (30.0–83.8)	2.7 (1.7–4.0)	1.1 (0.8–1.5)	0.5 (0.4–0.7)	1.2 (1.0–1.4)
Cleft lip and cleft palate	0.1 (0.0–0.2)	0.9 (0.4–1.4)	0 (0–0)	0 (0–0)	0 (0–0)	0 (0–0)
Down syndrome	0.8 (0.2–1.9)	5.6 (0.9–14.6)	0.1 (0.0–0.2)	0.1 (0.1–0.2)	0.3 (0.2–0.4)	0.1 (0.0–0.1)
Turner syndrome	0 (0–0)	0 (0–0)	0 (0–0)	0 (0–0)	0 (0–0)	0 (0–0)
Klinefelter syndrome	0 (0–0)	0 (0–0)	0 (0–0)	0 (0–0)	0 (0–0)	0 (0–0)
Other chromosomal abnormalities	0.5 (0.2–1.9)	4.2 (1.2–15.6)	0.0 (0.0–0.1)	0.0 (0.0–0.0)	0.0 (0.0–0.0)	0.0 (0.0–0.0)
Other congenital anomalies	2.7 (1.8–4.8)	20.5 (13.3–37.0)	0.5 (0.4–0.8)	0.2 (0.1–0.2)	0.5 (0.4–0.6)	1.4 (1.1–1.6)
Skin and subcutaneous diseases	0.9 (0.6–1.4)	1.0 (0.5–1.9)	0.1 (0.0–0.1)	0.2 (0.1–0.3)	1.3 (0.8–2.4)	10.9 (7.3–16.1)
Dermatitis	0 (0–0)	0 (0–0)	0 (0–0)	0 (0–0)	0 (0–0)	0 (0–0)
Psoriasis	0 (0–0)	0 (0–0)	0 (0–0)	0 (0–0)	0 (0–0)	0 (0–0)
Cellulitis	0.1 (0.1–0.2)	0.1 (0.0–0.3)	0.0 (0.0–0.0)	0.0 (0.0–0.1)	0.3 (0.2–0.6)	1.3 (0.9–2.1)
Pyoderma	0.3 (0.2–0.6)	0.8 (0.4–1.6)	0.0 (0.0–0.1)	0.1 (0.1–0.2)	0.6 (0.3–1.1)	2.6 (1.7–4.5)
Scabies	0 (0–0)	0 (0–0)	0 (0–0)	0 (0–0)	0 (0–0)	0 (0–0)
Fungal skin diseases	0 (0–0)	0 (0–0)	0 (0–0)	0 (0–0)	0 (0–0)	0 (0–0)
Viral skin diseases	0 (0–0)	0 (0–0)	0 (0–0)	0 (0–0)	0 (0–0)	0 (0–0)
Acne vulgaris	0 (0–0)	0 (0–0)	0 (0–0)	0 (0–0)	0 (0–0)	0 (0–0)
Alopecia areata	0 (0–0)	0 (0–0)	0 (0–0)	0 (0–0)	0 (0–0)	0 (0–0)
Pruritus	0 (0–0)	0 (0–0)	0 (0–0)	0 (0–0)	0 (0–0)	0 (0–0)
Urticaria	0 (0–0)	0 (0–0)	0 (0–0)	0 (0–0)	0 (0–0)	0 (0–0)
Decubitus ulcer	0.4 (0.2–0.5)	0.0 (0.0–0.0)	0.0 (0.0–0.0)	0.0 (0.0–0.1)	0.4 (0.2–0.7)	6.5 (3.6–9.1)
Other skin and subcutaneous diseases	0.0 (0.0–0.1)	0.1 (0.0–0.1)	0.0 (0.0–0.0)	0.0 (0.0–0.0)	0.1 (0.0–0.1)	0.5 (0.3–0.7)
Sense organ diseases	0 (0–0)	0 (0–0)	0 (0–0)	0 (0–0)	0 (0–0)	0 (0–0)
Glaucoma	0 (0–0)	0 (0–0)	0 (0–0)	0 (0–0)	0 (0–0)	0 (0–0)
Cataract	0 (0–0)	0 (0–0)	0 (0–0)	0 (0–0)	0 (0–0)	0 (0–0)
Macular degeneration	0 (0–0)	0 (0–0)	0 (0–0)	0 (0–0)	0 (0–0)	0 (0–0)
Refraction and accommodation disorders	0 (0–0)	0 (0–0)	0 (0–0)	0 (0–0)	0 (0–0)	0 (0–0)
Age-related and other hearing loss	0 (0–0)	0 (0–0)	0 (0–0)	0 (0–0)	0 (0–0)	0 (0–0)
Other vision loss	0 (0–0)	0 (0–0)	0 (0–0)	0 (0–0)	0 (0–0)	0 (0–0)
Other sense organ diseases	0 (0–0)	0 (0–0)	0 (0–0)	0 (0–0)	0 (0–0)	0 (0–0)
Oral disorders	0 (0–0)	0 (0–0)	0 (0–0)	0 (0–0)	0 (0–0)	0 (0–0)
Deciduous caries	0 (0–0)	0 (0–0)	0 (0–0)	0 (0–0)	0 (0–0)	0 (0–0)
Permanent caries	0 (0–0)	0 (0–0)	0 (0–0)	0 (0–0)	0 (0–0)	0 (0–0)
Periodontal diseases	0 (0–0)	0 (0–0)	0 (0–0)	0 (0–0)	0 (0–0)	0 (0–0)
Edentulism and severe tooth loss	0 (0–0)	0 (0–0)	0 (0–0)	0 (0–0)	0 (0–0)	0 (0–0)
Other oral disorders	0 (0–0)	0 (0–0)	0 (0–0)	0 (0–0)	0 (0–0)	0 (0–0)
Sudden infant death syndrome	0.5 (0.3–0.7)	4.1 (3.0–5.8)	0 (0–0)	0 (0–0)	0 (0–0)	0 (0–0)
Injuries	56.4 (49.1–60.7)	97.5 (76.6–117.6)	26.6 (23.4–28.8)	42.9 (36.8–45.7)	67.9 (60.3–71.6)	200.1 (178.5–210.3)
Transport injuries	12.7 (11.7–13.5)	15.7 (11.4–19.6)	7.2 (6.5–7.9)	10.8 (10.2–11.4)	18.2 (17.4–19.1)	33.4 (32.3–34.6)
Road injuries	12.0 (11.1–12.8)	14.6 (10.7–18.1)	6.9 (6.3–7.6)	10.3 (9.7–10.9)	17.2 (16.5–18.1)	31.9 (30.7–33.1)
Pedestrian road injuries	6.2 (5.5–6.7)	7.7 (5.7–9.5)	4.0 (3.6–4.3)	4.5 (4.1–4.9)	9.7 (8.8–10.4)	20.2 (18.8–21.3)
Cyclist road injuries	0.5 (0.4–0.6)	0.3 (0.2–0.6)	0.3 (0.3–0.4)	0.4 (0.4–0.6)	0.8 (0.7–0.9)	1.0 (0.8–1.1)
Motorcyclist road injuries	1.1 (0.9–1.3)	1.3 (0.6–2.3)	0.5 (0.4–0.6)	1.2 (1.0–1.4)	1.4 (1.2–1.7)	1.6 (1.3–2.2)

SR Table 13 - Global death rates (per 100,000) by age and cause for females in 1990

Cause	All ages	0-5 years	5-14 years	15-49 years	50-69 years	70+ years
Motor vehicle road injuries	4.1 (3.8–4.5)	5.0 (3.4–6.5)	2.0 (1.8–2.4)	4.0 (3.8–4.5)	5.1 (4.8–5.8)	8.8 (8.1–9.9)
Other road injuries	0.1 (0.1–0.2)	0.2 (0.1–0.4)	0.1 (0.1–0.1)	0.1 (0.1–0.1)	0.2 (0.1–0.2)	0.3 (0.2–0.4)
Other transport injuries	0.7 (0.5–0.8)	1.2 (0.5–1.6)	0.3 (0.2–0.4)	0.6 (0.4–0.7)	1.0 (0.8–1.2)	1.6 (1.3–1.8)
Unintentional injuries	29.2 (24.4–32.0)	75.4 (56.4–92.8)	15.7 (12.7–17.4)	14.7 (12.2–16.0)	28.5 (24.8–31.1)	132.1 (117.5–140.6)
Falls	5.4 (4.6–5.8)	6.1 (2.7–8.7)	1.3 (1.1–1.5)	1.3 (1.2–1.5)	6.3 (5.7–6.7)	63.1 (57.5–67.4)
Drowning	7.0 (3.9–8.2)	26.2 (10.1–33.8)	7.1 (4.5–8.2)	3.0 (1.9–3.4)	4.1 (2.6–4.9)	10.0 (7.0–11.8)
Fire, heat, and hot substances	4.9 (3.0–5.6)	9.3 (3.8–12.2)	1.8 (1.1–2.1)	4.3 (2.5–4.9)	4.4 (3.2–4.8)	15.0 (11.9–16.2)
Poisonings	1.9 (1.4–2.4)	5.4 (2.2–9.3)	0.9 (0.8–1.1)	1.3 (1.1–1.5)	2.0 (1.6–2.3)	4.5 (3.9–5.1)
Exposure to mechanical forces	2.2 (1.9–2.7)	7.9 (5.6–10.9)	1.0 (0.9–1.3)	1.0 (0.9–1.2)	2.1 (1.9–2.7)	7.7 (6.0–9.3)
Unintentional firearm injuries	0.3 (0.2–0.3)	0.3 (0.2–0.5)	0.1 (0.1–0.2)	0.2 (0.1–0.2)	0.3 (0.2–0.4)	2.1 (1.8–2.4)
Unintentional suffocation	0.7 (0.5–1.0)	4.6 (2.9–6.6)	0.2 (0.2–0.4)	0.1 (0.1–0.1)	0.2 (0.1–0.3)	1.1 (0.9–1.5)
Other exposure to mechanical forces	1.2 (1.1–1.6)	3.0 (1.9–4.6)	0.6 (0.5–0.8)	0.7 (0.6–0.9)	1.6 (1.4–2.2)	4.5 (3.2–5.7)
Adverse effects of medical treatment	1.8 (1.3–2.3)	4.2 (1.9–6.4)	0.5 (0.3–0.7)	0.8 (0.5–1.0)	2.4 (1.8–2.9)	11.0 (8.4–12.6)
Animal contact	1.8 (1.2–2.1)	3.6 (1.9–4.6)	1.5 (0.9–1.7)	1.3 (0.8–1.5)	2.6 (1.7–2.9)	3.1 (2.1–3.6)
Venomous animal contact	1.6 (1.0–1.8)	2.6 (1.4–3.5)	1.2 (0.8–1.5)	1.2 (0.7–1.3)	2.3 (1.4–2.6)	2.5 (1.5–3.0)
Non-venomous animal contact	0.3 (0.2–0.4)	0.9 (0.5–1.4)	0.2 (0.2–0.3)	0.1 (0.1–0.2)	0.3 (0.2–0.4)	0.7 (0.6–0.8)
Foreign body	2.2 (1.4–3.3)	8.9 (4.8–17.7)	0.7 (0.5–1.1)	0.6 (0.4–1.0)	1.7 (1.3–2.6)	9.0 (7.3–11.8)
Pulmonary aspiration and foreign body in airway	1.6 (1.0–2.9)	7.3 (3.0–16.2)	0.5 (0.3–0.9)	0.3 (0.2–0.5)	1.1 (0.9–1.5)	6.8 (6.0–7.6)
Foreign body in eyes	0 (0–0)	0 (0–0)	0 (0–0)	0 (0–0)	0 (0–0)	0 (0–0)
Foreign body in other body part	0.6 (0.2–1.3)	1.6 (0.4–2.8)	0.2 (0.1–0.6)	0.3 (0.2–0.8)	0.6 (0.3–1.6)	2.3 (1.3–5.1)
Environmental heat and cold exposure	0.6 (0.4–1.0)	1.0 (0.4–1.3)	0.1 (0.1–0.2)	0.2 (0.1–0.3)	1.2 (0.7–2.1)	5.1 (3.2–8.2)
Other unintentional injuries	1.3 (1.2–1.5)	2.8 (2.1–3.5)	0.8 (0.7–1.0)	0.8 (0.7–1.0)	1.8 (1.6–2.2)	3.4 (3.0–4.1)
Self-harm and interpersonal violence	13.0 (9.6–14.1)	3.6 (3.1–4.1)	2.2 (1.7–2.4)	16.0 (11.4–17.6)	19.6 (14.8–21.4)	32.4 (25.0–35.3)
Self-harm	10.0 (6.6–11.1)	0 (0–0)	1.0 (0.6–1.2)	12.7 (7.8–14.2)	16.7 (11.9–18.4)	28.0 (20.6–30.8)
Interpersonal violence	2.9 (2.8–3.1)	3.6 (3.1–4.1)	1.2 (1.1–1.3)	3.4 (3.2–3.6)	3.0 (2.8–3.1)	4.4 (4.2–4.6)
Assault by firearm	0.6 (0.5–0.6)	0.3 (0.2–0.4)	0.2 (0.2–0.2)	0.8 (0.7–0.9)	0.6 (0.5–0.7)	0.7 (0.6–0.8)
Assault by sharp object	0.7 (0.6–0.7)	0.4 (0.2–0.6)	0.2 (0.2–0.2)	0.9 (0.8–1.0)	0.7 (0.6–0.8)	1.1 (1.0–1.2)
Assault by other means	1.7 (1.6–1.8)	2.9 (2.4–3.4)	0.8 (0.7–0.9)	1.7 (1.5–1.8)	1.7 (1.6–1.9)	2.6 (2.4–2.8)
Forces of nature, war, and legal intervention	1.6 (1.1–2.2)	2.8 (1.4–4.9)	1.5 (0.8–2.3)	1.4 (0.9–1.9)	1.6 (0.9–2.3)	2.1 (1.1–4.4)
Exposure to forces of nature	0.7 (0.4–1.1)	0.9 (0.4–1.4)	0.9 (0.4–1.3)	0.6 (0.3–0.9)	0.8 (0.4–1.1)	0.9 (0.5–1.3)
Collective violence and legal intervention	0.9 (0.5–1.4)	1.9 (0.7–4.0)	0.6 (0.2–1.3)	0.8 (0.4–1.3)	0.8 (0.3–1.4)	1.2 (0.3–3.3)

SR Table 13 - Global death rates (per 100,000) by age and cause for females in 1995						
Cause	All ages	0-5 years	5-14 years	15-49 years	50-69 years	70+ years
All causes	825.8 (815.4–837.3)	1643.4 (1626.8–1662.1)	101.2 (99.8–102.7)	225.9 (220.1–232.1)	1324.9 (1296.9–1354.9)	7094.1 (7011.9–7175.7)
Communicable, maternal, neonatal, and nutritional diseases	257.6 (249.9–264.7)	1393.2 (1339.0–1432.7)	56.8 (53.9–60.4)	83.1 (79.8–87.8)	176.6 (163.5–187.9)	652.1 (611.3–684.5)
HIV/AIDS and tuberculosis	29.7 (25.8–33.9)	24.6 (21.7–30.0)	3.3 (2.8–4.5)	26.8 (24.1–30.1)	60.5 (47.3–68.6)	101.0 (74.2–120.1)
Tuberculosis	20.1 (16.1–24.0)	8.2 (5.4–13.5)	2.4 (2.0–3.7)	13.6 (10.9–16.9)	54.1 (40.7–62.4)	99.3 (72.6–118.2)
HIV/AIDS	9.6 (8.8–10.4)	16.5 (15.6–17.5)	0.9 (0.8–0.9)	13.2 (12.0–14.7)	6.3 (5.5–7.3)	1.7 (1.4–2.0)
HIV/AIDS - Tuberculosis	2.3 (1.9–2.8)	4.9 (4.2–5.6)	0.2 (0.2–0.3)	2.9 (2.4–3.5)	1.7 (1.5–2.0)	0.4 (0.4–0.5)
HIV/AIDS resulting in other diseases	7.3 (6.5–8.2)	11.6 (10.5–12.9)	0.6 (0.5–0.7)	10.3 (9.0–11.7)	4.6 (3.8–5.5)	1.2 (1.0–1.5)
Diarrhea, lower respiratory, and other common infectious diseases	124.8 (118.5–131.4)	670.3 (623.6–722.2)	39.6 (35.6–44.3)	20.6 (19.1–22.7)	88.4 (82.0–94.3)	475.2 (443.5–503.0)
Diarrheal diseases	36.1 (33.1–39.1)	176.8 (150.8–201.7)	9.7 (8.5–10.9)	7.4 (7.0–7.9)	37.8 (35.7–40.3)	133.6 (124.7–142.5)
Intestinal infectious diseases	4.1 (2.3–6.7)	10.7 (5.7–18.7)	7.9 (4.6–12.7)	2.1 (1.1–3.5)	1.2 (0.6–2.2)	1.4 (0.8–2.1)
Typhoid fever	3.3 (1.8–5.6)	8.6 (4.4–15.3)	6.6 (3.7–10.7)	1.7 (0.9–2.9)	1.0 (0.5–1.7)	1.0 (0.5–1.6)
Paratyphoid fever	0.7 (0.3–1.3)	1.6 (0.7–3.4)	1.3 (0.6–2.5)	0.4 (0.2–0.7)	0.2 (0.1–0.5)	0.2 (0.1–0.4)
Other intestinal infectious diseases	0.1 (0.0–0.2)	0.5 (0.1–1.1)	0.0 (0.0–0.1)	0.0 (0.0–0.0)	0.0 (0.0–0.0)	0.1 (0.1–0.3)
Lower respiratory infections	55.2 (50.7–58.9)	284.6 (253.9–312.4)	7.8 (5.8–9.0)	6.1 (5.1–7.0)	37.3 (30.2–41.2)	315.5 (280.9–339.0)
Upper respiratory infections	0.1 (0.1–0.1)	0.3 (0.2–0.4)	0.0 (0.0–0.0)	0.0 (0.0–0.0)	0.1 (0.1–0.1)	0.3 (0.3–0.4)
Otitis media	0.1 (0.1–0.1)	0.3 (0.3–0.4)	0.1 (0.0–0.1)	0.0 (0.0–0.1)	0.1 (0.1–0.1)	0.3 (0.2–0.6)
Meningitis	7.9 (6.6–9.2)	42.9 (32.9–54.4)	3.8 (3.0–4.4)	2.4 (2.0–2.8)	5.7 (4.9–6.9)	11.4 (10.2–13.7)
Pneumococcal meningitis	2.0 (1.6–2.5)	9.4 (6.4–13.4)	1.2 (0.8–1.7)	0.8 (0.6–1.0)	1.6 (1.2–2.1)	3.2 (2.5–4.0)
H influenzae type B meningitis	2.4 (1.8–3.0)	16.1 (11.5–21.6)	1.1 (0.7–1.5)	0.4 (0.3–0.5)	1.0 (0.7–1.3)	1.9 (1.5–2.5)
Meningococcal meningitis	1.4 (1.0–1.8)	7.8 (5.1–11.8)	0.6 (0.4–0.9)	0.4 (0.3–0.5)	0.9 (0.7–1.2)	1.7 (1.3–2.3)
Other meningitis	2.1 (1.7–2.6)	9.5 (6.7–13.3)	0.9 (0.6–1.2)	0.8 (0.7–1.0)	2.2 (1.8–2.8)	4.5 (3.7–5.4)
Encephalitis	2.6 (2.0–3.1)	9.6 (6.6–11.9)	1.7 (1.4–2.1)	0.9 (0.8–1.1)	2.9 (2.3–3.5)	7.7 (6.2–9.3)
Diphtheria	0.2 (0.1–0.4)	1.4 (0.6–3.2)	0.1 (0.1–0.2)	0.0 (0.0–0.0)	0.0 (0.0–0.1)	0 (0–0)
Whooping cough	2.6 (0.9–5.9)	22.5 (8.1–51.2)	0.7 (0.2–1.7)	0.1 (0.0–0.1)	0.0 (0.0–0.1)	0 (0–0)
Tetanus	3.7 (3.2–4.9)	23.4 (20.2–27.3)	0.8 (0.6–1.5)	0.9 (0.7–1.6)	2.8 (2.3–5.8)	3.5 (2.7–6.7)
Measles	12.1 (4.6–24.1)	97.2 (37.2–193.3)	6.7 (2.5–13.5)	0.5 (0.2–1.4)	0.1 (0.0–0.2)	0 (0–0)
Varicella and herpes zoster	0.2 (0.2–0.3)	0.6 (0.4–0.8)	0.1 (0.1–0.1)	0.1 (0.1–0.1)	0.3 (0.2–0.4)	1.5 (1.1–2.0)
Neglected tropical diseases and malaria	21.3 (16.9–25.7)	133.9 (96.3–173.9)	8.9 (6.6–11.5)	5.3 (4.1–6.9)	13.2 (10.0–17.4)	14.7 (10.9–19.9)
Malaria	18.8 (14.4–23.3)	128.6 (90.6–169.2)	6.9 (4.6–9.4)	3.8 (2.6–5.3)	9.5 (6.4–13.7)	10.2 (6.5–15.4)
Chagas disease	0.1 (0.1–0.1)	0.0 (0.0–0.0)	0.0 (0.0–0.0)	0.0 (0.0–0.0)	0.4 (0.4–0.4)	0.9 (0.8–1.0)
Leishmaniasis	0.2 (0.1–0.3)	0.5 (0.2–0.7)	0.1 (0.1–0.2)	0.2 (0.1–0.3)	0.1 (0.1–0.2)	0.1 (0.1–0.1)
Visceral leishmaniasis	0.2 (0.1–0.3)	0.5 (0.2–0.7)	0.1 (0.1–0.2)	0.2 (0.1–0.3)	0.1 (0.1–0.2)	0.1 (0.1–0.1)
Cutaneous and mucocutaneous leishmaniasis	0 (0–0)	0 (0–0)	0 (0–0)	0 (0–0)	0 (0–0)	0 (0–0)
African trypanosomiasis	0.5 (0.2–0.8)	0.3 (0.2–0.6)	0.3 (0.1–0.5)	0.5 (0.3–0.8)	0.7 (0.4–1.3)	0.5 (0.2–0.8)
Schistosomiasis	0.2 (0.2–0.2)	0.0 (0.0–0.1)	0.0 (0.0–0.0)	0.1 (0.1–0.1)	0.7 (0.5–0.8)	1.0 (0.7–1.3)
Cysticercosis	0.0 (0.0–0.0)	0.0 (0.0–0.0)	0.0 (0.0–0.0)	0.0 (0.0–0.0)	0.0 (0.0–0.0)	0.0 (0.0–0.0)
Cystic echinococcosis	0.0 (0.0–0.1)	0.0 (0.0–0.0)	0.0 (0.0–0.0)	0.0 (0.0–0.0)	0.1 (0.1–0.1)	0.2 (0.2–0.2)
Lymphatic filariasis	0 (0–0)	0 (0–0)	0 (0–0)	0 (0–0)	0 (0–0)	0 (0–0)
Onchocerciasis	0 (0–0)	0 (0–0)	0 (0–0)	0 (0–0)	0 (0–0)	0 (0–0)
Trachoma	0 (0–0)	0 (0–0)	0 (0–0)	0 (0–0)	0 (0–0)	0 (0–0)
Dengue	0.1 (0.1–0.2)	0.5 (0.2–1.0)	0.2 (0.1–0.3)	0.1 (0.0–0.1)	0.1 (0.1–0.1)	0.1 (0.1–0.2)
Yellow fever	0.1 (0.0–0.2)	0.1 (0.0–0.2)	0.1 (0.0–0.3)	0.1 (0.0–0.2)	0.1 (0.0–0.2)	0.0 (0.0–0.1)
Rabies	0.7 (0.6–0.9)	1.2 (0.9–1.7)	1.0 (0.7–1.2)	0.4 (0.3–0.5)	1.2 (0.9–1.4)	1.1 (0.8–1.5)
Intestinal nematode infections	0.1 (0.1–0.1)	0.8 (0.6–0.9)	0.1 (0.1–0.1)	0.0 (0.0–0.0)	0.0 (0.0–0.0)	0.0 (0.0–0.1)
Ascariasis	0.1 (0.1–0.1)	0.8 (0.6–0.9)	0.1 (0.1–0.1)	0.0 (0.0–0.0)	0.0 (0.0–0.0)	0.0 (0.0–0.1)
Trichuriasis	0 (0–0)	0 (0–0)	0 (0–0)	0 (0–0)	0 (0–0)	0 (0–0)
Hookworm disease	0 (0–0)	0 (0–0)	0 (0–0)	0 (0–0)	0 (0–0)	0 (0–0)
Food-borne trematodiases	0 (0–0)	0 (0–0)	0 (0–0)	0 (0–0)	0 (0–0)	0 (0–0)
Leprosy	0 (0–0)	0 (0–0)	0 (0–0)	0 (0–0)	0 (0–0)	0 (0–0)
Ebola	0.0 (0.0–0.0)	0.0 (0.0–0.0)	0.0 (0.0–0.0)	0.0 (0.0–0.0)	0.0 (0.0–0.0)	0.0 (0.0–0.0)
Other neglected tropical diseases	0.4 (0.3–0.6)	1.9 (1.3–2.8)	0.2 (0.1–0.3)	0.1 (0.1–0.2)	0.3 (0.2–0.6)	0.6 (0.4–1.1)
Maternal disorders	13.5 (12.7–14.4)	0 (0–0)	0.3 (0.3–0.4)	26.4 (24.8–28.2)	0.3 (0.2–0.5)	0 (0–0)
Maternal hemorrhage	3.8 (3.1–4.5)	0 (0–0)	0.1 (0.1–0.1)	7.3 (6.1–8.7)	0.1 (0.1–0.2)	0 (0–0)
Maternal sepsis and other maternal infections	1.0 (0.8–1.2)	0 (0–0)	0.0 (0.0–0.0)	1.9 (1.5–2.4)	0.0 (0.0–0.0)	0 (0–0)
Maternal hypertensive disorders	1.8 (1.5–2.3)	0 (0–0)	0.1 (0.0–0.1)	3.6 (2.9–4.4)	0.0 (0.0–0.0)	0 (0–0)
Maternal obstructed labor and uterine rupture	0.9 (0.7–1.1)	0 (0–0)	0.0 (0.0–0.0)	1.7 (1.3–2.2)	0.0 (0.0–0.0)	0 (0–0)
Maternal abortion, miscarriage, and ectopic pregnancy	1.8 (1.4–2.3)	0 (0–0)	0.1 (0.0–0.1)	3.5 (2.8–4.5)	0.1 (0.0–0.1)	0 (0–0)

SR Table 13 - Global death rates (per 100,000) by age and cause for females in 1995						
Cause	All ages	0-5 years	5-14 years	15-49 years	50-69 years	70+ years
Indirect maternal deaths	1.3 (1.0–1.7)	0 (0–0)	0.0 (0.0–0.1)	2.6 (2.0–3.3)	0.0 (0.0–0.1)	0 (0–0)
Late maternal deaths	0.3 (0.2–0.4)	0 (0–0)	0.0 (0.0–0.0)	0.6 (0.4–0.8)	0.0 (0.0–0.0)	0 (0–0)
Maternal deaths aggravated by HIV/AIDS	0.1 (0.0–0.1)	0 (0–0)	0.0 (0.0–0.0)	0.1 (0.1–0.2)	0.0 (0.0–0.0)	0 (0–0)
Other maternal disorders	2.5 (2.1–3.0)	0 (0–0)	0.1 (0.0–0.1)	5.0 (4.1–6.0)	0.1 (0.0–0.1)	0 (0–0)
Neonatal disorders	49.3 (47.7–50.9)	459.8 (445.2–475.3)	0 (0–0)	0 (0–0)	0 (0–0)	0 (0–0)
Neonatal preterm birth complications	24.6 (22.0–27.3)	229.4 (205.6–254.4)	0 (0–0)	0 (0–0)	0 (0–0)	0 (0–0)
Neonatal encephalopathy due to birth asphyxia and trauma	13.5 (11.6–15.5)	126.4 (108.7–144.5)	0 (0–0)	0 (0–0)	0 (0–0)	0 (0–0)
Neonatal sepsis and other neonatal infections	4.3 (2.1–6.5)	40.2 (19.2–60.7)	0 (0–0)	0 (0–0)	0 (0–0)	0 (0–0)
Hemolytic disease and other neonatal jaundice	1.8 (0.9–3.7)	16.8 (8.1–34.8)	0 (0–0)	0 (0–0)	0 (0–0)	0 (0–0)
Other neonatal disorders	5.0 (3.5–7.8)	46.9 (32.2–72.5)	0 (0–0)	0 (0–0)	0 (0–0)	0 (0–0)
Nutritional deficiencies	11.8 (10.0–13.8)	66.5 (52.8–80.8)	2.0 (1.7–2.4)	1.9 (1.7–2.1)	7.8 (6.8–9.0)	48.0 (40.7–55.9)
Protein-energy malnutrition	10.0 (8.1–12.0)	60.9 (48.1–75.1)	1.6 (1.3–1.9)	1.0 (0.8–1.3)	5.1 (3.9–6.4)	39.6 (31.8–48.1)
Iodine deficiency	0.1 (0.0–0.1)	0.2 (0.1–0.4)	0.0 (0.0–0.0)	0.0 (0.0–0.0)	0.1 (0.0–0.1)	0.2 (0.1–0.4)
Vitamin A deficiency	0 (0–0)	0 (0–0)	0 (0–0)	0 (0–0)	0 (0–0)	0 (0–0)
Iron-deficiency anemia	1.0 (0.6–1.2)	3.0 (1.2–4.3)	0.2 (0.1–0.3)	0.5 (0.4–0.6)	1.7 (1.2–2.1)	2.7 (1.8–4.1)
Other nutritional deficiencies	0.8 (0.4–1.9)	2.4 (0.5–6.7)	0.2 (0.1–0.4)	0.3 (0.1–0.6)	1.0 (0.4–2.2)	5.5 (3.2–9.7)
Other communicable, maternal, neonatal, and nutritional diseases	7.2 (5.9–8.9)	38.0 (27.1–52.8)	2.7 (2.2–3.5)	2.1 (2.0–2.3)	6.4 (5.8–6.8)	13.2 (11.9–14.3)
Sexually transmitted diseases excluding HIV	3.4 (2.1–5.1)	26.0 (14.8–40.9)	1.3 (0.7–2.0)	0.3 (0.2–0.4)	0.7 (0.4–0.8)	1.4 (0.9–1.8)
Syphilis	3.3 (2.0–5.0)	26.0 (14.8–40.9)	1.3 (0.7–2.0)	0.3 (0.2–0.4)	0.6 (0.3–0.7)	1.2 (0.7–1.5)
Chlamydial infection	0.0 (0.0–0.0)	0 (0–0)	0.0 (0.0–0.0)	0.0 (0.0–0.0)	0.0 (0.0–0.0)	0.0 (0.0–0.0)
Gonococcal infection	0.0 (0.0–0.0)	0 (0–0)	0.0 (0.0–0.0)	0.0 (0.0–0.0)	0.1 (0.0–0.1)	0.1 (0.1–0.2)
Trichomoniasis	0 (0–0)	0 (0–0)	0 (0–0)	0 (0–0)	0 (0–0)	0 (0–0)
Genital herpes	0 (0–0)	0 (0–0)	0 (0–0)	0 (0–0)	0 (0–0)	0 (0–0)
Other sexually transmitted diseases	0.0 (0.0–0.0)	0 (0–0)	0.0 (0.0–0.0)	0.0 (0.0–0.0)	0.0 (0.0–0.0)	0.0 (0.0–0.0)
Hepatitis	2.4 (2.3–2.6)	6.1 (5.2–6.9)	1.1 (0.9–1.2)	1.5 (1.4–1.6)	3.7 (3.5–3.9)	6.9 (6.6–7.3)
Acute hepatitis A	0.6 (0.4–0.8)	4.6 (3.3–5.7)	0.2 (0.0–0.3)	0.0 (0.0–0.1)	0.1 (0.0–0.2)	0.1 (0.0–0.3)
Hepatitis B	1.1 (0.9–1.2)	0.3 (0.1–0.8)	0.1 (0.1–0.2)	0.8 (0.6–0.9)	3.0 (2.6–3.4)	5.2 (4.3–6.0)
Hepatitis C	0.0 (0.0–0.1)	0 (0–0)	0.0 (0.0–0.0)	0.0 (0.0–0.1)	0.2 (0.0–0.4)	0.2 (0.0–0.5)
Acute hepatitis E	0.7 (0.5–1.0)	1.1 (0.4–2.2)	0.8 (0.6–1.0)	0.6 (0.5–0.8)	0.5 (0.2–0.8)	1.4 (0.7–2.3)
Other infectious diseases	1.4 (1.0–1.6)	6.0 (3.0–8.0)	0.4 (0.3–0.4)	0.3 (0.3–0.4)	2.0 (1.6–2.3)	4.9 (4.0–5.6)
Non-communicable diseases	515.1 (505.8–527.1)	170.2 (141.5–220.6)	20.9 (19.2–23.2)	100.5 (97.4–105.4)	1081.2 (1058.6–1106.4)	6245.6 (6168.1–6330.0)
Neoplasms	96.3 (94.4–98.5)	6.4 (5.6–7.9)	4.5 (4.2–4.9)	32.1 (31.0–33.5)	295.2 (288.1–303.0)	825.1 (809.8–840.7)
Lip and oral cavity cancer	1.0 (1.0–1.1)	0 (0–0)	0 (0–0)	0.3 (0.3–0.3)	3.4 (3.2–3.6)	9.2 (8.8–9.5)
Nasopharynx cancer	0.6 (0.5–0.6)	0 (0–0)	0.0 (0.0–0.0)	0.4 (0.4–0.4)	1.8 (1.7–1.9)	2.4 (2.2–2.5)
Other pharynx cancer	0.5 (0.4–0.5)	0 (0–0)	0 (0–0)	0.1 (0.1–0.2)	1.9 (1.7–2.0)	3.3 (3.1–3.5)
Esophageal cancer	4.6 (4.4–4.9)	0 (0–0)	0 (0–0)	0.9 (0.8–1.0)	16.3 (15.2–17.5)	42.7 (40.2–45.4)
Stomach cancer	10.3 (9.9–10.6)	0 (0–0)	0 (0–0)	2.9 (2.7–3.1)	29.9 (28.7–31.0)	102.0 (99.1–105.4)
Colon and rectum cancer	9.6 (9.4–9.8)	0 (0–0)	0 (0–0)	1.9 (1.8–2.0)	25.4 (24.8–26.1)	110.2 (107.4–112.8)
Liver cancer	6.5 (5.5–6.9)	0 (0–0)	0.1 (0.1–0.1)	2.3 (1.7–2.6)	21.8 (17.9–23.5)	50.6 (45.2–55.1)
Liver cancer due to hepatitis B	2.1 (1.7–2.3)	0 (0–0)	0.0 (0.0–0.1)	1.3 (0.9–1.4)	7.0 (5.6–8.0)	10.4 (8.6–11.8)
Liver cancer due to hepatitis C	1.7 (1.5–1.9)	0 (0–0)	0.0 (0.0–0.0)	0.2 (0.2–0.3)	5.4 (4.6–6.0)	18.5 (17.2–20.1)
Liver cancer due to alcohol use	1.0 (0.9–1.1)	0 (0–0)	0 (0–0)	0.2 (0.1–0.2)	4.1 (3.3–4.6)	8.7 (7.7–9.6)
Liver cancer due to other causes	1.6 (1.3–1.8)	0 (0–0)	0.1 (0.0–0.1)	0.6 (0.5–0.7)	5.3 (4.2–6.0)	13.0 (11.1–14.5)
Gallbladder and biliary tract cancer	2.4 (2.3–2.5)	0 (0–0)	0 (0–0)	0.3 (0.3–0.4)	7.1 (6.7–7.4)	27.8 (27.0–28.8)
Pancreatic cancer	4.2 (4.1–4.3)	0 (0–0)	0 (0–0)	0.5 (0.5–0.5)	11.9 (11.6–12.2)	49.0 (47.8–50.2)
Larynx cancer	0.6 (0.6–0.7)	0 (0–0)	0 (0–0)	0.2 (0.2–0.2)	2.2 (1.9–2.3)	4.9 (4.6–5.2)
Tracheal, bronchus, and lung cancer	11.3 (11.0–11.6)	0 (0–0)	0 (0–0)	2.4 (2.3–2.5)	37.1 (36.1–38.4)	107.6 (104.8–110.5)
Malignant skin melanoma	0.6 (0.6–0.7)	0 (0–0)	0 (0–0)	0.3 (0.3–0.3)	1.7 (1.6–1.9)	5.7 (5.2–6.3)
Non-melanoma skin cancer	0.3 (0.3–0.3)	0 (0–0)	0 (0–0)	0.1 (0.1–0.1)	0.6 (0.6–0.6)	3.9 (3.7–4.0)
Non-melanoma skin cancer (squamous-cell carcinoma)	0.3 (0.3–0.3)	0 (0–0)	0 (0–0)	0.1 (0.1–0.1)	0.6 (0.6–0.6)	3.9 (3.7–4.0)
Non-melanoma skin cancer (basal-cell carcinoma)	0 (0–0)	0 (0–0)	0 (0–0)	0 (0–0)	0 (0–0)	0 (0–0)
Breast cancer	12.8 (12.3–13.5)	0 (0–0)	0 (0–0)	5.9 (5.5–6.5)	43.1 (41.6–45.6)	86.2 (83.1–89.4)
Cervical cancer	6.8 (6.4–7.2)	0 (0–0)	0 (0–0)	3.8 (3.5–4.1)	24.3 (22.8–25.8)	36.2 (34.8–38.3)
Uterine cancer	2.5 (2.4–2.6)	0 (0–0)	0 (0–0)	0.7 (0.6–0.7)	9.1 (8.6–9.7)	20.2 (19.5–21.3)
Ovarian cancer	4.0 (3.9–4.1)	0 (0–0)	0 (0–0)	1.4 (1.4–1.5)	14.2 (13.9–14.7)	29.0 (28.2–29.8)
Prostate cancer	0 (0–0)	0 (0–0)	0 (0–0)	0 (0–0)	0 (0–0)	0 (0–0)
Testicular cancer	0 (0–0)	0 (0–0)	0 (0–0)	0 (0–0)	0 (0–0)	0 (0–0)

SR Table 13 - Global death rates (per 100,000) by age and cause for females in 1995						
**Cause**	**All ages**	**0-5 years**	**5-14 years**	**15-49 years**	**50-69 years**	**70+ years**
Kidney cancer	1.1 (1.1–1.2)	0.3 (0.2–0.4)	0.1 (0.1–0.1)	0.2 (0.2–0.2)	3.4 (3.3–3.5)	11.0 (10.7–11.4)
Bladder cancer	1.2 (1.2–1.3)	0 (0–0)	0 (0–0)	0.1 (0.1–0.2)	2.7 (2.6–2.8)	16.9 (16.4–17.4)
Brain and nervous system cancer	2.5 (2.3–2.6)	0.9 (0.7–1.1)	0.9 (0.7–0.9)	1.4 (1.2–1.5)	7.0 (6.7–7.3)	11.6 (11.1–12.0)
Thyroid cancer	0.5 (0.5–0.6)	0 (0–0)	0.0 (0.0–0.0)	0.1 (0.1–0.1)	1.5 (1.3–1.7)	5.6 (5.2–6.1)
Mesothelioma	0.2 (0.2–0.2)	0 (0–0)	0 (0–0)	0.1 (0.1–0.1)	0.6 (0.6–0.7)	2.0 (1.8–2.2)
Hodgkin lymphoma	0.4 (0.3–0.5)	0.1 (0.0–0.1)	0.1 (0.0–0.1)	0.3 (0.2–0.4)	1.0 (0.7–1.4)	2.2 (1.6–2.8)
Non-Hodgkin lymphoma	2.3 (2.0–2.5)	0.5 (0.3–0.8)	0.3 (0.2–0.4)	0.8 (0.7–0.9)	5.8 (5.1–6.5)	20.9 (18.1–22.8)
Multiple myeloma	1.1 (1.0–1.1)	0 (0–0)	0 (0–0)	0.2 (0.2–0.2)	3.3 (3.2–3.5)	11.7 (11.0–12.2)
Leukemia	4.2 (4.1–4.4)	2.6 (2.2–3.1)	2.2 (2.0–2.4)	2.4 (2.2–2.6)	8.2 (7.9–8.5)	25.3 (24.5–26.0)
Acute lymphoid leukemia	1.4 (1.3–1.6)	1.8 (1.5–2.3)	1.6 (1.4–1.8)	0.9 (0.8–1.1)	2.0 (1.8–2.2)	3.9 (3.6–4.1)
Chronic lymphoid leukemia	0.7 (0.7–0.8)	0 (0–0)	0 (0–0)	0.3 (0.2–0.4)	1.6 (1.4–1.8)	8.0 (7.6–8.5)
Acute myeloid leukemia	1.5 (1.4–1.5)	0.8 (0.5–1.0)	0.6 (0.5–0.7)	0.8 (0.8–0.9)	3.0 (2.9–3.1)	8.6 (8.3–8.9)
Chronic myeloid leukemia	0.6 (0.6–0.7)	0 (0–0)	0 (0–0)	0.4 (0.3–0.4)	1.6 (1.5–1.7)	4.8 (4.5–5.1)
Other neoplasms	4.0 (3.8–4.3)	2.0 (1.6–3.1)	0.9 (0.8–1.0)	2.1 (1.9–2.2)	9.9 (9.3–10.6)	27.2 (25.8–28.6)
Cardiovascular diseases	247.3 (243.3–252.0)	13.8 (12.4–15.2)	3.5 (3.2–3.7)	32.9 (31.7–34.5)	467.3 (457.3–478.3)	3483.6 (3421.0–3548.8)
Rheumatic heart disease	7.1 (6.7–7.7)	1.0 (0.7–1.3)	0.9 (0.8–1.1)	3.8 (3.4–4.2)	18.3 (17.1–19.8)	52.5 (49.6–55.8)
Ischemic heart disease	110.8 (109.0–112.9)	0.9 (0.7–1.1)	0.3 (0.2–0.3)	10.9 (10.5–11.6)	195.7 (190.9–200.5)	1656.6 (1626.1–1689.6)
Cerebrovascular disease	97.1 (94.7–100.1)	5.1 (4.4–5.9)	1.2 (1.1–1.4)	12.6 (12.1–13.4)	194.4 (188.6–202.3)	1341.5 (1307.1–1381.3)
Ischemic stroke	47.1 (44.2–50.4)	0.3 (0.2–0.3)	0.1 (0.1–0.1)	2.0 (1.7–2.4)	66.2 (59.3–74.4)	779.4 (740.5–825.4)
Hemorrhagic stroke	49.9 (47.4–52.8)	4.8 (4.2–5.7)	1.1 (1.0–1.3)	10.6 (10.1–11.4)	128.1 (121.2–136.3)	562.1 (525.8–591.5)
Hypertensive heart disease	13.5 (11.3–15.1)	0.1 (0.1–0.2)	0.0 (0.0–0.1)	2.0 (1.6–2.3)	29.7 (24.2–33.5)	179.5 (149.2–202.3)
Cardiomyopathy and myocarditis	4.6 (4.5–4.8)	2.2 (1.8–2.6)	0.3 (0.3–0.4)	1.2 (1.2–1.3)	7.5 (7.2–7.8)	56.5 (54.3–58.8)
Atrial fibrillation and flutter	2.2 (1.8–2.7)	0 (0–0)	0 (0–0)	0.0 (0.0–0.0)	1.0 (0.8–1.4)	43.3 (35.0–52.6)
Aortic aneurysm	1.3 (1.3–1.4)	0 (0–0)	0 (0–0)	0.2 (0.2–0.2)	2.2 (2.1–2.4)	19.9 (19.2–20.7)
Peripheral vascular disease	0.4 (0.4–0.4)	0 (0–0)	0 (0–0)	0.0 (0.0–0.0)	0.3 (0.3–0.4)	6.8 (6.4–7.2)
Endocarditis	1.1 (0.9–1.3)	0.7 (0.4–1.1)	0.1 (0.1–0.2)	0.3 (0.3–0.5)	1.6 (1.4–2.0)	12.0 (10.9–13.7)
Other cardiovascular and circulatory diseases	9.1 (8.8–9.5)	3.8 (3.2–4.5)	0.5 (0.5–0.6)	1.9 (1.7–2.0)	16.5 (15.7–17.5)	114.9 (110.3–120.4)
Chronic respiratory diseases	61.7 (59.4–64.1)	15.7 (13.7–17.8)	1.6 (1.4–1.8)	7.4 (7.0–7.9)	130.2 (124.4–136.9)	811.3 (778.9–842.6)
Chronic obstructive pulmonary disease	50.4 (45.4–54.9)	7.2 (4.7–9.2)	0.4 (0.3–0.7)	4.1 (3.4–5.1)	101.1 (87.0–115.1)	712.6 (654.4–759.2)
Pneumoconiosis	0.3 (0.2–0.4)	0 (0–0)	0 (0–0)	0.0 (0.0–0.1)	0.6 (0.3–0.9)	4.2 (2.9–5.8)
Silicosis	0.0 (0.0–0.0)	0 (0–0)	0 (0–0)	0.0 (0.0–0.0)	0.0 (0.0–0.0)	0.2 (0.1–0.3)
Asbestosis	0.0 (0.0–0.0)	0 (0–0)	0 (0–0)	0.0 (0.0–0.0)	0.0 (0.0–0.0)	0.1 (0.1–0.1)
Coal workers pneumoconiosis	0.0 (0.0–0.0)	0 (0–0)	0 (0–0)	0.0 (0.0–0.0)	0.0 (0.0–0.0)	0.1 (0.0–0.1)
Other pneumoconiosis	0.3 (0.2–0.4)	0 (0–0)	0 (0–0)	0.0 (0.0–0.1)	0.5 (0.3–0.8)	3.8 (2.6–5.4)
Asthma	9.6 (5.6–14.0)	4.8 (2.6–6.4)	1.0 (0.7–1.3)	2.9 (1.9–3.6)	26.4 (13.6–39.9)	82.1 (47.8–134.0)
Interstitial lung disease and pulmonary sarcoidosis	0.8 (0.6–0.9)	0.1 (0.0–0.1)	0.0 (0.0–0.0)	0.1 (0.1–0.2)	1.7 (1.4–2.1)	9.5 (7.6–11.9)
Other chronic respiratory diseases	0.7 (0.5–1.0)	3.7 (2.4–5.9)	0.1 (0.1–0.3)	0.1 (0.1–0.2)	0.5 (0.3–0.7)	3.0 (2.2–3.9)
Cirrhosis and other chronic liver diseases	13.0 (12.2–14.5)	3.3 (2.3–4.8)	1.1 (0.9–1.4)	5.8 (5.4–6.6)	40.9 (38.0–46.0)	85.1 (79.3–93.0)
Cirrhosis and other chronic liver diseases due to hepatitis B	3.4 (3.1–3.9)	0.0 (0.0–0.0)	0.0 (0.0–0.0)	1.5 (1.3–1.8)	11.3 (9.9–13.3)	24.3 (21.7–28.4)
Cirrhosis and other chronic liver diseases due to hepatitis C	2.8 (2.6–3.1)	0.0 (0.0–0.0)	0.0 (0.0–0.0)	1.1 (1.0–1.3)	9.2 (8.1–10.5)	21.5 (19.2–23.7)
Cirrhosis and other chronic liver diseases due to alcohol use	2.9 (2.6–3.2)	0 (0–0)	0 (0–0)	1.5 (1.4–1.8)	10.6 (9.4–12.0)	14.8 (13.2–16.5)
Cirrhosis and other chronic liver diseases due to other causes	3.8 (3.5–4.4)	3.2 (2.3–4.7)	1.0 (0.9–1.3)	1.6 (1.5–1.9)	9.8 (8.7–11.2)	24.5 (22.3–27.1)
Digestive diseases	17.0 (16.3–17.9)	7.5 (4.9–13.6)	2.2 (2.0–2.4)	5.9 (5.6–6.3)	35.6 (34.1–37.5)	169.1 (163.7–175.1)
Peptic ulcer disease	4.9 (4.3–5.6)	1.1 (0.6–2.3)	0.5 (0.4–0.7)	1.9 (1.6–2.2)	11.4 (9.8–13.1)	47.0 (41.8–52.6)
Gastritis and duodenitis	0.7 (0.3–1.0)	0.2 (0.1–0.4)	0.1 (0.0–0.1)	0.3 (0.1–0.4)	1.7 (0.6–2.5)	7.2 (3.1–10.2)
Appendicitis	0.8 (0.7–1.1)	1.4 (0.6–3.4)	0.4 (0.3–0.4)	0.5 (0.4–0.6)	1.4 (1.2–1.6)	3.6 (3.2–4.1)
Paralytic ileus and intestinal obstruction	3.3 (2.6–3.7)	2.2 (1.0–4.2)	0.6 (0.4–0.8)	1.2 (0.9–1.4)	6.6 (5.1–7.6)	29.8 (25.8–34.3)
Inguinal, femoral, and abdominal hernia	0.6 (0.4–0.8)	0.2 (0.1–0.3)	0.1 (0.0–0.1)	0.2 (0.1–0.2)	1.5 (1.0–1.8)	6.9 (5.2–8.1)
Inflammatory bowel disease	0.7 (0.6–0.8)	0.6 (0.2–1.5)	0.1 (0.0–0.1)	0.2 (0.2–0.3)	1.2 (1.0–1.4)	7.1 (6.2–7.7)
Vascular intestinal disorders	1.2 (1.1–1.4)	0.0 (0.0–0.1)	0.0 (0.0–0.0)	0.1 (0.1–0.1)	2.0 (1.7–2.4)	19.1 (17.6–20.5)
Gallbladder and biliary diseases	1.8 (1.7–2.0)	0.5 (0.3–1.2)	0.1 (0.1–0.2)	0.5 (0.4–0.5)	4.0 (3.7–4.3)	20.2 (19.0–21.4)
Pancreatitis	1.1 (1.0–1.2)	0.1 (0.0–0.1)	0.1 (0.0–0.1)	0.6 (0.5–0.7)	2.9 (2.6–3.3)	8.9 (8.3–9.5)
Other digestive diseases	1.7 (1.4–2.0)	1.1 (0.6–2.1)	0.2 (0.2–0.3)	0.4 (0.3–0.5)	2.9 (2.3–3.5)	19.4 (16.9–22.1)
Neurological disorders	26.9 (23.2–30.8)	3.1 (2.4–4.4)	1.2 (1.1–1.4)	2.2 (2.0–2.5)	15.1 (12.8–17.5)	485.3 (406.4–562.8)
Alzheimer disease and other dementias	23.2 (19.3–27.0)	0 (0–0)	0 (0–0)	0.0 (0.0–0.0)	9.3 (7.1–11.7)	458.2 (379.1–536.0)
Parkinson disease	0.9 (0.9–0.9)	0 (0–0)	0 (0–0)	0.0 (0.0–0.0)	0.9 (0.9–1.0)	15.9 (15.3–16.7)
Epilepsy	1.6 (1.4–1.9)	2.2 (1.6–3.4)	1.0 (0.9–1.2)	1.6 (1.4–1.8)	1.5 (1.4–1.6)	3.2 (3.1–3.4)

SR Table 13 - Global death rates (per 100,000) by age and cause for females in 1995

Cause	All ages	0-5 years	5-14 years	15-49 years	50-69 years	70+ years
Multiple sclerosis	0.3 (0.3–0.4)	0 (0–0)	0 (0–0)	0.2 (0.2–0.3)	1.0 (0.8–1.1)	1.3 (1.1–1.4)
Motor neuron disease	0.3 (0.3–0.4)	0.2 (0.1–0.4)	0.0 (0.0–0.0)	0.1 (0.1–0.1)	1.1 (1.0–1.3)	2.7 (2.6–2.8)
Migraine	0 (0–0)	0 (0–0)	0 (0–0)	0 (0–0)	0 (0–0)	0 (0–0)
Tension-type headache	0 (0–0)	0 (0–0)	0 (0–0)	0 (0–0)	0 (0–0)	0 (0–0)
Medication overuse headache	0 (0–0)	0 (0–0)	0 (0–0)	0 (0–0)	0 (0–0)	0 (0–0)
Other neurological disorders	0.6 (0.6–0.7)	0.6 (0.5–1.2)	0.2 (0.2–0.3)	0.3 (0.3–0.3)	1.2 (1.2–1.3)	4.0 (3.8–4.1)
Mental and substance use disorders	2.0 (1.9–2.1)	0.0 (0.0–0.0)	0.0 (0.0–0.0)	2.2 (2.0–2.3)	4.8 (4.6–5.0)	5.9 (5.6–6.1)
Schizophrenia	0.3 (0.3–0.3)	0 (0–0)	0 (0–0)	0.3 (0.2–0.3)	0.7 (0.7–0.8)	1.8 (1.7–2.0)
Alcohol use disorders	0.9 (0.8–0.9)	0 (0–0)	0 (0–0)	0.8 (0.7–0.8)	2.8 (2.6–3.0)	1.9 (1.8–1.9)
Drug use disorders	0.8 (0.7–0.9)	0.0 (0.0–0.0)	0 (0–0)	1.1 (0.9–1.2)	1.3 (1.2–1.4)	2.2 (2.0–2.3)
Opioid use disorders	0.5 (0.4–0.6)	0.0 (0.0–0.0)	0 (0–0)	0.6 (0.6–0.8)	0.8 (0.7–0.9)	1.1 (1.0–1.4)
Cocaine use disorders	0.0 (0.0–0.0)	0 (0–0)	0 (0–0)	0.0 (0.0–0.0)	0.0 (0.0–0.0)	0.0 (0.0–0.0)
Amphetamine use disorders	0.0 (0.0–0.0)	0 (0–0)	0 (0–0)	0.0 (0.0–0.1)	0.0 (0.0–0.0)	0.0 (0.0–0.0)
Cannabis use disorders	0 (0–0)	0 (0–0)	0 (0–0)	0 (0–0)	0 (0–0)	0 (0–0)
Other drug use disorders	0.3 (0.2–0.4)	0 (0–0)	0 (0–0)	0.4 (0.3–0.5)	0.5 (0.3–0.5)	1.0 (0.7–1.2)
Depressive disorders	0 (0–0)	0 (0–0)	0 (0–0)	0 (0–0)	0 (0–0)	0 (0–0)
Major depressive disorder	0 (0–0)	0 (0–0)	0 (0–0)	0 (0–0)	0 (0–0)	0 (0–0)
Dysthymia	0 (0–0)	0 (0–0)	0 (0–0)	0 (0–0)	0 (0–0)	0 (0–0)
Bipolar disorder	0 (0–0)	0 (0–0)	0 (0–0)	0 (0–0)	0 (0–0)	0 (0–0)
Anxiety disorders	0 (0–0)	0 (0–0)	0 (0–0)	0 (0–0)	0 (0–0)	0 (0–0)
Eating disorders	0.0 (0.0–0.0)	0 (0–0)	0.0 (0.0–0.0)	0.0 (0.0–0.0)	0 (0–0)	0 (0–0)
Anorexia nervosa	0.0 (0.0–0.0)	0 (0–0)	0.0 (0.0–0.0)	0.0 (0.0–0.0)	0 (0–0)	0 (0–0)
Bulimia nervosa	0.0 (0.0–0.0)	0 (0–0)	0.0 (0.0–0.0)	0.0 (0.0–0.0)	0 (0–0)	0 (0–0)
Autistic spectrum disorders	0 (0–0)	0 (0–0)	0 (0–0)	0 (0–0)	0 (0–0)	0 (0–0)
Autism	0 (0–0)	0 (0–0)	0 (0–0)	0 (0–0)	0 (0–0)	0 (0–0)
Asperger syndrome and other autistic spectrum disorders	0 (0–0)	0 (0–0)	0 (0–0)	0 (0–0)	0 (0–0)	0 (0–0)
Attention-deficit/hyperactivity disorder	0 (0–0)	0 (0–0)	0 (0–0)	0 (0–0)	0 (0–0)	0 (0–0)
Conduct disorder	0 (0–0)	0 (0–0)	0 (0–0)	0 (0–0)	0 (0–0)	0 (0–0)
Idiopathic developmental intellectual disability	0 (0–0)	0 (0–0)	0 (0–0)	0 (0–0)	0 (0–0)	0 (0–0)
Other mental and substance use disorders	0 (0–0)	0 (0–0)	0 (0–0)	0 (0–0)	0 (0–0)	0 (0–0)
Diabetes, urogenital, blood, and endocrine diseases	36.0 (35.1–37.1)	21.7 (16.7–28.4)	3.1 (2.8–3.4)	9.6 (9.3–10.0)	86.3 (83.7–89.0)	351.1 (342.3–360.1)
Diabetes mellitus	15.9 (15.5–16.4)	0.6 (0.5–0.7)	0.3 (0.3–0.4)	2.8 (2.6–2.9)	46.7 (45.1–48.3)	172.1 (166.6–177.3)
Acute glomerulonephritis	0.2 (0.2–0.3)	0.1 (0.1–0.2)	0.1 (0.0–0.1)	0.1 (0.1–0.2)	0.5 (0.4–0.7)	1.5 (1.2–2.5)
Chronic kidney disease	11.7 (11.4–12.1)	3.3 (2.9–3.7)	1.0 (0.9–1.1)	3.7 (3.6–3.9)	27.0 (26.1–27.9)	119.5 (116.1–123.3)
Chronic kidney disease due to diabetes mellitus	3.6 (3.4–3.8)	0.4 (0.3–0.5)	0.1 (0.1–0.1)	0.9 (0.8–0.9)	10.1 (9.4–11.0)	36.9 (34.8–39.1)
Chronic kidney disease due to hypertension	5.2 (5.0–5.4)	1.1 (0.9–1.3)	0.3 (0.2–0.3)	1.4 (1.3–1.5)	11.4 (10.5–12.1)	59.7 (56.7–62.6)
Chronic kidney disease due to glomerulonephritis	2.6 (2.4–2.7)	1.3 (1.1–1.5)	0.5 (0.4–0.5)	1.3 (1.2–1.4)	5.2 (4.8–5.6)	20.3 (18.8–22.0)
Chronic kidney disease due to other causes	0.3 (0.3–0.4)	0.5 (0.4–0.6)	0.1 (0.1–0.2)	0.2 (0.1–0.2)	0.3 (0.2–0.5)	2.7 (2.2–3.4)
Urinary diseases and male infertility	2.7 (2.6–3.0)	1.2 (0.9–1.5)	0.2 (0.2–0.2)	0.8 (0.7–0.9)	5.5 (5.1–6.3)	30.0 (28.4–33.4)
Interstitial nephritis and urinary tract infections	2.2 (2.1–2.4)	0.9 (0.7–1.1)	0.1 (0.1–0.1)	0.6 (0.5–0.7)	4.2 (3.8–4.9)	25.8 (24.3–28.8)
Urolithiasis	0.2 (0.2–0.2)	0 (0–0)	0.0 (0.0–0.0)	0.1 (0.1–0.1)	0.6 (0.5–0.7)	1.7 (1.3–2.0)
Benign prostatic hyperplasia	0 (0–0)	0 (0–0)	0 (0–0)	0 (0–0)	0 (0–0)	0 (0–0)
Male infertility	0 (0–0)	0 (0–0)	0 (0–0)	0 (0–0)	0 (0–0)	0 (0–0)
Other urinary diseases	0.3 (0.3–0.4)	0.3 (0.2–0.4)	0.0 (0.0–0.0)	0.1 (0.1–0.1)	0.7 (0.6–0.9)	2.5 (2.3–3.0)
Gynecological diseases	0.2 (0.2–0.3)	0 (0–0)	0 (0–0)	0.2 (0.2–0.3)	0.5 (0.4–0.7)	1.1 (0.9–1.4)
Uterine fibroids	0.1 (0.0–0.1)	0 (0–0)	0 (0–0)	0.1 (0.0–0.1)	0.2 (0.1–0.3)	0.3 (0.1–0.4)
Polycystic ovarian syndrome	0.0 (0.0–0.1)	0 (0–0)	0 (0–0)	0.0 (0.0–0.1)	0.1 (0.0–0.1)	0 (0–0)
Female infertility	0 (0–0)	0 (0–0)	0 (0–0)	0 (0–0)	0 (0–0)	0 (0–0)
Endometriosis	0.0 (0.0–0.0)	0 (0–0)	0 (0–0)	0.0 (0.0–0.0)	0.0 (0.0–0.0)	0 (0–0)
Genital prolapse	0.0 (0.0–0.1)	0 (0–0)	0 (0–0)	0.0 (0.0–0.0)	0.1 (0.0–0.2)	0.4 (0.2–0.7)
Premenstrual syndrome	0 (0–0)	0 (0–0)	0 (0–0)	0 (0–0)	0 (0–0)	0 (0–0)
Other gynecological diseases	0.1 (0.1–0.1)	0 (0–0)	0 (0–0)	0.1 (0.1–0.2)	0.2 (0.1–0.3)	0.4 (0.3–0.7)
Hemoglobinopathies and hemolytic anemias	3.5 (3.0–4.2)	13.9 (9.4–20.6)	1.3 (1.0–1.5)	1.3 (1.2–1.4)	3.2 (3.0–3.4)	14.4 (13.1–15.7)
Thalassemias	0.5 (0.3–0.6)	2.6 (1.5–4.0)	0.4 (0.3–0.5)	0.1 (0.1–0.1)	0.0 (0.0–0.0)	1.0 (0.8–1.3)
Thalassemias trait	0 (0–0)	0 (0–0)	0 (0–0)	0 (0–0)	0 (0–0)	0 (0–0)
Sickle cell disorders	1.7 (1.2–2.4)	11.1 (7.2–17.2)	0.7 (0.6–0.9)	0.5 (0.5–0.6)	0.2 (0.1–0.2)	1.1 (1.0–1.4)
Sickle cell trait	0 (0–0)	0 (0–0)	0 (0–0)	0 (0–0)	0 (0–0)	0 (0–0)

SR Table 13 - Global death rates (per 100,000) by age and cause for females in 1995						
**Cause**	**All ages**	**0-5 years**	**5-14 years**	**15-49 years**	**50-69 years**	**70+ years**
G6PD deficiency	0.2 (0.2–0.3)	0.1 (0.1–0.2)	0.1 (0.0–0.1)	0.3 (0.2–0.3)	0.5 (0.4–0.5)	0.3 (0.2–0.3)
G6PD trait	0 (0–0)	0 (0–0)	0 (0–0)	0 (0–0)	0 (0–0)	0 (0–0)
Other hemoglobinopathies and hemolytic anemias	1.2 (1.1–1.2)	0.1 (0.1–0.2)	0.1 (0.1–0.1)	0.4 (0.4–0.5)	2.6 (2.4–2.7)	12.0 (10.8–12.9)
Endocrine, metabolic, blood, and immune disorders	1.6 (1.6–1.7)	2.5 (2.1–2.9)	0.3 (0.3–0.4)	0.6 (0.6–0.6)	2.9 (2.7–3.0)	12.5 (12.0–13.1)
Musculoskeletal disorders	1.5 (1.3–1.7)	0 (0–0)	0.2 (0.2–0.2)	0.8 (0.6–1.0)	2.8 (2.5–3.1)	14.2 (12.7–14.9)
Rheumatoid arthritis	0.6 (0.5–0.7)	0 (0–0)	0.0 (0.0–0.0)	0.1 (0.1–0.2)	1.5 (1.3–1.7)	7.0 (6.1–8.0)
Osteoarthritis	0 (0–0)	0 (0–0)	0 (0–0)	0 (0–0)	0 (0–0)	0 (0–0)
Low back and neck pain	0 (0–0)	0 (0–0)	0 (0–0)	0 (0–0)	0 (0–0)	0 (0–0)
Low back pain	0 (0–0)	0 (0–0)	0 (0–0)	0 (0–0)	0 (0–0)	0 (0–0)
Neck pain	0 (0–0)	0 (0–0)	0 (0–0)	0 (0–0)	0 (0–0)	0 (0–0)
Gout	0 (0–0)	0 (0–0)	0 (0–0)	0 (0–0)	0 (0–0)	0 (0–0)
Other musculoskeletal disorders	0.9 (0.8–1.0)	0 (0–0)	0.2 (0.1–0.2)	0.6 (0.5–0.9)	1.3 (1.2–1.6)	7.2 (6.1–7.7)
Other non-communicable diseases	13.3 (9.8–18.7)	98.6 (70.0–145.6)	3.6 (2.6–5.0)	1.7 (1.4–2.1)	2.9 (2.3–3.9)	15.0 (10.6–20.2)
Congenital anomalies	11.9 (8.4–17.3)	94.0 (65.7–140.4)	3.5 (2.5–4.9)	1.5 (1.2–1.9)	1.5 (1.2–1.7)	2.9 (2.6–3.2)
Neural tube defects	1.9 (1.3–3.2)	16.7 (10.9–28.9)	0.3 (0.2–0.5)	0.1 (0.0–0.1)	0.1 (0.0–0.1)	0.1 (0.1–0.2)
Congenital heart anomalies	6.1 (4.0–9.3)	46.2 (28.7–71.8)	2.5 (1.7–3.6)	1.1 (0.8–1.4)	0.5 (0.4–0.6)	1.2 (1.0–1.4)
Cleft lip and cleft palate	0.1 (0.0–0.1)	0.8 (0.4–1.1)	0 (0–0)	0 (0–0)	0 (0–0)	0 (0–0)
Down syndrome	0.6 (0.2–1.5)	4.9 (0.9–12.2)	0.1 (0.0–0.2)	0.1 (0.1–0.2)	0.3 (0.2–0.4)	0.1 (0.0–0.1)
Turner syndrome	0 (0–0)	0 (0–0)	0 (0–0)	0 (0–0)	0 (0–0)	0 (0–0)
Klinefelter syndrome	0 (0–0)	0 (0–0)	0 (0–0)	0 (0–0)	0 (0–0)	0 (0–0)
Other chromosomal abnormalities	0.5 (0.2–1.5)	4.1 (1.3–14.1)	0.0 (0.0–0.1)	0.0 (0.0–0.0)	0.0 (0.0–0.0)	0.0 (0.0–0.0)
Other congenital anomalies	2.6 (1.9–4.2)	21.3 (14.8–35.6)	0.5 (0.4–0.8)	0.2 (0.2–0.3)	0.6 (0.4–0.7)	1.6 (1.3–1.8)
Skin and subcutaneous diseases	1.0 (0.6–1.5)	1.1 (0.5–1.9)	0.1 (0.0–0.1)	0.2 (0.1–0.3)	1.5 (0.9–2.5)	12.0 (7.9–17.2)
Dermatitis	0 (0–0)	0 (0–0)	0 (0–0)	0 (0–0)	0 (0–0)	0 (0–0)
Psoriasis	0 (0–0)	0 (0–0)	0 (0–0)	0 (0–0)	0 (0–0)	0 (0–0)
Cellulitis	0.2 (0.1–0.2)	0.1 (0.0–0.2)	0.0 (0.0–0.0)	0.0 (0.0–0.1)	0.4 (0.2–0.6)	1.5 (0.9–2.2)
Pyoderma	0.4 (0.2–0.7)	0.9 (0.4–1.6)	0.0 (0.0–0.1)	0.1 (0.1–0.2)	0.7 (0.4–1.3)	3.2 (2.1–5.3)
Scabies	0 (0–0)	0 (0–0)	0 (0–0)	0 (0–0)	0 (0–0)	0 (0–0)
Fungal skin diseases	0 (0–0)	0 (0–0)	0 (0–0)	0 (0–0)	0 (0–0)	0 (0–0)
Viral skin diseases	0 (0–0)	0 (0–0)	0 (0–0)	0 (0–0)	0 (0–0)	0 (0–0)
Acne vulgaris	0 (0–0)	0 (0–0)	0 (0–0)	0 (0–0)	0 (0–0)	0 (0–0)
Alopecia areata	0 (0–0)	0 (0–0)	0 (0–0)	0 (0–0)	0 (0–0)	0 (0–0)
Pruritus	0 (0–0)	0 (0–0)	0 (0–0)	0 (0–0)	0 (0–0)	0 (0–0)
Urticaria	0 (0–0)	0 (0–0)	0 (0–0)	0 (0–0)	0 (0–0)	0 (0–0)
Decubitus ulcer	0.4 (0.2–0.6)	0.0 (0.0–0.0)	0.0 (0.0–0.0)	0.0 (0.0–0.0)	0.4 (0.2–0.7)	6.9 (3.9–9.7)
Other skin and subcutaneous diseases	0.1 (0.0–0.1)	0.1 (0.0–0.1)	0.0 (0.0–0.0)	0.0 (0.0–0.0)	0.1 (0.0–0.1)	0.5 (0.3–0.7)
Sense organ diseases	0 (0–0)	0 (0–0)	0 (0–0)	0 (0–0)	0 (0–0)	0 (0–0)
Glaucoma	0 (0–0)	0 (0–0)	0 (0–0)	0 (0–0)	0 (0–0)	0 (0–0)
Cataract	0 (0–0)	0 (0–0)	0 (0–0)	0 (0–0)	0 (0–0)	0 (0–0)
Macular degeneration	0 (0–0)	0 (0–0)	0 (0–0)	0 (0–0)	0 (0–0)	0 (0–0)
Refraction and accommodation disorders	0 (0–0)	0 (0–0)	0 (0–0)	0 (0–0)	0 (0–0)	0 (0–0)
Age-related and other hearing loss	0 (0–0)	0 (0–0)	0 (0–0)	0 (0–0)	0 (0–0)	0 (0–0)
Other vision loss	0 (0–0)	0 (0–0)	0 (0–0)	0 (0–0)	0 (0–0)	0 (0–0)
Other sense organ diseases	0 (0–0)	0 (0–0)	0 (0–0)	0 (0–0)	0 (0–0)	0 (0–0)
Oral disorders	0 (0–0)	0 (0–0)	0 (0–0)	0 (0–0)	0 (0–0)	0 (0–0)
Deciduous caries	0 (0–0)	0 (0–0)	0 (0–0)	0 (0–0)	0 (0–0)	0 (0–0)
Permanent caries	0 (0–0)	0 (0–0)	0 (0–0)	0 (0–0)	0 (0–0)	0 (0–0)
Periodontal diseases	0 (0–0)	0 (0–0)	0 (0–0)	0 (0–0)	0 (0–0)	0 (0–0)
Edentulism and severe tooth loss	0 (0–0)	0 (0–0)	0 (0–0)	0 (0–0)	0 (0–0)	0 (0–0)
Other oral disorders	0 (0–0)	0 (0–0)	0 (0–0)	0 (0–0)	0 (0–0)	0 (0–0)
Sudden infant death syndrome	0.4 (0.3–0.6)	3.6 (2.5–5.2)	0 (0–0)	0 (0–0)	0 (0–0)	0 (0–0)
Injuries	53.1 (45.9–56.5)	80.0 (62.6–96.4)	23.5 (20.4–25.4)	42.2 (36.0–44.6)	67.1 (59.1–70.1)	196.5 (175.2–205.4)
Transport injuries	12.3 (11.6–12.9)	12.8 (9.8–15.9)	6.7 (6.2–7.3)	10.9 (10.4–11.5)	18.2 (17.6–18.9)	33.9 (32.9–34.9)
Road injuries	11.6 (11.0–12.2)	11.8 (9.2–14.6)	6.4 (5.9–6.9)	10.3 (9.9–10.9)	17.2 (16.6–17.9)	32.3 (31.3–33.3)
Pedestrian road injuries	6.0 (5.6–6.4)	6.1 (4.9–7.3)	3.7 (3.4–4.0)	4.6 (4.3–5.0)	9.7 (9.0–10.3)	20.1 (19.1–21.1)
Cyclist road injuries	0.5 (0.4–0.6)	0.3 (0.2–0.5)	0.3 (0.3–0.4)	0.5 (0.4–0.6)	0.8 (0.8–1.0)	1.0 (0.9–1.2)
Motorcyclist road injuries	1.0 (0.9–1.2)	1.0 (0.6–1.6)	0.4 (0.4–0.6)	1.1 (1.0–1.3)	1.4 (1.2–1.7)	1.7 (1.4–2.2)

SR Table 13 - Global death rates (per 100,000) by age and cause for females in 1995						
**Cause**	**All ages**	**0-5 years**	**5-14 years**	**15-49 years**	**50-69 years**	**70+ years**
Motor vehicle road injuries	4.0 (3.7–4.3)	4.1 (3.1–5.4)	1.9 (1.7–2.2)	4.0 (3.7–4.4)	5.1 (4.8–5.7)	9.1 (8.4–10.1)
Other road injuries	0.1 (0.1–0.1)	0.2 (0.1–0.3)	0.1 (0.1–0.1)	0.1 (0.1–0.1)	0.2 (0.1–0.2)	0.3 (0.3–0.4)
Other transport injuries	0.7 (0.5–0.8)	1.0 (0.5–1.4)	0.3 (0.2–0.4)	0.6 (0.4–0.7)	1.0 (0.8–1.2)	1.6 (1.4–1.8)
Unintentional injuries	26.7 (22.1–29.1)	62.9 (46.6–78.0)	14.1 (11.2–15.6)	14.1 (11.6–15.2)	28.0 (24.2–29.9)	128.6 (114.1–135.8)
Falls	5.2 (4.6–5.5)	5.0 (2.6–7.2)	1.2 (1.0–1.4)	1.3 (1.2–1.4)	6.1 (5.6–6.4)	60.8 (55.8–64.6)
Drowning	5.9 (3.3–6.7)	21.0 (7.6–26.6)	6.2 (3.8–7.0)	2.8 (1.7–3.0)	4.0 (2.5–4.6)	10.0 (7.2–11.2)
Fire, heat, and hot substances	4.7 (2.9–5.4)	8.2 (3.7–10.5)	1.7 (1.0–2.0)	4.3 (2.4–4.9)	4.7 (3.3–5.1)	15.3 (11.8–16.5)
Poisonings	1.6 (1.2–1.9)	4.2 (1.7–6.6)	0.8 (0.7–0.9)	1.2 (1.0–1.3)	1.8 (1.5–2.1)	4.0 (3.4–4.4)
Exposure to mechanical forces	2.0 (1.8–2.3)	6.7 (5.1–8.4)	0.9 (0.8–1.1)	0.9 (0.9–1.1)	2.1 (1.9–2.5)	7.5 (5.8–8.8)
Unintentional firearm injuries	0.3 (0.2–0.3)	0.3 (0.1–0.4)	0.1 (0.1–0.1)	0.2 (0.1–0.2)	0.3 (0.2–0.4)	2.1 (1.7–2.3)
Unintentional suffocation	0.6 (0.4–0.7)	3.8 (2.2–4.7)	0.2 (0.2–0.3)	0.1 (0.1–0.1)	0.2 (0.1–0.2)	1.1 (0.9–1.4)
Other exposure to mechanical forces	1.2 (1.0–1.4)	2.6 (1.8–3.8)	0.6 (0.5–0.7)	0.7 (0.6–0.8)	1.6 (1.5–2.1)	4.3 (3.1–5.4)
Adverse effects of medical treatment	1.7 (1.2–2.0)	3.6 (1.6–5.2)	0.4 (0.3–0.6)	0.7 (0.5–1.0)	2.2 (1.7–2.7)	10.6 (8.1–11.9)
Animal contact	1.7 (1.1–1.9)	3.2 (1.8–4.1)	1.4 (0.8–1.6)	1.2 (0.8–1.4)	2.5 (1.5–2.9)	3.1 (2.0–3.5)
Venomous animal contact	1.4 (0.9–1.6)	2.3 (1.2–3.0)	1.1 (0.7–1.4)	1.1 (0.7–1.2)	2.2 (1.3–2.5)	2.4 (1.4–2.8)
Non-venomous animal contact	0.3 (0.2–0.4)	0.9 (0.4–1.2)	0.2 (0.1–0.3)	0.1 (0.1–0.2)	0.3 (0.2–0.4)	0.7 (0.5–0.8)
Foreign body	2.0 (1.4–2.9)	8.0 (4.4–15.3)	0.7 (0.4–1.0)	0.6 (0.4–0.8)	1.7 (1.3–2.3)	9.5 (8.0–11.3)
Pulmonary aspiration and foreign body in airway	1.5 (1.0–2.5)	6.8 (3.0–14.3)	0.5 (0.3–0.9)	0.3 (0.2–0.5)	1.1 (0.9–1.5)	7.4 (6.7–8.1)
Foreign body in eyes	0 (0–0)	0 (0–0)	0 (0–0)	0 (0–0)	0 (0–0)	0 (0–0)
Foreign body in other body part	0.5 (0.2–0.9)	1.2 (0.4–2.0)	0.2 (0.1–0.4)	0.3 (0.1–0.6)	0.5 (0.3–1.2)	2.0 (1.3–4.0)
Environmental heat and cold exposure	0.6 (0.4–0.8)	0.7 (0.3–1.0)	0.1 (0.1–0.2)	0.2 (0.2–0.3)	1.1 (0.8–1.7)	4.5 (2.9–6.5)
Other unintentional injuries	1.3 (1.1–1.4)	2.4 (1.8–3.0)	0.8 (0.7–0.9)	0.9 (0.8–1.0)	1.9 (1.7–2.1)	3.4 (3.0–3.9)
Self-harm and interpersonal violence	13.4 (9.9–14.3)	3.2 (2.8–3.6)	2.1 (1.7–2.3)	16.6 (11.8–17.9)	19.9 (15.0–21.1)	32.5 (24.8–34.6)
Self-harm	10.2 (6.7–11.1)	0 (0–0)	1.0 (0.5–1.1)	12.8 (8.1–14.1)	16.3 (11.6–17.5)	27.4 (20.0–29.5)
Interpersonal violence	3.2 (3.1–3.4)	3.2 (2.8–3.6)	1.2 (1.1–1.2)	3.8 (3.6–4.0)	3.5 (3.4–3.7)	5.1 (4.8–5.3)
Assault by firearm	0.7 (0.6–0.7)	0.3 (0.2–0.4)	0.2 (0.2–0.2)	0.9 (0.7–1.0)	0.6 (0.6–0.7)	0.8 (0.7–0.9)
Assault by sharp object	0.8 (0.7–0.9)	0.3 (0.2–0.4)	0.2 (0.2–0.2)	1.0 (0.9–1.1)	0.8 (0.8–0.9)	1.3 (1.2–1.4)
Assault by other means	1.8 (1.7–1.9)	2.6 (2.2–2.9)	0.7 (0.7–0.8)	1.9 (1.7–2.0)	2.1 (1.9–2.2)	3.0 (2.7–3.2)
Forces of nature, war, and legal intervention	0.7 (0.6–1.0)	1.1 (0.8–1.4)	0.5 (0.4–0.7)	0.6 (0.5–0.8)	1.0 (0.7–1.5)	1.6 (0.8–3.8)
Exposure to forces of nature	0.3 (0.1–0.4)	0.2 (0.1–0.4)	0.2 (0.1–0.3)	0.2 (0.1–0.3)	0.4 (0.2–0.9)	0.8 (0.3–2.4)
Collective violence and legal intervention	0.5 (0.4–0.6)	0.9 (0.6–1.2)	0.3 (0.2–0.4)	0.4 (0.3–0.5)	0.6 (0.5–0.7)	0.8 (0.5–2.6)

SR Table 13 - Global death rates (per 100,000) by age and cause for females in 2000

Cause	All ages	0-5 years	5-14 years	15-49 years	50-69 years	70+ years
All causes	797.7 (787.9–807.3)	1478.2 (1462.4–1494.3)	90.8 (89.1–92.5)	226.8 (221.4–232.3)	1243.2 (1218.7–1268.3)	6778.2 (6713.6–6848.4)
Communicable, maternal, neonatal, and nutritional diseases	233.8 (227.4–239.9)	1253.9 (1208.6–1287.2)	51.3 (48.7–54.2)	91.2 (87.6–95.7)	167.2 (156.1–177.3)	618.8 (579.2–649.4)
HIV/AIDS and tuberculosis	37.9 (34.4–41.6)	34.7 (32.4–38.4)	5.0 (4.6–6.0)	39.9 (37.0–43.2)	60.4 (50.5–68.5)	90.1 (68.9–106.2)
Tuberculosis	17.9 (14.4–21.5)	6.9 (5.0–10.4)	2.1 (1.7–3.1)	11.8 (9.8–14.5)	45.8 (35.6–53.7)	86.0 (65.0–102.3)
HIV/AIDS	20.0 (18.7–21.3)	27.8 (26.8–28.9)	2.9 (2.7–3.1)	28.1 (25.7–30.3)	14.5 (13.1–16.3)	4.0 (3.5–4.7)
HIV/AIDS - Tuberculosis	4.2 (3.6–5.0)	7.7 (6.7–8.8)	0.8 (0.6–0.9)	5.3 (4.4–6.3)	4.0 (3.4–4.6)	1.1 (1.0–1.3)
HIV/AIDS resulting in other diseases	15.7 (14.4–17.2)	20.1 (18.6–21.7)	2.2 (1.9–2.4)	22.8 (20.5–25.0)	10.6 (9.1–12.3)	2.9 (2.4–3.5)
Diarrhea, lower respiratory, and other common infectious diseases	104.4 (99.3–109.6)	550.8 (509.4–593.6)	33.4 (30.0–37.3)	18.9 (17.4–20.9)	80.9 (74.7–86.3)	455.9 (424.4–481.6)
Diarrheal diseases	29.9 (27.5–32.3)	144.3 (122.9–166.2)	7.9 (6.9–8.8)	6.7 (6.3–7.2)	33.1 (31.2–35.2)	122.6 (114.9–130.9)
Intestinal infectious diseases	3.5 (2.0–5.8)	9.7 (5.1–17.1)	7.1 (4.1–11.6)	1.9 (1.0–3.2)	1.1 (0.6–1.9)	1.2 (0.7–1.9)
Typhoid fever	2.9 (1.6–4.9)	7.9 (4.1–14.5)	6.0 (3.3–9.6)	1.5 (0.8–2.7)	0.8 (0.4–1.5)	0.9 (0.5–1.5)
Paratyphoid fever	0.6 (0.3–1.1)	1.4 (0.6–2.9)	1.1 (0.5–2.1)	0.3 (0.1–0.7)	0.2 (0.1–0.4)	0.2 (0.1–0.4)
Other intestinal infectious diseases	0.0 (0.0–0.1)	0.3 (0.0–0.7)	0.0 (0.0–0.1)	0.0 (0.0–0.0)	0.0 (0.0–0.0)	0.1 (0.0–0.2)
Lower respiratory infections	48.3 (44.4–51.4)	235.8 (212.2–260.3)	6.9 (5.1–7.8)	5.9 (4.9–6.7)	36.4 (29.4–39.8)	310.5 (275.8–331.3)
Upper respiratory infections	0.1 (0.1–0.1)	0.2 (0.2–0.3)	0.0 (0.0–0.0)	0.0 (0.0–0.0)	0.1 (0.1–0.1)	0.3 (0.2–0.4)
Otitis media	0.1 (0.1–0.1)	0.3 (0.2–0.3)	0.0 (0.0–0.0)	0.0 (0.0–0.0)	0.1 (0.1–0.1)	0.2 (0.2–0.5)
Meningitis	6.8 (5.7–7.9)	38.3 (29.7–48.6)	3.4 (2.8–4.0)	2.3 (1.9–2.6)	5.3 (4.4–6.2)	10.7 (9.3–12.7)
Pneumococcal meningitis	1.8 (1.5–2.2)	8.6 (5.9–12.6)	1.1 (0.8–1.6)	0.7 (0.6–1.0)	1.5 (1.1–1.9)	3.0 (2.4–3.8)
H influenzae type B meningitis	2.0 (1.6–2.5)	14.4 (10.3–19.7)	1.0 (0.6–1.3)	0.4 (0.3–0.5)	0.9 (0.6–1.2)	1.8 (1.4–2.3)
Meningococcal meningitis	1.2 (0.9–1.6)	7.1 (4.6–10.5)	0.6 (0.4–0.8)	0.4 (0.3–0.5)	0.9 (0.6–1.1)	1.6 (1.2–2.1)
Other meningitis	1.8 (1.5–2.2)	8.2 (5.8–11.3)	0.8 (0.5–1.1)	0.7 (0.6–0.9)	2.1 (1.7–2.5)	4.3 (3.5–5.1)
Encephalitis	2.3 (1.9–2.7)	8.9 (6.3–10.8)	1.6 (1.3–1.9)	0.9 (0.8–1.1)	2.4 (2.0–3.3)	6.3 (5.3–8.6)
Diphtheria	0.2 (0.1–0.4)	1.2 (0.4–3.4)	0.1 (0.0–0.2)	0.0 (0.0–0.0)	0.0 (0.0–0.1)	0 (0–0)
Whooping cough	2.2 (0.7–4.9)	21.1 (7.1–46.8)	0.6 (0.2–1.5)	0.0 (0.0–0.1)	0.0 (0.0–0.1)	0 (0–0)
Tetanus	2.4 (2.1–3.5)	15.6 (13.1–18.5)	0.6 (0.4–1.1)	0.7 (0.5–1.3)	2.2 (1.7–4.6)	2.8 (2.1–5.4)
Measles	8.5 (3.2–16.6)	74.9 (28.3–145.5)	5.0 (1.8–10.0)	0.4 (0.1–0.9)	0.0 (0.0–0.1)	0 (0–0)
Varicella and herpes zoster	0.2 (0.2–0.2)	0.5 (0.4–0.7)	0.1 (0.1–0.1)	0.1 (0.0–0.1)	0.2 (0.2–0.3)	1.3 (1.0–1.7)
Neglected tropical diseases and malaria	21.3 (17.3–25.1)	147.0 (110.0–184.5)	8.6 (6.3–11.0)	5.4 (4.1–7.0)	12.7 (9.4–17.1)	14.8 (10.9–20.4)
Malaria	19.2 (15.2–23.0)	142.3 (105.2–179.9)	6.9 (4.6–9.3)	4.1 (2.8–5.7)	9.7 (6.5–14.1)	10.8 (6.9–16.3)
Chagas disease	0.1 (0.1–0.1)	0.0 (0.0–0.0)	0.0 (0.0–0.0)	0.0 (0.0–0.0)	0.3 (0.3–0.3)	0.9 (0.8–0.9)
Leishmaniasis	0.2 (0.1–0.3)	0.4 (0.3–0.7)	0.1 (0.1–0.2)	0.2 (0.1–0.3)	0.1 (0.1–0.2)	0.1 (0.1–0.1)
Visceral leishmaniasis	0.2 (0.1–0.3)	0.4 (0.3–0.7)	0.1 (0.1–0.2)	0.2 (0.1–0.3)	0.1 (0.1–0.2)	0.1 (0.1–0.1)
Cutaneous and mucocutaneous leishmaniasis	0 (0–0)	0 (0–0)	0 (0–0)	0 (0–0)	0 (0–0)	0 (0–0)
African trypanosomiasis	0.4 (0.2–0.6)	0.3 (0.2–0.5)	0.3 (0.1–0.4)	0.4 (0.2–0.7)	0.5 (0.3–1.0)	0.3 (0.2–0.6)
Schistosomiasis	0.2 (0.1–0.2)	0.0 (0.0–0.0)	0.0 (0.0–0.0)	0.1 (0.1–0.1)	0.5 (0.4–0.6)	0.8 (0.6–1.1)
Cysticercosis	0.0 (0.0–0.0)	0.0 (0.0–0.0)	0.0 (0.0–0.0)	0.0 (0.0–0.0)	0.0 (0.0–0.0)	0.0 (0.0–0.0)
Cystic echinococcosis	0.0 (0.0–0.0)	0.0 (0.0–0.0)	0.0 (0.0–0.0)	0.0 (0.0–0.0)	0.1 (0.1–0.1)	0.2 (0.1–0.2)
Lymphatic filariasis	0 (0–0)	0 (0–0)	0 (0–0)	0 (0–0)	0 (0–0)	0 (0–0)
Onchocerciasis	0 (0–0)	0 (0–0)	0 (0–0)	0 (0–0)	0 (0–0)	0 (0–0)
Trachoma	0 (0–0)	0 (0–0)	0 (0–0)	0 (0–0)	0 (0–0)	0 (0–0)
Dengue	0.1 (0.1–0.2)	0.5 (0.2–1.0)	0.2 (0.1–0.3)	0.1 (0.0–0.1)	0.1 (0.1–0.1)	0.1 (0.1–0.2)
Yellow fever	0.1 (0.0–0.2)	0.1 (0.0–0.2)	0.1 (0.0–0.3)	0.1 (0.0–0.2)	0.1 (0.0–0.2)	0.0 (0.0–0.1)
Rabies	0.6 (0.5–0.7)	1.0 (0.7–1.4)	0.7 (0.6–0.9)	0.3 (0.3–0.4)	0.9 (0.7–1.1)	0.9 (0.7–1.2)
Intestinal nematode infections	0.1 (0.1–0.1)	0.6 (0.5–0.7)	0.1 (0.0–0.1)	0.0 (0.0–0.0)	0.0 (0.0–0.0)	0.0 (0.0–0.1)
Ascariasis	0.1 (0.1–0.1)	0.6 (0.5–0.7)	0.1 (0.0–0.1)	0.0 (0.0–0.0)	0.0 (0.0–0.0)	0.0 (0.0–0.1)
Trichuriasis	0 (0–0)	0 (0–0)	0 (0–0)	0 (0–0)	0 (0–0)	0 (0–0)
Hookworm disease	0 (0–0)	0 (0–0)	0 (0–0)	0 (0–0)	0 (0–0)	0 (0–0)
Food-borne trematodiases	0 (0–0)	0 (0–0)	0 (0–0)	0 (0–0)	0 (0–0)	0 (0–0)
Leprosy	0 (0–0)	0 (0–0)	0 (0–0)	0 (0–0)	0 (0–0)	0 (0–0)
Ebola	0.0 (0.0–0.0)	0.0 (0.0–0.0)	0.0 (0.0–0.0)	0.0 (0.0–0.0)	0.0 (0.0–0.0)	0.0 (0.0–0.0)
Other neglected tropical diseases	0.3 (0.2–0.5)	1.8 (1.2–2.7)	0.2 (0.1–0.3)	0.1 (0.1–0.2)	0.3 (0.2–0.6)	0.6 (0.4–1.1)
Maternal disorders	12.3 (11.5–13.1)	0 (0–0)	0.3 (0.2–0.3)	23.6 (22.1–25.2)	0.3 (0.2–0.5)	0 (0–0)
Maternal hemorrhage	3.2 (2.7–3.7)	0 (0–0)	0.1 (0.1–0.1)	6.1 (5.2–7.1)	0.1 (0.1–0.2)	0 (0–0)
Maternal sepsis and other maternal infections	0.8 (0.7–1.0)	0 (0–0)	0.0 (0.0–0.0)	1.6 (1.3–2.0)	0.0 (0.0–0.0)	0 (0–0)
Maternal hypertensive disorders	2.1 (1.7–2.5)	0 (0–0)	0.1 (0.0–0.1)	4.0 (3.3–4.7)	0.0 (0.0–0.0)	0 (0–0)
Maternal obstructed labor and uterine rupture	1.0 (0.8–1.2)	0 (0–0)	0.0 (0.0–0.0)	1.9 (1.5–2.3)	0.0 (0.0–0.0)	0 (0–0)
Maternal abortion, miscarriage, and ectopic pregnancy	1.8 (1.5–2.2)	0 (0–0)	0.0 (0.0–0.1)	3.4 (2.8–4.2)	0.1 (0.0–0.1)	0 (0–0)

SR Table 13 - Global death rates (per 100,000) by age and cause for females in 2000						
**Cause**	**All ages**	**0-5 years**	**5-14 years**	**15-49 years**	**50-69 years**	**70+ years**
Indirect maternal deaths	1.5 (1.2–1.8)	0 (0–0)	0.0 (0.0–0.1)	2.8 (2.2–3.4)	0.0 (0.0–0.1)	0 (0–0)
Late maternal deaths	0.3 (0.2–0.4)	0 (0–0)	0.0 (0.0–0.0)	0.5 (0.4–0.8)	0.0 (0.0–0.0)	0 (0–0)
Maternal deaths aggravated by HIV/AIDS	0.1 (0.1–0.1)	0 (0–0)	0.0 (0.0–0.0)	0.2 (0.1–0.2)	0.0 (0.0–0.0)	0 (0–0)
Other maternal disorders	1.6 (1.3–1.9)	0 (0–0)	0.0 (0.0–0.1)	3.0 (2.5–3.6)	0.1 (0.0–0.1)	0 (0–0)
Neonatal disorders	42.0 (40.6–43.3)	432.1 (418.2–445.7)	0 (0–0)	0 (0–0)	0 (0–0)	0 (0–0)
Neonatal preterm birth complications	19.4 (17.5–21.7)	199.7 (180.1–223.3)	0 (0–0)	0 (0–0)	0 (0–0)	0 (0–0)
Neonatal encephalopathy due to birth asphyxia and trauma	12.7 (11.1–14.4)	130.6 (113.9–148.1)	0 (0–0)	0 (0–0)	0 (0–0)	0 (0–0)
Neonatal sepsis and other neonatal infections	4.1 (2.0–6.2)	42.2 (20.4–64.0)	0 (0–0)	0 (0–0)	0 (0–0)	0 (0–0)
Hemolytic disease and other neonatal jaundice	1.3 (0.6–2.6)	13.7 (6.6–26.4)	0 (0–0)	0 (0–0)	0 (0–0)	0 (0–0)
Other neonatal disorders	4.5 (3.0–6.7)	45.8 (30.8–68.7)	0 (0–0)	0 (0–0)	0 (0–0)	0 (0–0)
Nutritional deficiencies	10.0 (8.5–11.7)	56.5 (46.4–69.1)	1.8 (1.5–2.1)	1.6 (1.4–1.8)	7.0 (6.0–8.2)	45.0 (38.0–53.1)
Protein-energy malnutrition	8.4 (6.9–10.0)	51.9 (42.2–64.1)	1.4 (1.1–1.7)	0.9 (0.7–1.2)	4.7 (3.5–5.8)	37.6 (30.4–45.6)
Iodine deficiency	0.0 (0.0–0.1)	0.2 (0.1–0.4)	0.0 (0.0–0.0)	0.0 (0.0–0.0)	0.1 (0.0–0.1)	0.2 (0.1–0.3)
Vitamin A deficiency	0 (0–0)	0 (0–0)	0 (0–0)	0 (0–0)	0 (0–0)	0 (0–0)
Iron-deficiency anemia	0.9 (0.6–1.2)	2.8 (1.2–3.9)	0.2 (0.1–0.3)	0.4 (0.3–0.5)	1.5 (1.2–1.9)	2.6 (1.8–3.8)
Other nutritional deficiencies	0.7 (0.3–1.4)	1.6 (0.4–4.1)	0.2 (0.1–0.3)	0.3 (0.1–0.5)	0.8 (0.3–1.7)	4.6 (2.8–7.9)
Other communicable, maternal, neonatal, and nutritional diseases	6.0 (5.0–7.4)	32.9 (23.2–45.4)	2.2 (1.8–2.8)	1.8 (1.7–2.0)	5.8 (5.3–6.2)	12.9 (11.5–13.8)
Sexually transmitted diseases excluding HIV	2.7 (1.6–4.0)	22.6 (13.0–35.3)	1.1 (0.6–1.7)	0.3 (0.2–0.3)	0.5 (0.3–0.6)	1.1 (0.8–1.3)
Syphilis	2.6 (1.6–4.0)	22.6 (13.0–35.3)	1.1 (0.6–1.7)	0.2 (0.2–0.3)	0.4 (0.3–0.5)	1.0 (0.7–1.1)
Chlamydial infection	0.0 (0.0–0.0)	0 (0–0)	0.0 (0.0–0.0)	0.0 (0.0–0.0)	0.0 (0.0–0.0)	0.0 (0.0–0.0)
Gonococcal infection	0.0 (0.0–0.0)	0 (0–0)	0.0 (0.0–0.0)	0.0 (0.0–0.0)	0.0 (0.0–0.0)	0.1 (0.1–0.1)
Trichomoniasis	0 (0–0)	0 (0–0)	0 (0–0)	0 (0–0)	0 (0–0)	0 (0–0)
Genital herpes	0 (0–0)	0 (0–0)	0 (0–0)	0 (0–0)	0 (0–0)	0 (0–0)
Other sexually transmitted diseases	0.0 (0.0–0.0)	0 (0–0)	0.0 (0.0–0.0)	0.0 (0.0–0.0)	0.0 (0.0–0.0)	0.0 (0.0–0.0)
Hepatitis	2.1 (2.0–2.2)	4.4 (3.8–5.0)	0.8 (0.7–0.9)	1.2 (1.2–1.3)	3.4 (3.2–3.6)	6.9 (6.5–7.3)
Acute hepatitis A	0.4 (0.3–0.5)	3.4 (2.4–4.2)	0.1 (0.0–0.3)	0.0 (0.0–0.1)	0.1 (0.0–0.2)	0.2 (0.0–0.4)
Hepatitis B	1.0 (0.9–1.2)	0.2 (0.1–0.6)	0.1 (0.0–0.1)	0.7 (0.5–0.8)	2.8 (2.4–3.1)	5.2 (4.4–6.0)
Hepatitis C	0.0 (0.0–0.1)	0 (0–0)	0.0 (0.0–0.0)	0.0 (0.0–0.0)	0.2 (0.0–0.4)	0.2 (0.1–0.6)
Acute hepatitis E	0.6 (0.4–0.8)	0.8 (0.3–1.6)	0.6 (0.4–0.8)	0.5 (0.4–0.7)	0.4 (0.2–0.7)	1.3 (0.7–2.0)
Other infectious diseases	1.3 (0.9–1.5)	5.8 (3.0–8.0)	0.3 (0.3–0.4)	0.3 (0.3–0.3)	1.9 (1.5–2.1)	4.9 (3.7–5.5)
Non-communicable diseases	515.3 (507.5–524.4)	157.2 (131.3–196.7)	19.1 (17.6–21.0)	97.3 (94.7–101.0)	1013.4 (993.9–1035.3)	5971.8 (5909.2–6040.3)
Neoplasms	98.7 (96.7–100.8)	5.9 (5.1–7.2)	4.3 (4.0–4.6)	31.7 (30.6–32.9)	282.4 (275.8–289.0)	821.6 (805.4–836.3)
Lip and oral cavity cancer	1.1 (1.1–1.1)	0 (0–0)	0 (0–0)	0.3 (0.3–0.4)	3.3 (3.1–3.5)	9.2 (8.9–9.5)
Nasopharynx cancer	0.6 (0.5–0.6)	0 (0–0)	0.0 (0.0–0.0)	0.4 (0.3–0.4)	1.7 (1.6–1.9)	2.3 (2.2–2.5)
Other pharynx cancer	0.5 (0.4–0.5)	0 (0–0)	0 (0–0)	0.1 (0.1–0.2)	1.8 (1.6–1.9)	3.2 (3.0–3.4)
Esophageal cancer	4.7 (4.5–5.0)	0 (0–0)	0 (0–0)	0.9 (0.8–1.0)	15.4 (14.6–16.4)	41.8 (39.5–44.2)
Stomach cancer	9.7 (9.4–10.0)	0 (0–0)	0 (0–0)	2.6 (2.4–2.7)	26.2 (25.1–27.3)	93.4 (90.8–96.1)
Colon and rectum cancer	9.8 (9.6–10.0)	0 (0–0)	0 (0–0)	1.8 (1.8–1.9)	23.7 (23.2–24.3)	108.5 (106.0–111.0)
Liver cancer	6.9 (5.7–7.3)	0 (0–0)	0.1 (0.1–0.1)	2.2 (1.7–2.5)	21.7 (17.8–23.2)	52.8 (46.8–56.1)
Liver cancer due to hepatitis B	2.1 (1.7–2.3)	0 (0–0)	0.0 (0.0–0.1)	1.2 (0.9–1.4)	6.8 (5.4–7.7)	10.3 (8.5–11.6)
Liver cancer due to hepatitis C	1.9 (1.6–2.0)	0 (0–0)	0.0 (0.0–0.0)	0.2 (0.2–0.3)	5.3 (4.5–5.9)	19.6 (17.9–21.1)
Liver cancer due to alcohol use	1.1 (0.9–1.2)	0 (0–0)	0 (0–0)	0.2 (0.1–0.2)	4.0 (3.3–4.6)	9.0 (7.9–9.9)
Liver cancer due to other causes	1.8 (1.5–2.0)	0 (0–0)	0.0 (0.0–0.1)	0.6 (0.5–0.7)	5.5 (4.3–6.2)	13.9 (11.7–15.1)
Gallbladder and biliary tract cancer	2.3 (2.2–2.4)	0 (0–0)	0 (0–0)	0.3 (0.3–0.4)	6.2 (5.8–6.4)	25.6 (24.8–26.4)
Pancreatic cancer	4.4 (4.3–4.5)	0 (0–0)	0 (0–0)	0.5 (0.5–0.6)	11.6 (11.3–11.9)	49.5 (48.4–50.6)
Larynx cancer	0.6 (0.5–0.6)	0 (0–0)	0 (0–0)	0.2 (0.1–0.2)	1.9 (1.7–2.1)	4.4 (4.2–4.7)
Tracheal, bronchus, and lung cancer	12.3 (12.0–12.6)	0 (0–0)	0 (0–0)	2.5 (2.3–2.6)	37.1 (36.1–38.2)	115.4 (112.6–118.4)
Malignant skin melanoma	0.7 (0.6–0.7)	0 (0–0)	0 (0–0)	0.3 (0.3–0.3)	1.6 (1.5–1.8)	5.8 (5.2–6.2)
Non-melanoma skin cancer	0.3 (0.3–0.3)	0 (0–0)	0 (0–0)	0.1 (0.1–0.1)	0.6 (0.6–0.6)	3.9 (3.8–4.1)
Non-melanoma skin cancer (squamous-cell carcinoma)	0.3 (0.3–0.3)	0 (0–0)	0 (0–0)	0.1 (0.1–0.1)	0.6 (0.6–0.6)	3.9 (3.8–4.1)
Non-melanoma skin cancer (basal-cell carcinoma)	0 (0–0)	0 (0–0)	0 (0–0)	0 (0–0)	0 (0–0)	0 (0–0)
Breast cancer	13.2 (12.5–13.9)	0 (0–0)	0 (0–0)	6.0 (5.4–6.6)	41.8 (39.9–44.1)	84.6 (81.6–87.5)
Cervical cancer	6.9 (6.5–7.3)	0 (0–0)	0 (0–0)	3.8 (3.5–4.1)	23.3 (21.9–24.7)	34.8 (33.6–36.1)
Uterine cancer	2.6 (2.4–2.7)	0 (0–0)	0 (0–0)	0.7 (0.6–0.7)	8.6 (8.2–9.2)	19.9 (19.2–20.7)
Ovarian cancer	4.0 (4.0–4.2)	0 (0–0)	0 (0–0)	1.4 (1.4–1.6)	13.4 (13.1–13.9)	28.7 (28.0–29.5)
Prostate cancer	0 (0–0)	0 (0–0)	0 (0–0)	0 (0–0)	0 (0–0)	0 (0–0)
Testicular cancer	0 (0–0)	0 (0–0)	0 (0–0)	0 (0–0)	0 (0–0)	0 (0–0)

SR Table 13 - Global death rates (per 100,000) by age and cause for females in 2000						
Cause	All ages	0-5 years	5-14 years	15-49 years	50-69 years	70+ years
Kidney cancer	1.1 (1.1–1.2)	0.3 (0.2–0.4)	0.1 (0.1–0.1)	0.2 (0.2–0.2)	3.1 (3.0–3.2)	11.1 (10.8–11.4)
Bladder cancer	1.3 (1.2–1.3)	0 (0–0)	0 (0–0)	0.1 (0.1–0.1)	2.5 (2.5–2.6)	16.6 (16.1–17.1)
Brain and nervous system cancer	2.6 (2.4–2.7)	0.9 (0.7–1.0)	0.8 (0.7–0.9)	1.4 (1.3–1.5)	7.1 (6.7–7.4)	12.6 (11.9–13.1)
Thyroid cancer	0.5 (0.5–0.6)	0 (0–0)	0.0 (0.0–0.0)	0.1 (0.1–0.1)	1.3 (1.2–1.5)	5.2 (4.9–5.7)
Mesothelioma	0.2 (0.2–0.2)	0 (0–0)	0 (0–0)	0.1 (0.1–0.1)	0.6 (0.6–0.6)	1.9 (1.8–2.0)
Hodgkin lymphoma	0.4 (0.3–0.4)	0.1 (0.0–0.1)	0.1 (0.0–0.1)	0.3 (0.2–0.3)	0.9 (0.6–1.1)	1.9 (1.4–2.3)
Non-Hodgkin lymphoma	2.4 (2.1–2.7)	0.5 (0.3–0.8)	0.3 (0.2–0.3)	0.8 (0.7–0.9)	5.7 (4.9–6.3)	21.9 (18.0–23.3)
Multiple myeloma	1.2 (1.1–1.2)	0 (0–0)	0 (0–0)	0.2 (0.2–0.2)	3.3 (3.2–3.5)	12.1 (11.0–12.5)
Leukemia	4.2 (4.1–4.4)	2.3 (2.0–2.7)	2.0 (1.9–2.2)	2.3 (2.2–2.4)	7.9 (7.6–8.2)	26.1 (25.3–26.9)
Acute lymphoid leukemia	1.4 (1.3–1.5)	1.5 (1.3–2.0)	1.4 (1.3–1.6)	0.9 (0.8–1.0)	2.0 (1.9–2.2)	4.1 (3.8–4.3)
Chronic lymphoid leukemia	0.8 (0.7–0.8)	0 (0–0)	0 (0–0)	0.3 (0.2–0.3)	1.5 (1.3–1.6)	8.1 (7.7–8.5)
Acute myeloid leukemia	1.5 (1.5–1.6)	0.7 (0.5–0.9)	0.6 (0.5–0.7)	0.8 (0.8–0.9)	3.0 (2.9–3.2)	9.6 (9.3–10.0)
Chronic myeloid leukemia	0.6 (0.5–0.6)	0 (0–0)	0 (0–0)	0.3 (0.3–0.4)	1.3 (1.3–1.5)	4.3 (4.1–4.5)
Other neoplasms	4.3 (4.0–4.5)	1.9 (1.5–2.8)	0.9 (0.8–1.0)	2.1 (1.9–2.3)	10.0 (9.4–10.6)	28.4 (27.2–29.6)
Cardiovascular diseases	245.7 (241.8–250.0)	11.4 (10.4–12.5)	3.0 (2.8–3.3)	31.5 (30.5–32.9)	430.7 (421.3–440.9)	3278.7 (3223.1–3335.9)
Rheumatic heart disease	6.7 (6.2–7.2)	0.8 (0.6–1.0)	0.8 (0.7–0.9)	3.3 (2.9–3.7)	16.0 (14.8–17.5)	49.4 (46.6–52.7)
Ischemic heart disease	109.0 (107.3–111.1)	0.7 (0.6–0.8)	0.2 (0.2–0.3)	10.7 (10.2–11.2)	179.5 (175.2–184.1)	1531.4 (1503.6–1560.9)
Cerebrovascular disease	98.0 (96.0–100.5)	4.0 (3.5–4.7)	1.0 (1.0–1.2)	12.1 (11.6–12.7)	181.9 (176.8–188.0)	1287.9 (1261.3–1317.6)
Ischemic stroke	46.9 (44.4–49.5)	0.2 (0.2–0.2)	0.1 (0.1–0.1)	1.9 (1.6–2.1)	60.0 (54.3–66.3)	731.4 (699.6–762.1)
Hemorrhagic stroke	51.1 (48.9–53.9)	3.8 (3.3–4.5)	1.0 (0.9–1.1)	10.2 (9.7–10.9)	121.9 (115.9–129.2)	556.5 (527.0–584.4)
Hypertensive heart disease	13.4 (11.6–14.9)	0.1 (0.1–0.1)	0.0 (0.0–0.0)	1.9 (1.5–2.1)	27.1 (22.7–30.3)	170.0 (146.1–190.7)
Cardiomyopathy and myocarditis	4.3 (4.2–4.5)	2.1 (1.8–2.4)	0.3 (0.3–0.4)	1.3 (1.2–1.4)	6.5 (6.3–6.8)	48.6 (46.8–50.3)
Atrial fibrillation and flutter	2.3 (1.9–2.8)	0 (0–0)	0 (0–0)	0.0 (0.0–0.0)	1.0 (0.7–1.3)	42.3 (34.3–51.2)
Aortic aneurysm	1.4 (1.4–1.5)	0 (0–0)	0 (0–0)	0.2 (0.2–0.2)	2.2 (2.1–2.3)	20.3 (19.7–20.8)
Peripheral vascular disease	0.5 (0.5–0.5)	0 (0–0)	0 (0–0)	0.0 (0.0–0.0)	0.4 (0.3–0.4)	8.5 (8.0–9.0)
Endocarditis	1.1 (1.0–1.3)	0.7 (0.3–1.0)	0.1 (0.1–0.1)	0.4 (0.3–0.5)	1.6 (1.3–2.0)	12.4 (11.2–13.9)
Other cardiovascular and circulatory diseases	8.8 (8.5–9.1)	3.1 (2.7–3.6)	0.4 (0.4–0.5)	1.7 (1.6–1.8)	14.4 (13.8–15.3)	108.0 (104.1–112.2)
Chronic respiratory diseases	58.2 (56.3–60.1)	11.6 (10.1–13.0)	1.3 (1.2–1.4)	6.5 (6.2–6.8)	114.0 (109.2–119.3)	735.4 (711.2–759.1)
Chronic obstructive pulmonary disease	47.7 (43.9–51.3)	5.0 (3.3–6.5)	0.3 (0.2–0.5)	3.5 (2.9–4.2)	88.7 (78.2–99.8)	645.0 (601.7–681.0)
Pneumoconiosis	0.3 (0.2–0.4)	0 (0–0)	0 (0–0)	0.0 (0.0–0.1)	0.5 (0.3–0.8)	4.0 (2.9–5.5)
Silicosis	0.0 (0.0–0.0)	0 (0–0)	0 (0–0)	0.0 (0.0–0.0)	0.0 (0.0–0.0)	0.2 (0.1–0.2)
Asbestosis	0.0 (0.0–0.0)	0 (0–0)	0 (0–0)	0.0 (0.0–0.0)	0.0 (0.0–0.0)	0.1 (0.1–0.1)
Coal workers pneumoconiosis	0.0 (0.0–0.0)	0 (0–0)	0 (0–0)	0.0 (0.0–0.0)	0.0 (0.0–0.0)	0.1 (0.0–0.1)
Other pneumoconiosis	0.3 (0.2–0.4)	0 (0–0)	0 (0–0)	0.0 (0.0–0.1)	0.5 (0.2–0.7)	3.7 (2.6–5.1)
Asthma	8.7 (5.5–12.5)	3.5 (2.2–4.6)	0.9 (0.6–1.1)	2.7 (1.8–3.3)	22.5 (12.9–33.3)	72.0 (45.5–115.3)
Interstitial lung disease and pulmonary sarcoidosis	0.9 (0.7–1.1)	0.1 (0.0–0.1)	0.0 (0.0–0.0)	0.2 (0.1–0.2)	1.9 (1.5–2.2)	11.7 (8.5–13.3)
Other chronic respiratory diseases	0.6 (0.4–0.8)	3.0 (2.0–4.5)	0.1 (0.1–0.2)	0.1 (0.1–0.2)	0.4 (0.3–0.6)	2.7 (2.0–3.4)
Cirrhosis and other chronic liver diseases	12.7 (11.9–14.2)	2.7 (2.0–3.9)	1.0 (0.8–1.2)	5.6 (5.2–6.3)	38.2 (35.4–42.9)	79.9 (75.4–88.4)
Cirrhosis and other chronic liver diseases due to hepatitis B	3.3 (3.0–3.9)	0.0 (0.0–0.0)	0.0 (0.0–0.0)	1.4 (1.3–1.7)	10.4 (9.1–12.3)	22.6 (20.1–26.2)
Cirrhosis and other chronic liver diseases due to hepatitis C	2.8 (2.6–3.1)	0.0 (0.0–0.0)	0.0 (0.0–0.0)	1.1 (1.0–1.3)	8.7 (7.7–9.9)	19.9 (18.1–22.1)
Cirrhosis and other chronic liver diseases due to alcohol use	2.8 (2.6–3.1)	0 (0–0)	0 (0–0)	1.5 (1.3–1.7)	9.9 (8.7–11.2)	14.0 (12.5–15.7)
Cirrhosis and other chronic liver diseases due to other causes	3.7 (3.4–4.3)	2.6 (1.9–3.9)	0.9 (0.8–1.2)	1.6 (1.4–1.8)	9.2 (8.2–10.6)	23.4 (21.4–26.3)
Digestive diseases	16.4 (15.8–17.3)	6.2 (3.7–12.3)	1.8 (1.6–2.0)	5.4 (5.1–5.7)	32.3 (31.0–33.7)	160.7 (156.1–165.5)
Peptic ulcer disease	4.3 (3.8–4.8)	0.9 (0.4–2.0)	0.4 (0.3–0.5)	1.5 (1.3–1.7)	9.4 (8.2–10.6)	40.5 (36.4–44.8)
Gastritis and duodenitis	0.7 (0.3–0.9)	0.2 (0.1–0.4)	0.1 (0.0–0.1)	0.2 (0.1–0.3)	1.5 (0.7–2.1)	6.7 (3.3–9.3)
Appendicitis	0.8 (0.6–1.0)	1.3 (0.5–3.1)	0.3 (0.3–0.4)	0.5 (0.4–0.5)	1.3 (1.1–1.5)	3.5 (3.0–4.0)
Paralytic ileus and intestinal obstruction	3.4 (2.8–3.9)	2.0 (1.0–3.8)	0.6 (0.4–0.7)	1.2 (0.9–1.4)	6.5 (5.2–7.6)	30.5 (26.2–34.9)
Inguinal, femoral, and abdominal hernia	0.7 (0.4–0.8)	0.2 (0.1–0.3)	0.1 (0.0–0.1)	0.2 (0.1–0.2)	1.4 (0.9–1.7)	6.7 (5.0–7.9)
Inflammatory bowel disease	0.7 (0.6–0.8)	0.5 (0.2–1.2)	0.1 (0.0–0.1)	0.2 (0.2–0.3)	1.1 (0.9–1.2)	7.0 (6.3–7.5)
Vascular intestinal disorders	1.4 (1.2–1.5)	0.0 (0.0–0.1)	0.0 (0.0–0.0)	0.1 (0.1–0.2)	2.1 (1.7–2.4)	19.7 (18.3–21.4)
Gallbladder and biliary diseases	1.8 (1.7–1.9)	0.5 (0.2–1.0)	0.1 (0.1–0.2)	0.5 (0.4–0.5)	3.6 (3.3–4.0)	19.0 (17.9–20.2)
Pancreatitis	1.2 (1.1–1.3)	0.0 (0.0–0.1)	0.1 (0.0–0.1)	0.6 (0.5–0.7)	2.9 (2.6–3.3)	9.2 (8.5–9.8)
Other digestive diseases	1.5 (1.3–1.7)	0.7 (0.3–1.6)	0.1 (0.1–0.2)	0.3 (0.3–0.4)	2.5 (2.1–3.0)	17.9 (15.9–19.9)
Neurological disorders	28.3 (24.2–32.3)	2.9 (2.3–4.1)	1.1 (1.0–1.3)	2.1 (1.9–2.4)	14.9 (12.7–17.3)	478.2 (401.8–553.0)
Alzheimer disease and other dementias	24.4 (20.3–28.4)	0 (0–0)	0 (0–0)	0.0 (0.0–0.1)	9.2 (6.9–11.5)	449.3 (372.5–524.7)
Parkinson disease	1.0 (1.0–1.1)	0 (0–0)	0 (0–0)	0.0 (0.0–0.0)	0.9 (0.9–1.0)	17.3 (16.6–18.0)
Epilepsy	1.6 (1.4–1.8)	2.1 (1.6–3.1)	0.9 (0.8–1.1)	1.5 (1.4–1.7)	1.5 (1.5–1.7)	3.5 (3.4–3.7)

SR Table 13 - Global death rates (per 100,000) by age and cause for females in 2000

Cause	All ages	0-5 years	5-14 years	15-49 years	50-69 years	70+ years
Multiple sclerosis	0.3 (0.3–0.4)	0 (0–0)	0 (0–0)	0.2 (0.2–0.3)	1.0 (0.8–1.1)	1.3 (1.0–1.4)
Motor neuron disease	0.4 (0.3–0.4)	0.2 (0.1–0.3)	0.0 (0.0–0.0)	0.1 (0.1–0.1)	1.1 (1.0–1.2)	3.0 (2.9–3.1)
Migraine	0 (0–0)	0 (0–0)	0 (0–0)	0 (0–0)	0 (0–0)	0 (0–0)
Tension-type headache	0 (0–0)	0 (0–0)	0 (0–0)	0 (0–0)	0 (0–0)	0 (0–0)
Medication overuse headache	0 (0–0)	0 (0–0)	0 (0–0)	0 (0–0)	0 (0–0)	0 (0–0)
Other neurological disorders	0.6 (0.6–0.7)	0.6 (0.5–1.1)	0.2 (0.2–0.3)	0.3 (0.3–0.3)	1.2 (1.2–1.3)	3.9 (3.7–4.0)
Mental and substance use disorders	2.1 (2.0–2.1)	0.0 (0.0–0.0)	0.0 (0.0–0.0)	2.1 (2.0–2.2)	4.6 (4.4–4.8)	6.4 (6.1–6.6)
Schizophrenia	0.3 (0.3–0.3)	0 (0–0)	0 (0–0)	0.2 (0.2–0.3)	0.7 (0.7–0.8)	1.8 (1.7–2.0)
Alcohol use disorders	0.8 (0.8–0.9)	0 (0–0)	0 (0–0)	0.7 (0.7–0.8)	2.6 (2.4–2.7)	2.0 (1.9–2.1)
Drug use disorders	0.9 (0.8–0.9)	0.0 (0.0–0.0)	0 (0–0)	1.1 (1.0–1.2)	1.3 (1.2–1.4)	2.6 (2.3–2.7)
Opioid use disorders	0.6 (0.5–0.6)	0.0 (0.0–0.0)	0 (0–0)	0.7 (0.6–0.8)	0.8 (0.8–0.9)	1.5 (1.4–1.7)
Cocaine use disorders	0.0 (0.0–0.0)	0 (0–0)	0 (0–0)	0.1 (0.0–0.1)	0.1 (0.0–0.1)	0.1 (0.0–0.1)
Amphetamine use disorders	0.0 (0.0–0.0)	0 (0–0)	0 (0–0)	0.0 (0.0–0.1)	0.0 (0.0–0.0)	0.0 (0.0–0.0)
Cannabis use disorders	0 (0–0)	0 (0–0)	0 (0–0)	0 (0–0)	0 (0–0)	0 (0–0)
Other drug use disorders	0.3 (0.2–0.3)	0 (0–0)	0 (0–0)	0.3 (0.3–0.4)	0.4 (0.3–0.5)	1.0 (0.8–1.1)
Depressive disorders	0 (0–0)	0 (0–0)	0 (0–0)	0 (0–0)	0 (0–0)	0 (0–0)
Major depressive disorder	0 (0–0)	0 (0–0)	0 (0–0)	0 (0–0)	0 (0–0)	0 (0–0)
Dysthymia	0 (0–0)	0 (0–0)	0 (0–0)	0 (0–0)	0 (0–0)	0 (0–0)
Bipolar disorder	0 (0–0)	0 (0–0)	0 (0–0)	0 (0–0)	0 (0–0)	0 (0–0)
Anxiety disorders	0 (0–0)	0 (0–0)	0 (0–0)	0 (0–0)	0 (0–0)	0 (0–0)
Eating disorders	0.0 (0.0–0.0)	0 (0–0)	0.0 (0.0–0.0)	0.0 (0.0–0.0)	0 (0–0)	0 (0–0)
Anorexia nervosa	0.0 (0.0–0.0)	0 (0–0)	0.0 (0.0–0.0)	0.0 (0.0–0.0)	0 (0–0)	0 (0–0)
Bulimia nervosa	0.0 (0.0–0.0)	0 (0–0)	0.0 (0.0–0.0)	0.0 (0.0–0.0)	0 (0–0)	0 (0–0)
Autistic spectrum disorders	0 (0–0)	0 (0–0)	0 (0–0)	0 (0–0)	0 (0–0)	0 (0–0)
Autism	0 (0–0)	0 (0–0)	0 (0–0)	0 (0–0)	0 (0–0)	0 (0–0)
Asperger syndrome and other autistic spectrum disorders	0 (0–0)	0 (0–0)	0 (0–0)	0 (0–0)	0 (0–0)	0 (0–0)
Attention-deficit/hyperactivity disorder	0 (0–0)	0 (0–0)	0 (0–0)	0 (0–0)	0 (0–0)	0 (0–0)
Conduct disorder	0 (0–0)	0 (0–0)	0 (0–0)	0 (0–0)	0 (0–0)	0 (0–0)
Idiopathic developmental intellectual disability	0 (0–0)	0 (0–0)	0 (0–0)	0 (0–0)	0 (0–0)	0 (0–0)
Other mental and substance use disorders	0 (0–0)	0 (0–0)	0 (0–0)	0 (0–0)	0 (0–0)	0 (0–0)
Diabetes, urogenital, blood, and endocrine diseases	39.8 (38.7–41.0)	22.5 (17.5–32.0)	2.9 (2.7–3.2)	9.9 (9.6–10.3)	90.2 (87.7–92.9)	379.6 (371.0–388.2)
Diabetes mellitus	18.0 (17.5–18.5)	0.5 (0.4–0.6)	0.3 (0.2–0.3)	3.0 (2.8–3.1)	48.9 (47.2–50.7)	187.7 (182.9–193.1)
Acute glomerulonephritis	0.2 (0.2–0.3)	0.1 (0.1–0.1)	0.0 (0.0–0.1)	0.1 (0.1–0.1)	0.5 (0.3–0.5)	1.4 (1.1–1.9)
Chronic kidney disease	13.1 (12.8–13.5)	3.0 (2.6–3.3)	0.9 (0.8–1.0)	3.9 (3.7–4.1)	29.1 (28.2–30.1)	129.5 (125.8–133.3)
Chronic kidney disease due to diabetes mellitus	4.1 (3.9–4.3)	0.4 (0.3–0.5)	0.1 (0.1–0.1)	0.9 (0.8–1.0)	11.1 (10.4–12.0)	39.8 (37.8–41.9)
Chronic kidney disease due to hypertension	5.8 (5.6–6.1)	1.0 (0.9–1.2)	0.3 (0.2–0.3)	1.5 (1.4–1.6)	11.8 (11.0–12.6)	64.2 (61.4–67.3)
Chronic kidney disease due to glomerulonephritis	2.9 (2.7–3.0)	1.1 (1.0–1.3)	0.4 (0.4–0.5)	1.4 (1.3–1.5)	5.8 (5.4–6.3)	22.6 (21.0–24.3)
Chronic kidney disease due to other causes	0.4 (0.3–0.4)	0.4 (0.3–0.5)	0.1 (0.1–0.2)	0.2 (0.1–0.2)	0.3 (0.2–0.5)	2.9 (2.4–3.7)
Urinary diseases and male infertility	3.0 (2.8–3.2)	1.1 (0.8–1.3)	0.2 (0.1–0.2)	0.8 (0.8–0.9)	5.2 (4.8–5.9)	32.6 (30.9–35.2)
Interstitial nephritis and urinary tract infections	2.4 (2.3–2.6)	0.8 (0.6–1.0)	0.1 (0.1–0.1)	0.6 (0.6–0.7)	3.9 (3.6–4.4)	28.1 (26.5–30.5)
Urolithiasis	0.2 (0.2–0.2)	0 (0–0)	0.0 (0.0–0.0)	0.1 (0.1–0.1)	0.5 (0.4–0.6)	1.7 (1.3–1.9)
Benign prostatic hyperplasia	0 (0–0)	0 (0–0)	0 (0–0)	0 (0–0)	0 (0–0)	0 (0–0)
Male infertility	0 (0–0)	0 (0–0)	0 (0–0)	0 (0–0)	0 (0–0)	0 (0–0)
Other urinary diseases	0.3 (0.3–0.4)	0.2 (0.2–0.4)	0.0 (0.0–0.0)	0.1 (0.1–0.2)	0.8 (0.7–1.0)	2.8 (2.5–3.3)
Gynecological diseases	0.2 (0.2–0.3)	0 (0–0)	0 (0–0)	0.2 (0.2–0.3)	0.5 (0.4–0.6)	1.1 (0.9–1.4)
Uterine fibroids	0.1 (0.0–0.1)	0 (0–0)	0 (0–0)	0.1 (0.0–0.1)	0.2 (0.1–0.3)	0.3 (0.1–0.4)
Polycystic ovarian syndrome	0.0 (0.0–0.0)	0 (0–0)	0 (0–0)	0.0 (0.0–0.1)	0.1 (0.0–0.1)	0 (0–0)
Female infertility	0 (0–0)	0 (0–0)	0 (0–0)	0 (0–0)	0 (0–0)	0 (0–0)
Endometriosis	0.0 (0.0–0.0)	0 (0–0)	0 (0–0)	0.0 (0.0–0.0)	0.0 (0.0–0.0)	0 (0–0)
Genital prolapse	0.0 (0.0–0.1)	0 (0–0)	0 (0–0)	0.0 (0.0–0.0)	0.1 (0.0–0.2)	0.4 (0.2–0.6)
Premenstrual syndrome	0 (0–0)	0 (0–0)	0 (0–0)	0 (0–0)	0 (0–0)	0 (0–0)
Other gynecological diseases	0.1 (0.1–0.1)	0 (0–0)	0 (0–0)	0.1 (0.1–0.2)	0.2 (0.1–0.3)	0.4 (0.3–0.7)
Hemoglobinopathies and hemolytic anemias	3.6 (3.1–4.5)	15.7 (11.1–25.2)	1.2 (1.0–1.4)	1.3 (1.1–1.4)	3.0 (2.9–3.2)	13.7 (12.8–14.9)
Thalassemias	0.4 (0.3–0.5)	2.2 (1.5–3.3)	0.3 (0.3–0.4)	0.1 (0.1–0.1)	0.0 (0.0–0.0)	1.0 (0.9–1.3)
Thalassemias trait	0 (0–0)	0 (0–0)	0 (0–0)	0 (0–0)	0 (0–0)	0 (0–0)
Sickle cell disorders	1.8 (1.4–2.7)	13.3 (8.8–23.1)	0.8 (0.6–0.9)	0.5 (0.5–0.6)	0.2 (0.1–0.2)	1.2 (1.1–1.5)
Sickle cell trait	0 (0–0)	0 (0–0)	0 (0–0)	0 (0–0)	0 (0–0)	0 (0–0)

SR Table 13 - Global death rates (per 100,000) by age and cause for females in 2000

Cause	All ages	0-5 years	5-14 years	15-49 years	50-69 years	70+ years
G6PD deficiency	0.3 (0.2–0.3)	0.1 (0.1–0.2)	0.1 (0.0–0.1)	0.3 (0.2–0.3)	0.5 (0.4–0.6)	0.3 (0.3–0.4)
G6PD trait	0 (0–0)	0 (0–0)	0 (0–0)	0 (0–0)	0 (0–0)	0 (0–0)
Other hemoglobinopathies and hemolytic anemias	1.1 (1.1–1.2)	0.1 (0.1–0.1)	0.1 (0.0–0.1)	0.4 (0.3–0.4)	2.4 (2.3–2.5)	11.1 (10.3–12.0)
Endocrine, metabolic, blood, and immune disorders	1.7 (1.6–1.7)	2.1 (1.8–2.4)	0.3 (0.3–0.3)	0.6 (0.6–0.6)	3.0 (2.9–3.1)	13.6 (13.1–14.0)
Musculoskeletal disorders	1.6 (1.4–1.8)	0 (0–0)	0.2 (0.2–0.3)	0.8 (0.7–1.0)	2.8 (2.6–3.2)	14.8 (13.3–15.5)
Rheumatoid arthritis	0.6 (0.5–0.7)	0 (0–0)	0.0 (0.0–0.0)	0.1 (0.1–0.1)	1.4 (1.2–1.6)	6.7 (6.0–7.8)
Osteoarthritis	0 (0–0)	0 (0–0)	0 (0–0)	0 (0–0)	0 (0–0)	0 (0–0)
Low back and neck pain	0 (0–0)	0 (0–0)	0 (0–0)	0 (0–0)	0 (0–0)	0 (0–0)
Low back pain	0 (0–0)	0 (0–0)	0 (0–0)	0 (0–0)	0 (0–0)	0 (0–0)
Neck pain	0 (0–0)	0 (0–0)	0 (0–0)	0 (0–0)	0 (0–0)	0 (0–0)
Gout	0 (0–0)	0 (0–0)	0 (0–0)	0 (0–0)	0 (0–0)	0 (0–0)
Other musculoskeletal disorders	1.0 (0.9–1.1)	0 (0–0)	0.2 (0.1–0.2)	0.7 (0.6–0.9)	1.5 (1.3–1.7)	8.0 (6.8–8.6)
Other non-communicable diseases	12.0 (9.1–15.8)	94.0 (69.2–130.2)	3.4 (2.6–4.5)	1.7 (1.3–2.0)	3.1 (2.3–4.0)	16.4 (11.2–21.2)
Congenital anomalies	10.5 (7.7–14.3)	89.5 (65.1–125.1)	3.3 (2.5–4.4)	1.5 (1.2–1.7)	1.5 (1.2–1.6)	3.1 (2.5–3.4)
Neural tube defects	1.5 (1.0–2.5)	14.5 (9.5–24.4)	0.2 (0.1–0.4)	0.0 (0.0–0.1)	0.0 (0.0–0.1)	0.1 (0.0–0.1)
Congenital heart anomalies	5.3 (3.6–7.7)	42.7 (27.4–65.1)	2.4 (1.7–3.3)	1.0 (0.8–1.2)	0.5 (0.4–0.6)	1.1 (1.0–1.3)
Cleft lip and cleft palate	0.1 (0.0–0.1)	0.7 (0.4–1.0)	0 (0–0)	0 (0–0)	0 (0–0)	0 (0–0)
Down syndrome	0.6 (0.2–1.3)	4.8 (1.0–11.4)	0.1 (0.0–0.2)	0.1 (0.1–0.2)	0.3 (0.2–0.4)	0.1 (0.0–0.1)
Turner syndrome	0 (0–0)	0 (0–0)	0 (0–0)	0 (0–0)	0 (0–0)	0 (0–0)
Klinefelter syndrome	0 (0–0)	0 (0–0)	0 (0–0)	0 (0–0)	0 (0–0)	0 (0–0)
Other chromosomal abnormalities	0.4 (0.2–1.3)	4.2 (1.5–13.1)	0.0 (0.0–0.1)	0.0 (0.0–0.0)	0.0 (0.0–0.0)	0.0 (0.0–0.0)
Other congenital anomalies	2.6 (2.0–3.8)	22.6 (16.9–34.7)	0.6 (0.4–0.8)	0.2 (0.2–0.3)	0.6 (0.5–0.7)	1.8 (1.3–2.0)
Skin and subcutaneous diseases	1.2 (0.7–1.7)	1.1 (0.5–1.9)	0.1 (0.0–0.1)	0.2 (0.1–0.3)	1.6 (0.9–2.6)	13.3 (8.4–18.2)
Dermatitis	0 (0–0)	0 (0–0)	0 (0–0)	0 (0–0)	0 (0–0)	0 (0–0)
Psoriasis	0 (0–0)	0 (0–0)	0 (0–0)	0 (0–0)	0 (0–0)	0 (0–0)
Cellulitis	0.2 (0.1–0.3)	0.1 (0.0–0.2)	0.0 (0.0–0.0)	0.0 (0.0–0.1)	0.4 (0.2–0.6)	1.7 (1.0–2.4)
Pyoderma	0.5 (0.3–0.8)	0.9 (0.4–1.7)	0.1 (0.0–0.1)	0.1 (0.1–0.2)	0.8 (0.4–1.4)	4.0 (2.6–6.1)
Scabies	0 (0–0)	0 (0–0)	0 (0–0)	0 (0–0)	0 (0–0)	0 (0–0)
Fungal skin diseases	0 (0–0)	0 (0–0)	0 (0–0)	0 (0–0)	0 (0–0)	0 (0–0)
Viral skin diseases	0 (0–0)	0 (0–0)	0 (0–0)	0 (0–0)	0 (0–0)	0 (0–0)
Acne vulgaris	0 (0–0)	0 (0–0)	0 (0–0)	0 (0–0)	0 (0–0)	0 (0–0)
Alopecia areata	0 (0–0)	0 (0–0)	0 (0–0)	0 (0–0)	0 (0–0)	0 (0–0)
Pruritus	0 (0–0)	0 (0–0)	0 (0–0)	0 (0–0)	0 (0–0)	0 (0–0)
Urticaria	0 (0–0)	0 (0–0)	0 (0–0)	0 (0–0)	0 (0–0)	0 (0–0)
Decubitus ulcer	0.4 (0.2–0.6)	0.0 (0.0–0.0)	0.0 (0.0–0.0)	0.0 (0.0–0.0)	0.4 (0.2–0.6)	7.2 (3.8–9.5)
Other skin and subcutaneous diseases	0.1 (0.0–0.1)	0.0 (0.0–0.1)	0.0 (0.0–0.0)	0.0 (0.0–0.0)	0.1 (0.0–0.1)	0.5 (0.3–0.7)
Sense organ diseases	0 (0–0)	0 (0–0)	0 (0–0)	0 (0–0)	0 (0–0)	0 (0–0)
Glaucoma	0 (0–0)	0 (0–0)	0 (0–0)	0 (0–0)	0 (0–0)	0 (0–0)
Cataract	0 (0–0)	0 (0–0)	0 (0–0)	0 (0–0)	0 (0–0)	0 (0–0)
Macular degeneration	0 (0–0)	0 (0–0)	0 (0–0)	0 (0–0)	0 (0–0)	0 (0–0)
Refraction and accommodation disorders	0 (0–0)	0 (0–0)	0 (0–0)	0 (0–0)	0 (0–0)	0 (0–0)
Age-related and other hearing loss	0 (0–0)	0 (0–0)	0 (0–0)	0 (0–0)	0 (0–0)	0 (0–0)
Other vision loss	0 (0–0)	0 (0–0)	0 (0–0)	0 (0–0)	0 (0–0)	0 (0–0)
Other sense organ diseases	0 (0–0)	0 (0–0)	0 (0–0)	0 (0–0)	0 (0–0)	0 (0–0)
Oral disorders	0 (0–0)	0 (0–0)	0 (0–0)	0 (0–0)	0 (0–0)	0 (0–0)
Deciduous caries	0 (0–0)	0 (0–0)	0 (0–0)	0 (0–0)	0 (0–0)	0 (0–0)
Permanent caries	0 (0–0)	0 (0–0)	0 (0–0)	0 (0–0)	0 (0–0)	0 (0–0)
Periodontal diseases	0 (0–0)	0 (0–0)	0 (0–0)	0 (0–0)	0 (0–0)	0 (0–0)
Edentulism and severe tooth loss	0 (0–0)	0 (0–0)	0 (0–0)	0 (0–0)	0 (0–0)	0 (0–0)
Other oral disorders	0 (0–0)	0 (0–0)	0 (0–0)	0 (0–0)	0 (0–0)	0 (0–0)
Sudden infant death syndrome	0.3 (0.2–0.5)	3.4 (2.4–4.8)	0 (0–0)	0 (0–0)	0 (0–0)	0 (0–0)
Injuries	48.6 (42.9–51.4)	67.1 (52.5–81.0)	20.4 (17.8–22.1)	38.3 (33.6–40.3)	62.6 (55.8–65.2)	187.7 (171.1–195.2)
Transport injuries	12.1 (11.6–12.7)	10.6 (8.4–13.1)	6.0 (5.5–6.4)	10.8 (10.3–11.4)	18.3 (17.7–19.0)	35.3 (34.3–36.4)
Road injuries	11.5 (11.0–12.0)	9.7 (7.8–11.9)	5.7 (5.2–6.1)	10.3 (9.8–10.8)	17.3 (16.6–18.0)	33.5 (32.5–34.7)
Pedestrian road injuries	5.6 (5.3–6.0)	4.7 (3.8–5.7)	3.1 (2.8–3.3)	4.4 (4.1–4.7)	9.2 (8.7–9.7)	19.9 (19.0–20.9)
Cyclist road injuries	0.5 (0.5–0.6)	0.2 (0.2–0.4)	0.3 (0.2–0.3)	0.5 (0.5–0.6)	0.9 (0.8–1.0)	1.1 (1.0–1.2)
Motorcyclist road injuries	1.3 (1.1–1.4)	1.0 (0.7–1.4)	0.5 (0.4–0.6)	1.3 (1.2–1.5)	1.8 (1.6–2.1)	2.6 (2.2–3.1)

SR Table 13 - Global death rates (per 100,000) by age and cause for females in 2000						
Cause	All ages	0-5 years	5-14 years	15-49 years	50-69 years	70+ years
Motor vehicle road injuries	3.9<br>(3.7–4.3)	3.6<br>(2.8–4.7)	1.8<br>(1.6–2.0)	3.9<br>(3.7–4.3)	5.2<br>(4.8–5.6)	9.6<br>(8.8–10.3)
Other road injuries	0.1<br>(0.1–0.1)	0.2<br>(0.1–0.2)	0.1<br>(0.1–0.1)	0.1<br>(0.1–0.1)	0.2<br>(0.1–0.2)	0.3<br>(0.3–0.4)
Other transport injuries	0.7<br>(0.5–0.8)	0.9<br>(0.5–1.3)	0.3<br>(0.2–0.3)	0.6<br>(0.5–0.7)	1.1<br>(0.9–1.2)	1.8<br>(1.5–2.0)
Unintentional injuries	23.3<br>(19.6–25.3)	51.9<br>(38.3–64.3)	11.8<br>(9.4–13.0)	12.2<br>(10.2–13.1)	25.1<br>(21.6–26.5)	121.1<br>(109.0–126.9)
Falls	5.0<br>(4.5–5.3)	4.1<br>(2.4–5.9)	1.1<br>(0.9–1.2)	1.2<br>(1.1–1.3)	5.7<br>(5.3–6.0)	58.9<br>(54.3–62.5)
Drowning	4.9<br>(2.7–5.5)	16.5<br>(6.2–21.2)	5.0<br>(3.1–5.6)	2.4<br>(1.5–2.7)	3.7<br>(2.3–4.0)	9.9<br>(6.9–10.8)
Fire, heat, and hot substances	3.9<br>(2.6–4.3)	6.4<br>(3.4–8.2)	1.3<br>(0.9–1.6)	3.4<br>(2.2–3.9)	3.9<br>(2.9–4.2)	13.2<br>(10.7–14.3)
Poisonings	1.4<br>(1.0–1.7)	3.3<br>(1.4–5.8)	0.6<br>(0.6–0.7)	1.1<br>(0.8–1.1)	1.6<br>(1.3–1.8)	3.6<br>(3.1–4.0)
Exposure to mechanical forces	1.8<br>(1.6–2.0)	5.8<br>(4.5–6.9)	0.8<br>(0.7–1.0)	0.9<br>(0.8–1.0)	2.0<br>(1.9–2.3)	7.3<br>(5.6–8.3)
Unintentional firearm injuries	0.3<br>(0.2–0.3)	0.3<br>(0.1–0.4)	0.1<br>(0.1–0.1)	0.2<br>(0.1–0.2)	0.3<br>(0.2–0.3)	2.0<br>(1.6–2.2)
Unintentional suffocation	0.5<br>(0.3–0.5)	3.2<br>(1.9–3.9)	0.2<br>(0.1–0.3)	0.1<br>(0.1–0.1)	0.2<br>(0.1–0.2)	1.0<br>(0.8–1.2)
Other exposure to mechanical forces	1.1<br>(1.0–1.2)	2.4<br>(1.8–3.1)	0.5<br>(0.4–0.6)	0.6<br>(0.6–0.7)	1.6<br>(1.4–1.9)	4.3<br>(3.0–5.1)
Adverse effects of medical treatment	1.5<br>(1.1–1.8)	3.0<br>(1.4–4.3)	0.4<br>(0.2–0.5)	0.7<br>(0.4–0.9)	2.0<br>(1.5–2.4)	9.1<br>(7.3–10.3)
Animal contact	1.5<br>(0.9–1.7)	2.7<br>(1.5–3.5)	1.2<br>(0.7–1.4)	1.1<br>(0.7–1.2)	2.3<br>(1.3–2.5)	2.8<br>(1.8–3.2)
Venomous animal contact	1.3<br>(0.7–1.5)	2.0<br>(1.1–2.6)	1.0<br>(0.6–1.2)	1.0<br>(0.6–1.1)	2.0<br>(1.1–2.3)	2.2<br>(1.3–2.6)
Non-venomous animal contact	0.2<br>(0.2–0.3)	0.7<br>(0.4–1.1)	0.2<br>(0.1–0.2)	0.1<br>(0.1–0.1)	0.3<br>(0.2–0.3)	0.6<br>(0.5–0.7)
Foreign body	1.8<br>(1.4–2.6)	7.3<br>(4.2–13.5)	0.6<br>(0.4–0.9)	0.6<br>(0.4–0.7)	1.6<br>(1.3–2.0)	9.7<br>(8.7–10.9)
Pulmonary aspiration and foreign body in airway	1.4<br>(1.0–2.3)	6.3<br>(3.0–12.7)	0.4<br>(0.2–0.8)	0.3<br>(0.2–0.5)	1.1<br>(0.9–1.4)	7.9<br>(7.3–8.6)
Foreign body in eyes	0<br>(0–0)	0<br>(0–0)	0<br>(0–0)	0<br>(0–0)	0<br>(0–0)	0<br>(0–0)
Foreign body in other body part	0.4<br>(0.2–0.7)	0.9<br>(0.4–1.5)	0.2<br>(0.1–0.3)	0.2<br>(0.1–0.4)	0.5<br>(0.3–0.9)	1.8<br>(1.3–3.0)
Environmental heat and cold exposure	0.4<br>(0.3–0.6)	0.6<br>(0.2–0.8)	0.1<br>(0.1–0.1)	0.2<br>(0.1–0.2)	0.8<br>(0.5–1.1)	3.4<br>(2.1–4.4)
Other unintentional injuries	1.1<br>(1.0–1.2)	2.1<br>(1.6–2.6)	0.7<br>(0.6–0.8)	0.8<br>(0.7–0.8)	1.5<br>(1.4–1.7)	3.0<br>(2.7–3.3)
Self-harm and interpersonal violence	12.2<br>(9.4–12.9)	2.7<br>(2.4–2.9)	2.0<br>(1.6–2.1)	14.5<br>(11.0–15.5)	18.4<br>(14.3–19.5)	30.1<br>(24.0–31.8)
Self-harm	9.1<br>(6.5–9.8)	0<br>(0–0)	0.9<br>(0.6–1.0)	10.9<br>(7.4–11.8)	15.0<br>(10.9–15.9)	24.6<br>(18.6–26.1)
Interpersonal violence	3.1<br>(3.0–3.2)	2.7<br>(2.4–2.9)	1.1<br>(1.0–1.1)	3.6<br>(3.5–3.8)	3.4<br>(3.3–3.6)	5.6<br>(5.3–5.8)
Assault by firearm	0.7<br>(0.6–0.7)	0.3<br>(0.2–0.4)	0.2<br>(0.2–0.2)	0.9<br>(0.8–1.0)	0.7<br>(0.6–0.7)	0.9<br>(0.8–1.1)
Assault by sharp object	0.8<br>(0.7–0.8)	0.3<br>(0.2–0.3)	0.2<br>(0.2–0.2)	1.0<br>(0.9–1.1)	0.8<br>(0.8–0.9)	1.4<br>(1.3–1.6)
Assault by other means	1.7<br>(1.6–1.8)	2.1<br>(1.9–2.3)	0.7<br>(0.6–0.7)	1.7<br>(1.6–1.9)	2.0<br>(1.8–2.1)	3.2<br>(2.9–3.4)
Forces of nature, war, and legal intervention	0.9<br>(0.6–1.3)	1.9<br>(0.8–3.8)	0.7<br>(0.3–1.3)	0.8<br>(0.4–1.2)	0.8<br>(0.4–1.3)	1.1<br>(0.6–2.1)
Exposure to forces of nature	0.1<br>(0.1–0.2)	0.1<br>(0.1–0.2)	0.1<br>(0.1–0.2)	0.1<br>(0.1–0.2)	0.2<br>(0.1–0.2)	0.3<br>(0.2–0.4)
Collective violence and legal intervention	0.8<br>(0.5–1.2)	1.8<br>(0.7–3.7)	0.6<br>(0.2–1.2)	0.7<br>(0.3–1.1)	0.7<br>(0.3–1.1)	0.9<br>(0.3–1.7)

SR Table 13 - Global death rates (per 100,000) by age and cause for females in 2005						
Cause	All ages	0-5 years	5-14 years	15-49 years	50-69 years	70+ years
All causes	765.1 (756.5–774.1)	1246.0 (1231.5–1261.6)	82.0 (80.4–83.6)	216.8 (211.5–222.0)	1119.9 (1099.9–1142.2)	6476.9 (6417.5–6536.1)
Communicable, maternal, neonatal, and nutritional diseases	209.2 (203.4–214.3)	1054.8 (1020.9–1080.3)	47.6 (45.5–50.0)	92.8 (89.1–96.9)	148.0 (138.7–157.1)	586.2 (546.2–613.7)
HIV/AIDS and tuberculosis	42.6 (39.5–46.0)	35.5 (34.0–38.1)	8.0 (7.6–8.8)	48.0 (45.0–51.1)	56.1 (48.4–64.1)	80.2 (63.2–95.8)
Tuberculosis	15.4 (12.6–18.6)	5.3 (4.1–7.6)	1.8 (1.5–2.5)	9.8 (8.3–12.0)	36.3 (28.3–43.7)	74.2 (57.4–90.1)
HIV/AIDS	27.2 (25.8–28.7)	30.3 (29.3–31.3)	6.3 (6.0–6.6)	38.1 (35.8–40.8)	19.8 (18.3–21.6)	6.0 (5.4–6.9)
HIV/AIDS - Tuberculosis	5.2 (4.1–5.9)	8.0 (6.5–9.0)	1.5 (1.1–1.7)	6.3 (4.9–7.2)	5.3 (4.4–5.9)	1.8 (1.6–2.1)
HIV/AIDS resulting in other diseases	22.0 (20.5–23.6)	22.2 (20.8–24.0)	4.8 (4.5–5.3)	31.9 (29.3–34.6)	14.5 (13.0–16.3)	4.2 (3.7–5.0)
Diarrhea, lower respiratory, and other common infectious diseases	87.8 (83.7–92.0)	421.5 (393.3–453.0)	28.0 (25.4–31.1)	16.6 (15.3–18.1)	70.8 (65.3–75.8)	446.1 (410.6–469.2)
Diarrheal diseases	26.4 (24.6–28.3)	124.6 (108.7–141.5)	6.5 (5.8–7.3)	5.7 (5.3–6.2)	27.7 (25.8–29.6)	119.8 (112.4–127.9)
Intestinal infectious diseases	3.0 (1.7–5.0)	8.2 (4.4–14.2)	6.6 (3.8–10.6)	1.7 (0.9–2.9)	0.9 (0.5–1.6)	1.2 (0.7–1.8)
Typhoid fever	2.5 (1.4–4.2)	6.8 (3.5–12.1)	5.5 (3.1–8.9)	1.4 (0.7–2.4)	0.7 (0.4–1.3)	0.9 (0.5–1.4)
Paratyphoid fever	0.5 (0.2–0.9)	1.2 (0.5–2.5)	1.0 (0.5–2.0)	0.3 (0.1–0.6)	0.2 (0.1–0.4)	0.2 (0.1–0.4)
Other intestinal infectious diseases	0.0 (0.0–0.1)	0.2 (0.0–0.4)	0.0 (0.0–0.1)	0.0 (0.0–0.0)	0.0 (0.0–0.0)	0.1 (0.0–0.2)
Lower respiratory infections	42.4 (38.5–44.8)	179.5 (164.0–196.0)	6.3 (4.5–7.2)	5.6 (4.5–6.2)	33.6 (27.0–36.7)	305.2 (266.5–323.8)
Upper respiratory infections	0.1 (0.0–0.1)	0.2 (0.1–0.3)	0.0 (0.0–0.0)	0.0 (0.0–0.0)	0.1 (0.1–0.1)	0.3 (0.2–0.4)
Otitis media	0.1 (0.1–0.1)	0.2 (0.2–0.2)	0.0 (0.0–0.0)	0.0 (0.0–0.0)	0.0 (0.0–0.1)	0.2 (0.1–0.4)
Meningitis	5.9 (4.9–6.8)	32.6 (25.4–42.2)	3.1 (2.5–3.6)	2.0 (1.7–2.3)	4.6 (3.8–5.5)	10.2 (8.8–12.1)
Pneumococcal meningitis	1.6 (1.3–2.0)	7.6 (5.2–11.0)	1.1 (0.8–1.5)	0.7 (0.5–0.9)	1.3 (1.0–1.7)	3.0 (2.3–3.8)
H influenzae type B meningitis	1.6 (1.3–2.0)	11.8 (8.5–16.0)	0.8 (0.5–1.1)	0.3 (0.2–0.4)	0.7 (0.5–0.9)	1.6 (1.3–2.1)
Meningococcal meningitis	1.1 (0.8–1.4)	6.3 (4.1–9.4)	0.6 (0.4–0.8)	0.3 (0.2–0.4)	0.8 (0.5–1.0)	1.5 (1.1–2.0)
Other meningitis	1.6 (1.3–1.9)	6.9 (4.9–9.5)	0.7 (0.5–0.9)	0.7 (0.5–0.8)	1.8 (1.5–2.2)	4.1 (3.4–5.0)
Encephalitis	2.1 (1.8–2.4)	7.8 (5.9–9.2)	1.4 (1.2–1.7)	0.8 (0.7–1.0)	2.1 (1.9–2.9)	6.1 (5.3–8.1)
Diphtheria	0.1 (0.0–0.2)	0.7 (0.2–1.8)	0.1 (0.0–0.1)	0.0 (0.0–0.0)	0.0 (0.0–0.0)	0 (0–0)
Whooping cough	1.7 (0.6–3.8)	16.9 (6.2–38.3)	0.5 (0.2–1.2)	0.0 (0.0–0.1)	0.0 (0.0–0.1)	0 (0–0)
Tetanus	1.5 (1.2–2.2)	8.9 (7.4–10.8)	0.4 (0.3–0.7)	0.5 (0.3–0.9)	1.5 (1.0–3.2)	2.1 (1.5–4.2)
Measles	4.5 (1.7–9.4)	41.5 (15.6–87.2)	3.0 (1.1–6.4)	0.2 (0.1–0.5)	0.0 (0.0–0.0)	0 (0–0)
Varicella and herpes zoster	0.2 (0.1–0.2)	0.4 (0.3–0.6)	0.1 (0.0–0.1)	0.0 (0.0–0.1)	0.2 (0.1–0.2)	1.0 (0.8–1.4)
Neglected tropical diseases and malaria	20.0 (16.6–23.4)	144.6 (113.3–175.1)	8.0 (5.8–10.3)	5.1 (3.9–6.7)	11.4 (8.3–15.6)	14.3 (10.6–19.6)
Malaria	18.3 (15.0–21.7)	140.4 (109.3–171.2)	6.5 (4.2–8.9)	4.1 (2.9–5.7)	9.2 (6.2–13.3)	10.8 (7.1–15.9)
Chagas disease	0.1 (0.1–0.1)	0.0 (0.0–0.0)	0.0 (0.0–0.0)	0.0 (0.0–0.0)	0.3 (0.2–0.3)	0.8 (0.8–0.9)
Leishmaniasis	0.2 (0.1–0.3)	0.4 (0.3–0.6)	0.1 (0.1–0.2)	0.2 (0.1–0.3)	0.1 (0.1–0.2)	0.1 (0.1–0.1)
Visceral leishmaniasis	0.2 (0.1–0.3)	0.4 (0.3–0.6)	0.1 (0.1–0.2)	0.2 (0.1–0.3)	0.1 (0.1–0.2)	0.1 (0.1–0.1)
Cutaneous and mucocutaneous leishmaniasis	0 (0–0)	0 (0–0)	0 (0–0)	0 (0–0)	0 (0–0)	0 (0–0)
African trypanosomiasis	0.2 (0.1–0.3)	0.2 (0.1–0.3)	0.2 (0.1–0.3)	0.2 (0.1–0.4)	0.3 (0.1–0.4)	0.2 (0.1–0.3)
Schistosomiasis	0.1 (0.1–0.1)	0.0 (0.0–0.0)	0.0 (0.0–0.0)	0.1 (0.0–0.1)	0.3 (0.3–0.4)	0.6 (0.4–0.8)
Cysticercosis	0.0 (0.0–0.0)	0.0 (0.0–0.0)	0.0 (0.0–0.0)	0.0 (0.0–0.0)	0.0 (0.0–0.0)	0.0 (0.0–0.0)
Cystic echinococcosis	0.0 (0.0–0.0)	0.0 (0.0–0.0)	0.0 (0.0–0.0)	0.0 (0.0–0.0)	0.1 (0.0–0.1)	0.1 (0.1–0.1)
Lymphatic filariasis	0 (0–0)	0 (0–0)	0 (0–0)	0 (0–0)	0 (0–0)	0 (0–0)
Onchocerciasis	0 (0–0)	0 (0–0)	0 (0–0)	0 (0–0)	0 (0–0)	0 (0–0)
Trachoma	0 (0–0)	0 (0–0)	0 (0–0)	0 (0–0)	0 (0–0)	0 (0–0)
Dengue	0.2 (0.1–0.2)	0.7 (0.4–1.0)	0.3 (0.2–0.4)	0.1 (0.1–0.1)	0.1 (0.1–0.2)	0.2 (0.1–0.3)
Yellow fever	0.1 (0.0–0.1)	0.1 (0.0–0.2)	0.1 (0.0–0.2)	0.0 (0.0–0.1)	0.0 (0.0–0.1)	0.0 (0.0–0.1)
Rabies	0.4 (0.3–0.5)	0.6 (0.5–0.9)	0.5 (0.4–0.6)	0.2 (0.2–0.3)	0.6 (0.5–0.8)	0.7 (0.5–0.9)
Intestinal nematode infections	0.1 (0.1–0.1)	0.5 (0.4–0.5)	0.0 (0.0–0.1)	0.0 (0.0–0.0)	0.0 (0.0–0.0)	0.0 (0.0–0.0)
Ascariasis	0.1 (0.1–0.1)	0.5 (0.4–0.5)	0.0 (0.0–0.1)	0.0 (0.0–0.0)	0.0 (0.0–0.0)	0.0 (0.0–0.0)
Trichuriasis	0 (0–0)	0 (0–0)	0 (0–0)	0 (0–0)	0 (0–0)	0 (0–0)
Hookworm disease	0 (0–0)	0 (0–0)	0 (0–0)	0 (0–0)	0 (0–0)	0 (0–0)
Food-borne trematodiases	0 (0–0)	0 (0–0)	0 (0–0)	0 (0–0)	0 (0–0)	0 (0–0)
Leprosy	0 (0–0)	0 (0–0)	0 (0–0)	0 (0–0)	0 (0–0)	0 (0–0)
Ebola	0.0 (0.0–0.0)	0 (0–0)	0 (0–0)	0.0 (0.0–0.0)	0.0 (0.0–0.0)	0 (0–0)
Other neglected tropical diseases	0.3 (0.3–0.5)	1.7 (1.2–2.5)	0.2 (0.1–0.3)	0.1 (0.1–0.2)	0.4 (0.3–0.6)	0.7 (0.5–1.1)
Maternal disorders	10.8 (10.1–11.6)	0 (0–0)	0.3 (0.2–0.3)	20.5 (19.2–22.0)	0.3 (0.2–0.4)	0 (0–0)
Maternal hemorrhage	3.1 (2.7–3.5)	0 (0–0)	0.1 (0.1–0.1)	5.8 (5.1–6.6)	0.1 (0.1–0.1)	0 (0–0)
Maternal sepsis and other maternal infections	0.8 (0.6–0.9)	0 (0–0)	0.0 (0.0–0.0)	1.5 (1.2–1.8)	0.0 (0.0–0.0)	0 (0–0)
Maternal hypertensive disorders	2.0 (1.7–2.3)	0 (0–0)	0.1 (0.0–0.1)	3.7 (3.2–4.4)	0.0 (0.0–0.0)	0 (0–0)
Maternal obstructed labor and uterine rupture	0.8 (0.7–1.0)	0 (0–0)	0.0 (0.0–0.0)	1.6 (1.3–1.9)	0.0 (0.0–0.0)	0 (0–0)
Maternal abortion, miscarriage, and ectopic pregnancy	1.3 (1.1–1.5)	0 (0–0)	0.0 (0.0–0.1)	2.4 (2.0–2.9)	0.0 (0.0–0.1)	0 (0–0)

SR Table 13 - Global death rates (per 100,000) by age and cause for females in 2005

Cause	All ages	0-5 years	5-14 years	15-49 years	50-69 years	70+ years
Indirect maternal deaths	1.2 (1.0–1.4)	0 (0–0)	0.0 (0.0–0.0)	2.2 (1.8–2.7)	0.0 (0.0–0.0)	0 (0–0)
Late maternal deaths	0.2 (0.2–0.4)	0 (0–0)	0.0 (0.0–0.0)	0.5 (0.3–0.7)	0.0 (0.0–0.0)	0 (0–0)
Maternal deaths aggravated by HIV/AIDS	0.1 (0.1–0.1)	0 (0–0)	0.0 (0.0–0.0)	0.2 (0.1–0.2)	0.0 (0.0–0.0)	0 (0–0)
Other maternal disorders	1.4 (1.2–1.7)	0 (0–0)	0.0 (0.0–0.0)	2.7 (2.3–3.2)	0.0 (0.0–0.1)	0 (0–0)
Neonatal disorders	36.0 (35.0–37.1)	388.0 (376.8–399.7)	0 (0–0)	0 (0–0)	0 (0–0)	0 (0–0)
Neonatal preterm birth complications	15.3 (13.9–17.1)	165.2 (149.8–184.1)	0 (0–0)	0 (0–0)	0 (0–0)	0 (0–0)
Neonatal encephalopathy due to birth asphyxia and trauma	11.8 (10.3–13.4)	127.3 (111.2–144.1)	0 (0–0)	0 (0–0)	0 (0–0)	0 (0–0)
Neonatal sepsis and other neonatal infections	4.0 (1.9–6.0)	43.0 (20.8–64.9)	0 (0–0)	0 (0–0)	0 (0–0)	0 (0–0)
Hemolytic disease and other neonatal jaundice	1.0 (0.5–1.7)	10.9 (5.5–18.7)	0 (0–0)	0 (0–0)	0 (0–0)	0 (0–0)
Other neonatal disorders	3.9 (2.6–5.6)	41.7 (28.5–60.8)	0 (0–0)	0 (0–0)	0 (0–0)	0 (0–0)
Nutritional deficiencies	7.0 (6.3–7.8)	38.2 (31.8–45.8)	1.4 (1.2–1.6)	1.1 (1.1–1.3)	4.8 (4.5–5.3)	34.2 (32.1–36.6)
Protein-energy malnutrition	5.6 (5.0–6.4)	34.5 (28.5–41.9)	1.1 (0.9–1.3)	0.6 (0.5–0.7)	2.9 (2.5–3.4)	27.5 (24.5–30.6)
Iodine deficiency	0.0 (0.0–0.1)	0.1 (0.1–0.3)	0.0 (0.0–0.0)	0.0 (0.0–0.0)	0.1 (0.0–0.1)	0.2 (0.1–0.3)
Vitamin A deficiency	0 (0–0)	0 (0–0)	0 (0–0)	0 (0–0)	0 (0–0)	0 (0–0)
Iron-deficiency anemia	0.8 (0.5–1.1)	2.5 (1.2–3.7)	0.2 (0.2–0.3)	0.4 (0.3–0.5)	1.3 (1.0–1.6)	2.4 (1.6–3.5)
Other nutritional deficiencies	0.5 (0.3–0.9)	1.0 (0.4–1.9)	0.1 (0.1–0.2)	0.2 (0.1–0.3)	0.6 (0.3–1.1)	4.1 (2.6–6.6)
Other communicable, maternal, neonatal, and nutritional diseases	4.9 (4.1–6.1)	27.0 (18.6–37.7)	1.9 (1.4–2.4)	1.5 (1.4–1.6)	4.7 (4.3–5.0)	11.3 (9.8–12.1)
Sexually transmitted diseases excluding HIV	2.1 (1.3–3.2)	18.5 (10.4–29.1)	0.9 (0.5–1.5)	0.2 (0.2–0.2)	0.3 (0.2–0.4)	0.9 (0.7–1.1)
Syphilis	2.1 (1.2–3.1)	18.5 (10.4–29.1)	0.9 (0.5–1.5)	0.2 (0.1–0.2)	0.3 (0.2–0.3)	0.8 (0.6–0.9)
Chlamydial infection	0.0 (0.0–0.0)	0 (0–0)	0.0 (0.0–0.0)	0.0 (0.0–0.0)	0.0 (0.0–0.0)	0.0 (0.0–0.0)
Gonococcal infection	0.0 (0.0–0.0)	0 (0–0)	0.0 (0.0–0.0)	0.0 (0.0–0.0)	0.0 (0.0–0.0)	0.1 (0.1–0.1)
Trichomoniasis	0 (0–0)	0 (0–0)	0 (0–0)	0 (0–0)	0 (0–0)	0 (0–0)
Genital herpes	0 (0–0)	0 (0–0)	0 (0–0)	0 (0–0)	0 (0–0)	0 (0–0)
Other sexually transmitted diseases	0.0 (0.0–0.0)	0 (0–0)	0.0 (0.0–0.0)	0.0 (0.0–0.0)	0.0 (0.0–0.0)	0.0 (0.0–0.0)
Hepatitis	1.6 (1.5–1.7)	2.9 (2.5–3.4)	0.6 (0.5–0.7)	1.0 (0.9–1.1)	2.6 (2.5–2.8)	5.6 (5.3–5.9)
Acute hepatitis A	0.3 (0.2–0.3)	2.2 (1.6–2.8)	0.1 (0.0–0.2)	0.0 (0.0–0.1)	0.1 (0.0–0.1)	0.1 (0.0–0.2)
Hepatitis B	0.8 (0.7–1.0)	0.2 (0.1–0.4)	0.1 (0.0–0.1)	0.5 (0.4–0.6)	2.1 (1.8–2.4)	4.1 (3.4–4.8)
Hepatitis C	0.0 (0.0–0.1)	0 (0–0)	0.0 (0.0–0.0)	0.0 (0.0–0.0)	0.1 (0.0–0.3)	0.2 (0.0–0.5)
Acute hepatitis E	0.5 (0.3–0.6)	0.5 (0.2–1.0)	0.4 (0.3–0.6)	0.4 (0.3–0.5)	0.3 (0.2–0.6)	1.2 (0.6–1.9)
Other infectious diseases	1.3 (0.8–1.5)	5.6 (2.7–7.6)	0.3 (0.2–0.4)	0.3 (0.3–0.3)	1.8 (1.3–2.0)	4.9 (3.5–5.5)
Non-communicable diseases	510.7 (503.4–519.7)	137.4 (119.6–164.5)	16.9 (15.6–18.4)	89.6 (87.2–92.8)	912.7 (894.7–932.8)	5702.3 (5641.4–5769.8)
Neoplasms	100.1 (98.2–102.3)	5.3 (4.7–6.3)	3.8 (3.6–4.1)	29.6 (28.7–30.7)	264.8 (258.9–271.1)	805.2 (790.2–821.4)
Lip and oral cavity cancer	1.2 (1.1–1.2)	0 (0–0)	0 (0–0)	0.3 (0.3–0.4)	3.2 (3.1–3.4)	9.4 (9.0–9.7)
Nasopharynx cancer	0.5 (0.5–0.6)	0 (0–0)	0.0 (0.0–0.0)	0.3 (0.3–0.3)	1.6 (1.5–1.7)	2.2 (2.1–2.3)
Other pharynx cancer	0.5 (0.4–0.5)	0 (0–0)	0 (0–0)	0.1 (0.1–0.2)	1.6 (1.5–1.7)	3.0 (2.9–3.2)
Esophageal cancer	4.6 (4.3–4.8)	0 (0–0)	0 (0–0)	0.8 (0.7–0.8)	13.3 (12.6–14.0)	39.9 (38.0–41.8)
Stomach cancer	9.3 (8.9–9.6)	0 (0–0)	0 (0–0)	2.2 (2.0–2.3)	22.7 (21.6–23.7)	86.5 (84.0–89.1)
Colon and rectum cancer	9.9 (9.7–10.2)	0 (0–0)	0 (0–0)	1.7 (1.7–1.8)	22.1 (21.5–22.8)	104.7 (102.1–107.3)
Liver cancer	7.0 (5.9–7.4)	0 (0–0)	0.1 (0.1–0.1)	1.9 (1.5–2.1)	20.1 (16.7–21.5)	53.9 (47.4–56.8)
Liver cancer due to hepatitis B	2.1 (1.7–2.3)	0 (0–0)	0.0 (0.0–0.0)	1.0 (0.8–1.2)	6.4 (5.2–7.2)	10.4 (8.6–11.6)
Liver cancer due to hepatitis C	1.9 (1.7–2.1)	0 (0–0)	0.0 (0.0–0.0)	0.2 (0.2–0.3)	4.8 (4.0–5.3)	20.1 (18.2–21.4)
Liver cancer due to alcohol use	1.1 (0.9–1.2)	0 (0–0)	0 (0–0)	0.1 (0.1–0.2)	3.7 (3.0–4.2)	9.0 (7.8–9.9)
Liver cancer due to other causes	1.8 (1.5–2.0)	0 (0–0)	0.0 (0.0–0.0)	0.5 (0.4–0.6)	5.2 (4.2–5.8)	14.5 (12.2–15.8)
Gallbladder and biliary tract cancer	2.3 (2.2–2.4)	0 (0–0)	0 (0–0)	0.3 (0.3–0.3)	5.5 (5.2–5.8)	24.2 (23.4–25.0)
Pancreatic cancer	4.7 (4.6–4.8)	0 (0–0)	0 (0–0)	0.5 (0.5–0.6)	11.3 (11.0–11.6)	49.9 (48.7–51.0)
Larynx cancer	0.5 (0.5–0.6)	0 (0–0)	0 (0–0)	0.1 (0.1–0.2)	1.6 (1.5–1.7)	4.1 (3.8–4.3)
Tracheal, bronchus, and lung cancer	13.2 (12.9–13.6)	0 (0–0)	0 (0–0)	2.4 (2.2–2.5)	36.6 (35.5–37.6)	120.4 (117.3–124.1)
Malignant skin melanoma	0.7 (0.6–0.7)	0 (0–0)	0 (0–0)	0.3 (0.2–0.3)	1.6 (1.4–1.7)	5.7 (5.0–6.0)
Non-melanoma skin cancer	0.4 (0.3–0.4)	0 (0–0)	0 (0–0)	0.1 (0.1–0.1)	0.6 (0.6–0.6)	4.2 (4.0–4.4)
Non-melanoma skin cancer (squamous-cell carcinoma)	0.4 (0.3–0.4)	0 (0–0)	0 (0–0)	0.1 (0.1–0.1)	0.6 (0.6–0.6)	4.2 (4.0–4.4)
Non-melanoma skin cancer (basal-cell carcinoma)	0 (0–0)	0 (0–0)	0 (0–0)	0 (0–0)	0 (0–0)	0 (0–0)
Breast cancer	13.4 (12.8–14.1)	0 (0–0)	0 (0–0)	5.7 (5.2–6.2)	40.1 (38.2–42.1)	81.4 (78.2–84.2)
Cervical cancer	7.0 (6.6–7.3)	0 (0–0)	0 (0–0)	3.7 (3.4–3.9)	22.0 (20.7–23.3)	32.8 (31.7–34.0)
Uterine cancer	2.5 (2.4–2.6)	0 (0–0)	0 (0–0)	0.6 (0.6–0.6)	8.0 (7.6–8.4)	18.9 (18.2–19.7)
Ovarian cancer	4.1 (4.0–4.3)	0 (0–0)	0 (0–0)	1.4 (1.3–1.5)	12.8 (12.5–13.3)	27.6 (26.9–28.4)
Prostate cancer	0 (0–0)	0 (0–0)	0 (0–0)	0 (0–0)	0 (0–0)	0 (0–0)
Testicular cancer	0 (0–0)	0 (0–0)	0 (0–0)	0 (0–0)	0 (0–0)	0 (0–0)

SR Table 13 - Global death rates (per 100,000) by age and cause for females in 2005

Cause	All ages	0-5 years	5-14 years	15-49 years	50-69 years	70+ years
Kidney cancer	1.2 (1.2–1.2)	0.3 (0.2–0.4)	0.1 (0.1–0.1)	0.2 (0.2–0.2)	2.9 (2.8–3.0)	11.0 (10.7–11.3)
Bladder cancer	1.3 (1.3–1.3)	0 (0–0)	0 (0–0)	0.1 (0.1–0.1)	2.3 (2.3–2.4)	16.1 (15.7–16.6)
Brain and nervous system cancer	2.6 (2.5–2.7)	0.8 (0.7–0.9)	0.7 (0.7–0.8)	1.3 (1.2–1.4)	6.9 (6.6–7.1)	13.0 (12.3–13.4)
Thyroid cancer	0.5 (0.5–0.6)	0 (0–0)	0.0 (0.0–0.0)	0.1 (0.1–0.1)	1.2 (1.1–1.3)	4.9 (4.7–5.5)
Mesothelioma	0.2 (0.2–0.2)	0 (0–0)	0 (0–0)	0.1 (0.1–0.1)	0.5 (0.5–0.5)	1.8 (1.8–1.9)
Hodgkin lymphoma	0.3 (0.2–0.4)	0.0 (0.0–0.0)	0.0 (0.0–0.1)	0.2 (0.2–0.3)	0.6 (0.5–0.9)	1.6 (1.3–2.0)
Non-Hodgkin lymphoma	2.4 (2.1–2.7)	0.4 (0.3–0.7)	0.3 (0.2–0.3)	0.7 (0.7–0.9)	5.3 (4.7–5.8)	21.4 (17.7–22.8)
Multiple myeloma	1.2 (1.2–1.2)	0 (0–0)	0 (0–0)	0.2 (0.2–0.2)	3.1 (3.0–3.3)	11.7 (10.8–12.2)
Leukemia	4.1 (4.0–4.2)	2.0 (1.8–2.4)	1.7 (1.6–1.9)	2.1 (2.0–2.2)	7.4 (7.2–7.7)	25.8 (25.0–26.6)
Acute lymphoid leukemia	1.3 (1.2–1.4)	1.4 (1.1–1.7)	1.2 (1.1–1.4)	0.8 (0.7–0.9)	2.0 (1.9–2.2)	4.2 (4.0–4.5)
Chronic lymphoid leukemia	0.7 (0.7–0.8)	0 (0–0)	0 (0–0)	0.2 (0.2–0.3)	1.4 (1.2–1.5)	7.7 (7.4–8.1)
Acute myeloid leukemia	1.6 (1.5–1.6)	0.7 (0.5–0.8)	0.5 (0.5–0.6)	0.8 (0.7–0.9)	3.0 (2.9–3.1)	10.2 (9.9–10.6)
Chronic myeloid leukemia	0.5 (0.5–0.5)	0 (0–0)	0 (0–0)	0.2 (0.2–0.3)	1.1 (1.0–1.2)	3.6 (3.4–3.8)
Other neoplasms	4.5 (4.2–4.7)	1.8 (1.5–2.5)	0.9 (0.8–0.9)	2.1 (1.8–2.3)	9.9 (9.3–10.4)	29.2 (27.7–30.2)
Cardiovascular diseases	243.4 (239.2–247.7)	9.0 (8.3–9.7)	2.5 (2.3–2.7)	28.8 (27.8–29.9)	384.7 (375.9–393.9)	3100.1 (3046.0–3157.9)
Rheumatic heart disease	5.9 (5.4–6.4)	0.6 (0.4–0.7)	0.7 (0.6–0.8)	2.7 (2.4–3.0)	13.0 (11.8–14.1)	43.8 (41.2–46.6)
Ischemic heart disease	110.4 (108.5–112.7)	0.5 (0.4–0.5)	0.2 (0.2–0.2)	10.1 (9.7–10.6)	165.9 (161.8–170.1)	1468.0 (1440.6–1498.4)
Cerebrovascular disease	96.0 (94.1–98.2)	3.0 (2.7–3.4)	0.9 (0.8–1.0)	10.8 (10.3–11.4)	160.0 (155.4–165.0)	1207.3 (1182.1–1235.1)
Ischemic stroke	46.7 (44.5–48.9)	0.1 (0.1–0.2)	0.1 (0.0–0.1)	1.7 (1.5–1.9)	53.5 (49.1–58.2)	690.6 (661.8–716.4)
Hemorrhagic stroke	49.3 (47.3–51.9)	2.9 (2.6–3.3)	0.8 (0.7–0.9)	9.1 (8.6–9.7)	106.5 (101.6–112.6)	516.7 (494.2–544.5)
Hypertensive heart disease	13.2 (11.7–14.6)	0.1 (0.1–0.1)	0.0 (0.0–0.0)	1.7 (1.4–2.0)	23.4 (19.8–26.5)	160.6 (145.0–179.1)
Cardiomyopathy and myocarditis	4.4 (4.2–4.5)	1.9 (1.7–2.1)	0.3 (0.3–0.3)	1.5 (1.4–1.5)	6.3 (6.0–6.5)	44.7 (43.1–46.2)
Atrial fibrillation and flutter	2.5 (2.1–3.1)	0 (0–0)	0 (0–0)	0.0 (0.0–0.0)	0.9 (0.7–1.2)	43.8 (35.5–53.2)
Aortic aneurysm	1.5 (1.4–1.5)	0 (0–0)	0 (0–0)	0.2 (0.2–0.2)	2.1 (2.0–2.2)	19.6 (19.0–20.3)
Peripheral vascular disease	0.6 (0.6–0.6)	0 (0–0)	0 (0–0)	0.0 (0.0–0.0)	0.4 (0.4–0.5)	9.7 (9.1–10.4)
Endocarditis	1.2 (1.0–1.4)	0.6 (0.3–0.9)	0.1 (0.1–0.1)	0.4 (0.3–0.5)	1.5 (1.2–1.9)	12.4 (11.2–13.9)
Other cardiovascular and circulatory diseases	7.6 (7.4–7.9)	2.3 (2.0–2.6)	0.3 (0.3–0.4)	1.4 (1.3–1.5)	11.3 (10.8–11.9)	90.2 (86.9–94.0)
Chronic respiratory diseases	50.9 (49.2–52.7)	7.9 (7.0–8.8)	1.0 (0.9–1.1)	5.1 (4.9–5.4)	87.2 (83.4–91.3)	627.4 (604.9–648.7)
Chronic obstructive pulmonary disease	42.1 (39.4–44.4)	3.2 (2.2–4.0)	0.2 (0.1–0.3)	2.7 (2.4–3.2)	68.6 (62.7–74.6)	550.7 (518.8–576.8)
Pneumoconiosis	0.3 (0.2–0.4)	0 (0–0)	0 (0–0)	0.0 (0.0–0.1)	0.5 (0.3–0.7)	4.0 (2.9–5.3)
Silicosis	0.0 (0.0–0.0)	0 (0–0)	0 (0–0)	0.0 (0.0–0.0)	0.0 (0.0–0.0)	0.2 (0.1–0.2)
Asbestosis	0.0 (0.0–0.0)	0 (0–0)	0 (0–0)	0.0 (0.0–0.0)	0.0 (0.0–0.0)	0.1 (0.1–0.2)
Coal workers pneumoconiosis	0.0 (0.0–0.0)	0 (0–0)	0 (0–0)	0.0 (0.0–0.0)	0.0 (0.0–0.0)	0.1 (0.0–0.1)
Other pneumoconiosis	0.3 (0.2–0.4)	0 (0–0)	0 (0–0)	0.0 (0.0–0.0)	0.4 (0.2–0.6)	3.6 (2.6–4.9)
Asthma	6.9 (5.1–8.9)	2.4 (1.7–3.0)	0.7 (0.5–0.8)	2.1 (1.6–2.5)	15.8 (10.9–21.0)	57.1 (41.7–80.6)
Interstitial lung disease and pulmonary sarcoidosis	1.1 (0.8–1.2)	0.1 (0.0–0.1)	0.0 (0.0–0.0)	0.2 (0.1–0.2)	1.9 (1.5–2.2)	13.3 (9.3–14.9)
Other chronic respiratory diseases	0.5 (0.3–0.6)	2.3 (1.6–3.2)	0.1 (0.1–0.2)	0.1 (0.1–0.1)	0.4 (0.3–0.5)	2.4 (1.7–3.1)
Cirrhosis and other chronic liver diseases	12.7 (12.0–14.3)	2.1 (1.6–3.0)	0.8 (0.7–1.1)	5.4 (5.0–6.2)	35.6 (33.4–40.1)	77.1 (72.6–86.0)
Cirrhosis and other chronic liver diseases due to hepatitis B	3.3 (2.9–3.8)	0.0 (0.0–0.0)	0.0 (0.0–0.0)	1.3 (1.2–1.5)	9.3 (8.1–11.0)	21.6 (19.3–25.2)
Cirrhosis and other chronic liver diseases due to hepatitis C	2.8 (2.6–3.2)	0.0 (0.0–0.0)	0.0 (0.0–0.0)	1.1 (1.0–1.3)	8.2 (7.2–9.3)	19.3 (17.6–21.6)
Cirrhosis and other chronic liver diseases due to alcohol use	2.9 (2.7–3.2)	0 (0–0)	0 (0–0)	1.5 (1.3–1.7)	9.4 (8.4–10.7)	13.2 (11.8–14.9)
Cirrhosis and other chronic liver diseases due to other causes	3.7 (3.4–4.3)	2.0 (1.6–3.0)	0.8 (0.7–1.0)	1.5 (1.4–1.8)	8.7 (7.8–10.1)	23.0 (21.0–26.0)
Digestive diseases	16.0 (15.4–16.7)	5.4 (3.0–10.2)	1.5 (1.4–1.7)	4.7 (4.5–5.0)	28.2 (27.1–29.4)	155.5 (151.0–160.3)
Peptic ulcer disease	3.8 (3.4–4.1)	0.7 (0.3–1.6)	0.3 (0.2–0.3)	1.2 (1.0–1.3)	7.4 (6.5–8.2)	35.5 (32.5–39.0)
Gastritis and duodenitis	0.7 (0.4–0.9)	0.1 (0.0–0.3)	0.0 (0.0–0.1)	0.2 (0.1–0.3)	1.3 (0.7–1.8)	6.6 (3.9–8.9)
Appendicitis	0.8 (0.6–0.9)	1.1 (0.4–2.7)	0.3 (0.2–0.3)	0.4 (0.3–0.5)	1.2 (0.9–1.3)	3.6 (3.0–4.1)
Paralytic ileus and intestinal obstruction	3.4 (2.8–3.8)	1.8 (0.9–3.6)	0.5 (0.4–0.6)	1.1 (0.8–1.3)	5.7 (4.7–6.8)	30.6 (26.2–34.8)
Inguinal, femoral, and abdominal hernia	0.6 (0.4–0.8)	0.1 (0.0–0.3)	0.1 (0.0–0.1)	0.2 (0.1–0.2)	1.3 (0.8–1.5)	6.3 (4.7–7.6)
Inflammatory bowel disease	0.7 (0.6–0.8)	0.4 (0.2–1.1)	0.1 (0.0–0.1)	0.2 (0.2–0.3)	1.0 (0.9–1.2)	7.3 (6.3–7.8)
Vascular intestinal disorders	1.5 (1.3–1.6)	0.0 (0.0–0.1)	0.0 (0.0–0.0)	0.1 (0.1–0.2)	2.0 (1.7–2.3)	20.2 (18.6–21.9)
Gallbladder and biliary diseases	1.8 (1.6–1.9)	0.4 (0.2–0.9)	0.1 (0.1–0.1)	0.4 (0.4–0.5)	3.2 (2.9–3.5)	18.6 (17.6–19.8)
Pancreatitis	1.3 (1.2–1.4)	0.0 (0.0–0.1)	0.1 (0.0–0.1)	0.6 (0.6–0.7)	3.0 (2.7–3.3)	9.7 (9.0–10.4)
Other digestive diseases	1.5 (1.3–1.6)	0.6 (0.3–1.3)	0.1 (0.1–0.1)	0.3 (0.2–0.3)	2.2 (1.9–2.5)	17.1 (15.6–18.6)
Neurological disorders	30.8 (26.4–35.1)	2.6 (2.2–3.5)	1.0 (0.9–1.2)	1.9 (1.7–2.1)	14.4 (12.3–16.7)	497.3 (417.8–575.4)
Alzheimer disease and other dementias	27.0 (22.5–31.3)	0 (0–0)	0 (0–0)	0.0 (0.0–0.1)	8.9 (6.7–11.1)	467.2 (387.1–545.4)
Parkinson disease	1.2 (1.1–1.2)	0 (0–0)	0 (0–0)	0.0 (0.0–0.0)	0.9 (0.8–0.9)	18.5 (17.7–19.2)
Epilepsy	1.4 (1.3–1.6)	1.9 (1.6–2.6)	0.8 (0.7–0.9)	1.3 (1.2–1.5)	1.4 (1.4–1.6)	3.7 (3.5–3.9)

SR Table 13 - Global death rates (per 100,000) by age and cause for females in 2005						
Cause	All ages	0-5 years	5-14 years	15-49 years	50-69 years	70+ years
Multiple sclerosis	0.3 (0.3–0.3)	0 (0–0)	0 (0–0)	0.2 (0.2–0.2)	0.9 (0.8–1.0)	1.2 (0.9–1.3)
Motor neuron disease	0.4 (0.4–0.4)	0.2 (0.1–0.2)	0.0 (0.0–0.0)	0.1 (0.1–0.1)	1.1 (1.0–1.2)	3.1 (3.0–3.2)
Migraine	0 (0–0)	0 (0–0)	0 (0–0)	0 (0–0)	0 (0–0)	0 (0–0)
Tension-type headache	0 (0–0)	0 (0–0)	0 (0–0)	0 (0–0)	0 (0–0)	0 (0–0)
Medication overuse headache	0 (0–0)	0 (0–0)	0 (0–0)	0 (0–0)	0 (0–0)	0 (0–0)
Other neurological disorders	0.6 (0.6–0.7)	0.5 (0.5–0.9)	0.2 (0.2–0.3)	0.3 (0.3–0.3)	1.2 (1.2–1.3)	3.7 (3.5–3.8)
Mental and substance use disorders	2.2 (2.1–2.3)	0.0 (0.0–0.0)	0.0 (0.0–0.0)	2.1 (2.0–2.2)	4.7 (4.6–4.9)	6.9 (6.2–7.3)
Schizophrenia	0.3 (0.3–0.3)	0 (0–0)	0 (0–0)	0.2 (0.2–0.2)	0.7 (0.6–0.7)	1.8 (1.7–1.9)
Alcohol use disorders	0.8 (0.8–0.9)	0 (0–0)	0 (0–0)	0.7 (0.6–0.7)	2.5 (2.3–2.6)	1.9 (1.9–2.0)
Drug use disorders	1.1 (1.0–1.1)	0.0 (0.0–0.0)	0 (0–0)	1.2 (1.1–1.3)	1.6 (1.5–1.7)	3.2 (2.5–3.5)
Opioid use disorders	0.7 (0.6–0.7)	0.0 (0.0–0.0)	0 (0–0)	0.8 (0.7–0.9)	1.1 (0.9–1.1)	2.0 (1.5–2.3)
Cocaine use disorders	0.1 (0.1–0.1)	0 (0–0)	0 (0–0)	0.1 (0.1–0.1)	0.1 (0.1–0.1)	0.1 (0.1–0.1)
Amphetamine use disorders	0.0 (0.0–0.0)	0 (0–0)	0 (0–0)	0.0 (0.0–0.1)	0.1 (0.0–0.1)	0.1 (0.0–0.1)
Cannabis use disorders	0 (0–0)	0 (0–0)	0 (0–0)	0 (0–0)	0 (0–0)	0 (0–0)
Other drug use disorders	0.3 (0.2–0.3)	0 (0–0)	0 (0–0)	0.3 (0.2–0.4)	0.4 (0.4–0.5)	1.1 (0.8–1.3)
Depressive disorders	0 (0–0)	0 (0–0)	0 (0–0)	0 (0–0)	0 (0–0)	0 (0–0)
Major depressive disorder	0 (0–0)	0 (0–0)	0 (0–0)	0 (0–0)	0 (0–0)	0 (0–0)
Dysthymia	0 (0–0)	0 (0–0)	0 (0–0)	0 (0–0)	0 (0–0)	0 (0–0)
Bipolar disorder	0 (0–0)	0 (0–0)	0 (0–0)	0 (0–0)	0 (0–0)	0 (0–0)
Anxiety disorders	0 (0–0)	0 (0–0)	0 (0–0)	0 (0–0)	0 (0–0)	0 (0–0)
Eating disorders	0.0 (0.0–0.0)	0 (0–0)	0.0 (0.0–0.0)	0.0 (0.0–0.0)	0 (0–0)	0 (0–0)
Anorexia nervosa	0.0 (0.0–0.0)	0 (0–0)	0.0 (0.0–0.0)	0.0 (0.0–0.0)	0 (0–0)	0 (0–0)
Bulimia nervosa	0.0 (0.0–0.0)	0 (0–0)	0.0 (0.0–0.0)	0.0 (0.0–0.0)	0 (0–0)	0 (0–0)
Autistic spectrum disorders	0 (0–0)	0 (0–0)	0 (0–0)	0 (0–0)	0 (0–0)	0 (0–0)
Autism	0 (0–0)	0 (0–0)	0 (0–0)	0 (0–0)	0 (0–0)	0 (0–0)
Asperger syndrome and other autistic spectrum disorders	0 (0–0)	0 (0–0)	0 (0–0)	0 (0–0)	0 (0–0)	0 (0–0)
Attention-deficit/hyperactivity disorder	0 (0–0)	0 (0–0)	0 (0–0)	0 (0–0)	0 (0–0)	0 (0–0)
Conduct disorder	0 (0–0)	0 (0–0)	0 (0–0)	0 (0–0)	0 (0–0)	0 (0–0)
Idiopathic developmental intellectual disability	0 (0–0)	0 (0–0)	0 (0–0)	0 (0–0)	0 (0–0)	0 (0–0)
Other mental and substance use disorders	0 (0–0)	0 (0–0)	0 (0–0)	0 (0–0)	0 (0–0)	0 (0–0)
Diabetes, urogenital, blood, and endocrine diseases	42.1 (41.1–43.2)	18.5 (14.7–26.6)	2.7 (2.5–3.0)	9.6 (9.3–10.0)	87.2 (84.7–89.9)	401.0 (392.2–410.6)
Diabetes mellitus	19.1 (18.5–19.7)	0.4 (0.3–0.4)	0.3 (0.2–0.3)	3.0 (2.8–3.1)	46.5 (44.5–48.3)	193.4 (187.6–199.8)
Acute glomerulonephritis	0.2 (0.1–0.2)	0.1 (0.1–0.1)	0.0 (0.0–0.0)	0.1 (0.1–0.1)	0.4 (0.3–0.4)	1.3 (1.0–1.5)
Chronic kidney disease	14.4 (14.0–14.9)	2.4 (2.1–2.6)	0.8 (0.7–0.9)	3.7 (3.6–3.9)	29.0 (27.9–30.2)	142.0 (137.4–146.9)
Chronic kidney disease due to diabetes mellitus	4.6 (4.4–4.8)	0.4 (0.3–0.4)	0.1 (0.1–0.1)	0.9 (0.8–1.0)	11.5 (10.8–12.5)	44.0 (41.8–46.3)
Chronic kidney disease due to hypertension	6.5 (6.2–6.8)	0.8 (0.7–0.9)	0.3 (0.2–0.3)	1.5 (1.3–1.6)	11.6 (10.8–12.4)	70.6 (67.3–74.2)
Chronic kidney disease due to glomerulonephritis	2.9 (2.8–3.1)	0.9 (0.7–1.0)	0.4 (0.3–0.4)	1.2 (1.2–1.3)	5.5 (5.1–6.0)	24.2 (22.4–26.0)
Chronic kidney disease due to other causes	0.4 (0.3–0.4)	0.3 (0.3–0.4)	0.1 (0.1–0.1)	0.2 (0.1–0.2)	0.3 (0.2–0.5)	3.2 (2.7–4.0)
Urinary diseases and male infertility	3.2 (3.1–3.4)	0.9 (0.7–1.1)	0.2 (0.1–0.2)	0.8 (0.8–0.9)	5.0 (4.7–5.5)	35.4 (33.6–37.3)
Interstitial nephritis and urinary tract infections	2.6 (2.5–2.8)	0.7 (0.5–0.9)	0.1 (0.1–0.1)	0.6 (0.6–0.7)	3.7 (3.4–4.1)	30.8 (28.9–32.6)
Urolithiasis	0.2 (0.2–0.2)	0 (0–0)	0.0 (0.0–0.0)	0.1 (0.1–0.1)	0.5 (0.3–0.6)	1.7 (1.3–2.0)
Benign prostatic hyperplasia	0 (0–0)	0 (0–0)	0 (0–0)	0 (0–0)	0 (0–0)	0 (0–0)
Male infertility	0 (0–0)	0 (0–0)	0 (0–0)	0 (0–0)	0 (0–0)	0 (0–0)
Other urinary diseases	0.4 (0.3–0.4)	0.2 (0.1–0.3)	0.0 (0.0–0.0)	0.1 (0.1–0.2)	0.8 (0.7–1.0)	2.9 (2.5–3.5)
Gynecological diseases	0.2 (0.2–0.3)	0 (0–0)	0 (0–0)	0.2 (0.2–0.2)	0.5 (0.4–0.6)	1.1 (0.9–1.3)
Uterine fibroids	0.1 (0.0–0.1)	0 (0–0)	0 (0–0)	0.0 (0.0–0.1)	0.2 (0.1–0.2)	0.3 (0.1–0.4)
Polycystic ovarian syndrome	0.0 (0.0–0.0)	0 (0–0)	0 (0–0)	0.0 (0.0–0.1)	0.0 (0.0–0.1)	0 (0–0)
Female infertility	0 (0–0)	0 (0–0)	0 (0–0)	0 (0–0)	0 (0–0)	0 (0–0)
Endometriosis	0.0 (0.0–0.0)	0 (0–0)	0 (0–0)	0.0 (0.0–0.0)	0.0 (0.0–0.0)	0 (0–0)
Genital prolapse	0.0 (0.0–0.1)	0 (0–0)	0 (0–0)	0.0 (0.0–0.0)	0.1 (0.0–0.2)	0.4 (0.2–0.5)
Premenstrual syndrome	0 (0–0)	0 (0–0)	0 (0–0)	0 (0–0)	0 (0–0)	0 (0–0)
Other gynecological diseases	0.1 (0.1–0.1)	0 (0–0)	0 (0–0)	0.1 (0.1–0.2)	0.2 (0.1–0.2)	0.5 (0.3–0.7)
Hemoglobinopathies and hemolytic anemias	3.2 (2.8–3.9)	12.9 (9.5–21.2)	1.2 (1.0–1.4)	1.2 (1.1–1.3)	2.8 (2.7–2.9)	13.1 (12.4–13.9)
Thalassemias	0.3 (0.3–0.4)	1.7 (1.3–2.5)	0.3 (0.2–0.3)	0.1 (0.1–0.1)	0.0 (0.0–0.0)	1.1 (0.9–1.3)
Thalassemias trait	0 (0–0)	0 (0–0)	0 (0–0)	0 (0–0)	0 (0–0)	0 (0–0)
Sickle cell disorders	1.5 (1.2–2.3)	11.1 (7.7–19.2)	0.8 (0.6–1.0)	0.5 (0.4–0.6)	0.2 (0.1–0.2)	1.3 (1.1–1.6)
Sickle cell trait	0 (0–0)	0 (0–0)	0 (0–0)	0 (0–0)	0 (0–0)	0 (0–0)

SR Table 13 - Global death rates (per 100,000) by age and cause for females in 2005						
Cause	All ages	0-5 years	5-14 years	15-49 years	50-69 years	70+ years
G6PD deficiency	0.3 (0.2–0.3)	0.1 (0.1–0.1)	0.1 (0.0–0.1)	0.3 (0.2–0.3)	0.5 (0.4–0.6)	0.3 (0.3–0.4)
G6PD trait	0 (0–0)	0 (0–0)	0 (0–0)	0 (0–0)	0 (0–0)	0 (0–0)
Other hemoglobinopathies and hemolytic anemias	1.1 (1.0–1.1)	0.1 (0.0–0.1)	0.0 (0.0–0.0)	0.3 (0.3–0.3)	2.1 (2.0–2.2)	10.4 (9.7–10.9)
Endocrine, metabolic, blood, and immune disorders	1.8 (1.8–1.9)	1.8 (1.6–2.0)	0.3 (0.2–0.3)	0.6 (0.6–0.7)	3.1 (3.0–3.2)	14.8 (14.3–15.3)
Musculoskeletal disorders	1.7 (1.5–1.8)	0 (0–0)	0.2 (0.2–0.2)	0.8 (0.7–0.9)	2.7 (2.5–3.0)	14.6 (13.1–15.3)
Rheumatoid arthritis	0.6 (0.5–0.7)	0 (0–0)	0.0 (0.0–0.0)	0.1 (0.1–0.1)	1.2 (1.0–1.4)	6.3 (5.8–7.3)
Osteoarthritis	0 (0–0)	0 (0–0)	0 (0–0)	0 (0–0)	0 (0–0)	0 (0–0)
Low back and neck pain	0 (0–0)	0 (0–0)	0 (0–0)	0 (0–0)	0 (0–0)	0 (0–0)
Low back pain	0 (0–0)	0 (0–0)	0 (0–0)	0 (0–0)	0 (0–0)	0 (0–0)
Neck pain	0 (0–0)	0 (0–0)	0 (0–0)	0 (0–0)	0 (0–0)	0 (0–0)
Gout	0 (0–0)	0 (0–0)	0 (0–0)	0 (0–0)	0 (0–0)	0 (0–0)
Other musculoskeletal disorders	1.1 (0.9–1.2)	0 (0–0)	0.2 (0.1–0.2)	0.7 (0.6–0.8)	1.5 (1.3–1.7)	8.3 (7.0–8.9)
Other non-communicable diseases	10.9 (8.8–13.5)	86.7 (68.5–112.2)	3.3 (2.6–4.0)	1.6 (1.3–1.8)	3.1 (2.3–3.9)	17.2 (11.7–21.4)
Congenital anomalies	9.3 (7.4–11.9)	82.4 (64.6–107.8)	3.2 (2.5–3.9)	1.4 (1.1–1.6)	1.4 (1.2–1.6)	3.1 (2.5–3.4)
Neural tube defects	1.2 (0.8–1.9)	12.5 (8.2–20.1)	0.2 (0.1–0.3)	0.0 (0.0–0.1)	0.0 (0.0–0.1)	0.0 (0.0–0.1)
Congenital heart anomalies	4.6 (3.3–6.3)	38.4 (26.2–55.4)	2.2 (1.7–2.9)	1.0 (0.8–1.1)	0.5 (0.4–0.5)	1.0 (0.9–1.2)
Cleft lip and cleft palate	0.0 (0.0–0.1)	0.4 (0.3–0.6)	0 (0–0)	0 (0–0)	0 (0–0)	0 (0–0)
Down syndrome	0.5 (0.2–1.0)	4.3 (1.1–9.3)	0.1 (0.0–0.2)	0.1 (0.1–0.2)	0.3 (0.2–0.3)	0.1 (0.0–0.1)
Turner syndrome	0 (0–0)	0 (0–0)	0 (0–0)	0 (0–0)	0 (0–0)	0 (0–0)
Klinefelter syndrome	0 (0–0)	0 (0–0)	0 (0–0)	0 (0–0)	0 (0–0)	0 (0–0)
Other chromosomal abnormalities	0.4 (0.2–1.1)	4.2 (1.6–11.5)	0.0 (0.0–0.1)	0.0 (0.0–0.0)	0.0 (0.0–0.0)	0.0 (0.0–0.0)
Other congenital anomalies	2.5 (2.1–3.5)	22.6 (17.9–32.0)	0.6 (0.5–0.9)	0.2 (0.2–0.3)	0.6 (0.5–0.7)	2.0 (1.4–2.2)
Skin and subcutaneous diseases	1.3 (0.7–1.8)	1.1 (0.6–1.9)	0.1 (0.0–0.1)	0.2 (0.1–0.3)	1.6 (0.9–2.6)	14.0 (8.8–18.4)
Dermatitis	0 (0–0)	0 (0–0)	0 (0–0)	0 (0–0)	0 (0–0)	0 (0–0)
Psoriasis	0 (0–0)	0 (0–0)	0 (0–0)	0 (0–0)	0 (0–0)	0 (0–0)
Cellulitis	0.2 (0.1–0.3)	0.1 (0.0–0.2)	0.0 (0.0–0.0)	0.0 (0.0–0.1)	0.4 (0.2–0.6)	1.9 (1.0–2.6)
Pyoderma	0.5 (0.3–0.8)	0.9 (0.4–1.7)	0.1 (0.0–0.1)	0.1 (0.1–0.2)	0.8 (0.5–1.4)	4.5 (3.0–6.6)
Scabies	0 (0–0)	0 (0–0)	0 (0–0)	0 (0–0)	0 (0–0)	0 (0–0)
Fungal skin diseases	0 (0–0)	0 (0–0)	0 (0–0)	0 (0–0)	0 (0–0)	0 (0–0)
Viral skin diseases	0 (0–0)	0 (0–0)	0 (0–0)	0 (0–0)	0 (0–0)	0 (0–0)
Acne vulgaris	0 (0–0)	0 (0–0)	0 (0–0)	0 (0–0)	0 (0–0)	0 (0–0)
Alopecia areata	0 (0–0)	0 (0–0)	0 (0–0)	0 (0–0)	0 (0–0)	0 (0–0)
Pruritus	0 (0–0)	0 (0–0)	0 (0–0)	0 (0–0)	0 (0–0)	0 (0–0)
Urticaria	0 (0–0)	0 (0–0)	0 (0–0)	0 (0–0)	0 (0–0)	0 (0–0)
Decubitus ulcer	0.5 (0.3–0.6)	0.0 (0.0–0.0)	0.0 (0.0–0.0)	0.0 (0.0–0.0)	0.4 (0.2–0.6)	7.1 (3.8–9.1)
Other skin and subcutaneous diseases	0.1 (0.0–0.1)	0.0 (0.0–0.1)	0.0 (0.0–0.0)	0.0 (0.0–0.0)	0.1 (0.0–0.1)	0.5 (0.3–0.7)
Sense organ diseases	0 (0–0)	0 (0–0)	0 (0–0)	0 (0–0)	0 (0–0)	0 (0–0)
Glaucoma	0 (0–0)	0 (0–0)	0 (0–0)	0 (0–0)	0 (0–0)	0 (0–0)
Cataract	0 (0–0)	0 (0–0)	0 (0–0)	0 (0–0)	0 (0–0)	0 (0–0)
Macular degeneration	0 (0–0)	0 (0–0)	0 (0–0)	0 (0–0)	0 (0–0)	0 (0–0)
Refraction and accommodation disorders	0 (0–0)	0 (0–0)	0 (0–0)	0 (0–0)	0 (0–0)	0 (0–0)
Age-related and other hearing loss	0 (0–0)	0 (0–0)	0 (0–0)	0 (0–0)	0 (0–0)	0 (0–0)
Other vision loss	0 (0–0)	0 (0–0)	0 (0–0)	0 (0–0)	0 (0–0)	0 (0–0)
Other sense organ diseases	0 (0–0)	0 (0–0)	0 (0–0)	0 (0–0)	0 (0–0)	0 (0–0)
Oral disorders	0 (0–0)	0 (0–0)	0 (0–0)	0 (0–0)	0 (0–0)	0 (0–0)
Deciduous caries	0 (0–0)	0 (0–0)	0 (0–0)	0 (0–0)	0 (0–0)	0 (0–0)
Permanent caries	0 (0–0)	0 (0–0)	0 (0–0)	0 (0–0)	0 (0–0)	0 (0–0)
Periodontal diseases	0 (0–0)	0 (0–0)	0 (0–0)	0 (0–0)	0 (0–0)	0 (0–0)
Edentulism and severe tooth loss	0 (0–0)	0 (0–0)	0 (0–0)	0 (0–0)	0 (0–0)	0 (0–0)
Other oral disorders	0 (0–0)	0 (0–0)	0 (0–0)	0 (0–0)	0 (0–0)	0 (0–0)
Sudden infant death syndrome	0.3 (0.2–0.4)	3.2 (2.3–4.4)	0 (0–0)	0 (0–0)	0 (0–0)	0 (0–0)
Injuries	45.2 (40.5–47.4)	53.9 (42.5–64.3)	17.5 (15.5–18.8)	34.4 (30.7–35.8)	59.2 (52.9–61.4)	188.4 (168.9–195.8)
Transport injuries	11.9 (11.3–12.4)	9.2 (7.5–11.1)	5.1 (4.7–5.4)	10.3 (9.8–10.8)	18.3 (17.5–19.0)	36.1 (34.8–37.5)
Road injuries	11.2 (10.7–11.7)	8.4 (7.0–10.2)	4.8 (4.5–5.2)	9.8 (9.3–10.3)	17.2 (16.5–18.0)	34.2 (32.9–35.7)
Pedestrian road injuries	5.1 (4.9–5.4)	3.8 (3.1–4.5)	2.4 (2.2–2.6)	3.9 (3.7–4.1)	8.6 (8.1–9.2)	19.1 (18.2–20.4)
Cyclist road injuries	0.5 (0.5–0.6)	0.2 (0.1–0.3)	0.2 (0.2–0.3)	0.5 (0.4–0.5)	0.9 (0.9–1.0)	1.1 (1.0–1.2)
Motorcyclist road injuries	1.5 (1.4–1.6)	1.1 (0.9–1.4)	0.5 (0.4–0.6)	1.5 (1.4–1.6)	2.2 (2.0–2.4)	3.6 (3.2–4.2)

SR Table 13 - Global death rates (per 100,000) by age and cause for females in 2005						
Cause	All ages	0-5 years	5-14 years	15-49 years	50-69 years	70+ years
Motor vehicle road injuries	3.9 (3.7–4.2)	3.2 (2.5–4.1)	1.7 (1.5–1.8)	3.8 (3.6–4.1)	5.3 (4.9–5.6)	10.1 (9.0–10.7)
Other road injuries	0.1 (0.1–0.1)	0.1 (0.1–0.2)	0.1 (0.0–0.1)	0.1 (0.1–0.1)	0.2 (0.2–0.2)	0.4 (0.3–0.4)
Other transport injuries	0.7 (0.6–0.7)	0.7 (0.5–1.0)	0.3 (0.2–0.3)	0.5 (0.5–0.6)	1.1 (0.9–1.2)	1.9 (1.7–2.2)
Unintentional injuries	21.0 (17.5–22.5)	40.9 (30.1–50.1)	9.5 (7.6–10.4)	10.5 (8.7–11.3)	22.6 (19.2–23.8)	121.3 (104.7–127.0)
Falls	5.3 (4.5–5.6)	3.7 (2.2–5.1)	0.9 (0.8–1.0)	1.1 (1.0–1.2)	5.3 (4.8–5.6)	62.3 (53.2–65.8)
Drowning	3.9 (2.2–4.4)	12.1 (5.0–15.2)	3.7 (2.3–4.2)	2.1 (1.2–2.3)	3.2 (2.0–3.5)	9.7 (6.5–10.6)
Fire, heat, and hot substances	3.1 (2.3–3.4)	4.9 (3.1–6.0)	1.0 (0.8–1.2)	2.6 (1.8–2.9)	3.1 (2.4–3.4)	11.4 (9.4–12.3)
Poisonings	1.2 (0.8–1.4)	2.7 (1.1–4.9)	0.5 (0.4–0.6)	0.9 (0.7–1.0)	1.5 (1.1–1.6)	3.5 (2.8–3.7)
Exposure to mechanical forces	1.7 (1.5–1.8)	4.6 (3.8–5.3)	0.7 (0.7–0.9)	0.9 (0.7–0.9)	1.9 (1.7–2.1)	7.2 (5.3–8.0)
Unintentional firearm injuries	0.3 (0.2–0.3)	0.2 (0.1–0.4)	0.1 (0.1–0.1)	0.2 (0.1–0.2)	0.3 (0.2–0.3)	1.9 (1.4–2.0)
Unintentional suffocation	0.4 (0.3–0.4)	2.4 (1.6–2.8)	0.2 (0.1–0.2)	0.1 (0.1–0.1)	0.2 (0.1–0.2)	1.0 (0.7–1.2)
Other exposure to mechanical forces	1.1 (0.9–1.1)	2.0 (1.6–2.5)	0.5 (0.4–0.5)	0.6 (0.5–0.7)	1.5 (1.4–1.7)	4.4 (2.9–5.0)
Adverse effects of medical treatment	1.3 (1.0–1.6)	2.4 (1.3–3.3)	0.3 (0.2–0.4)	0.6 (0.4–0.7)	1.8 (1.3–2.1)	8.6 (6.9–9.7)
Animal contact	1.4 (0.8–1.5)	2.2 (1.2–2.8)	1.0 (0.6–1.2)	1.0 (0.6–1.2)	2.0 (1.1–2.3)	2.8 (1.6–3.2)
Venomous animal contact	1.2 (0.6–1.4)	1.7 (0.8–2.1)	0.8 (0.5–1.0)	0.9 (0.5–1.1)	1.8 (0.9–2.1)	2.3 (1.2–2.6)
Non-venomous animal contact	0.2 (0.1–0.2)	0.6 (0.3–0.8)	0.1 (0.1–0.2)	0.1 (0.1–0.1)	0.2 (0.1–0.3)	0.6 (0.4–0.6)
Foreign body	1.7 (1.4–2.4)	6.3 (3.9–11.2)	0.6 (0.4–0.9)	0.5 (0.4–0.7)	1.5 (1.3–1.8)	10.0 (9.0–10.7)
Pulmonary aspiration and foreign body in airway	1.4 (1.1–2.1)	5.6 (2.9–10.7)	0.4 (0.3–0.7)	0.3 (0.3–0.5)	1.2 (1.0–1.4)	8.3 (7.6–9.0)
Foreign body in eyes	0 (0–0)	0 (0–0)	0 (0–0)	0 (0–0)	0 (0–0)	0 (0–0)
Foreign body in other body part	0.3 (0.2–0.5)	0.7 (0.3–1.1)	0.2 (0.1–0.2)	0.2 (0.1–0.3)	0.4 (0.3–0.6)	1.6 (1.3–2.2)
Environmental heat and cold exposure	0.5 (0.3–0.6)	0.4 (0.2–0.6)	0.1 (0.0–0.1)	0.2 (0.2–0.2)	1.0 (0.7–1.2)	3.2 (2.1–3.8)
Other unintentional injuries	0.9 (0.9–1.0)	1.7 (1.4–2.0)	0.6 (0.5–0.7)	0.6 (0.6–0.7)	1.2 (1.1–1.3)	2.5 (2.3–2.7)
Self-harm and interpersonal violence	11.1 (8.7–11.7)	2.0 (1.8–2.2)	1.6 (1.4–1.7)	12.4 (9.7–13.2)	17.1 (13.3–18.0)	29.0 (22.6–30.7)
Self-harm	8.4 (6.1–8.9)	0 (0–0)	0.7 (0.5–0.8)	9.3 (6.7–9.9)	14.0 (10.2–14.9)	23.9 (17.6–25.5)
Interpersonal violence	2.7 (2.6–2.8)	2.0 (1.8–2.2)	0.9 (0.8–0.9)	3.1 (3.0–3.3)	3.1 (2.9–3.2)	5.2 (4.9–5.4)
Assault by firearm	0.6 (0.5–0.7)	0.3 (0.2–0.3)	0.2 (0.2–0.2)	0.8 (0.7–0.9)	0.6 (0.5–0.7)	0.9 (0.7–1.1)
Assault by sharp object	0.6 (0.6–0.7)	0.2 (0.2–0.2)	0.1 (0.1–0.2)	0.8 (0.8–0.9)	0.7 (0.7–0.8)	1.3 (1.2–1.5)
Assault by other means	1.4 (1.4–1.6)	1.5 (1.4–1.7)	0.5 (0.5–0.6)	1.5 (1.4–1.6)	1.8 (1.6–1.9)	3.0 (2.7–3.2)
Forces of nature, war, and legal intervention	1.2 (0.8–1.7)	1.7 (1.1–2.4)	1.3 (0.8–1.9)	1.1 (0.7–1.4)	1.3 (0.8–1.7)	1.9 (1.2–3.3)
Exposure to forces of nature	1.0 (0.6–1.4)	1.3 (0.7–1.8)	1.2 (0.7–1.7)	0.8 (0.5–1.2)	1.0 (0.6–1.4)	1.5 (0.9–2.2)
Collective violence and legal intervention	0.2 (0.2–0.3)	0.5 (0.3–0.6)	0.2 (0.1–0.2)	0.2 (0.2–0.3)	0.2 (0.2–0.3)	0.4 (0.2–1.7)

SR Table 13 - Global death rates (per 100,000) by age and cause for females in 2010

Cause	All ages	0-5 years	5-14 years	15-49 years	50-69 years	70+ years
All causes	713.4 (703.8–722.8)	1022.8 (1006.3–1039.9)	75.5 (72.6–78.7)	189.5 (183.8–195.2)	963.2 (941.1–984.4)	6057.0 (5995.3–6117.2)
Communicable, maternal, neonatal, and nutritional diseases	174.6 (169.7–179.5)	847.5 (822.1–870.3)	41.9 (39.9–44.3)	77.0 (73.3–80.9)	118.0 (109.9–125.9)	535.6 (494.3–562.5)
HIV/AIDS and tuberculosis	35.2 (32.6–38.1)	25.8 (24.5–27.6)	9.8 (9.3–10.4)	39.6 (37.2–42.5)	42.4 (36.4–48.5)	64.3 (49.7–76.7)
Tuberculosis	12.6 (10.3–15.2)	4.2 (3.3–5.9)	1.4 (1.2–2.0)	7.8 (6.5–9.5)	27.6 (21.6–33.1)	60.1 (45.8–72.4)
HIV/AIDS	22.6 (21.5–23.8)	21.6 (20.7–22.5)	8.3 (8.0–8.6)	31.8 (29.8–34.0)	14.8 (13.6–16.2)	4.2 (3.8–4.9)
HIV/AIDS - Tuberculosis	4.0 (3.0–4.5)	5.4 (4.1–6.0)	1.7 (1.2–1.9)	4.9 (3.6–5.5)	3.8 (3.1–4.2)	1.2 (1.1–1.4)
HIV/AIDS resulting in other diseases	18.6 (17.4–20.0)	16.2 (15.1–17.6)	6.6 (6.2–7.2)	27.0 (24.9–29.3)	11.0 (9.9–12.4)	3.0 (2.6–3.6)
Diarrhea, lower respiratory, and other common infectious diseases	73.8 (70.0–77.2)	315.1 (294.2–337.6)	23.1 (20.9–25.8)	14.0 (12.7–15.4)	59.0 (53.6–63.7)	418.0 (382.8–439.3)
Diarrheal diseases	21.8 (20.4–23.3)	100.3 (89.1–113.1)	5.1 (4.5–5.8)	4.4 (4.0–4.9)	21.1 (19.4–22.9)	104.3 (97.3–111.7)
Intestinal infectious diseases	2.5 (1.4–4.1)	6.7 (3.5–11.9)	5.7 (3.3–9.3)	1.5 (0.8–2.5)	0.8 (0.4–1.4)	1.0 (0.6–1.6)
Typhoid fever	2.1 (1.1–3.5)	5.6 (2.8–10.1)	4.8 (2.7–7.7)	1.2 (0.6–2.1)	0.6 (0.3–1.1)	0.8 (0.4–1.3)
Paratyphoid fever	0.4 (0.2–0.8)	1.0 (0.4–2.1)	0.9 (0.4–1.7)	0.3 (0.1–0.5)	0.2 (0.1–0.3)	0.2 (0.1–0.3)
Other intestinal infectious diseases	0.0 (0.0–0.0)	0.1 (0.0–0.2)	0.0 (0.0–0.0)	0.0 (0.0–0.0)	0.0 (0.0–0.0)	0.1 (0.0–0.1)
Lower respiratory infections	38.2 (34.3–40.4)	136.2 (123.8–149.5)	5.9 (4.2–7.1)	5.1 (4.0–5.8)	30.2 (23.9–33.4)	294.8 (255.9–312.9)
Upper respiratory infections	0.0 (0.0–0.1)	0.1 (0.1–0.2)	0.0 (0.0–0.0)	0.0 (0.0–0.0)	0.1 (0.0–0.1)	0.2 (0.2–0.3)
Otitis media	0.0 (0.0–0.1)	0.2 (0.1–0.2)	0.0 (0.0–0.0)	0.0 (0.0–0.0)	0.0 (0.0–0.0)	0.2 (0.1–0.4)
Meningitis	5.2 (4.4–6.1)	28.7 (22.0–37.8)	2.9 (2.4–3.4)	1.7 (1.4–2.0)	3.9 (3.2–4.7)	9.6 (8.1–11.1)
Pneumococcal meningitis	1.6 (1.3–1.9)	7.7 (5.3–11.0)	1.1 (0.8–1.5)	0.6 (0.5–0.8)	1.2 (0.9–1.5)	2.8 (2.2–3.5)
H influenzae type B meningitis	1.1 (0.9–1.4)	8.1 (5.8–11.3)	0.6 (0.4–0.8)	0.2 (0.2–0.3)	0.5 (0.4–0.7)	1.3 (1.0–1.7)
Meningococcal meningitis	1.0 (0.8–1.3)	6.2 (4.1–9.6)	0.6 (0.4–0.8)	0.3 (0.2–0.4)	0.7 (0.5–0.9)	1.4 (1.1–1.9)
Other meningitis	1.5 (1.3–1.8)	6.7 (4.8–9.4)	0.6 (0.4–0.9)	0.6 (0.5–0.7)	1.6 (1.3–2.0)	4.0 (3.3–4.9)
Encephalitis	1.9 (1.7–2.3)	7.1 (5.5–8.3)	1.3 (1.1–1.7)	0.8 (0.7–0.9)	1.9 (1.7–2.6)	5.8 (5.2–7.7)
Diphtheria	0.0 (0.0–0.1)	0.4 (0.1–1.1)	0.0 (0.0–0.1)	0.0 (0.0–0.0)	0.0 (0.0–0.0)	0 (0–0)
Whooping cough	1.2 (0.5–2.6)	12.3 (4.9–26.2)	0.4 (0.2–0.9)	0.0 (0.0–0.1)	0.0 (0.0–0.0)	0 (0–0)
Tetanus	0.9 (0.7–1.3)	5.0 (4.1–6.1)	0.2 (0.2–0.4)	0.3 (0.2–0.5)	0.9 (0.6–1.8)	1.3 (1.0–2.5)
Measles	1.9 (0.7–4.2)	17.9 (6.7–38.3)	1.4 (0.5–3.1)	0.1 (0.0–0.2)	0.0 (0.0–0.0)	0 (0–0)
Varicella and herpes zoster	0.1 (0.1–0.1)	0.3 (0.2–0.5)	0.1 (0.0–0.1)	0.0 (0.0–0.0)	0.1 (0.1–0.1)	0.8 (0.5–1.1)
Neglected tropical diseases and malaria	15.4 (12.9–17.9)	112.1 (88.4–136.1)	5.8 (4.0–8.2)	4.1 (3.2–5.4)	8.6 (6.5–11.7)	11.6 (8.9–15.6)
Malaria	14.0 (11.4–16.4)	108.4 (84.3–132.5)	4.6 (2.8–7.0)	3.3 (2.4–4.6)	7.0 (4.8–10.0)	8.5 (5.8–12.3)
Chagas disease	0.1 (0.1–0.1)	0.0 (0.0–0.0)	0.0 (0.0–0.0)	0.0 (0.0–0.0)	0.2 (0.2–0.2)	0.8 (0.8–0.9)
Leishmaniasis	0.2 (0.1–0.2)	0.4 (0.3–0.6)	0.1 (0.1–0.2)	0.2 (0.1–0.3)	0.1 (0.1–0.2)	0.1 (0.1–0.1)
Visceral leishmaniasis	0.2 (0.1–0.2)	0.4 (0.3–0.6)	0.1 (0.1–0.2)	0.2 (0.1–0.3)	0.1 (0.1–0.2)	0.1 (0.1–0.1)
Cutaneous and mucocutaneous leishmaniasis	0 (0–0)	0 (0–0)	0 (0–0)	0 (0–0)	0 (0–0)	0 (0–0)
African trypanosomiasis	0.1 (0.0–0.1)	0.1 (0.0–0.1)	0.1 (0.0–0.1)	0.1 (0.0–0.1)	0.1 (0.0–0.2)	0.1 (0.0–0.1)
Schistosomiasis	0.1 (0.1–0.1)	0.0 (0.0–0.0)	0.0 (0.0–0.0)	0.0 (0.0–0.0)	0.2 (0.2–0.3)	0.4 (0.3–0.5)
Cysticercosis	0.0 (0.0–0.0)	0.0 (0.0–0.0)	0.0 (0.0–0.0)	0.0 (0.0–0.0)	0.0 (0.0–0.0)	0.0 (0.0–0.0)
Cystic echinococcosis	0.0 (0.0–0.0)	0.0 (0.0–0.0)	0.0 (0.0–0.0)	0.0 (0.0–0.0)	0.0 (0.0–0.0)	0.1 (0.1–0.1)
Lymphatic filariasis	0 (0–0)	0 (0–0)	0 (0–0)	0 (0–0)	0 (0–0)	0 (0–0)
Onchocerciasis	0 (0–0)	0 (0–0)	0 (0–0)	0 (0–0)	0 (0–0)	0 (0–0)
Trachoma	0 (0–0)	0 (0–0)	0 (0–0)	0 (0–0)	0 (0–0)	0 (0–0)
Dengue	0.2 (0.2–0.3)	0.7 (0.4–1.0)	0.4 (0.2–0.5)	0.1 (0.1–0.1)	0.2 (0.1–0.2)	0.3 (0.2–0.4)
Yellow fever	0.0 (0.0–0.1)	0.1 (0.0–0.2)	0.1 (0.0–0.3)	0.0 (0.0–0.1)	0.0 (0.0–0.1)	0.0 (0.0–0.0)
Rabies	0.3 (0.2–0.3)	0.4 (0.3–0.7)	0.3 (0.2–0.4)	0.2 (0.1–0.2)	0.4 (0.3–0.5)	0.4 (0.3–0.5)
Intestinal nematode infections	0.0 (0.0–0.1)	0.4 (0.3–0.4)	0.0 (0.0–0.0)	0.0 (0.0–0.0)	0.0 (0.0–0.0)	0.0 (0.0–0.0)
Ascariasis	0.0 (0.0–0.1)	0.4 (0.3–0.4)	0.0 (0.0–0.0)	0.0 (0.0–0.0)	0.0 (0.0–0.0)	0.0 (0.0–0.0)
Trichuriasis	0 (0–0)	0 (0–0)	0 (0–0)	0 (0–0)	0 (0–0)	0 (0–0)
Hookworm disease	0 (0–0)	0 (0–0)	0 (0–0)	0 (0–0)	0 (0–0)	0 (0–0)
Food-borne trematodiases	0 (0–0)	0 (0–0)	0 (0–0)	0 (0–0)	0 (0–0)	0 (0–0)
Leprosy	0 (0–0)	0 (0–0)	0 (0–0)	0 (0–0)	0 (0–0)	0 (0–0)
Ebola	0 (0–0)	0 (0–0)	0 (0–0)	0 (0–0)	0 (0–0)	0 (0–0)
Other neglected tropical diseases	0.4 (0.3–0.5)	1.6 (1.1–2.3)	0.2 (0.1–0.3)	0.1 (0.1–0.2)	0.4 (0.2–0.7)	0.9 (0.5–1.4)
Maternal disorders	9.0 (8.2–9.9)	0 (0–0)	0.3 (0.2–0.3)	17.1 (15.7–18.8)	0.2 (0.2–0.3)	0 (0–0)
Maternal hemorrhage	2.6 (2.2–3.0)	0 (0–0)	0.1 (0.1–0.1)	4.9 (4.2–5.7)	0.1 (0.0–0.1)	0 (0–0)
Maternal sepsis and other maternal infections	0.7 (0.6–0.8)	0 (0–0)	0.0 (0.0–0.0)	1.3 (1.0–1.6)	0.0 (0.0–0.0)	0 (0–0)
Maternal hypertensive disorders	1.6 (1.3–1.9)	0 (0–0)	0.1 (0.0–0.1)	3.0 (2.5–3.6)	0.0 (0.0–0.0)	0 (0–0)
Maternal obstructed labor and uterine rupture	0.8 (0.6–0.9)	0 (0–0)	0.0 (0.0–0.0)	1.5 (1.2–1.8)	0.0 (0.0–0.0)	0 (0–0)
Maternal abortion, miscarriage, and ectopic pregnancy	1.1 (0.9–1.3)	0 (0–0)	0.0 (0.0–0.1)	2.1 (1.7–2.5)	0.0 (0.0–0.1)	0 (0–0)

SR Table 13 - Global death rates (per 100,000) by age and cause for females in 2010

Cause	All ages	0-5 years	5-14 years	15-49 years	50-69 years	70+ years
Indirect maternal deaths	1.0 (0.8–1.2)	0 (0–0)	0.0 (0.0–0.1)	1.9 (1.5–2.3)	0.0 (0.0–0.0)	0 (0–0)
Late maternal deaths	0.2 (0.1–0.3)	0 (0–0)	0.0 (0.0–0.0)	0.4 (0.3–0.6)	0.0 (0.0–0.0)	0 (0–0)
Maternal deaths aggravated by HIV/AIDS	0.1 (0.0–0.1)	0 (0–0)	0.0 (0.0–0.0)	0.1 (0.1–0.2)	0.0 (0.0–0.0)	0 (0–0)
Other maternal disorders	1.0 (0.9–1.2)	0 (0–0)	0.0 (0.0–0.0)	1.9 (1.6–2.3)	0.0 (0.0–0.1)	0 (0–0)
Neonatal disorders	31.0 (30.1–32.0)	340.3 (330.4–351.0)	0 (0–0)	0 (0–0)	0 (0–0)	0 (0–0)
Neonatal preterm birth complications	12.5 (11.2–13.9)	137.0 (123.0–152.2)	0 (0–0)	0 (0–0)	0 (0–0)	0 (0–0)
Neonatal encephalopathy due to birth asphyxia and trauma	10.5 (9.2–12.1)	115.5 (100.6–132.9)	0 (0–0)	0 (0–0)	0 (0–0)	0 (0–0)
Neonatal sepsis and other neonatal infections	3.8 (1.9–5.9)	42.2 (20.7–64.3)	0 (0–0)	0 (0–0)	0 (0–0)	0 (0–0)
Hemolytic disease and other neonatal jaundice	0.8 (0.4–1.3)	8.7 (4.7–14.5)	0 (0–0)	0 (0–0)	0 (0–0)	0 (0–0)
Other neonatal disorders	3.4 (2.4–4.7)	36.9 (26.1–51.5)	0 (0–0)	0 (0–0)	0 (0–0)	0 (0–0)
Nutritional deficiencies	6.0 (5.5–6.8)	31.8 (26.3–38.5)	1.3 (1.1–1.5)	0.9 (0.8–1.1)	3.9 (3.5–4.4)	31.2 (29.1–34.0)
Protein-energy malnutrition	4.8 (4.3–5.6)	28.8 (23.6–35.6)	1.0 (0.8–1.1)	0.4 (0.4–0.5)	2.3 (2.0–2.7)	24.9 (22.6–27.6)
Iodine deficiency	0.0 (0.0–0.1)	0.1 (0.0–0.3)	0.0 (0.0–0.0)	0.0 (0.0–0.0)	0.0 (0.0–0.1)	0.2 (0.1–0.3)
Vitamin A deficiency	0 (0–0)	0 (0–0)	0 (0–0)	0 (0–0)	0 (0–0)	0 (0–0)
Iron-deficiency anemia	0.7 (0.5–1.0)	2.3 (1.2–3.5)	0.2 (0.2–0.3)	0.3 (0.3–0.4)	1.1 (0.8–1.4)	2.5 (1.5–3.4)
Other nutritional deficiencies	0.4 (0.3–0.6)	0.6 (0.3–0.9)	0.1 (0.1–0.1)	0.1 (0.1–0.2)	0.4 (0.3–0.7)	3.6 (2.4–5.3)
Other communicable, maternal, neonatal, and nutritional diseases	4.2 (3.4–5.1)	22.5 (15.6–31.4)	1.6 (1.2–2.2)	1.3 (1.2–1.4)	3.9 (3.4–4.2)	10.4 (8.2–11.2)
Sexually transmitted diseases excluding HIV	1.7 (1.0–2.6)	15.2 (8.5–24.3)	0.9 (0.5–1.4)	0.2 (0.1–0.2)	0.3 (0.2–0.3)	0.8 (0.6–1.0)
Syphilis	1.7 (1.0–2.6)	15.2 (8.5–24.3)	0.9 (0.5–1.4)	0.1 (0.1–0.2)	0.2 (0.2–0.3)	0.7 (0.5–0.9)
Chlamydial infection	0.0 (0.0–0.0)	0 (0–0)	0.0 (0.0–0.0)	0.0 (0.0–0.0)	0.0 (0.0–0.0)	0.0 (0.0–0.0)
Gonococcal infection	0.0 (0.0–0.0)	0 (0–0)	0.0 (0.0–0.0)	0.0 (0.0–0.0)	0.0 (0.0–0.0)	0.1 (0.1–0.1)
Trichomoniasis	0 (0–0)	0 (0–0)	0 (0–0)	0 (0–0)	0 (0–0)	0 (0–0)
Genital herpes	0 (0–0)	0 (0–0)	0 (0–0)	0 (0–0)	0 (0–0)	0 (0–0)
Other sexually transmitted diseases	0.0 (0.0–0.0)	0 (0–0)	0.0 (0.0–0.0)	0.0 (0.0–0.0)	0.0 (0.0–0.0)	0.0 (0.0–0.0)
Hepatitis	1.3 (1.2–1.3)	2.1 (1.9–2.4)	0.5 (0.4–0.6)	0.8 (0.8–0.9)	2.0 (1.9–2.1)	4.4 (4.1–4.7)
Acute hepatitis A	0.2 (0.1–0.3)	1.6 (1.2–2.0)	0.1 (0.0–0.2)	0.0 (0.0–0.1)	0.0 (0.0–0.1)	0.1 (0.0–0.1)
Hepatitis B	0.7 (0.6–0.8)	0.1 (0.0–0.3)	0.1 (0.0–0.1)	0.4 (0.3–0.5)	1.6 (1.4–1.8)	3.2 (2.5–3.7)
Hepatitis C	0.0 (0.0–0.1)	0 (0–0)	0.0 (0.0–0.0)	0.0 (0.0–0.0)	0.1 (0.0–0.2)	0.1 (0.0–0.3)
Acute hepatitis E	0.4 (0.3–0.5)	0.4 (0.1–0.7)	0.3 (0.2–0.4)	0.3 (0.2–0.4)	0.3 (0.1–0.5)	1.0 (0.5–1.7)
Other infectious diseases	1.2 (0.8–1.4)	5.2 (2.5–7.3)	0.3 (0.2–0.3)	0.3 (0.2–0.3)	1.6 (1.1–1.8)	5.2 (3.1–5.9)
Non-communicable diseases	496.0 (488.6–504.7)	126.9 (112.7–145.9)	16.1 (14.9–17.4)	81.5 (79.0–84.6)	792.1 (774.3–810.6)	5338.1 (5281.7–5405.7)
Neoplasms	99.3 (97.4–101.1)	5.2 (4.6–6.1)	3.9 (3.6–4.1)	27.6 (26.7–28.6)	240.0 (234.8–245.4)	776.1 (760.5–791.7)
Lip and oral cavity cancer	1.2 (1.2–1.3)	0 (0–0)	0 (0–0)	0.4 (0.3–0.4)	3.1 (3.0–3.3)	9.6 (9.3–10.0)
Nasopharynx cancer	0.5 (0.5–0.5)	0 (0–0)	0.0 (0.0–0.0)	0.3 (0.3–0.3)	1.4 (1.3–1.5)	2.1 (2.0–2.2)
Other pharynx cancer	0.5 (0.5–0.5)	0 (0–0)	0 (0–0)	0.1 (0.1–0.1)	1.5 (1.4–1.6)	2.9 (2.8–3.1)
Esophageal cancer	3.7 (3.6–3.9)	0 (0–0)	0 (0–0)	0.6 (0.6–0.7)	9.6 (9.1–10.1)	31.8 (30.3–33.3)
Stomach cancer	8.2 (8.0–8.5)	0 (0–0)	0 (0–0)	1.8 (1.7–1.9)	18.0 (17.4–18.7)	75.8 (73.8–78.0)
Colon and rectum cancer	10.0 (9.7–10.2)	0 (0–0)	0 (0–0)	1.6 (1.6–1.7)	20.0 (19.5–20.6)	101.2 (98.4–104.2)
Liver cancer	6.6 (5.7–7.0)	0 (0–0)	0.1 (0.1–0.1)	1.6 (1.4–1.8)	17.1 (14.7–18.3)	51.2 (45.1–54.4)
Liver cancer due to hepatitis B	1.8 (1.6–2.0)	0 (0–0)	0.0 (0.0–0.0)	0.8 (0.7–0.9)	5.3 (4.5–5.9)	9.6 (7.9–10.7)
Liver cancer due to hepatitis C	1.9 (1.7–2.1)	0 (0–0)	0.0 (0.0–0.0)	0.2 (0.2–0.2)	4.3 (3.6–4.7)	19.8 (17.9–21.1)
Liver cancer due to alcohol use	1.1 (1.0–1.2)	0 (0–0)	0 (0–0)	0.1 (0.1–0.1)	3.3 (2.8–3.8)	8.8 (7.6–9.6)
Liver cancer due to other causes	1.7 (1.4–1.8)	0 (0–0)	0.0 (0.0–0.0)	0.4 (0.4–0.5)	4.2 (3.6–4.7)	13.1 (11.2–14.3)
Gallbladder and biliary tract cancer	2.3 (2.2–2.4)	0 (0–0)	0 (0–0)	0.3 (0.3–0.3)	4.9 (4.6–5.1)	22.9 (21.9–23.8)
Pancreatic cancer	5.0 (4.9–5.1)	0 (0–0)	0 (0–0)	0.5 (0.5–0.6)	11.1 (10.8–11.3)	51.1 (49.8–52.4)
Larynx cancer	0.5 (0.5–0.5)	0 (0–0)	0 (0–0)	0.1 (0.1–0.1)	1.4 (1.3–1.5)	3.8 (3.6–4.0)
Tracheal, bronchus, and lung cancer	13.7 (13.4–14.1)	0 (0–0)	0 (0–0)	2.2 (2.1–2.3)	34.9 (34.1–35.8)	120.1 (116.7–123.4)
Malignant skin melanoma	0.7 (0.6–0.7)	0 (0–0)	0 (0–0)	0.2 (0.2–0.3)	1.6 (1.4–1.6)	5.9 (5.0–6.3)
Non-melanoma skin cancer	0.4 (0.4–0.4)	0 (0–0)	0 (0–0)	0.1 (0.1–0.1)	0.6 (0.6–0.7)	4.7 (4.5–5.0)
Non-melanoma skin cancer (squamous-cell carcinoma)	0.4 (0.4–0.4)	0 (0–0)	0 (0–0)	0.1 (0.1–0.1)	0.6 (0.6–0.7)	4.7 (4.5–5.0)
Non-melanoma skin cancer (basal-cell carcinoma)	0 (0–0)	0 (0–0)	0 (0–0)	0 (0–0)	0 (0–0)	0 (0–0)
Breast cancer	13.7 (13.0–14.2)	0 (0–0)	0 (0–0)	5.6 (5.2–5.9)	38.1 (36.0–39.6)	80.7 (76.1–83.4)
Cervical cancer	6.6 (6.3–6.9)	0 (0–0)	0 (0–0)	3.4 (3.1–3.6)	19.5 (18.3–20.6)	30.2 (29.2–31.3)
Uterine cancer	2.5 (2.4–2.6)	0 (0–0)	0 (0–0)	0.5 (0.5–0.6)	7.1 (6.8–7.4)	18.1 (17.2–18.8)
Ovarian cancer	4.2 (4.1–4.3)	0 (0–0)	0 (0–0)	1.4 (1.3–1.5)	12.1 (11.8–12.5)	26.8 (26.1–27.5)
Prostate cancer	0 (0–0)	0 (0–0)	0 (0–0)	0 (0–0)	0 (0–0)	0 (0–0)
Testicular cancer	0 (0–0)	0 (0–0)	0 (0–0)	0 (0–0)	0 (0–0)	0 (0–0)

SR Table 13 - Global death rates (per 100,000) by age and cause for females in 2010

Cause	All ages	0-5 years	5-14 years	15-49 years	50-69 years	70+ years
Kidney cancer	1.2 (1.2–1.3)	0.3 (0.2–0.4)	0.1 (0.1–0.1)	0.2 (0.2–0.2)	2.7 (2.6–2.8)	11.2 (10.9–11.6)
Bladder cancer	1.3 (1.3–1.4)	0 (0–0)	0 (0–0)	0.1 (0.1–0.1)	2.1 (2.1–2.2)	15.7 (15.2–16.2)
Brain and nervous system cancer	2.7 (2.6–2.8)	0.7 (0.7–0.8)	0.8 (0.7–0.8)	1.3 (1.2–1.3)	6.6 (6.4–6.8)	13.4 (12.6–13.8)
Thyroid cancer	0.5 (0.5–0.5)	0 (0–0)	0.0 (0.0–0.0)	0.1 (0.1–0.1)	1.1 (1.0–1.2)	4.7 (4.5–5.2)
Mesothelioma	0.2 (0.2–0.3)	0 (0–0)	0 (0–0)	0.1 (0.1–0.1)	0.5 (0.5–0.6)	2.0 (2.0–2.1)
Hodgkin lymphoma	0.3 (0.2–0.3)	0.0 (0.0–0.0)	0.0 (0.0–0.0)	0.2 (0.1–0.2)	0.5 (0.4–0.7)	1.3 (1.2–1.7)
Non-Hodgkin lymphoma	2.5 (2.2–2.7)	0.4 (0.3–0.6)	0.3 (0.2–0.3)	0.7 (0.6–0.8)	5.1 (4.5–5.5)	21.4 (17.1–22.8)
Multiple myeloma	1.2 (1.2–1.3)	0 (0–0)	0 (0–0)	0.2 (0.2–0.2)	3.0 (2.9–3.1)	11.5 (10.5–12.0)
Leukemia	4.1 (4.0–4.2)	1.9 (1.7–2.3)	1.7 (1.6–1.8)	1.9 (1.8–2.0)	6.9 (6.7–7.1)	25.9 (25.0–26.7)
Acute lymphoid leukemia	1.3 (1.2–1.4)	1.3 (1.1–1.6)	1.2 (1.1–1.3)	0.7 (0.7–0.8)	1.9 (1.8–2.1)	4.4 (4.2–4.7)
Chronic lymphoid leukemia	0.7 (0.7–0.8)	0 (0–0)	0 (0–0)	0.2 (0.2–0.2)	1.2 (1.1–1.3)	7.5 (7.2–7.9)
Acute myeloid leukemia	1.6 (1.6–1.7)	0.7 (0.5–0.8)	0.5 (0.5–0.6)	0.8 (0.7–0.8)	2.9 (2.8–3.0)	10.8 (10.4–11.1)
Chronic myeloid leukemia	0.4 (0.4–0.5)	0 (0–0)	0 (0–0)	0.2 (0.2–0.2)	0.9 (0.8–0.9)	3.1 (3.0–3.3)
Other neoplasms	4.7 (4.3–4.9)	1.8 (1.5–2.4)	0.9 (0.8–1.0)	2.1 (1.8–2.3)	9.6 (8.8–10.1)	30.1 (26.5–31.2)
Cardiovascular diseases	233.7 (229.4–238.1)	7.8 (7.3–8.4)	2.3 (2.1–2.5)	25.7 (24.8–26.8)	322.0 (313.9–331.5)	2863.3 (2808.2–2920.9)
Rheumatic heart disease	5.3 (4.8–5.6)	0.5 (0.4–0.6)	0.6 (0.5–0.7)	2.3 (2.0–2.5)	10.4 (9.5–11.2)	38.3 (36.3–40.7)
Ischemic heart disease	109.1 (106.9–111.7)	0.4 (0.3–0.4)	0.2 (0.1–0.2)	9.3 (8.9–9.8)	143.4 (139.5–148.0)	1387.9 (1359.4–1419.3)
Cerebrovascular disease	87.7 (85.7–89.9)	2.5 (2.2–2.8)	0.8 (0.7–0.8)	9.3 (9.0–9.9)	127.6 (123.4–131.9)	1059.7 (1035.4–1085.3)
Ischemic stroke	43.5 (41.5–45.5)	0.1 (0.1–0.1)	0.0 (0.0–0.1)	1.5 (1.3–1.7)	42.0 (38.3–45.8)	614.0 (587.7–636.9)
Hemorrhagic stroke	44.2 (42.1–46.8)	2.3 (2.1–2.6)	0.7 (0.7–0.8)	7.8 (7.4–8.3)	85.6 (81.2–90.7)	445.7 (422.7–476.2)
Hypertensive heart disease	13.8 (11.7–15.3)	0.1 (0.1–0.1)	0.0 (0.0–0.0)	1.6 (1.3–1.8)	21.0 (17.9–23.7)	163.2 (139.3–181.0)
Cardiomyopathy and myocarditis	4.2 (4.1–4.4)	1.8 (1.6–2.0)	0.3 (0.3–0.3)	1.3 (1.2–1.3)	5.3 (5.1–5.5)	42.8 (41.2–44.5)
Atrial fibrillation and flutter	2.7 (2.3–3.3)	0 (0–0)	0 (0–0)	0.0 (0.0–0.0)	0.8 (0.6–1.1)	44.4 (36.1–54.1)
Aortic aneurysm	1.5 (1.5–1.6)	0 (0–0)	0 (0–0)	0.2 (0.2–0.2)	2.0 (1.9–2.1)	19.2 (18.6–19.9)
Peripheral vascular disease	0.7 (0.6–0.7)	0 (0–0)	0 (0–0)	0.0 (0.0–0.0)	0.4 (0.4–0.4)	10.1 (9.4–10.9)
Endocarditis	1.2 (1.0–1.4)	0.6 (0.3–0.8)	0.1 (0.1–0.1)	0.4 (0.3–0.5)	1.4 (1.2–1.7)	12.4 (11.0–13.9)
Other cardiovascular and circulatory diseases	7.4 (7.2–7.8)	2.0 (1.8–2.3)	0.3 (0.3–0.3)	1.3 (1.2–1.4)	9.7 (9.1–10.3)	85.3 (81.6–89.1)
Chronic respiratory diseases	44.5 (42.9–46.1)	6.2 (5.6–6.8)	0.9 (0.8–1.0)	4.3 (4.1–4.6)	68.7 (65.5–72.0)	522.3 (501.8–541.2)
Chronic obstructive pulmonary disease	36.6 (34.9–38.2)	2.4 (1.7–3.0)	0.2 (0.1–0.2)	2.3 (2.0–2.5)	53.9 (50.4–57.5)	453.6 (433.5–473.4)
Pneumoconiosis	0.3 (0.2–0.4)	0 (0–0)	0 (0–0)	0.0 (0.0–0.1)	0.4 (0.2–0.6)	4.1 (3.0–5.3)
Silicosis	0.0 (0.0–0.0)	0 (0–0)	0 (0–0)	0.0 (0.0–0.0)	0.0 (0.0–0.0)	0.1 (0.1–0.2)
Asbestosis	0.0 (0.0–0.0)	0 (0–0)	0 (0–0)	0.0 (0.0–0.0)	0.0 (0.0–0.0)	0.1 (0.1–0.1)
Coal workers pneumoconiosis	0.0 (0.0–0.0)	0 (0–0)	0 (0–0)	0.0 (0.0–0.0)	0.0 (0.0–0.0)	0.0 (0.0–0.1)
Other pneumoconiosis	0.3 (0.2–0.4)	0 (0–0)	0 (0–0)	0.0 (0.0–0.0)	0.4 (0.2–0.5)	3.8 (2.8–5.0)
Asthma	5.8 (4.8–6.8)	1.8 (1.4–2.2)	0.6 (0.5–0.7)	1.7 (1.4–2.0)	11.9 (9.5–14.1)	47.0 (38.7–56.1)
Interstitial lung disease and pulmonary sarcoidosis	1.3 (1.0–1.4)	0.1 (0.0–0.1)	0.0 (0.0–0.0)	0.2 (0.1–0.2)	2.0 (1.6–2.3)	14.9 (10.2–16.7)
Other chronic respiratory diseases	0.5 (0.3–0.6)	1.9 (1.4–2.7)	0.1 (0.1–0.2)	0.1 (0.1–0.1)	0.4 (0.3–0.5)	2.7 (1.7–3.5)
Cirrhosis and other chronic liver diseases	12.1 (11.5–13.7)	1.7 (1.3–2.4)	0.7 (0.6–1.0)	4.8 (4.5–5.6)	31.6 (29.8–35.7)	73.0 (69.1–81.2)
Cirrhosis and other chronic liver diseases due to hepatitis B	3.0 (2.7–3.6)	0.0 (0.0–0.0)	0.0 (0.0–0.0)	1.1 (1.0–1.4)	7.9 (6.9–9.5)	20.0 (17.8–23.3)
Cirrhosis and other chronic liver diseases due to hepatitis C	2.8 (2.6–3.1)	0.0 (0.0–0.0)	0.0 (0.0–0.0)	1.0 (0.9–1.2)	7.4 (6.6–8.4)	18.5 (16.9–20.6)
Cirrhosis and other chronic liver diseases due to alcohol use	2.8 (2.5–3.1)	0 (0–0)	0 (0–0)	1.3 (1.2–1.5)	8.5 (7.6–9.7)	12.4 (11.0–14.1)
Cirrhosis and other chronic liver diseases due to other causes	3.5 (3.3–4.1)	1.7 (1.3–2.4)	0.7 (0.6–0.9)	1.4 (1.2–1.6)	7.8 (6.9–9.0)	22.0 (20.2–24.8)
Digestive diseases	15.1 (14.5–15.7)	4.5 (2.5–8.6)	1.3 (1.2–1.5)	4.2 (3.9–4.4)	24.0 (22.9–25.1)	144.3 (139.9–149.2)
Peptic ulcer disease	3.2 (3.0–3.5)	0.5 (0.2–1.3)	0.2 (0.2–0.3)	0.9 (0.8–1.0)	5.7 (5.2–6.3)	30.1 (28.1–32.7)
Gastritis and duodenitis	0.6 (0.4–0.9)	0.1 (0.0–0.2)	0.0 (0.0–0.1)	0.2 (0.1–0.2)	1.0 (0.6–1.5)	5.8 (3.8–8.1)
Appendicitis	0.7 (0.5–0.8)	0.9 (0.3–2.2)	0.2 (0.2–0.3)	0.4 (0.3–0.4)	1.0 (0.8–1.2)	3.4 (2.7–3.8)
Paralytic ileus and intestinal obstruction	3.2 (2.8–3.7)	1.6 (0.9–2.9)	0.4 (0.4–0.6)	1.0 (0.8–1.2)	5.0 (4.1–5.9)	29.4 (25.3–33.2)
Inguinal, femoral, and abdominal hernia	0.6 (0.4–0.7)	0.1 (0.0–0.3)	0.0 (0.0–0.1)	0.1 (0.1–0.2)	1.1 (0.7–1.3)	5.9 (4.2–6.9)
Inflammatory bowel disease	0.7 (0.6–0.8)	0.4 (0.2–0.9)	0.0 (0.0–0.1)	0.2 (0.2–0.3)	0.9 (0.8–1.0)	6.6 (5.9–7.0)
Vascular intestinal disorders	1.5 (1.4–1.7)	0.0 (0.0–0.1)	0.0 (0.0–0.0)	0.1 (0.1–0.2)	1.8 (1.6–2.2)	19.8 (18.2–21.7)
Gallbladder and biliary diseases	1.7 (1.6–1.8)	0.3 (0.1–0.7)	0.1 (0.1–0.1)	0.4 (0.3–0.4)	2.7 (2.5–2.9)	17.7 (16.8–19.0)
Pancreatitis	1.3 (1.2–1.4)	0.0 (0.0–0.1)	0.0 (0.0–0.1)	0.6 (0.5–0.7)	2.7 (2.4–3.0)	9.4 (8.7–10.1)
Other digestive diseases	1.5 (1.4–1.6)	0.5 (0.2–1.1)	0.1 (0.1–0.1)	0.3 (0.2–0.3)	2.0 (1.8–2.2)	16.4 (15.2–17.7)
Neurological disorders	33.4 (28.4–38.2)	2.5 (2.2–3.2)	1.0 (0.9–1.1)	1.7 (1.6–1.9)	13.9 (11.8–16.1)	508.9 (426.8–590.0)
Alzheimer disease and other dementias	29.4 (24.4–34.3)	0 (0–0)	0 (0–0)	0.0 (0.0–0.1)	8.5 (6.5–10.7)	478.0 (395.4–559.6)
Parkinson disease	1.3 (1.2–1.3)	0 (0–0)	0 (0–0)	0.0 (0.0–0.0)	0.8 (0.8–0.9)	19.3 (18.5–20.0)
Epilepsy	1.3 (1.3–1.4)	1.8 (1.5–2.3)	0.8 (0.7–0.9)	1.2 (1.1–1.3)	1.3 (1.3–1.4)	3.7 (3.5–3.9)

SR Table 13 - Global death rates (per 100,000) by age and cause for females in 2010

Cause	All ages	0-5 years	5-14 years	15-49 years	50-69 years	70+ years
Multiple sclerosis	0.3 (0.3–0.3)	0 (0–0)	0 (0–0)	0.2 (0.2–0.2)	0.9 (0.7–1.0)	1.2 (0.9–1.3)
Motor neuron disease	0.4 (0.4–0.4)	0.2 (0.1–0.2)	0.0 (0.0–0.0)	0.1 (0.1–0.1)	1.1 (1.1–1.2)	3.1 (3.0–3.2)
Migraine	0 (0–0)	0 (0–0)	0 (0–0)	0 (0–0)	0 (0–0)	0 (0–0)
Tension-type headache	0 (0–0)	0 (0–0)	0 (0–0)	0 (0–0)	0 (0–0)	0 (0–0)
Medication overuse headache	0 (0–0)	0 (0–0)	0 (0–0)	0 (0–0)	0 (0–0)	0 (0–0)
Other neurological disorders	0.6 (0.6–0.7)	0.5 (0.5–0.9)	0.2 (0.2–0.3)	0.3 (0.3–0.3)	1.2 (1.2–1.2)	3.7 (3.6–3.9)
Mental and substance use disorders	2.1 (2.0–2.1)	0.0 (0.0–0.0)	0.0 (0.0–0.0)	1.9 (1.8–2.0)	4.3 (4.0–4.4)	7.1 (5.8–7.6)
Schizophrenia	0.2 (0.2–0.2)	0 (0–0)	0 (0–0)	0.1 (0.1–0.1)	0.5 (0.5–0.5)	1.4 (1.4–1.5)
Alcohol use disorders	0.6 (0.6–0.7)	0 (0–0)	0 (0–0)	0.5 (0.4–0.5)	1.8 (1.8–1.9)	1.8 (1.7–1.9)
Drug use disorders	1.2 (1.1–1.3)	0.0 (0.0–0.0)	0 (0–0)	1.3 (1.2–1.4)	1.9 (1.7–2.0)	3.9 (2.6–4.4)
Opioid use disorders	0.8 (0.7–0.8)	0.0 (0.0–0.0)	0 (0–0)	0.8 (0.8–0.9)	1.3 (1.0–1.4)	2.6 (1.6–3.0)
Cocaine use disorders	0.1 (0.1–0.1)	0 (0–0)	0 (0–0)	0.1 (0.1–0.1)	0.2 (0.1–0.2)	0.1 (0.1–0.1)
Amphetamine use disorders	0.0 (0.0–0.1)	0 (0–0)	0 (0–0)	0.1 (0.0–0.1)	0.1 (0.1–0.1)	0.1 (0.1–0.1)
Cannabis use disorders	0 (0–0)	0 (0–0)	0 (0–0)	0 (0–0)	0 (0–0)	0 (0–0)
Other drug use disorders	0.3 (0.2–0.4)	0 (0–0)	0 (0–0)	0.3 (0.2–0.4)	0.4 (0.4–0.5)	1.2 (0.8–1.5)
Depressive disorders	0 (0–0)	0 (0–0)	0 (0–0)	0 (0–0)	0 (0–0)	0 (0–0)
Major depressive disorder	0 (0–0)	0 (0–0)	0 (0–0)	0 (0–0)	0 (0–0)	0 (0–0)
Dysthymia	0 (0–0)	0 (0–0)	0 (0–0)	0 (0–0)	0 (0–0)	0 (0–0)
Bipolar disorder	0 (0–0)	0 (0–0)	0 (0–0)	0 (0–0)	0 (0–0)	0 (0–0)
Anxiety disorders	0 (0–0)	0 (0–0)	0 (0–0)	0 (0–0)	0 (0–0)	0 (0–0)
Eating disorders	0.0 (0.0–0.0)	0 (0–0)	0.0 (0.0–0.0)	0.0 (0.0–0.0)	0 (0–0)	0 (0–0)
Anorexia nervosa	0.0 (0.0–0.0)	0 (0–0)	0.0 (0.0–0.0)	0.0 (0.0–0.0)	0 (0–0)	0 (0–0)
Bulimia nervosa	0.0 (0.0–0.0)	0 (0–0)	0.0 (0.0–0.0)	0.0 (0.0–0.0)	0 (0–0)	0 (0–0)
Autistic spectrum disorders	0 (0–0)	0 (0–0)	0 (0–0)	0 (0–0)	0 (0–0)	0 (0–0)
Autism	0 (0–0)	0 (0–0)	0 (0–0)	0 (0–0)	0 (0–0)	0 (0–0)
Asperger syndrome and other autistic spectrum disorders	0 (0–0)	0 (0–0)	0 (0–0)	0 (0–0)	0 (0–0)	0 (0–0)
Attention-deficit/hyperactivity disorder	0 (0–0)	0 (0–0)	0 (0–0)	0 (0–0)	0 (0–0)	0 (0–0)
Conduct disorder	0 (0–0)	0 (0–0)	0 (0–0)	0 (0–0)	0 (0–0)	0 (0–0)
Idiopathic developmental intellectual disability	0 (0–0)	0 (0–0)	0 (0–0)	0 (0–0)	0 (0–0)	0 (0–0)
Other mental and substance use disorders	0 (0–0)	0 (0–0)	0 (0–0)	0 (0–0)	0 (0–0)	0 (0–0)
Diabetes, urogenital, blood, and endocrine diseases	44.0 (42.8–45.4)	17.2 (13.2–25.5)	2.6 (2.4–2.9)	9.1 (8.7–9.6)	82.2 (79.4–85.3)	411.7 (400.9–423.5)
Diabetes mellitus	19.6 (18.8–20.3)	0.3 (0.2–0.4)	0.2 (0.2–0.3)	2.8 (2.7–3.0)	42.8 (40.8–44.7)	191.4 (183.9–198.6)
Acute glomerulonephritis	0.2 (0.1–0.2)	0.0 (0.0–0.1)	0.0 (0.0–0.0)	0.1 (0.0–0.1)	0.3 (0.2–0.3)	1.1 (0.7–1.3)
Chronic kidney disease	15.6 (15.1–16.2)	2.1 (1.9–2.3)	0.7 (0.7–0.8)	3.5 (3.4–3.7)	28.2 (27.2–29.4)	152.3 (147.2–158.2)
Chronic kidney disease due to diabetes mellitus	5.2 (5.0–5.4)	0.4 (0.3–0.4)	0.1 (0.1–0.1)	0.9 (0.8–1.0)	11.8 (11.0–12.7)	47.9 (45.5–50.6)
Chronic kidney disease due to hypertension	7.1 (6.7–7.4)	0.7 (0.6–0.8)	0.2 (0.2–0.3)	1.4 (1.3–1.5)	11.2 (10.4–12.0)	75.5 (71.8–79.3)
Chronic kidney disease due to glomerulonephritis	3.0 (2.8–3.1)	0.7 (0.6–0.8)	0.3 (0.3–0.4)	1.1 (1.0–1.2)	4.9 (4.5–5.4)	25.4 (23.6–27.3)
Chronic kidney disease due to other causes	0.4 (0.3–0.5)	0.3 (0.2–0.3)	0.1 (0.1–0.1)	0.2 (0.1–0.2)	0.3 (0.2–0.5)	3.5 (3.0–4.4)
Urinary diseases and male infertility	3.5 (3.3–3.7)	0.9 (0.7–1.0)	0.2 (0.1–0.2)	0.8 (0.7–0.9)	4.9 (4.6–5.3)	37.5 (34.9–39.1)
Interstitial nephritis and urinary tract infections	2.9 (2.7–3.0)	0.7 (0.5–0.8)	0.1 (0.1–0.1)	0.6 (0.5–0.6)	3.6 (3.3–3.9)	32.5 (30.1–34.1)
Urolithiasis	0.2 (0.2–0.2)	0 (0–0)	0.0 (0.0–0.0)	0.1 (0.1–0.1)	0.4 (0.3–0.5)	1.7 (1.5–2.1)
Benign prostatic hyperplasia	0 (0–0)	0 (0–0)	0 (0–0)	0 (0–0)	0 (0–0)	0 (0–0)
Male infertility	0 (0–0)	0 (0–0)	0 (0–0)	0 (0–0)	0 (0–0)	0 (0–0)
Other urinary diseases	0.4 (0.4–0.5)	0.2 (0.1–0.3)	0.0 (0.0–0.0)	0.1 (0.1–0.2)	0.9 (0.8–1.0)	3.3 (2.9–4.0)
Gynecological diseases	0.2 (0.2–0.3)	0 (0–0)	0 (0–0)	0.2 (0.1–0.2)	0.4 (0.3–0.5)	1.1 (0.8–1.2)
Uterine fibroids	0.1 (0.0–0.1)	0 (0–0)	0 (0–0)	0.0 (0.0–0.1)	0.2 (0.1–0.2)	0.3 (0.1–0.4)
Polycystic ovarian syndrome	0.0 (0.0–0.0)	0 (0–0)	0 (0–0)	0.0 (0.0–0.0)	0.0 (0.0–0.1)	0 (0–0)
Female infertility	0 (0–0)	0 (0–0)	0 (0–0)	0 (0–0)	0 (0–0)	0 (0–0)
Endometriosis	0.0 (0.0–0.0)	0 (0–0)	0 (0–0)	0.0 (0.0–0.0)	0.0 (0.0–0.0)	0 (0–0)
Genital prolapse	0.0 (0.0–0.0)	0 (0–0)	0 (0–0)	0.0 (0.0–0.0)	0.1 (0.0–0.1)	0.3 (0.2–0.4)
Premenstrual syndrome	0 (0–0)	0 (0–0)	0 (0–0)	0 (0–0)	0 (0–0)	0 (0–0)
Other gynecological diseases	0.1 (0.1–0.1)	0 (0–0)	0 (0–0)	0.1 (0.1–0.1)	0.2 (0.1–0.2)	0.5 (0.4–0.6)
Hemoglobinopathies and hemolytic anemias	3.0 (2.6–3.7)	12.2 (8.4–20.5)	1.2 (1.0–1.4)	1.1 (1.0–1.2)	2.5 (2.4–2.7)	12.5 (11.8–13.1)
Thalassemias	0.3 (0.2–0.3)	1.4 (1.0–2.0)	0.3 (0.2–0.3)	0.1 (0.1–0.1)	0.0 (0.0–0.0)	1.1 (0.9–1.3)
Thalassemias trait	0 (0–0)	0 (0–0)	0 (0–0)	0 (0–0)	0 (0–0)	0 (0–0)
Sickle cell disorders	1.5 (1.1–2.2)	10.7 (7.0–18.7)	0.9 (0.7–1.1)	0.5 (0.4–0.5)	0.1 (0.1–0.2)	1.4 (1.2–1.6)
Sickle cell trait	0 (0–0)	0 (0–0)	0 (0–0)	0 (0–0)	0 (0–0)	0 (0–0)

SR Table 13 - Global death rates (per 100,000) by age and cause for females in 2010						
Cause	All ages	0-5 years	5-14 years	15-49 years	50-69 years	70+ years
G6PD deficiency	0.3 (0.2–0.3)	0.1 (0.1–0.1)	0.1 (0.0–0.1)	0.3 (0.2–0.3)	0.5 (0.4–0.6)	0.3 (0.3–0.4)
G6PD trait	0 (0–0)	0 (0–0)	0 (0–0)	0 (0–0)	0 (0–0)	0 (0–0)
Other hemoglobinopathies and hemolytic anemias	1.0 (1.0–1.1)	0.0 (0.0–0.1)	0.0 (0.0–0.0)	0.3 (0.3–0.3)	1.9 (1.8–2.0)	9.7 (9.0–10.1)
Endocrine, metabolic, blood, and immune disorders	1.9 (1.9–2.0)	1.7 (1.5–1.9)	0.3 (0.2–0.3)	0.6 (0.6–0.6)	3.1 (3.0–3.2)	15.8 (15.3–16.4)
Musculoskeletal disorders	1.6 (1.5–1.8)	0 (0–0)	0.2 (0.2–0.2)	0.7 (0.7–0.8)	2.5 (2.3–2.8)	13.9 (12.6–14.6)
Rheumatoid arthritis	0.6 (0.5–0.7)	0 (0–0)	0.0 (0.0–0.0)	0.1 (0.1–0.1)	1.0 (0.9–1.2)	5.8 (5.2–6.9)
Osteoarthritis	0 (0–0)	0 (0–0)	0 (0–0)	0 (0–0)	0 (0–0)	0 (0–0)
Low back and neck pain	0 (0–0)	0 (0–0)	0 (0–0)	0 (0–0)	0 (0–0)	0 (0–0)
Low back pain	0 (0–0)	0 (0–0)	0 (0–0)	0 (0–0)	0 (0–0)	0 (0–0)
Neck pain	0 (0–0)	0 (0–0)	0 (0–0)	0 (0–0)	0 (0–0)	0 (0–0)
Gout	0 (0–0)	0 (0–0)	0 (0–0)	0 (0–0)	0 (0–0)	0 (0–0)
Other musculoskeletal disorders	1.1 (1.0–1.2)	0 (0–0)	0.2 (0.2–0.2)	0.7 (0.6–0.7)	1.4 (1.3–1.6)	8.1 (6.6–8.7)
Other non-communicable diseases	10.3 (8.7–12.1)	81.8 (68.7–99.7)	3.2 (2.7–3.9)	1.5 (1.3–1.7)	3.0 (2.2–3.8)	17.4 (11.7–21.4)
Congenital anomalies	8.7 (7.3–10.4)	77.7 (65.0–95.7)	3.1 (2.6–3.8)	1.3 (1.1–1.5)	1.4 (1.1–1.5)	3.0 (2.3–3.3)
Neural tube defects	1.0 (0.6–1.5)	10.3 (6.6–15.4)	0.2 (0.1–0.3)	0.0 (0.0–0.0)	0.0 (0.0–0.0)	0.0 (0.0–0.0)
Congenital heart anomalies	4.3 (3.3–5.5)	36.5 (27.2–49.1)	2.2 (1.7–2.6)	0.9 (0.7–1.0)	0.4 (0.3–0.5)	0.9 (0.8–1.0)
Cleft lip and cleft palate	0.0 (0.0–0.0)	0.3 (0.2–0.4)	0 (0–0)	0 (0–0)	0 (0–0)	0 (0–0)
Down syndrome	0.5 (0.2–0.8)	3.6 (1.2–7.6)	0.1 (0.0–0.1)	0.1 (0.1–0.2)	0.3 (0.2–0.3)	0.1 (0.0–0.1)
Turner syndrome	0 (0–0)	0 (0–0)	0 (0–0)	0 (0–0)	0 (0–0)	0 (0–0)
Klinefelter syndrome	0 (0–0)	0 (0–0)	0 (0–0)	0 (0–0)	0 (0–0)	0 (0–0)
Other chromosomal abnormalities	0.4 (0.2–1.0)	4.3 (1.8–10.1)	0.0 (0.0–0.1)	0.0 (0.0–0.0)	0.0 (0.0–0.0)	0.0 (0.0–0.0)
Other congenital anomalies	2.5 (2.1–3.4)	22.8 (18.3–31.6)	0.7 (0.6–1.0)	0.3 (0.2–0.3)	0.6 (0.5–0.7)	2.0 (1.4–2.2)
Skin and subcutaneous diseases	1.3 (0.8–1.8)	1.1 (0.6–1.9)	0.1 (0.0–0.2)	0.2 (0.1–0.4)	1.6 (0.9–2.5)	14.4 (8.9–18.6)
Dermatitis	0 (0–0)	0 (0–0)	0 (0–0)	0 (0–0)	0 (0–0)	0 (0–0)
Psoriasis	0 (0–0)	0 (0–0)	0 (0–0)	0 (0–0)	0 (0–0)	0 (0–0)
Cellulitis	0.2 (0.1–0.3)	0.1 (0.0–0.2)	0.0 (0.0–0.0)	0.0 (0.0–0.1)	0.4 (0.2–0.6)	2.0 (1.1–2.7)
Pyoderma	0.6 (0.4–0.9)	1.0 (0.5–1.7)	0.1 (0.0–0.1)	0.1 (0.1–0.2)	0.8 (0.5–1.4)	4.9 (3.3–7.2)
Scabies	0 (0–0)	0 (0–0)	0 (0–0)	0 (0–0)	0 (0–0)	0 (0–0)
Fungal skin diseases	0 (0–0)	0 (0–0)	0 (0–0)	0 (0–0)	0 (0–0)	0 (0–0)
Viral skin diseases	0 (0–0)	0 (0–0)	0 (0–0)	0 (0–0)	0 (0–0)	0 (0–0)
Acne vulgaris	0 (0–0)	0 (0–0)	0 (0–0)	0 (0–0)	0 (0–0)	0 (0–0)
Alopecia areata	0 (0–0)	0 (0–0)	0 (0–0)	0 (0–0)	0 (0–0)	0 (0–0)
Pruritus	0 (0–0)	0 (0–0)	0 (0–0)	0 (0–0)	0 (0–0)	0 (0–0)
Urticaria	0 (0–0)	0 (0–0)	0 (0–0)	0 (0–0)	0 (0–0)	0 (0–0)
Decubitus ulcer	0.5 (0.3–0.6)	0.0 (0.0–0.0)	0.0 (0.0–0.0)	0.0 (0.0–0.0)	0.3 (0.2–0.5)	6.9 (3.8–8.7)
Other skin and subcutaneous diseases	0.1 (0.0–0.1)	0.0 (0.0–0.1)	0.0 (0.0–0.0)	0.0 (0.0–0.0)	0.1 (0.0–0.1)	0.5 (0.4–0.8)
Sense organ diseases	0 (0–0)	0 (0–0)	0 (0–0)	0 (0–0)	0 (0–0)	0 (0–0)
Glaucoma	0 (0–0)	0 (0–0)	0 (0–0)	0 (0–0)	0 (0–0)	0 (0–0)
Cataract	0 (0–0)	0 (0–0)	0 (0–0)	0 (0–0)	0 (0–0)	0 (0–0)
Macular degeneration	0 (0–0)	0 (0–0)	0 (0–0)	0 (0–0)	0 (0–0)	0 (0–0)
Refraction and accommodation disorders	0 (0–0)	0 (0–0)	0 (0–0)	0 (0–0)	0 (0–0)	0 (0–0)
Age-related and other hearing loss	0 (0–0)	0 (0–0)	0 (0–0)	0 (0–0)	0 (0–0)	0 (0–0)
Other vision loss	0 (0–0)	0 (0–0)	0 (0–0)	0 (0–0)	0 (0–0)	0 (0–0)
Other sense organ diseases	0 (0–0)	0 (0–0)	0 (0–0)	0 (0–0)	0 (0–0)	0 (0–0)
Oral disorders	0 (0–0)	0 (0–0)	0 (0–0)	0 (0–0)	0 (0–0)	0 (0–0)
Deciduous caries	0 (0–0)	0 (0–0)	0 (0–0)	0 (0–0)	0 (0–0)	0 (0–0)
Permanent caries	0 (0–0)	0 (0–0)	0 (0–0)	0 (0–0)	0 (0–0)	0 (0–0)
Periodontal diseases	0 (0–0)	0 (0–0)	0 (0–0)	0 (0–0)	0 (0–0)	0 (0–0)
Edentulism and severe tooth loss	0 (0–0)	0 (0–0)	0 (0–0)	0 (0–0)	0 (0–0)	0 (0–0)
Other oral disorders	0 (0–0)	0 (0–0)	0 (0–0)	0 (0–0)	0 (0–0)	0 (0–0)
Sudden infant death syndrome	0.3 (0.2–0.4)	3.0 (2.2–4.2)	0 (0–0)	0 (0–0)	0 (0–0)	0 (0–0)
Injuries	42.8 (37.9–45.8)	48.4 (39.0–56.3)	17.5 (14.7–20.4)	31.0 (27.7–33.4)	53.1 (47.5–56.4)	183.3 (162.9–191.6)
Transport injuries	10.7 (10.3–11.1)	8.1 (6.9–9.6)	4.5 (4.2–4.8)	8.9 (8.6–9.3)	16.3 (15.7–16.9)	33.6 (32.4–34.8)
Road injuries	10.1 (9.8–10.5)	7.4 (6.3–8.9)	4.3 (4.0–4.5)	8.4 (8.1–8.8)	15.4 (14.8–16.0)	31.8 (30.7–33.0)
Pedestrian road injuries	4.5 (4.4–4.8)	3.3 (2.8–3.9)	2.0 (1.9–2.2)	3.3 (3.1–3.4)	7.4 (7.1–7.9)	17.2 (16.4–18.3)
Cyclist road injuries	0.4 (0.4–0.5)	0.2 (0.1–0.2)	0.2 (0.2–0.2)	0.4 (0.4–0.4)	0.8 (0.8–0.9)	1.0 (0.9–1.1)
Motorcyclist road injuries	1.5 (1.4–1.6)	1.0 (0.8–1.2)	0.5 (0.4–0.6)	1.4 (1.3–1.6)	2.2 (2.0–2.5)	3.9 (3.5–4.5)

SR Table 13 - Global death rates (per 100,000) by age and cause for females in 2010						
Cause	All ages	0-5 years	5-14 years	15-49 years	50-69 years	70+ years
Motor vehicle road injuries	3.5 (3.3–3.7)	2.9 (2.4–3.6)	1.5 (1.4–1.6)	3.2 (3.0–3.5)	4.7 (4.2–5.0)	9.3 (8.3–9.9)
Other road injuries	0.1 (0.1–0.1)	0.1 (0.1–0.2)	0.1 (0.0–0.1)	0.1 (0.1–0.1)	0.2 (0.2–0.2)	0.4 (0.3–0.4)
Other transport injuries	0.6 (0.5–0.7)	0.6 (0.4–0.9)	0.2 (0.2–0.3)	0.5 (0.4–0.5)	0.9 (0.8–1.1)	1.8 (1.6–2.1)
Unintentional injuries	19.3 (15.7–20.5)	34.7 (25.6–41.8)	8.3 (6.7–9.1)	8.9 (7.1–9.6)	19.3 (16.1–20.7)	119.1 (100.3–124.9)
Falls	5.6 (4.6–5.9)	3.5 (2.1–5.0)	0.9 (0.8–1.1)	1.0 (0.9–1.1)	4.9 (4.4–5.2)	64.1 (53.2–67.9)
Drowning	3.2 (1.9–3.6)	9.2 (4.3–11.5)	3.1 (2.0–3.6)	1.7 (1.0–2.0)	2.7 (1.6–3.1)	9.1 (6.1–9.9)
Fire, heat, and hot substances	2.6 (1.9–2.9)	4.1 (2.8–4.9)	0.9 (0.7–1.0)	2.1 (1.4–2.4)	2.4 (1.8–2.7)	10.0 (8.1–11.0)
Poisonings	1.1 (0.7–1.3)	2.5 (1.0–4.6)	0.4 (0.3–0.5)	0.7 (0.5–0.8)	1.2 (0.9–1.3)	3.2 (2.5–3.4)
Exposure to mechanical forces	1.6 (1.3–1.7)	4.1 (3.3–4.8)	0.7 (0.6–0.8)	0.8 (0.6–0.9)	1.8 (1.5–1.9)	7.2 (5.0–7.8)
Unintentional firearm injuries	0.3 (0.2–0.3)	0.2 (0.1–0.3)	0.1 (0.1–0.1)	0.1 (0.1–0.2)	0.2 (0.2–0.3)	1.8 (1.4–1.9)
Unintentional suffocation	0.4 (0.2–0.4)	2.2 (1.4–2.6)	0.2 (0.1–0.2)	0.1 (0.1–0.1)	0.2 (0.1–0.2)	1.0 (0.6–1.3)
Other exposure to mechanical forces	1.0 (0.8–1.1)	1.7 (1.5–2.1)	0.4 (0.4–0.5)	0.6 (0.4–0.6)	1.4 (1.2–1.5)	4.4 (2.7–4.9)
Adverse effects of medical treatment	1.2 (0.9–1.4)	2.0 (1.2–2.7)	0.3 (0.2–0.4)	0.5 (0.3–0.6)	1.5 (1.1–1.8)	8.1 (6.5–9.1)
Animal contact	1.2 (0.6–1.5)	1.9 (1.0–2.3)	0.8 (0.5–1.1)	0.9 (0.5–1.1)	1.8 (0.9–2.2)	2.7 (1.5–3.1)
Venomous animal contact	1.1 (0.5–1.3)	1.4 (0.7–1.7)	0.7 (0.4–1.0)	0.8 (0.4–1.1)	1.6 (0.8–2.0)	2.2 (1.1–2.6)
Non-venomous animal contact	0.2 (0.1–0.2)	0.5 (0.3–0.7)	0.1 (0.1–0.2)	0.1 (0.1–0.1)	0.2 (0.1–0.2)	0.5 (0.4–0.6)
Foreign body	1.7 (1.4–2.2)	5.8 (3.7–9.6)	0.6 (0.4–0.8)	0.5 (0.4–0.6)	1.4 (1.2–1.6)	10.0 (9.0–10.7)
Pulmonary aspiration and foreign body in airway	1.4 (1.1–1.9)	5.2 (2.9–9.1)	0.4 (0.3–0.7)	0.3 (0.2–0.4)	1.1 (0.9–1.3)	8.5 (7.7–9.1)
Foreign body in eyes	0 (0–0)	0 (0–0)	0 (0–0)	0 (0–0)	0 (0–0)	0 (0–0)
Foreign body in other body part	0.3 (0.2–0.4)	0.6 (0.3–1.0)	0.2 (0.1–0.2)	0.2 (0.1–0.2)	0.3 (0.2–0.4)	1.5 (1.2–1.8)
Environmental heat and cold exposure	0.4 (0.3–0.5)	0.3 (0.2–0.4)	0.1 (0.0–0.1)	0.2 (0.1–0.2)	0.8 (0.6–0.9)	2.8 (1.9–3.3)
Other unintentional injuries	0.7 (0.7–0.8)	1.3 (1.1–1.5)	0.5 (0.4–0.6)	0.5 (0.4–0.5)	0.9 (0.8–0.9)	2.0 (1.8–2.2)
Self-harm and interpersonal violence	9.8 (7.9–10.4)	1.7 (1.5–1.9)	1.4 (1.2–1.5)	10.6 (8.6–11.3)	14.5 (11.5–15.4)	26.1 (21.1–27.6)
Self-harm	7.4 (5.6–7.9)	0 (0–0)	0.6 (0.5–0.7)	8.0 (5.9–8.6)	12.0 (9.0–12.9)	21.3 (16.4–22.7)
Interpersonal violence	2.3 (2.2–2.5)	1.7 (1.5–1.9)	0.8 (0.7–0.8)	2.7 (2.5–2.8)	2.5 (2.3–2.7)	4.8 (4.5–5.1)
Assault by firearm	0.6 (0.5–0.7)	0.3 (0.2–0.3)	0.2 (0.2–0.2)	0.8 (0.6–0.9)	0.5 (0.5–0.7)	0.9 (0.7–1.1)
Assault by sharp object	0.5 (0.5–0.6)	0.2 (0.1–0.2)	0.1 (0.1–0.1)	0.6 (0.6–0.7)	0.5 (0.5–0.6)	1.1 (1.0–1.3)
Assault by other means	1.2 (1.1–1.3)	1.3 (1.1–1.4)	0.5 (0.4–0.5)	1.2 (1.1–1.4)	1.4 (1.3–1.5)	2.7 (2.4–3.0)
Forces of nature, war, and legal intervention	3.0 (0.8–5.4)	3.9 (1.2–6.7)	3.4 (0.8–6.1)	2.6 (0.7–4.7)	3.0 (0.8–5.3)	4.6 (1.3–8.2)
Exposure to forces of nature	2.8 (0.6–5.2)	3.3 (0.7–6.2)	3.2 (0.6–6.0)	2.4 (0.5–4.5)	2.7 (0.6–5.0)	4.2 (1.0–7.9)
Collective violence and legal intervention	0.3 (0.2–0.3)	0.6 (0.4–0.7)	0.2 (0.1–0.2)	0.2 (0.2–0.3)	0.3 (0.2–0.3)	0.3 (0.2–1.1)

SR Table 13 - Global death rates (per 100,000) by age and cause for females in 2015						
Cause	All ages	0-5 years	5-14 years	15-49 years	50-69 years	70+ years
All causes	682.3 (670.2–696.2)	831.4 (809.6–853.8)	62.2 (60.6–64.1)	164.5 (157.8–172.2)	873.6 (848.4–902.9)	5832.3 (5750.5–5916.6)
Communicable, maternal, neonatal, and nutritional diseases	144.2 (138.9–149.3)	672.7 (651.5–694.9)	34.6 (32.7–36.8)	61.2 (57.3–65.7)	95.3 (87.2–103.5)	499.1 (458.5–526.0)
HIV/AIDS and tuberculosis	25.7 (23.4–28.3)	16.6 (15.6–18.2)	8.1 (7.7–8.7)	28.3 (26.3–30.6)	30.5 (25.8–35.0)	51.2 (39.0–61.7)
Tuberculosis	10.5 (8.3–12.9)	3.2 (2.4–4.6)	1.1 (0.9–1.6)	6.4 (5.3–7.9)	21.5 (16.6–25.9)	48.9 (36.7–59.5)
HIV/AIDS	15.2 (14.3–16.2)	13.4 (12.7–14.1)	7.0 (6.7–7.3)	21.8 (20.2–23.6)	9.0 (8.3–9.9)	2.3 (2.0–2.7)
HIV/AIDS - Tuberculosis	2.6 (1.9–3.0)	2.7 (2.1–3.1)	1.2 (0.9–1.5)	3.5 (2.6–4.1)	1.9 (1.5–2.2)	0.5 (0.4–0.6)
HIV/AIDS resulting in other diseases	12.6 (11.6–13.6)	10.7 (9.9–11.6)	5.7 (5.3–6.2)	18.4 (16.6–20.2)	7.1 (6.4–8.0)	1.8 (1.5–2.2)
Diarrhea, lower respiratory, and other common infectious diseases	64.4 (60.7–67.8)	238.6 (222.2–257.8)	19.6 (17.5–22.0)	12.6 (11.3–14.2)	51.0 (45.7–55.8)	397.9 (361.5–421.8)
Diarrheal diseases	17.5 (16.3–18.8)	74.5 (65.0–85.5)	3.9 (3.4–4.5)	3.6 (3.3–4.1)	16.5 (15.0–18.3)	88.2 (81.8–94.6)
Intestinal infectious diseases	2.3 (1.3–3.7)	6.0 (3.2–10.6)	5.1 (3.0–8.4)	1.4 (0.7–2.4)	0.7 (0.4–1.3)	1.0 (0.5–1.5)
Typhoid fever	1.9 (1.0–3.1)	5.1 (2.6–9.2)	4.3 (2.4–7.0)	1.1 (0.6–2.0)	0.6 (0.3–1.0)	0.8 (0.4–1.2)
Paratyphoid fever	0.4 (0.2–0.7)	0.9 (0.4–1.9)	0.8 (0.4–1.5)	0.2 (0.1–0.5)	0.1 (0.1–0.3)	0.2 (0.1–0.3)
Other intestinal infectious diseases	0.0 (0.0–0.0)	0.1 (0.0–0.1)	0.0 (0.0–0.0)	0.0 (0.0–0.0)	0.0 (0.0–0.0)	0.0 (0.0–0.1)
Lower respiratory infections	35.5 (31.6–38.0)	103.8 (94.4–114.9)	5.4 (3.8–6.6)	4.8 (3.8–5.6)	27.6 (22.0–31.1)	291.8 (254.2–313.1)
Upper respiratory infections	0.0 (0.0–0.0)	0.1 (0.1–0.2)	0.0 (0.0–0.0)	0.0 (0.0–0.0)	0.0 (0.0–0.1)	0.2 (0.2–0.3)
Otitis media	0.0 (0.0–0.1)	0.1 (0.1–0.2)	0.0 (0.0–0.0)	0.0 (0.0–0.0)	0.0 (0.0–0.0)	0.2 (0.1–0.4)
Meningitis	4.6 (3.9–5.7)	24.7 (18.8–35.2)	2.6 (2.1–3.1)	1.6 (1.4–1.9)	3.5 (2.8–4.2)	9.0 (7.4–10.2)
Pneumococcal meningitis	1.4 (1.1–1.8)	6.6 (4.4–10.3)	1.0 (0.7–1.3)	0.6 (0.4–0.7)	1.0 (0.7–1.3)	2.6 (2.0–3.2)
H influenzae type B meningitis	0.9 (0.7–1.2)	6.4 (4.4–9.5)	0.5 (0.3–0.7)	0.2 (0.1–0.3)	0.4 (0.3–0.6)	1.2 (0.9–1.5)
Meningococcal meningitis	0.9 (0.7–1.2)	5.3 (3.5–8.6)	0.5 (0.3–0.8)	0.3 (0.2–0.4)	0.6 (0.4–0.8)	1.3 (1.0–1.7)
Other meningitis	1.5 (1.2–1.8)	6.4 (4.4–9.2)	0.6 (0.4–0.9)	0.6 (0.5–0.7)	1.5 (1.2–1.9)	3.9 (3.1–4.7)
Encephalitis	1.9 (1.6–2.3)	6.6 (5.3–7.9)	1.2 (1.0–1.6)	0.7 (0.6–0.9)	1.8 (1.6–2.5)	6.0 (5.3–7.4)
Diphtheria	0.0 (0.0–0.1)	0.2 (0.1–0.8)	0.0 (0.0–0.1)	0.0 (0.0–0.0)	0.0 (0.0–0.0)	0 (0–0)
Whooping cough	0.9 (0.3–1.9)	9.3 (3.2–19.7)	0.3 (0.1–0.7)	0.0 (0.0–0.0)	0.0 (0.0–0.0)	0 (0–0)
Tetanus	0.6 (0.5–0.9)	3.4 (2.7–4.3)	0.2 (0.1–0.3)	0.2 (0.2–0.4)	0.6 (0.4–1.2)	1.0 (0.7–1.7)
Measles	1.0 (0.4–2.2)	9.5 (3.4–20.6)	0.8 (0.3–1.8)	0.0 (0.0–0.1)	0.0 (0.0–0.0)	0 (0–0)
Varicella and herpes zoster	0.1 (0.1–0.1)	0.3 (0.2–0.5)	0.0 (0.0–0.1)	0.0 (0.0–0.0)	0.1 (0.1–0.1)	0.6 (0.4–0.9)
Neglected tropical diseases and malaria	11.3 (8.9–13.7)	78.0 (56.7–100.8)	4.1 (2.7–6.2)	3.7 (2.8–5.1)	7.0 (5.2–9.7)	9.9 (7.5–13.3)
Malaria	10.1 (7.7–12.5)	74.9 (53.3–97.4)	3.1 (1.7–5.2)	3.0 (2.0–4.3)	5.6 (3.8–8.4)	7.0 (4.7–10.3)
Chagas disease	0.1 (0.1–0.1)	0.0 (0.0–0.0)	0.0 (0.0–0.0)	0.0 (0.0–0.0)	0.2 (0.2–0.2)	0.9 (0.8–1.0)
Leishmaniasis	0.2 (0.1–0.2)	0.4 (0.3–0.6)	0.1 (0.1–0.2)	0.2 (0.1–0.2)	0.1 (0.1–0.1)	0.1 (0.1–0.1)
Visceral leishmaniasis	0.2 (0.1–0.2)	0.4 (0.3–0.6)	0.1 (0.1–0.2)	0.2 (0.1–0.2)	0.1 (0.1–0.1)	0.1 (0.1–0.1)
Cutaneous and mucocutaneous leishmaniasis	0 (0–0)	0 (0–0)	0 (0–0)	0 (0–0)	0 (0–0)	0 (0–0)
African trypanosomiasis	0.0 (0.0–0.1)	0.0 (0.0–0.1)	0.0 (0.0–0.1)	0.0 (0.0–0.1)	0.0 (0.0–0.1)	0.0 (0.0–0.0)
Schistosomiasis	0.1 (0.0–0.1)	0.0 (0.0–0.0)	0.0 (0.0–0.0)	0.0 (0.0–0.0)	0.1 (0.1–0.2)	0.2 (0.2–0.3)
Cysticercosis	0.0 (0.0–0.0)	0.0 (0.0–0.0)	0.0 (0.0–0.0)	0.0 (0.0–0.0)	0.0 (0.0–0.0)	0.0 (0.0–0.0)
Cystic echinococcosis	0.0 (0.0–0.0)	0.0 (0.0–0.0)	0.0 (0.0–0.0)	0.0 (0.0–0.0)	0.0 (0.0–0.0)	0.1 (0.1–0.1)
Lymphatic filariasis	0 (0–0)	0 (0–0)	0 (0–0)	0 (0–0)	0 (0–0)	0 (0–0)
Onchocerciasis	0 (0–0)	0 (0–0)	0 (0–0)	0 (0–0)	0 (0–0)	0 (0–0)
Trachoma	0 (0–0)	0 (0–0)	0 (0–0)	0 (0–0)	0 (0–0)	0 (0–0)
Dengue	0.2 (0.1–0.3)	0.7 (0.4–1.1)	0.3 (0.2–0.5)	0.1 (0.1–0.2)	0.2 (0.1–0.2)	0.4 (0.2–0.5)
Yellow fever	0.0 (0.0–0.1)	0.0 (0.0–0.1)	0.1 (0.0–0.2)	0.0 (0.0–0.1)	0.0 (0.0–0.1)	0.0 (0.0–0.0)
Rabies	0.2 (0.1–0.2)	0.3 (0.2–0.5)	0.2 (0.1–0.3)	0.1 (0.1–0.2)	0.3 (0.2–0.3)	0.4 (0.3–0.4)
Intestinal nematode infections	0.0 (0.0–0.0)	0.3 (0.2–0.3)	0.0 (0.0–0.0)	0.0 (0.0–0.0)	0.0 (0.0–0.0)	0.0 (0.0–0.0)
Ascariasis	0.0 (0.0–0.0)	0.3 (0.2–0.3)	0.0 (0.0–0.0)	0.0 (0.0–0.0)	0.0 (0.0–0.0)	0.0 (0.0–0.0)
Trichuriasis	0 (0–0)	0 (0–0)	0 (0–0)	0 (0–0)	0 (0–0)	0 (0–0)
Hookworm disease	0 (0–0)	0 (0–0)	0 (0–0)	0 (0–0)	0 (0–0)	0 (0–0)
Food-borne trematodiases	0 (0–0)	0 (0–0)	0 (0–0)	0 (0–0)	0 (0–0)	0 (0–0)
Leprosy	0 (0–0)	0 (0–0)	0 (0–0)	0 (0–0)	0 (0–0)	0 (0–0)
Ebola	0.1 (0.1–0.1)	0.1 (0.1–0.1)	0.1 (0.0–0.1)	0.1 (0.1–0.1)	0.1 (0.1–0.1)	0.0 (0.0–0.1)
Other neglected tropical diseases	0.3 (0.2–0.4)	1.2 (0.9–1.8)	0.2 (0.1–0.3)	0.1 (0.1–0.2)	0.3 (0.2–0.5)	0.7 (0.4–1.1)
Maternal disorders	7.5 (6.7–8.6)	0 (0–0)	0.2 (0.2–0.3)	14.6 (12.9–16.8)	0.2 (0.1–0.3)	0 (0–0)
Maternal hemorrhage	2.3 (1.8–2.8)	0 (0–0)	0.1 (0.0–0.1)	4.4 (3.6–5.4)	0.1 (0.0–0.1)	0 (0–0)
Maternal sepsis and other maternal infections	0.5 (0.4–0.7)	0 (0–0)	0.0 (0.0–0.0)	1.0 (0.7–1.3)	0.0 (0.0–0.0)	0 (0–0)
Maternal hypertensive disorders	1.3 (1.0–1.6)	0 (0–0)	0.0 (0.0–0.1)	2.5 (2.0–3.2)	0.0 (0.0–0.0)	0 (0–0)
Maternal obstructed labor and uterine rupture	0.6 (0.5–0.8)	0 (0–0)	0.0 (0.0–0.0)	1.2 (0.9–1.6)	0.0 (0.0–0.0)	0 (0–0)
Maternal abortion, miscarriage, and ectopic pregnancy	0.9 (0.7–1.1)	0 (0–0)	0.0 (0.0–0.0)	1.7 (1.3–2.1)	0.0 (0.0–0.0)	0 (0–0)

SR Table 13 - Global death rates (per 100,000) by age and cause for females in 2015						
**Cause**	**All ages**	**0-5 years**	**5-14 years**	**15-49 years**	**50-69 years**	**70+ years**
Indirect maternal deaths	0.8 (0.6–1.1)	0 (0–0)	0.0 (0.0–0.0)	1.6 (1.2–2.2)	0.0 (0.0–0.0)	0 (0–0)
Late maternal deaths	0.2 (0.1–0.3)	0 (0–0)	0.0 (0.0–0.0)	0.4 (0.2–0.5)	0.0 (0.0–0.0)	0 (0–0)
Maternal deaths aggravated by HIV/AIDS	0.1 (0.0–0.1)	0 (0–0)	0.0 (0.0–0.0)	0.1 (0.1–0.2)	0.0 (0.0–0.0)	0 (0–0)
Other maternal disorders	0.9 (0.7–1.1)	0 (0–0)	0.0 (0.0–0.0)	1.7 (1.4–2.2)	0.0 (0.0–0.0)	0 (0–0)
Neonatal disorders	26.1 (25.2–27.0)	294.1 (284.2–304.5)	0 (0–0)	0 (0–0)	0 (0–0)	0 (0–0)
Neonatal preterm birth complications	9.9 (8.8–11.2)	111.9 (98.7–126.2)	0 (0–0)	0 (0–0)	0 (0–0)	0 (0–0)
Neonatal encephalopathy due to birth asphyxia and trauma	9.0 (7.7–10.5)	101.2 (86.8–118.5)	0 (0–0)	0 (0–0)	0 (0–0)	0 (0–0)
Neonatal sepsis and other neonatal infections	3.6 (1.8–5.5)	40.5 (20.1–61.9)	0 (0–0)	0 (0–0)	0 (0–0)	0 (0–0)
Hemolytic disease and other neonatal jaundice	0.6 (0.3–1.0)	6.9 (3.9–11.6)	0 (0–0)	0 (0–0)	0 (0–0)	0 (0–0)
Other neonatal disorders	3.0 (2.1–4.2)	33.5 (23.2–46.9)	0 (0–0)	0 (0–0)	0 (0–0)	0 (0–0)
Nutritional deficiencies	5.3 (4.8–5.9)	25.2 (20.8–30.6)	1.1 (0.9–1.3)	0.9 (0.8–1.0)	3.3 (2.9–3.9)	29.5 (27.2–32.8)
Protein-energy malnutrition	4.1 (3.6–4.7)	22.5 (18.2–27.8)	0.8 (0.7–0.9)	0.4 (0.3–0.5)	1.9 (1.6–2.3)	23.6 (21.2–26.5)
Iodine deficiency	0.0 (0.0–0.0)	0.1 (0.0–0.2)	0.0 (0.0–0.0)	0.0 (0.0–0.0)	0.0 (0.0–0.1)	0.2 (0.1–0.3)
Vitamin A deficiency	0 (0–0)	0 (0–0)	0 (0–0)	0 (0–0)	0 (0–0)	0 (0–0)
Iron-deficiency anemia	0.7 (0.5–1.0)	2.2 (1.1–3.3)	0.2 (0.2–0.3)	0.3 (0.3–0.4)	1.0 (0.8–1.3)	2.4 (1.5–3.3)
Other nutritional deficiencies	0.4 (0.2–0.5)	0.4 (0.2–0.6)	0.1 (0.0–0.1)	0.1 (0.1–0.2)	0.4 (0.2–0.5)	3.3 (2.3–4.7)
Other communicable, maternal, neonatal, and nutritional diseases	3.8 (3.2–4.6)	20.2 (13.9–28.3)	1.4 (1.1–1.9)	1.1 (1.0–1.2)	3.4 (2.9–3.7)	10.7 (7.5–12.2)
Sexually transmitted diseases excluding HIV	1.5 (0.9–2.3)	13.7 (7.6–21.9)	0.8 (0.4–1.2)	0.2 (0.1–0.2)	0.2 (0.2–0.3)	0.8 (0.6–1.0)
Syphilis	1.5 (0.9–2.3)	13.7 (7.6–21.9)	0.8 (0.4–1.2)	0.1 (0.1–0.2)	0.2 (0.1–0.3)	0.7 (0.5–0.8)
Chlamydial infection	0.0 (0.0–0.0)	0 (0–0)	0.0 (0.0–0.0)	0.0 (0.0–0.0)	0.0 (0.0–0.0)	0.0 (0.0–0.0)
Gonococcal infection	0.0 (0.0–0.0)	0 (0–0)	0.0 (0.0–0.0)	0.0 (0.0–0.0)	0.0 (0.0–0.0)	0.1 (0.0–0.1)
Trichomoniasis	0 (0–0)	0 (0–0)	0 (0–0)	0 (0–0)	0 (0–0)	0 (0–0)
Genital herpes	0 (0–0)	0 (0–0)	0 (0–0)	0 (0–0)	0 (0–0)	0 (0–0)
Other sexually transmitted diseases	0.0 (0.0–0.0)	0 (0–0)	0.0 (0.0–0.0)	0.0 (0.0–0.0)	0.0 (0.0–0.0)	0.0 (0.0–0.0)
Hepatitis	1.1 (1.1–1.2)	1.6 (1.4–1.9)	0.4 (0.3–0.5)	0.7 (0.6–0.8)	1.7 (1.6–1.9)	4.0 (3.8–4.4)
Acute hepatitis A	0.1 (0.1–0.2)	1.3 (0.9–1.6)	0.1 (0.0–0.2)	0.0 (0.0–0.1)	0.0 (0.0–0.1)	0.1 (0.0–0.1)
Hepatitis B	0.6 (0.5–0.7)	0.1 (0.0–0.2)	0.0 (0.0–0.1)	0.4 (0.3–0.5)	1.4 (1.2–1.6)	2.9 (2.3–3.4)
Hepatitis C	0.0 (0.0–0.1)	0 (0–0)	0.0 (0.0–0.0)	0.0 (0.0–0.0)	0.1 (0.0–0.2)	0.1 (0.0–0.3)
Acute hepatitis E	0.3 (0.2–0.4)	0.3 (0.1–0.5)	0.3 (0.2–0.3)	0.3 (0.2–0.4)	0.2 (0.1–0.4)	1.0 (0.5–1.6)
Other infectious diseases	1.2 (0.7–1.5)	4.8 (2.4–7.1)	0.3 (0.2–0.3)	0.3 (0.2–0.3)	1.4 (1.0–1.7)	5.9 (2.8–7.2)
Non-communicable diseases	499.8 (490.2–511.1)	116.6 (104.4–130.5)	14.6 (13.4–15.9)	77.1 (73.6–80.9)	731.8 (710.6–757.5)	5158.1 (5084.1–5243.9)
Neoplasms	100.8 (98.3–103.2)	5.1 (4.5–5.8)	3.7 (3.4–4.0)	26.8 (25.6–28.0)	226.8 (220.1–234.0)	749.5 (731.5–767.7)
Lip and oral cavity cancer	1.3 (1.3–1.4)	0 (0–0)	0 (0–0)	0.4 (0.3–0.4)	3.0 (2.8–3.2)	9.8 (9.4–10.3)
Nasopharynx cancer	0.5 (0.4–0.5)	0 (0–0)	0.0 (0.0–0.0)	0.2 (0.2–0.3)	1.3 (1.2–1.4)	2.0 (1.8–2.1)
Other pharynx cancer	0.5 (0.4–0.5)	0 (0–0)	0 (0–0)	0.1 (0.1–0.1)	1.3 (1.2–1.5)	2.8 (2.6–3.0)
Esophageal cancer	3.3 (3.1–3.5)	0 (0–0)	0 (0–0)	0.5 (0.5–0.6)	8.0 (7.4–8.6)	26.6 (25.1–28.2)
Stomach cancer	7.8 (7.5–8.0)	0 (0–0)	0 (0–0)	1.5 (1.4–1.6)	15.7 (15.0–16.4)	68.2 (66.0–70.7)
Colon and rectum cancer	10.3 (9.9–10.7)	0 (0–0)	0 (0–0)	1.6 (1.5–1.7)	19.0 (18.3–19.7)	99.4 (95.4–104.1)
Liver cancer	6.4 (5.6–7.0)	0 (0–0)	0.1 (0.0–0.1)	1.4 (1.2–1.6)	15.4 (13.2–17.1)	47.9 (42.4–51.2)
Liver cancer due to hepatitis B	1.8 (1.5–2.0)	0 (0–0)	0.0 (0.0–0.0)	0.7 (0.6–0.9)	4.7 (4.0–5.5)	8.8 (7.5–9.9)
Liver cancer due to hepatitis C	2.0 (1.7–2.1)	0 (0–0)	0.0 (0.0–0.0)	0.2 (0.2–0.2)	3.9 (3.3–4.3)	19.1 (17.3–20.5)
Liver cancer due to alcohol use	1.1 (1.0–1.3)	0 (0–0)	0 (0–0)	0.1 (0.1–0.1)	3.1 (2.6–3.6)	8.2 (7.2–9.1)
Liver cancer due to other causes	1.6 (1.4–1.7)	0 (0–0)	0.0 (0.0–0.0)	0.4 (0.3–0.4)	3.7 (3.1–4.2)	11.7 (10.2–12.9)
Gallbladder and biliary tract cancer	2.2 (2.0–2.4)	0 (0–0)	0 (0–0)	0.3 (0.2–0.3)	4.3 (3.9–4.7)	21.3 (19.0–23.1)
Pancreatic cancer	5.4 (5.2–5.6)	0 (0–0)	0 (0–0)	0.5 (0.5–0.6)	11.0 (10.6–11.4)	51.4 (49.6–53.1)
Larynx cancer	0.5 (0.5–0.6)	0 (0–0)	0 (0–0)	0.1 (0.1–0.1)	1.4 (1.3–1.4)	3.7 (3.5–3.9)
Tracheal, bronchus, and lung cancer	14.1 (13.6–14.7)	0 (0–0)	0 (0–0)	2.1 (2.0–2.2)	33.6 (32.0–35.4)	116.3 (112.1–120.6)
Malignant skin melanoma	0.8 (0.7–0.8)	0 (0–0)	0 (0–0)	0.2 (0.2–0.3)	1.5 (1.3–1.6)	6.0 (5.0–6.4)
Non-melanoma skin cancer	0.5 (0.5–0.5)	0 (0–0)	0 (0–0)	0.1 (0.1–0.1)	0.7 (0.6–0.7)	5.3 (4.9–5.7)
Non-melanoma skin cancer (squamous-cell carcinoma)	0.5 (0.5–0.5)	0 (0–0)	0 (0–0)	0.1 (0.1–0.1)	0.7 (0.6–0.7)	5.3 (4.9–5.7)
Non-melanoma skin cancer (basal-cell carcinoma)	0 (0–0)	0 (0–0)	0 (0–0)	0 (0–0)	0 (0–0)	0 (0–0)
Breast cancer	14.3 (13.5–14.9)	0 (0–0)	0 (0–0)	5.6 (5.2–6.0)	37.1 (34.8–38.7)	80.2 (75.1–83.3)
Cervical cancer	6.5 (6.2–6.9)	0 (0–0)	0 (0–0)	3.2 (2.9–3.4)	17.9 (16.7–19.1)	28.7 (27.6–30.1)
Uterine cancer	2.5 (2.4–2.6)	0 (0–0)	0 (0–0)	0.5 (0.5–0.5)	6.5 (6.2–6.9)	17.3 (16.3–18.1)
Ovarian cancer	4.4 (4.3–4.6)	0 (0–0)	0 (0–0)	1.4 (1.3–1.5)	11.8 (11.4–12.3)	26.3 (25.4–27.2)
Prostate cancer	0 (0–0)	0 (0–0)	0 (0–0)	0 (0–0)	0 (0–0)	0 (0–0)
Testicular cancer	0 (0–0)	0 (0–0)	0 (0–0)	0 (0–0)	0 (0–0)	0 (0–0)

SR Table 13 - Global death rates (per 100,000) by age and cause for females in 2015

Cause	All ages	0-5 years	5-14 years	15-49 years	50-69 years	70+ years
Kidney cancer	1.3 (1.3–1.4)	0.3 (0.2–0.4)	0.1 (0.1–0.1)	0.2 (0.2–0.2)	2.6 (2.5–2.7)	11.2 (10.8–11.7)
Bladder cancer	1.4 (1.3–1.5)	0 (0–0)	0 (0–0)	0.1 (0.1–0.1)	2.1 (2.0–2.2)	15.8 (15.1–16.5)
Brain and nervous system cancer	2.8 (2.6–2.9)	0.7 (0.7–0.8)	0.8 (0.7–0.8)	1.3 (1.2–1.3)	6.5 (6.2–6.9)	13.1 (12.4–13.7)
Thyroid cancer	0.5 (0.5–0.5)	0 (0–0)	0.0 (0.0–0.0)	0.1 (0.1–0.1)	1.0 (0.9–1.1)	4.7 (4.4–5.1)
Mesothelioma	0.3 (0.2–0.3)	0 (0–0)	0 (0–0)	0.1 (0.1–0.1)	0.5 (0.5–0.6)	2.0 (1.9–2.1)
Hodgkin lymphoma	0.2 (0.2–0.3)	0.0 (0.0–0.0)	0.0 (0.0–0.0)	0.2 (0.1–0.2)	0.5 (0.4–0.6)	1.2 (1.0–1.6)
Non-Hodgkin lymphoma	2.7 (2.2–2.8)	0.4 (0.3–0.6)	0.3 (0.2–0.4)	0.7 (0.6–0.8)	5.1 (4.4–5.5)	21.4 (16.9–23.0)
Multiple myeloma	1.3 (1.3–1.4)	0 (0–0)	0 (0–0)	0.2 (0.2–0.2)	3.0 (2.8–3.2)	11.5 (10.5–12.1)
Leukemia	4.1 (3.9–4.2)	1.9 (1.6–2.2)	1.5 (1.4–1.7)	1.8 (1.7–1.9)	6.7 (6.4–7.0)	25.3 (24.1–26.4)
Acute lymphoid leukemia	1.2 (1.2–1.3)	1.2 (1.0–1.5)	1.1 (0.9–1.2)	0.7 (0.6–0.8)	1.9 (1.8–2.1)	4.4 (4.1–4.7)
Chronic lymphoid leukemia	0.7 (0.7–0.8)	0 (0–0)	0 (0–0)	0.2 (0.2–0.2)	1.1 (1.1–1.2)	7.2 (6.8–7.7)
Acute myeloid leukemia	1.7 (1.6–1.8)	0.7 (0.6–0.8)	0.5 (0.4–0.5)	0.8 (0.7–0.8)	2.9 (2.7–3.0)	10.7 (10.2–11.2)
Chronic myeloid leukemia	0.4 (0.4–0.4)	0 (0–0)	0 (0–0)	0.2 (0.2–0.2)	0.8 (0.7–0.8)	2.9 (2.7–3.2)
Other neoplasms	5.0 (4.4–5.3)	1.8 (1.5–2.2)	0.9 (0.8–1.0)	2.2 (1.8–2.6)	9.4 (8.6–10.0)	30.2 (26.5–31.7)
Cardiovascular diseases	232.6 (227.1–238.6)	6.3 (5.8–6.9)	1.9 (1.7–2.1)	23.7 (22.4–25.1)	290.0 (279.9–302.0)	2724.3 (2659.9–2792.0)
Rheumatic heart disease	4.7 (4.3–5.1)	0.3 (0.3–0.4)	0.5 (0.4–0.5)	2.0 (1.7–2.2)	8.7 (7.8–9.5)	33.5 (31.4–36.1)
Ischemic heart disease	110.4 (107.8–113.4)	0.3 (0.2–0.3)	0.1 (0.1–0.2)	8.7 (8.2–9.3)	130.8 (126.3–136.3)	1339.1 (1307.2–1373.2)
Cerebrovascular disease	84.7 (82.1–87.6)	1.9 (1.7–2.2)	0.6 (0.6–0.7)	8.4 (7.9–9.1)	112.4 (107.4–118.1)	974.6 (946.9–1005.0)
Ischemic stroke	42.4 (40.4–44.3)	0.1 (0.1–0.1)	0.0 (0.0–0.0)	1.4 (1.2–1.6)	36.7 (33.8–40.1)	569.0 (544.6–590.5)
Hemorrhagic stroke	42.2 (40.3–45.0)	1.8 (1.6–2.1)	0.6 (0.5–0.7)	7.1 (6.6–7.6)	75.7 (71.0–81.3)	405.6 (386.6–435.8)
Hypertensive heart disease	14.1 (11.7–15.5)	0.1 (0.0–0.1)	0.0 (0.0–0.0)	1.5 (1.3–1.7)	19.5 (16.4–21.9)	160.4 (130.2–174.6)
Cardiomyopathy and myocarditis	4.2 (4.1–4.4)	1.5 (1.3–1.7)	0.3 (0.2–0.3)	1.1 (1.1–1.2)	4.8 (4.6–5.1)	42.7 (40.8–44.6)
Atrial fibrillation and flutter	3.0 (2.4–3.6)	0 (0–0)	0 (0–0)	0.0 (0.0–0.0)	0.8 (0.6–1.1)	45.7 (37.1–55.6)
Aortic aneurysm	1.7 (1.6–1.8)	0 (0–0)	0 (0–0)	0.2 (0.2–0.2)	2.0 (1.9–2.0)	20.0 (19.1–21.0)
Peripheral vascular disease	0.7 (0.7–0.8)	0 (0–0)	0 (0–0)	0.0 (0.0–0.0)	0.4 (0.4–0.4)	10.6 (9.7–11.6)
Endocarditis	1.3 (1.1–1.5)	0.5 (0.3–0.7)	0.1 (0.1–0.1)	0.4 (0.3–0.5)	1.4 (1.1–1.7)	12.2 (11.0–13.9)
Other cardiovascular and circulatory diseases	7.7 (7.4–8.1)	1.7 (1.5–1.9)	0.3 (0.2–0.3)	1.2 (1.1–1.3)	9.2 (8.6–10.0)	85.5 (81.5–89.6)
Chronic respiratory diseases	43.5 (41.2–45.7)	4.9 (4.4–5.8)	0.7 (0.6–0.8)	3.9 (3.6–4.1)	61.7 (58.3–65.5)	486.9 (458.1–511.0)
Chronic obstructive pulmonary disease	35.9 (33.9–37.9)	1.9 (1.3–2.4)	0.1 (0.1–0.2)	2.0 (1.8–2.2)	48.6 (45.4–52.5)	422.1 (395.3–445.4)
Pneumoconiosis	0.3 (0.2–0.4)	0 (0–0)	0 (0–0)	0.0 (0.0–0.0)	0.4 (0.2–0.6)	4.0 (2.9–5.1)
Silicosis	0.0 (0.0–0.0)	0 (0–0)	0 (0–0)	0.0 (0.0–0.0)	0.0 (0.0–0.0)	0.1 (0.1–0.2)
Asbestosis	0.0 (0.0–0.0)	0 (0–0)	0 (0–0)	0.0 (0.0–0.0)	0.0 (0.0–0.0)	0.1 (0.1–0.1)
Coal workers pneumoconiosis	0.0 (0.0–0.0)	0 (0–0)	0 (0–0)	0.0 (0.0–0.0)	0.0 (0.0–0.0)	0.0 (0.0–0.1)
Other pneumoconiosis	0.3 (0.2–0.4)	0 (0–0)	0 (0–0)	0.0 (0.0–0.0)	0.4 (0.2–0.5)	3.7 (2.7–4.7)
Asthma	5.4 (4.6–6.1)	1.4 (1.1–1.7)	0.5 (0.4–0.6)	1.5 (1.3–1.8)	10.2 (8.5–11.8)	41.9 (36.2–49.0)
Interstitial lung disease and pulmonary sarcoidosis	1.5 (1.1–1.6)	0.0 (0.0–0.1)	0.0 (0.0–0.0)	0.2 (0.2–0.2)	2.1 (1.8–2.4)	16.2 (11.0–18.0)
Other chronic respiratory diseases	0.4 (0.3–0.5)	1.6 (1.2–2.1)	0.1 (0.1–0.1)	0.1 (0.1–0.1)	0.4 (0.3–0.5)	2.7 (1.7–3.4)
Cirrhosis and other chronic liver diseases	11.8 (11.2–13.4)	1.4 (1.1–1.9)	0.6 (0.5–0.8)	4.4 (4.1–5.2)	28.6 (26.7–32.5)	69.4 (65.6–76.8)
Cirrhosis and other chronic liver diseases due to hepatitis B	2.9 (2.7–3.5)	0.0 (0.0–0.0)	0.0 (0.0–0.0)	1.1 (0.9–1.3)	7.1 (6.2–8.6)	18.6 (16.6–21.8)
Cirrhosis and other chronic liver diseases due to hepatitis C	2.8 (2.5–3.0)	0.0 (0.0–0.0)	0.0 (0.0–0.0)	0.9 (0.8–1.1)	6.7 (5.9–7.6)	17.8 (16.2–19.8)
Cirrhosis and other chronic liver diseases due to alcohol use	2.7 (2.5–3.1)	0 (0–0)	0 (0–0)	1.2 (1.1–1.4)	7.7 (6.8–8.8)	11.9 (10.6–13.5)
Cirrhosis and other chronic liver diseases due to other causes	3.4 (3.1–3.9)	1.4 (1.1–1.9)	0.6 (0.5–0.8)	1.2 (1.1–1.5)	7.1 (6.2–8.2)	21.1 (19.3–23.6)
Digestive diseases	14.9 (14.1–15.6)	3.7 (2.1–7.9)	1.1 (1.0–1.2)	3.8 (3.5–4.1)	21.7 (20.3–23.0)	139.3 (132.9–145.1)
Peptic ulcer disease	3.0 (2.8–3.3)	0.4 (0.2–1.0)	0.2 (0.1–0.2)	0.8 (0.7–0.9)	4.9 (4.5–5.4)	27.7 (25.7–29.8)
Gastritis and duodenitis	0.6 (0.4–0.8)	0.1 (0.0–0.2)	0.0 (0.0–0.1)	0.1 (0.1–0.2)	0.9 (0.6–1.3)	5.3 (3.6–7.3)
Appendicitis	0.6 (0.5–0.7)	0.7 (0.3–1.8)	0.2 (0.2–0.2)	0.3 (0.3–0.4)	0.9 (0.7–1.1)	3.2 (2.5–3.6)
Paralytic ileus and intestinal obstruction	3.2 (2.8–3.7)	1.4 (0.7–2.7)	0.4 (0.3–0.5)	0.9 (0.8–1.1)	4.5 (3.8–5.5)	28.7 (24.9–32.5)
Inguinal, femoral, and abdominal hernia	0.6 (0.4–0.7)	0.1 (0.0–0.2)	0.0 (0.0–0.1)	0.1 (0.1–0.2)	1.0 (0.6–1.2)	5.6 (4.0–6.5)
Inflammatory bowel disease	0.7 (0.6–0.7)	0.3 (0.1–0.7)	0.0 (0.0–0.1)	0.2 (0.2–0.2)	0.8 (0.8–0.9)	6.1 (5.6–6.7)
Vascular intestinal disorders	1.6 (1.5–1.8)	0.0 (0.0–0.1)	0.0 (0.0–0.0)	0.1 (0.1–0.2)	1.8 (1.5–2.1)	19.9 (18.3–21.8)
Gallbladder and biliary diseases	1.7 (1.6–1.8)	0.3 (0.1–0.6)	0.1 (0.1–0.1)	0.3 (0.3–0.4)	2.4 (2.2–2.7)	17.3 (16.1–18.8)
Pancreatitis	1.3 (1.2–1.4)	0.0 (0.0–0.1)	0.0 (0.0–0.1)	0.6 (0.5–0.6)	2.5 (2.2–2.7)	9.2 (8.5–10.0)
Other digestive diseases	1.5 (1.4–1.6)	0.4 (0.2–0.9)	0.1 (0.1–0.1)	0.3 (0.2–0.3)	1.9 (1.7–2.0)	16.3 (15.1–17.7)
Neurological disorders	36.2 (30.7–41.7)	2.3 (2.0–2.7)	0.9 (0.8–1.0)	1.7 (1.6–1.9)	13.7 (11.6–16.0)	525.7 (440.0–611.6)
Alzheimer disease and other dementias	32.2 (26.6–37.7)	0 (0–0)	0 (0–0)	0.0 (0.0–0.1)	8.6 (6.4–10.8)	494.1 (407.9–580.5)
Parkinson disease	1.4 (1.3–1.5)	0 (0–0)	0 (0–0)	0.0 (0.0–0.0)	0.8 (0.8–0.9)	19.9 (19.0–20.9)
Epilepsy	1.3 (1.2–1.4)	1.7 (1.5–1.9)	0.7 (0.6–0.8)	1.2 (1.1–1.3)	1.2 (1.2–1.3)	3.6 (3.4–3.8)

SR Table 13 - Global death rates (per 100,000) by age and cause for females in 2015						
**Cause**	**All ages**	**0-5 years**	**5-14 years**	**15-49 years**	**50-69 years**	**70+ years**
Multiple sclerosis	0.3 (0.3–0.3)	0 (0–0)	0 (0–0)	0.2 (0.2–0.2)	0.9 (0.7–0.9)	1.2 (0.9–1.3)
Motor neuron disease	0.4 (0.4–0.4)	0.1 (0.1–0.2)	0.0 (0.0–0.0)	0.1 (0.1–0.1)	1.1 (1.0–1.1)	3.0 (2.8–3.2)
Migraine	0 (0–0)	0 (0–0)	0 (0–0)	0 (0–0)	0 (0–0)	0 (0–0)
Tension-type headache	0 (0–0)	0 (0–0)	0 (0–0)	0 (0–0)	0 (0–0)	0 (0–0)
Medication overuse headache	0 (0–0)	0 (0–0)	0 (0–0)	0 (0–0)	0 (0–0)	0 (0–0)
Other neurological disorders	0.7 (0.6–0.7)	0.5 (0.4–0.8)	0.2 (0.2–0.3)	0.3 (0.2–0.3)	1.2 (1.1–1.2)	3.8 (3.6–4.0)
Mental and substance use disorders	2.1 (1.9–2.2)	0.0 (0.0–0.0)	0.0 (0.0–0.0)	1.8 (1.7–2.0)	4.1 (3.7–4.2)	7.2 (5.8–7.8)
Schizophrenia	0.2 (0.2–0.2)	0 (0–0)	0 (0–0)	0.1 (0.1–0.1)	0.4 (0.4–0.5)	1.3 (1.2–1.4)
Alcohol use disorders	0.6 (0.5–0.6)	0 (0–0)	0 (0–0)	0.4 (0.4–0.4)	1.5 (1.5–1.6)	1.7 (1.6–1.7)
Drug use disorders	1.3 (1.1–1.4)	0.0 (0.0–0.0)	0 (0–0)	1.3 (1.2–1.4)	2.1 (1.7–2.2)	4.2 (2.8–4.8)
Opioid use disorders	0.8 (0.7–0.9)	0.0 (0.0–0.0)	0 (0–0)	0.9 (0.8–1.0)	1.3 (1.1–1.5)	2.8 (1.8–3.2)
Cocaine use disorders	0.1 (0.1–0.1)	0 (0–0)	0 (0–0)	0.1 (0.1–0.1)	0.2 (0.1–0.2)	0.1 (0.1–0.1)
Amphetamine use disorders	0.1 (0.0–0.1)	0 (0–0)	0 (0–0)	0.1 (0.1–0.1)	0.1 (0.1–0.1)	0.1 (0.1–0.1)
Cannabis use disorders	0 (0–0)	0 (0–0)	0 (0–0)	0 (0–0)	0 (0–0)	0 (0–0)
Other drug use disorders	0.3 (0.3–0.4)	0 (0–0)	0 (0–0)	0.3 (0.2–0.4)	0.5 (0.4–0.5)	1.2 (0.8–1.6)
Depressive disorders	0 (0–0)	0 (0–0)	0 (0–0)	0 (0–0)	0 (0–0)	0 (0–0)
Major depressive disorder	0 (0–0)	0 (0–0)	0 (0–0)	0 (0–0)	0 (0–0)	0 (0–0)
Dysthymia	0 (0–0)	0 (0–0)	0 (0–0)	0 (0–0)	0 (0–0)	0 (0–0)
Bipolar disorder	0 (0–0)	0 (0–0)	0 (0–0)	0 (0–0)	0 (0–0)	0 (0–0)
Anxiety disorders	0 (0–0)	0 (0–0)	0 (0–0)	0 (0–0)	0 (0–0)	0 (0–0)
Eating disorders	0.0 (0.0–0.0)	0 (0–0)	0.0 (0.0–0.0)	0.0 (0.0–0.0)	0 (0–0)	0 (0–0)
Anorexia nervosa	0.0 (0.0–0.0)	0 (0–0)	0.0 (0.0–0.0)	0.0 (0.0–0.0)	0 (0–0)	0 (0–0)
Bulimia nervosa	0.0 (0.0–0.0)	0 (0–0)	0.0 (0.0–0.0)	0.0 (0.0–0.0)	0 (0–0)	0 (0–0)
Autistic spectrum disorders	0 (0–0)	0 (0–0)	0 (0–0)	0 (0–0)	0 (0–0)	0 (0–0)
Autism	0 (0–0)	0 (0–0)	0 (0–0)	0 (0–0)	0 (0–0)	0 (0–0)
Asperger syndrome and other autistic spectrum disorders	0 (0–0)	0 (0–0)	0 (0–0)	0 (0–0)	0 (0–0)	0 (0–0)
Attention-deficit/hyperactivity disorder	0 (0–0)	0 (0–0)	0 (0–0)	0 (0–0)	0 (0–0)	0 (0–0)
Conduct disorder	0 (0–0)	0 (0–0)	0 (0–0)	0 (0–0)	0 (0–0)	0 (0–0)
Idiopathic developmental intellectual disability	0 (0–0)	0 (0–0)	0 (0–0)	0 (0–0)	0 (0–0)	0 (0–0)
Other mental and substance use disorders	0 (0–0)	0 (0–0)	0 (0–0)	0 (0–0)	0 (0–0)	0 (0–0)
Diabetes, urogenital, blood, and endocrine diseases	46.4 (45.1–48.0)	15.5 (11.5–24.0)	2.4 (2.2–2.7)	8.8 (8.3–9.3)	79.9 (76.5–83.8)	422.9 (410.3–436.7)
Diabetes mellitus	21.1 (20.1–22.1)	0.3 (0.2–0.3)	0.2 (0.2–0.2)	2.8 (2.6–3.1)	42.1 (39.6–44.6)	198.3 (189.4–207.6)
Acute glomerulonephritis	0.1 (0.1–0.2)	0.0 (0.0–0.1)	0.0 (0.0–0.0)	0.1 (0.0–0.1)	0.3 (0.2–0.3)	1.0 (0.6–1.2)
Chronic kidney disease	16.3 (15.8–17.0)	1.8 (1.6–2.0)	0.7 (0.6–0.7)	3.3 (3.2–3.6)	27.0 (25.9–28.2)	155.5 (149.7–161.3)
Chronic kidney disease due to diabetes mellitus	5.5 (5.2–5.8)	0.3 (0.3–0.4)	0.1 (0.1–0.1)	0.9 (0.8–0.9)	11.5 (10.7–12.3)	48.6 (46.1–51.5)
Chronic kidney disease due to hypertension	7.5 (7.2–7.9)	0.6 (0.5–0.7)	0.2 (0.2–0.3)	1.4 (1.3–1.5)	10.8 (10.0–11.7)	78.1 (73.9–82.1)
Chronic kidney disease due to glomerulonephritis	2.9 (2.7–3.1)	0.6 (0.5–0.7)	0.3 (0.2–0.3)	0.9 (0.8–1.1)	4.4 (4.0–4.9)	25.1 (23.3–27.1)
Chronic kidney disease due to other causes	0.4 (0.3–0.5)	0.2 (0.2–0.3)	0.1 (0.1–0.1)	0.1 (0.1–0.2)	0.3 (0.2–0.4)	3.6 (3.0–4.5)
Urinary diseases and male infertility	3.8 (3.6–3.9)	0.8 (0.6–0.9)	0.1 (0.1–0.2)	0.8 (0.7–0.9)	4.8 (4.5–5.2)	39.1 (36.6–40.9)
Interstitial nephritis and urinary tract infections	3.1 (2.9–3.2)	0.6 (0.5–0.7)	0.1 (0.1–0.1)	0.6 (0.5–0.6)	3.6 (3.3–3.9)	33.9 (31.4–35.6)
Urolithiasis	0.2 (0.2–0.3)	0 (0–0)	0.0 (0.0–0.0)	0.1 (0.0–0.1)	0.4 (0.3–0.5)	1.8 (1.5–2.1)
Benign prostatic hyperplasia	0 (0–0)	0 (0–0)	0 (0–0)	0 (0–0)	0 (0–0)	0 (0–0)
Male infertility	0 (0–0)	0 (0–0)	0 (0–0)	0 (0–0)	0 (0–0)	0 (0–0)
Other urinary diseases	0.5 (0.4–0.5)	0.1 (0.1–0.2)	0.0 (0.0–0.0)	0.1 (0.1–0.2)	0.8 (0.7–1.0)	3.5 (3.1–4.2)
Gynecological diseases	0.2 (0.2–0.3)	0 (0–0)	0 (0–0)	0.2 (0.1–0.2)	0.4 (0.3–0.4)	1.0 (0.7–1.2)
Uterine fibroids	0.1 (0.0–0.1)	0 (0–0)	0 (0–0)	0.0 (0.0–0.1)	0.2 (0.1–0.2)	0.3 (0.1–0.4)
Polycystic ovarian syndrome	0.0 (0.0–0.0)	0 (0–0)	0 (0–0)	0.0 (0.0–0.0)	0.0 (0.0–0.1)	0 (0–0)
Female infertility	0 (0–0)	0 (0–0)	0 (0–0)	0 (0–0)	0 (0–0)	0 (0–0)
Endometriosis	0.0 (0.0–0.0)	0 (0–0)	0 (0–0)	0.0 (0.0–0.0)	0.0 (0.0–0.0)	0 (0–0)
Genital prolapse	0.0 (0.0–0.0)	0 (0–0)	0 (0–0)	0.0 (0.0–0.0)	0.0 (0.0–0.1)	0.2 (0.2–0.4)
Premenstrual syndrome	0 (0–0)	0 (0–0)	0 (0–0)	0 (0–0)	0 (0–0)	0 (0–0)
Other gynecological diseases	0.1 (0.1–0.1)	0 (0–0)	0 (0–0)	0.1 (0.1–0.1)	0.1 (0.1–0.2)	0.5 (0.4–0.6)
Hemoglobinopathies and hemolytic anemias	2.8 (2.4–3.6)	11.1 (7.1–20.0)	1.2 (1.0–1.4)	1.0 (0.9–1.1)	2.3 (2.1–2.4)	11.6 (10.5–12.6)
Thalassemias	0.2 (0.2–0.3)	1.1 (0.8–1.7)	0.2 (0.2–0.2)	0.1 (0.0–0.1)	0.0 (0.0–0.0)	1.1 (1.0–1.3)
Thalassemias trait	0 (0–0)	0 (0–0)	0 (0–0)	0 (0–0)	0 (0–0)	0 (0–0)
Sickle cell disorders	1.4 (1.0–2.2)	9.8 (6.0–18.7)	0.9 (0.7–1.2)	0.5 (0.4–0.6)	0.2 (0.1–0.2)	1.5 (1.3–1.7)
Sickle cell trait	0 (0–0)	0 (0–0)	0 (0–0)	0 (0–0)	0 (0–0)	0 (0–0)

SR Table 13 - Global death rates (per 100,000) by age and cause for females in 2015						
Cause	All ages	0-5 years	5-14 years	15-49 years	50-69 years	70+ years
G6PD deficiency	0.3 (0.2–0.3)	0.1 (0.0–0.1)	0.1 (0.0–0.1)	0.3 (0.2–0.3)	0.5 (0.5–0.6)	0.4 (0.3–0.4)
G6PD trait	0 (0–0)	0 (0–0)	0 (0–0)	0 (0–0)	0 (0–0)	0 (0–0)
Other hemoglobinopathies and hemolytic anemias	0.9 (0.8–1.0)	0.0 (0.0–0.1)	0.0 (0.0–0.0)	0.2 (0.2–0.2)	1.6 (1.5–1.7)	8.6 (7.7–9.4)
Endocrine, metabolic, blood, and immune disorders	2.0 (2.0–2.1)	1.6 (1.4–1.7)	0.2 (0.2–0.3)	0.6 (0.6–0.6)	3.1 (2.9–3.2)	16.5 (15.7–17.3)
Musculoskeletal disorders	1.7 (1.6–1.8)	0 (0–0)	0.2 (0.2–0.2)	0.7 (0.6–0.8)	2.5 (2.3–2.7)	14.3 (12.8–15.3)
Rheumatoid arthritis	0.6 (0.5–0.7)	0 (0–0)	0.0 (0.0–0.0)	0.1 (0.1–0.1)	1.0 (0.8–1.2)	5.7 (5.1–7.0)
Osteoarthritis	0 (0–0)	0 (0–0)	0 (0–0)	0 (0–0)	0 (0–0)	0 (0–0)
Low back and neck pain	0 (0–0)	0 (0–0)	0 (0–0)	0 (0–0)	0 (0–0)	0 (0–0)
Low back pain	0 (0–0)	0 (0–0)	0 (0–0)	0 (0–0)	0 (0–0)	0 (0–0)
Neck pain	0 (0–0)	0 (0–0)	0 (0–0)	0 (0–0)	0 (0–0)	0 (0–0)
Gout	0 (0–0)	0 (0–0)	0 (0–0)	0 (0–0)	0 (0–0)	0 (0–0)
Other musculoskeletal disorders	1.1 (1.0–1.2)	0 (0–0)	0.2 (0.1–0.2)	0.6 (0.6–0.7)	1.5 (1.3–1.7)	8.6 (6.7–9.4)
Other non-communicable diseases	9.8 (8.4–11.2)	77.4 (67.5–89.5)	3.1 (2.6–3.8)	1.5 (1.2–1.7)	2.9 (2.1–3.7)	18.3 (12.3–22.2)
Congenital anomalies	8.1 (6.9–9.3)	73.5 (63.8–85.5)	3.0 (2.5–3.6)	1.3 (1.0–1.5)	1.3 (1.0–1.4)	3.0 (2.2–3.3)
Neural tube defects	0.8 (0.5–1.2)	9.0 (5.5–12.9)	0.2 (0.1–0.3)	0.0 (0.0–0.0)	0.0 (0.0–0.0)	0.0 (0.0–0.0)
Congenital heart anomalies	3.9 (3.1–4.6)	33.5 (26.6–41.0)	2.0 (1.6–2.4)	0.9 (0.7–1.0)	0.4 (0.3–0.4)	0.9 (0.7–1.0)
Cleft lip and cleft palate	0.0 (0.0–0.0)	0.2 (0.1–0.3)	0 (0–0)	0 (0–0)	0 (0–0)	0 (0–0)
Down syndrome	0.4 (0.2–0.7)	3.4 (1.3–6.3)	0.1 (0.0–0.1)	0.1 (0.1–0.1)	0.3 (0.2–0.3)	0.1 (0.0–0.1)
Turner syndrome	0 (0–0)	0 (0–0)	0 (0–0)	0 (0–0)	0 (0–0)	0 (0–0)
Klinefelter syndrome	0 (0–0)	0 (0–0)	0 (0–0)	0 (0–0)	0 (0–0)	0 (0–0)
Other chromosomal abnormalities	0.4 (0.2–0.8)	4.5 (2.0–9.0)	0.0 (0.0–0.1)	0.0 (0.0–0.0)	0.0 (0.0–0.0)	0.0 (0.0–0.0)
Other congenital anomalies	2.5 (2.1–3.3)	22.9 (18.5–31.1)	0.7 (0.6–1.0)	0.3 (0.2–0.3)	0.6 (0.5–0.7)	2.0 (1.4–2.2)
Skin and subcutaneous diseases	1.5 (0.9–2.0)	1.2 (0.6–2.0)	0.1 (0.0–0.2)	0.2 (0.1–0.4)	1.6 (0.9–2.4)	15.3 (9.3–19.5)
Dermatitis	0 (0–0)	0 (0–0)	0 (0–0)	0 (0–0)	0 (0–0)	0 (0–0)
Psoriasis	0 (0–0)	0 (0–0)	0 (0–0)	0 (0–0)	0 (0–0)	0 (0–0)
Cellulitis	0.2 (0.1–0.3)	0.1 (0.0–0.2)	0.0 (0.0–0.0)	0.0 (0.0–0.1)	0.4 (0.2–0.5)	2.1 (1.1–2.8)
Pyoderma	0.7 (0.4–1.0)	1.0 (0.5–1.7)	0.1 (0.0–0.1)	0.1 (0.1–0.2)	0.8 (0.5–1.4)	5.4 (3.5–7.8)
Scabies	0 (0–0)	0 (0–0)	0 (0–0)	0 (0–0)	0 (0–0)	0 (0–0)
Fungal skin diseases	0 (0–0)	0 (0–0)	0 (0–0)	0 (0–0)	0 (0–0)	0 (0–0)
Viral skin diseases	0 (0–0)	0 (0–0)	0 (0–0)	0 (0–0)	0 (0–0)	0 (0–0)
Acne vulgaris	0 (0–0)	0 (0–0)	0 (0–0)	0 (0–0)	0 (0–0)	0 (0–0)
Alopecia areata	0 (0–0)	0 (0–0)	0 (0–0)	0 (0–0)	0 (0–0)	0 (0–0)
Pruritus	0 (0–0)	0 (0–0)	0 (0–0)	0 (0–0)	0 (0–0)	0 (0–0)
Urticaria	0 (0–0)	0 (0–0)	0 (0–0)	0 (0–0)	0 (0–0)	0 (0–0)
Decubitus ulcer	0.5 (0.3–0.7)	0.0 (0.0–0.0)	0.0 (0.0–0.0)	0.0 (0.0–0.0)	0.3 (0.2–0.5)	7.2 (3.9–8.9)
Other skin and subcutaneous diseases	0.1 (0.0–0.1)	0.0 (0.0–0.1)	0.0 (0.0–0.0)	0.0 (0.0–0.0)	0.1 (0.0–0.1)	0.6 (0.4–0.8)
Sense organ diseases	0 (0–0)	0 (0–0)	0 (0–0)	0 (0–0)	0 (0–0)	0 (0–0)
Glaucoma	0 (0–0)	0 (0–0)	0 (0–0)	0 (0–0)	0 (0–0)	0 (0–0)
Cataract	0 (0–0)	0 (0–0)	0 (0–0)	0 (0–0)	0 (0–0)	0 (0–0)
Macular degeneration	0 (0–0)	0 (0–0)	0 (0–0)	0 (0–0)	0 (0–0)	0 (0–0)
Refraction and accommodation disorders	0 (0–0)	0 (0–0)	0 (0–0)	0 (0–0)	0 (0–0)	0 (0–0)
Age-related and other hearing loss	0 (0–0)	0 (0–0)	0 (0–0)	0 (0–0)	0 (0–0)	0 (0–0)
Other vision loss	0 (0–0)	0 (0–0)	0 (0–0)	0 (0–0)	0 (0–0)	0 (0–0)
Other sense organ diseases	0 (0–0)	0 (0–0)	0 (0–0)	0 (0–0)	0 (0–0)	0 (0–0)
Oral disorders	0 (0–0)	0 (0–0)	0 (0–0)	0 (0–0)	0 (0–0)	0 (0–0)
Deciduous caries	0 (0–0)	0 (0–0)	0 (0–0)	0 (0–0)	0 (0–0)	0 (0–0)
Permanent caries	0 (0–0)	0 (0–0)	0 (0–0)	0 (0–0)	0 (0–0)	0 (0–0)
Periodontal diseases	0 (0–0)	0 (0–0)	0 (0–0)	0 (0–0)	0 (0–0)	0 (0–0)
Edentulism and severe tooth loss	0 (0–0)	0 (0–0)	0 (0–0)	0 (0–0)	0 (0–0)	0 (0–0)
Other oral disorders	0 (0–0)	0 (0–0)	0 (0–0)	0 (0–0)	0 (0–0)	0 (0–0)
Sudden infant death syndrome	0.2 (0.2–0.4)	2.8 (2.1–4.0)	0 (0–0)	0 (0–0)	0 (0–0)	0 (0–0)
Injuries	38.3 (34.1–40.6)	42.1 (34.5–47.9)	13.0 (11.7–14.3)	26.3 (23.5–28.2)	46.5 (41.5–49.9)	175.2 (155.9–183.3)
Transport injuries	9.8 (9.2–10.4)	7.0 (5.8–8.6)	3.8 (3.5–4.2)	7.8 (7.3–8.4)	14.9 (13.9–15.9)	31.7 (29.7–33.6)
Road injuries	9.2 (8.7–9.8)	6.4 (5.3–7.9)	3.6 (3.3–3.9)	7.4 (6.9–7.9)	14.0 (13.1–14.9)	30.0 (28.1–31.8)
Pedestrian road injuries	4.1 (3.8–4.4)	2.8 (2.3–3.4)	1.7 (1.5–1.8)	2.7 (2.5–3.0)	6.7 (6.1–7.2)	16.0 (14.8–17.2)
Cyclist road injuries	0.4 (0.4–0.4)	0.2 (0.1–0.2)	0.2 (0.1–0.2)	0.3 (0.3–0.4)	0.7 (0.7–0.8)	0.9 (0.8–1.0)
Motorcyclist road injuries	1.4 (1.2–1.6)	0.8 (0.6–1.1)	0.4 (0.4–0.5)	1.3 (1.1–1.4)	2.0 (1.8–2.3)	3.8 (3.2–4.4)

SR Table 13 - Global death rates (per 100,000) by age and cause for females in 2015

Cause	All ages	0-5 years	5-14 years	15-49 years	50-69 years	70+ years
Motor vehicle road injuries	3.3<br>(3.1–3.5)	2.5<br>(2.0–3.2)	1.3<br>(1.2–1.5)	2.9<br>(2.7–3.2)	4.4<br>(4.0–4.8)	9.1<br>(8.0–9.7)
Other road injuries	0.1<br>(0.1–0.1)	0.1<br>(0.1–0.1)	0.0<br>(0.0–0.1)	0.1<br>(0.1–0.1)	0.2<br>(0.1–0.2)	0.3<br>(0.3–0.4)
Other transport injuries	0.6<br>(0.5–0.7)	0.6<br>(0.4–0.8)	0.2<br>(0.2–0.2)	0.4<br>(0.4–0.5)	0.9<br>(0.7–1.1)	1.7<br>(1.5–2.1)
Unintentional injuries	18.3<br>(15.0–19.6)	29.8<br>(22.2–35.1)	7.1<br>(5.8–8.0)	8.0<br>(6.4–8.8)	17.6<br>(14.6–19.4)	118.2<br>(99.8–124.7)
Falls	5.8<br>(4.8–6.2)	3.4<br>(1.9–4.9)	0.9<br>(0.7–1.0)	1.0<br>(0.9–1.1)	4.7<br>(4.1–5.1)	64.8<br>(53.9–69.6)
Drowning	2.8<br>(1.7–3.3)	7.0<br>(3.5–9.2)	2.5<br>(1.7–3.1)	1.5<br>(0.8–1.9)	2.4<br>(1.5–3.0)	8.6<br>(5.8–9.7)
Fire, heat, and hot substances	2.4<br>(1.8–2.7)	3.6<br>(2.5–4.3)	0.8<br>(0.6–0.9)	1.9<br>(1.3–2.2)	2.2<br>(1.6–2.5)	9.6<br>(7.7–10.5)
Poisonings	0.9<br>(0.6–1.1)	2.3<br>(0.8–4.2)	0.3<br>(0.3–0.4)	0.6<br>(0.5–0.7)	1.1<br>(0.8–1.2)	3.0<br>(2.3–3.2)
Exposure to mechanical forces	1.5<br>(1.2–1.6)	3.7<br>(2.9–4.2)	0.6<br>(0.6–0.7)	0.7<br>(0.6–0.8)	1.6<br>(1.4–1.8)	7.0<br>(4.8–7.8)
Unintentional firearm injuries	0.3<br>(0.2–0.3)	0.2<br>(0.1–0.3)	0.1<br>(0.1–0.1)	0.1<br>(0.1–0.2)	0.2<br>(0.1–0.3)	1.7<br>(1.4–1.9)
Unintentional suffocation	0.3<br>(0.2–0.4)	1.9<br>(1.2–2.3)	0.2<br>(0.1–0.2)	0.1<br>(0.1–0.1)	0.2<br>(0.1–0.2)	1.1<br>(0.6–1.3)
Other exposure to mechanical forces	0.9<br>(0.7–1.0)	1.5<br>(1.3–1.8)	0.4<br>(0.3–0.4)	0.5<br>(0.4–0.6)	1.2<br>(1.1–1.4)	4.2<br>(2.6–4.8)
Adverse effects of medical treatment	1.2<br>(0.9–1.4)	1.7<br>(1.0–2.3)	0.3<br>(0.2–0.3)	0.5<br>(0.3–0.6)	1.4<br>(1.0–1.6)	7.9<br>(6.3–8.9)
Animal contact	1.1<br>(0.6–1.5)	1.5<br>(0.8–2.0)	0.7<br>(0.4–1.0)	0.8<br>(0.4–1.1)	1.5<br>(0.8–2.1)	2.4<br>(1.4–3.1)
Venomous animal contact	0.9<br>(0.5–1.3)	1.1<br>(0.6–1.6)	0.6<br>(0.3–0.9)	0.7<br>(0.3–1.1)	1.4<br>(0.7–1.9)	2.0<br>(1.0–2.6)
Non-venomous animal contact	0.2<br>(0.1–0.2)	0.4<br>(0.3–0.5)	0.1<br>(0.1–0.2)	0.1<br>(0.1–0.1)	0.2<br>(0.1–0.2)	0.5<br>(0.4–0.5)
Foreign body	1.7<br>(1.4–2.0)	5.3<br>(3.6–8.1)	0.6<br>(0.4–0.7)	0.5<br>(0.4–0.6)	1.3<br>(1.1–1.5)	10.2<br>(9.3–10.9)
Pulmonary aspiration and foreign body in airway	1.4<br>(1.1–1.7)	4.7<br>(2.9–7.6)	0.4<br>(0.3–0.6)	0.3<br>(0.2–0.4)	1.0<br>(0.9–1.2)	8.7<br>(7.9–9.4)
Foreign body in eyes	0<br>(0–0)	0<br>(0–0)	0<br>(0–0)	0<br>(0–0)	0<br>(0–0)	0<br>(0–0)
Foreign body in other body part	0.3<br>(0.2–0.4)	0.6<br>(0.3–0.9)	0.1<br>(0.1–0.2)	0.2<br>(0.1–0.2)	0.3<br>(0.2–0.4)	1.5<br>(1.2–1.8)
Environmental heat and cold exposure	0.4<br>(0.3–0.4)	0.3<br>(0.1–0.4)	0.1<br>(0.0–0.1)	0.1<br>(0.1–0.1)	0.6<br>(0.4–0.8)	2.6<br>(1.8–3.0)
Other unintentional injuries	0.6<br>(0.6–0.7)	1.0<br>(0.9–1.2)	0.4<br>(0.3–0.5)	0.4<br>(0.4–0.5)	0.7<br>(0.7–0.8)	2.1<br>(1.9–2.3)
Self-harm and interpersonal violence	9.0<br>(7.4–9.8)	1.4<br>(1.2–1.8)	1.2<br>(1.0–1.3)	9.5<br>(7.7–10.6)	13.1<br>(10.4–14.4)	24.2<br>(20.5–26.0)
Self-harm	6.8<br>(5.2–7.6)	0<br>(0–0)	0.5<br>(0.4–0.6)	7.1<br>(5.3–8.0)	10.8<br>(8.2–12.0)	19.6<br>(15.9–21.3)
Interpersonal violence	2.2<br>(2.0–2.3)	1.4<br>(1.2–1.8)	0.7<br>(0.6–0.8)	2.4<br>(2.2–2.6)	2.3<br>(2.1–2.5)	4.6<br>(4.2–4.9)
Assault by firearm	0.6<br>(0.5–0.7)	0.2<br>(0.2–0.3)	0.2<br>(0.1–0.2)	0.7<br>(0.6–0.8)	0.5<br>(0.4–0.6)	0.9<br>(0.7–1.2)
Assault by sharp object	0.5<br>(0.4–0.5)	0.1<br>(0.1–0.2)	0.1<br>(0.1–0.1)	0.6<br>(0.5–0.6)	0.5<br>(0.5–0.6)	1.1<br>(1.0–1.3)
Assault by other means	1.1<br>(1.0–1.2)	1.1<br>(0.9–1.3)	0.4<br>(0.3–0.5)	1.1<br>(1.0–1.3)	1.2<br>(1.1–1.4)	2.6<br>(2.2–2.8)
Forces of nature, war, and legal intervention	1.2<br>(0.7–1.7)	3.9<br>(2.5–5.3)	0.9<br>(0.5–1.4)	1.0<br>(0.5–1.4)	0.9<br>(0.5–1.3)	1.0<br>(0.6–1.4)
Exposure to forces of nature	0.1<br>(0.1–0.2)	0.1<br>(0.1–0.2)	0.1<br>(0.1–0.2)	0.1<br>(0.1–0.2)	0.1<br>(0.1–0.2)	0.2<br>(0.1–0.3)
Collective violence and legal intervention	1.1<br>(0.6–1.6)	3.8<br>(2.4–5.1)	0.8<br>(0.4–1.2)	0.9<br>(0.4–1.3)	0.7<br>(0.4–1.1)	0.8<br>(0.4–1.2)

SR Table 14: Global death rates (per 100,000) by cause and age group for males (with 95% uncertainty intervals), 1990						
Cause	All ages	0-5 years	5-14 years	15-49 years	50-69 years	70+ years
All causes	949.3 (934.8–963.6)	1939.7 (1920.5–1959.9)	127.5 (125.8–129.2)	325.9 (317.0–335.4)	2025.1 (1977.2–2070.7)	8766.0 (8643.1–8884.0)
Communicable, maternal, neonatal, and nutritional diseases	306.9 (297.4–318.9)	1622.2 (1563.4–1666.5)	58.5 (55.0–62.8)	72.1 (67.5–78.2)	250.8 (229.2–281.8)	886.0 (826.7–975.8)
HIV/AIDS and tuberculosis	42.0 (34.8–53.2)	15.8 (11.9–24.7)	2.7 (1.9–4.0)	35.0 (29.7–41.9)	117.3 (91.4–151.0)	234.9 (182.0–329.0)
Tuberculosis	35.3 (27.8–46.3)	10.3 (6.4–19.3)	2.5 (1.8–3.8)	25.0 (19.5–31.9)	110.6 (84.5–144.2)	232.5 (179.9–326.6)
HIV/AIDS	6.7 (6.1–7.4)	5.5 (5.1–6.1)	0.2 (0.1–0.2)	10.0 (9.0–11.1)	6.7 (5.9–7.7)	2.4 (2.0–2.9)
HIV/AIDS - Tuberculosis	1.7 (1.4–1.9)	1.8 (1.5–2.1)	0.0 (0.0–0.1)	2.3 (1.9–2.7)	1.8 (1.6–2.1)	0.7 (0.6–0.9)
HIV/AIDS resulting in other diseases	5.1 (4.5–5.8)	3.7 (3.2–4.2)	0.1 (0.1–0.1)	7.7 (6.8–8.8)	4.9 (4.2–5.7)	1.7 (1.3–2.1)
Diarrhea, lower respiratory, and other common infectious diseases	149.8 (142.6–158.8)	788.9 (737.3–847.5)	42.0 (37.2–47.6)	26.0 (23.6–28.7)	105.0 (97.0–115.3)	576.4 (540.1–622.3)
Diarrheal diseases	42.1 (38.0–45.9)	214.2 (183.1–243.3)	9.6 (8.3–10.9)	8.1 (7.4–9.0)	37.8 (34.7–41.2)	160.4 (148.4–174.1)
Intestinal infectious diseases	4.8 (2.6–7.8)	11.1 (5.9–19.4)	8.4 (4.8–13.3)	2.7 (1.4–4.6)	1.4 (0.7–2.5)	2.0 (1.2–3.1)
Typhoid fever	3.7 (2.0–6.3)	8.3 (4.1–15.2)	6.6 (3.6–10.7)	2.2 (1.2–3.8)	1.1 (0.6–2.1)	1.5 (0.8–2.4)
Paratyphoid fever	0.9 (0.4–1.7)	2.2 (0.9–4.4)	1.7 (0.8–3.2)	0.5 (0.2–1.0)	0.2 (0.1–0.5)	0.3 (0.1–0.5)
Other intestinal infectious diseases	0.1 (0.0–0.3)	0.6 (0.2–1.7)	0.0 (0.0–0.1)	0.0 (0.0–0.0)	0.0 (0.0–0.1)	0.2 (0.1–0.4)
Lower respiratory infections	64.3 (59.3–68.9)	327.5 (293.2–358.3)	8.1 (6.1–9.4)	8.5 (7.0–9.7)	50.8 (43.2–58.5)	381.5 (341.9–423.3)
Upper respiratory infections	0.1 (0.1–0.1)	0.3 (0.2–0.4)	0.0 (0.0–0.0)	0.0 (0.0–0.0)	0.2 (0.1–0.2)	0.5 (0.4–0.6)
Otitis media	0.1 (0.1–0.2)	0.6 (0.5–0.8)	0.1 (0.1–0.1)	0.0 (0.0–0.1)	0.1 (0.1–0.1)	0.2 (0.2–0.2)
Meningitis	9.8 (7.6–11.5)	50.9 (36.7–64.2)	3.8 (2.5–4.5)	2.9 (2.1–3.4)	6.4 (4.7–8.1)	13.3 (10.1–17.7)
Pneumococcal meningitis	2.4 (1.8–3.0)	10.7 (7.0–15.2)	1.2 (0.7–1.6)	0.9 (0.6–1.2)	1.8 (1.2–2.4)	3.7 (2.6–5.1)
H influenzae type B meningitis	3.0 (2.2–3.8)	18.8 (13.0–25.2)	1.1 (0.6–1.5)	0.5 (0.3–0.7)	1.1 (0.7–1.4)	2.3 (1.7–3.2)
Meningococcal meningitis	1.7 (1.2–2.3)	9.3 (5.8–13.6)	0.7 (0.4–1.0)	0.5 (0.3–0.6)	1.0 (0.7–1.4)	1.9 (1.4–2.6)
Other meningitis	2.7 (2.0–3.3)	12.1 (8.2–16.4)	0.9 (0.5–1.2)	1.0 (0.7–1.2)	2.6 (1.8–3.3)	5.4 (4.0–7.0)
Encephalitis	3.0 (2.7–3.4)	9.5 (7.9–11.2)	1.7 (1.5–2.0)	1.3 (1.2–1.5)	3.7 (3.3–4.3)	11.3 (9.8–13.3)
Diphtheria	0.3 (0.2–0.5)	1.8 (0.8–3.6)	0.2 (0.1–0.3)	0.0 (0.0–0.1)	0.1 (0.0–0.1)	0 (0–0)
Whooping cough	2.7 (1.0–6.4)	20.5 (7.2–48.6)	0.7 (0.2–1.6)	0.1 (0.0–0.2)	0.0 (0.0–0.1)	0 (0–0)
Tetanus	6.7 (5.8–8.2)	40.9 (35.4–46.9)	1.2 (0.7–2.1)	1.5 (0.8–2.4)	4.1 (2.5–7.9)	5.7 (3.5–10.9)
Measles	15.7 (6.2–31.0)	111.0 (44.1–217.4)	8.1 (3.1–16.3)	0.6 (0.2–1.5)	0.1 (0.0–0.3)	0 (0–0)
Varicella and herpes zoster	0.2 (0.2–0.3)	0.6 (0.5–0.9)	0.1 (0.1–0.1)	0.1 (0.1–0.1)	0.3 (0.2–0.4)	1.5 (1.1–2.0)
Neglected tropical diseases and malaria	20.7 (16.1–25.7)	109.6 (73.7–148.9)	9.4 (7.0–12.5)	6.4 (5.1–7.9)	12.0 (10.0–14.7)	15.6 (12.6–19.7)
Malaria	17.1 (12.7–22.0)	104.0 (68.3–143.9)	6.6 (4.0–9.5)	3.6 (2.6–4.8)	6.7 (4.9–9.1)	8.6 (5.8–12.6)
Chagas disease	0.2 (0.2–0.2)	0.0 (0.0–0.0)	0.0 (0.0–0.0)	0.1 (0.1–0.1)	0.8 (0.8–0.9)	1.7 (1.6–1.8)
Leishmaniasis	0.5 (0.3–0.8)	0.8 (0.4–1.3)	0.3 (0.1–0.5)	0.6 (0.3–0.9)	0.5 (0.3–0.7)	0.3 (0.2–0.4)
Visceral leishmaniasis	0.5 (0.3–0.8)	0.8 (0.4–1.3)	0.3 (0.1–0.5)	0.6 (0.3–0.9)	0.5 (0.3–0.7)	0.3 (0.2–0.4)
Cutaneous and mucocutaneous leishmaniasis	0 (0–0)	0 (0–0)	0 (0–0)	0 (0–0)	0 (0–0)	0 (0–0)
African trypanosomiasis	0.4 (0.2–0.8)	0.3 (0.1–0.5)	0.3 (0.1–0.4)	0.6 (0.3–1.0)	0.5 (0.2–0.9)	0.4 (0.2–0.7)
Schistosomiasis	0.3 (0.2–0.3)	0.0 (0.0–0.1)	0.0 (0.0–0.1)	0.2 (0.2–0.2)	0.9 (0.7–1.1)	1.4 (1.1–1.9)
Cysticercosis	0.0 (0.0–0.0)	0.0 (0.0–0.0)	0.0 (0.0–0.0)	0.0 (0.0–0.0)	0.0 (0.0–0.0)	0.0 (0.0–0.0)
Cystic echinococcosis	0.1 (0.0–0.1)	0.0 (0.0–0.0)	0.0 (0.0–0.0)	0.0 (0.0–0.0)	0.1 (0.1–0.1)	0.2 (0.2–0.2)
Lymphatic filariasis	0 (0–0)	0 (0–0)	0 (0–0)	0 (0–0)	0 (0–0)	0 (0–0)
Onchocerciasis	0 (0–0)	0 (0–0)	0 (0–0)	0 (0–0)	0 (0–0)	0 (0–0)
Trachoma	0 (0–0)	0 (0–0)	0 (0–0)	0 (0–0)	0 (0–0)	0 (0–0)
Dengue	0.1 (0.1–0.2)	0.4 (0.1–1.0)	0.1 (0.1–0.2)	0.0 (0.0–0.1)	0.1 (0.0–0.1)	0.1 (0.1–0.2)
Yellow fever	0.4 (0.1–1.0)	0.3 (0.1–0.8)	0.4 (0.1–1.2)	0.4 (0.1–1.0)	0.2 (0.0–0.7)	0.2 (0.0–0.5)
Rabies	1.1 (1.0–1.2)	1.2 (0.9–1.7)	1.4 (1.2–1.7)	0.7 (0.6–0.8)	1.7 (1.4–1.9)	1.8 (1.4–2.1)
Intestinal nematode infections	0.1 (0.1–0.1)	0.8 (0.6–1.0)	0.1 (0.1–0.1)	0.0 (0.0–0.0)	0.0 (0.0–0.0)	0.1 (0.0–0.1)
Ascariasis	0.1 (0.1–0.1)	0.8 (0.6–1.0)	0.1 (0.1–0.1)	0.0 (0.0–0.0)	0.0 (0.0–0.0)	0.1 (0.0–0.1)
Trichuriasis	0 (0–0)	0 (0–0)	0 (0–0)	0 (0–0)	0 (0–0)	0 (0–0)
Hookworm disease	0 (0–0)	0 (0–0)	0 (0–0)	0 (0–0)	0 (0–0)	0 (0–0)
Food-borne trematodiases	0 (0–0)	0 (0–0)	0 (0–0)	0 (0–0)	0 (0–0)	0 (0–0)
Leprosy	0 (0–0)	0 (0–0)	0 (0–0)	0 (0–0)	0 (0–0)	0 (0–0)
Ebola	0 (0–0)	0 (0–0)	0 (0–0)	0 (0–0)	0 (0–0)	0 (0–0)
Other neglected tropical diseases	0.4 (0.2–0.6)	1.6 (0.8–2.3)	0.2 (0.1–0.3)	0.1 (0.1–0.2)	0.4 (0.2–0.7)	0.7 (0.4–1.4)
Maternal disorders	0 (0–0)	0 (0–0)	0 (0–0)	0 (0–0)	0 (0–0)	0 (0–0)
Maternal hemorrhage	0 (0–0)	0 (0–0)	0 (0–0)	0 (0–0)	0 (0–0)	0 (0–0)
Maternal sepsis and other maternal infections	0 (0–0)	0 (0–0)	0 (0–0)	0 (0–0)	0 (0–0)	0 (0–0)
Maternal hypertensive disorders	0 (0–0)	0 (0–0)	0 (0–0)	0 (0–0)	0 (0–0)	0 (0–0)
Maternal obstructed labor and uterine rupture	0 (0–0)	0 (0–0)	0 (0–0)	0 (0–0)	0 (0–0)	0 (0–0)
Maternal abortion, miscarriage, and ectopic pregnancy	0 (0–0)	0 (0–0)	0 (0–0)	0 (0–0)	0 (0–0)	0 (0–0)

SR Table 14 - Global death rates (per 100,000) by age and cause for males in 1990						
Cause	All ages	0-5 years	5-14 years	15-49 years	50-69 years	70+ years
Indirect maternal deaths	0<br>(0–0)	0<br>(0–0)	0<br>(0–0)	0<br>(0–0)	0<br>(0–0)	0<br>(0–0)
Late maternal deaths	0<br>(0–0)	0<br>(0–0)	0<br>(0–0)	0<br>(0–0)	0<br>(0–0)	0<br>(0–0)
Maternal deaths aggravated by HIV/AIDS	0<br>(0–0)	0<br>(0–0)	0<br>(0–0)	0<br>(0–0)	0<br>(0–0)	0<br>(0–0)
Other maternal disorders	0<br>(0–0)	0<br>(0–0)	0<br>(0–0)	0<br>(0–0)	0<br>(0–0)	0<br>(0–0)
Neonatal disorders	73.5<br>(71.1–76.2)	597.6<br>(577.9–619.4)	0<br>(0–0)	0<br>(0–0)	0<br>(0–0)	0<br>(0–0)
Neonatal preterm birth complications	36.5<br>(32.8–40.6)	296.4<br>(266.7–329.6)	0<br>(0–0)	0<br>(0–0)	0<br>(0–0)	0<br>(0–0)
Neonatal encephalopathy due to birth asphyxia and trauma	19.6<br>(16.2–23.1)	159.6<br>(132.0–188.0)	0<br>(0–0)	0<br>(0–0)	0<br>(0–0)	0<br>(0–0)
Neonatal sepsis and other neonatal infections	7.9<br>(4.9–12.2)	63.8<br>(39.8–98.9)	0<br>(0–0)	0<br>(0–0)	0<br>(0–0)	0<br>(0–0)
Hemolytic disease and other neonatal jaundice	2.5<br>(1.6–4.6)	20.2<br>(13.0–37.6)	0<br>(0–0)	0<br>(0–0)	0<br>(0–0)	0<br>(0–0)
Other neonatal disorders	7.1<br>(4.8–9.6)	57.7<br>(39.2–77.8)	0<br>(0–0)	0<br>(0–0)	0<br>(0–0)	0<br>(0–0)
Nutritional deficiencies	11.5<br>(7.1–14.8)	67.4<br>(39.3–93.1)	1.8<br>(1.3–2.4)	1.6<br>(1.0–2.0)	6.8<br>(4.7–8.7)	39.6<br>(27.0–48.3)
Protein-energy malnutrition	10.1<br>(6.3–13.3)	62.8<br>(37.3–87.6)	1.5<br>(1.0–1.9)	1.0<br>(0.7–1.2)	4.8<br>(3.5–6.2)	31.9<br>(21.7–38.9)
Iodine deficiency	0.0<br>(0.0–0.1)	0.2<br>(0.1–0.4)	0.0<br>(0.0–0.0)	0.0<br>(0.0–0.0)	0.1<br>(0.0–0.1)	0.2<br>(0.1–0.3)
Vitamin A deficiency	0<br>(0–0)	0<br>(0–0)	0<br>(0–0)	0<br>(0–0)	0<br>(0–0)	0<br>(0–0)
Iron-deficiency anemia	0.7<br>(0.3–1.0)	2.3<br>(0.5–3.8)	0.1<br>(0.1–0.3)	0.3<br>(0.2–0.5)	1.1<br>(0.5–1.7)	2.9<br>(1.2–4.3)
Other nutritional deficiencies	0.7<br>(0.4–1.1)	2.1<br>(1.0–4.0)	0.2<br>(0.1–0.3)	0.3<br>(0.2–0.4)	0.8<br>(0.5–1.3)	4.6<br>(3.5–6.9)
Other communicable, maternal, neonatal, and nutritional diseases	9.3<br>(7.6–11.5)	43.0<br>(30.4–59.3)	2.6<br>(2.1–3.3)	3.3<br>(3.0–3.5)	9.7<br>(8.5–10.4)	19.5<br>(16.4–21.0)
Sexually transmitted diseases excluding HIV	4.0<br>(2.5–6.2)	28.3<br>(16.2–44.6)	1.2<br>(0.7–1.9)	0.3<br>(0.1–0.3)	0.9<br>(0.4–1.2)	2.1<br>(1.1–2.8)
Syphilis	4.0<br>(2.4–6.1)	28.3<br>(16.2–44.6)	1.2<br>(0.7–1.8)	0.2<br>(0.1–0.3)	0.8<br>(0.4–1.1)	1.8<br>(1.0–2.4)
Chlamydial infection	0<br>(0–0)	0<br>(0–0)	0<br>(0–0)	0<br>(0–0)	0<br>(0–0)	0<br>(0–0)
Gonococcal infection	0.0<br>(0.0–0.0)	0<br>(0–0)	0.0<br>(0.0–0.0)	0.0<br>(0.0–0.0)	0.1<br>(0.0–0.1)	0.2<br>(0.1–0.3)
Trichomoniasis	0<br>(0–0)	0<br>(0–0)	0<br>(0–0)	0<br>(0–0)	0<br>(0–0)	0<br>(0–0)
Genital herpes	0<br>(0–0)	0<br>(0–0)	0<br>(0–0)	0<br>(0–0)	0<br>(0–0)	0<br>(0–0)
Other sexually transmitted diseases	0.0<br>(0.0–0.0)	0<br>(0–0)	0.0<br>(0.0–0.0)	0.0<br>(0.0–0.0)	0.0<br>(0.0–0.0)	0.1<br>(0.0–0.1)
Hepatitis	3.2<br>(3.0–3.4)	6.3<br>(5.3–7.1)	1.0<br>(0.9–1.1)	2.4<br>(2.2–2.6)	5.9<br>(5.5–6.3)	10.1<br>(9.5–10.6)
Acute hepatitis A	0.7<br>(0.5–0.9)	4.8<br>(3.5–5.9)	0.1<br>(0.0–0.3)	0.1<br>(0.0–0.1)	0.1<br>(0.0–0.2)	0.1<br>(0.0–0.3)
Hepatitis B	1.7<br>(1.5–1.9)	0.4<br>(0.1–0.8)	0.1<br>(0.1–0.2)	1.6<br>(1.3–1.8)	4.8<br>(4.2–5.5)	7.6<br>(6.2–8.8)
Hepatitis C	0.1<br>(0.0–0.1)	0<br>(0–0)	0.0<br>(0.0–0.0)	0.0<br>(0.0–0.1)	0.2<br>(0.0–0.5)	0.3<br>(0.1–0.7)
Acute hepatitis E	0.8<br>(0.6–1.1)	1.1<br>(0.4–2.1)	0.7<br>(0.5–0.8)	0.7<br>(0.5–1.0)	0.7<br>(0.4–1.3)	2.1<br>(1.1–3.5)
Other infectious diseases	2.0<br>(1.2–2.6)	8.4<br>(3.3–12.6)	0.5<br>(0.3–0.6)	0.6<br>(0.5–0.7)	3.0<br>(2.0–3.4)	7.4<br>(5.1–8.6)
Non-communicable diseases	532.2<br>(519.2–546.2)	193.9<br>(162.2–250.5)	24.0<br>(22.2–25.8)	138.6<br>(133.5–144.0)	1629.8<br>(1586.2–1674.0)	7592.7<br>(7462.5–7710.1)
Neoplasms	119.4<br>(115.9–122.8)	8.7<br>(6.7–12.4)	6.0<br>(5.4–6.5)	32.3<br>(30.3–33.7)	464.9<br>(450.4–478.2)	1416.0<br>(1376.9–1459.0)
Lip and oral cavity cancer	1.9<br>(1.8–2.0)	0<br>(0–0)	0<br>(0–0)	0.7<br>(0.6–0.7)	8.6<br>(8.2–9.1)	16.9<br>(16.2–17.7)
Nasopharynx cancer	1.1<br>(0.8–1.2)	0<br>(0–0)	0.1<br>(0.0–0.1)	0.7<br>(0.4–0.8)	4.6<br>(3.3–5.0)	7.1<br>(5.6–7.6)
Other pharynx cancer	1.0<br>(1.0–1.0)	0<br>(0–0)	0<br>(0–0)	0.3<br>(0.3–0.3)	5.0<br>(4.8–5.2)	7.1<br>(6.8–7.4)
Esophageal cancer	8.7<br>(8.0–9.5)	0<br>(0–0)	0<br>(0–0)	1.8<br>(1.6–2.0)	40.1<br>(36.9–43.7)	92.8<br>(85.7–102.1)
Stomach cancer	17.4<br>(16.7–18.2)	0<br>(0–0)	0<br>(0–0)	3.9<br>(3.6–4.1)	71.6<br>(68.4–74.9)	217.1<br>(209.8–224.7)
Colon and rectum cancer	9.1<br>(8.9–9.4)	0<br>(0–0)	0<br>(0–0)	1.9<br>(1.8–2.0)	32.4<br>(31.5–33.5)	135.4<br>(132.1–139.3)
Liver cancer	13.2<br>(11.0–14.5)	0<br>(0–0)	0.2<br>(0.1–0.2)	6.1<br>(4.5–6.9)	56.7<br>(47.6–62.6)	98.7<br>(90.9–108.3)
Liver cancer due to hepatitis B	5.5<br>(4.4–6.2)	0<br>(0–0)	0.1<br>(0.1–0.1)	3.8<br>(2.8–4.3)	22.1<br>(18.1–25.0)	26.9<br>(23.5–31.1)
Liver cancer due to hepatitis C	1.9<br>(1.7–2.1)	0<br>(0–0)	0.0<br>(0.0–0.0)	0.4<br>(0.3–0.4)	8.3<br>(7.2–9.2)	22.3<br>(20.3–24.9)
Liver cancer due to alcohol use	3.9<br>(3.3–4.3)	0<br>(0–0)	0<br>(0–0)	0.9<br>(0.7–1.1)	18.9<br>(16.0–21.4)	35.7<br>(31.9–39.6)
Liver cancer due to other causes	1.9<br>(1.5–2.1)	0<br>(0–0)	0.1<br>(0.0–0.1)	1.1<br>(0.7–1.2)	7.4<br>(6.1–8.5)	13.9<br>(12.2–15.9)
Gallbladder and biliary tract cancer	1.4<br>(1.4–1.5)	0<br>(0–0)	0<br>(0–0)	0.2<br>(0.2–0.3)	5.1<br>(4.9–5.3)	22.5<br>(21.8–23.1)
Pancreatic cancer	4.3<br>(4.2–4.4)	0<br>(0–0)	0<br>(0–0)	0.8<br>(0.8–0.8)	17.6<br>(17.1–18.1)	56.0<br>(54.6–57.3)
Larynx cancer	2.5<br>(2.4–2.6)	0<br>(0–0)	0<br>(0–0)	0.6<br>(0.5–0.6)	12.0<br>(11.6–12.4)	24.6<br>(23.8–25.4)
Tracheal, bronchus, and lung cancer	28.1<br>(27.3–29.0)	0<br>(0–0)	0<br>(0–0)	4.6<br>(4.4–4.9)	126.1<br>(122.3–130.3)	332.3<br>(322.9–345.1)
Malignant skin melanoma	0.6<br>(0.4–0.9)	0<br>(0–0)	0<br>(0–0)	0.3<br>(0.2–0.4)	2.2<br>(1.5–3.3)	6.0<br>(4.3–10.0)
Non-melanoma skin cancer	0.7<br>(0.6–0.7)	0<br>(0–0)	0<br>(0–0)	0.2<br>(0.1–0.2)	2.2<br>(2.1–2.2)	10.4<br>(10.1–10.8)
Non-melanoma skin cancer (squamous-cell carcinoma)	0.7<br>(0.6–0.7)	0<br>(0–0)	0<br>(0–0)	0.2<br>(0.1–0.2)	2.2<br>(2.1–2.2)	10.4<br>(10.1–10.8)
Non-melanoma skin cancer (basal-cell carcinoma)	0<br>(0–0)	0<br>(0–0)	0<br>(0–0)	0<br>(0–0)	0<br>(0–0)	0<br>(0–0)
Breast cancer	0.1<br>(0.1–0.1)	0<br>(0–0)	0<br>(0–0)	0.0<br>(0.0–0.0)	0.5<br>(0.4–0.5)	1.7<br>(1.5–1.8)
Cervical cancer	0<br>(0–0)	0<br>(0–0)	0<br>(0–0)	0<br>(0–0)	0<br>(0–0)	0<br>(0–0)
Uterine cancer	0<br>(0–0)	0<br>(0–0)	0<br>(0–0)	0<br>(0–0)	0<br>(0–0)	0<br>(0–0)
Ovarian cancer	0<br>(0–0)	0<br>(0–0)	0<br>(0–0)	0<br>(0–0)	0<br>(0–0)	0<br>(0–0)
Prostate cancer	7.2<br>(5.8–8.9)	0<br>(0–0)	0<br>(0–0)	0.1<br>(0.1–0.2)	15.1<br>(12.1–18.9)	173.1<br>(136.0–215.1)
Testicular cancer	0.3<br>(0.3–0.3)	0<br>(0–0)	0<br>(0–0)	0.4<br>(0.4–0.5)	0.5<br>(0.4–0.5)	1.2<br>(1.1–1.3)

SR Table 14 - Global death rates (per 100,000) by age and cause for males in 1990

Cause	All ages	0-5 years	5-14 years	15-49 years	50-69 years	70+ years
Kidney cancer	1.6 (1.6–1.7)	0.5 (0.3–0.7)	0.1 (0.1–0.1)	0.3 (0.3–0.4)	6.5 (6.3–6.8)	19.1 (18.5–19.8)
Bladder cancer	3.0 (2.9–3.1)	0 (0–0)	0 (0–0)	0.3 (0.3–0.3)	9.6 (9.2–10.0)	56.4 (54.6–58.3)
Brain and nervous system cancer	2.9 (2.5–3.7)	1.3 (0.7–2.8)	1.1 (0.8–1.5)	1.8 (1.4–2.1)	9.6 (8.3–11.1)	15.3 (13.9–18.7)
Thyroid cancer	0.3 (0.2–0.3)	0 (0–0)	0.0 (0.0–0.0)	0.1 (0.1–0.1)	1.0 (0.9–1.1)	3.3 (3.1–3.7)
Mesothelioma	0.5 (0.4–0.5)	0 (0–0)	0 (0–0)	0.2 (0.1–0.2)	1.8 (1.6–1.9)	5.1 (4.7–5.8)
Hodgkin lymphoma	0.7 (0.5–1.0)	0.2 (0.1–0.3)	0.2 (0.1–0.2)	0.5 (0.4–0.7)	2.2 (1.5–2.9)	4.0 (3.0–5.5)
Non-Hodgkin lymphoma	2.6 (2.3–3.0)	0.7 (0.4–1.3)	0.5 (0.4–0.7)	1.1 (1.0–1.3)	8.1 (7.2–9.3)	26.7 (24.3–32.5)
Multiple myeloma	0.9 (0.9–1.0)	0 (0–0)	0 (0–0)	0.2 (0.2–0.2)	3.5 (3.4–3.7)	13.2 (12.8–13.7)
Leukemia	5.2 (5.0–5.4)	3.5 (3.0–4.1)	2.9 (2.7–3.1)	3.1 (2.9–3.3)	11.4 (11.0–11.8)	38.7 (37.6–39.8)
Acute lymphoid leukemia	1.9 (1.6–2.3)	2.3 (1.6–3.2)	2.1 (1.8–2.4)	1.3 (1.1–1.6)	2.9 (2.5–3.4)	5.6 (4.9–6.6)
Chronic lymphoid leukemia	0.8 (0.7–0.8)	0 (0–0)	0 (0–0)	0.2 (0.2–0.3)	2.1 (1.9–2.4)	12.4 (11.0–13.3)
Acute myeloid leukemia	1.8 (1.5–2.1)	1.2 (0.6–1.8)	0.9 (0.5–1.1)	1.1 (0.9–1.3)	4.3 (3.8–5.0)	13.1 (11.9–15.5)
Chronic myeloid leukemia	0.7 (0.6–0.8)	0 (0–0)	0 (0–0)	0.5 (0.4–0.6)	2.0 (1.8–2.3)	7.5 (6.9–8.2)
Other neoplasms	3.9 (3.5–4.2)	2.4 (1.4–4.3)	1.0 (0.8–1.1)	2.1 (1.7–2.3)	11.0 (9.4–12.3)	31.5 (28.7–36.1)
Cardiovascular diseases	231.5 (226.3–236.7)	19.3 (17.2–21.6)	4.1 (3.8–4.5)	50.6 (48.7–52.9)	709.5 (689.7–729.3)	3770.6 (3697.2–3838.1)
Rheumatic heart disease	5.3 (5.0–5.8)	1.1 (0.9–1.5)	0.9 (0.8–1.1)	3.1 (2.8–3.4)	15.2 (14.2–16.5)	50.8 (48.1–54.1)
Ischemic heart disease	116.9 (114.4–119.4)	1.3 (1.0–1.6)	0.3 (0.3–0.4)	24.7 (23.8–25.8)	370.4 (360.7–380.0)	1913.9 (1875.5–1949.2)
Cerebrovascular disease	80.2 (77.6–83.0)	7.9 (7.0–9.0)	1.6 (1.4–1.8)	15.3 (14.6–16.3)	243.6 (233.6–254.1)	1347.4 (1307.8–1393.5)
Ischemic stroke	34.0 (32.6–35.6)	0.3 (0.2–0.4)	0.1 (0.1–0.1)	2.4 (2.2–2.7)	85.5 (80.4–90.7)	729.6 (703.7–759.1)
Hemorrhagic stroke	46.2 (44.0–48.2)	7.6 (6.7–8.6)	1.5 (1.4–1.7)	12.8 (12.2–13.7)	158.2 (150.0–166.3)	617.8 (588.8–644.5)
Hypertensive heart disease	11.1 (10.5–12.8)	0.2 (0.1–0.2)	0.1 (0.0–0.1)	2.5 (2.3–2.8)	35.0 (32.5–40.5)	181.1 (169.3–208.3)
Cardiomyopathy and myocarditis	5.3 (5.0–5.6)	2.8 (2.1–3.6)	0.4 (0.4–0.5)	2.3 (2.1–2.5)	14.8 (13.6–15.8)	61.4 (58.0–64.4)
Atrial fibrillation and flutter	1.4 (1.1–1.7)	0 (0–0)	0 (0–0)	0.0 (0.0–0.0)	1.3 (1.0–1.7)	40.2 (31.9–50.0)
Aortic aneurysm	2.4 (2.3–2.5)	0 (0–0)	0 (0–0)	0.4 (0.3–0.4)	6.8 (6.5–7.3)	44.1 (42.5–46.0)
Peripheral vascular disease	0.3 (0.3–0.3)	0 (0–0)	0 (0–0)	0.0 (0.0–0.0)	0.7 (0.6–0.7)	7.2 (6.8–7.7)
Endocarditis	0.7 (0.6–0.9)	0.9 (0.4–1.5)	0.1 (0.1–0.1)	0.3 (0.3–0.4)	1.7 (1.4–2.0)	7.6 (6.6–9.1)
Other cardiovascular and circulatory diseases	7.8 (7.5–8.2)	5.1 (4.5–6.4)	0.6 (0.6–0.7)	2.0 (1.9–2.2)	20.0 (18.9–21.0)	116.8 (111.1–122.3)
Chronic respiratory diseases	70.5 (67.5–73.3)	19.5 (16.7–22.7)	1.7 (1.5–1.9)	8.9 (8.4–9.6)	197.3 (186.8–207.8)	1275.7 (1223.2–1322.2)
Chronic obstructive pulmonary disease	57.1 (51.6–61.7)	8.4 (5.9–11.2)	0.4 (0.3–0.5)	5.3 (4.4–6.1)	157.6 (138.5–174.2)	1112.0 (1022.7–1183.9)
Pneumoconiosis	0.8 (0.7–0.9)	0 (0–0)	0 (0–0)	0.2 (0.1–0.2)	2.4 (2.0–2.8)	13.3 (11.4–15.3)
Silicosis	0.4 (0.3–0.5)	0 (0–0)	0 (0–0)	0.1 (0.1–0.1)	1.3 (1.0–1.6)	6.0 (4.8–7.4)
Asbestosis	0.0 (0.0–0.1)	0 (0–0)	0 (0–0)	0.0 (0.0–0.0)	0.1 (0.1–0.2)	0.7 (0.5–1.0)
Coal workers pneumoconiosis	0.1 (0.1–0.2)	0 (0–0)	0 (0–0)	0.0 (0.0–0.0)	0.3 (0.3–0.5)	2.3 (1.9–2.9)
Other pneumoconiosis	0.2 (0.2–0.3)	0 (0–0)	0 (0–0)	0.1 (0.0–0.1)	0.7 (0.5–0.8)	4.3 (3.6–5.0)
Asthma	10.5 (6.6–14.0)	5.5 (3.7–7.3)	1.2 (0.9–1.4)	3.0 (2.1–3.7)	33.1 (19.9–45.8)	127.8 (75.4–179.4)
Interstitial lung disease and pulmonary sarcoidosis	0.8 (0.6–1.2)	0.1 (0.0–0.2)	0.0 (0.0–0.0)	0.1 (0.1–0.2)	2.4 (1.8–3.8)	14.7 (11.5–21.2)
Other chronic respiratory diseases	1.3 (0.8–1.7)	5.5 (3.4–8.1)	0.2 (0.1–0.3)	0.3 (0.2–0.4)	1.8 (1.1–2.4)	8.0 (5.6–10.4)
Cirrhosis and other chronic liver diseases	22.1 (21.2–24.2)	4.0 (3.0–4.8)	1.2 (1.0–1.3)	13.9 (13.2–15.3)	82.7 (79.1–91.5)	133.0 (127.3–138.4)
Cirrhosis and other chronic liver diseases due to hepatitis B	7.0 (6.5–7.9)	0.0 (0.0–0.1)	0.0 (0.0–0.1)	4.6 (4.1–5.1)	27.0 (24.2–31.0)	44.9 (40.8–50.0)
Cirrhosis and other chronic liver diseases due to hepatitis C	5.5 (5.1–5.9)	0.0 (0.0–0.0)	0.0 (0.0–0.0)	3.2 (2.8–3.6)	21.3 (19.1–23.9)	39.2 (34.7–42.8)
Cirrhosis and other chronic liver diseases due to alcohol use	6.1 (5.7–6.7)	0 (0–0)	0 (0–0)	4.3 (3.9–4.7)	24.7 (22.3–27.7)	28.6 (25.6–32.0)
Cirrhosis and other chronic liver diseases due to other causes	3.5 (3.2–3.9)	3.9 (3.0–4.8)	1.1 (1.0–1.3)	1.9 (1.7–2.1)	9.7 (8.5–11.3)	20.3 (18.3–22.4)
Digestive diseases	20.2 (18.9–21.6)	7.9 (6.0–9.9)	2.6 (2.3–2.9)	10.4 (9.5–11.2)	57.0 (53.0–61.3)	208.2 (196.6–225.5)
Peptic ulcer disease	7.2 (6.6–8.0)	0.9 (0.5–1.7)	0.5 (0.4–0.7)	3.7 (3.3–4.1)	22.9 (20.8–25.3)	74.7 (68.2–83.7)
Gastritis and duodenitis	1.0 (0.7–1.3)	0.3 (0.2–0.6)	0.1 (0.1–0.1)	0.6 (0.4–0.7)	3.2 (2.2–4.2)	10.1 (7.0–13.5)
Appendicitis	0.8 (0.7–1.0)	0.8 (0.5–1.2)	0.4 (0.3–0.5)	0.6 (0.5–0.7)	1.7 (1.4–2.2)	4.6 (4.0–5.8)
Paralytic ileus and intestinal obstruction	4.0 (3.1–4.6)	2.2 (1.2–3.5)	0.9 (0.6–1.0)	2.2 (1.6–2.6)	10.4 (7.8–12.2)	35.8 (30.7–44.6)
Inguinal, femoral, and abdominal hernia	1.1 (0.7–1.4)	0.5 (0.1–0.8)	0.1 (0.1–0.2)	0.5 (0.3–0.6)	3.0 (1.9–3.9)	13.3 (8.9–16.0)
Inflammatory bowel disease	0.6 (0.4–0.7)	0.4 (0.2–1.0)	0.1 (0.0–0.1)	0.3 (0.2–0.3)	1.5 (1.2–1.7)	6.2 (5.1–7.1)
Vascular intestinal disorders	0.9 (0.8–1.0)	0.2 (0.1–0.4)	0.0 (0.0–0.0)	0.2 (0.1–0.2)	2.6 (2.4–2.8)	15.7 (14.7–17.0)
Gallbladder and biliary diseases	1.3 (1.1–1.4)	0.3 (0.1–0.4)	0.1 (0.1–0.1)	0.4 (0.4–0.5)	3.4 (2.9–3.8)	18.5 (16.8–20.0)
Pancreatitis	1.5 (1.4–1.7)	0.1 (0.0–0.1)	0.0 (0.0–0.1)	1.3 (1.2–1.5)	4.1 (3.8–4.6)	9.2 (8.5–10.1)
Other digestive diseases	1.8 (1.4–2.4)	2.2 (1.3–3.2)	0.3 (0.2–0.5)	0.7 (0.5–1.0)	4.4 (3.1–6.0)	20.2 (15.3–25.5)
Neurological disorders	16.5 (14.6–18.5)	4.8 (2.9–6.3)	1.5 (1.4–1.7)	3.2 (3.1–3.4)	17.1 (14.7–19.6)	390.0 (331.0–448.5)
Alzheimer disease and other dementias	11.9 (10.0–13.8)	0 (0–0)	0 (0–0)	0.0 (0.0–0.0)	9.6 (7.2–12.0)	351.5 (292.3–410.4)
Parkinson disease	0.9 (0.9–1.0)	0 (0–0)	0 (0–0)	0.0 (0.0–0.0)	1.4 (1.2–1.5)	24.9 (23.4–27.1)
Epilepsy	2.4 (2.1–2.6)	3.6 (2.1–4.9)	1.2 (1.1–1.4)	2.4 (2.3–2.6)	2.5 (2.4–2.7)	4.3 (4.0–4.5)

SR Table 14 - Global death rates (per 100,000) by age and cause for males in 1990						
Cause	All ages	0-5 years	5-14 years	15-49 years	50-69 years	70+ years
Multiple sclerosis	0.2 (0.2–0.2)	0 (0–0)	0 (0–0)	0.2 (0.1–0.2)	0.8 (0.7–0.9)	1.2 (1.0–1.2)
Motor neuron disease	0.4 (0.3–0.4)	0.3 (0.2–0.3)	0.0 (0.0–0.0)	0.1 (0.1–0.1)	1.4 (1.3–1.5)	3.4 (3.2–3.6)
Migraine	0 (0–0)	0 (0–0)	0 (0–0)	0 (0–0)	0 (0–0)	0 (0–0)
Tension-type headache	0 (0–0)	0 (0–0)	0 (0–0)	0 (0–0)	0 (0–0)	0 (0–0)
Medication overuse headache	0 (0–0)	0 (0–0)	0 (0–0)	0 (0–0)	0 (0–0)	0 (0–0)
Other neurological disorders	0.8 (0.7–0.8)	1.0 (0.6–1.4)	0.3 (0.2–0.3)	0.5 (0.4–0.5)	1.4 (1.3–1.6)	4.8 (4.4–5.2)
Mental and substance use disorders	5.2 (4.9–5.4)	0.0 (0.0–0.0)	0.0 (0.0–0.0)	6.2 (5.7–6.4)	13.3 (12.8–13.8)	12.0 (11.4–12.5)
Schizophrenia	0.3 (0.3–0.3)	0 (0–0)	0 (0–0)	0.3 (0.2–0.3)	0.8 (0.7–0.9)	1.5 (1.3–1.7)
Alcohol use disorders	3.0 (2.9–3.1)	0 (0–0)	0 (0–0)	3.1 (2.9–3.2)	9.7 (9.3–10.2)	7.4 (7.1–7.7)
Drug use disorders	1.9 (1.6–2.0)	0.0 (0.0–0.0)	0 (0–0)	2.8 (2.3–3.0)	2.8 (2.5–3.0)	3.1 (2.8–3.4)
Opioid use disorders	1.4 (1.2–1.5)	0.0 (0.0–0.0)	0 (0–0)	2.2 (1.8–2.3)	2.1 (1.9–2.3)	2.1 (1.9–2.4)
Cocaine use disorders	0.1 (0.0–0.1)	0 (0–0)	0 (0–0)	0.1 (0.1–0.1)	0.1 (0.0–0.1)	0.0 (0.0–0.1)
Amphetamine use disorders	0.0 (0.0–0.0)	0 (0–0)	0 (0–0)	0.1 (0.0–0.1)	0.0 (0.0–0.1)	0.0 (0.0–0.0)
Cannabis use disorders	0 (0–0)	0 (0–0)	0 (0–0)	0 (0–0)	0 (0–0)	0 (0–0)
Other drug use disorders	0.3 (0.2–0.4)	0 (0–0)	0 (0–0)	0.5 (0.3–0.6)	0.5 (0.4–0.6)	0.9 (0.7–1.0)
Depressive disorders	0 (0–0)	0 (0–0)	0 (0–0)	0 (0–0)	0 (0–0)	0 (0–0)
Major depressive disorder	0 (0–0)	0 (0–0)	0 (0–0)	0 (0–0)	0 (0–0)	0 (0–0)
Dysthymia	0 (0–0)	0 (0–0)	0 (0–0)	0 (0–0)	0 (0–0)	0 (0–0)
Bipolar disorder	0 (0–0)	0 (0–0)	0 (0–0)	0 (0–0)	0 (0–0)	0 (0–0)
Anxiety disorders	0 (0–0)	0 (0–0)	0 (0–0)	0 (0–0)	0 (0–0)	0 (0–0)
Eating disorders	0.0 (0.0–0.0)	0 (0–0)	0.0 (0.0–0.0)	0.0 (0.0–0.0)	0 (0–0)	0 (0–0)
Anorexia nervosa	0.0 (0.0–0.0)	0 (0–0)	0.0 (0.0–0.0)	0.0 (0.0–0.0)	0 (0–0)	0 (0–0)
Bulimia nervosa	0.0 (0.0–0.0)	0 (0–0)	0.0 (0.0–0.0)	0.0 (0.0–0.0)	0 (0–0)	0 (0–0)
Autistic spectrum disorders	0 (0–0)	0 (0–0)	0 (0–0)	0 (0–0)	0 (0–0)	0 (0–0)
Autism	0 (0–0)	0 (0–0)	0 (0–0)	0 (0–0)	0 (0–0)	0 (0–0)
Asperger syndrome and other autistic spectrum disorders	0 (0–0)	0 (0–0)	0 (0–0)	0 (0–0)	0 (0–0)	0 (0–0)
Attention-deficit/hyperactivity disorder	0 (0–0)	0 (0–0)	0 (0–0)	0 (0–0)	0 (0–0)	0 (0–0)
Conduct disorder	0 (0–0)	0 (0–0)	0 (0–0)	0 (0–0)	0 (0–0)	0 (0–0)
Idiopathic developmental intellectual disability	0 (0–0)	0 (0–0)	0 (0–0)	0 (0–0)	0 (0–0)	0 (0–0)
Other mental and substance use disorders	0 (0–0)	0 (0–0)	0 (0–0)	0 (0–0)	0 (0–0)	0 (0–0)
Diabetes, urogenital, blood, and endocrine diseases	30.6 (28.2–32.3)	24.3 (16.4–31.6)	3.3 (2.8–3.6)	10.6 (9.4–11.5)	83.3 (77.8–88.4)	366.0 (346.8–387.4)
Diabetes mellitus	11.2 (10.8–11.6)	0.7 (0.5–0.8)	0.2 (0.2–0.3)	2.9 (2.7–3.0)	40.1 (38.6–41.8)	151.9 (147.2–156.9)
Acute glomerulonephritis	0.3 (0.2–0.3)	0.2 (0.1–0.2)	0.1 (0.1–0.1)	0.2 (0.1–0.2)	0.8 (0.6–1.0)	2.3 (1.7–2.8)
Chronic kidney disease	11.1 (9.5–12.4)	3.5 (2.4–4.1)	1.0 (0.7–1.1)	4.7 (3.7–5.1)	29.8 (25.6–34.3)	145.3 (132.6–162.9)
Chronic kidney disease due to diabetes mellitus	3.3 (3.0–3.7)	0.3 (0.2–0.4)	0.1 (0.1–0.1)	1.0 (0.8–1.1)	10.7 (9.3–12.1)	47.3 (43.5–52.2)
Chronic kidney disease due to hypertension	4.7 (4.0–5.2)	1.2 (0.8–1.4)	0.3 (0.2–0.3)	1.7 (1.4–1.9)	12.1 (10.3–14.0)	67.8 (61.5–76.1)
Chronic kidney disease due to glomerulonephritis	2.8 (2.3–3.2)	1.5 (1.0–1.8)	0.5 (0.3–0.5)	1.7 (1.3–1.9)	6.6 (5.5–7.8)	27.1 (23.6–31.8)
Chronic kidney disease due to other causes	0.3 (0.3–0.4)	0.5 (0.3–0.7)	0.2 (0.1–0.2)	0.2 (0.1–0.3)	0.4 (0.2–0.6)	3.0 (2.5–3.8)
Urinary diseases and male infertility	2.8 (2.4–3.6)	2.0 (1.3–2.7)	0.2 (0.2–0.3)	0.9 (0.7–1.2)	6.7 (5.2–9.3)	40.4 (34.5–54.0)
Interstitial nephritis and urinary tract infections	1.8 (1.5–2.3)	1.6 (0.9–2.1)	0.1 (0.1–0.2)	0.5 (0.4–0.7)	3.8 (2.8–5.3)	27.0 (22.2–34.9)
Urolithiasis	0.4 (0.2–0.5)	0 (0–0)	0.0 (0.0–0.0)	0.1 (0.1–0.2)	1.2 (0.7–1.5)	4.7 (2.9–6.1)
Benign prostatic hyperplasia	0 (0–0)	0 (0–0)	0 (0–0)	0 (0–0)	0 (0–0)	0 (0–0)
Male infertility	0 (0–0)	0 (0–0)	0 (0–0)	0 (0–0)	0 (0–0)	0 (0–0)
Other urinary diseases	0.7 (0.5–1.0)	0.4 (0.3–0.8)	0.1 (0.0–0.1)	0.2 (0.1–0.4)	1.7 (1.1–3.0)	8.8 (6.3–14.0)
Gynecological diseases	0 (0–0)	0 (0–0)	0 (0–0)	0 (0–0)	0 (0–0)	0 (0–0)
Uterine fibroids	0 (0–0)	0 (0–0)	0 (0–0)	0 (0–0)	0 (0–0)	0 (0–0)
Polycystic ovarian syndrome	0 (0–0)	0 (0–0)	0 (0–0)	0 (0–0)	0 (0–0)	0 (0–0)
Female infertility	0 (0–0)	0 (0–0)	0 (0–0)	0 (0–0)	0 (0–0)	0 (0–0)
Endometriosis	0 (0–0)	0 (0–0)	0 (0–0)	0 (0–0)	0 (0–0)	0 (0–0)
Genital prolapse	0 (0–0)	0 (0–0)	0 (0–0)	0 (0–0)	0 (0–0)	0 (0–0)
Premenstrual syndrome	0 (0–0)	0 (0–0)	0 (0–0)	0 (0–0)	0 (0–0)	0 (0–0)
Other gynecological diseases	0 (0–0)	0 (0–0)	0 (0–0)	0 (0–0)	0 (0–0)	0 (0–0)
Hemoglobinopathies and hemolytic anemias	3.7 (2.6–4.6)	14.7 (7.7–21.3)	1.4 (1.1–1.8)	1.5 (1.2–1.9)	3.3 (2.6–4.2)	15.0 (11.6–18.7)
Thalassemias	0.5 (0.3–0.7)	2.9 (1.6–4.3)	0.4 (0.3–0.6)	0.1 (0.0–0.1)	0.0 (0.0–0.0)	0.9 (0.5–1.3)
Thalassemias trait	0 (0–0)	0 (0–0)	0 (0–0)	0 (0–0)	0 (0–0)	0 (0–0)
Sickle cell disorders	1.8 (1.1–2.6)	11.3 (5.7–17.6)	0.7 (0.5–0.9)	0.5 (0.3–0.7)	0.1 (0.1–0.1)	1.0 (0.7–1.4)
Sickle cell trait	0 (0–0)	0 (0–0)	0 (0–0)	0 (0–0)	0 (0–0)	0 (0–0)

SR Table 14 - Global death rates (per 100,000) by age and cause for males in 1990						
Cause	All ages	0-5 years	5-14 years	15-49 years	50-69 years	70+ years
G6PD deficiency	0.5 (0.4–0.6)	0.3 (0.1–0.6)	0.2 (0.1–0.2)	0.5 (0.4–0.7)	0.9 (0.7–1.2)	0.7 (0.5–0.9)
G6PD trait	0 (0–0)	0 (0–0)	0 (0–0)	0 (0–0)	0 (0–0)	0 (0–0)
Other hemoglobinopathies and hemolytic anemias	0.9 (0.7–1.1)	0.2 (0.1–0.3)	0.1 (0.1–0.1)	0.4 (0.3–0.5)	2.3 (1.7–2.9)	12.4 (9.8–15.3)
Endocrine, metabolic, blood, and immune disorders	1.4 (1.4–1.5)	3.3 (2.8–3.9)	0.4 (0.4–0.4)	0.6 (0.5–0.6)	2.5 (2.3–2.8)	11.0 (10.4–11.8)
Musculoskeletal disorders	0.6 (0.5–0.7)	0 (0–0)	0.1 (0.1–0.2)	0.3 (0.3–0.3)	1.6 (1.3–1.7)	8.4 (6.6–9.0)
Rheumatoid arthritis	0.2 (0.2–0.3)	0 (0–0)	0.0 (0.0–0.0)	0.0 (0.0–0.1)	0.7 (0.5–0.8)	3.7 (2.7–4.1)
Osteoarthritis	0 (0–0)	0 (0–0)	0 (0–0)	0 (0–0)	0 (0–0)	0 (0–0)
Low back and neck pain	0 (0–0)	0 (0–0)	0 (0–0)	0 (0–0)	0 (0–0)	0 (0–0)
Low back pain	0 (0–0)	0 (0–0)	0 (0–0)	0 (0–0)	0 (0–0)	0 (0–0)
Neck pain	0 (0–0)	0 (0–0)	0 (0–0)	0 (0–0)	0 (0–0)	0 (0–0)
Gout	0 (0–0)	0 (0–0)	0 (0–0)	0 (0–0)	0 (0–0)	0 (0–0)
Other musculoskeletal disorders	0.4 (0.4–0.4)	0 (0–0)	0.1 (0.1–0.1)	0.2 (0.2–0.3)	0.9 (0.8–1.0)	4.7 (3.7–5.1)
Other non-communicable diseases	15.6 (11.8–22.5)	105.4 (76.8–158.4)	3.4 (2.6–4.4)	2.1 (1.9–2.6)	3.1 (2.5–3.9)	12.9 (9.9–17.2)
Congenital anomalies	14.3 (10.5–20.9)	100.0 (71.8–150.2)	3.4 (2.5–4.4)	1.9 (1.7–2.3)	1.6 (1.5–2.0)	3.8 (3.5–4.7)
Neural tube defects	2.7 (1.8–4.3)	20.4 (13.8–33.5)	0.4 (0.3–0.5)	0.1 (0.1–0.2)	0.1 (0.1–0.2)	0.3 (0.2–0.5)
Congenital heart anomalies	7.4 (5.3–10.9)	49.4 (34.1–76.4)	2.4 (1.7–3.1)	1.4 (1.2–1.6)	0.6 (0.5–0.7)	1.4 (1.2–1.8)
Cleft lip and cleft palate	0.1 (0.1–0.2)	0.9 (0.6–1.5)	0 (0–0)	0 (0–0)	0 (0–0)	0 (0–0)
Down syndrome	0.3 (0.2–0.8)	1.9 (0.8–5.8)	0.1 (0.0–0.1)	0.1 (0.1–0.2)	0.3 (0.2–0.4)	0.1 (0.0–0.1)
Turner syndrome	0 (0–0)	0 (0–0)	0 (0–0)	0 (0–0)	0 (0–0)	0 (0–0)
Klinefelter syndrome	0 (0–0)	0 (0–0)	0 (0–0)	0 (0–0)	0 (0–0)	0 (0–0)
Other chromosomal abnormalities	0.3 (0.1–0.8)	2.0 (0.7–6.3)	0.0 (0.0–0.0)	0.0 (0.0–0.0)	0.0 (0.0–0.1)	0.0 (0.0–0.1)
Other congenital anomalies	3.5 (2.5–5.1)	25.3 (17.6–38.2)	0.5 (0.4–0.7)	0.3 (0.2–0.3)	0.6 (0.5–0.7)	2.0 (1.7–2.5)
Skin and subcutaneous diseases	0.7 (0.4–1.1)	0.8 (0.3–1.5)	0.1 (0.0–0.1)	0.2 (0.1–0.4)	1.5 (0.9–2.3)	9.1 (6.0–13.1)
Dermatitis	0 (0–0)	0 (0–0)	0 (0–0)	0 (0–0)	0 (0–0)	0 (0–0)
Psoriasis	0 (0–0)	0 (0–0)	0 (0–0)	0 (0–0)	0 (0–0)	0 (0–0)
Cellulitis	0.1 (0.1–0.3)	0.1 (0.0–0.3)	0.0 (0.0–0.0)	0.1 (0.0–0.1)	0.4 (0.2–0.9)	1.3 (0.8–2.8)
Pyoderma	0.3 (0.2–0.5)	0.6 (0.3–1.2)	0.0 (0.0–0.1)	0.1 (0.1–0.2)	0.5 (0.3–0.8)	2.3 (1.6–3.7)
Scabies	0 (0–0)	0 (0–0)	0 (0–0)	0 (0–0)	0 (0–0)	0 (0–0)
Fungal skin diseases	0 (0–0)	0 (0–0)	0 (0–0)	0 (0–0)	0 (0–0)	0 (0–0)
Viral skin diseases	0 (0–0)	0 (0–0)	0 (0–0)	0 (0–0)	0 (0–0)	0 (0–0)
Acne vulgaris	0 (0–0)	0 (0–0)	0 (0–0)	0 (0–0)	0 (0–0)	0 (0–0)
Alopecia areata	0 (0–0)	0 (0–0)	0 (0–0)	0 (0–0)	0 (0–0)	0 (0–0)
Pruritus	0 (0–0)	0 (0–0)	0 (0–0)	0 (0–0)	0 (0–0)	0 (0–0)
Urticaria	0 (0–0)	0 (0–0)	0 (0–0)	0 (0–0)	0 (0–0)	0 (0–0)
Decubitus ulcer	0.3 (0.1–0.3)	0.0 (0.0–0.0)	0.0 (0.0–0.0)	0.0 (0.0–0.1)	0.6 (0.2–1.0)	5.1 (2.9–7.0)
Other skin and subcutaneous diseases	0.0 (0.0–0.1)	0.0 (0.0–0.1)	0.0 (0.0–0.0)	0.0 (0.0–0.0)	0.1 (0.0–0.1)	0.4 (0.2–0.6)
Sense organ diseases	0 (0–0)	0 (0–0)	0 (0–0)	0 (0–0)	0 (0–0)	0 (0–0)
Glaucoma	0 (0–0)	0 (0–0)	0 (0–0)	0 (0–0)	0 (0–0)	0 (0–0)
Cataract	0 (0–0)	0 (0–0)	0 (0–0)	0 (0–0)	0 (0–0)	0 (0–0)
Macular degeneration	0 (0–0)	0 (0–0)	0 (0–0)	0 (0–0)	0 (0–0)	0 (0–0)
Refraction and accommodation disorders	0 (0–0)	0 (0–0)	0 (0–0)	0 (0–0)	0 (0–0)	0 (0–0)
Age-related and other hearing loss	0 (0–0)	0 (0–0)	0 (0–0)	0 (0–0)	0 (0–0)	0 (0–0)
Other vision loss	0 (0–0)	0 (0–0)	0 (0–0)	0 (0–0)	0 (0–0)	0 (0–0)
Other sense organ diseases	0 (0–0)	0 (0–0)	0 (0–0)	0 (0–0)	0 (0–0)	0 (0–0)
Oral disorders	0 (0–0)	0 (0–0)	0 (0–0)	0 (0–0)	0 (0–0)	0 (0–0)
Deciduous caries	0 (0–0)	0 (0–0)	0 (0–0)	0 (0–0)	0 (0–0)	0 (0–0)
Permanent caries	0 (0–0)	0 (0–0)	0 (0–0)	0 (0–0)	0 (0–0)	0 (0–0)
Periodontal diseases	0 (0–0)	0 (0–0)	0 (0–0)	0 (0–0)	0 (0–0)	0 (0–0)
Edentulism and severe tooth loss	0 (0–0)	0 (0–0)	0 (0–0)	0 (0–0)	0 (0–0)	0 (0–0)
Other oral disorders	0 (0–0)	0 (0–0)	0 (0–0)	0 (0–0)	0 (0–0)	0 (0–0)
Sudden infant death syndrome	0.6 (0.4–1.4)	4.7 (3.5–11.7)	0 (0–0)	0 (0–0)	0 (0–0)	0 (0–0)
Injuries	110.3 (105.1–114.6)	123.6 (98.9–142.6)	45.0 (42.0–47.9)	115.3 (110.7–119.8)	144.5 (137.2–151.0)	287.3 (268.6–302.0)
Transport injuries	32.8 (31.2–34.2)	19.8 (14.5–24.8)	12.4 (11.4–13.3)	39.3 (37.5–41.1)	44.2 (41.8–46.6)	71.1 (67.6–75.0)
Road injuries	30.8 (29.1–32.3)	18.3 (13.4–22.9)	11.9 (10.9–12.7)	37.1 (35.4–38.7)	40.8 (38.5–43.0)	67.0 (63.7–70.6)
Pedestrian road injuries	13.3 (11.1–14.7)	10.4 (7.8–13.2)	6.9 (6.1–7.6)	13.6 (11.1–15.2)	20.4 (16.9–22.6)	37.4 (32.1–40.8)
Cyclist road injuries	1.2 (1.1–1.5)	0.5 (0.2–0.8)	0.7 (0.6–0.9)	1.2 (1.0–1.5)	2.3 (2.0–2.8)	4.0 (3.6–4.5)
Motorcyclist road injuries	4.6 (3.6–5.9)	1.5 (0.7–2.7)	0.9 (0.6–1.3)	6.9 (5.4–8.7)	4.4 (3.1–6.0)	5.0 (3.4–6.9)

SR Table 14 - Global death rates (per 100,000) by age and cause for males in 1990						
Cause	All ages	0-5 years	5-14 years	15-49 years	50-69 years	70+ years
Motor vehicle road injuries	11.3 (10.0–13.3)	5.6 (3.5–7.7)	3.2 (2.8–3.9)	15.0 (13.4–17.7)	13.2 (11.2–16.5)	19.7 (16.7–24.6)
Other road injuries	0.3 (0.2–0.5)	0.3 (0.1–0.7)	0.2 (0.1–0.3)	0.4 (0.2–0.5)	0.5 (0.3–0.6)	0.9 (0.6–1.3)
Other transport injuries	2.0 (1.7–2.3)	1.5 (0.8–2.1)	0.6 (0.4–0.7)	2.3 (1.9–2.5)	3.4 (2.8–3.9)	4.1 (3.3–4.6)
Unintentional injuries	47.6 (44.5–50.8)	97.3 (77.4–114.9)	27.8 (25.7–29.9)	35.4 (33.5–37.7)	57.3 (53.7–61.4)	151.2 (139.2–165.6)
Falls	7.9 (7.2–8.5)	6.3 (3.5–8.9)	2.2 (1.8–2.5)	5.5 (5.1–5.9)	15.7 (14.4–16.7)	63.3 (57.9–67.1)
Drowning	14.4 (13.2–15.7)	38.2 (31.1–45.3)	15.8 (14.5–17.0)	9.5 (8.7–10.3)	9.1 (8.3–10.0)	13.4 (12.2–14.7)
Fire, heat, and hot substances	3.9 (2.8–4.2)	7.7 (5.4–9.4)	1.3 (1.0–1.5)	3.2 (2.2–3.5)	4.8 (3.8–5.1)	14.1 (11.4–14.8)
Poisonings	3.3 (2.0–4.1)	8.1 (2.3–14.4)	1.1 (0.8–1.5)	2.5 (1.8–3.1)	4.5 (3.3–5.4)	6.2 (4.6–7.7)
Exposure to mechanical forces	5.4 (4.5–6.6)	9.9 (6.5–13.4)	2.1 (1.6–2.8)	5.0 (4.3–6.3)	6.4 (5.5–8.1)	11.7 (10.1–13.6)
Unintentional firearm injuries	1.0 (0.7–1.1)	0.9 (0.5–1.3)	0.4 (0.3–0.5)	1.1 (0.8–1.3)	0.9 (0.7–1.1)	2.4 (1.9–2.8)
Unintentional suffocation	1.0 (0.7–1.4)	6.1 (3.9–8.6)	0.4 (0.3–0.7)	0.2 (0.2–0.3)	0.2 (0.2–0.3)	1.8 (1.5–2.1)
Other exposure to mechanical forces	3.4 (2.7–4.5)	2.9 (1.5–4.7)	1.2 (0.9–1.8)	3.7 (3.0–4.9)	5.2 (4.3–7.0)	7.5 (6.2–9.3)
Adverse effects of medical treatment	2.1 (1.5–2.6)	5.1 (2.2–7.7)	0.6 (0.4–0.8)	1.1 (0.8–1.4)	3.1 (2.5–3.8)	11.6 (9.3–13.7)
Animal contact	2.4 (1.7–2.7)	3.7 (1.8–4.8)	1.8 (1.2–2.1)	1.8 (1.3–2.1)	3.7 (2.6–4.2)	5.7 (4.5–6.7)
Venomous animal contact	1.9 (1.3–2.2)	2.6 (1.2–3.3)	1.4 (0.9–1.7)	1.5 (1.0–1.8)	2.9 (2.0–3.5)	4.3 (3.0–5.4)
Non-venomous animal contact	0.5 (0.3–0.7)	1.1 (0.5–1.7)	0.4 (0.2–0.5)	0.3 (0.2–0.4)	0.8 (0.5–1.0)	1.4 (1.0–1.8)
Foreign body	3.6 (2.5–5.7)	13.8 (7.3–21.1)	1.3 (0.9–2.1)	1.7 (1.2–4.0)	3.4 (2.7–7.3)	11.7 (9.5–23.6)
Pulmonary aspiration and foreign body in airway	2.5 (1.7–3.6)	10.3 (4.7–17.1)	0.9 (0.6–1.2)	0.9 (0.8–1.1)	2.5 (2.1–2.9)	9.2 (8.0–10.2)
Foreign body in eyes	0 (0–0)	0 (0–0)	0 (0–0)	0 (0–0)	0 (0–0)	0 (0–0)
Foreign body in other body part	1.1 (0.5–3.7)	3.5 (1.4–6.4)	0.4 (0.2–1.4)	0.7 (0.3–3.1)	0.9 (0.4–4.7)	2.5 (1.2–13.8)
Environmental heat and cold exposure	1.2 (0.8–1.5)	1.3 (0.6–1.7)	0.2 (0.1–0.3)	0.9 (0.7–1.1)	2.6 (2.0–3.7)	5.6 (3.9–7.7)
Other unintentional injuries	3.5 (3.2–3.8)	3.2 (2.2–4.0)	1.4 (1.2–1.6)	4.1 (3.7–4.5)	4.0 (3.7–4.5)	7.9 (6.7–9.4)
Self-harm and interpersonal violence	25.9 (23.6–26.9)	3.9 (3.0–4.5)	2.8 (2.4–3.0)	35.1 (32.1–36.6)	40.0 (36.4–41.9)	62.0 (55.0–65.4)
Self-harm	15.8 (13.7–16.7)	0 (0–0)	1.1 (0.8–1.3)	19.9 (17.1–21.0)	30.7 (27.1–32.4)	53.7 (46.3–56.9)
Interpersonal violence	10.1 (9.6–10.5)	3.9 (3.0–4.5)	1.7 (1.6–1.9)	15.3 (14.6–15.9)	9.3 (8.8–10.0)	8.3 (7.8–9.4)
Assault by firearm	3.9 (3.7–4.2)	0.4 (0.3–0.5)	0.4 (0.3–0.4)	6.5 (6.2–7.0)	3.0 (2.7–3.4)	2.0 (1.8–2.2)
Assault by sharp object	2.6 (2.3–2.8)	0.5 (0.3–0.8)	0.3 (0.3–0.4)	4.1 (3.6–4.5)	2.2 (2.0–2.5)	1.9 (1.7–2.1)
Assault by other means	3.6 (3.1–4.1)	3.0 (2.2–3.6)	1.0 (0.9–1.2)	4.7 (4.0–5.2)	4.1 (3.8–4.7)	4.4 (4.0–5.3)
Forces of nature, war, and legal intervention	3.9 (2.7–5.4)	2.6 (1.3–4.4)	2.0 (1.1–3.1)	5.4 (3.3–7.9)	3.0 (1.8–4.6)	3.0 (1.8–4.4)
Exposure to forces of nature	1.2 (0.6–1.8)	0.9 (0.4–1.4)	1.2 (0.5–1.8)	1.3 (0.6–1.9)	1.3 (0.6–1.9)	1.6 (0.8–2.4)
Collective violence and legal intervention	2.7 (1.6–4.0)	1.7 (0.6–3.4)	0.8 (0.3–1.7)	4.1 (2.1–6.5)	1.8 (0.8–3.3)	1.4 (0.6–2.6)

SR Table 14 - Global death rates (per 100,000) by age and cause for males in 1995

Cause	All ages	0-5 years	5-14 years	15-49 years	50-69 years	70+ years
All causes	929.7 (917.8–941.9)	1723.6 (1706.1–1742.0)	114.9 (113.5–116.3)	350.0 (342.5–357.8)	2030.2 (1992.4–2068.4)	8564.3 (8463.0–8662.5)
Communicable, maternal, neonatal, and nutritional diseases	273.5 (265.9–284.8)	1448.7 (1402.8–1486.8)	52.2 (49.0–55.6)	81.7 (77.3–87.8)	241.2 (221.4–268.6)	830.7 (781.8–905.4)
HIV/AIDS and tuberculosis	48.0 (41.5–58.0)	24.4 (21.3–30.7)	3.0 (2.4–4.0)	46.0 (41.0–52.1)	113.7 (92.6–142.6)	205.2 (160.4–285.8)
Tuberculosis	31.8 (25.2–41.9)	8.5 (5.7–15.2)	2.1 (1.6–3.2)	23.1 (18.1–29.3)	97.0 (75.2–126.1)	198.7 (154.0–279.6)
HIV/AIDS	16.1 (15.0–17.7)	15.9 (15.0–16.8)	0.9 (0.8–0.9)	23.0 (21.1–25.4)	16.7 (15.0–19.1)	6.5 (5.6–8.2)
HIV/AIDS - Tuberculosis	3.4 (2.9–4.0)	4.8 (4.1–5.4)	0.2 (0.2–0.3)	4.3 (3.6–5.1)	4.1 (3.4–4.8)	1.8 (1.5–2.2)
HIV/AIDS resulting in other diseases	12.7 (11.6–14.2)	11.1 (10.1–12.3)	0.6 (0.5–0.7)	18.7 (16.9–20.8)	12.6 (11.1–14.9)	4.6 (3.9–6.0)
Diarrhea, lower respiratory, and other common infectious diseases	123.6 (116.9–130.3)	649.0 (603.3–695.9)	36.1 (32.1–40.9)	24.4 (22.3–27.0)	98.9 (92.3–110.2)	549.3 (514.8–597.4)
Diarrheal diseases	33.1 (30.3–36.1)	171.0 (147.6–194.2)	7.8 (6.8–8.8)	7.2 (6.6–7.9)	32.6 (30.1–35.5)	136.9 (127.4–147.9)
Intestinal infectious diseases	4.3 (2.4–7.0)	10.8 (5.8–19.1)	7.9 (4.6–12.5)	2.4 (1.2–4.1)	1.2 (0.6–2.2)	1.7 (1.0–2.7)
Typhoid fever	3.5 (1.9–5.9)	8.7 (4.5–15.8)	6.5 (3.6–10.5)	1.9 (1.0–3.3)	1.0 (0.5–1.7)	1.3 (0.7–2.1)
Paratyphoid fever	0.8 (0.3–1.4)	1.8 (0.7–3.5)	1.4 (0.6–2.6)	0.4 (0.2–0.8)	0.2 (0.1–0.5)	0.3 (0.1–0.6)
Other intestinal infectious diseases	0.1 (0.0–0.2)	0.4 (0.1–1.0)	0.0 (0.0–0.1)	0.0 (0.0–0.0)	0.0 (0.0–0.1)	0.2 (0.1–0.3)
Lower respiratory infections	55.8 (51.8–60.3)	274.6 (246.6–300.5)	7.3 (5.4–8.3)	8.8 (7.4–10.1)	51.6 (44.4–60.3)	381.3 (342.9–425.1)
Upper respiratory infections	0.1 (0.1–0.1)	0.3 (0.2–0.4)	0.0 (0.0–0.0)	0.0 (0.0–0.0)	0.1 (0.1–0.2)	0.4 (0.3–0.5)
Otitis media	0.1 (0.1–0.1)	0.4 (0.4–0.5)	0.1 (0.1–0.1)	0.0 (0.0–0.0)	0.1 (0.1–0.1)	0.2 (0.1–0.2)
Meningitis	8.6 (6.9–10.1)	45.6 (33.8–57.3)	3.5 (2.4–4.2)	2.9 (2.1–3.4)	6.2 (4.7–7.8)	12.7 (9.7–16.5)
Pneumococcal meningitis	2.1 (1.7–2.7)	9.8 (6.6–14.2)	1.1 (0.7–1.5)	0.9 (0.7–1.2)	1.7 (1.2–2.4)	3.5 (2.6–4.9)
H influenzae type B meningitis	2.6 (1.9–3.2)	17.0 (11.8–22.8)	1.0 (0.6–1.4)	0.5 (0.3–0.7)	1.0 (0.7–1.4)	2.1 (1.6–3.0)
Meningococcal meningitis	1.6 (1.1–2.0)	8.7 (5.6–12.6)	0.7 (0.4–1.0)	0.5 (0.3–0.6)	1.0 (0.7–1.4)	1.8 (1.3–2.5)
Other meningitis	2.3 (1.8–2.8)	10.1 (7.1–13.7)	0.8 (0.5–1.1)	1.0 (0.7–1.2)	2.5 (1.8–3.1)	5.2 (3.9–6.7)
Encephalitis	2.8 (2.5–3.1)	9.0 (7.7–10.6)	1.6 (1.4–1.9)	1.3 (1.2–1.5)	3.5 (3.1–4.0)	10.4 (9.2–12.0)
Diphtheria	0.2 (0.1–0.4)	1.3 (0.6–3.2)	0.1 (0.1–0.2)	0.0 (0.0–0.0)	0.0 (0.0–0.1)	0 (0–0)
Whooping cough	2.1 (0.7–4.9)	17.3 (6.1–40.2)	0.5 (0.2–1.3)	0.1 (0.0–0.1)	0.0 (0.0–0.1)	0 (0–0)
Tetanus	4.2 (3.7–5.4)	25.8 (22.8–29.3)	0.9 (0.6–1.6)	1.2 (0.7–2.1)	3.1 (2.1–6.2)	4.2 (2.8–8.6)
Measles	12.0 (4.5–24.1)	92.3 (35.3–184.3)	6.2 (2.3–12.7)	0.5 (0.1–1.2)	0.1 (0.0–0.2)	0 (0–0)
Varicella and herpes zoster	0.2 (0.2–0.2)	0.6 (0.4–0.8)	0.1 (0.1–0.1)	0.1 (0.1–0.1)	0.3 (0.2–0.4)	1.4 (1.1–1.9)
Neglected tropical diseases and malaria	20.8 (16.4–25.3)	120.4 (84.1–160.4)	9.0 (6.8–11.7)	6.4 (5.2–7.9)	11.8 (9.6–14.4)	15.5 (12.3–20.0)
Malaria	17.6 (13.3–22.0)	115.6 (79.0–155.2)	6.5 (4.1–9.2)	3.8 (2.8–5.2)	7.2 (5.3–9.7)	9.4 (6.3–13.7)
Chagas disease	0.2 (0.2–0.2)	0.0 (0.0–0.0)	0.0 (0.0–0.0)	0.1 (0.1–0.1)	0.7 (0.6–0.7)	1.5 (1.4–1.7)
Leishmaniasis	0.5 (0.3–0.8)	0.8 (0.4–1.2)	0.3 (0.1–0.5)	0.6 (0.3–0.9)	0.5 (0.3–0.7)	0.3 (0.2–0.4)
Visceral leishmaniasis	0.5 (0.3–0.8)	0.8 (0.4–1.2)	0.3 (0.1–0.5)	0.6 (0.3–0.9)	0.5 (0.3–0.7)	0.3 (0.2–0.4)
Cutaneous and mucocutaneous leishmaniasis	0 (0–0)	0 (0–0)	0 (0–0)	0 (0–0)	0 (0–0)	0 (0–0)
African trypanosomiasis	0.5 (0.3–0.8)	0.3 (0.2–0.5)	0.3 (0.1–0.5)	0.6 (0.3–1.1)	0.6 (0.3–1.0)	0.4 (0.2–0.7)
Schistosomiasis	0.2 (0.2–0.2)	0.0 (0.0–0.1)	0.0 (0.0–0.0)	0.1 (0.1–0.2)	0.7 (0.5–0.9)	1.1 (0.8–1.6)
Cysticercosis	0.0 (0.0–0.0)	0.0 (0.0–0.0)	0.0 (0.0–0.0)	0.0 (0.0–0.0)	0.0 (0.0–0.0)	0.0 (0.0–0.0)
Cystic echinococcosis	0.0 (0.0–0.0)	0.0 (0.0–0.0)	0.0 (0.0–0.0)	0.0 (0.0–0.0)	0.1 (0.1–0.1)	0.2 (0.2–0.2)
Lymphatic filariasis	0 (0–0)	0 (0–0)	0 (0–0)	0 (0–0)	0 (0–0)	0 (0–0)
Onchocerciasis	0 (0–0)	0 (0–0)	0 (0–0)	0 (0–0)	0 (0–0)	0 (0–0)
Trachoma	0 (0–0)	0 (0–0)	0 (0–0)	0 (0–0)	0 (0–0)	0 (0–0)
Dengue	0.1 (0.1–0.2)	0.4 (0.2–0.8)	0.2 (0.1–0.3)	0.1 (0.0–0.1)	0.1 (0.0–0.1)	0.1 (0.1–0.2)
Yellow fever	0.2 (0.1–0.6)	0.2 (0.0–0.5)	0.3 (0.1–0.8)	0.2 (0.1–0.7)	0.1 (0.0–0.4)	0.1 (0.0–0.3)
Rabies	1.0 (0.8–1.0)	1.1 (0.7–1.5)	1.2 (1.0–1.5)	0.6 (0.5–0.7)	1.5 (1.2–1.7)	1.6 (1.2–1.8)
Intestinal nematode infections	0.1 (0.1–0.1)	0.7 (0.5–0.8)	0.1 (0.1–0.1)	0.0 (0.0–0.0)	0.0 (0.0–0.0)	0.1 (0.0–0.1)
Ascariasis	0.1 (0.1–0.1)	0.7 (0.5–0.8)	0.1 (0.1–0.1)	0.0 (0.0–0.0)	0.0 (0.0–0.0)	0.1 (0.0–0.1)
Trichuriasis	0 (0–0)	0 (0–0)	0 (0–0)	0 (0–0)	0 (0–0)	0 (0–0)
Hookworm disease	0 (0–0)	0 (0–0)	0 (0–0)	0 (0–0)	0 (0–0)	0 (0–0)
Food-borne trematodiases	0 (0–0)	0 (0–0)	0 (0–0)	0 (0–0)	0 (0–0)	0 (0–0)
Leprosy	0 (0–0)	0 (0–0)	0 (0–0)	0 (0–0)	0 (0–0)	0 (0–0)
Ebola	0.0 (0.0–0.0)	0.0 (0.0–0.0)	0.0 (0.0–0.0)	0.0 (0.0–0.0)	0.0 (0.0–0.0)	0.0 (0.0–0.0)
Other neglected tropical diseases	0.3 (0.2–0.5)	1.3 (0.7–1.9)	0.2 (0.1–0.3)	0.1 (0.1–0.2)	0.3 (0.2–0.6)	0.6 (0.4–1.1)
Maternal disorders	0 (0–0)	0 (0–0)	0 (0–0)	0 (0–0)	0 (0–0)	0 (0–0)
Maternal hemorrhage	0 (0–0)	0 (0–0)	0 (0–0)	0 (0–0)	0 (0–0)	0 (0–0)
Maternal sepsis and other maternal infections	0 (0–0)	0 (0–0)	0 (0–0)	0 (0–0)	0 (0–0)	0 (0–0)
Maternal hypertensive disorders	0 (0–0)	0 (0–0)	0 (0–0)	0 (0–0)	0 (0–0)	0 (0–0)
Maternal obstructed labor and uterine rupture	0 (0–0)	0 (0–0)	0 (0–0)	0 (0–0)	0 (0–0)	0 (0–0)
Maternal abortion, miscarriage, and ectopic pregnancy	0 (0–0)	0 (0–0)	0 (0–0)	0 (0–0)	0 (0–0)	0 (0–0)

SR Table 14 - Global death rates (per 100,000) by age and cause for males in 1995

Cause	All ages	0-5 years	5-14 years	15-49 years	50-69 years	70+ years
Indirect maternal deaths	0 (0–0)	0 (0–0)	0 (0–0)	0 (0–0)	0 (0–0)	0 (0–0)
Late maternal deaths	0 (0–0)	0 (0–0)	0 (0–0)	0 (0–0)	0 (0–0)	0 (0–0)
Maternal deaths aggravated by HIV/AIDS	0 (0–0)	0 (0–0)	0 (0–0)	0 (0–0)	0 (0–0)	0 (0–0)
Other maternal disorders	0 (0–0)	0 (0–0)	0 (0–0)	0 (0–0)	0 (0–0)	0 (0–0)
Neonatal disorders	61.8 (59.7–63.9)	548.2 (530.0–567.2)	0 (0–0)	0 (0–0)	0 (0–0)	0 (0–0)
Neonatal preterm birth complications	28.9 (26.0–32.2)	256.0 (230.5–286.2)	0 (0–0)	0 (0–0)	0 (0–0)	0 (0–0)
Neonatal encephalopathy due to birth asphyxia and trauma	17.6 (15.0–20.4)	156.6 (132.8–181.4)	0 (0–0)	0 (0–0)	0 (0–0)	0 (0–0)
Neonatal sepsis and other neonatal infections	7.5 (4.6–11.2)	66.1 (41.0–99.2)	0 (0–0)	0 (0–0)	0 (0–0)	0 (0–0)
Hemolytic disease and other neonatal jaundice	2.0 (1.3–3.7)	18.0 (11.8–33.0)	0 (0–0)	0 (0–0)	0 (0–0)	0 (0–0)
Other neonatal disorders	5.8 (3.9–7.8)	51.4 (34.8–69.0)	0 (0–0)	0 (0–0)	0 (0–0)	0 (0–0)
Nutritional deficiencies	11.3 (7.8–14.6)	68.1 (43.5–94.3)	1.8 (1.4–2.4)	1.7 (1.3–2.2)	7.7 (5.8–9.8)	42.3 (30.1–51.1)
Protein-energy malnutrition	10.0 (6.8–13.2)	64.2 (40.7–89.9)	1.5 (1.1–2.0)	1.2 (0.8–1.5)	5.8 (4.3–7.5)	35.1 (24.9–42.4)
Iodine deficiency	0.0 (0.0–0.1)	0.1 (0.0–0.3)	0.0 (0.0–0.0)	0.0 (0.0–0.0)	0.0 (0.0–0.1)	0.1 (0.1–0.3)
Vitamin A deficiency	0 (0–0)	0 (0–0)	0 (0–0)	0 (0–0)	0 (0–0)	0 (0–0)
Iron-deficiency anemia	0.7 (0.3–1.0)	2.2 (0.6–3.7)	0.1 (0.1–0.3)	0.3 (0.2–0.5)	1.1 (0.5–1.7)	3.0 (1.2–4.4)
Other nutritional deficiencies	0.5 (0.4–0.9)	1.5 (0.8–2.8)	0.1 (0.1–0.2)	0.2 (0.2–0.3)	0.7 (0.5–1.1)	4.1 (3.1–6.0)
Other communicable, maternal, neonatal, and nutritional diseases	8.2 (6.7–10.0)	38.6 (26.9–53.7)	2.3 (1.9–2.9)	3.1 (2.9–3.3)	9.1 (8.1–9.7)	18.5 (16.0–19.8)
Sexually transmitted diseases excluding HIV	3.4 (2.1–5.2)	26.0 (14.9–40.6)	1.0 (0.6–1.5)	0.2 (0.1–0.3)	0.8 (0.4–1.1)	1.9 (1.0–2.5)
Syphilis	3.4 (2.1–5.2)	26.0 (14.9–40.6)	1.0 (0.6–1.5)	0.2 (0.1–0.3)	0.7 (0.4–1.0)	1.7 (0.8–2.2)
Chlamydial infection	0 (0–0)	0 (0–0)	0 (0–0)	0 (0–0)	0 (0–0)	0 (0–0)
Gonococcal infection	0.0 (0.0–0.0)	0 (0–0)	0.0 (0.0–0.0)	0.0 (0.0–0.0)	0.0 (0.0–0.1)	0.2 (0.1–0.2)
Trichomoniasis	0 (0–0)	0 (0–0)	0 (0–0)	0 (0–0)	0 (0–0)	0 (0–0)
Genital herpes	0 (0–0)	0 (0–0)	0 (0–0)	0 (0–0)	0 (0–0)	0 (0–0)
Other sexually transmitted diseases	0.0 (0.0–0.0)	0 (0–0)	0.0 (0.0–0.0)	0.0 (0.0–0.0)	0.0 (0.0–0.0)	0.1 (0.0–0.1)
Hepatitis	3.0 (2.9–3.2)	5.4 (4.6–6.1)	0.9 (0.8–1.0)	2.3 (2.2–2.5)	5.7 (5.4–6.0)	10.1 (9.6–10.6)
Acute hepatitis A	0.6 (0.4–0.7)	4.2 (3.1–5.1)	0.1 (0.0–0.3)	0.1 (0.0–0.2)	0.1 (0.0–0.2)	0.2 (0.0–0.4)
Hepatitis B	1.7 (1.5–1.9)	0.3 (0.1–0.7)	0.1 (0.1–0.2)	1.5 (1.2–1.8)	4.7 (4.1–5.3)	7.6 (6.4–8.8)
Hepatitis C	0.1 (0.0–0.1)	0 (0–0)	0.0 (0.0–0.0)	0.0 (0.0–0.1)	0.2 (0.0–0.5)	0.3 (0.1–0.7)
Acute hepatitis E	0.7 (0.5–1.0)	0.9 (0.4–1.7)	0.6 (0.5–0.8)	0.7 (0.5–0.9)	0.7 (0.3–1.1)	2.0 (1.1–3.2)
Other infectious diseases	1.7 (1.1–2.2)	7.2 (3.1–10.8)	0.4 (0.3–0.5)	0.6 (0.5–0.6)	2.6 (1.9–3.0)	6.5 (4.8–7.5)
Non-communicable diseases	547.3 (535.9–558.6)	173.9 (146.0–216.7)	22.4 (20.8–23.8)	149.7 (145.1–154.9)	1640.7 (1605.6–1676.8)	7449.8 (7344.1–7552.7)
Neoplasms	122.9 (119.7–125.7)	7.9 (6.3–11.1)	5.8 (5.3–6.2)	34.5 (32.6–35.8)	459.5 (447.9–470.0)	1414.7 (1374.8–1453.1)
Lip and oral cavity cancer	2.0 (2.0–2.1)	0 (0–0)	0 (0–0)	0.7 (0.7–0.8)	8.8 (8.5–9.1)	17.1 (16.5–17.7)
Nasopharynx cancer	1.1 (0.8–1.2)	0 (0–0)	0.0 (0.0–0.1)	0.7 (0.5–0.8)	4.4 (3.3–4.7)	6.8 (5.6–7.2)
Other pharynx cancer	1.0 (1.0–1.1)	0 (0–0)	0 (0–0)	0.3 (0.3–0.3)	5.0 (4.8–5.2)	7.1 (6.8–7.4)
Esophageal cancer	8.8 (8.4–9.3)	0 (0–0)	0 (0–0)	1.9 (1.8–2.1)	39.1 (36.9–41.2)	91.1 (87.2–95.6)
Stomach cancer	16.7 (16.2–17.3)	0 (0–0)	0 (0–0)	3.9 (3.7–4.1)	66.3 (64.0–68.9)	199.3 (194.3–204.7)
Colon and rectum cancer	9.8 (9.6–10.0)	0 (0–0)	0 (0–0)	2.1 (2.1–2.2)	33.8 (33.1–34.7)	137.1 (134.5–139.9)
Liver cancer	13.9 (11.5–14.8)	0 (0–0)	0.2 (0.1–0.2)	6.9 (5.0–7.5)	56.4 (47.2–60.1)	101.2 (94.0–108.3)
Liver cancer due to hepatitis B	5.7 (4.5–6.2)	0 (0–0)	0.1 (0.1–0.1)	4.2 (3.1–4.7)	21.1 (17.3–23.3)	26.4 (23.4–29.8)
Liver cancer due to hepatitis C	2.0 (1.9–2.2)	0 (0–0)	0.0 (0.0–0.0)	0.4 (0.3–0.5)	8.5 (7.5–9.4)	23.8 (22.0–26.0)
Liver cancer due to alcohol use	4.3 (3.6–4.6)	0 (0–0)	0 (0–0)	1.1 (0.8–1.3)	19.9 (16.8–22.0)	37.3 (33.8–40.6)
Liver cancer due to other causes	1.9 (1.5–2.1)	0 (0–0)	0.1 (0.0–0.1)	1.1 (0.8–1.3)	6.9 (5.7–7.8)	13.8 (12.3–15.5)
Gallbladder and biliary tract cancer	1.4 (1.4–1.5)	0 (0–0)	0 (0–0)	0.2 (0.2–0.3)	4.9 (4.8–5.1)	21.6 (21.0–22.3)
Pancreatic cancer	4.5 (4.4–4.5)	0 (0–0)	0 (0–0)	0.9 (0.9–0.9)	17.7 (17.2–18.1)	55.5 (54.3–56.7)
Larynx cancer	2.5 (2.5–2.6)	0 (0–0)	0 (0–0)	0.6 (0.6–0.6)	11.7 (11.3–12.0)	23.9 (23.3–24.6)
Tracheal, bronchus, and lung cancer	29.1 (28.5–29.8)	0 (0–0)	0 (0–0)	5.1 (5.0–5.4)	124.7 (122.0–127.5)	338.6 (331.4–345.8)
Malignant skin melanoma	0.7 (0.5–1.0)	0 (0–0)	0 (0–0)	0.3 (0.2–0.5)	2.3 (1.6–3.4)	6.6 (4.4–10.1)
Non-melanoma skin cancer	0.7 (0.7–0.7)	0 (0–0)	0 (0–0)	0.2 (0.1–0.2)	2.1 (2.0–2.2)	10.1 (9.8–10.5)
Non-melanoma skin cancer (squamous-cell carcinoma)	0.7 (0.7–0.7)	0 (0–0)	0 (0–0)	0.2 (0.1–0.2)	2.1 (2.0–2.2)	10.1 (9.8–10.5)
Non-melanoma skin cancer (basal-cell carcinoma)	0 (0–0)	0 (0–0)	0 (0–0)	0 (0–0)	0 (0–0)	0 (0–0)
Breast cancer	0.1 (0.1–0.1)	0 (0–0)	0 (0–0)	0.0 (0.0–0.0)	0.5 (0.5–0.6)	1.8 (1.5–1.8)
Cervical cancer	0 (0–0)	0 (0–0)	0 (0–0)	0 (0–0)	0 (0–0)	0 (0–0)
Uterine cancer	0 (0–0)	0 (0–0)	0 (0–0)	0 (0–0)	0 (0–0)	0 (0–0)
Ovarian cancer	0 (0–0)	0 (0–0)	0 (0–0)	0 (0–0)	0 (0–0)	0 (0–0)
Prostate cancer	7.8 (6.2–9.5)	0 (0–0)	0 (0–0)	0.1 (0.1–0.2)	16.0 (12.9–19.9)	176.7 (138.1–216.8)
Testicular cancer	0.3 (0.3–0.3)	0 (0–0)	0 (0–0)	0.4 (0.4–0.5)	0.5 (0.4–0.5)	1.1 (1.1–1.2)

SR Table 14 - Global death rates (per 100,000) by age and cause for males in 1995

Cause	All ages	0-5 years	5-14 years	15-49 years	50-69 years	70+ years
Kidney cancer	1.8 (1.7–1.8)	0.4 (0.2–0.7)	0.1 (0.1–0.1)	0.4 (0.4–0.4)	6.8 (6.6–7.0)	20.2 (19.6–20.8)
Bladder cancer	3.2 (3.1–3.2)	0 (0–0)	0 (0–0)	0.3 (0.3–0.3)	9.6 (9.4–9.9)	55.9 (54.5–57.3)
Brain and nervous system cancer	3.1 (2.6–3.7)	1.2 (0.8–2.3)	1.1 (0.8–1.4)	1.9 (1.6–2.2)	9.8 (8.4–11.1)	16.2 (14.6–18.7)
Thyroid cancer	0.3 (0.3–0.3)	0 (0–0)	0.0 (0.0–0.0)	0.1 (0.1–0.1)	1.0 (0.9–1.1)	3.3 (3.1–3.7)
Mesothelioma	0.4 (0.4–0.5)	0 (0–0)	0 (0–0)	0.1 (0.1–0.1)	1.7 (1.6–1.8)	5.2 (4.9–5.6)
Hodgkin lymphoma	0.7 (0.5–0.8)	0.2 (0.1–0.2)	0.2 (0.1–0.2)	0.5 (0.4–0.6)	1.9 (1.4–2.4)	3.6 (2.7–4.6)
Non-Hodgkin lymphoma	2.8 (2.4–3.1)	0.7 (0.4–1.2)	0.5 (0.4–0.6)	1.2 (1.1–1.4)	8.4 (7.4–9.4)	28.7 (24.7–32.5)
Multiple myeloma	1.0 (1.0–1.1)	0 (0–0)	0 (0–0)	0.2 (0.2–0.2)	3.7 (3.6–3.8)	14.3 (13.9–14.7)
Leukemia	5.2 (5.1–5.5)	3.2 (2.8–3.6)	2.8 (2.6–3.0)	3.1 (2.9–3.2)	11.3 (11.0–11.7)	39.6 (38.6–40.6)
Acute lymphoid leukemia	1.9 (1.6–2.2)	2.1 (1.5–2.7)	2.0 (1.7–2.3)	1.3 (1.1–1.5)	2.9 (2.5–3.4)	5.7 (5.0–6.7)
Chronic lymphoid leukemia	0.8 (0.7–0.9)	0 (0–0)	0 (0–0)	0.2 (0.2–0.3)	2.1 (1.9–2.3)	12.5 (11.3–13.3)
Acute myeloid leukemia	1.9 (1.6–2.2)	1.1 (0.6–1.6)	0.8 (0.5–1.1)	1.1 (0.9–1.3)	4.4 (3.9–5.0)	13.9 (12.8–15.9)
Chronic myeloid leukemia	0.7 (0.7–0.8)	0 (0–0)	0 (0–0)	0.5 (0.4–0.6)	1.9 (1.7–2.2)	7.4 (6.9–8.0)
Other neoplasms	4.0 (3.6–4.3)	2.3 (1.4–4.0)	0.9 (0.8–1.0)	2.1 (1.8–2.3)	11.1 (9.8–12.3)	32.1 (29.8–36.4)
Cardiovascular diseases	239.4 (235.0–244.0)	15.9 (14.4–17.4)	3.7 (3.4–4.0)	55.4 (53.7–57.6)	725.5 (709.9–740.4)	3640.9 (3576.9–3700.4)
Rheumatic heart disease	5.0 (4.7–5.4)	0.9 (0.7–1.1)	0.8 (0.8–0.9)	2.9 (2.7–3.2)	13.8 (13.0–14.9)	46.2 (43.9–49.0)
Ischemic heart disease	120.4 (118.2–122.7)	1.1 (0.9–1.3)	0.3 (0.3–0.4)	27.4 (26.6–28.5)	379.3 (370.4–387.6)	1815.6 (1784.7–1844.6)
Cerebrovascular disease	84.2 (81.8–86.6)	6.4 (5.8–7.2)	1.4 (1.3–1.5)	16.5 (15.8–17.3)	253.0 (244.6–262.1)	1331.0 (1296.7–1367.6)
Ischemic stroke	35.4 (34.2–36.7)	0.2 (0.2–0.3)	0.1 (0.1–0.1)	2.6 (2.4–2.9)	89.8 (85.7–94.3)	704.8 (683.3–727.7)
Hemorrhagic stroke	48.7 (47.0–50.5)	6.2 (5.6–6.9)	1.3 (1.2–1.4)	13.8 (13.3–14.6)	163.3 (156.8–169.9)	626.2 (603.8–650.9)
Hypertensive heart disease	11.0 (10.4–12.2)	0.1 (0.1–0.2)	0.0 (0.0–0.1)	2.5 (2.3–2.8)	33.2 (31.1–37.0)	170.9 (160.9–189.6)
Cardiomyopathy and myocarditis	5.7 (5.4–6.0)	2.5 (1.9–2.9)	0.4 (0.3–0.5)	3.0 (2.8–3.2)	15.6 (14.7–16.6)	56.6 (53.5–59.3)
Atrial fibrillation and flutter	1.5 (1.2–1.8)	0 (0–0)	0 (0–0)	0.0 (0.0–0.0)	1.4 (1.1–1.8)	40.7 (32.2–50.6)
Aortic aneurysm	2.5 (2.4–2.6)	0 (0–0)	0 (0–0)	0.4 (0.4–0.5)	6.9 (6.6–7.3)	44.8 (43.4–46.3)
Peripheral vascular disease	0.4 (0.3–0.4)	0 (0–0)	0 (0–0)	0.0 (0.0–0.0)	0.7 (0.7–0.8)	8.3 (7.9–8.6)
Endocarditis	0.8 (0.7–1.0)	0.8 (0.4–1.3)	0.1 (0.1–0.1)	0.4 (0.3–0.5)	1.7 (1.5–2.1)	8.2 (7.2–9.8)
Other cardiovascular and circulatory diseases	8.0 (7.7–8.3)	4.0 (3.6–4.8)	0.6 (0.5–0.7)	2.2 (2.1–2.3)	19.8 (19.0–20.7)	118.6 (114.3–123.1)
Chronic respiratory diseases	71.5 (69.2–74.0)	15.9 (13.6–18.5)	1.6 (1.4–1.7)	9.2 (8.8–9.8)	192.1 (184.7–200.9)	1256.9 (1219.5–1294.8)
Chronic obstructive pulmonary disease	58.8 (54.8–62.7)	6.5 (4.6–8.6)	0.3 (0.3–0.4)	5.6 (4.9–6.2)	155.2 (141.5–168.6)	1106.3 (1044.7–1165.7)
Pneumoconiosis	0.8 (0.7–0.9)	0 (0–0)	0 (0–0)	0.2 (0.2–0.2)	2.3 (1.9–2.6)	12.7 (11.1–14.2)
Silicosis	0.4 (0.3–0.5)	0 (0–0)	0 (0–0)	0.1 (0.1–0.1)	1.2 (1.0–1.5)	5.7 (5.0–6.8)
Asbestosis	0.1 (0.0–0.1)	0 (0–0)	0 (0–0)	0.0 (0.0–0.0)	0.1 (0.1–0.2)	0.8 (0.6–1.0)
Coal workers pneumoconiosis	0.1 (0.1–0.1)	0 (0–0)	0 (0–0)	0.0 (0.0–0.0)	0.3 (0.2–0.3)	2.0 (1.8–2.4)
Other pneumoconiosis	0.2 (0.2–0.3)	0 (0–0)	0 (0–0)	0.1 (0.0–0.1)	0.7 (0.5–0.8)	4.1 (3.4–4.6)
Asthma	9.7 (6.5–12.4)	4.5 (3.1–5.9)	1.1 (0.9–1.2)	2.9 (2.1–3.5)	30.2 (19.5–40.2)	113.4 (72.5–151.5)
Interstitial lung disease and pulmonary sarcoidosis	1.0 (0.7–1.4)	0.1 (0.0–0.2)	0.0 (0.0–0.0)	0.2 (0.1–0.3)	2.7 (2.0–4.1)	16.8 (12.9–22.3)
Other chronic respiratory diseases	1.2 (0.8–1.5)	4.8 (3.0–6.9)	0.2 (0.1–0.2)	0.4 (0.2–0.4)	1.7 (1.1–2.2)	7.7 (5.4–9.7)
Cirrhosis and other chronic liver diseases	22.5 (21.7–24.5)	3.3 (2.6–4.0)	1.1 (1.0–1.2)	14.7 (14.0–16.2)	81.7 (78.5–89.3)	127.4 (122.3–133.1)
Cirrhosis and other chronic liver diseases due to hepatitis B	7.2 (6.6–8.1)	0.0 (0.0–0.0)	0.0 (0.0–0.1)	4.8 (4.3–5.4)	26.3 (23.6–30.1)	43.4 (39.5–48.9)
Cirrhosis and other chronic liver diseases due to hepatitis C	5.6 (5.2–6.0)	0.0 (0.0–0.0)	0.0 (0.0–0.0)	3.4 (3.0–3.8)	21.1 (19.0–23.5)	37.2 (33.8–40.6)
Cirrhosis and other chronic liver diseases due to alcohol use	6.3 (5.9–6.8)	0 (0–0)	0 (0–0)	4.6 (4.2–5.1)	24.5 (22.1–27.4)	27.2 (24.4–30.5)
Cirrhosis and other chronic liver diseases due to other causes	3.4 (3.2–3.8)	3.3 (2.6–4.0)	1.0 (0.9–1.2)	2.0 (1.8–2.2)	9.8 (8.6–11.3)	19.7 (17.8–21.8)
Digestive diseases	19.6 (18.5–20.9)	6.0 (4.6–7.6)	2.3 (2.0–2.5)	10.3 (9.5–11.0)	54.1 (50.8–58.1)	195.4 (186.9–213.0)
Peptic ulcer disease	6.9 (6.3–7.6)	0.7 (0.4–1.3)	0.5 (0.4–0.6)	3.6 (3.3–4.0)	21.3 (19.4–23.2)	67.8 (63.3–75.2)
Gastritis and duodenitis	1.0 (0.7–1.2)	0.3 (0.1–0.5)	0.1 (0.0–0.1)	0.5 (0.4–0.7)	2.8 (2.1–3.7)	9.3 (6.9–12.1)
Appendicitis	0.8 (0.7–0.9)	0.6 (0.4–0.9)	0.3 (0.3–0.4)	0.6 (0.5–0.7)	1.6 (1.4–2.0)	4.3 (3.8–5.2)
Paralytic ileus and intestinal obstruction	3.8 (3.1–4.5)	1.8 (1.1–2.8)	0.8 (0.6–0.9)	2.1 (1.6–2.4)	9.9 (7.9–11.8)	34.6 (30.4–43.8)
Inguinal, femoral, and abdominal hernia	1.1 (0.7–1.3)	0.4 (0.1–0.6)	0.1 (0.1–0.2)	0.5 (0.3–0.6)	2.9 (1.9–3.6)	12.7 (8.3–15.0)
Inflammatory bowel disease	0.6 (0.5–0.7)	0.3 (0.2–0.8)	0.1 (0.0–0.1)	0.3 (0.2–0.3)	1.5 (1.3–1.8)	6.2 (5.2–7.1)
Vascular intestinal disorders	1.0 (0.9–1.0)	0.2 (0.1–0.4)	0.0 (0.0–0.0)	0.2 (0.2–0.2)	2.7 (2.5–2.9)	15.8 (14.7–17.0)
Gallbladder and biliary diseases	1.2 (1.0–1.3)	0.2 (0.1–0.3)	0.1 (0.1–0.1)	0.4 (0.3–0.5)	3.0 (2.7–3.4)	16.9 (15.5–18.4)
Pancreatitis	1.6 (1.5–1.8)	0.1 (0.0–0.1)	0.0 (0.0–0.1)	1.5 (1.4–1.7)	4.3 (4.0–4.8)	9.4 (8.7–10.2)
Other digestive diseases	1.7 (1.3–2.2)	1.4 (0.9–2.1)	0.3 (0.2–0.4)	0.7 (0.5–0.9)	4.0 (3.0–5.4)	18.6 (14.8–23.3)
Neurological disorders	17.6 (15.5–19.6)	4.3 (2.7–5.6)	1.5 (1.3–1.6)	3.3 (3.2–3.5)	17.6 (15.1–20.0)	396.2 (337.1–455.5)
Alzheimer disease and other dementias	12.8 (10.8–14.8)	0 (0–0)	0 (0–0)	0.0 (0.0–0.1)	10.0 (7.5–12.4)	356.2 (296.9–414.8)
Parkinson disease	1.0 (1.0–1.1)	0 (0–0)	0 (0–0)	0.0 (0.0–0.0)	1.3 (1.3–1.5)	26.0 (24.7–27.9)
Epilepsy	2.4 (2.2–2.5)	3.2 (2.0–4.3)	1.2 (1.0–1.3)	2.5 (2.4–2.6)	2.6 (2.5–2.7)	4.4 (4.2–4.6)

SR Table 14 - Global death rates (per 100,000) by age and cause for males in 1995

Cause	All ages	0-5 years	5-14 years	15-49 years	50-69 years	70+ years
Multiple sclerosis	0.2 (0.2–0.2)	0 (0–0)	0 (0–0)	0.2 (0.1–0.2)	0.8 (0.7–0.9)	1.2 (0.9–1.2)
Motor neuron disease	0.4 (0.4–0.4)	0.2 (0.1–0.3)	0.0 (0.0–0.0)	0.1 (0.1–0.1)	1.4 (1.4–1.6)	3.6 (3.4–3.8)
Migraine	0 (0–0)	0 (0–0)	0 (0–0)	0 (0–0)	0 (0–0)	0 (0–0)
Tension-type headache	0 (0–0)	0 (0–0)	0 (0–0)	0 (0–0)	0 (0–0)	0 (0–0)
Medication overuse headache	0 (0–0)	0 (0–0)	0 (0–0)	0 (0–0)	0 (0–0)	0 (0–0)
Other neurological disorders	0.8 (0.7–0.8)	0.9 (0.6–1.2)	0.3 (0.2–0.3)	0.5 (0.5–0.6)	1.5 (1.3–1.6)	4.8 (4.5–5.2)
Mental and substance use disorders	6.7 (6.1–7.0)	0.0 (0.0–0.0)	0.0 (0.0–0.0)	8.3 (7.5–8.6)	16.2 (15.2–16.9)	13.0 (12.5–13.5)
Schizophrenia	0.3 (0.3–0.4)	0 (0–0)	0 (0–0)	0.3 (0.3–0.4)	0.8 (0.7–0.8)	1.5 (1.4–1.6)
Alcohol use disorders	4.0 (3.7–4.2)	0 (0–0)	0 (0–0)	4.3 (3.9–4.6)	12.0 (11.1–12.7)	7.7 (7.4–8.0)
Drug use disorders	2.4 (2.2–2.5)	0.0 (0.0–0.0)	0 (0–0)	3.6 (3.2–3.8)	3.4 (3.2–3.6)	3.8 (3.6–4.1)
Opioid use disorders	1.9 (1.7–2.0)	0.0 (0.0–0.0)	0 (0–0)	2.8 (2.5–3.0)	2.6 (2.4–2.8)	2.7 (2.5–2.9)
Cocaine use disorders	0.1 (0.1–0.1)	0 (0–0)	0 (0–0)	0.1 (0.1–0.2)	0.1 (0.1–0.1)	0.1 (0.0–0.1)
Amphetamine use disorders	0.0 (0.0–0.1)	0 (0–0)	0 (0–0)	0.1 (0.1–0.1)	0.1 (0.1–0.1)	0.0 (0.0–0.1)
Cannabis use disorders	0 (0–0)	0 (0–0)	0 (0–0)	0 (0–0)	0 (0–0)	0 (0–0)
Other drug use disorders	0.4 (0.3–0.4)	0 (0–0)	0 (0–0)	0.6 (0.4–0.6)	0.6 (0.6–0.7)	1.0 (0.9–1.1)
Depressive disorders	0 (0–0)	0 (0–0)	0 (0–0)	0 (0–0)	0 (0–0)	0 (0–0)
Major depressive disorder	0 (0–0)	0 (0–0)	0 (0–0)	0 (0–0)	0 (0–0)	0 (0–0)
Dysthymia	0 (0–0)	0 (0–0)	0 (0–0)	0 (0–0)	0 (0–0)	0 (0–0)
Bipolar disorder	0 (0–0)	0 (0–0)	0 (0–0)	0 (0–0)	0 (0–0)	0 (0–0)
Anxiety disorders	0 (0–0)	0 (0–0)	0 (0–0)	0 (0–0)	0 (0–0)	0 (0–0)
Eating disorders	0.0 (0.0–0.0)	0 (0–0)	0.0 (0.0–0.0)	0.0 (0.0–0.0)	0 (0–0)	0 (0–0)
Anorexia nervosa	0.0 (0.0–0.0)	0 (0–0)	0.0 (0.0–0.0)	0.0 (0.0–0.0)	0 (0–0)	0 (0–0)
Bulimia nervosa	0.0 (0.0–0.0)	0 (0–0)	0.0 (0.0–0.0)	0.0 (0.0–0.0)	0 (0–0)	0 (0–0)
Autistic spectrum disorders	0 (0–0)	0 (0–0)	0 (0–0)	0 (0–0)	0 (0–0)	0 (0–0)
Autism	0 (0–0)	0 (0–0)	0 (0–0)	0 (0–0)	0 (0–0)	0 (0–0)
Asperger syndrome and other autistic spectrum disorders	0 (0–0)	0 (0–0)	0 (0–0)	0 (0–0)	0 (0–0)	0 (0–0)
Attention-deficit/hyperactivity disorder	0 (0–0)	0 (0–0)	0 (0–0)	0 (0–0)	0 (0–0)	0 (0–0)
Conduct disorder	0 (0–0)	0 (0–0)	0 (0–0)	0 (0–0)	0 (0–0)	0 (0–0)
Idiopathic developmental intellectual disability	0 (0–0)	0 (0–0)	0 (0–0)	0 (0–0)	0 (0–0)	0 (0–0)
Other mental and substance use disorders	0 (0–0)	0 (0–0)	0 (0–0)	0 (0–0)	0 (0–0)	0 (0–0)
Diabetes, urogenital, blood, and endocrine diseases	32.8 (30.2–34.5)	23.6 (15.9–31.7)	3.1 (2.6–3.4)	11.5 (10.4–12.3)	89.1 (83.6–93.5)	383.1 (363.6–402.1)
Diabetes mellitus	12.9 (12.5–13.2)	0.6 (0.5–0.6)	0.2 (0.2–0.3)	3.3 (3.2–3.4)	44.9 (43.3–46.4)	168.1 (163.5–172.9)
Acute glomerulonephritis	0.3 (0.2–0.3)	0.1 (0.1–0.1)	0.1 (0.0–0.1)	0.2 (0.1–0.2)	0.7 (0.5–0.8)	2.0 (1.5–2.4)
Chronic kidney disease	11.7 (10.0–13.0)	3.0 (2.2–3.5)	0.9 (0.7–1.0)	5.0 (4.0–5.4)	30.8 (26.3–35.1)	147.3 (132.4–164.0)
Chronic kidney disease due to diabetes mellitus	3.5 (3.2–3.9)	0.3 (0.2–0.4)	0.1 (0.1–0.1)	1.1 (0.9–1.2)	11.3 (9.8–12.8)	47.2 (43.2–51.6)
Chronic kidney disease due to hypertension	4.9 (4.2–5.5)	1.0 (0.7–1.2)	0.3 (0.2–0.3)	1.8 (1.4–2.0)	12.5 (10.6–14.1)	70.2 (63.5–78.3)
Chronic kidney disease due to glomerulonephritis	2.9 (2.4–3.2)	1.3 (0.9–1.5)	0.4 (0.3–0.5)	1.8 (1.5–2.0)	6.6 (5.6–7.8)	26.7 (23.4–31.0)
Chronic kidney disease due to other causes	0.3 (0.3–0.4)	0.4 (0.3–0.6)	0.2 (0.1–0.2)	0.2 (0.1–0.3)	0.4 (0.3–0.6)	3.2 (2.6–4.0)
Urinary diseases and male infertility	2.8 (2.4–3.5)	1.8 (1.2–2.4)	0.2 (0.2–0.3)	0.9 (0.7–1.1)	6.7 (5.4–8.7)	39.3 (34.3–49.7)
Interstitial nephritis and urinary tract infections	1.8 (1.5–2.2)	1.4 (0.9–1.9)	0.1 (0.1–0.1)	0.5 (0.4–0.7)	3.9 (3.0–5.0)	26.5 (22.1–33.2)
Urolithiasis	0.3 (0.2–0.4)	0 (0–0)	0.0 (0.0–0.0)	0.1 (0.1–0.2)	1.0 (0.7–1.3)	3.9 (2.6–5.0)
Benign prostatic hyperplasia	0 (0–0)	0 (0–0)	0 (0–0)	0 (0–0)	0 (0–0)	0 (0–0)
Male infertility	0 (0–0)	0 (0–0)	0 (0–0)	0 (0–0)	0 (0–0)	0 (0–0)
Other urinary diseases	0.7 (0.5–1.0)	0.4 (0.2–0.7)	0.1 (0.0–0.1)	0.2 (0.2–0.4)	1.8 (1.2–2.8)	8.9 (6.7–12.9)
Gynecological diseases	0 (0–0)	0 (0–0)	0 (0–0)	0 (0–0)	0 (0–0)	0 (0–0)
Uterine fibroids	0 (0–0)	0 (0–0)	0 (0–0)	0 (0–0)	0 (0–0)	0 (0–0)
Polycystic ovarian syndrome	0 (0–0)	0 (0–0)	0 (0–0)	0 (0–0)	0 (0–0)	0 (0–0)
Female infertility	0 (0–0)	0 (0–0)	0 (0–0)	0 (0–0)	0 (0–0)	0 (0–0)
Endometriosis	0 (0–0)	0 (0–0)	0 (0–0)	0 (0–0)	0 (0–0)	0 (0–0)
Genital prolapse	0 (0–0)	0 (0–0)	0 (0–0)	0 (0–0)	0 (0–0)	0 (0–0)
Premenstrual syndrome	0 (0–0)	0 (0–0)	0 (0–0)	0 (0–0)	0 (0–0)	0 (0–0)
Other gynecological diseases	0 (0–0)	0 (0–0)	0 (0–0)	0 (0–0)	0 (0–0)	0 (0–0)
Hemoglobinopathies and hemolytic anemias	3.7 (2.5–4.8)	15.3 (8.1–23.5)	1.3 (1.0–1.6)	1.5 (1.2–1.9)	3.3 (2.5–4.1)	14.9 (11.3–18.6)
Thalassemias	0.4 (0.3–0.5)	2.3 (1.4–3.3)	0.4 (0.3–0.5)	0.1 (0.0–0.1)	0.0 (0.0–0.0)	1.0 (0.6–1.5)
Thalassemias trait	0 (0–0)	0 (0–0)	0 (0–0)	0 (0–0)	0 (0–0)	0 (0–0)
Sickle cell disorders	1.9 (1.1–2.9)	12.6 (6.3–21.1)	0.7 (0.5–0.9)	0.5 (0.4–0.7)	0.1 (0.1–0.1)	1.1 (0.8–1.5)
Sickle cell trait	0 (0–0)	0 (0–0)	0 (0–0)	0 (0–0)	0 (0–0)	0 (0–0)

SR Table 14 - Global death rates (per 100,000) by age and cause for males in 1995						
Cause	All ages	0-5 years	5-14 years	15-49 years	50-69 years	70+ years
G6PD deficiency	0.5 (0.4–0.6)	0.3 (0.1–0.5)	0.2 (0.1–0.2)	0.6 (0.5–0.7)	1.0 (0.8–1.3)	0.8 (0.6–1.0)
G6PD trait	0 (0–0)	0 (0–0)	0 (0–0)	0 (0–0)	0 (0–0)	0 (0–0)
Other hemoglobinopathies and hemolytic anemias	0.9 (0.7–1.1)	0.1 (0.1–0.2)	0.1 (0.0–0.1)	0.4 (0.3–0.5)	2.2 (1.7–2.8)	12.1 (9.4–14.8)
Endocrine, metabolic, blood, and immune disorders	1.4 (1.4–1.5)	2.9 (2.5–3.3)	0.4 (0.4–0.4)	0.6 (0.6–0.7)	2.7 (2.5–2.9)	11.5 (10.9–12.1)
Musculoskeletal disorders	0.7 (0.6–0.7)	0 (0–0)	0.1 (0.1–0.2)	0.3 (0.3–0.3)	1.6 (1.3–1.7)	8.3 (6.7–8.9)
Rheumatoid arthritis	0.2 (0.2–0.2)	0 (0–0)	0.0 (0.0–0.0)	0.1 (0.0–0.1)	0.7 (0.5–0.8)	3.4 (2.6–3.9)
Osteoarthritis	0 (0–0)	0 (0–0)	0 (0–0)	0 (0–0)	0 (0–0)	0 (0–0)
Low back and neck pain	0 (0–0)	0 (0–0)	0 (0–0)	0 (0–0)	0 (0–0)	0 (0–0)
Low back pain	0 (0–0)	0 (0–0)	0 (0–0)	0 (0–0)	0 (0–0)	0 (0–0)
Neck pain	0 (0–0)	0 (0–0)	0 (0–0)	0 (0–0)	0 (0–0)	0 (0–0)
Gout	0 (0–0)	0 (0–0)	0 (0–0)	0 (0–0)	0 (0–0)	0 (0–0)
Other musculoskeletal disorders	0.4 (0.4–0.5)	0 (0–0)	0.1 (0.1–0.1)	0.3 (0.2–0.3)	0.9 (0.8–1.0)	4.9 (3.9–5.3)
Other non-communicable diseases	13.6 (10.6–18.2)	96.9 (72.3–135.6)	3.4 (2.6–4.2)	2.2 (1.9–2.6)	3.3 (2.5–4.2)	13.8 (10.5–18.3)
Congenital anomalies	12.4 (9.5–16.8)	92.1 (68.1–129.8)	3.3 (2.5–4.1)	1.9 (1.7–2.3)	1.6 (1.5–2.0)	4.0 (3.7–4.9)
Neural tube defects	2.0 (1.4–3.0)	16.2 (11.4–25.4)	0.3 (0.2–0.4)	0.1 (0.1–0.1)	0.1 (0.1–0.1)	0.2 (0.1–0.4)
Congenital heart anomalies	6.5 (4.8–8.9)	45.6 (32.2–66.0)	2.3 (1.7–2.9)	1.4 (1.3–1.7)	0.6 (0.5–0.7)	1.4 (1.2–1.8)
Cleft lip and cleft palate	0.1 (0.1–0.1)	0.8 (0.6–1.2)	0 (0–0)	0 (0–0)	0 (0–0)	0 (0–0)
Down syndrome	0.3 (0.2–0.7)	1.9 (0.9–5.1)	0.1 (0.0–0.1)	0.1 (0.1–0.2)	0.3 (0.2–0.4)	0.1 (0.0–0.1)
Turner syndrome	0 (0–0)	0 (0–0)	0 (0–0)	0 (0–0)	0 (0–0)	0 (0–0)
Klinefelter syndrome	0 (0–0)	0 (0–0)	0 (0–0)	0 (0–0)	0 (0–0)	0 (0–0)
Other chromosomal abnormalities	0.2 (0.1–0.7)	1.9 (0.8–5.5)	0.0 (0.0–0.0)	0.0 (0.0–0.0)	0.0 (0.0–0.1)	0.0 (0.0–0.1)
Other congenital anomalies	3.3 (2.4–4.7)	25.6 (18.3–37.4)	0.5 (0.4–0.7)	0.3 (0.2–0.3)	0.6 (0.5–0.8)	2.3 (2.0–2.7)
Skin and subcutaneous diseases	0.8 (0.5–1.2)	0.8 (0.3–1.4)	0.1 (0.0–0.1)	0.3 (0.2–0.4)	1.7 (0.9–2.6)	9.8 (6.4–14.0)
Dermatitis	0 (0–0)	0 (0–0)	0 (0–0)	0 (0–0)	0 (0–0)	0 (0–0)
Psoriasis	0 (0–0)	0 (0–0)	0 (0–0)	0 (0–0)	0 (0–0)	0 (0–0)
Cellulitis	0.2 (0.1–0.3)	0.1 (0.0–0.3)	0.0 (0.0–0.0)	0.1 (0.0–0.1)	0.5 (0.2–0.9)	1.4 (0.9–2.9)
Pyoderma	0.3 (0.2–0.5)	0.6 (0.3–1.2)	0.0 (0.0–0.1)	0.1 (0.1–0.2)	0.6 (0.4–1.0)	2.7 (1.9–4.4)
Scabies	0 (0–0)	0 (0–0)	0 (0–0)	0 (0–0)	0 (0–0)	0 (0–0)
Fungal skin diseases	0 (0–0)	0 (0–0)	0 (0–0)	0 (0–0)	0 (0–0)	0 (0–0)
Viral skin diseases	0 (0–0)	0 (0–0)	0 (0–0)	0 (0–0)	0 (0–0)	0 (0–0)
Acne vulgaris	0 (0–0)	0 (0–0)	0 (0–0)	0 (0–0)	0 (0–0)	0 (0–0)
Alopecia areata	0 (0–0)	0 (0–0)	0 (0–0)	0 (0–0)	0 (0–0)	0 (0–0)
Pruritus	0 (0–0)	0 (0–0)	0 (0–0)	0 (0–0)	0 (0–0)	0 (0–0)
Urticaria	0 (0–0)	0 (0–0)	0 (0–0)	0 (0–0)	0 (0–0)	0 (0–0)
Decubitus ulcer	0.3 (0.1–0.4)	0.0 (0.0–0.0)	0.0 (0.0–0.0)	0.0 (0.0–0.1)	0.6 (0.2–1.2)	5.3 (3.0–7.3)
Other skin and subcutaneous diseases	0.0 (0.0–0.1)	0.0 (0.0–0.1)	0.0 (0.0–0.0)	0.0 (0.0–0.0)	0.1 (0.0–0.2)	0.4 (0.2–0.6)
Sense organ diseases	0 (0–0)	0 (0–0)	0 (0–0)	0 (0–0)	0 (0–0)	0 (0–0)
Glaucoma	0 (0–0)	0 (0–0)	0 (0–0)	0 (0–0)	0 (0–0)	0 (0–0)
Cataract	0 (0–0)	0 (0–0)	0 (0–0)	0 (0–0)	0 (0–0)	0 (0–0)
Macular degeneration	0 (0–0)	0 (0–0)	0 (0–0)	0 (0–0)	0 (0–0)	0 (0–0)
Refraction and accommodation disorders	0 (0–0)	0 (0–0)	0 (0–0)	0 (0–0)	0 (0–0)	0 (0–0)
Age-related and other hearing loss	0 (0–0)	0 (0–0)	0 (0–0)	0 (0–0)	0 (0–0)	0 (0–0)
Other vision loss	0 (0–0)	0 (0–0)	0 (0–0)	0 (0–0)	0 (0–0)	0 (0–0)
Other sense organ diseases	0 (0–0)	0 (0–0)	0 (0–0)	0 (0–0)	0 (0–0)	0 (0–0)
Oral disorders	0 (0–0)	0 (0–0)	0 (0–0)	0 (0–0)	0 (0–0)	0 (0–0)
Deciduous caries	0 (0–0)	0 (0–0)	0 (0–0)	0 (0–0)	0 (0–0)	0 (0–0)
Permanent caries	0 (0–0)	0 (0–0)	0 (0–0)	0 (0–0)	0 (0–0)	0 (0–0)
Periodontal diseases	0 (0–0)	0 (0–0)	0 (0–0)	0 (0–0)	0 (0–0)	0 (0–0)
Edentulism and severe tooth loss	0 (0–0)	0 (0–0)	0 (0–0)	0 (0–0)	0 (0–0)	0 (0–0)
Other oral disorders	0 (0–0)	0 (0–0)	0 (0–0)	0 (0–0)	0 (0–0)	0 (0–0)
Sudden infant death syndrome	0.5 (0.3–1.1)	4.1 (2.9–9.8)	0 (0–0)	0 (0–0)	0 (0–0)	0 (0–0)
Injuries	109.0 (103.6–112.5)	101.0 (82.0–116.0)	40.3 (37.8–42.8)	118.7 (113.7–122.1)	148.3 (140.8–153.0)	283.8 (264.5–294.6)
Transport injuries	33.3 (31.9–34.8)	16.8 (12.7–20.3)	11.8 (10.8–12.6)	40.6 (39.0–42.2)	45.1 (43.1–47.1)	71.5 (68.7–74.4)
Road injuries	31.2 (29.8–32.6)	15.5 (11.7–18.9)	11.2 (10.3–11.9)	38.2 (36.6–39.8)	41.5 (39.4–43.5)	67.2 (64.5–70.1)
Pedestrian road injuries	13.8 (12.0–15.0)	8.5 (6.8–10.5)	6.5 (5.8–7.1)	14.7 (12.6–16.2)	21.2 (18.3–23.0)	37.8 (33.2–40.8)
Cyclist road injuries	1.3 (1.1–1.5)	0.4 (0.2–0.6)	0.7 (0.5–0.8)	1.3 (1.1–1.6)	2.5 (2.2–2.9)	3.8 (3.5–4.4)
Motorcyclist road injuries	4.6 (3.7–5.8)	1.2 (0.6–2.0)	0.9 (0.6–1.2)	7.0 (5.6–8.7)	4.4 (3.3–5.8)	5.0 (3.6–6.7)

SR Table 14 - Global death rates (per 100,000) by age and cause for males in 1995						
Cause	All ages	0-5 years	5-14 years	15-49 years	50-69 years	70+ years
Motor vehicle road injuries	11.1 (10.0–13.1)	5.0 (3.3–6.4)	3.0 (2.6–3.7)	14.7 (13.3–17.5)	13.0 (11.2–15.8)	19.7 (16.7–23.8)
Other road injuries	0.4 (0.2–0.5)	0.3 (0.1–0.8)	0.2 (0.1–0.3)	0.4 (0.2–0.5)	0.5 (0.3–0.7)	0.9 (0.7–1.4)
Other transport injuries	2.1 (1.8–2.4)	1.3 (0.8–1.6)	0.6 (0.5–0.7)	2.4 (2.1–2.7)	3.6 (3.1–4.2)	4.3 (3.7–5.0)
Unintentional injuries	44.5 (41.9–46.8)	79.7 (63.5–93.3)	25.0 (23.3–26.7)	35.3 (33.7–36.9)	57.2 (53.7–59.9)	148.3 (136.1–157.2)
Falls	8.0 (7.4–8.5)	5.4 (3.4–7.3)	2.1 (1.8–2.4)	5.8 (5.4–6.1)	15.8 (14.6–16.7)	62.7 (57.6–66.0)
Drowning	12.5 (11.8–13.3)	29.4 (24.6–34.5)	14.1 (13.2–14.9)	9.0 (8.6–9.6)	9.1 (8.7–9.7)	13.2 (12.5–14.0)
Fire, heat, and hot substances	3.9 (2.9–4.1)	6.6 (4.7–7.9)	1.2 (0.9–1.4)	3.4 (2.4–3.7)	5.3 (4.3–5.6)	13.8 (11.3–14.6)
Poisonings	2.8 (1.8–3.4)	6.1 (1.9–11.0)	1.0 (0.7–1.2)	2.4 (1.8–2.8)	4.2 (3.1–4.7)	5.4 (3.9–6.4)
Exposure to mechanical forces	5.0 (4.5–5.8)	8.6 (6.3–10.9)	1.8 (1.5–2.4)	4.9 (4.5–5.9)	6.0 (5.4–7.4)	11.4 (9.7–12.7)
Unintentional firearm injuries	0.9 (0.6–1.0)	0.7 (0.4–1.0)	0.3 (0.3–0.4)	1.1 (0.8–1.2)	0.9 (0.6–1.0)	2.3 (1.8–2.6)
Unintentional suffocation	0.9 (0.7–1.1)	5.4 (3.5–7.3)	0.4 (0.3–0.6)	0.2 (0.2–0.3)	0.3 (0.2–0.3)	2.0 (1.6–2.2)
Other exposure to mechanical forces	3.3 (2.8–4.1)	2.5 (1.5–3.7)	1.1 (0.8–1.5)	3.7 (3.2–4.6)	4.9 (4.4–6.3)	7.1 (5.9–8.3)
Adverse effects of medical treatment	2.0 (1.4–2.3)	4.6 (1.9–6.5)	0.6 (0.4–0.7)	1.1 (0.8–1.3)	3.0 (2.3–3.5)	11.2 (8.8–12.7)
Animal contact	2.2 (1.5–2.5)	3.2 (1.6–4.2)	1.6 (1.0–1.9)	1.8 (1.2–2.0)	3.5 (2.3–3.9)	5.5 (4.0–6.3)
Venomous animal contact	1.7 (1.1–2.0)	2.2 (1.1–2.9)	1.3 (0.8–1.5)	1.4 (0.9–1.6)	2.8 (1.8–3.2)	4.2 (2.9–5.0)
Non-venomous animal contact	0.5 (0.3–0.6)	1.0 (0.5–1.5)	0.3 (0.2–0.4)	0.3 (0.2–0.4)	0.7 (0.5–0.9)	1.3 (1.0–1.6)
Foreign body	3.2 (2.3–4.5)	11.9 (6.7–18.0)	1.2 (0.8–1.7)	1.6 (1.2–3.0)	3.3 (2.7–5.5)	12.0 (9.8–19.3)
Pulmonary aspiration and foreign body in airway	2.3 (1.7–3.2)	9.2 (4.4–15.0)	0.8 (0.5–1.1)	1.0 (0.8–1.1)	2.5 (2.1–2.9)	9.8 (8.3–10.7)
Foreign body in eyes	0 (0–0)	0 (0–0)	0 (0–0)	0 (0–0)	0 (0–0)	0 (0–0)
Foreign body in other body part	0.9 (0.4–2.5)	2.7 (1.2–4.7)	0.4 (0.2–1.0)	0.6 (0.3–2.1)	0.7 (0.4–3.0)	2.2 (1.1–9.3)
Environmental heat and cold exposure	1.2 (0.9–1.4)	1.1 (0.4–1.4)	0.2 (0.1–0.2)	1.0 (0.8–1.2)	2.8 (2.2–3.5)	5.2 (3.6–6.5)
Other unintentional injuries	3.6 (3.4–3.9)	2.8 (2.1–3.5)	1.4 (1.2–1.5)	4.3 (4.0–4.7)	4.2 (3.9–4.6)	8.0 (6.9–9.2)
Self-harm and interpersonal violence	29.1 (26.2–30.1)	3.5 (2.8–3.9)	2.8 (2.4–3.0)	39.9 (36.0–41.3)	44.3 (40.3–45.9)	62.2 (54.7–64.6)
Self-harm	17.6 (15.2–18.4)	0 (0–0)	1.1 (0.8–1.2)	22.3 (19.1–23.4)	33.3 (29.3–34.7)	53.3 (45.6–55.6)
Interpersonal violence	11.5 (10.7–11.9)	3.5 (2.8–3.9)	1.7 (1.5–1.9)	17.6 (16.5–18.3)	11.0 (10.3–11.5)	8.9 (8.4–9.5)
Assault by firearm	4.3 (4.1–4.6)	0.4 (0.3–0.5)	0.4 (0.4–0.4)	7.3 (6.8–7.6)	3.1 (2.8–3.4)	2.0 (1.8–2.2)
Assault by sharp object	3.0 (2.7–3.2)	0.4 (0.3–0.6)	0.3 (0.3–0.4)	4.8 (4.4–5.1)	2.8 (2.5–3.0)	2.1 (1.9–2.3)
Assault by other means	4.2 (3.7–4.5)	2.7 (2.1–3.1)	1.0 (0.8–1.1)	5.6 (4.8–6.0)	5.1 (4.6–5.6)	4.7 (4.3–5.3)
Forces of nature, war, and legal intervention	2.0 (1.6–2.5)	1.0 (0.7–1.3)	0.7 (0.5–0.9)	2.8 (2.2–3.5)	1.8 (1.3–2.7)	1.8 (1.1–3.4)
Exposure to forces of nature	0.4 (0.2–0.7)	0.2 (0.1–0.4)	0.3 (0.2–0.5)	0.4 (0.3–0.8)	0.6 (0.3–1.4)	0.9 (0.4–2.5)
Collective violence and legal intervention	1.6 (1.2–1.9)	0.7 (0.5–1.0)	0.4 (0.3–0.5)	2.4 (1.9–2.9)	1.2 (0.9–1.5)	0.9 (0.7–1.1)

SR Table 14 - Global death rates (per 100,000) by age and cause for males in 2000						
Cause	All ages	0-5 years	5-14 years	15-49 years	50-69 years	70+ years
All causes	902.8 (892.0–913.5)	1559.9 (1543.8–1575.9)	105.9 (104.5–107.7)	351.3 (344.1–358.2)	1890.3 (1857.7–1921.2)	8152.0 (8066.2–8235.2)
Communicable, maternal, neonatal, and nutritional diseases	247.4 (241.0–257.4)	1314.6 (1275.2–1348.8)	48.2 (45.6–51.3)	88.2 (83.6–94.3)	226.5 (209.5–252.7)	758.9 (715.2–829.7)
HIV/AIDS and tuberculosis	52.9 (46.9–61.8)	34.0 (31.6–38.0)	4.6 (4.2–5.5)	53.8 (48.9–59.6)	107.1 (89.5–133.4)	177.2 (141.3–245.2)
Tuberculosis	28.9 (22.9–38.1)	7.2 (5.1–11.4)	1.8 (1.4–2.7)	21.3 (16.7–27.1)	82.0 (64.5–108.5)	166.9 (131.2–234.6)
HIV/AIDS	24.0 (22.6–25.9)	26.8 (25.8–27.9)	2.8 (2.7–3.0)	32.5 (30.2–35.4)	25.1 (23.0–28.5)	10.2 (9.2–12.5)
HIV/AIDS - Tuberculosis	5.0 (4.3–5.8)	7.6 (6.6–8.6)	0.8 (0.6–0.9)	6.0 (5.1–7.1)	6.2 (5.2–7.1)	2.9 (2.5–3.3)
HIV/AIDS resulting in other diseases	19.0 (17.6–20.8)	19.3 (17.9–20.9)	2.1 (1.9–2.3)	26.5 (24.1–29.1)	18.9 (16.9–21.9)	7.4 (6.4–9.3)
Diarrhea, lower respiratory, and other common infectious diseases	104.3 (98.8–110.5)	537.7 (500.5–577.9)	31.3 (27.9–35.6)	23.5 (21.6–25.9)	92.7 (85.6–102.1)	511.8 (475.6–558.9)
Diarrheal diseases	27.2 (25.0–29.4)	139.7 (121.0–159.8)	6.5 (5.7–7.3)	6.7 (6.2–7.5)	28.6 (26.4–31.2)	118.8 (110.4–127.8)
Intestinal infectious diseases	3.9 (2.2–6.3)	10.0 (5.2–17.4)	7.3 (4.3–11.6)	2.1 (1.1–3.7)	1.1 (0.6–1.9)	1.5 (0.9–2.4)
Typhoid fever	3.2 (1.7–5.3)	8.2 (4.1–14.7)	6.1 (3.5–9.7)	1.7 (0.9–3.0)	0.8 (0.4–1.5)	1.1 (0.6–1.9)
Paratyphoid fever	0.6 (0.3–1.2)	1.5 (0.6–3.0)	1.2 (0.6–2.3)	0.4 (0.2–0.8)	0.2 (0.1–0.4)	0.3 (0.1–0.5)
Other intestinal infectious diseases	0.0 (0.0–0.1)	0.2 (0.1–0.6)	0.0 (0.0–0.0)	0.0 (0.0–0.0)	0.0 (0.0–0.0)	0.1 (0.0–0.2)
Lower respiratory infections	49.5 (45.5–53.5)	231.1 (210.0–253.1)	6.6 (5.0–7.4)	9.0 (7.5–10.2)	51.2 (43.2–58.6)	365.8 (326.8–407.2)
Upper respiratory infections	0.1 (0.1–0.1)	0.2 (0.2–0.3)	0.0 (0.0–0.0)	0.0 (0.0–0.0)	0.1 (0.1–0.2)	0.4 (0.3–0.5)
Otitis media	0.1 (0.1–0.1)	0.3 (0.3–0.4)	0.1 (0.1–0.1)	0.0 (0.0–0.0)	0.1 (0.1–0.1)	0.2 (0.1–0.2)
Meningitis	7.5 (6.1–8.9)	40.9 (31.1–51.8)	3.3 (2.3–4.0)	2.8 (2.1–3.4)	5.8 (4.4–7.3)	11.9 (9.1–15.5)
Pneumococcal meningitis	2.0 (1.5–2.5)	9.1 (6.1–13.2)	1.1 (0.7–1.5)	0.9 (0.7–1.2)	1.6 (1.1–2.2)	3.4 (2.4–4.6)
H influenzae type B meningitis	2.2 (1.7–2.8)	15.3 (10.7–20.4)	0.9 (0.6–1.3)	0.5 (0.3–0.6)	0.9 (0.6–1.3)	2.0 (1.5–2.8)
Meningococcal meningitis	1.4 (1.0–1.8)	7.9 (5.1–11.6)	0.6 (0.4–0.9)	0.5 (0.3–0.6)	1.0 (0.7–1.3)	1.7 (1.2–2.3)
Other meningitis	2.0 (1.6–2.4)	8.6 (6.1–11.6)	0.7 (0.5–1.0)	0.9 (0.7–1.2)	2.3 (1.7–3.0)	4.9 (3.7–6.2)
Encephalitis	2.6 (2.3–2.9)	8.6 (7.2–10.0)	1.6 (1.4–1.8)	1.3 (1.2–1.4)	3.0 (2.7–3.3)	8.6 (7.8–9.8)
Diphtheria	0.2 (0.1–0.4)	1.2 (0.4–3.2)	0.1 (0.0–0.2)	0.0 (0.0–0.0)	0.0 (0.0–0.1)	0 (0–0)
Whooping cough	1.8 (0.6–4.0)	16.2 (5.3–36.2)	0.5 (0.2–1.1)	0.0 (0.0–0.1)	0.0 (0.0–0.1)	0 (0–0)
Tetanus	2.9 (2.4–4.0)	17.5 (15.1–20.6)	0.7 (0.5–1.3)	1.0 (0.6–1.8)	2.5 (1.7–5.3)	3.4 (2.2–7.3)
Measles	8.4 (3.2–16.5)	71.4 (27.6–139.3)	4.6 (1.6–9.4)	0.3 (0.1–0.8)	0.0 (0.0–0.1)	0 (0–0)
Varicella and herpes zoster	0.2 (0.2–0.2)	0.5 (0.4–0.8)	0.1 (0.1–0.1)	0.1 (0.1–0.1)	0.2 (0.2–0.3)	1.3 (0.9–1.7)
Neglected tropical diseases and malaria	20.8 (16.9–25.2)	132.9 (96.4–171.0)	8.7 (6.5–11.2)	6.5 (5.2–8.0)	11.3 (9.1–14.1)	15.2 (11.9–20.0)
Malaria	18.1 (14.2–22.4)	128.6 (92.2–167.0)	6.5 (4.2–9.1)	4.3 (3.1–5.8)	7.5 (5.4–10.2)	9.9 (6.7–14.4)
Chagas disease	0.2 (0.1–0.2)	0.0 (0.0–0.0)	0.0 (0.0–0.0)	0.1 (0.1–0.1)	0.6 (0.5–0.6)	1.4 (1.3–1.5)
Leishmaniasis	0.5 (0.3–0.8)	0.8 (0.5–1.2)	0.3 (0.1–0.4)	0.6 (0.4–0.9)	0.5 (0.3–0.7)	0.3 (0.2–0.4)
Visceral leishmaniasis	0.5 (0.3–0.8)	0.8 (0.5–1.2)	0.3 (0.1–0.4)	0.6 (0.4–0.9)	0.5 (0.3–0.7)	0.3 (0.2–0.4)
Cutaneous and mucocutaneous leishmaniasis	0 (0–0)	0 (0–0)	0 (0–0)	0 (0–0)	0 (0–0)	0 (0–0)
African trypanosomiasis	0.4 (0.2–0.7)	0.3 (0.1–0.5)	0.3 (0.1–0.4)	0.5 (0.3–0.9)	0.4 (0.2–0.7)	0.3 (0.2–0.5)
Schistosomiasis	0.2 (0.1–0.2)	0.0 (0.0–0.0)	0.0 (0.0–0.0)	0.1 (0.1–0.1)	0.5 (0.4–0.7)	0.9 (0.6–1.2)
Cysticercosis	0.0 (0.0–0.0)	0.0 (0.0–0.0)	0.0 (0.0–0.0)	0.0 (0.0–0.0)	0.0 (0.0–0.0)	0.0 (0.0–0.0)
Cystic echinococcosis	0.0 (0.0–0.0)	0.0 (0.0–0.0)	0.0 (0.0–0.0)	0.0 (0.0–0.0)	0.1 (0.1–0.1)	0.1 (0.1–0.2)
Lymphatic filariasis	0 (0–0)	0 (0–0)	0 (0–0)	0 (0–0)	0 (0–0)	0 (0–0)
Onchocerciasis	0 (0–0)	0 (0–0)	0 (0–0)	0 (0–0)	0 (0–0)	0 (0–0)
Trachoma	0 (0–0)	0 (0–0)	0 (0–0)	0 (0–0)	0 (0–0)	0 (0–0)
Dengue	0.1 (0.1–0.2)	0.4 (0.2–0.8)	0.1 (0.1–0.2)	0.1 (0.0–0.1)	0.1 (0.0–0.1)	0.1 (0.1–0.2)
Yellow fever	0.2 (0.0–0.5)	0.2 (0.0–0.5)	0.2 (0.0–0.6)	0.2 (0.0–0.5)	0.1 (0.0–0.3)	0.1 (0.0–0.2)
Rabies	0.8 (0.7–0.9)	0.9 (0.6–1.2)	1.0 (0.8–1.3)	0.5 (0.4–0.6)	1.2 (1.0–1.3)	1.3 (1.0–1.5)
Intestinal nematode infections	0.1 (0.1–0.1)	0.5 (0.4–0.6)	0.1 (0.0–0.1)	0.0 (0.0–0.0)	0.0 (0.0–0.0)	0.0 (0.0–0.1)
Ascariasis	0.1 (0.1–0.1)	0.5 (0.4–0.6)	0.1 (0.0–0.1)	0.0 (0.0–0.0)	0.0 (0.0–0.0)	0.0 (0.0–0.1)
Trichuriasis	0 (0–0)	0 (0–0)	0 (0–0)	0 (0–0)	0 (0–0)	0 (0–0)
Hookworm disease	0 (0–0)	0 (0–0)	0 (0–0)	0 (0–0)	0 (0–0)	0 (0–0)
Food-borne trematodiases	0 (0–0)	0 (0–0)	0 (0–0)	0 (0–0)	0 (0–0)	0 (0–0)
Leprosy	0 (0–0)	0 (0–0)	0 (0–0)	0 (0–0)	0 (0–0)	0 (0–0)
Ebola	0.0 (0.0–0.0)	0.0 (0.0–0.0)	0.0 (0.0–0.0)	0.0 (0.0–0.0)	0.0 (0.0–0.0)	0.0 (0.0–0.0)
Other neglected tropical diseases	0.3 (0.2–0.5)	1.2 (0.6–1.7)	0.2 (0.1–0.2)	0.1 (0.1–0.2)	0.4 (0.2–0.6)	0.7 (0.4–1.2)
Maternal disorders	0 (0–0)	0 (0–0)	0 (0–0)	0 (0–0)	0 (0–0)	0 (0–0)
Maternal hemorrhage	0 (0–0)	0 (0–0)	0 (0–0)	0 (0–0)	0 (0–0)	0 (0–0)
Maternal sepsis and other maternal infections	0 (0–0)	0 (0–0)	0 (0–0)	0 (0–0)	0 (0–0)	0 (0–0)
Maternal hypertensive disorders	0 (0–0)	0 (0–0)	0 (0–0)	0 (0–0)	0 (0–0)	0 (0–0)
Maternal obstructed labor and uterine rupture	0 (0–0)	0 (0–0)	0 (0–0)	0 (0–0)	0 (0–0)	0 (0–0)
Maternal abortion, miscarriage, and ectopic pregnancy	0 (0–0)	0 (0–0)	0 (0–0)	0 (0–0)	0 (0–0)	0 (0–0)

SR Table 14 - Global death rates (per 100,000) by age and cause for males in 2000						
Cause	All ages	0-5 years	5-14 years	15-49 years	50-69 years	70+ years
Indirect maternal deaths	0 (0–0)	0 (0–0)	0 (0–0)	0 (0–0)	0 (0–0)	0 (0–0)
Late maternal deaths	0 (0–0)	0 (0–0)	0 (0–0)	0 (0–0)	0 (0–0)	0 (0–0)
Maternal deaths aggravated by HIV/AIDS	0 (0–0)	0 (0–0)	0 (0–0)	0 (0–0)	0 (0–0)	0 (0–0)
Other maternal disorders	0 (0–0)	0 (0–0)	0 (0–0)	0 (0–0)	0 (0–0)	0 (0–0)
Neonatal disorders	52.9 (51.1–54.7)	516.2 (499.4–534.5)	0 (0–0)	0 (0–0)	0 (0–0)	0 (0–0)
Neonatal preterm birth complications	22.7 (20.6–25.9)	221.8 (200.9–252.6)	0 (0–0)	0 (0–0)	0 (0–0)	0 (0–0)
Neonatal encephalopathy due to birth asphyxia and trauma	16.7 (14.3–19.0)	162.8 (140.0–185.4)	0 (0–0)	0 (0–0)	0 (0–0)	0 (0–0)
Neonatal sepsis and other neonatal infections	7.1 (4.5–10.5)	68.9 (43.5–102.1)	0 (0–0)	0 (0–0)	0 (0–0)	0 (0–0)
Hemolytic disease and other neonatal jaundice	1.4 (0.9–2.6)	14.1 (9.2–25.4)	0 (0–0)	0 (0–0)	0 (0–0)	0 (0–0)
Other neonatal disorders	5.0 (3.3–6.7)	48.5 (32.5–65.0)	0 (0–0)	0 (0–0)	0 (0–0)	0 (0–0)
Nutritional deficiencies	9.7 (6.9–12.6)	60.8 (40.5–85.8)	1.6 (1.3–2.2)	1.6 (1.2–2.1)	7.1 (5.3–9.4)	38.1 (27.5–46.8)
Protein-energy malnutrition	8.5 (6.0–11.2)	57.2 (37.6–81.0)	1.4 (1.0–1.8)	1.1 (0.8–1.4)	5.4 (3.8–7.2)	31.6 (22.8–39.0)
Iodine deficiency	0.0 (0.0–0.0)	0.1 (0.0–0.3)	0.0 (0.0–0.0)	0.0 (0.0–0.0)	0.0 (0.0–0.1)	0.1 (0.1–0.2)
Vitamin A deficiency	0 (0–0)	0 (0–0)	0 (0–0)	0 (0–0)	0 (0–0)	0 (0–0)
Iron-deficiency anemia	0.7 (0.3–1.0)	2.3 (0.6–3.9)	0.1 (0.1–0.3)	0.3 (0.2–0.5)	1.1 (0.5–1.7)	2.8 (1.1–4.2)
Other nutritional deficiencies	0.5 (0.3–0.7)	1.2 (0.6–2.0)	0.1 (0.1–0.2)	0.2 (0.1–0.3)	0.6 (0.4–1.0)	3.5 (2.7–5.0)
Other communicable, maternal, neonatal, and nutritional diseases	6.9 (5.7–8.3)	33.0 (22.9–45.9)	1.9 (1.5–2.4)	2.7 (2.6–2.9)	8.3 (7.6–8.7)	16.6 (14.9–17.6)
Sexually transmitted diseases excluding HIV	2.7 (1.6–4.1)	22.6 (13.0–35.4)	0.8 (0.5–1.2)	0.1 (0.1–0.2)	0.5 (0.3–0.6)	1.0 (0.8–1.3)
Syphilis	2.6 (1.6–4.0)	22.6 (13.0–35.4)	0.8 (0.5–1.2)	0.1 (0.1–0.2)	0.4 (0.3–0.5)	0.9 (0.7–1.2)
Chlamydial infection	0 (0–0)	0 (0–0)	0 (0–0)	0 (0–0)	0 (0–0)	0 (0–0)
Gonococcal infection	0.0 (0.0–0.0)	0 (0–0)	0.0 (0.0–0.0)	0.0 (0.0–0.0)	0.0 (0.0–0.0)	0.1 (0.1–0.1)
Trichomoniasis	0 (0–0)	0 (0–0)	0 (0–0)	0 (0–0)	0 (0–0)	0 (0–0)
Genital herpes	0 (0–0)	0 (0–0)	0 (0–0)	0 (0–0)	0 (0–0)	0 (0–0)
Other sexually transmitted diseases	0.0 (0.0–0.0)	0 (0–0)	0.0 (0.0–0.0)	0.0 (0.0–0.0)	0.0 (0.0–0.0)	0.0 (0.0–0.0)
Hepatitis	2.7 (2.5–2.8)	3.9 (3.3–4.5)	0.7 (0.6–0.8)	2.1 (2.0–2.2)	5.4 (5.1–5.6)	9.3 (8.9–9.8)
Acute hepatitis A	0.4 (0.3–0.5)	3.0 (2.2–3.8)	0.1 (0.0–0.2)	0.1 (0.0–0.1)	0.1 (0.0–0.3)	0.2 (0.0–0.4)
Hepatitis B	1.6 (1.4–1.8)	0.2 (0.1–0.5)	0.1 (0.0–0.2)	1.4 (1.2–1.6)	4.4 (3.9–4.9)	7.1 (6.0–8.1)
Hepatitis C	0.1 (0.0–0.1)	0 (0–0)	0.0 (0.0–0.0)	0.0 (0.0–0.1)	0.2 (0.0–0.5)	0.3 (0.1–0.7)
Acute hepatitis E	0.6 (0.4–0.8)	0.6 (0.3–1.2)	0.5 (0.4–0.6)	0.6 (0.4–0.8)	0.6 (0.3–1.0)	1.8 (0.9–2.9)
Other infectious diseases	1.6 (1.0–1.9)	6.4 (3.0–9.4)	0.3 (0.2–0.4)	0.5 (0.4–0.6)	2.4 (1.7–2.8)	6.2 (4.5–7.1)
Non-communicable diseases	551.3 (541.2–560.4)	161.8 (136.6–199.1)	21.1 (19.7–22.4)	149.2 (145.0–153.2)	1522.9 (1493.1–1550.5)	7117.9 (7029.8–7198.7)
Neoplasms	126.2 (123.2–128.8)	7.2 (5.8–9.8)	5.6 (5.2–6.0)	34.3 (32.4–35.4)	429.6 (419.1–438.7)	1416.5 (1379.0–1451.0)
Lip and oral cavity cancer	2.1 (2.0–2.1)	0 (0–0)	0 (0–0)	0.8 (0.7–0.8)	8.3 (8.1–8.6)	17.2 (16.6–17.7)
Nasopharynx cancer	1.2 (0.9–1.3)	0 (0–0)	0.0 (0.0–0.1)	0.7 (0.5–0.8)	4.3 (3.2–4.6)	6.8 (5.5–7.2)
Other pharynx cancer	1.1 (1.0–1.1)	0 (0–0)	0 (0–0)	0.3 (0.3–0.3)	4.8 (4.7–5.0)	7.3 (7.0–7.6)
Esophageal cancer	9.1 (8.7–9.5)	0 (0–0)	0 (0–0)	1.9 (1.8–2.1)	37.1 (35.3–39.0)	91.6 (87.8–95.4)
Stomach cancer	16.1 (15.7–16.6)	0 (0–0)	0 (0–0)	3.6 (3.4–3.7)	57.9 (56.0–59.7)	188.4 (183.8–193.0)
Colon and rectum cancer	10.2 (10.0–10.4)	0 (0–0)	0 (0–0)	2.1 (2.1–2.2)	31.9 (31.2–32.6)	137.4 (134.8–140.1)
Liver cancer	15.1 (12.3–15.9)	0 (0–0)	0.2 (0.1–0.2)	7.2 (5.2–7.8)	56.9 (47.2–60.1)	108.6 (96.5–113.8)
Liver cancer due to hepatitis B	6.0 (4.8–6.5)	0 (0–0)	0.1 (0.1–0.1)	4.4 (3.2–4.8)	21.3 (17.2–23.3)	27.1 (22.8–30.2)
Liver cancer due to hepatitis C	2.2 (2.0–2.4)	0 (0–0)	0.0 (0.0–0.0)	0.4 (0.3–0.5)	8.2 (7.2–9.0)	25.9 (23.9–27.9)
Liver cancer due to alcohol use	4.7 (3.9–5.0)	0 (0–0)	0 (0–0)	1.2 (0.9–1.4)	20.1 (16.6–22.0)	40.3 (35.3–43.4)
Liver cancer due to other causes	2.1 (1.7–2.3)	0 (0–0)	0.1 (0.0–0.1)	1.2 (0.9–1.3)	7.3 (5.9–8.1)	15.3 (13.1–16.8)
Gallbladder and biliary tract cancer	1.4 (1.4–1.5)	0 (0–0)	0 (0–0)	0.2 (0.2–0.2)	4.5 (4.3–4.6)	20.8 (20.3–21.5)
Pancreatic cancer	4.6 (4.6–4.7)	0 (0–0)	0 (0–0)	0.9 (0.9–0.9)	16.8 (16.5–17.2)	55.5 (54.4–56.8)
Larynx cancer	2.4 (2.3–2.5)	0 (0–0)	0 (0–0)	0.5 (0.5–0.6)	10.0 (9.7–10.3)	22.8 (22.2–23.5)
Tracheal, bronchus, and lung cancer	29.8 (29.2–30.3)	0 (0–0)	0 (0–0)	5.1 (4.9–5.3)	113.6 (111.5–116.0)	347.3 (340.8–353.9)
Malignant skin melanoma	0.7 (0.5–1.0)	0 (0–0)	0 (0–0)	0.3 (0.2–0.5)	2.3 (1.5–3.3)	6.8 (4.4–10.1)
Non-melanoma skin cancer	0.7 (0.7–0.7)	0 (0–0)	0 (0–0)	0.2 (0.1–0.2)	2.0 (2.0–2.1)	10.3 (10.0–10.5)
Non-melanoma skin cancer (squamous-cell carcinoma)	0.7 (0.7–0.7)	0 (0–0)	0 (0–0)	0.2 (0.1–0.2)	2.0 (2.0–2.1)	10.3 (10.0–10.5)
Non-melanoma skin cancer (basal-cell carcinoma)	0 (0–0)	0 (0–0)	0 (0–0)	0 (0–0)	0 (0–0)	0 (0–0)
Breast cancer	0.2 (0.1–0.2)	0 (0–0)	0 (0–0)	0.0 (0.0–0.0)	0.6 (0.5–0.6)	1.9 (1.6–2.0)
Cervical cancer	0 (0–0)	0 (0–0)	0 (0–0)	0 (0–0)	0 (0–0)	0 (0–0)
Uterine cancer	0 (0–0)	0 (0–0)	0 (0–0)	0 (0–0)	0 (0–0)	0 (0–0)
Ovarian cancer	0 (0–0)	0 (0–0)	0 (0–0)	0 (0–0)	0 (0–0)	0 (0–0)
Prostate cancer	8.1 (6.6–10.1)	0 (0–0)	0 (0–0)	0.1 (0.1–0.2)	15.2 (12.3–18.7)	169.9 (136.5–212.0)
Testicular cancer	0.3 (0.3–0.3)	0 (0–0)	0 (0–0)	0.4 (0.4–0.4)	0.4 (0.4–0.4)	1.0 (1.0–1.1)

SR Table 14 - Global death rates (per 100,000) by age and cause for males in 2000						
**Cause**	**All ages**	**0-5 years**	**5-14 years**	**15-49 years**	**50-69 years**	**70+ years**
Kidney cancer	1.9 (1.8–1.9)	0.4 (0.2–0.7)	0.1 (0.1–0.1)	0.4 (0.4–0.4)	6.5 (6.4–6.7)	20.9 (20.3–21.5)
Bladder cancer	3.2 (3.2–3.3)	0 (0–0)	0 (0–0)	0.3 (0.3–0.3)	8.7 (8.5–8.9)	54.6 (53.3–55.9)
Brain and nervous system cancer	3.2 (2.6–3.6)	1.1 (0.7–1.9)	1.1 (0.8–1.3)	1.9 (1.5–2.2)	9.6 (8.1–10.6)	17.1 (14.7–18.5)
Thyroid cancer	0.3 (0.3–0.3)	0 (0–0)	0.0 (0.0–0.0)	0.1 (0.1–0.1)	0.9 (0.8–1.0)	3.3 (3.1–3.7)
Mesothelioma	0.5 (0.5–0.5)	0 (0–0)	0 (0–0)	0.1 (0.1–0.1)	1.6 (1.5–1.7)	5.6 (5.4–5.8)
Hodgkin lymphoma	0.6 (0.4–0.7)	0.1 (0.1–0.1)	0.1 (0.1–0.2)	0.4 (0.3–0.5)	1.6 (1.2–1.9)	3.2 (2.5–3.9)
Non-Hodgkin lymphoma	2.9 (2.5–3.2)	0.6 (0.4–1.1)	0.5 (0.4–0.6)	1.2 (1.1–1.4)	8.3 (7.2–9.0)	29.9 (24.4–32.0)
Multiple myeloma	1.1 (1.1–1.1)	0 (0–0)	0 (0–0)	0.2 (0.2–0.2)	3.6 (3.5–3.7)	14.6 (14.2–15.0)
Leukemia	5.3 (5.1–5.5)	2.7 (2.3–3.1)	2.7 (2.6–2.9)	3.0 (2.9–3.1)	11.0 (10.7–11.3)	40.8 (39.8–41.9)
Acute lymphoid leukemia	1.8 (1.6–2.1)	1.7 (1.3–2.2)	1.9 (1.7–2.2)	1.3 (1.1–1.5)	2.9 (2.5–3.4)	6.1 (5.3–7.0)
Chronic lymphoid leukemia	0.8 (0.7–0.9)	0 (0–0)	0 (0–0)	0.2 (0.2–0.3)	1.9 (1.8–2.1)	12.3 (11.6–13.0)
Acute myeloid leukemia	2.0 (1.7–2.3)	1.0 (0.6–1.3)	0.8 (0.5–1.0)	1.1 (0.9–1.3)	4.5 (4.0–5.0)	15.6 (14.3–17.1)
Chronic myeloid leukemia	0.7 (0.6–0.8)	0 (0–0)	0 (0–0)	0.4 (0.4–0.5)	1.6 (1.5–1.8)	6.8 (6.4–7.4)
Other neoplasms	4.2 (3.7–4.5)	2.2 (1.4–3.7)	0.9 (0.8–1.0)	2.2 (1.8–2.3)	11.1 (9.9–12.0)	33.0 (30.5–36.6)
Cardiovascular diseases	240.5 (236.6–244.6)	13.3 (12.2–14.3)	3.3 (3.1–3.5)	55.3 (53.8–57.3)	668.7 (655.4–681.4)	3428.8 (3377.1–3479.2)
Rheumatic heart disease	4.7 (4.4–5.1)	0.8 (0.7–1.0)	0.8 (0.7–0.9)	2.7 (2.5–2.9)	12.1 (11.3–13.3)	41.2 (39.1–43.8)
Ischemic heart disease	119.6 (117.5–121.7)	0.9 (0.8–1.1)	0.3 (0.3–0.3)	27.4 (26.6–28.6)	346.0 (338.6–353.1)	1682.7 (1654.5–1708.7)
Cerebrovascular disease	87.2 (84.9–89.5)	5.2 (4.7–5.7)	1.3 (1.2–1.3)	16.9 (16.3–17.7)	240.4 (231.9–248.0)	1295.3 (1263.5–1326.3)
Ischemic stroke	36.1 (34.9–37.4)	0.2 (0.2–0.2)	0.1 (0.1–0.1)	2.7 (2.5–3.0)	82.1 (78.3–86.1)	671.6 (652.0–692.0)
Hemorrhagic stroke	51.1 (49.4–52.8)	5.0 (4.5–5.5)	1.2 (1.1–1.3)	14.2 (13.7–14.9)	158.4 (152.4–164.2)	623.7 (602.9–645.0)
Hypertensive heart disease	10.5 (10.0–11.5)	0.1 (0.1–0.1)	0.0 (0.0–0.1)	2.4 (2.2–2.6)	28.9 (27.3–31.9)	154.1 (145.7–168.5)
Cardiomyopathy and myocarditis	5.4 (5.1–5.7)	2.2 (1.7–2.6)	0.4 (0.3–0.4)	2.9 (2.7–3.2)	13.7 (12.9–14.6)	49.3 (46.6–51.7)
Atrial fibrillation and flutter	1.6 (1.3–2.0)	0 (0–0)	0 (0–0)	0.0 (0.0–0.0)	1.3 (1.0–1.7)	40.0 (31.7–49.7)
Aortic aneurysm	2.6 (2.5–2.7)	0 (0–0)	0 (0–0)	0.5 (0.4–0.5)	6.6 (6.3–6.9)	42.8 (41.4–44.2)
Peripheral vascular disease	0.5 (0.4–0.5)	0 (0–0)	0 (0–0)	0.0 (0.0–0.0)	0.8 (0.8–0.9)	9.5 (9.0–9.9)
Endocarditis	0.9 (0.7–1.0)	0.7 (0.4–1.1)	0.1 (0.1–0.1)	0.4 (0.3–0.5)	1.8 (1.5–2.1)	8.8 (7.7–10.2)
Other cardiovascular and circulatory diseases	7.5 (7.3–7.7)	3.3 (2.9–3.8)	0.5 (0.4–0.5)	2.0 (1.9–2.1)	17.1 (16.5–17.8)	105.4 (102.0–109.2)
Chronic respiratory diseases	68.9 (66.9–70.9)	12.2 (10.3–14.5)	1.3 (1.2–1.5)	8.7 (8.4–9.3)	167.6 (161.1–174.0)	1145.9 (1114.3–1176.0)
Chronic obstructive pulmonary disease	57.3 (54.4–60.5)	4.7 (3.3–6.6)	0.3 (0.2–0.3)	5.3 (4.8–5.9)	136.4 (127.0–147.0)	1014.1 (970.5–1060.3)
Pneumoconiosis	0.7 (0.7–0.8)	0 (0–0)	0 (0–0)	0.2 (0.1–0.2)	1.8 (1.6–2.0)	11.5 (10.4–12.5)
Silicosis	0.3 (0.3–0.4)	0 (0–0)	0 (0–0)	0.1 (0.1–0.1)	0.9 (0.8–1.1)	5.1 (4.6–5.7)
Asbestosis	0.1 (0.0–0.1)	0 (0–0)	0 (0–0)	0.0 (0.0–0.0)	0.1 (0.1–0.2)	1.1 (0.7–1.3)
Coal workers pneumoconiosis	0.1 (0.1–0.1)	0 (0–0)	0 (0–0)	0.0 (0.0–0.0)	0.2 (0.2–0.2)	1.6 (1.4–1.8)
Other pneumoconiosis	0.2 (0.2–0.3)	0 (0–0)	0 (0–0)	0.0 (0.0–0.1)	0.6 (0.5–0.7)	3.8 (3.2–4.3)
Asthma	8.6 (6.1–10.6)	3.5 (2.4–4.6)	0.9 (0.8–1.1)	2.7 (2.1–3.3)	24.8 (17.0–31.8)	93.8 (65.4–120.5)
Interstitial lung disease and pulmonary sarcoidosis	1.2 (0.9–1.6)	0.0 (0.0–0.1)	0.0 (0.0–0.0)	0.2 (0.1–0.3)	3.0 (2.1–4.2)	19.5 (13.9–24.0)
Other chronic respiratory diseases	1.1 (0.7–1.3)	4.0 (2.5–5.7)	0.1 (0.1–0.2)	0.3 (0.2–0.4)	1.6 (1.0–2.0)	7.0 (4.9–8.6)
Cirrhosis and other chronic liver diseases	22.6 (21.8–23.9)	2.7 (2.2–3.4)	1.0 (0.9–1.1)	14.9 (14.2–15.9)	77.7 (74.8–83.3)	119.1 (115.1–124.5)
Cirrhosis and other chronic liver diseases due to hepatitis B	7.1 (6.6–7.8)	0.0 (0.0–0.0)	0.0 (0.0–0.1)	4.7 (4.3–5.2)	24.5 (22.0–27.7)	40.5 (36.9–44.7)
Cirrhosis and other chronic liver diseases due to hepatitis C	5.7 (5.3–6.1)	0.0 (0.0–0.0)	0.0 (0.0–0.0)	3.5 (3.1–3.9)	20.5 (18.4–22.6)	34.2 (31.3–37.6)
Cirrhosis and other chronic liver diseases due to alcohol use	6.4 (6.0–6.9)	0 (0–0)	0 (0–0)	4.7 (4.3–5.2)	23.3 (21.0–25.8)	25.6 (22.9–28.7)
Cirrhosis and other chronic liver diseases due to other causes	3.4 (3.1–3.7)	2.7 (2.2–3.3)	0.9 (0.9–1.1)	2.0 (1.8–2.2)	9.4 (8.3–10.7)	18.8 (16.9–20.8)
Digestive diseases	18.7 (17.8–20.4)	4.6 (3.5–6.1)	1.9 (1.7–2.2)	9.8 (9.1–10.5)	48.2 (45.9–53.0)	179.9 (173.0–199.5)
Peptic ulcer disease	6.1 (5.6–6.7)	0.5 (0.3–0.9)	0.4 (0.3–0.4)	3.1 (2.8–3.4)	17.6 (16.2–19.2)	56.8 (52.8–63.3)
Gastritis and duodenitis	0.9 (0.7–1.1)	0.2 (0.1–0.4)	0.1 (0.0–0.1)	0.5 (0.3–0.6)	2.4 (1.9–3.1)	8.3 (6.6–10.7)
Appendicitis	0.7 (0.7–0.9)	0.5 (0.3–0.7)	0.3 (0.2–0.4)	0.6 (0.5–0.7)	1.5 (1.3–1.8)	4.1 (3.6–4.9)
Paralytic ileus and intestinal obstruction	3.8 (3.3–4.7)	1.5 (1.0–2.3)	0.7 (0.6–0.9)	2.1 (1.7–2.5)	9.3 (8.0–11.7)	34.1 (30.2–43.4)
Inguinal, femoral, and abdominal hernia	1.1 (0.7–1.3)	0.3 (0.1–0.5)	0.1 (0.1–0.1)	0.5 (0.3–0.6)	2.8 (1.8–3.4)	12.2 (7.9–14.4)
Inflammatory bowel disease	0.6 (0.5–0.6)	0.3 (0.1–0.6)	0.1 (0.0–0.1)	0.3 (0.2–0.3)	1.3 (1.1–1.5)	6.1 (5.3–6.9)
Vascular intestinal disorders	1.0 (1.0–1.1)	0.2 (0.1–0.3)	0.0 (0.0–0.0)	0.2 (0.2–0.2)	2.6 (2.4–2.9)	16.0 (15.0–17.3)
Gallbladder and biliary diseases	1.2 (1.1–1.3)	0.1 (0.1–0.2)	0.1 (0.1–0.1)	0.4 (0.3–0.5)	2.7 (2.5–3.1)	15.9 (14.7–17.6)
Pancreatitis	1.8 (1.7–1.9)	0.0 (0.0–0.1)	0.0 (0.0–0.1)	1.6 (1.5–1.8)	4.4 (4.0–4.8)	9.7 (9.0–10.6)
Other digestive diseases	1.5 (1.2–2.1)	0.9 (0.6–1.4)	0.2 (0.2–0.3)	0.6 (0.5–0.9)	3.5 (2.7–5.0)	16.6 (14.1–21.6)
Neurological disorders	18.5 (16.4–20.7)	3.9 (2.6–5.1)	1.4 (1.3–1.6)	3.2 (3.1–3.4)	17.4 (15.0–19.8)	387.3 (330.2–443.4)
Alzheimer disease and other dementias	13.7 (11.5–15.8)	0 (0–0)	0 (0–0)	0.0 (0.0–0.1)	9.8 (7.4–12.2)	345.1 (288.1–401.5)
Parkinson disease	1.2 (1.1–1.3)	0 (0–0)	0 (0–0)	0.0 (0.0–0.0)	1.3 (1.3–1.5)	28.0 (26.7–29.6)
Epilepsy	2.3 (2.1–2.4)	3.0 (1.9–3.9)	1.1 (1.0–1.2)	2.4 (2.3–2.5)	2.6 (2.5–2.7)	4.6 (4.4–4.8)

SR Table 14 - Global death rates (per 100,000) by age and cause for males in 2000

Cause	All ages	0-5 years	5-14 years	15-49 years	50-69 years	70+ years
Multiple sclerosis	0.2 (0.2–0.2)	0 (0–0)	0 (0–0)	0.2 (0.1–0.2)	0.7 (0.7–0.8)	1.1 (0.9–1.2)
Motor neuron disease	0.4 (0.4–0.4)	0.2 (0.1–0.3)	0.0 (0.0–0.0)	0.1 (0.1–0.1)	1.4 (1.4–1.6)	4.0 (3.8–4.1)
Migraine	0 (0–0)	0 (0–0)	0 (0–0)	0 (0–0)	0 (0–0)	0 (0–0)
Tension-type headache	0 (0–0)	0 (0–0)	0 (0–0)	0 (0–0)	0 (0–0)	0 (0–0)
Medication overuse headache	0 (0–0)	0 (0–0)	0 (0–0)	0 (0–0)	0 (0–0)	0 (0–0)
Other neurological disorders	0.8 (0.7–0.8)	0.8 (0.5–1.1)	0.3 (0.2–0.3)	0.5 (0.5–0.5)	1.4 (1.4–1.6)	4.6 (4.3–4.9)
Mental and substance use disorders	6.9 (6.3–7.1)	0.0 (0.0–0.0)	0.0 (0.0–0.0)	8.2 (7.6–8.5)	16.0 (14.5–16.6)	14.1 (13.3–14.5)
Schizophrenia	0.3 (0.3–0.4)	0 (0–0)	0 (0–0)	0.3 (0.3–0.4)	0.8 (0.7–0.9)	1.5 (1.4–1.6)
Alcohol use disorders	3.9 (3.6–4.0)	0 (0–0)	0 (0–0)	4.0 (3.8–4.2)	11.5 (10.6–12.0)	8.0 (7.6–8.3)
Drug use disorders	2.7 (2.4–2.8)	0.0 (0.0–0.0)	0 (0–0)	3.9 (3.5–4.0)	3.7 (3.3–3.9)	4.6 (4.2–4.9)
Opioid use disorders	2.1 (1.9–2.2)	0.0 (0.0–0.0)	0 (0–0)	3.0 (2.7–3.2)	2.8 (2.5–3.0)	3.5 (3.2–3.8)
Cocaine use disorders	0.1 (0.1–0.1)	0 (0–0)	0 (0–0)	0.2 (0.1–0.2)	0.2 (0.1–0.2)	0.1 (0.1–0.1)
Amphetamine use disorders	0.1 (0.1–0.1)	0 (0–0)	0 (0–0)	0.1 (0.1–0.1)	0.1 (0.1–0.1)	0.1 (0.1–0.1)
Cannabis use disorders	0 (0–0)	0 (0–0)	0 (0–0)	0 (0–0)	0 (0–0)	0 (0–0)
Other drug use disorders	0.4 (0.4–0.4)	0 (0–0)	0 (0–0)	0.5 (0.5–0.6)	0.6 (0.5–0.7)	0.9 (0.8–1.0)
Depressive disorders	0 (0–0)	0 (0–0)	0 (0–0)	0 (0–0)	0 (0–0)	0 (0–0)
Major depressive disorder	0 (0–0)	0 (0–0)	0 (0–0)	0 (0–0)	0 (0–0)	0 (0–0)
Dysthymia	0 (0–0)	0 (0–0)	0 (0–0)	0 (0–0)	0 (0–0)	0 (0–0)
Bipolar disorder	0 (0–0)	0 (0–0)	0 (0–0)	0 (0–0)	0 (0–0)	0 (0–0)
Anxiety disorders	0 (0–0)	0 (0–0)	0 (0–0)	0 (0–0)	0 (0–0)	0 (0–0)
Eating disorders	0.0 (0.0–0.0)	0 (0–0)	0.0 (0.0–0.0)	0.0 (0.0–0.0)	0 (0–0)	0 (0–0)
Anorexia nervosa	0.0 (0.0–0.0)	0 (0–0)	0.0 (0.0–0.0)	0.0 (0.0–0.0)	0 (0–0)	0 (0–0)
Bulimia nervosa	0.0 (0.0–0.0)	0 (0–0)	0.0 (0.0–0.0)	0.0 (0.0–0.0)	0 (0–0)	0 (0–0)
[truncated: 24,062,015 more chars]
